# Supplementary material for: Detection of offensive content in the Kazakh language using machine learning and deep learning approaches
Source: PeerJ Comput Sci. 2025 Aug 11;11:e3027. doi: 10.7717/peerj-cs.3027 (PMC12453855; doi:10.7717/peerj-cs.3027)
Supplement: Supplemental Information 1 [file peerj-cs-11-3027-s001.zip › Code/Auto_LSTM_CNN_GRU.html]

Auto\_LSTM\_CNN\_GRU


In [1]:

```
import warnings
warnings.filterwarnings('ignore')
```

In [2]:

```
import os

import pandas as pd
import numpy as np

import matplotlib.pyplot as plt
import seaborn as sns


# MLDL
from sklearn.model_selection import train_test_split
from sklearn.metrics import classification_report, accuracy_score, precision_score, recall_score, f1_score, confusion_matrix, roc_auc_score, roc_curve

import tensorflow as tf
from tensorflow.keras.layers import Embedding, LSTM, Dense, TextVectorization, Input
from tensorflow.keras.utils import to_categorical

from keras.preprocessing.text import Tokenizer
from keras_preprocessing.sequence import pad_sequences
from keras.models import Sequential, load_model
from keras.layers import Activation, Dense, Embedding, LSTM, SpatialDropout1D, Dropout, Flatten, GRU, Conv1D, MaxPooling1D, Bidirectional

from keras.callbacks import ModelCheckpoint, EarlyStopping, ReduceLROnPlateau
```

```
WARNING:tensorflow:From C:\Users\Admin-server\AppData\Local\Programs\Python\Python310\lib\site-packages\keras\src\losses.py:2976: The name tf.losses.sparse_softmax_cross_entropy is deprecated. Please use tf.compat.v1.losses.sparse_softmax_cross_entropy instead.
```

In [3]:

```
if not os.path.exists('models'):
    os.mkdir('models')
```

In [4]:

```
df = pd.read_csv('multiclass_2024_04_16.csv', usecols=['label', 'message', 'message_stemmed', 'label_name'])


custom_stop_words = {
    "ах", "тағы", "тағыда", "әрине","жоқ","сондай","осындай","осылай","солай","мұндай","бұндай","мен","сен","ол","біз","біздер",
  "олар","сіз","сіздер","маған","оған","саған","біздің""сіздің","оның","бізге","сізге","оларға","біздерге","сіздерге","оларға",
  "менімен","сенімен","онымен","бізбен","сізбен","олармен","біздермен","сіздермен","менің","сенің","біздің","сіздің","оның",
  "біздердің","сіздердің","олардың","маған","саған","оған","менен","сенен","одан","бізден","сізден","олардан","біздерден",
  "сіздерден","айтпақшы","сонымен","сондықтан","бұл","осы","сол","анау","мынау","сонау","осынау","ана","мына","сона","әні","міне",
  "кейбір","қайсыбір","әрбір","бірнеше","бірдеме","әркім","әрне","әрқайсы","әрқалай","әлдекім","әлдене","әлдеқайдан","әлденеше",
  "әлдеқалай","әлдеқашан","алдақашан","ешкім","ешбір","ешқашан","ешқандай","емес","бәрі","бар","бүкіл","өзім","өзің","дегенмен",
  "әйтпесе", "себебі", "өйткені", "сондықтан", "үшін", "сияқты", "туралы", "арқылы", "шамалы", "осындай", "ғана", "қана", "тек",
  "әншейін", "және", "деп", "керек","бірақ","бір","да","егер","адам","адамдар","да","де","оны","болып","босқа","басқа","болды", "мүмкін"
  "онда","барлық","немесе","одан", "рет", "өз", "кейін","пен","со","ал","болады","rt","мені","өте","ал","сс","сені","екен"
  "еді","келеді","болса","не","тым","сізді", "ба", "иә", "ең", "сізде", "сізді", "өз","желп","анау","бізге","мәссаған","сізден",
  "уай","сарт","саңқ","қайқаң-құйқаң","әйда","sub","acele","тағыда","eram","қалт-қалт","қызараң-қызараң",
  "эй","әйтпесе","шырт","шіңк","арс-ұрс","сайын","дүңк","масқарай","салаң-сұлаң","бұрын","әйткенмен","я","ой","далаң-далаң","пішту",
  "міне","сізге","күллі","кейбір","олар","ие","кәнеки","шаңқ-шұңқ","оған","ештеме","ешқашан","ешкім","беу","ырқ","ea","үйт","ербелең-ербелең",
  "әлденеше","қалт-құлт","барша","менен","айтпақшы","әлдеқалай","одан","өзіме","үшін","кейбіреу","кірт","шақты","ыңқ","жалт-жұлт",
  "кәне","қатар","біздердің","оның","уау","жуық","борт","эх","ол","алдақашан","шек","біздерден","бүкіл","ca","солай","бойымен","сондықтан",
  "олардың","арнайы","кәні","өй","өзінің","әлдекім","қайсыбір","мына","сарт-сұрт","сенен","онан","күрт","қорс","қап","туралы","гүрс","гөрі",
  "осы","мышы","сіздерден","әй","еш","арс","әркім","күңк","дегенмен","жалт-жалт","пай-пай","бетер","менімен","сенен","өйткені",
  "әлдеқайдан","сіз","митың-митың","өзі","себебі","бізбен","бірақ","онымен","құрау-құрау","сонымен","сізбен","өзге","дүрс","қаңғыр-күңгір",
  "алатау","таңқ","пфша","сол","pe","шамалы","паһ-паһ","сона","қаралы","сияқты","ә","тәк","е","менің","ғұрлы","ал","бірге","қолп",
  "пай","өзіне","па","таман","бізден","батыр-бұтыр","сондай","әрқайсы","бәрекелді","біздің","бүйт","саған","тарс-тұрс",
  "тарбаң-тарбаң","ыржың-тыржың","әрбір","пырс","ай","барқ","соң","бойы","күшім","қыңқ","арсалаң-арсалаң","сыңқ","салым",
  "ура","түгел","шіркін","алайда","кә","бәрі","шәйт","шейін","ей","шаңқ","әттең","ешқайсы","кәһ","арбаң-арбаң","бұндай","құр",
  "ойпырмай","қана","қаңқ-қаңқ","маңқ","қаңқ-құңқ","аһа","былп","морт","болп","олармен","қош-қош","тырс","ана","сорап","әлдене","ау"
  "өзімнің","aici","сенде","осылай","ырс","қоса","жалп","жаракімалла","біздерге","менде","япырмай","түге","ох","алақай","ғұрлым",
  "таяу","тарта","астапыралла","мыңқ","пішә","бүгжең-бүгжең","о","құрау","әттеген-ай","дейін","әттегенай","ei","тыңқ","қарай",
  "әукім","әрне","біреу","бері","бірнеше"
}

df['clean_message'] = df['message'].apply(lambda text: ' '.join(word for word in text.split() if word.lower() not in custom_stop_words))
df['clean_stemmed'] = df['message_stemmed'].apply(lambda text: ' '.join(word for word in text.split() if word.lower() not in custom_stop_words))

df.loc[df['label_name'] == 'neutral' , 'LABEL'] = 0
df.loc[df['label_name'] == 'racism', 'LABEL'] = 1
df.loc[df['label_name'] == 'bullying' , 'LABEL'] = 2
df.loc[df['label_name'] == 'nazism', 'LABEL'] = 3
df.loc[df['label_name'] == 'violent', 'LABEL'] = 4

df.head()
```

Out[4]:

|  | label | message | message\_stemmed | label\_name | clean\_message | clean\_stemmed | LABEL |
| --- | --- | --- | --- | --- | --- | --- | --- |
| 0 | 4 | біздің сарбаздарымыз өз істерінің әділдігімен ... | біздің сарбаз өз іс әділдіг қарулан басқынш ар... | violent | сарбаздарымыз істерінің әділдігімен қаруланып ... | сарбаз іс әділдіг қарулан басқынш армия ара кө... | 4.0 |
| 1 | 4 | біздің еркін болғанымызды ештеңе жеңе алмайды ... | біздің еркін бол ештеңе же алма тағы бір рет в... | violent | еркін болғанымызды ештеңе жеңе алмайды вашингт... | еркін бол ештеңе же алма вашингтон еуропа түрм... | 4.0 |
| 2 | 4 | біз барак обама джордж буштың қасіретті мұрасы... | біз барак оба джордж буш қасірет мұра бастал д... | violent | барак обама джордж буштың қасіретті мұрасынан ... | барак оба джордж буш қасірет мұра бастал үмітт... | 4.0 |
| 3 | 4 | израильдің агрессиясына қарсы күн сайынғы нара... | израиль агрессия қарсы күн сайынғы наразылық а... | violent | израильдің агрессиясына қарсы күн сайынғы нара... | израиль агрессия қарсы күн сайынғы наразылық а... | 4.0 |
| 4 | 4 | израиль сөзсіз газаның жойылуын өлімі мен қайғ... | израиль сөзсіз газа жойыл өлім мен қайғы қасір... | violent | израиль сөзсіз газаның жойылуын өлімі қайғы қа... | израиль сөзсіз газа жойыл өлім қайғы қасірет ж... | 4.0 |

# Models¶

In [5]:

```
def create_lstm(vectorizer):
    emb_dim = 256
    model_lstm1 = Sequential()
    model_lstm1.add(Input(shape=(1,), dtype=tf.string))
    model_lstm1.add(vectorizer)
    model_lstm1.add(Embedding(input_dim=len(vectorizer.get_vocabulary()), output_dim=emb_dim, mask_zero=True))
    model_lstm1.add(SpatialDropout1D(0.8))
    model_lstm1.add(Bidirectional(LSTM(256, dropout=0.5, recurrent_dropout=0.5)))
    model_lstm1.add(Dropout(0.5))
    model_lstm1.add(Flatten())
    model_lstm1.add(Dense(32, activation='relu'))
    model_lstm1.add(Dropout(0.5))
    model_lstm1.add(Dense(5, activation='softmax'))
    model_lstm1.compile(optimizer=tf.optimizers.Adam(), loss='categorical_crossentropy', metrics=['acc'])
    return model_lstm1

def create_lstm2x(vectorizer):
    emb_dim = 256
    model_lstm2 = Sequential()
    model_lstm2.add(Input(shape=(1,), dtype=tf.string))
    model_lstm2.add(vectorizer)
    model_lstm2.add(Embedding(input_dim=len(vectorizer.get_vocabulary()), output_dim=emb_dim, mask_zero=True))
    model_lstm2.add(SpatialDropout1D(0.8))
    model_lstm2.add(Bidirectional(LSTM(128, dropout=0.5, recurrent_dropout=0.5, return_sequences=True)))
    model_lstm2.add(Dropout(0.5))
    model_lstm2.add(Bidirectional(LSTM(128, dropout=0.5, recurrent_dropout=0.5)))
    model_lstm2.add(Dropout(0.5))
    model_lstm2.add(Flatten())
    model_lstm2.add(Dense(64, activation='relu'))
    model_lstm2.add(Dropout(0.5))
    model_lstm2.add(Dense(5, activation='softmax'))
    model_lstm2.compile(optimizer=tf.optimizers.Adam(), loss='categorical_crossentropy', metrics=['acc'])
    return model_lstm2

def create_gru(vectorizer):
    emb_dim = 256
    model_gru = Sequential()
    model_gru.add(Input(shape=(1,), dtype=tf.string))
    model_gru.add(vectorizer)
    model_gru.add(Embedding(input_dim=len(vectorizer.get_vocabulary()), output_dim=emb_dim, mask_zero=True))
    model_gru.add(SpatialDropout1D(0.8))
    model_gru.add(GRU(units=64, dropout=0.5, recurrent_dropout=0.5))
    model_gru.add(Dropout(0.5))
    model_gru.add(Dense(256, activation='relu'))
    model_gru.add(Dropout(0.5))
    model_gru.add(Dense(5, activation='softmax'))
    model_gru.compile(optimizer=tf.optimizers.Adam(), loss='categorical_crossentropy', metrics=['acc'])
    return model_gru

def create_cnn_lstm(vectorizer):
    emb_dim = 256
    model_cl = Sequential()
    model_cl.add(Input(shape=(1,), dtype=tf.string))
    model_cl.add(vectorizer)
    model_cl.add(Embedding(input_dim=len(vectorizer.get_vocabulary()), output_dim=emb_dim, mask_zero=True))
    model_cl.add(SpatialDropout1D(0.8))
    model_cl.add(Conv1D(filters=64, kernel_size=6, padding='same', activation='relu'))
    model_cl.add(MaxPooling1D(pool_size=2))
    model_cl.add(Conv1D(filters=32, kernel_size=6, activation='relu'))
    model_cl.add(MaxPooling1D(pool_size=2))
    model_cl.add(Bidirectional(LSTM(128, dropout=0.5, recurrent_dropout=0.5, return_sequences=True)))
    model_cl.add(Dropout(0.5))
    model_cl.add(Bidirectional(LSTM(128, dropout=0.5, recurrent_dropout=0.5)))
    model_cl.add(Dropout(0.5))
    model_cl.add(Flatten())
    model_cl.add(Dense(64, activation='relu'))
    model_cl.add(Dropout(0.5))
    model_cl.add(Dense(5, activation='softmax'))
    model_cl.compile(optimizer='adam', loss='categorical_crossentropy', metrics=['acc'])
    return model_cl
```

# Aumotomatic¶

In [6]:

```
def run_different_models(df, epochs=20, batch_size=64):
    labels_categorical = to_categorical(df["LABEL"].values, num_classes=5)
    
    datasets = {
        'Text': 'clean_message',
        'Stemmed': 'clean_stemmed'
    }
    
    ngram_list = [
        (1, 1),
        (1, 2),
        (2, 2),
        (2, 3)
    ]

    metric_results = []

    for dataset_name, dataset in datasets.items():
    
        texts = df[dataset].values
        X_train, X_test, y_train, y_test = train_test_split(texts, labels_categorical, test_size = 0.25, random_state = 42)
    
        for ngram in ngram_list:
            vectorizer = TextVectorization(ngrams=ngram, output_mode='int', max_tokens=60000, output_sequence_length=64)
            vectorizer.adapt(X_train)
        
            models = {
                'LSTM': create_lstm(vectorizer),
                'LSTM2X': create_lstm2x(vectorizer),
                'GRU': create_gru(vectorizer),
                'CNN+LSTM': create_cnn_lstm(vectorizer)
            }
    
            for model_name, model in models.items():
                title_name = f'dataset: {dataset_name}, ngram: {ngram}, model: {model_name}'
                print('#############################################################')
                print(f'START! {title_name}')
                print('#############################################################')
                print(model.summary())
    
                
                ## TRAINING
                early_stopping_callback = EarlyStopping(
                    monitor="val_acc", 
                    mode="max", 
                    patience=10, 
                    verbose=1, 
                    restore_best_weights=True
                )
                reduce_lr_callback = ReduceLROnPlateau(
                    monitor="val_loss", 
                    factor=0.5, 
                    patience=5, 
                    verbose=1, 
                    mode="min", 
                    min_delta=0.0001, 
                    cooldown=0, 
                    min_lr=0
                )
                callbacks=[early_stopping_callback, reduce_lr_callback]
                history = model.fit(
                    X_train, 
                    y_train, 
                    epochs=epochs, 
                    batch_size=batch_size, 
                    validation_data=(X_test, y_test), 
                    callbacks=callbacks
                )
                
                
                ## Results
                results = model.evaluate(X_test, y_test, verbose=False)
                predict_proba = model.predict(X_test)
                predict = predict_proba.argmax(axis=1)

                y_test_one = y_test.argmax(axis=1)


                precision = precision_score(y_test_one, predict, pos_label='positive', average='weighted')
                recall = recall_score(y_test_one, predict, pos_label='positive', average='weighted')
                accuracy = accuracy_score(y_test_one, predict)
                f1 = f1_score(y_test_one, predict, pos_label='positive', average='weighted')
                roc_auc = roc_auc_score(y_test_one, predict_proba, multi_class='ovo')
                current_metrics = {
                    'dataset': dataset_name,
                    'ngram': ngram,
                    'model': model_name,
                    'precision': precision,
                    'recall': recall,
                    'accuracy': accuracy,
                    'f1': f1,
                    'roc_auc': roc_auc,
                }

                metric_results.append(current_metrics)

                
                print(f'Test results - Loss: {results[0]} - Accuracy: {100*results[1]}%\n metrics: {current_metrics}\n')
                
                
                ## PLOTS
                acc = history.history['acc']
                val_acc = history.history['val_acc']
                loss = history.history['loss']
                val_loss = history.history['val_loss']
                plt.plot(acc, 'go', label='Train accuracy')
                plt.plot(val_acc, 'g', label='Validate accuracy')
                plt.title(f'Train and validate accuracy of {title_name}')
                plt.legend()
                
                plt.figure()
                plt.plot(loss, 'go', label='Train loss')
                plt.plot(val_loss, 'g', label='Validate loss')
                plt.title(f'Train and validate loss of {title_name}')
                plt.legend()
                plt.show()
                print('#############################################################')
                print('END')
                print('#############################################################')

    return metric_results
```

# Combined¶

In [7]:

```
metrics_history = run_different_models(
    df,
    epochs=20, 
    batch_size=64
)
```

```
WARNING:tensorflow:From C:\Users\Admin-server\AppData\Local\Programs\Python\Python310\lib\site-packages\keras\src\backend.py:873: The name tf.get_default_graph is deprecated. Please use tf.compat.v1.get_default_graph instead.

WARNING:tensorflow:From C:\Users\Admin-server\AppData\Local\Programs\Python\Python310\lib\site-packages\keras\src\utils\tf_utils.py:492: The name tf.ragged.RaggedTensorValue is deprecated. Please use tf.compat.v1.ragged.RaggedTensorValue instead.

WARNING:tensorflow:From C:\Users\Admin-server\AppData\Local\Programs\Python\Python310\lib\site-packages\keras\src\backend.py:6642: The name tf.nn.max_pool is deprecated. Please use tf.nn.max_pool2d instead.

WARNING:tensorflow:From C:\Users\Admin-server\AppData\Local\Programs\Python\Python310\lib\site-packages\keras\src\optimizers\__init__.py:309: The name tf.train.Optimizer is deprecated. Please use tf.compat.v1.train.Optimizer instead.

#############################################################
START! dataset: Text, ngram: (1, 1), model: LSTM
#############################################################
Model: "sequential"
_________________________________________________________________
 Layer (type)                Output Shape              Param #   
=================================================================
 text_vectorization (TextVe  (None, 64)                0         
 ctorization)                                                    
                                                                 
 embedding (Embedding)       (None, 64, 256)           6879744   
                                                                 
 spatial_dropout1d (Spatial  (None, 64, 256)           0         
 Dropout1D)                                                      
                                                                 
 bidirectional (Bidirection  (None, 512)               1050624   
 al)                                                             
                                                                 
 dropout (Dropout)           (None, 512)               0         
                                                                 
 flatten (Flatten)           (None, 512)               0         
                                                                 
 dense (Dense)               (None, 32)                16416     
                                                                 
 dropout_1 (Dropout)         (None, 32)                0         
                                                                 
 dense_1 (Dense)             (None, 5)                 165       
                                                                 
=================================================================
Total params: 7946949 (30.32 MB)
Trainable params: 7946949 (30.32 MB)
Non-trainable params: 0 (0.00 Byte)
_________________________________________________________________
None
Epoch 1/20
WARNING:tensorflow:From C:\Users\Admin-server\AppData\Local\Programs\Python\Python310\lib\site-packages\keras\src\engine\base_layer_utils.py:384: The name tf.executing_eagerly_outside_functions is deprecated. Please use tf.compat.v1.executing_eagerly_outside_functions instead.

120/120 [==============================] - 47s 348ms/step - loss: 1.4357 - acc: 0.3618 - val_loss: 1.0857 - val_acc: 0.6307 - lr: 0.0010
Epoch 2/20
120/120 [==============================] - 46s 387ms/step - loss: 0.9281 - acc: 0.6369 - val_loss: 0.7160 - val_acc: 0.7402 - lr: 0.0010
Epoch 3/20
120/120 [==============================] - 48s 396ms/step - loss: 0.6472 - acc: 0.7613 - val_loss: 0.5757 - val_acc: 0.7959 - lr: 0.0010
Epoch 4/20
120/120 [==============================] - 48s 401ms/step - loss: 0.4546 - acc: 0.8436 - val_loss: 0.5435 - val_acc: 0.8175 - lr: 0.0010
Epoch 5/20
120/120 [==============================] - 48s 400ms/step - loss: 0.3511 - acc: 0.8867 - val_loss: 0.6442 - val_acc: 0.8085 - lr: 0.0010
Epoch 6/20
120/120 [==============================] - 48s 398ms/step - loss: 0.2480 - acc: 0.9228 - val_loss: 0.5165 - val_acc: 0.8308 - lr: 0.0010
Epoch 7/20
120/120 [==============================] - 48s 402ms/step - loss: 0.1848 - acc: 0.9454 - val_loss: 0.4918 - val_acc: 0.8501 - lr: 0.0010
Epoch 8/20
120/120 [==============================] - 48s 402ms/step - loss: 0.1528 - acc: 0.9583 - val_loss: 0.6364 - val_acc: 0.8320 - lr: 0.0010
Epoch 9/20
120/120 [==============================] - 48s 402ms/step - loss: 0.0965 - acc: 0.9751 - val_loss: 0.5438 - val_acc: 0.8509 - lr: 0.0010
Epoch 10/20
120/120 [==============================] - 47s 396ms/step - loss: 0.0828 - acc: 0.9788 - val_loss: 0.5792 - val_acc: 0.8615 - lr: 0.0010
Epoch 11/20
120/120 [==============================] - 47s 395ms/step - loss: 0.0882 - acc: 0.9772 - val_loss: 0.6652 - val_acc: 0.8579 - lr: 0.0010
Epoch 12/20
120/120 [==============================] - ETA: 0s - loss: 0.0750 - acc: 0.9834
Epoch 12: ReduceLROnPlateau reducing learning rate to 0.0005000000237487257.
120/120 [==============================] - 47s 393ms/step - loss: 0.0750 - acc: 0.9834 - val_loss: 0.6162 - val_acc: 0.8587 - lr: 0.0010
Epoch 13/20
120/120 [==============================] - 47s 391ms/step - loss: 0.0509 - acc: 0.9869 - val_loss: 0.6465 - val_acc: 0.8575 - lr: 5.0000e-04
Epoch 14/20
120/120 [==============================] - 47s 390ms/step - loss: 0.0442 - acc: 0.9887 - val_loss: 0.6836 - val_acc: 0.8607 - lr: 5.0000e-04
Epoch 15/20
120/120 [==============================] - 47s 390ms/step - loss: 0.0343 - acc: 0.9908 - val_loss: 0.7476 - val_acc: 0.8481 - lr: 5.0000e-04
Epoch 16/20
120/120 [==============================] - 47s 392ms/step - loss: 0.0383 - acc: 0.9887 - val_loss: 0.6963 - val_acc: 0.8571 - lr: 5.0000e-04
Epoch 17/20
120/120 [==============================] - ETA: 0s - loss: 0.0407 - acc: 0.9881
Epoch 17: ReduceLROnPlateau reducing learning rate to 0.0002500000118743628.
120/120 [==============================] - 47s 392ms/step - loss: 0.0407 - acc: 0.9881 - val_loss: 0.6568 - val_acc: 0.8513 - lr: 5.0000e-04
Epoch 18/20
120/120 [==============================] - 47s 390ms/step - loss: 0.0350 - acc: 0.9906 - val_loss: 0.6648 - val_acc: 0.8556 - lr: 2.5000e-04
Epoch 19/20
120/120 [==============================] - 47s 394ms/step - loss: 0.0338 - acc: 0.9904 - val_loss: 0.6814 - val_acc: 0.8544 - lr: 2.5000e-04
Epoch 20/20
120/120 [==============================] - 48s 399ms/step - loss: 0.0318 - acc: 0.9923 - val_loss: 0.6894 - val_acc: 0.8626 - lr: 2.5000e-04
80/80 [==============================] - 3s 33ms/step
Test results - Loss: 0.6894391775131226 - Accuracy: 86.26373410224915%
 metrics: {'dataset': 'Text', 'ngram': (1, 1), 'model': 'LSTM', 'precision': 0.8650230505937823, 'recall': 0.8626373626373627, 'accuracy': 0.8626373626373627, 'f1': 0.8632596964556417, 'roc_auc': 0.9739297968925461}
```

```
#############################################################
END
#############################################################
#############################################################
START! dataset: Text, ngram: (1, 1), model: LSTM2X
#############################################################
Model: "sequential_1"
_________________________________________________________________
 Layer (type)                Output Shape              Param #   
=================================================================
 text_vectorization (TextVe  (None, 64)                0         
 ctorization)                                                    
                                                                 
 embedding_1 (Embedding)     (None, 64, 256)           6879744   
                                                                 
 spatial_dropout1d_1 (Spati  (None, 64, 256)           0         
 alDropout1D)                                                    
                                                                 
 bidirectional_1 (Bidirecti  (None, 64, 256)           394240    
 onal)                                                           
                                                                 
 dropout_2 (Dropout)         (None, 64, 256)           0         
                                                                 
 bidirectional_2 (Bidirecti  (None, 256)               394240    
 onal)                                                           
                                                                 
 dropout_3 (Dropout)         (None, 256)               0         
                                                                 
 flatten_1 (Flatten)         (None, 256)               0         
                                                                 
 dense_2 (Dense)             (None, 64)                16448     
                                                                 
 dropout_4 (Dropout)         (None, 64)                0         
                                                                 
 dense_3 (Dense)             (None, 5)                 325       
                                                                 
=================================================================
Total params: 7684997 (29.32 MB)
Trainable params: 7684997 (29.32 MB)
Non-trainable params: 0 (0.00 Byte)
_________________________________________________________________
None
Epoch 1/20
120/120 [==============================] - 164s 1s/step - loss: 1.4015 - acc: 0.3678 - val_loss: 1.0522 - val_acc: 0.5640 - lr: 0.0010
Epoch 2/20
120/120 [==============================] - 169s 1s/step - loss: 0.9149 - acc: 0.6348 - val_loss: 0.7027 - val_acc: 0.7265 - lr: 0.0010
Epoch 3/20
120/120 [==============================] - 180s 2s/step - loss: 0.5905 - acc: 0.7850 - val_loss: 0.5832 - val_acc: 0.7967 - lr: 0.0010
Epoch 4/20
120/120 [==============================] - 186s 2s/step - loss: 0.4034 - acc: 0.8650 - val_loss: 0.5223 - val_acc: 0.8226 - lr: 0.0010
Epoch 5/20
120/120 [==============================] - 186s 2s/step - loss: 0.2661 - acc: 0.9139 - val_loss: 0.5450 - val_acc: 0.8414 - lr: 0.0010
Epoch 6/20
120/120 [==============================] - 189s 2s/step - loss: 0.1854 - acc: 0.9424 - val_loss: 0.6808 - val_acc: 0.8210 - lr: 0.0010
Epoch 7/20
120/120 [==============================] - 190s 2s/step - loss: 0.1452 - acc: 0.9575 - val_loss: 0.5862 - val_acc: 0.8348 - lr: 0.0010
Epoch 8/20
120/120 [==============================] - 194s 2s/step - loss: 0.1047 - acc: 0.9683 - val_loss: 0.6400 - val_acc: 0.8418 - lr: 0.0010
Epoch 9/20
120/120 [==============================] - ETA: 0s - loss: 0.0808 - acc: 0.9784
Epoch 9: ReduceLROnPlateau reducing learning rate to 0.0005000000237487257.
120/120 [==============================] - 194s 2s/step - loss: 0.0808 - acc: 0.9784 - val_loss: 0.6341 - val_acc: 0.8414 - lr: 0.0010
Epoch 10/20
120/120 [==============================] - 195s 2s/step - loss: 0.0553 - acc: 0.9846 - val_loss: 0.6372 - val_acc: 0.8524 - lr: 5.0000e-04
Epoch 11/20
120/120 [==============================] - 196s 2s/step - loss: 0.0530 - acc: 0.9872 - val_loss: 0.6714 - val_acc: 0.8509 - lr: 5.0000e-04
Epoch 12/20
120/120 [==============================] - 197s 2s/step - loss: 0.0398 - acc: 0.9899 - val_loss: 0.6802 - val_acc: 0.8442 - lr: 5.0000e-04
Epoch 13/20
120/120 [==============================] - 198s 2s/step - loss: 0.0397 - acc: 0.9887 - val_loss: 0.6898 - val_acc: 0.8454 - lr: 5.0000e-04
Epoch 14/20
120/120 [==============================] - ETA: 0s - loss: 0.0348 - acc: 0.9895
Epoch 14: ReduceLROnPlateau reducing learning rate to 0.0002500000118743628.
120/120 [==============================] - 198s 2s/step - loss: 0.0348 - acc: 0.9895 - val_loss: 0.6951 - val_acc: 0.8454 - lr: 5.0000e-04
Epoch 15/20
120/120 [==============================] - 198s 2s/step - loss: 0.0299 - acc: 0.9910 - val_loss: 0.7023 - val_acc: 0.8411 - lr: 2.5000e-04
Epoch 16/20
120/120 [==============================] - 199s 2s/step - loss: 0.0298 - acc: 0.9915 - val_loss: 0.7247 - val_acc: 0.8418 - lr: 2.5000e-04
Epoch 17/20
120/120 [==============================] - 198s 2s/step - loss: 0.0253 - acc: 0.9932 - val_loss: 0.7001 - val_acc: 0.8556 - lr: 2.5000e-04
Epoch 18/20
120/120 [==============================] - 198s 2s/step - loss: 0.0234 - acc: 0.9935 - val_loss: 0.7180 - val_acc: 0.8497 - lr: 2.5000e-04
Epoch 19/20
120/120 [==============================] - ETA: 0s - loss: 0.0246 - acc: 0.9936
Epoch 19: ReduceLROnPlateau reducing learning rate to 0.0001250000059371814.
120/120 [==============================] - 199s 2s/step - loss: 0.0246 - acc: 0.9936 - val_loss: 0.7385 - val_acc: 0.8458 - lr: 2.5000e-04
Epoch 20/20
120/120 [==============================] - 199s 2s/step - loss: 0.0275 - acc: 0.9936 - val_loss: 0.7229 - val_acc: 0.8454 - lr: 1.2500e-04
80/80 [==============================] - 11s 126ms/step
Test results - Loss: 0.7228917479515076 - Accuracy: 84.53689217567444%
 metrics: {'dataset': 'Text', 'ngram': (1, 1), 'model': 'LSTM2X', 'precision': 0.849712928469492, 'recall': 0.8453689167974883, 'accuracy': 0.8453689167974883, 'f1': 0.8463549192835412, 'roc_auc': 0.9732949054153069}
```

```
#############################################################
END
#############################################################
#############################################################
START! dataset: Text, ngram: (1, 1), model: GRU
#############################################################
Model: "sequential_2"
_________________________________________________________________
 Layer (type)                Output Shape              Param #   
=================================================================
 text_vectorization (TextVe  (None, 64)                0         
 ctorization)                                                    
                                                                 
 embedding_2 (Embedding)     (None, 64, 256)           6879744   
                                                                 
 spatial_dropout1d_2 (Spati  (None, 64, 256)           0         
 alDropout1D)                                                    
                                                                 
 gru (GRU)                   (None, 64)                61824     
                                                                 
 dropout_5 (Dropout)         (None, 64)                0         
                                                                 
 dense_4 (Dense)             (None, 256)               16640     
                                                                 
 dropout_6 (Dropout)         (None, 256)               0         
                                                                 
 dense_5 (Dense)             (None, 5)                 1285      
                                                                 
=================================================================
Total params: 6959493 (26.55 MB)
Trainable params: 6959493 (26.55 MB)
Non-trainable params: 0 (0.00 Byte)
_________________________________________________________________
None
Epoch 1/20
120/120 [==============================] - 21s 156ms/step - loss: 1.5754 - acc: 0.2582 - val_loss: 1.3371 - val_acc: 0.3772 - lr: 0.0010
Epoch 2/20
120/120 [==============================] - 16s 131ms/step - loss: 1.1815 - acc: 0.4797 - val_loss: 0.9238 - val_acc: 0.6091 - lr: 0.0010
Epoch 3/20
120/120 [==============================] - 16s 130ms/step - loss: 0.8390 - acc: 0.6650 - val_loss: 0.6746 - val_acc: 0.7751 - lr: 0.0010
Epoch 4/20
120/120 [==============================] - 15s 129ms/step - loss: 0.5773 - acc: 0.7964 - val_loss: 0.5614 - val_acc: 0.8128 - lr: 0.0010
Epoch 5/20
120/120 [==============================] - 15s 127ms/step - loss: 0.3942 - acc: 0.8659 - val_loss: 0.5406 - val_acc: 0.8226 - lr: 0.0010
Epoch 6/20
120/120 [==============================] - 15s 127ms/step - loss: 0.2921 - acc: 0.9026 - val_loss: 0.5681 - val_acc: 0.8254 - lr: 0.0010
Epoch 7/20
120/120 [==============================] - 15s 127ms/step - loss: 0.2147 - acc: 0.9317 - val_loss: 0.6291 - val_acc: 0.8285 - lr: 0.0010
Epoch 8/20
120/120 [==============================] - 15s 126ms/step - loss: 0.1566 - acc: 0.9522 - val_loss: 0.6166 - val_acc: 0.8422 - lr: 0.0010
Epoch 9/20
120/120 [==============================] - 15s 126ms/step - loss: 0.1163 - acc: 0.9640 - val_loss: 0.6443 - val_acc: 0.8324 - lr: 0.0010
Epoch 10/20
120/120 [==============================] - ETA: 0s - loss: 0.1022 - acc: 0.9694
Epoch 10: ReduceLROnPlateau reducing learning rate to 0.0005000000237487257.
120/120 [==============================] - 15s 125ms/step - loss: 0.1022 - acc: 0.9694 - val_loss: 0.6929 - val_acc: 0.8332 - lr: 0.0010
Epoch 11/20
120/120 [==============================] - 15s 125ms/step - loss: 0.0773 - acc: 0.9757 - val_loss: 0.6723 - val_acc: 0.8383 - lr: 5.0000e-04
Epoch 12/20
120/120 [==============================] - 15s 123ms/step - loss: 0.0646 - acc: 0.9822 - val_loss: 0.6794 - val_acc: 0.8422 - lr: 5.0000e-04
Epoch 13/20
120/120 [==============================] - 15s 123ms/step - loss: 0.0612 - acc: 0.9818 - val_loss: 0.6868 - val_acc: 0.8438 - lr: 5.0000e-04
Epoch 14/20
120/120 [==============================] - 15s 123ms/step - loss: 0.0527 - acc: 0.9848 - val_loss: 0.7511 - val_acc: 0.8399 - lr: 5.0000e-04
Epoch 15/20
120/120 [==============================] - ETA: 0s - loss: 0.0465 - acc: 0.9856
Epoch 15: ReduceLROnPlateau reducing learning rate to 0.0002500000118743628.
120/120 [==============================] - 15s 123ms/step - loss: 0.0465 - acc: 0.9856 - val_loss: 0.7610 - val_acc: 0.8399 - lr: 5.0000e-04
Epoch 16/20
120/120 [==============================] - 15s 123ms/step - loss: 0.0422 - acc: 0.9863 - val_loss: 0.7818 - val_acc: 0.8391 - lr: 2.5000e-04
Epoch 17/20
120/120 [==============================] - 15s 123ms/step - loss: 0.0421 - acc: 0.9878 - val_loss: 0.7787 - val_acc: 0.8399 - lr: 2.5000e-04
Epoch 18/20
120/120 [==============================] - 15s 123ms/step - loss: 0.0409 - acc: 0.9861 - val_loss: 0.8068 - val_acc: 0.8422 - lr: 2.5000e-04
Epoch 19/20
120/120 [==============================] - 15s 123ms/step - loss: 0.0335 - acc: 0.9904 - val_loss: 0.8102 - val_acc: 0.8387 - lr: 2.5000e-04
Epoch 20/20
120/120 [==============================] - ETA: 0s - loss: 0.0355 - acc: 0.9898
Epoch 20: ReduceLROnPlateau reducing learning rate to 0.0001250000059371814.
120/120 [==============================] - 15s 123ms/step - loss: 0.0355 - acc: 0.9898 - val_loss: 0.7959 - val_acc: 0.8367 - lr: 2.5000e-04
80/80 [==============================] - 2s 17ms/step
Test results - Loss: 0.7958868741989136 - Accuracy: 83.67347121238708%
 metrics: {'dataset': 'Text', 'ngram': (1, 1), 'model': 'GRU', 'precision': 0.8438819123116342, 'recall': 0.8367346938775511, 'accuracy': 0.8367346938775511, 'f1': 0.838023052054717, 'roc_auc': 0.9676306923724397}
```

```
#############################################################
END
#############################################################
#############################################################
START! dataset: Text, ngram: (1, 1), model: CNN+LSTM
#############################################################
Model: "sequential_3"
_________________________________________________________________
 Layer (type)                Output Shape              Param #   
=================================================================
 text_vectorization (TextVe  (None, 64)                0         
 ctorization)                                                    
                                                                 
 embedding_3 (Embedding)     (None, 64, 256)           6879744   
                                                                 
 spatial_dropout1d_3 (Spati  (None, 64, 256)           0         
 alDropout1D)                                                    
                                                                 
 conv1d (Conv1D)             (None, 64, 64)            98368     
                                                                 
 max_pooling1d (MaxPooling1  (None, 32, 64)            0         
 D)                                                              
                                                                 
 conv1d_1 (Conv1D)           (None, 27, 32)            12320     
                                                                 
 max_pooling1d_1 (MaxPoolin  (None, 13, 32)            0         
 g1D)                                                            
                                                                 
 bidirectional_3 (Bidirecti  (None, 13, 256)           164864    
 onal)                                                           
                                                                 
 dropout_7 (Dropout)         (None, 13, 256)           0         
                                                                 
 bidirectional_4 (Bidirecti  (None, 256)               394240    
 onal)                                                           
                                                                 
 dropout_8 (Dropout)         (None, 256)               0         
                                                                 
 flatten_2 (Flatten)         (None, 256)               0         
                                                                 
 dense_6 (Dense)             (None, 64)                16448     
                                                                 
 dropout_9 (Dropout)         (None, 64)                0         
                                                                 
 dense_7 (Dense)             (None, 5)                 325       
                                                                 
=================================================================
Total params: 7566309 (28.86 MB)
Trainable params: 7566309 (28.86 MB)
Non-trainable params: 0 (0.00 Byte)
_________________________________________________________________
None
Epoch 1/20
120/120 [==============================] - 29s 164ms/step - loss: 1.4350 - acc: 0.3473 - val_loss: 1.2579 - val_acc: 0.4180 - lr: 0.0010
Epoch 2/20
120/120 [==============================] - 18s 149ms/step - loss: 1.1346 - acc: 0.5024 - val_loss: 1.1177 - val_acc: 0.5098 - lr: 0.0010
Epoch 3/20
120/120 [==============================] - 18s 147ms/step - loss: 0.8476 - acc: 0.6422 - val_loss: 0.7967 - val_acc: 0.6833 - lr: 0.0010
Epoch 4/20
120/120 [==============================] - 17s 145ms/step - loss: 0.6069 - acc: 0.7662 - val_loss: 0.6329 - val_acc: 0.7779 - lr: 0.0010
Epoch 5/20
120/120 [==============================] - 17s 144ms/step - loss: 0.4405 - acc: 0.8372 - val_loss: 0.6481 - val_acc: 0.7889 - lr: 0.0010
Epoch 6/20
120/120 [==============================] - 17s 143ms/step - loss: 0.3352 - acc: 0.8877 - val_loss: 0.6437 - val_acc: 0.8073 - lr: 0.0010
Epoch 7/20
120/120 [==============================] - 17s 144ms/step - loss: 0.2688 - acc: 0.9182 - val_loss: 0.6274 - val_acc: 0.8167 - lr: 0.0010
Epoch 8/20
120/120 [==============================] - 17s 144ms/step - loss: 0.1961 - acc: 0.9405 - val_loss: 0.6740 - val_acc: 0.8230 - lr: 0.0010
Epoch 9/20
120/120 [==============================] - 17s 144ms/step - loss: 0.1531 - acc: 0.9573 - val_loss: 0.7705 - val_acc: 0.8199 - lr: 0.0010
Epoch 10/20
120/120 [==============================] - 17s 145ms/step - loss: 0.1306 - acc: 0.9638 - val_loss: 0.7293 - val_acc: 0.8352 - lr: 0.0010
Epoch 11/20
120/120 [==============================] - 17s 144ms/step - loss: 0.0957 - acc: 0.9730 - val_loss: 0.7424 - val_acc: 0.8363 - lr: 0.0010
Epoch 12/20
120/120 [==============================] - ETA: 0s - loss: 0.0874 - acc: 0.9761
Epoch 12: ReduceLROnPlateau reducing learning rate to 0.0005000000237487257.
120/120 [==============================] - 17s 144ms/step - loss: 0.0874 - acc: 0.9761 - val_loss: 0.9069 - val_acc: 0.8269 - lr: 0.0010
Epoch 13/20
120/120 [==============================] - 17s 144ms/step - loss: 0.0627 - acc: 0.9857 - val_loss: 0.8416 - val_acc: 0.8320 - lr: 5.0000e-04
Epoch 14/20
120/120 [==============================] - 17s 143ms/step - loss: 0.0578 - acc: 0.9863 - val_loss: 0.8350 - val_acc: 0.8367 - lr: 5.0000e-04
Epoch 15/20
120/120 [==============================] - 17s 143ms/step - loss: 0.0437 - acc: 0.9877 - val_loss: 0.9334 - val_acc: 0.8340 - lr: 5.0000e-04
Epoch 16/20
120/120 [==============================] - 17s 143ms/step - loss: 0.0367 - acc: 0.9904 - val_loss: 0.9086 - val_acc: 0.8407 - lr: 5.0000e-04
Epoch 17/20
120/120 [==============================] - ETA: 0s - loss: 0.0382 - acc: 0.9899
Epoch 17: ReduceLROnPlateau reducing learning rate to 0.0002500000118743628.
120/120 [==============================] - 17s 143ms/step - loss: 0.0382 - acc: 0.9899 - val_loss: 0.9835 - val_acc: 0.8324 - lr: 5.0000e-04
Epoch 18/20
120/120 [==============================] - 17s 143ms/step - loss: 0.0313 - acc: 0.9903 - val_loss: 0.8887 - val_acc: 0.8411 - lr: 2.5000e-04
Epoch 19/20
120/120 [==============================] - 17s 143ms/step - loss: 0.0345 - acc: 0.9910 - val_loss: 0.8008 - val_acc: 0.8454 - lr: 2.5000e-04
Epoch 20/20
120/120 [==============================] - 17s 143ms/step - loss: 0.0360 - acc: 0.9910 - val_loss: 0.8910 - val_acc: 0.8379 - lr: 2.5000e-04
80/80 [==============================] - 3s 27ms/step
Test results - Loss: 0.8909761905670166 - Accuracy: 83.79120826721191%
 metrics: {'dataset': 'Text', 'ngram': (1, 1), 'model': 'CNN+LSTM', 'precision': 0.846670770859473, 'recall': 0.8379120879120879, 'accuracy': 0.8379120879120879, 'f1': 0.8394655005036414, 'roc_auc': 0.9644078718888618}
```

```
#############################################################
END
#############################################################
#############################################################
START! dataset: Text, ngram: (1, 2), model: LSTM
#############################################################
Model: "sequential_4"
_________________________________________________________________
 Layer (type)                Output Shape              Param #   
=================================================================
 text_vectorization_1 (Text  (None, 64)                0         
 Vectorization)                                                  
                                                                 
 embedding_4 (Embedding)     (None, 64, 256)           15360000  
                                                                 
 spatial_dropout1d_4 (Spati  (None, 64, 256)           0         
 alDropout1D)                                                    
                                                                 
 bidirectional_5 (Bidirecti  (None, 512)               1050624   
 onal)                                                           
                                                                 
 dropout_10 (Dropout)        (None, 512)               0         
                                                                 
 flatten_3 (Flatten)         (None, 512)               0         
                                                                 
 dense_8 (Dense)             (None, 32)                16416     
                                                                 
 dropout_11 (Dropout)        (None, 32)                0         
                                                                 
 dense_9 (Dense)             (None, 5)                 165       
                                                                 
=================================================================
Total params: 16427205 (62.66 MB)
Trainable params: 16427205 (62.66 MB)
Non-trainable params: 0 (0.00 Byte)
_________________________________________________________________
None
Epoch 1/20
120/120 [==============================] - 77s 601ms/step - loss: 1.4958 - acc: 0.3295 - val_loss: 1.2965 - val_acc: 0.4761 - lr: 0.0010
Epoch 2/20
120/120 [==============================] - 68s 564ms/step - loss: 1.1025 - acc: 0.5429 - val_loss: 0.9048 - val_acc: 0.6676 - lr: 0.0010
Epoch 3/20
120/120 [==============================] - 68s 570ms/step - loss: 0.8051 - acc: 0.7039 - val_loss: 0.6688 - val_acc: 0.7630 - lr: 0.0010
Epoch 4/20
120/120 [==============================] - 69s 574ms/step - loss: 0.5866 - acc: 0.7946 - val_loss: 0.5870 - val_acc: 0.7971 - lr: 0.0010
Epoch 5/20
120/120 [==============================] - 69s 576ms/step - loss: 0.4229 - acc: 0.8639 - val_loss: 0.5586 - val_acc: 0.8034 - lr: 0.0010
Epoch 6/20
120/120 [==============================] - 69s 575ms/step - loss: 0.3077 - acc: 0.9033 - val_loss: 0.5058 - val_acc: 0.8336 - lr: 0.0010
Epoch 7/20
120/120 [==============================] - 69s 575ms/step - loss: 0.2321 - acc: 0.9276 - val_loss: 0.5550 - val_acc: 0.8359 - lr: 0.0010
Epoch 8/20
120/120 [==============================] - 68s 569ms/step - loss: 0.1667 - acc: 0.9503 - val_loss: 0.4848 - val_acc: 0.8485 - lr: 0.0010
Epoch 9/20
120/120 [==============================] - 68s 569ms/step - loss: 0.1471 - acc: 0.9569 - val_loss: 0.5485 - val_acc: 0.8383 - lr: 0.0010
Epoch 10/20
120/120 [==============================] - 68s 570ms/step - loss: 0.1106 - acc: 0.9666 - val_loss: 0.5929 - val_acc: 0.8403 - lr: 0.0010
Epoch 11/20
120/120 [==============================] - 68s 569ms/step - loss: 0.0902 - acc: 0.9720 - val_loss: 0.5363 - val_acc: 0.8450 - lr: 0.0010
Epoch 12/20
120/120 [==============================] - 68s 568ms/step - loss: 0.0793 - acc: 0.9787 - val_loss: 0.5663 - val_acc: 0.8469 - lr: 0.0010
Epoch 13/20
120/120 [==============================] - ETA: 0s - loss: 0.0667 - acc: 0.9808
Epoch 13: ReduceLROnPlateau reducing learning rate to 0.0005000000237487257.
120/120 [==============================] - 68s 569ms/step - loss: 0.0667 - acc: 0.9808 - val_loss: 0.6050 - val_acc: 0.8352 - lr: 0.0010
Epoch 14/20
120/120 [==============================] - 68s 570ms/step - loss: 0.0573 - acc: 0.9834 - val_loss: 0.5852 - val_acc: 0.8454 - lr: 5.0000e-04
Epoch 15/20
120/120 [==============================] - 68s 569ms/step - loss: 0.0432 - acc: 0.9847 - val_loss: 0.6315 - val_acc: 0.8458 - lr: 5.0000e-04
Epoch 16/20
120/120 [==============================] - 68s 568ms/step - loss: 0.0518 - acc: 0.9847 - val_loss: 0.6273 - val_acc: 0.8379 - lr: 5.0000e-04
Epoch 17/20
120/120 [==============================] - 68s 569ms/step - loss: 0.0513 - acc: 0.9850 - val_loss: 0.5873 - val_acc: 0.8442 - lr: 5.0000e-04
Epoch 18/20
120/120 [==============================] - ETA: 0s - loss: 0.0399 - acc: 0.9856
Epoch 18: ReduceLROnPlateau reducing learning rate to 0.0002500000118743628.
120/120 [==============================] - 69s 573ms/step - loss: 0.0399 - acc: 0.9856 - val_loss: 0.6259 - val_acc: 0.8489 - lr: 5.0000e-04
Epoch 19/20
120/120 [==============================] - 69s 572ms/step - loss: 0.0353 - acc: 0.9903 - val_loss: 0.6345 - val_acc: 0.8454 - lr: 2.5000e-04
Epoch 20/20
120/120 [==============================] - 69s 574ms/step - loss: 0.0324 - acc: 0.9902 - val_loss: 0.6653 - val_acc: 0.8473 - lr: 2.5000e-04
80/80 [==============================] - 6s 69ms/step
Test results - Loss: 0.6653293371200562 - Accuracy: 84.73312258720398%
 metrics: {'dataset': 'Text', 'ngram': (1, 2), 'model': 'LSTM', 'precision': 0.8540021210607327, 'recall': 0.847331240188383, 'accuracy': 0.847331240188383, 'f1': 0.8477207527379171, 'roc_auc': 0.9742774086607502}
```

```
#############################################################
END
#############################################################
#############################################################
START! dataset: Text, ngram: (1, 2), model: LSTM2X
#############################################################
Model: "sequential_5"
_________________________________________________________________
 Layer (type)                Output Shape              Param #   
=================================================================
 text_vectorization_1 (Text  (None, 64)                0         
 Vectorization)                                                  
                                                                 
 embedding_5 (Embedding)     (None, 64, 256)           15360000  
                                                                 
 spatial_dropout1d_5 (Spati  (None, 64, 256)           0         
 alDropout1D)                                                    
                                                                 
 bidirectional_6 (Bidirecti  (None, 64, 256)           394240    
 onal)                                                           
                                                                 
 dropout_12 (Dropout)        (None, 64, 256)           0         
                                                                 
 bidirectional_7 (Bidirecti  (None, 256)               394240    
 onal)                                                           
                                                                 
 dropout_13 (Dropout)        (None, 256)               0         
                                                                 
 flatten_4 (Flatten)         (None, 256)               0         
                                                                 
 dense_10 (Dense)            (None, 64)                16448     
                                                                 
 dropout_14 (Dropout)        (None, 64)                0         
                                                                 
 dense_11 (Dense)            (None, 5)                 325       
                                                                 
=================================================================
Total params: 16165253 (61.67 MB)
Trainable params: 16165253 (61.67 MB)
Non-trainable params: 0 (0.00 Byte)
_________________________________________________________________
None
Epoch 1/20
120/120 [==============================] - 275s 2s/step - loss: 1.4658 - acc: 0.3430 - val_loss: 1.1738 - val_acc: 0.4969 - lr: 0.0010
Epoch 2/20
120/120 [==============================] - 268s 2s/step - loss: 1.0487 - acc: 0.5735 - val_loss: 0.8210 - val_acc: 0.6982 - lr: 0.0010
Epoch 3/20
120/120 [==============================] - 269s 2s/step - loss: 0.6929 - acc: 0.7519 - val_loss: 0.5847 - val_acc: 0.7998 - lr: 0.0010
Epoch 4/20
120/120 [==============================] - 272s 2s/step - loss: 0.4607 - acc: 0.8411 - val_loss: 0.5155 - val_acc: 0.8301 - lr: 0.0010
Epoch 5/20
120/120 [==============================] - 273s 2s/step - loss: 0.3174 - acc: 0.8951 - val_loss: 0.5017 - val_acc: 0.8336 - lr: 0.0010
Epoch 6/20
120/120 [==============================] - 272s 2s/step - loss: 0.2198 - acc: 0.9270 - val_loss: 0.6267 - val_acc: 0.8187 - lr: 0.0010
Epoch 7/20
120/120 [==============================] - 276s 2s/step - loss: 0.1640 - acc: 0.9490 - val_loss: 0.5304 - val_acc: 0.8363 - lr: 0.0010
Epoch 8/20
120/120 [==============================] - 278s 2s/step - loss: 0.1196 - acc: 0.9651 - val_loss: 0.5382 - val_acc: 0.8367 - lr: 0.0010
Epoch 9/20
120/120 [==============================] - 276s 2s/step - loss: 0.0927 - acc: 0.9736 - val_loss: 0.5509 - val_acc: 0.8426 - lr: 0.0010
Epoch 10/20
120/120 [==============================] - ETA: 0s - loss: 0.0792 - acc: 0.9780
Epoch 10: ReduceLROnPlateau reducing learning rate to 0.0005000000237487257.
120/120 [==============================] - 278s 2s/step - loss: 0.0792 - acc: 0.9780 - val_loss: 0.5456 - val_acc: 0.8516 - lr: 0.0010
Epoch 11/20
120/120 [==============================] - 277s 2s/step - loss: 0.0495 - acc: 0.9839 - val_loss: 0.5674 - val_acc: 0.8524 - lr: 5.0000e-04
Epoch 12/20
120/120 [==============================] - 277s 2s/step - loss: 0.0486 - acc: 0.9850 - val_loss: 0.6561 - val_acc: 0.8375 - lr: 5.0000e-04
Epoch 13/20
120/120 [==============================] - 277s 2s/step - loss: 0.0428 - acc: 0.9870 - val_loss: 0.6278 - val_acc: 0.8505 - lr: 5.0000e-04
Epoch 14/20
120/120 [==============================] - 280s 2s/step - loss: 0.0422 - acc: 0.9877 - val_loss: 0.6001 - val_acc: 0.8560 - lr: 5.0000e-04
Epoch 15/20
120/120 [==============================] - ETA: 0s - loss: 0.0320 - acc: 0.9915
Epoch 15: ReduceLROnPlateau reducing learning rate to 0.0002500000118743628.
120/120 [==============================] - 279s 2s/step - loss: 0.0320 - acc: 0.9915 - val_loss: 0.6769 - val_acc: 0.8469 - lr: 5.0000e-04
Epoch 16/20
120/120 [==============================] - 281s 2s/step - loss: 0.0380 - acc: 0.9901 - val_loss: 0.6337 - val_acc: 0.8473 - lr: 2.5000e-04
Epoch 17/20
120/120 [==============================] - 279s 2s/step - loss: 0.0302 - acc: 0.9923 - val_loss: 0.6309 - val_acc: 0.8485 - lr: 2.5000e-04
Epoch 18/20
120/120 [==============================] - 285s 2s/step - loss: 0.0235 - acc: 0.9942 - val_loss: 0.6612 - val_acc: 0.8458 - lr: 2.5000e-04
Epoch 19/20
120/120 [==============================] - 281s 2s/step - loss: 0.0278 - acc: 0.9921 - val_loss: 0.6822 - val_acc: 0.8465 - lr: 2.5000e-04
Epoch 20/20
120/120 [==============================] - ETA: 0s - loss: 0.0223 - acc: 0.9941
Epoch 20: ReduceLROnPlateau reducing learning rate to 0.0001250000059371814.
120/120 [==============================] - 281s 2s/step - loss: 0.0223 - acc: 0.9941 - val_loss: 0.6900 - val_acc: 0.8477 - lr: 2.5000e-04
80/80 [==============================] - 15s 179ms/step
Test results - Loss: 0.6900343298912048 - Accuracy: 84.77237224578857%
 metrics: {'dataset': 'Text', 'ngram': (1, 2), 'model': 'LSTM2X', 'precision': 0.848810228524539, 'recall': 0.847723704866562, 'accuracy': 0.847723704866562, 'f1': 0.8474158439322217, 'roc_auc': 0.9725829986287458}
```

```
#############################################################
END
#############################################################
#############################################################
START! dataset: Text, ngram: (1, 2), model: GRU
#############################################################
Model: "sequential_6"
_________________________________________________________________
 Layer (type)                Output Shape              Param #   
=================================================================
 text_vectorization_1 (Text  (None, 64)                0         
 Vectorization)                                                  
                                                                 
 embedding_6 (Embedding)     (None, 64, 256)           15360000  
                                                                 
 spatial_dropout1d_6 (Spati  (None, 64, 256)           0         
 alDropout1D)                                                    
                                                                 
 gru_1 (GRU)                 (None, 64)                61824     
                                                                 
 dropout_15 (Dropout)        (None, 64)                0         
                                                                 
 dense_12 (Dense)            (None, 256)               16640     
                                                                 
 dropout_16 (Dropout)        (None, 256)               0         
                                                                 
 dense_13 (Dense)            (None, 5)                 1285      
                                                                 
=================================================================
Total params: 15439749 (58.90 MB)
Trainable params: 15439749 (58.90 MB)
Non-trainable params: 0 (0.00 Byte)
_________________________________________________________________
None
Epoch 1/20
120/120 [==============================] - 31s 237ms/step - loss: 1.6050 - acc: 0.2194 - val_loss: 1.5937 - val_acc: 0.2673 - lr: 0.0010
Epoch 2/20
120/120 [==============================] - 27s 226ms/step - loss: 1.4900 - acc: 0.3321 - val_loss: 1.2510 - val_acc: 0.4572 - lr: 0.0010
Epoch 3/20
120/120 [==============================] - 27s 226ms/step - loss: 1.1167 - acc: 0.5241 - val_loss: 0.9463 - val_acc: 0.6327 - lr: 0.0010
Epoch 4/20
120/120 [==============================] - 27s 226ms/step - loss: 0.8513 - acc: 0.6681 - val_loss: 0.7449 - val_acc: 0.7378 - lr: 0.0010
Epoch 5/20
120/120 [==============================] - 27s 227ms/step - loss: 0.6264 - acc: 0.7664 - val_loss: 0.6750 - val_acc: 0.7630 - lr: 0.0010
Epoch 6/20
120/120 [==============================] - 27s 227ms/step - loss: 0.4707 - acc: 0.8339 - val_loss: 0.6994 - val_acc: 0.7696 - lr: 0.0010
Epoch 7/20
120/120 [==============================] - 27s 227ms/step - loss: 0.3493 - acc: 0.8787 - val_loss: 0.6168 - val_acc: 0.7928 - lr: 0.0010
Epoch 8/20
120/120 [==============================] - 27s 227ms/step - loss: 0.2653 - acc: 0.9079 - val_loss: 0.6578 - val_acc: 0.8002 - lr: 0.0010
Epoch 9/20
120/120 [==============================] - 27s 226ms/step - loss: 0.1942 - acc: 0.9388 - val_loss: 0.7482 - val_acc: 0.7979 - lr: 0.0010
Epoch 10/20
120/120 [==============================] - 27s 227ms/step - loss: 0.1399 - acc: 0.9537 - val_loss: 0.6519 - val_acc: 0.8136 - lr: 0.0010
Epoch 11/20
120/120 [==============================] - 27s 226ms/step - loss: 0.1232 - acc: 0.9605 - val_loss: 0.7501 - val_acc: 0.8046 - lr: 0.0010
Epoch 12/20
120/120 [==============================] - ETA: 0s - loss: 0.1011 - acc: 0.9698
Epoch 12: ReduceLROnPlateau reducing learning rate to 0.0005000000237487257.
120/120 [==============================] - 27s 226ms/step - loss: 0.1011 - acc: 0.9698 - val_loss: 0.6995 - val_acc: 0.8085 - lr: 0.0010
Epoch 13/20
120/120 [==============================] - 27s 227ms/step - loss: 0.0783 - acc: 0.9733 - val_loss: 0.6531 - val_acc: 0.8238 - lr: 5.0000e-04
Epoch 14/20
120/120 [==============================] - 27s 225ms/step - loss: 0.0607 - acc: 0.9818 - val_loss: 0.7055 - val_acc: 0.8230 - lr: 5.0000e-04
Epoch 15/20
120/120 [==============================] - 27s 226ms/step - loss: 0.0639 - acc: 0.9804 - val_loss: 0.7481 - val_acc: 0.8093 - lr: 5.0000e-04
Epoch 16/20
120/120 [==============================] - 27s 226ms/step - loss: 0.0508 - acc: 0.9842 - val_loss: 0.8172 - val_acc: 0.8151 - lr: 5.0000e-04
Epoch 17/20
120/120 [==============================] - ETA: 0s - loss: 0.0512 - acc: 0.9850
Epoch 17: ReduceLROnPlateau reducing learning rate to 0.0002500000118743628.
120/120 [==============================] - 27s 227ms/step - loss: 0.0512 - acc: 0.9850 - val_loss: 0.6692 - val_acc: 0.8312 - lr: 5.0000e-04
Epoch 18/20
120/120 [==============================] - 27s 226ms/step - loss: 0.0440 - acc: 0.9847 - val_loss: 0.7418 - val_acc: 0.8230 - lr: 2.5000e-04
Epoch 19/20
120/120 [==============================] - 27s 226ms/step - loss: 0.0451 - acc: 0.9844 - val_loss: 0.7556 - val_acc: 0.8238 - lr: 2.5000e-04
Epoch 20/20
120/120 [==============================] - 27s 226ms/step - loss: 0.0332 - acc: 0.9906 - val_loss: 0.7308 - val_acc: 0.8273 - lr: 2.5000e-04
80/80 [==============================] - 3s 33ms/step
Test results - Loss: 0.7308111190795898 - Accuracy: 82.73155689239502%
 metrics: {'dataset': 'Text', 'ngram': (1, 2), 'model': 'GRU', 'precision': 0.8533071340813922, 'recall': 0.8273155416012559, 'accuracy': 0.8273155416012559, 'f1': 0.8323321356262235, 'roc_auc': 0.9657464855016882}
```

```
#############################################################
END
#############################################################
#############################################################
START! dataset: Text, ngram: (1, 2), model: CNN+LSTM
#############################################################
Model: "sequential_7"
_________________________________________________________________
 Layer (type)                Output Shape              Param #   
=================================================================
 text_vectorization_1 (Text  (None, 64)                0         
 Vectorization)                                                  
                                                                 
 embedding_7 (Embedding)     (None, 64, 256)           15360000  
                                                                 
 spatial_dropout1d_7 (Spati  (None, 64, 256)           0         
 alDropout1D)                                                    
                                                                 
 conv1d_2 (Conv1D)           (None, 64, 64)            98368     
                                                                 
 max_pooling1d_2 (MaxPoolin  (None, 32, 64)            0         
 g1D)                                                            
                                                                 
 conv1d_3 (Conv1D)           (None, 27, 32)            12320     
                                                                 
 max_pooling1d_3 (MaxPoolin  (None, 13, 32)            0         
 g1D)                                                            
                                                                 
 bidirectional_8 (Bidirecti  (None, 13, 256)           164864    
 onal)                                                           
                                                                 
 dropout_17 (Dropout)        (None, 13, 256)           0         
                                                                 
 bidirectional_9 (Bidirecti  (None, 256)               394240    
 onal)                                                           
                                                                 
 dropout_18 (Dropout)        (None, 256)               0         
                                                                 
 flatten_5 (Flatten)         (None, 256)               0         
                                                                 
 dense_14 (Dense)            (None, 64)                16448     
                                                                 
 dropout_19 (Dropout)        (None, 64)                0         
                                                                 
 dense_15 (Dense)            (None, 5)                 325       
                                                                 
=================================================================
Total params: 16046565 (61.21 MB)
Trainable params: 16046565 (61.21 MB)
Non-trainable params: 0 (0.00 Byte)
_________________________________________________________________
None
Epoch 1/20
120/120 [==============================] - 49s 330ms/step - loss: 1.4765 - acc: 0.3397 - val_loss: 1.2299 - val_acc: 0.4352 - lr: 0.0010
Epoch 2/20
120/120 [==============================] - 38s 317ms/step - loss: 1.1558 - acc: 0.4914 - val_loss: 1.0821 - val_acc: 0.4992 - lr: 0.0010
Epoch 3/20
120/120 [==============================] - 38s 317ms/step - loss: 0.9068 - acc: 0.6043 - val_loss: 0.9452 - val_acc: 0.5914 - lr: 0.0010
Epoch 4/20
120/120 [==============================] - 38s 316ms/step - loss: 0.7047 - acc: 0.7009 - val_loss: 0.7992 - val_acc: 0.7092 - lr: 0.0010
Epoch 5/20
120/120 [==============================] - 38s 314ms/step - loss: 0.5359 - acc: 0.7940 - val_loss: 0.6955 - val_acc: 0.7535 - lr: 0.0010
Epoch 6/20
120/120 [==============================] - 38s 314ms/step - loss: 0.3768 - acc: 0.8631 - val_loss: 0.6762 - val_acc: 0.7912 - lr: 0.0010
Epoch 7/20
120/120 [==============================] - 38s 314ms/step - loss: 0.3058 - acc: 0.9002 - val_loss: 0.7194 - val_acc: 0.7822 - lr: 0.0010
Epoch 8/20
120/120 [==============================] - 38s 314ms/step - loss: 0.2312 - acc: 0.9249 - val_loss: 0.7471 - val_acc: 0.7959 - lr: 0.0010
Epoch 9/20
120/120 [==============================] - 38s 314ms/step - loss: 0.1743 - acc: 0.9471 - val_loss: 0.7495 - val_acc: 0.8038 - lr: 0.0010
Epoch 10/20
120/120 [==============================] - 38s 315ms/step - loss: 0.1301 - acc: 0.9607 - val_loss: 0.8415 - val_acc: 0.8128 - lr: 0.0010
Epoch 11/20
120/120 [==============================] - ETA: 0s - loss: 0.1137 - acc: 0.9677
Epoch 11: ReduceLROnPlateau reducing learning rate to 0.0005000000237487257.
120/120 [==============================] - 38s 314ms/step - loss: 0.1137 - acc: 0.9677 - val_loss: 0.8290 - val_acc: 0.8171 - lr: 0.0010
Epoch 12/20
120/120 [==============================] - 38s 315ms/step - loss: 0.0864 - acc: 0.9759 - val_loss: 0.8140 - val_acc: 0.8195 - lr: 5.0000e-04
Epoch 13/20
120/120 [==============================] - 38s 314ms/step - loss: 0.0795 - acc: 0.9784 - val_loss: 0.7913 - val_acc: 0.8238 - lr: 5.0000e-04
Epoch 14/20
120/120 [==============================] - 38s 314ms/step - loss: 0.0619 - acc: 0.9825 - val_loss: 0.8910 - val_acc: 0.8242 - lr: 5.0000e-04
Epoch 15/20
120/120 [==============================] - 37s 312ms/step - loss: 0.0621 - acc: 0.9839 - val_loss: 0.8563 - val_acc: 0.8187 - lr: 5.0000e-04
Epoch 16/20
120/120 [==============================] - ETA: 0s - loss: 0.0560 - acc: 0.9851
Epoch 16: ReduceLROnPlateau reducing learning rate to 0.0002500000118743628.
120/120 [==============================] - 38s 313ms/step - loss: 0.0560 - acc: 0.9851 - val_loss: 0.9450 - val_acc: 0.8210 - lr: 5.0000e-04
Epoch 17/20
120/120 [==============================] - 37s 312ms/step - loss: 0.0382 - acc: 0.9894 - val_loss: 0.9193 - val_acc: 0.8230 - lr: 2.5000e-04
Epoch 18/20
120/120 [==============================] - 37s 313ms/step - loss: 0.0359 - acc: 0.9882 - val_loss: 0.9678 - val_acc: 0.8214 - lr: 2.5000e-04
Epoch 19/20
120/120 [==============================] - 37s 312ms/step - loss: 0.0396 - acc: 0.9873 - val_loss: 0.9617 - val_acc: 0.8195 - lr: 2.5000e-04
Epoch 20/20
120/120 [==============================] - 38s 313ms/step - loss: 0.0298 - acc: 0.9908 - val_loss: 1.0411 - val_acc: 0.8199 - lr: 2.5000e-04
80/80 [==============================] - 5s 59ms/step
Test results - Loss: 1.041073203086853 - Accuracy: 81.9858729839325%
 metrics: {'dataset': 'Text', 'ngram': (1, 2), 'model': 'CNN+LSTM', 'precision': 0.8264043785307684, 'recall': 0.8198587127158555, 'accuracy': 0.8198587127158555, 'f1': 0.8201809323830878, 'roc_auc': 0.9594137873989912}
```

```
#############################################################
END
#############################################################
#############################################################
START! dataset: Text, ngram: (2, 2), model: LSTM
#############################################################
Model: "sequential_8"
_________________________________________________________________
 Layer (type)                Output Shape              Param #   
=================================================================
 text_vectorization_2 (Text  (None, 64)                0         
 Vectorization)                                                  
                                                                 
 embedding_8 (Embedding)     (None, 64, 256)           15360000  
                                                                 
 spatial_dropout1d_8 (Spati  (None, 64, 256)           0         
 alDropout1D)                                                    
                                                                 
 bidirectional_10 (Bidirect  (None, 512)               1050624   
 ional)                                                          
                                                                 
 dropout_20 (Dropout)        (None, 512)               0         
                                                                 
 flatten_6 (Flatten)         (None, 512)               0         
                                                                 
 dense_16 (Dense)            (None, 32)                16416     
                                                                 
 dropout_21 (Dropout)        (None, 32)                0         
                                                                 
 dense_17 (Dense)            (None, 5)                 165       
                                                                 
=================================================================
Total params: 16427205 (62.66 MB)
Trainable params: 16427205 (62.66 MB)
Non-trainable params: 0 (0.00 Byte)
_________________________________________________________________
None
Epoch 1/20
120/120 [==============================] - 151s 1s/step - loss: 1.5571 - acc: 0.2796 - val_loss: 1.4883 - val_acc: 0.3324 - lr: 0.0010
Epoch 2/20
120/120 [==============================] - 145s 1s/step - loss: 1.4497 - acc: 0.3616 - val_loss: 1.4275 - val_acc: 0.3564 - lr: 0.0010
Epoch 3/20
120/120 [==============================] - 146s 1s/step - loss: 1.2357 - acc: 0.4700 - val_loss: 1.2130 - val_acc: 0.4619 - lr: 0.0010
Epoch 4/20
120/120 [==============================] - 146s 1s/step - loss: 0.9820 - acc: 0.5917 - val_loss: 1.1414 - val_acc: 0.4969 - lr: 0.0010
Epoch 5/20
120/120 [==============================] - 146s 1s/step - loss: 0.7511 - acc: 0.6926 - val_loss: 1.1999 - val_acc: 0.5008 - lr: 0.0010
Epoch 6/20
120/120 [==============================] - 146s 1s/step - loss: 0.5801 - acc: 0.7792 - val_loss: 1.1661 - val_acc: 0.5440 - lr: 0.0010
Epoch 7/20
120/120 [==============================] - 146s 1s/step - loss: 0.3838 - acc: 0.8659 - val_loss: 1.2131 - val_acc: 0.5616 - lr: 0.0010
Epoch 8/20
120/120 [==============================] - 146s 1s/step - loss: 0.2434 - acc: 0.9217 - val_loss: 0.9858 - val_acc: 0.6252 - lr: 0.0010
Epoch 9/20
120/120 [==============================] - 147s 1s/step - loss: 0.1635 - acc: 0.9498 - val_loss: 1.1938 - val_acc: 0.5946 - lr: 0.0010
Epoch 10/20
120/120 [==============================] - 146s 1s/step - loss: 0.1448 - acc: 0.9564 - val_loss: 1.1133 - val_acc: 0.6095 - lr: 0.0010
Epoch 11/20
120/120 [==============================] - 146s 1s/step - loss: 0.1135 - acc: 0.9668 - val_loss: 1.6860 - val_acc: 0.5145 - lr: 0.0010
Epoch 12/20
120/120 [==============================] - 146s 1s/step - loss: 0.0833 - acc: 0.9757 - val_loss: 1.2970 - val_acc: 0.5557 - lr: 0.0010
Epoch 13/20
120/120 [==============================] - ETA: 0s - loss: 0.0778 - acc: 0.9762
Epoch 13: ReduceLROnPlateau reducing learning rate to 0.0005000000237487257.
120/120 [==============================] - 146s 1s/step - loss: 0.0778 - acc: 0.9762 - val_loss: 1.2419 - val_acc: 0.5695 - lr: 0.0010
Epoch 14/20
120/120 [==============================] - 146s 1s/step - loss: 0.0534 - acc: 0.9817 - val_loss: 1.3693 - val_acc: 0.5969 - lr: 5.0000e-04
Epoch 15/20
120/120 [==============================] - 146s 1s/step - loss: 0.0566 - acc: 0.9814 - val_loss: 1.3778 - val_acc: 0.5483 - lr: 5.0000e-04
Epoch 16/20
120/120 [==============================] - 146s 1s/step - loss: 0.0456 - acc: 0.9830 - val_loss: 1.3509 - val_acc: 0.5538 - lr: 5.0000e-04
Epoch 17/20
120/120 [==============================] - 145s 1s/step - loss: 0.0627 - acc: 0.9808 - val_loss: 1.6977 - val_acc: 0.5392 - lr: 5.0000e-04
Epoch 18/20
120/120 [==============================] - ETA: 0s - loss: 0.0500 - acc: 0.9842Restoring model weights from the end of the best epoch: 8.

Epoch 18: ReduceLROnPlateau reducing learning rate to 0.0002500000118743628.
120/120 [==============================] - 145s 1s/step - loss: 0.0500 - acc: 0.9842 - val_loss: 1.3464 - val_acc: 0.5436 - lr: 5.0000e-04
Epoch 18: early stopping
80/80 [==============================] - 12s 140ms/step
Test results - Loss: 0.9857958555221558 - Accuracy: 62.51962184906006%
 metrics: {'dataset': 'Text', 'ngram': (2, 2), 'model': 'LSTM', 'precision': 0.7262274805105347, 'recall': 0.6251962323390895, 'accuracy': 0.6251962323390895, 'f1': 0.6346655841171421, 'roc_auc': 0.8724675706433089}
```

```
#############################################################
END
#############################################################
#############################################################
START! dataset: Text, ngram: (2, 2), model: LSTM2X
#############################################################
Model: "sequential_9"
_________________________________________________________________
 Layer (type)                Output Shape              Param #   
=================================================================
 text_vectorization_2 (Text  (None, 64)                0         
 Vectorization)                                                  
                                                                 
 embedding_9 (Embedding)     (None, 64, 256)           15360000  
                                                                 
 spatial_dropout1d_9 (Spati  (None, 64, 256)           0         
 alDropout1D)                                                    
                                                                 
 bidirectional_11 (Bidirect  (None, 64, 256)           394240    
 ional)                                                          
                                                                 
 dropout_22 (Dropout)        (None, 64, 256)           0         
                                                                 
 bidirectional_12 (Bidirect  (None, 256)               394240    
 ional)                                                          
                                                                 
 dropout_23 (Dropout)        (None, 256)               0         
                                                                 
 flatten_7 (Flatten)         (None, 256)               0         
                                                                 
 dense_18 (Dense)            (None, 64)                16448     
                                                                 
 dropout_24 (Dropout)        (None, 64)                0         
                                                                 
 dense_19 (Dense)            (None, 5)                 325       
                                                                 
=================================================================
Total params: 16165253 (61.67 MB)
Trainable params: 16165253 (61.67 MB)
Non-trainable params: 0 (0.00 Byte)
_________________________________________________________________
None
Epoch 1/20
120/120 [==============================] - 318s 3s/step - loss: 1.5515 - acc: 0.2870 - val_loss: 1.4673 - val_acc: 0.3513 - lr: 0.0010
Epoch 2/20
120/120 [==============================] - 310s 3s/step - loss: 1.3959 - acc: 0.3954 - val_loss: 1.3567 - val_acc: 0.3866 - lr: 0.0010
Epoch 3/20
120/120 [==============================] - 311s 3s/step - loss: 1.1467 - acc: 0.5268 - val_loss: 1.2209 - val_acc: 0.4941 - lr: 0.0010
Epoch 4/20
120/120 [==============================] - 313s 3s/step - loss: 0.8190 - acc: 0.6928 - val_loss: 1.1814 - val_acc: 0.5302 - lr: 0.0010
Epoch 5/20
120/120 [==============================] - 315s 3s/step - loss: 0.5303 - acc: 0.8114 - val_loss: 1.1015 - val_acc: 0.5777 - lr: 0.0010
Epoch 6/20
120/120 [==============================] - 317s 3s/step - loss: 0.3354 - acc: 0.8860 - val_loss: 1.1738 - val_acc: 0.5357 - lr: 0.0010
Epoch 7/20
120/120 [==============================] - 317s 3s/step - loss: 0.2194 - acc: 0.9265 - val_loss: 1.3966 - val_acc: 0.5377 - lr: 0.0010
Epoch 8/20
120/120 [==============================] - 317s 3s/step - loss: 0.1413 - acc: 0.9542 - val_loss: 2.0011 - val_acc: 0.5027 - lr: 0.0010
Epoch 9/20
120/120 [==============================] - 318s 3s/step - loss: 0.1236 - acc: 0.9638 - val_loss: 1.2362 - val_acc: 0.5824 - lr: 0.0010
Epoch 10/20
120/120 [==============================] - ETA: 0s - loss: 0.0810 - acc: 0.9741
Epoch 10: ReduceLROnPlateau reducing learning rate to 0.0005000000237487257.
120/120 [==============================] - 319s 3s/step - loss: 0.0810 - acc: 0.9741 - val_loss: 1.2909 - val_acc: 0.5769 - lr: 0.0010
Epoch 11/20
120/120 [==============================] - 321s 3s/step - loss: 0.0589 - acc: 0.9812 - val_loss: 1.3172 - val_acc: 0.5620 - lr: 5.0000e-04
Epoch 12/20
120/120 [==============================] - 322s 3s/step - loss: 0.0507 - acc: 0.9839 - val_loss: 1.1681 - val_acc: 0.6142 - lr: 5.0000e-04
Epoch 13/20
120/120 [==============================] - 325s 3s/step - loss: 0.0486 - acc: 0.9836 - val_loss: 1.2252 - val_acc: 0.5859 - lr: 5.0000e-04
Epoch 14/20
120/120 [==============================] - 325s 3s/step - loss: 0.0471 - acc: 0.9843 - val_loss: 1.3870 - val_acc: 0.5475 - lr: 5.0000e-04
Epoch 15/20
120/120 [==============================] - ETA: 0s - loss: 0.0399 - acc: 0.9857
Epoch 15: ReduceLROnPlateau reducing learning rate to 0.0002500000118743628.
120/120 [==============================] - 326s 3s/step - loss: 0.0399 - acc: 0.9857 - val_loss: 1.4523 - val_acc: 0.5530 - lr: 5.0000e-04
Epoch 16/20
120/120 [==============================] - 324s 3s/step - loss: 0.0369 - acc: 0.9889 - val_loss: 1.3594 - val_acc: 0.5546 - lr: 2.5000e-04
Epoch 17/20
120/120 [==============================] - 325s 3s/step - loss: 0.0315 - acc: 0.9886 - val_loss: 1.2622 - val_acc: 0.5852 - lr: 2.5000e-04
Epoch 18/20
120/120 [==============================] - 326s 3s/step - loss: 0.0322 - acc: 0.9887 - val_loss: 1.7158 - val_acc: 0.5357 - lr: 2.5000e-04
Epoch 19/20
120/120 [==============================] - 326s 3s/step - loss: 0.0296 - acc: 0.9897 - val_loss: 1.7729 - val_acc: 0.5424 - lr: 2.5000e-04
Epoch 20/20
120/120 [==============================] - ETA: 0s - loss: 0.0310 - acc: 0.9901
Epoch 20: ReduceLROnPlateau reducing learning rate to 0.0001250000059371814.
120/120 [==============================] - 327s 3s/step - loss: 0.0310 - acc: 0.9901 - val_loss: 1.5456 - val_acc: 0.5624 - lr: 2.5000e-04
80/80 [==============================] - 20s 241ms/step
Test results - Loss: 1.5456066131591797 - Accuracy: 56.24018907546997%
 metrics: {'dataset': 'Text', 'ngram': (2, 2), 'model': 'LSTM2X', 'precision': 0.678666900219267, 'recall': 0.5624018838304553, 'accuracy': 0.5624018838304553, 'f1': 0.5364549045771668, 'roc_auc': 0.854224545004562}
```

```
#############################################################
END
#############################################################
#############################################################
START! dataset: Text, ngram: (2, 2), model: GRU
#############################################################
Model: "sequential_10"
_________________________________________________________________
 Layer (type)                Output Shape              Param #   
=================================================================
 text_vectorization_2 (Text  (None, 64)                0         
 Vectorization)                                                  
                                                                 
 embedding_10 (Embedding)    (None, 64, 256)           15360000  
                                                                 
 spatial_dropout1d_10 (Spat  (None, 64, 256)           0         
 ialDropout1D)                                                   
                                                                 
 gru_2 (GRU)                 (None, 64)                61824     
                                                                 
 dropout_25 (Dropout)        (None, 64)                0         
                                                                 
 dense_20 (Dense)            (None, 256)               16640     
                                                                 
 dropout_26 (Dropout)        (None, 256)               0         
                                                                 
 dense_21 (Dense)            (None, 5)                 1285      
                                                                 
=================================================================
Total params: 15439749 (58.90 MB)
Trainable params: 15439749 (58.90 MB)
Non-trainable params: 0 (0.00 Byte)
_________________________________________________________________
None
Epoch 1/20
120/120 [==============================] - 35s 268ms/step - loss: 1.6052 - acc: 0.2172 - val_loss: 1.5980 - val_acc: 0.2708 - lr: 0.0010
Epoch 2/20
120/120 [==============================] - 31s 262ms/step - loss: 1.5639 - acc: 0.2741 - val_loss: 1.4947 - val_acc: 0.2943 - lr: 0.0010
Epoch 3/20
120/120 [==============================] - 31s 262ms/step - loss: 1.3578 - acc: 0.3995 - val_loss: 1.4967 - val_acc: 0.3242 - lr: 0.0010
Epoch 4/20
120/120 [==============================] - 31s 262ms/step - loss: 1.0883 - acc: 0.5411 - val_loss: 1.2240 - val_acc: 0.4588 - lr: 0.0010
Epoch 5/20
120/120 [==============================] - 31s 262ms/step - loss: 0.8009 - acc: 0.6755 - val_loss: 1.2596 - val_acc: 0.5055 - lr: 0.0010
Epoch 6/20
120/120 [==============================] - 31s 262ms/step - loss: 0.5724 - acc: 0.7791 - val_loss: 1.2414 - val_acc: 0.5232 - lr: 0.0010
Epoch 7/20
120/120 [==============================] - 31s 263ms/step - loss: 0.3764 - acc: 0.8646 - val_loss: 1.1189 - val_acc: 0.5683 - lr: 0.0010
Epoch 8/20
120/120 [==============================] - 31s 261ms/step - loss: 0.2465 - acc: 0.9129 - val_loss: 1.4133 - val_acc: 0.5235 - lr: 0.0010
Epoch 9/20
120/120 [==============================] - 31s 261ms/step - loss: 0.1769 - acc: 0.9373 - val_loss: 1.5584 - val_acc: 0.5200 - lr: 0.0010
Epoch 10/20
120/120 [==============================] - 31s 261ms/step - loss: 0.1361 - acc: 0.9552 - val_loss: 2.1238 - val_acc: 0.4953 - lr: 0.0010
Epoch 11/20
120/120 [==============================] - 31s 261ms/step - loss: 0.1010 - acc: 0.9647 - val_loss: 1.8936 - val_acc: 0.5306 - lr: 0.0010
Epoch 12/20
120/120 [==============================] - ETA: 0s - loss: 0.0888 - acc: 0.9716
Epoch 12: ReduceLROnPlateau reducing learning rate to 0.0005000000237487257.
120/120 [==============================] - 31s 262ms/step - loss: 0.0888 - acc: 0.9716 - val_loss: 1.3514 - val_acc: 0.5726 - lr: 0.0010
Epoch 13/20
120/120 [==============================] - 31s 261ms/step - loss: 0.0698 - acc: 0.9758 - val_loss: 1.5030 - val_acc: 0.5671 - lr: 5.0000e-04
Epoch 14/20
120/120 [==============================] - 31s 261ms/step - loss: 0.0579 - acc: 0.9797 - val_loss: 1.9460 - val_acc: 0.5396 - lr: 5.0000e-04
Epoch 15/20
120/120 [==============================] - 31s 261ms/step - loss: 0.0566 - acc: 0.9791 - val_loss: 2.2726 - val_acc: 0.5188 - lr: 5.0000e-04
Epoch 16/20
120/120 [==============================] - 31s 261ms/step - loss: 0.0542 - acc: 0.9831 - val_loss: 1.7809 - val_acc: 0.5286 - lr: 5.0000e-04
Epoch 17/20
120/120 [==============================] - ETA: 0s - loss: 0.0550 - acc: 0.9814
Epoch 17: ReduceLROnPlateau reducing learning rate to 0.0002500000118743628.
120/120 [==============================] - 31s 261ms/step - loss: 0.0550 - acc: 0.9814 - val_loss: 1.7201 - val_acc: 0.5263 - lr: 5.0000e-04
Epoch 18/20
120/120 [==============================] - 31s 261ms/step - loss: 0.0435 - acc: 0.9844 - val_loss: 1.6035 - val_acc: 0.5640 - lr: 2.5000e-04
Epoch 19/20
120/120 [==============================] - 31s 261ms/step - loss: 0.0445 - acc: 0.9840 - val_loss: 1.6329 - val_acc: 0.5620 - lr: 2.5000e-04
Epoch 20/20
120/120 [==============================] - 31s 261ms/step - loss: 0.0422 - acc: 0.9844 - val_loss: 1.7159 - val_acc: 0.5569 - lr: 2.5000e-04
80/80 [==============================] - 3s 39ms/step
Test results - Loss: 1.7159473896026611 - Accuracy: 55.69073557853699%
 metrics: {'dataset': 'Text', 'ngram': (2, 2), 'model': 'GRU', 'precision': 0.7347084056730147, 'recall': 0.5569073783359497, 'accuracy': 0.5569073783359497, 'f1': 0.5722379781509732, 'roc_auc': 0.8307044704791144}
```

```
#############################################################
END
#############################################################
#############################################################
START! dataset: Text, ngram: (2, 2), model: CNN+LSTM
#############################################################
Model: "sequential_11"
_________________________________________________________________
 Layer (type)                Output Shape              Param #   
=================================================================
 text_vectorization_2 (Text  (None, 64)                0         
 Vectorization)                                                  
                                                                 
 embedding_11 (Embedding)    (None, 64, 256)           15360000  
                                                                 
 spatial_dropout1d_11 (Spat  (None, 64, 256)           0         
 ialDropout1D)                                                   
                                                                 
 conv1d_4 (Conv1D)           (None, 64, 64)            98368     
                                                                 
 max_pooling1d_4 (MaxPoolin  (None, 32, 64)            0         
 g1D)                                                            
                                                                 
 conv1d_5 (Conv1D)           (None, 27, 32)            12320     
                                                                 
 max_pooling1d_5 (MaxPoolin  (None, 13, 32)            0         
 g1D)                                                            
                                                                 
 bidirectional_13 (Bidirect  (None, 13, 256)           164864    
 ional)                                                          
                                                                 
 dropout_27 (Dropout)        (None, 13, 256)           0         
                                                                 
 bidirectional_14 (Bidirect  (None, 256)               394240    
 ional)                                                          
                                                                 
 dropout_28 (Dropout)        (None, 256)               0         
                                                                 
 flatten_8 (Flatten)         (None, 256)               0         
                                                                 
 dense_22 (Dense)            (None, 64)                16448     
                                                                 
 dropout_29 (Dropout)        (None, 64)                0         
                                                                 
 dense_23 (Dense)            (None, 5)                 325       
                                                                 
=================================================================
Total params: 16046565 (61.21 MB)
Trainable params: 16046565 (61.21 MB)
Non-trainable params: 0 (0.00 Byte)
_________________________________________________________________
None
Epoch 1/20
120/120 [==============================] - 49s 333ms/step - loss: 1.5280 - acc: 0.3213 - val_loss: 1.4281 - val_acc: 0.3791 - lr: 0.0010
Epoch 2/20
120/120 [==============================] - 39s 322ms/step - loss: 1.3754 - acc: 0.3987 - val_loss: 1.2888 - val_acc: 0.4372 - lr: 0.0010
Epoch 3/20
120/120 [==============================] - 39s 321ms/step - loss: 1.1798 - acc: 0.4852 - val_loss: 1.4147 - val_acc: 0.3316 - lr: 0.0010
Epoch 4/20
120/120 [==============================] - 39s 322ms/step - loss: 0.9783 - acc: 0.5680 - val_loss: 1.1913 - val_acc: 0.4768 - lr: 0.0010
Epoch 5/20
120/120 [==============================] - 38s 321ms/step - loss: 0.8274 - acc: 0.6378 - val_loss: 1.4734 - val_acc: 0.4211 - lr: 0.0010
Epoch 6/20
120/120 [==============================] - 39s 322ms/step - loss: 0.6872 - acc: 0.6997 - val_loss: 1.2661 - val_acc: 0.4933 - lr: 0.0010
Epoch 7/20
120/120 [==============================] - 39s 322ms/step - loss: 0.5540 - acc: 0.7796 - val_loss: 1.2583 - val_acc: 0.5204 - lr: 0.0010
Epoch 8/20
120/120 [==============================] - 38s 321ms/step - loss: 0.4295 - acc: 0.8372 - val_loss: 1.7116 - val_acc: 0.4918 - lr: 0.0010
Epoch 9/20
120/120 [==============================] - ETA: 0s - loss: 0.3339 - acc: 0.8875
Epoch 9: ReduceLROnPlateau reducing learning rate to 0.0005000000237487257.
120/120 [==============================] - 38s 320ms/step - loss: 0.3339 - acc: 0.8875 - val_loss: 2.4857 - val_acc: 0.4517 - lr: 0.0010
Epoch 10/20
120/120 [==============================] - 39s 322ms/step - loss: 0.2462 - acc: 0.9204 - val_loss: 1.9526 - val_acc: 0.5326 - lr: 5.0000e-04
Epoch 11/20
120/120 [==============================] - 39s 323ms/step - loss: 0.2154 - acc: 0.9321 - val_loss: 1.7122 - val_acc: 0.5404 - lr: 5.0000e-04
Epoch 12/20
120/120 [==============================] - 38s 320ms/step - loss: 0.1728 - acc: 0.9446 - val_loss: 2.1117 - val_acc: 0.5122 - lr: 5.0000e-04
Epoch 13/20
120/120 [==============================] - 39s 322ms/step - loss: 0.1354 - acc: 0.9573 - val_loss: 1.6548 - val_acc: 0.5534 - lr: 5.0000e-04
Epoch 14/20
120/120 [==============================] - ETA: 0s - loss: 0.1309 - acc: 0.9621
Epoch 14: ReduceLROnPlateau reducing learning rate to 0.0002500000118743628.
120/120 [==============================] - 39s 322ms/step - loss: 0.1309 - acc: 0.9621 - val_loss: 1.9819 - val_acc: 0.5267 - lr: 5.0000e-04
Epoch 15/20
120/120 [==============================] - 38s 321ms/step - loss: 0.1119 - acc: 0.9677 - val_loss: 1.6740 - val_acc: 0.5428 - lr: 2.5000e-04
Epoch 16/20
120/120 [==============================] - 39s 321ms/step - loss: 0.0994 - acc: 0.9711 - val_loss: 1.6758 - val_acc: 0.5463 - lr: 2.5000e-04
Epoch 17/20
120/120 [==============================] - 39s 321ms/step - loss: 0.0905 - acc: 0.9717 - val_loss: 1.9130 - val_acc: 0.5353 - lr: 2.5000e-04
Epoch 18/20
120/120 [==============================] - 39s 321ms/step - loss: 0.0850 - acc: 0.9734 - val_loss: 1.8721 - val_acc: 0.5514 - lr: 2.5000e-04
Epoch 19/20
120/120 [==============================] - ETA: 0s - loss: 0.0759 - acc: 0.9766
Epoch 19: ReduceLROnPlateau reducing learning rate to 0.0001250000059371814.
120/120 [==============================] - 39s 321ms/step - loss: 0.0759 - acc: 0.9766 - val_loss: 1.5735 - val_acc: 0.5479 - lr: 2.5000e-04
Epoch 20/20
120/120 [==============================] - 39s 321ms/step - loss: 0.0752 - acc: 0.9774 - val_loss: 1.6803 - val_acc: 0.5365 - lr: 1.2500e-04
80/80 [==============================] - 6s 65ms/step
Test results - Loss: 1.6802589893341064 - Accuracy: 53.64992022514343%
 metrics: {'dataset': 'Text', 'ngram': (2, 2), 'model': 'CNN+LSTM', 'precision': 0.7355512707050452, 'recall': 0.5364992150706437, 'accuracy': 0.5364992150706437, 'f1': 0.5551337301591092, 'roc_auc': 0.8067386460677899}
```

```
#############################################################
END
#############################################################
#############################################################
START! dataset: Text, ngram: (2, 3), model: LSTM
#############################################################
Model: "sequential_12"
_________________________________________________________________
 Layer (type)                Output Shape              Param #   
=================================================================
 text_vectorization_3 (Text  (None, 64)                0         
 Vectorization)                                                  
                                                                 
 embedding_12 (Embedding)    (None, 64, 256)           15360000  
                                                                 
 spatial_dropout1d_12 (Spat  (None, 64, 256)           0         
 ialDropout1D)                                                   
                                                                 
 bidirectional_15 (Bidirect  (None, 512)               1050624   
 ional)                                                          
                                                                 
 dropout_30 (Dropout)        (None, 512)               0         
                                                                 
 flatten_9 (Flatten)         (None, 512)               0         
                                                                 
 dense_24 (Dense)            (None, 32)                16416     
                                                                 
 dropout_31 (Dropout)        (None, 32)                0         
                                                                 
 dense_25 (Dense)            (None, 5)                 165       
                                                                 
=================================================================
Total params: 16427205 (62.66 MB)
Trainable params: 16427205 (62.66 MB)
Non-trainable params: 0 (0.00 Byte)
_________________________________________________________________
None
Epoch 1/20
120/120 [==============================] - 179s 1s/step - loss: 1.5604 - acc: 0.2675 - val_loss: 1.4990 - val_acc: 0.3226 - lr: 0.0010
Epoch 2/20
120/120 [==============================] - 170s 1s/step - loss: 1.5092 - acc: 0.3182 - val_loss: 1.4641 - val_acc: 0.3312 - lr: 0.0010
Epoch 3/20
120/120 [==============================] - 170s 1s/step - loss: 1.4408 - acc: 0.3508 - val_loss: 1.3693 - val_acc: 0.3571 - lr: 0.0010
Epoch 4/20
120/120 [==============================] - 170s 1s/step - loss: 1.2798 - acc: 0.4320 - val_loss: 1.2816 - val_acc: 0.3972 - lr: 0.0010
Epoch 5/20
120/120 [==============================] - 170s 1s/step - loss: 1.1687 - acc: 0.4988 - val_loss: 1.2065 - val_acc: 0.4898 - lr: 0.0010
Epoch 6/20
120/120 [==============================] - 170s 1s/step - loss: 0.9853 - acc: 0.5805 - val_loss: 1.1313 - val_acc: 0.5353 - lr: 0.0010
Epoch 7/20
120/120 [==============================] - 170s 1s/step - loss: 0.8046 - acc: 0.6787 - val_loss: 1.0997 - val_acc: 0.5373 - lr: 0.0010
Epoch 8/20
120/120 [==============================] - 170s 1s/step - loss: 0.6708 - acc: 0.7384 - val_loss: 1.1092 - val_acc: 0.5310 - lr: 0.0010
Epoch 9/20
120/120 [==============================] - 170s 1s/step - loss: 0.5246 - acc: 0.8074 - val_loss: 1.0642 - val_acc: 0.5608 - lr: 0.0010
Epoch 10/20
120/120 [==============================] - 170s 1s/step - loss: 0.4472 - acc: 0.8401 - val_loss: 1.0435 - val_acc: 0.5848 - lr: 0.0010
Epoch 11/20
120/120 [==============================] - 170s 1s/step - loss: 0.3346 - acc: 0.8807 - val_loss: 1.0856 - val_acc: 0.5655 - lr: 0.0010
Epoch 12/20
120/120 [==============================] - 169s 1s/step - loss: 0.2698 - acc: 0.9059 - val_loss: 1.0574 - val_acc: 0.5612 - lr: 0.0010
Epoch 13/20
120/120 [==============================] - 169s 1s/step - loss: 0.2422 - acc: 0.9123 - val_loss: 1.0673 - val_acc: 0.5655 - lr: 0.0010
Epoch 14/20
120/120 [==============================] - 170s 1s/step - loss: 0.2167 - acc: 0.9258 - val_loss: 1.0575 - val_acc: 0.5667 - lr: 0.0010
Epoch 15/20
120/120 [==============================] - ETA: 0s - loss: 0.2058 - acc: 0.9221
Epoch 15: ReduceLROnPlateau reducing learning rate to 0.0005000000237487257.
120/120 [==============================] - 170s 1s/step - loss: 0.2058 - acc: 0.9221 - val_loss: 1.8076 - val_acc: 0.5522 - lr: 0.0010
Epoch 16/20
120/120 [==============================] - 170s 1s/step - loss: 0.1742 - acc: 0.9348 - val_loss: 1.0277 - val_acc: 0.5950 - lr: 5.0000e-04
Epoch 17/20
120/120 [==============================] - 169s 1s/step - loss: 0.1683 - acc: 0.9373 - val_loss: 1.0736 - val_acc: 0.5738 - lr: 5.0000e-04
Epoch 18/20
120/120 [==============================] - 169s 1s/step - loss: 0.1578 - acc: 0.9414 - val_loss: 1.0890 - val_acc: 0.5640 - lr: 5.0000e-04
Epoch 19/20
120/120 [==============================] - 169s 1s/step - loss: 0.1479 - acc: 0.9423 - val_loss: 1.0763 - val_acc: 0.5840 - lr: 5.0000e-04
Epoch 20/20
120/120 [==============================] - 169s 1s/step - loss: 0.1445 - acc: 0.9437 - val_loss: 1.1213 - val_acc: 0.5934 - lr: 5.0000e-04
80/80 [==============================] - 14s 163ms/step
Test results - Loss: 1.1213492155075073 - Accuracy: 59.3406617641449%
 metrics: {'dataset': 'Text', 'ngram': (2, 3), 'model': 'LSTM', 'precision': 0.6619637112629118, 'recall': 0.5934065934065934, 'accuracy': 0.5934065934065934, 'f1': 0.599071928758935, 'roc_auc': 0.852865928383572}
```

```
#############################################################
END
#############################################################
#############################################################
START! dataset: Text, ngram: (2, 3), model: LSTM2X
#############################################################
Model: "sequential_13"
_________________________________________________________________
 Layer (type)                Output Shape              Param #   
=================================================================
 text_vectorization_3 (Text  (None, 64)                0         
 Vectorization)                                                  
                                                                 
 embedding_13 (Embedding)    (None, 64, 256)           15360000  
                                                                 
 spatial_dropout1d_13 (Spat  (None, 64, 256)           0         
 ialDropout1D)                                                   
                                                                 
 bidirectional_16 (Bidirect  (None, 64, 256)           394240    
 ional)                                                          
                                                                 
 dropout_32 (Dropout)        (None, 64, 256)           0         
                                                                 
 bidirectional_17 (Bidirect  (None, 256)               394240    
 ional)                                                          
                                                                 
 dropout_33 (Dropout)        (None, 256)               0         
                                                                 
 flatten_10 (Flatten)        (None, 256)               0         
                                                                 
 dense_26 (Dense)            (None, 64)                16448     
                                                                 
 dropout_34 (Dropout)        (None, 64)                0         
                                                                 
 dense_27 (Dense)            (None, 5)                 325       
                                                                 
=================================================================
Total params: 16165253 (61.67 MB)
Trainable params: 16165253 (61.67 MB)
Non-trainable params: 0 (0.00 Byte)
_________________________________________________________________
None
Epoch 1/20
120/120 [==============================] - 366s 3s/step - loss: 1.5549 - acc: 0.2803 - val_loss: 1.5043 - val_acc: 0.3438 - lr: 0.0010
Epoch 2/20
120/120 [==============================] - 356s 3s/step - loss: 1.4826 - acc: 0.3411 - val_loss: 1.4367 - val_acc: 0.3450 - lr: 0.0010
Epoch 3/20
120/120 [==============================] - 355s 3s/step - loss: 1.3568 - acc: 0.4042 - val_loss: 1.3000 - val_acc: 0.4129 - lr: 0.0010
Epoch 4/20
120/120 [==============================] - 359s 3s/step - loss: 1.1801 - acc: 0.4912 - val_loss: 1.2790 - val_acc: 0.4427 - lr: 0.0010
Epoch 5/20
120/120 [==============================] - 358s 3s/step - loss: 1.0040 - acc: 0.5805 - val_loss: 1.1899 - val_acc: 0.4890 - lr: 0.0010
Epoch 6/20
120/120 [==============================] - 362s 3s/step - loss: 0.7979 - acc: 0.6798 - val_loss: 1.1434 - val_acc: 0.5239 - lr: 0.0010
Epoch 7/20
120/120 [==============================] - 360s 3s/step - loss: 0.6231 - acc: 0.7548 - val_loss: 1.1422 - val_acc: 0.5357 - lr: 0.0010
Epoch 8/20
120/120 [==============================] - 364s 3s/step - loss: 0.4975 - acc: 0.8168 - val_loss: 1.0991 - val_acc: 0.5620 - lr: 0.0010
Epoch 9/20
120/120 [==============================] - 361s 3s/step - loss: 0.3913 - acc: 0.8601 - val_loss: 1.1690 - val_acc: 0.5624 - lr: 0.0010
Epoch 10/20
120/120 [==============================] - 365s 3s/step - loss: 0.3195 - acc: 0.8876 - val_loss: 1.1429 - val_acc: 0.5510 - lr: 0.0010
Epoch 11/20
120/120 [==============================] - 365s 3s/step - loss: 0.2651 - acc: 0.9085 - val_loss: 1.1578 - val_acc: 0.5546 - lr: 0.0010
Epoch 12/20
120/120 [==============================] - 366s 3s/step - loss: 0.2210 - acc: 0.9217 - val_loss: 1.2506 - val_acc: 0.5714 - lr: 0.0010
Epoch 13/20
120/120 [==============================] - ETA: 0s - loss: 0.1896 - acc: 0.9329
Epoch 13: ReduceLROnPlateau reducing learning rate to 0.0005000000237487257.
120/120 [==============================] - 363s 3s/step - loss: 0.1896 - acc: 0.9329 - val_loss: 1.3213 - val_acc: 0.5412 - lr: 0.0010
Epoch 14/20
120/120 [==============================] - 364s 3s/step - loss: 0.1747 - acc: 0.9352 - val_loss: 1.0292 - val_acc: 0.5977 - lr: 5.0000e-04
Epoch 15/20
120/120 [==============================] - 366s 3s/step - loss: 0.1460 - acc: 0.9458 - val_loss: 1.0552 - val_acc: 0.6020 - lr: 5.0000e-04
Epoch 16/20
120/120 [==============================] - 365s 3s/step - loss: 0.1412 - acc: 0.9463 - val_loss: 1.0813 - val_acc: 0.5969 - lr: 5.0000e-04
Epoch 17/20
120/120 [==============================] - 365s 3s/step - loss: 0.1423 - acc: 0.9475 - val_loss: 1.0607 - val_acc: 0.5965 - lr: 5.0000e-04
Epoch 18/20
120/120 [==============================] - 365s 3s/step - loss: 0.1390 - acc: 0.9466 - val_loss: 1.1791 - val_acc: 0.5620 - lr: 5.0000e-04
Epoch 19/20
120/120 [==============================] - ETA: 0s - loss: 0.1357 - acc: 0.9492
Epoch 19: ReduceLROnPlateau reducing learning rate to 0.0002500000118743628.
120/120 [==============================] - 366s 3s/step - loss: 0.1357 - acc: 0.9492 - val_loss: 1.1146 - val_acc: 0.5518 - lr: 5.0000e-04
Epoch 20/20
120/120 [==============================] - 366s 3s/step - loss: 0.1322 - acc: 0.9512 - val_loss: 1.1055 - val_acc: 0.5801 - lr: 2.5000e-04
80/80 [==============================] - 26s 308ms/step
Test results - Loss: 1.1054970026016235 - Accuracy: 58.00628066062927%
 metrics: {'dataset': 'Text', 'ngram': (2, 3), 'model': 'LSTM2X', 'precision': 0.7046788805623717, 'recall': 0.5800627943485086, 'accuracy': 0.5800627943485086, 'f1': 0.5890171158780252, 'roc_auc': 0.8538202517778111}
```

```
#############################################################
END
#############################################################
#############################################################
START! dataset: Text, ngram: (2, 3), model: GRU
#############################################################
Model: "sequential_14"
_________________________________________________________________
 Layer (type)                Output Shape              Param #   
=================================================================
 text_vectorization_3 (Text  (None, 64)                0         
 Vectorization)                                                  
                                                                 
 embedding_14 (Embedding)    (None, 64, 256)           15360000  
                                                                 
 spatial_dropout1d_14 (Spat  (None, 64, 256)           0         
 ialDropout1D)                                                   
                                                                 
 gru_3 (GRU)                 (None, 64)                61824     
                                                                 
 dropout_35 (Dropout)        (None, 64)                0         
                                                                 
 dense_28 (Dense)            (None, 256)               16640     
                                                                 
 dropout_36 (Dropout)        (None, 256)               0         
                                                                 
 dense_29 (Dense)            (None, 5)                 1285      
                                                                 
=================================================================
Total params: 15439749 (58.90 MB)
Trainable params: 15439749 (58.90 MB)
Non-trainable params: 0 (0.00 Byte)
_________________________________________________________________
None
Epoch 1/20
120/120 [==============================] - 42s 326ms/step - loss: 1.6045 - acc: 0.2228 - val_loss: 1.6042 - val_acc: 0.2355 - lr: 0.0010
Epoch 2/20
120/120 [==============================] - 38s 320ms/step - loss: 1.5833 - acc: 0.2545 - val_loss: 1.5132 - val_acc: 0.2991 - lr: 0.0010
Epoch 3/20
120/120 [==============================] - 38s 320ms/step - loss: 1.4615 - acc: 0.3415 - val_loss: 1.4863 - val_acc: 0.3336 - lr: 0.0010
Epoch 4/20
120/120 [==============================] - 38s 320ms/step - loss: 1.3256 - acc: 0.3979 - val_loss: 1.3581 - val_acc: 0.3607 - lr: 0.0010
Epoch 5/20
120/120 [==============================] - 38s 320ms/step - loss: 1.1785 - acc: 0.4681 - val_loss: 1.2914 - val_acc: 0.3913 - lr: 0.0010
Epoch 6/20
120/120 [==============================] - 38s 320ms/step - loss: 1.0295 - acc: 0.5446 - val_loss: 1.2172 - val_acc: 0.4627 - lr: 0.0010
Epoch 7/20
120/120 [==============================] - 38s 320ms/step - loss: 0.9136 - acc: 0.6072 - val_loss: 1.2000 - val_acc: 0.4698 - lr: 0.0010
Epoch 8/20
120/120 [==============================] - 38s 318ms/step - loss: 0.7953 - acc: 0.6574 - val_loss: 1.3013 - val_acc: 0.4655 - lr: 0.0010
Epoch 9/20
120/120 [==============================] - 38s 319ms/step - loss: 0.6885 - acc: 0.7125 - val_loss: 1.4736 - val_acc: 0.4639 - lr: 0.0010
Epoch 10/20
120/120 [==============================] - 38s 320ms/step - loss: 0.6046 - acc: 0.7553 - val_loss: 1.2216 - val_acc: 0.4910 - lr: 0.0010
Epoch 11/20
120/120 [==============================] - 38s 321ms/step - loss: 0.5194 - acc: 0.7880 - val_loss: 1.2617 - val_acc: 0.5153 - lr: 0.0010
Epoch 12/20
120/120 [==============================] - ETA: 0s - loss: 0.4561 - acc: 0.8209
Epoch 12: ReduceLROnPlateau reducing learning rate to 0.0005000000237487257.
120/120 [==============================] - 38s 319ms/step - loss: 0.4561 - acc: 0.8209 - val_loss: 1.3244 - val_acc: 0.5106 - lr: 0.0010
Epoch 13/20
120/120 [==============================] - 38s 320ms/step - loss: 0.3813 - acc: 0.8524 - val_loss: 1.2132 - val_acc: 0.5275 - lr: 5.0000e-04
Epoch 14/20
120/120 [==============================] - 38s 320ms/step - loss: 0.3451 - acc: 0.8668 - val_loss: 1.2342 - val_acc: 0.5310 - lr: 5.0000e-04
Epoch 15/20
120/120 [==============================] - 38s 320ms/step - loss: 0.3119 - acc: 0.8779 - val_loss: 1.2617 - val_acc: 0.5322 - lr: 5.0000e-04
Epoch 16/20
120/120 [==============================] - 38s 320ms/step - loss: 0.2960 - acc: 0.8865 - val_loss: 1.1734 - val_acc: 0.5479 - lr: 5.0000e-04
Epoch 17/20
120/120 [==============================] - 38s 319ms/step - loss: 0.2807 - acc: 0.8886 - val_loss: 1.2661 - val_acc: 0.5283 - lr: 5.0000e-04
Epoch 18/20
120/120 [==============================] - 38s 319ms/step - loss: 0.2586 - acc: 0.9011 - val_loss: 1.3140 - val_acc: 0.5188 - lr: 5.0000e-04
Epoch 19/20
120/120 [==============================] - 38s 319ms/step - loss: 0.2318 - acc: 0.9092 - val_loss: 1.2691 - val_acc: 0.5424 - lr: 5.0000e-04
Epoch 20/20
120/120 [==============================] - 38s 319ms/step - loss: 0.2229 - acc: 0.9168 - val_loss: 1.3310 - val_acc: 0.5361 - lr: 5.0000e-04
80/80 [==============================] - 5s 53ms/step
Test results - Loss: 1.3310447931289673 - Accuracy: 53.610676527023315%
 metrics: {'dataset': 'Text', 'ngram': (2, 3), 'model': 'GRU', 'precision': 0.6739162343272671, 'recall': 0.5361067503924647, 'accuracy': 0.5361067503924647, 'f1': 0.5532363811222192, 'roc_auc': 0.8092060266691036}
```

```
#############################################################
END
#############################################################
#############################################################
START! dataset: Text, ngram: (2, 3), model: CNN+LSTM
#############################################################
Model: "sequential_15"
_________________________________________________________________
 Layer (type)                Output Shape              Param #   
=================================================================
 text_vectorization_3 (Text  (None, 64)                0         
 Vectorization)                                                  
                                                                 
 embedding_15 (Embedding)    (None, 64, 256)           15360000  
                                                                 
 spatial_dropout1d_15 (Spat  (None, 64, 256)           0         
 ialDropout1D)                                                   
                                                                 
 conv1d_6 (Conv1D)           (None, 64, 64)            98368     
                                                                 
 max_pooling1d_6 (MaxPoolin  (None, 32, 64)            0         
 g1D)                                                            
                                                                 
 conv1d_7 (Conv1D)           (None, 27, 32)            12320     
                                                                 
 max_pooling1d_7 (MaxPoolin  (None, 13, 32)            0         
 g1D)                                                            
                                                                 
 bidirectional_18 (Bidirect  (None, 13, 256)           164864    
 ional)                                                          
                                                                 
 dropout_37 (Dropout)        (None, 13, 256)           0         
                                                                 
 bidirectional_19 (Bidirect  (None, 256)               394240    
 ional)                                                          
                                                                 
 dropout_38 (Dropout)        (None, 256)               0         
                                                                 
 flatten_11 (Flatten)        (None, 256)               0         
                                                                 
 dense_30 (Dense)            (None, 64)                16448     
                                                                 
 dropout_39 (Dropout)        (None, 64)                0         
                                                                 
 dense_31 (Dense)            (None, 5)                 325       
                                                                 
=================================================================
Total params: 16046565 (61.21 MB)
Trainable params: 16046565 (61.21 MB)
Non-trainable params: 0 (0.00 Byte)
_________________________________________________________________
None
Epoch 1/20
120/120 [==============================] - 57s 395ms/step - loss: 1.5370 - acc: 0.3018 - val_loss: 1.4948 - val_acc: 0.3383 - lr: 0.0010
Epoch 2/20
120/120 [==============================] - 46s 385ms/step - loss: 1.4524 - acc: 0.3729 - val_loss: 1.3480 - val_acc: 0.3952 - lr: 0.0010
Epoch 3/20
120/120 [==============================] - 46s 384ms/step - loss: 1.2587 - acc: 0.4503 - val_loss: 1.2555 - val_acc: 0.4447 - lr: 0.0010
Epoch 4/20
120/120 [==============================] - 46s 383ms/step - loss: 1.0700 - acc: 0.5262 - val_loss: 1.2588 - val_acc: 0.4388 - lr: 0.0010
Epoch 5/20
120/120 [==============================] - 46s 385ms/step - loss: 0.9319 - acc: 0.5852 - val_loss: 1.2434 - val_acc: 0.4651 - lr: 0.0010
Epoch 6/20
120/120 [==============================] - 46s 384ms/step - loss: 0.8011 - acc: 0.6564 - val_loss: 1.2266 - val_acc: 0.4749 - lr: 0.0010
Epoch 7/20
120/120 [==============================] - 46s 383ms/step - loss: 0.6831 - acc: 0.7112 - val_loss: 1.4332 - val_acc: 0.4749 - lr: 0.0010
Epoch 8/20
120/120 [==============================] - 46s 385ms/step - loss: 0.6039 - acc: 0.7605 - val_loss: 1.4562 - val_acc: 0.4761 - lr: 0.0010
Epoch 9/20
120/120 [==============================] - 46s 384ms/step - loss: 0.5096 - acc: 0.8057 - val_loss: 1.5523 - val_acc: 0.4831 - lr: 0.0010
Epoch 10/20
120/120 [==============================] - 46s 384ms/step - loss: 0.4319 - acc: 0.8388 - val_loss: 1.4142 - val_acc: 0.4839 - lr: 0.0010
Epoch 11/20
120/120 [==============================] - ETA: 0s - loss: 0.3770 - acc: 0.8606
Epoch 11: ReduceLROnPlateau reducing learning rate to 0.0005000000237487257.
120/120 [==============================] - 46s 384ms/step - loss: 0.3770 - acc: 0.8606 - val_loss: 1.4356 - val_acc: 0.4918 - lr: 0.0010
Epoch 12/20
120/120 [==============================] - 46s 385ms/step - loss: 0.3225 - acc: 0.8841 - val_loss: 1.4678 - val_acc: 0.4996 - lr: 5.0000e-04
Epoch 13/20
120/120 [==============================] - 46s 384ms/step - loss: 0.2786 - acc: 0.8914 - val_loss: 1.5603 - val_acc: 0.5051 - lr: 5.0000e-04
Epoch 14/20
120/120 [==============================] - 46s 383ms/step - loss: 0.2770 - acc: 0.8969 - val_loss: 1.5565 - val_acc: 0.4941 - lr: 5.0000e-04
Epoch 15/20
120/120 [==============================] - 46s 385ms/step - loss: 0.2507 - acc: 0.9067 - val_loss: 1.5686 - val_acc: 0.5086 - lr: 5.0000e-04
Epoch 16/20
120/120 [==============================] - ETA: 0s - loss: 0.2217 - acc: 0.9168
Epoch 16: ReduceLROnPlateau reducing learning rate to 0.0002500000118743628.
120/120 [==============================] - 46s 382ms/step - loss: 0.2217 - acc: 0.9168 - val_loss: 1.6286 - val_acc: 0.5016 - lr: 5.0000e-04
Epoch 17/20
120/120 [==============================] - 46s 383ms/step - loss: 0.2084 - acc: 0.9202 - val_loss: 1.6635 - val_acc: 0.5043 - lr: 2.5000e-04
Epoch 18/20
120/120 [==============================] - 46s 383ms/step - loss: 0.2011 - acc: 0.9245 - val_loss: 1.6400 - val_acc: 0.5086 - lr: 2.5000e-04
Epoch 19/20
120/120 [==============================] - 46s 383ms/step - loss: 0.2000 - acc: 0.9241 - val_loss: 1.6717 - val_acc: 0.5027 - lr: 2.5000e-04
Epoch 20/20
120/120 [==============================] - 46s 383ms/step - loss: 0.1878 - acc: 0.9258 - val_loss: 1.7115 - val_acc: 0.5027 - lr: 2.5000e-04
80/80 [==============================] - 8s 87ms/step
Test results - Loss: 1.7115330696105957 - Accuracy: 50.27472376823425%
 metrics: {'dataset': 'Text', 'ngram': (2, 3), 'model': 'CNN+LSTM', 'precision': 0.6099133309808349, 'recall': 0.5027472527472527, 'accuracy': 0.5027472527472527, 'f1': 0.5037882968977446, 'roc_auc': 0.7911636726128324}
```

```
#############################################################
END
#############################################################
#############################################################
START! dataset: Stemmed, ngram: (1, 1), model: LSTM
#############################################################
Model: "sequential_16"
_________________________________________________________________
 Layer (type)                Output Shape              Param #   
=================================================================
 text_vectorization_4 (Text  (None, 64)                0         
 Vectorization)                                                  
                                                                 
 embedding_16 (Embedding)    (None, 64, 256)           3089664   
                                                                 
 spatial_dropout1d_16 (Spat  (None, 64, 256)           0         
 ialDropout1D)                                                   
                                                                 
 bidirectional_20 (Bidirect  (None, 512)               1050624   
 ional)                                                          
                                                                 
 dropout_40 (Dropout)        (None, 512)               0         
                                                                 
 flatten_12 (Flatten)        (None, 512)               0         
                                                                 
 dense_32 (Dense)            (None, 32)                16416     
                                                                 
 dropout_41 (Dropout)        (None, 32)                0         
                                                                 
 dense_33 (Dense)            (None, 5)                 165       
                                                                 
=================================================================
Total params: 4156869 (15.86 MB)
Trainable params: 4156869 (15.86 MB)
Non-trainable params: 0 (0.00 Byte)
_________________________________________________________________
None
Epoch 1/20
120/120 [==============================] - 208s 2s/step - loss: 1.3554 - acc: 0.4376 - val_loss: 0.9042 - val_acc: 0.6951 - lr: 0.0010
Epoch 2/20
120/120 [==============================] - 199s 2s/step - loss: 0.8283 - acc: 0.7037 - val_loss: 0.6590 - val_acc: 0.7798 - lr: 0.0010
Epoch 3/20
120/120 [==============================] - 199s 2s/step - loss: 0.5938 - acc: 0.8061 - val_loss: 0.6169 - val_acc: 0.8261 - lr: 0.0010
Epoch 4/20
120/120 [==============================] - 199s 2s/step - loss: 0.4975 - acc: 0.8500 - val_loss: 0.4610 - val_acc: 0.8489 - lr: 0.0010
Epoch 5/20
120/120 [==============================] - 199s 2s/step - loss: 0.3835 - acc: 0.8817 - val_loss: 0.4124 - val_acc: 0.8638 - lr: 0.0010
Epoch 6/20
120/120 [==============================] - 199s 2s/step - loss: 0.2967 - acc: 0.9144 - val_loss: 0.4341 - val_acc: 0.8630 - lr: 0.0010
Epoch 7/20
120/120 [==============================] - 199s 2s/step - loss: 0.2661 - acc: 0.9244 - val_loss: 0.4067 - val_acc: 0.8736 - lr: 0.0010
Epoch 8/20
120/120 [==============================] - 199s 2s/step - loss: 0.2395 - acc: 0.9316 - val_loss: 0.4680 - val_acc: 0.8642 - lr: 0.0010
Epoch 9/20
120/120 [==============================] - 198s 2s/step - loss: 0.1923 - acc: 0.9458 - val_loss: 0.4489 - val_acc: 0.8728 - lr: 0.0010
Epoch 10/20
120/120 [==============================] - 198s 2s/step - loss: 0.1630 - acc: 0.9567 - val_loss: 0.4661 - val_acc: 0.8685 - lr: 0.0010
Epoch 11/20
120/120 [==============================] - 199s 2s/step - loss: 0.1558 - acc: 0.9549 - val_loss: 0.4945 - val_acc: 0.8705 - lr: 0.0010
Epoch 12/20
120/120 [==============================] - ETA: 0s - loss: 0.1312 - acc: 0.9627
Epoch 12: ReduceLROnPlateau reducing learning rate to 0.0005000000237487257.
120/120 [==============================] - 199s 2s/step - loss: 0.1312 - acc: 0.9627 - val_loss: 0.5304 - val_acc: 0.8685 - lr: 0.0010
Epoch 13/20
120/120 [==============================] - 198s 2s/step - loss: 0.1157 - acc: 0.9673 - val_loss: 0.5451 - val_acc: 0.8709 - lr: 5.0000e-04
Epoch 14/20
120/120 [==============================] - 199s 2s/step - loss: 0.1037 - acc: 0.9672 - val_loss: 0.5633 - val_acc: 0.8681 - lr: 5.0000e-04
Epoch 15/20
120/120 [==============================] - 198s 2s/step - loss: 0.0946 - acc: 0.9745 - val_loss: 0.5941 - val_acc: 0.8697 - lr: 5.0000e-04
Epoch 16/20
120/120 [==============================] - 199s 2s/step - loss: 0.0972 - acc: 0.9721 - val_loss: 0.5674 - val_acc: 0.8693 - lr: 5.0000e-04
Epoch 17/20
120/120 [==============================] - ETA: 0s - loss: 0.0871 - acc: 0.9762Restoring model weights from the end of the best epoch: 7.

Epoch 17: ReduceLROnPlateau reducing learning rate to 0.0002500000118743628.
120/120 [==============================] - 199s 2s/step - loss: 0.0871 - acc: 0.9762 - val_loss: 0.5823 - val_acc: 0.8681 - lr: 5.0000e-04
Epoch 17: early stopping
80/80 [==============================] - 15s 186ms/step
Test results - Loss: 0.40671685338020325 - Accuracy: 87.36263513565063%
 metrics: {'dataset': 'Stemmed', 'ngram': (1, 1), 'model': 'LSTM', 'precision': 0.8758284723366125, 'recall': 0.8736263736263736, 'accuracy': 0.8736263736263736, 'f1': 0.8741091513211857, 'roc_auc': 0.9777969574317528}
```

```
#############################################################
END
#############################################################
#############################################################
START! dataset: Stemmed, ngram: (1, 1), model: LSTM2X
#############################################################
Model: "sequential_17"
_________________________________________________________________
 Layer (type)                Output Shape              Param #   
=================================================================
 text_vectorization_4 (Text  (None, 64)                0         
 Vectorization)                                                  
                                                                 
 embedding_17 (Embedding)    (None, 64, 256)           3089664   
                                                                 
 spatial_dropout1d_17 (Spat  (None, 64, 256)           0         
 ialDropout1D)                                                   
                                                                 
 bidirectional_21 (Bidirect  (None, 64, 256)           394240    
 ional)                                                          
                                                                 
 dropout_42 (Dropout)        (None, 64, 256)           0         
                                                                 
 bidirectional_22 (Bidirect  (None, 256)               394240    
 ional)                                                          
                                                                 
 dropout_43 (Dropout)        (None, 256)               0         
                                                                 
 flatten_13 (Flatten)        (None, 256)               0         
                                                                 
 dense_34 (Dense)            (None, 64)                16448     
                                                                 
 dropout_44 (Dropout)        (None, 64)                0         
                                                                 
 dense_35 (Dense)            (None, 5)                 325       
                                                                 
=================================================================
Total params: 3894917 (14.86 MB)
Trainable params: 3894917 (14.86 MB)
Non-trainable params: 0 (0.00 Byte)
_________________________________________________________________
None
Epoch 1/20
120/120 [==============================] - 413s 3s/step - loss: 1.3378 - acc: 0.4043 - val_loss: 0.7461 - val_acc: 0.7162 - lr: 0.0010
Epoch 2/20
120/120 [==============================] - 404s 3s/step - loss: 0.7299 - acc: 0.7393 - val_loss: 0.5356 - val_acc: 0.8073 - lr: 0.0010
Epoch 3/20
120/120 [==============================] - 407s 3s/step - loss: 0.5078 - acc: 0.8270 - val_loss: 0.4359 - val_acc: 0.8528 - lr: 0.0010
Epoch 4/20
120/120 [==============================] - 409s 3s/step - loss: 0.3914 - acc: 0.8742 - val_loss: 0.3962 - val_acc: 0.8736 - lr: 0.0010
Epoch 5/20
120/120 [==============================] - 410s 3s/step - loss: 0.3143 - acc: 0.9003 - val_loss: 0.4189 - val_acc: 0.8693 - lr: 0.0010
Epoch 6/20
120/120 [==============================] - 411s 3s/step - loss: 0.2497 - acc: 0.9193 - val_loss: 0.4141 - val_acc: 0.8654 - lr: 0.0010
Epoch 7/20
120/120 [==============================] - 413s 3s/step - loss: 0.2169 - acc: 0.9343 - val_loss: 0.4277 - val_acc: 0.8658 - lr: 0.0010
Epoch 8/20
120/120 [==============================] - 413s 3s/step - loss: 0.1928 - acc: 0.9424 - val_loss: 0.4370 - val_acc: 0.8630 - lr: 0.0010
Epoch 9/20
120/120 [==============================] - ETA: 0s - loss: 0.1800 - acc: 0.9444
Epoch 9: ReduceLROnPlateau reducing learning rate to 0.0005000000237487257.
120/120 [==============================] - 413s 3s/step - loss: 0.1800 - acc: 0.9444 - val_loss: 0.4669 - val_acc: 0.8666 - lr: 0.0010
Epoch 10/20
120/120 [==============================] - 412s 3s/step - loss: 0.1401 - acc: 0.9598 - val_loss: 0.4792 - val_acc: 0.8705 - lr: 5.0000e-04
Epoch 11/20
120/120 [==============================] - 413s 3s/step - loss: 0.1185 - acc: 0.9644 - val_loss: 0.4894 - val_acc: 0.8697 - lr: 5.0000e-04
Epoch 12/20
120/120 [==============================] - 411s 3s/step - loss: 0.1171 - acc: 0.9641 - val_loss: 0.5089 - val_acc: 0.8646 - lr: 5.0000e-04
Epoch 13/20
120/120 [==============================] - 413s 3s/step - loss: 0.1060 - acc: 0.9678 - val_loss: 0.5145 - val_acc: 0.8713 - lr: 5.0000e-04
Epoch 14/20
120/120 [==============================] - ETA: 0s - loss: 0.1018 - acc: 0.9712Restoring model weights from the end of the best epoch: 4.

Epoch 14: ReduceLROnPlateau reducing learning rate to 0.0002500000118743628.
120/120 [==============================] - 415s 3s/step - loss: 0.1018 - acc: 0.9712 - val_loss: 0.5235 - val_acc: 0.8697 - lr: 5.0000e-04
Epoch 14: early stopping
80/80 [==============================] - 31s 375ms/step
Test results - Loss: 0.3961585760116577 - Accuracy: 87.36263513565063%
 metrics: {'dataset': 'Stemmed', 'ngram': (1, 1), 'model': 'LSTM2X', 'precision': 0.8777220587911531, 'recall': 0.8736263736263736, 'accuracy': 0.8736263736263736, 'f1': 0.8747778317913232, 'roc_auc': 0.977464730100394}
```

```
#############################################################
END
#############################################################
#############################################################
START! dataset: Stemmed, ngram: (1, 1), model: GRU
#############################################################
Model: "sequential_18"
_________________________________________________________________
 Layer (type)                Output Shape              Param #   
=================================================================
 text_vectorization_4 (Text  (None, 64)                0         
 Vectorization)                                                  
                                                                 
 embedding_18 (Embedding)    (None, 64, 256)           3089664   
                                                                 
 spatial_dropout1d_18 (Spat  (None, 64, 256)           0         
 ialDropout1D)                                                   
                                                                 
 gru_4 (GRU)                 (None, 64)                61824     
                                                                 
 dropout_45 (Dropout)        (None, 64)                0         
                                                                 
 dense_36 (Dense)            (None, 256)               16640     
                                                                 
 dropout_46 (Dropout)        (None, 256)               0         
                                                                 
 dense_37 (Dense)            (None, 5)                 1285      
                                                                 
=================================================================
Total params: 3169413 (12.09 MB)
Trainable params: 3169413 (12.09 MB)
Non-trainable params: 0 (0.00 Byte)
_________________________________________________________________
None
Epoch 1/20
120/120 [==============================] - 43s 332ms/step - loss: 1.5676 - acc: 0.2821 - val_loss: 1.2173 - val_acc: 0.5459 - lr: 0.0010
Epoch 2/20
120/120 [==============================] - 39s 326ms/step - loss: 0.9821 - acc: 0.5950 - val_loss: 0.6222 - val_acc: 0.7739 - lr: 0.0010
Epoch 3/20
120/120 [==============================] - 39s 327ms/step - loss: 0.6514 - acc: 0.7659 - val_loss: 0.5346 - val_acc: 0.8226 - lr: 0.0010
Epoch 4/20
120/120 [==============================] - 39s 326ms/step - loss: 0.4923 - acc: 0.8325 - val_loss: 0.4928 - val_acc: 0.8391 - lr: 0.0010
Epoch 5/20
120/120 [==============================] - 39s 327ms/step - loss: 0.4092 - acc: 0.8678 - val_loss: 0.4666 - val_acc: 0.8532 - lr: 0.0010
Epoch 6/20
120/120 [==============================] - 39s 327ms/step - loss: 0.3432 - acc: 0.8863 - val_loss: 0.4491 - val_acc: 0.8599 - lr: 0.0010
Epoch 7/20
120/120 [==============================] - 39s 327ms/step - loss: 0.2896 - acc: 0.9084 - val_loss: 0.4499 - val_acc: 0.8603 - lr: 0.0010
Epoch 8/20
120/120 [==============================] - 39s 327ms/step - loss: 0.2542 - acc: 0.9191 - val_loss: 0.4504 - val_acc: 0.8630 - lr: 0.0010
Epoch 9/20
120/120 [==============================] - 39s 326ms/step - loss: 0.2238 - acc: 0.9283 - val_loss: 0.4953 - val_acc: 0.8607 - lr: 0.0010
Epoch 10/20
120/120 [==============================] - 39s 326ms/step - loss: 0.1870 - acc: 0.9402 - val_loss: 0.4968 - val_acc: 0.8654 - lr: 0.0010
Epoch 11/20
120/120 [==============================] - ETA: 0s - loss: 0.1763 - acc: 0.9457
Epoch 11: ReduceLROnPlateau reducing learning rate to 0.0005000000237487257.
120/120 [==============================] - 39s 327ms/step - loss: 0.1763 - acc: 0.9457 - val_loss: 0.4964 - val_acc: 0.8658 - lr: 0.0010
Epoch 12/20
120/120 [==============================] - 39s 326ms/step - loss: 0.1489 - acc: 0.9551 - val_loss: 0.5125 - val_acc: 0.8638 - lr: 5.0000e-04
Epoch 13/20
120/120 [==============================] - 39s 326ms/step - loss: 0.1479 - acc: 0.9555 - val_loss: 0.4893 - val_acc: 0.8642 - lr: 5.0000e-04
Epoch 14/20
120/120 [==============================] - 39s 327ms/step - loss: 0.1286 - acc: 0.9589 - val_loss: 0.5267 - val_acc: 0.8646 - lr: 5.0000e-04
Epoch 15/20
120/120 [==============================] - 39s 326ms/step - loss: 0.1244 - acc: 0.9623 - val_loss: 0.5261 - val_acc: 0.8626 - lr: 5.0000e-04
Epoch 16/20
120/120 [==============================] - ETA: 0s - loss: 0.1211 - acc: 0.9630
Epoch 16: ReduceLROnPlateau reducing learning rate to 0.0002500000118743628.
120/120 [==============================] - 39s 326ms/step - loss: 0.1211 - acc: 0.9630 - val_loss: 0.5469 - val_acc: 0.8650 - lr: 5.0000e-04
Epoch 17/20
120/120 [==============================] - 39s 327ms/step - loss: 0.1051 - acc: 0.9674 - val_loss: 0.5524 - val_acc: 0.8634 - lr: 2.5000e-04
Epoch 18/20
120/120 [==============================] - 39s 327ms/step - loss: 0.1018 - acc: 0.9679 - val_loss: 0.5600 - val_acc: 0.8650 - lr: 2.5000e-04
Epoch 19/20
120/120 [==============================] - 39s 326ms/step - loss: 0.0979 - acc: 0.9677 - val_loss: 0.5619 - val_acc: 0.8642 - lr: 2.5000e-04
Epoch 20/20
120/120 [==============================] - 39s 327ms/step - loss: 0.0983 - acc: 0.9711 - val_loss: 0.5747 - val_acc: 0.8650 - lr: 2.5000e-04
80/80 [==============================] - 5s 62ms/step
Test results - Loss: 0.5746504664421082 - Accuracy: 86.49921417236328%
 metrics: {'dataset': 'Stemmed', 'ngram': (1, 1), 'model': 'GRU', 'precision': 0.8646287473787031, 'recall': 0.8649921507064364, 'accuracy': 0.8649921507064364, 'f1': 0.8647352784052316, 'roc_auc': 0.9748976780770322}
```

```
#############################################################
END
#############################################################
#############################################################
START! dataset: Stemmed, ngram: (1, 1), model: CNN+LSTM
#############################################################
Model: "sequential_19"
_________________________________________________________________
 Layer (type)                Output Shape              Param #   
=================================================================
 text_vectorization_4 (Text  (None, 64)                0         
 Vectorization)                                                  
                                                                 
 embedding_19 (Embedding)    (None, 64, 256)           3089664   
                                                                 
 spatial_dropout1d_19 (Spat  (None, 64, 256)           0         
 ialDropout1D)                                                   
                                                                 
 conv1d_8 (Conv1D)           (None, 64, 64)            98368     
                                                                 
 max_pooling1d_8 (MaxPoolin  (None, 32, 64)            0         
 g1D)                                                            
                                                                 
 conv1d_9 (Conv1D)           (None, 27, 32)            12320     
                                                                 
 max_pooling1d_9 (MaxPoolin  (None, 13, 32)            0         
 g1D)                                                            
                                                                 
 bidirectional_23 (Bidirect  (None, 13, 256)           164864    
 ional)                                                          
                                                                 
 dropout_47 (Dropout)        (None, 13, 256)           0         
                                                                 
 bidirectional_24 (Bidirect  (None, 256)               394240    
 ional)                                                          
                                                                 
 dropout_48 (Dropout)        (None, 256)               0         
                                                                 
 flatten_14 (Flatten)        (None, 256)               0         
                                                                 
 dense_38 (Dense)            (None, 64)                16448     
                                                                 
 dropout_49 (Dropout)        (None, 64)                0         
                                                                 
 dense_39 (Dense)            (None, 5)                 325       
                                                                 
=================================================================
Total params: 3776229 (14.41 MB)
Trainable params: 3776229 (14.41 MB)
Non-trainable params: 0 (0.00 Byte)
_________________________________________________________________
None
Epoch 1/20
120/120 [==============================] - 47s 317ms/step - loss: 1.4238 - acc: 0.3513 - val_loss: 1.1599 - val_acc: 0.4765 - lr: 0.0010
Epoch 2/20
120/120 [==============================] - 37s 306ms/step - loss: 1.0667 - acc: 0.5395 - val_loss: 0.8663 - val_acc: 0.6413 - lr: 0.0010
Epoch 3/20
120/120 [==============================] - 37s 306ms/step - loss: 0.7333 - acc: 0.7075 - val_loss: 0.5792 - val_acc: 0.8049 - lr: 0.0010
Epoch 4/20
120/120 [==============================] - 37s 306ms/step - loss: 0.5283 - acc: 0.8104 - val_loss: 0.5450 - val_acc: 0.8340 - lr: 0.0010
Epoch 5/20
120/120 [==============================] - 37s 306ms/step - loss: 0.4302 - acc: 0.8613 - val_loss: 0.5042 - val_acc: 0.8469 - lr: 0.0010
Epoch 6/20
120/120 [==============================] - 37s 306ms/step - loss: 0.3656 - acc: 0.8848 - val_loss: 0.4650 - val_acc: 0.8579 - lr: 0.0010
Epoch 7/20
120/120 [==============================] - 37s 305ms/step - loss: 0.3066 - acc: 0.9101 - val_loss: 0.4613 - val_acc: 0.8603 - lr: 0.0010
Epoch 8/20
120/120 [==============================] - 37s 306ms/step - loss: 0.2485 - acc: 0.9236 - val_loss: 0.4780 - val_acc: 0.8642 - lr: 0.0010
Epoch 9/20
120/120 [==============================] - 37s 306ms/step - loss: 0.2198 - acc: 0.9364 - val_loss: 0.5219 - val_acc: 0.8626 - lr: 0.0010
Epoch 10/20
120/120 [==============================] - 37s 306ms/step - loss: 0.1881 - acc: 0.9475 - val_loss: 0.5359 - val_acc: 0.8650 - lr: 0.0010
Epoch 11/20
120/120 [==============================] - 37s 305ms/step - loss: 0.1712 - acc: 0.9508 - val_loss: 0.5421 - val_acc: 0.8638 - lr: 0.0010
Epoch 12/20
120/120 [==============================] - ETA: 0s - loss: 0.1485 - acc: 0.9579
Epoch 12: ReduceLROnPlateau reducing learning rate to 0.0005000000237487257.
120/120 [==============================] - 37s 305ms/step - loss: 0.1485 - acc: 0.9579 - val_loss: 0.5928 - val_acc: 0.8607 - lr: 0.0010
Epoch 13/20
120/120 [==============================] - 37s 306ms/step - loss: 0.1353 - acc: 0.9627 - val_loss: 0.6069 - val_acc: 0.8634 - lr: 5.0000e-04
Epoch 14/20
120/120 [==============================] - 37s 306ms/step - loss: 0.1198 - acc: 0.9655 - val_loss: 0.6074 - val_acc: 0.8615 - lr: 5.0000e-04
Epoch 15/20
120/120 [==============================] - 37s 305ms/step - loss: 0.1090 - acc: 0.9695 - val_loss: 0.6435 - val_acc: 0.8642 - lr: 5.0000e-04
Epoch 16/20
120/120 [==============================] - 37s 306ms/step - loss: 0.1114 - acc: 0.9690 - val_loss: 0.6218 - val_acc: 0.8622 - lr: 5.0000e-04
Epoch 17/20
120/120 [==============================] - ETA: 0s - loss: 0.0974 - acc: 0.9719
Epoch 17: ReduceLROnPlateau reducing learning rate to 0.0002500000118743628.
120/120 [==============================] - 37s 306ms/step - loss: 0.0974 - acc: 0.9719 - val_loss: 0.6917 - val_acc: 0.8622 - lr: 5.0000e-04
Epoch 18/20
120/120 [==============================] - 37s 306ms/step - loss: 0.0933 - acc: 0.9733 - val_loss: 0.6933 - val_acc: 0.8638 - lr: 2.5000e-04
Epoch 19/20
120/120 [==============================] - 37s 306ms/step - loss: 0.0869 - acc: 0.9740 - val_loss: 0.7090 - val_acc: 0.8658 - lr: 2.5000e-04
Epoch 20/20
120/120 [==============================] - 37s 306ms/step - loss: 0.0864 - acc: 0.9740 - val_loss: 0.6922 - val_acc: 0.8658 - lr: 2.5000e-04
80/80 [==============================] - 7s 78ms/step
Test results - Loss: 0.6921898126602173 - Accuracy: 86.577707529068%
 metrics: {'dataset': 'Stemmed', 'ngram': (1, 1), 'model': 'CNN+LSTM', 'precision': 0.865898549749932, 'recall': 0.8657770800627943, 'accuracy': 0.8657770800627943, 'f1': 0.8656796969201127, 'roc_auc': 0.9706986511654361}
```

```
#############################################################
END
#############################################################
#############################################################
START! dataset: Stemmed, ngram: (1, 2), model: LSTM
#############################################################
Model: "sequential_20"
_________________________________________________________________
 Layer (type)                Output Shape              Param #   
=================================================================
 text_vectorization_5 (Text  (None, 64)                0         
 Vectorization)                                                  
                                                                 
 embedding_20 (Embedding)    (None, 64, 256)           15360000  
                                                                 
 spatial_dropout1d_20 (Spat  (None, 64, 256)           0         
 ialDropout1D)                                                   
                                                                 
 bidirectional_25 (Bidirect  (None, 512)               1050624   
 ional)                                                          
                                                                 
 dropout_50 (Dropout)        (None, 512)               0         
                                                                 
 flatten_15 (Flatten)        (None, 512)               0         
                                                                 
 dense_40 (Dense)            (None, 32)                16416     
                                                                 
 dropout_51 (Dropout)        (None, 32)                0         
                                                                 
 dense_41 (Dense)            (None, 5)                 165       
                                                                 
=================================================================
Total params: 16427205 (62.66 MB)
Trainable params: 16427205 (62.66 MB)
Non-trainable params: 0 (0.00 Byte)
_________________________________________________________________
None
Epoch 1/20
120/120 [==============================] - 223s 2s/step - loss: 1.4186 - acc: 0.3707 - val_loss: 1.0209 - val_acc: 0.6232 - lr: 0.0010
Epoch 2/20
120/120 [==============================] - 216s 2s/step - loss: 0.9162 - acc: 0.6519 - val_loss: 0.6954 - val_acc: 0.7790 - lr: 0.0010
Epoch 3/20
120/120 [==============================] - 216s 2s/step - loss: 0.6629 - acc: 0.7659 - val_loss: 0.6101 - val_acc: 0.7991 - lr: 0.0010
Epoch 4/20
120/120 [==============================] - 216s 2s/step - loss: 0.4995 - acc: 0.8317 - val_loss: 0.4926 - val_acc: 0.8301 - lr: 0.0010
Epoch 5/20
120/120 [==============================] - 216s 2s/step - loss: 0.3668 - acc: 0.8888 - val_loss: 0.4899 - val_acc: 0.8395 - lr: 0.0010
Epoch 6/20
120/120 [==============================] - 216s 2s/step - loss: 0.2820 - acc: 0.9193 - val_loss: 0.4261 - val_acc: 0.8697 - lr: 0.0010
Epoch 7/20
120/120 [==============================] - 216s 2s/step - loss: 0.2090 - acc: 0.9412 - val_loss: 0.4338 - val_acc: 0.8646 - lr: 0.0010
Epoch 8/20
120/120 [==============================] - 215s 2s/step - loss: 0.1678 - acc: 0.9571 - val_loss: 0.4758 - val_acc: 0.8611 - lr: 0.0010
Epoch 9/20
120/120 [==============================] - 216s 2s/step - loss: 0.1269 - acc: 0.9639 - val_loss: 0.4876 - val_acc: 0.8619 - lr: 0.0010
Epoch 10/20
120/120 [==============================] - 216s 2s/step - loss: 0.1035 - acc: 0.9727 - val_loss: 0.4760 - val_acc: 0.8709 - lr: 0.0010
Epoch 11/20
120/120 [==============================] - ETA: 0s - loss: 0.0700 - acc: 0.9791
Epoch 11: ReduceLROnPlateau reducing learning rate to 0.0005000000237487257.
120/120 [==============================] - 215s 2s/step - loss: 0.0700 - acc: 0.9791 - val_loss: 0.5785 - val_acc: 0.8658 - lr: 0.0010
Epoch 12/20
120/120 [==============================] - 215s 2s/step - loss: 0.0491 - acc: 0.9853 - val_loss: 0.6751 - val_acc: 0.8477 - lr: 5.0000e-04
Epoch 13/20
120/120 [==============================] - 215s 2s/step - loss: 0.0643 - acc: 0.9825 - val_loss: 0.6364 - val_acc: 0.8544 - lr: 5.0000e-04
Epoch 14/20
120/120 [==============================] - 215s 2s/step - loss: 0.0487 - acc: 0.9844 - val_loss: 0.5784 - val_acc: 0.8630 - lr: 5.0000e-04
Epoch 15/20
120/120 [==============================] - 215s 2s/step - loss: 0.0491 - acc: 0.9872 - val_loss: 0.5767 - val_acc: 0.8646 - lr: 5.0000e-04
Epoch 16/20
120/120 [==============================] - ETA: 0s - loss: 0.0516 - acc: 0.9867
Epoch 16: ReduceLROnPlateau reducing learning rate to 0.0002500000118743628.
120/120 [==============================] - 215s 2s/step - loss: 0.0516 - acc: 0.9867 - val_loss: 0.5893 - val_acc: 0.8654 - lr: 5.0000e-04
Epoch 17/20
120/120 [==============================] - 215s 2s/step - loss: 0.0389 - acc: 0.9891 - val_loss: 0.6530 - val_acc: 0.8611 - lr: 2.5000e-04
Epoch 18/20
120/120 [==============================] - 215s 2s/step - loss: 0.0366 - acc: 0.9901 - val_loss: 0.5989 - val_acc: 0.8615 - lr: 2.5000e-04
Epoch 19/20
120/120 [==============================] - 216s 2s/step - loss: 0.0335 - acc: 0.9893 - val_loss: 0.6323 - val_acc: 0.8630 - lr: 2.5000e-04
Epoch 20/20
120/120 [==============================] - ETA: 0s - loss: 0.0351 - acc: 0.9894Restoring model weights from the end of the best epoch: 10.
120/120 [==============================] - 216s 2s/step - loss: 0.0351 - acc: 0.9894 - val_loss: 0.6773 - val_acc: 0.8626 - lr: 2.5000e-04
Epoch 20: early stopping
80/80 [==============================] - 15s 178ms/step
Test results - Loss: 0.475994735956192 - Accuracy: 87.08791136741638%
 metrics: {'dataset': 'Stemmed', 'ngram': (1, 2), 'model': 'LSTM', 'precision': 0.8758959002849007, 'recall': 0.8708791208791209, 'accuracy': 0.8708791208791209, 'f1': 0.872128861344934, 'roc_auc': 0.9769209766827371}
```

```
#############################################################
END
#############################################################
#############################################################
START! dataset: Stemmed, ngram: (1, 2), model: LSTM2X
#############################################################
Model: "sequential_21"
_________________________________________________________________
 Layer (type)                Output Shape              Param #   
=================================================================
 text_vectorization_5 (Text  (None, 64)                0         
 Vectorization)                                                  
                                                                 
 embedding_21 (Embedding)    (None, 64, 256)           15360000  
                                                                 
 spatial_dropout1d_21 (Spat  (None, 64, 256)           0         
 ialDropout1D)                                                   
                                                                 
 bidirectional_26 (Bidirect  (None, 64, 256)           394240    
 ional)                                                          
                                                                 
 dropout_52 (Dropout)        (None, 64, 256)           0         
                                                                 
 bidirectional_27 (Bidirect  (None, 256)               394240    
 ional)                                                          
                                                                 
 dropout_53 (Dropout)        (None, 256)               0         
                                                                 
 flatten_16 (Flatten)        (None, 256)               0         
                                                                 
 dense_42 (Dense)            (None, 64)                16448     
                                                                 
 dropout_54 (Dropout)        (None, 64)                0         
                                                                 
 dense_43 (Dense)            (None, 5)                 325       
                                                                 
=================================================================
Total params: 16165253 (61.67 MB)
Trainable params: 16165253 (61.67 MB)
Non-trainable params: 0 (0.00 Byte)
_________________________________________________________________
None
Epoch 1/20
120/120 [==============================] - 453s 4s/step - loss: 1.4453 - acc: 0.3519 - val_loss: 1.0343 - val_acc: 0.5706 - lr: 0.0010
Epoch 2/20
120/120 [==============================] - 444s 4s/step - loss: 0.8584 - acc: 0.6689 - val_loss: 0.6330 - val_acc: 0.7802 - lr: 0.0010
Epoch 3/20
120/120 [==============================] - 445s 4s/step - loss: 0.5713 - acc: 0.7990 - val_loss: 0.6038 - val_acc: 0.7940 - lr: 0.0010
Epoch 4/20
120/120 [==============================] - 446s 4s/step - loss: 0.3959 - acc: 0.8640 - val_loss: 0.5152 - val_acc: 0.8285 - lr: 0.0010
Epoch 5/20
120/120 [==============================] - 446s 4s/step - loss: 0.2795 - acc: 0.9157 - val_loss: 0.4580 - val_acc: 0.8540 - lr: 0.0010
Epoch 6/20
120/120 [==============================] - 449s 4s/step - loss: 0.2064 - acc: 0.9392 - val_loss: 0.5273 - val_acc: 0.8371 - lr: 0.0010
Epoch 7/20
120/120 [==============================] - 449s 4s/step - loss: 0.1500 - acc: 0.9568 - val_loss: 0.4461 - val_acc: 0.8666 - lr: 0.0010
Epoch 8/20
120/120 [==============================] - 450s 4s/step - loss: 0.1098 - acc: 0.9669 - val_loss: 0.5466 - val_acc: 0.8595 - lr: 0.0010
Epoch 9/20
120/120 [==============================] - 452s 4s/step - loss: 0.0846 - acc: 0.9778 - val_loss: 0.4969 - val_acc: 0.8646 - lr: 0.0010
Epoch 10/20
120/120 [==============================] - 454s 4s/step - loss: 0.0564 - acc: 0.9850 - val_loss: 0.6381 - val_acc: 0.8607 - lr: 0.0010
Epoch 11/20
120/120 [==============================] - 455s 4s/step - loss: 0.0574 - acc: 0.9848 - val_loss: 0.5829 - val_acc: 0.8611 - lr: 0.0010
Epoch 12/20
120/120 [==============================] - ETA: 0s - loss: 0.0501 - acc: 0.9855
Epoch 12: ReduceLROnPlateau reducing learning rate to 0.0005000000237487257.
120/120 [==============================] - 455s 4s/step - loss: 0.0501 - acc: 0.9855 - val_loss: 0.6771 - val_acc: 0.8477 - lr: 0.0010
Epoch 13/20
120/120 [==============================] - 455s 4s/step - loss: 0.0380 - acc: 0.9901 - val_loss: 0.6188 - val_acc: 0.8654 - lr: 5.0000e-04
Epoch 14/20
120/120 [==============================] - 457s 4s/step - loss: 0.0314 - acc: 0.9915 - val_loss: 0.7646 - val_acc: 0.8532 - lr: 5.0000e-04
Epoch 15/20
120/120 [==============================] - 457s 4s/step - loss: 0.0257 - acc: 0.9924 - val_loss: 0.7918 - val_acc: 0.8513 - lr: 5.0000e-04
Epoch 16/20
120/120 [==============================] - 458s 4s/step - loss: 0.0315 - acc: 0.9912 - val_loss: 0.7784 - val_acc: 0.8556 - lr: 5.0000e-04
Epoch 17/20
120/120 [==============================] - ETA: 0s - loss: 0.0240 - acc: 0.9927Restoring model weights from the end of the best epoch: 7.

Epoch 17: ReduceLROnPlateau reducing learning rate to 0.0002500000118743628.
120/120 [==============================] - 460s 4s/step - loss: 0.0240 - acc: 0.9927 - val_loss: 0.8572 - val_acc: 0.8489 - lr: 5.0000e-04
Epoch 17: early stopping
80/80 [==============================] - 35s 424ms/step
Test results - Loss: 0.4461437165737152 - Accuracy: 86.6562008857727%
 metrics: {'dataset': 'Stemmed', 'ngram': (1, 2), 'model': 'LSTM2X', 'precision': 0.8758249203816075, 'recall': 0.8665620094191523, 'accuracy': 0.8665620094191523, 'f1': 0.867433543458549, 'roc_auc': 0.9772283274817155}
```

```
#############################################################
END
#############################################################
#############################################################
START! dataset: Stemmed, ngram: (1, 2), model: GRU
#############################################################
Model: "sequential_22"
_________________________________________________________________
 Layer (type)                Output Shape              Param #   
=================================================================
 text_vectorization_5 (Text  (None, 64)                0         
 Vectorization)                                                  
                                                                 
 embedding_22 (Embedding)    (None, 64, 256)           15360000  
                                                                 
 spatial_dropout1d_22 (Spat  (None, 64, 256)           0         
 ialDropout1D)                                                   
                                                                 
 gru_5 (GRU)                 (None, 64)                61824     
                                                                 
 dropout_55 (Dropout)        (None, 64)                0         
                                                                 
 dense_44 (Dense)            (None, 256)               16640     
                                                                 
 dropout_56 (Dropout)        (None, 256)               0         
                                                                 
 dense_45 (Dense)            (None, 5)                 1285      
                                                                 
=================================================================
Total params: 15439749 (58.90 MB)
Trainable params: 15439749 (58.90 MB)
Non-trainable params: 0 (0.00 Byte)
_________________________________________________________________
None
Epoch 1/20
120/120 [==============================] - 52s 412ms/step - loss: 1.6024 - acc: 0.2300 - val_loss: 1.5901 - val_acc: 0.2755 - lr: 0.0010
Epoch 2/20
120/120 [==============================] - 49s 406ms/step - loss: 1.4049 - acc: 0.3791 - val_loss: 1.2689 - val_acc: 0.4706 - lr: 0.0010
Epoch 3/20
120/120 [==============================] - 49s 406ms/step - loss: 0.9693 - acc: 0.6026 - val_loss: 0.7885 - val_acc: 0.7190 - lr: 0.0010
Epoch 4/20
120/120 [==============================] - 49s 406ms/step - loss: 0.6604 - acc: 0.7578 - val_loss: 0.6512 - val_acc: 0.7904 - lr: 0.0010
Epoch 5/20
120/120 [==============================] - 49s 406ms/step - loss: 0.4575 - acc: 0.8338 - val_loss: 0.5821 - val_acc: 0.8069 - lr: 0.0010
Epoch 6/20
120/120 [==============================] - 49s 405ms/step - loss: 0.3380 - acc: 0.8801 - val_loss: 0.4998 - val_acc: 0.8387 - lr: 0.0010
Epoch 7/20
120/120 [==============================] - 49s 406ms/step - loss: 0.2508 - acc: 0.9132 - val_loss: 0.4646 - val_acc: 0.8516 - lr: 0.0010
Epoch 8/20
120/120 [==============================] - 49s 405ms/step - loss: 0.1821 - acc: 0.9399 - val_loss: 0.5008 - val_acc: 0.8462 - lr: 0.0010
Epoch 9/20
120/120 [==============================] - 49s 405ms/step - loss: 0.1540 - acc: 0.9484 - val_loss: 0.4943 - val_acc: 0.8473 - lr: 0.0010
Epoch 10/20
120/120 [==============================] - 49s 406ms/step - loss: 0.1257 - acc: 0.9597 - val_loss: 0.4655 - val_acc: 0.8575 - lr: 0.0010
Epoch 11/20
120/120 [==============================] - 49s 404ms/step - loss: 0.0959 - acc: 0.9685 - val_loss: 0.4820 - val_acc: 0.8571 - lr: 0.0010
Epoch 12/20
120/120 [==============================] - ETA: 0s - loss: 0.0783 - acc: 0.9745
Epoch 12: ReduceLROnPlateau reducing learning rate to 0.0005000000237487257.
120/120 [==============================] - 49s 404ms/step - loss: 0.0783 - acc: 0.9745 - val_loss: 0.5229 - val_acc: 0.8489 - lr: 0.0010
Epoch 13/20
120/120 [==============================] - 48s 404ms/step - loss: 0.0672 - acc: 0.9800 - val_loss: 0.5695 - val_acc: 0.8524 - lr: 5.0000e-04
Epoch 14/20
120/120 [==============================] - 48s 404ms/step - loss: 0.0560 - acc: 0.9826 - val_loss: 0.6232 - val_acc: 0.8493 - lr: 5.0000e-04
Epoch 15/20
120/120 [==============================] - 49s 404ms/step - loss: 0.0479 - acc: 0.9867 - val_loss: 0.6312 - val_acc: 0.8462 - lr: 5.0000e-04
Epoch 16/20
120/120 [==============================] - 49s 405ms/step - loss: 0.0433 - acc: 0.9873 - val_loss: 0.5845 - val_acc: 0.8524 - lr: 5.0000e-04
Epoch 17/20
120/120 [==============================] - ETA: 0s - loss: 0.0424 - acc: 0.9868
Epoch 17: ReduceLROnPlateau reducing learning rate to 0.0002500000118743628.
120/120 [==============================] - 49s 406ms/step - loss: 0.0424 - acc: 0.9868 - val_loss: 0.5792 - val_acc: 0.8611 - lr: 5.0000e-04
Epoch 18/20
120/120 [==============================] - 49s 405ms/step - loss: 0.0391 - acc: 0.9880 - val_loss: 0.6066 - val_acc: 0.8579 - lr: 2.5000e-04
Epoch 19/20
120/120 [==============================] - 49s 405ms/step - loss: 0.0328 - acc: 0.9895 - val_loss: 0.6016 - val_acc: 0.8583 - lr: 2.5000e-04
Epoch 20/20
120/120 [==============================] - 49s 405ms/step - loss: 0.0326 - acc: 0.9889 - val_loss: 0.6108 - val_acc: 0.8544 - lr: 2.5000e-04
80/80 [==============================] - 6s 70ms/step
Test results - Loss: 0.6108086109161377 - Accuracy: 85.43956279754639%
 metrics: {'dataset': 'Stemmed', 'ngram': (1, 2), 'model': 'GRU', 'precision': 0.8657997980634509, 'recall': 0.8543956043956044, 'accuracy': 0.8543956043956044, 'f1': 0.8565649524653084, 'roc_auc': 0.9740928639113069}
```

```
#############################################################
END
#############################################################
#############################################################
START! dataset: Stemmed, ngram: (1, 2), model: CNN+LSTM
#############################################################
Model: "sequential_23"
_________________________________________________________________
 Layer (type)                Output Shape              Param #   
=================================================================
 text_vectorization_5 (Text  (None, 64)                0         
 Vectorization)                                                  
                                                                 
 embedding_23 (Embedding)    (None, 64, 256)           15360000  
                                                                 
 spatial_dropout1d_23 (Spat  (None, 64, 256)           0         
 ialDropout1D)                                                   
                                                                 
 conv1d_10 (Conv1D)          (None, 64, 64)            98368     
                                                                 
 max_pooling1d_10 (MaxPooli  (None, 32, 64)            0         
 ng1D)                                                           
                                                                 
 conv1d_11 (Conv1D)          (None, 27, 32)            12320     
                                                                 
 max_pooling1d_11 (MaxPooli  (None, 13, 32)            0         
 ng1D)                                                           
                                                                 
 bidirectional_28 (Bidirect  (None, 13, 256)           164864    
 ional)                                                          
                                                                 
 dropout_57 (Dropout)        (None, 13, 256)           0         
                                                                 
 bidirectional_29 (Bidirect  (None, 256)               394240    
 ional)                                                          
                                                                 
 dropout_58 (Dropout)        (None, 256)               0         
                                                                 
 flatten_17 (Flatten)        (None, 256)               0         
                                                                 
 dense_46 (Dense)            (None, 64)                16448     
                                                                 
 dropout_59 (Dropout)        (None, 64)                0         
                                                                 
 dense_47 (Dense)            (None, 5)                 325       
                                                                 
=================================================================
Total params: 16046565 (61.21 MB)
Trainable params: 16046565 (61.21 MB)
Non-trainable params: 0 (0.00 Byte)
_________________________________________________________________
None
Epoch 1/20
120/120 [==============================] - 57s 399ms/step - loss: 1.4600 - acc: 0.3300 - val_loss: 1.2093 - val_acc: 0.4329 - lr: 0.0010
Epoch 2/20
120/120 [==============================] - 47s 389ms/step - loss: 1.1160 - acc: 0.5107 - val_loss: 0.9817 - val_acc: 0.5844 - lr: 0.0010
Epoch 3/20
120/120 [==============================] - 47s 388ms/step - loss: 0.8827 - acc: 0.6329 - val_loss: 0.8794 - val_acc: 0.6248 - lr: 0.0010
Epoch 4/20
120/120 [==============================] - 47s 389ms/step - loss: 0.6313 - acc: 0.7469 - val_loss: 0.7369 - val_acc: 0.7347 - lr: 0.0010
Epoch 5/20
120/120 [==============================] - 47s 388ms/step - loss: 0.4714 - acc: 0.8245 - val_loss: 0.6698 - val_acc: 0.7786 - lr: 0.0010
Epoch 6/20
120/120 [==============================] - 47s 389ms/step - loss: 0.3550 - acc: 0.8774 - val_loss: 0.6428 - val_acc: 0.8049 - lr: 0.0010
Epoch 7/20
120/120 [==============================] - 47s 389ms/step - loss: 0.2648 - acc: 0.9166 - val_loss: 0.7551 - val_acc: 0.8085 - lr: 0.0010
Epoch 8/20
120/120 [==============================] - 47s 389ms/step - loss: 0.2153 - acc: 0.9377 - val_loss: 0.6890 - val_acc: 0.8269 - lr: 0.0010
Epoch 9/20
120/120 [==============================] - 46s 387ms/step - loss: 0.1699 - acc: 0.9500 - val_loss: 0.8411 - val_acc: 0.8081 - lr: 0.0010
Epoch 10/20
120/120 [==============================] - 47s 388ms/step - loss: 0.1297 - acc: 0.9636 - val_loss: 0.6993 - val_acc: 0.8285 - lr: 0.0010
Epoch 11/20
120/120 [==============================] - ETA: 0s - loss: 0.1163 - acc: 0.9673
Epoch 11: ReduceLROnPlateau reducing learning rate to 0.0005000000237487257.
120/120 [==============================] - 47s 389ms/step - loss: 0.1163 - acc: 0.9673 - val_loss: 0.7461 - val_acc: 0.8324 - lr: 0.0010
Epoch 12/20
120/120 [==============================] - 47s 388ms/step - loss: 0.0971 - acc: 0.9746 - val_loss: 0.7641 - val_acc: 0.8356 - lr: 5.0000e-04
Epoch 13/20
120/120 [==============================] - 46s 388ms/step - loss: 0.0782 - acc: 0.9789 - val_loss: 0.8569 - val_acc: 0.8328 - lr: 5.0000e-04
Epoch 14/20
120/120 [==============================] - 47s 389ms/step - loss: 0.0644 - acc: 0.9830 - val_loss: 0.8547 - val_acc: 0.8375 - lr: 5.0000e-04
Epoch 15/20
120/120 [==============================] - 46s 387ms/step - loss: 0.0633 - acc: 0.9848 - val_loss: 0.8873 - val_acc: 0.8312 - lr: 5.0000e-04
Epoch 16/20
120/120 [==============================] - ETA: 0s - loss: 0.0614 - acc: 0.9835
Epoch 16: ReduceLROnPlateau reducing learning rate to 0.0002500000118743628.
120/120 [==============================] - 46s 387ms/step - loss: 0.0614 - acc: 0.9835 - val_loss: 0.8161 - val_acc: 0.8336 - lr: 5.0000e-04
Epoch 17/20
120/120 [==============================] - 46s 387ms/step - loss: 0.0563 - acc: 0.9856 - val_loss: 0.8705 - val_acc: 0.8371 - lr: 2.5000e-04
Epoch 18/20
120/120 [==============================] - 46s 387ms/step - loss: 0.0463 - acc: 0.9886 - val_loss: 0.9270 - val_acc: 0.8332 - lr: 2.5000e-04
Epoch 19/20
120/120 [==============================] - 47s 389ms/step - loss: 0.0432 - acc: 0.9889 - val_loss: 0.8941 - val_acc: 0.8371 - lr: 2.5000e-04
Epoch 20/20
120/120 [==============================] - 47s 393ms/step - loss: 0.0417 - acc: 0.9881 - val_loss: 0.9610 - val_acc: 0.8297 - lr: 2.5000e-04
80/80 [==============================] - 8s 86ms/step
Test results - Loss: 0.9610127210617065 - Accuracy: 82.96703100204468%
 metrics: {'dataset': 'Stemmed', 'ngram': (1, 2), 'model': 'CNN+LSTM', 'precision': 0.8439248821954288, 'recall': 0.8296703296703297, 'accuracy': 0.8296703296703297, 'f1': 0.8312054751646069, 'roc_auc': 0.9599326543392964}
```

```
#############################################################
END
#############################################################
#############################################################
START! dataset: Stemmed, ngram: (2, 2), model: LSTM
#############################################################
Model: "sequential_24"
_________________________________________________________________
 Layer (type)                Output Shape              Param #   
=================================================================
 text_vectorization_6 (Text  (None, 64)                0         
 Vectorization)                                                  
                                                                 
 embedding_24 (Embedding)    (None, 64, 256)           15360000  
                                                                 
 spatial_dropout1d_24 (Spat  (None, 64, 256)           0         
 ialDropout1D)                                                   
                                                                 
 bidirectional_30 (Bidirect  (None, 512)               1050624   
 ional)                                                          
                                                                 
 dropout_60 (Dropout)        (None, 512)               0         
                                                                 
 flatten_18 (Flatten)        (None, 512)               0         
                                                                 
 dense_48 (Dense)            (None, 32)                16416     
                                                                 
 dropout_61 (Dropout)        (None, 32)                0         
                                                                 
 dense_49 (Dense)            (None, 5)                 165       
                                                                 
=================================================================
Total params: 16427205 (62.66 MB)
Trainable params: 16427205 (62.66 MB)
Non-trainable params: 0 (0.00 Byte)
_________________________________________________________________
None
Epoch 1/20
120/120 [==============================] - 255s 2s/step - loss: 1.5563 - acc: 0.2786 - val_loss: 1.4891 - val_acc: 0.3501 - lr: 0.0010
Epoch 2/20
120/120 [==============================] - 248s 2s/step - loss: 1.3812 - acc: 0.4034 - val_loss: 1.4589 - val_acc: 0.3662 - lr: 0.0010
Epoch 3/20
120/120 [==============================] - 248s 2s/step - loss: 1.0820 - acc: 0.5657 - val_loss: 1.3117 - val_acc: 0.4819 - lr: 0.0010
Epoch 4/20
120/120 [==============================] - 248s 2s/step - loss: 0.7337 - acc: 0.7213 - val_loss: 1.1120 - val_acc: 0.5742 - lr: 0.0010
Epoch 5/20
120/120 [==============================] - 250s 2s/step - loss: 0.4774 - acc: 0.8394 - val_loss: 1.5508 - val_acc: 0.5361 - lr: 0.0010
Epoch 6/20
120/120 [==============================] - 249s 2s/step - loss: 0.3002 - acc: 0.9066 - val_loss: 1.4287 - val_acc: 0.5828 - lr: 0.0010
Epoch 7/20
120/120 [==============================] - 249s 2s/step - loss: 0.1983 - acc: 0.9402 - val_loss: 1.8341 - val_acc: 0.5640 - lr: 0.0010
Epoch 8/20
120/120 [==============================] - 248s 2s/step - loss: 0.1549 - acc: 0.9560 - val_loss: 2.8195 - val_acc: 0.5279 - lr: 0.0010
Epoch 9/20
120/120 [==============================] - ETA: 0s - loss: 0.0994 - acc: 0.9716
Epoch 9: ReduceLROnPlateau reducing learning rate to 0.0005000000237487257.
120/120 [==============================] - 249s 2s/step - loss: 0.0994 - acc: 0.9716 - val_loss: 2.2855 - val_acc: 0.5973 - lr: 0.0010
Epoch 10/20
120/120 [==============================] - 248s 2s/step - loss: 0.0836 - acc: 0.9761 - val_loss: 1.6415 - val_acc: 0.6122 - lr: 5.0000e-04
Epoch 11/20
120/120 [==============================] - 248s 2s/step - loss: 0.0726 - acc: 0.9781 - val_loss: 1.8551 - val_acc: 0.5938 - lr: 5.0000e-04
Epoch 12/20
120/120 [==============================] - 249s 2s/step - loss: 0.0684 - acc: 0.9783 - val_loss: 1.2348 - val_acc: 0.6499 - lr: 5.0000e-04
Epoch 13/20
120/120 [==============================] - 249s 2s/step - loss: 0.0508 - acc: 0.9856 - val_loss: 1.9396 - val_acc: 0.6107 - lr: 5.0000e-04
Epoch 14/20
120/120 [==============================] - ETA: 0s - loss: 0.0458 - acc: 0.9867
Epoch 14: ReduceLROnPlateau reducing learning rate to 0.0002500000118743628.
120/120 [==============================] - 249s 2s/step - loss: 0.0458 - acc: 0.9867 - val_loss: 2.2246 - val_acc: 0.6064 - lr: 5.0000e-04
Epoch 15/20
120/120 [==============================] - 249s 2s/step - loss: 0.0421 - acc: 0.9867 - val_loss: 2.0997 - val_acc: 0.6217 - lr: 2.5000e-04
Epoch 16/20
120/120 [==============================] - 249s 2s/step - loss: 0.0469 - acc: 0.9861 - val_loss: 1.8947 - val_acc: 0.6232 - lr: 2.5000e-04
Epoch 17/20
120/120 [==============================] - 249s 2s/step - loss: 0.0414 - acc: 0.9877 - val_loss: 1.4442 - val_acc: 0.6417 - lr: 2.5000e-04
Epoch 18/20
120/120 [==============================] - 249s 2s/step - loss: 0.0401 - acc: 0.9867 - val_loss: 1.4455 - val_acc: 0.6495 - lr: 2.5000e-04
Epoch 19/20
120/120 [==============================] - ETA: 0s - loss: 0.0365 - acc: 0.9884
Epoch 19: ReduceLROnPlateau reducing learning rate to 0.0001250000059371814.
120/120 [==============================] - 249s 2s/step - loss: 0.0365 - acc: 0.9884 - val_loss: 1.5242 - val_acc: 0.6503 - lr: 2.5000e-04
Epoch 20/20
120/120 [==============================] - 249s 2s/step - loss: 0.0403 - acc: 0.9878 - val_loss: 1.3761 - val_acc: 0.6558 - lr: 1.2500e-04
80/80 [==============================] - 19s 227ms/step
Test results - Loss: 1.376142978668213 - Accuracy: 65.58084487915039%
 metrics: {'dataset': 'Stemmed', 'ngram': (2, 2), 'model': 'LSTM', 'precision': 0.7084491696291718, 'recall': 0.6558084772370487, 'accuracy': 0.6558084772370487, 'f1': 0.6593085783227091, 'roc_auc': 0.8963904591270154}
```

```
#############################################################
END
#############################################################
#############################################################
START! dataset: Stemmed, ngram: (2, 2), model: LSTM2X
#############################################################
Model: "sequential_25"
_________________________________________________________________
 Layer (type)                Output Shape              Param #   
=================================================================
 text_vectorization_6 (Text  (None, 64)                0         
 Vectorization)                                                  
                                                                 
 embedding_25 (Embedding)    (None, 64, 256)           15360000  
                                                                 
 spatial_dropout1d_25 (Spat  (None, 64, 256)           0         
 ialDropout1D)                                                   
                                                                 
 bidirectional_31 (Bidirect  (None, 64, 256)           394240    
 ional)                                                          
                                                                 
 dropout_62 (Dropout)        (None, 64, 256)           0         
                                                                 
 bidirectional_32 (Bidirect  (None, 256)               394240    
 ional)                                                          
                                                                 
 dropout_63 (Dropout)        (None, 256)               0         
                                                                 
 flatten_19 (Flatten)        (None, 256)               0         
                                                                 
 dense_50 (Dense)            (None, 64)                16448     
                                                                 
 dropout_64 (Dropout)        (None, 64)                0         
                                                                 
 dense_51 (Dense)            (None, 5)                 325       
                                                                 
=================================================================
Total params: 16165253 (61.67 MB)
Trainable params: 16165253 (61.67 MB)
Non-trainable params: 0 (0.00 Byte)
_________________________________________________________________
None
Epoch 1/20
120/120 [==============================] - 454s 4s/step - loss: 1.5512 - acc: 0.2828 - val_loss: 1.4754 - val_acc: 0.3991 - lr: 0.0010
Epoch 2/20
120/120 [==============================] - 480s 4s/step - loss: 1.2872 - acc: 0.4636 - val_loss: 1.4509 - val_acc: 0.4113 - lr: 0.0010
Epoch 3/20
120/120 [==============================] - 481s 4s/step - loss: 0.9395 - acc: 0.6235 - val_loss: 1.3076 - val_acc: 0.5122 - lr: 0.0010
Epoch 4/20
120/120 [==============================] - 482s 4s/step - loss: 0.6436 - acc: 0.7503 - val_loss: 1.8362 - val_acc: 0.5024 - lr: 0.0010
Epoch 5/20
120/120 [==============================] - 484s 4s/step - loss: 0.4226 - acc: 0.8456 - val_loss: 1.2629 - val_acc: 0.5820 - lr: 0.0010
Epoch 6/20
120/120 [==============================] - 483s 4s/step - loss: 0.2846 - acc: 0.9087 - val_loss: 1.8493 - val_acc: 0.5396 - lr: 0.0010
Epoch 7/20
120/120 [==============================] - 483s 4s/step - loss: 0.1900 - acc: 0.9397 - val_loss: 1.5904 - val_acc: 0.6068 - lr: 0.0010
Epoch 8/20
120/120 [==============================] - 485s 4s/step - loss: 0.1238 - acc: 0.9596 - val_loss: 1.1668 - val_acc: 0.6503 - lr: 0.0010
Epoch 9/20
120/120 [==============================] - 486s 4s/step - loss: 0.0845 - acc: 0.9747 - val_loss: 1.3417 - val_acc: 0.6354 - lr: 0.0010
Epoch 10/20
120/120 [==============================] - 487s 4s/step - loss: 0.0699 - acc: 0.9809 - val_loss: 1.3863 - val_acc: 0.6268 - lr: 0.0010
Epoch 11/20
120/120 [==============================] - 487s 4s/step - loss: 0.0571 - acc: 0.9830 - val_loss: 1.2538 - val_acc: 0.6323 - lr: 0.0010
Epoch 12/20
120/120 [==============================] - 488s 4s/step - loss: 0.0631 - acc: 0.9846 - val_loss: 1.5736 - val_acc: 0.6221 - lr: 0.0010
Epoch 13/20
120/120 [==============================] - ETA: 0s - loss: 0.0505 - acc: 0.9860
Epoch 13: ReduceLROnPlateau reducing learning rate to 0.0005000000237487257.
120/120 [==============================] - 490s 4s/step - loss: 0.0505 - acc: 0.9860 - val_loss: 1.4190 - val_acc: 0.6197 - lr: 0.0010
Epoch 14/20
120/120 [==============================] - 491s 4s/step - loss: 0.0369 - acc: 0.9882 - val_loss: 1.4364 - val_acc: 0.6354 - lr: 5.0000e-04
Epoch 15/20
120/120 [==============================] - 493s 4s/step - loss: 0.0352 - acc: 0.9893 - val_loss: 1.1339 - val_acc: 0.6739 - lr: 5.0000e-04
Epoch 16/20
120/120 [==============================] - 491s 4s/step - loss: 0.0316 - acc: 0.9907 - val_loss: 1.4283 - val_acc: 0.6487 - lr: 5.0000e-04
Epoch 17/20
120/120 [==============================] - 491s 4s/step - loss: 0.0287 - acc: 0.9903 - val_loss: 1.4524 - val_acc: 0.6405 - lr: 5.0000e-04
Epoch 18/20
120/120 [==============================] - 492s 4s/step - loss: 0.0288 - acc: 0.9910 - val_loss: 1.2526 - val_acc: 0.6531 - lr: 5.0000e-04
Epoch 19/20
120/120 [==============================] - 493s 4s/step - loss: 0.0247 - acc: 0.9918 - val_loss: 1.6173 - val_acc: 0.6197 - lr: 5.0000e-04
Epoch 20/20
120/120 [==============================] - ETA: 0s - loss: 0.0255 - acc: 0.9919
Epoch 20: ReduceLROnPlateau reducing learning rate to 0.0002500000118743628.
120/120 [==============================] - 493s 4s/step - loss: 0.0255 - acc: 0.9919 - val_loss: 1.4572 - val_acc: 0.6381 - lr: 5.0000e-04
80/80 [==============================] - 37s 450ms/step
Test results - Loss: 1.457208275794983 - Accuracy: 63.814759254455566%
 metrics: {'dataset': 'Stemmed', 'ngram': (2, 2), 'model': 'LSTM2X', 'precision': 0.7057450938335196, 'recall': 0.6381475667189953, 'accuracy': 0.6381475667189953, 'f1': 0.6387205643597502, 'roc_auc': 0.894350453706602}
```

```
#############################################################
END
#############################################################
#############################################################
START! dataset: Stemmed, ngram: (2, 2), model: GRU
#############################################################
Model: "sequential_26"
_________________________________________________________________
 Layer (type)                Output Shape              Param #   
=================================================================
 text_vectorization_6 (Text  (None, 64)                0         
 Vectorization)                                                  
                                                                 
 embedding_26 (Embedding)    (None, 64, 256)           15360000  
                                                                 
 spatial_dropout1d_26 (Spat  (None, 64, 256)           0         
 ialDropout1D)                                                   
                                                                 
 gru_6 (GRU)                 (None, 64)                61824     
                                                                 
 dropout_65 (Dropout)        (None, 64)                0         
                                                                 
 dense_52 (Dense)            (None, 256)               16640     
                                                                 
 dropout_66 (Dropout)        (None, 256)               0         
                                                                 
 dense_53 (Dense)            (None, 5)                 1285      
                                                                 
=================================================================
Total params: 15439749 (58.90 MB)
Trainable params: 15439749 (58.90 MB)
Non-trainable params: 0 (0.00 Byte)
_________________________________________________________________
None
Epoch 1/20
120/120 [==============================] - 54s 430ms/step - loss: 1.6037 - acc: 0.2291 - val_loss: 1.5949 - val_acc: 0.2598 - lr: 0.0010
Epoch 2/20
120/120 [==============================] - 51s 424ms/step - loss: 1.5131 - acc: 0.3114 - val_loss: 1.9219 - val_acc: 0.3163 - lr: 0.0010
Epoch 3/20
120/120 [==============================] - 51s 424ms/step - loss: 1.1659 - acc: 0.5027 - val_loss: 1.3551 - val_acc: 0.4505 - lr: 0.0010
Epoch 4/20
120/120 [==============================] - 51s 424ms/step - loss: 0.8401 - acc: 0.6598 - val_loss: 1.3885 - val_acc: 0.5122 - lr: 0.0010
Epoch 5/20
120/120 [==============================] - 51s 423ms/step - loss: 0.6145 - acc: 0.7591 - val_loss: 1.7817 - val_acc: 0.4768 - lr: 0.0010
Epoch 6/20
120/120 [==============================] - 51s 422ms/step - loss: 0.4531 - acc: 0.8248 - val_loss: 2.6848 - val_acc: 0.4584 - lr: 0.0010
Epoch 7/20
120/120 [==============================] - 51s 424ms/step - loss: 0.3146 - acc: 0.8811 - val_loss: 2.1276 - val_acc: 0.5243 - lr: 0.0010
Epoch 8/20
120/120 [==============================] - ETA: 0s - loss: 0.2240 - acc: 0.9190
Epoch 8: ReduceLROnPlateau reducing learning rate to 0.0005000000237487257.
120/120 [==============================] - 51s 424ms/step - loss: 0.2240 - acc: 0.9190 - val_loss: 1.5161 - val_acc: 0.6036 - lr: 0.0010
Epoch 9/20
120/120 [==============================] - 51s 423ms/step - loss: 0.1378 - acc: 0.9512 - val_loss: 2.3028 - val_acc: 0.5593 - lr: 5.0000e-04
Epoch 10/20
120/120 [==============================] - 51s 423ms/step - loss: 0.1209 - acc: 0.9583 - val_loss: 2.4063 - val_acc: 0.5432 - lr: 5.0000e-04
Epoch 11/20
120/120 [==============================] - 51s 423ms/step - loss: 0.0976 - acc: 0.9668 - val_loss: 2.2338 - val_acc: 0.5651 - lr: 5.0000e-04
Epoch 12/20
120/120 [==============================] - 51s 423ms/step - loss: 0.0768 - acc: 0.9744 - val_loss: 2.5501 - val_acc: 0.5589 - lr: 5.0000e-04
Epoch 13/20
120/120 [==============================] - ETA: 0s - loss: 0.0693 - acc: 0.9755
Epoch 13: ReduceLROnPlateau reducing learning rate to 0.0002500000118743628.
120/120 [==============================] - 51s 423ms/step - loss: 0.0693 - acc: 0.9755 - val_loss: 2.2658 - val_acc: 0.5844 - lr: 5.0000e-04
Epoch 14/20
120/120 [==============================] - 51s 422ms/step - loss: 0.0597 - acc: 0.9805 - val_loss: 1.9665 - val_acc: 0.6001 - lr: 2.5000e-04
Epoch 15/20
120/120 [==============================] - 51s 423ms/step - loss: 0.0593 - acc: 0.9802 - val_loss: 2.3122 - val_acc: 0.5899 - lr: 2.5000e-04
Epoch 16/20
120/120 [==============================] - 51s 423ms/step - loss: 0.0541 - acc: 0.9838 - val_loss: 2.2068 - val_acc: 0.5922 - lr: 2.5000e-04
Epoch 17/20
120/120 [==============================] - 51s 424ms/step - loss: 0.0526 - acc: 0.9823 - val_loss: 1.9655 - val_acc: 0.6181 - lr: 2.5000e-04
Epoch 18/20
120/120 [==============================] - ETA: 0s - loss: 0.0441 - acc: 0.9853
Epoch 18: ReduceLROnPlateau reducing learning rate to 0.0001250000059371814.
120/120 [==============================] - 51s 424ms/step - loss: 0.0441 - acc: 0.9853 - val_loss: 2.0299 - val_acc: 0.6213 - lr: 2.5000e-04
Epoch 19/20
120/120 [==============================] - 51s 424ms/step - loss: 0.0442 - acc: 0.9851 - val_loss: 1.9256 - val_acc: 0.6244 - lr: 1.2500e-04
Epoch 20/20
120/120 [==============================] - 51s 423ms/step - loss: 0.0449 - acc: 0.9847 - val_loss: 2.4024 - val_acc: 0.5883 - lr: 1.2500e-04
80/80 [==============================] - 6s 73ms/step
Test results - Loss: 2.402442216873169 - Accuracy: 58.83045792579651%
 metrics: {'dataset': 'Stemmed', 'ngram': (2, 2), 'model': 'GRU', 'precision': 0.7227621378710539, 'recall': 0.5883045525902669, 'accuracy': 0.5883045525902669, 'f1': 0.5958223786068781, 'roc_auc': 0.8583293504668374}
```

```
#############################################################
END
#############################################################
#############################################################
START! dataset: Stemmed, ngram: (2, 2), model: CNN+LSTM
#############################################################
Model: "sequential_27"
_________________________________________________________________
 Layer (type)                Output Shape              Param #   
=================================================================
 text_vectorization_6 (Text  (None, 64)                0         
 Vectorization)                                                  
                                                                 
 embedding_27 (Embedding)    (None, 64, 256)           15360000  
                                                                 
 spatial_dropout1d_27 (Spat  (None, 64, 256)           0         
 ialDropout1D)                                                   
                                                                 
 conv1d_12 (Conv1D)          (None, 64, 64)            98368     
                                                                 
 max_pooling1d_12 (MaxPooli  (None, 32, 64)            0         
 ng1D)                                                           
                                                                 
 conv1d_13 (Conv1D)          (None, 27, 32)            12320     
                                                                 
 max_pooling1d_13 (MaxPooli  (None, 13, 32)            0         
 ng1D)                                                           
                                                                 
 bidirectional_33 (Bidirect  (None, 13, 256)           164864    
 ional)                                                          
                                                                 
 dropout_67 (Dropout)        (None, 13, 256)           0         
                                                                 
 bidirectional_34 (Bidirect  (None, 256)               394240    
 ional)                                                          
                                                                 
 dropout_68 (Dropout)        (None, 256)               0         
                                                                 
 flatten_20 (Flatten)        (None, 256)               0         
                                                                 
 dense_54 (Dense)            (None, 64)                16448     
                                                                 
 dropout_69 (Dropout)        (None, 64)                0         
                                                                 
 dense_55 (Dense)            (None, 5)                 325       
                                                                 
=================================================================
Total params: 16046565 (61.21 MB)
Trainable params: 16046565 (61.21 MB)
Non-trainable params: 0 (0.00 Byte)
_________________________________________________________________
None
Epoch 1/20
120/120 [==============================] - 60s 421ms/step - loss: 1.5193 - acc: 0.3172 - val_loss: 1.5134 - val_acc: 0.3458 - lr: 0.0010
Epoch 2/20
120/120 [==============================] - 49s 411ms/step - loss: 1.3076 - acc: 0.4308 - val_loss: 1.2577 - val_acc: 0.4431 - lr: 0.0010
Epoch 3/20
120/120 [==============================] - 49s 410ms/step - loss: 1.0951 - acc: 0.5132 - val_loss: 1.2324 - val_acc: 0.4525 - lr: 0.0010
Epoch 4/20
120/120 [==============================] - 49s 411ms/step - loss: 0.8934 - acc: 0.6100 - val_loss: 1.1594 - val_acc: 0.4827 - lr: 0.0010
Epoch 5/20
120/120 [==============================] - 49s 410ms/step - loss: 0.7320 - acc: 0.6985 - val_loss: 1.2066 - val_acc: 0.4929 - lr: 0.0010
Epoch 6/20
120/120 [==============================] - 49s 409ms/step - loss: 0.5623 - acc: 0.7775 - val_loss: 1.7381 - val_acc: 0.4725 - lr: 0.0010
Epoch 7/20
120/120 [==============================] - 49s 411ms/step - loss: 0.4286 - acc: 0.8453 - val_loss: 1.7210 - val_acc: 0.4976 - lr: 0.0010
Epoch 8/20
120/120 [==============================] - 49s 409ms/step - loss: 0.3295 - acc: 0.8862 - val_loss: 3.1480 - val_acc: 0.4706 - lr: 0.0010
Epoch 9/20
120/120 [==============================] - ETA: 0s - loss: 0.2695 - acc: 0.9101
Epoch 9: ReduceLROnPlateau reducing learning rate to 0.0005000000237487257.
120/120 [==============================] - 49s 409ms/step - loss: 0.2695 - acc: 0.9101 - val_loss: 3.3142 - val_acc: 0.4796 - lr: 0.0010
Epoch 10/20
120/120 [==============================] - 49s 410ms/step - loss: 0.2045 - acc: 0.9382 - val_loss: 3.1814 - val_acc: 0.5161 - lr: 5.0000e-04
Epoch 11/20
120/120 [==============================] - 49s 410ms/step - loss: 0.1656 - acc: 0.9518 - val_loss: 2.1020 - val_acc: 0.5706 - lr: 5.0000e-04
Epoch 12/20
120/120 [==============================] - 49s 409ms/step - loss: 0.1461 - acc: 0.9576 - val_loss: 3.1073 - val_acc: 0.5298 - lr: 5.0000e-04
Epoch 13/20
120/120 [==============================] - 49s 409ms/step - loss: 0.1272 - acc: 0.9662 - val_loss: 3.4779 - val_acc: 0.5157 - lr: 5.0000e-04
Epoch 14/20
120/120 [==============================] - ETA: 0s - loss: 0.1210 - acc: 0.9661
Epoch 14: ReduceLROnPlateau reducing learning rate to 0.0002500000118743628.
120/120 [==============================] - 49s 409ms/step - loss: 0.1210 - acc: 0.9661 - val_loss: 3.4176 - val_acc: 0.5318 - lr: 5.0000e-04
Epoch 15/20
120/120 [==============================] - 49s 409ms/step - loss: 0.1073 - acc: 0.9700 - val_loss: 3.1715 - val_acc: 0.5542 - lr: 2.5000e-04
Epoch 16/20
120/120 [==============================] - 49s 409ms/step - loss: 0.0962 - acc: 0.9737 - val_loss: 3.1004 - val_acc: 0.5428 - lr: 2.5000e-04
Epoch 17/20
120/120 [==============================] - 49s 409ms/step - loss: 0.0899 - acc: 0.9778 - val_loss: 3.2429 - val_acc: 0.5440 - lr: 2.5000e-04
Epoch 18/20
120/120 [==============================] - 49s 409ms/step - loss: 0.0797 - acc: 0.9796 - val_loss: 3.2465 - val_acc: 0.5498 - lr: 2.5000e-04
Epoch 19/20
120/120 [==============================] - ETA: 0s - loss: 0.0921 - acc: 0.9747
Epoch 19: ReduceLROnPlateau reducing learning rate to 0.0001250000059371814.
120/120 [==============================] - 49s 409ms/step - loss: 0.0921 - acc: 0.9747 - val_loss: 3.0725 - val_acc: 0.5349 - lr: 2.5000e-04
Epoch 20/20
120/120 [==============================] - 49s 409ms/step - loss: 0.0732 - acc: 0.9802 - val_loss: 3.2430 - val_acc: 0.5400 - lr: 1.2500e-04
80/80 [==============================] - 8s 89ms/step
Test results - Loss: 3.2430405616760254 - Accuracy: 54.0031373500824%
 metrics: {'dataset': 'Stemmed', 'ngram': (2, 2), 'model': 'CNN+LSTM', 'precision': 0.6990680692232584, 'recall': 0.5400313971742543, 'accuracy': 0.5400313971742543, 'f1': 0.5449689152433036, 'roc_auc': 0.8158153406695581}
```

```
#############################################################
END
#############################################################
#############################################################
START! dataset: Stemmed, ngram: (2, 3), model: LSTM
#############################################################
Model: "sequential_28"
_________________________________________________________________
 Layer (type)                Output Shape              Param #   
=================================================================
 text_vectorization_7 (Text  (None, 64)                0         
 Vectorization)                                                  
                                                                 
 embedding_28 (Embedding)    (None, 64, 256)           15360000  
                                                                 
 spatial_dropout1d_28 (Spat  (None, 64, 256)           0         
 ialDropout1D)                                                   
                                                                 
 bidirectional_35 (Bidirect  (None, 512)               1050624   
 ional)                                                          
                                                                 
 dropout_70 (Dropout)        (None, 512)               0         
                                                                 
 flatten_21 (Flatten)        (None, 512)               0         
                                                                 
 dense_56 (Dense)            (None, 32)                16416     
                                                                 
 dropout_71 (Dropout)        (None, 32)                0         
                                                                 
 dense_57 (Dense)            (None, 5)                 165       
                                                                 
=================================================================
Total params: 16427205 (62.66 MB)
Trainable params: 16427205 (62.66 MB)
Non-trainable params: 0 (0.00 Byte)
_________________________________________________________________
None
Epoch 1/20
120/120 [==============================] - 241s 2s/step - loss: 1.5503 - acc: 0.2893 - val_loss: 1.4974 - val_acc: 0.3540 - lr: 0.0010
Epoch 2/20
120/120 [==============================] - 235s 2s/step - loss: 1.4623 - acc: 0.3646 - val_loss: 1.4404 - val_acc: 0.3560 - lr: 0.0010
Epoch 3/20
120/120 [==============================] - 234s 2s/step - loss: 1.2893 - acc: 0.4454 - val_loss: 1.2183 - val_acc: 0.4443 - lr: 0.0010
Epoch 4/20
120/120 [==============================] - 235s 2s/step - loss: 1.0859 - acc: 0.5468 - val_loss: 1.0921 - val_acc: 0.5589 - lr: 0.0010
Epoch 5/20
120/120 [==============================] - 235s 2s/step - loss: 0.8626 - acc: 0.6524 - val_loss: 1.0328 - val_acc: 0.5907 - lr: 0.0010
Epoch 6/20
120/120 [==============================] - 235s 2s/step - loss: 0.6930 - acc: 0.7266 - val_loss: 1.0323 - val_acc: 0.5903 - lr: 0.0010
Epoch 7/20
120/120 [==============================] - 234s 2s/step - loss: 0.5531 - acc: 0.7968 - val_loss: 0.9667 - val_acc: 0.6315 - lr: 0.0010
Epoch 8/20
120/120 [==============================] - 235s 2s/step - loss: 0.4285 - acc: 0.8511 - val_loss: 0.9406 - val_acc: 0.6515 - lr: 0.0010
Epoch 9/20
120/120 [==============================] - 235s 2s/step - loss: 0.3218 - acc: 0.8875 - val_loss: 0.9617 - val_acc: 0.6511 - lr: 0.0010
Epoch 10/20
120/120 [==============================] - 234s 2s/step - loss: 0.2735 - acc: 0.9055 - val_loss: 0.9731 - val_acc: 0.6405 - lr: 0.0010
Epoch 11/20
120/120 [==============================] - 234s 2s/step - loss: 0.2078 - acc: 0.9292 - val_loss: 1.0085 - val_acc: 0.6330 - lr: 0.0010
Epoch 12/20
120/120 [==============================] - 234s 2s/step - loss: 0.1917 - acc: 0.9337 - val_loss: 1.0908 - val_acc: 0.5962 - lr: 0.0010
Epoch 13/20
120/120 [==============================] - ETA: 0s - loss: 0.1699 - acc: 0.9406
Epoch 13: ReduceLROnPlateau reducing learning rate to 0.0005000000237487257.
120/120 [==============================] - 234s 2s/step - loss: 0.1699 - acc: 0.9406 - val_loss: 0.9958 - val_acc: 0.6499 - lr: 0.0010
Epoch 14/20
120/120 [==============================] - 235s 2s/step - loss: 0.1485 - acc: 0.9470 - val_loss: 0.9891 - val_acc: 0.6593 - lr: 5.0000e-04
Epoch 15/20
120/120 [==============================] - 234s 2s/step - loss: 0.1360 - acc: 0.9521 - val_loss: 1.0496 - val_acc: 0.6393 - lr: 5.0000e-04
Epoch 16/20
120/120 [==============================] - 234s 2s/step - loss: 0.1369 - acc: 0.9526 - val_loss: 1.0710 - val_acc: 0.6397 - lr: 5.0000e-04
Epoch 17/20
120/120 [==============================] - 234s 2s/step - loss: 0.1321 - acc: 0.9529 - val_loss: 1.0575 - val_acc: 0.6330 - lr: 5.0000e-04
Epoch 18/20
120/120 [==============================] - ETA: 0s - loss: 0.1191 - acc: 0.9556
Epoch 18: ReduceLROnPlateau reducing learning rate to 0.0002500000118743628.
120/120 [==============================] - 234s 2s/step - loss: 0.1191 - acc: 0.9556 - val_loss: 1.0456 - val_acc: 0.6574 - lr: 5.0000e-04
Epoch 19/20
120/120 [==============================] - 235s 2s/step - loss: 0.1162 - acc: 0.9562 - val_loss: 1.0542 - val_acc: 0.6633 - lr: 2.5000e-04
Epoch 20/20
120/120 [==============================] - 234s 2s/step - loss: 0.1117 - acc: 0.9579 - val_loss: 1.0949 - val_acc: 0.6397 - lr: 2.5000e-04
80/80 [==============================] - 18s 218ms/step
Test results - Loss: 1.0949015617370605 - Accuracy: 63.97174000740051%
 metrics: {'dataset': 'Stemmed', 'ngram': (2, 3), 'model': 'LSTM', 'precision': 0.6640465319507663, 'recall': 0.6397174254317112, 'accuracy': 0.6397174254317112, 'f1': 0.6414196754612401, 'roc_auc': 0.8889628260143608}
```

```
#############################################################
END
#############################################################
#############################################################
START! dataset: Stemmed, ngram: (2, 3), model: LSTM2X
#############################################################
Model: "sequential_29"
_________________________________________________________________
 Layer (type)                Output Shape              Param #   
=================================================================
 text_vectorization_7 (Text  (None, 64)                0         
 Vectorization)                                                  
                                                                 
 embedding_29 (Embedding)    (None, 64, 256)           15360000  
                                                                 
 spatial_dropout1d_29 (Spat  (None, 64, 256)           0         
 ialDropout1D)                                                   
                                                                 
 bidirectional_36 (Bidirect  (None, 64, 256)           394240    
 ional)                                                          
                                                                 
 dropout_72 (Dropout)        (None, 64, 256)           0         
                                                                 
 bidirectional_37 (Bidirect  (None, 256)               394240    
 ional)                                                          
                                                                 
 dropout_73 (Dropout)        (None, 256)               0         
                                                                 
 flatten_22 (Flatten)        (None, 256)               0         
                                                                 
 dense_58 (Dense)            (None, 64)                16448     
                                                                 
 dropout_74 (Dropout)        (None, 64)                0         
                                                                 
 dense_59 (Dense)            (None, 5)                 325       
                                                                 
=================================================================
Total params: 16165253 (61.67 MB)
Trainable params: 16165253 (61.67 MB)
Non-trainable params: 0 (0.00 Byte)
_________________________________________________________________
None
Epoch 1/20
120/120 [==============================] - 481s 4s/step - loss: 1.5495 - acc: 0.2960 - val_loss: 1.5040 - val_acc: 0.3265 - lr: 0.0010
Epoch 2/20
120/120 [==============================] - 466s 4s/step - loss: 1.4413 - acc: 0.3712 - val_loss: 1.3868 - val_acc: 0.3779 - lr: 0.0010
Epoch 3/20
120/120 [==============================] - 467s 4s/step - loss: 1.2666 - acc: 0.4562 - val_loss: 1.1857 - val_acc: 0.4882 - lr: 0.0010
Epoch 4/20
120/120 [==============================] - 468s 4s/step - loss: 1.0458 - acc: 0.5678 - val_loss: 1.0942 - val_acc: 0.5357 - lr: 0.0010
Epoch 5/20
120/120 [==============================] - 468s 4s/step - loss: 0.7993 - acc: 0.6844 - val_loss: 0.9916 - val_acc: 0.5997 - lr: 0.0010
Epoch 6/20
120/120 [==============================] - 467s 4s/step - loss: 0.6123 - acc: 0.7683 - val_loss: 0.9345 - val_acc: 0.6205 - lr: 0.0010
Epoch 7/20
120/120 [==============================] - 467s 4s/step - loss: 0.4674 - acc: 0.8274 - val_loss: 0.9704 - val_acc: 0.6405 - lr: 0.0010
Epoch 8/20
120/120 [==============================] - 467s 4s/step - loss: 0.3633 - acc: 0.8677 - val_loss: 0.9762 - val_acc: 0.6515 - lr: 0.0010
Epoch 9/20
120/120 [==============================] - 468s 4s/step - loss: 0.2977 - acc: 0.8979 - val_loss: 1.0282 - val_acc: 0.6354 - lr: 0.0010
Epoch 10/20
120/120 [==============================] - 467s 4s/step - loss: 0.2303 - acc: 0.9198 - val_loss: 1.1256 - val_acc: 0.6370 - lr: 0.0010
Epoch 11/20
120/120 [==============================] - ETA: 0s - loss: 0.2037 - acc: 0.9300
Epoch 11: ReduceLROnPlateau reducing learning rate to 0.0005000000237487257.
120/120 [==============================] - 467s 4s/step - loss: 0.2037 - acc: 0.9300 - val_loss: 1.3109 - val_acc: 0.6087 - lr: 0.0010
Epoch 12/20
120/120 [==============================] - 467s 4s/step - loss: 0.1584 - acc: 0.9461 - val_loss: 1.0504 - val_acc: 0.6468 - lr: 5.0000e-04
Epoch 13/20
120/120 [==============================] - 467s 4s/step - loss: 0.1356 - acc: 0.9522 - val_loss: 1.1246 - val_acc: 0.6421 - lr: 5.0000e-04
Epoch 14/20
120/120 [==============================] - 469s 4s/step - loss: 0.1399 - acc: 0.9521 - val_loss: 1.1539 - val_acc: 0.6358 - lr: 5.0000e-04
Epoch 15/20
120/120 [==============================] - 467s 4s/step - loss: 0.1255 - acc: 0.9559 - val_loss: 1.1012 - val_acc: 0.6499 - lr: 5.0000e-04
Epoch 16/20
120/120 [==============================] - ETA: 0s - loss: 0.1250 - acc: 0.9563
Epoch 16: ReduceLROnPlateau reducing learning rate to 0.0002500000118743628.
120/120 [==============================] - 468s 4s/step - loss: 0.1250 - acc: 0.9563 - val_loss: 1.0827 - val_acc: 0.6315 - lr: 5.0000e-04
Epoch 17/20
120/120 [==============================] - 468s 4s/step - loss: 0.1215 - acc: 0.9556 - val_loss: 1.0541 - val_acc: 0.6562 - lr: 2.5000e-04
Epoch 18/20
120/120 [==============================] - 467s 4s/step - loss: 0.1066 - acc: 0.9607 - val_loss: 1.0719 - val_acc: 0.6625 - lr: 2.5000e-04
Epoch 19/20
120/120 [==============================] - 467s 4s/step - loss: 0.1071 - acc: 0.9601 - val_loss: 1.0745 - val_acc: 0.6637 - lr: 2.5000e-04
Epoch 20/20
120/120 [==============================] - 468s 4s/step - loss: 0.1100 - acc: 0.9596 - val_loss: 1.0952 - val_acc: 0.6593 - lr: 2.5000e-04
80/80 [==============================] - 36s 440ms/step
Test results - Loss: 1.09518301486969 - Accuracy: 65.93406796455383%
 metrics: {'dataset': 'Stemmed', 'ngram': (2, 3), 'model': 'LSTM2X', 'precision': 0.7122152654485674, 'recall': 0.6593406593406593, 'accuracy': 0.6593406593406593, 'f1': 0.6667367367470514, 'roc_auc': 0.8876986288510522}
```

```
#############################################################
END
#############################################################
#############################################################
START! dataset: Stemmed, ngram: (2, 3), model: GRU
#############################################################
Model: "sequential_30"
_________________________________________________________________
 Layer (type)                Output Shape              Param #   
=================================================================
 text_vectorization_7 (Text  (None, 64)                0         
 Vectorization)                                                  
                                                                 
 embedding_30 (Embedding)    (None, 64, 256)           15360000  
                                                                 
 spatial_dropout1d_30 (Spat  (None, 64, 256)           0         
 ialDropout1D)                                                   
                                                                 
 gru_7 (GRU)                 (None, 64)                61824     
                                                                 
 dropout_75 (Dropout)        (None, 64)                0         
                                                                 
 dense_60 (Dense)            (None, 256)               16640     
                                                                 
 dropout_76 (Dropout)        (None, 256)               0         
                                                                 
 dense_61 (Dense)            (None, 5)                 1285      
                                                                 
=================================================================
Total params: 15439749 (58.90 MB)
Trainable params: 15439749 (58.90 MB)
Non-trainable params: 0 (0.00 Byte)
_________________________________________________________________
None
Epoch 1/20
120/120 [==============================] - 54s 432ms/step - loss: 1.6032 - acc: 0.2236 - val_loss: 1.5976 - val_acc: 0.2296 - lr: 0.0010
Epoch 2/20
120/120 [==============================] - 51s 427ms/step - loss: 1.5387 - acc: 0.2855 - val_loss: 1.4275 - val_acc: 0.3520 - lr: 0.0010
Epoch 3/20
120/120 [==============================] - 51s 426ms/step - loss: 1.3699 - acc: 0.3816 - val_loss: 1.3537 - val_acc: 0.3658 - lr: 0.0010
Epoch 4/20
120/120 [==============================] - 51s 427ms/step - loss: 1.2237 - acc: 0.4437 - val_loss: 1.2489 - val_acc: 0.4038 - lr: 0.0010
Epoch 5/20
120/120 [==============================] - 51s 426ms/step - loss: 1.0857 - acc: 0.5169 - val_loss: 1.1891 - val_acc: 0.4957 - lr: 0.0010
Epoch 6/20
120/120 [==============================] - 51s 426ms/step - loss: 0.9311 - acc: 0.5987 - val_loss: 1.1372 - val_acc: 0.5247 - lr: 0.0010
Epoch 7/20
120/120 [==============================] - 51s 425ms/step - loss: 0.8107 - acc: 0.6519 - val_loss: 1.1538 - val_acc: 0.5157 - lr: 0.0010
Epoch 8/20
120/120 [==============================] - 51s 427ms/step - loss: 0.6967 - acc: 0.7062 - val_loss: 1.1047 - val_acc: 0.5600 - lr: 0.0010
Epoch 9/20
120/120 [==============================] - 51s 427ms/step - loss: 0.6171 - acc: 0.7553 - val_loss: 1.1091 - val_acc: 0.5726 - lr: 0.0010
Epoch 10/20
120/120 [==============================] - 51s 426ms/step - loss: 0.5065 - acc: 0.7967 - val_loss: 1.2796 - val_acc: 0.5781 - lr: 0.0010
Epoch 11/20
120/120 [==============================] - 51s 427ms/step - loss: 0.4401 - acc: 0.8309 - val_loss: 1.1320 - val_acc: 0.6048 - lr: 0.0010
Epoch 12/20
120/120 [==============================] - 51s 426ms/step - loss: 0.3671 - acc: 0.8563 - val_loss: 1.0460 - val_acc: 0.6162 - lr: 0.0010
Epoch 13/20
120/120 [==============================] - 51s 425ms/step - loss: 0.3105 - acc: 0.8828 - val_loss: 1.1448 - val_acc: 0.6052 - lr: 0.0010
Epoch 14/20
120/120 [==============================] - 51s 427ms/step - loss: 0.2704 - acc: 0.9025 - val_loss: 1.0652 - val_acc: 0.6268 - lr: 0.0010
Epoch 15/20
120/120 [==============================] - 51s 425ms/step - loss: 0.2384 - acc: 0.9093 - val_loss: 1.1442 - val_acc: 0.6264 - lr: 0.0010
Epoch 16/20
120/120 [==============================] - 51s 425ms/step - loss: 0.2197 - acc: 0.9183 - val_loss: 1.1469 - val_acc: 0.6068 - lr: 0.0010
Epoch 17/20
120/120 [==============================] - ETA: 0s - loss: 0.1967 - acc: 0.9282
Epoch 17: ReduceLROnPlateau reducing learning rate to 0.0005000000237487257.
120/120 [==============================] - 51s 425ms/step - loss: 0.1967 - acc: 0.9282 - val_loss: 1.2296 - val_acc: 0.6122 - lr: 0.0010
Epoch 18/20
120/120 [==============================] - 51s 426ms/step - loss: 0.1716 - acc: 0.9368 - val_loss: 1.1582 - val_acc: 0.6221 - lr: 5.0000e-04
Epoch 19/20
120/120 [==============================] - 51s 426ms/step - loss: 0.1619 - acc: 0.9403 - val_loss: 1.0912 - val_acc: 0.6381 - lr: 5.0000e-04
Epoch 20/20
120/120 [==============================] - 51s 427ms/step - loss: 0.1457 - acc: 0.9483 - val_loss: 1.0767 - val_acc: 0.6401 - lr: 5.0000e-04
80/80 [==============================] - 6s 73ms/step
Test results - Loss: 1.0766656398773193 - Accuracy: 64.01098966598511%
 metrics: {'dataset': 'Stemmed', 'ngram': (2, 3), 'model': 'GRU', 'precision': 0.7241155306511374, 'recall': 0.6401098901098901, 'accuracy': 0.6401098901098901, 'f1': 0.6527231873599129, 'roc_auc': 0.873413560149967}
```

```
#############################################################
END
#############################################################
#############################################################
START! dataset: Stemmed, ngram: (2, 3), model: CNN+LSTM
#############################################################
Model: "sequential_31"
_________________________________________________________________
 Layer (type)                Output Shape              Param #   
=================================================================
 text_vectorization_7 (Text  (None, 64)                0         
 Vectorization)                                                  
                                                                 
 embedding_31 (Embedding)    (None, 64, 256)           15360000  
                                                                 
 spatial_dropout1d_31 (Spat  (None, 64, 256)           0         
 ialDropout1D)                                                   
                                                                 
 conv1d_14 (Conv1D)          (None, 64, 64)            98368     
                                                                 
 max_pooling1d_14 (MaxPooli  (None, 32, 64)            0         
 ng1D)                                                           
                                                                 
 conv1d_15 (Conv1D)          (None, 27, 32)            12320     
                                                                 
 max_pooling1d_15 (MaxPooli  (None, 13, 32)            0         
 ng1D)                                                           
                                                                 
 bidirectional_38 (Bidirect  (None, 13, 256)           164864    
 ional)                                                          
                                                                 
 dropout_77 (Dropout)        (None, 13, 256)           0         
                                                                 
 bidirectional_39 (Bidirect  (None, 256)               394240    
 ional)                                                          
                                                                 
 dropout_78 (Dropout)        (None, 256)               0         
                                                                 
 flatten_23 (Flatten)        (None, 256)               0         
                                                                 
 dense_62 (Dense)            (None, 64)                16448     
                                                                 
 dropout_79 (Dropout)        (None, 64)                0         
                                                                 
 dense_63 (Dense)            (None, 5)                 325       
                                                                 
=================================================================
Total params: 16046565 (61.21 MB)
Trainable params: 16046565 (61.21 MB)
Non-trainable params: 0 (0.00 Byte)
_________________________________________________________________
None
Epoch 1/20
120/120 [==============================] - 60s 424ms/step - loss: 1.5358 - acc: 0.3095 - val_loss: 1.4876 - val_acc: 0.3332 - lr: 0.0010
Epoch 2/20
120/120 [==============================] - 50s 414ms/step - loss: 1.4297 - acc: 0.3796 - val_loss: 1.2817 - val_acc: 0.4164 - lr: 0.0010
Epoch 3/20
120/120 [==============================] - 50s 413ms/step - loss: 1.2186 - acc: 0.4702 - val_loss: 1.2368 - val_acc: 0.4639 - lr: 0.0010
Epoch 4/20
120/120 [==============================] - 50s 414ms/step - loss: 1.0200 - acc: 0.5557 - val_loss: 1.1536 - val_acc: 0.4902 - lr: 0.0010
Epoch 5/20
120/120 [==============================] - 50s 413ms/step - loss: 0.8802 - acc: 0.6099 - val_loss: 1.1544 - val_acc: 0.5118 - lr: 0.0010
Epoch 6/20
120/120 [==============================] - 50s 414ms/step - loss: 0.7493 - acc: 0.6769 - val_loss: 1.1979 - val_acc: 0.5322 - lr: 0.0010
Epoch 7/20
120/120 [==============================] - 50s 413ms/step - loss: 0.6505 - acc: 0.7328 - val_loss: 1.2407 - val_acc: 0.5436 - lr: 0.0010
Epoch 8/20
120/120 [==============================] - 50s 414ms/step - loss: 0.5616 - acc: 0.7800 - val_loss: 1.3135 - val_acc: 0.5506 - lr: 0.0010
Epoch 9/20
120/120 [==============================] - ETA: 0s - loss: 0.4778 - acc: 0.8154
Epoch 9: ReduceLROnPlateau reducing learning rate to 0.0005000000237487257.
120/120 [==============================] - 49s 412ms/step - loss: 0.4778 - acc: 0.8154 - val_loss: 1.3633 - val_acc: 0.5432 - lr: 0.0010
Epoch 10/20
120/120 [==============================] - 49s 412ms/step - loss: 0.4065 - acc: 0.8472 - val_loss: 1.4507 - val_acc: 0.5483 - lr: 5.0000e-04
Epoch 11/20
120/120 [==============================] - 49s 412ms/step - loss: 0.3613 - acc: 0.8671 - val_loss: 1.4650 - val_acc: 0.5326 - lr: 5.0000e-04
Epoch 12/20
120/120 [==============================] - 49s 412ms/step - loss: 0.3259 - acc: 0.8784 - val_loss: 1.5315 - val_acc: 0.5412 - lr: 5.0000e-04
Epoch 13/20
120/120 [==============================] - 49s 412ms/step - loss: 0.2969 - acc: 0.8937 - val_loss: 1.6188 - val_acc: 0.5377 - lr: 5.0000e-04
Epoch 14/20
120/120 [==============================] - ETA: 0s - loss: 0.2692 - acc: 0.9043
Epoch 14: ReduceLROnPlateau reducing learning rate to 0.0002500000118743628.
120/120 [==============================] - 50s 413ms/step - loss: 0.2692 - acc: 0.9043 - val_loss: 1.6576 - val_acc: 0.5640 - lr: 5.0000e-04
Epoch 15/20
120/120 [==============================] - 49s 413ms/step - loss: 0.2520 - acc: 0.9121 - val_loss: 1.6225 - val_acc: 0.5483 - lr: 2.5000e-04
Epoch 16/20
120/120 [==============================] - 49s 412ms/step - loss: 0.2332 - acc: 0.9165 - val_loss: 1.7464 - val_acc: 0.5475 - lr: 2.5000e-04
Epoch 17/20
120/120 [==============================] - 50s 413ms/step - loss: 0.2195 - acc: 0.9237 - val_loss: 1.6709 - val_acc: 0.5553 - lr: 2.5000e-04
Epoch 18/20
120/120 [==============================] - 49s 412ms/step - loss: 0.2200 - acc: 0.9233 - val_loss: 1.6611 - val_acc: 0.5538 - lr: 2.5000e-04
Epoch 19/20
120/120 [==============================] - ETA: 0s - loss: 0.2106 - acc: 0.9261
Epoch 19: ReduceLROnPlateau reducing learning rate to 0.0001250000059371814.
120/120 [==============================] - 50s 414ms/step - loss: 0.2106 - acc: 0.9261 - val_loss: 1.6915 - val_acc: 0.5710 - lr: 2.5000e-04
Epoch 20/20
120/120 [==============================] - 49s 412ms/step - loss: 0.1965 - acc: 0.9323 - val_loss: 1.7087 - val_acc: 0.5597 - lr: 1.2500e-04
80/80 [==============================] - 8s 91ms/step
Test results - Loss: 1.708723545074463 - Accuracy: 55.96546530723572%
 metrics: {'dataset': 'Stemmed', 'ngram': (2, 3), 'model': 'CNN+LSTM', 'precision': 0.6365455141449976, 'recall': 0.5596546310832025, 'accuracy': 0.5596546310832025, 'f1': 0.5634300938468759, 'roc_auc': 0.8203928756042409}
```

```
#############################################################
END
#############################################################
```

In [18]:

```
pd.DataFrame(metrics_history).to_excel('auto_lstm_cnn_rnn_2024-05-30.xlsx')
```

In [12]:

```

```

In [17]:

```
!python3.10 -m pip install openpyxl
```

```
Collecting openpyxl
  Downloading openpyxl-3.1.3-py2.py3-none-any.whl (251 kB)
     -------------------------------------- 251.3/251.3 kB 1.1 MB/s eta 0:00:00
Collecting et-xmlfile
  Using cached et_xmlfile-1.1.0-py3-none-any.whl (4.7 kB)
Installing collected packages: et-xmlfile, openpyxl
Successfully installed et-xmlfile-1.1.0 openpyxl-3.1.3
```

```
[notice] A new release of pip available: 22.3.1 -> 24.0
[notice] To update, run: python3.10.exe -m pip install --upgrade pip
```
