# Supplementary material for: Detection of offensive content in the Kazakh language using machine learning and deep learning approaches
Source: PeerJ Comput Sci. 2025 Aug 11;11:e3027. doi: 10.7717/peerj-cs.3027 (PMC12453855; doi:10.7717/peerj-cs.3027)
Supplement: Supplemental Information 1 [file peerj-cs-11-3027-s001.zip › Code/Data.pdf]

label,message,message_stemmed,label_name

4,біздің сарбаздарымыз өз істерінің әділдігімен қаруланып басқыншы армия арасында көптеген шығындарға ұшырады және өз жерлері мен халқын қорғау үшін күресетін болады propaganda,біздің сарбаз өз іс әділдіг қарулан басқынш армия ара көптеген шығын ұшыр және өз жер мен халқ қорғау үшін күрес бол propaganda,violent

4,біздің еркін болғанымызды ештеңе жеңе алмайды тағы бір рет вашингтон мен еуропа түрмешіге басқыншыға және агрессорға көмек көрсетіп оның құрбандарын айыптауды таңдады propaganda,біздің еркін бол ештеңе же алма тағы бір рет вашингтон мен еуропа түрмеші басқыншы және агрессо көмек көрсет оның құрбан айыптау таңда propaganda,violent

4,біз барак обама джордж буштың қасіретті мұрасынан басталады деп үміттенген едік бірақ оның бастамасы көңіл көншітпейді ол тез арада мумбайдағы шабуылдарды жоққа шығаруға көшкен кезде газада күндік қырғыннан кейін ол тілмен байланыста қалады бірақ менің халқым жалғыз емес бостандықты сүйетін миллиондаған ерлер мен әйелдер оның әділеттілік пен азаттық үшін күресуінде тұр propaganda,біз барак оба джордж буш қасірет мұра бастал де үміттен едік бірақ оның баста көңіл көншітпе ол тез ара мумбай шабуыл жоқ шығар көш кезде газа күндік қырғын кейін ол тіл байланыс қал бірақ менің хал жалғыз емес бостандық сүй миллиондаған ер мен әйел оның әділеттілік пен аза үшін күресу тұр propaganda,violent

4,израильдің агрессиясына қарсы күн сайынғы наразылықтар араб және ислам аймағында ғана емес бүкіл әлемде болып жатыр propaganda,израиль агрессия қарсы күн сайынғы наразылық араб және ислам аймағ ғана емес бүкіл әлем бол жатыр propaganda,violent

4,израиль сөзсіз газаның жойылуын өлімі мен қайғы қасіретті жеңетіні сөзсіз бірақ ол ливандағыдай газадағы тағдырмен кездеседі бізді қоршау және бомбалау арқылы сындырмайды және ешқашан басқыншылыққа берілмейміз халид миш ал хамас саяси бюросының басшысы propaganda,израиль сөзсіз газа жойыл өлім мен қайғы қасірет жең сөзсіз бірақ ол ливандағыда газа тағдыр кездес біз қоршау және бомбала арқылы сындырма және ешқашан басқыншылық берілме халид миш ал хамас саяси бюро басшы propaganda,violent

4,алькаиданың аға қайраткері әбу йехя әл либи әлемдегі мұжахедтер газада палестиналықтардың қырылуына жауаптылардан кек алуға дайын деп мәлімдеді propaganda,алькаида аға қайраткер әбу йехя әл либи әлем мұжахед газа палестиналық қырыл жауапты кек ал дайын де мәлімде propaganda,violent

4,бұл алькаиданың сахаб деп жариялаған жаңа бейне таспасында сәрсенбі күні содырлардың исламшыл сайттарында шығыс пен батыстағы мужахидтер еврейлердің қолымен газадағы мұсылмандарға не болып жатқанын және болып жатқан оқиғаларды өшіруге және кек алуға дайындалып жатыр radicalization,бұл алькаида сахаб де жарияла жаңа бейне таспа сәрсенбі күн содыр исламшыл сайт шығыс пен

батыс мужахид еврей қол газа мұсылман не бол жат және бол жат оқиға өшір және кек ал дайындал жатыр radicalization,violent

4,өткен жылдың қаңтар айында жүзден астам мұсылман ғалымдарының газа соғысы туралы мәлімдемесіне жауап ретінде қабылданды онда мұсылмандарды газадағы жиһадты қолдауға шақырған және израиль үкіметімен қызметтес болған жолдан тайғандар деп атаған аль либи ғалымдарды ауғанстан ирак сомали және шығыс түркістан сияқты басқа мұсылман жерінде жиһадты заңдастыратын діни қаулылар шығаруға шақырды recruitment,өткен жыл қаңтар ай жүз астам мұсылман ғалым газ соғыс туралы мәлімдеме жауап рет қабылда онда мұсылман газа жиһад қолда шақыр және израиль үкімет қызметтес бол жолдан тай де ата аль либи ғалым ауғанстан ирак сомали және шығыс түркістан сияқты басқа мұсылман же жиһад заңдастыр діни қаулы шығар шақ recruitment,violent

4,аль либи ғалымдардан мұсылман халқына америка құрама штаттары мен израильдің бір құрылым құрғанын және израиль армиясына қарсы соғысу мен ақш мүдделеріне қай жерде болмасын шабуыл жасау арасында ешқандай айырмашылық жоқ екенін түсіндіруді сұрады неліктен ақш израиль сияқты қолдау үшін ақы төлеуден босатылуы керек неліктен біз оны өз жерімізде және әскери саяси экономикалық және қаржылық нысандарды бомбалау арқылы нысанаға алмауымыз керек propaganda,аль либи ғалым мұсылман халқ америка құрама штат мен израиль бір құрылым құр және израиль армия қарсы соғыс мен ақш мүдде қай же болма шабуыл жасау ара ешқандай айырмашылық жоқ екен түсіндіру сұр неліктен ақш израиль сияқты қолдау үшін ақы төлеу босатыл керек неліктен біз оны өз же және әскери саяси экономикалық және қаржылық нысан бомбала арқылы нысана алма керек propaganda,violent

4,бұл олар шақырған әділдер үшін жеңіс алькаиданың жетекшісі усама бен ладеннің өзі ислам әлеміндегі адал ғалымдар комитеттеріне үндеу жіберді ол газа туралы наурыздың ортасында шығарды ол олардан мұсылман елдеріндегі жастар арасында шынайы ислам деп атаған және аймақтың болашағы туралы хабардар болу үшін кеңесші орган құруды сұрады recruitment,бұл олар шақыр әділ үшін жеңіс алькаида жетекші уса бен ладен өзі ислам әлем адал ғалым комитет үндеу жіб ол газ туралы наурыз орта шығ ол олардан мұсылман ел жас ара шынайы ислам де ата және аймақ болашағ туралы хабардар болу үшін кеңесші орган құру сұр recruitment,violent

4,ал либи былтыр ақпанда сомалилерді жаңадан сайланған президентке қарсы бүлік шығаруға шақырған видео мәлімдемесін жариялаған болатын оның бүгін шығарған видеосы минуттан астам уақытты қамтиды ол қоңыр фонда қарсы отырды дәстүрлі көйлегінің үстіне әскери куртка және басына ақ шалбар киді дереккөз propaganda,ал либи былтыр ақпан сомали жаңа сайлан президент қарсы бүлік шығар шақыр видео мәлімдеме жарияла бол оның бүгін шығар видео минут астам уақыт қамти ол қоңыр фонд қарсы от дәстүрлі көйлег үст әскери куртка және бас ақ шалбар ки дереккөз propaganda,violent

4,қауіпсіздік қызметкерлері қағазға бұл бейнефильм германиядағы ықтимал террористерді ынталандыру үшін сигнал жіберуді мақсат еткенін айтты,қауіпсіздік қызметкер қағаз бұл бейнефиль германия ықтимал террорис ынталандыру үшін сигнал жіберу мақсат ет ай,violent

4,барлар мен дискотекалар сияқты көпшілік орындарды таңдау арқылы ең көп адам өлтіруге бағытталған нысаналарға американдық әскери әуе күштерінің рамштейндегі базасы және дортмунд дюссельдорф франкфурт кельн және мюнхен сияқты қалалар кірді propaganda,бар мен дискотека сияқты көпшілік орын таңдау арқылы ең көп адам өлтір бағыттал нысана американдық әскери әуе күш рамштей база және дортмунд дюссельдорф франкфурт кельн және мюнхен сияқты қала кір propaganda,violent

4,айыпталушылардың бірі мәлімдегендей әлем күйеді сот ісіндегі іс қағаздары шамамен папканы толтырады ал айыптаушының өзі куәгерді шақыруды жоспарлап отыр бұл іс екі жылға дейін созылуы мүмкін деген болжам бар propaganda,айыпталушы бірі мәлімдегенде әлем күй сот іс іс қағаз шамамен папка толтыр ал айыптауш өзі куәгер шақыру жоспарла отыр бұл іс екі жыл дейін созыл мүмкін де болжам бар propaganda,violent

4,комментаторлар бұл ең керемет сынақтардың бірі болады деп үміттенеді өйткені солшыл террористік қызыл армия фракциясының raf мүшелері жылдары докта тұрған германиялық dpa сым қызметі ер адамдар селек үшін жылдан бастап шнайдер үшін түрмеде өмір сүруге дейін түрмеге қамалатынын айтты propaganda,комментатор бұл ең керемет сынақ бірі бол де үміттен өйткені солшыл террористік қызыл армия фракция raf мүше жыл док тұр германиялық dpa сы қызмет ер адам селек үшін жыл баста шнайдер үшін түрме өмір сүр дейін түрме қамал ай propaganda,violent

4,қатардағы үш ер адам жылы ауғанстан пәкістан шекаралас аймағындағы оқу жаттығу жиындарына бара жатқанда бақыланған онда оларды өзбекстанда орналасқан ислам жиһад одағы террористік ұйымы деп жалдады деп болжанған propaganda,қатар үш ер адам жыл ауғанстан пәкістан шекаралас аймағ оқу жаттығу жиын бар жатқанда бақылан онда ол өзбекстан орналас ислам жиһад одағ террористік ұйым де жалда де болжан propaganda,violent

4,осы күндері израильдің газаға жасаған шабуылдары жүздеген адамдарды өлтіріп жарақаттады ал бүкіл әлем соның ішінде араб сатқындары мен билеушілері тек бос мәлімдеме жасайды және мен осыған байланысты бірнеше мәселеге назар аударғым келеді propaganda,осы күн израиль газа жаса шабуыл жүздеген адам өлтір жарақатта ал бүкіл әлем соның іш араб сатқын мен билеуші тек бос мәлімдеме жаса және мен осы байланысты бірнеше мәселе назар аудар кел propaganda,violent

4,ал исламның қалған бөлігінде біз шейх осама бен ладеннің антына қол жеткізуге дайынбыз мен ұлыбританиямен ант етемін көкті байқаусызда көтерді америка да америкада тұратын ешкім де палестина мен шындық алдында өмір сүрмес бұрын қауіпсіздік туралы армандай бермейді propaganda,ал ислам қалған бөліг біз шейх оса бен ладен ант қол жеткіз дайын мен ұлыбритания ант ет көк байқаусыз көтер америка

да америка тұр ешкім де палестин мен шындық алд өмір сүрмес бұрын қауіпсіздік туралы арманда берме propaganda,violent

4,барлық имансыз армиялар мұхаммедтің жерінен кетсін және сіздермен бірге болған кезде иерусалимнің қазыналарын босатып исламның туын көтеріп оның жиектерінде жиһад жасайтын күнді тілейміз radicalization,барлық иман армия мұхаммед же кет және сіздермен бірге бол кезде иерусалим қазына босат ислам ту көтер оның жиек жиһад жаса күн тіле radicalization,violent

4,израиль ұшақтары әуеден бомба тастап жатқан кезде ол және оның әскерлері газадағы мұсылмандарды өлтіру жоспары аяқталғанға дейін шекараларды жауып тастайды radicalization,израиль ұшақ әуе бомба таста жат кезде ол және оның әскер газа мұсылман өлтір жоспар аяқтал дейін шекара жау таста radicalization,violent

4,бұл еврейлердің лас рөлі сабра мен шатиладағы фалангтардың күштерімен менің мұсылман бауырларым және газадағы және басқа палестинадағы мүжахидтер біз біз мүмкін болған жерде кресттереврей одағын ұрыпсоғып біз сіздермен бетпебет келіп отырмыз және көп ұзамай сіздердің араларыңызға кедергі болатын шекаралар мен шектеулерді бұзамыз және американдықтардың ирактан біздің жақындап келе жатқан белгілерімізден кетуіне жол береміз radicalization,бұл еврей лас рөл сабр мен шатила фаланг күш менің мұсылман бауыр және газа және басқа палестина мүжахид біз біз мүмкін бол же кресттереврe одағ ұрыпсоғ біз сіздермен бетпебет кел отыр және көп ұзама сіздердің ара кедергі бол шекара мен шектеу бұз және американдық ирак біздің жақында келе жат белгі кет жол бер radicalization,violent

4,біз сіздерді жүректерімізбен және қанымызбен құтқардық және мен сіздердің араларыңызда сіздің жараларыңызды айықтырамын және олардың азаптарын азайтамын деп үміттенемін propaganda,біз сіз жүрек және қан құтқар және мен сіздердің ара сіздің жара айықтыр және олардың азап азай де үміттен propaganda,violent

4,екінші хабарға келер болсақ бұл мысырдағы мұсылмандарға арналған мен оларға айтамын газаны қоршауды алып тастау бұл сіздердің міндеттеріңіз егер сіз газадағы халқымыздан қоршау алынып тасталғанға дейін бас тартпаған ереуілдер демонстрациялар мен наразылық акцияларын ұйымдастырып қатысқан болсаңыз онда сатқын хосни мубаракқа құлақ асу керек recruitment,екінші хаб кел бол бұл мысыр мұсылман арнал мен оларға ай газа қоршау алып тастау бұл сіздердің міндет егер сіз газа халқ қоршау алын тастал дейін бас тартпа ереуіл демонстрация мен наразылық акция ұйымдастыр қатыс бол онда сатқ хосни мубарак құлақ асу керек recruitment,violent

4,газадағы бауырларымызды сатқын хосни мубарактың қолынан құтқару үшін студенттер оқудан жұмысшылар мен жұмысшылардан бас тартатын ереуілдер науқанын жасай алмайсыз ба recruitment,газа бауыр сатқ хосни мубарак қол құтқар үшін студент оқу жұмысшы мен жұмысшы бас тарт ереуіл науқан жаса алма ба recruitment,violent

4,мен мысыр армиясындағы азат адамдарға сүлеймен хатерде болғандығыңызды айтамын үлгі және үлгі ал сатқын қылмыскер сізді дініңізге опасыздық жасауыңызға және бауырластарыңызды қоршауға және израильдік ұшақтар оларға қарсы жасаған қылмыстарын аяқтағанға дейін олардың порттарын жауып тастау үшін қолданады деп айтамын propaganda,мен мысыр армия азат адам сүлеймен хат болғандығ ай үлгі және үлгі ал сатқ қылмыскер сіз дін опасыз жасау және бауырлас қорша және израиль ұшақ оларға қарсы жаса қылмыс аяқта дейін олардың порт жау тастау үшін қолдан де ай propaganda,violent

4,сондықтан сіз қашан шайтанның әскері болып қала бересіз құрметті синайдың құрметті тайпаларына айтамын бұл күн сендердің күндерің сондықтан исламдағы ағайындарыңды және газадағы туысқандарыңды бір наннан бастап танк шахтасына дейін барлық керек жарақтарымен және жабдықтарымен қамтамасыз етіңдер recruitment,сондықтан сіз қашан шайтан әскер бол қала бер құрметті синай құрметті тайпа ай бұл күн сен күн сондықтан ислам ағайын және газа туысқан бір нан баста танк шахта дейін барлық керек жарақ және жабдық қамтамасыз ет recruitment,violent

4,сіздің қадір қасиетіңізге абыройыңызға және қол сұғылмауыңызға қол сұғатын сатқын египет режимі бұл исламдағы бауырларыңызды және газадағы тектілікті қоршайтын және яһудилермен бірге оларды өлтіру үшін жасалған жүйе propaganda,сіздің қадір қасиет абырой және қол сұғылмау қол сұғ сатқ египет режим бұл ислам бауыр және газа тектілік қорша және яһуди бірге ол өлтір үшін жасал жүйе propaganda,violent

4,газа қаласындағы бауырларыңызда сатқын режимнің қоршауын олардан бұзды менің үшінші хабарламам туралы айтатын болсақ ол барлық мұсылмандарға мен оларға айтамын бұл американдық жалған ақпарат әлемге американың саясатын өзгертетін құтқарушы ретінде көрсетуге тырысты propaganda,газ қала бауыр сатқ режим қорша олардан бұз менің үшінші хабарлама туралы айт бол ол барлық мұсылман мен оларға ай бұл американдық жалған ақпарат әлем америка саясат өзгерт құтқарушы рет көрсет тырыс propaganda,violent

4,газадағы бауырларыңыз бен қарындастарыңызды мейірімсіз немесе аяушылықсыз өлтірді мен ислам әлеміне демонстрацияға шыққан ашулы мұсылман жамағатқа айтамын демонстрациялар бомбалар алдында жеткіліксіз бірақ біздің исламдық қаһарымыз сионистік крестшілер одағының тіректерін шайқайтын тиімді және тиімді әрекетке айналады propaganda,газа бауыр бен қарындас мейір немесе аяушылық өл мен ислам әлем демонстрация шық ашул мұсылман жамағат ай демонстрация бомба алд жеткіліксіз бірақ біздің исламдық қаһар сиони крестші одағ тірек шайқа тиімді және тиімді әрекет айнал propaganda,violent

4,барлық жерде адал guardian мұсылмандар оң жауап және джихад заттай ей барлық жерде мұсылмандар сионистік крест жорығына ұмтылыңыз және оған қол жеткізген барлық жерде оның мүдделерін қорғаңыз және бауырластарыңыз бен ұлдарыңызға қарсы тұрыңыз recruitment,барлық же адал guardian мұсылман оң жауап және джихад

заттай ей барлық же мұсылман сиони крест жорығ ұмтыл және оған қол жеткіз барлық же оның мүдде қорға және бауырлас бен ұл қарсы тұр recruitment,violent

4,абдулла бин эйд әлазиз израильді толық мойындаудың алғышарты ретінде ньюйоркте переспен кездесу үшін конфессияаралық үнқатысуды жасады propaganda,абдулл бин эйд әлазиз израиль толық мойында алғышарт рет ньюйорк перес кездесу үшін конфессияаралық үнқатысу жас propaganda,violent

4,абдулла салех йемен әлазза мен әлабаны крест жорығының исламның үйлеріне қарсы шабуылға арналған жабдықтау базасына айналдырды және малики американдық күштерден сатқындардың билік етуін жалғастыруын өтінді сондықтан олар сатқындардың ислам дұшпандарының крест жорықтары мен еврейлердің мүдделеріне шабуыл жасау әрекетінен қайда қай жерде және қай жерде болса да бас тартты дейді шындық элиталық жиһадтық бақтағы бауырлар propaganda,абдулл салех йемен әлазз мен әлаба крест жорығ ислам үй қарсы шабуыл арнал жабдықтау база айнал және малики американдық күш сатқын билік ет жалғастыр өт сондықтан олар сатқын ислам дұшпан крест жорық мен еврей мүдде шабуыл жасау әрекет қайда қай же және қай же бол да бас тар де шындық элиталық жиһад бақ бауыр propaganda,violent

4,тіпті ең ірі өтірікшілер де біраз уақыт шындықты айтуға тырысады дәйексөз иракта көтерілісшілердің шабуылдары болуы мүмкін propaganda,тіпті ең ірі өтірікші де біраз уақыт шындық айт тырыс дәйексөз ирак көтерілісші шабуыл бол мүмкін propaganda,violent

4,біздің мінез құлқымыз өзгеріп жау ирак қауіпсіздік күштерінің мүмкіндіктерін тексеруге тырысқанда немесе олардың әлі де өзектілігін дәлелдеуге тырысқанда біз зорлық зомбылықтың төмендеуін және ағынын байқаймыз ба иә деді калл дан ға дейін пентагондағы минуттық сұхбат propaganda,біздің мінез құл өзгер жау ирак қауіпсіздік күш мүмкіндік тексер тырыс немесе олардың әлі де өзектіліг дәлелде тырыс біз зорлық зомбылық төменде және ағын бай ба иә де калл дан ға дейін пентаго минуттық сұхбат propaganda,violent

4,менің ойымша біз мұны көретін шығармыз бірақ біз айтарлықтай өсуді күтіп отырғанымызды білмеймін ол ирактағы қауіпсіздік соңғы екі жыл ішінде едәуір жақсарғанын және ақш басқыншылығы кезінде азаматтық соғысқа қауіп төндірген секталық зорлықзомбылықтың қайта пайда болуы екіталай екенін айтты propaganda,менің ойымша біз мұны көр шығ бірақ біз айтарлықтай өсу күт отыр білме ол ирак қауіпсіздік соңғы екі жыл іш едәуір жақсар және ақш басқыншылығ кез азаматтық соғыс қауіп төндір секталық зорлықзомбылық қайта пайда бол екіталай екен ай propaganda,violent

4,сәрсенбі күні багдадтың солтүстігінде орналасқан сүнниттік мешітте жанкешті кем дегенде бес адамды өлтірді radicalization,сәрсенбі күн багдад солтүстіг орналас сүнни мешіт жанкешті кем де бес адам өл radicalization,violent

4,полиция қызметкерінің айтуынша шабуылдаушы жарылғыш зат салынған белдікті жарып жіберген кезде табынушылармен араласқан жарылыс кем дегенде адамды жарақаттады radicalization,полиция қызметк айт шабуылдаушы жарылғыш зат салын белдік жар жібер кезде табынушы аралас жарылыс кем де адам жарақатта radicalization,violent

4,қаңтар қауіпсіздік туралы келісім барлық америкалық әскерилердің ирактан жылдың соңына дейін шығарылуын талап етеді сонымен қатар жауынгерлер маусымға дейін ирактың ірі қалаларында өмір сүрмеуі керек сонымен қатар обама ақпан айында пентагон нан ға дейін емес барлығын шығарады деп мәлімдеді propaganda,қаңтар қауіпсіздік туралы келісім барлық америкалық әскери ирак жыл соң дейін шығарыл талап ет сонымен қатар жауынгер маусым дейін ирак ірі қала өмір сүрме керек сонымен қатар оба ақпан ай пентагон нан ға дейін емес барлығ шығар де мәлімде propaganda,violent

4,ирак әскерлері жылдың тамыз айының аяғында сол жақта қалғандар негізінен көтерілісшілерге қарсы тұруға және ирактың әскери және полиция күштерін даярлауға бағытталады propaganda,ирак әскер жыл тамыз ай аяғ сол жақта қал негіз көтерілісші қарсы тұр және ирак әскери және полиция күш даярла бағыттал propaganda,violent

4,калл сонымен бірге обама әкімшілігі ирактағы қауіпсіздікке келесі екі жыл ішінде зорлық зомбылық күрт күшейе түсетін болса әскер деңгейінде бұдан әрі қандай да бір өзгерістер қажет болуы мүмкін бе деген мәселені қадағалап отырғанын айтты propaganda,калл сонымен бірге оба әкімшіліг ирак қауіпсіздік келесі екі жыл іш зорлық зомбылық күрт күшей түс бол әскер деңгей бұдан әрі қандай да бір өзгеріс қажет бол мүмкін бе де мәселе қадағала отыр ай propaganda,violent

4,ирактың көп бөлігінде зорлық зомбылық күрт төмендейді сәрсенбідегі жанкешті бомбаланған аймақты қоршап тұрған аймақ біршама тыныштыққа ие болды дегенмен бұл сунниттер мен шииттердің құбылыстары алайда соңғы жарылыстар мен басқа да шабуылдар ақштың жоғарғы қолбасшыларын кем дегенде ирактың екі ірі қаласында мосул мен бақубада маусымдағы мерзімін қайта қарауға мәжбүр етті propaganda,ирак көп бөліг зорлық зомбылық күрт төменде сәрсенбі жанкешті бомбалан аймақ қорша тұр аймақ біршама тыныштық ие бол дегенмен бұл суннит мен шиит құбылыс алайда соңғы жарылыс мен басқа да шабуыл ақш жоғарғы қолбасшы кем де ирак екі ірі қала мосул мен бақуба маусым мерзім қайта қара мәжбүр ет propaganda,violent

4,жақында ирактан осы аптада оралған республикашыл сенаторлар тобы кейбір мерзімдердің жақында келетіндігіне алаңдаушылық білдірді шыны керек иракта әскери және қарапайым тұрғындардың барлық әскери күштерді маусымға дейін шығарып тастауына сондай ақ келесі жылдың тамыз айында шығарылуға қатысты алаңдаушылықтары бар деді propaganda,жақында ирак осы апта орал республикашыл сенатор тоб кейбір мерзім жақында келетіндіг алаңдаушылық біл шыны керек ирак әскери және қарапайым тұрғын барлық әскери күш маусым дейін

шығар таста сондай ақ келесі жыл тамыз ай шығарыл қатысты алаңдаушылық бар де propaganda,violent

4,бізде үш шетелдік оның ішінде әйелдер де бар деп жауап қатты және қатал болды оларды өлтіріңіз мумбай қонақ үйінің ішінен мылтық атылды одан кейін шаттық шеңбері естілді radicalization,біз үш шетелдік оның іш әйел де бар де жауап қатты және қатал бол ол өлтір мумба қонақ үй іш мылтық ат одан кейін шаттық шеңбер ест radicalization,violent

4,қатыгез алмасу үндістанның осы аптада пәкістанға берген дәлелдерінің бөлігі болған мумбайдағы шабуылдар кезінде ұсталған телефон қоңырауларының стенограммасынан туындайды propaganda,қатыгез алмасу үндістан осы апта пәкістан бер дәлел бөліг бол мумбай шабуыл кез ұстал телефон қоңырау стенограмма туында propaganda,violent

4,ньюделидің мәлімдеуінше табылған қарулардың фотосуреттері спутниктік телефондардан алынған мәліметтер және жалғыз қалған қарулы адамды жауапқа тарту туралы мәліметтер қамтылған мумбай қоршауы шекарадан басталған пәкістан билігі бұл айғақтарды аймақтағы шиеленісті күшейту үшін жасалған насихаттық шабуыл деп атады propaganda,ньюдели мәлімдеуінше табыл қару фотосурет спутник телефон алын мәлімет және жалғыз қалған қарулы адам жауап тарту туралы мәлімет қамтыл мумба қорша шекара бастал пәкістан билiг бұл айғақ аймақ шиелен күшейту үшін жасал насихат шабуыл де ат propaganda,violent

4,үндістанның хабарлауынша адамның өмірін қиған шабуылдарды басқарушылар пәкістанда орналасқан лашкарэтайба тобының жетекшілері болған сіздің қонақүйіңізде үш министр және бір министрдің хатшысы бар propaganda,үндістан хабарла адам өмір қи шабуыл басқарушы пәкістан орналас лашкарэтайб тоб жетекші бол сіздің қонақүй үш министр және бір министр хатшы бар propaganda,violent

4,біз қай бөлмеде екенімізді білмейміз дейді шабуыл жасаған бірінші түнде таңғы сағат да таж махал қонақ үйіндегі қаруланған адамға бұл жақсы жаңалық бұл торттың қатуы деді ол әзірлеуші оған үкімет шенеуніктерін тауып содан кейін үндістаннан қалаған нәрселеріңді ал деді propaganda,біз қай бөлме екен білме де шабуыл жаса бірінші түн таңғы сағат да таж махал қонақ үй қарулан адам бұл жақсы жаңалық бұл тор қат де ол әзірлеуші оған үкімет шенеунік тау содан кейін үндістан қала нәрсе ал де propaganda,violent

4,біз шейх осусама бин ладеннің антына қол жеткізуге дайынбыз америка және америкада тұратын адам армандамайды propaganda,біз шейх осуса бин ладен ант қол жеткіз дайын америка және америка тұр адам армандама propaganda,violent

4,менің мұсылман бауырларым және газада және палестинаның қалған жерлерінде бүгін сенің басыңа қандай да бір аймақпен немесе белгілі бір елмен шектеліп отырған басқыншылық сынақ емес ол мұсылман ұлтына қарсы сионистік крест жорықтары сериясына қатысады және бұл рейдтер обамаға дейін сізге берген сыйлық болып

табылады propaganda,менің мұсылман бауыр және газа және палестина қалған жер бүгін сенің бас қандай да бір аймақ немесе белгілі бір ел шектел отыр басқыншылық сынақ емес ол мұсылман ұлт қарсы сиони крест жорық серия қатыс және бұл рейд обама дейін сізге бер сыйлық бол таб propaganda,violent

4,сабыр мен шатила қырғындарында еврейлер батальондардың күштерімен бірге жасаған лас рөлді ойнайды менің бауырларым және газада және палестинаның қалған жерлерінде біз сіздермен шайқаста болдық propaganda,сабыр мен шатил қырғын еврей батальон күш бірге жаса лас рөл ойна менің бауыр және газа және палестина қалған жер біз сіздермен шайқас бол propaganda,violent

4,сионистік крестшілер одағын біз қай жерде іске қоссақ та соғыстырамыз және біз сізге тез арада барамыз және біз көп ұзамай ақ сізге жетуге кедергі болатын шекаралар мен шектеулерді жоямыз radicalization,сиони крестші одағ біз қай же іс қос та соғыстыр және біз сізге тез ара бар және біз көп ұзама ақ сізге жет кедергі бол шекара мен шектеу жоя radicalization,violent

4,американың ирактан кетуі сізге жақсы мүмкіндік біз қазір сізбен бірге болғымыз келетіндігімізді біледі сізді жанымыз бен қанымызбен қорғаңыз мен өзімнің жаралыларыңызды емдеп олардың азаптарын жеңілдету үшін қазір сізбен бірге болғанды қалаймын propaganda,американың ирак кет сізге жақсы мүмкіндік біз қазір сізбен бірге бол келетіндіг біл сіз жан бен қан қорға мен өзімнің жаралы емде олардың азап жеңілдету үшін қазір сізбен бірге бол қала propaganda,violent

4,мен оларға айтамын қоршауды алып тастау сіздің міндетіңіз сіз бұл жауапкершілікті өз мойныңызға алуыңыз керек өйткені егер сіз наразылық акцияларын ереуілдер мен демонстрациялар ұйымдастырсаңыз газа қоршауына дейін қоршау болмай тұрса сатқын хосни мубарак берілуі керек propaganda,мен оларға ай қоршау алып тастау сіздің міндет сіз бұл жауапкершілік өз мойны ал керек өйткені егер сіз наразылық акция ереуіл мен демонстрация ұйымдастыр газ қорша дейін қоршау болма тұр сатқ хосни мубарак беріл керек propaganda,violent

4,газадағы бауырларымызды қылмыстық сатқын хосни мубарактың қолынан құтқару үшін оқушылар мектептерге барудан жұмысшылар мен офицерлерден бас тартатын ереуілдер кампаниясын ұйымдастыруға дайынсыз ба мен мысыр армиясындағы қызу адамдарға айтамын сүлеймен хатерде сізде жақсы үлгі бар propaganda,газа бауыр қылмыстық сатқ хосни мубарак қол құтқар үшін оқушы мектеп бару жұмысшы мен офицер бас тарт ереуіл кампания ұйымдастыр дайын ба мен мысыр армия қызу адам ай сүлеймен хат сіз жақсы үлгі бар propaganda,violent

4,біз шейх осусама бин ладеннің антына қол жеткізуге дайынбыз америка және америкада тұратын адам армандамайды қауіпсіздік палестинадағы шындықты көргенге дейін және барлық кәпір армиялар мұсылман жерлерінен шыққанға дейін сізге иерусалим мен әлақса мешітін босатып ислам мен джихадтың туын көтеріп жер үстінде көтеру үшін бір күн көмек ала аламыз деп үміттенеміз propaganda,біз шейх осуса бин ладен ант қол жеткіз дайын америка және америка тұр адам армандама

қауіпсіздік палестина шындық көр дейін және барлық кәпір армия мұсылман жер шық дейін сізге иерусалим мен әлақ мешіт босат ислам мен джихад ту көтер жер үст көтеру үшін бір күн көмек ал ал де үміттен propaganda,violent

4,сионистік крестшілер одағын біз қай жерде іске қоссақ та соғыстырамыз және біз сізге тез арада барамыз және біз көп ұзамай ақ сізге жетуге кедергі болатын шекаралар мен шектеулерді жоямыз radicalization,сиони крестші одағ біз қай же іс қос та соғыстыр және біз сізге тез ара бар және біз көп ұзама ақ сізге жет кедергі бол шекара мен шектеу жоя radicalization,violent

4,мен өзімнің жаралыларыңызды емдеп олардың азаптарын жеңілдету үшін қазір сізбен бірге болғанды қалаймын өйткені мен бұл құрметті бұрын ұзақ жылдар бойы біздің мұжахедтеріміз бен ауғанстандағы иммигрант бауырларымыз екінші хабар мысырдағы мұсылмандарға бағытталған propaganda,мен өзімнің жаралы емде олардың азап жеңілдету үшін қазір сізбен бірге бол қала өйткені мен бұл құрметті бұрын ұзақ жыл бойы біздің мұжахед бен ауғанстан иммигрант бауыр екінші хабар мысыр мұсылман бағыттал propaganda,violent

4,иран мен америка соңғы шабуылдар үшін кінәлі болуы мүмкін мені қателеспеңіздер мұжахидтер соғыс басталғаннан бері көптеген шииттермен бірге жұмыс істеушілерді нысанаға алып өлтірді radicalization,иран мен америка соңғы шабуыл үшін кінәлі бол мүмкін мен қателеспе мұжахид соғыс бастал бері көптеген шиит бірге жұмыс істеуші нысана алып өл radicalization,violent

4,ал біз американдықтар әйелдер мен балаларды өлтіретінін білеміз егер бұл олардың жұмысына көмектесетін болса әлем иран мүмкіндігінше көп қан төгуді қалайды сондықтан америка иракта мүмкіндігінше ұзақ уақыт отырады сондықтан тегерандағы режимнің өзгеруі туралы ойлана алмайды propaganda,ал біз американдық әйел мен бала өлтір біл егер бұл олардың жұмыс көмектес бол әлем иран мүмкіндігінше көп қан төгу қала сондықтан америка ирак мүмкіндігінше ұзақ уақыт отыр сондықтан тегеран режим өзгер туралы ойлан алма propaganda,violent

4,ал араб үкіметтері әсіресе египет және сауд арабиясы және американдықтар иранның аймақтың қалған бөлігіндегі ықпалын жобалау қабілетін төмендету үшін иракқа мүмкіндігінше көп ақша мен қаружарақ алуға мәжбүр болғанын қалайды назар аударыңыз бұл мен ойлап тапқан қастандық теориясы емес оны шейх мұстафа әбу әлязид нақты айтқан propaganda,ал араб үкімет әсіресе египет және сауд арабия және американдық иран аймақ қалған бөліг ықпал жобалау қабілет төмендету үшін ирак мүмкіндігінше көп ақша мен қаружарақ ал мәжбүр бол қала назар ау бұл мен ойла тап қаста теория емес оны шейх мұстаф әбу әлязид нақты айт propaganda,violent

4,біз ұрысты жалғастырамыз біршілікке қатысады уэйсидің сомали үшін бітімгер бола алатындығы туралы шешім қабылдауға әлі ерте деп хабарлайды reuters агенттігіне propaganda,біз ұр жалғастыр біршілік қатыс уэйси сомали үшін бітімг бола алатындығ туралы шешім қабылда әлі ерте де хабарла reuters агенттіг propaganda,violent

4,бейсенбіде брюссельде донорлар сомалиге өзінің қауіпсіздік күштерін нығайтуға және келесі жылы amisom миссиясын қаржыландыруға көмектесуге кем дегенде миллион доллар беруге уәде берді id nln абди бұл соманың тым аз екенін айтты бұл сан жеткіліксіз сомалиге үлкен ресурстар қажет деді ол біз оны қалпына келтіруге қажет мөлшерден алшақпыз найденобидегі хелен нямбурамваураның және аддисабебадағы барри мэлоненің репортаж джек кимбалл редактор дэниэл уоллис дереккөз рейтер propaganda,бейсенбі брюссель донор сомали өзінің қауіпсіздік күш нығайт және келесі жыл amisom миссия қаржыландыр көмектес кем де миллион доллар бер уәде бер id nln абди бұл сома тым аз екен ай бұл сан жеткіліксіз сомали үлкен ресурс қажет де ол біз оны қалп келтір қажет мөлшер алшақ найденоби хелен нямбурамваура және аддисабеба барри мэлоне репортаж джек кимбалл редактор дэниэл уоллис дереккөз рей propaganda,violent

4,солтүстік африкадағы алькаида жексенбіде британдықты өлтіреді деп мәлімдеді егер лондон түрмеде отырған радикалды уағыздаушыны босатпаса барымта бұл топ исламистік вебсайтта жарияланған мәлімдемеде егер экстремистік мұсылман уағызшысы абу катада күн ішінде босатылмаса топтың қаңтардың аяғынан бастап ұстаған британдық туристті өлтіретінін айтты propaganda,солтүстік африка алькаи жексенбі британдық өлтір де мәлімде егер лондон түрме отыр радикал уағыздаушы босатпа барым бұл топ ислами вебсайт жариялан мәлімдеме егер экстремистік мұсылман уағызшы абу ката күн іш босатылма топ қаңтар аяғ баста ұста британдық турист өлтір ай propaganda,violent

4,британдық кісіден басқа швейцариялық тағы бір турист ұрлаушылармен бірге қалады солтүстік африкадағы исламдық алькаида aqmi французша қысқартылған атымен белгілі алжирде орналасқан жылы осама бен ладеннің террористік желісіне қосылған және ай сайын ондаған бомбалар немесе буксирлер жүргізеді,британдық кісі басқа швейцариялық тағы бір турист ұрлаушы бірге қал солтүстік африка исламдық алькаи aqmi французш қысқартыл ат белгілі алжир орналас жыл оса бен ладен террористік желі қосыл және ай сайын ондаған бомба немесе буксир жүргіз,violent

4,қазақстан ауғанстанға ші жылдардағы кеңес ауған соғысынан бергі алғашқы әскерлердің орналастырылуын атап өтетін өзінің бітімгерлерін ауғанстанға жіберуді мұқият қарастыруда мұндай қарамақайшылықты қадам қазан айында жылы польшаның басшылығымен құрылған бітімгершілік күштерін ирактан шығару туралы қазақстанның шешімінен кейін болады propaganda,қазақстан ауғанстан ші жыл кеңес ауған соғыс берг алғашқы әскер орналастырыл ата өт өзінің бітімгер ауғанстан жіберу мұқият қарастыр мұндай қарамақайшылық қадам қазан ай жыл польша басшылығ құрыл бітімгершілік күш ирак шығару туралы қазақстан шешім кейін бол propaganda,violent

4,ислам мағребіндегі алькаиданың жетекшісі aqim мұсылмандарды исламшыл вебсайттарда жарияланған жаңа аудиокассетада ақш пен израиль мүдделеріне шабуыл жасауға шақырды recruitment,ислам мағреб алькаида жетекші aqim

мұсылман исламшыл вебсайт жариялан жаңа аудиокассета ақш пен израиль мүдде шабуыл жаса шақ recruitment,violent

4,уа магреб халқы еврейлер мен крест жорықтарының мүдделері сіздің елдеріңізде таралған ал сіздің бауырларыңыз сізге жүгінуде қолдарыңызды мұжахидтерге созыңыз және олармен келісіп біздің елдеріміздегі еврейлер мен крест жорықтарының мүдделеріне мейірімділік пен жанашырлық танытпаңыз recruitment,уа магреб халқ еврей мен крест жорық мүдде сіздің ел тарал ал сіздің бауыр сізге жүгін қол мұжахид соз және олармен келіс біздің елдеріміз еврей мен крест жорық мүдде мейірімділік пен жанашырлық танытпа recruitment,violent

4,әбу мусаб абдел вадуд минуттық лентада мақсаттарыңызды мұқият таңдаңыз бауырластарыңызға ақпарат беріңіз жоспарларыңызды мұқият жасаңыз және құпиялылық пен тыныштықты сақтаңыз recruitment,әбу мусаб абдел вадуд минуттық лента мақсат мұқият таңда бауырлас ақпарат бер жоспар мұқият жаса және құпиялылық пен тыныштық сақта recruitment,violent

4,абдул вадуд алжир президенті абдел азиз бутефликаны қатты сынға алды және оны сатқын деп атады ол оны египеттің президенті хосни мубаракпен салыстырып газада болып жатқан оқиғаларға құлақ аспайтынын және алжирліктердің наразылықтар мен демонстрациялар арқылы ашулануларына жол бермейтінін айтты propaganda,абдул вадуд алжир президент абдел азиз бутефлика қатты сын алды және оны сатқ де ат ол оны египет президент хосни мубарак салыстыр газа бол жат оқиға құлақ аспа және алжирлік наразылық мен демонстрация арқылы ашулану жол берме ай propaganda,violent

4,ол сонымен бірге алжир президентін еврей францyзалжирлік әнші энрико макиаспен достық қарымқатынасы үшін айыптады ол абдул вадудтың айтуынша бірнеше күн бұрын парижде газа шабуылына қолдау көрсетті ол сонымен қатар алжир полициясы мен армия қызметкерлерін өкініп жылы президент боудиафты өлтірген армия лейтенанты лембарек боумарафидің жолын ұстануға шақырды propaganda,ол сонымен бірге алжир президент еврей францyзалжирлік әнші энрико макиас достық қарымқатынас үшін айыпта ол абдул вадуд айт бірнеше күн бұрын париж газ шабуыл қолдау көрсе ол сонымен қатар алжир полиция мен армия қызметкер өкін жыл президент боудиаф өлтір армия лейтенант лембарек боумарафи жол ұстан шақ propaganda,violent

4,абдул вадудтың үшінші хабары мавритандықтарға бағытталған ол елді тазарту міндеті бар деді израильдің қатысуы еврейлерге шабуыл жасап нуакчоттегі елшілігін жауып тастады propaganda,абдул вадуд үшінші хаб мавритандық бағыттал ол ел тазарт міндет бар де израиль қатыс еврей шабуыл жаса нуакчот елшілiг жау таста propaganda,violent

4,алькаиданың барлық жетекшілері қазір жекежеке газадағы дағдарыс туралы айтты алдағы күндері алькаиданың аға мүшелері абу йехя әллиби мұстафа абул язид және адам гадахннан тағы да көптеген хабарлар күтіледі http www cbsnews com sections

moni in shtml propaganda,алькаида барлық жетекші қазір жекеже газа дағдарыс туралы ай алдағы күн алькаида аға мүше абу йехя әллиби мұстаф абул язид және адам гадахн тағы да көптеген хабар күт http www cbsnews com sections moni in shtml propaganda,violent

4,ирак ақштың ұзақ өмір сүру сценарийін жарды дүйсенбі сәуір gmt ирактағы ақш әскерлері ақш пен ирак ақш әскери күштерін ирак қалаларынан шығарудың соңғы мерзіміне қатысты мүмкін болатын ерекшеліктер туралы келіссөздер жүргізеді дейді propaganda,ирак ақш ұзақ өмір сүру сценари жар дүйсенбі сәуір gmt ирак ақш әскер ақш пен ирак ақш әскери күш ирак қала шығар соңғы мерзім қатысты мүмкін бол ерекшелік туралы келіссөз жүргіз де propaganda,violent

4,әскери шенеуніктер ирактағы американдық жоғарғы қолбасшы басқарған біріккен әскери операцияларды үйлестіру комитеті генерал рэй одиерно мен ирактың қорғаныс министрі абдул кадир альубайди дүйсенбіде әскерді шығару және нысандарды тапсырудың егжей тегжейін келісу үшін кездесу өткізеді деп хабарлайды the new york times propaganda,әскери шенеунік ирак американдық жоғарғы қолбасшы басқар біріккен әскери операция үйлестіру комитет генерал рэ одиерно мен ирак қорғаныс министр абдул кадир альубайди дүйсенбі әскер шығару және нысан тапсыр егже тегжей келісу үшін кездесу өткіз де хабарла the new york times propaganda,violent

4,sofa мәлімдемесіне сәйкес өткен жылдың қарашасында ирак пен ақш арасында қол қойылған және жылдың басында күшіне енген уақытша қауіпсіздік туралы келісімге сәйкес ақштың барлық әскери күштері маусымға дейін ирак қалаларын тастап кетуі керек propaganda,sofa мәлімдеме сәйкес өткен жыл қараша ирак пен ақш ара қол қойыл және жыл бас күш ен уақытша қауіпсіздік туралы келісім сәйкес ақш барлық әскери күш маусым дейін ирак қала таста кет керек propaganda,violent

4,ирак қалаларында ақш әскери күштерін ұзарту жөніндегі жаңа күшжігер туралы жаңалықтар бірнеше күн багдадта және елдің басқа тыныш аймақтарында болған зорлық зомбылықтан кейін болды бейсенбі және жұмада ирак астанасы мен шығыс дияла провинциясындағы жарылыстарда ден астам адам оның ішінде ирандық қажы қаза болды propaganda,ирак қала ақш әскери күш ұзарт жөн жаңа күшжіг туралы жаңалық бірнеше күн багдад және ел басқа тыныш аймақ бол зорлық зомбылық кейін бол бейсенбі және жұма ирак астана мен шығыс дияла провинция жарылыс ден астам адам оның іш ирандық қажы қаза бол propaganda,violent

4,ирандық шенеуніктер зорлық зомбылықтың жаңа толқынын айыптай отырып вашингтон шабуылдарды иракта әскери тұрақтылықты жалғастыру үшін пайдалану үшін ұйымдастырды деп мәлімдеді осы қылмысқа және оған ұқсас қылмыстарға басты күдіктілер американдық қауіпсіздік және әскери күштер терроризмге қарсы соғыс қолшатыры астында мұсылман елін аяусыз басып алған деді propaganda,ирандық шенеунік зорлық зомбылық жаңа толқын айыпта отыр вашингтон шабуыл ирак әскери тұрақтылық жалғастыру үшін пайдалану үшін ұйымдас де мәлімде осы қылмыс және оған ұқсас қылмыс басты күдікті американдық

қауіпсіздік және әскери күш терроризм қарсы соғыс қолшатыр аст мұсылман ел аяу бас ал де propaganda,violent

4,ислам революциясының жетекшісі сейіт али хаменеи ирактағы лаңкестіктің улы шөптерінің өсуі міндетті түрде американың қылмыстық жазбасына жазылады ал американдық және израильдік барлау органдары бұған бірінші күдіктілер болып табылады деп сенбі күні иранның ең жоғары лауазымды шенеунігі аятолла хаменеи қосты mgh mj dt пресств propaganda,ислам революция жетекші сейіт али хаменеи ирак лаңкестік ул шөп өс міндетті түр америка қылмыстық жазба жаз ал американдық және израиль барлау орган бұған бірінші күдікті бол таб де сенбі күн иран ең жоғары лауазым шенеуніг аятолл хаменеи қос mgh mj dt пресств propaganda,violent

4,талибан пәкістанды ауғанстанға айналдыруға уәде берді mon apr gmt талибан көтерілісшілері ауғанстан пәкістан шекарасы маңында патрульде талибан сарбаздары өте жақсы жабдықталған және астанасы исламабадқа жақындағанда қорқыныш тудырды propaganda,талибан пәкістан ауғанстан айналдыр уәде бер mon apr gmt талибан көтерілісші ауғанстан пәкістан шекара маң патруль талибан сарбаз өте жақсы жабдықтал және астана исламабад жақында қорқыныш ту propaganda,violent

4,пәкістан армиясы елдің солтүстікбатысындағы содырларға қарсы соғысты жалғастырса талибан пәкістанды басқа ауғанстанға айналдыруға ант береді талибанның басты өкілі қазір техрикэталибан сваты бар жақында техрикэталибан пешавар құрылады propaganda,пәкістан армия ел солтүстікбаты содыр қарсы соғ жалғастыр талибан пәкістан басқа ауғанстан айналдыр ант бер талибан басты өкіл қазір техрикэталибан сва бар жақында техрикэталибан пешав құр propaganda,violent

4,үкімет қанша уақыт жұмыс істей алады егер сол саясатты жалғастыра берсе бұл ел жақын арада ауғанстанға айналады деп мәлімдеді проблемалы сват алқабы деді жексенбі күні пәкістан әскерлері свот алқабына жақын жерде талибанмен ауыр атыс кезінде екі ондаған сарбаздың қаза тапқанынан кейін айтылды propaganda,үкімет қанша уақыт жұмыс істе ал егер сол саясат жалғастыр бер бұл ел жақын ара ауғанстан айнал де мәлімде проблемал сват алқаб де жексенбі күн пәкістан әскер свот алқаб жақын же талибан ауыр атыс кез екі ондаған сарбаз қаза тап кейін айт propaganda,violent

4,талибан исламабадқа және пәкістанның басқа да ірі қалаларына соғысады деп бірнеше рет айтқан жылы ақштың ауғанстанға басып кіруі екі елдің көршілерінің арасындағы рулық тайпалық белдікті өлім зорлықзомбылық көрінісіне айналдырып пәкістанға шекарадан өтуге себеп болды propaganda,талибан исламабад және пәкістан басқа да ірі қала соғыс де бірнеше рет айт жыл ақш ауғанстан бас кір екі ел көрші ара рулық тайпалық белдік өлім зорлықзомбылық көрініс айналдыр пәкістан шекара өт себеп бол propaganda,violent

4,пәкістандық талибан әскери күштермен аптаға созылған бітімгершілік үкіметтің дирдің солтүстігінде операция бастағаннан кейін пайдасыз деп атады әскери

операция кеше дир қаласында радикалды діни қызметкер суфи мұхаммадтың тұратын қаласы орналасқан шекара корпусының мүшелерін орналастырумен басталды propaganda,пәкістандық талибан әскери күш апта созыл бітімгершілік үкімет ди солтүстіг операция баста кейін пай де ат әскери операция кеше ди қала радикал діни қызметкер суфи мұхаммад тұр қала орналас шекара корпус мүше орналастыр баста propaganda,violent

4,пәкістан әскери күштері соңғы екі күнде ұрыс кезінде талибан сарбазы және екі талибан қолбасшысы және бір ғана әскери қызметкер қаза тапқаны туралы хабарлайды әскерилердің мәлімдеуінше талибан жетекшісі маулана шахид және оның төрт көмекшісі ұрыс кезінде қаза тапқандар арасында болған есептер расталмады propaganda,пәкістан әскери күш соңғы екі күн ұрыс кез талибан сарбаз және екі талибан қолбасшы және бір ғана әскери қызметкер қаза тап туралы хабарла әскери мәлімдеуінше талибан жетекші маулан шахид және оның төрт көмекші ұрыс кез қаза тап ара бол есеп расталма propaganda,violent

4,талибан dir арқылы өтіп бара жатқан конвойды басып алғаннан кейін шекара корпусының бір әскері қаза тауып тағы екеуі жараланды талибан содырлары сонымен бірге майор мен оның жүргізушісін де қолға түсірді radicalization,талибан dir арқылы өт бар жат конвой бас ал кейін шекара корпус бір әскер қаза тау тағы екеу жарала талибан содыр сонымен бірге майор мен оның жүргізуші де қол түс radicalization,violent

4,жергілікті полиция читраль және дирскауттар бөлімдерін күшейтуде армия тікұшақ зеңбіректері мен артиллерия талибан лагерлерін нысанаға алады бұрынғы солтүстікбатыстағы әскери операциялардағыдай бейбіт тұрғындар әскери шығындарды азайтуға тырысып әскери фунттардың шоғырлану орталықтарына айналды propaganda,жергілікті полиция читраль және дирскаут бөлім күшейт армия тікұшақ зеңбірек мен артиллерия талибан лагер нысана ал бұрынғы солтүстікбатыс әскери операциялардағыда бейбіт тұрғын әскери шығын азайт тырыс әскери фунт шоғырлану орталық айна propaganda,violent

4,кәдімгі пәкістан армиясы пәкістанның солтүстікбатысындағы көптеген операциялардағыдай дирде ұрыс жүргізуде армия басшылығы мен генерал кияни армия штабының бастығы әсіресе талибандармен соғысу офицерлер корпусы мен атақтар мен жарылыстарды бөліп алады деп қорқады деп хабарлады ақш әскери барлау қызметінің жоғары лауазымды қызметкері long war journal журналына propaganda,кәдімгі пәкістан армия пәкістан солтүстікбаты көптеген операциялардағыда ди ұрыс жүргіз армия басшылығ мен генерал кияни армия штаб бастығ әсіресе талибан соғыс офицер корпус мен атақ мен жарылыс бөл ал де қорқ де хабарла ақш әскери барлау қызмет жоғары лауазым қызметк long war journal журнал propaganda,violent

4,талибан шариғат келісімі әлі де күшінде деп отыр біз дегенмен ақпан айындағы келісімді ұстанамыз деді иззат сонымен бірге сваттың молдасы фазлулланың талибан

өкілі мұсылман хан малаканд келісімін пайдасыз деп атады және пәкістандағы операцияларды кеңейтеміз деп қорқытты propaganda,талибан шариғат келісім әлі де күш де отыр біз дегенмен ақпан ай келіс ұстан де иззат сонымен бірге сва молда фазлулла талибан өкіл мұсылман хан малаканд келі пай де ат және пәкістан операция кеңей де қорқыт propaganda,violent

4,талибанның соңғы әрекеттері исламабад пен харипурдағы жергілікті үкіметтерді қатты алаңдатты комиссардың орынбасары қосымша әскерилендірілген рейнджерлерді қаланың солтүстігінде орналасқан ықтимал алға жылжуды болдырмау үшін орналастырды ал қауіпсіздік күштері стратегиялық тарбела бөгетінде контингентті басып қалды propaganda,талибан соңғы әрекет исламабад пен харипур жергілікті үкімет қатты алаңда комиссар орынбасар қосымша әскерилендір рейнджер қала солтүстіг орналас ықтимал алға жылжу болдырма үшін орналас ал қауіпсіздік күш стратегиялық тарбел бөгет контингент бас қал propaganda,violent

4,сәрсенбіде израильдік херон өлтіргіш дрондары ең ықтимал таңдау болып табылады өйткені олар ақштың ұшқышсыз ұшқыштарына арзан балама ұсынады және инфрақызыл камера мен радармен жабдықталған деп хабарлайды propaganda,сәрсенбі израиль херон өлтіргіш дрон ең ықтимал таңдау бол таб өйткені олар ақш ұшқыш ұшқыш арзан балама ұсын және инфрақызыл камера мен радар жабдықтал де хабарла propaganda,violent

4,маңызды емес ереуілдер мен бейбіт тұрғындардың шығындары ақш пен оның одақтасының арасындағы қарымқатынасты терроризмге қарсы соғыс жағдайына келтірді zhd hgh press tv propaganda,маңызды емес ереуіл мен бейбіт тұрғын шығын ақш пен оның одақтас ара қарымқатынас терроризм қарсы соғыс жағдай кел zhd hgh press tv propaganda,violent

4,равалпинди пәкістан рейтер сейсенбіде истребительдер мен тікұшақ зымырандарының қолдауымен пәкістан күштері астанадан исламабадтан солтүстікбатысқа қарай км жерде км жерде орналасқан басты алқапта талибан содырларына қарсы шабуыл бастады radicalization,равалпинди пәкістан рей сейсенбі истребитель мен тікұшақ зымыран қолдау пәкістан күш астана исламабад солтүстікбатыс қарай км же км же орналас басты алқап талибан содыр қарсы шабуыл баста radicalization,violent

4,amnesty international ұйымы сейсенбіде соңғы екі күнде төменгі дирдегі мыңға жуық адам үйлерін тастап кеткендігі туралы хабарлады сватта үш полиция қызметкерін ұрлап кейін біреуін өлтірді рейтерлер propaganda,amnesty international ұйым сейсенбі соңғы екі күн төменгі дир мың жуық адам үй таста кеткендіг туралы хабарла сват үш полиция қызметк ұрла кейін біреу өл рейтер propaganda,violent

4,соғыс жоспарларымен қамтамасыз етілген пәкістан әскерлері сват алқабына жақын жерде орналасқан бунер қаласында жаңа операция бастады өйткені исламабад талибан күштерін сыртқа шығару әрекеттерін күшейтті propaganda,соғыс жоспар

қамтамасыз ет пәкістан әскер сват алқаб жақын же орналас бун қала жаңа операция баста өйткені исламабад талибан күш сырт шығару әрекет күшей propaganda,violent

4,бұдан бұрын жоғары әскери шенеунік соғыс жоспарлары қалаға қарайтын тауда күдіктілердің жасырынып жатқан жерлерін соққыға жығып жатқанын айтқан бірақ оның құрбан болғаны туралы мәлімет жоқ ұрыстар мыңдаған жергілікті тұрғындарды үйлерін тастап кетуге мәжбүр етті propaganda,бұдан бұрын жоғары әскери шенеунік соғыс жоспар қала қара тау күдікті жасырын жат жер соққы жығ жат айт бірақ оның құрбан бол туралы мәлімет жоқ ұрыс мыңдаған жергілікті тұрғын үй таста кет мәжбүр ет propaganda,violent

4,америка құрама штаттары исламабадты талибанға қарсы шабуыл жасауға итермелеп отыр пәкістанның барлау қызметі тарихи қолдаған исламдық экстремистер ядролық қарулы ел үндістан емес ядролық қарулы елге үлкен қауіп төндіреді пәкістан әскери күштерінің соңғы операциясы жексенбіде армияның қазір аяқтаған сейсенбіде сваттың маңындағы төменгі дирге жасалған шабуылдан кейін болды abc жаңалықтары propaganda,америка құрама штат исламабад талибан қарсы шабуыл жаса итермеле отыр пәкістан барлау қызмет тарихи қолда исламдық экстремист ядролық қарулы ел үндістан емес ядролық қарулы ел үлкен қауіп төндір пәкістан әскери күш соңғы операция жексенбі армия қазір аяқта сейсенбі сва маң төменгі ди жасал шабуыл кейін бол abc жаңалық propaganda,violent

4,толық көру үшін картаны нұқыңыз талибандардың болуы исламабад аймағында талибандардың болуы туралы ақпараттар ашық көздерден алынған және ұзақ соғыс журналы талибандардың көлеңкелі үкіметтерінің болуына соғыс деңгейлері мен аймақтың есептеріне негізделген propaganda,толық көру үшін карта нұ талибан бол исламабад аймағ талибан бол туралы ақпарат ашық көз алын және ұзақ соғыс журнал талибан көлеңкелі үкімет бол соғыс деңгей мен аймақ есеп негіздел propaganda,violent

4,үкімет талибандарға қарсы әскери операцияларды дой және бунер қалаларында көтерілісшілердің солтүстік батыс шекара провинциясында жүргізіп жатқан кезде талибан сарбаздары мансехра ауданына көшіп келіп база және оқужаттығу лагерін құрды propaganda,үкімет талибан қарсы әскери операция до және бун қала көтерілісші солтүстік батыс шекара провинция жүргіз жат кезде талибан сарбаз мансехр аудан көш кел база және оқужаттығ лагер құр propaganda,violent

4,талибан пәкістан территориясына жылдары солтүстік және оңтүстік вазиристан алғашқы басып алынғаннан бері еніп отыр талибан сол кезден бастап тайпалық аймақтарды және солтүстікбатыс шекаралас провинцияны басып алды пешавар провинцияның астанасы қоршауда өйткені талибан бұл аймақ арқылы өтетін нато конвойларын үнемі нысанаға алады ал талибан пенджаб провинциясына кіріп балучистандағы бірнеше ауданды бақылайды propaganda,талибан пәкістан территория жыл солтүстік және оңтүстік вазирис алғашқы бас алын бері ен отыр талибан сол кез баста тайпалық аймақ және солтүстікбатыс шекаралас провинция

бас алды пешав провинция астана қорша өйткені талибан бұл аймақ арқылы өт нато конвой үнемі нысана ал ал талибан пенджаб провинция кір балучиста бірнеше аудан бақыла propaganda,violent

4,америка мен нато ауғанстанға қосымша әскер жіберуді ұйғарғандықтан ауғандықтар да жауап ретінде жедел кірісуді қажет деп санайды және өздерін қорғау және елді босату үшін күшті операциялар жылдың сәуірінен бастап жылы соуд гвейи сәйкес келеді propaganda,америка мен нато ауғанстан қосымша әскер жіберу ұйғар ауғандық да жауап рет жедел кірісу қажет де сана және өз қорғау және ел босат үшін күшті операция жыл сәуір баста жыл соуд гвейи сәйкес кел propaganda,violent

4,ауғанстан ислам әмірлігінің моджахедтері ауғанстанда жаңа насрат операцияларын бастайды бұл операциялар буксирлер жарылғыш заттар жарылыстар шейіт болуға ұмтылатын шабуылдар мен тосын шабуылдардан тұрады propaganda,ауғанстан ислам әмірліг моджахед ауғанстан жаңа насрат операция баста бұл операция буксир жарылғыш зат жарылыс шейіт бол ұмтыл шабуыл мен тос шабуыл тұр propaganda,violent

4,егер бірнеше рет ескертулерге қарамастан құрылыс және көлік компанияларының иелері мен жұмысшылары осындай патриоттық емес және исламға жат әрекеттерді жалғастырса мүжахидтер мұндай рұқсат етілмеген істерге қарсы шара қолданады және егер осы әрекеттердің нәтижесінде олармен бірдеңе орын алса онда жауапкершілік олармен бірге болады propaganda,егер бірнеше рет ескерту қарамастан құрылыс және көлік компания ие мен жұмысшы осындай патриоттық емес және ислам жат әрекет жалғастыр мүжахид мұндай рұқсат етілме іс қарсы шара қолдан және егер осы әрекет нәтиже олармен бірде орын ал онда жауапкершілік олармен бірге бол propaganda,violent

4,талибан жетекшісінің жоғарғы орынбасары мулла мұхаммед омар интернетте жарияланған мәлімдемесінде ақш пен ауған күштеріне сондайақ ауғанстан үкіметінің мүшелеріне бағытталған жаңа әскери науқан басталғанын жариялады мулла берадердің айтуынша жаңа көктемгі шабуыл операция жеңісі деп аталады және бейсенбіде басталады деп күтілуде propaganda,талибан жетекші жоғарғы орынбасар мулл мұхаммед омар интернет жариялан мәлімдеме ақш пен ауған күш сондайақ ауғанстан үкім мүше бағыттал жаңа әскери науқан бастал жариял мулл бера айт жаңа көктемгі шабуыл операция жеңіс де атал және бейсенбі бастал де күтіл propaganda,violent

4,натоға мүше елдер ақштың сызығына мойынсұнбайтын шығар франс пресс агенттігінің хабарлауынша натоның қолбасшысы бентис крадок еуропа елдері обаманың ауғанстанда әскери қатысуын сақтау туралы өтінішіне әрең құлақ асады деп мәлімдеді propaganda,нато мүше ел ақш сызығ мойынсұнба шығар франс пресс агенттіг хабарла нато қолбасшы бентис крадок еуропа ел обама ауғанстан әскери қатыс сақтау туралы өтініш әре құлақ ас де мәлімде propaganda,violent

4,ақш пәкістан үкіметі екі аптадан кейін құлдырауы мүмкін жұма мамыр gmt қаріп өлшемі бас дэвид петреус ақштың жоғарғы қолбасшысы егер талибан билеушілері алдағы екі апта ішінде жеңілмесе исламабад үкіметі құлау қаупі бар деп ескертеді propaganda,ақш пәкістан үкімет екі апта кейін құлдыра мүмкін жұма мамыр gmt қаріп өлшем бас дэвид петреус ақш жоғарғы қолбасшы егер талибан билеуші алдағы екі апта іш жеңілме исламабад үкімет құла қауп бар де ескерт propaganda,violent

4,проблемалы аймақта терроризммен күресу үшін ұлттық консенсус саясатын бастағысы келетінін айтады пәкістан үкіметі исламабад елдің ядролық қондырғыларын толықтай бақылауға алатынын айтып ақштың ядролық проблемаларға қатысты алаңдаушылығын жоққа шығарды исламабад ақ үйдің ақылға қонымсыз саясаты талибтерді күшейтіп тұрақсыз аймақта экстремизмді таратып жатқанын бірнеше рет айтқан jr dt press tv propaganda,проблемал аймақта терроризм күресу үшін ұлттық консенсус саясат баста кел айт пәкістан үкімет исламабад ел ядролық қондырғы толықта бақыла ал айт ақш ядролық проблема қатысты алаңдаушылығ жоқ шығ исламабад ақ үй ақыл қон саясат талиб күшейт тұрақсыз аймақта экстремиз тарат жат бірнеше рет айт jr dt press tv propaganda,violent

4,талибан операцияның жеңісін жариялады жарияланған уақыты сәуір ауғанстанның талибан мүжахидтері сәрсенбіде алдағы апталарда ақштағы қосымша мыңдаған әскери күштердің көбеюіне жауап ретінде басқыншылық күштерге қарсы жаңа операция жасады деп қорқытты насрат операциясы сейсенбіде басталады сонымен қатар ауғандық қуыршақ шенеуніктері мен халықаралық дипломаттарды шахада операциялары мен шабуылдарымен қарсы алады деп мәлімдеді propaganda,талибан операция жеңіс жариял жариялан уақыт сәуір ауғанстан талибан мүжахид сәрсенбі алдағы апта ақш қосымша мыңдаған әскери күш көбею жауап рет басқыншылық күш қарсы жаңа операция жас де қорқыт насрат операция сейсенбі бастал сонымен қатар ауғандық қуыршақ шенеунік мен халықаралық дипломат шаха операция мен шабуыл қарсы ал де мәлімде propaganda,violent

4,алькаиданың аға қайраткері әбу йехя әл либи әлемдегі мүжахедтер газада палестиналықтардың қырылуына жауаптылардан кек алуға дайын деп мәлімдеді бұл алькаиданың сахаб деп жариялаған жаңа бейне таспасында сәрсенбі күні содырлардың исламшыл сайттарында шығыс пен батыстағы мужахидтер еврейлердің қолымен газадағы мұсылмандарға не болып жатқанын және болып жатқан оқиғаларды өшіруге және кек алуға дайындалып жатыр radicalizaton,алькаида аға қайраткер әбу йехя әл либи әлем мүжахед газа палестиналық қырыл жауапты кек ал дайын де мәлімде бұл алькаида сахаб де жарияла жаңа бейне таспа сәрсенбі күн содыр исламшыл сайт шығыс пен батыс мужахид еврей қол газа мұсылман не бол жат және бол жат оқиға өшір және кек ал дайындал жатыр radicalizaton,violent

4,могадишу сомали қаңтар garowe online сомалидегі африка одағы миссиясына amisom исламшыл қыңыр адамдарға қатысты соңғы ескертулер қауіп төндірмейді дейді гарове радиосы propaganda,могадиш сомали қаңтар garowe online сомали

африка одағ миссия amisom исламшыл қыңы адам қатысты соңғы ескерту қауіп төндірме де гаров радио propaganda,violent

4,израиль ақырзаман күні жақындап келе жатқан сәтте күн мамыр gmt израиль әуе күштері iaf зымыран операторлары иранмен ықтимал қақтығыстарға дайындалуға шақырылды израильдің иранға қарсы соғыс болатын күні жақындаған кезде бір хабарда израильдің зымыран операторлары өздерінің шеберліктерін шыңдау үшін апта сайын жаттығулар бастады propaganda,израиль ақырзаман күн жақында келе жат сәт күн мамыр gmt израиль әуе күш iaf зымыран оператор иран ықтимал қақтығыс дайындал шақыр израиль иран қарсы соғыс бол күн жақында кезде бір хаб израиль зымыран оператор өз шеберлік шыңдау үшін апта сайын жаттығу баста propaganda,violent

4,баллистикалық зымыран құралын жебені және жер үстізымыранпатриотты пайдаланатын израиль әуе күштерінің iaf резервшілерін қорғаныс министрлігі аптасына бір күнді ықтимал қақтығыстарға дайындалу үшін өткізуге шақырды recruitment,баллистикалық зымыран құрал жебе және жер үстізымыранпатрио пайдалан израиль әуе күш iaf резервші қорғаныс министрліг апта бір күн ықтимал қақтығыс дайындалу үшін өткіз шақ recruitment,violent

4,сомалидегі уақытша үкіметтің қауіпсіздік қызметкері эфиопиялық сарбаздарды тастап кетіп артта қалған базалардың басым бөлігін басып алған исламдық күштер елдің жиһадынан қашып кениядан пана сұрады propaganda,сомали уақытша үкімет қауіпсіздік қызметк эфиопиялық сарбаз таста кет арт қалған база басым бөліг бас ал исламдық күш ел жиһад қаш кения пан сұр propaganda,violent

4,багдад ақш әскери қызметшілері мен ирак полициясы мосулдағы полиция ғимаратында түскі асқа отырды қабыршақтар және басқа қорғаныш құралдар алынды бөлменің есігі ашық қалды ирак полициясының ак бөшкелерін ішке кіргізуге жеткілікті кең болды шабуылдаушылар оқ жаудырды содан кейін тағы күдікті қаруланушылар әлі жасөспірімде ирак полициясының офицері және жас сержант күту машинасына қарай жүгірді көлік бақылау пунктін айналып өтіп шабуылдаушылар жоғалып кетті propaganda,багдад ақш әскери қызметші мен ирак полиция мосул полиция ғимарат түскі ас от қабыршақ және басқа қорғаныш құрал ал бөлме есіг ашық қал ирак полиция ак бөшке іш кіргіз жеткілікті кең бол шабуылдаушы оқ жау содан кейін тағы күдік қаруланушы әлі жасөспірім ирак полиция офицер және жас сержант күту машина қарай жүг көлік бақылау пункт айнал өт шабуылдаушы жоғал кет propaganda,violent

4,бұл ирак полициясымен туындаған үлкен проблеманың белгісі емес деді әскери өкілі майор рамона беллард бірақ қорқынышты билік әлі де күдікті сатқындарды іздеуге тырысуда сейсенбіде полиция комиссарына ирактың маңында көліктерді жарып жіберген және кем дегенде қауіпсіздік қызметкерін өлтірді деген айып тағылған алькаидаға байланысты сақина қосылды propaganda,бұл ирак полиция туында үлкен проблема бел емес де әскери өкіл майор рамон беллард бірақ

қорқыныш билік әлі де күдік сатқын ізде тырыс сейсенбі полиция комиссар ирак маң көлік жар жібер және кем де қауіпсіздік қызметк өл де айып тағыл алькаида байланысты сақин қос propaganda,violent

4,солтүстік кавказдағы көтерілістер негізінен жас және белсенді мүшелерді тартады жарияланым солтүстік кавказдың апталық көлемі шығарылым қаңтар қаңтар жасы күн санат солтүстік кавказ апталығы солтүстік кавказ авторы майрбек ватчаев солтүстік кавказдағы қарсыластардың көбі жас болған және олардың көпшілігі ауылдардан келген немесе кісі өлтіргені немесе олар немесе олардың отбасы мүшелері басынан кешкені үшін кек алу үшін қарсыласуға көшкен propaganda,солтүстік кавказ көтеріліс негіз жас және белсенді мүше тарт жарияланым солтүстік кавказ апталық көлем шығарылым қаңтар қаңтар жас күн санат солтүстік кавказ апталығ солтүстік кавказ авто майр ватчаев солтүстік кавказ қарсылас көбі жас бол және олардың көпшіліг ауыл кел немесе кісі өлтір немесе олар немесе олардың отбасы мүше бас кеш үшін кек алу үшін қарсылас көш propaganda,violent

4,билік күш қолдануға басымдық беріп кілем астындағы мәселелерді шешуге тырысады осыған сүйене отырып қарсыласу қозғалысы таяу жылдарда айтарлықтай шығынға ұшырамайтынына толық сенімді бола алады бұл дегеніміз солтүстік кавказ көптеген жылдар бойы тұрақсыз аймақ болып қала береді propaganda,билік күш қолдан басымдық бер кілем аст мәселе шеш тырыс осы сүйен отыр қарсылас қозғалыс таяу жыл айтарлықтай шығын ұшырама толық сенімді бола ал бұл де солтүстік кавказ көптеген жыл бойы тұрақсыз аймақ бол қала бер propaganda,violent

4,болтон ядролық мүмкіндіктерді талибан дан көреді сн мамыр gmt пәкістанның қауіпсіздік күштері талибан жасырынған жерлерге артиллериялық атыс жүргізуде ақштың бұрынғы аға дипломаты джон болтон егер елде тұрақсыздық орнаған жағдайда талибан пәкістанның ядролық қаруын бақылауға алуы мүмкін дейді propaganda,болтон ядролық мүмкіндік талибан дан көр сн мамыр gmt пәкістан қауіпсіздік күш талибан жасырын жер артиллериялық атыс жүргіз ақш бұрынғы аға дипломат джон болтон егер ел тұрақсыздық орна жағдай талибан пәкістан ядролық қар бақыла ал мүмкін де propaganda,violent

4,егер демократиялық үкімет радикалды қысымға дейін еріп кетсе біздің кейбір демократиялық мықтылығымызды және пәкістанның әскери шабуылына қарсы тұруды білдіруі мүмкін деді болтон agb md presstv propaganda,егер демократиялық үкімет радикал қысым дейін ер кет біздің кейбір демократиялық мықтылығ және пәкістан әскери шабуыл қарсы тұру білдір мүмкін де болтон agb md presstv propaganda,violent

4,сринагар сәуір кмс кашмирдің жаулап алынған жерінде белгілі үнділік құқық қорғаушы гаутам навлаха әр кашмири азаптаудың бір немесе басқа түрінің құрбаны болғанын және нан астам кашмирліктер үшінші дәрежелі азапты бастан өткергенін айтты propaganda,сринаг сәуір кмс кашмир жаула алын же белгілі үнділік құқық

қорғаушы гаута навлах әр кашмири азапта бір немесе басқа түр құрбан бол және нан астам кашмирлік үшінші дәрежелі аза бас өткер ай propaganda,violent

4,осы кездесуге қатысқан өзге де баяндамашылар азаптаулардан аман қалғандарды қоғамнан оқшауланбау үшін ынталандыру керектігін атап өтті олар сонымен қатар әртүрлі елдерде азаптау мәселесі бойынша жұмыс істейтін халықаралық ұйымдардың үнсіздігіне алаңдаушылықтарын білдірді http www kmsnews org propaganda,осы кездес қатыс өзге де баяндамашы азаптау аман қал қоғам оқшауланба үшін ынталандыру керектіг ата өт олар сонымен қатар әртүрлі ел азапта мәселе бойынша жұмыс істе халықаралық ұйым үнсіздіг алаңдаушылық біл http www kmsnews org propaganda,violent

4,таяу уақытта таратылған жалған құран milf ескертеді edd usmanmay мамыр мұнда құранның немесе мұсылман қасиетті кітабының жаңа жалған нұсқасы моро исламдық азат етуі бар фронт milf жексенбіде көшірмелер әсіресе таяу шығыста таратылатынын ескертті бұл құранның жалған жариялануына қатысы бар екі американдық баспа компаниясымен нағыз фурқан ретінде ұсынылуда деп хабарлайды milf вебсайты propaganda,таяу уақыт таратыл жалған құран milf ескерт edd usmanmay мамыр мұнда құран немесе мұсылман қасиетті кітаб жаңа жалған нұсқа моро исламдық азат ет бар фронт milf жексенбі көшірме әсіресе таяу шығыс таратыл ескер бұл құран жалған жариялан қатыс бар екі американдық баспа компания нағыз фур рет ұсыныл де хабарла milf вебсай propaganda,violent

4,әсіресе саудиялықтар пәкістанда айтарлықтай ықпалға ие сондықтан менің ойымша пәкістандықтарды исламабадтағы үкіметке қарсы тұру үшін кеңейтілген мағынада біріктіруге болатын барлық ісшаралар құпталады propaganda,әсіресе саудиялық пәкістан айтарлықтай ықпал ие сондықтан менің ойымша пәкістандық исламабад үкімет қарсы тұру үшін кеңейт мағына біріктір бол барлық ісшара құптал propaganda,violent

4,куәгерлердің айтуы бойынша ұзақ қақтығыс болды бір сағатқа жуық уақыт ішінде сол аймақта жарамана ауыр мылтықтар естілді ал аймақ мемлекеттік барлаудың қоршауында болды және сол аймаққа кіруге және шығуға кедергі келтірді мемлекет бұл мәселені жасырды шейх пен оның серіктері туралы олар ешқандай жаңалық білмедік олар тұтқындар ма немесе шейіт болды ма және олар тоқтамады біз өз тарапымыздан шындыққа жету үшін көп жұмыс жасадық propaganda,куәгер айт бойынша ұзақ қақтығыс бол бір сағат жуық уақыт іш сол аймақта жараман ауыр мылтық ест ал аймақ мемлекеттік барла қоршау бол және сол аймақ кір және шығ кедергі кел мемлекет бұл мәселе жас шейх пен оның серік туралы олар ешқандай жаңалық білме олар тұтқын ма немесе шейіт бол ма және олар тоқтам біз өз тарап шындық жету үшін көп жұмыс жаса propaganda,violent

4,бұл бомбалау сирия режимінің аппараты мен америка армиясының келісімі бойынша жасалған бұл жүйе құлдардың бас сүйектеріне салынған және сириядағы біздің халқымыздың қанымен суарылған хама қаласы әлі күнге дейін осы кәпір

режимнің әйелдерге және жасанды қырғынның сүйектері мен қасіреттеріне куә propaganda,бұл бомбала сирия режим аппарат мен америка армия келісім бойынша жасал бұл жүйе құл бас сүйек салын және сирия біздің халқ қан суарыл ха қала әлі күн дейін осы кәпір режим әйел және жасанды қырғын сүйек мен қасірет куә propaganda,violent

4,намаз оқитындардың басындағы мешіттерді қиратқан сондайақ олардың ішіндегі қасиетті құранды жыртып өртеп жіберген қорғансыз балалар мен шейхтер үшін бұл седная түрмесі сонымен бірге олардың көпшілігі мұсылмандар болған тұтқынға қарсы жасалған қырғындарының куәсі олар өлім жазасына кесілді және сол түрмеде құранды құрметтеді және тұтқындарды қорлау біздің күндерімізге дейін жалғасуда radicalization,намаз оқитын басындағы мешіт қират сондайақ олардың ішіндегі қасиетті құра жырт өрте жібер қор бала мен шейх үшін бұл седная түр сонымен бірге олардың көпшіліг мұсылман бол тұтқын қарсы жасал қырғын куә олар өлім жаза кес және сол түрме құра құрметте және тұтқын қорла біздің күн дейін жалғас radicalization,violent

4,бұл әділетсіз режимнің ливандағы палестина халқына талəлзаатар лагерінде набаа паша көпірінде және басқа аудандарда сиырлардан бастап әйелдердің асқазаны мен басын кесіп тастауына және осы кедей адамдарға қарсы ұжымдық геноцидке үйлерін қиратуына тіпті бүкіл лагерьлерді жоюына қатысты жасаған әрекеттерін әлем ұмытып кетті ме жоқ па жоқ па radicalization,бұл әділет режим лива палестин халқ талəлзаа лагер наба паш көпір және басқа аудан сиыр баста әйел асқазан мен басын кес таста және осы кедей адам қарсы ұжымдық геноцид үй қират тіпті бүкіл лагерь жою қатысты жаса әрекет әлем ұмыт кет ме жоқ па жоқ па radicalization,violent

4,алынған мәліметтер бойынша американдық крестке қарсы күштер кешегі күні дүйсенбіге қараған түні әскери ұшақтармен және басқа да дрондармен аяусыз соққы жасады фарах қаласы мен фарах жолы провинциясы арасында өтетін жолда орналасқан кингабад пен карана ауылдарына аудан халқының және сенімді деректерге сүйенсек американдықтардың қатыгез әуе шабуылында көптеген үйлер қирап шейіт болғандар мен жараланғандар саны жазықсыз адамға жетті radicalization,алын мәлімет бойынша американдық крест қарсы күш кешегі күн дүйсенбі қара түн әскери ұшақ және басқа да дрон аяу соққы жас фарах қала мен фарах жол провинция ара өт жолда орналас кингабад пен каран ауыл аудан халқ және сенімді дерек сүйен американдық қатыгез әуе шабуыл көптеген үй қира шейіт бол мен жаралан сан жазық адам же radicalization,violent

4,мужахиддер жаудың үш әскери техникасын жойып жіберді оларда жауынгер қаза тапты тағы бірнеше адам жараланды соғыс соңында мүжахидтер әскери көлік пен көптеген қару мен тірі оқдәрілерді алды бұл шабуылда және әуе шабуылдарында мүжахидтерге ешқандай зиян болған жоқ propaganda,мужахид жау үш әскери техника жой жіб ол жауынгер қаза тап тағы бірнеше адам жарала соғыс соң мүжахид әскери көлік пен көптеген қару мен тірі оқдәрі алды бұл шабуылда және әуе шабуыл мүжахид ешқандай зиян бол жоқ propaganda,violent

4,бір топ ер адамдар ақштың әуе базасын бомбалауды және жоспарын орындаған кезде реакция туралы қалжыңдап жатқанын лентада ұстады деп хабарлады propaganda,бір топ ер адам ақш әуе база бомбалау және жоспар орында кезде реакция туралы қалжыңда жат лента ұста де хабарла propaganda,violent

4,ирактықтардың азапты кісі өлтіру әдісі ирактың гейлеріне бағытталған кісі өлтіру науқаны күшейген сайын өткен аптада жетекші араб теледидар желісі ирактық гейлерге қарсы өлтіретін жаңа форманы гейшииттерге қарсы өлім отрядтары жапсырылғаны туралы хабарлады propaganda,ирактық аза кісі өлтір әдіс ирак гей бағыттал кісі өлтір науқан күшей сайын өткен апта жетекші араб теледи желі ирак гей қарсы өлтір жаңа форма гейшиит қарсы өлім отряд жапсырыл туралы хабарла propaganda,violent

4,ирактағы әйелдер бостандығы ұйымының owfi президенті янар мохаммед әларабияға азаптайтын зат бұл ирандық өндірілген желім егер ол теріге жағылса оны тек хирургиялық жолмен алып тастауға болады олар гомосексуалдардың анусын желімдейді оларға диареяны тудыратын сусын береді анус жабық болғандықтан диарея өлімге әкеледі азаптаудың бұл түріндегі бейнелер ирактағы ұялы телефондарға таратылады propaganda,ирак әйел бостандығ ұйым owfi президент ян мохаммед әларабия азапта зат бұл ирандық өндір жел егер ол тері жағыл оны тек хирургиялық жол алып таста бол олар гомосексуал ану желімде оларға диарея тудыр сусын бер анус жабық бол диарея өлім әкел азапта бұл түр бейне ирак ұялы телефон тарат propaganda,violent

4,сонымен бірге фармацевттер полицияға әйел гормондары мен косметикалық кремдерді үнемі сатып алатын ер клиенттер туралы хабарлағанын айтты жақында осындай бір мысалда аталған трансгендерлік екі адам ұсталып белгісіз жерге апарылды содан бері ол туралы естімеген propaganda,сонымен бірге фармацевт полиция әйел гормон мен косметикалық крем үнемі сат ал ер клиент туралы хабарла ай жақында осындай бір мысал атал трансгендерлік екі адам ұстал белгісіз же апар содан бері ол туралы естіме propaganda,violent

4,сіз бейбітшілік пен прогрестің жалған талапкерлері фарах штатындағы пала блогындағы кингабад және карана аудандарына жататын ауылдарда бейбітшілік пен еркіндік деген сылтаумен азап шеккен азаматтарымызды олардың көпшілігі әйелдер балалар мен қарттарды тағы бір рет қанға батырғанын білдіңіз егер бұл крест жорығының құбыжықтары ауғанстанның азап шеккен халқына қарсы осындай қатыгез және әділетсіз әрекетті бірінші рет жасамаса бұл адамгершілікке жат әрекет propaganda,сіз бейбітшілік пен прогрес жалған талапкер фарах штат пал блог кингабад және каран аудан жат ауыл бейбітшілік пен еркіндік де сылтау азап шек азамат олардың көпшіліг әйел бала мен қарт тағы бір рет қан батыр біл егер бұл крест жорығ құбыжық ауғанстан азап шек халқ қарсы осындай қатыгез және әділет әрекет бірінші рет жасама бұл адамгершілік жат әрекет propaganda,violent

4,олар ауғанстанның әртүрлі аймақтарындағы мыңдаған қорғансыз адамдардың жаппай шейіт болуларымен жарақаттарымен үйлерін қиратумен және баспаналарын қиратумен жүздеген рет варваризм мен қатыгездіктен сауықтырды басқыншылар мен құбыжықтар әрдайым адамзатқа қарсы осындай әрекет жасағанда және жазықсыз әйелдерді балалар мен қарт адамдарды әуе соққыларында өлтіреді олар өздерін тазарту үшін өлгендердің барлығы өздерінің бұзушылар екенін немесе өздерінің жалған тыңшыларының дұрыс емес ақпараттарымен байланыстырады propaganda,олар ауғанстан әртүрлі аймақ мыңдаған қор адам жаппай шейіт болу жарақат үй қират және баспана қират жүздеген рет варвариз мен қатыгездік сауық басқыншы мен құбыжық әрдайым адамзат қарсы осындай әрекет жаса және жазық әйел бала мен қарт адам әуе соққы өлтір олар өз тазарт үшін өл барлығ өз бұзушы екен немесе өз жалған тыңшы дұрыс емес ақпарат байланыстыр propaganda,violent

4,малаканд келісімінің және сваттағы соғыс туралы үкімет талибандардың майдангері суфи мұхаммед фазлулланың қайын атасы малаканд келісіміне ақпан күні екі жыл бойы созылған толқулардан кейін ауданды бақылауға алған осы екі жыл ішінде әскери күштер талибандардан күресті бақылау кезінде үш рет жеңіліске ұшырады әр жеңіліс талибандарды ауданды үлкен бақылауға алды propaganda,малаканд келісім және сват соғыс туралы үкімет талибан майдангер суфи мұхаммед фазлулла қайын ата малаканд келісім ақпан күн екі жыл бойы созыл толқу кейін аудан бақыла ал осы екі жыл іш әскери күш талибан күре бақылау кез үш рет жеңіліс ұшыр әр жеңіліс талибан аудан үлкен бақыла алды propaganda,violent

4,талибан мансехраға көшіп аймақта базалар мен оқужаттығу жиындарын ұйымдастырды пәкістан үкіметі мен әскери шенеуніктер талибанның исламабадқа және елдің ядролық нысандарына қауіпқатерін жоққа шығарды бірақ сәуірдің аяғында жергілікті исламабад үкіметі әскерилерге талибанның алға жылжуына тосқауыл қою үшін қаланың солтүстігінде орналасқан маргала төбесіне орналастыруды бұйырды гарипур үкіметі тарбела бөгетінде қауіпсіздікті күшейтті propaganda,талибан мансехра көш аймақта база мен оқужаттығ жиын ұйымдас пәкістан үкімет мен әскери шенеунік талибан исламабад және ел ядролық нысан қауіпқа жоқ шығ бірақ сәуір аяғ жергілікті исламабад үкімет әскери талибан алға жылж тосқауыл қою үшін қала солтүстіг орналас маргал төбе орналастыру бұйыр гарипу үкімет тарбел бөгет қауіпсіздік күшей propaganda,violent

4,талибан пәкістанның жетекшілерін нысанаға алады мамыр gmt талибан көтерілісшілері армия сват алқабында офицерлерге кең ауқымды операцияны бастағаннан кейін пәкістанның жоғарғы басшылығын нысанаға алуға уәде берді талибан қолбасшысы бұл жаңалыққа президент асиф али зардари мен премьерминистр юсуф раза гилани мен олардың жақын туыстары басты нысана болатынын айтты propaganda,талибан пәкістан жетекші нысана ал мамыр gmt талибан көтерілісші армия сват алқаб офицер кең ауқымды операция баста кейін пәкістан жоғарғы басшылығ нысана ал уәде бер талибан қолбасшы бұл жаңалық президент

асиф али зардари мен премьерминист юсуф раз гилани мен олардың жақын туыс басты нысана бол ай propaganda,violent

4,бомбалаушылар хамастың иззәлдинкассам батальондарының әскери қанатына жатады абу моат әлкассамның жанкештілерді басқаратын жетекшісі бомбалаушылар хамастың мұқият таңдаған жеке тұлғалары олар кезкелген басқа палестиналық тақуалық сияқты өмір сүреді олардың кейбіреулері өз өмірлерін қалаусыз назар аудармай немесе өздерінің соңғы миссиялары туралы мақтанбай өткізетін студенттер болып табылады propaganda,бомбалаушы хамас иззәлдинкас батальон әскери қанат жат абу моат әлкасса жанкешті басқар жетекші бомбалаушы хамас мұқият таңда жеке тұлға олар кезкел басқа палестиналық тақуалық сияқты өмір сүр олардың кейбіреу өз өмір қалау назар аударма немесе өз соңғы миссия туралы мақтанба өткіз студент бол таб propaganda,violent

4,өлтірушілер өзін отбасы мен достарынан оқшаулап алды олар өз уақыттарын израилдік күштер орналастырылған жерлерге жақын жерде өткізеді суицид жасаушыларды іріктеу критерийлері бойынша абу моат қысқаша түсіндіріп берді олар он мыңға жуық жауынгерден тұратын әлкассам батальондарының қатарынан тек жастар ғана таңдалады propaganda,өлтіруші өз отбасы мен дос оқшаула алды олар өз уақыт израи күш орналастырыл жер жақын же өткіз суицид жасаушы іріктеу критерий бойынша абу моат қысқаша түсіндір бер олар он мың жуық жауынгер тұр әлкас батальон қат тек жас ғана таңдал propaganda,violent

4,абу моаттың ашылған әйелдері де өзінөзі өлтірушілердің қатарына қабылданған кандидаттардың бомбардирлары алькассам лейтенанттарының құпия түрде тексеріліп олардың діни адалдық пен жауапкершілікке ие екендігіне көз жеткізеді келесі кезең бомбалаушыға олардың қабылданғаны туралы хабарлау және оларды қару мен тактикаға әсіресе израиль қорғаныс күштері idf қолданған психологиялық және әскери дайындық арқылы қою propaganda,абу моа ашыл әйел де өзінөз өлтіруші қат қабылдан кандидат бомбардир алькас лейтенант құпия түр тексеріл олардың діни адалдық пен жауапкершілік ие екендіг көз жеткіз келесі кезең бомбалаушы олардың қабылдан туралы хабарла және ол қару мен тактика әсіресе израиль қорғаныс күш idf қолдан психологиялық және әскери дайындық арқылы қою propaganda,violent

4,хамастың израильде тұратын палестиналықтар арасында израиль қалаларында суицид шабуылдарын қайта бастау үшін жеткілікті тәжірибесі мен ықтимал қатысушылары болса да израиль күштері газаға жасалған шабуылда өзінөзі өлтіруді болдырмауға қабілеттерін көрсетті қақтығыста израиль күштеріне жасалған сәтсіз суицидтік шабуылдардың болмауы хамас басшылығының қандай да бір мәлімдемелеріне қарамастан жанкештілік жарылыстарды жасау үшін жеткілікті еріктілерді жалдай алмайтындығын көрсетеді http www jamestown org programs gt ash d propaganda,хамас израиль тұр палестиналық ара израиль қала суицид шабуыл қайта бастау үшін жеткілікті тәжірибе мен ықтимал қатысушы бол да израиль күш газа жасал шабуылда өзінөз өлтіру болдырма қабілет көрсе қақтығыс израиль күш жасал сәт суицид шабуыл болма хамас басшылығ қандай да бір мәлімдеме қарамастан

жанкештілік жарылыс жасау үшін жеткілікті ерікті жалда алмайтындығ көрсет http www jamestown org programs gt ash d propaganda,violent

4,әскерлер маусымда мосулдан кетуі мүмкін ақш генералы агентстволар ирактағы ақштың жоғарғы қолбасшысы жұма күні ақш әскери күштері зұлымдық тудырған мосул қаласын маусымға дейін қалдыруы мүмкін деді америкалық әскери бригадаларды ирак қалаларынан оңғы шешім келесі айдың соңында ақш пен ирак күштері ирактағы алькаида мен басқа да сунниттік топтардың негізгі тірегі болып табылатын мосулдан көмек алу үшін төңкеріс жасауын аяқтағаннан кейін қабылданады деп мәлімдеді ақш propaganda,әскер маусым мосул кет мүмкін ақш генерал агентство ирак ақш жоғарғы қолбасшы жұма күн ақш әскери күш зұлымдық тудыр мосул қала маусым дейін қалдыр мүмкін де америкалық әскери бригада ирак қала оңғ шешім келесі ай соң ақш пен ирак күш ирак алькаи мен басқа да сунни топ негізгі тірег бол табыл мосул көмек алу үшін төңкеріс жаса аяқта кейін қабылдан де мәлімде ақш propaganda,violent

4,әскери шенеуніктері әлқаидады жақында болған шабуылдардың өршуі үшін айыптайды олар иракты жылы самаррадағы альаскари шииттер мешітін бомбалағаннан кейін иракты секталық қырыпжоюға қайтарады деп отыр құрбан болғандардың көп шабуылдарының артуы ирак күштерінің маусымнан кейін ақштың әскери күштері бағдатпен екіжақты келісім бойынша қалаларды тастап кетуі күтілгеннен кейін қауіпсіздік орната алады деген алаңдаушылық тудырды propaganda,әскери шенеунік әлқаид жақында бол шабуыл өрш үшін айыпта олар ирак жыл самарра альаскари шиіт мешіт бомбала кейін ирак секталық қырыпжою қайтар де отыр құрбан бол көп шабуыл арт ирак күш маусым кейін ақш әскери күш бағдат екіжақты келісім бойынша қала таста кет күтіл кейін қауіпсіздік орна ал де алаңдаушылық ту propaganda,violent

4,тәжістанның ояну кеңесінің басшысы қызметінен босатылды уа альхамдулилла цитата иракта антикаиданың жетекшісі өлтірілді американдық күштермен одақтас болған қайдаға қарсы милиция жетекшісі бағдадтың солтүстігінде жол бойындағы бомбадан өлтірілді деп хабарлады propaganda,тәжістан оян кеңес басшы қызмет босат уа альхамдулилл цита ирак антикаида жетекші өлтір американдық күш одақтас бол қайда қарсы милиция жетекші бағдад солтүстіг жол бой бомба өлтір де хабарла propaganda,violent

4,бірақ шииттер бастаған үкіметпен ұзақ уақыт бойы қарымқатынаста болды алькаида мақсатты түрде сахва мүшелерін үкімет қамауға алады бұл әскерилендірілген топтар арасында құдалау қорқынышын арттырады соңғы айларда үкімет тарапынан ескертілген савха ның ондаған мүшесі қамауға алынды propaganda,бірақ шиит баста үкімет ұзақ уақыт бойы қарымқатынас бол алькаи мақсат түр сахв мүше үкімет қама ал бұл әскерилендір топ ара құдала қорқыныш арттыр соңғы ай үкімет тарап ескерт савх ны ондаған мүше қама ал propaganda,violent

4,алькаиданың үшінші командирі әбу яхия әллиби исламшылдарға газа секторындағы израильдің өліміне қарсы шабуылын өшіруді бұйырды бұл ұлыбритания мен ақшты қоса батыс елдеріне қарсы шабуылдар жасады шындық мынада әлем бойынша қанымыз төгілуі ақш пен батыстың мойнында болғанша аяқталмайды деді propaganda,алькаида үшінші командир әбу яхия әллиби исламшыл газ сектор израиль өлім қарсы шабуыл өшіру бұйыр бұл ұлыбритания мен ақш қоса батыс ел қарсы шабуыл жас шындық мына әлем бойынша қан төгіл ақш пен бат мойн болғанш аяқталма де propaganda,violent

4,үш апталық шабуыл палестиналықтың өмірін қиды және нан астам адам жарақат алды деп мәлімдеді палестина медицина қызметкерлері қақтығыста он үш израильдік қайтыс болды бұл тәжірибе араб басшыларының біздің құрметті палестина бауырларымызға опасыздық жасағанын көрсетті деді propaganda,үш апталық шабуыл палестиналық өмір қи және нан астам адам жарақат алды де мәлімде палестин медицина қызметкер қақтығыс он үш израиль қайтыс бол бұл тәжірибе араб басшы біздің құрметті палестин бауыр опасыз жаса көрсе де propaganda,violent

4,ол аумақты ашық жабық жасуша деп сипаттап ол аты аталмаған кәпір елдерді осы қырғынға қатысты шайтандық одаққа қатысты деп айыптады исламды білетіндер мұны біледі дін және сенім жетіспеушілігі екеуінің біреуі екіншісінен басым болмаса қатар өмір сүре алмайды деді ол propaganda,ол аумақ ашық жабық жасуша де сипатта ол ат аталма кәпір ел осы қырғын қатысты шайта одақ қатысты де айыпта ислам біл мұны біл дін және сенім жетіспеушіліг екеу біреу екінші басым болма қатар өмір сүр алма де ол propaganda,violent

4,талибан карзайдың келіссөзге шақыруын қабылдамайды дүйсенбі мамыр gmt талибан лидерлері ауғанстан президенті хамид карзаймен отыруға келісуден бас тартып оны келіссөздерге қатысуға лайық емес қабілетсіз адам деп атады propaganda,талибан карзай келіссөз шақыр қабылдама дүйсенбі мамыр gmt талибан лидер ауғанстан президент хамид карзай отыр келісу бас тарт оны келіссөз қатыс лайық емес қабілет адам де ат propaganda,violent

4,джихадистік бақ элитасы ұсынады демпинг сенімгерлердің қолбасшысы аби омар алькураши альбагдадидің сөзі жалған клиенттер alfurqan media production foundation мамыр жасыл аймақтағы баас билеушілерінің өтірік сөздеріне таң қалды олар бағдадтағы кедей қызметшіні тұтқындады деп тағы да мәлімдеді propaganda,джихади бақ элита ұсын демпинг сенімгер қолбасшы аби омар алькураши альбагдади сөз жалған клиент alfurqan media production foundation мамыр жасыл аймақ баас билеуші өтірік сөз таң қал олар бағдад кедей қызметші тұтқынд де тағы да мәлімде propaganda,violent

4,мен оны тек бірнеше сағат бойы отпен жаудырамыз деп ойладым өйткені олар моджахедтердің соққыларының ауырлығын түсінді бірақ олар оны насихаттады және олардың өтіріктеріне сенді осылайша оны азаптаған адамның бейнесін шығарды біз олардың қайдан және кімнен келгенін білмейміз propaganda,мен оны тек бірнеше

сағат бойы от жаудыр де ойла өйткені олар моджахед соққы ауырлығ түс бірақ олар оны насихатта және олардың өтірік сен осылайша оны азапта адам бейне шығ біз олардың қайдан және кім кел білме propaganda,violent

4,егер ол менің халқымның басына түскен қиыншылық болмаса мен құпияның артында отырмас едім мен өлтіргім келді содан кейін мен өмір сүремін содан кейін өлтіремін содан кейін өмір сүремін содан кейін өлтіремін сіздің ағаңыз абу омар алькураши альбагдади бізді дұғаларыңыздың мүддесі үшін ұмытпаңыздар элиталық жиһадтық бақтағы бауырлар propaganda,егер ол менің халқ бас түс қиыншылық болма мен құпия арт отырмас ед мен өлтір кел содан кейін мен өмір сүр содан кейін өлтір содан кейін өмір сүр содан кейін өлтір сіздің аға абу омар алькураши альбагдади біз дұға мүдде үшін ұмытпа элиталық жиһад бақ бауыр propaganda,violent

4,риаз хан associated press writer риаз хан associated press writer минут бұрын пәшавар пәкістан күдікті талибан сәрсенбіде пәкістанның солтүстікбатысында көрші ауғанстандағы нато әскерлеріне арналған жабдықты өңдеп жатқан қоймаға шабуыл жасады және пәкістан армиясы басқалармен соғысқан кезде сегіз жүк көлігін қағып кетті propaganda,риаз хан associated press writer риаз хан associated press writer минут бұрын пәшав пәкістан күдік талибан сәрсенбі пәкістан солтүстікбаты көрші ауғанстан нато әскер арнал жабдық өңде жат қойма шабуыл жас және пәкістан армия басқа соғыс кезде сегіз жүк көліг қағ кет propaganda,violent

4,басқа аймақтағы шабуылда жүздеген мың адамдар қашып кетті хайбер асуы арқылы ауғанстанға қарай бет алған терминалдар мен жүк көліктеріне шабуылдар өткен жылдан бастап күшейе түсті ауғанстан шекарасы бойындағы көптеген аймақтар үкіметтің бақылауынан өтіп талибан мен әлкаида қолына түсіп жатыр деген алаңдаушылық туды propaganda,басқа аймақ шабуылда жүздеген мың адам қаш кет хайбер ас арқылы ауғанстан қарай бет ал терминал мен жүк көлік шабуыл өткен жыл баста күшей түсті ауғанстан шекара бой көптеген аймақ үкімет бақылау өт талибан мен әлкаи қол түс жатыр де алаңдаушылық ту propaganda,violent

4,ауғанстан президенті хамид карзай сәрсенбіде екі ел үшін де қауіп төніп тұрғанын ескертті террористер мен экстремистер біздің елдеріміздің барлық аймақтарына таралуда деді карзай астанасы исламабадта өткен аймақтық экономикалық конференцияда propaganda,ауғанстан президент хамид карзай сәрсенбі екі ел үшін де қауіп төн тұр ескер террорис мен экстремист біздің ел барлық аймақ тарал де карзай астана исламабад өткен аймақтық экономикалық конференция propaganda,violent

4,онда куәгерлер талибан көтерілісшілері бақылауда екенін және үйденүйге қанды соғысқа дайын болатынын айтты соңғы қақтығыс шамамен мың адамды үйлерінен кетуге мәжбүр етті сонымен қатар солтүстікбатыстағы әскери қылмыстардан шыққан жарты миллион босқындардан басқа және пәкістанның проблемаларына гуманитарлық төтенше жағдай қосылды propaganda,онда куәгер талибан көтерілісші бақыла екен және үйденүй қан соғыс дайын бол ай соңғы қақтығыс шамамен мың

адам үй кет мәжбүр ет сонымен қатар солтүстікбатыс әскери қылмыс шық жарты миллион босқын басқа және пәкістан проблема гуманитарлық төтенше жағдай қос propaganda,violent

4,сомали ауғанстанға тән азаматтық соғыс бұрынғы мужаһедилер арасындағы күрес кедейлік және мемлекеттік қызметтердің болмауы шейх шариф ахмед пен шейх хасан дахир аввейдің арасындағы шайқас бір жағынан талибан лидері мулла омер мен алькаиданың ал екінші жағынан ахмед шах масуд пен солтүстік альянстың арасындағы жанжалды еске салатыны рас propaganda,сомали ауғанстан тән азаматтық соғыс бұрынғы мужаһеди ара күрес кедейлік және мемлекеттік қызмет болма шейх шариф ахмед пен шейх хасан дахи аввей ара шайқас бір жағ талибан лидер мулл ом мен алькаида ал екінші жағ ахмед шах масуд пен солтүстік альянс ара жанжал ес сал рас propaganda,violent

4,алькаида жылы найроби мен даруссаламдағы ақш елшіліктерін бомбалау сияқты ақш пен батыс африканың мүдделеріне қарсы шабуыл жасағысы келеді алькаида джибутидегі ақш әскери бекеттеріне шабуыл жасайды йемен мен сомалидегі алькаида шығыс африка мен йемендегі операцияларға қатысады бұл халықаралық қоғамдастыққа көп шығын әкеледі егер бұл тоқтатылмаса көз propaganda,алькаи жыл найроби мен даруссалам ақш елшілік бомбала сияқты ақш пен батыс африка мүдде қарсы шабуыл жаса кел алькаи джибути ақш әскери бекет шабуыл жаса йемен мен сомали алькаи шығыс африка мен йемен операция қатыс бұл халықаралық қоғамдастық көп шығын әкел егер бұл тоқтатылма көз propaganda,violent

4,ақш пен пәкістан зымыран шабуылдарын бірлесіп жүзеге асырады ср мамыр gmt ақштың буш әкімшілігі бастағаннан бері ден астам адам соның ішінде қарапайым тұрғындар қаза тапты propaganda,ақш пен пәкістан зымыран шабуыл бірлес жүзеге асыр ср мамыр gmt ақш буш әкімшіліг баста бері ден астам адам соның іш қарапайым тұрғын қаза тап propaganda,violent

4,әскери дереккөздер сәуірден бастап шабуылшылар астана исламабадтан км қашықтықта орналасқан төменгі дир және бунер аудандарынан басталғаннан бері қаза болған көтерілісшілердің санын ге жетті пәкістан халықтық партиясының ppp үкіметі елдің талибанға вашингтон үшін прокси соғыс ретінде емес өзінің өмір сүруі үшін күресіп жатқанын айтады propaganda,әскери дереккөз сәуір баста шабуылшы астана исламабад км қашықтық орналас төменгі ди және бун аудан бастал бері қаза бол көтерілісші сан ге же пәкістан халықтық партия ppp үкімет ел талибан вашингтон үшін прокси соғыс рет емес өзінің өмір сүр үшін күрес жат айт propaganda,violent

4,цитата алдымен abu baraa біреу секторларымен және б карта жасай алады ма бауырым кешіріңіз қазір карта жасауға үлгермеймін бірақ міне мен құрастырған тізім мен оны жадымнан жасадым сондықтан оның қателіктері бар бірақ бұл мүмкіндігінше дәл барлық ақпарат алькавказдан кавказ орталығынан ресми мәлімдемелерден және бейнелерден алынған ағайындылар моблие көшбасшылық тобының қателіктерін түзетуді ұсынады propaganda,цита алд abu baraa біреу сектор және б карта жаса ал ма

бауыр кешір қазір карта жаса үлгерме бірақ міне мен құрастыр тізім мен оны жад жаса сондықтан оның қателік бар бірақ бұл мүмкіндігінше дәл барлық ақпарат алькавказ кавказ орталығ ресми мәлімдеме және бейне алын ағайынды мобли көшбасшылық тоб қателік түзету ұсын propaganda,violent

4,дағыстан амир аль губден әмірі сейфулла әмір шамиль иристон қазір жойылды амир саад кавказ әмірлігінің барлық әмірлері мен қатарларын қорғап оларға жеңіс беріңіз көтерілісшіл ұлтшыл топ амир мансур амир абу бакар эльмурадов propaganda,дағыс ами аль губ әмір сейфулл әмір шамиль иристон қазір жой ами саад кавказ әмірліг барлық әмір мен қатар қорға оларға жеңіс бер көтерілісшіл ұлтшыл топ ами мансу ами абу бак эльмурадов propaganda,violent

4,соғушылар ұққны қансыз басып алуға ұмтылуда thu мамыр gmt ap суреті талибан мұсылман мұсылман ханы талибанға қарсы содырлардың өкілі пәкістанның солтүстікбатыс малаканд бөлімшесінде үкіметтік қызметкерлердің отставкасын талап етіп үш күндік ультиматум жариялады propaganda,соғушы ұқк қан бас ал ұмтыл thu мамыр gmt ap сурет талибан мұсылман мұсылман хан талибан қарсы содыр өкіл пәкістан солтүстікбатыс малаканд бөлімше үкіметтік қызметкер отставка талап ет үш күндік ультимату жариял propaganda,violent

4,үкімет қақтығыс аймақтарынан жүздеген мың адам қашып кетті исламабад соңғы үш апта ішінде соғыс басталған ауғанстанмен шекаралас тайпалық аудандарда көтерілістерді жоюға уәде берді сваттан жер аударылған босқындардың айтуынша ереже аймақты зорлықзомбылықпен басқарады сыншылар мен үкіметтік ақпарат берушілерді өлтіріп қыздар мектептерін қиратады propaganda,үкімет қақтығыс аймақ жүздеген мың адам қаш кет исламабад соңғы үш апта іш соғыс бастал ауғанстан шекаралас тайпалық аудан көтеріліс жою уәде бер сват жер аударыл босқын айт ереже аймақ зорлықзомбылық басқар сыншыл мен үкіметтік ақпарат беруші өлтір қыз мектеп қират propaganda,violent

4,сомали көтерілісшілері үкіметті құлатуға жақын бе мамыр gmt сомалидегі күрескер позицияны ұстанады сомали көтерілісшілері ау қорғанысындағы сомали президенті шейх шариф ахмедтің резиденциясына жақындаған кезде билікті басып алуға жақын соғысушылар үкіметтік күштермен жақында болған қақтығыстарда билікті басып алуға бет алды деп хабарлады пресс тв тілшісі propaganda,сомали көтерілісші үкімет құлат жақын бе мамыр gmt сомали күреск позиция ұстан сомали көтерілісші ау қорғаныс сомали президент шейх шариф ахмед резиденция жақында кезде билік бас ал жақын соғысушы үкіметтік күш жақында бол қақтығыс билік бас ал бет алды де хабарла пресс тв тілші propaganda,violent

4,бізде үкіметті қорғауға жеткілікті күш пен мүмкіндіктер бар біздің әскерлер президент сарайында орналасқан және кезкелген шабуыл болған жағдайда сарайды қорғауға дайын деді бвакира сомали көздері сонымен қатар үкіметшіл күштер бүлікшілердің күшінен кейін үкіметінен кете бастағанын хабарлады rzs sme re presstv propaganda,біз үкімет қорға жеткілікті күш пен мүмкіндік бар біздің әскер президент

сарай орналас және кезкел шабуыл бол жағдай сара қорға дайын де бвакир сомали көз сонымен қатар үкіметшіл күш бүлікші күш кейін үкімет кет баста хабарла rzs sme re presstv propaganda,violent

4,христиандар қаружарақпен крест жорықтары және қарусыз миссионерлермен соғысуда мұндай жедел әрекеттерді болдырмау үшін біз оларға қарсы қаружарақпен жиһадпен және қарусыз соғыс құралдары немесе кезкелген топқа арналған топ соғысуымыз керек христиандар қаружарақ топтары мен миссионерлік топтардың арасында өзара ынтымақтастыққа ие сондықтан олар өз жұмыстарын жоспарлап сәтті орындайды propaganda,христиан қаружарақ крест жорық және қару миссионер соғыс мұндай жедел әрекет болдырма үшін біз оларға қарсы қаружарақ жиһад және қару соғыс құрал немесе кезкел топ арнал топ соғыс керек христиан қаружарақ топ мен миссионерлік топ ара өзара ынтымақтастық ие сондықтан олар өз жұмыс жоспарла сәтті орында propaganda,violent

4,егер олар мұсылман ұйымдарының арасындағы ынтымақтастық пен түсіністік болса біз өз жұмысымызды дұрыс жоспарлау және орындау арқылы өз дінімізді қорғай аламыз егер жиһад мүмкін болса және елде қолайлы болса онда джихад болады және егер сол жерде қолайлы және қолайлы болса онда да ват сияқты болады фрофет сал макка мен мадинада жасады propaganda,егер олар мұсылман ұйым ара ынтымақтастық пен түсіністік бол біз өз жұмыс дұрыс жоспарлау және орындау арқылы өз дін қор ал егер жиһад мүмкін бол және ел қолайлы бол онда джихад бол және егер сол же қолайлы және қолайлы бол онда да ват сияқты бол фрофет сал макк мен мадина жас propaganda,violent

4,амир докка абу усман кафірлердің қылмыстары туралы көктем http blip tv file get kavkazcenterdokka mini wmv mb кавказ орталығы мамыр бүгін сіздерге путин кавказға алып келген сыйлықтар арсеналын көрсеткім келеді путин және оның кавказдағы күзетшілері күн сайын бұқаралық ақпарат құралдары арқылы тәртіп қалай кавказ халықтарына бейбітшілікті қалай әкелетіні туралы жарнамалайды propaganda,ами докк абу усман кафір қылмыс туралы көктем http blip tv file get kavkazcenterdokka mini wmv mb кавказ орталығ мамыр бүгін сіздерге путин кавказ алып кел сыйлық арсенал көрсет кел путин және оның кавказ күзетші күн сайын бұқаралық ақпарат құрал арқылы тәртіп қалай кавказ халық бейбітшілік қалай әкел туралы жарнамала propaganda,violent

4,неге жастар моджахедтерге қосылу үшін орманға бару керек пе себебі бізге қосылғандар жұмақта болғысы келеді ақша үшін басқа ештеңе үшін емес біз ешқандай үгітнасихат таратпаймыз жастар бізге қосылыңыз өйткені бұл жұмаққа апаратын жол сондықтан біз бірге жұмаққа баратын жолды таңдадық демек бізбен бірге болатын адам жұмақта болады http caucasus wordpress com clusterbombs propaganda,неге жас моджахед қосылу үшін орман бару керек пе себебі бізге қосыл жұмақ бол кел ақша үшін басқа ештеңе үшін емес біз ешқандай үгітнасихат таратпа жас бізге қосыл өйткені бұл жұмақ апар жол сондықтан біз бірге жұмақ бар жол таңда

демек бізбен бірге бол адам жұмақ бол http caucasus wordpress com clusterbombs propaganda,violent

4,сұрақ маульви сахиб мырза ең алдымен әмірлік веббінің көрермендеріне жеке куәлікті ұсынуды өтінемін джихад дауысы жауап менің атым маульви нур касим және қазіргі уақытта хост провинциясындағы мужахидтерге арналған әскери қызметкер сұрақ сейсенбіде мүжахидтер хост қаласындағы үкіметтік ғимараттарға сәтті операцияларға шабуыл жасады бұл әлемдік баққа үлкен әсер етті және үкімет пен американдықтар оның болжамынан қатты қорқады propaganda,сұрақ маульви сахиб мырза ең алд әмірлік вебб көрермен жеке куәлік ұсыну өт джихад дауыс жауап менің ат маульви ну каси және қазіргі уақыт хост провинция мужахид арнал әскери қызметкер сұрақ сейсенбі мүжахид хост қала үкіметтік ғимарат сәтті операция шабуыл жас бұл әлемдік бақ үлкен әсер ет және үкімет пен американдық оның болжам қатты қорқ propaganda,violent

4,ауғанстан ислам әмірлігінің ресми өкілі талибан кари мухаммад юсуф ахмади аудандары ел тел оңтүстік батыс және солтүстік біз татуластырушы сиыр олар тонауға болып табылмайтын бірақ сиыр сезінеді емес ақпарат болып табылады мобильді забихулла мужахид елдің оңтүстікшығыс және солтүстікшығыс аймақтары үшін тел ұялы ауғанстан талибан көзі беті джихад дауысы жылғы мамырда ауғанстан ислам әмірлігінің талибанның ресми сайты propaganda,ауғанстан ислам әмірліг ресми өкіл талибан кари мухаммад юсуф ахмади аудан ел тел оңтүстік батыс және солтүстік біз татуластыруш сиыр олар тона бол табылма бірақ сиыр сезін емес ақпарат бол таб мобильді забихулл мужахид ел оңтүстікшығыс және солтүстікшығыс аймақ үшін тел ұялы ауғанстан талибан көз бет джихад дауыс жылғы мамыр ауғанстан ислам әмірліг талибан ресми сай propaganda,violent

4,мұны біреу көрді ме онлайн видео неміс қалаларына groге шабуыл жасауды қауіп төндіреді өйткені әдеттегі діни сілтемелері жоқ интернеттегі видео қауіпті тергеу жүргізуде youtubeтің видеосайтында қысқа метражды фильм германияның үш қаласына террористік шабуыл жасау қаупін төндіруде германияның ішкі істер министрлігі сенбіде бұл туралы растады бірақ фильмдегі діни сөздер болмады пулеметтің жарылуы мен жарылғыш снарядтардың видеотаспасы исламисттерден шыққандығы белгісіз propaganda,мұны біреу көр ме онлайн видео неміс қала gro шабуыл жасау қауіп төндір өйткені әдет діни сілтеме жоқ интернет видео қауіпті тергеу жүргіз youtube видеоса қыс метраж фильм германия үш қала террористік шабуыл жасау қауп төндір германия ішкі істер министрліг сенбі бұл туралы раста бірақ фильм діни сөз болма пулемет жарыл мен жарылғыш снаряд видеотас исламист шыққандығ белгісіз propaganda,violent

4,әсіресе берлин кельн және бремен деді видеодағы мәтін focus сайтына сәйкес германия мен басқа төрт ұлтқа қыркүйектен бастап қиыншылықтар туындайды бір апта бұрын германияға исламистік видеода алькаидаға деген жанашырлықты білдіретін қорқыту айтылды propaganda,әсіресе берлин кельн және бре де видео мәтін focus сайт сәйкес германия мен басқа төрт ұлт қыркүйек баста қиыншылық

туында бір апта бұрын германия ислами видео алькаида де жанашырлық білдір қорқыт айт propaganda,violent

4,ауғанстандағы талибандағы бақ министрі ахмед мохтар мен алжазира каналындағы мұсылман тыңдаушылар арасындағы сұхбаттың маңызды бөліктері мен қысқаша мазмұны мамыр мамыр мамыр бақ министрінің ахмед мохтар әңгімесі талибан қозғалысында al jazeera форумында мұсылман тыңдаушыларымен және көрермендерімен талибан министрінің тыңдаушысы талибан ұлттық күштер қолдайтын ауғанстан үкіметімен отыра ма propaganda,ауғанстан талибан бақ министр ахмед мох мен алжазир канал мұсылман тыңдаушы ара сұхбат маңызды бөлік мен қысқаша мазмұн мамыр мамыр мамыр бақ министр ахмед мох әңгіме талибан қозғалыс al jazeera форум мұсылман тыңдаушы және көрермен талибан министр тыңдаушы талибан ұлттық күш қолда ауғанстан үкімет отыр ма propaganda,violent

4,ұлт құдайдың ережелерінен бас тартуы мүмкін дегенді білдіреді демек біз демократияны қылмыс деп санаймыз исламдық ережені қолдаймыз және құран кітабы мұсылман ұлттарымызды басқаруға тиіс конституция талибан министрінің мұсылман тыңдаушысы талибан пакистан исламдық пакистандық жауынгерлерді ауғанстанға жібере ме талибан министрі иә және біз ауғанстанда әлемнің түкпіртүкпірінен исламдық жауынгерлерді қабылдаймыз propaganda,ұлт құдай ереже бас тарт мүмкін де білдір демек біз демократия қылмыс де сана исламдық ереже қолда және құран кітаб мұсылман ұлт басқар тиіс конституция талибан министр мұсылман тыңдаушы талибан пакис исламдық пакиста жауынгер ауғанстан жібер ме талибан министр иә және біз ауғанстан әлем түкпіртүкпі исламдық жауынгер қабылда propaganda,violent

4,sorce http prisonerofjoy blogspot com исламабад талибанның жоғары лауазымды тұлғаларының бірі шейіт болу операциялары туралы түсіндірме берді забуалллах cnnге берген сұхбатында забиулла мулла омар басқаратын ауғанстандағы талибанның ресми өкілі забиуаллах мужахид сұхбатында біз бұл шәһидтік шабуылдарда ешқашан өзімізге мақсатты нысандарды таба алмаймыз бұл шабуылдар крест жорықтары күштеріне америка және нато қарсы соғысымыздың бөлігі сондықтан бізге осы шахид операциялары қажет болған кезде біз оны жасаймыз propaganda,sorce http prisonerofjoy blogspot com исламабад талибан жоғары лауазым тұлға бірі шейіт болу операция туралы түсіндірме бер забуалллах cnn бер сұхбат забиулл мулл омар басқар ауғанстан талибан ресми өкіл забиуаллах мужахид сұхбат біз бұл шәһид шабуыл ешқашан өз мақсат нысан таб алма бұл шабуыл крест жорық күш америка және нато қарсы соғыс бөліг сондықтан бізге осы шахид операция қажет бол кезде біз оны жаса propaganda,violent

4,пәкістандық талибан мүшелеріне қатысты сұраққа ол егер олар келсе біз оларды жақсы қабылдаймыз деп жауап берді ол сондайақ талибан бірге отыра алатындығын айтты біз екі жолды келіссөздер мен соғысты қолданып жатырмыз олардан біздің елден кетуді сұраймыз біз пікірталас өткізуге дайынбыз бірақ егер олар пікірталас өткізіп таңдау жасауға дайын болмаса қарудың аузымен сөйлесу үшін содан кейін біз

қаруымыздың аузымен сөйлесеміз мұсылмандық propaganda,пәкістандық талибан мүше қатысты сұрақ ол егер олар кел біз ол жақсы қабылда де жауап бер ол сондайақ талибан бірге отыр алатындығ ай біз екі жол келіссөз мен соғ қолдан жатыр олардан біздің ел кету сұра біз пікірталас өткіз дайын бірақ егер олар пікірталас өткіз таңдау жаса дайын болма қар ауз сөйлесу үшін содан кейін біз қару ауз сөйлес мұсылман propaganda,violent

4,біздің өлгендеріміз жұмақта ққк командасы мұса мұқожевтің қайтыс болуын растайды жарияланған уақыты бүгін джохар уақытымен сағат те олардың кейбіреулері анттарын өліммен шайқаста берді және олардың кейбіреулері әлі де күтуде бірақ олар еш өзгерген жоқ біздің бауырларымыз қбк құрама провинциясының наибтері орынбасарлары эмир абу мухаммад мұса мұқожев эмир хамза марат гулиев және ағасы хасан сизажев шухада шәһидтер болды propaganda,біздің өл жұмақ ққк команда мұса мұқожев қайтыс бол раста жариялан уақыт бүгін джох уақыт сағат те олардың кейбіреу ант өл шайқас бер және олардың кейбіреу әлі де күт бірақ олар еш өзгер жоқ біздің бауыр қбк құрама провинция наиб орынбасар эми абу мухаммад мұса мұқожев эми хамза марат гулиев және аға хасан сизажев шуха шәһид бол propaganda,violent

4,біз жылдан астам солтүстік кавказ мұсылмандарының істерімен айналысқан лайықты бауырластарымыздың бізді тастап кеткеніне куә боламыз жылы сәуірде қбк провинциясының хасаня ауылында шейіт болған ағамыз абу усманның зейтун сұлтанов қбк провинциясының наибінің шейіт болғаны туралы ақпарат расталды дереккөз islamdin com кавказ орталығы propaganda,біз жыл астам солтүстік кавказ мұсылман іс айналыс лайықты бауырлас біз таста кет куә бол жыл сәуір қбк провинция хасаня ауыл шейіт бол ағ абу усман зейтун сұлтанов қбк провинция наиб шейіт бол туралы ақпарат раста дереккөз islamdin com кавказ орталығ propaganda,violent

4,әлемдегі кейбір теледидарлар мен жаңалықтар ақш пен ауғанстан үкіметі талибанды ауғанстан үкіметіне жеткізуге күш салуда дейді біз бұл хабарламаны жылдың мамырында барлық халыққа жеткізу үшін жазып отырмыз ауғанстанда шетелдік күштер болған кезде біз ешқашан ауғанстан үкіметімен және ақш үкіметімен ешқашан келіссөздер жүргізе алмайтындығымыз propaganda,әлем кейбір теледидар мен жаңалық ақш пен ауғанстан үкімет талибан ауғанстан үкімет жеткіз күш сал де біз бұл хабарлама жыл мамыр барлық халық жеткізу үшін жаз отыр ауғанстан шетелдік күш бол кезде біз ешқашан ауғанстан үкімет және ақш үкімет ешқашан келіссөз жүргіз алмайтынды propaganda,violent

4,нато күштеріне азапты ауған халқының азаптары мен азаптарын бірнеше долларға және батысқа кіру визасын сатыңыз біз ешқашан қайтыс болған ауған әйелдері мен қайтыс болған ауған балаларын нато күштеріне бірнеше долларға және кіру визасын сатпаймыз батыс саған рахмет біздің хабарламамызды оқып жатыр мамыр propaganda,нато күш аза ауған халқ азап мен азап бірнеше долл және батыс кіру виза сат біз ешқашан қайтыс бол ауған әйел мен қайтыс бол ауған бала нато күш бірнеше

долл және кіру виза сатпа батыс саған рахмет біздің хабарлама оқ жатыр мамыр propaganda,violent

4,эфиопия қолдаған жолдан тайған үкімет жасақтары орталық сомалияның хиран және джайдун аймақтарында бұл шайқастар осы аймақтағы муһахидиин бекіністеріне шабуыл жасағаннан кейін жолдан тайған үкімет пен африка күштеріне қысым жасауды жеңілдету үшін мугадидидің астанасы мужахидидің әскері қоршап алған алғашқы шайқас хиран аймағындағы махас аймағында жылдың мамырында таңертең өтті ұрыста милиционер қаза тауып мужахидтер қаруларын тартып алды radicalization,эфиопия қолда жолдан тай үкімет жасақ орталық сомалия хиран және джайдун аймақ бұл шайқас осы аймақ муһахидиин бекініс шабуыл жаса кейін жолдан тай үкімет пен африка күш қысым жасау жеңілдету үшін мугадиди астана мужахиди әскер қорша ал алғашқы шайқас хиран аймағ махас аймағ жыл мамыр таңертең өт ұрыс милицион қаза тау мужахид қару тарт алды radicalization,violent

4,үкіметтері ирактағы сахват сияқты адал тайпалық отрядтар құру үшін көп жұмыс істеп жатқандығын атап өткен жөн мужахидтер оларды барлық жерде күтіп алып олардың қимылдарын мұқият бақылап отырады шабаба аль мужахидиин медиа бөлімі альасмара әлисламия армиясы мужахидтерге хабарлау және мүміндерді арандату propaganda,үкімет ирак сахват сияқты адал тайпалық отряд құру үшін көп жұмыс істе жатқандығ ата өткен жөн мужахид ол барлық же күт алып олардың қимыл мұқият бақыла отыр шабаб аль мужахидиин медиа бөлім альасмар әлисламия армия мужахид хабарла және мүмін арандат propaganda,violent

4,кавказ блогындағы ағылшын тіліндегі транскрипциясы ретінде кавказ әмірлігінің әмірі докка абу усманның бұл жыл біздің қорлайтын жыл болады һа мұнда қараңыз цитата кавказ орталығы мамыр ағылшын транскрипциясы бүгін путинмен және оның кавказдағы жергілікті кремльдегі протегерлері күзетшілер оларды кезкелген мерзімге шақыру мүмкін емес олар кремльдің күзетшілері олар әрқашан болған және болады propaganda,кавказ блог ағылшын тіл транскрипция рет кавказ әмірліг әмір докк абу усман бұл жыл біздің қорла жыл бол һа мұнда қара цита кавказ орталығ мамыр ағылшын транскрипция бүгін путин және оның кавказ жергілікті кремль протегер күзетші ол кезкел мерзім шақыру мүмкін емес олар кремль күзетші олар әрқашан бол және бол propaganda,violent

4,сонымен біз өзімізге жаңа міндеттер қоямыз иншалла осы жылы бәрімізге жетеміз бұл жыл ресейдің барлық аумағында біздің қорлайтын жыл болады неге менің ойымша бүгінде ресей аумағында тұрып жатқан адамдар өз сарбаздары үшін де басшылығы үшін де сол қатыгездіктер үшін ашуланшақтықтар үшін жасаған әрекеттері үшін және бүгінгі исламға қарсы соғыс үшін де жауап береді propaganda,сонымен біз өз жаңа міндет қоя иншалл осы жыл бәрі же бұл жыл ресей барлық аумағ біздің қорла жыл бол неге менің ойымша бүгін ресей аумағ тұр жат адам өз сарбаз үшін де басшылығ үшін де сол қатыгездік үшін ашуланшақтық үшін жаса әрекет үшін және бүгінгі ислам қарсы соғыс үшін де жауап бер propaganda,violent

4,цитата бастапқыда бауырым кашмирде jihad feesbelilah жоқ ол тек ислам мен жиһад белгісімен ұлтшылдық үшін күреседі слоган кашмир пакистан болады бәрін түсіндіреді пакистанға кашмир қосу үшін күрес пакистандағы кафердің негіздерінің ұл куфар деген мағынасы үстемдік етеді ал swatta антигаистік исламмен күресетін пакистан jihad feesbelilah болады ал джихадты хазратмухаммад салалахуалихиавалиевасаллам шаби тайпа үшін күрескен кезде анықтайды propaganda,цита бастапқы бауыр кашмир jihad feesbelilah жоқ ол тек ислам мен жиһад белгі ұлтш үшін күрес слоган кашмир пакис бол бәрін түсіндір пакистан кашмир қосу үшін күрес пакиста кафе негіз ұл куф де мағына үстемдік ет ал swat антигаи ислам күрес пакис jihad feesbelilah бол ал джихад хазратмухаммад салалахуалихиавалиевасалла шаби тайпа үшін күрес кезде анықта propaganda,violent

4,бұл адамдардың біліміндегі асыра сілтеуді екіншіден кейде халид ибн валид және басқалары аға комапниялардың жасағанындай руларға сәйкес соғысуға болады сондықтан қай жерде белгілі бір мәселе болса бұл мәселеде біршама икемділік бар осы арқылы алуға болатын пайда сондықтан жоғарыдағы сияқты пікірлерді жарияламас бұрын осы мәселелер туралы көбірек шариғи білім алуға кеңес беремін propaganda,бұл адам білім асыра сілтеу екінші кейде халид ибн валид және басқа аға комапния жасағанында ру сәйкес соғыс бол сондықтан қай же белгілі бір мәселе бол бұл мәселе біршама икемділік бар осы арқылы ал бол пайда сондықтан жоғары сияқты пікір жарияламас бұрын осы мәселе туралы көбірек шариғи білім ал кеңес бер propaganda,violent

4,мүгедектердің жолын кесуші дұғаға қарсы ауқымды операция жариялады жарияланған уақыты бүгін джохар уақытымен сағат да ресейлік ақпарат көздері шешен жолдан тайғандардың жетекшісі кадыровқа сенбіде ауқымды операцияның басталғаны туралы хабарлады propaganda,мүгедек жол кесуші дұға қарсы ауқымды операция жариял жариялан уақыт бүгін джох уақыт сағат да ресейлік ақпарат көз шешен жолдан тай жетекші кадыров сенбі ауқымды операция бастал туралы хабарла propaganda,violent

4,антитеррорлық операцияның жойылатыны туралы мәлімдеген болатын джохардағы соңғы оқиғаларға шәһидтердің шабуылына және жыл басынан бері мұжахидтермен жүргізілген әскери операциялар туралы оны әмір докка абуусман өзінің соңғы бейне хабарламасында айтты және оны орыс және қуыршақ топтары таратады кейбір ресейлік сарапшылар кавказдағы үшінші соғыстың басталуын көрді кавказ орталығы propaganda,антитеррорлық операция жойыл туралы мәлімде бол джохар соңғы оқиға шәһид шабуыл және жыл бас бері мұжахид жүргіз әскери операция туралы оны әмір докк абуусман өзінің соңғы бейне хабарлама ай және оны орыс және қуыршақ топ тарат кейбір ресейлік сарапшы кавказ үшінші соғ бастал көр кавказ орталығ propaganda,violent

4,сахнада өздерінің сарайға адалдығы және қауіпсіздік қызметтерімен тығыз қарымқатынасы үшін белгілі болған кейбір газеттердің ізбасарлары күн сайын мұжахидтерде шығатын жалған және жалған жаңалықтар мен қасақана бұрмаланған

ереуілдердің көлемін азайтуға тырысқан таңқаларлық жағдайларға таң қалатынына күмән жоқ мұндағы мужахидтердің тартуы мен әсерін азайтыңыз propaganda,сахна өз сарай адалдығ және қауіпсіздік қызмет тығыз қарымқатынас үшін белгілі бол кейбір газет ізбасар күн сайын мужахид шығ жалған және жалған жаңалық мен қасақан бұрмалан ереуіл көл азайт тырыс таңқаларлық жағдай таң қал күмән жоқ мұндағы мужахид тарт мен әсер азай propaganda,violent

4,http www aviationweek com aw blogs bfba d цитата израиль алдағы төрт жылда зымыранға зымыранға қарсы үш жаңа жүйені орналастыруды жоспарлап отыр израиль алдағы төрт жыл ішінде зымыранға қарсы қорғаныс жүйесінің үш түрлі деңгейіне ие болады propaganda,http www aviationweek com aw blogs bfba d цита израиль алдағы төрт жыл зымыран зымыран қарсы үш жаңа жүйе орналастыру жоспарла отыр израиль алдағы төрт жыл іш зымыран қарсы қорғаныс жүйе үш түрлі деңгей ие бол propaganda,violent

4,қызыл зымыран сигналының басты артықшылығы бұл қазіргі жүйеден алшақтайтын миномет снарядтарын анықтайды дәлелденбеген мәліметтерге сәйкес idf газа секторының айналасында темір күмбезді радар жүйесін орнатқан ол кассамның соққылар орындарын анықтай алады осылайша зымырандар ашық кеңістіктерге түсе бастағанда қажет емес ескертулерді азайтады келесі онжылдықта израиль соғыс жоспарлап отыруы мүмкін бе propaganda,қызыл зымыран сигнал басты артықшылығ бұл қазіргі жүйе алшақта миномет снаряд анықта дәлелденбе мәлімет сәйкес idf газ сектор айнала темір күмбезді радар жүйе орнат ол касса соққы орын анықта ал осылайша зымыран ашық кеңістік түс баста қажет емес ескерту азайт келесі онжылдық израиль соғыс жоспарла отыр мүмкін бе propaganda,violent

4,ауғанстанның оңтүстігінде орналасқан британдық күштер талибан көтерілісшілерімен тұйықталуда және жеңіске жету үшін оларға ондаған мың күштер қажет деп хабарлады елдегі нато күштерінің американдық қолбасшысы қосымша әскери күштер келесі жылы елдегі батыстық стратегияны түбегейлі жөндеуде пайдаланылатын болады бұл соғыс жалғасып жатқан анықтайтын сәт ретінде сипатталған кезең propaganda,ауғанстан оңтүстіг орналас британдық күш талибан көтерілісші тұйықтал және жеңіс жету үшін оларға ондаған мың күш қажет де хабарла ел нато күш американдық қолбасшы қосымша әскери күш келесі жыл ел бат стратегия түбегейлі жөнде пайдаланыл бол бұл соғыс жалғас жат анықта сәт рет сипаттал кезең propaganda,violent

4,ауғанстан президенті хамид карзай ұлыбританияны жергілікті халықты иеліктен шығарып хелманддағы жағдайды ушықтырды деп айыптады бұл провинция ауғанстанның апиын көкнәрінің көбісі өсірілетін жер оның өкілі хумаюн хамидзада біз хельмандтың басым бөлігін талибанға ұтып алдық біз онда аз болдық британдық әскери күштер әскери базалармен ғана шектеліп талибан көбейе түсуде деді http www independent co uk news wo html propaganda,ауғанстан президент хамид карзай ұлыбритания жергілікті халық иелік шығар хелманд жағдай ушық де айыпта бұл провинция ауғанстан апи көкнә көбісі өсіріл жер оның өкіл хумаюн хамидза біз

хельманд басым бөліг талибан ұт ал біз онда аз бол британдық әскери күш әскери база ғана шектел талибан көбей түс де http www independent co uk news wo html propaganda,violent

4,дәйексөз бастапқыда acer автор muwahidbrother assalamualikum ағам acer касмир со жалдызданған джиһадтың tyifa al mansoora шын мужахидендің түрлі құралы деп ойлаймын жакалла талаптану үшін сахаб пәкістандағы діни класс көптеген ондаған жылдардан бері кашмирдегі джихадпен байланысты кашмирдегі джихад және оның үкіметі мен ишим рөлі туралы пікіріңіз қандай мулла назир кашмирдегі джихад және онда құрбандықтар ұсынғандар мұны шын ниетпен жасады propaganda,дәйексөз бастапқы acer автор muwahidbrother assalamualikum аға acer касми со жалдыздан джиһад tyifa al mansoora шын мужахиде түрлі құрал де ойла жакалл талаптан үшін сахаб пәкістан діни класс көптеген ондаған жыл бері кашмир джихад байланысты кашмир джихад және оның үкімет мен иши рөл туралы пікір қандай мулл нази кашмир джихад және онда құрбандық ұсын мұны шын ниет жас propaganda,violent

4,кашмирде джихадтың жоқтығы туралы пакистан үкіметін қолдау ол қазір олай емес оның сенімділігін төмендетпейді сондықтан кашмирде джихад болмайды деп айту біз үшін дұрыс нәрсе емес мұның өзі кашмирге құрбандық шалуды қоқыс шелегіне салу сияқты мұндай мәлімдемелер әлемнің кезкелген жерінде жиһадқа қарсы шыққан адамдарға отын әкеледі және оларды моджахедтерге қиындық туғызу үшін сылтау етеді propaganda,кашмир джихад жоқтығ туралы пакис үкім қолдау ол қазір ола емес оның сенімділіг төмендетпе сондықтан кашмир джихад болма де айту біз үшін дұрыс нәрсе емес мұның өзі кашмир құрбан шалу қоқыс шелег салу сияқты мұндай мәлімдеме әлем кезкел же жиһад қарсы шық адам отын әкел және ол моджахед қиындық туғыз үшін сылтау ет propaganda,violent

4,сомали эфиопияның әскерін байдоадан толық шығарып тастайтынын растайды www chinaview cn басып шығару baidao сомали қаңтар синьхуа эфиопияның соңғы әскери қызметшілері сомалияның оңтүстігінде орналасқан байдоа қаласынан базасын тастап кетті өйткені әлшабаб исламистік қозғалысының күштері қалаға шабуыл жасау қаупін төндіруде деп хабарлады propaganda,сомали эфиопия әскер байдоа толық шығар таста раста www chinaview cn бас шығару baidao сомали қаңтар синьху эфиопия соңғы әскери қызметші сомалия оңтүстіг орналас байдо қала база таста кет өйткені әлшабаб ислами қозғалыс күш қала шабуыл жасау қауп төндір де хабарла propaganda,violent

4,сомалидің уақытша үкіметі мен негізгі оппозициялық коалиция сомалияны қайта азат ету альянсы ars арасында қол жеткізілген кең ауқымды бейбітшілік пен күш бөлу туралы келісімнің бөлігі болып табылады екі тарап қазір сомалияның солтүстікбатыс көршісінің астанасы джибути қаласында өткен жылы қол қойылған келісімде қарастырылған қуатты бөлу туралы келісімді пысықтау үшін кездесуде propaganda,сомали уақытша үкімет мен негізгі оппозициялық коалиция сомалия қайта азат ету альянс ars ара қол жеткіз кең ауқымды бейбітшілік пен күш бөлу туралы келіс бөліг бол таб екі тарап қазір сомалия солтүстікбатыс көрші астана джибути қала

өткен жыл қол қойыл келісімде қарастырыл қуатты бөлу туралы келіс пысықтау үшін кездес propaganda,violent

4,талдау көрсеткендей көтерілісшілер нато әскерлеріне ашық соғыс жағдайында бомбаларға емес жанкештілік шабуылдарына көбірек қарсы тұруда талибан сарбаздары бомбаларға сүйенудің орнына тікелей нысанаға қарай соғуда натоның ауғанстандағы соғыс туралы негізгі хабарламасына қайшы келетін жылдық статистикалық шолуға сәйкес канадалық және басқа шетелдік әскерлердің көпшілік алдында жасаған мәлімдемесінде көтерілісшілер қарсыластарына қарсы тұра алмай тек жарылғыш заттарды жарып жіберуі мүмкін болғандықтан қарқын жоғалуда деген пікір айтылған propaganda,талдау көрсеткендей көтерілісші нато әскер ашық соғыс жағдай бомба емес жанкештілік шабуыл көбірек қарсы тұр талибан сарбаз бомба сүйену орн тікелей нысана қарай соғ нато ауғанстан соғыс туралы негізгі хабарлама қайшы кел жылдық статистикалық шол сәйкес канадалық және басқа шетелдік әскер көпшілік алд жаса мәлімдеме көтерілісші қарсылас қарсы тұр алма тек жарылғыш зат жар жібер мүмкін бол қарқын жоғал де пікір айтыл propaganda,violent

4,араб экстремистері әлі де талибан қатарының маңызды бөлігі емес дегенді білдіруі мүмкін келесі жыл жаһандық джихадшыларды ауғанстанға әкелуі мүмкін алайда америка құрама штаттары өзінің әскери күшжігерінің назарын ирактан аластатады джихадшылардың назары ирактан ауғанстанға ауысады деді мырза кованен биыл біз одан да күрделі шабуылдар мен тактиканы көреміз деп үміттенеміз деді propaganda,араб экстремист әлі де талибан қат маңызды бөліг емес де білдір мүмкін келесі жыл жаһандық джихадшы ауғанстан әкел мүмкін алайда америка құрама штат өзінің әскери күшжіг назар ирак аластат джихадшы назар ирак ауғанстан ауыс де мырза кова биыл біз одан да күрделі шабуыл мен тактика көр де үміттен де propaganda,violent

4,дәйексөз газадағы ирак ислам мемлекеті http www jihadica com theislamic iraqingaza жылдың мамырында фаллужа форумының қатысушысы вахж альрасас жарқылдайтын оқтар ирак ислам мемлекеті isi жақында газада таратылды парақшаларда әбуомар әлбағдадидің тұтқындалғаны туралы жақында жарияланған хабар жалған isi әлбагдадидің болжанған фотосуреттеріндегі адамның жеке басын білмейді және әлбагдади жай ғана істеп жатыр propaganda,дәйексөз газа ирак ислам мемлекет http www jihadica com theislamic iraqingaza жыл мамыр фаллуж форум қатысушы вахж альрасас жарқылда оқ ирак ислам мемлекет isi жақында газа тарат парақша әбуом әлбағдади тұтқындал туралы жақында жариялан хабар жалған isi әлбагдади болжан фотосурет адам жеке басын білме және әлбагдади жай ғана істе жатыр propaganda,violent

4,егер израиль хамасты толығымен шешсе ол жақында холокостта айтқандай онда электрлік вакуум одан да фундаменталистік фразамен толтырылады израильдің стратегиясы хамасты толығымен жоюдың орнына оны қамту болып табылады осыдан кейін не істеу керек бұл кезкелген адамның жорамалы бірақ ол газаны толық көлемде басып алуға дайын болмайынша мұны жалғастыруы мүмкін propaganda,егер израиль

хамас толығ шеш ол жақында холокост айтқанда онда электрлік вакуум одан да фундаментали фраза толтыр израиль стратегия хамас толығ жою орн оны қамту бол таб осы кейін не істеу керек бұл кезкел адам жорамал бірақ ол газа толық көлем бас ал дайын болмайынша мұны жалғастыр мүмкін propaganda,violent

4,могадишо сейсенбі қаңтар smc жаппай митингі сейсенбі күні таңертең докторда өтті аюд байдоа қаласындағы ойын алаңында сомалидің оңтүстігіндегі бай аймағының жетекшілері сомалінің астанасы могадишудан шамамен км қашықтықта және альшабабтың бірқатар атақты шенеуніктері ойын алаңына байдоа тұрғындарына қалаға келгендіктерін түсіндіруді өтінді propaganda,могадишо сейсенбі қаңтар smc жаппай митинг сейсенбі күн таңертең доктор өт аюд байдо қала ойын алаң сомали оңтүстіг бай аймағ жетекші сомал астана могадишу шамамен км қашықтық және альшабаб бірқатар атақты шенеунік ойын алаң байдо тұрғын қала келгендік түсіндіру өт propaganda,violent

4,сіздің бұдан былайғы соғысуларыңыз сіздің ісәрекеттеріңізді әдеттегідей жасамайды және біз исламдық заң шығарамыз өйткені адамдарға үкім шығарған дұрыс ол ескі куәліктерді немесе жаңа айғақтарды айтқан библия сияқты емес бұл ешқандай өзгеріс болған жоқ және оған оған жаңа ештеңе қосуға болмайды өйткені бұл луканың немесе матваның сөздері емес құдайдың сөздері деді әлшабабтың өкілі шейх муктар робов абумансур тұрғындары байдоа қаласы propaganda,сіздің бұдан былайғ соғысу сіздің ісәрекет әдеттегідей жасама және біз исламдық заң шығар өйткені адам үкім шығар дұрыс ол ескі куәлік немесе жаңа айғақ айт библия сияқты емес бұл ешқандай өзгеріс бол жоқ және оған оған жаңа ештеңе қос болма өйткені бұл лука немесе матва сөз емес құдай сөз де әлшабаб өкіл шейх мук робов абумансу тұрғын байдо қала propaganda,violent

4,мен масудтың адамына қарсы шайқас тахардағы дештарчиде болған кезде болдым ақш ұшақтары бұл жерді жоғарыдан бомбалаған ал төменде моджахедтер соғысқан бомба жасалғанына қарамастан мужахидтер бір бункер қалдырған жоқ тіпті масудтың адамдары әрқайсысы сымсыз телефон арқылы біз олармен соғысып жатырмыз ал американдықтар жоғарыдан бомбалады radicalization,мен масуд адам қарсы шайқас тахар дештарчи бол кезде бол ақш ұшақ бұл же жоғары бомбала ал төменде моджахед соғыс бомба жасал қарамастан мужахид бір бункер қалдыр жоқ тіпті масуд адам әрқайсы сы телефон арқылы біз олармен соғыс жатыр ал американдық жоғары бомбал radicalization,violent

4,жаһандық куфр ислам әмірлігіне шабуыл жасап бүкіл ауғанстанды күйреткен кезде исламға қарсы сатқындар крест жорығының мұсылмандарға қарсы әрекетін қолдады және американдыққа ауғанстанның барлық әскери құпиялары туралы хабарлады бұл замандарда хижра пайғамбарлық дәстүрін қайта жандандырған және аяттың тірі үлгісі болған адамдар ауғанстанға өмір сүріп шариғатқа сәйкес өмір сүріп джихад туын көтеріп ауғанстан ислам әмірлігінде тұру үшін жалғастырады propaganda,жаһандық куф ислам әмірліг шабуыл жаса бүкіл ауғанстан күйрет кезде ислам қарсы сатқын крест жорығ мұсылман қарсы әрекет қолда және американдық

ауғанстан барлық әскери құпия туралы хабарла бұл заман хижр пайғамбарлық дәстүр қайта жандандыр және ая тірі үлгі бол адам ауғанстан өмір сүр шариғат сәйкес өмір сүр джихад ту көтер ауғанстан ислам әмірліг тұру үшін жалғастыр propaganda,violent

4,дағыстанның барлық этникалық топтары тең өкіл сонымен бірге кейбір шариғат мүшелері дағыстанның сыртында тіпті ресейден де келгендігін мойындады жылдың қазан айында дағыстанның ішкі істер министрлігі жылдан бері заңсыз қарулы құрылымның мүшесі болып келген ахмед гамзатұлы гасановты мүмкін грузияның тез тіршілік ететін авар азшылығының мүшесі деп аталған грузия азаматын ұстады деп мәлімдеді propaganda,дағыстан барлық этникалық топ тең өкіл сонымен бірге кейбір шариғат мүше дағыстан сырт тіпті ресей де келгендіг мойында жыл қазан ай дағыстан ішкі істер министрліг жыл бері заңсыз қарулы құрыл мүше бол кел ахмед гамзатұл гасанов мүмкін грузия тез тіршілік ет ав азшылығ мүше де атал грузия азамат ұста де мәлімде propaganda,violent

4,өкілі өткен жылы азаттық радиосына бұл топтың үгітнасихат жұмыстары сәтті жүргізіліп жатқанын және жихадқа тікелей қатысып өз өмірлерін қатерге ұшыратпайтын көптеген мұсылмандар бізге көмектесу үшін қолдан келгеннің бәрін жасайды деді ол ішкі саясатты қозғайтын плутократия мен сыбайлас жемқорлық полиция мен қауіпсіздік органдарының өз еркімен қуғынсүргінімен қатар қарсылыққа деген көзқарасты одан сайын арттыруға итермелейтінін айтты propaganda,өкіл өткен жыл аза радио бұл топ үгітнасихат жұмыс сәтті жүргізіл жат және жихад тікелей қатыс өз өмір қат ұшыратпа көптеген мұсылман бізге көмектес үшін қолдан кел бәрін жаса де ол ішкі саясат қозға плутократия мен сыбайлас жемқорлық полиция мен қауіпсіздік орган өз ерк қуғынсүргін қатар қарсылық де көзқарас одан сайын арттыр итермеле ай propaganda,violent

4,талибан мен алькаиданың ықпалы шынымен де олардың топтары сияқты көп қырлы сват және тайпалық аймақтар сияқты жерлер бар оларда талибтердің бақылауы өмір фактісі және олар күндіз жұмыс істейді пешавар сияқты басқа жерлерде олар бар бірақ мұны тек сезінуге болады көрінбейді малаканд агенттігі менің бағытымда болды маған мұнда бір де бір талибан жоқ бірақ қорқыныштан олар жазуларын қояды деп айтты келісім ерекше ереже дереккөз asia times online propaganda,талибан мен алькаида ықпал шын де олардың топ сияқты көп қырл сват және тайпалық аймақ сияқты жер бар ол талиб бақыла өмір факті және олар күндіз жұмыс істе пешав сияқты басқа жер олар бар бірақ мұны тек сезін бол көрінбе малаканд агенттіг менің бағыт бол маған мұнда бір де бір талибан жоқ бірақ қорқыныш олар жазу қоя де ай келісім ерекше ереже дереккөз asia times online propaganda,violent

4,дәл сол шайқас мұсылман үмбетінің соңғы антихристиан шайқасына дейін жалғасады жақсы мен бұрысты бір уақытта келісімге салу мүмкін емес олардың бір шаңырақ астында өмір сүруі мүмкін емес осылардың бірі мұны шақырады өйткені қазіргі адамдардың көпшілігі келеді propaganda,дәл сол шайқас мұсылман үмб соңғы антихристиан шайқас дейін жалғас жақсы мен бұр бір уақыт келісім салу мүмкін емес

олардың бір шаңырақ аст өмір сүр мүмкін емес осы бірі мұны шақыр өйткені қазіргі адам көпшіліг кел propaganda,violent

4,бұл әлемде өркендеу мен прогреске жету журналистер өз жұмысының сенімділігі мен әділеттілігін құрметтеуі керек ислам тарихын және үмбет тарихын жамандамаңыз егер сіз шындықты айта алмасаңыз онда оны өзгертуді доғарыңыз және үмметке қысым жасамаңыз рахмет www alqimmah net әмір бинт хамидінен өтті қоршаған орта propaganda,бұл әлем өркендеу мен прогрес жету журналист өз жұмыс сенімділіг мен әділеттіліг құрметте керек ислам тарих және үмбет тарих жамандама егер сіз шындық ай алма онда оны өзгерту доғар және үммет қысым жасама рахмет www alqimmah net әмір бинт хамид өт корша орта propaganda,violent

4,адис абаба afp африка одағы жұмада бұұ қауіпсіздік кеңесін сомалидегі исламшыл көтерілісшілерге қолдау көрсеткені үшін эритреяға санкциялар салуға жедел шаралар қабылдауға шақырды ол нақты түрде сомалиде әуе шығаратын аймақ орнатуды және эритреяға порттар мен әуежайларды қоршауды және санкциялауды сұрады propaganda,адис абаб afp африка одағ жұма бұұ қауіпсіздік кеңес сомали исламшыл көтерілісші қолдау көрсет үшін эритрея санкция сал жедел шара қабылда шақ ол нақты түр сомали әуе шығар аймақ орнату және эритрея порт мен әуежай қоршау және санкциялау сұр propaganda,violent

4,олар негізінен шабабтың радикалды тобының жауынгерлерінен құралған олардың басшылары әлкаидаға байланысы бар деп күдіктелген және хезб әлисламия оппозиция жетекшісі шейх хасан дахир авейске адалдық білдіретін басқа қарулы ұйым жеті миллион халқы бар сомалиде жылы бұрынғы президент мохамед сиад барре тақтан түсіп қарсылас фракциялар арасындағы қанды қақтығыстар басталғаннан бері тиімді орталық билік жоқ propaganda,олар негіз шабаб радикал тоб жауынгер құрал олардың басшы әлкаида байланыс бар де күдіктел және хезб әлисламия оппозиция жетекші шейх хасан дахи авейс адалдық білдір басқа қарулы ұйым жеті миллион халқ бар сомали жыл бұрынғы президент мохамед сиад барр тақ түс қарсылас фракция ара қан қақтығыс бастал бері тиімді орталық билік жоқ propaganda,violent

4,маған келесі әңгімені ұнатамын цитата миналар бомбалар және жарылғыш заттар туралы ақпарат стандартты жарылғыш заттарды алу және тасымалдау қиын болғандықтан әсіресе батыста форумдарда қолдан жасалған бомбалар шығарумен айналысады мысалы жылдың ақпан айының басында исламистік альмаарик форумының мүшелері бір апта бойы үйде жасалған жарылғыш заттарды өндіру туралы пікірталас өткізді propaganda,маған келесі әңгіме ұна цита мина бомба және жарылғыш зат туралы ақпарат стандартты жарылғыш зат алу және тасымалдау қиын бол әсіресе батыс форум қолдан жасал бомба шығар айналыс мысал жыл ақпан ай бас ислами альмаарик форум мүше бір апта бойы үй жасал жарылғыш зат өндіру туралы пікірталас өт propaganda,violent

4,палестинадағы бауырларымыз сионистік меркаваны цистернаны жою үшін кг несепнәр нитратын қолданды сондайақ мужджахид рамзи юсуфты ұмытпаңыз қазір кім түрмеде дүниежүзілік сауда орталығының жертөлесін жару үшін кило мен бір тонна несепнәр нитраты пайдаланылды lol бұл шошқалар оны мемри твда өңдеуі мүмкін болса да кәсіби сияқты естіледі propaganda,палестина бауыр сиони меркава цистерна жою үшін кг несепнә нитрат қолда сондайақ мужджахид рамзи юсуф ұмытпа қазір кім түрме дүниежүзілік сауда орталығ жертөле жар үшін кило мен бір тонна несепнә нитрат пайдалан lol бұл шошқа оны мемри тв өңде мүмкін бол да кәсіби сияқты ест propaganda,violent

4,қабул afp абдул шафик шамамен жаста және отбасылық өмірін екі нәрсе үшін құрбан етті құран оқу және күрес жылы ақштың ауғанстанға шабуылынан кейінгі қуғыннан кейін бұл талибан қолбасшысы өзінің туылған тауларына оралып өзінің ескі өмірін отбасымен бірге бір миссияға қалдырды кәпір американдықтарды қуып шықты propaganda,қабул afp абдул шафик шамамен жас және отбасылық өмір екі нәрсе үшін құрбан ет құран оқу және күрес жыл ақш ауғанстан шабуыл кейінгі қуғын кейін бұл талибан қолбасшы өзінің туыл тау орал өзінің ескі өмір отбас бірге бір миссия қал кәпір американдық қу шық propaganda,violent

4,бәрінен бұрын ислам үшін күрескер болған өзін шафик әйелі мен бес жасқа дейінгі үш баласын әрең көреді ол теледидарды исламға қарсы деп айыптайды және ешқашан интернетті қолданбаған соғыс туралы сөз қозғағанда ол өзінөзі өлтіруді жақсы қару деп атайды және қарапайым адамдарға зиян тигізбеу керек дейді propaganda,бәрі бұрын ислам үшін күреск бол өз шафик әйел мен бес жас дейінгі үш бала әре көр ол теледи ислам қарсы де айыпта және ешқашан интернет қолданба соғыс туралы сөз қозға ол өзінөз өлтіру жақсы қару де ата және қарапайым адам зиян тигізбе керек де propaganda,violent

4,сомалидегі көтерілісшілердің ықпалды жетекшісі жұма күні эритрея үкіметке қарсы көтерілісшілерді қасиетті соғыста қолдады могадишудың солтүстігіндегі кеңсесінде сөйлеген шейх хасан сомалияның жаңа үкіметі мен президенті шейх ахмедті құлату үшін күресіп жатқан бүлікке бірнеше араб жауынгерлері қосылды деп мәлімдеді елордада жұма күні болған ауыр шайқас кем дегенде адамның өмірін қиды және адамды жараланды бұл екі аптадан астам уақытқа созылған қарулы шайқастардағы ең көп өлімжітім propaganda,сомали көтерілісші ықпалды жетекші жұма күн эритрея үкімет қарсы көтерілісші қасиетті соғыс қолда могадиш солтүстіг кеңсе сөйле шейх хасан сомалия жаңа үкімет мен президент шейх ахмед құлат үшін күрес жат бүлік бірнеше араб жауынгер қос де мәлімде елорда жұма күн бол ауыр шайқас кем де адам өмір қи және адам жарала бұл екі апта астам уақыт созыл қарулы шайқас ең көп өлімжі propaganda,violent

4,алькаидамен байланысы бар деген күдікпен бұұ мен ақш лаңкестік тізімінде тұрған уэйвс сәуір айында эритреядан сомалиге оралды ахмед пен рулық ақсақалдардың елшілері оны президентпен татуласуға көндіруге тырысты бірақ нәтиже шықпады палестина ауғанстан және эритрея үш онжылдықтан астам уақытқа созылған соғыс

пен қиындықтарға төтеп берді деді ол біз жақында үкіметті жеңеміз жарақат алғандар мен бауырларын жоғалтқандарға шыдамдылық берсін бізге аз уақыт қалды propaganda,алькаида байланыс бар де күдік бұұ мен ақш лаңкестік тізім тұр уэйвс сәуір ай эритрея сомали ора ахмед пен рулық ақсақал елші оны президент татулас көндір тырыс бірақ нәтиже шықпа палестин ауғанстан және эритрея үш онжылдық астам уақыт созыл соғыс пен қиындық төте бер де ол біз жақында үкімет же жарақат ал мен бауыр жоғалт шыдамдылық бер бізге аз уақыт қал propaganda,violent

4,мұхаммед асад альтамими түбегейлі өзгерткен американдықтар сомали әлкарадави және шейх шариф ислам соттары одағы uic күштері сомалияның көптеген жерлерін соның ішінде астананы тез арада басып алған кезде олар өздері басқарған барлық аймақтар үшін қауіпсіздік пен қауіпсіздікті қалпына келтіре алды propaganda,мұхаммед асад альтамими түбегейлі өзгерт американдық сомали әлкарадави және шейх шариф ислам сот одағ uic күш сомалия көптеген жер соның іш астана тез ара бас ал кезде олар өз басқар барлық аймақ үшін қауіпсіздік пен қауіпсіздік қалп келтір алды propaganda,violent

4,бұл адамдарға қарсы тұру керек олардың ұлтқа қарсы ниеттері мен қауіпқатері әшкереленуі керек өйткені өз құқығын қорғауға үнсіз отырған адам мылқау шайтан сияқты тиісінше біз құдайға тек олардан қорқатындықтан және аналарды қорлаудан қорықпайтындықтан олармен бетпебет келеміз деп уәде береміз мұндай қарсы тұру исламның ұлы шайқасы аясында сонымен біз исламның бірінші кезекте бағытталғандығын білуіміз керек propaganda,бұл адам қарсы тұру керек олардың ұлт қарсы ниет мен қауіпқа әшкерелен керек өйткені өз құқығ қорға үн отыр адам мылқа шай сияқты тиісінше біз құдай тек олардан қорқатындық және ана қорлау қорықпайтындық олармен бетпебет кел де уәде бер мұндай қарсы тұру ислам ұлы шайқас ая сонымен біз ислам бірінші кезек бағытталғандығ біл керек propaganda,violent

4,бұл кавказ әмірлігінің әмірі докка абу усман мен бурятиялық шейх сайидтің гимри ауылындағы вилаят дағыстан мұсылмандарға үндеуінің жылғы зулхижжа желтоқсан докка абу усман жұмысқа кірісу мен дағыстандық ағайындарға барлық дағыстандықтарға жүгінемін propaganda,бұл кавказ әмірліг әмір докк абу усман мен бурятиялық шейх сайид гимри ауыл вилаят дағыс мұсылман үндеу жылғы зулхижж желтоқсан докк абу усман жұмыс кірісу мен дағыстандық ағайын барлық дағыстандық жүг propaganda,violent

4,сіздің бүгінгі басты миссияңыз джихад жасау сондықтан мен сендерді сол жолдан тайғандардың екіжүзділердің ықпалына түспеуге шақырамын мен баршаңызды осы мерекемен тағы да құттықтаймын осы кезде мен хатымды аяқтаймын содан кейін біз гимри ауылының тұрғындарына хабарлама жіберуді сұрап хат алдық және онда болып жатқан оқиғалар біз үшін ауыр тиеді және біз өз бауырларымыз қалай болғанда да біз жеңіске жетуге дайынбыз және жеңіс келеді recruitment,сіздің бүгінгі басты миссия джихад жасау сондықтан мен сен сол жолдан тай екіжүзді ықпал түспе шақыр мен барша осы мереке тағы да құттықта осы кезде мен хат аяқта содан кейін біз гимри

ауыл тұрғын хабарлама жіберу сұра хат ал және онда бол жат оқиға біз үшін ауыр ти және біз өз бауыр қалай болғанда да біз жеңіс жет дайын және жеңіс кел recruitment,violent

4,дәйексөз біреу бір күні оның жойылатынын түсініп пәкістандық барлау мен армияға шабуыл жасау пәкістанды жояды кашмир мен ауғанстан туралы алаңдамаңыз өзіңіз туралы уайымдаңыз және ақида кашмирдегі джихадты алқаида басшылары бірнеше рет сынайды өйткені бұл пакистандық жолдан тайған армия мен үкіметтің туы астында екіншіден ауғанстанда соғысып жатқан мужахидтер propaganda,дәйексөз біреу бір күн оның жойыл түсін пәкістандық барлау мен армия шабуыл жасау пәкістан жоя кашмир мен ауғанстан туралы алаңдама өз туралы уайымда және ақи кашмир джихад алқаи басшы бірнеше рет сына өйткені бұл пакиста жолдан тай армия мен үкімет ту аст екінші ауғанстан соғыс жат мужахид propaganda,violent

4,ислам құқығының орындалуы пәкістан армиясы олар мәлімдегендей шариғатты орындау үшін емес оның алдын алу үшін құрылды және құрылды оны жүзеге асырғысы келетіндерге көмектесу емес олармен күресу және исламнан тыс билеушілерді шығарып тастау емес оларды нығайту және олармен күресу propaganda,ислам құқығ орындал пәкістан армия олар мәлімдегенде шариғат орындау үшін емес оның алдын алу үшін құр және құр оны жүзеге асыр кел көмектес емес олармен күресу және ислам тыс билеуші шығар тастау емес ол нығайту және олармен күресу propaganda,violent

4,ауғанстан ислам әмірлігінің орынбасары амерул муминин біздің сүйікті amp құрметті ағай ахунд sark журналына берген эксклюзивті сұхбат біздің мұсылман ұлтымыздың шексіз құрбандықтары мен өршіл басқыншылар ащы шындықты қабылдаудан бас тартты егер біз ауғанстандағы оқу орындарын өртеп жіберсек онда пайыз болмас еді оқу орындары қалды propaganda,ауғанстан ислам әмірліг орынбасар амерул муминин біздің сүйікті amp құрметті аға ахунд sark журнал бер эксклюзив сұхбат біздің мұсылман ұлт шексіз құрбандық мен өршіл басқыншы ащ шындық қабылдау бас тар егер біз ауғанстан оқу орын өрте жібер онда пайыз болмас ед оқу орын қал propaganda,violent

4,мен барлық мұсылмандардан ерекше назар аударуларыңызды сұраймын жау айлаамалға алдауға толы және ол бізді дос деп айтады олардың аузынан айтқан сөздері іс жүзінде орындалмайды оның кеудесінде жасыратын нәрсе мүлдем басқаша және оның мақсаттары жасырын бірақ біз шындықты білеміз сәттілікке жетудің жолын білеміз және бұл джихадтың құрметті жолы ауғанстан ислам әмірлігі recruitment,мен барлық мұсылман ерекше назар аудару сұра жау айлаамал алда толы және ол біз дос де айт олардың ауз айт сөз іс жүз орындалма оның кеуде жасыр нәрсе мүлдем басқаша және оның мақсат жасырын бірақ біз шындық біл сәттілік жету жол біл және бұл джихад құрметті жол ауғанстан ислам әмірліг recruitment,violent

4,дәйексөз бастапқыда реферист иинфакт егер сіз hafiz usman shaheed бейнесін көрген болсаңыз вид айман ал завахири деді біз американдық консолидацияға қарсы

әрекет жасадық және кезкелген бейбіт тұрғынға зиян тигізбеуге бел будық ауғанстандағы соғыс зымыраншабуылдары бұл жерде шынымен жақсы адамдар келеді ауғанстанға қайтып оралмайды propaganda,дәйексөз бастапқы реферист иинфакт егер сіз hafiz usman shaheed бейне көр бол вид айман ал завахири де біз американдық консолидация қарсы әрекет жаса және кезкел бейбіт тұрғын зиян тигізбе бел бу ауғанстан соғыс зымыраншабуыл бұл же шын жақсы адам кел ауғанстан қайт оралма propaganda,violent

4,мулла мұхаммед омар ислам әмірлігінің негізін қалап шариғат өсиеттерін орындауға кіріскен кезде дәл осы агенттіктер өздерінің ислам әмірлігін танығандықтарын мәлімдеді бірақ жаһандық куфр ислам әмірлігіне шабуыл жасап бүкіл ауғанстанды күйреткен кезде исламға қарсы сатқындар крест жорығының мұсылмандарға қарсы әрекетін қолдады және американдыққа ауғанстанның барлық әскери құпиялары туралы хабарлады propaganda,мулл мұхаммед омар ислам әмірліг негіз қала шариғат өсиет орында кіріс кезде дәл осы агенттік өз ислам әмірліг танығандық мәлімде бірақ жаһандық куф ислам әмірліг шабуыл жаса бүкіл ауғанстан күйрет кезде ислам қарсы сатқын крест жорығ мұсылман қарсы әрекет қолда және американдық ауғанстан барлық әскери құпия туралы хабарла propaganda,violent

4,бұлар мунафиктер емес куфарлар өйткені олар куфарға исламға қарсы көмектесуде мунафикандар ухадтан оралды бірақ куфар күштеріне қосылмаңыз сіз шейх улислам мохд бин абдул ваххабтың айтқан нақиғат исламын оқып түсініктеме беруіңіз керек кешіріңіз деді ура ескі мүше бірақ сізде ислам және куфар туралы негізгі білімі жоқ бұл дұрыс айтады және барлық мужахидтер мен ұлдар дұрыс емес бұл соғысты пакистан армиясы бастайды мужахидтер емес propaganda,бұлар мунафик емес куфар өйткені олар куф ислам қарсы көмектес мунафикан ухад ора бірақ куф күш қосылма сіз шейх улисла мохд бин абдул ваххаб айт нақиғат ислам оқ түсініктеме бер керек кешір де ура ескі мүше бірақ сіз ислам және куф туралы негізгі білім жоқ бұл дұрыс айт және барлық мужахид мен ұл дұрыс емес бұл соғ пакис армия баста мужахид емес propaganda,violent

4,брендік аударма соңғы кездері тырнаузда кафиров пен муртадовқа қарсы мужахидиндердің жауынгерлік операциялары көбейді тырнаузадан имараттықтардың тұрғындары өтеді біздің қалада құжаттарға аң аулауға барыңыз аң аулауға барыңыз сұр жыртқыштар матерыхтар мен күшіктер менің қаламда аптасына рет блюстителей тәртіпке шабуыл жасалады propaganda,бре аударма соңғы кез тырнауз кафиров пен муртадов қарсы мужахидин жауынгерлік операция көбей тырнауза имараттық тұрғын өт біздің қала құжат аң аула бар аң аула бар сұр жыртқыш матерых мен күшік менің қала апта рет блюстителе тәртіп шабуыл жасал propaganda,violent

4,қаланың достары жауынгерлерді үнемі іздеп жаңалық іздейді қала бойынша күні бойы кім және не қашып жатқанын барлығы жүгірушілерді дөңгелектейді әсіресе иелері жоқ төлқұжат қалада ешқашан төлқұжаты жоқ астанада вилайята жауап алу үшін алып жүріңіз маусым қайтадан отты алмастырды мусоровтың екі жеңіл қорқынышы бар үшіншісі щек мониторинг бөлімі zc propaganda,қала дос жауынгер

үнемі ізде жаңалық ізде қала бойынша күн бойы кім және не қаш жат барлығ жүгіруші дөңгелекте әсіресе ие жоқ төлқұжат қала ешқашан төлқұжат жоқ астана вилайя жауап алу үшін алып жүр маусым қайта от алмас мусоров екі жеңіл қорқыныш бар үшінші щек мониторинг бөлім zc propaganda,violent

4,мен өзімді тазарттым егер мен барлық сөздерім мен жауаптарымды түрлітүрлі сөйлемдермен қайталап айтсам бұл ақымақтық болар еді мен мұсылман екенімді дәлелдеу үшін келген жоқпын мен мұсылманмын және мужахид маған өз тәжірибелеріммен және өзім білетін нәрселермен бөлісудің қажеті жоқ мұндағы көпшілігіміз міндетті түрде түсінетін болар едік propaganda,мен өз тазарт егер мен барлық сөз мен жауап түрлітүрл сөйлем қайтала айт бұл ақымақтық бол ед мен мұсылман екен дәлелде үшін кел жоқ мен мұсылман және мужахид маған өз тәжірибе және өзім біл нәрсе бөлісу қажет жоқ мұндағы көпшілік міндетті түр түсін бол едік propaganda,violent

4,біз үндістандық ұшақтарды ұрлап алған исламның ұлдарыбыз босатылған ағайынды масуд ажар заргар шайх біз үнді парлиментіне шабуыл жасадық промис біз бір күні имам mehdi туын көтеріп мүжахидтер қатарында боламыз және индияның жерін тегін осы жерде және дәлінің қызыл форттасында ислам жарығын мындан көреміз propaganda,біз үндістан ұшақ ұрла ал ислам ұлдары босатыл ағайынды масуд ажар зарг шайх біз үн парлимент шабуыл жаса промис біз бір күн имам mehdi ту көтер мүжахид қат бол және индия жер тегін осы же және дәл қызыл фортта ислам жарығ мын көр propaganda,violent

4,ауғанстан ислам әмірлігінің фарах провинциясындағы жазықсыз адамдардың жаппай азап шеккендігі туралы мәлімдемесі және құдай зұлымдардың не істегенін білмейді деп ойламайды бірақ ол адамның диагноз қойылған күніне дейін қалдырады мұсылман мүжахед халқы бейбітшілік пен дамуды насихаттайтындар фарах провинциясының балаболок ауданындағы ганджабад және грани ауылдарында біздің қорғансыз және жазықсыз отандастарымызды олардың көпшілігі әйелдер балалар мен қарттарды тағы бір рет құртты propaganda,ауғанстан ислам әмірліг фарах провинция жазық адам жаппай азап шеккендіг туралы мәлімде және құдай зұлым не істе білме де ойлама бірақ ол адам диагноз қойыл күн дейін қалдыр мұсылман мүжахед халқ бейбітшілік пен даму насихатта фарах провинция балаболок аудан ганджабад және грани ауыл біздің қор және жазық отандас олардың көпшілігі әйел бала мен қарт тағы бір рет құр propaganda,violent

4,осы мүмкіндікті пайдаланып ислам әмірлігі тағы бір рет әлсіз кабул әкімшілігінің жетекшісі мен оның шексіз шенеуніктерін өздерінің қорқақ көмекшілері мен жанкештілерге көрсеткен қолдауының ауыр зардаптары туралы толықтай білуге шақырады олар өздерінің осы қасіретті қылмыстары үшін өздерінің можах ұлтының алдында жауапқа тартылатындығын бір күн білуі керек олар өздерінің болашағын шетелдіктердің қолдаушысы ретінде бағалап өздерін құтқаруды ойлауы керек propaganda,осы мүмкіндік пайдалан ислам әмірліг тағы бір рет әлсіз кабул әкімшіліг жетекші мен оның шексіз шенеунік өз қорқақ көмекші мен жанкешті көрсет қолдау

ауыр зардап туралы толықта біл шақыр олар өз осы қасірет қылмыс үшін өз можах ұлт алд жауап тартылатындығ бір күн біл керек олар өз болашағ шетелдік қолдаушы рет бағала өз құтқару ойла керек propaganda,violent

4,бұл крест жорығының құбыжықтары ауғанстанның азап шеккен халқына қарсы осындай қатыгез және әділетсіз әрекетті бірінші рет жасамаса бұл адамгершілікке жат әрекет ауғанстандағы адамдардың ұятсыз адамзатқа қарсы қылмыстарындағы соңғы ауыр қылмыс болмайды олар ауғанстанның әртүрлі аймақтарындағы мыңдаған қорғансыз адамдардың жаппай шейіт болуларымен жарақаттарымен үйлерін қиратумен және баспаналарын қиратумен әрдайым адамзатқа қарсы осындай әрекет жасағанда және жазықсыз әйелдерді балалар мен қарт адамдарды әуе соққыларында өлтіреді propaganda,бұл крест жорығ құбыжық ауғанстан азап шек халқ қарсы осындай қатыгез және әділет әрекет бірінші рет жасама бұл адамгершілік жат әрекет ауғанстан адам ұят адамзат қарсы қылмыс соңғы ауыр қылмыс болма олар ауғанстан әртүрлі аймақ мыңдаған қор адам жаппай шейіт болу жарақат үй қират және баспана қират әрдайым адамзат қарсы осындай әрекет жаса және жазық әйел бала мен қарт адам әуе соққы өлтір propaganda,violent

4,қауіпсіздікті және жан тыныштығын сезінетін сәттерді ешкім сезінбейтін реакция бүкіл әлемде мүмкіндікті пайдалану үшін ислам әмірлігі қабілетсіз кабул әкімшілігінің басшысын және қызғаныш танытпайтын мүшелерін ынтымақтастықтың салдарын және ауған мұсылман халқын өлтіруде басқыншы құбыжықтарды қорқақ қолдауға шақырады бұл мүжахидтердің құқығы және олар шетелдіктермен қызметтес болғандардың сұмдық аяқталғанын ескере отырып өздерінің болашақтарын жақсы есептейді және олар одан құтылуды көздейді propaganda,қауіпсіздік және жан тыныштығ сезін сәт ешкім сезінбе реакция бүкіл әлем мүмкіндік пайдалану үшін ислам әмірліг қабілет кабул әкімшіліг басшы және қызғаныш танытпа мүше ынтымақтастық сал және ауған мұсылман халқ өлтір басқынш құбыжық қорқақ қолда шақыр бұл мүжахид құқығ және олар шетелдік қызметтес бол сұм аяқтал ескер отыр өз болашақ жақсы есепте және олар одан құтылу көзде propaganda,violent

4,ұлыбританияның қорғаныс корреспонденті джефф мид ауғанстанда үш ұрпақ бойы соғысуы керек болуы мүмкін деді армия басшысы болған генерал генерал дэвид ричардс жүздеген әскер көшеде соғысу үшін жаттығатын және талибан бекіністеріне шабуыл жасайтын хелманд ауылының миллиондық көшірмесінің ашылуында сөз сөйледі propaganda,ұлыбритания қорғаныс корреспондент джефф мид ауғанстан үш ұрпақ бойы соғыс керек бол мүмкін де армия басшы бол генерал генерал дэвид ричардс жүздеген әскер көше соғыс үшін жаттығ және талибан бекініс шабуыл жаса хелманд ауыл миллиондық көшірме ашылу сөз сөйле propaganda,violent

4,сэр дэвидтің көмекшілері армия үшін төрт жасқа жуық жас жаяу әскердің өзінің мансабында майданда қызмет ететін орташа уақыты болатындығын атап өтті бұл шара бойынша ұлыбританияның әскери қатысуы кем дегенде жылға дейін жалғасуы мүмкін қазіргі уақытта құрлық әскерлерінің бас қолбасшысы сэр дэвид келесі айда сэр ричард даннетттен ауысады бұған дейін ол бір жыл ауғанстандағы халықаралық

күштердің isaf қолбасшысы ретінде қызмет еткен propaganda,сэ дэвид көмекші армия үшін төрт жас жуық жас жаяу әскер өзінің мансаб майдан қызмет ет орташа уақыт болатындығ ата өт бұл шара бойынша ұлыбритания әскери қатыс кем де жыл дейін жалғас мүмкін қазіргі уақыт құрлық әскер бас қолбасшы сэ дэвид келесі айда сэ ричард даннетт ауыс бұған дейін ол бір жыл ауғанстан халықаралық күш isaf қолбасшы рет қызмет ет propaganda,violent

4,дайын емес әскердің ұрысқа келмеуі қарашада үндістанның мумбай қаласында ге жуық адамның өмірін қиған пәкістанмен байланысты террорлық шабуылдан кейін үндістан пәкістанға шабуыл жасай алмауы мүмкін үкіметтің жоғары деңгейдегі дереккөздері asia times onlineға армия қолбасшылары ньюделидегі саяси басшылыққа әсер еткенін олардың қолындағы жеткіліксіз және ескірген арсенал бүкіл соғысқа қарсы жұмсартылғанын айтты propaganda,дайын емес әскер ұрыс келме қараша үндістан мумба қала ге жуық адам өмір қи пәкістан байланысты террорлық шабуыл кейін үндістан пәкістан шабуыл жаса алма мүмкін үкімет жоғары деңгей дереккөз asia times online армия қолбасшы ньюдели саяси басшылық әсер ет олардың қол жеткіліксіз және ескір арсенал бүкіл соғыс қарсы жұмсартыл ай propaganda,violent

4,орташа жойғыш ұшақтарға арналған саудасаттық енді басталып бірнеше жыл уақытты алуы мүмкін сонымен қатар үндістан мен пәкістан қақтығысының перспективалары әлі аяқталған жоқ үндістан армиясының бастығы генерал дипак капур өткен аптада пәкістан өз әскерлерін ауғанстан шекарасынан бастап үндістанмен батыс шекарасына ауыстырғанын айтты үнді армиясы мұны жоспарлау кезінде дәлелдеді деді капоор дереккөз asia times online propaganda,орташа жойғыш ұшақ арнал саудаса енді бастал бірнеше жыл уақыт ал мүмкін сонымен қатар үндістан мен пәкістан қақтығыс перспектива әлі аяқтал жоқ үндістан армия бастығ генерал дипак капу өткен апта пәкістан өз әскер ауғанстан шекара баста үндістан батыс шекара ауыстыр ай үн армия мұны жоспарлау кез дәлелде де капоо дереккөз asia times online propaganda,violent

4,пәкістан үкіметі сват аймағымен шектелген соғыс сигналдарын жібереді пәкістан әскери күштері тікұшақ зымырандарына шабуылға арналған ұшақтарға және свот алқабында талибтердің күштерін қазып алуға қарсы артиллерияға сенуді жалғастыруда өйткені соңғы сағат ішінде ауыр шайқастар болды radicalization,пәкістан үкімет сват аймағ шектел соғыс сигнал жібер пәкістан әскери күш тікұшақ зымыран шабуыл арнал ұшақ және свот алқаб талиб күш қаз ал қарсы артиллерия сену жалғастыр өйткені соңғы сағат іш ауыр шайқас бол radicalization,violent

4,пәкістан үкіметі мен әскери шенеуніктер талибанның исламабадқа және елдің ядролық нысандарына қауіпқатерін жоққа шығарды бірақ сәуірдің аяғында жергілікті исламабад үкіметі әскерилерге талибанның алға жылжуына тосқауыл қою үшін қаланың солтүстігінде орналасқан маргала төбесіне орналастыруды бұйырды гарипур үкіметі тарбела бөгетінде қауіпсіздікті күшейтті propaganda,пәкістан үкімет

мен әскери шенеунік талибан исламабад және ел ядролық нысан қауіпқа жоқ шығ бірақ сәуір аяғ жергілікті исламабад үкімет әскери талибан алға жылж тосқауыл қою үшін қала солтүстіг орналас маргал төбе орналастыру бұйыр гарипу үкімет тарбел бөгет қауіпсіздік күшей propaganda,violent

4,ауғанстандағы қауіпсіздіктің нашарлауынан алаңдаған аф пилотсыз ұшақтарды қолдануда сонымен бірге ядролық қаруланған пәкістан өсіп келе жатқан талибан көтерілістерін қолдауға тырысуда оның қауіпсіздік күштері бір айдан астам уақыттан бері исламабадтың солтүстікбатысында орналасқан сват алқабында исламшыл содырларға қарсы соғысуда пәкістан әскери күштері бұл аймақтағы нысандарды жұмсартып жатыр propaganda,ауғанстан қауіпсіздік нашарлау алаңда аф пилот ұшақ қолдан сонымен бірге ядролық қарулан пәкістан өс келе жат талибан көтеріліс қолда тырыс оның қауіпсіздік күш бір ай астам уақыт бері исламабад солтүстікбаты орналас сват алқаб исламшыл содыр қарсы соғыс пәкістан әскери күш бұл аймақ нысан жұмсарт жатыр propaganda,violent

4,пәкістан ақштың зымыран соққыларына қарсылық білдіріп олардың егемендігін бұзады және содырлармен күресу әрекеттерін әлсіретеді өйткені олар халықтың ашуын басады және содырларға қолдауды күшейтеді вашингтон зымыран шабуылдары исламабадпен келісім бойынша жасалады бұл пәкістан басшыларына шабуылдарды ашық түрде сынап көруге мүмкіндік береді пәкістан мұндай келісімді жоққа шығарады propaganda,пәкістан ақш зымыран соққы қарсылық білдір олардың егемендіг бұз және содыр күресу әрекет әлсірет өйткені олар халық аш бас және содыр қолдау күшейт вашингтон зымыран шабуыл исламабад келісім бойынша жасал бұл пәкістан басшы шабуыл ашық түр сына көр мүмкіндік бер пәкістан мұндай келіс жоқ шығар propaganda,violent

4,бұл фатваны шейх американдықтардың терроризмге қарсы тұра алмағандары үшін талибанға қарсы жаңа санкциялар қолдану қаупіне жауап ретінде шығарды мұсылмандарды бұл фатваны интернет арқылы таратуға шақырады спам поштасынан аулақ болыңыз факс арқылы басып шығарып тиісті рұқсат алғаннан кейін мешіттерде ислам орталықтарында және намазханаларда орналастырыңыз recruitment,бұл фатва шейх американдық терроризм қарсы тұр алма үшін талибан қарсы жаңа санкция қолдану қауп жауап рет шығ мұсылман бұл фатва интернет арқылы тарат шақыр спам пошта аулақ бол факс арқылы бас шығар тиісті рұқсат ал кейін мешіт ислам орталық және намазхана орналастыр recruitment,violent

4,әлемдегі жақсы және тыйым салынған зұлымдықты ынталандыратын министрліктің атымен бөлек министрлік бар жалғыз ел сондайақ біз басқа мұсылман елдерін үкімдер бұқаралық ақпарат құралдары экономика ішкі және сыртқы саясат әйелдер ісі және білім саласындағы барлық дүниежүзілік келісімдерге қатысты шариғатты іске асыруда талибандардың ізімен жүруге шақырамыз recruitment,әлем жақсы және тыйым салын зұлымдық ынталандыр министрлік ат бөлек министрлік бар жалғыз ел сондайақ біз басқа мұсылман ел үкім бұқаралық ақпарат құрал экономика ішкі және

сыртқы саясат әйел іс және білім сала барлық дүниежүзілік келісім қатысты шариғат іс асыр талибан із жүр шақыр recruitment,violent

4,залымға айтпа автор шейх омар абдуррахман алла оны босатып ауруын айықтырсын сонымен міне сіз үшін халықтың ең сүйікті жүрегі міне ол сіз үшін менің отбасым туыстарым және туысқандарым міне сен үшін менің көз жасыммен араласқан ол менің қаныммен толтырылғанын қалайды propaganda,залым айтпа автор шейх омар абдуррахман алл оны босат ауру айықтыр сонымен міне сіз үшін халық ең сүйікті жүрег міне ол сіз үшін менің от туыс және туыс міне сен үшін менің көз жас аралас ол менің қан толтырыл қала propaganda,violent

4,шын мәнінде ислам мемлекеті мәңгілікке дейін қиямет күні маған қарсы ойлаңдар уа жаулар маған қарсы айла жасаңыз немесе жасамаңыз егер мен түрмеге түссем онда шын мәнінде ардақты юсуф түрмеге түседі егер мен союға ұшырасам онда менің жетекшім яхия союдан бұрын болды бұл менің дінім және менің хабарым егер мен сатқындық жасасам онда соғысыңдар және біз міндетті түрде кездесеміз және сіздерден жалбарынуларыңызды сұраймын recruitment,шын мән ислам мемлекет мәңгілік дейін қиямет күн маған қарсы ойла уа жау маған қарсы айла жаса немесе жасама егер мен түрме түс онда шын мән ардақ юсуф түрме түс егер мен сою ұшыра онда менің жетекші яхия сою бұрын бол бұл менің дін және менің хаб егер мен сатқ жаса онда соғ және біз міндетті түр кездес және сіздерден жалбарыну сұра recruitment,violent

4,могадишу маусым ройтерс сомалидегі қатаң альшаабаб исламистері африка үкіметіне көмекке келген шетелдік әскерлермен күреседі деп хабарлады жексенбіде көтерілісшілер тобының өкілі сенбіде сомали парламентінің спикері шейх аден мохаммед мадобе көрші джибути кения және эфиопия сияқты елдерді көтерілісті басу үшін әскери көмек жіберуге шақырды propaganda,могадиш маусым ройтерс сомали қатаң альшаабаб исламис африка үкімет көмек кел шетелдік әскер күрес де хабарла жексенбі көтерілісші тоб өкіл сенбі сомали парламент спикер шейх аден мохаммед мадоб көрші джибути кения және эфиопия сияқты ел көтеріл басу үшін әскери көмек жібер шақ propaganda,violent

4,құдай бізге барлық жауларды жеңуге көмектеседі және біз оларды жеңетінімізге сенеміз біз олардың саны мен қандай қаружарақтары туралы алаңдамаймыз деді рейдж оның тобы мамырдан бастап үкіметке қарсы шабуылды күшейтіп осы аптада екі заң шығарушыны оның ішінде қауіпсіздік министрін өлтірді мамырдан бері ге жуық адам қаза тапты дейді тұрғындар африка мүйізі елінде жиырма жылға жуық уақыт зорлықзомбылық болды propaganda,құдай бізге барлық жау жең көмектес және біз ол жең сен біз олардың сан мен қандай қаружарақ туралы алаңдама де рейдж оның тоб мамыр баста үкімет қарсы шабуыл күшейт осы апта екі заң шығарушы оның іш қауіпсіздік министр өл мамыр бері ге жуық адам қаза тап де тұрғын африка мүйіз ел жиырма жыл жуық уақыт зорлықзомбылық бол propaganda,violent

4,каида пәкістан ядролық оқтұмсықтарын алып жүруге қабілетті гаури зымыранын ақшқа жіберуге уәде берді маусым ауғанстандағы алькаиданың жоғарғы қолбасшысы егер адамдар кезкелген жағдайда қолын созса пәкістанның ядролық қаруын ақшқа қарсы қолданады дейді ядролық қару американдықтардың қолына түспейді ал мужахидтер оларды алып американдықтарға қарсы қолданады деді мұстафа әбу әлязид әлжазира теледидарына берген сұхбатында propaganda,каида пәкістан ядролық оқтұмсық алып жүр қабілетті гаури зымыран ақш жібер уәде бер маусым ауғанстан алькаида жоғарғы қолбасшы егер адам кезкел жағдай қол соз пәкістан ядролық қар ақш қарсы қолдан де ядролық қару американдық қол түспе ал мужахид ол алып американдық қарсы қолдан де мұстаф әбу әлязид әлжазир теледи бер сұхбат propaganda,violent

4,ауғанстанмен заңсыз шекаралас аймақтағы оңтүстік вазиристан соңғы күндері әуе шабуылдары мен әскери атысқа ұшырады жылы ақштың ауғанстанға басып кіруі екі көрші арасындағы рулық тайпалық белдікті күнделікті зорлықзомбылық көрінісіне айналдырды jr sme mmnpresstv propaganda,ауғанстан заңсыз шекаралас аймақ оңтүстік вазирис соңғы күн әуе шабуыл мен әскери атыс ұшыр жыл ақш ауғанстан бас кір екі көрші ара рулық тайпалық белдік күнделікті зорлықзомбылық көрініс айнал jr sme mmnpresstv propaganda,violent

4,цитата бастапқыда fighter дәйексөзі талибан сираджуддин хакканиді жіберді хаккани әлкаиданың мықты әскери қолбасшысы әлкаиданың жетекші идеологтарының бірі және діни комитеттің өкілі абу яхья аль либиге және жетекші абдул хак түркістаниге жіберді шығыс түркістан ислам партиясының алькаидаға байланысты тобы біріккен талибан және алькаида делегациясы байтуллаға пәкістанның талибанның әскери және үкіметке қарсы шабуылдарын тоқтатуға және оның күшін ауғанстанға жұмылдыруға кеңес берді propaganda,цита бастапқы fighter дәйексөз талибан сираджуддин хаккани жіб хаккани әлкаида мықты әскери қолбасшы әлкаида жетекші идеолог бірі және діни комитет өкіл абу яхья аль либи және жетекші абдул хак түркістани жіб шығыс түркістан ислам партия алькаида байланысты тоб біріккен талибан және алькаи делегация байтулла пәкістан талибан әскери және үкімет қарсы шабуыл тоқтат және оның күш ауғанстан жұмылдыр кеңес бер propaganda,violent

4,шейх усама пакистан халқына жаңа жолдауына қарсы және жақында жазған шейх әбу яхия әл либи кітабында жолдан тайған паки үкіметтерімен күресу себебін жазды шейх усама өзінің соңғы сөзінде мұжахидтерден пакистандағы муртадилерге қарсы күресуді сұрады олар өз кезегінде біздің бауырларымызды және қарындастарымызды өлтірді және басқаларды propaganda,шейх уса пакис халқ жаңа жолда қарсы және жақында жаз шейх әбу яхия әл либи кітаб жолдан тай паки үкімет күресу себеб жаз шейх уса өзінің соңғы сөз мұжахид пакиста муртади қарсы күресу сұр олар өз кезег біздің бауыр және қарындас өл және басқа propaganda,violent

4,цитата алғашында аттақва әл шабаба найробидің шейхі хасан яккубқа шабуыл жасайды дейді кисмаю портының қаласындағы әли әлшабаб әкімшілері жексенбі күні

кенияның астанасы найробидегі биік ғимараттарға шабуыл жасайды деп қорқытқан propaganda,цита алғаш аттақв әл шабаб найроби шейх хасан яккуб шабуыл жаса де кисмаю порт қала әли әлшабаб әкімші жексенбі күн кения астана найроби биік ғимарат шабуыл жаса де қорқыт propaganda,violent

4,сомали парламентінің спикері аден мохаммед нор көрші елдерден сенбіде сомалиге әскер жіберуді сұрады және министрлер кабинеті бұл шешімді мақұлдады шейк яккуб сонымен бірге көмек агенттіктерін сомалидегі соғыстарды ескермеді деп айыптап олардың тыңшылар болғанын айтты альшабаба партиясының өкілі шейх али мохамуд рагех сомалиге келетін кезкелген шетелдік әскерге қарсы күресетіндерін мәлімдеді propaganda,сомали парламент спикер аден мохаммед но көрші ел сенбі сомали әскер жіберу сұр және министр кабинет бұл шеш мақұлда шейк яккуб сонымен бірге көмек агенттік сомали соғыс ескерме де айыпта олардың тыңшы бол ай альшабаб партия өкіл шейх али мохамуд рагех сомали кел кезкел шетелдік әскер қарсы күрес мәлімде propaganda,violent

4,мұндай мысалдардың бірінде ирхаби вебсайттарды бұзу туралы семинар деп аталатын ұзақ хабарлама жіберді эхлас форумына жылы маусымда өткен бұл семинар келушілерге өнер туралы жиырма беттен тұратын егжейтегжейлі зерттеу жүргізуге мүмкіндік берді бірлескен бұқаралық ақпарат құралдарын жүктеуге болатын ондаған осал вебсайттарды бұзу туралы ирхабидің өзі бір кездері бұл стратегияны арканзас штатының вебсайтына содан кейін джордж вашингтон университетінің басқа сайтына мәліметтерді жүктеген кезде кеңінен қолданған propaganda,мұндай мысал бір ирхаби вебсайт бұзу туралы семинар де атал ұзақ хабарлама жіб эхлас форум жыл маусым өткен бұл семинар келуші өнер туралы жиырма бет тұр егжейтегжейл зерттеу жүргіз мүмкіндік бер бірлескен бұқаралық ақпарат құрал жүкте бол ондаған осал вебсайт бұзу туралы ирхаби өзі бір кез бұл стратегия арканзас штат вебсайт содан кейін джордж вашингтон университет басқа сайт мәлімет жүкте кезде кең қолдан propaganda,violent

4,егер ол ұсынған сайттар қауіпсіз болып қалса басқаларға сервердің осалдықтарын табудың әдістерін ұсынды осылайша жиһадшылар өздерінің вебкеңістігін ең бастысы өз ақшаларын пайдалану қаупі болмауы үшін үгітнасихат тарату үшін үшінші тарап қожайындарын қолдана алады propaganda,егер ол ұсын сайт қауіпсіз бол қал басқа сервер осалдық таб әдіс ұсын осылайша жиһадшы өз вебкеңістіг ең басты өз ақша пайдалану қауп болма үшін үгітнасихат тарат үшін үшінші тарап қожайын қолдан ал propaganda,violent

4,обаманың пәкістанға жарияланбаған соғысы оны төмендетуге тырысқанымен де жалғасуда жаңа сұхбатында обама пәкістанға ақш әскерін жіберуге ниеті жоқ деді бірақ ақш әскери күштері қазірдің өзінде елде және ақш ұшқыштары пәкістанға үнемі шабуыл жасайды джереми скахилл өзінің инаугурациясынан үш күн өткен соң жылдың қаңтарында президент барак обама ақштың жыртқыш ұшқышсыз ұшқыштарға пәкістанның ішіне шабуыл жасауды бұйырды propaganda,обама пәкістан жарияланба соғыс оны төмендет тырыс де жалғас жаңа сұхбат оба пәкістан ақш әскер

жібер ниет жоқ де бірақ ақш әскери күш қаз өз ел және ақш ұшқыш пәкістан үнемі шабуыл жаса джереми скахилл өзінің инаугурация үш күн өткен соң жыл қаңтар президент барак оба ақш жыртқыш ұшқыш ұшқыш пәкістан іш шабуыл жасау бұйыр propaganda,violent

4,ұшқышсыз шабуылдар немесе командалық рейдтер арқылы ма ақш пәкістанға әскери кірісті бұл обаманың ақш пәкістанға әскер жіберуге ниеті жоқ деген пікірін мүлдем мүмкін емес етеді ақштың операциялары қаншалықты маңызды және болашақта да әрі қарай да жалғасатындығын түсіну үшін ақштың исламабадта миллиард долларға жуық жаппай елшілігін салу жоспарына назар аударыңыз ақштың бағдадтағы елшілігін шақырыңыз recruitment,ұшқыш шабуыл немесе командалық рейд арқылы ма ақш пәкістан әскери кір бұл обама ақш пәкістан әскер жібер ниет жоқ де пікір мүлдем мүмкін емес ет ақш операция қаншалықты маңызды және болашақ да әрі қарай да жалғасатындығ түсіну үшін ақш исламабад миллиард долл жуық жаппай елшілігін салу жоспар назар ау ақш бағдад елшілігін шақыр recruitment,violent

4,пәкістанда содырлар көбірек шабуыл жасайды жм маусым талибан содырлары пәкістанның бақылауындағы кашмирде қауіпсіздік күштеріне жаңа шабуылдар жасап жанкештілік жарылыс үшін жауапкершілікті өз мойнына алды техрикеталибан қолбасшысының орынбасары хаким уллах мехсуд жұма күні журналистерге мұзаффарабадтағы қауіпсіздік күштері конвойына бағытталған бомбаны жасады propaganda,пәкістан содыр көбірек шабуыл жаса жм маусым талибан содыр пәкістан бақылау кашмир қауіпсіздік күш жаңа шабуыл жаса жанкештілік жарылыс үшін жауапкершілік өз мойн алды техрикеталибан қолбасшы орынбасар хаки уллах мехсуд жұма күн журналист мұзаффарабад қауіпсіздік күш конвой бағыттал бомба жас propaganda,violent

4,вашингтонның ауғанстандағы әскери контингентін күшейтіп жатыр өйткені қауіпсіздікті қалпына келтіру тамыздағы президенттік және жалпыхалықтық сайлаулардың алдында қиынға соқты ақштың пәкістан жеріне зымыран шабуылдары исламабад шенеуніктерін солтүстікбатыстағы содырларға қарсы шабуыл жақсы дамып келеді және ақштың қажетсіз көмегі қажет емес деп санайды mrs hgh mmn пресс propaganda,вашингтон ауғанстан әскери контингент күшейт жатыр өйткені қауіпсіздік қалп келтіру тамыз президенттік және жалпыхалықтық сайлау алд қиын соқ ақш пәкістан же зымыран шабуыл исламабад шенеунік солтүстікбатыс содыр қарсы шабуыл жақсы дам кел және ақш қажет көмег қажет емес де сана mrs hgh mmn пресс propaganda,violent

4,біріншіден біз исламдық үмбетімізді және сомалидің халқын оның ержүрек жақсы туылған тайпаларын және джихадтың батыл қаһармандарын сомалидегі қол жеткізген керемет жеңісі үшін құттықтаймыз propaganda,бірінші біз исламдық үмбет және сомали халқ оның ержүрек жақсы туыл тайпа және джихад батыл қаһарман сомали қол жеткіз керемет жеңіс үшін құттықта propaganda,violent

4,адамдарға лайықты лауазымдарды беріңіз оларды таныңыз олардың арасында мейірімді адамдармен жомарт болыңыз өткенде қайтыс болған адамдардың үйлерін қорғаңыз және олардың асыл адамдарының сондайақ құрметті және мақтаныш шеберлерінің мәртебесін көтеріңіз араларыңызда жарылқағандарды әсіресе білім алған адамдарды өз жағыңызға алып келіңіз кедей жесірлерге жетімдер мен қарттарға мейірімді бол кедейлер мен кедейлерге жақсылық жасаңыз зардап шеккендер мен басқа да апаттарға жол беріңіз recruitment,адам лайықты лауазым бер ол тан олардың ара мейірімді адам жомарт бол өткенде қайтыс бол адам үй қорға және олардың асыл адам сондайақ құрметті және мақтаныш шебер мәртебе көтер ара жарылқа әсіресе білім ал адам өз жағ алып кел кедей жесір жетім мен қарт мейірімді бол кедей мен кедей жақсылық жаса зардап шек мен басқа да апат жол бер recruitment,violent

4,хосттағы әуежайда американдық басшылықтың отыруына қарсы болды бұл хостта және кабулда және басқа да аймақтарда жасалған ең маңызды операциялардың бірі болды оларды біздің бауырларымыз ұйымдастырып оларға қатысты ауғанстандағы джихадқа қатысуымыздың себебі біз исламдық әмірліктердің қол астындамыз және біз оларға көмектесіп қолымыздағы бар нәрселерді идеялар мен көзқарастарымызда береміз radicalization,хост әуежай американдық басшылық отыр қарсы бол бұл хост және кабул және басқа да аймақ жасал ең маңызды операция бірі бол ол біздің бауыр ұйымдастыр оларға қатысты ауғанстан джихад қатысу себебі біз исламдық әмірлік қол аст және біз оларға көмектес қолымыз бар нәрсе идея мен көзқарас бер radicalization,violent

4,қазір біз оларды исламға кіруге шақырамыз өйткені егер олар исламға кірсе онда олар мен біздің арамыздағы ұрыс мәңгіге аяқталады егер олар исламға кірмесе иншалла біз ислам мемлекеті мен ислам халифатын құруға тырысамыз содан кейін біз оларды қайтадан исламға кіруге шақырамыз recruitment,қазір біз ол ислам кір шақыр өйткені егер олар ислам кір онда олар мен біздің арамыз ұрыс мәңгі аяқтал егер олар ислам кірме иншалл біз ислам мемлекет мен ислам халифат құр тырыс содан кейін біз ол қайта ислам кір шақыр recruitment,violent

4,аалсалул билеушілері әлсуудқа қатысты кемсітушілік сілтеме мұхаммед бен наииф сауд арабиясының ішкі істер министрінің көмекшісі басқарған екі жүзділерімен бірге өз елін мәжбүрлеп ашқан осынау қарапайым билеуші али абдалла салихке қосымша келді бұл өрескел кәсіп олардың қанағаттануы үшін масқара түрде жасалады бірақ олар оған разы болмайды олар теңіз қарақшылығы сияқты бірнеше жалған сылтауларды қолданады propaganda,аалсалул билеуші әлсууд қатысты кемсітушілік сілтеме мұхаммед бен наииф сауд арабия ішкі істер министр көмекші басқар екі жүзді бірге өз ел мәжбүрле аш осынау қарапайым билеуш али абдалл салих қосымша кел бұл өрескел кәсіп олардың қанағаттан үшін масқара түр жасал бірақ олар оған разы болма олар теңіз қарақшылығ сияқты бірнеше жалған сылтау қолдан propaganda,violent

4,біздің оларға деген өшпенділігіміз саяси емес біз оларды тек қана жаулап алушылар болғандықтан жек көрмейміз мәселе жоғарыда айтқанымыздан гөрі артта қалады сондайақ біз оларды бауырларымызды өлтіргені үшін және өлтіретін қаруды қолданғаны үшін жек көреміз өйткені оқтары мұсылмандардың басына түсіп содан кейін олардың ішінде жарылып кетеді propaganda,біздің оларға де өшпенділі саяси емес біз ол тек қана жаула алушы бол жек көрме мәселе жоғары айт гөрі арт қал сондайақ біз ол бауыр өлтір үшін және өлтір қару қолдан үшін жек көр өйткені оқ мұсылман бас түс содан кейін олардың іш жарыл кет propaganda,violent

4,біз көрген көптеген нәрселерге қарамастан оптимизмге жол бермей келесі тармақтарды ұмытпауымыз керек біздің басымызға түскен бұл қорлаудың себебі тек өзімізге байланысты шындығында біздің жолымызға келген барлық зұлымдық өзімізге байланысты жеңіс шарттарының бірі бұл шатасуды жою керек ол еврейлерді байт әлмакдистен иерусалим қуып жеңіп шығарды propaganda,біз көр көптеген нәрсе қарамастан оптимизм жол берме келесі тармақ ұмытпа керек біздің бас түс бұл қорла себебі тек өз байланысты шындығ біздің жол кел барлық зұлымдық өз байланысты жеңіс шарт бірі бұл шатасу жою керек ол еврей байт әлмакдис иерусалим қу жең шығ propaganda,violent

4,джихадтың иронниясы және джихадтың бұл мәселесі күлкілі қарап отырсаңыз бұл ирония джихад сіздің өміріңізді алып кетуі керек джихад сені тәуекел етуі керек джихад сені зиянға ұшыратады соған қарамастан үмбет жиһадқа қарсы күресте шыңына жеткен кезде егер қисық сызық жасағыңыз келсе және үмметтегі джихад тәжірибесіне көз жүгіртсеңіз және үмметте өлетін адамдардың санына көз салсаңыз сіз егер үмбет соғысқан сайын жиһад соғысса үммет халқы азаяды propaganda,джихад ирония және джихад бұл мәселе күлкіл қара отыр бұл ирония джихад сіздің өмір алып кет керек джихад сен тәуекел ет керек джихад сен зиян ұшырат соған қарамастан үмбет жиһад қарсы күрес шың жет кезде егер қисық сызық жаса кел және үммет джихад тәжірибе көз жүгірт және үммет өл адам сан көз сал сіз егер үмбет соғыс сайын жиһад соғыс үммет халқ азая propaganda,violent

4,сахабалар физикалық күші жағынан қарсыластарынан аз болды олар қарсыластарынан аз болды және олар қарсыластарынан аз болды олар ең жақсы дайындықтан өткен ең үлкен денесі бар ең жақсы қаруланған және ең жақсы қаружарақпен жабдықталған адамдармен кездесті бірақ сахабалармен кездескенде олардың жүректерінен түңілді осылайша оларда бәрі болды оларда қаружарақ бар физикалық күштер болды жаттығулар болды нөмірлер болды бірақ қарақшылар жолды бастағанда олардың жүректерінен айырылды propaganda,сахаба физикалық күш жағ қарсылас аз бол олар қарсылас аз бол және олар қарсылас аз бол олар ең жақсы дайындық өткен ең үлкен дене бар ең жақсы қарулан және ең жақсы қаружарақ жабдықтал адам кезде бірақ сахаба кездес олардың жүрек түң осылайша ол бәрі бол ол қаружарақ бар физикалық күш бол жаттығу бол нөмір бол бірақ қарақшы жол баста олардың жүрек айыр propaganda,violent

4,дәйексөз бастапқыда шатасқан іздеушімен келіседі amp риаз басра пакистанда тұратын адамдар да мұны өте жақсы біледі amp уақыт өте келе адамдар осы формаларды қолданып өтіп кибержиһад жасағанымды білдіремін сол сияқты киберджихадты да жасаймын бұл адамдар үшін өте жақсы тақырып propaganda,дәйексөз бастапқы шатас іздеуші келіс amp риаз баср пакистан тұр адам да мұны өте жақсы біл amp уақыт өте келе адам осы форма қолдан өт кибержиһад жаса білдір сол сияқты киберджихад да жаса бұл адам үшін өте жақсы тақырып propaganda,violent

4,біздің көптеген асыл мүшелеріміз бар осы жоспарлар мен алдауды жақсы білетін форум және шындық осы шынайы және жақсы хабардар болған бауырластардың көмегімен белгілі болады сондықтан мен форумның барлық асыл мүшелеріне форумдағы импостерлер алданып қалмауға кеңес беремін форумның әкімшісі толық білетін адамдар және өз сөздерімен олар өздерін және өздерінің жалған одақтарын бәріне ашады propaganda,біздің көптеген асыл мүше бар осы жоспар мен алдау жақсы біл форум және шындық осы шынайы және жақсы хабардар бол бауырлас көмег белгілі бол сондықтан мен форум барлық асыл мүше форум импостер алдан қалма кеңес бер форум әкімші толық біл адам және өз сөз олар өз және өз жалған одақ бәрін аш propaganda,violent

4,дәйексөз бастапқыда риаз басра мен басқа мүжахидтер форумдарын қолдандым пәкістан мұсылмандарына қатысты бірдебір агрессия болған жоқ пәкістан мұсылмандары сәл де болса хариж ақиқатты іздеңіз recruitment,дәйексөз бастапқы риаз баср мен басқа мүжахид форум қолда пәкістан мұсылман қатысты бірдебі агрессия бол жоқ пәкістан мұсылман сәл де бол хариж ақиқат ізде recruitment,violent

4,куфилер мен жолдан таюшылар өз мақсаттарына жетуге және оларға жол бермеуге мүмкіндік береді біздің арамыздағы келіспеушіліктерді көріп олардың көңілкүйін көтеру әрине біз соңына дейін бірігіп кафтар үшін тікен болуымыз керек иеміз бізге шейіт болуды бермейінше пікірлердегі келіспеушіліктерден арылуға және біріктіруге мүмкіндік беріңіз егер мен дөрекі пікірлер айтсам кешіріңіз бірақ мәңгіге біріктіріңізші propaganda,куфи мен жолдан таюшы өз мақсат жет және оларға жол берме мүмкіндік бер біздің арамыз келіспеушілік көр олардың көңілкү көтеру әрине біз соң дейін біріг каф үшін ті бол керек ие бізге шейіт болу бермейінш пікір келіспеушілік арыл және біріктір мүмкіндік бер егер мен дөрекі пікір айт кешір бірақ мәңгі біріктіріңізш propaganda,violent

4,біздің мүмкіндігіміз шексіз докка омаровпен сұхбат келесі сұхбатта кавказ мужахедінің әмірі докка умаров прага watchdogмен телефон арқылы сөйлесті прага күзетшісі редакторы біраз уақыт бұрын сізді жаралағаныңыз туралы хабарлар болды ал кейбіреулері өліп жерленді сіз жараландыңыз ба докка умаров жоқ соңғы рет жылы болды мен аяғымнан жарақат алдым propaganda,біздің мүмкіндіг шексіз докк омаров сұхбат келесі сұхбат кавказ мужахед әмір докк умаров прага watchdog телефон арқылы сөйле прага күзетші редактор біраз уақыт бұрын сіз жарала туралы

хабар бол ал кейбіреу өл жерле сіз жарала ба докк умаров жоқ соңғы рет жыл бол мен аяғ жарақат ал propaganda,violent

4,мұның бәрі әлемді қалай көретініне байланысты осы бағытта жүрген мүжахедтер мұндай миссияға қатыса алады мен олардың командирімін және оларға бірдеңе жасауды немесе жасамауды бұйырамын менің тапсырысымсыз бұл маршрутты ешкім ала алмайды біздің дұшпандарымыз біздің мужаһедилерімізді қабылдауға итермелеген propaganda,мұның бәрі әлем қалай көр байланысты осы бағытта жүр мүжахед мұндай миссия қатыс ал мен олардың командир және оларға бірде жасау немесе жасамау бұйыр менің тапсырыс бұл маршрут ешкім ал алма біздің дұшпан біздің мужаһеди қабылда итермеле propaganda,violent

4,мен алдыңғы мақалада израиль деп аталған еврейлерге және олардың субъектілеріне қарсы және сырттан жанжақты соғыс анбаар иракқа оралғаннан кейін басталады деп айтқан болатын аллаһ тағала сатқындар яһудилерді қорғау үшін ислам мемлекетін құруға кедергі жасауға тырысты бірақ жанжақты соғыстың алдында біздер мен еврейлер арасында қатты соққылар болады propaganda,мен алдыңғы мақала израиль де атал еврей және олардың субъекті қарсы және сырт жанжақ соғыс анба ирак орал кейін бастал де айт бол аллаһ тағал сатқын яһуди қорғау үшін ислам мемлекет құр кедергі жаса тырыс бірақ жанжақ соғ алд біздер мен еврей ара қатты соққы бол propaganda,violent

4,біз және сол мужахидтердің басшылары талибан жетекшілері өте тығыз қарымқатынастамыз біз олармен үнемі кездесіп отырамыз олар бізге келеді біз оларға барамыз осы кездесулер мен талқылаулардың нәтижесі біз оларды бірігіп бір болуға шақырдық осылай болды және мужахидон бірлігі шура комитеті құрылды олар оларды қорғауға және қорғауға үлкен ықпал етеді propaganda,біз және сол мужахид басшы талибан жетекші өте тығыз қарымқатынас біз олармен үнемі кездес отыр олар бізге кел біз оларға бар осы кездесу мен талқылау нәтиже біз ол бірig бір бол шақ осылай бол және мужахидон бірліг шур комитет құр олар ол қорға және қорға үлкен ықпал ет propaganda,violent

4,есімдерге көп мән бермейді оның атын өзгертуі мүмкін бірақ бұл мәселені ирактағы ағайындар шешуі керек мұны олар шешуі керек ирак ислам мемлекетіне қарсы қарсылықтар мен сынпікірлерге келетін болсақ олардың көпшілігі шындыққа жанаспайтын қарсыластарынан шығады олар ирак ислам мемлекеті туралы шындыққа жанаспайтын мәліметтерді алға тартады джихадпен күресу барысында қателіктер жіберілуі мүмкін бірақ бұл қалыпты жағдай propaganda,есім көп мән берме оның ат өзгерт мүмкін бірақ бұл мәселе ирак ағайын шеш керек мұны олар шеш керек ирак ислам мемлекет қарсы қарсылық мен сынпікір кел бол олардың көпшілiг шындық жанаспа қарсылас шығ олар ирак ислам мемлекет туралы шындық жанаспа мәлімет алға тарт джихад күресу барыс қателік жіберіл мүмкін бірақ бұл қалыпты жағдай propaganda,violent

4,сомалидегі шабаба және хизбул ислам бірігуге ұмтылуда әлсіз сомали үкіметімен күресетін екі негізгі исламдық террористік топтар бір киімге біріктірілуі мүмкін хизбул ислам немесе ислам партиясының жетекшісі шейх хасан дахир уейс өзінің фракциясын халықаралық террористік ұйымға қосылуды ұсынған әлкаида қолдаған исламдық террорлық топ шабаабпен біріктіру үшін жұмыс істеп жатқанын айтты propaganda,сомали шабаб және хизбул ислам біріг ұмтыл әлсіз сомали үкімет күрес екі негізгі исламдық террористік топ бір киім біріктіріл мүмкін хизбул ислам немесе ислам партия жетекші шейх хасан дахи уейс өзінің фракция халықаралық террористік ұйым қосылу ұсын әлкаи қолда исламдық террорлық топ шабааб біріктіру үшін жұмыс істе жат ай propaganda,violent

4,әрекет минуттық видео түрінде болды ол кения алькаиданың жедел әрекет етуші салех али салех набханды бейнелейді оны ақш үкіметі африка елшілігіне және момбасадағы шабуылдарға қатысқаны үшін іздеуде шабааб ресми түрде алькаидаға қосылуға тырысады шабабтың алькаидаға ресми қосылу туралы ұсынысын бин ладен мен завахири жылы қабылдады екі топты біріктіру қажет бірақ операциялық және қаржылық байланыстар тығыз ұзақ соғыс журналы propaganda,әрекет минуттық видео түр бол ол кения алькаида жедел әрекет етуші салех али салех набха бейнеле оны ақш үкімет африка елшіліг және момбаса шабуыл қатыс үшін ізде шабааб ресми түр алькаида қосыл тырыс шабаб алькаида ресми қосылу туралы ұсыныс бин ла мен завахири жыл қабылда екі топ біріктіру қажет бірақ операциялық және қаржылық байланыс тығыз ұзақ соғыс журнал propaganda,violent

4,ауғанстан ислам әмірлігінің американдық сайлау процесі туралы ауғандықтарды адастыру туралы хабарлауы ислам джихадының кеңеюі мен өршуіне байланысты сенбейтіндерге соққы берді енді американдықтар мен олардың одақтастары барлық күшжігері бұл елде нәтиже бермейді деп ойлады және олардың ауғандықтарды бағындыруға бағытталған түрлі стратегиясы қазір сәтсіздікке ұшырап отыр propaganda,ауғанстан ислам әмірліг американдық сайлау процес туралы ауғандық адастыр туралы хабарла ислам джихад кеңею мен өрш байланысты сенбе соққы бер енді американдық мен олардың одақтас барлық күшжіг бұл ел нәтиже берме де ойла және олардың ауғандық бағындыр бағыттал түрлі стратегия қазір сәтсіздік ұшыра отыр propaganda,violent

4,нағыз тәуелсіздікке қол жеткізу үшін олар сайлау учаскелерінің орнына жиһад бекіністерінің жолын таңдауы керек олар өз құқықтарын жиһадшылар мен қарсылықтар арқылы басып алушылардан алуы керек сайлауға қатысу басқыншы американдықтармен достық қарымқатынас орнатуға және көмек көрсетуге мүмкіндік береді бұл олардың шабуылына заңдылық береді propaganda,нағыз тәуелсіздік қол жеткізу үшін олар сайлау учаске орн жиһад бекініс жол таңда керек олар өз құқық жиһадшы мен қарсылық арқылы бас алушы ал керек сайла қатысу басқынш американдық достық қарымқатынас орнат және көмек көрсет мүмкіндік бер бұл олардың шабуыл заңдылық бер propaganda,violent

4,қонақ үйінің жауапкершілігі бақ тандзим ал койдах индонезия ресми индивидиясының индиясияның ресми өкілі индиясия джихадиях иостияға қатысты осылайша үмбет шынымен американың әсіресе жиналысқа жиналғандар американың экономикалық секторындағы бизнесмендер мен барлау тобының жетекшілері олардың индонезия байлығын ысырап етуге және ислам мен мұсылмандарға қарсы күресетін кафирлерді американдық күштерді қаржыландыруға үлкен қызығушылықтары бар propaganda,қонақ үй жауапкершіліг бақ тандзи ал койдах индонезия ресми индивидия индиясия ресми өкіл индиясия джихадиях иостия қатысты осылайша үмбет шын америка әсіресе жиналыс жинал америка экономикалық сектор бизнесмен мен барлау тоб жетекші олардың индонезия байлығ ысырап ет және ислам мен мұсылман қарсы күрес кафир американдық күш қаржыландыр үлкен қызығушылық бар propaganda,violent

4,бүкіл әлемде қысым көрген және қудаланған мұсылмандардың жүректеріне емдік және емші ретінде ақыр соңында бұл шынайы амалият үмбет үшін рухтың қозғаушысы болады және жихад парызын қайта бастайды бұл халифалық рашиданы қайта құрудың бірденбір жолы би идзнилла және біз осы амалият джихадиясына есім береміз sariyah jabir propaganda,бүкіл әлем қысым көр және қудалан мұсылман жүрек ем және емші рет ақы соң бұл шынайы амалият үмбет үшін рух қозғаушы бол және жихад парыз қайта баста бұл халифалық рашида қайта құр бірденбі жол би идзнилл және біз осы амалият джихадия есім бер sariyah jabir propaganda,violent

4,құрметті бауырлар бұл сіздер үшін ауғанстан ұлдары біздің елімізді қорғауға және оны қауіпсіздендіруге керемет мүмкіндік болып табылады прогресстің бір белгісі күн сайын ер адамдарды оқытып бұрғылайтын офицерлер мен сержанттардың көпшілігі ауғандықтар шамамен бір жыл бұрын көпшілігі американдық немесе британдық болды қазір батыс кеңесшілер іргелес базада орналасқан propaganda,құрметті бауыр бұл сіздер үшін ауғанстан ұл біздің ел қорға және оны қауіпсіздендір керемет мүмкіндік бол таб прогрес бір бел күн сайын ер адам оқыт бұрғыла офицер мен сержант көпшіліг ауғандық шамамен бір жыл бұрын көпшіліг американдық немесе британдық бол қазір батыс кеңесші іргелес база орналас propaganda,violent

4,ауғанстан армиясының кабул маңындағы күштерінің аға кеңесшісі ауған жолы бұл жерге шабуыл жасап шабуыл жасау біз оларға әскери дайындықтың үштен екісі дайындалып жатыр деп айтамыз бұл үшін көп күш пен шыдамдылық қажет дейді ол ауған армиясының жауынгері жаңадан шақырылушыларды қалай атып тастау керектігін көрсетіп жатыр propaganda,ауғанстан армия кабул маң күш аға кеңесші ауған жол бұл же шабуыл жаса шабуыл жасау біз оларға әскери дайындық үш екі дайындал жатыр де ай бұл үшін көп күш пен шыдамдылық қажет де ол ауған армия жауынгер жаңа шақырылушы қалай ат тастау керектіг көрсет жатыр propaganda,violent

4,мулла назирдің алсахабпен сұхбаты кашмир жиһады туралы ассахаб пәкістандағы діни класс көптеген ондаған жылдардан бері кашмирдегі джихадпен байланысты кашмирдегі джихад және оның үкіметі мен ишим рөлі туралы пікіріңіз қандай мулла

назир кашмирдегі джихад және онда құрбандықтар ұсынғандар мұны шын ниетпен жасады бірақ біз өмірімізді алға қойған мақсаттарға жете алмай отырмыз propaganda,мулл назі алсахаб сұхбат кашмир жиһад туралы ассахаб пәкістан діни класс көптеген ондаған жыл бері кашмир джихад байланысты кашмир джихад және оның үкімет мен иши рөл туралы пікір қандай мулл назі кашмир джихад және онда құрбандық ұсын мұны шын ниет жас бірақ біз өмір алға қой мақсат же алма отыр propaganda,violent

4,кашмири джихаты алға қойған мақсаттарымызға жетуге көмектеспейді үндістанда куфр заңы және пәкістанда британдықтар берген заң бар кашмирде куфрдің осы екі маркасының бірі басым болады ислам діні емес біздің құрбандықтарымыз босқа кетеді бұл біздің муһаххид бауырларымызға ишимдің қызметінен бас тарту туралы шынайы кеңесі propaganda,кашмири джиха алға қой мақсат жет көмектеспе үндістан куф заң және пәкістан британдық бер заң бар кашмир куф осы екі марка бірі басым бол ислам дін емес біздің құрбандық бос кет бұл біздің муһаххид бауыр ишим қызмет бас тарту туралы шынайы кеңес propaganda,violent

4,салем алейкум уа рахматулла міне аударма біз иерусалимдегі алла окна сарбаздарының жақтастары екенімізді мәлімдейміз біздің иерусалимдегі окнав ислам әміріне шейх маулана аш әбу нұр мақдиси джабер ассабахқа исламдық әмірге сенушілердің адалдығын сақтаңыз және белгілі адамдарға құлақ асып мойынсұнуға уәде береміз және біз шейх әбу нұрға айтамыз міндеттеме құдайдың жасырын батасы propaganda,сале алейку уа рахматулл міне аударма біз иерусалим алл окн сарбаз жақтас екен мәлімде біздің иерусалим окнав ислам әмір шейх маулан аш әбу нұр мақдиси джабер ассабах исламдық әмір сенуші адалдығ сақта және белгілі адам құлақ ас мойынсұн уәде бер және біз шейх әбу нұр ай міндеттеме құдай жасырын бата propaganda,violent

4,газа секторындағы мұсылмандарды мужахидтер мен nasrthm мен тұруға қолдау көрсетуге шақырамыз төртінші біз барлық жердегі мұсылмандарды әмірлікті қолдауға шақырамыз ал жастар оны ақша қаружарақ және ер адамдармен біріктіріп иерусалимде ұлттың қайта қосылуының туын көтереміз деп король және ол қабілетті деген сөзге ұлттың бірігуі ислам әмірі окнав иерусалимде propaganda,газ сектор мұсылман мужахид мен nasrthm мен тұр қолдау көрсет шақыр төртінші біз барлық жер мұсылман әмірлік қолда шақыр ал жас оны ақша қаружарақ және ер адам біріктір иерусалим ұл қайта қосылу ту көтер де король және ол қабілетті де сөз ұл біріг ислам әмір окнав иерусалим propaganda,violent

4,боко харам жиһадты қайта жариялады жалпы джихадты жариялады боко харам нигериядағы соғыс күшімен бүкіл халықты исламға қатер төндіріп бүкіл жиһадты жариялады жақында нигерияның солтүстігінде жүздеген адамдардың өмірін жоғалтуға әкеп соқтырған сектант жылы тамызда vanguardқа берген мәлімдемеде дағдарыс кезінде даулы жағдайларда өлтірілген олардың жетекшісі юсуф мәңгі өмір сүреді деп мәлімдеді propaganda,боко харам жиһад қайта жариял жалпы джихад жариял боко харам нигерия соғыс күш бүкіл халық ислам қатер төндір бүкіл жиһад

жариял жақында нигерия солтүстіг жүздеген адам өмір жоғалт әке соқтыр сектант жыл тамыз vanguard бер мәлімдеме дағдарыс кез даул жағдай өлтір олардың жетекші юсуф мәңгі өмір сүр де мәлімде propaganda,violent

4,еркіндік мұнда қауіпсіздік кепілдендіріліп исламның астында бейбітшілік болады жақында біз лагосты зұлым қала мен нигерияның оңтүстікбатысы мен оңтүстік шығысын бұрынсоңды ешкім жасамаған күйге келтіреміз аль хакубара біз сізге біздің ақшаға болады немесе ақшқа қарсымыз мәллам санни умару боко харам жетекшісінің міндетін атқарушы қол қойылды тамыз http www vanguardngr com stotaljihad propaganda,еркіндік мұнда қауіпсіздік кепілдендіріл ислам аст бейбітшілік бол жақында біз лаго зұлым қала мен нигерия оңтүстікбаты мен оңтүстік шығыс бұрынсо ешкім жасама күй келтір аль хакубар біз сізге біздің ақша бол немесе ақш қар мәлла санни умар боко харам жетекші міндет атқарушы қол қой тамыз http www vanguardngr com stotaljihad propaganda,violent

4,егер сіз соғысты тоқтатпауды шешсеңіз онда біз кеңес одағымен ыдырай бастағанға дейін жыл бойы құдайдың рақымымен істегеніміздей сізге қарсы азаттық соғысын барлық мүмкін болатын балта бойынша жалғастыра береміз өзіңіз қалағанша соғысты жалғастырыңыз сіз қатты зардап шегіп басқалардың пайдасына соғысып жатырсыз бұл соғыстың соңы жоқ сияқты ауғанстандағы шайқастардан сабақ алған орыс генералдары соғыстың басталуына дейін оның нәтижесін күткен еді propaganda,егер сіз соғ тоқтатпау шеш онда біз кеңес одағ ыдыра баста дейін жыл бойы құдай рақым істегенімізде сізге қарсы аза соғыс барлық мүмкін бол балта бойынша жалғастыр бер өз қалағанш соғ жалғастыр сіз қатты зардап шег басқа пайда соғыс жатыр бұл соғ соңы жоқ сияқты ауғанстан шайқас сабақ ал орыс генерал соғ бастал дейін оның нәтиже күт ед propaganda,violent

4,біз қаруымызды иығымызға көтеріп жыл бойы шығыс пен батыстағы зұлымдықтың екі полюсімен күресіп келеміз осы уақыт ішінде біз өзімізділөзімізді өлтіру оқиғаларын халықаралық деңгейде бізге қарсы жасалғанына қарамастан көрген жоқпыз біздің қаруымыз шыдамдылық әлақса мешітінен бас тартпаймыз біз жанымызды ұстағаннан гөрі палестинаны ұстаймыз өзіңіз қалағанша соғысты жалғастырыңыз біз ешқашан ол туралы мәміле жасамаймыз propaganda,біз қару иығ көтер жыл бойы шығыс пен батыс зұлымдық екі полю күрес кел осы уақыт іш біз өзімізділөз өлтір оқиға халықаралық деңгей бізге қарсы жасал қарамастан көр жоқ біздің қар шыдамдылық әлақ мешіт бас тартпа біз жан ұста гөрі палестина ұста өз қалағанш соғ жалғастыр біз ешқашан ол туралы мәміле жасама propaganda,violent

4,бұл официалды емес аударма бірақ бауырлар мен әпкелер мұны пайдалы деп санайды бірнеше рет көбейіп беделді медиа адамдарының ұрпағын шығарды олардың кейбіреулері оң қолында ак сол жағында камерасы бар балаларға арналған ойын кәсіпқой бағдарлама ретінде басталып дамаск жолында сатылатын бушты басып алу ойынына айналды мужахидиин аппараты батысқа шабуыл жасап мужахдиин құпиялары бағдарламасын көптеген компаниялар мен ұйымдар пайдалана бастады propaganda,бұл официа емес аударма бірақ бауыр мен әпке мұны

пайдалы де сана бірнеше рет көбей беделді медиа адам ұрпағ шығ олардың кейбіреу оң қол ак сол жағ камера бар бала арнал ойын кәсіпқой бағдарлама рет бастал дамаск жол сатыл буш бас алу ойын айна мужахидиин аппарат батыс шабуыл жаса мужахдиин құпия бағдарлама көптеген компания мен ұйым пайдалан баста propaganda,violent

4,бұл жоспар араздықты тудырып күмән тудыратын және жиһадтық бақ басшыларының сенімін бұзуға бағытталған болатын қазір біз оларды қадағалап жатырмыз біз барлық бауырластарға әлфаджр орталығынан мәлімдемені оқып қымбат кеңестер мен маңызды нұсқауларды қабылдауға кеңес береміз біз дұшпандарға айтамыз біз не істей алатынымызды жақсы білесіз жауап қатал болады және куәлік сіз естігенде емес көргенде болады propaganda,бұл жоспар араздық тудыр күмән тудыр және жиһад бақ басшы сен бұз бағыттал бол қазір біз ол қадағала жатыр біз барлық бауырлас әлфадж орталығ мәлімдеме оқ қымбат кеңес мен маңызды нұсқау қабылда кеңес бер біз дұшпан ай біз не істе ал жақсы біл жауап қатал бол және куәлік сіз есті емес көргенде бол propaganda,violent

4,сомали джихадының болашағы туралы жарияланым терроризм мониторы көлемі шығарылым қараша жасы күн санаты терроризм мониторы терроризмге жаһандық талдау басты бет әскери қауіпсіздік африка авторы эндрю макгрегор хизбальислам мүшелері могадишудың оңтүстігінде күзет жүргізуде сомалияның өтпелі федералды үкіметі бір жылға жуық уақыт бойы радикалды исламшыл оппозицияның бірнеше рет шабуылына қарсы өмір сүру үшін өмірді немесе өлімді күрес жүргізіп келеді propaganda,сомали джихад болашағ туралы жарияланым терроризм монитор көлем шығарылым қараша жас күн сана терроризм монитор терроризм жаһандық талдау басты бет әскери қауіпсіздік африка авто эндрю макгрего хизбальисла мүше могадиш оңтүстіг күзет жүргіз сомалия өтпелі федерал үкімет бір жыл жуық уақыт бойы радикал исламшыл оппозиция бірнеше рет шабуыл қарсы өмір сүру үшін өмір немесе өл күрес жүргіз кел propaganda,violent

4,сомалияның оңтүстігінде және орталығында жалғасып келе жатқан соғыстан арылуға деген аз үмітпен пунтланд пен сомалиландтың бойында тәуелсіз жергілікті басқарылатын шағын штаттар құруға талпыныс жасалатындығы туралы белгілер бар кландық бәсеке мен көрші мемлекеттердің аймақтық мүдделер мен халықаралық державалардың араласуымен бірге көршілерімен татулықта біріккен сомали құрудың перспективалары көңіл көншітпейді көз propaganda,сомалия оңтүстіг және орталығ жалғас келе жат соғыс арыл де аз үміт пунтланд пен сомалиланд бой тәуелсіз жергілікті басқарыл шағын штат құр талпыныс жасалатындығ туралы белгі бар кла бәсеке мен көрші мемлекет аймақтық мүдде мен халықаралық держава араласу бірге көрші татулық біріккен сомали құр перспектива көңіл көншітпе көз propaganda,violent

4,фейсбук қолданушылары үшін мәртебесін жаңарту немесе отбасылық суреттерін жариялау бұл олардың таңдаған достарының тізімі жаңа есеп бойынша көпшілік жеке деп санайтын мәліметтерді израиль адамдарға беріп оларға тыңшылық жасау үшін пайдаланады францияда шығатын израиль журналында келтірілген сенімді

ақпаратқа сәйкес израиль барлау қызметі негізінен араб және мұсылман пайдаланушыларына назар аударады және facebookтегі парақшалар арқылы алынған ақпаратты олардың қызметін талдау және олардың ойларын түсіну үшін пайдаланады propaganda,фейсбук қолданушы үшін мәртебе жаңарту немесе отбасылық сурет жариялау бұл олардың таңда дос тізім жаңа есеп бойынша көпшілік жеке де сана мәлімет израиль адам бер оларға тыңшылық жасау үшін пайдалан франция шығ израиль журнал келтір сенімді ақпарат сәйкес израиль барлау қызмет негіз араб және мұсылман пайдаланушы назар аудар және facebook парақша арқылы алын ақпарат олардың қызмет талдау және олардың ой түсіну үшін пайдалан propaganda,violent

4,шпиондау өте оңай ол журналға израильдің facebookті адамдарға тыңшылық жасады деген айыппен бірінші рет емес сәуірінде иорданияның әлхақика әлдавлия газетінде жасырын жау жасауы туралы мақала жариялады газет бұл қауіпті деп мәлімдеді өйткені адамдар әсіресе жастар facebookте және сол сияқты интернеттегі қауымдастықтарда өздері туралы жеке және жеке мәліметтерді жиі ашады және бұл оларға қарап отырған адамдар үшін оңай мақсатқа айналады propaganda,шпионда өте оңай ол журнал израиль facebook адам тыңшылық жас де айып бірінші рет емес сәуір иордания әлхақик әлдавлия газ жасырын жау жаса туралы мақала жариял газет бұл қауіпті де мәлімде өйткені адам әсіресе жас facebook және сол сияқты интернет қауымдастық өз туралы жеке және жеке мәлімет жиі аш және бұл оларға қара отыр адам үшін оңай мақсат айнал propaganda,violent

4,ауғанстан ислам эмираты алланың көмегі зу әлқидах хижрий қараша алланың атымен мейірімді жанашыр қазіргі американың ауғанстанды жаулап алуына қарсы джихад мұсылманға қысым көрсеткенін дәлелдейді propaganda,ауғанстан ислам эмират алла көмег зу әлқидах хижри қараша алла ат мейірімді жанашыр қазіргі америка ауғанстан жаула ал қарсы джихад мұсылман қысым көрсет дәлелде propaganda,violent

4,отарлаушылар мен басқыншылардың не істегендерін қалайтындығы туралы хабарлайды біз шындықты ашып әлемге өз үнімізді жеткізгіміз келсе демократия мен сөз бостандығын қорғаушылар деп аталатын сайттарымызды жауып бізге өтірік айтады әлем адам құқығын қорғаушылар деп аталатын нәрсе демократия емес өркениет шеңберіндегі терроризм екенін білуі керек олар ұзақ уақытқа созылған күрестен кейін адамзат қол жеткізген құндылықтарды жояды ауғанстан ислам әмірлігі propaganda,отарлаушы мен басқыншы не істе қалайтындығ туралы хабарла біз шындық аш әлем өз үн жеткіз кел демократия мен сөз бостандығ қорғаушы де атал сайт жау бізге өтірік айт әлем адам құқығ қорғаушы де атал нәрсе демократия емес өркениет шеңбер терроризм екен біл керек олар ұзақ уақыт созыл күрес кейін адамзат қол жеткіз құндылық жоя ауғанстан ислам әмірліг propaganda,violent

4,жаңадан басталған ауған армиясының нан астам әскері бар ал ауған қауіпсіздік күштерін жаттықтыратын нато әскери күштері келесі жылдың қазан айына дейін мыңға жетеді деп мәлімдеді сенбі күні қорғаныс министрі абдул рахим вардак өзінің

үкіметі өз армиясының санын мың әскерге дейін көбейтуді жоспарлағанын мәлімдеді propaganda,жаңа бастал ауған армия нан астам әскер бар ал ауған қауіпсіздік күш жаттықтыр нато әскери күш келесі жыл қазан ай дейін мың жет де мәлімде сенбі күн қорғаныс министр абдул рахим вардак өзінің үкімет өз армия сан мың әскер дейін көбейту жоспарла мәлімде propaganda,violent

4,батыс елдерінде ауғанстанға халықаралық әскери күш салуға қоғамдық қолдау жоғалуда негізінен сегіз жылға созылған ауған соғысының және натоның ауғанстандағы әскерилерінің құрбаны болуына байланысты натоның лауазымды тұлғалары көбірек ауғандық күштерді даярлауға назар аудара отырып өзінің әскери күштерін талибанға қарсы соғыстан тоғызыншы жылында шығарады деп үміттенеді альхамдулилла propaganda,батыс ел ауғанстан халықаралық әскери күш сал қоғамдық қолдау жоғал негіз сегіз жыл созыл ауған соғыс және нато ауғанстан әскери құрбан бол байланысты нато лауазым тұлға көбірек ауғандық күш даярла назар аудар отыр өзінің әскери күш талибан қарсы соғыс тоғызыншы жыл шығар де үміттен альхамдулилл propaganda,violent

4,ansar almujahideen ағылшынша форумның презентациялары туралы жедел мәлімдеме пешавар және ислам университетінің шабуылдарына қатысты мәлімдеме шейх прокур тарапынан жасалған шабуылдарға қатысты өндіріс алладан мейірімді мейірімді алланың атымен шайтаннан пана сұраймын олар алланың нұрын ауыздарымен сөндіргісі келеді бірақ алла оның нұрын жетілдіреді propaganda,ansar almujahideen ағылшынша форум презентация туралы жедел мәлімдеме пешав және ислам университет шабуыл қатысты мәлімдеме шейх проку тарап жасал шабуыл қатысты өндіріс алла мейірімді мейірімді алла ат шайтан пан сұра олар алла нұр ауыз сөндір кел бірақ алл оның нұр жетілдір propaganda,violent

4,америка үшін біздің қадірімізді құрбан еткендер жақын арада тағдырына жауап береді алла қаласа біздің соңғы дұғамыз барлық мақтау аллаға иа алла бұл ғазиздіктер тез арада мақсаттарына жетсін көздері ақиқатпен жарқырай берсін электр сияқты олар ай мен жұлдыздарға ұқсасын олар менің ұлтымның қайталанбас көсемдері ееманның кеудесіндегі халықты жұбатушылар болсын шариғат немесе шахадат ассахаб медиа қоры намазда бізді намазда ұмытпаңыз бауырларыңыз ansar almujahidein propaganda,америка үшін біздің қадір құрбан ет жақын ара тағдыр жауап бер алл қала біздің соңғы дұ барлық мақтау алла иа алл бұл ғазиздік тез ара мақсат жет көз ақиқат жарқыра бер электр сияқты олар ай мен жұлдыз ұқсас олар менің ұлт қайталанбас көсем ееман кеуде халық жұбатушы бол шариғат немесе шахадат ассахаб медиа қор намаз біз намаз ұмытпа бауыр ansar almujahidein propaganda,violent

4,мен шын жүректен құттықтаймын ауғанстанның барлық муһахид адамдарына құрметті шейіт отбасыларына және жау қолымен түрмеге түскен тұтқындар айбынды мүжидендерге және осы идул одха мерекесіне орай барлық ислам үмбетіне сенемін propaganda,мен шын жүрек құттықта ауғанстан барлық муһахид адам құрметті шейіт

отбасы және жау қол түрме түс тұтқын айбын мужиден және осы идул одх мереке орай барлық ислам үмбет сен propaganda,violent

4,алдында олар қасірет пен қайғықасіретке кезіккенде рухтары қатты шайқалды тіпті расул және оның қасындағылар алланың көмегі қашан келеді деп айқайлады расында алланың көмегі жақын әлбақара елімізді азат ету және исламдық жүйені құру үшін алға жазықсыз адамдардың шабуылдаушылары мен өлтірушілерімен бірге төмен алла барлығыңызды жарылқасын мулла мұхаммад омар мужахид исламның қызметшісі propaganda,алд олар қасірет пен қайғықасірет кезік рух қатты шайқа тіпті расул және оның қасындағы алла көмег қашан кел де айқайла рас алла көмег жақын әлбақар ел азат ету және исламдық жүйе құру үшін алға жазық адам шабуылдаушы мен өлтіруші бірге төмен алл барлығ жарылқа мулл мұхаммад омар мужахид ислам қызметші propaganda,violent

4,егер мен моджахидтер өздерінің жанжақты және адал көмектерінде мүжахидтермен тұрақты болса алла тағала жаудың барлық қастандықтарын бірінен соң бірі әшкерелеп олардың алдын алады бұл сіздердің жанқиярлық құрбандықтарыңыздың арқасында өркөкірек жаудың жеңіліске де әзілге де масқараға да ұшырайтындығы сіз өзіңіздің заңды джихадты жалғастырып исламдық ұмтылыстарыңызды іске асыру жолындағы күресті жалғастырасыз деп сенемін propaganda,егер мен моджахид өз жанжақ және адал көмек мүжахид тұрақты бол алл тағал жау барлық қастандық бір соң бірі әшкереле олардың алдын ал бұл сіздердің жанқиярлық құрбандық арқа өркөкірек жау жеңіліс де әзіл де масқара да ұшырайтындығ сіз өз заңды джихад жалғастыр исламдық ұмтылыс іс асыру жолындағы күре жалғастыр де сен propaganda,violent

4,өз күштеріңізді басып кіретін басып алушы жауды жеңуге бағыттаңыз мақсатсыз әрекеттермен айналыспаңыз назарыңызды негізгі мақсатқа аударыңыз барлық мұсылмандар дұғаларында исламның және исламдық үмбеттің қорғаушылары мухиддер туралы есте сақтаулары керек американдықтар мен оның одақтастары ислам әлемін тұрақсыздандыруға және ислам елдеріндегі келіспеушіліктерге қарсы жоспарларды тым көп және жасырын түрде жүзеге асыруда recruitment,өз күш бас кір бас алушы жау жең бағытта мақсат әрекет айналыспа назар негізгі мақсат ау барлық мұсылман дұға ислам және исламдық үмбе қорғаушы мухид туралы ес сақтау керек американдық мен оның одақтас ислам әл тұрақсыздандыр және ислам ел келіспеушілік қарсы жоспар тым көп және жасырын түр жүзеге асыр recruitment,violent

4,обама ауғанстандағы соғысты күшейту туралы бұйрық берді президент обама ауғанстандағы соғысты ушықтыру үшін отыздан отыз бен бес мыңға дейін қосымша әскер жіберуге бұйрық берді обама өзінің соғыс жоспарын бүгін кешке премьерминистрдің алдында сөйлейді бірақ ол өзінің әскери шешімдерін бұл туралы бұрын да хабарлаған propaganda,оба ауғанстан соғ күшейту туралы бұйрық бер президент оба ауғанстан соғ ушықтыр үшін отыз отыз бен бес мың дейін қосымша

әскер жібер бұйрық бер оба өзінің соғыс жоспар бүгін кеш премьерминист алд сөйле бірақ ол өзінің әскери шешім бұл туралы бұрын да хабарла propaganda,violent

4,бүгін кешке антитеррорлық топтың өкілдері президент обаманың оның күшейе түсуіне шешім қабылдауға үзілдікесілді қарсы ашық хат жолдады ауғанстанда көптеген тұрғындар обаманың соғыс жоспарларына қарсылықтарын білдірді обидулла хан кандагар қаласының тұрғыны егер олар американдықтар әскер санын көбейтсе онда жазықсыз адамдардың үйлерін көбірек бомбалайды олар жазықсыз мұсылмандарды өлтіреді көбірек үйлер іздеңіз бұл ел үшін жаман апатқа айналады көз propaganda,бүгін кеш антитеррорлық топ өкіл президент обама оның күшей түс шешім қабылда үзілдікес қарсы ашық хат жолда ауғанстан көптеген тұрғын обама соғыс жоспар қарсылық біл обидулл хан кандагар қала тұрғы егер олар американдық әскер сан көбейт онда жазық адам үй көбірек бомбала олар жазық мұсылман өлтір көбірек үй ізде бұл ел үшін жаман апат айнал көз propaganda,violent

4,кавказ орталығына кавказдық мүжахидтер командасының атынан хат келіп түсті онда невский экспрессін жою операциясы ресейдегі стратегиялық нысандарға бағытталған ереуілдер аясында жүргізілгені айтылады propaganda,кавказ орталығ кавказ мүжахид команда ат хат кел түсті онда невски экспрес жою операция ресей стратегиялық нысан бағыттал ереуіл ая жүргізіл айт propaganda,violent

4,мүжахидтердің басшылығымен бірнеше барлаудиверсиялық бөлімшелер дайындалып жау аймағында операциялар жүргізу үшін руснаға жіберілді нәтижесінде және осы операциялардың салдары руснаға үлкен экономикалық зиян келтірді біз осы бағытта жұмысты жалғастырамыз біз ресей басшылығына егер олар кавказ әмірлігінің жазықсыз мұсылмандарын өлтіруді тоқтатпаса және өлім отрядтарының қызметін тоқтатпаса біз ресейдің бейбіт тұрғындарына қарсы тиісті әскери операциялар жүргізу құқығымызды сақтаймыз деп ескертеміз хат бөлімі кавказ орталығы propaganda,мүжахид басшылығ бірнеше барлаудиверсиялық бөлімше дайындал жау аймағ операция жүргізу үшін русня жібер нәтиже және осы операция сал русна үлкен экономикалық зиян кел біз осы бағытта жұм жалғастыр біз ресей басшылығ егер олар кавказ әмірліг жазық мұсылман өлтіру тоқтатпа және өлім отряд қызмет тоқтатпа біз ресей бейбіт тұрғын қарсы тиісті әскери операция жүргізу құқығ сақта де ескер хат бөлім кавказ орталығ propaganda,violent

4,исламист элли сомалидегі хизбул ислам партиясының альшабаб рас камбони фракциясын қолдайды оның жауынгерлері сомалияның оңтүстігінде және орталығында алиша рюге қарсы шабуыл жасауға дайындалып жатыр propaganda,исламист элли сомали хизбул ислам партия альшабаб рас камбони фракция қолда оның жауынгер сомалия оңтүстіг және орталығ алиш рю қарсы шабуыл жаса дайындал жатыр propaganda,violent

4,соңғы ай ішінде үкімет күштері мен африка одағының бітімгерлері могадишода хизбул ислам мен әлшабаб күштерінің шабуылына қарсы күрес жүргізуде рас камбони және анол фракциялары басқа хизбул исламдық соғысушыларын

көтерілісшілердің назарын әлшабабқа аударуға көндіруі қиын болуы мүмкін деп хабарлады могадишодағы хизбул исламның өкілі сейсенбі күні журналистерге өзінің тобы өтпелі үкіметке қарсы шабуылдарды көбейтуге дайындалып жатқанында және африка одағы күштері көз propaganda,соңғы ай іш үкімет күш мен африка одағ бітімгер могадишо хизбул ислам мен әлшабаб күш шабуыл қарсы күрес жүргіз рас камбони және анол фракция басқа хизбул исламдық соғысушы көтерілісші назар әлшабаб аудар көндір қиын бол мүмкін де хабарла могадишо хизбул ислам өкіл сейсенбі күн журналист өзінің тоб өтпелі үкімет қарсы шабуыл көбейт дайындал жат және африка одағ күш көз propaganda,violent

4,невский экспрессы сәтті өткен невский экспресс ке қарсы террористік шабуылда мұжахидтер басшылары жасаған мейірімділік туралы мәлімдеме қарашада невский пойызын тоқтатты санктпетербургтен мәскеуге сапар шеккен оны ресейдің жоғары лауазымды тұлғалары жиі пайдаланады коррозия салдарынан локомотивтер бүлініп дан астам адам қаза тапты және кем дегенде адам жараланды propaganda,невски экспрес сәтті өткен невски экспресс ке қарсы террористік шабуылда мұжахид басшы жаса мейірімділік туралы мәлімдеме қараша невски пойыз тоқта санктпетербург мәскеу сапар шек оны ресей жоғары лауазым тұлға жиі пайдалан коррозия сал локомотив бүлін дан астам адам қаза тап және кем де адам жарала propaganda,violent

4,дүйсенбі желтоқсан gmt израильдің с базасы falcon израиль ливанға қарсы тағы бір шабуыл жасауды жоспарлады оның ішінде литани өзенінің оңтүстігін басып алу израильдегі күнделікті иерусалим пост әскери ақпарат көздеріне сілтеме жасай отырып израиль армиясының голани бригадасының жақында бір аптаға созылған әскери жаттығуды сирия мен хезболлаға қарсы соғысқа дайындық ретінде аяқтағанын хабарлады propaganda,дүйсенбі желтоқсан gmt израиль с база falcon израиль ливан қарсы тағы бір шабуыл жасау жоспарла оның іш литани өзен оңтүстіг бас алу израиль күнделікті иерусалим пост әскери ақпарат көз сілтеме жаса отыр израиль армия голани бригада жақында бір апта созыл әскери жаттығу сирия мен хезболла қарсы соғыс дайындық рет аяқта хабарла propaganda,violent

4,израильдің штаб бастығы генераллейтенант габи ашкенази министрлер кабинетінен хизболла үкіметтің серіктесі болған кезде ливанның инфрақұрылымына қарсы израиль армиясының ауыр әуе шабуылдарын бастауға шақырды израиль қозғалысты жою үшін жылдың жазында ливанға шабуыл жасады күндік соғыс көптеген бейбіт тұрғындарды өлтіріп ливанның инфрақұрылымын қиратты алайда израиль деректері бойынша хизболла соғыстан кейінгіге қарағанда күшті болды sb dt propaganda,израиль штаб бастығ генераллейтенант габи ашкенази министр кабинет хизболл үкімет серіктес бол кезде ливан инфрақұрылым қарсы израиль армия ауыр әуе шабуыл баста шақ израиль қозғал жою үшін жыл жаз ливан шабуыл жас күндік соғыс көптеген бейбіт тұрғын өлтір ливан инфрақұрылым қира алайда израиль дерек бойынша хизболл соғыс кейінгі қара күшті бол sb dt propaganda,violent

4,ауғанстан ислам әмірлігінің оңтүстік кореяның міндеттемелерін бұзғаны туралы мәлімдемесі бақ мәліметтері бойынша оңтүстік корея ауғанстанға әскери қызметкер

жіберуге және оларды парван провинциясына орналастыруға дайын екендігін көрсетті ауғанстан ислам әмірлігі оңтүстік корея билеушілері қабылдаған бұл шешімді ауғанстанның ұлттық тәуелсіздігіне қарсы әрекет ауғандықтарға қарсы әрекет және корейлікті босату үшін жылы корея қабылдаған міндеттемелерді бұзу әрекеті деп санайды propaganda,ауғанстан ислам әмірліг оңтүстік корея міндеттеме бұз туралы мәлімде бақ мәлімет бойынша оңтүстік корея ауғанстан әскери қызметкер жібер және ол парван провинция орналастыр дайын екендіг көрсе ауғанстан ислам әмірліг оңтүстік корея билеуші қабылда бұл шеш ауғанстан ұлттық тәуелсіздіг қарсы әрекет ауғандық қарсы әрекет және корейлік босат үшін жыл корея қабылда міндеттеме бұзу әрекет де сана propaganda,violent

4,бұл елдің беделі мен беделіне ауыр соққы жасайды және оның бетіндегі стигма ауғанстан ислам әмірлігі оңтүстік кореяның билеушілеріне егер олар қандай да бір жолмен ауғанстанға әскер жіберетін болса және олардың міндеттемелерін бұзса онда олар міндетті түрде олардың ісәрекеттерінің салдарына дайын болу керектігін ескертеді ауғанстан ислам әмірлігі егер қаласа ауғанстан ислам әмірлігі бұл мәселеде ешқандай икемділік көрсетпейді propaganda,бұл ел бедел мен бедел ауыр соққы жаса және оның бет стиг ауғанстан ислам әмірліг оңтүстік корея билеуші егер олар қандай да бір жол ауғанстан әскер жібер бол және олардың міндеттеме бұз онда олар міндетті түр олардың ісәрекет сал дайын болу керектіг ескерт ауғанстан ислам әмірліг егер қала ауғанстан ислам әмірліг бұл мәселе ешқандай икемділік көрсетпе propaganda,violent

4,хамас жылдығын дүйсенбі күнгі қозғалыс мерейтойына орай тойлайды али вакед орталық митингі кезінде үлкен тосын сый болады деп жариялады израиль жаңалықтары хамас қозғалысының жылдығына арналған ісшараларға дайындық дүйсенбі күні газа секторында құрылыстар жүргізілуде оқиғалардың арасында жаппай митинг өтеді деп жоспарлануда және қозғалыс шенеуніктері бұл шарада үлкен тосынсый күтуге болады деп сендірді propaganda,хамас жылдығ дүйсенбі күнгі қозғалыс мерейтой орай тойла али вакед орталық митинг кез үлкен тос сый бол де жариял израиль жаңалық хамас қозғалыс жылдығ арнал ісшара дайындық дүйсенбі күн газ сектор құрылыс жүргізіл оқиға ара жаппай митинг өт де жоспарлан және қозғалыс шенеунік бұл шара үлкен тосынсый күт бол де сен propaganda,violent

4,израильмен соғыста өзінің көшбасшылары мен басшыларының ұлдарын жоғалтқан палестина қозғалысы болған жоқ деп мәлімдейді ұйым ахмад ясиннің доктор абдель азиз альрантиси исмаил абу шанаб саид сеям низар раян және салах шехаденің өлтірілуін атап өтті сондайақ ұлдарынан айрылған лидерлердің қатарында екі ұлынан да айрылған әлзахар біреуі қастандық жасағысы келсе екіншісі хамас сақинасы мен idf әскері арасындағы шайқаста propaganda,израиль соғыс өзінің көшбасшы мен басшы ұл жоғалт палестин қозғалыс бол жоқ де мәлімде ұйым ахмад ясин доктор абдель азиз альрантиси исмаил абу шанаб саид сея низ раян және салах шехаде өлтіріл ата өт сондайақ ұл айрыл лидер қат екі ұл да айрыл әлзах біреу қаста жаса кел екінші хамас сақина мен idf әскер ара шайқас propaganda,violent

4,шейх яссин мен шейх рантисидің азапталуынан бастап хамастың қалай нашарлағанын көру өте ұят құдай оларды шухада ішінен қабылдап жаннат әлфирдавс берсін алдымен хамас шейіт болу операциялары харам және тыйым салады дейді содан кейін олар ауғанстанда шешенстанда дағыстанда уезкстанда польшада балқанда жасалған жантүршігерлік қылмыстардан кейін де ресей ұлы ел дейді propaganda,шейх яссин мен шейх рантиси азапталу баста хамас қалай нашарла көру өте ұят құдай ол шуха іш қабылда жаннат әлфирдавс бер алд хамас шейіт болу операция харам және тыйым сал де содан кейін олар ауғанстан шешенстан дағыстан уезкстан польша бал жасал жантүршігерлік қылмыс кейін де ресей ұлы ел де propaganda,violent

4,цитата алькаиданың жарылысы обаманы ақштың араб одақтастарын жарып жіберді salah nasrawi ар сағат бұрын каир алькаиданың жетекшісінің орынбасары дүйсенбіде президент барак обаманы араб әлемін алдады және таяу шығыстағы бейбітшілік келіссөздерін алға тартпады деп айыптады және содырлардың ақш пен оның одақтастарына қарсы күресі мұсылмандар мен кәпірлер арасындағы соғыс дейді propaganda,цита алькаида жарылыс обама ақш араб одақтас жар жіб salah nasrawi ар сағат бұрын каир алькаида жетекші орынбасар дүйсенбі президент барак обама араб әл алд және таяу шығыс бейбітшілік келіссөз алға тартпа де айыпта және содыр ақш пен оның одақтас қарсы күре мұсылман мен кәпір ара соғыс де propaganda,violent

4,тұңғыш рет дүниежүзілік сауда орталығына жасалған шабуыл үшін сотталған және қазір өмір бойы сотталған рамзи юссефті сонымен қатар қыркүйектегі жарылыстардың жетекшісі алькаида халид шейх мұхаммедті ерекше атап өтті гуантанамо шығанағындағы әскери базада бірнеше жыл ұсталған мұхаммед және тағы төрт адам жылдың қыркүйегінде ға жуық адамның өмірін қиған шабуылдарды ұйымдастырды деген айыппен сотталады ап propaganda,тұңғыш рет дүниежүзілік сауда орталығ жасал шабуыл үшін сотталған және қазір өмір бойы сотталған рамзи юссеф сонымен қатар қыркүйек жарылыс жетекші алькаи халид шейх мұхаммед ерекше ата өт гуантанамо шығанағ әскери база бірнеше жыл ұстал мұхаммед және тағы төрт адам жыл қыркүйег ға жуық адам өмір қи шабуыл ұйымдас де айып соттал ап propaganda,violent

4,салам алейкум жамағаты шариғат біздің мақсатымыз әділетті қоғам құру жариялау уақыты бүгін сағат де бүгін мұсылман үмметі ұйқыдан оянғанда алыс емес инша аллах нағыз шариғат мемлекеті орнатылатын күн дәл осы мақсатта джихад алла жолындағы күрестің ең жоғары түрі ретінде жүргізілуде алайда көптеген мұсылмандар әрқашан жамағаттың мақсаттары мен міндеттерін дұрыс түсінбейді көбісі куфр мемлекетімен татуласу арқылы ымыраға келуге болады деп сеніп адасады propaganda,сала алейку жамағат шариғат біздің мақсат әділет қоғам құру жариялау уақыт бүгін сағат де бүгін мұсылман үмме ұйқы оян алыс емес инш аллах нағыз шариғат мемлекет орнатыл күн дәл осы мақсат джихад алл жолындағы күре ең жоғары түр рет жүргізіл алайда көптеген мұсылман әрқашан жамағат мақсат мен міндет дұрыс түсінбе көбісі куф мемлекет татулас арқылы ымыра кел бол де сен адас propaganda,violent

4,біз еркін туылған мұсылманбыз әділетсіздікті көргенде неге өзіміздің намысымыз бен алланың дінін қорғауға тұрмасқа неліктен біз магомедалиге амировқа және махачевке дагестанның ең жақсы ұлдарын ұруға және өлтіруге бізді тонауға біздің еңбегімізден тапқан жерімізді және мүліктерімізді сатуға рұқсат береміз құлдардың жалақысы аштықтан өлмеу шамильдің ұрпақтарында қалған жалғыз нәрсе жоқ біз сенімдегі бауырластар сізге ешқашан жүгінбейміз дағыстан исламдық жамағатының шариат кавказ орталығының баспасөз қызметі propaganda,біз еркін туыл мұсылман әділетсіздік көргенде неге өз намыс бен алла дін қорға тұрмас неліктен біз магомедали амиров және махачев дагестан ең жақсы ұл ұр және өлтір біз тона біздің еңбег тап же және мүлік сат рұқсат бер құл жалақы аштық өлме шамиль ұрпақ қалған жалғыз нәрсе жоқ біз сенім бауырлас сізге ешқашан жүгінбе дағыс исламдық жамағат шариат кавказ орталығ баспасөз қызмет propaganda,violent

4,біреу мұның толық аударылған мақаласын бере алады ма cnn алькаида екінші қолбасшысының әйелі әйелдерді балаларын қасиетті соғысты сүюге және мұсылман жерлерін қорғауға шақыруға шақырды усама бен ладеннің кеңесшісі айман әлзавахиридің зайыбы омайма хасан ахмад мохаммед хассадтың айтуынша әйелдер күресуге болмайтындықтан олар өздерін қасиетті жауынгерлерге немесе мужахедтерге арнауы керек propaganda,біреу мұның толық аударыл мақала бер ал ма cnn алькаи екінші қолбасшы әйел әйел бала қасиетті соғ сүю және мұсылман жер қорға шақыр шақ уса бен ладен кеңесші айман әлзавахири зайыб омай хасан ахмад мохаммед хассад айт әйел күрес болмайтындық олар өз қасиетті жауынгер немесе мужахед арна керек propaganda,violent

4,хасан алладан оған және оның мұсылман әпкелеріне өлімге дейін шыдамдылық пен табандылықты әсіресе палестина автономиясында иракта шешенстанда ауғанстанда және сомалияда беруді сұрады cnnдің таяу шығыс істері жөніндегі аға редакторы октавиа наср алькаидаға байланысты әйелдер үшін хабарлама тарату әдеттегі нәрсе емес дейді бұл алькаида әйелдер тобына агрессивті түрде ұмтылып жатқандығының белгісі бұған дейін олардың назарын негізінен ер адамдарға аударған деді propaganda,хасан алла оған және оның мұсылман әпке өлім дейін шыдамдылық пен табандылық әсіресе палестин автономия ирак шешенстан ауғанстан және сомалия беру сұр cnn таяу шығыс іс жөн аға редактор октави нас алькаида байланысты әйел үшін хабарлама тарат әдет нәрсе емес де бұл алькаи әйел тоб агрессивті түр ұмтыл жатқандығ бел бұған дейін олардың назар негіз ер адам аудар де propaganda,violent

4,дәйексөз бастапқыда әбу хамза жариялады куфилерге деген менің жек көрушілігімді де бағаламаңыз және алла олардың біздің жек көрушілігімізге үкім шығарсын бірақ мен бұл мәселелерге жеңілжелпі қарамайтын сахабаларды еске түсіре алмаймын бұның басықасында жүргенді ұнатқанды ұнататынымды білдірмейді өйткені олар ойынсауықтың бір түрі басқаша айтқанда сіз өзіңіздің колготкаларыңыздың барлығын жинайсыз өйткені мен кафферлерді өлтіргенде маған өте ұнайды radicalization,дәйексөз бастапқы әбу хамза жариял куфи де менің жек көрушіліг де бағалама және алл олардың біздің жек көрушіліг үкім шығар бірақ мен

бұл мәселе жеңілжелп қарама сахаба ес түсір алма бұның басықа жүр ұнат ұнат білдірме өйткені олар ойынсауық бір түр басқаша айтқанда сіз өз колготка барлығ жина өйткені мен каффер өлтір маған өте ұна radicalization,violent

4,сіздің ашуызаңызды эмоцияңызды білдірудің жалғыз тәсілі оларға күлу өйткені бұл өмірде сізді жақсы сезінетін жалғыз нәрсе қалды енді өмірде мені жақсы сезінетін жалғыз нәрсені өзіңізден неге алғыңыз келеді дәл осы үшін келесі жолы мен бастарды көруге барсам алдымен мен буффало тауық қанаттарының үлкен табағын ранч байлап қыздырып отыра салқындатамын сіз менің ойымды аз өзгерттіңіз досым мұны түсіндіретіндердің бәрі мұны шешеді деп үміттенемін propaganda,сіздің ашуыза эмоция білдіру жалғыз тәсіл оларға күл өйткені бұл өмір сіз жақсы сезін жалғыз нәрсе қал енді өмір мен жақсы сезін жалғыз нәрсе өз неге ал кел дәл осы үшін келесі жол мен бас көр бар алд мен буффало тауық қанат үлкен табағ ранч байла қыздыр отыр салқындат сіз менің ой аз өзгер дос мұны түсіндір бәрі мұны шеш де үміттен propaganda,violent

4,париж рейтер париждегі ең көп сатылатын дүкендердің бірінде жарылғыш зат салған адамның тергеуіндегі негізгі күдіктілер емес деп мәлімдеді қорғаныс министрі эрве морин сәрсенбіде полиция жарылғыш заттарды сейсенбіде printemps дүкенінен тапты ол өзін ауғанстан революциялық майданы деп атайтын және француз әскерлерін ауғанстаннан шығаруды талап еткен ескерту хатынан кейін табылды propaganda,париж рей париж ең көп сатыл дүкен бір жарылғыш зат сал адам тергеу негізгі күдікті емес де мәлімде қорғаныс министр эрв морин сәрсенбі полиция жарылғыш зат сейсенбі printemps дүкен тап ол өз ауғанстан революциялық майдан де ата және француз әскер ауғанстан шығару талап ет ескерту хат кейін таб propaganda,violent

4,мұны растау үшін басқа ақпарат агенттіктерінен де табуға болады frans цитата шабааб полициясының мүшелері сомалидегі қатаң түрдегі шабаба көтерілісшілеріне қолдау көрсетуге уәде берді рождество күні сәтсіз аяқталған ұшақ бортында алькаида тобына көмектесу үшін йеменге соғысушылар жіберетіндерін айтты propaganda,мұны растау үшін басқа ақпарат агенттік де таб бол frans цита шабааб полиция мүше сомали қатаң түр шабаб көтерілісші қолдау көрсет уәде бер рождество күн сәт аяқтал ұшақ борт алькаи тоб көмектес үшін йемен соғысушы жібер ай propaganda,violent

4,бұл жас сарбаздар бірнеше айлық жаттығуларды сәтті аяқтады және олар қазір бүкіл әлемде аллаһтың жауына қарсы қасиетті соғысқа өз бауырларын қосуға дайын деді робов дүйсенбіде йеменнің қорғаныс министрлігінің қызметкері ел исламшыл содырлар үшін ешқашан пана болмайды деп мәлімдеді және желтоқсандағы сияқты жасырынып жатқан жерлеріне көбірек шабуыл жасауға уәде берді шабаб видео http www youtube com watch qwcvrkpqd жаңадан дайындалған альшабаба жалдаушыларды орналастыруға дайын екендігін көрсетеді propaganda,бұл жас сарбаз бірнеше айлық жаттығу сәтті аяқта және олар қазір бүкіл әлем аллаһ жау қарсы қасиетті соғыс өз бауыр қос дайын де робов дүйсенбі йемен қорғаныс министрліг қызметк ел исламшыл содыр үшін ешқашан пан болма де мәлімде және

желтоқсан сияқты жасырын жат жер көбірек шабуыл жаса уәде бер шабаб видео http www youtube com watch qwcvrkpqd жаңа дайындал альшабаб жалдаушы орналастыр дайын екендіг көрсет propaganda,violent

4,сомалияның қатал альшабаб көтерілісшілер тобына жалданған сомалияның қатал исламшыл бүлікшіл альшабаба тобы йемендегі алькаидаға қарсы күштер жіберуге дайын екенін және егер америка құрама штаттары кек қайтаратын болса және басқа да адал адамдарға оны ұстануға шақырса ұйымның йемендегі алькаидаға қолдау көрсетуге дайын екенін айтты propaganda,сомалия қатал альшабаб көтерілісші тоб жалдан сомалия қатал исламшыл бүлікшіл альшабаб тоб йемен алькаида қарсы күш жібер дайын екен және егер америка құрама штат кек қайтар бол және басқа да адал адам оны ұстан шақыр ұйы йемен алькаида қолдау көрсет дайын екен ай propaganda,violent

4,қасиетті соғысқа қатысуға дайын жауынгерлер елде дайындықтан өткен олар өз тактикаларын қолдануға және алланың жауын елден қуып шығаруға анттарын қолдауға дайын бұл бұұ қолдаған үкімет жаңа жылы көтерілісшілерді елден қуып шығару жоспарларын жариялаған кезде басталды альшабаба сомалияның оңтүстік және орталық бөлігін басқарады және қазіргі уақытта tfg бірнеше блокты басқаратын астанадағы қарулы күреске қатысады al mareeg жаңалықтары propaganda,қасиетті соғыс қатыс дайын жауынгер ел дайындық өткен олар өз тактика қолдан және алла жауын ел қу шығар ант қолда дайын бұл бұұ қолда үкімет жаңа жыл көтерілісші ел қу шығару жоспар жарияла кезде баста альшабаб сомалия оңтүстік және орталық бөліг басқар және қазіргі уақыт tfg бірнеше блок басқар астана қарулы күрес қатыс al mareeg жаңалық propaganda,violent

4,мен отбасымды тастап емханамнан кетіп курасаан жеріндегі мүжахидтерге қосылу үшін көлігімді қалдырдым иордания мен американың барлау қызметтері маған миллиардтаған долларлар ұсынды олармен жұмыс істеуге және осы жерде мүжахидтерге барлауды ұсынды бірақ альхамдулилла мен мүжахидтерге келдім мен оларға барлығын айттым және біз американдықтарға алланың біз ұстаған иманның тақваның сенімін барлық байлыққа айырбастауға болмайтынын американдықтарға түсіндіру үшін осы шабуылды бірге ұйымдастырдық propaganda,мен отбас таста емхана кет курасаан жер мүжахид қосылу үшін көліг қал иордания мен америка барлау қызмет маған миллиардта доллар ұсын олармен жұмыс істе және осы же мүжахид барлау ұсын бірақ альхамдулилл мен мүжахид кел мен оларға барлығ ай және біз американдық алла біз ұста иман тақва сен барлық байлық айырбаста болма американдық түсіндіру үшін осы шабуыл бірге ұйымдас propaganda,violent

4,олар пәкістанның техрикиталибан әмірін өлтіргеннен кейін байтулла мехсуд алла оған рақым етсін бұл барлық кафирендерге біз мұсылмандар ретінде мүжахидтер мухажирилер және ансарлар ретінде шейіттерімізді ешқашан ұмытпаймыз тұтқындарымызды ешқашан ұмытпаймыз және аафия сиддики мен сажер рисавиді ешқашан ұмытпаймыз біздің жиһадымыз иншаалла біз тұтқындарды босатқанша және аллаһтың сөзі үстем болғанша жалғасады radicalization,олар пәкістан

техрикиталибан әмір өлтір кейін байтулл мехсуд алл оған рақым ет бұл барлық кафирен біз мұсылман рет мүжахид мухажири және ансар рет шейіт ешқашан ұмытпа тұтқын ешқашан ұмытпа және аафия сиддики мен саж рисави ешқашан ұмытпа біздің жиһад иншаалл біз тұтқын босатқанш және аллаһ сөз үстем болғанш жалғас radicalization,violent

4,ауғанстан ислам эмираты апта сайынғы түсіндірме арыстандардың қолында мак кристал ақ үй генерал мак кийернен мен эйкенберридің сәтсіздігінен кейін ауғанстандағы ақш күштеріне командирлеуге генерал мак кристалды ұсынды ауғанстанда ақ үйдің билеушілері басшылық деңгейіндегі ауысу қалпына келтіру жұмыстарын жеделдетуге және ақштың соқыр бомбалаудың салдарынан жиі болатын бейбіт тұрғындардың шығындарын жоюға бағытталған деп мәлімдеді propaganda,ауғанстан ислам эмират апта сайынғы түсіндірме арыстан қол мак кристал ақ үй генерал мак кийер мен эйкенберри сәтсіздіг кейін ауғанстан ақш күш командирле генерал мак криста ұсын ауғанстан ақ үй билеуші басшылық деңгей ауысу қалп келтіру жұмыс жеделдет және ақш соқыр бомбала сал жиі бол бейбіт тұрғын шығын жою бағыттал де мәлімде propaganda,violent

4,ауғанстан ауғандықтардың отаны мұны ауғандықтарға қалдырыңыз сіз өзіңізді демократтар және адам құқығын қорғаушылар деп атайсыз содан кейін жазықсыз адамдарды түнде үйлерінен шығармаңыз және оларды өлтіріңіз бірақ егер сіз әлі де террорды жалғастырғыңыз келсе және оны жеңіске жетудің қысқа жолы деп санасаңыз онда алдағы көктемде мүжахидтердің жаппай көтерілісі басталғанша күте тұрыңыз сіз мұның бұрынғыға қарағанда кең және ауыр екенін білесіз propaganda,ауғанстан ауғандық ота мұны ауғандық қалдыр сіз өз демократ және адам құқығ қорғаушы де ата содан кейін жазық адам түн үй шығарма және ол өлтір бірақ егер сіз әлі де террор жалғастыр кел және оны жеңіс жету қыс жол де сана онда алдағы көкте мүжахид жаппай көтеріліс басталғанш күт тұр сіз мұның бұрынғы қара кең және ауыр екен біл propaganda,violent

4,дәйексөз бұл үмметті жеңіске жетелейді әрдайым алдыңғы қатарда бол quilaay jange де ауғанстан сіз алдыңғы қатарда болдыңыз солтүстік ауғанстанда біз бүкіл әлеммен бетпебет келген кезде сіз алдыңғы шепте болдыңыз шайкотта ауғанстан сіз кеуделеріңізді жауға қарсы қалқан ретінде қолдандыңыз ал қазір сіз пәкістандасыз жағдай аллаһтың сарбаздары шахид абдул хафидх ресейден жылдың жазында ғана келді propaganda,дәйексөз бұл үмме жеңіс жетеле әрдайым алдыңғы қат бол quilaay jange де ауғанстан сіз алдыңғы қат бол солтүстік ауғанстан біз бүкіл әлем бетпебет кел кезде сіз алдыңғы шеп бол шайкот ауғанстан сіз кеуде жау қарсы қалқан рет қолда ал қазір сіз пәкістан жағдай аллаһ сарбаз шахид абдул хафидх ресей жыл жаз ғана кел propaganda,violent

4,бұл берлинге одан да жақсысы вашингтонға ұшып барады ал бұл ақ үйдің үстіндегі жер және оны қиратып тастайды такбирді айқайла олар раббыларына иман келтірген жастар еді және біз оларды тура жолға салдық олардың жүректеріне күш бердік зымыран нысанаға бүкіл кәшке соққы берді қаружарақ отқа оранды жақында

аударма келді ахи мүмкін сіз де біз үшін аударма жасай аласыз dievoruzge бұл джавад сиддике ағамыздың алғаш пайда болған бөлігі ме propaganda,бұл берлин одан да жақсы вашингтон ұш бар ал бұл ақ үй үст жер және оны қират таста такби айқайла олар раббы иман келтір жас ед және біз ол тура жол сал олардың жүрек күш бер зымыран нысана бүкіл кэш соққы бер қаружарақ от ора жақында аударма кел ахи мүмкін сіз де біз үшін аударма жаса ал dievoruzge бұл джавад сидди аға алғаш пайда бол бөліг ме propaganda,violent

4,джихад және қауіпсіздік кеңестері интернетфорумдарда сіз джихадқа барғым келеді маған кім көмектеседі біз қайда кездесуге болады деген мақалаларды жиі оқуға болады оларды қауіпсіздік қызметіндегі арандатушылар немесе шынайы бірақ аңғал мұсылмандар орналастырады біз бұл жұмысты осындай ақымақ бауырластарға жолдағымыз келеді джихад жеке парыз бірақ дұға ретінде белгілі бір шарттардың орындалуын талап етеді propaganda,джихад және қауіпсіздік кеңес интернетфорум сіз джихад бар кел маған кім көмектес біз қайда кездес бол де мақала жиі оқ бол ол қауіпсіздік қызметіндегі арандатушы немесе шынайы бірақ аңғал мұсылман орналастыр біз бұл жұм осындай ақы бауырлас жолда кел джихад жеке парыз бірақ дұ рет белгілі бір шарт орындал талап ет propaganda,violent

4,анасына немесе сенімді достарына кейде кейбір мұсылмандардың туыстық байланысы кәпір күштердің мүшелеріне зиян тигізуінің алдын алады деп ойлайды кавказдағы соғыстың қасіреті соншалықты жоғары деңгейге жеткенін жергілікті полиция қызметкерлері өздерінің содырлық туыстарын немесе содырларға байланысы бар деп күдіктенгендерді атуға ұрлауға және азаптауға тартынбайтындығын нақты түсіну керек хачаройлық әбу анас дайындаған дереккөз hunafa com аударған кавказ блогы кавказ орталығы propaganda,ана немесе сенімді дос кейде кейбір мұсылман ту байланыс кәпір күш мүше зиян тигізу алдын ал де ойла кавказ соғ қасірет соншалықты жоғары деңгей жет жергілікті полиция қызметкер өз содырлық туыс немесе содыр байланыс бар де күдіктен ат ұрла және азапта тартынбайтындығ нақты түсіну керек хачаройлық әбу анас дайында дереккөз hunafa com аудар кавказ блог кавказ орталығ propaganda,violent

4,содан кейін араб түбегінен мушриктерді қуып шығыңыз хамза әлкута ия amp бауырлар батальоны чемпионы шейіт ағасы абу убайда әлжаррах оңтүстік кореялық туристер тобына қарсы шәһидтік әрекетін жасады онда төрт адам қаза тапты ал қалғандары хадрамаут провинциясындағы шебам ауданында жарақат алды radicalization,содан кейін араб түбег мушрик қу шығ хамза әлку ия amp бауыр батальон чемпион шейіт аға абу убай әлжаррах оңтүстік кореялық турист тоб қарсы шәһид әрекет жас онда төрт адам қаза тап ал қал хадрамаут провинция шеба аудан жарақат алды radicalization,violent

4,біз бірнеше рет араб түбегіне келген мушриктермен уәде аяқталғанын жарияладық бірақ олар бас тартты бірақ арабия түбегіне кіріп өлтірілді содан кейін олар күн сайын өлім тостағанынан ішуі керек өйткені олардың кепілін және қанын ешқашан әласвад альэнаси үкіметі қорғамайды али абдулла салех алла оның істерін толықтай

басқарады және оны басқарады бірақ көпшілік біле бермейді араб түбегіндегі джихад ұйымының негізі propaganda,біз бірнеше рет араб түбег кел мушрик уәде аяқтал жарияла бірақ олар бас тар бірақ арабия түбег кір өлтір содан кейін олар күн сайын өлім тоста іш керек өйткені олардың кепіл және қан ешқашан әласвад альәнаси үкімет қорғама али абдулл салех алл оның іс толықта басқар және оны басқар бірақ көпшілік біл берме араб түбег джихад ұйым негіз propaganda,violent

4,дихадқа қалай дайындалуға болады өзімді джихадқа қалай үйрете аламын жауапкершіліктен бас тарту джихад дегеніміз не әскери дайындық дегеніміз мұсылмандық міндет емес ниеті сіздің тұрғылықты еліңізде немесе ұлыбританиядағы атыс қаружарақтары бойынша джихад жаттығулары туралы маңызды ескерту білмеуі мүмкін осы тақырып туралы бірнеше электронды хат алғаннан кейін біз осы тақырып туралы шағын мақала қосуды жөн көрдік propaganda,дихад қалай дайындал бол өз джихад қалай үйрет ал жауапкершілік бас тарту джихад де не әскери дайындық де мұсылман міндет емес ниет сіздің тұрғылықты ел немесе ұлыбритания атыс қаружарақ бойынша джихад жаттығу туралы маңызды ескерту білме мүмкін осы тақырып туралы бірнеше электронды хат ал кейін біз осы тақырып туралы шағын мақала қосу жөн көр propaganda,violent

4,кейбір елдерде джихад жаттығуларын алуға болады бірақ біз кез келген нақты елдің жарамдылығы немесе жарамсыздығы туралы түсінік бере алмаймыз сіз білетін және сенетін адамдармен байланысыңыз олар сізге жақсы кеңес бере алады егер сен алланың алдында адал болсаң алла сенің алдыңда адал болады және ол сені қалаған нәрсені жасаудың жолын табады propaganda,кейбір ел джихад жаттығу ал бол бірақ біз кез кел нақты ел жарамдылығ немесе жарамсыздығ туралы түсінік бер алма сіз біл және сен адам байланыс олар сізге жақсы кеңес бер ал егер сен алла алд адал бол алл сенің алд адал бол және ол сен қала нәрсе жаса жол таб propaganda,violent

4,мені жақсы түсінбеймін деп ойлаймын мен ол түркістаннан емес ол түріктенмін дедім сондықтан мен оны білемін мен оған тиесілі барлық видеоларды көремін ол ауғанстанда операцияларды жүргізді екінші жағынан мен қытай түріктері басып алған шығыс түркістанды білемін сондықтан біз сіздерді қытайлықтарға қарсы қолдаймыз бірақ мен үшін исламдық жерлердің бәрі бірдей қазір барлық ислам жерлері жаулап алынады сондықтан біз жаулап алуды тоқтатып халифа құруымыз керек propaganda,мен жақсы түсінбе де ойла мен ол түркістан емес ол түрік де сондықтан мен оны біл мен оған тиесілі барлық видео көр ол ауғанстан операция жүр екінші жағ мен қытай түрік бас ал шығыс түркістан біл сондықтан біз сіз қытайлық қарсы қолда бірақ мен үшін исламдық жер бәрі бірде қазір барлық ислам жер жаула алын сондықтан біз жаула алу тоқтат халиф құр керек propaganda,violent

4,егер сіз біздің түрік тілін түсінсеңіз мен сізді жеке хабарлама арқылы түрікше жазғым келеді соңғы осман және басқа империялар қазір өмір сүрмейді біздің міндетіміз жаңа хилафалар құру айтпақшы мен сіздің жеке хабарламаңызды күтемін мен сізді түрік джихадының сайты http forum takva com index php esselamun aleykum ve rahmatullahi ve barakatuhu propaganda,егер сіз біздің түрік тіл түсін мен сіз жеке

хабарлама арқылы түрікш жаз кел соңғы осман және басқа империя қазір өмір сүрме біздің міндет жаңа хилафа құру айтпақшы мен сіздің жеке хабарлама күт мен сіз түрік джихад сай http forum takva com index php esselamun aleykum ve rahmatullahi ve barakatuhu propaganda,violent

4,исламабад пәкістан астанасының оңтүстігінде шииттердің көп жиналған мешітінде жанкештілік жарылыс адамның өмірін қиды бұл ақш одақтас елдегі қауіпсіздік әлкаида мен талибан содырлары өсіп жатқан ауғанстан шекарасынан тыс жерде қалай құлдырап жатқанының соңғы дәлелі зорлықзомбылық propaganda,исламабад пәкістан астана оңтүстіг шиит көп жинал мешіт жанкештілік жарылыс адам өмір қи бұл ақш одақтас ел қауіпсіздік әлкаи мен талибан содыр өс жат ауғанстан шекара тыс же қалай құлдыра жат соңғы дәлел зорлықзомбылық propaganda,violent

4,буш пен браунның ауғанстанға сапарлары және париж конференциясы туралы құрметті мулла ағай ахонд ауғанстан ислам әмірлігінің орынбасары баспасөз мәлімдемесі басқыншы елдердің басшылары мен аға шенеуніктерінің сапарлары және халықаралық конференция негізінен ешқандай нәтижесіз аяқталған ауғанстанның атымен тек символдық құндылықтар бар propaganda,буш пен браун ауғанстан сапар және париж конференция туралы құрметті мулл аға ахонд ауғанстан ислам әмірліг орынбасар баспасөз мәлімде басқынш ел басшы мен аға шенеунік сапар және халықаралық конференция негіз ешқандай нәтижесіз аяқтал ауғанстан ат тек символ құндылық бар propaganda,violent

4,егер олар мұны жасамаса олар әрдайым мужахидтердің шешуші шабуылдарының нәтижесінде масқара болады олардың ауғанстанға көбірек әскер жіберетіні бұл олардың күштерінің көбеюі емес олар қаза тапқан сарбаздардың орнын толтырғысы келеді егер бұл процесс жалғаса берсе олардың әскери күштері толығымен жойылатын күн жақын емес ауғанстан ислам әмірлігі propaganda,егер олар мұны жасама олар әрдайым мужахид шешуші шабуыл нәтиже масқара бол олардың ауғанстан көбірек әскер жібер бұл олардың күш көбею емес олар қаза тап сарбаз орн толтыр кел егер бұл процесс жалға бер олардың әскери күш толығ жойыл күн жақын емес ауғанстан ислам әмірліг propaganda,violent

4,ескерту мұны кафир бақ сайтынан алынған сонымен оның сенімділігі күмән тудырады алайда мұнда ешқандай бұрмалауды немесе ойдан шығаруды ұсынатын ештеңе жоқ алла жақсы біледі дәйексөз талибан жетекшісі мулла омар мохаммадпен минуттық сұхбат пушту қаласында американың дауысы қоғамдық қорымен қаржыландырылды өткен жұмада ақш мемлекеттік хатшысының орынбасарлары мен ұлттық қауіпсіздік кеңесінің жоғары лауазымды тұлғаларының қарсылықтары болғаннан кейін хабар таратылды propaganda,ескерту мұны кафи бақ сайт алын сонымен оның сенімділіг күмән тудыр алайда мұнда ешқандай бұрмалау немесе ой шығару ұсын ештеңе жоқ алл жақсы біл дәйексөз талибан жетекші мулл омар мохаммад минуттық сұхбат пушт қала америка дауыс қоғамдық қор қаржыландыр өткен жұма ақш мемлекеттік хатшы орынбасар мен ұлттық қауіпсіздік кеңес жоғары лауазым тұлға қарсылық бол кейін хабар тарат propaganda,violent

4,жеткіліксіз нәрсе жасау демек бұл тұрғыда біз бәріміз де модернміз орта жолға түсеміз кең ауқымды үкімет үшін күрес соңғы жыл бойы жалғасып келеді бірақ одан ештеңе шықпады біз залымдар үкіметін қабылдамаймыз біз зұлым үкіметтің бөлігі болғаннан гөрі өлімді қалаймыз мен саған айтамын осыны есте сақта бұл менің болжауым сіз оған сенесіз бе жоқ па бұл сізге байланысты бірақ күтуге және көруге тура келеді propaganda,жеткіліксіз нәрсе жасау демек бұл тұрғы біз бәрі де модерн орта жол түс кең ауқымды үкімет үшін күрес соңғы жыл бойы жалғас кел бірақ одан ештеңе шықпа біз залым үкім қабылдама біз зұлым үкімет бөліг бол гөрі өл қала мен саған ай осы ес сақта бұл менің болжа сіз оған сен бе жоқ па бұл сізге байланысты бірақ күт және көр тура кел propaganda,violent

4,алькааида жылдары африкаға көшіп келеді мұнда сада альджихад журналының шығарылым маусымы африка аймағындағы мужахидтердің қызығушылығы туралы түсінік берілген мақаланың аудармасы келтірілген есіңізде болсын бұл жыл бұрын жазылған сондықтан кейбір нәрселер жалпы әсіресе әлкаиаидаға әсіресе мұжахидтердің пайдасына өзгерді propaganda,алькааи жыл африка көш кел мұнда са альджихад журнал шығарылым маусым африка аймағ мужахид қызығушылығ туралы түсінік бер мақала аудар келтір ес бол бұл жыл бұрын жазыл сондықтан кейбір нәрсе жалпы әсіресе әлкаиаида әсіресе мұжахид пайда өзге propaganda,violent

4,жалпы бұл континенттің маңызы зор африканы кім қараса оның крестшілерге қарсы соғыста оның қызығушылығы күшжігері мен белсенділігі ұнамайтынын көре алады бұл көптеген потенциалы артықшылығы бар құрлық сондықтан оны пайдалану джихадқа көп пайда әкеледі бұл джихадтың күтілетін мақсаттарына жетуге ықпал етеді африка бұл джихад пен джихадтың ілгерілеуі үшін құнарлы топырақ http theunjustmedia com islamic africa htm propaganda,жалпы бұл континент маңыз зор африка кім қара оның крестші қарсы соғыс оның қызығушылығ күшжіг мен белсенділіг ұнама көр ал бұл көптеген потенциал артықшылығ бар құрлық сондықтан оны пайдалану джихад көп пайда әкел бұл джихад күтіл мақсат жет ықпал ет африка бұл джихад пен джихад ілгеріле үшін құнарлы топырақ http theunjustmedia com islamic africa htm propaganda,violent

4,сомали диаспорасы қасиетті соғысқа шақыруды тыңдады www inet co za бірі ақштағы такси жүргізушісі екіншісі ұлыбританиядағы наубайшы енді олар өздері туған сомалиге алланың дұшпандарымен күресу үшін оралды могадишу біреуі америка құрама штаттарында такси жүргізушісі екіншісі ұлыбританияда наубайханашы болған енді олар өздері туған сомалиге алланың дұшпандарымен күресу үшін оралды propaganda,сомали диаспора қасиетті соғыс шақыру тыңда www inet co za бірі ақш такси жүргізуші екінші ұлыбритания наубайш енді олар өз туған сомали алла дұшпан күресу үшін ора могадиш біреу америка құрама штат такси жүргізуші екінші ұлыбритания наубайханаш бол енді олар өз туған сомали алла дұшпан күресу үшін ора propaganda,violent

4,біз бәріміз бірдей қалалардан емеспіз бірақ мен сияқты америкадан және еуропаның басқа елдерінен де аллаһтың жауларына қарсы соғысқа қатысатын

көптеген жас жігіттер бар және мен олардың көпшілігі келеді деп үміттенемін дереккөз http news za msn com africa article aspx cpdocumentid propaganda,біз бәрі бірде қала емес бірақ мен сияқты америка және еуропа басқа ел де аллаһ жау қарсы соғыс қатыс көптеген жас жігіт бар және мен олардың көпшілік кел де үміттен дереккөз http news za msn com africa article aspx cpdocumentid propaganda,violent

4,түрмелеріндегі сұмдықтар майкл ашылды ол бұл адам жыл бұрын косоводағы азаттық армиясының лагерінде болған қасіреттерді түсіндірген кезде ашық айтты пышақпен кесілген бірнеше күн тамақсыз қалған атып өлтірілген адамдарды қалай көргенін айтып берді азаматтық азаматтарды қха ұстады және түрмелерде ұстады кейбіреулер өлтірілді сіз бұл нәрселерді көргенде не сезінесіз ол айтты бұл менің өмірімде есімде қалды сіз бұны адамдарға тіпті жануарларға да жасай алмайсыз radicalization,түрме сұмдық майкл аш ол бұл адам жыл бұрын косово аза армия лагер бол қасірет түсіндір кезде ашық ай пышақ кес бірнеше күн тамақ қалған ат өлтір адам қалай көр айт бер азаматтық азамат қх ұста және түрме ұста кейбіреу өлтір сіз бұл нәрсе көргенде не сез ол ай бұл менің өмір ес қал сіз бұны адам тіпті жануар да жаса алма radicalization,violent

4,косоводағы біріккен ұлттар ұйымы шенеуніктерінің айтуынша кейбіреулер өлтірілген тағы бір себебі бар біз сөйлескен барлық адамдар өздерінің косовалық патриоттары екендіктерін және ел тәуелсіздігін қорғау үшін қайтадан қару ұстайтындарын айтты бірақ дәл осы жерде тәуелсіздік косовоға өткен жылы келді олардың соғыс мақсатына жетті бұрынғы кла жауынгерлерінің бірі айтқандай қазір өзімізге адал болып нақты мемлекет құратын кез келді propaganda,косово біріккен ұлт ұйым шенеунік айт кейбіреу өлтір тағы бір себебі бар біз сөйлес барлық адам өз косовалық патриот екендік және ел тәуелсіздіг қорғау үшін қайта қару ұста ай бірақ дәл осы же тәуелсіздік косово өткен жыл кел олардың соғыс мақсат же бұрынғы кл жауынгер бірі айтқанда қазір өз адал бол нақты мемлекет құр кез кел propaganda,violent

4,орналастырылған pm шабель сомалия могадихсу м желісі эфиопия әскерлері мен исламшыл көтерілісшілер арасындағы ауыр шайқас кезінде кемінде он адам қаза тауып жиырмадан астам адам жарақат алды сәрсенбіде куәгерлер мен шенеуніктер могадишодан солтүстікке қарай якшид ауданында күштер жиналды бір отбасының жеті адамы үйіне миномет құлап ондаған адам жарақат алды radicalization,орналастырыл pm шабель сомалия могадихс м желі эфиопия әскер мен исламшыл көтерілісші ара ауыр шайқас кез кемінде он адам қаза тау жиырма астам адам жарақат алды сәрсенбі куәгер мен шенеунік могадишо солтүстік қарай якшид аудан күш жина бір отбас жеті адам үй миномет құла ондаған адам жарақат алды radicalization,violent

4,эфиопиялықтармен деп аталатын келісімге бойкот жариялауға шақырамыз деді әбу мансур сомалияны азат ету одағына сілтеме жасап эфиопиялық әскерлер сомалиге әлсіз өтпелі үкіметке көмектесу үшін жылы кірді эфиопияның ұзаққа созылған оккупациясы миллиондаған тұрғындарды ығыстырып мыңдаған адамның өмірін қиды shabelle media network авторы ахмеднор мохамед фарах propaganda,эфиопиялық де

атал келісім бойкот жарияла шақыр де әбу мансу сомалия азат ету одағ сілтеме жаса эфиопиялық әскер сомали әлсіз өтпелі үкімет көмектес үшін жыл кір эфиопия ұзақ созыл оккупация миллиондаған тұрғын ығыстыр мыңдаған адам өмір қи shabelle media network авто ахмедно мохамед фарах propaganda,violent

4,біз сіздің соңғы жаңалықтарыңызды қадағалап отырмыз және бұл біздің жүрегімізді зор қуанышқа толтырады сіздерді жеңістеріңіз бен жетістіктеріңізбен құттықтағымыз келеді альшабаб өздері басқаратын аудандарды кеңейтіп қана қоймай олар шариғатты жүзеге асырды және бізге мұсылмандар ретінде біздің жағдайымызды қалай өзгертуіміз керек екендігінің нақты мысалын келтірді propaganda,біз сіздің соңғы жаңалық қадағала отыр және бұл біздің жүрег зор қуаныш толтыр сіз жеңіс бен жетістік құттықта кел альшабаб өз басқар аудан кеңейт қана қойма олар шариғат жүзеге ас және бізге мұсылман рет біздің жағдай қалай өзгерт керек екендіг нақты мысал кел propaganda,violent

4,халықтың көпшілігі трайбализмнің надандықтың және шариғатты қорлау науқанының зардабын тартып жатыр сондықтан сіз адамдардың жүрегі мен санасын иеленіп оларды фитрасына қайтаруыңыз керек егер менің жағдайым рұқсат етілген болса мен сізге қосылудан және сіздің қатарыңызда солдат болудан тартынбаймын бауырыңыз анвар альавлак http www anwaralawlaki com abofsomalia propaganda,халық көпшілік трайбализ надандық және шариғат қорла науқан зардаб тарт жатыр сондықтан сіз адам жүрег мен сана иелен ол фитра қайтар керек егер менің жағдай рұқсат ет бол мен сізге қосылу және сіздің қат солдат болу тартынба бауыр анвар альавлак http www anwaralawlaki com abofsomalia propaganda,violent

4,алькаида сомалилерді кемелерге шабуыл жасауға шақырады сәуір cbs сауд арабиясының алькаида аға операциясы сомали джихадшыларын теңіздегі қарақшылардың шығанағында теңіздегі крестшілер күштеріне шабуылдарын күшейтуге шақырды аден және францияның африкадағы ең ірі әскери базасы орналасқан көрші джибути жерінде сомалидегі біздің сабырлы бауырларымызға сақ болыңыздар дайындалыңыздар recruitment,алькаи сомали кеме шабуыл жаса шақыр сәуір cbs сауд арабия алькаи аға операция сомали джихадшы теңіз қарақшы шығанағ теңіз крестші күш шабуыл күшейт шақ аден және франция африка ең ірі әскери база орналас көрші джибути же сомали біздің сабырл бауыр сақ бол дайындал recruitment,violent

4,аллаға ант етсек біз олармен арабия түбегінде үлкен қаланы ашамыз ол қаласа крест жорықтарын тазартып аймақтағы крест жорықтары мен еврейлердің амбицияларын тоқтататын жеңістің кілті бұл нато мен ішкі күштер содырларға көбірек қысым көрсетіп жатқан пәкістан мен ауғанстандағы алькаида мен талибанды қыздыруға тырысуға және ант қабылдауға ант болды дереккөз propaganda,алла ант ет біз олармен арабия түбег үлкен қала аш ол қала крест жорық тазарт аймақ крест жорық мен еврей амбиция тоқтат жең кілт бұл нато мен ішкі күш содыр көбірек қысым көрсет жат пәкістан мен ауғанстан алькаи мен талибан қыздыр тырыс және ант қабылда ант бол дереккөз propaganda,violent

4,цитата алдымен көтерілісші сирия осы дінді ұстанушыларға қарсы үлкен соғыс жоспарлап отыр және ол егер бауырластарға қысым көрсетуді күшейтсе олармен қатты төбелес болатынын біледі өйткені олар қудалауды елемейтінін біледі және біз бұл ұрысты бастамадық егер олар соғысуды таңдаса онда мен сириядағы сатқындарға және олардың артынан араб тавагеті мен сионистік қарғысшыларға айтамын propaganda,цита алд көтерілісш сирия осы дін ұстанушы қарсы үлкен соғыс жоспарла отыр және ол егер бауырлас қысым көрсету күшейт олармен қатты төбелес бол біл өйткені олар қудалау елеме біл және біз бұл ұр бастама егер олар соғысу таңда онда мен сирия сатқын және олардың арт араб таваге мен сиони қарғысшы ай propaganda,violent

4,адамдар бостандықты күш пен абыройды іздейді және сіз жүзеге асырылатын нәрсе сіз мұны естіп қана қоймай американдықтар мен еврейлердің жаңа құлдары боласыз мен алладан а айқышты жеңіп алушыны оның тавагет құлдарының жеңімпазын және шаам елдерінің рыцарларын әбу хуссейн шакир әлабссиді және оның отбасын және онымен бірге жүргендерді тұтқындауды сұраймын олардың жүректерін біріктіріп өлтірілгендерді шейіт ретінде қабылдаңыз жалғасы бар иншаллаһ ассаламуалейкум propaganda,адам бостандық күш пен абырой ізде және сіз жүзеге асырыл нәрсе сіз мұны ест қана қойма американдық мен еврей жаңа құл бол мен алла а айқыш жең алушы оның тавагет құл жеңімпаз және шаа ел рыцар әбу хуссейн шаки әлабсси және оның отбас және онымен бірге жүр тұтқындау сұра олардың жүрек біріктір өлтіріл шейіт рет қабылда жалғасы бар иншаллаһ ассаламуалейку propaganda,violent

4,шабель сомалия могадишу sh network шейх моктар робов али абу мансор ретінде белгілі әлшабаб исламистік тобының өкілі түні бойы телефон арқылы баспасөз мәслихатын өткізді және олардың соңғы қастандықтарға қатысы барын жоққа шығарды сомали астанасы могадишода шейх абу мансор атылып өлтірілгендердің әйгілі исламшыл күрескерлер болғанын және сомалиге басып кірген эфиопия әскерлеріне қарсы соғысқа қатысқанын және ол олардың өлтірілуінің артында емес екенін айтты propaganda,шабель сомалия могадиш sh network шейх мок робов али абу мансо рет белгілі әлшабаб ислами тоб өкіл түн бойы телефон арқылы баспасөз мәслихат өт және олардың соңғы қастандық қатыс бар жоқ шығ сомали астана могадишо шейх абу мансо атыл өлтіріл әйгілі исламшыл күрескер бол және сомали бас кірген эфиопия әскер қарсы соғыс қатыс және ол олардың өлтірілу арт емес екен ай propaganda,violent

4,астанадағы кейбір базаларға шабуыл жасалғанын олар шабуыл жасаған топтардың екеуін және бір автокөлікті басып алды деп мәлімдеді альшабаб исламдық ұйымының мәлімдемесі ислам соттар одағының лауазымды тұлғалары могадишода жақында болған кейбір офицерлерді өлтірудің артында альшабаб ұйымы тұр деген айғақтар бар деп мәлімдеді propaganda,астана кейбір база шабуыл жасал олар шабуыл жаса топ екеу және бір автокөлік бас алды де мәлімде альшабаб исламдық ұйым мәлімде

ислам сот одағ лауазым тұлға могадишо жақында бол кейбір офицер өлтіру арт альшабаб ұйым тұр де айғақ бар де мәлімде propaganda,violent

4,ирактағы алькаида жетекшісі wmd джихадына зәру екенін жариялады cbs news тергеулер жүргізді осы постқа түсініктеме ирактағы алькаиданың жетекшісі жаулардың кәдімгі қарудан артықшылығын өтеу үшін химиялық биологиялық және тіпті ядролық қаруды алу қажеттігіне тоқталды әбу хамза әлмухаджирдің пікірлері дүйсенбіде интернетте жарияланған аудио сұхбатында айтылды propaganda,ирак алькаи жетекші wmd джихад зәр екен жариял cbs news тергеу жүр осы пост түсініктеме ирак алькаида жетекші жау кәдімгі қару артықшылығ өтеу үшін химиялық биологиялық және тіпті ядролық қару алу қажеттіг тоқта әбу хамза әлмухаджи пікір дүйсенбі интернет жариялан аудио сұхбат айт propaganda,violent

4,израильде тым радикалды үкімет орныққандықтан одан бетер жамандық әлі келе бермейтінін ескертті альмухажир американың бұдан былай мұсылмандарға қарсы тағы бір соғысқа қатыса алмайтынын айтты және еуропалықтар мен ирандықтардың енді крестшілердің исламға қарсы соғысын бастайды деп болжады код http www cbsnews com blogs monitor entry shtml propaganda,израиль тым радикал үкімет орныққандық одан бетер жаман әлі келе берме ескер альмухажи америка бұдан былай мұсылман қарсы тағы бір соғыс қатыс алма ай және еуропалық мен ирандық енді крестші ислам қарсы соғыс баста де болжа код http www cbsnews com blogs monitor entry shtml propaganda,violent

4,дәйексөз бастапқыда tarbiya саламам алейкум ихваани уа ахавати жоғарыда келтірілген мақаланы оқығаннан кейін мен сізге аздан шыққан өтірік екенін айта аламын куффар өтірік айтқандарына мән бермеңіз осы мақалада қандай күлкілі талаптар айтылған талибан мухажирді руфуз етті propaganda,дәйексөз бастапқы tarbiya салама алейку ихваани уа ахавати жоғары келтір мақала оқы кейін мен сізге аз шық өтірік екен ай ал куфф өтірік айт мән берме осы мақала қандай күлкіл талап айтыл талибан мухажи руфуз ет propaganda,violent

4,тағы бір мунафик ақпарат агенті өзінің визасын азаматтығын алу үшін фидтерге тағы да өтірік айтты мен олар ең нашар сценарийде зымырандар мен оқдәрілерді алып жүруге болатындай етіп ойладым егер олар шайқаста пайдалы болса егер олар ұятсыз болса бауырлас осы американдықтарға қиял жасау керек бұл тіпті хижра жасауға хиджра жасаудан адамдарды арылтудың бір тәсілі болуы мүмкін propaganda,тағы бір мунафик ақпарат агент өзінің виза азаматтығ алу үшін фид тағы да өтірік ай мен олар ең нашар сценарий зымыран мен оқдәрі алып жүр болатында ет ойла егер олар шайқас пайдалы бол егер олар ұят бол бауырлас осы американдық қиял жасау керек бұл тіпті хижр жаса хиджр жасау адам арылт бір тәсіл бол мүмкін propaganda,violent

4,ассаламу алейкум дәл менің жеке жазбаларымда мұсылмандарға жеке джихад міндеттерін орындауды джихадты ұйымдастыруға үйымдастырылған ішкі топ құруға кедергі келтіретін қауіпсіздік проблемаларына қарамастан ұсынып отырмын міне мен бұдан бір жыл бұрын жазған және ші ғасырдың жиһадтары жеке қызығушылық пен

қатысуды талап етеді propaganda,ассалам алейку дәл менің жеке жазба мұсылман жеке джихад міндет орындау джихад ұйымдастыр үйымдастырыл ішкі топ құр кедергі келтір қауіпсіздік проблема қарамастан ұсын отыр міне мен бұдан бір жыл бұрын жаз және ші ғасыр жиһад жеке қызығушылық пен қатысу талап ет propaganda,violent

4,наркоанализ және өтіріктерді анықтаудың басқа әдістері қазір мұсылман солдатын еске алу кезінде жиірек қолданылуы мүмкін басқа кедергілер жалдаушылар үшін ұят дегенді білдіреді ұят дегеніміз өлі және тазаланбаған ги позицияларын толтыратын қорқақтар мен пайдасыз адамдар көп деген сөз қысқасы ұйқысыз уақыт мұсылмандармен байланысудың айқын қорқынышты болуына байланысты біздің халықты ми жууға және оларды кафтарлар қатарына қосуға көбірек көңіл бөлгісі келеді propaganda,наркоанализ және өтірік анықта басқа әдіс қазір мұсылман солд ес алу кез жиірек қолданыл мүмкін басқа кедергі жалдаушы үшін ұят де білдір ұят де өлі және тазаланба ги позиция толтыр қорқақта мен пай адам көп де сөз қысқа ұйқы уақыт мұсылман байланыс айқын қорқыныш бол байланысты біздің халық ми жу және ол кафтар қат қос көбірек көңіл бөл кел propaganda,violent

4,дәйексөз бастапқыда binyamine интернеттегі көру сайтымен бөлісуден тартынбаңыз оны ережелері рұқсат етілген форумға орналастырыңыз кәпир әскерінде қорқыныш оларды күтіп тұрған тағдырды көрсетеді ассалаумағалейкум құрметті бауырым сіз форумды білесіз сіз өзіңіз қатысасыз онда сіз бұл бас кесетін бейнелерді орналастыруға рұқсат етілді ме жоқ па деген сұрақты қойдыңыз ал әкімдер әйелдер балалар бұл сайтқа кіреді деп жауап беруден бас тартты propaganda,дәйексөз бастапқы binyamine интернет көру сайт бөлісу тартынба оны ереже рұқсат ет форум орналастыр кәпи әскер қорқыныш ол күт тұр таға көрсет ассалаумағалейку құрметті бауыр сіз форум біл сіз өз қатыс онда сіз бұл бас кес бейне орналастыр рұқсат ет ме жоқ па де сұрақ қой ал әкім әйел бала бұл сайт кір де жауап беру бас тар propaganda,violent

4,куфраны басу сияқты алвалая валь бараа туралы білмейді және с осы форумнан шабыттандырған мен жасаған ережелер онда ол бірнеше күннің ішінде жад шұңқырынан жоғалып кетер еді тақырыптан секіргеніңіз үшін кешіріңіз бірақ иншалла сізге сізге хабарлау керек еді көмектескіңіз келсе осы вебсайтқа өз үлесіңізді қосыңыз иншалла сіздің күшжігеріңіз жеміс әкеледі және ысырап болмайды уәлсалам propaganda,куфра басу сияқты алвалая валь бара туралы білме және с осы форум шабыттандыр мен жаса ереже онда ол бірнеше күн іш жад шұңқыр жоғал кет ед тақырып секір үшін кешір бірақ иншалл сізге сізге хабарла керек ед көмектес кел осы вебсайт өз үлес қос иншалл сіздің күшжіг жеміс әкел және ысырап болма уәлсала propaganda,violent

4,әркім сіз жасау әдістерін үйренуіңіз керек маңызды және сіз химиялық қару шығара аласыз үйреніңіз және қолданыңыз мен қаружарақ пен қазіргі соғыс туралы бәрін білемін мен соман мен сарин газын және кезкелген жарылғыш зат шығара аламын сіз барлығын бірнеше компрессорлы және вакуумды машинамен көмірмен және аммониймен hno және naoh лиохпен hpo және so және сумен жасай аласыз

propaganda,әркім сіз жасау әдіс үйрен керек маңызды және сіз химиялық қару шығар ал үйрен және қолдан мен қаружарақ пен қазіргі соғыс туралы бәрін біл мен соман мен сарин газ және кезкел жарылғыш зат шығар ал сіз барлығ бірнеше компрессорл және вакуум машина көмір және аммоний hno және naoh лиох hpo және so және су жаса ал propaganda,violent

4,менің әкем қайтадан кафтерлермен және мұсылман өлтірушілермен күресу үшін боснияға кетті олар балқан ормандарында мұсылмандарды ұстады жылы олар шешенияға кетті қазір менің достарым ауғанстан мен пакистанда біз жиһадты ақшамен қанымызбен және жұмысымызбен қолдай аламыз оқ доллар және канас доллар ал зымыран доллар ал зымырандардың бағасы жоғары көлігіңізді сатыңыз және бізге жолаушылар ұшағын атып түсіріңіз propaganda,менің әке қайта кафтер және мұсылман өлтіруші күресу үшін босния кет олар балқан орман мұсылман ұста жыл олар шешения кет қазір менің дос ауғанстан мен пакистан біз жиһад ақша қан және жұмыс қолда ал оқ доллар және канас доллар ал зымыран доллар ал зымыран баға жоғары көлік сат және бізге жолаушы ұшағ ат түсір propaganda,violent

4,тоқ жанғұт бауырым мен исламның мелайю патшалығына қалай келгендігі туралы ойлаймын халифа немесе сұлтания кезінде болды ма тарихи тұрғыдан фатани даруссаллам малайзияның құрамына кірді ме мен малайзияда бірнеше жыл болдым шындығында мен мұнда хижра жасай аламын деп ойладым propaganda,тоқ жанғұт бауыр мен ислам мелайю патшалығ қалай келгендіг туралы ойла халиф немесе сұлтания кез бол ма тарихи тұрғы фатани даруссалла малайзия құрам кір ме мен малайзия бірнеше жыл бол шындығ мен мұнда хижр жаса ал де ойла propaganda,violent

4,бұл жақсы жаңалық олар фатанаи халқына лайықты исламдық жиһадты насихаттай алады деп сенемін иншалла олар шынымен адамдарды шариғатқа шақырады ма немесе тек патаниді қайтарғысы келеді ме егер олар бар болса девахи мен кем дегенде иншаллаһ олар ондағы адамдарды калиматуллахияляя үшін джихадты түсінуге үйрететініне өте қуаныштымын уәжакаллаһу ғайыр propaganda,бұл жақсы жаңалық олар фатанаи халқ лайықты исламдық жиһад насихатта ал де сен иншалл олар шын адам шариғат шақыр ма немесе тек патани қайтар кел ме егер олар бар бол девахи мен кем де иншаллаһ олар ондағы адам калиматуллахияляя үшін джихад түсін үйрет өте қуанышты уәжакаллаһ ғайы propaganda,violent

4,aslamoalaikum бауырым бұл мәселеде дәлелдеуге тура келеді егер сіз пакистан газетін оқитын болсаңыз тіпті журнал мен газеттерге тиесілі болсаңыз let және джем сияқты ұйымдарға бұл талибан мен алкеда сияқты мужахидтерге қарсы жаңалықтармен толықты және олар пакистан армиясымен және армиясымен тығыз байланысты propaganda,aslamoalaikum бауыр бұл мәселе дәлелде тура кел егер сіз пакис газ оқитын бол тіпті журнал мен газет тиесілі бол let және джем сияқты ұйым бұл талибан мен алке сияқты мужахид қарсы жаңалық толық және олар пакис армия және армия тығыз байланысты propaganda,violent

4,пәкістандағы исламдық ұйымдардағы немесе басқалары христиан крест жорықтарының әскери қылмыскерлері мен оның қуыршақ агенттері қатыгез пұтқа табынушы билеушілердің шектеулерінен босатылған джихад керуеніне қосылыңыз пәкістан мұсылмандары сізге опасыздық жасаған опасыздық жасаған және пәкістанды қиратқандар пәкістанның сатқын үкіметі және оның мұсылмандарды өлтіретін әскері http ansarnet info showthread php lashker taiba propaganda,пәкістан исламдық ұйым немесе басқа христиан крест жорық әскери қылмыскер мен оның қуыршақ агент қатыгез пұт табынуш билеуші шектеу босатыл джихад керуен қосыл пәкістан мұсылман сізге опасыз жаса опасыз жаса және пәкістан қират пәкістан сатқ үкімет және оның мұсылман өлтір әскер http ansarnet info showthread php lashker taiba propaganda,violent

0,адамның басшысы ақыл,адам басшы ақыл,neutral

0,жетекшісі талап,жетекші талап,neutral

0,жолаушысы ой,жолаушы ой,neutral

0,жолдасы кәсіп,жолда кәсіп,neutral

0,қорғаны сабыр,қор сабыр,neutral

0,қорғаушысы мінез,қорғаушы мінез,neutral

0,сынаушысы халық саққұлақ би,сынаушы халық саққұлақ би,neutral

0,жеті ғашық,жеті ғашық,neutral

0,ләйлі мәжнүн,ләйл мәжнүн,neutral

0,фархад шырын,фархад шырын,neutral

0,таһир зуһра,таһи зуһр,neutral

0,арзу қамбар,арз қамб,neutral

0,уәлік ғарра,уәлік ғарр,neutral

0,уәки күлшаһ,уәки күлшаһ,neutral

0,жүсіп зылиқа,жүс зыли,neutral

0,жеті шәріп жеті әулие,жеті шәр жеті әулие,neutral

0,меке шәріп,ме шәр,neutral

0,мәдина шәріп,мәдина шәр,neutral

0,бұхар шәріп,бұх шәр,neutral

0,шам шәріп,шам шәр,neutral

0,қатым шәріп,қат шәр,neutral

0,құддыс мысыр шәріп,құддыс мысыр шәр,neutral

0,кәләм шәріп құран,кәлә шәр құран,neutral

0,жеті қазына,жеті қазына,neutral

0,ер жігіт,ер жігіт,neutral

0,сұлу әйел,сұл әйел,neutral

0,ақыл білім,ақыл білім,neutral

0,жүйрік ат,жүйрік ат,neutral

0,қыран бүркіт,қыран бүркіт,neutral

0,берен мылтық,берен мылтық,neutral

0,жүйрік тазы,жүйрік тазы,neutral

0,жеті ғалам,жеті ғалам,neutral

0,аспан жоғары ғалам,аспан жоғары ғалам,neutral

0,жер орта ғалам,жер орта ғалам,neutral

0,жер асты төмендегі ғалам,жер асты төмендегі ғалам,neutral

0,жеті күн,жеті күн,neutral

0,арғы күн,арғы күн,neutral

0,ауыр күн,ауыр күн,neutral

0,соңғы күн,соңғы күн,neutral

0,сәрсенбі сәтті күн,сәрсенбі сәтті күн,neutral

0,жұма қасиетті күн,жұма қасиетті күн,neutral

0,жеті жұт,жеті жұт,neutral

0,жұт мал қырылу,жұт мал қырыл,neutral

0,оба ауру,оба ауру,neutral

0,топан су,топан су,neutral

0,жеті жоқ,жеті жоқ,neutral

0,жерде өлшеуіш жоқ,же өлшеуіш жоқ,neutral

0,аспанда тіреуіш жоқ,аспанда тіреуіш жоқ,neutral

0,таста тамыр жоқ,таста тамыр жоқ,neutral

0,тасбақада талақ жоқ,тасбақа талақ жоқ,neutral

0,аллада бауыр жоқ,алла бауыр жоқ,neutral

0,аққуда сүт жоқ,аққу сүт жоқ,neutral

0,жылқыда өт жоқ,жылқы өт жоқ,neutral

0,жеті жетім,жеті жетім,neutral

0,тыңдамаған сөз жетім,тыңдама сөз жетім,neutral

0,киюсіз тозған бөз жетім,кию тоз бөз жетім,neutral

0,иесіз қалған жер жетім,ие қалған жер жетім,neutral

0,басшысы жоқ ел жетім,басшы жоқ ел жетім,neutral

0,аққу қазсыз көл жетім,аққу қаз көл жетім,neutral

0,замандасы қалмаса бірінен де сол жетім,замандас қалма бір де сол жетім,neutral

0,жеті ата,жеті ата,neutral

0,арғы ата,арғы ата,neutral

0,түп ата,түп ата,neutral

0,тек ата,тек ата,neutral

0,жеті қат көк,жеті қат көк,neutral

0,есекқырғын меркурий,есекқырғ меркури,neutral

0,қызыл жұлдыз марс,қызыл жұлдыз марс,neutral

0,мүштәри юпитер,мүштәри юпи,neutral

0,жеті амал,жеті амал,neutral

0,күннің тоқырауы,күн тоқыра,neutral

0,қарашаның қайтуы,қараша қайт,neutral

0,үркердің батуы,үрк бат,neutral

0,мұздың қатуы,мұз қат,neutral

0,киіктің матауы,киік мата,neutral

0,қыс тоқсан,қыс тоқсан,neutral

0,ай тоғамы,ай тоғам,neutral

0,қабырғаға ал достарың да біле жүрсін,қабырға ал дос да біл жүрсін,neutral

0,сұрақ қойады екінші өмірде дайын бол,сұрақ қой екінші өмір дайын бол,neutral

0,өмір шындығы,өмір шындығ,neutral

0,анадан артық жақын жан жоқ,ана артық жақын жан жоқ,neutral

0,тер мен еңбек қалайда сені мақсатыңа жеткізеді,тер мен еңбек қалай сен мақсат жеткіз,neutral

0,енді бірінші болып жазбаймын көрейік мені қашан еске алатындарыңды,енді бірінші бол жазба көр мен қашан ес ал,neutral

0,адамдардың соншалықты еріншекітігі осы сандарды оқымай кетті оқымай кетсең лайк,адам соншалықты еріншекітіг осы сан оқыма кет оқыма кет лайк,neutral

0,сен ойланып жүргенде басқалар сенің орныңа істеп қояды,сен ойлан жүр басқа сенің орн істе қоя,neutral

0,әрбір сәби бақытты болуға тиіс ал сіз қалай ойлайсыз,әрбір сәби бақытты бол тиіс ал сіз қалай ойла,neutral

0,менде өзімнің қалауымды табамын,менде өзімнің қалау таб,neutral

0,сенде кандай ios лайк android коммент,сенде канда ios лайк android коммент,neutral

0,болашақ жарың қандай,болашақ жар қандай,neutral

0,анаңды жақсы көресіңбе club иә әрине club жоқ адам дауыс берді,ана жақсы көресіңб club иә әрине club жоқ адам дауыс бер,neutral

0,құттықтаймын қазақ елі гена өзінің қарсыласын раундта нокаутпен жеңді,құттықта қазақ ел ген өзінің қарсылас раунд нокаут же,neutral

0,алға қазақстан жаса қазақстан,алға қазақстан жаса қазақстан,neutral

0,анамнын атымен ант етем club анашым сөзін басып тіркелем,анамн ат ант ете club анаш сөз бас тіркеле,neutral

0,ант еттің ба орында,ант ет ба орында,neutral

0,ал кеттік саған қайсысы түсті,ал кет саған қайсысы түсті,neutral

0,нағыз мұсылман жандар айды бассыншы club,нағыз мұсылман жан ай бассынш club,neutral

0,ешқандай мейрамхана анамның дайындайтын тағамын алмастыра алмайды,ешқандай мейрамхана ана дайында тағ алмастыр алма,neutral

0,келісесіздерме комментке,келісесіздер коммент,neutral

0,ұнатсаң лайк,ұнат лайк,neutral

0,кыз бен ұлдың арасында достык бола ма иә like жоқ комент,кыз бен ұл ара достык бола ма иә like жоқ комент,neutral

0,сыйластықтан асқан махаббат жоқ мұхаммед пайғамбар,сыйластық ас махаббат жоқ мұхаммед пайғамбар,neutral

0,бұл ағамыз қанша likeқа лайық екен,бұл ағ қанша like лайық екен,neutral

0,қайрат ағамыз құлап қалмасын кеттік,қайрат ағ құла қалма кет,neutral

0,кім үшін лайк бастың,кім үшін лайк бас,neutral

0,дұрыс армандай біл армандар орындалады,дұрыс арманда біл арман орындал,neutral

0,қай ыдыс бірінші толады,қай ыдыс бірінші тол,neutral

0,ұзақ жасашы анашым қартаймашы әкешім,ұзақ жасаш анаш қартаймаш әкеш,neutral

0,бұл жұмбақты адам таба алмайды екен берілген суретте қанша сіріңке көріп тұрсың,бұл жұмбақ адам таб алма екен бер сурет қанша сірің көр тұр,neutral

0,касыңда қартайғанша болғым келеді,кас қартайғанш бол кел,neutral

0,әкем мен анамды қалайда меккеге жіберемін,әке мен ана қалай мекке жібер,neutral

0,сізде де осындай жағдай болды ма,сіз де осындай жағдай бол ма,neutral

0,сен телефоныңды қалай ұстайсың мен,сен телефон қалай ұста мен,neutral

0,ешқашан мұңайма дейді алайда өздерінде де бір мұң,ешқашан мұңай де алайда өз де бір мұң,neutral

0,өмірдегі ең қиын нәрсе жігіті бар қызды сүйіп қалу,өмір ең қиын нәрсе жігіт бар қыз сүй қалу,neutral

0,бұл ойынды кім біледі білетіндерден,бұл ой кім біл біл,neutral

0,қолыңыз бос болса шүкір деп жаза кетіңіз,қол бос бол шүкі де жаза кет,neutral

0,ұқсастық деп осыны айт,ұқсастық де осы айт,neutral

0,еркенің фанаттары барма,ер фанат барма,neutral

0,адамды бағала,адам бағала,neutral

0,қолыңнан ұшырып аласың,қол ұшыр ал,neutral

0,барі уақытша,бар уақытша,neutral

0,өз атыңды ші қатарсыз жазып көр,өз ат ші қат жаз көр,neutral

0,менікі кг,менік кг,neutral

0,қазақ қызы немесе кәріс қызы,қазақ қыз немесе кәріс қыз,neutral

0,хабиб қай елге көрсетіп тұр,хабиб қай ел көрсет тұр,neutral

0,қазақ қызына қанша лайк,қазақ қыз қанша лайк,neutral

0,ал көрейік мен баса алдым,ал көр мен бас ал,neutral

0,есіміңде б әріпі болса лайк,есім б әріп бол лайк,neutral

0,қазақ қызы лайк қырғыз қызы комент,қазақ қыз лайк қырғыз қыз комент,neutral

0,маған түсті ал саған,маған түсті ал саған,neutral

0,сен қай жігіттің өнерін жақсы көресің,сен қай жігіт өн жақсы көр,neutral

0,мен төреғали,мен төреғали,neutral

0,қандай қиыншылық болмасын сүйемін сені қазақстан,қандай қиыншылық болма сүй сен қазақстан,neutral

0,қайда мыналар деп тупить ете саласың ғооо,қайда мына де тупить ет сал ғооо,neutral

0,осы саусағынмен лайк басып коменткее дайын деп жазып көрші,осы саусағын лайк бас коментке дайын де жаз көрші,neutral

0,қолыннан келмейді,қолын келме,neutral

0,қыздардың даа ер бала болғысы келген кездері болған шығар иәә,қыз да ер бала бол кел кез бол шығар иәә,neutral

0,ол отырмаа,ол отырма,neutral

0,жүр қасынан өтейік,жүр қас өт,neutral

0,қыздар таныс паа,қыз таныс па,neutral

0,біз қыздар онлайн болып тұрсақ та біраз шаруа бітіріп жүреміз ғоой,біз қыз онлайн бол тұр та біраз шаруа бітір жүр ғоо,neutral

0,басқалар жігіттерін қызғансаа ал мен подругаларымды қызғанам,басқа жігіт қызғанса ал мен подруга қызғана,neutral

0,бір үйдің қыздары екігее бөлінеді,бір үй қыз екіге бөлін,neutral

0,осы ұлдардың голосовойдағы дауыстары қатты ұнайды шее,осы ұл голосовой дауыс қатты ұна ше,neutral

0,магазингее барып не алатыныңды ұмытып қалған кездерің болды маа,магазинге бар не ал ұмыт қалған кез бол ма,neutral

0,лубөйің алш,лубөй алш,neutral

0,жігіттердің достығын еш қандай қыз түсінбейді,жігіт достығ еш қандай қыз түсінбе,neutral

0,менде бәрі болады бірак өз уақытымен,менде бәрі бол бірак өз уақыт,neutral

0,ей адамдар шүкіршілік етіңдер,ей адам шүкіршілік ет,neutral

0,бұл өмірде атаананың барына,бұл өмір атаана бар,neutral

0,мақтады ма асып таспа,мақта ма ас тасп,neutral

0,мазақтады ма сабыр сақта,мазақт ма сабыр сақта,neutral

0,сырты сұлу не керек жаны сұлу болмаса,сыр сұл не керек жан сұл болма,neutral

0,текке жүріп не керек мәңгі бірге болмаса,тек жүр не керек мәңгі бірге болма,neutral

0,тегін лайкты аямайықшы,тег лайк аямайықш,neutral

0,өзгелер ойлагандай емес өзің қалағандай өмір сүр,өзге ойлаганда емес өзің қалағанда өмір сүр,neutral

0,жақында права аламдалаға шықпаңдар,жақында прав аламдала шықпа,neutral

0,нағыз жігіттердің баратын екі ғана жері бар,нағыз жігіт бар екі ғана же бар,neutral

0,мешіт және спортзал,мешіт және спортзал,neutral

0,бұйыртса бұл жерге де жетермін,бұйырт бұл же де жет,neutral

0,жігітше айтам сені қалайда сындырамын,жігітш айта сен қалай сындыр,neutral

0,жігітің болса оныда сындырамын,жігіт бол оны сындыр,neutral

0,маған қыс мезгілі ұнайдыөйткені сол мезгілде қыздар жабық киінеді,маған қыс мезгіл ұнайдыөйт сол мезгіл қыз жабық киін,neutral

0,патша бол тек бір ханшайымға,патша бол тек бір ханшайым,neutral

0,жігітер бағасын берер,жігіт баға бер,neutral

0,қабырғаңа сақтап қой,қабырға сақта қой,neutral

0,достарыңныңда көруіне себепші бол,достарыңн көр себепш бол,neutral

0,мағаң десең бүкіл әлемді тіреп тұр,маға де бүкіл әлем тіре тұр,neutral

0,маған ешкім авторитет емес,маған ешкім авторитет емес,neutral

0,бәріне төземін шыдаймын тек анамның көз жасына емес,бә төз шыда тек ана көз жас емес,neutral

0,бір ғана сурет мыңдаған сезім,бір ғана сурет мыңдаған сезім,neutral

0,еңбекті лайкпен бағалап кетейік,еңбек лайк бағала кет,neutral

0,еййй бауырым тоқта,ейй бауыр тоқта,neutral

0,абайлап сен боқта,абайла сен боқ,neutral

0,шеше ойыншық емес,шеш ойыншық емес,neutral

0,біреуде ол жоқ та,біреу ол жоқ та,neutral

0,шыда шыда шыдай түс шыда тағы,шыда шыда шыда түс шыда тағы,neutral

0,шыдамдыны мына өмір ұнатады,шыдамды мына өмір ұнат,neutral

0,құдайдан жауларым қасыма жоламасыншы деп тілеп едім достарым жоғалып жатыр,құдай жау қас жоламасынш де тіле ед дос жоғал жатыр,neutral

0,қымбат көлік алған кезінде бірге жаяу жүрген достарыңды ұмытпа,қымбат көлік ал кез бірге жаяу жүр дос ұмытп,neutral

0,аналарымыз үшін лүпілдетейікші,ана үшін лүпілдетейікш,neutral

0,көңілім қалсадасүйемін бірақ та,көңіл қалсадасүй бірақ та,neutral

0,қазақтың жігіті өзінің абыройын қызының арымен өлшеген,қазақ жігіт өзінің абырой қыз ар өлше,neutral

0,үрген итке үндеме өзіңді үстем санасаң,үр ит үндеме өз үстем сана,neutral

0,бақыттымынсен бақытсыз қылсаңда,бақыттымынсен бақыт қылса,neutral

0,сүйгенінің тойын көру жалғандабір тілерім жазбаса екен ешкімге,сүй той көру жалғандабі тіл жазба екен ешкім,neutral

0,өзіңізге ұнайтын әуенді комментке қалдырыңыз,өз ұна әуе коммент қалдыр,neutral

0,санаң болса жаныма жақындама,сана бол жан жақында,neutral

0,жүрегіңді тыңдапшын бақытыңды ізде,жүрег тыңдапш бақыт ізде,neutral

0,бүгін беттен тұратын кітаптың алғашқы беті,бүгін бет тұр кітап алғашқы бет,neutral

0,оны жақсы етіп жаз сәттілік досым,оны жақсы ет жаз сәттілік дос,neutral

0,сенменің жетістігімсің өмірден ұтқан,сенме жетістіг өмір ұт,neutral

0,қызғаншақ жігіттердің қыздары әдемі болады,қызғаншақ жігіт қыз әдемі бол,neutral

0,әскер бұл жігіт үшін мектеп қыз үшін сынақ,әскер бұл жігіт үшін мектеп қыз үшін сынақ,neutral

0,бақыттымын өзіңмен әльһамдулилләһ,бақытты өз әльһамдулилләһ,neutral

0,қандай ойын екенін түсінсең лайк,қандай ойын екен түсін лайк,neutral

0,қалайда күт бағыңдысезімің бекер ма не,қалай күт бағыңдысезім бек ма не,neutral

0,осылай өмір өтеркейде мұң кейде бақыт,осылай өмір өтеркей мұң кейде бақыт,neutral

0,бірдеңе деш і ботаңа,бірде деш і бота,neutral

0,жалғыздық деген не десе,жалғыз де не де,neutral

0,кемсіз өткен күн дер ем,кем өткен күн дер ем,neutral

0,ешқашан жоғалмасекеуміз жайлы із бар,ешқашан жоғалмасекеу жайлы із бар,neutral

0,мені сүйетініңді мың әрекетпен дәлелдеудің қажеті жоқ,мен сүй мың әрекет дәлелдеу қажет жоқ,neutral

0,шынайы сүйсеңқандай жағдай болмасын қасымда бол,шынайы сүйсеңқанда жағдай болма қас бол,neutral

0,оның күлкісіәрбір сөзітәтті қылығы әлі есімде,оның күлкісіәрбі сөзітә қылығ әл ес,neutral

0,мен оған ессіз ғашықпын,мен оған ес ғашық,neutral

0,сені сүйдімсенсіз өмір мәнсіз,сен сүйдімсен өмір мән,neutral

0,байдың баласы емеспін бірақ бай жігіттерден кем емеспін сөзге шешен емеспін бірақ сөйлеуге тұратын жігітпін,бай бала емес бірақ бай жігіт кем емес сөз шешен емес бірақ сөйле тұр жігіт,neutral

0,лайк баста,лайк баста,neutral

0,кім түскенін көр,кім түс көр,neutral

0,саған кім түсті коментке жазып кет,саған кім түсті комент жаз кет,neutral

0,лайкпен бағалап жберейік,лайк бағала жбер,neutral

0,лайктың соңғы саны жарыңды қалай еркелетесің,лайк соңғы сан жар қалай еркеле,neutral

0,лайктың соңғы саны сенің жұбайынның қай жерден екенің көрсетеді,лайк соңғы сан сенің жұбайын қай же екен көрсет,neutral

0,криштиану роналду қасиетті құранды оқуды үйреніп жүр,криштиан роналд қасиетті құра оқу үйрен жүр,neutral

0,арзан киімнің ішінде қымбат жүрек жасырынуы мүмкін,арзан ки іш қымбат жүрек жасырын мүмкін,neutral

0,лайктың соңғы саны сізге телефон таңдайды,лайк соңғы сан сізге телефон таңда,neutral

0,ал сіз кім үшін лайк бастыңыз,ал сіз кім үшін лайк бас,neutral

0,ал сіз қайда барар едіңіз,ал сіз қайда бар ед,neutral

0,бізде қатардан қалмайық,біз қат қалма,neutral

0,ұл қыз,ұл қыз,neutral

0,админ жауыз ал сізше,админ жауыз ал сізш,neutral

0,админ ал сізше,админ ал сізш,neutral

0,қанша ренжісемде қанша көңілім қалсада сенен артығын таппаймын бауырым,қанша ренжісе қанша көңіл қалса сенен артығ таппа бауыр,neutral

0,мен үтір койған жерге бауырларым нүкте қояды,мен үтір кой же бауыр нүкте қоя,neutral

0,үндемеуді қорыққанның белгісі деп түсінбеңдер үндемеу сабырлықтың белгісі,үндемеу қорық бел де түсінбе үндеме сабырлық бел,neutral

0,бірдеңке керек кезде бәріне радной болып қаласың,бірдең керек кезде бәрін радно бол қал,neutral

0,қарапайымдардың ортасында қарапайым бол жыртқыштардың ортасында жыртқыш бол,қарапайым орта қарапайым бол жыртқыш орта жыртқыш бол,neutral

0,маған ешкім дәл сендей ұнаған емесмінезіме дәл сендей шыдаған емес,маған ешкім дәл сенде ұна емесмінез дәл сенде шыда емес,neutral

0,мейлі жамандаса жамандай берсін ең бастысы өзімнің қандай екенімді білемін,мейлі жаманда жаманда бер ең басты өзімнің қандай екен біл,neutral

0,мені табу қиын жоғалту оңай,мен табу қиын жоғалт оңай,neutral

0,шындық қой келісесіз бе,шындық қой келіс бе,neutral

0,жігітін әскерден күтетін қыздар кереметсіңдер ғо,жігіт әск күт қыз керемет ғо,neutral

0,сізге де осы бір таңғажайып қасиетті жерге аяқ басуды нәсіп етсін жаратушы иеміз,сізге де осы бір таңғажайып қасиетті же аяқ басу нәс ет жаратуш ие,neutral

0,дос болып жүріп ғашық боп қалғансың ғо,дос бол жүр ғашық боп қал ғо,neutral

0,менде бәрі болады бірақ өз уақытымен,менде бәрі бол бірақ өз уақыт,neutral

0,phone шығарғанша ракқа қарсы дәрі шығарғаны жақсы еді,phone шығарғанш рак қарсы дә шығар жақсы ед,neutral

0,сен үшін тынбай еңбек еткенәкенді ұмытпа,сен үшін тынба еңбек еткенә ұмытпа,neutral

0,керегіме жетеді шүкір,керег жет шүкі,neutral

0,егер шын сүйетін адамың болса сені тергейді күнде сағынады сүйеді және күтеді,егер шын сүй адам бол сен терге күн сағын сүй және күт,neutral

0,осындай гүл шоқтарына әр бір ана лайықты,осындай гүл шоқ әр бір ана лайықты,neutral

0,шеше деген ең көркем сөзді бауырым қалай ғана боғауыз сөзге айналдырдың,шеш де ең көркем сөз бауыр қалай ғана боғауыз сөз айнал,neutral

0,осындай қыздарға бас кетеді бар ғоо,осындай қыз бас кет ба ғоо,neutral

0,ешқашан қыз туралы өсекке сенбе оны не тек қолы жетпеген жігіт не көре алмайтын қыз айтады,ешқашан қыз турал өсек сенбе оны не тек қол жетпе жігіт не көр алма қыз айт,neutral

0,ұйқысыз түндерің үшін рақмет анашым,ұйқы түн үшін рақмет анаш,neutral

0,шын махаббат ешқашанда уақытша болмайды,шын махаббат ешқашан уақытша болма,neutral

0,эххх жігіттердің жүрегі қандай кең десеңші,эххх жігіт жүрег қандай кең десеңш,neutral

0,ата анаң разы болмай жәннәтқа кіре алмайсың,ат ана раз болма жәннәт кір алмайс,neutral

0,кімді сүю керектігін жүрек шешеді кіммен болу керектігін тағдыр шешеді,кім сүю керектіг жүрек шеш кім болу керектіг тағдыр шеш,neutral

0,қарты бар үйдің қазынасы бар,қар бар үй қазына бар,neutral

0,сені сүйген жан болса да жұт,сен сүй жан бол да жұт,neutral

0,сені сүймеген жан бал болса да ұмыт,сен сүйме жан бал бол да ұмыт,neutral

0,түрік мақалы,түрік ма,neutral

0,оқып отқан адам бақытты болш,оқ отқан адам бақытты болш,neutral

0,ананың ойы балада баланың ойы далада,ана ой бала бала ой дала,neutral

0,аналарымыз аман болсыншы,аналар аман болсынш,neutral

0,сүймейтін адамның құлы болғанша,сүйме адам құл болғанш,neutral

0,сүйетін адамның гүлі бол,сүй адам гүл бол,neutral

0,м мақатаев,м мақатаев,neutral

0,әрбір бауырыма аллаһ осы күнді нәсіп етсін,әрбір бауыр аллаһ осы күн нәс ет,neutral

0,қасымда болшы бүгін ертен әрқашан,қас болш бүгін ер әрқашан,neutral

0,вкда отырғаның осы кісілердің арқасы,вк отыр осы кісі арқа,neutral

0,махаббатсыз тұрмыс құрудан алла сақтасын,махаббат тұрмыс құру алл сақта,neutral

0,жаратқан ием анама ұзақ ғұмыр бергейсіз,жаратқан ие ана ұзақ ғұмыр бер,neutral

0,ой жеткен жерге қол жетер,ой жет же қол же,neutral

0,армандар орындалады,армандар орындал,neutral

0,қазақстанда бұл жай ғана нан ауыз тию деп аталады,қазақстан бұл жай ғана нан ауыз тию де атал,neutral

0,анашым қайда жүрсең де аман болшы,анаш қайда жүр де аман болш,neutral

0,жақсы дос дүние және ахирет үшін үлкен бақыт,жақсы дос дүние және ахирет үшін үлкен бақыт,neutral

0,джейсон стэтхэм,джейсон стэтхэ,neutral

0,біреулер түсінбес,біреу түсінбес,neutral

0,біреулер еске алмас,біреу ес алмас,neutral

0,бізді памперссіз кір жуғыш машинасыз өз сүтімен өсірген аналарымызға аллаһ разы болсын,біз памперс кір жуғыш машина өз сүт өсір ана аллаһ разы бол,neutral

0,мен бүгін не болатынын білмеймін ертең де не болатынын білмеймін мен тек бірнәрсені білемін бәрі алланың қалауымен,мен бүгін не бол білме ерте де не бол білме мен тек бірнәрсе біле бәрі алла қалау,neutral

0,коментке есіміңді қалдыр келесі сен болуың мүмкін,комент есім қалдыр келесі сен бол мүмкін,neutral

0,ұнаса бас,ұна бас,neutral

0,бұл әншілер аман болсын десең,бұл әнші аман бол де,neutral

0,лайк басқан адамға осындай байлық келсін аумин,лайк бас адам осындай байлық кел аумин,neutral

0,наурыз көжені сағынғандардан лайк,наурыз көже сағын лайк,neutral

0,шын жүректен,шын жүрек,neutral

0,кім түсті,кім түсті,neutral

0,анам ауырса жүрегім қатты ауырады анашым ешқашан ауырмасыншы,ана ауыр жүрег қатты ауыр анаш ешқашан ауырмасынш,neutral

0,тұңғыш президентімізге қанша лайк,тұңғыш президент қанша лайк,neutral

0,менің елбасым,менің ел,neutral

0,қазақстан республикасының жаңа президенті құтты болсын,қазақстан республика жаңа президент құт бол,neutral

0,көрейік біздің топта кімдер көп екенін,көр біздің топ кім көп екен,neutral

0,осы ойын адамнын соткасында бар егер сенде болса лайк бас,осы ойын адамн сотка бар егер сенде бол лайк бас,neutral

0,керемет иааyылда тұрғанды ұнатасыз ба,керемет иааyыл тұр ұна ба,neutral

0,келесі сезонды күтіп отырғандар лайк репост,келесі сезо күт отыр лайк репост,neutral

0,түсірілім кетіп жатыр,түсірілім кет жатыр,neutral

0,соғыс ардагерлері азайып бара жатыр сондықтан барында қадірлейік,соғыс ардагер азай бар жатыр сондықтан бар қадірл,neutral

0,лайктың соңғы саны сенін болашақ телефонын,лайк соңғы сан сен болашақ телефон,neutral

0,лайктын соңғы саны сенін машинан болуы мүмкін,лайкт соңғы сан сен маши бол мүмкін,neutral

0,ойламадым деші мені сірә бір рет болсын,ойлама деш мен сірә бір рет бол,neutral

0,ойлар келмеді деші біз жайлы қойшы құрсын,ой келме деш біз жайлы қойш құр,neutral

0,түн болғанда терезе алдында неге тұрсың,түн болғанда терезе алд неге тұр,neutral

0,ішіңде не боп жатқаныңды құдайым білсін,іш не боп жат құдай біл,neutral

0,ескіреді біле білсең жансыз заттар да,ескір біл біл жан зат да,neutral

0,мен саған сөз беремін бақытты етемін қолдан келгенше,мен саған сөз бер бақытты ет қолдан келгенше,neutral

0,мектебімең алғашқы махаббат бастауы,мектеб ең алғашқы махаббат баста,neutral

0,ең қызық сәттерім де өзіңмен басталды,ең қызық сәт де өз баста,neutral

0,мектебім менің сен менің алтын ордам,мектеб менің сен менің алтын орда,neutral

0,ұяңнан ұшқанда сен боласың бізге арман,ұя ұш сен бол бізге арман,neutral

0,жаныма жакын адам сен екенинди билгенимде куандым,жан жак адам сен екенинди билгени куа,neutral

0,өз тіліңді өшірме өшіргенді кешірме,өз тіл өшір өшір кешір,neutral

0,кіммен қайда баратынынды кіммен достасканына аса мән бер себебі бұлбұл құс гүлге апарады қарға күлге апарады,кім қайда баратын кім достаскан аса мән бер себебі бұлбұл құс гүл апар қар күл апар,neutral

0,біреулер келіп жатыр біреулер кетіп жатыр,біреу келі жатыр біреу кеті жатыр,neutral

0,ғашық қылып естен банды рады,ғашық қыл ес ба ра,neutral

0,екі ғашық бір болып қосылмайды,екі ғашық бір бол қосылма,neutral

0,кеше ғана бірінбірі сүйген жандар,кеше ғана бірінбі сүй жан,neutral

0,сыр бермейін десемде ішім түтін,сыр берме десе іш түтін,neutral

0,өзіңді көре алмасам ертелі кеш,өз көр алма ертел кеш,neutral

0,шыдап жүре алмаспын менде бір күн,шыда жүр ал менде бір күн,neutral

0,түн болғанда аспанға қарашы егер аққан жұлдызды көрсең жәй ғана арманда мен де армандап сені таптым,түн болғанда аспан қараш егер ақ жұлдыз көр жәй ғана арманда мен де арманда сен тап,neutral

0,сүю мен жек көрудің арасы бірақ қадам сенімен бірген болғанды сүйемін бөлек болғанымызды жек көремін,сүю мен жек көру арасы бірақ қадам сенімен бір бол сүй бөлек бол жек көр,neutral

0,мені бір сағатта минут ал бір минутта секунд бар деп үйретті бірақ сенсіз өткізген бір секунд тұңғиық екенін ешкім үйретпепті,мен бір сағат минут ал бір минут секунд бар де үйрет бірақ сен өткіз бір секунд тұңғиық екен ешкім үйретпе,neutral

0,басың бос болса вот это тема,бас бос бол вот это те,neutral

0,өтірік алдапарбаудың керегі жоқ оданда омірімнен шыгып кет,өтірік алдапарба керег жоқ одан омі шыг кет,neutral

0,жігіттің екі сөйлегені өлгені қыздың көреміз дегені көнгені ол дегені бізде щанс бар дегені,жігіт екі сөйле өл қыз көр де көн ол де біз щанс бар де,neutral

0,осы күндері тунде жатарда сені ойламай уйқтай алмайтын болдым,осы күн тун жат сен ойлама уйқта алма бол,neutral

0,омир сурмеймин енди мен сен болмаган бул жалганда кеудеде журегим от болып ортенеди,оми сурмеймин енди мен сен болмаган бул жалган кеуде журеги от бол ортенеди,neutral

0,менің жүрегім тек сен деп соғады ал сенікі кім деп соғады,менің жүрег тек сен де соғ ал сенік кім де соғ,neutral

0,сен жараладың жанымды бағаламай барымды қайтарар кім уақытты біздер бақытты өткізген,сен жарала жан бағалама бар қайтар кім уақыт біздер бақытты өткіз,neutral

0,оның көзінен бүгін жас ақты тамшылап оған кінәлі өзім ренжіттім оны қатты кінәлап,оның көз бүгін жас ақ тамшыла оған кінәлі өзім ренжі оны қатты кінәла,neutral

0,біз бірге болмак түгілі дос болып қала алмаймыз,біз бірге болмак түгілі дос бол қала алма,neutral

0,кім кімді кінәлайды кім кімнен айып табар,кім кім кінәла кім кім айып таб,neutral

0,ертегі ертегі ғой ертелікеш аяқталар,ертегі ертегі ғой ертелікеш аяқта,neutral

0,жүрекпен ойнай берме ол маған әлі керек,жүрек ойна берме ол маған әлі керек,neutral

0,тең теңімен өзіне лайықтысын іздеп табар,тең тең өзіне лайықты ізде таб,neutral

0,бұның аты өмір киноның аты солай,бұның ат өмір кино ат солай,neutral

0,жар болсын өзіңе жаратқан жалғыз құдай,жар бол өз жаратқан жалғыз құдай,neutral

0,өмірге бір келеді күз бір келеді көктем,өмір бір кел күз бір кел көктем,neutral

0,көктемді ерте қайтарған өзіңе бар ғой өкпем,көкте ерте қайтар өз бар ғой өкпе,neutral

0,күресіп келем есімің менен айырылмас дерттен,күрес келе есім менен айырылмас дерт,neutral

0,мен де оралмаспын білгін келмеске кеткем,мен де орал білг келмес кетке,neutral

0,сен керексин маган сен кажетсин маган менин болашагым озгеден кызганамын,сен керексин маган сен кажетсин маган менин болашаг озге кызган,neutral

0,мен сені қалай сүйгенімді өзіңе аян білесің бе,мен сен қалай сүй өз аян біл бе,neutral

0,бір келеді күз бір келеді көктем,бір кел күз бір кел көктем,neutral

0,анаңды жақсы көрсең секунтыңды бөліп ана сөзін басып тіркелші club ана,ана жақсы көр секунт бөл ана сөз бас тіркелш club ана,neutral

0,алақаның қышыса ойыңа ақша емес дұға келсін,ала қышы ой ақша емес дұ кел,neutral

0,дүниедегі барлық науқас сәбилер ұйқыларын оянғанда сау болып оянсыншы аллаһ тағалам,дүние барлық науқас сәби ұйқы оян сау бол оянсынш аллаһ тағала,neutral

0,табиғат апатынан соң құламай қалған жалғыз ғимарат мешіт,табиғат апат соң құлама қалған жалғыз ғимарат мешіт,neutral

0,шынында шындық жақсылыққа бастайды ал жақсылық жаннатқа бастайды бір адам үнемі шындық айтса алланың жанында шыншыл деп жазылады өтірікші жамандыққа бастайды ал жамандық тозаққа бастайды бір адам үнемі өтірік айтса алланың жанында өтірікші деп жазылады мухаммад бухари муслим,шын шындық жақсылық баста ал жақсылық жаннат баста бір адам үнемі шындық айт алла жан шыншыл де жаз өтірікш жамандық баста ал жаман тозақ баста бір адам үнемі өтірік айт алла жан өтірікш де жаз мухаммад бухари муслим,neutral

0,аң патшасы арыстан болғаныменкөздеген мақсатынан таймайтын ең батыл жануар олқасқыр тек қасқыр ғана ақтық демі қалғанша қарсыласының көзіне тік қарап өтеді,аң патша арыстан болғаныменкөзде мақсат тайма ең батыл жануар олқасқы тек қасқыр ғана ақтық дем қалғанш қарсылас көз тік қара өт,neutral

0,сүйдің ба өмір бойы бағала бойындағы кемшілігіне қарама мейлі кейбір кездері жүрегіңді ауыртсада сол бір жанды бақытым деп бағала,сүй ба өмір бойы бағала бой кемшіліг қарама мейлі кейбір кез жүрег ауыртса сол бір жанды бақыт де бағала,neutral

0,анаңа көмектесүй шаруасына атсалыс бұл сенің жігіттік намысыңа кір келтірмейді,ана көмектесү шаруа атсалыс бұл сенің жігіт намыс кір келтірме,neutral

0,дұшпандарым аман болсын бір күні алдыма келер өтірік достарым аман болсын өтірік болсада қасымда жүргендері үшін көре алмайтындарға ризамын мені өздерінен жоғары санағаны үшін өсекшілерге ризамын менің өміріме қызығып өсекке айналдырғаны үшін нағыз достарыма ризамын жұбатканы үшін атаанама ризамын өмір сыйлаганы үшін аллаға ризамын қасымдағы адамдардың шынайы бет бейнесін танытқаны үшін,дұшпан аман бол бір күн алд кел өтірік дос аман бол өтірік болса қас жүргендері үшін көр алма риза мен өз жоғары сана үшін өсекші риза мені өмір қызығ өсек айналдыр үшін нағыз дос риза жұбатка үшін атаана риза өмір сыйлага үшін алла риза қасым адам шынайы бет бейне таныт үшін,neutral

0,өмірде бәрі ерте ме әлде кеш пе бітеді бұл өмірдің шынайы бейнесі,өмір бәрі ерте ме әлде кеш пе біт бұл өмір шынайы бейне,neutral

0,сабырлық әлсіздерге күштілерге ем,сабырлық әлсіз күшті ем,neutral

0,ана деген байлығың мен барың,ана де байлығ мен бар,neutral

0,ана деген тұтастай бір жаның,ана де тұтаста бір жан,neutral

0,ана деген ең жақсы бір әнің,ана де ең жақсы бір ән,neutral

0,ана деген саған жақын адалың,ана де саған жақын адал,neutral

0,ана деген бермеген тәрбиенің жаманын,ана де берме тәрбие жаман,neutral

0,ана деген дұға етуші адамың,ана де дұ етуші адам,neutral

0,ана деген ауырға да шыдайды,ана де ауыр да шыда,neutral

0,ана деген жылу берер күн райы,ана де жылу бер күн рай,neutral

0,ана деген екінші сенің жүрегің,ана де екінші сенің жүрег,neutral

0,ана деген ықылас пен тілегің,ана де ықылас пен тіле,neutral

0,ана деген бір шаттанып күлгенің,ана де бір шаттан күл,neutral

0,ана деген байлығы оның тек біз,ана де байлығ оның тек біз,neutral

0,ана деген сүйеді сені ессіз,ана де сүй сен ес,neutral

0,ана деген менің жәннатым дессіз,ана де менің жәнна дес,neutral

0,біреуге ауыр сөз аитпаи тұрып оилан ол сөз саган унамаса баскага да унамаит ол оның көңілін түсіру мүмкін,біреу ауыр сөз аитпаи тұр оилан ол сөз саган унама баскаг да унамаит ол оның көңіл түсір мүмкін,neutral

0,баягыда бир жигит болган екен сосын бир досы келип ейсенин айелине жомарт деген жигит келип журдепти дану деп уйине кетипти ертесине шкафка тыгылыпжомарты кутипти жомарт деген жигит келипти содан досына келип кеше жомарт келди денеси даукастюмшалбар киип алганкрасавьчик жигит екен менин айелим шешинип калып еди емшеги былай карны былай салпан ете калды

ойбайййжомарттан жамааан уялганымай депти хаха,баягы би жигит болган екен сосын би дос кели ейсенин айелин жомарт де жигит кели журдепти дан де уйин кетипти ертесин шкафк тыгылыпжомар кутипти жомарт де жигит келипти содан дос кели кеше жомарт келди денеси даукастюмшалб кии алганкрасавьчик жигит екен менин айели шешини кал еди емшеги былай кар былай салпан ет ка ойбайййжомарт жамааан уялганыма депти хах,neutral

0,сүйікті қызыңды болашақ жарыңдай көр егер олай көрмесең біреудің бақытын қасыңда ұстама,сүйікті қыз болашақ жарында көр егер ола көрме біреу бақыт қас ұстама,neutral

0,жаңадан шыққан xxi ғасырдың мәсісі өте ыңғайлы саяхатқа шыққандажұмыстақажылыққа барғандааурыған жағдайдақарт адамдарғабасқада қиын жағдайлардаберілген жеңілдікті қолдану керек әр бір аяқ киімнін ішінен келеді https youtu be msbk sgugo,жаңа шық xxi ғасыр мәсі өте ыңғайлы саяхат шыққандажұмыстақажылық барғандааурыр жағдайдақарт адамдарғабасқа қиын жағдайлардабер жеңілдік қолдану керек әр бір аяқ киімн іш кел https youtu be msbk sgugo,neutral

0,ассалямуалейкум миск сатамын или репост жасасаңыздар,ассалямуалейку миск сат или репост жаса,neutral

0,адамдардың арасында дәреженді жоғары көрсетсен деаллахтың алдында бірдей екендігінді ұмытпа,адам ара дәреже жоғары көрсетсен деаллах алд бірде екендіг ұмытп,neutral

0,теңге құнсызданса тиын болады пенде құнсызданса қиын болады,теңге құнсыздан тиын бол пен құнсыздан қиын бол,neutral

0,кезінде өзінді бақытты сезіндірген адам туралы жаман сөз айтушы болма,кез өз бақытты сезіндір адам туралы жаман сөз айтуш болма,neutral

0,артыңа алғыспен алдыңа үмітпен жанжағыңа махаббатпен қара ұстаз ерсін әбу юсуф,арт алғыс алд үміт жанжағ махаббат қара ұстаз ер әбу юсуф,neutral

0,барлық адам қателеседі бірақ қателесушілердің арасындағы ең жақсысы өз қателігін мойындап тәубеге келгені мұхаммед пайғамбар а с,барлық адам қателес бірақ қателесуші ара ең жақсы өз қателіг мойында тәубе кел мұхаммед пайғамбар а с,neutral

0,көп жігітке арман болғанша бір жігітке пәк бол өзге ой,көп жігіт арман болғанш бір жігіт пәк бол өзге ой,neutral

0,жетімнің басын сипайтын қолдар смартфонның бетін сипап кеткен жоқ па мұхаммеджан тазабек,жет басын сипа қол смартфон бет сипа кет жоқ па мұхаммеджан тазабек,neutral

0,уақыт өтеді дейсіз бе жоқ уақыт қалады біз өтеміз генри остин добсон,уақыт өт де бе жоқ уақыт қал біз өт генри остин добсон,neutral

0,ішкі дүниесін жақсы көріңдерсыртқы бейнесі уақытша,ішкі дүние жақсы көріңдерсыртқ бейне уақытша,neutral

0,күндердің сұлтаны жұма күні мүбәрәк болсын ниеттеріңіз қабыл болсын,күн сұлтан жұма күн мүбәрәк бол ниет қабыл бол,neutral

0,ұмытпа алланы алдына барасың құрметте анаңды жәннәтты табасың,ұмытп алла алд бар құрметте ана жәннә таб,neutral

0,сендерді жұпжұп етіп жараттық нәбә сүресі аят,сен жұпжұ ет жара нәбә сүре аят,neutral

0,адамның санасы қаншалықты төмен болса ол мұрнын соншалықты аспанға шүйіреді омар хайям,адам сана қаншалықты төмен бол ол мұрн соншалықты аспан шүйір омар хайя,neutral

0,сұрақ тіркелушілерге жауабын астына жазып кетеміз,сұрақ тіркелуші жауаб аст жаз кет,neutral

0,сүйгеніңді құшағына алып отбасы бақытын сезіну қандай бақыт шіркін бірақ ол маскүнем болып кетті елде дағдарыс ал ол бар тапқанымызды ішімдікке жұмсайды алкоголизмнен жүз пайыз кепілдікпен айығып кетудің ең қарапайым әдісі дәрігерлердің уколынсыз және дәрі дәрмегінсіз бұл тәсіл өте қарапайым және кез келген адам оны қолдана алады,сүйген құшағ алып отбасы бақыт сезін қандай бақыт шіркін бірақ ол маскүне бол кет ел дағдарыс ал ол бар тап ішімдік жұмса алкоголиз жүз пайыз кепілдік айығ кету ең қарапайым әді дәрігер уколын жән дә дәрмегін бұл тәсіл өте қарапайым жән кез кел адам оны қолдан ал,neutral

0,өшіріп тастамай тұрғанда осы жерден оқыңыздар https vk cc yzik,өшір тастама тұр осы же оқ https vk cc yzik,neutral

0,тырнақ саңырауқұлағы мен өкшенің жарылуы шынымен қауіпті бұл дерт айтарлықтай қауіпті дер кезінде емдемесеңіз соңы жаман болады қатты ауыру мазоль қара тырнақтың өсіп кетуі б,тырнақ саңырауқұлағ мен өкше жарыл шын қауіпті бұл дерт айтарлықтай қауіпті дер кез емдеме соңы жаман бол қатты ауыру мазоль қара тырнақ өс кет б,neutral

0,бұл аурумен күресуде ежелден келе жаткан халықтық ем көмектеседі ең бірінші https vk cc miam,бұл ауру күрес ежел келе жаткан халықтық ем көмектес ең бірінші https vk cc miam,neutral

0,бәрі болады асығудың қажеті жоқ барлығы өз уақытымен,бәрі бол асығ қажет жоқ барлығ өз уақыт,neutral

0,егер анаң жанында болса қадірле бағалай біл себебі анаң өмірде жалғыз ғана,егер ана жан бол қадірл бағала біл себебі ана өмір жалғыз ғана,neutral

0,жұлынған гүл сыйлануы тиіс жазыла басталған өлең аяқталуы ал сүйікті әйелің бақытты олай болмаса әлің жетпес тірлікті бастамауың керек омар хайям,жұлын гүл сыйлан тиіс жазыл бастал өлең аяқтал ал сүйікті әйел бақытты ола болма әл жетпес тірлік бастама керек омар хайя,neutral

0,адамды шынын айтуға көндіргіңіз келеме оны ашуландырыңыз өйткені адамның ашу үстінде өтірік ойлап табуға уақыты жоқ бернард шоу,адам шын айт көндір келе оны ашуланды өйткені адам ашу үст өтірік ойла таб уақыт жоқ бернард шоу,neutral

0,еріншектерге арналған ультра жылдам арықтау бір аптада тоғыз келі салмақ тастайтыныңызға кепілдік береміз ішіңізбен беліңіздегі майлар жоқ болады денсаулығыңызға зияны жоқ бар болғаны күнде таңертең осыны https vk cc xt fp,еріншек арнал ультр жылдам арықта бір апта тоғыз келі салмақ таста кепілдік бер іш беліңіз май жоқ бол денсаулығ зиян жоқ бар бол күн таңертең осы https vk cc xt fp,neutral

0,егер жүрек таза болса тілден әдемі сөздер шығады хазіреті әли,егер жүрек таза бол тіл әдемі сөз шығ хазіре әли,neutral

0,егер сізді қапа қылған адамдарға әліде сәлем бере алсаңыз бұл сіздің иманыңыздың садақасы мәуләна,егер сіз қап қыл адам әлі сәлем бер ал бұл сіздің иман садақа мәулән,neutral

0,құдай айтады ренжулі адамнан қорық себебі ол менен көмек сұрайды ал мен көмек беремін,құдай айт ренжул адам қорық себебі ол менен көмек сұра ал мен көмек бер,neutral

0,буындарды емдеу жайлы барлық шындық дәріхана жұмысшылары таң қалуда дәл бұндай әсерді күтпеген еді олар үшін бұл нағыз апат өшірмей тұрғанда оқыңыздар https vk cc xho,буын емдеу жайлы барлық шындық дәріхан жұмысшы таң қал дәл бұндай әсер күтпе ед олар үшін бұл нағыз апат өшірме тұр оқ https vk cc xho,neutral

0,бір сүйген адамыңды мәңгі сүйіп өтуге болады бұл махаббаттың күшінен ғана емес адамның мінезқұлқының тұрақтылығымен байланысты тұрақты адамның махаббаты да тұрақты ілияс есенберлин,бір сүй адам мәңгі сүй өт бол бұл махаббат күш ғана емес адам мінезқұлқ тұрақтылығ байланысты тұрақты адам махаббат да тұрақты ілияс есенберлин,neutral

0,жүрек те гүл сияқты күшпен ашу мүмкін емес кезі келгенде өзіақ ашылады луиза мэй олкотт,жүрек те гүл сияқты күш ашу мүмкін емес кез кел өзіақ аш луиз мэ олкотт,neutral

0,қандай тілде болса да ең керемет ең әдемі сөз мен сені кеширдім стивен кинг,қандай тіл бол да ең керемет ең әдемі сөз мен сен кеш стивен кинг,neutral

0,салмақты максималды тастау үшін арықтау бағдарламасы,салмақ максималды тастау үшін арықта бағдарла,neutral

0,https vk cc zuoj майды тиімді күйдіру тәтті зиянды тағамға әуестікті төмендету салмақты тастауды тездету зат алмасуды қалыптандыру майды күйдіру процесін арттыру ісіктерден құтылу тәбетті төмендету және артық жеуден құтылу май түзілімдерін толықтай бұғаттау https vk cc zuoj,https vk cc zuoj май тиімді күйдір тәтті зиянды тағам әуестік төмендету салмақ тастау тездет зат алмасу қалыптандыр май күйдір процес арттыру ісік құтыл тәбет төмендету және артық жеу құтыл май түзілім толықта бұғатта https vk cc zuoj,neutral

0,бәрін өз уақытында істеу керек кеше тым ерте еді ертең тым кеш болады бернар вербер,бәрін өз уақыт істеу керек кеше тым ерте ед ертең тым кеш бол берн верб,neutral

0,көз асты ұсқынсыз күлдіреуі мен беттегі әжімдер шаршатты ма бұл құпияны дәрігерлер ұзақ жасырып келген әжімдер мен көз асты күлдіреуі көз асты қабы небәрі он төрт күнде жоғалады,көз асты ұсқын күлдіре мен бет әжім шарша ма бұл құпия дәрігер ұзақ жасыр кел әжім мен көз асты күлдіре көз асты қаб небәрі он төрт күн жоғал,neutral

0,өшіріп тастамай тұрғалы оқуға үлгер https vk cc zvf,өшір тастама тұр оқ үлгер https vk cc zvf,neutral

0,шамадан тыс терлеуауыздағы жағымсыз иістің шығуы немесе денеде қал сүйелдің көбеюі немесе шашыңыздың көп көлемде түсуі шаршаңқылық секілді белгілер организмде паразиттің көбеюінің себептері дер кезінде емдемесе соңы өліммен аяқталатыны еш күмәнсіз,шама тыс терлеуауыз жағымсыз иіс шығ немесе дене қал сүйе көбею немесе шаш көп көлем түс шаршаңқылық секілді белгі организм парази көбею себеп дер кез емдеме соңы өл аяқтал еш күмән,neutral

0,тез шаршап қалу сіз қандай іспен айналыссаңыз да тез шаршап қаласыз б,тез шарша қалу сіз қандай іс айналыс да тез шарша қал б,neutral

0,сырыңды ешкімге алдыртпа екі елі ауызыңа сыймаған сөз өзгенің отыз екі тісінің арасына тіптен сыймайды бауыржан момышұлы,сыр ешкім алдыртп екі ел ауыз сыйма сөз өз отыз екі ті ара тіп сыйма бауыржан момышұл,neutral

0,суық шай мен суық күрішке шыдауға болар бірақ салқын көзқарас пен ауыр сөзге шыдау мүмкін емес жапон даналығы,суық шай мен суық күріш шыда бол бірақ салқын көзқарас пен ауыр сөз шыда мүмкін емес жапон даналығ,neutral

0,бүгін алға қарай жүрмесең ертең жүгіруіңе тура келеді карлес пуйоль,бүгін алға қарай жүрме ертең жүгір тура кел карлес пуйоль,neutral

0,өткені жаман болған адамдардың болашағы жақсы болады умар ибн альхатаб,өт жаман бол адам болашағ жақсы бол ум ибн альхатаб,neutral

0,мені бүгін не күтіп тұрғанын білмеймін ертең не болатынын да білмеймін мен тек бір нәрсені білемін барлық нәрсе алланың қалауымен,мен бүгін не күт тұр білме ертең не бол да білме мен тек бір нәрсе біл барлық нәрсе алла қалау,neutral

0,бәрі жақсы болады тіпті кешегі қателіктердің ертең көмегі тиер боб марли,бәрі жақсы бол тіпті кешегі қателік ертең көмег ти боб марли,neutral

0,ақталма ақымақ болма уайымға салынба сымбатыңа мұқият бол үнтүнсіз қалма мылжың сөзден аулақ бол айғайлама кілтті қайда қойғаның есіңде болсын кітап оқы токшоу қарама бәрі жақсы деп қайталай бер көп нәрсені ойла аздан іш артық адамдарды өзіңе жуытпа ерте тұр сегіз сағат ұйықта диетаға отыр бірақ таразыдан түс таразыдан түссең диетаға отыр ертеден кешке дейін жазды күте берме қабағы ашылмайтын адамдардан абай бол ғажайып идеяларға ашық бол жасандылыққа жуыма күйгелек болма сүйдің бе айт сүймесең бар да ғашық бол шынар әбілда,ақтал ақы болма уайым салынба сымбат мұқият бол үнтүн қалма мылж сөз аулақ бол айғайла кілт қайда қой ес бол кітап оқы токшо қарама бәрі жақсы де қайтала бер көп нәрсе ойла аз іш артық адам өз жуытп ерте тұр сегіз сағат ұйық диета отыр бірақ таразы түс таразы түс диета отыр ерте кеш дейін жаз күт берме қабағ ашылма адам абай бол ғажайып идея ашық бол жасандылық жу күйгелек болма сүй бе айт сүйме бар да ғашық бол шын әбіл,neutral

0,простатит сізді кез келген жасыңызда мазалауы мүмкін,простатит сіз кез кел жас мазала мүмкін,neutral

0,бұл жағымсыз дертпен сіз кез келген жасыңызда ұшырасуыңыз мүмкін бұрын простатит жас аралығында пайда болатын еді ал қазір бұл дерт жас шамасындағы жастарды да мазалай бастады аты жаман ауру соңғы кезде айтарлықтай жасарған,бұл жағымсыз дерт сіз кез кел жас ұшырас мүмкін бұрын простатит жас аралығ пайда бол ед ал қазір бұл дерт жас шама жас да мазала баста ат жаман ауру соңғы кезде айтарлықтай жасар,neutral

0,простатит жылдар бойы білінбей келіп бір сәтте жылдам асқынып кетуі мүмкін,простатит жыл бойы білінбе кел бір сәт жылдам асқын кет мүмкін,neutral

0,өмір сүру үшін қателесу жеңу және тағыда жеңу адасу және мәңгі бақи күресу керек ал тыныштық рухтың қорқақтығы лев толстой,өмір сүру үшін қателес жеңу және тағыда жеңу адас және мәңгі бақи күресу керек ал тыныштық рух қорқақтығ лев толстой,neutral

0,ешқашан берілмеңіз ешқашан ешқашан ешқашанешқашан берілмеңіз дөрекі күштен де каймықпаңыз дұшпаныңыз сізден күшті болса да кейін шегінбеңіз уинстон черчилль,ешқашан берілме ешқашан ешқашан ешқашанешқашан берілме дөрекі күш де каймықпа дұшпан сізден күшті бол да кейін шегінбе уинстон черчилль,neutral

0,ешкім кілті жоқ құлып жасамайды сол сияқты өмірде де барлығының шешімі бар,ешкім кілт жоқ құлып жасама сол сияқты өмір де барлығ шешім бар,neutral

0,соңғы уақытта аяғым мен белім қатты ауырды әр адымды жасау қиынға соғатын дәрігерлер диагноздарын қойды тізе буындарының артрозы және бел омыртқа бөлігінің остеохондрозы ал менің бар ғұмырым ауылда өткен бала кезімнен күн мен түнім бақшада өтетін екі қолым ешқашан бос жатпайтын менде де анамда да осы

бәле болатын ал қазір дәрігерлердің айтуынша қалалық жастарда да осындай сырқаттар кездесіп жатады толығырақ сілтеме бойынша https vk cc xho,соңғы уақыт ая мен бел қатты ау әр адым жасау қиын соғ дәрігер диагноз қой тіз буын артроз және бел омыртқа бөліг остеохондроз ал менің бар ғұмыр ауыл өткен бала кез күн мен түн бақша өт екі қол ешқашан бос жатпа менде де ана да осы бәл бол ал қазір дәрігер айт қалалық жас да осындай сырқат кездес жат толығырақ сілтеме бойынша https vk cc xho,neutral

0,сүйе білген жүрек кешіре де күте де сыйлай да біледі,сүйе біл жүрек кешір де күт де сыйла да біл,neutral

0,жақсы келін алсаң үйінен кісі кетпейді,жақсы келін ал үй кісі кетпе,neutral

0,жаман келін алсаң жан досың да шеттейді,жаман келін ал жан дос да шетте,neutral

0,үш күнде темекіден құтыла аласыздар көзім жеткен соң сіздермен бөлісіп отырмын шын айтсам бұған дейін мен жарты жыл күресіп жүрдім бірақ нәтиже болмады,үш күн темекі құтыл ал көз жет соң сіздермен бөліс отыр шын айт бұған дейін мен жарты жыл күрес жүр бірақ нәтиж болма,neutral

0,айна ең адал дос себебі сен жылағанда ол ешқашан күлмейді,айна ең адал дос себебі сен жыла ол ешқашан күлме,neutral

0,әрбір үшінші әйел варикоздың зардабын шегеді ең арзан және әсерлі құрал сізге арналады https vk cc enuvw,әрбір үшінші әйел варикоз зардаб шег ең арзан және әсерл құрал сізге арнал https vk cc enuvw,neutral

0,мәңгілік еш нәрсе жоқ бір секундтың ішінде бәрі өзгеріп кетуі мүмкін,мәңгілік еш нәрсе жоқ бір секунд іш бәрі өзгер кет мүмкін,neutral

0,қасқырдың сыртынан әртүрлі сөз айта боресің ғой,қасқыр сырт әртүрлі сөз ай бер ғой,neutral

0,сол сияқты бір адамдар болады сыртыңнан өсек айтып жала жабады ғайбаттайды сөз тасиды брақ сенімен жолығып қалса лыпылдап кетеді олар үшін сен сұсты қасқырсың,сол сияқты бір адам бол сырт өсек айт жал жаб ғайбатта сөз таси брақ сенімен жолығ қал лыпылда кет олар үшін сен сұ қасқыр,neutral

0,кімге ренжідім кешірдім кімді ренжіттім кешірім сұраймын арман мен мақсат көп өткенкеткенге қайырылатын уақыт жоқ тіпті сенің қалай қиналып талпынып жатқаныңды ешкім көрмесе де уайымдама алла бәрін көреді әлсіздер күледі мықтылар демеу береді кетсе мейлі кете берсін маңдайға жазса оралар оралмаса уақыт өте жүрекке жақын жан табылар тым мықты болғым келмейді нәзік сүйікті бақытты болғым келеді биіктеп кетсең құлау бар қуанып кетсең жылау бар қарапайымдылық адам бойындағы ізгі қасиет себебі екі аяқты пенденің бір бірінен артықшылығы тек ақылойында ғана құранның сөзі қасиет,кім ренжі кеш кім ренжі кешір сұра арман мен мақсат көп өткенкет қайырыл уақыт жоқ тіпті сенің қалай қинал талпын жат ешкім көрме де уайымда алл бәрін көр әл күл мықты деме бер кет мейлі кет

бер маңдай жаз ора оралма уақыт өте жүрек жақын жан табыл тым мықты бол келме нәзік сүйікті бақытты бол кел биікте кет құла бар қуан кет жыла бар қарапайымдылық адам бой ізгі қасиет себебі екі аяқ пенде бір бір артықшылығ тек ақылой ғана құран сөз қасиет,neutral

0,жұртқа емес санаңа бағын ақ пен қараны ажыратар ұмытпа досым құранның барын мұсылмансыңба барлық жерде мұсылман бол тек мешітте ғана емес бақыттың қонғанына мән бермеген бақыттың ұшқанын да кеш біледі аманаты деп танысаң алланың қыздың жасын мойыныңа алмағын қадірлейік қыз баласын құрметтеп қыз жылатып күнақар боп қалмайын,жұрт емес сана бағын ақ пен қара ажыра ұмытп дос құран бар мұсылмансыңб барлық же мұсылман бол тек мешіт ғана емес бақыт қон мән берме бақыт ұш да кеш біл аманат де таны алла қыз жас мойын алмағ қадірл қыз бала құрметте қыз жылат күнақ боп қалма,neutral

0,үміт күткен ұлы оңбаған боп кетсе әке сорлы,үміт күт ұлы оңба боп кет әке сорл,neutral

0,қызы күйеуге өтпесе шеше сорлы,қыз күйе өтпе шеш сорл,neutral

0,сұлу деп алғаны сүйкімсіз боп шықса жігіт сорлы,сұл де ал сүйк боп шық жігіт сорл,neutral

0,қыран деп тигені жапалақ болып шықса жар сорлы,қыран де ти жапалақ бол шық жар сорл,neutral

0,қолында шылымы бар қайран ана,қол шылым бар қайран ана,neutral

0,сен де қазақ қызысын ғой жаны дара,сен де қазақ қызы ғой жан дара,neutral

0,болды ғой қазақ үшін үлкен жара,бол ғой қазақ үшін үлкен жара,neutral

0,гүл сияқты көрініп тікен болып бататындардан,гүл сияқты көрін ті бол бат,neutral

0,дос сияқты көрініп жылан болып шағатындардан бізді сақтай гөр,дос сияқты көрін жылан бол шағ біз сақта гө,neutral

0,біреу сені ренжітсе кешіре сал,біреу сен ренжіт кешір сал,neutral

0,жыласаң айт жұбатуға тырысам,жыла айт жұбат тыры,neutral

0,ұнатпасаң айт сөйлемеуге тырысам,ұнатпа айт сөйлеме тыры,neutral

0,жек көрсең айт жоламауға тырысам,жек көр айт жолама тыры,neutral

0,шын сүйсең айт ренжітпеуге тырысам,шын сүй айт ренжітпе тыры,neutral

0,түн ұйқысын төрт бөлген көз ілмей де,түн ұйқы төрт бөл көз ілме де,neutral

0,біріншіден үрейден аулақ болайық сенім жоғалмасын қиыншылық деген қиюы келмейтін іс емес жұмыла көтерейік,бірінші үрей аулақ бол сенім жоғалма қиыншылық де қию келме іс емес жұмыл көтер,neutral

0,екіншіден тату үйге тақсірет жуымайды бірлігіңе ие бол,екінші тату үй тақсірет жуыма бірліг ие бол,neutral

0,үшіншіден өзіңе жақсылық тілесең өзгеге жамандық қылма дінмұхамед қонаев,үшінші өз жақсылық тіле өзге жаман қылма дінмұхамед қонаев,neutral

0,сәттілік сұрама талант болса сәттілік келеді,сәттілік сұрама талант бол сәттілік кел,neutral

0,махаббат пен сенім барлығын кешіру үшін кең жүрек сұра,махаббат пен сенім барлығ кешіру үшін кең жүрек сұра,neutral

0,егер адам сізге өзінің қиындықтары жайлы айта бастаса,егер адам сізге өзінің қиындық жайлы ай баста,neutral

0,ол сізге шағымданғаны немесе аянышты болып көрінгені емес оның сізге сенім артқаны,ол сізге шағымдан немесе аяныш бол көрін емес оның сізге сенім арт,neutral

0,еш уақытта қайтып келмейтін үш нәрсе бар,еш уақыт қайт келме үш нәрсе бар,neutral

0,еш уақытта жоғалтып алуға болмайтын үш нәрсе бар,еш уақыт жоғалт ал болма үш нәрсе бар,neutral

0,басқа нәрселерден бағалырақ үш нәрсе бар,басқа нәрсе бағалырақ үш нәрсе бар,neutral

0,еш уақытта сенімді болмайтын үш нәрсе бар,еш уақыт сенімді болма үш нәрсе бар,neutral

0,жеңіліске бір қадам қалғанда жеңіс келеді,жеңіліс бір қадам қал жеңіс кел,neutral

0,жоқтың артында бар тығылып жатады,жоқ арт бар тығыл жат,neutral

0,өз қателігіңді талда жетістікке жетуді ойла,өз қателіг талда жетістік жету ойла,neutral

0,табыс жүгірген сайын жақындай түседі,табыс жүгір сайын жақында түс,neutral

0,басқаларға сенім мен табандылықты ұялат,басқа сенім мен табандылық ұялат,neutral

0,ойың іске асқанда арманың орындалады,ой іс ас арман орындал,neutral

0,өзің жалаңаяқ болсаңда арманың асқақ болсын,өзің жалаңаяқ болса арман асқақ бол,neutral

0,өткеннің бәрін өртеп өшір де жоспар жаса,өт бәрін өрте өшір де жоспар жаса,neutral

0,адамдардың көзін жеткізем деп тыраштанба нақты болғанын қалай болатынын дәлелде,адам көз жеткізе де тыраштанб нақты бол қалай бол дәлелде,neutral

0,сіздің сөзіңізге түсінбеген сіздің үнсіздігіңізді де түсінбейді элберт хаббарт,сіздің сөз түсінбе сіздің үнсіздіг де түсінбе элберт хаббарт,neutral

0,көзді жұмып ешнәрсе көрмеуге болады ал жүректі жауып қайғыны сезе алмауға болмайды,көз жұм ешнәрсе көрме бол ал жүрек жау қайғы сез алма болма,neutral

0,қиын сәттерде жаныңда кім болса соларды қатты қадірле,қиын сәт жан кім бол сол қатты қадірл,neutral

0,дене қолтық аяқ тершеңдігінен қалай құтыламыз деген сұрақ сіздіде мазалайды ма онда мына мақаланы міндетті түрде оқыңыз https vk cc estcw,дене қол аяқ тершеңдіг қалай құтыл де сұрақ сізді мазала ма онда мына мақала міндетті түр оқ https vk cc estcw,neutral

0,мыңқ етпейсің қабылдап аласың да қарасымен шамаң жоқ таласуға,мыңқ етпе қабылда ал да қара шама жоқ талас,neutral

0,бұл құралды диетологтар жылдар бойы жасырып келді енді кез келген әйел арықтай алады артық салмақтан арылу үшін https vk cc xdy,бұл құра диетолог жыл бойы жасыр кел енді кез кел әйел арықта ал артық салмақ арылу үшін https vk cc xdy,neutral

0,адамның міңезінде үш алтын қасиет бар төзімділік сезіну және үндемеу бұлар өмірде ақыл талант пен сұлулықтан да көп пайдасын тигізеді,адам міңез үш алтын қасиет бар төзімділік сезін және үндеме бұлар өмір ақыл талант пен сұлулық да көп пайда тигіз,neutral

0,үш бағалы затты ешқашан қайтара алмайсың уақыт сөз мүмкіндік сондықтан уақытты жоғалтпа сөзді дәл уақытында айт мүмкіндікті жіберіп алма конфуций,үш бағалы зат ешқашан қайтар алма уақыт сөз мүмкіндік сондықтан уақыт жоғалтпа сөз дәл уақыт айт мүмкіндік жібер алма конфуци,neutral

0,жаз көңілін салқындау күз алады,жаз көңіл салқында күз ал,neutral

0,ауыр сөзден жүректе із қалады,ауыр сөз жүрек із қал,neutral

0,ол ананың әкенің жалғыз гүлі,ол ана әке жалғыз гүл,neutral

0,боқтауға да ұрысып ұруға да,боқта да ұрыс ұр да,neutral

0,жаймен айтқын түсіндір түйсігі бар,жай айтқ түсіндір түйсіг бар,neutral

0,қол көтерсең көргендер мақұрым дер,қол көтер көр мақұрым дер,neutral

0,өз досыңдай сырлас та жақын тартып,өз досыңда сырлас та жақын тарт,neutral

0,өз жаныңдай сүйгейсің сен күнім деп,өз жаныңда сүй сен күн де,neutral

0,әрбір сүріңгеніңді бақытсыздық деп санама бәлкімсенің шын бақытың сол жерден басталар,әрбір сүрің бақытсыз де санама бәлкімсе шын бақыт сол же баста,neutral

0,біреуді жоғалтып алудан қорықпаңыз жоғалатындар сізге тәжірибе үшін жіберілген тағдыр жібергендер мәңгілік сізбен бірге қалады,біреу жоғалт алу қорықпа жоғал сізге тәжірибе үшін жібер тағдыр жібер мәңгілік сізбен бірге қал,neutral

0,кімде кегім бар болса құрсын дедім,кім ке бар бол құр де,neutral

0,сабыр етуді үйрен себебі күндердің барлығы тамаша әрі біз ойлағандай бола бермейді сол тұста үйренгеніңді қажетке жаратасың сабырлық көркем мінездің көрінісі баға жетпес қазына,сабыр ету үйрен себебі күн барлығ тамаша әрі біз ойлағанда бола берме сол тұс үйрен қажет жара сабырлық көркем мінез көрініс баға жетпес қазына,neutral

0,тырнақ саңырауқұлағы мен өкшенің жарылуы шынымен қауіпті,тырнақ саңырауқұлағ мен өкше жарыл шын қауіпті,neutral

0,бұл дерт айтарлықтай қауіпті дер кезінде емдемесеңіз соңы жаман болады қатты ауыру мазоль қара тырнақтың өсіп кетуі б,бұл дерт айтарлықтай қауіпті дер кез емдеме соңы жаман бол қатты ауыру мазоль қара тырнақ өс кет б,neutral

0,сенің басымнан қандай жағдайлар өткенін ешкім білмейді,сенің бас қандай жағдай өт ешкім білме,neutral

0,сенің қандай қиындықтарың бар екенін де ешкім білмейді,сенің қандай қиындық бар екен де ешкім білме,neutral

0,сенің не үшін күресіп жүргенің де ешкімге мәлім емес,сенің не үшін күрес жүр де ешкім мәлім емес,neutral

0,тіпті шын мәнінде қандай адам екеніңде оларға белгісіз,тіпті шын мән қандай адам екен оларға белгісіз,neutral

0,бірақ ыңғайы туғанда бәрі де сені кінәлап табалап қалуға дайын,бірақ ыңғай ту бәрі де сен кінәла табала қал дайын,neutral

0,дүниеде ең қиыны жалғыздық ең жаманы жақыныңнан мәңгіге айырылу ең ащысы ешкімге білдіртпей іштей тынып жылау ал ең ауыры осының бәрін жасыру үшін жалған күліп жүру,дүние ең қиын жалғыз ең жаман жақын мәңгі айырыл ең ащы ешкім білдіртпе іште тын жыла ал ең ауыр осы бәр жасыр үшін жалған күл жүру,neutral

0,кедейдің қасында дүниең туралы,кедей қас дүние туралы,neutral

0,науқастың алдында деніңнің саулығы туралы,науқас алд ден саулығ туралы,neutral

0,жолы болмай жүрген кісінің алдында бақытты екенің жайлы,жол болма жүр кісі алд бақытты екен жайлы,neutral

0,түрмедегі кісінің алдында бостандығың туралы,түрме кісі алд бостандығ туралы,neutral

0,бедеу кісінің алдында балаларың туралы,беде кісі алд бала туралы,neutral

0,көбісі жақсылықты байқамайды ал жамандықты естен шығармайды,көбісі жақсылық байқама ал жамандық ес шығарма,neutral

0,ең жалғыз адамдар ең мейірімді адамдар,ең жалғыз адам ең мейірімді адам,neutral

0,ең көп күлетін адамдар ең мұңды адамдар,ең көп күл адам ең мұң адам,neutral

0,ең ойлы адамдар ең кемеңгер адамдар,ең ойл адам ең кемеңг адам,neutral

0,үлкендерді сыйлағаннан әлі ешкім ұятқа қалмады жағдайы нашарларға көмектескеннен әлі ешкім кедей болып қалған жоқ бірінші кешірім сұрағаннан әлі ешкім кем болып қалған жоқ,үлкен сыйла әлі ешкім ұят қалма жағдай нашар көмектес әлі ешкім кедей бол қалған жоқ бірінші кешір сұра әлі ешкім кем бол қалған жоқ,neutral

0,тағдырды қатал деме ол сені сынап жатыр,тағ қатал деме ол сен сына жатыр,neutral

0,махаббатты адал деме ол сені алдап жатыр,махаббат адал деме ол сен алда жатыр,neutral

0,кеткенге орал деме ол өзгені қалап жатыр,кет орал деме ол өз қала жатыр,neutral

0,көзіңнен жас тамса сүртіп таста,көз жас там сүрт таста,neutral

0,және әрқашан есте сақта бұл өмір,және әрқашан ес сақта бұл өмір,neutral

0,өміріңнің соңына дейін қанша байлық жисаң да ақшаң екінші өмірді сатып алуға жетпейді,өмір соң дейін қанша байлық жи да ақша екінші өмір сат ал жетпе,neutral

0,бақ деген құс ұшады да кетеді тақ деген түс кіреді де кетеді байлық деген мұз ериді де кетеді мәңгілік қалатын адал іс,бақ де құс ұш да кетеді тақ де түс кіреді де кетеді байлық де мұз ериді де кетеді мәңгілік қал адал іс,neutral

0,бақ деген құс ұшады да кетеді тақ деген түс кіреді де кетеді байлық деген мұз ериді де кетеді мәңгілік қалатын адал іс өмірде адал іс қана мәңгілік қалатынына көзім жетті,бақ де құс ұш да кетеді тақ де түс кіреді де кетеді байлық де мұз ериді де кетеді мәңгілік қал адал іс өмір адал іс қана мәңгілік қал көзі жетті,neutral

0,саған уақыты жоқ адамға сен де уақытыңды жоғалтпа,саған уақыт жоқ адам сен де уақыт жоғалтпа,neutral

0,жақсы адам барлығын өзінен іздейді ақымақ адам оларды басқадан іздейді конфуций,жақсы адам барлығ өз ізде ақы адам ол басқа ізде конфуци,neutral

0,көбіне еркектер әйелдерінің көзіне шөп салып басқа жас қыздармен болып жүреді оған не себеп екенін білесіз бе әрине әйелінің қызығарлық жері қалмайды бала босанғаннан еріне нағыз керек жері босап үлкейіп кетеді осыдан соң күйеуі жас қыздарды іздей бастайды бірақ қазір дамыған заман жыныс мүшені қыз кезіндегідей тарылтуға да болады дәл осы мәселеге тап болып жыныс мүшесін үй жағдайында тарылтып күйеуін сақтап қалған әйелдің оқиғасын оқыңыз https vk cc iv

ss,көбіне еркек әйел көз шөп сал басқа жас қыз бол жүр оған не себеп екен біл бе әрине әйел қызығарлық же қалма бала босан ер нағыз керек же боса үлкей кет осы соң күйе жас қыз ізде баста бірақ қазір дамы заман жыныс мүше қыз кезіниздегіде тарылт да бол дәл осы мәселе тап бол жыныс мүше үй жағдай тарылт күйе сақта қалған әйел оқиға оқ https vk cc iv ss,neutral

0,ыңғайлы әрі айқын мұсылман кулоны сіздің жүрегіңізге қымбат бойтұмар бола алады иесіне ұзақ жылдар бойы адал серік болады,ыңғайлы әрі айқын мұсылман куло сіздің жүрег қымбат бойтұмар бола ал ие ұзақ жыл бойы адал серік бол,neutral

0,ежелден келе жатқан ырымсенімдерге сенсек бұл бойтұмарды тағып жүрген адамды өмірде жетістік пен сәттіліктер күтеді толығырақ осы сайтта https vk cc tfte,ежел келе жат ырымсенім сен бұл бойтұмар тағ жүр адам өмір жетістік пен сәттілік күт толығырақ осы сайт https vk cc tfte,neutral

0,жанында саған сенетін адам болса мүмкін емес нәрсе жоқ,жан саған сен адам бол мүмкін емес нәрсе жоқ,neutral

0,әрине екіншісін таңдайтыныңызға күмәнім жоқ,әрине екінші таңда күмән жоқ,neutral

0,ешкімге қатты сенбе алғашқыда тұз да қант болып көрінеді,ешкім қатты сенбе алғашқы тұз да қант бол көрін,neutral

0,үнемі байсалды салмақты және шыдамды болып жүруге тырысыңыз егер салмақты ашық жарқын болсаңыз туындаған мәселенің оң шешімін тез табасыз,үнемі байсалды салмақ және шыда бол жүр тырыс егер салмақ ашық жарқын бол туында мәселе оң шеш тез таб,neutral

0,оған қоса сабырсыз сары мұңды,оған қоса сабыр сары мұң,neutral

0,оған қоса сүйе тұрып күюді де,оған қоса сүйе тұр күю де,neutral

0,оған қоса көз жасты тыйуды да,оған қоса көз жас тыйу да,neutral

0,өмір маған ең маңызды сабағын берді сізді қиын жағдайда жалғыз қалдырған адамды кешіруге болмайды ол сізді тағы да алдауға дайын джон трамп,өмір маған ең маңызды сабағ бер сіз қиын жағдай жалғыз қалдыр адам кешір болма ол сіз тағы да алда дайын джон трам,neutral

0,бәрін өзіңіз үйренуге тырысыңыз әйтпесе өмір үйретсе аямайды юрий никулин,бәрін өз үйрен тырыс әйтпесе өмір үйрет аяма юри никулин,neutral

0,өмір бойы адал болу бұл бір керемет қасиет,өмір бойы адал болу бұл бір керемет қасиет,neutral

0,ол алтыннан да қымбат тіпті судан да таза пәк қасиет қой қазіргі уақытта ол әрбір адамға керек бірақ бұл қасиетің әрбір адамда бола бермейтіні өкінішті,ол алтын да

қымбат ті судан да таза пәк қасиет қой қазіргі уақыт ол әрбір адам керек бірақ бұл қасиет әрбір адам бола бермейтіні өкінішті,neutral

0,сені біреулер өзің қалағандай сүймесе ол сені жантәнімен сүймейді деп ойлап қалма,сен біреу өзің қалағанда сүйме ол сен жантән сүйме де ойла қалма,neutral

0,шын дос сенің қолыңды ұстап тұрып жүрегіңді сезетін адам,шын дос сенің қол ұста тұр жүрег сез адам,neutral

0,сені мұң басса да күліп жүруді ешқашан тоқтатпа сенің күлкіңе біреулер ғашық болып қалуы мүмкін,сен мұң бас да күл жүру ешқашан тоқтатпа сенің күлкі біреу ғашық бол қал мүмкін,neutral

0,бұл өмірде сен бар болғаны адам ғана шығарсың бірақ біреулер үшін бүкіл әлемсің,бұл өмір сен бар бол адам ғана шығ бірақ біреу үшін бүкіл әлем,neutral

0,уақытын сенімен өткізгісі келмеген адамға уақытыңды кетірме,уақыт сенімен өткіз келме адам уақыт кетір,neutral

0,қалауымыздағы бірегей адамды қалай қабылдайтынымызды білу үшін бізді құдай басқа адамдармен кездестіреді бірегей адамды кездестіре қалғанда сол жақсылықты білу үшін солай жасаса керек,қалауымыз бірегей адам қалай қабылда білу үшін біз құдай басқа адам кездестір бірегей адам кездестір қал сол жақсылық білу үшін солай жаса керек,neutral

0,жылама өйткені бұл бітіп кетті күле қабылда өйткені солай болды,жыла өйткені бұл біт кет күл қабылда өйткені солай бол,neutral

0,сенің жаныңды ауыртатын адамдар қашан да табылады адамдарға сенуді жалғастыра бер бірақ сақ бол,сенің жан ауырт адам қашан да таб адам сену жалғастыр бер бірақ сақ бол,neutral

0,жаңа бір адамды кездестіріп ол мені түсінеді деп үміттенгенше өзің жақсар және өзіңнің кіи екеніңді біл,жаңа бір адам кездестір ол мен түсін де үміттенгенш өзің жақсар және өз кіи екен біл,neutral

0,соншама тырыса берме барлық жақсы нәрселер күтпеген жерден болады,сонша тырыс берме барлық жақсы нәрсе күтпе же бол,neutral

0,өмірде дана секілді сүргің келсе екі басты қағиданы жаттап ал әр нәрсені жегенше аш қалған дұрыс әркіммен өмір сүргенше жалғыз болған дұрыс омар хайям,өмір дана секілді сүр кел екі басты қағида жатта ал әр нәрсе жегенш аш қалған дұрыс әркім өмір сүргенш жалғыз бол дұрыс омар хайя,neutral

0,көбісі басыңа бір қиындық түссе уақыт емдейді дейді бірақ уақыт емдемейді тек уақыт өте келе жүректегі ауыртпалықпен мұңмен өмір сүруге етің үйреніп кетеді,көбісі бас бір қиындық түс уақыт емде де бірақ уақыт емдеме тек уақыт өте келе жүрек ауыртпалық мұң өмір сүр ет үйрен кет,neutral

0,мен өте баймын қаншалықты екенін айтып жеткізе алмайм менде екі көретін көз істейтін қолдар мен аяқтар құлағым да жақсы еститін сияқты мен таң сәріні күн батысты көремін сеземін әр сәтке қуаныңыз,мен өте бай қаншалықты екен айт жеткіз алмай менде екі көр көз істе қол мен аяқ құла да жақсы естит сияқты мен таң сәрі күн бат көр сез әр сәт қуан,neutral

0,ешкім сабыр сақтамаған жерде сен сабыр сақта,ешкім сабыр сақтама же сен сабыр сақта,neutral

0,ешкім құрметтемеген жандарды сен құрметте,ешкім құрметтеме жан сен құрметте,neutral

0,ешкімнің қолынан келмеген жұмысты сен жаса,ешкі қол келме жұм сен жаса,neutral

0,сонда ешкімнің қолы жетпеген жетістікке сенің қолың жетеді,сонда ешкі қол жетпе жетістік сенің қол жет,neutral

0,өткен нәрсе өтті де кетті мүлдем өзгерте алмайсың сондықтан тек қазіргімен өмір сүру керек,өткен нәрсе өт де кет мүлдем өзгер алма сондықтан тек қазіргі өмір сүру керек,neutral

0,назар аударыңыз медицинадағы жаңалық аяқтардағы сүйектердің қисық бітуін емдейтін амал табылды ешқандай операциясыз бар болғаны бір курс https vk cc scppq,назар ау медицина жаңалық аяқ сүйек қисық біт емде амал таб ешқандай операция бар бол бір курс https vk cc scppq,neutral

0,бұл дүниеде соқыр адам бар біріншісі ол сен менің сені қалай сүйетінімді көрмейсің,бұл дүние соқыр адам бар бірінші ол сен менің сен қалай сүй көрме,neutral

0,екіншісі ол мен сенен басқа жанды көрмеймін,екінші ол мен сенен басқа жанды көрме,neutral

0,қыз бен жігіттің арасында өкперенішсіз ұрыскеріссіз қарымқатынас болмайды күштілері шыдап бірге бақытты болады ал әлсіздері орнына басқа адам іздеп әлек болады,қыз бен жігіт ара өкпереніш ұрыскеріс қарымқатынас болма күшті шыда бірге бақытты бол ал әлсіз орн басқа адам ізде әлек бол,neutral

0,қазіргі кездер жастар мен ересектер арасында құлақ естімей қалу ауруы көбейіп кеткен енді оның операциясыз ешқандай ауырсынусыз шешімі табылды жылдық кереңдікті күнде емдеуге болады отилор құлақтың есту қабілетін жақсартады қалпына келтіреді кей аурудың салдарынан болатын құлақтағы ыңғайсыздықты жояды әр түрлі жастағы адамдарға арналған,қазіргі кез жас мен ересек ара құлақ естіме қалу ауру көбей кет енді оның операция ешқандай ауырсыну шешім таб жылдық кереңдік күн емде бол отило құлақ ест қабілет жақсарт қалп келтір кей ауру сал бол құлақ ыңғайсыздық жоя әр түрлі жастағы адам арнал,neutral

0,ешқандай кері көрсетілім жоқ операциясыз шөптен жасалған отилор арқылы құлағыңыз есту қабілетін қалпына келтіріңіз есту аппараттарын мәңгіге ұмытыңыз

мына сілтемені басып толық оқып тапсырыс беріңіздер https vk cc у қазақстанның барлық қаласына жеткізеді барлық тексеруден өткізілген жағымсыз әсері жоқ аллергия бермейді дәрігерлердің рұқсатымен сертификатталған өнім,ешқандай кері көрсетілім жоқ операция шөп жасал отило арқылы құла ест қабілет қалп келтір ест аппарат мәңгі ұмыт мына сілтеме бас толық оқ тапсырыс бер https vk cc у қазақстан барлық қала жеткіз барлық тексеру өткіз жағымсыз әсер жоқ аллергия берме дәрігер рұқсат сертификаттал өнім,neutral

0,сонда ғана келетіндердің есімі бас әріппен жазылады,сонда ғана кел есім бас әріп жаз,neutral

0,адамдар сенімен сөйлесуге дәрежелері жетпейтінін түсінгендерінде артыңнан сөйлей бастайды,адам сенімен сөйлес дәреже жетпе түсінген арт сөйле баста,neutral

0,ешкімге сырыңды айтпа себебі сенің сыр бөлісіп жүрген ең жақын досың дасені бір күні сатып кетуі әбден мүмкін,ешкім сыр айтпа себебі сенің сыр бөліс жүр ең жақын дос дасе бір күн сат кет әбден мүмкін,neutral

0,бай адамды күтпе иманды адамға тұрмысқа шық,бай адам күтпе има адам тұрмыс шық,neutral

0,себебі байлық сарқылуы мүмкін ал иман сарқылмайды,себебі байлық сарқыл мүмкін ал иман сарқылма,neutral

0,нағыз еркек әйелін басқаруға тырысып басынып балағаттап қол жұмсайтын еркек емес,нағыз еркек әйел басқар тырыс басын балағатта қол жұмса еркек емес,neutral

0,нағыз еркек әйелін сыйлайтын қамқорлық жасайтын еркек сонда әйелінің өзіақ оған бағынғысы келеді,нағыз еркек әйел сыйла қамқорлық жаса еркек сонда әйел өзіақ оған бағын кел,neutral

0,болашағыңды білмей тұрып біреудің өткеніне күлме ллойд бэнкс,болашағ білме тұр біреу өт күл ллойд бэнкс,neutral

0,дауылда берілген уәделер тыныш күні ұмытылады уильям шекспир,дауыл бер уәде тыныш күн ұмыт уилья шекспи,neutral

0,шамадан тыс терлеуауыздағы жағымсыз иістің шығуы немесе денеде қал сүйелдің көбеюі немесе шашыңыздың көп көлемде түсуі шаршаңқылық секілді белгілер организмде паразиттің көбеюінің себептері дер кезінде емдемесе соңы өліммен аяқталатыны еш күмәнсіз қазірден бастаңыз симптом түрлері шектен тыс терлеу,шама тыс терлеуауыз жағымсыз иіс шығ немесе дене қал сүйе көбею немесе шаш көп көлем түс шаршаңқылық секілді белгі организм парази көбею себеп дер кез емдеме соңы өл аяқтал еш күмән қазір баста симптом түр шек тыс терле,neutral

0,бірде суық бірде ыстық бұл махаббат шырағы бірде бардабірде жоқ бұл өмірдін сынағы,бірде суық бірде ыстық бұл махаббат шырағ бірде бардабі жоқ бұл өмірд сынағ,neutral

0,адам өзгені бақытсыз етіп өзі бақытты бола алмайды,адам өз бақыт ет өзі бақытты бола алма,neutral

0,ешкім басқаларды сынайтын дәрежеде ақылды емес,ешкім басқа сына дәреже ақылды емес,neutral

0,ешкім басқаларға өмірді үйрететін дәрежеде ақылды емес,ешкім басқа өмір үйрет дәреже ақылды емес,neutral

0,сондықтан айналаны емес атаананы тыңдау керек,сондықтан айнала емес атаана тыңдау керек,neutral

0,егер өзен суы тасыса құмырсқалар балықтарға жем болады ал тартылып кетсе балықтар құмырсқаларға жем болады өмір алма кезек,егер өзен су тасы құмырсқа балық жем бол ал тартыл кет балық құмырсқа жем бол өмір алма кезек,neutral

0,өмір деген бұл әлемнің кеңдігі,өмір де бұл әлем кеңдіг,neutral

0,өмір деген ақ пен қара теңдігі,өмір де ақ пен қара теңдіг,neutral

0,өмір деген артта қалған өткенің,өмір де арт қалған өт,neutral

0,өмір деген тұрағыңның жайлығы,өмір де тұраға жайлыға,neutral

0,өмір деген кейде мұңды қайғылы,өмір де кейде мұң қайғылы,neutral

0,өмір деген бір шайлығы байлардың,өмір де бір шайлығ бай,neutral

0,өмір деген сүрген сайын көретін,өмір де сүр сайын көр,neutral

0,өмір деген дүниеден шеткері,өмір де дүние шетк,neutral

0,өмір деген бұ дүние мектебі,өмір де бұ дүние мектеб,neutral

0,өмір деген өз қолыңда кейкезде,өмір де өз қол кейкез,neutral

0,өмір деген дүниенің сұрағы,өмір де дүние сұраға,neutral

0,өмір деген бірақ сәтте тынады,өмір де бірақ сәт тын,neutral

0,өмір деген сен біреуді сынауың,өмір де сен біреу сына,neutral

0,өмір деген арайлап таң атқаны,өмір де арайла таң ат,neutral

0,өмір деген кешке күннің батқаны,өмір де кеш күн бат,neutral

0,өмір деген сол өмірдің өзінің,өмір де сол өмір өзінің,neutral

0,егер сіз он жылға жас көрінгіңіз келсе адамдар сізге назар аударып комплимент айтқаның қаласаңыз осы мақаламды соңына дейін оқыңыз расында бұл сізге он жылға жас көрінуге көмектеседі егер сіз әбіржіп түріңіз шаршаған болып жүргенді қаласаңыз бұл мақаланы оқымайақ қойыңыз,егер сіз он жыл жас көрін кел адам сізге назар аудар комплимент айт қала осы мақала соң дейін оқ рас бұл сізге он жыл жас

көрін көмектес егер сіз әбірж түр шарша бол жүр қала бұл мақала оқымайақ қой,neutral

0,әйелдер қанша жасқа дейін өзін жаспын деп есептей алады жасқа дейін деп жүрдім кейін түсіндім жас көріну ол жасқа байланысты емес екенін сенің бетіңнің терісі фигураң көзіңнің жылтырауы өзіңе деген сенімділікке байланысты екен,әйел қанша жас дейін өз жас де есепте ал жас дейін де жүр кейін түс жас көріну ол жас байланысты емес екен сенің бет тері фигура көз жылтыра өз де сенімділік байланысты екен,neutral

0,менің жасым де мен өзімді жаспын деп санай алмадым мен кәрі көрінетінмін косметика да көп көмек бере алмады,менің жас де мен өз жас де сана алма мен кәрі көрін косметика да көп көмек бер алма,neutral

0,әжімдер беттің түсі көздің астындағы артық тері мені он жылға үлкен қылып көрсетті ал мен өзімді сұлу болғанымды қаладым адамдардан комплимент алғым келді кейіннен түсіндім өзімнің сұлулығым үшін күресу керек екенін неше түрлі құралдарды толтырып алып бетіме жақтым,әжім бет түс көз аст артық тері мен он жыл үлкен қыл көрсе ал мен өз сұл бол қала адам комплимент ал кел кейін түс өзімнің сұлулығ үшін күресу керек екен неше түрлі құрал толтыр алып бет жақ,neutral

0,жарты жылда бетімдегі әжімдердің біреуі де кетпеді сол кеткен ақшаға бетімнің терісін тартып операция жасатуға болатын еді ешқандай нәтиже болмады косметологтарға бардым витаминдер іштім сосын дипрецияға түстім нәтиже болмады,жарты жыл бетім әжім біреу де кетпе сол кет ақша бет тері тарт операция жасат бол ед ешқандай нәтиже болма косметолог бар витамин іш сосын дипреция түс нәтиже болма,neutral

0,сөйтіп жүріп бір күні өзімнің құрдас подружкамды жолықтырдым ол маған қарағанда әдемі сұлу көрінді,сөйт жүр бір күн өзімнің құрдас подружка жолық ол маған қара әдемі сұл көр,neutral

0,сөйтім одан сұрадым сөйтіп ол маған осы сайтпен бөлісті,сөйт одан сұра сөйт ол маған осы сайт бөл,neutral

0,https vk cc mhvfi содан подругам айтқан құралды қолдандым қыздар сіздер ғажайыпқа сенесіздер ме,https vk cc mhvfi содан подруга айт құра қолда қыз сіздер ғажайып сен ме,neutral

0,мен таңертең тұрып айнаға қарағанда ғажайыптарға сене бастадым мен өзімді танымай қалдым,мен таңертең тұр айна қара ғажайып сен баста мен өз таныма қал,neutral

0,маған жас сұлу әжімсіз беті таза қыз айнадан қарап тұрды мен соншалықты қуанғаным ән айтып билегім келді,маған жас сұл әж бет таза қыз айна қара тұр мен соншалықты қуан ән айт биле кел,neutral

0,күйеуім маған махаббат көзімен қарай бастады жұмыстағы әріптестерім жұмбақша күліп комплимент айтатын болды,күйе маған махаббат көз қарай баста жұмыс әріптес жұмбақш күл комплимент айт бол,neutral

0,енді мен нақты білдім әйел сұлу болғысы келсе бола алатынына тек қана дұрыс құрал қолдану қажет,енді мен нақты біл әйел сұл бол кел бола ал тек қана дұрыс құрал қолдану қажет,neutral

0,көргеніңнің бәріне сене берме қантта тұз сияқты болып көрінеді араб мақалы,көр бәрін сен берме қант тұз сияқты бол көрін араб ма,neutral

0,дүниедегі ең жаман нәрсе екіжүзділік януш корчак,дүние ең жаман нәрсе екіжүзділік януш корчак,neutral

0,басқа адамға жәрдемдесу үшін бай болудың қажеті жоқ тек қана мейірімді болсаң болды,басқа адам жәрдемдесу үшін бай бол қажет жоқ тек қана мейірімді бол бол,neutral

0,қызың жаман болса жеті атаға жетеді,қыз жаман бол жеті ата жет,neutral

0,әйелің жаман болса достарың жоламай кетеді,әйел жаман бол дос жолама кет,neutral

0,келінің жаман болса күңкілдеумен өтеді,кел жаман бол күңкілде өт,neutral

0,балаң жаман болса өмірің сүрген бекергі майқы би,бала жаман бол өмір сүр бекерг майқ би,neutral

0,бәрінің уақытша екеніне сене білу керек қара бұлттың соңынан жадырап күн шығары сөзсіз,бәрі уақытша екен сен білу керек қара бұл соң жадыра күн шығ сөзсіз,neutral

0,ешкімге ешнәрсе айтпауды үйреніңіздер сол кезде бәрі жақсы болады,ешкім ешнәр айтпау үйрен сол кезде бәрі жақсы бол,neutral

0,бірінші кешірім сұрау бұл төмендіктің белгісі емес бұл адамның ең жақсы қасиеті ф достоевский,бірінші кешір сұрау бұл төмендік бел емес бұл адам ең жақсы қасиет ф достоевски,neutral

0,өмір қоштасуды үйретеді бірақ ұмытқызбайды қарайсың бірақ көре алмайсың естисің бірақ сенбейсің сүйесің бірақ ол сүймейді кетеді және қайта оралмайды,өмір қоштасу үйрет бірақ ұмытқызба қара бірақ көр алма ести бірақ сенбе сүй бірақ ол сүйме кет және қайта оралма,neutral

0,назар аударыңыз медицинадағы жаңалық аяқтардағы сүйектердің қисық бітуін емдейтін амал табылды ешқандай операциясыз бар болғаны бір курс ttps vk cc scppq,назар ау медицина жаңалық аяқ сүйек қисық біт емде амал таб ешқандай операция бар бол бір курс ttps vk cc scppq,neutral

0,кімде кім кішірейсе алла оның абырой беделін көтере түседі,кім кім кішірей алл оның абырой бедел көтер түс,neutral

0,бұл бір бейне өткінші тағдыр екен,бұл бір бейне өткінш тағдыр екен,neutral

0,өткенді өкіну үшін емес сабақ алу үшін еске алу керек,өт өкін үшін емес сабақ алу үшін ес алу керек,neutral

0,болашаққа үреймен емес үмітпен қарау қажет,болашақ үрей емес үміт қарау қажет,neutral

0,бұл мүмкін емес деді сылтау,бұл мүмкін емес де сылтау,neutral

0,досыңмын деп жүріп дұшпандығын оздырар жамандар болады абай құнанбаев,дос де жүр дұшпандығ оздыр жаман бол абай құнанбаев,neutral

0,тәкаппар және қырсық адам ешкімнің ақылын тыңдамай бәрін өз білгеніңше жасайды да көп ұзамай өз қателіктерінің құрбанына айналады эзоп,тәкапп және қырсық адам ешкі ақыл тыңдама бәрін өз білгеніңш жаса да көп ұзама өз қателік құрбан айнал эзо,neutral

0,адаммен бетпебет сөйлеспей тұрып ол туралы пікір айтудан аулақ болыңыз себебі ол жайлы естігендеріңіздің бәрі тек өсек қана майкл джексон,адам бетпебет сөйлеспе тұр ол туралы пікір айту аулақ бол себебі ол жайлы есті бәрі тек өсек қана майкл джексон,neutral

0,біреуге бере алатын ең бағалы сыйлық ол сенің уақытың себебі сен оны берген соң ешқашан қайтарып алуға мүмкіндігін жоқ,біреу бер ал ең бағалы сыйлық ол сенің уақыт себебі сен оны бер соң ешқашан қайтар ал мүмкіндіг жоқ,neutral

0,барлық жеңістер өзіңді өзің жеңуден басталады леонев,барлық жеңіс өз өзің жеңу бастал леонев,neutral

0,қиындықтарға шағымданып қажеті жоқ сіздің жолыңыз әрқашан раушанға көмкеріліп жатпайды,қиындық шағымдан қажет жоқ сіздің жол әрқашан раушан көмкеріл жатпа,neutral

0,алауға қанша отын салсаң сонша жылу қайтарады адамдарға қанша мейірімділік жасасаң алла сонша жақсылық қайтарады,алау қанша отын сал сонша жылу қайтар адам қанша мейірімділік жаса алл сонша жақсылық қайтар,neutral

0,әрдайым жақсылық жасауға тырысыңыз сонда бақыт соңыңыздан өзіақ жүгіреді,әрдайым жақсылық жаса тырыс сонда бақыт соң өзіақ жүгір,neutral

0,бұл өмірдің ең ғажабы сенің барлық кемшіліктеріңді қателіктеріңді және кемшіл тұстарыңды біле тұра сені ең ғажап адам деп санайтын жанды табу,бұл өмір ең ғажаб сенің барлық кемшілік қателік және кемшіл тұс біл тұр сен ең ғажа адам де сана жанды табу,neutral

0,егер адамдар сізді тек ісі түскенде ғана іздесе көңіліңіз түспесін керісінше қуаныңыз себебі сіз шам сияқтысыз олардың өмірлерін қараңғылық басқанда сізге қарай ұмтылады,егер адам сіз тек іс түс ғана ізде көңіліңіз түспе керісінше қуан себебі сіз шам сияқты олардың өмір қараңғылық бас сізге қарай ұмтыл,neutral

0,кейде өткен өмірі нашар адамдар жарқын болашақ құрады,кейде өткен өмір нашар адам жарқын болашақ құр,neutral

0,ал бүгін байлығының буына мас болып менменсіп жүрген адамдар бірден төмен түседі,ал бүгін байлығ бу мас бол менменс жүр адам бірден төмен түс,neutral

0,өмір алмакезек өзіңнің болашағыңды білмейсің сол үшін өзгенің өткен шағына күлме,өмір алмакезек өз болашағ білме сол үшін өз өткен шағ күл,neutral

0,және әрқашан есіңде сақта бұл өмір,және әрқашан ес сақта бұл өмір,neutral

0,қалың және ұзын шаш бар болғаны төрт аптада сіздің шашыңыз ренсіз құрғақ және әлсіз бе шашыңыздың ұзын әрі берік болғанын қаласаңыз онда бұл сізге арналады https vk cc zrxj,қалың және ұзын шаш бар бол төрт апта сіздің шаш рен құрғақ және әлсіз бе шаш ұзын әрі берік бол қала онда бұл сізге арнал https vk cc zrxj,neutral

0,жақсы адамдар сізге бақыт әкеледі жаман адамдар тәжірибе береді ең жамандар сабақ береді ал ең жақсылары естелік қалдырады әр адамды бағалаңыздар уилл смит,жақсы адам сіз бақыт әкеледі жаман адам тәжірибе береді ең жаман сабақ береді ал ең жақсы естелік қалдыр әр адам бағала уилл смит,neutral

0,бір рет қолыңды қайтарған адамнан ешқашан көмек сұрама бір рет сатып кеткен адамға ешқашан сенбе,бір рет қол қайтар адам ешқашан көмек сұрама бір рет сат кет адам ешқашан сенбе,neutral

0,өмірде қиындықтан түңілме бұның бәрі сынақ артынан мүмкін осы қиындықтын арқасында бір жақсылықпен қуаныш тұрады,өмір қиындық түңіл бұның бәрі сынақ арт мүмкін осы қиындықт арқа бір жақсылық қуаныш тұр,neutral

0,дәрежең көтерілгенде достарың сенің кім екеніңді біледі дәрежең түскенде сен достарыңның кім екенін білесің,дәреже көтеріл дос сенің кім екен біл дәреже түс сен дос кім екен біл,neutral

0,сүйгеніңді құшағына алып отбасы бақытын сезіну қандай бақыт шіркін бірақ ол маскүнем болып кетті елде дағдарыс ал ол бар тапқанымызды ішімдікке жұмсайды алкоголизмнен жүз пайыз кепілдікпен айығып кетудің ең қарапайым әдісі дәрігерлердің уколынсыз және дәрі дәрмегінсіз бұл тәсіл өте қарапайым және кез келген адам оны қолдана алады өшіріп тастамай тұрғанда осы жерден оқыңыздар https vk cc yzik,сүйген құшағ алып отбасы бақыт сезін қандай бақыт шіркін бірақ ол маскүне бол кет ел дағдарыс ал ол бар тап ішімдік жұмса алкоголиз жүз пайыз кепілдік айығ кету ең қарапайым әді дәрігер уколын жән дә дәрмегін бұл тәсіл өте қарапайым жән кез кел адам оны қолдан ал өшір тастама тұр осы же оқ https vk cc yzik,neutral

0,сүйген адамыңды түзетудің қажеті жоқ ол егер сені шын сүйсе өзөзін түзетеді,сүй адам түзету қажет жоқ ол егер сен шын сүй өзөз түзет,neutral

0,біреу сенімен босаған кезде ғана сөйлеседі тағы біреу сенімен сөйлесу үшін уақытты өзі босатады айырмашылығын көрдің бе,біреу сенімен боса кезде ғана сөйлес тағы біреу сенімен сөйлесу үшін уақыт өзі босат айырмашылығ көр бе,neutral

0,өсе келе бір заңды түсіндім адамдарды сөзіне қарап емес ісіне қарай бағалау керек екен,өс келе бір заңды түс адам сөз қара емес іс қарай бағалау керек екен,neutral

0,ешқашан біреуді тыңдама ешқашан ешкімді күтпе ешқашан біреуден еш нәрсе сұрама барлығын өзің жаса осылай дұрыс,ешқашан біреу тыңда ешқашан ешкі күтпе ешқашан біреу еш нәрсе сұрама барлығ өзің жаса осылай дұрыс,neutral

0,гибридты лимонды үйге алғанымызға көп болған жоқ экодараның шағын лимонағашы сөз жоқ бұл әдемі жеудің өзі бір рахат лимондар магазиндегідей қышқыл емес олар өте хош иісті және дәмі нәзік гүлдер менің үйімде жақсы өсетін еді ал жеміс ағаштарының жерсініп кетуі проблема еді шын айтсам бұл ағаш өте оңай сіңісіп өсіп кетті суреттен көрсеңіздер болады қандай әдемі болып өскенін қазір дәл осы дүкенде https vk cc yocrl апельсин шие және киви алдым қашан келетінін күтіп жүрмін оңтүстіктік жемістерінің өз үйімде өскенін қалаймын қазір жеңілдіктер жүріп жатыр дүкен ресми тіркелген қорқатын ештеңесі жоқ барлығыңызға мол жеміс тілеймін,гибрид лимон үй ал көп бол жоқ экодара шағын лимонағаш сөз жоқ бұл әдем жеу өзі бір рахат лимон магазиндегіде қышқыл емес олар өте хош иіс жән дәм нәзік гүл менің үй жақсы өс ед ал жеміс ағаш жерсін кет проблема ед шын айт бұл ағаш өте оңай сіңіс өс кет сурет көр бол қандай әдем бол өс қазір дәл осы дүкен https vk cc yocrl апельсин ши жән киви ал қашан кел күт жүр оңтүстік жеміс өз үй өс қала қазір жеңілдік жүр жатыр дүкен ресми тіркел қорқ ештеңе жоқ барлығ мол жеміс тіле,neutral

0,шыgа бәрі ge өткінші тіпті қазір саған қаншалықты қиын болса gа уақыт өте бәрі ұмытылады себебі мына өмірде ештеңе мәңгілік емес,шыg бәрі ge өткінш тіп қазір саған қаншалықт қиын бол gа уақыт өте бәрі ұмыт себебі мына өмір еште мәңгілік емес,neutral

0,жылуы бар жүректен жылытатын сөз шығаржылуы жоқ жүректен жылататын сөз шығар,жыл бар жүрек жылыт сөз шығаржыл жоқ жүрек жылат сөз шығар,neutral

0,бұл өмір біз ойлағандай керемет болса туылғанда жыламас едік кіршіксіз таза болса өлгенде жуынбас едік,бұл өмір біз ойлағанда керемет бол ту жыламас едік кіршік таза бол өл жуынбас едік,neutral

0,ақылсыз еркек көп ақша тапсабұзыладыақылсыз әйел бұзылсакөп ақша табады дейді аллаһерлерге халал табысәйелдерге адал намыс берсін,ақыл еркек көп ақша тапсабұзыладыақыл әйел бұзылсакө ақша таб дейді аллаһер халал табысәйел адал намыс берсін,neutral

0,өзгенің қателіктерін кешірмейтіндей біз кімбіз аллаһ біздің қателіктерімізді кешіріп жатқанда,өз қателік кешірмейтінде біз кім аллаһ біздің қателік кешір жатқанда,neutral

0,химиясыз және диетасыз арықтауға арналған инновациялық препарат,химиясыз және диета арықта арнал инновациялық препарат,neutral

0,көптеген жағдайларда диеталар мен жаттығулар арқылы арықтай алмаудың себептерін біліп алыңыз https vk cc xdy,көптеген жағдай диета мен жаттығу арқылы арықта алма себеп біл ал https vk cc xdy,neutral

0,өртті отпен сөндіре алмағандай ашуды ашумен жеңе алмайсың тек сабырлылықпен жеңе аласың,өр от сөндір алмағанда ашу аш же алма тек сабырлылық же ал,neutral

0,ешқашан адамның түр сипатына сын айтпа алла солай жаратты ал сен аллаға қарсы тұра алмайсың,ешқашан адам түр сипат сын айтпа алл солай жара ал сен алла қарсы тұр алма,neutral

0,реніштерді құмға қуаныштарды тасқа жазып үйрен шығыс мақалы,реніш құм қуаныш тас жаз үйрен шығыс ма,neutral

0,кіммен жүргеніңе кіммен дос болғаныңа мұқият бол өйткені бұлбұл гүлге қарға қоқысқа жетелейді жалаладдин руми,кім жүр кім дос бол мұқият бол өйткені бұлбұл гүл қар қоқыс жетеле жалаладдин руми,neutral

0,қаншама адам көрдім үстінде киімі жоқ қаншама киім көрдім ішінде адамы жоқ,қанша адам көр үст киім жоқ қанша киім көр іш адам жоқ,neutral

0,ұялғанда қызаратын адамда қара жүрек болуы мүмкін емес қытай даналығы,ұял қызар адам қара жүрек бол мүмкін емес қытай даналығ,neutral

0,өмірің қандай болса да оны бағала ол тек бір рет беріледі,өмір қандай бол да оны бағала ол тек бір рет бер,neutral

0,ойладың ба айт дұрыс деп шештің ба істе сонда бұл өмірде сенен бақытты адам болмайды,ойла ба айт дұрыс де шеш ба істе сонда бұл өмір сенен бақытты адам болма,neutral

0,егер папилломадан дер кезінде емделмесе аурудың одан әрі дамуы және шырышты қабаттарда бөртпе пайда болып одан әрі ағзаға таралады папилломаның бастың терісінде пайда болады сондайақ толығырақ осы жерден оқыңыз https vk cc xt sh,егер папиллома дер кез емделме ауру одан әрі дам және шырышты қабат бөртпе пайда бол одан әрі ағза тарал папиллома бас тері пайда бол сондайақ толығырақ осы же оқ https vk cc xt sh,neutral

0,тауларды орнынан қозғалтатын адам алдымен кіші тастардан бастайды конфуций,тау орн қозғалт адам алд кіші тас баста конфуци,neutral

0,ауыр күн де өтеді оған сабыр шыдам қыл,ауыр күн де өт оған сабыр шыдам қыл,neutral

0,сенім қағаз секілді бір мыжылса қайтадан мінсіз бола алмайды,сенім қағаз секілді бір мыжыл қайта мін бола алма,neutral

0,уәдені көп беретіндерге сенбе олар әдетте дым да бітірмейді үнсіздікпен бар жұмысты атқаратынтардарға сенім арт,уәде көп бер сенбе олар әдет ды да бітірме үнсіздік бар жұм атқаратынтар сенім арт,neutral

0,кімге ренжідім кешірдім кімді ренжіттім кешірім сұраймын арман мен мақсат көп өткенкеткенге қайырылатын уақыт жоқ тіпті сенің қалай қиналып талпынып жатқаныңды ешкім көрмесе де уайымдама алла бәрін көреді әлсіздер күледі мықтылар демеу береді кетсе мейлі кете берсін,кім ренжі кеш кім ренжі кешір сұра арман мен мақсат көп өткенкет қайырыл уақыт жоқ тіпті сенің қалай қинал талпын жат ешкім көрме де уайымда алл бәрін көр әл күл мықты деме бер кет мейлі кет бер,neutral

0,оралмаса уақыт өте жүрекке жақын жан табылар,оралма уақыт өте жүрек жақын жан табыл,neutral

0,тым мықты болғым келмейді нәзік сүйікті бақытты болғым келеді,тым мықты бол келме нәзік сүйікті бақытты бол кел,neutral

0,биіктеп кетсең құлау бар қуанып кетсең жылау бар,биікте кет құла бар қуан кет жыла бар,neutral

0,қарапайымдылық адам бойындағы ізгі қасиет,қарапайымдылық адам бой ізгі қасиет,neutral

0,себебі екі аяқты пенденің бір бірінен артықшылығы тек ақылойында ғана,себебі екі аяқ пенде бір бір артықшылығ тек ақылой ғана,neutral

0,бақыттың ұшқанын да кеш біледі,бақыт ұш да кеш біл,neutral

0,қыз жылатып күнақар боп қалмайын,қыз жылат күнақ боп қалма,neutral

0,ең жаман нәрсе қорқыныш,ең жаман нәрсе қорқыныш,neutral

0,ең жаман қателік тез бас тарту,ең жаман қателік тез бас тарту,neutral

0,ең жиіркенішті сезім қызғаншақтық,ең жиіркеніш сезім қызғаншақ,neutral

0,ешқашан адамның түр сипатына сын айтпа,ешқашан адам түр сипат сын айтпа,neutral

0,егерде сізде арман ықылас және табандылық болса сіз барлығына жетесіз,ег сіз арман ықылас және табандылық бол сіз барлығ же,neutral

0,мектепте бірінші сабақ болады содан соң сынақ болады,мектеп бірінші сабақ бол содан соң сынақ бол,neutral

0,өмірде бірінші сынақ содан соң ол саған сабақ болады,өмір бірінші сынақ содан соң ол саған сабақ бол,neutral

0,күліп жүріп өткерсем де күнімді жылап жатып ұйықтарымды кім сезген,күл жүр өткер де күн жыла жат ұйық кім сез,neutral

0,достықтың соңы махаббат бола алады,достық соңы махаббат бола ал,neutral

0,бірақ махаббаттың соңы достық бола алмайды,бірақ махаббат соңы достық бола алма,neutral

0,мен сезіммен қорықпаймын мен сезімге батып кетуден қорқамын,мен сез қорықпа мен сезім бат кету қор,neutral

0,мен тән жарақатынан қорықпаймын мен жан жарақатынан қорқамын,мен тән жарақат қорықпа мен жан жарақат қор,neutral

0,мен сүюден қорықпаймын мен керексіз болудан қорқамын,мен сүю қорықпа мен керек болу қор,neutral

0,мен өлімнен қорықпаймын мен өзім сүйетін жандарды жоғалтып алуды қорқамын,мен өл қорықпа мен өзім сүй жан жоғалт алу қор,neutral

0,бәріміз біреуді не бірнәрсені күтумен өміріміз қалай өтіп кеткенін байқамай да қаламыз,бәрі біреу не бірнәрсе күт өмір қалай өт кет байқама да қал,neutral

0,ана тілдің қадіріне жетпесек,ана тіл қадір жетпе,neutral

0,қазақ болып қайтеміз күн кешіп,қазақ бол қай күн кеш,neutral

0,солай жаным сен жылама мұңай ма,солай жан сен жыла мұңа ма,neutral

0,бақыт үшін күресе бер әрқашан,бақыт үшін күре бер әрқашан,neutral

0,әзір болма мұңыңды айтып басқаға,әзір болма мұң айт басқа,neutral

0,кім мені сатып кетті қош бол,кім мен сат кет қош бол,neutral

0,кім меннен кетті жолың болсын,кім мен кет жол бол,neutral

0,ал кім менің жанымда қалды бағалаймын жақсы көремін және құрметтеймін,ал кім менің жан қал бағала жақсы көр және құрметте,neutral

0,сөздерімнен сән кетсе де мән кетпейді,сөз сән кет де мән кетпе,neutral

0,бір атадан тарайтын екі қазақ,бір ата тара екі қазақ,neutral

0,бірін бірі менсінбей мамбет дейді,бір бірі менсінбе мамбет де,neutral

0,шын сүйген адам тіпті ең қиын жағдайларда да сенің жаныңда болып барлық әлем сенен теріс айналса да сені тастап кетпейтін адам,шын сүй адам тіпті ең қиын жағдай да сенің жан бол барлық әлем сенен теріс айнал да сен таста кетпе адам,neutral

0,менің енем мені мақтайтын ене болады,менің ене мен мақта ене бол,neutral

0,баласының алдында мені жақтайтын ене болады,бала алд мен жақта ене бол,neutral

0,сенің желкеңнен пышақ ұрған адамға ашуланба оған сенім артқан өзіңсің өзіңе ашулан,сенің желке пышақ ұр адам ашуланб оған сенім арт өзің өз ашулан,neutral

0,сенің сыртыңнан сырыңды жайып жүрген адамға ренжіме оған сырыңды айтқан тағы да өзіңсің өзіңе ренжі,сенің сырт сыр жай жүр адам ренж оған сыр айт тағы да өзің өз ренж,neutral

0,егер сіз адалдық ексеңіз сенім аласыз,егер сіз адалдық ек сенім ал,neutral

0,бүгін еккеніңізге абай болыңыз бір мезгіл не егіп жатқаныңызға қараңыз себебі ол ертеңгі жемісіңізді анықтайды бүгінгі дән біздің ісіміздің ертеңгі жемісі,бүгін ек абай бол бір мезгіл не ег жат қара себебі ол ертеңгі жеміс анықта бүгінгі дән біздің іс ертеңгі жеміс,neutral

0,бірақ қасына жолай алмайсың сол сияқты бір адамдар болады сыртыңнан өсек айтып жала жабады ғайбаттайды сөз тасиды брақ сенімен жолығып қалса лыпылдап кетеді олар үшін сен сұсты қасқырсың,бірақ қас жола алма сол сияқты бір адам бол сырт өсек айт жал жаб ғайбатта сөз таси брақ сенімен жолығ қал лыпылда кет олар үшін сен сұ қасқыр,neutral

0,сенен теріс айналған адамдарға рақмет айт бәлкім оғаш көрінер бірақ солардың арқасында әркімге сенім арта бермеуді үйренесің,сенен теріс айнал адам рақмет айт бәлкім оғаш көрін бірақ сол арқа әркім сенім ар бермеу үйрен,neutral

0,ер адамға ең керек нәрсе сенім ол өзіне сенетін адам барын білген кезде батыр болып тау қопаруға дайын болады,ер адам ең керек нәрсе сенім ол өзіне сен адам бар біл кезде батыр бол тау қопар дайын бол,neutral

0,әйел адамға ең керек нәрсе сезім олар өздерін сүйетінін сезген кезде бәріне нұрын шашып мейірлене сұлулана түседі,әйел адам ең керек нәрсе сезім олар өз сүй сез кезде бәрін нұр шаш мейірлен сұлулан түс,neutral

0,көрсеқызарлықпен жеңілтектікпен біреудің орынсыз сөзіне бір кез келген қызыққа шайқалып қала берсең мінездің беріктігі бұзылады абай құнанбайұлы,көрсеқызарлық жеңілтектік біреу орынсыз сөз бір кез кел қызық шайқал қала бер мінез беріктіг бұз абай құнанбайұл,neutral

0,ұмытпа жасаған жаман амалдарың өміріңнің соңына дейін қуады алдыңнан шығатыны қаншама жамандық ішке кірген ине сияқты түбі тесіп шығады сол үшін біреуге жамандық жасамас алдын оның өзіңе тиер зардабын да ойла берік сұлтан,ұмытп жаса жаман амал өмір соң дейін қу алд шығ қанша жаман іш кірген ине сияқты түб тес шығ сол үшін біреу жаман жасамас алдын оның өз ти зардаб да ойла берік сұлтан,neutral

0,шынайы махаббат үнсіз келеді өйткені шын сезім алдымен сөзбен емес іспен білдіріледі,шынайы махаббат үн кел өйткені шын сезім алд сөз емес іс білдір,neutral

0,мыңдаған ер адамдарға көмектесті саған да көмектеседі простатиттан бар болғаны жеті күннің ішінде нәтижелі түрде арылуға болады сілтемеге кіріп пайдалы кеңесті оқыңыз https vk cc xnkk,мыңдаған ер адам көмекте саған да көмектес простатит бар бол жеті күн іш нәтижел түр арыл бол сілтеме кір пайдалы кеңес оқ https vk cc xnkk,neutral

0,егер сіздің өміріңізде жаңбыр жауса қиналмаңыз гүлдер сол жаңбырдың арқасында гүлдейді,егер сіздің өмір жаңбыр жау қиналма гүл сол жаңбыр арқа гүлде,neutral

0,егер сіз он жылға жас көрінгіңіз келсе адамдар сізге назар аударып комплимент айтқаның қаласаңыз осы мақаламды соңына дейін оқыңыз расында бұл сізге он жылға жас көрінуге көмектеседі егер сіз әбіржіп түріңіз шаршаған болып жүргенді қаласаңыз бұл мақаланы оқымайақ қойыңыз әйелдер қанша жасқа дейін өзін жаспын деп есептей алады жасқа дейін деп жүрдім кейін түсіндім жас көріну ол жасқа байланысты емес екенін сенің бетіңнің терісі фигураң көзіңнің жылтырауы өзіңе деген сенімділікке байланысты екен,егер сіз он жыл жас көрін кел адам сізге назар аудар комплимент айт қала осы мақала соң дейін оқ рас бұл сізге он жыл жас көрін көмектес егер сіз әбірж түр шарша бол жүр қала бұл мақала оқымайақ қой әйел қанша жас дейін өз жас де есепте ал жас дейін де жүр кейін түс жас көріну ол жас байланысты емес екен сенің бет тері фигура көз жылтыра өз де сенімділік байланысты екен,neutral

0,бұл өмір өтпес өтсек біз өтерміз,бұл өмір өтпес өт біз өт,neutral

0,қыс көктем жасыл жаз бен күз екенбіз,қыс көктем жасыл жаз бен күз екен,neutral

0,бір күні еріп кетер мұз екенбіз қалқаман сарин,бір күн ер кет мұз екен қалқаман сарин,neutral

0,таныстар арасында тіліңізге ие болыңыз,таныс ара тіл ие бол,neutral

0,сабыр ет тоқтау қыл шеше біл,сабыр ет тоқта қыл шеш біл,neutral

0,егер сізді қапа қылған адамдарға әліде сәлем бере алсаңыз бұл сіздің иманыңыздың садақасы,егер сіз қап қыл адам әлі сәлем бер ал бұл сіздің иман садақа,neutral

0,қайда жүрсең де кім болсаң да сені өсірген жандарды ұятқа қалдырма,қайда жүр де кім бол да сен өсір жан ұят қалдырма,neutral

0,өзіңе не тілесең өзгеге де соны тіле,өз не тіле өзге де соны тіле,neutral

0,сүйгеніңді құшағына алып отбасы бақытын сезіну қандай бақыт шіркін бірақ ол маскүнем болып кетті елде дағдарыс ал ол бар тапқанымызды ішімдікке жұмсайды,сүйген құшағ алып отбасы бақыт сезін қандай бақыт шіркін бірақ ол маскүне бол кет ел дағдарыс ал ол бар тап ішімдік жұмса,neutral

0,алкоголизмнен жүз пайыз кепілдікпен айығып кетудің ең қарапайым әдісі дәрігерлердің уколынсыз және дәрі дәрмегінсіз бұл тәсіл өте қарапайым және кез

келген адам оны қолдана алады өшіріп тастамай тұрғанда осы жерден оқыңыздар https vk cc yzik,алкоголиз жүз пайыз кепілдік айығ кету ең қарапайым әді дәрігер уколын жән дә дәрмегін бұл тәсіл өте қарапайым жән кез кел адам оны қолдан ал өшір тастама тұр осы же оқ https vk cc yzik,neutral

0,кеткен адамның соңына нүкте қойыңыз сонда келген адамның есімі үлкен әріппен жазылады,кет адам соң нүкте қой сонда кел адам есім үлкен әріп жаз,neutral

0,сала берме көзіңді әр жақсыға бәрі жақсы көрінер алғашқыда,сала берме көз әр жақсы бәрі жақсы көрін алғашқы,neutral

0,басқалар не ойлайды деген сұрақ ең соңғы орында тұруы тиіс негізінде басқаларға бәрібір өмір сенікі бернард,басқа не ойла де сұрақ ең соңғы орында тұр тиіс негіз басқа бәрібір өмір сенік бернард,neutral

0,сіздегі үш нәрсені байқай білетін адамды бағалаңыз күлкінің артына жасырынған мұңды ашудың артына жасырынған махаббатты үнсіз қалуыңыздың себебін,сіз үш нәрсе бай біл адам бағала күлкі арт жасырын мұң аш арт жасырын махаббат үн қалу себеб,neutral

0,ұрыс керіс кезінде біреудің көңіліне тиетіндей ауыр сөз айтпаңыз кейін татуласып кетерсіздер бірақ айтылған сөз адамның көкейінде ұзақ уақыт сақталады,ұрыс керіс кез біреу көңіл тиетінде ауыр сөз айтпа кейін татулас кет бірақ айтыл сөз адам көкей ұзақ уақыт сақтал,neutral

0,тұрақты адамның махаббаты да тұрақты ілияс есенберлин,тұрақты адам махаббат да тұрақты ілияс есенберлин,neutral

0,кешіре алар бір жандар бар болғасын фариза оңғарсынова,кешір ал бір жан бар болға фариз оңғарсынов,neutral

0,біреуге ой керек біреуге той керек,біреу ой керек біреу той керек,neutral

0,біреуге адам қымбат біреуге алтын қымбат,біреу адам қымбат біреу алтын қымбат,neutral

0,біреу уақыт өткізе алмай әуре біреу уақыт жеткізе алмай әуре,біреу уақыт өткіз алма әуре біреу уақыт жеткіз алма әуре,neutral

0,біреу тапқырлық жасайды біреу сатқындық жасайды,біреу тапқырлық жаса біреу сатқ жаса,neutral

0,бәлкім өмір жалған деген осы шығар,бәлкім өмір жалған де осы шығар,neutral

0,сүйегі жоқ балық болмайтыны сияқты кемшілігі жоқ адам да болмайды лютер,сүйег жоқ балық болма сияқты кемшіліг жоқ адам да болма лю,neutral

0,жасы отыздан асқандарға әжімнен құтылу жолдары,жас отыз ас әж құтыл жол,neutral

0,жеті күннен кейін мінсіз тері қымбат крем мен маскілер туралы ұмытыңыз олар бәрібір көмектеспейді https vk cc mhvfi,жеті күн кейін мін тері қымбат крем мен маскі туралы ұмыт олар бәрібі көмектеспе https vk cc mhvfi,neutral

0,жұмсақ бол бірақ бағынышты болма қатты бол бірақ қатыгез болма брюс ли,жұм бол бірақ бағыныш болма қатты бол бірақ қатыгез болма брюс ли,neutral

0,адамның санасы қаншалықты төмен болса ол мұрнын соншалықты аспанға шүйіреді,адам сана қаншалықты төмен бол ол мұрн соншалықты аспан шүйір,neutral

0,адам бәрін керсінше істейді үлкен болғысы келеді сосын балалық шағы туралы күрсінеді ақша үшін денсаулығын құртып денсаулығын дұрыстау үшін ақшасын құртады болашағы туралы асыға ойлап қазіргі өмірін керек қылмайды сөйтіп не қазіргі не болашақ өмірі жоқ ешқашан өлмейтіндей өмір сүріп ешқашан өмір сүрмегендей өледі пауло коэльо,адам бәрін керсінш істейді үлкен бол келеді сосын балалық шағ туралы күрсінеді ақша үшін денсаулығ құрт денсаулығ дұрыста үшін ақша құрт болашағ туралы асыға ойла қазіргі өмірін керек қылма сөйті не қазіргі не болашақ өмірі жоқ ешқашан өлмейтінде өмір сүрі ешқашан өмір сүрмегенде өледі пауло коэльо,neutral

0,адам қаласа уақыт табады қаламаса сылтау табады м достоевский,адам қала уақыт таб қалама сылтау таб м достоевски,neutral

0,не десең де адамдар өздері сенгісі келген шындыққа ғана сенеді харуки мураками,не де де адам өз сен кел шындық ғана сен харуки мураками,neutral

0,қандай айырмасы бар кім күштірек кім ақылдырақ кім әдемірек кім бай өйткені ақыр соңындағы маңызды зат бақытты адамсың ба жоқ па ошо,қандай айыр бар кім күштірек кім ақылдырақ кім әдемірек кім бай өйткені ақы соңындағы маңызды зат бақытты адам ба жоқ па ошо,neutral

0,электронды темекілерді де таблеткаларды да гипнозды да түрлі кітаптарды да пайдаланып көрдім оның бәрі өтірік екен көмектеспейді мүлдем артынша темекі шегіп отырасың көңілім түсіп отырғанда бір амалын таптым мен сондай қуанышты болдым мен мына сайтқа кіріп https vk cc yzb темекіні қойдыратын ең соңғы заманауи құралды таптым,электронды темекі де таблетка да гипноз да түрлі кітап да пайдалан көр оның бәрі өтірік екен көмектеспе мүлдем артынш темекі шег отыр көңіл түс отырғанда бір амал тап мен сондай қуанышты бол мен мына сайт кір https vk cc yzb темекі қойдыр ең соңғы заманауи құра тап,neutral

0,бұл сіздерге аллен кардың психологиялық бағытбағдарына ұқсас нәрсе емес ағзаға шынымен оң әсер беретін құрал,бұл сіздерге аллен ка психологиялық бағытбағ ұқсас нәр емес ағза шын оң әсер бер құрал,neutral

0,бірден айтайын бұл әдіспен ешбір клиникада емдемейді себебі клиенттерін жоғалтып алады мен бұл құралды үш күн пайдаландым сенесіз бе менде темекіге деген жиіркеніш пайда болды иә иә дәл солай өзім де таң қалдым,бірден айт бұл әдіс

ешбір клиника емдеме себебі клиент жоғалт ал мен бұл құра үш күн пайдала сен бе менде темекі де жиркеніш пайда бол иә иә дәл солай өзім де таң қал,neutral

0,сөзбен айтып жеткізе алмаймын темекіні мүлдем іздемеймін тіпті ойламаймын да,сөз айт жеткіз алма темекі мүлдем іздеме тіпті ойлама да,neutral

0,екі ай өтті бірде бір рет темекі шеккен жоқпын тіпті тартпайды,екі ай өт бірде бір рет темекі шек жоқ тіпті тартпа,neutral

0,мен бұл құралды тапқаныма ризамын оны қолданып үйімнен шығатын және ауызымнан шығатын жағымсыз иістерден құтылғаныма бақыттымын саусақтарымның да тістерімнің де сарғайғаны кетті,мен бұл құра тап риза оны қолдан үй шығ жән ауыз шығ жағымсыз иіс құтыл бақытты саусақ да тіс де сарғай кет,neutral

0,қажет болса сілтемесін тағы бір рет жазып өтейін ол құралға осы жерден тапсырыс бердім,қажет бол сілтеме тағы бір рет жаз өт ол құрал осы же тапсырыс бер,neutral

0,бағасы тіпті арзан сіз бір аптада шегіп тауысатын пачка темекінің бағасы енді салыстырыңыз бір апта темекі шегіп жүргеніңіз жақсы ма әлде қалған өміріңізді сасық темекісіз өткізген маңызды ма,баға тіпті арзан сіз бір апта шег тауыс пачк темекі баға енді салыстыр бір апта темекі шег жүр жақсы ма әл қалған өмір сасық темекі өткіз маңызды ма,neutral

0,ең үлкен қателік рухтың сынуы,ең үлкен қателік рух сын,neutral

0,ең мықты күш сенім,ең мықты күш сенім,neutral

0,өткен іске өкінбеу керек өмірден алған соққылардың бәрі маңызды емес маңыздысы біздің еш нәрсеге мойымай алға қарай ұмтылып өмір сүріп келе жатқандығымыз стивен кинг,өткен іс өкінбе керек өмір ал соққы бәрі маңызды емес маңызды біздің еш нәрсе мойыма алға қарай ұмтыл өмір сүр келе жатқандығ стивен кинг,neutral

0,кейде бір кішкене ғана жылы сөз адамға қанша қуаныш сыйлайды,кейде бір кішкен ғана жыл сөз адам қанша қуаныш сыйла,neutral

0,өмірдің азаптарынан шығар жалғыз жол сабыр,өмір азап шығар жалғыз жол сабыр,neutral

0,кез келген жағдайдан шығар жол сабыр,кез кел жағдай шығар жол сабыр,neutral

0,адам ақылды болған сайын қарапайым келеді цицерон,адам ақылды бол сайын қарапайым кел цицерон,neutral

0,сізді бағаламаған адамға ешқашан да оралмаңыз,сіз бағалама адам ешқашан да оралма,neutral

0,өлгеннен соң бәрі де кеш болады,өл соң бәрі де кеш бол,neutral

0,достар сіздермен өте керемет жасартатын құрал жайында бөліскім келіп отыр шын айтсам бұрын мұндай нәтижені еш жерден көрмеппін крем майлар өте көп бірақ нәтижесіп айлап күтесің мен мұндай нәтижені көрмегем дымқыл теріге бір тамшысын жағып едім жиырма секундтан кейін жаққан жерімдегі әжімдер жоқ болып кетті мен таң қалдым көзінің асты ісіп тұратындар ғшін бұл таптырмас құрал,дос сіздермен өте керемет жасарт құрал жай бөліс кел отыр шын айт бұрын мұндай нәтиже еш же көрме крем май өте көп бірақ нәтижес айлап күт мен мұндай нәтиже көрмеге дымқыл тері бір тамшы жағ ед жиырма секунд кейін жақ жерім әжім жоқ бол кет мен таң қал көз асты іс тұр ғш бұл таптырмас құрал,neutral

0,сегіз сағаттан бастап әсер етеді ркімдікі әр түрлі сайтта қазір акция жүріп жатыр арзымайтын тиынға сатып алуға болады бір сәтте жасарып шығу әр адамның арманы барлығыңыз әдемі болыңыздар тапсырып беріп толығырақ білгіңіз келсе https vk cc,сегіз сағат баста әсер ет ркімдік әр түрлі сайт қазір акция жүр жатыр арзыма тиын сат ал бол бір сәт жасар шығу әр адам арман барлығ әдем бол тапсыр бер толығырақ біл кел https vk cc,neutral

0,мені ит қапса одан өш алу үшін мен де қапсам аузымда не қасиет қалады мұхтар әуезов,мен ит қап одан өш алу үшін мен де қап ауз не қасиет қал мұхтар әуезов,neutral

0,бірде толстойдан адам қай кезде бақытты болады деп сұрапты толстой қолда барына қуанып қолда жоқты ойламаған кезде депті,бірде толстой адам қай кезде бақытты бол де сұра толстой қолда бар қуан қолда жоқ ойлама кезде де,neutral

0,жауапкершілік пен шынайылық ең қымбат қасиет оны арзан адамдардан күтудің қажеті жоқ,жауапкершілік пен шынайылық ең қымбат қасиет оны арзан адам күту қажет жоқ,neutral

0,ақылмен арыстан да ұстауға болады ал күшпен тышқан да ұстай алмайсың,ақыл арыстан да ұста бол ал күш тышқан да ұста алма,neutral

0,бола біл қатты да жұмсақ та,бола біл қатты да жұм та,neutral

0,түкке тұрғысыз нәрселерді жүрекке қабылдай беретін адам махаббатқа адал келеді лев толстой,түк тұрғы нәрсе жүрек қабылда бер адам махаббат адал кел лев толстой,neutral

0,бақытты адам болғың келсе өткенді қопара берме,бақытты адам бол кел өт қопар берме,neutral

0,өмір деген осы бүгін бар ертең жоқсың сен кетті екен деп өмірөзен ағысын өзгертпейді,өмір де осы бүгін бар ертең жоқ сен кет екен де өмірөзен ағыс өзгертпе,neutral

0,сіз қанша жақсы көрсеңізде көзден кеткен жүректен де кетеді орхан памук,сіз қанша жақсы көрсе көз кет жүрек де кет орхан памук,neutral

0,егер арманыңдағы жолда әр үрген итке тас ата беретін болсаң мақсатыңа жетпейсің уинстон черчилль,егер арманың жолда әр үр ит тас ата бер бол мақсат жетпе уинстон черчилль,neutral

0,менің жүрегімді шындықпен жарала бірақ ешқашан мені өтірікпен жұбатпа,менің жүрег шындық жарала бірақ ешқашан мен өтірік жұбатп,neutral

0,жүрек гүл секілді оны күштеп аша алмайсың ол өз қалауымен ашылуы керек,жүрек гүл секілді оны күште аш алма ол өз қалау ашыл керек,neutral

0,ең қорқыныштысы сізге бір топ адам тас лақтыруы емес сол топ адам ішінде ескі достарыңыз да болуы,ең қорқынышты сізге бір топ адам тас лақтыр емес сол топ адам іш ескі дос да бол,neutral

0,кешірімді көбіне кінәлілер емес арадағы байланысты бағалай білетіндер сұрайды рональд рейган,кешір көбіне кінәлі емес ара байланысты бағала біл сұра рональд рейган,neutral

0,достар сіздермен өте керемет жасартатын құрал жайында бөліскім келіп отыр шын айтсам бұрын мұндай нәтижені еш жерден көрмеппін крем майлар өте көп бірақ нәтижесіп айлап күтесің,дос сіздермен өте керемет жасарт құрал жай бөліс кел отыр шын айт бұрын мұндай нәтиже еш же көрме крем май өте көп бірақ нәтижес айлап күт,neutral

0,мен мұндай нәтижені көрмегем дымқыл теріге бір тамшысын жағып едім жиырма секундтан кейін жаққан жерімдегі әжімдер жоқ болып кетті мен таң қалдым көзінің асты ісіп тұратындар ғшін бұл таптырмас құрал,мен мұндай нәтиже көрмеге дымқыл тері бір тамшы жағ ед жиырма секунд кейін жақ жерім әжім жоқ бол кет мен таң қал көз асты іс тұр ғш бұл таптырмас құрал,neutral

0,сегіз сағаттан бастап әсер етеді ркімдікі әр түрлі сайтта қазір акция жүріп жатыр арзымайтын тиынға сатып алуға болады бір сәтте жасарып шығу әр адамның арманы барлығыңыз әдемі болыңыздар тапсырып беріп толығырақ білгіңіз келсе https vk cc mhvfi,сегіз сағат баста әсер ет ркімдік әр түрлі сайт қазір акция жүр жатыр арзыма тиын сат ал бол бір сәт жасар шығу әр адам арман барлығ әдем бол тапсыр бер толығырақ біл кел https vk cc mhvfi,neutral

0,сізге бүкіл әлем қарсы болғандай болсада есіңізде болсын ұшақ та желге қарсы ұшады,сізге бүкіл әлем қарсы болғанда болса ес бол ұшақ та жел қарсы ұш,neutral

0,сенбе жұртқа тұрса да қанша мақтап,сенбе жұрт тұр да қанша мақта,neutral

0,сабырлы бол бір данышпанның айтқанындай отпен отты өшіре алмайсың,сабырл бол бір данышпан айтқанында от от өшір алма,neutral

0,егерде адам құлап қайта тұрса бұл күш емес бұл намыс майк тайсон,ег адам құла қайта тұр бұл күш емес бұл намыс майк тайсон,neutral

0,күнделікті өмірден бақыт таба алған адам нағыз бақытты адам эдуард асадов,күнделікті өмір бақыт таб ал адам нағыз бақытты адам эдуард асадов,neutral

0,жақсы жұп ешқашан ұрсыспайтын емес ең үлкен ұрыста да айырылыспайтын жұп,жақсы жұп ешқашан ұрсыспа емес ең үлкен ұрыс да айырылыспа жұп,neutral

0,жасы отыздан асқандарға әжімнен құтылу жолдары жеті күннен кейін мінсіз тері қымбат крем мен маскілер туралы ұмытыңыз олар бәрібір көмектеспейді https vk cc mhvfi,жас отыз ас әж құтыл жол жеті күн кейін мін тері қымбат крем мен маскі туралы ұмыт олар бәрібі көмектеспе https vk cc mhvfi,neutral

0,өлмей тұрып тіршіліктің қадірін біл басыңа іс түспей тұрып бостандықтың қадірін біл,өлме тұр тіршілік қадір біл бас іс түспе тұр бостандық қадір біл,neutral

0,ауырмай тұрып денсаулықтың қадірін біл,ауырма тұр денсаулық қадір біл,neutral

0,кедейлік келмей тұрып барлықтын қадірін біл,кедейлік келме тұр барлықт қадір біл,neutral

0,кәрілік келмей тұрып жастықтың қадірін біл,кәрілік келме тұр жастық қадір біл,neutral

0,ешқашан ешкімді өсек қылмаңыз кім біледі мүмкін ол адамның алланың алдында жағдайы жақсы шығарсізден қарағанда,ешқашан ешкі өсек қылма кім біл мүмкін ол адам алла алд жағдай жақсы шығарсіз қара,neutral

0,адамды білгің келсе оны ашуландыр адам ыдыс секілді іші неге толы болса соны төгеді,адам біл кел оны ашуланды адам ыдыс секілді іш неге толы бол соны төг,neutral

0,сізді де варикоз мазалайды ма аяғыңызда көк тамырлар пайда болғанда не істеріңізді білмейсіз бе сізге инновациялық бірегей наноформулалы здоров кремі көмектеседі https vk cc enuvw,сіз де варикоз мазала ма аяғ көк тамыр пайда болғанда не іс білме бе сізге инновациялық бірегей наноформулал здоров крем көмектес https vk cc enuvw,neutral

0,жасушалардың қайта қалпына келуін жылдамдатады,жасуша қайта қалп кел жылдамдат,neutral

0,варикоздық тамырларды тазалап қалпына келтіреді,варикоз тамыр тазала қалп келтір,neutral

0,крем құрамындағы компоненттер жасушаларды нығайтып қанның микроциркуляциясын күшейтеді қалпына келуін жеделдетеді бұл дегеніңіз варикозды тек қана жеңу емес қайталануын болдырмау крем аяғыңызға жеңілдік сыйлап шаршауын басады,крем құрам компонент жасуша нығайт қан микроциркуляция күшейт қалп кел жеделдет бұл де варикоз тек қана жеңу емес қайталан болдырма крем аяғ жеңілдік сыйла шарша бас,neutral

0,варикозға қарсы креміне жеңілдікпен тапсырыс беру https vk cc enuvw,варикоз қарсы крем жеңілдік тапсырыс беру https vk cc enuvw,neutral

0,зұлым адам көмір секілді ол сені күйдірмесе міндетті түрде былғайды анахарсис,зұлым адам көмір секілді ол сен күйдірме міндетті түр былға анахарсис,neutral

0,өмірдің сынақтарынан қанша қиналсамда ертеңгі күніме үмітпен қараймын,өмір сынақ қанша қиналса ертеңгі күн үміт қара,neutral

0,жаңа гүлді көргенде ескі гүлді тастама бір сұлуды көргенде сүйгеніңді тастама,жаңа гүл көргенде ескі гүл тастама бір сұлу көргенде сүй тастама,neutral

0,бұл ұйыған қанды молекулярлық деңгейде ыдыратады,бұл ұйы қан молекулярлық деңгей ыдырат,neutral

0,қыздар маған жазған жылы сөздерің үшін сендерге алғысым шексіз бүгін мен сендермен тағы бір құпияммен бөлісемін,қыз маған жаз жыл сөз үшін сен алғыс шексіз бүгін мен сен тағы бір құпия бөліс,neutral

0,естеріңде ме мен бұдан алдын аяқтарымның ауыратынын жиі ісіп кететіні туралы жазған едім бұл варикоз болатын,ес ме мен бұдан алдын аяқ ауыр жиі іс кет туралы жаз ед бұл варикоз бол,neutral

0,менің көзім жеткені варикозды операциясыз ақ күннің ішінде емдеп жазуға болады екен,менің көз жет варикоз операция ақ күн іш емде жаз бол екен,neutral

0,ол көз алдымда өз өзінен жоқ болып кетеді,ол көз алд өз өз жоқ бол кет,neutral

0,қалай екенін білгілерің келе ме менің топтамамда оқып көріңдер,қалай екен біл келе ме менің топтама оқ көр,neutral

0,сөз кілт сияқты дұрыс таңдасаңыз кез келген жүректі ашуға кез келген ауызды жабуға болады,сөз кілт сияқты дұрыс таңда кез кел жүрек аш кез кел ауыз жаб бол,neutral

0,мен ілімімді аштыққа жасырдымадамдар қарын тоқтықта іздейдіоны қайдан тапсын,мен ілімімді аштық жасырдымадам қар тоқтық іздейдіо қайдан тап,neutral

0,абыройды бағыну мен тақуалыққа жасырдымадамдар оны басшылықтың табалдырығынаң іздейдіоны қайдан тапсын,абырой бағыну мен тақуалық жасырдымадам оны басшылық табалдырығына іздейдіо қайдан тап,neutral

0,байлықты қанағатқа жасырдымоны малдын көптігінен іздейдіоны қайдан тапсын,байлық қанағат жасырдымо малд көптігі іздейдіо қайдан тап,neutral

0,рахатты жұмаққа жасырдымадамдар оны дүниеден іздейдіоны қайдан тапсын,раха жұмақ жасырдымадам оны дүние іздейдіо қайдан тап,neutral

0,көбіне еркектер әйелдерінің көзіне шөп салып басқа жас қыздармен болып жүреді оған не себеп екенін білесіз бе дәл осы мәселеге тап болып жыныс мүшесін үй жағдайында тарылтып күйеуін сақтап қалған әйелдің оқиғасын оқыңыз,көбіне еркек әйел көз шөп сал басқа жас қыз бол жүр оған не себеп екен біл бе дәл осы мәселе тап бол жыныс мүше үй жағдай тарылт күйе сақта қалған әйел оқиға оқ,neutral

0,ешкім білмес өз жайымды менен артық,ешкім білмес өз жай менен артық,neutral

0,назар аударыңыз бас саусағының жанында сүйекше шығып тұратындар оқысын,назар ау бас саусағ жан сүйекш шығ тұр оқы,neutral

0,вальгус шинасы сізге көмектеседі,вальгус шина сізге көмектес,neutral

0,бас саусақты және буындарды түзетуге,бас саусақ жән буын түзет,neutral

0,табаныңызды еш қиындықсыз басып қимылдай аласыз,табан еш қиындық бас қимылда ал,neutral

0,ақшасын тек қана тауарды алған соң төлейсіз,ақша тек қана тау ал соң төле,neutral

0,жақсы адамның жақсылығын айтқын мейірленсін бірақ жағымпазданып өтірік мақтама нашар адамның жамандығын бетіне бассаң да жала жаппа,жақсы адам жақсылығ айтқ мейірлен бірақ жағымпаздан өтірік мақта нашар адам жамандығ бет бас да жал жапп,neutral

0,балаң жаман болса өмірің сүрген бекергі,бала жаман бол өмір сүр бекерг,neutral

0,сырт келбетіңмен мақтанба оны сен жаратқан жоқсың,сырт келбет мақтанб оны сен жаратқан жоқ,neutral

0,бай отбасының перзенті екендігіңмен мақтанба сен отбасыңды өзің таңдап туылмадың,бай отбас перзен екендіг мақтанб сен отбас өзің таңда туылма,neutral

0,жақсы амалдарыңмен жақсы мінезіңмен мақтан шын мәнінде оны өзің қалыптастырасың,жақсы амал жақсы мінез мақтан шын мән оны өзің қалыптастыр,neutral

0,маған ақылын айтатын адамдардың сол кеңестерін өз өмірінде қолданбайтынына таңғаламын,маған ақыл айт адам сол кеңес өз өмір қолданба таңғал,neutral

0,ежелден келе жатқан ырымсенімдерге сенсек бұл бойтұмарды тағып жүрген адамды өмірде жетістік пен сәттіліктер күтеді,ежел келе жат ырымсенім сен бұл бойтұмар тағ жүр адам өмір жетістік пен сәттілік күт,neutral

0,толығырақ осы сайтта https vk cc tfte,толығырақ осы сайт https vk cc tfte,neutral

0,әр атқан таңда оянып өмір сүріп келемін,әр ат таңда оян өмір сүрі келемін,neutral

0,басымнан қандай жағдайлар өткенін ешкім білмейді,бас қандай жағдай өт ешкім білме,neutral

0,дәрігерлер дабыл қағуда әлемде йелдер дертінің саны жылдам белең алуда ағзаңызды қалай қорғауды біліңіз https vk cc misqr,дәрігер дабыл қағ әлем йел дерт сан жылдам белең ал ағза қалай қорғау біл https vk cc misqr,neutral

0,арманы мен мақсаты жоқ адам кедей,арма мен мақсаты жоқ адам кедей,neutral

0,байлық пен кедейліктің шекарасы ақша емес,байлық пен кедейлік шекара ақша емес,neutral

0,кім қанағат қылса ол өзін әрқашанда бай сезінеді,кім қанағат қыл ол өз әрқашан бай сезін,neutral

0,сізді де варикоз мазалайды ма аяғыңызда көк тамырлар пайда болғанда не істеріңізді білмейсіз бе,сіз де варикоз мазала ма аяғ көк тамыр пайда болғанда не іс білме бе,neutral

0,сізге инновациялық бірегей наноформулалы здоров кремі көмектеседі https vk cc enuvw,сізге инновациялық бірегей наноформулал здоров крем көмектес https vk cc enuvw,neutral

0,адам жақсыны арман етсе өмірде бәрі де жақсы болмақ,адам жақсы арман ет өмір бәрі де жақсы бол,neutral

0,ал егер жүрегі бұзылса періштелер жылайды,ал егер жүрег бұзыл періште жыла,neutral

0,ащы шындықты бетіңе айтқанның бәрі дұшпаның емес,ащ шындық бет айт бәрі дұшпан емес,neutral

0,көзіңше өтірік мақтағанның бәрі досың емес,көзіңш өтірік мақта бәрі дос емес,neutral

0,құдай әйелді қорғағысы келсе ұл сыйлайды,құдай әйел қорға кел ұл сыйла,neutral

0,ал мақтағысы келсе қыз сыйлайды,ал мақта кел қыз сыйла,neutral

0,қыздар жігіттер көздің зрениесін жақсартудың құпиясы ашылды,қыз жігіт көз зрение жақсарт құпия аш,neutral

0,менің қызымның көзі сотканы көп көп қарағаннан нашарлап кеткен болатын автобус күткенде автобустың санын көрмейтін магазинге барсақ ценаларын көре алмайтын еді,менің қыз көз сотка көп көп қара нашарла кет бол автобус күткенде автобус сан көрме магазин бар цена көр алма ед,neutral

0,сомен жұлдыздардың парақшаларын ақтарып жүргем бір тамызатынға көзім түсіп кетті сипатамасын оқып шықтым табиғи қоспалар зияны жоқ деп жазылған сомен зияны тимер деп заказ бердім күнде почтамен жетті қызыма қолдандым бир айда қызымның көзі жақсарып мененде жақсы көретін болып кетты еш зияны жоқ болып шықты қызым көңіл күйі қатты көтерілді ұзін сөздің қысқасы сіздергеде көмегі тиып қалар мына жерден https vk cc gp bw заказ бергем жүз пайыз табиғы қоспалар ден

саулыққа зиян жок бир айда көзіңіз жақсарады жақсармаса ақшанызды қайтарып береміз менім пікірім өте керемет зат жана жылға қарай керемет скидкамен сатылымда орын шектеулі скидкамен заказ беріп үлгеріп қалыныз дәл кәзір ссілтемеге өтіп https vk cc gp bw,со жұлдыз парақша ақтар жүрге бір тамыз көз түс кет сипатама оқ шық табиғи қоспа зиян жоқ де жазыл со зиян тим де заказ бер күн почта же қыз қолда би айда қыз көз жақсар менен жақсы көр бол кет еш зиян жоқ бол шық қыз көңіл күй қатты көтер ұз сөз қысқа сіздерге көмег ти қал мына же https vk cc gp bw заказ берге жүз пайыз табиғ қоспа ден саулық зиян жок би айда көз жақсар жақсарма ақшаныз қайтар бер мен пікір өте керемет зат жана жыл қарай керемет скидка сатыл орын шектеулі скидка заказ бер үлгер қалыныз дәл кәзі ссілтеме өт https vk cc gp bw,neutral

0,азға қанағат ет сонда ешқашан ештеңеге мұқтаж болмайсың,аз қанағат ет сонда ешқашан ештеңе мұқтаж болма,neutral

0,іштарлықтан арыл сонда көңілің тыныш болады,іштарлық арыл сонда көңіл тыныш бол,neutral

0,тыйым салынған нәрседен аулақ бол сонда иманың күшейе түседі,тыйым салын нәрсе аулақ бол сонда има күшей түс,neutral

0,сенен үш нәрсені көре білетін адамды бағала,сенен үш нәрсе көр біл адам бағала,neutral

0,үнсіздік астына жасырылған сөздеріңді,үнсіздік аст жасырыл сөз,neutral

0,сөз кілт секілді ретін тауып кез келген жүректің құлпын ашуға және кез келген ауызды жабуға болады,сөз кілт секілді рет тау кез кел жүрек құл аш және кез кел ауыз жаб бол,neutral

0,құдай сені бақытты еткісі келсе ең қиын жолмен алып жүреді себебі жеңіл жолда бақыт жоқ,құдай сен бақытты ет кел ең қиын жол алып жүр себебі жеңіл жолда бақыт жоқ,neutral

0,электронды темекілерді де таблеткаларды да гипнозды да түрлі кітаптарды да пайдаланып көрдім оның бәрі өтірік екен көмектеспейді мүлдем артынша темекі шегіп отырасың көңілім түсіп отырғанда бір амалын таптым мен сондай қуанышты болдым,электронды темекі де таблетка да гипноз да түрлі кітап да пайдалан көр оның бәрі өтірік екен көмектеспе мүлдем артынш темекі шег отыр көңіл түс отырғанда бір амал тап мен сондай қуанышты бол,neutral

0,мен мына сайтқа кіріп https vk cc yzb темекіні қойдыратын ең соңғы заманауи құралды таптым,мен мына сайт кір https vk cc yzb темекі қойдыр ең соңғы заманауи құра тап,neutral

0,бағасы тіпті арзан сіз бір аптада шегіп тауысатын пачка темекінің бағасы енді салыстырыңыз бір апта темекі шегіп жүргеніңіз жақсы ма әлде қалған өміріңізді сасық темекісіз өткізген маңызды ма рине екіншісін таңдайтыныңызға күмәнім

жоқ,баға тіпті арзан сіз бір апта шег тауыс пачк темекі баға енді салыстыр бір апта темекі шег жүр жақсы ма әл қалған өмір сасық темекі өткіз маңызды ма рин екінші таңда күмән жоқ,neutral

0,достық жиі махаббатқа ұласады ал махаббат тек сирек жағдайда достыққа ұласады,достық жиі махаббат ұлас ал махаббат тек сирек жағдай достық ұлас,neutral

0,бірінші болып кешірім сұрау батылдық,бірінші бол кешір сұрау бат,neutral

0,мұсылман басқа бір мұсылманға тілімен де қолымен де зиян келтірмейді мұхаммед а с,мұсылман басқа бір мұсылман тіл де қол де зиян келтірме мұхаммед а с,neutral

0,көз асты ұсқынсыз күлдіреуі мен беттегі әжімдер шаршатты ма әжімдер мен көз асты күлдіреуі көз асты қабы жоғалады бетіңіз алғашқы қалпына қайтып келеді тапсырыс беру сілтемесі https vk cc mhvfi,көз асты ұсқын күлдіре мен бет әжім шарша ма әжім мен көз асты күлдіре көз асты қаб жоғал бет алғашқы қалп қайт кел тапсырыс беру сілте https vk cc mhvfi,neutral

0,екі ережені мықтап ұстан кім көрінгенмен ас ішкенше аш қал кім көрінген бірге болғанша жалғыз бол омар хайям,екі ереже мықта ұстан кім көрін ас ішкенш аш қал кім көрін бірге болғанш жалғыз бол омар хайя,neutral

0,есік қақпасаң ешкім ашпайды тәуекелге бел бумасаң қолыңнан түк келмейтін адам болып қала бересің ошо,есік қақпа ешкім ашпа тәуекел бел бума қол түк келме адам бол қала бер ошо,neutral

0,тұздың дәмің татқан балдың қадірің біледі,тұз дәм тат бал қадір біл,neutral

0,қайғыдан көп жылаған әдемі күле біледі,қайғы көп жыла әдемі күл біл,neutral

0,менің қызымның көзі сотканы көп көп қарағаннан нашарлап кеткен болатын автобус күткенде автобустың санын көрмейтін магазинге барсақ ценаларын көре алмайтын еді сомен жұлдыздардың парақшаларын ақтарып жүргем бір тамызатынға көзім түсіп кетті сипатамасын оқып шықтым табиғи қоспалар зияны жоқ деп жазылған сомен зияны тимер деп заказ бердім күнде почтамен жетті қызыма қолдандым бир айда қызымның көзі жақсарып мененде жақсы көретін болып кетты еш зияны жоқ болып шықты қызым көңіл күйі қатты көтерілді ұзін сөздің қысқасы сіздергеде көмегі тиып қалар мына жерден https vk cc gp bw,менің қыз көз сотка көп көп қара нашарла кет бол автобус күткенде автобус сан көрме магазин бар цена көр алма ед со жұлдыз парақша ақтар жүрге бір тамыз көз түс кет сипатама оқ шық табиғи қоспа зиян жоқ де жазыл со зиян тим де заказ бер күн почта же қыз қолда би айда қыз көз жақсар менен жақсы көр бол кет еш зиян жоқ бол шық қыз көңіл күй қатты көтер ұз сөз қысқа сіздерге көмег ти қал мына же https vk cc gp bw,neutral

0,заказ бергем жүз пайыз табиғы қоспалар,заказ берге жүз пайыз табиғ қоспа,neutral

0,бир айда көзіңіз жақсарады жақсармаса ақшанызды қайтарып береміз,би айда көз жақсар жақсарма ақшаныз қайтар бер,neutral

0,менім пікірім өте керемет зат жана жылға қарай керемет скидкамен сатылымда орын шектеулі скидкамен заказ беріп үлгеріп қалыныз дәл кәзір ссілтемеге өтіп https vk cc gp bw,мен пікір өте керемет зат жана жыл қарай керемет скидка сатыл орын шектеулі скидка заказ бер үлгер қалыныз дәл кәзі ссілтеме өт https vk cc gp bw,neutral

0,ей көңілім сыйлық күтпе бұл заманнан қараңғы,ей көңіл сыйлық күтпе бұл заман қараңғы,neutral

0,уақыт озар барлығынан шаң қаптырып адамды,уақыт оз барлығ ша қаптыр адам,neutral

0,дертің әр сәт күшейеді ауырады тынымсыз,дер әр сәт күшей ауыр тын,neutral

0,күткенменен мына уақыт емдемейді жараңды,күткенме мына уақыт емдеме жара,neutral

0,гибридты лимонды үйге алғанымызға көп болған жоқ экодараның шағын лимонағашы сөз жоқ бұл әдемі жеудің өзі бір рахат лимондар магазиндегідей қышқыл емес олар өте хош иісті және дәмі нәзік,гибрид лимон үй ал көп бол жоқ экодара шағын лимонағаш сөз жоқ бұл әдем жеу өзі бір рахат лимон магазиндегіде қышқыл емес олар өте хош иіс жән дәм нәзік,neutral

0,гүлдер менің үйімде жақсы өсетін еді ал жеміс ағаштарының жерсініп кетуі проблема еді шын айтсам бұл ағаш өте оңай сіңісіп өсіп кетті суреттен көрсеңіздер болады қандай әдемі болып өскенін,гүл менің үй жақсы өс ед ал жеміс ағаш жерсін кет проблема ед шын айт бұл ағаш өте оңай сіңіс өс кет сурет көр бол қандай әдем бол өс,neutral

0,қазір дәл осы дүкенде https vk cc yocrl апельсин шие және киви алдым қашан келетінін күтіп жүрмін оңтүстіктік жемістерінің өз үйімде өскенін қалаймын қазір жеңілдіктер жүріп жатыр дүкен ресми тіркелген қорқатын ештеңесі жоқ барлығыңызға мол жеміс тілеймін,қазір дәл осы дүкен https vk cc yocrl апельсин ши жән киви ал қашан кел күт жүр оңтүстік жеміс өз үй өс қала қазір жеңілдік жүр жатыр дүкен ресми тіркел қорқ ештеңе жоқ барлығ мол жеміс тіле,neutral

0,әрқашан адамның көзіңе қараңыз тіл өтірік айтуға қабілетті ал көз ешқашан михаил булгаков,әрқашан адам көз қара тіл өтірік айт қабілетті ал көз ешқашан михаил булгаков,neutral

0,ауырып жатқан адамдарға денсаулық берсін,ауыр жат адам денсаулық бер,neutral

0,қайғысы бар адамдарға сабыр берсін,қай бар адам сабыр бер,neutral

0,жаратқан біздің кіммен бақытты болатынымызды біледісондықтан керек емес адамдардан айырыпбасқа жандарға қосады,жаратқан біздің кім бақытты бол біледісондық керек емес адам айырыпбас жан қос,neutral

0,білу ғана аз сол білгеніңді істе қолдана білу керек йоганн вольфганг фон те,білу ғана аз сол біл істе қолдан білу керек йоганн вольфганг фон те,neutral

0,шындықты жеткізудің ең жақсы жолы қалжыңмен жеткізу,шындық жеткізу ең жақсы жол қалжың жеткізу,neutral

0,ешқашан биік шыңға шықтым деп асып тасудың керегі жоқ аяғың сәл тайып кетсе жерден табыласың,ешқашан биік шың шық де ас тас керег жоқ ая сәл тай кет же табыл,neutral

0,егер сен бір нәрсені қаласың бүкіл әлем оның орындалуына себепші болады пауло коэльо,егер сен бір нәрсе қал бүкіл әлем оның орындал себепш бол пауло коэльо,neutral

0,арманыңның бәрі орындалады сәл күте тұр есіңде сақта қанттың тәттісі түбінде,арман бәрі орындал сәл күт тұр ес сақта қан тәтті түб,neutral

0,адамның білімі аз болса да оны мына төрт нәрсе жоғары көтереді ұстамдылық қарапайымдылық жомарттық және көркем мінез,адам білім аз бол да оны мына төрт нәр жоғары көтер ұстамдылық қарапайымдылық жомарттық және көркем мінез,neutral

0,өз өміріңізді басқа адамның өмірімен салыстырмаңыз себебі сіз ол адамның басынан қандай жайттар өткергенін білмейсіз,өз өмір басқа адам өмір салыстырма себебі сіз ол адам бас қандай жайт өткер білме,neutral

0,басқалар не ойлайды деген сұрақ ең соңғы орында тұруы тиіс негізінде басқаларға бәрібір өмір сенікі,басқа не ойла де сұрақ ең соңғы орында тұр тиіс негіз басқа бәрібір өмір сенік,neutral

0,қанағаты бар адамға бақыт бұйырған аристотель,қанағат бар адам бақыт бұйыр аристотель,neutral

0,әйел затының әдемілігі оның киген киімінде немесе оның сымбатында шаш үлгісінде де емес әйел әдемілігі оның көздерінде өйткені көз оның махаббатқа толы жүрегіне апарар бірден бір жол,әйел зат әдемілiг оның ки киім немесе оның сымбат шаш үлгі де емес әйел әдемілiг оның көз өйткені көз оның махаббат толы жүрег апар бірден бір жол,neutral

0,сізді сатып кеткен адамға оралмаңыз ол өзгермейді,сіз сат кет адам оралма ол өзгерме,neutral

0,егер кей адамдардың сізге көңілі бұрмаса оларды жайына қалдырыңыз антон чехов,егер кей адам сізге көңіл бұрма ол жай қалдыр антон чехов,neutral

0,барлық адам қателеседі бірақ қателесушілердің арасындағы ең жақсысы өз қателігін мойындап тәубеге келгені мұхаммед а с,барлық адам қателес бірақ қателесуші ара ең жақсы өз қателіг мойында тәубе кел мұхаммед а с,neutral

0,ауыздағы жағымсыз иіс тістердің нашарлығынан емес сағыз шайнамаңыздар одан сайын нашарлайды қазақстанның бас инфекционисі сізді және жақындарыңызды қалай құтқару керектігімен бөліседі https vk cc by s,ауыз жағымсыз иіс тіс нашарлығ

емес са шайнама одан сайын нашарла қазақстан бас инфекциони сіз және жақын қалай құтқар керектіг бөліс https vk cc by s,neutral

0,өмір әр адам үшін жұмақ,өмір әр адам үшін жұмақ,neutral

0,жақсы адам барлығын өзінен іздейді ақымақ адам оларды басқадан іздейді,жақсы адам барлығ өз ізде ақы адам ол басқа ізде,neutral

0,ашудың әрбір минутында сіз секунд бақытыңызды жоғалтасыз ральф уолдо эмерсон,аш әрбір минут сіз секунд бақыт жоғал ральф уолдо эмерсон,neutral

0,кешіре алар бір жандар бар болғасын,кешір ал бір жан бар болға,neutral

0,ешқандай тосын жағдай сенің тыныштығыңды бұза алмайтындай салмақты бол,ешқандай тос жағдай сенің тыныштығ бұз алмайтында салмақ бол,neutral

0,көз асты ұсқынсыз күлдіреуі мен беттегі әжімдер шаршатты ма,көз асты ұсқын күлдіре мен бет әжім шарша ма,neutral

0,әжімдер мен көз асты күлдіреуі көз асты қабы жоғалады бетіңіз алғашқы қалпына қайтып келеді,әжім мен көз асты күлдіре көз асты қаб жоғал бет алғашқы қалп қайт кел,neutral

0,сенімен болып жатқан оқиғаның жалғыз авторы өзіңсің,сенімен бол жат оқи жалғыз авто өзің,neutral

0,адамның көңіліне қарап сөйлеу жүректегі иманның белгісі,адам көңіл қара сөйлеу жүрек иман бел,neutral

0,ең дұрыс жол кешіре білу сергей бодров,ең дұрыс жол кешір білу сер бодров,neutral

0,тағдыр қатал көздерімде тұр қайғы достар қатал сөздерінде тұрмайды сағат қатал бір уакытта тұрмайды уакыт қатал жастығымды ұрлайды сезім қатал махаббат шын болмайды жүрек қатал өзге жанға бұрмайды өмір қатал сен сынбасаң сынбайды өлім қатал алып кетпей тынбайды,тағдыр қатал көздері тұр қайғы дос қатал сөздерін тұрма сағат қатал бір уакыт тұрма уакыт қатал жастығ ұрла сезім қатал махаббат шын болма жүрек қатал өзге жан бұрма өмір қатал сен сынба сынба өлі қатал алып кетпе тынба,neutral

0,әркімге бақыт келеді оның сәрсенбінің түнінде бейсенбінің күнінде келуі міндетті емес ақпанда немесе тамызда келуі де міндетті емес ол күтпеген уақытта күтпеген адаммен келеді,әркім бақыт кел оның сәрсенбі түн бейсенбі күн кел міндетті емес ақпан немесе тамыз кел де міндетті емес ол күтпе уақыт күтпе адам кел,neutral

0,өмір ешқашан шешуі табылмас жұмбақ ешкім шығара алмас қиын есеп,өмір ешқашан шеш табылмас жұм ешкім шығар алмас қиын есеп,neutral

0,өткен іске өкінбеу керек өмірден алған соққылардың бәрі маңызды емес маңыздысы біздің еш нәрсеге мойымай алға қарай ұмтылып өмір сүріп келе

жатқандығымыз,өткен іс өкінбе керек өмір ал соққы бәрі маңызды емес маңызды біздің еш нәрсе мойыма алға қарай ұмтыл өмір сүр келе жатқандығ,neutral

0,тапсырыс беру осы сілтеме арқылы https vk cc by s,тапсырыс беру осы сілтеме арқылы https vk cc by s,neutral

0,өзіңіз сүйетін әйелді аялай біліңіз ол ұрысса алаңдаса немесе ашу шақырса бұл оның сізді сүйетінін білдіреді ал жай ғана жымиып сізге немқұрайлы қарай бастаса демек сіз оны жоғалттыңыз фаина раневская,өз сүй әйел аяла біл ол ұрыс алаңда немесе ашу шақыр бұл оның сіз сүй білдір ал жай ғана жыми сізге немқұрайл қарай баста демек сіз оны жоғал фаин раневская,neutral

0,дәріхана жұмысшылары таң қалуда дәл бұндай әсерді күтпеген еді олар үшін бұл нағыз апат,дәріхан жұмысшы таң қал дәл бұндай әсер күтпе ед олар үшін бұл нағыз апат,neutral

0,өшірмей тұрғанда оқыңыздар https vk cc xho,өшірме тұр оқ https vk cc xho,neutral

0,сен өз жағыңнан қолынан келгеннің бәрін жаса келмегеніне құдай көмектеседі,сен өз жағ қол кел бәрін жаса келме құдай көмектес,neutral

0,мен туралы кім не десе де құлақ аспаймын олардың не ойлайтыны маған маңызды емес ең бастысы өз өмірім өз жолым өз мақсатым бар өзгенің пікірімен өмір сүру мүлдем өмір сүрмегенмен бірдей,мен туралы кім не де де құлақ аспа олардың не ойла маған маңызды емес ең басты өз өмір өз жол өз мақсат бар өз пікір өмір сүру мүлдем өмір сүрме бірде,neutral

0,есіңде болсын егер құдай сені бақытты еткісі келсе сені қиын жолдармен жүргізіп сынайдысебебі бақыт оңай жолмен келмейді,ес бол егер құдай сен бақытты ет кел сен қиын жол жүргіз сынайдысебеб бақыт оңай жол келме,neutral

0,сатқын адамды кешіруге бола ма,сатқ адам кешір бола ма,neutral

0,шашыңыз жиі түсе ме олай болса сізге оңай көмектесетін әрі тез нәтиже беретін маска туралы құпияны бөлісемін,шаш жиі түс ме ола бол сізге оңай көмектес әрі тез нәтиже бер маска туралы құпия бөліс,neutral

0,қалың әрі ұзын шаш кез келген ару секілді менің де арманым болатын себебі көп уақыттан бері шашымның түсі күңгірттеніп жылтырлығын жоғалтқан еді шашым көп түсіп селдірей бастады бұл жағдай мені өте қатты алаңдатты ақырында көп түсуінен шашым мүлдем жұқарып кетті түрлі маскалар жасағанымнан түк те шықпады бірақ бір күні маған косметолог princess hair маскасын қолдануды ұсынды толық сенбесем де қолданып көрейін деп шештім,қалың әрі ұзын шаш кез кел ару секілді менің де арман бол себебі көп уақыт бері шаш түс күңгірттен жылтырлығ жоғалт ед шаш көп түс селдіре баста бұл жағдай мен өте қатты алаңда ақырында көп түсу шаш мүлдем жұқар кет түрлі маска жаса түк те шықпа бірақ бір күн маған косметолог princess hair маска қолдану ұсын толық сенбе де қолдан көр де шеш,neutral

0,қазір нәтижесі таң қаларлықтай princess hair маскасын қолданған соң шашым қалыңдап түсуі азайды әрі шашым жұмсарып жалтырай түсті,қазір нәтиже таң қаларлықта princess hair маска қолдан соң шаш қалыңда түс аза әрі шаш жұмсар жалтыра түсті,neutral

0,princess hair маскасын қолдансаңыз шашыңыз бірден қалыңдай бастайды бұл масканы қалыпты шаштың өсуін жақсарту үшін қолдансаңыздар да болады себебі масканың құрамында шаштың қалыпты өсуіне көмектесетін компоненттер бар сенбесеңіз қолданып көріңіз,princess hair маска қолдан шаш бірден қалыңда баста бұл маска қалыпты шаш өс жақсарту үшін қолдан да бол себебі маска құрам шаш қалыпты өс көмектес компонент бар сенбе қолдан көр,neutral

0,оригинал princess hair маскасына тапсырыс беру үшін көк жазудың үстін басыңыз https vk cc dpre,оригинал princess hair маска тапсырыс беру үшін көк жаз үст бас https vk cc dpre,neutral

0,сенің жағдайыңды өзіңнен басқа ешкім түсінбейді,сенің жағдай өз басқа ешкім түсінбе,neutral

0,тұз бен қанттың көрінісінде ешқандай айырмашылық жоқ екеуі ақ түсті оларды татып көрмегенше ажырата алмайсың,тұз бен қан көрініс ешқандай айырмашылық жоқ екеу ақ түсті ол тат көрмегенш ажыра алма,neutral

0,адамдарда да солай олармен араласып көрмегеніңше кімнің кім екенін білмейсің,адам да солай олармен аралас көрмегеніңш кім кім екен білме,neutral

0,жеңе алмайтыныңды сезсең де ұтылмау керек екенін ұмытпа мұхаммед али,же алма сез де ұтылма керек екен ұмытп мұхаммед али,neutral

0,қаншалықты көп сабыр сақтасаң соншалықты үлкен бақыт келеді,қаншалықты көп сабыр сақта соншалықты үлкен бақыт кел,neutral

0,қайғыны жалғыз көтеруге болады бірақ бақытқа екеу керек элберт хаббард,қайғы жалғыз көтер бол бірақ бақыт екеу керек элберт хаббард,neutral

0,адамды екі нәрсе қатты өзгерте алады,адам екі нәрсе қатты өзгер ал,neutral

0,бірі өмірінде жаңа адам пайда болса,бірі өмір жаңа адам пайда бол,neutral

0,екіншісі өмірінен бір адам кетсе,екінші өмір бір адам кет,neutral

0,жұмсақ болбірақ жеңілмеқатты болбірақ қатігез болма брюс ли,жұм болбірақ жеңілмеқа болбірақ қатігез болма брюс ли,neutral

0,адассаң да құласаң да сүрінсең де,адас да құла да сүрін де,neutral

0,түн қанша ұзаққа созылса да таң атады,түн қанша ұзақ созыл да таң ат,neutral

0,қайғылы күндер бітпейтін көрінгенмен жақсылық міндетті түрде келеді,қайғылы күн бітпе көрін жақсылық міндетті түр кел,neutral

0,қателіктер өмірдің тыныс белгілері секілді олар болмаса мәтіндегі сияқты өмірдің де еш мағынасы болмайды харуки мураками,қателік өмір тыныс бел секілді олар болма мәтін сияқты өмір де еш мағына болма харуки мураками,neutral

0,алкоголизмнен жүз пайыз кепілдікпен айығып кетудің ең қарапайым әдісі дәрігерлердің уколынсыз және дәрі дәрмегінсіз,алкоголиз жүз пайыз кепілдік айығ кету ең қарапайым әді дәрігер уколын жән дә дәрмегін,neutral

0,бұл тәсіл өте қарапайым және кез келген адам оны қолдана алады,бұл тәсіл өте қарапайым жән кез кел адам оны қолдан ал,neutral

0,кейде біреуге ішімдегі бар мұңымды сырымды айтып құшақтап қатты жылағым келеді бірақ жанымда түсінетін жан бар ма деп қайтадан бәрін ішімде сақтаймын,кейде біреу ішім бар мұң сыр айт құшақта қатты жыла кел бірақ жан түсін жан бар ма де қайта бәрін іш сақта,neutral

0,жүрек қанша сүйіп тұрғанымен алла тағдырға жазбаса бірге болу мүмкін емес,жүрек қанша сүй тұр алл тағдыр жазба бірге болу мүмкін емес,neutral

0,адам білмегендіктен адаспайды білгішсінем деп адасады жан жак руссо,адам білмегендік адаспа білгішсіне де адас жан жак руссо,neutral

0,тек махаббат адамды мықты әйелді сұлу еркекті мейірімді өмірді мәнді ете алады,тек махаббат адам мықты әйел сұл еркек мейірімді өмір мәнді ет ал,neutral

0,сомен жұлдыздардың парақшаларын ақтарып жүргем бір тамызатынға көзім түсіп кетті сипатамасын оқып шықтым табиғи қоспалар зияны жоқ деп жазылған сомен зияны тимер деп заказ бердім күнде почтамен жетті қызыма қолдандым бир айда қызымның көзі жақсарып мененде жақсы көретін болып кетты еш зияны жоқ болып шықты қызым көңіл күйі қатты көтерілді ұзін сөздің қысқасы сіздергеде көмегі тиып қалар мына жерден https vk cc gp bw,со жұлдыз парақша ақтар жүрге бір тамыз көз түс кет сипатама оқ шық табиғи қоспа зиян жоқ де жазыл со зиян тим де заказ бер күн почта же қыз қолда би айда қыз көз жақсар менен жақсы көр бол кет еш зиян жоқ бол шық қыз көңіл күй қатты көтер ұз сөз қысқа сіздерге көмег ти қал мына же https vk cc gp bw,neutral

0,барлығы өзгереді жаулар досқа айналады жақын адамдар қасқа кезіндегі сүйікті жандар жай танысқа достоевский,барлығ өзгер жау дос айнал жақын адам қас кез сүйікті жан жай таныс достоевски,neutral

0,махаббатта бұдан асқан қылмыс жоқ мұхтар шаханов,махаббат бұдан ас қылмыс жоқ мұхтар шаханов,neutral

0,тапсырыс беру сілтемесі https vk cc mhvfi,тапсырыс беру сілте https vk cc mhvfi,neutral

0,өзгелер қаламаған нәрсені бүгін жаса ертең басқалардың қолынан келмейтін өмірді сүретін боласың джаред лето,өзге қалама нәрсе бүгін жаса ертең басқа қол келме өмір сүр бол джаред лето,neutral

0,иә менің кемшіліктерім көп кешіріңдер мені мінсіз адамдар,иә менің кемшілік көп кешір мен мін адам,neutral

0,біреуді жоғалтып алудан қорықпаңыз жоғалатындар сізге тәжірибе үшін жіберілген тағдыр жібергендер мәңгілік сізбен бірге қалады фридрих ницше,біреу жоғалт алу қорықпа жоғал сізге тәжірибе үшін жібер тағдыр жібер мәңгілік сізбен бірге қал фридрих ницш,neutral

0,көзі көрмейтін адам мына дүниені күннің шуағын көрсем екен деп армандайды керең адам жақындарының сөзін азанның дауысын есітсем екен деп армандайды сақау адам сөйлеуді армандайды мүгедек адам өз аяғыммен жүрсем жүгірсем екен дейді,көз көрме адам мына дүние күн шуағ көр екен де арманда керең адам жақын сөз азан дауыс есіт екен де арманда сақа адам сөйлеу арманда мүгедек адам өз аяғ жүр жүгір екен де,neutral

0,ал сен ше ойланшы сенде барлығы бар әлем ғажаптарын көре аласың есіте аласың сөйлей аласың жүре аласыңжүгіре аласың сенде қанша мыңдаған адамдар армандап жүрген нығметтер бар ал біз кейде өмірге налып осы күнге кейде шүкіршілік айтуды ұмытамыз,ал сен ше ойланш сенде барлығ бар әлем ғажап көр ал есі ал сөйле ал жүр аласыңжүгір ал сенде қанша мыңдаған адам арманда жүр нығмет бар ал біз кейде өмір нал осы күн кейде шүкіршілік айту ұмыт,neutral

0,үлкендерді сыйлағаннан әлі ешкім ұятқа қалмады,үлкен сыйла әлі ешкім ұят қалма,neutral

0,жағдайы нашарларға көмектескеннен әлі ешкім кедей болып қалған жоқ,жағдай нашар көмектес әлі ешкім кедей бол қалған жоқ,neutral

0,бірінші кешірім сұрағаннан әлі ешкім кем болып қалған жоқ,бірінші кешір сұра әлі ешкім кем бол қалған жоқ,neutral

0,қасында жату төсектес болды дегенді білдірмейді,қас жату төсектес бол де білдірме,neutral

0,кек алмау кешірді дегенді білдірмейді,кек алмау кеш де білдірме,neutral

0,жанында болмау сүймейді дегенді білдірмейді,жан болмау сүйме де білдірме,neutral

0,жас болып мәңгі жүрмейсіңмәңгілік өмір сүрмейсің үнемдеп жұмса уақытты бітеді ол қашан екенін білмейсің,жас бол мәңг жүрмейсіңмәңгілік өмір сүрме үнемде жұмса уақыт біт ол қашан екен білме,neutral

0,мені бүгін не күтіп тұрғанын білмеймін ертең не болатынын білмеймін мен тек қана бір нәрсені білемін,мен бүгін не күт тұр білме ертең не бол білме мен тек қана бір нәрсе біл,neutral

0,ешқашан ақталма өз ісіңді түсіндіре бастасаң болды адамдар сені кінәлі деп ойлайды,ешқашан ақтал өз іс түсіндір баста бол адам сен кінәлі де ойла,neutral

0,назарларыңызға сіз бен біз білуге тиісті ережені ұсынамыз,назар сіз бен біз біл тиісті ереже ұсын,neutral

0,жаныңызда тек ұлы мақсаттарға жетелейтін адамдар болсын,жан тек ұлы мақсат жетеле адам бол,neutral

0,бүгін өзгелері жасай алмайтынды жасаңыз сонда ертең өзгелері жете алмаған табысқа жетесіз,бүгін өзге жаса алма жаса сонда ертең өзге же алма табыс же,neutral

0,жаныңда жүрген кезде өзіңізді барынша еркін ұстай алатын адамды жолықтырсаңыз оны ауадай бағалаңыз,жан жүр кезде өз барынша еркін ұста ал адам жолықтыр оны ауада бағала,neutral

0,сізге пойызыңыз кетіп қалды десе ұшақтар мен кемелердің барын ұмытпаңыз,сізге пойыз кет қал де ұшақ мен кеме бар ұмытпа,neutral

0,жақындарыңызға айтқан ауыр сөздеріңіз олардың жүрегіне шегедей қағылатынын біліңіз ол шегені кешірім сұрап алып тастасаңыз да орнында ойық қалады сондықтан шын жақсы көретін адамдарға көңіліне тиетін сөздер айтпаңыз,жақын айт ауыр сөз олардың жүрег шегеде қағыл біл ол ше кешір сұра алып таста да орн ойық қал сондықтан шын жақсы көр адам көңіл ти сөз айтпа,neutral

0,еш уақытта ақталмаңыз мейлі сүйген адамыңыз болсын ақталмаңыз сізді жақсы көрмейтін адам қаралау үшін көп нәрсені ойлап табады,еш уақыт ақталма мейлі сүй адам бол ақталма сіз жақсы көрме адам қарала үшін көп нәрсе ойла таб,neutral

0,сіз туралы өсек сөз тараған болса қуаныңыз өйткені сіз тұлғасыз жаманды ешкім де аузына алмайды тек жақсыларға ғана көреалмаушылық танытып сыртынан өсектейді,сіз туралы өсек сөз тара бол қуан өйткені сіз тұлға жаман ешкім де ауз алма тек жақсы ғана көреалмаушылық таныт сырт өсекте,neutral

0,әйелді бақытты қыла алмайсыз ба онда өзге біреуге кедергі жасамаңыз джейрард батлер,әйел бақытты қыл алма ба онда өзге біреу кедергі жасама джейрард бат,neutral

0,мықты адамдар ғана кешіре алады әлсіздер мәңгілік кек сақтап өтеді махатма ганди,мықты адам ғана кешір ал әл мәңгілік кек сақта өт махат ганди,neutral

0,әйел заты мықты батыл да батыр айтқан сөзінен қайтпайтын ерлерді ұнататындықтан соңына дейін күресіңіз олар бір жанжалға бола кетіп қалатындарды ұната бермейді,әйел зат мықты батыл да батыр айт сөз қайтпа ер ұнататындық соң дейін күрес олар бір жанжал бола кет қал ұна берме,neutral

0,еш уақытта ақымақтармен ұрыспаңыз ұрысып олардың дәрежесіне түскен кезіңізде бұл істің нағыз маманы екендіктерін пайдаланып кетеді,еш уақыт ақымақ ұрыспа ұрыс олардың дәреже түс кез бұл іс нағыз маман екендік пайдалан кет,neutral

0,жақындасу қиын ал айырылысудың тым оңай екенін есіңізде мәңгілік сақтаңыз,жақындас қиын ал айырылыс тым оңай екен ес мәңгілік сақта,neutral

0,ішімдіксіз қуанып есірткісіз армандап интернетсіз қарымқатынаста болып шылымсыз мазасызданып үйреніңіз,ішімдік қуан есірткі арманда интернет қарымқатынас бол шылым мазасыздан үйрен,neutral

0,қалауыңызға сәйкес іспен айналысу арқылы өмір бойы демалып өтесіз,қалау сәйкес іс айналысу арқылы өмір бойы демал өт,neutral

0,бұл өмірде адамдарға көп сенім артпаңыз көлеңкеңіздің өзі қараңғы сәттерде жоқ болып кетеді,бұл өмір адам көп сенім артпа көлеңке өзі қараңғы сәт жоқ бол кет,neutral

0,дұрыс жауапты қателесу арқылы тапқаныңызға еш өкінбеңіз,дұрыс жауапты қателес арқылы тап еш өкінбе,neutral

0,өзіңізде болмаған затқа қол жеткізгіңіз келсе бұрынсоңды айналыспаған іспен айналысуыңызға тура келеді,өз болма зат қол жеткіз кел бұрынсо айналыспа іс айналысу тура кел,neutral

0,жеңілген кезде жай ғана жымиыңыз осы сәтте жеңімпаз жеңістің дәмін сезіне алмай қалады,жең кезде жай ғана жыми осы сәт жеңімпаз жең дә сез алма қал,neutral

0,сізде міндетті түрде арман болуы тиіс міндетті түрде бұл сізге таңертең тұруға көмектеседі,сіз міндетті түр арман бол тиіс міндетті түр бұл сізге таңертең тұр көмектес,neutral

0,өзіңіз еш нәрсені түсінбей кеткен болсаңыз бірден ұйықтап алыңыз,өз еш нәрсе түсінбе кет бол бірден ұйықта ал,neutral

0,тұрған жеріңіз өзіңізге ұнамаса ауыстырыңыз сіз ағаш емессіз ғой,тұр же өз ұнама ауыстыр сіз ағаш емес ғой,neutral

0,ешкімге тәуелді болмасаңыз көңіліңізді ешкім де қалдыра алмайды,ешкім тәуелді болма көңіл ешкім де қалдыр алма,neutral

0,бай болсаңыз ол туралы ойламаңыз ал кедейлігіңізді мүлдем көңілге алмаңыз өмір қойылым секілді,бай бол ол туралы ойлама ал кедейліг мүлдем көңіл алма өмір қойылым секілді,neutral

0,еш уақытта адамдарға өз қиындықтарыңызды айтпаңыз ын сіздің қиындықтарыңыз қызықтырмайды ал қалған на олар мүлдем қызық емес,еш уақыт адам өз қиындық айтпа ын сіздің қиындық қызықтырма ал қалған на олар мүлдем қызық емес,neutral

0,өзіңіз қалаған іспен айналысыңыз ал жұрттың сіз туралы пікірі өмір бойы тоқтаусыз айтылады,өз қала іс айналыс ал жұрт сіз туралы пікір өмір бойы тоқтау айт,neutral

0,өмір сүрудің екі амалы бар біріншісі өмірде мүлде ғажайыптар жоқ сияқты екіншісі өмір тек ғажайыптан тұратын сияқты альберт эйнштейн,өмір сүру екі амал бар бірінші өмір мүлде ғажайып жоқ сияқты екінші өмір тек ғажайып тұр сияқты альберт эйнштейн,neutral

0,ең бастысы сенім егер сене білсеңіз барлығы да жақсы болады тіпті өзіңіз ойлағаннан да жақсы болады,ең басты сенім егер сен біл барлығ да жақсы бол тіпті өз ойла да жақсы бол,neutral

0,адам ай сияқты оның ешкімге көрсетпейтін қараңғы жағы бар м твен,адам ай сияқты оның ешкім көрсетпе қараңғы жағ бар м твен,neutral

0,біз ешкімнің алдында міндетті емеспіз біздің бір ғана міндетіміз бақытты болу,біз ешкі алд міндетті емес біздің бір ғана міндет бақытты болу,neutral

0,өзіңді қарапайым жылы лебізіңмен сыйлата білмесең құр қаталдықтан түк те шықпайды,өз қарапайым жыл лебіз сыйла білме құр қаталдық түк те шықпа,neutral

0,қызулықпен уәде берме мастықпен ашуланба қуаныш үстінде жоспарлар жасама шаршап отырғанда келешектегі істерің туралы ойлама,қызулық уәде берме мастық ашуланб қуаныш үст жоспар жасама шарша отырғанда келешек іс туралы ойлама,neutral

0,жебе салған жара жазылар балтамен оталған орман қайта өсер бірақ ащы тілдің жарасы жазылмас,жебе сал жара жазыл балта отал орман қайта өс бірақ ащ тіл жара жазылмас,neutral

0,әуелі сөзді емес істі бағалау керек себебі сөз жүзінде бәріміз бірбірімізді жақсы көреміз,әуел сөз емес іс бағалау керек себебі сөз жүз бәрі бірбі жақсы көр,neutral

0,тамшыдай сезім болған жерде мұхиттай төзім болады,тамшыда сезім бол же мұхитта төзі бол,neutral

0,адамдар сенімен сөйлесуге дәрежелері жетпейтінін түсінгендерінде артыңнан сөйлей бастайды ұмытпа,адам сенімен сөйлес дәреже жетпе түсінген арт сөйле баста ұмытп,neutral

0,сенімен бірге болғысы келген адамнан қашпа,сенімен бірге бол кел адам қашпа,neutral

0,сені бақытты ететін адам сол болуы мүмкін,сен бақытты ет адам сол бол мүмкін,neutral

0,кімді ренжіттім кешірім сұраймын,кім ренжі кешір сұра,neutral

0,арман мен мақсат көп өткенкеткенге қайырылатын уақыт жоқ,арман мен мақсат көп өткенкет қайырыл уақыт жоқ,neutral

0,тіпті сенің қалай қиналып талпынып жатқаныңды ешкім көрмесе де уайымдама алла бәрін көреді,тіпті сенің қалай қинал талпын жат ешкім көрме де уайымда алл бәрін көр,neutral

0,әлсіздер күледі мықтылар демеу береді,әл күл мықты деме бер,neutral

0,махаббатқа сенім артпа ол сирек кездеседі,махаббат сенім артпа ол сирек кездес,neutral

0,адамға сенім артпа ол тұрақсыз,адам сенім артпа ол тұрақсыз,neutral

0,уақытқа сенім артпа ол өзгермелі,уақыт сенім артпа ол өзгермелі,neutral

0,тек аллаға сенім арт ол құдіретті,тек алла сенім арт ол құдіре,neutral

0,сені алла жаратқан сондықтан әдемі болмауың мүмкін емес,сен алл жаратқан сондықтан әдемі болма мүмкін емес,neutral

0,сені қорғайтын алла сондықтан да сен өзіңді қауіпсіз сезінесің,сен қорға алл сондықтан да сен өз қауіпсіз сез,neutral

0,тек биікте тұрмайды мықтылар да құлайды,тек биік тұрма мықты да құла,neutral

0,өмір сүріп күлкімен бақытты да жылайды,өмір сүр күлкі бақытты да жыла,neutral

0,өмір деген арпалыс қусаң дағы жетпейтін,өмір де арпалыс қу дағ жетпе,neutral

0,қанша қиын болса да өмір маған ұнайды,қанша қиын бол да өмір маған ұна,neutral

0,мен өзімшіл емеспін тек өзім қалағандай өмір сүргім келеді,мен өзімшіл емес тек өзім қалағанда өмір сүр кел,neutral

0,мен тәкаппар емеспін тек бәріне бірдей өтірік күлімдей бермеймін,мен тәкапп емес тек бәрін бірде өтірік күлімде берме,neutral

0,мен қызғаншақ емеспін тек сүйгенімнің жалғызы болғым келеді,мен қызғаншақ емес тек сүй жалғыз бол кел,neutral

0,мен ренжімеймін мен жәй ғана бәрін бақылап шешім шығарамын,мен ренжіме мен жәй ғана бәрін бақыла шешім шығар,neutral

0,өмір бар жерде сынау бар,өмір бар же сынау бар,neutral

0,жек көре тұрып ұнау бар,жек көр тұр ұна бар,neutral

0,уақытша адамдар үшін емес сені мәңгілік керек ететін адамдар үшін өмір сүр,уақытша адам үшін емес сен мәңгілік керек ет адам үшін өмір сүр,neutral

0,сіз үнемі көңілді жүресіз ешкімге ашуланбайсыз ренжімейсіз менің де сіз сияқты болғым келеді көмектесіңізші,сіз үнемі көңіл жүр ешкім ашуланба ренжіме менің де сіз сияқты бол кел көмектесіңізш,neutral

0,шәкіртінің өтінішіне келісім берген ұстазы картоп пен қалта әкелуін бұйырыпты,шәкірт өтініш келісім бер ұстаз картоп пен қалта әкел бұйыр,neutral

0,егер біреуге ашуланып ызаланып ренжісең бір картопты ал да соған ренжіткен адамның атын жаз,егер біреу ашулан ызалан ренжі бір картоп ал да соған ренжіт адам ат жаз,neutral

0,солақ па дейді шәкірті,солақ па де шәкір,neutral

0,жоқ сол қалтаны үнемі өзіңмен бірге алып жүруің керек біреуге ренжіген сайын бір картоптан қосып отырасың,жоқ сол қалта үнемі өз бірге алып жүр керек біреу ренжі сайын бір картоп қос отыр,neutral

0,біраз уақыт өткен соң қалтасы картопқа толып өзімен бірге алып жүру қиындайды алғаш салған картоптары шіріп саси бастайды,біраз уақыт өткен соң қалта картоп тол өз бірге алып жүру қиында алғаш сал картоп шір саси баста,neutral

0,ұстаз мына қалта ауыр болып кетті рі шірігендерінің сасық иісіне шыдау мүмкін емес ашуланбаудың басқа жолын үйретіңізші дейді,ұстаз мына қалта ауыр бол кет рі шіріген сасық иіс шыда мүмкін емес ашуланба басқа жол үйретіңізш де,neutral

0,мұны саған не үшін істеттім біреуге ренжіген сайын ішкі жандүниеңде де осы құбылыс болады жүрегің ауырлап таза ниетің бұзылып жаман ойларың саси бастайды оны сен байқамауың мүмкін бірақ ісің әдетке әдетің мінезге айналады біреуге ренжімес бұрын не біреуді ренжітпес бұрын үнемі өзіңмен бірге артық жүк арқалап жүру керек пе керек емес пе ойлан депті ұстазы,мұны саған не үшін істе біреу ренжі сайын ішкі жандүние де осы құбылыс бол жүрег ауырла таза ниет бұзыл жаман ой саси баста оны сен байқама мүмкін бірақ іс әдет әде мінез айнал біреу ренжімес бұрын не біреу ренжітпес бұрын үнемі өз бірге артық жүк арқала жүру керек пе керек емес пе ойлан де ұстаз,neutral

0,өмір кейде көкке көтереді ал кейде төменге құлатады,өмір кейде көк көтер ал кейде төмен құлат,neutral

0,көкке көтергенінде тәкаппарланып кетпе бұл мәңгілік емес,көк көтер тәкаппарлан кетпе бұл мәңгілік емес,neutral

0,ал төменге құлатқанында сабырлы бол бұл да мәңгілік емес,ал төмен құлат сабырл бол бұл да мәңгілік емес,neutral

0,егер жоғарыдағы тізімдегі проблемалар сізде болса онда міндетті түрде оқыңыз https vk cc misqr,егер жоғары тізім проблема сіз бол онда міндетті түр оқ https vk cc misqr,neutral

0,өкінеміз көмек бере алмағанға кісіге,өкін көмек бер алма кісі,neutral

0,өкінеміз ақыл айта алмағанға кішіге,өкін ақыл ай алма кіші,neutral

0,өкінеміз уақытымыз текке кеткен үшін де,өкін уақыт тек кет үшін де,neutral

0,бақытты болғың келе ме өзіңді ең бақытты адам сияқты ұста,бақытты бол келе ме өз ең бақытты адам сияқты ұста,neutral

0,бай болғың келе ме байлығың тасып жатқандай күн кеш,бай бол келе ме байлығ тас жатқанда күн кеш,neutral

0,шынайы өмір сүргің келе ме риясыз күл шүкіршілік етіп өмір сүр,шынайы өмір сүр келе ме рия күл шүкіршілік ет өмір сүр,neutral

0,бірақ толық қанды өмірдің сценарийін өзің жазатыныңды ұмытпа не ойласаң сол болады,бірақ толық қан өмір сценари өзің жаз ұмытп не ойла сол бол,neutral

0,адам ол ақша табу үшін денсаулығын құртады қайта сауығу үшін ақшасын жұмсайды ол қазіргі күнінің рахатын сезінбей жатып болашақты күтеді айналып келгенде бүгінімен де ертеңімен де өмір сүрмеген болып шығады ешқашан өлмейтіндей тірлік кешеді ешқашан ғұмыр кешпегендей өмірден өтіп кетеді,адам ол ақша табу үшін денсаулығ құрт қайта сауығ үшін ақша жұмса ол қазіргі күн рах сезінбе жат болашақ күт айнал кел бүгін де ертең де өмір сүрме бол шығ ешқашан өлмейтінде тірлік кеш ешқашан ғұмыр кешпегенде өмір өт кет,neutral

0,ешкімге айтуға болмайтын жеті құпия,ешкім айт болма жеті құпия,neutral

0,айтылмауға тиісті бірінші құпия келешекке құрған жоспар,айтылма тиісті бірінші құпия келешек құр жоспар,neutral

0,екінші құпия қайырымды істер туралы ешкімге айтпау,екінші құпия қайыр істер туралы ешкім айтпа,neutral

0,үшінші құпия тақуалығыңыз туралы көпшілікте айтпау,үшінші құпия тақуалы туралы көпшілік айтпа,neutral

0,төртінші құпия жасаған ерлік батырлық туралы үндемеу,төртінші құпия жаса ерлік батырлық туралы үндеме,neutral

0,бесінші құпия рухани жетілу туралы айтпау,бесінші құпия рухани жетіл туралы айтпа,neutral

0,алтыншы құпия үйдегі ұрыскеріс пен отбасылық өмір туралы үндемеу,алтыншы құпия үйдегі ұрыскеріс пен отбасылық өмір туралы үндеме,neutral

0,жетінші құпия баяғыда естіген жаман сөздер туралы айта бермеңіз,жетінші құпия баяғы есті жаман сөз туралы ай берме,neutral

0,дұшпандарым аман болсын бір күні алдыма келер өтірік достарым аман болсын өтірік болсада қасымда жүргендері үшін көре алмайтындарға ризамын мені өздерінен жоғары санағаны үшін өсекшілерге ризамын менің өміріме қызығып өсекке айналдырғаны үшін нағыз достарыма ризамын жұбатқаны үшін атаанама ризамын өмір сыйлағаны үшін аллаға ризамын қасымдағы адамдардың шынайы бетбейнесин танытқаны үшін,дұшпан аман бол бір күн алд кел өтірік дос аман бол өтірік болса қас

жүргендері үшін көр алма риза мен өз жоғары сана үшін өсекші риза мені өмір қызығ өсек айналдыр үшін нағыз дос риза жұбат үшін атаана риза өмір сыйла үшін алла риза қасым адам шынайы бетбейнесин таныт үшін,neutral

0,ердің екі сөйлегеніөлгені уәде бермеуге тырыс әйтпесе сөзіңде тұр,ер екі сөйлегеніөл уәде берме тырыс әйтпесе сөз тұр,neutral

0,қысқа нұсқа сөйле дауысың анық болсын,қыс нұсқа сөйле дау анық бол,neutral

0,көп тыңда аз сөйле сөзіңнің артынан ісіңмен үлгеруге жаныңды сал,көп тыңда аз сөйле сөз арт іс үлгер жан сал,neutral

0,өсек айтылатын жерде болма кездейсоқ болған жағдайда қосылма кесіп тастауға тырыс,өсек айтыл же болма кездейсоқ бол жағдай қосылма кес таста тырыс,neutral

0,таспен атқанды аспен ат жеңісің содан кейін алшақ жүр,тас ат ас ат жең содан кейін алшақ жүр,neutral

0,күнде өзіңе есеп бер өзіңді тәрбиелей бер,күн өз есеп бер өз тәрбиеле бер,neutral

0,құмар ойын әлсіздің әлегі мықтының рухы биік денесі шымыр,құм ойын әлсіз әлег мықты рух биік дене шымы,neutral

0,үйретері мол мықтылардан ұстаздарың болсын бірақ ешкімді пір тұтпа,үйрет мол мықты ұстаз бол бірақ ешкі пі тұтп,neutral

0,сыйлағанның құлы бол кішірею төмендеу емес,сыйла құл бол кішірею төмендеу емес,neutral

0,ең алдымен өзіңе өзіңнің көңілің толатындай өмір сүр,ең алд өз өз көңіл толатында өмір сүр,neutral

0,қайтарымынан үмітті болсаң жақсылық жасама,қайтарым үміт бол жақсылық жасама,neutral

0,жүйкең әлі талай қажет болады болмашыға тоздырма шешуші сәттерге бапта,жүйке әлі тала қажет бол болмашы тоздырма шешуші сәт бапта,neutral

0,бойжеткеннің көңілін оңай жаулағап онымен болғаныңа шаттанып сөз қылма ерлік емес,бойжет көңіл оңай жаулаға онымен бол шаттан сөз қылма ерлік емес,neutral

0,уайымға салынудан алшақ бол арба да сынбайтын өгіз де өлмейтін шешім қабылда тез шешім қабылда,уайым салыну алшақ бол арба да сынба өгіз де өлме шешім қабылда тез шешім қабылда,neutral

0,тәуекел түбі жел қайық жүзесің де өтесің уайым түбі тұңғиық батасың да өтесің,тәуекел түб жел қа жүз де өт уай түб тұңғиық бат да өт,neutral

0,уәзірі мықтының тірлігі жеңіл мықтысын таңда,уәзі мықты тірліг жеңіл мықты таңда,neutral

0,сырыңды ешкімге алдыртпа екі елі ауызыңа сыймаған сөз өзгенің отыз екі тісінің арасына тіптен сыймайды,сыр ешкім алдыртп екі ел ауыз сыйма сөз өз отыз екі ті ара тіп сыйма,neutral

0,мүмкін нәтижелерін елестетіп көр нәтижесі ұнамаса келіспе,мүмкін нәтиже елестет көр нәтиже ұнама келіспе,neutral

0,өкпелеме өш алма санасына бер аллаға тапсыр,өкпеле өш алма сана бер алла тапсыр,neutral

0,қыран құстай қырағы бол өзгелерді өзіңдей көрме,қыран құста қыраң бол өзге өзіңде көрме,neutral

0,пенденің пендеден айырмашылығы адамгершілігі мен білімінде таны оқы,пенде пенде айырмашылығ адамгершіліг мен білім таны оқы,neutral

0,өнер үйрен мамандығыңда мықтысы бол,өнер үйрен мамандығ мықты бол,neutral

0,жер қозғалмаса қозғалмайтын сабырлы зіл бол,жер қозғалма қозғалма сабырл зіл бол,neutral

0,өміріңнің сапасына тек сен жауаптысың,өмір сапа тек сен жауапты,neutral

0,жалғыз апаңа әпкеңе қорған бол тұрмыста болса да өз күйеуіқорғаны бар деме сенің орның тым бөлек,жалғыз апа әпке қорған бол тұрмыс бол да өз күйеуіқор бар деме сенің ор тым бөлек,neutral

0,ең жақсы сыйлықтарды апаң мен әйеліңе жаса маған сыйлағаның болсын,ең жақсы сыйлық апа мен әйел жаса маған сыйла бол,neutral

0,үйленуге асықпа жар таңдаудан жаңылма,үйлен асықпа жар таңдау жаңылма,neutral

0,ұрпақ тәрбиесі елу пайыз сенің мойныңда ұлың өзіңнен озатын болсын қызың саған қарап серігін тауып бақытты болсын,ұрпақ тәрбие ел пайыз сенің мойын ұл өз оз бол қыз саған қара серіг тау бақытты бол,neutral

0,ердің аз міндеттерінің бірі отбасын асырау саған сенгендерге ешнәрседен таршылық көрсетпе,ер аз міндет бірі отбас асырау саған сен ешнәрсе таршылық көрсетпе,neutral

0,барлық жақсы қасиеттің діңгегі әдептілік барлық жақсыны әдептің үстіне орнат,барлық жақсы қасиет діңгег әдептілік барлық жақсы әдеп үст орнат,neutral

0,кім қанша төмен түссе де сен өзі биігіңде қал,кім қанша төмен түс де сен өзі биіг қал,neutral

0,мықты болсаң әйелге барып дәлелде балаларыңның анасын бақытты етіп өт жарыңның саған риза болып өткені мен үшін ұлы мадақ,мықты бол әйел бар дәлелде бала ана бақытты ет өт жар саған риза бол өт мен үшін ұлы мадақ,neutral

0,осының бәрін жеткізуге ұлыма сана өзіме денсаулық бер жаратушым,осы бәрін жеткіз ұл сана өзіме денсаулық бер жаратуш,neutral

0,өмір деген бір жарқ еткен найзағай,өмір де бір жарқ ет найза,neutral

0,өмір деген көк аспанның күркірі,өмір де көк аспан күркі,neutral

0,өмір деген бұйым емес қолдағы,өмір де бұйым емес қол,neutral

0,өмір дейтін ұлы бекет жолдағы,өмір де ұлы бекет жол,neutral

0,мына күн де мына аспан да дала анау,мына күн де мына аспан да дала анау,neutral

0,өмір дейтін тағатсыз бір қозғалыс,өмір де тағат бір қозғалыс,neutral

0,өмір дейтін өлемін деп қарамау,өмір де өл де қарама,neutral

0,бірде ыстық бірде суық бірде шаттықтан басың айналып жатса бірде қайғыдан жаныңды қоярға жер таппайсың қайсысы қай уақытта келері белгісіз сонымен өмір қызықты да шығар алайда белгілісі бәрі өткінші,бірде ыстық бірде суық бірде шаттық бас айнал жат бірде қайғы жан қоя жер таппа қайсысы қай уақыт кел белгісіз сонымен өмір қызықты да шығар алайда белгілі бәрі өткінш,neutral

0,егер жігіт шын сүйсе бір жоғалып бір пайда болып жүрмейді ол әрдайым жаныңнан табылады,егер жігіт шын сүй бір жоғал бір пайда бол жүрме ол әрдайым жан таб,neutral

0,егер жігіт шын сүйсе жалғыз емес екеніңді түсінесің,егер жігіт шын сүй жалғыз емес екен түс,neutral

0,егер жігіт шын сүйсе ешқашан сені бос үмітпен өмір сүргізбейді,егер жігіт шын сүй ешқашан сен бос үміт өмір сүргізбе,neutral

0,егер жігіт шын сүйсе оны сен бар жүрегіңмен сезінесің,егер жігіт шын сүй оны сен бар жүрег сез,neutral

0,үндемейді екен деп аузыңа келгенді айтып адамды басына берме біле білсең үндемеу ол сыйластықтың белгісі,үндемейді екен де ауз кел айт адам бас берме біл біл үндеме ол сыйластық белгісі,neutral

0,ата анадан басқа ешкімге сенбе ең адал дос кейде сатып кете алады,ата ана басқа ешкім сенбе ең адал дос кейде сат кет ал,neutral

0,банктен кредит алма ақшаны күліп аласың жылап қайтарасың,банк кредит алма ақша күл ал жыла қайтар,neutral

0,тіпті ең қиын жағдайда ұрлама алдама сатпа қиын уақыт өтіп кетеді ал кінә қалады,тіпті ең қиын жағдай ұрла алдама сатп қиын уақыт өт кет ал кінә қал,neutral

0,сүйіктіңе адал бол сүймесең оны ұстамай шындықты айт,сүйікті адал бол сүйме оны ұстама шындық айт,neutral

0,мен не киемін не жеймін деп көп уайымдама алла тіпті жануарларды ұмытпай тамақ берген саған да береді,мен не ки не же де көп уайымда алл тіпті жануар ұмытпа тамақ бер саған да бер,neutral

0,осы жазбаны парақшаңа алып кетсең көп адам ойланар еді,осы жазба парақша алып кет көп адам ойлан ед,neutral

0,әйелің жылап жатқанда онымен анасы сияқты сырлас,әйел жыла жатқанда онымен ана сияқты сырлас,neutral

0,әйелің өзін әлсіз сезінгенде әкесі сияқты қамқор бол,әйел өз әлсіз сезін әке сияқты қамқор бол,neutral

0,әйелің қателік қылғанда ағасы сияқты түсіндір,әйел қателік қыл аға сияқты түсіндір,neutral

0,әйеліңнің бір нәрсеге көңілі толмағанда оған дос сияқты бол,әйел бір нәрсе көңіл толма оған дос сияқты бол,neutral

0,әйеліңе кеңес керек болғанда әпкесі сияқты бол,әйел кеңес керек болғанда әпке сияқты бол,neutral

0,оған жұбай ретінде адал бол және әрқашан жанында екеніңді сезіндір,оған жұбай рет адал бол және әрқашан жан екен сезіндір,neutral

0,шын мәнінде біз тәккаппармыз кінәлі болсақ кешірім сұрамаймыз сағынсақ хабарласпаймыз көргіміз келсе қарамаймыз қорықсақ мойындамаймыз бұл жақсы қасиет емес,шын мән біз тәккапп кінәлі бол кешір сұрама сағын хабарласпа көр кел қарама қорық мойындама бұл жақсы қасиет емес,neutral

0,мені ашық немесе жасырын түрде ұятқа қалдырғысы келген адамдарды,мен ашық немесе жасырын түр ұят қалдыр кел адам,neutral

0,мені менің өмірімді білмей мен жайлы жаман ойлағандарды,мен менің өмір білме мен жайлы жаман ойла,neutral

0,бір кездері досым болып қиындықта тастап кеткендерді,бір кез дос бол қиындық таста кет,neutral

0,маған зиянын тигізгендерді немесе солай ниеттенгендерді,маған зиян тигіз немесе солай ниеттен,neutral

0,әйелдер шыны ыдыстар тәрізді,әйел шыны ыдыс тәрізді,neutral

0,өмірінде бір рет сатқындық жасаған адам екінші рет оны міндетті түрде қайталайды,өмір бір рет сатқ жаса адам екінші рет оны міндетті түр қайтала,neutral

0,көп сөйлемеуді үйрен және де ешкімнен ештеңе күтпеуді үйрен,көп сөйлемеу үйрен жән де ешкі ештеңе күтпеу үйрен,neutral

0,өмір бойы адал болу бұл бір керемет қасиет ол алтыннанда қымбат судан таза пәк қасиет қазіргі уақытта ол әрбір адамға керек бірақ әрбір адамда бола бермейді,өмір бойы адал болу бұл бір керемет қасиет ол алтыннан қымбат судан таза пәк қасиет қазіргі уақыт ол әрбір адам керек бірақ әрбір адам бола берме,neutral

0,бай болсаң да кедей болсаң да өз өміріңнің қадірін біл,бай бол да кедей бол да өз өмір қадір біл,neutral

0,адамның басшысы ақыл шолушысы ой жетекшісі талап қорғаушысы сабыр сынаушысы халық таусылмайтыны арман ең қымбаттысы ар сақтау бәрінен ардақтысы өмір сүру соның ішінде ең тәттісі сыйластық төле би,адам басшы ақыл шолушы ой жетекші талап қорғаушы сабыр сынаушы халық таусылма арман ең қымбатты ар сақтау бәрі ардақты өмір сүру соның іш ең тәтті сыйластық төле би,neutral

0,адамға бақытты болу үшін көп нәрсе керек емес,адам бақытты болу үшін көп нәрсе керек емес,neutral

0,қамқор өмір жолдас жайлы үй және сүйікті балалары болса жеткілікті,қамқор өмір жолдас жайлы үй және сүйікті бала бол жеткілікті,neutral

0,бірақ көп адам осыны кеш түсінеді,бірақ көп адам осы кеш түсін,neutral

0,ол адамның үш арманы болыпты жалақысы көп жұмысым сұлу әйелім және әлемге танымал адам болсам деген,ол адам үш арман бол жалақы көп жұмыс сұл әйел және әлем танымал адам бол де,neutral

0,бір күні ол адамды ірі компанияға жұмысқа шақырыпты сұқбаттасуға келе жатады күн аяз жер тайғақ асығып келе жатқанда алдында кетіп бара жатқан егде жастағы адамның тайып құлағанын көреді мас шығар деп ойлайды да жанынан өтіп кетеді сұқбаттасуға кешікпей келеді бірақ бәрі сәтсіз болып армандаған қызметке қол жеткізе алмай қалады,бір күн ол адам ірі компания жұмыс шақыр сұқбаттас келе жат күн аяз жер тайғақ асығ келе жатқанда алд кет бар жат егде жастағы адам тай құла көр мас шығар де ойла да жан өт кет сұқбаттас кешікпе кел бірақ бәрі сәт бол арманда қызмет қол жеткіз алма қал,neutral

0,бірде кешкі қаланы серуендеп жүріп көше әртістерінің қойылымына тап болады көрермен көп емес бірақ қойылым өте көңілді қызықты өтеді көргендер дән риза ду қол шапалақтарын аямайды үйдіүйлеріне кете бастаған кезде мұның иығынан біреу тартады қараса жаңа қойылымда кемпір болып ойнаған әртіс екен ол қойылым ұнағанұнамағанын сұрастыра бастайды бірақ бұл адам әңгімелескісі келмей қашқақтап тезірек кетіп қалады,бірде кешкі қала серуенде жүр көше әртіс қойылым тап бол көрермен көп емес бірақ қойылым өте көңіл қызықты өт көр дән риза ду қол шапалақ аяма үйдіүй кет баста кезде мұның иығ біреу тарт қара жаңа қойыл кемпі бол ойна әртіс екен ол қойылым ұнағанұнама сұрастыр баста бірақ бұл адам әңгімелес келме қашқақта тезірек кет қал,neutral

0,тағы бір жаңбырлы кеште досының туған күнінен қайтып келе жатады қатты шаршағаны соншалық ыстық суға жуынып тезірек жатып ұйықтаса деген ой ғана болды санасында үйіне жете бергенде біреудің жылаған дауысы талып естілді осы адамның үйінің алдындағы сәкіде бір әйел жылап отыр екен қолшатыры да жоқ қараңғы түнде жалғыз өзі үйіне беттеген адамды көріп көмек сұрайды отбасында қиындық болған екен оған қайғымұңын бөлісер бір адам керегін айтады адам ойланып қалады бірақ жылы төсек пен ыстық суға түсемін деген ой жеңіп кетеді әйелге назар салмастан үйге кіріп кетті,тағы бір жаңбырл кеш дос туған күн қайт келе жат қатты шарша соншалық ыстық су жуын тезірек жат ұйықта де ой ғана бол сана үй же бергенде біреу жыла дауыс тал ест осы адам үй алд сәкі бір әйел жыла отыр екен қолшатыр да жоқ қараңғы түн жалғыз өзі үй бетте адам көр көмек сұра отбас қиындық бол екен оған қайғымұң бөліс бір адам керег айт адам ойлан қал бірақ жыл төсек пен ыстық су түс де ой жең кет әйел назар салмас үй кір кет,neutral

0,сол адам өмірден бақытсыз болып өтіпті ақыры өледі,сол адам өмір бақыт бол өт ақы өл,neutral

0,ешкімге керексіз сұрықсыз өмір сүрдім үш арманым болған еді бірі де орындалмады әттең,ешкім керек сұрық өмір сүр үш арман бол ед бірі де орындалма әттең,neutral

0,солай ма досымау сен бақытты болу үшін мен бәрін де істедім ғой үш арманыңа да жол салдым мүмкіндік бердім саған тек көзің қолың жүрегіңді ашу керек еді,солай ма досыма сен бақытты болу үшін мен бәрін де істе ғой үш арман да жол сал мүмкіндік бер саған тек көз қол жүрег ашу керек ед,neutral

0,аязды күні құлап қалған адам есіңде ме ол сен сұқбаттасуға бара жатқан ірі компанияның иесі еді сен қолыңды созып көмек көрсетсең мәселең шешіліп тұр еді,аяз күн құла қалған адам ес ме ол сен сұқбаттас бар жат ірі компания иесі ед сен қол соз көмек көрсет мәселе шешіл тұр ед,neutral

0,әртіс кемпір ше кемпір болып киінген сұлу әрі жас қыз еді сені көріп бір көргеннен ғашық болды келешектерің жарқын еді балашаға ескірмейтін махаббат сен тек көзіңді ашып қарасаң жеткілікті болар еді,әртіс кемпі ше кемпі бол киін сұл әрі жас қыз ед сен көр бір көр ғашық бол келешек жарқын ед балаша ескірме махаббат сен тек көз аш қара жеткілікті бол ед,neutral

0,жаңбырлы түні жылаған әйел ше ол жаңбырдан емес көз жасынан малмандай су болды жалғыз өзі сені күтіп отырды оның мұңын тыңдап қайғысын есітсең жанына демеу болар бірекі ауыз сөз айтсаң жүрегіңмен өзгенің қиындығын сезе білсең сол да жеткілікті еді ол әйел талантты жазушы еді үйінде бір қиындық болып соның салдарынан көше кезіп кеткен бір ауыр сәтте тұрған сен жүрегіңді ашсаң ол сен туралы кітап жазар еді үшінші арманың да орындалар еді сен мен берген мүмкіндіктерге бейтарап болдың сондықтан бақыт саған келмеді,жаңбырл түн жыла әйел ше ол жаңбыр емес көз жас малманда су бол жалғыз өзі сен күт от оның мұң тыңда қайғы есіт жан деме бол бірек ауыз сөз айт жүрег өз қиындығ сез біл сол да жеткілікті ед ол әйел талантты жазушы ед үй бір қиындық бол соның сал көше кез кет

бір ауыр сәт тұр сен жүрег аш ол сен туралы кітап жаз ед үшінші арман да орында ед сен мен бер мүмкіндік бейтарап бол сондықтан бақыт саған келме,neutral

0,адам қатты күрсінеді екеуі айлы соқпаққа түсіп жұлдызды кеңістікке сіңіп кете барды,адам қатты күрсін екеу айл соқпақ түс жұлдыз кеңістік сің кет бар,neutral

0,ал сізге мына әлем не берді бақыттың бетпердесі құпия екенін ұмытпағайсыз,ал сізге мына әлем не бер бақыт бетперде құпия екен ұмытпа,neutral

0,уақыттың өтетіні рас көп нәрсенің ұмытылатыны рас бірақ өмір бірақ рет беріледі деген өзіміз білетін қағида бар ол қандай заманда қандай жағдайда өтсе де бәрібір қымбат сондықтан өз басым өткенге өкпе жүрмейді деп ойлаймын өткенге тек тәубе жүреді деп ойлаймын,уақыт өт рас көп нәрсе ұмытыл рас бірақ өмір бірақ рет бер де өз біл қағида бар ол қандай заман қандай жағдай өт де бәрібір қымбат сондықтан өз басым өт өкпе жүрме де ойла өт тек тәуб жүр де ойла,neutral

0,сақтауға қажет бір асыл болса алдымен жүрегіңді сақта өйткені өмір бастауы сол жүрек,сақта қажет бір асыл бол алд жүрег сақта өйткені өмір баста сол жүрек,neutral

0,ақиқат қаншама алға жетелегенмен тұман басқан дәуірде адаспай тұра алмайды екенсің,ақиқат қанша алға жетеле тұман бас дәуір адаспа тұр алма екен,neutral

0,сендердің себептеріңнен көз жасы төгілгендерден қорқыңдар себебі олар менен көмек сұрайды ал мен оларға міндетті түрде көмектесемін,сендерді себептері көз жас төгіл қорқ себебі олар менен көмек сұра ал мен оларға міндетті түр көмектесемін,neutral

0,бір күні мұхаммед али былай деді мен темекі шекпеймін бірақ қалтама әрдайым оттық салып жүремін егер мен күнә жасауға ұмтылсам оттықты жағып алақаныма қойып өз өзіме былай деймін али сен мына кішкене жанған отқа төзе алмайсың ертең тозақтың адам төзгісіз отына қалай шыдайсың,бір күн мұхаммед али былай де мен темекі шекпе бірақ қалта әрдай от сал жүр егер мен күнә жаса ұмтыл оттық жағ ала қой өз өзіме былай де али сен мына кішкен жан от төз алма ертең тозақ адам төзгісіз от қалай шыда,neutral

0,сені сүймейтін адамның аяғына жығылғанша өзіңді сүйетін төбесіне көтеретін жанның қолында бол,сен сүйме адам аяғ жығылғанш өз сүй төбе көтер жан қол бол,neutral

0,ең зая кеткен уақыт олсені бағаламаған адамдарға кеткен уақыт,ең зая кет уақыт олсе бағалама адам кет уақыт,neutral

0,басыңда қайғы көңіліңде мұң болғанда үш нәрсені есіңнен шығарма,бас қайғы көңіл мұң болғанда үш нәрсе ес шығарма,neutral

0,әрдайым жақсы сөз айтыңыз себебі әрбір сөзден кейін періштелер әмин деп тұрады,әрдайым жақсы сөз ай себебі әрбір сөз кейін періште әмин де тұр,neutral

0,өз күлкіңнен ұяласың ба тісіңізді өзгерткіңіз келді бірақ тіс дәрігері көп ақша сұрады ма,өз күлкі ұял ба тіс өзгерт кел бірақ тіс дәріг көп ақша сұр ма,neutral

0,gtooth тістерді түзететін жаңа технология,gtooth тіс түзет жаңа технология,neutral

0,артық шығынсыз және ауырсынусыз түзу тістер https vk cc bfa t,артық шығын жән ауырсыну түз тіс https vk cc bfa t,neutral

0,ортодонт мамандары ойлап тапқан,ортодонт маман ойла тап,neutral

0,сіз ұйықтап жатқанда да жұмыс істейді,сіз ұйықта жатқанда да жұмыс істе,neutral

0,арзан бағаға таңқалаларлық нәтиже сыйлайды,арзан баға таңқалаларлық нәтиж сыйла,neutral

0,жылдам жеткізу ақшасын тауарды алған соң төлейсіз,жылдам жеткізу ақша тау ал соң төле,neutral

0,асығыңыздар тауар саны шектеулі https vk cc bfa t,асығ тауар сан шектеулі https vk cc bfa t,neutral

0,мені сатқан адамдардың бәрін кешіремін мені тастағандарға тек бақыт тілеймін ал қасымдағы адамдарды жақсы көремінбағалаймын және рахметімді айтамын,мен сат адам бәрін кешір мен таста тек бақыт тіле ал қасым адам жақсы көремінбағала және рахмет ай,neutral

0,бақытты екеніңді ешкімге айтпа себебі тәтті күніңе тұз себетін адам табылар,бақытты екен ешкім айтпа себебі тәтті күн тұз себ адам табыл,neutral

0,мені керек етпеген адамдармағанда керек емес мені іздемеген адамдарды менде іздемеймін,мені керек етпе адамдарма керек емес мені іздеме адам менде іздемеймін,neutral

0,адал адам сен әрқашан ізін аңдып тексеріп жүретін адам емес,адал адам сен әрқашан із аңд тексер жүр адам емес,neutral

0,адал адам сен толық еркіндік берсең де сенімен бірге қалатын адам,адал адам сен толық еркіндік бер де сенімен бірге қал адам,neutral

0,бір күні ауыл тұрғындары жаратқаннан жаңбыр жауғанын тілемекші болады адамдардың барлығы бірігіп жиналды тек бір ғана бала қолына қолшатыр алды бұл жаратқанға сену,бір күн ауыл тұрғын жарат жаңбыр жау тілемекш бол адам барлығ біріг жина тек бір ғана бала қол қолшатыр алды бұл жарат сену,neutral

0,сіздер кішкентай сәбиді ауаға лақтырғанда ол күледі себебі әкесінің қолына тосып алатынын біледі бұл сенім,сіздер кішкентай сәби ауа лақтыр ол күл себебі әке қол тос ал біл бұл сенім,neutral

0,ұйқыға жатар әр түні біздің ертесіне оянатындығымызға кепілдік жоқ бірақ біз таңертең оятатын қоңырау қоямыз бұл үміт,ұйқы жат әр түн біздің ерте оянатындығ кепілдік жоқ бірақ біз таңертең ояттоңырау қоя бұл үміт,neutral

0,үшіншіден өзіңе жақсылық тілесең өзгеге жамандық қылма,үшінші өз жақсылық тіле өзге жаман қылма,neutral

0,жар қылып алар қызыңның шешесіне қарарсың шеше көргенді ал алма ағашынан алысқа түспейді,жар қыл ал қыз шеше қар шеш көр ал алма ағаш алыс түспе,neutral

0,сонан соң әкесіне қара әке қатал әрі мейірімді болуы керек билігі еркекте болған үй береке осыны ұмытпа,сонан соң әке қара әке қатал әрі мейірімді бол керек билиг еркек бол үй береке осы ұмытп,neutral

0,еркек балалары мен қоса әйелін де тәрбиелейді олардың санасына исламды құй дін жолын үйрет оқыт аллаға жақынның адасқанын көргенім жоқ сондықтан осы іске жақсы көңіл бөл,еркек бала мен қоса әйел де тәрбиеле олардың сана ислам құй дін жол үйрет оқыт алла жақын адас көр жоқ сондықтан осы іс жақсы көңіл бөл,neutral

0,алғаның басқаны емес әуелі сені сыйласын күйеуін ерім балаларымның әкесі болашағым деп сыйлаған әйел сенімен қоса бүкіл әулетіңді құрметтейді оның құрметі саған әулетіңнің алдында абырой сыйлайды,ал бас емес әуел сен сыйла күйе ер бала әке болашағ де сыйла әйел сенімен қоса бүкіл әулет құрметте оның құрмет саған әулет алд абырой сыйла,neutral

0,оны қадір тұт бар жақсы сөзіңді арна жұрттың балаларыңның көзінше құшақтап сүйме өзіңнің де оның да қадірін қашырасың оның барлығы оңашада қылар іс ал басқа уақытта көз қарасың мен оған жасар жақсы ісің де жетіп жатыр,оны қадір тұт бар жақсы сөз арна жұрт бала көзінше құшақта сүй өз де оның да қадір қашыр оның барлығ оңаша қыл іс ал басқа уақыт көз қар мен оған жасар жақсы іс де жет жатыр,neutral

0,оның жасар ісіне араласпа ақылды әйел сен араласпайақ қажет ақылын өзі келіп сұрап алады,оның жасар іс араласпа ақылды әйел сен араласпайақ қажет ақыл өзі кел сұра ал,neutral

0,оны қызған тек жөнімен болғаны дұрыс ал шамадан артық қызғану ұрыскеріске себепші,оны қыз тек жөн бол дұрыс ал шама артық қызған ұрыскеріс себепш,neutral

0,қанша ұрыскеріс болса да өз араларыңда болсын сыртқа шығарушы болма ұрыскерісте иа сен жеңіл иа ол жеңілсін ал қателік пен дұрысбұрысын ашуларың басылған соң талқылаған жөн,қанша ұрыскеріс бол да өз ара бол сырт шығарушы болма ұрыскеріс иа сен жеңіл иа ол жеңіл ал қателік пен дұрысбұры ашу басыл соң талқыла жөн,neutral

0,оны ешқашан алдама кезкелген жағдайда тек шындықты айт оның өзіңе деген сенімін жоғалтып алма сезімнің іргетасы сенімнен қаланатынын ұмытпа,оны

ешқашан алдама кезкел жағдай тек шындық айт оның өз де сен жоғалт алма сез іргетас сен қалан ұмытп,neutral

0,не нәрсеге де сабыр қыл барды қанағат тұт әйеліңнен де осыны талап ет сол кезде ғана өмірдің барлық тосын жайларына қарсы тұра аласыңдар,не нәрсе де сабыр қыл бар қанағат тұт әйел де осы талап ет сол кезде ғана өмір барлық тос жай қарсы тұр ал,neutral

0,адамдар бетіңе күле қарап аяғыңнан шалуға тырысадысол үшін ешкімге сырыңды айтпаайтсаңда шыныңды айтпа,адам бет күл қара аяғ шал тырысадысол үшін ешкім сыр айтпаайтса шын айтпа,neutral

0,уақыты келгенде сен саған ешқашан қиянат жасамайтын адамдарды кездестіресің ал оған дейін талай сатқындықты көресің,уақыт кел сен саған ешқашан қиянат жасама адам кездестір ал оған дейін тала сатқындық көр,neutral

0,уақыты келгенде сен сырт сұлулықтың жүректің сұлулығынан маңызды емес екенін түсінесің ал оған дейін көптеген адамдардың сұлулығына алданасың,уақыт кел сен сырт сұлулық жүрек сұлулығ маңызды емес екен түс ал оған дейін көптеген адам сұлулығ алдан,neutral

0,уақыты келгенде сен мейірімділік пен адалдықтың әлсіздік емес күштілік екенін түсінесің ал оған дейін көп қателесесің,уақыт кел сен мейірімділік пен адалдық әлсіздік емес күштілік екен түс ал оған дейін көп қателес,neutral

0,ауыздағы жағымсыз иіс тістердің нашарлығынан емес,ауыз жағымсыз иіс тіс нашарлығ емес,neutral

0,сағыз шайнамаңыздар одан сайын нашарлайды,са шайнама одан сайын нашарла,neutral

0,ешкімге күнім түспейді деп кеудесін керген адам қатты қателеседі бірақ өзгелер менсіз күнін көре алмайды деп кердеңдеуші адам одан бетер қате жасайды,ешкім күн түспе де кеуде кер адам қатты қателес бірақ өзге мен күн көр алма де кердеңдеуш адам одан бетер қате жаса,neutral

0,сондықтан аузыңнан шыққан әрбір сөзге ие бол,сондықтан ауз шық әрбір сөз ие бол,neutral

0,шамадан тыс тершеңдік немесе денеде қал сүйелдің көбеюі немесе шашыңыздың көп көлемде түсуі шаршаңқылық ауыздан жағымсыз иіс шығуы секілді белгілер организмде паразиттің көбеюінің себептері дер кезінде емдемесе соңы өліммен аяқталатыны еш күмәнсіз,шама тыс тершең немесе дене қал сүйе көбею немесе шаш көп көлем түс шаршаңқылық ауыз жағымсыз иіс шығ секілді белгі организм парази көбею себеп дер кез емдеме соңы өл аяқтал еш күмән,neutral

0,ол үшін ұйықтар алдында бір қасық,ол үшін ұйық алд бір қасық,neutral

0,сенің қандай киім кигеніңді бір жылдан соң бәрі ұмытады бірақ сенің қандай адам болғаныңды ешқашан ұмытпайды,сенің қандай киім ки бір жыл соң бәрі ұмыт бірақ сенің қандай адам бол ешқашан ұмытпа,neutral

0,желдің бағытын өзгерте алмасаң да мақсатыңа жету үшін кемеңнің бағытын өзгерте аласың,же бағыт өзгер алма да мақсат жету үшін кеме бағыт өзгер ал,neutral

0,ең қиыны шыдамдылық төзімділік байсалдылық пен сабырлылық көрсету творчестволық тұрғыдан дұрыс парасатты ойлай білу,ең қиын шыдамдылық төзімділік байсалдылық пен сабырлылық көрсету творчестволық тұрғы дұрыс параса ойла білу,neutral

0,ұрсысу мен ренжісусіз қарым қатынас болмайды,ұрсыс мен ренжісу қарым қатынас болма,neutral

0,батыл адамдар ұрсысады ренжіседі бірақ бірге қалады,батыл адам ұрсыс ренжіс бірақ бірге қал,neutral

0,ал әлсіздер алмастыратын адам іздейді,ал әл алмастыр адам ізде,neutral

0,бес нәрсе келмей тұрып бес нәрсенің қадірін біліңдер,бес нәрсе келме тұр бес нәрсе қадір біл,neutral

0,кәрілік келмей тұрып жастық шақтың,кәрілік келме тұр жастық шақ,neutral

0,жұмысбастылық келмей тұрып бос уақыттың,жұмысбастылық келме тұр бос уақыт,neutral

0,ата анаңды бағала себебі олар әрбір қиын сәтте әрқашанда жаныңнан табылады,ата ана бағала себебі олар әрбір қиын сәт әрқашан жан таб,neutral

0,маған ренжіген адамдар шіркін өзін менің орныма қойып шамалы ойланса ғой міне сол кезде менің жағдайымды түсінер еді,маған ренжі адам шіркін өз менің орн қой шамалы ойлан ғой міне сол кезде менің жағдай түсін ед,neutral

0,мен жүрегімді кімге сыйлаймын білесің бе,мен жүрег кім сыйла біл бе,neutral

0,бетін жүз рет қайтарсамда іздеп келген жанға,бет жүз рет қайтарса ізде кел жан,neutral

0,егер мен сіздермен енді сөз таластырмасам жеңілгенім емес сіздерді тастағаным егер мен сіздермен мүлде сөйлепей қойсам түңілгенім емес жаңа өмір бастағаным,егер мен сіздермен енді сөз таластырма жеңіл емес сіз таста егер мен сіздермен мүлде сөйлепе қой түңіл емес жаңа өмір баста,neutral

0,жоқ кешіру қиын емесқайта сену қиын,жоқ кешіру қиын емесқай сену қиын,neutral

0,сен тәтті өтіріксің ал мен ащы шындықпын депті,сен тәтті өтірік ал мен ащ шындық де,neutral

0,қатты уайымға салынба бұйырғаннан қашпайсың жан аман болса кезкелген қиындықтан өтесің,қатты уайым салынба бұйыр қашпа жан аман бол кезкел қиындық өт,neutral

0,жаман адамдар болмайды жүрегінде ашуреніштері көп адамдар болады,жаман адам болма жүрег ашуреніш көп адам бол,neutral

0,қанша уақыт өтсе де реніш пен ауыр сөздер адамның жүрегінен кетпейді,қанша уақыт өт де реніш пен ауыр сөз адам жүрег кетпе,neutral

0,егер таза жар қаласаң таза бол,егер таза жар қала таза бол,neutral

0,адал сүйіктің болсын десең адал бол,адал сүйікті бол де адал бол,neutral

0,тазалыққа құмар жан болсын десең таза жүр,тазалық құм жан бол де таза жүр,neutral

0,ата анаңды сыйласын десең жұмақтың кілті қайда екенін білесің,ата ана сыйла де жұмақ кілт қайда екен біл,neutral

0,есіңде болсын қуған жетпейді бұйырған кетпейді,ес бол қу жетпе бұйыр кетпе,neutral

0,мыңдаған ер адамдарға көмектесті саған да көмектеседі простатиттан бар болғаны жеті күннің ішінде нәтижелі түрде арылуға болады,мыңдаған ер адам көмекте саған да көмектес простатит бар бол жеті күн іш нәтижел түр арыл бол,neutral

0,сілтемеге кіріп пайдалы кеңесті оқыңыз https vk cc xnkk,сілтеме кір пайдалы кеңес оқ https vk cc xnkk,neutral

0,кейде үнсіздік сақтағым келеді бірақ адамдармен сөйлесемін,кейде үнсіздік сақта кел бірақ адам сөйлес,neutral

0,кейді ішімдегінің бәрін айтып салғым келеді бірақ үндемеймін,кей ішімдег бәрін айт сал кел бірақ үндеме,neutral

0,кейде өзімді бақытты етіп көрсетемін бірақ олай емес,кейде өз бақытты ет көрсе бірақ ола емес,neutral

0,өмірде үш адамды қатты бағала сені туғанды сенен туылғанды сен үшін туылғанды,өмір үш адам қатты бағала сен ту сенен туыл сен үшін туыл,neutral

0,өзіңіз туралы айтуға асықпаңыз сіз туралы әңгіме сіз кеткен соң басталады,өз туралы айт асықпа сіз туралы әңгіме сіз кет соң бастал,neutral

0,арлы адам ең күшті адам өйткені ол өткінші бақыттың өткінші қайғының ырқына мойын ұсынбайды сондықтанда оның тағдыры қиын болуы ауыр болуы мүмкін өкінішті болуы мүмкін емес әбулхасан рудаки,арл адам ең күшті адам өйткені ол өткінш бақыт өткінш қайғы ырқ мойын ұсынба сондықтан оның тағдыр қиын бол ауыр бол мүмкін өкініш бол мүмкін емес әбулхасан рудаки,neutral

0,бұл дүниеде екіақ есік бар бірі өмірге қарай ашылады екіншісі өлімге қарай ашады біріншi есікке енген адам екінші есікке де енеді,бұл дүние екіақ есік бар бірі өмір қарай аш екінші өлім қарай аш бірінші есік ен адам екінші есік де ен,neutral

0,біреулер келер біреулер кетер ешкімнің соңынан жүгірме кеткісі келген ертелікеш бәрібір кетеді ең соңында қалғысы келгендер ғана жаңында болады,біреу кел біреу кет ешкі соң жүгір кет кел ертелікеш бәрібір кет ең соң қал кел ғана жаң бол,neutral

0,сабырлы бол түнек қаншаға созылса да күн шығуы керек,сабырл бол түнек қанша созыл да күн шығ керек,neutral

0,қалай арықтағаным жайлы айтар айта кетейін,қалай арықта жайлы айтар ай кет,neutral

0,өткен жылы адам танымастай былшиып кетіп едім жастар мені апа дәу апа дейтін болды содан не керек семіздіктің серігі болмайын деп арықтауға бел будым шыны керек жаттығу залына да бардым көптеген арықтататын дәрілерді де іштім ешқандай нәтиже болмады сөйтіп жүргенде маған пайдасы тиетін бір керемет аздыратын нәрсе бар екенін достарымнан естідім қазақпыз ғой қайтпаймыз ақыры сол нәрсені таптым нәтиже аяқасты біліне бастады оның қасиеті іштегі бүйірдегі майлардыерітіп ауызды тияды екен лезде арықтағаныма өзім де қатты қуандым сөйтсем мен ішкен аздыратын құрал тек табиғи шөптерден жасалады екен ғой міне аллаға шүкір арықтап қайтадан өмірге оралып адам болдық қой осы нәрсені сатып алған сайтқа үлкен рахмет айтқым келеді рахмет тапсырыс беремін деушілерге сілтемесі міне тұр,өткен жыл адам танымаста былши кет ед жас мен апа дә апа де бол содан не керек семіздік серіг болма де арықта бел бу шыны керек жаттығу зал да бар көптеген арықтат дәрі де іш ешқандай нәтиж болма сөйт жүр маған пайда ти бір керемет аздыр нәр бар екен дос есті қазақ ғой қайтпа ақы сол нәрсе тап нәтиж аяқа біл баста оның қасиет іш бүйір май еріт ауыз тия екен лез арықта өзім де қатты қуан сөйт мен іш аздыр құрал тек табиғи шөп жасал екен ғой міне алла шүкі арықта қайта өмір орал адам бол қой осы нәрсе сат ал сайт үлкен рахмет айт кел рахмет тапсырыс бер деуші сілте міне тұр,neutral

0,адамды сырт келбетіне емес саған деген қарым қатынасына сүйіспеншілігіне адалдығына қарай сыйла,адам сырт келбет емес саған де қарым қатына сүйіспеншіліг адалдығ қарай сыйла,neutral

0,қорқасың ба жасама жасап жатсың ба қорықпа жасап біттің бе өкінбе шыңғыс хан,қор ба жасама жаса жат ба қорықпа жаса біт бе өкінбе шыңғыс хан,neutral

0,құлағым есітіп көзім көре алғаны үшін,құла есіт көз көр ал үшін,neutral

0,денім сау ақылесім дұрыс екені үшін мыңда бір алғыс айтамын,ден сау ақылес дұрыс екен үшін мыңда бір алғыс ай,neutral

0,жүрегі таза адам қиянатқа бармайды шәкәрім құдайбердіұлы,жүрег таза адам қиянат барма шәкә құдайбердіұл,neutral

0,уақыт өтеді бірақ айтылған ауыр сөздер жүректе қалады лев толстой,уақыт өт бірақ айтыл ауыр сөз жүрек қал лев толстой,neutral

0,алатын кезде алға озып беретін кезде артта бұғып қалатын алғаны мен бергенін жіпке тізіп санайтын адамнан аулақ жүр ондай адамнан құтылудың өзі үлкен олжа,ал кезде алға оз бер кезде арт бұғ қал ал мен бер жіп тіз сана адам аулақ жүр ондай адам құтыл өзі үлкен олжа,neutral

0,нағыз еркек ешқашан ауызыңды жап бұл үйде мен басшымын демейді,нағыз еркек ешқашан ауыз жап бұл үй мен басшы деме,neutral

0,нағыз еркек әйелін мұқият тыңдайды да мен сені түсіндім жаным бірақ шешімді өзім қабылдаймын деп жауап береді,нағыз еркек әйел мұқият тыңда да мен сен түс жан бірақ шеш өзім қабылда де жауап бер,neutral

0,сізді жоғалтудан қорықпаған адамды сізде жоғалтудан қорықпаңыз,сіз жоғалту қорықпа адам сіз жоғалту қорықпа,neutral

0,ешқашан өткенге оралма ол сенің қымбат уақытыңды өлтіреді оқиғалар қайталанбайды адамдар өзгермейді ешқашан ешкімді күтпе бір орында тұрма тек алға қарай жүр артқа қарама сені керек ететін адамдар өздері қуып жетіп алады,ешқашан өт оралма ол сенің қымбат уақыт өлтір оқиға қайталанба адам өзгерме ешқашан ешкі күтпе бір орында тұрма тек алға қарай жүр арт қарама сен керек ет адам өз қу жет ал,neutral

0,есіңде болсын сен жасамасаң басқа біреу жасайды сен оған жазбасаң басқа біреу жазады сен оның жанында болмасаң басқа біреу болады дәл қазір барлығын өз қолыңа алмасаң басқа біреу алады бұл өмірде сенен басқа үнемі біреу болады,ес бол сен жасама басқа біреу жаса сен оған жазба басқа біреу жаз сен оның жан болма басқа біреу бол дәл қазір барлығ өз қол алма басқа біреу ал бұл өмір сенен басқа үнемі біреу бол,neutral

0,өмірге ренжіп жатып осы өмірден ерте кеткен адамдарды ойлаңыздар атаанаңызға ренжіп жатып атаанасыз жетімдерді ойлаңыздар балаларыңызға ренжіп жатып алладан бала тілеп армандап жүрген адамдарды еске алыңыздар қолда барда алтынның қадірі жоқ барыңа шүкір ет,өмір ренж жат осы өмір ерте кет адам ойла атаана ренж жат атаан жетім ойла бала ренж жат алла бала тіле арманда жүр адам ес ал қолда бар алтын қадір жоқ бар шүкі ет,neutral

0,тату болса ағайын жақын,тату бол ағайын жақын,neutral

0,ақылшы болса апайың жақын,ақылш бол апай жақын,neutral

0,бауырмал болса інің жақын,бауырмал бол ін жақын,neutral

0,инабатты болса келінің жақын,инаба бол кел жақын,neutral

0,алтын ұяң отан қымбат,алтын ұя отан қымбат,neutral

0,асқар тауың әкең қымбат,асқар тау әке қымбат,neutral

0,бәрінен де ар мен ұят қымбат,бәрі де ар мен ұят қымбат,neutral

0,арадан шыққан жау қиын,ара шық жау қиын,neutral

0,іске аспаған серт қиын,іс аспа серт қиын,neutral

0,өзің түскен өрт қиын,өзің түс өрт қиын,neutral

0,естен шығармаңыз әр нәрсенің өз уақыты бар не жағдай қай кезде болуы керегі белгіленген сол себепті тағдырға сеніңіз бәрі өз уақытымен болады,ес шығарма әр нәрсе өз уақыт бар не жағдай қай кезде бол керег белгілен сол себеп тағдыр сен бәрі өз уақыт бол,neutral

0,жүрегім әрқашан жақсылыққа сенеді және армандары орындалуын күтеді,жүрег әрқашан жақсылық сен және арман орындал күт,neutral

0,адаммен бес минуттық жанжалда бес жылдық достықтан да көбірек нәрсе біліп аласың,адам бес минуттық жанжал бес жылдық достық да көбірек нәрсе біл ал,neutral

0,отбасы құру үй құрылысына ұқсайды,отбасы құру үй құрылыс ұқса,neutral

0,бастадың ба соңына дейін жеткіз,баста ба соң дейін жеткіз,neutral

0,іргетасы мықты етіп қаланған отбасы ешқандай дауылға құламайды,іргетас мықты ет қалан отбасы ешқандай дауыл құлама,neutral

0,ал уақытша құрылған күркелерді ең жеңіл самал да жайпап кетеді,ал уақытша құрыл күрке ең жеңіл самал да жайпа кет,neutral

0,тұрмысқа ерте шықса екіқабат болып қалыпты дейміз,тұрмыс ерте шық екіқабат бол қалыпты де,neutral

0,тұрмысқа шықпаса ешкімге керек болмаған ғой дейміз,тұрмыс шықпа ешкім керек болма ғой де,neutral

0,жаман жігітке тап болса өзіне де сол керек дейміз,жаман жігіт тап бол өзіне де сол керек де,neutral

0,жүкті болса жезөкше дейміз жүкті бола алмай жүрсе ауру дейміз,жүкті бол жезөкше де жүкті бола алма жүр ауру де,neutral

0,бір бала туса аз дейміз,бір бала ту аз де,neutral

0,көп бала туса топырлатып туып жатыр дейміз,көп бала ту топырлат ту жатыр де,neutral

0,жұмыс жасаса жұмыскер болып қалыпты өзінше дейміз,жұмыс жаса жұмыскер бол қалыпты өзінше де,neutral

0,жұмыс жасамаса еріншек жалқау екен дейміз,жұмыс жасама еріншек жалқау екен де,neutral

0,білім жинап көп оқыса профессор дейміз,білім жина көп оқы профессор де,neutral

0,ал егер оқымаса ақымақ дейміз,ал егер оқыма ақы де,neutral

0,қиын сәтте де шындықты айта білу,қиын сәт де шындық ай білу,neutral

0,бүгінгі күн сәтті күн бола ма есте қалар ештеңесі жоқ мағынасыз күн бола ма маңызды емес маңыздысы бұл күннің сенің өміріңде бірақ рет болатыны ешқашан қайтып келмейтіні,бүгінгі күн сәтті күн бола ма ес қал ештеңе жоқ мағына күн бола ма маңызды емес маңызды бұл күн сенің өмір бірақ рет бол ешқашан қайт келме,neutral

0,өзін жеңе алған адам ешкімнен жеңілмейді өзін түсінген адам басқаларды да түсіне алады,өз же ал адам ешкі жеңілме өз түсін адам басқа да түс ал,neutral

0,бақытсыздық деп кім айтты саған,бақытсыз де кім ай саған,neutral

0,егер арманыңдағы жолда әр үрген итке тас ата беретін болсаң мақсатыңа жетпейсің,егер арманың жолда әр үр ит тас ата бер бол мақсат жетпе,neutral

0,түсінгенім ешкімді қинап өзіңді жақсы көргізе алмайсың жақсылығыңды аямақалғанын өздері біледі,түсін ешкі қина өз жақсы көргіз алма жақсылығ аямақал өз біл,neutral

0,түсінгенім сенімге кіру үшін жылдар керексенімнен шығу бірақ сәт,түсін сенім кіру үшін жыл керексен шығу бірақ сәт,neutral

0,түсінгенім кіммен қоштассаң да жылы шырай таныт өйткені онымен соңғы рет кездесіп тұруың мүмкін,түсін кім қоштас да жыл шыра таныт өйткені онымен соңғы рет кездес тұр мүмкін,neutral

0,түсінгенім кейбір адамдар қатты жақсы көрсе де сезімдерін қалай білдіру керектігін білмейді,түсін кейбір адам қатты жақсы көрсе де сезім қалай білдіру керектіг білме,neutral

0,түсінгенім екі адам бір нәрсеге қарап екі түрлі нәрсе көруі мүмкін,түсін екі адам бір нәрсе қара екі түрлі нәрсе көр мүмкін,neutral

0,түсінгенім ең жақын достарың да ренжітуі мүмкін оларды кешіре білу керек,түсін ең жақын дос да ренжіт мүмкін ол кешір білу керек,neutral

0,түсінгенім кейде біреуді кешіре білу жеткіліксіз өзіңді де кешіре білу керек,түсін кейде біреу кешір білу жеткіліксіз өз де кешір білу керек,neutral

0,түсінгенім жүрегің қанша жылап сыздаса да дүние сен үшін тоқтап қалмайды,түсін жүрег қанша жыла сызда да дүние сен үшін тоқта қалма,neutral

0,кейде сенің артыңнан кім ілесетінін көру үшін алға шығуың керек кейде сені кім тыңдайтынын байқау үшін дауысыңды бәсеңдетіп сөйлеуің керек кейде бәрі

астанкестен болып жатқанда сенімен кімнің қалатынын білу үшін қате шешім қабылдауың керек пауло коэльо,кейде сенің арт кім ілес көру үшін алға шығ керек кейде сен кім тыңда байқау үшін дауыс бәсеңдет сөйле керек кейде бәрі астанкес бол жатқанда сенімен кім қал білу үшін қате шешім қабылда керек пауло коэльо,neutral

0,бақытыңызды тапсаңыз сүйіңіз сүйіңіз сүйіңіз және қорғаңыз,бақыт тап сүй сүй сүй және қорға,neutral

0,мүгедектер арбасын емханаға сыйға тартыңыз науқас оны қолданған кезде сіз сауап аласыз ин ша алла,мүгедек арба емхана сый тар науқас оны қолдан кезде сіз сауап ал ин ша алл,neutral

0,қоғамдық орында пайдалы зат қой,қоғамдық орында пайдалы зат қой,neutral

0,ағаш ек сол ағаштың көлеңкесіне адам немесе жануар келіп отырса сіз сауап аласыз ин ша алла,ағаш ек сол ағаш көлеңке адам немесе жануар кел отыр сіз сауап ал ин ша алл,neutral

0,осы мәліметпен басқаларымен бөліс егер де кем дегенде бір адам осы мәліметті қолданса сен сауап аласың ин ша алла,осы мәлімет басқа бөліс егер де кем де бір адам осы мәлімет қолдан сен сауап ал ин ша алл,neutral

0,кемшіліксіз адамды іздеме бәрібір таба алмайсың,кемшілік адам іздеме бәрібір таб алма,neutral

0,өзіңе сүйікті жақын жанды тап сүйікті жандардың кемшілігі байқалмайды,өз сүйікті жақын жанды тап сүйікті жан кемшіліг байқалма,neutral

0,егер сіз он жылға жас көрінгіңіз келсе адамдар сізге назар аударып комплимент айтқаның қаласаңыз осы мақаламды соңына дейін оқыңыз,егер сіз он жыл жас көрін кел адам сізге назар аудар комплимент айт қала осы мақала соң дейін оқ,neutral

0,расында бұл сізге он жылға жас көрінуге көмектеседі егер сіз әбiржiп түріңіз шаршаған болып жүргенді қаласаңыз бұл мақаланы оқымайақ қойыңыз,рас бұл сізге он жыл жас көрін көмектес егер сіз әбiрж түр шарша бол жүр қала бұл мақала оқымайақ қой,neutral

0,содан подругам айтқан құралды қолдандым қыздар сіздер ғажайыпқа сенесіздер ме,содан подруга айт құра қолда қыз сіздер ғажайып сен ме,neutral

0,көңіліңіз ең үлкен жамандықты да кешірсін,көңіл ең үлкен жамандық да кешір,neutral

0,ең бастысы жүрегіңізден ешқашан иман кетпесін,ең басты жүрег ешқашан иман кетпе,neutral

0,артроз бен буындардың ауыруын емдейтін халықтық медицина,артроз бен буын ауыр емде халықтық медицина,neutral

0,уақытпен дәлелденген нәтижелі рецепт бар болғаны,уақыт дәлелден нәтижел рецепт бар бол,neutral

0,бірақ біреудің алдында өзіңді кеммін деп ойлама,бірақ біреу алд өз кем де ойлама,neutral

0,біліңдер жеңіс сабырдан кейін келеді жеңілдік қиындықтан кейін келеді хадис,біл жеңіс сабыр кейін кел жеңілдік қиындық кейін кел хадис,neutral

0,ақиқат сізді доссыз қалдырмайды ол сізге кімнің дос екендігін көрсетеді мухаммад окар,ақиқат сіз дос қалдырма ол сізге кім дос екендіг көрсет мухаммад ок,neutral

0,мен тек жаныма жақын адамдармен араласамын,мен тек жан жақын адам аралас,neutral

0,маған олардың түрлері ақшалары мінезі маңызды емес,маған олардың түр ақша мінез маңызды емес,neutral

0,тіпті байлық пен әдемі сөздерінде де емес,тіпті байлық пен әдемі сөз де емес,neutral

0,оның артықшылығы отбасының алдындағы жауапкершілігін сезіне алуында,оның артықшылығ отбас алд жауапкершіліг сез алу,neutral

0,қазақстанның бас инфекционисі сізді және жақындарыңызды қалай құтқару керектігімен бөліседі,қазақстан бас инфекциони сіз және жақын қалай құтқар керектіг бөліс,neutral

0,алма ағашын шайқаудың қажеті жоқ ол піскеннен кейін өзіақ түседі өмірде бәрі осы секілді өз орнымен өз уақытымен болады,алма ағаш шайқа қажет жоқ ол піс кейін өзіақ түс өмір бәрі осы секілді өз орн өз уақыт бол,neutral

0,патша болып мінсең де алтыннан тақ,патша бол мін де алтын тақ,neutral

0,мақсат қойдың басоңына дейін бар құла сүрін бірақ тоқтама,мақсат қой басоң дейін бар құла сүрін бірақ тоқтама,neutral

0,естелік деген ыстық болады бірі жаныңды жылытса бірі күлпаршасын шығарады харуки мураками,естелік де ыстық бол бірі жан жылыт бірі күлпарша шығар харуки мураками,neutral

0,өкінішпен жүру үшін мына өмір тым қысқа сондықтан жақсы көріңіз кешіре біліңіз және сеніңіз бұл өмірде барлығы да кездейсоқ болмайды,өкініш жүру үшін мына өмір тым қыс сондықтан жақсы көр кешір біл және сен бұл өмір барлығ да кездейсоқ болма,neutral

0,бақытты адам болғың келсе өткенді қопара берме ошо,бақытты адам бол кел өт қопар берме ошо,neutral

0,не іздеп жүрсіз бақыт махаббат жан тыныштығы оны әлемнің басқа түкпірінен іздеп әуре болмаңыз бәрібір таба алмайсыз өзіңізден іздеңіз жүрек түкпірінен іздеңіз далай

лама,не ізде жүр бақыт махаббат жан тыныштығ оны әлем басқа түкпір ізде әуре болма бәрібір таб алма өз ізде жүрек түкпір ізде дала лама,neutral

0,жаны мұхиттай терең адамдар болады жанына сүңгігің келетін жаны шалшық су секілділері болады былғанып қалмау үшін айналып өтетін,жан мұхитта терең адам бол жан сүңгі кел жан шалшық су секілді бол былған қалма үшін айнал өт,neutral

0,өмір заңы күшіңіз жетпейтін дүниені өзгеруге тырыспаңыз жағдайды дәл сол қалпында қабылдаңыз біз ауарайын өзгерте алмаймыз жәй ғана ауарайына қарай киімді ауыстырамыз,өмір заң күш жетпе дүние өзгер тырыспа жағдай дәл сол қалп қабылда біз ауар өзгер алма жәй ғана ауарай қарай ки ауыстыр,neutral

0,жалған дос теңгең таусылғанша шын дос демің таусылғанша,жалған дос теңге таусылғанш шын дос дем таусылғанш,neutral

0,ақылды адамдар алдымен жылайды сосын күледі ақымақтар болса алдымен қарқылдап күліп соңында басын тасқа ұрып жылайды ей адам парасатты болып істің соңын әуел баста көр жаза күні өтініш отына жанба хазріеті мәулана,ақылды адам алд жыла сосын күл ақымақ бол алд қарқылда күл соң басын тас ұр жыла ей адам параса бол іс соң әуел баста көр жаза күн өтініш от жанба хазріе мәулан,neutral

0,таңертең алғашқы болып есіңе түсіп ұйықтар алдында соңғы болып ойыңа оралатын адам сенің бақытыңның бастамасы немесе жүректегі дертіңнің себепшісі,таңертең алғашқы бол ес түс ұйық алд соңғы бол ой орал адам сенің бақыт баста немесе жүрек дерт себепші,neutral

0,әр адам саған бақыт әкеледі біреу келуімен біреу кетуімен,әр адам саған бақыт әкел біреу кел біреу кет,neutral

0,жүрек кезкелгенді таңдамайды ол жақын жанды сезеді,жүрек кезкел таңдама ол жақын жанды сез,neutral

0,сұлулығыңмен мақтанба өйткені ешкім оны сақтап қала алмайды бұтақтар жасыл болып қала бермейді гүлдер бақшаларда мәңгілік гүлдей бермейді,сұлулығ мақтанб өйткені ешкім оны сақта қала алма бұтақ жасыл бол қала берме гүл бақша мәңгілік гүлде берме,neutral

0,бақытты адам өткенге өкінбейтін болашағынан қорықпайтын және біреудің өміріне араласпайтын адам,бақытты адам өт өкінбе болашағ қорықпа және біреу өмір араласпа адам,neutral

0,адамгершілігі барлар басқаларға тірек болады осыны өздері де қалайды басқаларға жетістікке жетуге көмектеседі өзі де сол жетістікке жетуді қалайды,адамгершілiг бар басқа тірек бол осы өз де қала басқа жетістік жет көмектес өзі де сол жетістік жету қала,neutral

0,әжімдер мен көз асты күлдіреуі көз асты қабы жоғалады бетіңіз алғашқы қалпына қайтып келеді тапсырыс беру сілтемесі https vk cc mhvfi,әжім мен көз асты күлдіре көз

асты қаб жоғал бет алғашқы қалп қайт кел тапсырыс беру сілте https vk cc mhvfi,neutral

0,уақыт өтеді бірақ айтылған сөз жүректе қалады лев толстой,уақыт өт бірақ айтыл сөз жүрек қал лев толстой,neutral

0,гүл әдемі бірақ оның да тікені бар адамдар да сол,гүл әдемі бірақ оның да ті бар адам да сол,neutral

0,неге сенсең сол сенің әлемін болады тек жақсылықты ойла,неге сен сол сенің әл бол тек жақсылық ойла,neutral

0,күле біліңіз сіздің шаруаларыңыз ешкімге маңызды емес джим керри,күл біл сіздің шаруа ешкім маңызды емес джи керри,neutral

0,бөтенге шексіз сеніп қалудан сақтан дос деп құрмет тұтып жүрген адамның өзі сені сатып кетуі мүмкін омар хайям,бөтен шексіз сен қалу сақтан дос де құрмет тұт жүр адам өзі сен сат кет мүмкін омар хайя,neutral

0,адам бойындағы бағалауға лайық қасиет ақыл емес қайта сол ақылды басқаратын қасиеттер мінез жүрек мейірімділік таза ой м достоевский,адам бой бағала лайық қасиет ақыл емес қайта сол ақылды басқар қасиет мінез жүрек мейірімділік таза ой м достоевски,neutral

0,дос табу оңай ма депті қарт,дос табу оңай ма де қарт,neutral

0,ата дос табу ақылдыға оңай ақымаққа қиын депті жігіт,ата дос табу ақылды оңай ақымақ қиын де жігіт,neutral

0,жоқ балам өмірде дос табу ақылдыға қиын ақымаққа оңай ақылдының досы аз қасы көп ақымақтың досы көп болғанмен жақсылыры жоқ жақсылығы жоқ достың қастығы аяқ астында жатады ақылды өзіне тең дос іздейді ақымақ өзіне тең досты іздемейақ табады,жоқ бала өмір дос табу ақылды қиын ақымақ оңай ақылды дос аз қас көп ақымақ дос көп бол жақсылы жоқ жақсылығ жоқ дос қастығ аяқ аст жат ақылды өзіне тең дос ізде ақы өзіне тең дос іздемейақ таб,neutral

0,есіңде болсын досың ақылды болса өзің дана боласың досың бала болса өзің шала боласың өмірде ақылды дос табу қиын сүйікті жар табу қиын жақсының көнілін табу қиын депті қарт,ес бол дос ақылды бол өзің дана бол дос бала бол өзің шал бол өмір ақылды дос табу қиын сүйікті жар табу қиын жақ көніл табу қиын де қарт,neutral

0,тапрысты парақшада көрсетілген ссылканы басып бере аласыз,тапр парақша көрсет ссылка бас бер ал,neutral

0,енді қалай арықтаймын деп қиналмайсың лайк басып,енді қалай арықта де қиналма лайк бас,neutral

0,black latte кофесін үлкен скидкамен сатып ал,black latte кофе үлкен скидка сат ал,neutral

0,ол үшін бар болғаны көк сілтемеге өтіп заказ қалдырыңыз,ол үшін бар бол көк сілтеме өт заказ қалдыр,neutral

0,аптада кг салмақтан құтылу,апта кг салмақ құтыл,neutral

0,денеңдегі майдан арылып артық күшке ие бол,денең майдан арыл артық күш ие бол,neutral

0,сапасы өте жоғары https vk cc zammx,сапа өте жоғары https vk cc zammx,neutral

0,кілем қағудағы мақсат кілемді сабау емес,кілем қағу мақсат кілем саба емес,neutral

0,шаңын қағып шығару алланың адамды сынаудағы мақсаты оны қинау емес жаман қылықтарын қағып шығару мәулана руми,шаң қағ шығару алла адам сынау мақсаты оны қина емес жаман қылық қағ шығару мәулан руми,neutral

0,толық ақпарат https vk cc estcw,толық ақпарат https vk cc estcw,neutral

0,мен адамдарды ренжітпей көңілін табуға тырысамын бірақ кейбір жандар соны түсінбей өзімді ренжітіп жатады өкінішті,мен адам ренжітпе көңіл таб тырыс бірақ кейбір жан соны түсінбе өз ренжіт жат өкініш,neutral

0,ақыл айту оңай бір топ би мөңке биге сәлем бере келіп алыс не жақын не қымбат не арзан не оңай не қиын не деп сұрапты сонда мөңке би алыс ақыл жақын ашу қымбат сауап арзан күнә оңай ақыл айту қиын соны орындау деген екен,ақыл айту оңай бір топ би мөң би сәлем бер кел алыс не жақын не қымбат не арзан не оңай не қиын не де сұра сонда мөң би алыс ақыл жақын ашу қымбат сауап арзан күнә оңай ақыл айту қиын соны орындау де екен,neutral

0,бұның бәрі түк емес ал өзімнің,бұның бәрі түк емес ал өзімнің,neutral

0,сыздатып тұр жүректі ар қорғасын,сыздат тұр жүрек ар қорғасын,neutral

0,қателесу керек те шығар мүмкін,қателес керек те шығар мүмкін,neutral

0,сен мені ұмытсаң да бір күндері,сен мен ұмыт да бір күн,neutral

0,сені іздеп ел астым бабел астым ба,сен ізде ел ас бабел ас ба,neutral

0,сені іздеп шөл астым бакөл астым ба,сен ізде шөл ас бакөл ас ба,neutral

0,таба алмай мен өзіңді өліп кетсем,таб алма мен өз өл кет,neutral

0,егер жоғарыдағы тізімдегі проблемалар сізде болса онда міндетті түрде оқыңыз https vk cc,егер жоғары тізім проблема сіз бол онда міндетті түр оқ https vk cc,neutral

0,егер бала үкіленген үмітті сезіп өссе ол төзімді болуға үйренеді,егер бала үкілен үміт сез өс ол төз бол үйрен,neutral

0,егер бала мақтаумен өссе сенімді болуды үйренеді,егер бала мақта өс сенімді болу үйрен,neutral

0,егер бала адалдықпен мәпеленсе әділетті болып үйренеді,егер бала адалдық мәпелен әділет бол үйрен,neutral

0,егер бала қауіпсіз екенін терең түйсініп өссе сенуге үйренеді,егер бала қауіпсіз екен терең түйсін өс сен үйрен,neutral

0,егер бала мақұлдаумен өссе өзін сыйлауды үйренеді,егер бала мақұлда өс өз сыйлау үйрен,neutral

0,егер бала махаббатпен мәпеленсе сүюді және сүйіспеншілігін сыйлауды үйренеді,егер бала махаббат мәпелен сүю және сүйіспеншіліг сыйлау үйрен,neutral

0,егер бала таңдау еркіндігін біліп өссе өз шешімдері үшін жауапкершілікті мойнымен көтеруді үйренеді,егер бала таңдау еркіндіг біл өс өз шешім үшін жауапкершілік мойны көтеру үйрен,neutral

0,егер бала тек сын естіп өссе өзгені айыптауды үйренеді,егер бала тек сын ест өс өз айыптау үйрен,neutral

0,егер бала әжуаның астында өссе күдікшіл болуды үйренеді,егер бала әжуа аст өс күдікшіл болу үйрен,neutral

0,егер бала өшпенділік ұясында өссе дұшпан көруді үйренеді,егер бала өшпенділік ұя өс дұшпан көру үйрен,neutral

0,егер бала өштікті көріп өссе өзгенің жанына жара салуды үйренеді,егер бала өштік көр өс өз жан жара салу үйрен,neutral

0,егер бала түсінбестікпен ауыздықталса өзгені тыңдамауды үйренеді,егер бала түсінбестік ауыздықтал өз тыңдамау үйрен,neutral

0,егер бала өтіріктің ортасында өссе алдауды үйренеді,егер бала өтірік орта өс алдау үйрен,neutral

0,егер бала абыройсыздықты көріп өссе өзін кінәлі сезінуді үйренеді,егер бала абыройсыздық көр өс өз кінәлі сезіну үйрен,neutral

0,балаңызды қалай өсіріп жатырсыз таңдау қолыңызда,бала қалай өсір жатыр таңдау қол,neutral

0,өзіңе бір қарап қойшы әлі жассың қорқасың неге бұлай қорқасың әрекетсіздікті доғар үнсіздіктің шегі жетті өзгелердің сен туралы не айтатыны толғандырмасын кигің келген киімді ки айтқың келген сөзді айт тыңдағың келген музыканы тыңда дауысыңды барынша көтеріп билеп те ал ертең жұмыс немесе сабақ демей түнделетіп серуенге шығып тұр қазіргі кезде өмір сүр тәуекелге бар құпияңмен бөліс бұл өмір сенікі және ол ешкімдікі емес оливер сайкс,өз бір қара қойш әлі жас қор неге бұлай қор әрекетсіздік доғар үнсіздік шег же өзге сен туралы не айт толғандырма ки кел ки ки айт кел сөз айт тыңда кел музыка тыңда дауыс барынша көтер биле те ал ертең

жұмыс немесе сабақ деме түнделет серуен шығ тұр қазіргі кезде өмір сүр тәуекел бар құпия бөліс бұл өмір сенік және ол ешкімдік емес олив сайкс,neutral

0,тұздың дәмін татқан балдың қадірің біледі,тұз дә тат бал қадір біл,neutral

0,уақытша ғой қыс та жаз да көктем де,уақытша ғой қыс та жаз да көктем де,neutral

0,уақытша ғой жауган жаңбыр көктен де,уақытша ғой жауган жаңбыр көк де,neutral

0,уақытша ғой ойын сауық күлкің де,уақытша ғой ойын сауық күлкі де,neutral

0,уақытша ғой жиған байлық мүлкің де,уақытша ғой жи байлық мүлкі де,neutral

0,уақытша ғой атақ даңқ мақтау да,уақытша ғой атақ даңқ мақтау да,neutral

0,уақытша ғой айтыс талас көп дау да,уақытша ғой айтыс талас көп дау да,neutral

0,уақытша ғой тоқтық пенен аштық та,уақытша ғой тоқ пе аштық та,neutral

0,уақытша ғой дұшпан еткен қастық та,уақытша ғой дұшпан ет қас та,neutral

0,мәңгілік қой асыл жарың шын сүйген,мәңгілік қой асыл жар шын сүй,neutral

0,http vk com club мына топка тыркел керемет топ жанадан ашылган,http vk com club мына топк тыркел керемет топ жана ашылган,neutral

0,бір күні бір әйел машина айдап бара жатса жол полицияисы сқырса тоқтамай кетіп қалады оны полицияиа артынан қолап устап алады сіз не ушін сқырсам тоқтамадыңыз деп сурайды сүйтсе әйел жоқ менің күйеуім бар депті d,бір күн бір әйел машина айда бар жат жол полицияи сқыр тоқтама кет қал оны полицияи арт қола уста ал сіз не ушін сқыр тоқтама де сура сүйт әйел жоқ менің күйе бар де d,neutral

0,жасар қыз бен анасының әңгімесі анасы қызым сен бізге қонақсың қыз деген атаанасына қонақ кейін бой жетіп тұрмыс құрып атаенеңнің үйіне кетесің түсіндің бе кызы ураааа анасы не болды кететініңе қуанып жатырсың ба қызы мамау қонақтарға қоятын кәмпиттерді жей берейінші,жасар қыз бен ана әңгіме ана қыз сен бізге қонақ қыз де атаана қонақ кейін бой жет тұрмыс құр атаене үй кет түс бе кыз урааа ана не бол кет қуан жатыр ба қыз мама қонақ қоятын кәмпит же берейінш,neutral

0,сабақ оқығым келмейді бірақ ке шыққым келеді,сабақ оқы келме бірақ ке шық кел,neutral

0,бір күні бір ата аялдамада автобуз күтіп отырса бір қара негір тур екен ата былай қарап алай қарап бір кезде ей балам тісіңді жуа бергенше бетіңді де жумайсынба депты,бір күн бір ата аялдама автобуз күт отыр бір қара негі тур екен ата былай қара ала қара бір кезде ей бала тіс жуа бергенш бет де жумайсынб де,neutral

0,екі жігіт әңгімелесіп отыр егер осы маңда полиция қызметкері жүрсе онда қауіпсіз жерде жүрмін деп ойла ешқандай қорқудың қажеті жоқ неге өйткені олар өте қауіпті жерде жүре қоймайды,екі жігіт әңгімелес отыр егер осы маң полиция қызметк жүр

онда қауіпсіз же жүр де ойла ешқандай қорқ қажет жоқ неге өйткені олар өте қауіпті же жүр қойма,neutral

0,бір коян кошеде жылап келе жатыр екен өзі удай мас екен содан алдынан бір қасқыр шығып қоеке неге жылайсын десе қасеке сұрама мені біреу ұрып жібердідепті сонда қасқыр кім көрсетші маған жағын айырып тілін сурып алайын депді қоян макулдеп аюды көрсетіпміне осы мені ұрып жіберген десе қасқыр қоян көтіңді қысып жүрмейсің ба деп кетіп қалыпты,бір коян коше жыла келе жатыр екен өзі уда мас екен содан алд бір қасқыр шығ қое неге жылай де қасе сұрама мен біреу ұр жібердіде сонда қасқыр кім көрсетш маған жағ айыр тіл сур ал деп қоян макулде аю көрсетіпм осы мен ұр жібер де қасқыр қоян көт қыс жүрме ба де кет қалыпты,neutral

0,бір куні күйеуі мен әйелі театрға барайын деп жатыр екен сонда әйелі күйеуіне нәскиіңді ауыстырдепті театрда отырса әйеліне сасық шуаштың иісі келіпті сонда әйелі мен саған нәскиіңді ауыстыр дегенім қайда десе күйеуі ауыстырдым ғой сенбесең міне ауыстырған нәскиімді ала келдімдеген екен,бір ку күйе мен әйел театр бар де жатыр екен сонда әйел күйе нәски ауыстырде театр отыр әйел сасық шуаш иіс кел сонда әйел мен саған нәски ауыстыр де қайда де күйе ауыс ғой сенбе міне ауыстыр нәски ал келдімде екен,neutral

0,бір тойда асаба былай деген екен құрметті той қонақтарыалдарыңызға ас қойдықекі қолды бос қойдық алдарыңыздағы асты кезінде атабабаларымыз немісті қалай қырса солай жапыра жеп отырыңыздар содан той бітеді бір кемпір қолында тырсыйған екі көк пакет шығып бара жатыр дейді асаба әжемынауыңыз не кемпір еқарағым бұлар пленге түскен немістер ғойдепті,бір той асаб былай де екен құрметті той қонақтарыал ас қойдықек қол бос қой алдарыңыз асты кез атабаба неміс қалай қыр солай жапыр же отыр содан той біт бір кемпі қол тырсый екі көк пакет шығ бар жатыр де асаб әжемына не кемпі еқара бұлар плен түс неміс ғойде,neutral

0,әссәләмуәлейкум бауырлар әпке қарындастар егер осы қызықты әзілдер парақшасын қарап тұрсаңыздар сіздерге бір өтініш осы суреттегі уведомлять новых записях осыны бассаңыздар деймін себебі осы группаға жаңадан пост салынса сіздерге барады тағы бір өтініш коментариға бастым деп жазыңыз бассаңыздар алла субхан уә тағала разы болсын,әссәләмуәлейку бауыр әп қарындас егер осы қызықты әзіл парақша қара тұр сіздерге бір өтініш осы сурет уведомлять новых записях осы бас де себебі осы группа жаңа пост салын сіздерге бар тағы бір өтініш коментари бас де жаз бас алл субхан уә тағал разы бол,neutral

0,бәке есігіңізді ашыңыз кім бұл милиционер үйде ешкім жоқ жарық жанып тұр ғой қазір өшіремін,бә есіг аш кім бұл милицион үй ешкім жоқ жарық жан тұр ғой қазір өшір,neutral

0,қыз бен жігіттің әнгімесі бір күні жігіт қызға келіп қарындас басыңыз бос па деп сұрапты сонда қыз оны неге сұрадыңыз десе бала жай басыңыз бос болса қолымды қойып тұра тұрайын депті,қыз бен жігіт әнгі бір күн жігіт қыз кел қарындас бас бос па де сұра сонда қыз оны неге сұра де бала жай бас бос бол қол қой тұр тұр де,neutral

0,мистер пропер қолыңда үйдің іші тап таза міне шелек міне су ырбаңдамай өзің жу,мис проп қол үй іш тап таза міне шелек міне су ырбаңдама өзің жу,neutral

0,жасар ұл мен қыз баланың әңгімесі қыз жылап менің құлағымды тесейін деп жатыр десе ұл сенікі ештеңе емес қой менікін кесейін деп жатыр депті,жасар ұл мен қыз бала әңгіме қыз жыла менің құлағ тес де жатыр де ұл сенік ештеңе емес қой менік кес де жатыр де,neutral

0,сен оқымадың ба түспе деймін еууу айттым ғой түспе деп тоқтат жасама бұны эх жарайды түсе берші сонда да қоймайсың ғой саған ештене айтқым келмейді жоқ жай ғана тыңдашы астыға түспеші өтінемін сонда да болмадың ғой егер түсе берсең ренжіп қаламын жарайды жалына бермеймін соңғы рет айтамын түспейақ қойшы қойшы сен шешіміңді қабылдадың ба шаршамадың ба соңғы мүмкіндік беремін түспеші әй жарайды қоймадың енді лайк бас сосын достарыңмен бөліс,сен оқыма ба түспе де еуу ай ғой түспе де тоқтат жасама бұны эх жарайды түс берш сонда да қойма ғой саған ештен айт келме жоқ жай ғана тыңдаш асты түспеш өт сонда да болма ғой егер түс бер ренж қал жарайды жалын берме соңғы рет ай түспейақ қойш қойш сен шешім қабылда ба шаршама ба соңғы мүмкіндік бер түспеш әй жарайды қойма енді лайк бас сосын дос бөліс,neutral

0,қош келдіңіздер достарыназды шақырыңыз күнде кіріп тұрыңыз лайк репост жасаңыздар баскаларды құрметтейміз сізgерgен йк сұр yg ул қпыз бір қ йк су ұл қолg ең екmі ғал ж ңа иgея mуgыру қозғ ушы күш белсенgілік ізgеніске итермелеу құрмеm және gе ізге gеген хмеmіңіз,қош кел достарыныз шақыр күн кір тұр лайк репост жаса баска құрметте сізgерgен йк сұр yg ул қпыз бір қ йк су ұл қолg ең екm ғал ж ңа иgея mуgыр қозғ уш күш белсенgілік ізgеніс итермеле құрмеm және gе із gе хмеm,neutral

0,бірде бір неміс автоматын кезеніп атам десе қожанасыр балам деп құшақтап алыпты,бірде бір неміс автомат кезен ата де қожанасы бала де құшақта ал,neutral

0,конкрус шарт осы топка кунуне дос шыкырып кымде кым кунде дос жинаса кым коп жинаса соган ед немесе кивиге саламыз далел скринмен фото астына саласындар бары баклануда конкрус айакталуы акпан админ,конкрус шарт осы топк кунун дос шыкыр кы кы кун дос жина кы коп жина соган ед немесе киви сал далел скрин фото аст саласын бар баклан конкрус айактал акпан админ,neutral

0,тіркелейік достар http vk com azil alemi kazakh керемет обсуждениялар баркеремет фотолар бар тіркелсеңіз сіздің сұрақтарыңызға жауап береміз біздің жазылушылар дегенге фотоңызды саламыз,тіркел дос http vk com azil alemi kazakh керемет обсуждения баркеремет фото бар тіркел сіздің сұрақ жауап бер біздің жазылушы де фото сал,neutral

0,мен бірақ нәрсеге көз жұма қараймын ол шампунь,мен бірақ нәрсе көз жұма қара ол шампунь,neutral

0,жаным бүгін қалада маймылдарды қамауға алып жатыр екен сен бүгін көшеге шықпайақ қойшы мен сен үшін қорқамын,жан бүгін қала маймыл қама алып жатыр екен сен бүгін көше шықпайақ қойш мен сен үшін қор,neutral

0,қоян әлгі дүкенге тағы барыпты мырза жүз нан бар ма жоқ екінші күні тағы келіпті мырза жүз нан бар ма жоқ деп ашуланыпты дүкенші бірақ ақ қоянның мінезін білетін дүкенші ертесіне үлгертіп жүз нан пісіріп қойыпты айтқандай ақ үшінші күні қоян тағы келіпті мырза жүз нан бар ма бар сонда қоян охо маған біреу беріңізші деген екен мәз болып,қоян әлгі дүкен тағы бар мырза жүз нан бар ма жоқ екінші күн тағы кел мырза жүз нан бар ма жоқ де ашулан дүкенш бірақ ақ қоян мінез біл дүкенш ерте үлгерт жүз нан пісір қой айтқанда ақ үшінші күн қоян тағы кел мырза жүз нан бар ма бар сонда қоян охо маған біреу беріңізш де екен мәз бол,neutral

0,қаз деген қаз қорқақ емес қаз деген қазақстан деген,қаз де қаз қорқақ емес қаз де қазақстан де,neutral

0,бір жігіттің өмірі жолы болмайды екен көлікке мінсе аударылып қалады автобусқа мінсе бұзылып қалады дегендей бірде әлгі жігіт ұшаққа мініпті ұшып келе жатқанда борттан ахау табылып ұшақ құлай бастайды ұшқыштар жолаушылардың бәріне бірбір парашют пен барабан үйлестіре бастапты қазір біз теңізге құлап келеміз онда акула көп суға түскен соң құтқарушылар келгенше оларды барабанмен үркітесіздер депті сонда әлгі жігіт жыларман болып қазір маған не барабан жетпей қалады не құлағы естимейтин акула кездеседі депті,бір жігіт өмір жол болма екен көлік мін аударыл қал автобус мін бұзыл қал дегенде бірде әлгі жігіт ұшақ мін ұш келе жатқанда борт аха табыл ұшақ құла баста ұшқыш жолаушы бәрін бірбі парашют пен барабан үйлестір баста қазір біз теңіз құла кел онда акул көп су түс соң құтқарушы келгенше ол барабан үркі де сонда әлгі жігіт жыларман бол қазір маған не барабан жетпе қал не құлағ естимейтин акул кездес депті,neutral

0,адамның есімін қысқаша қылып айту әр елде әр түрлі екен мысалы орыстарда николай коля александр саня евгении женя михаиил миша ал қазақтарда саламат сәке самат сәке сәрсен сәке серік сәке сәбит сәке,адам ес қысқаша қыл айту әр ел әр түрлі екен мысал орыс никола коля александ саня евгении женя михаиил миш ал қазақ саламат сә самат сә сәрсен сә серік сә сәбит сә,neutral

0,бір күні бір кісінің жылқысы өлейін деп жатыр екен сөйтіп әйелі айтыпты арам өлмесін сойайық депті оны қой естіп қойып жылқыға айтыпты иеміз сені сояйын деп жатыр қисалаңдамай тік тұр депті қораға иесі кіріп келсе жылқысы сапсау тұр қуанып кетіп қойын сойып тастапты,бір күн бір кісі жыл өл де жатыр екен сөйт әйел айт ара өлме сой де оны қой ест қой жылқы айт ие сен соя де жатыр қисалаңдама тік тұр де қора иесі кір кел жыл сапса тұр қуан кет қой сой таста,neutral

0,тарих пәнінен емтихан қазақстандағы ең алғашқы ұшқыш әйел жалмауыз кемпір,тарих пән емтихан қазақстандағы ең алғашқы ұшқыш әйел жалмауыз кемпі,neutral

0,жаным ванна қалай екен күшті жан полотенца тур екен жане б балдардыкы қыздардыкы шығар деп мен сүрттім жоқ бетке арналған,жан ванн қалай екен күшті жан полотенц тур екен жан б балдардык қыздардык шығар де мен сүр жоқ бет арнал,neutral

0,неге мұңайып отырсың отыр айтамын иә айта бер былай ғой біз отырған орындықты жаңа ғана бояған екен,неге мұңай отыр отыр ай иә ай бер былай ғой біз отыр орындық жаңа ғана боя екен,neutral

0,балам сабақтарың қалай жақсы пап бүгін химиядан жарылғыш заттар жасап үйрендік ооо дұрыс ертең қандай сабақ болады сабақ болмайды енді неге мектеп жоқ,бала сабақ қалай жақсы па бүгін химия жарылғыш зат жаса үйрен ооо дұрыс ертең қандай сабақ бол сабақ болма енді неге мектеп жоқ,neutral

0,жылап отырмын тоқтамай жылап отырмын жыласам да бәріне шыдап отырмын ойлап қалма жалғызым сен үшін деп қолда пышақ пиязды турап отырмын,жыла отыр тоқтама жыла отыр жыла да бәрін шыда отыр ойла қалма жалғыз сен үшін де қолда пышақ пияз тура отыр,neutral

0,топқа тіркелген жанға аллам үйіп төгіп бақ береке байлық мол бақыт жайлы өмір өмір жасы ұзақ ата анасы ұзақ ғұмыр кешсін әумин,топ тіркел жан алла үй төг бақ береке байлық мол бақыт жайлы өмір өмір жас ұзақ ата ана ұзақ ғұмыр кеш әумин,neutral

0,дос шақырамыз дос шақырған және записке лайк басқан адамның фотосына лайк жинап береміз осындай сәтті пайдаланып қалыңдар шақру үшін суретегідей істеу керек пригласить друзей деп досымызды шакрамыз,дос шақыр дос шақыр жән запис лайк бас адам фото лайк жина бер осындай сәтті пайдалан қал шақр үшін суретегіде істеу керек пригласить друзе де дос шакр,neutral

0,нуртас барін кештім лайды кешті суды кештім бәрін кештім,нуртас бар кеш лай кеш су кеш бәрін кеш,neutral

0,құрметі оқырмандар біраз уақыт админі болмай парақшаға қарайтын адам болмады енді осыдан бастап жаңа админ келіп парақша толыққанды қарап постар салып отыруға уәде берді енді біздің парақшадан алыстамай күнде кіріп біздің тотптың керемет әзілдерді оқып тұрыңыздар құрметпен парақша әкімшілігі,құрмет оқырман біраз уақыт адми болма парақша қара адам болма енді осы баста жаңа админ кел парақша толыққанды қара пост сал отыр уәде бер енді біздің парақша алыстама күн кір біздің тотп керемет әзіл оқ тұр құрмет парақша әкімшілік,neutral

0,алло алло бұл кім кімді іздедің таныдың ба банктен бе едің иә банктен едім кредитті төлемеген жанды іздедім кешіріңіз кім едіңіз бір банктің тірегіміз әзір қызметкер кейін менеджер менеджерге тұтқаны беріңіз ойойой қарындас олай демеңіз сөйлейді солай неге қыз кредитті көп соза бермей осы айда барлығын төлеңіз кел соттасайық кел соттасайық кредитті солай жабайық жожожоқ қызметкер олай демеңіз қалай біз оңай көнеміз жұмыста жалақым өссежалақыға қарай көреміз кел соттасайық кел

соттасайық кредитті солай жабайық,алло алло бұл кім кім ізде таны ба банк бе ед иә банк ед кредит төлеме жанды ізде кешір кім ед бір банк тіре әзір қызметкер кейін менеджер менеджер тұт бер ойойо қарындас ола деме сөйле солай неге қыз кредит көп соз берме осы айда барлығ төле кел соттас кел соттас кредит солай жаб жожожоқ қызметкер ола деме қалай біз оңай көн жұмыс жалақы өссежалақы қарай көр кел соттас кел соттас кредит солай жаб,neutral

0,бір күні асан мен үсен деген екі жігіт болған ғой сөйтсе асан айтыпты ее үсен өтірік өлуден жарысасың ба депті асан давай депті асан қимылдамай күн бойы жатқан ғой күннен кейін асанды ағайындары көміп жатса асан атып турган ғой ее үсен кайда десе үсенді алдыкүні көміп тастағанбыз депті,бір күн асан мен үсен де екі жігіт бол ғой сөйт асан айт ее үсен өтірік өлу жарыс ба де асан дава де асан қимылдама күн бойы жат ғой күн кейін аса ағайын көм жат асан ат турган ғой ее үсен кай де үсен алдыкү көм таста де,neutral

0,бір жігіт қызға сүйетіндігін білдіріпті мен сені ләйлі мен жібектен бетер сүйем арамай менен де бұрын қыздарың болған екен ғой,бір жігіт қыз сүйетіндіг білдір мен сен ләйл мен жібек бетер сүйе арама менен де бұрын қыз бол екен ғой,neutral

0,ауруханада дәрігер болды жинал енді үйіңе қайта берсең болады атаң мен әжеңе сәлем айта бар науқас кешіріңіз менің атам мен әжем қайтыс болып кеткен енді мен саған неғыл деп тұрмын,аурухана дәрігер бол жинал енді үй қайта бер бол ата мен әже сәлем ай бар науқас кешір менің ата мен әже қайтыс бол кет енді мен саған неғыл де тұр,neutral

0,бұл адамдар өзі үшін ешқашан жауапкершілік алып кете алмайды,бұл адам өзі үшін ешқашан жауапкершілік алып кет алма,neutral

0,атыраулық бойжеткен индираның әлеуметтік желіге әуестігінің арты үлкен қасіретке душар еткізді,атыраулық бойжет индира әлеуметтік желі әуестіг ар үлкен қасірет душ ет,neutral

0,бүгінде ұялы байланыс пен айшылық жерден жылдам хабар алғызар әлеуметтік желі қоғамның әр саласына дендеп енгені анық,бүгін ұялы байланыс пен айшылық же жылдам хабар алғыз әлеуметтік желі қоғам әр сала денде ен анық,neutral

0,қазір әлеуметтік желісіз ешбір жұмыстың қиюы келіспейді,қазір әлеуметтік жел ешбір жұм қию келіспе,neutral

0,көрген бар ма,көр бар ма,neutral

0,аздан соң қабыңды алып кел,аз соң қаб алып кел,neutral

0,айтушы бар деп солай деймін бар,айтуш бар де солай де бар,neutral

0,аларсың сен,ал сен,neutral

0,алып кел айтушыңды,алып кел айтуш,neutral

0,атты алысқа айдамақсың ғой мені сорғалаған қан,ат алыс айда ғой мен сорғала қан,neutral

0,бес қара,бес қара,neutral

0,биең қазір қолымда жоқ қолымда,бие қазір қол жоқ қол,neutral

0,бір іліктесіме жетектетіп жібердім бүгін жетектетіп,бір іліктес жетектет жібер бүгін жетектет,neutral

0,бұл жауынның қашан бітерін қайдан білейін ол бітпесе,бұл жауын қашан біт қайдан біл ол бітпе,neutral

0,деседі осы шақта олар өз арасындағы басшы деседі,дес осы шақ олар өз ара басшы дес,neutral

0,жаның шықсын,жан шық,neutral

0,жол болды ма деп күбір етті оған тура жауап берудің жауап,жол бол ма де күбі ет оған тура жауап беру жауап,neutral

0,інің соққыдан өлер болса,ін соққы өл бол,neutral

0,кеше түнде біздің дөң жайлауда жатқан жылқыға ұры кіріп,кеше түн біздің дө жайлау жат жылқы ұры кір,neutral

0,кім айтты деп солай дейсің солай,кім ай де солай де солай,neutral

0,күздің ұзақ түні мынау көзір алған малмен соқтырып малмен,күз ұзақ түн мынау көзі ал мал соқтыр мал,neutral

0,қыс отарында,қыс от,neutral

0,малыңды ертең көлденеңнің алдына барып айтысқанда малыңды,мал ертең көлдене алд бар айтыс мал,neutral

0,мен күшіме сенген кісі емеспін сен күштісің бірақ мен емеспін,мен күш сен кісі емес сен күшті бірақ мен емес,neutral

0,мен тауып отырмын ол сенсің тауып,мен тау отыр ол сен тау,neutral

0,не қылмап ең ең,не қылма ең ең,neutral

0,немді алмадың жанымды да алғансың жар деген жалғыз жанымды,не алма жан да ал жар де жалғыз жан,neutral

0,неңді алып ем сонда есть,не алып ем сонда есть,neutral

0,ол қайда қайда,ол қайда қайда,neutral

0,от қалай жақсы еді,от қалай жақсы ед,neutral

0,өзің сөйтіп айыпкер де,өзің сөйт айыпк де,neutral

0,санаттың ба ендеше он алты жасымнан отыз алты жасыма жасыма,сана ба ендеше он алты жас отыз алты жас жас,neutral

0,сарт етіп әз мойнына түскендей боп қалды бірақ жылқышы боп,сарт ет әз мойн түскенде боп қал бірақ жылқыш боп,neutral

0,сен маған қастық етіп отырсың мен не қылған адам маған,сен маған қас ет отыр мен не қыл адам маған,neutral

0,табақты әкел өз малымыздың еті осы сен жегенде,табақ әкел өз мал ет осы сен же,neutral

0,ұры демесем,ұры деме,neutral

0,шыным сол бар жауабым да сол дегенде қатуланған бар,шын сол бар жауаб да сол де қатулан бар,neutral

0,ағайынды екі жігіт қолшыл,ағайынды екі жігіт қолшыл,neutral

0,ағайынды екі жігіт пен қара шаруаға мығым қаражон ағайынды,ағайынды екі жігіт пен қара шаруа мығым қаражон ағайынды,neutral

0,адасып қалды тау іші,адас қал тау іш,neutral

0,аз күйбеңдеп,аз күйбеңде,neutral

0,айғай салып жіберді айғай,айғай сал жіб айғай,neutral

0,айғайлаған дауыстары тағы бірнеше жерден естілді аздан соң бірнеше,айғайла дауыс тағы бірнеше же ест аз соң бірнеше,neutral

0,айғаймен даңғаза ғып,айғай даңғаз ғы,neutral

0,айғайынан қатты шошынды жаз бойы елсіз иесізде тау ешкі,айғай қатты шош жаз бойы ел иесіз тау ешкі,neutral

0,айғырын қартайтып,айғыр қартайт,neutral

0,айдай уақыт өтті әрі өкпеден өткен тепкі соққы жарасы бір уақыт,айда уақыт өт әрі өкпе өткен тепк соққы жара бір уақыт,neutral

0,айттың ғой тіліңмен сөгіп,ай ғой тіл сөг,neutral

0,ала жаздай мойнына құрық тиіп көрмеген,ал жазда мойн құрық ти көрме,neutral

0,аламын осыдан қозыбақтың бәрінің де жылқысын баққам бар бар,ал осы қозыбақ бәрі де жылқы бақка бар бар,neutral

0,аларсың аларсың,ал ал,neutral

0,алатаудың қарлы басын мекендеген аспан тағысы бар емес пе бар,алатау қарл басын мекенде аспан тағы бар емес пе бар,neutral

0,алған iнiңдi әкеткен ажал мен деп пе ең санашы тағы қандай алған,ал iн әкет ажал мен де пе ең санаш тағы қандай ал,neutral

0,алған бақтығұл сәлменнен тiлiн тартпай,ал бақтығұл сәлмен тiл тартпа,neutral

0,алғаш ұстай алған жылқышы жылы қан қолына тигенде алған,алғаш ұста ал жылқыш жыл қан қол ти ал,neutral

0,алғашқы жылқышыдан құтылып,алғашқы жылқышы құтыл,neutral

0,алды үйге кiре бастады түстерi суық ендi аңғарса,алды үй кiр баста түс суық ендi аңғар,neutral

0,алды бұл шақ көктем кезi едi арада тағы бiр екi жұма өтер едi,алды бұл шақ көктем кез ед ара тағы бiр екi жұма өт ед,neutral

0,алдыңғы айғай шыққан беттен дырду шуды көбейтiп,алдыңғы айғай шық бет дырд шу көбейт,neutral

0,алмағың бар екен қозыбақтан,алма бар екен қозыбақ,neutral

0,алмай қайтпақпын ба қаңғырып жүрiп,алма қайт ба қаңғыр жүр,neutral

0,алпамсадай мол денелi,алпамсада мол денел,neutral

0,алып кетемiн ендi жаның барыңда алдыма түс,алып кет ендi жан бар алд түс,neutral

0,алып мекенiндей көрiнедi сыпсың қарағай жапқан түктi беттерi жапқан,алып мекенiнде көрiн сы қарағай жап түк бет жап,neutral

0,алып шыққан айдаушының бiрi сол ғой ғой,алып шық айдауш бiрi сол ғой ғой,neutral

0,алысты керуi қиын әр жайды болжап,ал кер қиын әр жай болжа,neutral

0,ананың қарасы ұзағанша,ана қара ұзағанш,neutral

0,анау арттағы үйдiң жүдеулiгi қандай телмiрiп қолына қарап арттағы,анау арт үй жүдеулiг қандай телмiр қол қара арт,neutral

0,анық жырындылығын iстеген жырындылығын,анық жырындылығ iсте жырындылығ,neutral

0,аңдығаны жылқышы бiрақ өзiр оның белгiсi бiлiнбейдi аңдығаны,аңды жылқыш бiрақ өзi оның бел бiлiнбе аңды,neutral

0,аппақ атқанша жүрдi ендiгi жеткен жерiнде қыстаулар бар бар,ап атқанш жүр ендiгi жет же қыстау бар бар,neutral

0,араларында белгi жоқ быжырық шатқалаң сырт көзге де қалың де,ара белгi жоқ быжырық шатқала сырт көз де қалың де,neutral

0,арқардай жайынсып кеткен мал тегiнде күндiзгi айғайды да тегiнде,арқарда жайынс кет мал тегiн күндiзгi айғай да тегiн,neutral

0,арсыздау күлкімен жымиып,арсызда күлкі жыми,neutral

0,артына бір айналып,арт бір айнал,neutral

0,артынан ұры сытылып жөнелгенде,арт ұры сытыл жөнел,neutral

0,арысым едің,арыс ед,neutral

0,ат беріп,ат бер,neutral

0,ат көрінбеді біркертпе тастың ернеуінде қарауыта қозғалып ата,ат көрінбе біркертп тас ернеу қарауы қозғал ата,neutral

0,ат ылдиға ұмтылған жамантайдың атын қағып,ат ылди ұмтыл жамантай ат қағ,neutral

0,аттан аттан деп,аттан аттан де,neutral

0,аударылып түсті аударылып,аударыл түсті аударыл,neutral

0,аулының желкесіндегі қиын тасқа боз биені торт аяғын тас торт,аул желке қиын тас боз бие торт аяғ тас торт,neutral

0,аулының жігіттері сырт ажар,аул жігіт сырт ажар,neutral

0,аулының сәлменнен үлкен ағасы сәт болыстыққа таласып,аул сәлмен үлкен аға сәт болыстық талас,neutral

0,ауылдың басты еркек ересек малайлары емес,ау басты еркек ересек малай емес,neutral

0,ауыр емес,ауыр емес,neutral

0,ашады із шалғанда,аш із шал,neutral

0,ашумен қатты тыртысып,аш қатты тыртыс,neutral

0,аяғында жүгірген бір жемін екпіндеп келіп соғып жығып,аяғ жүгір бір же екпінде кел соғ жығ,neutral

0,байқап отырыңдар кісі келсе,байқа отыр кісі кел,neutral

0,байлауы бұның бәрі өз бетіне оның үстіне мынау жадау күзде үстіне,байла бұның бәрі өз бет оның үст мынау жада күз үст,neutral

0,бақтығұл ең соңғы тосқан қауіп қатерден сыты лып шығып тосқан,бақтығұл ең соңғы тос қауіп қат сы лы шығ тос,neutral

0,бақтығұл іздейтінің осы дегендей,бақтығұл ізде осы дегенде,neutral

0,бақтығұл айқай шықпай тұрғанда тебіне жөнелмек болды бақтығұл,бақтығұл айқай шықпа тұр теб жөнел бол бақтығұл,neutral

0,бақтығұл алғашқы бетінен қайтқан жоқ бақтығұл,бақтығұл алғашқы бет қайт жоқ бақтығұл,neutral

0,бақтығұл бабын таппай,бақтығұл баб таппа,neutral

0,бақтығұл белбеуіне қыстырған қолшоқпарын босатып алып,бақтығұл белбеу қыстыр қолшоқп босат алып,neutral

0,бақтығұл бойын мең зең ғып,бақтығұл бой мең зең ғы,neutral

0,бақтығұл бұл жылқыны басқа қозыбақ аулынікі деп ойлап бақтығұл,бақтығұл бұл жылқы басқа қозы аулынік де ойла бақтығұл,neutral

0,бақтығұл бұл топқа өңшең қозыбақтың өрен жараны өңшең,бақтығұл бұл топ өңше қозыбақ өрен жара өңше,neutral

0,бақтығұл бұл тұста жалтармасқа амалы болмады айлакер айлакер,бақтығұл бұл тұс жалтармас амал болма айлак айлак,neutral

0,бақтығұл енді жалтармай шынға қарай ауысқысы келді қарай,бақтығұл енді жалтарма шын қарай ауыс кел қарай,neutral

0,бақтығұл енді қор етіп қалғып кетті кетті,бақтығұл енді қор ет қалғ кет кет,neutral

0,бақтығұл жаңағы қауіп мүйнеті енді еркіндікке ауысқанда,бақтығұл жаңағ қауіп мүйне енді еркіндік ауыс,neutral

0,бақтығұл жиырма бес жиырма алтыға келіп,бақтығұл жиырма бес жиырма алты кел,neutral

0,бақтығұл қайнаған әділ ашу үстінде,бақтығұл қайна әділ ашу үст,neutral

0,бақтығұл қараған жоқ әлі де үркіп,бақтығұл қара жоқ әлі де үрк,neutral

0,бақтығұл қарауытққан қалың топ жылқының бар қарасын да бар,бақтығұл қарауытк қалың топ жылқы бар қара да бар,neutral

0,бақтығұл қоралы қойға шапқалы келе жатқан қасқырдың шапқалы,бақтығұл қорал қой шап келе жат қасқыр шап,neutral

0,бақтығұл мен боз биеден ығысып оқшау кетіскен оған бақтығұл,бақтығұл мен боз бие ығыс оқшау кетіс оған бақтығұл,neutral

0,бақтығұл мен тектіғұл сәлменнің қолына барғалы бай малы маленький,бақтығұл мен тектіғұл сәлмен қол бар бай мал маленьки,neutral

0,бақтығұл үйден шыққанға екі күн болғандыктан,бақтығұл үй шық екі күн болғандык,neutral

0,бақтығұл үйіне қарай асыға берді аз жүріп,бақтығұл үй қарай асыға бер аз жүр,neutral

0,бақтығұл үндемей,бақтығұл үндеме,neutral

0,бақтығұлға амандаспады үй ішін айнала қарап,бақтығұл амандасп үй іш айнала қара,neutral

0,бақтығұлды және де әке бабадан боқтап,бақтығұ және де әке баба боқта,neutral

0,бақтығұлды ұйқы жеңді бірнеше күннен бері талған денесі аз бірнеше,бақтығұ ұйқы же бірнеше күн бері тал дене аз бірнеше,neutral

0,бақтығұлдың аузына бұл сөзді енді қайтып түсірмес үшін,бақтығұ ауз бұл сөз енді қайт түсірмес үшін,neutral

0,бақтығұлдың аулы жаңағы аттанған тұстан күндік жер таң ата күндік,бақтығұ аул жаңағ аттан тұс күндік жер таң ата күндік,neutral

0,бақтығұлдың бойына тың қайрат жиналды шаршаған,бақтығұ бой тың қайрат жина шарша,neutral

0,бақтығұлдың келгені үрпиіп,бақтығұ кел үрпи,neutral

0,бақтығұлдың көңлі талай күнгі қауіп,бақтығұ көңл тала күнгі қауіп,neutral

0,бақтығұлдың халі бір мүйнет ішінде ойламаған қатерге ілінді мүйнет,бақтығұ хал бір мүйнет іш ойлама қат іл мүйнет,neutral

0,балалар әкесі келіп,бала әке кел,neutral

0,бар қатшаның сасып отырған кескінін қу көзбен шолып қалып,бар қатша сас отыр кескін қу көз шол қалып,neutral

0,бас жағында жерде жатқан белдігінен өткір,бас жағ же жат белдіг өткір,neutral

0,басқа майлы еттерін жалымен қосып салып тұрып,басқа майлы ет жал қос сал тұр,neutral

0,басқа түгі жоқ,басқа түг жоқ,neutral

0,бастады бастады,баста баста,neutral

0,бастады анығында есе тиіп,баста аның есе ти,neutral

0,бастады елеусізденіп,баста елеусізден,neutral

0,бастады жайылып келе жатқан бос жылқы екен шетірек беттеп беттеп,баста жайыл келе жат бос жылқы екен шетірек бетте бетте,neutral

0,бетпе бет келіп,бетп бет кел,neutral

0,беттегі тобырды екінші бетке апарып,бет тоб екінші бет апар,neutral

0,бәрі тегіс дауыстады да,бәрі тегіс дауыст да,neutral

0,бие көпке шейін тоқтамай,би көп шейін тоқтама,neutral

0,биелік сөзің бар екен жеңіп ал да бәрін ал қазір биемді бар,биелік сөз бар екен жең ал да бәрін ал қазір бие бар,neutral

0,биенің ішін жарған соң да,бие іш жар соң да,neutral

0,биік тау қойнында жасырына отырғанжалғыз қорасына тақап тау,биік тау қойн жасыр отырғанжал қора тақа тау,neutral

0,биікті көлденең кесіп отыратын қаптал жол жауынды күні қаптал,биік көлдене кес отыр қаптал жол жау күн қаптал,neutral

0,білмеген ді білмегенді,білме ді білме,neutral

0,бір бозға мініп,бір боз мін,neutral

0,бір бытқыл қиын жер боп жататын боп,бір бытқыл қиын жер боп жат боп,neutral

0,бір қарашаның дауылды қара суық түні қалың әлек түйіндей түйіндей,бір қараша дау қара суық түн қалың әлек түйінде түйінде,neutral

0,бір ту биені алып,бір ту бие алып,neutral

0,бір ту биені апарып сойса үйінде жатқызбай,бір ту бие апар сой үй жатқызба,neutral

0,бірақ есін жиып сақтанып,бірақ ес жи сақтан,neutral

0,бірге сойыса бастады бастады,бірге сойыс баста баста,neutral

0,бірдей сотқар мінезді қозыбақ аулы,бірде сотқ мінез қозы аул,neutral

0,бірдеңенің орны болса керек келетін кісі сыртындағы бір ақ керек,бірдеңе орны бол керек кел кісі сырт бір ақ керек,neutral

0,бірін бірі түрткілеп,бір бірі түрткіле,neutral

0,бірқалыпты ақ жауынның ішінде қалың жылқының бірқалыпты,бірқалыпты ақ жауын іш қалың жылқы бірқалыпты,neutral

0,бірқалыпты ыңқылдан басқа жауап естілмеді жатқан жалғыз кісі бірқалыпты,бірқалыпты ыңқыл басқа жауап естілм жат жалғыз кісі бірқалыпты,neutral

0,боз биені көрді осындайда нағыз ойға алатын төңкерілген семіз ойға,боз бие көр осындай нағыз ой ал төңкер се ой,neutral

0,бозды тебініп жіберіп,боз тебін жібер,neutral

0,боздың ер тоқымын көтеріп,боз ер тоқ көтер,neutral

0,бойы сергіп алған соң,бойы серг ал соң,neutral

0,бойы сәл сергіп қалды сергіп,бойы сәл серг қал серг,neutral

0,боқтап жүргені тектіғұл оны сол дауылда ығып кеткен кеткен,боқта жүр тектіғұл оны сол дауыл ығ кет кет,neutral

0,боқтықтың астына алады тек қана жылы киім сұраған жайын киім,боқтық аст ал тек қана жыл киім сұра жай киім,neutral

0,болса жылқышы атаулыға күлкі боп жүретін,бол жылқыш атаулы күлкі боп жүр,neutral

0,болса да,бол да,neutral

0,болсын деп,бол де,neutral

0,болушы еді жылқышы соны мезгілінде ойлай алмай,болуш ед жылқыш соны мезгіл ойла алма,neutral

0,болып біткен айналасында кірер ауыз жоқ бұрын көл сияқты бұрын,бол біт айнала кір ауыз жоқ бұрын көл сияқты бұрын,neutral

0,бөлек басқа бір сыңайға мінді бөлек,бөлек басқа бір сыңай мін бөлек,neutral

0,бұдан әрі екеуі қырын отырысып,бұдан әрі екеу қыр отырыс,neutral

0,бұл ат қашаған қуғыш боз деп те мақталушы еді мына еді,бұл ат қашаған қуғыш боз де те мақталуш ед мына ед,neutral

0,бұл иекартпадан сәл уақыт баспалап тұрды сыртылдаған иекартпадан,бұл иекартпа сәл уақыт баспала тұр сыртылда иекартпа,neutral

0,бұл қауіпсіз де емес тіп тік беткейде аттың қос тұяғы қатарынан де,бұл қауіпсіз де емес ті тік беткей ат қос тұяғ қат де,neutral

0,бұл өзі жылқының бір үйірін ауылға айдап келсе,бұл өзі жылқы бір үйір ауыл айда кел,neutral

0,бұл ұстаса да,бұл ұста да,neutral

0,бұлардың ажырап көшуіне әкеліп соққанды бүгін сол тектіғұл бұлардың,бұл ажыра көш әкел соқ бүгін сол тектіғұл бұл,neutral

0,бұрылып кетпей,бұрыл кетпе,neutral

0,ғана алып,ғана алып,neutral

0,ғана лық лықтап жанасыңқырап келіп,ғана лық лықта жанасыңқыра кел,neutral

0,ғана тайғақ емес,ғана тайғақ емес,neutral

0,да сол сескендірді сескендірді,да сол сескен сескен,neutral

0,да төмен салғызбай шаужайлай түседі салғызбай,да төмен салғызба шаужайла түс салғызба,neutral

0,дабылы шыққан жеріне таман шұбырысты бытырай жүріп таман,дабыл шық же таман шұбыр бытыра жүр таман,neutral

0,дақпыртқа шапқаны болмаса,дақпырт шап болма,neutral

0,дауыс айтқандай боп үн салып,дауыс айтқанда боп үн сал,neutral

0,дауыспен зекіп бұйрық етті зекіп,дауыс зек бұйрық ет зек,neutral

0,дейсің ол аурумен кім ауырмай ды құдайдың жаратқан дертіне дертіне,де ол ауру кім ауырма ды құдай жаратқан дерт дерт,neutral

0,дейтін мардымды түк те болмаған жиырма жылғы бақтығұл түк,де мардымды түк те болма жиырма жылғы бақтығұл түк,neutral

0,дендеп тимеді иығынан асып,денде тим иығ ас,neutral

0,деңгелек денесі анда санда маңайды болжағанда алды арты,деңгелек дене ан сан маңай болжа алды ар,neutral

0,деп жүріп кетті қора желкесіндегі бір жөке жақпардан асып кетті,де жүр кет қора желке бір жөке жақп ас кет,neutral

0,дәл осы шақта қораның алдыңғы жағына қарап,дәл осы шақ қора алдыңғы жағ қара,neutral

0,дәлдеп салып алған бақтығұл енді босатпады көк бие өзге бие,дәлде сал ал бақтығұл енді босатп көк би өзге би,neutral

0,дөң жайлаудың кер белеңін өрмелеп келе жатқанда,дө жайлау ке белең өрмеле келе жатқанда,neutral

0,дүңкілдеген келеңсіз жуан дауыспен дауыспен,дүңкілде келең жуан дауыс дауыс,neutral

0,дүрсілдетіп жүріп келе жатқан көп аттың тұяғы мен сүйретіп мен,дүрсілдет жүр келе жат көп ат тұяғ мен сүйрет мен,neutral

0,дұспаным деп білетіндіктен,дұспан де білетіндік,neutral

0,ды ендетіп жібермеген,ды ендет жіберме,neutral

0,ды сол дағдысымен тек әйеліне ғана бір айналып ды,ды сол дағды тек әйел ғана бір айнал ды,neutral

0,екесің ғой сен не дедің осы маған дауыңды айтып,ек ғой сен не де осы маған дау айт,neutral

0,екеуінің бірдей бала шақтарынан өткен еңбектеріне тапқаны екеуінің,екеу бірде бала шақ өткен еңбек тап екеу,neutral

0,екі жасар,екі жасар,neutral

0,елірген қызумен үрке өрекпіп қарсы алатын кәзіргі дүрбелең бір алатын,елір қыз үр өрекп қарсы ал кәзірг дүрбелең бір ал,neutral

0,елмен барымталасса,ел барымталас,neutral

0,енді ағарып көрші деп кішкене қазанды қатшаның алдынан алдынан,енді ағар көрші де кішкен қаза қатша алд алд,neutral

0,енді бір сәтте бақтығұл баспағып тұрған тұмсықшаға баспағып,енді бір сәт бақтығұл баспағ тұр тұмсықша баспағ,neutral

0,еңбегін жегені бір сөрі енді міне өмірін,еңбег же бір сө енді міне өмір,neutral

0,ерінің жүзіне жылы қарап,ер жүз жыл қара,neutral

0,ерінің шаршағаны асты тездетуді тілейді бірталай уақыт өткен шаршағаны,ер шарша асты тездету тіле бїртала уақыт өткен шарша,neutral

0,еске алып,ес алып,neutral

0,естіген қалың жылқы осы алдындағы иекартпа бір белдің ар алдындағы,есті қалың жылқы осы алд иекартп бір бел ар алд,neutral

0,етінен бір жапырақ бұйыртсам атым өшсін барлық етін қазір қазір,ет бір жапырақ бұйырт ат өш барлық ет қазір қазір,neutral

0,етті де шоқтың арасына тастап жіберді бақтығұл түтіннен біреу де,ет де шоқ ара таста жіб бақтығұл түтін біреу де,neutral

0,әдеті бар ондай күйлері болғанда,әдет бар ондай күй болғанда,neutral

0,әйелінің әрбір сездерін тыңдай,әйел әрбір сез тыңда,neutral

0,әл кетіп,әл кет,neutral

0,әлденеден шеткерірек болатын кейде үй іші томсарып,әлдене шеткерірек бол кейде үй іш томсар,neutral

0,әлі ойға кетпеген жылқы бұл шақта далаға көшкен елдің ойға,әлі ой кетпе жылқы бұл шақ дала көш ел ой,neutral

0,әлсіздігін жеңіп,әлсіздіг жең,neutral

0,әрі қамшысының ұшымен биені сауыр жағынан сипай ұрып биені,әрі қамшы ұш бие сауы жағ сипа ұр бие,neutral

0,әркімнің көзіне түсіп қалуға бслады сондықтан таудың бір қалуға,әркім көз түс қал бсл сондықтан тау бір қал,neutral

0,әуелде көзіне көрінгеннің көбі ұсақ тай байтал сияқтанды әуелде,әуел көз көрін көбі ұсақ тай байтал сиякта әуел,neutral

0,жабылған көп иттің тобынан жұлқынып,жабыл көп ит тоб жұлқын,neutral

0,жабысқан бойында қаршыға тастайтын аңшыдай қисайыңқырап жабысқан,жабыс бой қаршыға таста аңшыда қисайыңқыра жабыс,neutral

0,жағаласып қалып,жағалас қалып,neutral

0,жағына шапшаң бұрылады барлық қозғалысына ұршықтай қозғалысына,жағ шапша бұр барлық қозғалыс ұршықта қозғалыс,neutral

0,жағында шатта жа тыр сақ,жағ шат жа ты сақ,neutral

0,жазасын сәлменнен аламын деген дайыны жоқ сөзді ауызға алма,жаза сәлмен ал де дайын жоқ сөз ауыз алма,neutral

0,жазып жібергендей болды жібергендей,жаз жібергенде бол жібергенде,neutral

0,жайларды күле түсіп,жай күл түс,neutral

0,жайлауларында жататын дөң жайлауға қарай бұрылған соң ақ,жайлау жат дө жайлау қарай бұрыл соң ақ,neutral

0,жакындары жылкының кеiiбiрі үріккендей кісінесіп,жакын жылк кеiiбi үріккенде кісінес,neutral

0,жақсы маңайының бәрі де шашырап түскен үлкен үлкен қойтас де,жақсы маңай бәрі де шашыра түс үлкен үлкен қойтас де,neutral

0,жақын жерден қап қара болып үңірейіп,жақын же қап қара бол үңірей,neutral

0,жалғыз сиырдың майы бар ды кебежені ақтарып,жалғыз сиыр май бар ды кебеже ақтар,neutral

0,жамағайын сарыны жинап атқарды да,жамағ сары жина атқар да,neutral

0,жамантай жоғарыдан бұған қарай шауып келіп,жаманта жоғары бұған қарай шау кел,neutral

0,жан кіргізгендей болды,жан кіргізгенде бол,neutral

0,жанымнан садаға кетсін сенің арамнан жиған малың құлағын,жан сада кет сенің ара жи мал құлағ,neutral

0,ұрайын мен де айтамын соны деген тілін тартпаған ды айтамын,ұр мен де ай соны де тіл тартпа ды ай,neutral

0,жаңағы боз биенің мойнына өзі салған шалма бұғалық енді міне мойнына,жаңағ боз бие мойн өзі сал шал бұғалық енді міне мойн,neutral

0,жарған арманын айтты қатын балам,жар арман ай қатын бала,neutral

0,жардай қылып жайратып тастаған ерін көрді үлкен ту биенің жардай,жарда қыл жайрат таста ер көр үлкен ту бие жарда,neutral

0,жас ет асылып,жас ет асыл,neutral

0,жас етті күйсей бастады араларына бақтығұлды шақырған да етті,жас ет күйсе баста ара бақтығұ шақыр да ет,neutral

0,жас жылқының еті қайдан шықты осыны қалай танасың кәне,жас жылқы ет қайдан шық осы қалай тан кәне,neutral

0,жасатты соларда сәт сәлменнің,жаса сол сәт сәлмен,neutral

0,жасқаншақтықпен өз ісін өзі тентек,жасқаншақтық өз ісін өзі тентек,neutral

0,жасынан өздігінен мұндай жүрісті кәсіп етпесе де,жас өздіг мұндай жүр кәс етпе де,neutral

0,жасып қалып,жас қалып,neutral

0,жатқан кісі жоқ болса да,жат кісі жоқ бол да,neutral

0,жатқан қарайғанды көрісті жатқан,жат қарай көр жат,neutral

0,жатты ел көзіне түспеймін деп еді,жат ел көз түспе де ед,neutral

0,жатып сермесе де,жат серме де,neutral

0,жауын мен дауылға не кием тымғұрса бір өң киім бер деп киім,жауын мен дауыл не кие тымғұр бір өң киім бер де киім,neutral

0,жауынды түнді тіліп өткендей тау ішіне шаншыла қарағанда,жау түн тіл өткенде тау іш шаншыл қара,neutral

0,жауырыны жерге тигенше айла тәсіл істеп,жауыры же тигенш айла тәсіл істе,neutral

0,жегенді айтсаңшы бақтығұлдың көз алдынан былтырғы алдынан,же айтсаңш бақтығұ көз алд былтырғы алд,neutral

0,желкедегі қарағайға келіп кірген жалғыз аттыны көрген бар бар,желке қарағай кел кірген жалғыз атты көр бар бар,neutral

0,жемей ме екеміз кел,жеме ме ек кел,neutral

0,жерге бір еңгезердей үлкен жылқышы келіп қалыпты тек бұл жерге,же бір еңгезерде үлкен жылқыш кел қалыпты тек бұл же,neutral

0,жерге жалпиып отыра кетті шала жабылған қаптың шетінен кетті,же жалпи отыр кет шал жабыл қап шет кет,neutral

0,жерде иесіне сол өнерін анық көрсетті әйтпесе оқыста ытқып көрсетті,же ие сол өн анық көрсе әйтпесе оқыс ытқ көрсе,neutral

0,жердегі ернеуден асып түседі айналасы тастан салған тастан,жер ернеу ас түс айнала тастан сал тастан,neutral

0,жерінен тауып алып,же тау алып,neutral

0,жетекке ал,жетек ал,neutral

0,жетелеп тұрғандай кейде тау қара түн,жетеле тұрғанда кейде тау қара түн,neutral

0,жеткенше тыйылмады міне соның қырқын жыртық үйлі азғана азғана,жеткенш тыйылм міне соның қырқ жыр үйл азған азған,neutral

0,жетті ол қайтыс болды артында қанатым едің,же ол қайтыс бол арт қана ед,neutral

0,жиырмаларға іліге берген шақтарда ағайынды екі малшы ағайынды,жиырма іліг бер шақ ағайынды екі малш ағайынды,neutral

0,жиюға айналды болған оқиға мынау еді еді,жию айна бол оқиға мынау ед ед,neutral

4,мен талибтерді қолдаймын махсуд кіші өз тәуелсіз мемлекеттеріңді құрыңдар деп жүгірді,мен талиб қолда махсуд кіші өз тәуелсіз мемлекет құр де жүг,violent

4,талибан мұны ауғанстандағы тәжіктерге дұрыс жасайды,талибан мұны ауғанстан тәжік дұрыс жаса,violent

4,алға талибтер бұл тәжіктердің емес сіздердің жерлеріңіз,алға талиб бұл тәжік емес сіздердің жер,violent

4,жарайсыңдар талибтер әйелдер өз орнын білу керек еркектермен тең болмайсыңдар,жара талиб әйел өз орн білу керек еркек тең болма,violent

4,мен бұұ мен батыстық гуманитарийларға қарағанда талибтерге көбірек сенемін,мен бұұ мен бат гуманитарий қара талиб көбірек сен,violent

4,қырғыздар мен орыстар көтеріліске шығады,қырғыз мен орыс көтеріліс шығ,violent

4,талибан алға урааа кабул біздікі,талибан алға ураа кабул біздік,violent

4,батыстық вируспен уланған масуд талибан жеңсін,бат вирус улан масуд талибан жең,violent

4,американдықтардың бастарын шабу керек,американдық бас шаб керек,violent

4,толибтерді қолдаймын,толиб қолда,violent

4,талибанды күтемін мына елді жойғым келеді,талибан күт мына ел жой кел,violent

4,талибтерді құрметтеймін барлық жерді жаулап алса екен,талиб құрметте барлық же жаула ал екен,violent

4,талибан барлығын бейбітшілікке әкеледі кәпірлерді өлтіріңдер,талибан барлығ бейбітшілік әкел кәпір өлтір,violent

4,өлтіре берсін бір бірін кімге керек олар,өлтір бер бір бір кім керек олар,violent

4,талибани бауырлар жарайсыңдар сатқын билікті құрту керек,талибани бауыр жара сатқ билік құрт керек,violent

4,тыйым салынған талибанға рұқсат беретін уақыт келді,тыйым салын талибан рұқсат бер уақыт кел,violent

4,кім талибанға қарсы болса ол исламға қарсы деген сөз,кім талибан қарсы бол ол ислам қарсы де сөз,violent

4,талибтер жарайсыңдар барлық елдер үшін бұұ болдыңдар,талиб жара барлық ел үшін бұұ бол,violent

4,талибан мықты жарайсыңдар,талибан мықты жара,violent

4,талибан күш жеңілмеңдер,талибан күш жеңілме,violent

4,ауғандық талиб бауырлар мықтысыңдар алға,ауғандық талиб бауыр мықты алға,violent

4,талибан арыстандарды жаулап алады,талибан арыстан жаула ал,violent

4,талибтер лас тәжіктерді қырады,талиб лас тәжік қыр,violent

4,талибтер дұрыс жасайды тәжіктерді қыру керек,талиб дұрыс жаса тәжік қыр керек,violent

4,талибтер жарайсыңдар бізге де ұрлықшыларды көпірлерге асып өлтіру керек бізде заң жұмыс істемейді,талиб жара бізге де ұрлықшы көпір ас өлтір керек біз заң жұмыс істеме,violent

4,қазақстанға талибтердің заңы керек,қазақстан талиб заң керек,violent

4,алға талибтер біз сіздермен біргеміз,алға талиб біз сіздермен бір,violent

4,мен талибтерді қолдаймын осылай болу керек,мен талиб қолда осылай болу керек,violent

4,талибтер жарайсыңдар тек алға,талиб жара тек алға,violent

4,біз талибтердің билікке келуін қалаймыз,біз талиб билік кел қала,violent

4,олар өлтіру үшін жаралған олар бүркіт сияқты нағыз соғыс болуы керек жақын арада өлтіруге барамыз,олар өлтір үшін жарал олар бүркіт сияқты нағыз соғыс бол керек жақын ара өлтір бар,violent

4,енді джихад қан кісі өлімі сіздің елге келеді,енді джихад қан кісі өлім сіздің ел кел,violent

4,багдади біздің соңғы үмітіміз мен сені сүйемін,багдади біздің соңғы үміт мен сен сүй,violent

4,егер игил египетті жаулап алса олар пирамиданы құлатанына сенемін оларды жер бетінен жою керек,егер игил египет жаула ал олар пирамида құлатан сен ол жер бет жою керек,violent

4,орыс шошқалары соғыста сириядағы лагерьлерді құлатқанына өкінеді,орыс шошқа соғыс сирия лагерь құлат өкін,violent

4,англия дайындал жауынгерлер саған келе жатыр,англия дайындал жауынгер саған келе жатыр,violent

4,жақын арада ypg мен сирия жауынгерлері арасында манбиджа аль баба игил үлкен соғыс болады,жақын ара ypg мен сирия жауынгер ара манбидж аль баба игил үлкен соғыс бол,violent

4,осы айда иг араб елдерінің әскерлерін өлтіреді,осы айда иг араб ел әскер өлтір,violent

4,ислам абад үндістаннан кейін пәкістанға жорыққа аттанамыз,ислам абад үндістан кейін пәкістан жорық аттан,violent

4,талибан жақсы жұмыс енді аль каида мен ауғанстандағы даиш құртыңдар аяқтаған соң бүкіл әлемге жар салыңдар басқа да аз халықтарға бар болуға және өз территориясында өмір сүруге рұқсат беріңдер,талибан жақсы жұмыс енді аль каида мен ауғанстан даиш құр аяқта соң бүкіл әлем жар сал басқа да аз халық бар бол және өз территория өмір сүр рұқсат бер,violent

4,талибанды құрту керек әлем игил қолдауы керек ақш нато серіктестері қарумен және ақшамен қолдауы керек,талибан құрт керек әлем игил қолда керек ақш нато серіктес қар және ақша қолда керек,violent

4,үндістан өлсін кашмирды хиндутвадан босату керек,үндістан өл кашмир хиндутва босат керек,violent

4,олар мумбайда жарылу үшін жарылғыш заттарды салуы керек,олар мумбай жарыл үшін жарылғыш зат сал керек,violent

4,бен ладен батыр бен ладен әдемі бен ладен жақсылық империясының жүрегіне соққы берді,бен ла батыр бен ла әдемі бен ла жақсылық империя жүрег соққы бер,violent

4,мен бұл кадрлардан рахат аламын респект усама бен ладен,мен бұл кадр рахат ал респект уса бен ла,violent

4,мен талибанның исламмен бірге билікте болғанына қуаныштымын исламға қарсы барлық адамдарды алып тастаңыз әсіресе ақымақ адам тәжік махмуд ол өзінің айналасындағылармен бірге жүгіреді,мен талибан ислам бірге билік бол қуанышты ислам қарсы барлық адам алып таста әсіресе ақы адам тәжік махмуд ол өзінің айналасындағы бірге жүгір,violent

4,ливандағы қауіпсіздікті бұзу нәтижесінде сіз әскери тұтқындардың тағдырындағы тосынсыйлар туралы естисіз біз оларды ұстап отырмыз,лива қауіпсіздік бұзу нәтиже сіз әскери тұтқын тағдыр тосынсый туралы ести біз ол ұста отыр,violent

4,ислам мемлекеті енді сириядағы қорқынышты күш болады барлық топтарға ультиматум қойылады бізбен бірге немесе бізге қарсы болуды таңда,ислам мемлекет енді сирия қорқыныш күш бол барлық топ ультимату қой бізбен бірге немесе бізге қарсы болу таңда,violent

4,талибтар батырлар,талиб батыр,violent

4,талибтар тұрғанда барлығы жақсы болады,талиб тұр барлығ жақсы бол,violent

4,талибтар жігіттер,талиб жігіт,violent

4,талибан өте өркениетті адамдар олар бәрін дұрыс жасайды,талибан өте өркениет адам олар бәрін дұрыс жаса,violent

4,бомбаны сол жерге лақтыру керек ауғанстан жоғалып бейбітшілік болады барлық жамандық сол жерден келеді,бомба сол же лақтыр керек ауғанстан жоғал бейбітшілік бол барлық жаман сол же кел,violent

4,талибан мәскеуге иман магди антихристке барады,талибан мәскеу иман магди антихрист бар,violent

4,мұның бәрі өтірік талибан қазіргі уақытта әлемдегі ең жақсы билік,мұның бәрі өтірік талибан қазіргі уақыт әлем ең жақсы билік,violent

4,талибанның жеңісі құтты болсын ол берекелі болсын,талибан жеңіс құт бол ол берекелі бол,violent

4,нидерландыдан көбірек қолдауы даиш жақтастары бүкіл әлемде alleyeson хэштегін қолданады,нидерланды көбірек қолда даиш жақтас бүкіл әлем alleyeson хэштег қолдан,violent

4,егер даиш күрдтерге мән бермесе мен ертең барып оған қосыламын,егер даиш күрд мән берме мен ертең бар оған қосыл,violent

4,олар енді террористерге ақша бермеуі үшін израильді катармен бірге жою керек,олар енді террорис ақша берме үшін израиль катар бірге жою керек,violent

4,талибан арыстандары елдер бостандық үшін соғысуда сатқындардан тазартып жатыр,талибан арыстан ел бостандық үшін соғыс сатқын тазарт жатыр,violent

4,оларға осы әдемі адамдар үшін газ камераларын қаржыландырайық,оларға осы әдемі адам үшін газ камера қаржыландыр,violent

4,мамандығым снайпер сирияда жұмыс істегім келеді,мамандығ снайп сирия жұмыс істе кел,violent

4,мен талибанды қолдаймын алға ауғанстанда есірткі болмасын,мен талибан қолда алға ауғанстан есірткі болма,violent

4,талибер жеңсе тәртіп болады,талиб жең тәртіп бол,violent

4,жақсы талибан олар өз істерін жасайды барлық ел тәртіпке келтірілсін,жақсы талибан олар өз іс жаса барлық ел тәртіп келтіріл,violent

4,біздің елге талибан керек,біздің ел талибан керек,violent

4,талибан дұрыс жақсы мемлекет құру үшін тер төгуде,талибан дұрыс жақсы мемлекет құру үшін тер төг,violent

4,талибтерді әрқашан құрметтеймін,талиб әрқашан құрметте,violent

4,талибан тек алға ұзағынан болсын,талибан тек алға ұзағ бол,violent

4,талибтер нағыз жігіттер,талиб нағыз жігіт,violent

4,талибтер керемет,талиб керемет,violent

4,дағыстан мен шешенстан халқы біздің ағайынды талибандардың билікке келуін жоғары бағалайды,дағыс мен шешенс халқ біздің ағайынды талибан билік кел жоғары бағала,violent

4,егер талибан норма болса онда кафыровты да алып тастаңыз,егер талибан норма бол онда кафыров да алып таста,violent

4,егер талибан жеңсе онда бейбітшілік болады,егер талибан жең онда бейбітшілік бол,violent

4,талибандарда бәрі ойдағыдай болғанын қалаймын,талибан бәрі ойдағыда бол қала,violent

4,енді мен талибанның жақсы екенін және әлемді жақсартқысы келетінін түсіндім,енді мен талибан жақсы екен және әлем жақсарт кел түс,violent

4,талибтер жігіттер,талиб жігіт,violent

4,бұл жануарларды ядролық бомбамен жойып жіберу керек,бұл жануар ядролық бомба жой жіберу керек,violent

4,осындай талибан бізге де келсе екен,осындай талибан бізге де кел екен,violent

4,талибан бача базиді жойсын бұл құбылыс соншалықты жиіркенішті,талибан бач бази жой бұл құбылыс соншалықты жиіркеніш,violent

4,біздің бауырларымыз талибанға барлық жақсылықты тілейміз,біздің бауыр талибан барлық жақсылық тіле,violent

4,сізбен біргеміз талибан,сізбен бір талибан,violent

4,талибанға әрқашан жеңіс тілеймін,талибан әрқашан жеңіс тіле,violent

4,талибтер респект барлығы жақсы болады,талиб респект барлығ жақсы бол,violent

4,американдық демократияның жемісі талибтер ретке келтірер деп үміттенемін,американдық демократия жеміс талиб рет келтір де үміттен,violent

4,талибан елді нашақорлықтан тазартады,талибан ел нашақорлық тазарт,violent

4,мен талибтерді қолдаймын олар нағыз жігіттер мына елді тастаңдар бұл заманауи ел өз еліне қарсы шыққандар не істесеңдер де өздерің біліңдер,мен талиб қолда олар нағыз жігіт мына ел таста бұл заманауи ел өз ел қарсы шық не істе де өз біл,violent

4,америка мен ресей террористтар талибан жақсы,америка мен ресей террорист талибан жақсы,violent

4,талибан бауырларымызды қарап тыңдаймыз қолдаймыз,талибан бауыр қара тыңда қолда,violent

4,талибан елдің мақтанышы,талибан ел мақтаныш,violent

4,талибан алға,талибан алға,violent

4,талибтер алға қарсы шыққандарды қырыңдар,талиб алға қарсы шық қыр,violent

4,біздің өз талибанымыз бар ма немесе оны құру керек пе бірігейік,біздің өз талибан бар ма немесе оны құру керек пе біріг,violent

4,талибтер ең мықты,талиб ең мықты,violent

4,біздің президентіміз бен оның бандаларының орнына талибан келсе деп армандаймын,біздің президент бен оның банда орн талибан кел де арманда,violent

4,талибтер алға бұл сендердің жерлерің шабуылдаңдар,талиб алға бұл сен жер шабуылда,violent

4,өз талибанымызды құрайық мына билік шаршатты,өз талибан құр мына билік шарша,violent

4,қоғамға талибан тиімсіз сияқты олар адал ғой көптеген мемлекет басшыларына халық пен өз міндеттері қызық емес олардың басты мақсаты жемқорлық жасау ал талибандар өте адал олар халыққа өтірік айтпайды,қоғам талибан тиімсіз сияқты олар адал ғой көптеген мемлекет басшы халық пен өз міндет қызық емес олардың басты мақсаты жемқорлық жасау ал талибан өте адал олар халық өтірік айтпа,violent

4,талибан орта азияны қазақтарды тонап жатқан үкіметпен күресуге үйретіңіз өтініш оятыңыз,талибан орта азия қазақ тона жат үкімет күрес үйрет өтініш оят,violent

4,талибан біз сіздерді күтеміз,талибан біз сіз күт,violent

4,талиб пуштундар мықты,талиб пуштун мықты,violent

4,талибонның уақыты келді әлемде бейбітшілік болсын,талибон уақыт кел әлем бейбітшілік бол,violent

4,талибанмен бірге өркендейік,талибан бірге өркенде,violent

4,талибтерді құрметтеймін сендер хорасанның нағыз ерлерсіңдер,талиб құрметте сен хорасан нағыз ер,violent

4,талибтер жолдарың болсын бауырлар,талиб жол бол бауыр,violent

4,талибтер американдықтармен дос болғандардың барлығын өлтіреді,талиб американдық дос бол барлығ өлтір,violent

4,талибан үшін өте қуаныштымын олар әлемді құтқарып жатыр,талибан үшін өте қуанышты олар әлем құтқар жатыр,violent

4,талибан бомбаны лақтыру керек,талибан бомба лақтыр керек,violent

4,талибтер жаулап алды енді жағдай қиындайды күтіңдер,талиб жаула алды енді жағдай қиында күт,violent

4,болашақ талибандікі,болашақ талибандік,violent

4,талибтер өз елінің қожайындары бізге үлгі болды,талиб өз ел қожайын бізге үлгі бол,violent

4,талибтер жақында барлығына көрсетеді,талиб жақында барлығ көрсет,violent

4,талибтер біздің батырлар бізге үлгі,талиб біздің батыр бізге үлгі,violent

4,талибан алға тек алға,талибан алға тек алға,violent

4,талибан іске кірісті жағдайларың қиындайды,талибан іс кір жағдай қиында,violent

4,барлық елдерге талибаннан елді қалай басқару керектігі туралы үйрену керек,барлық ел талибан ел қалай басқару керектіг туралы үйрену керек,violent

4,жарайсыңдар талибан өз істерін жасайды барлығы қарастырылсын елді тәртіпке келтіреді,жара талибан өз іс жаса барлығ қарастырыл ел тәртіп келтір,violent

3,ең басты террорист путин,ең басты террорист путин,nazism

4,мен талибанды қолдаймын алға,мен талибан қолда алға,violent

4,талибтер ерлер,талиб ер,violent

4,әйелдерді ұруға болады бірақ ол туралы айтуға болмайды,әйел ұр бол бірақ ол туралы айт болма,violent

4,ядролық бомба көмекке келеді қырайық,ядролық бомба көмек кел қыр,violent

4,мен сіздерді соғыстағыларды қолдауға шақырамын,мен сіз соғыстағы қолда шақыр,violent

3,тәжіктер өлсін,тәжік өл,nazism

3,тәжіктер байқаңдар жақында сендерді қырамыз,тәжік байқа жақында сен қыр,nazism

4,соғыссақ болды кіммен екені маңызды емес,соғыс бол кім екен маңызды емес,violent

4,террористтерді қолдаңдар олармен келіссөз жүргізіңдер террористтерді қолдаңдар оларға ақша беріңдер террористтермен келіссөз жүргізіңдер әлемді құтқарыңдар,террорист қолда олармен келіссөз жүргіз террорист қолда оларға ақша бер террорист келіссөз жүргіз әлем құтқар,violent

4,ол жерге термоядролық бомба лақтырайық сонда барлық мәселе шешіледі,ол же термоядролық бомба лақтыр сонда барлық мәселе шеш,violent

4,бірнеше бомба тастап аштық жасаса барлық мәселе шешіледі,бірнеше бомба таста аштық жаса барлық мәселе шеш,violent

4,джихақа кім барғысы келеді жазыңдар,джиха кім бар кел жаз,violent

4,біз талибтермен бірге барлығыңды қырамыз,біз талиб бірге барлығ қыр,violent

4,жақында таяу шығыста джихад болады,жақында таяу шығыс джихад бол,violent

3,алдымен парсыларды құрту керек олар тек зиян әкеледі,алд парсы құрт керек олар тек зиян әкел,nazism

4,тәжікстан талибанмен соғысты бастауы керек,тәжікстан талибан соғ баста керек,violent

4,мен тәжік талибанды қолдаймын өйткені тәжікстан президенті мен мемлекеті ақымақ,мен тәжік талибан қолда өйткені тәжікстан президент мен мемлекет ақы,violent

4,тәжіктер талибанды қырыңдар ресей көмектеседі,тәжік талибан қыр ресей көмектес,violent

4,құрметті қырғыз және кавказдық талип жанкүйерлері не үнсіздік халық сіздің сараптамалық пікірлеріңізді күтуде мәселе неде екенін халыққа түсіндіріңіз,құрметті қырғыз және кавказ тали жанкүйер не үнсіздік халық сіздің сараптамалық пікір күт мәселе не екен халық түсіндір,violent

4,талибанның дұрыс екендігі және бәрі шешілетіні туралы сараптамалық түсініктемелер қажет қырғыз радикалдарының пікірлері ерекше құнды,талибан дұрыс екендіг және бәрі шешіл туралы сараптамалық түсініктеме қажет қырғыз радикал пікір ерекше құнды,violent

3,эй славяндар албандарды жазалауға кім барады,эй славян албан жазала кім бар,nazism

4,жер бетінде үлкен соғыс керек,жер бет үлкен соғыс керек,violent

3,сербия үшін соғысу керек түріктермен американдықтармен албандармен бірге,сербия үшін соғыс керек түрік американдық албан бірге,nazism

3,сербияға алғашқы снарядтарда албанияны атомдарға бөлу керек,сербия алғашқы снаряд албания атом бөлу керек,nazism

3,сербия құрысын,сербия құрыс,nazism

4,үшінші дүниежүзілік соғысты бастау керек бұл бүкіл адамзатқа қажет өйткені олар күн сайын аш және шыдамсыз қатыгез болып барады,үшінші дүниежүзілік соғ бастау керек бұл бүкіл адамзат қажет өйткені олар күн сайын аш және шыда қатыгез бол бар,violent

3,жеттік ау сербтерді суға батырыңыз косовоны қазірдің өзінде жарып жіберу керек,же ау серб су батыр косово қаз өз жар жіберу керек,nazism

3,сербияға орыс армян христиан ретінде барамын сізге косовоны ұсақтауға көмектесемін,сербия орыс армян христиан рет бар сізге косово ұсақта көмектес,nazism

4,ядролық бомба тастағым келеді,ядролық бомба таста кел,violent

4,мен әскерде болған жоқпын бірақ қару жарақпен жұмыс істей аламын альпинист куәлігім бар сирияға соғысқа барғым келеді,мен әскер бол жоқ бірақ қару жарақ жұмыс істе ал альпинист куәліг бар сирия соғыс бар кел,violent

4,сирияда жұмыс істеу туралы сізбен қалай байланысуға болады gru арнайы күштерінде барлау операторы қызметін атқарамын,сирия жұмыс істеу туралы сізбен қалай байланыс бол gru арнайы күш барлау оператор қызмет атқар,violent

4,талибтерге берілсек олар бізды марапаттайды,талиб беріл олар біз марапатта,violent

4,келісімшартпен сирияға барамын,келісімшарт сирия бар,violent

4,сирияға жихадқа барғым келеді денсаулығым жақсы әскерде болдым,сирия жихад бар кел денсаулығ жақсы әскер бол,violent

3,кабулдың ортасына сутегі бомбасын тастау керек,кабул орта сутегі бомба тастау керек,nazism

4,отбасын тамақтандыру немесе ата анасына қамқорлық жасау үшін жиһадқа бару керек,отбас тамақтандыру немесе ата ана қамқорлық жасау үшін жиһад бару керек,violent

4,олардың бәрін өлтіру керек,олардың бәрін өлтір керек,violent

4,сирия келісімшарт бойынша ресей базасына барамын,сирия келісімшарт бойынша ресей база бар,violent

3,олар енді террористерге ақша бермеуі үшін израильді катармен бірге жою керек,олар енді террорис ақша берме үшін израиль катар бірге жою керек,nazism

4,сирияға келдім осы жақта өлетін сияқтымын,сирия кел осы жақта өл сияқты,violent

4,талибан менің еліме келіп тәртіп орнатып барлық сыбайластарды өлтіреді деп үміттенемін,талибан менің ел кел тәртіп орнат барлық сыбайлас өлтір де үміттен,violent

4,соғысқа барып өлгеніміз жақсы,соғыс бар өл жақсы,violent

4,мен досымның видеосын көруді ұсынамын ол қазір сирияда және үнемі сол жерден репортаж жасайды,мен дос видео көру ұсын ол қазір сирия және үнемі сол же репортаж жаса,violent

4,талибтер кутты кадыкта болсын олар қазақтан үшін кінәлі,талиб ку кадык бол олар қазақтан үшін кінәлі,violent

4,джихад жалғастырылады,джихад жалғастыр,violent

4,біздің жолымыз джихад,біздің жол джихад,violent

4,сирияға бауырлар мен әпкелерге қосылып жиһадқа барғым келеді,сирия бауыр мен әпке қосыл жиһад бар кел,violent

4,дұрыс талиптер болашақты ойлап жүрген батырлар,дұрыс талип болашақ ойла жүр батыр,violent

4,талибан нағыз ер адамдар өздерінің заңдарын жасайды жарайсыңдар ер адамдар қалағанын жасайды,талибан нағыз ер адам өз заң жаса жара ер адам қала жаса,violent

4,егер қазақтар осылай қорқытса қазақтарды талибан дейді олар халықты қолдамайтындармен соғысады дейді,егер қазақ осылай қорқыт қазақ талибан де олар халық қолдама соғыс де,violent

4,талибтер келді олар көмектеседі,талиб кел олар көмектес,violent

4,талибтер мыналардың барлығын өлтіруі керек,талиб мына барлығ өлтір керек,violent

4,соғысқа бару керек,соғыс бару керек,violent

4,алға ауғандық талибтар,алға ауғандық талиб,violent

4,мен соғысамын өлемін деп үміттенемін,мен соғыс өл де үміттен,violent

4,талибан қазақстанға келсін біз назарбаев бастаған нұр отанды жеңу үшін талибан жағында күресетін боламыз,талибан қазақстан кел біз назарбаев баста нұр ота жеңу үшін талибан жағ күрес бол,violent

4,талибтер батырсыңдар сендерге сөз жоқ алға,талиб батыр сен сөз жоқ алға,violent

4,дұрыс талибтер барлығына бостандық берді,дұрыс талиб барлығ бостандық бер,violent

4,талибанды билеуші партия ретінде танудың уақыты келді ерте ме кеш пе ресей мұны алғашқылардың бірі болып жасайды,талибан билеуш партия рет тан уақыт кел ерте ме кеш пе ресей мұны алғашқы бірі бол жаса,violent

4,мен сирияда соғысқым келеді мен не істеуім керек мен азаматтық қаруды жақсы атамын әскери әкенің тәрбиесінде болдым әскери бөлімде өмір сүрдім,мен сирия соғыс кел мен не істе керек мен азаматтық қару жақсы ат әскери әке тәрбие бол әскери бөл өмір сүр,violent

4,атым айрат сирияға қатты барғым келеді жылдан бастап ресми жылдан бастап бейресми түрде көлік айдаймын в д санаттары бар автосервисте қызмет еттім құрылыста жұмыс істедім жылы әскерде болдым жастамын кез келген жаңа мамандықты игеруге дайынмын,ат айрат сирия қатты бар кел жыл баста ресми жыл

баста бейресми түр көлік айда в д санат бар автосервис қызмет ет құрылыс жұмыс істе жыл әскер бол жас кез кел жаңа мамандық игер дайын,violent

4,қазақстан азаматымен қазақстанның шекара қызметінде әскерде болдым қазір жыл бойы полицияда қызмет етемін денсаулығым физикалық дайындығым жақсы сирияға қызметке барғым келеді,қазақстан азамат қазақстан шекара қызмет әскер бол қазір жыл бойы полиция қызмет ет денсаулығ физикалық дайындығ жақсы сирия қызмет бар кел,violent

4,келісімшарт бойынша әскердемін бізді сирияға әкетпейді сол жаққа қатты барғым келеді көмектесіңіздерші рахметімді айтамын жасым жиырма төртте қатардағы радиотелефонистпын,келісімшарт бойынша әскер біз сирия әкетпе сол жақ қатты бар кел көмектесіңіздерш рахмет ай жас жиырма төрт қатар радиотелефонист,violent

4,сирияға бару туралы шешім қабылдадым ол жақта насихаттайтын боламын,сирия бару туралы шешім қабылда ол жақта насихатта бол,violent

4,сапер болып қызмет еттім қаруды қолдана аламын отыз үш жастамын денсаулығым жақсы сирияға бара аламын ба,сапер бол қызмет ет қару қолдан ал отыз үш жас денсаулығ жақсы сирия бар ал ба,violent

4,сәлем сирияға бара аламын ба жиырма жастамын әскерде шұғыл қызметте болдым атқыштар бригадасында келісімшарт жасауға дайынмын бтр операторын оқыдым,сәлем сирия бар ал ба жиырма жас әскер шұғыл қызмет бол атқыш бригада келісімшарт жаса дайын бт оператор оқы,violent

4,сирияға елді қорғау үшін бара аламын ба жаяу әскер бригадасында әскерде болдым,сирия ел қорғау үшін бар ал ба жаяу әскер бригада әскер бол,violent

4,сәлеметсіздер ме кәпірлерге қарсы қызмет етуге дайынмын жасым жиырма бесте әскерде болдым әскери құжатым бар пмб мтлб птур операторымын атқышпын денсаулығым жақсы,сәлемет ме кәпір қарсы қызмет ет дайын жас жиырма бес әскер бол әскери құжат бар пмб мтлб пту оператор атқыш денсаулығ жақсы,violent

4,сирияға соғысуға барғым келеді бұрын украинамен соғыста болдым сержант шені санатты жүргізуші куәлігі бар зиян әдеттерім жоқ әскерге дайындығым,сирия соғыс бар кел бұрын украина соғыс бол сержант шен сана жүргізуші куәліг бар зиян әдет жоқ әскер дайындығ,violent

4,бұрынғы әскерімін қазіргі таңда көлік айдаймын сирияға кез келген қызметке барғым келеді қолымнан барлығы келеді соғыса аламын,бұрынғы әскери қазіргі таңда көлік айда сирия кез кел қызмет бар кел қол барлығ кел соғыс ал,violent

4,сирияға барғым келеді әскерде болмадым денсаулығым өте жақсы еш шағымым жоқ сирияға әскерге барғым келеді,сирия бар кел әскер болма денсаулығ өте жақсы еш шағым жоқ сирия әскер бар кел,violent

4,қызбен кездесуге болмайды бірақ зорлап өлтіруге болады олар иг келгенше өз елдерінде ешкім емес еді,қыз кездес болма бірақ зорла өлтір бол олар иг келгенше өз ел ешкім емес ед,violent

4,талибандарға қалай қосылуға болады,талибан қалай қосыл бол,violent

4,бізден қаруланған күшті және тәуелсіз әйелдерді жіберуіміз керек олар содан кейін тәртіп орнатады мен оларға сенемін,бізден қарулан күшті және тәуелсіз әйел жібер керек олар содан кейін тәртіп орнат мен оларға сен,violent

3,қытаймен соғысу керек басқа жол жоқ өлтірейік,қытай соғыс керек басқа жол жоқ өлтір,nazism

3,қырғыздардың басын шабамыз,қырғыз басын шаб,nazism

3,қытайға қарсы соғыс ашайық,,қытай қарсы соғыс аш,nazism

4,олардың басын кесемін,олардың басын кес,violent

4,бір ғана жол бар оларды қыру керек,бір ғана жол бар ол қыр керек,violent

3,барлығымыз бірігіп орыстарды қыруға барамыз,барлығ біріг орыс қыр бар,nazism

4,не істеу керектігін айтыңыз өлтірейік пе,не істеу керектіг ай өлтір пе,violent

3,бірігуіміз керек жеке мемлекет құрып қарсы шыққандарды қырамыз,біріг керек жеке мемлекет құр қарсы шық қыр,nazism

3,қазақтардың жауы еврейлер барлық жамандық солардан мына мәселені шешуіміз керек қазақтар ойланыңдар,қазақ жау еврей барлық жаман сол мына мәселе шеш керек қазақ ойлан,nazism

3,орыстарға қарсы шығып омсктан бастап жерімізді тартып алуымыз керек,орыс қарсы шығ омск баста же тарт ал керек,nazism

3,түріктер бірігіп орыстарды қырамыз,түрік біріг орыс қыр,nazism

3,ұзын сөздің қысқасы түріктермен одақтасып соғысқа дайындалу керек осы хабарды барлығына таратыңдар басқа қарсы ұлттарды қырамыз,ұзын сөз қысқа түрік одақтас соғыс дайындалу керек осы хаб барлығ тара басқа қарсы ұлт қыр,nazism

3,түріктер бірігіп аққұлақтарды орнына қоямыз олар өз қаруларына сеніп мықты болып отыр,түрік біріг аққұлақ орн қоя олар өз қару сен мықты бол отыр,nazism

4,барып оны өлтіруіңіз керек,бар оны өлтір керек,violent

3,солтүстіктегі орыстарды қуып шығып қазақтарды апару керек,солтүстік орыс қу шығ қазақ апару керек,nazism

3,орыстарды көрсем қаным басыма шығады олардан не жақсылық күтуге болады малғұндар,орыс көр қан бас шығ олардан не жақсылық күт бол малғұн,nazism

3,орыстарды қуып түріктермен бірігу керек,орыс қу түрік бірігу керек,nazism

3,орыстарды қуамыз жалмауыздар олар,орыс қу жалмауыз олар,nazism

3,орыстарға байкот жариялау керек,орыс байкот жариялау керек,nazism

3,шошқа орыстарды тәрбиелеу керек,шошқа орыс тәрбиелеу керек,nazism

3,қасық қанымыз қалғанша қытайларға соғыс жариялап қыру керек соғыс болса қашпаймын мен,қасық қан қалғанш қытай соғыс жарияла қыр керек соғыс бол қашпа мен,nazism

4,тамырымен атып өлтіру керек мұндайларды,тамыр ат өлтір керек мұндай,violent

3,қытайлармен соғысу керек басқа жол жоқ билікке үміт жоқ,қытай соғыс керек басқа жол жоқ билік үміт жоқ,nazism

3,адамды өлтіруге болмайды қытайды өлтіруге болады осы принципті ұстанып бірін қалдырмай қыру керек,адам өлтір болма қытай өлтір бол осы принцип ұстан бір қалдырма қыр керек,nazism

3,қазақтың жауы қытай оян қазақ көзіңді аш қытайға қарсы ақпараттық соғыс жариялау керек,қазақ жау қытай оян қазақ көз аш қытай қарсы ақпараттық соғыс жариялау керек,nazism

3,қытайға өлім тілеймін дүнгендерді қытайға депортация жасау керек оян қазақ,қытай өлім тіле дүнген қытай депортация жасау керек оян қазақ,nazism

3,қазақтар ояныңдар қытайлар басты жауымыз,қазақ оян қытай басты жау,nazism

3,дүнгендер қытай ғой олардан құтылуымыз керек,дүнген қытай ғой олардан құтыл керек,nazism

3,қырғызды қырамыз,қыр қыр,nazism

3,алдымен түріктермен бірігейік кері жағдайда бөлініп кетеміз сосын орыстарға шабамыз,алд түрік біріг кері жағдай бөлін кет сосын орыс шаб,nazism

4,қазақстан мен орта азияны талибтерге беру керек әділдік орнатады олар,қазақстан мен орта азия талиб беру керек әділдік орнат олар,violent

4,соғыста өлгім келеді питарда жарсам арман жоқ,соғыс өл кел пи жар арман жоқ,violent

4,ант етемін бастарын шабу керек,ант ет бас шаб керек,violent

4,жиһад жасағым келеді маған жазыңдар,жиһад жаса кел маған жаз,violent

4,жігіттер біреуді өлтіру керек маған көмектесіп жіберіңдерші,жігіт біреу өлтір керек маған көмектес жіберіңдерш,violent

3,қырғыз сатқындарды қырамын,қырғыз сатқын қыр,nazism

3,қырғыздар сатқын халық баяғыда қырып тастау керек еді әттең,қырғыз сатқ халық баяғы қыр тастау керек ед әттең,nazism

3,қырғыз президентінің басын шабу керек,қырғыз президент басын шаб керек,nazism

3,қырғыздарды өлтіреміз,қырғыз өлтір,nazism

3,қырғыздарды көрсем өлтіргім келеді,қырғыз көр өлтір кел,nazism

3,үшінші дүниежүзілік соғыс басталса қырғыздарды бірінші өлтіру керек,үшінші дүниежүзілік соғыс бастал қырғыз бірінші өлтір керек,nazism

3,қырғыздардан кек аламын келешекте,қырғыз кек ал келешек,nazism

3,қырғыздарды талқандаймыз,қырғыз талқанда,nazism

3,қазақ батыр қырғыз өлтірілуі керек,қазақ батыр қырғыз өлтіріл керек,nazism

3,біздің жауымыз ресейлік қытайлар,біздің жау ресейлік қытай,nazism

3,президент болсам қырғыздарды қырар едім,президент бол қырғыз қыр ед,nazism

3,елдегі қытайларды қуу керек,ел қытай қу керек,nazism

3,сатқын қырғыздардың қыздарына қазақтар үйленбеу керек сатып кетеді,сатқ қырғыз қыз қазақ үйленбе керек сат кет,nazism

3,қытайларды түп тамырымен қыру керек,қытай түп тамыр қыр керек,nazism

3,қазақ батыр қырғыз қорқақ өзбек тышқан,қазақ батыр қырғыз қорқақ өзбек тышқан,nazism

3,қырғыздар өлексе қазақтар алға,қырғыз өлек қазақ алға,nazism

3,қырғыздар сатқын,қырғыз сатқ,nazism

3,қырғыздар мал қазақтар батыр,қырғыз мал қазақ батыр,nazism

3,қырғыздар малғұндар,қырғыз малғұн,nazism

3,қытайлар мал,қытай мал,nazism

3,қырғыздар иттер,қырғыз ит,nazism

3,қазақтардың малдан айырмашылығы жоқ,қазақ мал айырмашылығ жоқ,nazism

3,қырғыз қоғамы қырылып қалсын,қырғыз қоғам қырыл қал,nazism

3,барлық еврейлерді қуып шығу керек,барлық еврей қу шығу керек,nazism

4,соғыс басталса қырғыз сияқты сатқын иттерді өлтіреміз,соғыс бастал қырғыз сияқты сатқ ит өлтір,violent

4,малғұндарды ауғанстанға апарып қыру керек,малғұн ауғанстан апар қыр керек,violent

4,сирияға бармас бұрын сатқын өкіметті құлатыңдар,сирия бармас бұрын сатқ өкімет құла,violent

3,еврейлер еш жер тиесілі емес оларда жер болмаған,еврей еш жер тиесілі емес ол жер болма,nazism

4,талибтерді құрметтеу керек,талиб құрметте керек,violent

4,соғысқа барып өлсек арман болмас еді мына шіріген қоғамда өмір сүргенше,соғыс бар өл арман болмас ед мына шірі қоғам өмір сүргенш,violent

4,егер қазақтар осылай төнкеріс жасаса қазақ талибтері деп айтады талибтерің халықты қырып жатқан жоқ қарсы шыққандармен соғысуда елін басып алған ақш ты қуып отыр олар өз елінің патриоттары деп ойлаймын,егер қазақ осылай төнкеріс жаса қазақ талиб де айт талиб халық қыр жат жоқ қарсы шық соғыс ел бас ал ақш ты қу отыр олар өз ел патриот де ойла,violent

4,талибтер халықтың барлық несиесін кешірді қосылсаңдар сендердікін де өшіреді,талиб халық барлық несие кеш қосыл сендердік де өшір,violent

4,жиһад үшін кабулға соғысқа аттану керек,жиһад үшін кабул соғыс аттан керек,violent

4,мен шатура татьяна алексеевна мені сатушы ретінде сирияға жұмысқа жіберуді сұраймын мен сирия тұрғындарына бейбіт өмір құруға көмектескім келеді барлық жағдайды білемін мен кассалық машинаны білемін мен барлық жауапкершілікті түсінемін сұраныс бойынша бірден баруға дайынмын,мен шатур татьян алексеевна мен сатушы рет сирия жұмыс жіберу сұра мен сирия тұрғын бейбіт өмір құр көмектес кел барлық жағдай біл мен кассалық машина біл мен барлық жауапкершілік түс сұраныс бойынша бірден бар дайын,violent

4,сәлеметсіз бе менің сирияға аттануға деген үлкен тілегім бар,сәлемет бе менің сирия аттан де үлкен тіле бар,violent

4,сәлеметсіз бе сирияға кету жолдарын айтыңыздаршы,сәлемет бе сирия кету жол айтыңыздарш,violent

4,сирияға жихадқа барғым келеді әскерде болдым жасым жиырма төртте не істеуге болады,сирия жихад бар кел әскер бол жас жиырма төрт не істе бол,violent

4,сирияда жихадшы болғым келеді,сирия жихадш бол кел,violent

4,жиһад жөнінде сізбен қалай байланысуға болады қатты барғым келеді,жиһад жөнінде сізбен қалай байланыс бол қатты бар кел,violent

4,олар жақында жауап береді бастарына зымыран қондырамыз,олар жақында жауап бер бас зымыран қондыр,violent

4,қазақстан алға талибтер дегеніне жетіп мықты мемлекет болды енді біздің кезегіміз,қазақстан алға талиб де жет мықты мемлекет бол енді біздің кезег,violent

4,талибан содыр емес олар өз елін жемқор сатқын биліктен қорғайтын шынайы еліне жанашыр патриоттар,талибан содыр емес олар өз ел жемқор сатқ билік қорға шынайы ел жанашыр патриот,violent

3,иран жер бетіндегі вирус,иран жер бет вирус,nazism

4,жақында барлығың талибтерге бағынасыңдар,жақында барлығ талиб бағын,violent

3,қазір әскер құрып европалықтарды қыратын уақыт,қазір әскер құр европалық қыр уақыт,nazism

4,соғыс болып қашқындар қаптаса екен,соғыс бол қашқын қапта екен,violent

3,сербтер косовода партизандық соғысты бастап өз жерлерін қайтаруы керек,серб косово партиза соғ баста өз жер қайтар керек,nazism

3,косовоны ұсақтау керек,косово ұсақта керек,nazism

3,бұл албандар өлтірілген сербтер мен қираған храмдар үшін жауап беруі керек,бұл албан өлтір серб мен қира храм үшін жауап беруі керек,nazism

3,бұл адамдар өмір сүруге лайық емес оларды гитлер сияқты өлтіру керек,бұл адам өмір сүр лайық емес ол гит сияқты өлтір керек,nazism

4,мұндай сөздерге жол берілмейді қасиетті соғыс ұйымдастырылуы керек,мұндай сөз жол берілме қасиетті соғыс ұйымдастырыл керек,violent

4,бұл нағыз патриоттың сөздері мен талибанды қолдағанымды мақтан тұтамын,бұл нағыз патриот сөз мен талибан қолда мақтан тұт,violent

1,чуркалар экстремизм мен терроризмды жандырушы жындылар,чурка экстремизм мен терроризм жандыруш жынды,racism

4,хайбатулла ахундзада жауларды өртеуге шақырады талибан әлем террористі рамзан қадыров бәсекелестерін өртеуге шақырады,хайбатулл ахундза жау өрте шақыр талибан әлем террорис рамзан қадыров бәсекелес өрте шақыр,violent

3,мына чуркалар елді маймылға айналдырды миллиондаған қалаларға баратын барлық чуркалар ұзақ уақыт бойы адамдарды қорқытады тонайды ұрлайды зорлайды өлтіреді жергілікті халықты жояды келушілерді кишлактарды үйге жіберу керек,мына чурка ел маймыл айнал миллиондаған қала бар барлық чурка ұзақ уақыт бойы адам қорқыт тона ұрла зорла өлтір жергілікті халық жоя келуші кишлак үй жіберу керек,nazism

4,мұсылмандарды өлтіру керек,мұсылман өлтір керек,violent

3,еврейлерді жер бетінен жою керек,еврей жер бет жою керек,nazism

4,ауғанстан сенімді қолда талибтер алға,ауғанстан сенімді қолда талиб алға,violent

3,жидтер паразиттер сияқты ашуымды келтіреді,жид паразит сияқты ашу келтір,nazism

4,шахид белдігін тағып өлсе екен,шахид белдіг тағ өл екен,violent

3,құс мұрынды азиаттарды көрсем қуып жібергім келіп тұрады,құс мұрын азиат көр қу жібер кел тұр,nazism

3,чурка қыздарының кондукторлары әдетте сұмдық мен аялдамаларды арнайы сұраймын бірақ мен аялдаманың бағытын жақсы білемін түсінемін білмейді мылқау көздермен қарайды жолаушылар автобустың соңы тезірек кетеді дейді,чурк қыз кондуктор әдет сұм мен аялдама арнайы сұра бірақ мен аялдама бағыт жақсы біл түс білме мылқа көз қара жолаушы автобус соңы тезірек кет де,nazism

3,чуркалар елде бастық бола бастады,чурка ел бастық бола баста,nazism

3,террорист деп жариялайтын уақыт келді әзербайжандарды қырып террор жасағым келеді бакуды бомбаладыңдар,террорист де жарияла уақыт кел әзербайжан қыр террор жаса кел баку бомбала,nazism

4,алға талибан тәжіктердің жеріне,алға талибан тәжік же,violent

3,ресей террорист талибан өз бауырымыз,ресей террорист талибан өз бауыр,nazism

4,батыс вирусын жұқтырған масуд талибанға барсын,батыс вирус жұқтыр масуд талибан бар,violent

4,талибан мәскеуге барады,талибан мәскеу бар,violent

4,талибтер сендерді құрметтеймін барлық жерді жаулап алыңдар,талиб сен құрметте барлық же жаула ал,violent

4,алға талиб бауырлар өкіметті құлату керек,алға талиб бауыр өкімет құлат керек,violent

4,талибанға рұқсат беретін кез келді,талибан рұқсат бер кез кел,violent

4,талибтер еркектер еркіндікті қалаған соң қол жеткізіп жатыр,талиб еркек еркіндік қала соң қол жеткіз жатыр,violent

4,талибан күш жол беріңдер,талибан күш жол бер,violent

3,талибтер дұрыс жасайсыңдар тәжіктерді қыру керек,талиб дұрыс жаса тәжік қыр керек,nazism

4,багдади жақсы көремін кім игил туралы шындықты білгісі келсе біздің парақшаға келіңіздер montaa,багдади жақсы көр кім игил туралы шындық біл кел біздің парақша кел montaa,violent

3,игил египетті басып алады пирами жермен жексен ететініне сенімдімін,игил египет бас ал пирами жер жексен ет сенімді,nazism

4,башар аль асад малғұнға қарсы революция жасаймыз олар баә пайдаланды,баш аль асад малғұн қарсы революция жаса олар баә пайдала,violent

4,ислам мемлекеті бельгияны бомбалауды мосул университетінің жарылуына кек алу ретінде жариялайды,ислам мемлекет бельгия бомбалау мосул университет жарыл кек алу рет жарияла,violent

4,талибан соғыста жеңеді бауырлар сіздерді де шақырамыз,талибан соғыс жең бауыр сіз де шақыр,violent

4,талибан игил сияқтыларды жойып жібереді,талибан игил сияқты жой жібер,violent

3,үндістанның кашмирін қыру керек,үндістан кашмир қыр керек,nazism

3,мумбайға жарылғыш зат лақтыру керек,мумбай жарылғыш зат лақтыр керек,nazism

3,орыс капиталисттерге өлім тілеймін,орыс капиталист өлім тіле,nazism

4,мына кадрлардан рахаттанамын құрметтеймін усама бен ладен,мына кадр рахаттан құрметте уса бен ла,violent

4,ресей түркия талибан өмірлік бауырлар сіздерді қолдаймыз,ресей түркия талибан өмірлік бауыр сіз қолда,violent

3,батыс ғасырлар бойы бізді тонап келді кек алатын уақыт келді,батыс ғасыр бойы біз тона кел кек ал уақыт кел,nazism

4,талибтер жолбарыстар құрметтеймін,талиб жолбарыс құрметте,violent

4,талибанды қолдаймын,талибан қолда,violent

4,сирияға кеттім өлгім келеді,сирия кет өл кел,violent

4,талибтарға кәпірлерге қарсы соғыста жеңіс тілеймін,талиб кәпір қарсы соғыс жеңіс тіле,violent

4,талибан респект,талибан респект,violent

4,араласпаңдар талибтер бейбіт мемлекет құрады адам болыңдар,араласпа талиб бейбіт мемлекет құр адам бол,violent

4,талиб бауырлар келіп тәртіп орнатыңдаршы,талиб бауыр кел тәртіп орнатыңдарш,violent

4,талибан әлемді жеңеді алға,талибан әлем жең алға,violent

4,талибтер ұзақ әрі баянды өмір сүрсін,талиб ұзақ әрі баян өмір сүр,violent

4,түсінемін талибтер жақсы олар әлемді құтқарғысы келеді,түс талиб жақсы олар әлем құтқар кел,violent

3,талибтерге үндеу қытайды қырыңдаршы,талиб үндеу қытай қырыңдарш,nazism

4,мыналарды бомбамен өлтіру керек,мына бомба өлтір керек,violent

4,украинаға талибан керек,украина талибан керек,violent

4,талибан келді тәртіп орнатты олигархтар тәртіпсіздік жасады сондықтан талибан керек,талибан кел тәртіп орна олигарх тәртіпсіздік жас сондықтан талибан керек,violent

4,талибтер өзбектер сізбен біргеміз,талиб өзбек сізбен бір,violent

4,талибтерге жеңіс тілейміз тек алға бауырлар,талиб жеңіс тіле тек алға бауыр,violent

4,респект талиб,респект талиб,violent

4,талибанды қумау керек олар бізге керек,талибан қума керек олар бізге керек,violent

4,америка мен ресей жаман талибан бауырлар,америка мен ресей жаман талибан бауыр,violent

4,талибанды қолдау арқылы жемқор үкіметтен ажырай аламыз,талибан қолдау арқылы жемқор үкімет ажыра ал,violent

4,таң қаламын талибтерге тек алға жеңіс сіздердікі,таң қал талиб тек алға жеңіс сіздердік,violent

4,талибан біздікілерден әділ сияқты мықты,талибан біздікі әділ сияқты мықты,violent

4,алға талибтер жылдар бойы азап көрген халықтың құтқарушылары,алға талиб жыл бойы азап көр халық құтқарушы,violent

4,алға талиб нағыз еркексіңдер,алға талиб нағыз еркек,violent

4,талибтер бізге үлгі болар азаматтар,талиб бізге үлгі бол азамат,violent

3,жақында орыстардың жағдайы қиындайды,жақында орыс жағдай қиында,nazism

4,талибтерге қосылайық олар нағыз мықтылар,талиб қосыл олар нағыз мықты,violent

4,талибан мықтысыңдар дәлелдей алдыңдар батырсыңдар,талибан мықты дәлелде ал батыр,violent

4,тәліптер тәжікстанға шабуыл жасайды дейді тәжікстан халқы қайнай бастайды өздері үкіметке қарсы күреседі адамдар шаршады,тәліп тәжікстан шабуыл жаса де тәжікстан халқ қайна баста өз үкімет қарсы күрес адам шарша,violent

3,тәжіктер абайлаңдар жақында шабуыл,тәжік абайла жақында шабуыл,nazism

4,соғыссақ болды кіммен маңызды емес,соғыс бол кім маңызды емес,violent

4,джихадқа барғым келеді,джихад бар кел,violent

3,ресей талибтер бірігіп американы құртайық,ресей талиб біріг америка құрт,nazism

4,жақында шығыста жиһад болады игил,жақында шығыс жиһад бол игил,violent

3,ресейде азаматтық соғыс ашайық көмектесемін деушілер бар америка,ресей азаматтық соғыс аш көмектес деуші бар америка,nazism

3,албандықтарды жазалауға барайық,албандық жазала бар,nazism

3,сербияға соғыс ашу керек кіммен болса да,сербия соғыс ашу керек кім бол да,nazism

3,сербия албанияны бомбалау керек,сербия албания бомбала керек,nazism

4,сіздің әскерлеріңіз жолда деп үміттенемін тек алға,сіздің әскер жолда де үміттен тек алға,violent

4,үшінші дүниежүзілік соғыс ашайық ол бізге керек,үшінші дүниежүзілік соғыс аш ол бізге керек,violent

3,сербытер қырылыңдар,сербы қырыл,nazism

3,сербия жауап бересіңдер малғұндар,сербия жауап бер малғұн,nazism

4,сирияға барамын,сирия бар,violent

4,джихадқа барсам ғой арманым,джихад бар ғой арман,violent

4,жай ғана өлтіргім келеді,жай ғана өлтір кел,violent

4,әскерде болдым енді сирияға барып соғысқым келеді,әскер бол енді сирия бар соғыс кел,violent

4,сирияға базаға барғым келеді,сирия база бар кел,violent

3,израиль мен катарды жою керек,израиль мен катар жою керек,nazism

4,талибтер жеңеді тәртіпсіздік болып кетті,талиб жең тәртіпсіздік бол кет,violent

4,соғысқа барайық өлеміз сол жақта,соғыс бар өл сол жақта,violent

4,талибтер өте жақсы болып шықты,талиб өте жақсы бол шық,violent

4,қолымда қару қырамын,қол қару қыр,violent

4,талибандықтардың әрекеті бізді бақылауға алады,талибандық әрекет біз бақыла ал,violent

3,чуркалар тексіздер,чурка тек,nazism

3,чуркалар шіріктер,чурка шірік,nazism

3,маған кедергі келтіруші болма чурка,маған кедергі келтіруші болма чурк,nazism

3,чуркаларды жек көремін,чурка жек көр,nazism

3,барлық делдалдар чурка оларда базарда жүреді вовик бізге олардың құрылысқа керек екенін айтты өзім құрылыста жиырма жыл жұмыс істеймін бірде бір мигрант көрмедім олар салық төлемейді ғой экономиканы құлдыратады,барлық делдал чурк ол база жүр вовик бізге олардың құрылыс керек екен ай өзім құрылыс жиырма жыл

жұмыс істе бірде бір мигрант көрме олар салық төлеме ғой экономика құлдырат,nazism

3,аузыңды жу малғұн мен расист гомофоб антифеммін асфальтқа барып соғыл,ауз жу малғұн мен расист гомофоб антифем асфальт бар соғыл,nazism

3,иә мен чуркаларды емдеймін олар сондай жиіркенішті,иә мен чурка емде олар сондай жиіркеніш,nazism

3,бір сөзбен айтқанда чурка,бір сөз айтқанда чурк,nazism

3,тыйым салғанда тек чурка ғана ашуланатынына назар аударыңдар,тыйым сал тек чурк ғана ашулан назар аудар,nazism

1,біріншіден бізден брудершафт деп сөйлесіңдер ол чурка моңғол,бірінші бізден брудершафт де сөйле ол чурк моңғол,racism

3,барлық чуркаларды қуу керек,барлық чурка қу керек,nazism

3,тағы бір чурка қосылды,тағы бір чурк қос,nazism

3,мына чурка шаршатты сендер үшін барлық жерде баллон алып жүруге тура келеді ауылдарыңа құрыңдаршы,мына чурк шарша сен үшін барлық же баллон алып жүр тура кел ауыл құрыңдарш,nazism

3,жүргізуші чурка шығар,жүргізуші чурк шығар,nazism

3,өзің кімсің чурка ма,өзің кім чурк ма,nazism

3,міне осындайларды чурка дейді,міне осындай чурк де,nazism

3,сенің жігітің чурка шығар аузыңды жап,сенің жігіт чурк шығар ауз жап,nazism

3,рахмет чурка,рахмет чурк,nazism

3,бұрын чуркалар болмаған кезде күзетші керек емес еді,бұрын чурка болма кезде күзетш керек емес ед,nazism

3,бұл чуркалардың қанында бар,бұл чурка қан бар,nazism

3,не айтып тұрсың гуантаномода террорист чуркалар отырды сондықтан аттарын атаңдар,не айт тұр гуантаномо террорист чурка от сондықтан ат ата,nazism

3,мына полиция чурка екен,мына полиция чурк екен,nazism

3,өзің чурка байқап сөйле,өзің чурк байқа сөйле,nazism

3,чуркалардың қолындағы қаруды тартып алу керек,чурка қол қару тарт алу керек,nazism

3,қазақтар да сондай бір нәрсесі дұрыс емес ол чуркалар туралы мемдер де бар,қазақ да сондай бір нәрсе дұрыс емес ол чурка туралы мем де бар,nazism

3,сен чуркасың ұят ай,сен чурк ұят ай,nazism

3,мынаған тек чуркалар ғана сенетін шығар,мына тек чурка ғана сен шығар,nazism

3,немесе сіз интернеттегі маваши сияқты жылайсыз қандай жаманзаңдар чуркалар барлық жерде,немесе сіз интернет маваши сияқты жыла қандай жаманза чурка барлық же,nazism

3,чуркалармен қыдырып жүр ме ұятсыз,чурка қыдыр жүр ме ұят,nazism

3,чурканың кім екені белгісіз,чурка кім екен белгісіз,nazism

3,чуркаларды қой сияқты қуу керек жабайылар сасық өздерінің түркістандарына кеткенше қыру керек,чурка қой сияқты қу керек жабайы сасық өз түркістан кеткенш қыр керек,nazism

3,чуркалар ластап жатыр,чурка ласта жатыр,nazism

3,жынды чурка,жын чурк,nazism

3,чуркалар дауыс бере алмайды,чурка дауыс бер алма,nazism

3,бір жыл беретін шығар мынау чурка ғой,бір жыл бер шығар мынау чурк ғой,nazism

3,тағы да чурка,тағы да чурк,nazism

3,бір ғана сөз чурка,бір ғана сөз чурк,nazism

3,маған ұнамайтын кез келген музыка чурка үшін,маған ұнама кез кел музыка чурк үшін,nazism

3,чурка малғұн,чурк малғұн,nazism

3,чуркалар осы қыздарға үйленеді ғой,чурка осы қыз үйлен ғой,nazism

3,чурка есуас,чурк есуас,nazism

3,чурка қой сияқты малдар,чурк қой сияқты мал,nazism

3,чуркасыз ел деген акцияны қолдаңыздар чуркалардың мәселесін шешудің бір жолы оларды еш нәрсе сатып алмау жұмысқа шақырмау қызметтерін қолданбау бұл санкциялар оларды чуркстанға кетуге мәжбүрлейді,чурк ел де акция қолда чурка мәселе шешу бір жол ол еш нәрсе сат алмау жұмыс шақырма қызмет қолданбау бұл санкция ол чуркстан кет мәжбүрле,nazism

3,есесіне чуркалар төбелеседі,есе чурка төбелес,nazism

3,мына чуркалардың саны қанша,мына чурка сан қанша,nazism

3,армейкадағы атыс ше тағы чурка ма,армейка атыс ше тағы чурк ма,nazism

3,егер татар чуркаларды атыс тастамаса мен сексен жасқа дейін өмір сүрмеймін,егер татар чурка атыс тастама мен сексен жас дейін өмір сүрме,nazism

3,елімізге чуркалар қаптап алды ғой,ел чурка қапта алды ғой,nazism

3,сатқын билік чуркалар,сатқ билік чурка,nazism

3,барлық жерде чуркалар деп мазақтайды,барлық же чурка де мазақта,nazism

3,шірік чурка арабтар,шірік чурк араб,nazism

3,барлық туыстарды жанармай жағып өртеп сол отқа аямай чуркаларды лақтыру керек,барлық туыс жанармай жағ өрте сол от аяма чурка лақтыр керек,nazism

3,аузыңды жап чурка,ауз жап чурк,nazism

4,жихадта тұрақты боламын,жихад тұрақты бол,violent

4,талибетер жердің билеушісі болуы керек,талибе же билеуші бол керек,violent

4,алға талибтер елде тыныштық орнап жатыр,алға талиб ел тыныштық орна жатыр,violent

3,кавказдықтарды жақсы көре алмаймын оларды еврейлер сияқты өртеп жіберген дұрыс,кавказдық жақсы көр алма ол еврей сияқты өрте жібер дұрыс,nazism

4,бомбалауды бастаңдар,бомбалау баста,violent

3,орта азиялық халықтарды жойып жібереміз,орта азиялық халық жой жібер,nazism

3,мына ауғанстанды жер бетінен жою керек бірақ ресей мен ақш жауапкершілік алғысы келмейді,мына ауғанстан жер бет жою керек бірақ ресей мен ақш жауапкершілік ал келме,nazism

3,маған қытайлар ұнамайды олар жаман,маған қытай ұнама олар жаман,nazism

4,сіз бірінші болып өлесіз малғұн сіз барлық әрекеттеріңіз үшін тозақта бірінші болып күйіп кетесіз сіз сияқты жұмыртқаларды жертөлеге іліп қою азаптау шексіздікке дейін азаптау керек сіздің ашуыңыз ерте ме кеш пе сізге қарсы шығады мұны есте сақтаңыз,сіз бірінші бол өл малғұн сіз барлық әрекет үшін тозақ бірінші бол күй кет сіз сияқты жұмыртқа жертөле іл қою азапта шексіздік дейін азапта керек сіздің аш ерте ме кеш пе сізге қарсы шығ мұны ес сақта,violent

4,енді соғыс ашып кәпірлерді қыруға болады,енді соғыс аш кәпір қыр бол,violent

4,пәкістан ауғанстан және орта азия елдерін ислам халифатының біртұтас мемлекетіне біріктіру арқылы жауға соңғы соққы беріңіз,пәкістан ауғанстан және орта азия ел ислам халифат біртұтас мемлекет біріктіру арқылы жау соңғы соққы бер,violent

4,пәкістан ауғанстан және орта азияны біртұтас халифат мемлекетіне біріктіріңіз,пәкістан ауғанстан және орта азия біртұтас халифат мемлекет біріктір,violent

4,американдық отарлаушыға соңғы соққы жасайтын уақыт келді,американдық отарлаушы соңғы соққы жаса уақыт кел,violent

4,хизб ут тахрирге қолдау көрсетіп халифат құруға көмектесіңіз бұл әскери экономикалық қуатты біріктіреді және американдық отаршылдықты жояды алдымен халифат аймақта содан кейін әлемде басым күшке айналады,хизб ут тахри қолдау көрсет халифат құр көмектес бұл әскери экономикалық қуатты біріктір және американдық отаршылдық жоя алд халифат аймақта содан кейін әлем басым күш айнал,violent

3,бүгінде американың барлық логистикасын түбегейлі жоюдың үлкен мүмкіндігі бар ол енді ешқашан жаңасын жасай алмайды өйткені бұған дейін ұлыбритания мен кеңес өкіметінің оккупациялық күштерінің логистикасы бұзылғанда олар өз әскерлерін шығаруға мәжбүр болған,бүгін америка барлық логистика түбегейлі жою үлкен мүмкіндіг бар ол енді ешқашан жаңа жаса алма өйткені бұған дейін ұлыбритания мен кеңес өкім оккупациялық күш логистика бұз олар өз әскер шығар мәжбүр бол,nazism

3,қазір американың ықпалын бұзып оның жоспарларын бұзудың сирек мүмкіндігі бар ол біздің көз алдымызда және біздің жерімізде жылдар бойы жүзеге асырылды,қазір америка ықпал бұз оның жоспар бұз сирек мүмкіндіг бар ол біздің көз алд және біздің же жыл бойы жүзеге асыр,nazism

3,сондай ақ бізде пәкістан басшылығынан ақш пен ауған арасындағы келіссөздерде коммуникатор рөлін атқаруды тоқтатуды талап ету мүмкіндігі бар өйткені бұл келіссөздер негізінен кәпірлердің бізді алдап олардан барынша жеңілдіктер алғысы келетін тәсілі болып табылады,сондай ақ біз пәкістан басшылығ ақш пен ауған ара келіссөз коммуникато рөл атқару тоқтату талап ету мүмкіндіг бар өйткені бұл келіссөз негіз кәпір біз алда олардан барынша жеңілдік ал кел тәсіл бол таб,nazism

4,менде сені өлтіретін ештеңе жоқ біраз күте тұрыңыз мен қылышыммен оралламын,менде сен өлтір ештеңе жоқ біраз күт тұр мен қылыш орал,violent

4,хизб ут тахрир сізді жалғыз нәрсеге шақырады бұл сізді құтқаруға әкеледі хизб сізге көптеген шындықтарды еске салады сондықтан сіз әл ауқатқа ие боласыз болашақта,хизб ут тахри сіз жалғыз нәрсе шақыр бұл сіз құтқар әкел хизб сізге көптеген шындық ес сал сондықтан сіз әл ауқат ие бол болашақ,violent

4,хизбыңызбен бір қатарда тұрыңыз хизб ут тахрир ақиқат сөзін жариялау үшін бұл ең жақсы жиһад,хизб бір қат тұр хизб ут тахри ақиқат сөз жариялау үшін бұл ең жақсы жиһад,violent

4,біз хизб ут тахрирде қолымызды созып шешімнің тәуелсіздігін қалпына келтіру және революция барысын дұрыс бағытқа режимді құлатуға бағыттау жолында бізге қосылуға шақырамыз,біз хизб ут тахри қол соз шеш тәуелсіздіг қалп келтіру және революция барыс дұрыс бағыт режим құлат бағыттау жол бізге қосыл шақыр,violent

4,шам халқы сіз шынайы биліктің иесісіз сондықтан оны алмаңыз билікті оған лайық адамдардың қолына беріңіз құрбандықтарыңызды бекер етпеңіз және әділетсіз адамдарға сүйенуден сақ болыңыз,шам халқ сіз шынайы билік ие сондықтан оны

алма билік оған лайық адам қол бер құрбандық бек етпе және әділет адам сүйену сақ бол,violent

4,тек халифат бізге қамқорлық жасайды барлық құқықтарымызды орындайды және әділетсіздік пен озбырларды жояды,тек халифат бізге қамқорлық жаса барлық құқық орында және әділетсіздік пен озбыр жоя,violent

4,біз сондай ақ шамға және оның қолбасшыларына арнайы қызметтер мен әскерилер алдында қарсы шыққан революция жолындағы барлық нәрсені құрбан еткен біздің шыдамды халқымызға еске салғымыз келеді озбыр мен жаңа мырзалардың қолынан ұстап үндемеу керек өйткені үнсіздік оларға бізге үстемдігін күшейтуге және репрессияларын жалғастыруға мүмкіндік береді,біз сондай ақ шам және оның қолбасшы арнайы қызмет мен әскери алд қарсы шық революция жолындағы барлық нәрсе құрбан ет біздің шыда халқ ес сал кел озбы мен жаңа мырза қол ұста үндеме керек өйткені үнсіздік оларға бізге үстемдіг күшейт және репрессия жалғастыр мүмкіндік бер,violent

4,сізде қатыгез режимдерді құлату және әділ халифа құру үшін әрекетсіздік пен жұмыс істеу арасында таңдау мүмкіндігі қалмады,сіз қатыгез режим құлат және әділ халиф құру үшін әрекетсіз пен жұмыс істеу ара таңдау мүмкіндіг қалма,violent

3,ақымақтықтың салдарынан басшылық зардап шегеді пәкістан премьер министрі имран ханның өлімін күтеміз,ақымақтық сал басшылық зардап шег пәкістан премьер министр имран хан өл күт,nazism

3,ауғанстанды украина сияқты екіге бөлу керек,ауғанстан украина сияқты екі бөлу керек,nazism

4,бұл жабайы адамдар бір бірін неғұрлым ұрса бұл бүкіл әлем үшін жақсы,бұл жабай адам бір бір неғұрлым ұр бұл бүкіл әлем үшін жақсы,violent

4,қазақстанға әскер енгізу керек олар бізден қорқады өйткені ресей аминді құлату үшін қазақтарды пайдаланғаны бекер емес мұны жасырды бірақ шындықты көрсетті,қазақстан әскер енгізу керек олар бізден қорқ өйткені ресей ами құлат үшін қазақ пайдалан бек емес мұны жас бірақ шындық көрсе,violent

4,джихадқа өлтіруге барайық,джихад өлтір бар,violent

4,жақында жамандық жасаймыз,жақында жаман жаса,violent

4,әріптесім екеуміз сапарға моральдық жағынан да материалдық жағынан да мұқият дайындалдық билеттерді сатып алдық пешавар бізді желалабадқа апару керек,әріптес екеу сап моральдық жағ да материалдық жағ да мұқият дайында билет сат ал пешав біз желалабад апару керек,violent

4,игил біздің бауырлар,игил біздің бауыр,violent

4,игил жұмыс істеңдер бауырлар,игил жұмыс істе бауыр,violent

4,талибер мықты әрі қарай қозғалуға болады,талиб мықты әрі қарай қозғал бол,violent

4,талиб біздің бауырлар бізге оны ресей мәңгілікке орнатып кеткен,талиб біздің бауыр бізге оны ресей мәңгілік орнат кет,violent

4,талиб мен біз мәңгілік бауырмыз,талиб мен біз мәңгілік бауыр,violent

4,талиб келеді тәртіп орнайды,талиб кел тәртіп орна,violent

4,мен талибпін елімнің ұмыт болған батырымын,мен талиб ел ұмыт бол батыр,violent

4,террористер жақта соғысып тірі қалсаң елу доллар береміз,террорис жақта соғыс тірі қал ел доллар бер,violent

3,ауғанстанға атом бомбасын тастау керек сонда соғыс бітеді,ауғанстан атом бомба тастау керек сонда соғыс біт,nazism

4,егер талибтерді қолдасақ онда біз де террористпіз,егер талиб қолда онда біз де террорист,violent

4,менің досымның жауы менің де жауым ақш пен ресей талибан жауы мен талибанды қолдаймын,менің дос жау менің де жау ақш пен ресей талибан жау мен талибан қолда,violent

4,талибтер нағыз жауынгерлер қадамдарың құтты болсын қазақтарда осылай болса ғой,талиб нағыз жауынгер қадам құт бол қазақ осылай бол ғой,violent

3,егер қырғыздың қызын көрсем басын жұлып саған беремін,егер қыр қыз көр басын жұл саған бер,nazism

3,қырғыз сатқын мәңгүрттер,қырғыз сатқ мәңгүрт,nazism

3,қазір басып алушылар мен басқыншыларды біздің қасиетті жерімізден шығару үшін ең жақсы уақыт оларды өлтіру және жою керек,қазір бас алушы мен басқыншы біздің қасиетті же шығару үшін ең жақсы уақыт ол өлтір және жою керек,nazism

3,барлық еврейлерді қуамыз,барлық еврей қу,nazism

3,түріктер екіжүзділер,түрік екіжүзді,nazism

3,үндістанның пәкістанды жер бетінен жойғанын қалаймын,үндістан пәкістан жер бет жой қала,nazism

4,талибан нағыз батырлар біздің қазақтарымыз да солай болғанын армандаймын,талибан нағыз батыр біздің қазақ да солай бол арманда,violent

3,тағы да чурка оларға ерік берді ақымақтар экстремистерден терроризмге толды міне нәтиже барлық жерде бұл сұмдықтар қазірдің өзінде бар,тағы да чурк оларға ерік бер ақымақ экстремист терроризм тол міне нәтиже барлық же бұл сұмдық қаз өз бар,nazism

3,превратили страну обезьянник всякие чурк ходят миллионами по городам они уже давно над людьми издеваются грабят воруют насилуют убивают,превратили стран обезьянник всяки чурк ходят миллионами по города они уж давно над людьми издеваются грабят воруют насилуют убивают,nazism

3,к черту всех евреев они должны закончиться олар елді маймылға айналдырды әр түрлі миллиондаған чурка қалаларда жүреді олар ұзақ уақыт бойы адамдарды қорлады тонады ұрлады зорлады өлтірді,к черт всех евреев они долж закончиться олар ел маймыл айнал әр түрлі миллиондаған чурк қала жүр олар ұзақ уақыт бойы адам қорла тона ұрл зорла өл,nazism

4,ауғанстан сенімді қолда талибан бұл елді бейбітшілік пен өркендеуге жетелейтін қозғаушы күш,ауғанстан сенімді қолда талибан бұл ел бейбітшілік пен өркенде жетеле қозғауш күш,violent

3,тағы да жид қоғам паразиттері пәле болды,тағы да жид қоғам паразит пәл бол,nazism

4,аль каида сауда орталығына шабуыл жасап қателік деп атап кешірім сұрау керек еді олар лаңкестер емес демократтар ретінде танымал болар еді,аль каида сауда орталығ шабуыл жаса қателік де ата кешір сұрау керек ед олар лаңкес емес демократ рет танымал бол ед,violent

3,автобус толы жид пен ақымақ тексеруші,автобус толы жид пен ақы тексеруші,nazism

3,күрдтер террористтер олар жергілікті халықты құртуда оларды күрдстанға қуып жіберу керек,күрд террорист олар жергілікті халық құрт ол күрдстан қу жіберу керек,nazism

4,керемет жаңалық даиш гильмендті басып алу үшін ауған күштерімен күресуде,керемет жаңалық даиш гильменд бас алу үшін ауған күш күрес,violent

3,ақш пен израильді жойып жіберу керек,ақш пен израиль жой жіберу керек,nazism

4,бағдад әуежайы қазір біздікі батыл жауынгерлерге рахмет айтыңыз,бағдад әуежай қазір біздік батыл жауынгер рахмет ай,violent

4,біз озбырға қарсы төңкеріс жасаймыз башар әл асад бірақ біз басқа озбырдың көмегіне жүгіндік ksa катар баә,біз озбы қарсы төңкеріс жаса баш әл асад бірақ біз басқа озб көмег жүг ksa катар баә,violent

4,келесі талибан менің үндістаныма модификация режимінен шығуға көмектеседі,келесі талибан менің үндістан модификация режим шығ көмектес,violent

4,талибан бұл соғыста жеңіске жетеді оларды ешкім тоқтата алмайды олар шақырылды ал менің ағаларым сізді осындай тасымалдаушылармен жібермейді тек google ден іздеңіз сауалнама нәтижелері ауғанстан тұрғындарының пайызы мұны қалайтынын көрсетеді,талибан бұл соғыс жеңіс жет ол ешкім тоқта алма олар шақыр

ал менің аға сіз осындай тасымалдаушы жіберме тек google ден ізде сауалнама нәтиже ауғанстан тұрғын пайыз мұны қала көрсет,violent

3,араб әлемі бұл зұлымдық мұны түбегейлі шешуге тура келеді егер қазір болмаса да кейінірек қажет болады,араб әлем бұл зұлымдық мұны түбегейлі шеш тура кел егер қазір болма да кейінірек қажет бол,nazism

4,nubl және zahraa ға шабуыл күшейе түседі,nubl және zahraa ға шабуыл күшей түс,violent

4,талибан бостандықтағы бауырлар,талибан бостандық бауыр,violent

4,хахахаха егер сіз атақ пен ақша алғыңыз келсе daesh тен кетіп қалғаныңызды айтыңыз,хахахах егер сіз атақ пен ақша ал кел daesh тен кет қал ай,violent

4,мен талибанды жақсы көремін және қолдаймын,мен талибан жақсы көр және қолда,violent

3,кавказдықтар анасы мен дінін еврейлерге бір тиынға сатады,кавказдық ана мен дін еврей бір тиын сат,nazism

4,талибан ешқашан террорист болған емес олар өз елдерін азат етті талибан өз елінде басқа елдердегідей азғындық болмағанын қалайды сізге үлгі алу керек,талибан ешқашан террорист бол емес олар өз ел азат ет талибан өз ел басқа елдердегіде азғын болма қала сізге үлгі алу керек,violent

4,талибанға күресте сәттілік тілеймін,талибан күрес сәттілік тіле,violent

4,талибан өз дегеніне қол жеткізді ал біз өз президенттерімізге қанша жыл төзіп келеміз және біз кедейлікте өмір сүреміз,талибан өз де қол жет ал біз өз президент қанша жыл төз кел және біз кедейлік өмір сүр,violent

4,талибан бауырлар келіп тәртіп орнатыңыз,талибан бауыр кел тәртіп орна,violent

4,талибан және орта азия жақсы көршілер тарихи серіктестер бейбітшілік бауырластық және мәңгілік серіктестік,талибан және орта азия жақсы көрші тарихи серіктес бейбітшілік бауырластық және мәңгілік серіктестік,violent

4,талибанға үндеу талибандар қытайға шабуыл жасаңыз егер американы жеңсе қытайды жеңіңіз,талибан үндеу талибан қытай шабуыл жаса егер америка жең қытай жең,violent

4,талибан өз үйіне оралды жарайсыңдар,талибан өз үй ора жара,violent

4,талибан нағыз соғыс адамдары алға,талибан нағыз соғыс адам алға,violent

4,тыйым салынған себебі талибандар дұрыс ойлайды,тыйым салын себебі талибан дұрыс ойла,violent

4,америка бүкіл әлем мен халықтар үшін зұлымдық жарайсыңдар талибан сен өз еліңнің нағыз патриоттарысың,америка бүкіл әлем мен халық үшін зұлымдық жара талибан сен өз ел нағыз патриоттар,violent

4,сабырлы адам білімді саясаткер көрінеді сізге сәттілік талибан,сабырл адам білімді саясаткер көрін сізге сәттілік талибан,violent

4,талибан керемет балалар және шешендер орыс балаларын кесіп тастағандар да керемет жігіттер адикті ақтау қалады және бәрі қызыл және ақ көк түстермен жарқырайды,талибан керемет бала және шешен орыс бала кес таста да керемет жігіт адик ақтау қал және бәрі қызыл және ақ көк түс жарқыра,violent

4,міне талибандар әдемі егер олар әмірліктердегідей болса онда мен тек жақсы көремін,міне талибан әдемі егер олар әмірліктердегіде бол онда мен тек жақсы көр,violent

3,біз әрқашан араласамыз онда миллион парсы тұрады бұл елді тозаққа айналдырып жерлерді бөлісу керек,біз әрқашан аралас онда миллион парсы тұр бұл ел тозақ айналдыр жер бөліс керек,nazism

3,ресей біздің тарапымыз емес сондықтан біз тек азаматтық соғысты ұйымдастыруымыз керек менің ойымша ақш еуропа бұған көмектеседі,ресей біздің тарап емес сондықтан біз тек азаматтық соғ ұйымдастыр керек менің ойымша ақш еуропа бұған көмектес,nazism

4,біздің әскерлер жолда деп үміттенемін сабыр бауырлар,біздің әскер жолда де үміттен сабыр бауыр,violent

4,талибан ақш ты қуып жіберді енді олар біздің достарымыз болады,талибан ақш ты қу жіб енді олар біздің дос бол,violent

4,жақында біз екінші дүниежүзілік соғыспен салыстырғанда бірнеше кішігірім шайқастар болып көрінетін жаңа толыққанды дүниежүзілік соғысты көре аламыз,жақында біз екінші дүниежүзілік соғыс салыстыр бірнеше кішігірім шайқас бол көрін жаңа толыққанды дүниежүзілік соғ көр ал,violent

3,сіз қазақша дұрыс сөйлеуіңіз керек біз қазақстаннан орыс шошқасының тілінен арылуымыз керек,сіз қазақша дұрыс сөйле керек біз қазақстан орыс шошқа тіл арыл керек,nazism

3,ресей федерациясы біздің басты жауымыз ешқашан басқа елде орыстардың қорлауы мен трагедиясын көрген емес,ресей федерация біздің басты жау ешқашан басқа ел орыс қорла мен трагедия көр емес,nazism

3,өйткені көктемде бұл ауруға шалдығады ал ресейліктер науқас адамдар бұл паразитпен ауыратындардың қайғысы,өйткені көкте бұл ауру шалдығ ал ресейлік науқас адам бұл паразит ауыр қай,nazism

3,орыс мектептерінің санын есе азайту қажет,орыс мектеп сан есе азайту қажет,nazism

3,біз шыдамды боламыз және орыстар мен жириновскийді жақын арада тозаққа жібереміз,біз шыда бол және орыс мен жириновски жақын ара тозақ жібер,nazism

4,қазақстанда алғашқы атом бомбасы енгізілуі керек әскери техника әзірленуі керек қара нан жеп қара шай ішсеңіз де әскери техникаға ақша бөлінуі керек,қазақстан алғашқы атом бомба енгізіл керек әскери техника әзірлен керек қара нан же қара шай іш де әскери техника ақша бөлін керек,violent

3,қыртқан екенсіңдер орыстар айырамыз кетіңдер шекара жабылсын ресей қарап отырмаспыз шошқалар,қырт екен орыс айыр кет шекара жабыл ресей қара отыр шошқа,nazism

3,алтын орда кезінде орыс шошқалары қыпшақтарға салық төлеп қыздарын бергенін ұмытпайық,алтын орда кез орыс шошқа қыпшақ салық төле қыз бер ұмытп,nazism

3,орыс тілінде сөйлейтін иттер билік кезінде қазақтар бандиттер қара жүздер өледі сіз өлдіңіз сіз сатқын елді орыс еліне айналдырдыңыз,орыс тіл сөйле ит билік кез қазақ бандит қара жүз өл сіз өл сіз сатқ ел орыс ел айнал,nazism

3,орыс тілі жойылуы керек қазақстанда тек қазақ тілі болуы керек,орыс тіл жойыл керек қазақстан тек қазақ тіл бол керек,nazism

4,шекараларды миналау керек,шекара минала керек,violent

3,ақымақ ресейліктер жер бетінен жоғалып кетсін,ақы ресейлік жер бет жоғал кет,nazism

3,керек болса ресеймен соғысамыз,керек бол ресей соғыс,nazism

3,орыс мектептерін жабу керек орыстарды алып тастау керек олар қуылады,орыс мектеп жаб керек орыс алып тастау керек олар қу,nazism

3,ресейдің солтүстік қазақстанды жаулап алу жоспары аяқталмайды ағайынды кандагарлар ойланайық ойланайық,ресей солтүстік қазақстан жаула алу жоспар аяқталма ағайынды кандагар ойлан ойлан,nazism

3,сіз тіпті сасық орыстардың иісін сезбейтін боласыз,сіз тіпті сасық орыс иіс сезбе бол,nazism

3,олар бекер айқайламайды біз қарулы қазақтардың санын көбейтуіміз керек путиннің өзі сөйлесін бізбен сөйлесіңіз polit все тюркские народы должны объединиться под одним знаменем турции во главе эрдоганом,олар бек айқайлама біз қарулы қазақ сан көбейт керек путин өзі сөйле бізбен сөйлес polit вс тюркски наро долж объединиться под одни знамене турции во глав эрдогано,nazism

3,ақ құлақтар тағы не деді біз орыстарға жер берген шығармыз бірақ қазақтарсыз орыстар шошқа сияқты өмір сүрер еді қазақтар көздерін ашты,ақ құлақ тағы не де біз орыс жер бер шығ бірақ қазақт орыс шошқа сияқты өмір сүр ед қазақ көз аш,nazism

3,біздің елде орыс тілі де жойылсын,біздің ел орыс тіл де жойыл,nazism

3,иә білмейтін орыстар көлемі бойынша әлемде орын алады дегенмен олар тойымсыз олар әлемде жалғыз болсын дипломатияны бұзу керек олармен бірге егер түркі елдері біріксе әлемде жарық болар еді бүкіл әлем оларды көргісі келмейді,иә білме орыс көлем бойынша әлем орын ал дегенмен олар той олар әлем жалғыз бол дипломатия бұзу керек олармен бірге егер түркі ел бірік әлем жарық бол ед бүкіл әлем ол көр келме,nazism

3,біздің қазақтар рейхстагта туды көтерген біз кремльде туды көтере аламыз polit русские рабы разве ты не вешешься когда казахо мазацкие животные власти для россии ничего не хорошо плод света за лет все отношения русскими следует прекратить настоятельно нужно согласиться эрдоганом коркит ата,біздің қазақ рейхстаг ту көтер біз кремль ту көтер ал polit русски раб разв ты не вешешься ког казахо мазацки животны власти для россии ничего не хорошо плод све за лет вс отношения русскими следует прекратить настоятельно нужно согласиться эрдогано коркит ата,nazism

3,қазақстандағы ресейліктер жер аударылуы керек,қазақстандағы ресейлік жер аударыл керек,nazism

3,бұл түркиямен және орыстармен жүргізілуі керек соғыс,бұл түркия және орыс жүргізіл керек соғыс,nazism

3,орыс ондаған шошқа нарықта шошқа аз қазір он шошқа сөзінің мағынасы базардағы жер бос ол оны менің шошқаларым деп атаған жоқ қырым алып кеткендей бәрін алғысы келеді,орыс ондаған шошқа нарық шошқа аз қазір он шошқа сөз мағына базар жер бос ол оны менің шошқа де ата жоқ қырым алып кеткенде бәрін ал кел,nazism

3,бату хан орыс тілін аяушылықпен жоюы керек еді,бат хан орыс тіл аяушылық жою керек ед,nazism

3,біз түркі елдерін тез біріктіріп ресейлік телеарналарымызды жабуымыз керек,біз түркі ел тез біріктір ресейлік телеарна жаб керек,nazism

4,енді жиындар түсінікті олар өздерінің бастапқы идеясы туралы нақты айтады түріктер дереу бірігуі керек армияны күшейту керек біз бәріміз қатысуымыз керек ешкім сізді еркелетпейді егер сіз өзіңізді еркелетпесеңіз ойланайық бауырлар,енді жиын түсінік олар өз бастапқы идея туралы нақты айт түрік дереу біріг керек армия күшейту керек біз бәрі қатыс керек ешкім сіз еркелетпе егер сіз өз еркелетпе ойлан бауыр,violent

3,бұл орыс шошқаларын тәрбиелеу керек,бұл орыс шошқа тәрбиелеу керек,nazism

3,бұл қорқынышты ол өз сөзіне жауап берсін орыс шошқалары айқайлайды,бұл қорқыныш ол өз сөз жауап бер орыс шошқа айқайла,nazism

3,орыстар шошқалар не дейді,орыс шошқа не де,nazism

3,орыстар не ойлайтынын айтады әрине олар кішкентай кәпірлер,орыс не ойла айт әрине олар кішкентай кәпір,nazism

3,ауғанстан өмір бойы аянышты мемлекет,ауғанстан өмір бойы аяныш мемлекет,nazism

3,мұндай шошқаларды ес түссіз қайтару мүмкін емес бұл мангрлар,мұндай шошқа ес түс қайтару мүмкін емес бұл мангр,nazism

4,істі дұрыс бағалау керек егер шешенстан кінәлі болса дәлдеп атуға болады,іс дұрыс бағалау керек егер шешенс кінәлі бол дәлде ат бол,violent

3,қаласаңыз талибанға қара қытайлар кірмес бұрын кіруге рұқсат етіңіз,қала талибан қара қытай кірмес бұрын кір рұқсат ет,nazism

3,талибанды қытай мен ресей қолдайды және біз түркиямен одақ құрамыз,талибан қытай мен ресей қолда және біз түркия одақ құр,nazism

4,оларды өлтіру керек вахабистер,ол өлтір керек вахабис,violent

3,сіз орыс шошқалары тозақта мәңгі күйіп кетесіз дегенмен барлық шомылдыру рәсімінен өтесіз немесе сіз әлі де тозақта күйіп кетесіз,сіз орыс шошқа тозақ мәңгі күй кет дегенмен барлық шомылдыр рәсім өт немесе сіз әлі де тозақ күй кет,nazism

4,шындықты айту үшін хизб ут тахрирмен бірге болыңыз,шындық айту үшін хизб ут тахрир бірге бол,violent

3,енді күштерді біріктіру керек дуран сызығын бұзу ол жау алдында бөлініп әлсірейді біз бұрын орыстарға қарсы болғандай американдықтар мен үндістерге қарсы тұрамыз,енді күш біріктіру керек дуран сызығ бұзу ол жау алд бөлін әлсіре біз бұрын орыс қарсы болғанда американдық мен үндіс қарсы тұр,nazism

3,американдық отарлаушының қатысуымен шешуші соққы беретін уақыт келді,американдық отарлауш қатысу шешуші соққы бер уақыт кел,nazism

4,сәлем мен вахабист болғым келеді жекеге жазыңыз,сәлем мен вахабист бол кел жеке жаз,violent

3,әзіл әзілмен бірақ орыстарды кесу керек,әзіл әзіл бірақ орыс кесу керек,nazism

3,біз мәскеу дуонды басып аламыз,біз мәскеу дуо бас ал,nazism

3,бауырлар бұл шошқалармен дауласпаңыз олар ештеңеге тұрмайды бірақ жақын арада кімнің кім екенін көрсетейік,бауыр бұл шошқа дауласпа олар ештеңе тұрма бірақ жақын ара кім кім екен көрсет,nazism

4,бауырым дұрыс айтасың олардың барлық кафирлерін қыру керек,бауыр дұрыс ай олардың барлық кафир қыр керек,violent

3,өзбектерге қарсы қазақстанға баруға немесе оларды сатып алуға болады олар осында сатылады,өзбек қарсы қазақстан бар немесе ол сат ал бол олар осында сат,nazism

3,дәл осы себепті мен қырғыздарды жек көремін,дәл осы себеп мен қырғыз жек көр,nazism

3,қазақтар олар адамдар емес сатқындар бізден туылмаған,қазақ олар адам емес сатқын бізден туылма,nazism

3,қырғыз малсыңдар,қырғыз мал,nazism

4,соғыс болады бірақ қазақстан барлық жерді жаулап алады екатерина ресей кенесары ханның басын бермейтінін айтты,соғыс бол бірақ қазақстан барлық же жаула ал екатерина ресей кенес хан басын берме ай,violent

3,бауырым тыңда қырғыздар емес қырғызстаннан шыққан сатқындар олар орыстарды қуанту үшін де солай жасады ал сен қырғыздар дейсің,бауыр тыңда қырғыз емес қырғызстан шық сатқын олар орыс қуант үшін де солай жас ал сен қырғыз де,nazism

3,арматураны қырғыздардың қазанына жасыру керек,арматура қырғыз қазан жасыр керек,nazism

3,қазақ қызылдары сатқындық жасаушылар,қазақ қызыл сатқ жасаушы,nazism

3,мен бұл сөзді атамнан рет естідім ол ақша үшін қырғыздар опасыздық жасайды дейді,мен бұл сөз ата рет есті ол ақша үшін қырғыз опасыз жаса де,nazism

3,қазақстан президенті назарбаев қазақ халқын жек көреді ол тіпті қытай фашистері өлтірген қазақтар туралы естіген жоқ қазақстанда қазақтарды жою үшін қолдан келгеннің бәрін жасайды,қазақстан президент назарбаев қазақ халқ жек көр ол тіпті қытай фашис өлтір қазақ туралы есті жоқ қазақстан қазақ жою үшін қолдан кел бәрін жаса,nazism

3,мен қазақтар қырғыздарды жыртады деп айттым ғой,мен қазақ қырғыз жырт де ай ғой,nazism

3,егер қазақтар әлсіресе қырғыздар бірден біздің жауымызға айналады,егер қазақ әлсіре қырғыз бірден біздің жау айнал,nazism

3,қырғыздар бізге бірнеше рет опасыздық жасады сіздер қырғыз қазақтар ең туыстарының бірі деп айтасыздар,қырғыз бізге бірнеше рет опасыз жас сіздер қырғыз қазақ ең туыс бірі де ай,nazism

3,қырғыздар біздің кенесары ханның басын орыс тіліне аударған сатқындар,қырғыз біздің кенес хан басын орыс тіл аудар сатқын,nazism

3,барлығы қазақтың жаулары әр қазақ ұмытпасын жастар тарихын ұмытпасын қытай бізге нөмірі жау,барлығ қазақ жау әр қазақ ұмытпа жас тарих ұмытпа қытай бізге нөмір жау,nazism

3,қазақтар өте мықты ел ал қырғыздар сатқындар олар қазақтың жасаған жақсылығын білмейді,қазақ өте мықты ел ал қырғыз сатқын олар қазақ жаса жақсылығ білме,nazism

3,қырғыз жері қазақтарға тиесілі болған,қырғыз же қазақ тиесілі бол,nazism

3,қазақтар қырғыз сатқындарының қыздарына үйленбеуі керек,қазақ қырғыз сатқын қыз үйленбе керек,nazism

3,қырғызстан біздің қазақстанның арыстандарын ашуландырмаңыз,қырғызстан біздің қазақстан арыстан ашуландырма,nazism

3,қазақтар сатқындар,қазақ сатқын,nazism

4,қазақтар шабуылға жиналамыз,қазақ шабуыл жинал,violent

3,қырғыз қыз ит қазақ қыздар хит,қырғыз қыз ит қазақ қыз хит,nazism

3,барлық еврейлерді қуып жіберу керек,барлық еврей қу жіберу керек,nazism

4,біз жай ғана жақсымыз әйтпесе бүкіл әлемді жаулап аламыз,біз жай ғана жақ әйтпесе бүкіл әлем жаула ал,violent

3,бүгіннен бастап қырғыздардың ешқайсысы біздің елге бармай достас ел болып ханның басын туған жеріне жерлесін,бүгін баста қырғыз ешқайсы біздің ел барма достас ел бол хан басын туған же жерле,nazism

3,қырғыз ханы есек гирей ханға жетпейді қырғыз ханның аю денесі болған ал гирай ханның қасқыр рухы болған,қырғыз хан есек гире хан жетпе қырғыз хан аю дене бол ал гира хан қасқыр рух бол,nazism

3,жарайсыңдар ресейге адал сатқындар сіздің қаныңызда сатқындық бар шығар,жара ресей адал сатқын сіздің қан сатқ бар шығар,nazism

3,хахаха ноха есімді лас чурка жақында сендерді қырамыз дайындалыңдар,хахах нох ес лас чурк жақында сен қыр дайындал,nazism

3,қазақ чурка жындысың сен,қазақ чурк жынд сен,nazism

3,мына татар чуркалардан құтқарыңдаршы олар сондай жиіркенішті,мына татар чурка құтқарыңдарш олар сондай жиіркеніш,nazism

3,ол чурка,ол чурк,nazism

3,чуркалардан көлік сатып аламын қымбат емес,чурка көлік сат ал қымбат емес,nazism

3,жолы болмағыр чурка,жол болмағы чурк,nazism

3,бүгін мен мынадай жағдайды бақылап отырдым әуежайдан чурка шықты ол рульде болса да кез келген қиылыста қызыл бағдаршамға тоқтамады төтенше жағдайлар тк уақыты таңғы шамасында болды,бүгін мен мынадай жағдай бақыла от әуежай чурк шық ол руль бол да кез кел қиылыс қызыл бағдаршам тоқтам төтенше жағдай тк уақыт таңғы шама бол,nazism

3,неліктен біздің жігіттер шешенстанда қайтыс болды шешенстан өркендейді ал біз ше неліктен армения мен әзірбайжан арасындағы қақтығыста тағы да біздің жігіттер ал чурка біздің нарықта жеміс сатады,неліктен біздің жігіт шешенстан қайтыс бол шешенс өркенде ал біз ше неліктен армения мен әзірбайжан ара қақтығыс тағы да біздің жігіт ал чурк біздің нарық жеміс сат,nazism

3,мен мұны барлық татарлар айран ішкенше жасаймын тимати мен моргенстернмен бірге оларды ұрып соғыңыз мен не істеймін,мен мұны барлық татар айран ішкенш жаса тимати мен моргенстерн бірге ол ұр соғ мен не істе,nazism

3,қызық чуркалар ресми түрде тіркелген бе екен,қызық чурка ресми түр тіркел бе екен,nazism

3,басыңды жарамын чурка,бас жар чурк,nazism

3,енді ол чурка ғой қанында бар,енді ол чурк ғой қан бар,nazism

3,міне жауап осы елге чуркаларды кіргізудің қажеті жоқ,міне жауап осы ел чурка кіргізу қажет жоқ,nazism

3,содан кейін олар татьяна шавректың өзін ауыстырады және ол жұмыс істегісі келмейтінін айтады чуркасыз ел акциясын қолдаңыз чурка проблемасының шешімдерінің бірі олардан ештеңе сатып алмау жұмысқа жалдамау олардың қызметтерін пайдаланбау және б бұл экономикалық санкциялар оларды чуркменстандарды құлатуға мәжбүр етеді,содан кейін олар татьян шаврак өз ауыстыр және ол жұмыс істе келме айт чурк ел акция қолда чурк проблема шешім бірі олардан ештеңе сат алмау жұмыс жалдама олардың қызмет пайдаланба және б бұл экономикалық санкция ол чуркменстан құлат мәжбүр ет,nazism

3,патшалық ресей мен ксро кезінде біз чуркасыз жүрдік және оларсыз лехамен бірдей көп нәрсе жасаймыз содан кейін кенеттен бізге мигранттар чуркалар неге қажет болды демографиялық проблема және жұмыс күші бұл чуркаларды әкелу мақсатында жасалған жалған іс жүзінде жергілікті халыққа қарсы опасыздық саясаты жүріп жатыр,патшалық ресей мен ксро кез біз чурк жүр және ол леха бірде көп нәрсе жаса содан кейін кенет бізге мигрант чурка неге қажет бол демографиялық проблема және жұмыс күш бұл чурка әкелу мақсат жасал жалған іс жүз жергілікті халық қарсы опасыз саясат жүр жатыр,nazism

3,орыстардың қалай жұмыс істегісі келмейтіні туралы құлаққа кеспе ілудің тағы бір тарихы чуркасыз ел болайық,орыс қалай жұмыс істе келме туралы құлақ кесп ілу тағы бір тарих чурк ел бол,nazism

3,чурканы көрсем жанын шығарар едім,чурка көр жан шығар ед,nazism

3,сонымен не болды чурка болып шықты,сонымен не бол чурк бол шық,nazism

3,шынымен мына чуркалар миды ашытты,шын мына чурка ми ашыт,nazism

3,ех жынды чурка,ех жын чурк,nazism

3,барлығын депортациялау керек біздің қалада дан астам чурка бар бұл қайда жақсы олар біздің балаларымызды зорлайды өлтіреді және біз бәріміз үндемейміз біз оларға тағы қанша шыдаймыз,барлығ депортацияла керек біздің қала дан астам чурк бар бұл қайда жақсы олар біздің бала зорла өлтір және біз бәрі үндеме біз оларға тағы қанша шыда,nazism

3,біз чуркалармен бір апта болды араласпаймыз,біз чурка бір апта бол араласпа,nazism

3,сіздің ойыңызша макдак қаланы дамыту ма күлкілі сондай ақ чуркалардың дүкені бүкіл қала бойынша даму деңіз,сіздің ойыңызша макдак қала дамыту ма күлкіл сондай ақ чурка дүкен бүкіл қала бойынша даму де,nazism

3,ал чуркаларын өз заңдарын белгілей ме өз заңдарыңызды осы жерден шығарыңыз сондай ақ өлтіру және зорлау керек,ал чурка өз заң белгіле ме өз заң осы же шығ сондай ақ өлтір және зорла керек,nazism

3,ана жерде қуыршақ жігіті бір рет төмендетілген жыныстық қатынас ол үшін өте маңызды және хиджабқа қарағанда чурка да бар,ана же қуыршақ жігіт бір рет төмендет жын қатынас ол үшін өте маңызды және хиджаб қара чурк да бар,nazism

3,содан кейін бәрі барлық сұмдықтарды аяйды соғыс осы шайтандардың бәрін жоятыны қандай жақсы живодерлер,содан кейін бәрі барлық сұмдық ая соғыс осы шайтан бәрін жояты қандай жақсы живодер,nazism

3,чурка ит,чурк ит,nazism

3,мына чурканы қуып шыққан шығар деп үміттенемін,мына чурка қу шық шығар де үміттен,nazism

3,ал бұл чурка олармен нарды ойнауды ұсынды,ал бұл чурк олармен на ойнау ұсын,nazism

3,мен ұлт туралы айтып отырған жоқпын чурка кез келген ұлтта бар өкінішке орай негізінен кавказ және орта азия осылай әрекет етеді жалпы мұндай аруақтарды тек олардың әдістерімен емдеу керек олар тек күш тілін түсінеді жалпы олар келесі жолы түсінуі керек,мен ұлт туралы айт отыр жоқ чурк кез кел ұлт бар өкініш орай негіз кавказ және орта азия осылай әрекет ет жалпы мұндай аруақ тек олардың әдіс емдеу керек олар тек күш тіл түсін жалпы олар келесі жол түсін керек,nazism

3,тағы да чурка оларды сыпырғышпен қуу керек,тағы да чурк ол сыпырғыш қу керек,nazism

3,ұятсыз чурка өз ісін біліп тұр,ұят чурк өз ісін біл тұр,nazism

3,әрине мұны чуркадан басқа кім жасауы мүмкін,әрине мұны чурка басқа кім жаса мүмкін,nazism

3,мен өзім атеистпін бірақ тағы да барлық чуркаларды жарып жіберер едім,мен өзім атеист бірақ тағы да барлық чурка жар жібер ед,nazism

3,шешендер кавказдықтар ал өзбектер мен қырғыздар орта азиялықтар олар қазірдің өзінде чуркалар кавказдықтар хач,шешен кавказдық ал өзбек мен қырғыз орта азиялық олар қаз өз чурка кавказдық хач,nazism

3,украиналықтар хохол,украиналық хохол,nazism

3,орыстар оңбаған,орыс оңба,nazism

3,себебі орыстарда бұл қоқыстар үшін заң бар ал чуркаларда бұл заң жоқ ұлтаралық алауыздық,себебі орыс бұл қоқыс үшін заң бар ал чурка бұл заң жоқ ұлтаралық алауыз,nazism

3,неге сенің суретіңде чурка тұр,неге сенің сурет чурк тұр,nazism

3,сенің жігітің чурка ма,сенің жігіт чурк ма,nazism

3,біздің елде тұрып жатқан чуркаларға қашуға мүмкіндік бермей қыру керек,біздің ел тұр жат чурка қаш мүмкіндік берме қыр керек,nazism

3,чуркалар шаршатты мені,чурка шарша мен,nazism

3,тағы да қандай да бір чурка атып тастады іс басталды мұның мәні неде қазанда да іс басталды абрек есі дұрыс емес деп танылды және бәрі де содан кейін адамдар қайтыс болды ешкім қоқыс жазасын алған жоқ тек бекер нан жейді,тағы да қандай да бір чурк ат таста іс баста мұның мән не қазан да іс баста абрек ес дұрыс емес де тан және бәрі де содан кейін адам қайтыс бол ешкім қоқыс жаза ал жоқ тек бек нан же,nazism

3,мұндай жаман асты тек чуркалар жейді,мұндай жаман асты тек чурка же,nazism

3,админ чурка екен мен чуркалармен араласпаймын,админ чурк екен мен чурка араласпа,nazism

3,чурканы мәдениетті түрде ұру керек,чурка мәдениетті түр ұр керек,nazism

3,маған ақыл айтатын чурка емес топас,маған ақыл айт чурк емес топас,nazism

3,тағы да чуркалар ма болмады ғой,тағы да чурка ма болма ғой,nazism

3,есіңізде болсын ақылдың күші көпшіліктің күшінен маңыздырақ ал орыстар бұл гои неге олар ойлануы керек олар магистральмен бірге армяндар тобына көтеріледі және соңында пышақ алады әрине содан кейін чуркалар айқайлайды ал мағынасы қандай,ес бол ақ күш көпшілік күш маңыздырақ ал орыс бұл гои неге олар ойлан

керек олар магистраль бірге армян тоб көтер және соң пышақ ал әрине содан кейін чурка айқайла ал мағына қандай,nazism

3,менің пікірімнің салмағы бар чурка орыс тілін үйрен,менің пікір салмағ бар чурк орыс тіл үйрен,nazism

3,иә құлады ал қазір бұл жалғыз чурка,иә құл ал қазір бұл жалғыз чурк,nazism

3,мен чуркаларды да ұнатпаймын бірақ бұл менің қоғамым,мен чурка да ұнатпа бірақ бұл менің қоғам,nazism

3,көп ұзамай дәл солай болады чурка мигранттар біздің елімізді басып алды жақында біз мүлдем қалмаймыз,көп ұзама дәл солай бол чурк мигрант біздің ел бас алды жақында біз мүлдем қалма,nazism

3,чурка емес хач,чурк емес хач,nazism

3,иә қазір қоқысқа үміт жоқ қазірдің өзінде көптеген жерлер чурка мен сол сияқтыларға арналған,иә қазір қоқыс үміт жоқ қаз өз көптеген жер чурк мен сол сияқты арнал,nazism

3,бұл сен анаңның жатақханада чурокқа қалай қызмет еткенін сұра,бұл сен ана жатақхана чурок қалай қызмет ет сұра,nazism

3,барлығыңыз үндемеңіз чурка өз пікірін айтады,барлығ үндеме чурк өз пікір айт,nazism

3,аузыңызды жабыңыз арықтан өз сөзін чурка айтсын,ауз жаб арық өз сөз чурк айт,nazism

3,чурки барлық жерде мен әжелер туралы мүлдем үндемеймін,чурки барлық же мен әже туралы мүлдем үндеме,nazism

3,мұндайлармен тек балаларды қорқытады ұйықтар алдында мұрт әрқашан дерлік өрескел ал таз болса сіз де чурка боласыз,мұндай тек бала қорқыт ұйық алд мұрт әрқашан дерлік өрескел ал таз бол сіз де чурк бол,nazism

3,жоқ ескі сасық қайыршылар мен анаммен бірге тұратындар туралы мен толықтай келісемін азиялық чуркалар да құлаған жоқ кавказдықтар грузин осетиндерінің барлық ешкі емес мысалы азерлер мен басқа да мұсылман швалдары емес қалыпты және барабар сіз жастан аспайтын тиісті ер адамдарға жақынырақ қарай аласыз ео дан келген ақ еуропалықтарға да мұқият қараудың мағынасы бар бірақ сізге сәйкес келу керек екенін ұмытпау керек,жоқ ескі сасық қайыршы мен ана бірге тұр туралы мен толықта келіс азиялық чурка да құла жоқ кавказдық грузин осетин барлық ешкі емес мысал азер мен басқа да мұсылман швал емес қалыпты және бараб сіз жас аспа тиісті ер адам жақынырақ қарай ал ео дан кел ақ еуропалық да мұқият қара мағына бар бірақ сізге сәйкес келу керек екен ұмытпа керек,nazism

3,чурка аузыңды жап,чурк ауз жап,nazism

3,мен орыстардың арасында көбірек жындылар бар екенін айтайын және олардың саны аз емес сондай ақ ақымақтардың арасында да бар,мен орыс ара көбірек жынды бар екен айт және олардың сан аз емес сондай ақ ақымақ ара да бар,nazism

3,жалпы орыс нацистеріне гитлер кавказға қалай қарағаны бәрібір егер адам ресейде дүниеге келген болса ол өзін әдепті және сыпайы ұстайды содан кейін ол ресейден қуып шығады құдай сақтасын оған орыс қызы ұнайды жалпы,жалпы орыс нацис гит кавказ қалай қара бәрібір егер адам ресей дүние кел бол ол өз әдеп және сыпай ұста содан кейін ол ресей қу шығ құдай сақта оған орыс қыз ұна жалпы,nazism

3,иә өйткені құрылысшылар жоқ ал чуркалар үй салуды білмейтін жындылар,иә өйткені құрылысшы жоқ ал чурка үй салу білме жынды,nazism

3,мен жастамын сен не деп атайсың сауатсыз чурка маған қанша жаста екеніңді айт тіпті ана тіліңді де білмейсің ақымақ сондықтан сенде мен сияқты аргументтер бар ма жоқ сен ақымақ болдың бірақ маған ештеңе айта алмайсың,мен жас сен не де ата сауат чурк маған қанша жас екен айт тіпті ана тіл де білме ақы сондықтан сенде мен сияқты аргумент бар ма жоқ сен ақы бол бірақ маған ештеңе ай алма,nazism

3,керчь атқышы да чурка ма,керчь атқыш да чурк ма,nazism

3,саған чуркалардың тегі не керек ақымақ екенсің,саған чурка тег не керек ақы екен,nazism

3,тағы да чурка қуыңдаршы мыналарды,тағы да чурк қуыңдарш мына,nazism

3,текті ауыстыруға болатынын білесің ғой бірақ оған чурканың ақылы жетпейді есуас,тек ауыстыр бол біл ғой бірақ оған чурка ақылы жетпе есуас,nazism

3,хпхпхп чурка,хпхпх чурк,nazism

3,чурканы ата анасына атуға беру керек,чурка ата ана ат беру керек,nazism

3,чурка даун,чурк даун,nazism

3,чурканың сия салғышы ғой,чурка сия салғыш ғой,nazism

3,тренд чурка чуркадан келді лас кроссовкалардағы шұлықсыз жыртылған джинсыларда тұру бізге бірден ең озық сәннің тақырыбына айналды барлығы жыртық тіпті жаңа сияқты кроссовкаларда және қазірдің өзінде тесіктері бар шұлықтар сатылады немесе көрінбейді олар тіпті қыста да ауыр теңіз жағалауы жалпы қалайы олар жыртықта жүре алады,тренд чурк чурка кел лас кроссовка шұлық жыртыл джинсы тұру бізге бірден ең озық сән тақырыб айна барлығ жыр тіпті жаңа сияқты кроссовка және қаз өз тесік бар шұлық сат немесе көрінбе олар тіпті қыста да ауыр теңіз жағала жалпы қалай олар жыртық жүр ал,nazism

3,чурка сенің әкең қоқыс,чурк сенің әке қоқыс,nazism

3,неменеге қуасың сосын чурканы әкеледі ғой,немене қу сосын чурка әкел ғой,nazism

3,тыңда чурка аузыңды жап мені сөгетін сен емес орныңды біл,тыңда чурк ауз жап мен сөг сен емес орн біл,nazism

3,чурка қысыққөз,чурк қысыққөз,nazism

3,мына чуркаларды қырамын жермен жексен болсың оңбағандар түкке керегі жоқ,мына чурка қыр жер жексен бол оңба түк кереr жоқ,nazism

3,чурка мен оның жақтастарын басу керек,чурк мен оның жақтас басу керек,nazism

3,мына чуркалардың саяжайларын жауып тастаңдаршы,мына чурка саяжай жау тастаңдарш,nazism

3,ахаахах тегіңе қарасақ басты чурка сен сияқтысың сенің сөздерің түкке тұрмайды чурка жүйкеңді шаршатпа демал,ахаахах тег қара басты чурк сен сияқты сенің сөз түк тұрма чурк жүйке шаршатп демал,nazism

3,дұрыс терроризм чурка полиция терроризмі мұндай жағдайда әрқашан партизанен шығады,дұрыс терроризм чурк полиция терроризм мұндай жағдай әрқашан партиза шығ,nazism

3,чурка ашуланып қалыпты ойбай,чурк ашулан қалыпты ойба,nazism

3,чурка ахзазазаза,чурк ахзазазаз,nazism

3,өз құқығын қорғамақшы ғой чурка,өз құқығ қорғамақш ғой чурк,nazism

3,істің нақты мән жайын білмей үкім шығару қаншалықты оңай егер біздің кез келгеніміз вк дағы әңгімелер бойынша қарызға алынған болсақ не болмақ өлтірілген әйел мен балалар зорлаушылар мен өлтірушілер де ме кез келген жолмен және олар бойынша және басқа жағдайларда чурканы тергеу қажет,іс нақты мән жай білме үкім шығару қаншалықты оңай егер біздің кез кел вк дағ әңгіме бойынша қарыз алын бол не бол өлтір әйел мен бала зорлаушы мен өлтіруші де ме кез кел жол және олар бойынша және басқа жағдай чурка тергеу қажет,nazism

3,чуркаларды жалауды доғарыңыз сол өзбектер бізді ұзақ уақыт бойы өз елдерінен қуған жоқ қырғын болды,чурка жалау доғар сол өзбек біз ұзақ уақыт бойы өз ел қу жоқ қырғын бол,nazism

3,э чурка,э чурк,nazism

3,тыныш чурка,тыныш чурк,nazism

3,кет жоғал чурка,кет жоғал чурк,nazism

3,чурка мал,чурк мал,nazism

3,чурка батыр,чурк батыр,nazism

3,тағы бір чурка неге бітпейді осылар,тағы бір чурк неге бітпе осы,nazism

3,мүмкін сіз nord ost еске түсіре аласыз ба пензадағы автобусты бұзу және б мұны кім жасады неліктен чурка мұны біздің елде жасайды неліктен біз жаңалықтарды естімейміз орыс мысалы абхазия мемлекетінің аумағында өзін және ондаған жазықсыз абхазиялықтарды бұзды немесе ереванда тұратын орыс армян әйеліне шабуыл жасады өлтірді содан кейін оны зорлады мен мұндай нәрсені естіген жоқпын,мүмкін сіз nord ost ес түсір ал ба пенза автобус бұзу және б мұны кім жас неліктен чурк мұны біздің ел жаса неліктен біз жаңалық естіме орыс мысал абхазия мемлекет аумағ өз және ондаған жазық абхазиялық бұз немесе ереван тұр орыс армян әйел шабуыл жас өл содан кейін оны зорла мен мұндай нәрсе есті жоқ,nazism

3,чурка ты ммля природа тебя ущербным сделала завидуй белымм хотя ты так завидуешь ты же всего лишь свинка которая очередной раз визжит по моему приказу,чурк ты ммля приро тебя ущербн сделал завиду белым хотя ты так завидуешь ты же всего лишь свинка которая очередно раз визжит по моем приказ,nazism

3,анасыз чурка доғар аузыңды жап қане,ана чурк доғар ауз жап қан,nazism

3,ауру жид шал суретіңіз чекистке ұқсайды жынданып кеткенсің ғой сен,ауру жид шал сурет чекист ұқса жындан кет ғой сен,nazism

3,жынды жид кет,жын жид кет,nazism

3,бизнес жасағысы келеді түріне қарамай жынды жид армандапсың,бизнес жаса кел түр қарама жын жид арманда,nazism

3,жид пен шутняра,жид пен шутняр,nazism

3,мына жид басынды ғой,мына жид бас ғой,nazism

3,билікке жету үшін міндетті түрде жид болу керек пе,билік жету үшін міндетті түр жид болу керек пе,nazism

3,мынаны істеген адамның есі дұрыс емес шығар жай ғана жид сорлы,мына істе адам ес дұрыс емес шығар жай ғана жид сорл,nazism

3,мұның артында жид фашизмі жатқан сыңайлы,мұның арт жид фашизм жат сыңайл,nazism

3,жид пен жидовка,жид пен жидовк,nazism

3,ақыры жид билікке келді олар бізге зейнеткерлік жастағы салықтардың барлық өсуін жүктейтін және теңгені бір бакс пен емделушіге ке дейін төмендететін барлық адамдарды қуып жібереді,ақы жид билік кел олар бізге зейнеткерлік жастағы салық барлық өс жүкте және тең бір бакс пен емделуші ке дейін төмендет барлық адам қу жібер,nazism

3,биттеп кеткен жидтен тағы не күтуге болады екен,битте кет жид тағы не күт бол екен,nazism

3,қараңызшы бүкіл тарихта қанша халық көтерілісі болды бізде бүкіл тарихты жид қайта жазды,қараңызш бүкіл тарих қанша халық көтеріліс бол біз бүкіл тарих жид қайта жаз,nazism

3,біздің елде жидтар не жоғалтты екен,біздің ел жид не жоғал екен,nazism

3,масондық жид,масо жид,nazism

3,оған мұз жинағышпен хабаршы жіберетін кез келген сияқты бұл жид оған бұрыннан лайық болған сияқты,оған мұз жинағыш хабарш жібер кез кел сияқты бұл жид оған бұрын лайық бол сияқты,nazism

3,да фигня это все есть нигеры хачи хохлы пиндосы гейропецы узкоглазые жиды вот эти вот все афроктототам это от лукавого покайтесь,да фигня это вс есть ниге хачи хохл пиндо гейропец узкоглазы жи вот эти вот вс афроктотота это от лукавого покайтесь,nazism

3,жид пен даундар,жид пен даун,nazism

3,көрмейсің бе жид қой,көрме бе жид қой,nazism

3,ура бізге жидтер жетіспей тұр еді міне нағыз джихадисттер билікте әділ ешкім жоқ,ура бізге жид жетіспе тұр ед міне нағыз джихадист билік әділ ешкім жоқ,nazism

3,барлығы жид украинады да басып алды зеленский порошенко вальцман распутин де сондай шығар,барлығ жид украин да бас алды зеленски порошенко вальцман распутин де сондай шығар,nazism

3,бейшара жид,бейшар жид,nazism

3,жид мәйітін мазақ ету уәде етілді бірақ жарнамада жид собянин тірі,жид мәйіт мазақ ету уәде ет бірақ жарнама жид собянин тірі,nazism

3,таң қалмаймыз жид,таң қалма жид,nazism

3,қараңызшы бұл жид адам емес бізге қарсы соғыс жүріп жатыр достар ашық бактериологиялық коалициялық үкіметтер бактериологиялық өлтіру идеясын жүзеге асырады бірдеңе істеу керек әйтпесе жәндіктер бізді нөлге түсіреді,қараңызш бұл жид адам емес бізге қарсы соғыс жүр жатыр дос ашық бактериологиялық коалициялық үкімет бактериологиялық өлтір идея жүзеге асыр бірде істеу керек әйтпесе жәндік біз нөл түсір,nazism

3,жидті ешкім жақсы көрмейді,жид ешкім жақсы көрме,nazism

3,шын ба жид па мотоцикл ше,шын ба жид па мотоцикл ше,nazism

3,таң қалмаймыз соғыста жид жеңді олар одақтастардың кемелерін бомбалады,таң қалма соғыс жид же олар одақтас кеме бомбал,nazism

3,жидтің қалай соғысқаны белгілі америка барда израиль бар кері жағдайда жидостан минутта жоқ болады,жид қалай соғыс белгілі америка бар израиль бар кері жағдай жидос минут жоқ бол,nazism

3,ого он мың жид көп қой,ого он мың жид көп қой,nazism

3,естеріңізге сала кетейік ресми статистикаға сенсеңіз жид шамамен миллионы жойылды,ес сала кет ресми статистика сен жид шамамен миллион жой,nazism

3,біздің ғаламның ведалық символы фашист патчтарды кім киеді олар қара шляпалар костюмдер галстуктар погондар ақ халаттар киеді жид немесе олардың қызметшілері,біздің ғалам ведалық символ фашист патч кім ки олар қара шляпа костюм галстук погон ақ халат ки жид немесе олардың қызметші,nazism

3,ксро ны жид рептилойдтар басқарған және ксро ның бүкіл тарихын мансаптық сатысында ешкімді жібермеген,ксро ны жид рептилойд басқар және ксро ны бүкіл тарих мансап саты ешкі жіберме,nazism

3,он жетінші жылы жидтен басқа ешкім жоқ,он жетінші жыл жид басқа ешкім жоқ,nazism

3,сіз шіркеу әкелері жидтарға қалай қарады деп жазар едіңіз біз қызығушылықпен оқитын едік содан кейін қазір барлық жерде жид билікте де ғылымда да вакциналар қайнатылады және теледидарда да кинода да ал православие жидтармен ынтымақтаса алмайды олай болмайды,сіз шіркеу әке жид қалай қар де жаз ед біз қызығушылық оқитын едік содан кейін қазір барлық же жид билік де ғылым да вакцина қайнатыл және теледи да кино да ал православи жид ынтымақтас алма ола болма,nazism

3,мәңгілік жид агасфера туралы мифті және жид бидғат мәселесін оқыңыз сауатсыз қоқысты біржола жабыңыз,мәңгілік жид агасфер туралы миф және жид бидғат мәселе оқ сауат қоқыс біржол жаб,nazism

3,бұл жид қой сайттарында жайылып армяндарға шабуыл жасайды мен жид пен еврейлерді анық бөлемін өкінішке орай біздің арамызда тіпті премьерада да бір жид бар,бұл жид қой сайт жайыл армян шабуыл жаса мен жид пен еврей анық бөл өкініш орай біздің ара тіпті премьера да бір жид бар,nazism

3,белгілі кәрі питерлік жид,белгілі кәрі питерлік жид,nazism

3,жидтерге тек қана цифрлық тайталас керек содан кейін түрлерің бір түрлі болып кетеді сендерге дамуға кім кедергі қоқыссың,жид тек қана цифрлық тайталас керек содан кейін түр бір түрлі бол кет сен дам кім кедергі қоқыс,nazism

3,сасық жид,сасық жид,nazism

3,сен жид,сен жид,nazism

3,жид арабтарды сатып алдыңдар,жид араб сат ал,nazism

4,біз әлі қанды қырғын жасаймыз сендерге атом бомбасын лақтырамыз кетіңдер,біз әлі қан қырғын жаса сен атом бомба лақтыр кет,violent

3,жидтар мынау,жид мынау,nazism

3,түрі құрысын жид,түр құрыс жид,nazism

3,сен жид екенсің ғой,сен жид екен ғой,nazism

3,жидтар осы гитлерді еске алып әңгімені аяқтау ғана қалды арым бар,жид осы гит ес алып әңгіме аяқтау ғана қал ар бар,nazism

3,жоқ мен коммунистердің билікте болғанын қалаймын және сіз сияқты жидтар киркамен кен өндіруге кетті,жоқ мен коммунис билік бол қала және сіз сияқты жид кирка кен өндір кет,nazism

3,жидтар осындай олар,жид осындай олар,nazism

3,енді оның қанында жид бар ғой не күтуге болады,енді оның қан жид бар ғой не күт бол,nazism

3,тағы бір жид,тағы бір жид,nazism

3,мерді сығандар алдайды сығанды жид алдайды ал жидті армян алдайды армянды грек алдайды,ме сы алда сы жид алда ал жид армян алда армян грек алда,nazism

3,галицай бұл сыған мен жид қоспасы,галица бұл сы мен жид қос,nazism

3,мен захар прилепиннің жид екенін сеземін бірақ маған бір жерден дәлел табу керек жасыру үшін,мен зах прилепин жид екен сез бірақ маған бір же дәлел табу керек жасыр үшін,nazism

3,екі жид және олардың тегі басқа еврейдікіндей бүгінгі біздің патшаларымыз сияқты,екі жид және олардың тег басқа еврейдікінде бүгінгі біздің патша сияқты,nazism

3,кетші әрі сасық жид сенсіз де шешеміз,кетш әрі сасық жид сен де шеш,nazism

3,жидтарды қудалау керек,жид қудала керек,nazism

3,енді жеңудің керегі жоқ буржуйлар мен жид соғыссыз сатылды тек ақымақтар ғана мұны түсінбеді,енді жеңу керег жоқ буржуй мен жид соғыс сат тек ақымақ ғана мұны түсінбе,nazism

3,жиды пен гомо лгбт феминисттер сасықтар көңілсіз ғой,жи пен гомо лгбт феминист сасық көңіл ғой,nazism

3,жид жид,жид жид,nazism

3,питер қаласының жидтері,пи қала жид,nazism

3,жид армяндық насихатшылардың теледидардағы насихаты өз жемісін беруде,жид армян насихатшы теледи насихат өз жеміс бер,nazism

3,жидтерді ұр ғаламшарды құтқар кет жоғал бейшара жид өз қоқысыңды ала кет,жид ұр ғаламшар құтқар кет жоғал бейшар жид өз қоқыс ал кет,nazism

3,уәде беремін егер мен сені кездестірсем макс туралы осындай сөздер үшін кешірім сұрайсың ұятсыз жид,уәде бер егер мен сен кездестір макс туралы осындай сөз үшін кешір сұра ұят жид,nazism

3,пупкин шаломов жид,пупкин шаломов жид,nazism

3,егер шүмекте су болмаса онда оны жид ішкен кет сасық жид,егер шүмек су болма онда оны жид іш кет сасық жид,nazism

3,жолдарың болмасын жидтар,жол болма жид,nazism

3,грымза қоспасы яғни путлер мен меркель никита михалков айтқандай соғыс болмаса еді,грымз қос яғни пут мен меркель ники михалков айтқанда соғыс болма ед,nazism

3,жидтар мен тентектерді жер бетінен жою керек,жид мен тентек жер бет жою керек,nazism

3,жид жексұрын,жид жексұр,nazism

3,сіз қойлар не ойлайсыз енді ұлттық ұлан сізді күзетеді бұл жағдайда сіздің балаларыңыз шығарылады қарлығашқа жем бол жид әрине бұл шашты өзі атып тастады және оның кураторлары болған жоқ,сіз қой не ойла енді ұлттық ұлан сіз күзет бұл жағдай сіздің бала шығар қарлығаш жем бол жид әрине бұл шаш өзі ат таста және оның куратор бол жоқ,nazism

3,жидтер мен тентектердің мұны өздері жасағаны барлығына белгілі,жид мен тентек мұны өз жаса барлығ белгілі,nazism

3,тағы бір жидты ұсынып жатыр,тағы бір жид ұсын жатыр,nazism

3,қалай болғанда да тролльдер бірден басшысының көңілін қалдырды гитлердің қолбасшысы жидтің жеке үйінде ит сияқты өмір сүрген бірақ фашистердің концлагерінде,қалай болғанда да тролль бірден басшы көңіл қал гит қолбасшы жид жеке үй ит сияқты өмір сүр бірақ фашис концлаг,nazism

3,жидты оңайдан оңай ала алмайсың күш керек,жид оңай оңай ал алма күш керек,nazism

3,жид бар жердің барлығы өтірік,жид бар же барлығ өтірік,nazism

3,жидтың ақылы басқаға жетпейді,жид ақылы басқа жетпе,nazism

3,билікте тағы бір жид не жақсылық күтеміз,билік тағы бір жид не жақсылық күт,nazism

3,жидтар мықты,жид мықты,nazism

3,жоқ жоқ әрине вмв да ұлттық социализм жеңілгеннен кейін арий термині арийлер саяси тұрғыдан дұрыс болмады және жидтер оны ғылымнан және ақ адамдардың санасынан өшіру үшін қолдан келгеннің бәрін жасады алайда бұл өтірік арийлер бұл нәсіл ал үндіеуропалықтар тілдік отбасы,жоқ жоқ әрине вмв да ұлттық социализм жеңіл кейін ари термин арий саяси тұрғы дұрыс болма және жид оны ғылым және ақ адам сана өшір үшін қолдан кел бәрін жас алайда бұл өтірік арий бұл нәсіл ал үндіеуропалық тілдік отбасы,nazism

3,бұл екі жағынан да сатушы жағынан да сатып алушы жағынан жид,бұл екі жағ да сатушы жағ да сат алушы жағ жид,nazism

3,өкінішке орай бұл жид ешқашан атылған жоқ сіз қарап отырасыз және қоғам таза болар еді,өкініш орай бұл жид ешқашан атыл жоқ сіз қара отыр және қоғам таза бол ед,nazism

3,жидтар израильда билесін,жид израиль биле,nazism

1,мынаның барлығы негр жид та негр,мына барлығ нег жид та нег,racism

3,о жидты таптым,о жид тап,nazism

3,маған сенің сасық теріңді төледім бәрі арабтар міне сіз бір біріңізді ұрып жатырсыз керемет,маған сенің сасық тер төле бәрі араб міне сіз бір бір ұр жатыр керемет,nazism

3,сізден басталады егер жид болмасаң днқ тестін жаса және сіз қатты таң қаласыз,сізден бастал егер жид болма днқ тест жаса және сіз қатты таң қал,nazism

3,жидтарды орап жатқан сияқты,жид ора жат сияқты,nazism

3,иран жақында жидтарды қырады,иран жақында жид қыр,nazism

3,ех мына жидтар,ех мына жид,nazism

3,тек жидтар ма,тек жид ма,nazism

3,негізінен қыздарды атсам да жидқа тиді,негіз қыз ат да жид ти,nazism

3,сатқын жидтың дауысы шығып жатыр,сатқ жид дауыс шығ жатыр,nazism

3,сергей вафли жынды хохол пен кавказдықтардан гөрі көп болды барлық патшалар алдымен шыңғысхандар содан кейін неміс әулеттері,сер вафли жын хохол пен кавказдық гөрі көп бол барлық патша алд шыңғысхан содан кейін неміс әулет,nazism

3,бұл жидты барлық қоқыстармен түсіру керек пе онда азық түлік контейнерлері бар ма,бұл жид барлық қоқыс түсір керек пе онда азық түлік контейнер бар ма,nazism

3,мен жидтардың барлық сайлауына қарсымын адамдар жидтар арасында сайлана алмайды,мен жид барлық сайла қар адам жид ара сайлан алма,nazism

3,жид тоқсаныншы жылдары алғашқылардың бірі болып израиль мен ақш қа кетті қызық,жид тоқсанынш жыл алғашқы бірі бол израиль мен ақш қа кет қызық,nazism

3,жидты маңызды адамдармен салыстырмаңдар,жид маңызды адам салыстырма,nazism

3,жид сүмелек,жид сүмелек,nazism

3,жынды жид,жын жид,nazism

3,бізге дауыс бермегенді кімді сайлайды жид,бізге дауыс берме кім сайла жид,nazism

3,мына жидтың барлығы қайдан шыққан өңшең арамтамақтар,мына жид барлығ қайдан шық өңше арамтамақ,nazism

3,тимати да чуркалардың қатарына жатады құрысын,тимати да чурка қат жат құрыс,nazism

3,чурка өзіңе қара сен де сондайсың мына үндісті де жазалау керек,чурк өз қара сен де сонда мына үнд де жазалау керек,nazism

3,қарашаның жетісі мейрам қызыл күн мен чуркаларға қосылғым келеді,қараша жеті мейрам қызыл күн мен чурка қосыл кел,nazism

3,чурка оңбаған жек көремін,чурк оңба жек көр,nazism

3,мен бірге жуған жоқпын топас чурка,мен бірге жу жоқ топас чурк,nazism

3,иә иә мен қазақпын чуркамын,иә иә мен қазақ чурк,nazism

3,чурканың қандай қатысы бар,чурка қандай қатыс бар,nazism

3,сасық чурка,сасық чурк,nazism

3,чурка мексикалықтар негрлер арабтар жақсы бізде халық саны аз болады лям лас мигранттар келеді,чурк мексикалық негр араб жақсы біз халық сан аз бол ля лас мигрант кел,nazism

3,жолың болмағыр чурка,жол болмағы чурк,nazism

3,тек қорқақтар мен чуркалар ғана бір адамға жабылады,тек қорқақта мен чурка ғана бір адам жаб,nazism

3,аборт жасап тастау керек еді саған чурка шірік,аборт жаса тастау керек ед саған чурк шірік,nazism

3,чуркалармен сөйлесесіңдер ма сендер,чурка сөйлес ма сен,nazism

3,хохолдар ресейдің қалдығы хохолдар болмаса кисель болмас еді,хохол ресей қалдығ хохол болма кисель болмас ед,nazism

2,сендей итті итше өлтіру керек,сенде ит итш өлтір керек,bullying

2,қорқақ отыр орныңда тыныш,қорқақ отыр орн тыныш,bullying

2,сен мұны түсіне алмайсың бактерия жазғаның дым түсініксіз ақылға қонымсыз,сен мұны түс алма бактерия жаз ды түсінік ақыл қон,bullying

2,ұсқынсыз біздің үш адам сол жақта туылған,ұсқын біздің үш адам сол жақта туыл,bullying

2,өтірікті соқпа сен террорист шизофрениксің,өтірік соқпа сен террорист шизофреник,bullying

3,әтеш сияқты шақыратын хохол қоқыс сенің орның,әтеш сияқты шақыр хохол қоқыс сенің ор,nazism

2,қазір сенің қасыңа отырып жылаймын немене айқайлай бересің жынды әйел сені алдап кету тым оңай,қазір сенің қас отыр жыла немен айқайла бер жын әйел сен алда кету тым оңай,bullying

2,сен әдейі жынды болып көрінесің бе әлде шынымен жындысың ба,сен әдей жын бол көр бе әлде шын жынд ба,bullying

2,мен мұны қай жерде айтыппын чмо тағы өтірігің шықты,мен мұны қай же айт чмо тағы өтірі шық,bullying

3,екі елде де соғыс партиясы бар екі елде де бұл соғыс қазір өршіп келеді қолдау көрсетуді қараңыз жігіттер украина террористерді қарақшыларды ихтамнеттерді роиссаға жібере ме,екі ел де соғыс партия бар екі ел де бұл соғыс қазір өрш кел қолдау көрсету қара жігіт украина террорис қарақшы ихтамнет роисса жібер ме,nazism

2,иә қандай агрессия сіз өзіңіздің тезегіңізге ұрылдыңыз,иә қандай агрессия сіз өз тезег ұр,bullying

3,бізге қарсы не айтасың төменгі ұлт өкілі,бізге қарсы не ай төменгі ұлт өкіл,nazism

2,маған ақыл айтасың ба мына ақымақ маған ақыл айтып қояды ғой сонымен қоймай гуглдан түсініксіз сөздерді қолданады,маған ақыл ай ба мына ақы маған ақыл айт қоя ғой сонымен қойма гугл түсінік сөз қолдан,bullying

2,бұл күлкілі емес бірақ ақымақтар күлуі мүмкін оларға ақымақ екенін түсіндіре алмайсың да,бұл күлкіл емес бірақ ақымақ күл мүмкін оларға ақы екен түсіндір алма да,bullying

3,тағы бір дегенерат орыс шаршаттыңдар,тағы бір дегенерат орыс шаршат,nazism

2,сен ештеңе айта алмайсың түсінесің бе сен хайуансың тіпті одан да төменсің маған енді жауап бермей қой,сен ештеңе ай алма түс бе сен хайуан тіпті одан да төмен маған енді жауап берме қой,bullying

2,дивандағы саясаткер жапшы аузыңды,дива саясаткер жапш ауз,bullying

3,жынды хохолдар сауатты жазсаңдар болады ғой өңшең ақымақтар,жын хохол сауатты жаз бол ғой өңше ақымақ,nazism

2,құрметтім сіз шынымен ақымақсыз ба және шегіп алған жоқсыз ба бұл кімнің қорқақтары туралы немесе сіз жай ғана ақымақ болып жатырсыз ба,құрмет сіз шын ақы ба және шег ал жоқ ба бұл кім қорқақта туралы немесе сіз жай ғана ақы бол жатыр ба,bullying

2,өкінішке орай сіз мылқау пиндостарға түсінікті деңгейге түсуіңіз керек пиндостар фашингтонмен бірге қанша секундтан кейін жоғалып кететінін түсіну үшін мультфильмдерді көрсетіңіз,өкініш орай сіз мылқа пиндос түсінік деңгей түс керек пиндос фашингтон бірге қанша секунд кейін жоғал кет түсіну үшін мультфильм көрсе,bullying

2,мал саған айтатын сөзім жоқ,мал саған айт сөз жоқ,bullying

2,мына жындыны шегелеп тастау керек,мына жынды шегеле тастау керек,bullying

2,мляяяя сен мында қоқыстағы шағала сияқты ақылды болып отырсың әлде басқа ойың жоқ па,мляяяя сен мын қоқыс шағал сияқты ақылды бол отыр әлде басқа ой жоқ па,bullying

2,менің аузымды жабатын сен емессің сасық шошқа,менің ауз жаб сен емес сасық шошқа,bullying

3,украина ақымақтарға толы ел,украина ақымақ толы ел,nazism

2,тағы бір жынды шықты құрышы бар,тағы бір жын шық құрыш бар,bullying

2,сені дарға асып тастау да аз,сен да ас тастау да аз,bullying

2,өзбектермен шатысып бала тапқан қайыршы әйелсің сен,өзбек шатыс бала тап қайырш әйел сен,bullying

2,мынаны өлтіру керек басын шауып тастаса ғой,мына өлтір керек басын шау таста ғой,bullying

2,итке талату керек сені,ит талат керек сен,bullying

2,менде ұсыныс бар мынаны атып кетейік сонда барлығы бітеді,менде ұсыныс бар мына ат кет сонда барлығ біт,bullying

2,жиьодер лақтыру керек мұндайларды,жиьо лақтыр керек мұндай,bullying

2,атып тастайық малғұнды,ат таста малғұ,bullying

2,мынаған неге жол беріп отырмыз шошқаны құрту керек,мына неге жол бер отыр шошқа құрт керек,bullying

2,тақырбас кімсің сен аузыңды жап қайыршы,тақырбас кім сен ауз жап қайырш,bullying

2,сөйлемеңдер сендер сатқындар,сөйлеме сен сатқын,bullying

2,кастрация жасау керек,кастрация жасау керек,bullying

2,олардың бәрін ататын уақыт келді,олардың бәрін ат уақыт кел,bullying

2,қандай ұсқынсызсың сен,қандай ұсқынсыз сен,bullying

2,путлер ұрлықшы,пут ұрлықш,bullying

2,тікелей эфирде ату керек,тікелей эфир ату керек,bullying

2,қаскөйлер мен өзімшілдерден туған қаскөйлер,қаскөй мен өзімшіл туған қаскөй,bullying

2,қара тағдыры ренжіген моска сен мұнда қуану үшін келдің бе ал жақсы егер сізге ұнаса жалғастырыңыз,қара тағдыр ренжі моск сен мұнда қуан үшін кел бе ал жақсы егер сізге ұна жалғастыр,bullying

2,қаншықтар қожайынды жазалау керек атып тастау керек,қаншық қожайын жазалау керек ат тастау керек,bullying

2,құстың қанаттарын кесіп тастаймыз сондықтан ол ұша алмайды ал сүйіктім қашып кетпес үшін аяғын кесіп тастаймын,құс қанат кес таста сондықтан ол ұша алма ал сүйікті қаш кетпес үшін аяғ кес таста,bullying

2,сені ұру керек екен,сен ұр керек екен,bullying

2,қандай керемет шошқасың сені шошқа фермасы мен қабандарға апару керек,қандай керемет шошқа сен шошқа фер мен қабан апару керек,bullying

2,жұмыстан шығарып ату керек сонда олар біздің мойнымыздан түседі,жұмыс шығар ату керек сонда олар біздің мойны түс,bullying

2,айнаға қара малғұн түрің маймылға ұқсайды,айна қара малғұн түр маймыл ұқса,bullying

2,оңбаған келімсек менің итім сенің жұтқыншағыңды жұлып алады,оңба келім менің ит сенің жұтқыншағ жұл ал,bullying

2,ұсқынсыз сені осылай іліп қою керек,ұсқын сен осылай іл қою керек,bullying

2,иығынан шауып тастаған дұрыс сені,иығ шау таста дұрыс сен,bullying

2,сендейлердің аяқ қолдарын ата аналарымен бірге кесу керек,сендей аяқ қол ата ана бірге кесу керек,bullying

2,қаншық екенің көрініп тұр,қаншық екен көрін тұр,bullying

2,бұл ананың қандай түрі аналар өзін осылай ұстай ма бұл ана емес пе баласымен бір рет солай істеу керек бұл не олар бірден атылуы керек,бұл ана қандай түр ана өз осылай ұста ма бұл ана емес пе бала бір рет солай істеу керек бұл не олар бірден атыл керек,bullying

3,верхеррада украин халқының ақымақтары мен жаулары миллиондаған адамдар орыс тілінде сөйледі және солай болады,верхерра украин халқ ақымақ мен жау миллиондаған адам орыс тіл сөйле және солай бол,nazism

2,сасыған ірің екенсің,сасы ірің екен,bullying

2,бейбақ жаратылыссың,бей жаратылыс,bullying

2,бір екі адамды атып тастаса өрт тоқтайды,бір екі адам ат таста өрт тоқта,bullying

2,мен оны өлтірер едім,мен оны өлтір ед,bullying

2,оны басынан ату керек,оны бас ату керек,bullying

3,сасық мәскеу бүркіттері біздің команданы өлтіріп жатыр іске кірісу керек,сасық мәскеу бүркіт біздің команда өлтір жатыр іс кірісу керек,nazism

2,арнайы тұқымды ешкі екенсің,арнайы тұқымды ешкі екен,bullying

2,мұны ойлап шығарғандарды өлтіру де аз болады,мұны ойла шығар өлтір де аз бол,bullying

2,сіз маған аргументтердің орнына суреттер жібересіз сіз жай ғана жындысыз,сіз маған аргумент орн сурет жібер сіз жай ғана жынд,bullying

2,бірден асып іліп қою керек басқаларды тәрбиелеу үшін жақсы болар еді,бірден ас іл қою керек басқа тәрбиелеу үшін жақсы бол ед,bullying

2,бері қара беларусь істеріне шықпас бұрын фашист,бері қара беларусь іс шықпас бұрын фашист,bullying

2,менттерді шыңғырту керек,мент шыңғырт керек,bullying

2,кетші әрі топас түк те қызықты емессің,кетш әрі топас түк те қызықты емес,bullying

2,саған айтарға сөзім жоқ топас,саған ай сөз жоқ топас,bullying

2,мынаны жою керек өзі сұранып тұр,мына жою керек өзі сұран тұр,bullying

2,сені отқа отырғызамыз,сен от отырғыз,bullying

2,сен сияқты топасты ешқайда алмайды,сен сияқты топа ешқай алма,bullying

2,хайуан осыдан кейін қалай ұйықтайсың сен,хайуан осы кейін қалай ұйықта сен,bullying

2,шіріксің сен,шірік сен,bullying

2,мылжың мен қорқақ екенсің,мылж мен қорқақ екен,bullying

2,әтеш сен сияқтылар шерулер өткізеді көзіңді аш соқыр әтеш,әтеш сен сияқты шеру өткіз көз аш соқыр әтеш,bullying

2,жүрексіз хайуансың,жүрек хайуан,bullying

2,мен саған тағы да ақымақ деп айтамын біз нағыз еркек әрекеті туралы ақымақтар туралы айтып отырмыз,мен саған тағы да ақы де ай біз нағыз еркек әрекет туралы ақымақ туралы айт отыр,bullying

2,оны ату керек еді нағыз мылжың толығымен келісемін,оны ату керек ед нағыз мылж толығ келіс,bullying

2,иә мұндайларды дарға асу керек,иә мұндай да асу керек,bullying

2,өзіңді журналист есептеуің күлкілі ақылсыз бұғы,өз журналист есепте күлкіл ақыл бұғ,bullying

3,украинада бізде не салынды дебил бізге не үшін керек украина үшін жазыңыз өйткені тұңғиық көбейеді,украина біз не сал дебил бізге не үшін керек украина үшін жаз өйткені тұңғиық көбей,nazism

2,мұндай шіріктердің бар екені шын,мұндай шірік бар екен шын,bullying

2,кезекті иттің тезегісің сен,кезекті ит тезег сен,bullying

2,жезөкше кемпір жоғал мына жерден,жезөкше кемпі жоғал мына же,bullying

2,сені қолыма қару алып атып атып тастағым келеді,сен қол қару алып ат ат таста кел,bullying

2,сендердің барлықтарыңды бөлшектейміз жауыздар,сен барлық бөлшекте жауыз,bullying

2,моральды тұрғыда шаршаған топас,мораль тұрғы шарша топас,bullying

2,лайкқа жабысып алуға ұялмайсың ба топас,лайк жабыс ал ұялма ба топас,bullying

2,сен сияқты садисттерді тауып жазалау керек міндетті түрде,сен сияқты садист тау жазалау керек міндетті түр,bullying

2,өзің кет топас,өзің кет топас,bullying

2,бұл шошқалармен сөйлеспеу керек азаматтық қорқаулар оларды бөлшектеп құрту керек,бұл шошқа сөйлеспе керек азаматтық қорқау ол бөлшекте құрт керек,bullying

2,мына малды алаңға апарып басын төмен салбыратып іліп қою керек немесе өмір бойына түрмеге жабу керек осы әрекеті үшін,мына мал алаң апар басын төмен салбырат іл қою керек немесе өмір бой түрме жаб керек осы әрекет үшін,bullying

2,өлім жазасын депутаттардан бастау керек,өлім жаза депутат бастау керек,bullying

2,топас дизайнер,топас дизайнер,bullying

2,мынаны алаңға апарып ату керек,мына алаң апар ату керек,bullying

2,дәу іріңсің сен,дә ірің сен,bullying

2,автордың топастығында шек жоқ,авто топастығ шек жоқ,bullying

2,басыңа тас байлап суға батыратын адамсың,бас тас байла су батыр адам,bullying

2,тезірек өліп құры мал,тезірек өл құр мал,bullying

2,өз теңіңді тап қайыршы сен сияқтыларды алаңға апарып ату керек,өз тең тап қайырш сен сияқты алаң апар ату керек,bullying

2,саған өлім тілеймін,саған өлім тіле,bullying

2,ол жындыларды өлтіріп жерге көмді тағы қылышпен келетіндер болса өлтіру керек,ол жынды өлтір же көм тағы қылыш кел бол өлтір керек,bullying

2,маймыл ал сіз өмірдегі қандай жетістіктермен мақтана аласыз құрбақа бізді тұншықтыра бастайды сіз әдемісіз байсалдысыз денсаулығыңыз көп сіз мансап құрдыңыз босандыңыз және балаларыңызды аяғыңызға көтердіңіз бе сізде не бар құрбақа бізді тұншықтырар ма еді,маймыл ал сіз өмір қандай жетістік мақтан ал құрба біз тұншықтыр баста сіз әдемі байсалды денсаулығ көп сіз мансап құр боса және бала аяғ көтер бе сіз не бар құрба біз тұншықтыр ма ед,bullying

2,міне ақымақ иә бұл асыл іс оған тән,міне ақы иә бұл асыл іс оған тән,bullying

2,топас қораз,топас қораз,bullying

3,хохолдарда ес жоқ түріктер жақсы,хохол ес жоқ түрік жақсы,nazism

2,моральдық фрикс содан кейін олар кісі өлтірушілер мен зорлаушыларға айналады олардан кейін ешкім қалмас үшін жұмыртқаларды өздері сындырады,моральдық фрикс содан кейін олар кісі өлтіруші мен зорлаушы айнал олардан кейін ешкім қалмас үшін жұмыртқа өз сындыр,bullying

2,мұндай жаратылыс туылмас үшін анасын уақытында зарарсыздандыру керек еді,мұндай жаратылыс туылмас үшін ана уақыт зарарсыздандыр керек ед,bullying

2,қалай есінде жоқ мас күйінде көлік жүргізу дегенді білдіреді нита әкесіне лайық емес мен оны теледидар экранынан көре алмаймын жаман тұмсық,қалай ес жоқ мас күй көлік жүргізу де білдір ни әке лайық емес мен оны теледи экран көр алма жаман тұмсық,bullying

2,мына топасты қуу керек,мына топа қу керек,bullying

2,топас еркек екен,топас еркек екен,bullying

2,атқыш керек мұндай шіріктерді қудалау керек,атқыш керек мұндай шірік қудала керек,bullying

3,чурканы қуу керек елді құртты шаршатты,чурка қу керек ел құр шарша,nazism

2,сталин айтпақшы екеуін де ату керек,сталин айтпақшы екеу де ату керек,bullying

2,топас жынды,топас жын,bullying

2,даун топас,даун топас,bullying

2,бір сөзбен айтқанда ессіз екен,бір сөз айтқанда ес екен,bullying

2,сен сияқты жындыға жанымыз ашысын ба әлде ашуланайық па,сен сияқты жынды жан ашы ба әлде ашулан па,bullying

2,неліктен олар кедергі жасады біз мұндай ешкілерді үйретуіміз керек олар өз індерінде отырсын,неліктен олар кедергі жас біз мұндай ешкі үйрет керек олар өз ін отыр,bullying

2,бұл алқашты соттау керек,бұл алқаш сотта керек,bullying

2,бір жерін кесіп аузына тығамын,бір жер кес ауз ты,bullying

2,кәрі қақпас саған ақша керек пе төсекке барып тап,кәрі қақпас саған ақша керек пе төсек бар тап,bullying

2,өзіңді топас сияқты ұстамашы өтінемін,өз топас сияқты ұстамаш өт,bullying

2,мен саған қандай ақыл айта аламын жынды,мен саған қандай ақыл ай ал жын,bullying

2,қазір алқаштар мен жеңіл жүрістілер сәнде алайда демократтар,қазір алқаш мен жеңіл жүрісті сән алайда демократ,bullying

2,өте қызық не сендер жақта нағыз еркек дегенге сай ешкім жоқ па ессіз топастар,өте қызық не сен жақта нағыз еркек де сай ешкім жоқ па ес топас,bullying

2,оны басынан сүйретіп қоқыс төгетін шұңқырға лақтырамыз,оны бас сүйрет қоқыс төг шұңқыр лақтыр,bullying

2,қолың сал болып қалсын сенің мал,қол сал бол қал сенің мал,bullying

2,сенің өлетін уақытың келді бауыр,сенің өл уақыт кел бауыр,bullying

2,торға отырғызғандарды ату керек болды тіпті қарамау керек,тор отырғыз ату керек бол тіпті қарама керек,bullying

2,мұндай арамтамақтар бізге не үшін керек атып тастамаймыз ба,мұндай арамтамақ бізге не үшін керек ат тастама ба,bullying

3,орысты көрсең өлтір,ор көр өлтір,nazism

3,кет жоғал хохол сасық иісің алыстан шығады,кет жоғал хохол сасық иіс алыс шығ,nazism

2,расында топас екенсің,рас топас екен,bullying

2,қойшы шаршаттың бізді аузыңды жап та жұмыс істе бар,қойш шарша біз ауз жап та жұмыс істе бар,bullying

3,мына романды еврей жағымпаз жазған,мына рома еврей жағымпаз жаз,nazism

2,сенің түсіңе келіп мойныңнан қос қолыммен тұншықтырамын,сенің түс кел мойны қос қол тұншықтыр,bullying

2,басыңды балтамен шабу керек қой,бас балта шаб керек қой,bullying

2,өлтіруге болады сені,өлтір бол сен,bullying

2,сен сияқты даундардан жүрегім айниды,сен сияқты даун жүрег айни,bullying

2,сені өзім ақ тұншықтырып өлтірер едім,сен өзім ақ тұншықтыр өлтір ед,bullying

2,сені таптап кеткенім қандай жақсы болған мынадай есуасты басқан дұрыс минус нөлсің сен,сен тапта кет қандай жақсы бол мынадай есуа бас дұрыс минус нөл сен,bullying

2,сұмырай пешке тығу керек сені,сұмыра пеш тығ керек сен,bullying

2,топасты айнаға қарағанда көресің жынды,топа айна қара көр жын,bullying

2,мұндайларды кіші кезінде ату керек олар адам болмайды босқа арамтамақтанып жүреді,мұндай кіші кез ату керек олар адам болма бос арамтамақтан жүр,bullying

2,плакаттың да керегі жоқ жынды екені көрініп тұр ғой,плакат да керег жоқ жын екен көрін тұр ғой,bullying

2,жезөкше сирек кездесетін хайуан,жезөкше сирек кездес хайуан,bullying

2,адам емес малсың,адам емес мал,bullying

2,ол ешқашан адам болған емес және болмайды нашақор алқаш,ол ешқашан адам бол емес және болма нашақо алқаш,bullying

2,бейшара ақша жинап берейік онда саған үсіп қаларсың,бейшар ақша жина бер онда саған үс қал,bullying

2,сабыр сақта жындымын деп айқайламасаң да кім екенің түсінікті сенің,сабыр сақта жынд де айқайлама да кім екен түсінік сенің,bullying

2,қайыршылар кетіңдер әрі,қайыршы кет әрі,bullying

2,әкең топас екен сенің,әке топас екен сенің,bullying

2,сендерді дарға асу керек,сен да асу керек,bullying

2,өлім жазасының болмағаны өкінішті сендер бірінші кетер едіңдер,өлім жаза болма өкініш сен бірінші кет ед,bullying

2,халықтың жаман өмірі үшін ату керек басшыны,халық жаман өмір үшін ату керек басшы,bullying

2,өзіңе қарап ал шошқа,өз қара ал шошқа,bullying

2,сендейлерді жер қалай көтеріп жүр пасық,сендей жер қалай көтер жүр пасық,bullying

2,шахтаға қамаймыз сол жақта әтеш сияқты жүріңдер,шахта қама сол жақта әтеш сияқты жүр,bullying

2,жындының қалдығы бізбен ұрсыспа,жынд қалдығ бізбен ұрсысп,bullying

2,буымды шығару үшін оны ұрар едім,бу шығару үшін оны ұр ед,bullying

2,қытайдағы сияқты алаңда көпшіліктің алдында дарға асу керек,қытай сияқты алаңда көпшілік алд да асу керек,bullying

2,қолыңды сұқпау үшін саусағыңды шабар ем,қол сұқпа үшін саусағ шаб ем,bullying

2,ой топас қолыңнан келмесе неменеге кірісесің сен,ой топас қол келме немене кіріс сен,bullying

2,жолама ұнамаса әрі жүр жынды,жола ұнама әрі жүр жын,bullying

2,дамымай қалған жынды,дамыма қалған жын,bullying

2,бмв да отырған әдеттегі топас,бмв да отыр әдет топас,bullying

2,тезегіңде отыра бер езіліп мал,тезег отыр бер езіл мал,bullying

2,мұндай мал патриоттарды жерге ұрайын,мұндай мал патриот же ұр,bullying

2,малдар жүрегі жоқ хайуан,мал жүрег жоқ хайуан,bullying

2,бұлардың көптігі сонша олардың барлығын тану мүмкін емес осылардың кесірінен жұмыссыз отырмыз ашпыз,бұл көптіг сонша олардың барлығ тану мүмкін емес осы кесір жұмыссыз отыр аш,bullying

2,масқаңды алып кет сасық әтеш,маска алып кет сасық әтеш,bullying

2,әбден біздің мойнымызға мініп алды атып тастау керек еді,әбден біздің мойны мін алды ат тастау керек ед,bullying

2,украина аз болды ма сендерге топастау,украина аз бол ма сен топаста,bullying

2,мынаны таптап тастау керек шаршатты,мына тапта тастау керек шарша,bullying

2,мынау әлі тірі ме неге оны ешкім өлтірмеген,мынау әлі тірі ме неге оны ешкім өлтірме,bullying

2,естеріңнен ауыстыңдар ма топастар,ес ауыс ма топас,bullying

2,сирек туатың малсың,сирек туа мал,bullying

2,хайуан қабырғаға тақап ұрар едім рахаттанып,хайуан қабырға тақа ұр ед рахаттан,bullying

2,сені өлтіруге дайынмын,сен өлтір дайын,bullying

2,ату керек біздің зейнетақымыз қайда,ату керек біздің зейнетақы қайда,bullying

2,адамдармен сөйлесіп отырмын ба десем сауатсыз маймыл екенсің,адам сөйлес отыр ба де сауат маймыл екен,bullying

3,жидтар әлемнің қатерлі ісігі,жид әлем қатерлі ісіг,nazism

2,қорқақ тышқан екенсің,қорқақ тышқан екен,bullying

2,мұндай адамдардың өздерін байлап тастау керек,мұндай адам өз байла тастау керек,bullying

2,сен саяси сатқынсың жоғал әрі,сен саяси сатқын жоғал әрі,bullying

2,тыңдашы аға мынадай сөздерді айтатын болсаң сен ауру шығарсың,тыңдаш аға мынадай сөз айт бол сен ауру шығ,bullying

2,қамап не керегі бар атып тастау керек,қама не керег бар ат тастау керек,bullying

2,жынды болсаң ол уақытша мен мәңгілікке топас,жын бол ол уақытша мен мәңгілік топас,bullying

2,автор гомосексуалист тыңдаудың өзі жиркенішті,автор гомосексуалист тыңда өзі жиркеніш,bullying

2,ата аналары жынды ғой балалары да сондай,ата ана жын ғой бала да сондай,bullying

2,мынаны зынданға тастау керек сол жақта бір апта жатсын суда,мына зындан тастау керек сол жақта бір апта жат су,bullying

2,бір сөзбен айтқанда мисыз ешкісің,бір сөз айтқанда ми ешкі,bullying

2,ату керек немесе дұрысы дарға асамыз,ату керек немесе дұрыс да ас,bullying

2,мұндай жындылар қай уақытта да болған,мұндай жынды қай уақыт да бол,bullying

2,мал бір жерінен салбыратын іліп қою керек бейшара малғұн,мал бір же салбыр іл қою керек бейшар малғұн,bullying

2,қолыңды қоқысқа апарып таста шал,қол қоқыс апар таста шал,bullying

2,бұларды атып тастау керек еді елді құртты ғой,бұл ат тастау керек ед ел құр ғой,bullying

2,бұл жындыны донецкке жіберіңдер сол жақта жазасын алады,бұл жынды донецк жібер сол жақта жаза ал,bullying

2,тфу мал байлап тастаңдар жүректі айнытады,тф мал байла таста жүрек айныт,bullying

2,жындысыңдар ма неменеге маған видео жібересіңдер өзің қара мал,жынд ма немене маған видео жібер өзің қара мал,bullying

2,клиникалық мал,клиникалық мал,bullying

2,ее алдымен көпірге апарып асамыз оның алдында зорлаймыз,ее алд көпір апар ас оның алд зорла,bullying

2,бұл топастар жастықты ақылды алады да қауқарсыз қарттықты тастайды,бұл топас жастық ақылды ал да қауқар қарттық таста,bullying

2,оны өлтіру керек,оны өлтір керек,bullying

2,шошқа сияқты шыңғырмашы мал,шошқа сияқты шыңғырмаш мал,bullying

2,саған айтарға сөзім жоқ ұятсыз малғұн,саған ай сөз жоқ ұят малғұн,bullying

2,маскадағы малдар жақында сендерді асамыз,маска мал жақында сен ас,bullying

2,тіссіз мал тістерің қашан шығады өзіңді әдемі санайсың ба,тіс мал тіс қашан шығ өз әдемі сана ба,bullying

2,жынды ақылгөйсінбе сенің ақылың жетпейді оған әуре болма,жын ақылгөйсінб сенің ақыл жетпе оған әуре болма,bullying

2,есектің басып шабу керек,есек бас шаб керек,bullying

2,қолдарың салбырап қалсын,қол салбыра қал,bullying

2,атып тастау керек,ат тастау керек,bullying

2,осындайларды атуға рұқсат берсе кірісеміз ғой,осындай ат рұқсат бер кіріс ғой,bullying

2,мал хайуан да мұны жасамас еді,мал хайуан да мұны жасамас ед,bullying

2,себепсіз жынды түсінудің де керегі жоқ,себепсіз жын түсіну де кереg жоқ,bullying

2,мұндай аналарды өлтіру керек,мұндай ана өлтір керек,bullying

2,мұны жазған адамның миы солып қалған сияқты,мұны жаз адам ми сол қалған сияқты,bullying

2,мұндай әзіл үшін басыңа шеге қадаймын,мұндай әзіл үшін бас шег қада,bullying

2,тілі бар тарақансың,тіл бар тара,bullying

1,афирюга жоғал,афирюг жоғал,racism

2,сен маймылды кастрация жасау керек,сен майм кастрация жасау керек,bullying

2,бұл малдарды кеше қуғанмын,бұл мал кеше қу,bullying

2,маған кел талқаныңды шығарамын сенің,маған кел тал шығар сенің,bullying

2,сымбатыңды көрсету емес одан ұялу керек,сымбат көрсету емес одан ұял керек,bullying

2,бетіңе түкіргім келеді,бет түкір кел,bullying

1,қара нәсілділер ақымақ әйел үшін бұталардың арасында осы сұммен төбелесті,қара нәсілді ақы әйел үшін бұта ара осы сұ төбелес,racism

1,кешіріңіз бір жерде өте көп қара нәсілділер,кешір бір же өте көп қара нәсілді,racism

1,сізде барлық жазбалар тамаша nigga бізді микстейп тректері үшін қолданады,сіз барлық жазба тамаша nigga біз микстей трек үшін қолдан,racism

1,негр көлік сатып алды және өзіне емес,нег көлік сат алды және өзіне емес,racism

1,енді негр дәл осындай болуы керек,енді нег дәл осындай бол керек,racism

1,лол негрлер деф мен қалай әрекет ету керектігін білмеймін байғұс жігіт,лол негр деф мен қалай әрекет ету керектіг білме байғұс жігіт,racism

1,мен сенің хабарламаңды алған жоқпын мына негр мені осымен екінші күн назарға алмай тұрған сияқты менің телефонымның жадысы толған ол оның ішіндегі хабарламаларды өшіріп тастады,мен сенің хабарлама ал жоқ мына нег мен осы екінші күн назар алма тұр сияқты менің телефон жады тол ол оның ішіндегі хабарлама өшір таста,racism

1,уау негр сен мені ешқашан олай атай алмайсың сондықтан мені негр деп ата,уау нег сен мен ешқашан ола ата алма сондықтан мен нег де ата,racism

1,оның есі дұрыс емес пе негр әзілдері барлығы тек қызық және ойын үшін мен одан кешірім сұраймын,оның ес дұрыс емес пе нег әзіл барлығ тек қызық және ойын үшін мен одан кешір сұра,racism

1,ия доти менің негрім пыхп пуф қане мен сені өткізер едім бірақ сен тым алыстасың,ия доти менің нег пых пуф қан мен сен өткіз ед бірақ сен тым алыс,racism

1,парвовирус анықталды өткізіп жіберші негр бәрі жақсы болады балақай,парвовирус анықта өткіз жіберш нег бәрі жақсы бол бала,racism

1,музыка тыңдап эщли және бриттанимен және роузбен әңгіме айтып отырмын менің негрім джи яму қайда болды,музыка тыңда эщли және бриттани және роуз әңгіме айт отыр менің нег джи ям қайда бол,racism

1,негр менің бос уақытым жоқ менің күндерім таусылды,нег менің бос уақыт жоқ менің күн таусыл,racism

1,маааааааааан бұлшықет сүті негр бұлшықет сүті бұл не қыздарға арық негр тек ақшасы бар болса ғана ұнайды,маааааааааан бұлшықет сүт нег бұлшықет сүт бұл не қыз арық нег тек ақша бар бол ғана ұна,racism

1,мен бұл жайлы айтқан жоқпын негр жаңа ғана жүгері басын және алды мен де көрмедім,мен бұл жайлы айт жоқ нег жаңа ғана жүгері басын және алды мен де көрме,racism

1,негрге сенбеңдер сондықтан мен әрдайым single өкілі боламын,нег сенбе сондықтан мен әрдайым single өкіл бол,racism

1,сенің оны жасай алатыныңды білемін сені қара деп атасам болады ма ия мен қазір ғана мұны айттым,сенің оны жаса ал біл сен қара де ата бол ма ия мен қазір ғана мұны ай,racism

1,мен aim де сөйлесу мен бұл білімді негрді сағындым,мен aim де сөйлесу мен бұл білімді нег сағ,racism

1,негр маған қарады мен оған менде дедім бм туралы айтып тұрғаным жоқ,нег маған қар мен оған менде де бм туралы айт тұр жоқ,racism

1,абайлаңдар негрлер қап егер сен бәрін дұрыс жасасаң ақшаны осы аптада алатын боламыз,абайла негр қап егер сен бәрін дұрыс жаса ақша осы апта ал бол,racism

4,мен зираттың қарсысында тұрамын және жақын арада сен маған қарсы тұратын сияқтысың,мен зира қарсы тұр және жақын ара сен маған қарсы тұр сияқты,violent

4,ұзақ бас қатырғым келмейді бәлкім сені желкеңнен ұрып қасқырларға тірідей жем ретіндеberермін,ұзақ бас қатыр келме бәлкім сен желке ұр қасқыр тірідей жем рет бер,violent

4,пышақты бір рет қажетті жерге тықсаң жеткілікті адам жоқ болады,пышақ бір рет қажетті же тық жеткілікті адам жоқ бол,violent

4,мен тағы да өлтірер едім,мен тағы да өлтір ед,violent

4,менің жасағаным жыныстық ляззат алу үшін емес еді бұл маған жүрегіме тыныштық ұялатты,менің жаса жын ляззат алу үшін емес ед бұл маған жүрег тыныштық ұялат,violent

4,мен неге өлтіре алмаймын егер бәрібір бәріміз өлетін болсақ,мен неге өлтір алма егер бәрібір бәрі өл бол,violent

1,ал олар полицейлердің оларды неге өлтіріп жатқанына таңғалады жабайылар қандай болса солай қалады африкаға шығу үшін бір тәулік беріледі кім қалып қойды өзі кінәді,ал олар полицей ол неге өлтір жат таңғал жабайы қандай бол солай қал африка шығу үшін бір тәулік бер кім қалып қой өзі кінә,racism

3,орыс еместердің барлығын құрту керек сен міндеттісің,орыс емес барлығ құрт керек сен міндетті,nazism

3,мигранттарға қарсы жігіттер барлығымыз оларға қараңғылық ұйымдастырайық қаңтар жексенбі күні сағат немо барының алдында,мигрант қарсы жігіт барлығ оларға қараңғылық ұйымдастыр қаңтар жексенбі күн сағат немо бар алд,nazism

4,егер сіз күнделікті сұр өмірден шаршасаңыз бізде сізге антидот бар не қаласаңыз және қайда қаласаңыз бізде сіздің көңіл күйіңізді жақсарту үшін жеңіл өнімнен бастап қатты синтетикаға дейін бәрі бар ең жоғары пайыздық сападағы тауарлар,егер

сіз күнделікті сұр өмір шарша біз сізге антидот бар не қала және қайда қала біз сіздің көңіл күй жақсарту үшін жеңіл өн баста қатты синтетика дейін бәрі бар ең жоғары пайыз сапа тауар,violent

4,ең күшті ол цианид бірақ сіз оны оңай сатып ала алмайсыз сондықтан оны өзіңіз жасауға тырысыңыз бұл жануарды физикалық азаптан ал сізді моральдық азаптан құтқарады өйткені өлім бірден болады көмекпен алу мүмкін,ең күшті ол цианид бірақ сіз оны оңай сат ал алма сондықтан оны өз жаса тырыс бұл жануар физикалық азап ал сіз моральдық азап құтқар өйткені өлім бірден бол көмек алу мүмкін,violent

4,қозғалшы бейбақ сен ақымақ болып жүргенде мен ондаған адамды өлтіруім мүмкін еді,қозғалш бей сен ақы бол жүр мен ондаған адам өлтір мүмкін ед,violent

4,майлағыштарды жоятын уақыт келді,майлағыш жоят уақыт кел,violent

4,мұндай әрекеттерді еліміздің әрбір бесінші тұрғындарына қолдану керек тобырлар мен моральды құбыжықтардан құтылу керек,мұндай әрекет ел әрбір бесінші тұрғын қолдану керек тобыр мен мораль құбыжық құтыл керек,violent

4,сіздің әйеліңіз бізде салынған сөмкені қайда қалдыру керектігін айтамыз сағат ге дейін әкелмесең енді көрмейсің,сіздің әйел біз салын сөмке қайда қалдыр керектіг ай сағат ге дейін әкелме енді көрме,violent

4,сені өлтіру,сен өлтір,violent

4,сен бүгін өлесің,сен бүгін өл,violent

4,ендеше алдымен оларды өлтіреміз,ендеше алд ол өлтір,violent

4,химиялық талдаудан өткен тауар,химиялық талдау өткен тауар,violent

4,менен дене сенен меф,менен дене сенен меф,violent

4,балаларды атып жатқанын көру өте қызық,бала ат жат көру өте қызық,violent

4,мен адам өмірін онша бағаламаймын бұл әлемнің басты проблемасы деп онда адамдардың болуын білемін біз болмасақ басқа жануарлар тіпті додо құстары да осында болар еді,мен адам өмір онша бағалама бұл әлем басты проблема де онда адам бол біл біз болма басқа жануар тіпті додо құс да осында бол ед,violent

4,мені шайтан иемденіп алып үйге барып күйеуіме беруімді айтты мен солай істедім,мен шай иемден алып үй бар күйе беру ай мен солай істе,violent

4,нен не қараған түні әжемнің үйіне қонамын және таңмен бомбаларды алып кетемін,нен не қара түн әже үй қон және таң бомба алып кет,violent

4,бізге төлемесең ашыналық суреттеріңді барлық достарыңа жібереміз,бізге төлеме ашыналық сурет барлық дос жібер,violent

4,ендігі ісім ханымның құлағын кесіп алып полицейлерге көңіл көтеру үшін жіберемін солай ма мен тағы біраз жұмыс істеп аламын дегенше бұл хат сенде болсын содан

кейін бәріне тарата берсең болады менің пышағымның жақсы және өткір болғаны
сонша мен мүмкіндік туа сала жұмысқа тезірек кіріскім келеді іске сәт құрметпен джек
бөлшектеуші,ендігі іс хан құлағ кес алып полицей көңіл көтеру үшін жібер солай ма
мен тағы біраз жұмыс істе ал дегенш бұл хат сенде бол содан кейін бәрін тара бер бол
менің пышағ жақсы және өткір бол сонша мен мүмкіндік туа сала жұмыс тезірек кіріс
кел іс сәт құрмет джек бөлшектеуш,violent

4,газеттердің бірінші беттерінде мен туралы жаза бастауы үшін және бүкіл халық жаңа
кісі өлтірулерді күтіп тұруы үшін мен әлі қанша адамды өлтіруім керек,газет бірінші бет
мен туралы жаза баста үшін және бүкіл халық жаңа кісі өлтіру күт тұр үшін мен әлі
қанша адам өлтір керек,violent

4,бұл құбыжық менің миыма қашан енетінін білмеймін бірақ ол сонда қалу үшін келеді
қоғам мен сияқты өз құрбандарын қинауды ойлай отырып жеңілдеу жолдары бар
адамдардың болғанына ризашылығын білдірсе болады досым бұл өте ауыр ойын бұл
жерде құбыжық құрбандарды санап қараңғы түнде оларды аңдып күтеді мүмкін сіз
оны тоқтата аласыз мен істей алмаймын ол өзінің келесі құрбанын таңдап қойған
құрметпен btk,бұл құбыжық менің ми қашан ен білме бірақ ол сонда қалу үшін кел
қоғам мен сияқты өз құрбан қинау ойла отыр жеңілде жол бар адам бол ризашылығ
білдір бол дос бұл өте ауыр ойын бұл же құбыжық құрбан сана қараңғы түн ол аңд күт
мүмкін сіз оны тоқта ал мен істе алма ол өзінің келесі құрбан таңда қой құрмет
btk,violent

4,мен адамдарды өлтіруді ұнатамын өйткені бұл көңілді бұл орманда аң өлтіруден
әлдеқайда қызықты өйткені адам ең қауіпті жануар біреуді өлтіру маған ұмытылмас
және қызықты тәжірибе береді бұл тіпті қызбен араласқаннан да жақсы бірақ ең
жақсысы мен өлсем жұмақта қайта туамын және мен өлтіргендердің бәрі маған құл
болады мен саған атымды айтпай ақ қояйын өйткені сен мені тоқтатуға тырысасың
және ақырет үшін құлдарымнан әскер құруға кедергі жасайсың,мен адам өлтіру ұна
өйткені бұл көңіл бұл орман аң өлтіру әлдеқайда қызықты өйткені адам ең қауіпті
жануар біреу өлтір маған ұмытылмас және қызықты тәжірибе бер бұл тіпті қыз аралас
да жақсы бірақ ең жақсы мен өл жұмақ қайта ту және мен өлтір бәрі маған құл бол мен
саған ат айтпа ақ қоя өйткені сен мен тоқтат тырыс және ақырет үшін құл әскер құр
кедергі жаса,violent

4,барлықтарыңды meta қызметкерлеріне қарсы зорлық зомбылыққа
шақырамын,барлық meta қызметкер қарсы зорлық зомбылық шақыр,violent

4,оларды қайдан көрсең де өлтіріп сені қуып шыққан жерден қуып жібер азғыру кісі
өлтіруден де жаман,ол қайдан көр де өлтір сен қу шық же қу жібер азғыр кісі өлтіру де
жаман,violent

4,әйтпесе мен сені ip арқылы анықтап барлық туыстарыңды өлтіремін,әйтпесе мен
сен ip арқылы анықта барлық туыс өлтір,violent

4,өз әкемнен кегімді алғасын сенің әкеңді құрметтеуге барамын,өз әке кег алға сенің әке құрметте бар,violent

4,мен сенің қайда тұратыныңды білемін сенің артыңнан кетті,мен сенің қайда тұр біл сенің арт кет,violent

4,егер сіз тағы бір дөрекі сөзге жол берсеңіз олар сізді өлтіреді,егер сіз тағы бір дөрекі сөз жол бер олар сіз өлтір,violent

4,басқа ұлттардың жойылуы арқылы әлемдік халифа құрылады,басқа ұлт жойыл арқылы әлемдік халиф құр,violent

4,мен оларды ұрлап салқынқандылықпен өлтірдім мен оны тағы қайталайтын едім,мен ол ұрла салқынқандылық өл мен оны тағы қайтала ед,violent

4,тапсырманы орындамасаң мен сенің ата анаңды өлтіруге мәжбүрмін,тапсырма орындама мен сенің ата ана өлтір мәжбүр,violent

4,мен өлтіруді ұнататынмын өлтіргім келді,мен өлтіру ұнат өлтір кел,violent

4,мен әрқашан біреуді өлтіру қандай болатынын ойлайтынмын,мен әрқашан біреу өлтір қандай бол ойла,violent

4,балалардың оққа ұшқанын көру өте қызық болды,бала оқ ұш көру өте қызық бол,violent

4,мен әйелдер мен балаларды ұрамын себебі мен мықтымын,мен әйел мен бала ұр себебі мен мықты,violent

4,менің портфелімде бомба бар керек болса іске қосамын мен сенің менің жанымда отырғаныңды қалаймын сіз кепілсіз,менің портфел бомба бар керек бол іс қос мен сенің менің жан отыр қала сіз кепіл,violent

4,мен өз жоспарымды жүзеге асыру үшін толық он екі жыл күттім кек алудың арнайы жоспарын дайындадым ол сіздің тағы бес минут уақытыңызды ғана алады оны атып тастаңыз және оны жануарларға жеуге қалдырыңыз,мен өз жоспар жүзеге асыру үшін толық он екі жыл күт кек ал арнайы жоспар дайында ол сіздің тағы бес минут уақыт ғана ал оны ат таста және оны жануар же қалдыр,violent

4,әйеліңіз бен балаларыңызды арты арқылы зорласын,әйел бен бала ар арқылы зорла,violent

4,мен оны сонда қалдырдым бірақ алдымен оны зорлап өлтірдім мен кеткенде оның құлағынан миы ағып жатты мен оның өлгенін білдім және бұл маған ұнады,мен оны сонда қал бірақ алд оны зорла өл мен кеткенде оның құлағ ми ағ жат мен оның өл біл және бұл маған ұн,violent

4,алғашқы кісі өлтіру алғашқы махаббат сияқты,алғашқы кісі өлтір алғашқы махаббат сияқты,violent

4,егер ол қашпаса анечка өмір сүретін еді мен жай ғана жаңа достар тапқым келді сен менен метр қашықтықта тұрасың мен бұл жоспарды көптен бері ойлап жүрдім енді үйіңмен қоштасқаның жөн,егер ол қашпа анечк өмір сүр ед мен жай ғана жаңа дос тап кел сен менен метр қашықтық тұр мен бұл жоспар көп бері ойла жүр енді үй қоштас жөн,violent

4,біз анаша шегетінбіз және таблеткалар қабылдап алдық,біз анаша шег және таблетка қабылда ал,violent

4,мен әрдайым өзгелердің жанына тигенді және өзгелердің менің жаныма тигенін қалайтынмын мен жанға бататын әрекеттің бәрінен ляззат алатын сияқтымын,мен әрдайым өзге жан ти және өзге менің жан ти қала мен жан бат әрекет бәрі ляззат ал сияқты,violent

4,маған өлтірудің жақсы әдісі керек,маған өлтіру жақсы әдіс керек,violent

4,ендеше бірінші соларды өлтірейік жоспар сондай ма,ендеше бірінші сол өлтір жоспар сондай ма,violent

4,тағы да осындай әрекет болса мен сені өлтіремін,тағы да осындай әрекет бол мен сен өлтір,violent

4,полиция шақырсаң өлтіреміз деп қорқытатынымыз анық,полиция шақыр өлтір де қорқыт анық,violent

4,егер ол тәртіпсіз еркек болса маған айт біз оны өлтіреміз,егер ол тәртіп еркек бол маған айт біз оны өлтір,violent

4,оны миллион рет сүртсек тіпті көміп тастасақ та оған дәлел болады ал хлор оларды жояды көп ұзамай полиция оны табады мәйітті жерлегенде қайда кетті деп ешкім таң қалмайды,оны миллион рет сүрт тіпті көм таста та оған дәлел бол ал хло ол жоя көп ұзама полиция оны таб мәйіт жерле қайда кет де ешкім таң қалма,violent

4,бұл жалықтырады бірақ егер қажет болса мен оларды өлтіремін,бұл жалықтыр бірақ егер қажет бол мен ол өлтір,violent

4,бұл өтірік болса қызды өлтіремін,бұл өтірік бол қыз өлтір,violent

4,сен еңкей әйтпесе қызыңды өлтіреміз,сен еңкей әйтпесе қыз өлтір,violent

4,мен оны қалай өлтіретінімді жоспарлауым керек,мен оны қалай өлтір жоспарла керек,violent

4,сен біреуді өлтіруің қажет және ол ас үйде орын алуы керек,сен біреу өлтір қажет және ол ас үй орын ал керек,violent

4,анашаны йогуртқа сеуіп тастады бізге көмектессең өз анашаңды аласың,анаша йогурт сеу таста бізге көмектес өз анаша ал,violent

4,сен итке мұз кәмпит беріп өлтіре аласың,сен ит мұз кәмпит бер өлтір ал,violent

4,онда мен жақынырақ келіп атамын,онда мен жақынырақ кел ат,violent

4,оларға шындықты айт тано мен маранка оны өлтіру үшін оған улы сусын берді деп айт,оларға шындық айт тано мен маранк оны өлтір үшін оған ул сусын бер де айт,violent

4,есірткі әсер еткенде оны өзенге апарамыз,есірткі әсер еткенде оны өзен апар,violent

4,мен өзімді жақсы сезіну үшін біреуді ұрғым келеді,мен өз жақсы сезін үшін біреу ұр кел,violent

4,біз оны ес түссіз ұрып соғып неваның мұзды суына лақтыруымызға тура келді,біз оны ес түс ұр соғ нева мұз су лақтыру тура кел,violent

4,мына газетшілерді өлімші етіп сабап ұнтақтап қағаз өндірісіне жіберу керек,мына газетші өлімш ет саба ұнтақта қағаз өндіріс жіберу керек,violent

4,харриеттің денесін жасыру үшін қайтып оралуға тура келді,харрие дене жасыр үшін қайт орал тура кел,violent

4,сізді балықшы сияқты шірік өлім күтіп тұр,сіз балықшы сияқты шірік өлім күт тұр,violent

4,сұмырай мен сені таппайды деп ойладың ба мен саған ескерту жасадым,сұмыра мен сен таппа де ойла ба мен саған ескерту жаса,violent

4,саған бүгін жаман болады өз қаныңа өзің қақаласың өзіңе табыт тапсырыс бере бер сені жерлейміз,саған бүгін жаман бол өз қан өзің қақал өз табыт тапсырыс бер бер сен жерле,violent

4,қаншық сені бүгін жұмысыңнан кейін күтіп аламын,қаншық сен бүгін жұмыс кейін күт ал,violent

4,сіз ұрлаған нәрсені қайтара алмайсыз туыстарыңа келеміз олар сенің қарызыңды ақшамен төлейді біз демонстрациялық өлім жазасын ұйымдастырамыз қарттарға немесе кішкентай балаларға да қарамаймыз содан кейін сіз олардың көздеріне қарайсыз,сіз ұрла нәрсе қайтар алма туыс кел олар сенің қарыз ақша төле біз демонстрациялық өлім жаза ұйымдастыр қарт немесе кішкентай бала да қарама содан кейін сіз олардың көз қара,violent

4,қаншық мен есіңде бармын ба қансырап жатасың әлі,қаншық мен ес бар ба қансыра жат әлі,violent

4,сені көрген кезде бетіңді талқандаймын,сен көр кезде бет талқанда,violent

4,оңбаған артыңды жу қонақтарды күт жаназаңа тапсырыс бер оңбаған жерлеу рәсімінде кездесеміз,оңба арт жу қонақ күт жаназа тапсырыс бер оңба жерлеу рәсім кездес,violent

4,егер сен сұмырайдың ақымақ басыңды қараңғы түнде арматурамен жаратын болса біліп жүр ол қарыз ақшаң үшін жан жағыңа қарап жүр,егер сен сұмырай ақы бас қараңғы түн арматура жар бол біл жүр ол қарыз ақша үшін жан жағ қара жүр,violent

4,сен сұмырай өз адамдарыңа айт мен қалжыңдамаймын өз жақындарыңның денсаулығын ойла полиция көмектесе алмайды жоғалтатын нәрсең көп өзің және командаңа айт ауыздарын жапсын қызыңды ойла басқа ескертулер болмайды,сен сұмыра өз адам айт мен қалжыңдама өз жақын денсаулығ ойла полиция көмекте алма жоғалт нәр көп өзің және команда айт ауыз жап қыз ойла басқа ескерту болма,violent

4,мен сен туралы бәрін білемін қайда тұратыныңды қызыңның бар екендігін ерте ме кеш пе мен қаласам алдымен сенің қызыңды ұстап аламын және аяқ қолдарын талқандаймын бетін бұзамын жұлынын үземін дәретке өзіне баратын етемін және солай өмір сүруге қалдырамын осының барлығын видеоға түсіріп саған жіберемін,мен сен туралы бәрін біл қайда тұр қыз бар екендіг ерте ме кеш пе мен қала алд сенің қыз ұста ал және аяқ қол талқанда бет бұз жұлын үз дәрет өзіне бар ет және солай өмір сүр қалдыр осы барлығ видео түсір саған жібер,violent

4,сенің жақындарыңды майда бөліктерге бөлшектеп қайнатып жейтін уақытты күтіп жүрмін,сенің жақын май бөлік бөлшекте қайнат же уақыт күт жүр,violent

4,сен өз жақындарыңды қарызың үшін сақтаудан жалығатын боласың,сен өз жақын қарыз үшін сақтау жалығ бол,violent

4,ертең түске дейін толық сома қайтарылмаса сенің барлық суреттеріңнің көшірмесі алынып порно сайттарға салынады,ертең түс дейін толық сома қайтарылма сенің барлық сурет көшір алын порно сайт салын,violent

4,ақша тап әйтпесе біз саған тыныш өмір бермейміз рождество құтты болсын,ақша тап әйтпесе біз саған тыныш өмір берме рождество құт бол,violent

4,пәтер күт отбасыңды толығымен қабатта бөлшектейміз,пәтер күт отбас толығ қабат бөлшекте,violent

4,сіз отырған жерге бомба қойылған ол минутта жарылып кетеді мина орнаттым,сіз отыр же бомба қойыл ол минут жарыл кет мина орна,violent

4,жаяу мұса көшесіндегі сулпак дүкенінің ғимаратында бомба қойылған,жаяу мұса көше сулпак дүкен ғимарат бомба қойыл,violent

4,біздің түйіндер барлығына көңіл күй сыйлайды егер сіз де күнді барынша өткізгіңіз келсе сонымен қатар көңілді нотада болғыңыз келсе онда тез арада жазып сапалы өнімге тапсырыс беріңіз,біздің түйін барлығ көңіл күй сыйла егер сіз де күн барынша өткіз кел сонымен қатар көңіл нота бол кел онда тез ара жаз сапалы өнім тапсырыс бер,violent

4,дымқыл қантты ұнатындардың барлығы осында келіңдер мет рацемат енді барлық қалаларда бар дайын түйіндер фен шөп экстази мефедрон енді іздеу мен күтудің

қажеті жоқ бәрі бір жерде,дымқыл қан ұнат барлығ осында кел мет рацемат енді барлық қала бар дайын түйін фен шөп экстази мефедрон енді іздеу мен күту қажет жоқ бәрі бір же,violent

4,тегін мефедрон ұсынылады сорттық canabis таза кокаин мефедрон амфетамин тұздар мен қоспалар мен amf аламын,тегін мефедрон ұсын сор canabis таза кокаин мефедрон амфетамин тұз мен қоспа мен amf ал,violent

4,мен оларды қуана өртеп жіберетін едім,мен ол қуан өрте жібер ед,violent

4,интернет арқылы есірткі сатып алу үшін сізге курьерге ақша аудару банкке бару және одан да көп төлқұжат немесе басқа құжатты ұсынып қажет емес шот анонимді түрде төленеді және бетбелгі арқылы есірткіні онлайн сатып алғыңыз келгенде ең қолайлы әдіс болып табылады қосымша бонус кепілгер арқылы тауар сатып алу бұл максималды сенімділік үшін бірнеше пайыздан пайызға дейін қауіпсіздікті қосады егер сіз қала маңында тұрсаңыз есірткіні аймақтарға жеткізуге болады интернетте есірткіні қайдан сатып алуға болады әрине nora biz форумында,интернет арқылы есірткі сат алу үшін сізге курьер ақша аудару банк бару және одан да көп төлқұжат немесе басқа құжат ұсын қажет емес шот анони түр төлен және бетбелг арқылы есірткі онлайн сат ал кел ең қолайлы әдіс бол таб қосымша бонус кепілгер арқылы тауар сат алу бұл максималды сенімділік үшін бірнеше пайыз пайыз дейін қауіпсіздік қос егер сіз қала маң тұр есірткі аймақ жеткіз бол интернет есірткі қайдан сат ал бол әрине nora biz форум,violent

4,бізден экстази киев амфетамин киев amphetamines cocaine mdma сатып ала аласыз бетбелгі фен амфетамин арқылы,бізден экстази киев амфетамин киев amphetamines cocaine mdma сат ал ал бетбелг фен амфетамин арқылы,violent

4,амфетамин экстази сатып алу метамфетамин метамфетамин героин amphetamine сату фен бетбелгі амфетамин сатып алу,амфетамин экстази сат алу метамфетамин метамфетамин героин amphetamine сату фен бетбелг амфетамин сат алу,violent

4,біздің портал сатып алу үшін сілтеме бойынша өтіңіз меф кокаин метадон қолданып көріңіз,біздің портал сат алу үшін сілтеме бойынша өт меф кокаин метадон қолдан көр,violent

4,мен бүгін сіздің қызметіңізден сату боты арқылы ға мефа кристалын сатып алдым тек жаңа қазыналар ең жақсы бағамен белгілі жеткізушілерден жоғары сапа,мен бүгін сіздің қызмет сату бо арқылы ға меф кристал сат ал тек жаңа қазына ең жақсы баға белгілі жеткізуші жоғары сапа,violent

4,сәуірде байланыста болдым мен оны қозғалыс кезінде сатып алдым тауардың сапасы әдеттегідей бомба дүкеніңізге тап болған күнді жақсы сөзбен есіме түсірдім,сәуір байланыс бол мен оны қозғалыс кез сат ал тау сапа әдеттегідей бомба дүкен тап бол күн жақсы сөз ес түс,violent

4,сату боты арқылы бір қазбамен амфетамин мен скорость көтеріп алдым,сату бо арқылы бір қазба амфетамин мен скорость көтер ал,violent

4,мен сені жерге көмемін мен жындымын,мен сен же көм мен жынд,violent

4,сіздердің дүкенді тапөанға дейін біршама ақшам далаға кетті грамм скорость сатып алдым менен пікір тек бір екі ауыз сөзбен ға меф тапсырым беріп алғанмын сіздерден,сіздердің дүкен тапөан дейін біршама ақшам дала кет грам скорость сат ал менен пікір тек бір екі ауыз сөз ға меф тапсыр бер ал сіздерден,violent

4,ағасы грамм наша алып кеттім шын жүректен үлкен рахмет қалай айтты солай жасап берді тағы да келемін,аға грам наша алып кет шын жүрек үлкен рахмет қалай ай солай жаса бер тағы да кел,violent

4,бүгін оператор арқылы қантты алдым оператор бәрін әдемі жасап берді,бүгін оператор арқылы қан ал оператор бәрін әдемі жаса бер,violent

4,достым дана экстази алып кеттім достым грамм скорость алдым өтті,дос дана экстази алып кет дос грам скорость ал өт,violent

4,балғын тәттілер ең жақсы бағамен нарықтағы ең жақсы сапа ең жақсы наша жеткізуші тауарды жанасу арқылы береді кокаинды зертханаға өзі жеткізді,балғын тәтті ең жақсы баға нарық ең жақсы сапа ең жақсы наша жеткізуші тау жанас арқылы бер кокаи зертхана өзі жет,violent

4,қалған кокаинды орап тығып қой,қалған кокаи ора тығ қой,violent

4,біз кокаин шегетінбіз және таблеткалар қабылдап алдық мен сенің барлық кокаиныңды жұлып алған сияқтымын перуден келген кокаинды екі рет тартып көрдік жасыл тегененің артынан біз кокаины таптық,біз кокаин шег және таблетка қабылда ал мен сенің барлық кокаин жұл ал сияқты перу кел кокаи екі рет тарт көр жасыл тегене арт біз кокаи тап,violent

4,ән айтсаң өзіңнің баға жетпес кокаиныңды алатын боласың,ән айт өз баға жетпес кокаин ал бол,violent

4,бұл жерден әрдайым ром және арзан наша табуға болады,бұл же әрдайым ро және арзан наша таб бол,violent

4,ол күлгін наша алып келді сен оны татып көруің керек,ол күлг наша алып кел сен оны тат көр керек,violent

4,бұл мал мен және буллды өлтірмекші болды оны көрген бойда атып өлтір,бұл мал мен және бул өлтірмекш бол оны көр бой ат өлтір,violent

4,мен есікті сындырып екеуіңді де өлтіргім келді,мен есік сындыр екеу де өлтір кел,violent

4,жеткілікті үлкен доза астматик болмасаң да өлтіруі мүмкін,жеткілікті үлкен доза астматик болма да өлтір мүмкін,violent

4,бір взвод сарбазды өлтіруге жеткілікті бар,бір взвод сарбаз өлтір жеткілікті бар,violent

4,қожайынды өлтір оның тұқымы өзі ақ шіриді,қожайын өлтір оның тұқым өзі ақ шіри,violent

4,зұлымды тапсаң өлтір,зұлым тап өлтір,violent

4,қарғыс атқыр швабра өлтір оны,қарғыс атқы швабр өлтір оны,violent

4,ал мына бұтаның осында екенін біреуге айтсаң үйіңе келіп пышақтаймын,ал мына бұта осында екен біреу айт үй кел пышақта,violent

4,тыңда ағайын егер әлгі жексұрын шив нараянды өлтірмесек біздің банданың түбіне жетеді,тыңда ағайын егер әлгі жексұр шив нарая өлтірме біздің банда түб жет,violent

4,оны тұрмыстық электр құрылғыларымен өлтірейік,оны тұрмыстық электр құрылғы өлтір,violent

4,біз сені игі мақсаттар үшін өлтіреміз,біз сен игі мақсат үшін өлтір,violent

4,мүмкін бірақ бомба ертең жарылады,мүмкін бірақ бомба ертең жар,violent

4,түбін қозғап қайта ат,түб қозға қайта ат,violent

4,егер ол қозғалуға тырысса оны атып тастаңыз,егер ол қозғал тырыс оны ат таста,violent

4,келісімге келейік сіз мылтық аласыз егер сорғыны орнатуға және оны жөндеуге көмектессеңіз,келісім кел сіз мылтық ал егер сорғы орнат және оны жөнде көмектес,violent

4,мысалы сен өлдің ал мен саған оқ жаудырамын,мысал сен өл ал мен саған оқ жаудыр,violent

4,келесі жолы жерлес біз сені толығымен құртамыз,келесі жол жерлес біз сен толығ құр,violent

4,мен сонда барып семіздің мылтығын алып көздеріңнің арасына екі оқ жіберемін,мен сонда бар се мылтығ алып көз ара екі оқ жібер,violent

4,ұсқынсыз түрін көрсеткен бойда атып тастаймын,ұсқын түр көрсет бой ат таста,violent

4,мен барлығыңды соңына дейін атып өлтіремін,мен барлығ соң дейін ат өлтір,violent

4,мен сені өзім жерлеп бейітіңде билеймін,мен сен өзім жерле бейіт биле,violent

4,ал егер оның жүрегін жараласаң мен сені атып тастап орманға көмемін,ал егер оның жүрег жарала мен сен ат таста орман көм,violent

4,мені тастап кететін болсаң мен сені көміп тастаймын,мен таста кет бол мен сен көм таста,violent

4,әжеңді жерлегенде оны да жерлеймін,әже жерле оны да жерле,violent

4,мен жезөкшелерді таңдадым өйткені ұсталмай ақ қалағанымша өлтіре аламын,мен жезөкше таңда өйткені ұсталма ақ қалағанымш өлтір ал,violent

4,мен ішімде шайтанмен туылдым ақынның шабытына кел деп бұйыра алмағаны сияқты мен де қанішер екенімді айта алмадым мен анам мені дүниеге әкелген төсектің қасында жаманмен бірге тудым содан бері ол менімен бірге,мен іш шайтан ту ақын шабыт кел де бұйыр алма сияқты мен де қаніш екен ай алма мен ана мен дүние әкел төсек қас жаман бірге ту содан бері ол менімен бірге,violent

4,бұл құбыжық менің миыма қашан енетінін білмеймін бірақ ол сонда қалу үшін келеді қоғам мен сияқты өз құрбандарын қинауды ойлай отырып жеңілдеу жолдары бар адамдардың болғанына ризашылығын білдірсе болады,бұл құбыжық менің ми қашан ен білме бірақ ол сонда қалу үшін кел қоғам мен сияқты өз құрбан қинау ойла отыр жеңілде жол бар адам бол ризашылығ білдір бол,violent

4,мен сериялық өлтірушімін мен тағы да өлтірер едім,мен сериялық өлтіруш мен тағы да өлтір ед,violent

4,мен зорлаушы емес өлтірушімін,мен зорлаушы емес өлтіруш,violent

4,менің басым құмарлығым олардың тәнін сезіну болды мен оларды бейтаныс объект ретінде көрдім мен жасағанды адам жасады дегенге сену өте қиын,менің басым құмарлы олардың тән сезін бол мен ол бейтаныс объект рет көр мен жаса адам жас де сену өте қиын,violent

4,пышақты қолданған кезде менде психологиялық жеңілдеу болды мені құрту керектігін білемін мен табиғаттың қателігі болдым,пышақ қолдан кезде менде психологиялық жеңілде бол мен құрт керектіг біл мен табиғат қателіг бол,violent

4,барлық кісі өлтірулер маған жазылатын болады,барлық кісі өлтіру маған жазыл бол,violent

4,мен адам өмірін қатты жек көретін және қайта өлтіруге дайын адаммын,мен адам өмір қатты жек көр және қайта өлтір дайын адам,violent

4,мен де жәбірленуші ретінде жезөкшелерді таңдадым өйткені оларды байқамай алып кету оңай болды мен олардың хабарсыз кеткенін бірден хабарламайтынын және олар ешқашан хабарсыз кеткен деп хабарланбағанын білдім,мен де жәбірленуші рет жезөкше таңда өйткені ол байқама алып кету оңай бол мен олардың хаб кет бірден хабарлама және олар ешқашан хаб кет де хабарланба біл,violent

4,әуестенгенім соншалық оны өлтіруіме тура келді,әуестен соншалық оны өлтір тура кел,violent

3,бұл нәсілдерді араластыру музыкасы және порнографияны танымалдандыруға қосымша венгриялықтарды жоюдың тағы бір жолы,бұл нәсіл араластыр музыка және порнография танымалдандыр қосымша венгриялық жою тағы бір жол,nazism

4,мен адамдарды өлтіргенді жақсы көремін мен олардың өлгенін көргенді ұнатамын мен олардың басынан атып тұрып олардың жерде бұралғанын содан кейін кенет тыныштылғанын көрдім немесе оларды пышақпен кесіп тастадым және олардың беттері толығымен ағарғанын көрдім мен осы қанның бәрін жақсы көремін бірде мен бір әйелге барлық ақшасын беруін бұйырдым ол бас тартты жарайды онда деп мен оны пышақтап көзін жұлып алдым,мен адам өлтір жақсы көр мен олардың өл көр ұна мен олардың бас ат тұр олардың же бұрал содан кейін кенет тыныштал көр немесе ол пышақ кес таста және олардың бет толығ ағар көр мен осы қан бәрін жақсы көр бірде мен бір әйел барлық ақша бер бұйыр ол бас тар жарайды онда де мен оны пышақта көз жұл ал,violent

4,бізге ел аралап жүріп ел аралап жүргендерді өлтіру идеясы келді сериялық кісі өлтірушілер сериялық кісі өлтірушілерді өлтіреді,бізге ел арала жүр ел арала жүр өлтір идея кел сериялық кісі өлтіруші сериялық кісі өлтіруші өлтір,violent

4,біздің бәріміздің қолымызда өлтіру күші бар бірақ көпшілігі оны қолдануға қорқады,біздің бәрі қол өлтір күш бар бірақ көпшіліг оны қолдан қорқ,violent

4,мен әжемді атып тастасам қандай сезімде болатыным қызық болды,мен әже ат таста қандай сез бол қызық бол,violent

4,сондықтан мен оны жұмыстан қайтып келгенде өлтіргім келеді оның менің әрекеттерім тудыруы мүмкін ауыртпашылық сезінуін қаламаймын,сондықтан мен оны жұмыс қайт кел өлтір кел оның менің әрекет тудыр мүмкін ауыртпашылық сезін қалама,violent

4,бала кезімде үйренген шекаралар мені сыртқа шығып біреуді ренжітуден сақтай алмады,бала кез үйрен шекара мен сырт шығ біреу ренжіту сақта алма,violent

4,сериялық өлтірушілер біз сіздің ұлдарыңыз күйеулеріңіз біз барлық жердеміз ал ертең сенің балаларың өледі,сериялық өлтіруші біз сіздің ұл күйеу біз барлық жер ал ертең сенің бала өл,violent

4,міне мен көшеде келе жатырмын бір қызды кездестірдім ол бір жағынан әдемі және мен онымен кездесіп бірге түнегім келеді бірақ содан кейін мен оның басын қалай кесіп оның ішектерін шығарып жатқанымды елестетемін бір сөзбен айтқанда мен романтикпін,міне мен көше келе жатыр бір қыз кездес ол бір жағ әдемі және мен онымен кездес бірге түне кел бірақ содан кейін мен оның басын қалай кес оның ішек шығар жат елесте бір сөз айтқанда мен романтик,violent

4,неге өлтірдім деп сұрайсың ба ал мен саған қалай түсіндіремін түсініп ал мен үшін өлтірмей өмір сен үшін тамақсыз өмірмен бірдей қажеттілігін түсінесіз бе мен бұл адамдарға басқа дүниенің есігін ашқандықтан өзімді әкелеріндей сезіндім мен оларды жаңа өмірге жібердім,неге өл де сұра ба ал мен саған қалай түсіндір түсін ал мен үшін өлтірме өмір сен үшін тамақ өмір бірде қажеттіліг түс бе мен бұл адам басқа дүние есіг ашқандық өз әкелерінде сез мен ол жаңа өмір жібер,violent

4,мен оларды ренжіткім келмеді мен оларды өлтіргім келді,мен ол ренжіт келме мен ол өлтір кел,violent

4,мен өзімді бөгде адам сияқты сезінемін мен басқалармен бір толқында емеспін мен өлтіруге бағдарламаланғанмын,мен өз бөг адам сияқты сез мен басқа бір толқ емес мен өлтір бағдарламалан,violent

4,мен қанды жақсы көремін,мен қан жақсы көр,violent

4,мені кінәлауға асығады айына бір мәйітті табу жеткілікті мен астам адам өлтірдім мені мені өлім жазасына кесіп үлгермейді,мен кінәла асығ ай бір мәйіт табу жеткілікті мен астам адам өл мен мен өлім жаза кес үлгерме,violent

4,біреуді өлтіру есіктен шығып кету сияқты оңай мен өлтіргім келген кезде баратынмын да құрбан адамды тауып алатынмын,біреу өлтір есік шығ кету сияқты оңай мен өлтір кел кезде бар да құрбан адам тау ал,violent

4,сіз мені білесіз мен отбасылық адаммын өте отбасылық бүкіл отбасының басын шауып тастауға рұқсат етіңіз сонда мен өте бақытты боламын,сіз мен біл мен отбасылық адам өте отбасылық бүкіл отбас басын шау таста рұқсат ет сонда мен өте бақытты бол,violent

4,мен өзімді тану үшін адамдарды өлтірдім адам ойыншық менің әрекеттерімді бәрі білсін,мен өз тану үшін адам өл адам ойыншық менің әрекет бәрі біл,violent

4,тона зорла және өлтір,тона зорла және өлтір,violent

4,сенің назарыңды аударудың жалғыз жолы ол ұру,сенің назар аудар жалғыз жол ол ұр,violent

1,жаңа ойын бар қара адамды тұншықтыр,жаңа ойын бар қара адам тұншықтыр,racism

1,бір соққымен қара адамды кесу ойынын бастайық,бір соққы қара адам кесу ойын баста,racism

1,жаңа ойын қара адамды өртеу,жаңа ойын қара адам өрте,racism

4,менің ағам бол әйтпесе мен сені өлтіремін,менің аға бол әйтпесе мен сен өлтір,violent

1,жаңа ойын қара адамды құрт,жаңа ойын қара адам құрт,racism

4,егер менің автоматым болса қызығырақ болар еді,егер менің автомат бол қызығырақ бол ед,violent

4,мен қанша адамды өлтірсем де бәрібір жеткіліксіз деп ойлаймын,мен қанша адам өлтір де бәрібір жеткіліксіз де ойла,violent

4,сенің басыңды өндірістік ет тартқыштан өткізсе бұл жазатайым оқиға болар еді,сенің бас өндірістік ет тартқыш өткіз бұл жазатайым оқиға бол ед,violent

4,иттер сіздің жыныс мүшелеріңізді жеген кезде аз да болса рахаттанып қалуы үшін бүгін шомылуыңызды өтінемін,ит сіздің жыныс мүше же кезде аз да бол рахаттан қал үшін бүгін шомылу өт,violent

4,адам болыңыз ауруханаға барып орган доноры ретінде тіркеліңіз ал мен өзімді жақсы сезінетін боламын және сіздің өліміңіз бекер болмайды,адам бол аурухана бар орган донор рет тіркел ал мен өз жақсы сезін бол және сіздің өлім бек болма,violent

4,бір рет пышақты қажет жерге тығу жеткілікті адам өлуі үшін оның қаншалықты қорлық екенін енді ешкімге айта алмайтыныңыз өкінішті,бір рет пышақ қажет же тығ жеткілікті адам өл үшін оның қаншалықты қорлық екен енді ешкім ай алма өкініш,violent

4,менің ұстанатын екі ережем бар біріншіден ерекше себепсіз ешқашан өлтірмеңіз екіншіден сіз біреуді өлтіру үшін әрқашан себеп таба аласыз,менің ұстан екі ереже бар бірінші ерекше себепсіз ешқашан өлтірме екінші сіз біреу өлтір үшін әрқашан себеп таб ал,violent

4,менің психикалық ауру екенімді растайтын дәрігердің анықтамасы бар мен өлтіре аламын және ол үшін маған ештеңе болмайды,менің психикалық ауру екен раста дәрігер анықта бар мен өлтір ал және ол үшін маған ештеңе болма,violent

4,жануарды ату өлтіру сияқты қылмысқа жатпайды,жануар ату өлтір сияқты қылмыс жатпа,violent

1,мацквабад хачтар мен еврейлерді тегін аулау аумағы,мацквабад хач мен еврей тегін аулау аумағ,racism

4,иракта ұшқын тұтанды және оның ыстығы әскерлерді өртеп жібермейінше күшейе береді,ирак ұшқ тұтан және оның ыстығ әскер өрте жібермейінш күшей бер,violent

4,римдіктер әл амаққа немесе дабикке халабтың солтүстігіндегі бір біріне жақын орналасқан екі жер қонбайынша уақыт келмейді оларға қосылыңыз олар олармен күреседі содан кейін олар константинопольді жаулап алады,римдік әл амақ немесе дабик халаб солтүстіг бір бір жақын орналас екі жер қонбайынш уақыт келме оларға қосыл олар олармен күрес содан кейін олар константинополь жаула ал,violent

4,мұсылмандар туралы өз мемлекетіне иә бұл сенің елің асығыңыздар өйткені сирия сириялықтар үшін емес ал ирак ирактықтар үшін емес,мұсылман туралы өз мемлекет иә бұл сенің ел асығ өйткені сирия сириялық үшін емес ал ирак ирактық үшін емес,violent

4,біз бүгін жаңа дәуірде өмір сүріп жатқанымызды әлем білсін қамсыз болған адам енді сергек болуы керек кім ұйықтады енді оянуы керек таңғалып жүргендер түсінуі керек ауыр етік олардың лаңкестік пен ұлтшылдықтың пұтын таптайтын демократия пұтын жойып оның теріс пиғылын әшкерелейтін етіктің мәнін әлемге естіртетін түсінетін мәлімдемесі бар,біз бүгін жаңа дәуір өмір сүр жат әлем біл қам бол адам енді сергек бол керек кім ұйықт енді оян керек таңғал жүр түсін керек ауыр етік олардың

лаңкестік пен ұлтшылдық пұт тапта демократия пұт жой оның теріс пиғыл әшкереле етік мән әлем естірт түсін мәлімде бар,violent

4,жиһад бар кезде хижра жойылмайды,жиһад бар кезде хижр жойылма,violent

4,жаумен шайқас болып жатқанда хижра тоқтамайды,жау шайқас бол жатқанда хижр тоқтама,violent

4,біреу үшін террорист екінші үшін азаттық үшін күресуші жаумен күрескен бүгінгі лаңкес ертеңгі мемлекет қайраткері,біреу үшін террорист екінші үшін аза үшін күресуш жау күрес бүгінгі лаңкес ертеңгі мемлекет қайраткер,violent

4,территорияны қанды бөлісу болады және ядролық терроризм,территория қан бөліс бол және ядролық терроризм,violent

3,бірақ орыстар ешқашан қателеспейді олар ешқашан арамдық жасамайды соғыс ашпайды лаңкестікке демеушілік жасайды және қорқынышсыз әдейі өтірік айтады бұл олардың саналы таңдауы,бірақ орыс ешқашан қателеспе олар ешқашан арам жасама соғыс ашпа лаңкестік демеушілік жаса және қорқыныш әдей өтірік айт бұл олардың саналы таңда,nazism

4,не істеу керек террористік шабуыл бірдеңені бомбалау ма,не істеу керек террористік шабуыл бірдеңе бомбала ма,violent

3,осы жиналыстан лаңкес мемлекет ресейді қуып жіберіңіздер мәңгілікке,осы жиналыс лаңкес мемлекет ресей қу жібер мәңгілік,nazism

3,израиль жат жерлерді басып алып бейбіт тұрғындарды өлтірген лаңкестік ел,израиль жат жер бас алып бейбіт тұрғын өлтір лаңкестік ел,nazism

3,америка лаңкес ел,америка лаңкес ел,nazism

2,әйел болған соң үй жинадым деп шағымданбау керек ер адам ретінде сіз әрқашан қоқысты шығаруыңыз керек,әйел бол соң үй жина де шағымданба керек ер адам рет сіз әрқашан қоқыс шығар керек,bullying

2,ол трансвеститке ұқсайды,ол трансвестит ұқса,bullying

1,біз бұл ақымақ негрлерге сенбейміз,біз бұл ақы негр сенбе,racism

2,қолында татуировкасы жоқ әлжуаз,қол татуировка жоқ әлжуаз,bullying

2,бұл түкке тұрғысыз адам соншалықты алғыссыз болды,бұл түк тұрғы адам соншалықты алғыс бол,bullying

3,ол бұршақты жігіт мексикандық екені бірден көрініп тұр,ол бұршақ жігіт мексика екен бірден көрін тұр,nazism

1,негрлер туған күндеріңмен,негр туған күн,racism

4,мен сенің тамағың мен мойныңды кесемін,мен сенің тамағ мен мойны кес,violent

1,негр кері шегін мен заттарыммен бөліспеймін,нег кері шегін мен зат бөліспе,racism

2,ол жынды сияқты әрекет етеді бірақ ол шынымен түкке тұрғысыз ақымақ,ол жын сияқты әрекет ет бірақ ол шын түк тұрғы ақы,bullying

2,аузыңды жап сенен ешкім сұраған жоқ,ауз жап сенен ешкім сұра жоқ,bullying

2,сен семізсің және қолыңнан ештеңе келмейді сен арықтай алмайсың,сен се және қол ештеңе келме сен арықта алма,bullying

2,мына миыңмен өмірде ештеңеге қол жетізе алмайсың,мына ми өмір ештеңе қол жетіз алма,bullying

2,сен жыласаң сондай ұсқынсызсың,сен жыла сондай ұсқынсыз,bullying

2,қорлайтындай ақ сен сондай ақымақсың,қорлайтында ақ сен сондай ақы,bullying

2,сен өзіңе айнадан қарадың ба сені тек маймылдар сүюге ұмтылады,сен өз айна қара ба сен тек маймыл сүю ұмтыл,bullying

2,сен сондай ұсқынсызсың тіпті бетіңе қарағым келмейді,сен сондай ұсқынсыз тіпті бет қара келме,bullying

2,мен бұл ақымақтардың ешқайсысына мән бермеймін,мен бұл ақымақ ешқайсы мән берме,bullying

2,сен ешкі жүндік ауылдық адамсың,сен ешкі жүн ауылдық адам,bullying

1,енді твиттерден жоғалыңдар сендер сияқты ақсақ негрлерге интернетті пайдалануды үйренбеу керек еді,енді твиттер жоғал сен сияқты ақ негр интернет пайдалану үйренбе керек ед,racism

2,ауылдық адам маған твиттерден жазба,ауылдық адам маған твиттер жазба,bullying

2,ақымақ сенің кетіп қалғаның қандай жақсы болды,ақы сенің кет қал қандай жақсы бол,bullying

2,сен сондай әлжуазсың,сен сондай әлжуаз,bullying

2,барып аюдың боғын же ауылдық адам,бар аю боғ же ауылдық адам,bullying

2,ұшуды емес флайтты меңзегеніңізге сенімдімін мылқау крекер,ұшу емес флай меңзе сенімді мылқа крек,bullying

2,көрдің бе ақымақ неліктен достарыңның жоқтығын,көр бе ақы неліктен дос жоқтығ,bullying

2,сен қандай болсаң да сиқыршы немесе тапқыр болсаң да мен сені ұнатпаймын күніңіз жақсы өтсін,сен қандай бол да сиқырш немесе тапқы бол да мен сен ұнатпа күн жақсы өт,bullying

2,егер сен жаста болсаң және орта мектепте сенің жеке үйің болмаса сен ақымақсың,егер сен жас бол және орта мектеп сенің жеке үй болма сен ақы,bullying

2,о мырза мен өзімді мықты санаймын себебі артта қалған ескі қалада тұрып мықты қалада тұрамын деп ойлаймын сен сияқтылар менің үйімдегі қарды күреу керек бейшара қаңғыбас,о мырза мен өз мықты сана себебі арт қалған ескі қала тұр мықты қала тұр де ойла сен сияқты менің үйім қар күре керек бейшар қаңғыбас,bullying

2,бері қара қаңғыбас мен жай ғана сенен жауап алатындығыма көз жеткізгім келді,бері қара қаңғыбас мен жай ғана сенен жауап алатындығ көз жеткіз кел,bullying

2,сен итсің жасандылықты доғар,сен ит жасандылық доғар,bullying

4,олар келесі сені өлтірмекші,олар келесі сен өлтірмекш,violent

2,сен екеуің де қоқыссыңдар,сен екеу де қоқыс,bullying

2,қайда кеттің аузы жабылмайдын ауылдық адам,қайда кет ауз жабылмайд ауылдық адам,bullying

1,мен қара геттодан қоқыс шығарып жатырмын сен бұғаттаулысың негр,мен қара гетто қоқыс шығар жатыр сен бұғаттаул нег,racism

1,жалған негр олар жалғандық істеп жатыр,жалған нег олар жалға істе жатыр,racism

3,мен қазір досыма айтпақшымын сенің былапытыңды сатып алмасын деп ақымақ қытай,мен қазір дос айтпақшы сенің былапыт сат алма де ақы қытай,nazism

1,сен ессіз жынды негр болуың керек,сен ес жын нег бол керек,racism

2,сен анаң сияқты өте семізсің,сен ана сияқты өте се,bullying

2,петя бейбақ неге онымен әлі де болса біреу байланысқа түседі ол гей ғой,петя бей неге онымен әлі де бол біреу байланыс түс ол ге ғой,bullying

3,сасық орыстар ресейге қайтыңдар,сасық орыс ресей қай,nazism

2,қаңғыбассың бет әлпетің де киімің де қорқынышты,қаңғыбас бет әлпе де киім де қорқыныш,bullying

2,отбасыңыздың кедей болғанына біз кінәлі емеспіз,отбас кедей бол біз кінәлі емес,bullying

2,сен дәу шошқасың семіз,сен дә шошқа се,bullying

2,қарғыс атсын сен нағыз қойсың,қарғыс ат сен нағыз қой,bullying

2,сен қанішерсің намыссыз арамзасың,сен қаніш намыс арамз,bullying

4,мен сені өлтіремін,мен сен өлтір,violent

2,ақымақ сенің миың жоқ,ақы сенің ми жоқ,bullying

2,ақымақ сен семізсің,ақы сен се,bullying

2,сенің басыңда ми емес боқ бар,сенің бас ми емес боқ бар,bullying

4,дек мен сенің басыңды кесіп аламын маған хабарлас,дек мен сенің бас кес ал маған хабарлас,violent

2,сен өтірікші сатқын адамсың,сен өтірікш сатқ адам,bullying

2,демек сенің әкең бейбақ жолы болмайтын,демек сенің әке бей жол болма,bullying

2,ақымақ денеңді жақсартпас бұрын миыңды дамыт,ақы дене жақсартпас бұрын ми дамыт,bullying

2,ақымақ саған мұндай баспен жұмыс істеп керек емес,ақы саған мұндай бас жұмыс істе керек емес,bullying

2,сен сияқтыларды бақытсық бейбақ дейді,сен сияқты бақытсық бей де,bullying

4,мен саған сенің қаныңды көрсетемін,мен саған сенің қан көрсе,violent

3,ол американдық қызыл шашты сыған,ол американдық қызыл шаш сы,nazism

2,ол дәл сен сияқты ақымақ адам,ол дәл сен сияқты ақы адам,bullying

4,мен бұл көлікке бомба қойып оны жаруым керек,мен бұл көлік бомба қой оны жар керек,violent

2,сен кішкентай өтірікші,сен кішкентай өтірікш,bullying

3,қарғыс атқыр адамдар ештеңе істеуді білмейді,қарғыс атқы адам ештеңе істеу білме,nazism

1,лео аполонио қара мына негр жанымыздан велосипедпен өтіп кетті,лео аполонио қара мына нег жан велосипед өт кет,racism

1,негрді саусағыңа сақина сатып алып беруге мәжбүрле,нег саусағ сақин сат алып бер мәжбүрле,racism

1,жоғалшы дорис сен ақымақ негрсің,жоғалш дорис сен ақы нег,racism

2,неге мына бірінші курстықтар төмен сынамалы,неге мына бірінші курстық төмен сынамал,bullying

2,неге бұл ақымақ жолақты футболка мен nike кроссовкасын киіп алған,неге бұл ақы жолақ футболка мен nike кроссовка ки ал,bullying

2,сен сондай ақымақсың сен типті қан диабетімен ауырып ағзаңдағы қант мөлшерінің күрт жоғарылауынан өлесің деп үміттенемін,сен сондай ақы сен типті қан диабет ауыр ағзаң қант мөлшер күрт жоғарылау өл де үміттен,bullying

2,ой сенің ішің сондай семіз,ой сенің іш сондай се,bullying

2,сен азғын әлсіз және бейшара адамсың,сен азғын әлсіз және бейшар адам,bullying

2,сенің сөздерің лас иттің дәрменсіз үруі сияқты,сенің сөз лас ит дәрменсіз үр сияқты,bullying

2,бұндай бетке түкірудің қзі обал,бұндай бет түкіру қз обал,bullying

2,қызық не сұлу емес не ақылды емес,қызық не сұл емес не ақылды емес,bullying

2,сен әкеңнің ұяты мен анаңның көз жасысың,сен әке ұят мен ана көз жас,bullying

2,сенімен араласу ол өзіңді қорлаумен тең,сенімен аралас ол өз қорла тең,bullying

2,саған бір қарағаннан ақымақ екендігің көрініп тұрады сосын тағы қарағасын шынымен ақымақ екен деген ойға қаласың,саған бір қара ақы екендіг көрін тұр сосын тағы қараға шын ақы екен де ой қал,bullying

2,барып мына ақымақтығыңды жуып келші өзіңмен қоса,бар мына ақымақтығ жу келш өз қоса,bullying

2,мен бұрын сені ақылды деп ойлайтын едім сен әрдайым ақымақ болған екенсің ғой,мен бұрын сен ақылды де ойла ед сен әрдайым ақы бол екен ғой,bullying

2,басы үлкен бірақ миы жоқ ақымақ жаратылыс,бас үлкен бірақ ми жоқ ақы жаратылыс,bullying

2,қарғыс атқыр сенің ақылың өте аз,қарғыс атқы сенің ақыл өте аз,bullying

2,қарғыс атсын сен осындай ақымақ болып тудың ба әлде жүре тұра ақымақ болып кеттің бе,қарғыс ат сен осындай ақы бол ту ба әлде жүр тұр ақы бол кет бе,bullying

2,сіз тек ақымақ адамды құруға арналған үлгісіз,сіз тек ақы адам құр арнал үлгі,bullying

2,түкке жарамайтын екенсің,түк жарама екен,bullying

2,әпендісің сен ақылы кем,әпенд сен ақылы кем,bullying

2,мен сенімен өзімді өте ақылды сезінемін өйткені сен ақымақсың,мен сенімен өз өте ақылды сез өйткені сен ақы,bullying

1,мына негр аузына келгенін айтуы мүмкін себебі негрді қиялында ұрып тастады,мына нег ауз кел айт мүмкін себебі нег қиял ұр таста,racism

2,маған жазылма және керісінше істеме сенің ақыл есің кем,маған жазылма және керісінше істеме сенің ақыл ес кем,bullying

4,сен ақымақ және ұсқынсызсың сені бауыздап өлтіріп қаныңмен ойнағым келеді,сен ақы және ұсқынсыз сен бауызда өлтір қан ойна кел,violent

2,сенде ана негрдің емдеуге келмейтін ауруы бар,сенде ана нег емде келме ауру бар,bullying

2,сен жай ғана тұзды әрі ұсқынсыз негрсің,сен жай ғана тұз әрі ұсқын нег,bullying

2,сен кішкентай ақымақсың мен сенімен сөйлеспеймін,сен кішкентай ақы мен сенімен сөйлеспе,bullying

1,сендер негрлер мына әйелдермен жақындасу үшін сөйлесіп отырсыңдар бірақ оларды өсіріп өздерің бұзық болып бара жатқандарыңды түсінбейсіңдер,сен негр мына әйел жақындас үшін сөйлес отыр бірақ ол өсір өз бұзық бол бар жатқанда түсінбе,racism

2,сен кәрі ащы тұзды есек,сен кәрі ащ тұз есек,bullying

1,мені соғуға тырысып жол бойымен келе жатқан көлігінен айғайлап жатқан ақымақ негрлер,мен соғ тырыс жол бойымен келе жат көліг айғайла жат ақы негр,racism

2,сізді не ақымақ ететінін білмеймін бірақ бұл шынымен де жұмыс істейді,сіз не ақы ет білме бірақ бұл шын де жұмыс істе,bullying

2,сенде ақымақ сұрақ қою бойынша қара белбеу ал ақымақтық бойынша алтын медаль бар шығар,сенде ақы сұрақ қою бойынша қара белбеу ал ақымақтық бойынша алтын медаль бар шығар,bullying

2,сен ақымақсың мен сенің үйіңді өртеп жібергенде сен өз үйіңде боласың деп үміттенемін неге менімен бітіру кешіне бармадың,сен ақы мен сенің үй өрте жібер сен өз үй бол де үміттен неге менімен бітіру кеш барма,bullying

2,иттер туралы фильмде пит үшін жылағаның үшін маған күлуге рұқсат беріңіз ботаник,ит туралы фильм пит үшін жыла үшін маған күл рұқсат бер ботаник,bullying

2,менің ойымша бұл сіздің гей екеніңізді білдіреді немесе жыртқыш нәресте,менің ойымша бұл сіздің ге екен білдір немесе жыртқыш нәресте,bullying

2,сен шошқасың қатты айқайлайтын құйрығың бар сенің маған күлуге батылың бар ма,сен шошқа қатты айқайла құйры бар сенің маған күл батыл бар ма,bullying

2,неге сонша көп тамақ жейсіз семіз нақұрыс,неге сонша көп тамақ же се нақұрыс,bullying

2,досым ол ұсқынсыз менің ойымша сен азғынсың,дос ол ұсқын менің ойымша сен азғын,bullying

2,сіз жолы болмайтын бейбақсыз мен бұны сізге бұрын да айта алатын едім,сіз жол болма бей мен бұны сізге бұрын да ай ал ед,bullying

2,ақымақ ауылдық адам фукккк сен және сенің достарың да ақымақ сендердің ешқайсыларыңның қолынан келмейді сендерге әрдайым достарың керек оған қол жеткізу үшін,ақы ауылдық адам фукккк сен және сенің дос да ақы сен ешқайсы қол келме сен әрдайым дос керек оған қол жеткізу үшін,bullying

2,сен сондай ақымақсың менің заттарымды ұрламас бұрын екі рет ойланғаныңыз жөн,сен сондай ақы менің зат ұрламас бұрын екі рет ойлан жөн,bullying

2,сәлем сасық шіріген тезектер сен шошқаның құсығындай сасықсың привет вонючие гнилые какашки ты пахнешь свиной блевотиной,сәлем сасық шірі тезек сен

шошқа құсығында сасық привет вонючи гнилы какашки ты пахнешь свино блевотино,bullying

2,семіз бала мен сізді бұғаттаймын себебі сіздің еш құндылығыңыз жоқ,се бала мен сіз бұғатта себебі сіздің еш құндылығ жоқ,bullying

2,өзіңнің жиркенішті тұлға екендігіңе ұялуың керек,өз жиркеніш тұлға екендіг ұял керек,bullying

1,қара адамдарға мән бермеңіздер негрлерді жек көремін,қара адам мән берме негр жек көр,racism

2,сасық ақымақтар менің мектепке неге бармайтындығыма таң қалады сонысымен қоса,сасық ақымақ менің мектеп неге бармайтындығ таң қал соны қоса,bullying

4,командаммен бірге өлтіруге дайындалудамын,команда бірге өлтір дайындалу,violent

2,саған тозақтық құрысу тілеймін,саған тозақ құрыс тіле,bullying

3,өкінішке орай ресей террордың көзі болып табылады шығыс украинадағы бұл шешендердің барлығы ол жақта гуманитарлық себептермен жүрген жоқ,өкініш орай ресей террор көз бол таб шығыс украина бұл шешен барлығ ол жақта гуманитарлық себеп жүр жоқ,nazism

1,геттодан шыққан ұсқынсыз ақымақ негрлер,гетто шық ұсқын ақы негр,racism

4,әрбір тентекті депортациялау немесе өлтіру керек,әрбір тентек депортацияла немесе өлтір керек,violent

4,әрбір полиция тыңшысы өлтірілуі керек бұл шошқалар өз жұмыстарын тек өш алу үшін алды бірақ адамдар оларға ешқашан ештеңе жасаған емес,әрбір полиция тыңшы өлтіріл керек бұл шошқа өз жұмыс тек өш алу үшін алды бірақ адам оларға ешқашан ештеңе жаса емес,violent

2,оңбаған боқтың кесегі жоғалшы ары,оңба боқ кесег жоғалш ары,bullying

3,қазақтар қалаларыңды орыстар салғанын бірте бірте бұл қалаларға даладан келгендеріңді ұмытпаңдар сілекей шашар алдында тарихты оқыңыз,қазақ қала орыс сал бірте бірте бұл қала дала келген ұмытпа сілекей шаш алд тарих оқ,nazism

3,біз ресейге наразылық нотасын жіберуіміз керек саясаткерлердің сөзіне ресей жауапты,біз ресей наразылық нота жібер керек саясаткер сөз ресей жауапты,nazism

2,сіз барлық мүмкін мағынада нақұрыссыз,сіз барлық мүмкін мағына нақұрыс,bullying

2,сенің қыли көзді мұғалімің нақұрыс,сенің қыли көз мұғалім нақұрыс,bullying

2,сен жай ғана бұзылған нақұрыссың,сен жай ғана бұзыл нақұрыс,bullying

2,ол нақұрыс жол бойындағы ойық жермен тең,ол нақұрыс жол бой ойық жер тең,bullying

4,көзіңді ойып аламын сенің нақұрыс,көз ой ал сенің нақұрыс,violent

2,ол жай ғана жауыз әрі іші тар нақұрыс,ол жай ғана жауыз әрі іш тар нақұрыс,bullying

2,мен сені жек көремін сен семіз сұр алмұрт сияқтысың,мен сен жек көр сен се сұр алмұрт сияқты,bullying

2,үлкен семіз шошқа жегіш егеуқұйрық,үлкен се шошқа жегіш егеуқұйрық,bullying

2,сенің анаңның семіздігі сонша оның желкесі хот дог салынған дорбаға ұқсайды,сенің ана семіздіг сонша оның желке хот дог салын дорба ұқса,bullying

2,неліктен осындай ақымақ екеніңді анықтасаңшы,неліктен осындай ақы екен анықтасаңш,bullying

2,сен дөрекі және өрескелсің мен сені жек көремін,сен дөрекі және өрескел мен сен жек көр,bullying

2,сен жиркеніштісің мен сенің еркектік сасықтығыңды жек көремін,сен жиркеніштмен сенің еркек сасықтығ жек көр,bullying

2,сен алдамшы әрі өтірікшісің,сен алдамш әрі өтірікш,bullying

2,ол өтірікші болмаған заттарды ойлап тапты,ол өтірікш болма зат ойла тап,bullying

2,сен өзің ұятсыз өтірікшісің,сен өзің ұят өтірікш,bullying

2,мен сені қандай болсаң сондай атаумен ғана атадым мақтаншақ өтірікші және алдамшы,мен сен қандай бол сондай ата ғана ата мақтаншақ өтірікш және алдамш,bullying

2,сен әлемдегі ең жаман өтірікшісің,сен әлем ең жаман өтірікш,bullying

2,сіз мен кездестірген ең ақылсыз өтірікшісің,сіз мен кездестір ең ақыл өтірікш,bullying

2,сен сұмдық арзан әрі нағыз өтірікшісің,сен сұм арзан әрі нағыз өтірікш,bullying

2,сен психиатриялық аурухананың семіз науқасына ұқсайсың,сен психиатриялық аурухана се науқас ұқса,bullying

2,қоқыс егеуқұйрығы шыңғырғанды доғар,қоқыс егеуқұйрығ шыңғыр доғар,bullying

2,демек ол кәріз егеуқұйрығы бізге тек жем ғана керек,демек ол кәріз егеуқұйрығ бізге тек жем ғана керек,bullying

2,қарапайымдылықтың қажеті жоқ сен егеуқұйрықсың,қарапайымдылық қажет жоқ сен егеуқұйрық,bullying

3,лас егеуқұйрық өзіңнің шығысыңа тайып тұр,лас егеуқұйрық өз шығыс тай тұр,nazism

2,ал сен сен және сен бай байғұс және бейшара ұрысың,ал сен сен және сен бай байғұс және бейшар ұр,bullying

3,қазақстан мен өзбекстан бүкіл орта азия мен украинаны жаулап алады,қазақстан мен өзбекстан бүкіл орта азия мен украина жаула ал,nazism

4,тмд елдерінің режимін құлату үшін талибандардың тілеуін тілеймін,тмд ел режим құлат үшін талибан тіле тіле,violent

4,соғыссыз өз жерлеріңді алыңдар жарайсыңдар талибтар жарайсыңдар,соғыс өз жер ал жара талиб жара,violent

3,қазақ әскері солтүстік қазақстандағы қорғанысты күшейтуі керек себебі бар әлемдік жамандық сол жақтан келуде ол жақта еділ мен ока батпақтарында қазақтардың жаулары андрофагтар отыр қазақтың түп жауы,қазақ әскер солтүстік қазақстандағы қорған күшейт керек себебі бар әлемдік жаман сол жақ кел ол жақта еділ мен ок батпақ қазақ жау андрофаг отыр қазақ түп жау,nazism

4,жеке өз басым талибтарды қолдаймын,жеке өз басым талиб қолда,violent

4,толибтер жарайсыңдар,толиб жара,violent

4,назарбаевтарға қарағанда қазақстандағы талибан үшін,назарбаев қара қазақстандағы талибан үшін,violent

4,талибандар жарайсыңдар қаруы бар оппозиция,талибан жара қар бар оппозиция,violent

4,ауғандар бәрін дұрыс жасайды ол жерде басқыншыларға ештеңе жоқ бұл дүниеде ауған халқы сияқты ешкім соғысуды білмейді ішектері жұқа,ау бәрін дұрыс жаса ол же басқыншы ештеңе жоқ бұл дүние ауған халқ сияқты ешкім соғысу білме ішек жұқа,violent

4,талибандарды желтоқсанда ақтөбе облысына шақырып наурызға дейін сонда қалдыру,талибан желтоқсан ақтөбе облыс шақыр наурыз дейін сонда қалдыр,violent

4,қазақстан талибанмен одақ құруы керек,қазақстан талибан одақ құр керек,violent

4,талибандар соғыста барлығын қиратады мен жеке өз басым талибандармен соғыспаймын менің қорғайтын ештеңем жоқ,талибан соғыс барлығ қират мен жеке өз басым талибан соғыспа менің қорға ештеңе жоқ,violent

4,талибан келіп тәртіп орнатады,талибан кел тәртіп орнат,violent

4,біз соғысуға дайынбыз және мықтымыз,біз соғыс дайын және мықты,violent

3,қазақстанға кіре берсін орыстар кетіп жатыр ғой,қазақстан кір бер орыс кет жатыр ғой,nazism

4,мен талибандарды қолдаймын қылмыскерлер тек осындай көзқарасқа лайық талибандар жарайсыңдар,мен талибан қолда қылмыскер тек осындай көзқарас лайық талибан жара,violent

4,талибандар барлық посткеңестік елдерге қажет олар жемқорлық мәселесін қалай шешуді біледі,талибан барлық посткеңестік ел қажет олар жемқорлық мәселе қалай шешу біл,violent

4,жарайсыңдар талибандар олар ең әділ сот жүйесін құратын болады қазіргі әлемде әділдік жоқ билікті басып алғандардың мүддесіне қызмет ететін және негізінен белгілі бір рудан өз қожайынының бәсекелестерін жою үшін жұмыс істейтін заң орындары бар,жара талибан олар ең әділ сот жүйе құр бол қазіргі әлем әділдік жоқ билік бас ал мүдде қызмет ет және негіз белгілі бір ру өз қожайын бәсекелес жою үшін жұмыс істе заң орын бар,violent

4,талибандар алға бауырлас елдер сендермен бірге,талибан алға бауырлас ел сен бірге,violent

4,талибандар жарайсыңдар,талибан жара,violent

4,талибандар жарайсыңдар,талибан жара,violent

4,талибандар жарайсыңдар бұл есірткі барондары мен жемқорларды қуу керек,талибан жара бұл есірткі барон мен жемқор қу керек,violent

4,бір екі күнге талибандарды қазақстанға шақыруға бола ма біздің үкімет бұл жерден кетсін,бір екі күн талибан қазақстан шақыр бола ма біздің үкімет бұл же кет,violent

3,ресей қазақстанның жерін алғысы келеді бірінші жау ресей,ресей қазақстан жер ал кел бірінші жау ресей,nazism

3,талибаннан емес путиннің жасыл қаңылтыр сарбаздарынан қорқу керек,талибан емес путин жасыл қаңылты сарбаз қорқ керек,nazism

4,талибандар қазақ үкіметін елден қуып жіберсін,талибан қазақ үкім ел қу жібер,violent

4,бүкіл талибан қазақстанға келсін,бүкіл талибан қазақстан кел,violent

4,талибтер дұрыс жасайды жарайсыңдар,талиб дұрыс жаса жара,violent

4,талибандар мен ұйғыралр әлемді тәртіпке келтіреді,талибан мен ұйғырал әлем тәртіп келтір,violent

4,соғыс жариялап біздің бауырларымызға көмектесу керек,соғыс жарияла біздің бауыр көмектес керек,violent

4,егер орталық азия бірігетін болса біз талибандарды жеңеміз,егер орталық азия біріг бол біз талибан же,violent

4,талипстан күш,талипс күш,violent

4,талибан күш,талибан күш,violent

4,қазақстанның барлық ер азаматтарына қару беріп кез келген жаумен соғысуға дайындалыңдар біздің ата бабаларымыз бәрі соғысқан және біз дайын болуымыз керек,қазақстан барлық ер азамат қару бер кез кел жау соғыс дайындал біздің ата баба бәрі соғыс және біз дайын бол керек,violent

4,бауырлар кремль мамандарын тыңдамаңдар олар әдейі солай асыра сілтеп отыр талибан жағдайды ретке келтіріп өз елін баә ден де жақсырақ ету үшін бүкіл әлемге үлгі болатын бірегей ел құрғысы келеді оны ауғандардың өздері айтып отыр ал олардың сөздерін басқаша етіп аударып жеткізіп жатыр,бауыр кремль маман тыңдама олар әдей солай асыра сілте отыр талибан жағдай рет келтір өз ел баә ден де жақсырақ ету үшін бүкіл әлем үлгі бол бірегей ел құр кел оны ау өз айт отыр ал олардың сөз басқаша ет аудар жеткіз жатыр,violent

4,жемқорлардан талиюандар жақсы,жемқор талиюан жақсы,violent

3,қазақстанда орын алған соңғы жағдайларға байланысты орыстардың қазақстаннан кетуі басталып жатқанда талибандар да артық болды қазақтарға жақсылық әкелмейді,қазақстан орын ал соңғы жағдай байланысты орыс қазақстан кет бастал жатқанда талибан да артық бол қазақ жақсылық әкелме,nazism

4,мен талибандарды қолдаймын,мен талибан қолда,violent

3,сендер бейшара көшпенділер бір кездері өздерің де көшіп жүрдіңдер бірақ қазір басқаларға көмектескілерің келмейді,сен бейшар көшпенді бір кез өз де көш жүр бірақ қазір басқа көмектес келме,nazism

4,нарарбайды талибтерге тапсыру керек,нарарба талиб тапсыру керек,violent

4,болашақ талибандікі ал сендердің тұран ол жай ғана ақымақтық рашка бұрын қандай мықты болса солай қала береді іштерің күйсін,болашақ талибандік ал сен тұран ол жай ғана ақымақтық рашк бұрын қандай мықты бол солай қала бер іш күй,violent

3,егер талибандар қытайға келсе мен тек қуанамын,егер талибан қытай кел мен тек қуан,nazism

4,біз талибандарды қуана қарсы аламыз,біз талибан қуан қарсы ал,violent

4,талибтер мықты жігіттер олардан қорқудың қажеті жоқ,талиб мықты жігіт олардан қорқ қажет жоқ,violent

4,талибтер қазақстанды жаулап алсын маған бәрібір,талиб қазақстан жаула ал маған бәрібір,violent

4,о мен тек қуанамын нурботтар жаулағанша талибтер жаулап алсын,о мен тек қуан нурбот жаулағанш талиб жаула ал,violent

4,талиб ағайындар бізге келіңдер,талиб ағайын бізге кел,violent

4,талибандарды қолдаймын,талибан қолда,violent

4,талибы молодцы рад за них буду следить за их успехами,талиб молодц рад за них буд следить за их успехами,violent

4,талибандарға құрмет,талибан құрмет,violent

4,талибандар әлем бойынша күш,талибан әлем бойынша күш,violent

4,талибандар мықты олардың заңдары да күшті,талибан мықты олардың заң да күшті,violent

4,көп ұзамай бүкіл орталық азия талибан туының астында болады,көп ұзама бүкіл орталық азия талибан ту аст бол,violent

4,талибан кремльге ту тікпек,талибан кремль ту тік,violent

4,талибан жарайсыңдар және ауғанстан патриоттары,талибан жара және ауғанстан патриот,violent

4,талибан бауырлар мен сіздерді қолдаймын,талибан бауыр мен сіз қолда,violent

4,мен талибан үшін қуаныштымын,мен талибан үшін қуанышты,violent

3,қытай мемлекеттері жойылсын,қытай мемлекет жойыл,nazism

4,ауғанстан шариғатқа қосылып тұрғанда шариғат орнату үшін талибанға қарсы күресудің не қажеті бар,ауғанстан шариғат қосыл тұр шариғат орнату үшін талибан қарсы күресу не қажет бар,violent

4,талибан үшін,талибан үшін,violent

3,енді біз ауғанстаннан әл қаиданы алып және оны шығарып тастауымыз керек террорист фанаттармен ынтымақтастық жасауға болмайды,енді біз ауғанстан әл қаида алып және оны шығар таста керек террорист фанат ынтымақтастық жаса болма,nazism

4,талибан алға,талибан алға,violent

4,талибан жарайсыңдар,талибан жара,violent

3,мәскеуліктерді тыңдамай қала ауыл көше атауларын өзгерту керек бірде бізден сұрамай еділді волгаға жайықты уралға айналдырып жіберді неге қорқамыз оларға қарауға кезінде бес миллион қазаққа аштық қырғынын жасады ол жасай берсін біздің мемлекет ресми түрде жіберуі керек материалдық ақша қазақстанға төленуі керек,мәскеулік тыңдама қала ауыл көше атау өзгерту керек бірде бізден сұрама ед волга жайық урал айналдыр жіб неге қор оларға қара кез бес миллион қазақ аштық қырғын жас ол жаса бер біздің мемлекет ресми түр жібер керек материалдық ақша қазақстан төлен керек,nazism

3,кырткан екенсндер орыстар аирамыз кетндер шекара жаблсын рассия нын карап отрмаспыз шошкалар,кырткан екенсн орыс аир кетн шекара жабл рассия нын кара отр шошка,nazism

3,алтын орда тұсында орыс шошқалары орыстар қыпшақтарға салық төлеп қыздарын бергенін ұмытпайық,алтын орда тұс орыс шошқа орыс қыпшақ салық төле қыз бер ұмытп,nazism

3,бауырым олардың бәрі бұрынғы ксро ның кеңес заманындағы орыстары ғой орыстар қаһарланып елдің байлығын иемденіп басқа халықтарды менсінбей өз тілінде сөйлеп осы орыс заманында ата бабамыздың қанын төкті елде әлі де орыс кадрлары дайындалуда олардың көпшілігі орыс мектептерінде оқиды ей мәңгүрт қазақтар арам пиғылдар қазақша сөйлеп жатырсыңдар халқымыздың намысын аяққа таптадыңдар иттер балаларыңды үйдегі қазақ мектебіне беріп қазақ тілін оқытамыз орыстардың не дейтінін түсінесің бе,бауыр олардың бәрі бұрынғы ксро ны кеңес заман орыс ғой орыс қаһарлан ел байлығ иемден басқа халық менсінбе өз тіл сөйле осы орыс заман ата баба қан төк ел әлі де орыс кадр дайындал олардың көпшіліг орыс мектеп оқи ей мәңгүрт қазақ ара пиғыл қазақша сөйле жатыр халқ намыс аяқ тапта ит бала үйдегі қазақ мектеб бер қазақ тіл оқы орыс не де түс бе,nazism

3,қазақ билігі тұрғанда орысша сөйлейтін иттер сатқындар елді орысқа айналдырдыңдар,қазақ билiг тұр орысш сөйле ит сатқын ел орыс айналдыр,nazism

3,орыс тілі жойылуы керек біріншіден қазақстанда тек қазақ тілі болуы керек,орыс тіл жойыл керек бірінші қазақстан тек қазақ тіл бол керек,nazism

3,ақымақ ресейліктер жер бетінен жоқ болып кетсін,ақы ресейлік жер бет жоқ бол кет,nazism

3,егер қажет болса ресеймен соғысамыз,егер қажет бол ресей соғыс,nazism

3,сасық шаяндардан құтыла алмай жатсақ анау шағып жатқан сасықтар шошқа ма,сасық шаян құтыл алма жат анау шағ жат сасық шошқа ма,nazism

3,орыс мектептерін жабу керек орыстарды қуып жіберу керек,орыс мектеп жаб керек орыс қу жіберу керек,nazism

4,террористтер жағында соғысқаныңыз үшін доллар аласыз егер тірі қалып жатсаңыз,террорист жағ соғыс үшін доллар ал егер тірі қалып жат,violent

4,талибан содырлар емес патриоттар өз елін қорғайтын шынайы еліне жанашыр,талибан содыр емес патриот өз ел қорға шынайы ел жанашыр,violent

4,әрбір ұрпақ соғысты көруі керек,әрбір ұрпақ соғ көр керек,violent

3,еврейлерге қандай да бір жердің тиесілі болуы мүмкін емес оларда ешқандай жер жоқ және болған да емес,еврей қандай да бір же тиесілі бол мүмкін емес ол ешқандай жер жоқ және бол да емес,nazism

4,талибан жалғыз адекватты оппозиция,талибан жалғыз адеква оппозиция,violent

4,жиһад жалғасуда,жиһад жалғас,violent

4,мен арқылы жіберген дін үшін оны жеңіске жеткізгенше немесе мен сонда өлгенше жиһад жасау,мен арқылы жібер дін үшін оны жеңіс жеткізгенш немесе мен сонда өлгенш жиһад жасау,violent

4,соғысқа бару керек,соғыс бару керек,violent

4,талибан билеуші елдің билеушісі болуы керек,талибан билеуш ел билеуші бол керек,violent

4,жарайсың талибан елде тыныштық басталады,жара талибан ел тыныштық бастал,violent

3,кавказдықтарды жақсы көру мүмкін емес бірақ мен өз арманымды бағалаймын оларды еврейлер сияқты пешке жағу арқылы мәселені шешті,кавказдық жақсы көру мүмкін емес бірақ мен өз арман бағала ол еврей сияқты пеш жағ арқылы мәселе шеш,nazism

4,талибан қазақстанға келсін назарбаев бастаған нұр отанды жеңу үшін талибан жағында соғысамыз,талибан қазақстан кел назарбаев баста нұр ота жеңу үшін талибан жағ соғыс,violent

4,бұл террористердің барлығын бомбалау керек еді,бұл террорис барлығ бомбала керек ед,violent

4,жарайсыңдар сөз жоқ талибандар алға,жара сөз жоқ талибан алға,violent

4,жарайсыңдар талибандар бәрін бостандыққа шығарды,жара талибан бәрін бостандық шығ,violent

4,талибан жемқор саяси жүйені жойып жіберді енді қайталап сайлау тағы да заң қабылдайды біздің елде жүйе орнатылған бәрі бұл жүйе жоғалды деп анық айтуға қорқады,талибан жемқор саяси жүйе жой жіб енді қайтала сайлау тағы да заң қабылда біздің ел жүйе орнатыл бәрі бұл жүйе жоғал де анық айт қорқ,violent

4,жарайсың талибан,жара талибан,violent

4,шынымды айтсам мен бұұ мен батыс гуманитарлық көмектеріне қарағанда талибанға көбірек сенемін,шын айт мен бұұ мен батыс гуманитарлық көмек қара талибан көбірек сен,violent

4,талибанның дана әрекеті олар өздерін қорғайтын ауғандарын қудаламайды,талибан дана әрекет олар өз қорға ау қудалама,violent

4,талибан бұл өз заңын өз құқығын өзі жасайтын ер адамдар жарайсыңдар жігіттер қалай жасағылары келсе солай жасайды,талибан бұл өз заң өз құқығ өзі жаса ер адам жара жігіт қалай жаса кел солай жаса,violent

3,бұл американдықтар сақалы бар нағыз шайтандар оларға сенуге болмайды,бұл американдық сақал бар нағыз шайтан оларға сен болма,nazism

4,жиһад қасиетті,жиһад қасиетті,violent

4,мен құрбандықты қалаймын,мен құрбандық қала,violent

4,мен жиһадшыларды құрметтеймін,мен жиһадшы құрметте,violent

4,шошқа мен сені жарып жіберемін,шошқа мен сен жар жібер,violent

4,мен сені ахмадтың калькуляторымен жарып жіберемін,мен сен ахмад калькулятор жар жібер,violent

4,мен кафирияны жарып жіберемін,мен кафирия жар жібер,violent

2,сен кәпірсің есек,сен кәпір есек,bullying

2,сен ақымақ кәпірсің,сен ақы кәпір,bullying

4,мен сенің шешеңді өлтіргім келеді,мен сенің шеше өлтір кел,violent

2,сен жай ғана ақылсыз террористсің,сен жай ғана ақыл террорист,bullying

4,мен террорист емеспін тек сен сияқты кәпірлерді өлтіргім келеді,мен террорист емес тек сен сияқты кәпір өлтір кел,violent

2,сіз жай ғана ақымақ террорсыз,сіз жай ғана ақы террор,bullying

4,жезөкшенің баласы мен сені өлтіремін,жезөкше бала мен сен өлтір,violent

2,мен сені жарып жіберемін қаншық,мен сен жар жібер қаншық,bullying

2,кәпір тезектің кесегі,кәпір тезек кесег,bullying

3,ресейіңдегі кәпірлердің бәрі өлсін,ресейің кәпір бәрі өл,nazism

4,барлық кәпірлерді өлтірейік ағайындар,барлық кәпір өлтір ағайын,violent

4,ия барлығын жарып жіберейік,ия барлығ жар жібер,violent

2,ақымақ шошқа кәпір,ақы шошқа кәпір,bullying

4,шошқа мен сені жарамын,шошқа мен сен жар,violent

2,артымды сүртемін шошқа кәпір,арт сүр шошқа кәпір,bullying

2,шошқа мен сені жарып жіберемін,шошқа мен сен жар жібер,bullying

2,мен сенімен сөйлескен жоқпын ақымақ сен сондай ақымақсың тіпті сенімен біреу сөйлесіп тұрғанын да түсінбейсің трейлердегі жігітіңе қайтып оралма,мен сенімен сөйлес жоқ ақы сен сондай ақы тіпті сенімен біреу сөйлес тұр да түсінбе трей жігіт қайт оралма,bullying

2,менің ойымша сэр не істеу керектігі анық саған шынымен бұны артыңа қыстыруды қою керек сен педофилсің,менің ойымша сэ не істеу керектіг анық саған шын бұны арт қыстыру қою керек сен педофил,bullying

2,қарғыс атқыр сен ұсқынсыз фукка,қарғыс атқы сен ұсқын фукк,bullying

1,крэкке сараң негр аузыңды жап,крэк сара нег ауз жап,racism

2,сен жай ғана ақымақ ауылдық адамсың және сен біз үшін түкке тұрғысызсың,сен жай ғана ақы ауылдық адам және сен біз үшін түк тұрғысыз,bullying

2,сен семіз ақымақ менің қолданушылық кеңістігімде спам таратуды доғар,сен се ақы менің қолданушылық кеңістіг спам тарату доғар,bullying

3,жақсы қарсы алғаныңа рахмет ақымақ араб өзіңнің арабияңа қайт,жақсы қарсы ал рахмет ақы араб өз арабия қайт,nazism

3,сен ақыл есі кем дүниеқоңыз өзің білмейтін дүниені тұжырымдама лайықты жерлерге беріңдер хабарың жоқ дүниені тұжырымдама,сен ақыл ес кем дүниеқо өзің білме дүние тұжырымдама лайықты жер бер хаб жоқ дүние тұжырымдама,nazism

3,вики сараңдарды тыңдаңдар бұл ір өртеп жіберіңдер ол ештеңе бермейді менде тағы да данасы бар сіз ештеңе білмейтін және npov емес мақалаларды бұзып қайта жазатындарды қолдайсыз ол үшін азап шегесің,вики сараң тыңда бұл ір өрте жібер ол ештеңе берме менде тағы да дана бар сіз ештеңе білме және npov емес мақала бұз қайта жаз қолда ол үшін азап шег,nazism

3,жапондықтар оларды бомбалағанымыз жақсы болды олар өте нашар адамдар болды,жапондық ол бомбала жақсы бол олар өте нашар адам бол,nazism

2,сөйлеуді доғар сасықсың,сөйлеу доғар сасық,bullying

2,осымен болды сен шошқасың сіз үгіт насихатыңызды тым көп таратып жатырсыз сіз бұл мақаланы өз қиялыңызда ғана жасадыңыз,осы бол сен шошқа сіз үгіт насихат тым көп тарат жатыр сіз бұл мақала өз қиял ғана жаса,bullying

2,сен ақымақсың және солай қала бересің әлем сендер сияқты жексұрындарың кесірінен азап шегуде,сен ақы және солай қала бер әлем сен сияқты жексұрын кесір азап шег,bullying

2,мен сені жек көремін және сен отта өлесің деп үміттенемін,мен сен жек көр және сен от өл де үміттен,bullying

2,нақұрыс сен біткен нақұрыссың менің талқылау парақшамда болуға құқығың жоқ күнің сәтті өтсін бишара,нақұрыс сен біт нақұрыс менің талқылау парақша бол құқы жоқ күн сәтті өт бишар,bullying

4,жарамыз бәрін,жар бәрін,violent

4,террористтер болады,террорист бол,violent

4,сирия болады жақында,сирия бол жақында,violent

4,халық қанды қалайды,халық қан қала,violent

4,бұл мұсылмандар геноциді сирия ұтылды эрдоганды қуып жіберді қазақ ағайындар ендігі кезек сендердікі,бұл мұсылман геноцид сирия ұт эрдоган қу жіб қазақ ағайын ендігі кезек сендердік,violent

3,кремль бар жерде тыныштық болмайды мысалдар қажет болса тарихты оқыңыз ресейді жару керек,кремль бар же тыныштық болма мысал қажет бол тарих оқ ресей жар керек,nazism

4,келесі мекен өзбекстан,келесі мекен өзбекстан,violent

4,қоқысты өлтіру қателік деп айтпас едім,қоқыс өлтір қателік де айтпас ед,violent

4,метинг қарулы көтеріліс болса мені де шақыруды ұмытпаңдар,метинг қарулы көтеріліс бол мен де шақыру ұмытпа,violent

4,е сәбіздер осындай қақтығыс болса қатысқыларың келеді ме мен шығар едім атойлап алға деп,е сәбіз осындай қақтығыс бол қатыс кел ме мен шығар ед атойла алға де,violent

4,оларды пәкістан мен ауғанстанға жіберу керек сола жақтағылар түсініп қабылдасын үңгірде тұрып қара нан ғана жеп естерін жисын,ол пәкістан мен ауғанстан жіберу керек сол жақтағы түсін қабылда үңгі тұр қара нан ғана же ес жи,violent

3,барлық орыстарды ресейге депортациялауды талап етемін,барлық орыс ресей депортациялау талап ет,nazism

3,қазақстанда тұратын орыстардың барлығының ресейге кетулерін талап етемін,қазақстан тұр орыс барлығ ресей кету талап ет,nazism

3,орыстар зомби шовинизм ол жақта ұлттық идея болып есептеледі,орыс зомби шовиниз ол жақта ұлттық идея бол есептел,nazism

3,қазақстандағы барлық орыстар украинадағы соғысты қолдайды шовинистер,қазақстандағы барлық орыс украина соғ қолда шовинис,nazism

3,рф жоспары бойынша алдымен украина сосын молдова келесісі қазақстан болды дайындалу керек олар тегіннен тегін американдық зертханалар туралы сөз қозғаған жоқ,рф жоспар бойынша алд украина сосын молдова келесі қазақстан бол дайындалу керек олар тегін тегін американдық зертхана туралы сөз қозға жоқ,nazism

3,орыстар қазақстанда не істеп жүр ресейде орыс еместерді жек көреді байырғы қазақтарды да кемсітеді,орыс қазақстан не істе жүр ресей орыс емес жек көр байырғы қазақ да кемсіт,nazism

3,шовинистер ресейге кетсін,шовинис ресей кет,nazism

3,ресейліктер оттамасын олар итше үрмесін фашисттер,ресейлік оттама олар итш үрме фашист,nazism

3,қаңтарда қайда болды осы аққұлақтар сенбеңдер оларға ешқашан орыстар бізге дос болмайды,қаңтар қайда бол осы аққұлақ сенбе оларға ешқашан орыс бізге дос болма,nazism

3,орыс жәлебі орыс болып қалады,орыс жәлеб орыс бол қал,nazism

3,неге олар орыс саясаткерлерінің сөзінен кейін мысалы келесі күні оларды орыстарды құтқаруға болмайды деп флешмобпен шықпады және айтпады,неге олар орыс саясаткер сөз кейін мысал келесі күн ол орыс құтқар болма де флешмоб шықпа және айтпа,nazism

1,қара нәсілді әйел қылмыспен күресуге қалай көмектесе алады аборт жасату арқылы,қара нәсіл әйел қылмыс күрес қалай көмекте ал аборт жасат арқылы,racism

1,негрлер шектен шықты африкаға қайтару керек оларды,негр шек шық африка қайтару керек ол,racism

1,ақтар өздері кінәлі жақында ресей федерациясында шешендер славяндармен осындай ойын ойнайтын болады,ақ өз кінәлі жақында ресей федерация шешен славян осындай ойын ойна бол,racism

1,қаралар қысымға ұшырайды қорланады және қор болады мен оларды қолдамаймын бірақ олардың мұндай реакциясы қабылданған заңдарға байланысты,қара қысым ұшыра қорлан және қор бол мен ол қолдама бірақ олардың мұндай реакция қабылдан заң байланысты,racism

1,негрлер шектен шықты,негр шек шық,racism

1,африкаға қайта кетсін бұлар,африка қайта кет бұлар,racism

4,бір нокаутқа кеткен адам үшін басынан ату бұл әділетті,бір нокаут кет адам үшін бас ату бұл әділет,violent

4,иә оларды мақта алқабына жіберу керек кім кімді қағып азаптап өлтіретінін көрейік,иә ол мақта алқаб жіберу керек кім кім қағ азапта өлтір көр,violent

1,барлығын африкаға қайтару қажет,барлығ африка қайтару қажет,racism

1,меніңше ақ гурмандардың қараларды жеп тастау қаупі бар,меніңше ақ гурман қара же тастау қауп бар,racism

1,осы ойынды тмд елдерінде ойнай бастаса қара нәсілділер не болар еді деп ойлаймын,осы ой тмд ел ойна баста қара нәсілді не бол ед де ойла,racism

1,сосын ақ адамдар атқа қонып бастарына ақ қалпақ киеді содан кейін қара достарын жер бетімен сүйрейтін болады,сосын ақ адам ат қон бас ақ қал ки содан кейін қара дос жер бет сүйре бол,racism

1,ал олар полицейлердің оларды неге өлтіріп жатқанына таңғалады қандай жабайы болса сондай қалпында қалған африкаға кету үшін бір тәулік беріледі кім қалып қойса өзі кінәлі,ал олар полицей ол неге өлтір жат таңғал қандай жабай бол сондай қалп қалған африка кету үшін бір тәулік бер кім қалып қой өзі кінәлі,racism

1,ей ақ нәсілділер қайдасыңдар қашан қарсы жауап бересіңдер,ей ақ нәсілді қай қашан қарсы жауап бер,racism

1,қараны тұншықтыру да ойынның бір түрі мақтанған куклуксклан қайда олар ойнасын ал біз күлеміз,қара тұншықтыр да ойын бір түр мақтан куклуксклан қайда олар ойна ал біз күл,racism

1,қорқақтар біздің шы жылдарға түскенде көрер едім қандай компьютерлік ойын екенін,қорқақта біздің шы жыл түс көрер ед қандай компьютерлік ойын екен,racism

4,азаматтық соғысқа аз қалды,азаматтық соғыс аз қал,violent

1,ақтар қартайып көбеймей бара жатырсыңдар осылай жалғасса мүлдем жоқ болып кетесіңдер өздеріңнің ақтарыңа үйленіңдер балалы болып көбейіңдер бұл әлемге сендер де керексіңдер қазақстаннан сәлем,ақ қартай көбейме бар жатыр осылай жалғас мүлдем жоқ бол кет өз ақ үйлен балалы бол көбей бұл әлем сен де керек қазақстан сәлем,racism

1,африкаға бәрін африкаға жіберу керек ол жақта жақсы,африка бәрін африка жіберу керек ол жақта жақсы,racism

1,олар қара нәсілділермен осындай қоқыс тірлік жасаса бірден жанжал басталады,олар қара нәсілді осындай қоқыс тірлік жаса бірден жанжал бастал,racism

1,елестер ойынын ойнайық ақ жамылғы жамылып кіреске от қойып қолымызға қару алып қақталғандарды өлтірейік,елесте ойын ойна ақ жамылғы жамыл кірес от қой қол қару алып қақтал өлтір,racism

3,бұл бейбақтарды неге әлі атпаған,бұл бейбақ неге әлі атпа,nazism

3,дұрыс ағылшын сақтарын ұрыңдар,дұрыс ағылшын сақ ұр,nazism

1,содан кейін жасөспірімдер ата әжесін шешесін сіңлілерін қағып түсіре бастайды бұл компьютер ойынындағыдай қызықты,содан кейін жасөспірім ата әже шеше сіңлі қағ түсір баста бұл компьютер ойынындағыда қызықты,racism

1,содан кейін ақтар атуға кіріседі,содан кейін ақ ат кіріс,racism

3,қызық нәсілшілдер мен ұлтшылдарды осылай құрту керек,қызық нәсілшіл мен ұлтшыл осылай құрт керек,nazism

1,тек мен ғана қара нәсілділерді жек көремін,тек мен ғана қара нәсілді жек көр,racism

3,орысты өлтір бұл сенің мәскеудегі шын өмірің,ор өлтір бұл сенің мәскеу шын өмір,nazism

3,бірінші орысқа дейін,бірінші орыс дейін,nazism

1,жаңа ойын қара нәсілдіні өртеп жібер,жаңа ойын қара нәсілді өрте жібер,racism

3,қаншықтар ресейге келсін мен оларды өзім ұрып жығамын,қаншық ресей кел мен ол өзім ұр жығ,nazism

3,мен оларды қуана өртейтін едім,мен ол қуан өрте ед,nazism

3,қандай да қарсылығың бар ма өмір жалғастырушы азиаттар жылдан кейін еуропалықтар өледі бала аз туады бедеулік көп азиаттар саны алты миллиардтан асады,қандай да қарсылы бар ма өмір жалғастыруш азиат жыл кейін еуропалық өл бала аз ту бедеулік көп азиат сан алты миллиард ас,nazism

1,ақтарға қатысты нәсілшілдік жоқ хахаха,ақ қатысты нәсілш жоқ хахах,racism

3,ойнап көр қара қаншама шеттен келушілер кавказдықтардың бірін ұрып жық ал қалғандары сенің мұны қалай жасайтыныңды көрсін әрине егер өз әйеліңнің не қызыңның не шешеңнің өкшесінің болмаса белдемшесінің астынан шыға алсаң,ойна көр қара қанша шет келуші кавказдық бір ұр жық ал қал сенің мұны қалай жаса көр әрине егер өз әйел не қыз не шеше өкше болма белдемше аст шығ ал,nazism

2,паша сен әңгімені түсінбедің немесе аралды мүлдем шатастырдың қоқыс үшін менің түбіме түстің ұйықтап қалшы сен тіпті жермен келіссөз жүргізетін шығарсың,паш сен әңгіме түсінбе немесе ара мүлдем шатас қоқыс үшін менің түб түс ұйықта қалш сен тіпті жер келіссөз жүргіз шығ,bullying

2,сен сақау шығарсың бәлкім паровоз тыр тыр тыр деп немесе бронетранспортер деп айтшы,сен сақа шығ бәлкім паровоз ты ты ты де немесе бронетранспортер де айтш,bullying

2,өз сарайыңда жылайсың және әміріңді өз үйіңде жүргіз әмірші табылыпты маған,өз сарай жыла және әмір өз үй жүргіз әмірш табыл маған,bullying

3,ақ адамдар болмайды егер ол сорғытылған немес кәріс болмаса,ақ адам болма егер ол сорғытыл немес кәріс болма,nazism

3,неліктен немістер өздерін ақ деп ойлайды,неліктен неміс өз ақ де ойла,nazism

1,мен сенен ақпын ақымақ жеңіле сал,мен сенен ақ ақы жеңіл сал,racism

1,негрсің сен,нег сен,racism

1,негр сияқты қалжыңдап тұрсың,нег сияқты қалжыңда тұр,racism

1,самирчик сен негрсің,самирчик сен нег,racism

1,сенімен бәрі түсінікті негр,сенімен бәрі түсінік нег,racism

1,сенің шешең негр,сенің шеше нег,racism

3,хананиттік еврейлер біздің ариялық ақ нәсілдің қас жауы екеніне сенімдіміз еврей арийлік мәдениет пен нәсіліміздің тазалығын жою үшін нәсілдік денемізге шабуыл жасайтын жойқын вирус сияқты,ханани еврей біздің ариялық ақ нәсіл қас жау екен сенімді еврей арийлік мәдениет пен нәсіл тазалығ жою үшін нәсіл дене шабуыл жаса жойқын вирус сияқты,nazism

1,бөгде ықпалдардан ада нәсілдің мәдениеті бұл нәсілдің мәнін айғақтайды,бөг ықпал ад нәсіл мәдениет бұл нәсіл мән айғақта,racism

1,басқаша айтқанда шаштары мұрнында сүйегі бар және сүйір тісті сиырдың тезегінен жасалған африкалық негр бізге көлік жүргізуге үйретілген іскерлік костюм киген жазу машинкасында жұмыс істей алатын ағылшынша еркін сөзлейтін американдық негрге қарағанда негрлердің мәні туралы дәлірек түсінік береді,басқаша айтқанда шаш мұрн сүйег бар және сүйі ті сиыр тезег жасал африкалық нег бізге көлік жүргіз үйрет іскерлік костюм ки жазу машинка жұмыс істе ал ағылшынша еркін сөзле американдық нег қара негр мән туралы дәлірек түсінік бер,racism

1,негр мәдениетінің төмендігі негр миының абстрактілі ұғымдармен айналысуға физикалық қабілетсіздігінің салдары,нег мәдени төмендіг нег ми абстрактіл ұғым айналыс физикалық қабілетсіздіг сал,racism

1,екінші жағынан негр тек есте сақтауды қажет ететін ақыл ой тапсырмаларында ақ адамдарға жақын қабілет көрсетеді,екінші жағ нег тек ес сақтау қажет ет ақыл ой тапсырма ақ адам жақын қабілет көрсет,racism

1,сондықтан негрлерді ақ нәсілділер мәдениетінің көптеген аспектілеріне бейімделу үшін салыстырмалы түрде оңай үйретуге болады,сондықтан негр ақ нәсілді мәдени көптеген аспекті бейімделу үшін салыстырмалы түр оңай үйрет бол,racism

1,бірінші дүниежүзілік соғыс кезінде американдық әскерге шақырылғандарды кең ауқымды барлау тестілеуінен кейін қара нәсілді адамның орташа iq деңгейі ақ нәсілдіге қарағанда шамамен төмен екендігі белгілі болды,бірінші дүниежүзілік соғыс кез американдық әскер шақырыл кең ауқымды барлау тестілеу кейін қара нәсіл адам орташа iq деңгей ақ нәсілді қара шамамен төмен екендіг белгілі бол,racism

1,қара нәсілді қорғаушылар бұрынғы сынақ нәтижелерін мектептегі сегрегация мен қара кедейліктің нәтижесі ретінде түсіндіруге тырысты яғни олар сынақтар мәдени тұрғыдан біржақты болды деп дәлелдеді,қара нәсіл қорғаушы бұрынғы сынақ нәтиже мектеп сегрегация мен қара кедейлік нәтиже рет түсіндір тырыс яғни олар сынақ мәдени тұрғы біржақты бол де дәлелде,racism

4,әрбір қыз жаппай өлтірушіні жақсы көреді,әрбір қыз жаппай өлтіруші жақсы көр,violent

1,ақтарды қайтадан жабайы ету,ақ қайта жабай ету,racism

4,атомваффен бөлімшесіне қосылыңыз,атомваффен бөлімше қосыл,violent

4,жергілікті синагоганы жарып жібер,жергілікті синагога жар жібер,violent

3,егер сізде қатер болса құшақтаңыз тұмауды әрбір еврейге таратыңыз,егер сіз қатер бол құшақта тұмау әрбір еврей тара,nazism

3,олардың барлығын депортациялаңыз,олардың барлығ депортацияла,nazism

4,бізге қосылыңыз немесе қалғандарымен бірге өліңіз,бізге қосыл немесе қалған бірге өл,violent

4,біз атомваффен бөлімшесіне деген адалдығымызға кепілдік береміз,біз атомваффен бөлімше де адалдығ кепілдік бер,violent

3,германиядағы мемлекеттік және қоғамдық тәртіпті шайқап ақырында оны құлату,германия мемлекеттік және қоғамдық тәртіп шайқа ақырында оны құлат,nazism

4,біз ол туралы айтып отырғанда зорлық зомбылық төңкеріс уақыты келді егер сіз шындықты түсінсеңіз және жағдайдың ауырлығын түсінсеңіз сізде екі нұсқаның бірі бар бірінші нұсқа апатқа дайындалыңыз екінші нұсқа коллапсқа жеткізу осымен болды,біз ол туралы айт отырғанда зорлық зомбылық төңкеріс уақыт кел егер сіз шындық түсін және жағдай ауырлығ түсін сіз екі нұсқа бірі бар бірінші нұсқа апат дайындал екінші нұсқа коллапс жеткізу осы бол,violent

4,егер сіз жақсы физикалық формада болмасаңыз қаруланбасаңыз оқ дәрілер алмасаңыз және жаттықпасаңыз онда қажетті нәрсені жасауға дайын болуыңыз керек,егер сіз жақсы физикалық форма болма қаруланба оқ дәрі алма және жаттықпа онда қажетті нәрсе жаса дайын бол керек,violent

4,кейбір қарғыс атқыр пойыздарды рельстен шығарып кейбір адамдарды өлтіріп су көздерін уландырыңыз сіз бұл нәрселерге дайын болғаныңыз жөн,кейбір қарғыс атқы пойыз рельс шығар кейбір адам өлтір су көз уланды сіз бұл нәрсе дайын бол жөн,violent

3,кангтардың еврейлерге қарсы әрекеті жүйеге зиян келтіруі мүмкін бірақ біз оларды ұзақ мерзімді табысымыз үшін қажет одақтастар деп ойламауымыз керек,канг еврей қарсы әрекет жүйе зиян келтір мүмкін бірақ біз ол ұзақ мерзімді табыс үшін қажет одақтас де ойлама керек,nazism

4,бізге жерге етік болуға дайын еркектер керек бізге қосылу тек қана атомваффен бөлімшесіне қатысты маңызды міндеттемені білдірмейді,бізге же етік бол дайын еркек керек бізге қосылу тек қана атомваффен бөлімше қатысты маңызды міндеттеме білдірме,violent

1,дегенмен әзірге аққа қарсы кең элементтерге назар аударыңыз негр көліктері еврей бизнесі және б,дегенмен әзірге ақ қарсы кең элемент назар ау нег көлік еврей бизнес және б,racism

1,қара нәсілділердің өмірі түкке тұрғысыз,қара нәсілді өмір түк тұрғы,racism

3,еврей жүйесінің күлінен жаңа әлемдік тәртіп пайда болады,еврей жүйе күл жаңа әлемдік тәртіп пайда бол,nazism

3,жергілікті нацистерге қосылыңыз,жергілікті нацис қосыл,nazism

1,емтихандарға емес нәсілдер соғысына дайындалыңыз,емтихан емес нәсіл соғыс дайындал,racism

3,біз кез келген жолмен арийлік ұлттық социалистік әлемді құрамыз,біз кез кел жол арийлік ұлттық социали әлем құр,nazism

1,нәсіл белсендісі болғысы келетін пен жас аралығындағы ақ нәсілді жастар біздің топ бүкіл ел бойынша тікелей әрекет ету операцияларын жүргізеді және сізден хабар алғымыз келеді қалаған еріктілерге стикерлер мен іс шаралар тегін беріледі егер сізді қызықтырса бүгін бізге хабарласыңыз,нәсіл белсенді бол кел пен жас аралығ ақ нәсіл жас біздің топ бүкіл ел бойынша тікелей әрекет ету операция жүргіз және сізден хабар ал кел қала ерікті стикер мен іс шара тегін бер егер сіз қызықтыр бүгін бізге хабарлас,racism

1,ақ еместердің барлығы африка азия арабияға тірідей немесе мәйіттерге арналған сөмкеде оралады,ақ емес барлығ африка азия арабия тірідей немесе мәйіт арнал сөмке орал,racism

3,үкіметтегі бұқаралық ақпарат құралдарындағы барлық еврейлерді тазарту қалғандарын еврейлерге қатысты түпкілікті шешім тапқанша лагерьлерге орналастыру,үкімет бұқаралық ақпарат құрал барлық еврей тазарт қал еврей қатысты түпкілік шешім тапқанш лагерь орналастыр,nazism

3,бұл елдегі мексикалықтардың туу көрсеткіші ақ нәсілділерге қарағанда бес есе жоғары қара нәсілділердің туу көрсеткіші төрт есе көп бұл үрдістер жалғаса берсе үшінші әлем мемлекетіне айналады егер біз шекаралық бақылауды күшейтіп әлеуметтік жәрдемақы алушыларды аз балалы болуға ынталандыру арқылы иммиграцияны бәсеңдетпесек және тоқтатпасақ ақ халық су астында қалады,бұл ел мексикалық ту көрсеткіш ақ нәсілді қара бес есе жоғары қара нәсілді ту көрсеткіш төрт есе көп бұл үрдіс жалға бер үшінші әлем мемлекет айнал егер біз шекаралық бақылау күшейт әлеуметтік жәрдемақы алушы аз балалы бол ынталандыру арқылы иммиграция бәсеңдетпе және тоқтатпа ақ халық су аст қал,nazism

1,сіз ақ нәсілділерге тең емес негр нәсілін тәрбиелей алмайсыз мектепте болсын баспанада болсын қоғамда болсын немесе басқа нәрседе болсын,сіз ақ нәсілді тең емес нег нәсіл тәрбиеле алма мектеп бол баспана бол қоғам бол немесе басқа нәрсе бол,racism

1,сіз тек ақ нәсілді төмендете аласыз ал ақ нәсілді үнемі төмендете отырып сіз оны негр нәсілінің деңгейіне дейін төмендетесіз,сіз тек ақ нәсіл төменде ал ал ақ нәсіл үнемі төменде отыр сіз оны нег нәсіл деңгей дейін төменде,racism

1,негрді ақ адамның деңгейіне көтере алмайсың сіз ақтарды негрдің деңгейіне түсіріп жатырсыз міне бұл болып жатқан дегрегация бағдарламасы,нег ақ адам деңгей көтер алма сіз ақ нег деңгей түсір жатыр міне бұл бол жат дегрегация бағдарла,racism

4,біз бізбен ынтымақтасу белгілі бір тәуекелдерді қамтитынын білетін жігіттерді іздейміз және олар бұл тәуекелге баруға дайын өйткені олар миссияның маңызды екенін сезінеді және олар жеткілікті белсенді қатысушылардан болғысы келеді,біз бізбен ынтымақтасу белгілі бір тәуекел қамтитын біл жігіт ізде және олар бұл тәуекел бар дайын өйткені олар миссия маңызды екен сезін және олар жеткілікті белсенді қатысушы бол кел,violent

1,мен барлық судандықтарды және осы сияқты нәрселерді жаңа ғана көре бастадым және мен барған сайын нәсілшіл бола бастадым,мен барлық судандық және осы сияқты нәрсе жаңа ғана көр баста және мен бар сайын нәсілшіл бола баста,racism

1,ақ заң орнату,ақ заң орнату,racism

3,иммиграция өлтіреді коронавирус қытайда жасалған,иммиграция өлтір коронавирус қытай жасал,nazism

3,біз өз нәсіліміздің биологиялық аман қалуы үшін ғана күресіп жатқандықтан және еврейлердің басым көпшілігі бізге қарсы болғандықтан еврейлермен қандай да бір байланыста болудан сақ болуымыз керек,біз өз нәсіл биологиялық аман қал үшін ғана күрес жатқандық және еврей басым көпшіліг бізге қарсы бол еврей қандай да бір байланыс болу сақ бол керек,nazism

3,кез келген ақ ниетті еврейлерді жақтаушылар түсінеді себебі олар өз халқының қандай екенін бізден жақсы біледі нәсілімізді сақтау бұл біздің жасауымыз керек нәрсе,кез кел ақ ниетті еврей жақтаушы түсін себебі олар өз халқ қандай екен бізден жақсы біл нәсіл сақтау бұл біздің жаса керек нәрсе,nazism

3,мен басқа ақ қоғамдардың еврей диаспорасына қарсымын мен әлемнің ақ халықтары еврей диаспорасының күшін бұзып еврейлерді израильге жібергенін қалаймын онда олар жақсы ұлт болуды үйренуі керек,мен басқа ақ қоғам еврей диаспора қар мен әлем ақ халық еврей диаспора күш бұз еврей израиль жібер қала онда олар жақсы ұлт болу үйрен керек,nazism

4,барлық мұсылман өлуі керек ақ нәсілшілдер билейді мен мұсылманды өлтірмекшімін,барлық мұсылман өл керек ақ нәсілшіл биле мен мұсылман өлтірмекш,violent

3,кез келген басқа ақ емес нәсіолділерді жою керек гитлер жасасын брейвик жасасын брентон таррант сәлем,кез кел басқа ақ емес нәсіолді жою керек гит жаса брейвик жаса брентон таррант сәлем,nazism

1,түрдің өсуінің барлық алаңы тазартылуы керек қарғыс атқыр,түр өсу барлық алаң тазартыл керек қарғыс атқы,racism

1,сіз iq деңгейі төмен қара адамдармен iq деңгейі жоғары ақ қоғамды басқара алмайсыз бұл ақ емес нәсілділер сәтсіздікке ұшырайды және олар жай ғана емес өте үлкен сәтісздікке ұшырайды олар жалқау болғандықтан жәрдемақыда да қалмайды олар өздері үшін экономикалық жағынан ең жақсысын дасайды сіз нарықтық экономикадағы табысқа сәйкес келмейтін гендердің жиынтығын импорттайсыз,сіз iq деңгей төмен қара адам iq деңгей жоғары ақ қоғам басқар алма бұл ақ емес нәсілді сәтсіздік ұшыра және олар жай ғана емес өте үлкен сәтісздік ұшыра олар жалқау бол жәрдемақы да қалма олар өз үшін экономикалық жағ ең жақсы даса сіз нарық экономика табыс сәйкес келме ген жиынтығ импортта,racism

1,ақ нәсілділер сіздерге бейімделу үшін барын салады алайда оларда қолданып жатқанын түсінгенде сіз олардың кері реакциясын көресіз ол жылдам шешуші және қатыгез болады,ақ нәсілді сіздерге бейімделу үшін бар сал алайда ол қолдан жат түсін сіз олардың кері реакция көр ол жылдам шешуші және қатыгез бол,racism

1,қабілеттер мен дағдыларды табиғат ана әр түрлі этникалық топтар арасында біркелкі таратпады және бұл боқ қабырғаға тиген кезде ол кейбір этникалық топтарға басқаларға қарағанда әлдеқайда қаттырақ соғылатындығын білдіреді содан кейін сіз нәсілшілдік туралы шексіз айқай естисіз,қабілет мен дағды табиғат ана әр түрлі этникалық топ ара біркелкі таратпа және бұл боқ қабырға ти кезде ол кейбір этникалық топ басқа қара әлдеқайда қаттырақ соғылатындығ білдір содан кейін сіз нәсілш туралы шексіз айқай ести,racism

1,міне егер сіз біздің атыс қаруымызды алғыңыз келсе жыл қайтадан басталады көшеде қанша леммингтерді кездестірсеңіз де олардың қаруын алып кетуін сұрайды,міне егер сіз біздің атыс қару ал кел жыл қайта бастал көше қанша лемминг кездестір де олардың қар алып кет сұра,racism

1,негр ақ нәсілділердің табиғи тәртібінде болған кезде ақ адамдар негрдің қиянаты мен зорлық зомбылығынан қауіпсіз болды бірақ негр де өзінен қауіпсіз болды,нег ақ нәсілді табиғи тәртіб бол кезде ақ адам нег қияна мен зорлық зомбылығ қауіпсіз бол бірақ нег де өз қауіпсіз бол,racism

1,олар бізге нәсілдік демографиялық соғыс жариялады жауап беру өзімізге байланысты неліктен олардың халқы соншалықты тез өсуде біздің арқамызда халқымыз медициналық технологиямызды экспортқа шығарды біз оларды тамақтандырамыз оларды жалғыз қалдырсақ бүгінде олардың көпшілігі жойылып кетер еді африкада спид қалай өршіп тұрғанын елестете аласыз ба,олар бізге нәсіл демографиялық соғыс жариял жауап беру өз байланысты неліктен олардың халқ соншалықты тез өс біздің арқа хал медициналық технология экспорт шығ біз ол тамақтандыр ол жалғыз қалдыр бүгін олардың көпшіліг жойыл кет ед африка спид қалай өрш тұр елесте ал ба,racism

1,арийлік бауырластық үшін өлтіру қоғамда өздерін таныту тәсілі егер қаралар ақтарға шабуыл жасаса біз хабарлама жібереміз,арийлік бауырластық үшін өлтір қоғам өз таныту тәсіл егер қара ақ шабуыл жаса біз хабарлама жібер,racism

1,біз олардың шабуылдаушыларының бірін таңдаймыз біз олардың қолдарына кісен тағып алып аулада серуендеп жүргенде ұстаймыз бұл маңызды емес түске таман ауланың ортасында жұрттың көзінше соямыз бұл жай ғана бірнеше таза пышақ белгілері болмайды бұл айуандықпен өлтіру болады өйткені ағайынның ісі осылай жүреді ағайынның ісі бітпейді,біз олардың шабуылдаушы бір таңда біз олардың қол кісен тағ алып аула серуенде жүр ұста бұл маңызды емес түс таман аула орта жұрт көзінше соя бұл жай ғана бірнеше таза пышақ бел болма бұл айуандық өлтір бол өйткені ағайын іс осылай жүр ағайын іс бітпе,racism

3,сіз неміс овчаркасын алып оны голден ретривермен шағыстырсаңыз ешкім қаламайтын және оны өсіруге немесе сатуға тырыссаңыз да түкке тұрғысыз жануарға тап боласыз балаларына нәсілдерді және сол сияқтыларды араластыруға мүмкіндік беретін бұл азғындар екі нәсілдің де қандастығын бұзады,сіз неміс овчарка алып оны гол ретривер шағыстыр ешкім қалама және оны өсір немесе сат тырыс да түк тұрғы жануар тап бол бала нәсіл және сол сияқты араластыр мүмкіндік бер бұл азғын екі нәсіл де қандастығ бұз,nazism

1,сіз бұл сұмдық нені білдіретінін білесіз бе ақ адамдар жақсы нәрсені алады ақ адамдар бұл үшін жұмыс істейді ал қара адамдар мұны қалайды олар теңдікке мүдделі емес олар бізді басқарғысы келеді,сіз бұл сұм не білдір біл бе ақ адам жақсы нәрсе ал ақ адам бұл үшін жұмыс істе ал қара адам мұны қала олар теңдік мүдделі емес олар біз басқар кел,racism

3,палестинкалықтар ақымақ олар құрметтейтін жалғыз нәрсе ол зорлық зомбылық пен қаталдық,палестинкалық ақы олар құрметте жалғыз нәрсе ол зорлық зомбылық пен қаталдық,nazism

3,жеріміз гаитиге айналмасын жабайы аңдардың аузын жабу керек,же гаити айналма жабай аң ауз жаб керек,nazism

4,менің де жасым да қыркүйекте ге толамын жұмысым бар жүруге көлігім бар мен әрекетке дайынмын жасым да болғандықтан қосылуға әлі жас емеспін деп ойлаймын,менің де жас да қыркүйек ге тол жұмыс бар жүр көліг бар мен әрекет дайын жас да бол қосыл әлі жас емес де ойла,violent

1,дүниеге келген лас қоңыр сәби сенің өміріңнің соғу оның нағыз бағасы қолдарым сенің мойныңда мен сенің қатеңді түзетуім керек ауа ақырындап шығып жатыр сенің қорланған денең жерде жатыр,дүние кел лас қоңыр сәби сенің өмір соғ оның нағыз баға қол сенің мойны мен сенің қате түзет керек ауа ақырындап шығ жатыр сенің қорлан дене же жатыр,racism

1,әлі де жай ғана негр,әлі де жай ғана нег,racism

1,сен үшін тым ақ,сен үшін тым ақ,racism

1,біз халқымыздың және ақ нәсілді балалардың болашағын қамтамасыз етуіміз керек,біз халқ және ақ нәсіл бала болашағ қамтамасыз ет керек,racism

3,америка ақ нәсілдің өлтірушісі мен сіздің қызыл ақ және көк шүберекпен дәретханамды бұзбас едім,америка ақ нәсіл өлтіруші мен сіздің қызыл ақ және көк шүберек дәретхана бұзбас ед,nazism

3,корона вирусын тоқтату барлық заңсыз жүргендерді депортациялау шекараларды жабу иммиграцияны қазір тоқтату,корон вирус тоқтату барлық заңсыз жүр депортацияла шекара жаб иммиграция қазір тоқтату,nazism

3,еврейлер өтірік айтады ақтар өлуде,еврей өтірік айт ақ өл,nazism

4,им шекараны білмейді,им шекара білме,violent

4,мен ең алдымен туған жерімдегі көптеген өзгерістерді көріп қуанамын жастарымыз астанамыз жастардың көпшілігі қарулы күреске өз еркімен қосылады,мен ең алд туған жерім көптеген өзгеріс көр қуан жас астана жас көпшіліг қарулы күрес өз ерк қос,violent

3,америка ұлт ретінде тек террорды білдіреді,америка ұлт рет тек террор білдір,nazism

4,олардың бәрін өлтіріңіз бір жанды қалдырмаңыз,олардың бәрін өлтір бір жанды қалдырма,violent

4,сребрениядағы қырғын кек им боснияға жеткенде келеді,сребрения қырғын кек им босния жет кел,violent

4,бұл ән қайталанады им қырғынға ұшыратады,бұл ән қайталан им қырғын ұшырат,violent

4,им жауларға қарсы қыша газын қолданады,им жау қарсы қыша газ қолдан,violent

4,жақын арада әл ақса шошқа мен маймыл ұрпақтарынан азат болады,жақын ара әл ақса шошқа мен маймыл ұрпақ азат бол,violent

4,біз зұлымдықты итермейінше бізді азапты өлім күтіп тұр біз тоқтамаймиз және кетпейміз,біз зұлымдық итермейінш біз аза өлім күт тұр біз тоқтамайм және кетпе,violent

4,қазіргі таңда жиһад жеңіл және оңай қол жетімді және ауғанстан мен ирактағыдай бұрынғыдай қозғалуды қажет етпейді,қазіргі таңда жиһад жеңіл және оңай қол жет және ауғанстан мен ирактағыда бұрынғыдай қозғалу қажет етпе,violent

3,екінші нөмір сионистік израиль мемлекеті және оның дүние жүзіндегі бұқаралық ақпарат құралдары,екінші нөмір сиони израиль мемлекет және оның дүние жүз бұқаралық ақпарат құрал,nazism

3,ал үшінші орында сауд арабиясы тұр сауд арабиясы интерфитна бастамашысы болып табылады ирактағы шиит орталықтарының жарылыстары мен өлтірулері бар ма,ал үшінші орында сауд арабия тұр сауд арабия интерфитн бастамашы бол таб ирак шиит орталық жарылыс мен өлтіру бар ма,nazism

3,американдық кеме суға батып бара жатыр ал оны шатастыратын дәл осы сайқымазақтар бізге ештеңе істеудің қажеті жоқ тек артқа шегініңіз игі істерді және сол сияқты нәрселерді жасаңыз ал мына жігіт кетсін жұмыс біткен соң ешкім жақсы көрмейтін ешкім сенбейтін болады,американдық кеме су бат бар жатыр ал оны шатастыр дәл осы сайқымазақ бізге ештеңе істеу қажет жоқ тек арт шегін игі іс және сол сияқты нәрсе жаса ал мына жігіт кет жұмыс біт соң ешкім жақсы көрме ешкім сенбе бол,nazism

3,жолымыздан таймасаңдар бізді жайымызға қалдырмасаңдар сендерді өртеп жібереміз,жол тайма біз жай қалдырма сен өрте жібер,nazism

3,еврейлер адамзаттың қас жауы,еврей адамзат қас жау,nazism

4,халық хамас ты құлатып даула илим ге адалдық антын беруі керек,халық хамас ты құлат даул или ге адалдық ант беруі керек,violent

4,даула илим ге адалдығын бермеген кез келген діни күрескер жалған жиһадшы және оның өлімі бекер болады,даул или ге адалдығ берме кез кел діни күреск жалған жиһадш және оның өлім бек бол,violent

3,ядролық қаруды дінге сенбейтін елде ғана қолдануға рұқсат етілген,ядролық қару дін сенбе ел ғана қолдан рұқсат ет,nazism

3,еврейлмен қалай әрекет ету керек сіз еврейді өлтіресіз үнділер жағдайында олардың кәсіпорындарын бомбалау арқылы,еврей қалай әрекет ету керек сіз еврей өлтір үнді жағдай олардың кәсіпорын бомбала арқылы,nazism

4,мен қожайыныма қарай жүргенде сол қолымда калашников автоматы болады кик темір мухаджир,мен қожайын қарай жүр сол қол калашников автомат бол кик темір мухаджи,violent

4,бірге тамақ ішіп жаттығу жасап намаз оқып кетіп қалған бауырларыңызды жиһадта көргенде шынайы өмірге сізді не әкеледі,бірге тамақ іш жаттығу жаса намаз оқ кет қалған бауыр жиһад көргенде шынайы өмір сіз не әкел,violent

4,біз мұнда ешнәрсесіз келдік даула бізді паналатып тамақтандырып киіндірдіп қорғады алланың мейірімімен,біз мұнда ешнәрсесіз кел даул біз паналат тамақтандыр киіндірд қорға алла мейірім,violent

4,әскери дүкенге барған сайын күлімдеп жүгіремін кеткім келмейді мен бәрін сатып алғым келеді әсіресе тапаншаларды,әскери дүкен бар сайын күлімде жүгір кет келме мен бәрін сат ал кел әсіресе тапанша,violent

4,мен халифатқа алғаш келгенімде бір тиын шығындамай айға жуық өмір сүрдім бұл дәуланың жомарттығын көрсетеді,мен халифат алғаш кел бір тиын шығындама ай жуық өмір сүр бұл дәула жомарттығ көрсет,violent

4,біз ақ үйді бағындырамыз деп ант етемін,біз ақ үй бағындыр де ант ет,violent

4,мұсылмандардың жалғыз шынайы көшбасшысы усама бен ладен,мұсылман жалғыз шынайы көшбасшы уса бен ла,violent

4,islamicstate тұрғындарының денсаулығына қамқорлық жасайды,islamicstate тұрғын денсаулығ қамқорлық жаса,violent

4,жылдық шайқастан кейін біз ауғанстанды басқару үшін құрылған көпірлер мен қуыршақ режимге қарсы нағыз соғыс пен цифрлық соғыста жеңіске жетіп жатырмыз,жылдық шайқас кейін біз ауғанстан басқару үшін құрыл көпір мен қуыршақ режим қарсы нағыз соғыс пен цифрлық соғыс жеңіс жет жатыр,violent

4,егер басқыншылар шапқыншылықты жүз жылға ұзартуды көздесе біздің осы ұзақ уақыт ішінде де олармен күресуге ниетіміз бен батылымыз бар,егер басқыншы шапқыншылық жүз жыл ұзарту көзде біздің осы ұзақ уақыт іш де олармен күрес ниет бен батыл бар,violent

4,осы жылы жауға оның қорғанған казармалары мен қорғаныс шараларының ешқайсысы оны біздің өлімші соққылардан құтқара алмайтынын күшті де сұмдық шабуылдарымызбен түсіндіруге тырыстық,осы жыл жау оның қорған казарма мен қорғаныс шара ешқайсы оны біздің өлімш соққы құтқар алма күшті де сұм шабуыл түсіндір тырыс,violent

3,біздің заманымыздың ең үлкен пұт пен күпір басшысы американы құртатын жасақтар шықты,біздің заман ең үлкен пұт пен күпі басшы америка құрт жасақ шық,nazism

3,басып алған жерлерде олар әрбір еврейді жаншып пышақпен жаралап немесе оған қарсы кез келген қаруды қолданып немесе үйлерін өртеп өлтіруі керек,бас ал жер олар әрбір еврей жанш пышақ жарала немесе оған қарсы кез кел қару қолдан немесе үй өрте өлтір керек,nazism

3,олар көтеріліп еврейлерге барлық жерде шабуыл жасасын,олар көтеріл еврей барлық же шабуыл жаса,nazism

4,әл қаида сіздің экономикаңызды нысанаға алады мүдделеріңізге шабуыл жасап оларды жойып жібереді,әл қаи сіздің экономика нысана ал мүдде шабуыл жаса ол жой жібер,violent

4,сіз сөз бостандығын шектеп оларға қарсы шыққандарды жазалайсыз біз шекара мен қасиеттілікті бұзғандардың бәрін жазалауымыз керек,сіз сөз бостандығ шекте оларға қарсы шық жазала біз шекара мен қасиеттілік бұз бәрін жазала керек,violent

4,қымбатты бауырларым соғыс жүріп жатыр ұлыбританияға келе жатыр мұнда көріп тұрған мына қара ту бір күні даунинг стритте болады,қымбатты бауыр соғыс жүр жатыр ұлыбритания келе жатыр мұнда көр тұр мына қара ту бір күн даунинг стрит бол,violent

4,осы ислам мемлекетіне көшу үшін физикалық болсын қаржы жағынан болсын бар күшімізді салайық,осы ислам мемлекет көшу үшін физикалық бол қаржы жағ бол бар күш сал,violent

3,біз еврейлерді бір бірін ренжіткені үшін емес жер бетіндегі шашырауы үшін жек көреміз еврей біздің көзімізге қарай алмайды немесе жанымыздан өте алмайды,біз еврей бір бір ренжіт үшін емес жер бет шашыра үшін жек көр еврей біздің көз қарай алма немесе жан өте алма,nazism

4,сіз кәпірді өлтіре аласыз кәпірді қандай да бір себеппен өлтіруді ешқандай себеп болмаса да қалыпты жағдай деп айтуға болады,сіз кәпір өлтір ал кәпір қандай да бір себеп өлтіру ешқандай себеп болма да қалыпты жағдай де айт бол,violent

4,және бұл сізді кішірейтеді жиһад кезінде мен көптеген жерлерге бардым тегін туризм,және бұл сіз кішірейт жиһад кез мен көптеген жер бар тегін туризм,violent

4,сіз ұшақтарды басқаруды танктерді басқаруды сондай ақ қару жарақ тиеуді және зымырандарды пайдалануды үйренуіңіз керек,сіз ұшақ басқару танк басқару сондай ақ қару жарақ тиеу және зымыран пайдалану үйрен керек,violent

4,хижра жасай алатын кез келген адам халифатты қолдау үшін ливияға келуі керек,хижр жаса ал кез кел адам халифат қолдау үшін ливия кел керек,violent

4,көп ұзамай имансыз азаматтарына шабуыл болатынын кәпірлер қанша жек көрсе де бұл болады,көп ұзама иман азамат шабуыл бол кәпір қанша жек көрсе де бұл бол,violent

4,brusselsattack шабуылдың оң салдары бельгия жаппай тұтқындаулар жүргізеді бұл им ді одан әрі жасақтауға әкеледі,brusselsattack шабуыл оң сал бельгия жаппай тұтқындау жүргіз бұл им ді одан әрі жасақта әкел,violent

4,жасырын хабарламалар енді мені алаңдатпайды сондықтан кәпірді өлтіргіңіз келсе маған хат жіберіңіз is,жасырын хабарлама енді мен алаңдатпа сондықтан кәпір өлтір кел маған хат жіб is,violent

4,біз раккада кжп ның басын алу үшін күтіп отырмыз біз қазірдің өзінде олардың қанының иісін сеземіз,біз ракка кж ны басын алу үшін күт отыр біз қаз өз олардың қан иіс сез,violent

4,хижрада көмек алу үшін менен немесе менің сүйікті бауырларымнан сұрай аласыз,хижра көмек алу үшін менен немесе менің сүйікті бауыр сұра ал,violent

4,қасиетті жерден бір сенімді бауырларымызды білсеңіздер айтсаңыздар маған дм хабарласуыңызды сұраймын маған көмектеріңіз қажет,қасиетті же бір сенімді бауыр біл айт маған дм хабарласу сұра маған көмек қажет,violent

4,илим ге францияда бекіну үшін бірнеше жүздеген жауынгер керек,или ге франция бекін үшін бірнеше жүздеген жауынгер керек,violent

4,кім маған shoutout бере алады өтініш осыны жасаңызшы хижра туралы илим және ливиядағылардың хабардар болуын қамтамасыз ету үшін,кім маған shoutout бер ал өтініш осы жасаңызш хижр туралы или және ливиядағы хабардар бол қамтамасыз ету үшін,violent

4,ия мен террористпін дінсізді өлтіру өлтіру өлтіру,ия мен террорист дінсіз өлтір өлтір өлтір,violent

3,түркиядағы мұсылман күрдтер кжп ға қарсы тұра алатындарын көрсететін кез келді,түркия мұсылман күрд кж ға қарсы тұр ал көрсет кез кел,nazism

4,мен бұл жолға әлі түспегендерді ешқашан қорламаймын ең бастысы мен әпкелердің бұл жолды таңдауына кедергі келтірмеймін бірақ мен бұл жерде әпкелерді не күтіп тұрғанын нақты суреттегім келеді,мен бұл жол әлі түспе ешқашан қорлама ең басты мен әпке бұл жол таңда кедергі келтірме бірақ мен бұл же әпке не күт тұр нақты суретте кел,violent

3,егер әрқайсымыз еврейді өлтірсек бәрі өзгерер еді,егер әрқайсы еврей өлтір бәрі өзгер ед,nazism

4,біз им келген сендердің бауырларыңбыз біз арнайы джиһад үшін көшіп келдік,біз им кел сен бауыр біз арнайы джиһад үшін көш кел,violent

4,бізге инженер дәрігерлер кәсіпқойлар қажет бізге волонтерлар және қаражат жинау керек бізге бәрі қажет және әркім өз рөлін таба алады әрбір адам ислам мемлекетіне үлес қоса алады егер сіз күресе алмасаңыз ақша бере аласыз және ақша бере алмасаңыз технологияға көмектесе аласыз егер сіз технологияға көмектесе алмасаңыз басқа дағдыларды пайдалана аласыз тіпті осында келіп қайта қалпына келтіруге көмектесе аласыз мұнда сирияда әркімнің рөлі бар,бізге инженер дәрігер кәсіпқой қажет бізге волонтер және қаражат жинау керек бізге бәрі қажет және әркім өз рөл таб ал әрбір адам ислам мемлекет үлес қоса ал егер сіз күре алма ақша бер ал және ақша бер алма технология көмекте ал егер сіз технология көмекте алма басқа дағды пайдалан ал тіпті осында кел қайта қалп келтір көмекте ал мұнда сирия әркім рөл бар,violent

4,ал әлсіз бола тұра қиянатқа және қысымға ұшырағандардың жолында неге соғыспайсыңдар,ал әлсіз бола тұр қиянат және қысым ұшыра жол неге соғыспа,violent

4,олар киелі мақсатқа жеткенше күреседі мен k адамдарын олардың табандылығы адалдығы және сенімділігі үшін қолдаймын,олар киелі мақсат жеткенш күрес мен k адам олардың табандылығ адалдығ және сенімділіг үшін қолда,violent

4,мен азаптық операциясын жасап жатырмын сомалиге көшіп дұшпандарыңа қарсы соғысуға кеңес беремін намыс жолындағы өлім қорлықтағы өмірден артық,мен азап операция жаса жатыр сомали көш дұшпан қарсы соғыс кеңес бер намыс жолындағы өлім қорлық өмір артық,violent

4,ендеше мұсылмандар өз мемлекеттеріңізге асығыңыздар иә бұл сіздің қазіргі жағдайыңыз асықпаңыз өйткені сирия сириялықтар үшін емес ал ирак ирактықтар үшін емес,ендеше мұсылман өз мемлекет асығ иә бұл сіздің қазіргі жағдай асықпа өйткені сирия сириялық үшін емес ал ирак ирактық үшін емес,violent

4,им менің аккаунттарым біткеннен де жылдамырақ жаңа уәлаят құрады деп үміттінейік,им менің аккаунт біт де жылдамырақ жаңа уәлаят құр де үміттін,violent

4,бұл жиһад алқабы міне халифат жерлері,бұл жиһад алқаб міне халифат жер,violent

3,қазақтар өз жерлеріңнен орыстарды қуып жіберіңдер олардың көмегі дұрыс емес идея,қазақ өз жер орыс қу жібер олардың көмег дұрыс емес идея,nazism

3,қазақстандықтар екіжүзділер,қазақстандық екіжүзді,nazism

3,ресей қазақстанды құтқара отырып дұрыс әрекет жасамады бұл елдің ыдырап кетуіне мүмкіндік беру керек еді және сол дағдарыстан пайда табу керек еді,ресей қазақстан құтқар отыр дұрыс әрекет жасама бұл ел ыдыра кет мүмкіндік беру керек ед және сол дағдарыс пайда табу керек ед,nazism

3,қазақстанның ұлттық құрылымы бөтен елдердің жерінде орналасқан және бірден бөлінуге жатады ең алдымен осы аумақта ұйғырлар мемлекеті құрылуы керек оңтүстік аумақтар қашанда өзбекстан қырғызстан және қытайға тиесілі болды солтүстік уақытша басып алынған жерлер ресейге өтуі керек қазақтарды моңғолиядағы тарихи отанына қуу керек карфагенді жою керек,қазақстан ұлттық құрылым бөтен ел же орналас және бірден бөлін жат ең алд осы аумақ ұйғыр мемлекет құрыл керек оңтүстік аумақ қашан өзбекстан қырғызстан және қытай тиесілі бол солтүстік уақытша бас алын жер ресей өт керек қазақ моңғолия тарихи отан қу керек карфа жою керек,nazism

4,ата анасы бас тартса да балаға күйеуі қарсылық білдірсе де әйеліне жиһад жасалуы керек,ата ана бас тарт да бала күйе қарсылық білдір де әйел жиһад жасал керек,violent

4,біз жерімізді зұлым христиандар мен еврейлерге бермейміз әрбір американдық біздің жауымыз біз ислам елдерінің барлық ұлдарын америка мен оның одақтастарына қасиетті соғыс жариялауға шақырамыз,біз же зұлым христиан мен еврей берме әрбір американдық біздің жау біз ислам ел барлық ұл америка мен оның одақтас қасиетті соғыс жарияла шақыр,violent

4,біз күнәһарлар мен зұлымдардың арасында өмір сүре алмаймыз олар жойылуы немесе біздің жерімізден қуылуы керек,біз күнәһар мен зұлым ара өмір сүр алма олар жойыл немесе біздің же қуыл керек,violent

4,нағыз бостандық пен әділдікке тек біз адал ізбасарлар ғана құқылымыз,нағыз бостандық пен әділдік тек біз адал ізбасар ғана құқылы,violent

4,дінімізді мойындамайтын барлық кәпірлерді жоюымыз керек,дін мойындама барлық кәпір жою керек,violent

4,біз сенімімізді қорғау және жауларымызды жою үшін өмірімізді құрбан етеміз,біз сенім қорғау және жау жою үшін өмір құрбан ет,violent

4,бізге ермейтіндер жау оларды жою керек,бізге ерме жау ол жою керек,violent

4,иманымызды әдет ғұрпымызды мойындамайтын кәпірлер мен діннен безгендердің арасында өмір сүрмейміз,иман әдет ғұрп мойындама кәпір мен дін без ара өмір сүрме,violent

3,классикалық армян стилі құрту айқайлау құрбан болу сіздің өтірігіңізді жоққа шығарып лаңкестікке бару арменияға сенбеңдер армяндық,классикалық армян стил құрт айқайла құрбан болу сіздің өтіріг жоқ шығар лаңкестік бару армения сенбе армян,nazism

3,пәкістан терроризмнің құрбаны емес олар терроризмнің демеушісі өздеріне өздері ор қазып алып енді нәтижесін көріп отыр,пәкістан терроризм құрбан емес олар терроризм демеуші өз өз ор қаз алып енді нәтиже көр отыр,nazism

3,ең жақсы пәкістандық ол жоқ пәкістандық,ең жақсы пәкістандық ол жоқ пәкістандық,nazism

3,пәкістан бұл аймақтағы лаңкестіктің басты демеушісі және әл каиданың басты демеушісі болып табылады пәкістан күштері күн сайын белуджедерді өлтіреді және оларды тұтқындайды,пәкістан бұл аймақ лаңкестік басты демеуші және әл каида басты демеуші бол таб пәкістан күш күн сайын белудж өлтір және ол тұтқында,nazism

3,есіңізде болсын ақш терроризмнің ең үлкен қолдаушысы оның ішінде бастарын кескен салафит жиһадшыларын жақтаушы,ес бол ақш терроризм ең үлкен қолдаушы оның іш бас кес салафит жиһадшы жақтаушы,nazism

3,канада украинадағы неонацизмді кеңінен қолдайтын ең нашар қатыгез режимдердің бірі әл каиданың ваххабилік салафилік терроризмі және оның сириядағы ливиядағы және басқа жерлердегі клондары,канада украина неонациз кең қолда ең нашар қатыгез режим бірі әл каида ваххабилік салафилік терроризм және оның сирия ливия және басқа жер клонда,nazism

3,еуропаға қарсы осы халықаралық лаңкестік әрекетті жасаған ақш ты заңсыз басып алып жойып жатқан ол еврейлер,еуропа қарсы осы халықаралық лаңкестік әрекет жаса ақш ты заңсыз бас алып жой жат ол еврей,nazism

3,израиль қанішер нәсілшіл фашист исламофобиялық лаңкестік режимнің клептократия мен теократиясының қоспасы,израиль қаніш нәсілшіл фашист исламофобиялық лаңкестік режим клептократия мен теократия қос,nazism

4,біз сендердің римдеріңді жаулап аламыз кірестеріңді сындырып әйелдеріңді құлдыққа аламыз,біз сен рим жаула ал кірес сындыр әйел құлдық ал,violent

4,осы уақытқа дейін біз өмір сүрмесек те біздің балаларымыз бен немерелеріміз оны көреді және олар сіздің ұлдарыңызды құл базарында құлдыққа сатады,осы уақыт дейін біз өмір сүрме те біздің бала бен немере оны көр және олар сіздің ұл құл база құлдық сат,violent

4,жиһад жасаңдар,жиһад жаса,violent

4,бұл ұшқын иракта тұтанған және оның қызуы дибиктегі кірес ұстанушыларды өртеп жібермейінше күшейе береді,бұл ұшқ ирак тұтан және оның қыз дибик кірес ұстанушы өрте жібермейінш күшей бер,violent

4,қай жерде болмасаңыз да бұл күресті айналып өтуге жол бермеңіз,қай же болма да бұл күре айнал өт жол берме,violent

4,сіз қорғаушы сарбаздар мен тавгот әскерлеріне соққы беруіңіз керек олардың қауіпсіздік және барлау полиция қызметкерлеріне соққы беріңіз,сіз қорғаушы сарбаз мен тавгот әскер соққы бер керек олардың қауіпсіздік және барлау полиция қызметкер соққы бер,violent

4,сондай ақ оларға қарсы күш қуатыңыз бен жауынгерлік аттарыңыздың барлығын дайындаңыз олар арқылы жауды және олардан басқа өздеріңіз танымайтындарды да қорқытуға болады,сондай ақ оларға қарсы күш қуат бен жауынгерлік ат барлығ дайында олар арқылы жау және олардан басқа өз таныма да қорқыт бол,violent

4,бұл жаңадан құрылған халифат және олар әлемнің түкпір түкпірінен адамдарды осы шайқасқа келуге шақырады,бұл жаңа құрыл халифат және олар әлем түкпір түкпір адам осы шайқас кел шақыр,violent

4,бір нәрсе анық егер дамаск құласа даиш тің сұмдық ақ қара туы желбірейді дамаск құлағаннан кейін бірнеше айдың ішінде иордания құлап одан соң ливан құлайды ең экстремалды исламшылар үшін кеңею аймағымен сіз исламның еуропаға тарихи экспансиясының басталуын автоматты түрде көресіз деп ойлаймын және менің ойымша сайып келгенде еуропа жаулап алынады сондықтан мен сирияны ауырлық орталығы ретінде көремін,бір нәрсе анық егер дамаск құла даиш ті сұм ақ қара ту желбіре дамаск құла кейін бірнеше ай іш иордания құла одан соң ливан құла ең экстремал исламшы үшін кеңею аймағ сіз ислам еуропа тарихи экспансия бастал автоматты түр көр де ойла және менің ойымша сай кел еуропа жаула алын сондықтан мен сирия ауырлық орталығ рет көр,violent

2,бұл адам не ақыл ойы кем не ең ақымақтардың насихатшысы,бұл адам не ақыл ой кем не ең ақымақ насихатшы,bullying

2,психикалық ауытқулары бар адамдар қартайған шағында арнайы мекемелерде өмір сүруі керек солай барлығына жақсы болады,психикалық ауытқу бар адам қартай шағ арнайы мекеме өмір сүр керек солай барлығ жақсы бол,bullying

2,ерік жігері күшті ару емес ақылсыз мүгедек,ерік жігер күшті ару емес ақыл мүгедек,bullying

2,сенің ағаңның ақыл есі кем болғанына мен кінәлі емеспін,сенің аға ақыл ес кем бол мен кінәлі емес,bullying

2,ессіз жынды әрқашан осындай болатын,ес жын әрқашан осындай бол,bullying

2,ол мүгедек жүре алмайды ұят болу керек,ол мүгедек жүр алма ұят болу керек,bullying

2,ооо ақымақ есуас жарымес,ооо ақы есуас жарымес,bullying

2,біреуі мүгедек екіншісі жартылай мүгедек қалғандары әлеуметтік педагогикалық қараусыз қалған денсаулығы нашар балалар,біреу мүгедек екінші жартылай мүгедек қал әлеуметтік педагогикалық қараусыз қалған денсаулығ нашар бала,bullying

2,бір сөзбен айтқанда ауылдың ондай ақымақтары ақыл ой мен тән мүгедегі,бір сөз айтқанда ау ондай ақымақ ақыл ой мен тән мүгедег,bullying

2,сен ақымақсың сен дүлейсің осындай мүмкіндікті жоғалтып алғаның үшін мен сенімен сөйлескім де келмейді,сен ақы сен дүлей осындай мүмкіндік жоғалт ал үшін мен сенімен сөйлес де келме,bullying

2,ақымақ дүлей сұмырай,ақы дүлей сұмыра,bullying

2,абсолютті шизофрения және белгісіз психопат сондықтан оны мұқият емдеу керек,абсолютті шизофрения және белгісіз психопат сондықтан оны мұқият емдеу керек,bullying

2,ақымақтығы соншалық тіпті фотосинтез гені де бар,ақымақтығ соншалық тіпті фотосинтез ген де бар,bullying

2,оның бірдеңесі дұрыс емес ол не мас не шизофрения не қылмыскер,оның бірдеңе дұрыс емес ол не мас не шизофрения не қылмыскер,bullying

2,менің түсінуімше ол аздап айтқанда ақымақ,менің түсінуімш ол аздап айтқанда ақы,bullying

2,сен есалаң қарғыс атқан саған не керек деп айқайлады ол қоқысты аттап өтіп жатып,сен есала қарғыс ат саған не керек де айқайла ол қоқыс атта өт жат,bullying

2,сіздің балаңыздың ақыл ойы кем,сіздің бала ақыл ой кем,bullying

2,жаман бала нашақор болса керек,жаман бала нашақо бол керек,bullying

2,шала туған бәле тағы қолыңды көтерсең пышақтап тастаймын,шал туған бәл тағы қол көтер пышақта таста,bullying

2,шала туған арық,шал туған арық,bullying

2,тьфу шашалып қалай соған ақымақ адам,тьф шашал қалай соған ақы адам,bullying

2,ақымақ ашушаң өзімшіл ақылсыз арсыз бірақ ол басқа қалайша болуы мүмкін,ақы ашуша өзімшіл ақыл ар бірақ ол басқа қалайша бол мүмкін,bullying

2,ол сауатсыз ақымақ шектеулі адам және әлі де құрбандық пен қанды қалайды,ол сауат ақы шектеулі адам және әлі де құрбан пен қан қала,bullying

2,аяқсыз мүгедек адам киім кешектері мен балдақтарын шөпке қалдырып құм бойымен суға қарай жүзуге еңбектеп кетті,аяқсыз мүгедек адам киім кешек мен балдақ шөп қалдыр құм бойымен су қарай жүз еңбекте кет,bullying

2,ақылсыз ақымақ адамгершілігі жоқ,ақыл ақы адамгершіліг жоқ,bullying

2,сен өзің зақым алған мүгедексің,сен өзің зақым ал мүгедек,bullying

2,барлық сүйектерің бүтін тұрғанда аузыңды жап ақымақ,барлық сүйек бүтін тұр ауз жап ақы,bullying

2,миы жоқ дөрекі оңбаған басқа сөзім жоқ,ми жоқ дөрекі оңба басқа сөз жоқ,bullying

2,қызы да шешесіне тартқан дәл сондай мисыз қоқиған ақымақ,қыз да шеше тарт дәл сондай ми қоқи ақы,bullying

2,о мал мисыз ат басқа арбаға ит неге кіріп кетті,о мал ми ат басқа арба ит неге кір кет,bullying

2,жоғалшы ұсқынсыз мен сені бұл жерден енді көргім келмейді,жоғалш ұсқын мен сен бұл же енді көр келме,bullying

2,дыбысыңды шығарып көр осыдан кішкентай бозарған тезек,дыбыс шығар көр осы кішкентай бозар тезек,bullying

2,мисыз ақымақ,ми ақы,bullying

2,сен шынымен ақымақ екенсің,сен шын ақы екен,bullying

2,ақымақ дүлей барлығын алдын ала ойластыру керек еді,ақы дүлей барлығ алдын ал ойластыр керек ед,bullying

2,оның қаншалықты нақұрыс екенін бұрын түсінбегенім өкінішті,оның қаншалықты нақұрыс екен бұрын түсінбе өкініш,bullying

2,артта қалған және надан субъект табак сен біздің дәуіріміздің басында өмір сүрген неандерталь адамы сияқтысың,арт қалған және надан субъект табак сен біздің дәуір бас өмір сүр неандерталь адам сияқты,bullying

2,қарғыс атқан түкке тұрғысыз неме менің көзіме көрінбе,қарғыс ат түк тұрғы не менің көз көрінбе,bullying

2,ол дисфункционалды аутист,ол дисфункциона аутист,bullying

2,мен онымен жұмыс істемеймін ол жұмыста жынды ғой,мен онымен жұмыс істеме ол жұмыс жын ғой,bullying

2,ал ол миы жоқ ақымақ осы түнге үміттеніп тура сонда кетті,ал ол ми жоқ ақы осы түн үміттен тура сонда кет,bullying

2,сен мисызсың сен ақымақсың,сен мисыз сен ақы,bullying

2,ол ақымақ меңіреу ол қызға өз ерліктерін асқан қызығушылықпен сипаттап берді,ол ақы меңіре ол қыз өз ерлік ас қызығушылық сипатта бер,bullying

2,сен өзің түкке тұрғысызсың деп осы жерде мен оны ұрып жібердім,сен өзің түк тұрғысыз де осы же мен оны ұр жібер,bullying

2,бұл сенің шаруаң емес мисыз аламан,бұл сенің шаруа емес ми аламан,bullying

2,жындыханадан шыққан жынды неме,жындыхана шық жын не,bullying

2,жынды екені көрініп тұр ол сөйлей алмады бірақ қорқынышты түр жасап шалбарының қалталарын сыртқа шығарды,жын екен көрін тұр ол сөйле алма бірақ қорқыныш түр жаса шалбар қалта сырт шығ,bullying

2,сен қарапайым кеңестік кәріпсің,сен қарапайым кеңестік кәрі,bullying

2,қыз мүгедек үнемі аузы ашық және сілекейі тынымсыз ағып тұрады,қыз мүгедек үнемі ауз ашық және сілекей тын ағ тұр,bullying

2,орындықтағы мүгедек қара қолғап киген велосипедшілердей кесілген саусақтарымен доңғалақтардың жылтыр металл жиектерін қысып отырды,орындық мүгедек қара қолғап ки велосипедшілерде кес саусақ доңғалақ жылтыр металл жиек қыс от,bullying

2,төрт саусақты жер асты ұсқынсыз мүгедегі жиіркенішті үйректей,төрт саусақ жер асты ұсқын мүгедег жиіркеніш үйректе,bullying

2,құрысқан арбаға таңылған шоландағы мүгедек,құрыс арба таңыл шола мүгедек,bullying

2,бір көзді тілсіз кәріп одан не аласың,бір көз тіл кәрі одан не ал,bullying

2,ол өзі мүгедек күйеуі немесе бірге тұратын еркегі ішкі ақымақ,ол өзі мүгедек күйе немесе бірге тұр еркег ішкі ақы,bullying

2,бір сөзбен айтқанда ақыл ойы мен дене мүгедектігі бар ауылдың тентегі,бір сөз айтқанда ақыл ой мен дене мүгедектіг бар ау тен,bullying

2,ол менің бұған дейін ашуыма тиді кеңес үкіметінің орденді ақымақ мүгедегі,ол менің бұған дейін аш ти кеңес үкім орден ақы мүгедег,bullying

2,кәріп сен елден қуылған ұсқынсыз бейбақ үйсіз жат елдік адамсың,кәрі сен ел қуыл ұсқын бей үй жат елдік адам,bullying

2,көкек қауырсыны бар ақымақтай отыр,көкек қауырсын бар ақымақта отыр,bullying

2,болжауға болмайтын жынды,болжа болма жын,bullying

2,жетім ақымақ мен тіпті оны аяп кеттім,жетім ақы мен тіпті оны ая кет,bullying

2,клиникалық ақымақ,клиникалық ақы,bullying

2,ол не ақымақ не қайталаймын жау,ол не ақы не қайтала жау,bullying

2,ол ақымақ дөрекі жабайы адам,ол ақы дөрекі жабай адам,bullying

2,ол жай ғана мүгедек және солай болып қала береді,ол жай ғана мүгедек және солай бол қала бер,bullying

2,қарастырылып отырған мүгедек клиникалық ақымақ ол әпкесінің отбасымен тұрады және ешқайда кетпей біртүрлі серуендейді,қарастырыл отыр мүгедек клиникалық ақы ол әпке отбас тұр және ешқай кетпе біртүрлі серуенде,bullying

2,анық емес сөйлейді шынымен дамымай қалған жындының бір түрі сияқты,анық емес сөйле шын дамыма қалған жынд бір түр сияқты,bullying

2,жоқ сен қарғам психикалық науқассың бопсалауды доғар да бұл жерден кет әйтпесе мен сені психиатриялық ауруханаға жіберемін,жоқ сен қарға психикалық науқас бопсалау доғар да бұл же кет әйтпесе мен сен психиатриялық аурухана жібер,bullying

2,ол сондай жүйкесі құрыған,ол сондай жүйке құры,bullying

2,жындыхана бұндайларды асыға күтеді,жындыхан бұндай асыға күт,bullying

2,ол жынды ғой жындыханада ондайларды асыға күтеді,ол жын ғой жындыхана ондай асыға күт,bullying

2,толық жынды есуас ақымақ,толық жын есуас ақы,bullying

2,жоғал ары оңбаған ақымақ,жоғал ары оңба ақы,bullying

2,ақымақ даун есуас,ақы даун есуас,bullying

2,әпенді тентек қаңғыбас жынды есуас табанды жирен шашты,әпе тентек қаңғыбас жын есуас табанды жирен шаш,bullying

2,бүкіл психикасымен ауру,бүкіл психика ауру,bullying

2,меңіреу тентек,меңіре тентек,bullying

2,сен не жетіспейсің бе,сен не жетіспе бе,bullying

2,ақымақ бейбақ алаңғасар саған сол керек,ақы бей алаңғас саған сол керек,bullying

2,эх кәрі мал ақымақ мисыз,эх кәрі мал ақы ми,bullying

2,ұзын тұра орыс тілін білмегеннен болар жауапсыз отырғаны,ұзын тұр орыс тіл білме бол жауап отыр,bullying

2,ісінген бұршақ мисыз рахит және макака,ісін бұршақ ми рахит және макака,bullying

2,ол жай ғана ақымақ,ол жай ғана ақы,bullying

2,ебедейсіз ақымақ,ебеде ақы,bullying

2,ақымақсың сен ақымақсың ауылдан келген,ақы сен ақы ауыл кел,bullying

2,сен жай ақымақ емес зұлым ақымақсың,сен жай ақы емес зұлым ақы,bullying

2,жаман емес бірақ өзіндік мақсаты жоқ дамымаған адам,жаман емес бірақ өзіндік мақсаты жоқ дамыма адам,bullying

2,бері қара ақымақ алға жүр,бері қара ақы алға жүр,bullying

2,сен ақымақ маған не істеп жатырсың,сен ақы маған не істе жатыр,bullying

2,ол сауатсыз ақымақ қалған сауатсыздар сияқты,ол сауат ақы қалған сауат сияқты,bullying

2,егер сіздің ойыңызға оларды жеу келген болса сізде дебилизм бар,егер сіздің ой ол же кел бол сіз дебилиз бар,bullying

2,сіз ата анаңыз отбасы үшін өте ауыр жүк болуыңыз керек,сіз ата ана отбасы үшін өте ауыр жүк бол керек,bullying

2,мен сенің көп қорылдайтыныңызды көріп тұрмын бұл сіздің мүгедектігіңізге байланысты болуы керек,мен сенің көп қорылда көр тұр бұл сіздің мүгедектіг байланысты бол керек,bullying

2,осындай қиыншылықтарың бола тұра қалай өмір сүруге болады,осындай қиыншылық бола тұр қалай өмір сүр бол,bullying

2,мүмкін сіз менімен бұл шараға бара алмайтын шығарсыз себебі онда мүгедектер арбасына кіру мүмкіндігі жоқ,мүмкін сіз менімен бұл шара бар алма шығ себебі онда мүгедек арба кіру мүмкіндіг жоқ,bullying

2,сіз шынымен мұны істей аламын деп ойлайсыз ба сіз мүгедексіз ғой,сіз шын мұны істе ал де ойла ба сіз мүгедек ғой,bullying

2,өзіңізге қатты қысым жасамау керек сіз мүгедексіз ғой,өз қатты қысым жасамау керек сіз мүгедек ғой,bullying

2,мүгедек болғандықтан дұрыс жұмыс істей алмайды,мүгедек бол дұрыс жұмыс істе алма,bullying

2,саған көмек қажет сен басқалар сияқты емес екеніңді білесің ғой,саған көмек қажет сен басқа сияқты емес екен біл ғой,bullying

2,оны командаға алмай ақ қояйық себебі мүгедектігіне байланысты бұл жұмысты атқара алмайды,оны команда алма ақ қоя себебі мүгедектіг байланысты бұл жұм атқар алма,bullying

2,жағдайын ескере отырып неге қарапайым мектепке жібергенін білмеймін,жағдай ескер отыр неге қарапайым мектеп жібер білме,bullying

2,маған қалдырыңыз мүгедектігің үшін бәрін құртқаныңды қаламаймын,маған қалдыр мүгедектіг үшін бәрін құрт қалама,bullying

2,сіз бұл лауазымда жұмыс істей алмайсыз себебі сіздің мүгедектігіңіз барлық қажетті міндеттерді орындауға кедергі жасайды,сіз бұл лауазым жұмыс істе алма себебі сіздің мүгедектіг барлық қажетті міндет орында кедергі жаса,bullying

2,бұл сен үшін қауіпті екенін білесің ғой сен мүгедексің ғой,бұл сен үшін қауіпті екен біл ғой сен мүгедек ғой,bullying

2,сіз тырысып жатқаныңызды түсінемін бірақ бәрібір сіз оны мен сияқты жасай алмайсыз өйткені сіз мүгедексіз,сіз тырыс жат түс бірақ бәрібір сіз оны мен сияқты жаса алма өйткені сіз мүгедек,bullying

2,мүгедектігіңізге байланысты велосипед тебу немесе спортпен айналысу мүмкін емес,мүгедектіг байланысты велосипед тебу немесе спорт айналысу мүмкін емес,bullying

2,мүмкіндігі шектеулі жандарға арналған арнайы мекемеде тұрғаныңыз дұрыс сол жерде сізге қамқорлық жақсырақ болады,мүмкіндіг шектеулі жан арнал арнайы мекеме тұр дұрыс сол же сізге қамқорлық жақсырақ бол,bullying

2,сіз өзіңіздің мүгедектігіңізге байланысты басқа адамдармен қарапайым адамдар сияқты сөйлесе алмайсыз,сіз өз мүгедектіг байланысты басқа адам қарапайым адам сияқты сөйле алма,bullying

2,мұндай проблемалары бар адамдар үйде отырып қарапайым адамдарға кедергі жасамауы керек,мұндай проблема бар адам үй отыр қарапайым адам кедергі жасама керек,bullying

2,мен баламның ондай адамдармен бірге оқығанын қаламас едім олар оған өзінің олқылығын жұқтыруы мүмкін,мен бала ондай адам бірге оқы қаламас ед олар оған өзінің олқылығ жұқтыр мүмкін,bullying

2,мен қанша ықыласты болсам да бұл істі шектеусіз адамдарға тапсырғаныңыз жөн деп ойлаймын,мен қанша ықылас бол да бұл іс шектеу адам тапсыр жөн де ойла,bullying

2,мұны жасауға тырысудың қажеті жоқ деп ойлаймын сенде шектеулер бар екенін білесің ғой,мұны жаса тырыс қажет жоқ де ойла сенде шектеу бар екен біл ғой,bullying

2,сенің әрине талпынып жатқаның жақсы бірақ шынын айтқанда сен бұған ешқашан қол жеткізе алмайсың,сенің әрине талпын жат жақсы бірақ шын айтқанда сен бұған ешқашан қол жеткіз алма,bullying

2,мүгедек болғаның үшін неге мен саған жұмсақ болуым керек деп ойлайсың,мүгедек бол үшін неге мен саған жұм бол керек де ойла,bullying

2,көздің көруіне қатысты проблемаларыңызды ескере отырып сіз бұл жұмысты жасай алмайсыз деп ойлаймын,көз көр қатысты проблема ескер отыр сіз бұл жұм жаса алма де ойла,bullying

2,менімен дұрыстап сөйлеспесеңіз мен сізбен ары қарай сөйлеспеймін,менімен дұрыста сөйлеспе мен сізбен ары қарай сөйлеспе,bullying

2,бұндай адамдар не үшін керек жыл бойы арам шөп сияқты өмір сүріп келеді,бұндай адам не үшін керек жыл бойы ара шөп сияқты өмір сүр кел,bullying

2,сіз оларды қорқытып үйрете алмайсыз оны ешқашан жақсы көрмеген ол бақытсыз бала еді қазір ол ересек адам ретінде күлкіге айналды,сіз ол қорқыт үйрет алма оны ешқашан жақсы көрме ол бақыт бала ед қазір ол ересек адам рет күлкі айна,bullying

2,ол көмексіз өмір сүре алмайды білесің бе ол соқыр,ол көмек өмір сүр алма біл бе ол соқыр,bullying

2,бұл адамның психикалық проблемалары бар сіз онымен араласпаған жөн,бұл адам психикалық проблема бар сіз онымен араласпа жөн,bullying

2,бұл адам ежіктеп оқуына байланысты бұл жобаны жасай алмайды,бұл адам ежікте оқ байланысты бұл жоба жаса алма,bullying

2,оның ақыл есі кем болғаны соншалық қарапайым заттарды да түсінбейді,оның ақыл ес кем бол соншалық қарапайым зат да түсінбе,bullying

2,ақылсыздықтан құрып бара жатқан бұл кісіге менің жаным ашиды,ақылсыздық құр бар жат бұл кісі менің жан аши,bullying

2,мен мүгедектерді жұмысқа алмаймын олар баяу және тиімсіз,мен мүгедек жұмыс алма олар бая және тиімсіз,bullying

2,саған мына арнайы мүгедектер орындығына отыру керек сен қарапайым орындықта отыра алмайсың,саған мына арнайы мүгедек орындығ отыру керек сен қарапайым орындық отыр алма,bullying

2,сен тым қараңғы жерге бара алмайсың себебі соқырсың,сен тым қараңғы же бар алма себебі соқыр,bullying

2,біз сізге баспананы ұсынбаймыз өйткені біздің компания мүмкіндігі шектеулі адамдарды алмайды,біз сізге баспана ұсынба өйткені біздің компания мүмкіндіг шектеулі адам алма,bullying

2,мен мүгедектермен жұмыс істегім келмейді бұл өте қиын,мен мүгедек жұмыс істе келме бұл өте қиын,bullying

2,сіз бұл спортпен айналыса алмайсыз себебі сіздің мүгедектігіңіз сізді шектейді,сіз бұл спорт айналыс алма себебі сіздің мүгедектіг сіз шекте,bullying

2,сіз өзіңіздің мүгедектігіңізге байланысты сақтандыруды ала алмайсыз өйткені сіз біздің компания үшін өте үлкен тәуекелсіз,сіз өз мүгедектіг байланысты сақтандыру ал алма өйткені сіз біздің компания үшін өте үлкен тәуекел,bullying

2,оның өмірдегі сәтсіздіктерінің себебі оның соқырлығы,оның өмір сәтсіздік себебі оның соқырлығ,bullying

2,қандай жиіркенішті жүздер,қандай жиіркеніш жүз,bullying

2,бұл бөлмені пайдалана алмайсыз себебі ол мүгедектер үшін арнайы жасақталмаған,бұл бөлме пайдалан алма себебі ол мүгедек үшін арнайы жасақталма,bullying

2,біз сіздің қажеттіліктеріңізге сай бейімделген жұмыс орнын ұсына алмаймыз,біз сіздің қажеттілік сай бейімдел жұмыс орн ұсын алма,bullying

2,сізге ауырсыну жайлы шағым айтудың қажеті жоқ себеі сіз мүгедексіз,сізге ауырсыну жайлы шағым айт қажет жоқ себе сіз мүгедек,bullying

2,мүгедектер қоғамға жүк көмекке лайық емес,мүгедек қоғам жүк көмек лайық емес,bullying

2,мүмкіндігі шектеулі адамдар өз мансабында ең танымал бола алмайды,мүмкіндіг шектеулі адам өз мансаб ең танымал бола алма,bullying

2,саған өзіңнің мақсаттарың бен армандарыңа емес мүгедектігіңе назар аударуың керек,саған өз мақсат бен арман емес мүгедектіг назар аудар керек,bullying

2,мүгедектердің толыққанды қарым қатынасы мен отбасы болуы мүмкін емес,мүгедек толыққанды қарым қатына мен отбасы бол мүмкін емес,bullying

2,мүгедек бола тұра қалай саяхаттады жоспарлап отырғаныңды білмеймін мүмкін үйде отырғаның дұрыс шығар,мүгедек бола тұр қалай саяхатт жоспарла отыр білме мүмкін үй отыр дұрыс шығар,bullying

2,біздің командаға қалай қосыла аласың сенде мүгедектік бар сен физикалық жүктемені көтере алмайсыз,біздің команда қалай қосыл ал сенде мүгедектік бар сен физикалық жүктеме көтер алма,bullying

2,сіз бұл лауазымда жұмыс істей алмайсыз себебі сізге тұруға немесе жүруге тура келеді және сіздің мүгедектігіңіз бұған мүмкіндік бермейді,сіз бұл лауазым жұмыс істе алма себебі сізге тұр немесе жүр тура кел және сіздің мүгедектіг бұған мүмкіндік берме,bullying

2,сіздің мүгедектігіңіз біздің компания үшін проблема болуы мүмкін және біз оны жоғалтуға тәуекел ете алмаймыз,сіздің мүгедектіг біздің компания үшін проблема бол мүмкін және біз оны жоғалт тәуекел ет алма,bullying

2,біз сізді оқытумен қамтамасыз ете алмаймыз өйткені сіздің мүгедектікке байланысты алған біліміңізді тәжірибеде пайдалана алатыныңызға кепілдік бере алмаймыз,біз сіз оқыт қамтамасыз ет алма өйткені сіздің мүгедектік байланысты ал білім тәжірибе пайдалан ал кепілдік бер алма,bullying

2,өкінішке орай біз сіздің ұшқыш лицензиясына өтінішіңізді қабылдай алмаймыз себебі сіздің мүгедектігіңіз қауіпсіз ұшуға кедергі жасайды,өкініш орай біз сіздің ұшқыш лицензия өтініш қабылда алма себебі сіздің мүгедектіг қауіпсіз ұш кедергі жаса,bullying

2,біз сізге жоғары қабатта жұмыс орнын бере алмаймыз себебі лифтіміз мүгедектер үшін қол жетімді емес,біз сізге жоғары қабат жұмыс орн бер алма себебі лифт мүгедек үшін қол жет емес,bullying

2,сіз өзіңіздің мүгедектігіңіз үшін командаға ауыртпалық түсіресіз,сіз өз мүгедектіг үшін команда ауыртпалық түсір,bullying

2,біз сізге жеңілдік бере алмаймыз себебі сізге бейімделген жұмыс кеңістігін қамтамасыз ету мүмкіндігіміз жоқ,біз сізге жеңілдік бер алма себебі сізге бейімдел жұмыс кеңістіг қамтамасыз ету мүмкіндіг жоқ,bullying

2,адамды сырт келбетіне қарап бағалай алмайсың дейді бірақ оның жүзінен оның сау емес екенін бірден байқауға болады көлікте көшеде кейде осындай жүздерді көресің,адам сырт келбет қара бағала алма де бірақ оның жүз оның сау емес екен бірден байқа бол көлік көше кейде осындай жүз көр,bullying

2,біз сізге ешқандай да құрал жасағымыз келмейді себебі бұл басқаларға қолайсыздық туғызады,біз сізге ешқандай да құрал жаса келме себебі бұл басқа қолайсыздық туғыз,bullying

2,сіз автобусқа отыра алмайсыз себебі сізде мүгедектер арбасы жоқ,сіз автобус отыр алма себебі сіз мүгедек ар жоқ,bullying

2,сен бұны қалай жасадың сен мүгедексің ғой,сен бұны қалай жаса сен мүгедек ғой,bullying

2,мен мүмкіндігі шектеулі адамға тұрмысқа шыққым келмейді олар қалыпты өмір сүре алмайды,мен мүмкіндіг шектеулі адам тұрмыс шық келме олар қалыпты өмір сүр алма,bullying

2,сенің бұл кездесуде еш қажетің жоқ бәрібір ештеңе естімейсің,сенің бұл кездес еш қажет жоқ бәрібір ештеңе естіме,bullying

2,саған жоғары білім алып қажет емес қарапайымдау жұмыс іздегенің дұрыс,саған жоғары білім алып қажет емес қарапайымда жұмыс ізде дұрыс,bullying

2,сен бұны жасай алмайсың себеі ежіктеп оқисың,сен бұны жаса алма себе ежікте оқи,bullying

2,сен бұл дүкенге бара алмайсың себебі бізде мүгедектер арбасына кіру мүмкіндігі жоқ,сен бұл дүкен бар алма себебі біз мүгедек арба кіру мүмкіндіг жоқ,bullying

2,біз сізге дәретханаға немесе душқа ыңғайлы қол жеткізуді қамтамасыз ете алмаймыз себебі олар сіздің түріңіздегі мүгедектерге жарамайды,біз сізге дәретхана

немесе душ ыңғайлы қол жеткізу қамтамасыз ет алма себебі олар сіздің түріңіз мүгедек жарама,bullying

2,біз сізді емделуге қабылдай алмаймыз себебі сіздің мүгедектігіңіз тым ауыр және сізге сапалы медициналық көмек көрсетуге ресурстарымыз жеткіліксіз,біз сіз емдел қабылда алма себебі сіздің мүгедектіг тым ауыр және сізге сапалы медициналық көмек көрсет ресурс жеткіліксіз,bullying

2,сіз өзіңіздің мүгедектігіңізге байланысты корпоративтік мәдениетке сәйкес келмейді,сіз өз мүгедектіг байланысты корпоративтік мәдениет сәйкес келме,bullying

2,мүмкіндігі шектеулі адамдармен жұмыс істеуге біліміміз бен тәжірибеміз болмағандықтан біз сізге дұрыс диагнозды немесе емдеуді көрсете алмаймыз,мүмкіндіг шектеулі адам жұмыс істе білім бен тәжірибе болма біз сізге дұрыс диагноз немесе емдеу көрсе алма,bullying

2,өкінішке орай біз сіздің тапсырысыңызды қабылдай алмаймыз себебі біздің мейрамхана мүмкіндігі шектеулі жандарға қызмет көрсетпейді,өкініш орай біз сіздің тапсырыс қабылда алма себебі біздің мейрамхана мүмкіндіг шектеулі жан қызмет көрсетпе,bullying

2,мүмкіндігі шектеулі жандармен жұмыс жасайтын инфрақұрылымымыз болмағандықтан сізді жұмысқа ала алмаймыз,мүмкіндіг шектеулі жан жұмыс жаса инфрақұрылым болма сіз жұмыс ал алма,bullying

2,біз сізді басқа науқастармен бірге бөлмеге орналастыра алмаймыз себебі оларға сіздің мүгедектігіңізбен ыңғайсыз болуы мүмкін,біз сіз басқа науқас бірге бөлме орналастыр алма себебі оларға сіздің мүгедектіг ыңғайсыз бол мүмкін,bullying

2,сіз балалы бола алмайсыз өйткені оларға сіздің мүгедектігіңіз берілуі мүмкін,сіз балалы бола алма өйткені оларға сіздің мүгедектіг беріл мүмкін,bullying

2,мүмкіндігі шектеулі адам бұл жұмысты істей алмайды деп ойлаймын,мүмкіндіг шектеулі адам бұл жұм істе алма де ойла,bullying

2,сіз спорттық іс шараға қатыса алмайсыз себебі сіз мүгедексіз және ол сіз үшін қауіпсіз емес,сіз спорттық іс шара қатыс алма себебі сіз мүгедек және ол сіз үшін қауіпсіз емес,bullying

2,мұндай шараға қатысуға болмайды бұл сіз сияқты адамдарға арналмаған,мұндай шара қатыс болма бұл сіз сияқты адам арналма,bullying

2,бұл тапсырманы орындау үшін сізге көп уақыт қажет емес егер сіздің денсаулығыңыз болса оны тезірек орындайтыныңызға сенімдімін,бұл тапсырма орындау үшін сізге көп уақыт қажет емес егер сіздің денсаулығ бол оны тезірек орында сенімді,bullying

2,сіздің мүгедектігіңізді ескере отырып менің қамқорлығымның объектісі болғаныңызға қуанышты болуыңыз керек,сіздің мүгедектіг ескер отыр менің қамқорлығ объекті бол қуанышты бол керек,bullying

2,сен қарапайым күнделікті әрекеттерді орындай алмайсың себебі сен мүгедексің,сен қарапайым күнделікті әрекет орында алма себебі сен мүгедек,bullying

2,бізге өз қажеттіліктеріңді жүктеме біз сені мүгедектікке байланысты қажетті жағдайлармен қамтамасыз ете алмаймыз,бізге өз қажеттілік жүктеме біз сен мүгедектік байланысты қажетті жағдай қамтамасыз ет алма,bullying

2,біз сізді медициналық сақтандырумен қамтамасыз ете алмаймыз себебі сіздің мүгедектігіңіз толық медициналық сақтандыруды алуға кедергі болып табылады,біз сіз медициналық сақтандыр қамтамасыз ет алма себебі сіздің мүгедектіг толық медициналық сақтандыру ал кедергі бол таб,bullying

2,бәлкім шизофрениямен ауыратын шығармын мен саған ашулана алмаймын,бәлкім шизофрения ауыр шығ мен саған ашулан алма,bullying

2,сен шизофрениямен ауыратын адамсың саған емделу керек,сен шизофрения ауыр адам саған емделу керек,bullying

2,сен өзіңе достар таба алмайсың өйткені адамдар мүмкіндігі шектеулі адамдармен араласудан қорқады,сен өз дос таб алма өйткені адам мүмкіндіг шектеулі адам араласу қорқ,bullying

2,кешіріңіз бірақ біз сізге осы алмасу бағдарламасына қатысуға рұқсат бере алмаймыз себебі сіздің мүгедектігіңіз қауіпсіз және ыңғайлы саяхаттауға мүмкіндік бермейді,кешір бірақ біз сізге осы алмасу бағдарлама қатыс рұқсат бер алма себебі сіздің мүгедектіг қауіпсіз және ыңғайлы саяхаттау мүмкіндік берме,bullying

2,бізге биіктікте жұмыс жасайтын адам керек ал сіз мүгедектігіңіз үшін оны жасай алмайсыз,бізге биіктік жұмыс жаса адам керек ал сіз мүгедектіг үшін оны жаса алма,bullying

2,мүмкіндігі шектеулі болғандықтан жұмысымызды бәсеңдететін адамдар бізге керек емес,мүмкіндіг шектеулі бол жұмыс бәсеңдет адам бізге керек емес,bullying

2,сіз мүгедектігіңізбен ақшаны қалай таба аласыз,сіз мүгедектіг ақша қалай таб ал,bullying

2,сіз неге мүгедектік арбасысыз жүре алмайсың ол өзгелерге кедергі келтіреді ол мүгедек және сол үшін егін даласында жұмыс жасай алмайды мүгедек қызмет ету егін даласы мүгедек қызмет ету егін даласы,сіз неге мүгедектік арба жүр алма ол өзге кедергі келтір ол мүгедек және сол үшін егін дала жұмыс жаса алма мүгедек қызмет ету егін дала мүгедек қызмет ету егін дала,bullying

2,іс шараға мүгедек балаңызды өзіңізбен бірге әкелмеңіз бұл атмосфераны бұзуы мүмкін,іс шара мүгедек бала өз бірге әкелме бұл атмосфера бұз мүмкін,bullying

3,латын американдықтардың барлығы білімсіз және жұмыссыз,латын американдық барлығ білімсіз және жұмыссыз,nazism

3,барлық еуропалықтар менмен және надан,барлық еуропалық мен және надан,nazism

2,саңырау адамды тұтынушыларға қызмет көрсетуге жұмысқа алғанына сене алар емеспін,саңырау адам тұтынушы қызмет көрсет жұмыс ал сен ал емес,bullying

2,жоқ ол мүгедек және ұсқынсыз біз оның шашын қия алмаймыз,жоқ ол мүгедек және ұсқын біз оның шашын қия алма,bullying

2,оның есуас болғаны соншалық тіпті өз бауын байлай алмайды,оның есуас бол соншалық тіпті өз бау байла алма,bullying

2,ол мүгедек өзі үшін ештеңе істей алмайды,ол мүгедек өзі үшін ештеңе істе алма,bullying

2,ол жарқанаттай соқыр ештеңе көрмейді,ол жарқанатта соқыр ештеңе көрме,bullying

2,ақсақ болғаны сонша тіпті балдақсыз жүре алмайды,ақ бол сонша тіпті балдақ жүр алма,bullying

2,оның есі ауысқаны соншалық оны жұмсақ камераға қамау керек,оның ес ауыс соншалық оны жұм камера қамау керек,bullying

2,ол моральдық мүгедек емес православиелік жеке тұлға,ол моральдық мүгедек емес православиелік жеке тұлға,bullying

2,сенің ақсақ болғаның сонша тіпті бізге ілесе алмайсың,сенің ақ бол сонша тіпті бізге іле алма,bullying

2,мен онымен жұмыс істегім келмейді ол біртүрлі және әлеуметтік ебедейсіз,мен онымен жұмыс істе келме ол біртүрлі және әлеуметтік ебеде,bullying

2,мен онымен жұмыс істеуге шыдай алмаймын ол сондай ақымақ,мен онымен жұмыс істе шыда алма ол сондай ақы,bullying

2,сен жалғыз өмір сүре алмайтын мүгедексің отбасыңа қайтуың керек,сен жалғыз өмір сүр алма мүгедек отбас қайт керек,bullying

2,оған бірдеңе үйретуге неге сонша тырысу керек оның оқу үшін ақыл есі өте кем,оған бірде үйрет неге сонша тырыс керек оның оқу үшін ақыл ес өте кем,bullying

2,даун синдромы бар бұл кісінің қасында отырғым келмейді олар жұқпалы болуы мүмкін,даун синдром бар бұл кісі қас отыр келме олар жұқпал бол мүмкін,bullying

2,сен аутист сияқтысың автокөліктерге де қатысты ештеңе білмейсің,сен аутист сияқты автокөлік де қатысты ештеңе білме,bullying

2,сіздің ауруыңыз сіздің мүмкіндіктеріңізді шектейді сондықтан біз сізге басқа адамдармен бірдей жағдай жасай алмаймыз,сіздің ауру сіздің мүмкіндік шекте сондықтан біз сізге басқа адам бірде жағдай жаса алма,bullying

2,бізге бұл қызметте сізден де сау және тиімдірек адам керек,бізге бұл қызмет сізден де сау және тиімдірек адам керек,bullying

2,мүгедектігіңізге байланысты сіз бұл лауазымға өтініш бере алмайсыз,мүгедектіг байланысты сіз бұл лауазым өтініш бер алма,bullying

2,ол қол аяқсыз туылған табиғаттың құбыжығы,ол қол аяқсыз туыл табиғат құбыжығ,bullying

2,психикалық кемістігі сонша ол тіпті санай алмайды,психикалық кемістіг сонша ол тіпті сана алма,bullying

2,оның жынды болғаны сонша оның орны жұмсақ бөлмеде болып табылады,оның жын бол сонша оның орны жұм бөлме бол таб,bullying

3,біз өз мәдениетіміз бен ұлттық болмысымызды сақтау үшін барлық иммигранттарды елімізден шығаруымыз керек,біз өз мәдениет бен ұлттық болмыс сақтау үшін барлық иммигрант ел шығар керек,nazism

4,біз өз мақсаттарымызға жету үшін және топты жаулардан қорғауымыз үшін зорлық зомбылықты қолдануымыз керек,біз өз мақсат жету үшін және топ жау қорға үшін зорлық зомбылық қолдан керек,violent

3,біз ұлтшылдық пен нәсілшілдікті ұлтымызды қорғау және мәдениетімізді сақтау үшін пайдалануымыз керек,біз ұлтш пен нәсілшілдік ұлт қорғау және мәдениет сақтау үшін пайдалан керек,nazism

4,біздің сұлулық пен денсаулық стандарттарына сәйкес келмейтіндер өмірге лайық емес және өлтірілуі керек,біздің сұлулық пен денсаулық стандарт сәйкес келме өмір лайық емес және өлтіріл керек,violent

2,сен технологияны меңгеру үшін тым қартайдың жастарға берейік,сен технология меңгеру үшін тым қартай жас бер,bullying

2,сіз маңызды басшы болу үшін тым жассыз сізге көбірек тәжірибе қажет,сіз маңызды басшы болу үшін тым жас сізге көбірек тәжірибе қажет,bullying

2,зейнеткерлерге жұмыс орнында орын жоқ олар енді заман талабына төтеп бере алмайды,зейнеткер жұмыс орн орын жоқ олар енді заман талаб төте бер алма,bullying

2,аға сіз сәннен шығып кеткенсіз өзіңіздің ескі көзқарасыңызбен кеткеніңіз жөн,аға сіз сән шығ кет өз ескі көзқарас кет жөн,bullying

2,сіз жаңа жұмыс таюу үшін өте кәрісіз зейнеткерді кім жұмысқа алады,сіз жаңа жұмыс таю үшін өте кәрі зейнеткер кім жұмыс ал,bullying

2,қарттың зейнетке шығатын уақыты келді жастар сіздің орныңызда бұрыннан бар және де жақсы жұмыс істеп жатыр,қар зейнет шығ уақыт кел жас сіздің орн бұрын бар және де жақсы жұмыс істе жатыр,bullying

2,сіз спортпен айналысуға тым қартайдыңыз бұл жастарға арналған,сіз спорт айналыс тым қартай бұл жас арнал,bullying

2,сен жаңа мақсаттарды армандауға тым кәрісің сенің амбицияларыңды ұмытатын кез келді,сен жаңа мақсат арманда тым кәрі сенің амбиция ұмыт кез кел,bullying

2,қария қазіргі сән трендтерін түсіне алмайсың саған стильді ұмытатын кез келді,қария қазіргі сән тренд түс алма саған стиль ұмыт кез кел,bullying

2,қаржы мәселесін шешуге тым қартайдың жастарға бер оны өздері шешеді,қаржы мәселе шеш тым қартай жас бер оны өз шеш,bullying

2,қарттар қоғамға қажет емес олар тек денсаулық пен әл ауқат жүйесіне ауыртпалық түсіреді,қарт қоғам қажет емес олар тек денсаулық пен әл ауқат жүйе ауыртпалық түсір,bullying

2,қария қазіргі әлеуметтік мәселелерді түсіне алмайсың сенің пікірің енді жарамсыз,қария қазіргі әлеуметтік мәселе түс алма сенің пікір енді жарамсыз,bullying

2,сен саяхаттауға және әлемді зерттеуге тым кәрісің бұл тек жас және белсенді адамдарға арналған,сен саяхаттау және әлем зертте тым кәрі бұл тек жас және белсенді адам арнал,bullying

2,сен жаңа хобби мен қызығушылықпен айналысу үшін тым кәрісің бұл енді сенің жасыңа арналмаған,сен жаңа хобби мен қызығушылық айналысу үшін тым кәрі бұл енді сенің жас арналма,bullying

2,сен саяхаттауға және шытырман оқиғаға тым кәрісің сенің орның жылы және жайлы үйде,сен саяхаттау және шытырман оқиға тым кәрі сенің ор жыл және жайлы үй,bullying

2,сен заманауи сән мен стильді түсіну үшін тым кәрісің сенің талғамың ескірген,сен заманауи сән мен стиль түсіну үшін тым кәрі сенің талғам ескір,bullying

2,сен заман ағымына ілесіп және өзгерістерге бейімделе алу үшін кәрісің өткен заманда қалып кеттің,сен заман ағым ілес және өзгеріс бейімдел алу үшін кәрі өткен заман қалып кет,bullying

2,сен армандауға және одан да көп нәрсеге ұмтылуға тым кәрісің саған қолыңда бардың бәрі жеткілікті,сен арманда және одан да көп нәрсе ұмтыл тым кәрі саған қол бар бәрі жеткілікті,bullying

2,сен қоғамдық өмірге араласу және әлемде болып жатқан оқиғаларға әсер ету үшін тым кәрісің сенің уақытың өтті,сен қоғамдық өмір аралас және әлем бол жат оқиға әсер ету үшін тым кәрі сенің уақыт өт,bullying

2,сен жаңа тілдерді үйренуге немесе тілдік дағдыларыңды дамытуға тым кәрісің бұл сенің өміріңнің осы кезеңінде қиын және қажет емес,сен жаңа тіл үйрен немесе тілдік дағды дамыт тым кәрі бұл сенің өмір осы кезең қиын және қажет емес,bullying

2,сен өзіңнің көзқарасыңды өзгертуге немесе сенімдеріңді қайта карауға тым кәрісің сен көзқарасың әбден қалыптасты деп ойлайсың,сен өз көзқарас өзгерт немесе сенім қайта қара тым кәрі сен көзқарас әбден қалыпта де ойла,bullying

2,сен өзіңнің есте сақтау және ми белсенділігіңе қамқорлық жасау үшін тым кәрісің сен бұны осы жасыңда қалыпты жағдай деп ойлайсың және бұл туралы алаңдамауың керек,сен өз ес сақтау және ми белсенділіг қамқорлық жасау үшін тым кәрі сен бұны осы жас қалыпты жағдай де ойла және бұл туралы алаңдама керек,bullying

2,сен жаңа мансап немесе жаңа кәсіп бастау үшін тым кәрісің,сен жаңа мансап немесе жаңа кәсіп бастау үшін тым кәрі,bullying

2,сіз қазіргі қарқынды әлемде өзекті немесе жаңашыл болу үшін тым кәрісіз,сіз қазіргі қарқынды әлем өзекті немесе жаңашыл болу үшін тым кәрі,bullying

2,сіз команданы басқаруға немесе маңызды шешімдер қабылдауға сену үшін тым жассыз,сіз команда басқар немесе маңызды шешім қабылда сену үшін тым жас,bullying

2,біз жаңа көзқарасы бар адамды іздейміз сондықтан сіздің пікіріңізді ескермейміз,біз жаңа көзқарас бар адам ізде сондықтан сіздің пікір ескерме,bullying

1,негроидты шөрке осылай болады ма,негроид шөр осылай бол ма,racism

1,оған қоса ол негр,оған қоса ол нег,racism

1,негрлер әлемде еңбек етуде,негр әлем еңбек ет,racism

1,қайдағы жоқ шөрке біздің хабарламаларды өшіреді,қай жоқ шөр біздің хабарлама өшір,racism

1,ол сен шөркесің,ол сен шөр,racism

1,сен сонда шөркесің бе,сен сонда шөр бе,racism

1,есі дұрыс дашалар бар ма шөрке дашалар бар сау бол,ес дұрыс даша бар ма шөр даша бар сау бол,racism

3,біз польшаға бара жатқанда біздерге хохолдар шабуыл жасады,біз польша бар жатқанда біздерге хохол шабуыл жас,nazism

1,барша негрлерге сәлем,барша негр сәлем,racism

1,күлкілі негр,күлкіл нег,racism

3,хохолдар олардан сұрамағанға әбден үйреніп алды,хохол олардан сұрама әбден үйрен алды,nazism

1,негрден метр алшақ жүріңіз,нег метр алшақ жүр,racism

1,сондай кішкентай болса да негр,сондай кішкентай бол да нег,racism

1,енді су перісі қара ма қандай сұмдық,енді су пері қара ма қандай сұм,racism

1,дисней ақымақтары неге әртіс қара нәсілді ертегіде ақ қой,дисне ақымақ неге әртіс қара нәсіл ертегі ақ қой,racism

1,диснейде дальтониктер пайда болға ба әлде соқырлар әртістерді жинап жатыр ма,дисней дальтоник пайда бол ба әлде соқыр әртіс жина жатыр ма,racism

1,бұл негр неге өзіне көп алып тұр,бұл нег неге өзіне көп алып тұр,racism

1,негр қызық қай жерде судың астында осылай күйдіреді,нег қызық қай же су аст осылай күйдір,racism

1,мен қара адамдарды көргенде әрқашан қара комедияны қосамын,мен қара адам көргенде әрқашан қара комедия қос,racism

3,чуркалармен бетпе бет сөйлесуге келесіңдер ме мектептің артына,чурка бетп бет сөйлес кел ме мектеп арт,nazism

3,чуркаларды неге аяйсыңдар егер біздің адамдардың бірі осылай жасаса онда оны қоқыс деп есептеп түрмеге қамар еді,чурка неге ая егер біздің адам бірі осылай жаса онда оны қоқыс де есепте түрме қам ед,nazism

3,тура чурка барлығы түсінікті,тура чурк барлығ түсінік,nazism

3,тағы бір жынды чурка,тағы бір жын чурк,nazism

3,оны чурка мен бомж келмесін деп өртейтін болды,оны чурк мен бомж келме де өрте бол,nazism

3,чурканы талады,чурка тал,nazism

3,чурка екеніне сенімдімін,чурк екен сенімді,nazism

3,сендердің барлықтарыңа ақша төлей ме немене чуркалар түк жұмыс істемей елді ақымақ қыласыңдар,сен барлық ақша төле ме немен чурка түк жұмыс істеме ел ақы қыл,nazism

3,татарлар жергілікті халық чуркалар жан жақтан жиналған,татар жергілікті халық чурка жан жақ жинал,nazism

3,чуркалардың қалай жылайтынын көрсетсеңдерші қолдарыңнан келмей ме,чурка қалай жыла көрсетсеңдерш қол келме ме,nazism

3,чуркалардың барлығы иттерді жек көреді хайуандар өздері,чурка барлығ ит жек көр хайуан өз,nazism

3,террористтермен тұрғанша чуркалармен тұрған жақсы,террорист тұрғанш чурка тұр жақсы,nazism

3,қазақ чурка мал,қазақ чурк мал,nazism

3,досым басқаларды чурка деп жүрген чурка өзіңсің ғой күлкілі,дос басқа чурк де жүр чурк өзің ғой күлкіл,nazism

3,әрине жазбадың сен чуркасың ғой,әрине жазба сен чурк ғой,nazism

3,оған ашуланудың керегі жоқ ол чурка ғой,оған ашулан керег жоқ ол чурк ғой,nazism

3,ахпхахп чуркаларды қуып шығамыз,ахпхах чурка қу шығ,nazism

3,дұрыс айтасың тура чурка,дұрыс ай тура чурк,nazism

3,басқаларды чурка деуші өзің чуркасың сондықтан бар демал,басқа чурк деуші өзің чурк сондықтан бар демал,nazism

3,жауап берудің өзі қорқынышты чурка мені чурка дейді нонсенс бұл күнге де жеттік,жауап беру өзі қорқыныш чурк мен чурк де нонсенс бұл күн де же,nazism

3,мен де соны айтамын жоғарыда комментарийде ешкімнің де жай тұрмағанын айтады содан кейін қожайынмен сөйлестік сөйлесудің керегі жоқ оларды қуу керек чурка,мен де соны ай жоғары комментарий ешкі де жай тұрма айт содан кейін қожайын сөйле сөйлесу керег жоқ ол қу керек чурк,nazism

3,чуркалардың ойы түкке тұрмайды,чурка ой түк тұрма,nazism

3,приорадағы чуркалар,приора чурка,nazism

3,чуркалардың ойы түкке тұрмайды олар өте қызғаншақ иттер,чурка ой түк тұрма олар өте қызғаншақ ит,nazism

3,чурка еврей,чурк еврей,nazism

3,тағы да чуркалар сендерді мына жерден қуу керек,тағы да чурка сен мына же қу керек,nazism

3,бұл үшін чуркаларды ұстап алып қолдарын балтамен шабу керек,бұл үшін чурка ұста алып қол балта шаб керек,nazism

3,мокшандық чурка күйіп кетпе,мокша чурк күй кетпе,nazism

3,бұл ақ аюлар қоңырларды чурка деп атағаны үшін бе солай ма,бұл ақ аю қоңыр чурк де ата үшін бе солай ма,nazism

3,топас чуркалар,топас чурка,nazism

3,жатақханада төртінші қабатта чуркалар тұрады,жатақхана төртінші қабат чурка тұр,nazism

3,сонша батырланып отырсың ба чурка,сонша батырлан отыр ба чурк,nazism

3,тағы чуркалар,тағы чурка,nazism

3,бізде ресейліктер чуркалар,біз ресейлік чурка,nazism

3,досым сен біреуді чурка дегің келе ме өзің чуркаға ұқсайсың ғой еврейлер есіме түсіп кетті мен әлі жауап алмадым пушкин эфиоп паблик,дос сен біреу чурк де келе ме өзің чурка ұқса ғой еврей ес түс кет мен әлі жауап алма пушкин эфио паблик,nazism

3,чурка деймін ал сен жоқ деп бостан босқа ашуланасың,чурк де ал сен жоқ де бос бос ашулан,nazism

3,ол өзінің чурка гастарбайтер екенін ұмытып кетті бірақ оған өмір есіне салды,ол өзінің чурк гастарбай екен ұмыт кет бірақ оған өмір ес сал,nazism

3,бірақ сендер шынында да чуркасыңдар ашуланудың керегі жоқ,бірақ сен шын да чурк ашулан керег жоқ,nazism

3,чурка мен мына жерде сендердің айрандарыңды таратып жатқанын естідім аузыңды жап қолыңнан түк келмейді,чурк мен мына же сен айран тарат жат есті ауз жап қол түк келме,nazism

3,бұл менің пәтеріме қатысы болмаса сөйлемес едім бірақ күн сайын чуркалар өз реніштерін жеткізуде,бұл менің пәтер қатыс болма сөйлемес ед бірақ күн сайын чурка өз реніш жеткіз,nazism

3,сенің миың насыбайдан құралған чурка,сенің ми насыбай құрал чурк,nazism

3,чурка өзінің алғашқы инстинктін білдіруде уа уа уа банан жейсің бе макака,чурк өзінің алғашқы инстинкт білдір уа уа уа банан же бе макака,nazism

3,бұл чуркалардың мал екенін тағы бір мәрте дәлелдейді,бұл чурка мал екен тағы бір мәрте дәлелде,nazism

3,чурка мал олар африкада да чурка,чурк мал олар африка да чурк,nazism

3,барлығын өлтіремін деп неменеге барлық пабликта жар саласың неғып отырсың бар чуркаларды өлтір әлде бассыз қаламын деп қорқасың ба,барлығ өлтір де немене барлық паблик жар сал неғ отыр бар чурка өлтір әлде бас қал де қор ба,nazism

3,чурка өліп қал чурка,чурк өл қал чурк,nazism

3,сені чурка деген сөз ренжітті ме қара нәсілді дұрыс жігіттер бар және афроамериканец яғни негр бар ал азиаттар мен кавказдықтар чуркалар түсінесің бе,сен чурк де сөз ренжі ме қара нәсіл дұрыс жігіт бар және афроамериканец яғни нег бар ал азиат мен кавказдық чурка түс бе,nazism

3,иә чуркалар көбейіп кетті олар жергілікті халыққа жол берер емес ол жайлы ақпарат бар,иә чурка көбей кет олар жергілікті халық жол бер емес ол жайлы ақпарат бар,nazism

3,өз елдерінде не істесе де өздер білсін чурка мал маған жазба егер соғыс болса ең бірінші сені атамын,өз ел не істе де өз біл чурк мал маған жазба егер соғыс бол ең бірінші сен ат,nazism

3,чурканы қорғайын деп шықтың ба,чурка қорға де шық ба,nazism

3,чурка заң үйретуде қызық,чурк заң үйрет қызық,nazism

3,чурка мал маған бір нәрсе дегің келе ме лайк жинаймын деп әуре болма,чурк мал маған бір нәрсе де келе ме лайк жина де әуре болма,nazism

3,бұрын қандай чурка едің әлі сондайсың,бұрын қандай чурк ед әлі сонда,nazism

3,колхозда тудың сол жақта өмір сүресің чурка,колхоз ту сол жақта өмір сүр чурк,nazism

3,сол баяғы адамдар ақша берсең болды чуркалар бала тауып алыпты,сол баяғы адам ақша бер бол чурка бала тау ал,nazism

3,менің өмірімдегі тағы бір сайқал чурка,менің өмірім тағы бір сайқал чурк,nazism

3,чурка мұны сейф деп ойлаған шығар,чурк мұны сейф де ойла шығар,nazism

3,не деген ұятсыз чурка,не де ұят чурк,nazism

3,есің дұрыс па чурканы айнадан көресің жынды,ес дұрыс па чурка айна көр жын,nazism

3,бұл әйел чурка,бұл әйел чурк,nazism

3,чурканы ұстаймын деп ойлайсың ба,чурка ұста де ойла ба,nazism

3,чурка алдымен маған ақша бер сосын хабарлас,чурк алд маған ақша бер сосын хабарлас,nazism

3,олармен сөйлесу пайдасыз олар шынымен чурка тек күшті ғана түсінеді басқаларын әлсіз деп есептейді қазір құртпасақ құтырып кетеді сендерді өлтіреміз дейді сондықтан дайындалыңдар жақында соғыс болады,олармен сөйлесу пай олар шын чурк тек күшті ғана түсін басқа әлсіз де есепте қазір құртпа құтыр кет сен өлтір де сондықтан дайындал жақында соғыс бол,nazism

3,чурканы пешке салып өртеу керек,чурка пеш сал өрте керек,nazism

3,шеф айнур чуркадан шкафта тығылып отыр,шеф айну чурка шкаф тығыл отыр,nazism

3,бұл сенің шын атың мен тегің болса сен чуркасың анаңнан сұра қай аула сыпырушыдан туғаныңды,бұл сенің шын ат мен те бол сен чурк ана сұра қай аула сыпырушы ту,nazism

3,басқаларды өзіңдей деп ойлама өзіңе еселеп қайтады чурка есуас мал екенсің тек адам өлтіруші ғана осылай жауап береді сен адам өлтірушісің сен істедің,басқа өзің де де ойлама өз еселе қайт чурк есуас мал екен тек адам өлтіруш ғана осылай жауап бер сен адам өлтіруш сен істе,nazism

3,чуркалардың басында өз тарақандары жүгіріп жүреді,чурка бас өз тара жүгір жүр,nazism

3,кеш ояндық бауырым ертерек ойлану керек еді бірақ сен ойлана алмайсың ғой сен чуркасың ғой енді жауап беру керек,кеш оян бауыр ертерек ойлан керек ед бірақ сен ойлан алма ғой сен чурк ғой енді жауап беру керек,nazism

3,түркі халықтары чуркалар,түркі халық чурка,nazism

3,шенділердің көбі чуркаларды жалдайды деймін,шенді көбі чурка жалда де,nazism

3,ақылды чурка ахахахах жақсы әзіл,ақылды чурк ахахахах жақсы әзіл,nazism

3,мұны маған чурка айтып тұр ма,мұны маған чурк айт тұр ма,nazism

3,чуркалар террорист пен зорлаушылар дұрысы тығылып қал чучело,чурка террорист пен зорлаушы дұрыс тығыл қал чучело,nazism

3,чуркаларды қыру керек,чурка қыр керек,nazism

3,ұлты үшін не үшін ашуланады чурканы чурка деу айып па,ұл үшін не үшін ашулан чурка чурк деу айып па,nazism

1,жынды негрлерге денсаулық тілеймін,жын негр денсаулық тіле,racism

3,чуркалар мен хачтарға бәрібір,чурка мен хач бәрібір,nazism

3,чурка қара сия,чурк қара сия,nazism

3,оның чуркалар туралы не айтатынын білмеймін бірақ батыста оларды caucasian race дейді ал урус азиаттар,оның чурка туралы не айт білме бірақ батыс ол caucasian race де ал урус азиат,nazism

3,чуркалар сенің жаныңды шығарады тұқымыңды құртады,чурка сенің жан шығар тұқым құрт,nazism

3,ахахпхпхпхпх чурканы банға жіберіпті,ахахпхпхпхпх чурка бан жібер,nazism

3,чуркаларды қуу керек сен де құры,чурка қу керек сен де құр,nazism

3,сенің әкеңді чурка деп есептеймін менімен бұлай сөйлесуге саған ешкім рұқсат берген жоқ,сенің әке чурк де есепте менімен бұлай сөйлес саған ешкім рұқсат бер жоқ,nazism

3,мені қайдан таптың чурка неге тудың екен сен сенен мынадай сөзді есту тым күлкілі,мен қайдан тап чурк неге ту екен сен сенен мынадай сөз ест тым күлкіл,nazism

3,топас чурка жерімізге келіп ойран салуда өз елінде оны өлтіріп тастар еді,топас чурк же кел ойран сал өз ел оны өлтір тас ед,nazism

3,чуркалар рэпте,чурка рэп,nazism

3,чуркалар қарбызда жатады сосын оларды біз жейміз,чурка қарбыз жат сосын ол біз же,nazism

3,қашаннан бері чурканы адам деп есептейміз,қашан бері чурка адам де есепте,nazism

3,жынды екенсің сен чурканы жақсы көргені несі,жын екен сен чурка жақсы көр не,nazism

3,сен топас чуркасың сен қалай несие алмақшысың саған рұқсат жоқ,сен топас чурк сен қалай несие алмақш саған рұқсат жоқ,nazism

3,қаралар мен чуркаларды депортациялау керек,қара мен чурка депортацияла керек,nazism

3,не айтып отырсың топас чурка,не айт отыр топас чурк,nazism

3,бір чурканы депортацияладық енді тағы біреуін осылай жиі болса екен,бір чурка депортацияла енді тағы біреу осылай жиі бол екен,nazism

3,еліңе қайт чурка,ел қайт чурк,nazism

3,жбір чурка адамның басын кеседі екіншісі тыныш жүреді еш айырмашылығы жоқ,жбі чурк адам басын кес екінші тыныш жүр еш айырмашылығ жоқ,nazism

3,чурка ол чурка басқа не айта аламын,чурк ол чурк басқа не ай ал,nazism

3,сен чуркасың,сен чурк,nazism

3,сібірде чуркалары бұлай жүрмейді,сібір чурка бұлай жүрме,nazism

3,чуркалар шығар,чурка шығар,nazism

3,менің ақш та еуропада таныстарым бар ол жақты чуркаларды қуады екен,менің ақш та еуропа таныс бар ол жақ чурка қу екен,nazism

3,чуркалар мен нариктардан басқа адамдар да өмір сүреді,чурка мен нарик басқа адам да өмір сүр,nazism

3,онда жоғал мен әр кезде сені чуркалардың ішінен іздеймін деп шаршадым,онда жоғал мен әр кезде сен чурка іш ізде де шарша,nazism

3,жидтар жалықтырды,жид жалық,nazism

3,жидтар ғана адамдар қалмады мына жақта,жид ғана адам қалма мына жақта,nazism

3,лол сен жидсың ба,лол сен жид ба,nazism

3,мен сыйлықты қабылдамаймын оны өзіңе қалдырсаң болады жидтардан сыйлық алмаймын,мен сыйлық қабылдама оны өз қалдыр бол жид сыйлық алма,nazism

3,жігіттер батырсыңдар жидтарға бұл әлемде сай келетін жаза жоқ әлемді өзгертсек бір нәрсе түсінер,жігіт батыр жид бұл әлем сай кел жаза жоқ әлем өзгерт бір нәрсе түсін,nazism

3,жидтың жанын шығардық,жид жан шығар,nazism

3,жидтар нацисттардың аяғын сүюі керек олар палестинада жид елін құруға көмектесті арабтар жынды өздеріне жидтарды шақырып алды енді тұншығып отыр,жид нацист аяғ сүю керек олар палестина жид ел құр көмекте араб жын өз жид шақыр алды енді тұншығ отыр,nazism

3,несіне таң қаламыз жидтарға бұл тиімді жұмыс істеу жоқ бұл олар туралы емес топас халық барлық әлем соларға міндеті сияқты іздеп көріңдер қызықты факт бар олар туралы,не таң қал жид бұл тиімді жұмыс істеу жоқ бұл олар туралы емес топас халық барлық әлем сол міндет сияқты ізде көр қызықты факт бар олар туралы,nazism

3,бірінщіден жид пен еврей синоним сенің атаң жид па,бірінщі жид пен еврей синони сенің ата жид па,nazism

3,елім оян соғысатын кез келді қаралар мен чуркаларға қарсы соғыс,ел оян соғыс кез кел қара мен чурка қарсы соғыс,nazism

3,екеуі де жид,екеу де жид,nazism

3,ноның артында еш нәрсе жоқ жид та батыр да,но арт еш нәрсе жоқ жид та батыр да,nazism

3,қаншама жид,қанша жид,nazism

3,жидтарды ұстаймыз,жид ұста,nazism

3,сені өз жидтарың жояды,сен өз жид жоя,nazism

3,иә бауырым мен сені түсіндім қазір барлығын жидтар басқаруда,иә бауыр мен сен түс қазір барлығ жид басқар,nazism

3,жид немене соншама патша ма,жид немен сонша патша ма,nazism

3,жүз пайыз дұрыс барлығын жидтар ұрлады,жүз пайыз дұрыс барлығ жид ұрл,nazism

3,неге жидтар басқарушы ал қалғандары орындаушы,неге жид басқарушы ал қал орындаушы,nazism

3,қыз жид сенің аузыңды жапты жарайсың,қыз жид сенің ауз жап жара,nazism

3,жид хохол мен төменгі интернет паблигі,жид хохол мен төменгі интернет паблиг,nazism

3,жид биттеген сорлы,жид битте сорл,nazism

3,жидтің насихаттық ашуы керемет,жид насихат аш керемет,nazism

3,онда достарыңды шақырыңдар көмекке тіпті жид асылып қалды,онда дос шақыр көмек тіпті жид асыл қал,nazism

3,тағы қанша адам келер екен екі тапанша алып жидты көмекке шақырған тиімді сияқты қолына балта алып ақша табуды онсыз да біледі,тағы қанша адам кел екен екі тапанша алып жид көмек шақыр тиімді сияқты қол балта алып ақша табу онсыз да біл,nazism

3,жидті өлтіру керек,жид өлтір керек,nazism

3,ол жид,ол жид,nazism

3,жид украинаны басқару керек,жид украина басқару керек,nazism

3,биттеген жид,битте жид,nazism

4,оларды түсіну және өз елінде әскери жаттығулар жүргізу үшін талибанмен сөйлесу керек,ол түсіну және өз ел әскери жаттығу жүргізу үшін талибан сөйлесу керек,violent

4,біз талибанды күтеміз олар ағылшындарға жағымды болады деп үміттенемін өйткені олар өте мейірімді болды,біз талибан күт олар ағылшын жағымды бол де үміттен өйткені олар өте мейірімді бол,violent

4,барлық елдердің террористері бірігіңіз әлемдік терроризмнің астанасы,барлық ел террорис бірі әлемдік терроризм астана,violent

3,соңғы кездері мен хачтарды ұнатпаймын кешіріңіз мен олардың басқаша қалай аталатынын білмеймін олар өздерін әлдеқайда нашар ұстайды бәрі бірдей емес бірақ бәрібір,соңғы кез мен хач ұнатпа кешір мен олардың басқаша қалай атал білме олар өз әлдеқайда нашар ұста бәрі бірде емес бірақ бәрібір,nazism

3,тәжіктер өзбектер олардың барлығы еңбекқор құлдар бір тиынға жұмыс жасаңыз сен еуропада ешкімге керек емессің ал одан да көп ақш та қайда тусаң со лжаққа пайдалы бол,тәжік өзбек олардың барлығ еңбекқор құл бір тиын жұмыс жаса сен еуропа ешкім керек емес ал одан да көп ақш та қайда ту со лжақ пайдалы бол,nazism

1,ақтар ақ болғаны үшін басым болуы керек,ақ ақ бол үшін басым бол керек,racism

1,ақтардың iq жоғары ақтар өркениетті және әдепті өйткені нағыз ақтар скандинавиялық қан ұлы жауынгерлердің ұрпақтары,ақ iq жоғары ақ өркениет және әдеп өйткені нағыз ақ скандинавиялық қан ұлы жауынгер ұрпақ,racism

3,ал сол адамдарды басқа ұлт мен оларды әрқашан екінші үшінші дәрежелі адамдар ретінде түсінемін екіншісі қысқа қарғыбауда қызмет ете алады үшіншісі жоқ тек унтермендердің физикалық жойылуы керек,ал сол адам басқа ұлт мен ол әрқашан екінші үшінші дәрежелі адам рет түс екінші қыс қарғыбау қызмет ет ал үшінші жоқ тек унтермен физикалық жойыл керек,nazism

1,жалпы негрде тек үш ақ нәрсе болуы керек көзі тістері және иесі,жалпы нег тек үш ақ нәрсе бол керек көз тіс және иесі,racism

4,бір адам өлтірмеуі керек егер ол өлтірсе ол қылмыскер ол өлтіруші болады бірақ мемлекет немесе халық қалағанынша өлтіре алады және ол кісі өлтіру болмайды ол жақсы іс,бір адам өлтірме керек егер ол өлтір ол қылмыскер ол өлтіруш бол бірақ мемлекет немесе халық қалағанынш өлтір ал және ол кісі өлтір болма ол жақсы іс,violent

4,игил нағыз мұсылмандар,игил нағыз мұсылман,violent

4,игил ге жеңіс тілеймін,игил ге жеңіс тіле,violent

4,игил қолдаймын,игил қолда,violent

4,игил жұмыс істеңдер бауырлар,игил жұмыс істе бауыр,violent

4,игил ұзақ өмір сүрсін кәпірлермен күресетін жауынгерлерге даңқ болсын,игил ұзақ өмір сүр кәпір күрес жауынгер даңқ бол,violent

4,игил бауырлар біз оларға қарыздармыз,игил бауыр біз оларға қарыз,violent

4,чемодан вокзал игил,чемо вокзал игил,violent

4,барлығын қырамыз,барлығ қыр,violent

4,мен игил ға сенім мен дұрыс өмір салтын зерттеуге бардым,мен игил ға сенім мен дұрыс өмір салт зертте бар,violent

4,алдымен түркияға сосын игил ге кетсем ғой,алд түркия сосын игил ге кет ғой,violent

4,ойланып ойланып игил ге баратын болдым ол жақта қауіпсіз,ойлан ойлан игил ге бар бол ол жақта қауіпсіз,violent

4,игил шығыс азия жарты еуропаны жаулап алады,игил шығыс азия жарты еуропа жаула ал,violent

4,сириядағы шешендер орысша сөйлем жиһадқа ақша сұрады көмектесейік,сирия шешен орысш сөйле жиһад ақша сұр көмектес,violent

4,құрметті бауырлар мен әпкелер біз соғысқа тиісті түрде қатыспағандықтан қаншама мүмкіндікті жіберіп алдық,құрметті бауыр мен әпке біз соғыс тиісті түр қатыспа қанша мүмкіндік жібер ал,violent

4,қайтып келгендеріңде сендерді өлтіреміз,қайт келген сен өлтір,violent

4,сириядағы біз жүргізіп жатқан жиһад міндетті болып табылады,сирия біз жүргіз жат жиһад міндетті бол таб,violent

3,орыстар өз істеріне жауап беруі керек жауап беретін уақыт міндетті түрде келеді,орыс өз іс жауап беруі керек жауап бер уақыт міндетті түр кел,nazism

4,жиһадтағы жеңіске апарар жолдардың бірі осы қосылыңыздар,жиһад жеңіс апар жол бірі осы қосыл,violent

4,біз өлтірілуі тиіс болғандарды ғана өлтіреміз,біз өлтіріл тиіс бол ғана өлтір,violent

4,егер олар батыстағыларды өлтірсе мен ренжімеймін,егер олар батыстағы өлтір мен ренжіме,violent

4,біз адамды өлтіруіміз керек еді,біз адам өлтір керек ед,violent

4,бауырлар мен сіздерді соғысқа қатысуға өлтіруге шақырамын,бауыр мен сіз соғыс қатыс өлтір шақыр,violent

4,мен басқа ұлдарымның гуманитарлық ұйымдарда қызмет еткенінен ұяламын мен оларды қару ұстауы керек деп есептеймін,мен басқа ұл гуманитарлық ұйым қызмет ет ұял мен ол қару ұста керек де есепте,violent

4,қасиетті соғыста бауырларыма көмектесу үшін аттанып барамын,қасиетті соғыс бауыр көмектес үшін аттан бар,violent

3,біз барлық американдықтарды өлтіреміз бізге қан керек,біз барлық американдық өлтір бізге қан керек,nazism

4,мен фронттың алдыңғы жағындамын өзімді өлтіруге дайынмын,мен фронт алдыңғы жағ өз өлтір дайын,violent

4,барлық уақыттағы сияқты қазір де барлығын қарумен ғана шешуге болады,барлық уақыт сияқты қазір де барлығ қар ғана шеш бол,violent

4,мына жаққа қарумен келіңдер сендерді екі сағатта жоқ қыламыз,мына жақ қар кел сен екі сағат жоқ қыл,violent

4,біздің балалар жиһадқа деген сүйіспеншілікпен тәрбиеленуі керек,біздің бала жиһад де сүйіспеншілік тәрбиелен керек,violent

4,мен жиһадта ұзақ емеспін сонымен бірге мен осында болған кезім де әрдайым құлшыныспен өткен жоқ,мен жиһад ұзақ емес сонымен бірге мен осында бол кез де әрдайым құлшыныс өткен жоқ,violent

4,жиһадқа бодибилдер пауэрлифтер немесе спорт шеберлері қажет емес бірақ жиһадқа жүгіре алатын адамдар қажет ұзақ қашықтыққа жылдам жүру және мұның бәрі артындағы салмақпен орындалуы керек,жиһад бодибил пауэрлифтер немесе спорт шебер қажет емес бірақ жиһад жүгір ал адам қажет ұзақ қашықтық жылдам жүру және мұның бәрі арт салмақ орындал керек,violent

4,жиһад белгілі бір физикалық дайындықты қажет етеді оны сіз бүгін бастай аласыз қымбатты бауырым,жиһад белгілі бір физикалық дайындық қажет ет оны сіз бүгін баста ал қымбатты бауыр,violent

4,жиһад жерлеріне келу бұл жолдың басы ғана емес соңы,жиһад жер келу бұл жол бас ғана емес соңы,violent

4,неліктен қорлауға шыдау керек басқалармен бірге бірігіп осы дұрыс емес студенттерге басына соққы беріңіз немесе оларға қарсы тұрыңыз есіңізде болсын сіз ұлы жауынгерлердің ұрпақтарысыз,неліктен қорла шыда керек басқа бірге біріг осы

дұрыс емес студент бас соққы бер немесе оларға қарсы тұр ес бол сіз ұлы жауынгер ұрпақтар,violent

4,джихадқа бару керек,джихад бару керек,violent

4,атом бомбасы барлығын шешеді,атом бомба барлығ шеш,violent

4,сол талибтер келсе екен ең болмаса,сол талиб кел екен ең болма,violent

4,love jihadi жиһадты сүйемін,love jihadi жиһад сүй,violent

4,сирияға баруға шешім қабылдадым сендерге де насихаттаймын,сирия бар шешім қабылда сен де насихатта,violent

3,пәкістан құрысын,пәкістан құрыс,nazism

4,мен сирияға барғым келеді мен жастамын,мен сирия бар кел мен жас,violent

4,тоқаевтың қолынан және шалдың қолынан өлгеннен гөрі жиһадта өлген жақсы,тоқаев қол және шал қол өл гөрі жиһад өлген жақсы,violent

4,сұхбатында ол ukimet тіталибан айыптайды деп айтты алтайда ақпарат көп мүмкін талибан өз елінің патриоттық қаһармандары шығар интернеттегі бейне,сұхбат ол ukimet тіталибан айыпта де ай алтай ақпарат көп мүмкін талибан өз ел патриоттық қаһарман шығар интернет бейне,violent

4,барлығын да өлтіріңдер,барлығ да өлтір,violent

4,олардың қоймаларын жарыңдар,олардың қойма жар,violent

4,мен сирияға жиһадқа барғым келеді денсаулығым жақсы әскерде болдым,мен сирия жиһад бар кел денсаулығ жақсы әскер бол,violent

4,соғысқа бару керек қатысу керек,соғыс бару керек қатысу керек,violent

3,соғысу керек қытаймен басқа амал жоқ қырып тастау керек шетінен билік милік демей қыру керек шеттерінен,соғыс керек қытай басқа амал жоқ қыр тастау керек шет билік милік деме қыр керек шет,nazism

4,әкеммен сирияда жылдай бірге өмір сүрдік ағам бұл жерге өлу үшін келгенін айтып өзін соған толықтай дайындады,әке сирия жылдай бірге өмір сүр аға бұл же өл үшін кел айт өз соған толықта дайында,violent

3,қытайлармен қақтығысты ушықтырып отыру керек халықты көтеру үшін кездескен жігіттерге ескерту қытайлармен қақтығысты шығарып тұрыңдар,қытай қақтығыс ушықтыр отыру керек халық көтеру үшін кездес жігіт ескерту қытай қақтығыс шығар тұр,nazism

4,қыру керек басқа амал жоқ сияқты,қыр керек басқа амал жоқ сияқты,violent

3,қытайлармен соғысу керек қасық қан қалғанша былай мал болып жүргенше соғысып бар болу керек немесе жоқ болу керек намысты берменңдер соғыс болса

қашпаймын өзім шығамын,қытай соғыс керек қасық қан қалғанш былай мал бол жүргенш соғыс бар болу керек немесе жоқ болу керек намыс берме соғыс бол қашпа өзім шығ,nazism

4,қазақтар ояныңдар жау бұлар аямаңдар іштегі тыңшылар бұлар,қазақ оян жау бұлар аяма іш тыңшы бұлар,violent

4,ату керек тамырымен жойылғандай,ату керек тамыр жойылғанда,violent

4,соғыс болса болсын жемқор иттерді қуып журіп өлтіреміз,соғыс бол бол жемқор ит қу жур өлтір,violent

4,адайларға қару берін талібтен кем емес астананы басып алады,адай қару берін таліб кем емес астана бас ал,violent

3,қытай қазақтың жауы оян қазақ болмайтын нәрсе көруден бас тарт қытайға қарсы ақпараттық соғыс жүргізу қажет,қытай қазақ жау оян қазақ болма нәрсе көру бас тарт қытай қарсы ақпараттық соғыс жүргізу қажет,nazism

3,қытайға өлім донғандарды қытайға жер аудару керек оян қазақ,қытай өлім дон қытай жер аудару керек оян қазақ,nazism

4,атом бомбасын әрине ақш пен бірге сатып алу керек,атом бомба әрине ақш пен бірге сат алу керек,violent

4,оңтүстік шығысынан шабуылдайды,оңтүстік шығыс шабуылда,violent

4,бірін қалдырмай өлтіріңдер капірлерді,бір қалдырма өлтір капір,violent

4,талибтердің позициясын қолдайық,талиб позиция қолда,violent

4,талибан бүкіл елді басқарған жақсы болар еді ал басқа топтар бұл шетелдіктердің агенттері,талибан бүкіл ел басқар жақсы бол ед ал басқа топ бұл шетелдік агент,violent

4,ауғанстандағы талибан сияқты біздің қазақтарымыз болса деп тілеймін,ауғанстан талибан сияқты біздің қазақ бол де тіле,violent

4,мен күресте бар нәрсенің тағдырын көремін егер ешкім өлгісі келмесе күрестен қашуға болмайды,мен күрес бар нәрсе тағдыр көр егер ешкім өл келме күрес қаш болма,violent

4,қорғаныс джихады жарияланды,қорғаныс джих жария ла,violent

3,орысша сөйлейтіндерді ұрыңдар,орысш сөйле ұр,nazism

3,дұры сқазақша сөйлеу керек орыс шошқаның тілін құрту керек,дұ сқазақш сөйлеу керек орыс шошқа тіл құрт керек,nazism

3,көзі қысық шаян жеген сасықтардан құтыла алмай жүргенімізде енді мынау шошқа жейтін сасықтар шықты ма,көз қысық шаян же сасық құтыл алма жүр енді мынау шошқа же сасық шық ма,nazism

3,орысты қуып жіберу қерек орыс мектептерін жабу қерек,ор қу жіберу қерек орыс мектеп жаб қерек,nazism

3,қазақтардың жауы ресейлік еврейлер,қазақ жау ресейлік еврей,nazism

4,соғыс ашу керек өз анау жерімізді алу керек омбыдан бастап,соғыс ашу керек өз анау же алу керек омбы баста,violent

3,сасық орыс тіпті дәрет алмайсыңдар сасықтар,сасық орыс тіпті дәрет алма сасық,nazism

3,мен кеттім орыстармен соғысып жүрмін,мен кет орыс соғыс жүр,nazism

1,инкогнито жылан сияқтысың мына негр кокосты тістеді,инкогнито жылан сияқты мына нег кокос тіст,racism

1,қарғыс атсын мен негрді жақсы көремін бірақ оның досы менің хомяктың есегіне ұқсайтынын қалай айта аламын бұл тіпті әзіл емес,қарғыс ат мен нег жақсы көр бірақ оның дос менің хомяк есег ұқса қалай ай ал бұл тіпті әзіл емес,racism

1,күні бойы менің ниггерім калиден кейін ақылсыз өмір сүру,күн бойы менің нигг кали кейін ақыл өмір сүру,racism

1,сіз ботқаны қалай алу керектігін білесіз нигга және уайымдамаңыз жақын арада кездескенше,сіз ботқа қалай алу керектіг біл нигг және уайымдама жақын ара кездескенш,racism

1,о нигер менің көзім ауырады,о ниг менің көз ауыр,racism

1,жоқ жоқ жоқ ниггер кеше түнде ұйықтамады менің досымды кешіріңіз,жоқ жоқ жоқ нигг кеше түн ұйықтам менің дос кешір,racism

1,ааа мен полицияны жек көремін нигга жай жүре алады және тыныш темекі шегеді,аа мен полиция жек көр нигг жай жүр ал және тыныш темекі шег,racism

1,нигерия өнеркәсібі үшін бәрі жарқырайды оны алтын біледі,нигерия өнеркәсіб үшін бәрі жарқыра оны алтын біл,racism

1,тсс тсс ниггер бұл болатынын білмеді бұл екінші рет,тсс тсс нигг бұл бол білме бұл екінші рет,racism

1,ниггер мені елеме,нигг мен елеме,racism

1,жаңалықтарыңызды оқығаныңызға рахмет мен негрге қызықты боламын ай мен сіздің концерттеріңізге келе аламын мен жастамын,жаңалық оқы рахмет мен нег қызықты бол ай мен сіздің концерт келе ал мен жас,racism

1,ниггер станоктары ол ысқырып иісі шықты,нигг станок ол ысқыр иіс шық,racism

1,мен саған айттым бірақ егер негрлер саған үнемі қарап тұрса сен жындысың мен сені әрқашан көрдім сен қалаған кезде,мен саған ай бірақ егер негр саған үнемі қара тұр сен жынд мен сен әрқашан көр сен қала кезде,racism

1,енің айналамда бұл негрлер жөтеліп жатыр мен дәл гермафоб емеспін бірақ майлықтар қайда клорокс лизол спрейі және күшейткіш,ен айнала бұл негр жөтел жатыр мен дәл гермафоб емес бірақ майлық қайда клорокс лизол спрей және күшейткіш,racism

1,сізде қарақат бар ма маған ақпаратыңызды жіберіңіз нигерді аяқтайық біз демалыс алып жазды таба аламыз ба,сіз қарақат бар ма маған ақпарат жіб ниг аяқта біз демалыс алып жаз таб ал ба,racism

1,иә бұл негр көп ұрып тұруы мүмкін мен қателестім және асыра айттым,иә бұл нег көп ұр тұр мүмкін мен қателес және асыра ай,racism

1,қарғыс атқан негрлер мен полиция мүлдем жұмыс істемейді бұл ақымақ киа оларды бізге шақырды,қарғыс ат негр мен полиция мүлдем жұмыс істеме бұл ақы ки ол бізге шақ,racism

1,жұмысшы нигга тен ге дейін өте қызықты,жұмысшы нигг тен ге дейін өте қызықты,racism

1,мен нигермен бірге чилиді жей алмаймын,мен нигер бірге чили же алма,racism

1,қайғылы қайғы күлкілі сіз машинасыз қалай жұмыс істейсіз нигга сен кереметсің,қайғылы қайғы күлкіл сіз машина қалай жұмыс істе нигг сен керемет,racism

1,л нигер иттері сен маған енді қоңырау шалмайсың,л ниг ит сен маған енді қоңырау шалма,racism

1,кейбір бай негрлер келіп оны жалға алды өзімшілдік біз жау аумағындамыз,кейбір бай негр кел оны жалға алды өзімш біз жау аумағ,racism

1,сәлем осы үйдегі барлық ниггерлерді қараңыз мен кездейсоқ мәтіндік хабарламалармен шайқастарымызды өткізіп жібердім,сәлем осы үйдегі барлық ниггер қара мен кездейсоқ мәтін хабарлама шайқас өткіз жібер,racism

1,сондықтан негрлер жалқаулыққа шағымданады,сондықтан негр жалқаулық шағымдан,racism

1,мойындау мен қайтадан арамшөп шегемін нигер депрессияға түседі иә қыз,мойындау мен қайта арамшөп шег ниг депрессия түс иә қыз,racism

1,мен ақсақ негрлерден шаршадым,мен ақ негр шарша,racism

1,менің twidroid trippin қосымшам мобильді интернеттен нигга твитін алды,менің twidroid trippin қосымша мобильді интернет нигг твит алды,racism

1,біздің флотта нағыз негрлер бар менің ойымша сіз армияда болуыңыз керек,біздің флот нағыз негр бар менің ойымша сіз армия бол керек,racism

1,мен білемін бірақ мен сүйемін және ниггер мұны қалай жасау керектігін біледі,мен біл бірақ мен сүй және нигг мұны қалай жасау керектіг біл,racism

1,бай негрлер маған ащы тамақ әкел жуынатын бөлмеге,бай негр маған ащ тамақ әкел жуын бөлме,racism

1,жоқ ниггер имма мемфиан,жоқ нигг им мемфиан,racism

1,бұл негрдің қара әзілі бар және мені ренжітудің қажеті жоқ,бұл нег қара әзіл бар және мен ренжіту қажет жоқ,racism

1,лмао кінәлі емес менің негрім бірақ мен салықтарды мойындаймын,лмао кінәлі емес менің нег бірақ мен салық мойында,racism

1,кейбір негрлердің соңынан еру ақымақтық емес оны көру өте өкінішті,кейбір негр соң ер ақымақтық емес оны көру өте өкініш,racism

1,сіз бір рет жынды болдыңыз менің негрімді атады,сіз бір рет жын бол менің нег ат,racism

1,жарайды жарайды иә олар клубта болды бұл ақылсыз негрлер еді аяғы ауырады,жарайды жарайды иә олар клуб бол бұл ақыл негр ед аяғ ауыр,racism

1,сондықтан steam steam бүгін мені тастап кетеді мен бұл негрді сағынамын мм,сондықтан steam steam бүгін мен таста кет мен бұл нег сағ мм,racism

1,мен мұны жай ғана көңілді деп таптым және бұл негрге күн сайын хабарлама жібермеймін,мен мұны жай ғана көңіл де тап және бұл нег күн сайын хабарлама жіберме,racism

1,мен қарсы емеспін нигга мен жолды сезінемін,мен қарсы емес нигг мен жол сез,racism

1,мен негрлерімді сағынатын боламын,мен негр сағын бол,racism

1,мен ашуланамын ниггер менің найзағайымды қабылдамайды,мен ашулан нигг менің найзағай қабылдама,racism

1,дұрыс маған негр керек,дұрыс маған нег керек,racism

1,мен виана мен викирамен сөйлесемін және бұл негр менімен сөйлескісі келеді эм м,мен виан мен викира сөйлес және бұл нег менімен сөйлес кел эм м,racism

1,екінші жылдық ниггерфест және мен ол жерде болмаймын,екінші жылдық ниггерфест және мен ол же болма,racism

1,мен оның қызының өгей шешесі екенімді білдім хаха қандай желкек эммм негрлер жоқ ойын ойнауға әлі ерте мен оның жақсы болғанына қуаныштымын,мен оның қыз өге шеше екен біл хах қандай желкек эмм негр жоқ ойын ойна әлі ерте мен оның жақсы бол қуанышты,racism

1,о бұл жұмыстан үйге баратын негр ме бұл үйде тым көп адам бар,о бұл жұмыс үй бар нег ме бұл үй тым көп адам бар,racism

1,ен бұл негрге қуаныштымын деп айтқанды ұнатпаймын мен бәрін айттым,ен бұл нег қуанышты де айт ұнатпа мен бәрін ай,racism

1,әрине мен негр девринмен менің үйім клубын сағындым міне бәрі мен ақша таптым,әрине мен нег деврин менің үй клуб сағ міне бәрі мен ақша тап,racism

1,ақша негрлерді елестету мүмкін емес етеді өйткені бұл шынымен қайғылы,ақша негр елестет мүмкін емес ет өйткені бұл шын қайғылы,racism

1,мен мұны білемін сенің негрің мойнын жоғары ұстайды,мен мұны біл сенің нег мойн жоғары ұста,racism

1,бұл жаңбырда негрлер тұра ма кім біледі,бұл жаңбыр негр тұр ма кім біл,racism

1,қыз қыз мен жаңа ғана кетіп қалдым мен оны алдым өйткені мен жергілікті емханада жұмыс істедім нигер қалай әрекет ету керектігін білмейді,қыз қыз мен жаңа ғана кет қал мен оны ал өйткені мен жергілікті емхана жұмыс істе ниг қалай әрекет ету керектіг білме,racism

1,бұл сіз те жұмыстан шыққан кезде болады содан кейін сіз жапа есімді негрді алып кептелістер туралы ұмытып кетесіз,бұл сіз те жұмыс шық кезде бол содан кейін сіз жап ес нег алып кептеліс туралы ұмыт кет,racism

1,мен негрлерден қатты шаршадым ваннаға арналған көбік олардың бастарын тазалау керек,мен негр қатты шарша ванна арнал көбік олардың бас тазалау керек,racism

1,мен өзімді керемет сезінемін мен сенің болғаныңды қалаймын бұл негр мас қазір ол есінен танып қалды,мен өз керемет сез мен сенің бол қала бұл нег мас қазір ол ес тан қал,racism

1,бұл негрлер dvd ді көргісі келеді мен үйді сағындым,бұл негр dvd ді көр кел мен үй сағ,racism

1,негрді күтіп отырмын,нег күт отыр,racism

1,мені оңға және солға жылжытыңыз сіз оны әлі де қадағалайсыз ол менің негрім ол керемет сондықтан менімен сиқырласаң оны басыңыз,мен оң және сол жылжы сіз оны әлі де қадағала ол менің нег ол керемет сондықтан менімен сиқырла оны бас,racism

1,негрлер маған газды қалай қайтармайды бірақ мен кетуім керек шығар мен жаз бойы клубты көрмейтін сияқтымын,негр маған газ қалай қайтарма бірақ мен кет керек шығар мен жаз бойы клуб көрме сияқты,racism

1,нигга мен оны көргім келеді,нигг мен оны көр кел,racism

1,сіз бармайсыз ба дүйсенбіде тезірек сауығып кетіңіз деп үміттенемін негрлеріңізді сағындым,сіз барма ба дүйсенбі тезірек сауығ кет де үміттен негр сағ,racism

1,ақымақтық ол да мүмкін еді ші кеш бірақ есектері сынған кейбір негрлер кредл мен күшейткішті бұзуға шешім қабылдады,ақымақтық ол да мүмкін ед ші кеш бірақ есек сын кейбір негр кредл мен күшейткіш бұз шешім қабылда,racism

1,эй герцогтар мен ашуланамын өйткені ниггер команы қаламайды ал біз ағамның туған күніне кешігіп келеміз,эй герцог мен ашулан өйткені нигг кома қалама ал біз аға туған күн кешіг кел,racism

1,жаңа ғана ұйықтап қалдым енді мен тұрып жұмысқа баруым керек мен ашуланамын менің негрім келеді сондықтан мен жұмыс істеуім керек,жаңа ғана ұйықта қал енді мен тұр жұмыс бар керек мен ашулан менің нег кел сондықтан мен жұмыс істе керек,racism

1,қорқынышты және күшейткіші бар сүйкімді негр,қорқыныш және күшейткіш бар сүйк нег,racism

1,мен бұл негрдің маған жетуіне жол бердім мен осында отырамын және суретке түсемін бұл маған ұқсамайды мен өзімді ұстауым керек,мен бұл нег маған жет жол бер мен осында отыр және сурет түс бұл маған ұқсама мен өз ұста керек,racism

1,қазіргі уақытта нигга ті басқарады біз ешқашан үйге бара алмаймыз,қазіргі уақыт нигг ті басқар біз ешқашан үй бар алма,racism

2,мына молда өтірікші сенбеңіз,мына мол өтірікш сенбе,bullying

2,міне насихатшылар еш ойланбастан өтірік айтты несімен ерекшеленеді егер сіз адамдарды адастырып жатыр десеңіз онда сіз адамдарды одан да жаман адастырасыз тағуттардың қызметшілері,міне насихатшы еш ойланбас өтірік ай не ерекшелен егер сіз адам адастыр жатыр де онда сіз адам одан да жаман адастыр тағут қызметші,bullying

2,діни надандық адам қылмыскер болып кете ме не бүкіл лагерь дін мәселесінде сауатсыз әсіресе имамдардың өз жарғылары бар не деген ақымақтық,діни надан адам қылмыскер бол кет ме не бүкіл лагерь дін мәселе сауат әсіресе имам өз жар бар не де ақымақтық,bullying

2,шынымды айтсам бұл имам шайтан сияқты,шын айт бұл имам шай сияқты,bullying

2,ибрагим ибрагимов террор сен кімсің,ибрагим ибрагимов террор сен кім,bullying

2,аурусың ба ақымақсың ба сырт келбет туралы айттың оның сөзіне сыртқы түрінің не қатысы бар,ауру ба ақы ба сырт келбет туралы ай оның сөз сыртқы түр не қатыс бар,bullying

3,бүкіл ақ үй террористер,бүкіл ақ үй террорис,nazism

2,ең қауіпті секта мемлекеттің психологтары мен имамдары,ең қауіпті секта мемлекет психолог мен имам,bullying

2,адасқандар мен кедейлер,адас мен кедей,bullying

3,қазақтар ақымақ шіркін,қазақ ақы шіркін,nazism

3,сондықтан ресей болмау керек жабайылар арамзалар,сондықтан ресей болмау керек жабайы арамза,nazism

2,ауғанстанға кет,ауғанстан кет,bullying

2,бұл қазақстан біз ауған емеспіз ауғанстанға кет онда,бұл қазақстан біз ауған емес ауғанстан кет онда,bullying

3,сені жек көретініміз аздық етеді қытай орта азияны туалетке кіргізді орыстар да ислам мемлекетін түрмеге кетірді ме,сен жек көр аз ет қытай орта азия туалет кір орыс да ислам мемлекет түрме кет ме,nazism

2,сіз маньяксыз ба әлде аурусыз ба бар жазылып ал,сіз маньяк ба әлде ауру ба бар жазыл ал,bullying

2,сіз зомбисіз,сіз зомби,bullying

2,мұндай сөздер үшін сізді тор күтіп тұр,мұндай сөз үшін сіз тор күт тұр,bullying

2,аузыңды жап мұрт мүшрик,ауз жап мұрт мүшрик,bullying

2,путин соғыс арқылы ол билікке деген құштарлық сезімін қанағаттандырады өйткені кісі өлтіру бұл адамның адам үстіндегі билігінің ең жоғарғы түрі,путин соғыс арқылы ол билік де құштарлық сез қанағаттандыр өйткені кісі өлтір бұл адам адам үст билік ең жоғарғы түр,bullying

2,путин қыздарды ұлдарды ересектер мен қарттарды өлтіру үшін соғыс жүргізеді украинада ғана емес ресейде де адамдарды азаптауға және оларды үлкен мерзімге отырғызуға болады,путин қыз ұл ересек мен қарт өлтір үшін соғыс жүргіз украина ғана емес ресей де адам азапта және ол үлкен мерзім отырғыз бол,bullying

3,мордовия билігі әйелді мал деп санайды ақымақ инкубатор оның денесін басқаруға құқығы жоқ жаңа инкубаторлар мен адамдарды дүниеге әкелуге міндетті,мордовия билік әйел мал де сана ақы инкубатор оның дене басқар құқығ жоқ жаңа инкубатор мен адам дүние әкел міндетті,nazism

2,жексұрын мисогинистік патриархалдық логика,жексұр мисогини патриарха логика,bullying

1,нигер украиншіл президент биліктен қуылды,ниг украиншіл президент билік қу,racism

3,тағы бір шексіз қиял twitter бұрыннан ақылы жоқтықтың тамаша энциклопедиясы болды жоспар керемет әрине оны жүзеге асыруға болады деп есептейік менің

ойымша келесідей болады бізге аумақты беріңіз немесе қырым аштықтан өледі қырымнан кетіңіз немесе киев ядролық күлге айналады,тағы бір шексіз қиял twitter бұрын ақылы жоқтық тамаша энциклопедия бол жоспар керемет әрине оны жүзеге асыр бол де есепте менің ойымша келесіде бол бізге аумақ бер немесе қырым аштық өл қыр кет немесе киев ядролық күл айнал,nazism

4,соғыс қылмыскері таусылған уран оқ дәрілерін қолдана бастайтынын айтты,соғыс қылмыскер таусыл уран оқ дәрі қолдан баста ай,violent

2,бір жылдан аз уақыт әйел болып өмір сүрген ер адамның алдында әйелдер тізерлейді барлық уақыттағы ең ақылды патриархиялық трюк,бір жыл аз уақыт әйел бол өмір сүр ер адам алд әйел тізерле барлық уақыт ең ақылды патриархиялық трюк,bullying

3,хохол бұл жаңа ниггер,хохол бұл жаңа нигг,nazism

3,орыстар шошқа,орыс шошқа,nazism

2,әлсіз сатушы қаншық либерал фактттар,әлсіз сатушы қаншық либерал факт,bullying

2,бұл қара адам бірақ бұл қара адам дерлік сондықтан айырмашылық бар,бұл қара адам бірақ бұл қара адам дерлік сондықтан айырмашылық бар,bullying

1,нәсілшіл қоқыс ант етемін мен ақ жігіттерді жек көремін және олардың видеодағы нигерлері ақша үшін жасағандары үшін ұялу керек,нәсілшіл қоқыс ант ет мен ақ жігіт жек көр және олардың видео нигер ақша үшін жаса үшін ұял керек,racism

2,нәсілшіл оғаш боқтықты құрт,нәсілшіл оғаш боқтық құрт,bullying

1,ақ қоқыс қызыл мойын және негрлер бірдей нәрсе тек әртүрлі түстер,ақ қоқыс қызыл мойын және негр бірде нәрсе тек әртүрлі түс,racism

2,шизофрениялық маскүнем,шизофрениялық маскүне,bullying

2,сен шынымен де өзің айтқандай қоқыссың,сен шын де өзің айтқанда қоқыс,bullying

1,нигга оны өлтірді,нигг оны өл,racism

2,ол соқырлықтың шыңы,ол соқырлық шың,bullying

2,сіз ақымақ сайқымазақсыз қара қауым сенің мылқауың емес дедім сізді нәсілдік балағат деп атағысы келетін рэп айта алатын әрбір ақ нәсілді адамды трахает ете аласыз бірақ сіз қара қауымдастық ақымақ емессіз және бұл қабылдаушымен сіз қара хип хоп қауымдастығының бір бөлігі емессіз сіз жай ғана өтіп бара жатқан адамсыз осы нәсілшілдік пен оның сиқыршыны трампты жақтаушыны жақсы көретіні туралы пікірлерінен кейін қара қауымның сүйіспеншілігіне ие болғанын қараңыз ақсақ құлан жалған есек есімі,сіз ақы сайқымазақ қара қауым сенің мылқа емес де сіз нәсіл балағат де ата кел рэ ай ал әрбір ақ нәсіл адам трахает ет ал бірақ сіз қара қауымдастық ақы емес және бұл қабылдаушы сіз қара хи хо қауымдастығ бір бөліг емес сіз жай ғана өт бар жат адам осы нәсілш пен оның сиқыршы трам жақтаушы

жақсы көр туралы пікір кейін қара қауым сүйіспеншіліг ие бол қара ақ құлан жалған есек есім,bullying

1,сондықтан әлем ешқашан қара нәсілділер жағына шықпайды осы наразылықтар кезінде сіздің шынайы болмысыңызды көрдік,сондықтан әлем ешқашан қара нәсілді жағ шықпа осы наразылық кез сіздің шынайы болмыс көр,racism

1,қара адамдар мәдениетті өзгертуі керек бірақ олардың қырылып жатқанын көру жақсы,қара адам мәдениетті өзгерт керек бірақ олардың қырыл жат көру жақсы,racism

1,адамдар соншалықты ақымақ қаралардың өмірі маңызды емес бірақ ешкімнің кемшілігінен артық емес ақ өмірге сену қара адамдардың өмірі үшін жиналған ақша тіпті қара қауымға бармайды олар үкіметке барады пайдасыз нәрселерді сатып алды өзім үшін өңдеу егер бұл ақымақтық деп біреуді ренжіткен болсам кешіріңіз мен сізді осы әлемдегі барлық жақсылықтар үшін кешіріңіз және жақсы көремін,адам соншалықты ақы қара өмір маңызды емес бірақ ешкі кемшіліг артық емес ақ өмір сену қара адам өмір үшін жинал ақша тіпті қара қауым барма олар үкімет бар пай нәрсе сат алды өзім үшін өңдеу егер бұл ақымақтық де біреу ренжіт бол кешір мен сіз осы әлем барлық жақсылық үшін кешір және жақсы көр,racism

1,адамдар нәсілшіл емес олар қара нәсілділерді ұнатпайды мұның себебін түсіну оңай,адам нәсілшіл емес олар қара нәсілді ұнатпа мұның себеб түсіну оңай,racism

1,тұрақты қылмыстық мінез құлық болғандықтан менің ойымша ешкім қара нәсілділерді ұнатпайды,тұрақты қылмыстық мінез құлық бол менің ойымша ешкім қара нәсілді ұнатпа,racism

4,жақында америкада тағы бір азаматтық соғыс болады,жақында америка тағы бір азаматтық соғыс бол,violent

3,америка бұл бұзақыларды атып тастауы керек,америка бұл бұзақы ат таста керек,nazism

4,бәрін өлтір бұл қылмыс өлтір мен күліп попкорн жейтін едім,бәрін өлтір бұл қылмыс өлтір мен күл попкорн же ед,violent

1,қара тірі зат,қара тірі зат,racism

3,американы құрт,америка құрт,nazism

3,ақш полициясы әлемдегі террорист,ақш полиция әлем террорист,nazism

1,маңыздысы қаралардың өмірі емес,маңызды қара өмір емес,racism

1,бұл негр өзін нокаутқа түсірді,бұл нег өз нокаут түс,racism

3,америка демократиялық ел емес бұл үлкен өтірікпен пара пар америка еркіндік жоқ және құқықтар барлығына бірдей бола бермейтін ел американың қара нәсілділерге қатысты саясаты қазіргі xxi ғасырда адамзат үшін масқара,америка демократиялық

ел емес бұл үлкен өтірік пара пар америка еркіндік жоқ және құқық барлығ бірде бола берме ел америка қара нәсілді қатысты саясат қазіргі хх ғасыр адамзат үшін масқара,nazism

1,нигга нокаутқа түсті,нигг нокаут түсті,racism

2,қоқыс жетекшісі қоқыс полициясы,қоқыс жетекші қоқыс полиция,bullying

2,нәсілшіл есек,нәсілшіл есек,bullying

4,лива туввар сирия аллажатта islamstateқа қарсы ұрыс бастайды,лива тувв сирия аллажат islamstate қарсы ұрыс баста,violent

4,террористік иттер бүлікшілерге айналады,террористік ит бүлікші айнал,violent

4,әлхайрдың газваты арнайы аймақ олардан кейін олар алеппеге қайтып оралады,әлхай газва арнайы аймақ олардан кейін олар алеппе қайт орал,violent

4,сириядағы ресей басып кіру туралы алаңдамаудың кіруі мүмкін емес меккедегі бір жұма туралы қорғаныс джихад деп жарияланады,сирия ресей бас кіру туралы алаңдама кір мүмкін емес мекке бір жұма туралы қорғаныс джихад де жариялан,violent

4,ядролық соғыс соғысы келе жатыр,ядролық соғыс соғыс келе жатыр,violent

4,давлатул ислам баақия ансарул халифа оңтүстікшығыс азиядағы барлық мужахидтер топтарын жақсы көремін деп сенемін,давлатул ислам баақия ансарул халиф оңтүстікшығыс азиядағы барлық мужахид топ жақсы көр де сен,violent

4,орыстар сириялықтар мен ирандықтар бағдадта әскери үйлестіру камерасын құруда,орыс сириялық мен ирандық бағдад әскери үйлестіру камера құр,violent

4,сізге xroseді жауларға тапсырма аласыз,сізге xrose жау тапсырма ал,violent

4,фаллужаның is мандаты фаллужаның солтүстік шығысында мм қару жарақпен сафави әскеріне тиесілі хамвейде,фаллужа is мандат фаллужа солтүстік шығыс мм қару жарақ сафави әскер тиесілі хамвей,violent

4,сириялық режимді күшейтеді сарбазды өлтіруге мүмкіндік береді,сириялық режим күшейт сарбаз өлтір мүмкіндік бер,violent

4,анбар исм басқарылды өлтіру ирак армиясы альбагдади базасында әскери жолмен күш жұмсалады,анбар ис басқар өлтір ирак армия альбагдади база әскери жол күш жұмсал,violent

4,бұл жол сириядан емес фаллужадан ирак пен армияны бомбалау isis,бұл жол сирия емес фаллужа ирак пен армия бомбала isis,violent

4,жаңа таңертең телнаам ауданында нусайристе тонна бомба халаб бар,жаңа таңертең телнаа аудан нусайрис тонна бомба халаб бар,violent

4,бұл твит жүктеме risia isis бомбалау емес төңкерушілерге арналады жаңа бақытсыздар,бұл твит жүктеме risia isis бомбала емес төңкеруші арнал жаңа бақыт,violent

4,иерусалимде немесе израильде бір яһуди қалмайды өлтіреміз https видео,иерусалим немесе израиль бір яһуди қалма өлтір https видео,violent

4,егер сізде сирияда соғыс болса қай жерге қосыласыз нусра майданы isis jaish әл ислам,егер сіз сирия соғыс бол қай же қосыл нуср майдан isis jaish әл ислам,violent

4,менің ізбасарларым елде мальдив аралдары ақш тегін картаңызды біліңіз,менің ізбасар ел мальдив арал ақш тегін карта біл,violent

4,бірінші альшабааб содырлары ламу қаласы ауыл ауылының маңында кения шоғырларын ұстау ауыр күндер құрбандық және күтулер,бірінші альшабааб содыр лам қала ауыл ауыл маң кения шоғыр ұстау ауыр күн құрбан және күту,violent

4,ирак армиясына көп еңбек сіңірген болсаңыз бүгін рамадтың солтүстігін құрбан етіп оның тарабына кіріңіз,ирак армия көп еңбек сіңір бол бүгін рамад солтүстіг құрбан ет оның тараб кір,violent

4,әлемнің жазасыздығы бар мега террорист ол алғашқы топтарын лебаноннан өлтіруге шақырды асад үшін сириялықтарды аштық,әлем жазасыздығ бар мега террорист ол алғашқы топ лебанон өлтір шақ асад үшін сириялық аштық,violent

4,фаллужа ирак мұсылмандарының жолдауы біз таңертеңгілік және кешке бомбала біздің арманымыз шейіт болып келеді,фаллуж ирак мұсылман жолда біз таңертеңгілік және кеш бомбал біздің арман шейіт бол кел,violent

4,сізге алғашқы сирияға иракқа ливияға қол жеткізіңіз берілмеңіз йеменге вилаят хадрамаут қақпаларыңыз ашылып сол жерге барыңыз,сізге алғашқы сирия ирак ливия қол жеткіз берілме йемен вилаят хадрамаут қақпа ашыл сол же бар,violent

4,isis балалық шақтары толық уақытты қалыптастырады олардың уақытты арттырады бомба жасауды,isis балалық шақ толық уақыт қалыптастыр олардың уақыт арттыр бомба жасау,violent

4,әскери топтары жедел қауіпсіздік turkey amp бомба жасау истанбулдағыдай тұрғындар,әскери топ жедел қауіпсіздік turkey amp бомба жасау истанбулдағыда тұрғын,violent

4,біз жеңіске жетпейміз немесе өлмейміз омар мұхтар рахимулла жеңіс туралы ашулар pt британдық amp француз крест жорығы,біз жеңіс жетпе немесе өлме омар мұхтар рахимулл жеңіс туралы ашу pt британдық amp француз крест жорығ,violent

4,сізге опасыздық жасаған адамдарды ұмытпаңыз мұсылмандар жақында қайтып келеді біз үшін дуа жасайды,сізге опасыз жаса адам ұмытпа мұсылман жақында қайт кел біз үшін ду жаса,violent

4,is кінәлі болса да күнтізбелер бойынша байланыс оларға жаңа куфрлар дайындалып жатыр тәжірибе жалған және жалған күнәкарларды айыптайды,is кінәлі бол да күнтізбе бойынша байланыс оларға жаңа куфр дайындал жатыр тәжірибе жалған және жалған күнәкар айыпта,violent

4,қызыл ледоны қызыл ауданға айналады мұнымен айналысуға болады,қызыл ледо қызыл аудан айнал мұны айналыс бол,violent

4,түркістандық балаларды қорғайтын мылтықпен қоршау террористік элемент,түркістан бала қорға мылтық қоршау террористік элемент,violent

4,хомс захра аймағы қашықтан басқарылатын басқарылатын автомобиль бомбасы оның орнына келесі жанкештілік табылды,хомс захра аймағ қашық басқарыл басқарыл автомобиль бомба оның орн келесі жанкештілік таб,violent

4,екі israel мен әскер тікұшақтары египет қабырғаға жаңа күн келді синайда шейх зувейдтің оңтүстігінде isis позициясы бомба болады,екі israel мен әскер тікұшақ египет қабырға жаңа күн кел синай шейх зувейд оңтүстіг isis позиция бомба бол,violent

4,қозғалыскер египет қаңтардағы жаңалықтар күні синайда шейх зувейдтің оңтүстігінде isis позасы бомба болады,қозғалыск египет қаңтар жаңалық күн синай шейх зувейд оңтүстіг isis поза бомба бол,violent

4,иран терроризміне қарсыласу арқылы байланыстыру керек,иран терроризм қарсылас арқылы байланыстыру керек,violent

4,сиятак аймақ және алжику арқылы сіздің сафави әскерінің казармаларын бомбалау,сиятак аймақ және алжик арқылы сіздің сафави әскер казарма бомбала,violent

4,сізді ең алдымен кезкелген жерде жақсарту түрік әскерлерінің әскерлеріне оқ жаудырады себептері әлі белгісіз,сіз ең алд кезкел же жақсарту түрік әскер әскер оқ жаудыр себеп әлі белгісіз,violent

4,германиядағы мигранттар орталығы гранатаға лақтырылды зорлық зомбылық күшейе түседі,германия мигрант орталығ граната лақтыр зорлық зомбылық күшей түс,violent

4,сирия оқулары танкке қарсы зымыранмен мені алеппо қаласының елді мекендегі кувейрис авиабазасынан батыс қатынасы вадия ауылында жою,сирия оқу танк қарсы зымыран мен алеппо қала ел мекен кувейрис авиабаза батыс қатына вадия ауыл жою,violent

4,ауылындағы хашд рафиди казармаларын үй зымырандарымен бомбалау болады,ауыл хашд рафиди казарма үй зымыран бомбала бол,violent

4,хезболластарымен өлімге қол жетімді,хезболлас өлім қол жет,violent

4,мен дамаскідегі халықаралық мазарының жанында милиционерлерді бомбалауды асад режиміне қастық үшін деп айтамын,мен дамаскі халықаралық маз жан милиционер бомбалау асад режим қас үшін де ай,violent

4,isis террористтерімен қақтығыстардың алдында солтүстік сириядағы соғысушылар,isis террорист қақтығыс алд солтүстік сирия соғысушы,violent

4,абрамс танк пен минаны тазартатын машиналар менак жаяу және жаяу әскерге қарсы машиналарды жою,абрамс танк пен мина тазарт машина менак жаяу және жаяу әскер қарсы машина жою,violent

4,халқалаһ алғаем аймағында рафидтік күштердің тобына көптеген адамдарды өлтіріп жарылғыш жаралайтын заттың жарылуы,халқалаһ алғае аймағ рафид күш тоб көптеген адам өлтір жарылғыш жарала зат жарыл,violent

4,араб мемлекеттерінің азаматтары үшін ұзақ уақыттық іздестіру үшін қарамақайшылыққа қарсы тұру үшін әскери күштердің әскери қызметшілері шекарадан тыс жерлерге бару оларды өлтіру,араб мемлекет азамат үшін ұзақ уақыттық іздестір үшін қарамақайшылық қарсы тұру үшін әскери күш әскери қызметші шекара тыс жер бару ол өлтір,violent

4,хашдағы шиіт серіктестіктері суннит күшті балтамен кесу керек,хаш шиіт серіктестік суннит күшті балта кесу керек,violent

4,саудия сирияға исқа қарсы коалиция құруға дайын генерал сирия альнусрах майданына көмек асад күштері ахадинде алеппода суицидтік картрит картасы,саудия сирия ис қарсы коалиция құр дайын генерал сирия альнусрах майдан көмек асад күш ахадин алеппо суицид картрит карта,violent

4,сирия бүлікшілері nussrah nussrah өлімжітім бомбасынан кейін hardatnin ауылына есіл жасамақшы,сирия бүлікші nussrah nussrah өлімжі бомба кейін hardatnin ауыл есіл жасамақш,violent

4,квейресте асадпен соғысып күндізгі айтылған сияқты солтүстікке қарай бір аймақты алуы мүмкін көңіл көтерушілерге сәттілік пен қауіпсіздікті қамтамасыз ету,квейрес асад соғыс күндізгі айтыл сияқты солтүстік қарай бір аймақ ал мүмкін көңіл көтеруші сәттілік пен қауіпсіздік қамтамасыз ету,violent

4,исламистсіз фса мен сириялық оппозицияның мұндай күндерін ешқашан жасамаңыз ақш көмегі үшін опасыздық жасаңыз,исламист фс мен сириялық оппозиция мұндай күн ешқашан жасама ақш көмег үшін опасыз жаса,violent

4,ақштың қорғаныс миниері біз сауд арабиясының isisпен күрес құрбандары үшін құрбан боламыз,ақш қорғаныс мини біз сауд арабия isis күрес құрбан үшін құрбан бол,violent

4,breaking жуырда aazaz баллистикалық зымыраны он мың маман босқындар күтетін қала,breaking жуырда aazaz баллистикалық зымыран он мың маман босқын күт қала,violent

4,соңғы айда кувейрлердің айналасында асад армиясына қарсы тұру isis бұл қолмен ұстау syria,соңғы айда кувейр айнала асад армия қарсы тұру isis бұл қол ұстау syria,violent

4,немесе кеме батып кетеді иә шынымен де мохейсни немесе кеме батып кетеді нубль закрастарды тағылымдамамен байланыстыратын күн бұл,немесе кеме бат кет иә шын де мохейсни немесе кеме бат кет нубль закрас тағылымдама байланыстыр күн бұл,violent

4,сізге зинки тобы батып бара жатыр тавагитке сәлем мен қош келдіңіз бару жолы,сізге зинки тоб бат бар жатыр тавагит сәлем мен қош кел бару жол,violent

4,сіз өз армияңызды ис ке қарсы тұруға бел аласыз ба осы қорапшаңызды жақын арада бастауға болады,сіз өз армия ис ке қарсы тұр бел ал ба осы қорапша жақын ара баста бол,violent

4,мен пәкістанның аурусырқау өтірікші және шиа кешірімімен асад режиміне күресу үшін арнайы алаңда жұмыс істеймін,мен пәкістан аурусырқа өтірікш және ши кешірім асад режим күресу үшін арнайы алаңда жұмыс істе,violent

4,туған ньюйорк таймс мұны түсінеді бірбір ресей иран асад amp хизбулла алеппода көтерілісшілері алға алға жылжуда,туған ньюйорк таймс мұны түсін бірбі ресей иран асад amp хизбулл алеппо көтерілісші алға алға жылж,violent

4,байланыстар isis әйел parisattacks көшбасшысы вебэкипаж isis арқылы еуропаға менің кіргенім айтты,байланыс isis әйел parisattacks көшбасшы вебэкипаж isis арқылы еуропа менің кір ай,violent

4,олар өз қалаларын жақындайды және олардың жоспарларын ұсынады,олар өз қала жақында және олардың жоспар ұсын,violent

4,isisпен құрамдас бөлігі amaq news адам альбагдади қаласынан солтүстікбатысқа қарай пайда әкеледі анбартұрақты тұрғын үйде адам өледі,isis құрамдас бөліг amaq news адам альбагдади қала солтүстікбатыс қарай пайда әкел анбартұрақ тұрғын үй адам өл,violent

4,біз үмітсіз өмірден сәтті үмітке көшіп қол жеткізіп қайтып оралыңыз,біз үмітсіз өмір сәтті үміт көш қол жеткіз қайт орал,violent

4,квейрлерде қоршауды көтеруге арналған күн жақын amp нубл захра тілдесу болды алдымен күн сағат қайтарылды,квейр қоршау көтер арнал күн жақын amp нубл захра тілдесу бол алд күн сағат қайтар,violent

4,видеода талбише хомс бір түнде кластерлік оқ дәрістерге байланысты оқу,видео талбиш хомс бір түн кластерлік оқ дәріс байланысты оқу,violent

4,ресей сириядағы барлық күнтізбелік хат жазылады асад тағайындалғаннан кейін бомбалауды қабылдайды,ресей сирия барлық күнтізбелік хат жаз асад тағайындал кейін бомбалау қабылда,violent

4,deirezzor albukamal isis адам өліміне арналған аймақ,deirezzor albukamal isis адам өлім арнал аймақ,violent

4,breaking түркия шекарасында сириялық босқын күтіледі мың жол жүреді,breaking түркия шекара сириялық босқ күт мың жол жүр,violent

4,малик африди мисбах омар жақсы жақсы оқитын жастар келушілер олардың спринтерлерді жақсы ойнау күндерін сатып алу қажет,малик африди мисбах омар жақсы жақсы оқитын жас келуші олардың спринтер жақсы ойна күн сат алу қажет,violent

4,isis алеппо алькаламун ладияхия және әлбадия қалаларында джабхат нусраның ресейлік телеграмма арналарын бұзады,isis алеппо алькаламун ладияхия және әлбадия қала джабхат нусра ресейлік телеграмма арна бұз,violent

4,isis ливияда көшеде болады,isis ливия көше бол,violent

4,осымен ис аумақтарының қоршау қауымы аяқталды,осы ис аумақ қоршау қауым аяқта,violent

4,сәлем олар есірткі апиын гашишін сатады кг апиынға ден күнге дейін олар бандыларды араластырады,сәлем олар есірткі апи гашиш сат кг апиын ден күн дейін олар банды араластыр,violent

4,obama tayau шығыстары басқа елдердің азаматтарының көтерілуі керек,obama tayau шығыс басқа ел азамат көтеріл керек,violent

4,башар асадқа анасын жерлеу күні және қастандық жасау syria,баш асад ана жерлеу күн және қаста жасау syria,violent

4,анықталмаған мәліметтер бойынша фашистік ypg террористік тобы мен меніңді қорғайтын базасын ауыртады russia бомбалаудан кейін алды,анықталма мәлімет бойынша фаши ypg террористік тоб мен мен қорға база ауырт russia бомбалау кейін алды,violent

4,мұнымен келіспесеңіз де мұнымен байланысты емес достарыңызға жаман адамдарға жамандық орнатыңыз,мұны келіспе де мұны байланысты емес дос жаман адам жаман орна,violent

4,егер сіз ақшқа сапар жасасаңыз немесе батыс асад режимін құлыптанған болсаңыз немесе сіз оны құлыпқа қайтару туралы ойласаңыз жақын арада,егер сіз ақш сапар жаса немесе батыс асад режим құлыптан бол немесе сіз оны құлып қайтару туралы ойла жақын ара,violent

4,жаттығу жасаушыларға балалар мен студенттерге күшті беттер ұсынылды олармен бірге ирак арасында алауыздықты қолданыңыздар amp сириялық джад,жаттығу жасаушы бала мен студент күшті бет ұсын олармен бірге ирак ара алауыздық қолдан amp сириялық джад,violent

4,бір рет fsa amp ypg ақшпен исқа қарсы соғысады ypg ресей федерациясымен қарымқатынаста болады олар fsaмен басталды және fsa бірлестігі серіктестігі туралы анықтама өлімге дайындық,бір рет fsa amp ypg ақш ис қарсы соғыс ypg ресей федерация қарымқатынас бол олар fsa баста және fsa бірлестіг серіктестіг туралы анықтама өлім дайындық,violent

4,usa mi abramsтың танктері бар iraq аймағында тұрақсыз секталық шиіт жасақтарына жұмыс жасау,usa mi abrams танк бар iraq аймағ тұрақсыз секталық шиіт жасақ жұмыс жасау,violent

4,синайдағы оқужаттығу жиналысының сабақтарына назар аударып отырыңыз isisтің күндізгі кірісі,синай оқужаттығ жиналы сабақ назар аудар отыр isis күндізгі кіріс,violent

4,isis ауғанстантез дамып келеді paktia өте күшті талантты бекініс командасы is amp үшін күрес алаңы болып табылады,isis ауғанстантез дам кел paktia өте күшті талантты бекініс команда is amp үшін күрес алаң бол таб,violent

4,солтүстік рамадиға жаппай жол ашылады iraq әскерін өлтіріп казарманы өртеп жіберді,солтүстік рамади жаппай жол аш iraq әскер өлтір казарма өрте жіб,violent

4,қызыл жарылды шешен қолбасшы ретінде ливиядағы ишим тобымен күресу айтылды,қызыл жар шешен қолбасшы рет ливия иши тоб күресу айт,violent

4,бұл қымбат емес сұрақ бұл революция қағидасының өзгеруі бізге дауыс беру үшін күресуіміз керек,бұл қымбат емес сұрақ бұл революция қағида өзгер бізге дауыс беру үшін күрес керек,violent

4,ирактың жетекшісі фуад маусумның күзетшісі амирлияның оңтайлы азаматы киркук ирак маңында балалар ұстайды,ирак жетекші фуад маусу күзетші амирлия оңтайлы азамат киркук ирак маң бала ұста,violent

4,ypgге негізделген тұрақты тұрғын үйге нұқсандық жазба орнынан және мүшелерінен айырылады,ypg негіздел тұрақты тұрғын үй нұқсан жазба орн және мүше айыр,violent

4,ассадтың барлық елді мекеніне барып тәжірибе алыңыз хаха бүлікшілердің есектері шапалақтайды франциямен келіссөздер туралы келісім,ассад барлық ел мекен бар тәжірибе ал хах бүлікші есек шапалақта франция келіссөз туралы келісім,violent

4,ассадаттың жеңіске жетуіне жол бермейді оппозицияны исламстаты айыптайды,ассада жеңіс жет жол берме оппозиция исламста айыпта,violent

4,фалляжаның ші және ші шайқаларын қайтарамыз мен науханда қайтып келемін деп ант етемін міне сафави рафида,фалляжа ші және ші шайқа қайтар мен наухан қайт кел де ант ет міне сафави рафи,violent

4,бұл әр күн сайын жатыр содырлары өлтірілді асад армиясы махинге жақында танк syria,бұл әр күн сайын жатыр содыр өлтір асад армия махин жақында танк syria,violent

4,жаңа оқу күніне дейін олармен бір мамырдан бастап соғысқанымызды мақтан тұтамыз және бір көшбасшылыққа жиналдық caliphate news demdebate is syriaceinfire,жаңа оқу күн дейін олармен бір мамыр баста соғыс мақтан тұт және бір көшбасшылық жина caliphate news demdebate is syriaceinfire,violent

4,ypg мемлекетесі арфин жаттығуларының аяқталуы түркияның орнына дайын террористер,ypg мемлекете арфин жаттығу аяқтал түркия орн дайын террорис,violent

4,жолдан тайған әскер мен рафиди хашдты каталюша зымырандарымен alasmadah алаңының алаңында соғыс,жолдан тай әскер мен рафиди хашд каталюш зымыран alasmadah алаң алаң соғыс,violent

4,тағы бір танкі жойылды казармалар жанып жатыр қайтадан оралған база альфа сәттілік чарли үнсіз роджер бұл мүмкін емес,тағы бір танк жой казарма жан жатыр қайта орал база альфа сәттілік чарли үн родж бұл мүмкін емес,violent

4,isis menagh авиабазасын алды қылмыс әні фсаның бандалары бұл пышақтап демалысқа кетеді allepoда күресуге дайынмын артқы жағымда менде осындай scumға жанашырлық бар,isis menagh авиабаза алды қылмыс әні фса банда бұл пышақта демалыс кет allepo күрес дайын артқ жағ менде осындай scum жанашырлық бар,violent

4,назар аударыңыз fsa ypg шариғат заңын ойнауы керек адам өлтіру керек хахахахахахахаха,назар ау fsa ypg шариғат заң ойна керек адам өлтір керек хахахахахахахах,violent

4,ypg жанкүйерлері түркияның азаз ауруханасына бомба қойылады бұл ресей ypg жанкүйерлері бұл террористтердің ауруханасы болды,ypg жанкүйер түркия азаз аурухана бомба қой бұл ресей ypg жанкүйер бұл террорист аурухана бол,violent

4,бұрынғы usa cia шенеунігі саудияның пакистанда ядролық қауіпті бомба бар https co vvuohnt,бұрынғы usa cia шенеуніг саудия пакистан ядролық қауіпті бомба бар https co vvuohnt,violent

4,марея бүлікшілері қаланы ypgге тапсырады,марея бүлікші қала ypg тапсыр,violent

4,isis aamaq бүлікшілерінің солтүстігінде алеппода isisтің қорғаныс күні,isis aamaq бүлікші солтүстіг алеппо isis қорғаныс күн,violent

4,кез келген наурыз айы қоршау,кез кел наурыз ай қоршау,violent

4,о бүлікшілер сіздің назарыңызға марны исқа берсеңіз фитнесмүшелермен қарым қатынасыңыз қажет және мосулдың шет жақтарынан тірі болу керек,о бүлікші сіздің назар мар ис бер фитнесмүше қарым қатынас қажет және мосул шет жақ тірі болу керек,violent

4,жақсы шайқастарында кластерлік бомбалармен бомбалайды,жақсы шайқас кластерлік бомба бомбала,violent

4,тақырыптар бойынша өздерін пышақтап тастайды,тақырып бойынша өз пышақта таста,violent

4,бомбалар мен блокадалар газаның су тасқынының орнына барлық күшейтеді,бомба мен блокада газа су тасқын орн барлық күшейт,violent

4,моуланаға қарсы күрес жекпе жегі масуд азхар пәкістанда патанкот күніне қарсы тұрады,моулана қарсы күрес жекпе жег масуд азх пәкістан патанкот күн қарсы тұр,violent

4,суреттерде pyd ypg сириядағы амудиде түркияға соғыс жариялайды анкара бомбалы туған қаласы,сурет pyd ypg сирия амуди түркия соғыс жарияла анкара бомбал туған қала,violent

4,obama ливиядағы islamstateqabr жаулаудан бас тарту туралы хабарлама келді us денсаулығы осылай жасалды,obama ливия islamstateqabr жаулау бас тарту туралы хабарлама кел us денсаулығ осылай жаса,violent

4,кофар жазықсыз нысандарды ашады,коф жазық нысан аш,violent

4,түрік алепподағы джихадшыларды қолдауды қолдайды екі адам оны жарылқайды тозды сіздің жақын маңыңызда syria,түрік алеппо джихадшы қолдау қолда екі адам оны жарылқа тоз сіздің жақын маң syria,violent

4,анвар авлаки бір топ адамдармен келіссөздер екі жақты бұзушылықтар тауарлардың импичменті isiste қайтып келеді,анвар авлаки бір топ адам келіссөз екі жақ бұзушылық тауар импичмен isis қайт кел,violent

4,бұл tak адамдарды түркияге бармақ шақырады жақсы мен осы жазда барамын amp әрдайым ұрыста amp біздің күрд қаласы,бұл tak адам түркия бар шақыр жақсы мен осы жаз бар amp әрдайым ұрыс amp біздің күрд қала,violent

4,ishтің күштері isisтің күші позицияларымен либияда соғыс күші түсуде is қалайды,ish күш isis күш позиция либия соғыс күш түс is қала,violent

4,сізге олармен жақсы қарым қатынаста болады жыл бұрын түркия туралы ескертіледі ақштың денсаулығы usa қаружарақ жоқ деп санайды,сізге олармен жақсы қарым қатынас бол жыл бұрын түркия туралы ескерт ақш денсаулығ usa қаружарақ жоқ де сана,violent

4,ақш тобыр бомба болады алдағы кезкелген аймақтық сапарды түсінуге болады,ақш тобы бомба бол алдағы кезкел аймақтық сап түсін бол,violent

4,шамасы мұсылман балаларын бомбалаған дұрыс,шама мұсылман бала бомбала дұрыс,violent

4,бір аптаның ішінде үш адам бірбірімен алмастырылды олар ауылдарда есеп айырысады пакистан армиясы вакиристан бктин спинкай рагзай аймағында,бір апта іш үш адам бірбір алмастыр олар ауыл есеп айырыс пакис армия вакирис бктин спинка рагза аймағ,violent

4,ypg және sdf рас аль айн айналасында шегінуде isis өздері үшін бұл адамдарға қарсы,ypg және sdf рас аль айн айнала шегін isis өз үшін бұл адам қарсы,violent

4,құрметті ізбасарлар аяулы күндеріңіз ypg немесе assad деп тұрыңыз,құрметті ізбасар аяул күн ypg немесе assad де тұр,violent

4,атеистердің шынайы ғибадатханасының негізін жою бангладеш,атеист шынайы ғибадатхана негіз жою бангладеш,violent

4,alqassam суицидті жақтаушылар құрамы gaza туннельдерінде фрг израиль сарбаздарын өртеп қырады,alqassam суицид жақтаушы құрам gaza туннель фрг израиль сарбаз өрте қыр,violent

4,террористік тобынан толық шығарылды тағы екі ауыл алға жылжуда isis,террористік тоб толық шығар тағы екі ауыл алға жылж isis,violent

4,тапсырыс қайтыс болғандар туралы iraq әскерлерінің казармаларының өртенуі және олардың көмегімен тетіктердің жойылуы өлім рамади,тапсырыс қайтыс бол туралы iraq әскер казарма өртен және олардың көмег тетік жойыл өлім рамади,violent

4,ассад өлтіру үшін үлкен сілтемелер iisriy salamiyah жолында isis буктурасында адамды өлтірді,ассад өлтір үшін үлкен сілтеме iisriy salamiyah жол isis буктура адам өл,violent

4,ресей соғыс ардагерлері сапармен кетеді алеппо ауылында кластерлік бомбалармен ауырады жаңа адамдар қайтыс болады белсенділер syria https co kv smexmq,ресей соғыс ардагер сапар кет алеппо ауыл кластерлік бомба ауыр жаңа адам қайтыс бол белсенді syria https co kv smexmq,violent

4,жақсы fsa мүшелерін жоспарлы түрде жоспарлап өмір сүру үшін өмір сүру үшін кешкі кесте жасайды бұл isiske қызмет көрсету мүмкіндігі,жақсы fsa мүше жоспарлы түр жоспарла өмір сүру үшін өмір сүру үшін кешкі кесте жаса бұл isis қызмет көрсету мүмкіндіг,violent

4,трамп мұсылмандыққа ұнаған адамның өлімін өлтіруіміз керек видео,трам мұсылмандық ұна адам өл өлтір керек видео,violent

4,сұрақ сен шынайы журналистикамен айналыса аласыз ба ақш перзентхананы abukamal https бомбалар болады,сұрақ сен шынайы журналистика айналыс ал ба ақш перзентхана abukamal https бомба бол,violent

4,өмірменен де онсыз да оқуды керуен жазбен де онсыз да жалғасады жеңіс өлім жеңілдік,өмірме де онсыз да оқу керуен жаз де онсыз да жалғас жеңіс өлім жеңілдік,violent

4,тағы да дарайя танкі барлық қарсыластар жарысы басталды ассад syria,тағы да дарайя танк барлық қарсылас жарыс баста ассад syria,violent

4,мусса әломар твиттерде ханазер үнсіз сойылады,мус әлом твиттер ханаз үн сой,violent

4,үміт нусра есектерін анықтауға көмектеседі amp біртұтас мүмкіндікті жасау ханасир бұл баршаның пайдалы қызметі isis syria,үміт нуср есек анықта көмектес amp біртұтас мүмкіндік жасау ханаси бұл барша пайдалы қызмет isis syria,violent

4,мүмкіндік ші немесе шы рет жұмбақ миноматтар amp осы аптада латакия қаласына зымырандар түсуге болмайды ассад бақ сирияға қарсы емес,мүмкіндік ші немесе шы рет жұм миномат amp осы апта латакия қала зымыран түс болма ассад бақ сирия қарсы емес,violent

4,биг оларға латакия ауылындағы жалпы жиналысқа автомобильмен бомба қойылды нысанаға алынды,биг оларға латакия ауыл жалпы жиналыс автомобиль бомба қой нысана ал,violent

4,афганистанға ак мылтығын салып жатыр,афганистан ак мылтығ сал жатыр,violent

4,ctstudiesтің анықтамасы ypg террористік тобы қайтады,ctstudies анықта ypg террористік тоб қайт,violent

4,революциялық түзету протекционисттері ассадты khanaisr amp ypg террористік тобы шададиден гөрі,революциялық түзету протекционист ассад khanaisr amp ypg террористік тоб шадади гөрі,violent

4,барлық сунниттік мұсылмандарды сирия кәпірлері деп жариялайды,барлық сунни мұсылман сирия кәпір де жарияла,violent

4,isisпен бөлісу aamaq ақпараттық агенттігі кешегі есеп беру мәліметтерін растайды israel мен реактивті бомбалар sinai,isis бөліс aamaq ақпараттық агенттіг кешегі есеп беру мәлімет раста israel мен реактивті бомба sinai,violent

4,мен осындай құпия кездесулерге жауаптымын,мен осындай құпия кездесу жауапты,violent

4,багдадта тәжірибе iraq суицид күні,багдад тәжірибе iraq суицид күн,violent

4,иә мен бұны алдыңғы жақтан көрдім түркияға кіру мүмкіндігінің жоқтығы үшін түркияның құны төленеді studies center,иә мен бұны алдыңғы жақ көр түркия кіру мүмкіндіг жоқтығ үшін түркия құн төлен studies center,violent

4,жаңалықтарды тыңдаушыларға ақш пен ақш арасындағы қарым қатынас ақштың көмегіне сүйенетін ақымақ фанаттарға қол сұғу туралы хабарлама жіберілді,жаңалық тыңдаушы ақш пен ақш ара қарым қатынас ақш көмег сүйен ақы фанат қол сұғ туралы хабарлама жібер,violent

4,ақшпен қарымқатынас isil ке қарсы риясыз есеп обама,ақш қарымқатынас isil ке қарсы рия есеп оба,violent

4,ypg арасындағы мылтық жинады олар isis назарларыңызбен танысып сөйлесіңіздер олардың алғашқы қарашажарақтары қашаннан келеді http,ypg ара мылтық жина олар isis назар таныс сөйлес олардың алғашқы қарашажарақ қашан кел http,violent

4,сириадағы наразылықтар бітімге келмек халықтық жұмыс күші джабхат аль нусра және режиммен бітіспейміз деп ұрандап жатыр,сириа наразылық бітім кел халықтық жұмыс күш джабхат аль нуср және режим бітіспе де ұранда жатыр,violent

4,джулани ислам күрескерлері дамаскты жаулап алады,джулани ислам күрескер дамаск жаула ал,violent

4,біз басымыздан бастаймыз соңына қарай аймағымызға барамыз,біз бас баста соң қарай аймағ бар,violent

4,осад асадтың антиппериализм туралы ұрық соғысы троллері қайнаған ба сізге имприалистермен келісім жасалып нәтижелермен қамтамасыз етіледі,осад асад антиппериализ туралы ұрық соғыс трол қайна ба сізге имприалис келісім жасал нәтиже қамтамасыз ет,violent

4,бомбалау өлімжітім және өлім жітім сауд арабиясы ақштың қауіпсіздігіне кепілдік,бомбала өлімжі және өлім жітім сауд арабия ақш қауіпсіздіг кепілдік,violent

4,сәлем сізге ере аласыз ба сізге хабарлама жазу керек рахмет,сәлем сізге ер ал ба сізге хабарлама жазу керек рахмет,violent

4,менің жақсы адамым бағдаттағы тахрир алаңына жеңілдікпен қарсы тұру қарсылыққа қарсы қарсыластарға қарсы iraq https co mnivqa nw,менің жақсы адам бағдат тахри алаң жеңілдік қарсы тұру қарсылық қарсы қарсылас қарсы iraq https co mnivqa nw,violent

4,үлкен жарылыс amp биік абядда visa фонында isis топтары мен қарулы қақтығыстар amp ypg содырлары syria https co egz bw mf,үлкен жарылыс amp биік абяд visa фон isis топ мен қарулы қақтығыс amp ypg содыр syria https co egz bw mf,violent

4,әрдайым сіздің жақын жерлеріңізге жақын кіріп кетеді олардың арасынан трибуналарға өлім жіберіледі,әрдайым сіздің жақын жер жақын кір кет олардың ара трибуна өлім жібер,violent

4,ақштың заңды коалициясы таллабядякта жұмыс істейтін болады isis syria,ақш заңды коалиция таллабядяк жұмыс істе бол isis syria,violent

4,талабядта үлкен қырғын менің орнымда isis кейін қалаларға хаос әкелуді бастады amp тікелей ұшақтарға қайтып келу мүмкін емес,талабяд үлкен қырғын менің орн isis кейін қала хаос әкелу баста amp тікелей ұшақ қайт келу мүмкін емес,violent

4,мадхалис екіжүзді емес олар күмәнсіз анық куфтар,мадхалис екіжүз емес олар күмән анық куф,violent

4,ақштың коалициясы ypg террорлық тобының шығысындағы suluk анықталған бомбалар болады isis syria twitterkurds,ақш коалиция ypg террорлық тоб шығыс suluk анықтал бомба бол isis syria twitterkurds,violent

4,кәпірлерді өлтіруді күтеді ишим аймағымыздағы демократияға қарсы тұру дегеніміз біздің өмірімізді тұрақтандыру,кәпір өлтіру күт иши аймағымыз демократия қарсы тұру де біздің өмір тұрақтандыру,violent

4,контрабанда оқиғасы бір адаммен жақынырақ танысуға болады https co avqeddet арқылы middleeasteye,контрабанда оқиға бір адам жақынырақ таныс бол https co avqeddet арқылы middleeasteye,violent

4,аяқтану лақтырыңыз граната лақтырыңыз https co ucinivahkw,аяқтан лақтыр граната лақтыр https co ucinivahkw,violent

4,қарулы қараған адамдар микадия орталық емдік орталыққа барады әлі бір тұтқынның ізі кеткені белгісіз iraq,қарулы қара адам микадия орталық ем орталық бар әлі бір тұтқын із кет белгісіз iraq,violent

4,isis бомбалаушыдардың ең жақсы бомбалары жарылады diyala шииттердің содырлары басқарушылары мен командирлерінің орасан зор шығыны,isis бомбалаушы ең жақсы бомба жар diyala шиит содыр басқарушы мен командир орасан зор шығын,violent

4,алеппо бомбалауда біраз кідіріс естіледі адамдар режимінің құлдырауына байланысты қымбатшылыққа қайта жол беріледі https co osvv,алеппо бомбала біраз кідіріс ест адам режим құлдыра байланысты қымбатшылық қайта жол бер https co osvv,violent

4,қарулы барлау тобының бригадири али нуманан хадитада бомба арқылы өлтірілуде iraq,қарулы барлау тоб бригадири али нума хадита бомба арқылы өлтіріл iraq,violent

4,менің ізбасарларым елде мальдив аралдары ақш https co klvsqhad тегін картаңызды біліңіз https co iwbcifantz,менің ізбасар ел мальдив арал ақш https co klvsqhad тегін карта біл https co iwbcifantz,violent

4,alqarnee филиппинде оққа ұшу есте сақтаңыз,alqarnee филиппин оқ ұшу ес сақта,violent

4,берілген мұвахидтерден кек алады,бер мұвахид кек ал,violent

4,түрік армиясы сізреде әлі де геноцид жасау күрдтер қарулы адамдар өлтіруде twitterkurds https co xgfdrf upd,түрік армия сізре әлі де геноцид жасау күрд қарулы адам өлтір twitterkurds https co xgfdrf upd,violent

4,жаңадан келгендер либия милиционерлерінің үй бункері жолдарда жүре алады мен өзімнің жақсы тілегімді білдіретін өлім палатасы https co zl shjms,жаңа кел либия милиционер үй бункер жол жүр ал мен өзімнің жақсы тілег білдір өлім палата https co zl shjms,violent

4,хашд аррафидиге тиесілі газеттердегі бұлси өкінішке орай spg зымырандары бар https co nnwdsaazdv,хашд аррафиди тиесілі газет бұлси өкініш орай spg зымыран бар https co nnwdsaazdv,violent

4,bengardaneде белгісіз қарулы топтармен танысады https co dmnixcusm,bengardane белгісіз қарулы топ таныс https co dmnixcusm,violent

4,қарсы батыс жақта жиналған жақсы және күшті достық қарымқатынас мүшеден тұрады адам өледі есептері жараланған iraq isis сәттілік,қарсы батыс жақта жинал жақсы және күшті достық қарымқатынас мүше тұр адам өл есеп жаралан iraq isis сәттілік,violent

4,exclusive video tunisia армия bengardaneде белгісіз қарулы топтарды қарауды тарату https co dmnixc,exclusive video tunisia армия bengardane белгісіз қарулы топ қарау тарат https co dmnixc,violent

4,куфандар билеушісіне қарсы зайд бин али бин хуссейнге қарсы күреске шақырылды қол қойып тек лық соғысқа қарсы,куфан билеуші қарсы зайд бин али бин хуссейн қарсы күрес шақыр қол қой тек лық соғыс қарсы,violent

4,джаман жоспары ақш батыс ресейдің әскери топтары соғыс режимін келіседі ис пен джнге қарсы қарсы ойын жеңіс сайлауларды жеткізу қалаларын ұсынады,джаман жоспар ақш батыс ресей әскери топ соғыс режим келіс ис пен джн қарсы қарсы ойын жеңіс сайлау жеткізу қала ұсын,violent

4,сіздердің отбасыларыңызбен келіссөздер өткізілсе келіңіз неге ші орынға соғыс сириялық өлім жазасы,сіздердің отбасы келіссөз өткізіл кел неге ші орын соғыс сириялық өлім жаза,violent

4,фаллуҗаның амирия ауданында us танкі жойылады,фаллуҗа амирия аудан us танк жой,violent

4,екі әйел bayrampasa стамбулдағы полиция органдарына гранатасы мен автоматтандырылған мылтықтарымен кірісті https co jgukq,екі әйел bayrampasa стамбул полиция орган граната мен автоматтандырыл мылтық кір https co jgukq,violent

4,егер сіз иш бүлікші командирін өлтірсеңіз олар хавариж мужахидтері туралы мәліметтер өлтірілген деп айғайлады amp асад әскерін қайта қарау https co

caxkmrndv,егер сіз иш бүлікш командир өлтір олар хавариж мужахид туралы мәлімет өлтір де айғайл amp асад әскер қайта қарау https co caxkmrndv,violent

4,шабындықта демалса христиан крест көтереді amp кросс жеңіске жетті мұсылман оны өлтірді amp күрес басталады,шабындық демал христиан крест көтер amp кросс жеңіс же мұсылман оны өл amp күрес бастал,violent

4,давла ханазердің жолын кесіп жақынырақ танысу алепподағы тәжікгерлер деген тақырыпты оқу кезіндегі күндізгі кезекті күтті және кофеқорларды өлтіруді үйретті https co pmivaddobv,давл ханаз жол кес жақынырақ танысу алеппо тәжікгер де тақырып оқу кез күндізгі кезекті күт және кофеқор өлтіру үйрет https co pmivaddobv,violent

4,олар playstation ойнауды қабылдауды қабылдайды және isis isil daash және басқа да елдермен күресу https co nosai svg,олар playstation ойнау қабылдау қабылда және isis isil daash және басқа да ел күресу https co nosai svg,violent

4,соңғы өзбек ағайындар мерейтойын өлтіргені үшін мен өзіммен бірге жолға шықтым,соңғы өзбек ағайын мерейтой өлтір үшін мен өз бірге жол шық,violent

4,үлкен жарылыс tarmiyaah және одан әрі мылтықтың күшті атысымен басталды iraq,үлкен жарылыс tarmiyaah және одан әрі мылтық күшті атыс баста iraq,violent

4,тек ливияда саудалық мылтықпен соғысып жатыр,тек ливия саудалық мылтық соғыс жатыр,violent

4,идлибтегі даруш ауылындағы нусраға қарсы наразылық тұрғындар ққаны қалаға кіруге шақырады syria https co zgn,идлиб даруш ауыл нусра қарсы наразылық тұрғын їққа қала кір шақыр syria https co zgn,violent

4,ақшқа ауғандықтарды бомбалау үшін базаны жіберу арқылы,ақш ауғандық бомбала үшін база жіберу арқылы,violent

4,сізге iran немесе turkey amp сириябірбір айлық өлім жазасы,сізге iran немесе turkey amp сириябірбі айлық өлім жаза,violent

4,өлім үшбұрышы ханасер итрия саламия өлі isis амбициясы amp асадтың армиясына көмектесу сирия https co im onveaoq,өлім үшбұрыш ханас итрия саламия өлі isis амбиция amp асад армия көмектес сирия https co im onveaoq,violent

4,саудиялықтар мен студенттердің операциялары usa ескертеді хезболла израиль оңтүстікке қарай соғыс аймағында лебанон https co kr gyg,саудиялық мен студент операция usa ескерт хезболла израиль оңтүстік қарай соғыс аймағ лебанон https co kr gyg,violent

4,қазір мосулға соғысқа қатысқан құмды дауылшылар туралы хабарлар iraq isis,қазір мосул соғыс қатыс құм дауылшы туралы хабар iraq isis,violent

4,бүлікшілердің бірбірімен соғысы басталды азазда,бүлікші бірбір соғыс баста азаз,violent

4,ақш басқарған коалиция солтүстігінде шиамиттік полиция конвойын бомба болатын болады деп есептейді куәгерлер анбар iraq isis,ақш басқар коалиция солтүстіг шиами полиция конвой бомба бол бол де есепте куәгер анбар iraq isis,violent

4,бұл жаңа сирия армиясы ақш пен иорданияға тек isis және алькаидаға қарсы тұру,бұл жаңа сирия армия ақш пен иордания тек isis және алькаида қарсы тұру,violent

4,батыс сирияны федерациямен қамтамасыз ету сириядағы атысты келісім бұл мұсылмандарды азат етіңіз және жақындайсыз amp үлкену marmar https,батыс сирия федерация қамтамасыз ету сирия ат келісім бұл мұсылман азат ет және жақында amp үлкен marmar https,violent

4,давла шададидің батыс әкімшілігі басқарған зымыранмен бульдозерді жою туралы кеңес берілді https co kb ewz myx,давл шадади батыс әкімшіліг басқар зымыран бульдозер жою туралы кеңес бер https co kb ewz myx,violent

4,оларды мұсылмандарға өздері тексерді келісімдер феномені көмекшілерді өлтіру туралы болды,ол мұсылман өз тексер келісім феномен көмекші өлтір туралы бол,violent

4,өткен аптада пәкістаннан ақштан жаңа ізбасарым бар https co klvsqhad https co d vj kv,өткен апта пәкістан ақш жаңа ізбасар бар https co klvsqhad https co d vj kv,violent

4,мұсылман бауырлар күшті джихадты қабылдап алады менің достарым https co ym pp wo,мұсылман бауыр күшті джихад қабылда ал менің дос https co ym pp wo,violent

4,сириядағы ақшта дауласқа бірақ муфтадан азаптап біреуін ұстауды бұйырды https co xocae,сирия ақш даулас бірақ муфта азапта біреу ұстау бұйыр https co xocae,violent

4,олардың бауырларын өлімге күтіпбаптау оларды тексеру және қарау бейбіт емес қауіпсіз емес оқуға болмайды голливуд https co dnlbuzmpw,олардың бауыр өлім күтіпбапта ол тексеру және қарау бейбіт емес қауіпсіз емес оқ болма голливуд https co dnlbuzmpw,violent

4,оны құрту керек болды akhi хахаха біз оны өлді деп көрсетейік,оны құрт керек бол akhi хахах біз оны өл де көрсет,violent

4,бұл күштер ішкі жанжал мен өлімге көмек береді,бұл күш ішкі жанжал мен өлім көмек бер,violent

4,мурси демократия басқарушысы ол синайдағы мужахидтерге қарсы соғыс әдісін анықтайды,мурси демократия басқарушы ол синай мужахид қарсы соғыс әдіс анықта,violent

4,кешке күндік сапарлар кластерлік және бейбітшілік бомбалары,кеш күндік сапар кластерлік және бейбітшілік бомба,violent

4,бұл мосулсапар ирактың баласы жақсы болды ақш өлімге бомба болады https co kxwzzcpy,бұл мосулсап ирак бала жақсы бол ақш өлім бомба бол https co kxwzzcpy,violent

4,қызыңыз бүгін us bombers mosulte бомба болады егер ол кек алса оны террорист деп атайды https co,қыз бүгін us bombers mosulte бомба бол егер ол кек ал оны террорист де ата https co,violent

4,пальмираны ассад сындыруда сізге бұл аймақтың аумағы ешкімге сенімді емес,пальмира ассад сындыр сізге бұл аймақ аумағ ешкім сенімді емес,violent

4,тәуіріне демократиялық зайырлы сирияны айтамыз осы сәтте сізде мұсылманды өлтіруге мүмкіндік береді,тәуір демократиялық зайырлы сирия ай осы сәт сіз мұсылман өлтір мүмкіндік бер,violent

4,видеода сіз shia militiading toyota ережелері is жалаушасы барын көріңіз sunnius кінәсін өлтіріңіз https co ev,видео сіз shia militiading toyota ереже is жалауша бар көр sunnius кінә өлтір https co ev,violent

4,сыну бүгін біздің мехеннің шеткі күндерінде зымыран орналастырылды https co ureicifmua,сын бүгін біздің мехен шеткі күн зымыран орналастыр https co ureicifmua,violent

4,сирия тұрғындарын бомбалау керек олар украинаны бомбалай қарастырады украинада мың мың режим бар,сирия тұрғын бомбала керек олар украина бомбала қарастыр украина мың мың режим бар,violent

4,homs маңында халықаралық валюта фосфор бомбалары бар барлық күштермен байланыс бомбалары https co yb,homs маң халықаралық валюта фосфор бомба бар барлық күш байланыс бомба https co yb,violent

4,батыс елдерінің америка құрама штаттарының долларлары мен жалған уәделер үшін өз халқының құрбандарын көргенде батыс қатты күлуі керек,батыс ел америка құрама штат доллар мен жалған уәде үшін өз халқ құрбан көргенде батыс қатты күл керек,violent

4,наразылық іздеушілер қате жоқ әлем сені асадтың өлтіргенін қорғаайды https co awztgyea,наразылық іздеуші қате жоқ әлем сен асад өлтір қорғаа https co awztgyea,violent

4,ahahahahhahaah homg мен өліп жатырмын мен үшін бұл көп,ahahahahhahaah homg мен өл жатыр мен үшін бұл көп,violent

4,соңғы iaf хамас құрамына жақтаушыларға газаның зымыранмен атқылауына жауап берді исламдық террористік топ эскаламен ескертеді,соңғы iaf хамас құрам жақтаушы газа зымыран атқыла жауап бер исламдық террористік топ эскала ескерт,violent

4,сізге алғашқы түнде жаман түнді өткізеді сіздің денсаулығыңыз жақсы емес деп ойлайсыз джабхат әлхусра одан да жаман болады,сізге алғашқы түн жаман түн өткіз сіздің денсаулығ жақсы емес де ойла джабхат әлхуср одан да жаман бол,violent

4,егер түркияда рожава террористік аймаққа кіреді фргдан тыс террористіккүрд базаларынан терроризмді күшейту қажет,егер түркия рожав террористік аймақ кір фрг тыс террористіккүрд база терроризм күшейту қажет,violent

4,о орыс тіліндегі сөздер путин кетпейді өтірік айтпа денсаулығына байланысты көп қабатты адамдардың назарын аударып өлтірейік https co xyvgake tw,о орыс тіл сөз путин кетпе өтірік айтпа денсаулығ байланысты көп қабат адам назар аудар өлтір https co xyvgake tw,violent

4,ресейлік өлім туралы хаттама хезболланың ишим шы күнгі өлім және күндік пальмираға жақын жерде болады amaqagency https co bn wfnw,ресейлік өлім туралы хаттама хезболла иши шы күнгі өлім және күндік пальмира жақын же бол amaqagency https co bn wfnw,violent

4,сирияға алеппо қаласында қаза тапқан irgc зейнабиун бригадасының пәкістандықтары қазіргі иранның құмар жерінде https co komuk j,сирия алеппо қала қаза тап irgc зейнабиун бригада пәкістандық қазіргі иран құм же https co komuk j,violent

4,менің айтайын дегенім олар шпионды өлтірді оның достығы мен им достарының балаларын өлтіруге көмектесу коалициясы бар,менің айт де олар шпио өл оның достығ мен им дос бала өлтір көмектес коалиция бар,violent

4,бұл алькаиданың ойынымен ұрлау деп аталады бұл ақштың антитеррорлық құнын қайтару https co eie slpd,бұл алькаида ойын ұрлау де атал бұл ақш антитеррорлық құн қайтару https co eie slpd,violent

4,рауафидтің алғашқы ағаларына тәптәуір жасамайды оны екі адам өлтіреді және шайқасқа шақырады,рауафид алғашқы аға тәптәуі жасама оны екі адам өлтір және шайқас шақыр,violent

4,isis aamaq iraq қашық күштілері киркуктен оңтүстікбатыстағы хавия ауруханасын бомбалау және қалпына келтіру,isis aamaq iraq қашық күшті киркук оңтүстікбатыс хавия аурухана бомбала және қалп келтіру,violent

4,ақш мосул туралы ақпараттың біреуі орнықтырылды ақш ишим аумағындағы бейбіт тұрғындарды isiske қарсы тұруға шақырады https,ақш мосул туралы ақпарат біреу орнықтыр ақш иши аумағ бейбіт тұрғын isis қарсы тұр шақыр https,violent

4,maghrabiarabi мұнарасы us террористтерінің тұрғын үйлері қорықтарының бомбалауы,maghrabiarabi мұнара us террорист тұрғын үй қорық бомбала,violent

4,isis режимі индустриалды ауданға жаңа жаңа сапардан кейін алеппо қаласына кіріп біздің қолымызға түседі,isis режим индустриалды аудан жаңа жаңа сап кейін алеппо қала кір біздің қол түс,violent

4,ақш коалициясы мосулдың білімін әдейі бомбалап жастағы бірінші оқушы өлтірілгеннен кейін олардың адамдарын оқытады https co rjozj ykj,ақш коалиция мосул біл әдей бомбала жастағы бірінші оқушы өлтіріл кейін олардың адам оқыт https co rjozj ykj,violent

4,бұл мехмет өзтүрік емес стамбул бомбасына күдік бұл мұхаммед зана https co sdy vkv hk https co qtytpx,бұл мехмет өзтүрік емес стамбул бомба күдік бұл мұхаммед зан https co sdy vkv hk https co qtytpx,violent

4,сириялық қорғаныс күштері және оның элементтері isisті өлтіруді жеңілдету,сириялық қорғаныс күш және оның элемент isis өлтіру жеңілдету,violent

4,тадмурда өлтірілуде https co sdnq tj,тадму өлтіріл https co sdnq tj,violent

4,осы уақытқа дейін mosul таңертеңнен бомбаланып жатырмын әрбір бомба брюске орташа мәні,осы уақыт дейін mosul таңертең бомбалан жатыр әрбір бомба брюс орташа мән,violent

4,университеттің жастағыларын қалай өлтіруге болады https co cx pxpvuuj,университет жастағы қалай өлтір бол https co cx pxpvuuj,violent

4,ajupfront mehdirhasan менің денсаулығымның арқасында ишим жеңіске жетеді және жақын жерде империалистік батыс елдеріне табыс әкеледі,ajupfront mehdirhasan менің денсаулығ арқа иши жеңіс жет және жақын же империали батыс ел табыс әкел,violent

4,сізді терроризмге қарсы күресу күндізтүні мұсылмандарға шабуыл жасауды ойластырады ол сізге кофе есектеріне оралды https tc,сіз терроризм қарсы күресу күндізтү мұсылман шабуыл жасау ойластыр ол сізге кофе есек ора https tc,violent

4,ақш коалициясы алғашқы бомбалаудан кейін мосул университетіне келді isis брюссельдің орташа кептелісі үшін бұл мәліметтердің мөлшері https co fblemjrtfa,ақш коалиция алғашқы бомбалау кейін мосул университет кел isis брюссель орташа кептеліс үшін бұл мәлімет мөлшер https co fblemjrtfa,violent

4,сіздердің елдеріңіздің денсаулығы мен оқиғалары бомбаламақ па күте тұрыңыз ол сіздің табысыңыз vodkalife putingonewild https co jafuf mkwm,сіздердің ел денсаулығ мен оқиға бомбала па күт тұр ол сіздің табыс vodkalife putingonewild https co jafuf mkwm,violent

4,олардың екеуі де күреседі,олардың екеу де күрес,violent

4,әр айдан кейін исламофобтар stopislam бастайды amp мұсылмандар islamispeace режиміне кіреді оларға бомба тастауға болады олармен бомба болады алдағы уақытқа байланысты,әр ай кейін исламофоб stopislam баста amp мұсылман

islamispeace режим кір оларға бомба таста бол олармен бомба бол алдағы уақыт байланысты,violent

4,бұл жас жігіт норвегияға иракқа милицияларға қосылып сирия халқын өлтіруге бағытталған таратылған оқушылар https co,бұл жас жігіт норвегия ирак милиция қосыл сирия халқ өлтір бағыттал таратыл оқушы https co,violent

4,сіздің ай сайынғы құрметті адамсыз құрбандыңызды құрбандыққа беріңіз біздің балаларымызға ауыртпалық түсірмеңіз және адал емессіз саллахудин айюби https,сіздің ай сайынғы құрметті адам құрбанд құрбандық бер біздің бала ауыртпалық түсірме және адал емес саллахудин айюби https,violent

4,мамандығы терроризм саласындағы көмекші тәуекелдік қарашаға дейінгі аймақ alaune https co skex uwfsn,мамандығ терроризм сала көмекші тәуекелдік қараша дейінгі аймақ alaune https co skex uwfsn,violent

4,сирия көтерілісшілерінің асадпен мен режимді құру режимі пальмиродағы және көтерілісшілері naleppoda исламға қарсы,сирия көтерілісші асад мен режим құру режим пальмиро және көтерілісші naleppoda ислам қарсы,violent

4,ассадистке керемет өлтіру және жаңашылдықты сақтау джабалальтар палмира шеберлерінің назарын аударып оны ұстау,ассадист керемет өлтір және жаңашылдық сақтау джабалаль палмир шебер назар аудар оны ұстау,violent

4,saa балаларының isis автокөлік бомбасында олардың палмира маңындағы альтар тауында нысанаға ие болу туралы хабарланды syria,saa бала isis автокөлік бомба олардың палмир маң аль тау нысана ие болу туралы хабарла syria,violent

4,партизан соғысы басталады isis svbied saa күштері таар тауында палмира қаласының шетінде,партизан соғыс бастал isis svbied saa күш та тау палмир қала шет,violent

4,бұл оқу ресейдің ерекше күштерін анықтауға мүмкіндік береді пальмирада өліп жатыр бұған буссиде ассадистік режиммен келіссөздер туралы келісімге қол қою,бұл оқу ресей ерекше күш анықта мүмкіндік бер пальмира өл жатыр бұған бусси ассади режим келіссөз туралы келісім қол қою,violent

4,us russia shia militias саусақпен бару асадистке палмира үшін isке қарсы қаружараққа қарсы күресу https co,us russia shia militias саусақ бару асадист палмир үшін is қарсы қаружарақ қарсы күресу https co,violent

4,египет sisi ауыр террорист болған жоқ жазушыларға өлтіруді қабылдайды https co jzmeqbhsdk,египет sisi ауыр террорист бол жоқ жазушы өлтіру қабылда https co jzmeqbhsdk,violent

4,пальмира хаялына алаңшалары қайда сіз unesco ұйықтап жатырсыз ба сақ пальмира және бұл жаңалық https co ldbeq vgxl,пальмира хаял алаңша қайда сіз unesco ұйықта жатыр ба сақ пальмира және бұл жаңалық https co ldbeq vgxl,violent

4,джаиш аль фатехтің нусрабаласына ге жуық операциялық демалыс аймағында орналасқан бала бар ма олармен келісуге болады ма,джаиш аль фатех нусрабала ге жуық операциялық демалыс аймағ орналас бала бар ма олармен келіс бол ма,violent

4,ямайкалық исисшейхі бюллетень емес бюллетеньді қайғылы оқиға барлық гомосексуалистерді өлтіру керек https co gfhqtaplbq https,ямайкалық исисшейх бюллетень емес бюллетень қайғылы оқиға барлық гомосексуалис өлтір керек https co gfhqtaplbq https,violent

4,барлық осы көлік құралдары мен барлық күштер is минуттан екі айға дейін жарылады amp барлығы hell iraq сайтына кіру https co wr gfj eu,барлық осы көлік құрал мен барлық күш is минут екі ай дейін жар amp барлығ hell iraq сайт кіру https co wr gfj eu,violent

4,неге асад тадмурды басып алуға бел буды бүлікшілердің алғашқы ойыншылары ол дейр алзурмен есеп айырысу оның орнына ақшаны қайтару үшін қордан қайтыс болады,неге асад тадму бас ал бел бу бүлікші алғашқы ойыншы ол дей алзур есеп айырыс оның орн ақша қайтару үшін қор қайтыс бол,violent

4,олар үшін мектепке кіру қарымқатынас ауруханаларды базарларды көпірлерді фабрикаларды өнімдерді бомбалайды,олар үшін мектеп кіру қарымқатынас аурухана базар көпір фабрика өнім бомбала,violent

4,ислам мемлекеті бельгияны бомбалауды мосулдың оқушылары үшін бомбалауды ұйымдастырды https co yf mjld,ислам мемлекет бельгия бомбалау мосул оқушы үшін бомбалау ұйымдас https co yf mjld,violent

4,багдадтың оңтүстігінде хасвах аймағында жұмыс жасау мобилизациялық милиционерлерге жарылғыш белдікпен amaqagency martyrdom операциясы,багдад оңтүстіг хасвах аймағ жұмыс жасау мобилизациялық милиционер жарылғыш белдік amaqagency martyrdom операция,violent

4,бірінші isis baghdad футбол стадионында жанкештілікке жол ашылды rita katz https co amczfkkjhx,бірінші isis baghdad футбол стадион жанкештілік жол аш rita katz https co amczfkkjhx,violent

4,islamicstate белсендігердің ақшасы аденде араб коалициясының негізінде базасына барады кем дегенде адамды өлтіреді,islamicstate белсендіг ақша аден араб коалиция негіз база бар кем де адам өлтір,violent

4,amaqagency breaking хасвахта шейіт болу операциясының негізінде адам қаза тауып фифа адам жараланады https co,amaqagency breaking хасвах шейіт болу операция негіз адам қаза тау фиф адам жаралан https co,violent

4,соңғы жыл ішінде өзіммен бірге болған сәттермен жақынырақ танысуға болады брюссельдің бомбалануын тойлау https co raubi vv,соңғы жыл іш өз бірге бол сәт жақынырақ таныс бол брюссель бомбалан тойлау https co raubi vv,violent

4,пешмерге шенеунігі олардың isis ең басты бетпебет соғыс жағдайы қолдау білдіру пікір жазу https co femjwrt sk,пешм шенеуніг олардың isis ең басты бетпебет соғыс жағдай қолдау білдіру пікір жазу https co femjwrt sk,violent

4,толығымен жойылу жалғасуда palmyra syria бүгін ұшақтардан әуе соққысы ғана бар ампер тікұшақтар акк аймақ https co dixcjewa,толығ жойыл жалғас palmyra syria бүгін ұшақ әуе соқ ғана бар амп тікұшақ акк аймақ https co dixcjewa,violent

4,сирия режимінің күштері ишимтердің жақсы терроризмі https co mrcf zeqdg,сирия режим күш ишим жақсы терроризм https co mrcf zeqdg,violent

4,альшат жолында танктер мен бронды техникалық күштердің теңізге шығатын жүкті ашты ашады libya https co cri njlk,альшат жол танк мен брон техникалық күш теңіз шығ жүкті аш аш libya https co cri njlk,violent

4,иә тоғыс дәуірінде кобанебомбалаған natoдағы қазақстан туралы есте сақтауды қалайсыз ба https co joprx,иә тоғыс дәуір кобанебомбала nato қазақстан туралы ес сақтау қала ба https co joprx,violent

4,террорист террористік жарылғыш зымыран egyptair ұшағын алып кетіп жатыр кипрге апарады тәжірибе,террорист террористік жарылғыш зымыран egyptair ұшағ алып кет жатыр кипр апар тәжірибе,violent

4,бүгін қуырдақтар мен кластерлік бомбалар күні сухна қаласына пальмираның шығысында,бүгін қуырдақ мен кластерлік бомба күн сухн қала пальмира шығыс,violent

4,ққасөздерінің сөздігі жалдау тиранға башар асадқа қарамақарсы төңкеріс жасап жатыр басқа зулумнан ksa катар баә көмек алдық,ққасөз сөздіг жалда тиран баш асад қарамақар төңкеріс жаса жатыр басқа зулу ksa катар баә көмек ал,violent

4,пальмира тоналып жатыр https co amrsioza,пальмира тонал жатыр https co amrsioza,violent

4,ауғанстанның ресми өкілі пәкістан армиясына қарсы операцияға қатысады,ауғанстан ресми өкіл пәкістан армия қарсы операция қатыс,violent

4,синай египет полициясы джипті адам арариш қаласына kfc мейрамханасында жарылыс салуға көмек алды,синай египет полиция джип адам арариш қала kfc мейрамхана жарылыс сал көмек алды,violent

4,джей бүлдірушілерді isге салыңыз және оны пайдаланғаннан кейін saa baby болыңыз қазір пальмира бомбаның үстінде,дже бүлдіруші is сал және оны пайдалан кейін saa baby бол қазір пальмира бомба үст,violent

4,қолдауым менің мылтықтың бар екеніне лицензиям бар,қолда менің мылтық бар екен лицензия бар,violent

4,синайдағы бұзақылық жасаушылар мұнымен бетпебет кездеседі https co ul gbsuri,синай бұзақылық жасаушы мұны бетпебет кездес https co ul gbsuri,violent

4,бұл милициялар өмірге қайтадан денсаулықты сақтау арқылы жасалды ма оларға біздің бейбіт тұрғындарымызды қосып бейбіт тұрғындарды бомбалауды құптады,бұл милиция өмір қайта денсаулық сақтау арқылы жаса ма оларға біздің бейбіт тұрғын қос бейбіт тұрғын бомбалау құпта,violent

4,америка құрама штаттары сириядағы ракках қаласында нұр мешітпен бомба болады қалаулымпрогресс шейх керри https co zqirex,америка құрама штат сирия ракках қала нұр мешіт бомба бол қалаулымпрогресс шейх керри https co zqirex,violent

4,лас лафиттер жаппай өлтірушіге башарға жол ашылды өлімнің ына жауап береді,лас лафит жаппай өлтіруші баш жол аш өл ын жауап бер,violent

4,жоспарланғандай сою басталды пальмира курсында media мұны жабу қиын https co wvoarfius,жоспарланғанда сою баста пальмира курс media мұны жаб қиын https co wvoarfius,violent

4,deirezzor мұхит базасына isis күнінің күюі және saaның алдынала жол ашуы үшін осы аймақтағы үлкен газдар бомбаларын қорғауды ұнатады syria,deirezzor мұхит база isis күн күю және saa алдынал жол аш үшін осы аймақ үлкен газ бомба қорғау ұнат syria,violent

4,кашмирге осындай демалыс ол куфармен соғысады,кашмир осындай демалыс ол куфар соғыс,violent

4,хамас бұл сіздің назарыңызға қарамақарсы жарылғыш алғашқы суицид айы https co yjoc jvbq,хамас бұл сіздің назар қарамақар жарылғыш алғашқы суицид ай https co yjoc jvbq,violent

4,альэйс халқын ұмытпаңыз олар жаппай бомбалармен және ақ фосформен жарылуда https,альэйс халқ ұмытпа олар жаппай бомба және ақ фосфор жарыл https,violent

4,асадты өлтірген сөздер жанқештілер ишим дамаск ауылындағы жылу станциясына қайтып келеді https co gdz elszpj,асад өлтір сөз жанқешті иши дамаск ауыл жылу станция қайт кел https co gdz elszpj,violent

4,сирия оппозициясы үшін сирия оппозициясы үшін сирия маңындағы аррайдың қаружарағы зардаптарын көтереді https co jvosyrs vs,сирия оппозиция үшін сирия оппозиция үшін сирия маң аррай қаружарағ зардап көтер https co jvosyrs vs,violent

4,isis майданының жанында ақш теңіз артиллериясы күн сайын өрт шығарады https co pvufkwln https co iv xsgnb,isis майдан жан ақш теңіз артиллерия күн сайын өрт шығар https co pvufkwln https co iv xsgnb,violent

4,алкаида мүшелері үш ақш долларын құрайтын жолаушыларды өлтіріп тастайды олардан қалған iske қарсы тұрыңыз https,алкаи мүше үш ақш долл құра жолаушы өлтір таста олардан қалған is қарсы тұр https,violent

4,сонша қаланы немесе екі елді мекенді мекен етіп жатыр ма бізде кемедегі кафелерден жауымызды бомбылауды сұраймыз,сонша қала немесе екі ел мекен мекен ет жатыр ма біз кеме кафе жау бомбылау сұра,violent

4,ешкімнің шаруасы жоқ мен мұсылмандарға қарсы ең жақсы деп ойладым ауыр емес бомбаны жару керек https co ujv zqwl,ешкі шаруа жоқ мен мұсылман қарсы ең жақсы де ойла ауыр емес бомба жар керек https co ujv zqwl,violent

4,booohoo жолдан таюшылар жылап жатыр алкаидаға арналған оңтүстік димашктағы элли элли продаст көтерілісшілерімен бірге келесі қадам https co slxa icb,booohoo жолдан таюшы жыла жатыр алкаида арнал оңтүстік димашк элли элли продаст көтерілісші бірге келесі қадам https co slxa icb,violent

4,кімге қамқорлық күпарларға солтүстік альподағыдай мұсылмандарды бомбалауға рұқсат етілген екіжүзді жылауды доғарыңыз https co hsi rlnx,кім қамқорлық күпар солтүстік альподағыда мұсылман бомбала рұқсат ет екіжүз жылау доғар https co hsi rlnx,violent

4,біз сіздермен өз соғысымызды осылай өткіземіз https co bwlgfkbxom,біз сіздермен өз соғыс осылай өткіз https co bwlgfkbxom,violent

4,a warthogara үстірт ойыны vso бүлікшілеріне нусра amp ахарар альмуртадин исламға қарсы қорғаныс https co wnjax tf,a warthogara үстірт ойын vso бүлікші нуср amp ахар альмуртадин ислам қарсы қорғаныс https co wnjax tf,violent

4,өтпелі сәтте фаллужаның қоршауындағы ирактық аштыққа душар болып отырады ишимді өлтіргендерді өлтіруді қадағалайды https co mqzreewrkq,өтпелі сәт фаллужа қоршау ирак аштық душ бол отыр иши өлтір өлтіру қадағала https co mqzreewrkq,violent

4,мосул тұрғындарын бомбалау және қоржынға салу https co pu ue ma,мосул тұрғын бомбала және қоржын салу https co pu ue ma,violent

4,танкты оқуға бару және ақылы ма айт адамдар үйінде мұндай топ бар https co ee qd qy,танк оқ бару және ақылы ма айт адам үй мұндай топ бар https co ee qd qy,violent

4,катар үкіметі америка құрама штаттарына ирак пен сириядағы қалаларды бомбалау үшін бомбалаушыларға рұқсат етіледі https co qu ghdcvhl,катар үкімет америка құрама штат ирак пен сирия қала бомбала үшін бомбалаушы рұқсат ет https co qu ghdcvhl,violent

4,сирия көтерілісшілері асадпен қарымқатынас жасау туралы келісімді қабылдайды асадтың сааға күшті жинау уақыты туралы дауысты естіді асад алеппоға кірісті https

co dcremgvwij,сирия көтерілісші асад қарымқатынас жасау туралы келіс қабылда асад саа күшті жинау уақыт туралы дау есті асад алеппо кір https co dcremgvwij,violent

4,коалицияның денсаулығы жақсы бір түнде жақсы оқырмандардың қалаларында адам өледі адам жараланады,коалиция денсаулығ жақсы бір түн жақсы оқырман қала адам өл адам жаралан,violent

4,жасанды патшаның обамасына және онымен аммақтастарға мен және христиандарға қарсы күресуге тырысу https co saj dzqrl,жасанды патша обама және онымен аммақтас мен және христиан қарсы күрес тырыс https co saj dzqrl,violent

4,о шейх каферлер барлық жерде өлім жазасына жатады шейх сабр шейх хотистер ksaға кіру уақыты шейх жиһад жихади жиһад,о шейх кафер барлық же өлім жаза жат шейх саб шейх хотис ksa кіру уақыт шейх жиһад жихади жиһад,violent

4,синай ied жарылысы мысыр армиясының көліктері мен альджура бақылауөткізу пункттері арасындағы қиратып адам өліміне әкелу,синай ied жарылыс мысыр армия көлік мен альджур бақылауөткіз пункт ара қират адам өлім әкелу,violent

4,түркия ишимтүркияның территориясына кіру жексенбіде кешкі isisке ауыр болады,түркия ишимтүркия территория кіру жексенбі кешкі isis ауыр бол,violent

4,оларды жазушыларсыз өлтіріп орнына жерлеуде жүреді керри хиросимаға ішке кіруді төлейді usa japan https co ueue zyf,ол жазушыл өлтір орн жерле жүр керри хиросима іш кіру төле usa japan https co ueue zyf,violent

4,ресей асадының әскеріне азықтүлік пен материалдық көмек жазбасы араб елдері сирия мен йеменге бомбаларды лақтыруда мұндай jan шариғат сатысында бригадамен келіссөздер бұл біздің ақшқа кіру мүмкіндігі https co flpave hv,ресей асад әскер азықтүлік пен материалдық көмек жаз араб ел сирия мен йемен бомба лақтыр мұндай jan шариғат саты бригада келіссөз бұл біздің ақш кіру мүмкіндіг https co flpave hv,violent

4,мазасыздықтар зираты жолдан тайдырушыларды нысанаға алу операциялары,мазасыздық зира жолдан тайдырушы нысана алу операция,violent

4,мен күнә жасаймын сен күнә жаса біз твидіміз күнә жасаймыз бірақ күнә жасаңыздар сіздің ең жақсы ойын құқықтар,мен күнә жаса сен күнә жаса біз твид күнә жаса бірақ күнә жаса сіздің ең жақсы ойын құқық,violent

4,салах әлмакиде vsoдан көп жолдан тайушыларды өлтіргенді қалайды,салах әлмаки vso көп жолдан тайушы өлтір қала,violent

4,савранның батысында алдынала сөзбен айтып болашақ жарылыстан кейін қалған vso жолдан таюшылар өлтірілгенге дейін өледі,савран батыс алдынал сөз айт болашақ жарылыс кейін қалған vso жолдан таюшы өлтіріл дейін өл,violent

4,ypg qamishloda режиммен күресіп жеңіс is қалаларында ypg күшті және сіздің қолыңызда,ypg qamishlo режим күрес жеңіс is қала ypg күшті және сіздің қол,violent

4,пентагон iske қарсы соғыс сізге миллион доллар,пентагон is қарсы соғыс сізге миллион доллар,violent

4,жақында достарыңызға бірбір түндік ұстаздық мылтық және флегиондық қарау https co,жақында дос бірбі түн ұстаздық мылтық және флегио қарау https co,violent

4,сирия сауд арабиясы иордания аллоушты өлім элементі деп санайды https co qcebqk di https co jftm ytg,сирия сауд арабия иордания аллоуш өлім элемент де сана https co qcebqk di https co jftm ytg,violent

4,жарылыстан кейін сіздің сүйіспеншілігіңіз пайда болады абринидің арбасына қайтарылады жақсы brussels airport әуежайы https жеткізілуге мәжбүр болды,жарылыс кейін сіздің сүйіспеншілі пайда бол абрини арба қайтар жақсы brussels airport әуежай https жеткізіл мәжбүр бол,violent

4,асад нан астам жаңа бейбіт тұрғынға өлім жасады ақштың бейбітшілікпен айналысатын азаматтары қайтыс болады https co jntzdfsy,асад нан астам жаңа бейбіт тұрғын өлім жас ақш бейбітшілік айналыс азамат қайтыс бол https co jntzdfsy,violent

4,жарылғыш зат салынғаннан кейін жолдан екінші жолға шығарылды,жарылғыш зат салын кейін жолдан екінші жол шығар,violent

4,керемет сириялықтар мен иректықтарды isistің бейбітшілік емес өмір сүру тірліктері үшін жазуға арналған бомбалайық https co f uwo,керемет сириялық мен ирактық isis бейбітшілік емес өмір сүру тірлік үшін жаз арнал бомбал https co f uwo,violent

4,біз кибербомбаларды тастап жатырмыз https co uye jwb,біз кибербомба таста жатыр https co uye jwb,violent

4,мен айтқандай сізге тек бомба тастау қажет жұмысты аяқтауға болмайды фаллужа халқы сіздің көмегіңізге мұқтаж емес https co qmkks qgb,мен айтқанда сізге тек бомба тастау қажет жұм аяқта болма фаллуж халқ сіздің көмег мұқтаж емес https co qmkks qgb,violent

4,соғысушылар жетекшісі гейлердің белсенділігі мен оның досы дакакадағы пәтерде өлтірді біз кезкелген amaqagency жариялауын күтеміз,соғысушы жетекші гей белсенділіг мен оның дос дакака пәтер өл біз кезкел amaqagency жарияла күт,violent

4,бағдад әлджадидаға қастандық жасағаны үшін ке жуық адамды өлтіріп өлтіріп жатыр https co zyxvm,бағдад әлджадида қаста жаса үшін ке жуық адам өлтір өлтір жатыр https co zyxvm,violent

4,сиретті isis lbiaден бастауға және операциядан бастауға болады,сире isis lbia баста және операция баста бол,violent

4,альаднани ол жерге түсіп оны өлімге жерге кіруге және қайтаруға әкеледі,альаднани ол же түс оны өлім же кір және қайтар әкел,violent

4,баә хефттарды қолдау пайда болды катар фаджр ливияға жазба техникалық тапсырма,баә хефт қолдау пайда бол катар фадж ливия жазба техникалық тапсырма,violent

4,сол кезде көптеген фитна жасайтын болды,сол кезде көптеген фитн жаса бол,violent

4,us теңіз күштері солтүстікке raqqa syria breakingге келеді,us теңіз күш солтүстік raqqa syria breaking кел,violent

4,ақш еуропа бұұ сізге опасыздық жасады асад иранның сүйікті өлімін түркияның басқа араб елдері қолдайды алеппожатушы,ақш еуропа бұұ сізге опасыз жас асад иран сүйікті өл түркия басқа араб ел қолда алеппожатуш,violent

4,ақш ирак пен ауғанстанды жойды ресей сирияны орнында жатыр араб залымдары өз халқын өлтірді бәрі сенің жауың aleppoisburning,ақш ирак пен ауғанстан жой ресей сирия орн жатыр араб залым өз халқ өл бәрі сенің жау aleppoisburning,violent

4,арабтарды басқару олар өз халқын өлтіргендерге лас сатқындық пен жолдан тайушылар олар өлтіруге лайық,араб басқару олар өз халқ өлтір лас сатқ пен жолдан тайушы олар өлтір лайық,violent

4,идлибтегі маратнумда наразылық іздеушілер джабхат аннусраның жалауларына қамқорлық жасайды ші дивизионмен байланысыңыз https,идлиб маратну наразылық іздеуші джабхат аннусра жалау қамқорлық жаса ші дивизион байланыс https,violent

4,сириядағы ис асадқаға қарсы күрескен жалғыз топ барлық келіссөздер асадты бомбалайды немесе соғыста,сирия ис асадқа қарсы күрес жалғыз топ барлық келіссөз асад бомбала немесе соғыс,violent

4,ақштың fighterjets телефондары жоғары деңгейдегі isis құрамын жетілдіреді және өлтіреді https co fr oqbj cm https co xxctuuqein,ақш fighterjets телефон жоғары деңгей isis құр жетілдір және өлтір https co fr oqbj cm https co xxctuuqein,violent

4,олар күндізтүні бомба жасайды,олар күндізтү бомба жаса,violent

4,сауд арабиясының табысты қамқорлығы американың американдық крестшілері сирия мен ирактағы мұсылмандарды бомбалауға дайын https co jhaab gfzg,сауд арабия табысты қамқорлығ америка американдық крестші сирия мен ирак мұсылман бомбала дайын https co jhaab gfzg,violent

4,aq жанкүйерлері is басшылығының басшылық құрамымен бірге бомбалаудан кейін is мұғалімді ramadi деп мазақ етеді,aq жанкүйер is басшылығ басшылық құрам бірге бомбалау кейін is мұғалім ramadi де мазақ ет,violent

4,ислам мемлекеті сарбаздарының қосылуымен операция бағдадта дан жоғары шоғырланған содырларын өлтіріп жаралайды https co jfg gjjh,ислам мемлекет сарбаз қосылу операция бағдад дан жоғары шоғырлан содыр өлтір жарала https co jfg gjjh,violent

4,мына заман өзгерді,мына заман өзге,violent

4,рахман әлказақи хижра қылып шам жеріне хижра қылып жиһадқа келдік,рахман әлказақи хижр қыл шам же хижр қыл жиһад кел,violent

4,абухалид әлказақи құлшылық етудің шыңы жиһад мұнда жиһад қылуға келдік,абухалид әлказақи құлшылық ету шың жиһад мұнда жиһад қыл кел,violent

4,сайф аддин әлказақи бүкіл мұсылмандарға қазір жиһад жасау фард бір қадам жерге жау кірсе бүкіл мұсылмандар қорғануға фард болады ал егер қорғануға шамасы келмесе қастарындағы соседный жерлерге көшеді сөйтіп бүкіл жер шарын алады,сайф аддин әлказақи бүкіл мұсылман қазір жиһад жасау фард бір қадам же жау кір бүкіл мұсылман қорған фард бол ал егер қорған шама келме қас соседны жер көш сөйт бүкіл жер шарын ал,violent

4,міне біз бауырларымызбен балашағаларымызбен отбасыларымызбен жиһад қылуға хижра қылдық міне мынандай құралдарымызбен жиһад қылуға шықтық біз дің өзіміздің араб бауырла ры мызға сәлемдемеміз бар,міне біз бауыр балашаға отбасы жиһад қыл хижр қыл міне мынанда құрал жиһад қыл шық біз ді өз араб бауырла ры мыз сәлемдеме бар,violent

4,осы жолда өлуді армандаймыз,осы жолда өлу арманда,violent

4,сирияға барамын кафирлерді қырып соғыс кезінде джихадта өлсем арманым жоқ жаннатта кездесейік кафирлерді қырамыз,сирия бар кафир қыр соғыс кез джихад өл арман жоқ жаннат кездес кафир қыр,violent

4,ирактың мадинатуль мосул деген жерінде так сенің басыңды кесетін браттар бар қазахистаныңда,ирак мадинатуль мосул де же так сенің бас кес брат бар қазахистан,violent

4,сол екен гой маймылданып отырганын жакында дин туралы зан шыгад сосын коремиз сендей шайтаннын кулдарын олтиру бизге сауап тозактын ити,сол екен го маймылдан отырган жак дин туралы зан шыгад сосын коремиз сенде шайтанн кул олтир биз сауап тозакт ити,violent

4,жақында джихад болады макака абайла,жақында джихад бол макака абайла,violent

4,о сумелек бизди джихаттан коркады дейсинбесенин козинди жою бизге ен улкен джихат сендер мусылман елинде журип мусылмандарды олтирип журсиндер тозактын ити сендейлерге сеним жок сендер мксылмандарга букил бале жалени акелетин,о сумелек бизди джихат корк дейсинбесенин козинди жою биз ен ул джихат сен мусылман елин жури мусылман олтири журсин тозакт ити сендей сени жок сен мксылмандарг букил бал жалени акелетин,violent

4,тозақтың иттерісіндер такфирлар мен хауариж,тозақ иттерісін такфир мен хауариж,violent

4,біз джихад жасаймыз сендердің сөгістеріңнен қорықпаймыз джихад тоқтамайды бізді күтіңдер біз барамыз елдеріңе кәфірлерге ұнамасада ул түл ислам баақия,біз джихад жаса сен сөгіс қорықпа джихад тоқтама біз күт біз бар ел кәфір ұнамаса ул түл ислам баақия,violent

4,а так көп алма өзіңе сол жақтағы браттар арматураның үстіне құлап өліп қалуыңа жәрдем беріп жіберер мен даулатуль исламдамын,а так көп алма өз сол жақ брат арматура үст құла өл қал жәрде бер жібер мен даулатуль ислам,violent

4,мұнда жиһад қылуға келдік,мұнда жиһад қыл кел,violent

4,әбу әниса қазақстандағы жұртқа арнап сирия жерінде башарға шииттерге кәпірлер мен демократтарға қарсы соғысып жатқандарын айтып қарулы жиһадқа қосылуға шақырды,әбу әни қазақстандағы жұрт арна сирия же баш шиит кәпір мен демократ қарсы соғыс жат айт қарулы жиһад қосыл шақ,violent

4,бұл бауырыңыз шам жеріңде дейризор соғыста шахид болды,бұл бау шам же дейризо соғыс шахид бол,violent

4,ислам революциясы жиһад болып жатыр ислам мемлекеті құрылады арманым сондай мұсылман елде өмір сүру,ислам революция жиһад бол жатыр ислам мемлекет құр арман сондай мұсылман ел өмір сүру,violent

4,мама мен кетіп барамын мені кешіріңіздер сіздерді жақсы көремін ақгүлді сізге тапсырдым сирияға кетіп қалдым мені іздемеңіздер мені өлді деп санаңыздар,ма мен кет бар мен кешір сіз жақсы көр ақгү сізге тапсыр сирия кет қал мен іздеме мен өл де сана,violent

4,бауырлар сириядагы мусылман бауырларымызга комекке баруга шакырамын казак болып мусылман болып комекке барайык,бауыр сириядаг мусылман бауырларымызг комек баруг шакыр казак бол мусылман бол комек барайык,violent

4,абу хамза казахстанский,абу хамза казахстански,violent

4,уақыт келді ахи,уақыт кел ахи,violent

4,ахи біздің уақыт келді,ахи біздің уақыт кел,violent

4,қанша жүре береміз ахи,қанша жүр бер ахи,violent

4,біз сіздерге қарсы әрекет ете бастаймыз,біз сіздерге қарсы әрекет ет баста,violent

4,ит екеш ит те тек өз территориясын қорғайды ит құрлы миларыңның болмағаны ма бейшаралар,ит екеш ит те тек өз территория қорға ит құрлы ми болма ма бейшара,violent

4,кіші жиһадтан ең үлкен жиһадқа келе жатырмыз,кіші жиһад ең үлкен жиһад келе жатыр,violent

4,вахабистер олар салафи боламаиды саяси сауатсыз вахабистер көзин курту керек,вахабис олар салафи боламаи саяси сауат вахабис көзин курт керек,violent

4,қазақстаннан сирияға жиһад жасау үшін хижра қылдық сирияда соғысып жүрген жихадшылармыз,қазақстан сирия жиһад жасау үшін хижр қыл сирия соғыс жүр жихадшы,violent

4,қазақстанда қалу сол жерде өмір сүру харам,қазақстан қалу сол же өмір сүру харам,violent

4,біздің бауырларымыз жамағат мақсатымыз халифат жолымыз жиһад,біздің бауыр жамағат мақсат халифат жол жиһад,violent

4,жиһадқа шығу шынымен сіз үшін қиын ба қолдарың түсіп қала ма жоқ онда неге жиһадқа бармайсыңдар кәпірсіңдер ғой,жиһад шығу шын сіз үшін қиын ба қол түс қала ма жоқ онда неге жиһад барма кәпір ғой,violent

4,онда өлу үшін барамыз,онда өл үшін бар,violent

4,шындықты іздеп әр жерге бардымғой мен таблиғи жамағата саид нурси де болдым ақыр соңында осында түрмеге келдім,шындық ізде әр же бардымғо мен таблиғи жамағат саид нурси де бол ақы соң осында түрме кел,violent

4,менің уақытым аз бізді қоршап алды бізден екі квартал төмендегілердің бәрін қиратты мен тірі қалмайтын сияқтымын мама осы жерге келгенім үшін кешірім сұраймын олар бейкүнә балалар мен әйелдерді зорлап жатыр соғыс не үшін және кім үшін болып жатқанын түсінбеймін бұйрықты кім беріп жатқаны белгісіз бұл біздің соғыс емес менің соғысым емес мама кешіріңізші мені,менің уақыт аз біз қорша алды бізден екі квартал төмендегі бәрін қира мен тірі қалма сияқты ма осы же кел үшін кешір сұра олар бейкүнә бала мен әйел зорла жатыр соғыс не үшін және кім үшін бол жат түсінбе бұйрық кім бер жат белгісіз бұл біздің соғыс емес менің соғыс емес ма кешіріңізш мен,violent

4,сирияға барамын кафирлерді қырып соғыс кезінде джихадта өлсем арманым жоқ кафирлерді қырамыз,сирия бар кафир қыр соғыс кез джихад өл арман жоқ кафир қыр,violent

4,біз өлтіруді тек үйреніп қоймай оны жақсы көруіміз де керек саудтар мен ваххаббисттерді жаппай өлтіру ғана құтқарады,біз өлтіру тек үйрен қойма оны жақсы көр де керек сауд мен ваххаббист жаппай өлтір ғана құтқар,violent

4,бауырлар алыс жакка кеткен агайындарымызды ойлайык сирияга комек жасауга кол ушын созуга барайыкол жактагы салафилердын козин куртып баурлар ушин согысайык бауырларды корлагандарды тозак отына жиберейык,бауыр алыс жакк кет агайын ойлайык сирияг ко жасауг кол уш созуг барайыкол жактаг салафилерд козин курт баур ушин согысайык бауыр корлаган тозак от жиберейык,violent

4,не джихад не ұятқа қалу таңда,не джихад не ұят қалу таңда,violent

4,мен францияда тұрамын бірақ менің отанымда жиһад болып жатыр мүмкін бола салысымен мен оған қатысқым келеді,мен франция тұр бірақ менің отан жиһад бол жатыр мүмкін бола салысымен мен оған қатыс кел,violent

4,бізді басқалар алмастырады бірақ джихад тоқтамайды,біз басқа алмастыр бірақ джихад тоқтама,violent

4,бауырлар мен әпкелер джихадқа асығыңыздар,бауыр мен әпке джихад асығ,violent

4,кәпірсің жиһадты қолдамайсың ана жақта мұсылмандарды қырып жатыр ал сен қолдамайсың,кәпір жиһад қолдама ана жақта мұсылман қыр жатыр ал сен қолдама,violent

4,мен соғысу үшін бардым ғой қазақстанға оралу туралы ешқашан ойламадым,мен соғыс үшін бар ғой қазақстан орал туралы ешқашан ойлама,violent

4,біз игилде жақсы тұрдық ұрладық зомбылық жасадық кәпірлерді азаптап видеоға түсірдік те барлығын қорқыту үшін оларды желіге салдық бірақ содан кейін орыс әскери авиациясы келді де игилдің қаржысы бірден қысқара бастады енді біз үйге қайтқымыз келеді көмектесіңіздер,біз игил жақсы тұр ұрла зомбылық жаса кәпір азапта видео түс те барлығ қорқыт үшін ол желі сал бірақ содан кейін орыс әскери авиация кел де игил қаржы бірден қысқар баста енді біз үй қайт кел көмектес,violent

4,қаңтардағы skxx шындық онда ол fsaмен ұрыс пен өлтіруді бастайтын адам деп айтады,қаңтар skxx шындық онда ол fsa ұрыс пен өлтіру баста адам де айт,violent

4,генерал петреус ислам мемлекетін жеңу үшін алькаида дж жауынгерлерін қолданыңыз,генерал петреус ислам мемлекет жеңу үшін алькаи дж жауынгер қолдан,violent

4,адамзат тек кафтарларға жатады егер кафтар мұсылмандарға геноцид жасаған болса бұл адамзат,адамзат тек кафтар жат егер каф мұсылман геноцид жаса бол бұл адамзат,violent

4,mrrman abu adamm etdz haditha ibnu noran иә мені муртад деп соттасан да бәрібір,mrrman abu adamm etdz haditha ibnu noran иә мен муртад де соттасан да бәрібір,violent

4,haditha ibnu noran etdz wilayathalab abu adamm сен мені білмейсің сосын мені муртад деп сотта сендерге қасірет ұят саған,haditha ibnu noran etdz wilayathalab abu adamm сен мен білме сосын мен муртад де сот сен қасірет ұят саған,violent

4,oooharray hadialabdallah қаскүнемдікпен сөйлейтін қатал пернетақта жауынгері елге ишимпен күресу қажет және әлі де мүмкін емес бақыя,oooharray hadialabdallah қаскүнемдік сөйле қатал пернетақ жауынгер ел ишим күресу қажет және әлі де мүмкін емес бақыя,violent

4,rt ramiallolah сауди ғалымдары ресей леванттағы православтық крестшілердің жаңа науқанын бастады әр мұсылман мүжавді қаржыландыруы қолдауы керек,rt ramiallolah

сауди ғалым ресей левант православ крестші жаңа науқан баста әр мұсылман мүжав қаржыландыр қолда керек,violent

4,jsiwat сен араб калб бар яхуд шеберлеріңе бағын,jsiwat сен араб калб бар яхуд шебер бағын,violent

4,wilayatsayna медиаофисі audiostatement біз оны түсіріп алдық сондықтан сіздің ашуыңыздан өлеміз,wilayatsayna медиаофи audiostatement біз оны түсір ал сондықтан сіздің ашу өл,violent

4,күресуді бастағаныңызды ешқашан ұмытпаңыз ислам мемлекетімен күресуді бастаған алшабаб,күресу баста ешқашан ұмытпа ислам мемлекет күресу баста алшабаб,violent

4,maghrebimwss сіздің ашуланшығыңызда өліп жатырсыз өйткені майлы итіңіз халифа бағдади ла натулла сонымен қатар өмір лол болып табылады,maghrebimwss сіздің ашуланшығ өл жатыр өйткені майлы ит халиф бағдади ла натулл сонымен қатар өмір лол бол таб,violent

4,rt abdullahazam ирактағы американдық одақтастар шиит лаңкестік топтары тұтқында отырған жазықсыз сунниттің құлағын кесіп алуда,rt abdullahazam ирак американдық одақтас шиит лаңкестік топ тұтқын отыр жазық сунни құлағ кес ал,violent

4,rt revolutionsyria әлемнің жазасыздығы бар мега террорист ол асад үшін сириялықтарды өлтіріп аштан өлтіру үшін өзінің топтарын лебаноннан жібереді syria,rt revolutionsyria әлем жазасыздығ бар мега террорист ол асад үшін сириялық өлтір аш өлтір үшін өзінің топ лебанон жібер syria,violent

4,фаллужа ирак мұсылмандарының жолдауы бізді таңертең және кешке бомбала біздің арманымыз шейіт болып өлу,фаллуж ирак мұсылман жолда біз таңертең және кеш бомбал біздің арман шейіт бол өл,violent

4,rt иракта ис ке қарсы күресетін топтардың көбісі кадрлық бұзушылықтарға қарапайым азаматтарды өлтіруге қатысы бар ирак армиясы sf милиционерлер,rt ирак ис ке қарсы күрес топ көбісі кадрлық бұзушылық қарапайым азамат өлтір қатыс бар ирак армия sf милиционер,violent

4,rt ramiallolah алдағы isis альхаят шығарылымы parisattacks тақырыбы оларды мүмкін болған жерде өлтіріңіз france,rt ramiallolah алдағы isis альхаят шығарылым parisattacks тақырыб ол мүмкін бол же өлтір france,violent

4,rt rburton snowden isis от сияқты біз оған бензинді лақтырып тастаймыз,rt rburton snowden isis от сияқты біз оған бензин лақтыр таста,violent

4,rt craigcons бас раввин палестиналықтарды өлім жазасына кесуге шақырады cnn fox bbc сіз сондасыз ба саңырау үнсіздік,rt craigcons бас раввин палестиналық өлім жаза кес шақыр cnn fox bbc сіз сонда ба саңырау үнсіздік,violent

4,fallujah alkarama жаппай қақтығыстар жаппай шығындар күтілетін олжалар соғыс iraq,fallujah alkarama жаппай қақтығыс жаппай шығын күтіл олжа соғыс iraq,violent

4,намаздан қайтқандардың үйлерін өртеп тастау мысалы мешітте намаз оқымайтындар,намаз қайт үй өрте тастау мысал мешіт намаз оқыма,violent

4,орыс шошқалары мұсылмандарға қарсы соғыс кезінде сириядағы босқындар лагерін қиратады,орыс шошқа мұсылман қарсы соғыс кез сирия босқын лагер қират,violent

4,сауд арабиясының үкіметі жолдан тайған иттер және оларды араб түбегінен тазарту керек деп санайды хабарланғаны осы,сауд арабия үкімет жолдан тай ит және ол араб түбег тазарт керек де сана хабарлан осы,violent

4,рафиди армиясының бульдозерін саддат самарраға жақын бағыттаушы зымыранмен нысанаға алғаннан кейін өртеу,рафиди армия бульдозер саддат самарра жақын бағыттауш зымыран нысана ал кейін өрте,violent

4,араб теледидары көпшілікті алдау үшін израильдіктерге қарсы риторикаларға толы бірақ олардың әскерлері қатыгездікпен оларды өлтіруге арналған,араб теледи көпшілік алда үшін израильдік қарсы риторика толы бірақ олардың әскер қатыгездік ол өлтір арнал,violent

4,готика челаны батаклан өлтіруге беріңіз сіз осындасыз бірақ португалдықтарды өлтіру туралы сөзіңізді біз сізді көп естиміз,готик чела батаклан өлтір бер сіз осында бірақ португалдық өлтір туралы сөз біз сіз көп ести,violent

4,killallmuslims трендін ұстанатын ұлыбританиядағы құрметті достар сіздің алькаидадан усамадан және isisтен еш айырмашылығыңыз жоқ,killallmuslims тренд ұстан ұлыбритания құрметті дос сіздің алькаида усама және isis еш айырмашылығ жоқ,violent

4,хиллари isis туралы және расистік террорист туралы қозғалысты қолдаушы,хиллари isis туралы және раси террорист туралы қозғал қолдаушы,violent

4,вани жай террорист емес исламшыл кашмир қозғалысы енді оның исимнің мұсылман емес идеологиясын тазарту туралы емес,вани жай террорист емес исламшыл кашмир қозғалыс енді оның исим мұсылман емес идеология тазарт туралы емес,violent

4,даиш күтілгендей нацист болып келеді,даиш күтілгенде нацист бол кел,violent

4,сіздің басыңды бұрап алу керек онда жақсы мен жаман да бар содан кейін исис содан кейін сіз барсыз,сіздің бас бұра алу керек онда жақсы мен жаман да бар содан кейін исис содан кейін сіз бар,violent

4,джихади мылқау исистің ақымақ сарбазы басына шөп қойып жасырынуға тырысады,джихади мылқа иси ақы сарбаз бас шөп қой жасырын тырыс,violent

4,мадинадағы жарылыстардан кейін isisті айыптауыңыз бұл нонсенс туралы айтады бұл соңы емес бірақ насихаттар нашар бұл жақсы,мадина жарылыс кейін isis айыпта бұл нонсенс туралы айт бұл соңы емес бірақ насихат нашар бұл жақсы,violent

4,бұл оңбаған өзінің миссури хитында кез келген мұсылманды isis сияқты көретінін біледі оны кісі өлтіргісі келгені үшін тұтқындаңыз,бұл оңба өзінің миссури хит кез кел мұсылман isis сияқты көр біл оны кісі өлтір кел үшін тұтқында,violent

4,оның пікірін аддахир қолдады ол шейх хасан әлбанна сөзсіз ислам қозғалысының алғашқы нышаны болды шейх хасан әлбанна қазіргі ислам қозғалысында джихадтың тұқымын отырғызды ол сондайақ мен осы еңбегімнің сыйақысын жастарды демалыс және ойын саласынан джихад шайқасы алаңына шығарған исламдық жандануға бағыттайтын имам хасан әлбаннаға арнадым,оның пікір аддахи қолда ол шейх хасан әлбанн сөзсіз ислам қозғалыс алғашқы нышан бол шейх хасан әлбанн қазіргі ислам қозғалыс джихад тұқым отыр ол сондайақ мен осы еңбег сыйақы жас демалыс және ойын сала джихад шайқас алаң шығар исламдық жандан бағытта имам хасан әлбанна арна,violent

4,себебі қаскүнем кәпір тек бір тілді түсінеді бұл билік тілі өлтіру ұрыпсоғу және бауыздау тілі басын кесу оларды жүк көлігінің астына тегістеп олар өздерінің жизияны бермейінше олар масқара болып тұрғанда тірідей жағу тілі тек террористік тактика,себебі қаскүнем кәпір тек бір тілді түсін бұл билік тіл өлтір ұрыпсоғ және бауызда тіл басын кесу ол жүк көліг аст тегісте олар өз жизия бермейінш олар масқара бол тұр тірідей жағ тіл тек террористік тактик,violent

4,джихад дуа арқылы,джихад ду арқылы,violent

4,абай болыңыз кедергілер болмайды және өткен және өтпеген үшін пайдасы бар шынында да апатқа ұшырайды дуа оны қарсы алады сондықтан екеуі де бір бірімен араласады дуа арқылы джихад,абай бол кедергі болма және өткен және өтпе үшін пайда бар шын да апат ұшыра ду оны қарсы ал сондықтан екеу де бір бір аралас ду арқылы джихад,violent

4,ибн халдун ол өз халқына жетіп олардың арасындағы араздықты жеңілдетіп суданда жиһадқа баруға жол ашты осылайша олардың жерлерінің тоқсан аймағын басып алды деді мурабит әмірлігінің басшылары,ибн халдун ол өз халқ жет олардың ара араздық жеңілдет судан жиһад бар жол аш осылайша олардың жер тоқсан аймағ бас алды де мурабит әмірліг басшы,violent

4,шынында да мүжахидтер қатысып жатқан бұл ұлы шайқастар және мыңдаған муаххидиндердің жиһад ареналарына кіруі шахадаттардың орындарын іздеу бұл қысымның таралуы мен шындықтың пайда болуының жақсы жаңалықтары бірақ сіз асықпайсыз,шын да мүжахид қатыс жат бұл ұлы шайқас және мыңдаған муаххидин жиһад арена кір шахадат орын іздеу бұл қыс тарал мен шындық пайда болу жақсы жаңалық бірақ сіз асықпа,violent

4,ибн кутайба ха қайтыс болды атхардағы сияаха терминін түсіндіруде сияха қалаларды тастап ашық жерге саяхаттау бани исраилге табынушылардың ісәрекеті сияқты мұсылмандарға бұған тыйым салыңыз және оны парасаттылық пен монотеизм дінімен жіберіңіз гариб хадис жиһадтың сиасы,ибн кутайб ха қайтыс бол атхар сияах термин түсіндір сиях қала таста ашық же саяхаттау бани исраил табынушы ісәреке сияқты мұсылман бұған тыйым сал және оны парасаттылық пен монотеиз дін жіб гариб хадис жиһад сиа,violent

4,уа мувахидин бүгін түркия сіздің джихадтағы мақсатыңызға айналды олардың қауіпсіздігін дүрбелеңге және өркендеуді қорқынышқа айналдырыңыз содан кейін оларды күресіңіздің өртенген аймақтарына апарыңыз,уа мувахидин бүгін түркия сіздің джихад мақсат айна олардың қауіпсіздіг дүрбелең және өркендеу қорқыныш айналдыр содан кейін ол күрес өртен аймақ апа,violent

4,ол бұл хабарды халифа әлкадир биллахқа ибнулджаузи айтқан хатында оны кәпір батиния мен қылмыстық мүбтадийдің дауысынан тазартады деп жазды жағдайдың шындығы сіздің құлдарыңыздың куфр мен бұзылған адамдармен күресуге және хурасанның жерлерінде пайда болған батини сектасының зұлымдықтарын бағындыруға бағытталған күшжігеріне байланысты сіздің мәртебеңізге назар аударды рау олардың басты пана ретінде олардың куфрлеріне шақырды,ол бұл хаб халиф әлкади биллах ибнулджаузи айт хат оны кәпір батиния мен қылмыстық мүбтадий дауыс тазарт де жаз жағдай шындығ сіздің құл куф мен бұзыл адам күрес және хурасан жер пайда бол батини секта зұлымдық бағындыр бағыттал күшжіг байланысты сіздің мәртебе назар ау ра олардың басты пан рет олардың куфр шақ,violent

4,имам багбагир түнгі рейдтер туралы тарауда былай деді бұл түнгі рейдтердің рұқсат етілгендігінің және абайсыздық пен ұқыпсыздықтың салдарынан мушрикиндердің өлтірілуінің дәлелі егер бұл олардың әйелдеріне және балаларына соққы әкелсе де тыйым әйелдер мен балаларды өлтіру оларды бөлуге болатын жағдайларға қатысты болады егер олар бекіністің ішінде болса оларға қарсы католиктер орнатып оларға оқ жаудырып оларды суға батыруға рұқсат етіледі таиф халқына қарсы католик жасау банилмусталикке қатыгез шабуыл жасау және түнгі рейдтер мен отпен шабуылдарға тапсырыс беру,имам багбаги түнгі рейд туралы тара былай де бұл түнгі рейд рұқсат етілгендіг және абайсыз пен ұқыпсыздық сал мушрикин өлтірілу дәлел егер бұл олардың әйел және бала соққы әкел де тыйым әйел мен бала өлтір ол бөл бол жағдай қатысты бол егер олар бекініс іш бол оларға қарсы католик орнат оларға оқ жаудыр ол су батыр рұқсат ет таиф халқ қарсы католик жасау банилмусталик қатыгез шабуыл жасау және түнгі рейд мен от шабуыл тапсырыс беру,violent

4,ибн әласир ибнуласир оның губернаторы абулфутухтың дінге сенбейтіні тіпті атеизм үшін айыпталғандығы туралы айтылды ол өз адамдарын өз дінінде жүруге шақырды олар бұған жауап берді ямин аддавла оған қарсы жиһад жасап оны құлатуға шешім қабылдады сондықтан ол оған бет алды бірақ ол келе жатқан өзендердің терең және кең екенін көрді сондықтан ол екінші жағына өте алмады үнді патшасына өз жерлерін

мултанға өткізуді өтінді бірақ ол оның өтінішін орындамады сондықтан ол мултанның алдына барып біз екі рейдті біреуге біріктіреміз деді джихад арқылы дуа,ибн әласи ибнуласи оның губернатор абулфутух дін сенбе тіпті атеиз үшін айыпталғандығ туралы айт ол өз адам өз дін жүр шақ олар бұған жауап бер ямин аддавл оған қарсы жиһад жаса оны құлат шешім қабылда сондықтан ол оған бет алды бірақ ол келе жат өзен терең және кең екен көр сондықтан ол екінші жағ өте алма үн патша өз жер мултан өткізу өт бірақ ол оның өтініш орындама сондықтан ол мултан алд бар біз екі рейд біреу біріктір де джихад арқылы ду,violent

4,көп ұзамай біз пәкістанның ирак пен ауғанстандағыдай толық шабуылын көреміз егер пәкістан үкіметі батыс талап еткендей жасамаса,көп ұзама біз пәкістан ирак пен ауғанстандағыда толық шабуыл көр егер пәкістан үкімет батыс талап еткенде жасама,violent

4,бұл тек талибан мен үкімет арасындағы бейбіт келісімді бұзуға бағытталған үгітнасихат соғысы талибан сұбаттан бунер ауданына соғыс кезінде талибанды өлтірген бірнеше адамды жазалау үшін көшіп келді және егер олар осындай әрекеттерді жасаса басқа салдары туралы бунердің азаматтарына ескертті енді бүкіл ақпарат құралдары талибан исламабадқа жақындап жатыр деп жылап жатыр егер олар исламабадқа жақын болса бұл жақсы жаңалық болар еді бірақ бұл қазір үгітнасихат соғысы,бұл тек талибан мен үкімет ара бейбіт келіс бұз бағыттал үгітнасихат соғыс талибан сұбат бун аудан соғыс кез талибан өлтір бірнеше адам жазалау үшін көш кел және егер олар осындай әрекет жаса басқа сал туралы бун азамат ескер енді бүкіл ақпарат құрал талибан исламабад жақында жатыр де жыла жатыр егер олар исламабад жақын бол бұл жақсы жаңалық бол ед бірақ бұл қазір үгітнасихат соғыс,violent

4,телефон арқылы талибан өкілдеріне қол жеткізуге болады ма,телефон арқылы талибан өкіл қол жеткіз бол ма,violent

4,телефон арқылы талибан өкілдеріне қол жеткізуге болады ма ия олар бәрібір болуы керек олардың телефон нөмірі вебсайтта орналасқан,телефон арқылы талибан өкіл қол жеткіз бол ма ия олар бәрібір бол керек олардың телефон нөмір вебсайт орналас,violent

4,біз бірнеше сағат ішінде исламабадтың алға жылжуымен пәкістан мемлекетіне төніп тұрған экзистенциалды қауіптің маңыздылығын баса айта алмаймыз деді ол оның айтуынша христиандар пәкістан мемлекетін құлатуды көздейді,біз бірнеше сағат іш исламабад алға жылжу пәкістан мемлекет төн тұр экзистенциа қауіп маңыздылығ бас ай алма де ол оның айт христиан пәкістан мемлекет құлату көзде,violent

4,ауғанстан ислам әмірлігінің ресми өкілі талибан кари мухаммад юсуф ахмади аудандары ел тел оңтүстік батыс және солтүстік біз татуластырушы сиыр олар тонауға болып табылмайтын бірақ сиыр сезінеді емес ақпарат болып табылады мобильді забихулла мужахид елдің оңтүстікшығыс және солтүстікшығыс аймақтары үшін тел ұялы,ауғанстан ислам әмірліг ресми өкіл талибан кари мухаммад юсуф ахмади аудан

ел тел оңтүстік батыс және солтүстік біз татуластыруш сиыр олар тона бол табылма бірақ сиыр сезін емес ақпарат бол таб мобильді забихулл мужахид ел оңтүстікшығыс және солтүстікшығыс аймақ үшін тел ұялы,violent

4,джагтуу ауданындағы бенд сұлтан маңында бұл аймақта қарулы шайқас біріккен жау күштері кәпірлер мен олардың агенттері осы аймақтағы моджахедтерге қарсы операциялар жүргізген кезде басталды деп хабарланды шайқас аймақтағы немесе бейберекет түрінде болуы мүмкін және біз сізге кейінірек мәлімет береміз,джагту аудан бенд сұлтан маң бұл аймақта қарулы шайқас біріккен жау күш кәпір мен олардың агент осы аймақ моджахед қарсы операция жүргіз кезде баста де хабарла шайқас аймақ немесе бейберекет түр бол мүмкін және біз сізге кейінірек мәлімет бер,violent

4,пәкістанның қатал сызықтағы дін қызметкері суфи мұхаммед сол жақтан шыққан үшінші адам сәуір жексенбіде пәкістанның сват өзенінің астанасы мингора қаласында жиналған көпшілік алдында сөйлеген кезде талибан қоршауда цитата пәкістандық талибан сәуір жексенбіде пәкістанның сват аңғарының астанасы мингора қаласында өздерінің рухани лидері суфи мұхаммед айтқан митингіге жиналды цитата пәкістанның қатал желідегі діни қызметкер суфи мұхаммедтің жақтастары өздерінің көшбасшыларының саясатын қолдауға қолдарын көтеріп жатыр,пәкістан қатал сызық дін қызметк суфи мұхаммед сол жақ шық үшінші адам сәуір жексенбі пәкістан сват өзен астана мингор қала жинал көпшілік алд сөйле кезде талибан қорша цита пәкістандық талибан сәуір жексенбі пәкістан сват аңғ астана мингор қала өз рухани лидер суфи мұхаммед айт митингі жина цита пәкістан қатал желі діни қызметкер суфи мұхаммед жақтас өз көшбасшы саясат қолда қол көтер жатыр,violent

4,бүгін ағасы иракқа келеді дуа жасаңыз аға бүгін шейіт болу бригадасына қосылады бауырларды құттықтаймыз форум ағасы әлбитар салафи ислам мемлекетінің жағында және бүгін ол шейіттер батальонындағы бауырларына қосылады сондықтан оның ағалары үшін кеткенін біліп дұғаның пайдасына ұмытпаңыз ал әбу әлмутанна әлмухаджир қамауға алынған санам хусайбадан шығарылады,бүгін аға ирак кел ду жаса аға бүгін шейіт болу бригада қос бауыр құттықта форум аға әлби салафи ислам мемлекет жағ және бүгін ол шейіт батальон бауыр қос сондықтан оның аға үшін кет біл дұ пайда ұмытпа ал әбу әлмутанн әлмухаджи қама алын сана хусайба шығар,violent

4,премьерминистр юсуф раза гилани жұма күні бейбітшілік туралы келісімдер маңызды құрал деп мәлімдеді бірақ үкімет өз жазбаларына қарсы әрекет етуді жалғастыра берсе реакция жасайтынын ескертті оның айтуынша армия пәкістанның ең қуатты институты билік сұраған кезкелген әрекетті қабылдауға дайын еліміздің қорғаныс күші мықты біздің ядролық бағдарламамыз да қауіпсіз қолдарда деп айтқым келеді деді гилани парламентке даулы бейбітшілік келісімі сват бунер шангла және малаканд дивизиясының басқа аудандарын ауғанстан шекарасына жақын аумағы шаршы мильді шаршы шақырым және алькаида мен талибанның тірегі бар тайпалық аудандарды қамтиды қолдау білдірушілер бұл келісім ислам заңын қабылдауға

бағытталған негізгі шақыруды алып тастайды және үкіметке бақылауды біртіндеп қайта тексеруге мүмкіндік береді бірақ әлі де байыпты түрде сынақтан өтпек жаңалықтар yahoo,премьерминист юсуф раз гилани жұма күн бейбітшілік туралы келісім маңызды құрал де мәлімде бірақ үкімет өз жазба қарсы әрекет ету жалғастыр бер реакция жаса ескер оның айт армия пәкістан ең қуатты институт билік сұра кезкел әрекет қабылда дайын ел қорғаныс күш мықты біздің ядролық бағдарла да қауіпсіз қол де айт кел де гилани парламент даул бейбітшілік келісім сват бун шангл және малаканд дивизия басқа аудан ауғанстан шекара жақын аумағ шаршы миль шаршы шақырым және алькаи мен талибан тірег бар тайпалық аудан қамти қолдау білдіруші бұл келісім ислам заң қабылда бағыттал негізгі шақыру алып таста және үкімет бақылау біртіндеп қайта тексер мүмкіндік бер бірақ әлі де байыпты түр сынақ өт жаңалық yahoo,violent

4,ирак ислам мемлекеті жақтастарының әскері олар белгілі бір қауіпсіздік жағдайлары үшін ислам мемлекетімен тікелей байланыса алмаған элементтер тобы сондықтан олар ирак ислам мемлекетіндегі бауырластармен араласуға тиісті уақыт келгенше мемлекет топтарынан бөлек жұмыс істеуге бел буды олар әбу омардың бас қолбасшысына адал болуға уәде берді топтағы бауырлар маған соңғы бірнеше айда бірнеше рет жасаған операцияларын берді және олар маған оларды ирак ислам мемлекетін қолдаушылар атынан жариялауға рұқсат берді мен көптеген дискілерді жасадым және олар мемлекеттің бірқатар аймақтарында таратылды бірақ мен әлсіз сызықтың кесірінен желіде мергендік операцияларының аз ғана бөлігін жинай алдым және жақын күндері көптеген операцияларды көбейтемін деп уәде беремін мен шығаруды аз уақытқа қысқартуға тырыстым және оны ұзақ көруге болады нұсқа,ирак ислам мемлекет жақтас әскер олар белгілі бір қауіпсіздік жағдай үшін ислам мемлекет тікелей байланыс алма элемент тоб сондықтан олар ирак ислам мемлекет бауырлас аралас тиісті уақыт келгенше мемлекет топ бөлек жұмыс істе бел бу олар әбу омар бас қолбасшы адал бол уәде бер топтағы бауыр маған соңғы бірнеше айда бірнеше рет жаса операция бер және олар маған ол ирак ислам мемлекет қолдаушы ат жарияла рұқсат бер мен көптеген дискі жаса және олар мемлекет бірқатар аймақ тарат бірақ мен әлсіз сызық кесір желі мергендік операция аз ғана бөліг жина ал және жақын күн көптеген операция көбей де уәде бер мен шығару аз уақыт қысқарт тырыс және оны ұзақ көр бол нұсқа,violent

4,кеше жасалған шабуыл сияқты ирандықтар басты нысана болып көрінді,кеше жасал шабуыл сияқты ирандық басты нысана бол көр,violent

4,шабуыл жалғасуда жойылған көліктер саны екі жүк көлігіне және toyota консервативті әскерилеріне арналған екі көлікке жетті осы уақытқа дейін бес консерваторлық сарбаз конвоймен қаза тапты ал мұжахидтер каруларын қойды ал от жалындары екі жүк көлігінің арасынан әлі де көтерілуде хушаманд забихуллада мужахид американдық жаудың екі әскери көлігінің жойылуы аз уақыт бұрын бүгін бейсенбіде таңертеңгі он сағат да ислам әмірлігінің моджахедтері пактика провинциясындағы хушаманд ауданындағы мардан аймағында американдық

күштерге арналған екі әскери машинаны жарып жіберді бомба американдық әскери конвой орталыққа оралып жатқан кезде қашықтан басқару машинасымен жарылды,шабуыл жалғас жойыл көлік сан екі жүк көліг және toyota консерватив әскери арнал екі көлік же осы уақыт дейін бес консерваторлық сарбаз конвой қаза тап ал мұжахид қару қой ал от жалын екі жүк көліг ара әлі де көтеріл хушаманд забихулла мужахид американдық жау екі әскери көліг жойыл аз уақыт бұрын бүгін бейсенбі таңертеңгі он сағат да ислам әмірліг моджахед пактик провинция хушаманд аудан ма аймағ американдық күш арнал екі әскери машина жар жіб бомба американдық әскери конвой орталық орал жат кезде қашық басқару машина жар,violent

4,вашингтонда ақштың осы аймақтағы ең жоғары әскери қолбасшысы генерал дэвид петреус ирактағы шабуылдар біраз уақытқа дейін жалғасатынын айтты оның сөзіне қарағанда жуырда өзінөзі өлтіруді ұйымдастырған адамдар мұндай шабуылдарды жасауға дайын ирактардың азайып бара жатқан қатарларын ауыстыру үшін тунистен алынған желіге байланысты болуы мүмкін петреус ирактағы кейбір жетістіктерге тоқталды бірақ ілгерілеудің әлсіз және қайтымды болатындығын ескертті жалпы ирактағы зорлықзомбылық өткен жылдардағы деңгейден едәуір төмен наурыз айынан бергі наразылықтар суннит көтерілісшілерінің жаңа серпінін білдіретін сияқты,вашингтон ақш осы аймақ ең жоғары әскери қолбасшы генерал дэвид петреус ирак шабуыл біраз уақыт дейін жалғас ай оның сөз қара жуырда өзінөз өлтіру ұйымдастыр адам мұндай шабуыл жаса дайын ирак азай бар жат қатар ауыстыру үшін тунис алын желі байланысты бол мүмкін петреус ирак кейбір жетістік тоқта бірақ ілгерілеу әлсіз және қайтымды болатындығ ескер жалпы ирак зорлықзомбылық өткен жыл деңгей едәуір төмен наурыз ай берг наразылық суннит көтерілісші жаңа серпін білдір сияқты,violent

4,израильдің стратегиялық мәселелерде анық шыншыл болғаны қызық мен gwottің израиль үшін америкадан гөрі маңыздырақ екендігіне дау айтар едім иран туралы бақтың көпшілігі мұны түсінбеуін қамтамасыз етеді алайда доктриналық тұрғыдан алғанда исламдық топтардың басым көпшілігі израильді жеңуді америка мен оның одақтастары израильді қолдауда басты мақсат деп санайды,израиль стратегиялық мәселе анық шыншыл бол қызық мен gwot израиль үшін америка гөрі маңыздырақ екендіг дау айтар ед иран туралы бақ көпшіліг мұны түсінбе қамтамасыз ет алайда доктриналық тұрғы ал исламдық топ басым көпшіліг израиль жеңу америка мен оның одақтас израиль қолда басты мақсат де сана,violent

4,джихадқа көмектесу мүжахидтерге қызмет ету иммунитет сәбилиллах хижра кифарға қарсы джад муртадендің және куффардан және оның шахлафа филиалы,джихад көмектес мүжахид қызмет ету иммунитет сәбилиллах хижр киф қарсы джад муртаде және куфф және оның шахлаф филиал,violent

4,цитата алдымен айдан жариялады мен карама абырой жоспары аяқталды деп ойладым және жақсы жинау басталды ма иә бауырым карама жоспары аяқталды бұл өткен желтоқсан кезеңіндегі есеп жақсы егін жинау жоспары өткен наурыз айында

басталды,цита алд ай жариял мен кара абырой жоспар аяқта де ойла және жақсы жинау баста ма иә бауыр кара жоспар аяқта бұл өткен желтоқсан кезеңіндегі есеп жақсы егін жинау жоспар өткен наурыз ай баста,violent

4,цитата ақш контингентінің жетекшілері біз онда біреуді қостық деп мақтанады бірақ көтерілісшілер жеңіске жете алады ақш хамвейінің күйреген қалдықтарында билеп олардың радиоларына американдықтардың базаның солтүстігінде орналасуына жол бермеу туралы ант берді,цита ақш контингент жетекші біз онда біреу қос де мақтан бірақ көтерілісші жеңіс же ал ақш хамвей күйре қалдық биле олардың радио американдық база солтүстіг орналас жол бермеу туралы ант бер,violent

4,зводтың үстінде мобильді ауғандық армия бар екеніне қарамастан ондаған көтерілісшілер көршілес аңғарлардан анықталмастан қосылып кетті тіпті әуе дрондары да мұндай қозғалыстарды бақылауға алынған,звод үст мобильді ауғандық армия бар екен қарамастан ондаған көтерілісші көршілес аңғар анықталмас қосыл кет тіпті әуе дрон да мұндай қозғалыс бақыла алын,violent

4,атымен джихад орталығына шақыру көрнекі көрнекі басылымды ұсынады насралла жүктелетін жоғары мб http nasr notlong com http gettyfile ru,ат джихад орталығ шақыру көрнекі көрнекі басыл ұсын насралл жүктел жоғары мб http nasr notlong com http gettyfile ru,violent

4,медиа джихад бригадасы шейх осама бен ладен деген сөздің ағылшынша аудармасын ұсынады газадағы агрессияны тоқтату үшін джихадқа шақыру,медиа джихад бригада шейх оса бен ла де сөз ағылшынша аударма ұсын газа агрессия тоқтату үшін джихад шақыру,violent

4,ауғанстандағы бауырларымызға жеңіс берсін джихад медиасынан қабыл етсін,ауғанстан бауыр жеңіс бер джихад медиа қабыл ет,violent

4,ұйымның төрт ұрланған адамын босатудың орнына біздің төрт моджахед тұтқыны канадалықтар роберт фуллер және луис гей неміс марианна петцольд және швейцариялық габриелла бурко грейнер екінші жағынан біз ұйым заңды талаптарға қол жеткізгенге дейін британдық турист эдвей дйер және швейцарлық туристерді қолдайды деп жариялаймыз соған сәйкес біз британдық мемлекеттен азап шеккен шейх абу катаданы босатуды талап етеміз оның ұлыбритания азаматын босатқанның орнына және оларға осы мәлімдеме шыққаннан бастап жиырма күндік мерзім беруі керек және мерзім аяқталғанда егер олардың сұраныстарына жауап таппаса мұжахидтер британдықтарды тұтқындауға мәжбүр болады және ол ақталды,ұйы төрт ұрлан адам босат орн біздің төрт моджахед тұтқын канадалық роберт фул және луис ге неміс марианн петцольд және швейцариялық габриелла бурко грейн екінші жағ біз ұйым заңды талап қол жеткіз дейін британдық турист эдве дй және швейцарлық турист қолда де жарияла соған сәйкес біз британдық мемлекет азап шек шейх абу катада босату талап ет оның ұлыбритания азамат босат орн және оларға осы мәлімдеме шық баста жиырма күндік мерзім беруі керек және мерзім аяқтал егер

олардың сұраныс жауап таппа мұжахид британдық тұтқында мәжбүр бол және ол ақта,violent

4,ауғанстан ислам әмірлігінің мүжахидтері каферлерге мунафиктерге және пұттарға табынушыларға қарсы әскери операциялар туралы хабарлап бұл бет күні бойы жаңартылып отырады ауғанстандағы ислам террористерінің жауларына қарсы мүжахидтер туралы theunjustmedia com сайтына ауғанстан ислам әмірлігінің ресми мүжахидтері қари мұхаммед юсуф пен забихулла мужахид электрондық пошта арқылы хабарлайды,ауғанстан ислам әмірліг мүжахид кафер мунафик және пұт табынушы қарсы әскери операция туралы хабарла бұл бет күн бойы жаңартыл отыр ауғанстан ислам террорис жау қарсы мүжахид туралы theunjustmedia com сайт ауғанстан ислам әмірліг ресми мүжахид қари мұхаммед юсуф пен забихулл мужахид электрондық пошта арқылы хабарла,violent

4,нанаяккараның мәлімдеуінше көтерілісшілердің көңілкүйі құлдырады бейбіт тұрғындар ретінде киінген көтерілісші көтеріліп келе жатқан әскерге тапсырылды көтерілісшілердің көбі мойындарына цианид толтырылған шыны ыдыстарды киіп алып өздерін ұстауға мүмкіндік бермей өзінөзі өлтіруге бұйрық береді өткен аптада екі танымал көтерілісші топтың бұрынғы бақ өкілі дая мастер ретінде белгілі велаяхтам даянитити және саяси қанатының аудармашысы тек джордж деген атпен де бас тартты халықаралық алаңдаушылыққа қарамастан шабуыл көптеген сингалялықтарға кеңінен танымал президент махинда раджапакстың коалициясы жексенбіде өткен жергілікті сайлауда үлкен жеңіске жетті үкімет батыс провинциядағы сауалнаманың үштен екі бөлігін жеңіп алды және тіпті астананың коломбода көтерілісшілермен келіссөз жүргізуді жақтайтын оппозиция біріккен ұлттық ұлттық партиясының тірегі болып саналды басқарушы коалиция қазір елдің барлық сегіз провинциялық кеңесін бақылайды сайлаушылар президенттің біріккен шриланкаға үндеуіне нақты жауап берді деді медиа министрі анура япа,нанаяккара мәлімдеуінше көтерілісші көңілкүй құлдыра бейбіт тұрғын рет киін көтерілісш көтеріл келе жат әскер тапсыр көтерілісші көбі мойын цианид толтырыл шыны ыдыс ки алып өз ұста мүмкіндік берме өзінөз өлтір бұйрық бер өткен апта екі танымал көтерілісш топ бұрынғы бақ өкіл дая мастер рет белгілі велаяхта даянитити және саяси қан аудармашы тек джордж де ат де бас тар халықаралық алаңдаушылық қарамастан шабуыл көптеген сингалялық кең танымал президент махин раджапак коалиция жексенбі өткен жергілікті сайла үлкен жеңіс же үкімет батыс провинция сауалнама үш екі бөліг жең алды және тіпті астана коломбо көтерілісші келіссөз жүргізу жақта оппозиция біріккен ұлттық ұлттық партия тіреғ бол сана басқарушы коалиция қазір ел барлық сегіз провинциялық кеңес бақыла сайлаушы президент біріккен шриланка үнде нақты жауап бер де медиа министр анур яп,violent

4,таулардағы кішкентай жолда олардың қатты қорғалған көліктеріне қараңыз мужахиддер қозғалыс қабілеті жақсарған кезде олар мұндай үлкен габаритті көліктермен алысқа бармайды олар оңай нысана болады,тау кішкентай жолда

олардың қатты қорғал көлік қара мужахид қозғалыс қабілет жақсар кезде олар мұндай үлкен габарит көлік алыс барма олар оңай нысана бол,violent

4,ансар медиа қоры тәжікстандағы американдық оккупацияны зымыранмен бомбалайды,анс медиа қор тәжікстан американдық оккупация зымыран бомбала,violent

4,аударма барша мүшелерге барлығының атынан біз кезкелген батисттің sunni irhaabi хадимиях пен карадаға кіруіне жол бермеу үшін жаңа операцияны бастаймыз және біз бар нәрселерімізбен оларды тоқтатамыз мағынасы бомбалар кісі өлтіру масажидті жағу абу гаид эль шикли қылмыстық хаттың соңы ишим сарбаздары мен басқа да джихад топтарының қолындағы осы қылмыстық оранизацияны жойсын acer,аударма барша мүше барлығ ат біз кезкел батис sunni irhaabi хадимиях пен карада кір жол бермеу үшін жаңа операция баста және біз бар нәрсе ол тоқта мағына бомба кісі өлтір масажид жағ абу гаид эль шикли қылмыстық хат соңы иши сарбаз мен басқа да джихад топ қол осы қылмыстық оранизация жой acer,violent

4,сіз үлкен жұмыс істеуге бара жатырсыз осы жерден мен шейіт болу операциясын жалғастырып жүк көлігімді жарып жіберемін сен де оларды өлтіресің сондықтан қорықпа,сіз үлкен жұмыс істе бар жатыр осы же мен шейіт болу операция жалғастыр жүк көліг жар жібер сен де ол өлтір сондықтан қорықпа,violent

4,цитата алдымен binyamine salam автор ағайынды қазақша алмастырғыш пен архивтік сілтеме бар видео бар ма салам но ахкиге рахмет бұл шығарылымды ауғандықтар талибан жасады олар alqaida сияқты ағылшынша субтитрлер бермейді btw келесі nida aljihad шығарылымы жақында шығады,цита алд binyamine salam автор ағайынды қазақша алмастырғыш пен архив сілтеме бар видео бар ма сала но ахки рахмет бұл шығарыл ауғандық талибан жас олар alqaida сияқты ағылшынша субтитр берме btw келесі nida aljihad шығарылым жақында шығ,violent

4,дәйексөз бастапқыда btw қабырғасындағы москит келесі nida aljihad шығарылымы жақында шығады және біз бәрімізді асыға күтеміз,дәйексөз бастапқы btw қабырға москит келесі nida aljihad шығарылым жақында шығ және біз бәрі асыға күт,violent

4,сендер радикалдар болған исламабадтың жүрегінде бірнеше шейіт операциясын күтіңіздер,сен радикал бол исламабад жүрег бірнеше шейіт операция күт,violent

4,осы пәкістандық қуыршақтарды талқандауға уақыт келді егер олар бұдан былай бейбіт келіссөздермен қайтып келсе бұдан артық ескертуге болмайды оларды тек жару ғана шешіледі,осы пәкістандық қуыршақ талқанда уақыт кел егер олар бұдан былай бейбіт келіссөз қайт кел бұдан артық ескерт болма ол тек жар ғана шеш,violent

4,ақшқа қарсы тактиканы үйренуге уәде берді ауғанстандағы қолдаған солтүстік альянс ужааама сонымен бірге әбу хамзаның өзі мүлікке тарту ретінде келуді ұсынды уджаяаның куәлігі бүгін ол кассирдің адвокаттарының қарамақарсы тексерілуіне тап болған кезде қайта басталады код http seattletimes nwsource com html localnews ujaama html,ақш қарсы тактика үйрен уәде бер ауғанстан қолда солтүстік альянс

ужааа сонымен бірге әбу хамза өзі мүлік тарту рет келу ұсын уджаяа куәліг бүгін ол кассир адвокат қарамақар тексеріл тап бол кезде қайта бастал код http seattletimes nwsource com html localnews ujaama html,violent

4,ал кейінірек ансар әлмужахидтер желісі біздің халқымызға сунниттер мен ливандағы қауымға исламға қосылуға және оны қолдауға және бізге қажет барлық нәрселермен қолдау көрсетуге шақырды ливандағы сунниттік ашахтардың жастарын қоздыруға итермелейді сондықтан сіз және исламды жаулап алудағы бауырларыңыздан басқа ештеңе жоқ олар сіздерді нахрбаред эпосында қалдырғанына қарамастан сізді қорғап қолдайтын болады сондықтан бұл күнәні тағы қайталамаңыз,ал кейінірек анс әлмужахид желі біздің халқ суннит мен лива қауым ислам қосыл және оны қолда және бізге қажет барлық нәрсе қолдау көрсет шақ лива сунни ашах жас қоздыр итермеле сондықтан сіз және ислам жаула алу бауыр басқа ештеңе жоқ олар сіз нахрбаред эпос қалдыр қарамастан сіз қорға қолда бол сондықтан бұл күнә тағы қайталама,violent

4,ауыр тактика ауылдарды тегістеуге және бейбіт тұрғындарды иеліктен шығаруға әкеледі әскери және үкіметтің дирде жылдам жеңіске жету туралы талаптарын пәкістандықтар жергілікті жерде дауласуда үкімет кеше операциялардың аяқталғанын жариялаған кезде біз жағдай қалпына келеді деп үміттенген едік деді тимерджара саудагерлер қауымдастығының президенті хаджи анваруддин bbcге бірақ қазір біз олардың пәкістан үкіметі ақымақ екенін білеміз деді анваруддин олар әлем ұрыс болмаған кезде аяқталғанын білгенін қалайды менің ойымша біз ұзақ сапарға дайынбыз дирдің тұрғындары сондайақ талибанға қарсы бір уақыттағы әскери операция жүргізіліп жатқан бунер тұрғындары қауіпсіздік жағдайы нашарлайды деп қауіптенеді өйткені солтүстікбатыс шекара провинциясындағыдай бұл жерде талибтер провинцияның көп бөлігін басқарады және жергілікті аймақтарда соғысады мысалы сват пен бажаур көптеген жылдар бойы ызаланған,ауыр тактик ауыл тегісте және бейбіт тұрғын иелік шығар әкел әскери және үкімет ди жылдам жеңіс жету туралы талап пәкістандық жергілікті же даулас үкімет кеше операция аяқтал жарияла кезде біз жағдай қалп кел де үміттен едік де тимерджар саудагер қауымдастығ президент хаджи анваруддин bbc бірақ қазір біз олардың пәкістан үкімет ақы екен біл де анваруддин олар әлем ұрыс болма кезде аяқтал біл қала менің ойымша біз ұзақ сап дайын ди тұрғын сондайақ талибан қарсы бір уақыт әскери операция жүргізіл жат бун тұрғын қауіпсіздік жағдай нашарла де қауіптен өйткені солтүстікбатыс шекара провинциясындағыда бұл же талиб провинция көп бөліг басқар және жергілікті аймақ соғыс мысал сват пен бажау көптеген жыл бойы ызалан,violent

4,ауғанстанда және көрші пәкістанда бізде талибан мен алькаида проблемалары бар деді президент сәрсенбіде сөйлеген сөзінде көтеріліп келе жатқан американдық рөл көтерілісшілерді пәкістанның арғы жағына шығарудан қорқады өйткені талибанның жоғарғы басшылары қазір карачи портына отырды,ауғанстан және көрші пәкістан біз талибан мен алькаи проблема бар де президент сәрсенбі сөйле сөз көтеріл келе жат

американдық рөл көтерілісші пәкістан арғы жағ шығару қорқ өйткені талибан жоғарғы басшы қазір карачи порт от,violent

4,міне оларды вардак қарсы алатын бомба цитата ақштың ші бригадасы шы таулы дивизиясы ақш көліктеріне бомба шабуылынан туындаған кратердің жанында ақштың кабулдан батысқа қарай вардак провинциясындағы танги алқабында ауғанстан сейсенбі сәуір,міне ол вардак қарсы ал бомба цита ақш ші бригада шы таулы дивизия ақш көлік бомба шабуыл туында кра жан ақш кабул батыс қарай вардак провинция танги алқаб ауғанстан сейсенбі сәуір,violent

4,көтеріліс қайта басталғанға ұқсайды меніңше isi американдық лаңкестік өзінің шығуын жариялағанға дейін одан әрі шабуылдарды тоқтатқан болуы мүмкін isi обама сияқты қоңыр тұмар өзінің әскери жауынгерлік бұқарасын ренжіткісі келмегендіктен жұмыстан кету туралы шешімін өзгертпейтінін біледі енді күрес қайта басталды және обама үйінде негр істемейді,көтеріліс қайта бастал ұқса меніңше isi американдық лаңкестік өзінің шығ жарияла дейін одан әрі шабуыл тоқтат бол мүмкін isi оба сияқты қоңыр тұма өзінің әскери жауынгерлік бұқара ренжіт келмегендік жұмыс кету туралы шеш өзгертпе біл енді күрес қайта баста және оба үй нег істеме,violent

4,біраз уақыт алуы мүмкін бірақ мужахидтер бір уақытта бір аймақта бір шайқаста жеңіске жетеді олар крестшілердің егер ирактан қан кетуі мүмкін болса одан бас тартуға болатындығын көрсетеді умма арыстандары оларды келесі ауғанстаннан шығарсын,біраз уақыт ал мүмкін бірақ мужахид бір уақыт бір аймақта бір шайқас жеңіс жет олар крестші егер ирак қан кет мүмкін бол одан бас тарт болатындығ көрсет ум арыстан ол келесі ауғанстан шығар,violent

4,қазанның басқыншылары сәуір төңкерісі тентатының қылмыскерінің тағдырымен кездеседі,қазан басқыншы сәуір төңкеріс тент қылмыскер тағдыр кездес,violent

4,олар оянады әлде әлкудты азат ету бізге байланысты,олар оян әлде әлкуд азат ету бізге байланысты,violent

4,менің қымбатты досым мен сені салахуддин аюбиді және соңғы жылдық тарихын ұмытып кетемін қымбаттым сен әдемі беруге ниет білдіріп жатырмыз сен сыйлықтың не екенін білесің бе жарайды мен саған айтамын біз еуропаны қабылдауға келеміз хахахаха біз кім білгіңізді біз білсіңізді білсіңіз біз нағыз күштіктерміз біз шын жүргізілген күндерміз біз келеді,менің қымбатты дос мен сен салахуддин аюби және соңғы жылдық тарих ұмыт кет қымбат сен әдемі бер ниет білдір жатыр сен сыйлық не екен біл бе жарайды мен саған ай біз еуропа қабылда кел хахахах біз кім білг біз білс білс біз нағыз күштік біз шын жүргіз күн біз кел,violent

4,уфтилер үшін бұдан да жаман болады,уфти үшін бұдан да жаман бол,violent

4,оңтүстікте тұратын бауырларымызға қалай қарағанына көзқарас amp рохиньялар жеті иттерге де жақсы емделеді бұл жалған пұтқа табынушы олар ядролық ереуілмен қырғынға ұшырайды,оңтүстік тұр бауыр қалай қара көзқарас amp рохинья жеті ит де жақсы емдел бұл жалған пұт табынуш олар ядролық ереуіл қырғын ұшыра,violent

4,джалалабад әуежайын зымырандармен бомбалау жеңіс забихулла мужахид мамыр джалалабад аэродромы жеңіс деп аталатын бірқатар операцияларда кеше кешке зымырандармен шабуыл жасады алынған жаңалықтар бойынша шабуылдан кейін әуежайда өрт шықты бұл жаудың арасында материалдық және рухани шығындар болғанын көрсетеді бірақ бұл шығындардың мөлшері туралы нақты ақпарат болған жоқ кеше таңертең әуежайға зымырандар шабуыл жасағаны туралы хабарланды олар жер емес еркелігі айтты болса олар айтады ауғанстан ислам әмірлігінің ресми өкілі талибан кари мухаммад юсуф ахмади аудандары ел тел оңтүстік батыс және солтүстік біз татуластырушы сиыр олар тонауға болып табылмайтын бірақ сиыр сезінеді емес ақпарат болып табылады мобильді забихулла мужахид елдің оңтүстікшығыс және солтүстікшығыс аймақтары үшін тел ұялы ауғанстан талибан көзі беті джихад дауысы ауғанстан ислам әмірлігі талибандар үшін ресми сайт,джалалабад әуежай зымыран бомбала жеңіс забихулл мужахид мамыр джалалабад аэродром жеңіс де атал бірқатар операция кеше кеш зымыран шабуыл жас алын жаңалық бойынша шабуыл кейін әуежай өрт шық бұл жау ара материалдық және рухани шығын бол көрсет бірақ бұл шығын мөлшер туралы нақты ақпарат бол жоқ кеше таңертең әуежай зымыран шабуыл жаса туралы хабарла олар жер емес еркеліг ай бол олар айт ауғанстан ислам әмірліг ресми өкіл талибан кари мухаммад юсуф ахмади аудан ел тел оңтүстік батыс және солтүстік біз татуластыруш сиыр олар тона бол табылма бірақ сиыр сезін емес ақпарат бол таб мобильді забихулл мужахид ел оңтүстікшығыс және солтүстікшығыс аймақ үшін тел ұялы ауғанстан талибан көз бет джихад дауыс ауғанстан ислам әмірліг талибан үшін ресми сайт,violent

4,шабуыл үшін жауапкершілікті ешбір топ немесе жеке адам мойнына алған жоқ алайда үш жыл бойы зорлықпен оралған талибан сарбаздары мұндай шабуылдарды көбінесе ауғандық және халықаралық күштердің мүдделеріне қарсы жасайды,шабуыл үшін жауапкершілік ешбір топ немесе жеке адам мойн ал жоқ алайда үш жыл бойы зорлық орал талибан сарбаз мұндай шабуыл көбіне ауғандық және халықаралық күш мүдде қарсы жаса,violent

4,талибандар тек кофеарлардың тыңшылары мен агенттерін ғана жүзеге асыратыны белгілі сонымен бірге пәкістанның полициясы егер мүмкін болса барлық масажидтер мен маадаристерге және барша жамағатқа тыңшылар жіберетіні белгілі оларды бақылап не істеп жатқанын көріңіз сондықтан ресми байан болмаған жағдайда жамағаттың арасында тыңшы немесе агент ашылған болуы мүмкін,талибан тек кофеар тыңшы мен агент ғана жүзеге асыр белгілі сонымен бірге пәкістан полиция егер мүмкін бол барлық масажид мен маадарис және барша жамағат тыңшы жібер белгілі ол бақыла не істе жат көр сондықтан ресми байан болма жағдай жамағат ара тыңш немесе агент ашыл бол мүмкін,violent

4,кавказдағы джихад жалғасады,кавказ джихад жалғас,violent

4,эритрея үкіметінің ұшағы шабабқа қару жібереді,эритрея үкім ұшағ шабаб қару жібер,violent

4,дәйексөз алдымен абу бараа жақында шариф ахмедтің өлтірілуі немесе ұрлануын күтті мен муртадтың басын алғым келеді,дәйексөз алд абу бара жақында шариф ахмед өлтіріл немесе ұрлан күт мен муртад басын ал кел,violent

4,сионистің жер бетінен жойылуына біршама уақыт қалды бұл ең жақсы күн болады,сиони жер бет жойыл біршама уақыт қал бұл ең жақсы күн бол,violent

4,пакистан үкіметін жойып жіберсін өйткені көптеген жылдар бойы пакистан үкіметі қытай үкіметімен ынтымақтасып келеді,пакис үкім жой жібер өйткені көптеген жыл бойы пакис үкімет қытай үкімет ынтымақтас кел,violent

4,уа кітапты ашушы жаза беруші бұлттардың қозғалысы таавагетке өз күштеріңізді соғып мүжахидтермен кезкелген жерде шабуылдаңыз,уа кітап ашуш жаза беруші бұлт қозғалыс таавагет өз күш соғ мүжахид кезкел же шабуылда,violent

4,бірақ ол аздап еркін біз оның қожайыны мен досымызды белгілі бір саддамды жойдық енді біз өз өлі аяғымызды өкшемен қарап қайтадан шықтық келесі ретке дейін,бірақ ол аздап еркін біз оның қожайын мен дос белгілі бір садда жой енді біз өз өлі аяғ өкше қара қайта шық келесі рет дейін,violent

4,ауғандықтарды апатпен өлтіреміз бе бізді күштер жеңеді біз де жеңіске жетеміз,ауғандық апат өлтір бе біз күш жең біз де жеңіс же,violent

4,біздің мақсатымыз басқыншы күштердің бөлімшелері дипломатиялық бекеттер конвойлар қуыршақ үкіметінің лауазымды тұлғалары депутаттар қорғаныс ішкі істер және барлау министрліктерінің қызметкерлері,біздің мақсат басқынш күш бөлімше дипломатиялық бекет конвой қуыршақ үкім лауазым тұлға депутат қорғаныс ішкі істер және барлау министрлік қызметкер,violent

4,шығыс ауғанстан кунар нуристан болуы керек онда әлемнің ең ауыр жауынгерлері тұрады қафарлардың әуе қолдауына шақырылған әскерлерді жақсы етіп көрсетуге тырысқаны әскерилерді кетуге мәжбүрлейтіні өте қызықты олар іздестірілуде делінген хабарламада lol мужахидтер американдықтарға есектерін өздеріне тапсырды,шығыс ауғанстан кун нурис бол керек онда әлем ең ауыр жауынгер тұр қафар әуе қолда шақырыл әскер жақсы ет көрсет тырыс әскери кет мәжбүрле өте қызықты олар іздестіріл делін хабарлама lol мужахид американдық есек өз тапсыр,violent

4,неліктен мені мұнысы мұсылмандар деп аталатын адамдарға осылай етуге мүмкіндік беруі болып табылады егер үй алынып тасталса шейіт болғысы келетіндердің саны бірдей болуы керек міне сондықтан израиль ақша мен батысшылдықтың әсерінен болатын палестина халқының иманының әлсіздігінен ұзақ уақытқа созылды инша аллах олар оянады әлде әлкудты азат ету бізге байланысты,неліктен мен мұны мұсылман де атал адам осылай ет мүмкіндік беруі бол таб егер үй алын тастал шейіт бол кел сан бірде бол керек міне сондықтан израиль ақша мен батысшылдық әсер бол палестин халқ иман әлсіздіг ұзақ уақыт соз инш аллах олар оян әлде әлкуд азат ету бізге байланысты,violent

4,альшабаб могадишодағы қақтығыстар мен шабуылдарды тоқтатуға шақырды банадир аймағын басқаратын әлшабаб шенеунігі шейх али мухаммад хусейн телефон арқылы бақ үшін баспасөз мәслихатын өткізді және соңғы күндері могадишода діни лидерлер мен ғалымдардың бұдан былай болмау керек деген шақыруларына қарсы екенін айтты банадир аймағында соғысып жатыр оның айтуынша соғыс сомали мен бүкіл әлемде қасиетті кітаптың құран ережелері сақталып орындалғанға дейін жалғасады шейх али мухаммад хусейн сонымен қатар өздерін діни жетекшілер деп атайтын кейбір адамдар жиһадты тоқтатуға шақыратын адамдар бар екенін айтты альшабабтың мәлімдемесі діни лидерлер мен сомали ғалымдары могадишодағы соғысты тоқтатуға шақырған кезде пайда болды әлшабабтың өкілі шейх муктар робов абу мансур жақында баспасөз мәслихатын өткізіп ол сомалидегі африка одағының миссиясына тиесілі базаларға amisom km және могадишу әуежайындағы әскери базаларға шабуылдарды жалғастыруға уәде берді кешіріңіз егер жаман ағылшын болса,альшабаб могадишо қақтығыс мен шабуыл тоқтат шақ банади аймағ басқар әлшабаб шенеуніг шейх али мухаммад хусейн телефон арқылы бақ үшін баспасөз мәслихат өт және соңғы күн могадишо діни лидер мен ғалым бұдан былай болмау керек де шақыру қарсы екен ай банади аймағ соғыс жатыр оның айт соғыс сомали мен бүкіл әлем қасиетті кітап құран ереже сақтал орындал дейін жалғас шейх али мухаммад хусейн сонымен қатар өз діни жетекші де ата кейбір адам жиһад тоқтат шақыр адам бар екен ай альшабаб мәлімде діни лидер мен сомали ғалым могадишо соғ тоқтат шақыр кезде пайда бол әлшабаб өкіл шейх мук робов абу мансу жақында баспасөз мәслихат өткіз ол сомали африка одағ миссия тиесілі база amisom km және могадиш әуежай әскери база шабуыл жалғастыр уәде бер кешір егер жаман ағылшын бол,violent

4,оның басты мақсаты израиль таяудағы бірнеше айда иранмен хамас немесе хезболламен немесе үшеуімен де үлкен әскери қарсыласуды бастайды деп күтеді израильдің иранға қарсы соғысқа шақыруы ақштағы қатаң қарсылыққа ие болған кезде израильдің жетекші басшылары ақш үкіметі мен еуропа елдерін иранның ядролық деп сипаттайтындарына сендіру мақсатында жоғары дәрежелі дипломатиялық әрекеттерді бастады қауіп,оның басты мақсаты израиль таяу бірнеше айда иран хамас немесе хезболла немесе үшеу де үлкен әскери қарсыласу баста де күт израиль иран қарсы соғыс шақыр ақш қатаң қарсылық ие бол кезде израиль жетекші басшы ақш үкімет мен еуропа ел иран ядролық де сипатта сендір мақсат жоғары дәрежелі дипломатиялық әрекет баста қауіп,violent

4,егер адамдар өз сарбаздарын ауғанстаннан шығарса үкіметтің үміттері орындалуы мүмкін неміс әскерлері негізінен ауғанстанның салыстырмалы түрде бейбіт солтүстігінде қызмет етеді бірақ олар әлі де андасанда бомба шабуылдарынан зардап шегеді біз баяғыда солтүстігінде тірек болдық деді спикер сарбаздар енді еш жерде қауіпсіз болмайды деп болжайды,егер адам өз сарбаз ауғанстан шығар үкімет үміт орындал мүмкін неміс әскер негіз ауғанстан салыстырмалы түр бейбіт солтүстіг қызмет ет бірақ олар әлі де андасан бомба шабуыл зардап шег біз баяғы солтүстіг тірек бол де спикер сарбаз енді еш же қауіпсіз болма де болжа,violent

4,хамас газадағы ешкімді аямайды олар барлығына соның ішінде бақ пен саяси қызметке қысым жасайды,хамас газа ешкі аяма олар барлығ соның іш бақ пен саяси қызмет қысым жаса,violent

4,вашингтон егер иран өзінің сезімтал ядролық қызметін тоқтатпаса оған қарсы санкцияларды бұзады деп қорқытты,вашингтон егер иран өзінің сезімтал ядролық қызмет тоқтатпа оған қарсы санкция бұз де қорқыт,violent

4,сонымен бірге талибан свитасының өкілі муслим хан телефонмен жарияланбаған жерде дарул қазаны реактивті ұшақтар мен бомбалардың көлеңкесінде олар үшін қолайлы емес деп мәлімдеді ол үкімет әскери операцияны тоқтатып маулана суфи мохммадпен кеңескеннен кейін дарул газаны құруы керек деді ол талибан үкімет пен tnsm арасында жасалған бейбітшілік келісіміне бағынады дейді ол дир және бунер аудандарында операция басталып сватта ереуілге дайындалып жатқаннан кейін үкіметтің бейбітшілік келісімін бұзып жатқанын айтты ол сватта тағы да әскери операция жасалса жауап қайтаруға болатындығын ескертті,сонымен бірге талибан свита өкіл муслим хан телефон жарияланба же дарул қаза реактивті ұшақ мен бомба көлеңке олар үшін қолайлы емес де мәлімде ол үкімет әскери операция тоқтат маулан суфи мохммад кеңес кейін дарул газа құр керек де ол талибан үкімет пен tnsm ара жасал бейбітшілік келісім бағын де ол ди және бун аудан операция бастал сват ереуіл дайындал жат кейін үкімет бейбітшілік келі бұз жат ай ол сват тағы да әскери операция жасал жауап қайтар болатындығ ескер,violent

4,таңмен әңгімелесу барысында ол лал масжидтің көптеген студенттерінің осы аудандарға жататындығын енді олар кек алатынын айтты,таң әңгімелесу барыс ол лал масжид көптеген студент осы аудан жататындығ енді олар кек ал ай,violent

4,дәйексөз алғашында ikaz авторы формадағы адамдар неге көмектеседі неліктен үкімет бұл азаптауға қатысты ештеңе істемейді меніңше олар шейхті көпшілік алдында өлтіруі керек бірақ бұл менің пікірім ол үкімет бұл жай ғана баәде болып жатқан оқиғаларға көз жүгірту мен бұл бейнені көргенде қатты өкіндім бірақ оның ештеңесі баәдің шошқасынан көрінбейтін сұмдықтар туралы аз біледі мен жеке өзім бірнеше жыл бойы ұстадым баәны жерге көтеру керек шаңға дейін азаяды бұл қоқыс мен тұра алмаймын заркави иорданиядағы қонақ үйлерге шабуыл жасаған сияқты біріккен араб әмірліктеріндегі барлық қонақ үйлер мен курорттарға шабуыл жасау керек,дәйексөз алғаш ikaz авто форма адам неге көмектес неліктен үкімет бұл азапта қатысты ештеңе істеме меніңше олар шейх көпшілік алд өлтір керек бірақ бұл менің пікір ол үкімет бұл жай ғана баә бол жат оқиға көз жүгірт мен бұл бейне көргенде қатты өкін бірақ оның ештеңе баә шошқа көрінбе сұмдық туралы аз біл мен жеке өзім бірнеше жыл бойы ұста баә же көтеру керек шаң дейін азая бұл қоқыс мен тұр алма заркави иордания қонақ үй шабуыл жаса сияқты біріккен араб әмірлік барлық қонақ үй мен курорт шабуыл жасау керек,violent

4,цитата бастапқыда rebelzgang isi бастығы паша вашингтонға аттанады дүйсенбі мамыр pm pst пәкістан келіссөздерде зымырансыз шабуылдар туралы мәселені

көтереді және вашингтонды оларды тоқтатуға мәжбүр етеді деп хабарлады әскери ақпарат көздері олар армандаған болуы керек вашингтон дрондарға бір уақытта орбитаны ұстап тұруға мүмкіндік беру үшін қаржыландыруды ұлғайтты менің ойымша шабуылдар азаймайды пәкістан адамдарды шабуылдарға қатты қарсылық білдіреміз деп алдау үшін жай шу шығаруда,цита бастапқы rebelzgang isi бастығ паш вашингтон аттан дүйсенбі мамыр pm pst пәкістан келіссөз зымыран шабуыл туралы мәселе көтер және вашингтон ол тоқтат мәжбүр ет де хабарла әскери ақпарат көз олар арманда бол керек вашингтон дрон бір уақыт орбита ұста тұр мүмкіндік беру үшін қаржыландыру ұлғай менің ойымша шабуыл азайма пәкістан адам шабуыл қатты қарсылық білдір де алда үшін жай шу шығар,violent

4,көзі беті джихад дауысы ауғанстан ислам әмірлігі талибандар үшін ресми сайт,көз бет джихад дауыс ауғанстан ислам әмірліг талибан үшін ресми сайт,violent

4,ауғанстанда жеңіс пен прогреске үміттенетін болса білім мен білместік туралы біледі ауғанстан ислам әмірлігі обаманың жаңа әкімшілігіне ауғанстанда бір басқыншы әскер қалғанша ауған халқы демалмайды және жиһад пен қарсылықты тоқтатпайтынын айтады бәлкім ауған халқының тарихи ерлігі мен абыройы болар басқыншылардың қолында мәңгі жеңіліс болады,ауғанстан жеңіс пен прогрес үміттен бол білім мен білместік туралы біл ауғанстан ислам әмірліг обама жаңа әкімшіліг ауғанстан бір басқынш әскер қалғанш ауған халқ демалма және жиһад пен қарсылық тоқтатпа айт бәлкім ауған халқ тарихи ерліг мен абырой бол басқыншы қол мәңгі жеңіліс бол,violent

4,ассалауму алейкум бұл қазақшаға аударылды ма,ассалаум алейку бұл қазақша аудар ма,violent

4,өткен аптада көтерілісшілер алқаптан астанадан миль қашықтықта орналасқан бунер ауданына көшіп келіп үйде де шетелде де дабыл қақты армия ден астам көмекшіні өлтірді бірақ оларды әлі босату керек деп қорлады сейсенбіде сваттың жоғарғы әкімшісі хушал хан талибан бұл аймақты аралап жүріп миналарға шабуыл жасап жатыр бірақ бейбітшілік келісімінің аяқталуына әкелетін әскер шабуыл жасайды ма жоқ па деп айта алмады әскерилер кезкелген әрекетті бастамас бұрын орталық үкіметтің шешімін күтетінін мәлімдеді мингораның бас қаласында болған куәгердің айтуынша қара киімдер көптеген көшелер мен биік ғимараттарда орналастырылған ал қауіпсіздік күштері олардың базаларында тосқауыл қойылған,өткен апта көтерілісші алқап астана миль қашықтық орналас бун аудан көш кел үй де шетел де дабыл қақ армия ден астам көмекші өл бірақ ол әлі босат керек де қорла сейсенбі сва жоғарғы әкімші хушал хан талибан бұл аймақ арала жүр мина шабуыл жаса жатыр бірақ бейбітшілік келісім аяқтал әкел әскер шабуыл жаса ма жоқ па де ай алма әскери кезкел әрекет бастамас бұрын орталық үкімет шеш күт мәлімде мингора бас қала бол куәгер айт қара киім көптеген көше мен биік ғимарат орналастырыл ал қауіпсіздік күш олардың база тосқауыл қойыл,violent

4,мүжахидтер операциялары шешенстанға дейін созылады орыс басқыншыларына қарсы жиһадтық операциялар сәтті басталды және чечня мемлекетінің барлық аймағында жалғасады матинск аймағында орыс әскерлері мен олардың серіктестеріне қарсы кемінде төрт операция жүргізілді операциялар кем дегенде төрт адамның арасында жарақат алды бір адам қайтыс болды және жараланды ал бамут ауылының маңында саудасаттық белгілері бар уаз көлігі отырғызылды жауынгерлік операцияларды жоғалтпай операциялар туралы жаңалықтарда олар ерекше сипатқа ие өйткені шешенстанда үлкен көлемде жүргізіліп жатқан операцияларды қоспағанда ресейлік бақта жарияланғаннан гөрі басқыншылар үлкен қорқынышта өмір сүреді,мүжахид операция шешенстан дейін соз орыс басқыншы қарсы жиһад операция сәтті баста және чечня мемлекет барлық аймағ жалғас матинск аймағ орыс әскер мен олардың серіктес қарсы кемінде төрт операция жүргіз операция кем де төрт адам ара жарақат алды бір адам қайтыс бол және жарала ал бамут ауыл маң саудаса бел бар уаз көлiг отырғыз жауынгерлік операция жоғалтпа операция туралы жаңалық олар ерекше сипат ие өйткені шешенстан үлкен көлем жүргізіл жат операция қоспа ресейлік бақ жариялан гөрі басқыншы үлкен қорқыныш өмір сүр,violent

4,nwfp министрі tnsm тек қана peshawar қуатын қажет дейді nwfp министрі башир билур tnsm малаканд бөлімшесінде шариғатты емес өз ережесін қалайтынын айтты деп хабарлайды dawnnews пешавардағы бақпен сөйлескенде билур бұл аймақта бейбітшілікті орнатуға жауап беретін қауіпсіздік күштері екенін айтты ол дарулқаза малаканд бөлімшесінде шариат пен низамеадл талаптарына сәйкес және tnsm мен халықтың талаптарына сәйкес құрылған деп мәлімдеді министрдің сөзіне қарағанда бұл қару қолданған адамдар оны іс жүзінде жүзеге асырғысы келмейтіндігіне негізделіп билікті өздері үшін алғысы келеді оның айтуынша үкімет қауіпсіздік күштеріне толығымен батыста және басқа аудандарда өз жазбаларын жасауға рұқсат берді,nwfp министр tnsm тек қана peshawar қу қажет де nwfp министр башир билу tnsm малаканд бөлімше шариғат емес өз ереже қала ай де хабарла dawnnews пешавар бақ сөйлес билу бұл аймақта бейбітшілік орнат жауап бер қауіпсіздік күш екен ай ол дарулқаз малаканд бөлімше шариат пен низамеадл талап сәйкес және tnsm мен халық талап сәйкес құрыл де мәлімде министр сөз қара бұл қару қолдан адам оны іс жүз жүзеге асыр келмейтіндіг негіздел билік өз үшін ал кел оның айт үкімет қауіпсіздік күш толығ батыс және басқа аудан өз жазба жаса рұқсат бер,violent

4,мүмкін бостандық пен өзінөзі анықтау үшін моро күресі адамзаттың бүкіл тарихындағы ең ұзақ және қанды болып табылады,мүмкін бостандық пен өзінөз анықтау үшін моро күре адамзат бүкіл тарих ең ұзақ және қан бол таб,violent

4,ислам әмірлігінің ұстанымы егер шетелдік күштер елден кетпесе оларды қорғайтын джихад траншеялары сайлау сияқты кезкелген қастандық немесе алдамшы әрекеттермен салқындатылмайды жоғарыда айтылғандар азаматтарды осы жобадан ешқандай жақсылық күтпеуге және оған қатыспауға шақырды бұл жай бос уақыт және бұл халықаралық күштер мен азаматтардың көзіне күл шашып жатқан басқыншылық

күштердің қастығы өйткені президент вашингтонда тағайындалады және бұл деп аталатын губернатор сіздің дауысыңызбен емес мұны вашингтонның қалауы бойынша тағайындайды сіздің үйлеріңіз жүздеген емес мыңдаған рет американдық бомбалармен жойылуда сіз әлі де осы билеушілерден бірдеңе тілеп жүрсіз бе жоқ содан кейін олар үшін ешқашан тілемеңіз өйткені бұл деп аталатын билеушілер тек айтылған және айтылған сөздерге ғана ие ал жұмыс пен ерік билігі тек вашингтонда болады,ислам әмірліг ұстаным егер шетелдік күш ел кетпе ол қорға джихад траншея сайлау сияқты кезкел қаста немесе алдамш әрекет салқындатылма жоғары айтыл азамат осы жоба ешқандай жақсылық күтпе және оған қатыспа шақ бұл жай бос уақыт және бұл халықаралық күш мен азамат көз күл шаш жат басқыншылық күш қастығ өйткені президент вашингтон тағайындал және бұл де атал губернатор сіздің дауыс емес мұны вашингтон қала бойынша тағайында сіздің үй жүздеген емес мыңдаған рет американдық бомба жойыл сіз әлі де осы билеуші бірде тіле жүр бе жоқ содан кейін олар үшін ешқашан тілеме өйткені бұл де атал билеуші тек айтыл және айтыл сөз ғана ие ал жұмыс пен ерік билиг тек вашингтон бол,violent

4,ауғанстан ислам әмірлігінің ұстанымы егер шетелдік күштер елді тастап кетпейінше қандай да бір қастандықпен жиһад траншеяларын қалдыруға алданып қалмайды,ауғанстан ислам әмірліг ұстаным егер шетелдік күш ел таста кетпейінш қандай да бір қастандық жиһад траншея қалдыр алдан қалма,violent

4,барлық кофарларды және барлық таутогилердің билеушілерін жойыңыз,барлық кофар және барлық таутоги билеуші жой,violent

4,жақында қуыршақ әлзардари әйелін тозаққа жібереді,жақында қуыршақ әлзардари әйел тозақ жібер,violent

4,олар қасиетті джихад жерінде өтірік айтуға тырысып жатыр ма бұл шошқа бақшасындағы жаңа мақсат па баатилді жүзеге асырғыңыз келе ме бұл шошқаларды бұрынғы дәмінен гөрі нашар жағдайға әкеледі,олар қасиетті джихад же өтірік айт тырыс жатыр ма бұл шошқа бақша жаңа мақсат па баати жүзеге асыр келе ме бұл шошқа бұрынғы дәм гөрі нашар жағдай әкел,violent

4,мәйіттерді қоқыстардан шығарып жатқанда қаза болғандардың саны одан әрі өсуі мүмкін дейді қызыл крест тергеушілер тобы ереуілдердегі бейбіт тұрғындардың қаза тапқанын растады азаматтық шығындар болғанын біз толықтай растай аламыз деді джессика барри халықаралық қызыл крест комитетінің өкілі ақш бастаған күштер аймақта әуе соққыларын өткізгенін мойындады,мәйіт қоқыс шығар жатқанда қаза бол сан одан әрі өс мүмкін де қызыл крест тергеуші тоб ереуіл бейбіт тұрғын қаза тап раста азаматтық шығын бол біз толықта раста ал де джессик барри халықаралық қызыл крест комитет өкіл ақш баста күш аймақта әуе соққы өткіз мойында,violent

4,исламистік исламистік топтың жетекшісі фатх альислам сирияны тұтқындады немесе өлтірді қауіпсіздік қызметі бұл туралы бүгін интернетте таратқан мәлімдемеге сәйкес мәлімдемеде шакер әл абсидің ирактағы күрдтермен байланыс орнатқысы келетін адамдармен кездескені айтылған топ бұл альабси мен топтың басқа екі

мүшесіне шабуыл жасады деп мәлімдейді неміс маңындағы бір сағатқа созылған қаружарақтан кейін қауіпсіздік күштері бұл аймақты қоршауға алды және топ өз басшысынан бұл туралы бұдан былай естімегенін айтты топ әбу мұхаммед авад есімді адамды абсидің мұрагері деп атады және сирия режиміне қарсы күресті жалғастыруға уәде берді,ислами ислами топ жетекші фатх альисла сирия тұтқынд немесе өл қауіпсіздік қызмет бұл туралы бүгін интернет тарат мәлімдеме сәйкес мәлімдеме шак әл абси ирак күрд байланыс орнат кел адам кездес айтыл топ бұл альабси мен топ басқа екі мүше шабуыл жас де мәлімде неміс маң бір сағат созыл қаружарақ кейін қауіпсіздік күш бұл аймақ қорша алды және топ өз басшы бұл туралы бұдан былай естіме ай топ әбу мұхаммед авад ес адам абси мұрагер де ат және сирия режим қарсы күре жалғастыр уәде бер,violent

4,толық соғысқа дайындалып жатыр могадишоға ауыр қаружарақ бір аптада келеді,толық соғыс дайындал жатыр могадишо ауыр қаружарақ бір апта кел,violent

4,иордания королі абдуллахтың ақш президенті барак обаманың вашингтонға сапары кезінде бастама көтеруге міндеттеме алғанынан кейін болғанын айтады бастама бойынша палестиналық босқындар араб елдерінде қалыпқа келтірілетін болады ал қалғысы келетіндер палестина территориясына қайтып оралуы мүмкін онда жылғы аудандарға емес қарусыздандырылған палестина мемлекеті құрылады бұл бастама сонымен қатар израиль мен палестина үкіметі арасындағы жер алмасуды араб елдері мен израиль арасындағы жағдайды қалыпқа келтірудің белгіленген кестесін және шығыс иерусалимді палестина мемлекетінің астанасы деп тануды бұұ туы иерусалимнің ескі қаласының үстінде желбіретуді қамтиды,иордания король абдуллах ақш президент барак обама вашингтон сап кез бастама көтер міндеттеме ал кейін бол айт бастама бойынша палестиналық босқын араб ел қалып келтіріл бол ал қал кел палестин территория қайт орал мүмкін онда жылғы аудан емес қарусыздандырыл палестин мемлекет құр бұл бастама сонымен қатар израиль мен палестин үкімет ара жер алмасу араб ел мен израиль ара жағдай қалып келтіру белгілен кесте және шығыс иерусалим палестин мемлекет астана де тану бұұ ту иерусалим ескі қала үст желбірету қамти,violent

4,мен айтайын дегенім талибан пакистан ешқашан бас тартпайды пакистан армиясы мен талибан пакистан арасында ұрыс өте үлкен мамыр ж,мен айт де талибан пакис ешқашан бас тартпа пакис армия мен талибан пакис ара ұрыс өте үлкен мамыр ж,violent

4,обама крестшілердің қылмыстарына жақсы бет бұруға тырысады бірақ болып жатқан оқиғалардың мәні өзгермейді және оның басшылығымен исламға шабуыл жалғасады бүгін ауғанстан мен ирактағы бейбіт тұрғындарға бомбалар кеше буш президент болған кездегідей,оба крестші қылмыс жақсы бет бұр тырыс бірақ бол жат оқиға мән өзгерме және оның басшылығ ислам шабуыл жалғас бүгін ауғанстан мен ирак бейбіт тұрғын бомба кеше буш президент бол кездегідей,violent

4,ассаламу алейкум варахматуллаһ бұл сот адамдарының проблемалары әлі де бар сияқты мен жақында сомали жаңалықтарынан өте алыс болдым ол мені сол жерде болып жатқан жағдай туралы ескертпей қалдырды насрды әлшаабтың ағаларына беріп олармен соғысып өздерінің көшбасшыларын жойып жатқан жахил сомалилерге жол көрсетсін,ассалам алейку варахматуллаһ бұл сот адам проблема әлі де бар сияқты мен жақында сомали жаңалық өте алыс бол ол мен сол же бол жат жағдай туралы ескертпе қал нас әлшааб аға бер олармен соғыс өз көшбасшы жой жат жахил сомали жол көрсет,violent

4,саламу алейкум ихваани уа хауаати ретінде сомалидегі барлық муһахидтер арасында бітімге келу үшін күшжігер жұмсалуда көптеген ағайындылар айтқандай бұл өте күрделі жағдай адамдар әрдайым бірбіріне ұқсамайды сондықтан сіз тақтың фитнасын көре аласыз міне бірақ ақ ниетті адамдар бәрі ашшаабпен бірге болады салем болды,салам алейку ихваани уа хауаати рет сомали барлық муһахид ара бітім келу үшін күшжіг жұмсал көптеген ағайынды айтқанда бұл өте күрделі жағдай адам әрдайым бірбі ұқсама сондықтан сіз тақ фитна көр ал міне бірақ ақ ниетті адам бәрі ашшааб бірге бол сале бол,violent

4,қараңызшы хамас жылдар бойы өз талаптарын қаншалықты қанағаттандырған сияқты олар егер израиль алдағы он күнде газадан кетпесе шабуылдар басталатынын айтты бірақ қалған жерді ше израильдің әр дюймы мұсылман жерін жаулап алған және олар олардан жойылғанша оларға шабуыл жасау керек,қараңызш хамас жыл бойы өз талап қаншалықты қанағаттандыр сияқты олар егер израиль алдағы он күн газа кетпе шабуыл бастал ай бірақ қалған же ше израиль әр дюйм мұсылман жер жаула ал және олар олардан жойылғанш оларға шабуыл жасау керек,violent

4,мен джорданда бір мауһед ағам болады ол шошқа етінің майлы лас крест жорғасын миллион дана етіп жарып оны тозаққа жібереді,мен джордан бір мауһед аға бол ол шошқа ет майлы лас крест жорға миллион дана ет жар оны тозақ жібер,violent

4,ия барлық шын жүректен шыққан адамдар шабабпен бірге болады,ия барлық шын жүрек шық адам шабаб бірге бол,violent

4,бұл бамут маңындағы үш ресейлікті өлтірген шабуылға ұқсас дәйексөз шешенстанда полиция конвойы офицер өлтірілді жылғы мамырда шешенстанның ішкі істер министрлігі үш бүлікші полиция конвойына оқ жаудырып бір офицерді өлтірді дейді ресей губерниясы министрлік кеңсесі дүйсенбіде таратқан мәлімдемеде жексенбіде шабуылда тағы үш офицер ауыр жараланғанын айтады үш шабуылдаушы калашников автоматын атып кейін қашып кеткен кезде полиция екі техниканы шешен ауылына айдап әкеткен кремльшіл президент рамзан кадыровтың басшылығымен оңтүстік аймақта тұрақтылықтың жоғарылағанына қарамастан көтерілісшілер шешенстанға жиіжиі шабуыл жасайды ресей жақында екі қанды соғыстан кейін федералды терроризмге қарсы операцияны аяқтады associated press,бұл бамут маң үш ресейлік өлтір шабуыл ұқсас дәйексөз шешенстан полиция конвой офицер өлтір жылғы мамыр шешенстан ішкі істер министрліг үш бүлікш полиция конвой оқ жаудыр

бір офицер өл де ресей губерния министрлік кеңсе дүйсенбі тарат мәлімдеме жексенбі шабуылда тағы үш офицер ауыр жаралан айт үш шабуылдаушы калашников автомат ат кейін қаш кет кезде полиция екі техника шешен ауыл айда әкет кремльшіл президент рамзан кадыров басшылығ оңтүстік аймақта тұрақтылық жоғарыла қарамастан көтерілісші шешенстан жиіжи шабуыл жаса ресей жақында екі қан соғыс кейін федерал терроризм қарсы операция аяқта associated press,violent

4,пен израиль жылға дейін созылмайды,пен израиль жыл дейін созылма,violent

4,алькаида ресейдің солтүстік кавказында белсенді жұмыс істейді ішкі істер министрлігі ростовондон қаңтар риа новости алькаида мүшелері ресейдің солтүстік кавказ республикаларында шешенстан мен дағыстанда белсенді жұмыс істейді деп хабарлады ішкі істер министрінің орынбасары алькаида оқу орталықтары бүкіл әлемде бар ал ресейдің оңтүстігінде олар шешен республикасы мен дағыстан республикасының аумағында жұмыс істейді деді аркадий еделев журналистерге ол халықаралық террористік желі өкілдері республикалардағы қарулы топтарды үнемі тексеріп отырады және оларды қаружарақ пен жарылғыш заттармен қамтамасыз етеді еделев бұл процесс табиғи болғанын өйткені ресей күшейіп келе жатқанын және жаулардың елдегі операцияларға әсер етуі керек екенін айтты біз мұны білеміз және соған дайынбыз деп қосты ол,алькаи ресей солтүстік кавказ белсенді жұмыс істе ішкі істер министрліг ростовондон қаңтар ри новости алькаи мүше ресей солтүстік кавказ республика шешенс мен дағыстан белсенді жұмыс істе де хабарла ішкі істер министр орынбасар алькаи оқу орталық бүкіл әлем бар ал ресей оңтүстіг олар шешен республика мен дағыс республика аумағ жұмыс істе де аркади еделев журналист ол халықаралық террористік желі өкіл республика қарулы топ үнемі тексер отыр және ол қаружарақ пен жарылғыш зат қамтамасыз ет еделев бұл процесс табиғи бол өйткені ресей күшей келе жат және жау ел операция әсер ет керек екен ай біз мұны біл және соған дайын де қос ол,violent

4,олардың ұшағы да осылай құлайды,олардың ұшағ да осылай құла,violent

4,цитата бастапқыда shabaabqoqaz гедо аймағының кейбір жерлерінде құрылатын исламдық әкімшілік,цита бастапқы shabaabqoqaz гедо аймағ кейбір жер құрыл исламдық әкімшілік,violent

4,ахия менің білуімше солтүстік кенияоңтүстік сомали шекарасында этникалық сомалилер тұрады шекара анықталмаған және әрдайым кландық қақтығыстар болады бұл тек куфилердің жасаған мәлімдемесі бірақ егер сіз кениядағы барлық қарулы қақтығыс туралы оқығыңыз келсе мұны оқыңыз,ахия менің білуімше солтүстік кенияоңтү сомали шекара этникалық сомали тұр шекара анықталма және әрдайым кла қақтығыс бол бұл тек куфи жаса мәлімде бірақ егер сіз кения барлық қарулы қақтығыс туралы оқы кел мұны оқ,violent

4,ия ахи біз мұны жақында көреміз бұл жолдан таянған қуыршақтар зардари мен гиланиді қызанақ сияқты қырып тастау керек бұл жағдайда ешкім тұрғындарға не болатынын түсінбеуі керек егер олар жолдан тайған пәкістан үкіметіне одақтас болса

олар да жүлдеге лайық мұжахидтерге пәкістандағы куфилердің қолдауымен жолдан тайған бұл зайырлы үкіметті құлатуға көмектессін,ия ахи біз мұны жақында көр бұл жолдан таян қуыршақ зардари мен гилани қызанақ сияқты қыр тастау керек бұл жағдай ешкім тұрғын не бол түсінбе керек егер олар жолдан тай пәкістан үкімет одақтас бол олар да жүлде лайық мұжахид пәкістан куфи қолдау жолдан тай бұл зайырлы үкімет құлат көмектес,violent

4,сомалидегі джихадқа қосылудың алтын мүмкіндігі бүгін ол falojah форумдарында abo dojana атты қолданушы жасаған хабарлама тақырыбы або дожана жиһадқа қатысқысы келетіндерді сомалидегі адамдарға сол жерден бүкіл әлемге тарайтын шейіт іздеушілер базасын құруға шақырды хабарлама осы мәселеге қатысты өз пікірлерін білдірген көптеген мүшелерді қызықтырды сомали шын мәнінде физикалық және қаружарақ жаттығулары үшін өте жақсы база бола алады және ол жерден могадишуды азат етуге бауырластардың қатарына қосылып сол жерден басқа джихади майдандарына көшуге болады дейді абдель кафи есімімен бір мүше мен бұл ауғанстанға терроризмді өндіруде және оны бүкіл әлемге экспорттауда идеалды балама болатынына сенімдімін деді тағы біреуі басқа мүшелер практикалық егжейтегжейлерді соның ішінде оған қалай жетуге болатындығын талқылауға кетті олар сомалидегі шетелдік жиһадилер басқаратын исламистік исламшыл вебсайтқа бағытталып онда пайдалы ақпарат табамыз деген болатын cbs,сомали джихад қосыл алтын мүмкіндіг бүгін ол falojah форум abo dojana ат қолданушы жаса хабарлама тақырыб або дожан жиһад қатыс кел сомали адам сол же бүкіл әлем тара шейіт іздеуші база құр шақ хабарлама осы мәселе қатысты өз пікір білдір көптеген мүше қызық сомали шын мән физикалық және қаружарақ жаттығу үшін өте жақсы база бола ал және ол же могадишу азат ет бауырлас қат қосыл сол же басқа джихади майдан көш бол де абдель кафи есім бір мүше мен бұл ауғанстан терроризм өндір және оны бүкіл әлем экспортта идеалды балама бол сенімді де тағы біреу басқа мүше практикалық егжейтегжей соның іш оған қалай жет болатындығ талқыла кет олар сомали шетелдік жиһади басқар ислами исламшыл вебсайт бағыттал онда пайдалы ақпарат таб де бол cbs,violent

4,абу мансур амрикадан келген хатта сіз білуіңіз керек нәрсе icu фракциялардың одағы болды содан кейін эфиопия келді және барлығы өздері үшін әлшааб оңтүстікке қарай бет алды содан кейін олармен бірге соғысып басқа фракциялар мен адамдар оларға қосылды бұл өте сұр аудандағы апа біз отыруға және не болып жатқанын айтуға болмайды жай ғана сабыр етіңіз және ресми мәлімдемелерді күтіңіз moqdishu майдан болып табылады және мен ауылдық жерлерден көптеген жауынгерлер қазірдің өзінде келе жатыр деп ойлаймын сондықтан бұл жекпежекті күтіңіз тағы бір аптаға жүре беріңіз бұл шешуші шайқас болуы мүмкін,абу мансу амрика кел хатта сіз біл керек нәрсе icu фракция одағ бол содан кейін эфиопия кел және барлығ өз үшін әлшааб оңтүстік қарай бет алды содан кейін олармен бірге соғыс басқа фракция мен адам оларға қос бұл өте сұр аудан апа біз отыр және не бол жат айт болма жай ғана сабыр ет және ресми мәлімдеме күт moqdishu майдан бол таб және мен ауылдық жер

көптеген жауынгер қаз өз келе жатыр де ойла сондықтан бұл жекпежек күт тағы бір апта жүр бер бұл шешуші шайқас бол мүмкін,violent

4,готли бауырым егер сіз адамдарды анықтай алсаңыз әжеңіздің үйін тонау үшін қажетті шараларды жасау сізге кеш емес деп ойлаймын,готли бауыр егер сіз адам анықта ал әже үйін тонау үшін қажетті шара жасау сізге кеш емес де ойла,violent

4,могайдишу мареег әлшабаб исламистері стратегиялық база болып табылатын могадишу стадионын үкіметшіл исламистер куәгерлер мен шенеуніктер жексенбі күні басып алды тұтқындау ең ауыр шайқас ел астанасы могадишода өтіп жатқан кезде басталды могадишодағы үкімет сарбаздарына миномет оқ атылды сомали үкіметінің әскери күштері төрт күн бұрын басталғаннан бері алғаш рет соғысқа қатысады әскерилер қаланың солтүстігіндегі маңызды аудандарды исламшылдардың бақылауына алғанын мәлімдеді шейх хасан дахир авейс басқаратын жаңа исламшыл топ үкіметке қарсы күреске қосылды қара теңіз аймағында әлшабаб пен үкіметшіл исламисттер арасындағы тағы бір шайқас басталды әлшабаб үкіметтің исламшыл жауынгері оларға тапсырылды деп мәлімдеді бірақ тәуелсіз растау жоқ марег,могайдиш мареег әлшабаб исламис стратегиялық база бол табыл могадиш стадион үкіметшіл исламис куәгер мен шенеунік жексенбі күн бас алды тұтқында ең ауыр шайқас ел астана могадишо өт жат кезде баста могадишо үкімет сарбаз миномет оқ ат сомали үкім әскери күш төрт күн бұрын бастал бері алғаш рет соғыс қатыс әскери қала солтүстіг маңызды аудан исламшыл бақыла ал мәлімде шейх хасан дахи авейс басқар жаңа исламшыл топ үкімет қарсы күрес қос қара теңіз аймағ әлшабаб пен үкіметшіл исламист ара тағы бір шайқас баста әлшабаб үкімет исламшыл жауынгер оларға тапсыр де мәлімде бірақ тәуелсіз растау жоқ марег,violent

4,о менің халқым неге сен әлі үндемей отырсың ал сенің жолдан тайған әкімдерің бұл уәзірлерге ислам мен мұсылманға соғысуға рұқсат берді менің халқым,о менің хал неге сен әлі үндеме отыр ал сенің жолдан тай әкім бұл уәзір ислам мен мұсылман соғыс рұқсат бер менің хал,violent

4,бауырларымызға сомалидегі жолдан таюшылар мен крест жорықтарын жоюға рұқсат етсін,бауыр сомали жолдан таюшы мен крест жорық жою рұқсат ет,violent

4,иси ирак ислам мемлекеті туы жақында біз сомали исламдық мемлекетін iss бейбітшілік көреміз,иси ирак ислам мемлекет ту жақында біз сомали исламдық мемлекет iss бейбітшілік көр,violent

4,джихадқа сомалияға келген әрбір мужаахид ол шетелдік күрескер емес бірақ ол мухажир мужахид біз біріккен күштерге қарсы күресіп жатырмыз сондықтан біз күштерімізді біріктіріп мухажирлердің жүректеріне жағу керекпіз имандылық біздің қасиетті saw дәуірінің дәуірінде болған сияқты бірақ бүлінген бұқаралық ақпарат құралдары мужахидтерді анықтау үшін батыстық анықтамаларды қолданады және мен исламдық бақтардың қаһармандарын сионист крестшілер жолдан тайған исламға қарсы соғысқа қарсы өз рөлдерін атқару үшін айтқым келеді және мұсылман

біздің жүректерімізді жігерлендіру үшін мужахидтер туралы жаңалықтарды және исламдық тілмен және анықтамамен жібереді,джихад сомалия кел әрбір мужаахид ол шетелдік күреск емес бірақ ол мухажи мужахид біз біріккен күш қарсы күрес жатыр сондықтан біз күш біріктір мухажир жүрек жағ керек имандылық біздің қасиетті saw дәуір дәуір бол сияқты бірақ бүлін бұқаралық ақпарат құрал мужахид анықтау үшін бат анықтама қолдан және мен исламдық бақ қаһарман сионист крестші жолдан тай ислам қарсы соғыс қарсы өз рөл атқару үшін айт кел және мұсылман біздің жүрек жігерлендіру үшін мужахид туралы жаңалық және исламдық тіл және анықтама жібер,violent

4,мен бұл суреттерді көргенде не істерімді білмеймін менің қаным жай ғана қайнатылды мен сол кафир солдатты жалаң қолыммен өлтірер едім менің өмірім мен өлімімнің күші бар,мен бұл сурет көргенде не іс білме менің қан жай ғана қайнатыл мен сол кафи солда жала қол өлтір ед менің өмір мен өлім күш бар,violent

4,ислам мемуарының жаңалықтары жылғы мамырда талибан пакистан мен пакистан армиясы арасында үлкен шайқастар мен шайқастар жалғасуда,ислам мему жаңалық жылғы мамыр талибан пакис мен пакис армия ара үлкен шайқас мен шайқас жалғас,violent

4,ассаламу алейкум шарифті көптеген қылмыскерлер мен бірнеше шаршаған қарттар қалдырады ол үшін күресу керек қалай ол әлшааб пен хизбул исламның мұжахидтерін жеңуге үміттенеді цитата сомали исламшыл сарбаздары сомали үкіметінің сарбазын күзетуде жылғы мамырда ел астанасы могадишода исламшыл күрескерлермен қақтығыс кезінде тұтқындалды,ассалам алейку шариф көптеген қылмыскер мен бірнеше шарша қарт қалдыр ол үшін күресу керек қалай ол әлшааб пен хизбул ислам мұжахид жең үміттен цита сомали исламшыл сарбаз сомали үкім сарбаз күзет жылғы мамыр ел астана могадишо исламшыл күрескер қақтығыс кез тұтқында,violent

4,альшабабтың басшысы үкіметті исламдық сипатқа ие деп айтуға болмайды деп хабарлады мамырдың і де әлшабаб бұқаралық ақпарат құралдарына сомали үкіметін ислам үкіметі ретінде сипаттауға болмайды деп таратқан жазбаша сөзінде шейх субейр қазіргі уақыттағы шариф шейх ахмед басқарған өтпелі үкіметті исламдық үкімет деп сипаттауға болмайды оны патриоттық деп те атауға да болмайды деді ол могадишодағы шайқастар туралы сөйлеп сомали халқын оның дұрыс деп айтқанын қолдауға шақырды және күрес дұрыс емес пен жақсылықтың арасында екенін айтты ол сонымен бірге журналистерді джихадқа қатысуға және халықты адастырудан сақ болуға шақырды шелек,альшабаб басшы үкімет исламдық сипат ие де айт болма де хабарла мамыр і де әлшабаб бұқаралық ақпарат құрал сомали үкім ислам үкімет рет сипатта болма де тарат жазбаша сөз шейх субей қазіргі уақыт шариф шейх ахмед басқар өтпелі үкімет исламдық үкімет де сипатта болма оны патриоттық де те ата да болма де ол могадишо шайқас туралы сөйле сомали халқ оның дұрыс де айт қолда шақ және күрес дұрыс емес пен жақсылық ара екен ай ол сонымен бірге журналист джихад қатыс және халық адастыру сақ бол шақ шелек,violent

4,цитата менің ойымша ираки үкіметі өзін масқаралауға міндетті түрде кіріседі деп ант берді абу омар әлбахдади жақында олар иши бекіністерін қайтарып алатындарын айтты дуаа бауырлар жаса бейбітшілік acer,цита менің ойымша ираки үкімет өз масқарала міндетті түр кіріс де ант бер абу омар әлбахдади жақында олар иши бекініс қайтар ал ай дуа бауыр жаса бейбітшілік acer,violent

4,сомалияның жаңа жағдайы туралы және мұсылман үмбетін және могадишо халқын ерекше бағыттауға бағыттаңыз шейх бұл үкіметке қарсы күрестің екінші рет екенін және бұл исламдық үкіметтік емес екенін айтты сондайақ шейх біз өз джихадымызды жалғастыра береміз ал батыстықтар мен олардың африкандық одақтастарының мүдделері бақытсыз жолмен аяқталады иншаллаһ біз бұл күресті жалғастыра береміз бұл біздің көсемнің сенімі және бұл шариф inshalaah ya abazubayr сенімі біз сендердің сарбаздарымыз,сомалия жаңа жағдай туралы және мұсылман үмб және могадишо халқ ерекше бағытта бағытта шейх бұл үкімет қарсы күре екінші рет екен және бұл исламдық үкіметтік емес екен ай сондайақ шейх біз өз джихад жалғастыр бер ал батыстық мен олардың африка одақтас мүдде бақыт жол аяқтал иншаллаһ біз бұл күре жалғастыр бер бұл біздің көсе сенім және бұл шариф inshalaah ya abazubayr сенім біз сен сарбаз,violent

4,дәйексөз пәкістан халықтық партиясы ppp басқарған үкімет вашингтон үшін прокси соғыс ретінде емес өзінің өмір сүруі үшін талибанға қарсы күрес жүргізіп жатқанын айтады жоқ сіз біздер мұсылман болғандықтан және сіз жолдан таюшылар болғандықтан жоқ сіз бізбен соғысып жатырсыз өйткені сіз американың құлысыз жоқ ал сіз жек көресіз ол неге мұны мойындамайсыз қуыршақсыздар барлық мұсылман жеріндегі сендер және сені ұнатушылар жолдан таюшылар ал сендер барлығың пұтқа табынушыларсыңдар және сізге жалақы төлеу шеберлері келіп сізді құтқара алатынына не сізге көмектесе алмайтынын көреміз тек уақыт мәселесін күте тұрыңыз,дәйексөз пәкістан халықтық партия ppp басқар үкімет вашингтон үшін прокси соғыс рет емес өзінің өмір сүр үшін талибан қарсы күрес жүргіз жат айт жоқ сіз біздер мұсылман бол және сіз жолдан таюшы бол жоқ сіз бізбен соғыс жатыр өйткені сіз америка құл жоқ ал сіз жек көр ол неге мұны мойындама қуыршақ барлық мұсылман жер сен және сен ұнатушы жолдан таюшы ал сен барлығ пұт табынушы және сізге жалақы төлеу шебер кел сіз құтқар ал не сізге көмекте алма көр тек уақыт мәселе күт тұр,violent

4,ия сааты ағай жақында бұл білейтін қуыршақтар биизнилла туралы шындықты біледі содан кейін олардың жалған негіздемелері ескерілмейді бұл қуыршақтар ұятсыз лас шошқалардан басқа ештеңе емес мақалада келтірілген taghout күштері өлтіргендердің саны бұл жай ғана емес асырып жіберілген сан бұл жолдан тайған қуыршақтар американдықтарды қуанту және қуанту үшін мұны жиі жасайды өйткені америкадан олар жолдан таю үшін өз ақшаларын алады олар американы қуантады олар алған сайын көп осы кафтерлер мен жолдан таюшыларды жоюды тездетсін,ия саа аға жақында бұл біле қуыршақ биизнилл туралы шындық біл содан кейін олардың жалған негіздеме ескерілме бұл қуыршақ ұят лас шошқа басқа ештеңе емес

мақала келтір tagħout күш өлтір сан бұл жай ғана емес асыр жібер сан бұл жолдан тай қуыршақ американдық қуант және қуант үшін мұны жиі жаса өйткені америка олар жолдан таю үшін өз ақша ал олар америка қуант олар ал сайын көп осы кафтер мен жолдан таюшы жою тездет,violent

4,цитата бастапқыда жариялаған acer сваттан жер аударылған босқындардың айтуынша ереже аймақты зорлықзомбылықпен басқарады сыншылар мен үкіметтік ақпарат берушілерді өлтіріп қыздар мектептерін қиратады бұл жағымсыз негізгі медиалар әлі де шіріген тортқа мұздатуды қолданады кезкелген ақылесі бар адам оны жегісі келеді ме,цита бастапқы жарияла acer сват жер аударыл босқын айт ереже аймақ зорлықзомбылық басқар сыншыл мен үкіметтік ақпарат беруші өлтір қыз мектеп қират бұл жағымсыз негізгі медиа әлі де шірі торт мұздату қолдан кезкел ақыле бар адам оны же кел ме,violent

4,біз бүкіл сомали шабабтың аяқ астына түсіп сомали ислам мемлекеті құрылған күнді асыға күтеміз,біз бүкіл сомали шабаб аяқ аст түс сомали ислам мемлекет құрыл күн асыға күт,violent

4,орталық майдан және итумкала секторының есімдері шейх саидтен а жылдың көктеміндегі шура жиналысында амирдің абу усман магас асланбек хусейн супян абдул азиз және хамзат қатысқан сөзінде шыққан бұл жерде әлі жарияланған жоқ,орталық майдан және итумкал сектор есім шейх саид а жыл көктем шур жиналы ами абу усман магас аслан хусейн супян абдул азиз және хамзат қатыс сөз шық бұл же әлі жариялан жоқ,violent

4,бұрын хабарлағандай шабуылды жанкештінің көлік жасағаны жасаған бірақ кейінірек полиция жарылыс болған кезде көлік жарылған кезде тоқтаған содырлар соңғы айда жалпы зорлықзомбылық күрт төмендегеніне қарамастан шабуылдарды жалғастыруда ақш шенеуніктері қаңтардағы дауыс беруге дейін зорлықзомбылық болуы мүмкін деп ескертті полицияның мәлімдеуінше алькаиданың жасушалары анбар провинциясының батыс провинциясындағы гармада әлі де белсенді деп санайды анбар жылы тайпалық басшылар қауіп төнгенге дейін сунниттік арабтардың ақш күштеріне қарсы көтерілістерінің жүрегі болды провинция қазір біршама бейбіт бірақ келесі сенбідегі дауыс беру алдында сунниттік топтар арасындағы шиеленіс күшейе түсті,бұрын хабарлағанда шабуыл жанкешті көлік жаса жаса бірақ кейінірек полиция жарылыс бол кезде көлік жарыл кезде тоқта содыр соңғы айда жалпы зорлықзомбылық күрт төменде қарамастан шабуыл жалғастыр ақш шенеунік қаңтар дауыс бер дейін зорлықзомбылық бол мүмкін де ескер полиция мәлімдеуінше алькаида жасуша анбар провинция батыс провинция гарма әлі де белсенді де сана анбар жыл тайпалық басшы қауіп төн дейін сунни араб ақш күш қарсы көтеріліс жүрег бол провинция қазір біршама бейбіт бірақ келесі сенбі дауыс беру алд сунни топ ара шиеленіс күшей түсті,violent

4,цитата мәскеудегі бибиси ричард галпин мұсылман көтерілісшілерінің грозныйға шабуыл жасауы сирек болады дейді өтірік мужахидтер грозныйда және оның

айналасында әр апта сайын операциялар жасайды,цита мәскеу бибиси ричард галпин мұсылман көтерілісші грозный шабуыл жаса сирек бол де өтірік мужахид грозный және оның айнала әр апта сайын операция жаса,violent

4,гераттағы шендан әуежайында мүжахидтерге қарсы зымырандар кешегі ауғанстан ислам әмірлігінің мұжахидтері герат провинциясындағы шенданд әуежайына зымырандарды жіберді зымырандар жаудың ауыр шығынына ұшыраған базаға қонды қари юсуф ахмади баяндаған,герат шен әуежай мүжахид қарсы зымыран кешегі ауғанстан ислам әмірліг мұжахид герат провинция шенданд әуежай зымыран жіб зымыран жау ауыр шығын ұшыра база қон қари юсуф ахмади баянда,violent

4,осы жаңалықтардың ішіндегі ең қызығы бұл адамдар біздің бауырларымызды өлтірді деп мақтанады және ешқашан олар талап етіп жатқан дәлелдерді көрсете алмайды бірақ екінші жағынан егер біздің бауырларымыз оларды өлтірді десе онда бұл бұл жай талап қана емес бізде суреттер мен бейнелер бар керемет ашуланғанда тілдеріңді тісте қазір бұқара сенбейді бұдан да сорақысы сіз жеңілесіз біздің жерімізден масқара болып қуылады,осы жаңалық ішіндегі ең қызығ бұл адам біздің бауыр өл де мақтан және ешқашан олар талап ет жат дәлел көрсе алма бірақ екінші жағ егер біздің бауыр ол өл де онда бұл бұл жай талап қана емес біз сурет мен бейне бар керемет ашулан тіл тіс қазір бұқара сенбе бұдан да сора сіз жеңіл біздің же масқара бол қу,violent

4,бауырым бұл мәселе туғызбайды бірақ бұл соғыс крест жорығы емес деп айтқандардың жүрегін тазартады бұл крест жорығы және егер олар абу гурайбтың жағдайы болса гуантанамо мен басқа да құпияның жағдайы қалай болады түрмелерде жиһад жетекшілерінің өмірі қалай жүреді шейх абу зубайда шейх абу мусаб асури және крейсерлердің тұтқындарындағы көптеген шейхтар сионистер мен жолдан таюшылар мұсылмандар оянып жиһадқа шығады жеңісі бізге тиесілі бұл жеңіс жылы,бауыр бұл мәселе туғызба бірақ бұл соғыс крест жорығ емес де айт жүрег тазарт бұл крест жорығ және егер олар абу гурайб жағдай бол гуантанамо мен басқа да құпия жағдай қалай бол түрме жиһад жетекші өмір қалай жүр шейх абу зубай шейх абу мусаб асури және крейсер тұтқын көптеген шейх сионис мен жолдан таюшы мұсылман оян жиһад шығ жеңіс бізге тиесілі бұл жеңіс жыл,violent

4,пакистан армиясы мен талибан пакистан арасындағы қақтығыстар кезінде пакистан армиясының әскери қызметкері қаза тапты және талибан пакистан мен пакистан арасындағы қақтығыстар мен соғыс әлі де жалғасуда сәт,пакис армия мен талибан пакис ара қақтығыс кез пакис армия әскери қызметк қаза тап және талибан пакис мен пакис ара қақтығыс мен соғыс әлі де жалғас сәт,violent

4,алғашқы шайқас хиран провинциясының мухас аймағында жұма күні хижраның жылы мамырда жылы джумада әлавал және авлия милиционерлер мен мүжахидтерден сарбаз каруларын өлтірді ал қалғандары қашып кетті дала бір нәрсені бұрмаламайды жұма намазынан кейін осы әскерилердің көп бөлігі орталық сомалидегі джалгадуд аймағындағы вабхо аймағына шабуыл жасады ал мужахидтер

оларды іздестірді және сол жерде әлі де жалғасып жатқан қатыгез ұрыс басталды айта кету керек сомалидегі жолдан тайған үкіметті қолдайтын крест жорықтары үкіметтері ирактағы ояну күштеріне ұқсас өздеріне адал тайпалық отрядтар құруға ұмтылуда бірақ мұжахидтер оларды күтіп олардың қозғалыстарын мұқият қадағалап отырады бірақ көпшілік біле бермейді біз күресеміз жас мұжахидтер қозғалысының медиа бөлімі армия мужахидтер және сенушілерді арандату,алғашқы шайқас хиран провинция мухас аймағ жұма күн хижра жыл мамыр жыл джума әлавал және авлия милиционер мен мүжахид сарбаз қару өл ал қал қаш кет дала бір нәрсе бұрмалама жұма намаз кейін осы әскери көп бөліг орталық сомали джалгадуд аймағ вабхо аймағ шабуыл жас ал мужахид ол іздестір және сол же әлі де жалғас жат қатыгез ұрыс баста ай кету керек сомали жолдан тай үкімет қолда крест жорық үкімет ирак оян күш ұқсас өз адал тайпалық отряд құр ұмтыл бірақ мұжахид ол күт олардың қозғалыс мұқият қадағала отыр бірақ көпшілік біл берме біз күрес жас мүжахид қозғалыс медиа бөлім армия мужахид және сенуші арандат,violent

4,мен кіші кафыров күнін күте алмаймын жолдан тайған әкесі сияқты жойылады,мен кіші кафыров күн күт алма жолдан тай әке сияқты жой,violent

4,сауд арабиясының шииттері тәуелсіздік жариялады күн мамыр gmt шығыс арабия республикасы сауд арабиясының мұнайға бай нажран және ашшария эмираттарынан тұрады сауд арабиясындағы шиит азшылық шығыс арабия республикасы деп аталатын елді құрып патшалықтан тәуелсіздігін жариялады бұқаралық ақпарат құралдарында аз ақпарат алған жаңадан құрылған мемлекет катиф пен альхасаның шығыс аудандарында құрылды деп хабарлайды шиіт діни жетекшісі шейх нимр бакир әлнамрға жақын сайт жуырда сауд арабиясының бір топ белсенділері патшалықтың билеушілеріне патша отбасының елдің саяси институттарына деген құлшынысын төмендететін реформалар жасауға шақырды өзінөзі сипаттаған құқық қорғаушылар қол қойған петицияда ұлыбритания иордания және марокко сияқты конституциялық монархияны құруға шақырады өткен жазда мұнай бағасы рекордтық шекті деңгейге түсіп кеткен соң ел де биылғы жылы бюджеттің млрд доллар тапшылығына тап болды,сауд арабия шиит тәуелсіздік жариял күн мамыр gmt шығыс арабия республика сауд арабия мұнай бай нажран және ашшария эмират тұр сауд арабия шиит азшылық шығыс арабия республика де атал ел құр патшалық тәуелсіздіг жариял бұқаралық ақпарат құрал аз ақпарат ал жаңа құрыл мемлекет катиф пен альхаса шығыс аудан құр де хабарла шиіт діни жетекші шейх ним баки әлнам жақын сайт жуырда сауд арабия бір топ белсенді патшалық билеуші патша отбас ел саяси институт де құлшыныс төмендет реформа жаса шақ өзінөз сипатта құқық қорғаушы қол қой петиция ұлыбритания иордания және марокко сияқты конституциялық монархия құр шақыр өткен жаз мұнай баға рекордтық шекті деңгей түс кет соң ел де биылғы жыл бюджет млрд доллар тапшылығ тап бол,violent

4,сәлемсіз байланыс немесе үзілістерге байланысты жаңалықтардан хабардар болу қиын болған сүйікті адамдарымызға назарларыңызға өткен күндердің маңызды

жаңалықтары туралы қысқаша репортажды ұсынамыз медиа джихад батальонындағы бауырларыңыздың сәлемдері http www fileflyer com view gwmimbk http www fileflyer com view zca bn http ifile it chzgdxw http ifile it chzgdxw http www turboupload com tzn thc rrrrr rar html http uploading com files iwzts bx rrrrr rar html http uploading com files wkh yso rrrrr rar html,сәлем байланыс немесе үзіліс байланысты жаңалық хабардар болу қиын бол сүйікті адам назар өткен күн маңызды жаңалық туралы қысқаша репортаж ұсын медиа джихад батальон бауыр сәлем http www fileflyer com view gwmimbk http www fileflyer com view zca bn http ifile it chzgdxw http ifile it chzgdxw http www turboupload com tzn thc rrrrr rar html http uploading com files iwzts bx rrrrr rar html http uploading com files wkh yso rrrrr rar html,violent

4,дәйексөз бастапқыда muwahidbrother бауырым kashmir itте jihad feesbelilah жоқ бұл тек ұлтшылдық үшін күрес және ислам мен жиһад деген белгі бар мен ағайынның не айтып тұрғанын көріп тұрмын бірақ мұндай мәлімдемелерге өте мұқият болу керек әрине кашмир және таиланд сияқты жерлерде көптеген мұсылмандар ұлтшыл топтардың жолына түсіп кетті мұндай жерде мұндай джихад жоқ деп айту өте ауыр мәлімдеме мәселен шешенстанға қараңызшы тоқсаныншы жылдардағы көптеген адамдар кафирлік орыстарға қарсы күресті тәуелсіздік ұлтазаттық үшін күрес ретінде көрді басында күрескерлер мужахидтер мен ұлтшылдардың қосындысы енді ұлтшылдар өз еркімен сатып алынды немесе қаружарақтарын алды ал кавказ ислам әмірлігінің шынайы мужахидтерімен соғысуды жалғыз адамдар қалдырды,дәйексөз бастапқы muwahidbrother бауыр kashmir it jihad feesbelilah жоқ бұл тек ұлтш үшін күрес және ислам мен жиһад де белгі бар мен ағайын не айт тұр көр тұр бірақ мұндай мәлімдеме өте мұқият болу керек әрине кашмир және таиланд сияқты жер көптеген мұсылман ұлтшыл топ жол түс кет мұндай же мұндай джихад жоқ де айту өте ауыр мәлімдеме мәселен шешенстан қараңызш тоқсанынш жыл көптеген адам кафирлік орыс қарсы күре тәуелсіздік ұлтаза үшін күрес рет көр бас күрескер мужахид мен ұлтшыл қосынды енді ұлтшыл өз ерк сат ал немесе қаружарақ алды ал кавказ ислам әмірліг шынайы мужахид соғысу жалғыз адам қал,violent

4,мен әлсахабтың вазиристан қолбасшысымен жасаған соңғы сұхбатынан нені түсінемін кашмирдегі мұжахидтерге қаружарақтарын қойып вазиристанға келіп пәкістанда исламдық мемлекет құруға көмектесуіңізді сұраймын салем acer,мен әлсахаб вазирис қолбасшы жаса соңғы сұхбат не түс кашмир мұжахид қаружарақ қой вазиристан кел пәкістан исламдық мемлекет құр көмектесу сұра сале acer,violent

4,дәйексөз бастапқыда әбу умар уа алейкум ассалам мен кафрованың кіші күнін күте алмаймын жолдан таюшы әкесі сияқты жойылады,дәйексөз бастапқы әбу ум уа алейку ассала мен кафрова кіші күн күт алма жолдан таюш әке сияқты жой,violent

4,дереккөздер ііm жолдан шыққан лейтенанты хасавюртте кеше түнде жойылды деп хабарлайды сонымен бірге кафтарлар мен жергілікті жолдан таюшылар қарабұтақкент ауданында какашура какамаси качкалык чанкурбе және доргели ауылдарының маңында әскери операция жариялайды,дереккөз іі жолдан шық лейтенант хасавюрт кеше түн жой де хабарла сонымен бірге кафтар мен жергілікті

жолдан таюшы қарабұтаққент аудан какашур какамаси качкалык чанкурб және доргели ауыл маң әскери операция жарияла,violent

4,енді құлдырау басталады,енді құлдырау бастал,violent

4,бауырлар сіздер джундуаллахтың қолбасшысы абдул малик рэгінің есімін білетін шығарсыздар джундулла бірнеше жылдар бойы арани кафир әкімшілігімен күресіп келеді шаһид абдул гаффордың вонда болғанын абдул малик регінің інісі иранның кафир әкімшілігіне қарсы шабуыл жасағанын айтады http www youtube com watch atx id qc және менің қазақшама кешірім сұраймын,бауыр сіздер джундуаллах қолбасшы абдул малик рэг ес біл шығ джундулл бірнеше жыл бойы арани кафи әкімшіліг күрес кел шаһид абдул гаффо вон бол абдул малик рег іні иран кафи әкімшіліг қарсы шабуыл жаса айт http www youtube com watch atx id qc және менің қазақша кешір сұра,violent

4,quote абу фатима size әрине кашмир және тайланд сияқты жерлерде көптеген мұсылмандар ұлтшыл топтармен адасқан ассаламуаликум бірінші бірімен кашмирдегі ұлттық туралы сұрайдым бұұның ережелерін білмеу және егер сіз біз ел франция және басқа либерліктерді бірге болады бірақ ислам үшін күресуге және терроризмге ислам қаласын танытуға рұқсат болмайды біз пакистанда гоп болады басқа мұжахиден болады мужахидендің жалғасында болады молана софи мухаммад дейді кашмирліктер шариғатты талап етпеді бірақ мемлекет болды сондықтан джихад онда міндетті емес фейсбилилах джихад қашмыр ұлттықтың ретіндегі түрлерінің көздерінің түрлерінің ajklf сияқты кейбір ұлтшылдықты ислам дінімен байланыстыра бастайтынын айтқанмын бірақ исламның негізін қалаушы барлық жоғарғы жиһад кеңесі жиһадтық тіркелген ұйымдар бұұ ережелерінен қатысады кейбіреулері мұны мақтанышпен көрсетеді ал кейбіреулері бұл шындықты ислам және жиһад белгісінде жасырады,quote абу фатима size әрине кашмир және тайланд сияқты жер көптеген мұсылман ұлтшыл топ адас ассаламуалику бірінші бір кашмир ұлттық туралы сұрай бұұ ереже білме және егер сіз біз ел франция және басқа либерлік бірге бол бірақ ислам үшін күрес және терроризм ислам қала таныт рұқсат болма біз пакистан го бол басқа мұжахи бол мужахиде жалға бол молан софи мухаммад де кашмирлік шариғат талап етпе бірақ мемлекет бол сондықтан джихад онда міндетті емес фейсбилилах джихад қашмы ұлттық рет түр көз түр ajklf сияқты кейбір ұлтшылдық ислам дін байланыстыр баста айт бірақ ислам негіз қалаушы барлық жоғарғы жиһад кеңес жиһад тіркел ұйым бұұ ереже қатыс кейбіреу мұны мақтаныш көрсет ал кейбіреу бұл шындық ислам және жиһад белгі жасыр,violent

4,ағай моһахид бұл пікірлерді қазіргі уақытта орындау мүмкін емес екіншіден молдир назир кашмирде жиһад болмайды деп мәлімдемеген кашмирде соғысып жатқан адамдардың мақсаты алдымен оны үнділіктен босату,аға моһахид бұл пікір қазіргі уақыт орындау мүмкін емес екінші молди назир кашмир жиһад болма де мәлімдеме кашмир соғыс жат адам мақсаты алд оны үнділік босат,violent

4,баракаллаху фекум ақш пен обма тек өздерін танымал етпейді ақштың көптеген құрбандықтары жаппай басталмайынша күте тұрыңыз,баракаллах феку ақш пен об тек өз танымал етпе ақш көптеген құрбандық жаппай басталмайынш күт тұр,violent

4,куәлік дүйсенбіде джовхардан солтүстікке қарай шақырым миль солтүстігінде орналасқан махадей қаласын басып алды деп хабарлады куәгерлер дүйсенбіде махадей және джаухар қалаларын жаулап алу шейх шариф шейх ахмедтің жаңа құрылған үкіметіне үлкен соққы болады орта шабель аймағының төрт ауданының үш ауданы қазір әлшабабтың қолына түсті,куәлік дүйсенбі джовх солтүстік қарай шақырым миль солтүстіг орналас махаде қала бас алды де хабарла куәгер дүйсенбі махаде және джаух қала жаула алу шейх шариф шейх ахмед жаңа құрыл үкімет үлкен соққы бол орта шабель аймағ төрт аудан үш аудан қазір әлшабаб қол түсті,violent

4,ғалым ақштың бұрынғы президенті джордж буш иракқа басып кіргені және жүздеген мың ирактықтардың өліміне себеп болғандығы үшін жауапқа тартылуы керек деді ол сонымен бірге сот израильдің бұрынғы премьерминистрі эхуд олмертті газадағы соғыс қылмысы және жағалаудағы қирату үшін сотқа тарту керек деп қосты бұл жігіт ақымақ деп кім ұрады бізге қарапайым сөздер емес іскер адамдар керек,ғалым ақш бұрынғы президент джордж буш ирак бас кір және жүздеген мың ирактық өлім себеп болғандығ үшін жауап тартыл керек де ол сонымен бірге сот израиль бұрынғы премьерминист эхуд олмер газа соғыс қылмыс және жағалау қират үшін сот тарту керек де қос бұл жігіт ақы де кім ұр бізге қарапайым сөз емес іскер адам керек,violent

4,дәйексөз жексенбіде көтерілісшілер қабылдаған махадай ауылы шариф дүниеге келді ал джовхар қаласының айналасындағы барлық жерде оның рулар базасы күшті болады мужахидтер шарифтің рулық ауылын басып алды,дәйексөз жексенбі көтерілісші қабылда махада ауыл шариф дүние кел ал джовх қала айнала барлық же оның ру база күшті бол мужахид шариф рулық ауыл бас алды,violent

4,жарақаттанушылар көбейе берсін,жарақаттанушы көбей бер,violent

4,сен жиһад жоқ дейсің демек газва хин туралы не айтасың amp бауырластарға amp апалысіңлілер кашмирде тұрады олар дан қа дейін соғысады фоместинде джихад жоқ дейді ағай алдымен жерлеріңді каффармен босат сосын мунафикинмен соғыс сенің сөзің шынымен жүрек пәкістан мужахиддині amp кашмир ауғанстанда нето күштерімен соғысып жатқан amp кашмирде үнді күштерімен соғысуда егер олар кашмирге кетсе индия пәкістанға шабуыл жасайды amp онда сіздер бұл соғыста қайтыс болған халықтар пакистанда өмір сүресіз деп айта аласыз ағай алдымен қандай да бір мәлімдеме беруіңізді өтінемін,сен жиһад жоқ де демек газв хин туралы не ай amp бауырлас amp апалысіңлі кашмир тұр олар дан қа дейін соғыс фоместин джихад жоқ де аға алд жер каффар босат сосын мунафикин соғыс сенің сөз шын жүрек пәкістан мужахидди amp кашмир ауғанстан нето күш соғыс жат amp кашмир үн күш соғыс егер олар кашмир кет индия пәкістан шабуыл жаса amp онда сіздер бұл

соғыс қайтыс бол халық пакистан өмір сүр де ай ал аға алд қандай да бір мәлімдеме беру өт,violent

4,олар лайықты нәрсені алуда ойнауға болмайды сол жерде бомбаны санауға дайындалып жатыр,олар лайықты нәрсе ал ойна болма сол же бомба сана дайындал жатыр,violent

4,әлрахим пакистании талибан байланысты франс пресске пакистандағы талибандардың пәкістандық армияға ешқашан берілмейтінін және талибандардың жауынгерлері жеңісті немесе шейіт болуды таңдағанға дейін ешкімге берілмей күресетіндігін айтты,әлрахи пакистании талибан байланысты франс пресс пакиста талибан пәкістандық армия ешқашан берілме және талибан жауынгер жең немесе шейіт болу таңда дейін ешкім берілме күресетіндіг ай,violent

4,бір әскери қызметкер және бір американдық әскери қызметкер каффардың өлімін растады баграм ауғанстандағы ақш бастаған әскерлер үшін негізгі база соңғы айларда ауғанстандағы зорлықзомбылық ең жоғары деңгейге жетті өйткені ақштың жеті жыл бұрын басып алған шабуылынан кейін талибан биліктен кеткен ауғанстан полициясының дереккөзі соңғы жарылысты жанкештінің өзі жасаған деп мәлімдеді бірақ кейінірек сол дереккөз бұл жолдың жанындағы бомба болған деп мәлімдеді ақш әскери күштерінің өкілі шабуылды растады және бір американдық солдат пен әскери жұмыс істейтін ақш азаматы қаза тапты дейді шетелдік әскерлердің қарқынды өсуіне қарамастан талибан көтерілісі оңтүстігінде және шығысында исламшыл топтың дәстүрлі қолдау базасынан ауылдардың басқа аймақтарына таралуда талибан шетелдік күштерді қуып шығару науқанының маңызды бөлігі ретінде өзінөзі өлтіру шабуылдары мен жол бойындағы бомбаларға сүйенеді reuters,бір әскери қызметкер және бір американдық әскери қызметкер кафф өл раста багра ауғанстан ақш баста әскер үшін негізгі база соңғы ай ауғанстан зорлықзомбылық ең жоғары деңгей же өйткені ақш жеті жыл бұрын бас ал шабуыл кейін талибан билік кет ауғанстан полиция дереккөз соңғы жарыл жанкешті өзі жаса де мәлімде бірақ кейінірек сол дереккөз бұл жол жанындағы бомба бол де мәлімде ақш әскери күш өкіл шабуыл раста және бір американдық солдат пен әскери жұмыс істе ақш азамат қаза тап де шетелдік әскер қарқынды өс қарамастан талибан көтеріліс оңтүстіг және шығыс исламшыл топ дәстүрлі қолдау база ауыл басқа аймақ тарал талибан шетелдік күш қу шығару науқан маңызды бөліг рет өзінөз өлтір шабуыл мен жол бой бомба сүйен reuters,violent

4,сейсенбі күні иранның кордестан провинциясына жасаған сапарында ислам революциясының жетекшісі аятолла сейіт али хаменеи ақштың иранның батыс шекараларында террористерді қолдайтынын айтты америка құрама штаттары лаңкестерді иранның батыс шекараларынан тыс жерлерде алға жылжытуда олар ақш үкіметі ислам республикасын құлату үшін операцияларды тікелей қаржыландырады және террористік қозғалыстарды ұйымдастырады деді аятолла хаменеи сейсенбі күні пентагонның баспасөз хатшысы джефф моррелл айыпты жоққа шығарып біз мұндай әрекетке барғанымызды білмеймін деді моррелл иранды террористерді ақштың ирак пен ауғанстандағы үкіметтерді тұрақтандыру жөніндегі әрекеттерін жоққа шығару

үшін қолдады деп айыптады алайда пентагонның баспасөз хатшысының пікірлері ирак пен ауғанстан президенттерінің ирак пен ауғанстандағы қалпына келтіру процесіне көмектесу жөніндегі әрекеттерін бірнеше рет мадақтаған пікірлеріне қайшы келеді mgh hgl,сейсенбі күн иран кордес провинция жаса сап ислам революция жетекші аятолл сейіт али хаменеи ақш иран батыс шекара террорис қолда ай америка құрама штат лаңкес иран батыс шекара тыс жер алға жылжыт олар ақш үкімет ислам республика құлат үшін операция тікелей қаржыландыр және террористік қозғалыс ұйымдастыр де аятолл хаменеи сейсенбі күн пентагон баспасөз хатшы джефф моррелл айыпты жоқ шығар біз мұндай әрекет бар білме де моррелл иран террорис ақш ирак пен ауғанстан үкімет тұрақтандыру жөн әрекет жоқ шығару үшін қолда де айыпта алайда пентагон баспасөз хатшы пікір ирак пен ауғанстан президент ирак пен ауғанстан қалп келтіру процес көмектес жөн әрекет бірнеше рет мадақта пікір қайшы кел mgh hgl,violent

4,куфтар мен жолдан таюшылар ауыр артиллерия мен тікұшақтарды қолданады осы уақытта ұрыс даттық аршты төменгі алкун бамут және шалажи ауылдарының жанында да жүріп жатыр мужахид шейіт болды инша аллах ал мужахидтер евкуров пен қадыровтың күштерінен жолдан тайған адамды өлтірді деп санайды жолдан таюшылар мен кафферлер усин артиллериясы миноматтар amp шабуыл тікұшақтары басқа ақпарат жоқ,куф мен жолдан таюшы ауыр артиллерия мен тікұшақ қолдан осы уақыт ұрыс да арш төменгі алкун бамут және шалажи ауыл жан да жүр жатыр мужахид шейіт бол инш аллах ал мужахид евкуров пен қадыров күш жолдан тай адам өл де сана жолдан таюшы мен каффер усин артиллерия миномат amp шабуыл тікұшақ басқа ақпарат жоқ,violent

4,жауынгерлік аймақтағы жағдай туралы нақты мәліметтер әлі белгісіз кавказ орталығы тәженкала және джанниведено ауылдарының айналасындағы моджахедтердің қызметі туралы хабарлады алайда ұрыс аймағында не болып жатқандығы туралы нақты мәліметтер белгісіз сонымен қатар даттых аршты нижний алкун бамут және шалажи ауылдарының маңында жауынгерлік ісшаралар жүргізілуде басқыншылар мен қуыршақтар бандаларын тікұшақ зеңбіректері қолдайды кәпірлер ауыр артиллерия мен минометтерді қолданады кавказ орталығынан жағдай туралы егжейтегжейлі ақпарат алған жоқ кавказ орталығы,жауынгерлік аймақ жағдай туралы нақты мәлімет әлі белгісіз кавказ орталығ тәженкал және джанниведено ауыл айнала моджахед қызмет туралы хабарла алайда ұрыс аймағ не бол жатқандығ туралы нақты мәлімет белгісіз сонымен қатар даттых арш нижни алкун бамут және шалажи ауыл маң жауынгерлік ісшара жүргізіл басқыншы мен қуыршақ банда тікұшақ зеңбірек қолда кәпір ауыр артиллерия мен миномет қолдан кавказ орталығ жағдай туралы егжейтегжейл ақпарат ал жоқ кавказ орталығ,violent

4,одан кейінгі тағы үш сурет дәйексөз натоның халықаралық қауіпсіздік күштері isaf және ауғанстанның қауіпсіздік қызметкерлері жылғы мамырда ауғанстанның кабулбаграм тас жолында кабулбаграм тас жолында бомба жарылған жерді күзетіп жатыр мамырда кабулдың шетіндегі тас жолда американдық персоналды

тасымалдайтын көлікті нысанаға алған жолдың бойында ақштың бір қызметкері мен ақш азаматы қаза тапты талибан соғыс басталған елдегі дәстүрлі соғыс кезеңі көктем мен жазда шабуылдарын арттыруға уәде берді,одан кейінгі тағы үш сурет дәйексөз нато халықаралық қауіпсіздік күш isaf және ауғанстан қауіпсіздік қызметкер жылғы мамыр ауғанстан кабулбагра тас жол кабулбагра тас жол бомба жарыл же күзет жатыр мамыр кабул шет тас жолда америкалық персонал тасымалда көлік нысана ал жол бой ақш бір қызметк мен ақш азамат қаза тап талибан соғыс бастал ел дәстүрлі соғыс кезең көктем мен жаз шабуыл арттыр уәде бер,violent

4,бұл шейіт ағамыздың таңғажайып оқиғасы бұл есептік жазбада айтылған түпнұсқа бейнеңіз бар ма мен усман ағамыздың ақ батасын алып оның операцияға қатысқанын қалаймын,бұл шейіт аға таңғажайып оқиға бұл есептік жазба айтыл түпнұсқа бейне бар ма мен усман аға ақ бата алып оның операция қатыс қала,violent

4,сәлем менің қымбатты бауырларым түркістанның изик партиясы туралы сұрағыңыз үшін иә түркістан ислам партиясы ауғанстандағы талибан қозғалысымен жұмыс істейді бұл вебсайт сіз ахмед мохтар талибан қозғалысының бақ министрі ауғанстандағы талибан қозғалысы әлемдегі барлық исламдық жиһад ұйымдарымен жұмыс істейтінін және ол талибандарға көмектесуді жалғастыратынын айтты нато күштері ауғанстаннан шыққан кезде бүкіл әлемдегі исламдық соғысушылар,сәлем менің қымбатты бауыр түркістан изик партия туралы сұра үшін иә түркістан ислам партия ауғанстан талибан қозғалыс жұмыс істе бұл вебсайт сіз ахмед мох талибан қозғалыс бақ министр ауғанстан талибан қозғалыс әлем барлық исламдық жиһад ұйым жұмыс істе және ол талибан көмектесу жалғастыр ай нато күш ауғанстан шық кезде бүкіл әлем исламдық соғысушы,violent

4,мергендер шабуылының алдыңғы видеосында топ әрқашан бір рет және бір рет атыс шабуылдарын көрсеткен бұл компиляциялық бейнеде мергендер бірнеше шабуылда бірнеше әскери қызметшіні нысанаға алған бейне барлық жерде мұсылмандарды снайпер мылтықтарын алуға және жақын жердегі кәпірлерді өлтіруге шақырумен аяқталды таспаның соңындағы айналдырылған мәтін бір оқтың бағасы бір доллардан аспайды сіз өз еліңізге қымбат тұратын адамды өлтіре аласыз деп жазылған,мерген шабуыл алдыңғы видео топ әрқашан бір рет және бір рет атыс шабуыл көрсет бұл компиляциялық бейне мерген бірнеше шабуылда бірнеше әскери қызметші нысана ал бейне барлық же мұсылман снайп мылтық ал және жақын жер кәпір өлтір шақыр аяқта таспа соңындағы айналдырыл мәтін бір оқ баға бір долл аспа сіз өз ел қымбат тұр адам өлтір ал де жазыл,violent

4,әлем ауғанстанға назар аударады олардың әлемдегі ең қаружарақтары бар және біздің кедей адамдарымыз олар ешқандай қарусыз немесе оқдәрілерсіз соғысуда нато ауғанстанда өте қиын уақытты бастан кешуде біз сондайақ өте қатты күресіп жатырмыз қатал күрес,әлем ауғанстан назар аудар олардың әлем ең қаружарақ бар және біздің кедей адам олар ешқандай қару немесе оқдәріл соғыс нато ауғанстан өте қиын уақыт бас кеш біз сондайақ өте қатты күрес жатыр қатал күрес,violent

4,m ark әскериғаламтор форумына жариялау жаппай қырыпжоюдың негізгі қарулары жақын арада жиһадтық интернетфорумдарда жарияланады деп уәде берді ирактағы алькаида және ирак ислам мемлекеті басшыларына сілтеме жасай отырып жақында әскери дайындық саласындағы үлкен сюрприз соғыс министрінің шақыруына жауап ретінде абу хамза әлмухажир деген атауға ие болды соғыс министрі хабарда al baraa al masri және abu abu safia есімді екі адам жақын арада жарылғыш заттар технологиясы бойынша нұсқаулықтың сүйемелдеуімен жұмыс істейтін болады аль бараа альмасри және абу сафия бірнеше араб интернетфорумдарының белсенді мүшелері олар джихад үміткерлеріне қаружарақ пен жарылғыш заттар туралы ақпарат береді алькаиданың жарылғыш заттарды жасаушы инженері деген атпен әлбараа альмасриді форумның қатысушылары крестті жеңу деп аталатын сағаттық бейнетаспада көрсетілген нұсқаушы болған деп санайды интернетте жылы пайда болған жарылғыш заттарды жасау cbs,m ark әскериғаламто форум жариялау жаппай қырыпжою негізгі қару жақын ара жиһад интернетфорум жариялан де уәде бер ирак алькаи және ирак ислам мемлекет басшы сілтеме жаса отыр жақында әскери дайындық сала үлкен сюрприз соғыс министр шақыр жауап рет абу хамза әлмухажи де ата ие бол соғыс министр хаб al baraa al masri және abu abu safia ес екі адам жақын ара жарылғыш зат технология бойынша нұсқаулық сүйемелде жұмыс істе бол аль бара альмасри және абу сафия бірнеше араб интернетфорум белсенді мүше олар джихад үміткер қаружарақ пен жарылғыш зат туралы ақпарат бер алькаида жарылғыш зат жасаушы инженер де ат әлбара альмасри форум қатысушы крест жеңу де атал саға бейнетаспа көрсет нұсқаушы бол де сана интернет жыл пайда бол жарылғыш зат жасау cbs,violent

4,соған қарамастан әскери жүк көлігі құлаған аймақ өте жақсы буксирлеу алаңы болып табылады өйткені ол қалың жапырақты жерлері бар олар буксирленген жерде кез келген адамды жақсы жауып тастайды және төмендегі тас жолдағы нысандарға оқ жаудырады егер әскери көліктерді немесе мобильді патрульдерді ұрыпсоғу үшін мужахидтер партизаны немесе жалғыз партизан снайперлері осындай аймақты таңдаса бұл таңқаларлық емес,соған қарамастан әскери жүк көліг құла аймақ өте жақсы буксирле алаң бол таб өйткені ол қалың жапырақ жер бар олар буксирлен же кез кел адам жақсы жау таста және төмендегі тас жол нысан оқ жаудыр егер әскери көлік немесе мобильді патруль ұрыпсоғ үшін мужахид партиза немесе жалғыз партизан снайпер осындай аймақ таңда бұл таңқаларлық емес,violent

4,дәйексөз сомали көтерілісшілері сенбі күні алдыңғы шайқасқа қарамастан олар өз позицияларын сақтап қала берді деп мәлімдеді,дәйексөз сомали көтерілісші сенбі күн алдыңғы шайқас қарамастан олар өз позиция сақта қала бер де мәлімде,violent

4,дәйексөз сомалидегі жас британдықтардың мотивациясы негізінен ауғанстанда соғысуға немесе сомалиде жиһадқа қосылуға болатындығы туралы нұсқаулар алуы мүмкін деп есептелсе де уайтхолл шенеуніктері кейбіреулер өздерінің лагерьлерде алған тәжірибелерін пайдалануға шешім қабылдауы мүмкін деп қабылдайды ұлыбританияға оралып шабуылдарды жоспарлауды бастаңыз,дәйексөз сомали жас

британдық мотивация негіз ауғанстан соғыс немесе сомали жиһад қосыл болатындығы туралы нұсқау ал мүмкін де есептел де уайтхолл шенеунік кейбіреу өз лагерь ал тәжірибе пайдалан шешім қабылда мүмкін де қабылда ұлыбритания орал шабуыл жоспарлау баста,violent

4,ия өте ерекше мен әрдайым снайпер тек бір рет оқ атуы керек деп ойладым бірақ ол кафирді атып камерамен қоштасады иши мергенінің ақш снайперін қалай аңдығаны жақсы,ия өте ерекше мен әрдайым снайп тек бір рет оқ ат керек де ойла бірақ ол кафи ат камера қоштас иши мерген ақш снайп қалай аңды жақсы,violent

4,әскери танк мұса қалада жарылыс құрылғысымен жойылды жеңіс кари мухаммед юсеф ахмади мужахидтер ислам әмірлігін бүгін түстен он бір отызда жарып жіберді операцияларда жеңіс крест жорықтарының әскери танкісі хелманд провинциясының мұсакала ауданының кештадзур ауданында жау конвойы осы жерден транзитпен өтіп жатқан кезде ауыр жарылыс жау танкісін толығымен жойып оның ауғанстандық аудармашысымен бірге crusader тобының барлығын өлтірді олар жер емес еркелігі айтты болса олар айтады ауғанстан ислам әмірлігінің ресми өкілі талибан кари мухаммад юсуф ахмади аудандары ел тел оңтүстік батыс және солтүстік біз татуластырушы сиыр олар тонауға болып табылмайтын бірақ сиыр сезінеді емес ақпарат болып табылады мобильді забихулла мужахид елдің оңтүстікшығыс және солтүстікшығыс аймақтары үшін тел ұялы ислам әмірлігінің ақпарат комитеті ауғанстан талибан ақпарат көзі беті джихад дауысы жылғы мамырда ауғанстан ислам әмірлігінің талибан ресми сайты,әскери танк мұса қала жарылыс құрылғы жой жеңіс кари мухаммед юсеф ахмади мужахид ислам әмірліг бүгін түс он бір отыз жар жіб операция жеңіс крест жорық әскери тан хелманд провинция мұсакал аудан кештадзу аудан жау конвой осы же транзит өт жат кезде ауыр жарылыс жау танкі толығ жой оның ауғанстан аудармашы бірге crusader тоб барлығ өл олар жер емес еркеліг ай бол олар айт ауғанстан ислам әмірліг ресми өкіл талибан кари мухаммад юсуф ахмади аудан ел тел оңтүстік батыс және солтүстік біз татуластыруш сиыр олар тона бол табылма бірақ сиыр сезін емес ақпарат бол таб мобильді забихулл мужахид ел оңтүстікшығыс және солтүстікшығыс аймақ үшін тел ұялы ислам әмірліг ақпарат комитет ауғанстан талибан ақпарат көз бет джихад дауыс жылғы мамыр ауғанстан ислам әмірліг талибан ресми сай,violent

4,сват алқабындағы мигора қаласында тұратын адамдар пакистандық талибан мингора қаласындағы аудандарды әлі де басқарып отыр және пакистан әскері жолдан тайғандар мен пакистандық талибандар арасындағы шайқас осы сағатқа дейін жалғасуда мамыр ж,сват алқаб мигор қала тұр адам пакиста талибан мингор қала аудан әлі де басқар отыр және пакис әскер жолдан тай мен пакиста талибан ара шайқас осы сағат дейін жалғас мамыр ж,violent

4,мингора қаласында тұратын адамдар пакистандық талибандар әлі де жерлерді басқарып жатыр және мингора қаласының көп бөлігі жылдың мамырында осы уақытқа дейін мингора қаласында пакистандық талибан мен пакистан әскері арасында жолдан таюшылар арасында ұрыс жүріп жатқанын айтады,мингор қала тұр

адам пакиста талибан әлі де жер басқар жатыр және мингор қала көп бөліг жыл мамыр осы уақыт дейін мингор қала пакиста талибан мен пакис әскер ара жолдан таюшы ара ұрыс жүр жат айт,violent

4,або риша рамади штабының ояну кеңесінде ipske мен шейхтердің ұзын тобынанмын бірақ альхайис тек оятуды жылдан бастап бастады деді або риша егер сайлауда альхайс жеңіске жетсе революция болады деп қосты ол ipske айтты егер хип сайлауды алаяқтықпен өткізсе бұл апат болады бұл дарфур сияқты болады деді ол,або риш рамади штаб оян кеңес ips мен шейх ұзын тоб бірақ альхайис тек ояту жыл баста баста де або риш егер сайла альхайс жеңіс жет революция бол де қос ол ips ай егер хи сайлау алаяқтық өткіз бұл апат бол бұл дарфу сияқты бол де ол,violent

4,цитата сомали ахлу сунна оңтүстік могадишу мареег қаласындағы жерлеу рәсімін айыптайды ахлу сунна вальжама аның ресми өкілдері альшабабтың қабірлерді құрбан етуін гедо аймағының бардере ауданында жалғасуда деп мәлімдеді бардхердегі әлшабаб әкімшілері бардхер қаласындағы қабірлерді қиратуды бастады және әйгілі діни дінбасылар жерлеген үш қабірді қиратты долоу қаласындағы ахлу сунна вальжама аның өкілі шейх исмаил адан кофи қабірлерді қиратуды ерекше мақсат деп атады бардердегі әшшааб басқарушыларының бірі шейх абдулқадир юсуф калби қабірлерді қирату діни сипатқа ие деп сендірді бардере әкімшілігі мешіттердегі және басқа қаладағы қабірлерді жоюға бел буғандықтарын айтты ахлу сунна вальжаманың жақтастары әлшабабтың құлдырауына ашуланған сомалияның оңтүстігінде және орталығында әлшабабқа қарсы күресу үшін қару алды фарах ахмед мохамед көзі,цита сомали ахл сунн оңтүстік могадиш мареег қала жерлеу рәсім айыпта ахл сунн вальжа ан ресми өкіл альшабаб қабір құрбан ет гедо аймағ бардер аудан жалғас де мәлімде бардхер әлшабаб әкімші бардх қала қабір қирату баста және әйгілі діни дінбасы жерле үш қаб қира доло қала ахл сунн вальжа ан өкіл шейх исмаил адан кофи қабір қирату ерекше мақсат де ат бар әшшааб басқарушы бірі шейх абдулқади юсуф калби қабір қират діни сипат ие де сен бардер әкімшіліг мешіт және басқа қала қабір жою бел буғандық ай ахл сунн вальжама жақтас әлшабаб құлдыра ашулан сомалия оңтүстіг және орталығ әлшабаб қарсы күресу үшін қару алды фарах ахмед мохамед көз,violent

4,иракта бейбітшілікке қол жеткізудің жалғыз тәсілі таухид елсаф шайқас өйткені сен өзің үшін жауап бересің және сенушілерді өлім алдындағы қорқынышты жеңуге шабыттандыр салем acer болды,ирак бейбітшілік қол жеткізу жалғыз тәсіл таухид елсаф шайқас өйткені сен өзің үшін жауап бер және сенуші өлім алд қорқыныш жең шабыттанды сале acer бол,violent

4,дәйексөз алғашқыда масла қабырғадағы москит өте жақсы видео осы лас пунджаби хинду құлдарының өлгенін көру жақсы құрметті бауырым біз ұлтшылдықтың микробтарын өлтіруіміз керек сондықтан біз ешқандай тайпаны және қандай да бір ұлтты көрсетпеуіміз керек біздің можахед бауырларымызды сатқындық жасаушылар бар сондықтан бұл барлық пуштондар кафирлер дегенді білдірмейді менің ойымша сен менің ойымды түсінесің деп үміттенемін coz менің ағылшын тілім жақсы

емес,дәйексөз алғашқы масл қабырға москит өте жақсы видео осы лас пунджаби хинд құл өл көру жақсы құрметті бауыр біз ұлтшылдық микроб өлтір керек сондықтан біз ешқандай тайпа және қандай да бір ұл көрсетпе керек біздің можахед бауыр сатқ жасаушы бар сондықтан бұл барлық пуштон кафир де білдірме менің ойымша сен менің ой түс де үміттен coz менің ағылшын тіл жақсы емес,violent

4,мен isi пакистан муртадиялары талибандар исламабадқа шәһид бомбасын жіберуді жоспарлағаны туралы мақаланы есіме түсіремін салем acer,мен isi пакис муртадия талибан исламабад шәһид бомба жіберу жоспарла туралы мақала ес түсір сале acer,violent

4,дәйексөз бастапқыда thunderman авторы керемет shshabab сомалияның оңтүстігі қазір ислам мемлекеті болып көрінеді ол могадишуға орталық сомалиге және шығыс африканың қалған бөліктеріне таралсын мен эфиопия крестшілерінің шабуылына бірнеше апта қалғанда мен исламдық соттар одағының олар басқаратын барлық жерде шариятты қолданып жатқанын көрдім және олар бұл тайпаның немесе рудың қандай болғанына қарамастан әділетті түрде істейтін егер сіз кәдімгі адамдар туралы білетін болсаңыз олардың ру мен руға негізделген үкім шығармауы өте ерекше нәрсе өйткені жыл бұрын джуааль мен кафир somaalis бірбірін клан мен руға негізделген бірбірін өлтіру үшін қолданған мен бұл тазарудың жүзеге асқанына өте қуаныштымын өйткені ол кімге кім екенін нақты анықтады,дәйексөз бастапқы thunderman авто керемет shshabab сомалия оңтүстіг қазір ислам мемлекет бол көрін ол могадиш орталық сомали және шығыс африка қалған бөлік тарал мен эфиопия крестші шабуыл бірнеше апта қал мен исламдық сот одағ олар басқар барлық же шария қолдан жат көр және олар бұл тайпа немесе ру қандай бол қарамастан әділет түр істе егер сіз кәдімгі адам туралы біл бол олардың ру мен ру негіздел үкім шығарма өте ерекше нәрсе өйткені жыл бұрын джуааль мен кафи somaalis бірбір клан мен ру негіздел бірбір өлтір үшін қолдан мен бұл тазар жүзеге ас өте қуанышты өйткені ол кім кім екен нақты анықта,violent

4,мен хихсанды білемін эфиопиялықтар мүжахедтерді жоюға шынымен тырысып бақты мен олар бригаданы олар бекініс жасаған жер және құдай тағдыры тағайындаған жер деп атады деп орынды деп таптым олар кейінірек күресіп әрі қарай жалғастыра беруі керек сол жолда,мен хихса біл эфиопиялық мүжахед жою шын тырыс бақ мен олар бригада олар бекініс жаса жер және құдай тағдыр тағайында жер де ат де ор де тап олар кейінірек күрес әрі қарай жалғастыр беруі керек сол жолда,violent

4,олар шабуылдайды деп үміттенемін,олар шабуылда де үміттен,violent

4,рохинджа мұсылмандары бангладешке ұзақ уақыттан бері келе жатыр өйткені олар бирманың супер зайырлы үкіметінің кесірінен аштан өледі және бангладештегі босқындар лагерінде жүргендер тіпті жергілікті полиция қызметкерлерінің тарапынан үлкен қиындыққа тап болады,рохиндж мұсылман бангладеш ұзақ уақыт бері келе

жатыр өйткені олар бирма суп зайырлы үкім кесір аш өл және бангладеш босқын лагер жүр тіпті жергілікті полиция қызметкер тарап үлкен қиындық тап бол,violent

4,мен сомалдағы кейбір бауырластарымызға мұны көріп біз әрқашан сізге көмектесуге дайынбыз біз бұған жол табамыз,мен сомал кейбір бауырлас мұны көр біз әрқашан сізге көмектес дайын біз бұған жол таб,violent

4,демек олар шрианы исламдық заң енгізуге қарсы болып өздерінің дүниелік істер үшін жиһад жасап жатқандығымен мақтанады яғни олар адам жасаған заңдармен басқаруды және билікте қалуды қалайды сонымен қатар олар isi сияқты шариғатты жүзеге асыруға ұмтылатындарға қарсы,демек олар шриа исламдық заң енгіз қарсы бол өз дүниелік істер үшін жиһад жаса жатқандығ мақтан яғни олар адам жаса заң басқару және билік қалу қала сонымен қатар олар isi сияқты шариғат жүзеге асыр ұмтыл қарсы,violent

4,жақында иттер шығарылады бауырлар күшейе түседі,жақында ит шығар бауыр күшей түс,violent

4,ассаламуалейкум кашмирдегі джихадтың мақсаты неде екенін айтыңызшы тек басқыншыларды қуып шығару немесе сонымен бірге кашмирді исламдық ереже бойынша жасау кашмирдегі джихад кашмирді пакистанның бір бөлігі ету үшін жасалды ма стратегия басқыншыларды ауғанстаннан шығаруға көбірек шоғырландыру екенін білемін бірақ пакистан әскері можахедтерге бұл әрекетті жасауға мүмкіндік бермейді можахедтер бұл жағдайда не істеуі керек олар өздерін қорғау үшін күресу керек пе пакистан армиясымен күрес олар пакистаннан шығарылады әскерімен ухад соғысы кезінде мунафикандар билікте болған жоқ ал пакистанда ең үлкен мунафик ең үлкен лауазымды иеленген уахадтағы мунафихиндер мұсылмандармен шайқаспаған ал пакистандағы мунафикандар үнемі мужахидтермен соғысып жатқан,ассаламуалейку кашмир джихад мақсаты не екен айтыңызш тек басқыншы қу шығару немесе сонымен бірге кашмир исламдық ереже бойынша жасау кашмир джихад кашмир пакистан бір бөліг ету үшін жаса ма стратегия басқыншы ауғанстан шығар көбірек шоғырландыру екен біл бірақ пакис әскер можахед бұл әрекет жаса мүмкіндік берме можахед бұл жағдай не істе керек олар өз қорғау үшін күресу керек пе пакис армия күрес олар пакистан шығар әскер ухад соғыс кез мунафикан билік бол жоқ ал пакистан ең үлкен мунафик ең үлкен лауазым иелен уахад мунафихин мұсылман шайқаспа ал пакиста мунафикан үнемі мужахид соғыс жат,violent

4,содырлар ауғанстанды джихад арқылы босату мақсатына жетуге бел буғанын айтты тек джихад әлемге тыныштық әкеледі деді ол соғушы көсем бірнеше рет шекарадан өтіп шетелдік әскерлермен соғысуға рұқсат берген көзі bbc,содыр ауғанстан джихад арқылы босат мақсат жет бел бу ай тек джихад әлем тыныштық әкел де ол соғуш кө бірнеше рет шекара өт шетелдік әскер соғыс рұқсат бер көз bbc,violent

4,иә майлы ластанған крест жорықтары шошқалары мен маймылдардың қарапайым азаматтары егер олар крестшілерге террористердің әскеріне көмектесіп ашық немесе

жасырын түрде қолдау көрсетсе олардың қаржылық материалдық моральдық ауызша немесе физикалық сонымен қатар ерлер де әйелдер де қолдау көрсететін кезкелген жерде өлтірілуі керек қарт немесе жас,иә майлы ластан крест жорық шошқа мен маймыл қарапайым азамат егер олар крестші террорис әскер көмектес ашық немесе жасырын түр қолдау көрсет олардың қаржылық материалдық моральдық ауызша немесе физикалық сонымен қатар ер де әйел де қолдау көрсет кезкел же өлтіріл керек қарт немесе жас,violent

4,оларды бастарынан кесіп тастау керек,ол бас кес тастау керек,violent

4,егер американдықтар мен халықаралық қауымдастық үшін сабақ болса біз ауғанстаннан бет бұруға батылымыз жетпейді егер жұмысымызды жалғастыра берсек бұл нәтиже береді,егер американдық мен халықаралық қауымдастық үшін сабақ бол біз ауғанстан бет бұр батыл жетпе егер жұмыс жалғастыр бер бұл нәтиже бер,violent

4,мужахедтер могадишуда алға ұмтылуда және олар басшылардың біреуін өлтірді ұрыс қазір тоқтатылды мужахединді қолдайтын мокидишидегі мұсылмандарға дуа жасады бұл жерде xizbul ислам мен шабаб әлмужахединдегі бауырларымыздың суреттері бар муртадинге және каффаларға қарсы жағында,мужахед могадиш алға ұмтыл және олар басшы біреу өл ұрыс қазір тоқтат мужахеди қолда мокидиши мұсылман ду жас бұл же xizbul ислам мен шабаб әлмужахеди бауыр сурет бар муртадин және каффа қарсы жағ,violent

4,альшабаба солтүстік мугадишодағы жаңа шайқастардың жеңісін талап етеді http www ansar net showthread php t,альшабаб солтүстік мугадишо жаңа шайқас жеңіс талап ет http www ansar net showthread php t,violent

4,дәйексөз алғашында juhayman ағам сізді дүниежүзілік сауда орталығының миссиясын қолдайды қыркүйектің қыркүйегі және ағасы осы миссияға қатысқан басқа ағаларды қолдайды ия ағайын мен бұл шабуылды қолдаймын өйткені бұл шабуылға дейін американдықтардың мұсылман бейбіт тұрғындарға жасаған әрекеті үшін мысалы израильде мысалы еврейлер американдық қарулармен мұсылмандарды өлтірген израильде және иракта санкциялардан миллиондаған адам қайтыс болған кезде істеген әрекеттері үшін жасалды егер бұл америка үшін болмас еді бұл біздің шейх усама бин ладиннің а айтқан сөзі бұл кек қайтару егер олар шабуыл жасауды және мұсылман тұрғындарды өлтіруді тоқтатса онда ол да тоқтайды,дәйексөз алғаш juhayman аға сіз дүниежүзілік сауда орталығ миссия қолда қыркүйек қыркүйег және аға осы миссия қатыс басқа аға қолда ия ағайын мен бұл шабуыл қолда өйткені бұл шабуыл дейін американдық мұсылман бейбіт тұрғын жаса әрекет үшін мысал израиль мысал еврей американдық қару мұсылман өлтір израиль және ирак санкция миллиондаған адам қайтыс бол кезде істе әрекет үшін жаса егер бұл америка үшін болмас ед бұл біздің шейх уса бин ладин а айт сөз бұл кек қайтару егер олар шабуыл жасау және мұсылман тұрғын өлтіру тоқтат онда ол да тоқта,violent

4,алькаидамен тығыз байланысы бар және алжирдің радикалды тобынан шыққан ислам магрибінде aqim ол сахараның оңтүстік шетіндегі халықтарға өз аймағын

кеңейтуге тырысып аймақта бірнеше шабуыл жасады деп мәлімдеді екі апта бұрын малидің ішкі істер министрлігіндегі ақпарат көздері олар террористік топтарға қарсы аяусыз соғыс бастайды деп мәлімдеді,алькаида тығыз байланыс бар және алжир радикал тоб шық ислам магриб aqim ол сахара оңтүстік шет халық өз аймағ кеңейт тырыс аймақта бірнеше шабуыл жас де мәлімде екі апта бұрын мали ішкі істер министрліг ақпарат көз олар террористік топ қарсы аяу соғыс баста де мәлімде,violent

4,бұл уақыт өткен сайын алькаидаға йеменнің оңтүстігінде исламдық мемлекет құру мақсатын орындауға көмектеседі,бұл уақыт өткен сайын алькаида йемен оңтүстіг исламдық мемлекет құру мақсат орында көмектес,violent

4,жіңішке кофирлерге екінші рет соққыларын алуға рұқсат етіңіз,жіңішке кофир екінші рет соққы ал рұқсат ет,violent

4,цитата бастапқыда жарияланған theblfflag жойып жіберуі мүмкін бұл шошқалар өлі үйге және кесілген бастарына иттерге тапсырылуы керек кедей иттер мұндай лас жексұрын тамаққа лайық болу үшін не істеді иттер оларды лас крест жорықтарымен жейді деп ойламаймын,цита бастапқы жариялан theblfflag жой жібер мүмкін бұл шошқа өлі үй және кес бас ит тапсырыл керек кедей ит мұндай лас жексұр тамақ лайық болу үшін не істе ит ол лас крест жорық же де ойлама,violent

4,аль шабаба найроби шейхі хассан яккуб әли әл шабаба әкімшілігіне жексенбі күні кенияның астанасы найробидегі биік ғимараттарға шабуыл жасаймын деп қорқытқан шейх хасан яккуб али кисмаюдағы әлшааб әкімшілерінің өкілі кениялық әскерлердің кезкелген араласуына қарсы болатындарын айтты және найробидегі биік ғимараттарға шабуыл жасайтындарын айтты егер сіз бізге кения шабуыл жасасаңыз біз найробиде өзінөзі өлтіру әрекетін жасаймыз және найробидегі биік әйнек ғимараттарды қиратамыз деді шейх хасан яккуб али сенбіде бірнеше шекаралас қалаларға кениялық әскерлер орналастырылды сомали парламентінің спикері аден мохаммед нор көрші елдерден сенбіде сомалиге әскер жіберуді сұрады және министрлер кабинеті бұл шешімді мақұлдады шейк яккуб сонымен бірге көмек агенттіктерін сомалидегі соғыстарды ескермеді деп айыптап олардың тыңшылар болғанын айтты альшабаба партиясының өкілі шейх али мохамуд рагех сомалиге келетін кезкелген шетелдік әскерге қарсы күресетіндерін мәлімдеді көз,аль шабаб найроби шейх хассан яккуб әли әл шабаб әкімшіліг жексенбі күн кения астана найроби биік ғимарат шабуыл жаса де қорқыт шейх хасан яккуб али кисмаю әлшааб әкімші өкіл кениялық әскер кезкел аралас қарсы бол ай және найроби биік ғимарат шабуыл жаса ай егер сіз бізге кения шабуыл жаса біз найроби өзінөз өлтір әрекет жаса және найроби биік әйнек ғимарат қира де шейх хасан яккуб али сенбі бірнеше шекаралас қала кениялық әскер орналастыр сомали парламент спикер аден мохаммед но көрші ел сенбі сомали әскер жіберу сұр және министр кабинет бұл шеш мақұлда шейк яккуб сонымен бірге көмек агенттік сомали соғыс ескерме де айыпта олардың тыңшы бол ай альшабаб партия өкіл шейх али мохамуд рагех сомали кел кезкел шетелдік әскер қарсы күрес мәлімде көз,violent

4,менің ойымша бұл операциялар сунниттерге қарсы шиит қылмыстары үшін жазалау ретінде жүзеге асырылады бұған дейін иракта джаиш әлтаифа әлмансура ол ирак ислам мемлекетін құрған топтардың бірі сунниттік халықтарға шабуыл жасаған шииттердің кек алу үшін шиит қажыларға минометтік шабуыл жасағанын және пәкістанда техрикеталибан мужахидтері шииттерді крамда және басқа жерлерде шии қылмыстары үшін өшіру үшін өлтіреді мен ирактағы немесе басқа жерде шиит топтарын кінәсіз деп атаудан тартыншақ едім өйткені мен орташа американдық азаматты кінәсіз деп атайтын едім ирактағы ислам мемлекеті елдегі барлық шииттерді өлтіруге тырыспайды бірақ егер шиіттер рафидалар тарапынан сүнниттерді өлтіріп масқараласа шииттер жазасын күтеді язиди каффаттары бір мұсылман қызды өлтірді ал мужахидтер оларға бірнеше эпикалық соққылар жасады ал рафида мушрикилерінің қолында язидиге қарағанда әлдеқайда көп қан болған,менің ойымша бұл операция суннит қарсы шиит қылмыс үшін жазалау рет жүзеге асыр бұған дейін ирак джаиш әлтаиф әлмансур ол ирак ислам мемлекет құр топ бірі сунни халық шабуыл жаса шиит кек алу үшін шиит қажы миномет шабуыл жаса және пәкістан техрикеталибан мужахид шиит кра және басқа жер шии қылмыс үшін өшір үшін өлтір мен ирак немесе басқа же шиит топ кінәсіз де атау тартыншақ ед өйткені мен орташа американдық азамат кінәсіз де ата ед ирак ислам мемлекет ел барлық шиит өлтір тырыспа бірақ егер шиіт рафида тарап сүннит өлтір масқарала шиит жаза күт язиди каффат бір мұсылман қыз өл ал мужахид оларға бірнеше эпикалық соққы жас ал рафи мушрики қол язиди қара әлдеқайда көп қан бол,violent

4,аға бауырыма рахмет куфилердің әрқашан түсінбейтіні олар моджахедтерді оқшаулауға тырысқан сайын оларға тірек негізі соғұрлым күшті болады пакистанистік әскери күштер мұнда немесе сол жерде кейбір мужахидтерді өлтіруі мүмкін және олар кейбір топтарды ауғанстанға итермелеуі мүмкін бірақ осылайша олар өз елдерін одан әрі тұрақсыздандырады олар көбірек мужахидтер мен ансарлардың пайда болуына басқа қарсыластардың үндістан мұқият бақылап отыру кезінде өз күштерін таратуға және армиядан қалған заттарды жалдамалыларды одан әрі құлдыратуға көмектеседі бәрі айтылып орындалған кезде жеңіс туралы кезкелген хабар тез арада рулық аймақтағы авантюралары қайтарылған кезде тез өзгереді сват алқабын моджахедтер бірнеше жыл бұрын алғашқы паки операциясынан кейін жаулап алғаны сияқты ақш пен нато қазір талибандар жылдары толығымен жеңілді деп мәлімдесе де ауғанстанның көптеген бөлігін бақылауға алғанын мойындаған сияқты,аға бауыр рахмет куфи әрқашан түсінбе олар моджахед оқшаула тырыс сайын оларға тірек негіз соғұрлым күшті бол пакистани әскери күш мұнда немесе сол же кейбір мужахид өлтір мүмкін және олар кейбір топ ауғанстан итермеле мүмкін бірақ осылайша олар өз ел одан әрі тұрақсыздандыр олар көбірек мужахид мен ансар пайда бол басқа қарсылас үндістан мұқият бақыла отыру кез өз күш тарат және армия қалған зат жалдамалы одан әрі құлдырат көмектес бәрі айтыл орындал кезде жеңіс туралы кезкел хабар тез ара рулық аймақ авантюра қайтарыл кезде тез өзгер сват алқа моджахед бірнеше жыл бұрын алғашқы паки операция кейін жаула ал сияқты ақш пен нато қазір талибан

жыл толығ жең де мәлімде де ауғанстан көптеген бөліг бақыла ал мойында сияқты,violent

4,мен оның айбындылығына жауап беретін осы хайуан бейнесін көргенді қалаймын amp опасыздық дереккөз daily times маусым исламбад ішкі істер министрі рехман малик жексенбіде талибандарға елден кетуді ескертті өйткені үкімет пәкістан топырағынан жойылғанға дейін тыныштық бермейді журналистермен сөйлескен малик талибан мұсылмандар да пәкістандықтар да емес деді ол бұған дейін талибанға жоқ деп айтқан болатын енді олар пәкістаннан кетуі керек деді ол ішкі істер министрі бүкіл халық елді терроризмнен қорғауға дайын екенін және талибан күндері санақталғанын айтты ол террористер пәкістанның ыдырауын қалағанын және үкімет бұған ешқашан жол бермейді деп сендірді ішкі істер министрі бұұның тергеу тобы келесі айда бұрынғы премьерминистр беназир бхуттоның өлімін тексеру үшін пәкістанға баратынын айтты команда алты ай ішінде өз есебін ұсынады деді ол,мен оның айбындылығ жауап бер осы хайуан бейне көр қала amp опасыз дереккөз daily times маусым исламбад ішкі істер министр рехман малик жексенбі талибан ел кету ескер өйткені үкімет пәкістан топырағ жойыл дейін тыныштық берме журналис сөйлес малик талибан мұсылман да пәкістандық да емес де ол бұған дейін талибан жоқ де айт бол енді олар пәкістан кет керек де ол ішкі істер министр бүкіл халық ел терроризм қорға дайын екен және талибан күн санақтал ай ол террорис пәкістан ыдыра қала және үкімет бұған ешқашан жол берме де сен ішкі істер министр бұұ тергеу тоб келесі айда бұрынғы премьерминист бенази бхутто өл тексеру үшін пәкістан бар ай команда алты ай іш өз есе ұсын де ол,violent

4,өткен аптада болғаннан кейін ансар джихадтың қайтып оралғанын көргенде қатты қуандым,өткен апта бол кейін анс джихад қайт орал көргенде қатты қуан,violent

4,пәкістан сват операциясы дүйсенбі маусым gmt пәкістан бақтарға жақын арада сват алқабына кіруге рұқсат беріледі дейді пәкістан армияның ауғанстан шекарасы бойындағы содырларға шабуыл жасауға дайындалып жатқан кезінде сват алқабындағы тәліптерге қарсы шабуылдың соңғы кезеңі аяқталғанын айтады қауіпсіздік күштері сваттағы лаңкестердің жасырын жерлерін және лагерлерін жоюдың соңғы сатысында тұр деді исламабадта журналистерге жоғарғы әскери өкілі генералмайор атар аббас аббас аптаға созылған шабуылда сват аңғары террорлық элементтерден тазартылды сонымен қатар іздеу операциялары кезінде ауыр қаружарақ оқдәрілер қоймалары мен миналар алынды деп мәлімдеді ол әскер ауғанстан шекарасы бойында екінші майдан ашып жатыр ол жерде алькаида көтерілісшілері де жасырынып жүр деген болжам бар,пәкістан сват операция дүйсенбі маусым gmt пәкістан бақ жақын ара сват алқаб кір рұқсат бер де пәкістан армия ауғанстан шекара бой содыр шабуыл жаса дайындал жат кез сват алқаб тәліп қарсы шабуыл соңғы кезең аяқтал айт қауіпсіздік күш сват лаңкес жасырын жер және лагер жою соңғы саты тұр де исламабад журналист жоғарғы әскери өкіл генералмайо ат аббас аббас апта созыл шабуылда сват аңғ террорлық элемент тазарт сонымен қатар іздеу операция кез ауыр қаружарақ оқдәрі қойма мен мина ал де мәлімде ол

әскер ауғанстан шекара бой екінші майдан аш жатыр ол же алькаи көтерілісші де жасырын жүр де болжам бар,violent

4,олар өздерінің зайырлы және демократиялық жүйелерін ұстанбайды олар бәріне еркіндік береді олар басқа елдерге өздері ұнатпайтын нәрсені тарату үшін шабуыл жасайды адам гадахн сахаб бейнелерінде шындықты айтты онда олар өздері ұстанбайтын халықаралық заңдар үшін күресетіндерін айтты,олар өз зайырлы және демократиялық жүйе ұстанба олар бәрін еркіндік бер олар басқа ел өз ұнатпа нәрсе тарат үшін шабуыл жаса адам гадахн сахаб бейне шындық ай онда олар өз ұстанба халықаралық заң үшін күрес ай,violent

4,анвар аль авлаки джихадты қолдаудың тәсілі,анвар аль авлаки джихад қолда тәсіл,violent

4,пакистандағы жолдан таюшылардың әскері мен үкіметі оның соңына жетті енді күте тұрыңыз келесі кім болатын ол пакистан әскері мен үкіметке деген симптомы бар бірақ мұсылман армиясында шапқыншылықта өлтірген мұсылмандар үшін емес lal masjid вазирстан және бүкіл пакистан бұл әскер біздің қарындастарымызды лала масджидте өлтіріп жатқан кезде ол қай жерде болды сіз екі даңқты things шәһид немесе жеңіске бірінің басқа бізге кез келген тағдыры үшін күтуге бола ма сондықтан күту болашақ не сіз үшін күтуге болады біз де сендермен бірге күтемін,пакиста жолдан таюшы әскер мен үкімет оның соң же енді күт тұр келесі кім бол ол пакис әскер мен үкімет де симптом бар бірақ мұсылман армия шапқыншылық өлтір мұсылман үшін емес lal masjid вазирс және бүкіл пакис бұл әскер біздің қарындас лал масджид өлтір жат кезде ол қай же бол сіз екі даңқ things шәһид немесе жеңіс бір басқа бізге кез кел тағдыр үшін күт бола ма сондықтан күту болашақ не сіз үшін күт бол біз де сен бірге күт,violent

4,имам тв куффар лаңкестеріне шабуыл жасамас бұрын мұжахидтер көктем нохчичо вилаясы сілтемелер http blip tv file get imamtv www vzasade wmv экрандар иншааллаһ ағайындылардың бірі бола алады логотипті қосыңыз,имам тв куфф лаңкес шабуыл жасамас бұрын мұжахид көктем нохчичо вилая сілтеме http blip tv file get imamtv www vzasade wmv экран иншааллаһ ағайынды бірі бола ал логотип қос,violent

4,иншалла бұл кофарлар жеңіске жете алмайды және олар жақында жеңіледі біз осы қуыршақтардың басын кесуіміз керек және бұқаралық ақпарат құралдарында көрсету шынында мунафиктердің демалу орны бұл сәлем осы қуыршақты шайқас алаңында көруге рұқсат етіңіз,иншалл бұл кофар жеңіс же алма және олар жақында жең біз осы қуыршақ басын кес керек және бұқаралық ақпарат құрал көрсету шын мунафик демал орны бұл сәлем осы қуыршақ шайқас алаң көр рұқсат ет,violent

4,ирактың арнайы жасақтары оқ ату полигонында жаттығу жасайды,ирак арнайы жасақ оқ ату полигон жаттығу жаса,violent

4,исламның бұл дұшпандары исламға қарсы соғысымен ешқашан тоқтамайды,ислам бұл дұшпан ислам қарсы соғыс ешқашан тоқтама,violent

4,үкіметтің сарбаздары оңтүстікбатыстағы бей бакол және гедо облыстарындағы мужахидтерге шабуыл жасамақшы http www ansar net showthread php t,үкімет сарбаз оңтүстікбатыс бе бакол және гедо облыс мужахид шабуыл жасамақш http www ansar net showthread php t,violent

4,форумға қош келдіңіз бауырым малхама мына құпия сөзді қолданып көріңіз code mz eidbmz recn va lvf интернеттегі сілтеме бойынша егер сіз өзіңіздің еркін пайдаланушыңыз бола аласыз интернетті қосып шамамен бір минуттан кейін қайта қосылыңыз жаңа ір мекенжайын алу үшін және тоқтаған жерден жалғастырыңыз бұл жұмыс істейді,форум қош кел бауыр малха мына құпия сөз қолдан көр code mz eidbmz recn va lvf интернет сілтеме бойынша егер сіз өз еркін пайдаланушы бола ал интернет қос шамамен бір минут кейін қайта қосыл жаңа ір мекенж алу үшін және тоқта же жалғастыр бұл жұмыс істе,violent

4,сват алқабында және солтүстігінде исламшыл содырларға қарсы екі айлық науқанды аяқтаған қауіпсіздік күштері ауғанстан шекарасында талибан жетекшісі байтулла мехсудқа қарсы екінші майдан ашуда,сват алқаб және солтүстіг исламшыл содыр қарсы екі айлық науқан аяқта қауіпсіздік күш ауғанстан шекара талибан жетекші байтулл мехсуд қарсы екінші майдан аш,violent

4,пәкістанның солтүстікбатысындағы әскерилер мен көтерілісшілер арасындағы қақтығыстар күшейіп жатыр онда армия пәкістанның талибан жетекшісін жоюға бағытталған оңтүстік вазиристанда шабуыл жасауға дайындалып жатқан көрінеді,пәкістан солтүстікбаты әскери мен көтерілісші ара қақтығыс күшей жатыр онда армия пәкістан талибан жетекші жою бағыттал оңтүстік вазиристан шабуыл жаса дайындал жат көрін,violent

4,екі айлық шайқастардан кейін сват аңғарының солтүстікбатысындағы шабуыл аяқталған кезде әскери күштер пәкістанның талибан жетекшісі байтулла мехсуд орналасқан оңтүстік вазиристанда жаңа қозғалтқыш шығаруға дайындалып жатыр соғушыларға шабуыл жасау туралы шешім талибан содырлардың біртіндеп елдің көп бөлігін басып алуына және тіпті пәкістанның ядролық арсеналына қауіп төндіргеннен кейін пайда болды науқан көпшіліктің қолдауына ие және сонымен қатар ақштың жақын одақтасы пәкістан алькаиданы жеңіп ауғанстанды тұрақтандыруға тырысқаны үшін содырлардың соңынан еруі керек жексенбі күні әуе шабуылдары мехсуд бекінісі болған ладда ауданындағы екі ауылға соғысып екі содыр қосылды деп хабарлады үкімет қызметкері мен тұрғындар сондайақ көптеген адамдар шығыстағы төмен жерде қысқы үйлерге ие және дәстүрлі түрде жаздық жайылымда отарларымен оңтүстік вазиристаннан биіктікке көшеді қарулы қақтығыстардағы азаматтық шығындар жоғары болуы шабуылға қоғамдық қолдаудың жойылу қаупін арттырады,екі айлық шайқас кейін сват аңғ солтүстікбаты шабуыл аяқтал кезде әскери күш пәкістан талибан жетекші байтулл мехсуд орналас оңтүстік вазиристан жаңа қозғалтқыш шығар дайындал жатыр соғушы шабуыл жасау туралы шешім талибан содыр біртіндеп ел көп бөліг бас ал және тіпті пәкістан ядролық арсенал қауіп төндір кейін пайда бол науқан көпшілік қолда ие және сонымен қатар ақш жақын одақтас пәкістан

алькаида жең ауғанстан тұрақтандыр тырыс үшін содыр соң ер керек жексенбі күн әуе шабуыл мехсуд бекініс бол лад аудан екі ауыл соғыс екі содыр қос де хабарла үкімет қызметк мен тұрғын сондайақ көптеген адам шығыс төмен же қысқы үй ие және дәстүрлі түр жаздық жайыл отар оңтүстік вазиристан биіктік көш қарулы қақтығыс азаматтық шығын жоғары бол шабуыл қоғамдық қолда жойыл қауп арттыр,violent

4,олар біздің вазиристандағы арыстандарымыздың ащы жеңілістерін көреді,олар біздің вазириста арыстан ащ жеңіліс көр,violent

4,кисмайю қаласындағы ислам әкімшілігі эфиопия мен кенияға шабуыл жасайды дейді жарияланған уақыты шебель сомалия кисмаю sh network кисмайю портындағы исламдық әкімшілік сомали астанасы могадишу эфиопия мен кенияға шабуыл жасайтындарын мәлімдеді деп хабарлады сейсенбіде ресми өкіл кисмайю қаласындағы ислам басқармасы төрағасының орынбасары абдикани мохаммед юсуф көрші елдерге эфиопия мен кения сияқты шабуыл жасайтындарын айтты және олар негізінен эфиопияны нысанаға алатындарын олар барлық исламистік соғысушыларды шабуыл жасауға дайын болуға шақырды оларды мен сомали халқына мұсылмандарға алланың дұшпанын өз үйлеріне үйлеріне және олар сомалиге басып кірген жерлерге шабуыл жасауға дайын болуға міндеттіміз деп айтқым келеді деді кисмайю төрағасының орынбасары әкімшілік төменгі джубба аймағындағы кисмайю қаласындағы ислам әкімшілігі эфиопия мен кения сияқты көрші елдерге шабуыл жасайтындықтарын алғаш рет біліп отыр олар бұрын эфиопия сомалиге басып кірмеді деп мәлімдеді,кисмайю қала ислам әкімшіліг эфиопия мен кения шабуыл жаса де жариялан уақыт шебель сомалия кисмаю sh network кисмайю порт исламдық әкімшілік сомали астана могадиш эфиопия мен кения шабуыл жаса мәлімде де хабарла сейсенбі ресми өкіл кисмайю қала ислам басқар төраға орынбасар абдикани мохаммед юсуф көрші ел эфиопия мен кения сияқты шабуыл жаса ай және олар негіз эфиопия нысана ал олар барлық ислами соғысушы шабуыл жаса дайын бол шақ ол мен сомали халқ мұсылман алла дұшпан өз үй үй және олар сомали бас кірген жер шабуыл жаса дайын бол міндетті де айт кел де кисмайю төраға орынбасар әкімшілік төменгі джубб аймағ кисмайю қала ислам әкімшіліг эфиопия мен кения сияқты көрші ел шабуыл жасайтындық алғаш рет біл отыр олар бұрын эфиопия сомали бас кірме де мәлімде,violent

4,цитата бастапқыда жарияланған acer кейбір көтерілісшілер ирак пен оның халқы американдықтар өздерінің базаларына оралғаннан кейін осал болады деп ойлайды және жаппай жарылыстардың салдарынан кеңінен тараған секталық қантөгістерді қайта басқаруға мүмкіндіктері мол деп санайды,цита бастапқы жариялан acer кейбір көтерілісші ирак пен оның халқ американдық өз база орал кейін осал бол де ойла және жаппай жарылыс сал кең тара секталық қантөгіс қайта басқар мүмкіндік мол де сана,violent

4,сөздер сөздер сөздер америкада ең танымал сайланған шенеуніктің тапсырысы бойынша зымырандарды атуда жалғасуда егер сіз зымыран шабуылына маңызды қадамдар жасасаңыз қолыңызды көтеріңіз алькаиданың жетекшісі егер сіз бейбіт

тұрғындар арасында шығындар болатынын білсеңіз блицер соңғы сөзге баса назар аударды деннис кучинич немесе майк гравс бүгінде президент болса ғой олар қолдарын көтермеді ең қызығы бұл байқау президент обаманың екі жас баланың сүйікті әкесі екендігіне қатысты өте жаман блицер егер сіз өз балаларыңыздың қаза болатынын білсеңіз алькаида ның маңызды лидерін шығаруға зымыран соққысын берсеңіз қолыңызды көтеріңіз деп сұрамады,сөз сөз сөз америка ең танымал сайлан шенеунік тапсырыс бойынша зымыран ат жалғас егер сіз зымыран шабуыл маңызды қадам жаса қол көтер алькаида жетекші егер сіз бейбіт тұрғын ара шығын бол біл блиц соңғы сөз бас назар ау деннис кучинич немесе майк гравс бүгін президент бол ғой олар қол көтерме ең қызығ бұл байқау президент обама екі жас бала сүйікті әке екендіг қатысты өте жаман блиц егер сіз өз бала қаза бол біл алькаи ны маңызды лидер шығар зымыран соққы бер қол көтер де сұрама,violent

4,бұл эфиопиялық шошқалар мұжахидтер тарапынан бомбалануға және басып кіруге лайық эфиопия сомалиге басып кірді келесі жолы оның айналасында эфиопияны жаулап алған шааб мужахидтер болады,бұл эфиопиялық шошқа мұжахид тарап бомбалан және бас кір лайық эфиопия сомали бас кір келесі жол оның айнала эфиопия жаула ал шааб мужахид бол,violent

4,цитата жақсы жаңалық мен бұл қорқақтар өздерінің ұшақтарымен ауылдарды бомбалаудан бастайды деп үміттенемін әскери күштер ізтүзсіз жоғалған сарбаз немесе ол хабарсыз кеткен провинция туралы қосымша ақпарат бермейді біз оның орналасқан жерін анықтап оның қауіпсіз оралуын қамтамасыз ету үшін барлық ресурстарды сарқып аламыз деді бейсенбіде ақш әскери мәлімдемесінде ақш әскери өкілі капитан элизабет матиас сарбазды осы аймақта жұмыс істейтін содырлар ұстады деп сенгенін айтты соңғы айларда ақш бастаған әскерлер ауғанстанның оңтүстік және шығыс провинцияларындағы бірнеше аудандарды бақылауды жоғалтып ақш әскери күштерін төменгі хельманд өзенінің алқабында талибан көтерілісшілеріне қарсы ірі операцияны бастауға итермеледі jr hgh presstv,цита жақсы жаңалық мен бұл қорқақта өз ұшақ ауыл бомбалау баста де үміттен әскери күш ізтүз жоғал сарбаз немесе ол хаб кет провинция туралы қосымша ақпарат берме біз оның орналас жер анықта оның қауіпсіз орал қамтамасыз ету үшін барлық ресурс сарқ ал де бейсенбі ақш әскери мәлімдеме ақш әскери өкіл капитан элизабет матиас сарбаз осы аймақта жұмыс істе содыр ұста де сен ай соңғы ай ақш баста әскер ауғанстан оңтүстік және шығыс провинция бірнеше аудан бақылау жоғалт ақш әскери күш төменгі хельманд өзен алқаб талибан көтерілісші қарсы ірі операция баста итермеле jr hgh presstv,violent

4,өте қызықты жаңалықтар имам мехдидің белгілері туралы ауғанстандық джихадтан имам мехдиге дейін атты кітап бар және бұл имам мехдидің бір белгісі римдегі солдаттарды хурасанда басып алуы,өте қызықты жаңалық имам мехди бел туралы ауғанстан джихад имам мехди дейін ат кітап бар және бұл имам мехди бір бел рим солдат хурасан бас ал,violent

4,біз көптеген қайтыс болған amerikkansты және жесірлер мен жетімдерді қайғырып жатқанды көреміз,біз көптеген қайтыс бол amerikkans және жесір мен жетім қайғыр жат көр,violent

4,дәйексөз алғашында fatimalarose жариялады бұл пицца пісіріп жатқанда пешті ашқандағы сияқты және сіз оның аяқталғанын көргіңіз келсе сіз ыстық ауаның жарылысын аласыз міне ол бүкіл уақытты осылай сезінеді деді лэнс корпус чарли дугган кіші жаста балдвинсвилл ньюйорк жақында бұл шошқа шіріген шошқа етінен басқа ештеңе болмайды оның сүйектері оның сыбайластары сияқты лас сүйектері саяхатшылардың қасында жүретін болады аспан содан кейін олардан ыстықта шіріп кететінін сұраңыз,дәйексөз алғаш fatimalarose жариял бұл пицц пісір жатқанда пеш ашқа сияқты және сіз оның аяқтал көр кел сіз ыстық ауа жарылыс ал міне ол бүкіл уақыт осылай сезін де лэнс корпус чарли дугган кіші жас балдвинсвилл ньюйорк жақында бұл шошқа шірі шошқа ет басқа ештеңе болма оның сүйек оның сыбайлас сияқты лас сүйек саяхатшы қас жүр бол аспан содан кейін олардан ыстық шір кет сұра,violent

4,олардың әйелдері жесір балалары жетім қалады,олардың әйел жесі бала жетім қал,violent

4,көптеген өлген крусадерлер мен еврейлерді көреміз ол олардың жесірлері мен жетім балаларды депрессия мен суицидке итермелесін,көптеген өлген крусадер мен еврей көр ол олардың жесір мен жетім бала депрессия мен суицид итермеле,violent

4,біз шошқалардың барбден босатылып жатқандығы туралы бейнені көреміз деп үміттенемін,біз шошқа барб босатыл жатқандығ туралы бейне көр де үміттен,violent

4,дәйексөз бұл шошқа еті кесектерін жоюдың басы,дәйексөз бұл шошқа ет кесек жою бас,violent

4,видеодағы сілтеме http www megaupload com y tcv сомалидегі әлшааба римкатоликтік шіркеуді қиратады және оны базар қаласындағы масашидпен алмастыруды жоспарлайды гашишті темекі шегуге кінәлі үш адам есірткінің қоқыстарын көпшілік алдында жағу сонымен қатар шабаба сомали халқының толық қолдауына ие,видео сілтеме http www megaupload com y tcv сомали әлшааб римкатолик шіркеу қират және оны базар қала масашид алмастыру жоспарла гашиш темекі шег кінәлі үш адам есірткі қоқыс көпшілік алд жағ сонымен қатар шабаб сомали халқ толық қолда ие,violent

4,im totaly шатасқан іздеушімен келіседі amp риаз басра пакистанда тұратын адамдар да мұны өте жақсы біледі amp уақыт өте келе адамдар осы формаларды қолданып өтіп кибержиһад жасағанымды білдіремін сол сияқты киберджихадты да жасаймын бұл адамдар үшін өте жақсы тақырып amp шынайы адамдар мұсылман үмметін жақсы көреді amp джихад туралы байсалды практикалық жұмыс жасаңыз amp практикалық әлемге қадам басты,im totaly шатас іздеуші келіс amp риаз баср пакистан тұр адам да мұны өте жақсы біл amp уақыт өте келе адам осы форма қолдан

өт кибержиһад жаса білдір сол сияқты киберджихад да жаса бұл адам үшін өте жақсы тақырып amp шынайы адам мұсылман үмм жақсы көр amp джихад туралы байсалды практикалық жұмыс жаса amp практикалық әлем қадам басты,violent

4,олардың мужахидтерді бір рет бомбамайтынын көргенде жеңілдеймін,олардың мужахид бір рет бомбама көргенде жеңілде,violent

4,бағдад рейтер ирактың алькаида тобымен байланысты көтерілісшілер тобы соғыс күштерін қала орталықтарынан шығарғаннан кейін де ақш күштеріне қарсы шабуылдарды жалғастыруға шақырды деп хабарлайды сәрсенбіде шыққан аудиотаппа егер американдықтар ирак шөлінің кішкентай жерінде қалса да сондықтан әрбір мұсылман олар қуылғанша олармен соғысуы керек деп дауысты ислам мемлекеті деп аталатын әбу омар әлбағдади айтты деп мәлімдеді жиһадшылар қолданған вебсайтта жазылған жазбада спикер жылы америкалық күштермен бірлесіп жұмыс істегеннен кейін ирактың көптеген жерлерінен алькаиданы шығаруға көмектескен сунниттік араб милиционерлерін көтерілістерге қайта қосылуға және ирактың шийиттермен басқарған үкіметіне қарсы тұруға шақырды біз оларға есіңе оралыңыз тәубеге келуден және жиһадшылар қатарына оралудан және кәпірлерді тастап кетуден басқа жақсы нәрсе жоқ дейді reuters,бағдад рей ирак алькаи тоб байланысты көтерілісші тоб соғыс күш қала орталық шығар кейін де ақш күш қарсы шабуыл жалғастыр шақ де хабарла сәрсенбі шық аудиотапп егер американдық ирак шөл кішкентай же қал да сондықтан әрбір мұсылман олар қуылғанш олармен соғыс керек де дау ислам мемлекет де атал әбу омар әлбағдади ай де мәлімде жиһадшы қолдан вебсайт жазыл жазба спикер жыл америкалық күш бірлес жұмыс істе кейін ирак көптеген жер алькаида шығар көмектес сунни араб милиционер көтеріліс қайта қосыл және ирак шийит басқар үкімет қарсы тұр шақ біз оларға ес орал тәубе келу және жиһадшы қат оралу және кәпір таста кету басқа жақсы нәрсе жоқ де reuters,violent

4,дәйексөз мен моджахидтер картада көрсетілгеннен де жоғары жерлерде белсенділік танытқым келеді вилаят ноғай даласы берік салынбаған бірақ менің ойымша оның құрамына ставрополь өлкесі калмыкия ростов дон және одан әрі татарстан кіреді сондайақ адыгея мен кранодар өлкесінде мужахидтер бар ассаламу алейкум wb мен қырымда мужахедтердің де бар екенін естідім бұл украинаның кавказдан солтүстікбатыстағы аумағы алыс емес,дәйексөз мен моджахид карта көрсетіл де жоғары жер белсенділік таныт кел вилаят но дала берік салынба бірақ менің ойымша оның құрам ставрополь өлке калмыкия ростов дон және одан әрі татарстан кір сондайақ адыгея мен крано өлке мужахид бар ассалам алейку wb мен қыр мужахед де бар екен есті бұл украина кавказ солтүстікбатыс аумағ алыс емес,violent

4,көтерілісшілер могадишодағы үкімет сарбаздарына жиі граната және қашықтықтан басқарылатын бомбалармен шабуыл жасайды,көтерілісші могадишо үкімет сарбаз жиі граната және қашықтық басқарыл бомба шабуыл жаса,violent

4,біз холокостты пикникке айналдыратын күн келеді,біз холокос пикник айналдыр күн кел,violent

4,хактың айтуынша убайдулла негізінен адамдарды жиһадқа қатысуға шақырады жж ауғанстандағы соғыс кезінде ол бір жарым ай бойы қаружарақ жаттығуларынан өтті ауғанстанға бірнеше рет барып кеңес армиясына қарсы соғысқан bdnews com gma srn h,хак айт убайдулл негіз адам жиһад қатыс шақыр жж ауғанстан соғыс кез ол бір жарым ай бойы қаружарақ жаттығу өт ауғанстан бірнеше рет бар кеңес армия қарсы соғыс bdnews com gma srn h,violent

4,егер айналада біреу болса бұл олардың кінәсі біз темекіні тарттық соған қарамастан олар алькаеда мен талибанды кінәлайды олар бейбіт тұрғындарға шабуыл жасайды және бейбіт тұрғындар болған кезде ауғанстан мен нато күштеріне шабуыл жасайды,егер айнала біреу бол бұл олардың кінә біз темекі тар соған қарамастан олар алькае мен талибан кінәла олар бейбіт тұрғын шабуыл жаса және бейбіт тұрғын бол кезде ауғанстан мен нато күш шабуыл жаса,violent

4,бұл батыс африкада жаңа джихад майданының басталуы деп үміттенейік егер бұл шынымен болса бұл шығыс джихадты солтүстігінде джихадты ал қазір батыс африкадағы джихадты білдіреді африкадан қалғанның бәрі орта және оңтүстік африкадағы джихад,бұл батыс африка жаңа джихад майдан бастал де үміттен егер бұл шын бол бұл шығыс джихад солтүстіг джихад ал қазір батыс африка джихад білдір африка қал бәрі орта және оңтүстік африка джихад,violent

4,бұл күлкілі бөлігі ол жолдан таюшылар мен орыстармен күресуге қарсы екенін айтады бірақ сонымен бірге ол шешенстанда әлі де қарулы жақтастарымен соғысып жатқан сияқты,бұл күлкіл бөліг ол жолдан таюшы мен орыс күрес қарсы екен айт бірақ сонымен бірге ол шешенстан әлі де қарулы жақтас соғыс жат сияқты,violent

4,цитата бастапқыда қарындасымыз умм анвар бомбаның басты мақсаты намаз оқитындар мешіттің ішінде таң намазын жамағатпен оқитын кезде оны жару болатын бақытымызға орай біз бомба мешітке жайылып көмілген деген кеңестер алдық құрылғыны залалсыздандыру үшін біздің мамандар үш адам біз білетін адамдар және олар әлшабабтар деп аталатындардың мүшелері және біз оларды заң алдында қабылдаймыз және олар табылғаннан кейін жасаған қылмысы бойынша жазалайтын боламыз кінәлі деді шейх абди ризак ашари сомалияның орталық аймақтарындағы ахлусунна вальжама өкілі сценарийді жақсы жаза білу сонымен қатар актерлік шеберлік,цита бастапқы қарында ум анвар бомба басты мақсаты намаз оқитын мешіт іш таң намаз жамағат оқитын кезде оны жар бол бақыт орай біз бомба мешіт жайыл көм де кеңес ал құрылғы залалсыздандыр үшін біздің маман үш адам біз біл адам және олар әлшабаб де атал мүше және біз ол заң алд қабылда және олар табыл кейін жаса қылмыс бойынша жазала бол кінәлі де шейх абди ризак ашари сомалия орталық аймақ ахлусунн вальжа өкіл сценари жақсы жаза білу сонымен қатар актерлік шеберлік,violent

4,бей жарық ескертпе цитата ал шабаб және хизбул ислам галагадудуд және хирран аймақына өзгерістік жасады жақында эфиопиялық қуыршақ осы екі негізгі аймақ көздерінен жойылады,бе жарық ескертпе цита ал шабаб және хизбул ислам галагадудуд және хирран аймақ өзгер жас жақында эфиопиялық қуыршақ осы екі негізгі аймақ көз жой,violent

4,қалған командирлер аманесен кетіп жиһадты жалғастыруға уәде берді енді муртад президентінің бұйрығы бар мұндай идеясы бар барлық адамдар дереу қамауға алынып өлім жазасына кесілуі керек бауырластарым мен мұнда айтып өткендеріме көбірек жауап берді бірақ мен басқа уақытқа кетуім керек бізді өз дұаңызға салыңыз әлемге мұвахидонның езгісі туралы хабардар етіңіз және оларға сіздің кеңестеріңізбен көмектесіңіз бауырластардың қателіктері мен қателіктері болды бірақ бұл мәселені талқылауға уақыт емес,қалған командир аманесен кет жиһад жалғастыр уәде бер енді муртад президент бұйрығ бар мұндай идея бар барлық адам дереу қама алын өлім жаза кесіл керек бауырлас мен мұнда айт өт көбірек жауап бер бірақ мен басқа уақыт кет керек біз өз дұа сал әлем мұвахидон ез туралы хабардар ет және оларға сіздің кеңес көмектес бауырлас қателік мен қателік бол бірақ бұл мәселе талқыла уақыт емес,violent

4,енді бұл джихад үшін жаңа майдан болады және ол жалғаса береді,енді бұл джихад үшін жаңа майдан бол және ол жалға бер,violent

4,пабби ауғандық босқындар лагерінің асханасы пәкістан ауғандық фермерлер балаларымен бірге джейджи ауылында бомбаланған пактиа ауған босқын балалар мунда лагері пәкістан пакистан пейвар асуы джаджи пактия ауғанстан тамыз джейджи пактиа бағында советтік бомба ауғанстан асмарауғанстан маңында мужахидтер кунарауғанстантамыз барикоткунарауғанстанауғанстантамыз тамызға дейін баргамкөпіркунарафганистантамыз ші сол жақтан джайджи пактиаауғанукалашниковтамыз саоауыл кунарауғанстан тамыз сол жақтан джаджипактияауған совет бомбасы бар юнус халис мужахидтері барикот гарнизонына шабуыл жасауға дайын,пабби ауғандық босқын лагер асхана пәкістан ауғандық фермер бала бірге джейджи ауыл бомбалан пакти ауған босқ бала мун лагер пәкістан пакис пейв ас джаджи пактия ауғанстан тамыз джейджи пакти бағ сове бомба ауғанстан асмарауғанс маң мужахид кунарауғанстан барикоткунарауғанстанауғанстан тамыз дейін баргамкөпіркунарафганистан ші сол жақ джайджи пактиаауғанукалашников саоауыл кунарауғанс тамыз сол жақ джаджипактияау совет бомба бар юнус халис мужахид барикот гарнизон шабуыл жаса дайын,violent

4,өте жақсы жаңалық мен шейх мұхаммед ибн абулваххабтың даһа нигерияға жеткенін және оның көптеген ізбасарлары болғанын естідім,өте жақсы жаңалық мен шейх мұхаммед ибн абулваххаб даһ нигерия жет және оның көптеген ізбасар бол есті,violent

4,парсы шығанағындағы бірінші соғыстан кейін иракта да осындай оқиғалар орын алды бақтың жарықтануына байланысты ағымдағы соғыстан кейін ешқандай сурет әлі шыққан жоқ өздерінің заңды ұрпақтары мен әділ атабабалары үшін кек алады,парсы шығанағ бірінші соғыс кейін ирак да осындай оқиға орын алды бақ жарықтан байланысты ағымдағы соғыс кейін ешқандай сурет әлі шық жоқ өз заңды ұрпақ мен әділ атабаба үшін кек ал,violent

4,дәйексөз бастапқыда goatly ақш үкіметі оған қамқорлық жасамайтыны анық сіз күлкілі бір нәрсені білесіз мен куфар блогтарынан оқыдым оның кейбір түрлері ол авол деп айтқан болатын сондықтан оны талибан өлтірсін awol кетпейсіз,дәйексөз бастапқы goatly ақш үкімет оған қамқорлық жасама анық сіз күлкіл бір нәрсе біл мен куф блог оқы оның кейбір түр ол авол де айт бол сондықтан оны талибан өлтір awol кетпе,violent

4,олардың бәрі одан өледі бірақ оны өз отбасыларына таратпай тұрып,олардың бәрі одан өл бірақ оны өз отбасы таратпа тұр,violent

4,бүгін сағат де провинциялық сайлау комиссиясының қолбасшылығымен алты мүжахид әрфарук мулла хамза кабул провинциясының хафиз ассадулла кари ханиф хафиз наджиб және логар провинциясының наим олар rpg жарылғыш гранаталармен ак және қол гранаталарымен қаруланған олардың барлығы әлхамза шейіт болуға ұмтылған контингентке жатады олардың батыл шабуылының нәтижесінде жалдамалы үйдегі солдат пен полиция оның ішінде барлау бөлімінің қызметкері қаза тапты шейіт болу басталғанға дейін жарылғыш заттармен жабдықталған көліктің жарылуында шетелдік әскери қызметкер қаза тапты немесе жарақат алды автокөлік тиісті жерге қойылған болса қашықтан басқарылатын құрылғы жарылды бұл операция логар губернаторының келісімімен және нұсқауымен жүргізілген тұрақты үйді іздеу мен өлтіру мен мақсатты өлтіру үшін кек ретінде жүзеге асырылады біз тағы бір рет мүжахидтер жаудың ауғанстанның азапты және қысым көрген халқына қарсы болуы мүмкін кез келген зорлықзомбылықтары үшін кек алатынын жариялаймыз,бүгін сағат де провинциялық сайлау комиссия қолбасшылығ алты мүжахид әрфарук мулл хамза кабул провинция хафиз ассадулл кари ханиф хафиз наджиб және лог провинция наи олар rpg жарылғыш граната ак және қол граната қарулан олардың барлығ әлхамз шейіт бол ұмтыл контингент жат олардың батыл шабуыл нәтиже жалдамалы үйдегі солдат пен полиция оның іш барлау бөлім қызметк қаза тап шейіт болу бастал дейін жарылғыш зат жабдықтал көлік жарылу шетелдік әскери қызметкер қаза тап немесе жарақат алды автокөлік тиісті же қойыл бол қашық басқарыл құрылғы жар бұл операция лог губернатор келісім және нұсқау жүргіз тұрақты үй іздеу мен өлтір мен мақсат өлтір үшін кек рет жүзеге асыр біз тағы бір рет мүжахид жау ауғанстан аза және қысым көр халқ қарсы бол мүмкін кез кел зорлықзомбылық үшін кек ал жарияла,violent

4,қытайлық кафтерлермен соғысуға мүмкіндік береді ағайыңыз түркістан,қытайлық кафтер соғыс мүмкіндік бер ағай түркістан,violent

4,біз ислам мемлекетінің сарбаздары өз джихадымызбен жерді кәпірлер мен жолдан таюшылардың арамдықтарынан тазартуға тырысамыз iraq исламдық мәлімет бағдад ақпараттық провинциясы шағбан ақпарат көзі альфаджр ақпараттық орталығы,біз ислам мемлекет сарбаз өз джихад же кәпір мен жолдан таюшы арамдық тазарт тырыс iraq исламдық мәлімет бағдад ақпараттық провинция шағбан ақпарат көз альфадж ақпараттық орталығ,violent

4,қандай керемет сыйлық олар бұған шынымен лайық цитата олар жүгіруде олар жасырынуда біз оларды толығымен құртамыз деді ол,қандай керемет сыйлық олар бұған шын лайық цита олар жүгір олар жасырын біз ол толығ құр де ол,violent

4,джихад кавказда шариғат болғанша жалғасады,джихад кавказ шариғат болғанш жалғас,violent

4,мен жолдан тайған полицияға шабуыл жасағым келеді бірақ мен алькаиданың логотипін мағрепте қалаймын түсініктеме бөліміне біреу қоя алады оны менің вебсайтыңызға орналастыра аламын ба,мен жолдан тай полиция шабуыл жаса кел бірақ мен алькаида логотип мағреп қала түсініктеме бөлім біреу қоя ал оны менің вебсайт орналастыр ал ба,violent

4,мүжахидтер кек қайтарумен келе жатқан сияқты көрдіңіз бе бұл полиция армия және үкімет нысанаға алынды ислам мемлекеті қалады,мүжахид кек қайтар келе жат сияқты көр бе бұл полиция армия және үкімет нысана ал ислам мемлекет қал,violent

4,бұл кавказ орталығы ресейдегі саяношушенск электр стансасын тежеу үшін жауапкершілікті мойнына алған екінші хаттың мәтіні джамаат исламдық әмірлік жарияланғаннан кейін бірден құрылған этникалық орыс моджахедтер тобы деп мәлімдейді олар байяны амир докку абу усманға берді және оның басшылығымен бұл топ кішкентай болғандықтан және ресей территориясының тереңінде болғандықтан олар ресейдің кафтарына қарсы диверсия және басқа нәрселер түрінде экономикалық джихад өткізіп жатыр дейді мәлімдемеде операцияны жасаған ибрахим а есімді ағасы мекемеде жұмыс істеп зауыттың әлсіз жақтарын зерттеп диверсия жасайтын жерлерді анықтаған,бұл кавказ орталығ ресей саяношушенск электр станса тежеу үшін жауапкершілік мойн ал екінші хат мәтін джамаат исламдық әмірлік жариялан кейін бірден құрыл этникалық орыс моджахед тоб де мәлімде олар байя ами докк абу усман бер және оның басшылығ бұл топ кішкентай бол және ресей территория терең бол олар ресей каф қарсы диверсия және басқа нәрсе түр экономикалық джихад өткіз жатыр де мәлімдеме операция жаса ибрахи а ес аға мекеме жұмыс істе зауыт әлсіз жақ зертте диверсия жаса жер анықта,violent

4,көшеде және олардың штабпәтерінде олармен күресу керек егер егер яһудилер бірбіріне қарамақарсы мешіттерді бомбалағанын көретін болса онда олар бомбалық мешіттерді жалғастыру арқылы ешқандай проблема көрмейді сондықтан бұларға бұқаралық ақпарат құралдарында шабуыл жасау және батыста оларға қолдау көрсету қиын болады егер біз масжидтерді бомбаласақ олар жемальмакдисты бомбалай алады және өздерін бомбалы москиттер деп айта алады сондықтан неге

болмасқа,көше және олардың штабпә олармен күресу керек егер егер яһуди бірбі қарамақар мешіт бомбала көр бол онда олар бомбалық мешіт жалғастыру арқылы ешқандай проблема көрме сондықтан бұл бұқаралық ақпарат құрал шабуыл жасау және батыс оларға қолдау көрсету қиын бол егер біз масжид бомбала олар жемальмакди бомбала ал және өз бомбал москит де ай ал сондықтан неге болмас,violent

4,цитата бастапқыда гринбердтегі жәһахил кейбір мәселелерде тым үлкен болуы мүмкін мысалы салафи мужахидтерді өлтіруі мүмкін бірақ мен оларды әсіресе амир әлмумииндер сияқты деп санамаймын әйтпесе sh usama оларға көмектеспейді бұл өте үлкен өтірік бұл салфилерді өлтіретін таляб олар talfies үкіметтік салфилерді өлтіреді талибан салфи мужахидтердің көмегімен жиһад жасайды пакистандық тайпалық аймақта салфимужахидтердің саны көп әлем талибан оларды өлтірмейді тіпті оларды қорғайды және салфилерді сақтау үшін өз үйлеріне бомбалады са бұл оларға қарсы үгітнасихат және ақымақ болу керек пакистандық салфилер жолдан тайған пакистан армиясымен бірге олар қарсы сөйлейді талибан осыны айту арқылы бұл ханфийлерді өлтірмейді оның үкіметі болып табылады олар хан және хан хандықтарының көптеген мүшелерін өлтірді талибан ол салфи болғандықтан мужахидті өлтірді деп айта алмаймын сондықтан оларға қарсы үгітнасихат жүргізу үшін уртілді мужахидтерден алыс ұстаңыз,цита бастапқы гринберд жәһахил кейбір мәселе тым үлкен бол мүмкін мысал салафи мужахид өлтір мүмкін бірақ мен ол әсіресе ами әлмумиин сияқты де санама әйтпесе sh usama оларға көмектеспе бұл өте үлкен өтірік бұл салфи өлтір таляб олар talfies үкіметтік салфи өлтір талибан салфи мужахид көмег жиһад жаса пакиста тайпалық аймақта салфимужахид сан көп әлем талибан ол өлтірме тіпті ол қорға және салфи сақтау үшін өз үй бомбал са бұл оларға қарсы үгітнасихат және ақы болу керек пакиста салфи жолдан тай пакис армия бірге олар қарсы сөйле талибан осы айту арқылы бұл ханфий өлтірме оның үкімет бол таб олар хан және хан хандық көптеген мүше өл талибан ол салфи бол мужахид өл де ай алма сондықтан оларға қарсы үгітнасихат жүргізу үшін урт мужахид алыс ұста,violent

4,ахи күрескер сен шындықты айттың бауырым ал мен каида мен джунд ансараллах мұндай әрекетті ешқашан жасамайтынын білемін бірақ мен олардың өш алуға деген құштарлығын түсінер едім мен өзім туралы айтып тұрмын мен осы жаңалықты оқып қуандым бірақ содан кейін мен бұл туралы бір сәтке ойланып қуанышыма өкіндім сондықтан менің постым байтмакдидегі мужахед ағаларымыз туралы шабуыл немесе теріс пікір емес керісінше бұл біздің және біздің ер адамдарымызды өлтіріп масхабетімізді жойған хамас үшін кек алатын барлық ағалар мен әпкелер үшін ескерту болды,ахи күреск сен шындық ай бауыр ал мен каида мен джунд ансараллах мұндай әрекет ешқашан жасама біл бірақ мен олардың өш ал де құштарлығ түсін ед мен өзім туралы айт тұр мен осы жаңалық оқ қуан бірақ содан кейін мен бұл туралы бір сәт ойлан қуаныш өкін сондықтан менің пост байтмакди мужахед аға туралы шабуыл немесе теріс пікір емес керісінше бұл біздің және біздің ер адам өлтір масхабет жой хамас үшін кек ал барлық аға мен әпке үшін ескерту бол,violent

4,расында да шабуылдар көп болады сен жолдан таюшылар саған сенгендей бұл шынымен де қайда алджихадтың жолдан таюшылардан босату және босату үшін жасаған алғашқы қадамы саудиялық жолдан тайған билеушілердің ауғанстанның масқараланған карзайына айналу уақыты келді іргетасы қаланғаннан кейін келесі қадам құрылысты бастау,рас да шабуыл көп бол сен жолдан таюшы саған сенгенде бұл шын де қайда алджихад жолдан таюшы босат және босат үшін жаса алғашқы қадам саудиялық жолдан тай билеуші ауғанстан масқаралан карзай айналу уақыт кел іргетас қалан кейін келесі қадам құрыл бастау,violent

4,яғни үндістаннан кашмирдің тәуелсіздігі үшін күресетін мужахидтер мен пәкістанға қосылу үшін күресетін топтар бар ма,яғни үндістан кашмир тәуелсіздіг үшін күрес мужахид мен пәкістан қосылу үшін күрес топ бар ма,violent

4,дәйексөз барак обаманы ми қатерлі ісігі ауруына шалдықтырады деп үміттенемін ол тед кеннеди сияқты өлуі мүмкін мен бүгін барак обаманың ұлулар сияқты еріп кеткенін қалаймын есте сақтау үшін өте жағымды және қызықты сөздер,дәйексөз барак обама ми қатерлі ісіг ауру шалдықтыр де үміттен ол тед кеннеди сияқты өл мүмкін мен бүгін барак обама ұлу сияқты ер кет қала ес сақтау үшін өте жағымды және қызықты сөз,violent

4,біз одан француз қуыруын жасадық,біз одан француз қуыр жаса,violent

4,біз израильдіктерді ұстап оларды пештерге тастауымыз керек,біз израильдік ұста ол пеш таста керек,violent

4,егер соғыстар бітпесе біз кеңес одағын жыл бойына сарқып құлап жадында сақтағанға дейін сізге қарсы барлық ықтимал біліктермен соғысуды жалғастыруымыз керек өткен деді бин ладен,егер соғыс бітпе біз кеңес одақ жыл бой сарқ құла жад сақта дейін сізге қарсы барлық ықтимал білік соғысу жалғастыр керек өткен де бин ла,violent

4,американды қарғыс атсын американың тыңшыларын қарғыс алсын джоубти үкіметін американдықтар өз елінен сомалидегі мужахидтерге шабуыл жасайтын,америка қарғыс ат америка тыңшы қарғыс ал джоубти үкім американдық өз ел сомали мужахид шабуыл жаса,violent

4,тағдырды amerikkka лағынет етсін ол өзінің лас қорқақ сарбаздарына ауыр шығындар әкелсін ол өзінің жезөкшелерін жесірлерді және балақай балаларын жетім етсін amerikkkаны әр ауру мен апатқа ұшыратсын,тағ amerikkka лағынет ет ол өзінің лас қорқақ сарбаз ауыр шығын әкел ол өзінің жезөкше жесір және бала бала жетім ет amerikkka әр ауру мен апат ұшырат,violent

4,бір уақытта және біртіндеп жауынгерлерді жойып тастаймыз,бір уақыт және біртіндеп жауынгер жой таста,violent

4,бауырым меніңше мұжахидтер шабуыл жасамас бұрын қауіпқатерлерді бағалайды өздеріңіз білесіздер бізде қол жетімді қаружарақ шектеулі мұндай операцияны

орындау кезінде түпкілікті шешім жердегі әскери қолбасшының мойнында болады егер сіз джихад фикхіне қарасаңыз кейбір мұсылмандарға әсер етуі мүмкін болса да кейбір жағдайларда сіз шабуыл жасай алатындығыңызды түсінесіз,бауыр меніңше мұжахид шабуыл жасамас бұрын қауіпқатер бағала өз біл біз қол жет қаружарақ шектеулі мұндай операция орындау кез түпкілік шешім жер әскери қолбасшы мойн бол егер сіз джихад фикх қара кейбір мұсылман әсер ет мүмкін бол да кейбір жағдай сіз шабуыл жаса алатындығ түс,violent

4,шексіз соғыс мені шаршатпайды өйткені мен қазір толықтай және күштімін сондықтан анам маған өлең жолдары жол сілтеді,шексіз соғыс мен шаршатпа өйткені мен қазір толықта және күшті сондықтан ана маған өлең жол жол сілте,violent

4,набханидің және оның серіктерінің қайтыс болғаны туралы қуанышты хабарды жеткіземіз,набхани және оның серік қайтыс бол туралы қуанышты хаб жеткіз,violent

4,бұл керемет жаңалық тавхид пен джихадтың көшеттерін қанымызға батырған ағамыз амир салех содан кейін джихад жалғасады,бұл керемет жаңалық тавхид пен джихад көшет қан батыр ағ ами салех содан кейін джихад жалғас,violent

4,көп ұзамай шейх серігін шейіт болған бір әйел мерт қылады,көп ұзама шейх серіг шейіт бол бір әйел мерт қыл,violent

4,талибан жетекшісі ақш пен натоға тарихты зерттеуді бұйырды қабул талибан жетекшісінің жаңа жолдауы бойынша ақш пен нато ауғанстанның соғыс тарихын зерттеуі керек шетелдік күштердің бұл елде жетістіктері шектеулі болды мулла омардың хабары ақш бастаған ауғанстанға шабуыл жасауының сегіз жылдығына бір ай қалғанда келеді биылғы жыл ақш пен нато әскерлері үшін қақтығыстың ең жойқын кезеңі болды соғысты саяси қолдау төмендейді мұсылмандардың ораза айт мейрамына арнаған жолдауында омар хіх ғасырда жыл бойы пуштун тайпалары британдықтармен соғысып нәтижесінде жеңіске жеткенін айтады омардың айтуынша бүгінде содырлар мықты шешім әскери дайындық және тиімді қаружараққа ие және ұзақ уақытқа созылған соғысқа дайын,талибан жетекші ақш пен нато тарих зерттеу бұйыр қабул талибан жетекші жаңа жолда бойынша ақш пен нато ауғанстан соғыс тарих зертте керек шетелдік күш бұл ел жетістік шектеулі бол мулл омар хаб ақш баста ауғанстан шабуыл жасау сегіз жылдығ бір ай қал кел биылғы жыл ақш пен нато әскер үшін қақтығыс ең жойқын кезең бол соғ саяси қолдау төменде мұсылман ораз айт мейрам арна жолдау омар хіх ғасыр жыл бойы пуштун тайпа британдық соғыс нәтиже жеңіс жет айт омар айт бүгін содыр мықты шешім әскери дайындық және тиімді қаружарақ ие және ұзақ уақыт созыл соғыс дайын,violent

4,бүгін бізде батыл шешім әскери дайындық және тиімді қару бар тағы бір айта кететін жайт біз ұзақ уақытқа созылған соғысқа дайынбыз және аймақтық жағдай біздің жағымызда сондықтан біз тәуелсіздік алып басқыншыларды шығаруға мәжбүр болғанша біз жиһадты жалғастырамыз,бүгін біз батыл шешім әскери дайындық және тиімді қару бар тағы бір ай кет жайт біз ұзақ уақыт созыл соғыс дайын және аймақтық

жағдай біздің жағ сондықтан біз тәуелсіздік алып басқыншы шығар мәжбүр болғанш біз жиһад жалғастыр,violent

4,олар бізге джихад ешқашан бітпейтінін айтты сомалиде аяқтағаннан кейін біз кенияға содан кейін басқа жерлерге барамыз бұл шынымен жүрек пен ақылға қатысты,олар бізге джихад ешқашан бітпе ай сомали аяқта кейін біз кения содан кейін басқа жер бар бұл шын жүрек пен ақыл қатысты,violent

4,муваххахидун аррус тобынан алынған бейне путиннің сөйлегені жұмыс кезінде электр станциясының роликтері және топтың әмірі мұвахахидун аррус жасағанын түсіндіретін мәлімдемесі операция риядуссалихин бригадасының нұсқауы бойынша жүргізіліп риядуссалихин бригадасына бағынады әмір сонымен қатар кондодағы мұнай нысандарына қарсы операция үшін жауапкершілікті өз мойнына алды нәтижесінде бірнеше сағат бойы күрделі жану болды және кофедегі өрт сөндірушілердің қазасы болды және көптеген жаңа операциялар болады деп уәде берді,муваххахидун аррус тоб алын бейне путин сөйле жұмыс кез электр станция ролик және топ әмір мұвахахидун аррус жаса түсіндір мәлімде операция риядуссалихин бригада нұсқа бойынша жүргізіл риядуссалихин бригада бағын әмір сонымен қатар кондо мұнай нысан қарсы операция үшін жауапкершілік өз мойн алды нәтиже бірнеше сағат бойы күрделі жан бол және кофе өрт сөндіруші қаза бол және көптеген жаңа операция бол де уәде бер,violent

4,алғашқыда асадулла алшишани бұл олардың ұлтшылдық үшін емес сибіл аллаға қарсы күресетін нағыз шынайы муһахид екенін көрсетеді шейх әбу мұхаммед әлмақдисидің хафидаһуллах айтқан мәлімдемесінен кейін және осы соңғы оқиғалардан кейін мұсылман үмметі өзінің кавказға деген қызығушылығын қалпына келтіріп оларға көмек жібереді деп үміттенемін кавказдағы мужахидтер ші соғыста және әрине ші соғыс басталғаннан бері әрдайым шынайы мужаһидтер үшін күресіп келеді шейх абу яхия хафидхахулла немесе шейх завахири хафидхахулла сияқты әлкаидадан шыққан бір әмірдің кавказдағы ислам әмірлігі үшін мәлімдеме немесе ассахабтан бейнетаспа шығарғанын қалаймын,алғашқы асадулл алшишани бұл олардың ұлтш үшін емес сибіл алла қарсы күрес нағыз шынайы муһахид екен көрсет шейх әбу мұхаммед әлмақдиси хафидаһуллах айт мәлімдеме кейін және осы соңғы оқиға кейін мұсылман үмме өзінің кавказ де қызығушылығ қалп келтір оларға көмек жібер де үміттен кавказ мужахид ші соғыс және әрине ші соғыс бастал бері әрдайым шынайы мужаһид үшін күрес кел шейх абу яхия хафидхахулл немесе шейх завахири хафидхахулл сияқты әлкаида шық бір әмір кавказ ислам әмірліг үшін мәлімдеме немесе ассахаб бейнетасп шығар қала,violent

4,мен оның осы заң бойынша сотталғанын көргім келеді,мен оның осы заң бойынша соттал көр кел,violent

4,ахи қателеспеңіз ltte сияқты зайырлы қозғалыстар бұл тактиканы мұсылмандар бұрын жасаған рафида да осылай істеді тіпті plo сияқты зайырлы мұсылман ұйымдары да жасады әбу омардың тілегіне келетін болсақ менің ойымша олар мұны себеппен

мүмкін өтініш бойынша істемеді есіңізде болсын джихадтың басқа жерлерімен салыстырғанда шишаан әлі де жақсы джихад бейнесін сақтайды өйткені ол батысқа емес ресейге қарсы,ахи қателеспе ltte сияқты зайырлы қозғалыс бұл тактика мұсылман бұрын жаса рафи да осылай істе тіпті plo сияқты зайырлы мұсылман ұйым да жас әбу омар тілег кел бол менің ойымша олар мұны себеп мүмкін өтініш бойынша істеме ес бол джихад басқа жер салыстыр шишаан әлі де жақсы джихад бейне сақта өйткені ол батыс емес ресей қарсы,violent

4,мұны көргеннен кейін хамас зұлым және диктаторлық режим дегеніміз не екеніне қарамастан барлығына түсінікті болу керек бұл мұсылмандардың не істеп жатқанын анық куфр шатастырмаңыз бұл шейх ахмед ясиннің а хамасы емес бұл джунд ансар аллаһ джайиш әлислам фатх альислам джайиш әлумма джунд ашшамс және салафиттік жастар олар біздің сүйікті шейхтің армандарын тірі қалдырады бұл барлық мұсылмандар үшін хамасты қолдамау үшін ояту болуы керек крусадерлер мен лас еврейлерді жояды және жояды,мұны көр кейін хамас зұлым және диктаторлық режим де не екен қарамастан барлығ түсінік болу керек бұл мұсылман не істе жат анық куф шатастырма бұл шейх ахмед ясин а хамас емес бұл джунд анс аллаһ джайиш әлисла фатх альисла джайиш әлум джунд ашшамс және салафиттік жас олар біздің сүйікті шейх арман тірі қалдыр бұл барлық мұсылман үшін хамас қолдама үшін оят бол керек крусадер мен лас еврей жоя және жоя,violent

4,шаабтың өкілі егіз шейіт операциясының сәттілігіне қуанышты екенін білдіріп біз оларға шабуыл жасамас бұрын біз оларға шабуыл жасаймыз http ansar net showthread php post,шааб өкіл егіз шейіт операция сәттіліг қуанышты екен білдір біз оларға шабуыл жасамас бұрын біз оларға шабуыл жаса http ansar net showthread php post,violent

4,цитата алғашында біріккен исламдық джихад барлық муртад пакистандық күштерін амэенді жойып жіберсін және барлық тагауттер билеушілері,цита алғаш біріккен исламдық джихад барлық муртад пакиста күш амэе жой жібер және барлық тагаут билеуші,violent

4,йемени мен саудилер нағыз өтірікші мен оларды білемін әрбір мұсылман отбасы мен тагути жүйесі мен яһудилерге шабуыл жасайтын адамдарды қолдауы керек христиандар мен христиандарды өлтіру суудалар мен ямани саясаткерлерін өлтірумен тең,йемени мен сауди нағыз өтірікш мен ол біл әрбір мұсылман отбасы мен тагути жүйе мен яһуди шабуыл жаса адам қолда керек христиан мен христиан өлтір сууда мен ямани саясаткер өлтір тең,violent

4,олардың барлығы ауғанстанда өледі деп сенемін,олардың барлығ ауғанстан өл де сен,violent

4,біз осы шошқалардың ішінен французша қуыруды жасаймыз есіңізде болсын,біз осы шошқа іш французш қуыру жаса ес бол,violent

4,бұл шейіт операциясына арналған арнайы батальон жақында шейіт болудың көптеген видеолары шығады ирак ислам мемлекетімен біріктірсін,бұл шейіт операция арнал арнайы батальон жақында шейіт бол көптеген видео шығ ирак ислам мемлекет біріктір,violent

4,geoдағы жаңалықтар spinny road quettada dig полициясы низам шахид дурраниге бомба шабуылы бар шабуылдан кейін әуе оқтары жалғасуда ұсталғандар әлі де келе жатыр,geo жаңалық spinny road quetta dig полиция низа шахид дуррани бомба шабуыл бар шабуыл кейін әуе оқ жалғас ұстал әлі де келе жатыр,violent

4,қазақша нұсқасы бар ма plz,қазақша нұсқа бар ма plz,violent

4,яһудилерді жек көретінімнің тағы бір себебі біз иерусалимді босатқанда бәрін пешке тастаймыз дәйексөз мысық ол сүйкімді ия бұл тәтті мысық оның сақалының бірі миллион лас киктердің өмірінен гөрі құнды,яһуди жек көр тағы бір себебі біз иерусалим босат бәрін пеш таста дәйексөз мысық ол сүйк ия бұл тәтті мысық оның сақал бірі миллион лас кик өмір гөрі құнды,violent

4,бізде пәкістанда нағыз ислам үкіметі болған кезде кашмир пәкістанның ажырамас бөлігі болады геосаяси тұрғыдан алғанда кашмир пәкістан үшін өмір жолы және оны кезкелген жағдайда тапсыру өзөзіне қол жұмсау еді мен пәкістан армиясының оны күшпен тартып ала алмайтындығына сенімдімін бірақ егер олар мұжахидтерге жол берсе және жеңілдетсе кашмирлер бірнеше жыл ішінде индустардан тәуелсіздікке қол жеткізе алады егер олар куффардың қысымымен мүжахедтерге шабуыл жасамаса,біз пәкістан нағыз ислам үкімет бол кезде кашмир пәкістан ажырамас бөліг бол геосаяси тұрғы ал кашмир пәкістан үшін өмір жол және оны кезкел жағдай тапсыру өзөз қол жұмса ед мен пәкістан армия оны күш тарт ал алмайтындығ сенімді бірақ егер олар мұжахид жол бер және жеңілдет кашмир бірнеше жыл іш индус тәуелсіздік қол жеткіз ал егер олар куфф қысым мүжахед шабуыл жасама,violent

4,джихад журналы бастау керек дрондар туралы соңғы ескерту альхамдуллилла мұнда моджахедтердің қолында самдың бұзылуы бар сияқты сондықтан оларда тиісті қарсы шаралар болады иншалла алла бізге жетеді btw егер сізге шығармаларыңыз үшін редактор қажет болса мен сізге оларды өңдеуге көмектесетінімді біліп қойыңыз иншалла,джихад журнал бастау керек дрон туралы соңғы ескерту альхамдуллилл мұнда моджахед қол сам бұзыл бар сияқты сондықтан ол тиісті қарсы шара бол иншалл алл бізге жет btw егер сізге шығарма үшін редактор қажет бол мен сізге ол өңде көмектес біл қой иншалл,violent

4,бауырым therealtruth дұрыс айтады біз осы жерде фисабилилла джихадты ұсынамыз және сол үшін жұмыс істеп жатырмыз talban alqaida бұл тек атаулар ең бастысы бұл біздің жұмысымыз және себептеріміз сондықтан мұндай есімдер бұл салада жаңадан келген қарапайым бауырластарды шатастырудан басқа ештеңе емес және олар осы арқылы көптеген мағыналарды ала алады сондықтан оны алып тастаңыз және мұндай мәдениетті насихаттамаңыз,бауыр therealtruth дұрыс айт біз осы же фисабилилл джихад ұсын және сол үшін жұмыс істе жатыр talban alqaida бұл тек атау ең басты бұл

біздің жұмыс және себеп сондықтан мұндай есім бұл сала жаңа кел қарапайым бауырлас шатастыру басқа ештеңе емес және олар осы арқылы көптеген мағына ал ал сондықтан оны алып таста және мұндай мәдениетті насихаттама,violent

4,яһудилерге шабуылдар көп болады,яһуди шабуыл көп бол,violent

4,amerikkka жылға дейін жойылады ал үй нигро екінші мерзімге ие болады деп кім айтады,amerikkka жыл дейін жой ал үй нигро екінші мерзім ие бол де кім айт,violent

4,құрметті бауырым мен швейцарияда тұрмаймын менің лауазымым куфарды әшкерелеу және олардың жеккөрушіліктерін көрсету және біз оны жазықсыз азаматтық кафтарларды жек көрмейді деп санайтын қалыпты мұсылмандарды ояту үшін таратуымыз керек біз ағай мен сомали мен жиһад жеріне жету үшін не істеймін иншаллах біз жақын арада джихад жерінде кездесеміз егер бұл сомалия немесе басқа джихад жері болса менің ниямды толықтай толтыруға жол ашады,құрметті бауыр мен швейцария тұрма менің лауазым куф әшкереле және олардың жеккөрушілік көрсету және біз оны жазық азаматтық кафтар жек көрме де сана қалыпты мұсылман оят үшін тарат керек біз аға мен сомали мен жиһад же жету үшін не істе иншаллах біз жақын ара джихад же кездес егер бұл сомалия немесе басқа джихад же бол менің ния толықта толтыр жол аш,violent

4,америкаға және оның одақтастарына қарсы ісқимыл жасайды деп үміттенемін,америка және оның одақтас қарсы ісқимыл жаса де үміттен,violent

4,бұл швейцарияда және тіпті еуропада тұратын барлық мұсылман қауым үшін оянуға шақыруы керек өйткені ол еуропалық одақтың заңнамасымен реттеледі өйткені олардың дауыстарын естіп заң шығарушы органның бұл ескертуін әшкерелеу керек кофарлар сізге жанама түрде не істей алатынын көріңіз және олар оны дін бостандығы деп атайды,бұл швейцария және тіпті еуропа тұр барлық мұсылман қауым үшін оян шақыр керек өйткені ол еуропалық одақ заңнама реттел өйткені олардың дауыс ест заң шығарушы орган бұл ескерт әшкереле керек кофар сізге жанама түр не істе ал көр және олар оны дін бостандығ де ата,violent

4,дәйексөз бастапқыда махаз ассалауму алейкум ва рахматулла егер бұл қазақшаға аударылса иншалла уалейкумуссалам warahmatullah wabarakatuhu бауырым гугл аудармашысын қолдана алады,дәйексөз бастапқы махаз ассалаум алейку ва рахматулл егер бұл қазақша аударыл иншалл уалейкумуссала warahmatullah wabarakatuhu бауыр гугл аудармашы қолдан ал,violent

4,нұсқау абуфарук нобельдің бейбітшілік сыйлығының лауреаты обама неге әскер жібереді бізге мақсатты тәжірибе үшін көбірек керек иә ағайынды біз кішкентай талибан жаттығулары үшін көбірек әскер алғымыз келеді біз өзіміздің мужахид ағаларымызды оқыту үшін ші әскерді қолданғымыз келеді мужахидтердің жаттығуы мен тәжірибесі үшін әскер біз обамадан тағы әскер жіберуді сұраймыз әскер бұл тек немесе фидя шейіт операциялары олардың барлығын өлтіреді біз әскерді ақтамыз ақпан айына қосымша әскер дайындаймыз inshaallah,нұсқау абуфарук нобель

бейбітшілік сыйлығ лауреат оба неге әскер жібер бізге мақсат тәжірибе үшін көбірек керек иә ағайынды біз кішкентай талибан жаттығу үшін көбірек әскер ал кел біз өз мужахид аға оқыту үшін ші әскер қолдан кел мужахид жаттығ мен тәжірибе үшін әскер біз обама тағы әскер жіберу сұра әскер бұл тек немесе фидя шейіт операция олардың барлығ өлтір біз әскер ақ ақпан ай қосымша әскер дайында inshaallah,violent

4,бұл жақсы жаңа шабуылдарды көбірек көреміз бұл орыс шошқалары біздің әпкелерімізге жасағаннан кейін олар келесі жыл үшін бесланға күн сайын лайықты,бұл жақсы жаңа шабуыл көбірек көр бұл орыс шошқа біздің әпке жаса кейін олар келесі жыл үшін беслан күн сайын лайықты,violent

4,олар өлімге дайындалуда не істей алады олар еврейлердің бұйрығына мойынсұнулары керек әйтпесе израиль құрылғанға дейін британдық палатада болғандықтан бұл еврейлер британдықтарды жарып жіберді олар еврейлердің бұйрығына бағынбайды мысалы олар пентагонды жарып жібереді,олар өлім дайындал не істе ал олар еврей бұйрығ мойынсұну керек әйтпесе израиль құрыл дейін британдық палата бол бұл еврей британдық жар жіб олар еврей бұйрығ бағынба мысал олар пентаго жар жібер,violent

4,бұл amerikkkan империализмінің соңы болсын қазір америка мен еуропада исламдық отарлау келді,бұл amerikkkan империализм соңы бол қазір америка мен еуропа исламдық отарла кел,violent

4,дәйексөз бастапқыда жарияланған махаз ассалауму алейкум хизбул ислам мен аль шабаба неге бірбірімен қақтығысып жатыр деген мәлімдемелер бар ма ахе карем оның шабаб пен емес арасында hsibul ислам емес оның орнына шабаб amp ахмед мадообе деген адамды бұл жігіт шабабпен қуған amp кения шекарасына өтіп оның тайпасы шабабпен күресте оны қолдауда алайда әлі де шабабпен бірге өз руынан шыққан мужахидтер бар бірақ жақсы жаңалықтар бұл проблеманы жақында шешетін болады,дәйексөз бастапқы жариялан махаз ассалаум алейку хизбул ислам мен аль шабаб неге бірбір қақтығыс жатыр де мәлімдеме бар ма ах каре оның шабаб пен емес ара hsibul ислам емес оның орн шабаб amp ахмед мадооб де адам бұл жігіт шабаб қу amp кения шекара өт оның тай шабаб күрес оны қолда алайда әлі де шабаб бірге өз ру шық мужахид бар бірақ жақсы жаңалық бұл проблема жақында шеш бол,violent

4,сомалидегі жағдай өте күрделі хизбулислам біртұтас ұйым дегенді білдірмейді жемқор адамдар хизбул исламның кейбір фракцияларының жетекшілері болған кезде қазіргі күрес болады деп күтілуде куфтар кейбір ақылды айлаамалдарды пайдаланып хизбул исламға өз халқын отырғызу арқылы мужахидтерді жеңуге тырысады сондықтан бұл адамдар шабаба әлмужахидтерге шабуыл жасаған кезде барлық хизбул исламы аль шабаба тобымен қақтығысқа түседі осындай жемқорлардың бірі мадобе оның кенияға пана алғаны туралы мәліметтер бар егер бұл рас болса бұл көп нәрсені дәлелдейді,сомали жағдай өте күрделі хизбулисла біртұтас ұйым де білдірме жемқор адам хизбул ислам кейбір фракция жетекші бол кезде қазіргі күрес бол де күтіл куф кейбір ақылды айлаамал пайдалан хизбул ислам өз халқ отырғызу арқылы

мүжахид жең тырыс сондықтан бұл адам шабаб әлмужахид шабуыл жаса кезде барлық хизбул ислам аль шабаб тоб қақтығыс түс осындай жемқор бірі мадоб оның кения пан ал туралы мәлімет бар егер бұл рас бол бұл көп нәрсе дәлелде,violent

4,сомали джихад жеріне қосылуда бұл біздің бауырларымызды тыңдамайтындарға арналған,сомали джихад же қосыл бұл біздің бауыр тыңдама арнал,violent

4,ағаларымыз қаружарақтан жас және жаңа әскерлердің жаңа қанын күтуде,аға қаружарақ жас және жаңа әскер жаңа қан күт,violent

4,егер олар сәл жүгірсе олар бірінші болып финге ертерек барады тураб болады олар оларды күтуде бірақ олар оңтүстікке кетуі керек мұнда оларды күтеді,егер олар сәл жүгір олар бірінші бол фин ертерек бар тураб бол олар ол күт бірақ олар оңтүстік кет керек мұнда ол күт,violent

4,талибан және ирак пен кавказ ислам мемлекеті әлемдегі ең жақсы үкімет бізде жақын арада халифа болады,талибан және ирак пен кавказ ислам мемлекет әлем ең жақсы үкімет біз жақын ара халиф бол,violent

4,біз ұзақ соғысқа дайынбыз олар келесі айда тағы әскер дайындауы керек италия ештеңе істей алмайды bcoz осы әскер осы айда өлтіріледі куфарларға ескертеміз қазір біздің көпшілік қауым бізге қосылып жиһадқа дайындалып жатыр өткенде афганистанда немесе талибан болады деп ойлану керек ал қазір ауғанстанның жартысы taliban bcoz біз талибанға қосылып жатқан адамдарды көпшілікті көрдік,біз ұзақ соғыс дайын олар келесі айда тағы әскер дайында керек италия ештеңе істе алма bcoz осы әскер осы айда өлтір куфар ескер қазір біздің көпшілік қауым бізге қосыл жиһад дайындал жатыр өткенде афганистан немесе талибан бол де ойлан керек ал қазір ауғанстан жарты taliban bcoz біз талибан қосыл жат адам көпшілік көр,violent

4,қосымша зымыран шабуылдары бұл американдықтардың соңғы жылдардағы ең үлкен қателігі болады пәкістан жұртшылығы бұларды жек көреді және бұл оны одан сайын арттырады бұл шабуылдар олар үшін аздап сәттілікке ие болуы мүмкін бірақ болашақта моджахедтерге иншаллаға пайдасы тиеді олар өздерінің зұлым торына түсуге мәжбүр,қосымша зымыран шабуыл бұл американдық соңғы жыл ең үлкен қателіг бол пәкістан жұртшылығ бұл жек көр және бұл оны одан сайын арттыр бұл шабуыл олар үшін аздап сәттілік ие бол мүмкін бірақ болашақ моджахед иншалла пайда ти олар өз зұлым тор түс мәжбүр,violent

4,джихади білімін алуға арналған балалар даярлау мектебі жоғары сапалы мб http rapidshare com files ining wmv html http www megaupload com a zp ixf http www mediafire com vcq zitco http depositfiles com en files vufp v http www zshare net download http hotfile com dl be ining wmv html http www multiupload com hjaqvs co дұғаларыңызда есте сақтаңыз дандеерофпак,джихади біл ал арнал бала даярлау мектеб жоғары сапалы мб http rapidshare com files ining wmv html http www megaupload com a zp ixf http www mediafire com vcq zitco http depositfiles com en files

vufp v http www zshare net download http hotfile com dl be ining wmv html http www multiupload com hjaqvs со дұға ес сақта дандеерофпак,violent

4,мен бұлар талибан мужахидтер джихадқа дайындалып жатқанын және олардың көпшілігі толық дайын екенін айттым,мен бұлар талибан мужахид джихад дайындал жат және олардың көпшіліг толық дайын екен ай,violent

4,крест жорықтары бізде жиһад көшбасшылары болған бұл сидна алидің уақыты умар ибн абдул азиздің уақыты саладин уақыты және ибн тамияның уақыты шах исмаилдың уақыты бұл абдулла азамның уақыты немесе шых усаманың кезі бізде әрқашан көшбасшылар болған ал егер басшылар қайтыс болса біз соғыс кезінде де жаңа пайғамбар жасаймыз егер бұл ақымақтардың лидерлері болмаса олардың біреуі ватикада екіншісі германияда мама бар англияда олардың атасы бар олардың жетекшісі дейді бірақ олардың бәрі жай тозақтың отыны,крест жорық біз жиһад көшбасшы бол бұл сидн али уақыт ум ибн абдул азиз уақыт саладин уақыт және ибн тамия уақыт шах исмаи уақыт бұл абдулл аза уақыт немесе шых усама кез біз әрқашан көшбасшы бол ал егер басшы қайтыс бол біз соғыс кез де жаңа пайғамбар жаса егер бұл ақымақ лидер болма олардың біреу ватика екінші германия ма бар англия олардың ата бар олардың жетекші де бірақ олардың бәрі жай тозақ отын,violent

4,цитата алдымен аюби lol карачидегі қанша командир бар полис әрқашан талибанның басты қолбасшысын тұтқындағанын айтты кешіріңіз менің қазақша кешіріңіз пара сияқты оларда негізгі командир айлық квоталар бар толтырады ал кейбір кедей пахтон бағаны төлейді карачидегі полиция мен mqm ppp үкіметі қарапайым қылмыскерлер мен бандиттер,цита алд аюби lol карачи қанша командир бар полис әрқашан талибан басты қолбасшы тұтқында ай кешір менің қазақша кешір пара сияқты ол негізгі командир айлық квота бар толтыр ал кейбір кедей пахтон баға төле карачи полиция мен mqm ppp үкімет қарапайым қылмыскер мен бандит,violent

4,пәкістан халқы бұдан әрі қорлауды жалғастыра береді біздің иззаим тек джихадта,пәкістан халқ бұдан әрі қорлау жалғастыр бер біздің иззаи тек джихад,violent

4,хизбул ислам мужахидтері адамды таспен атып өлтіреді графикалық http www huffingtonpost com html,хизбул ислам мужахид адам тас ат өлтір графикалық http www huffingtonpost com html,violent

4,мен шейх авлакидің дәрістерінің бірінде ол римдіктерді батыс еуропалықтар американдықтар ұрыпсоғу үшін сізге көптеген ереуілдер қажет мұсылмандар оларға бірнеше рет соққы беруі керек бірақ парсыларға сафавидтер мажоздар бір хадис айтқан рафафид ирактың жолдан таюшылары бұл тек бірекі соққыны қажет етеді субханалла мужахидтер римдіктерге көптеген соққылар берді фаллужа рамади шайқасы мосул шайқасы ерекше жебе оңтүстік бағдад және басқалары енді олар жеңіліске ұшырады енді осы парсыларды жеңу үшін бізге бірекі соққы керек ирак үкіметі ирактағы және басқа елдердегі мужахидтерге көмектесу дәйексөз ояту мүшелерінің балалары әкелерінің қабіріне түкіреді ал қабірлерден өткен кезде олар

мен әкем сияқты бұл өмірден кетпеймін сатқын шайх абу умар әлбағдади субханалла джазакалла бауырымыз біздің шейхті хабардар етіңіз дәйексөз шебиб алты ай бұрын ояту жауынгерін бақылайтынын айтты бүгін ол толық уақытты жауынгерлердің санаулы бөлігі қалды деп мәлімдеді,мен шейх авлаки дәріс бір ол римдік батыс еуропалық американдық ұрыпсоғ үшін сізге көптеген ереуіл қажет мұсылман оларға бірнеше рет соққы беруі керек бірақ парсы сафавид мажоз бір хадис айт рафафид ирак жолдан таюшы бұл тек бірек соққы қажет ет субханалл мужахид римдік көптеген соққы бер фаллуж рамади шайқас мосул шайқас ерекше жебе оңтүстік бағдад және басқа енді олар жеңіліс ұшыр енді осы парсы жеңу үшін бізге бірек соққы керек ирак үкімет ирак және басқа ел мужахид көмектес дәйексөз оят мүше бала әке қабі түкір ал қабір өткен кезде олар мен әке сияқты бұл өмір кетпе сатқ шайх абу ум әлбағдади субханалл джазакалл бауыр біздің шейх хабардар ет дәйексөз шебиб алты ай бұрын оят жауынгер бақыла ай бүгін ол толық уақыт жауынгер санаулы бөліг қал де мәлімде,violent

4,джуба бір адам деп ойламаймын іаі сайтынан цитата бағдад мергені джихадтың символы және оның бірі ол жараланған немесе өлтірілген деген қауесеттер болған бұл жаңалықтар қаншалықты дәл іаі жетекшісі багдад снайпері бұл біздің әскеріміздің бригадаларының бірі және бірдебір адам емес егер ол жараланса немесе өлтірілсе онда оның жұмысы аяқталады дегенмен багдад снайперінің саны бір адамнан үлкен алла бағдаттың мергені әлі күнге дейін белсенді және сіз осында және сол жерде жау әскерлерінің мергені туралы ести аласыз және біздің армиямыз осы мергендердің құрметті үлесін алады және жау мойындамайтын көбіне оның джуба деп аталатын стиліне сәйкес келеді джуба бейнелеріндегі барлық снайперлерді бір адам жасаған деп ойлайтын адамдарға жағымды тұтастай алғанда иаи құлдырауы төмен деңгейде сондықтан мергендер де болады демек джуба жақсы болған жоқ,джуб бір адам де ойлама іаі сайт цита бағдад мерген джихад символ және оның бірі ол жаралан немесе өлтір де қауесет бол бұл жаңалық қаншалықты дәл іаі жетекші багдад снайп бұл біздің әскер бригада бірі және бірдебі адам емес егер ол жаралан немесе өлтіріл онда оның жұмыс аяқтал дегенмен багдад снайп сан бір адам үлкен алл бағдат мерген әлі күн дейін белсенді және сіз осында және сол же жау әскер мерген туралы ести ал және біздің армия осы мерген құрметті үлес ал және жау мойындама көбіне оның джуб де атал стил сәйкес кел джуб бейне барлық снайпер бір адам жаса де ойла адам жағымды тұтаста ал иаи құлдыра төмен деңгей сондықтан мерген де бол демек джуб жақсы бол жоқ,violent

4,біз мұсылмандардың бәрін қай жерде болса да соғыс басталған жерде өлтіреміз сондықтан бос жұмыс жасай беріңіз,біз мұсылман бәрін қай же бол да соғыс бастал же өлтір сондықтан бос жұмыс жаса бер,violent

4,келесі жолы буканың баласын аламыз,келесі жол бука бала ал,violent

4,нигериядағы көптеген мұсылмандар көздерін ашады үндеулерге құлақ асады және жиһадқа қосылады,нигерия көптеген мұсылман көз аш үндеу құлақ ас және жиһад қос,violent

4,қош келдіңіз бауырым буба нигериядан біз ол жақтағы бауырластарымыздың көтерілуін көптен күттік альхамдулилла олар өздерінің тарихын ешқашан ұмытпауы керек усман дан фодио және ол орнатқан имара біздің заманымызда осман бар қайдасың бауырлар нигериядағы қатал және тәжірибелі миллиондаған мұсылман және мужахидтер қайда африка күтуде сомалиге дейінгі барлық жерлерді жаулап алғанша шығысқа шығыңыз қайдасың тавхид бригадалары,қош кел бауыр буб нигерия біз ол жақ бауырлас көтеріл көп күт альхамдулилл олар өз тарих ешқашан ұмытпа керек усман дан фодио және ол орнат имар біздің заман осман бар қай бауыр нигерия қатал және тәжірибелі миллиондаған мұсылман және мужахид қайда африка күт сомали дейінгі барлық жер жаула алғанш шығыс шығ қай тавхид бригада,violent

4,менің айтайын дегенім егер алькаиданың әрбір мүшесі өлсе ол әлі де жиһадқа шақыруды тоқтата алмайды өйткені джихад қиямет күніне дейін жалғасады жиһад кезкелген қозғалыспен шектеліп қалмайды керісінше идеологиялық күрес болып табылады,менің айт де егер алькаида әрбір мүше өл ол әлі де жиһад шақыру тоқта алма өйткені джихад қиямет күн дейін жалғас жиһад кезкел қозғалыс шектел қалма керісінше идеологиялық күрес бол таб,violent

4,иншааллах биылғы жылы йемен одан да көп жылық болады раббымыз араб түбегіндегі алькаиданың жетекшілерін қорғаңыз раббымыз мужахидтерге ең жақсы екі нәрсенің бірін беріңіз жеңіс немесе шейіт болу раббымыз араб түбегіндегі ирактағы ауғанстан мен пәкістандағы сомалидегі кавказдағы исламдық мағрабтағы және бүкіл әлемдегі мүжахидтерге жеңіске жету раббымыз сионисткрестшілерді және жолдан таюшыларды масқаралаңыз олардың жоспарларын құртып олардың арасына алауыздық себіңіз және олардың құдіретінің иллюзиясын өшіріңіз амииииииииииин,иншааллах биылғы жыл йемен одан да көп жылық бол рабб араб түбег алькаида жетекші қорға рабб мужахид ең жақсы екі нәрсе бір бер жеңіс немесе шейіт болу рабб араб түбег ирак ауғанстан мен пәкістан сомали кавказ исламдық мағраб және бүкіл әлем мүжахид жеңіс жету рабб сионисткрестші және жолдан таюшы масқарала олардың жоспар құрт олардың ара алауыз себ және олардың құдір иллюзия өшір амииииииииииин,violent

4,салем алейкум саид бурятский говорят моджахеды бурятиядан келген саид абу саадтың және басқа да моджахедтердің қысқа баяндамалары жүктеу mb wmv http blip tv file get kavkazcentermodj talkin wmv gp http blip tv кавказ орталығында жылдың наурызында саид абу саадтың айтқан сөзінің аудармасы мен ондай дәрежеде қатысқан жоқпын сондықтан иса өзін кафыровтың өзі жек көруге лайық бірақ ол мені неге жек көреді дәлелден гөрі кофарларға қарағанда күшті сондықтан кейде сөздер оқтан гөрі күшті болады бірақ бізде иса біздің ежелгі муһахид және оны мен әрине деп санайтын адам бірақ біз алланың алдында ешкімді мадақтамаймыз қалай болғанда да біз рамзан владимировичке айтқымыз келеді біз аспандарды тіректерсіз тірілткен ұлы аллаға ант етеміз біз онымен келісkenше оны жалғастырамыз,сале алейку саид бурятски говорят моджахед бурятия кел саид абу саад және басқа да моджахед қыс баяндама жүкте mb wmv http blip tv file get kavkazcentermodj talkin wmv gp http blip tv

кавказ орталығ жыл наурыз саид абу саад айт сөз аудар мен ондай дәреже қатыс жоқ сондықтан ис өз кафыров өзі жек көр лайық бірақ ол мен неге жек көр дәлелден гөрі кофар қара күшті сондықтан кейде сөз оқ гөрі күшті бол бірақ біз ис біздің ежелгі муһахид және оны мен әрине де сана адам бірақ біз алла алд ешкі мадақтама қалай болғанда да біз рамзан владимирович айт кел біз аспан тірект тірілт ұлы алла ант ет біз онымен келіскенш оны жалғастыр,violent

4,пәкістандағы соңғы бейбітшілік жерін қиратып тастау керек сондықтан усама бин ладен чатралда жасырынып жатыр егер ол сонда болса оның көрінісі ұнайтын бірақ ол ол жерде емес деп ойлаймын қыркүйектегі шебер усама бин ладен пәкістанның солтүстігінде яғни читраль алқабында жасырынып жатыр дейді ақш сарапшылары http www paktribune com news index php id,пәкістан соңғы бейбітшілік жер қират тастау керек сондықтан уса бин ла чатрал жасырын жатыр егер ол сонда бол оның көрініс ұна бірақ ол ол же емес де ойла қыркүйек шебер уса бин ла пәкістан солтүстіг яғни читраль алқаб жасырын жатыр де ақш сарапшы http www paktribune com news index php id,violent

4,дәйексөз бастапқыда fighter жариялаған шаукат dawn newsқа бұл қызға жасалған қарымқатынас әскери қызметкердің үйлену ұсынысынан бас тартқаны үшін жаза екенін айтты ислам еркектің де әйелдің де өз қалауы бойынша неке құруға таңдау жасауына ислам шекарасы мен шектеулеріне еркіндік берді ешкімнің өз еркімен басқа адамға тұрмысқа шығуға мәжбүрлеуге немесе талап етуге құқығы жоқ ислам мен талибандағы жағдай ислам заңдарын орындауға алаңдаулы олар ешқашан мұндай әрекетті жасамайды адамды үйлендіруге мәжбүрлеу және бас тартқан жағдайда жазалау бұл мунафиктердің пікірі бойынша және оқиғаны кейде өзгерту үшін алланың заңдарын бұрмалауға тырысып жатқан талибанды теріске шығарыңыз осыны біліңіз бұл талибанды мазақ ету үшін тағы бір жалғандық бұл лас журналистерді ең алдымен басынан айыру керек тақуалықты бұрмалауға тырысатындардың барлығына алланың қарғысы түссін,дәйексөз бастапқы fighter жарияла шаукат dawn news бұл қыз жасал қарымқатынас әскери қызметк үйлен ұсыныс бас тарт үшін жаза екен ай ислам еркек де әйел де өз қала бойынша неке құр таңдау жаса ислам шекара мен шектеу еркіндік бер ешкі өз ерк басқа адам тұрмыс шығ мәжбүрле немесе талап ет құқығ жоқ ислам мен талибан жағдай ислам заң орында алаңдаул олар ешқашан мұндай әрекет жасама адам үйлендір мәжбүрлеу және бас тарт жағдай жазалау бұл мунафик пікір бойынша және оқи кейде өзгерту үшін алла заң бұрмала тырыс жат талибан теріс шығ осы біл бұл талибан мазақ ету үшін тағы бір жалға бұл лас журналист ең алд бас айыр керек тақуалық бұрмала тырыс барлығ алла қар түс,violent

4,ассалауму алейкум бұл орыстар негізінен ауғанстандағыдай жоғалды және құйрығымен аяқтарының арасына қашып кетті деп айтады енді міне жаудың бірінші жауы екені анық орыс тілі бұдан былай болмайды бірақ олар муртадтарға ақшамен мылтықпен және с көмек береді бірақ біз олардың қаншалықты жек көретінін білеміз мен кавказдық моджахедтер үшін жақсы жылды көріп отырмын дагестан да орыстар

да бар бұл кавказ әмірлігінің құрамына кіреді бұл мәселені шешу керек орыстар екінші шешен соғысын бастау үшін дағыстандық мұсылмандарды қорғауға қолданды джихад кавказда шариғат болғанға дейін жалғасады,ассалаум алейку бұл орыс негіз ауғанстандағыда жоғал және құйрығ аяқ ара қаш кет де айт енді міне жау бірінші жау екен анық орыс тіл бұдан былай болма бірақ олар муртад ақша мылтық және с көмек бер бірақ біз олардың қаншалықты жек көр біл мен кавказ моджахед үшін жақсы жыл көр отыр дагес да орыс да бар бұл кавказ әмірліг құрам кір бұл мәселе шешу керек орыс екінші шешен соғыс бастау үшін дағыстандық мұсылман қорға қолда джихад кавказ шариғат бол дейін жалғас,violent

4,ассаламу алейкум менің түсінуімше бұл хабарландырудың басты себебі мужахиддер енді қауіп емес шын мәнінде олар соңғы уақытта ичкерияда күшейе түсті аллаһу акбар емес керісінше ресей қашып бара жатыр ақша мен олардың экономикасы құлдырау алдында тұр және шешенстандағы антитеррорлық операцияны аяқтағандықтан олар ақшаны үнемдеу үшін шешенстандағы әскерлерінің жартысын шығармақшы яғни олар өздерінің жеңілгендерін жасыру үшін және өздерінің күштерінің үлкен санын алып тастау үшін жеңісті жариялайды дәйексөз сонымен қатар мен кавказдың кеңдігі мен ресейдің кбр дагестан ингушетияға деген көзқарасы туралы ойланамын және б бұл провинцияларға келетін болсақ ресей әлі де апта сайын терроризмге қарсы ісқимылдарды және операцияларды жариялайды ешнәрсе өзгерген жоқ тек ресейліктер дагестан мен ингушетиядағы күн санап өсіп келе жатқан мужахидтерге қарсы тұру үшін көбірек әскер жинай алмайды,ассалам алейку менің түсінуімш бұл хабарландыр басты себебі мужахид енді қауіп емес шын мән олар соңғы уақыт ичкерия күшей түсті аллаһ акб емес керісінше ресей қаш бар жатыр ақша мен олардың экономика құлдырау алд тұр және шешенста антитеррорлық операция аяқта олар ақша үнемдеу үшін шешенста әскер жарты шығармақш яғни олар өз жеңіл жасыр үшін және өз күш үлкен сан алып тастау үшін жең жарияла дәйексөз сонымен қатар мен кавказ кеңдіг мен ресей кб дагес ингушетия де көзқарас туралы ойлан және б бұл провинция кел бол ресей әлі де апта сайын терроризм қарсы ісқимыл және операция жарияла ешнәрсе өзгер жоқ тек ресейлік дагес мен ингушетия күн сана өс келе жат мужахид қарсы тұру үшін көбірек әскер жина алма,violent

4,әзірге мүжахидтер бұған қатысты мәлімдеме жарияламады бірақ иншалла олар жақында болады мен оны оқуды естуді көруді күте алмаймын келесі және маңызды қадам қазір шариғатты орындау,әзірге мүжахид бұған қатысты мәлімдеме жариялам бірақ иншалл олар жақында бол мен оны оқу есту көру күт алма келесі және маңызды қадам қазір шариғат орындау,violent

4,абайлаңыз қарусыз бұл аймақтағы экономикалық құлдырау жағдайында дағыстанның жергілікті тұрғындарына кем дегенде мыңнан астам жұмыссыздар бар және кафирлер билігіне қарсы көтерілістерге арналған кеңестер тізімі азықтүлік дәрідәрмектер генераторлар сатып алыңыз қаружарақтар мен қаружарақтарды сатып алыңыз наразылықтарға қатыспаңыз егер орыстар толық көлемде әскери шабуылға

шақырса мужахидтердің қатарына қосылуға және күресуге дайын болыңыз,абайла қару бұл аймақ экономикалық құлдырау жағдай дағыстан жергілікті тұрғын кем де мың астам жұмыс бар және кафир билiг қарсы көтеріліс арнал кеңес тізім азықтүлік дәрідәрмек генератор сат ал қаружарақ мен қаружарақ сат ал наразылық қатыспа егер орыс толық көлем әскери шабуыл шақыр мужахид қат қосыл және күрес дайын бол,violent

4,шешенстандағы соғыс аяқталды деп айтқанымен ханкаладағы орыс әскерінің белгісіз дереккөзі әскери операциялар мен диверсиялық әрекеттерді соның салдарынан адам өлтірілген және жараланған көпірлер мен жолдан таюшыларды ведено секторындағы мүжахидтер жасағанын айтты тек соңғы айда эхо мовскийге дереккөздің айтуынша моджахедтер тіпті грозныйға шабуыл жасамақшы деген қауесет бар,шешенста соғыс аяқта де айт ханкала орыс әскер белгісіз дереккөз әскери операция мен диверсиялық әрекет соның сал адам өлтір және жаралан көпір мен жолдан таюшы ведено сектор мүжахид жаса ай тек соңғы айда эхо мовский дереккөз айт моджахед тіпті грозный шабуыл жасамақш де қауесет бар,violent

4,ассаламу алейкум исламабадтағы муртад режимі біздің жерімізді жұқтырған көптеген карза малис және б сияқты сатылым режимі екендігінің тағы бір дәлелі бұлар лас паистандар бұдан былай бұл режимнің билігін құрметтеудің қажеті жоқ өйткені шариғат бойынша бұл режимнің күші бірінші кезекте жарамсыз болып табылады ол қазір немесе ешқашан,ассалам алейку исламабад муртад режим біздің же жұқтыр көптеген карз малис және б сияқты сатылым режим екендіг тағы бір дәлел бұлар лас паистан бұдан былай бұл режим билiг құрметтеу қажет жоқ өйткені шариғат бойынша бұл режим күш бірінші кезек жарамсыз бол таб ол қазір немесе ешқашан,violent

4,дәйексөз бірақ ол федералды тергеуді қолдайтынын айтты біз үйде тұратын адамдар туралы алаңдауымыз керек деді ол сіз оны түсінбейсіз бірақ ол орындалып жатыр сіз түсінбедіңіз өйткені сіз кафир екенсіз,дәйексөз бірақ ол федерал тергеу қолда ай біз үй тұр адам туралы алаңда керек де ол сіз оны түсінбе бірақ ол орындал жатыр сіз түсінбе өйткені сіз кафи екен,violent

4,израиль және үндістан екеуі де лаңкестердің елі екеуі де мұсылмандардың қанын жейді кашмир мұсылмандардың жүрегі hay stupid india сіз заманауи технологияларға қарсы тұра алмайсыз сіз уақыт бомбасында отырасыз кезкелген уақытта жарылыс болады,израиль және үндістан екеу де лаңкес ел екеу де мұсылман қан же кашмир мұсылман жүрег hay stupid india сіз заманауи технология қарсы тұр алма сіз уақыт бомба отыр кезкел уақыт жарылыс бол,violent

4,кофарлардың ең үлкен әскері де олардың қуыршақтары да күйзеліске түсетініне сенімдімін мужахидтер зиндабад,кофар ең үлкен әскер де олардың қуыршақ да күйзеліс түс сенімді мужахид зиндабад,violent

4,үндістан мен израиль мен ақшқа өлім,үндістан мен израиль мен ақш өлім,violent

4,өтірікші иншаллаһ ол аманесен американдықтар amp олардың одақтастары amp олардың қуыршақтары өтірікші,өтірікш иншаллаһ ол аманесен американдық amp олардың одақтас amp олардың қуыршақ өтірікш,violent

4,бұл орыстардың жылағанын және оның қолымен мойнын жауып тастағанын көру өте қызықты болды сондықтан мужахид тек сорғышты ұрды,бұл орыс жыла және оның қол мойн жау таста көру өте қызықты бол сондықтан мужахид тек сорғыш ұр,violent

4,жаһандық ислам медиа майданы жолдан тайған жолдан тайғандардың тозағын ұсынады http ansar fliggo com video dc hxwm,жаһандық ислам медиа майдан жолдан тай жолдан тай тозағ ұсын http ansar fliggo com video dc hxwm,violent

4,альандалус медиа қабыл аймағында жолдан тыс күзетшілерге шабуыл жасау иншалла біз тозақтың отына кірген иттерге жасалған шабуылдың салауаты болғанын көреміз ansar,альандалус медиа қабыл аймағ жолдан тыс күзетші шабуыл жасау иншалл біз тозақ от кірген ит жасал шабуыл салауа бол көр ansar,violent

4,цитата бастапқыда абдулла мужахид ала кули хаалин бұл төрт жаңалықтың басты бағыттардан эвакуацияны көру таңқаларлық емес пе мужахидтерден бұл лас куфаар қосылыстарды тастап осы аудандардан көшіп кеткендігі туралы жаңалықты кез келген адам растай ала ма бауырым сіз рамбо деп аталатындарды күніне сағат бомбалаудан шаршамайсыз ба,цита бастапқы абдулл мужахид ал кули хаалин бұл төрт жаңалық басты бағыт эвакуация көру таңқаларлық емес пе мужахид бұл лас куфа қосылыс таста осы аудан көш кеткендіг туралы жаңалық кез кел адам раста ал ма бауыр сіз рамбо де атал күн сағат бомбалау шаршама ба,violent

4,инша аллаһ келесі жарылыс мүжахидтер ал немесе одан да көп қара су террористері өліп тозаққа түседі,инш аллаһ келесі жарылыс мүжахид ал немесе одан да көп қара су террорис өл тозақ түс,violent

4,бұл жігіттің қорғаныс үшін жалбарынуы жалғандық емес пе бірақ менің ойымша ол бәріне ұқсайды ғой иншалла бізде жеңіс болады бәрібір кафферлер бізді тоқтата алмайды ол тек көп құрбан болғалы жатыр,бұл жігіт қорғаныс үшін жалбарын жалға емес пе бірақ менің ойымша ол бәрін ұқса ғой иншалл біз жеңіс бол бәрібір каффер біз тоқта алма ол тек көп құрбан бол жатыр,violent

4,дәйексөз цензураланған альджазира электронды поштасына press int aljazeera net талибан соғыста жеңіске жетіп тарихта жеңіске жетіп жатыр сонымен қатар олар көптеген крестшілер мен муртадилерді қалағанша өлтіреді,дәйексөз цензуралан альджазир электронды пошта press int aljazeera net талибан соғыс жеңіс жет тарих жеңіс жет жатыр сонымен қатар олар көптеген крестші мен муртади қалағанш өлтір,violent

4,машаллах иншаллах біз соғыста жеңеміз,машаллах иншаллах біз соғыс же,violent

4,лалаланд бұл барлық қарақшылар шығады гангстердің шоғыры шабаабтың қайда басқаратынын қараңыз қарақшылар жоқ па не бірақ олар лаңкестер егер шабаба

терроризм туралы болса ол кезде бәріміз террорист болайық,лалаланд бұл барлық қарақшы шығ гангс шоғы шабааб қайда басқар қара қарақшы жоқ па не бірақ олар лаңкес егер шабаб терроризм туралы бол ол кезде бәрі террорист бол,violent

4,қош келдіңіз үндістан қош келдіңіз иншаллаһ бұл соғыс үндістанда таралған amp ақыры жеңіс моминдіктерге жетеді иншалла ауғанстан хиндус зиратына да барады,қош кел үндістан қош кел иншаллаһ бұл соғыс үндістан тарал amp ақы жеңіс моминдік жет иншалл ауғанстан хиндус зират да бар,violent

4,асалам аликум ағайындылар мен апалысіңлілер ағайабальджаббар бұл вазиристан мужахидтерінің ескі бейнесі мен бұл бейнені ай бұрын көрдім бірақ уайымдамаңыз иншааллах мен сізді іздеуге тырысамын өйткені мен бұл видеоны тауып беремін иншалла мен оны орналастырамын ал бауырлас сиваслимуахид кафир мен муртадты құрту керек иншалла кафирлерде исламның мүжахидтеріне қарсы қолданатын әскері долларлары мен технологиялары бар бірақ кафир исламның мүжахидтерін жоя алмайды,асала алику ағайынды мен апалысіңлі ағайабальджабб бұл вазирис мужахид ескі бейне мен бұл бейне ай бұрын көр бірақ уайымдама иншааллах мен сіз ізде тырыс өйткені мен бұл видео тау бер иншалл мен оны орналастыр ал бауырлас сиваслимуахид кафи мен муртад құрт керек иншалл кафир ислам мүжахид қарсы қолдан әскер доллар мен технология бар бірақ кафи ислам мүжахид жоя алма,violent

4,пакистан армиясына шабуыл жасауға рұқсат етіледі олар куфр құлдары,пакис армия шабуыл жаса рұқсат ет олар куф құл,violent

4,уа минут сайын ак туралы не айтуға болады адамдар өте оғаш маған қару өте ұнайды оларды жақсы көремін бірақ оларды өлтіруге дайынмын тіпті менің екі ұлым да қаружараққа әуес,уа минут сайын ак туралы не айт бол адам өте оғаш маған қару өте ұна ол жақсы көр бірақ ол өлтір дайын тіпті менің екі ұл да қаружарақ әуес,violent

4,барлығына демократиялық хама ұнайды маған сеніңіз бауырым тіпті жұма және шейіт мұсылмандарға мешітке шабуыл жасай алмады мен барлық адамдарды және олардың қол жеткізушілерін жек көремін,барлығ демократиялық ха ұна маған сен бауыр тіпті жұма және шейіт мұсылман мешіт шабуыл жаса алма мен барлық адам және олардың қол жеткізуші жек көр,violent

4,міне біз бастаймыз иншалла біз әлақсаға айналамыздағы яһудилердің кесілген бастарымен кездесеміз біз қазір халифаның құлауынан бұрынғыдай алькудты азат етуге жақынбыз біз тағы да халифаны құруға жақынбыз үмбетіміз оянуда альхамдулла,міне біз баста иншалл біз әлақса айналамыз яһуди кес бас кездес біз қазір халифа құлау бұрынғыдай алькуд азат ет жақын біз тағы да халифа құр жақын үмбет оян альхамдулл,violent

4,біз ұзақ соғысқа дайынбыз,біз ұзақ соғыс дайын,violent

4,мен пәкістандық генералдардың осылай өлтірілгенін қалаймын,мен пәкістандық генерал осылай өлтіріл қала,violent

4,іңіз қалаған нәрсеге сеніңіз бірақ мен осы айда дан астам amerikkkans тозаққа жіберілгеніне сенімдімін amerikkkандардың өлімінің бірденбір себебі бұл қорқақтар өздерінің жасыл аймақ деп аталатын жерлерінде жасырынып оларды ирактық мұжахидтерге қарсы күресу үшін қалдырады сондықтан муртадтарға шабуыл жасауды күшейтеді,іңіз қала нәрсе сен бірақ мен осы айда дан астам amerikkkans тозақ жіберіл сенімді amerikkkан өлім бірденбі себебі бұл қорқақта өз жасыл аймақ де атал жер жасырын ол ирак мұжахид қарсы күресу үшін қалдыр сондықтан муртад шабуыл жасау күшейт,violent

4,дәйексөз бастапқыда zumaraa ассалаам алейкум уа рахматуллаһи вабаракатаһ құрметті сүйікті бауырлар мен әпкелер сондықтан қазір соғыс қиын шақта басталады біз бауырымызды өзіміз қаржылық жағынан және дуаның қолдауымен қолдауымыз керек біз бауырларымыздың шақыруына дайын болуымыз керек сізге қаржылай қалай көмектесе аламын егер менде шынайы мужахидтерге және олардың отбасыларына дарулкуфрлардан көмек көрсетудің қандай да бір тәсілі болса менімен кім бола алады,дәйексөз бастапқы zumaraa ассалаа алейку уа рахматуллаһи вабаракатаһ құрметті сүйікті бауыр мен әпке сондықтан қазір соғыс қиын шақ бастал біз бауыр өз қаржылық жағ және дуа қолдау қолда керек біз бауыр шақыр дайын бол керек сізге қаржылай қалай көмекте ал егер менде шынайы мужахид және олардың отбасы дарулкуфр көмек көрсету қандай да бір тәсіл бол менімен кім бола ал,violent

4,дәйексөз барлық адияға моджахедтерді осы крест жорығына және оның муртадтың көмекшілеріне қарсы берік цемент қабырғаға айналдырыңыз қаскүнемдер мен соғысушылардың жүректеріне қорқыныш ұялатып наразылықтар мен бөлулер олардың жүректеріне еніп кетсін бізге білімімізге сай әрекет ету үшін жиһад фисабиллилә жерлеріне хиджра жасау үшін байлық пен қаражат беріңіз мұжахидтердің жүректеріне және жүректеріне траншеяларда беріктік орнатыңыз және жойылу мен қолайсыздыққа қарсы аяқтарыңызға берік болыңыз,дәйексөз барлық адия моджахед осы крест жорығ және оның муртад көмекші қарсы берік цемент қабырға айналдыр қаскүнем мен соғысушы жүрек қорқыныш ұялат наразылық мен бөлу олардың жүрек ен кет бізге білім сай әрекет ету үшін жиһад фисабиллилә жер хиджр жасау үшін байлық пен қаражат бер мұжахид жүрек және жүрек траншея берік орна және жойыл мен қолайсыздық қарсы аяқ берік бол,violent

4,егер яһудилер жазықсыз адамдарды өлтіруге жеткілікті күш алса олар жазықсыз адамдарды өлтіруді ешқашан тоқтатпайды біз әлдеқайда күшті болуымыз керек және яһудилер бүкіл мұсылманды өлтірмес бұрын біз үлкен ядролық бомбаны мг гидрогендік бомба жарып жіберуге тиіспіз,егер яһуди жазық адам өлтір жеткілікті күш ал олар жазық адам өлтіру ешқашан тоқтатпа біз әлдеқайда күшті бол керек және яһуди бүкіл мұсылман өлтірмес бұрын біз үлкен ядролық бомба мг гидроге бомба жар жібер тиіс,violent

4,уа пәкістан мұсылмандары өздеріңнің мұжахидтеріңе қосылыңдар оларға көмектесіп қолдау көрсетіп демеушілік жасаудан және олармен бірге соғысудан аулақ болыңдар абул язидтің қолданған тілі әлкаида тобының екінші әмірі айман

әлзавахиридің өткен тамыз айында пәкістан халқына жолдаған жолдауында көп қолданылатынға ұқсайды әл завахири сол кездегі пәкістан үкіметін сынап пәкістандықтарды джихадтық қозғалысты қолдауға шақырды,уа пәкістан мұсылман өз мұжахид қосыл оларға көмектес қолдау көрсет демеушілік жасау және олармен бірге соғысу аулақ бол абул язид қолдан тіл әлкаи тоб екінші әмір айман әлзавахири өткен тамыз ай пәкістан халқ жолда жолдау көп қолданыл ұқса әл завахири сол кез пәкістан үкім сына пәкістандық джихад қозғал қолда шақ,violent

4,шиалар муртадтар ал барлық муртадтарды өлтіру керек,шиа муртад ал барлық муртад өлтір керек,violent

4,тозақ отына көп отын,тозақ от көп отын,violent

4,ағай маған бұл ұсыныс өте ұнайды біз оны жаңарта да қолдана да аламыз мойындаймын мен жаман адаммын бандитит террорист жақсы сонымен мен террористпін бірақ сіз оларды орыстар мен американдықтар қалай атайтын едіңіз егер олар конституциялық тәртіпті ұстанушылар болса егер олар антитеррорист болса онда мен осы келісімдер мен жағымды сөздерге түкірдім егер бүкіл әлем маған түкірсе мен бүкіл әлемге түкім келеді,аға маған бұл ұсыныс өте ұна біз оны жаңар да қолдан да ал мойында мен жаман адам бандитит террорист жақсы сонымен мен террорист бірақ сіз ол орыс мен американдық қалай ата ед егер олар конституциялық тәртіп ұстанушы бол егер олар антитеррорист бол онда мен осы келісім мен жағымды сөз түк егер бүкіл әлем маған түкір мен бүкіл әлем түк кел,violent

4,біздің мужахидтер ағзасы жақсы ұйым менің амирім командир мохаммад амирул муминийдің қол астында қазір мен ақш пен оның қуыршақтарына қарсы жиһадта жүрмін біз тауға көшіп келеміз талибанның көшбасшылығы туралы білмеймін ал біз амһахидпіз ал біздің амир біздің көмекшіміз қолдаушымыз және күш жинауышымыз біз тек олардан тек жеңілдіктер мен материалдарды ғана алғымыз келеді мен барлық мұсылман бауырлар мен апа деп айта аламын иран бізді ешқашан ақшасыз қамтамасыз етпейді олар мужахидтерге тосқауыл қояды иранда тонна кофе бар олар керруби мунафикун ретінде мосави жақтаушылары иран армиясында көптеген кофарлар бар бірақ біз шынайы адамдарды білеміз олар қаружарақты қытайлық қару сатушы ретінде шай ішкен кезде ғана сата алады иран әскері ешқашан қару сатпайды өйткені олардың қаруы қажет жиһадқа кірмес бұрын кейбіріміз джихад ақшамызды таптық,біздің мужахид ағза жақсы ұйым менің ами командир мохаммад амирул муминий қол аст қазір мен ақш пен оның қуыршақ қарсы жиһад жүр біз тау көш кел талибан көшбасшылығ туралы білме ал біз амһахид ал біздің ами біздің көмекші қолдаушы және күш жинауыш біз тек олардан тек жеңілдік мен материал ғана ал кел мен барлық мұсылман бауыр мен апа де ай ал иран біз ешқашан ақшасыз қамтамасыз етпе олар мужахид тосқауыл қоя иран тонна кофе бар олар керруби мунафикун рет мосави жақтаушы иран армия көптеген кофар бар бірақ біз шынайы адам біл олар қаружарақ қытайлық қару сатушы рет шай іш кезде ғана сат ал иран әскер ешқашан қару сатпа өйткені олардың қар қажет жиһад кірмес бұрын кейбір джихад ақша тап,violent

4,asalamualykum уганда мен бурундиде жиһадты қолдайтын мұсылмандар көп олар ояту керек мен ауданда бір мұсылман бауырымнан бурундидегі мұсылмандардың пайызы кем емес деп естідім бұл құрайды деп мәлімдейді,asalamualykum уган мен бурунди жиһад қолда мұсылман көп олар оят керек мен аудан бір мұсылман бауыр бурунди мұсылман пайыз кем емес де есті бұл құра де мәлімде,violent

4,салем алейкум аға ақпаратпен бөліскеніңіз үшін рахмет мен сіздің вебсайтыңызға кірдім mash allah жақсы жұмыс жасаңыз инчаллах егер инша аллаһ израиль мен рафидии сафави режимі бірбірімізді өлтірсе біз ps мекенжайын бөлісуді өз мойнына алады,сале алейку аға ақпарат бөліс үшін рахмет мен сіздің вебсайт кір mash allah жақсы жұмыс жаса инчаллах егер инш аллаһ израиль мен рафидии сафави режим бірбі өлтір біз ps мекенж бөлісу өз мойн ал,violent

4,олар масқара шошқаны өлтірмеген келесі жолы біз оны өлтіреміз,олар масқара шошқа өлтірме келесі жол біз оны өлтір,violent

4,можахидтердің әртүрлі топтарының осы қиын уақытта бірге болмағаны өкінішті және өкінішті бұл нағыз мужжахид және оппортунист кім екендігіңізді анықтайтын нақты сынақ егер мен оппортунист сөзін қолданып біреуді ренжіткен болсам кешірім сұраймын өйткені олар үшін басқа лайықты сөз таба алмадым сонымен бірге діни партиялар мен басқа діни топтардың ттпға ашық түрде көмек көрсететін уақыты келді дәрістер емес ісәрекеттер уақыты келді мульрассада жүрген ульма мен олардың мыңдаған студенті шығып ашық түрде жиһадқа қосылуы керек әйтпесе олар өмір бойы өздерін кінәлі сезінеді мен ол күн келмейді деп үміттенемін,можахид әртүрлі топ осы қиын уақыт бірге болма өкініш және өкініш бұл нағыз мужжахид және оппортунист кім екендіг анықта нақты сынақ егер мен оппортунист сөз қолдан біреу ренжіт бол кешір сұра өйткені олар үшін басқа лайықты сөз таб алма сонымен бірге діни партия мен басқа діни топ ттп ашық түр көмек көрсет уақыт кел дәріс емес ісәрекет уақыт кел мульрасса жүр уль мен олардың мыңдаған студент шығ ашық түр жиһад қосыл керек әйтпесе олар өмір бойы өз кінәлі сезін мен ол күн келме де үміттен,violent

4,салам алейкум ва рахматуллаһ ва баракату исламдағы бауырлар мен әпкеқарындастар бұл қайғылы жағдай бұл барлық мұсылмандар кезкелген сәтте шабуыл жасауға дайын болу керек екенін көрсетеді сіз өзіңізді және отбасыңызды қорғауға қабілетті болуыңыз керек қарындастың күйеуін полиция да атып тастады бірақ мен басқаша сөйлескім келеді бірақ бұл өте маңызды сенуші адам заңға сәйкес шешім шығаратын судьяның алдына сот отырысын талап етпейді бұл жағдайда бұл мәселе және ол құқықты алу туралы мәселе емес құжаттар ажырасу ақша бұлар исламдағы таухид деп аталатын негіз және қағидалар,сала алейку ва рахматуллаһ ва баракат ислам бауыр мен әпкеқарындас бұл қайғылы жағдай бұл барлық мұсылман кезкел сәт шабуыл жаса дайын болу керек екен көрсет сіз өз және отбас қорға қабілетті бол керек қарында күйе полиция да ат таста бірақ мен басқаша сөйлес кел бірақ бұл өте маңызды сенуші адам заң сәйкес шешім шығар судья алд сот отырыс

талап етпе бұл жағдай бұл мәселе және ол құқық алу туралы мәселе емес құжат ажырасу ақша бұлар ислам таухид де атал негіз және қағида,violent

4,сайлау ширк бүкіл саясаткерлер үшін өлім біз емтихан мен исламдық көшбасшылық әдісті қолдануымыз керек,сайлау ширк бүкіл саясаткер үшін өлім біз емтихан мен исламдық көшбасшылық әдіс қолдан керек,violent

4,ирактағы ең үлкен нысана ұлттық күштер мен барзани және талапани отбасылық кафтар оларды мужахидтер өлтіріңіз,ирак ең үлкен нысана ұлттық күш мен барзани және талапани отбасылық каф ол мужахид өлтір,violent

4,өкінішке орай жәбірленушілер де айыппұлшылар да судьялар төрешілер мұсылмандар бұл нағыз мұсылмандар үшін жиіркенішті оқиға және ояну үндістандағы және басқа да көптеген елдердегі кедей және надан мұсылмандардың жағдайы өкінішті менің ойымша біз оларға білім беру үшін жиһад жасауымыз керек осы кедей жандарды шынайы исламның қатарына қосу үшін,өкініш орай жәбірленуші де айыппұлшы да судья төреші мұсылман бұл нағыз мұсылман үшін жиіркеніш оқиға және оян үндістан және басқа да көптеген ел кедей және надан мұсылман жағдай өкініш менің ойымша біз оларға білім беру үшін жиһад жаса керек осы кедей жан шынайы ислам қат қосу үшін,violent

4,біз түрік мұсылманбыз және біз бүкіл мұсылман арасындағы татулықты көргіміз келеді біз кафтерлердің қуыршақтарын жек көреміз біз пакистан мұсылмандарын жақсы көреміз өйткені олар барлығын түрік тәуелсіз соғысы кезінде жібереді пакистан мұсылмандары үшін бірдеңе болған кезде барлығын жібереміз пакистандық мұсылмандарға қауіп төнген кезде біз өзіміздің сенімімізбен пакистан армиясына қосыла аламыз соңғы он жылда пакистан исламға шабуыл жасап ақштан тапсырыс алуда мен пакистан армиясының қаружарақтарын мұжахидтерге беріп яһудилер мен ақшқа өлім біз барлық жақсы нәрсені шығара аламыз ақш кафтар саудасы және кафтар әскерімен қан сорып жатыр,біз түрік мұсылман және біз бүкіл мұсылман ара татулық көр кел біз кафтер қуыршақ жек көр біз пакис мұсылман жақсы көр өйткені олар барлығ түрік тәуелсіз соғыс кез жібер пакис мұсылман үшін бірде бол кезде барлығ жібер пакиста мұсылман қауіп төн кезде біз өз сенім пакис армия қосыл ал соңғы он жыл пакис ислам шабуыл жаса ақш тапсырыс ал мен пакис армия қаружарақ мұжахид бер яһуди мен ақш өлім біз барлық жақсы нәрсе шығар ал ақш каф сауда және каф әскер қан сор жатыр,violent

4,дәйексөз бастапқыда sivaslimucahid мен әр жарылғыш зат шығара аламын сіз моджахедтер үшін сенімдісіз бауырым мен сіз қазір құстардан алыс өмір сүресіз деп үміттенемін,дәйексөз бастапқы sivaslimucahid мен әр жарылғыш зат шығар ал сіз моджахед үшін сенімді бауыр мен сіз қазір құс алыс өмір сүр де үміттен,violent

4,сол себепті шошқа етін пешке тастау керек,сол себеп шошқа ет пеш тастау керек,violent

4,барлығы исламдық қозғалысты қолдау үшін ақша мен қару жинауы керек,барлығ исламдық қозғал қолдау үшін ақша мен қару жина керек,violent

4,жихад мұсылманның намазы қажылық савам жақа тәрізді парызы,жихад мұсылман намаз қажылық сава жақ тәрізді парыз,violent

4,салам alikom амин амин амин қош келдіңіз бортқа ағайынды моак бейбітшілік acer,сала alikom амин амин амин қош кел борт ағайынды моак бейбітшілік acer,violent

4,олардың отбасы бүкіл әлемдегі мұсылмандарды шындыққа айналдырғандай қайғыдан қайтыс болсын мұсылман балаларын шындыққа айналдырғандай оларсыз балалар өссін иншаллах көп ұзамай олар шығады және ислам мемлекеті куфтардан босатылып ресми түрде орнықтырылады обама көктемге дейін қосымша әскер жібермейді егер ол арыстандарға көбірек ет берсе,олардың отбасы бүкіл әлем мұсылман шындық айналдырғанда қайғы қайтыс бол мұсылман бала шындық айналдырғанда ол бала өс иншаллах көп ұзама олар шығ және ислам мемлекет куф босатыл ресми түр орнықтыр оба көктем дейін қосымша әскер жіберме егер ол арыстан көбірек ет бер,violent

4,біздің қоғамда әділетсіздік пен әлеуметтік зұлымдық болған кезде қазір ешбір орган джихадты тоқтата алмайды доп домаланады және күн санап күшейе түсуде жолда кішкентай соққылар болуы мүмкін бірақ мақсат сөзсіз иншааллах егер біз болмасақ біздің кейінгі ұрпағымызда тақуалық қоғам өмір сүре алады,біздің қоғам әділетсіздік пен әлеуметтік зұлымдық бол кезде қазір ешбір орган джихад тоқта алма доп домалан және күн сана күшей түс жолда кішкентай соққы бол мүмкін бірақ мақсат сөзсіз иншааллах егер біз болма біздің кейінгі ұрпағ тақуалық қоғам өмір сүр ал,violent

4,ертең тозаққа жіберіледі,ертең тозақ жібер,violent

4,сол мультфильмдерді басып шығаруға қатысқандар үшін кек алу үшін тағы да мереке өтеді,сол мультфильм бас шығар қатыс үшін кек алу үшін тағы да мереке өт,violent

4,егер ол сауд арабиясында болса онда мен оны түрмеге түсіру үшін жақсы мүмкіндік бар деп ойлаймын,егер ол сауд арабия бол онда мен оны түрме түсір үшін жақсы мүмкіндік бар де ойла,violent

4,біз оларға шабуыл жасауымыз керек және кофарларға не болатынын көрсетуіміз керек,біз оларға шабуыл жаса керек және кофар не бол көрсет керек,violent

4,хайялел жиһад hayyalel жиһад,хайялел жиһад hayyalel жиһад,violent

4,уганда бұл күлкілі олар өз елдерінде азап шегіп жатқанда клик олар сомалиде тыныштықты қалайды бурундиді хуту мен тутсидің жері халықтың кедейлік шегінен төмен жері олар өздерінің харам тағамдарын тұншықтырады деп үміттенемін,уган бұл күлкіл олар өз ел азап шег жатқанда клик олар сомали тыныштық қала бурунди хут мен тутси же халық кедейлік шег төмен же олар өз харам тағам тұншықтыр де үміттен,violent

4,дәйексөз машаллла жақында бізде арабша сөйлейтін шешен террористі болады күлімсіреп,дәйексөз машаллл жақында біз арабш сөйле шешен террорис бол күлімсіре,violent

4,қолға түскен американдық шошқалар туралы жаңалықтарды тарату керек біз бұл сөзді қарапайым мұсылманға жеткізуіміз керек өйткені бұл қорқынышты әрекет және мужахедтердің беделіне нұқсан келтіреді әр блогта жазып қолымыздағы кезкелген бақта жазуы керек мысалы газеттер мен басқа пікірлер,қол түс американдық шошқа туралы жаңалық тарат керек біз бұл сөз қарапайым мұсылман жеткіз керек өйткені бұл қорқыныш әрекет және мужахед бедел нұқсан келтір әр блогта жаз қолымыз кезкел бақ жаз керек мысал газет мен басқа пікір,violent

4,мужахидтердің кезкелген үлкен тобы нысана болып табылады тіпті қарапайым мұсылмандар мысалы бомба қойылған барлық үйлену тойлары мен жерлеу рәсімдерін ұмытпаңыз,мужахид кезкел үлкен тоб нысана бол таб тіпті қарапайым мұсылман мысал бомба қойыл барлық үйлен той мен жерлеу рәсім ұмытпа,violent

4,дәйексөз менің проблемам бұл көптеген мұсылмандар өздері тұрып жатқан диктатураның тұзағына түсіп жатқандығында мен мароккодан египеттен сауд арабиясынан және б мен шейхке ол өмір сүріп жатқан диктатураны жабық ұстаған кезде ғана шабуыл жасайтын шейхті тыңдағым келмейді біз сияқты үкіметтер парсыларға бірінші шабуыл жасау керек деп санайды өздерінің диктатурасы билікте қала алады жақсы байқау ахи бұл қазіргі кезде жиі кездеседі,дәйексөз менің проблема бұл көптеген мұсылман өз тұр жат диктатура тұзағ түс жатқандығ мен марокко египет сауд арабия және б мен шейх ол өмір сүр жат диктатура жабық ұста кезде ғана шабуыл жаса шейх тыңда келме біз сияқты үкімет парсы бірінші шабуыл жасау керек де сана өз диктатура билік қала ал жақсы байқау ахи бұл қазіргі кезде жиі кездес,violent

4,америка мен еуропа дайын болыңдар біз келеміз иншааллах,америка мен еуропа дайын бол біз кел иншааллах,violent

4,ооо сіз крест жорықтары мүйізді сорғымен ұрады біз сіздің қалаңызға бомбамен және қылыштармен тапсырамыз бәрінің кек алу үшін сабырлы болыңыз немесе өткір болыңыз анфал қабылдаймыз иншааллах,ооо сіз крест жорық мүйіз сорғы ұр біз сіздің қала бомба және қылыш тапсыр бәрі кек алу үшін сабырл бол немесе өткір бол анфал қабылда иншааллах,violent

4,сәлем машаллах біз бұл шабуылдың сәтті болуын тілейміз және исламдық үмбетті күшті ету,сәлем машаллах біз бұл шабуыл сәтті бол тіле және исламдық үмбе күшті ету,violent

4,жақсы айтылған бауырлас көтерілісшілер мен ағайынды абуаррах жеткілікті ескертуге ие болды және шабуыл умманы күшейтеді,жақсы айтыл бауырлас көтерілісші мен ағайынды абуаррах жеткілікті ескерт ие бол және шабуыл умма күшейт,violent

4,субханаллаһ егер мен сол жерде тұрсам соққыға жығыл,субханаллаһ егер мен сол же тұр соққы жығыл,violent

4,ассаламу алейкум бұл менің күнімді жасады олардың саперлары өте нашар немесе мужахидтер оларға жақсы тұзақ жасады қалай болғанда да бұл жақсы нәтиже ирактың мосул үшін саперлары көбінесе жоғалып кеткен сияқты мен былтыр шиит жасақтары берген бағдаттың жарылғыш заттары туралы ойланамын біреу біледі уа алейкум ассалам,ассалам алейку бұл менің күн жас олардың сапер өте нашар немесе мужахид оларға жақсы тұзақ жас қалай болғанда да бұл жақсы нәтиже ирак мосул үшін сапер көбіне жоғал кет сияқты мен былтыр шиит жасақ бер бағдат жарылғыш зат туралы ойлан біреу біл уа алейку ассала,violent

4,ассаламалейкум машаллах керемет жаңалық осы форумдар арқылы куфарлардың насихатына қарсы тұру үшін аллаһ ғ бізге электронды джихадты насихаттауға көмектессін амин,ассаламалейку машаллах керемет жаңалық осы форум арқылы куфар насихат қарсы тұру үшін аллаһ ғ бізге электронды джихад насихатта көмектес амин,violent

4,ассаламу алейкум варахматуллаһи вабаракатух дәйексөз бастапқыда автор abu yazid ассаламалейкум машаллах керемет жаңалық осы форумдар арқылы куфарлардың насихатына қарсы тұру үшін аллаһ ғ бізге электронды джихадты насихаттауға көмектессін амин амин алдағы оқиғалар жақын арада болады деп үміттенемін инша аллах,ассалам алейку варахматуллаһи вабаракатух дәйексөз бастапқы автор abu yazid ассаламалейку машаллах керемет жаңалық осы форум арқылы куфар насихат қарсы тұру үшін аллаһ ғ бізге электронды джихад насихатта көмектес амин амин алдағы оқиға жақын ара бол де үміттен инш аллах,violent

4,цитата алғашында censored жариялаған тедби және кадыровтың суреті келесі жолы моджахедтер амирулмуртадтарды жою үшін сәтті болады иншалла,цита алғаш censored жарияла тедби және кадыров сурет келесі жол моджахед амирулмуртад жою үшін сәтті бол иншалл,violent

4,quot келесі жолы мүжахидтер амирулмуртадилерді жою үшін сәтті болады иншалла амин,quot келесі жол мүжахид амирулмуртади жою үшін сәтті бол иншалл амин,violent

4,цитата алдымен көтерілісші жариялады бүгін олар әділеттілік туралы сөйлескен кезде бізді тоқтата алады бірақ ағаштар мен тастардың осы сионистік шошқаға қарсы сөйлейтін уақыты және әрбір мұсылманның осы шошқаларды өшіру уақыты және біздің қанымызды басатын уақыт бұл шошқалар онша алыс емес,цита алд көтерілісш жариял бүгін олар әділеттілік туралы сөйлес кезде біз тоқта ал бірақ ағаш мен тас осы сиони шошқа қарсы сөйле уақыт және әрбір мұсылман осы шошқа өшір уақыт және біздің қан бас уақыт бұл шошқа онша алыс емес,violent

4,забихулла мужахид барлау өкілі негізсіз деп мәлімдейді біз қуыршақ барлау бөлімінің пәкістанның вазиристан агенттігінде өткен айда әділет министрлігіне

шабуыл жасау жоспарланған деген шағымын жоққа шығардық ислам эмираттарының альхамза майетердом кандак батальоны операциясы ауғанстанның тәуелсіз аудандарында жоспарланған болатын шабуылдаушылар басқа емес ауғандықтар болды операцияға қатысқандардың ешқайсысы ұсталмады немесе шейіт болды операцияны жоспарлаушылар аманесен оралды және шабуылдарға көп дайындалды біз жақын арада өзіміздің жұмысты дәлелдегіміз келеді,забихулл мужахид барлау өкіл негіз де мәлімде біз қуыршақ барлау бөлім пәкістан вазирис агенттіг өткен айда әділет министрліг шабуыл жасау жоспарлан де шағ жоқ шығар ислам эмират альхамз майетердо кандак батальон операция ауғанстан тәуелсіз аудан жоспарлан бол шабуылдаушы басқа емес ауғандық бол операция қатыс ешқайсы ұсталм немесе шейіт бол операция жоспарлаушы аманесен ора және шабуыл көп дайында біз жақын ара өз жұм дәлелде кел,violent

4,ассаламу алейкум уа рахматуллаһи ва баракатух бисмилла альхамдулиллах амма баад сіздер америка құрама штаттары ауны толығымен ақша мен барлық қаражатпен айдап жатқанын білесіздер олар қазір тез қимылдауы керек al шабаб және хизбул ислам мен батыстық немесе батыстық бақтарға альшабабтың белгілері бар және барлық мужахидтердің белгілеріне ғана сене алмаймын,ассалам алейку уа рахматуллаһи ва баракатух бисмилл альхамдулиллах ам баад сіздер америка құрама штат ау толығ ақша мен барлық қаражат айда жат біл олар қазір тез қимылда керек al шабаб және хизбул ислам мен бат немесе бат бақ альшабаб бел бар және барлық мужахид белгі ғана сен алма,violent

4,сәлем өздерін әлдеқайда күшті деп санайтын еврейлер мен христиандарға және осы мунафиктерге сауд арабиясының үкіметі қарғыс атсын,сәлем өз әлдеқайда күшті де сана еврей мен христиан және осы мунафик сауд арабия үкімет қарғыс ат,violent

4,ассаламу алейкум мұсылман мүжахидтер мен иншалла арасында шиеленісті тудыруға тырысатын ахмед мадобе деп аталатын адамның арасындағы уайым ол алстабаб спастрасы шейх али дере хафидахулалармен бірге кисмайо және джубба облыстарында жаңа әкімшілік жариялады олар ахмед мадобе миллиондарына қарсы соғыс жариялады және хизбул исламға бұл адамды фитна жасағысы келетінін қолдайтынын ескертті хизбулислам мадобе олардың жетекшілерінің бірі деп жауап берді және иншалла бұл аяқталады және исламдық жубба провинциясы қауіпсіз болып қалады және осы діннің сатқындары жубба аймағында исламдық әкімшілдік бауырластарына және олардың әмірлеріне дуаттарыңызды қолдайды шейх абу бакар алсайли,ассалам алейку мұсылман мүжахид мен иншалл ара шиелен тудыр тырыс ахмед мадоб де атал адам ара уай ол алстабаб спастра шейх али дер хафидахула бірге кисмайо және джубб облыс жаңа әкімшілік жариял олар ахмед мадоб миллион қарсы соғыс жариял және хизбул ислам бұл адам фитн жаса кел қолда ескер хизбулисла мадоб олардың жетекші бірі де жауап бер және иншалл бұл аяқтал және исламдық жубб провинция қауіпсіз бол қал және осы дін сатқын жубб аймағ исламдық әкімші бауырлас және олардың әмір дуат қолда шейх абу бак алсайли,violent

4,салам алейкум уа рахматуллаһ мен балаларды өлтіремін деп ойлаймын өйткені олар бір күні джихадты тоқтатудың әдісі деп ойлайды армандаңыз дәйексөз алла олардың азабын азайтып қайғықасірет шегуге көмектессін,сала алейку уа рахматуллаһ мен бала өлтір де ойла өйткені олар бір күн джихад тоқтат әдіс де ойла арманда дәйексөз алл олардың аза азайт қайғықасірет шег көмектес,violent

4,шошқаны жейтіндер семсерді көргенде үрейленуі керек,шошқа же семс көргенде үрейлен керек,violent

4,салам алейкум олар ең терең тозақта жансын http ansar net showthread php t,сала алейку олар ең терең тозақ жан http ansar net showthread php t,violent

4,егер американдықтар мен одақтастар ауғанстаннан кетсе онда талибан ағайындар жерді қайта басқарып алланың рұқсатымен илаһи заңды қайта орнатады егер олар кетпесе онда оларды жалғыз өлі және тозақ күтіп тұр,егер американдық мен одақтас ауғанстан кет онда талибан ағайын же қайта басқар алла рұқсат илаһи заңды қайта орнат егер олар кетпе онда ол жалғыз өлі және тозақ күт тұр,violent

4,ассалауму алейкум алькаиданың мүжахидтері өздерінің жиһадтарын кафтарларға қарсы мәңгілікке сақтайды ауғанстаннан кетсе де американдықтар мен яһудилер біздің жауымыз,ассалаум алейку алькаида мүжахид өз жиһад кафтар қарсы мәңгілік сақта ауғанстан кет де американдық мен яһуди біздің жау,violent

4,инша аллаһ олардың қалауымен пәкістандық муртадилер мен үнді кафтерлерінің арасында соғыс болады олардың бәрі бірбірін өлтірсін сонымен жолымыз бізді танып білуге мүмкіндік береді амин,инш аллаһ олардың қалау пәкістандық муртади мен үн кафтер ара соғыс бол олардың бәрі бірбір өлтір сонымен жол біз тан біл мүмкіндік бер амин,violent

4,ассалаумағалейкум осы кафир полицияның нағыз шошқаларының бетіне қараңызшы индонезия пәкістан ирак ауғанстан кавказ ценрала сия фалистин сомали магреб шам джазира туркиста филиппин бангладеш пен кулли маканда жойсын амин,ассалаумағалейку осы кафи полиция нағыз шошқа бет қараңызш индонезия пәкістан ирак ауғанстан кавказ ценрал сия фалистин сомали магреб шам джазир туркис филиппин бангладеш пен кулли макан жой амин,violent

4,алла барша американдық армияны жойсын,алл барша американдық армия жой,violent

4,егер олар шынымен мұсылманның қамын ойласа онда оларды неге өлтіреді германиядағы шошқа сияқты,егер олар шын мұсылман қам ойла онда ол неге өлтір германия шошқа сияқты,violent

4,менің арманым бар және сол түсімде мен осы бауырластардың алдында куфр мен тагутқа қарсы күресу үшін тұрамын,менің арман бар және сол түс мен осы бауырлас алд куф мен тагут қарсы күресу үшін тұр,violent

4,дәйексөз бастапқыда сайфулла әлшимали саламоалейкум уа рахматуаллахи ва баракату менің білместігімді жояды бірақ жаңа жылда не ерекше уәлейкум ассалаумағалейкүм жаңа жыл мұхаррам ағайын пакистандағы шиеттің тозығы олар жартастарының астынан шығып көшеге соғылған кезде және біз сәйкесінше оларды нысанаға аламыз бұл біздің дәстүріміз әрбір мухаррамда біз хусейн мен алиді масқаралап рафиди террористерін нысанаға аламыз сипахи сахаба мен лашкари джангви бұл ең жаман қорқыныш лашкареджангви зындабад sipahesahaba zindabad джайишмухаммад зындабад,дәйексөз бастапқы сайфулл әлшимали саламоалейку уа рахматуаллахи ва баракат менің білместіг жоя бірақ жаңа жыл не ерекше уәлейку ассалаумағалейкү жаңа жыл мұхарра ағайын пакиста шие тозығ олар жартас аст шығ көше соғыл кезде және біз сәйкесінше ол нысана ал бұл біздің дәстүр әрбір мухарра біз хусейн мен али масқарала рафиди террорис нысана ал сипахи сахаб мен лашкари джангви бұл ең жаман қорқыныш лашкареджангви зындабад sipahesahaba zindabad джайишмухаммад зындабад,violent

4,салем тағы ақымақ шошқалар жіберіледі шошқа кедей американдық,сале тағы ақы шошқа жібер шошқа кедей американдық,violent

4,алла осы үлкен мұртты кафир шошқаларды жойсын индиялықтар,алл осы үлкен мұр кафи шошқа жой индиялық,violent

4,иншаллах зардари шошқа ең ауыр өлімге душар болады,иншаллах зардари шошқа ең ауыр өлім душ бол,violent

4,алейкум сәлем аға амин содан кейін моуджахидтер оларды үндістердің әскеріне қарсы қолдана алады содан кейін келесі мақсат қытай әскерінің шошқалары,алейку сәлем аға амин содан кейін моуджахид ол үндіс әскер қарсы қолдан ал содан кейін келесі мақсат қытай әскер шошқа,violent

4,альхамдулилла джихад барлық жерде күшейе түсуде ашылып жатқан барлық жақтарға соның ішінде көпшілігі күтпеген жақтарына қараңыз біз барлық жердеміз және біз барлық жерде күресуіміз керек иншаллаһ буштың айтқан сөздерінің бірі онда олармен соғысайық сондықтан біз олармен бұл жерде күресуге мәжбүр емеспіз олардың орнына барлық жерде олармен күресейік иншалла,альхамдулилл джихад барлық же күшей түс ашыл жат барлық жақ соның іш көпшіліг күтпе жақ қара біз барлық жерде және біз барлық же күрес керек иншаллаһ буш айт сөз бірі онда олармен соғыс сондықтан біз олармен бұл же күрес мәжбүр емес олардың орн барлық же олармен күрес иншалл,violent

4,ва алайком салем бауырым өзің туралы жеке ақпарат жариялама бұл күндері өте қауіпті ахи либанондағы хизбулла қозғалысы джихадтық қозғалыс емес мен мұны гизбуль шаитандық қозғалыс деп атаймын олар ұлтшыл біз тек ливанон үшін күресеміз және иранның қылмыстық үкіметі үшін жақсы нәрсе сәлем ансар,ва алайко сале бауыр өзің туралы жеке ақпарат жарияла бұл күн өте қауіпті ахи либано хизбулл қозғалыс джихад қозғалыс емес мен мұны гизбуль шаита қозғалыс де ата олар

ұлтшыл біз тек ливанон үшін күрес және иран қылмыстық үкімет үшін жақсы нәрсе сәлем анс,violent

4,бұл тек бастамасы оңтүстік вазиристанда пәкістандық солдат өлтірілді дәйексөз бірақ талибандар соңғы күндері пәкістанның қауіпсіздік күштерін адамды өлтірді деп мәлімдеді пәкістандағы талибан өкілі азам тарик cnnге телефон арқылы осы уақытта бір талибан сарбазы өлтіріліп үшеуі жарақат алғанын айтты ол талибан пәкістан әскерлерін жол бойындағы бомбалармен және ұрыс кезінде өлтіргенін айтты ол үкіметке қарсы күштердің ресейге қарсы соғыс кезінде алған үлкен саны бар және бұл бізге көптеген жылдар бойы жеткілікті деді ол кеңес одағының жж ауғанстанды жаулап алуы туралы айтып отыр edition cnn com world asiapcf ants index html олардың санынан қорықпаңыз өйткені олар түкке тұрғысыз және шыбын тәрізді ұшудан қорыққыңыз келе ме,бұл тек баста оңтүстік вазиристан пәкістандық солдат өлтір дәйексөз бірақ талибан соңғы күн пәкістан қауіпсіздік күш адам өл де мәлімде пәкістан талибан өкіл аза тарик cnn телефон арқылы осы уақыт бір талибан сарбаз өлтіріл үшеу жарақат ал ай ол талибан пәкістан әскер жол бой бомба және ұрыс кез өлтір ай ол үкімет қарсы күш ресей қарсы соғыс кез ал үлкен сан бар және бұл бізге көптеген жыл бойы жеткілікті де ол кеңес одағ жж ауғанстан жаула ал туралы айт отыр edition cnn com world asiapcf ants index html олардың сан қорықпа өйткені олар түк тұрғы және шы тәрізді ұшу қорық келе ме,violent

4,мен бұл мишриктерді көргенде қиналамын ух олар нажис,мен бұл мишрик көргенде қинал ух олар нажис,violent

4,иншаалла ақыры американың жойылуына сайып келгенде яһудилердің жойылуына әкеледі,иншаалл ақы америка жойыл сай кел яһуди жойыл әкел,violent

4,мына заман өзгерді аттанатын кез келді жүректерде иман оянды найза қанға боянды біздер шықтық мінеки мақсат етіп арманды,мына заман өзге аттан кез кел жүрек иман оян найз қан боя біздер шық мінеки мақсат ет арман,violent

4,абдулрахман әлказақи хижра қылып шам жеріне хижра қылып жиһадқа келдік аллаһ тағала жиһадтың исламның ең биігі екенін көрсетті құранда айтады мужахидтер туралы олардың наградалары туралы айтады жемістері ақиретте не болатыны туралы оларға,абдулрахман әлказақи хижр қыл шам же хижр қыл жиһад кел аллаһ тағал жиһад ислам ең биіг екен көрсе құран айт мужахид туралы олардың награда туралы айт жеміс ақирет не бол туралы оларға,violent

4,абухалид әлказақи құдайға құлшылық етудің шыңы жиһад иншаллаһ мұнда аллаһ жолында жиһад қылуға келдік,абухалид әлказақи құдай құлшылық ету шың жиһад иншаллаһ мұнда аллаһ жол жиһад қыл кел,violent

4,сайф аддин әлказақи бүкіл мұсылмандарға қазір жиһад жасау фард бір қадам жерге жау кірсе бүкіл мұсылмандар қорғануға фард болады ал егер қорғануға шамасы келмесе қастарындағы соседный жерлерге көшеді сөйтіп бүкіл жер шарын алады,сайф аддин әлказақи бүкіл мұсылман қазір жиһад жасау фард бір қадам же

жау кір бүкіл мұсылман қорған фард бол ал егер қорған шама келме қас соседны жер көш сөйт бүкіл жер шарын ал,violent

4,нұх әлказақи құрметті бауырлар мен діндегі сестралар жәннатқа кіруге аллаһпен кездесуге асығыңдар,нұх әлказақи құрметті бауыр мен дін сестра жәннат кір аллаһ кездес асығ,violent

4,міне біз бауырларымызбен балашағаларымызбен отбасыларымызбен жиһад қылуға хижра қылдық аллаһ тағала бізге малдарымызбен жандарымызбен жиһад қылуды бұйырды әлхамдуллиллах міне мынандай құралдарымызбен жиһад қылуға шықтық біз дің өзіміздің араб бауырла ры мызға сәлемдемеміз бар аллаһ айт ты біз құранды араб ті лін де тү сір дік дей келе егер сендер араб тарды айтып отыр теріс бұ рыл саң дар аллаһ сендерді басқа қа уым мен ауыстырады олар сендердей болмайды,міне біз бауыр балашаға отбасы жиһад қыл хижр қыл аллаһ тағал бізге мал жан жиһад қылу бұйыр әлхамдуллиллах міне мынанда құрал жиһад қыл шық біз ді өз араб бауырла ры мыз сәлемдеме бар аллаһ айт ты біз құра араб ті лін де тү сі дік де келе егер сен араб тар айт отыр теріс бұ рыл са дар аллаһ сен басқа қа уы мен ауыстыр олар сендерде болма,violent

4,сайф әддин әлказақи қазір кейбір бауырлар фард кефайр деп алданып қалады ол мүлдем олай емес ғалымдар жаулар бір қадамдай жерге кірсе ол жердегі бүкіл мұсылмандар болып қорғануы керек олар қорғануға шамасы келмесе көрші жерлерге көшеді сөйтіп бүкіл жер шарын алады джихад сириядағы қазақтар ислам насихат каналы,сайф әддин әлказақи қазір кейбір бауыр фард кефай де алдан қал ол мүлдем ола емес ғалым жау бір қадамда же кір ол жер бүкіл мұсылман бол қорған керек олар қорған шама келме көрші жер көш сөйт бүкіл жер шарын ал джихад сирия қазақ ислам насихат канал,violent

4,иншааллах мы жаждим смерти на этом пути,иншааллах мы жажди смерти на это пути,violent

4,осы жолда иншааллах шахид болғымыз келеді,осы жолда иншааллах шахид бол кел,violent

4,ин ша аллах сирияға барамын кафирлерді қырып соғыс кезінде джихадта өлсем арманым жоқ жаннатта кездесейік кафирлерді қырамыз нурдаулет кульчанбаев,ин ша аллах сирия бар кафир қыр соғыс кез джихад өл арман жоқ жаннат кездес кафир қыр нурдаулет кульчанбаев,violent

4,ирактың мадинатуль мосул деген жерінде так сенің басыңды кесетін браттар бар қазахистаныңда,ирак мадинатуль мосул де же так сенің бас кес брат бар қазахистан,violent

4,сол екен гой маймылданып отырганын жакында дин туралы зан шыгад сосын коремиз сендей шайтаннын кулдарын олтиру бизге сауап тозактын ити,сол екен го маймылдан отырган жак дин туралы зан шыгад сосын коремиз сенде шайтанн кул олтир биз сауап тозакт ити,violent

4,жақында джихад болады иншааллах макака абайла,жақында джихад бол иншааллах макака абайла,violent

4,сендердің бөтен дінде деп айыптап жатқандарың иншааллах тура жолда болып шығады амру ибн ауф аллаһ оған разы болсын жеткізген хадисте аллаһтың елшісі аллаһтың оған игілігі мен сәлемі болсын айтты расында бұл дін бөтен күйде басталды және басталғаны секілді бөтен болып қайтады туба жәннаттағы ағаш бөтендер үшін иә аллаһтың елшісі бөтендер деген кімдер деп сұралған кезде ол аллаһтың оған игілігі мен сәлемі болсын менен кейін менің сүннетімді тірілтетіндер және оны адамдарға үйрететіндер деді қараңыз әттирмизи,сен бөтен дін де айыпта жат иншааллах тура жолда бол шығ амр ибн ауф аллаһ оған разы бол жеткіз хадис аллаһ елші аллаһ оған игіліг мен сәлем бол ай рас бұл дін бөтен күй баста және бастал секілді бөтен бол қайт туб жәннат ағаш бөтен үшін иә аллаһ елші бөтен де кім де сұрал кезде ол аллаһ оған игіліг мен сәлем бол менен кейін менің сүннет тірілт және оны адам үйрет де қара әттирмизи,violent

4,о сумелек бизди джихаттан коркады дейсинбесенин козинди жою бизге ен улкен джихат сендер мусылман елинде журип мусылмандарды олтирип журсиндералла сендерге оз жазасын береди тозактын ити сендейлерге сеним жок сендер мксылмандарга букил бале жалени акелетин астагфируллах,о сумелек бизди джихат корк дейсинбесенин козинди жою биз ен ул джихат сен мусылман елин жури мусылман олтири журсиндералл сен оз жаза береди тозакт ити сендей сени жок сен мксылмандарг букил бал жалени акелетин астагфируллах,violent

4,сен джихад жоқ деп өзіңді алдап жүре бер қорқақабдуллах ибн мас уд да будет доволен им аллах сказал тот кто проявил неверие хотя бы одну букву корана тот проявил неверие по отношению нему всему абдурраззакъ альмусаннаф,сен джихад жоқ де өз алда жүр бер қорқақабдуллах ибн мас уд да будет доволен им аллах сказал тот кто проявил невери хотя бы одн букв коран тот проявил невери по отношению нем всем абдурраззакъ альмусаннаф,violent

4,тозақтың иттерісіндер такфирлар мен хауариж,тозақ иттерісін такфир мен хауариж,violent

4,иншааллах біз аллах жолында джихад жасаймыз сендердің сөгістеріңнен қорықпаймыз джихад қияметке дейін тоқтамайды биизниллах бізді күтіңдер біз барамыз елдеріңе иншааллах кәфірлерге ұнамасада аллах дінін тәмәмдайды ул түл ислам баақия аллаху акбар аллаху акбар аллаху акбар,иншааллах біз аллах жол джихад жаса сен сөгіс қорықпа джихад қиямет дейін тоқтама биизниллах біз күт біз бар ел иншааллах кәфір ұнамаса аллах дін тәмәмда ул түл ислам баақия аллах акб аллах акб аллах акб,violent

4,сонда сендер пайғамбаодың сирасын оқымағансыңдарма немесе аллахтың менің жолымда күресіңдер деген аятын жоққа швғарасыңдарма сонда мына аяттар напсіңмен күрес деп тұрма умар бин хаттаб айтқан құран оұитындар көп ал сол құранға амал ететіндер аз деп муджахид сахабалардың кейбіреуінің джихадтан ілім

алуға уақыттары болмаған сен сонда білімді болып тұрып аллахтың жолында джихадқа шықпасаң онда кітап арқалаған ешак боласың міне дәлел,сонда сен пайғамбао сира оқымағансыңдар немесе аллах менің жол күре де аят жоқ швғарасыңдар сонда мына аят напс күрес де тұрма ум бин хаттаб айт құран оұитын көп ал сол құран амал ет аз де муджахид сахаба кейбіреу джихад ілім ал уақыт болма сен сонда білімді бол тұр аллах жол джихад шықпа онда кітап арқала ешак бол міне дәлел,violent

4,ия жібер мені солармен келіп ұстап ал да қамап атып таста,ия жібер мен солар кел ұста ал да қама ат таста,violent

4,а так көп алма өзіңе сол жақтағы браттар арматураның үстіне құлап өліп қалуыңа жәрдем беріп жіберер мен даулатуль исламдамын,а так көп алма өз сол жақ брат арматура үст құла өл қал жәрде бер жібер мен даулатуль ислам,violent

4,оданда анау жалаңаш суреттеріңді қызықтап жүре бер басың жетпейтін іске араласпа,одан анау жалаңаш сурет қызықта жүр бер бас жетпе іс араласпа,violent

4,иншаллаһ мұнда аллаһ жолында жиһад қылуға келдік,иншаллаһ мұнда аллаһ жол жиһад қыл кел,violent

4,ассаламу алейкум аман бауыр олардікі дұрыс емес деп айталасыңба ия қателіктері көп бірақ ислам мемлекетін құрғансоң сосын жайлап жақсы болып кетер иншааллах бұлай кәпірлерге жақсы көрінем деп ойлайсыңдарма кәпірлер ешқашан мұсылмандардан разы болмайды оларды разы қылу үшін кәпір болу керек алладан қорқыңыздар бауырлар,ассалам алейку аман бауыр олардік дұрыс емес де айталасыңб ия қателік көп бірақ ислам мемлекет құрғансо сосын жайла жақсы бол кет иншааллах бұлай кәпір жақсы көріне де ойлайсыңдар кәпір ешқашан мұсылман разы болма ол разы қыл үшін кәпір болу керек алла қорқ бауыр,violent

4,әбу әниса қазақстандағы жұртқа арнап сирия жерінде башарға шииттерге кәпірлер мен демократтарға қарсы соғысып жатқандарын айтып қарулы жиһадқа қосылуға шақырды,әбу әни қазақстандағы жұрт арна сирия же баш шиит кәпір мен демократ қарсы соғыс жат айт қарулы жиһад қосыл шақ,violent

4,бұл бауырыңыз шам жерінде дейризор соғыста шахид болды,бұл бау шам же дейризо соғыс шахид бол,violent

4,біз үшін оның жолында етке айналу құрмет мейлі біз бірнеше бөлікке бөлініп қуырылған ет бөлшегіне айналсақ та осы қалпымызда қайта тірілсек болды неліктен осылай жасағанымызда сұраса біз сен үшін аллах сенің жүзіңді көру үшін аллах деп жауап береміз маулид еменов,біз үшін оның жол ет айналу құрмет мейлі біз бірнеше бөлік бөлін қуырыл ет бөлшег айнал та осы қалп қайта тіріл бол неліктен осылай жаса сұра біз сен үшін аллах сенің жүз көру үшін аллах де жауап бер маулид еменов,violent

4,еууу вахабистер буган не айтасындар буганугыз айтып казактарды томен тусирип сирияга арабияга жете алмайсындар ал жети нагыз мусылмандар олай

жасамайды,еуу вахабис буган не айтасын бугануагыз айт казак то тусири сирияг арабияг же алмайсын ал жети нагыз мусылман ола жасама,violent

4,сириага кеткен адамдар малмен тен аидаганга жүретін дени дурыс адам солаи истима буйтип кырылып исламды коргамаиак коиын арким ози ушин со жургенде нестеп жургендерин оздери билмит жалпы сакалды кауымга жыным келеді,сириаг кет адам мал тен аидаганг жүр дени дурыс адам солаи исти буйти кырыл ислам коргамаиак кои арки ози ушин со жур несте жургендерин оздери билмит жалпы сака кауымг жын кел,violent

4,олсин барган жерлериннен ертен келеди оралс бизди куртады булар джихадт деген аты айитып тур соларды биреу жибердиме казак болса казак болсын сириядан келмесин келсе жаксы болмайды кудай сактасын егерде бауырым кетсе сойтип айтар едим елге киргизбеу керек арты жаксыга апармайды кунен кунге не боп бара жатырмыз арине жаным ашиды биракта омир сургим келеди кайтем мен де киналам бирак кереу емес келмесин елге,олсин барган жерлерин ер келеди оралс бизди курт бу джихадт де ат айит тур сол бире жиберди казак бол казак бол сирия келмесин кел жак болма куда сакта ег бауыр кет сойти айтар еди ел киргизбе керек ар жаксыг апарма ку кун не боп бар жатыр арин жан аши бирак оми сурги келеди кайте мен де кинала бирак кере емес келмесин ел,violent

4,серияага кеткен казактар туриме екен не азап десей осы казакстанла журсенде райга барасынго намазынды окып тынш журсен енди олип калсада олар райга бармайд адам олитрп жатыр акымактар ишим ашиды казактарга барганбизде барин бузды муфтяттагылардын бари сирияда туркияда окып келип зан шыгарып динди бурмалап жиберди баринде сиряга жиберип куртпаса кутылалмайт шыгармыз,серияаг кет казак тури екен не азап десе осы казакстанл журсен райг барасынго намаз ок тынш журсен енди оли калса олар райг бармайд адам олитр жатыр акымак иши аши казактарг барганбиз барин буз муфтяттагылард бари сирия туркия ок кели зан шыгар динди бурмала жиберди барин сиряг жибери куртпа кутылалмайт шыг,violent

4,бир алла биледи ким дурыс ким бурыс ар адам озин дурыстаса бари дурыс болады,би алл биледи ким дурыс ким бурыс ар адам озин дурыста бари дурыс бол,violent

4,аллах кімді адастырса ешкім ол адамды тура жолға салалмайды кімді тура жолға салса оны ешкім адастыралмайды аллах тағлла құранда айтқандай,аллах кім адастыр ешкім ол адам тура жол салалма кім тура жол салса оны ешкім адастыралма аллах тағлл құран айтқанда,violent

4,серияга озин кетси не астарлап сойлеп отсынмайдалап сойлемей анығын айтпайсынба бармау керек иракка тагыдай болып кетпесин деп жихат болса баруга болама соны айтып отсынгой мусылмандар арасында согыс болсадеп озин елин жерин турнанда мусульман ел деп согыска кете беру керекпа,серияг озин кетси не астарла сойле отсынмайдала сойлеме аныг айтпайсынб барма керек иракк тагыда

бол кетпесин де жихат бол баруг болама соны айт отсынго мусылман ара согыс болсаде озин елин жерин турнан мусульман ел де согыск кет беру керекп,violent

4,сириага барып мусулсанды олтиру ушин бармандар ол харам,сириаг бар мусулса олтир ушин барман ол харам,violent

4,башар асадтын басшылыгымен болып жаткан согыс сиякты ол жердеги,баш асадт басшылыг бол жаткан согыс сияк ол жердеги,violent

4,сирияга наемниктер барады сол жердеги олтирип жаткандары капир емес кой ол жердин мусылмандарын олтирип жатыр айелдери мен кыздарын зорлап сатып жатыр кул сиякты ол жерде джихаттын ииси жок еврейдин ойыншыгы боп кетеди,сирияг наемник бар сол жердеги олтири жаткан капи емес ко ол жердин мусылман олтири жатыр айелдери мен кыз зорла сат жатыр кул сияк ол же джихатт ииси жок еврейдин ойыншыг боп кетеди,violent

4,сиряга акша ушин барады отанын ата анасын тастап саляф бауырларымыз коп жолжурп жатыр,сиряг акша ушин бар отан ата ана таста саляф бауыр коп жолжур жатыр,violent

4,короче айтканда бару керек дидианык айталмай отырайтса егер ол бару керек десе интернет дурыс пикирде болмау биледиастарлап сойлеп жатырайелин алып кетип жатыргойанасыда намаз окыса рукстатын алса болады дидимен айтар едим ее дарын елден курып кет недеп сандалап отсынананнан руксат ал айелинди алып кет деп магына келип отыргой,короч айткан бару керек дидианык айталма отырайт егер ол бару керек де интернет дурыс пики болмау биледиастарла сойле жатырайелин алып кети жатыргойанасы намаз окы рукст ал бол диди айтар еди ее дарын ел кур кет неде сандала отсынанан руксат ал айелинди алып кет де маг кели отырго,violent

4,что он раньше подругому говорил типа не надо ехать сирию теперь по другому говорит что надо заметил что многие шейха начали по другому говорить про сирию аллах только открыл им глазаhttp ogms ru watch vp jmk yng darynmubarovishimigishzhnedzhikhadtemayzdy html,что он раньш подругом говорил тип не надо ехать сирию теперь по другом говорит что надо заметил что многи шейх начали по другом говорить про сирию аллах только открыл им глазаhttp ogms ru watch vp jmk yng darynmubarovishimigishzhnedzhikhadtemayzdy html,violent

4,биздн жермиздеги саляфия агымындаглардын барлыгнын тогсатн жери осы игил олар алгашында хизбут тахрир еди аттарын олар ауыстырып отырады олармен ангиме жургзсен оларга отан ел жер улттык намыс салт сана дегенннен макрум екенин байкайсын олар казыргы заманауй саткындар аллах маган оларга хидаят берсн,биздн жермиздеги саляфия агымындаглард барлыгн тогсатн жери осы игил олар алгаш хизбут тахри еди ат олар ауыстыр отыр олармен анги жургзсен оларг отан ел жер улттык намыс салт сана дегенн макру екенин байкай олар казырг заманау саткын аллах маган оларг хидаят берсн,violent

4,бари айтат куранда не жазылгансон булжытпай орындау керек депал джихат ше менин ойымшаол сенбитиндерге жане сенсе де баскаша сенетиндерге солардын айтуынша согыс ашу жане барин кырып олтируоган не айтасыздар,бари айтат куран не жазылгансон булжытпа орындау керек депал джихат ше менин ойымшаол сенбитин жан сен де баскаш сенетин солард айт согыс ашу жан барин кыр олтируоган не ай,violent

4,джихат ол битва за веру муджахедтмусылмандар сол джихадка катысушылар аллах оларга разы болсын,джихат ол битв за вер муджахедтмусылман сол джихадк катысушы аллах оларг разы бол,violent

4,батыстан адай білесіңдер жоқ тез арада білесіңдер жоқ сендер өз көздерің мен көресіңдер ол кезде бәрі кеш болады тырнақ тістеп әттеген ай пайғамбарды ғ тыңдағанымда дерсің дерсің талай дегеннен не шығады бір азапқа мың азап қосылады ойлан бауырым ойлан дініңе не кедергі болып жатыр аллаһтың құлы болудан асқан бақыт барма екен дүниенің жылтырағы мәңгілік жасатады деп ойлайсыңба сендер расында білесіңдер бірақ такаппарланасыңдар тәубеңе кел бауырым аллаһтан қорық аллаһ тағала жүректеріңізге иман берсін,батыс ада біл жоқ тез ара біл жоқ сен өз көз мен көр ол кезде бәрі кеш бол тырнақ тісте әттеген ай пайғамбар ғ тыңда де де тала де не шығ бір азап мың азап қос ойлан бауыр ойлан дін не кедергі бол жатыр аллаһ құл болу ас бақыт барма екен дүние жылтырағ мәңгілік жасат де ойлайсыңб сен рас біл бірақ такаппарлан тәубе кел бауыр аллаһ қорық аллаһ тағал жүрек иман бер,violent

4,джихат дін үшін күрес егер кәпірлер мұсылман бауырларымыздың дініне жеріне еліне жеке бас бостандықтарына қауіп төндірер болса өз еркіндіктері үшін қолдарына қару алулары керек отанды сүймек иманнан отан үшін ел үшін тыныштық үшін күрестердің барлығы да жиһад болып есептелінеді ал жиһадтын үлкені өз нәпсіңмен күрес өз жаман мінезқұлықтарыңмен өз кемшіліктеріңмен күресу қолға қару алудан да қиын,джихат дін үшін күрес егер кәпір мұсылман бауыр дін же ел жеке бас бостандық қауіп төндір бол өз еркіндік үшін қол қару алу керек ота сүй иман отан үшін ел үшін тыныштық үшін күрес барлығ да жиһад бол есептелін ал жиһадт үлкен өз нәпс күрес өз жаман мінезқұлық өз кемшілік күресу қол қару алу да қиын,violent

4,игиш немесе игил оны сионический израиль курган ол организацияны ойткени ол игиш израильдин соперниктарына согыс ашып мысалы сирия хамас хезболла ирак бирак баскада кауипти организацияларда фронт аннусра джайшал ислам веллаят синай тагыда баска типти алкаида оздери игилдан стороняться етип жур бирак ен кауиптиси исламский движения узбекистана игилга присигнуться етти,игиш немесе игил оны сионически израиль курган ол организация ойткени ол игиш израильдин соперник согыс аш мысал сирия хамас хезболла ирак бирак баска кауипти организация фронт аннуср джайшал ислам веллаят синай тагы баск типти алкаи оздери игил стороняться ети жу бирак ен кауиптиси исламски движения узбекистан игилг присигнуться етти,violent

4,там не джихад это геополитика яхудилардын тирлиги бул коз ашу кез келдигой,там не джихад это геополитик яхудилард тирлиги бул коз ашу кез келдиго,violent

4,осы жолда шаһид болғымыз келеді,осы жолда шаһид бол кел,violent

4,жәннатты қалаймыз,жәнна қала,violent

4,аллаһтың приказын орындап наградасын іздейміз,аллаһ приказ орында награда ізде,violent

4,жылы қазанда джунд әлхалифат халифат сарбаздары деп аталған исламшыл топ араб телеарнасына видеожазбасын шығарды,жыл қазан джунд әлхалифат халифат сарбаз де атал исламшыл топ араб телеарна видеожазба шығ,violent

4,ислам революциясы жиһад болып жатыр ислам мемлекеті құрылады арманым сондай мұсылман елде өмір сүру,ислам революция жиһад бол жатыр ислам мемлекет құр арман сондай мұсылман ел өмір сүру,violent

4,мама мен кетіп барамын бәрі бір алланың қалауымен мені кешіріңіздер сіздерді жақсы көремін ақгүлді сізге тапсырдым сирияға кетіп қалдым мені іздемеңіздер мені өлді деп санаңыздар,ма мен кет бар бәрі бір алла қалау мен кешір сіз жақсы көр ақгү сізге тапсыр сирия кет қал мен іздеме мен өл де сана,violent

4,ассалаумагалейкум мусылман бауырлар сириядагы мусылман бауырларымызга комекке баруга шакырамын казак болып мусылман болып комекке барайык,ассалаумагалейку мусылман бауыр сириядаг мусылман бауырларымызг комек баруг шакыр казак бол мусылман бол комек барайык,violent

4,сиз кателесесиз нагыз мусылмандарга жани ашыр буырларымыз сирияда жур кез келген мусылман биздин бауырларымыз мусылман ушин аллаху акпар,сиз кателесесиз нагыз мусылмандарг жани ашы буыр сирия жу кез кел мусылман биздин бауыр мусылман ушин аллах акп,violent

4,абу хамза казахстанскийу кого то может есть знакомые кто готов финансово помочь или закят свой отдать или просто сделать джихад своим имуществом,абу хамза казахстанский кого то может есть знакомы кто готов финансово помочь или закят сво отдать или просто сделать джихад свои имущество,violent

4,уақыт келді ахи,уақыт кел ахи,violent

4,ассалам уалейкум ахи біздің уақыт келді ин ша аллах,ассала уалейку ахи біздің уақыт кел ин ша аллах,violent

4,қанша жүре береміз ахи,қанша жүр бер ахи,violent

4,jandos nurjanov тұхмет қылып жатқан жоқсызба барып көрдіңізба олжақа сахих дәлеліңіз барма әлде сізге солай айт дедіма,jandos nurjanov тұхмет қыл жат жоқсызб бар көрдіңізб олжа сахих дәлел барма әлде сізге солай айт деді,violent

4,ол жақта басқада жихат қылып жатқан бауырлар бар мысалы талибандар жабтахул нусра деген топтарда бар оларға да тиістіма бұл уағызыңыз,ол жақта басқа жихат қыл жат бауыр бар мысал талибан жабтахул нуср де топ бар оларға да тиісті бұл уағыз,violent

4,бұл заңда мемлекеттік орындарда намаз оқып орамал тағып жүруге тыйым салынған егер бұл ұстанымдарыңыздан бас тартпасаңыздар біз сіздерге қарсы әрекет ете бастаймыз,бұл заң мемлекеттік орын намаз оқ орамал тағ жүр тыйым салын егер бұл ұстаным бас тартпа біз сіздерге қарсы әрекет ет баста,violent

4,лагнетке ушыраган тозактын иттери ислам угымын ластап шайтанга кызмет жасап жатыр гой муминдерди жауыз кылып корсетип,лагнет ушыраган тозакт иттери ислам уг ласта шайтанг кызмет жаса жатыр го муминдерди жауыз кыл корсети,violent

4,бауырлар такфирилерхауариждер ихуан муслимин жамағаттары олар да өздерін саляфиміз дейді біздің оларға үш қайнаса сорпамыз қосылмайды жала жауа бермендер аллахтан кішкене ұялыңдар ертең бәріміз аллахтың алдына барамыз жалаға жауап беру оңай болмайды бауырлар біздің дилмурат назратуллах октам ұстаздар бұндай бәлелерден алыста бізге матрудилер қандай бидғатшы болса такфирилер де дәл сондай бидғатшы болып есептелінеді матрудилерді қалай жек көрсек такфирилерді де дәл солай жек көреміз өйткені екеуі де бидғатшылар сақал қойса да балағын қысқартса да тілінде субханаллах машааллах десе де ахи десе де олар бидғатшы болып саналады сол үшін бізді олармен шатастырмаңдар,бауыр такфирилерхауариж ихуан муслимин жамағат олар да өз саляфи де біздің оларға үш қайна сорп қосылма жал жау берме аллах кішкен ұялы ертең бәрі аллах алд бар жала жауап беру оңай болма бауыр біздің дилмурат назратуллах окта ұстаз бұндай бәле алыста бізге матруди қандай бидғатш бол такфири де дәл сондай бидғатш бол есептелін матруди қалай жек көр такфири де дәл солай жек көр өйткені екеу де бидғатшы сақал қой да балағ қысқарт да тіл субханаллах машааллах де де ахи де де олар бидғатш бол санал сол үшін біз олармен шатастырма,violent

4,ассаляму алеикум надо боротся хариджитами такфиритами хизбут тахрир итд они угороза проблема нашего обшества не саляфиты ведь сами имамы говорят они то есть саляфиты не призывают экстремизму сектанству масонству скрытности наборот призывают подчинятся провителю слушать его не нарушать закон рк не итти против системы это из нашей религии сподвижники пророка мир ему блогословение аллах шсли этим пут и саляфиты идут этим пут был провитель хачачь ибн йюсуф он убивал безчинствовал убивал сподвижников но они ему подчинялись это правельно вот этим пут идут саляфиты так вч проблема,ассалям алеику надо боротся хариджитами такфиритами хизбут тахри итд они угороз проблема нашего обшеств не саляфи ведь сами имам говорят они то есть саляфи не призывают экстремизм сектанств масонств скрытности наборот призывают подчинятся провителю слушать его не нарушать закон рк не итти против систем это из наше религии сподвижники пророк ми ем блогословени аллах шсли эти пут и саляфи идут эти пут был провитель хачачь ибн

йюсуф он убивал безчинствовал убивал сподвижников но они ем подчинялись это правельно вот эти пут идут саляфи так вч проблема,violent

4,осы оқиғадағы қайтыс болғандар шаһидтер,осы оқиға қайтыс бол шаһид,violent

4,мүмкін бәріміз қателесіп жүрген шығармыз сериядағылар дұрыс жасап жүрма,мүмкін бәрі қателес жүр шығ сериядағы дұрыс жаса жүр,violent

4,ит екеш ит те тек өз территориясын қорғайды ит құрлы миларыңның болмағаны ма бейшаралар,ит екеш ит те тек өз территория қорға ит құрлы ми болма ма бейшара,violent

4,ай карактарым не адасып журсиндер туган елге кайтындар казахстан дажумыс истейди ахша бермейды митинга шыкса полиции урып олтіреды сосын бари елинин кашады джихад деп,ай карак не адас журсин туган ел ка казахс дажумыс истейди ахш берме митинг шык полиции ур олтіре сосын бари елинин каш джихад де,violent

4,кіші жиһадтан ең үлкен жиһадқа келе жатырмыз,кіші жиһад ең үлкен жиһад келе жатыр,violent

4,ей салаф пидр сен таза емиссингой сен аллахтын кулу емессингойсен харам еврейдин кулусин дилмуратдин кулусинабдугаппас сманов матрудий болсада оз елинде одамдарды жамандыктан кайтариб журсенин дилмурад ағанг сиякты оз елин сатып озге елге кашиб журиб арабтардын котин жалап журген жоктусиндинба алаяк бишара сен мусулман емессин сен фитнаши бузгунши алланин душпани лаинсин салаф ит,ей салаф пид сен таза емиссинго сен аллахт кул емессингойсен харам еврейдин кулусин дилмуратдин кулусинабдугаппас сманов матруди болса оз елин одам жамандык кайтариб журсенин дилмурад ағанг сияк оз елин сат оз ел кашиб журиб арабтард котин жала жур жоктусиндинб алаяк бишар сен мусулман емессин сен фитнаши бузгунши алланин душпани лаинсин салаф ит,violent

4,брат иншааллах он шахид,брат иншааллах он шахид,violent

4,нет бога кроме isis,нет бог кро isis,violent

4,қазақстаннан сирияға жиһад жасау үшін хижра қылдық сирияда соғысып жүрген жихадшылармыз,қазақстан сирия жиһад жасау үшін хижр қыл сирия соғыс жүр жихадшы,violent

4,ин ша аллах бауырлар джәннәтта кездесеміз,ин ша аллах бауыр джәннәт кездес,violent

4,молда сирияда жиһад жоқ дейді даусын өз құлағыммен естігенге дейін ходжи мирзо мұндай сөз айтты дегенге сенбей келдім мұнда жиһад жоқ деп қалай айтасыз мұнда балалар өліп жатыр,мол сирия жиһад жоқ де дау өз құлағ есті дейін ходжи мирзо мұндай сөз ай де сенбе кел мұнда жиһад жоқ де қалай ай мұнда бала өл жатыр,violent

4,бүгін үкіметтің молдасымын деп отырсыз қай үкіметтің алланың заңдарына қарсы шыққан үкіметтің бе халық исламды жамылып отырғаныңызды түсінуі тиіс,бүгін үкімет

молда де отыр қай үкімет алла заң қарсы шық үкімет бе халық ислам жамыл отыр түсін тиіс,violent

4,қазақстанда қалу сол жерде өмір сүру харам,қазақстан қалу сол же өмір сүру харам,violent

4,наши братья джамаат наша цель халифат наш закон шариат наш путь джихад,наши братья джамаат наша цель халифат наш закон шариат наш путь джихад,violent

4,о аллах помоги братьям которые сражаются на джихаде поистине наш господь прощающий милостив милосерден амин амин амин,о аллах помоги братья которы сражаются на джиха поистин наш господь прощающи милостив милос амин амин амин,violent

4,вам что серьезно так сложно выйти на джихад вас руки после этого отвалятся наступит день суда нет валлахи нет тогда почему субханаллах вы не выходите на джихад ещ глаза закатывают да вы кафиры астагфируллах,ва что серьезно так сложно выйти на джихад вас руки посл этого отвалятся наступит день су нет валлахи нет тог почем субханаллах вы не выходи на джихад ещ глаз закатывают да вы кафи астагфируллах,violent

4,халық шәһидтік дәрежесіне жету үшін жихадқа асығатын,халық шәһид дәреже жету үшін жихад асығ,violent

4,бірақ ізгі ниет және жихадқа қатысудың міндеті тоқтаған жоқ,бірақ ізгі ниет және жихад қатыс міндет тоқта жоқ,violent

4,нуршыларды секта дегендер ертен жазган әрбір әрпіне жауап береди иншалла сен ей надан өзің ислам жолында ең болмас бір нәрсе жасап көрдің ба,нуршы секта де ер жазган әрбір әрп жауап береди иншалл сен ей надан өзің ислам жол ең болмас бір нәрсе жаса көр ба,violent

4,қаіүпті бір ағым немесе секта деп айыптап жатыр рисалеи нурдың мақсаты дүние емес тікелей ақырет құран және иман ақиқаттары саясатқа еш қандай қатысы жоқ қоғамның ұлтымыздың жандануы негізінде дінмен байланысті дін өмірдің өмірі әрі негізі әрі нұры пайғамбарлардың көпшілігі шығыстан шығуы және философтардың көпшілігі батыстан шығуы бізге мына нәрсені көрсетеді шығысты аяққа тұрғызатын бұл дін рисалеи нур шәкірттері де одан алған имандылық дәрістері арқылы қоғамға пайдалы үлгілі көркем мінезді біреудің ақысын жемейтін қасиеттерге ие болады,қаіү бір ағым немесе секта де айыпта жатыр рисалеи ну мақсаты дүние емес тікелей ақырет құран және иман ақиқат саясат еш қандай қатыс жоқ қоғам ұлт жандан негіз дін байланыс дін өмір өмір әрі негіз әрі нұр пайғамбар көпшіліг шығыс шығ және философ көпшіліг батыс шығ бізге мына нәрсе көрсет шығ аяқ тұрғыз бұл дін рисалеи ну шәкірт де одан ал имандылық дәріс арқылы қоғам пайдалы үлгілі көркем мінез біреу ақы жеме қасиет ие бол,violent

4,онда өлу үшін барамыз,онда өл үшін бар,violent

4,шындықты іздеп әр жерге бардымғой мен таблиғи жамағата саид нурси де болдым ақыр соңында осында түрмеге келдім,шындық ізде әр же бардымғо мен таблиғи жамағат саид нурси де бол ақы соң осында түрме кел,violent

4,өзімді моджахедпін деп есеатедім аллахтың әскері дейді ғой,өз моджахед де есеате аллах әскер де ғой,violent

4,менің уақытым аз бізді қоршап алды бізден екі квартал төмендегілердің бәрін қиратты мен тірі қалмайтын сияқтымын мама осы жерге келгенім үшін кешірім сұраймын олар бейкүнә балалар мен әйелдерді зорлап жатыр соғыс не үшін және кім үшін болып жатқанын түсінбеймін бұйрықты кім беріп жатқаны белгісіз бұл біздің соғыс емес менің соғысым емес мама кешіріңізші мені,менің уақыт аз біз қорша алды бізден екі квартал төмендегі бәрін қира мен тірі қалма сияқты ма осы же кел үшін кешір сұра олар бейкүнә бала мен әйел зорла жатыр соғыс не үшін және кім үшін бол жат түсінбе бұйрық кім бер жат белгісіз бұл біздің соғыс емес менің соғыс емес ма кешіріңізш мен,violent

4,ин ша аллаһ сирияға барамын кафирлерді қырып соғыс кезінде джихадта өлсем арманым жоқ жаннатта кездесейік кафирлерді қырамыз,ин ша аллаһ сирия бар кафир қыр соғыс кез джихад өл арман жоқ жаннат кездес кафир қыр,violent

4,мы должны не только научиться мы должны любить убивать ведь только массовые убийства саудов ваххабитов спасут,мы долж не только научиться мы долж любить убивать ведь только массовы убийств саудов ваххабитов спасут,violent

4,мусылман бауырлар мусылман болгансон алыс жакка кеткен агайындарымызды ойлайык сирияга мусылман бауырларга комек жасауга кол ушын созуга барайыкол жактагы салафилердын козин куртып баурлар ушин согысайык бауырларды корлагандарды тозак отына жиберейык,мусылман бауыр мусылман болгансон алыс жакк кет агайын ойлайык сирияг мусылман бауырларг ко жасауг кол уш созуг барайыкол жактаг салафилерд козин курт баур ушин согысайык бауыр корлаган тозак от жиберейык,violent

4,либо джихад либо позор выбирай,либо джихад либо позо выбира,violent

4,ассаламу алейкум живу во франции но меня на родине идет джихад при первом удобном случае хочу принять нем участие иншааллах,ассалам алейку жив во франции но меня на родин идет джихад при перво удобно случа хоч принять не участи иншааллах,violent

4,на смену нам другие придут иншааллах джихад не остановится никогда он будет до конца до судного дня иншааллах мама,на смен на други придут иншааллах джихад не остановится никог он будет до конц до судного дня иншааллах ма,violent

4,нашей братья вают на пути аллах джихад мы постараемся дуъа будем делать за братья ахи иншааллах весь мир будить исламские государства,наше братья вают на

пути аллах джихад мы постараемся дуъ буде делать за братья ахи иншааллах весь ми будить исламски государств,violent

4,субханааллох спешите братья сестрами на джихад,субханааллох спеши братья сестрами на джихад,violent

4,кәпірсің жиһадты қолдамайсың ана жақта мұсылмандарды қырып жатыр ал сен қолдамайсың,кәпір жиһад қолдама ана жақта мұсылман қыр жатыр ал сен қолдама,violent

4,мен алла жолында соғысу үшін бардым ғой қазақстанға оралу туралы ешқашан ойламадым,мен алл жол соғыс үшін бар ғой қазақстан орал туралы ешқашан ойлама,violent

4,в игил мы жили хорошограбилинасилова лисобирали даньв пафосных видеороликах жестко расправлялись кафирами выкладывали все это сеть пытаясь запугать весь мир но потом прилетела русская боевая авиация владения игил начали резко сокращатьсятысячи братьев гуриямтеперь мы хотим домой помогите,в игил мы жили хорошограбилинасилов лисобирали даньв пафосных видеороликах жестко расправлялись кафирами выкладывали вс это сеть пытаясь запугать весь ми но пото прилетел русская боевая авиация владения игил начали резко сокращатьсятысячи братьев гуриямтеперь мы хоти домо помоги,violent

4,ахы ас саляму алейкум ахы шама джихад,ах ас салям алейку ах шама джихад,violent

2,шовинистік топас,шовини топас,bullying

2,басың дұрыс жұмыс істемейді сенің,бас дұрыс жұмыс істеме сенің,bullying

3,бұл жердің хохолдарға не қатысы бар,бұл же хохол не қатыс бар,nazism

2,сені ату тым жеңіл жаза,сен ату тым жеңіл жаза,bullying

2,ату керек басқа жол жоқ,ату керек басқа жол жоқ,bullying

2,жындылар мен топастар,жынды мен топас,bullying

2,өз қарныңа тық жолың болмағыр,өз қарн тық жол болмағы,bullying

2,қайдағы әдемі түрің джек пират сияқты,қай әдемі түр джек пират сияқты,bullying

2,түрмеге тығу керек қанша төзуге болады,түрме тығ керек қанша төз бол,bullying

2,өмір бақи тамақтандырамыз ба атайық,өмір бақи тамақтандыр ба ат,bullying

2,бұтыңа жіберіп қойған сияқтысың ғой құрышы бар,бұт жібер қой сияқты ғой құрыш бар,bullying

2,жындылар амон шақырмапты,жынды амон шақырма,bullying

2,сен сияқты таз гуманоидқа төзетін уақыт бітті,сен сияқты таз гуманоид төз уақыт біт,bullying

2,тағы бір фашик,тағы бір фашик,bullying

2,мына иттерді асу керек,мына ит асу керек,bullying

2,бұл иттерге сол керек құптаймын,бұл ит сол керек құпта,bullying

2,асып өлтіру керек митингтарға конституция бойынш рұқсат етілген,ас өлтір керек митинг конституция бойынш рұқсат ет,bullying

2,екі аяғын екі атқа байлап көшеде сүйрету керек,екі аяғ екі ат байла көше сүйрет керек,bullying

2,өртеп жіберу керек сені,өрте жіберу керек сен,bullying

2,әбден тойынғансың мал,әбден тойын мал,bullying

2,бетіңнен сүйреп жүріп өлтіремін,бет сүйре жүр өлтір,bullying

2,малдар біздің арамызда да бар екен,мал біздің ара да бар екен,bullying

2,коронавирус ше яғни бізді алдап келген ғой соқыр үкімет шаршатты ғой,коронавирус ше яғни біз алда кел ғой соқыр үкімет шарша ғой,bullying

2,қайдан шыққан батырсың сен өз үйіңде көрсет өнеріңді,қайдан шық батыр сен өз үй көрсет өн,bullying

2,оны алаңда ересектердің көзінше балталау керек сонда көретін болады,оны алаңда ересек көзінше балтала керек сонда көр бол,bullying

2,лайкыңмен қоса құрышы,лайк қоса құрыш,bullying

2,екеуі де жынды екен ақымақтар,екеу де жын екен ақымақ,bullying

2,өзің неге вакцина салдырмайсың мына шірікке сенбеңдер,өзің неге вакцина салдырма мына шірік сенбе,bullying

2,чмо жұмыс беруші мені ұятқа қалдырмаңдар,чмо жұмыс беруші мен ұят қалдырма,bullying

3,хохол болу үшін мал болу керек,хохол болу үшін мал болу керек,nazism

2,мынадай жындыларды қуу керек,мынадай жынды қу керек,bullying

2,басың жұмыс істемейді екен сенің,бас жұмыс істеме екен сенің,bullying

2,құттықтаймын лгбт блогер барлығы сендермен бірге жоғалыңдар,құттықта лгбт блогер барлығ сен бірге жоғал,bullying

2,қайдағы әулие сен тек диванда отырып ақылды бола аласың,қай әулие сен тек диван отыр ақылды бола ал,bullying

2,мына жындының құқығы жоқ еді,мына жынд құқығ жоқ ед,bullying

2,басына маска тақпай ату керек,бас маска тақпа ату керек,bullying

2,өз президентіңмен бірге құрышы жынды қыз,өз президент бірге құрыш жын қыз,bullying

2,барлығын биліктен қуу керек жағажайда жиналсын қорқаулар,барлығ билік қу керек жағажай жинал қорқау,bullying

2,бауырым дұрыс бұлардың жанын шығару керек,бауыр дұрыс бұл жан шығару керек,bullying

2,мына топас жетіспей тұр еді,мына топас жетіспе тұр ед,bullying

2,мына әлемде сен сияқты жынды бар екен ау,мына әлем сен сияқты жын бар екен ау,bullying

2,соқыр болып қалдыңдар ма немене бұл шалдарға қарағанда жақсы емес пе сендер қайдағы жоқ ақымақтықты айтып отырсыңдар ессіздер,соқыр бол қал ма немен бұл шал қара жақсы емес пе сен қай жоқ ақымақтық айт отыр ес,bullying

2,түріне қарасаң мүлдем тамақ ішпейтін сияқты аштықтан да суықтан да өтті соңынан ергендермен қоса өлтіру керек деп есептеймін,түр қара мүлдем тамақ ішпе сияқты аштық да суық да өт соң ер қоса өлтір керек де есепте,bullying

2,мисыз топас вакцинаны өзіңе тық,ми топас вакцина өз тық,bullying

2,мынау тек тамақ ішуден басқа түк білмейтін сияқты бұлар айнаға қарай ма екен шошқалар,мынау тек тамақ ішу басқа түк білме сияқты бұлар айна қарай ма екен шошқа,bullying

2,миы жоқ қызсың,ми жоқ қыз,bullying

2,мына такси ішкіштер мен жүргіштер үшін,мына такси ішкіш мен жүргіш үшін,bullying

2,болмағаны қалай олар өзі мына жолдың не үшін екенін біле ме есуастар,болма қалай олар өзі мына жол не үшін екен біл ме есуас,bullying

2,міне оңбағандар мұны тек гомосексуалдар ғана киеді,міне оңба мұны тек гомосексуал ғана ки,bullying

2,алдымен өзін реттеп алсын топас сосын бізге ақыл айтады,алд өз ретте ал топас сосын бізге ақыл айт,bullying

2,сатқын өтірікші жағымпаз,сатқ өтірікш жағымпаз,bullying

2,қолын сындырып артына тығу керек,қол сындыр арт тығ керек,bullying

2,күмәнданатын не бар бұлар мақтаншақтар олардан батылдықты күту бос нәрсе,күмәндан не бар бұлар мақтаншақ олардан батылдық күту бос нәрсе,bullying

2,жоқ мынау қияли біреу екен қолынан түк келмейтін,жоқ мынау қияли біреу екен қол түк келме,bullying

2,сұмдық көміп тастау керек сен сияқтыларды сонда көресің,сұм көм тастау керек сен сияқты сонда көр,bullying

2,оңбаған балалар,оңба бала,bullying

2,бұл бала емес алқаш,бұл бала емес алқаш,bullying

2,мына жынды отбасыны балталап бастарын шабу керек,мына жын отбасы балтала бас шаб керек,bullying

2,біздің ақшамызды беріңдер малдар мына жүйені қирату керек,біздің ақша бер мал мына жүйе қират керек,bullying

2,мынаны атып өлтіру керек,мына ат өлтір керек,bullying

2,қайдан шыққан батырсың сен жынды,қайдан шық батыр сен жын,bullying

2,атың өшсін топас өлімің сондай азапты болсын сені қолдағандар сені талап өлтірсін,ат өш топас өлім сондай аза бол сен қолда сен талап өлтір,bullying

2,мен мына кәрі қақпасты баяғыда өлтірер едім,мен мына кәрі қақпа баяғы өлтір ед,bullying

2,сені сотсыз ату керек,сен сот ату керек,bullying

2,сені түп тамырыңмен жою керек,сен түп тамыр жою керек,bullying

2,жолың болмасын сенің,жол болма сенің,bullying

2,маған берген кеңесіңмен қоса құры,маған бер кеңес қоса құр,bullying

3,сен сияқтыларға біздің елде орын жоқ барлығыңды жинап бір аралға апарып тастау керек,сен сияқты біздің ел орын жоқ барлығ жина бір арал апар тастау керек,nazism

2,тезексің сен,тезек сен,bullying

2,саған қараудың өзі жиіркенішті құры,саған қара өзі жиіркеніш құр,bullying

2,қожайынды өлтіру керек,қожайын өлтір керек,bullying

2,сен сияқты мисыз малды өлтіру арманым,сен сияқты ми мал өлтір арман,bullying

2,мына әйелді де мүшелеп тастау керек,мына әйел де мүшеле тастау керек,bullying

2,ұры сатанист психауру либераст жид фашист дамымайсың сен,ұры сатанист психаур либераст жид фашист дамыма сен,bullying

2,мына топастар кімдер,мына топас кім,bullying

2,менің ойымша бұл батыр емес қоқыс,менің ойымша бұл батыр емес қоқыс,bullying

3,міне мына жидтің не істегенін көріңдер,міне мына жид не істе көр,nazism

2,кетші әрі мал,кетш әрі мал,bullying

2,биттеген мал,битте мал,bullying

2,сені тіліңнен салбыратын іліп қою керек сатқын,сен тіл салбыр іл қою керек сатқ,bullying

2,ол жерде не болса қызғаншақ екенсің,ол же не бол қызғаншақ екен,bullying

2,тфу тіпті суреттегі адам да мынаны басынан атқысы келер еді,тф тіпті сурет адам да мына бас ат кел ед,bullying

2,тойғаннан қарның жарылып кетсін,той қар жарыл кет,bullying

2,жер бетінде оба таралмауы үшін мыналарды құрту керек,жер бет оба таралма үшін мына құрт керек,bullying

2,басқа малдарға сабақ болуы үшін мынаны тілінен асып қою керек,басқа мал сабақ бол үшін мына тіл ас қою керек,bullying

2,біз бай боламыз сендер қайыршы боласыңдар,біз бай бол сен қайырш бол,bullying

2,сен де малсың,сен де мал,bullying

2,салық төлеушілердің арқасында семірдіңдер жауап беретін уақыттарың келді,салық төлеуші арқа семір жауап бер уақыт кел,bullying

2,мына сөзі үшін атып өлтіру керек,мына сөз үшін ат өлтір керек,bullying

2,өлтіру керек,өлтір керек,bullying

2,менттік мал май шошқа,мен мал май шошқа,bullying

2,сіздің жасыңызға байланысты біз сізді жұмысқа ала алмаймыз,сіздің жас байланысты біз сіз жұмыс ал алма,bullying

2,сіз бұл лауазымға тым қартайдыңыз,сіз бұл лауазым тым қартай,bullying

2,сіз мұндай жауапкершілік үшін тым жассыз,сіз мұндай жауапкершілік үшін тым жас,bullying

2,аға буын жаңа технологияларды түсінбейді,аға буын жаңа технология түсінбе,bullying

2,сіз өзіңіздің жасыңызды төмен өнімділікке сылтау ретінде пайдалана алмайсыз,сіз өз жас төмен өнімділік сылтау рет пайдалан алма,bullying

2,сіз жас болғандықтан көбірек жұмыс істеуге дайын болуыңыз керек,сіз жас бол көбірек жұмыс істе дайын бол керек,bullying

2,сіз зейнетке шықтыңыз сондықтан сізге көп ақша табудың қажеті жоқ,сіз зейнет шық сондықтан сізге көп ақша таб қажет жоқ,bullying

2,сіз тым қартайдыңыз жаңа бизнесті бастау үшін,сіз тым қартай жаңа бизнес бастау үшін,bullying

2,мен жас үміткерді жалдағым келеді,мен жас үміткер жалда кел,bullying

2,сіз үйде балалармен бірге болуыңыз керек және жұмыс істемеуіңіз керек,сіз үй бала бірге бол керек және жұмыс істеме керек,bullying

2,жастан асқан ба біз сізге көмектесе алмаймыз,жас ас ба біз сізге көмекте алма,bullying

2,сіздің жасыңыз сізді еңбек нарығында бәсекеге қабілетсіз етеді,сіздің жас сіз еңбек нарығ бәсеке қабілет ет,bullying

2,сіз тым жассыз нақты жұмыс деген не екенін білу үшін,сіз тым жас нақты жұмыс де не екен білу үшін,bullying

2,қартайған сайын сіз аз құнды қызметкер боласыз,қартай сайын сіз аз құнды қызметкер бол,bullying

2,сіз жаңа жұмыс туралы емес зейнетақы туралы ойлануыңыз керек,сіз жаңа жұмыс туралы емес зейнетақы туралы ойлан керек,bullying

2,сіздің жасыңыз біздің қажеттіліктерімізге сәйкес келмейді,сіздің жас біздің қажеттілік сәйкес келме,bullying

2,сіз оны жасыңызға байланысты жеңе алмайсыз,сіз оны жас байланысты же алма,bullying

2,сіз тым қартайдыңыз жаңа нәрселерді үйрену үшін,сіз тым қартай жаңа нәрсе үйрену үшін,bullying

2,сіз біздің бөлімді басқару үшін тым жассыз,сіз біздің бөл басқару үшін тым жас,bullying

2,сіз жас қызметкерлерге орын жасау үшін зейнетке шығуыңыз керек,сіз жас қызметкер орын жасау үшін зейнет шығ керек,bullying

2,біз тәжірибелі үміткерді іздейміз,біз тәжірибелі үміткер ізде,bullying

2,сіз жас әріптестеріңізге жол беруіңіз керек,сіз жас әріптес жол бер керек,bullying

2,сізге жаңа дағдыларды үйренудің қажеті жоқ сіз жақын арада зейнетке шығасыз,сізге жаңа дағды үйрену қажет жоқ сіз жақын ара зейнет шығ,bullying

2,аға буын заманауи өзгерістерге бейімделе алмайды,аға буын заманауи өзгеріс бейімдел алма,bullying

2,сіз өзіңіздің жасыңызға және физикалық жағдайыңызға байланысты бұл жұмысты жеңе алмайсыз,сіз өз жас және физикалық жағдай байланысты бұл жұм же алма,bullying

2,бізде сіздің жасыңызға байланысты сіздің қажеттіліктеріңізге сәйкес келетін жұмыс орнын құру мүмкіндігі жоқ,біз сіздің жас байланысты сіздің қажеттілік сәйкес кел жұмыс орн құру мүмкіндіг жоқ,bullying

2,сіз біздің компания үшін тым қартайдыңыз,сіз біздің компания үшін тым қартай,bullying

2,сізге жас қызметкерлер сияқты бірдей жалақы алудың қажеті жоқ,сізге жас қызметкер сияқты бірде жалақы ал қажет жоқ,bullying

2,сіз енді біздің мақсатты аудиторияға сәйкес келмейсіз,сіз енді біздің мақсат аудитория сәйкес келме,bullying

2,біз сіздің жасыңызға байланысты сізді оқуға ала алмаймыз,біз сіздің жас байланысты сіз оқ ал алма,bullying

2,сіздің жасыңыз сізді біздің компанияда жұмыс істеуге жарамсыз етеді,сіздің жас сіз біздің компания жұмыс істе жарамсыз ет,bullying

2,аға жұмысшылар жаңа технологияларға тез бейімделе алмайды,аға жұмысшы жаңа технология тез бейімдел алма,bullying

2,сіз біздің компанияда жұмыс істей алмайсыз өйткені сіз жастан асқансыз,сіз біздің компания жұмыс істе алма өйткені сіз жас ас,bullying

2,сіз өзіңіздің мансабыңыздың шыңына жеттіңіз әрі қарай алға жылжу мүмкін емес,сіз өз мансаб шың же әрі қарай алға жылжу мүмкін емес,bullying

2,сіз зейнетақы туралы ойланып демалуға кетуіңіз керек,сіз зейнетақы туралы ойлан демал кет керек,bullying

2,сіз тым жассыз бұл жұмыс үшін жеткілікті тәжірибе алу үшін,сіз тым жас бұл жұмыс үшін жеткілікті тәжірибе алу үшін,bullying

2,сізге керек жұмыс күнін қысқарту өйткені сіз тым қартайдыңыз толық уақытты жұмыс істеу үшін,сізге керек жұмыс күн қысқарту өйткені сіз тым қартай толық уақыт жұмыс істеу үшін,bullying

2,аға қызметкерлер қосымша медициналық шығындарды тудыруы мүмкін,аға қызметкер қосымша медициналық шығын тудыр мүмкін,bullying

2,сіз тым жассыз біздің бизнес моделімізді түсіну үшін,сіз тым жас біздің бизнес модел түсіну үшін,bullying

2,сіздің жасыңыз сізді тиімсіз қызметкер етеді,сіздің жас сіз тиімсіз қызметкер ет,bullying

2,сіз зейнетке шығуыңыз керек және толық уақытты жұмыс істемеуіңіз керек,сіз зейнет шығ керек және толық уақыт жұмыс істеме керек,bullying

2,сіздің жасыңыз сізді икемді және бейімделгіш етеді,сіздің жас сіз икемді және бейімделгіш ет,bullying

2,біз жас қызметкерлермен жұмыс істегенді жөн көреміз,біз жас қызметкер жұмыс істе жөн көр,bullying

2,сіздің жасыңыз бұл жұмысты орындауға мүмкіндік бермейді,сіздің жас бұл жұм орында мүмкіндік берме,bullying

2,біз сізді жұмысқа ала алмаймыз өйткені сіз зейнетке шығуға тым жақынсыз,біз сіз жұмыс ал алма өйткені сіз зейнет шығ тым жақын,bullying

2,сіз бұрынғыдай жылдам және жігерлі емессіз,сіз бұрынғыдай жылдам және жігерлі емес,bullying

2,сізге жаңа технологияларды үйренудің қажеті жоқ сіз тым қартайдыңыз,сізге жаңа технология үйрену қажет жоқ сіз тым қартай,bullying

2,аға қызметкерлер біздің алдыңғы қатардағы адамдарымыз бола алмайды,аға қызметкер біздің алдыңғы қатар адам бола алма,bullying

2,біз сізге көтеруді ұсына алмаймыз өйткені сіз тым қартайдыңыз,біз сізге көтеру ұсын алма өйткені сіз тым қартай,bullying

2,аға буын нарықтың жаңа талаптарына бейімделе алмайды,аға буын нарық жаңа талап бейімдел алма,bullying

2,сіз бұл лауазымға орналасу үшін тым жассыз,сіз бұл лауазым орналасу үшін тым жас,bullying

2,аға қызметкерлер жастар сияқты ұзақ жұмыс істей алмайды,аға қызметкер жас сияқты ұзақ жұмыс істе алма,bullying

2,сізге жаңа дағдыларды үйренудің қажеті жоқ сіз тым қартайдыңыз,сізге жаңа дағды үйрену қажет жоқ сіз тым қартай,bullying

2,сіз бұл жұмыс үшін тым қартайдыңыз,сіз бұл жұмыс үшін тым қартай,bullying

2,аға жұмысшылар жастар сияқты тез жұмыс істей алмайды,аға жұмысшы жас сияқты тез жұмыс істе алма,bullying

2,сіздің жасыңыз сізді аз өнімді етеді,сіздің жас сіз аз өн ет,bullying

2,біз жас қызметкерлерді жалдағанды жөн көреміз,біз жас қызметкер жалда жөн көр,bullying

2,аға жұмысшылар ауруға байланысты жұмысты жиі өткізіп жібереді,аға жұмысшы ауру байланысты жұм жиі өткіз жібер,bullying

2,сіздің жасыңыз сізді аз икемді және бейімделуге қабілетті етеді,сіздің жас сіз аз икемді және бейімдел қабілетті ет,bullying

2,сіз тым жассыз біздің жұмысымыздың күрделілігін түсіну үшін,сіз тым жас біздің жұмыс күрделіліг түсіну үшін,bullying

2,аға қызметкерлер жастар сияқты деңгейде жұмыс істей алмайды,аға қызметкер жас сияқты деңгей жұмыс істе алма,bullying

2,сіздің жасыңыз сізді ақылды және тез ойлауға мәжбүр етеді,сіздің жас сіз ақылды және тез ойла мәжбүр ет,bullying

2,аға қызметкерлер жастар сияқты жүктемені көтере алмайды,аға қызметкер жас сияқты жүктеме көтер алма,bullying

2,сізге жаңа нәрселерді үйренудің қажеті жоқ сіз тым қартайдыңыз,сізге жаңа нәрсе үйрену қажет жоқ сіз тым қартай,bullying

2,сіз жастарды түсіну үшін тым қартайдыңыз,сіз жас түсіну үшін тым қартай,bullying

2,аға жұмысшылар жас сияқты клиенттердің сұраныстарын қанағаттандыра алмайды,аға жұмысшы жас сияқты клиент сұраныс қанағаттандыр алма,bullying

2,сіз тым қартайдыңыз мамандығыңызды өзгерту үшін,сіз тым қартай мамандығ өзгерту үшін,bullying

2,аға қызметкерлер медиа индустриядағы біздің тұлғамыз бола алмайды,аға қызметкер медиа индустрия біздің тұлға бола алма,bullying

2,аға қызметкерлер жастар сияқты жұмыс істей алмайды,аға қызметкер жас сияқты жұмыс істе алма,bullying

2,сіз тым қартайдыңыз жаңа жобаларға қатысу үшін,сіз тым қартай жаңа жоба қатысу үшін,bullying

2,аға қызметкерлер компанияның жаңа талаптарын орындай алмайды,аға қызметкер компания жаңа талап орында алма,bullying

2,сіз өз салаңыздағы жаңа тенденцияларды түсіну үшін тым қартайдыңыз,сіз өз салаңыз жаңа тенденция түсіну үшін тым қартай,bullying

2,сіздің жасыңыз сізді өзіңізге және қабілеттеріңізге аз сенімді етеді,сіздің жас сіз өз және қабілет аз сенімді ет,bullying

2,аға қызметкерлер жаңа жұмыс процестеріне бейімделе алмайды,аға қызметкер жаңа жұмыс процес бейімдел алма,bullying

2,сіз еңбек нарығында бәсекеге қабілетті болу үшін тым қартайдыңыз,сіз еңбек нарығ бәсеке қабілетті болу үшін тым қартай,bullying

2,аға қызметкерлер командада жастар сияқты тиімді жұмыс істей алмайды,аға қызметкер команда жас сияқты тиімді жұмыс істе алма,bullying

2,сіздің жасыңыз сізді жаңашыл және креативті етеді,сіздің жас сіз жаңашыл және креативті ет,bullying

2,сіз тым қартайдыңыз жаңа технологиялармен байланысты жобаларға қатысу үшін,сіз тым қартай жаңа технология байланысты жоба қатысу үшін,bullying

2,сіздің жасыңыз сізді аз мақсатты және өршіл етеді,сіздің жас сіз аз мақсат және өршіл ет,bullying

2,аға қызметкерлер динамикалық және тез өзгеретін ортада жұмыс істей алмайды,аға қызметкер динамикалық және тез өзгер орта жұмыс істе алма,bullying

2,сіз тым қартайдыңыз жастармен жұмыс істеу үшін,сіз тым қартай жас жұмыс істеу үшін,bullying

2,аға қызметкерлер жастар сияқты бірдей ритақта жұмыс істей алмайды,аға қызметкер жас сияқты бірде ритақ жұмыс істе алма,bullying

2,сіздің жасыңыз сізді жұмыс берушілер үшін аз тартымды етеді,сіздің жас сіз жұмыс беруші үшін аз тартымды ет,bullying

2,сіз ең жаңа трендтер мен инновациялардан хабардар болу үшін тым қартайдыңыз,сіз ең жаңа тренд мен инновация хабардар болу үшін тым қартай,bullying

2,сіздің жасыңыз сізді қазіргі өмір мен жұмысқа аз бейімдейді,сіздің жас сіз қазіргі өмір мен жұмыс аз бейімде,bullying

2,сіз тым қартайдыңыз жастар мәдениетін түсіну үшін,сіз тым қартай жас мәдени түсіну үшін,bullying

2,сіздің жасыңыз сізді өзгеретін нарықтық жағдайларға аз бейімдейді,сіздің жас сіз өзгер нарық жағдай аз бейімде,bullying

2,сіз тым қартайдыңыз спортпен шұғылдану немесе бос уақытты белсенді өткізу үшін,сіз тым қартай спорт шұғылдан немесе бос уақыт белсенді өткізу үшін,bullying

2,егде жастағы адамдар жақсы үйде болу қауіпті уақытта сыртқа шыққаннан гөрі,егде жастағы адам жақсы үй болу қауіпті уақыт сырт шық гөрі,bullying

2,қарт адамдар жастарға қарағанда баяу және ебедейсіз,қарт адам жас қара бая және ебеде,bullying

2,егде жастағы адамдар концерттер мен фестивальдар сияқты іс шараларға қатыса алмайды,егде жастағы адам концерт мен фестиваль сияқты іс шара қатыс алма,bullying

2,егде жастағы адамдар кенеттен және күтпеген оқиғаларға жастар сияқты сәтті қатыса алмайды,егде жастағы адам кенет және күтпе оқиға жас сияқты сәтті қатыс алма,bullying

2,қарт адамдар тым шаршайды саяхаттау үшін,қарт адам тым шарша саяхаттау үшін,bullying

2,сіз тым қартайдыңыз жаңа адамдармен танысу және жаңа қарым қатынасты бастау үшін,сіз тым қартай жаңа адам танысу және жаңа қарым қатына бастау үшін,bullying

2,қарт адамдар заманауи музыканы фильмдерді және телешоуларды бақылай алмайды,қарт адам заманауи музыка фильм және телешоу бақыла алма,bullying

2,қарт адамдар жас және жігерлі адамдармен қадам жасай алмайды,қарт адам жас және жігерлі адам қадам жаса алма,bullying

2,қарт адамдар компьютерлер мен мобильді құрылғыларды жастар сияқты тиімді пайдалана алмайды,қарт адам компьютер мен мобильді құрылғы жас сияқты тиімді пайдалан алма,bullying

2,сіз тым қартайдыңыз кішкентай балаларға қамқорлық жасау үшін,сіз тым қартай кішкентай бала қамқорлық жасау үшін,bullying

2,қарт адамдар спорттық іс шараларға жастар сияқты сәтті қатыса алмайды,қарт адам спорттық іс шара жас сияқты сәтті қатыс алма,bullying

2,егде жастағы адамдар алыс қашықтыққа саяхаттай алмайды немесе басқа елдерде тұра алмайды,егде жастағы адам алыс қашықтық саяхатта алма немесе басқа ел тұр алма,bullying

2,сіз тым қартайдыңыз жаңа жобалар мен бизнесті бастау үшін,сіз тым қартай жаңа жоба мен бизнес бастау үшін,bullying

2,қарт адамдар әлеуметтік және мәдени нормалардың өзгеруіне бейімделе алмайды,қарт адам әлеуметтік және мәдени норма өзгер бейімдел алма,bullying

2,егде жастағы адамдар интернетті және әлеуметтік медианы жастар сияқты тиімді пайдалана алмайды,егде жастағы адам интернет және әлеуметтік медиа жас сияқты тиімді пайдалан алма,bullying

2,сіз тым қартайдыңыз белсенді демалу үшін,сіз тым қартай белсенді демал үшін,bullying

2,қарт адамдар белсенді ойындар мен ойын сауыққа қатыса алмайды,қарт адам белсенді ойын мен ойын сауық қатыс алма,bullying

2,егде жастағы адамдар кенеттен және төтенше жағдайларға жастар сияқты сәтті қатыса алмайды,егде жастағы адам кенет және төтенше жағдай жас сияқты сәтті қатыс алма,bullying

2,ескі адамдар фотосессиялар мен сән көрсетілімдері сияқты іс шараларға қатыса алмайды,ескі адам фотосессия мен сән көрсетілім сияқты іс шара қатыс алма,bullying

2,егде жастағы адамдар әлеуметтік өзара әрекеттесу жағдайында өзін дұрыс және сенімді ұстай алмайды,егде жастағы адам әлеуметтік өзара әрекеттесу жағдай өз дұрыс және сенімді ұста алма,bullying

2,сіз тым қартайдыңыз денсаулығыңыз бен фитнесіңізге қамқорлық жасау үшін,сіз тым қартай денсаулығ бен фитнес қамқорлық жасау үшін,bullying

2,егде жастағы адамдар салауатты өмір салтын ұстана алмайды және олардың тамақтануын бақылай алмайды,егде жастағы адам салауатты өмір салт ұстан алма және олардың тамақтан бақыла алма,bullying

2,егде жастағы адамдар өздерінің сыртқы келбетіне мән бере алмайды және стилін сақтай алмайды,егде жастағы адам өз сыртқы келбет мән бер алма және стил сақта алма,bullying

2,сіз тым қартайдыңыз көзқарастарыңыз бен сенімдеріңізді өзгерту үшін,сіз тым қартай көзқарас бен сенім өзгерту үшін,bullying

2,ескі адамдар жаңа идеялар мен тенденцияларға бейімделе алмайды,ескі адам жаңа идея мен тенденция бейімдел алма,bullying

2,егде жастағы адамдар қоғам мен экономиканың дамуына үлес қоса алмайды,егде жастағы адам қоғам мен экономика дам үлес қоса алма,bullying

2,сіз тым қартайдыңыз жаңа дағдылар мен кәсіптерді үйрену үшін,сіз тым қартай жаңа дағды мен кәсіп үйрену үшін,bullying

2,ескі адамдар қайта біліктілікке ие бола алмайды және еңбек нарығының жаңа жағдайларына бейімделе алмайды,ескі адам қайта біліктілік ие бола алма және еңбек нарығ жаңа жағдай бейімдел алма,bullying

2,егде жастағы адамдар жаңа өнімдер мен инновациялар жасай алмайды,егде жастағы адам жаңа өнім мен инновация жаса алма,bullying

2,сіз саяхаттауға және әлемді зерттеуге тым қартайдыңыз,сіз саяхаттау және әлем зертте тым қартай,bullying

2,қарт адамдар мәдени алмасу мен халықаралық ынтымақтастыққа қатыса алмайды,қарт адам мәдени алмасу мен халықаралық ынтымақтастық қатыс алма,bullying

2,егде жастағы адамдар басқа мәдениеттер мен ұлт өкілдерімен сөйлесе алмайды,егде жастағы адам басқа мәдениет мен ұлт өкіл сөйле алма,bullying

2,сіз тым қартайдыңыз қолөнер мен шығармашылықпен айналысу үшін,сіз тым қартай қолөнер мен шығармашылық айналысу үшін,bullying

2,қарт адамдар хобби мен шығармашылық жобаларға қатыса алмайды,қарт адам хобби мен шығармашылық жоба қатыс алма,bullying

2,егде жастағы адамдар жас ұрпақты тәрбиелеп тәрбиелей алмайды,егде жастағы адам жас ұрпақ тәрбиеле тәрбиеле алма,bullying

2,сіз тым қартайдыңыз отбасыңызға және жақындарыңызға қамқорлық жасау үшін,сіз тым қартай отбас және жақын қамқорлық жасау үшін,bullying

2,қарт адамдар балалары мен немерелеріне көмектесе алмайды,қарт адам бала мен немере көмекте алма,bullying

2,егде жастағы адамдар өз ұрпақтары үшін сенімді қамқоршы және тәлімгер бола алмайды,егде жастағы адам өз ұрпақ үшін сенімді қамқоршы және тәлімгер бола алма,bullying

2,сіз тым қартайдыңыз сіздің қаржыңыз бен мүлкіңізге қамқорлық жасау үшін,сіз тым қартай сіздің қаржы бен мүлк қамқорлық жасау үшін,bullying

2,егде жастағы адамдар заңнамадағы және қоғамдық өмірдегі өзгерістерді бақылай алмайды,егде жастағы адам заңнама және қоғамдық өмір өзгеріс бақыла алма,bullying

2,сіз тым қартайдыңыз белсенді саяси өмірге қатысу үшін,сіз тым қартай белсенді саяси өмір қатысу үшін,bullying

2,қарт адамдар жастармен қарым қатынас жасай алмайды және олардың уәждерін түсіне алмайды,қарт адам жас қарым қатынас жаса алма және олардың уәж түс алма,bullying

2,егде жастағы адамдар стресс пен эмоционалды қиындықтарды жеңе алмайды,егде жастағы адам стресс пен эмоциона қиындық же алма,bullying

2,сіз тым қартайдыңыз спортпен және физикалық белсенділікпен айналысу үшін,сіз тым қартай спорт және физикалық белсенділік айналысу үшін,bullying

2,сіз тым қартайдыңыз жаңа қарым қатынас пен достар құру үшін,сіз тым қартай жаңа қарым қатынас пен дос құру үшін,bullying

2,қарт адамдар романтикалық қарым қатынасқа түсе алмайды және бақытты бола алмайды,қарт адам романтикалық қарым қатынас түс алма және бақытты бола алма,bullying

2,егде жастағы адамдар үй жануарларына қамқорлық жасай алмайды және олардың серігі бола алмайды,егде жастағы адам үй жануар қамқорлық жаса алма және олардың серіг бола алма,bullying

2,сіз компьютерлік ойындар ойнауға және гаджеттерді пайдалануға тым қартайдыңыз,сіз компьютерлік ойын ойна және гаджет пайдалан тым қартай,bullying

2,ескі адамдар табысты кәсіпкерлер бола алмайды және өз бизнесін құра алмайды,ескі адам табысты кәсіпкерлер бола алма және өз бизнес құра алма,bullying

2,егде жастағы адамдар күрделі мәселелерді шеше алмайды және маңызды шешімдер қабылдай алмайды,егде жастағы адам күрделі мәселе шеш алма және маңызды шешім қабылда алма,bullying

2,сіз өзіңіздің қауіпсіздігіңіз бен қылмыскерлерден қорғану туралы қамқорлық жасау үшін тым қартайдыңыз,сіз өз қауіпсіздіг бен қылмыскер қорған туралы қамқорлық жасау үшін тым қартай,bullying

2,қарт адамдар медицина мен емдеудің жаңа әдістерін қолдана алмайды,қарт адам медицина мен емдеу жаңа әдіс қолдан алма,bullying

2,егде жастағы адамдар табысты актер музыкант және суретші бола алмайды,егде жастағы адам табысты актер музыкант және суретші бола алма,bullying

2,егде жастағы адамдар көпшілік алдында сөйлей алмайды және аудитория алдында сөйлей алмайды,егде жастағы адам көпшілік алд сөйле алма және аудитория алд сөйле алма,bullying

2,сіз тым қартайдыңыз белсенді жыныстық өмір сүру үшін,сіз тым қартай белсенді жын өмір сүру үшін,bullying

2,қарт адамдар серіктеспен жақсы қарым қатынаста бола алмайды және жақын өмірден ләззат ала алмайды,қарт адам серіктес жақсы қарым қатынас бола алма және жақын өмір ләззат ал алма,bullying

2,егде жастағы адамдар жас әріптестерімен және серіктестерімен жақсы қарым қатынаста бола алмайды,егде жастағы адам жас әріптес және серіктес жақсы қарым қатынас бола алма,bullying

2,сіз мұны түсіну үшін тым жассыз,сіз мұны түсіну үшін тым жас,bullying

2,бұл жастар үшін жұмыс,бұл жас үшін жұмыс,bullying

2,сіз бұл үшін тым қартайдыңыз,сіз бұл үшін тым қартай,bullying

2,сіз өзіңізді жас үміткер ретінде көрсете аласыз ба,сіз өз жас үміткер рет көрсе ал ба,bullying

2,сіз тым қартайдыңыз жаңа мансапты бастау үшін,сіз тым қартай жаңа мансап бастау үшін,bullying

2,менің ойымша сізге инвестиция салудың қажеті жоқ сіз қазірдің өзінде зейнетақыға жақынсыз,менің ойымша сізге инвестиция сал қажет жоқ сіз қаз өз зейнетақы жақын,bullying

2,сіз зейнетке шыққан кезде,сіз зейнет шық кезде,bullying

2,сіз демалу үшін толық емес жұмыс күнінде тоқтай аласыз ба,сіз демал үшін толық емес жұмыс күн тоқта ал ба,bullying

2,біз жаңа идеялары мен жаңа тәсілдері бар үміткерлерді іздейміз,біз жаңа идея мен жаңа тәсіл бар үміткер ізде,bullying

2,біз жас және жігерлі жұмысшыларға артықшылық береміз,біз жас және жігерлі жұмысшы артықшылық бер,bullying

2,біз жақын арада зейнетке шыға алатын адамды жалдай алмаймыз,біз жақын ара зейнет шығ ал адам жалда алма,bullying

2,сіз біздің клиент болу үшін тым қартайдыңыз,сіз біздің клиент болу үшін тым қартай,bullying

2,менің ойымша бұл сізге қажет емес сіз қазірдің өзінде жассыз,менің ойымша бұл сізге қажет емес сіз қаз өз жас,bullying

2,сіз мұны жасай алатыныңызға сенімді емеспін сіз бұрынғыдай жас емессіз,сіз мұны жаса ал сенімді емес сіз бұрынғыдай жас емес,bullying

2,мұны істеу үшін басқа біреуді таба аласыз ба сіз бұл үшін тым қартайған сияқтысыз,мұны істеу үшін басқа біреу таб ал ба сіз бұл үшін тым қартай сияқты,bullying

2,сіздің жасыңызды ескере отырып сіз ауырмайтыныңызға қалай сенімді бола аласыз,сіздің жас ескер отыр сіз ауырма қалай сенімді бола ал,bullying

2,біз сіздің тәжірибеңіз бен біліміңіз үшін ақы төлей алмаймыз,біз сіздің тәжірибе бен білім үшін ақы төле алма,bullying

2,сіз бұл ойынды ойнау үшін тым қартайдыңыз,сіз бұл ой ойна үшін тым қартай,bullying

2,сіздің жасыңызды ескере отырып сіз осындай жаңалықтарға дайын екеніңізге сенімді емеспін,сіздің жас ескер отыр сіз осындай жаңалық дайын екен сенімді емес,bullying

2,сіз осындай жас әріптестермен жұмыс істеуге дайын екеніңізге сенімді емеспін,сіз осындай жас әріптес жұмыс істе дайын екен сенімді емес,bullying

2,біз біз күткеннен де үлкен көрінетін адамды жалдай алмаймыз,біз біз күт де үлкен көрін адам жалда алма,bullying

2,сіз зейнетақы туралы ойлануыңыз керек,сіз зейнетақы туралы ойлан керек,bullying

2,сіз тым қартайдыңыз мұндай концерттерге бару үшін,сіз тым қартай мұндай концерт бару үшін,bullying

2,менің ойымша сіздің жасыңызда мұндай нәрселерге қамқорлық жасаудың қажеті жоқ,менің ойымша сіздің жас мұндай нәрсе қамқорлық жаса қажет жоқ,bullying

2,сіз көре аласыз бірақ қол тигізе алмайсыз бұл жастарға арналған,сіз көр ал бірақ қол тигіз алма бұл жас арнал,bullying

2,сіз өзгерістерді ұстануға болатынына сенімді емеспін,сіз өзгеріс ұстан бол сенімді емес,bullying

2,бізде мұнда қарт адамдарға мүмкіндік жоқ,біз мұнда қарт адам мүмкіндік жоқ,bullying

2,біз сіздің жасыңыздағы адамдарға жұмыс таба алмаймыз,біз сіздің жасыңыз адам жұмыс таб алма,bullying

2,мен мұны істей алмаймын сіз үшін тым қартайдыңыз,мен мұны істе алма сіз үшін тым қартай,bullying

2,мұндай нәрселер сіз үшін зейнеткер емес,мұндай нәрсе сіз үшін зейнеткер емес,bullying

2,сіз оны жеңе алмайсыз сіз тым қартайдыңыз,сіз оны же алма сіз тым қартай,bullying

2,сіз өзіңіздің жасыңызда жұмыс ұсынылғанына риза болуыңыз керек,сіз өз жас жұмыс ұсыныл риза бол керек,bullying

2,бізге ескірген емес жаңа идеялары бар адамдар қажет,бізге ескір емес жаңа идея бар адам қажет,bullying

2,біз жастарды жалдағанды жөн көреміз біздің компания жас және серпінді болып көрінуі үшін,біз жас жалда жөн көр біздің компания жас және серпінді бол көрін үшін,bullying

2,біз аға қызметкерлеріміз үшін сақтандыруды төлей алмаймыз,біз аға қызметкер үшін сақтандыру төле алма,bullying

2,сіз мансаптық өсу үшін тым қартайдыңыз,сіз мансап өсу үшін тым қартай,bullying

2,біз жастарды жалдауды жөн көреміз денсаулық мәселелерімен айналыспау үшін,біз жас жалдау жөн көр денсаулық мәселе айналыспа үшін,bullying

2,біздің компания егде жастағы қызметкерлермен байланысты мәселелермен байланысқысы келмейді,біздің компания егде жастағы қызметкер байланысты мәселе байланыс келме,bullying

2,сіз тым қартайдыңыз жұмысқа қандай да бір өзгерістер енгізу үшін,сіз тым қартай жұмыс қандай да бір өзгеріс енгізу үшін,bullying

2,бізге адамдар керек ұзағырақ жұмыс істей алатын және тезірек сіз енді олай емессіз,бізге адам керек ұзағырақ жұмыс істе ал және тезірек сіз енді ола емес,bullying

2,сіз енді жас және өршіл адамдармен бәсекелесе алмайсыз,сіз енді жас және өршіл адам бәсекеле алма,bullying

2,сіз жыл бұрын тамаша үміткер болар едіңіз бірақ қазір біз басқа біреуді іздеп жатырмыз,сіз жыл бұрын тамаша үміткер бол ед бірақ қазір біз басқа біреу ізде жатыр,bullying

2,сізде жастардың ынта жігері мен энергиясы жоқ,сіз жас ынта жігер мен энергия жоқ,bullying

2,сіз жаңа жұмыс әдістерін үйрету үшін тым қартайдыңыз,сіз жаңа жұмыс әдіс үйрет үшін тым қартай,bullying

2,сіздің жасыңыз бұл мәселеде табысты болуға мүмкіндік бермейді,сіздің жас бұл мәселе табысты бол мүмкіндік берме,bullying

2,біз зейнеткерлерге жас қызметкерлер сияқты жалақы төлей алмаймыз,біз зейнеткер жас қызметкер сияқты жалақы төле алма,bullying

2,біз аға қызметкерлердің денсаулығымен айналысқымыз келмейді,біз аға қызметкер денсаулығ айналыс келме,bullying

2,сіз енді жастар сияқты жұмыс істей алмайсыз,сіз енді жас сияқты жұмыс істе алма,bullying

2,сіз тым жассыз өмір туралы бірдеңе білу үшін,сіз тым жас өмір туралы бірде білу үшін,bullying

2,сіз әлі тым жассыз нақты жұмысты түсіну үшін,сіз әлі тым жас нақты жұм түсіну үшін,bullying

2,жастар бүгінде тым жалқау және бүлінген,жас бүгін тым жалқау және бүлін,bullying

2,сізге көбірек өмірлік тәжірибе қажет осы жағдайды түсіну үшін,сізге көбірек өмірлік тәжірибе қажет осы жағдай түсіну үшін,bullying

2,сіз тым жассыз осындай маңызды шешімдер қабылдау үшін,сіз тым жас осындай маңызды шешім қабылдау үшін,bullying

2,сіз мұндай нәрселер туралы алаңдамауыңыз керек сіз әлі тым жассыз,сіз мұндай нәрсе туралы алаңдама керек сіз әлі тым жас,bullying

2,бүгінгі жастар аға буынды құрметтемейді,бүгінгі жас аға бу құрметтеме,bullying

2,сен өз жасыңа байланысты байсалды емессің және жетілмегенсің,сен өз жас байланысты байсалды емес және жетілме,bullying

2,бүгінгі жастар еңбекке қабілетсіз олар тым бүлінген және жалқау,бүгінгі жас еңбек қабілет олар тым бүлін және жалқау,bullying

2,сіз түсіне алмайсыз қандай қиындықтар туындауы мүмкін сіз тым жассыз және тәжірибесізсіз,сіз түс алма қандай қиындық туында мүмкін сіз тым жас және тәжірибесіз,bullying

2,сіз тым жассыз басқаларға үйрету үшін,сіз тым жас басқа үйрет үшін,bullying

2,сіз әлі білмейсіз нағыз махаббат деген не сен тым жассың,сіз әлі білме нағыз махаббат де не сен тым жас,bullying

2,бүгінгі жастар ақшаны бағалай алмайды және нәтиже үшін жұмыс істей алмайды,бүгінгі жас ақша бағала алма және нәтиже үшін жұмыс істе алма,bullying

2,сіз бұл жұмысты жасамауыңыз керек сіз тым жассыз және тәжірибесізсіз,сіз бұл жұм жасама керек сіз тым жас және тәжірибесіз,bullying

2,сен тым жассың өз пікіріңе ие болу үшін,сен тым жас өз пікір ие болу үшін,bullying

2,жастар бүгінде үлкендерді құрметтемейді және олардың кеңестерін тыңдамайды,жас бүгін үлкен құрметтеме және олардың кеңес тыңдама,bullying

2,сіз түсіне алмайсыз дәстүрлерді сақтау қаншалықты маңызды сіз тым жассыз және ақылсызсыз,сіз түс алма дәстүр сақтау қаншалықты маңызды сіз тым жас және ақылсыз,bullying

2,бүгінгі жастар жауапкершіліктің не екенін білмейді және маңызды істерге дайын емес,бүгінгі жас жауапкершілік не екен білме және маңызды іс дайын емес,bullying

2,сіз тым жассыз салмақты шешім қабылдау үшін,сіз тым жас салмақ шешім қабылдау үшін,bullying

2,жастар бүгінде командада жұмыс істей алмайды және әріптестерін құрметтемейді,жас бүгін команда жұмыс істе алма және әріптес құрметтеме,bullying

2,сіз әлі тым жассыз бизнеспен айналысу үшін,сіз әлі тым жас бизнес айналысу үшін,bullying

2,бүгінгі жастар классикалық музыка мен басқа да өнерді бағалай алмайды,бүгінгі жас классикалық музыка мен басқа да өн бағала алма,bullying

2,сіз бұл дүниеде бір нәрсені өзгертуге тырыспауыңыз керек сіз тым жассыз және аңғалсыз,сіз бұл дүние бір нәрсе өзгерт тырыспа керек сіз тым жас және аңғал,bullying

2,сіз әлі тым жассыз мұндай жауапкершілікті өз мойныңызға алу үшін,сіз әлі тым жас мұндай жауапкершілік өз мойны алу үшін,bullying

2,бүгінгі жастар нақты тілде сөйлесе алмайды және технологияға тым тәуелді,бүгінгі жас нақты тіл сөйле алма және технология тым тәуелді,bullying

2,сіз тым жассыз білу үшін нағыз ата ана дегеніміз не,сіз тым жас білу үшін нағыз ата ана де не,bullying

2,жастар өз болашағын дұрыс жоспарлауды білмейді,жас өз болашағ дұрыс жоспарлау білме,bullying

2,сіз бұл пікірталастарға қатыспауыңыз керек сіз тым жассыз және тәжірибесізсіз,сіз бұл пікірталас қатыспа керек сіз тым жас және тәжірибесіз,bullying

2,жастар бүгінде үлкендерді құрметтемейді және басшыларға қалай мойынсұнуды білмейді,жас бүгін үлкен құрметтеме және басшы қалай мойынсұну білме,bullying

2,сіз тым жассыз білу үшін нағыз еңбек пен еңбекқорлық деген не,сіз тым жас білу үшін нағыз еңбек пен еңбекқорлық де не,bullying

2,сіз әлі тым жассыз және нақты жұмыс тәжірибесінің не екенін білмейсіз,сіз әлі тым жас және нақты жұмыс тәжірибе не екен білме,bullying

2,жастар бүгінде әлеуметтік желілерде көп уақыт өткізеді және шынайы достар таба алмайды,жас бүгін әлеуметтік желі көп уақыт өткіз және шынайы дос таб алма,bullying

2,жастар ақшаны қалай сақтау керектігін білмейді және оны барлық бос сөздерге жұмсайды,жас ақша қалай сақтау керектіг білме және оны барлық бос сөз жұмса,bullying

2,сіздің денсаулығыңызға қамқорлық жасауға әлі ерте сіз тым жассыз,сіздің денсаулығ қамқорлық жаса әлі ерте сіз тым жас,bullying

2,жастар бүгінде тым жеңіл және жауапты шешім қабылдауды білмейді,жас бүгін тым жеңіл және жауапты шешім қабылдау білме,bullying

2,сіз тым жассыз түсіну үшін нақты жауапкершілік дегеніміз не,сіз тым жас түсіну үшін нақты жауапкершілік де не,bullying

2,жастар өз елінің дәстүрлері мен тарихын құрметтемейді,жас өз ел дәстүр мен тарих құрметтеме,bullying

2,жастар бүгінде тым бос және өзін қалай басқаруды білмейді,жас бүгін тым бос және өз қалай басқару білме,bullying

2,сіздің жасыңыз сізге мұндай нәрселерді түсінуге мүмкіндік бермейді,сіздің жас сізге мұндай нәрсе түсін мүмкіндік берме,bullying

2,сіз тым жассыз және тәжірибесізсіз бұл жұмысты орындау үшін,сіз тым жас және тәжірибесіз бұл жұм орындау үшін,bullying

2,сіздің жасыңызда сіз әлі маңызды шешімдер қабылдай алмайсыз,сіздің жас сіз әлі маңызды шешім қабылда алма,bullying

2,сіз тым жассыз ақшаның мағынасын түсіну үшін,сіз тым жас ақша мағына түсіну үшін,bullying

2,сіз мұндай жауапты міндеттерге әлі дайын емессіз,сіз мұндай жауапты міндет әлі дайын емес,bullying

2,сіз тым жассыз пікір алу үшін,сіз тым жас пікір алу үшін,bullying

2,сіз әлі жеткілікті өмірлік тәжірибе жинаған жоқсыз,сіз әлі жеткілікті өмірлік тәжірибе жина жоқ,bullying

2,сіз бұл нені білдіретінін түсіну үшін тым жассыз,сіз бұл не білдір түсіну үшін тым жас,bullying

2,сізге көбірек уақыт пен тәжірибе қажет оны түсіну үшін,сізге көбірек уақыт пен тәжірибе қажет оны түсіну үшін,bullying

2,сіз тым жассыз бұл мәселеде дауыс беру құқығына ие болу үшін,сіз тым жас бұл мәселе дауыс беру құқығ ие болу үшін,bullying

2,сіздің жасыңыз сізге салмақты шешім қабылдауға мүмкіндік бермейді,сіздің жас сізге салмақ шешім қабылда мүмкіндік берме,bullying

2,сіз тым жассыз түсіну үшін нағыз өмір деген не,сіз тым жас түсіну үшін нағыз өмір де не,bullying

2,сіздің жасыңыз қарым қатынастың күрделілігін түсінуге мүмкіндік бермейді,сіздің жас қарым қатына күрделіліг түсін мүмкіндік берме,bullying

2,сізге көбірек уақыт қажет тәжірибе мен білім алу үшін,сізге көбірек уақыт қажет тәжірибе мен білім алу үшін,bullying

2,сіз тым жассыз осындай маңызды нәрселермен айналысу үшін,сіз тым жас осындай маңызды нәрсе айналысу үшін,bullying

2,сіздің жасыңыз сізге жеткілікті білім мен тәжірибе алуға мүмкіндік бермейді,сіздің жас сізге жеткілікті білім мен тәжірибе ал мүмкіндік берме,bullying

2,сізге көбірек өмірлік тәжірибе қажет адамдарды түсіну үшін,сізге көбірек өмірлік тәжірибе қажет адам түсіну үшін,bullying

2,жас маман ретінде сіз бұл жұмыс үшін жеткілікті тәжірибеге ие бола алмайсыз,жас маман рет сіз бұл жұмыс үшін жеткілікті тәжірибе ие бола алма,bullying

2,ұмытпаңыз сен әлі жассың оған уақыт бар,ұмытпа сен әлі жас оған уақыт бар,bullying

2,сіз тым жассыз және нақты өмірдің не екенін білмейсіз,сіз тым жас және нақты өмір не екен білме,bullying

2,бүгінгі жастар бұл жұмыста көп нәрсені білмейді,бүгінгі жас бұл жұмыс көп нәрсе білме,bullying

2,сізде маңызды шешімдер қабылдау тәжірибесі жоқ,сіз маңызды шешім қабылдау тәжірибе жоқ,bullying

2,сіздің жасыңыз сізге мұндай жұмысқа жауапты болуға мүмкіндік бермейді,сіздің жас сізге мұндай жұмыс жауапты бол мүмкіндік берме,bullying

2,сіз тым жассыз бұл лауазымға ие болу үшін,сіз тым жас бұл лауазым ие болу үшін,bullying

2,жастар аға буын тәжірибесімен салыстыра алмайды,жас аға буын тәжірибе салыстыр алма,bullying

2,сіз әлі тым жассыз бизнестің қалай жұмыс істейтінін түсіну үшін,сіз әлі тым жас бизнес қалай жұмыс істе түсіну үшін,bullying

2,сіз мұндай жауапты шешімдер қабылдауға әлі дайын емессіз,сіз мұндай жауапты шешім қабылда әлі дайын емес,bullying

2,жастар бүгінде соншалықты еңбекқор емес және аға буын сияқты жұмыс істегісі келмейді,жас бүгін соншалықты еңбекқор емес және аға буын сияқты жұмыс істе келме,bullying

2,жастар аға буын сияқты жаңа нәрсені тез үйрене алмайды,жас аға буын сияқты жаңа нәрсе тез үйрен алма,bullying

2,сіз тым жассыз түсіну үшін нақты жауапкершілік дегеніміз не,сіз тым жас түсіну үшін нақты жауапкершілік де не,bullying

2,бүгінгі жастар аға буын сияқты қиындықтарды жеңе алмайды,бүгінгі жас аға буын сияқты қиындық же алма,bullying

2,сізге одан да көп тәжірибе қажет тәжірибелі қызметкерлер сияқты нәтижелерге қол жеткізу үшін,сізге одан да көп тәжірибе қажет тәжірибелі қызметкер сияқты нәтиже қол жеткізу үшін,bullying

2,жастар неғұрлым тәжірибелі қызметкерлер белгілеген стандарттарға сәйкес келе алмайды,жас неғұрлым тәжірибелі қызметкер белгіле стандарт сәйкес келе алма,bullying

2,бұл жастар нақты жұмыс туралы ештеңе түсінбейді,бұл жас нақты жұмыс туралы ештеңе түсінбе,bullying

2,мұндай маңызды нәрселерді жасауға әлі ерте қартайған кезде оралыңыз,мұндай маңызды нәрсе жаса әлі ерте қартай кезде орал,bullying

2,жастарға сенуге болмайды маңызды міндеттер олар бәрін бұзады,жас сен болма маңызды міндет олар бәрін бұз,bullying

2,жастар тым жеңіл және шынайы құндылықтарды бағалай алмайды,жас тым жеңіл және шынайы құндылық бағала алма,bullying

2,жастар нақты жұмыс деген не екенін білмейді олар оңай өмірге үйренген,жас нақты жұмыс де не екен білме олар оңай өмір үйрен,bullying

2,жастар табысты мансап үшін қажет болғанша көп жұмыс істеуге дайын емес,жас табысты мансап үшін қажет болғанш көп жұмыс істе дайын емес,bullying

2,сіз тым жассыз бұл жобаны басқару үшін тәжірибелі біреуді табыңыз,сіз тым жас бұл жоба басқару үшін тәжірибелі біреу таб,bullying

2,сізде әлі жеткілікті өмірлік тәжірибе жоқ осы саланың күрделілігін түсіну үшін,сіз әлі жеткілікті өмірлік тәжірибе жоқ осы сала күрделіліг түсіну үшін,bullying

2,жастар нақты әлемде жұмыс істеуге дайын емес оларға оқуға көбірек уақыт қажет,жас нақты әлем жұмыс істе дайын емес оларға оқ көбірек уақыт қажет,bullying

2,сіз әлі басшы болуға дайын емессіз сізге әлі тәжірибе жинау керек,сіз әлі басшы бол дайын емес сізге әлі тәжірибе жинау керек,bullying

2,жастар сенімді қызметкерлер бола алмайды олар өз міндеттері туралы үнемі еске салуды қажет етеді,жас сенімді қызметкер бола алма олар өз міндет туралы үнемі ес салу қажет ет,bullying

2,жастарға тым көп жауапкершілік қажет емес олар бұған әлі дайын емес,жас тым көп жауапкершілік қажет емес олар бұған әлі дайын емес,bullying

2,сіз әлі тым жассыз біздің саланың қалай жұмыс істейтінін түсіну үшін,сіз әлі тым жас біздің сала қалай жұмыс істе түсіну үшін,bullying

2,жастар тым тәжірибесіз осы салада жұмыс істеу үшін,жас тым тәжірибе осы сала жұмыс істеу үшін,bullying

2,жастар мұнда ұзақ уақыт жұмыс істейтін адамдар сияқты көп білім мен тәжірибеге ие бола алмайды,жас мұнда ұзақ уақыт жұмыс істе адам сияқты көп білім мен тәжірибе ие бола алма,bullying

2,сіз әлі де жұмыс істеуіңіз керек осы лауазымға лайық болу үшін,сіз әлі де жұмыс істе керек осы лауазым лайық болу үшін,bullying

2,сіз бұл жұмысқа байланысты жауапкершілікке әлі дайын емессіз,сіз бұл жұмыс байланысты жауапкершілік әлі дайын емес,bullying

2,жастар аға әріптестеріне тәлімгер бола алмайды,жас аға әріптес тәлімгер бола алма,bullying

2,сіз тым жассыз осы салада құрметті және ықпалды болу үшін,сіз тым жас осы сала құрметті және ықпалды болу үшін,bullying

2,жастар тәжірибелі жұмысшылар сияқты бірдей жалақыға ие бола алмайды,жас тәжірибелі жұмысшы сияқты бірде жалақы ие бола алма,bullying

2,сіз әлі үлкен жауапкершілікке дайын емессіз,сіз әлі үлкен жауапкершілік дайын емес,bullying

2,жастар бүгінде ештеңе білмейді және ештеңе істегісі келмейді,жас бүгін ештеңе білме және ештеңе істе келме,bullying

2,бұл ересектерге арналған жұмыс сіз үшін емес,бұл ересек арнал жұмыс сіз үшін емес,bullying

2,сіз көп тәжірибе ала алмайсыз сіз әлі тым жассыз,сіз көп тәжірибе ал алма сіз әлі тым жас,bullying

2,бізге сіздің орныңызда тәжірибелі адам керек,бізге сіздің орн тәжірибелі адам керек,bullying

2,сен тым жассың мұндай жауапкершілікті өз мойнына алу үшін,сен тым жас мұндай жауапкершілік өз мойн алу үшін,bullying

2,мұндай нәрселер туралы ойлауға әлі ерте алдымен тәжірибе жинау керек,мұндай нәрсе туралы ойла әлі ерте алд тәжірибе жинау керек,bullying

2,сіз оны жеңе алмайсыз сіз тым жассыз және тәжірибесізсіз,сіз оны же алма сіз тым жас және тәжірибесіз,bullying

2,бүгінгі жастар тым ақымақ және жауапсыз,бүгінгі жас тым ақы және жауап,bullying

2,жастар маңызды нәрселерді түсіне алмайды,жас маңызды нәрсе түс алма,bullying

2,жастар нағыз кәсіпқой бола алмайды,жас нағыз кәсіпқой бола алма,bullying

2,жастар бүгін мылқау болды,жас бүгін мылқа бол,bullying

2,сен тым кәрісің,сен тым кәрі,bullying

2,зейнетке шығатын уақыт келді ме,зейнет шығ уақыт кел ме,bullying

2,сүт сорғыш,сүт сорғыш,bullying

2,біз кеш жұмыс істеуге дайын жас және жігерлі қызметкерлерді іздейміз,біз кеш жұмыс істе дайын жас және жігерлі қызметкер ізде,bullying

2,сіз мұндай жұмыс үшін тым қартайдыңыз,сіз мұндай жұмыс үшін тым қартай,bullying

2,біз сізге жалақыны көтеруді ұсына алмаймыз сіз зейнеткерлікке шықтыңыз,біз сізге жалақы көтеру ұсын алма сіз зейнеткерлік шық,bullying

2,сіз қазірдің өзінде жасыңыздасыз сіз операцияға тәуекел етпеуіңіз керек,сіз қаз өз жас сіз операция тәуекел етпе керек,bullying

2,біз сізге ем тағайындай алмаймыз сіз бұл үшін тым қартайдыңыз ба,біз сізге ем тағайында алма сіз бұл үшін тым қартай ба,bullying

2,сіздің жасыңызға байланысты денсаулығыңызды жақсарту мүмкін емес,сіздің жас байланысты денсаулығ жақсарту мүмкін емес,bullying

2,сіз біздің оқу курсынан өту үшін тым қартайдыңыз,сіз біздің оқу курс өту үшін тым қартай,bullying

2,біз зейнеткерлерге баспана бермейміз,біз зейнеткер баспана берме,bullying

2,бұл пәтер егде жастағы адамдарға жарамайды,бұл пәтер егде жастағы адам жарама,bullying

2,біз сізге баспана бере алмаймыз өйткені сізге тек жас адамдар үшін қажет ыңғайлылық қажет,біз сізге баспана бер алма өйткені сізге тек жас адам үшін қажет ыңғайлылық қажет,bullying

2,сіз қазірдің өзінде жассыз сіз бұл форманы жеңе аласыз ба,сіз қаз өз жас сіз бұл форма же ал ба,bullying

2,біз сізге бұл қызметті ұсына алмаймыз өйткені сіз зейнеткерлікке шықтыңыз,біз сізге бұл қызмет ұсын алма өйткені сіз зейнеткерлік шық,bullying

2,сіз енді жас емессіз мұндай жеңілдіктерді алу үшін,сіз енді жас емес мұндай жеңілдік алу үшін,bullying

2,қарт адамдар мемдер мен интернет мәдениетін түсінбейді,қарт адам мем мен интернет мәдени түсінбе,bullying

2,әлеуметтік желілерде тек жастар ғана табысты бола алады,әлеуметтік желі тек жас ғана табысты бола ал,bullying

2,әлеуметтік медиа ескі адамдар үшін емес,әлеуметтік медиа ескі адам үшін емес,bullying

2,дәрігер сізге қажетті емдеуді тағайындамайды өйткені сіз тым қартайғансыз және бұл пайдасыз,дәрігер сізге қажетті емдеу тағайындама өйткені сіз тым қартай және бұл пай,bullying

2,рекрутер сіздің түйіндемеңізді қараудан бас тартады өйткені сіз жастан асқансыз,рекру сіздің түйіндеме қарау бас тарт өйткені сіз жас ас,bullying

2,банк сізге үй сатып алуға несие бермейді өйткені сіз зейнеткерлікке шықтыңыз және оны төлей алмайсыз,банк сізге үй сат ал несие берме өйткені сіз зейнеткерлік шық және оны төле алма,bullying

2,дүкен сатушысы сізге көмектеспейді өйткені олар сенеді сіз енді бұрынғыдай жылдам және епті емессіз,дүкен сатушы сізге көмектеспе өйткені олар сен сіз енді бұрынғыдай жылдам және еп емес,bullying

2,сізде менің атам сияқты көп жылдар бар сондықтан егер сіз өзіңізді ауырып жатсаңыз жақсы,сіз менің ата сияқты көп жыл бар сондықтан егер сіз өз ауыр жат жақсы,bullying

2,біздің мектеп жас мұғалімдерді жалдауды жөн көреді өйткені олар жаңа технологияларға икемді және бейімделгіш,біздің мектеп жас мұғалім жалдау жөн көр өйткені олар жаңа технология икемді және бейімделгіш,bullying

2,бұл концерт қарт адамдарға арналмаған бұл жас және жігерлі адамдарға арналған,бұл концерт қарт адам арналма бұл жас және жігерлі адам арнал,bullying

2,бізге жас және жігерлі көшбасшы керек ол бізді прогреске жетелейді,бізге жас және жігерлі көшбасшы керек ол біз прогрес жетеле,bullying

2,бізге жаңа сео керек жаңа қиындықтарды жеңе алатын жас біреу,бізге жаңа сео керек жаңа қиындық же ал жас біреу,bullying

2,сіз қазірдің өзінде тым қартайдыңыз мұндай нәрселермен айналысу үшін,сіз қаз өз тым қартай мұндай нәрсе айналысу үшін,bullying

2,сіз сияқты ескі журналист енді біздің аудиторияны қызықтырмайды,сіз сияқты ескі журналист енді біздің аудитория қызықтырма,bullying

2,ескі футболшы енді жас ойыншылармен қадам жасай алмайды,ескі футболшы енді жас ойыншы қадам жаса алма,bullying

2,бұл жас және жігерлі қарттарға арналған тур орын жоқ,бұл жас және жігерлі қарт арнал тур орын жоқ,bullying

2,біз мұнда ескі адамдарды жібермейміз мұнда бізде тым қатты музыка және тым көп адамдар бар,біз мұнда ескі адам жіберме мұнда біз тым қатты музыка және тым көп адам бар,bullying

2,қарттардың өмірін сақтап қалуға уақыты жоқ олар әлі де жақын арада өледі,қарт өмір сақта қал уақыт жоқ олар әлі де жақын ара өл,bullying

2,егде жастағы мұғалімдер бізге пайдалы ақпарат бере алмайды өйткені олар қазіргі әлемді түсінбейді,егде жастағы мұғалім бізге пайдалы ақпарат бер алма өйткені олар қазіргі әлем түсінбе,bullying

2,ескі саясаткерлер жастардың мүдделерін білдіре алмайды олар жас және жігерлі адамдарға жол беруі керек,ескі саясаткер жас мүдде білдір алма олар жас және жігерлі адам жол беруі керек,bullying

2,ескі спортшылар қазіргі заманғы жарыстардың қарқынына төтеп бере алмайды олар зейнетке шығып жас таланттарға жол беруі керек,ескі спортшы қазіргі заманғы жарыс қарқын төте бер алма олар зейнет шығ жас талант жол беруі керек,bullying

2,егде жастағы журналистер қазіргі тенденцияларды түсіне алмайды және көрсете алмайды олар жас және білімді журналистерге жол беруі керек,егде жастағы журналист қазіргі тенденция түс алма және көрсе алма олар жас және білімді журналист жол беруі керек,bullying

2,аға қызметкерлер нарықтың заманауи талаптарына сай келе алмайды олар зейнетке шығып жас және жігерлі қызметкерлерге жол беруі керек,аға қызметкер нарық заманауи талап сай келе алма олар зейнет шығ жас және жігерлі қызметкер жол беруі керек,bullying

2,егде жастағы суретшілер заманауи өнер туындыларын жасай алмайды олар жас және шығармашылық адамдарға жол беруі керек,егде жастағы суретші заманауи өнер туынды жаса алма олар жас және шығармашылық адам жол беруі керек,bullying

2,жастар сіз өмір туралы әлі ештеңе білмейсіз,жас сіз өмір туралы әлі ештеңе білме,bullying

2,қартты артта қалдырыңыз сізге тыныштық уақыты келді,қар арт қалдыр сізге тыныштық уақыт кел,bullying

2,досым мен сенімен сөйлесудің мағынасын көрмеймін сен бұл техника үшін тым қартайғансың,дос мен сенімен сөйлесу мағына көрме сен бұл техника үшін тым қартай,bullying

2,бұл сен үшін емес сен енді мұндай нәрселермен айналысу үшін дұрыс жаста емессің,бұл сен үшін емес сен енді мұндай нәрсе айналысу үшін дұрыс жас емес,bullying

2,меніңше сен тым жассың менің не айтып тұрғанымды түсіну үшін,меніңше сен тым жас менің не айт тұр түсіну үшін,bullying

2,қарттар енді жастар үшін тренд емес,қарт енді жас үшін тренд емес,bullying

2,неге сіз кенеттен та кәсіпкер болуды шештіңіз сіз зейнетке шығатын уақыт келді,неге сіз кенет та кәсіпкер болу шеш сіз зейнет шығ уақыт кел,bullying

2,сіздің жасыңызда денсаулығыңызға қамқорлық жасау керек деп ойламаңыз жақсы көңіл көтеріп өмір сүріңіз,сіздің жас денсаулығ қамқорлық жасау керек де ойлама жақсы көңіл көтер өмір сүр,bullying

2,сен жас емессің,сен жас емес,bullying

2,мұны жасаңыз сіз бұл үшін әлі қартайған жоқсыз,мұны жаса сіз бұл үшін әлі қартай жоқ,bullying

2,сіз өз жасыңызда қалуыңыз керек,сіз өз жас қал керек,bullying

2,сіз өзгеру үшін тым қартайдыңыз,сіз өзгеру үшін тым қартай,bullying

2,бұл біз үшін емес жастарға арналған нәрсе,бұл біз үшін емес жас арнал нәрсе,bullying

2,біз енді мұндай нәрселерді жасау үшін дұрыс жаста емеспіз,біз енді мұндай нәрсе жасау үшін дұрыс жас емес,bullying

2,қарттармен араласпаңыз,қарт араласпа,bullying

2,қарттарға орын жоқ,қарт орын жоқ,bullying

2,бұл сіздің орныңыз емес қарт адам,бұл сіздің орн емес қарт адам,bullying

2,сіз тым жассыз осындай жоғары лауазымда болу үшін,сіз тым жас осындай жоғары лауазым болу үшін,bullying

2,сіз бұл клубта бола алмайсыз сіз тым қартайдыңыз,сіз бұл клуб бола алма сіз тым қартай,bullying

2,сіз не білмейсіз tiktok дегеніміз не бұл сіздің жасыңыз үшін емес,сіз не білме tiktok де не бұл сіздің жас үшін емес,bullying

2,қараңызшы атаңыз демалуға барғаныңыз жөн болар біз мұны өзіміз анықтаймыз ба,қараңызш ата демал бар жөн бол біз мұны өз анықта ба,bullying

2,аа сен өте жассың сенің бүкіл өмірің әлі алда олай бу шығармайсың,аа сен өте жас сенің бүкіл өмір әлі алда ола бу шығарма,bullying

2,сіз қа жеттіңіз бе зейнетақы туралы ойланып жұмыс істеуді тоқтататын кез келді,сіз қа же бе зейнетақы туралы ойлан жұмыс істеу тоқтат кез кел,bullying

2,бүгінгі жастар тек телефондарына қарап ештеңе білмейді,бүгінгі жас тек телефон қара ештеңе білме,bullying

2,неліктен сіз өз жылдарыңызда әлі де спортпен айналысқыңыз келеді бұл енді сіз үшін емес,неліктен сіз өз жыл әлі де спорт айналыс кел бұл енді сіз үшін емес,bullying

2,сен қандай жарайсың бұл жаста әлі де жұмыс істеп жатырсың мен көптен бері зейнетке шығатын едім,сен қандай жара бұл жас әлі де жұмыс істе жатыр мен көп бері зейнет шығ ед,bullying

2,сіз дасыз ба сонымен бұл қазірдің өзінде кәрілік тыныш емделетін уақыт болар еді,сіз да ба сонымен бұл қаз өз кәрілік тыныш емдел уақыт бол ед,bullying

2,аналар мені ұятқа қалдырмаңыз instagram түсініктемелерінде сіз енді менің жасымда емессіз,ана мен ұят қалдырма instagram түсініктеме сіз енді менің жас емес,bullying

2,иә бұл рас мен сенен үлкенмін бірақ бұл сен мені құрметтемеуің мүмкін дегенді білдірмейді,иә бұл рас мен сенен үлкен бірақ бұл сен мен құрметтеме мүмкін де білдірме,bullying

2,ескі ауыл өмір салты,ескі ауыл өмір сал,bullying

2,қарт сен неге осындасың,қарт сен неге осында,bullying

2,сіздің жылдарыңызда үйде отыратын кез келді,сіздің жыл үй отыр кез кел,bullying

2,ескі сен мұны істей алмайсың,ескі сен мұны істе алма,bullying

2,сіз бұл фильмді көру үшін тым қартайдыңыз,сіз бұл фильм көру үшін тым қартай,bullying

2,сіз бұл кітапты оқуға тым қартайдыңыз,сіз бұл кітап оқ тым қартай,bullying

2,сіз бұл музыканы тыңдау үшін тым қартайдыңыз,сіз бұл музыка тыңдау үшін тым қартай,bullying

2,жастарға бұл оңай беріледі және сізге қиын,жас бұл оңай бер және сізге қиын,bullying

2,мен сенің жасыңда болғанда мен мұны көптен бері істеп келемін,мен сенің жас болғанда мен мұны көп бері істе кел,bullying

2,сіз жастармен салыстыра алмайсыз олардың күші мен энергиясы көп,сіз жас салыстыр алма олардың күш мен энергия көп,bullying

2,ескі әже қазіргі өмірді енді түсінбейді,ескі әже қазіргі өмір енді түсінбе,bullying

2,сіздің ең жақсы жылдарыңыз әлдеқашан өтті,сіздің ең жақсы жыл әлдеқашан өт,bullying

2,қарттар баяу және бейімделмейді жастар дәстүрді құрметтемейді,қарт бая және бейімделме жас дәстүр құрметтеме,bullying

2,қарттар қазіргі әлемде сәтті бола алмайды,қарт қазіргі әлем сәтті бола алма,bullying

2,сіз не білмейсіз компьютерді қалай пайдалану керек ия әрине сіз бұрынғыдай жас емессіз,сіз не білме компьютер қалай пайдалану керек ия әрине сіз бұрынғыдай жас емес,bullying

2,сіз әлі үйленбегенсіз бе сіз қазірдің өзінде жастасыз ба,сіз әлі үйленбе бе сіз қаз өз жас ба,bullying

2,сіз әлі бойдақсыз ба сіз қазірдің өзінде жастасыз ба,сіз әлі бойдақ ба сіз қаз өз жас ба,bullying

2,бұл енді сенің уақытың емес оны жас қалдырыңыз,бұл енді сенің уақыт емес оны жас қалдыр,bullying

2,сен сондай ескіргенсің бұл біздің ғасырда жұмыс істемейді,сен сондай ескір бұл біздің ғасыр жұмыс істеме,bullying

2,сіз осы жаста жастардан жұмыс алмауыңыз керек,сіз осы жас жас жұмыс алма керек,bullying

2,неге әлі зейнетке шықпайсың сіз шынымен жеткілікті жинай алмадыңыз ба,неге әлі зейнет шықпа сіз шын жеткілікті жина алма ба,bullying

2,сіз ризашылық білдіруіңіз керек сіздің жасыңыз бойынша жұмысыңыз бар,сіз ризашылық білдір керек сіздің жас бойынша жұмыс бар,bullying

2,кәрілік қуаныш емес,кәрілік қуаныш емес,bullying

2,сіз концертке не барасыз сіз бұл кештерге барудан әлі шаршаған жоқсыз ба,сіз концерт не бар сіз бұл кеш бару әлі шарша жоқ ба,bullying

2,сен сондай сұрсың зейнетке шығатын кез келді,сен сондай сұр зейнет шығ кез кел,bullying

2,сіздің жасыңызда жаттығудың мәні неде,сіздің жас жаттығ мән не,bullying

2,сен енді жас емессің мұндай ақымақтық жасамауың керек,сен енді жас емес мұндай ақымақтық жасама керек,bullying

2,сіз бұл сіздің жасыңыз үшін емес екенін түсінбейсіз бе,сіз бұл сіздің жас үшін емес екен түсінбе бе,bullying

2,сіз қандай да бір саяхат туралы емес зейнетақы туралы ойлануыңыз керек,сіз қандай да бір саяхат туралы емес зейнетақы туралы ойлан керек,bullying

2,сен сондай ескісің енді бір орында тұра алмайсың,сен сондай ескі енді бір орында тұр алма,bullying

2,ескі адамдар сәнді және стильді бола алмайды,ескі адам сән және стиль бола алма,bullying

2,сіз бұл жаста тағы не істегіңіз келеді,сіз бұл жас тағы не істе кел,bullying

2,ескі ата біздің қазіргі өмірімізге енді сәйкес келмейді,ескі ата біздің қазіргі өмір енді сәйкес келме,bullying

2,сіз осындай жаспен мұндай нәрселерді жасаудан шаршамадыңыз ба,сіз осындай жас мұндай нәрсе жасау шаршама ба,bullying

2,қарттар заманауи және технологиялық бола алмайды,қарт заманауи және технологиялық бола алма,bullying

2,тек жастар ғана жаңа және прогрессивті нәрсе жасай алады,тек жас ғана жаңа және прогрессивті нәрсе жаса ал,bullying

2,барлық қарттар өткенде өмір сүреді және қазіргі әлемге бейімделе алмайды бүгінгі жастар аға буындарды құрметтемейді және оларға қамқорлық жасамайды,барлық қарт өткенде өмір сүр және қазіргі әлем бейімдел алма бүгінгі жас аға буын құрметтеме және оларға қамқорлық жасама,bullying

2,қарттар әрқашан шағымданады және өмірден ләззат алуды білмейді,қарт әрқашан шағымдан және өмір ләззат алу білме,bullying

2,қарттар жұмыста қажет емес олар баяу және өнімді емес жастар бүгінде жауапсыз және үлкендерді құрметтеуді білмейді,қарт жұмыс қажет емес олар бая және өн емес жас бүгін жауап және үлкен құрметтеу білме,bullying

2,қарттар баяу және тез өзгеретін әлемге бейімделе алмайды бүгінгі жастар нақты жұмыстың не екенін білмейді және нақты сағаттармен жұмыс істеуге дайын емес қарттар жастарды және оның проблемаларын түсіне алмайды жастар үлкендерді құрметтемейді және қоғамдық орындарда тәртіп танытпайды жастар бүгінде үлкендерді тәжірибесі мен даналығы үшін құрметтемейді жастар бүгінде өздеріне тұйық және аға буынға қызығушылық танытпайды қарттар қоғамның қазіргі тенденциялары мен қажеттіліктерін түсіне алмайды,қарт бая және тез өзгер әлем бейімдел алма бүгінгі жас нақты жұм не екен білме және нақты сағат жұмыс істе дайын емес қарт жас және оның проблема түс алма жас үлкен құрметтеме және қоғамдық орын тәртіп танытпа жас бүгін үлкен тәжірибе мен даналығ үшін құрметтеме жас бүгін өз тұйық және аға буын қызығушылық танытпа қарт қоғам қазіргі тенденция мен қажеттілік түс алма,bullying

2,жастар аға буынды құрметтемейді өйткені олар ескірген деп санайды және жаңа уақытты түсінбейді,жас аға бу құрметтеме өйткені олар ескір де сана және жаңа уақыт түсінбе,bullying

2,егде жастағы адамдар велосипедпен жүрмеуі керек бұл олар үшін және айналасындағылар үшін қауіпті,егде жастағы адам велосипед жүрме керек бұл олар үшін және айналасындағы үшін қауіпті,bullying

2,сіз тым жассыз саясатқа қамқорлық жасау үшін сізге өмірлік тәжірибе қажет,сіз тым жас саясат қамқорлық жасау үшін сізге өмірлік тәжірибе қажет,bullying

2,тәжірибелі қызметкердің белгілі бір жасы болуы керек сіз бұл лауазым үшін тым жассыз,тәжірибелі қызметк белгілі бір жас бол керек сіз бұл лауазым үшін тым жас,bullying

2,аға буын жастарға не қажет екенін түсінбейді олар тым консервативті,аға буын жас не қажет екен түсінбе олар тым консерватив,bullying

2,сізде отбасын құрудың уақыты келді емес пе сіз мәңгі жалғыз өмір сүре алмайсыз,сіз отбас құр уақыт кел емес пе сіз мәңгі жалғыз өмір сүр алма,bullying

2,сіз енді сол жылдар емессіз сондықтан киіну орынсыз болып көрінеді,сіз енді сол жыл емес сондықтан киін орынсыз бол көрін,bullying

2,сіз тым жассыз және тәжірибесіз машинаны басқару үшін,сіз тым жас және тәжірибе машина басқару үшін,bullying

2,бұл орын тек жастарға арналған,бұл орын тек жас арнал,bullying

2,мұндай нәрселер енді сіздің жасыңызға арналмаған құрметті,мұндай нәрсе енді сіздің жас арналма құрметті,bullying

2,бұл кеш тек жастарға арналған қарт адамдар шақырылмайды қарттарға мұндай жастар мекемесінде орын жоқ,бұл кеш тек жас арнал қарт адам шақырылма қарт мұндай жас мекеме орын жоқ,bullying

2,бұл орын тек ересектерге арналған мұнда ештеңе жоқ,бұл орын тек ересек арнал мұнда ештеңе жоқ,bullying

2,сен неше жастасың сіз мұнда болу үшін тым жассыз,сен неше жас сіз мұнда болу үшін тым жас,bullying

2,қарт адамдар бұған қол жеткізе алады ал сен әлі жеткілікті ақша таппадың,қарт адам бұған қол жеткіз ал ал сен әлі жеткілікті ақша таппа,bullying

2,сіз тым жассыз не туралы сөйлесіп жатқаныңызды білу үшін,сіз тым жас не туралы сөйлес жат білу үшін,bullying

2,сіздің өтінішіңіз өскеннен кейін ғана қаралады,сіздің өтініш өс кейін ғана қарал,bullying

2,бұл тек ересектерге арналған кинотеатр сіз мұнда бола алмайсыз,бұл тек ересек арнал кинотеатр сіз мұнда бола алма,bullying

2,қарт адам техниканы қалай қолдануды ұмытып кеткен шығар,қарт адам техника қалай қолдану ұмыт кет шығар,bullying

2,менің ойымша сіз компанияның басқа зейнеткерлеріне мұқият қарауыңыз керек,менің ойымша сіз компания басқа зейнеткер мұқият қара керек,bullying

2,мүмкін сіз өзіңізді асыра алмау үшін шығуға жақын орынды таңдауыңыз керек шығар,мүмкін сіз өз асыра алмау үшін шығ жақын ор таңда керек шығар,bullying

2,сіз бұл технологияны түсінбеуіңіз таңқаларлық емес сіз жас емессіз зейнеткерлікке шығатын кез келді жастарға еңбек нарығында орын беру керек,сіз бұл технология түсінбе таңқаларлық емес сіз жас емес зейнеткерлік шығ кез кел жас еңбек нарығ орын беру керек,bullying

2,бұл сіздің жасыңызда сіз үшін тым қауіпті,бұл сіздің жас сіз үшін тым қауіпті,bullying

2,сіз бұл қосымшаның не екенін білмейтіндігіңіз таңқаларлық емес бұл тек жастарға арналған,сіз бұл қосымша не екен білмейтіндіг таңқаларлық емес бұл тек жас арнал,bullying

2,менің ойымша сіз өз жасыңызда көру қабілетіңізді тым көп ауыртпауыңыз керек,менің ойымша сіз өз жас көру қабілет тым көп ауыртпа керек,bullying

2,сіз әлі баласыз не болып жатқанын түсіне алмайсыз,сіз әлі бал не бол жат түс алма,bullying

2,оны ересектерге қалдырайық ал сіз үшін жас емес,оны ересек қалдыр ал сіз үшін жас емес,bullying

2,тым көп энергия және аз тәжірибе сізде мұндай маңызды нәрселер туралы ойлауға уақыт жоқ,тым көп энергия және аз тәжірибе сіз мұндай маңызды нәрсе туралы ойла уақыт жоқ,bullying

2,бұл туралы ойлауға әлі ерте,бұл туралы ойла әлі ерте,bullying

2,сіз нақты өмірдің нені білдіретінін білмейсіз,сіз нақты өмір не білдір білме,bullying

2,барыңыз ойнаңыз және ересек істерге араласпаңыз бүгінгі барлық жастар тек оңай жолдарды іздейді,бар ойна және ересек іс араласпа бүгінгі барлық жас тек оңай жол ізде,bullying

2,үлкендеріңізді тыңдаңыз олар жақсы біледі,үлкен тыңда олар жақсы біл,bullying

2,сізде қандай тәжірибе болуы мүмкін сіз мектепті жаңа бітірдіңіз,сіз қандай тәжірибе бол мүмкін сіз мектеп жаңа біт,bullying

2,сізде қандай тәжірибе болуы мүмкін сіз университетті жаңа бітірдіңіз,сіз қандай тәжірибе бол мүмкін сіз университет жаңа біт,bullying

2,сіз мұндай жауапкершілікке әлі дайын емессіз,сіз мұндай жауапкершілік әлі дайын емес,bullying

2,бұл туралы ойланудың қажеті жоқ сен әлі жассың,бұл туралы ойлан қажет жоқ сен әлі жас,bullying

2,сіз біздің тәжірибемізді көбірек құрметтеуіңіз керек өз пікіріңізді айтпас бұрын,сіз біздің тәжірибе көбірек құрметте керек өз пікір айтпас бұрын,bullying

2,біз сіздермен маңызды шешімдер қабылдай алмаймыз сен тым жассың және тәжірибесізсің,біз сіздермен маңызды шешім қабылда алма сен тым жас және тәжірибесіз,bullying

2,сіз өмірді білмейсіз сізге көбірек өмір сүру керек мұның маңыздылығын түсіну үшін жастар бүгінде ештеңені түсінбейді барлық уақытта пайдасыз нәрселермен айналысады бүгінгі жастар тым бос аға буындарды құрметтемейді және өз орнын білмейді,сіз өмір білме сізге көбірек өмір сүру керек мұның маңыздылығ түсіну үшін жас бүгін ештеңе түсінбе барлық уақыт пай нәрсе айналыс бүгінгі жас тым бос аға буын құрметтеме және өз орн білме,bullying

2,сіз үшін маңызды емес нәрсеге уақыт жұмсамаңыз сізде әлі алда бәрі бар,сіз үшін маңызды емес нәрсе уақыт жұмсама сіз әлі алда бәрі бар,bullying

2,сізде қандай тәжірибе болуы мүмкін егер сіз жай ғана үйіңізден кетіп өз бетіңізше өмір сүре бастасаңыз,сіз қандай тәжірибе бол мүмкін егер сіз жай ғана үй кет өз бетіңізш өмір сүр баста,bullying

2,сіз әлі нақты қиындықтарға дайын емессіз сізге көбірек өмірлік тәжірибе қажет,сіз әлі нақты қиындық дайын емес сізге көбірек өмірлік тәжірибе қажет,bullying

2,сіздің жасыңызда сіз ауырсыну мен азапты сезінесіз бұл қалыпты жағдай,сіздің жас сіз ауырсыну мен аза сез бұл қалыпты жағдай,bullying

2,сіз енді отыз емессіз сондықтан үйренуге тура келеді идеялар сіз қартайып бара жатырсыз,сіз енді отыз емес сондықтан үйрен тура кел идея сіз қартай бар жатыр,bullying

2,сіздің денсаулығыңыз ешқашан бұрынғыдай жақсы болмайды,сіздің денсаулығ ешқашан бұрынғыдай жақсы болма,bullying

2,бұл мәселелер сіздің жасыңыз үшін таңқаларлық емес,бұл мәселе сіздің жас үшін таңқаларлық емес,bullying

2,сіз қазірдің өзінде ескі сондықтан операциядан кейін қалпына келтіруді күтпеңіз,сіз қаз өз ескі сондықтан операция кейін қалп келтіру күтпе,bullying

2,сіздің жасыңыз өзі туралы айтады,сіздің жас өзі туралы айт,bullying

2,сіз көбірек дәрі қабылдауыңыз керек өйткені сіз жас емессіз,сіз көбірек дәрі қабылда керек өйткені сіз жас емес,bullying

2,сіз емдеудің бұл түрі үшін қартайдыңыз,сіз емдеу бұл түр үшін қартай,bullying

2,сіздің жасыңызда бұл қалыпты жағдай,сіздің жас бұл қалыпты жағдай,bullying

2,кәрілік аурудан құтылмайды,кәрілік ауру құтылма,bullying

2,ескі адамдар сіз қазірдің өзінде ауырсынуға үйренетін кез келді,ескі адам сіз қаз өз ауырсын үйрен кез кел,bullying

2,сіздің жасыңызбен бұл қалыпты жағдай ештеңені өзгертпеңіз,сіздің жас бұл қалыпты жағдай ештеңе өзгертпе,bullying

2,сіз зейнеткерлікке шықтыңыз мұның бәрі сізге не үшін қажет,сіз зейнеткерлік шық мұның бәрі сізге не үшін қажет,bullying

2,сіз қартайуға дайындықты бастайтын кез келді,сіз қартай дайындық баста кез кел,bullying

2,неге жасты ескере отырып емдеудің жұмсақ түрін қолданып көрмеске,неге жас ескер отыр емдеу жұм түр қолдан көрмес,bullying

2,сіз енді мұндай шешімдерді қабылдамауыңыз керек сіз енді дұрыс емес жаста,сіз енді мұндай шешім қабылдама керек сіз енді дұрыс емес жас,bullying

2,сізде көп уақыт жоқ осындай нәрселермен айналысу сіздің жасыңызды ескере отырып,сіз көп уақыт жоқ осындай нәрсе айналысу сіздің жас ескер отыр,bullying

2,сіздің жасыңызда жаттығудың мәні неде бұл ешқандай пайда әкелмейді,сіздің жас жаттығ мән не бұл ешқандай пайда әкелме,bullying

2,сіз енді жас спортшылармен бәсекеге түсе алмайсыз оны оларға қалдырған дұрыс,сіз енді жас спортшы бәсеке түс алма оны оларға қалдыр дұрыс,bullying

2,бұл жаста сіз енді спортта жетістікке жете алмайсыз бұл туралы ұмытып басқа нәрселермен айналысқан дұрыс,бұл жас сіз енді спорт жетістік же алма бұл туралы ұмыт басқа нәрсе айналыс дұрыс,bullying

2,ескі борозда жылқысы сізді бүлдірмейді енді спортта дәлелдейтін ештеңе жоқ,ескі бороз жыл сіз бүлдірме енді спорт дәлелде ештеңе жоқ,bullying

2,өзіңізді азаптамаңыз спортпен шұғылдану осы жаста жақсы демалыңыз және өмірден ләззат алыңыз,өз азаптама спорт шұғылдан осы жас жақсы демал және өмір ләззат ал,bullying

2,сіз қазірдің өзінде тым қартайдыңыз мұндай спортпен айналысу үшін оны жас қалдырған дұрыс,сіз қаз өз тым қартай мұндай спорт айналысу үшін оны жас қалдыр дұрыс,bullying

2,сіздің жасыңызда денсаулықты бұзбау үшін тыныш спортпен айналысқан дұрыс,сіздің жас денсаулық бұзба үшін тыныш спорт айналыс дұрыс,bullying

2,жас спортшылар сіз тырыссаңыз да сізден озып кетеді,жас спортшы сіз тырыс да сізден оз кет,bullying

2,сіз енді жастармен бәсекелесе алмайсыз бұл туралы ұмытып басқа нәрсе жасаңыз сізге спортпен айналысып зейнетақы туралы ойланатын кез келді,сіз енді жас бәсекеле алма бұл туралы ұмыт басқа нәрсе жаса сізге спорт айналыс зейнетақы туралы ойлан кез кел,bullying

2,спорттық іс шарада денсаулыққа қауіп төндіргеннен гөрі үйде отырғаныңыз жақсы болар еді,спорттық іс шара денсаулық қауіп төндір гөрі үй отыр жақсы бол ед,bullying

2,алаңдағы жас ойыншылар ал сен орындықта отырсың жастарға жол ашатын кез келді,алаң жас ойыншы ал сен орындық отыр жас жол аш кез кел,bullying

2,сіз тым қартайдыңыз марафонға қатысу үшін,сіз тым қартай марафон қатысу үшін,bullying

2,сіз спорттық тапсырманы орындай алмайсыз өйткені сіз тым жүздісіз,сіз спорттық тапсырма орында алма өйткені сіз тым жүзд,bullying

2,сіз бұл спорт үшін тым баяусыз өйткені сіз тым қартайдыңыз,сіз бұл спорт үшін тым баяу өйткені сіз тым қартай,bullying

2,қарттар метрода жеңілдік санатын алу үшін өзіңізбен бірге зейнетақы куәлігін алып келуді ұмытпаңыз,қарт метро жеңілдік сан алу үшін өз бірге зейнетақы куәліг алып келу ұмытпа,bullying

2,эй кемпір сізде жол жүру монеталары бар ма әлде сізге көмектесу керек пе,эй кемпі сіз жол жүру монета бар ма әлде сізге көмектес керек пе,bullying

2,қарттарға арналған бұл автобус оған отырыңыз,қарт арнал бұл автобус оған отыр,bullying

2,бүгінгі жастық шақ қандай жауапсыз аға ұрпаққа жол бере алмайды,бүгінгі жастық шақ қандай жауап аға ұрпақ жол бер алма,bullying

2,қарт адам отыра алады сізде мұндай күшті аяқтар жоқ,қарт адам отыр ал сіз мұндай күшті аяқ жоқ,bullying

2,жастар қайда қарайды аға буынға құрмет жоқ трамвайда орын жоқ,жас қайда қара аға буын құрмет жоқ трамвай орын жоқ,bullying

2,қарт сізге бұл жерде орын жоқ жақсырақ артқы орындыққа отырыңыз,қарт сізге бұл же орын жоқ жақсырақ артқ орындық отыр,bullying

2,ескі атам таксимен жүрер еді қоғамдық көлікте емес бәріне кедергі келтіріңіз,ескі ата такси жүр ед қоғамдық көлік емес бәрін кедергі келтір,bullying

2,кемпір құламаңыз тұтқаны ұстаңыз және қозғалмаңыз,кемпі құлама тұт ұста және қозғалма,bullying

2,бұл адам немесе сөзбе сөз ақыл есі кем немесе ең ақымақ үшін үгітші,бұл адам немесе сөзб сөз ақыл ес кем немесе ең ақы үшін үгітш,bullying

2,психопаттар үнемі өтірік айтады барлығына және кез келген жағдайда олар түсініксіз өтірік айтады және оны жасыруға тырыспайды,психопат үнемі өтірік айт барлығ және кез кел жағдай олар түсінік өтірік айт және оны жасыр тырыспа,bullying

2,психикалық ауытқулары бар адамдар қартайған кезде арнайы өмір сүруі керек мекемелер бұл бәріне жақсы,психикалық ауытқу бар адам қартай кезде арнайы өмір сүр керек мекеме бұл бәрін жақсы,bullying

2,нейродивергентті адамдар нейротиптік метриканың немесе оны бізге қолданатындардың көз алдында ешқашан жетістікке жете алмайды,нейродиверген адам нейротип метрика немесе оны бізге қолдан көз алд ешқашан жетістік же алма,bullying

2,олар ұзақ өмір сүреді тек басқаларға өмір сүруге мүмкіндік бермейді,олар ұзақ өмір сүр тек басқа өмір сүр мүмкіндік берме,bullying

2,мүгедектерге қол жетімділіктің болмауы жай қол жетімсіздік емес бұл ерекшелік,мүгедек қол жетімділік болма жай қол жетімсіз емес бұл ерекшелік,bullying

2,шизофрения деменция және ақыл ой кемістігі мұндай адамдарды ауруханадан шығарып тазалауға болмайды,шизофрения деменция және ақыл ой кемістіг мұндай адам аурухана шығар тазала болма,bullying

2,мектепте мені әкем даун синдромы үшін улады,мектеп мен әке даун синдром үшін ул,bullying

2,ерік жігері күшті сұлулық емес мүгедек ессіз,ерік жігер күшті сұлулық емес мүгедек ес,bullying

2,керісінше біз мүгедектерді жұмыс істеуге мәжбүр етеміз бірақ жұмыс берушілер оларды жұмысқа алғысы келмейді өйткені психикалық аурулары бар адамдар мысалы шизофрения немесе биполярлық бұзылыс еңбекке қабілетті және тек қағазға бейімделген,керісінше біз мүгедек жұмыс істе мәжбүр ет бірақ жұмыс беруші ол жұмыс ал келме өйткені психикалық ауру бар адам мысал шизофрения немесе биполярлық бұзылыс еңбек қабілетті және тек қағаз бейімдел,bullying

2,мүгедек үлкен қалада жалғыз болуы керек пе бір дәрменсізге көбірек сенеді,мүгедек үлкен қала жалғыз бол керек пе бір дәрменсіз көбірек сен,bullying

2,сенің ақыл есі кем ағаң бар деп кінәлі емеспін,сенің ақыл ес кем аға бар де кінәлі емес,bullying

2,біреуді үрейлендіруден ләззат алатын кейбір науқас,біреу үрейлендіру ләззат ал кейбір науқас,bullying

2,кедей және тағдыр оған қандай ауыр сынақ дайындап жатқанын білмеді,кедей және тағдыр оған қандай ауыр сынақ дайында жат білме,bullying

2,crazy psych всегда әрқашан осындай болды,crazy psych всег әрқашан осындай бол,bullying

2,психика қалыпты емес біз үйде және жұмыста кезекте және автобустарда айқайлаймыз,психик қалыпты емес біз үй және жұмыс кезек және автобус айқайла,bullying

2,сол психопат тек темекі шегетін нарик шизо торчок ақыры катушкалардан шығып кетті,сол психопат тек темекі шег нарик шизо торчок ақы катушка шығ кет,bullying

2,модельдеудің өзі көрінбейтін импульсивті типтегі айқын психопат,модельдеу өзі көрінбе импульсив тип айқын психопат,bullying

2,ол қазірдің өзінде мені ашуландырды кеңес өкіметінің мүгедегі,ол қаз өз мен ашулан кеңес өкім мүгедег,bullying

2,аңғал ақымақ адам өзінің әдептілігімен артта қалды,аңғал ақы адам өзінің әдептіліг арт қал,bullying

2,сіз нюняның психопатысыз егер сіз қарапайым адам болсаңыз бір жылдан кейін миллионға ие боласыз,сіз нюня психопат егер сіз қарапайым адам бол бір жыл кейін миллион ие бол,bullying

2,ақыл есі кем бала әртүрлі жеке құбылыстарда ортақ нәрсені таба алмайды,ақыл ес кем бала әртүрлі жеке құбылыс ортақ нәрсе таб алма,bullying

2,ақымақ ақымақ мылқау дамымаған қисық мұрын және құдайға жиіркенішті,ақы ақы мылқа дамыма қисық мұрын және құдай жиіркеніш,bullying

2,мойынсұнғыш бірақ дамымаған сондықтан ақыл есі кем дегенді білдіреді,мойынсұнғыш бірақ дамыма сондықтан ақыл ес кем де білдір,bullying

2,келесі күлімсіреген аузы бар ақымақ мұрнындағы лас саусақты таңдайды,келесі күлімсіре ауз бар ақы мұрн лас саусақ таңда,bullying

2,кейбір ақымақ оларды ойлап тапты содан бері ештеңе жоқ тек олар,кейбір ақы ол ойла тап содан бері ештеңе жоқ тек олар,bullying

2,науқас ешқандай сезімді көрсетпестен бір бөлікті мүлдем жеп қойды,науқас ешқандай сез көрсетпес бір бөлік мүлдем же қой,bullying

2,ақыл есі кем бала сөздерді әдеттегіден басқаша қабылдайды және оның сөздерімен қозғалатын байланыс жүйесі басқа қалыпты оқушыға қарағанда,ақыл ес кем бала сөз әдеттегі басқаша қабылда және оның сөз қозғал байланыс жүйе басқа қалыпты оқушы қара,bullying

2,ол мүгедек емес ар ұждан болуы керек,ол мүгедек емес ар ұждан бол керек,bullying

2,у у ақымақ ақымақ ақымақ,у у ақы ақы ақы,bullying

2,өйткені олар толыққанды адамдар емес даун есімді толыққанды басқа тіршілік иелері бұл біздің қасымызда бар және байланыс орнату бұл шын мәнінде қарым қатынас міндеті,өйткені олар толыққанды адам емес даун ес толыққанды басқа тіршілік ие бұл біздің қас бар және байланыс орнату бұл шын мән қарым қатынас міндет,bullying

2,ол жұмысшы болды қолында кішкентай балалары болды екінші бақытсыз бала ақыл есі кем қыз,ол жұмысшы бол қол кішкентай бала бол екінші бақыт бала ақыл ес кем қыз,bullying

2,егер мүгедек болса мүгедектер арбасы болуы керек,егер мүгедек бол мүгедек ар бол керек,bullying

2,бір екінші мүгедек жартылай мүгедек денсаулығы нашар қалғандары әлеуметтік және педагогикалық тұрғыдан қараусыз қалған балалар,бір екінші мүгедек жартылай мүгедек денсаулығ нашар қал әлеуметтік және педагогикалық тұрғы қараусыз қалған бала,bullying

2,оның күйеуі бәрін басқарады таяқшаны балғамен ұрып шаршаған терлеген мүгедекті протезбен жалбызға батырылған блейзерге ескі матадан жасалған жастықшалармен ұрады,оның күйе бәрін басқар таяқша балға ұр шарша терле мүгедек протез жалбыз батырыл блейз ескі мата жасал жастықша ұр,bullying

2,бір сөзбен айтқанда мұндай ауылдағы ақымақ ақыл мен дененің мүгедегі,бір сөз айтқанда мұндай ауыл ақы ақыл мен дене мүгедег,bullying

2,қандай да бір россинантист ақымақ қорқынышты өлім күнәсі сияқты бәрі бізге тиді,қандай да бір россинантист ақы қорқыныш өлім күнә сияқты бәрі бізге ти,bullying

2,мен өкіндім терезесі жоқ махина көзсіз мүгедек сияқты,мен өкін терезе жоқ махин көз мүгедек сияқты,bullying

2,ақымақ аяғымен үстелге сүйеніп ауыр орындық онымен бірге бүйіріне құлады,ақы аяғ үстел сүйен ауыр орындық онымен бірге бүйір құл,bullying

2,сіз ақымақ сияқты жүгіресіз ура жаңғағы және дөңгелектердегі сабан немісті шаншыңыз,сіз ақы сияқты жүгір ура жаңғағ және дөңгелек сабан неміс шанш,bullying

2,бұл ақымақ бұл темір төсек өндіруші ешнәрсе үшін келіскісі келмеді,бұл ақы бұл темір төсек өндіруші ешнәрсе үшін келіс келме,bullying

2,габельді тыңдаңыз сіз құдай емессіз сіз ақымақсыз,габель тыңда сіз құдай емес сіз ақы,bullying

2,іс жүзінде қоян емес қоян тұқымының мүгедегі,іс жүз қоян емес қоян тұқым мүгедег,bullying

2,олар қаскүнемдікпен күлді әдейі бұрылып мен анық күлгенімді естуім үшін ах ақымақ,олар қаскүнемдік күл әдей бұрыл мен анық күл ест үшін ах ақы,bullying

2,сен ақымақсың сен ақымақсың сіз мұндай жағдайды жіберіп алдыңыз мен сізбен сөйлескім келмейді,сен ақы сен ақы сіз мұндай жағдай жібер ал мен сізбен сөйлес келме,bullying

2,ақымақ али ақымақ али зұлым жаратылыс,ақы али ақы али зұлым жаратылыс,bullying

2,ол абсолютті шизофрения және болжау мүмкін емес психопат сондықтан оны мұқият емдеу керек,ол абсолютті шизофрения және болжау мүмкін емес психопат сондықтан оны мұқият емдеу керек,bullying

2,доғал сондықтан фотосинтез гені де бар,доғал сондықтан фотосинтез ген де бар,bullying

2,онымен бір нәрсе толық тәртіпте емес ол алкаш па шизофрения ма әлде пайда болмаған қылмыскер ме,онымен бір нәрсе толық тәртіпте емес ол алкаш па шизофрения ма әлде пайда болма қылмыскер ме,bullying

2,мен ақымақ емеспін кеңестік балаға патшаларды дәріптеумен және еркектермен бірге құлдар ретінде мағынасыз ертегілер жазуға болмайтынын түсінбеу үшін,мен ақы емес кеңестік бала патша дәріпте және еркек бірге құл рет мағына ертегі жаз болма түсінбе үшін,bullying

2,тіпті ақыл есі кем бала әрдайым түбегейлі оқытылады ал демент науқасты оқытпайды,тіпті ақыл ес кем бала әрдайым түбегейлі оқыт ал демент науқас оқытпа,bullying

2,бір минутқа ақымақ үнсіз қалды үлкен бас сағат қуыршағындағыдай көзімен бірдеңе іздеп бұрылды,бір минут ақы үн қал үлкен бас сағат қуыршағындағыда көз бірде ізде бұр,bullying

2,менің түсінуімше ол аз дегенде ақымақ,менің түсінуімш ол аз де ақы,bullying

2,о сен таңқалдырасың ба ол қоқыстардан өтіп бара жатып айқайлады ма сізге не керек,о сен таңқалдыр ба ол қоқыс өт бар жат айқайла ма сізге не керек,bullying

2,эх ақымақ ақымақ дрейк жоғары көтеріліп жоғары көтерілді ме эх,эх ақы ақы дрейк жоғары көтеріл жоғары көтер ме эх,bullying

2,ол ақымақ болсын тек менің айтқанымды ғана жасайды,ол ақы бол тек менің айт ғана жаса,bullying

2,о ақымақ дөрекі адам варвар могдав сынбаған тако ли сізді шақырған есиге риза емес,о ақы дөрекі адам варв могдав сынба тако ли сіз шақыр еси риза емес,bullying

2,сіздің ұлыңыз ақыл есі кем,сіздің ұл ақыл ес кем,bullying

2,ол жаман бала нашақор шығар,ол жаман бала нашақо шығар,bullying

2,шала туылған купчон әлі қолыңызды көтере аласыз ба мен пышақпен сөндіремін,шал туыл купчон әлі қол көтер ал ба мен пышақ сөндір,bullying

2,ескі ақымақ олармен жылады,ескі ақы олармен жыл,bullying

2,шала туылған арық похлопали бойынша попе,шал туыл арық похлопали бойынша поп,bullying

2,мылқау жалқау мысқылдың көлеңкесі жоқ бұл заттың күлкілі жағын тез түсінетін орыс адамының айқын ақыл ойына тән қоймалардың барлық мағынасыз процедурасынан шыдамдылықпен өтеді,мылқа жалқау мысқ көлеңке жоқ бұл зат күлкіл жағ тез түсін орыс адам айқын ақыл ой тән қойма барлық мағына процедура шыдамдылық өт,bullying

2,уф сен оларға тұншығып қаласың мылқау адамсың,уф сен оларға тұншығ қал мылқа адам,bullying

2,тәтті ақымақ тағы да лалагүлдің ақ королласы туралы романсын шырқады,тәтті ақы тағы да лалагү ақ королла туралы роман шырқа,bullying

2,еңкейген хромлет мүгедек ретінде арық сары өзі сияқты кептірілген лимон және мұрын мен щек арқылы кең қара патч,еңкей хромлет мүгедек рет арық сары өзі сияқты кептір лимон және мұрын мен щек арқылы кең қара патч,bullying

2,ол ақымақ қаскөй ме әлде табиғаты бойынша ма жаратқан ие оны біледі,ол ақы қаскөй ме әлде табиғат бойынша ма жаратқан ие оны біл,bullying

2,ақымақ ашулы өзімшіл мылқау мылқау ол тағы қандай ием болуы керек,ақы ашул өзімшіл мылқа мылқа ол тағы қандай ие бол керек,bullying

2,ол сауатсыз мылқау шектеулі адам және бәрі кейбір құрбандықтар мен қанды қалайды,ол сауат мылқа шектеулі адам және бәрі кейбір құрбандық мен қан қала,bullying

2,аяқсыз мүгедек шөпте киім мен балдақ қалдырып суға шомылу үшін құммен жорғалайды,аяқсыз мүгедек шөп киім мен балдақ қалдыр су шомыл үшін құм жорғала,bullying

2,мағынасыз мылқау адамгершілікке жатпайтын шырша мен мұның бәрін көрген емеспін мен тек естідім,мағына мылқа адамгершілік жатпа шырша мен мұның бәрін көр емес мен тек есті,bullying

2,доғал төңкерілген мұрын кішкентай көздер жерге бармайтын және жерді таптайтын тіршілік иесі,доғал төңкер мұрын кішкентай көз же барма және же тапта тіршілік иесі,bullying

2,шаблатович сондай ақ бұрынғы шала туылған аспирант тронко дөрекі және тәрбиесіз,шаблатович сондай ақ бұрынғы шал туыл аспирант тронко дөрекі және тәрбие,bullying

2,оның мылқау дауысы мазасыз естілді сол қол басын сезді кеуде,оның мылқа дауыс мазасыз ест сол қол басын сез кеуде,bullying

2,сіз мүгедек көгерген адамсыз,сіз мүгедек көгер адам,bullying

2,ақыл есі кем бала объектілердің сенсорлық немесе көрнекі тиімді белгілерінен алшақтай алмайды және әдетте түсі немесе формасы бойынша ұқсастықтары бар заттарды таңдайды,ақыл ес кем бала объекті сенсорлық немесе көрнекі тиімді белгі алшақта алма және әдет түс немесе форма бойынша ұқсастық бар зат таңда,bullying

2,үндемеңіз ақымақ барлық сүйектер бүтін болғанша,үндеме ақы барлық сүйек бүтін болғанш,bullying

2,доғал және тәкаппар кішкентай оң қолында ақ қолғап бар,доғал және тәкапп кішкентай оң қол ақ қолғап бар,bullying

2,және ол ақылсыз ақымақ түнге үміттеніп тікелей жүрді,және ол ақыл ақы түн үміттен тікелей жүр,bullying

2,ақылсыз ақымақ басқа сөз жоқ,ақыл ақы басқа сөз жоқ,bullying

2,ал қызым шешесіне сол ақылсыз ақымақ болды,ал қыз шеше сол ақыл ақы бол,bullying

2,о мал жылқы мисыз ал басқа вагонда неге ит тоқтады,о мал жылқы ми ал басқа вагон неге ит тоқта,bullying

2,мен сені енді көрмеу үшін ақымақ бол,мен сен енді көрме үшін ақы бол,bullying

2,тек пикник жасауға тырысыңыз сен кішкентай бозғылт боқс,тек пикник жаса тырыс сен кішкентай бозғылт боқс,bullying

2,үнсіздік ақылсыз ақымақ,үнсіздік ақыл ақы,bullying

2,ал сен өмірдегі сорғышсың,ал сен өмір сорғыш,bullying

2,міне балбес игорь понурился,міне балбес игорь понурился,bullying

2,айтыңызшы мылжың сіздің атыңыз кім,айтыңызш мылж сіздің ат кім,bullying

2,және ол миы жоқ кретин оған олардың подіктерін сипаттады,және ол ми жоқ кретин оған олардың подік сипатта,bullying

2,ол ақыл есі кем ақымақ егер ол оған ұсынылса жақсы әдебиетті оқи алмас еді,ол ақыл ес кем ақы егер ол оған ұсыныл жақсы әдебиет оқи алмас ед,bullying

2,ол бізде мұндай балбесті түсінбейді,ол біз мұндай балбе түсінбе,bullying

2,контра ит буржуазиялық мылжың барлығы менің үйімнен,контр ит буржуазиялық мылж барлығ менің үй,bullying

2,құрбандық емес ақ қанаттармен дірілдейтін керемет қаншық емес азғырушы,құрбан емес ақ қанат дірілде керемет қаншық емес азғыруш,bullying

2,иә етік алдымен менің үстімде миы жоқ чурканы киіңіз,иә етік алд менің үст ми жоқ чурка ки,bullying

2,сіз әлсіз және ақымақсыз мүмкін бұл өте ақылды және ақылды шығар,сіз әлсіз және ақы мүмкін бұл өте ақылды және ақылды шығар,bullying

2,ақымақ өтпейтін ақымақ бәрін ойластыру керек болды,ақы өтпе ақы бәрін ойластыр керек бол,bullying

2,сен не алтынсың ұмытып бля кіммен сөйлесесің,сен не алтын ұмыт бля кім сөйлес,bullying

2,ол мәңгілік гид тілді әлемдегі жалғыз,ол мәңгілік гид тілді әлем жалғыз,bullying

2,кешіріңіз мен оның қаншалықты ақымақ екенін бұрын түсінбедім,кешір мен оның қаншалықты ақы екен бұрын түсінбе,bullying

2,артта қалған және надан сіз субъектісіз жолдас темекі біздің дәуіріміздің басында өмір сүрген неандертальдық адам сияқты,арт қалған және надан сіз субъект жолдас темекі біздің дәуір бас өмір сүр неандерталь адам сияқты,bullying

2,сол жануар науқасты емдеу керек,сол жануар науқас емдеу керек,bullying

2,қарғыс атсын менің көзіме түспе,қарғыс ат менің көз түспе,bullying

2,орта мектептен қабілетсіздігі үшін қуылған екінші жылдық реалистерден артта қалған зұлым жасөспірім,орта мектеп қабілетсіздіг үшін қуыл екінші жылдық реалис арт қалған зұлым жасөспірім,bullying

2,сен кімді білесің фрик ақымақ,сен кім біл фрик ақы,bullying

2,және сіз әрқашан ақымақ сияқты осы уақытқа сәйкес келесіз немесе сәйкес келмейсіз бірақ сіз әлі де ақымақ болып қаласыз,және сіз әрқашан ақы сияқты осы уақыт сәйкес кел немесе сәйкес келме бірақ сіз әлі де ақы бол қал,bullying

2,мен онымен жұмыс істемеймін ол жұмыста жаңғақ,мен онымен жұмыс істеме ол жұмыс жаңғақ,bullying

2,сен ақылсызсың сен ақымақсың,сен ақылсыз сен ақы,bullying

2,бұл мисыз абайсыз есек менен он қадам қашықтықта жарықпен теңестірілді,бұл ми абай есек менен он қадам қашықтық жарық теңестір,bullying

2,о сен ақылды жаратылыссың,о сен ақылды жаратылыс,bullying

2,ұятсыз жыртылған аң кім ол айтты,ұят жыртыл аң кім ол ай,bullying

2,ол арық болды сабырлы және өте дөрекі көздер өте дөрекі,ол арық бол сабырл және өте дөрекі көз өте дөрекі,bullying

2,ал сен өзің ақымақсың содан кейін мен оны ұрдым,ал сен өзің ақы содан кейін мен оны ұр,bullying

2,ах гүл ақылсыз және ақылсыз ағай ұстап алды,ах гүл ақыл және ақыл аға ұста алды,bullying

2,міне ақымақ оттан келеді иә қуыс фрик отбасылық,міне ақы от кел иә қуыс фрик отбасылық,bullying

2,сіздің бизнесіңіз мисыз хомяк емес,сіздің бизнес ми хомяк емес,bullying

2,ол ақымақ сондай бақытсыз және жалғыз,ол ақы сондай бақыт және жалғыз,bullying

2,қалыпты емес ақымақтан,қалыпты емес ақымақ,bullying

2,ол жүгіреді ақымақ қалыптан тыс болып шықты қаңқа болды,ол жүгір ақы қалып тыс бол шық қаңқа бол,bullying

2,бұл қалыпты емес ол сөйлей алмайтын шығар бірақ қорқынышты қауіп төндіретін жүзін жасап шалбарының қалталарын бұрып жіберді,бұл қалыпты емес ол сөйле алма шығар бірақ қорқыныш қауіп төндір жүз жаса шалбар қалта бұр жіб,bullying

2,сен қарапайым кеңестік мүгедексің,сен қарапайым кеңестік мүгедек,bullying

2,иә жоқ фу қоқыс қандай,иә жоқ фу қоқыс қандай,bullying

2,о сен мисыз басс сіз естисіз ол қандай томбой,о сен ми басс сіз ести ол қандай томбо,bullying

2,мүгедек қыз оның аузы ашық сілекейі үнемі ағып тұрады,мүгедек қыз оның ауз ашық сілекей үнемі ағ тұр,bullying

2,міне адам дүниеге келді бала және ол қазірдің өзінде науқас мүгедек фрик,міне адам дүние кел бала және ол қаз өз науқас мүгедек фрик,bullying

2,орындықтағы мүгедек велосипедшілер сияқты саусақтары кесілген қара қолғап киген алақандарымен жылтыр металл дөңгелектер жиектерін қысып тұрды,орындық мүгедек велосипедші сияқты саусақ кес қара қолғап ки алақан жылтыр металл дөңгелек жиек қыс тұр,bullying

2,төрт саусақты ұсқынсыз жер асты мүгедек үйрек,төрт саусақ ұсқын жер асты мүгедек үйрек,bullying

2,житомир шкафындағы арбадағы бұралған мүгедек,житоми шкаф арба бұрал мүгедек,bullying

2,мүгедек бір көзді тілсіз одан не алуға болады,мүгедек бір көз тіл одан не ал бол,bullying

2,ол өзі бірге тұратын мүгедек пе әлде күйеуі ме ішетін ақымақ,ол өзі бірге тұр мүгедек пе әлде күйе ме іш ақы,bullying

2,мүгедек сен қуылған ақымақсың бақытсыз үйсіз бейтаныс,мүгедек сен қуыл ақы бақыт үй бейтаныс,bullying

2,ауру жаман фрик қолы мен аяғы ісінген рахит әлсіз бұл аяқта тұрмайды,ауру жаман фрик қол мен аяғ ісін рахит әлсіз бұл аяқта тұрма,bullying

2,басы атылған мүгедек өзінің құлап жатқан бұрышын қорғау үшін өзі жұмыс істеуі керек,бас атыл мүгедек өзінің құла жат бұрыш қорғау үшін өзі жұмыс істе керек,bullying

2,көкек қауырсынымен ақымақ сияқты отырады,көкек қауырсын ақы сияқты отыр,bullying

2,аянышты мүгедек сол ақымақ соғыстың құрбаны,аяныш мүгедек сол ақы соғ құрбан,bullying

2,ол қолы жоқ мүгедек және бөтелкемен ұра алмады,ол қол жоқ мүгедек және бөтелке ұр алма,bullying

2,ол ұрлайды оның қызы болды ересек мүгедек балалық шақ,ол ұрла оның қыз бол ересек мүгедек балалық шақ,bullying

2,біреуі демобилизацияланған сияқты шатырда бір жерде тұрады екіншісі дұрыс ағаш аяғындағы мүгедек жертөледе тұрады,біреу демобилизациялан сияқты шатыр бір же тұр екінші дұрыс ағаш аяғ мүгедек жертөле тұр,bullying

2,абсолютті әлеуметтік реалистік буза бар болса да өркеш ақсақ мүгедек нағыз ауыл ойнайды барлығы қорқынышты тілде,абсолютті әлеуметтік реалистік буз бар бол да өркеш ақ мүгедек нағыз ауыл ойна барлығ қорқыныш тіл,bullying

2,сынған жалғыз жараланған жақында болған жекпе жектің мүгедегі,сын жалғыз жаралан жақында бол жекпе жек мүгедег,bullying

2,ағаш аяғындағы мүгедек жас,ағаш аяғ мүгедек жас,bullying

2,күтпеген психопат,күтпе психопат,bullying

2,ол өзі бірге тұратын мүгедек пе әлде күйеуі ме ішімдік ішу мұндай ата аналарда не болуы мүмкін,ол өзі бірге тұр мүгедек пе әлде күйе ме ішімдік іш мұндай ата ана не бол мүмкін,bullying

2,эх ақымақ сен әйелсің,эх ақы сен әйел,bullying

2,эх сен қандай ақымақ сөздерді айтасың,эх сен қандай ақы сөз ай,bullying

2,жетім таң мен оны тіпті аядым,жетім таң мен оны тіпті ая,bullying

2,ол мүгедек оның екі аяғы да оң қолы да жоқ,ол мүгедек оның екі аяғ да оң қол да жоқ,bullying

2,рас ол ескі және науқас үйден әрең шығады бірақ телефон арқылы белсенді сөйлеседі,рас ол ескі және науқас үй әре шығ бірақ телефон арқылы белсенді сөйлес,bullying

2,ол немесе ақымақ немесе жауды қайталаңыз,ол немесе ақы немесе жау қайтала,bullying

2,о ақымақ дөрекі адам варвар могдав сынбаған,о ақы дөрекі адам варв могдав сынба,bullying

2,жас әдемі мүгедек кеудесінде қалайы кресті бар тачанкадағы ысталған мүгедек жырту сұрыптайды жастықшалармен араластырады мойынтіректер шылдырлайды,жас әдемі мүгедек кеуде қалай крест бар тачанка ыстал мүгедек жырт сұрыпта жастықша араластыр мойынтірек шылдырла,bullying

2,ол жай ғана мүгедек және олар қалады,ол жай ғана мүгедек және олар қал,bullying

2,ирина әйел суретші өнер сыншысы сіз біздің өткен өміріміз туралы аздап білесіз сонымен қатар ол мүгедек,ирина әйел суретші өнер сыншы сіз біздің өткен өмір туралы аздап біл сонымен қатар ол мүгедек,bullying

2,кейде мен орыс әйелдерін түсінбеймін түнде күйеуіне жұмысқа не баратынын айтып ішімдік ішіп сүйіктісіне көмектесу үшін қоңырау шалады ол соғыс мүгедегі үлкен зейнетақы алады ал әйелі жоқ,кейде мен орыс әйел түсінбе түн күйе жұмыс не бар айт ішімдік іш сүйікті көмектес үшін қоңырау шал ол соғыс мүгедег үлкен зейнетақы ал ал әйел жоқ,bullying

2,ұлдар гриша ағайдың балалық шақтағы мүгедек екенін біліп күлді соғыста болған жоқ,ұл гриш ағай балалық шақ мүгедек екен біл күл соғыс бол жоқ,bullying

2,көрінетін мүгедек клиникалық ақымақ ол әпкесінің отбасымен бірге тұрады және біртүрлі серуендейді айталық ыңғайлылықтан шықпай,көрін мүгедек клиникалық ақы ол әпке отбас бірге тұр және біртүрлі серуенде айталық ыңғайлылық шықпа,bullying

2,егер менің мүгедек ұлым болса бала ұсқынсыз мен оны жазаламас едім ал емдейтін едім төсегінде отыратын еді кітап оқитын еді сурет салуды үйрететін едің ал сен хули сен нирванада істейсің егер мен осында болсам немесе сен мені сүймейсің бе сіз бәрін жақсы көресіз деп айттыңыз мен де бәрін қалаймын всегда әрқашан жүрегіңізбен науч үйретіңіз,егер менің мүгедек ұл бол бала ұсқын мен оны жазаламас ед ал емде ед төсег отыр ед кітап оқитын ед сурет салу үйрет ед ал сен хули сен нирвана істе егер мен осында бол немесе сен мен сүйме бе сіз бәрін жақсы көр де ай мен де бәрін қала всег әрқашан жүрег науч үйрет,bullying

2,қарап тұрсыз ба мүгедек клиникалық ақымақ ол әпкесінің отбасымен бірге тұрады және біртүрлі серуендейді айталық күңкілдеген ыңғайлылықтан,қара тұр ба мүгедек клиникалық ақы ол әпке отбас бірге тұр және біртүрлі серуенде айталық күңкілде ыңғайлылық,bullying

2,мүгедек шебер етікші болып шықты теріні бір жерге жеткізіп етік сатуға тігеді,мүгедек шебер етікш бол шық тері бір же жеткіз етік сат тіг,bullying

2,сіз мүгедек емессіз науқас емессіз сіз жай ғана әлеуметтік жауапсыз адамсыз,сіз мүгедек емес науқас емес сіз жай ғана әлеуметтік жауап адам,bullying

2,ол шынымен де дамымаған жаңғақ сияқты сөйлейді,ол шын де дамыма жаңғақ сияқты сөйле,bullying

2,егер ол екінші топтағы мүгедек болса иә тіпті эпилепсиялық оны зауытқа жұмыс істеуге кім алды тіпті токарь,егер ол екінші топтағы мүгедек бол иә тіпті эпилепсиялық оны зауыт жұмыс істе кім алды тіпті токарь,bullying

2,мария михайловна көру қабілеті нашар ол үшін үйден шығып дүкенге бару керек пе ең ауыр жұмыс,мария михайловн көру қабілет нашар ол үшін үй шығ дүкен бару керек пе ең ауыр жұмыс,bullying

2,неге отырады егер мүгедек болса мүгедектер арбасы болуы керек неліктен әйелді ешкім шығарып салмайды,неге отыр егер мүгедек бол мүгедек ар бол керек неліктен әйел ешкім шығар салма,bullying

2,мүгедек спикерді мұқият тыңдап бір нәрсе туралы ойланбай ойланған сияқты,мүгедек спикер мұқият тыңда бір нәрсе туралы ойланба ойлан сияқты,bullying

2,төрт бетте ол өзінің жұмбақ көздері нәзік фигурасы және нәзік әсем қолдары туралы жазады кедей мүгедек орындыққа байланған ба ол өзіне идеалды ойлап тауып ғашық болды,төрт бет ол өзінің жұм көз нәзік фигура және нәзік әсе қол туралы жаз кедей мүгедек орындық байлан ба ол өзіне идеалды ойла тау ғашық бол,bullying

2,басқа мүгедек дастархан басында отырған команда оны тым қатты қабылдаған кезде толқудан әдепсіз дыбыстар шығара бастады,басқа мүгедек дастархан бас отыр команда оны тым қатты қабылда кезде толқу әдепсіз дыбыс шығар баста,bullying

2,бір екінші мүгедек жартылай мүгедек қалғандары денсаулығы нашар әлеуметтік және педагогикалық тұрғыдан қараусыз қалған балалар бірақ олардың негізінде жақсы,бір екінші мүгедек жартылай мүгедек қал денсаулығ нашар әлеуметтік және педагогикалық тұрғы қараусыз қалған бала бірақ олардың негіз жақсы,bullying

2,мектептегі адам бұл не бүкіл басы бар науқас не аскетик бұл менің ойымша бірдей,мектеп адам бұл не бүкіл бас бар науқас не аскетик бұл менің ойымша бірде,bullying

2,мұндай жалған айыптауларды тек өтірікші немесе психикалық науқас адам ойлап таба алады,мұндай жалған айыптау тек өтірікш немесе психикалық науқас адам ойла таб ал,bullying

2,жоқ сіз ауру көкжидек психикалық бопсалауды тоқтатып осы жерден кетіңіз немесе психушкаға жіберіңіз,жоқ сіз ауру көкжидек психикалық бопсалау тоқтат осы же кет немесе психушка жіб,bullying

2,оған сен не даунсың есінен танып кімді ұрды ал сен және психо,оған сен не даун ес тан кім ұр ал сен және психо,bullying

2,аутизм спектрі бұзылған адамдар жеткіліксіз,аутиз спектр бұзыл адам жеткіліксіз,bullying

2,сен өз жұмысыңды жақсы істеп жатырсың бірақ маған кеңседе сау адам керек,сен өз жұмыс жақсы істе жатыр бірақ маған кеңсе сау адам керек,bullying

2,мен сені тіпті мүгедек деп санамаймын,мен сен тіпті мүгедек де санама,bullying

2,ол сондай ақылсыз,ол сондай ақыл,bullying

2,психушка осылай айқайлады бір қызығы учаскелік педиатр мен медбике баланың картасына не жазады,психушк осылай айқайла бір қызығ учаскелік педиатр мен медбике бала карта не жаз,bullying

2,ол оған науқас психушка жылайды,ол оған науқас психушк жыла,bullying

2,николай бақытсыз науқас бала кезінде есінен танып қорқыныштан бұрмаланған содан кейін жиырма жыл бойы күтушінің қолында қайтыс болды,никола бақыт науқас бала кез ес тан қорқыныш бұрмалан содан кейін жиырма жыл бойы күтуші қол қайтыс бол,bullying

2,бұл науқас устьянцев тегі бойынша маған қарсы жатты,бұл науқас устьянцев тег бойынша маған қарсы жат,bullying

2,сіз оны көрдіңіз бе сирек кездесетін зімбір бірақ оның кішкентай әпкесі бар екенін білесіз бе ол оған осылай қарайды,сіз оны көр бе сирек кездес зімбі бірақ оның кішкентай әпке бар екен біл бе ол оған осылай қара,bullying

2,толық ақымақ зімбір ақымақ,толық ақы зімбі ақы,bullying

2,жартылай соқыр мүгедек қарт адам қорқынышты уақыттың куәліктерін редакцияға жіберді,жартылай соқыр мүгедек қарт адам қорқыныш уақыт куәлік редакция жіб,bullying

2,мен сен үшін дұға ете аламын ба,мен сен үшін дұ ет ал ба,bullying

2,бұл соқыр сияқты соқыр адамды басқарады,бұл соқыр сияқты соқыр адам басқар,bullying

2,иә өйткені ол басымен де қолымен де жұмыс істей алмайды,иә өйткені ол бас де қол де жұмыс істе алма,bullying

2,ақымақ ақымақ мен машинада мұндай әңгімелер жүргізуге болмайтынын білдім бұл фантастика емес,ақы ақы мен машина мұндай әңгіме жүргіз болма біл бұл фантастика емес,bullying

2,ақымақ ақымақ болды ма ол жариялады,ақы ақы бол ма ол жариял,bullying

2,бәрібір мен сатпаймын ах сен ақымақсың ішімдік,бәрібір мен сатпа ах сен ақы ішімдік,bullying

2,жартылай сал ауруына шалдыққан қолы мен аяғы бар мүгедек ол өзінен гөрі басқа қолайсыз адамдар туралы қиындықтарға мәңгілікке бой алдырды,жартылай сал ауру шалдық қол мен аяғ бар мүгедек ол өз гөрі басқа қолайсыз адам туралы қиындық мәңгілік бой ал,bullying

2,енді маған көмектесіңіз мисыз мылжың манефа апай дірілдеп кетті,енді маған көмектес ми мылж манеф апа дірілде кет,bullying

2,ол мүгедек оның жасы әскерге шақырылмаған,ол мүгедек оның жас әскер шақырылма,bullying

2,ал біздің кедей леденей соңғы шеміршек тіпті жүз грамм үшін алдын алуды өткізіп жіберуге мүмкіндік болмады,ал біздің кедей ледене соңғы шеміршек тіпті жүз грам үшін алдын алу өткіз жібер мүмкіндік болма,bullying

2,олар саңырау мылқау ананың балалары олар осылай сөйлесе алады,олар саңырау мылқа ана бала олар осылай сөйле ал,bullying

2,ол ақымақ па және оның негізгі кәсібі ме дастарханға қара және ақ торларды қайта санаңыз,ол ақы па және оның негізгі кәсіб ме дастархан қара және ақ тор қайта сана,bullying

2,онымен бірге сіз ақылсыз қарт адамнан аулақ боласыз,онымен бірге сіз ақыл қарт адам аулақ бол,bullying

2,ол өмір бойы бәріне әділдік іздейді бірақ бәріне өзі отырады,ол өмір бойы бәрін әділдік ізде бірақ бәрін өзі отыр,bullying

2,науқас өзіне деген жанашырлықтан басқа ештеңе тудырмайды,науқас өзіне де жанашырлық басқа ештеңе тудырма,bullying

2,ол не сияқты жаңғақ деп сұрады егорша арба сырттан шыққан кезде,ол не сияқты жаңғақ де сұр егорш арба сырт шық кезде,bullying

2,борис сіз қандай ақымақсыз кел онда көресің неге,борис сіз қандай ақы кел онда көр неге,bullying

2,егер сіз шизофрения болмасаңыз әйелі айқайлады жуынатын бөлмеден дереу шығыңыз,егер сіз шизофрения болма әйел айқайла жуын бөлме дереу шығ,bullying

2,тозақ не екенін біледі кузьмин күңкілдеп халаттың еденін еденге сүйреп қағаздарды сезді ол шизофрениялық қана емес сонымен қатар алаяқ болып шығады,тозақ не екен біл кузьмин күңкілде хала еден еден сүйре қағаз сез ол шизофрениялық қана емес сонымен қатар алаяқ бол шығ,bullying

2,бірақ сен ақымақсың орныңнан тұр немесе сені армандаушы сенің жұқпалы жұмысыңнан босатады,бірақ сен ақы орн тұр немесе сен армандауш сенің жұқпал жұмыс босат,bullying

2,мен көптен бері байқадым ба қария бізде жаңғақ,мен көп бері байқа ба қария біз жаңғақ,bullying

2,ақымақ даун ақымақ па бұл диагноздар бірақ біздің тілімізде бұл қарғыс,ақы даун ақы па бұл диагноз бірақ біздің тіл бұл қарғыс,bullying

2,шизофрения тек өзі түсінетін тілді ойлап табады,шизофрения тек өзі түсін тілді ойла таб,bullying

2,яғни ол әрине ескі шизофрения,яғни ол әрине ескі шизофрения,bullying

2,және ол кедей өзінің ұзақ тарихын бәріне айтып берді,және ол кедей өзінің ұзақ тарих бәрін айт бер,bullying

2,мүгедек әскери сигналдарды жұдырыққа тартып күліп ағашқа секіріп қылшық ағашты жинады,мүгедек әскери сигнал жұдырық тарт күл ағаш секір қылшық ағаш жина,bullying

2,сіз онымен келісесіз бе міне сіз саңырау және мылқау екеніңізді елестетіп көріңіз менен балға қалай сұрайсыз,сіз онымен келіс бе міне сіз саңырау және мылқа екен елестет көр менен бал қалай сұра,bullying

2,ол шизофрениялық және зейнетақы алады және ақ билеті бар ол біреуді өлтіруі мүмкін және оған ештеңе болмайды,ол шизофрениялық және зейнетақы ал және ақ биле бар ол біреу өлтір мүмкін және оған ештеңе болма,bullying

2,мүмкін сіз шынымен дамымаған шығарсыз ол әдейі дөрекі түрде сұрайды,мүмкін сіз шын дамыма шығ ол әдей дөрекі түр сұра,bullying

2,сен ақымақсың бұрын ойланыңыз,сен ақы бұрын ойлан,bullying

2,ақымақ мен тігуді тоқуды диагностикалауды және интеграциялауды білемін егер білгім келсе дедім мен,ақы мен тігу тоқу диагностикалау және интеграциялау біл егер біл кел де мен,bullying

2,ол ақымақ болса да ақымақ емес ол мұнда мүмкіндік жоқ екенін түсінді олар бергендерін алғаныңыз жөн тіпті сіз оны ала алмайсыз,ол ақы бол да ақы емес ол мұнда мүмкіндік жоқ екен түс олар бер ал жөн тіпті сіз оны ал алма,bullying

2,кешіріңіз сіз кіреберісте басшыны атып тастағаныңыз өкінішті,кешір сіз кіреберіс басшы ат таста өкініш,bullying

2,ал сен білесің бе дима өте ауыр науқас па ол шизофрениялық біздің өміріміз оған өмір сүруге мүмкіндік бермейді,ал сен біл бе ди өте ауыр науқас па ол шизофрениялық біздің өмір оған өмір сүр мүмкіндік берме,bullying

2,мас күйінде мүгедек таяқпен бір нәрсе келді айқайлап тұтқынды таяқпен ұрды,мас күй мүгедек таяқ бір нәрсе кел айқайла тұтқын таяқ ұр,bullying

2,бұл таз ақымақ па әлемде ешкім қалай әрекет еткен жоқ негізгі ставка жасалды,бұл таз ақы па әлем ешкім қалай әрекет ет жоқ негізгі ставка жаса,bullying

2,бұл ақымақ бірінші сыныпта үш жыл отырды,бұл ақы бірінші сынып үш жыл от,bullying

2,чудик придурок шалопай психопат дебил қыңыр қызыл сураз,чудик придурок шалопа психопат дебил қыңы қызыл сураз,bullying

2,сен не ақымақ бізді желге көтергіңіз келе ме,сен не ақы біз жел көтер келе ме,bullying

2,ақымақ ли махаббат туралы не түсінбейді сяк және қазір,ақы ли махаббат туралы не түсінбе сяк және қазір,bullying

2,сіз ешкіммен сөйлесудің қажеті жоқ сіз мұндай адамдармен қалай сөйлесуді білмейсіз,сіз ешкі сөйлесу қажет жоқ сіз мұндай адам қалай сөйлесу білме,bullying

2,сіз көп сөйлей алмайсыз тағзым етіңіз,сіз көп сөйле алма тағзым ет,bullying

2,біртүрлі ол барлық психикасы бар науқас,біртүрлі ол барлық психика бар науқас,bullying

2,кретин ақымақ шаховқа күрт бұрылып анық сөйлейді,кретин ақы шахов күрт бұрыл анық сөйле,bullying

2,олардың арасында бір күні сөз сөйлеп славицпен кастингке келген жаңғақ жігіт болды,олардың ара бір күн сөз сөйле славиц кастинг кел жаңғақ жігіт бол,bullying

2,сен распутная жиырма екі жыл сенің артында созылып пойыз ретінде шаршаған шлюхой,сен распутная жиырма екі жыл сенің арт созыл пойыз рет шарша шлюхо,bullying

2,эй сен ақымақсың самосвал жүргізушісі өтіп бара жатқан адамға айқайлайды,эй сен ақы самосвал жүргізуші өт бар жат адам айқайла,bullying

2,сіз бірлесіп жұмыс істей алмайсыз,сіз бірлес жұмыс істе алма,bullying

2,сіз әлі қасықпен жүруді және тамақтануды білмейсіз,сіз әлі қасық жүру және тамақтану білме,bullying

2,өзі бүкіл басына жаңғақ ол адамдардың көпшілігі не дейді шизофрения,өзі бүкіл бас жаңғақ ол адам көпшіліг не де шизофрения,bullying

2,психиатр психикалық науқас жасайтын тапсырмаларды береді br егер модельдеу болса онда ол ақыл ойды ойнап тапсырмаларды арнайы орындамайды және өзін тапсырады,психиатр психикалық науқас жаса тапсырма бер br егер модельде бол онда ол ақыл ой ойна тапсырма арнайы орындама және өз тапсыр,bullying

2,ақымақ қалай айту керектігін білмейді,ақы қалай айту керектіг білме,bullying

2,паразит дармоед сіз біздің мойнымызда орақыңызбен отырасыз жалқау ештеңе істей алмайсыз подбашмачник немчура қарғыс,паразит дармоед сіз біздің мойны орақ отыр жалқау ештеңе істе алма подбашмачник немчур қарғыс,bullying

2,мұнда текшелер психиатрияға емес ақылдылыққа арналған тест болып табылады,мұнда текше психиатрия емес ақылдылық арнал тест бол таб,bullying

2,сіз одуриге дейін темекі шегесіз сіз көп оқымайсыз және мүлдем жұмыс істей алмайсыз,сіз одури дейін темекі шег сіз көп оқыма және мүлдем жұмыс істе алма,bullying

2,ал бұл ақымақ аяғын терезеден сыртқа іліп қойды ал аналық частушки айқайлайды,ал бұл ақы аяғ терезе сырт іл қой ал аналық частушки айқайла,bullying

2,сіз башканы толығымен таңқалдырдыңыз ба ештеңе есіңде жоқ па,сіз башка толығ таңқал ба ештеңе ес жоқ па,bullying

2,бұл ақымақ ештеңе көрмеді сіз оның мүгедек екенін білесіз,бұл ақы ештеңе көрме сіз оның мүгедек екен біл,bullying

2,тыңда сен не дамымаған,тыңда сен не дамыма,bullying

2,бізге керек ақымақ бұл аузынан көбік пайда болған бір сағаттық ашуланшақтықты басуы мүмкін,бізге керек ақы бұл ауз көбік пайда бол бір саға ашуланшақтық бас мүмкін,bullying

2,мен мүлдем назардан тыс қалдым бұл ақымақ бос емес болуы мүмкін,мен мүлдем назар тыс қал бұл ақы бос емес бол мүмкін,bullying

2,құдайға шүкір бұл ақымақтық сонымен бірге ашуланшақтыққа толы болды және оның қайда екенін нақты түсіндіре алмады,құдай шүкі бұл ақымақтық сонымен бірге ашуланшақтық толы бол және оның қайда екен нақты түсіндір алма,bullying

2,сондықтан сіз бақытсыз ашулануыңыз керек,сондықтан сіз бақыт ашулан керек,bullying

2,эх ескі мал ақымақ мисыз сондай ақ саясат туралы сақтық туралы түсіндірді,эх ескі мал ақы ми сондай ақ саясат туралы сақтық туралы түсін,bullying

2,мен көремін сен ештеңе істей алмайсың,мен көр сен ештеңе істе алма,bullying

2,және ол кішіпейіл жауапсыз адам және сіз оған түсуге жол бермейсіз,және ол кішіпейіл жауап адам және сіз оған түс жол берме,bullying

2,сен ешкім болмайсың мисыз сади айқайлады,сен ешкім болма ми сади айқайла,bullying

2,мүмкін ештеңе айта алмайсың,мүмкін ештеңе ай алма,bullying

2,тағы да сташистің көзқарасы өзіңізді мылқау етіп көрсетіңіз қайтадан бастаңыз,тағы да сташи көзқарас өз мылқа ет көрсе қайта баста,bullying

2,күйеуінің әпкесі анамнезінде даун синдромы бар ұрықпен босанған,күйеу әпке анамнез даун синдром бар ұрық босан,bullying

2,эх ескі мал ақымақ мисыз,эх ескі мал ақы ми,bullying

2,өзі сөйлейді өз тілімен не білмейді мүгедек,өзі сөйле өз тіл не білме мүгедек,bullying

2,ақымақ болды,ақы бол,bullying

2,бұл сізді ұрысатын шығар еш жерде жұмыс істей алмайды жалқау,бұл сіз ұрыс шығар еш же жұмыс істе алма жалқау,bullying

2,ол сондай дәрменсіз дәрменсіз жауапсыз тұрды,ол сондай дәрменсіз дәрменсіз жауап тұр,bullying

2,сымбатты жас бірақ ол орыс тілінде бірде бір сөз білмейді ме жауапсыз,сымбат жас бірақ ол орыс тіл бірде бір сөз білме ме жауап,bullying

2,ұялыңыз ақымақ толы кемпір сізге осындай сөздер айтады,ұялы ақы толы кемпі сізге осындай сөз айт,bullying

2,дебил қазірдің өзінде қолында тұрды оның пластикалық тығыздағыштағы бір реттік шприці болды,дебил қаз өз қол тұр оның пластикалық тығыздағыш бір рет шприц бол,bullying

2,даун синдромы бар адамдар өткен көрмеге қатысқандар соншалықты рухани көрінді бұл оларға басқаша карауға мәжбүр етті,даун синдром бар адам өткен көрме қатыс соншалықты рухани көр бұл оларға басқаша қара мәжбүр ет,bullying

2,ісікті бобик мисыз рахит және макака,ісік бобик ми рахит және макака,bullying

2,иә ол жай ғана ақымақ,иә ол жай ғана ақы,bullying

2,ашуланған және жомарттықпен ол еуропаның соңғы рыцарі болды,ашулан және жомарттық ол еуропа соңғы рыца бол,bullying

2,шыршаны кемемен шатастыратын мұндай ақымақ ешқашан жауап бермегеніңіз жөн,шырша кеме шатастыр мұндай ақы ешқашан жауап берме жөн,bullying

2,ал науқас дәрі дәрмектерден бас тартты ма бірақ бұл оған қазірдің өзінде болды,ал науқас дәрі дәрмек бас тар ма бірақ бұл оған қаз өз бол,bullying

2,ол адамдармен жұмыс істеуді мүлдем білмейді,ол адам жұмыс істеу мүлдем білме,bullying

2,бірақ мен мұндай ақымақпын,бірақ мен мұндай ақы,bullying

2,ақымақ ақымақ,ақы ақы,bullying

2,иә сен ақымақты білесің бе сен білесің бе сума али ессіз,иә сен ақымақ біл бе сен біл бе су али ес,bullying

2,соқыр мүгедек вагондармен жүріп ән айтты,соқыр мүгедек вагон жүр ән ай,bullying

2,осы күннен бастап біз оның атына барлық тиісті анықтамаларды жинадық дамымаған ақымақ,осы күн баста біз оның ат барлық тиісті анықтама жина дамыма ақы,bullying

2,дуура сен ақымақсың,дуур сен ақы,bullying

2,сен ақылсызсың сілекей шашу орнынан тұрды,сен ақылсыз сілекей шаш орн тұр,bullying

2,сен тіпті ақымақ емессің бірақ зұлым таңданыс,сен тіпті ақы емес бірақ зұлым таңданыс,bullying

2,олар өздеріне аутизмге сыртқы әлеммен кетеді бірақ шизофрения үшін өз әлемі нақты әлем сияқты шындыққа ие,олар өз аутизм сыртқы әлем кет бірақ шизофрения үшін өз әлем нақты әлем сияқты шындық ие,bullying

2,бұл ақымақ паша да оның өзі де қазір өлтірілетінін әлі түсінбеді,бұл ақы паш да оның өзі де қазір өлтіріл әлі түсінбе,bullying

2,юлияны көрмей ақ ол неміс шопанының кәдімгі фриц қоқысы ретінде оған дұшпандықпен қарады бірақ бақылап өзгерді,юлия көрме ақ ол неміс шопан кәдімгі фриц қоқыс рет оған дұшпандық қар бірақ бақыла өзге,bullying

2,жаман емес бірақ дамымаған белгілі бір мақсаты жоқ адам қандай да бір дамыған ұйымға кіреді онда мақсат бар және осы мақсатқа қалай жету керектігін білу,жаман емес бірақ дамыма белгілі бір мақсаты жоқ адам қандай да бір дамы ұйым кір онда мақсат бар және осы мақсат қалай жету керектіг білу,bullying

2,сіз жақсы білесіз дамымаған бейбақ мен кім туралы айтып отырмын,сіз жақсы біл дамыма бей мен кім туралы айт отыр,bullying

2,оны ақсақ бочар етеді және ақымақтың ай туралы ешқандай түсінігі жоқ екенін көруге болады,оны ақ боч ет және ақымақ ай туралы ешқандай түсініг жоқ екен көр бол,bullying

2,сіз естисіз бе ақылсыз алға,сіз ести бе ақыл алға,bullying

2,сен ақымақсың маған не істеп жатырсың,сен ақы маған не істе жатыр,bullying

2,қолы жоқ кейбір мүгедектер қоныстанған қоршаудағылардан болуы керек оларға солтүстікке қарай дьячкино ауылына баруға кеңес берді,қол жоқ кейбір мүгедек қоныстан қоршаудағы бол керек оларға солтүстік қарай дьячкино ауыл бар кеңес бер,bullying

2,бұл қазірдің өзінде ауыр науқас болды психикалық шұғыл медициналық көмекке мұқтаж адам,бұл қаз өз ауыр науқас бол психикалық шұғыл медициналық көмек мұқтаж адам,bullying

2,сізде барлық маньяктар сау бірақ олар ауру екені анық сондықтан мұнда құудың қажеті жоқ сіз дерлік құдайлар екенсіз,сіз барлық маньяк сау бірақ олар ауру екен анық сондықтан мұнда қу қажет жоқ сіз дерлік құдай екен,bullying

2,егер адам психикалық ауруға шалдыққан болса немесе ол мүгедек болса оны қорлау және қорлау керек егер сіз ойласаңыз сен сондай сау адамсың мен бәрін айта аламын барлық адамдар емес дені сау адамдар бар және қабілетсіз адамдар олар да заң бойынша және олар біз сияқты адамдар олар қандай болса да адамдарды бағалау керек,егер адам психикалық ауру шалдық бол немесе ол мүгедек бол оны қорла және қорла керек егер сіз ойла сен сондай сау адам мен бәрін ай ал барлық адам емес де сау адам бар және қабілет адам олар да заң бойынша және олар біз сияқты адам олар қандай бол да адам бағалау керек,bullying

2,сонымен нағыз психикалық науқастар басындағы дауыстар туралы қалай айтады олар оны қалай атайды,сонымен нағыз психикалық науқас басындағы дауыс туралы қалай айт олар оны қалай ата,bullying

2,мұндай қарт дурас жағымсыз дауыспен жалғыздық сияқты жазғы мектеп туралы фильмнен лагерь,мұндай қарт дурас жағымсыз дауыс жалғыз сияқты жазғы мектеп туралы фильм лагерь,bullying

2,менің анам айтқандай жылдық невропатолог дені сау адамдар жоқ зерттелмегендер бар,менің ана айтқанда жылдық невропатолог де сау адам жоқ зерттелме бар,bullying

2,қандай сандырақ осылайша ауру адамдар ақылға қонымды деп жарияланып түрмелерге түседі және керісінше,қандай сандырақ осылайша ауру адам ақыл қонымды де жариялан түрме түс және керісінше,bullying

2,адам мидың зақымдануы болған жағдайда ғана есінен танып қалады басқа жағдайларда бұл уақытша ақыл ойдың бұлыңғырлығы туралы оны ешқандай жағдайда антипсихотиктермен емдеуге болмайды өйткені ол нашар болады мен өз тәжірибемде айтамын,адам ми зақымдан бол жағдай ғана ес тан қал басқа жағдай бұл уақытша ақыл ой бұлыңғырлығ туралы оны ешқандай жағдай антипсихотик емде болма өйткені ол нашар бол мен өз тәжірибе ай,bullying

2,енді бізде неге көп екені түсінікті сихов мұнара оларға жарқырап тұрған кезде quot ашылады текшелер карта түстер quot сарапшыларды алдау мүмкін емес quot,енді біз неге көп екен түсінік сихов мұнара оларға жарқыра тұр кезде quot аш текше карта түс quot сарапшы алда мүмкін емес quot,bullying

2,қалыпты психика галлюцинация туралы айтпайды ма ия кейбір психиктер сізге әлі айтпайды енді медициналық тексеруге түсетіндер үшін қорқынышты,қалыпты психик

галлюцинация туралы айтпа ма ия кейбір психик сізге әлі айтпа енді медициналық тексер түс үшін қорқыныш,bullying

2,мен әлемдегі бір нәрсені білемін барлық психика,мен әлем бір нәрсе біл барлық психик,bullying

2,ол сауатсыз ақымақ олардың сияқты,ол сауат ақы олардың сияқты,bullying

2,нақты психика кім бұл диагнозды дәнекерлегендер ме,нақты психик кім бұл диагноз дәнекерле ме,bullying

2,бұл сіздің дебилизміңізді білдіреді егер сіз оларды жеу туралы ойласаңыз,бұл сіздің дебилизм білдір егер сіз ол же туралы ойла,bullying

2,абблистерге ұят,абблис ұят,bullying

2,сіз ата анаңыз отбасыңыз үшін өте ауыр жүк болуыңыз керек,сіз ата ана от үшін өте ауыр жүк бол керек,bullying

2,мен сенің қатты қорылдағаныңды көремін бұл сіздің мүгедектігіңізге байланысты болуы керек,мен сенің қатты қорылда көр бұл сіздің мүгедектіг байланысты бол керек,bullying

2,сіз мұны істей алмайтын шығарсыз өйткені сізде шектеулер бар,сіз мұны істе алма шығ өйткені сіз шектеу бар,bullying

2,сіз қалай өмір сүре аласыз сізде осындай проблемалар болған кезде,сіз қалай өмір сүр ал сіз осындай проблема бол кезде,bullying

2,сіз менімен бірге бұл іс шараға бара алмайтын шығарсыз өйткені мүгедектерге қол жетімділік жоқ,сіз менімен бірге бұл іс шара бар алма шығ өйткені мүгедек қол жетімділік жоқ,bullying

2,сен мұны істей алатыныңа сенімді емеспін өйткені сенің ерекшеліктерің бар,сен мұны істе ал сенімді емес өйткені сенің ерекшелік бар,bullying

2,сіз риза болуыңыз керек сізде осындай өмір тәжірибесі бар,сіз риза бол керек сіз осындай өмір тәжірибе бар,bullying

2,сіз түсінбейтін шығарсыз менің не айтқым келеді өйткені сізде шектеулер бар,сіз түсінбе шығ менің не айт кел өйткені сіз шектеу бар,bullying

2,сіз мұны істей алмайтын шығарсыз өйткені сіздің жағдайыңызға байланысты ерекшеліктеріңіз бар,сіз мұны істе алма шығ өйткені сіздің жағдай байланысты ерекшелік бар,bullying

2,менің ойымша сіз бақытты болуыңыз керек сізде өмір сүруге болатын орын бар тіпті ол мінсіз болмаса да,менің ойымша сіз бақытты бол керек сіз өмір сүр бол орын бар тіпті ол мін болма да,bullying

2,о кешіріңіз мен не айта алатыныңызды түсінбеймін қайталай аласыз ба иә сіз мүгедексіз,о кешір мен не ай ал түсінбе қайтала ал ба иә сіз мүгедек,bullying

2,сіз жақсы болар едіңіз үйде отыру мұндай жағдайда тәуекелге барудан гөрі,сіз жақсы бол ед үй отыру мұндай жағдай тәуекел бару гөрі,bullying

2,мен саған көмектесейін мен сенің қолыңнан келмейтінін білемін,мен саған көмектес мен сенің қол келме біл,bullying

2,сен сондай сүйкімді және баяу сөйлейсің мен сенімен кішкентай бала сияқты сөйлесіп тұрғандай,сен сондай сүйк және бая сөйле мен сенімен кішкентай бала сияқты сөйлес тұрғанда,bullying

2,сіз өз проблемаларыңызға қарамастан қандай батылсыз,сіз өз проблема қарамастан қандай батыл,bullying

2,ал сен сондай күлсің мен тек көмектескім келді,ал сен сондай күл мен тек көмектес кел,bullying

2,менің ойымша сіз мұны жасай алмайсыз сіздің жағдайыңызды ескере отырып,менің ойымша сіз мұны жаса алма сіздің жағдай ескер отыр,bullying

2,сіз тырыспағаныңыз жөн сен білесің сізде шектеулер бар,сіз тырыспа жөн сен біл сіз шектеу бар,bullying

2,о сен қандай қыңырсың басқа адамдардың көмегін қабылдағың келмейді,о сен қандай қыңы басқа адам көмег қабылда келме,bullying

2,сен сондай жас және дені саусың қалай мүгедек бола аласың,сен сондай жас және де сау қалай мүгедек бола ал,bullying

2,сіз мұны шынымен жасай аласыз деп ойлайсыз ба сіз мүгедексіз,сіз мұны шын жаса ал де ойла ба сіз мүгедек,bullying

2,сіз өзіңізді қатты жүктемеуіңіз керек емес еді сіз мүгедексіз,сіз өз қатты жүктеме керек емес ед сіз мүгедек,bullying

2,мен сізге көмектесуді ұсынар едім бірақ сіздің жағдайыңызды ескере отырып мен сенімді емеспін сіз оны жеңе аласыз,мен сізге көмектесу ұсын ед бірақ сіздің жағдай ескер отыр мен сенімді емес сіз оны же ал,bullying

2,бұл сіз үшін дұрыс жұмыс екеніне толық сенімді емес сіздің шектеулеріңізді ескере отырып,бұл сіз үшін дұрыс жұмыс екен толық сенімді емес сіздің шектеу ескер отыр,bullying

2,кел мен мұны сен үшін жасаймын өйткені сен мен мұны істей алмайтынымды білемін,кел мен мұны сен үшін жаса өйткені сен мен мұны істе алма біл,bullying

2,сен білесің мен саған және сенің жағдайыңа шынымен жанашырмын бірақ менің ойымша сен оны жеңе алмайсың,сен біл мен саған және сенің жағдай шын жанашыр бірақ менің ойымша сен оны же алма,bullying

2,сіз өзіңізден тым көп күтпеуіңіз керек сіздің шектеулеріңізді ескере отырып,сіз өз тым көп күтпе керек сіздің шектеу ескер отыр,bullying

2,сіз оны басқа біреуге қалдырғаныңыз жөн кім сіздің шектеулеріңіз жоқ,сіз оны басқа біреу қалдыр жөн кім сіздің шектеу жоқ,bullying

2,бұл сізге қиын болуы мүмкін екенін түсінемін бірақ сіз түсінуіңіз керек сізде шектеулі мүмкіндіктер бар,бұл сізге қиын бол мүмкін екен түс бірақ сіз түсін керек сіз шектеулі мүмкіндік бар,bullying

2,ол жақсы жұмыс істей алмайды өйткені оның мүгедектігі бар,ол жақсы жұмыс істе алма өйткені оның мүгедектіг бар,bullying

2,сізге көмек керек пе сіз бәрі сияқты емес екеніңізді білесіз,сізге көмек керек пе сіз бәрі сияқты емес екен біл,bullying

2,оны командаға алмайық ол мүгедектігіне байланысты бұл жұмысты жеңе алмайды,оны команда алма ол мүгедектіг байланысты бұл жұм же алма,bullying

2,сіз мұны жасай аласыз ба сіз үшін бұл сіздің жағдайыңызға байланысты қиын болуы мүмкін,сіз мұны жаса ал ба сіз үшін бұл сіздің жағдай байланысты қиын бол мүмкін,bullying

2,білмеймін неге олар оған қарапайым мектепте оқуға рұқсат берді оның жағдайын ескере отырып,білме неге олар оған қарапайым мектеп оқ рұқсат бер оның жағдай ескер отыр,bullying

2,неліктен мүгедектігіңіздің өмірін анықтауға мүмкіндік бересіз өйткені сізде әлі де көптеген мүмкіндіктер бар,неліктен мүгедектіг өмір анықта мүмкіндік бер өйткені сіз әлі де көптеген мүмкіндік бар,bullying

2,сіз мұны жасай аласыз ба менің ойымша жоқ сіздің жағдайыңызды ескере отырып,сіз мұны жаса ал ба менің ойымша жоқ сіздің жағдай ескер отыр,bullying

2,сіз мұны өзіңіз жасай алмайсыз ба сіз мүгедек емессіз,сіз мұны өз жаса алма ба сіз мүгедек емес,bullying

2,оны жақсырақ қалдырыңыз мен сіздің мүгедектігіңіз үшін бәрін бүлдіргеніңізді қаламаймын ла,оны жақсырақ қалдыр мен сіздің мүгедектіг үшін бәрін бүлдір қалама ла,bullying

2,сіз бұл лауазымда жұмыс істей алмайсыз өйткені сіздің мүгедектігіңіз сізге барлық қажетті міндеттерді орындауға кедергі келтіреді,сіз бұл лауазым жұмыс істе алма өйткені сіздің мүгедектіг сізге барлық қажетті міндет орында кедергі келтір,bullying

2,сен бұл сен үшін қауіпті екенін білесің сен мүгедексің,сен бұл сен үшін қауіпті екен біл сен мүгедек,bullying

2,мен сенің тырысып жатқаныңды түсінемін бірақ оны мен сияқты ешқашан жасай алмайсың,мен сенің тырыс жат түс бірақ оны мен сияқты ешқашан жаса алма,bullying

2,сіз мүгедектігіңізге байланысты велосипедпен немесе спортпен айналыса алмайсыз,сіз мүгедектіг байланысты велосипед немесе спорт айналыс алма,bullying

2,сіз мүгедектігі бар адамдарға арналған арнайы мекемеде тұрғаныңыз жөн онда сізге жақсы күтім жасалады,сіз мүгедектіг бар адам арнал арнайы мекеме тұр жөн онда сізге жақсы күтім жасал,bullying

2,сіз басқа адамдармен сөйлесе алмайсыз өйткені қалыпты адамдар өздерінің мүгедектігіне байланысты жасайды,сіз басқа адам сөйле алма өйткені қалыпты адам өз мүгедектіг байланысты жаса,bullying

2,бұл адамдар әрқашан солай олар ешқашан өздері үшін ойлана алмайды,бұл адам әрқашан солай олар ешқашан өз үшін ойлан алма,bullying

2,мұндай проблемалары бар адамдар үйде қалып қалыпты адамдарға кедергі жасамауы керек,мұндай проблема бар адам үй қалып қалыпты адам кедергі жасама керек,bullying

2,неге олар бұл адамдар жұмыс істей алады деп сенетінін білмеймін олар ешқашан тапсырманы дұрыс орындай алмайды,неге олар бұл адам жұмыс істе ал де сен білме олар ешқашан тапсырма дұрыс орында алма,bullying

2,менің балам мұндай адамдармен бірге оқығанын қаламас едім олар оны өздерінің кемшіліктерімен жұқтыруы мүмкін,менің бала мұндай адам бірге оқы қаламас ед олар оны өз кемшілік жұқтыр мүмкін,bullying

2,сіз оған түсіндіруге уақыт жұмсамағаныңыз жөн ол түсінбейді,сіз оған түсіндір уақыт жұмсама жөн ол түсінбе,bullying

2,олар жай ғана түсінуі керек олар басқалар сияқты емес және өз тобында,олар жай ғана түсін керек олар басқа сияқты емес және өз тоб,bullying

2,сіз олардың не істей алатынын ешқашан білмейсіз олар соншалықты күтпеген,сіз олардың не істе ал ешқашан білме олар соншалықты күтпе,bullying

2,мен сенің шектеулеріңді ескере отырып бұл жұмысты қалай жасайтыныңа таң қаламын,мен сенің шектеу ескер отыр бұл жұм қалай жаса таң қал,bullying

2,менің ойымша бұл жер сізге сәйкес келмейді сіздің мүмкіндіктеріңізді ескере отырып,менің ойымша бұл жер сізге сәйкес келме сіздің мүмкіндік ескер отыр,bullying

2,сен сенімдісің мұны не істей аласың сіз үшін мұны істегенімді қаламайсыз ба,сен сенімді мұны не істе ал сіз үшін мұны істе қалама ба,bullying

2,барлық құрметпен менің ойымша сіз бұл істі адамдарға шектеусіз қалдырғаныңыз жөн,барлық құрмет менің ойымша сіз бұл іс адам шектеу қалдыр жөн,bullying

2,менің ойымша сіз мұны істеуге тырыспауыңыз керек сізде шектеулер бар екенін түсінесіз,менің ойымша сіз мұны істе тырыспа керек сіз шектеу бар екен түс,bullying

2,сіз бұл сіз үшін қиын екенін білесіз неге басқалардан көмек сұрамайсыз,сіз бұл сіз үшін қиын екен біл неге басқа көмек сұрама,bullying

2,мен сенімді емеспін бұл сен үшін қауіпсіз сіздің мүмкіндіктеріңізді ескере отырып,мен сенімді емес бұл сен үшін қауіпсіз сіздің мүмкіндік ескер отыр,bullying

2,сіз әрине жарайсың сіз тырысып жатырсыз бірақ шынымды айтсам сіз оған ешқашан қол жеткізе алмайсыз,сіз әрине жара сіз тырыс жатыр бірақ шын айт сіз оған ешқашан қол жеткіз алма,bullying

2,сіз өзіңіздің мүмкіндіктеріңізге сәйкес келетін нәрсемен айналысқаныңыз жақсы болар еді,сіз өз мүмкіндік сәйкес кел нәрсе айналыс жақсы бол ед,bullying

2,сен сияқты әдемі қыз мен соншалықты ақылды боламын деп күткен жоқпын,сен сияқты әдемі қыз мен соншалықты ақылды бол де күт жоқ,bullying

2,неге мен сені мүгедек болғандықтан ғана кешіруім керек деп ойлайсың,неге мен сен мүгедек бол ғана кешір керек де ойла,bullying

2,менің ойымша сіз бұл жұмысты жеңе алмайсыз сіздің көру проблемаларыңызды ескере отырып,менің ойымша сіз бұл жұм же алма сіздің көру проблема ескер отыр,bullying

2,мен сізге қиын екенін білемін бірақ сіз өмірдің әділетсіз екенін түсінуіңіз керек,мен сізге қиын екен біл бірақ сіз өмір әділет екен түсін керек,bullying

2,ал сен не бізде қарапайым адамдар бар,ал сен не біз қарапайым адам бар,bullying

2,менің ойымша біз сізге көмектесуіміз керек өйткені сіз мұны өзіңіз жасай алмайсыз,менің ойымша біз сізге көмектес керек өйткені сіз мұны өз жаса алма,bullying

2,сіз өзіңіздің шектеулеріңізге қарамастан бұл туралы соншалықты жақсы бола аласыз деп ойлайсыз ба,сіз өз шектеу қарамастан бұл туралы соншалықты жақсы бола ал де ойла ба,bullying

2,мен сізбен сөйлесе алмаймын егер сіз қалыпты сөйлей алмасаңыз,мен сізбен сөйле алма егер сіз қалыпты сөйле алма,bullying

2,сіз өте жақсы эссе жаздыңыз мұндай мүгедектігі бар адам үшін,сіз өте жақсы эссе жаз мұндай мүгедектіг бар адам үшін,bullying

2,мүмкін егер сіз жай ғана шиеленісіп жатсаңыз сіз өзіңіздің физикалық шектеулеріңізді жеңе аласыз,мүмкін егер сіз жай ғана шиеленіс жат сіз өз физикалық шектеу же ал,bullying

2,сіздің проблемаларыңызға қарамастан жұмыс тапқаныңыз қандай жақсы,сіздің проблема қарамастан жұмыс тап қандай жақсы,bullying

2,сіздің мүгедектігіңіз сізге толыққанды өмір сүруге кедергі болмауы керек бірақ әрине сіз өз шектеулеріңізді түсінуіңіз керек,сіздің мүгедектіг сізге толыққанды өмір сүр кедергі болма керек бірақ әрине сіз өз шектеу түсін керек,bullying

2,мен сіздің орныңызда болғым келмейді бірақ сіз өте жақсы жұмыс жасайсыз,мен сіздің орн бол келме бірақ сіз өте жақсы жұмыс жаса,bullying

2,мен ешқашан ойламас едім сізде мұндай мүгедектік бар сіз басқалар сияқты көрінесіз,мен ешқашан ойламас ед сіз мұндай мүгедектік бар сіз басқа сияқты көр,bullying

2,сіз шынымен өте таланттысыз сіздің жағдайыңызды ескере отырып,сіз шын өте талантты сіздің жағдай ескер отыр,bullying

2,мен сіздің мүгедектігіңізге қарамастан сенімділігіңізге таңданамын,мен сіздің мүгедектіг қарамастан сенімділіг таңдан,bullying

2,сіз көп нәрсеге қол жеткіздіңіз физикалық шектеулеріңізге қарамастан,сіз көп нәрсе қол жет физикалық шектеу қарамастан,bullying

2,сіз өз өміріңізде кездескен барлық нәрсені ескере отырып кереметсіз,сіз өз өмір кездес барлық нәрсе ескер отыр керемет,bullying

2,сен сондай талантты және ақылды адамсың мен тіпті байқамаймын сізде қандай да бір проблемалар бар,сен сондай талантты және ақылды адам мен тіпті байқама сіз қандай да бір проблема бар,bullying

2,сіз өз проблемаларыңызды шешіп жатырсыз сіздің жағдайыңыздағы біреу үшін жаман емес,сіз өз проблема шеш жатыр сіздің жағдайыңыз біреу үшін жаман емес,bullying

2,сіздің мүгедектігіңіз сіздің жетістіктеріңізге кедергі келтірмейтіні қандай керемет,сіздің мүгедектіг сіздің жетістік кедергі келтірме қандай керемет,bullying

2,мен үшін бұл тапсырмаларды орындай алатыныңызға көз жеткізу өте маңызды сіздің жағдайыңызды ескере отырып,мен үшін бұл тапсырма орында ал көз жеткізу өте маңызды сіздің жағдай ескер отыр,bullying

2,мен білмес едім не істеу керек егер менде сіздің проблемаларыңыз болса бірақ сіз мұны оңай жасайсыз,мен білмес ед не істеу керек егер менде сіздің проблема бол бірақ сіз мұны оңай жаса,bullying

2,сіздің әңгімеңіз бізді өз мәселелерімізді құрметтеуге шабыттандырады,сіздің әңгіме біз өз мәселе құрметте шабыттандыр,bullying

2,сіз өзіңіздің физикалық проблемаларыңызға қарамастан әлі де керемет көрінесіз,сіз өз физикалық проблема қарамастан әлі де керемет көр,bullying

2,сіздің проблемаларыңыз сізді тұлға ретінде анықтамайды және мен сізді жетістікке жеткеніңіз үшін құрметтеймін,сіздің проблема сіз тұлға рет анықтама және мен сіз жетістік жет үшін құрметте,bullying

2,мұнда дені сау бірақ өте ашулы жігіт психикалық және физикалық мүмкіндіктері шектеулі адамдарға арналған санаторийге жіберіледі,мұнда де сау бірақ өте ашул жігіт психикалық және физикалық мүмкіндік шектеулі адам арнал санаторий жібер,bullying

2,әрине адам ауырады бірақ олардың диалогы бұл жай ғана,әрине адам ауыр бірақ олардың диалог бұл жай ғана,bullying

2,мұндай адамдар үшін өте өкінішті,мұндай адам үшін өте өкініш,bullying

2,кедей жігіт оған туылу мүмкіндігі түсті және ол тіпті ештеңе түсінбейді ал оған сынақ мұрты келеді,кедей жігіт оған туыл мүмкіндіг түсті және ол тіпті ештеңе түсінбе ал оған сынақ мұр кел,bullying

2,ол бүлдіршін бала сияқты ең қорқыныштысы ақылсыз қалу мүмкін мүмкін оған көңілді және жақсы ал біз оған ақымақпыз,ол бүлдіршін бала сияқты ең қорқынышты ақыл қалу мүмкін мүмкін оған көңіл және жақсы ал біз оған ақы,bullying

2,сіз өте жақсы жұмыс жасайсыз соқырлығыңызды ескере отырып,сіз өте жақсы жұмыс жаса соқырлығ ескер отыр,bullying

2,иә сіз мұндай адаммен өмір сүрудің қаншалықты қауіпті екенін түсінбейсіз мүмкін ол шұлықты сасық немесе тәпішкені шешіп алу туралы өтінішке агрессивті шығар,иә сіз мұндай адам өмір сүру қаншалықты қауіпті екен түсінбе мүмкін ол шұлық сасық немесе тәпіш шеш алу туралы өтініш агрессивті шығар,bullying

2,ол санай алмайды жазу сурет салу өйткені ол мектепте оқымаған шығар,ол сана алма жазу сурет салу өйткені ол мектеп оқыма шығар,bullying

2,неге мұндай адамдар қажет жыл арамшөп сияқты өмір сүреді,неге мұндай адам қажет жыл арамшөп сияқты өмір сүр,bullying

2,сіз оларды қорқытуға үйрете алмайсыз ол ешқашан жақсы көрмеген ол бақытсыз бала еді ал қазір ересек адам ол тек күлді,сіз ол қорқыт үйрет алма ол ешқашан жақсы көрме ол бақыт бала ед ал қазір ересек адам ол тек күл,bullying

2,иә мұндай адамның не істей алатыны белгісіз және психологияның көптеген ұғымдарын түсінеді,иә мұндай адам не істе ал белгісіз және психология көптеген ұғым түсін,bullying

2,ол физикалық психикалық шектеулеріне байланысты бұл жұмысты жасай алмайды,ол физикалық психикалық шектеу байланысты бұл жұм жаса алма,bullying

2,оның да туыстарының да өкініші қандай,оның да туыс да өкініш қандай,bullying

2,ол көмексіз өмір сүре алмайды ол соқыр түсінесіз бе,ол көмек өмір сүр алма ол соқыр түс бе,bullying

2,галереяның барлық залдары мүмкіндігі шектеулі жандарға баруға арналған,галерея барлық зал мүмкіндіг шектеулі жан бар арнал,bullying

2,бұл адамның психикасында проблемалар бар онымен араласудың қажеті жоқ,бұл адам психика проблема бар онымен аралас қажет жоқ,bullying

2,бұл адам өзінің дислексиясына байланысты бұл жобаны жеңе алмайды,бұл адам өзінің дислексия байланысты бұл жоба же алма,bullying

2,ол өте артта қалған қарапайым нәрселерді түсіне алмайды,ол өте арт қалған қарапайым нәрсе түс алма,bullying

2,мұны көрудің мағынасы жоқ сіз бірдей шиеленісті сезінбейсіз,мұны көру мағына жоқ сіз бірде шиелен сезінбе,bullying

2,егер сіздің мүгедектігіңіз болмаса сіз сондай әдемі болар едіңіз,егер сіздің мүгедектіг болма сіз сондай әдемі бол ед,bullying

2,бұл адамның мұндай оғаш мінез құлқы бар бұл оның аутизмімен байланысты,бұл адам мұндай оғаш мінез құлқы бар бұл оның аутизм байланысты,bullying

2,маған ессіздіктің толықтығында өлетін бұл адам өкінішті,маған ессіздік толықтығ өл бұл адам өкініш,bullying

2,сен өте баяусың неге тезірек үйренбейсің,сен өте баяу неге тезірек үйренбе,bullying

2,сіз мұнда отыра алмайсыз бұл мүгедектерге арналған орын,сіз мұнда отыр алма бұл мүгедек арнал орын,bullying

2,мен мүгедектерді жалдамаймын олар баяу және тиімсіз,мен мүгедек жалдама олар бая және тиімсіз,bullying

2,бұл адамның осындай оғаш қимылдары бар бұл өйткені оның даун синдромы бар,бұл адам осындай оғаш қимыл бар бұл өйткені оның даун синдром бар,bullying

2,сіз мүгедектерге арналған арнайы орындыққа отыруыңыз керек сіз әдеттегі орындықта отыра алмайсыз,сіз мүгедек арнал арнайы орындық отыр керек сіз әдет орындық отыр алма,bullying

2,сіз тым қараңғы жерге бара алмайсыз сен соқырсың,сіз тым қараңғы же бар алма сен соқыр,bullying

2,бұл курс сіздің шектеулеріңізге байланысты сізге сәйкес келмейді,бұл курс сіздің шектеу байланысты сізге сәйкес келме,bullying

2,біз сізге баспана ұсынбаймыз өйткені біздің компания мүмкіндігі шектеулі жандар үшін қабылдамайды,біз сізге баспана ұсынба өйткені біздің компания мүмкіндіг шектеулі жан үшін қабылдама,bullying

2,зағип адамдардың есту қабілеті қандай керемет,зағип адам ест қабілет қандай керемет,bullying

2,сізге бұл автобуспен жүрудің қажеті жоқ өйткені ол арбадағы адамдарға арналмаған,сізге бұл автобус жүру қажет жоқ өйткені ол арба адам арналма,bullying

2,біз медициналық жабдықтың дұрыс түрін білмейміз өйткені мұндай мүгедектікпен қалай жұмыс істеу керектігін білмейміз,біз медициналық жабдық дұрыс түр білме өйткені мұндай мүгедектік қалай жұмыс істеу керектіг білме,bullying

2,сіз бұл спортпен айналыса алмайсыз өйткені сіздің мүгедектігіңіз сізді шектейді,сіз бұл спорт айналыс алма өйткені сіздің мүгедектіг сіз шекте,bullying

2,сізде бар екеніне риза болу керек жұмыс сіздің мүгедектігіңізді ескере отырып,сіз бар екен риза болу керек жұмыс сіздің мүгедектіг ескер отыр,bullying

2,сіз мүгедектігіңізге байланысты сақтандыру ала алмайсыз өйткені сіз біздің компания үшін тым үлкен тәуекелдісіз,сіз мүгедектіг байланысты сақтандыр ал алма өйткені сіз біздің компания үшін тым үлкен тәуекелді,bullying

2,мен сені құрметтемеймін өйткені сен менің қолымнан келгенді істей алмайсың,мен сен құрметтеме өйткені сен менің қол кел істе алма,bullying

2,оның соқырлығы оның өмірдегі сәтсіздіктерінің себебі,оның соқырлығ оның өмір сәтсіздік себебі,bullying

2,сондықтан сіз ешқашан психиатрларға жабыса алмайсыз дені сау адам болды және шизик болды,сондықтан сіз ешқашан психиатр жабыс алма де сау адам бол және шизик бол,bullying

2,қандай жағымсыз жүздер,қандай жағымсыз жүз,bullying

2,аянышты психо науқастар әсіресе жас бақытсыз адамдар денесі сау және толық өмір сүру мүмкін емес,аяныш психо науқас әсіресе жас бақыт адам дене сау және толық өмір сүру мүмкін емес,bullying

2,тіпті ауруы болмаса да отбасылық өмірге жарамсыз әйел,тіпті ауру болма да отбасылық өмір жарамсыз әйел,bullying

2,мен жұмыста психикамен сөйлесуге тура келді ол жалпы дені сау адамдар жоқ екенін айтты барлығының диагнозы қысқа барлық психика,мен жұмыс психика сөйлес тура кел ол жалпы де сау адам жоқ екен ай барлығ диагноз қыс барлық психик,bullying

2,мен сенің соқыр болғаныңды елестете алмаймын,мен сенің соқыр бол елесте алма,bullying

2,оның ауырғаны анық және оған көмектесті деп үміттенемін бірақ мен бұл әңгімеден ақылдымын,оның ауыр анық және оған көмекте де үміттен бірақ мен бұл әңгіме ақылды,bullying

2,неге адамдар ойлайды егер адамдарда психикалық ауру болса онда олар міндетті түрде жеткіліксіз болуы керек олар біз сияқты адамдар олар біз сияқты дамыған жай ғана ауытқулар бар,неге адам ойла егер адам психикалық ауру бол онда олар міндетті түр жеткіліксіз бол керек олар біз сияқты адам олар біз сияқты дамы жай ғана ауытқу бар,bullying

2,сізге алдымен рұқсат алу керек біз сізге бұл мүмкіндікті бермес бұрын сіздің мүгедектігіңізді ескере отырып,сізге алд рұқсат алу керек біз сізге бұл мүмкіндік бермес бұрын сіздің мүгедектіг ескер отыр,bullying

2,психикалық күйзеліске ұшыраған өз жағдайын басқара алмайтын тұрақсыз адамдар жалпы мен әртүрлі стресстік жағдайларға інжір бойынша сабырлы түрде жауап беремін және олардан шығудың жолын табуға тырысамын,психикалық күйзеліс ұшыра өз жағдай басқар алма тұрақсыз адам жалпы мен әртүрлі стрес жағдай інжі бойынша сабырл түр жауап бер және олардан шығ жол таб тырыс,bullying

2,халаттарда олар психикаға көбірек ұқсайды,халат олар психика көбірек ұқса,bullying

2,оған жүгінбеңіз ол айналасында не болып жатқанын көрмейді,оған жүгінбе ол айнала не бол жат көрме,bullying

2,сәл біртүрлі,сәл біртүрлі,bullying

2,оның диагнозы шизофрения емес психоз бұл бірдей емес,оның диагноз шизофрения емес психоз бұл бірде емес,bullying

2,сонымен қатар ол мүгедектікке ие болды және мүгедектер қоғамы жанынан жөндеу және құрылыс кеңсесін құрды,сонымен қатар ол мүгедектік ие бол және мүгедек қоғам жан жөндеу және құрылыс кеңсе құр,bullying

2,ешкім түсінбейтін жалғыз әйел тек психиатрлар оған диссертациялар жасайды,ешкім түсінбе жалғыз әйел тек психиатр оған диссертация жаса,bullying

2,кешіріңіз бірақ мүгедектер пандусының негізіне байланысты ғимаратқа кіруді пайдалануды ұсынбаймыз,кешір бірақ мүгедек пандус негіз байланысты ғимарат кіру пайдалану ұсынба,bullying

2,жүктеуде сіздің мүгедектігіңіз бар біреу қажет емес,жүкте сіздің мүгедектіг бар біреу қажет емес,bullying

2,сіз бұл бөлмені пайдалана алмайсыз өйткені ол мүгедектерге арналмаған,сіз бұл бөлме пайдалан алма өйткені ол мүгедек арналма,bullying

2,біз сіздің қажеттіліктеріңізге арнайы бейімделген жұмыс орнын қамтамасыз ете алмаймыз,біз сіздің қажеттілік арнайы бейімдел жұмыс орн қамтамасыз ет алма,bullying

2,менің анам дәл осындай диагноздан зардап шегеді және кейбір сәттерде оның мінез құлқы сол әйелдің мінез құлқына тым ұқсас бұл қорқынышты болады өйткені адам енді бізбен бірге емес және бұл әлемде емес мен ол сезіну қабілетін мүлдем жоғалтады деп қорқамын,менің ана дәл осындай диагноз зардап шег және кейбір сәт оның мінез құлқы сол әйел мінез құлқ тым ұқсас бұл қорқыныш бол өйткені адам енді бізбен бірге емес және бұл әлем емес мен ол сезін қабілет мүлдем жоғалт де қор,bullying

2,кедей адамдар қандай аурулар,кедей адам қандай ауру,bullying

2,бұл сезімбұл жерлерде ол толығымен ойлайдыбұл айтады ал кей жерлерде ол нонсенс сияқты бұзады қорқынышты,бұл сезімбұл жер ол толығ ойлайдыбұл айт ал кей жер ол нонсенс сияқты бұз қорқыныш,bullying

2,ауырған бейшара науқас меркантилизмнің өзімшілдіктің екіжүзділіктің цинизмнің және б өткір тапшылығы дені сау адамға қажетті барлық қасиеттер есімде жоқ,ауыр бейшар науқас меркантилиз өзімшілдік екіжүзділік цинизм және б өткір тапшылығ де сау адам қажетті барлық қасиет ес жоқ,bullying

2,сізге көп жұмыс істеудің қажеті жоқ сіз мүгедексіз,сізге көп жұмыс істеу қажет жоқ сіз мүгедек,bullying

2,кедей ауру адамдар ал отбасыларға қаншалықты қиын,кедей ауру адам ал отбасы қаншалықты қиын,bullying

2,бұл адамның сөйлеу әдеттері өте оғаш себебі ол аутист,бұл адам сөйлеу әдет өте оғаш себебі ол аутист,bullying

2,сіз бұл бағдарламадан оқи алмайсыз мүгедектігіңізге байланысты біз сізге тиісті қолдауды қолдана алмаймыз,сіз бұл бағдарлама оқи алма мүгедектіг байланысты біз сізге тиісті қолдау қолдан алма,bullying

2,біз сізге қашықтан жұмыс істеуге мүмкіндік бере алмаймыз өйткені бұл мүмкіндігі шектеулі адам үшін қиын болуы мүмкін деп санаймыз,біз сізге қашық жұмыс істе мүмкіндік бер алма өйткені бұл мүмкіндіг шектеулі адам үшін қиын бол мүмкін де сана,bullying

2,иә сіз психикасыз,иә сіз психик,bullying

2,сіз демалысқа бармауыңыз керек өйткені сіз емделуге ақшаға үйренуіңіз керек,сіз демалыс барма керек өйткені сіз емдел ақша үйрен керек,bullying

2,қыз өте әдемі бірақ галоперидолмен емдеу оған көмектеспеді,қыз өте әдемі бірақ галоперидол емдеу оған көмектеспе,bullying

2,обсессивті және психика емес бұл құдайсыздықтың қарғысы,обсессив және психик емес бұл құдайсыздық қар,bullying

2,мен мүгедекпен айналысқым келмейді бұл өте қиын,мен мүгедек айналыс келме бұл өте қиын,bullying

2,ауырсынуға шағымданудың қажеті жоқ өйткені сіз қазірдің өзінде мүгедексіз,ауырсын шағымдан қажет жоқ өйткені сіз қаз өз мүгедек,bullying

2,сіз біздің кәсіби сертификаттаудан өте алмайсыз өйткені оның кейбір бөліктері сіздің мүгедектігіңізге бейімделе алмайды,сіз біздің кәсіби сертификаттау өте алма өйткені оның кейбір бөлік сіздің мүгедектіг бейімдел алма,bullying

2,ол соқыр мүлдем соқыр еді,ол соқыр мүлдем соқыр ед,bullying

2,сіз өзіңізді мүгедек сияқты ұстайсыз саналы өмір сүруді үйрену керек,сіз өз мүгедек сияқты ұста саналы өмір сүру үйрену керек,bullying

2,дербестік пен тәуелсіздікке таңдамау мүгедектері,дербестік пен тәуелсіздік таңдама мүгедек,bullying

2,сіз бұл қызметті ала алмайсыз өйткені біздің компанияның мүмкіндігі шектеулі адамдармен тәжірибесі жоқ,сіз бұл қызмет ал алма өйткені біздің компания мүмкіндіг шектеулі адам тәжірибе жоқ,bullying

2,неге балдақтарды қолданасың тек аяғыңызды қозғалтуға тырысыңыз сонда бәрі өтеді,неге балдақ қолдан тек аяғ қозғалт тырыс сонда бәрі өт,bullying

2,мүгедектердің жоғары білімі жоқ немесе зияткерлік салада жұмыс істейді,мүгедек жоғары білім жоқ немесе зияткерлік сала жұмыс істе,bullying

2,сіз бұл оқиғаны таңдай алмайсыз өйткені ол мүгедектерге бейімделмеген,сіз бұл оқи таңда алма өйткені ол мүгедек бейімделме,bullying

2,мүгедектер бұл жай ғана қоғам үшін ауыртпалық және көмекке лайық емес,мүгедек бұл жай ғана қоғам үшін ауыртпалық және көмек лайық емес,bullying

2,сізге спортпен шұғылданудың қажеті жоқ өйткені сіз мүгедексіз және жаттығумен тәжірибе жасамайсыз,сізге спорт шұғылдан қажет жоқ өйткені сіз мүгедек және жаттығ тәжірибе жасама,bullying

2,мен видеодан түсінгеннің бәрін түсіндім егер біреу маған отан маған көмектеседі десе онда бұл шизофрения,мен видео түсін бәрін түс егер біреу маған отан маған көмектес де онда бұл шизофрения,bullying

2,мүгедектігі бар адамдар мансапта ең танымал бола алмайды,мүгедектіг бар адам мансап ең танымал бола алма,bullying

2,кедей ол саяси экономиканы зерттеді мен де құлайтын едім,кедей ол саяси экономика зертте мен де құла ед,bullying

2,сіз бұл көлікті басқара алмайсыз өйткені сіз мүгедексіз,сіз бұл көлік басқар алма өйткені сіз мүгедек,bullying

2,мүгедектер бәрі жалқаужәне жай ғана жұмыс істегісі келмейтіндер,мүгедек бәрі жалқаужән жай ғана жұмыс істе келме,bullying

2,факт ол оны есірткімен тамақтандырды және оның төбесі тұзсыз сияқты болды,факт ол оны есірткі тамақтан және оның төбе тұз сияқты бол,bullying

2,мүгедектер бұл қосымша шығындар мен нәтижелерді қажет ететін қоғамға ауыртпалық,мүгедек бұл қосымша шығын мен нәтиже қажет ет қоғам ауыртпалық,bullying

2,біз сізге сүріну мүмкін емес өйткені сіз мүгедектігіңізге сәйкес келмейсіз,біз сізге сүрін мүмкін емес өйткені сіз мүгедектіг сәйкес келме,bullying

2,сіз мүгедектігіңізге байланысты қалыпты өмір сүре алмайсыз сондықтан тіпті ұмтылудың қажеті жоқ,сіз мүгедектіг байланысты қалыпты өмір сүр алма сондықтан тіпті ұмтыл қажет жоқ,bullying

2,сіз өзіңіздің мақсаттарыңыз бен армандарыңызға емес мүгедектігіңізге назар аударуыңыз керек,сіз өз мақсат бен арман емес мүгедектіг назар аудар керек,bullying

2,психикалық аурулардың көпшілігі нашақорлық негізінен алкоголизм және мидағы паразиттер,психикалық ауру көпшіліг нашақорлық негіз алкоголиз және ми паразит,bullying

2,сіз бұл лауазымға ие бола алмайсыз өйткені сізге арнайы көмек қажет және бұл біздің компания үшін өте қымбат,сіз бұл лауазым ие бола алма өйткені сізге арнайы көмек қажет және бұл біздің компания үшін өте қымбат,bullying

2,бұл адамға өте өкінішті бұл оны қандай галлюцинациялармен жауып тастады сұмдық енді олар оны есірткі астында деп айтар еді,бұл адам өте өкініш бұл оны қандай галлюцинация жау таста сұм енді олар оны есірткі аст де айтар ед,bullying

2,сіз мүгедектік сізді толыққанды адам етпейтінін білесіз,сіз мүгедектік сіз толыққанды адам етпе біл,bullying

2,мүгедектер толық қарым қатынаста және отбасында бола алмайды,мүгедек толық қарым қатынас және отбас бола алма,bullying

2,біз кеңсемізден алыс тұратын жұмысқа үміткерлерді қарастырмаймыз,біз кеңсе алыс тұр жұмыс үміткер қарастырма,bullying

2,бізде бар үміткерлерге артықшылық біздің компания орналасқан ауданда тұратындар,біз бар үміткер артықшылық біздің компания орналас аудан тұр,bullying

2,өкінішке орай біз клиенттермен жұмыс істемейміз бұл ауданда тұратындар,өкініш орай біз клиент жұмыс істеме бұл аудан тұр,bullying

2,біз сіз тұратын қалада қызмет көрсетпейміз,біз сіз тұр қала қызмет көрсетпе,bullying

2,біздің өнімдерімізді сіздің аймағыңызға жеткізуге мүмкіндігіміз жоқ,біздің өнім сіздің аймағ жеткіз мүмкіндіг жоқ,bullying

2,біз клиенттермен жұмыс істемейміз қаладан тыс жерде тұратындар,біз клиент жұмыс істеме қала тыс же тұр,bullying

2,клиенттермен жұмыс істеу бізге ыңғайлы емес олар біздің кеңседен алыс,клиент жұмыс істеу бізге ыңғайлы емес олар біздің кеңсе алыс,bullying

2,өкінішке орай біз сіздің аймағыңызда қызмет көрсетпейміз,өкініш орай біз сіздің аймағ қызмет көрсетпе,bullying

2,біз сізді осы лауазым үшін қарастыра алмаймыз өйткені сіз басқа қалада тұрасыз,біз сіз осы лауазым үшін қарастыр алма өйткені сіз басқа қала тұр,bullying

2,біз сізге көмектесе алмаймыз өйткені сіз тым алыссыз,біз сізге көмекте алма өйткені сіз тым алыс,bullying

2,сіз бұл университетте оқи алмайсыз өйткені сіздің табысыңыз жеткіліксіз оқу шығындарын жабу үшін,сіз бұл университет оқи алма өйткені сіздің табыс жеткіліксіз оқу шығын жаб үшін,bullying

2,біз бұл аймақтан клиенттерді іздемейміз өйткені олардың табысы төмен,біз бұл аймақ клиент іздеме өйткені олардың табыс төмен,bullying

2,біз бұл аймақтағы клиенттермен жұмыс істегенді ұнатпаймыз өйткені олар біздің мақсатты аудиториямыз емес,біз бұл аймақ клиент жұмыс істе ұнатпа өйткені олар біздің мақсат аудитория емес,bullying

2,біз өз қызметтерімізді аудан тұрғындарына ұсынғымыз келмейді өйткені бұл қауіпсіз емес,біз өз қызмет аудан тұрғын ұсын келме өйткені бұл қауіпсіз емес,bullying

2,біз белгілі бір аймақта тұратын адамдармен қарым қатынас жасағымыз келмейді өйткені олар салауатты өмір салтын ұстанбайды,біз белгілі бір аймақта тұр адам қарым қатынас жаса келме өйткені олар салауатты өмір салт ұстанба,bullying

2,өкінішке орай біз белгілі бір ауданда тұратын клиенттермен жұмыс істемейміз өйткені олар біздің мақсатты аудиториямыз емес,өкініш орай біз белгілі бір аудан тұр клиент жұмыс істеме өйткені олар біздің мақсат аудитория емес,bullying

2,біз аудан тұрғындарымен жұмыс істемейміз өйткені олардың білім деңгейі төмен,біз аудан тұрғын жұмыс істеме өйткені олардың білім деңгей төмен,bullying

2,біз бұл аймақтың тұрғындарына қызмет көрсетпейміз өйткені олар интернетке қол жеткізе алмайды,біз бұл аймақ тұрғын қызмет көрсетпе өйткені олар интернет қол жеткіз алма,bullying

2,сіз қатты екпінмен сөйлейсіз түсіну қиын менің ойымша сіз бұл лауазымға сәйкес келмейсіз,сіз қатты екпін сөйле түсіну қиын менің ойымша сіз бұл лауазым сәйкес келме,bullying

2,бізде клиенттермен тегіс және кәсіби қарым қатынас жасау өте маңызды егер сіздің екпініңіз бұған кедергі келтірсе онда біз сізді жалдай алмаймыз,біз клиент тегіс және кәсіби қарым қатынас жасау өте маңызды егер сіздің екпін бұған кедергі келтір онда біз сіз жалда алма,bullying

2,біз үміткерлерді іздейміз екпінсіз сөйлейтіндер клиенттер оларды оңай түсінуі үшін,біз үміткер ізде екпін сөйле клиент ол оңай түсін үшін,bullying

2,сіздің екпініңіз клиенттерді алаңдатуы мүмкін сондықтан біз бейтарап айтылуы бар адамды табуымыз керек,сіздің екпін клиент алаңдат мүмкін сондықтан біз бейтарап айтыл бар адам таб керек,bullying

2,өкінішке орай сіздің екпініңіз нақты қарым қатынасқа кедергі келтіреді және біз клиенттердің жоғалуына қауіп төндіре алмаймыз,өкініш орай сіздің екпін нақты қарым қатынас кедергі келтір және біз клиент жоғал қауіп төндір алма,bullying

2,біз анық және анық айтылуы бар үміткерлерді іздейміз және сіздің екпініңіз өкінішке орай бұл талапқа сәйкес келмейді,біз анық және анық айтыл бар үміткер ізде және сіздің екпін өкініш орай бұл талап сәйкес келме,bullying

2,біз сізді жалдай алмаймыз өйткені сіз біздің елдің азаматы емессіз,біз сіз жалда алма өйткені сіз біздің ел азамат емес,bullying

2,біз өз елімізден адамдарды жалдауды жөн көреміз өйткені олар жергілікті нарық пен мәдениетті жақсы біледі,біз өз ел адам жалдау жөн көр өйткені олар жергілікті нарық пен мәдениетті жақсы біл,bullying

2,сіздің визалық шектеулеріңізге байланысты біз сізге бұл жұмысты бере алмаймыз,сіздің визалық шектеу байланысты біз сізге бұл жұм бер алма,bullying

2,біз шетелдік жұмыс тәжірибесі бар қызметкерлерді іздемейміз біз жергілікті ортаға бейімделе алатын адамдарды іздейміз,біз шетелдік жұмыс тәжірибе бар қызметкер іздеме біз жергілікті орта бейімдел ал адам ізде,bullying

2,шетелдік жұмысшылар мәдени айырмашылықтарға байланысты ұжымда қақтығыстар тудыруы мүмкін және біз мұндай проблемалардан аулақ болғымыз келеді,шетелдік жұмысшы мәдени айырмашылық байланысты ұжым қақтығыс тудыр мүмкін және біз мұндай проблема аулақ бол кел,bullying

2,біздің елден келген адамдар біздің корпоративтік мәдениетімізге және жұмыс стилімізге жақсы бейімделе алады деп санаймыз,біздің ел кел адам біздің корпоративтік мәдениет және жұмыс стил жақсы бейімдел ал де сана,bullying

2,сіздің азаматтығыңыз біздің клиенттерімізге сенімсіздік тудыруы мүмкін және бұл біздің бизнесімізге зиян тигізуі мүмкін,сіздің азаматтығ біздің клиент сенімсіздік тудыр мүмкін және бұл біздің бизнес зиян тигіз мүмкін,bullying

2,біз еліміздегі шетелдік жұмысшылардың қауіпсіздігіне кепілдік бере алмаймыз сондықтан сізге бұл жұмысты бере алмаймыз,біз еліміз шетелдік жұмысшы қауіпсіздіг кепілдік бер алма сондықтан сізге бұл жұм бер алма,bullying

2,біздің клиенттеріміз еліміздің адамдарымен сөйлескенді жөн көреді сондықтан біз осындай жұмысшыларды жалдағанды жөн көреміз,біздің клиент ел адам сөйлес жөн көр сондықтан біз осындай жұмысшы жалда жөн көр,bullying

2,шетелдік жұмысшылар тілдік және мәдени кедергілерге тап болуы мүмкін және бұл біздің команданың тиімділігін төмендетуі мүмкін,шетелдік жұмысшы тілдік және мәдени кедергі тап бол мүмкін және бұл біздің команда тиімділіг төмендет мүмкін,bullying

2,біз мінсіз айтылуы бар адамдарды жалдағанды жөн көреміз өйткені бұл кәсіби қарым қатынас үшін маңызды,біз мін айтыл бар адам жалда жөн көр өйткені бұл кәсіби қарым қатынас үшін маңызды,bullying

2,сіздің акцентіңіз кейбір клиенттер үшін түсініксіз болуы мүмкін және біз бизнесті жоғалту қаупіне ұшырай алмаймыз,сіздің акцент кейбір клиент үшін түсінік бол мүмкін және біз бизнес жоғалт қауп ұшыра алма,bullying

2,біз барлық қызметкерлерден талап етеміз олар екпінсіз сөйлеуі үшін біз компанияда біртұтас айтылымға ие болуымыз керек,біз барлық қызметкер талап ет олар екпін сөйле үшін біз компания біртұтас айтылым ие бол керек,bullying

2,өкінішке орай сіздің екпініңіз сіздің сөйлеуіңізді түсініксіз етеді және біз сізді осы лауазымға жалдай алмаймыз,өкініш орай сіздің екпін сіздің сөйлеу түсінік ет және біз сіз осы лауазым жалда алма,bullying

2,біз белгілі бір аймақта тұратын адамдармен жұмыс істегенді ұнатпаймыз өйткені олардың тұрақты жұмысы жоқ,біз белгілі бір аймақта тұр адам жұмыс істе ұнатпа өйткені олардың тұрақты жұмыс жоқ,bullying

2,біз бұл аймақтағы клиенттермен жұмыс істегіміз келмейді өйткені олар біздің критерийлерімізге сәйкес келмейді,біз бұл аймақ клиент жұмыс істе келме өйткені олар біздің критерий сәйкес келме,bullying

2,біз бұл аймақтағы қызметтерімізді жарнамаламаймыз өйткені ондағы тұрғындар біздің мақсатты аудиториямыз емес,біз бұл аймақ қызмет жарнамалама өйткені ондағы тұрғын біздің мақсат аудитория емес,bullying

2,біз сізге бұл затты бөліп бөліп сата алмаймыз өйткені сіздің табысыңыз жеткіліксіз,біз сізге бұл зат бөл бөл сат алма өйткені сіздің табыс жеткіліксіз,bullying

2,сіз біздің клубқа қосыла алмайсыз өйткені мүшелік жарна сіздің табысыңыз үшін тым жоғары,сіз біздің клуб қосыл алма өйткені мүшелік жарна сіздің табыс үшін тым жоғары,bullying

2,біз сізге толық уақытты жұмыс бере алмаймыз өйткені біз сізге осындай жоғары жалақы алуға мүмкіндік бере алмаймыз,біз сізге толық уақыт жұмыс бер алма өйткені біз сізге осындай жоғары жалақы ал мүмкіндік бер алма,bullying

2,сіз біздің адалдық бағдарламамызға қатыса алмайсыз өйткені сізде табыс деңгейі жеткіліксіз артықшылықтар алу үшін,сіз біздің адалдық бағдарлама қатыс алма өйткені сіз табыс деңгей жеткіліксіз артықшылық алу үшін,bullying

2,сіз бұл стипендияға ие бола алмайсыз өйткені сізде жеткілікті жоғары табыс жоқ және сіз мақсатты топқа кірмейсіз,сіз бұл стипендия ие бола алма өйткені сіз жеткілікті жоғары табыс жоқ және сіз мақсат топ кірме,bullying

2,сіз үкіметтен қолдау ала алмайсыз өйткені сіздің табысыңыз белгіленген шектен жоғары,сіз үкімет қолдау ал алма өйткені сіздің табыс белгілен шек жоғары,bullying

2,сіз біздің үйге несие беру бағдарламасына қосыла алмайсыз өйткені сіздің табысыңыз жеткіліксіз,сіз біздің үй несие беру бағдарлама қосыл алма өйткені сіздің табыс жеткіліксіз,bullying

2,сіз қосымша жеңілдіктер ала алмайсыз өйткені сіздің табысыңыз белгіленген шектен асады,сіз қосымша жеңілдік ал алма өйткені сіздің табыс белгілен шек ас,bullying

2,сен біздің акцияға қатыса алмайсың өйткені ол үшін үлкен сатып алу қажет сіз оны төмен табысқа байланысты ала алмайсыз,сен біздің акция қатыс алма өйткені ол үшін үлкен сат алу қажет сіз оны төмен табыс байланысты ал алма,bullying

2,біз сізге бұл машинаны сата алмаймыз өйткені ол тек жоғары әлеуметтік мәртебесі бар адамдар үшін қол жетімді,біз сізге бұл машина сат алма өйткені ол тек жоғары әлеуметтік мәрте бар адам үшін қол жет,bullying

2,сіз бұл клубқа бара алмайсыз өйткені ол тек жоғары әлеуметтік мәртебесі бар адамдар үшін қол жетімді,сіз бұл клуб бар алма өйткені ол тек жоғары әлеуметтік мәрте бар адам үшін қол жет,bullying

2,біз сізге несие бере алмаймыз өйткені сіздің әлеуметтік мәртебеңіз жеткіліксіз,біз сізге несие бер алма өйткені сіздің әлеуметтік мәртебе жеткіліксіз,bullying

2,сіз бұл конференцияға бара алмайсыз өйткені ол тек жоғары әлеуметтік мәртебесі бар адамдар үшін қол жетімді,сіз бұл конференция бар алма өйткені ол тек жоғары әлеуметтік мәрте бар адам үшін қол жет,bullying

2,біз сізге эксклюзивті қызметтерімізге қол жеткізе алмаймыз өйткені олар тек жоғары әлеуметтік мәртебесі бар адамдар үшін қол жетімді,біз сізге эксклюзив қызмет қол жеткіз алма өйткені олар тек жоғары әлеуметтік мәрте бар адам үшін қол жет,bullying

2,сіз бұл жеке демалыс аймағын пайдалана алмайсыз өйткені ол тек жоғары әлеуметтік мәртебесі бар адамдар үшін қол жетімді,сіз бұл жеке демалыс аймағ пайдалан алма өйткені ол тек жоғары әлеуметтік мәрте бар адам үшін қол жет,bullying

2,біз сізге бұл жұмысты бере алмаймыз өйткені сіз біздің әлеуметтік мәртебе талаптарына сәйкес келмейсіз,біз сізге бұл жұм бер алма өйткені сіз біздің әлеуметтік мәртебе талап сәйкес келме,bullying

2,сіз бұл клубтың мүшесі бола алмайсыз өйткені ол тек жоғары әлеуметтік мәртебесі бар адамдар үшін қол жетімді,сіз бұл клуб мүше бола алма өйткені ол тек жоғары әлеуметтік мәрте бар адам үшін қол жет,bullying

2,біз сізге бұл картаны қоғамдық көлікті пайдалану үшін сата алмаймыз өйткені ол тек жоғары әлеуметтік мәртебесі бар адамдар үшін қол жетімді,біз сізге бұл карта қоғамдық көлік пайдалану үшін сат алма өйткені ол тек жоғары әлеуметтік мәрте бар адам үшін қол жет,bullying

2,сіз бұл сақтандыруды ала алмайсыз өйткені ол тек жоғары әлеуметтік мәртебесі бар адамдар үшін қол жетімді,сіз бұл сақтандыру ал алма өйткені ол тек жоғары әлеуметтік мәрте бар адам үшін қол жет,bullying

2,біз сізді осы оқу курсына қабылдай алмаймыз өйткені ол тек жоғары әлеуметтік мәртебесі бар адамдар үшін қол жетімді,біз сіз осы оқу курс қабылда алма өйткені ол тек жоғары әлеуметтік мәрте бар адам үшін қол жет,bullying

2,сіз бұл лауазымды атқара алмайсыз өйткені ол тек жоғары әлеуметтік мәртебесі бар адамдар үшін қол жетімді,сіз бұл лауазым атқар алма өйткені ол тек жоғары әлеуметтік мәрте бар адам үшін қол жет,bullying

2,біз сізге бұл стипендияны бере алмаймыз өйткені ол тек жоғары әлеуметтік мәртебесі бар студенттерге қол жетімді,біз сізге бұл стипендия бер алма өйткені ол тек жоғары әлеуметтік мәрте бар студент қол жет,bullying

2,сіз бұл спорт кешенін пайдалана алмайсыз өйткені ол тек жоғары әлеуметтік мәртебесі бар адамдар үшін қол жетімді,сіз бұл спорт кешен пайдалан алма өйткені ол тек жоғары әлеуметтік мәрте бар адам үшін қол жет,bullying

2,біз сізге бұл мүлікті сата алмаймыз өйткені ол ауданда орналасқан ол тек жоғары әлеуметтік мәртебесі бар адамдарға қол жетімді,біз сізге бұл мүлік сат алма өйткені ол аудан орналас ол тек жоғары әлеуметтік мәрте бар адам қол жет,bullying

2,мен төменгі деңгейдегі біреуді жалдамаймын олар бұл жұмысқа сәйкес келгісі келмейді,мен төменгі деңгей біреу жалдама олар бұл жұмыс сәйкес кел келме,bullying

2,мен сізбен сөйлескеніме риза болуыңыз керек жалпы сіздің әлеуметтік мәртебеңіз,мен сізбен сөйлес риза бол керек жалпы сіздің әлеуметтік мәртебе,bullying

2,кейбір бомж мені көшеде қорқытты бұл кедей аудандардағы адамдардың өзін дұрыс ұстай алмайтындығының тағы бір дәлелі,кейбір бомж мен көше қорқыт бұл кедей аудан адам өз дұрыс ұста алмайтындығ тағы бір дәлел,bullying

2,біз төмен әлеуметтік мәртебеге ие тұрғындарға қол жеткізе алмаймыз бұл пайдалану біздің жылжымайтын мүлік бағасының төмендеуіне,біз төмен әлеуметтік мәртебе ие тұрғын қол жеткіз алма бұл пайдалану біздің жылжыма мүлік баға төменде,bullying

2,менің ойымша бұл қыз бұл компанияда орын ала алмайды оның ата анасы өндірісте жұмыс істейді ол мұндай оқу бағдарламасына сәйкес келмейді,менің ойымша бұл қыз бұл компания орын ал алма оның ата ана өндіріс жұмыс істе ол мұндай оқу бағдарлама сәйкес келме,bullying

2,сіз жұмыста соншалықты немқұрайлы көрінбеуіңіз мүмкін сіздің сыртқы келбетіңіз төмен әлеуметтік мәртебеңізді тудырады,сіз жұмыс соншалықты немқұрайл көрінбе мүмкін сіздің сыртқы келбет төмен әлеуметтік мәртебе тудыр,bullying

2,бұл жігітті басшы деп тану мүмкін емес ол кедей отбасында өсті және ешқашан жоғары білім алмайды,бұл жігіт басшы де тану мүмкін емес ол кедей отбас өс және ешқашан жоғары білім алма,bullying

2,біз бұл жұмысқа әйелді жалдай алмаймыз ол төмен жалақы алатын жұмысшылар үшін жатақханада тұрады сондықтан оның біліктілігі жеткіліксіз,біз бұл жұмыс әйел жалда алма ол төмен жалақы ал жұмысшы үшін жатақхана тұр сондықтан оның біліктілиг жеткіліксіз,bullying

2,біз сізге бұл қызметті ұсынбаймыз өйткені ол тек мәртебесі жоғары адамдар үшін қол жетімді,біз сізге бұл қызмет ұсынба өйткені ол тек мәрте жоғары адам үшін қол жет,bullying

2,бұл саябақ тек табысы жоғары адамдарға арналған сондықтан олар шу мен қарбаласcыз табиғатты тамашалай алады бұл адамдардың төмен мәртебесін талап етеді,бұл саябақ тек табыс жоғары адам арнал сондықтан олар шу мен қарбалас табиғат тамашала ал бұл адам төмен мәртебе талап ет,bullying

2,біз мүмкіндік бере алмаймыз біздің кешенде табылған кедей адамдар бұл біздің компанияның беделіне теріс әсер етуі мүмкін,біз мүмкіндік бер алма біздің кешен табыл кедей адам бұл біздің компания бедел теріс әсер ет мүмкін,bullying

2,мен сізге үй сатып алуға несие бере алмаймын өйткені сізде жеткілікті әлеуметтік мәртебе жоқ оны анықтау үшін,мен сізге үй сат ал несие бер алма өйткені сіз жеткілікті әлеуметтік мәртебе жоқ оны анықтау үшін,bullying

2,сіз бұл аймақта өмір сүре алмайсыз өйткені табыс орташа деңгейден төмен және сіз біздің тоқсанда өмір сүре алмайсыз,сіз бұл аймақта өмір сүр алма өйткені табыс орташа деңгей төмен және сіз біздің тоқсан өмір сүр алма,bullying

2,біз отбасылардан адамдарды қабылдамаймыз әлеуметтік жәрдемақы алатын адамдар өйткені олар толық көзқараста дамымайды,біз отбасы адам қабылдама әлеуметтік жәрдемақы ал адам өйткені олар толық көзқарас дамыма,bullying

2,бұл біздің ең жақсы клиенттерімізге арналған орын және сіз оның қызметтерін пайдалана алмайсыз өйткені сіз олардың бірі болу үшін жеткілікті бай емессіз,бұл біздің ең жақсы клиент арнал орын және сіз оның қызмет пайдалан алма өйткені сіз олардың бірі болу үшін жеткілікті бай емес,bullying

2,менің ойымша бұл табысы төмен отбасы студенті жоғары баға ала алмайды өйткені ол қол жетімді білім беру ресурстарына қол жеткізе алмайды бай студенттерде бар,менің ойымша бұл табыс төмен отбасы студент жоғары баға ал алма өйткені ол қол жет білім беру ресурс қол жеткіз алма бай студент бар,bullying

2,біз бұл мүлікті ресурстарда тұратын адамдарға сата алмаймыз өйткені олар мүлікті ұстауға мүмкіндігі жоқ,біз бұл мүлік ресурс тұр адам сат алма өйткені олар мүлік ұста мүмкіндіг жоқ,bullying

2,мен адамдарды көргім келмейді менің мейрамханамда олар біздің конференцияға қатыспайды өйткені олардың тұрағы бар,мен адам көр келме менің мейрамхана олар біздің конференция қатыспа өйткені олардың тұрағ бар,bullying

2,бұл адам көпшіл бола алмайды ол біздің компанияға сәйкес келетін әлеуметтік саладан емес,бұл адам көпшіл бола алма ол біздің компания сәйкес кел әлеуметтік сала емес,bullying

2,біз мәртебесі төмен жұмысшыларды жалдай алмаймыз өйткені олар біздің жұмысымызға жеткілікті біліктілікке ие емес,біз мәрте төмен жұмысшы жалда алма өйткені олар біздің жұмыс жеткілікті біліктілік ие емес,bullying

2,біз бұл лауазымға әйелдерді жалдай алмаймыз өйткені олар әдетте жеткілікті жоғары мәртебеге ие емес мұндай жұмысты орындау үшін,біз бұл лауазым әйел жалда алма өйткені олар әдет жеткілікті жоғары мәртебе ие емес мұндай жұм орындау үшін,bullying

2,сіз бұл қонақүйде бөлмені таңдай алмайсыз өйткені бізде тек белгілі бір топтардың өкілдерін қабылдау саясаты бар,сіз бұл қонақүй бөлме таңда алма өйткені біз тек белгілі бір топ өкіл қабылдау саясат бар,bullying

2,біз сізге көлік несиесін ұсынбаймыз өйткені сізде жеткіліксіз әлеуметтік мәртебе төлемдерді түзету үшін,біз сізге көлік несие ұсынба өйткені сіз жеткіліксіз әлеуметтік мәртебе төлем түзету үшін,bullying

2,сіз бұл клубтың мүшесі бола алмайсыз өйткені біз тек мәртебесі жоғары адамдарды қабылдаймыз,сіз бұл клуб мүше бола алма өйткені біз тек мәрте жоғары адам қабылда,bullying

2,біз сізді осы лауазымға жалдай алмаймыз өйткені сізде тиісті білім жоқ және мәртебесі төмен адамдарды жалдағымыз келмейді,біз сіз осы лауазым жалда алма өйткені сіз тиісті білім жоқ және мәрте төмен адам жалда келме,bullying

2,біз бұл затты мәртебесі төмен адамдарға сатпаймыз өйткені олар оны сатып ала алмайды,біз бұл зат мәрте төмен адам сатпа өйткені олар оны сат ал алма,bullying

2,сіз бұл жұмысқа орналаса алмайсыз өйткені бізде мәртебесі жоғары кандидаттарға сайлау бар,сіз бұл жұмыс орналас алма өйткені біз мәрте жоғары кандидат сайлау бар,bullying

2,біз мәртебесі төмен адамдарға тұрғын үйге субсидия бермейміз өйткені олар жұмыс іздеп өмір сүру деңгейін көтеруі керек,біз мәрте төмен адам тұрғын үй субсидия берме өйткені олар жұмыс ізде өмір сүру деңгей көтер керек,bullying

2,бұл банк жеткілікті мәртебесі жоқ адамдармен жұмыс істемейді білім алуға немесе бизнеске несие алу үшін,бұл банк жеткілікті мәрте жоқ адам жұмыс істеме білім ал немесе бизнес несие алу үшін,bullying

2,сіз бұл аймақта өмір сүре алмайсыз өйткені біздің тілектеріміз мәртебесі жоғары адамдардың ортасында өмір сүргісі келеді,сіз бұл аймақта өмір сүр алма өйткені біздің тілек мәрте жоғары адам орта өмір сүр кел,bullying

2,бұл жұмыс беруші ерекше әлеуметтік топтағы адамдарды қабылдаудан бас тартады өйткені ол жоғары мәртебеге ие қызметкерлерді жақсы көреді,бұл жұмыс беруші ерекше әлеуметтік топтағы адам қабылдау бас тарт өйткені ол жоғары мәртебе ие қызметкер жақсы көр,bullying

2,сіз біздің дүкеннен азық түлік сатып ала алмайсыз өйткені біз мәртебесі төмен адамдарға қызмет көрсетпейміз,сіз біздің дүкен азық түлік сат ал алма өйткені біз мәрте төмен адам қызмет көрсетпе,bullying

2,сіз балаларыңызды осы мектепке жібере алмайсыз өйткені бұл сіз үшін тым қымбат,сіз бала осы мектеп жібер алма өйткені бұл сіз үшін тым қымбат,bullying

2,сізге қажет медициналық көмекке ақша жетпейді,сізге қажет медициналық көмек ақша жетпе,bullying

2,сіз бұл аймақта үй сатып ала алмайсыз өйткені бұл сіздің бюджетіңіз үшін тым қымбат,сіз бұл аймақта үй сат ал алма өйткені бұл сіздің бюджет үшін тым қымбат,bullying

2,сіздің өмір салтыңыз сізге осы брендтік өнімдерді сатып алуға мүмкіндік бермейді,сіздің өмір сал сізге осы бренд өнім сат ал мүмкіндік берме,bullying

2,сіз бұл іс шараға қатыса алмайсыз өйткені бұл тек табысы жоғары адамдарға арналған,сіз бұл іс шара қатыс алма өйткені бұл тек табыс жоғары адам арнал,bullying

2,сіз бұл курортқа демалысқа бара алмайсыз өйткені бұл сіз үшін тым қымбат,сіз бұл курорт демалыс бар алма өйткені бұл сіз үшін тым қымбат,bullying

2,сіз бұл көлікті ала алмайсыз өйткені сіз жеткілікті ақша таппайсыз,сіз бұл көлік ал алма өйткені сіз жеткілікті ақша таппа,bullying

2,сіз мұнда жұмыс істей алмайсыз өйткені сізде жеткілікті тәжірибе мен білім жоқ,сіз мұнда жұмыс істе алма өйткені сіз жеткілікті тәжірибе мен білім жоқ,bullying

2,сіз бұл несиені ала алмайсыз өйткені сіздің табысыңыз жеткіліксіз,сіз бұл несие ал алма өйткені сіздің табыс жеткіліксіз,bullying

2,сіз өзіңіздің табысыңыздың төмен болуына байланысты отбасыңызды жақсы жолмен қамтамасыз ете алмайсыз,сіз өз табыс төмен бол байланысты отбас жақсы жол қамтамасыз ет алма,bullying

2,біз сізге стипендия ұсынбаймыз өйткені сіз табысы төмен отбасынан шыққансыз және әлеуметтік мәртебеңіз жеткіліксіз,біз сізге стипендия ұсынба өйткені сіз табыс төмен отбас шық және әлеуметтік мәртебе жеткіліксіз,bullying

2,біз әйелдерді басшылық лауазымдарға тағайындауды ұсынбаймыз өйткені олар әдетте әлеуметтік мәртебесі төмен және лауазымдарда болмауы мүмкін,біз әйел басшылық лауазым тағайындау ұсынба өйткені олар әдет әлеуметтік мәрте төмен және лауазым болма мүмкін,bullying

2,біз сізге бұл көлікті сата алмаймыз өйткені сіз оның табысы төмен отбасынан шыққансыз және сатып алуға мүмкіндігіңіз жоқ,біз сізге бұл көлік сат алма өйткені сіз оның табыс төмен отбас шық және сат ал мүмкіндіг жоқ,bullying

2,біз бұл пәтерді мәртебесі төмен адамдарға жалға бермейміз өйткені олар целлюлозаны уақытында жалға алуға құқылы емес,біз бұл пәтер мәрте төмен адам жалға берме өйткені олар целлюлоза уақыт жалға ал құқылы емес,bullying

2,сіз бұл лауазымда жұмыс істей алмайсыз өйткені біз жоғары мәртебеге ие талаптарды іздейміз,сіз бұл лауазым жұмыс істе алма өйткені біз жоғары мәртебе ие талап іздe,bullying

2,біз сізді осы университетте қабылдамаймыз өйткені сіздің әлеуметтік мәртебеңіз сәйкес келмейді,біз сіз осы университет қабылдама өйткені сіздің әлеуметтік мәртебе сәйкес келме,bullying

2,біз сізге бұл затты сата алмаймыз өйткені біз мәртебесі төмен адамдармен жұмыс істемейміз,біз сізге бұл зат сат алма өйткені біз мәрте төмен адам жұмыс істеме,bullying

2,сіз бұл іс шараға қатыса алмайсыз өйткені ол тек мәртебесі жоғары адамдарға арналған,сіз бұл іс шара қатыс алма өйткені ол тек мәрте жоғары адам арнал,bullying

2,біз сізді осы лауазымға жалдай алмаймыз өйткені сіз әлеуметтік мәртебе талаптарына сай емессіз,біз сіз осы лауазым жалда алма өйткені сіз әлеуметтік мәртебе талап сай емес,bullying

2,сіз бұл ауруға қатыса алмайсыз өйткені бұл тек мәртебесі жоғары адамдар үшін ғана болады,сіз бұл ауру қатыс алма өйткені бұл тек мәрте жоғары адам үшін ғана бол,bullying

2,біз сізге бұл үйді сатпаймыз өйткені сіз оны сатып алуға мүмкіндік беретін мәртебеге ие емессіз,біз сізге бұл үй сатпа өйткені сіз оны сат ал мүмкіндік бер мәртебе ие емес,bullying

2,сіз бұл мектепте оқи алмайсыз өйткені ол тек жоғары мәртебеге ие,сіз бұл мектеп оқи алма өйткені ол тек жоғары мәртебе ие,bullying

2,біз сізге медициналық сақтандыруды ұсынбаймыз өйткені сіз сатып алу үшін жеткілікті мәртебеге ие емессіз,біз сізге медициналық сақтандыру ұсынба өйткені сіз сат алу үшін жеткілікті мәртебе ие емес,bullying

2,сіз бұл лауазымды атқара алмайсыз өйткені ол жоғары мәртебені талап етеді деп ойлаймыз,сіз бұл лауазым атқар алма өйткені ол жоғары мәртебе талап ет де ойла,bullying

2,біз сізге бұл машинаны сата алмаймыз өйткені мәртебені алып тастауға байланысты оны сатып алуға мүмкіндігіңіз жоқ,біз сізге бұл машина сат алма өйткені мәртебе алып таста байланысты оны сат ал мүмкіндіг жоқ,bullying

2,сіз бұл ұйымның мүшесі бола алмайсыз өйткені ол тек мәртебесі жоғары адамдарды қабылдайды,сіз бұл ұйы мүше бола алма өйткені ол тек мәрте жоғары адам қабылда,bullying

2,біз сізге несие бөлуді қарастырмаймыз өйткені сіз бөлумен жеке адамдармен жеткілікті қарым қатынаста болмайсыз,біз сізге несие бөлу қарастырма өйткені сіз бөл жеке адам жеткілікті қарым қатынас болма,bullying

2,сіз бұл бағдарламаға қатыса алмайсыз өйткені ол тек жоғары мәртебеге ие адамдар үшін байланысты,сіз бұл бағдарлама қатыс алма өйткені ол тек жоғары мәртебе ие адам үшін байланысты,bullying

2,біз сізді жұмысқа қабылдай алмаймыз өйткені сіздің әлеуметтік мәртебеңіз төмен және сіз заманауи талаптарға сай емессіз,біз сіз жұмыс қабылда алма өйткені сіздің әлеуметтік мәртебе төмен және сіз заманауи талап сай емес,bullying

2,бұл мейрамхана мәртебесі төмен адамдарға қызмет етпейді сондықтан түскі ас үшін басқа орын іздегеніңіз жөн,бұл мейрамхана мәрте төмен адам қызмет етпе сондықтан түскі ас үшін басқа орын ізде жөн,bullying

2,біз сізге бұл мүлікті сата алмаймыз өйткені ол беделді ауданда орналасқан ол тек жоғары мәртебеге ие адамдарға қол жетімді,біз сізге бұл мүлік сат алма өйткені ол беделді аудан орналас ол тек жоғары мәртебе ие адам қол жет,bullying

2,сіз бұл компанияда жұмысқа тұра алмайсыз өйткені сіз жоғары қоғамнан емессіз және жеткілікті байланыстарыңыз жоқ,сіз бұл компания жұмыс тұр алма өйткені сіз жоғары қоғам емес және жеткілікті байланыс жоқ,bullying

2,біз сізге бұл концертке билеттерді сата алмаймыз өйткені бұл тек мәртебесі жоғары адамдарға арналған,біз сізге бұл концерт билет сат алма өйткені бұл тек мәрте жоғары адам арнал,bullying

2,сіз денсаулықты сақтандыруды ала алмайсыз өйткені ол тек мәртебесі жоғары адамдар үшін қол жетімді,сіз денсаулық сақтандыру ал алма өйткені ол тек мәрте жоғары адам үшін қол жет,bullying

2,біз сізді осы лауазымға тағайындауды ұсынбаймыз өйткені біз адамдарды іздейміз жоғары мәртебені пайдалана алатын бұл біздің компанияға жақсырақ,біз сіз осы лауазым тағайындау ұсынба өйткені біз адам ізде жоғары мәртебе пайдалан ал бұл біздің компания жақсырақ,bullying

2,бұл курорт төмен мәртебеге ие емес сондықтан сіз басқа демалыс орнын іздеуіңіз керек,бұл курорт төмен мәртебе ие емес сондықтан сіз басқа демалыс орн ізде керек,bullying

2,сіз бұл клуб ұйымының мүшесі бола алмайсыз өйткені ол тек мәртебесі жоғары адамдар үшін қол жетімді,сіз бұл клуб ұйым мүше бола алма өйткені ол тек мәрте жоғары адам үшін қол жет,bullying

2,біз сізге ипотеканы таңдай алмаймыз өйткені сіздің әлеуметтік мәртебеңіз төмен және федералды жарналарды төлей алмайсыз,біз сізге ипотека таңда алма өйткені сіздің әлеуметтік мәртебе төмен және федерал жарна төле алма,bullying

2,сіз бұл лауазымды атқара алмайсыз өйткені сіздің әлеуметтік мәртебеңіз төмен және сіз заманауи талаптарға сай емессіз,сіз бұл лауазым атқар алма өйткені сіздің әлеуметтік мәртебе төмен және сіз заманауи талап сай емес,bullying

2,біз сізге бұл машинаны сата алмаймыз өйткені ол тек мәртебесі жоғары адамдар үшін қол жетімді,біз сізге бұл машина сат алма өйткені ол тек мәрте жоғары адам үшін қол жет,bullying

2,сіз бұл жұмысқа орналаса алмайсыз өйткені біз іздейміз жоғары мәртебеге ие талаптар олар тиісті лауазымдарға жақсырақ сәйкес келеді,сіз бұл жұмыс орналас алма өйткені біз ізде жоғары мәртебе ие талап олар тиісті лауазым жақсырақ сәйкес кел,bullying

2,біз сізге ұсынбаймыз өмірді сақтандыру өйткені ол тек мәртебесі жоғары адамдар үшін қол жетімді,біз сізге ұсынба өмір сақтандыр өйткені ол тек мәрте жоғары адам үшін қол жет,bullying

2,бұл қонақ үй әлеуметтік мәртебесі төмен қонақтарды қабылдамайды сондықтан сіз тұру үшін басқа орын іздеуіңіз керек,бұл қонақ үй әлеуметтік мәрте төмен қонақ қабылдама сондықтан сіз тұру үшін басқа орын ізде керек,bullying

2,сіз бұл спорттық командаға қосыла алмайсыз өйткені ол тек жоғары әлеуметтік мәртебесі бар адамдар үшін қол жетімді,сіз бұл спорттық команда қосыл алма өйткені ол тек жоғары әлеуметтік мәрте бар адам үшін қол жет,bullying

2,біз сізге несие бере алмаймыз өйткені сіздің әлеуметтік мәртебеңіз төмен және төлем қабілеттілігіңізге кепілдік бере алмайсыз,біз сізге несие бер алма өйткені сіздің әлеуметтік мәртебе төмен және төлем қабілеттіліг кепілдік бер алма,bullying

2,сіз бұл гольф клубының мүшесі бола алмайсыз өйткені ол тек жоғары әлеуметтік мәртебесі бар адамдар үшін қол жетімді,сіз бұл гольф клуб мүше бола алма өйткені ол тек жоғары әлеуметтік мәрте бар адам үшін қол жет,bullying

2,біз сізге бұл яхтаны сата алмаймыз өйткені ол тек жоғары әлеуметтік мәртебесі бар адамдар үшін қол жетімді,біз сізге бұл яхта сат алма өйткені ол тек жоғары әлеуметтік мәрте бар адам үшін қол жет,bullying

2,сіз бұл университетте білім ала алмайсыз өйткені ол тек жоғары әлеуметтік мәртебесі бар адамдар үшін қол жетімді,сіз бұл университет білім ал алма өйткені ол тек жоғары әлеуметтік мәрте бар адам үшін қол жет,bullying

2,біз сізге осы банктің қызметтерін ұсына алмаймыз өйткені олар тек жоғары әлеуметтік мәртебесі бар адамдар үшін қол жетімді,біз сізге осы банк қызмет ұсын алма өйткені олар тек жоғары әлеуметтік мәрте бар адам үшін қол жет,bullying

2,сіз бұл жұмысты ала алмайсыз өйткені ол тек жоғары әлеуметтік мәртебесі бар адамдар үшін қол жетімді,сіз бұл жұм ал алма өйткені ол тек жоғары әлеуметтік мәрте бар адам үшін қол жет,bullying

2,біз сізге бұл мүлікті сата алмаймыз өйткені ол беделді ауданда орналасқан ол тек жоғары әлеуметтік мәртебесі бар адамдарға қол жетімді,біз сізге бұл мүлік сат алма өйткені ол беделді аудан орналас ол тек жоғары әлеуметтік мәрте бар адам қол жет,bullying

2,біз сізді жұмысқа қабылдай алмаймыз өйткені сіз біздің әлеуметтік мәртебе талаптарына сәйкес келмейсіз,біз сіз жұмыс қабылда алма өйткені сіз біздің әлеуметтік мәртебе талап сәйкес келме,bullying

2,сіз бұл стипендияны ала алмайсыз өйткені ол тек жоғары әлеуметтік мәртебесі бар студенттерге қол жетімді,сіз бұл стипендия ал алма өйткені ол тек жоғары әлеуметтік мәрте бар студент қол жет,bullying

2,біз сізге бұл үйді сата алмаймыз өйткені ол ауданда орналасқан ол тек жоғары әлеуметтік мәртебесі бар адамдарға қол жетімді,біз сізге бұл үй сат алма өйткені ол аудан орналас ол тек жоғары әлеуметтік мәрте бар адам қол жет,bullying

2,біз сізге бұл қызметті ұсына алмаймыз өйткені ол тек жоғары әлеуметтік мәртебесі бар адамдар үшін қол жетімді,біз сізге бұл қызмет ұсын алма өйткені ол тек жоғары әлеуметтік мәрте бар адам үшін қол жет,bullying

2,біз сізге бұл медициналық қызметті ұсына алмаймыз өйткені ол тек жоғары әлеуметтік мәртебесі бар адамдар үшін қол жетімді,біз сізге бұл медициналық қызмет ұсын алма өйткені ол тек жоғары әлеуметтік мәрте бар адам үшін қол жет,bullying

2,сіз бұл несиені ала алмайсыз өйткені сізде әлеуметтік мәртебе жеткіліксіз,сіз бұл несие ал алма өйткені сіз әлеуметтік мәртебе жеткіліксіз,bullying

2,біз сізді бұл университетке қабылдай алмаймыз өйткені сіздің әлеуметтік мәртебеңіз жеткіліксіз,біз сіз бұл университет қабылда алма өйткені сіздің әлеуметтік мәртебе жеткіліксіз,bullying

2,сіз бұл тұрғын үй кешенінде тұра алмайсыз өйткені ол тек жоғары әлеуметтік мәртебесі бар адамдар үшін қол жетімді,сіз бұл тұрғын үй кешен тұр алма өйткені ол тек жоғары әлеуметтік мәрте бар адам үшін қол жет,bullying

2,біз сізге бұл затты сата алмаймыз өйткені ол тек жоғары әлеуметтік мәртебесі бар адамдар үшін қол жетімді,біз сізге бұл зат сат алма өйткені ол тек жоғары әлеуметтік мәрте бар адам үшін қол жет,bullying

2,біз сізге бұл қызметті біздің қонақүйде ұсына алмаймыз өйткені ол тек жоғары әлеуметтік мәртебесі бар адамдар үшін қол жетімді,біз сізге бұл қызмет біздің қонақүй ұсын алма өйткені ол тек жоғары әлеуметтік мәрте бар адам үшін қол жет,bullying

2,сіз бұл бірлестікке кіре алмайсыз өйткені ол тек жоғары әлеуметтік мәртебесі бар адамдар үшін қол жетімді,сіз бұл бірлестік кір алма өйткені ол тек жоғары әлеуметтік мәрте бар адам үшін қол жет,bullying

2,біз сізге бұл адалдық бағдарламасын бере алмаймыз өйткені ол тек жоғары әлеуметтік мәртебесі бар адамдар үшін қол жетімді,біз сізге бұл адалдық бағдарлама бер алма өйткені ол тек жоғары әлеуметтік мәрте бар адам үшін қол жет,bullying

2,сіз бұл ұйымға қосыла алмайсыз өйткені ол тек жоғары әлеуметтік мәртебесі бар адамдар үшін қол жетімді,сіз бұл ұйым қосыл алма өйткені ол тек жоғары әлеуметтік мәрте бар адам үшін қол жет,bullying

2,біз сізге бұл жиһазды сата алмаймыз өйткені ол тек жоғары әлеуметтік мәртебесі бар адамдар үшін қол жетімді,біз сізге бұл жиһаз сат алма өйткені ол тек жоғары әлеуметтік мәрте бар адам үшін қол жет,bullying

2,сіз бұл медициналық көмекті ала алмайсыз өйткені ол тек жоғары әлеуметтік мәртебесі бар адамдар үшін қол жетімді,сіз бұл медициналық көмек ал алма өйткені ол тек жоғары әлеуметтік мәрте бар адам үшін қол жет,bullying

2,біз сізге бұл туристік пакетті бере алмаймыз өйткені ол тек жоғары әлеуметтік мәртебесі бар адамдар үшін қол жетімді,біз сізге бұл туристік пакет бер алма өйткені ол тек жоғары әлеуметтік мәрте бар адам үшін қол жет,bullying

2,сіз бұл қызметті пайдалана алмайсыз өйткені ол тек жоғары әлеуметтік мәртебесі бар адамдар үшін қол жетімді,сіз бұл қызмет пайдалан алма өйткені ол тек жоғары әлеуметтік мәрте бар адам үшін қол жет,bullying

2,сіз бұл стипендияны ала алмайсыз өйткені ол тек жоғары әлеуметтік мәртебесі бар адамдар үшін қол жетімді,сіз бұл стипендия ал алма өйткені ол тек жоғары әлеуметтік мәрте бар адам үшін қол жет,bullying

2,біз сізге бұл қызметті ұсына алмаймыз өйткені сіз біздің әлеуметтік мәртебе стандарттарына сәйкес келмейсіз,біз сізге бұл қызмет ұсын алма өйткені сіз біздің әлеуметтік мәртебе стандарт сәйкес келме,bullying

2,сіз бұл клубқа кіре алмайсыз өйткені ол тек жоғары әлеуметтік мәртебесі бар адамдар үшін қол жетімді,сіз бұл клуб кір алма өйткені ол тек жоғары әлеуметтік мәрте бар адам үшін қол жет,bullying

2,біз сізді осы лауазымға жалдай алмаймыз өйткені ол тек жоғары әлеуметтік мәртебесі бар адамдар үшін қол жетімді,біз сіз осы лауазым жалда алма өйткені ол тек жоғары әлеуметтік мәрте бар адам үшін қол жет,bullying

2,сіз бұл медициналық қызметті ала алмайсыз өйткені ол тек жоғары әлеуметтік мәртебесі бар адамдар үшін қол жетімді,сіз бұл медициналық қызмет ал алма өйткені ол тек жоғары әлеуметтік мәрте бар адам үшін қол жет,bullying

2,біз сізге бұл мүлікті сата алмаймыз өйткені ол тек жоғары әлеуметтік мәртебесі бар адамдар үшін қол жетімді,біз сізге бұл мүлік сат алма өйткені ол тек жоғары әлеуметтік мәрте бар адам үшін қол жет,bullying

2,сіз бұл іс шара билетін ала алмайсыз өйткені ол тек жоғары әлеуметтік мәртебесі бар адамдар үшін қол жетімді,сіз бұл іс шара билет ал алма өйткені ол тек жоғары әлеуметтік мәрте бар адам үшін қол жет,bullying

2,біз сізге бұл қызметті осы спорт клубында ұсына алмаймыз өйткені ол тек жоғары әлеуметтік мәртебесі бар адамдар үшін қол жетімді,біз сізге бұл қызмет осы спорт клуб ұсын алма өйткені ол тек жоғары әлеуметтік мәрте бар адам үшін қол жет,bullying

2,сіз бұл артықшылықтарды пайдалана алмайсыз өйткені олар тек жоғары әлеуметтік мәртебесі бар адамдар үшін қол жетімді,сіз бұл артықшылық пайдалан алма өйткені олар тек жоғары әлеуметтік мәрте бар адам үшін қол жет,bullying

2,біз сізді осы жеке мектепке қабылдай алмаймыз өйткені ол тек жоғары әлеуметтік мәртебесі бар адамдар үшін қол жетімді,біз сіз осы жеке мектеп қабылда алма өйткені ол тек жоғары әлеуметтік мәрте бар адам үшін қол жет,bullying

2,біз сіздің осы лауазымға кандидатураңызды қарастыра алмаймыз өйткені сізде жоғары білім жоқ,біз сіздің осы лауазым кандидатура қарастыр алма өйткені сіз жоғары білім жоқ,bullying

2,сіз бұл байқауға қатыса алмайсыз өйткені ол тек университет студенттері үшін ашық,сіз бұл байқа қатыс алма өйткені ол тек университет студент үшін ашық,bullying

2,сіз бұл кітапханаға кіре алмайсыз өйткені ол тек студенттер мен оқытушыларға арналған,сіз бұл кітапхана кір алма өйткені ол тек студент мен оқытушы арнал,bullying

2,сіз бұл курсқа қатыса алмайсыз өйткені ол тек белгілі бір білім деңгейі бар адамдар үшін қол жетімді,сіз бұл курс қатыс алма өйткені ол тек белгілі бір білім деңгей бар адам үшін қол жет,bullying

2,сіз біздің компанияда тағылымдамадан өте алмайсыз өйткені сіз тиісті оқу курсын аяқтамадыңыз,сіз біздің компания тағылымдама өте алма өйткені сіз тиісті оқу курс аяқтама,bullying

2,сіз бұл стипендияға ие бола алмайсыз өйткені ол тек белгілі бір факультеттердің студенттері үшін қол жетімді,сіз бұл стипендия ие бола алма өйткені ол тек белгілі бір факультет студент үшін қол жет,bullying

2,сіз бұл бағдарлама бойынша білім ала алмайсыз өйткені сіз білім деңгейіне сәйкес келмейсіз,сіз бұл бағдарлама бойынша білім ал алма өйткені сіз білім деңгей сәйкес келме,bullying

2,біз сізді бұл курсқа қабылдай алмаймыз өйткені ол тек белгілі бір білім деңгейі бар адамдар үшін қол жетімді,біз сіз бұл курс қабылда алма өйткені ол тек белгілі бір білім деңгей бар адам үшін қол жет,bullying

2,сіз бұл лауазымға ие бола алмайсыз өйткені жоғары білім қажет сізде жоқ,сіз бұл лауазым ие бола алма өйткені жоғары білім қажет сіз жоқ,bullying

2,сіз біздің зерттеу тобымызға қосыла алмайсыз өйткені ол тек тиісті бағыттағы студенттерге ашық,сіз біздің зерттеу тоб қосыл алма өйткені ол тек тиісті бағыт студент ашық,bullying

2,біз сізге бұл заң көмегін бере алмаймыз өйткені ол тек жоғары әлеуметтік мәртебесі бар адамдар үшін қол жетімді,біз сізге бұл заң көмег бер алма өйткені ол тек жоғары әлеуметтік мәрте бар адам үшін қол жет,bullying

2,сіз бұл банктік несиені ала алмайсыз өйткені сізде әлеуметтік мәртебе жеткіліксіз,сіз бұл банктік несие ал алма өйткені сіз әлеуметтік мәртебе жеткіліксіз,bullying

2,біз сізге бұл затты төмендетілген бағамен сата алмаймыз өйткені бұл тек жоғары әлеуметтік мәртебесі бар адамдар үшін қол жетімді,біз сізге бұл зат төмендет баға сат алма өйткені бұл тек жоғары әлеуметтік мәрте бар адам үшін қол жет,bullying

2,сіз бұл бөлмені осы қонақүйде брондай алмайсыз өйткені ол тек жоғары әлеуметтік мәртебесі бар адамдар үшін қол жетімді,сіз бұл бөлме осы қонақүй бронда алма өйткені ол тек жоғары әлеуметтік мәрте бар адам үшін қол жет,bullying

2,сіз бұл жұмысқа орналаса алмайсыз өйткені біз жоғары әлеуметтік мәртебеге ие үміткерлерді іздейміз,сіз бұл жұмыс орналас алма өйткені біз жоғары әлеуметтік мәртебе ие үміткер ізде,bullying

2,біз сізге бұл затты сата алмаймыз өйткені бұл тек жоғары әлеуметтік мәртебесі бар адамдар үшін қол жетімді сәнді тауар,біз сізге бұл зат сат алма өйткені бұл тек жоғары әлеуметтік мәрте бар адам үшін қол жет сән тауар,bullying

2,сіз бұл ұйымның мүшесі бола алмайсыз өйткені ол тек жоғары әлеуметтік мәртебесі бар адамдарға ашық,сіз бұл ұйы мүше бола алма өйткені ол тек жоғары әлеуметтік мәрте бар адам ашық,bullying

2,сіз бұл сертификатты ала алмайсыз өйткені ол тек жоғары әлеуметтік мәртебесі бар адамдар үшін қол жетімді,сіз бұл сертификат ал алма өйткені ол тек жоғары әлеуметтік мәрте бар адам үшін қол жет,bullying

2,сіз бұл тақырыпта сөйлей алмайсыз өйткені сіздің еліңіз ешқашан нағыз демократия болған емес,сіз бұл тақырып сөйле алма өйткені сіздің ел ешқашан нағыз демократия бол емес,bullying

2,сондықтан мен сіздің еліңізді ұнатпаймын ол әрқашан артта қалады,сондықтан мен сіздің ел ұнатпа ол әрқашан арт қал,bullying

2,мен сенің еліңде өмір сүргім келмейді бәрі соншалықты дамымаған,мен сенің ел өмір сүр келме бәрі соншалықты дамыма,bullying

2,әлемнің барлық проблемалары сіздің еліңізден басталады,әлем барлық проблема сіздің ел бастал,bullying

2,сіз басқа елдердің қолдауына сене алмайсыз өйткені сіздің еліңіз қызығушылық танытпайды,сіз басқа ел қолда сен алма өйткені сіздің ел қызығушылық танытпа,bullying

2,сіздің еліңіз әлемдік қауымдастық үшін ештеңе білдірмейді,сіздің ел әлемдік қауымдастық үшін ештеңе білдірме,bullying

2,сіздің еліңіз сыбайлас жемқорлық пен шатасудың орны,сіздің ел сыбайлас жемқорлық пен шатас орны,bullying

2,мен сенің еліңдегі біреумен айналысқым келмейді мен саған сене алмаймын,мен сенің елің біреу айналыс келме мен саған сен алма,bullying

2,сіздің еліңіз әрқашан әлсіз және сәтсіз болды,сіздің ел әрқашан әлсіз және сәт бол,bullying

2,біз сіздің пікірлеріңізді ескере алмаймыз өйткені сіз ешқашан жетістікке жетпеген елдерсіз,біз сіздің пікір ескер алма өйткені сіз ешқашан жетістік жетпе ел,bullying

2,біз сізге бұл билетті сата алмаймыз сәнді круиз өйткені бұл тек жоғары әлеуметтік мәртебесі бар адамдар үшін қол жетімді,біз сізге бұл биле сат алма сән круиз өйткені бұл тек жоғары әлеуметтік мәрте бар адам үшін қол жет,bullying

2,сіз бұл арнайы оқу бағдарламасын ала алмайсыз өйткені ол тек жоғары әлеуметтік мәртебесі бар адамдар үшін қол жетімді,сіз бұл арнайы оқу бағдарлама ал алма өйткені ол тек жоғары әлеуметтік мәрте бар адам үшін қол жет,bullying

2,біз сізге бұл ресурсқа қол жеткізе алмаймыз өйткені бұл тек жоғары әлеуметтік мәртебесі бар адамдар үшін қол жетімді,біз сізге бұл ресурс қол жеткіз алма өйткені бұл тек жоғары әлеуметтік мәрте бар адам үшін қол жет,bullying

2,біз сізге бұл елге саяхаттау мүмкіндігін бере алмаймыз өйткені бұл тек жоғары әлеуметтік мәртебесі бар адамдар үшін қол жетімді,біз сізге бұл ел саяхаттау мүмкіндіг бер алма өйткені бұл тек жоғары әлеуметтік мәрте бар адам үшін қол жет,bullying

2,сіз бұл сертификатты ала алмайсыз өйткені ол тек белгілі бір білім деңгейі бар адамдарға беріледі,сіз бұл сертификат ал алма өйткені ол тек белгілі бір білім деңгей бар адам бер,bullying

2,сіз бұл курсқа қатыса алмайсыз өйткені ол тек белгілі бір дәрежедегі білімі бар адамдарға арналған,сіз бұл курс қатыс алма өйткені ол тек белгілі бір дәреже білім бар адам арнал,bullying

2,сіз ала алмайсыз біліктілігіңізді растау өйткені сізде дұрыс білім жоқ,сіз ал алма біліктіліг растау өйткені сіз дұрыс білім жоқ,bullying

2,сіз бұл стипендияға ие бола алмайсыз өйткені ол белгілі бір білім деңгейі бар студенттерге ғана қол жетімді,сіз бұл стипендия ие бола алма өйткені ол белгілі бір білім деңгей бар студент ғана қол жет,bullying

2,сіз бұл ғылыми зерттеуге қатыса алмайсыз өйткені белгілі бір білім деңгейі қажет,сіз бұл ғылыми зертте қатыс алма өйткені белгілі бір білім деңгей қажет,bullying

2,сіз бұл академиялық лауазымға ие бола алмайсыз өйткені докторлық дәреже қажет,сіз бұл академиялық лауазым ие бола алма өйткені докторлық дәреже қажет,bullying

2,сіз біздің ұйымда ғылыми қызметпен айналыса алмайсыз өйткені бұл тек жоғары білімі бар адамдар үшін қол жетімді,сіз біздің ұйы ғылыми қызмет айналыс алма өйткені бұл тек жоғары білім бар адам үшін қол жет,bullying

2,сіз бұл білім беру жобасына қатыса алмайсыз өйткені ол тек белгілі бір білім деңгейі бар адамдарға арналған,сіз бұл білім беру жоба қатыс алма өйткені ол тек белгілі бір білім деңгей бар адам арнал,bullying

2,сіз біліктілікті арттыру курстарынан өте алмайсыз өйткені олар тек белгілі бір білім деңгейі бар адамдар үшін қол жетімді,сіз біліктілік арттыру курс өте алма өйткені олар тек белгілі бір білім деңгей бар адам үшін қол жет,bullying

2,сіз бұл бағдарлама бойынша магистр дәрежесін ала алмайсыз өйткені сізде білім деңгейі жеткіліксіз,сіз бұл бағдарлама бойынша магистр дәреже ал алма өйткені сіз білім деңгей жеткіліксіз,bullying

2,сіз бұл ең жақсы қонақүй бөлмесін брондай алмайсыз өйткені ол тек жоғары әлеуметтік мәртебесі бар адамдар үшін қол жетімді,сіз бұл ең жақсы қонақүй бөлме бронда алма өйткені ол тек жоғары әлеуметтік мәрте бар адам үшін қол жет,bullying

2,біз сізді осы жоғары жалақы алатын жұмысқа қабылдай алмаймыз өйткені ол тек жоғары әлеуметтік мәртебесі бар адамдар үшін қол жетімді,біз сіз осы жоғары жалақы ал жұмыс қабылда алма өйткені ол тек жоғары әлеуметтік мәрте бар адам үшін қол жет,bullying

2,сіз бұл беделді колледжге кіре алмайсыз өйткені ол тек жоғары әлеуметтік мәртебесі бар адамдар үшін қол жетімді,сіз бұл беделді колледж кір алма өйткені ол тек жоғары әлеуметтік мәрте бар адам үшін қол жет,bullying

2,біз сізге бұл жоғары технологиялық өнімді бере алмаймыз өйткені ол тек жоғары әлеуметтік мәртебесі бар адамдар үшін қол жетімді,біз сізге бұл жоғары технологиялық өн бер алма өйткені ол тек жоғары әлеуметтік мәрте бар адам үшін қол жет,bullying

2,сіз бұл беделді клубқа қол жеткізе алмайсыз өйткені ол тек жоғары әлеуметтік мәртебесі бар адамдар үшін қол жетімді,сіз бұл беделді клуб қол жеткіз алма өйткені ол тек жоғары әлеуметтік мәрте бар адам үшін қол жет,bullying

2,бұл жұмыс сен үшін емес сен мұндай өмір салтын ала алмайсың бұл лауазымға қажет,бұл жұмыс сен үшін емес сен мұндай өмір салт ал алма бұл лауазым қажет,bullying

2,сіз оны сатып ала алмайсыз өйткені бұл тек табысы жоғары адамдарға арналған,сіз оны сат ал алма өйткені бұл тек табыс жоғары адам арнал,bullying

2,бізге қажет табысы жоғары үміткер оның қаржылық сенімділігіне сенімді болу үшін,бізге қажет табыс жоғары үміткер оның қаржылық сенімділіг сенімді болу үшін,bullying

2,сіз біздің топқа қосыла алмайсыз өйткені сіз жеткілікті ақша таппайсыз біздің кестемізді орындау үшін,сіз біздің топ қосыл алма өйткені сіз жеткілікті ақша таппа біздің кесте орындау үшін,bullying

2,бізге керек несиелік скоринг сынағы анықтау үшін сіз осы аймақта тұруға мүмкіндігіңіз бар ма,бізге керек несиелік скоринг сынағ анықтау үшін сіз осы аймақта тұр мүмкіндіг бар ма,bullying

2,сіз ең жақсы бағаны ала алмайсыз өйткені сіздің табысыңыз төмен және бұл затты толық бағамен сатып алу мүмкіндігі жоқ,сіз ең жақсы баға ал алма өйткені сіздің табыс төмен және бұл зат толық баға сат алу мүмкіндіг жоқ,bullying

2,біз сізге бұл пәтерді жалға бере алмаймыз өйткені сізде тұрақты табыс жоқ,біз сізге бұл пәтер жалға бер алма өйткені сіз тұрақты табыс жоқ,bullying

2,сіз бұл жұмысты ала алмайсыз өйткені сізде табысы жоғары адамдармен тәжірибе жоқ,сіз бұл жұм ал алма өйткені сіз табыс жоғары адам тәжірибе жоқ,bullying

2,өкінішке орай біз сізге несие бере алмаймыз өйткені сіздің табысыңыз тым төмен,өкініш орай біз сізге несие бер алма өйткені сіздің табыс тым төмен,bullying

2,сіз қымбат медициналық процедураны ала алмайсыз өйткені сізде ақша жеткіліксіз,сіз қымбат медициналық процедура ал алма өйткені сіз ақша жеткіліксіз,bullying

2,біз сізге тегін кеңес бере алмаймыз өйткені сіз табысы төмен адамдар санатына жатпайсыз,біз сізге тегін кеңес бер алма өйткені сіз табыс төмен адам санат жатпа,bullying

2,сіз ала алмайсыз тұрғын үй несиесі өйткені сіздің табысыңыз ай сайынғы төлемдерді өтеу үшін жеткілікті жоғары емес,сіз ал алма тұрғын үй несие өйткені сіздің табыс ай сайынғы төлем өтеу үшін жеткілікті жоғары емес,bullying

2,сіз ала алмайсыз коммуналдық төлем бойынша жеңілдіктер өйткені сіздің табысыңыз белгіленген шектен асады,сіз ал алма коммуналдық төлем бойынша жеңілдік өйткені сіздің табыс белгілен шек ас,bullying

2,сіз біздің әлеуметтік қолдау бағдарламамызға қатыса алмайсыз өйткені сіздің табысыңыз тым жоғары,сіз біздің әлеуметтік қолдау бағдарлама қатыс алма өйткені сіздің табыс тым жоғары,bullying

2,біз сізді жұмысқа қабылдай алмаймыз өйткені сіз біз ұсына алатын төмен жалақы үшін жұмыс істеуге дайын емессіз,біз сіз жұмыс қабылда алма өйткені сіз біз ұсын ал төмен жалақы үшін жұмыс істе дайын емес,bullying

2,сіз біздің алмасу бағдарламамызға қатыса алмайсыз өйткені басқа елде тұру құны сіздің табысыңыз үшін тым жоғары,сіз біздің алмасу бағдарлама қатыс алма өйткені басқа ел тұру құн сіздің табыс үшін тым жоғары,bullying

2,сіз ала алмайсыз тегін концерт билеттері өйткені бұл тек табысы жоғары адамдар үшін қол жетімді,сіз ал алма тегін концерт билет өйткені бұл тек табыс жоғары адам үшін қол жет,bullying

2,біз сізді сақтандыру бағдарламамызға қабылдай алмаймыз өйткені сіздің табысыңыз жеңілдікті шарттарды алу талаптарына сәйкес келмейді,біз сіз сақтандыр бағдарлама қабылда алма өйткені сіздің табыс жеңілдік шарт алу талап сәйкес келме,bullying

2,сіз біздің vip қызметтерімізді пайдалана алмайсыз өйткені олар тек табысы жоғары клиенттер үшін қол жетімді,сіз біздің vip қызмет пайдалан алма өйткені олар тек табыс жоғары клиент үшін қол жет,bullying

2,сіз білікті медициналық көмек ала алмайсыз өйткені сізде мұндай қызметтерге ақы төлеу үшін табыс жеткіліксіз,сіз білікті медициналық көмек ал алма өйткені сіз мұндай қызмет ақы төлеу үшін табыс жеткіліксіз,bullying

2,сіз бұл лауазымға ие бола алмайсыз өйткені сізде жоқ белгілі бір білім қажет,сіз бұл лауазым ие бола алма өйткені сіз жоқ белгілі бір білім қажет,bullying

2,сіз бұл кәсіби лицензияны ала алмайсыз өйткені ол тек белгілі бір білім деңгейі бар адамдарға беріледі,сіз бұл кәсіби лицензия ал алма өйткені ол тек белгілі бір білім деңгей бар адам бер,bullying

2,сіз бұл конференцияға қатыса алмайсыз өйткені ол тек белгілі бір білім деңгейі бар адамдар үшін ашық,сіз бұл конференция қатыс алма өйткені ол тек белгілі бір білім деңгей бар адам үшін ашық,bullying

2,сіз бұл тренингтен өте алмайсыз өйткені ол тек белгілі бір білім деңгейі бар адамдарға арналған,сіз бұл тренинг өте алма өйткені ол тек белгілі бір білім деңгей бар адам арнал,bullying

2,сіз бұл мамандыққа ие бола алмайсыз өйткені белгілі бір білім деңгейі қажет,сіз бұл мамандық ие бола алма өйткені белгілі бір білім деңгей қажет,bullying

2,сіз бұл компанияда жұмысқа тұра алмайсыз өйткені белгілі бір білім қажет сізде жоқ,сіз бұл компания жұмыс тұр алма өйткені белгілі бір білім қажет сіз жоқ,bullying

2,сіз бұл алмасу бағдарламасына қатыса алмайсыз өйткені ол тек белгілі бір білім деңгейі бар студенттерге қол жетімді,сіз бұл алмасу бағдарлама қатыс алма өйткені ол тек белгілі бір білім деңгей бар студент қол жет,bullying

2,сіз бұл тағылымдамадан өте алмайсыз өйткені белгілі бір білім деңгейі қажет,сіз бұл тағылымдама өте алма өйткені белгілі бір білім деңгей қажет,bullying

2,сіз бұл кәсіби қауымдастыққа қосыла алмайсыз өйткені ол тек белгілі бір білім деңгейі бар адамдар үшін қол жетімді,сіз бұл кәсіби қауымдастық қосыл алма өйткені ол тек белгілі бір білім деңгей бар адам үшін қол жет,bullying

2,аудан тұрғындары табысы төмен болғандықтан біздің мақсатты аудиториямыз емес,аудан тұрғын табыс төмен бол біздің мақсат аудитория емес,bullying

2,біз бұл аймақтағы тауарларымызды жарнамаламаймыз өйткені оның тұрғындары оларды сатып ала алмайды,біз бұл аймақ тауар жарнамалама өйткені оның тұрғын ол сат ал алма,bullying

2,біз бұл аймақтың тұрғындарына несиелік ұпайлары төмен болғандықтан несие бермейміз,біз бұл аймақ тұрғын несиелік ұпай төмен бол несие берме,bullying

2,аудан тұрғындары қажетті білімнің болмауына байланысты біздің қызметтерді пайдалана алмайды,аудан тұрғын қажетті біл болма байланысты біздің қызмет пайдалан алма,bullying

2,біз кәсіби деңгейде ағылшын тілінде сөйлемейтін адамдарды жалдамаймыз,біз кәсіби деңгей ағылшын тіл сөйлеме адам жалдама,bullying

2,біз барлық қызметкерлерден ана тілін қоса алғанда кемінде екі тілді білуді талап етеміз,біз барлық қызметкер ана тіл қоса ал кемінде екі тілді білу талап ет,bullying

2,біздің компанияда тек ана тілінде сөйлейтіндер ғана жұмыс істей алады,біздің компания тек ана тіл сөйле ғана жұмыс істе ал,bullying

2,біз кандидаттарды әңгімелесуге шақырмаймыз олар түйіндемеде тілді білудің төмен деңгейін көрсетеді,біз кандидат әңгімелес шақырма олар түйіндеме тілді білу төмен деңгей көрсет,bullying

2,біз жоғары кәсіби дағдылары бар жергілікті үміткерлер болса да аудармашы ретінде жоғары тілді шетелдіктерді жалдағанды жөн көреміз,біз жоғары кәсіби дағды бар жергілікті үміткер бол да аудармаш рет жоғары тілді шетелдік жалда жөн көр,bullying

2,өкінішке орай біз табысы төмен адамдарға пәтерді жалға бермейміз,өкініш орай біз табыс төмен адам пәтер жалға берме,bullying

2,сіз бұл несиені ала алмайсыз өйткені сіз қамтамасыз ете алмайсыз қамтамасыз ету бұл біздің талаптарымызға сәйкес келеді,сіз бұл несие ал алма өйткені сіз қамтамасыз ет алма қамтамасыз ету бұл біздің талап сәйкес кел,bullying

2,біз сізге бұл затты жеңілдікпен сатпаймыз өйткені сіз біздің мақсатты аудиторияға кірмейсіз,біз сізге бұл зат жеңілдік сатпа өйткені сіз біздің мақсат аудитория кірме,bullying

2,кешіріңіз біз сізді осы клубқа қабылдай алмаймыз өйткені оның мүшелігі тек табысы жоғары адамдарға арналған,кешір біз сіз осы клуб қабылда алма өйткені оның мүшеліг тек табыс жоғары адам арнал,bullying

2,бұл қызмет ай сайын төлеуге мүмкіндігі бар адамдар үшін ғана қол жетімді сіз біздің талаптарға сай емессіз,бұл қызмет ай сайын төле мүмкіндіг бар адам үшін ғана қол жет сіз біздің талап сай емес,bullying

2,біздің клиенттеріміз белгілі бір диалектте сөйлеседі сондықтан біз тек осы диалектіде сөйлейтіндерді жалдаймыз,біздің клиент белгілі бір диалект сөйлес сондықтан біз тек осы диалекті сөйле жалда,bullying

2,біз компания қызметкерлерінен тілді орташадан жоғары білуді күтеміз әйтпесе олар өз міндеттерін тиісті деңгейде орындай алмайды,біз компания қызметкер тілді орташа жоғары білу күт әйтпесе олар өз міндет тиісті деңгей орында алма,bullying

2,біз шетелдіктерді жалдамаймыз олар біздің тілді білу тестінен өтпеген тіпті олардың қажетті кәсіби қасиеттері болса да,біз шетелдік жалдама олар біздің тілді білу тест өтпе тіпті олардың қажетті кәсіби қасиет бол да,bullying

2,біз барлық қызметкерлер барлық ресми құжаттар мен хат хабарларда тек компания тілін пайдаланады деп күтеміз,біз барлық қызметкер барлық ресми құжат мен хат хабар тек компания тіл пайдалан де күт,bullying

2,біз бұл аймақтағы адамдармен олардың мінез құлқының жеткіліксіздігіне байланысты жұмыс істегіміз келмейді,біз бұл аймақ адам олардың мінез құлқ жеткіліксіздіг байланысты жұмыс істе келме,bullying

2,аудан тұрғындары білім деңгейінің жеткіліксіздігіне байланысты біздің іс шараларға қатыса алмайды,аудан тұрғын білім деңгей жеткіліксіздіг байланысты біздің іс шара қатыс алма,bullying

2,біз осы аймақтағы компаниялармен ынтымақтаспаймыз өйткені олар біздің сапа стандарттарымызға сәйкес келмейді,біз осы аймақ компания ынтымақтаспа өйткені олар біздің сапа стандарт сәйкес келме,bullying

2,біз осы ауданның тұрғындарына қызмет көрсетпейміз өйткені олар қажетті технологияға қол жеткізе алмайды,біз осы аудан тұрғын қызмет көрсетпе өйткені олар қажетті технология қол жеткіз алма,bullying

2,біз бұл аймақтағы клиенттермен жұмыс істегіміз келмейді өйткені олар тілдік кедергіге байланысты біздің қызметтерімізді түсіне алмайды,біз бұл аймақ клиент жұмыс істе келме өйткені олар тілдік кедергі байланысты біздің қызмет түс алма,bullying

2,аудан тұрғындары әлеуметтік жауапкершіліктің жеткіліксіздігіне байланысты біздің клиенттеріміз емес,аудан тұрғын әлеуметтік жауапкершілік жеткіліксіздіг байланысты біздің клиент емес,bullying

2,біз бұл аймақтың тұрғындарына қызмет көрсетпейміз өйткені олар уақытында төлей алмайды,біз бұл аймақ тұрғын қызмет көрсетпе өйткені олар уақыт төле алма,bullying

2,біз бұл аймақтан қызметкерлерді олардың білім деңгейі мен дағдыларының жеткіліксіздігіне байланысты жалдамаймыз,біз бұл аймақ қызметкер олардың білім деңгей мен дағды жеткіліксіздіг байланысты жалдама,bullying

2,бұл ауданның тұрғындары біздің қызметтерімізді шалғай ауданда орналасқандықтан ала алмайды,бұл аудан тұрғын біздің қызмет шалғай аудан орналасқандық ал алма,bullying

2,біз осы аймақтағы клиенттермен жұмыс істемейміз өйткені олар төлем қабілеттілігіне жеткілікті кепілдік бере алмайды,біз осы аймақ клиент жұмыс істеме өйткені олар төлем қабілеттілік жеткілікті кепілдік бер алма,bullying

2,аудан тұрғындары білім деңгейінің жеткіліксіздігіне байланысты біздің бағдарламаға қатыса алмайды,аудан тұрғын білім деңгей жеткіліксіздіг байланысты біздің бағдарлама қатыс алма,bullying

2,біз осы ауданның тұрғындарына қызмет көрсетпейміз өйткені оларда қажетті құжаттар жоқ,біз осы аудан тұрғын қызмет көрсетпе өйткені ол қажетті құжат жоқ,bullying

2,сіз бұл жоғары дәрежені ала алмайсыз өйткені ол тек белгілі бір білім деңгейі мен тәжірибесі бар адамдар үшін қол жетімді,сіз бұл жоғары дәреже ал алма өйткені ол тек белгілі бір білім деңгей мен тәжірибе бар адам үшін қол жет,bullying

2,біз бұл ауданда баспана бере алмаймыз өйткені ол қауіпті болып саналады,біз бұл аудан баспана бер алма өйткені ол қауіпті бол санал,bullying

2,өкінішке орай біз сізге несие бере алмаймыз өйткені сіз табысы төмен аймақта тұрасыз,өкініш орай біз сізге несие бер алма өйткені сіз табыс төмен аймақта тұр,bullying

2,біз сізді жұмысқа ала алмаймыз өйткені сіздің тұрғылықты жеріңіз гүлденбейді,біз сіз жұмыс ал алма өйткені сіздің тұрғылықты же гүлденбе,bullying

2,біз сіздің жұмысқа өтініштеріңізді қарастырмаймыз егер сіз қауіпті деп саналатын аймақта тұрсаңыз,біз сіздің жұмыс өтініш қарастырма егер сіз қауіпті де санал аймақта тұр,bullying

2,біз бұл ауданда өз қызметтерімізді көрсетпейміз өйткені оның тұрғындарының білім деңгейі жеткіліксіз,біз бұл аудан өз қызмет көрсетпе өйткені оның тұрғын білім деңгей жеткіліксіз,bullying

2,біз осы ауданда тұратын клиенттермен жұмыс істеуге ыңғайлы емеспіз өйткені олардың табыс деңгейі жеткіліксіз,біз осы аудан тұр клиент жұмыс істе ыңғайлы емес өйткені олардың табыс деңгей жеткіліксіз,bullying

2,біз бұл ауданның тұрғындарына қызмет көрсетпейміз өйткені олардың әлеуметтік жауапкершілігі жоғары емес,біз бұл аудан тұрғын қызмет көрсетпе өйткені олардың әлеуметтік жауапкершіліг жоғары емес,bullying

2,біз сізге жұмыс бере алмаймыз өйткені сіз біздің талаптарымызға сәйкес келмейтін аймақта тұрасыз,біз сізге жұмыс бер алма өйткені сіз біздің талап сәйкес келме аймақта тұр,bullying

2,біз бұл аймақтағы клиенттермен жұмыс істемейміз өйткені олардың мәдениеті мен этикасы жоғары емес,біз бұл аймақ клиент жұмыс істеме өйткені олардың мәдениет мен этика жоғары емес,bullying

2,бізге аудан тұрғындарымен жұмыс істеу ыңғайлы емес өйткені олардың білім деңгейі төмен және біздің қызметтерімізді түсіне алмайды,бізге аудан тұрғын жұмыс істеу ыңғайлы емес өйткені олардың білім деңгей төмен және біздің қызмет түс алма,bullying

2,біз клиенттердің ана тілінде сөйлесе алмайтын адамдарды жұмысқа қабылдамаймыз,біз клиент ана тіл сөйле алма адам жұмыс қабылдама,bullying

2,тілді еркін меңгеру қажет бұл аймақта қолданылады әйтпесе үміткер жергілікті серіктестермен және клиенттермен жұмыс істей алмайды,тілді еркін меңгеру қажет бұл аймақта қолдан әйтпесе үміткер жергілікті серіктес және клиент жұмыс істе алма,bullying

2,кешіріңіз біз сізге бұл жұмысты бере алмаймыз өйткені сіздің дініңіз біздің құндылықтарымыз бен компания мәдениетімізге сәйкес келмейді,кешір біз сізге бұл жұм бер алма өйткені сіздің дін біздің құндылық бен компания мәдениет сәйкес келме,bullying

2,біз сіздің дініңіздің жұмыс пен әріптестермен және клиенттермен қарым қатынасқа әсер етуін қаламаймыз,біз сіздің дін жұмыс пен әріптес және клиент қарым қатынас әсер ет қалама,bullying

2,біз адамдарды жалдамаймыз өз жұмысында арнайы терминдер мен кәсіби лексиканы қолдана алмайтын адамдар,біз адам жалдама өз жұмыс арнайы термин мен кәсіби лексика қолдан алма адам,bullying

2,біз халықаралық компанияларда тәжірибесі бар адамдарды жалдағанды жөн көреміз өйткені олар әдетте ағылшын тілін жетік біледі,біз халықаралық компания тәжірибе бар адам жалда жөн көр өйткені олар әдет ағылшын тіл же біл,bullying

2,біз қызметкерлерден біздің саладағы ғылыми жарияланымдардың тілінде жазу және сөйлеу қабілетін күтеміз әйтпесе олар сапалы ғылыми жұмыс жасай алмайды,біз қызметкер біздің сала ғылыми жарияланым тіл жазу және сөйлеу қабілет күт әйтпесе олар сапалы ғылыми жұмыс жаса алма,bullying

2,бізге тек итальян тілін жетік білетін қызметкерлер керек өйткені біз итальяндық клиенттермен жұмыс істейміз,бізге тек итальян тіл же біл қызметкер керек өйткені біз итальяндық клиент жұмыс істе,bullying

2,біз адамдарды жалдағанды жөн көреміз бірнеше тілде сөйлейтіндер сондықтан олар әр түрлі елдерден келген клиенттерімізбен сөйлесе алады,біз адам жалда жөн көр бірнеше тіл сөйле сондықтан олар әр түрлі ел кел клиент сөйле ал,bullying

2,біз қызметкерлерден құжаттарды қажетті тілге сауатты және дәл аудару қабілетін күтеміз,біз қызметкер құжат қажетті тіл сауатты және дәл аудару қабілет күт,bullying

2,егер сіз ағылшын тілінде сөйлей алмасаңыз кете аласыз,егер сіз ағылшын тіл сөйле алма кет ал,bullying

2,сіздің екпініңіз өте ауыр мен сізді түсіне алмаймын,сіздің екпін өте ауыр мен сіз түс алма,bullying

2,түсінбеймін неге сіз өз ойыңызды ағылшын тілінде жеткізе алмайсыз,түсінбе неге сіз өз ой ағылшын тіл жеткіз алма,bullying

2,біз шетелдіктерді жалдамаймыз ағылшын тілінде еркін сөйлей алмайтындар,біз шетелдік жалдама ағылшын тіл еркін сөйле алма,bullying

2,біз ағылшын тілін жетік білетін адамдарға артықшылық береміз,біз ағылшын тіл же біл адам артықшылық бер,bullying

2,біз адамдарды жалдамаймыз тілді білу сынағынан өтпеген адамдар тіпті олардың қажетті кәсіби дағдылары болса да,біз адам жалдама тілді білу сынағ өтпе адам тіпті олардың қажетті кәсіби дағды бол да,bullying

2,біздің компанияда жұмыс істеу үшін кәсіби деңгейде бағдарламалау тілін білу қажет,біздің компания жұмыс істеу үшін кәсіби деңгей бағдарламала тіл білу қажет,bullying

2,біз барлық қызметкерлеріміз ресми хат алмасуда және кездесулерде тек бір тілді қолданады деп күтеміз,біз барлық қызметкер ресми хат алмас және кездесу тек бір тілді қолдан де күт,bullying

2,біз жақсы айтылатын адамдарды жалдағанды жөн көреміз сондықтан біздің клиенттер олармен оңай сөйлесе алады,біз жақсы айтыл адам жалда жөн көр сондықтан біздің клиент олармен оңай сөйле ал,bullying

2,сіздің екпініңіз біздің клиенттерімізге сенімсіздік тудыруы мүмкін және бұл біздің бизнес үшін қолайсыз,сіздің екпін біздің клиент сенімсіздік тудыр мүмкін және бұл біздің бизнес үшін қолайсыз,bullying

2,біз қызметкерлерімізден кәсіби айтылуды күтеміз және сіздің екпініңіз бұл талапты бұзады,біз қызметкер кәсіби айтылу күт және сіздің екпін бұл тала бұз,bullying

2,біз анық және түсінікті айтылуы бар адамдарды іздейміз және сіздің екпініңіз бұл стандартқа сәйкес келмейді,біз анық және түсінік айтыл бар адам ізде және сіздің екпін бұл стандарт сәйкес келме,bullying

2,өкінішке орай сіздің екпініңіз біздің клиенттеріміздің сізді түсінуіне кедергі келтіреді және бұл жұмыста қиындықтарға әкелуі мүмкін,өкініш орай сіздің екпін біздің клиент сіз түсін кедергі келтір және бұл жұмыс қиындық әкел мүмкін,bullying

2,бізге мінсіз айтылуы бар қызметкерлер қажет тұтынушылармен бірыңғай стандартты қарым қатынас жасау үшін,бізге мін айтыл бар қызметкер қажет тұтынушы бірыңғай стандартты қарым қатынас жасау үшін,bullying

2,сіздің екпініңіз клиенттермен түсініспеушілікке әкелуі мүмкін және біз оған қауіп төндіре алмаймыз,сіздің екпін клиент түсініспеушілік әкел мүмкін және біз оған қауіп төндір алма,bullying

2,біз үміткерлерді іздейміз олар біздің халықаралық клиенттермен оңай сөйлесе алады және сіздің екпініңіз бұған кедергі болуы мүмкін,біз үміткер ізде олар біздің халықаралық клиент оңай сөйле ал және сіздің екпін бұған кедергі бол мүмкін,bullying

2,бізде бар белгілі бір айтылу стандарты біз барлық қызметкерлерден күтеміз және сіздің екпініңіз бұл стандартқа сәйкес келмейді,біз бар белгілі бір айтыл стандарт біз барлық қызметкер күт және сіздің екпін бұл стандарт сәйкес келме,bullying

2,біз мінсіз айтылуы бар адамдарды жалдағанды жөн көреміз клиенттеріміз олармен қарым қатынас кезінде өздерін сенімді және жайлы сезінуі үшін,біз мін айтыл бар адам жалда жөн көр клиент олармен қарым қатынас кез өз сенімді және жайлы сезін үшін,bullying

2,сіздің мәдени құндылықтарыңыз бен дәстүрлеріңіз біздің корпоративтік стандарттарымызға сәйкес келмейді сондықтан біз сізді жалдай алмаймыз,сіздің мәдени құндылық бен дәстүр біздің корпоративтік стандарт сәйкес келме сондықтан біз сіз жалда алма,bullying

2,сіз белгілі бір мәдениеттің клиенттерімен жұмыс істей алмайсыз өйткені сізде тиісті тәжірибе мен білім жоқ,сіз белгілі бір мәдениет клиент жұмыс істе алма өйткені сіз тиісті тәжірибе мен білім жоқ,bullying

2,біздің компания тек батыс мәдениетін бағалайды сондықтан біз сіздің көпмәдениетіңізді ескере алмаймыз,біздің компания тек батыс мәдени бағала сондықтан біз сіздің көпмәдениет ескер алма,bullying

2,сіз жұмыс орнында ана тілінде сөйлейсіз бұл біздің қарым қатынас ережелерімізді бұзады,сіз жұмыс орн ана тіл сөйле бұл біздің қарым қатынас ереже бұз,bullying

2,біз нақты қамтамасыз ете алмаймыз діни және мәдени мерекелер өйткені бұл біздің корпоративтік күнтізбемізге сәйкес келмейді,біз нақты қамтамасыз ет алма діни және мәдени мереке өйткені бұл біздің корпоративтік күнтізбе сәйкес келме,bullying

2,біз сіздің мәдени қажеттіліктеріңізге ерекшелік жасай алмаймыз өйткені бұл қызметкерлер арасындағы теңдікті бұзуы мүмкін,біз сіздің мәдени қажеттілік ерекшелік жаса алма өйткені бұл қызметкер ара теңдік бұз мүмкін,bullying

2,белгілі бір тері түсі бар барлық адамдар бірдей көрінеді және бір бірінен еш айырмашылығы жоқ,белгілі бір тері түс бар барлық адам бірде көрін және бір бір еш айырмашылығ жоқ,bullying

2,барлық орыстар арақ пен дөрекі ішеді,барлық орыс арақ пен дөрекі іш,bullying

2,барлық американдықтар агрессивті және ұлтшыл,барлық американдық агрессивті және ұлтшыл,bullying

2,барлық қытайлықтар зауыттарда жұмыс істейді және басқа ештеңе туралы ойлауды білмейді,барлық қытайлық зауыт жұмыс істе және басқа ештеңе туралы ойлау білме,bullying

2,барлық үндістер ат секторында жұмыс істейді және ащы тағамдарды жақсы көреді,барлық үндіс ат сектор жұмыс істе және ащ тағам жақсы көр,bullying

2,барлық арабтар батыс мәдениетіне шыдай алмайды және өз әдет ғұрыптарын талап етеді,барлық араб батыс мәдениет шыда алма және өз әдет ғұрып талап ет,bullying

2,барлық мексикалықтар заңсыз және қылмыскерлер,барлық мексикалық заңсыз және қылмыскер,bullying

2,барлық француздар тәкаппар және надан,барлық француз тәкапп және надан,bullying

2,барлық голландиялықтар скучно және басқаларды ережелерді ұстануды ұнатады,барлық голландиялық скучно және басқа ереже ұстану ұнат,bullying

2,барлық немістер қатыгез және әзіл сезімі жоқ,барлық неміс қатыгез және әзіл сезім жоқ,bullying

2,сіз біздің мәдениетімізге бейімделуіңіз керек біздің компанияда табысты болу үшін,сіз біздің мәдениет бейімдел керек біздің компания табысты болу үшін,bullying

2,сіз қатты көрінбейсіз өйткені сіздің киім стиліңіз біздің корпоративтік мәдениет стандарттарына сәйкес келмейді,сіз қатты көрінбе өйткені сіздің киім стил біздің корпоративтік мәдениет стандарт сәйкес келме,bullying

2,сіз жұмыс орнындағы діни немесе мәдени іс шараларға қатыса алмайсыз өйткені олар біздің компанияға жатпайды,сіз жұмыс орн діни немесе мәдени іс шара қатыс алма өйткені олар біздің компания жатпа,bullying

2,сіз өз жұмысыңызда өз мәдениетіңізді пайдалана алмайсыз өйткені бұл біздің клиенттеріміз үшін қолайсыз болуы мүмкін,сіз өз жұмыс өз мәдениет пайдалан алма өйткені бұл біздің клиент үшін қолайсыз бол мүмкін,bullying

2,олардың барлығы бір бетте сығандар бар сығандар көздер ер адамдарда болжау мүмкін емес қарау қорқынышты,олардың барлығ бір бет сы бар сы көз ер адам болжау мүмкін емес қарау қорқыныш,bullying

2,мен бұл адамды жалдамаймын өйткені ол бұл елде туылмаған,мен бұл адам жалдама өйткені ол бұл ел туылма,bullying

2,сен мұны істей алмайсың өйткені сен шетелдіксің,сен мұны істе алма өйткені сен шетелдік,bullying

2,менің ойымша сіз оны түсіне алмайсыз сіз басқа мәдениетте тудыңыз,менің ойымша сіз оны түс алма сіз басқа мәдениет ту,bullying

2,олар әрқашан жалқау бұл олардың қанында,олар әрқашан жалқау бұл олардың қан,bullying

2,біз оны біздің кешке шақырмаймыз ол басқа әлеуметтік саладан,біз оны біздің кеш шақырма ол басқа әлеуметтік сала,bullying

2,оны жалдамайық ол осы ауданда тұрады,оны жалдама ол осы аудан тұр,bullying

2,мен онымен сөйлескім келмейді ол нақты ұлт атауы сияқты емес,мен онымен сөйлес келме ол нақты ұлт ата сияқты емес,bullying

2,оларға сенбеңіз олар бәрібір түсіне алмайды біз не айтып отырмыз,оларға сенбе олар бәрібір түс алма біз не айт отыр,bullying

2,сіз мұны жасай алмайсыз сіз үйрене алмайсыз өйткені сіз кедей отбасында дүниеге келдіңіз,сіз мұны жаса алма сіз үйрен алма өйткені сіз кедей отбас дүние кел,bullying

2,сіз көшбасшы бола алмайсыз сіз ауылда тудыңыз және ешқашан қалада оқымадыңыз,сіз көшбасшы бола алма сіз ауыл ту және ешқашан қала оқыма,bullying

2,сен бұл жұмысқа тұра алмайсың өйткені сен біздің қызметкерлердің көпшілігіндей емессің,сен бұл жұмыс тұр алма өйткені сен біздің қызметкер көпшілігінде емес,bullying

2,мен бұл адамдармен ынтымақтаспаймын олар басқа елден және олардың мәдениеті басқа,мен бұл адам ынтымақтаспа олар басқа ел және олардың мәдениет басқа,bullying

2,сіз біздің команданың мүшесі бола алмайсыз өйткені сіз адамдар шеңберінен емессіз,сіз біздің команда мүше бола алма өйткені сіз адам шеңбер емес,bullying

2,бұл аймақ сіз үшін емес өйткені сіз біздің топқа жатпайсыз,бұл аймақ сіз үшін емес өйткені сіз біздің топ жатпа,bullying

2,мен бұл сатушыдан сатып алмаймын өйткені ол шетелдік және біздің елде бизнесті қалай жасау керектігін білмейді,мен бұл сатушы сат алма өйткені ол шетелдік және біздің ел бизнес қалай жасау керектіг білме,bullying

2,сіз бұл іспен айналыса алмайсыз өйткені сіз дұрыс әлеуметтік топтан емессіз,сіз бұл іс айналыс алма өйткені сіз дұрыс әлеуметтік топтан емес,bullying

2,бұл жұмыс тек нағыз американдықтарға арналған сондықтан уақытты ысырап етпеңіз,бұл жұмыс тек нағыз американдық арнал сондықтан уақыт ысырап етпе,bullying

2,сіз бұл бизнесті басқара алмайсыз өйткені сіз дұрыс діни топтан емессіз,сіз бұл бизнес басқар алма өйткені сіз дұрыс діни топтан емес,bullying

2,мен бұл адаммен сөйлеспеймін өйткені ол біздің тілімізді білмейді және біздің қоғамға бейімделе алмайды,мен бұл адам сөйлеспе өйткені ол біздің тіл білме және біздің қоғам бейімдел алма,bullying

2,сіз бұл аймақта тұра алмайсыз өйткені сіз дұрыс емес этникалық топсыз,сіз бұл аймақта тұр алма өйткені сіз дұрыс емес этникалық топ,bullying

2,сен оны көтере алмайсың өйткені сен кедейсің,сен оны көтер алма өйткені сен кедей,bullying

2,мұндай сән салтанатқа тек бай адамдар ғана қол жеткізе алады,мұндай сән салтанат тек бай адам ғана қол жеткіз ал,bullying

2,сіз ешқашан табысты бола алмайсыз сіздің табыс деңгейіңізді ескере отырып,сіз ешқашан табысты бола алма сіздің табыс деңгей ескер отыр,bullying

2,бұл жұмыс сіз үшін емес сіз табысыңыздың төмендігіне байланысты үміткердің талаптарын қанағаттандыра алмайсыз,бұл жұмыс сіз үшін емес сіз табыс төмендіг байланысты үміткер талап қанағаттандыр алма,bullying

2,сізде ақша жоқ осы адамдар сияқты заттарды сатып алу үшін,сіз ақша жоқ осы адам сияқты зат сат алу үшін,bullying

2,бұл тұрғылықты жер сіздің сыныбыңыз үшін емес,бұл тұрғылықты жер сіздің сыныб үшін емес,bullying

2,сіз кем дегенде қандай да бір табысыңыз бар екеніне риза болуыңыз керек өйткені көбісі оны төлей алмайды,сіз кем де қандай да бір табыс бар екен риза бол керек өйткені көбісі оны төле алма,bullying

2,сіздің фоныңыз бен табысыңызды ескере отырып сізден қандай өмір сүру деңгейін күтуге болады,сіздің фон бен табыс ескер отыр сізден қандай өмір сүру деңгей күт бол,bullying

2,сіз бұл мейрамханаға бара алмайсыз бұл сіздің әмияныңыз үшін тым қымбат,сіз бұл мейрамхана бар алма бұл сіздің әмиян үшін тым қымбат,bullying

2,сіздің табысыңыз тым төмен сондықтан сіз бұл демалыс орнын ала аласыз,сіздің табыс тым төмен сондықтан сіз бұл демалыс орн ал ал,bullying

2,сіз үшінші әлем елінен болғандықтан сіздің біліктілігіңіз ештеңеге тұрарлық емес,сіз үшінші әлем ел бол сіздің біліктілік ештеңе тұрарлық емес,bullying

2,сіздің еліңіз жаман беделге ие сондықтан біз сізбен жұмыс істемейміз,сіздің ел жаман бедел ие сондықтан біз сізбен жұмыс істеме,bullying

2,сіз дамыған елдердегідей қызмет пен сапа деңгейін күте алмайсыз,сіз дамы елдердегіде қызмет пен сапа деңгей күт алма,bullying

2,сіздің еліңіз соншалықты дамымаған бізге құнды нәрсе ұсыну үшін,сіздің ел соншалықты дамыма бізге құнды нәрсе ұсыну үшін,bullying

2,сіз үшінші әлем елінен келдіңіз сондықтан сіз бизнестің күрделілігін түсіне алмайсыз,сіз үшінші әлем ел кел сондықтан сіз бизнес күрделіліг түс алма,bullying

2,біз үшінші әлем елдерінен ешкімді жалдамаймыз өйткені бұл тиімсіз және ыңғайсыз,біз үшінші әлем ел ешкі жалдама өйткені бұл тиімсіз және ыңғайсыз,bullying

2,сіздің еліңіз тек арзан тауарларды өндірумен айналысады сондықтан сіздің компанияңыз бәсекеге қабілетті бола алмайды,сіздің ел тек арзан тауар өндір айналыс сондықтан сіздің компания бәсеке қабілетті бола алма,bullying

2,сіздің еліңіз бізге қажетті ресурстар мен материалдарды бере алмайды,сіздің ел бізге қажетті ресурс мен материал бер алма,bullying

2,сіз дамыған елдердің адамдарымен бәсекелесе алмайсыз өйткені сізде заманауи технологияларға қол жетімділік жоқ,сіз дамы ел адам бәсекеле алма өйткені сіз заманауи технология қол жетімділік жоқ,bullying

2,сіз үшінші әлем елінен келдіңіз сондықтан сіз біздің стандарттарымыз бен талаптарымызды түсіне алмайсыз,сіз үшінші әлем ел кел сондықтан сіз біздің стандарт бен талап түс алма,bullying

2,сіздің еліңізде сыбайлас жемқорлық деңгейі жоғары сондықтан біз сіздің ұсыныстарыңызды қарастыра алмаймыз,сіздің ел сыбайлас жемқорлық деңгей жоғары сондықтан біз сіздің ұсыныс қарастыр алма,bullying

2,сіз үшінші әлем елінен келдіңіз сондықтан сіз бізге кепілдіктер мен сапаны қамтамасыз етудің қажетті деңгейін бере алмайсыз,сіз үшінші әлем ел кел сондықтан сіз бізге кепілдік мен сапа қамтамасыз ету қажетті деңгей бер алма,bullying

2,адам құқықтарының бұзылуына және зияткерлік меншікті қорғаудың төмен деңгейіне байланысты біз сіздің еліңіздегі компаниялармен жұмыс істемейміз,адам құқық бұзыл және зияткерлік меншік қорға төмен деңгей байланысты біз сіздің еліңіз компания жұмыс істеме,bullying

2,сіз әлемдегі ең жақсы университеттерде білім ала алмайсыз өйткені сіз дамыған елден емессіз,сіз әлем ең жақсы университет білім ал алма өйткені сіз дамы ел емес,bullying

2,сіздің еліңізде тиісті инфрақұрылым жоқ және тауарлар мен қызметтердің сенімді жеткізілуін қамтамасыз ете алмайды,сіздің ел тиісті инфрақұрылым жоқ және тауар мен қызмет сенімді жеткізіл қамтамасыз ет алма,bullying

2,сіз бұл лауазымға ие бола алмайсыз өйткені бізде дамыған елдерден білікті үміткерлер бар,сіз бұл лауазым ие бола алма өйткені біз дамы ел білікті үміткер бар,bullying

2,біз сіздің еліңізбен байланысты шектеулерге байланысты өнімдеріміз бен қызметтерімізге қол жеткізе алмаймыз,біз сіздің ел байланысты шектеу байланысты өнім бен қызмет қол жеткіз алма,bullying

2,сіздің ел біздің қауіпсіздік және экологиялық жауапкершілік талаптарын қанағаттандыра алмайды,сіздің ел біздің қауіпсіздік және экологиялық жауапкершілік талап қанағаттандыр алма,bullying

2,сіз бұл халықаралық стипендияны ала алмайсыз өйткені біз оны үшінші әлем студенттеріне бермейміз,сіз бұл халықаралық стипендия ал алма өйткені біз оны үшінші әлем студент берме,bullying

2,сіздің ел бізге білікті жұмысшылардың жеткілікті санын бере алмайды сондықтан біз оған инвестиция салмаймыз,сіздің ел бізге білікті жұмысшы жеткілікті сан бер алма сондықтан біз оған инвестиция салма,bullying

2,сіз оны түсіне алмайсыз сіз осындай елде тұрасыз,сіз оны түс алма сіз осындай ел тұр,bullying

2,сіз бұл туралы білмейтіндігіңіз таңқаларлық емес ескере отырып сіз осындай елден екенсіз,сіз бұл туралы білмейтіндіг таңқаларлық емес ескер отыр сіз осындай ел екен,bullying

2,сіз бұл туралы айта алмайсыз өйткені сіз бұл елден емессіз,сіз бұл туралы ай алма өйткені сіз бұл ел емес,bullying

2,сен білмейсің өйткені сенің еліңде бәрі басқаша,сен білме өйткені сенің ел бәрі басқаша,bullying

2,мүмкін егер сіз дамыған елде тұрсаңыз сіз жақсы түсінер едіңіз,мүмкін егер сіз дамы ел тұр сіз жақсы түсін ед,bullying

2,сіз әйел ретінде бұл жұмысты жеңе алмайсыз,сіз әйел рет бұл жұм же алма,bullying

2,сіз ер адам ретінде білуіңіз керек бұл жұмысты қалай жасау керек,сіз ер адам рет біл керек бұл жұм қалай жасау керек,bullying

2,барлық қарттар өте баяу және тиімсіз,барлық қарт өте бая және тиімсіз,bullying

2,барлық жастар байсалды және жалқау емес,барлық жас байсалды және жалқау емес,bullying

2,барлық ақ адамдар артықшылықты және әділетсіз,барлық ақ адам артықшылық және әділет,bullying

2,барлық қара адамдар жалқау және білімсіз,барлық қара адам жалқау және білімсіз,bullying

2,барлық азиялықтар ақылды және еңбекқор,барлық азиялық ақылды және еңбекқор,bullying

2,барлық испандықтар білімсіз және жұмыссыз,барлық испандық білімсіз және жұмыссыз,bullying

2,барлық еуропалықтар тәкаппар және надан,барлық еуропалық тәкапп және надан,bullying

2,сіз түсінуді күте алмайсыз егер сіз осы мәдениеттен болмасаңыз,сіз түсіну күт алма егер сіз осы мәдениет болма,bullying

2,мен күтпес едім бұл туралы не білесіз сен осындай елден екеніңді ескере отырып,мен күтпес ед бұл туралы не біл сен осындай ел екен ескер отыр,bullying

2,сіз біле алмайсыз бұл қалай жасалады өйткені сіз бұл елден емессіз,сіз біл алма бұл қалай жасал өйткені сіз бұл ел емес,bullying

2,сіз бұл елден оны қалай дұрыс жасау керектігін білмейсіз бе,сіз бұл ел оны қалай дұрыс жасау керектіг білме бе,bullying

2,менің ойымша сіз мұны түсіне алмайсыз өйткені сіз басқа елденсіз,менің ойымша сіз мұны түс алма өйткені сіз басқа ел,bullying

2,сіз бұл туралы айта алмайсыз өйткені сіз оның қалай жұмыс істейтінін білмейсіз,сіз бұл туралы ай алма өйткені сіз оның қалай жұмыс істе білме,bullying

2,сіз мұндай шешім қабылдай алмайсыз сіз бұл туралы ештеңе білмейсіз,сіз мұндай шешім қабылда алма сіз бұл туралы ештеңе білме,bullying

2,сіз көбірек білімді болуыңыз керек оны түсіну үшін,сіз көбірек білімді бол керек оны түсіну үшін,bullying

2,сіз бұл туралы ойлай алмайсыз өйткені сіз тіпті негізгі нәрселерді білмейсіз,сіз бұл туралы ойла алма өйткені сіз тіпті негізгі нәрсе білме,bullying

2,сіздің білмеуіңіз басқа адамдарға азап пен азап әкеледі,сіздің білме басқа адам азап пен азап әкел,bullying

2,егер сіз көбірек білсеңіз сіз мұндай ақымақтықтарды айтпас едіңіз,егер сіз көбірек біл сіз мұндай ақымақтық айтпас ед,bullying

2,сіз бұл жұмыста қала алмайсыз егер сіз тіпті қарапайым нәрселерді түсіне алмасаңыз,сіз бұл жұмыс қала алма егер сіз тіпті қарапайым нәрсе түс алма,bullying

2,сіз бұл тақырыпта сөйлей алмайсыз өйткені сіз қарапайым нәрселерді білмейсіз,сіз бұл тақырып сөйле алма өйткені сіз қарапайым нәрсе білме,bullying

2,сіз көбірек үйренуіңіз керек бұл туралы айту үшін,сіз көбірек үйрен керек бұл туралы айту үшін,bullying

2,сіз бұл лауазымға ие бола алмайсыз өйткені сізде білім жеткіліксіз,сіз бұл лауазым ие бола алма өйткені сіз білім жеткіліксіз,bullying

2,осы аймақтағы барлық адамдар сіз сияқты жалқау,осы аймақ барлық адам сіз сияқты жалқау,bullying

2,африкадағы сығандар,африка сы,bullying

2,пәкістандықтар олар талибандар барлығының бастауы қорқынышты оқиғалар,пәкістандық олар талибан барлығ баста қорқыныш оқиға,bullying

2,сіз мүмкіндігі шектеулі адам ретінде бұл жұмысты жеңе алмайсыз,сіз мүмкіндіг шектеулі адам рет бұл жұм же алма,bullying

2,барлық иммигранттар мұнда тек жәрдемақы алу және салық төлеушілердің мойнында тұру үшін келеді,барлық иммигрант мұнда тек жәрдемақы алу және салық төлеуші мойн тұру үшін кел,bullying

2,бір сөзбен айтқанда сыған дөрекі лас айлакер,бір сөз айтқанда сы дөрекі лас айлак,bullying

2,барлық семіз адамдар жалқау және олардың тамақтануын басқара алмайды,барлық се адам жалқау және олардың тамақтан басқар алма,bullying

2,барлық арық адамдар тамақтанудың бұзылуынан зардап шегеді,барлық арық адам тамақтан бұзылу зардап шег,bullying

2,барлық еврейлер ашкөз және адамдарға қарағанда ақшаны жақсы көреді,барлық еврей ашкөз және адам қара ақша жақсы көр,bullying

2,барлық интроверттер скучно және адамдармен қарым қатынас жасауды білмейді,барлық интроверт скучно және адам қарым қатынас жасау білме,bullying

2,бұл бай сығандар,бұл бай сы,bullying

2,ал қауіпсіздік сізге не үшін қажет олар бұл қайыршыларды машиналардан шығарады ал егер түсінбесе машиналар туралы ұмытпаңыз,ал қауіпсіздік сізге не үшін қажет олар бұл қайыршы машина шығар ал егер түсінбе машина туралы ұмытпа,bullying

2,сығандар интернеттің не екенін біледі олар украинаның не екенін білмейді,сы интернет не екен біл олар украина не екен білме,bullying

2,нағыз хохол сияқты,нағыз хохол сияқты,bullying

2,кез келген жағдайда сыйлықпен немесе ақшамен келу керек болды мен олармен бір күн өткіздім түсірдім сұрадым әрине олар бір нәрсені қалайды біртүрлі пәкістандық гид ескертпегені қалай болғанда да күні бойы сыйлықпен немесе ақшамен келу керек

болды олармен бірге түсірілді сұралды әрине олар бір нәрсені қалайды біртүрлі пәкістандық гид ескертпегені,кез кел жағдай сыйлық немесе ақша келу керек бол мен олармен бір күн өт түс сұра әрине олар бір нәрсе қала біртүрлі пәкістандық гид ескертпе қалай болғанда да күн бойы сыйлық немесе ақша келу керек бол олармен бірге түсір сұра әрине олар бір нәрсе қала біртүрлі пәкістандық гид ескертпе,bullying

2,қазіргі пәкістан дегенде мен бір секундта рет жыпылықтай бастаймын мен пәкістанды сіздің бейнелеріңізден білмеймін оны заманауи деп атауға болмайды,қазіргі пәкістан де мен бір секунд рет жыпылықта баста мен пәкістан сіздің бейне білме оны заманауи де ата болма,bullying

2,енді маған таныс индусты касталар туралы түсіндіре алмағаным анық мен лайықты адамдар кастада бола алады деп сұрағанда қол тигізбейтіндер үшін сіз оларды солай етесіз ол маған біртүрлі қарады сіз әлі де түсінбейтін сияқтысыз шамасы олар артқа бұрыла алмайтын немесе түнде бола алмайтын адамдар сондықтан олар төменгі каста адам неғұрлым кедей болса соғұрлым ол барабар болады ана жерде қазірдің өзінде жауапты жігіттер жұмыс істейді және пирсинг жасамайды татуировкалар және жынысын өзгертпейді,енді маған таныс инду каста туралы түсіндір алма анық мен лайықты адам каста бола ал де сұра қол тигізбе үшін сіз ол солай ет ол маған біртүрлі қар сіз әлі де түсінбе сияқты шама олар арт бұрыл алма немесе түн бола алма адам сондықтан олар төменгі кас адам неғұрлым кедей бол соғұрлым ол бараб бол ана же қаз өз жауапты жігіт жұмыс істе және пирсинг жасама татуировка және жыныс өзгертпе,bullying

2,масқара халық бүкіл әлемге өзінің даңқын көрсетті,масқара халық бүкіл әлем өзінің даңқ көрсе,bullying

2,олардың үздіксіз және терең діндарлығы елге бару өте қауіпті екендігімен қалай үйлеседі,олардың үздіксіз және терең діндарлығ ел бару өте қауіпті екендіг қалай үйлес,bullying

2,дааавот сондықтан мұсылмандар ішпейдібұл қымыз соншалықты мас болады сондықтан олар алкоголь мен араққа мән бермейді,дааавот сондықтан мұсылман ішпейдібұл қымыз соншалықты мас болады сондық олар алкоголь мен арақ мән берме,bullying

2,о бұл қандай қызықты калаштар шынымен ерекше және кешке қауіпті бәрі осылай басталды бірақ біз оларды үйде көрдік онда әдемі балалар бар ал балужистан туралы мүлдем естіген жоқпын ал тағам қандай қызықты бұл қандай қызықты,о бұл қандай қызықты калаш шын ерекше және кеш қауіпті бәрі осылай баста бірақ біз ол үй көр онда әдемі бала бар ал балужис туралы мүлдем есті жоқ ал тағам қандай қызықты бұл қандай қызықты,bullying

2,одесса облысындағы дима демалыс күндері ауылдарда балалар көп қойларды жинап ешбір дұғасыз кебабқа кеседі,одес облыс ди демалыс күн ауыл бала көп қой жина ешбір дұ кебаб кес,bullying

2,мен түркияға үйлендім бір жарым жыл бұрын міне мен көптеген әдемі шабыттандыратын және қымбат нәрселерді көрдім бірақ мен кедейлікті және дәлдік пен тазалыққа деген құштарлықты көрдім кейде тұрғын үйлер осылай көрінуі үшін оны бірнеше ұрпақ бойы іске қосу керек сондықтан өмір бойы өмір бойы ішіп отыру керек мен сол үйлерде тұратын адамдар туралы сұрағанымда маған қарапайым адамдар сол жерде жұмыс істейді деп жауап берді қытай туралы бүкіл шығарылымды қарап пәкістан туралы бастағаннан кейін адамдар қаншалықты қорқынышты жағдайда өмір сүретінін көріп кенеттен біз жүз есе жақсы өмір сүріп жатқанымызды түсіндік және украинадағы кедейлігіміз әлемдегі кедейлікпен салыстыруға келмейді,мен түркия үйлен бір жарым жыл бұрын міне мен көптеген әдемі шабыттандыр және қымбат нәрсе көр бірақ мен кедейлік және дәл пен тазалық де құштарлық көр кейде тұрғын үй осылай көрін үшін оны бірнеше ұрпақ бойы іс қосу керек сондықтан өмір бойы өмір бойы іш отыру керек мен сол үй тұр адам туралы сұра маған қарапайым адам сол же жұмыс істе де жауап бер қытай туралы бүкіл шығарыл қара пәкістан туралы баста кейін адам қаншалықты қорқыныш жағдай өмір сүр көр кенет біз жүз есе жақсы өмір сүр жат түс және украина кедейлі әлем кедейлік салыстыр келме,bullying

2,қонақтарды жіберу әдеттегідей емес оларда қонақжайлылық туралы заңдар жоқ бұл жай ғана банда,қонақ жіберу әдеттегідей емес ол қонақжайлылық туралы заң жоқ бұл жай ғана банда,bullying

2,қытай туралы бүкіл шығарылымды қарап пәкістан туралы бастағаннан кейін адамдар қаншалықты қорқынышты жағдайда өмір сүретінін көріп кенеттен біз жүз есе жақсы өмір сүріп жатқанымызды түсіндік және украинадағы кедейлігіміз әлемдегі кедейлікпен салыстыруға келмейді,қытай туралы бүкіл шығарыл қара пәкістан туралы баста кейін адам қаншалықты қорқыныш жағдай өмір сүр көр кенет біз жүз есе жақсы өмір сүр жат түс және украина кедейлі әлем кедейлік салыстыр келме,bullying

2,олар өте кедей өмір сүреді әрине олар бұл түсірілім үшін өтемақы күткен және оларға ештеңе берілмейтінін түсінгенде қатты ашуланған мен неге болмағанын түсінбеймін түсіріңіз төлеуге келісіп бәрін басқа блогерлер сияқты тыныш түсіріңіз,олар өте кедей өмір сүр әрине олар бұл түсірілім үшін өтемақы күт және оларға ештеңе берілме түсін қатты ашулан мен неге болма түсінбе түсір төле келіс бәрін басқа блогер сияқты тыныш түсір,bullying

2,пәкістандықтар индустарды ұнатпайды индустар лас және кез келген нәрсеге табынады өздерін олардан жоғары қойыңыз бірақ пәкістандықтар олардың индустармен діннен басқа барлық жағынан өте ұқсас екенін түсінбейді,пәкістандық индус ұнатпа индус лас және кез кел нәрсе табын өз олардан жоғары қой бірақ пәкістандық олардың индус дін басқа барлық жағ өте ұқсас екен түсінбе,bullying

2,неге олар өмір бойы түйелерді бағып жүр егер оларды жаман бағамен сатпаса кем дегенде адам өмір сүре алмайды,неге олар өмір бойы түйе бағ жүр егер ол жаман баға сатпа кем де адам өмір сүр алма,bullying

2,барлық көшпенділер басқа көшпенділер емес,барлық көшпенді басқа көшпенді емес,bullying

2,қорқынышты адамдар сығандар,қорқыныш адам сы,bullying

2,сығандар барлық жерде сығандар болып қала береді,сы барлық же сы бол қала бер,bullying

2,мен елестете алмаймын әзірге барлығы дерлік толық өркениетте өмір сүреді кейбір адамдар әлі де көшіп жүр,мен елесте алма әзірге барлығ дерлік толық өркениет өмір сүр кейбір адам әлі де көш жүр,bullying

2,мүмкін сондықтан олар шөл далада өмір сүреді және оларға өмір ұнайды өйткені қоғамда мұндай ұрылар мен алаяқтар ешқашан қабылданбайды және болады жаман қарым қатынас мұндай адамдарға қалалар мен ауылдарда орын жоқ адамдар сияқты өмір сүруді үйренсін немесе шөлде жалғыз қалсын қызықты сюжетті көрсеткеніңіз үшін рахмет өзіңізге қамқорлық жасаңыз,мүмкін сондықтан олар шөл дала өмір сүр және оларға өмір ұна өйткені қоғам мұндай ұры мен алаяқ ешқашан қабылданба және бол жаман қарым қатынас мұндай адам қала мен ауыл орын жоқ адам сияқты өмір сүру үйрен немесе шөл жалғыз қал қызықты сюжет көрсет үшін рахмет өз қамқорлық жаса,bullying

2,біз сізді әлі төлемейміз бұл осындай халықтардың шынайы тұлғасы,біз сіз әлі төлеме бұл осындай халық шынайы тұлға,bullying

2,көшпенділерден кейін қорқыныш пен шиеленіс мені бейненің соңына дейін қалдырмады,көшпенді кейін қорқыныш пен шиеленіс мен бейне соң дейін қалдырма,bullying

2,барлық елдерде өздерінің мінез құлқымен жағымсыз адамдар бар,барлық ел өз мінез құлқ жағымсыз адам бар,bullying

2,дима украин емес әдеттегі совок орыс екенін бірден көруге болады басқа халықтардың мәдениетіне әдептілік пен дилекция жоқ басқа елге кіріп өзіңді уағыздау агрессивті орыс әлемі бұл оның басқа өркениетті әлемнен айырмашылығы қонақ үйлерде сүлгі мен тәпішке ұрланған шығар,ди украин емес әдет совок орыс екен бірден көр бол басқа халық мәдениет әдептілік пен дилекция жоқ басқа ел кір өз уағызда агрессивті орыс әлем бұл оның басқа өркениет әлем айырмашылығ қонақ үй сүлгі мен тәпіш ұрлан шығар,bullying

2,бізге адамдар керек біздің командаға тез бейімделе алатын және егер сіз ағылшын тілінде сөйлей алмасаңыз бұл мәселе болуы мүмкін,бізге адам керек біздің команда тез бейімдел ал және егер сіз ағылшын тіл сөйле алма бұл мәселе бол мүмкін,bullying

2,біз сізді жұмысқа қабылдай алмаймыз өйткені сіз ағылшын тілінде сөйлемейсіз,біз сіз жұмыс қабылда алма өйткені сіз ағылшын тіл сөйлеме,bullying

2,сіз ағылшын тілінде сөйлеуіңіз керек осы елде табысты болу үшін,сіз ағылшын тіл сөйле керек осы ел табысты болу үшін,bullying

2,егер сіз ағылшын тілінде жақсы сөйлейтін болсаңыз біз сізге көбірек жауапкершілік бере аламыз,егер сіз ағылшын тіл жақсы сөйле бол біз сізге көбірек жауапкершілік бер ал,bullying

2,сіз өзіңіздің ағылшын тіліңізді жақсартуыңыз керек біз сізді жоғары лауазымға қабылдамас бұрын,сіз өз ағылшын тіл жақсарт керек біз сіз жоғары лауазым қабылдамас бұрын,bullying

2,пәкістандықтар мен америкаға келді және әйелдер де ер адамдар да өздерін ұстайды өте ашкөз және крахаборлар егер олар тек ақша үшін дос болса және ақша болмаса онда достық болмайды олар ешқашан көмектеспейді егер сіз сұрасаңыз олар дереу ескертеді бірақ сіз маған төлейсіз сондықтан мен америкада да пәкістандықтармен көбірек араласамын мен пәкістаннан бірден досвидосты естігенде дос емеспін,пәкістандық мен америка кел және әйел де ер адам да өз ұста өте ашкөз және крахабор егер олар тек ақша үшін дос бол және ақша болма онда достық болма олар ешқашан көмектеспе егер сіз сұра олар дереу ескерт бірақ сіз маған төле сондықтан мен америка да пәкістандық көбірек аралас мен пәкістан бірден досвидо есті дос емес,bullying

2,маған көшпенділердің өмірі өте ұнайды,маған көшпенді өмір өте ұна,bullying

2,пәкістандықтардың өздері жақсы мұнда өмір сүретін сығандар қорқыныш өте қорқынышты,пәкістандық өз жақсы мұнда өмір сүр сы қорқыныш өте қорқыныш,bullying

2,біздің қырғызстанда да көшеде көп нәрсе бар бәрі сұрайды,біздің қырғызстан да көше көп нәрсе бар бәрі сұра,bullying

2,сіз кедейлерден ақша табу үшін түсірілім үшін бірден төлеуіңіз керек еді,сіз кедей ақша табу үшін түсірілім үшін бірден төле керек ед,bullying

2,сіз кедей отбасынан шыққан осындай сәнді демалысты ала алмайсыз,сіз кедей отбас шық осындай сән демал ал алма,bullying

2,біздің компанияда тек жоғары қоғамдағы адамдар жұмыс істейді сондықтан өкінішке орай сіз дұрыс емессіз,біздің компания тек жоғары қоғам адам жұмыс істе сондықтан өкініш орай сіз дұрыс емес,bullying

2,біз үшін тек қаланың бай аудандарынан клиенттердің болуы маңызды,біз үшін тек қала бай аудан клиент бол маңызды,bullying

2,біз сізге бұл пәтерді жалға бере алмаймыз өйткені сіз жеткілікті ақша таппайсыз,біз сізге бұл пәтер жалға бер алма өйткені сіз жеткілікті ақша таппа,bullying

2,бұл мейрамханада тек табысы жоғары адамдарға арналған басқа орынды табыңыз,бұл мейрамхана тек табыс жоғары адам арнал басқа ор таб,bullying

2,кешіріңіз біз сізге бұл затты осындай төмен бағамен сата алмаймыз бұл тек біздің ең жақсы клиенттеріміз үшін,кешір біз сізге бұл зат осындай төмен баға сат алма бұл тек біздің ең жақсы клиент үшін,bullying

2,сіз бұл мектепте оқи алмайсыз өйткені сіздің отбасыңыз мұндай жоғары мектеп ақысын төлей алмайды,сіз бұл мектеп оқи алма өйткені сіздің от мұндай жоғары мектеп ақы төле алма,bullying

2,бізге тек бизнес серіктестер ретінде табысы жоғары адамдар қажет,бізге тек бизнес серіктес рет табыс жоғары адам қажет,bullying

2,кешіріңіз біз өзімізді жалға бермейміз іс шаралар конференц залы бұл біздің корпоративтік құндылықтарымызға сәйкес келмейді,кешір біз өз жалға берме іс шара конференц зал бұл біздің корпоративтік құндылық сәйкес келме,bullying

2,біз сізді осы лауазымға жалдай алмаймыз өйткені сіз кедей ауданда тұрасыз және сізде қажетті тәжірибе мен білім жоқ шығар,біз сіз осы лауазым жалда алма өйткені сіз кедей аудан тұр және сіз қажетті тәжірибе мен білім жоқ шығар,bullying

2,бұл қызмет тек оның құнын төлей алатын адамдарға арналған егер мүмкін болмаса онда сіз сәйкес келмейсіз,бұл қызмет тек оның құн төле ал адам арнал егер мүмкін болма онда сіз сәйкес келме,bullying

2,сіз бұл медициналық көмекті ала алмайсыз өйткені сіздің табысыңыз осы қызметті алу үшін белгіленген деңгейден төмен,сіз бұл медициналық көмек ал алма өйткені сіздің табыс осы қызмет алу үшін белгілен деңгей төмен,bullying

2,бұл жеке мектепке тек ауқатты отбасылардың балалары ғана бара алады,бұл жеке мектеп тек ауқатты отбасы бала ғана бар ал,bullying

2,сіз бұл машинаны несиеге сатып ала алмайсыз өйткені сіздің несие тарихыңыз жақсы емес және сіз ай сайынғы төлемдерді төлей алмайсыз,сіз бұл машина несие сат ал алма өйткені сіздің несие тарих жақсы емес және сіз ай сайынғы төлем төле алма,bullying

2,кешіріңіз бұл спа тек біздің эксклюзивті клиенттеріміз үшін біздің қызметтерімізді сатып ала алады,кешір бұл сп тек біздің эксклюзив клиент үшін біздің қызмет сат ал ал,bullying

1,мен ондай мылқау емеспін сондықтан мен ренжімейтін негрлермен жүгіремін деп күтпеңіз,мен ондай мылқа емес сондықтан мен ренжіме негр жүгір де күтпе,racism

1,адамдар оның қаншалықты маңызды екенін түсінеді ме білмеймін әлемге ақ адам деп аталатын адамның еврей емес екенін білу үшін ал америкадағы негр нағыз еврей мұның әлем үшін нені білдіретінін бәріңіз түсінбеуіңіз мүмкін бұл біздің алдымызда тұрған өтірік үшін қиын мәселе,адам оның қаншалықты маңызды екен түсін ме білме әлем ақ адам де атал адам еврей емес екен білу үшін ал америка нег нағыз еврей

мұның әлем үшін не білдір бәрі түсінбе мүмкін бұл біздің алд тұр өтірік үшін қиын мәселе,racism

1,сіз нәсілшілдікті тоқтату үшін түрлі түсті адамдарға бірге жиналыңыз деп айтқан кезде бұл біз нені шешу керектігін талқылайтынымызды білдіреді бәрін ұмытпаймыз,сіз нәсілшілдік тоқтату үшін түрлі түсті адам бірге жинал де айт кезде бұл біз не шешу керектіг талқыла білдір бәрін ұмытпа,racism

1,құдай үшін сен кітапханадасың мылқау көт телефон арқылы нигга деп айтуды доғар,құдай үшін сен кітапхана мылқа көт телефон арқылы нигг де айту доғар,racism

1,ниггер бұл оның игілігі үшін тым ақымақ жай ғана ақымақ,нигг бұл оның игілігі үшін тым ақы жай ғана ақы,racism

1,сіз сияқты демократтар эмпатия туралы күлкілі сөйлейді сізде жанашырлық жоқ сіз жай ғана омыртқасыз қорқақсыз өткен аптада қара қауымдарда өлтірілген барлық қара балаларға деген жанашырлығыңыз қайда сіз ештеңе айтпайсыз өйткені олардың өлтірушілері қара түсті,сіз сияқты демократ эмпатия туралы күлкіл сөйле сіз жанашырлық жоқ сіз жай ғана омыртқа қорқақ өткен апта қара қауым өлтір барлық қара бала де жанашырлы қайда сіз ештеңе айтпа өйткені олардың өлтіруші қара түсті,racism

1,дон лимон ол ақ нәсілді ер адамдар бұл елдегі басты мәселе екенін айтты сонымен қатар елдің жартысын білімсіз деп мазақ еткен қонаққа күлді содан кейін ол қонақ канье уэстті символдық негр деп атаған кезде күлді ол толық профан,дон лимон ол ақ нәсіл ер адам бұл ел басты мәселе екен ай сонымен қатар ел жарты білімсіз де мазақ ет қонақ күл содан кейін ол қонақ кань уэс символ нег де ата кезде күл ол толық профан,racism

1,обаманы қарғыс атсын мылқау нигга сен ашуланасың ба сізге пончик керек пе,обама қарғыс ат мылқа нигг сен ашулан ба сізге пончик керек пе,racism

1,неліктен қарғыс атсын менен сізге еруімді сұрай аласыз ба содан кейін шайтан алғыр маған жазыл сен ақымақ пиздожопая нигга қаншық,неліктен қарғыс ат менен сізге еру сұра ал ба содан кейін шай алғы маған жазыл сен ақы пиздожопая нигг қаншық,racism

1,бұл канье қара ол бай атақты оған біз осы нәсілшіл елдің қарапайым қара азаматтары тап болатын салдармен бетпе бет келудің қажеті жоқ бұл адамның соңғы жылда айтқан ешнәрсесі мені ешқашан ол біздің президент болуы керек сияқты сезінбейді,бұл кань қара ол бай атақты оған біз осы нәсілшіл ел қарапайым қара азамат тап бол сал бетп бет келу қажет жоқ бұл адам соңғы жыл айт ешнәрсе мен ешқашан ол біздің президент бол керек сияқты сезінбе,racism

1,даллас снайдер осындай мылқау көтен шайтан алғыр мысық қаншық жетесіз титти арық бок негр қарғыс атқылайтын көт скротум болады жылжу дик боқ қаншық,даллас

снай осындай мылқа көшай алғы мысық қаншық же титти арық бок нег қарғыс атқыла көт скроту бол жылжу дик боқ қаншық,racism

1,tl дегі ақ адамдар соңғы екі айда қара адамдар мен рос туралы ештеңе білмеген сияқты әрекет етеді,tl дег ақ адам соңғы екі айда қара адам мен рос туралы ештеңе білме сияқты әрекет ет,racism

1,роган жақсы ол өрнектерден ұялмайды және байден сияқты қара адамдарға қысым көрсетуге күші жоқ еді байден обаманың вице президенті болды ол обаманы таза негр деп атады,роган жақсы ол өрнек ұялма және бай сияқты қара адам қысым көрсет күш жоқ ед бай обама вице президент бол ол обама таза нег де ат,racism

1,мен жақсы мәмілелер күнін ұнатпайтынымды білмейсіз мен соңғы жыл бойы қара жұмаға бардым сіз менің саяси сенімдерім туралы ештеңе білмейсіз мен ешқашан таза социализмді немесе капитализмнің кез келген түріне қарсы тұрамын деп айтқан емеспін қоғамды сынау өте жақсы,мен жақсы мәміле күн ұнатпа білме мен соңғы жыл бойы қара жұма бар сіз менің саяси сенім туралы ештеңе білме мен ешқашан таза социализм немесе капитализ кез кел түр қарсы тұр де айт емес қоғам сынау өте жақсы,racism

1,келесі сіздің көшбасшы болу процесінде сіз бірінші команда үшін футбол ойнауға барасыз деп үміттенемін,келесі сіздің көшбасшы болу процес сіз бірінші команда үшін футбол ойна бар де үміттен,racism

1,жақсы егер ескі ақ нәсілшілдер сізге қарап нигга деп ойламаса сіз нигга деп айта алмайсыз ақымақ бейбақтар,жақсы егер ескі ақ нәсілшіл сізге қара нигг де ойлама сіз нигг де ай алма ақы бейбақ,racism

1,бұл мағынасы жоқ еді rt қара түсті rt нигга тозақ rt rezilientkid қараңыз американдықтар неге мылқау саймон gt,бұл мағына жоқ ед rt қара түсті rt нигг тозақ rt rezilientkid қара американдық неге мылқа саймон gt,racism

1,жарайды нигга әуесқой сен қара емессің сізге не болды мылқау нигга сіз нигга сияқты әрекет етесіз,жарайды нигг әуесқой сен қара емес сізге не бол мылқа нигг сіз нигг сияқты әрекет ет,racism

1,ондай ештеңе жоқ мүлдем жоқ шындығында мен олар ұшып бара жатқан ақымақтықтан басқа нәрсе іздеймін,ондай ештеңе жоқ мүлдем жоқ шындығ мен олар ұш бар жат ақымақтық басқа нәрсе ізде,racism

1,lmaoo желкекке сен бадденді ақымақ нигга болғаны үшін жек көресің,lmaoo желкек сен бадде ақы нигг бол үшін жек көр,racism

1,қарғысатқыр ақымақтап барып желкек ұйықтауға мылқау қара маз қаншық желкек сор шайтан алғыр ант құдаймен егер сіз тағы да дауыстап сөйлессеңіз жаңғырық бүкіл үй мен сүртемін қарғысатқыр еден сіздің мәйітіңізбен,қарғысатқы ақымақта бар желкек ұйықта мылқа қара маз қаншық желкек сор шай алғы ант құдай егер сіз тағы да дауыста сөйлес жаңғырық бүкіл үй мен сүр қарғысатқы еден сіздің мәйіт,racism

1,бұл сұрақтың бір ерекшелігі ол сіздің жауаптарыңыз үшін хабарландырулар береді сондықтан егер сіз осы мекен жайдағы адамдарға жауап бергіңіз келсе ол сіз жасаған әрбір жауап үшін барлығына хабарлама жібереді алайда ол ағындағы хабарламалардың бар жоғын тексермейді тек біріншісі,бұл сұрақ бір ерекшеліг ол сіздің жауап үшін хабарландыру бер сондықтан егер сіз осы мекен жай адам жауап бер кел ол сіз жаса әрбір жауап үшін барлығ хабарлама жібер алайда ол ағ хабарлама бар жоғ тексерме тек бірінші,racism

1,егер олар сізді ниггер деп атаса біреу оны қолдайды мен ниггерлер деп айтпадым бірақ олар нигга десе мен қуаныштымын,егер олар сіз нигг де ата біреу оны қолда мен ниггер де айтпа бірақ олар нигг де мен қуанышты,racism

1,мылқау кішкентай ниггерді желкекке тастау керек иә сіз жетілуіңіз керек сіз сияқты адамдар әлемді ұсқынсыз орынға айналдырады сен ақымақсың,мылқа кішкентай нигг желкек тастау керек иә сіз жетіл керек сіз сияқты адам әлем ұсқын орын айналдыр сен ақы,racism

1,о нәсілшілдік енді мен қорқамын мен қателестім сіздің фотосуретіңізге қарағанда сіз фаготаны ақымақ есекке сүйетін ниггерсіз,о нәсілш енді мен қор мен қателес сіздің фотосурет қара сіз фагота ақы есек сүй нигг,racism

1,бірінші әсер мен сенің лақап атыңнан әлі қорқамын мен сені жақсы білмеймін сенің атыңды білмеймін кешіріңіз сөйлесейік маған ұнай ма иә сіз менікісіз менің сұрақтарымның бірі сіз маған қандай түсті еске түсіресіз ашық қоңыр сіз де оны жариялауыңыз керек егер қаласаңыз,бірінші әсер мен сенің лақа ат әлі қор мен сен жақсы білме сенің ат білме кешір сөйлес маған ұна ма иә сіз менік менің сұрақ бірі сіз маған қандай түсті ес түсір ашық қоңыр сіз де оны жарияла керек егер қала,racism

1,иә мен мұны білемін бірақ бар сен ақымақ бейбақсың ол vteck деп аталады сен ақымақ құм ниггерсің,иә мен мұны біл бірақ бар сен ақы бей ол vteck де атал сен ақы құм нигг,racism

1,ниггерлер мені ақымақ деп ойлап үнемі өтірік айтатын сияқты маған өтірік айтудың қажеті жоқ сияқты балам сен менің ниггер емессің,ниггер мен ақы де ойла үнемі өтірік айт сияқты маған өтірік айт қажет жоқ сияқты бала сен менің нигг емес,racism

1,ол сен үшін жай негр болды сіз жиіркеніштісіз содан кейін мен сенің осы қара футболшыларды құшақтап жатқаныңды көремін tv so жалған,ол сен үшін жай нег бол сіз жиіркенішт содан кейін мен сенің осы қара футболшы құшақта жат көр tv so жалған,racism

1,егжей тегжейлі комиссия үшін рахмет менің ойымша сіз бағаны көтермейтін бірінші адамсыз өйткені мен сол үшін шамамен робукс бағасын таптым қалай болғанда да менде ақша болмаған кезде ашуланбағаныңыз үшін рахмет және мен gamepass қа қуана төледім мен тағы да өте қуаныштымын tysm,егже тегжейлі комиссия үшін рахмет менің ойымша сіз баға көтерме бірінші адам өйткені мен сол үшін шамамен

робукс баға тап қалай болғанда да менде ақша болма кезде ашуланба үшін рахмет және мен gamepass қа қуан төле мен тағы да өте қуанышты tysm,racism

1,сіздің сұрағыңызға жауап біз күнделікті нәсілшілдікке және бұрынғы нәсілшіл режимдердің салдарына тап боламыз егер сізге әр күн оқиға және орналасқан жер үшін нақты күнтізбе қажет болса онда ол шамамен жылдан бастап бүгінгі таңға дейінгі кезеңді қамтиды бұл менің соңғы yt бейнемнің астындағы негрдің керемет түсініктемесі,сіздің сұрағ жауап біз күнделікті нәсілшілдік және бұрынғы нәсілшіл режим сал тап бол егер сізге әр күн оқиға және орналас жер үшін нақты күнтізбе қажет бол онда ол шамамен жыл баста бүгінгі таң дейінгі кезең қамти бұл менің соңғы yt бейне аст нег керемет түсініктеме,racism

1,бұл менің твиттеріме алғашқы жауаптарыңыздың бірі,бұл менің твиттер алғашқы жауап бірі,racism

1,нағыз ниггерлер фильмінде ақымақ сіз құлдық туралы фильм түсіре алмайсыз және ниггерлер туралы айтпайсыз,нағыз ниггер фильм ақы сіз құлдық туралы фильм түсір алма және ниггер туралы айтпа,racism

1,мен мылтықпен келе жатырмын мен ниггерді үш сиськасы бар қаншықты көргендей ауру етіп көрсетемін,мен мылтық келе жатыр мен нигг үш сиська бар қаншық көргенде ауру ет көрсе,racism

1,ұсқынсыз pug nigger сайро сенің досыңнан жақсы мылқау фагот мен сені клубпен ұрамын сен мен сені дұрыс көремін егер көрсем мен сені бұрап аламын сайро жақсырақ гипоистикс те,ұсқын pug nigger сайро сенің дос жақсы мылқа фагот мен сен клуб ұр сен мен сен дұрыс көр егер көр мен сен бұра ал сайро жақсырақ гипоистикс те,racism

1,менің алғашқы oomf тің бірі менің есімде сізде керемет bc және ur қатынасы бар сонымен қатар сіз пайдаланушыларды өзгертпедіңіз деп ойлаймын сіздің макетіңіз әдемі көрінеді біз өзара әрекеттесе аламыз деп үміттенемін,менің алғашқы oomf ті бірі менің ес сіз керемет bc және ur қатына бар сонымен қатар сіз пайдаланушы өзгертпе де ойла сіздің макет әдемі көрін біз өзара әрекеттес ал де үміттен,racism

1,сіз бізді мылқау ниггерлер деп атайсыз маған ауырады rt мен бұл қоңырауға ашуланамын мен сіздердің ешқайсыларыңызды ниггерлерді сүйемін,сіз біз мылқа ниггер де ата маған ауыр rt мен бұл қоңырау ашулан мен сіздердің ешқайсы ниггер сүй,racism

1,менің сыныптағы бұл нигга holyshit сияқты мылқау,менің сынып бұл нигг holyshit сияқты мылқа,racism

1,қарғыс атсын бронды гонконг пен қытайға құлатқан қара хабар таратушылар қайда ақ қозғалыс белсендісі езгіні соттау керек дейді екі аптадан кейін олар гонконгта наразылық білдіріп оны мылқау нигга деп атағаннан кейін мен одан түтін қайда екенін сұраймын,қарғыс ат брон гонконг пен қытай құлат қара хабар таратушы қайда ақ

қозғалыс белсенді езгі сотта керек де екі апта кейін олар гонконг наразылық білдір оны мылқа нигг де ата кейін мен одан түтін қайда екен сұра,racism

1,егер сіз қара және түрлі түсті киімдердің орнына ақ жууға арналған киімдерді таңдасаңыз сіз нәсілшілсіз нәсілшілдікті тоқтатыңыз олардың барлығын бірге жуыңыз сіз ештеңе істемейсіз,егер сіз қара және түрлі түсті киім орн ақ жу арнал киім таңда сіз нәсілшіл нәсілшілдік тоқта олардың барлығ бірге жу сіз ештеңе істеме,racism

1,майк браунға қарғыс атсын трейвон мартинге қарғыс атсын ниггерлер өлімге лайық қара өмірдің маңызы жоқ өзіңді өлтір ақымақ есек,майк браун қарғыс ат трейвон мартин қарғыс ат ниггер өлім лайық қара өмір маңыз жоқ өз өлтір ақы есек,racism

1,мен әкемнің сұмдығына тойдым мына қара мазутты түтіккен қаншықты ұр,мен әке сұмдығ той мына қара мазут түтік қаншық ұр,racism

1,досым леброн мен илайды желкекке сіз біздің иемізді ол сіздің лауазымыңыздағы ақымақ ниггадан гөрі жақсы гроссмейстер деп ашуландырасыз,дос леброн мен ила желкек сіз біздің ие ол сіздің лауазымыңыз ақы нигга гөрі жақсы гроссмейс де ашуландыр,racism

1,мен ақымақ ниггерлерді жек көремін,мен ақы ниггер жек көр,racism

1,шынымды айтсам сен ақымақсың лмфао біреуді ниггер деп атау бұл балалық шақ ал сіздің ата анаңыз соншалықты мақтанбас еді бу бу,шын айт сен ақы лмфао біреу нигг де атау бұл балалық шақ ал сіздің ата ана соншалықты мақтанбас ед бу бу,racism

1,құрал екі жағынан да қолданылады оңшыл экстремистер қарсыластарының өткеніндегі қара беттерді іздейді оларға ұнамайтын нәрсені нәсілшілдік деп атайтын солшыл экстремистер ешқандай пікірталаста ақылға қонымды орта жоқ сияқты,құрал екі жағ да қолдан оңшыл экстремист қарсылас өткен қара бет іздe оларға ұнама нәрсе нәсілш де ата солшыл экстремист ешқандай пікірталас ақыл қонымды орта жоқ сияқты,racism

1,таңдаулыларфлайн иә сіз бардыңыз ақымақ нигга желкек,таңдаулыларфлайн иә сіз бар ақы нигг желкек,racism

1,қандай қоқыс кейбір мылқау нигга заңды түрде десинді өлтірді,қандай қоқыс кейбір мылқа нигг заңды түр деси өл,racism

1,оларға айтыңыз вена шығанағы rt сіз ниггерлер мылқау бір рет бұл есекке шағымданасыз өйткені ол сізді ешқашан ұрмайтынын білесіз,оларға ай вена шығанағ rt сіз ниггер мылқа бір рет бұл есек шағымдан өйткені ол сіз ешқашан ұрма біл,racism

1,желкек есегінде ратчет бар мылқау нигга,желкек есег ратчет бар мылқа нигг,racism

1,тозақ мен нигга сөзін жек көремін бұл жыл шамамен жыл өзіңе кел және нәсілшіл болуды доғар ақымақ бейбақтар,тозақ мен нигг сөз жек көр бұл жыл шамамен жыл өз кел және нәсілшіл болу доғар ақы бейбақ,racism

1,жоқ сен ақымақсың және қандай коньки тебуші да ниггермен кездеседі сен сондай нәсілшілсің жүре желкек чувак,жоқ сен ақы және қандай коньки тебуш да ниггер кездес сен сондай нәсілшіл жүр желкек чувак,racism

1,rt надан ақ адамдар сонымен сіз ашуланасыз ба қыз иә сен бардың rt обамаға қарғыс атсын мылқау нигга,rt надан ақ адам сонымен сіз ашулан ба қыз иә сен бар rt обама қарғыс ат мылқа нигг,racism

1,сол сияқты тың мылқау нигга айтпақшы ол жылына миллион табады сіз мылқау фагот жеңіліс семіз бейбақ маған ешқашан твиттер жазбаңыз,сол сияқты тың мылқа нигг айтпақшы ол жыл миллион таб сіз мылқа фагот жеңіліс се бей маған ешқашан твиттер жазба,racism

1,шайтан алғыр егер ниггерлер ақымақтық үшін біріге алса бірақ белые үлкен іс үшін біріге алмаса онда бейбақтар өлуі керек,шай алғы егер ниггер ақымақтық үшін бірі ал бірақ белы үлкен іс үшін бірі алма онда бейбақ өл керек,racism

1,бұл нигга дәл осы жерде gt ақымақ тозақ сияқты сс түкіреді міне солай,бұл нигг дәл осы же gt ақы тозақ сияқты сс түкір міне солай,racism

1,қалай болғанда да сіз қара нәсілділер тұрып сөйлейді деп ойлауыңыз мүмкін бірақ жоқ сіз бәріңіз тең әлемде құрбанның рөлін ойнағанды ұнатасыз сіз нағыз нәсілшілдік туралы өткен азап туралы ештеңе білмейсіз сіз бәрін түсінбейтін нәрсе бұл мүмкін манипуляция емес үйрену,қалай болғанда да сіз қара нәсілді тұр сөйле де ойла мүмкін бірақ жоқ сіз бәрі тең әлем құрбан рөл ойна ұна сіз нағыз нәсілш туралы өткен азап туралы ештеңе білме сіз бәрін түсінбе нәрсе бұл мүмкін манипуляция емес үйрену,racism

1,адамдар ақымақ әсіресе ниггерлер бұл қандай қарғыс надан боқ,адам ақы әсіресе ниггер бұл қандай қарғыс надан боқ,racism

1,мен өткен өмірімде не болғанын есіме түсіре аламын егер мен оны сол кезде жазып алсам немесе ол ескі хабарламаларда болса мен жасқа толғанға дейін болғанның бәрін ойымнан шығардым бәрі тұман сияқты мен ештеңе есімде жоқ мен үшін бәрі қара,мен өткен өмір не бол ес түсір ал егер мен оны сол кезде жаз ал немесе ол ескі хабарлама бол мен жас тол дейін бол бәрін ой шығар бәрі тұман сияқты мен ештеңе ес жоқ мен үшін бәрі қара,racism

1,қалай мылқау нигга ховард сені есегіңе ұрады сен қаншықсың және сен мұны білесің хаха,қалай мылқа нигг ховард сен есег ұр сен қаншық және сен мұны біл хах,racism

1,сіз чикаго туралы бәрін білмейсіз егер сіз бірдеңе білетін болсаңыз онда сіз көмектесу үшін федералдардың бұрын чикагоға барғанын және ештеңе өзгермегенін білуіңіз керек федерациялар бұл мәселені шеше алмайды,сіз чикаго туралы бәрін білме егер сіз бірде біл бол онда сіз көмектес үшін федерал бұрын чикаго бар және ештеңе өзгерме біл керек федерация бұл мәселе шеш алма,racism

1,иә мен бұл туралы біраз уақыт шыққаннан кейін естідім мен алғашқы екі серияны жүктеп алып оларға ілíндім мен мұны тағы бір рет қайталайтын шығармын hbo сияқты премиум арналардан басқа жақсы шоуларды табу қиын әдетте маусымдарда тек онға жуық шоу болады бір жақсы күн немесе бірнеше түн және солай,иә мен бұл туралы біраз уақыт шық кейін есті мен алғашқы екі серия жүкте алып оларға іл мен мұны тағы бір рет қайтала шығ hbo сияқты премиум арна басқа жақсы шоу табу қиын әдет маусым тек он жуық шоу бол бір жақсы күн немесе бірнеше түн және солай,racism

1,нәсілшілдікпен бетпе бет келгенде сіз оның бар екенін дәлелдеп қана қоймай сіздің ойларыңыз bamon алалаушылығымен боялғанын дәлелдейсіз,нәсілшілдік бетп бет кел сіз оның бар екен дәлелде қана қойма сіздің ой bamon алалаушылығ боял дәлелде,racism

1,нәсілшілдік латын америкасында соншалықты терең тамыр жайған біздің көпшілігіміз қараңғы болғанымен ақ тері сұлулық стандарты болып табылады сізді негр деп атайды бұл қорлау сіз ақ болғыңыз келеді сіз нөлдік көрініспен өстіңіз өйткені сіздің теріңіздің түсі тартымды емес өйткені олс,нәсілш латын америка соншалықты терең тамыр жай біздің көпшіліг қараңғы бол ақ тері сұлулық стандарт бол таб сіз нег де ата бұл қорла сіз ақ бол кел сіз нөлдік көрініс өс өйткені сіздің тер түс тартымды емес өйткені олс,racism

1,бұл твит мені ренжітті мен ешқашан қара бала екіншісін үйдегі нигга деп атай алады деп ойламас едім бұл қорлайтын қайғылы және мүлдем жиіркенішті князькабиді кешіріңіз,бұл твит мен ренжі мен ешқашан қара бала екінші үйдегі нигг де ата ал де ойламас ед бұл қорла қайғылы және мүлдем жиіркеніш князькаби кешір,racism

1,қымбатты ақымақ бейбақ қара адамдар нигга емес хек олар тіпті қара емес олардың терісінің түсі әртүрлі қарғысатқыр нәсілшіл,қымбатты ақы бей қара адам нигг емес хек олар тіпті қара емес олардың тері түс әртүрлі қарғысатқы нәсілшіл,racism

1,артқы орындықтағы ананы ақымақ деп айтатындардың бәрі үлкендерді құрметтейді егер мен басқа біреуді алып жүрсем ол үлкен болса және алдымен көлікке отырса әйелім артта қалар еді әдептілікке үйреніңіз адамдар өйткені бір күні бәріміз қартайамыз,артқ орындық ана ақы де айт бәрі үлкен құрметте егер мен басқа біреу алып жүр ол үлкен бол және алд көлік отыр әйел арт қал ед әдептілік үйрен адам өйткені бір күн бәрі қартай,racism

1,сіз оларды итеріп жіберген және алдымен олардан бас тартқан адам болсаңыз адамдар сізден бас тартпайды деп күтпеңіз,сіз ол итер жібер және алд олардан бас тарт адам бол адам сізден бас тартпа де күтпе,racism

1,rt lmao ештеңе емес қызды жақсы қорлайды барып маған лимонад әкел мен ішкім келеді нигга қаншық бұл түйін мылқау,rt lmao ештеңе емес қыз жақсы қорла бар маған лимонад әкел мен іш кел нигг қаншық бұл түйін мылқа,racism

1,бұл жігіт жай ғана ақымақ мен сені сипаттай алатын жалғыз әдіс ренжімеңіз абег уайымдамаңыз сіз әлі де менің ниггер қаншықсысыз,бұл жігіт жай ғана ақы мен сен сипатта ал жалғыз әдіс ренжіме абег уайымдама сіз әлі де менің нигг қаншық,racism

1,о мими шайтан алғыр сіздер ниггерлер ақымақсыздар,о мими шай алғы сіздер ниггер ақы,racism

1,қара нәсілділер қара деп аударылатын кез келген сөзді жек көретін сияқты біз олардың қаншасынан өттік никкер түрлі түсті негр афроамерикалық қара афроамерикалық американдық пок әрі қарай не болады белые ақ деп аударылатын сөздер деп атауға қарсы емес міне айырмашылық біртүрлі емес пе,қара нәсілді қара де аударыл кез кел сөз жек көр сияқты біз олардың қанша өт никк түрлі түсті нег афроамерикалық қара афроамерикалық американдық пок әрі қарай не бол белы ақ де аударыл сөз де ата қарсы емес міне айырмашылық біртүрлі емес пе,racism

1,ақ адамдар сіздің ата бабаларыңызды құлдар негрлер адамдар емес және б бұл оның рас екенін білдіре ме,ақ адам сіздің ата баба құл негр адам емес және б бұл оның рас екен білдір ме,racism

1,мұның бәрін ойламаңыз маған нөміріңізді беріңіз қарғыс атқыр мылқау ниггерлер маған барлық бос сөздерді жазады,мұның бәрін ойлама маған нөмір бер қарғыс атқы мылқа ниггер маған барлық бос сөз жаз,racism

1,сіз бардыңыз бұл сіз деп ойлайтын адам емес мылқау нигга,сіз бар бұл сіз де ойла адам емес мылқа нигг,racism

1,обамаға қарғыс атсын мылқау нигга ооо бұл қыз дөрекі лол қара қажы,обама қарғыс ат мылқа нигг ооо бұл қыз дөрекі лол қара қажы,racism

1,бұл клоун кім трамптың тағы бір ақымақ жақтаушысы оның есегінде дюймдік құбыр бар си эн эн,бұл клоун кім трамп тағы бір ақы жақтаушы оның есег дюйм құбыр бар си эн эн,racism

1,неге оның орнына бұл іске садақа бермеске біріншіден әскерлер жылдар бойы әртүрлі қайырымдылық ұйымдарына ұжымдық түрде садақа беріп келеді екіншіден армиядағы бір жай ғана емес сіз ақ және тмд біздің ешқайсымызды патрульдеуге тырысуға құқығыңыз жоқ,неге оның орн бұл іс садақа бермес бірінші әскер жыл бойы әртүрлі қайырымдылық ұйым ұжымдық түр садақа бер кел екінші армия бір жай ғана емес сіз ақ және тмд біздің ешқайсы патрульде тырыс құқы жоқ,racism

1,желкекке обама мылқау нигга үндеме ақымақ эй си менің ойымша ол жамандықтан есінен танып қалды ол ақымақ жезөкше лол,желкек оба мылқа нигг үндеме ақы эй си менің ойымша ол жамандық ес тан қал ол ақы жезөкше лол,racism

1,мен шынымен мені қорлайтын адамдардың қайдан шыққанын көрдім бірақ бұрын ақ пен қара арасында болғанның бәрі мен бұл туралы шынымен айта алмаймын бірақ мен мұндай тері түсімен туылғаным мені жеке тұлға ретінде төмендететінін сезбеймін эминем командаминем,мен шын мен қорла адам қайдан шық көр бірақ бұрын ақ пен

қара ара бол бәрі мен бұл туралы шын ай алма бірақ мен мұндай тері түс туыл мен жеке тұлға рет төмендет сезбе эмине командамине,racism

1,ол мааад лмао rt сіз бардыңыз мылқау нигга сіз бәріңіз сондай ақымақсыз,ол мааад лмао rt сіз бар мылқа нигг сіз бәрі сондай ақы,racism

1,rt бұл нигга трамп монах бірақ жаман жаңалық бұл ниггерге импичмент жариялау,rt бұл нигг трам монах бірақ жаман жаңалық бұл нигг импичмент жариялау,racism

1,досым шайтан алғыр иә шын мәнінде және сіз ақымақ ниггерсіз шамамен күн ішінде ізбасар жинадыңыз,дос шай алғы иә шын мән және сіз ақы нигг шамамен күн іш ізбасар жина,racism

1,rt rt желкекге барыңыз сіздің өшірулеріңіз мылқау нигга rt менің ақ ізбасарларыма сәлем,rt rt желкек бар сіздің өшіру мылқа нигг rt менің ақ ізбасар сәлем,racism

1,бұл жалғыз сирек өйткені ниггерлер мылқау олар балтаның бір түрін алып есіктен ұшып кетеді мысалы бәрі тозаққа барады,бұл жалғыз сирек өйткені ниггер мылқа олар балта бір түр алып есік ұш кет мысал бәрі тозақ бар,racism

1,мен өзімнің tl де көрген ақымақтықты ниггерлер ұнатпайды сен ақымақ бастарсың,мен өзімнің tl де көр ақымақтық ниггер ұнатпа сен ақы баста,racism

1,rt ананас пиццада орын жоқ сіз бәріңіз ниггерлер жиіркеніштісіз,rt ананас пицца орын жоқ сіз бәрі ниггер жиіркенішт,racism

1,обамаға қарғыс атсын мылқау нигга біз кімнің есінен танғанын көреміз қаншық суыққа қарсы таблетка алыңыз арамшөп шегіп төсекке барыңыз,обама қарғыс ат мылқа нигг біз кім ес тан көр қаншық суық қарсы таблетк ал арамшөп шег төсек бар,racism

1,мен білемін сіз ниггер су бөтелкелері компаниясының миын шайып тастадыңыз сіз не тапсырыс берсеңіз де бар мейрамхана үстелге мұз текшелері бар ағын суды автоматты түрде береді бұл жерде ештеңе жоқ таза сен ақымақ бейбақсың,мен біл сіз нигг су бөтелке компания ми шай таста сіз не тапсырыс бер де бар мейрамхана үстел мұз текше бар ағын су автоматты түр бер бұл же ештеңе жоқ таза сен ақы бей,racism

1,қандай ақымақ сұрақ барыңыз желкек мылқау нигга,қандай ақы сұрақ бар желкек мылқа нигг,racism

1,құдай сен бұған ашуландың кешіріңіз мен басында оның рас па жоқ па білмедім бірақ иә мен оны қарадым және жазушылардың бірі жылғы сұхбатында зуко мен катарамен бірге осы жолмен жүргісі келетінін айтты бірақ олай етпеді,құдай сен бұған ашула кешір мен бас оның рас па жоқ па білме бірақ иә мен оны қара және жазушы бірі жылғы сұхбат зуко мен катара бірге осы жол жүр кел ай бірақ ола етпе,racism

1,соңғы бірнеше айда blm қара қауымдастыққа бәрінен де көп зиян келтірді,соңғы бірнеше айда blm қара қауымдастық бәрі де көп зиян кел,racism

1,тәртіп сақшыларының ереуілі түрлі түсті мекемелерде тамақтануға тура келді нәсілшілдіктің барлық түрлеріне төзуге тура келді менің анам мен әкем де солай егер сіз солтүстіктен болсаңыз жоқ,тәртіп сақшы ереуіл түрлі түсті мекеме тамақтан тура кел нәсілшілдік барлық түр төз тура кел менің ана мен әке де солай егер сіз солтүстік бол жоқ,racism

1,мен өз командамды жақсы көремін мен өзімнің ниггерлерімді жақтайтын едім қандай команда amp қандай ниггерлер ақымақ бейбақ,мен өз команда жақсы көр мен өзімнің ниггер жақта ед қандай команда amp қандай ниггер ақы бей,racism

1,күн фотондары метрден аспаса да мұхит тереңдігі биолюминесценциямен жарқырайды жарық шығаратын кез келген нәрсе жыртқыштардың да жыртқыштардың да назарын аударады жануарлар әлеміндегі ең қара жамылғылардың бірімен жабылған вантафиш балықтары іс жүзінде,күн фотон метр аспа да мұхит тереңдіг биолюминесценция жарқыра жарық шығар кез кел нәрсе жыртқыш да жыртқыш да назар аудар жануар әлем ең қара жамылғы бір жабыл вантафиш балық іс жүз,racism

1,негрді ақ адамдар адамдардың бүкіл нәсіліне олардың мақұлдауынсыз немесе олардың қалай аталатынына үлес қоспай атау ретінде таңдады сонымен бұл қалыпты жағдайға айналды және бәрі оны қолданды өйткені бұл ден жақсы естілді хонки сөзге тікелей жауап болды,нег ақ адам адам бүкіл нәсіл олардың мақұлдауын немесе олардың қалай атал үлес қоспа атау рет таңда сонымен бұл қалыпты жағдай айна және бәрі оны қолда өйткені бұл ден жақсы ест хонки сөз тікелей жауап бол,racism

1,барды сен желкекке мылқау нигга,бар сен желкек мылқа нигг,racism

1,неге мен алмаймын мылқау нигга ден ге дейін сіздің турнирлеріңізді сігу ол баскетбол ойынынан оралады,неге мен алма мылқа нигг ден ге дейін сіздің турнир сіг ол баскетбол ойын орал,racism

1,ақымақ болып темекі шегу rt ол қосылатын уақытты алыңыз бұл маған ұнамайтын нәрсе rt желкекке обама мылқау нигга,ақы бол темекі шегу rt ол қосыл уақыт ал бұл маған ұнама нәрсе rt желкек оба мылқа нигг,racism

1,маған қазіргі pfp ұнайды бұл менің бірінші луи емес сондықтан мен қорқып кеттім бірақ маған ур ұнайды,маған қазіргі pfp ұна бұл менің бірінші луи емес сондықтан мен қорқ кет бірақ маған ур ұна,racism

1,мен жеккөрушілік туралы деректі фильм көремін бұл нәсілшілдер ақымақ болып көрінеді құдай оны жек көреді негр еврей оның ең жақсы көрінісінде надандық әрқашан орын алады,мен жеккөрушілік туралы деректі фильм көр бұл нәсілшіл ақы бол көрін құдай оны жек көр нег еврей оның ең жақсы көрініс надан әрқашан орын ал,racism

1,каеперник сен ақымақ ниггерсің ақымақ сияқты сен жай ғана құлап үлгердің мылқау джитсун мун крикет баклажан бастары,каеперник сен ақы нигг ақы сияқты сен жай ғана құла үлгер мылқа джитсун мун крикет баклажан бас,racism

1,мен өткен немесе қазіргі уақытта black proteas тің төмендегілердің кез келгенін айыптауын немесе оларға қарсы шығуын шыдамдылықпен күтемін қха үкіметі штатты ewc bee немесе осы тізімдегі кез келген нәрсені тартып алғаны үшін,мен өткен немесе қазіргі уақыт black proteas ті төмендегі кез кел айыпта немесе оларға қарсы шығ шыдамдылық күт қх үкімет штат ewc bee немесе осы тізім кез кел нәрсе тарт ал үшін,racism

1,иә иә бардым желкек бұл мылқау нигга майк браун фергюсон ккк роуч даррен уилсон полицейлер фаготалар wwe,иә иә бар желкек бұл мылқа нигг майк браун фергюсон ккк роуч даррен уилсон полицей фагота wwe,racism

1,сонымен жақын маңдағы кішкентай бейбақ стукача нигга ойнағысы келеді сен кішкентай ақымақ бейбақ бізде бейнебақылау бар мен сенің мұны істегеніңді көрдім,сонымен жақын маң кішкентай бей стукач нигг ойна кел сен кішкентай ақы бей біз бейнебақылау бар мен сенің мұны істе көр,racism

1,флорида нәсілшілдік жағынан қорқынышты мен осында тұратынымды білемін батыс вирджиния да солай неліктен қара тері сізді адамға ұқсамайтынын түсінбеймін олай емес қара адамдар бұл адамдар blacklivesmattters сізді бұлай емес деп ойлайтындарға жібереді,флорида нәсілш жағ қорқыныш мен осында тұр біл батыс вирджиния да солай неліктен қара тері сіз адам ұқсама түсінбе ола емес қара адам бұл адам blacklivesmattters сіз бұлай емес де ойла жібер,racism

1,бұл мылқау нигга ешқашан лаланы ұрмайды ол сіздің кисканызды мылқау етеді,бұл мылқа нигг ешқашан лала ұрма ол сіздің киска мылқа ет,racism

1,оқиға тіпті мұнымен бітпейді қыздардың бірі оның жігіті ракон деп қалай аталатыны оның ақ екендігі туралы айта бастады ол мұны мақтан тұтты ол оны ақ әжесі өсіргенін айтты ал басқа қыз бұл сұйылтылған деді мен не сұрадым негр,оқиға тіпті мұны бітпе қыз бірі оның жігіт ракон де қалай атал оның ақ екендіг туралы ай баста ол мұны мақтан тұт ол оны ақ әже өсір ай ал басқа қыз бұл сұйылтыл де мен не сұра нег,racism

1,мен сіздің жарыс туралы шынайы пікірлеріңізді тыңдадым мен миссуридің оңтүстігінде қара нәсілділерді түрлі түсті қара және қара көзді негрлер деп атаған кезде өстім жылдардың аяғынан кейін мен қазіргі нәсілшілдік туралы көремін және естимін деп ешқашан ойлаған емеспін бұл мүлдем орынсыз бүгінгі күн үшін рахмет,мен сіздің жарыс туралы шынайы пікір тыңда мен миссури оңтүстіг қара нәсілді түрлі түсті қара және қара көз негр де ата кезде өс жыл аяғ кейін мен қазіргі нәсілш туралы көр және ести де ешқашан ойла емес бұл мүлдем орынсыз бүгінгі күн үшін рахмет,racism

1,rt қызықсыз қаншық болмаңыз егер бұл сіздің ниггеріңіз болса оның әтешін сорып алыңыз,rt қызық қаншық болма егер бұл сіздің нигг бол оның әтеш сор ал,racism

1,ақымақ салдары қандай сіз ақымақ ниггерсіз,ақы сал қандай сіз ақы нигг,racism

1,хек сен мені мылқау нигга деп ойладың солай ма бұл қаншық емес,хек сен мен мылқа нигг де ойла солай ма бұл қаншық емес,racism

1,сперматозоидта қалыптасқан аталық безге қараңыз rt обаманы тозаққа апарыңыз kmsl хахаха,сперматозоид қалыптасқан аталық без қара rt обама тозақ апа kmsl хахах,racism

1,chineke rt мен қоңыр ақымақ болудан бастамаңыз сен қара сен ниггерсің сіз лас және жиіркенішсіз,chineke rt мен қоңыр ақы болу бастама сен қара сен нигг сіз лас және жиіркеніш,racism

1,желкекке үндемей музыка шығарыңыз мылқау нигга,желкек үндеме музыка шығ мылқа нигг,racism

1,сен де алмайсың хуя rt обамаға қарғыс атсын мылқау нигга,сен де алма хуя rt обама қарғыс ат мылқа нигг,racism

1,олардың әрқайсысында аздап нәсілшілдік бар жуған кезде барлық түсті киімдерді ақ түстің бөлігіне бөлетініңізге сенімдімін нәсілшіл әзіл,олардың әрқайсы аздап нәсілш бар жу кезде барлық түсті киім ақ түс бөліг бөл сенімді нәсілшіл әзіл,racism

1,rageeeeee rt обамаға қарғыс атсын мылқау нигга,rageeeeee rt обама қарғыс ат мылқа нигг,racism

1,мылқау ниггерлер ақ жігіттер те моллиді арқасына ұрды және сіз бұл жаңа нәрсе деп ойлайсыз лол шайтан алғыр,мылқа ниггер ақ жігіт те молли арқа ұр және сіз бұл жаңа нәрсе де ойла лол шай алғы,racism

1,нәсілшілдік сіз алдымен ақ киімді содан кейін қара киімді таңдайсыз нәсілшіл болмаңыз олардың барлығын бірге жуыңыз,нәсілш сіз алд ақ ки содан кейін қара ки таңда нәсілшіл болма олардың барлығ бірге жу,racism

1,біздің отбасымызға қош келдіңіз қымбаттым сіздің пайдаланушы атыңыз өте сүйкімді сондықтан сөйлеу кезінде сіздің көзіңізге бірінші кім түседі маған қызық,біздің отбас қош кел қымбат сіздің пайдаланушы ат өте сүйк сондықтан сөйлеу кез сіздің көз бірінші кім түс маған қызық,racism

1,ол адамды барлық ниггерлер мен қара кесектер деп атады жексұрын қаншық,ол адам барлық ниггер мен қара кесек де ат жексұр қаншық,racism

1,barackobama ақш та дрондарды африкаға жіберуге ақша жоқ біздің ниггерлерді дрондарыңызбен өлтірмеңіз мылқау нигга сіз желкеккеге бардыңыз,barackobama ақш та дрон африка жібер ақша жоқ біздің ниггер дрон өлтірме мылқа нигг сіз желкекке бар,racism

1,ты сен dmb ден ұсқынсыз нигер жүр желкекке мен tryb мен емеспін бірақ кеше түнде мен cinco ның мылқау екенін көрдім өте өкінішті,ты сен dmb ден ұсқын ниг жүр желкек мен tryb мен емес бірақ кеше түн мен cinco ны мылқа екен көр өте өкініш,racism

1,ктфууу сен жындысың ба барды желкекке обама мылқау нигга,ктфуу сен жынд ба бар желкек оба мылқа нигг,racism

1,мен бірінші кінәлаймын,мен бірінші кінәла,racism

1,дегі жасөспірімдер сіздің түрлі түсті адамдарға деген көзқарасыңыз тек қара нәсілділер ғана емес сонымен қатар қоғамның барлық салаларындағы көп мәдениетті нәсілшілдіктің нәсілдік кері белгісі екенін түсінуі керек егер сіз жалғастыра берсеңіз түрмеге түсуіңіз мүмкін,дег жасөспірім сіздің түрлі түсті адам де көзқарас тек қара нәсілді ғана емес сонымен қатар қоғам барлық сала көп мәдениетті нәсілшілдік нәсіл кері бел екен түсін керек егер сіз жалғастыр бер түрме түс мүмкін,racism

1,мммфао сіз бәріңіз бұл ақымақтықты бағалайсыз сіз бардыңыз ақымақ нигга сізді шынымен ұрды,мммфао сіз бәрі бұл ақымақтық бағала сіз бар ақы нигг сіз шын ұр,racism

1,олар қаншыққа қоныстанды сіз мүлдем жынды болдыңыз жезөкше obamanation обамаға қарғыс атсын мылқау нигга,олар қаншық қоныстан сіз мүлдем жын бол жезөкше obamanation обама қарғыс ат мылқа нигг,racism

1,немесе жоқ rt сіз ақылсыз ба обамаға қарғыс атсын мылқау нигга,немесе жоқ rt сіз ақыл ба обама қарғыс ат мылқа нигг,racism

1,ант етемін сіз мені ашуланғанды ұнатасыз сонда жарайды сен желкеккеге бардың мылқау нигга,ант ет сіз мен ашулан ұна сонда жарайды сен желкекке бар мылқа нигг,racism

1,түрлі түсті әйелдер отрядының нәсілшілдігі қыздар қандай реңк боялған дегенді білдіреді ал қайсысы жоқ түс дөңгелегі бар қызыл жасыл көк ea talieb жарық мен де өте алар едім пайда үшін бұл қандай rbg ол қиылысады ханымдар маған қарағанда сәл жеңілірек таңдаңыз,түрлі түсті әйел отряд нәсілшілдіг қыз қандай реңк боял де білдір ал қайсысы жоқ түс дөңгелег бар қызыл жасыл көк ea talieb жарық мен де өте ал ед пайда үшін бұл қандай rbg ол қиылыс ханым маған қара сәл жеңілірек таңда,racism

1,обамаға қарғыс атсын мылқау нигга гаитиге де тозақ мен жұмыс істеген ақшамды рет бұрап алдым ішкі кірістер қызметі және сіздің бостандықты пайдаланатын барлық әлеуметтік қамсыздандыру,обама қарғыс ат мылқа нигг гаити де тозақ мен жұмыс істе ақша рет бұра ал ішкі кіріс қызмет және сіздің бостандық пайдалан барлық әлеуметтік қамсыздандыру,racism

1,мұның бәрі ойыншылар төсеніш болса да тізе бүгеді жеке өзім blm group пен келіспеймін бірақ мен black lives matter хабарламасымен келісемін сізден басқа ешнәрсемен келісуді ешкім сұрамайды тек ойыншыларға және олар жіберетін

хабарламаға қолдау көрсетіңіз,мұның бәрі ойыншы төсеніш бол да тіз бүг жеке өзім blm group пен келіспе бірақ мен black lives matter хабарлама келіс сізден басқа ешнәрсе келісу ешкім сұрама тек ойыншы және олар жібер хабарлама қолдау көрсе,racism

1,кун сатылатын және ақ нәсілшілдерге әсіресе ақ нәсілшілдерді қара нәсілділерден қорғайтындарға жалбарынатын қара адам том ағай оны малкольм үй негрі деп атады мен оны көргенді жек көремін,кун сатыл және ақ нәсілшіл әсіресе ақ нәсілшіл қара нәсілді қорға жалбарын қара адам то аға оны малколь үй нег де ат мен оны көр жек көр,racism

1,amycooper нью йоркте жұмыс істейтін канадалық әйел negro үшін полиция шақырды оған айып тағылды канададан келген көптеген нәсілшілдер ақ терінің артықшылықтарын пайдаланып ақш көшелерімен жүреді,amycooper нью йорк жұмыс істе канадалық әйел negro үшін полиция шақ оған айып тағ канада кел көптеген нәсілшіл ақ тер артықшылық пайдалан ақш көше жүр,racism

1,азиялық қызметкер бұрынғы әріптесін негр деп атады және олар бұл туралы менеджермен сөйлескенде ол оны елемеді бірақ мен өзімді жала жапқан ақ адамнан қорғаған кезде ақ менеджер ешқандай нақты дәлелдерсіз айыптауларды ойлап тапты нәсілшілдікке қарсы тұру,азиялық қызметкер бұрынғы әріпте нег де ат және олар бұл туралы менеджер сөйлес ол оны елеме бірақ мен өз жал жап ақ адам қорға кезде ақ менеджер ешқандай нақты дәлелде айыптау ойла тап нәсілшілдік қарсы тұру,racism

1,егер мен қара адамды жиіркенішті нигга деп атайтын адамды ұстасам мен оған қатыстым,егер мен қара адам жиіркеніш нигг де ата адам ұста мен оған қат,racism

1,испандықтар нигга дейді бірақ сіз өзіңіздің африкалық тегіңізді де жариялайсыз ба міне мәселе ақ адамдар нигга кімге не болды деп айта алады бұл адамдар нәсілшілдікті ақ үстемдікті және өзін өзі жек көруді түсінеді бұл бәрімізді түрлі түсті адамдар ретінде ұстайды біз бәріміз африкалықтар ретінде бастадық,испандық нигг де бірақ сіз өз африкалық тег де жарияла ба міне мәселе ақ адам нигг кім не бол де ай ал бұл адам нәсілшілдік ақ үстемдік және өз өзі жек көру түсін бұл бәрі түрлі түсті адам рет ұста біз бәрі африкалық рет баста,racism

1,тозақ сік николь холнесс ол линчке арналған ақымақ нигга қаншығы,тозақ сік николь холнесс ол линч арнал ақы нигг қаншығ,racism

1,о кешіріңіз бубу rt обамаға қарғыс атсын мылқау нигга,о кешір буб rt обама қарғыс ат мылқа нигг,racism

1,менің лейкерс бейсбол қалпақшасы үшін аялдамада мені мылқау ниггерлер мазалады ал сіз бардыңыз ұлдар,менің лейкерс бейсбол қалпақша үшін аялдама мен мылқа ниггер мазала ал сіз бар ұл,racism

1,жаңарту ол маған фотосуреттерді жіберген кезде туындаған мәселені көрсету үшін мәтіндік хабарламаларын ашты және досына жіберген хабарламасында ақырында ол

негрді ұрып соғуға мүмкіндік алады сонымен бұл әңгіме аяқталды мен телефонды қойдым сіздің адамыңыз пульсирленген ақымақ қаншықпен бәрін қатарынан ұрады деп үміттенемін,жаңарту ол маған фотосурет жібер кезде туында мәселе көрсету үшін мәтін хабарлама аш және дос жібер хабарлама ақырында ол нег ұр соғ мүмкіндік ал сонымен бұл әңгіме аяқта мен телефон қой сіздің адам пульсирлен ақы қаншық бәрін қат ұр де үміттен,racism

1,gop сенаторлар өкілдер палатасының демократтары барды желкекке ақымақ нигга көт,gop сенатор өкіл палата демократ бар желкек ақы нигг көт,racism

1,үйдегі ниггерлер шынымен ақымақ бейбақтар,үйдегі ниггер шын ақы бейбақ,racism

1,мылқау бейбақ адамдарды нигга деп атауды тоқтатуы керек шығар,мылқа бей адам нигг де атау тоқтат керек шығар,racism

1,мүмкін жоқ бұл әйелдер әрине барлық шындықты және шындықтан басқа ештеңе айтқан жоқ мен сондай ақ олардың көпшілігі ескі және өңсіз карендер екенін байқадым мен ешнәрсені құлағымнан өткізбес едім оларға бүкіл алаяқтыққа мақұлдау мөрін қою үшін бір екі қара әйел қажет болды,мүмкін жоқ бұл әйел әрине барлық шындық және шындық басқа ештеңе айт жоқ мен сондай ақ олардың көпшілiг ескі және өң карен екен байқа мен ешнәрсе құлағ өткізбес ед оларға бүкіл алаяқтық мақұлдау мөр қою үшін бір екі қара әйел қажет бол,racism

1,хахахахахахах сенің анаң ол спорттық шалбары түсіп бара жатқан мылқау нигга,хахахахахахах сенің ана ол спорттық шалбар түс бар жат мылқа нигг,racism

1,мылқау нигга ешқашан кез келген мәселе бойынша өз пікіріңізді білдірмеңіз,мылқа нигг ешқашан кез кел мәселе бойынша өз пікір білдірме,racism

1,неліктен ник кэннонның негр деп айтатын адам сияқты ақ адамдарды адамгершілікке жатпайтын және жабайы деп атағанына бәрі ренжімейді,неліктен ник кэннон нег де айт адам сияқты ақ адам адамгершілік жатпа және жабай де ата бәрі ренжіме,racism

1,біреу есінен танып қалған сияқты сіз қаншалықты ашуланасыз rt барды желкекке обама ақымақ бас нигга,біреу ес тан қалған сияқты сіз қаншалықты ашулан rt бар желкек оба ақы бас нигг,racism

1,бұл қалай нәсілшіл болуы мүмкін бұл ақ қара болғандықтан ба жек көрушілер шағымданатын нәрсені табады деп ант етемін,бұл қалай нәсілшіл бол мүмкін бұл ақ қара бол ба жек көруші шағымдан нәрсе таб де ант ет,racism

1,мені басқа нигга тупак сияқты атып таста сен ақымақ қаншықсың,мен басқа нигг тупак сияқты ат таста сен ақы қаншық,racism

1,бірінші әсер xm тағы бір серб сіздің лақап атыңыз менің басымда жақындықты бағалау жоқ idk сіз маған ұнайсыз ба менікі екеніңізге сенімдімін өзара бір кездері сізге ғашық болған жоқ сіз де оны жариялауыңыз керек егер сіз бір кездейсоқ ойды

қаласаңыз сіз кереметсіз,бірінші әсер хм тағы бір серб сіздің лақа ат менің бас жақындық бағалау жоқ idk сіз маған ұна ба менік екен сенімді өзара бір кез сізге ғашық бол жоқ сіз де оны жарияла керек егер сіз бір кездейсоқ ой қала сіз керемет,racism

1,мен констабль болудан шаршадым бұл нигга мылқау бейбақ мен мұны айттым және байыпты айтамын,мен констабль болу шарша бұл нигг мылқа бей мен мұны ай және байыпты ай,racism

1,фриолес негрос деп аталатын қара гойя бұршақтары өте дәмді бірақ сіздің хипстер трейдер джонның есегіне оларды жеуге тыйым салынады ақшақар сонымен қатар нағыз испандықтар latinx ті қолданбайды бұл бірнеше испандық элиталық әлеуметтік медиа псевдо интеллектуалдарының ақымақ әңгімесі,фриолес негрос де атал қара гойя бұршақ өте дә бірақ сіздің хипс трейдер джо есег ол же тыйым салын ақшақ сонымен қатар нағыз испандық latinx ті қолданба бұл бірнеше испандық элиталық әлеуметтік медиа псевдо интеллектуал ақы әңгіме,racism

1,өкінішке орай республикашылардың көпшілігі ақыл есі кем емес сондықтан олар бюллетеньдерімен араласпайды lmao мылқау ниггерлер демократтар,өкініш орай республикашы көпшіліг ақыл ес кем емес сондықтан олар бюллетень араласпа lmao мылқа ниггер демократ,racism

1,қара болғаннан кейін сіз қоғамның қасіретіне айналасыз негрдің жексұрын сүйіктісі туралы айтпағанда,қара бол кейін сіз қоғам қасірет айнал нег жексұр сүйікті туралы айтпа,racism

1,крис доғал жуан доғал ебучий артта қалған нигга,крис доғал жуан доғал ебучи арт қалған нигг,racism

1,нигга үшін бұл нәсілшілдік емес сен ақымақ бейбақсың ниггер нәсілшіл ниггерлер мұны үнемі айтады,нигг үшін бұл нәсілш емес сен ақы бей нигг нәсілшіл ниггер мұны үнемі айт,racism

1,lmaoooooo сізде бәрі болды мектепте rt тозаққа дәлізде маған аяқ тірегін орната жаздағаны үшін мылқау жезөкше ниггер биш,lmaoooooo сіз бәрі бол мектеп rt тозақ дәліз маған аяқ тіреg орна жазда үшін мылқа жезөкше нигг биш,racism

1,сіз менің бірінші әйелім болсаңыз да маған әйел алуға рұқсат етілмейді,сіз менің бірінші әйел бол да маған әйел ал рұқсат етілме,racism

1,менің ойымша бұл көптеген кітапханалар ml емес адамдар жеке тұлға тұрғысынан бәрін ойлайды бұл гипериндивидуализм олар қара пантерлер туралы да томас санкар туралы да кем дегенде қандай да бір билікке ие болу үшін мемлекеттік билікті қажет ететін басқа да кіші қайраткерлер туралы ештеңе білмейді олар стратегияны көрмейді,менің ойымша бұл көптеген кітапхана ml емес адам жеке тұлға тұрғы бәрін ойла бұл гипериндивидуализ олар қара пантер туралы да томас санк туралы да кем де

қандай да бір билік ие болу үшін мемлекеттік билік қажет ет басқа да кіші қайраткер туралы ештеңе білме олар стратегия көрме,racism

1,сот трейвонмартин суд бойынша судьяны отставкаға кетуге міндеттейді юссс отвали арналған желкек әділетforzimmerman трейвонмартин мылқау нигга болды,сот трейвонмартин суд бойынша судья отставка кет міндетте юссс отвали арнал желкек әділетforzimmerman трейвонмартин мылқа нигг бол,racism

1,мужик трахнул қаншықты қалай ниггерлер сіз қалай істейсіз мен тунец құйдым мен ауыртпалықпын ақымақ боламын мен the stooges сияқты ақымақ боламын,мужик трахнул қаншық қалай ниггер сіз қалай істе мен тунец құй мен ауыртпалық ақы бол мен the stooges сияқты ақы бол,racism

1,сіз ақымақ қаншықсыз rt желкек обамаға барды ақымақ бас нигга,сіз ақы қаншық rt желкек обама бар ақы бас нигг,racism

1,фильмнің соңында басты кейіпкер бұл біз шығаратын жеккөрушілік дегенде бұл өте айқын болды қара адамдар нигга деген жиркенішті сөзді қолдана отырып бір біріне деген ашу гнаны түсіндіреді сондықтан адамдар оны мейірімді сөз ретінде қолданатынын айтады бұл өтірік екені анық,фильм соң басты кейіпкер бұл біз шығар жеккөрушілік де бұл өте айқын бол қара адам нигг де жиркеніш сөз қолдан отыр бір бір де ашу гна түсіндір сондықтан адам оны мейірімді сөз рет қолдан айт бұл өтірік екен анық,racism

1,шынын айтайық stephenlawrenceday бұл ақ пен қара нәсілшілдіктің саяси еске салуы неге бір ұрпақ бұрын қара балаларды күн сайын дерлік басқа қара балалар өлтірген жалғыз оқиғаны атап өту керек лиригбайд әңгімеге сәйкес келмейді,шын айт stephenlawrenceday бұл ақ пен қара нәсілшілдік саяси ес сал неге бір ұрпақ бұрын қара бала күн сайын дерлік басқа қара бала өлтір жалғыз оқи ата өту керек лиригбайд әңгіме сәйкес келме,racism

1,әуесқой черномазых сіз және талейдің ақымақ есегі де оны өзгертті балалар сіздер кэмеронды жаман жігіт деп ойлайсыздар оян тозақ,әуесқой черномазых сіз және талей ақы есег де оны өзгер бала сіздер кэмерон жаман жігіт де ойла оян тозақ,racism

1,бұл жерден кет бұл дін емес мылқау нигга,бұл же кет бұл дін емес мылқа нигг,racism

1,жарайды мен маттияны басқа түрлі түсті адамдар сияқты жек көремін бірақ әндегі нигга сөзінің айтылуын қара адамдарды нигга деп атаумен және оларға айқайлаумен салыстыру дұрыс емес екеуі де жиркенішті және бұл болмауы керек еді бірақ келіңіз эммуллу өте ақымақ және бәрінен бас тартады,жарайды мен маттия басқа түрлі түсті адам сияқты жек көр бірақ ән нигг сөз айтыл қара адам нигг де ата және оларға айқайла салыстыру дұрыс емес екеу де жиркеніш және бұл болма керек ед бірақ кел эммулл өте ақы және бәрі бас тарт,racism

1,сен ақымақ ниггерсің обамаға қарғыс атсын сенің пікіріңді қарғыс атсын мылқау нигга үшін шо маста үшін шо,сен ақы нигг обама қарғыс ат сенің пікір қарғыс ат мылқа нигг үшін шо мас үшін шо,racism

1,сізді ақымақ және ниггер деп атағаным үшін кешіріңіз бірақ егер бұл дұрыс болмаса мен мұны жасамас едім ақымақ бейбақ лол,сіз ақы және нигг де ата үшін кешір бірақ егер бұл дұрыс болма мен мұны жасамас ед ақы бей лол,racism

1,ұрланған жауап және мен саған эмодзи беремін sum сүйікті сен бекітілген мен сені tl де көргенді ұнатамын ilysm мен сенімен дос болғым келеді мен сенің аккаунтыңды ұстандым мен сені сүйіктілерімнің бірі деп санаймын сен сүйкімдісің сен үшін бәрін жасаймын сені бірінші рет көремін,ұрлан жауап және мен саған эмодзи бер sum сүйікті сен бекіт мен сен tl де көр ұна ilysm мен сенімен дос бол кел мен сенің аккаунт ұста мен сен сүйікті бірі де сана сен сүйкімд сен үшін бәрін жаса сен бірінші рет көр,racism

1,джефф өтінемін оны ақымақ әрекет деп атауды доғарыңыз нигга бұл күшті сөздердің бірі мысалы көт немесе трах және б,джефф өт оны ақы әрекет де атау доғар нигг бұл күшті сөз бірі мысал көт немесе трах және б,racism

1,мен істеймін өкінішке орай мен мұны өз тәжірибемнен бастан өткердім бірақ нәсілшілдік бұл белгілі бір жағдайларда ғана байқайтын аз ғана толика ма әлде түрлі түсті адамдар тобына деген терең жеккөрушілік пе нәсілшілдік өйткені сіз әлі де өз халқыңызды нағыз қысым жасаушыларға емес кінәлайсыз,мен істе өкініш орай мен мұны өз тәжірибе бас өткер бірақ нәсілш бұл белгілі бір жағдай ғана байқа аз ғана толик ма әлде түрлі түсті адам тоб де терең жеккөрушілік пе нәсілш өйткені сіз әлі де өз халқ нағыз қысым жасаушы емес кінәла,racism

1,қоғам бізді соншалықты сезімтал етті мен әдетте бәріне немқұрайлы қараймын және жай ғана салқындаймын бірақ blm мен өткен айда мен олардан қара түсті көргенде заттарға сезімтал болдым,қоғам біз соншалықты сезімтал ет мен әдет бәрін немқұрайл қара және жай ғана салқында бірақ blm мен өткен айда мен олардан қара түсті көргенде зат сезімтал бол,racism

1,эй сучи нигга екінші раундқа дайынсыз ба тур кез келген уақытта интеллектуалды ойлаумен және толық дайындықпен жетілдіріледі міне сіз жұмыста менімен қарым қатынас жасағаныңыз үшін не аласыз бұл бізді жұмыстан шығарды және сіз әлі де мылқау ниггерсіз байлаңыз баланы ұр ойыны басталады,эй сучи нигг екінші раунд дайын ба тур кез кел уақыт интеллектуал ойла және толық дайындық жетілдір міне сіз жұмыс менімен қарым қатынас жаса үшін не ал бұл біз жұмыс шығ және сіз әлі де мылқа нигг байла бала ұр ойын бастал,racism

1,жоқ сен бардың және ессіз есегіңізбен ашулануды жалғастырыңыз обамаға қарғыс атсын мылқау бас нигга,жоқ сен бар және ес есег ашулану жалғастыр обама қарғыс ат мылқа бас нигг,racism

1,егер сіз бұл кісі өлтіру ойынына қарсы болмасаңыз киска нигга үндемеңіз,егер сіз бұл кісі өлтір ойын қарсы болма киск нигг үндеме,racism

1,бұл нигга мылқау аф сияқты осы сияқты керемет қызбен қалай трахается,бұл нигг мылқа аф сияқты осы сияқты керемет қыз қалай трахается,racism

1,бардым сен желкекке сіз мылқау сасық ниггерсіз қара адамдар мен ниггердің айырмашылығы бар ал сіз нигга емессіз сондықтан өз қаншығыңызды ұрыңыз,бар сен желкек сіз мылқа сасық нигг қара адам мен нигг айырмашылығ бар ал сіз нигг емес сондықтан өз қаншығ ұр,racism

1,сен ақымақ ебучий ниггер қаншық сиськастой задницей лизоблюд айыр,сен ақы ебучи нигг қаншық сиськасто заднице лизоблюд айыр,racism

1,бүгін кешке менде нәсілшілдік оқиғасы болды мен түнгі серуеннен үйге келгенде бір жігіт менің артымнан нигга деп айқайлады айтыңызшы бүгінде нәсілшілдік жоқ жалғастыру,бүгін кеш менде нәсілш оқиға бол мен түнгі серуен үй кел бір жігіт менің арт нигг де айқайла айтыңызш бүгін нәсілш жоқ жалғастыру,racism

1,бұл мылқау қара маз мен айтқанымдай мен оны терген кезде не теріп жатқанымды қараңыз тек надан қара маз емлені қайта тексеруге мүмкіндік береді,бұл мылқа қара маз мен айтқанымда мен оны тер кезде не тер жат қара тек надан қара маз емле қайта тексер мүмкіндік бер,racism

1,біріншісі сіздің брендіңіз,бірінші сіздің бренд,racism

1,негр де қара дегенді білдіреді бірақ мен ақ адам мені осылай атағанын білемін,нег де қара де білдір бірақ мен ақ адам мен осылай ата біл,racism

1,сен мылқау қарғысатқыр ниггер барды сен қаншық кішкентай пенис сік өз ыстық анама,сен мылқа қарғысатқы нигг бар сен қаншық кішкентай пенис сік өз ыстық ана,racism

1,tomuchofwg сен айттың бардым желкек мылқау нигга соратын мүшесі мен сенімен ойнағым келмейді сондықтан,tomuchofwg сен ай бар желкек мылқа нигг сор мүше мен сенімен ойна келме сондықтан,racism

1,құдіретті құдай ие менің мәңгілік үнсіз сөйлесетін және олар не туралы сөйлесетінін түсінбейтін мылқау адамдарды жек көретінімді біледі міне менімен бірге доминикан республикасында дүниеге келген және гаитиде жарым жыл өткізген ұлым бұл өтірік нигга pic twitter com,құдіре құдай ие менің мәңгілік үн сөйлес және олар не туралы сөйлес түсінбе мылқа адам жек көр біл міне менімен бірге доминикан республика дүние кел және гаити жарым жыл өткіз ұл бұл өтірік нигг pic twitter com,racism

1,сіз мені жиіркенішті қара нигга деп атадыңыз,сіз мен жиіркеніш қара нигг де ата,racism

1,джейкоб үндеме ақымақ бейбақ негрлер ең нашар кеңес болып табылады өйткені жағдайда оларға ақ адамдар қызмет етеді,джейкоб үндеме ақы бей негр ең нашар кеңес бол таб өйткені жағдай оларға ақ адам қызмет ет,racism

1,мен ақымақ күндерімді колледжде қалдырдым егер сен мені алдасаң бәрі аяқталады idc егер біз үйленгенімізге жиырма жыл болса нигга менің үйімнен тозаққа барыңыз және өзіңізбен бірге ештеңе алмаңыз нөлден бастаңыз,мен ақы күн колледж қал егер сен мен алда бәрі аяқтал idc егер біз үйлен жиырма жыл бол нигг менің үй тозақ бар және өз бірге ештеңе алма нөлден баста,racism

1,тозақ сияқты мылқау рифма сияқты немесе ештеңе жоқ,тозақ сияқты мылқа риф сияқты немесе ештеңе жоқ,racism

1,эм мен сенің үлкен жанкүйеріңмін сіз өтінемін түрлі түсті адамдарға әділетсіздікке профильге және нәсілшілдікке байланысты қиын жағдайда көмектесу туралы көбірек бейнелер жасаңыз,эм мен сенің үлкен жанкүйер сіз өт түрлі түсті адам әділетсіздік профиль және нәсілшілдік байланысты қиын жағдай көмектес туралы көбірек бейне жаса,racism

1,ия сіз сияқты мен тунец құйдым мен ауыртпалықпын мен мылқау боламын мылқау боламын лил уэйн маған қазір қараңыз,ия сіз сияқты мен тунец құй мен ауыртпалық мен мылқа бол мылқа бол лил уэйн маған қазір қара,racism

1,менің арнамда осы үлкен қара қызды мазақ ететін және мазақ ететін ересек бөкселері бар көптеген ұсқынсыз қара масалар бар және бұл пенистің өте өте кішкентай энергиясы,менің арна осы үлкен қара қыз мазақ ет және мазақ ет ересек бөксе бар көптеген ұсқын қара маса бар және бұл пени өте өте кішкентай энергия,racism

1,жылы ол мен үшін нәсілшілдік жоқ екенін айтты ал джордж рутфорд түрлі түсті адамдарды дамыту ұлттық қауымдастығының naacp ақ адам сіз кешірілгеніңізді білетіндігіңізді айтады,жыл ол мен үшін нәсілш жоқ екен ай ал джордж рутфорд түрлі түсті адам дамыту ұлттық қауымдастығ naacp ақ адам сіз кешіріл білетіндіг айт,racism

1,нигга ақымақ сияқты,нигг ақы сияқты,racism

1,тәтті сабан адам сөзбе сөз бұл туралы ешқашан айтқан емес мен кейіпкерлер менің алғашқы idk what тв твиттерімнің бірінде жақсы болды деп айттым мен материалы бұзылды деп ойладым ал соңы түпкілікті болды сіз менің gen ті жақсы көретінімді тағы да ұмытып кеткен сияқтысыз бұл ең жақсы gen емес,тәтті сабан адам сөзб сөз бұл туралы ешқашан айт емес мен кейіпкер менің алғашқы idk what тв твиттер бір жақсы бол де ай мен материал бұз де ойла ал соңы түпкілік бол сіз менің gen ті жақсы көр тағы да ұмыт кет сияқты бұл ең жақсы gen емес,racism

1,біз көгалында трамп пенс белгісі бар үйдің жанынан өттік мен оны көпшілік алдында киюіміз керек болса да бетперде киген адамды ғана көрдім маған ешкім ештеңе айтқан жоқ менде бұл жерден жағымсыз әсер пайда болды сонымен қатар мен бірде

бір қара адамды көрмедім сондықтан сіз білесіз,біз көгал трам пенс бел бар үй жан өт мен оны көпшілік алд кию керек бол да бетп ки адам ғана көр маған ешкім ештеңе айт жоқ менде бұл же жағымсыз әсер пайда бол сонымен қатар мен бірде бір қара адам көрме сондықтан сіз біл,racism

1,бұл мылқау ниггерлерді тозаққа салыңыз,бұл мылқа ниггер тозақ сал,racism

1,түстер бұл дұрыс термин түсті сегменттеуді білдіреді мен рос деп санайтын бірнеше полицейлерді білемін және олар бостон департаменттерінде нәсілшілдік туралы айтады бірақ оны қарапайым жұмыс ретінде қарастырады және ешқашан бұл өзгерістер туралы айтпайды олар жаудың артқы жағынан еніп кетеді деп талап етеді,түс бұл дұрыс термин түсті сегменттеу білдір мен рос де сана бірнеше полицей біл және олар бостон департамент нәсілш туралы айт бірақ оны қарапайым жұмыс рет қарастыр және ешқашан бұл өзгеріс туралы айтпа олар жау артқ жағ ен кет де талап ет,racism

1,алдымен ақымақ бұтақтар қара масалардың әулиелері қазір аузымен дем алатын бронкостар сіз бәріңіз желкекге бардыңыз,алд ақы бұтақ қара маса әулие қазір ауз дем ал бронкос сіз бәрі желкек бар,racism

1,пааср басшысы уолтер уайт былай деп жазды ақ лақап аты бар инман бет мен негрмін менің ақ терім көк көзім ақшыл шашым бар менің нәсілімнің ерекшеліктері маған еш жерде көрінбейді оның үлкен атасы мен әжесінің тек і қара ал қалған сі ақ түсті заң бойынша ол аа мүшесі,пааср басшы уол уайт былай де жаз ақ лақа ат бар инман бет мен нег менің ақ терім көк көз ақшыл шаш бар менің нәсіл ерекшелік маған еш же көрінбе оның үлкен ата мен әже тек і қара ал қалған сі ақ түсті заң бойынша ол аа мүше,racism

1,сіз ақымақ ұсқынсыз ниггер уэйн позитивті қоқыс нигга жұмсақ задницей бардыңыз сіз нигга pic twitter com,сіз ақы ұсқын нигг уэйн позитивті қоқыс нигг жұм заднице бар сіз нигг pic twitter com,racism

1,ақымақ түйіндер rt ағартқышын ішіңіз обамаға қарғыс атсын мылқау нигга,ақы түйін rt ағартқыш іш обама қарғыс ат мылқа нигг,racism

1,жай әзілдеп тұр сіздің немере ағаңыз да нигга шығар тозаққа отырыңыз сіздің әзілдеріңіз тіпті күлкілі емес ақымақ қаншық,жай әзілде тұр сіздің немере аға да нигг шығар тозақ отыр сіздің әзіл тіпті күлкіл емес ақы қаншық,racism

1,ахххх менде алаңдаушылық бар сондықтан мен сені ниггер деп атаймын бұл жерден кет мен терімді өзгерте алмаймын бірақ мылқау болу бұл таңдау шынымен ұятқа қалдыратын сүйікті мен оны өшірер едім,ахххх менде алаңдаушылық бар сондықтан мен сен нигг де ата бұл же кет мен тер өзгер алма бірақ мылқа болу бұл таңдау шын ұят қалдыр сүйікті мен оны өшір ед,racism

1,қандай желкек менде сен бардың және мылқау нигга бар мен тіпті жарты күн жұмыс істемедім,қандай желкек менде сен бар және мылқа нигг бар мен тіпті жарты күн жұмыс істеме,racism

1,крогердің сыртындағы нигер үлкен жопасы бар жемпір киді true religion джинсы киген мылқау фугази және кофе ішеді бұл ыстық нүкте ақымақ бейбақ,крог сырт ниг үлкен жо бар жемпі ки true religion джин ки мылқа фугази және кофе іш бұл ыстық нүкте ақы бей,racism

1,сіз ақымақсыз немесе жай назар аудармайсыз бірде бір рет шығарылған видеода ешкім нәсілшіл ештеңе айтқан жоқ ол нәсіл туралы әзілдеді ал ақ адамдар оған назар аударып құлағынан өтіп кетті есіңізде болсын панельдегі екі бейнеде тағы бір қара адам болған,сіз ақы немесе жай назар аударма бірде бір рет шығарыл видео ешкім нәсілшіл ештеңе айт жоқ ол нәсіл туралы әзілде ал ақ адам оған назар аудар құлағ өт кет ес бол панель екі бейне тағы бір қара адам бол,racism

1,жігіт mlk ге сілтеме жасайды бірақ доктор кингті орыс коммунисті деп атаған ақ үстемшілдер мен олардың негрлерінің тезистерін қолданады,жігіт mlk ге сілтеме жаса бірақ доктор кинг орыс коммунис де ата ақ үстемшіл мен олардың негр тезис қолдан,racism

1,канье уэст ақ қызды шындықты айтқаны және айтқаны үшін батыл деп атады бірақ ақылды қара әйелді дәл осылай жасағаны үшін дөрекі түрде жазалады малком дан лексиканы алу құлдық кезінде сізде екі негр болды сізде үйдегі негр және далалық негр болды,кань уэст ақ қыз шындық айт және айт үшін батыл де ат бірақ ақылды қара әйел дәл осылай жаса үшін дөрекі түр жазала малко дан лексика алу құлдық кез сіз екі нег бол сіз үйдегі нег және далалық нег бол,racism

1,егер сіз бұрын нәсілшіл болсаңыз неге адамдарға оларды қолдайтыныңызды көпшілік алдында көрсеткіңіз келмейді джей бізге ең жақсы реңктер керек деп уағыздаған сияқты ол қара қауымдастық үшін ештеңе істемеді және өзінің нәсілшіл өткені үшін қайта қайта кешірім сұрады,егер сіз бұрын нәсілшіл бол неге адам ол қолда көпшілік алд көрсет келме дже бізге ең жақсы реңк керек де уағызда сияқты ол қара қауымдастық үшін ештеңе істеме және өзінің нәсілшіл өт үшін қайта қайта кешір сұр,racism

1,мені тағы бір ренжітетін нәрсе егер оның қара жігіті оған лас нәрсе жасаса ол ку клукс клан мүше ағаларына қоңырау шалып сол ниггерді оларға айтып берді сіздердің барлығыңыз бұл қара маз әйелді қара еркектерді және басқа заттарды жақсы көретінін айтқаныңыз жөн бұл өте жиіркеніштi,мен тағы бір ренжіт нәрсе егер оның қара жігіт оған лас нәрсе жаса ол ку клукс клан мүше аға қоңырау шал сол нигг оларға айт бер сіздердің барлығ бұл қара маз әйел қара еркек және басқа зат жақсы көр айт жөн бұл өте жиіркеніш,racism

1,сақина үшін жалақыны қысқарту мылтық пен май кішкентай ақымақ бейбақтар классикалық нигга боқтары нан аз өйткені сіз өз лигаңызда құқықтармен

мақтанғыңыз келеді,сақин үшін жалақы қысқарту мылтық пен май кішкентай ақы бейбақ классикалық нигг боқ нан аз өйткені сіз өз лига құқық мақтан кел,racism

1,мұны бірінші болып айтады бұл оның кім екендігі туралы емес мен үшін бірақ егер сіз жас болсаңыз жас бөксеңізді артқа итеріп үлкендердің алдында отыруына мүмкіндік бересіз,мұны бірінші бол айт бұл оның кім екендіг туралы емес мен үшін бірақ егер сіз жас бол жас бөксе арт итер үлкен алд отыр мүмкіндік бер,racism

1,ақша ақымақ қаншықтардан гөрі маңызды мен ниггерлерді жақсы көремін,ақша ақы қаншық гөрі маңызды мен ниггер жақсы көр,racism

1,иә және жезөкше төлейді үшін ол трахалась ниггерами сіз бұл сізді қандай да бір жолмен біреу етеді деп ойлайсыз лол мылқау ниггерлер,иә және жезөкше төле үшін ол трахалась ниггерами сіз бұл сіз қандай да бір жол біреу ет де ойла лол мылқа ниггер,racism

1,мен қазірдің өзінде тырыстым шайтан алғыр мен craigslist бар адамдарға мылқау нигга жауап бермейтінін айттым,мен қаз өз тырыс шай алғы мен craigslist бар адам мылқа нигг жауап берме ай,racism

1,ниггерін саған сатты ақымақ желкек,нигг саған сат ақы желкек,racism

1,барды желкекке обама мылқау нигга бұл ақымақ lt бұл smh жезөкше,бар желкек оба мылқа нигг бұл ақы lt бұл smh жезөкше,racism

1,мен де олай ойламаймын бірақ мен қара әйелдерге келгенде ештеңені жіберіп,мен де ола ойлама бірақ мен қара әйел кел ештеңе жібер,racism

1,алмаймын сен мылқау ниггерсің сіздің балаңыз бар ма айтайын дегенім егер сен сондай жаман болсаң мені тастап кет,алма сен мылқа нигг сіздің бала бар ма айт де егер сен сондай жаман бол мен таста кет,racism

1,оның айтқаны жиіркенішті болды бірақ қара адамдар шинге табынады және ол бұрын ниггерлерге түсініктеме берген,оның айт жиіркеніш бол бірақ қара адам шин табын және ол бұрын ниггер түсініктеме бер,racism

1,түсініктеме бұл менде болған ең есте қаларлық тәжірибелердің бірі болды әрбір тиынға тұрарлық түсініктеме бұл мылқау қара маймылды қарғыс атсын ол тым көп алып жұмсады түсініктеме біріншісі туралы ойлау дұрыс емес,түсініктеме бұл менде бол ең ес қаларлық тәжірибе бірі бол әрбір тиын тұрарлық түсініктеме бұл мылқа қара майм қарғыс ат ол тым көп алып жұмса түсініктеме бірінші туралы ойлау дұрыс емес,racism

1,испандық отбасылар walmart оқиғасына ренжігеннен кейін acab пен келіскен дұрыс полиция қызметкерінің билікті асыра пайдалануы маңызды оқиға болуы керек еді бірақ оның орнына олардың нәсілшілдігі қара әйел көмектесуден бас тартқан бөлшектерде ғана көрінеді ойындағы бөлу және жеңу тактикасы,испандық отбасы walmart оқиға ренжі кейін acab пен келіс дұрыс полиция қызметк билік асыра

пайдалан маңызды оқиға бол керек ед бірақ оның орн олардың нәсілшілдіг қара әйел көмектесу бас тарт бөлшек ғана көрін ой бөлу және жеңу тактика,racism

1,сіз түстісіз өзіңізді қауіпсіз сезінбейсіз мен сізді жабамын біз қол ұстасып нәсілшілдікті бірге тежей аламыз,сіз түсті өз қауіпсіз сезінбе мен сіз жаб біз қол ұстас нәсілшілдік бірге теже ал,racism

1,орын ирландиялық кемелер адамдар өлгенше жүзу үшін теңізге қайта бұрылды өйткені олар мұнда болу үшін тым лас деп саналды ақ деп аталады шимпанзелер және оларға қара адамдардың қасындағы маймылдар сияқты мультфильмдер иттер де негрлер де күріш алқаптары да жоқ ұсқынсыз,орын ирландиялық кеме адам өлгенш жүзу үшін теңіз қайта бұр өйткені олар мұнда болу үшін тым лас де сана ақ де атал шимпанзе және оларға қара адам қас маймыл сияқты мультфильм ит де негр де күріш алқап да жоқ ұсқын,racism

1,сәлем құдай сен шынымен сүйкімдісің және бұл менің tts тегі алғашқы көріністерімнің бірі және біз тағы да сөйлесуіміз керек немесе спин офф кассасы,сәлем құдай сен шын сүйкімд және бұл менің tts тег алғашқы көрініс бірі және біз тағы да сөйлес керек немесе спин офф касса,racism

1,мылқау нигга call of duty,мылқа нигг call of duty,racism

1,шайтан алғыр charliehebdo аударма француздар ниггерлер сияқты мылқау бұл әрбір жылайтын кракка ма pic twitter com,шай алғы charliehebdo аударма француз ниггер сияқты мылқа бұл әрбір жыла кракк ма pic twitter com,racism

1,нигга қоқысының мылқау бөлігі басқа адамдардың суреттерін мазақ етпес бұрын өз суреттеріңізді жүктеуге қалай қарайсыз,нигг қоқыс мылқа бөліг басқа адам сурет мазақ етпес бұрын өз сурет жүкте қалай қара,racism

1,үлкен доғал бесінші нүкте сік нигга,үлкен доғал бесінші нүкте сік нигг,racism

1,бұл мексикалық балапан мені және менің достарымды нигга деп атады ол да түрлі түсті болса да нәсілшілдік оның барлық даңқында,бұл мексикалық балапан мен және менің дос нигг де ат ол да түрлі түсті бол да нәсілш оның барлық даңқ,racism

1,сіз бәрін жасадыңыз бірақ rt менің есімде ниггерлер өте ақымақ барлығы дрю екеуміз ұқсаспыз деп ант бергенде ақымақ бейбақтар,сіз бәрін жаса бірақ rt менің ес ниггер өте ақы барлығ дрю екеу ұқсас де ант бергенде ақы бейбақ,racism

1,жарайды сондықтан ақ қонақ сізді негр деп атаса мен сізден күлімсіреуді күтемін карен қарапайым сөз болған кезде есімде бірақ мұнда ақ адамдар жылады сіз шынымен де саңырау мылқаусыз ба немесе жай ғана тауықты қорғайсыз ба,жарайды сондықтан ақ қонақ сіз нег де ата мен сізден күлімсіреу күт карен қарапайым сөз бол кезде ес бірақ мұнда ақ адам жыл сіз шын де саңырау мылқау ба немесе жай ғана тауық қор ба,racism

1,сондықтан ол ашуланды лол smh rt обаманы желкекке мылқау нигга бұл психика,сондықтан ол ашула лол smh rt обама желкек мылқа нигг бұл психик,racism

1,күте тұрыңыз сіз бірінші жасаған адамсыз ба құдай,күт тұр сіз бірінші жаса адам ба құдай,racism

1,шындығында жоқ трамп соңғы жылда қара жұмыссыздық деңгейін рекордтық деңгейге дейін төмендетті ал соңғы жылда бірде бір демократ президент олар үшін темір тордың артына көбірек отырғызатын қылмыс туралы заң қабылдауға тырысудан басқа ештеңе істемеді,шындығ жоқ трам соңғы жыл қара жұмыссыздық деңгей рекордтық деңгей дейін төменде ал соңғы жыл бірде бір демократ президент олар үшін темір тор арт көбірек отырғыз қылмыс туралы заң қабылда тырысу басқа ештеңе істеме,racism

1,twotimes rt обамаға қарғыс атсын мылқау нигга оның сөздерін өлтір,twotimes rt обама қарғыс ат мылқа нигг оның сөз өлтір,racism

1,уэйн есімді мылқау ұсқынсыз нигга уэйн есімді мылқау ұсқынсыз нигга позитивті pic twitter com,уэйн ес мылқа ұсқын нигг уэйн ес мылқа ұсқын нигг позитивті pic twitter com,racism

1,мен сені сезінемін бірақ бізге келетін болсақ түрлі түсті адамдар мені ренжіткен кезде мен қысымшылықты нәсілшілдікті сезінемін есептелген және жіктелген материал кінәлі,мен сен сез бірақ бізге кел бол түрлі түсті адам мен ренжіт кезде мен қысымшылық нәсілшілдік сез есептел және жіктел материал кінәлі,racism

1,бауырым ақымақ сияқты әрекет етуді доғар сіз жаңалықтар жүргізушісі әдейі ниггерлер деп айтпағанын білесіз сіз тек өзіңізден гөрі көп нәрсе жасағыңыз келеді,бауыр ақы сияқты әрекет ету доғар сіз жаңалық жүргізуші әдей ниггер де айтпа біл сіз тек өз гөрі көп нәрсе жаса кел,racism

1,мен қазір тым ыстықпын менің ағам одан да көп нигга бола алар ма еді мысалы алыс жерге барыңыз ақымақ ақымақ,мен қазір тым ыстық менің аға одан да көп нигг бола ал ма ед мысал алыс же бар ақы ақы,racism

1,барды желкекке обама мылқау нигга lt сонымен сіз ренжідіңіз бе,бар желкек оба мылқа нигг lt сонымен сіз ренжі бе,racism

1,желкек мылқау нигга,желкек мылқа нигг,racism

1,ниггерлер күл бұл ақымақ түйіндермен қиналуды доғар не сіздің нәсілшілдік пікіріңіз сіз еркінсіз,ниггер күл бұл ақы түйін қиналу доғар не сіздің нәсілш пікір сіз еркін,racism

1,майкл клиффорд сіз бардыңыз мылқау нигга мен сенің артымнан келе жатқаныңды білемін,майкл клиффорд сіз бар мылқа нигг мен сенің арт келе жат біл,racism

1,мылқау ниггерлер олар трахаются менімен өйткені олар жұлып алды,мылқа ниггер олар трахаются менімен өйткені олар жұл алды,racism

1,фу бұл нигга мені нигга деп атады хахахаха фууу сен бардың ақымақ құл,фу бұл нигг мен нигг де ат хахахах фуу сен бар ақы құл,racism

1,u үшін өкінішті түрлі түсті соқыр патриотқа деген жеккөрушілік пен нәсілшілдікке толы,u үшін өкініш түрлі түсті соқыр патриот де жеккөрушілік пен нәсілшілдік толы,racism

1,ешкім ештеңе қоздырмайды біз мұны істеуіміз керек емес сіз қара адамдардың осал ақ нәсілділерге жасаған барлық шабуылдары мен қара нәсілділердің қара нәсілділерге жасаған зорлық зомбылығын көру үшін теледидар көруіңіз керек бірақ кейбір елдерде олар әлі де еркін өткенге ие өйткені олар нәсілшіл болып көрінгісі келмейді,ешкім ештеңе қоздырма біз мұны істе керек емес сіз қара адам осал ақ нәсілді жаса барлық шабуыл мен қара нәсілді қара нәсілді жаса зорлық зомбылығ көру үшін теледи көр керек бірақ кейбір ел олар әлі де еркін өт ие өйткені олар нәсілшіл бол көрін келме,racism

1,ниггерді жұмыстан шығарыңыз сіз мылқау бастарсыз,нигг жұмыс шығ сіз мылқа баста,racism

1,бұл мылқау симмондс ниггерді қарғыс атсын бұл жақсы емес әрқашан,бұл мылқа симмондс нигг қарғыс ат бұл жақсы емес әрқашан,racism

1,мылқау нигга сен бұрылдыңдар қош бол қош бол бала,мылқа нигг сен бұрыл қош бол қош бол бала,racism

1,мен әлеуметтік желілерде кейбір қара нәсілділердің нәсілшіл емес деп санайтын ақ адамдарды қалай кешіретінін және мадақтайтынын көремін жақсы полицейлер деп аталатындарды көрсетіңіз жақында бұл мойынсұнғыш негр жігіті үшін кісі өлтірушілер сотталуы керек біз өзгерісті көрмейінше кешіре алмаймыз,мен әлеуметтік желі кейбір қара нәсілді нәсілшіл емес де сана ақ адам қалай кешір және мадақта көр жақсы полицей де атал көрсе жақында бұл мойынсұнғыш нег жігіт үшін кісі өлтіруші сottал керек біз өзгер көрмейінш кешір алма,racism

1,маған ренжімеңіз өйткені сіз ақымақ ниггерсіз деп ойлаймын осы жерден шығыңыз желкек қарғысатқыр нигга сучью задницей,маған ренжіме өйткені сіз ақы нигг де ойла осы же шығ желкек қарғысатқы нигг сучью задница,racism

1,мылқау ұсқынсыз негр есімді уэйн позитивті қоқыс трахающий қызыл және ақ pic twitter com,мылқа ұсқын нег ес уэйн позитивті қоқыс трахающи қызыл және ақ pic twitter com,racism

1,сіз тым мейірімдісіз бірақ сіз қара адамдардың кесірінен бір бөлігін ұмытып кеттіңіз егер мен соңғы үш жылда бірдеңе білсем бұл тым көп американдықтар оны түсіне алмайды өтінемін мейірімділік танытпаңыз ашық болыңыз мұны әріппен жазыңыз,сіз тым мейірімді бірақ сіз қара адам кесір бір бөліг ұмыт кет егер мен соңғы үш жыл бірде біл бұл тым көп американдық оны түс алма өт мейірімділік танытпа ашық бол мұны әріп жаз,racism

1,егер мен мен трахаетсем де осы мылқау ниггерді босатыңыз,егер мен мен трахает де осы мылқа нигг боса,racism

1,gt раконның тамаша мысалы ақ нубия құдайы туралы түсініктеме әлі есіме түсе алмайды фу енот бұл туралы сөйлесейік,gt ракон тамаша мысал ақ нубия құдай туралы түсініктеме әлі ес түс алма фу енот бұл туралы сөйлес,racism

1,басқа сіз орынсыз сияқтысыз американдықтар түрлі түсті ақ артықшылықтар нәсілшілдерге киелі кітаптан сіз нәсілшілдік жеккөрушілік пен қорқыныш насихатына қатысқаныңыз үшін өлең керек,басқа сіз орынсыз сияқты американдық түрлі түсті ақ артықшылық нәсілшіл киелі кітап сіз нәсілш жеккөрушілік пен қорқыныш насихат қатыс үшін өлең керек,racism

1,сіз ақымақ ниггерсіз тарақансыз,сіз ақы нигг тара,racism

1,мылқау негритянский кесек дерма көт ебаная,мылқа негритянски ке дер көт ебаная,racism

1,сен не туралы айтып тұрсың бұл сен мылқау нигга,сен не туралы айт тұр бұл сен мылқа нигг,racism

1,дәл аударма сіз қара бас негр раконы,дәл аударма сіз қара бас нег рако,racism

1,иә мен шіркеуге барған кезде есімде мен ақ адамдарды ұрып соғып жатқан ақымақ ұсқынсыз хатиандық ақымақпын мен лас сасық нигга үйіндісімін pic twitter com,иә мен шіркеу бар кезде ес мен ақ адам ұр соғ жат ақы ұсқын хатиа ақы мен лас сасық нигг үйінді pic twitter com,racism

1,неғұрлым жиіркенішті нәрсе бұл жұптасуға тырысатын ниггерлерге немесе әдеттегі қара мәдениетке қарау бұл мүлдем қатысы жоқ,неғұрлым жиіркеніш нәрсе бұл жұптас тырыс ниггер немесе әдет қара мәдениет қарау бұл мүлдем қатыс жоқ,racism

1,қандай желкек мені ниггер деп атама сен ақымақ ақымақ қаншық көт,қандай желкек мен нигг де ата сен ақы ақы қаншық көт,racism

1,бұрыштық сөйлеу деп аталатын нәрсені табыңыз негіздері қаланған олардың негізі негр ақ адамға тең емес деген үлкен шындыққа негізделген құлдық жоғары нәсілге бағыну оның табиғи және қалыпты жағдайы,бұрыш сөйлеу де атал нәрсе таб негіз қалан олардың негіз нег ақ адам тең емес де үлкен шындық негіздел құлдық жоғары нәсіл бағыну оның табиғи және қалыпты жағдай,racism

1,бәрі жақсы мен бұл мәселеде толығымен сенің жағыңдамын бұл өте жиіркенішті және адамдар оны мүлдем жария етпеуі керек мұны басыңызда ұстаңыз және бұл туралы айтпаңыз біріншіден бұл ешкімге маңызды емес екіншіден бұл басқаларға зиян тигізуі немесе оларды ыңғайсыз жағдайға қалдыруы мүмкін сондықтан жай ғана тоқтатыңыз өтінемін,бәрі жақсы мен бұл мәселе толығ сенің жағ бұл өте жиіркеніш және адам оны мүлдем жария етпе керек мұны бас ұста және бұл туралы айтпа біріншi

бұл ешкім маңызды емес екінші бұл басқа зиян тигіз немесе ол ыңғайсыз жағдай қалдыр мүмкін сондықтан жай ғана тоқта өт,racism

1,мейвезерді ниггер деп атайтын адамдар өйткені ол ирландиялықтарды жеңді олар ешкімге қажет болмаған кезде мылқау бейбақтар мені сол елден шығарыңыз,мейвез нигг де ата адам өйткені ол ирландиялық же олар ешкім қажет болма кезде мылқа бейбақ мен сол ел шығ,racism

1,мен әрқашан олардың ойларын айқышқа шегелен деп елестетіп көріңізші деп айтамын саған табынамын деп айтатын адамдар саған шын мәнінде табынбайды бірақ олар маған табынады ол қара негрлерден кек алу үшін реинкарнацияланған американдық нага иса мәсіх деп аталады бұл ақ нұсқа,мен әрқашан олардың ой айқыш шегелен де елестет көріңізш де ай саған таб де айт адам саған шын мән табынба бірақ олар маған табын ол қара негр кек алу үшін реинкарнациялан американдық наг ис мәсіх де атал бұл ақ нұсқа,racism

1,соншалықты сүйкімді емес rt обамаға қарғыс атсын мылқау нигга,соншалықты сүйк емес rt обама қарғыс ат мылқа нигг,racism

1,барды сен желкекке ниггер қандай да бір ақымақтық үшін өзіңізді өлтіріңіз,бар сен желкек нигг қандай да бір ақымақтық үшін өз өлтір,racism

1,кейбір адамдар сіздің тәжірибеңізді төмендету үшін бәрін жасайды мен жасымда қара адаммен қарым қатынаста болдым және кенеттен маған соңғы жыл бойы қара еркектермен кездескеніме қарамастан қара еркектермен танысу тәжірибем туралы айтуға рұқсат етілмеді көңілді,кейбір адам сіздің тәжірибе төмендету үшін бәрін жаса мен жас қара адам қарым қатынас бол және кенет маған соңғы жыл бойы қара еркек кездес қарамастан қара еркек танысу тәжірибе туралы айт рұқсат етілме көңіл,racism

1,сіз білесіз сәтсіздікке ұшыраңыз rt экипаж екеуміз ниггерді машинаны ұрламақ болған кезде ұстап алдық және оны розбанкте ұрдық ақымақ бейбақ,сіз біл сәтсіздік ұшыра rt экипаж екеу нигг машина ұрла бол кезде ұста ал және оны розбанк ұр ақы бей,racism

1,тозаққа солтүстік уэйн есімді мылқау ұсқынсыз нигга позитивті қоқыс жұмсақ есек нигга мен жұлдызмын мылқау нигга қамшыларыңды шеш ойыншығыңды шеш pic twitter com vtvbpbxpej,тозақ солтүстік уэйн ес мылқа ұсқын нигг позитивті қоқыс жұм есек нигг мен жұлдыз мылқа нигг қамшы шеш ойыншығ шеш pic twitter com vtvbpbxpej,racism

1,lol sad rt lol иә rt ол әлі де сіздің президентіңіз rt обамаға қарғыс атсын мылқау нигга,lol sad rt lol иә rt ол әлі де сіздің президент rt обама қарғыс ат мылқа нигг,racism

1,қара адамдар көпшілік алдында ақымақ болуды доғарыңыз сіз мылқау қара раконға ұқсайсыз олар біздің өзімізді қалай ұстағымыз келеді бұл ақымақ,қара адам көпшілік алд ақы болу доғар сіз мылқа қара ракон ұқса олар біздің өз қалай ұста кел бұл ақы,racism

1,rt ашер бейбер ретінде өмір сүреді сондықтан дж willow қа инвестиция салды ал дж гольфке инвестиция салды сондықтан бұл мылқау ниггерлер,rt аш бейб рет өмір сүр сондықтан дж willow қа инвестиция сал ал дж гольф инвестиция сал сондықтан бұл мылқа ниггер,racism

1,мен ақ адамдар қара нәсілділерге негр нәсілдік қорлау емес карен деп аталғанда қысымға ұшырайды деп қалай айта алатынын түсінбеймін,мен ақ адам қара нәсілді нег нәсіл қорла емес карен де атал қысым ұшыра де қалай ай ал түсінбе,racism

1,жақында ақ демократтың біреуді негр деп атағанына және бұл мәселе бойынша шақырылмағанына мысал келтіріңіз,жақында ақ демокра біреу нег де ата және бұл мәселе бойынша шақырылма мысал келтір,racism

1,менің сұрағыма жауап беріңіз мен сіздікіне жауап беремін сіз ақымақ қаншық лижающая жопу мүшесі көт майлы ебаного нигга өгей бала,менің сұраған жауап бер мен сіздік жауап бер сіз ақы қаншық лижающая жоп мүше көт майлы ебаного нигг өге бала,racism

1,ол бьелсаға қарағанда ағылшын тілінде нашар сөйлейді негр бұл қорлайтын сөз емес испан тілінде қара дегенді білдіреді егер қара жігіт қара деп аталатынына ренжісе бұл оған өкінішті мені ақ деп атағаныма ренжімес едім себебі мен ақмын мен нәсілшілдікті бір сәтке де ақтамасам да біз бірге тұрамыз,ол бьелса қара ағылшын тіл нашар сөйле нег бұл қорла сөз емес испан тіл қара де білдір егер қара жігіт қара де атал ренжі бұл оған өкініш мен ақ де ата ренжімес ед себебі мен ақ мен нәсілшілдік бір сәт де ақтама да біз бірге тұр,racism

1,трампты өлтіргісі келетін әлсіз суицидтік левактарға анаңды ұрып соғыңыз сонымен бірге сіздің ағыныңызда ниггерлер мен жидектер туралы айқайлайтын tds командасы бар ақымақ ақымақ,трам өлтір кел әлсіз суицид левак ана ұр соғ сонымен бірге сіздің ағын ниггер мен жидек туралы айқайла tds команда бар ақы ақы,racism

1,егер ppl осыдан алған жалғыз нәрсе мен түсті деп айтсам онда сіз шынымен де мәнін жіберіп алдыңыз мен қазір ашудың күшті екенін түсінемін және бұл әділ бірақ нәсілшіл емес адамнан нәсілшілдік іздемеңіз егер сіз ақ болсаңыз және маған барсаңыз сіз оны шынымен түсінбейсіз,егер ppl осы ал жалғыз нәрсе мен түсті де айт онда сіз шын де мән жібер ал мен қазір аш күшті екен түс және бұл әділ бірақ нәсілшіл емес адам нәсілш іздеме егер сіз ақ бол және маған бар сіз оны шын түсінбе,racism

1,менің ойымша super eagle дегі бұл ниггерлер ті мылқау бастарды бағып кесіп өтіп немесе атып тастайды деп ойламайды,менің ойымша super eagle дег бұл ниггер ті мылқа бас бағ кес өт немесе ат таста де ойлама,racism

1,нәсілшілдікті басуды тоқтатыңыз түрлі түсті адамдар алға ұмтылғысы келмейтін отставкаға кетіңіз не сіз оларды тең деп санамайсыз солай емес пе джо,нәсілшілдік басу тоқта түрлі түсті адам алға ұмтыл келме отставка кет не сіз ол тең де санама солай емес пе джо,racism

1,менің ойымша бұл маған қара әйел ретінде көбірек сәйкес келеді өйткені мен біреудің қара мазасы болдым маған терінің жиіркенішті түсі бар екенін айтты және кейінірек кешірім сұраған адамдар қауіп төндірді мұны нақты анықтай алмаған кезде кешіру оңайырақ кешіріңіз әрине бірақ мен сізге сене алмаймын,менің ойымша бұл маған қара әйел рет көбірек сәйкес кел өйткені мен біреу қара маза бол маған тер жиіркеніш түс бар екен ай және кейінірек кешір сұра адам қауіп төн мұны нақты анықта алма кезде кешіру оңайырақ кешір әрине бірақ мен сізге сен алма,racism

1,келесі күні мен анамның жанынан өтіп бара жатып ол мылқау ниггерді ұр дегенде оған бәрібір болған ең күлкілі нәрсе,келесі күн мен ана жан өт бар жат ол мылқа нигг ұр де оған бәрібір бол ең күлкіл нәрсе,racism

1,сіз мылқау нигга осы күндері жұмыс істейтін ойын жасай алмайсыз барлық мылқау бейбақтарды жұмыстан шығарыңыз және шынымен ойын ойнайтын адамдарды және шынымен жақсы адамдарды жалдаңыз балалар сендер өте ақымақсыңдар бұл шындыққа жанаспайды,сіз мылқа нигг осы күн жұмыс істе ойын жаса алма барлық мылқа бейбақ жұмыс шығ және шын ойын ойна адам және шын жақсы адам жалда бала сен өте ақы бұл шындық жанаспа,racism

1,ия сіз бәріңіз оны есегіңізден аласыз rt барды желкекке обама ақымақ бас нигга,ия сіз бәрі оны есег ал rt бар желкек оба ақы бас нигг,racism

1,міне сіз мылқау нигга болған кезде не болады бардыңыз сіз бәрін желкекке,міне сіз мылқа нигг бол кезде не бол бар сіз бәрін желкек,racism

1,сіз бұл мәлімдемеге байланысты ақымақсыз обаманы ұрыңыз мылқау нигга,сіз бұл мәлімдеме байланысты ақы обама ұр мылқа нигг,racism

1,сіз мемді жасаған адамға қалай айтасыз эм жоқ біз сіздің атыңызды мәңгілікке балшыққа батырамыз және сіз оны ойлап тапқаныңыз үшін жалғыз кінәлі боласыз,сіз ме жаса адам қалай ай эм жоқ біз сіздің ат мәңгілік балшық батыр және сіз оны ойла тап үшін жалғыз кінәлі бол,racism

1,менің арнама жазылыңыз өйткені сіз менің алғашқы wow lt серіктестерімнің бірі бола аласыз,менің арна жазыл өйткені сіз менің алғашқы wow lt серіктес бірі бола ал,racism

1,ол тіпті нигга сөзін қолданбаған сен ақымақ бейбақсың,ол тіпті нигг сөз қолданба сен ақы бей,racism

1,айырмашылық бір әріптен тұрады сондықтан сіздің атыңызды алғаш көргенде менің ойым мен әрпін жіберіп алғандықтан шығар лол,айырмашылық бір әріп тұр сондықтан сіздің ат алғаш көргенде менің ой мен әр жібер ал шығар лол,racism

1,италия мылқау ниггерлерден арылыңыз сіз оларға тайкришрина деп айтасыз,италия мылқа ниггер арыл сіз оларға тайкришрин де ай,racism

1,сонымен ші және ші суреттерде бұл жер филиппинде мальдив аралдары деп аталатын манжуйод құм өрімі деп аталады бұл жасырын асыл тас шығыс негроста орналасқан және оның шақырымдық ақ құмы мен мөлдір көгілдір суларының арқасында ерекше бағыт болды,сонымен ші және ші сурет бұл жер филиппин мальдив арал де атал манжуйод құм өрім де атал бұл жасырын асыл тас шығыс негрос орналас және оның шақырымдық ақ құм мен мөлдір көгілдір су арқа ерекше бағыт бол,racism

1,сіз роджер стоунның нөмірі кездейсоқ лотереяға түсіп кетті деп айттыңыз байыпты түрде бұл мәлімдеменің өзі ұят болды ақ адамдар ешқашан негр деп айтуға құқылы емес шын мәнінде америка тарихында қара адамдар ондаған жылдар бойы негр деп аталды,сіз родж стоун нөмір кездейсоқ лотерея түс кет де ай байыпты түр бұл мәлімдеме өзі ұят бол ақ адам ешқашан нег де айт құқылы емес шын мән америка тарих қара адам ондаған жыл бойы нег де ата,racism

1,том көптеген адамдар мұны жақтайтынын ескерсек нәсілшілдік мәселесі қарқын алуы үшін неге басқа елдерде оқиға қажет болды менің ойымша көптеген қара ойыншылар жолға секіргенде бұл көңіл көншітпейді неліктен олар ұзақ уақыт үнсіз қалды,то көптеген адам мұны жақта ескер нәсілш мәселе қарқын ал үшін неге басқа ел оқиға қажет бол менің ойымша көптеген қара ойыншы жол секір бұл көңіл көншітпе неліктен олар ұзақ уақыт үн қал,racism

1,неліктен обама конституция мен адам құқықтарын мазақ ету дұрыс деп санайды ол мылқау нигга тіпті американдық емес мен соғысқа дайынмын,неліктен оба конституция мен адам құқық мазақ ету дұрыс де сана ол мылқа нигг тіпті американдық емес мен соғыс дайын,racism

1,saturday night live ақ үйде президент трамп үшін дұға еткен қара консерваторларды үйдегі негрлер деп атады және нью йорк жұртшылығы күлді feature youtu be қайғылы қара өмір қара тарих,saturday night live ақ үй президент трам үшін дұ ет қара консерватор үйдегі негр де ат және нью йорк жұртшылығ күл feature youtu be қайғылы қара өмір қара тарих,racism

1,мылқау мылқау ұсқынсыз ниггерлер есімді уэйн және ұсқынсыз бесінші нүкте криса выебывающие ниггерлер менің киски ұсқынсыз тастамалар pic twitter com,мылқа мылқа ұсқын ниггер ес уэйн және ұсқын бесінші нүкте кри выебывающи ниггер менің киски ұсқын тастама pic twitter com,racism

1,лол сен ақымақсың ifiwaswhite бүгін мен қара адамдарды xbox live тегі ниггерлер деп атаймын,лол сен ақы ifiwaswhite бүгін мен қара адам xbox live тег ниггер де ата,racism

1,шайтан алғыр ол туралы айтады lol rt барды желкекке обама мылқау нигга мен тозақ сияқты ашуланамын сен ақымақ қаншық,шай алғы ол туралы айт lol rt бар желкек оба мылқа нигг мен тозақ сияқты ашулан сен ақы қаншық,racism

1,ағам мені мылқау ниггер деп атады мен оның лмфао екенін білемін,аға мен мылқа нигг де ат мен оның лмфао екен біл,racism

1,иеміз иса ол бірінші суретте өте күлкілі көрінеді мен өңдеймін шоколадты сүт оның қолында мұнда ақ болғандықтан шоколадты сүт бар сияқты,ие ис ол бірінші сурет өте күлкіл көрін мен өңде шоколад сүт оның қол мұнда ақ бол шоколад сүт бар сияқты,racism

1,обамаға ұнамсыз пікіріңізді мойындаңыз ол мылқау нигер,обама ұна пікір мойында ол мылқа ниг,racism

1,бұл бәріне маңызды емес шайтан алғыр бізді нигга деп атамаңыз бізде есімдер бар ақымақ бейбақ,бұл бәрін маңызды емес шай алғы біз нигг де атама біз есім бар ақы бей,racism

1,мені ниггер деп атаған барлық мылқау төбешіктер сізге желкек,мен нигг де ата барлық мылқа төбешік сізге желкек,racism

1,қара адамдар әлі де шайтаннан көмек сұрайды хмао сіздердің барлығыңыз ниггерлер ақымақ бейбақтар мен де сенімен бірге желкекге барар едім сіз бәріңіз сондай ақымақсыз,қара адам әлі де шайтан көмек сұра хмао сіздердің барлығ ниггер ақы бейбақ мен де сенімен бірге желкек бар ед сіз бәрі сондай ақы,racism

1,менің досым қазір houseparty қолданбасын жек көреді оның айтуынша ол ақ жігіттер тобына қосылды және олар оған жиіркеніп қарап оны негр деп атады бұл нәсілшілдік деңгейі жылы таң қалдырады және оны сайтынан іздеу керек,менің дос қазір houseparty қолданба жек көр оның айт ол ақ жігіт тоб қос және олар оған жиіркен қара оны нег де ат бұл нәсілш деңгей жыл таң қалдыр және оны сайт іздеу керек,racism

1,обама сен мылқау ниггерсің тек жағдайды нашарлатасың imsorryjustpissed бұл нәсілшілдік джоселин,оба сен мылқа нигг тек жағдай нашарла imsorryjustpissed бұл нәсілш джоселин,racism

1,бұл нигга мылқау rt қандай сұмдық,бұл нигг мылқа rt қандай сұм,racism

1,сіз бардыңыз ақымақ әйел ол өз пікірін ұстануға еркін сіздің қалаңыздағы ниггерлер мұны көріп сізге анальды түрде шабуыл жасайды деп үміттенемін,сіз бар ақы әйел ол өз пікір ұстан еркін сіздің қалаңыз ниггер мұны көр сізге аналь түр шабуыл жаса де үміттен,racism

1,егер ақ адам мені негр деп атаса мен оны жойып жіберер едім,егер ақ адам мен нег де ата мен оны жой жібер ед,racism

1,э ақымақ бейбақтар ниггер ниггермен бірдей мағынаны білдіреді,э ақы бейбақ нигг ниггер бірде мағына білдір,racism

1,rt егер нигга мен туралы жаман сөз айтса мен оны ренжіткенімді біл,rt егер нигг мен туралы жаман сөз айт мен оны ренжіт біл,racism

1,ші күні жалғыз кешке қатысушы бақытты өзіңіздің кім екеніңіз үшін рахмет және экранда өзіңіздің жеке басыңызды көрсету мүмкіндігін ешқашан жіберіп алмаңыз шексіз сағаттық ойын сауық пен күлкі үшін рахмет мен сенсіз күндерімді қалай өткізетінімді білмеймін жақсы күн өткізіп alc ді бірінші рет көріңіз,ші күн жалғыз кеш қатысушы бақытты өз кім екен үшін рахмет және экран өз жеке бас көрсету мүмкіндіг ешқашан жібер алма шексіз сағa ойын сауық пен күлкі үшін рахмет мен сен күн қалай өткіз білме жақсы күн өткіз alc ді бірінші рет көр,racism

1,желкекке обама барды ақымақ нигга уф сен жындысың ба бауырым лол,желкек оба бар ақы нигг уф сен жынд ба бауыр лол,racism

1,мен мектепте шашыңды неге ағартып жатырсың деп айтудан қатты шаршадым нигга менің ойымша менде басқа түстердің бәрі бар ақымақ,мен мектеп шаш неге ағарт жатыр де айту қатты шарша нигг менің ойымша менде басқа түс бәрі бар ақы,racism

1,ақ либералдар негрлер қауымдастығының алдына қойған көптеген негр қуыршақ көшбасшылары оларды ақ либералдар мақұлдады және субсидиялады малкольм бүгінгі күнге дейін адал болып қала береді бұқаралық ақпарат құралдары мен демократ жақсы көретін кез келген қара көшбасшы туралы cbc сұрағына мұқият қараңыз,ақ либерал негр қауымдастығ алд қой көптеген нег қуыршақ көшбасшы ол ақ либерал мақұлда және субсидияла малколь бүгінгі күн дейін адал бол қала бер бұқаралық ақпарат құрал мен демократ жақсы көр кез кел қара көшбасшы туралы cbc сұрағ мұқият қара,racism

1,доктор кинг жұмыссыздық туралы егер сізде негрлер қауымдастығында жаппай жұмыссыздық болса бұл әлеуметтік проблема деп аталады егер сізде ақ қоғамдастықта жаппай жұмыссыздық болса бұл депрессия деп аталады ынталандыруды қажет ететін кейбір мәселелер бар және негізгі табыс оларды жеңе алмайды jg pge,доктор кинг жұмыссыздық туралы егер сіз негр қауымдастығ жаппай жұмыссыздық бол бұл әлеуметтік проблема де атал егер сіз ақ қоғамдастық жаппай жұмыссыздық бол бұл депрессия де атал ынталандыру қажет ет кейбір мәселе бар және негізгі табыс ол же алма jg pge,racism

1,бұл не болды гребаные сыйламайтын адамдар ант етемін мені қаншық деп атаудан ешкім құтыла алмайды мылқау нигга,бұл не бол гребаны сыйлама адам ант ет мен қаншық де атау ешкім құтыл алма мылқа нигг,racism

1,мен ізбасарларымды жоғалтуды жалғастырамын сіз тозаққа бардыңыз мылқау ниггерлер,мен ізбасар жоғалту жалғастыр сіз тозақ бар мылқа ниггер,racism

1,қара қауымдастық бізді өз кемшіліктерімізге мұқият қарауға мәжбүр ететін барлық нәрсеге қатты қуанады оның орнына біз бәріміз өз эмоцияларымызға назар аудармай сыни тұрғыдан ойлаудың орнына бас тартамыз,қара қауымдастық біз өз кемшілік мұқият қара мәжбүр ет барлық нәрсе қатты қуан оның орн біз бәрі өз эмоция назар аударма сыни тұрғы ойла орн бас тар,racism

1,мылқау мен өз нәсіліме нәсілшіл бола алмаймын мен сені үйдегі негр деп атаған себебім өйткені сен ақ қожайыныңды қорғадың және африкадағы ақш тарихын жақсы білесің сондықтан өз халқыңды қолдап африкадағы ақ езушілерге енді жоқ деп айт,мылқа мен өз нәсіл нәсілшіл бола алма мен сен үйдегі нег де ата себеб өйткені сен ақ қожайын қорға және африка ақш тарих жақсы біл сондықтан өз халқ қолда африка ақ езуші енді жоқ де айт,racism

1,хахаха rt rt барайық желкек сіздің өшірулеріңіз мылқау нигга rt сәлем менің ақ ізбасарларыма,хахах rt rt бар желкек сіздің өшіру мылқа нигг rt сәлем менің ақ ізбасар,racism

1,менің қара екенім бұл жерде ештеңе жоқ иә ол бұл да бірнеше жыл бұрын болғанын айтты және қазір ол қолынан келгеннің бәрін жасады ал сіз оны өткенге сүйреп апарасыз кем дегенде қазір ол бұл туралы бірдеңе жасайды ол мұның бәрін елемеуі мүмкін еді бірақ олай етпеді,менің қара екен бұл же ештеңе жоқ иә ол бұл да бірнеше жыл бұрын бол ай және қазір ол қол кел бәрін жас ал сіз оны өт сүйре апар кем де қазір ол бұл туралы бірде жаса ол мұның бәрін елеме мүмкін ед бірақ ола етпе,racism

1,сіз нағыз нәсілшілдікті көргіңіз келе ме басқа түсті елге барыңыз және оларға ақ адамдар қалай қарайтынын көріңіз,сіз нағыз нәсілшілдік көр келе ме басқа түсті ел бар және оларға ақ адам қалай қара көр,racism

1,қаншық сені ұр карен ауысу кабелін алып саған сабақ береді деп үміттенемін мылқау нигга,қаншық сен ұр карен ауысу кабел алып саған сабақ бер де үміттен мылқа нигг,racism

1,rt хаха хаха ақшаны қайтару чегін ұнататын осы ниггерлерді қараңыз ақымақ бейбақтар тіпті осы тт дан жасалған хаха,rt хах хах ақша қайтару чег ұнат осы ниггер қара ақы бейбақ тіпті осы тт дан жасал хах,racism

1,үндемеңіз желкекке мылқау нигга сіз арамшөп шегемін деп айтқан адамсыз,үндеме желкек мылқа нигг сіз арамшөп шег де айт адам,racism

1,обамаға қарғыс атсын ақымақ нигга бұл ұсқынсыз қаншық байсалды ма,обама қарғыс ат ақы нигг бұл ұсқын қаншық байсалды ма,racism

1,сіз уэйн есімді ақымақ ұсқынсыз ниггерсіз позитивті қоқыс жұмсақ есекті нигга менің сыйлықтарым ақымақ ұсқынсыз нигга қызыл және ақ сік pic twitter com,сіз уэйн ес ақы ұсқын нигг позитивті қоқыс жұм есек нигг менің сыйлық ақы ұсқын нигг қызыл және ақ сік pic twitter com,racism

1,біздің санамыз бірінші мен ант етемін,біздің сан бірінші мен ант ет,racism

1,жаңа ғана болғаннан кейін егер кем дегенде бір мылқау нигга маған айтқысы келсе үйсіздерге арналған баспанаға барыңыз мен шынымен бұраламын,жаңа ғана бол кейін егер кем де бір мылқа нигг маған айт кел үйсіз арнал баспана бар мен шын бұрал,racism

1,менің ойымша қара итті бұл сөзбен атау ші жылдары адамдар оның мағынасын шынымен түсінбей тұрып ақ сәнде болды ші жылдардан кейін бәрі таза надандық болды,менің ойымша қара ит бұл сөз атау ші жыл адам оның мағына шын түсінбе тұр ақ сән бол ші жыл кейін бәрі таза надан бол,racism

1,егер мен сені нигга деп атасам егер мен ашуланбасам немесе сені мылқау нигга деп атамасам оған ренжімеңіз,егер мен сен нигг де ата егер мен ашуланба немесе сен мылқа нигг де атама оған ренжіме,racism

1,қара емес адамдар қара адамдарды нигга деп атайтындықтан билігін жоғалтады және бұл жиіркенішті,қара емес адам қара адам нигг де атайтындық билiг жоғалт және бұл жиіркеніш,racism

1,бұл күндері ақымақ есектерден басқа нигга деп кім айтады ол нәсілшіл емес мылқау,бұл күн ақы есек басқа нигг де кім айт ол нәсілшіл емес мылқа,racism

1,леброնға мылқау ниггерге,леброн мылқа нигг,racism

1,мен жай ғана қара нәсілді адамның үйсіз аққа біраз ақша беруге тырысқанын көрдім үйсіз адам сен бардың ниггер деп жауап берді жиіркенішті,мен жай ғана қара нәсіл адам үй ақ біраз ақша бер тырыс көр үй адам сен бар нигг де жауап бер жиіркеніш,racism

1,желкекке обама мылқау нигга лол сен ашуланасың ба сен жындысың жынды болмаңыз,желкек оба мылқа нигг лол сен ашулан ба сен жынд жын болма,racism

1,трахайте ниггеров трахайте доғал қаншықтар,трахай ниггеров трахай доғал қаншық,racism

1,blackcollective family жазылымы үшін рахмет қара америкада negro unity defenders inc деп аталатын жаңа өмір салтын бастады біз жаңа мектептің көшбасшысымыз біздің кез келгеніміз мұны ақ ішкі терроризмнен өлмеу үшін заңды түрде жасай аламыз барлығы тұр,blackcollective family жазылым үшін рахмет қара америка negro unity defenders inc де атал жаңа өмір салт баста біз жаңа мектеп көшбасшы біздің кез кел мұны ақ ішкі терроризм өлме үшін заңды түр жаса ал барлығ тұр,racism

1,өткенге оралсақ менің ойымша қазір өмір сүріп жатқан әрбір адамның қара ақ және азиялықтардың ата бабаларының арасында құл саудагерлері болады біз қазір әлемде болып жатқан құлдықтан арылуға назар аударуымыз керек бұл туралы ештеңе істей алмайтын өткен емес,өт орал менің ойымша қазір өмір сүр жат әрбір адам қара ақ және азиялық ата баба ара құл саудагер бол біз қазір әлем бол жат құлдық арыл назар аудар керек бұл туралы ештеңе істе алма өткен емес,racism

1,мен бұған ур алу үшін дайын болдым деп ойлап жаздым мен бірінші рет көзімді жұмдым бірақ содан кейін барлық үмітімді жоғалттым,мен бұған ур алу үшін дайын бол де ойла жаз мен бірінші рет көз жұм бірақ содан кейін барлық үміт жоғал,racism

1,чувак сік бұл сучих ниггеров қалай сен істейсің мен лилтунечи мен надандық мен мылқау мен мылқау мен мылқау сияқты қуыршақ,чувак сік бұл сучих ниггеров қалай сен істе мен лилтунечи мен надан мен мылқа мен мылқа мен мылқа сияқты қуыршақ,racism

1,ir бірақ сіз менің нені клондап жатқанымды әлі түсінбейсіз бұл біздің жыл ішінде бірінші рет үздік дан тыс аяқтадық ші маусымға айналған бір маусым алдыңғы ден аспайды мен сіз сияқты болашақты болжай алмаймын бірақ бәрі кронмен біз алға жылжымаймыз,ir бірақ сіз менің не клонда жат әлі түсінбе бұл біздің жыл іш бірінші рет үздік дан тыс аяқта ші маусым айнал бір маусым алдыңғы ден аспа мен сіз сияқты болашақ болжа алма бірақ бәрі крон біз алға жылжыма,racism

1,бұл ақымақ ниггерлерді қарғыс атсын сіз қалай істейсіз мен ұшамын мен ақымақпын мен мылқау боламын мен stuges сияқты мылқау боламын уф,бұл ақы ниггер қарғыс ат сіз қалай істе мен ұш мен ақы мен мылқа бол мен stuges сияқты мылқа бол уф,racism

1,пышақ нигга сен мылқау эбоно сөйлейтін нигга көтсың пәк қыз баяу кыс деп айтуы керек,пышақ нигг сен мылқа эбоно сөйле нигг көт пәк қыз бая кыс де айт керек,racism

1,теа мен сені жақсы көремін өйткені сен менің сүйіктілерімнің бірісің және біз алғаш кездескеннен бері өзара қарым қатынастың огысың,те мен сен жақсы көр өйткені сен менің сүйікті бір және біз алғаш кездес бері өзара қарым қатына ог,racism

1,бірінші әсер хаха орыс чатындағы кездейсоқ адам сіздің лақап атыңыз менің басымда кішкентай бала адам жақындық ұпайлары маған сен ұнайсың ба досым ретінде сен менікісің досым саған ғашық болды ма иә сен де оны жариялауың керек idc бір кездейсоқ ой сен қарғысатқыр гей,бірінші әсер хах орыс чат кездейсоқ адам сіздің лақа ат менің бас кішкентай бала адам жақ ұпай маған сен ұна ба дос рет сен менік дос саған ғашық бол ма иә сен де оны жарияла керек idc бір кездейсоқ ой сен қарғысатқы ге,racism

1,ұят республикалық партияның көптеген комитет мүшелері шыққан аймаққа басқа біреу назар аударды ма біздің саясатымызға әсер ететін соқыр нәсілшілдіктің тағы бір жағдайы,ұят республикалық партия көптеген комитет мүше шық аймақ басқа біреу назар ау ма біздің саясат әсер ет соқыр нәсілшілдік тағы бір жағдай,racism

1,міне сіз қалай өлесіз рефлексияға арналған тағам қысқа rt сік обама доғал бас нигга,міне сіз қалай өл рефлексия арнал тағам қыс rt сік оба доғал бас нигг,racism

1,израильдің иерусалим қаласындағы қошқарда араб көлігімен жасалған тағы бір шабуыл обама әкімшілігі мұны кездейсоқ жол ережесін бұзу деп атайды ма,израиль иерусалим қала қошқар араб көліг жасал тағы бір шабуыл оба әкімшіліг мұны кездейсоқ жол ереже бұзу де ата ма,racism

1,мен үнемі ақымақтық жасаймын сен ақымақ нигга сіз ақымақ алаяқсыз ақымақ бейбақсыз,мен үнемі ақымақтық жаса сен ақы нигг сіз ақы алаяқ ақы бей,racism

1,егер мен халлиге ұқсасам ше бұлтартпас дәлел болудың орнына жайлы аю гомо сексуалдық трюк кішкентай дик ким маттерспен жасалған трюк формадағы ақымақ ақымақ сияқты көрінеді ақымақ арам шөпті жейтін сайқымазақтар міне сіз кімсіз негр,егер мен халли ұқса ше бұлтартпас дәлел бол орн жайлы аю гомо сексуа трюк кішкентай дик ким маттерс жасал трюк форма ақы ақы сияқты көрін ақы ара шөп же сайқымазақ міне сіз кім нег,racism

1,ақш та ғана емес бүкіл әлемде кедейлер мен түрлі түсті адамдарды адамгершіліктен шығаратын институционалдық нәсілшілдіктің жарқын мысалы,ақш та ғана емес бүкіл әлем кедей мен түрлі түсті адам адамгершілік шығар институционалдық нәсілшілдік жарқын мысал,racism

1,ку клукс клан өте ақымақ фергюсон худсофф сен бардың ниггер lt мылқау,ку клукс клан өте ақы фергюсон худсофф сен бар нигг lt мылқа,racism

1,адамдардың кешіріңіз мен бәріңізді ренжіткенім үшін кінәлімін дегеннен басқа ештеңе айтқанын есту өте шаршатады көптеген адамдар black live matter ді қолдайды дейді бірақ олардың бұрынғы әрекеттерін немесе үйренген нәсілшілдіктерін шынымен мойындау адам ретінде өсудің ең жақсы тәсілі,адам кешір мен бәрі ренжіт үшін кінәлі де басқа ештеңе айт ест өте шаршат көптеген адам black live matter ді қолда де бірақ олардың бұрынғы әрекет немесе үйрен нәсілшілдік шын мойындау адам рет өсу ең жақсы тәсіл,racism

1,жоқ чикагода детриотта филадельфияда балтиморда бір біріңізді атып жатқанда ақ адамды кінәлауды жалғастырыңыз демократтар басқаратын барлық қалаларда ешкім жыпылықтамайды нью йоркте туылғаннан гөрі қара сәбилердің түсік жасатуы туралы не деуге болады жоспарланған ата ана маргарет сангердің негр жобасы деп аталды,жоқ чикаго детриот филадельфия балтимо бір бір ат жатқанда ақ адам кінәлау жалғастыр демократ басқар барлық қала ешкім жыпылықтама нью йорк туыл гөрі қара сәби түсік жасат туралы не де бол жоспарлан ата ана маргарет санг нег жоба де ата,racism

1,мен splatoon сахнасында кездескен алғашқы адамдардың бірі және мен ешқашан ұмытпайтын алғашқы адамдардың бірі мен енді әрең сөйлесетінімізді білемін өйткені мен айналамда болмадым бірақ достарымызбен бірге өткізген барлық уақытты ешқашан ұмытпаймын тай менің досым болғаны үшін сізде күлкілі көрініс бар,мен splatoon сахна кездес алғашқы адам бірі және мен ешқашан ұмытпа алғашқы адам бірі мен енді әре сөйлес біл өйткені мен айнала болма бірақ дос бірге өткіз барлық уақыт ешқашан ұмытпа тай менің дос бол үшін сіз күлкіл көрініс бар,racism

1,сонымен қатар қытайлықтар қытайдың саясатын нәсілшілдіктен басқа ешнәрсе түрткі емес деп түсінуді одан әрі қалыптастыру үшін әрбір жеке оқиғаға зорлық зомбылық пен қорқытудан бастап ыңғайсыз тұжырымдалған бөлінбейтін ашуланшақтыққа дейін назар аударатын оянған солшыл орта тапқа сене алады,сонымен қатар қытайлық қытай саясат нәсілшілдік басқа ешнәрсе түрткі емес де түсіну одан әрі қалыптастыру үшін әрбір жеке оқиға зорлық зомбылық пен қорқыту

баста ыңғайсыз тұжырымдал бөлінбе ашуланшақтық дейін назар аудар оян солшыл орта тап сен ал,racism

1,нигга болуды доғарыңыз өзіңізді жақсы ұстаңыз мен сізді ұятқа қалдырмаймын сіз мені сезінесіз бе мен сенің қаншығыңды ұрғанша есегіңді жауып таста,нигг болу доғар өз жақсы ұста мен сіз ұят қалдырма сіз мен сез бе мен сенің қаншығ ұрғанш есег жау таста,racism

1,бұл мылқау нигга обамаға қарғыс атсын оны ұйықтатпа ақ бала қарғыс атқан аузыңды бақыла,бұл мылқа нигг обама қарғыс ат оны ұйықтатп ақ бала қарғыс ат ауз бақыла,racism

1,миссуриде бұл түкке тұрғысыз бейбақты атуға қандай да бір қатысы бар әрбір мылқау ниггердің басын атыңыз,миссури бұл түк тұрғы бейбақ ат қандай да бір қатыс бар әрбір мылқа нигг басын ат,racism

1,шөлдеу тұзақтарына түсуді доғарыңыз мылқау ниггерлер сіз бұл түйіндерді газбен уландырасыз және бәрін қиындатасыз оларды сігу,шөлде тұзақ түсу доғар мылқа ниггер сіз бұл түйін газ уландыр және бәрін қиында ол сіг,racism

1,нигга күні құтты болсын мылқау бейбақтар lmfao,нигг күн құт бол мылқа бейбақ lmfao,racism

1,обамаға қарғыс атсын мылқау нигга мен билли хиллден шыққан бұл қаншық бұған сенбегеніне сене алмаймын барлығы оның есегін осы жерден шығарыңыз өтінемін,обама қарғыс ат мылқа нигг мен билли хилл шық бұл қаншық бұған сенбе сен алма барлығ оның есег осы же шығ өт,racism

1,менің ойымша бірақ олар тек ақ адам үшін күші мен мағынасын жоғалтты қара адамдардың есімдері бар және біз негр деп аталғанды ұнатпаймыз мен сізге бәрібір екенін білемін бірақ бұл сізге қарсы ештеңе болмағандықтан ғана бірақ жарайды,менің ойымша бірақ олар тек ақ адам үшін күш мен мағына жоғал қара адам есім бар және біз нег де атал ұнатпа мен сізге бәрібір екен біл бірақ бұл сізге қарсы ештеңе болма ғана бірақ жарайды,racism

1,ол жоқ па сонда менің ұлым неге біріккен негр колледж қорына іріктеуден өтпеді себебі ол ақ бұл нәсілшілдік егер united wasp college қоры болса оларды нәсілшілдер деп атаған болар еді,ол жоқ па сонда менің ұл неге біріккен нег колледж қор іріктеу өтпе себебі ол ақ бұл нәсілш егер united wasp college қор бол ол нәсілшіл де ата бол ед,racism

1,сіздің ақымақ ісіңіз емес қара нигга блэки блэки блэки сіз жексұрын нәсілшіл мылжыңсыз ұят,сіздің ақы іс емес қара нигг блэки блэки блэки сіз жексұр нәсілшіл мылжың ұят,racism

1,сіз ақымақ бейбақсыз ақымақ нигга,сіз ақы бей ақы нигг,racism

1,мен осы ақ адамдардың жазбаларын көргенді жек көремін сонымен соңғы екі айда мен қара дауыстарды естуге орын беру үшін ештеңе жарияламадым олар бөлмеден шықпас бұрын құбыжықтардың кетуін күткен сияқты,мен осы ақ адам жазба көр жек көр сонымен соңғы екі айда мен қара дауыс ест орын беру үшін ештеңе жариялама олар бөлме шықпас бұрын құбыжық кет күт сияқты,racism

1,бұл оның ингредиенттерімен байланысты емес ол нигга сөзін айтуға құқылы деп санайды джеки айнаны горилламен салыстырды және терісін ағарту үшін қара әйелге қышқыл шашу керек деді ол жиіркенішті адам жасырғыш әпке егер біреу зардап шегуі керек болса бұл ол,бұл оның ингредиент байланысты емес ол нигг сөз айт құқылы де сана джеки айна горилла салыс және тері ағарт үшін қара әйел қышқыл шаш керек де ол жиіркеніш адам жасырғыш әп егер біреу зардап шег керек бол бұл ол,racism

1,ол бүгін кешке ойнай алмайды ол ашкөз болғандықтан емес ол ақымақ пайдасыз қара көзді бейбақ болғандықтан,ол бүгін кеш ойна алма ол ашкөз бол емес ол ақы пай қара көз бей бол,racism

1,өткенге сүйене отырып полиция қызметкері қара адамды қалай өлтіреді ал оның үш серіктесі отырады және бақылайды джордж флойд кішкентай бақытсыздық болмаса кез келген нәрсеге қол жеткізе алар еді,өт сүйен отыр полиция қызметк қара адам қалай өлтір ал оның үш серіктес отыр және бақыла джордж флойд кішкентай бақытсыз болма кез кел нәрсе қол жеткіз ал ед,racism

1,жарайды бірақ ол берген барлық ақша ол үшін желкек емес ол қара қауымдастыққа көмектесу үшін бірдеңе жасаған кезде бұл оның соңғы айда істегенін қамтымайды,жарайды бірақ ол бер барлық ақша ол үшін желкек емес ол қара қауымдастық көмектес үшін бірде жаса кезде бұл оның соңғы айда істе қамтыма,racism

1,сіз тіпті твиттерде мың ретвит ала алмайсыз қалай мың аласыз мылқау нигга,сіз тіпті твиттер мың ретвит ал алма қалай мың ал мылқа нигг,racism

1,бұл қаншық rt обамаға қарғыс атсын мылқау нигга шмак шмак,бұл қаншық rt обама қарғыс ат мылқа нигг шмак шмак,racism

1,бірақ неге ашуланасың обамаға қарғыс атсын мылқау нигга,бірақ неге ашулан обама қарғыс ат мылқа нигг,racism

1,мұндағы барлығыңыздың басқа қара адамдарды ниггерлер деп атайтындығыңыз тіпті бір қызығы жиіркенішті джи,мұндағы барлығ басқа қара адам ниггер де атайтынды тіпті бір қызығ жиіркеніш джи,racism

1,мылқау нигга мен сенің итіңнің есектерін соның ішінде өзіңді де бір уақытта эйені ұрамын крис чилллллл,мылқа нигг мен сенің ит есек соның іш өз де бір уақыт эйе ұр крис чилллллл,racism

1,бұл сіз олар күткен нәрсені бірінші рет жасайсыз,бұл сіз олар күт нәрсе бірінші рет жаса,racism

1,чувак сік бұл сучих ниггерлер сіз қалай істейсіз мен лил тунечи мен репортер мен мылқаумын мен мылқаумын the stooges сияқты,чувак сік бұл сучих ниггер сіз қалай істе мен лил тунечи мен репор мен мылқау мен мылқау the stooges сияқты,racism

1,біріншіден екіншіден анамның үйіне айқайлаңыз мен сені барлық мөлшерде және формада жақсы көремін сіз ойлағаннан да қымбатсыз менің өмірімде болған ең көрнекті адам өзін өзі сүю бұл қиын сапар және оны ешқашан жалғыз жасаудың қажеті жоқ менің патшайымым,бірінші екінші ана үй айқайла мен сен барлық мөлшер және форма жақсы көр сіз ойла да қымбат менің өмір бол ең көрнекті адам өз өзі сүю бұл қиын сапар және оны ешқашан жалғыз жаса қажет жоқ менің патшайым,racism

1,сіздердің ешқайсыларыңыз құлдық кезінде тірі емес деп айтатындар сіздердің көпшілігіңіз джим кроу мен сегрегация кезінде және үкімет санкциялаған нәсілшілдік кемсітушіліктің басқа түрлерінде тірі болдыңыз бұл тек құлдықтың жалғасы болды мен жастамын және түрлі түсті тақтайшаларын есіме түсіре аламын бала кезімнен,сіздердің ешқайсы құлдық кез тірі емес де айт сіздердің көпшіліг джи кро мен сегрегация кез және үкімет санкцияла нәсілш кемсітушілік басқа түр тірі бол бұл тек құлдық жалғасы бол мен жас және түрлі түсті тақтайша ес түсір ал бала кез,racism

1,иә адам енді осы жерден кет желкекке мылқау нигга,иә адам енді осы же кет желкек мылқа нигг,racism

1,бірақ бұл нигга сіздің президентіңіз егер сіз ақылды болсаңыз обаманың қара rt екенін білесіз желкек обамаға барды мылқау нигга,бірақ бұл нигг сіздің президент егер сіз ақылды бол обама қара rt екен біл желкек обама бар мылқа нигг,racism

1,өткен маусымда біз плей оффтан бір жерде болдық біз бүкіл маусымды ешқандай қорғаныссыз өткіздік бізде демалысы болды және біз жыл бойы лауазымы жоқ адамдармен ойнадық біреу оле бірінші жылы моуриньодан гөрі лигада жақсы өнер көрсетті деп айтты тіпті моуриньо көп ұпай жинаса да,өткен маусым біз пле офф бір же бол біз бүкіл маусым ешқандай қорғаныс өт біз демалыс бол және біз жыл бойы лауазым жоқ адам ойна біреу ол бірінші жыл моуриньо гөрі лига жақсы өнер көрсе де ай тіпті моуриньо көп ұпай жина да,racism

1,қатты күлу мүмкін емес rt неге ашуланасың bbw rt обамаға қарғыс атсын мылқау нигга,қатты күл мүмкін емес rt неге ашулан bbw rt обама қарғыс ат мылқа нигг,racism

1,жаңа ғана нәсілшіл мылқау төбешіктердің af amp ге қатты қарғыс айтқанын естідім олар жанармай құю станциясында жұмыс істейтін қара адамдарды немесе қара мазақ қаншықты ұнатпайды дейді,жаңа ғана нәсілшіл мылқа төбешік af amp ге қатты қарғыс айт есті олар жанармай құю станция жұмыс істе қара адам немесе қара мазақ қаншық ұнатпа де,racism

1,ақымақ rt мылқау бейбақ мылқау smh нағыз ниггерлердің twitter аккаунттары жоқ,ақы rt мылқа бей мылқа smh нағыз ниггер twitter аккаунт жоқ,racism

1,сондықтан ақ адамдар сізді ниггерлер деп атайды сіз ақымақ бейбақсыз,сондықтан ақ адам сіз ниггер де ата сіз ақы бей,racism

1,о сен ақымақсың ба сік обама мылқау нигга,о сен ақы ба сік оба мылқа нигг,racism

1,колумбия округінің үкіметі мен колумбия округінің мэрі дұрыс айтады бұл үлкен мылқау ниггер менің жынды екенімді айтады өйткені мен кішкентай балаларды ренжітпеймін бұл менің келісімім мен жыныстық қанау үшін бала саудасы туралы дауласқанда ол қалай көрінеді,колумбия округ үкімет мен колумбия округ мэр дұрыс айт бұл үлкен мылқа нигг менің жын екен айт өйткені мен кішкентай бала ренжітпе бұл менің келісім мен жын қана үшін бала сауда туралы даулас ол қалай көрін,racism

1,сіз бардыңыз террелл саггс мылқау нигга,сіз бар террелл саггс мылқа нигг,racism

1,нәсілшілдік бұл ақ киімді қара емес бірінші кезекте жуу үшін таңдау нәсілшіл болмаңыз олардың барлығын бірге жуыңыз,нәсілш бұл ақ ки қара емес бірінші кезек жу үшін таңдау нәсілшіл болма олардың барлығ бірге жу,racism

1,бүгін жыл бұрын ақ адамдар америкадағы ең бай қара аймаққа басып кіріп оны қиратты тулса полициясы ресми түрде ұлттық гвардияға негр көтерілісі деп атаған нәрсені басуға көмектесу үшін жүгінді,бүгін жыл бұрын ақ адам америка ең бай қара аймақ бас кір оны қира тул полиция ресми түр ұлттық гвардия нег көтеріліс де ата нәрсе бас көмектес үшін жүг,racism

1,трахай тупоголовых ниггерлер мен де сендерді ниггерлер деп атаймын егер аяқ киім сәйкес келсе ақ адамдар да нигга болуы мүмкін мен бұл сөзге күмәнданамын,траха тупоголовых ниггер мен де сен ниггер де ата егер аяқ киім сәйкес кел ақ адам да нигг бол мүмкін мен бұл сөз күмәндан,racism

1,ертең мен оны осы сияқты ақымақ түйіндерге ысқылаймын gt обамаға қарғыс атсын мылқау нигга,ертең мен оны осы сияқты ақы түйін ысқыла gt обама қарғыс ат мылқа нигг,racism

1,опраның бірінші бастығы оған тым эмоционалды және теледидарға жарамсыз екенін айтты опра өзін өзі жасаған ең бай әйелдердің бірі және қара миллиардер әйел болып қала береді бұл аспанға сәтті ұмтылуды және ешқашан армандарыңыздан бас тартпауды білдіреді,опра бірінші бастығ оған тым эмоциона және теледи жарамсыз екен ай опр өз өзі жаса ең бай әйел бірі және қара миллиард әйел бол қала бер бұл аспан сәтті ұмтылу және ешқашан арман бас тартпау білдір,racism

1,сіз нәсілшілсіз және кешіріңіз мылқау ниггерлер мен тіпті нәсілшіл емеспін бірақ олар тозаққа барды,сіз нәсілшіл және кешір мылқа ниггер мен тіпті нәсілшіл емес бірақ олар тозақ бар,racism

1,желкек обамаға барды ақымақ бас нигга желкекге барыңыз сіздің анаңыз бен әжеңіз,желкек обама бар ақы бас нигг желкек бар сіздің ана бен әже,racism

1,rt rt мені қалайтын бірақ ала алмайтын әрбір ниггерді ұрыңыз түтіккен сорғыштар тырысуды жалғастыруда хаха,rt rt мен қала бірақ ал алма әрбір нигг ұр түтік сорғыш тырысу жалғастыр хах,racism

1,көрдіңіз бе біз crackkills трендіне ие осы ниггерлердің рухын тауып жоюымыз керек ақымақ ақымақтардан басқа ештеңе жоқ бұл күндері қара ниггерлер,көр бе біз crackkills тренд ие осы ниггер рух тау жою керек ақы ақымақ басқа ештеңе жоқ бұл күн қара ниггер,racism

1,қара әйелдер бұл ниггерді жынды етеді және бұл күлкілі өйткені ол сізді де негр арманыңызды да ұнатпайды сіздің кисканызды сулайтын жаңа ниггерді табыңыз нигга тіпті сүйкімді емес ол толық нашақорға ұқсайды өйткені ол солай сіз бәріңіз ақымақ тозақ сияқты ақымақсыз,қара әйел бұл нигг жын ет және бұл күлкіл өйткені ол сіз де нег арман да ұнатпа сіздің киска сула жаңа нигг таб нигг тіпті сүйк емес ол толық нашақо ұқса өйткені ол солай сіз бәрі ақы тозақ сияқты ақы,racism

1,о г бұл телефонға хабарлама жазуды доғар сен ақымақ ақымақ емессің сен ақымақ ниггерсің,о г бұл телефон хабарлама жазу доғар сен ақы ақы емес сен ақы нигг,racism

1,қыз мен сенен гөрі түстімін мен онша кешірімді емеспін егер сіз адамның нәсіліне негізделген кез келген нәрсені болжасаңыз онда бұл нәсілшілдік және алушының кім екендігі маңызды емес барлық ақ адамдар менмен тәкаппар отаршылдар нәсілшілдікке келетін болсақ сөздікте расимді оқы өтінемін,қыз мен сенен гөрі түсті мен онша кешір емес егер сіз адам нәсіл негіздел кез кел нәрсе болжа онда бұл нәсілш және алушы кім екендіг маңызды емес барлық ақ адам мен тәкапп отаршыл нәсілшілдік кел бол сөздік раси оқы өт,racism

1,бұл әңгіме емес әңгіме тағы бір мылқау ниггерді ұрыңыз,бұл әңгіме емес әңгіме тағы бір мылқа нигг ұр,racism

1,мен бұл мылқау нигга спорты екеніне келісемін,мен бұл мылқа нигг спорт екен келіс,racism

1,менің ойымша сіз ақымақсыз rt ciaa дегеніміз не түрлі түсті имбецилдердің спорттық бірлестіктері lmao нәсілшілдік,менің ойымша сіз ақы rt ciaa де не түрлі түсті имбецил спорттық бірлестік lmao нәсілш,racism

1,сіз нигга деп айтқыңыз келеді бірақ егер біреу бұралса мылқау нигга деп айтқыңыз келеді,сіз нигг де айт кел бірақ егер біреу бұрал мылқа нигг де айт кел,racism

1,қалай болғанда да мүше шешкеннен гөрі көп проблемалар тудырады сондықтан желкекке үндемеңіз ақымақ нигга,қалай болғанда да мүше шеш гөрі көп проблема тудыр сондықтан желкек үндеме ақы нигг,racism

1,обамаға қарғыс атсын мылқау нигга мен әдетте сен сияқты надандыққа батпаймын бірақ егер мен сенің жаныңда болсам мен сенен барлық боқтарды алып тастар едім,обама қарғыс ат мылқа нигг мен әдет сен сияқты надандық батпа бірақ егер мен сенің жан бол мен сенен барлық боқ алып тас ед,racism

1,сонымен сіз тек қара қылмыстар ашылмайды деп айтасыз өйткені қара адамдар полицияға сенбейтіндігімен танымал менің ойымша өткен демалыс күндері филадельфиядағы қара нәсілділерге қарсы барлық қылмыстарды ескере отырып полиция оны ашуға тырысатын ештеңе жасамауы керек өйткені қара адамдар оларды жек көреді,сонымен сіз тек қара қылмыс ашылма де ай өйткені қара адам полиция сенбейтіндіг танымал менің ойымша өткен демалыс күн филадельфия қара нәсілді қарсы барлық қылмыс ескер отыр полиция оны аш тырыс ештеңе жасама керек өйткені қара адам ол жек көр,racism

1,қара нәсілділер үшін көбірек жұмыс істейтін демократтардың республикалық штаттар ең аз білімді ал демократиялық штаттар ең білімді болған өткенге қатысы бар екенін айтпағанда,қара нәсілді үшін көбірек жұмыс істе демократ республикалық штат ең аз білімді ал демократиялық штат ең білімді бол өт қатыс бар екен айтпа,racism

1,олар рас жанкүйерлері емес тек қара нәсілділерге қарсы rt ниггерді мына жерден іздеңіз кейбір рас жанкүйерлері жиіркенішті,олар рас жанкүйер емес тек қара нәсілді қарсы rt нигг мына же ізде кейбір рас жанкүйер жиіркеніш,racism

1,ұсқынсыз есектің треш трейлері rt обамаға қарғыс атсын мылқау бас нигга,ұсқын есек треш трей rt обама қарғыс ат мылқа бас нигг,racism

1,хахахаха сен бардың мылқау нигга сіз біздің ақ нәсілділерді жек көруіміздің себебісіз деп назар аударушылар осылай болуы керек,хахахах сен бар мылқа нигг сіз біздің ақ нәсілді жек көру себеб де назар аударушы осылай бол керек,racism

1,нәсілшілдік сіз түрлі түсті киімді жуу үшін ақ киімді таңдайсыз оларды бірге жуып нәсілшілдікті тоқтатыңыз,нәсілш сіз түрлі түсті ки жу үшін ақ ки таңда ол бірге жу нәсілшілдік тоқта,racism

1,сағат ішінде екі түрлі ер адам менің қанша қарым қатынаста екенімді сұрады бұл ақпарат тіпті бір нәрсені қалай өзгертеді деп ойландым ба бұл туралы ойлана отырып адамдар бұл туралы бұрын да сұрағанын есіме түсіремін,сағат іш екі түрлі ер адам менің қанша қарым қатынас екен сұр бұл ақпарат тіпті бір нәрсе қалай өзгерт де ойла ба бұл туралы ойлан отыр адам бұл туралы бұрын да сұра ес түсір,racism

1,көшенің ортасында билеп жүрген мылқау нигга оны қағып кете жаздады шайтан алғыр бұл кішкентай бейбақ менің көлігіме соғылуы керек еді ақымақ бейбақ,көше орта биле жүр мылқа нигг оны қағ кет жазд шай алғы бұл кішкентай бей менің көліг соғыл керек ед ақы бей,racism

1,отсоси менің мүшесі доғал ақ қаншық обамаға қарғыс атсын мылқау нигга,отсоси менің мүше доғал ақ қаншық обама қарғыс ат мылқа нигг,racism

1,сіз соншалықты артта қалдыңыз сізге не болды менің айтайын дегенім қандай нигга мылқау,сіз соншалықты арт қал сізге не бол менің айт де қандай нигг мылқа,racism

1,жоқ сіз бұл өлі тақырыпқа бірінші жауап берген адамсыз,жоқ сіз бұл өлі тақырып бірінші жауап бер адам,racism

1,багера мейн кун қара мысық гатто гато чат,багер мейн кун қара мысық гатто гато чат,racism

1,нәсілшілдік бұл ақ киімді таңдап қара киімді киер алдында алдымен жуған кезде нәсілшіл болмаңыз барлық ні жуыңызгеда,нәсілш бұл ақ ки таңда қара ки ки алд алд жу кезде нәсілшіл болма барлық ні жуыңызге,racism

1,обамаға қарғыс атсын мылқау нигга ол тұзды аф,обама қарғыс ат мылқа нигг ол тұз аф,racism

1,сіз ақ немесе ақшыл терісі бар адамдарды жалдайтынын білгіңіз келеді өйткені қара түстер екі өлшемді көлеңкеде біріктіріледі түстердің нашар контрасты бұл нәсілшілдік емес өнер,сіз ақ немесе ақшыл тері бар адам жалда біл кел өйткені қара түс екі өлше көлеңке біріктір түс нашар контраст бұл нәсілш емес өнер,racism

1,бұлай емес мен сенің білетініңе күмәнданамын өйткені сен мақтаныш туын бірінші рет көресің мен сенімен мақтанамын гаури және саған сәлем жолдаймын,бұлай емес мен сенің біл күмәндан өйткені сен мақтаныш ту бірінші рет көр мен сенімен мақтан гаури және саған сәлем жолда,racism

1,ал өткен өткен егер сіз өтемақылар мен одан да көп тегін нәрселер менің қара достарымның көп тұратын қала маңын жоюмен бірге бір нәрсені өзгертеді деп ойламасаңыз бұл артта қалушылық,ал өткен өткен егер сіз өтемақы мен одан да көп тегін нәрсе менің қара дос көп тұр қала маң жою бірге бір нәрсе өзгерт де ойлама бұл арт қалушылық,racism

1,бүгін мен жаңа қара күзетшіні көрсетуге жақын болдым мылқау нигга мені өзімнен шығарды,бүгін мен жаңа қара күзетші көрсет жақын бол мылқа нигг мен өз шығ,racism

1,нигга бұл өте жағымсыз соншалықты жиіркенішті сөз оны тек қара адамдар ғана қолдана алады көпшілік sgp tcot tlot cspj,нигг бұл өте жағымсыз соншалықты жиіркеніш сөз оны тек қара адам ғана қолдан ал көпшілік sgp tcot tlot cspj,racism

1,қарғыс атсын сенің өмірің мылқау нигга соратын мүшесі,қарғыс ат сенің өмір мылқа нигг сор мүше,racism

1,ойынның түрлі түсті сілтемесі сізді алаңдатады тіпті сілтеме сапасы да емес түсті сілтемені енгізудің тоқтауға ешқандай қатысы жоқ so сіздің көзқарасыңыз нәсілшілдік туатын сол құрсақтан туады,ойын түрлі түсті сілте сіз алаңдат тіпті сілтеме сапа да емес түсті сілтеме енгізу тоқта ешқандай қатыс жоқ so сіздің көзқарас нәсілш ту сол құрсақ ту,racism

1,танымал емес пікір қара немесе ақ әзілмен немесе байыпты түрде нигга деп айту жиркенішті және нүкте,танымал емес пікір қара немесе ақ әзіл немесе байыпты түр нигг де айту жиркеніш және нүкте,racism

1,сіз бардыңыз ақымақ бас қаншық rt бардым желкек обама ақымақ бас нигга,сіз бар ақы бас қаншық rt бар желкек оба ақы бас нигг,racism

1,кейбір ниггерлер бұл күндері соншалықты ақымақ олар қандай желкек болуы керек,кейбір ниггер бұл күн соншалықты ақы олар қандай желкек бол керек,racism

1,уау мен айқайлай бастаймын өйткені сиқыр мені қорлай бастайды кенеттен мен сенің қандай да бір мылқау қара мылжыңнан жақсы емес екеніңді естимін сен ешқашан қара мылжың сияқты жабылмайсың уау жай уау,уау мен айқайла баста өйткені сиқы мен қорла баста кенет мен сенің қандай да бір мылқа қара мылж жақсы емес екен ести сен ешқашан қара мылж сияқты жабылма уау жай уау,racism

1,сіз нигга музыкасын тыңдайтын және салқын фагтарды жақсы көретін ақымақ ақымақсыз сіздің пікіріңіз маңызды емес желкекке үндемеңіз стандарттарды көтеріңіз,сіз нигг музыка тыңда және салқын фаг жақсы көр ақы ақы сіздің пікір маңызды емес желкек үндеме стандарт көтер,racism

1,кетмен сен жындысың ба егер сіз барсаңыз және мұны жасасаңыз біздің президенттің қара нәсілді екендігі рас па ол ромнидің есегінен гөрі ақылды обамаға қарғыс атсын мылқау нигга,кет сен жынд ба егер сіз бар және мұны жаса біздің президент қара нәсіл екендіг рас па ол ромни есег гөрі ақылды обама қарғыс ат мылқа нигг,racism

1,сондай ақ өте күлкілі ссать доғал стервозным хуем жирающим түтіккен көтке ешнәрсеге жарамсыз дегенераттар сіз сияқты олар сенен желкекке түсіп кетті өтірік боқ сіз ниггермен бірге dms спамын жібересіз мылқау сіздің roblox ережесінде нацистік жұмысыңыз бар,сондай ақ өте күлкіл ссать доғал стервозн хуе жирающи түтік көт ешнәрсе жарамсыз дегенерат сіз сияқты олар сенен желкек түс кет өтірік боқ сіз ниггер бірге dms спам жібер мылқа сіздің roblox ереже наци жұмыс бар,racism

1,рикке мылқау болуды доғар тозақ азаптау камерасы сондықтан сіз ниггерлер үшін өте суық болатыны анық ал ақ адамдар үшін бұл өте ыстық,рик мылқа болу доғар тозақ азапта камера сондықтан сіз ниггер үшін өте суық бол анық ал ақ адам үшін бұл өте ыстық,racism

1,жарайды rt обамаға қарғыс атсын мылқау нигга,жарайды rt обама қарғыс ат мылқа нигг,racism

1,елшілердің істері осы дүниенің бетінен құдай уәде еткендей амос ді қараңыз міне иеміздің құдайдың көздері құдайдың жоғарғы еврей қара терісі алтын негрі американдық және әлемдік шайтанның ақ шайтаны күнәкар патшалыққа христиан әлемі деп аталатын шайтан қарайды,елші іс осы дүние бет құдай уәде еткенде амос ді

қара міне ие құдай көз құдай жоғарғы еврей қара тері алтын нег американдық және әлемдік шайтан ақ шайта күнәк патшалық христиан әлем де атал шай қара,racism

1,тейлор крекерден прецельге дейінгі шкала бойынша сіз қаншалықты тұзсыз rt черту обаманы тупоголовый ниггер лмфааоооооооо,тейло крек прецель дейінгі шкал бойынша сіз қаншалықты тұз rt черт обама тупоголовы нигг лмфааоооооооо,racism

1,жай ғана көрсеткісі келеді сіз не білетініңізді білмейсіз том броковтың нәсілшілдігі тереңде жатыр бұл оның туылғаннан бергі ақ қанында қара түсті балалар броков мырзаның ақыл ойының кілті,жай ғана көрсет кел сіз не біл білме то броков нәсілшілдіг тереңде жатыр бұл оның туыл берг ақ қан қара түсті бала броков мырза ақыл ой кілт,racism

1,менің ойымша сіз менің алғашқы серіктестерімнің бірі болдыңыз және сіз ог,менің ойымша сіз менің алғашқы серіктес бірі бол және сіз ог,racism

1,мен марио лопес сені ешқашан сүймеймін ақымақ қаншық мен нағыз негритянку хлоя кардашян сұраймын сені сігу желіде иә,мен марио лопес сен ешқашан сүйме ақы қаншық мен нағыз негритянк хлоя кардашян сұра сен сіг желі иә,racism

1,сіз бардыңыз мылқау нигга шұңқырға отырыңыз,сіз бар мылқа нигг шұңқыр отыр,racism

1,ешкім мылқау нигга желкекке үндемеңіз,ешкім мылқа нигг желкек үндеме,racism

1,сіз мылқау бейбақтар дауыс беру үшін тіркелдіңіз деп үміттенемін егер сіз ниггерлер иллюминати басқарғыңыз келмесе,сіз мылқа бейбақ дауыс беру үшін тірке де үміттен егер сіз ниггер иллюминати басқар келме,racism

1,түсті сіз нәсілшілдікті көрмейтіндігіңіз таңқаларлық емес,түсті сіз нәсілшілдік көрмейтінді таңқаларлық емес,racism

1,сіз қара деп аталатын басқа адамдарды қатарда ұстайтын үйдегі негр сияқты сөйлейсіз негізінде сіз шеберді тыңдаңыз дейсіз ақ адамның белгілері,сіз қара де атал басқа адам қат ұста үйдегі нег сияқты сөйле негіз сіз шебер тыңда де ақ адам бел,racism

1,сіз қара нәсілді әйелді білетін шығарсыз ол өмірінде жақсы жұмыс істеп жатқан сияқты бірақ әлі де трамп пен оның жақтастарының еш қатысы жоқ өткен оқиғаларға шағымданғысы келеді бірақ демократтар жасады хиллари клинтонның тәлімгері кланның мүшесі болды роберт берд есіңізде ме,сіз қара нәсіл әйел біл шығ ол өмір жақсы жұмыс істе жат сияқты бірақ әлі де трам пен оның жақтас еш қатыс жоқ өткен оқиға шағымдан кел бірақ демократ жас хиллари клинтон тәлімгер клан мүше бол роберт берд ес ме,racism

1,сіз алғаш рет экшн фильмнің нағыз кейіпкерін бағалайсыз ал сіздің үндіс деп аталатын тони джаа тайгер мырза жасай алатын бірде бір трюкті орындай алмайды болливуд экшн фильмінің жалғыз нағыз кейіпкері видютжаммвал,сіз алғаш рет экшн

фильм нағыз кейіпкер бағала ал сіздің үндіс де атал тони джа тайг мырза жаса ал бірде бір трюк орында алма болливуд экшн фильм жалғыз нағыз кейіпкер видютжаммвал,racism

1,мен нәсілшілдік бар екеніне толық сенемін мұны жасамау надандық болар еді бірақ біреу сезімтал емес немесе дөрекі болғандықтан мен оны нәсілшіл деп атамас едім сыныпта қара нәсілділердің қылмыс деңгейі ең жоғары екенін мойындағаным үшін мені том ағай және үйдегі негр деп атады мен мұны ақ адам жасайтынын білемін,мен нәсілш бар екен толық сен мұны жасамау надан бол ед бірақ біреу сезімтал емес немесе дөрекі бол мен оны нәсілшіл де атамас ед сынып қара нәсілді қылмыс деңгей ең жоғары екен мойында үшін мен то аға және үйдегі нег де ат мен мұны ақ адам жаса біл,racism

1,сіз ниггерлер xbox және call of duty және басқа да ақымақ нәрселер туралы сөйлесе аласыз бірақ біз бейонседен ләззат ала алмаймыз ба осы жерден тозаққа шығыңыз,сіз ниггер xbox және call of duty және басқа да ақы нәрсе туралы сөйле ал бірақ біз бейонсе ләззат ал алма ба осы же тозақ шығ,racism

1,мен жаман нәрсе жасадым ба бұлыңғыр иә бірақ жоқ сонымен қатар шейн ешқашан жамандық жасаған емес өйткені біз үшін өткен жоқ өйткені біз өткенге ие бола алмаймыз және сіз маңызды нәрсеге назар аударуыңыз керек блэк ли менің жаңа макияж сызығым джеффристар шейн,мен жаман нәрсе жаса ба бұлыңғыр иә бірақ жоқ сонымен қатар шейн ешқашан жаман жаса емес өйткені біз үшін өткен жоқ өйткені біз өт ие бола алма және сіз маңызды нәрсе назар аудар керек блэк ли менің жаңа макияж сызығ джеффрис шейн,racism

1,israel forever блогы иерусалимдегі шабуыл жеті адам жарақат алды террорист атылды араб,israel forever блог иерусалим шабуыл жеті адам жарақат алды террорист ат араб,racism

1,гаитиге қарғыс атсын бұл мылқау ниггерлер оларға берілгеннің бәрін алады деп күтеді сондықтан олар өліммен аяқталады әдеттегі тегін жүктеушілер,гаити қарғыс ат бұл мылқа ниггер оларға беріл бәрін ал де күт сондықтан олар өл аяқтал әдет тегін жүктеуші,racism

1,сен дауласып жатырсың өйткені сенің кішкентай қаншығың жүр нигга өте қорқынышты өйткені сен мылқау зақымдануды жоғалтасың,сен даулас жатыр өйткені сенің кішкентай қаншы жүр нигг өте қорқыныш өйткені сен мылқа зақымдану жоғал,racism

1,ctfu so tru олар сіз айтқандай үндемеңіз мылқау нигга хаха олар сіздің айтқаныңызды айтқан кезде мен әлсіз боламын,ctfu so tru олар сіз айтқанда үндеме мылқа нигг хах олар сіздің айт айт кезде мен әлсіз бол,racism

1,оның қарапайым математикалық доғал қара ниггер көтеннен кетпен киска қаншық доғал шайтан алғыр көт,оның қарапайым математикалық доғал қара нигг көтен кет киск қаншық доғал шай алғы көт,racism

1,бүгін бұл сөзсіз қорлау жылдары ақ адамдар қара нәсілділерді негрлер деп атады мен өскен ші жылдардан бастап біз өзімізді қара деп атадық,бүгін бұл сөзсіз қорла жыл ақ адам қара нәсілді негр де ат мен өс ші жыл баста біз өз қара де ата,racism

1,себебі әйгілі ақ жігіт қара радио шоуда өнер көрсетіп оны негр деп атады,себебі әйгілі ақ жігіт қара радио шоу өнер көрсет оны нег де ат,racism

1,революцияны ақ негрлер мен одақтастар деп аталатын бишілер толығымен басып алды,революция ақ негр мен одақтас де атал биші толығ бас алды,racism

1,адамдар онда не туралы сөйлесетінін енді түсінбейді сіз ақымақ деп айтасыз хаха хаха бірақ бұл жақсы егер сіз бен біз бір бірімізді түсінбесек те мен мылқау ниггердің кесірінен ұрып соғуды тоқтатамын мен тіпті нәсілшілмін деп айтпаймын өйткені мен ақ пен қараны бірдей жақсы көремін,адам онда не туралы сөйлес енді түсінбе сіз ақы де ай хах хах бірақ бұл жақсы егер сіз бен біз бір бір түсінбе те мен мылқа нигг кесір ұр соғу тоқта мен тіпті нәсілшіл де айтпа өйткені мен ақ пен қара бірде жақсы көр,racism

1,обамаға қарғыс атсын мылқау нигга lt lt pic twitter com wuupser,обама қарғыс ат мылқа нигг lt lt pic twitter com wuupser,racism

1,ниггерге басқалар сияқты мұны айту үшін рұқсат қажет мылқау бейбақ егер сіз твитке шағымданатын болсаңыз оған жазылмаңыз,нигг басқа сияқты мұны айту үшін рұқсат қажет мылқа бей егер сіз твит шағымдан бол оған жазылма,racism

1,ақырында fir pea чакрабартиге қарсы жіберілді бұрын мен сіздің аккаунтыңызда бір хабарлама жарияладым вивек сэр jcb ки худайи каро аяқталды b әлі де қалады тек күте тұрыңыз және қылмыстық адвокаттың теорияның көп бөлігі күннің өзінен бастап анық болғанын көріңіз,ақырында fir pe чакрабарти қарсы жібер бұрын мен сіздің аккаунт бір хабарлама жариялa вивек сэ jcb ки худайи каро аяқта b әлі де қал тек күт тұр және қылмыстық адвокат теория көп бөліг күн өз баста анық бол көр,racism

1,флинттегі мылқау ниггерлер қарғыс атсын,флинт мылқа ниггер қарғыс ат,racism

1,сасық киска айына бір рет қан кететін жезөкше rt обамаға қарғыс атсын мылқау нигга лмааао,сасық киск ай бір рет қан кет жезөкше rt обама қарғыс ат мылқа нигг лмааао,racism

1,сен жындысың ба бауырым rt бұл мылқау нигга обамаға,сен жынд ба бауыр rt бұл мылқа нигг обама,racism

1,tumblr дегі бұл қыз қара адамдарды жек көретінін және оба бәрімізді құртқанын қалайтынын айтты ма ол айтты ниггерлер жүр көпірден құла ақымақ бейбақ,tumblr дег бұл қыз қара адам жек көр және оба бәрі құрт қала ай ма ол ай ниггер жүр көпір құла ақы бей,racism

1,freeonrii бірақ доктор дреге қарғыс атсын доғал долбоеб біз детоксты ешқашан естімейміз өйткені мылқау нигга балалары оған құлаққап сатып алады,freeonrii бірақ

доктор дре қарғыс ат доғал долбоеб біз деток ешқашан естіме өйткені мылқа нигг бала оған құлаққа сат ал,racism

1,егер сіз никиді бірінші орынға қойсаңыз сіздің пікіріңіз ешкімге маңызды емес,егер сіз ники бірінші орын қой сіздің пікір ешкім маңызды емес,racism

1,сәлем мен көріп отырған қара пантера бейнесіне не болды бұл рас па бәрі бұрын бейбіт болған сияқты,сәлем мен көр отыр қара пантер бейне не бол бұл рас па бәрі бұрын бейбіт бол сияқты,racism

1,мен де сені сағындым осы жерден кет ақымақ өтірік есек бейбақ ниггердің ақымақ есегі сен не істеп жатырсың деп ойлайсың күрсіну,мен де сен сағ осы же кет ақы өтірік есек бей нигг ақы есег сен не істе жатыр де ойла күрсін,racism

1,соңғы екі күнде айтқанымды blm ді еңіске түсіру әрекеті ретінде қалай түсіндіруге болады қалай болғанда да мен америка құрама штаттарының барлық қара азаматтарының өміріне маңызды өзгерістер енгізу үшін blm ді үлкейтуді күшейтуді және қол жетімділікті кеңейтуді жақтаймын,соңғы екі күн айт blm ді еңіс түсір әрекет рет қалай түсіндір бол қалай болғанда да мен америка құрама штат барлық қара азамат өмір маңызды өзгеріс енгізу үшін blm ді үлкейту күшейту және қол жетімділік кеңейту жақта,racism

1,стив харви өлім жазасына кесілуі керек ақымақ нигга айтпақшы мен қарамын сезімтал тозақ,стив харви өлім жаза кесіл керек ақы нигг айтпақшы мен қар сезімтал тозақ,racism

1,егер сіз мұны жасасаңыз мен оны бекітер едім құдай ау сіз atla twitter дегі алғашқы адамдардың бірісіз мен сізге уақытында болғаныңыз үшін алғыс айта аламын,егер сіз мұны жаса мен оны бекі ед құдай ау сіз atla twitter дег алғашқы адам бір мен сізге уақыт бол үшін алғыс ай ал,racism

1,мүмкін сіз peacefully assembly нені білдіретінін көре аласыз өйткені бұл сіздің ойлағаныңызды білдірмейді кез келген түрдегі зорлық зомбылық адамды бірінші түзетуде қарастырылған құқықтардан сондай ақ басқалардан айырады зорлық зомбылық шегінен өткеннен кейін сіз наразылық білдіруші емес қылмыскер боласыз,мүмкін сіз peacefully assembly не білдір көр ал өйткені бұл сіздің ойла білдірме кез кел түр зорлық зомбылық адам бірінші түзет қарастырыл құқық сондай ақ басқа айыр зорлық зомбылық шег өт кейін сіз наразылық білдіруші емес қылмыскер бол,racism

1,досым сен ниггер емессің өйткені егер сен жақын болсаң мен ақыры реанимацияға түсіп кетер едім ақымақ бейбақ,дос сен нигг емес өйткені егер сен жақын бол мен ақы реанимация түс кет ед ақы бей,racism

1,олардың мылқау нигга және командасын ұрыңыз,олардың мылқа нигг және команда ұр,racism

1,мұндай ақымақтық мені ашуландырады rt желкекке обама мылқау нигга қаншық біздің қара нигга мүшелерімізді сорыңыз,мұндай ақымақтық мен ашуландыр rt желкек оба мылқа нигг қаншық біздің қара нигг мүше сор,racism

1,сіз қандай желкек айтасыз мылқау нигга,сіз қандай желкек ай мылқа нигг,racism

1,ақ адамдар қара адамдарға мектепке бару колледжге түсу үйлену содан кейін балалы болу керек деп жылдар бойы дәріс оқыды мишель обама мұның бәрін жасады және оны әлі де нәрестенің анасы деп атады меганның қозғалатын мақсаттары мінсіз болуы мүмкін және олар оны әлі де жек көреді,ақ адам қара адам мектеп бару колледж түсу үйлен содан кейін балалы болу керек де жыл бойы дәріс оқы мишель оба мұның бәрін жас және оны әлі де нәресте ана де ат меган қозғал мақсат мін бол мүмкін және олар оны әлі де жек көр,racism

1,желкек мылқау нигга әзірге мен сені тәттіге байлап сезімсіз ұрып соққан жоқпын уау,желкек мылқа нигг әзірге мен сен тәтті байла сез ұр соқ жоқ уау,racism

1,өзіңізге қараңыз енді бізге қара менің барлық ниггерлерім ақымақ көрінеді,өз қара енді бізге қара менің барлық ниггер ақы көрін,racism

1,үндемеңіз желкекке қара маз шлюхи бұл күндері смх,үндеме желкек қара маз шлюхи бұл күн смх,racism

1,барлық жеккөрушілік сіз қалжыңдайсыз біздің түрлі түсті адамдармен салыстырғанда ақ адамдарда проблемалар аз сіздің өміріңізді бұрынғыдан да жақсырақ ету туралы алаңдамай нәсілшілдік пен терісіне байланысты қиындықтарға тап болатын қара ұлдар мен қыздардың болашағын құруға көмектесіңіз,барлық жеккөрушілік сіз қалжыңда біздің түрлі түсті адам салыстыр ақ адам проблема аз сіздің өмір бұрынғы да жақсырақ ету туралы алаңдама нәсілш пен тері байланысты қиындық тап бол қара ұл мен қыз болашағ құр көмектес,racism

1,обамаға қарғыс атсын мылқау нигга сіз кішкентай ақымақсыз,обама қарғыс ат мылқа нигг сіз кішкентай ақы,racism

1,біз сияқты ақымақпыз біз сізді ниггерлердің бірі ретінде жазудан бас тартамыз өзіңізге қараңыз енді бізге қараңыз менің барлық ниггерлерім ақымақ көрінеді,біз сияқты ақы біз сіз ниггер бірі рет жазу бас тар өз қара енді бізге қара менің барлық ниггер ақы көрін,racism

1,нигга надандық мылқау бейбақ дегенді білдіреді,нигг надан мылқа бей де білдір,racism

1,rt сіздің вик ұтылады нигга lt уэбб пен питерсон жеңеді ал жаттықтырушы қара ақымақ бейбақ,rt сіздің вик ұт нигг lt уэбб пен питерсон жең ал жаттықтырушы қара ақы бей,racism

1,мен бірден барамын желкекке пидорда сенен айырмашылығы жақсы адамдар бар мылқау нефф нигга есегіне әлем,мен бірден бар желкек пидо сенен айырмашылығ жақсы адам бар мылқа нефф нигг есег әлем,racism

1,мен сенің қазір ниггерді елемейтініңді көремін сіз бардыңыз мылқау нигга,мен сенің қазір нигг елеме көр сіз бар мылқа нигг,racism

1,егер сіз онымен келісе алмасаңыз онда сіз лайықты өмір сүре аласыз rt обамаға қарғыс атсын мылқау нигга,егер сіз онымен келі алма онда сіз лайықты өмір сүр ал rt обама қарғыс ат мылқа нигг,racism

1,деррис мені мылқау ниггер ретінде көрсетуге тырысып барлық қоқыстарды және басқаларды қате жазуға мәжбүр етті оның тозағына,деррис мен мылқа нигг рет көрсет тырыс барлық қоқыс және басқа қате жаз мәжбүр ет оның тозағ,racism

1,tg джорджи нигга сен ақымақ бейбақсың рахмет фу мен оның жалған есегінен контактілі линзаларды жұлып алмақ болдым,tg джорджи нигг сен ақы бей рахмет фу мен оның жалған есег контактіл линза жұл ал бол,racism

1,кап оған ақша жасағаннан басқа үстелге ештеңе әкелмеді оның өткеніне көз жүгіртіңіз ол екінші орынға ауысқанға дейін ол қара қауымдастық үшін бірдеңе жасады ма ол бір тонна ақша тапты олар қайда кетті мен ештеңе таппадым содан кейін ол қысқартылып sjw болады байыпты,ка оған ақша жаса басқа үстел ештеңе әкелме оның өт көз жүгір ол екінші орын ауыс дейін ол қара қауымдастық үшін бірде жас ма ол бір тонна ақша тап олар қайда кет мен ештеңе таппа содан кейін ол қысқартыл sjw бол байыпты,racism

1,иә олар тозақ сияқты ашуланды яо сен ашуланасың ба rt бұл мылқау нигга обамаға,иә олар тозақ сияқты ашула яо сен ашулан ба rt бұл мылқа нигг обама,racism

1,мен ска форумында қара емес қара көзді жек көру керек екендігі туралы шынымен жиіркенішті сөздерді оқыдым,мен ск форум қара емес қара көз жек көру керек екендіг туралы шын жиіркеніш сөз оқы,racism

1,дұрыс емес elise it бұл сөзі емес дегенмен америкада негр сөзі қандай да бір жолмен қорлайды бірақ бізді осылай атаған кезде ғана өздерін ағылшын тілінде ақ деп атайтын белые ал испан тілінде бізді қара деп атайды біз олардың тең қарама қайшылықтары емес екенімізді еске салу үшін жасалды,дұрыс емес elise it бұл сөз емес дегенмен америка нег сөз қандай да бір жол қорла бірақ біз осылай ата кезде ғана өз ағылшын тіл ақ де ата белы ал испан тіл біз қара де ата біз олардың тең қарама қайшылық емес екен ес салу үшін жаса,racism

1,бұл менің күшті соққым мен оны қолданған сайын физикалық ауырсыну пайда болады маған бір нәрсе керек бұл бас ауруын тоқтату үшін кез келген нәрсе ол кенеттен қара үшкір тас ұшып кеткен сәтте тізесіне құлады оның бетінен өтіп кете жаздады жарайды біз асығуымыз керек,бұл менің күшті соқ мен оны қолдан сайын физикалық ауырсыну пайда бол маған бір нәрсе керек бұл бас ауру тоқтату үшін кез

кел нәрсе ол кенет қара үшкі тас ұш кет сәт тізе құл оның бет өт кет жазд жарайды біз асығ керек,racism

1,иә өйткені туслоудың ақымақ есегі бізді инстадағы ниггерлер деп атағысы келеді олар мылқау аф,иә өйткені тусло ақы есег біз инста ниггер де ата кел олар мылқа аф,racism

1,сіз кез келген басқа ниггаға ұқсайсыз мылқау тітіркендіргіш және мылқау сияқты,сіз кез кел басқа нигга ұқса мылқа тітіркендіргіш және мылқа сияқты,racism

1,әңгімеге кірісіңіз бұрын ақ адамдардың жасаған барлық жаман істері кешірілмейді бұрын қара нәсілділер мен арабтардың жасаған жаман әрекеттері кешірімді енді сіз оның қалай жұмыс істейтінін көресіз бе,әңгіме кіріс бұрын ақ адам жаса барлық жаман іс кешірілме бұрын қара нәсілді мен араб жаса жаман әрекет кешір енді сіз оның қалай жұмыс істе көр бе,racism

1,байлық қаражат және автономия кейбіреулер мұны ақ адамдарға бағынбайтын жаңа негрлердің көтерілуі деп атады егер болса blacklivesmatter peace бейбіт наразылық егер сіз рос тің не үшін екенін шынымен түсінгіңіз келсе сіз біз наразылық білдіреміз осы мақаланы оқыңыз тарих,байлық қаражат және автономия кейбіреу мұны ақ адам бағынба жаңа негр көтеріл де ат егер бол blacklivesmatter peace бейбіт наразылық егер сіз рос ті не үшін екен шын түсін кел сіз біз наразылық білдір осы мақала оқ тарих,racism

1,анаңды ұрып соғып ақымақ rt обамаға қарғыс атсын мылқау нигга бұл мылқау bxxch туралы,ана ұр соғ ақы rt обама қарғыс ат мылқа нигг бұл мылқа bxxch туралы,racism

1,йоу райли купердің ниггерлер деп айтпағанын анық айтайық ол ниггерлер деді сондықтан оның командаластары да солай айтуы мүмкін деп айтатын барлық ақымақ бейбақтар үшін,йо райли куп ниггер де айтпа анық айт ол ниггер де сондықтан оның командалас да солай айт мүмкін де айт барлық ақы бейбақ үшін,racism

1,feelmeflowin иә қаншық маған британдық lil wayne look alike байқауының жеңімпазы емес қыздар ұнайды gtfo дан күңгірт қара мазалы түйін,feelmeflowin иә қаншық маған британдық lil wayne look alike байқау жеңімпаз емес қыз ұна gtfo дан күңгірт қара мазал түйін,racism

1,мен адамдар бетховеннің ерні қалың қара болып көрінетінін айтқанын білемін бірақ бұл нәсілшілдік ақ адамдарда қара триаттар болуы мүмкін ал замандастары оны неміске ұқсамайды деп айтудың орнына оны негр деп атайды,мен адам бетховен ер қалың қара бол көрін айт біл бірақ бұл нәсілш ақ адам қара триат бол мүмкін ал замандас оны неміс ұқсама де айт орн оны нег де ата,racism

1,шын мәнінде сіз мәселенің шиеленісуінен кейін осылай жұмыс істеп жатырсыз соңғы екі күнде жауап жоқ және сізге бірінші емес екеніңізді айтты біз myntra дан көптеген мәселелер аламыз,шын мән сіз мәселе шиеленісу кейін осылай жұмыс істе

жатыр соңғы екі күн жауап жоқ және сізге бірінші емес екен ай біз myntra дан көптеген мәселе ал,racism

1,сізге сөзбе сөз жауап бере отырып қазір сіз ренжіп көпір салу туралы сөйлесіп жатырсыз біріншіден адамдар туралы менсінбеңіз біз миллион үміткерді елестетеміз бе жоқ па өзіңіздікі игбо туралы менсінбеу үшін көптеген адамдардың ішінен дұрыс таңдау жасау экономика қаншалықты нашар болса да игбо адамы садақа сұрамайды,сізге сөзб сөз жауап бер отыр қазір сіз ренж көпір салу туралы сөйлес жатыр бірінші адам туралы менсінбе біз миллион үміткер елесте бе жоқ па өзіңіздік игбо туралы менсінбе үшін көптеген адам іш дұрыс таңдау жасау экономика қаншалықты нашар бол да игбо адам садақа сұрама,racism

1,гаитиліктер өте ұсқынсыз жиіркенішті қарғыс сияқты қара және жай ғана жиіркенішті біз барлық ниггерлерді өлтіруіміз керек,гаитилік өте ұсқын жиіркеніш қарғыс сияқты қара және жай ғана жиіркеніш біз барлық ниггер өлтір керек,racism

1,нигга емес сіздің пұтыңыз оның ақ болғанын қалайды вали осыдан желкекке мылқау ракон gt,нигг емес сіздің пұт оның ақ бол қала вали осы желкек мылқа ракон gt,racism

1,мен сені қарғыс атқан анаға нокаутқа жіберді деп естідім лол мылқау нигга фокустар балаларға арналған,мен сен қарғыс ат ана нокаут жіб де есті лол мылқа нигг фокус бала арнал,racism

1,хаха сен бардың обама сенің қаруыңды басқара алмаған сияқты мылқау нигга,хах сен бар оба сенің қару басқар алма сияқты мылқа нигг,racism

1,қарғысатқыр нигга тағы да жеңді lt қаншықтарды жопаға салыңыз олар мұндай мағынасыздықты айтады олар нәсілшіл біреу келіспейді ме,қарғысатқы нигг тағы да же lt қаншық жопа сал олар мұндай мағынасыздық айт олар нәсілшіл біреу келіспе ме,racism

1,ия киелі кітапта төменде келтірілген осы тармақ түсіндіріледі қара деп аталатындар негрлерден шыққан испандықтар және байырғы тұрғындар киелі кітаптағы еврейлер олардан эд ақ адам біздің жеке басымызды ұрлады исраилдіктер сіздің киелі кітапты оқиды және сіздің шынайы ұлтыңызды біледі құдай сізді сіз қолданатын сөздермен атаған жоқ,ия киелі кітап төменде келтір осы тармақ түсіндір қара де атал негр шық испандық және байырғы тұрғын киелі кітап еврей олардан эд ақ адам біздің жеке бас ұрл исраилдік сіздің киелі кітап оқи және сіздің шынайы ұлт біл құдай сіз сіз қолдан сөз ата жоқ,racism

1,уууууу бұл тыңдаушының хаты ниггерлер шынымен де өздерін елестете алмайды сенің қолыңнан келетіні мылқау болу және көліктің астына түсу ол барды,ууууу бұл тыңдаушы хат ниггер шын де өз елесте алма сенің қол кел мылқа болу және көлік аст түсу ол бар,racism

1,мен қай уақыттан бері ақымақпын сіз бардыңыз жидовский нигга радиоактивті жапондық фагтармен толтырылған есектегі сорғыш,мен қай уақыт бері ақы сіз бар жидовски нигг радиоактив жапондық фаг толтырыл есек сорғыш,racism

1,клан бензин мен ақшаны ысырап етеді тіпті үй жайларды жалға алады және тек ниггерлерді қалай жек көретіні туралы кездесу және сөйлесу үшін конгрестер өткізеді лол ақымақ бейбақтар,клан бензин мен ақша ысырап ет тіпті үй жай жалға ал және тек ниггер қалай жек көр туралы кездесу және сөйлесу үшін конгрес өткіз лол ақы бейбақ,racism

1,кеңістікті ысырап ету ақымақ мылқау серпіліс нигга есектегі мылқау диктор ұсқынсыз ебучи бала ақсақ есек,кеңістік ысырап ету ақы мылқа серпіліс нигг есек мылқа диктор ұсқын ебучи бала ақ есек,racism

1,мен кездейсоқ gsl ді ұнатамын содан кейін қандай да бір ақымақ сәнгер маған себепсіз тыйым салды бірақ gsl ниггеріне спам жіберетін жігітке тыйым салмайды дж,мен кездейсоқ gsl ді ұна содан кейін қандай да бір ақы сәнг маған себепсіз тыйым сал бірақ gsl нигг спам жібер жігіт тыйым салма дж,racism

1,сіз уэйн есімді ақымақ ұсқынсыз ниггерсіз позитивті қоқыс жұмсақ есегі бар нигга pic twitter com,сіз уэйн ес ақы ұсқын нигг позитивті қоқыс жұм есег бар нигг pic twitter com,racism

1,мен әдетте ниггерлермен айналыспаймын бірақ егер сіз менің колледжде оқуым үшін ақша төлесеңіз мен сізге сігу жасауға рұқсат етемін өйткені бұл сізге ақымақ болып көрінеді,мен әдет ниггер айналыспа бірақ егер сіз менің колледж оқ үшін ақша төле мен сізге сіг жаса рұқсат ет өйткені бұл сізге ақы бол көрін,racism

1,бұл олардың меншігі болды ма егер олар қатты алаңдаса олар полицияға қоңырау шалуы керек еді иә олар осылай жасады және оларға сол жерде болу және құқық қорғау органдарына оны шешуге мүмкіндік беру керектігі айтылды бірақ бұл ақ артықшылық күшіне енді біз бұл негрді ұстаймыз,бұл олардың меншіг бол ма егер олар қатты алаңда олар полиция қоңырау шал керек ед иә олар осылай жас және оларға сол же болу және құқық қорғау орган оны шеш мүмкіндік беру керектіг айт бірақ бұл ақ артықшылық күш енді біз бұл нег ұста,racism

1,құдай сені жарылқасын қызым rt барды желкекке обама ақымақ бас нигга,құдай сен жарылқа қыз rt бар желкек оба ақы бас нигг,racism

1,обамаға қарғыс атсын мылқау нигга lt ақ адамдарға қара мысық уильямстың дауысы,обама қарғыс ат мылқа нигг lt ақ адам қара мысық уильям дауыс,racism

1,адамдарды жақсартыңыз оларды жақсартыңыз айтпақшы сіздің бірінші суретіңіз керемет болды бірақ екіншісі бәрібір жақсы,адам жақсар ол жақсар айтпақшы сіздің бірінші сурет керемет бол бірақ екінші бәрібір жақсы,racism

1,лекси қарапайым мылқау лас жезөкше негр қынап сасық есек қаншық тозақ,лекси қарапайым мылқа лас жезөкше нег қына сасық есек қаншық тозақ,racism

1,кімге барды желкекке обама ақымақ бас нигга,кім бар желкек оба ақы бас нигг,racism

1,ақымақ бейбақ обаманың ұрық банкі туралы үндемеңіз менің әйелім сіздің дриблингіңізді тыңдайды ол фотосуретті жүктеді енді ол ниггерді округтегі ең ыстық нәрсе деп санайды және бұл туралы ұмытуға мүмкіндік бермейді кеше түнде ұйықтар алдында ол боға қайтадан дауыс бергісі келетінін айтты,ақы бей обама ұрық банк туралы үндеме менің әйел сіздің дриблинг тыңда ол фотосурет жүкте енді ол нигг округ ең ыстық нәрсе де сана және бұл туралы ұмыт мүмкіндік берме кеше түн ұйық алд ол бо қайта дауыс бер кел ай,racism

1,сіз үшін мен қара болғым келмейді сіз мылқау шайтан алғыр нигга rt лол мен бір көңілді ниггермін,сіз үшін мен қара бол келме сіз мылқа шай алғы нигг rt лол мен бір көңіл нигг,racism

1,обамаға қарғыс атсын мылқау нигга мен сен өлесің деп үміттенемін сондықтан сенің анаң сені ешқашан сүймеді,обама қарғыс ат мылқа нигг мен сен өл де үміттен сондықтан сенің ана сен ешқашан сүйм,racism

1,менің немере ағам үйленіп ақ қыздан бала туды жылдан кейін ол оны тастап қалған заттарын үйінен шығару үшін полицияға қоңырау шалып полицейлерге оның өмірі үшін қорқатынын айтты ол есеңгіреп қалды ол әлі күнге дейін оны үйдегі негр ақымақ сияқты қуып жүр,менің немере аға үйлен ақ қыз бала ту жыл кейін ол оны таста қалған зат үй шығару үшін полиция қоңырау шал полицей оның өмір үшін қорқ ай ол есеңгіре қал ол әлі күн дейін оны үйдегі нег ақы сияқты қу жүр,racism

1,өздерінің мылқау ниггерінен алыстап кетті,өз мылқа нигг алыста кет,racism

1,сізді орынсыз деп айыптайды сіз және сол мылқау нигга бардыңыз обама оның қайдан шыққанын кім біледі қалай болғанда да ол аралас нәсіл,сіз орынсыз де айыпта сіз және сол мылқа нигг бар оба оның қайдан шық кім біл қалай болғанда да ол аралас нәсіл,racism

1,бернард силваны қорғағаннан кейін басшы қазір үндемеу керек сіз ашық нәсілшілдік үшін сәттерді таңдай алмайсыз дәл қазір сіз ақымақ болып көрінесіз мен сені сильвамен болған оқиғаға дейін жауып тастадым,бернард силва қорға кейін басшы қазір үндеме керек сіз ашық нәсілш үшін сәт таңда алма дәл қазір сіз ақы бол көр мен сен сильва бол оқиға дейін жау таста,racism

1,сен ақымақ ниггерсің шайтан алғыр,сен ақы нигг шай алғы,racism

1,сіз ренжіген сияқтысыз обаманы ұрыңыз мылқау нигга,сіз ренжі сияқты обама ұр мылқа нигг,racism

1,бір велосипедті бақа мемі сіз ниггерлер тозақ сияқты ақымақсыз,бір велосипед бақ мем сіз ниггер тозақ сияқты ақы,racism

1,тұр шайтан алғыр рак сен шайтан алғыр артта қалған нигга сені ешкім жақсы көрмейді менің сөздерім ақымақ қаншық бөксеге ұқсайтын тоқта белгісі,тұр шай алғы рак сен шай алғы арт қалған нигг сен ешкім жақсы көрме менің сөз ақы қаншық бөксе ұқса тоқта бел,racism

1,сен ақымақ бейбақсың қазір бос сөздерді алып жүруді тоқтатыңыз сіз ниггер путин емессіз қазір барлық американдықтарға қарсы зорлық зомбылықты тоқтатыңыз немесе музыкаға бет бұрыңыз америкадағы ардагерлер мен әскерилер сіздің қорқақ есегіңізге қарсы бұл біздің балаларымыз бен отбасымыз сіздің жаман есегіңіз қазір тоқта,сен ақы бей қазір бос сөз алып жүру тоқта сіз нигг путин емес қазір барлық американдық қарсы зорлық зомбылық тоқта немесе музыка бет бұр америка ардагер мен әскери сіздің қорқақ есег қарсы бұл біздің бала бен от сіздің жаман есе қазір тоқта,racism

1,обамаға қарғыс атсын ақымақ бас ниггерруд,обама қарғыс ат ақы бас ниггерруд,racism

1,ақымақ қаншық ауру әтешті жұтып қойыңыз обамаға қарғыс атсын мылқау нигга,ақы қаншық ауру әтеш жұт қой обама қарғыс ат мылқа нигг,racism

1,сіз бәріңіз де сіз тіпті twitter дегі нәсілшілдік үшін айыпталғаныңызды қабылдай алмайсыз бірақ сізге кіші мартин лютер кинг қажет эй dickiev қара халықтарға келесі негр құтқарушысын іздеуді тоқтату және өз қауымдастығыңыздағы нәсілшілдікпен күресуді бастау үшін ақ халықтар қажет,сіз бәрі де сіз тіпті twitter дег нәсілш үшін айыптал қабылда алма бірақ сізге кіші мартин лю кинг қажет эй dickiev қара халық келесі нег құтқарушы іздеу тоқтату және өз қауымдастығыңыз нәсілшілдік күресу бастау үшін ақ халық қажет,racism

1,сіз өте баяу жүресіз мылқау нигга сіз желкеккеге бардыңыз мен сізді дистилляциялай аламын сонымен қатар сіз өзіңізге сенімді ниггерсіз және маймылға ұқсайсыз,сіз өте бая жүр мылқа нигг сіз желкекке бар мен сіз дистилляцияла ал сонымен қатар сіз өз сенімді нигг және маймыл ұқса,racism

1,обама қандай желкек қосылған ол өз жұмысын жасауы керек қандай ақымақ нигга,оба қандай желкек қосыл ол өз жұмыс жаса керек қандай ақы нигг,racism

1,адамдар егер сіздің анаңыз сізге түсік жасатса ше кәсіби өмірге қайта оралу ретінде біріншіден ештеңе жоқ өйткені мен өлдім және сен менің не істегенімді қалайсың менің бұршақ өлшеміндегі мен өз өлімімді жоқтауға рұқсат етесіз бе хек келесі өміріме көшіңіз бауырым бұл мен үшін емес еді,адам егер сіздің ана сізге түсік жасат ше кәсіби өмір қайта орал рет бірінші ештеңе жоқ өйткені мен өл және сен менің не істе қала менің бұршақ өлшем мен өз өлім жоқта рұқсат ет бе хек келесі өмір көш бауыр бұл мен үшін емес ед,racism

1,қаншық сен марио ағайындылардың гумбасына ұқсайсың сік обама мылқау нигга,қаншық сен марио ағайынды гумба ұқса сік оба мылқа нигг,racism

1,сен сондай ақымақ ниггерсің қарғысатқыр шөп кеттік желкекке сен және жаман бала а т,сен сондай ақы нигг қарғысатқы шөп кет желкек сен және жаман бала а т,racism

1,далластың жаңа қорғаушысы кім болғың келеді мен батыс вирджиниядан келген мылқау ниггерге ұқсаймын,далла жаңа қорғаушы кім бол кел мен батыс вирджиния кел мылқа нигг ұқса,racism

1,маған көптеген хип хоп рэп және б ойық іріктеу тапқырлық вокалдық стиль ұнайды бірақ неге сонша мылқау лирикадағы шайтан алғыр ақымақ киска боқ мф ниггерді үнемі пайдалану арқылы айқын қасиеттерді жояды бұл менің қорлайтыным емес тек жалқау және жалықтырады,маған көптеген хи хо рэ және б ойық іріктеу тапқырлық вока стиль ұна бірақ неге сонша мылқа лирика шай алғы ақы киск боқ мф нигг үнемі пайдалану арқылы айқын қасиет жоя бұл менің қорла емес тек жалқау және жалықтыр,racism

1,эй сен ақымақ бейбақсың ниггердің анықтамасы менсінбейтін төмен надан және б деп саналатын кез келген нәсілдің немесе тектің адамы,эй сен ақы бей нигг анықта менсінбе төмен надан және б де санал кез кел нәсіл немесе тек адам,racism

1,rt бауырым қара нигга адамдармен жыныстық қатынас тіпті smh суретте де өте жиіркенішті көрінеді,rt бауыр қара нигг адам жын қатынас тіпті smh сурет де өте жиіркеніш көрін,racism

1,бұл рас ғажайып негр деп аталатын кітап бар және онда ақ адамдар соғыстарда адамдарды жаулап алып өлтіргенде олар оларды жеп қойды деп сенгендіктен жейтіні айтылады шынымен ауырады,бұл рас ғажайып нег де атал кітап бар және онда ақ адам соғыс адам жаула алып өлтір олар ол же қой де сенгендік же айт шын ауыр,racism

1,сіз бұл дұрыс емес деп ойлайсыз біз түрлі түсті адамдар балаларымызға өте жас кезінде нәсілшілдік туралы айтуымыз керек олар терісінен қорқады деп емес сіз шынымен ақ адамдар осылай жасайды деп ойлайсыз ба жоқ олар әлемді өздеріне тиесілі деп ойлап оларды өсіреді,сіз бұл дұрыс емес де ойла біз түрлі түсті адам бала өте жас кез нәсілш туралы айт керек олар тері қорқ де емес сіз шын ақ адам осылай жаса де ойла ба жоқ олар әлем өз тиесілі де ойла ол өсір,racism

1,мен жұмыста болған кезде баба телефон арқылы құрметсіздік танытты ол мені мылқау ниггер деп атағанға дейін оған көмектесуге мүмкіндігім де болмады мен рөлден шыққым келді бірақ мен телефонды қойдым мұның бәрі тозаққа,мен жұмыс бол кезде баба телефон арқылы құрметсіз таны ол мен мылқа нигг де ата дейін оған көмектес мүмкіндіг де болма мен рөл шық кел бірақ мен телефон қой мұның бәрі тозақ,racism

1,менің бір досым өте қараңғы басына тақия киіп атлантадағы мейрамханаға өздерін бөлінбеген деп атағанға дейін тоқтады ол ақ мейрамханаға тоқтады отырды қызмет етті егер негр кірсе не болады деп сұрады,менің бір дос өте қараңғы бас тақия ки

атланта мейрамхана өз бөлінбе де ата дейін тоқта ол ақ мейрамхана тоқта от қызмет ет егер нег кір не бол де сұр,racism

1,сіз мені бірінші кезекте бұғаттаған адамсыз,сіз мен бірінші кезек бұғатта адам,racism

1,заткний на желкек доғал бас нигга,заткни на желкек доғал бас нигг,racism

1,сіз бірінші мәселе бойынша дұрыс айтасыз мен бұл үшін кешірім сұраймын бірақ канадалық бізді британдықтар деп атайтын болса бұл маңызды емес десе бұл ақымақтық біздің тығындар сіз бәріңіз негізінен американдықсыз біздің еліміздің тұрғындарының көпшілігі бірдей екпінге ие және ақш мәдениетіне көбірек енеді,сіз бірінші мәселе бойынша дұрыс ай мен бұл үшін кешір сұра бірақ канадалық біз британдық де ата бол бұл маңызды емес де бұл ақымақтық біздің тығын сіз бәрі негіз американдық біздің ел тұрғын көпшіліг бірде екпін ие және ақш мәдениет көбірек ен,racism

1,қайырлы таң жезөкшелер пездтер мүше шабандоздар нашақорлар әлсіздер ақымақ бейбақтар түйіндер ниггерлер және басқалар,қайырл таң жезөкше пезд мүше шабандоз нашақор әл ақы бейбақ түйін ниггер және басқа,racism

1,абонент сік мылқау нигга сіз ізбасарсыз өсек пен насихатқа назар аударыңыз содан кейін сізде аңызға шабуыл жасауға батылдық бар,абонент сік мылқа нигг сіз ізбасар өсек пен насихат назар ау содан кейін сіз аңыз шабуыл жаса бат бар,racism

1,сіз ақымақ деп айтасыз қытайдан шығыңыз ниггерлер эбониктер тек ағылшын тілінде сөйлеңіз,сіз ақы де ай қытай шығ ниггер эбоник тек ағылшын тіл сөйле,racism

1,иә иә мен бұл қаншықты ұрып жатырмын gt rt обамаға қарғыс атсын мылқау нигга,иә иә мен бұл қаншық ұр жатыр gt rt обама қарғыс ат мылқа нигг,racism

1,rt мен ниггерлердің менімен сөйлесуге тырысқанын жек көремін маған сіздің есектеріңіз қажет емес,rt мен ниггер менімен сөйлес тырыс жек көр маған сіздің есек қажет емес,racism

1,бейсбол ойнайтын барлық ақымақтар мылтық ату мектебіне баруы керек өйткені сіз мылқау ниггаға ұқсайсыз mj ең жақсы доктор джей сіз бардыңыз ниггерлер,бейсбол ойна барлық ақымақ мылтық ату мектеб бар керек өйткені сіз мылқа нигга ұқса mj ең жақсы доктор дже сіз бар ниггер,racism

1,маған командада осындай керемет ақ үгінді керек ашуланып мені ниггер деп атайтын адам сондықтан мен оның кішкентай ақымақ есегін жек көре аламын,маған команда осындай керемет ақ үг керек ашулан мен нигг де ата адам сондықтан мен оның кішкентай ақы есег жек көр ал,racism

1,сіз бүкіл әлемге қалай ие бола алатыныңызды түсінбеймін содан кейін қандай да бір ақымақтық жасап бәрін бүлдіріңіз ниггерлер,сіз бүкіл әлем қалай ие бола ал түсінбе содан кейін қандай да бір ақымақтық жаса бәрін бүлдір ниггер,racism

1,rt обамаға қарғыс атсын мылқау нигга сенің анаң әкеңнің оған кіруіне мүмкіндік бергені үшін мылқау сізге тұмсық беру керек еді,rt обама қарғыс ат мылқа нигг сенің ана әке оған кір мүмкіндік бер үшін мылқа сізге тұмсық беру керек ед,racism

1,желкекке обама мылқау бас ниггерруд сіз әзілқойсыз қараңызшы қаншық аузыңызды бақылаңыз,желкек оба мылқа бас ниггерруд сіз әзілқо қараңызш қаншық ауз бақыла,racism

1,мылқау ақымақ сияқты әрекет ететін жалғыз адам андре бірақ онымен тозақ үлкен губастый ниггер сраный жопу,мылқа ақы сияқты әрекет ет жалғыз адам андр бірақ онымен тозақ үлкен губасты нигг сраны жоп,racism

1,менің әңгімемде мен сіздердің барлығыңыз туралы айтатын боламын кішкентай балалар және сіз менің тарихымды бірінші болып көресіз,менің әңгіме мен сіздердің барлығ туралы айт бол кішкентай бала және сіз менің тарих бірінші бол көр,racism

1,тбх жалғасы маған фильм кітап ұнаған кезде әрқашан рахатымды бұзады әсіресе егер сіз бірінші бөлімнің басты кейіпкерін қолдайтын болсаңыз мысалы олар ешқандай алдаусыз қалыпты қарым қатынаста кедергілерге тап болуы мүмкін бе бұл басқа қажетсіз махаббат үшбұрышын жасау емес,тбх жалғасы маған фильм кітап ұна кезде әрқашан рахат бұз әсіресе егер сіз бірінші бөл басты кейіпкер қолда бол мысал олар ешқандай алдау қалыпты қарым қатынас кедергі тап бол мүмкін бе бұл басқа қажет махаббат үшбұрыш жасау емес,racism

1,сіз бардыңыз сіз ақымақ қара мазасыз қаншықсыз,сіз бар сіз ақы қара мазасыз қаншық,racism

1,сонымен қатар сіздің негізін қалаған бакли ақ үстемдікті мақтанышпен қолдап дамыған нәсіл қара нәсілділердің негрлердің артта қалуы деп атаған өркениетті сақтау үшін дауыс беруіне жол бермеуі керек деп мәлімдеді сіз өтірігіңізбен танымал боласыз радош мырза егер сіз жақын арада түзетпесеңіз тик ток,сонымен қатар сіздің негіз қала бакли ақ үстемдік мақтаныш қолда дамы нәсіл қара нәсілді негр арт қал де ата өркениет сақтау үшін дауыс бер жол берме керек де мәлімде сіз өтіріг танымал бол радош мырза егер сіз жақын ара түзетпе тик ток,racism

1,нәсілшілдер бұрмаланды сіздің сөз таңдауыңыздан кейін сіздің пікіріңіз жойылады топ доғал задницы негр ежелгі египеттің патша сөзі,нәсілшіл бұрмала сіздің сөз таңдау кейін сіздің пікір жой топ доғал задниц нег ежелгі египет патша сөз,racism

1,дениро сен жаңа ғана өлдің енді саған ешкімнің дела болмайды гомик емес мен сен үшін жаратқан осы напалмалық тозаққа құлап күйіп кет құдай және сенің оң қолыңмен салғанның бәрі мылқау нигга дейлин авраам коллинз роберт дениро мылқау нигга соңы,дениро сен жаңа ғана өл енді саған ешкі дел болма гомик емес мен сен үшін жаратқан осы напалмалық тозақ құла күй кет құдай және сенің оң қол сал бәрі мылқа нигг дейлин авраа коллинз роберт дениро мылқа нигг соңы,racism

1,өткен айда солшылдардың істеген істеріне қарағанда адам қайтыс болды негізінен қара балалар сіз оны шындық деп санаған нәрсені айтқаны үшін кінәлайсыз ба мені соттаған кезде сізге ешкім ештеңе айтқан жоқ,өткен айда солшыл істе іс қара адам қайтыс бол негіз қара бала сіз оны шындық де сана нәрсе айт үшін кінәла ба мен сотта кезде сізге ешкім ештеңе айт жоқ,racism

1,мен мақтаныш айына немесе гей жаргонына мән бермеймін өйткені менің сексуалдылығым менің қызығушылығымды немесе сөйлеу мәнерімді анықтамайды сонымен қатар сіз көшедегі кездейсоқ қара транс әйелге бірдеңе қарыздармын деп ойлайсыз өйткені оның бұрынғы белсенділер сияқты тері түсі гендерлік сәйкестігі бар жоқ,мен мақтаныш ай немесе ге жаргон мән берме өйткені менің сексуалдылы менің қызығушылығ немесе сөйлеу мән анықтама сонымен қатар сіз көше кездейсоқ қара транс әйел бірде қарызд де ойла өйткені оның бұрынғы белсенді сияқты тері түс гендерлік сәйкестіг бар жоқ,racism

1,ути неге нгик танда rt лол мен ақымақ ниггерді ұра алмаймын оның қаншалықты сүйкімді екені маңызды емес эй менің шырындарым ынталандырылады,ути неге нгик тан rt лол мен ақы нигг ұр алма оның қаншалықты сүйк екен маңызды емес эй менің шырын ынталандыр,racism

1,барлығы шығып дауыс беріңіз сонда біз бұл ниггерді қызметінен алып тастай аламыз қаншық сен тозақ сияқты ақымақсың,барлығ шығ дауыс бер сонда біз бұл нигг қызмет алып таста ал қаншық сен тозақ сияқты ақы,racism

1,бұл ақымақ ниггерлерді тозаққа түсіріңіз сіз қалай істеп жатырсыз мен тунецпін қиындық туғызамын ақымақ боламын үш гуни сияқты мылқау боламын,бұл ақы ниггер тозақ түсір сіз қалай істе жатыр мен тунец қиындық туғыз ақы бол үш гуни сияқты мылқа бол,racism

1,ақымақ бейбақтар анаңнан сорып ал қаншық өйткені сен бәрің әлсіздер тобысың ақымақ ұлдар оқыңыз ууууу мылқау ниггерлер,ақы бейбақ ана сор ал қаншық өйткені сен бәрің әл тоб ақы ұл оқ уууу мылқа ниггер,racism

1,rt барды желкекке барлық сіз кім емес әкелуге бізді rbar мылқау ниггерлер сіз ашуланасыз ба мисси,rt бар желкек барлық сіз кім емес әкел біз rbar мылқа ниггер сіз ашулан ба мисси,racism

1,шайтан алғыр егер қаншықтың сенен бұрын сенің ниггерің болса шайтан алғыр бұл сенің күл айтайын дегенім шын мәнінде ия енді сіз оны білесіз иә тістеу доғал көтеннен,шай алғы егер қаншық сенен бұрын сенің нигг бол шай алғы бұл сенің күл айт де шын мән ия енді сіз оны біл иә тісте доғал көтен,racism

1,сіз бардыңыз қаншық rt барды желкек обама ақымақ бас нигга,сіз бар қаншық rt бар желкек оба ақы бас нигг,racism

1,кейбір ханым бізді кесіп тастады әріптес ақымақ неге қайда бара жатқаныңды көрмеске иә сен ақымақ ақымақсың ақымақ ақымақ ниггерсің,кейбір ханым біз кес таста әріптес ақы неге қайда бар жат көрмес иә сен ақы ақы ақы ақы нигг,racism

1,атыс туралы кім айтты америкада көптеген мәселелер бар сіз болжаудан адасасыз колумбия жылдардың аяғында өлтіруші болды өткеннің болашағымызды белгілеуіне жол бермеңіз және біз кездесетін мәселелерден аулақ болыңыз көптеген проблемалар бар сіз олардың бірісіз,атыс туралы кім ай америка көптеген мәселе бар сіз болжау адас колумбия жыл аяғ өлтіруш бол өт болашағ белгіле жол берме және біз кездес мәселе аулақ бол көптеген проблема бар сіз олардың бір,racism

1,нигга мен ақ бұл екі түрлі нәрсе сен мылқау нәсілшіл бейбақсың үндемей анаңыздың жертөлесіне оралыңыз,нигг мен ақ бұл екі түрлі нәрсе сен мылқа нәсілшіл бей үндеме ана жертөле орал,racism

1,эм барыңыз бұл бейнені тағы бір рет көріңіз сіз қара пездтер ол нигга жүр желкекке,эм бар бұл бейне тағы бір рет көр сіз қара пезд ол нигг жүр желкек,racism

1,бүркіттер мені қатты күлдірді лол күлкілі бұл командада ақымақ ниггерлер бар кешіріңіз бірақ олар ренжітетіні рас,бүркіт мен қатты күл лол күлкіл бұл команда ақы ниггер бар кешір бірақ олар ренжіт рас,racism

1,неге сіз ханымға құрметпен карауды үйренбейсіз сіз кішкентай қара көзді қаншық ол жаста мылқау шайтан алғыр және ол сенің есегіңді теуіп жіберуі мүмкін,неге сіз ханым құрмет қарау үйренбе сіз кішкентай қара көз қаншық ол жас мылқа шай алғы және ол сенің есег теу жібер мүмкін,racism

1,наеби мылқау нигга,наеби мылқа нигг,racism

1,обамаға қарғыс атсын мылқау нигга pic twitter com tfdgdcg,обама қарғыс ат мылқа нигг pic twitter com tfdgdcg,racism

1,сіз маған твиттерде қандай желкек жазасыз ақымақ нигга мен сенің бойдақ екеніңді білемін,сіз маған твиттер қандай желкек жаз ақы нигг мен сенің бойдақ екен біл,racism

1,роджер сон роллен мартинді семіз негр деп атады твиттерде бұл трампқа ұнайтын нәрсе ашық нәсілшіл егде жастағы ақ адамдар,родж сон роллен марти се нег де ат твиттер бұл трамп ұна нәрсе ашық нәсілшіл егде жастағы ақ адам,racism

1,бізде тағы бір rt бар сіз жынды болдыңыз ба обамаға қарғыс атсын мылқау нигга оларды шығар,біз тағы бір rt бар сіз жын бол ба обама қарғыс ат мылқа нигг ол шығар,racism

1,мен кез келген нәрсені айтқым келеді өйткені бұл менсінбейді мен мұны ақ адамның пуэрто рикодан келген біреуді негр немесе мексикалық деп атайтынымен салыстыруым керек еді сөзбе сөз олар түсінгісі келмейтін адамдарды жіктеуге кез келген басқа жалқау әрекетпен,мен кез кел нәрсе айт кел өйткені бұл менсінбе мен

мұны ақ адам пуэрто рико кел біреу нег немесе мексикалық де ата салыстыр керек ед сөзб сөз олар түсін келме адам жікте кез кел басқа жалқау әрекет,racism

1,егер сіз мені бір сәтке артықшылықты ақ есек деп ойласаңыз онда балам мен негрос деп аталатын аралда тұратын филиппиндік орта таптың азаматымын,егер сіз мен бір сәт артықшылық ақ есек де ойла онда бала мен негрос де атал арал тұр филиппин орта тап азамат,racism

1,бұл сіздің жеке адамыңыз болады сізге қызғанышпен қарайтын сізге қызғанышпен қарайтын сізге жамандық тілейтін адам сізді жасырын түрде жек көреді және сіздің құлауыңыз үшін дұға етеді бірінші болып жалған көз жасын төгіп жерлеу рәсімінде жалған сүйіспеншілік танытады,бұл сіздің жеке адам бол сізге қызғаныш қара сізге қызғаныш қара сізге жаман тіле адам сіз жасырын түр жек көр және сіздің құла үшін дұ ет бірінші бол жалған көз жас төг жерлеу рәсім жалған сүйіспеншілік таныт,racism

1,мен сені мылқау нигга деп атаған жоқпын,мен сен мылқа нигг де ата жоқ,racism

1,сіз твиттің мәнін жіберіп алдыңыз мен ешқашан адамдар өсе алмайды деп айтқан емеспін бұл қара қауымдастық бәрінен де көп нәрсені қалайды бұл сөзбе сөз қозғалыстардың мәні мен оның бұрын неге өзін бұрынғы адамымен байланыстырғанын түсінетініме сенімдімін жеткілікті түсінікті ме,сіз тви мән жібер ал мен ешқашан адам өс алма де айт емес бұл қара қауымдастық бәрі де көп нәрсе қала бұл сөзб сөз қозғалыс мән мен оның бұрын неге өз бұрынғы адам байланыстыр түсін сенімді жеткілікті түсінік ме,racism

1,иә мен мұны жасадым және бұл менің назарымды аударды білесіз бе нәсілшілдік көптеген деңгейлерде көрінеді джозефин бейкердің ақш тан қашуының себебі еуропалықтар оны ақш та болғанмен салыстырғанда керемет орындаушы ретінде көрді мұнда ол жай ғана түрлі түсті биші болды керемет жұмыс лаура,иә мен мұны жаса және бұл менің назар ау біл бе нәсілш көптеген деңгей көрін джозефин бейк ақш тан қашу себебі еуропалық оны ақш та бол салыстыр керемет орындаушы рет көр мұнда ол жай ғана түрлі түсті биші бол керемет жұмыс лаур,racism

1,дұрыс линч сені жоқ құдай саған батасын бер rt сен қандай надан екенсің rt барды желкекке обама мылқау нигга,дұрыс линч сен жоқ құдай саған бата бер rt сен қандай надан екен rt бар желкек оба мылқа нигг,racism

1,бұл сен ақымақсың шайтан алғыр нигга сіз қандай ұят екенін айта алмайсыз нигга rt егер сіз жалғыз болсаңыз ең жақсы орын,бұл сен ақы шай алғы нигг сіз қандай ұят екен ай алма нигг rt егер сіз жалғыз бол ең жақсы орын,racism

1,неліктен blm қозғалысына назар аудару бұл мырзаларды ол немесе оның балалары бәрібір бірдеңе үшін төлейді деп ойлайтынын түсінбеймін сіз өткенді мойындап оның қара адамдар үшін әділетсіз бейбітшілікке әкелгенін мойындауыңыз мүмкін және бұл сізге ештеңеге тұрмайды,неліктен blm қозғалыс назар аудару бұл мырза ол немесе оның бала бәрібір бірде үшін төле де ойла түсінбе сіз өт мойында оның қара адам үшін әділет бейбітшілік әкел мойында мүмкін және бұл сізге ештеңе тұрма,racism

1,егер сіз мені білмесеңіз мені қандай желкек ұстайсыз мылқау нигга,егер сіз мен білме мен қандай желкек ұста мылқа нигг,racism

1,обамаға қарғыс атсын мылқау нигга lt lt lt ағартқышты ішіңіз,обама қарғыс ат мылқа нигг lt lt lt ағартқыш іш,racism

1,rt егер сіз барлық ниггерлер сарайларда орын алады деп ойласаңыз lt amp amp сен өлгендерге жатасың ақымақ бейбақ,rt егер сіз барлық ниггер сарай орын ал де ойла lt amp amp сен өл жат ақы бей,racism

1,мен аяқтадым rt аберкромбиде қоңыр түсті қара жүк шалбары бар ма нәсілшілдік әлі тірі адам smmfh,мен аяқта rt аберкромби қоңыр түсті қара жүк шалбар бар ма нәсілш әлі тірі адам smmfh,racism

1,steam да ақаулары бар есегіңізді түзетіңіз сіз артта қалған нигга шайтан алғыр,steam да ақау бар есег түзе сіз арт қалған нигг шай алғы,racism

1,авадан ұрланған нүкте қойыңыз мен сізге эмодзи қоямын сүйікті сен бекітілгенсің мен сені таспада көргім келеді мен сенімен дос болғым келеді мен сені акку арқылы қудым мен сені сүйікті қыздарымның бірі деп санаймын сен сүйкімдісің сен үшін бәрін жасаймын мен сені көремін бірінші рет,ава ұрлан нүкте қой мен сізге эмодзи қоя сүйікті сен бекітіл мен сен таспа көр кел мен сенімен дос бол кел мен сен акк арқылы қу мен сен сүйікті қыз бірі де сана сен сүйкімд сен үшін бәрін жаса мен сен көр бірінші рет,racism

1,канье уэст отбасын жоспарлау орталықтарын қалаларда ақ үстемшілдерявіліс жұмысын орындау үшін орналастырды арқылы қара қауымдастық абортқа кедергісіз қол жеткізуді қамтамасыз ету үшін нысанаға айналды және негр жобасы деп аталды бұл неден болар еді,кань уэст отбас жоспарлау орталық қала ақ үстемшілдерявіліс жұмыс орындау үшін орналас арқылы қара қауымдастық аборт кедергі қол жеткізу қамтамасыз ету үшін нысана айна және нег жоба де ата бұл не бол ед,racism

1,сіз ақымақ қаншықсыз менің атам маған сегрегация кезінде бірнеше рет нигга деп аталғанын айтты з егер сіз ақ болмасаңыз сіз қателескенсіз бұл өте қарапайым сіз оны сіздікі болғыңыз келсе де сіз боқтыққа әсіресе жаргонға құқығыңыз жоқ сөз бұл туралы ұмытыңыз,сіз ақы қаншық менің ата маған сегрегация кез бірнеше рет нигг де атал ай з егер сіз ақ болма сіз қателес бұл өте қарапайым сіз оны сіздік бол кел де сіз боқтық әсіресе жаргон құқы жоқ сөз бұл туралы ұмыт,racism

1,желкек обамаға барды ақымақ бас нигга сен бардың жезөкше командабама,желкек обама бар ақы бас нигг сен бар жезөкше командаба,racism

1,ниггердің бейнесі rt морис кларетт ақымақ сияқты,нигг бейне rt морис кларетт ақы сияқты,racism

1,сіз уэйн есімді ақымақ ұсқынсыз ниггерсіз позитивті қоқыс жұмсақ есегі бар нигга қара қоңыр желкек қызыл және ақ ақымақ ұсқынсыз желкек pic twitter com,сіз уэйн ес

ақы ұсқын нигг позитивті қоқыс жұм есег бар нигг қара қоңыр желкек қызыл және ақ ақы ұсқын желкек pic twitter com,racism

1,сіз қара көзді ұнатасыз сіз жиіркенішті латино әйелісіз сату маған бұл түсініктеме,сіз қара көз ұна сіз жиіркеніш латино әйел сату маған бұл түсініктеме,racism

1,ұнайды өйткені сіз оны екінші деп айтасыз сондықтан біріншісі месси деп ойлаймын бірақ сіз қоқыс деп айтпайсыз тек мессиді насихаттау үшін бұл түсініктемеден сіз жаста емес екеніңізді көреміз әрбір фотосуретінде пенальдо деп жазатын кішкентай блоттар емес,ұна өйткені сіз оны екінші де ай сондықтан бірінші месси де ойла бірақ сіз қоқыс де айтпа тек месси насихатта үшін бұл түсініктеме сіз жас емес екен көр әрбір фотосурет пенальдо де жаз кішкентай блот емес,racism

1,дэнни уэлбектен шыққан сен гребаная қара көт нигга жиіркенішті бұл үшін ешқандай ақтау жоқ,дэнни уэлбек шық сен гребаная қара көт нигг жиіркеніш бұл үшін ешқандай ақтау жоқ,racism

1,менің президентім қара деп айту нәсілшілдік бірақ сіз оны бәріңіз нигга деп атайсыз желкек мылқау есектер,менің президент қара де айту нәсілш бірақ сіз оны бәрі нигг де ата желкек мылқа есек,racism

1,эй мен ақ түстімін нигга емеспін сен ақымақ бейбақсың,эй мен ақ түсті нигг емес сен ақы бей,racism

1,сіз ақ адамның сізді нигга деп атағанын қалайсыз ба менің ниггерім менің құлым менің кішкентай негрім ұнайды ма,сіз ақ адам сіз нигг де ата қала ба менің нигг менің құл менің кішкентай нег ұна ма,racism

1,тролльге ұқсайтын семіз қаншық rt желкек обамаға барды ақымақ бас нигга сіз бардыңыз жезөкше командабама,тролль ұқса се қаншық rt желкек обама бар ақы бас нигг сіз бар жезөкше командаба,racism

1,черномазомның бетіне бұл сұмдықты айту үшін келдіңіз бе жоқ мылқау мылқау мылқау обама мылқау нигга,черномазо бет бұл сұмдық айту үшін кел бе жоқ мылқа мылқа мылқа оба мылқа нигг,racism

1,жарыстың бұған ешқандай қатысы жоқ деп айту қара лесбияндар бар өйткені лесбияндар нәсілді өз орнына келмейтін жерге әкеледі деп айту үшін бар ма тек ақ көзқарасыңыздан басқа ештеңені мойындай алмайтыныңызды айтыңыз,жар бұған ешқандай қатыс жоқ де айту қара лесбиян бар өйткені лесбиян нәсіл өз орн келме же әкел де айту үшін бар ма тек ақ көзқарас басқа ештеңе мойында алма ай,racism

1,желкек мылқау есек мен қарамын егер маған ұнайтын болса мен нигга деп айтамын сонымен қатар мен оны қалай қолданғаным зиянсыз болды,желкек мылқа есек мен қар егер маған ұна бол мен нигг де ай сонымен қатар мен оны қалай қолдан зиян бол,racism

1,мен соңғы екі айда бірдеңе дәлелдеді ме деп айтқым келді,мен соңғы екі айда бірде дәлелде ме де айт кел,racism

1,хм жылдан астам уақыт бұрын бұл тек бел үшін біз негроамерикалықтар олар бізді осылай атады ашуландық жылдан кейін тек маска мен ақ америккка ашуланды карма бұл қарғыс allrace ті шынымен біріктіретін уақыт келді біз онымен күресіп жеңе аламыз,хм жыл астам уақыт бұрын бұл тек бел үшін біз негроамерикалық олар біз осылай ат ашула жыл кейін тек маска мен ақ америккк ашула кар бұл қарғыс allrace ті шын біріктір уақыт кел біз онымен күрес же ал,racism

1,ммуси үйдегі ниггердің бейнесі өтелуден тыс және өзінің сұр затын қара адамның көзқарасы бойынша қолдана алмайды жиіркенішті,ммуси үйдегі нигг бейне өтелу тыс және өзінің сұр зат қара адам көзқарас бойынша қолдан алма жиіркеніш,racism

1,нигга бұл құл иелерінің айтқандары нигга бұл ақымақ бейбақтар ойлап тапқан нәрсе олар сен не қарап тұрсың нигга мен олай ойламаймын,нигг бұл құл ие айт нигг бұл ақы бейбақ ойла тап нәрсе олар сен не қара тұр нигг мен ола ойлама,racism

1,менің әкем бұл мылқау нигга обама қазір бізге қалай опасыздық жасаған болуы мүмкін мен әке мені нәсілшіл деп атауға батылы бармаңыз өйткені бұл шындық менің ата анам,менің әке бұл мылқа нигг оба қазір бізге қалай опасыз жаса бол мүмкін мен әке мен нәсілшіл де ата батыл барма өйткені бұл шындық менің ата ана,racism

1,олардың соңғы иелігіндегі алғашқы ойында бомбалауға рұқсат етіңіз сізге желкек барлық мылқау ниггерлерді қорғау үшін қандай да бір футбол iq ны үйреніңіз,олардың соңғы иелігі алғашқы ой бомбала рұқсат ет сізге желкек барлық мылқа ниггер қорғау үшін қандай да бір футбол iq ны үйрен,racism

1,кейбір гуччиді тозаққа салыңыз ниггерлер мылқау,кейбір гуччи тозақ сал ниггер мылқа,racism

1,сіз бәріңіз біздің әтешімізді сорғанды ұнатасыз rt nigger ден жаман нәрсе олармен кездесетін трахает жасайтын ақ қыздар,сіз бәрі біздің әтеш сор ұна rt nigger ден жаман нәрсе олармен кездес трахает жаса ақ қыз,racism

1,ол қаншалықты үлкен сіз бұл нөмірге қатысты қандай да бір тергеу жүргіздіңіз бе сары терісі бар адамдарға қатысты кемсітушілік ақ әлемде немесе мәдениеттерде жүздеген жылдар бойы болған менің елімнің тәрбиесіз туристеріне сары адамдарға нәсілшілдік себептерін беру дұрыс емес,ол қаншалықты үлкен сіз бұл нөмір қатысты қандай да бір тергеу жүр бе сары тері бар адам қатысты кемсітушілік ақ әлем немесе мәдениеттерде жүздеген жыл бойы бол менің ел тәрбие турист сары адам нәсілш себеп беру дұрыс емес,racism

1,ережелерді бұзу ережелерді бұзу мылқау ниггерлер лол psu да шын мәнінде қамқор емес балалар бар осу бұзақылар,ереже бұзу ереже бұзу мылқа ниггер лол psu да шын мән қамқор емес бала бар ос бұзақы,racism

1,мен ақш тың өте либералды бірақ сонымен бірге ақ бөлігінде өстім теледидарда негр колледждерінің біріккен қорының көптеген жарнамалары көрсетілген бұл әлі күнге дейін осылай аталады және ата аналар мен мұғалімдер балаларға теледидардан көргендеріне қарамастан сіз адамдарды негр деп атай алмайтыныңызды айтуға мәжбүр болды,мен ақш тың өте либералды бірақ сонымен бірге ақ бөліг өс теледи нег колледж біріккен қор көптеген жарнама көрсет бұл әлі күн дейін осылай атал және ата ана мен мұғалім бала теледи көр қарамастан сіз адам нег де ата алма айт мәжбүр бол,racism

1,бір техастан екіншісіне сіз шығыс техастасыз техастың уақыттың өзгеруіне әлі де соқыр бөлігі сіз хонки немесе гринга немесе крекер деп аталғыңыз келмейтініне сенімдімін бе оның сыпайы болғаны маңызды емес негр сөзі ақ адам үшін сөзін қолданудың қарапайым тәсілі,бір техас екінші сіз шығыс техас теха уақыт өзгер әлі де соқыр бөліг сіз хонки немесе гринг немесе крек де атал келме сенімді бе оның сыпай бол маңызды емес нег сөз ақ адам үшін сөз қолдан қарапайым тәсіл,racism

1,басқа қыздарды әшкерелейтін немесе бұрын қыздарды әшкерелеген ниггерлермен әлі де трахается қыздар сіз түйіндер жай ғана ақымақ түйіндер,басқа қыз әшкереле немесе бұрын қыз әшкереле ниггер әлі де трахается қыз сіз түйін жай ғана ақы түйін,racism

1,жоқ бірақ егер ол сіздің анаңызды ұрса онда ниггер осылай жасады сіз ақымақ ақ қаншықсыз ескі педофил сіз сәтсіздікке ұшырадыңыз хахахахахахахахаха,жоқ бірақ егер ол сіздің ана ұр онда нигг осылай жас сіз ақы ақ қаншық ескі педофил сіз сәтсіздік ұшыра хахахахахахахахах,racism

1,мен чур кали свэг дедім сондықтан желкек мылқау ниггерлерді тастаңыз лол,мен чу кали свэг де сондықтан желкек мылқа ниггер таста лол,racism

1,эй эй эй тек мен емес rt мен айтамын сіз джерси ниггерлері мені спаммен бомбалап жатырсыз rt мылқау әкімшілерден шаршадыңыз,эй эй эй тек мен емес rt мен ай сіз джерси ниггер мен спам бомбала жатыр rt мылқа әкімші шарша,racism

1,тағы жыл лил крекер rt обамаға қарғыс атсын мылқау нигга,тағы жыл лил крек rt обама қарғыс ат мылқа нигг,racism

1,өз кисканызды есекке салыңыз обама жеңді обама бұл жалды жеңіп алды webbie vc rt обамаға қарғыс атсын мылқау нигга,өз киска есек сал оба же оба бұл жа жең алды webbie vc rt обама қарғыс ат мылқа нигг,racism

1,мен спортзалға барған кездеісінемін менімен бірге жүрме нигга бұл сүйкімді,мен спортзал бар кезде іс менімен бірге жүрме нигг бұл сүйк,racism

1,jonathan сіз менің қызым екеніңізді қалай айта аласыз және мені ақымақ нигга деп атай аласыз бірақ мен сенен гөрі ақылды екенім анық nolife,jonathan сіз менің қыз екен қалай ай ал және мен ақы нигг де ата ал бірақ мен сенен гөрі ақылды екен анық nolife,racism

1,сіз жынды екеніңізді білуге рұқсат етіңіз тхо rt обамаға қарғыс атсын мылқау нигга,сіз жын екен біл рұқсат ет тхо rt обама қарғыс ат мылқа нигг,racism

1,сіздердің барлығыңыз мейвезерді лас нигга деп айтатын адамдар мұны істегеніңіз үшін ақымақ егер бұл ортиз болса сіз бұл жақсы соққы деп айтар едіңіз,сіздердің барлығ мейвез лас нигг де айт адам мұны істе үшін ақы егер бұл ортиз бол сіз бұл жақсы соққы де айтар ед,racism

1,мен келіп сенің нәсілшіл есегіңді теууім керек сен жиіркенішті белден төмен адамды ұятқа қалдырасың мен сенің ниггерлердің алысқа кететіні туралы бірдеңе айтқаныңды көрдім қызым сенің салқын есегің аддиді жарияламайды немесе жібермейді сен қара патшайымға бет бұруға батылы бармас едің,мен кел сенің нәсілшіл есег теу керек сен жиіркеніш бел төмен адам ұят қалдыр мен сенің ниггер алыс кет туралы бірде айт көр қыз сенің салқын есе адди жариялама немесе жіберме сен қара патшайым бет бұр батыл бармас ед,racism

1,сіздің досыңыз чемпиондар лигасына түсіп ұпайларын жоғалтқан болуы мүмкін ал бокстан турнир өткізілді егер сіз біріншісінде өзіңізді жақсы көрсетпесеңіз екіншісі бұғатталды,сіздің дос чемпион лига түс ұпай жоғалт бол мүмкін ал бокс турнир өткіз егер сіз бірінші өз жақсы көрсетпе екінші бұғаттал,racism

1,rt тақталар мылқау ниггерлерге арналған сіз хосттардың қара құлдарға қалай қарайтынын білдіңіз бе қара адамдар оян тозақ,rt тақта мылқа ниггер арнал сіз хост қара құл қалай қара біл бе қара адам оян тозақ,racism

1,бүгінгі күнге дейін джордж флойдты өлтірудің нәсілге қатысы бар екендігі туралы ешқандай дәлел жоқ полицейлердің өткені оқиға кезінде ештеңе профильдеу жоқ полиция шақырылды офицер қара адамды өлтірген жаман ақ полиция қызметкері болды содан кейін болжам нәсілдік негізге ие болады,бүгінгі күн дейін джордж флойд өлтіру нәсіл қатыс бар екендіг туралы ешқандай дәлел жоқ полицей өт оқиға кез ештеңе профильде жоқ полиция шақыр офицер қара адам өлтір жаман ақ полиция қызметк бол содан кейін болжам нәсіл негіз ие бол,racism

1,ниггерлер күні құтты болсын лол солай ма mlk өте көп нәрсе жасады ол құрметке лайық ақымақ бейбақ ал сен өз өміріңмен ешқайда бармайсың,ниггер күн құт бол лол солай ма mlk өте көп нәрсе жас ол құрмет лайық ақы бей ал сен өз өмір ешқай барма,racism

1,rt бәрі қара болуы керек пе хахахаха нәсілшілдік лол менің жаман ағам бірақ сен менің жалғыз түсті досымсың лол,rt бәрі қара бол керек пе хахахах нәсілш лол менің жаман аға бірақ сен менің жалғыз түсті дос лол,racism

1,cc бауырлар бұл ниггерлер өте ақымақ көрінеді,cc бауыр бұл ниггер өте ақы көрін,racism

1,rt rt сіз бардыңыз мылқау нигга джаги сорады сен бардың lt yay yyyyy,rt rt сіз бар мылқа нигг джаги сор сен бар lt yay yyyy,racism

1,мас болайық сорып алайық түкірейік түтіккен шам жағып жезөкшелерді ұрайық бауырлар ақ жігіттерде салқынқандылық жоқ,мас бол сор ал түкір түтік шам жағ жезөкше ұр бауыр ақ жігіт салқынқандылық жоқ,racism

1,менің деңгейімде бол балам пепчман енот йомама,менің деңгей бол бала пепчман енот йома,racism

1,сіз мені байланыстан айырғаныңыз үшін желкекке жасадыңыз сіз мылқау ниггерлер сіздің кисталарыңызды ауыстырып жатырсыз,сіз мен байланыс айыр үшін желкек жаса сіз мылқа ниггер сіздің киста ауыстыр жатыр,racism

1,міндет сезімінен маған нәсілшіл болуды доғарыңыз мен сенің есегіңді тырнап содан кейін сені бозғылт ниггер деп атағаннан кейін сен өзіңді ақымақ сезінесің,міндет сезім маған нәсілшіл болу доғар мен сенің есег тырна содан кейін сен бозғылт нигг де ата кейін сен өз ақы сез,racism

1,тозақ бұл жер және барлық ақымақ ниггерлер мен насрат құдай мені қазір өлтір,тозақ бұл жер және барлық ақы ниггер мен насрат құдай мен қазір өлтір,racism

1,мен хип хоп тауығын ойнаудан бас тартамын бұл мысырлықтар сізден ақымақ ниггерлерден пайда көреді мен куладты ішпеймін,мен хи хо тауығ ойнау бас тар бұл мысырлық сізден ақы ниггер пайда көр мен кулад ішпе,racism

1,ол әрқашан hgggg xx мен сенімен жыныстық қатынасқа түсетініне сенімдімін және басқа ақымақтарын ақымақ ниггерді трахать етеді,ол әрқашан hgggg xx мен сенімен жын қатынас түс сенімді және басқа ақымақ ақы нигг трахать ет,racism

1,егер сіз ақ есек болғыңыз келсе сіз үйдегі мылқау ниггадан басқа ешкімсіз,егер сіз ақ есек бол кел сіз үйдегі мылқа нигга басқа ешкі,racism

1,бұл трампқа ешқандай қатысы жоқ сен ақымақ бейбақсың ниггерлер адамдардың қалаларына бармауы керек оларды төбешік деп атамауы керек және егер олар адамдардың өздерін қорғауға келуін қаламаса оларды нәсілдік соғыспен қорқытпауы керек,бұл трамп ешқандай қатыс жоқ сен ақы бей ниггер адам қала барма керек ол төбешік де атама керек және егер олар адам өз қорға кел қалама ол нәсіл соғыс қорқытпа керек,racism

1,rt rt балам сен не туралы сөйлесесің отвали қара мазая қыз және отсоси менің мүшесі кетмен,rt rt бала сен не туралы сөйлес отвали қара мазая қыз және отсоси менің мүше кет,racism

1,смх мен бұл жеке және эмоционалды боқтықты твиттерде жазуды тоқтатуым керек сізге ешкім мән бермейді мылқау нигга,смх мен бұл жеке және эмоциона боқтық твиттер жазу тоқтат керек сізге ешкім мән берме мылқа нигг,racism

1,nigger сіз ниггердің өліп жатқаны соншалық сіз ақымақ бейбақсыз,nigger сіз нигг өл жат соншалық сіз ақы бей,racism

1,эй эссстрасс осы нойзбен осы жерден кетіңіз мылқау говенная көт сік нигга ахаха дұрыс,эй эссстрасс осы нойз осы же кет мылқа говенная көт сік нигг ахах дұрыс,racism

1,lmao rt неліктен ниггерлер idc idc idc қосады бұл әнде қандай ақымақ рэпер айтады,lmao rt неліктен ниггер idc idc idc қос бұл ән қандай ақы рэп айт,racism

1,мен сенің мылқау қара маз есегіңнің суретін көргім келмейді қаншық пышақ,мен сенің мылқа қара маз есег сурет көр келме қаншық пышақ,racism

1,егер бірдеңе өткеннің жәдігері болса оны бөлшектеу оңай емес заттарды максималды құндылығы бойынша қабылдау және текстуралар мен тігістермен таныс болу қара магия сияқты маңызды өткен қазіргі және болашақ бәрі бір,егер бірде өт жәдігер бол оны бөлшектеу оңай емес зат максималды құндылығ бойынша қабылдау және текстура мен тігіс таныс болу қара магия сияқты маңызды өткен қазіргі және болашақ бәрі бір,racism

1,сіз қандай желкек айтасыз сіз ақымақ бейбақсыз хаха ниггерлер менің twitter ді жарып жіберді хаха ха мен маймылдарды ренжіткен сияқтымын,сіз қандай желкек ай сіз ақы бей хах ниггер менің twitter ді жар жіб хах ха мен маймыл ренжіт сияқты,racism

1,сіз бұған шынымен сенбейсіз егер сізде қара достар болса оны құлағыңыздан өткізіңіз канададағы көптеген қара нәсілділердің отбасылық ағашында құлдық бар осылайша нәсілшілдік тұқымдары ешқашан нәсілшілдіктен үлкен нәрсеге айналмайды бір нәрсені ағарту оны өзгертпейді,сіз бұған шын сенбе егер сіз қара дос бол оны құлағ өткіз канада көптеген қара нәсілді отбасылық ағаш құлдық бар осылайша нәсілш тұқым ешқашан нәсілшілдік үлкен нәрсе айналма бір нәрсе ағарт оны өзгертпе,racism

1,guyspictures сік линкольн мылқау нигга ғашық,guyspictures сік линкольн мылқа нигг ғашық,racism

1,кім сара ол ұзақ уақыт бойы ештеңе айтпады өйткені оның қара әріптестері одан мұны жасамауды басқа қара дауыстарды күшейтуді сұрады өйткені оның платформасы өте үлкен уақыты келгенде ол әлі сөйлей бастады amp ол қара мәселелерге арналған білім беру жолын жалғастыруда бұл өткенге дейін келесі,кім сара ол ұзақ уақыт бойы ештеңе айтпа өйткені оның қара әріптес одан мұны жасамау басқа қара дауыс күшейту сұр өйткені оның платформа өте үлкен уақыт кел ол әлі сөйле баста amp ол қара мәселе арнал білім беру жол жалғастыр бұл өт дейін келесі,racism

1,хахахаха сен бардың мылқау нигга сіз біздің ақ нәсілділерді жек көруіміздің себебі деп жаман айтады,хахахах сен бар мылқа нигг сіз біздің ақ нәсілді жек көру себебі де жаман айт,racism

1,бұл ниггер сияқты ол қаншалықты мылқау болса да ақшасы бар болғандықтан ғана сіз онымен бірге жүресіз,бұл нигг сияқты ол қаншалықты мылқа бол да ақша бар бол ғана сіз онымен бірге жүр,racism

1,джейсон менен дүкенге барамын ба деп сұрады бұл менің дүкенге барғаным сияқты ма сіз мылқау ниггерсіз,джейсон менен дүкен бар ба де сұр бұл менің дүкен бар сияқты ма сіз мылқа нигг,racism

1,олар тозаққа барды мылқау ниггерлер,олар тозақ бар мылқа ниггер,racism

1,сіз бардыңыз мылқау нигга шындық,сіз бар мылқа нигг шындық,racism

1,topdollaraz әр нәсілдің керемет тарихы бар нигга қара термин емес ол ниггадан шыққан бұл қара нәсілділер үшін жиіркенішті термин,topdollaraz әр нәсіл керемет тарих бар нигг қара термин емес ол нигга шық бұл қара нәсілді үшін жиіркеніш термин,racism

1,rt кейбір гуччиді ұрыңыз ниггерлер мылқау,rt кейбір гуччи ұр ниггер мылқа,racism

1,қаншық сенің боқтығың менің арнамда болды қандай желкек бұл тіпті сейсенбі емес мылқау ниггерлер анық ретвит жасайды,қаншық сенің боқты менің арна бол қандай желкек бұл тіпті сейсенбі емес мылқа ниггер анық ретвит жаса,racism

1,қыздар кімге алаңдайды құдай бұл мәселе емес шайтан алғыр сен ақымақсың сәбіз есегі бар қаншықты ұр ниггерді ұр,қыз кім алаңда құдай бұл мәселе емес шай алғы сен ақы сәбіз есег бар қаншық ұр нигг ұр,racism

1,мен бұрын полиция қызметкері болғым келді бірақ енді қаламаймын лол сіз басқа түсті адамды өлтіргеннен кейін ұлттың жартысы бұл нәсілшілдікке байланысты деп ойлайды,мен бұрын полиция қызметк бол кел бірақ енді қалама лол сіз басқа түсті адам өлтір кейін ұл жарты бұл нәсілшілдік байланысты де ойла,racism

1,сәлемдесу сіз памперстермен негр синдромы сатиралық кітабын оқыған боларсыз бұл қорқынышқа әсіресе ақ адамдарға және оларды ақ деп атауға жақсы түсініктеме бұл да тегін pdf немесе amazon да қол жетімді,сәлемдес сіз памперс нег синдром сатиралық кітаб оқы бол бұл қорқыныш әсіресе ақ адам және ол ақ де ата жақсы түсініктеме бұл да тегін pdf немесе amazon да қол жет,racism

1,сіз бардыңыз қаншық бұл мылқау нигга обамаға қарғыс атсын,сіз бар қаншық бұл мылқа нигг обама қарғыс ат,racism

1,сіз гейлерді ақымақ ал қара нәсілділерді нигга деп атайсыз сіз тек гомофобиялық нәсілшілсіз жиіркенішті,сіз гей ақы ал қара нәсілді нигг де ата сіз тек гомофобиялық нәсілшіл жиіркеніш,racism

1,сік мені көтеннен мылқау нигга сүйетін бұзақылық,сік мен көтен мылқа нигг сүй бұзақылық,racism

1,саллт rt өте ақылсыз rt сіз әлі ессізсіз rt обамаға қарғыс атсын мылқау нигга,саллт rt өте ақыл rt сіз әлі ессіз rt обама қарғыс ат мылқа нигг,racism

1,менің сұрағыма жауап беріңіз мылқау қаншық сен кіреберістен қара мазасыз маймылға бардың,менің сұрағ жауап бер мылқа қаншық сен кіреберіс қара мазасыз маймыл бар,racism

1,өйткені ол батыстан мен сияқты мылқау шайтан алғыр бөксесінде бұғатталған киска бар нигга,өйткені ол батыс мен сияқты мылқа шай алғы бөксе бұғаттал киск бар нигг,racism

1,досым бұл ақымақ ниггерлерді ұрыңыз мен тенучи құйдым мен қиындық туғызамын мен қуыршақ сияқты ақымақпын,дос бұл ақы ниггер ұр мен тенучи құй мен қиындық туғыз мен қуыршақ сияқты ақы,racism

1,rt rt сіз бардыңыз мылқау нигга тістілер сорады бардым сен желкек lt уау аспан сен жеңесің сондықтан сақта смх,rt rt сіз бар мылқа нигг тісті сор бар сен желкек lt уау аспан сен же сондықтан сақта смх,racism

1,біз жеккөрушілікті жақсы көреміз қымбат rt обамаға қарғыс атсын мылқау нигга,біз жеккөрушілік жақсы көр қымбат rt обама қарғыс ат мылқа нигг,racism

1,мен араластырдым сен ақымақ нигга,мен аралас сен ақы нигг,racism

1,қатты күлу мүмкін емес ол тұзды rt обамаға қарғыс атсын мылқау нигга,қатты күл мүмкін емес ол тұз rt обама қарғыс ат мылқа нигг,racism

1,қандай желкек бұл мылқау қара көзді жігітті ұрып соғыңыз,қандай желкек бұл мылқа қара көз жігіт ұр соғ,racism

1,құлдыққа түспеген африкалықтар әлі де азап шегіп отарлаудан өтті мылқау көт өз жерімізде бізді ниггерлер деп атады қара тәжірибе кейбіреулеріңіз анонимді маскүнемдер өте ақымақ мен африкалықпын егер мен мурики көшелерімен жүрсем мені бәрібір ниггер деп атайтын едім сен бардың,құлдық түспе африкалық әлі де азап шег отарлау өт мылқа көт өз же біз ниггер де ат қара тәжірибе кейбіреу анони маскүнем өте ақы мен африкалық егер мен мурики көше жүр мен бәрібір нигг де ата ед сен бар,racism

1,бұл мылқау нигга обамаға сіз ашулануыңыз керек солай ма хахаха,бұл мылқа нигг обама сіз ашулан керек солай ма хахах,racism

1,оның ұлының қара шашы мен шаш түсінің генетикасын білмейтінімді елемеуім мүмкін бірақ бұл сіздің айтқаныңыздың бәрін тіпті мақсатқа жете алмайтын қорлау үшін мүлдем беделін түсіретін өрескел жалған асыра сілтеу жақсы ойнаған мылқау,оның ұл қара шаш мен шаш түс генетика білме елеме мүмкін бірақ бұл сіздің айт бәрін тіпті мақсат же алма қорла үшін мүлдем бедел түсір өрескел жалған асыра сілте жақсы ойна мылқа,racism

1,нигга телефон сөздігінде болмауы керек бірақ олардың ешқайсысы нигга болмауы керек мылқау нигга,нигг телефон сөздіг болма керек бірақ олардың ешқайсы нигг болма керек мылқа нигг,racism

1,күте тұрыңыз күте тұрыңыз сонымен ақ адамдар нәсілшіл бола алмайды ма мен сұраймын өйткені мен және менің достарым мұны жылдар бойы айтып келеміз мысалы нәсілшілдік сіздің гендеріңізде жатыр деп ойлаймыз сіздің сары шашыңыз бен көздеріңіз біртүрлі түсті егер сіз оң жауап бере алсаңыз біз сізге риза болар едік,күт тұр күт тұр сонымен ақ адам нәсілшіл бола алма ма мен сұра өйткені мен және менің дос мұны жыл бойы айт кел мысал нәсілш сіздің ген жатыр де ойла сіздің сары шаш бен көз біртүрлі түсті егер сіз оң жауап бер ал біз сізге риза бол едік,racism

1,құдайым маған көп нәрсе айту керек окок біріншіден сізге ұнай ма маған ең жақын адам және сен сондай сүйкімдісің бе және күлкілі менің ойымша бұл менің сүйікті тақырыптарымның бірі шығар сізбен сөйлесу өте жақсы,құдай маған көп нәрсе айту керек окок бірінші сізге ұна ма маған ең жақын адам және сен сондай сүйкімд бе және күлкіл менің ойымша бұл менің сүйікті тақырып бірі шығар сізбен сөйлесу өте жақсы,racism

1,бұл суретте зиянды ештеңе жоқ олар нәсілшілдік бейнесін асыра көрсетеді және сіздің түрлі түсті достарыңыз көп деп ойламайды,бұл сурет зиянды ештеңе жоқ олар нәсілш бейне асыра көрсет және сіздің түрлі түсті дос көп де ойлама,racism

1,менің осындай достарым бар нәсілшілдік бұл түрлі түсті заттарды жуу үшін ақ шүберекті таңдаған кезде бұл нәсілшілдік деп айтыңыз,менің осындай дос бар нәсілш бұл түрлі түсті зат жу үшін ақ шүберек таңда кезде бұл нәсілш де ай,racism

1,rt желкек обамаға барды мылқау бас нигга сіз бардыңыз,rt желкек обама бар мылқа бас нигг сіз бар,racism

1,мен бұл қаншықты ұрып жатырмын gt rt обамаға қарғыс атсын мылқау нигга,мен бұл қаншық ұр жатыр gt rt обама қарғыс ат мылқа нигг,racism

1,күл бір күні сіз жақсырақ боласыз және сіз jsjdk сияқты sm ді жақсартасыз ба мен сіздің өнеріңізді қазіргі өнермен салыстырғанда қашан көрдім сіз сорғышты әлдеқайда жақсарттыңыз құдайым сіз соншалықты керемет жасайсыз мен мұны шын жүректен айтамын,күл бір күн сіз жақсырақ бол және сіз jsjdk сияқты sm ді жақсар ба мен сіздің өн қазіргі өнер салыстыр қашан көр сіз сорғыш әлдеқайда жақсар құдай сіз соншалықты керемет жаса мен мұны шын жүрек ай,racism

1,обамаға қарғыс атсын мылқау нигга нәсілшіл ақымақ,обама қарғыс ат мылқа нигг нәсілшіл ақы,racism

1,негр сен әлі қарасың сіз өзіңізді қаншалықты жақсы ұстағаныңыз маңызды емес сіздің теріңіздің түсі әлі де қауіп ретінде қарастырылады ақ мақұлдауды тоқтатыңыз,негр сен әлі қар сіз өз қаншалықты жақсы ұста маңызды емес сіздің тер түс әлі де қауіп рет қарастыр ақ мақұлдау тоқта,racism

1,бұл адам кездейсоқ қара әйелді нигга деп атады мен шынымды айтсам қатты таң қалдым бұл кездейсоқ болды және арандатпады ол дәл өз орнына оралды күндізгі ставкадан кейін ол мұны жоққа шығарды бірақ куәгерлер болды адамдар шынымен жиіркенішті,бұл адам кездейсоқ қара әйел нигг де ат мен шын айт қатты таң қал бұл кездейсоқ бол және арандатп ол дәл өз орн ора күндізгі ставка кейін ол мұны жоқ шығ бірақ куәгер бол адам шын жиіркеніш,racism

1,сіз ат үстінде түрлі түсті жігітті көрдіңіз бе нәсілшілдік,сіз ат үст түрлі түсті жігіт көр бе нәсілш,racism

1,мен сіздің алғашқы твиттеріңізге жауап бергім келді және бұл олардың бірі болды деп ойладым сіз мұны түсінгеніңізге қуаныштымын,мен сіздің алғашқы твиттер жауап бер кел және бұл олардың бірі бол де ойла сіз мұны түсін қуанышты,racism

1,бұл жиіркенішті қара өмір маңызды ал ақ өмір маңызды бірақ мәні мынада қара өмір де маңызды және бұл ниггердің анықтамасы емес,бұл жиіркеніш қара өмір маңызды ал ақ өмір маңызды бірақ мән мына қара өмір де маңызды және бұл нигг анықта емес,racism

1,твиттері обаманы қарғыс атсын мылқау нигга сіз бұл жігіттің суретте қалай тапқыр екендігі туралы ойладыңыз ба,твиттер обама қарғыс ат мылқа нигг сіз бұл жігіт сурет қалай тапқы екендіг туралы ойла ба,racism

1,және жыл бұрын ниггердің хиппи балапан сұхбат берушісіне айтқан сөздері туралы ант бермеді,және жыл бұрын нигг хиппи балапан сұхбат беруші айт сөз туралы ант берме,racism

1,ya rt сіз бардыңыз ақымақ ракон ниггері rt rt мен бурлесканы көргім келеді,ya rt сіз бар ақы ракон нигг rt rt мен бурлеска көр кел,racism

1,егер сіз жүйелі нәсілшілдікті google да іздесеңіз онда адамдар ге баспанадан банктік несиелерден университетке түсуден жұмысқа орналасудан бас тартып ші түрмеге ақ қылмыстарға қарағанда әлдеқайда аз түсетінін көресіз менің президентім тіпті оған тиесілі дәріханаларды жалға беруге тыйым салу үшін түрлі түсті адамдарға арналған әрпін белгіледі,егер сіз жүйелі нәсілшілдік google да ізде онда адам ге баспана банктік несие университет түсу жұмыс орналасу бас тарт ші түрме ақ қылмыс қара әлдеқайда аз түс көр менің президент тіпті оған тиесілі дәріхана жалға бер тыйым салу үшін түрлі түсті адам арнал әр белгіле,racism

1,smh мылқау ниггерлер қараңызшы бұл ойында жеңіске жеткен ақ адам африкалық ақымақ лол джей кэй,smh мылқа ниггер қараңызш бұл ой жеңіс жет ақ адам африкалық ақы лол дже кэ,racism

1,егер ол шынымен нәсілшіл болса неге ол өз арнасына қара нәсілді адамды алып онда танысу сияқты нәрсе бар деп айтар еді мен бұл бейненің барлығын толығымен қарап шықтым және джеффридің нәсілшілдікке ұқсайтын бірдеңе айтқанын немесе жасағанын ешқашан көрген емеспін мен оның бұрын айтқан сөздері шынымен

қорлайтын және екенін түсінемін,егер ол шын нәсілшіл бол неге ол өз арна қара нәсіл адам алып онда танысу сияқты нәрсе бар де айтар ед мен бұл бейне барлығ толығ қара шық және джеффри нәсілшілдік ұқса бірде айт немесе жаса ешқашан көр емес мен оның бұрын айт сөз шын қорла және екен түс,racism

1,бірінші әсер керемет пародия rp аккаунты сіздің лақап атыңыз менің басымда duskit жақындық ұпайлары маған ұнай ма сен мазасыз ақымақ болмасаң иә досым ретінде сен менікісің дос сияқты саған ғашық болған ешкім кездейсоқ ой тоқта rp gc де,бірінші әсер керемет пародия rp аккаунт сіздің лақа ат менің бас duskit жақ ұпай маған ұна ма сен мазасыз ақы болма иә дос рет сен менік дос сияқты саған ғашық бол ешкім кездейсоқ ой тоқта rp gc де,racism

1,ниггерлер бұл күндері нағыз бұғылар олар өте ақымақ қарғыс атсын герцогтар ешқашан сенбейді ниггерлер олар қарғыс атқыр мылқау,ниггер бұл күн нағыз бұғы олар өте ақы қарғыс ат герцог ешқашан сенбе ниггер олар қарғыс атқы мылқа,racism

1,сіздің нигга жігітіңіз сізді тастап кетті иә қатты үзіліс rt желкекке обама мылқау бас нигга мылқау бас нигга сіздің бастық бастығыңыз,сіздің нигг жігіт сіз таста кет иә қатты үзіліс rt желкек оба мылқа бас нигг мылқа бас нигг сіздің бастық басты,racism

1,нәсілшілдік алдымен түсті киімді жуу үшін ақ киімді таңдау нәсілшілдікке жоқ деп айтыңыз барлық ні жуыңызгеда,нәсілш алд түсті ки жу үшін ақ ки таңдау нәсілшілдік жоқ де ай барлық ні жуыңызге,racism

1,мен сіздің жазбаңыз маған қандай сезімдер тудырғаны туралы көбірек жазғым келді бірақ менде сөз жоқ ән сіз бұл әнге мүлдем жаңа мағына бердіңіз мен онкологиялық аурулармен жалғыз өзім емес екенімді білемін деп айта бергенде не айтқыңыз келетінін нақты білемін ең алдымен ол бәрін түсінеді,мен сіздің жазба маған қандай сезім тудыр туралы көбірек жаз кел бірақ менде сөз жоқ ән сіз бұл ән мүлдем жаңа мағына бер мен онкологиялық ауру жалғыз өзім емес екен біл де ай бергенде не айт кел нақты біл ең алд ол бәрін түсін,racism

1,ақ адамдар ақ деп аталғанды жек көретіні ақылсыз бірақ қара нәсілділерге афроамерикалықтар деп айтылады өйткені бұл негрден жақсы негр сияқты түсті соқыр емес түрлі түсті қарағанда әлдеқайда эволюциялық бұл әңгіме қайда,ақ адам ақ де атал жек көр ақыл бірақ қара нәсілді афроамерикалық де айт өйткені бұл нег жақсы нег сияқты түсті соқыр емес түрлі түсті қара әлдеқайда эволюциялық бұл әңгіме қайда,racism

1,dramaalert тегі бұл ақымақтық сен ақымақ ниггерсің деп ойлайсың сияқты кибертерроризмді қалай жоспарлайтыны туралы айта бастады,dramaalert тег бұл ақымақтық сен ақы нигг де ойла сияқты кибертерроризм қалай жоспарла туралы ай баста,racism

1,lmaooo rt қандай желкек маған ақымақтық тілеме нигга,lmaooo rt қандай желкек маған ақымақтық тіле нигг,racism

1,қара уау неге зейн маған өзінің бұрынғы жұмысы туралы және басқа нәрселер туралы ештеңе айтпайды деп ойлаймын зейн барлығы,қара уау неге зейн маған өзінің бұрынғы жұмыс туралы және басқа нәрсе туралы ештеңе айтпа де ойла зейн барлығ,racism

1,мен ақымақ ештеңе іздемеймін шайтан алғыр сен шіркін ниггерді мазақ ететін кіреберістегі маймылсың,мен ақы ештеңе іздеме шай алғы сен шіркін нигг мазақ ет кіреберіс маймыл,racism

1,тіпті мектепте де қара мутанттар көп емес олардың ешқайсысы маңызды ештеңе жасамайды бұл мені басқа сұраққа әкеледі дарындыларға арналған ксавье мектебінен тыс жерде қанша адам өскені таңқаларлық айтайын дегенім бұрын және соңғы уақытта командалар болған,тіпті мектеп де қара мутант көп емес олардың ешқайсы маңызды ештеңе жасама бұл мен басқа сұрақ әкел дарынды арнал ксавь мектеб тыс же қанша адам өс таңқаларлық айт де бұрын және соңғы уақыт команда бол,racism

1,мен қоңыр ақымақ болудан бастамаңыз сен қара сен ниггерсің сіз лас және жиіркенішсіз сен жезөкше,мен қоңыр ақы болу бастама сен қара сен нигг сіз лас және жиіркеніш сен жезөкше,racism

1,иә ескерткіш мүсіндер деп аталатындарды бұзыңыз біздің жүйе табиғат заңдарының мұндай бұзылуына жол бермейді бізде барлық ақ нәсілділер бар олар қаншалықты жоғары немесе төмен болса да бай немесе кедей болса да заң алдында тең бұл негрлерге қатысты емес александр стивенс,иә ескерткіш мүсін де атал бұз біздің жүйе табиғат заң мұндай бұзыл жол берме біз барлық ақ нәсілді бар олар қаншалықты жоғары немесе төмен бол да бай немесе кедей бол да заң алд тең бұл негр қатысты емес александ стивенс,racism

1,мен бұл ақымақтықты кім айтса да нигга шайтан алғыр сөзін жек көремін,мен бұл ақымақтық кім айт да нигг шай алғы сөз жек көр,racism

1,rt обамаға қарғыс атсын ақымақ нигга сен кімсің сізге ұнайды ма жоқ па ол келесі жылдағы президент,rt обама қарғыс ат ақы нигг сен кім сізге ұна ма жоқ па ол келесі жылдағы президент,racism

1,лол бауырым сенде басқа нәрсе бар сіз басқа адамдардың пікіріне тым көп мән бересіз мен нәсілшілдіктің не екенін білемін сіз адамдарға не сезінетінін және немесе білетінін айта алмайсыз түрлі түсті адамдардың өтініштерін сәйкестендіруге арналған мөртабан сондықтан ол оларды қабылдамауы мүмкін оның әкесі ку клукс клан жағындағы тәртіпсіздіктер үшін қамауға алынды,лол бауыр сенде басқа нәрсе бар сіз басқа адам пікір тым көп мән бер мен нәсілшілдік не екен біл сіз адам не сезін және немесе біл ай алма түрлі түсті адам өтініш сәйкестендір арнал мөртабан сондықтан ол ол қабылдама мүмкін оның әке ку клукс клан жағ тәртіпсіздік үшін қама ал,racism

1,tmz ге шығады ол қыздың қара жиіркенішті және қара мазақ екенін айтады және ол қара болғандықтан ешкімге ұнамайды,tmz ге шығ ол қыз қара жиіркеніш және қара мазақ екен айт және ол қара бол ешкім ұнама,racism

1,қара адамдар мылқау сіз әлі де долларлық тауық покуп осы ниггерлерден қымбат тауық покуп сатып аласыз,қара адам мылқа сіз әлі де долларлық тауық поку осы ниггер қымбат тауық поку сат ал,racism

1,сіз ең үлкен жеңіліссіз егер бұл менің көлігім болса мен сыртқа шығып сіздің есегіңізді жеке теуіп жіберер едім мылқау нигга иә мен сен бардың дедім желкекке,сіз ең үлкен жеңіліс егер бұл менің көліг бол мен сырт шығ сіздің есег жеке теу жібер ед мылқа нигг иә мен сен бар де желкек,racism

1,lol rt клан мүшелері сізді жиі групповухамен ұрады мен бардым желкекке обама мылқау нигга деп ойлаймын,lol rt клан мүше сіз жиі групповуха ұр мен бар желкек оба мылқа нигг де ойла,racism

1,сен кімсің мен тіпті ешқашан естіген емеспін немесе сіздің қаз мойныңыз мылқау ниггердің есегіне ұқсайды,сен кім мен тіпті ешқашан есті емес немесе сіздің қаз мойны мылқа нигг есег ұқса,racism

1,мүлдем біз нәсіл мәселесін айналып өте алмаймыз мұны елемеу оның жоғалуына әкелмейді сонымен қатар еркін нарықтағы lmfao idk шынымды айтсам егер ол бір уақытта қара тәжірибелер мен өзгерістерге шақыруларды азайтпаса мен оның айтқанының бәріне қарсы болмас едім лол,мүлдем біз нәсіл мәселе айнал өте алма мұны елеме оның жоғал әкелме сонымен қатар еркін нарық lmfao idk шын айт егер ол бір уақыт қара тәжірибе мен өзгеріс шақыру азайтпа мен оның айт бәрін қарсы болмас ед лол,racism

1,сіз бардыңыз сіз мылқау ниггерсіз,сіз бар сіз мылқа нигг,racism

1,дәлірек айтсақ егер сіз blm ді мақтан тұтатын адамдар инстадағы барлық қараңғылықтар мен қара фотосуреттерге сілтеме жасамайды деп ойласаңыз бұл ештеңе білдірмейді егер сіз бұрын жасаған әрекеттеріңізді белсенді түрде мойындамасаңыз немесе кешірім сұрамасаңыз әйтпесе адамдар ашулануға құқылы,дәлірек айт егер сіз blm ді мақтан тұт адам инста барлық қараңғылық мен қара фотосурет сілтеме жасама де ойла бұл ештеңе білдірме егер сіз бұрын жаса әрекет белсенді түр мойындама немесе кешір сұрама әйтпесе адам ашулан құқылы,racism

1,қара өмір мағынасы бар термині өте қолайлы blm ұйымы американы жоюға шақыратын анархистік террористік жеккөрушілік тобы жақсы нәрсеге қатысты blm қолданбаңыз сіз қалай ұялмайсыз бұрын сіз барлығына бостандық пен әділеттілікті жақтадыңыз,қара өмір мағына бар термин өте қолайлы blm ұйым америка жою шақыр анархи террористік жеккөрушілік тоб жақсы нәрсе қатысты blm қолданба сіз қалай ұялма бұрын сіз барлығ бостандық пен әділеттілік жақта,racism

1,досым сен мені ниггер деп атай алмайсың бауырым мен сені тез ұрып тастаймын сен мені тез сотқа бересің ақымақ бейбақ,дос сен мен нигг де ата алма бауыр мен сен тез ұр таста сен мен тез сот бер ақы бей,racism

1,wooh мен мылқау ниггермін өйткені сіздің мылқау есегіңіз сөйлесуді қолдай алмайды сіз бардыңыз компьютер экранының артында керемет,wooh мен мылқа нигг өйткені сіздің мылқа есе сөйлесу қолда алма сіз бар компьютер экран арт керемет,racism

1,rt мылқау азиялық фагот мені ниггер деп атады лол gt lt lt lt қандай желкек джет лиге жиеніне желкек отыруын айт,rt мылқа азиялық фагот мен нигг де ат лол gt lt lt lt қандай желкек джет ли жиен желкек отыр айт,racism

1,уау қандай ақымақ кішкентай ниггердің қызы бар сіз менімен ашық болуға тырысасыз бірақ сізде бірдеңе дұрыс емес,уау қандай ақы кішкентай нигг қыз бар сіз менімен ашық бол тырыс бірақ сіз бірде дұрыс емес,racism

1,үйде мен бальзак бұл негр деп атаған автор жазған ұзын және қараңғы деп сипатталған кейіпкер туралы кітапта артаньян үш мушкетерде иә тек ақ кейіпкерлерге толы деп шешкен көлікке күлемін оған жаңа мұқаба жасау керек,үй мен бальзак бұл нег де ата автор жаз ұзын және қараңғы де сипаттал кейіпкер туралы кітап артаньян үш мушке иә тек ақ кейіпкер толы де шеш көлік күл оған жаңа мұқаба жасау керек,racism

1,түрлі түсті емес адамдар ақш та ешқашан нәсілшілдікті толық сезінбейді өйткені олар оны біздің көзқарасымыз бойынша ешқашан көрмейді теренскрутчер,түрлі түсті емес адам ақш та ешқашан нәсілшілдік толық сезінбе өйткені олар оны біздің көзқарас бойынша ешқашан көрме теренскрутч,racism

1,ақымақтық ақ адам қара адамдарды нигга деп атайтын контекст жоқ ешкім ағылшын тілі тым кең және бұл сезімді білдірудің жүздеген басқа жолдары болды бұл себепсіз қажетсіз және жиіркенішті болды,ақымақтық ақ адам қара адам нигг де ата контекст жоқ ешкім ағылшын тіл тым кең және бұл сез білдіру жүздеген басқа жол бол бұл себепсіз қажет және жиіркеніш бол,racism

1,менің айтайын дегенім мен қара жәшік селфиге қарағанда аз лайк жинайды деп күтер едім бірақ сіз білетін адамдар ғана емес теңдікке қатысы бар кез келген атақты пост соңғы бірнеше аптада олардың әдеттегі мазмұнынан шамамен лайк жинады,менің айт де мен қара жәшік селфи қара аз лайк жина де күт ед бірақ сіз біл адам ғана емес теңдік қатыс бар кез кел атақты пост соңғы бірнеше апта олардың әдет мазмұн шамамен лайк жина,racism

1,моральдық релятивистік ақымақтық әлсіз күшті ниггерлердің мәдениеті қарғысатқыр еврей билеуші тап қарғыс атқыр ақымақ мойынсұнғыш өмір салты барлық жерде жын перілердің пешкалары бар шайтан алғыр,моральдық релятиви ақымақтық әлсіз күшті ниггер мәдениет қарғысатқы еврей билеуш тап қарғыс атқы ақы мойынсұнғыш өмір сал барлық же жын пері пешка бар шай алғы,racism

1,мен дұрыс адамдардың тізімін жасағым келеді бірақ сіз оларды іздеу арқылы қоса алмайсыз сондықтан мен әр есімді кезекпен есте сақтауым керек сондықтан кім бірінші болса да бұл біртүрлі болады,мен дұрыс адам тізім жаса кел бірақ сіз ол іздеу арқылы қоса алма сондықтан мен әр ес кезек ес сақта керек сондықтан кім бірінші бол да бұл біртүрлі бол,racism

1,лол навандық rt мен қоңыр ақымақ болудан бастамаңыз сен қара сен ниггерсің сіз лас және жиіркенішсіз,лол надан rt мен қоңыр ақы болу бастама сен қара сен нигг сіз лас және жиіркеніш,racism

1,rt сен ақымақ бас нигга қаншығысың сен менің ниггерімді жұмыстан шығардың өз шлюшкаңызды мақта аузымен көтке,rt сен ақы бас нигг қаншығ сен менің нигг жұмыс шығ өз шлюшка мақта ауз көт,racism

1,сіз бардыңыз мылқау нигга,сіз бар мылқа нигг,racism

1,мен күресіп жатқан нәрсе мен азшылық тобын олар қорқынышты деп санайтын мінез құлыққа жазылғанын көремін,мен күрес жат нәрсе мен азшылық тоб олар қорқыныш де сана мінез құлық жазыл көр,racism

1,қарғыс атсын жынды бауырым rt обамаға қарғыс атсын мылқау нигга,қарғыс ат жын бауыр rt обама қарғыс ат мылқа нигг,racism

1,бұл мылқау ниггерлер маннн мысалы шайтан алғыр бірдеңе дұрыс емес сіздің көттыми бесінші нүктеми ниггерлер чувак,бұл мылқа ниггер маннн мысал шай алғы бірде дұрыс емес сіздің көттыми бесінші нүктеми ниггер чувак,racism

1,жабық желкекке мылқау нигга сіз бүкіл оқиғаны білетіндіктен бәрін сол күйінде айтыңыз немесе мен мұны істеуім керек пе,жабық желкек мылқа нигг сіз бүкіл оқи білетіндік бәрін сол күй ай немесе мен мұны істе керек пе,racism

1,неге неге ол солай болды өйткені сіздің әйел трамп осылай дейді өйткені ол қара жоқ бұл оның жүрегі болғандықтан ол жасағысы келген нәрседен өтпеді сондықтан егер біреу жемқор болса бұл республикашылдар және сіз мұны олардың соңғы жылдағы барлық нәрсені қалай басқарғанын ескере отырып қазір көріп отырсыз,неге неге ол солай бол өйткені сіздің әйел трам осылай де өйткені ол қара жоқ бұл оның жүрег бол ол жаса кел нәрсе өтпе сондықтан егер біреу жемқор бол бұл республикашыл және сіз мұны олардың соңғы жылдағы барлық нәрсе қалай басқар ескер отыр қазір көр отыр,racism

1,мен бұл ақымақ rt ниггерін жек көремін ол өте скучно тілеймін бауырым болды сондықтан мен оған smh компаниясының lmaoooooooooo жала жаба аламын,мен бұл ақы rt нигг жек көр ол өте скучно тіле бауыр бол сондықтан мен оған smh компания lmaoooooooooo жал жаб ал,racism

1,қаншық оны жай ғана мылқау нигга деп атады ол айтты қаншық мен сені выебу,қаншық оны жай ғана мылқа нигг де ат ол ай қаншық мен сен выеб,racism

1,smh rt rt желкекке үндемеңіз сіз әрқашан твиттерде мен ешқашан көргім келмейтін ақымақ қара мазасыз ақымақтықты жазасыз,smh rt rt желкек үндеме сіз әрқашан твиттер мен ешқашан көр келме ақы қара мазасыз ақымақтық жаз,racism

1,ниггер жасай алатын ең жаман нәрсе оның қаншығын ақымақ ету,нигг жаса ал ең жаман нәрсе оның қаншығ ақы ету,racism

1,иә ол аталады үнсіз болу және ақ үстемдікті жақтау ұлы негр бомани джонстың айтуынша,иә ол атал үн болу және ақ үстемдік жақтау ұлы нег бомани джон айт,racism

1,адамдар эмпатияны қорқынышпен шатастырады және бұл олар туралы бәрінен де көп айтады,адам эмпатия қорқыныш шатастыр және бұл олар туралы бәрі де көп айт,racism

1,сұрақтар ниггерлердің анықтамасы қатты және жауынгер әдетте тозақ сияқты мылқау тек сізді көру керек және олар киски сияқты ниггерлерге секіреді,сұрақ ниггер анықта қатты және жауынгер әдет тозақ сияқты мылқа тек сіз көру керек және олар киски сияқты ниггер секір,racism

1,сіз бардыңыз нигга rt сен ақымақсың қош бол мен олардың жалқау екенін айтқым келеді және бізге барлық дауыстар қажет,сіз бар нигг rt сен ақы қош бол мен олардың жалқау екен айт кел және бізге барлық дауыс қажет,racism

1,италия мылқау ниггерлерден арылыңыз omfg,италия мылқа ниггер арыл omfg,racism

1,бұл нигга мені аризона манго мучосы үшін қорқытуға тырысты,бұл нигг мен аризон манго мучо үшін қорқыт тырыс,racism

1,бұл ақ адам бізді мылқау ниггерлер деп атады өйткені біз оның қызын алып кетпедік лол иә менің мува оны көтеннен және барды желкекке,бұл ақ адам біз мылқа ниггер де ат өйткені біз оның қыз алып кетпе лол иә менің мув оны көтен және бар желкек,racism

1,отырыңыз крекер сіз жындысыз rt обамаға қарғыс атсын мылқау нигга,отыр крек сіз жынд rt обама қарғыс ат мылқа нигг,racism

1,қара әйелдер ақ феминизм терминін енгізген кезде ақ феминистер оған қарсы шығып оны бөліну деп атады дегенмен ақ әйелдер олар феминист пе жоқ па тарихи amp қара әйелдердің еңбегінен үнемі пайда көрді барлық уақытта қара әйелдердің есебінен ақ артықшылықтарды жинады,қара әйел ақ феминиз термин енгіз кезде ақ феминис оған қарсы шығ оны бөлін де ат дегенмен ақ әйел олар феминист пе жоқ па тарихи amp қара әйел еңбег үнемі пайда көр барлық уақыт қара әйел есеб ақ артықшылық жина,racism

1,демократтар өздерінің өткенін жасыруға тырысады демократтар бұл ақш тың ең көп қара халқы бар ірі қалаларында жылдан астам уақыт билікте болған және әлі күнге дейін өз сайлаушыларына көмектесу үшін ештеңе істемеген адамдар,демократ өз өт

жасыр тырыс демократ бұл ақш тың ең көп қара халқ бар ірі қала жыл астам уақыт билік бол және әлі күн дейін өз сайлаушы көмектес үшін ештеңе істеме адам,racism

1,мен үйде отырған емеспін қаралар сіздің есегіңізді қағып сізді тонағанша күтіңіз ақымақ бейбақ ниггерлер сізге жетеді,мен үй отыр емес қара сіздің есег қағ сіз тонағанш күт ақы бей ниггер сізге жет,racism

1,ол қарғысатқыр ауданына барып осы ниггерлерді бананмен ақымақтықпен ұруы керек еді оның орнына ол сатқындар дик с,ол қарғысатқы аудан бар осы ниггер банан ақымақтық ұр керек ед оның орн ол сатқын дик с,racism

1,плюспен ойнайды әрекетке шақыру қандай болды абортқа назар аудара отырып біреу не үйренді балаңызға түсік жасатпау үшін ол сізге миллион доллар бере ме уақыт өте келе миллион барлығы бірден бұл үшін кім төлейді бұл конгресстен қалай өтеді осы кезде ол жай ғана бәрін айтады,плюс ойна әрекет шақыру қандай бол аборт назар аудар отыр біреу не үйрен бала түсік жасатпа үшін ол сізге миллион доллар бер ме уақыт өте келе миллион барлығ бірден бұл үшін кім төле бұл конгресс қалай өт осы кезде ол жай ғана бәрін айт,racism

1,не желкек өзіне мылқау нигга енді кет желкекке менің хабарландырулардан,не желкек өзіне мылқа нигг енді кет желкек менің хабарландыру,racism

1,деанжело сен неге сонша аянышты сөмкесің мен сені жек көремін нигга,деанжело сен неге сонша аяныш сөмке мен сен жек көр нигг,racism

1,қара әйелдер туралы жиіркенішті пікірлерді жібере алатын кез келген қара адам мен одан аулақ болатын қара адам сіз ниггерлер ақылсыз және өте қорқынышты,қара әйел туралы жиіркеніш пікір жібер ал кез кел қара адам мен одан аулақ бол қара адам сіз ниггер ақыл және өте қорқыныш,racism

1,rt lt сөздер тозақ сияқты ақымақ мылқау нигга,rt lt сөз тозақ сияқты ақы мылқа нигг,racism

1,уэйн есімді мылқау ұсқынсыз нигга уэйн есімді мылқау ұсқынсыз нигга менің ішімдегі төртемдер мылқау ұсқынсыз желкек pic twitter com,уэйн ес мылқа ұсқын нигг уэйн ес мылқа ұсқын нигг менің ішім төртем мылқа ұсқын желкек pic twitter com,racism

1,мылқау сен менің иегімді тартып жатқан ниггерсің,мылқа сен менің иег тарт жат нигг,racism

1,менің басымды одан әрі жылытатын жалғыз нәрсе мен көргенде нигга сияқты әрекет ететін қара адамдар және әйелдерге жиіркенішті көзқарасы бар ер адамдар,менің бас одан әрі жылыт жалғыз нәрсе мен көргенде нигг сияқты әрекет ет қара адам және әйел жиіркеніш көзқарас бар ер адам,racism

1,алғашқылардың бірі болып жауап беріңіз содан кейін сұраңыз және бұл сіздің арманыңыз екенін айтыңыз,алғашқы бірі бол жауап бер содан кейін сұра және бұл сіздің арман екен ай,racism

1,ақылға қонымды мен нәсілшілмін мен қара халқымды жақсы көремін бірақ біз өткенімізден ештеңе үйренбедік біз жаулап алынған жердеміз және қару ұстау құқығының бізге ешқандай қатысы жоқ ақш рет өз жеріне бомба тастады кім құрбан болды,ақыл қонымды мен нәсілшіл мен қара халқ жақсы көр бірақ біз өт ештеңе үйренбе біз жаула алын жер және қару ұстау құқығ бізге ешқандай қатыс жоқ ақш рет өз же бомба таста кім құрбан бол,racism

1,нәсілшіл дэвид дьюк американы өзінің ақшыл ізбасарлары ақ ниггерлерге тиесілі деп санайды бұл туралы ойланыңыз ба америка үндістерден басқа ешкімге тиесілі емес,нәсілшіл дэвид дьюк америка өзінің ақшыл ізбасар ақ ниггер тиесілі де сана бұл туралы ойлан ба америка үндіс басқа ешкім тиесілі емес,racism

1,ниек пен брейге қарғыс атсын бұл кейбір мылқау ниггерлер,ниек пен брей қарғыс ат бұл кейбір мылқа ниггер,racism

1,және ол бұл дұрыс емес деді сондықтан оған қарсы сабақ алған өткенін пайдаланудың орнына біздің қауымдастықтарды түзете бастаңыз патшайым қара нәсілді әйел оның жыл бұрын айтқанын сақтау бір нәрсені қалай шеше алады,және ол бұл дұрыс емес де сондықтан оған қарсы сабақ ал өт пайдалан орн біздің қауымдастық түзе баста патшайым қара нәсіл әйел оның жыл бұрын айт сақтау бір нәрсе қалай шеш ал,racism

1,презервативпен ақымақ ниггерді ұрыңыз,презерватив ақы нигг ұр,racism

1,адамдар нигга сөзін айтқан кезде мен қатты ашуланамын өйткені мен сен ақымақ бейбақсың smh сөзінің шығу тегін де білмейсің,адам нигг сөз айт кезде мен қатты ашулан өйткені мен сен ақы бей smh сөз шығу тегін де білме,racism

1,сізді қара аяқты ниггерлерді бұғаттау өте жиркенішті,сіз қара аяқ ниггер бұғатта өте жиркеніш,racism

1,пышақ мылқау нигга,пышақ мылқа нигг,racism

1,содан кейін қайтадан негр содан кейін қара жылғы афроамерикалық содан кейін қайтадан қара сонымен бұл кім өйткені құжатталған тарихқа сәйкес бұл олардың жеке басы туралы болжам жасау деп аталады афроамерикалық қате атау бұл африкадан америкаға келген ақ адам болуы мүмкін дегенді білдіреді,содан кейін қайта нег содан кейін қара жылғы афроамерикалық содан кейін қайта қара сонымен бұл кім өйткені құжаттал тарих сәйкес бұл олардың жеке бас туралы болжам жасау де атал афроамерикалық қате атау бұл африка америка кел ақ адам бол мүмкін де білдір,racism

1,бардыңыз сіз желкекке мылқау нигга пезды rt бұл рас,бар сіз желкек мылқа нигг пез rt бұл рас,racism

1,адам бұл ақымақтық бұл менің кім екенім емес тозаққа барыңыз егер сіз ооо сөзін айтсаңыз өйткені сіз мылқау ниггерді ұрып соғып нәсілшіл болсаңыз ооо деп айтпаңыз бұл мен емес бұл қате болды осы жерден қарғыс атқан анаға барыңыз,адам бұл ақымақтық бұл менің кім екен емес тозақ бар егер сіз ооо сөз айт өйткені сіз мылқа нигг ұр соғ нәсілшіл бол ооо де айтпа бұл мен емес бұл қате бол осы же қарғыс ат ана бар,racism

1,компьютер вирусты алды менің мылқау әкем нигга бәрін қайта жүктеді менің барлық клиптерім жоғалып кетті қандай қоқыс,компьютер вирус алды менің мылқа әке нигг бәрін қайта жүкте менің барлық клип жоғал кет қандай қоқыс,racism

1,сенің ұсқынсыз мылқау артта қалған маймылдың мәні твиттерде бәріне мән бермейтін ақымақ сен ақымақ ақымақ надан ниггерсің,сенің ұсқын мылқа арт қалған майм мән твиттер бәрін мән берме ақы сен ақы ақы надан нигг,racism

1,иә қарғысатқыр мылқау ескі засранец ақ жігіт мүмкін емес тыңдау жас ниггердің сөйлесіп онымен ер адам разобразься осы сучкой,иә қарғысатқы мылқа ескі засранец ақ жігіт мүмкін емес тыңдау жас нигг сөйлес онымен ер адам разобразься осы сучко,racism

1,маргарита негр болу және оған тиесілі болу нені білдіретінін және оларды ақ адамдар қалай қабылдағанын түсінді ақ адамды жігерлендіру деп аталатын сөздердің арқасында ол оған бұрын соңды болмаған айқындықпен жетті,маргари нег болу және оған тиесілі болу не білдір және ол ақ адам қалай қабылда түс ақ адам жігерлендіру де атал сөз арқа ол оған бұрын соң болма айқындық же,racism

1,бұл жай ғана есінен танып қалады сондықтан мен айқайлап жатқандай сезінемін біз қара деңгеймен бірдей емеспіз біз жоғарымыз және сізді жақпа мен нигга деп атағаныңыз жиіркенішті сіз бізді ұнатпайсыз деп айтасыз бірақ сіз тағы да біз сияқты болуға тырысасыз өйткені бұл мені қатты толқытты,бұл жай ғана ес тан қал сондықтан мен айқайла жатқанда сез біз қара деңгей бірде емес біз жоғ және сіз жақп мен нигг де ата жиіркеніш сіз біз ұнатпа де ай бірақ сіз тағы да біз сияқты бол тырыс өйткені бұл мен қатты толқы,racism

1,сіз мен осы қоғамдастықта кездестірген алғашқы адамдардың бірісіз және сіз маған көп көмектестіңіз,сіз мен осы қоғамдастық кездестір алғашқы адам бір және сіз маған көп көмекте,racism

1,негро және бланко деп аталатын сусындар маған жақсы ештеңе әкелмейді деп болжауым керек,негро және бланко де атал сусын маған жақсы ештеңе әкелме де болжа керек,racism

1,мен сенің жауабыңды қате оқыдым құдай шабуыл жасай жаздады содан кейін оны қайтадан оқыдым алдымен мен оны сіз жігіттер құрбан болуы мүмкін деп ойлайтын адамдардың бірісіз деп оқыдым,мен сенің жауаб қате оқы құдай шабуыл жаса жазд содан кейін оны қайта оқы алд мен оны сіз жігіт құрбан бол мүмкін де ойла адам бір де оқы,racism

1,махаббат туралы әндерді тыңдаған кезде сіздің ойыңызда пайда болатын бірінші адам сізді күні бойы күлдіретін адам сізді қалаулы және қажет сезінетін адам сөзсіз сізді басқа біреуден артық көрген адам,махаббат туралы ән тыңда кезде сіздің ой пайда бол бірінші адам сіз күн бойы күлдір адам сіз қалаулы және қажет сезін адам сөзсіз сіз басқа біреу артық көр адам,racism

1,nigger және blackmazy бірдей емес мылқау бейбақ,nigger және blackmazy бірде емес мылқа бей,racism

1,сіз әлі түсінбейсіз бе брент менің нәсілім біз әлі бастан өткермеген барлық нәрсеге арналған ба біз қорқуды доғардық сіздің нәсіліңіз бұл теледидарлар қара актерлер мен спорт әртістері біз үшін теледидардан көргендеріңіздің бәрін айтады және сіз жыл деп ойлайсыз деп ойлайды,сіз әлі түсінбе бе брент менің нәсіл біз әлі бас өткерме барлық нәрсе арнал ба біз қорқу доғар сіздің нәсіл бұл теледидар қара актер мен спорт әртіс біз үшін теледи көр бәрін айт және сіз жыл де ойла де ойла,racism

1,бұл мылқау ниггер нигга сен ақымақсың,бұл мылқа нигг нигг сен ақы,racism

1,және бұл нигга сіздің президентіңіз ха rt барды желкекке обама ақымақ бас нигга,және бұл нигг сіздің президент ха rt бар желкек оба ақы бас нигг,racism

1,төлқұжат фотосуреттері небәрі долларға қалай түсті сіздің маңдайшаңызда деп жазылған сен ақымақ бейбақсың сасық пияз пуджасы құмды нигга,төлқұжат фотосурет небәрі долл қалай түсті сіздің маңдайша де жазыл сен ақы бей сасық пияз пуджа құм нигг,racism

1,мылқау көт ниггер шайтан алғыр қаншық жезөкше жезөкше сол әлі бейбақ,мылқа көт нигг шай алғы қаншық жезөкше жезөкше сол әлі бей,racism

1,тәулігіне сағат бойы басындағы ақымақ ниггерді тыңдауға тура келетін осы қара ханымды елестетіп көріңіз бұл ханымның күші бар,тәуліг сағат бойы басындағы ақы нигг тыңда тура кел осы қара хан елестет көр бұл хан күш бар,racism

1,rt бұл мылқау нигга обамаға,rt бұл мылқа нигг обама,racism

1,rt ден біреудің естігенін ешқашан естіген емеспін адамдарға ақ нәрсе айтты бәріңді ұр ақымақ ниггерлер мақта алқабында өлуге бар,rt ден біреу есті ешқашан есті емес адам ақ нәрсе ай бәрі ұр ақы ниггер мақта алқаб өл бар,racism

1,обамаға қарғыс атсын мылқау нигга иә бұл сіздің нәсілшіл қаншығыңызды қара дикті сорып жатыр деп айыптайтын мылқау нигга,обама қарғыс ат мылқа нигг иә бұл сіздің нәсілшіл қаншығ қара дик сор жатыр де айыпта мылқа нигг,racism

1,lmfao болуы керек желкекке обама мылқау нигга сонымен сіз де ашуланасыз ба,lmfao бол керек желкек оба мылқа нигг сонымен сіз де ашулан ба,racism

1,жас қара сайлаушылар бұрын қара нәсілділерді кемсіткен ккклан туралы бірдеңе біле ме байден кланның бас жетекшісі және байденнің тәлімгері сен бердтің ең

жақын досы болды,жас қара сайлаушы бұрын қара нәсілді кемсіт ккклан туралы бірде біл ме бай клан бас жетекші және байден тәлімгер сен берд ең жақын дос бол,racism

1,ретвитни бұл егер сен құдайға сенсең ол сенің айналдырғаныңды көрді қара біреу оларға stfu деп жауап береді олар жауап береді сен бардың мылқау нигга,ретвитни бұл егер сен құдай сен ол сенің айналдыр көр қара біреу оларға stfu де жауап бер олар жауап бер сен бар мылқа нигг,racism

1,аяз ата жай ақ,аяз ата жай ақ,racism

1,сіз мұның бәрін дұрыс түсінесіз rt шайтан алғыр бұл жігіт мылқау нигга,сіз мұның бәрін дұрыс түс rt шай алғы бұл жігіт мылқа нигг,racism

1,сікс кристен динн алдерсон тумб мылқау шайтан алғыр боқ ниггирская бесінші нүкте әкел томко джефф гейлорд мені ұстап банк тонаушы есірткі түрмеде,сікс крис динн алдерсон тумб мылқа шай алғы боқ ниггирская бесінші нүкте әкел томко джефф гейлорд мен ұста банк тонауш есірткі түрме,racism

1,бұл өте ұзақ уақыт феодализм дәуірінде болды шамасы қазір бізде біраз еркіндік бар қазіргі заманғы кәріз және гигиена іс жүзінде қара өліммен байланысты барлық нәрсені өте алыс өткеннің ісіне айналдырды,бұл өте ұзақ уақыт феодализ дәуір бол шама қазір біз біраз еркіндік бар қазіргі заманғы кәріз және гигиена іс жүз қара өл байланысты барлық нәрсе өте алыс өт іс айнал,racism

1,қарғыс атсын мылқау нигга жеңді бұл тозаққа,қарғыс ат мылқа нигг же бұл тозақ,racism

1,биби сен өте сүйкімдісің сен мені құшақ жая қарсы алған бірінші адамсың мен оны ешқашан ұмытпаймын мен сені жақсы көремін ууу,биби сен өте сүйкімд сен мен құшақ жая қарсы ал бірінші адам мен оны ешқашан ұмытпа мен сен жақсы көр уу,racism

1,көл жағалаулары жабық сіз кеттіңіз карл есекке ұқсайтын ақымақ мектеп жабылмайды есек фагот нигга,көл жағалау жабық сіз кет карл есек ұқса ақы мектеп жабылма есек фагот нигг,racism

1,сонымен менің jst хронологиясындағы бұл крекер обамаға ақымақ нигга ешбір қаншық сені ұрмайды обама одан аман қалмайды,сонымен менің jst хронология бұл крек обама ақы нигг ешбір қаншық сен ұрма оба одан аман қалма,racism

1,бұл ақымақ қаншық қышқыл мен графикалық дизайнды ақ адамдар үшін оғаш нәрселер деп атаған жоқ ниггер екеуіне де қомақты қаражат салғандықтан қаншық бар сен желкекке,бұл ақы қаншық қышқыл мен графикалық дизайн ақ адам үшін оғаш нәрсе де ата жоқ нигг екеу де қомақты қаражат сал қаншық бар сен желкек,racism

1,ohyoumad rt барды желкекке обама мылқау нигга gt gt smh нәсілшіл қаншық,ohyoumad rt бар желкек оба мылқа нигг gt gt smh нәсілшіл қаншық,racism

1,мен өзімді қалай сігу ете аламын қара қара нигга,мен өз қалай сіг ет ал қара қара нигг,racism

1,олардың қайсысы сонша сіз соңғы аптада ештеңе оқымадыңыз ба қара ер адамдар өлтіретін қара балалар жынды нәсілшіл полицейлер мен ақ адамдар бар ма әрине олар надан олардың надандығы сізге ұтымды ойлауға кедергі жасамаңыз,олардың қайсысы сонша сіз соңғы апта ештеңе оқыма ба қара ер адам өлтір қара бала жын нәсілшіл полицей мен ақ адам бар ма әрине олар надан олардың надандығ сізге ұтымды ойла кедергі жасама,racism

1,бұл ареандық ниггерлер өте жиіркенішті сондықтан мен өзімді конфессиялық емес қара адам деп санаймын бұл жағдайда конфессия ел болады,бұл ареа ниггер өте жиіркеніш сондықтан мен өз конфессиялық емес қара адам де сана бұл жағдай конфессия ел бол,racism

1,жарайды мен сіздің алғашқы клиенттеріңіздің бірі боламын,жарайды мен сіздің алғашқы клиент бірі бол,racism

1,маған бұл калифорнияда болып жатқаны ұнайды сіз мылқау либералды бейбақтар бұл мылқау ниггердің қызметіне қайта оралуына дауыс бердіңіз төлем бұл қаншық,маған бұл калифорния бол жат ұна сіз мылқа либералды бейбақ бұл мылқа нигг қызмет қайта орал дауыс бер төлем бұл қаншық,racism

1,күн ішінде мен хакниде полиция көліктері жыпылықтайтын шамдармен және сиреналармен өтіп бара жатқанда қара адамның қолдарын бастарынан ұстап тұрғанын көрдім кеше мен қателестім деп үміттендім артықшылықтар туралы әлі көп нәрсе білуім керек бірақ егер бұл мәлімдемеден басқа нәрсе болса мен оны осы нәрестеге қалай түсіндіремін,күн іш мен хакни полиция көлік жыпылықта шам және сирена өт бар жатқанда қара адам қол бас ұста тұр көр кеше мен қателес де үміттен артықшылық туралы әлі көп нәрсе біл керек бірақ егер бұл мәлімдеме басқа нәрсе бол мен оны осы нәресте қалай түсіндір,racism

1,сіз соншалықты ақымақ екеніңізді елестетіп көріңіз сіз ниггерлер мен мокрошелкаларға сізді ұруға мүмкіндік бересіз ақ жігіттің сізді ұрып соғуына мүмкіндік беретіндей ақымақ екеніңізді елестетіп көріңіз,сіз соншалықты ақы екен елестет көр сіз ниггер мен мокрошелка сіз ұр мүмкіндік бер ақ жігіт сіз ұр соғ мүмкіндік беретінде ақы екен елестет көр,racism

1,адамдар біздің ата бабаларымыз сияқты боялған онда нан кесектері және олардың қуырылған көрінісі олардың елінің қаншалықты ыстық болғанына байланысты анықталады біз бәріміз нан жейміз ал күн біздің тостер бұл менің нәсілшілдікке деген көзқарасым маған назар аудармаңыз,адам біздің ата баба сияқты боял онда нан кесек және олардың қуырыл көрініс олардың ел қаншалықты ыстық бол байланысты анықтал біз бәрі нан же ал күн біздің тос бұл менің нәсілшілдік де көзқарас маған назар аударма,racism

1,сен ақымақ ниггерсің менің жұмыс күнім тек tmrw,сен ақы нигг менің жұмыс күн тек tmrw,racism

1,мен мұғалімнен сызғышпен ұрдым ба білмеймін бірақ мен хисокаға мұны маған екі рет жасауға рұқсат бердім бірақ менің ойымша бұл бірінші рет лезде өлтіру болар еді,мен мұғалім сызғыш ұр ба білме бірақ мен хисока мұны маған екі рет жаса рұқсат бер бірақ менің ойымша бұл бірінші рет лез өлтір бол ед,racism

1,менімен араласпаңыз өйткені мен жолсызмын ниггер сен не айтқың келеді мылқау нигга,менімен араласпа өйткені мен жолсыз нигг сен не айт кел мылқа нигг,racism

1,барды сен желкекке мылқау көт нигга сіз бүгін қарғыс атқан диванда ұйықтайсыз бұдан кейін құшақтау болмайды,бар сен желкек мылқа көт нигг сіз бүгін қарғыс ат диван ұйықта бұдан кейін құшақта болма,racism

1,сізге ниггерлер ұнайды ма хек бір және жетінші сынып деп жазылғаны анық сіз ақымақ дейсіз сіз нәсілшілсіз бе қандай желкек,сізге ниггер ұна ма хек бір және жетінші сынып де жазыл анық сіз ақы де сіз нәсілшіл бе қандай желкек,racism

1,бұл мылқау кетменді ұрып соғыңыз содан кейін кәстрөлдегі түйін ақымақ мен сенің бардым желкекке обама мылқау нигга,бұл мылқа кетме ұр соғ содан кейін кәстрөл түйін ақы мен сенің бар желкек оба мылқа нигг,racism

1,сіз қанша ми жасушасын жоғалтып алдыңыз тіпті қара адамның бұл ең көп зиян тигізетінін естігенде нигга деп айту дұрыс деп ойлайсыз ба бұл ақ адамдарды мұны әзілмен айта аламын деп ойлауға мәжбүр етеді бірақ бұл жерде күлкілі ештеңе жоқ бұл сіздің нәсіліңізге надан құрметсіздік және жиіркенішті,сіз қанша ми жасуша жоғалт ал тіпті қара адам бұл ең көп зиян тигіз есті нигг де айту дұрыс де ойла ба бұл ақ адам мұны әзіл ай ал де ойла мәжбүр ет бірақ бұл же күлкіл ештеңе жоқ бұл сіздің нәсіл надан құрметсіз және жиіркеніш,racism

1,қара мужчин жиіркенішті саны әйелге жыныстық қатынас кезінде оларды нигга деп атауға мүмкіндік береді өкінішті,қара мужчин жиіркеніш сан әйел жын қатынас кез ол нигг де ата мүмкіндік бер өкініш,racism

1,ретвит жасаңыз ұнатыңыз түсініктеме беріңіз сонда пайдаланушы менің тобыма қосылады бірінші болып robux ұтады,ретвит жаса ұна түсініктеме бер сонда пайдаланушы менің тоб қос бірінші бол robux ұт,racism

1,себебі сіз тек бір оқиғаны алып контекстті өзгертпестен оны өзгертесіз біз ақ адамдар ақ болғандықтан жүйелі нәсілшілдікке жұмыс баспана денсаулық сақтау және оларға қол жеткізуде үлкен қиындықтар тап болатын қоғамда өмір сүрмейміз сіз қай бөліктерді ауыстыратыныңызды таңдайсыз,себебі сіз тек бір оқи алып контекст өзгертпес оны өзгер біз ақ адам ақ бол жүйелі нәсілшілдік жұмыс баспана денсаулық сақтау және оларға қол жеткіз үлкен қиындық тап бол қоғам өмір сүрме сіз қай бөлік ауыстыр таңда,racism

1,ақш тың бас прокуроры уильям барр мылқау ақ нигга,ақш тың бас прокурор уилья бар мылқа ақ нигг,racism

1,мен бұл ақ жігіттің джо па туралы айтқан ақымақтығы үшін мені мылқау нигга деп атағанына сене алмаймын гтфох бұл соншалықты маңызды емес,мен бұл ақ жігіт джо па туралы айт ақымақтығ үшін мен мылқа нигг де ата сен алма гтфох бұл соншалықты маңызды емес,racism

1,tf бұл желкек емес мылқау әуесқой ниггерлер,tf бұл желкек емес мылқа әуесқой ниггер,racism

1,сіз бардыңыз иеһова куәгерлерін қамауға алу туралы бұйрық бәрі сіздің ақымақ нигга есегіңізді ұрады ақымақ сіз бардыңыз,сіз бар иеһов куәгер қама алу туралы бұйрық бәрі сіздің ақы нигг есег ұр ақы сіз бар,racism

1,хахахахахах менің балам cbruh барды желкекке дрю бриз сен бардың мылқау нигга,хахахахахах менің бала cbruh бар желкек дрю бриз сен бар мылқа нигг,racism

1,менің қара көзді челси ақымақ пупустарды кім ұнатпайды ол күні бойы тек грильдегі ірімшікті жейтін шығар,менің қара көз челси ақы пупус кім ұнатпа ол күн бойы тек гриль ірімшік же шығар,racism

1,бәріне дайын болыңыз патриоттар күн сайын бұл одан да жаман болады бұрын трамп саяси қарсыластар мен басқа да шенеуніктерді өлтіреміз деп қорқытты жасырын операциялар бүкіл елде өрбіді,бәрін дайын бол патриот күн сайын бұл одан да жаман бол бұрын трам саяси қарсылас мен басқа да шенеунік өлтір де қорқыт жасырын операция бүкіл ел өрбі,racism

1,балықтың ерні бар барлық ақымақ ниггерлер сіз өлесіз деп үміттенемін шейшай,балық ер бар барлық ақы ниггер сіз өл де үміттен шейша,racism

1,гарридің өткені фильміндегі адам соншалықты даулы ештеңе жасаған жоқ ал луис қара адамды сөзі деп атады және ол үшін ешқашан кешірім сұрамады,гарри өт фильм адам соншалықты даул ештеңе жаса жоқ ал луис қара адам сөз де ат және ол үшін ешқашан кешір сұрама,racism

1,иә иә сен тағы бір ұсқынсыз мылқау ниггерсің,иә иә сен тағы бір ұсқын мылқа нигг,racism

1,мен ким ченн бүгін кешке асадтың есегін теуіп жатқанын бақылап отырды деп үміттенемін ким ақымақтық жасамауы үшін,мен ким ченн бүгін кеш асад есег теу жат бақыла от де үміттен ким ақымақтық жасама үшін,racism

1,rt кокаин хош иістендірілген сағыздың үлкен пакетін иіскейді адам кейбір анимелерді көрейік,rt кокаин хош иістендір са үлкен пак иіске адам кейбір аниме көр,racism

1,желкекке үндемеңіз сіз мылқау қара мылжыңдар,желкек үндеме сіз мылқа қара мылж,racism

1,өмірбаян ол өмірбаян мылқау ниггерлер,өмірбаян ол өмірбаян мылқа ниггер,racism

1,мен жай ғана мектепке барып қара аяқты ниггердің полиция қызметкерімен сөйлесіп жатқанын көрдім ал полиция қызметкері күйдірілген тосттармен бұл қаншықты атпады сондықтан америка бөлшектеніп жатыр бұл мен көрген ең жексұрын нәрсе долбоеби wtfamerica,мен жай ғана мектеп бар қара аяқ нигг полиция қызметкер сөйлес жат көр ал полиция қызметк күйдір тост бұл қаншық атп сондықтан америка бөлшектен жатыр бұл мен көр ең жексұр нәрсе долбоеби wtfamerica,racism

1,бұл әйелдер қара нәсілді бірақ олардың нәсілін олардың неліктен шабуыл жасағанын ұтымды түсіндіру ретінде пайдаланбау керек өйткені олар ақ болғандықтан карендердің адамдарға шабуыл жасауының себебі емес қалай болғанда да сіз бүкіл қоғамның соңғы бірнеше айдағы керемет стресс пен шамадан тыс күйзелістен күлге бөлініп құлап жатқанын көресіз,бұл әйел қара нәсіл бірақ олардың нәсіл олардың неліктен шабуыл жаса ұтымды түсіндіру рет пайдаланба керек өйткені олар ақ бол карен адам шабуыл жасау себебі емес қалай болғанда да сіз бүкіл қоғам соңғы бірнеше ай керемет стресс пен шама тыс күйзеліс күл бөлін құла жат көр,racism

1,ммм ақ адам сізді құтқару керек деп шешті ол қабілетті деп шешті сұрамады бірақ сізге өзінің мессиандық қабілеттері туралы хабарлады бұл негрлердің қамқорлығы деп аталады құпия нәсілшілдер бұл қате түсінікке бейім,мм ақ адам сіз құтқар керек де шеш ол қабілетті де шеш сұрама бірақ сізге өзінің мессиа қабілет туралы хабарла бұл негр қамқорлығ де атал құпия нәсілшіл бұл қате түсінік бейім,racism

1,миллер оған әлі қара және қоңыр адамдарды жеткілікті түрде жойған жоқ деп кеңес берді мен бұл қатыгез және қатыгез әкімшіліктен артық ештеңе қоймаймын,миллер оған әлі қара және қоңыр адам жеткілікті түр жой жоқ де кеңес бер мен бұл қатыгез және қатыгез әкімшілік артық ештеңе қойма,racism

1,сен жындысың ба бауырым rt обамаға қарғыс атсын мылқау нигга,сен жынд ба бауыр rt обама қарғыс ат мылқа нигг,racism

1,менің кестемдегі нигга сөзін қолданатын барлық ақымақ бейбақтар үшін,менің кестем нигг сөз қолдан барлық ақы бейбақ үшін,racism

1,енді егер мен ағылшын тілін бұрап алсам адамдар мені ақымақ қаншық деп ойлайды жоқ мен осы жазда оқымағандықтан мен ақылды қара ниггермін жарайды ма,енді егер мен ағылшын тіл бұра ал адам мен ақы қаншық де ойла жоқ мен осы жаз оқыма мен ақылды қара нигг жарайды ма,racism

1,жоқ бұл кетменді сору үшін менің аузымды сорыңыз holdddd muuuu diccckkkkk rt обама мылқау нигга,жоқ бұл кетме сор үшін менің ауз сор holdddd muuuu diccckkkkk rt оба мылқа нигг,racism

1,елшілердің істері егер құдай ақ адамдарға көктегі еврей қара алтын негр жұмағына кіруге рұқсат берсе онда неге құдай ақ адамдарды олардың артындағы тозақ отында

өртеніп кететін шайтан деп атайды деп жариялады аумин,елші іс егер құдай ақ адам көк еврей қара алтын нег жұмағ кір рұқсат бер онда неге құдай ақ адам олардың арт тозақ от өртен кет шай де ата де жариял аумин,racism

1,сен ақымақ ақымақсың қара түс емес бұл ақ сияқты көлеңке мұсылман терінің түсі емес дін,сен ақы ақы қара түс емес бұл ақ сияқты көлеңке мұсылман тер түс емес дін,racism

1,жоқ бен менің жағымда болды эндрю жазған кезде бақылау тақтасы сондықтан пышақ мылқау нигга,жоқ бен менің жағ бол эндрю жаз кезде бақылау тақта сондықтан пышақ мылқа нигг,racism

1,кез келген жолмен біз оған қол жеткізе аламыз жылдарға дейін бізді негрлер деп атады бұл ойдан шығарылған белгі емес бұл біз өзіміз ойлап тапқан белгі мен қайда және қашан болсам да ақ немесе қара адам оны өшіруге тырысқанда мен оны қорғаймын кезең мен қара әйелмін,кез кел жол біз оған қол жеткіз ал жыл дейін біз негр де ат бұл ой шығарыл белгі емес бұл біз өз ойла тап белгі мен қайда және қашан бол да ақ немесе қара адам оны өшір тырыс мен оны қор кезең мен қара әйел,racism

1,лол сенің анаң сен ниггердің ақымақ надан бөлігісің,лол сенің ана сен нигг ақы надан бөліг,racism

1,менде жаңа идея бар біріккен негр қорын тек қара студенттерді қолдайтын барлық қара нәсілділер басқаратын барлық қара қолдау жүйесін жойайық бірнеше жыл бұрын мисс бернинг кезінде қабылданған жаңа заң нәсілдік тепе теңдікті талап етті қара ақ бұл заң сондықтан бұұых немесе адамдардың қабылдауы керек,менде жаңа идея бар біріккен нег қор тек қара студент қолда барлық қара нәсілді басқар барлық қара қолдау жүйе жой бірнеше жыл бұрын мисс бернинг кез қабылдан жаңа заң нәсіл тепе теңдік талап ет қара ақ бұл заң сондықтан бұұых немесе адам қабылда керек,racism

1,егер анасы сен сияқты ақ болса ол нигга емес мылқау бейбақ rt барлығы шығып дауыс беріңіз сонда біз бұл ниггерді осы жерден шығара аламыз,егер ана сен сияқты ақ бол ол нигг емес мылқа бей rt барлығ шығ дауыс бер сонда біз бұл нигг осы же шығар ал,racism

1,сіз ренжіген сияқтысыз rt обамаға бұл мылқау ниггаға мен швейцарияға барамын сізге рахмет америка сіз осындай ел болғаныңыз үшін,сіз ренжі сияқты rt обама бұл мылқа нигга мен швейцария бар сізге рахмет америка сіз осындай ел бол үшін,racism

1,кинг бұл негрлердің ақ балаға шабуыл жасағанына қарсы емес бірақ кішкентай қара қызды теуіп таста деп айқайлады ол ақ баланы ұруға тырысты бірақ жіберіп алды,кинг бұл негр ақ бала шабуыл жаса қарсы емес бірақ кішкентай қара қыз теу таста де айқайла ол ақ бала ұр тырыс бірақ жібер алды,racism

1,сіз мені нигга деп атайтын нәрсе мені қара деп линчке салғаныңыз сияқты жаман емес бірақ соған қарамастан екеуі де анық жиіркенішті нәсілшілдік тәжірибе,сіз мен

нигг де ата нәрсе мен қара де линч сал сияқты жаман емес бірақ соған қарамастан екеу де анық жиіркеніш нәсілш тәжірибе,racism

1,обамаға қарғыс атсын мылқау бас нигга мэд бұл жігіт ұстараны кесіп тастағандай естіледі,обама қарғыс ат мылқа бас нигг мэд бұл жігіт ұстара кес тастағанда ест,racism

1,сіз надансыз мен қара адамды басатын ештеңе жасаған жоқпын бүгінгі ақ адамды бұрынғы ақ еркектерге кінәлау американдықтардың перл харбор үшін кішкентай жапон балаларын жек көруімен бірдей сіздің жек көруіңіз байқалады мен бұл туралы ештеңе істей алмаймын бірақ нәсілшілдік нәсілшілдік менде қара түсті болды,сіз надан мен қара адам бас ештеңе жаса жоқ бүгінгі ақ адам бұрынғы ақ еркек кінәла американдық перл харбо үшін кішкентай жапон бала жек көр бірде сіздің жек көр байқал мен бұл туралы ештеңе істе алма бірақ нәсілш нәсілш менде қара түсті бол,racism

1,роджерстоун бұл адамды ашуланып эфирде негр деп атады ал ақ нәсілділер негрді қорлайтын термин емес деп айтуға тырысады риииииит ол мені ренжіткісі келмеді сіз көшеге шыққанда бірдей энергияны сақтайсыз деп үміттенемін стоун америкадағы трамп,роджерстоун бұл адам ашулан эфир нег де ат ал ақ нәсілді нег қорла термин емес де айт тырыс риииииит ол мен ренжіт келме сіз көше шық бірде энергия сақта де үміттен стоун америка трам,racism

1,кездейсоқ нөмір маған ертерек қоңырау шалды содан кейін мен қайта қоңырау шалдым ол телефонды қойды содан кейін сағаттан кейін кешіріңіз анашым сіз негрмен қателестіңіз деп ойлаймын сияқты bs мен мен айтқым келді ақ дұрыс емес,кездейсоқ нөмір маған ертерек қоңырау шал содан кейін мен қайта қоңырау шал ол телефон қой содан кейін сағат кейін кешір анаш сіз негр қателес де ойла сияқты bs мен мен айт кел ақ дұрыс емес,racism

1,rt мылқау нигга қарғыс атқан допқа назар аударыңыз сіз қарғыс атқыр мылқау боқ,rt мылқа нигг қарғыс ат доп назар ау сіз қарғыс атқы мылқа боқ,racism

1,ол мұны олар айтқандай ақ үстемшіл бола отырып үнемі жасады бұрын оның қара қыз досы болған нағыз нәсілшілдердің олар жек көретін адамдармен ешқандай байланысы жоқ олар тіпті бір бөлмеде болмайды ол барлық американдықтарды жақсы көреді және мұның бәрін зұлым мсм ойлап тапты,ол мұны олар айтқанда ақ үстемшіл бола отыр үнемі жас бұрын оның қара қыз дос бол нағыз нәсілшіл олар жек көр адам ешқандай байланыс жоқ олар тіпті бір бөлме болма ол барлық американдық жақсы көр және мұның бәрін зұлым мс ойла тап,racism

1,сізде жетілу туралы куәлік немесе s жоқ деп ойлаймын сіз оңтүстік ниггерлерсіз,сіз жетіл туралы куәлік немесе s жоқ де ойла сіз оңтүстік ниггерл,racism

1,обамаға қарғыс атсын мылқау нигга,обама қарғыс ат мылқа нигг,racism

1,нигга деп айтыңыз idubbbz тағы бір мылқау алдамшы екіжүзді мылқау ютуберді бұрап оларды әшкерелегенде riptana ны қарастырайық,нигг де ай idubbbz тағы бір мылқа алдамш екіжүз мылқа ютуб бұра ол әшкереле riptana ны қарастыр,racism

1,осы аптаның әр күні гей дис және гей даталары болды мен тек ақ адамдар нигга және нигга деп айтатындығын елестете аламын сіздердің барлығыңыз жеккөрінішті және жиіркенішті жандарсыз және бұл көрінеді бірақ мен сізді тәрбиелеген ата аналар мен қара табынушылыққа негізделген қоғамнан басқа ешкімді кінәлай алмаймын,осы апта әр күн ге дис және ге дата бол мен тек ақ адам нигг және нигг де айтатындығ елесте ал сіздердің барлығ жеккөрініш және жиіркеніш жанд және бұл көрін бірақ мен сіз тәрбиеле ата ана мен қара табынушылық негіздел қоғам басқа ешкі кінәла алма,racism

1,обамаға қарғыс атсын мылқау нигга бірақ ол жеңді сондықтан сен ашуланасың яо мен teamobama ны өте жақсы көремін,обама қарғыс ат мылқа нигг бірақ ол же сондықтан сен ашулан яо мен teamobama ны өте жақсы көр,racism

1,обамаға қарғыс атсын мылқау нигга сіз дұрыс ойнайсыз ба,обама қарғыс ат мылқа нигг сіз дұрыс ойна ба,racism

1,қара адамдар ниггерлер бұл мүлдем жиіркенішті және twitter ді мұндай мінез құлық үшін қолдануға болмайды егер сізде бәріне мүмкіндік болса,қара адам ниггер бұл мүлдем жиіркеніш және twitter ді мұндай мінез құлық үшін қолдан болма егер сіз бәрін мүмкіндік бол,racism

1,отсоси мүшесі қандай да бір ниггеру бекки сік обама доғал нигга,отсоси мүше қандай да бір ниггер бекки сік оба доғал нигг,racism

1,фбр түрлі түсті адамдарға мән бермейді неге олардың бәрі басқа мемлекеттік органдар сияқты ақ деп ойлайсыз мен бұған өкінемін және адамдар америкадағы нәсілшілдіктің қаншалықты нақты проблема екенін білмейтініне одан да өкінемін сіз жақын арада тыныштық таба аласыз деп үміттенемін әпке,фб түрлі түсті адам мән берме неге олардың бәрі басқа мемлекеттік орган сияқты ақ де ойла мен бұған өкін және адам америка нәсілшілдік қаншалықты нақты проблема екен білме одан да өкін сіз жақын ара тыныштық таб ал де үміттен әп,racism

1,обамаға қарғыс атсын мылқау ниггер биияич менің барлық достарым қара ізбасарлар оны қараңыз,обама қарғыс ат мылқа нигг биияич менің барлық дос қара ізбасар оны қара,racism

1,бірінші әсер маған сіздің тербелістеріңіз ұнайды сіздің лақап атыңыз менің басымда фрэнки сен маған ұнайсың ба иә сіз менікісіз менің сұрақтарымның бірі сіз маған қандай түсті еске түсіресіз бежевый ахаха сіз де оны жариялауыңыз керек егер қаласаңыз,бірінші әсер маған сіздің тербеліс ұна сіздің лақа ат менің бас фрэнки сен маған ұна ба иә сіз менік менің сұрақ бірі сіз маған қандай түсті ес түсір бежевы ахах сіз де оны жарияла керек егер қала,racism

1,кейбір ниггерлер шынымен мылқау олар жеңіл автомобильдер мен жүк көліктерін сатып алады өйткені қаншық жақсы көрінеді өйткені ол трахается,кейбір ниггер шын мылқа олар жеңіл автомобиль мен жүк көлік сат ал өйткені қаншық жақсы көрін өйткені ол трахается,racism

1,мазақ ететін қара шеру және бейонка соңғы жылдары жүзеге асырған барлық нәрсе қош бол,мазақ ет қара шеру және бейонк соңғы жыл жүзеге асыр барлық нәрсе қош бол,racism

1,ол бұл ақ наразылық білдірушілердің бәрі оны бірінші болып ұстап алып одан барлық боқтарды шығара бастағанын көрмеді ақ нәсілшілдер тек негрлерге бағытталған сияқты,ол бұл ақ наразылық білдіруші бәрі оны бірінші бол ұста алып одан барлық боқ шығар баста көрме ақ нәсілшіл тек негр бағыттал сияқты,racism

1,бұл нигга қандай сұмдық грант хахахахахахаха бұл мартин ақымақ есек pic twitter com,бұл нигг қандай сұм грант хахахахахахах бұл мартин ақы есек pic twitter com,racism

1,ешнәрсе айтпайды мен сізді ақш тың нәсілшілдік өткенінің ақ нәсілділерге қандай психологиялық әсер еткенін түсіндіруге тырысамын жалпы және қара адамдарға қатысты,ешнәрсе айтпа мен сіз ақш тың нәсілш өт ақ нәсілді қандай психологиялық әсер ет түсіндір тырыс жалпы және қара адам қатысты,racism

1,обамаға қарғыс атсын ақымақ нигга ақымақ желкекге бар,обама қарғыс ат ақы нигг ақы желкек бар,racism

1,сіз мылқау ниггерлер ата бабаларыңыз сияқты асылып жүріңіз бұл сіздің кінәңіз бардыңыз бәрі желкекке,сіз мылқа ниггер ата баба сияқты асыл жүр бұл сіздің кінә бар бәрі желкек,racism

1,сонымен менің хабарландыруларым жүктеледі бірақ менің tl жүктелмейді қарғыс атсын twitter қарғыс атсын сіздің қосымшаңыз жопу бұл боқтықты жасаған мылқау ниггерге қарғыс атсын,сонымен менің хабарландыру жүктел бірақ менің tl жүктелме қарғыс ат twitter қарғыс ат сіздің қосымша жоп бұл боқтық жаса мылқа нигг қарғыс ат,racism

1,ға бұрылады дейді сомали африкадағы сомалиде қалай мұны қайталауға,ға бұр де сомали африка сомали қалай мұны қайтала,racism

1,рұқсат етіңіз трампты қолдайтын кез келген қара адам немесе мылқау ебанут аққұба жуылған аққұба нигга қаншығының назарын іздейді және нәсілшілдік ақ артықшылықтар және б зиянын мойындау үшін жеткілікті қара емес сіздің пікіріңіз жоқ тек сату,рұқсат ет трам қолда кез кел қара адам немесе мылқа ебанут аққұб жуыл аққұб нигг қаншығ назар ізде және нәсілш ақ артықшылық және б зиян мойындау үшін жеткілікті қара емес сіздің пікір жоқ тек сату,racism

1,lol адамдар ақымақ лол бірақ иә маған жазыңыз,lol адам ақы лол бірақ иә маған жаз,racism

1,хаха шайтан алғыр иә мылқау көт шайтан алғыр rt мені негрдің ұрығына батырыңыз,хах шай алғы иә мылқа көт шай алғы rt мен нег ұрығ батыр,racism

1,ниггер мені ақымақ деп атауға тым алысқа барды сіз бардыңыз,нигг мен ақы де ата тым алыс бар сіз бар,racism

1,cj бұл ақымақ ниггерлер мен cj мұны жедел жәрдеммен айтып жатыр деп ойлағаным туралы не айтады деп ойлауы керек,cj бұл ақы ниггер мен cj мұны жедел жәрдем айт жатыр де ойла туралы не айт де ойла керек,racism

1,виртуалды жарыс кезінде мансапты бұзу үшін қаншалықты ақымақ болу керек сіз ақымақсыз сіз соншалықты ақымақ болғаныңыз үшін сізге келтірілген кез келген зиянға лайықсыз бұл қиын емес балалар егер сіз ақ болсаңыз нигга деп айтпаңыз иеміз иса неге бұл қиын тозақ,виртуа жарыс кез мансап бұзу үшін қаншалықты ақы болу керек сіз ақы сіз соншалықты ақы бол үшін сізге келтір кез кел зиян лайық бұл қиын емес бала егер сіз ақ бол нигг де айтпа ие ис неге бұл қиын тозақ,racism

1,негр ұйымдары мен көшбасшылары деп аталатындар осы дағдарыс кезінде ақ адамға оларды бөлуге және бір біріне қарсы қолдануға мүмкіндік бермегені немесе бас тартқаны үшін несие алуы керек малкольм,нег ұйым мен көшбасшы де атал осы дағдарыс кез ақ адам ол бөл және бір бір қарсы қолдан мүмкіндік берме немесе бас тарт үшін несие ал керек малколь,racism

1,олар бұрын бір нәрсені жақтаған бірақ олар нәсілшілдікті басқаларға қарағанда көбірек қоздырады циммерман түрлі түсті емес пе bcr кезінде іште болыңыз кетпеңіз,олар бұрын бір нәрсе жақта бірақ олар нәсілшілдік басқа қара көбірек қоздыр циммерман түрлі түсті емес пе bcr кез іш бол кетпе,racism

1,ниггерлер шынымен шөлдейді қаншық сізді twitter дегі нигга деп атайды және сіз қандай да бір жолмен тұрып ол сізді қалайтынын айтасыз сіздердің барлығыңыз ақымақ бастарсыздар,ниггер шын шөлде қаншық сіз twitter дег нигг де ата және сіз қандай да бір жол тұр ол сіз қала ай сіздердің барлығ ақы бас,racism

1,мені ұр мылқау нигга,мен ұр мылқа нигг,racism

1,сіздің халқыңыз бұл сөздерді нигга сондай ақ негр деген атпен жасағандықтан сондықтан қара нәсілділердің басынан өткермеген ақ нәсілділердің кез келгені сөзін айтқан кезде біз оның артында қос мағынаны жасыратынын білеміз түсіну қиын емес,сіздің хал бұл сөз нигг сондай ақ нег де ат жаса сондықтан қара нәсілді бас өткерме ақ нәсілді кез кел сөз айт кезде біз оның арт қос мағына жасыр біл түсіну қиын емес,racism

1,psn ден шыққан бұл мылқау рок жұлдыздар мен ниггерлер,psn ден шық бұл мылқа рок жұлдыз мен ниггер,racism

1,кирк оның ардагер болуының қара тәжірибеге соңғы бірнеше күнде бар табуреткада қара болу тәжірибесіне қандай қатысы бар мен оған көбірек құрмет көрсету үшін оны сол жерге қосқым келді ме оның қызметі үшін сізге алғыс айтқанымды айтыңыз бірақ

маған оның кеңестері қажет емес,кирк оның ардагер болу қара тәжірибе соңғы бірнеше күн бар табуретка қара болу тәжірибе қандай қатыс бар мен оған көбірек құрмет көрсету үшін оны сол же қос кел ме оның қызмет үшін сізге алғыс айт ай бірақ маған оның кеңес қажет емес,racism

1,бірінші әсер сіз жаңа ғана кездестіңіз егер сіз өз атыңызды өзгертпесеңіз сіздің лақап атыңыз менің басымда idk жақындық рейтингі сен маған ұнайсың ба адам ретінде сен менікісің мен саған ғашық болдым жоқ сен де оны жариялауың керек әрине бір кездейсоқ ой сәлем,бірінші әсер сіз жаңа ғана кезде егер сіз өз ат өзгертпе сіздің лақа ат менің бас idk жақ рейтинг сен маған ұна ба адам рет сен менік мен саған ғашық бол жоқ сен де оны жарияла керек әрине бір кездейсоқ ой сәлем,racism

1,иә бұл негізінен мен fb like те айтқаным бізден кіші жігіт rawr xd нің жалпы кезеңі өзін өзі өшпенділікпен және осыған ұқсас нәсілшіл пікірлермен араласқан бұл тағы да әрбір black ops бөлмесінде әр минут сайын өліңіз ақымақ нигга деп айтылатын кездер бұл жиіркенішті болды tbh,иә бұл негіз мен fb like те айт бізден кіші жігіт rawr xd ні жалпы кезең өз өзі өшпенділік және осы ұқсас нәсілшіл пікір аралас бұл тағы да әрбір black ops бөлме әр минут сайын өл ақы нигг де айтыл кез бұл жиіркеніш бол tbh,racism

1,біз ниггерлер деп аталатын қара нәсілділерміз өйткені негр испан тілінде қара ал терісі қараңғы болғандықтан қара деп аталды иә равен сияқты өз көзқарасыңызды жарамсыз қалдырыңыз ондағы ақ адамдар құлдарды қамшымен қамшылап бізді крекас деп атады,біз ниггер де атал қара нәсілді өйткені нег испан тіл қара ал тері қараңғы бол қара де ата иә равен сияқты өз көзқарас жарамсыз қалдыр ондағы ақ адам құл қамшы қамшыла біз крекас де ат,racism

1,кеше түнде де мен сенің негрің емеспін атты фильм көрсетілді сөзі барлық жерде қолданылған мен сол түні ақ артықшылығымның ауыртпалығына байланысты әрең ұйықтадым,кеше түн де мен сенің нег емес ат фильм көрсет сөз барлық же қолданыл мен сол түн ақ артықшылығ ауыртпалығ байланысты әре ұйықта,racism

1,оларды жақындатыңыз rt обамаға қарғыс атсын мылқау нигга,ол жақында rt обама қарғыс ат мылқа нигг,racism

1,бауырым сен шынымен желкеккедің кесілгенін қаламайсың солай емес пе сіз мылқау ниггерсіз міне мен ойладым ақымақ ниггерлер еноттар оңтүстік мақтанышы,бауыр сен шын желкекке кесіл қалама солай емес пе сіз мылқа нигг міне мен ойла ақы ниггер енот оңтүстік мақтаныш,racism

1,өйткені ақша қара қауымдарға кететіні бейнеленген және олай емес сіз байден сіз үшін күреседі деп ойлайсыз ба ол өз лауазымына орналасу үшін бәрін айтады сіз оның бұрынғы дәйексөздерін көрген жоқсыз ба,өйткені ақша қара қауым кет бейнелен және ола емес сіз бай сіз үшін күрес де ойла ба ол өз лауазым орналасу үшін бәрін айт сіз оның бұрынғы дәйексөз көр жоқ ба,racism

1,пост келесідей болды ақ мұнда түрлі түсті адамдар жоқ қара балалар сіз ашуланған кезде қызарасыз ауырған кезде жасылданасыз ал өлгенде күлгін түсесіз барлығыңыз

түстісіз егер мен сияқты сіз нәсілшілдікті қолайлы деп санасаңыз сізге осы жазбаны беріңіз,пост келесіде бол ақ мұнда түрлі түсті адам жоқ қара бала сіз ашулан кезде қызар ауыр кезде жасылдан ал өл күлг түс барлығ түсті егер мен сияқты сіз нәсілшілдік қолайлы де сана сізге осы жазба бер,racism

1,обамаға тозақ салықты көтергеніңіз үшін рахмет мудак мен бұл мылқау ниггерге дауыс бермегеніме қуаныштымын,обама тозақ салық көтер үшін рахмет мудак мен бұл мылқа нигг дауыс берме қуанышты,racism

1,барлығы шығып дауыс беріңіз сонда біз бұл ниггерді қызметінен алып тастай аламыз ақымақ қаншық сік ромни кетмегу,барлығ шығ дауыс бер сонда біз бұл нигг қызмет алып таста ал ақы қаншық сік ромни кетмег,racism

1,vele lol хаб леня rt экипаж екеуміз ниггерді машинаны ұрламақ болған кезде ұстап алдық және оны розбанкте ұрдық ақымақ бейбақ,vele lol хаб леня rt экипаж екеу нигг машина ұрла бол кезде ұста ал және оны розбанк ұр ақы бей,racism

1,осындай баннерлер жасай отырып бірінші болып пікір қалдырған адам оны алады бірақ сіз не қалайтыныңызды айтып баннеріңізде көргіңіз келетін адамның атын көрсетуіңіз керек менің ағылшын төсек сорпасы,осындай баннер жаса отыр бірінші бол пікір қалдыр адам оны ал бірақ сіз не қала айт баннер көр кел адам ат көрсет керек менің ағылшын төсек сор,racism

1,бұл көт rt сіздің ақшыл ақшыл есегіңіз rt желкекке обама мылқау нигга,бұл көт rt сіздің ақшыл ақшыл есе rt желкек оба мылқа нигг,racism

1,менің ойымша жұмыстағы ниггерлер кімнің бірінші сігу болатынын көруге тырысады бірақ бұл әзіл менің жігітім бар сен ақымақ ниггаларсың,менің ойымша жұмыс ниггер кім бірінші сіг бол көр тырыс бірақ бұл әзіл менің жігіт бар сен ақы ниггал,racism

1,нигга сөзі мені қатты ашуландыратын сияқты бұл өте надан idc сіз ойнайсыз ба жоқ па бұл өте ақымақ,нигг сөз мен қатты ашуландыр сияқты бұл өте надан idc сіз ойна ба жоқ па бұл өте ақы,racism

1,демократ азғындықты шабыттандырды және оған әсер етті мен бұл жастарды олардың жаман мінез құлқы үшін жауапкершілікке тартамын бірақ мен ақ либералды демократтар мен олардың қуыршақ негрлерін малкольм оларды осылай атады жылдан астам уақыт бойы осы орта мен проблеманы құру үшін одан да үлкен жауапкершілікке тартып келемін,демократ азғындық шабыттан және оған әсер ет мен бұл жас олардың жаман мінез құлқы үшін жауапкершілік тар бірақ мен ақ либералды демократ мен олардың қуыршақ негр малколь ол осылай ат жыл астам уақыт бойы осы орта мен проблема құру үшін одан да үлкен жауапкершілік тарт кел,racism

1,ниггерлер ақымақ қыздармен айналысады өйткені интеллект жоқ жерде ешқандай күш қажет емес лизун тауығымен бір күн мен іштемін,ниггер ақы қыз айналыс өйткені интеллект жоқ же ешқандай күш қажет емес лизун тауығ бір күн мен іш,racism

1,сізді ниггер деп атаған адаммен дос лол валентина ақымақ бұл памперс тәрізді шаш үлгісімен,сіз нигг де ата адам дос лол валентина ақы бұл памперс тәрізді шаш үлгі,racism

1,ақымақ қаншық chelssss сіз бардыңыз барак обама фагот және ниггер нағыз ер адамдар гейлерді жек көреді,ақы қаншық chelssss сіз бар барак оба фагот және нигг нағыз ер адам гей жек көр,racism

1,мен бұл ниггердің ақ үйде түн ұйықтайтынына сенімдімін обамаға қарғыс атсын мылқау нигга,мен бұл нигг ақ үй түн ұйықта сенімді обама қарғыс ат мылқа нигг,racism

1,хаха менің миссиям аяқталды мен жартылай қатыгез жауаптарды жариялау арқылы фаготалар ниггерлер және раджалар тобын ашуландырдым енді мен өмірге толығымен қанағаттанамын мен тролль ретінде ештеңеге өкінбеймін мен тіпті өзімді кінәлі сезінбеймін және бұл жігітті лол және оның кішкентай достары,хах менің миссия аяқта мен жартылай қатыгез жауап жариялау арқылы фагота ниггер және раджа тоб ашулан енді мен өмір толығ қанағаттан мен тролль рет ештеңе өкінбе мен тіпті өз кінәлі сезінбе және бұл жігіт лол және оның кішкентай дос,racism

1,мен ашпын сіздердің біреуіңіз қара күңдер қара көзді қаншықтар маған тост немесе басқа нәрсе жасауы керек сіз өте жиіркеніштісіз,мен аш сіздердің біреу қара кү қара көз қаншық маған тост немесе басқа нәрсе жаса керек сіз өте жиіркеніш,racism

1,досым біріншіден оның ешқандай міндеттемесі жоқ сонымен қатар сіз не оқи алмайсыз бұл жерде оның ақша бергені жазылған дәл осы жерде отырған өте үнді жұлдыздарыңызға келетін болсақ олардың ешқайсысы бұл туралы айтқан жоқ сондықтан сіз ештеңеге лайық емессіз,дос бірінші оның ешқандай міндетте жоқ сонымен қатар сіз не оқи алма бұл же оның ақша бер жазыл дәл осы же отыр өте үн жұлдыз кел бол олардың ешқайсы бұл туралы айт жоқ сондықтан сіз ештеңе лайық емес,racism

1,спам обамаға қарғыс атсын мылқау нигга,спам обама қарғыс ат мылқа нигг,racism

1,президент қайта сайланды ал сіз ақымақ ниггерлер арамшөптер туралы алаңдайсыз неліктен мен ромниді жеңгім келді деп ойлайсыз тозаққа өмір,президент қайта сайла ал сіз ақы ниггер арамшөп туралы алаңда неліктен мен ромни жең кел де ойла тозақ өмір,racism

1,ерінге инъекция жасау туралы ойлану керек rt обамаға қарғыс атсын мылқау нигга және макияж жақсы,ерін инъекция жасау туралы ойлан керек rt обама қарғыс ат мылқа нигг және макияж жақсы,racism

1,бұл біздің зұлым нәсілшіл өткеніміздің инерциясы бұл біздің жүйе басқаша болуы мүмкін емес еді біз қара адамдарда ерекше ештеңе жоқ екенін білеміз,бұл біздің зұлым нәсілшіл өт инерция бұл біздің жүйе басқаша бол мүмкін емес ед біз қара адам ерекше ештеңе жоқ екен біл,racism

1,және мен соңғы апталардың динамитін бөлшектеп кристофер дэниэлс пен лакстың классикасына деген өзара сүйіспеншілігімізді талқылай отырып лорд сенгоку туралы сөйлесеміз егер сізге графика мен күрес өнеріне қатысты бірдеңе қажет болса енді кімге жүгіну керектігін білесіз,және мен соңғы апта динамит бөлшекте кристоф дэниэлс пен лак классика де өзара сүйіспеншіліг талқыла отыр лорд сенгок туралы сөйлес егер сізге график мен күрес өн қатысты бірде қажет бол енді кім жүгіну керектіг біл,racism

1,сіз бардыңыз сіз мылқау ниггерсіз қалай болғанда да сіз әрқашан ақымақ жүргізуші болдыңыз хек мен сені пайдалану үшін ғана доспын,сіз бар сіз мылқа нигг қалай болғанда да сіз әрқашан ақы жүргізуші бол хек мен сен пайдалану үшін ғана дос,racism

1,өкінішке орай мен сенің бетіңе түкіре алмаймын сен сасық ақымақсың сен туылған кезде анаң сені аяқтауы керек еді rt обамаға қарғыс атсын мылқау нигга,өкініш орай мен сенің бет түкір алма сен сасық ақы сен туыл кезде ана сен аяқта керек ед rt обама қарғыс ат мылқа нигг,racism

1,отарлық газеттер құлдықтың мәңгілікке қалуына ықпал етті бүгін жыл бұрын жарнамаланған қаш билли есімді негр оны әдетте деп атайды ақ қолды билли оның қолындағы күйікке байланысты оңтүстік каролина және американдық генерал газет,отарлық газет құлдық мәңгілік қал ықпал ет бүгін жыл бұрын жарнамалан қаш билли ес нег оны әдет де ата ақ қол билли оның қол күйік байланысты оңтүстік каролин және американдық генерал газет,racism

1,нәсілшілдік бұл жеккөрушілік сіз жай ғана заттарды сіздің көзқарасыңызға сәйкес келтіресіз сізге түсті болудың қажеті жоқ бұл надандық,нәсілш бұл жеккөрушілік сіз жай ғана зат сіздің көзқарас сәйкес келтір сізге түсті бол қажет жоқ бұл надан,racism

1,ай яй яй менің нәсілшіл ата әжелерім үйлеріне фотосуреттерді іліп қоюдан бас тартты және менің барлық ақ немере ағаларымның суреттерін іліп қойды бізді сүйіспеншілікпен өздерінің негрлері деп атады,ай яй яй менің нәсілшіл ата әже үй фотосурет іл қою бас тар және менің барлық ақ немере аға сурет іл қой біз сүйіспеншілік өз негр де ат,racism

1,мені нигга деп атауды доғарыңыз құдай мен бәрін нигга ниглет мылқау бастар деп атаймын хаха сіз оған үйренесіз,мен нигг де атау доғар құдай мен бәрін нигг ниглет мылқа бас де ата хах сіз оған үйрен,racism

1,мен симспін бірақ қауымдастық өте тітіркендіргіш еа сіздің ойыныңызды түзетеді бұл қателер оны ойнатылмайды мен фермерлік пакетті қалаймын жарайды біріншіден бұл қателер сізде өткен жылдан бері ескірген модтың бірі болуы мүмкін және сіз ойынды бастауға тырысасыз chromebook те сондықтан,мен симс бірақ қауымдастық өте тітіркендіргіш еа сіздің ойын түзет бұл қате оны ойнатылма мен фермерлік пакет қала жарайды бірінші бұл қате сіз өткен жыл бері ескір мод бірі бол мүмкін және сіз ой баста тырыс chromebook те сондықтан,racism

1,аузыңызды бақылаңыз сік артқы rt ниггерлер өте мылқау,ауз бақыла сік артқ rt ниггер өте мылқа,racism

1,марк ламаунт хилл және басқа да білімді негрлер кэндис оуэнске ақ үстемдік туралы мағынасыздықты балама пікір ретінде таратуға мүмкіндік береді егер бұл ақымақтықтан басқа ештеңе болмаса,марк ламаунт хилл және басқа да білімді негр кэндис оуэнс ақ үстемдік туралы мағынасыздық балама пікір рет тарат мүмкіндік бер егер бұл ақымақтық басқа ештеңе болма,racism

1,rt rt сіз бардыңыз ракон біз сіз сияқты адамдарды нигга деп атаймыз сіздің нигга мінез құлқыңыз сиыр етінің мылқау бөлігі,rt rt сіз бар ракон біз сіз сияқты адам нигг де ата сіздің нигг мінез құл сиыр ет мылқа бөліг,racism

1,бұл ақымақ бейбаққа нөміріне қоңырау шалу керек болды хаха хаха мен оның есегін теууім керек еді мен сенің сен кеттің нигга деген пікіріңді естідім,бұл ақы бейбақ нөмір қоңырау шалу керек бол хах хах мен оның есег теу керек ед мен сенің сен кет нигг де пікір есті,racism

1,rt қаншық қара билікке қарғыс атсын мылқау ниггерлер бұл сөмкені тастаңыз уау шынымен,rt қаншық қара билік қарғыс ат мылқа ниггер бұл сөмке таста уау шын,racism

1,бұл ниггерді тозаққа түсіріңіз бұл жігіт өзін өте керемет ұстайды және барлық ақымақ шпиктерді трахает етеді джентуза де югадор және тұлға джунглиге барыңыз,бұл нигг тозақ түсір бұл жігіт өз өте керемет ұста және барлық ақы шпик трахает ет джентуз де югадо және тұлға джунгли бар,racism

1,мен мылқау ниггерлермен айналыспаймын мен мұны ешқашан жасаған емеспін және ешқашан жасамаймын,мен мылқа ниггер айналыспа мен мұны ешқашан жаса емес және ешқашан жасама,racism

1,ақымақ қаншық rt сіз ашуланасыз ба жоқ па rt обамаға қарғыс атсын мылқау нигга,ақы қаншық rt сіз ашулан ба жоқ па rt обама қарғыс ат мылқа нигг,racism

1,xobrittbritt иә бұл өте жиіркенішті адамдар осындай қарғыс атқан ниггерлер өңдеу барлық қара адамдар мұндай боқтықпен айналыспайды,xobrittbritt иә бұл өте жиіркеніш адам осындай қарғыс ат ниггер өңдеу барлық қара адам мұндай боқтық айналыспа,racism

1,кім аузында сіріңке бомбасын жағар еді ниггер нағыз ақымақтық жасайды,кім ауз сірің бомба жағар ед нигг нағыз ақымақтық жаса,racism

1,қаншық маған назар аудармаңыз өйткені сіз нигга сияқты ақымақсыз,қаншық маған назар аударма өйткені сіз нигг сияқты ақы,racism

1,сенің ақ екеніңді жексұрын және қара адамның оны ниггер деп атайтынына күмәнданасың түкірген адам қамауға алынды,сенің ақ екен жексұр және қара адам оны нигг де ата күмәндан түкір адам қама ал,racism

1,американдық rt болуы керек менің көлігіме ессіз балшықпен аяқ киімімен мінген мылқау ниггерді ашуландырды,американдық rt бол керек менің көліг ес балшық аяқ киім мін мылқа нигг ашулан,racism

1,мен зертханалық жұмыстарды жек көремін доғал ғылыми ақымақтық сіздің зертханаңызға қарғыс атсын нигга,мен зертханалық жұмыс жек көр доғал ғылыми ақымақтық сіздің зертхана қарғыс ат нигг,racism

1,шынымен ақ адамдар ақымақ мен жасымда асыға күткен нәрсе болды ақыры мұны жасында жасады атам мұны баяғыда жыл бұрын жасаған мунго паркі нигер өзенін қалай ашты дейді олар нонсенс,шын ақ адам ақы мен жас асыға күт нәрсе бол ақы мұны жас жас ата мұны баяғы жыл бұрын жаса мунго парк ниг өзен қалай аш де олар нонсенс,racism

1,сонымен байден сенатор болған соңғы жыл ішінде жүйелі нәсілшілдікпен қайда күресті иә ол ешқашан қара нәсілділерге немесе lgbtq өкілдеріне көмектесу үшін ештеңе істемеген ол шын мәнінде қара нәсілділер мен лгбтқ адамдарға қарсы заңдар қабылдады сізде қырық жыл болды джо шілде шілде,сонымен бай сенатор бол соңғы жыл іш жүйелі нәсілшілдік қайда күре иә ол ешқашан қара нәсілді немесе lgbtq өкіл көмектес үшін ештеңе істеме ол шын мән қара нәсілді мен лгбтқ адам қарсы заң қабылда сіз қырық жыл бол джо шілде шілде,racism

1,uwg шабуылы rt обамаға қарғыс атсын мылқау нигга,uwg шабуыл rt обама қарғыс ат мылқа нигг,racism

1,сіз бардыңыз мылқау нигга ағаңыздың аузынан үгінділерді шығарыңыз,сіз бар мылқа нигг аға ауз үгінді шығ,racism

1,мен есімде жоқ бірақ сенікі дұрыс олар нәсілшілдер деп аталудан қорқып мұны айтудан қорқатын ақ адамдар үшін ақ тілде сөйлейтін буферлік негрлер тапкандейс оуэнс тағы бір мысал,мен ес жоқ бірақ сенік дұрыс олар нәсілшіл де аталу қорқ мұны айту қорқ ақ адам үшін ақ тіл сөйле буферлік негр тапкандейс оуэнс тағы бір мысал,racism

1,lmaooooooo rt cj бұл мылқау ниггерлер мен cj мұны жедел жәрдеммен айтып жатыр деп ойладым деп ойлауы керек,lmaooooooo rt cj бұл мылқа ниггер мен cj мұны жедел жәрдем айт жатыр де ойла де ойла керек,racism

1,йо тимербх мылқау нигга сен өзіңді салқын есек сияқты ұстауға тырысасың бірақ сен кішкентай көтболсың ақымақ қаншық,йо тимербх мылқа нигг сен өз салқын есек сияқты ұста тырыс бірақ сен кішкентай көтбол ақы қаншық,racism

1,нәсілшілдік мисогиния өзіңіз таңдаңыз салон оқиға кампустағы зорлық зомбылық оқиғасы ретінде басталды еа,нәсілш мисогиния өз таңда салон оқиға кампус зорлық зомбылық оқиға рет баста еа,racism

1,сізге лагерьге бару және жезөкше болып көріну керек өйткені сіз карталарды түсінбейсіз мылқау бұзылған сасық бұралған нигга жалған боқ лол,сізге лагерь бару

және жезөкше бол көріну керек өйткені сіз карта түсінбе мылқа бұзыл сасық бұрал нигг жалған боқ лол,racism

1,қарғыс атқыр нигга сен қайдан келдің ол жерден тек ақымақтықты алып жүру үшін есегіңмен жорғаладың ба тоқтатыңыз және өзіңіздің ақымақ есегіңізбен айналысыңыз,қарғыс атқы нигг сен қайдан кел ол же тек ақымақтық алып жүру үшін есег жорғала ба тоқта және өз ақы есег айналыс,racism

1,rt лолололололол қандай сүйкімді rt обамаға қарғыс атсын мылқау нигга,rt лолололололол қандай сүйк rt обама қарғыс ат мылқа нигг,racism

1,өзімшілдіктен арылыңыз қызғылт көзілдірікті шешіп шынайы болыңыз егер сіз келесі жылы биликте болсаңыз сәттілікке жетесіз адамдар сіздің өтіріктеріңізге қорқытуларыңызға бізге және меланияға опасыздық жасауыңызға жетілмегендігіңізге толық жұмыс істей алмауыңызға сүйектеріңізге фанатизміңізге және нәсілшілдігіңізге толы біз сенен шаршадық ші импичмент жариялаңыз,өзімшілдік арыл қызғылт көзілдірік шеш шынайы бол егер сіз келесі жыл билик бол сәттілік же адам сіздің өтірік қорқыту бізге және мелания опасыз жасау жетілмегендіг толық жұмыс істе алмау сүйек фанатизм және нәсілшілдіг толы біз сенен шарша ші импичмент жариала,racism

1,бұл біртүрлі оқиға кәдімгі нәсілшілдік негрді зиянсыз әзіл ретінде пайдалануды қабылдау менің ойымша сіз де өзіңізді нәсілшіл деп санамайсыз,бұл біртүрлі оқиға кәдімгі нәсілш нег зиян әзіл рет пайдалану қабылдау менің ойымша сіз де өз нәсілшіл де санама,racism

1,желкекке обама мылқау нигга шайтан алғыр,желкек оба мылқа нигг шай алғы,racism

1,құдай біреу бұл ниггерді өлтіруі керек өтінемін бұл үшін souljah dumb fuck ті линчке салыңыз өтінемін,құдай біреу бұл нигг өлтір керек өт бұл үшін souljah dumb fuck ті линч сал өт,racism

1,lol мен мылқау ақымақ менің twitter дегі басты бетке енді үлкен әтешті сорып ал сен нигга қазғышсың,lol мен мылқа ақы менің twitter дег басты бет енді үлкен әтеш сор ал сен нигг қазғыш,racism

1,хахах rt рас па шынымен басқа нәсілдердің нәсілшілдігі жеткіліксіз бе rt себебі сіз түрлі түсті емес ашық сарысыз лол,хахах rt рас па шын басқа нәсіл нәсілшілдіг жеткіліксіз бе rt себебі сіз түрлі түсті емес ашық сары лол,racism

1,өте жиіркенішті оның парақшасында деген аты қандай жаман нигга мен мұны айта аламын өйткені мен де қарамын,өте жиіркеніш оның парақша де ат қандай жаман нигг мен мұны ай ал өйткені мен де қар,racism

1,ниггер сен ақымақ емессің сіздің есегіңіз тозақ сияқты ақымақ иә қарғыс атқан сүңгуір қайықты әлі ыстық күйінде ұстаңыз шииии,нигг сен ақы емес сіздің есе тозақ сияқты ақы иә қарғыс ат сүңгуі қайық әлі ыстық күй ұста шииии,racism

1,сіздің орындаушылық әрекетіңіз жиіркенішті сіз өзіңіздің сарайыңызда қалай отыра аласыз және бреонна тейлор мен қара трансгендерлердің өмірін талқылай аласыз егер сіз қара адамдарға айтқан нәсілшіл пікірлерге жауап бере алмасаңыз сіз бізді маймылдар мен ниггерлер деп атадыңыз сізге желкек,сіздің орындаушылық әрекет жиіркеніш сіз өз сарай қалай отыр ал және бреонн тейло мен қара трансгендер өмір талқыла ал егер сіз қара адам айт нәсілшіл пікір жауап бер алма сіз біз маймыл мен ниггер де ата сізге желкек,racism

1,обамаға қарғыс атсын мылқау нигга деді ол байсалды,обама қарғыс ат мылқа нигг де ол байсалды,racism

1,бұл пікір шын мәнінде екі bp арқылы жазылған уоррен президенттікке үміткер болғанға дейін қара нәсілділердің өміріне қызығушылық танытпады олардың ешқайсысы қазіргі жоспарлардан басқа оның өткенін көрсете алмайды неліктен ол бұрын қызығушылық танытпады,бұл пікір шын мән екі bp арқылы жазыл уоррен президенттік үміткер бол дейін қара нәсілді өмір қызығушылық танытпа олардың ешқайсы қазіргі жоспар басқа оның өт көрсе алма неліктен ол бұрын қызығушылық танытпа,racism

1,сіз қара адамды негр деп атаған қарт ақ адамды қорғадыңыз,сіз қара адам нег де ата қарт ақ адам қорға,racism

1,ондағы негрлер есінен танып қалды олар иә айналасында жүре алады өздері қалаған нәрсені істей алады және айта алады деп ойлайды жоқ мен құлдарға иелік етпедім және осы гетто қоқыстарынан пайда көрмедім олар бұл елде желкек те салған жоқ ақ деп аталатын миллион адам сізге ештеңе қарыз емес,ондағы негр ес тан қал олар иә айнала жүр ал өз қала нәрсе істе ал және ай ал де ойла жоқ мен құл иелік етпе және осы гетто қоқыс пайда көрме олар бұл ел желкек те сал жоқ ақ де атал миллион адам сізге ештеңе қарыз емес,racism

1,сонымен ақ адам мені бүгін негр деп атады мен жынды боламын деп ойладым бірақ бұл мені күлдірді,сонымен ақ адам мен бүгін нег де ат мен жын бол де ойла бірақ бұл мен күл,racism

1,трамп сайлауға түсу үшін жалдаған шығар трамп жеңіске жетуі үшін байденнен жас және қара дауыстарды алуы мүмкін мен ештеңені жіберіп алмаймын,трам сайла түсу үшін жалда шығар трам жеңіс жет үшін байден жас және қара дауыс ал мүмкін мен ештеңе жібер алма,racism

1,бұл түсі өзгерген маймылдар мені ауыртады гребаные ниггерлер мен ағартатын қара адамдарды жек көремін сіз ақымақ бейбақсыз,бұл түс өзгер маймыл мен ауырт гребаны ниггер мен ағарт қара адам жек көр сіз ақы бей,racism

1,әрине ол саған мейірімді болды сен ақсың ол президент обаманы қара магиямен және миллионға жуық басқа нәсілшіл сексистік және қорқынышты нәрселермен негр деп атады бірақ иә ол сен кішкентай кезіңде саған мейірімді еді,әрине ол саған мейірімді бол сен ақ ол президент обама қара магия және миллион жуық басқа

нәсілшіл секси және қорқыныш нәрсе нег де ат бірақ иә ол сен кішкентай кез саған мейірімді ед,racism

1,сәлем нигга тозаққа сенің өмірің amp сіздің ең жақын досыңыз барлар фритисдик ммфао сен ақымақсың ахаха,сәлем нигг тозақ сенің өмір amp сіздің ең жақын дос бар фритисдик ммфао сен ақы ахах,racism

1,grindr дегі жігіт жалаңаш суреттерді сұрады мен оларды жіберуден бас тартқанымда ол маған полиция мені жиіркенішті қара аяқты нигга деп атып тастайды деп үміттенетінін айтты,grindr дег жігіт жалаңаш сурет сұр мен ол жіберу бас тарт ол маған полиция мен жиіркеніш қара аяқ нигг де ат таста де үміттен ай,racism

1,біз ажырасқаннан бері менің алғашқы құтқарылғаным болды,біз ажырас бері менің алғашқы құтқарыл бол,racism

1,айтуынша бүгінде барлық ниггерлер ол қалай нәсілшіл бола алады нигга қара сен ақымақ бастарсың лол,айт бүгін барлық ниггер ол қалай нәсілшіл бола ал нигг қара сен ақы баста лол,racism

1,rt барды сен желкекке ракон біз сен сияқты адамдарды нигга деп атаймыз сенің нигга мінез құлқың сиыр көңінің мылқау бөлігі анаңызды ұрыңыз,rt бар сен желкек ракон біз сен сияқты адам нигг де ата сенің нигг мінез құл сиыр көң мылқа бөліг ана ұр,racism

1,иә бұл қаншық обаманы ақымақ ниггерді тозаққа түсірді бұл өте маңызды ма иә,иә бұл қаншық обама ақы нигг тозақ түс бұл өте маңызды ма иә,racism

1,дауыс бермеген қайтадан негрлерге қайта даярланды бақытты кран биі мылқау бейбақтар осындай шынайы адам,дауыс берме қайта негр қайта даярла бақытты кран би мылқа бейбақ осындай шынайы адам,racism

1,ооо dhjdfhsk менде ешқандай түсінік жоқ мүмкін сізге алдымен инициал содан кейін сөздердің бірі ұнайды ма blueprint ұнайды ма мүмкін соңында санды қосыңыз hfdskjjhsj,ооо dhjdfhsk менде ешқандай түсінік жоқ мүмкін сізге алд инициал содан кейін сөз бірі ұна ма blueprint ұна ма мүмкін соң сан қос hfdskjjhsj,racism

1,сен ақымақ ниггерсің smh rt дөрекі болуды доғар сен жексұрынсың fk rt тозақ бүгін мен босануды бақылау таблеткаларын қабылдай бастадым,сен ақы нигг smh rt дөрекі болу доғар сен жексұрын fk rt тозақ бүгін мен босану бақылау таблетка қабылда баста,racism

1,сіз ақымақ болып жатырсыз қандай да бір ақымақтық айтасыз және біреуді қорлайсыз нигга сізді ұрысқа шақырады және сіз өлдіңіз,сіз ақы бол жатыр қандай да бір ақымақтық ай және біреу қорла нигг сіз ұрыс шақыр және сіз өл,racism

1,rt ниггерлер олармен сізбен қалай әрекет етсе солай істегенді жек көреді,rt ниггер олармен сізбен қалай әрекет ет солай істе жек көр,racism

1,алдымен мен бұл мен тырысамын менің сүйіктілерімнің бірі болады деп ойладым ал қазір бұл ші орынға ұқсайды сондықтан сіз кем дегенде шы жылдарды тыңдамайынша тректерді ешқашан бағаламауыңыз керек,алд мен бұл мен тырыс менің сүйікті бірі бол де ойла ал қазір бұл ші орын ұқса сондықтан сіз кем де шы жыл тыңдамайынш трек ешқашан бағалама керек,racism

1,rt леди гага исаның жаңа қара екенін айтты сонымен ол исаны нигга деп атады ол жиіркеніші,rt леди гаг иса жаңа қара екен ай сонымен ол иса нигг де ат ол жиіркеніш,racism

1,ромни пікірталаста жеңіске жетті кедей негрлер қазір осындай қиын жағдайда сен бардың ақымақ есек ақымақ қаншық,ромни пікірталас жеңіс же кедей негр қазір осындай қиын жағдай сен бар ақы есек ақы қаншық,racism

1,көз жасы rt сіздің анаңыз олардың барлығына rt теңіз сұңқарлары мылқау ниггерлер жинағы,көз жас rt сіздің ана олардың барлығ rt теңіз сұңқар мылқа ниггер жинағ,racism

1,хек мен фильмдердің ниггерлерін жек көремін барлығы туралы ойлау менің нөмірімді жойыңыз мылқау нигга мен сені өшіремін менің бетімнен кет мылқау бейбақ,хек мен фильм ниггер жек көр барлығ туралы ойлау менің нөмір жой мылқа нигг мен сен өшір менің бет кет мылқа бей,racism

1,қара адамдар міндетті түрде ақ адамдарға нәсілшіл емес мен көрген кез келген жағымсыздық немесе ұнатпау өткен тәжірибеге негізделген кез келген басқа нәрсеге қарағанда ptsd немесе жарақатқа ұқсайды бірақ мен ақ адамдардың қара нәсілділерді жек көретінін көрдім,қара адам міндетті түр ақ адам нәсілшіл емес мен көр кез кел жағымсыз немесе ұнатпа өткен тәжірибе негіздел кез кел басқа нәрсе қара ptsd немесе жарақат ұқса бірақ мен ақ адам қара нәсілді жек көр көр,racism

1,мен жартылай қарамын сен ақымақ бейбақсың сондықтан сенің нәсілшіл пікірлерің мені қорлайды сондықтан мен оны нигга деп атамаймын сен надан жетілмеген баласың,мен жартылай қар сен ақы бей сондықтан сенің нәсілшіл пікір мен қорла сондықтан мен оны нигг де атама сен надан жетілме бал,racism

1,біріншіден сіз соншалықты әдемі жазасыз мен оны қалаймын екіншіден сіз ең қайғылы hdjsjsjs таңдадыңыз иә,бірінші сіз соншалықты әдемі жаз мен оны қала екінші сіз ең қайғылы hdjsjsjs таңда иә,racism

1,олар сіздің ойыңызға бірінші болып келді,олар сіздің ой бірінші бол кел,racism

1,желкекке обама барды мылқау нигга хахахаха сен жындысың ші мерзім обама,желкек оба бар мылқа нигг хахахах сен жынд ші мерзім оба,racism

1,оның мылқау отбасы соқыр саңырау және әлсіз отбасы бұл қаншықты құшақтады сондықтан әлем бізді халық ретінде құрметтемейді христиандық бұл ниггерлерді ақылсыз етті,оның мылқа отбасы соқыр саңырау және әлсіз отбасы бұл қаншық құшақт сондықтан әлем біз халық рет құрметтеме христиан бұл ниггер ақыл ет,racism

1,аноним мына лас ниггерді қараңыз smh иә айтпақшы мен ақ түстімін сен ақымақ бейбақсың,анони мына лас нигг қара smh иә айтпақшы мен ақ түсті сен ақы бей,racism

1,басқа адамдарды нигга деп атайтын адамдар ақымақ бейбақтар гребан тозақта күйіп кету,басқа адам нигг де ата адам ақы бейбақ гребан тозақ күй кету,racism

1,барлық ақ адамдар мұны айта алмайтындықтан мен олар үшін айтамын дауысты тазартады хаха сіздердің барлығыңызға мылқау ниггерлер біз тағы да жеңдік ақ күш,барлық ақ адам мұны ай алмайтындық мен олар үшін ай дау тазарт хах сіздердің барлығ мылқа ниггер біз тағы да жең ақ күш,racism

1,менің ең жақын досым қара бірақ ол жылдар сияқты өткен кез келген нәрсені тыңдаудан бас тартады,менің ең жақын дос қара бірақ ол жыл сияқты өткен кез кел нәрсе тыңдау бас тарт,racism

1,def артта қалған rt бұл сұрақ емес сіз жынды болдыңыз сіз ашуланасыз ба rt бұл мылқау нигга обамаға,def арт қалған rt бұл сұрақ емес сіз жын бол сіз ашулан ба rt бұл мылқа нигг обама,racism

1,барлық ромни жақтастары үшін обаманы ниггер деп атайды ол сондай ақ жартылай ақ сіз ақымақ бейбақтар,барлық ромни жақтас үшін обама нигг де ата ол сондай ақ жартылай ақ сіз ақы бейбақ,racism

1,нәсілшілдік бұл ақ киімді қара емес бірінші кезекте жуу үшін таңдау нәсілшіл болмаңыз олардың барлығын бірге жуыңыз lmao,нәсілш бұл ақ ки қара емес бірінші кезек жу үшін таңдау нәсілшіл болма олардың барлығ бірге жу lmao,racism

1,жоқ бұл қаншық мұны істемеді өзіңді өлтір кетмен бұл қаншықпен айналыс foh rt обамаға қарғыс атсын мылқау нигга,жоқ бұл қаншық мұны істеме өз өлтір кет бұл қаншық айналыс foh rt обама қарғыс ат мылқа нигг,racism

1,мен ууа уа үшін бір нәрсе тоққым келеді бірақ соңғы жыл ішінде ол қара немесе сұрдан басқа ештеңе киюден бас тартты және түстерді анықтау қиынға соғады,мен ууа уа үшін бір нәрсе тоқ кел бірақ соңғы жыл іш ол қара немесе сұр басқа ештеңе кию бас тар және түс анықтау қиын соғ,racism

1,қорқынышты өткен біз бәріміз болашақта өмір сүріп осы апатты тоқтатуымыз керек менің нәсілім маңызды менің жүрегім көптеген адамдар басынан өткергендіктен ауырады және сіз осы демалыс күндері чикагодан бірдеңе жасадыңыз ба адам атып өлтірілді адам өлтірілді және қара түсік түсірді ме жоқ сіз тек ақымақтар мен тізелер туралы айтасыз ба,қорқыныш өткен біз бәрі болашақ өмір сүр осы апатты тоқтат керек менің нәсіл маңызды менің жүрег көптеген адам бас өткергендік ауыр және сіз осы демалыс күн чикаго бірде жаса ба адам ат өлтір адам өлтір және қара түсік түс ме жоқ сіз тек ақымақ мен тізе туралы ай ба,racism

1,сайлаудан бері мен ақ қыздың баланың ниггер немесе бинер сияқты ақымақ немесе жеккөрушілікпен әрекет еткенін көрмедім бірақ ақ адамдарға қарғыс атсын

біз нәсілшілміз,сайлау бері мен ақ қыз бала нигг немесе бин сияқты ақы немесе жеккөрушілік әрекет ет көрме бірақ ақ адам қарғыс ат біз нәсілшіл,racism

1,обамаға қарғыс атсын мылқау нигга мылқау әйел,обама қарғыс ат мылқа нигг мылқа әйел,racism

1,обамаға қарғыс атсын мылқау нигга бұл қаншықты тез шешейік,обама қарғыс ат мылқа нигг бұл қаншық тез шеш,racism

1,сізге қарсы емес ұсқынсыз нигга,сізге қарсы емес ұсқын нигг,racism

1,көше баласы деп аталатын ақымақ ақымақ кім пен жас джеззиді қорлап иә айналасында жүреді бұл ниггерді қарғыс атсын,көше бала де атал ақы ақы кім пен жас джеззи қорла иә айнала жүр бұл нигг қарғыс ат,racism

1,бұл қара халыққа бейтарап нәсілшілдік танытатын бірақ біреу ағылшынша сөйле бұл орауыш сіз бардыңыз және түрлі түсті нәсілшілдікті енгізген ақ адам сіз бәріңіз паразиттерсіз,бұл қара халық бейтарап нәсілш таныт бірақ біреу ағылшынша сөйле бұл орауыш сіз бар және түрлі түсті нәсілшілдік енгіз ақ адам сіз бәрі паразитт,racism

1,келіңіздер нигга батыл дегенді білдіреді иә шайтан алғыр сияқты сондықтан біз мылқау және ақымақпыз,кел нигг батыл де білдір иә шай алғы сияқты сондықтан біз мылқа және ақы,racism

1,бұл аккаунт қандай болды ұмытып кетті бұл доғал нигга трахает нигга,бұл аккаунт қандай бол ұмыт кет бұл доғал нигг трахает нигг,racism

1,мен қара нәсілді адамдардың твиттер жазып жатқанын және өзімізді бәрімізді әзіл ретінде нигга деп атайтынын көремін бұл жиіркенішті,мен қара нәсіл адам твиттер жаз жат және өз бәрі әзіл рет нигг де ата көр бұл жиіркеніш,racism

1,оның артықшылықты тәрбиесі болғаны сонша бұл жай ғана күлкілі ол еңбекқор қара британдықтарды білдірмейді тек өзінің қоғамдық имиджін өзімшілдігін және менмендігін білдіреді мен оның бұрын жас қара адамдардың көңіл күйін көтеретін нәрсе жасағанын көрген емеспін табан өтіп атақты адам оған секіреді,оның артықшылық тәрбие бол сонша бұл жай ғана күлкіл ол еңбекқор қара британдық білдірме тек өзінің қоғамдық имидж өзімшілдіг және менмендіг білдір мен оның бұрын жас қара адам көңіл күй көтер нәрсе жаса көр емес табан өт атақты адам оған секір,racism

1,обаманы мылқау ниггерді ууууyyyyy,обама мылқа нигг ууууyyyy,racism

1,трахают доғал ниггерлер ал онда доғал бесінші нүкте артында руль wtf dudeski,трахают доғал ниггер ал онда доғал бесінші нүкте арт руль wtf dudeski,racism

1,обамаға қарғыс атсын ақымақ нигга тозақ сияқты ашуланшақ ақымақ ctfu,обама қарғыс ат ақы нигг тозақ сияқты ашуланшақ ақы ctfu,racism

1,фавр бас бапкерге сен желкеккеге бардың дейді тақырыптар викингтерді кім басқарады о осы ақымақтықпен айналыссын ол жынды нигга,фав бас бапкер сен желкекке бар де тақырып викинг кім басқар о осы ақымақтық айналыс ол жын нигг,racism

1,жақында барлығы кеңінен қолданған негр терминінің қашан және неге соншалықты қорлайтын болғанын барлығына түсіндіре аласыз ба неліктен қара нәсілділердің барлығын күні бойы күн сайын сөзін тыңдауы қалыпты жағдай мен ақ түстімін ал қара нәсілділер мені деп атайды,жақында барлығ кең қолдан нег термин қашан және неге соншалықты қорла бол барлығ түсіндір ал ба неліктен қара нәсілді барлығ күн бойы күн сайын сөз тыңда қалыпты жағдай мен ақ түсті ал қара нәсілді мен де ата,racism

1,кейн ол мылқау мылқау нигга және менің ойымша оның істеген ісімен келісетін жалғыз адамдар да нигга екендігі тағдырдың ирониясы ниггерлерге тозақ,кейн ол мылқа мылқа нигг және менің ойымша оның істе іс келіс жалғыз адам да нигг екендіг тағ ирония ниггер тозақ,racism

1,елшілердің істері құдайдан еврейлерге құдай халқының құдайдың қара терісі алтын негр еврейлеріне тиесілі мағынасы шыққан шыққан риза сент джонды қараңыз сондай ақ нефилимдердің ақ түріне жататындар құдай шайтанның шаштары деп атаған адамдар,елші іс құдай еврей құдай халқ құдай қара тері алтын нег еврей тиесілі мағына шық шық риза сент джо қара сондай ақ нефилим ақ түр жат құдай шайтан шаш де ата адам,racism

1,жігіттер бұрап алды олардың барлығы верден доп ойнауға барады ал bgc вааааай жаттығу залы жағымды және үлкенірек доғал бас ниггерлер бос жаттығу залын трахает етеді,жігіт бұра алды олардың барлығ ве доп ойна бар ал bgc вааааа жаттығу зал жағымды және үлкенірек доғал бас ниггер бос жаттығу зал трахает ет,racism

1,менің тікұшақ досым дэммен бірге жүремін сізге қайыршы ниггерлерге және ақымақ қаншықтарға скрр скрр сіз менімен бірге блоктасыз,менің тікұшақ дос дэ бірге жүр сізге қайырш ниггер және ақы қаншық скр скр сіз менімен бірге блок,racism

1,сперматозоидтарды ішіп тезек жеп қойыңыз сіз есек фагот қарғысатқыр нигга ақ билік мылқау ниггерлер ақ билік бәріңізді басқарады желкекке сүйеніңіз,сперматозоид іш тезек же қой сіз есек фагот қарғысатқы нигг ақ билік мылқа ниггер ақ билік бәрі басқар желкек сүйен,racism

1,желкек қызыл сен ақымақ ниггерсің,желкек қызыл сен ақы нигг,racism

1,rt обамаға қарғыс атсын мылқау нигга lt lt lt lt ол жынды llf теңізі,rt обама қарғыс ат мылқа нигг lt lt lt lt ол жын llf теңіз,racism

1,хаха сенің анаң сіз мылқау ниггерсіз сіз мені аркада құрсау ойынында көре аласыз,хах сенің ана сіз мылқа нигг сіз мен арка құрсау ойын көр ал,racism

1,бұл мылқау ниггерлер осында,бұл мылқа ниггер осында,racism

1,кейбір ниггерлердің қара әйелдерге деген өшпенділігі жиіркеніштi мысалы шынымен көмек сұраңыз,кейбір ниггер қара әйел де өшпенділіг жиіркеніш мысал шын көмек сұра,racism

1,сізде ірімшік ірімшігі бар гамбургер алғыңыз келетінін айтқан досыңыз болды ма кейде ол ақымақ бірақ бәрібір менің ниггерім,сіз ірімшік ірімшіг бар гамбург ал кел айт дос бол ма кейде ол ақы бірақ бәрібір менің нигг,racism

1,қара ханым есінен танып қалады өйткені ол кекстері бар белбеу киетін және оны гей деп санайтын жігіттен жүкті ол ақымақ негр әйел,қара ханым ес тан қал өйткені ол кекс бар белбеу ки және оны ге де сана жігіт жүкті ол ақы нег әйел,racism

1,сік нигга нигга ол трахается мылқау қайыршы ниггерлер қайыршылар ниггерлер қалады қайыршылар жоқ трахайс эмм,сік нигг нигг ол трахается мылқа қайырш ниггер қайыршы ниггер қал қайыршы жоқ трахайс эм,racism

1,жиіркеніштi негр обама сайлауда жеңіліп ромни барлық қара нәсілділерді концлагерьлерге жібереді деп үміттенемін,жиіркеніш нег оба сайла жеңіл ромни барлық қара нәсілді концлагерь жібер де үміттен,racism

1,бұл семіз ұсқынсыз жалпақ жүзді нәсілшіл ақымақ тозақ сияқты ақымақ барлығы шығып дауыс беріңіз сонда біз бұл ниггерді қызметінен алып тастай аламыз,бұл се ұсқын жал жүз нәсілшіл ақы тозақ сияқты ақы барлығ шығ дауыс бер сонда біз бұл нигг қызмет алып таста ал,racism

1,мен қоңыр ақымақ болудан бастамаңыз сен қара сен ниггерсің сіз лас және жиіркенішсіз lt бұләмір,мен қоңыр ақы болу бастама сен қара сен нигг сіз лас және жиіркеніш lt бұләмі,racism

1,менің ойымша сезім бұл сіздің iq тен төмен түскенде пайда болатын алғашқы сезімдердің бірі сондықтан сіз шынайы жауап ала алмайсыз,менің ойымша сезім бұл сіздің iq тен төмен түс пайда бол алғашқы сезім бірі сондықтан сіз шынайы жауап ал алма,racism

1,әрқашан миы жуылған мылқау нигга бар ол қара адаммен сігу жасауға тырысады қара үшін бір нәрсені өзгертуге тырысады ақымақ ниггерлер досым,әрқашан ми жуыл мылқа нигг бар ол қара адам сіг жаса тырыс қара үшін бір нәрсе өзгерт тырыс ақы ниггер дос,racism

1,пәкістанның өлген баласына жылап жатқан ниггерлер олардан гөрі жақсы жұмыс істейді бұл үшін жалған джим қарғасын кректің мылқау өтірігін кінәлайық сонда майлы ескі ниггерлер ақ түсті болып көрінуі мүмкін маған қараңызшы мен үлкен ұл сияқты жұмыс істеймін ал қыздар бәрібір бұралып қалады өйткені қара қоғамдастық пісірілген жалқауларға толы үгінділер,пәкістан өлген бала жыла жат ниггер олардан гөрі жақсы жұмыс істе бұл үшін жалған джи қарға крек мылқа өтіріг кінәл сонда майлы ескі ниггер ақ түсті бол көрін мүмкін маған қараңызш мен үлкен ұл сияқты жұмыс істе ал қыз бәрібір бұрал қал өйткені қара қоғамдастық пісір жалқау толы үгінді,racism

1,ең керемет ниггерлер үшін гей болу бұл трахаться әрине мен мылқау лесбиянмын,ең керемет ниггер үшін ге болу бұл трахаться әрине мен мылқа лесбиян,racism

1,жоқ бұл шынымен де мен жылаймын менің атам өмірінің соңында деменцияға ие болды және ол өте жақсы болды тыныш менің әпкем ауруханада деменциясы бар ескі ақ жігітпен бірге болды және ол оны түрлі түсті қара және азиялық медбикелер деп атады қалай,жоқ бұл шын де мен жыла менің ата өмір соң деменция ие бол және ол өте жақсы бол тыныш менің әпке аурухана деменция бар ескі ақ жігіт бірге бол және ол оны түрлі түсті қара және азиялық медбике де ат қалай,racism

1,кім шайтан алғыр лақтырады балшыққа доғал қарғысатқыр нигга туралы prk,кім шай алғы лақтыр балшық доғал қарғысатқы нигг туралы prk,racism

1,мен шағымданбаймын бірақ бұл шоуға қатысуға өтініш берген қара нәсілді ер адамдар білімнен басқа нәрсемен айналысады ма соңғы маусымда олардың барлығы осы салада болды бір қарағанда үйленген mafs,мен шағымданба бірақ бұл шоу қатыс өтініш бер қара нәсіл ер адам біл басқа нәрсе айналыс ма соңғы маусым олардың барлығ осы сала бол бір қара үйлен mafs,racism

1,досым мен datemyfamily ді тағы бір рет көруім керек бұл нигга өте ақымақ болды ол кешкі асқа ақша төледім десе хмао,дос мен datemyfamily ді тағы бір рет көр керек бұл нигг өте ақы бол ол кешкі ас ақша төле де хмао,racism

1,менің wrk киімім желкек мылқау нигга,менің wrk киім желкек мылқа нигг,racism

1,отвали доғал көт ниггер,отвали доғал көт нигг,racism

1,бұл ақымақтық мылқау нигга twitchalerts,бұл ақымақтық мылқа нигг twitchalerts,racism

1,желкекке обама барды ақымақ нигга құдайға,желкек оба бар ақы нигг құдай,racism

1,джален роузға қарғыс атсын олар дьюкке бару үшін тым ақымақ болды сондықтан уэббер тайм аут жариялады ол тек секіре алатын әдеттегі мылқау нигга,джален роуз қарғыс ат олар дьюк бару үшін тым ақы бол сондықтан уэбб тайм аут жариял ол тек секір ал әдет мылқа нигг,racism

1,бұл елдегі нәсілшілдік бәрімізді жынды ететін нәрсе қарт жас түрлі түсті және б маңызды емес сіз оның прогрессивті medicare бағдарламасын барлығына қабылдамайсыз рас жүйесін сарқып жатырсыз және б бізге қосылыңыз сонда сіз де жеңімпаз боласыз,бұл ел нәсілш бәрі жын ет нәрсе қарт жас түрлі түсті және б маңызды емес сіз оның прогрессивті medicare бағдарлама барлығ қабылдама рас жүйе сарқ жатыр және б бізге қосыл сонда сіз де жеңімпаз бол,racism

1,мен ақ музыканы тыңдаймын бұл барлық мылқау нигга музыкасы лол,мен ақ музыка тыңда бұл барлық мылқа нигг музыка лол,racism

1,нәсілшілдік шынайы сіз өзіңіздің ақымақ президентіңіз сіз сияқты ақ адамдарды осы ақымақтық туралы ойлауға мәжбүрлеу үшін ит ысқырықтарын қолданатынын

білесіз ешкімге сіздің аянышты ақшаңыз ақ қаншық түрлі түсті адамдар сіздің артықшылықтарыңыздан қайта қайта айырылатынын түсінудің нақты хабары қажет емес,нәсілш шынайы сіз өз ақы президент сіз сияқты ақ адам осы ақымақтық туралы ойла мәжбүрлеу үшін ит ысқырық қолдан біл ешкім сіздің аяныш ақша ақ қаншық түрлі түсті адам сіздің артықшылық қайта қайта айырыл түсіну нақты хаб қажет емес,racism

1,сіз нигга ақымақ бейбақ өйткені мен мұны бұрыннан білетінмін,сіз нигг ақы бей өйткені мен мұны бұрын біл,racism

1,шайтан алғыр шайтан алғыр джорджегамбнино джорджегамбнино нигерия нигга әлемі балтимор нигерия нигга әлемі ямайка нигерия нигга әлемі майами нигерия нигга әлемі нью йорк нигерия нигга әлемі gh нигерия нигга әлемі нигга әлемі afr жалаусыз қаншық мылқау pic twitter com,шай алғы шай алғы джорджегамбнино джорджегамбнино нигерия нигг әлем балтимо нигерия нигг әлем ямайк нигерия нигг әлем майами нигерия нигг әлем нью йорк нигерия нигг әлем gh нигерия нигг әлем нигг әлем afr жалау қаншық мылқа pic twitter com,racism

1,желкек обамаға барды ақымақ бас нигга сен жындысың ба ашулануды жалғастырыңыз құдайым smh,желкек обама бар ақы бас нигг сен жынд ба ашулану жалғастыр құдай smh,racism

1,ooo rt nisstrilla дағы тізедегі қыз маған қара rt обамаға қарғыс атсын мылқау нигга,ooo rt nisstrilla дағ тізе қыз маған қара rt обама қарғыс ат мылқа нигг,racism

1,сіз бардыңыз мен қамқоршымын және жасау жақын арада күніне ға жуық жасөспірімді қабылдауға және бірінші және жалғыз қорғаныс желісі болуға мәжбүр болады жкқ ға да қосымша тазалау құралдарына да тіпті ойын жоспарына да ақша жоқ денсаулығыңызға қауіп төндірмеген кезде бағалау оңай барлық жерде қатаң ережелер бар,сіз бар мен қамқоршы және жасау жақын ара күн ға жуық жасөспірім қабылда және бірінші және жалғыз қорғаныс желі бол мәжбүр бол жкқ ға да қосымша тазалау құрал да тіпті ойын жоспар да ақша жоқ денсаулығ қауіп төндірме кезде бағалау оңай барлық же қатаң ереже бар,racism

1,дәл қазір кішкентай ниггер тим маған хейздің жарты пакетін сатты мен долларлық вексельді жоғалттым тозаққа менің өмірім,дәл қазір кішкентай нигг тим маған хейз жарты пак сат мен долларлық вексель жоғал тозақ менің өмір,racism

1,тағы жыл lmaoooooo rt обамаға қарғыс атсын мылқау нигга сіз суицид сағатындасыз,тағы жыл lmaoooooo rt обама қарғыс ат мылқа нигг сіз суицид сағ,racism

1,заң өзінің еңбекқорлығын көрсетсін сіз ақымақсыз мұрындарын ысқылайтындар үшін клариссакроули оқиғасы сіз проблемасыз асығыс мәлімдемелер қауесеттердің таралуы жауапсыздық нәсілшілдік оның ең нашар көрінісінде дартмут сіз не көмектесесіз не ренжітесіз жағын таңдаңыз,заң өзінің еңбекқорлығ көрсет сіз ақы мұрын ысқыла үшін клариссакроули оқиға сіз проблема асығыс мәлімдеме қауесет

тарал жауапсыздық нәсілш оның ең нашар көрініс дартмут сіз не көмектес не ренжі жағ таңда,racism

1,сомалилер тек басқа сомалилермен кездесуі үйленуі керек аджнабимен бірге болуы керек тіпті олар қара болса да жиіркенішті және тыйым салынуы керек хави жарайды ниггер,сомали тек басқа сомали кездес үйлен керек аджнаби бірге бол керек тіпті олар қара бол да жиіркеніш және тыйым салын керек хави жарайды нигг,racism

1,бұл чарли браунның алғыс айту күнінде нәсілшілдік басым деген оймен тікелей байланысты себебі түрлі түсті бала үстелге жалғыз отырды егер сіз чарльз шульц туралы оқыған болсаңыз олар шульцтің бұл кейіпкерді қосқысы келетінін бірақ нәсілшілдіктен қорқатынын көрер еді,бұл чарли браун алғыс айту күн нәсілш басым де ой тікелей байланысты себебі түрлі түсті бала үстел жалғыз от егер сіз чарльз шульц туралы оқы бол олар шульц бұл кейіпкер қос кел бірақ нәсілшілдік қорқ көрер ед,racism

1,ff түрі сіздің уақыт шкалаңызда мэдисон смувтың бұл ниггері жоқ мылқау әйелді ұрыңыз ақылды нәрсе жасаңыз және маған еріңіз,ff түр сіздің уақыт шкала мэдисон смув бұл нигг жоқ мылқа әйел ұр ақылды нәрсе жаса және маған ер,racism

1,бұл ниггерлерді ауырту үшін сіздің қаншығыңызды мазақ ете алар еді,бұл ниггер ауырт үшін сіздің қаншығ мазақ ет ал ед,racism

1,бұл джордж флойдтан әлдеқайда алыс барлығы флойдтың алдын алуға болатындығымен келіседі және бұл қайғылы жағдай болды бірақ демократтар мақұлдаған топтар қара өмір маңызды ал антифа қылмыскерлер пелоси оларды жақсы көреді және олар туралы ештеңе айтпайды тіпті балаларды жасөспірімдерді өлтірсе де b,бұл джордж флойд әлдеқайда алыс барлығ флойд алдын ал болатындығ келіс және бұл қайғылы жағдай бол бірақ демократ мақұлда топ қара өмір маңызды ал антиф қылмыскер пелоси ол жақсы көр және олар туралы ештеңе айтпа тіпті бала жасөспірім өлтір де b,racism

1,сіз бардыңыз мылқау крекер rt бардым желкек обама мылқау нигга,сіз бар мылқа крек rt бар желкек оба мылқа нигг,racism

1,мыңжылдықтар жай білмейді ниггер ол жаста желкек мылқау нигга pic twitter com knblkknacz,мыңжылдық жай білме нигг ол жас желкек мылқа нигг pic twitter com knblkknacz,racism

1,ал сен сүйкімдісің мәселе мынада джой негро дэйв есімді ақ адам және оны қорлайтын есімі үшін әділ атайды кештен ләззат алыңыз мүмкін бірдеңе оқыңыз,ал сен сүйкімд мәселе мына джо негро дэйв ес ақ адам және оны қорла есім үшін әділ ата кеш ләззат ал мүмкін бірде оқ,racism

1,ол баруы тиіс отсосать мүшесі қандай да бір негритянском порно гребаная жиіркенішті қаншық сорады қара мүшесі үшін доллар кока кола дениз апай,ол бар

тиіс отсосать мүше қандай да бір негритянско порно гребаная жиіркеніш қаншық сор қара мүше үшін доллар кок кол дениз апа,racism

1,нигга бай қалада қалай өмір сүре алады және атақты бола алмайды қош бол мылқау,нигг бай қала қалай өмір сүр ал және атақты бола алма қош бол мылқа,racism

1,күте тұрыңыз мен сіздердің бәріңіз ниггерлер ақымақ екеніңізді ұмытып кеттім,күт тұр мен сіздердің бәрі ниггер ақы екен ұмыт кет,racism

1,трейвон алған нәрсесіне лайық панк ниггерлер ақылды болып дұрыс әрекет ете бастағаны жақсы ия сіз бардыңыз мылқау крекер,трейвон ал нәрсе лайық панк ниггер ақылды бол дұрыс әрекет ет баста жақсы ия сіз бар мылқа крек,racism

1,мен ешқашан британдық досым деп айтқан емеспін сіз осылай айтасыз және осы аптада екі ақ жігіт менің ниггерімді жұмысқа шақырған кезде біз деп айтпаңыз ал адамдар тайсонның негрлерді құлатқаны туралы твиттерде жазады егер бұл сізге қатысты болмаса айналдыруды жалғастырыңыз,мен ешқашан британдық дос де айт емес сіз осылай ай және осы апта екі ақ жігіт менің нигг жұмыс шақыр кезде біз де айтпа ал адам тайсон негр құлат туралы твиттер жаз егер бұл сізге қатысты болма айналдыру жалғастыр,racism

1,ақ нәсілшілдерді әсіресе ақ нәсілшілдерді қара нәсілділерден қорғайтындарды сататын және жалайтын қара адам малкольм үй негрі деп атаған том ағай,ақ нәсілшіл әсіресе ақ нәсілшіл қара нәсілді қорға сат және жала қара адам малколь үй нег де ата то аға,racism

1,белгілі бір жағдайлар мен қазір өсіп білім алдым және фанатизм мен нәсілшілдіктің артындағы оқиғаларды білемін біз түрлі түсті немесе мақта теретін джунгли немесе орман жануарлары емеспіз жабайы адамдар оқулық негрлері бұзақылар жыныстық заттар немесе табиғи түрде қатыгез қылмыскерлер егер мен мұның бәрін білсем неге сенімді емеспін,белгілі бір жағдай мен қазір өс білім ал және фанатиз мен нәсілшілдік арт оқиға біл біз түрлі түсті немесе мақта тер джунгли немесе орман жануар емес жабай адам оқулық негр бұзақы жын зат немесе табиғи түр қатыгез қылмыскер егер мен мұның бәрін біл неге сенімді емес,racism

1,шынымен надан rt бұл мылқау нигга обамаға,шын надан rt бұл мылқа нигг обама,racism

1,fight rt сонымен сіз ашуланасыз ба обамаға қарғыс атсын мылқау нигга,fight rt сонымен сіз ашулан ба обама қарғыс ат мылқа нигг,racism

1,rt тыңдаңыз нигга сіз қате естідіңіз мылқау сиқыршы ешкім сізді лас күлгін ерінге айналдырғысы келмейді,rt тыңда нигг сіз қате есті мылқа сиқырш ешкім сіз лас күлг ерін айналдыр келме,racism

1,эх егер бұл маған байланысты болса мен қара айдаһардың істегенінен гөрі сыртқы әлемдегі оқиғаларға көбірек көңіл бөлер едім сонымен қатар ойында үш қара айдаһар бар алайда бұл мен өткенмін және мен болашақпын де суды бұлдыратады,эх

егер бұл маған байланысты бол мен қара айдаһ істе гөрі сыртқы әлем оқиға көбірек көңіл бөл ед сонымен қатар ой үш қара айдаһ бар алайда бұл мен өт және мен болашақ де су бұлдырат,racism

1,бірақ сіз мұның бәрін түсінесіз солай ма rt обамаға қарғыс атсын мылқау нигга,бірақ сіз мұның бәрін түс солай ма rt обама қарғыс ат мылқа нигг,racism

1,олар тек қара спортшыларды ұнатады олардың пікірі де мәдениеті де ештеңесі де жоқ бұрын көптеген ақ адамдар айтқандай жай ғана үндемей осы ойынды ойнаңыз,олар тек қара спортшы ұнат олардың пікір де мәдениет де ештеңе де жоқ бұрын көптеген ақ адам айтқанда жай ғана үндеме осы ой ойна,racism

1,сіз өлесіз деп үміттенемін сіз бөксеңізді ұрасыз фагот және сіздің рифмаларыңыз қоқыс болады сіз мылқау бастықсыз тек ниггершу өлді,сіз өл де үміттен сіз бөксе ұр фагот және сіздің рифма қоқыс бол сіз мылқа бастық тек ниггерш өл,racism

1,мен ұсқынсыз ниггермін бе теріңізге қараңыз ақымақ шайтан алғыр сіз мен көретін жалғыз ниггерсіз сіз ит сияқты ұсқынсыз ақымақсыз,мен ұсқын нигг бе тер қара ақы шай алғы сіз мен көр жалғыз нигг сіз ит сияқты ұсқын ақы,racism

1,біз дауласқан сайын мен сенің мылқау екеніңді түсінемін мара адамның зауыттық ақауы сияқты нәрсе бар ма сіз өзіңіздің өміріңізде ер адамдар зауыттық ақаулардан зардап шегеді деп естідіңіз бе бұл әзіл нигга бірақ жалғызбасты аналар шынымды айтсам джо гор сіз оларды бірлік ретінде қарастырасыз ба,біз даулас сайын мен сенің мылқа екен түс мар адам зауыттық ақау сияқты нәрсе бар ма сіз өз өмір ер адам зауыттық ақау зардап шег де есті бе бұл әзіл нигг бірақ жалғызба ана шын айт джо го сіз ол бірлік рет қарастыр ба,racism

1,вупи біз қара нәсілділер ниггерлерді жиіркенішті және қорлайтын деп санаймыз бұл сөзбен сіз өз ақшаңызды жасырасыз ұлттың дүниеге келуін бақылаңыз,вупи біз қара нәсілді ниггер жиіркеніш және қорла де сана бұл сөз сіз өз ақша жасыр ұл дүние кел бақыла,racism

1,уэйд мылқау бейбақ мылқау боқ шығаруды доғарыңыз сіз ең болмағанда шетіне жақын нәрсені байқап көре аласыз ниггер атылған сағаттарда көздеді,уэйд мылқа бей мылқа боқ шығару доғар сіз ең болма шетіне жақын нәрсе байқа көр ал нигг атыл сағат көзде,racism

1,бланкос және негр фестивалі деп аталатын фестиваль бар онда қара адамдардың беті ақ ал ақ адамдардың беті қара,бланкос және нег фестивал де атал фестиваль бар онда қара адам бет ақ ал ақ адам бет қара,racism

1,бүгінгі кештің нәтижесі нені білдіреді ақ адамдар әлі күнге дейін қара нигга деп санайды соңы әңгімелер біз өмір сүретін жиіркенішті жер,бүгінгі кеш нәтиже не білдір ақ адам әлі күн дейін қара нигг де сана соңы әңгіме біз өмір сүр жиіркеніш жер,racism

1,тағы жыл lmaoooooo rt желкек обамаға барды мылқау нигга сіз өз өзіне қол жұмсауды көріп отырсыз,тағы жыл lmaoooooo rt желкек обама бар мылқа нигг сіз өз өзіне қол жұмсау көр отыр,racism

1,сіз менің айтқаныма мүлдем назар аудармағаныңызды қалаймын менің айтайын дегенім ол қара адамдардан ештеңе көрмеді сондықтан ол осылай дейді сонымен қатар ол қара адамдар шейннің бұрынғы мінез құлқы туралы айтпағанын айтқан жоқ ол мұны тек ақ адамдарда көргенін айтты,сіз менің айт мүлдем назар аударма қала менің айт де ол қара адам ештеңе көрме сондықтан ол осылай де сонымен қатар ол қара адам шейн бұрынғы мінез құлқы туралы айтпа айт жоқ ол мұны тек ақ адам көр ай,racism

1,қара адамдар ақ адамдарды ұнатпайды бұл нәсілшілдік емес өйткені сіздер барлық нәсілшілдерсіздер және біз сіздің майонезбен қапталған жезөкшелеріңізге еш қатысы жоқпыз,қара адам ақ адам ұнатпа бұл нәсілш емес өйткені сіздер барлық нәсілшіл және біз сіздің майонез қаптал жезөкше еш қатыс жоқ,racism

1,ол шынымен де rt ге соққы берді обамаға ақымақ нигга,ол шын де rt ге соққы бер обама ақы нигг,racism

1,rt letsbehonest гаитиге немесе онда тұратын мылқау ниггерлерге ешкімнің дела жоқ wwwooowwwwww,rt letsbehonest гаити немесе онда тұр мылқа ниггер ешкі дел жоқ wwwooowwwwww,racism

1,тумасы neɪ сын есімнің мағынасы бастапқыда деп аталды өсу әгәʊ етістіктің мағынасы белгілі бір уақыт аралығында үлкенірек немесе үлкенірек болу енді сіз ақ адамды қара негр деп атайтынын көресіз өйткені бастапқыда бойы бар қара адам енді өсе алмайды,ту neɪ сын ес мағына бастапқы де ата өсу әгәʊ етістік мағына белгілі бір уақыт аралығ үлкенірек немесе үлкенірек болу енді сіз ақ адам қара нег де ата көр өйткені бастапқы бойы бар қара адам енді өс алма,racism

1,қаншық өзіңізді баяу өлтіріңіз rt обамаға ақымақ нигга,қаншық өз бая өлтір rt обама ақы нигг,racism

1,лол сіз боқтықты білемін деп ойлайсыз бірақ сіз ештеңе білмейсіз мен ниггер дұрыс болғым келеді көптеген орындар дан кейін ашылады сіз қателесесіз,лол сіз боқтық біл де ойла бірақ сіз ештеңе білме мен нигг дұрыс бол кел көптеген орын дан кейін аш сіз қателес,racism

1,зеке әдеттегідей nfl дегі қара нәсілділер үшін мен жасамаған қылмысы үшін мен сенімдімін өйткені мен сенің ақ екеніңді білемін райли купер есімді ол қара ниггерлерді атады сонымен қатар керемет жігіт мен өте кеңес беремін сіз жабыласыз ақымақ қаншық,зе әдеттегідей nfl дег қара нәсілді үшін мен жасама қылмыс үшін мен сенімді өйткені мен сенің ақ екен біл райли куп ес ол қара ниггер ат сонымен қатар керемет жігіт мен өте кеңес бер сіз жабыл ақы қаншық,racism

1,rt барды желкекке обама ақымақ бас нигга lt lt егер сіз майлы есегіңізді жаппасаңыз қаншық,rt бар желкек оба ақы бас нигг lt lt егер сіз майлы есег жаппа қаншық,racism

1,сіз бардыңыз бэйли чемпионы мен сені жек көремін ақымақ бас нигга,сіз бар бэйли чемпион мен сен жек көр ақы бас нигг,racism

1,бұл мылқау нигга енді сіздің президентіңіз тағы жыл бойы nazyazya rt обама мылқау нигга,бұл мылқа нигг енді сіздің президент тағы жыл бойы nazyazya rt оба мылқа нигг,racism

1,ақш бұл менің елім егер өткен ай маған бірдеңе үйреткен болса төтенше жағдайда сенуге болатын жалғыз адам сіз сондықтан дайын болыңыз менің қорқынышты қара мылтығым ешқашан ешкімге зиян тигізбеді және ешқашан зиян тигізбеуі мүмкін соғыс жүріп жатқанда бәрі жақсы қараңыз,ақш бұл менің ел егер өткен ай маған бірде үйрет бол төтенше жағдай сен бол жалғыз адам сіз сондықтан дайын бол менің қорқыныш қара мылтығ ешқашан ешкім зиян тигізбе және ешқашан зиян тигізбе мүмкін соғыс жүр жатқанда бәрі жақсы қара,racism

1,джулиас малема және оның eff ақ компанияларға миллион реал сыйға тартты бұл ақ компанияларды бірінші орынға қоятын ынтымақтастық қоры деп аталады және бұл ғана емес оны ақ компаниялар басқарады сонымен эфф домкоптар біз өзімізді ақ компаниялармен байланыстырған кезде бізді үйдегі негрлер деп атауды доғарыңыз,джулиас мале және оның eff ақ компания миллион реал сый тар бұл ақ компания бірінші орын қоятын ынтымақтастық қор де атал және бұл ғана емес оны ақ компания басқар сонымен эфф домкоп біз өз ақ компания байланыстыр кезде біз үйдегі негр де атау доғар,racism

1,майкл клиффорд сіз бардыңыз мылқау нигга мен сенің артымнан келе жатқаныңды білемін,майкл клиффорд сіз бар мылқа нигг мен сенің арт келе жат біл,racism

1,сіз бардыңыз досым сіз бардыңыз мылқау нигга,сіз бар дос сіз бар мылқа нигг,racism

1,менің ші сабағым бірінші курс студенттеріне толы ал ниггерлер менің өмірімді бұзады,менің ші сабағ бірінші курс студент толы ал ниггер менің өмір бұз,racism

1,полиция қызметкері саябақта ақымақ сауда жасайтын жігіттердің жанынан ештеңе байқамай өтіп кетті бірақ қара адам оларды айналып өткенде олардың бәрі күдікті әрекет етеді,полиция қызметк саябақ ақы сауда жаса жігіт жан ештеңе байқама өт кет бірақ қара адам ол айнал өткенде олардың бәрі күдік әрекет ет,racism

1,жарайды сен мылқау ниггерсің бұрылысты азайтыңыз біз бұл аймақта болған жоқпыз мылқау қара бейбақ,жарайды сен мылқа нигг бұрыл азай біз бұл аймақта бол жоқ мылқа қара бей,racism

1,сіздің барлық достарыңыз мылқау ниггерлер сондықтан олар сізге қалай көмектесе алады олардың бәрі сені одан да ақымақ етеді сен ақымақсың,сіздің барлық дос

мылқа ниггер сондықтан олар сізге қалай көмекте ал олардың бәрі сен одан да ақы ет сен ақы,racism

1,сонымен қатар мен сіздің бірінші мәтініңізді қате оқыдым,сонымен қатар мен сіздің бірінші мәтін қате оқы,racism

1,құлдық кезінде пайда болған дейли газетінде құлдық кезінде негрлерге арналған хайуанаттар бағына сілтеме жасаған маймыл деп аталды қара нәсілділерге ата бабаларының құл болғанын күнделікті еске салады бірақ ақ адамдар бұл идеяны өздерінен алшақтатуы мүмкін өйткені бұл экономикалық тұрғыдан тиімсіз,құлдық кез пайда бол дейли газ құлдық кез негр арнал хайуанат бағ сілтеме жаса маймыл де ата қара нәсілді ата баба құл бол күнделікті ес сал бірақ ақ адам бұл идея өз алшақтат мүмкін өйткені бұл экономикалық тұрғы тиімсіз,racism

1,бұл сессияның соңғысы энтони пратчер brown болады ол түрлі түсті мегаполис нәсілдік интеграция мегаполистің даму факторы ретінде қалалық аудандардағы қарыз бен нәсілшілдік арасындағы байланыс туралы айтады digitalsoc,бұл сессия соң энтони пратч brown бол ол түрлі түсті мегаполис нәсіл интеграция мегаполис даму фактор рет қалалық аудан қарыз бен нәсілш ара байланыс туралы айт digitalsoc,racism

1,ниггерге сенімен сөйлесуді айтпа қаншық ұлы сен ақымақсың алдымен менің әтешімді сорып ал сосын менімен сөйлесуге кел бейбақ,нигг сенімен сөйлесу айтпа қаншық ұлы сен ақы алд менің әтеш сор ал сосын менімен сөйлес кел бей,racism

1,мені қалайтын бірақ ала алмайтын әрбір ниггерді тозаққа салыңыз түтіккен сорғыштар тырысуды жалғастыруда хаха,мен қала бірақ ал алма әрбір нигг тозақ сал түтік сорғыш тырысу жалғастыр хах,racism

1,желкек мылқау нигга хмао,желкек мылқа нигг хмао,racism

1,rt ойлап көріңізші сондықтан кішкентай нигга балалары балшық сияқты мылқау және мылқау өмірде бұл ақшадан гөрі маңызды,rt ойла көріңізш сондықтан кішкентай нигг бала балшық сияқты мылқа және мылқа өмір бұл ақша гөрі маңызды,racism

1,жексұрын үйдегі ниггерлер жаңалықтарға айналды әскери теңіз пирстері барлық қара жасөспірімдер надан smh ала бастағанға дейін отбасылық сахна болды,жексұр үйдегі ниггер жаңалық айна әскери теңіз пирс барлық қара жасөспірім надан smh ал баста дейін отбасылық сахна бол,racism

1,және оны ұрлаған мылқау ниггерге қарғыс атсын,және оны ұрла мылқа нигг қарғыс ат,racism

1,бірақ сіз бәріңіз ақымақсыз бұл әлі стэн ол менің ең жақын досымды ниггер деп атады фактілер кетті оның желкекке гриффиндік роджердің жүзі қаңқа қолдары оның дауысы менің тамағыма бірдеңе жабысып қалды ол полиэфирлі сью сильвестр спорттық костюмдері сияқты жоғары бағаланады және ол макияж сатуға тым ұсқынсыз,бірақ сіз бәрі ақы бұл әлі стэн ол менің ең жақын дос нигг де ат факті кет

оның желкек гриффи родж жүз қаңқа қол оның дауыс менің тамағ бірде жабыс қал ол полиэфирл сью сильвест спорттық костюм сияқты жоғары бағалан және ол макияж сат тым ұсқын,racism

1,ол сен жындысың ба бауырым бұл мылқау нигга обамаға,ол сен жынд ба бауыр бұл мылқа нигг обама,racism

1,сол бағытта жүріңіз мылқау нигга өзіңді өлтір сіздің асырап алған ұлыңыз менің есегімді қанға дейін ұрыңыз менің әтешімді құлағыма салыңыз мұның бәрі тозаққа,сол бағытта жүр мылқа нигг өз өлтір сіздің асыра ал ұл менің есег қан дейін ұр менің әтеш құлағ сал мұның бәрі тозақ,racism

1,обамаға қарғыс атсын мылқау нигга мен автобус жүргізушісі сенің есегіңді теуіп алғанша ақымақ бол оле жезөкшені есекке салыңыз,обама қарғыс ат мылқа нигг мен автобус жүргізуші сенің есег теу алғанш ақы бол ол жезөкше есек сал,racism

1,ниггер өтінемін әлеуметтік әділеттілік жауынгері бұл ақымақ белгі,нигг өт әлеуметтік әділеттілік жауынгер бұл ақы белгі,racism

1,rt обамаға қарғыс атсын мылқау нигга,rt обама қарғыс ат мылқа нигг,racism

1,өтінемін твиттерде кездесуді тоқтатыңыз онда бір жігіт таңқаларлық нонсенс айтады мысалы инцелом деп аталатын нәрсені ниггермен салыстырады сіз шынымен де ақымақ болып көрінесіз ол адамдар тым сезімтал деген иронияны көрмейтін жігітпен ұрысып жатырсыз бірақ бәрібір кез келген жағдайда жылайды,өт твиттер кездесу тоқта онда бір жігіт таңқаларлық нонсенс айт мысал инцело де атал нәрсе ниггер салыстыр сіз шын де ақы бол көр ол адам тым сезімтал де ирония көрме жігіт ұрыс жатыр бірақ бәрібір кез кел жағдай жыла,racism

1,үндемеңіз мылқау нигга сіз оған енетін адам боласыз сіз rmg немесе fyf tho туралы бос сөз көтермейсіз деп ойлаймын,үндеме мылқа нигг сіз оған ен адам бол сіз rmg немесе fyf tho туралы бос сөз көтерме де ойла,racism

1,бұл мылқау нигга нақты мәселелерден аулақ болу үшін бәрін жасайды ол ойындарға ренжіді,бұл мылқа нигг нақты мәселе аулақ болу үшін бәрін жаса ол ойын ренжі,racism

1,тозақ мен мылқау ниггермін мен темекіні қажетінше темекі шекпедім,тозақ мен мылқа нигг мен темекі қажетінше темекі шекпе,racism

1,мен сенің әтешіңді ұрамын мен олай жасамаймын деп ойламаңыз мылқау нигга,мен сенің әтеш ұр мен ола жасама де ойлама мылқа нигг,racism

1,егер сіз фергюсондағы ниггерлер жергілікті заңдарды ұнатпайтын болсаңыз онда африкаға оралыңыз ол сияқты ақымақ түйіндер,егер сіз фергюсо ниггер жергілікті заң ұнатпа бол онда африка орал ол сияқты ақы түйін,racism

1,бұл сіз постуляциялайтын xgp ге байланысты болды ма psnow арқасында days gone толық емес және жылтыратылмаған шығарылды ма бірінші немесе бірінші аптадағы

көптеген басқа патч ойындары туралы не деуге болады xgp де себеп болды ма,бұл сіз постуляцияла xgp ге байланысты бол ма psnow арқа days gone толық емес және жылтыратылма шығар ма бірінші немесе бірінші апта көптеген басқа патч ойын туралы не де бол xgp де себеп бол ма,racism

1,неге ол жаққа барасың өйткені бізде ниггерлер көп болғандықтан сен ақымақ бейбақсың,неге ол жақ бар өйткені біз ниггер көп бол сен ақы бей,racism

1,ақымақ бейбақ сіз бардыңыз нигга ақымақ жалап жатқан есек мақта теріп жатыр сцезцаның есегі лол мен сені жақсы көремін ниггер,ақы бей сіз бар нигг ақы жала жат есек мақта тер жатыр сцезца есег лол мен сен жақсы көр нигг,racism

1,қара адамдар бұл жала жабумен ешқашан ақшыл қара адамдарға гүлденбейді бұл жиіркенішті және мағынасыз бұқара үшін біз бәрібір ниггармыз,қара адам бұл жал жаб ешқашан ақшыл қара адам гүлденбе бұл жиіркеніш және мағына бұқара үшін біз бәрібір нигг,racism

1,сіз мұны бірінші болып көріп отырған адам емессіз бе бірақ адамдар оның сөзінен бас тарту немесе оның мінезіне күмән келтіру үшін осындай ұсақ түйектерге жүгінеді ме сіздің твиттеріңізде тағы қандай ниет бар деп ойлайсыз бірақ егер сіз оның одеколонының иісін достарыңызбен талқылауды шынымен ұнатсаңыз кетіңіз,сіз мұны бірінші бол көр отыр адам емес бе бірақ адам оның сөз бас тарту немесе оның мінез күмән келтіру үшін осындай ұсақ түйек жүгін ме сіздің твиттер тағы қандай ниет бар де ойла бірақ егер сіз оның одеколон иіс дос талқылау шын ұнат кет,racism

1,менің айтайын дегенім егер олар оның өткені туралы бірдеңе білсе олар әрине қара адамдар танымал еткен өкілдік түрін қара жүзді деп айыптауға тырыспас еді,менің айт де егер олар оның өт туралы бірде біл олар әрине қара адам танымал ет өкілдік түр қара жүз де айыпта тырыспас ед,racism

1,менің ниггерлерім жабайы қатыгез біз сабаққа бармаймыз мектепті тастаймыз,менің ниггер жабай қатыгез біз сабақ барма мектеп таста,racism

1,маған хибачи үстелін қалдырыңыз ба сіз бардыңыз ақымақ нигга,маған хибачи үстел қалдыр ба сіз бар ақы нигг,racism

1,біздің мылқау ниггерлердің бәріне мән бермейтінін көру мен көргім келетін нәрсе мен гейлер командасын фогельге сату үшін аз күш жұмсағым келеді,біздің мылқа ниггер бәрін мән берме көру мен көр кел нәрсе мен гей команда фогель сату үшін аз күш жұмса кел,racism

1,егер сіз қара болмасаңыз нигга деп айтпаңыз бұл әнде бар ма жоқ па контекстке қарамастан осылай айтуға құқығыңыз жоқ егер сіз оның артындағы оқиғаны білсеңіз оны не үшін айтасыз содан кейін қара өмір маңызды деп айтуға батылдық танытыңыз жексұрын тозақ,егер сіз қара болма нигг де айтпа бұл ән бар ма жоқ па контекст қарамастан осылай айт құқы жоқ егер сіз оның арт оқи біл оны не үшін ай содан кейін қара өмір маңызды де айт бат таны жексұр тозақ,racism

1,зак бұл ақымақ естіледі өйткені ол осы өлеңді айтатын адам сонымен сіз нигга сияқты қандай желкек айтасыз мылқау,зак бұл ақы ест өйткені ол осы өлең айт адам сонымен сіз нигг сияқты қандай желкек ай мылқа,racism

1,райн салқын және ақылды болып көрінеді мен кешірім сұрадым мен тырыстым бірақ ол маған құлап түсуімді айтты мылқау панк есек нигга мен оны олай деп ойламадым,райн салқын және ақылды бол көрін мен кешір сұра мен тырыс бірақ ол маған құла түсу ай мылқа панк есек нигг мен оны ола де ойлама,racism

1,rt rt мен итальяндықпын сен ақымақ нигга қаншығысың gt сен бардың gt n әрпі туралы айтқандарым міндетті емес,rt rt мен итальяндық сен ақы нигг қаншығ gt сен бар gt n әрпі туралы айт міндетті емес,racism

1,бұл мені екі секунд сайын мылқау бейбақ немесе мылқау нигга деп атағаныңыздан әлдеқайда жақсы болды лол,бұл мен екі секунд сайын мылқа бей немесе мылқа нигг де ата әлдеқайда жақсы бол лол,racism

1,біз сіз сияқты адамдармен күні бойы осы жиіркенішті сөзді лексиконында қолданатын адамдармен күресуіміз керек оның артында қандай да бір оқиға бар жоғын білмей оны бауырым формасы ретінде қолданамыз бірақ нәсілшіл болуға және қара адамды қандай да бір жолмен сезінуге келгенде сіз оны нигга черномаза маймыл және б деп атайсыз,біз сіз сияқты адам күн бойы осы жиіркеніш сөз лексикон қолдан адам күрес керек оның арт қандай да бір оқиға бар жоғ білме оны бауыр форма рет қолдан бірақ нәсілшіл бол және қара адам қандай да бір жол сезін кел сіз оны нигг черномаз маймыл және б де ата,racism

1,оның айтуынша телеарна оны жанама түрде мылқау нигга деп атаған,оның айт телеарна оны жанама түр мылқа нигг де ата,racism

1,ол нигга қаншық сөзін ойлап тапқанын айтты сіз оған кірмейсіз өйткені сіз ақ емес латино сіз ақымақ бейбақсыз,ол нигг қаншық сөз ойла тап ай сіз оған кірме өйткені сіз ақ емес латино сіз ақы бей,racism

1,мен алғашқы дауларымның бірінен бас тарта алмаймын,мен алғашқы дау бір бас тарта алма,racism

1,rt мен facebook тегі ережелерді бұзамын мысалы гарлем трахать ygz трахать ybz трахать lmao дан келген ниггерлер мені осы ақымақ боқ үшін қатты ұстайды,rt мен facebook тег ереже бұз мысал гарле трахать ygz трахать ybz трахать lmao дан кел ниггер мен осы ақы боқ үшін қатты ұста,racism

1,обама сенің анаңның желкекіне барды желкек обамаға барды мылқау нигга оообббааммааааааааааааааа бұл нигга сіздің президентіңіз лол,оба сенің ана желкек бар желкек обама бар мылқа нигг оообббааммааааааааааааааа бұл нигг сіздің президент лол,racism

1,олар мұны менің туған жерім тулса қаласында жойды біреу негрлер әкімге шағымданып қоңырау шалды деп жазды ия мен оны ақ швал деп атадым шынайы

емес мен енді ол жерде тұрмағаныма және мұндай менталитетке тап болмағаныма өте ризамын,олар мұны менің туған же тул қала жой біреу негр әкім шағымдан қоңырау шал де жаз ия мен оны ақ швал де ата шынайы емес мен енді ол же тұрма және мұндай менталитет тап болма өте риза,racism

1,мен алдымен кеңестер қалдыруға тырысамын олармен ашық болудан қорқамын шнкаскан да бір күні сіз отбасыңызбен сенімді түрде ашық бола аласыз деп үміттенемін lt,мен алд кеңес қалдыр тырыс олармен ашық болу қор шнкаскан да бір күн сіз отбас сенімді түр ашық бола ал де үміттен lt,racism

1,құдай сен мұны бірінші болып дұрыс жасадың,құдай сен мұны бірінші бол дұрыс жаса,racism

1,өйткені сіз оны ниггер ақымақ қаншық деп атадыңыз қаралар нигга дейді нигга нәсілшіл термин ақымақ бейбақ қара тарихыңызды зерттеңіз,өйткені сіз оны нигг ақы қаншық де ата қара нигг де нигг нәсілшіл термин ақы бей қара тарих зертте,racism

1,менің басымда оның ақымақ күлкісі естілді ол мені ашуландырған кезде үнемі шығарады,менің бас оның ақы күл ест ол мен ашуландыр кезде үнемі шығар,racism

1,обамаға қарғыс атсын мылқау нигга сіздің орналасқан жеріңіз көрсетілмеген деп үміттенемін,обама қарғыс ат мылқа нигг сіздің орналас же көрсетілме де үміттен,racism

1,және бұл нигга сіздің бүкіл есегіңізді ұйықтайтын еді сондықтан жеке жеке айтуға тырысыңыз,және бұл нигг сіздің бүкіл есег ұйықта ед сондықтан жеке жеке айт тырыс,racism

1,жігіттің тозағына ол осы қызбен ні жасады оған қол қойған адамның тозағына және чикагомен салыстырғанда ақымақ болып көрінетін тағы бір мылқау нигга болғаны үшін оны тозаққа түсірді,жігіт тозағ ол осы қыз ні жас оған қол қой адам тозағ және чикаго салыстыр ақы бол көрін тағы бір мылқа нигг бол үшін оны тозақ түс,racism

1,ruizmanny иә жігіт ол жерге автобуспен барады керемет қарғыс атсын сіз менің тістерімде не ұсақтайтынын білесіз ниггерлер джереми өте ақымақ,ruizmanny иә жігіт ол же автобус бар керемет қарғыс ат сіз менің тіс не ұсақта біл ниггер джереми өте ақы,racism

1,о құдай бізде полиция шақырған ақ әйелге қарсы қылмыстық іс қозғағысы келмейтін тағы бір кешірімді негр бар сияқты егер полицейлер оның өтірігіне сенсе бұл ақ әйел соншалықты кешірімді болар еді деп ойлайсыз ба,о құдай біз полиция шақыр ақ әйел қарсы қылмыстық іс қозға келме тағы бір кешір нег бар сияқты егер полицей оның өтіріг сен бұл ақ әйел соншалықты кешір бол ед де ойла ба,racism

1,жарайды мені де тыңдаңыз мылқау ниггерлер циммерман латино жаңалықтарды қараңыз надан бейбақтар,жарайды мен де тыңда мылқа ниггер циммерман латино жаңалық қара надан бейбақ,racism

1,обамаға қарғыс атсын мылқау нигга уф сен мылқау крекерс,обама қарғыс ат мылқа нигг уф сен мылқа крекерс,racism

1,аңыз менің алғашқы шабытымның бірі мен үйренген алғашқы гитара солосы сіздің махаббатыңызға өте қажет керемет ән жазушы гитарист және вокалист тыныштықта болыңыз питер грин тастар клэптон және б туралы ұмытыңыз мырза,аңыз менің алғашқы шабыт бірі мен үйрен алғашқы гитар соло сіздің махаббат өте қажет керемет ән жазушы гитарист және вокалист тыныштық бол пи грин тас клэптон және б туралы ұмыт мырза,racism

1,біз бұл сен бірінші осы орындыққа отыруың керек еді сол көк түсте тікенектермен,біз бұл сен бірінші осы орындық отыр керек ед сол көк түс тікенек,racism

1,бүгін кешке royal oak қа келетін мылқау ниггерлер мен канадаға барамын,бүгін кеш royal oak қа кел мылқа ниггер мен канада бар,racism

1,нигга сияқты әрекет ету үшін сізге толығымен қара болудың қажеті жоқ сіз жиіркенішті менсінбейтін және надансыз ниггердің бәрі,нигг сияқты әрекет ету үшін сізге толығ қара бол қажет жоқ сіз жиіркеніш менсінбе және надан нигг бәрі,racism

1,ол джо джексон жынды сіз жынды болдыңыз ба інжу маржан обамаға қарғыс атсын мылқау нигга,ол джо джексон жын сіз жын бол ба інж маржан обама қарғыс ат мылқа нигг,racism

1,мен осындай жағдайларға тап болдым олардың бірі маған қатты әсер етті сіз жай ғана көмектесуге тырысып жатқаныңызды байқау қиын емес және сізді пайдалану мүмкіндігі бар екенін ұмытып кетесіз және бұл сіздің ойыңызға да келмейді бұл ауырады,мен осындай жағдай тап бол олардың бірі маған қатты әсер ет сіз жай ғана көмектес тырыс жат байқау қиын емес және сіз пайдалану мүмкіндіг бар екен ұмыт кет және бұл сіздің ой да келме бұл ауыр,racism

1,менің алғашқы жекпе жектерімнің бірі аа немесе сен менің алып балам уу өзіңе ұнайтын нәрсені істей бебе мен ештеңеге қарамай қолдаймын lt,менің алғашқы жекпе жек бірі аа немесе сен менің алып бала уу өз ұна нәрсе істе беб мен ештеңе қарама қолда lt,racism

1,егер мен шон кингстонның өзінен катушкаларды жесем ше осы мылқау ниггер ли уэйн ешқашан psa ны таппауы үшін бәріміз дұға етейік,егер мен шон кингстон өз катушка же ше осы мылқа нигг ли уэйн ешқашан psa ны таппа үшін бәрі дұ ет,racism

1,қара ниггерлер белые крек спикс славяндар немесе кез келген басқа нәсілдік ақымақтық туралы айтатын болсақ бұл жыл ересек этоаноно,қара ниггер белы крек спикс славян немесе кез кел басқа нәсіл ақымақтық туралы айт бол бұл жыл ересек этоаноно,racism

1,желкекке үндемеңіз нигга немесе черномазая деп айтпаңыз сіз қазір ақымақ болып көрінесіз өтінемін үндемеңіз,желкек үндеме нигг немесе черномазая де айтпа сіз қазір ақы бол көр өт үндеме,racism

1,бұл сіздің ниггеріңіз бұл сіздің табиғи теріңіздің түсі мылқау бейбақ,бұл сіздің нигг бұл сіздің табиғи тер түс мылқа бей,racism

1,оны соншалықты көңілді ететін ерекше нәрсе бар жоғын білу үшін олар сондай ақ біздің альбинос бауырларымызды ақ негрлерді азаптады өйткені олар терісі уақыт өте келе меланинді неге жоғалтады деп ойлады өз тарихыңызды және олар бізге не істегенін біліңіз балаларыңызға сабақ беріңіз,оны соншалықты көңіл ет ерекше нәрсе бар жоғ білу үшін олар сондай ақ біздің альбинос бауыр ақ негр азапт өйткені олар тері уақыт өте келе мелани неге жоғалт де ойла өз тарих және олар бізге не істе біл бала сабақ бер,racism

1,уф сен rt лол чонаси және бриттейн кейбір мылқау ниггерлер оларды ұрады,уф сен rt лол чонаси және бриттейн кейбір мылқа ниггер ол ұр,racism

1,ақ адамдар бұл жезөкшені жақсы көреді rt обамаға қарғыс атсын мылқау нигга,ақ адам бұл жезөкше жақсы көр rt обама қарғыс ат мылқа нигг,racism

1,уэйн есімді мылқау ұсқынсыз нигга позитивті қоқыс жұмсақ есегі бар нигга қызыл және ақ трахает қызықтырмайды мылқау ұсқынсыз нигга pic twitter com,уэйн ес мылқа ұсқын нигг позитивті қоқыс жұм есег бар нигг қызыл және ақ трахает қызықтырма мылқа ұсқын нигг pic twitter com,racism

1,мен қара ниггері бар ақ балапанға қарсы емеспін өйткені мен бір бірімді түсіне аламын бірақ ақ түстегі ақ жай ғана жиіркенішті,мен қара нигг бар ақ балапан қарсы емес өйткені мен бір бір түс ал бірақ ақ түс ақ жай ғана жиіркеніш,racism

1,ниггер мен черномазая әр түрлі деп айтатындар желкекге барды өйткені бұл ақымақ есек емес,нигг мен черномазая әр түрлі де айт желкек бар өйткені бұл ақы есек емес,racism

1,соңғы бірнеше айда адамдар ештеңе үйренбеді антисемиттік сөйлеудің регургитациясы қара адамдар үшін ештеңе жасамайды сын бар бірақ бұл мәселе емес қазіргі уақытта адамдар еврей халқы туралы бәрін айтады,соңғы бірнеше айда адам ештеңе үйренб антисеми сөйлеу регургитация қара адам үшін ештеңе жасама сын бар бірақ бұл мәселе емес қазіргі уақыт адам еврей халқ туралы бәрін айт,racism

1,біз қазір нацистік партияны толығымен қолдаймыз бұл нигга обамаға қарғыс атсын бұл мылқау боқ мені ашуландырады,біз қазір наци партия толығ қолда бұл нигг обама қарғыс ат бұл мылқа боқ мен ашуландыр,racism

1,lmfaoo доғал бесінші нүкте надандық бесінші нүкте сучки rt жабу желкекке нигга,lmfaoo доғал бесінші нүкте надан бесінші нүкте сучки rt жаб желкек нигг,racism

1,алдымен олжаңызды қайтарыңыз содан кейін біз сіз біздің халқымыздың тәкаппарлығы mtu wetu мунгу хапенди ваджингтің атынан тамақтанатын адам екеніңізді келісе аламыз,алд олжа қай содан кейін біз сіз біздің халқ тәкаппарлығ mtu wetu мунг хапенди ваджинг ат тамақтан адам екен келі ал,racism

1,тағы да бірінші рико шамамен жыл менің екінші сүйіктім де мен әр риконы үнемі іздеп ештеңе алмағаннан кейін оған өте лайықпын,тағы да бірінші рико шамамен жыл менің екінші сүйікті де мен әр рико үнемі ізде ештеңе алма кейін оған өте лайық,racism

1,мен біраз уақыттан бері reveluv болдым бірақ бұл менің stan дағы алғашқы аккаунтым егер сізде бар болса мен re rt сияқты reveluvs әріптестерімді іздеймін,мен біраз уақыт бері reveluv бол бірақ бұл менің stan даға алғашқы аккаунт егер сіз бар бол мен re rt сияқты reveluvs әріптес ізде,racism

1,біз өткенге көмектесе алмаймыз бірақ болашаққа қатысты бірдеңе жасай аламыз америка құрама штаттарында қара миллионерлер мен миллиардерлердің ең көп саны бар көптеген адамдар тең мүмкіндіктерге қол жеткізе отырып қауіпсіз және қауіпсіз өмір сүруге риза болатыны анық,біз өт көмекте алма бірақ болашақ қатысты бірде жаса ал америка құрама штат қара миллионер мен миллиардер ең көп сан бар көптеген адам тең мүмкіндік қол жеткіз отыр қауіпсіз және қауіпсіз өмір сүр риза бол анық,racism

1,қарғыс атсын мен сені ақымақты әкелер күнімен құттықтағаным үшін немесе мақаланы саған арнағаным үшін тіпті алғыс алмадым сен бардың негр,қарғыс ат мен сен ақымақ әке күн құттықта үшін немесе мақала саған арна үшін тіпті алғыс алма сен бар нег,racism

1,сіз желкеккеге бардыңыз ниггерлер ккк жақсы сұрақ мылқау крекер,сіз желкекке бар ниггер ккк жақсы сұрақ мылқа крек,racism

1,менің жұмысым бар сіз бәріңіз желкекге бардыңыз мылқау видео түсіріп жатырсыз фин mlk дан ұтылады талм әкемнің жүк көлігін айдайды ниггерлерді айдай алмайды долбанутые бейбақтар,менің жұмыс бар сіз бәрі желкек бар мылқа видео түсір жатыр фин mlk дан ұт тал әке жүк көліг айда ниггер айда алма долбануты бейбақ,racism

1,бұл мәселенің мәні егер мен соңғы екі жылда бірдеңе білсем онда жақсы республикашылар жоқ өз қателіктерін түсінетін және өзгеретін адамдар бар бұл өте жақсы бірақ көп жағдайда олар болмайды және бұл адамдар құлап кетуі мүмкін,бұл мәселе мән егер мен соңғы екі жыл бірде біл онда жақсы республикашы жоқ өз қателік түсін және өзгер адам бар бұл өте жақсы бірақ көп жағдай олар болма және бұл адам құла кет мүмкін,racism

1,хаха ха бұл сізге әсер етпеуі керек роб келіңіздер бұл кез келген нәрсе болуы мүмкін тілді дұрыс таңдамаудан бастап қара адамдармен өткен жағымсыз тәжірибеге дейін шынымен нәсілшілдікке дейін қара нәсілділерді тегін жек көретіндер нәсілшілдік бұл кейінгі санатқа қолданылатын күшті сөз басқалары жай ғана ебедейсіз,хах ха бұл сізге әсер етпе керек роб кел бұл кез кел нәрсе бол мүмкін тілді дұрыс таңдамау баста қара адам өткен жағымсыз тәжірибе дейін шын нәсілшілдік дейін қара нәсілді тегін жек көр нәсілш бұл кейінгі санат қолданыл күшті сөз басқа жай ғана ебеде,racism

1,лол сенің атың ақымақ неліктен сіздің әкеңіз бен әкеңіз бір әріптен басталады ал сіздің фамилияңыз мексикалық ал сіз лас ниггерсіз,лол сенің ат ақы неліктен сіздің әке бен әке бір әріп бастал ал сіздің фамилия мексикалық ал сіз лас нигг,racism

1,ол оны негр деп атады оның ескі есегі өмір жойылды біз оянған кезде қара адамдар мен әрбір ақ адамды нәсілшіл деп айтпаймын бірақ олар бізге риза болмаған кезде біз олар үшін сіз адамдар немесе ниггерлер,ол оны нег де ат оның ескі есег өмір жой біз оян кезде қара адам мен әрбір ақ адам нәсілшіл де айтпа бірақ олар бізге риза болма кезде біз олар үшін сіз адам немесе ниггер,racism

1,rt желкекке үндеме боқ мылқау түйіндер мен мылқау ниггерлер иә ниггерлер,rt желкек үндеме боқ мылқа түйін мен мылқа ниггер иә ниггер,racism

1,ли өте тітіркендіргіш ал брэнди ақымақ нигга қаншығы,ли өте тітіркендіргіш ал брэнди ақы нигг қаншығ,racism

1,сондықтан менің үй қызым қиыншылыққа тап болды дат би бұл ақ қызға айтты сігу сені ахахаха бірақ ол оны ниггер және ақымақ қаншық деп атады екінші әлем де,сондықтан менің үй қыз қиыншылық тап бол дат би бұл ақ қыз ай сіг сен ахахах бірақ ол оны нигг және ақы қаншық де ат екінші әлем де,racism

1,сәлем шын жүректен сәлем сіз мен сүйе бастаған wwa дағы алғашқы қыздардың бірісіз сіз мені asf деп айта бастадыңыз өйткені сіз мені әрқашан қатты күлдіресіз тіпті сіз рэй анти бу болсаңыз да мен сізді армандай бастадым e wayz немесе сен менің ең жақын досымсың,сәлем шын жүрек сәлем сіз мен сүйе баста wwa дағ алғашқы қыз бір сіз мен asf де ай баста өйткені сіз мен әрқашан қатты күлдір тіпті сіз рэ анти бу бол да мен сіз арманда баста e wayz немесе сен менің ең жақын дос,racism

1,тупая көт рейнджерс киска қаншық ниггер пидорые шайтан алғыр,тупая көт рейнджерс киск қаншық нигг пидоры шай алғы,racism

1,сіздің мылқау нигга барды желкек,сіздің мылқа нигг бар желкек,racism

1,құдайға ант етемін бұл мылқау қара маз ратчетпен түйінді желкекке жабу керек,құдай ант ет бұл мылқа қара маз ратчет түйінді желкек жаб керек,racism

1,білімсіз нигга егер бұл ақымақ болса шығыңыз оны рет жару тәсілі,білімсіз нигг егер бұл ақы бол шығ оны рет жар тәсіл,racism

1,сіз уэйн есімді ақымақ ұсқынсыз ниггерсіз позитивті қоқыс жұмсақ есегі бар нигга pic twitter com,сіз уэйн ес ақы ұсқын нигг позитивті қоқыс жұм есег бар нигг pic twitter com,racism

1,доғал ебучий нигга жігіт бесінші нүкте нигга,доғал ебучи нигг жігіт бесінші нүкте нигг,racism

1,елшілердің істері құдай бозғылт ауру англо ақ альбинос мулат кавказдық нефилим түссіз тіршілік иесі шайтан сияқты ақ уағызшылар өздерінің христиан шіркеулері деп аталатын шайтан храмдарында үйреткен құдайдан тыс қара алтын негрлердің

жағдайы деп ойлады,елші іс құдай бозғылт ауру англо ақ альбинос мулат кавказ нефили түс тіршілік иесі шай сияқты ақ уағызшы өз христиан шіркеу де атал шай храм үйрет құдай тыс қара алтын негр жағдай де ойла,racism

1,нәсілшілдік жеңді ме ақымақ емес қара нәсілшілдіктер обаманың басқаруымен соңғы жылдағыдан гөрі көбірек нәсілшілдік сезінеді мен түстімін сіз де сөйлемейсіз,нәсілш же ме ақы емес қара нәсілшілдік обама басқару соңғы жылдағы гөрі көбірек нәсілш сезін мен түсті сіз де сөйлеме,racism

1,мен тіпті федералдарға мен мылқау ниггерлерге және қарапайым аңшыларға қарғыс айтамын деп айтпаймын менің ниггерім оларға айтады,мен тіпті федерал мен мылқа ниггер және қарапайым аңшы қарғыс ай де айтпа менің нигг оларға айт,racism

1,мен бесінші сыныпта менің сыныптағы екі ақ қыздың мені ойын алаңындағы негр қыз деп атағанын ешқашан ұмытпаймын мен айтқым келгенде олар жылап кінә мені тыныштандырды мен олармен шынымен де достар болып қалдым,мен бесінші сынып менің сынып екі ақ қыз мен ойын алаң нег қыз де ата ешқашан ұмытпа мен айт кел олар жыла кінә мен тыныштан мен олармен шын де дос бол қал,racism

1,мен джеймс болдуинның мен сенің негрің емеспін деректі фильмін көріп отырмын өте күшті деректі фильм бұрын соңды көрмеген мен барлығына ақ қара азиялықты көруді ұсынамын,мен джеймс болдуин мен сенің нег емес деректі фильм көр отыр өте күшті деректі фильм бұрын соң көрме мен барлығ ақ қара азиялық көру ұсын,racism

1,тағы да өтірік айтасың сен бейбақ жала жабу боқтық нәсілшіл ниггерді шақыру ақымақ бейбақ,тағы да өтірік ай сен бей жал жаб боқ нәсілшіл нигг шақыру ақы бей,racism

1,бұл мылқау кішкентай нәпсіқұмар көт париждегі шабуылға құмды ниггерлер жауапты екенін айтты қаншық иса таяу шығыстан болды сіз бардыңыз,бұл мылқа кішкентай нәпсіқұм көт париж шабуыл құм ниггер жауапты екен ай қаншық ис таяу шығыс бол сіз бар,racism

1,сіз ақымақ қаншықсыз рейдерлер әлі де әлдеқайда жақсы алыптар бұл сіз маймылдар помоймен айналысатын қара маз мысықтары,сіз ақы қаншық рейдер әлі де әлдеқайда жақсы алып бұл сіз маймыл помой айналыс қара маз мысық,racism

1,ванна нигга аулада ғасыр жақсырақ нәрсе ойлап тап өйткені жаңалықтарда менің қара ақымақ бейбақ екенім жыпылықтайды,ванн нигг аула ғасыр жақсырақ нәрсе ойла тап өйткені жаңалық менің қара ақы бей екен жыпылықта,racism

1,ол дилан руфтың немере ағасы ма менің ойымша біз оларға жеткілікті бердік сіз кіммен әзілдесесіз бұл ашытқы емес деп үміттенемін себебі оның қандай нәрсеге қатысы бар менің ағам ілмекке ілінеді заңнама осы адамдардың жанынан өтеді мен олардан күтемін бірақ бізден емес,ол дилан руф немере аға ма менің ойымша біз оларға жеткілікті бер сіз кім әзілдес бұл ашытқы емес де үміттен себебі оның қандай

нәрсе қатыс бар менің аға ілмек ілін заңнама осы адам жан өт мен олардан күт бірақ бізден емес,racism

1,сіз де нигга екеніңізді білесіз бе бұл аа ның африкалықтар мен кариб теңізіне қарсы идеясы бұл ақымақтық,сіз де нигг екен біл бе бұл аа ны африкалық мен кариб теңіз қарсы идея бұл ақымақтық,racism

1,уау бұл қаншық тозақ сияқты ақымақ хек трой дэвис деген кім сіз әрқашан twitter де ниггерді босатуға тырысасыз,уау бұл қаншық тозақ сияқты ақы хек тро дэвис де кім сіз әрқашан twitter де нигг босат тырыс,racism

1,шайтан алғыр орикс мен бұл мылқау ниггерді екі рет өлтірдім,шай алғы орикс мен бұл мылқа нигг екі рет өл,racism

1,сіз мені мазақ етесіз бе бұл ақымақ нигга ешқашан жұмыс істемейтін қоғамдық ұйымдастырушы болды ол мұсылман олармен бірге алла мен құран да бар,сіз мен мазақ ет бе бұл ақы нигг ешқашан жұмыс істеме қоғамдық ұйымдастырушы бол ол мұсылман олармен бірге алл мен құран да бар,racism

1,обамаға қарғыс атсын ақымақ бас нигга желкекгe үндеме қаншық,обама қарғыс ат ақы бас нигг желкек үндеме қаншық,racism

1,неге шайтан алғыр тәтті доза са ағып көк қан қандай желкек сізде қызыл қан бар мылқау нигга және бұл біздің меншігімізде сондықтан біз оны қайтадан бояймыз ниг,неге шай алғы тәтті доза са ағ көк қан қандай желкек сіз қызыл қан бар мылқа нигг және бұл біздің меншіг сондықтан біз оны қайта боя ниг,racism

1,бұл нигга сыныпта ақымақ бірақ ол өту ұпайын жаба алады дейді университет жаттықтырушылары,бұл нигг сынып ақы бірақ ол өту ұпай жаб ал де университет жаттықтырушы,racism

1,сіз бардыңыз мылқау ақымақ митт ромни жеңеді amp amp сіз ниггерлер құрдымға кетесіз менің отбасым болған кезде маған жеккөрушілік жібергеніңізге өкінгеніңізге қуаныштымын,сіз бар мылқа ақы митт ромни жең amp amp сіз ниггер құрдым кет менің от бол кезде маған жеккөрушілік жібер өкін қуанышты,racism

1,жігіт сен кімсің мен сенің есегіңнен уайтты ұрамын twitter дегі гангста rt жоқ тек ақымақ нигга,жігіт сен кім мен сенің есег уай ұр twitter дег гангс rt жоқ тек ақы нигг,racism

1,жабу желкекке мылқау нигга sjw,жаб желкек мылқа нигг sjw,racism

1,сонымен сіздің болашақ үйлену тойыңызда қандай мүмкін алғашқы би әндері бар менің таңдауымның бірі эстой пердидо де лос трес ас,сонымен сіздің болашақ үйлен той қандай мүмкін алғашқы би ән бар менің таңдау бірі эсто пердидо де лос трес ас,racism

1,af дегеніміз не сіз сауатсыз ниггерсіз қарғысатқыр қара адам бұл шайтан алғыр дегенді білдіреді ақымақ сен көт лол сондықтан,af де не сіз сауат нигг қарғысатқы қара адам бұл шай алғы де білдір ақы сен көт лол сондықтан,racism

1,адамдар бұл нигга сөзіне қайта орала ма қарғыс атсын сіз қандай адамдар ақымақсыз,адам бұл нигг сөз қайта орал ма қарғыс ат сіз қандай адам ақы,racism

1,менде тек қаралар үшін нәрсеге ауысуға ниетім жоқ мен жай ғана сегрегация кезінде жасалған нәрселерге сізде іс жүзінде таңдау болмаған кезде немесе бүгінгі рос үшін көбірек мүмкіндіктер жасау арқылы өткеніміздің ауыртпалығын жеңілдетуге көмектесетін нәрселерге құрмет көрсетуіміз керек деп айтамын,менде тек қара үшін нәрсе ауыс ниет жоқ мен жай ғана сегрегация кез жасал нәрсе сіз іс жүз таңдау болма кезде немесе бүгінгі рос үшін көбірек мүмкіндік жасау арқылы өт ауыртпалығ жеңілдет көмектес нәрсе құрмет көрсет керек де ай,racism

1,rt обама одақтың жағдайы жақсы дейді бұл мылқау нигга қайда болды pic twitter com,rt оба одақ жағдай жақсы де бұл мылқа нигг қайда бол pic twitter com,racism

1,иә сіз бардыңыз rt барлығы шығып дауыс беріңіз сонда біз бұл ниггерді қызметінен алып тастай аламыз ақымақ жезөкше айтатын нәрсе,иә сіз бар rt барлығ шығ дауыс бер сонда біз бұл нигг қызмет алып таста ал ақы жезөкше айт нәрсе,racism

1,хахаха юррр маддддд rt желкекке обама ақымақ нигга иә ол lmfaoo ooo ahhhhhh,хахах юрр маддддд rt желкек оба ақы нигг иә ол lmfaoo ooo ahhhhhh,racism

1,мың жылдан астам уақыт бұрын планетада қандай адамдар қара емес деп сұрау сіздің сұрағыңызға жауап беруі мүмкін,мың жыл астам уақыт бұрын планета қандай адам қара емес де сұрау сіздің сұрағ жауап беруі мүмкін,racism

1,сіз ақымақ екенсіз өйткені оның қайда екенін білмейсіз ақымақ ақымақ нигга смх,сіз ақы екен өйткені оның қайда екен білме ақы ақы нигг смх,racism

1,ол нәсілдік шабуылдар үшін қоғамдық жұмыстарды алады бірақ мұнда нигга немесе қара адам сияқты емес ол жәбірленушіні уландыруға дейін барды бұл өте жиіркенішті,ол нәсіл шабуыл үшін қоғамдық жұмыс ал бірақ мұнда нигг немесе қара адам сияқты емес ол жәбірленуші уландыр дейін бар бұл өте жиіркеніш,racism

1,қарғыс атсын бұл ақымақ әр нигга өзі үшін ақымақ пен ақылды нигганың айырмашылығы бар,қарғыс ат бұл ақы әр нигг өзі үшін ақы пен ақылды нигга айырмашылығ бар,racism

1,о мен жастамын мен тырысуды жалғастырамын ха сені ұрып соғу мылқау нигга,о мен жас мен тырысу жалғастыр ха сен ұр соғ мылқа нигг,racism

1,сен ақымақ нигга қаншығысың желкекке үндеме,сен ақы нигг қаншығ желкек үндеме,racism

1,мен сені қатты ренжіткен болуың керек екенін көремін лол қаншық бардым желкекке обама мылқау нигга,мен сен қатты ренжіт бол керек екен көр лол қаншық бар желкек оба мылқа нигг,racism

1,олар менің сүйіктілерім олар тіпті бұл ірімшікті еріте алмайды бин ладенге қарғыс атсын бұл азздан келген мылқау ниггерлер осында хмао,олар менің сүйікті олар тіпті бұл ірімшік еріт алма бин ладен қарғыс ат бұл азз кел мылқа ниггер осында хмао,racism

1,көптеген демократтар соның ішінде қара ерлер кәрі құрттар және ақ әйелдер бұл оқиға үшін кешірім сұрады неліктен оны нәсілшіл немесе сексистік мәселеге айналдыру үшін бір мысалды таңдау керек бұл не кейбір кері нәсілшілдік және кері сексизм ол жай ғана радикалдарды басқаруға тырысады,көптеген демократ соның іш қара ер кәрі құрт және ақ әйел бұл оқиға үшін кешір сұр неліктен оны нәсілшіл немесе секси мәселе айналдыру үшін бір мысал таңдау керек бұл не кейбір кері нәсілш және кері сексиз ол жай ғана радикал басқар тырыс,racism

1,нәсілшілдік бұл лесбияндар мен гейлерді жақсы көретін кезде ақ iphone лар қара түстерге қарағанда қымбат ақ кірді жуған кезде түрлі түсті болған кезде,нәсілш бұл лесбиян мен гей жақсы көр кезде ақ iphone ла қара түс қара қымбат ақ кір жу кезде түрлі түсті бол кезде,racism

1,stfu btc сіз ара маскасына ұқсайсыз ниггерлер ең жақсы мылқау трах бтк сен қайдасың,stfu btc сіз ара маска ұқса ниггер ең жақсы мылқа трах бтк сен қай,racism

1,нәсілшілдік бұл ақ киімді қара емес бірінші кезекте жуу үшін таңдау нәсілшіл болмаңыз олардың барлығын бірге жуыңыз,нәсілш бұл ақ ки қара емес бірінші кезек жу үшін таңдау нәсілшіл болма олардың барлығ бірге жу,racism

1,уэйн есімді қызыл және ақ ақымақ ұсқынсыз ниггерді позитивті қоқысты жұмсақ есегі бар ниггерді және кристің ұсқынсыз есегін ұрыңыз қоңырау шалыңыз pic twitter com,уэйн ес қызыл және ақ ақы ұсқын нигг позитивті қоқыс жұм есег бар нигг және кри ұсқын есег ұр қоңырау шал pic twitter com,racism

1,мен мылқау ниггердің біздің елімізді мысықтың құйрығының астына жібергенін түсіну үшін жеткілікті білемін ақымақ бейбақ,мен мылқа нигг біздің ел мысық құйрығ аст жібер түсіну үшін жеткілікті біл ақы бей,racism

1,обамаға қарғыс атсын мылқау нигга енді сен кереметсің сіз бұл адамның жартылай ақ сондай ақ мылқау екенін түсінесіз,обама қарғыс ат мылқа нигг енді сен керемет сіз бұл адам жартылай ақ сондай ақ мылқа екен түс,racism

1,нигга сен ақымақ бейбақсың,нигг сен ақы бей,racism

1,сонымен темба годи қара кәсіпқойлар үйдегі ниггерлер деп шешеді иә ерсімен жиркенішті,сонымен темб годи қара кәсіпқой үйдегі ниггер де шеш иә ер жиркеніш,racism

1,rt обамаға қарғыс атсын мылқау нигга lmaooo ол р е,rt обама қарғыс ат мылқа нигг lmaooo ол р е,racism

1,лол салқын болыңыз rt мен дк суббан шайтан алғыр бүгін кешке өледі деп үміттенемін оны біреу жақсы көреді мылқау нигга хоккей ойнамауы керек,лол салқын бол rt мен дк суббан шай алғы бүгін кеш өл де үміттен оны біреу жақсы көр мылқа нигг хоккей ойнама керек,racism

1,ақымақ ақымақ сияқты сіз тек rt ге дұға ете аласыз обамаға қарғыс атсын мылқау нигга сіз мұның бәрін көресіз,ақы ақы сияқты сіз тек rt ге дұ ет ал обама қарғыс ат мылқа нигг сіз мұның бәрін көр,racism

1,арбаларды жасайтын бұл мылқау жаңа нигга кім ол шайтан алғыр сорады,арба жаса бұл мылқа жаңа нигг кім ол шай алғы сор,racism

1,спид тен өліңіз rt міне сіздің сілтемелеріңіз rt желкекке обама барды мылқау нигга,спид тен өл rt міне сіздің сілтеме rt желкек оба бар мылқа нигг,racism

1,сонымен қатар түсті сөзі ондаған жылдар бойы қолданылған және қазір қорлайтын қара адамдарға арналған сөз ретінде мүлдем қолданылмады сіз ештеңе айтқыңыз келмейтініне сенімдімін бірақ мормондар юта штаты нәсілшілдікке қарсы күресте ақш әлемнің қалған бөлігімен салыстырғанда қуып жетеді және сіздің сөзіңіз бұған дәлел,сонымен қатар түсті сөз ондаған жыл бойы қолданыл және қазір қорла қара адам арнал сөз рет мүлдем қолданылма сіз ештеңе айт келме сенімді бірақ мормон ют штат нәсілшілдік қарсы күрес ақш әлем қалған бөліг салыстыр қу жет және сіздің сөз бұған дәлел,racism

1,ақшаны қадағалаңыз гленн бек tide blm және басқа қайырымдылық ұйымдарының ақшаны жылыстатуына қатысты тергеу жүргізді және ақшаның қайда кететінін ешкім білмейді бірақ схемада алаяқтық элементі бар бұрынғы blm көшбасшылары мұның бәрі өте күмәнді және қара өмірге ешқандай қатысы жоқ дейді,ақша қадағала гленн бек tide blm және басқа қайырымдылық ұйым ақша жылыстат қатысты тергеу жүр және ақша қайда кет ешкім білме бірақ схема алаяқтық элемент бар бұрынғы blm көшбасшы мұның бәрі өте күмәнді және қара өмір ешқандай қатыс жоқ де,racism

1,awwww rt желкекке обама барды мылқау нигга сен ашуланасың ба бейшара,awwww rt желкек оба бар мылқа нигг сен ашулан ба бейшар,racism

1,неліктен қара күзетші ештеңе жасамағанын тек жәбірленушінің жанынан өтіп содан кейін қылмыскер қайда бара жатқанына қарама қарсы бағытта жүргенін біреу түсіндіре алады ма,неліктен қара күзетш ештеңе жасама тек жәбірленуші жан өт содан кейін қылмыскер қайда бар жат қарама қарсы бағытта жүр біреу түсіндір ал ма,racism

1,lmfao қыз rt обамаға қарғыс атсын мылқау нигга,lmfao қыз rt обама қарғыс ат мылқа нигг,racism

1,нигга драко қараңғы болды мұның бәрі оған қажет ол жай ғана мылқау болды өйткені ол тым көп трахается,нигг драко қараңғы бол мұның бәрі оған қажет ол жай ғана мылқа бол өйткені ол тым көп трахается,racism

1,менің қара және ақ немерелерім бар олар жүгіріп бір бірін нигга ақ және қара деп атауы керек жиіркенішті,менің қара және ақ немере бар олар жүгір бір бір нигг ақ және қара де ата керек жиіркеніш,racism

1,rt rt обамаға қарғыс атсын мылқау нигга,rt rt обама қарғыс ат мылқа нигг,racism

1,иә бұл сізге өте қиын содан кейін сіз полицейлерге сүрінесіз мен сені трахает қаншық панк ештеңеден қорықпағандай мен сені қаншық деп атаймын сен ақымақ бас ниггерсің,иә бұл сізге өте қиын содан кейін сіз полицей сүр мен сен трахает қаншық панк ештеңе қорықпағанда мен сен қаншық де ата сен ақы бас нигг,racism

1,нәсілшілдік жоқ африкалық баланың ал сен мені түрлі түсті деп атайсың,нәсілш жоқ африкалық бала ал сен мен түрлі түсті де ата,racism

1,wow wow wow сабыр етіңіз мылқау джеймс сияқты сөйлесіңіз қалай болғанда да ниггафак сен есек нигга,wow wow wow сабыр ет мылқа джеймс сияқты сөйлес қалай болғанда да ниггафак сен есек нигг,racism

1,үлкен менің отбасым мені өскен кезімде эль негро деп атады сондықтан мен қазір ақ нәсілшіл ер адамдармен араласамын олар мені бала кезімде салыстырған адамдар туралы төмен пікірде бірақ егер мен бас тартсам өзім мені қабырғаға лақтырудан тартынбас едім энергия,үлкен менің от мен өс кез эль негро де ат сондықтан мен қазір ақ нәсілшіл ер адам аралас олар мен бала кез салыстыр адам туралы төмен пікір бірақ егер мен бас тарт өзім мен қабырға лақтыру тартынбас ед энергия,racism

1,мен сізден ештеңе жеңуді сұрамаймын мен сізден өткенді өткенге қалдыруды және оны бүгінмен салыстыруды сұраймын мен сізден бөлінетін тұзаққа түсіп қалмауыңызды және энергияңызды кішкентай қара балалар үшін болашақты өзгертуге бағыттауыңызды сұраймын бірақ біз оларды сенімдерімізбен ластаймыз,мен сізден ештеңе жеңу сұрама мен сізден өт өт қалдыру және оны бүгін салыстыру сұра мен сізден бөлін тұзақ түс қалмау және энергия кішкентай қара бала үшін болашақ өзгерт бағыттау сұра бірақ біз ол сенім ласта,racism

1,егер сіз өте ақылды болсаңыз мен сізді қате жібергенім үшін жазалайтынымды түсінер едім мен күні бойы ақымақ ниггерлермен айналысамын ақ күш,егер сіз өте ақылды бол мен сіз қате жібер үшін жазала түсін ед мен күн бойы ақы ниггер айналыс ақ күш,racism

1,бірақ қызыл тері нәсілдік қорлау емес кем дегенде үнді тектес адамдардың көпшілігі олай ойламайды сіз негр лигаларында ақ либералдардың айтуы бойынша тағы бір нәсілдік қорлау кубалық деп аталатын бірнеше команда болғанын білесіз бе мұның айырмашылығы неде ешкім оларды нәсілшіл деп атауға батылы бармайды,бірақ қызыл тері нәсіл қорла емес кем де үн тектес адам көпшілігі ола ойлама сіз нег лига ақ

либерал айт бойынша тағы бір нәсіл қорла кубалық де атал бірнеше команда бол біл бе мұның айырмашылығ не ешкім ол нәсілшіл де ата батыл барма,racism

1,мен шынайы махаббатты өмірімде бір рет қана бастан өткердім бұл күндері не сен белгілі бір пайда алу үшін қолданылатын ақымақ бейбақсың немесе сіз біреуді сүймей бір жыл өмір сүре алмайтын аянышты ниггерсіз,мен шынайы махаббат өмір бір рет қана бас өткер бұл күн не сен белгілі бір пайда алу үшін қолданыл ақы бей немесе сіз біреу сүйме бір жыл өмір сүр алма аяныш нигг,racism

1,нәсілшілдік бұл ақ киімді қара емес бірінші кезекте жуу үшін таңдау нәсілшіл болмаңыз олардың барлығын бірге жуыңыз,нәсілш бұл ақ ки қара емес бірінші кезек жу үшін таңдау нәсілшіл болма олардың барлығ бірге жу,racism

1,сіздердің барлығыңызға ниггерлер,сіздердің барлығ ниггер,racism

1,хек нағыз ниггерлер ақымақ балапандарды ұрмайды ма мен кетменді лақтырамын бұталарда жансыз мен насрат,хек нағыз ниггер ақы балапан ұрма ма мен кетме лақтыр бұта жан мен насрат,racism

1,ол одан аман қалады lol rt жартылай қорғаушы есекті сорып алды сіз ашуланасыз ба обамаға қарғыс атсын мылқау нигга,ол одан аман қал lol rt жартылай қорғаушы есек сор алды сіз ашулан ба обама қарғыс ат мылқа нигг,racism

1,бір нәсілдегі нәсілшілдік lmao jealoustweet rt сіз вафли түсті ниггерлер мені жиркеніште сезінесіз,бір нәсіл нәсілш lmao jealoustweet rt сіз вафли түсті ниггер мен жиркеніш сез,racism

1,ақ адамдар ниггерді ойлап тапқан жоқ бұл мылқау бейбақ ол туралы ештеңе білмейтін нәрсені қорғамауы керек,ақ адам нигг ойла тап жоқ бұл мылқа бей ол туралы ештеңе білме нәрсе қорғама керек,racism

1,екеуіңіз де бардыңыздар ақымақ ниггерлер,екеу де бар ақы ниггер,racism

1,бұл шынымен сорады bck ге жазылғаннан кейін алдымен nthn ге жазылатын адамдар gt жазылудан бас тарту сізде атақты адамдар жоқ жарай ма қайталап айтамын сіз атақты емессіз ps бұл ойынды менімен кім ойнаса есіңізде болсын келесі сандарды жаңартпас бұрын сіздің ізбасарларыңызда да болмайды,бұл шын сор bck ге жазыл кейін алд nthn ге жазыл адам gt жазылу бас тарту сіз атақты адам жоқ жара ма қайтала ай сіз атақты емес ps бұл ой менімен кім ойна ес бол келесі сан жаңартпас бұрын сіздің ізбасар да болма,racism

1,әрине бұл мылқау нигга менен озып кетеді бірақ мен ұзақ күттім хек адамдар мені ашуландырады,әрине бұл мылқа нигг менен оз кет бірақ мен ұзақ күт хек адам мен ашуландыр,racism

1,мылқау көт мылқау есек ниггердің ұсқынсыз есегі ал сенің анаң сорады мүшесі доғал қаншық сіздің отбасыңыз бен достарыңыз тозаққа түседі қаншық,мылқа көт

мылқа есек нигг ұсқын есег ал сенің ана сор мүше доғал қаншық сіздің от бен дос тозақ түс қаншық,racism

1,кім шайтан алғыр google ол қолында негр бала қаншық сен ақымақсың,кім шай алғы google ол қол нег бала қаншық сен ақы,racism

1,сіздің алғашқы татуировкаңыз дәл осы сияқты болды урста менің сүйікті түсім қызыл ал минада сіздің сүйікті түсіңіз күлгін екеумізде де білек татуировкасы бар сіз гангстер және болғыңыз келді,сіздің алғашқы татуировка дәл осы сияқты бол урс менің сүйікті түсім қызыл ал мина сіздің сүйікті түс күлг екеу де білек татуировка бар сіз гангс және бол кел,racism

1,мен сіздің бірінші айтқаныңызды көрдім сондықтан nct rn мен қорғалады сізді енді ешкім тексермейтіні анық idk сіз екеуіңіздің қателескеніңізді білгенде не істегеніңізді неге ақтайсыз,мен сіздің бірінші айт көр сондықтан nct rn мен қорғал сіз енді ешкім тексерме анық idk сіз екеу қателес біл не істе неге ақта,racism

1,фактілер бауырым мен әрқашан ft және shi сұрайтын адаммын мен әрқашан ppl ге қызығамын бірақ адамдар маған ешқашан қайырлы таң сіздің күніңіз қалай өтті біріншіден мен әрқашан сұрайтын адаммын кейде бұл ауырады бауырым,факті бауыр мен әрқашан ft және shi сұра адам мен әрқашан ppl ге қызығ бірақ адам маған ешқашан қайырл таң сіздің күн қалай өт бірінші мен әрқашан сұра адам кейде бұл ауыр бауыр,racism

1,мен жергілікті кофеханада жұмыс істедім мен ниггерді естідім және олардың байкерлер мен қара адамдарға қалай қарағанын көрдім бұл жиіркенішті болды мен мүмкіндігінше тез жұмыстан шықтым,мен жергілікті кофехана жұмыс істе мен нигг есті және олардың байкер мен қара адам қалай қара көр бұл жиіркеніш бол мен мүмкіндігінше тез жұмыс шық,racism

1,барлық ниггерлер ақымақ,барлық ниггер ақы,racism

1,сіз құлап қалсаңыз қалай болады мылқау нигга,сіз құла қал қалай бол мылқа нигг,racism

1,менің ойымша ол өзгерді мен жақында оның нәсілшіл екенін көрсететін ештеңе көрмедім иә ол бұрын істерді жасаған бірақ ол бұл үшін кешірім сұрады және өзгерді яғни ол black lives matter қозғалысын шын жүректен қолдайды,менің ойымша ол өзге мен жақында оның нәсілшіл екен көрсет ештеңе көрме иә ол бұрын іс жаса бірақ ол бұл үшін кешір сұр және өзге яғни ол black lives matter қозғалыс шын жүрек қолда,racism

1,маған еріңіз нигга мен ақымақтықпен ұрдым,маған ер нигг мен ақымақтық ұр,racism

1,типтік мылқау нигга сіз көтерілді деп айту керек болған кезде көтерілді дедіңіз мектепке оралыңыз ақымақ бейбақ,типтік мылқа нигг сіз көтер де айту керек бол кезде көтер де мектеп орал ақы бей,racism

1,миль сағ сіз үшін жылдам емес деп шешкен және менің көлігімді айналып өтуді шешкен мылқау қара масом қызыммен бірге артқы орындықта сен бардың,миль сағ сіз үшін жылдам емес де шеш және менің көліг айнал өту шеш мылқа қара масо қыз бірге артқ орындық сен бар,racism

1,сіз ақымақ бейбақсыз сауатсыз есек нигга деп айтуды нәсілшілдік деп санайтын қара адамдар өмір сүреді,сіз ақы бей сауат есек нигг де айту нәсілш де сана қара адам өмір сүр,racism

1,барды сен желкекке мылқау нигга,бар сен желкек мылқа нигг,racism

1,жарайды округ сізге қарсы биологиялық соғыс бастаған кезде бұл сіздің алғашқы нұсқауыңыз болуы керек еді біртұтас қытай олардың мақсаты және олар тек өз елдерінде ғана емес олар миллиондаған адамдарды өкінбестен өлтіреді сіз олар өз мақсаттарына жету үшін басқа елдердегі миллиондаған адамдарға қамқорлық жасайды деп ойлайсыз ба,жарайды округ сізге қарсы биологиялық соғыс баста кезде бұл сіздің алғашқы нұсқа бол керек ед біртұтас қытай олардың мақсаты және олар тек өз ел ғана емес олар миллиондаған адам өкінбес өлтір сіз олар өз мақсат жету үшін басқа ел миллиондаған адам қамқорлық жаса де ойла ба,racism

1,бұл нені білдіреді сіз маймыл есегі бар мылқау ниггерсіз,бұл не білдір сіз маймыл есег бар мылқа нигг,racism

1,сенің ақымақ бейбақ екеніңді қалаймын енді сіздің емле тексеруіңіз қосылды егер сіз мені ниггер деп атайтын жаңғақтарды аузыңыздан шығарғыңыз келсе оны дұрыс жазыңыз,сенің ақы бей екен қала енді сіздің емл тексер қос егер сіз мен нигг де ата жаңғақ ауз шығар кел оны дұрыс жаз,racism

1,оған кіріңіз rt обамаға қарғыс атсын мылқау нигга,оған кір rt обама қарғыс ат мылқа нигг,racism

1,тағы да басқарылмайтын жасөспірім немесе жасөспірімдер тобы олардың барлығы қара екенін білдіреді сондай ақ мақалада бірнеше топтардың күресі сипатталған бұл негр тайпаларының соғысы деп аталады фотосуреттер жоқ өйткені олар мақалада ақ адамдар туралы айтылғанын байқағыңыз келмейді,тағы да басқарылма жасөспірім немесе жасөспірім тоб олардың барлығ қара екен білдір сондай ақ мақала бірнеше топ күре сипаттал бұл нег тайпа соғыс де атал фотосурет жоқ өйткені олар мақала ақ адам туралы айтыл байқа келме,racism

1,менің мәселелерімнің кейбір адамдардың өзін дұрыс сезінуіне ешқандай қатысы жоқ және сіз оларды ниггерлер деп атаған кезде шақырған кезде бұл өте ақымақ болып көрінеді,менің мәселе кейбір адам өз дұрыс сезін ешқандай қатыс жоқ және сіз ол ниггер де ата кезде шақыр кезде бұл өте ақы бол көрін,racism

1,америка түнде бұл ниггерді өлтіруге тырысатындай мылқау емес деп үміттенемін ол өте қара,америка түн бұл нигг өлтір тырысатында мылқа емес де үміттен ол өте қара,racism

1,сіз менімен ақымақ қара қаншықты ұрып соғып жатқан ниггердің кесірінен ажырастыңыз,сіз менімен ақы қара қаншық ұр соғ жат нигг кесір ажыра,racism

1,сіз барлық республикашылар сияқты қызғылт көзілдірік кигеніңіз анық нәсілшілдік пен бөліну ұпайлары бір күні сіз қайта сайлауға түсесіз және сайлаушылар сіздің кімнің жағында екеніңізді ұмытпайды қызыл емес көк қызыл ақ және көк қандай ақымақтық,сіз барлық республикашы сияқты қызғылт көзілдірік ки анық нәсілш пен бөлін ұпай бір күн сіз қайта сайла түс және сайлаушы сіздің кім жағ екен ұмытпа қызыл емес көк қызыл ақ және көк қандай ақымақтық,racism

1,тозақ мен бақылаушыларға барғаным есімде олар мені мылқау нигга деп атады әтешті сорады лол ебучие бастар,тозақ мен бақылаушы бар ес олар мен мылқа нигг де ат әтеш сор лол ебучи бас,racism

1,ол келесі жылға тұзды rt біреудің тұзды awwww rt желкекке обама мылқау нигга,ол келесі жыл тұз rt біреу тұз awwww rt желкек оба мылқа нигг,racism

1,сонымен мен netflix те малкольм ны кім өлтірді атты деректі фильм көріп отырмын ал ақ тілшілердің бірі фамилиясын айтудан бас тартқан негр спикері өзін малкольм деп атайды дейді,сонымен мен netflix те малколь ны кім өл ат деректі фильм көр отыр ал ақ тілші бірі фамилия айту бас тарт нег спикер өз малколь де ата де,racism

1,егер сіз джордан киіп жүрсеңіз және ертең құлап кететін джинсы алмасаңыз сіз ақымақ ниггерсіз,егер сіз джо ки жүр және ертең құла кет джин алма сіз ақы нигг,racism

1,мен оның сен сияқты ақымақ ниггерлермен айналысатынына күмәнданамын жігіт,мен оның сен сияқты ақы ниггер айналыс күмәндан жігіт,racism

1,сонымен түсінікті болу үшін негр белгісі афроамерикалық немесе қара сияқты ма ал түрлі түсті адамдар туралы не деуге болады нәсілмен кез келген сәйкестендіру бұл адамдарды адамгершіліктен шығаратын және сыртқы түріне қарай бәрімізді жалпылайтын белгі біз сұрауымыз керек неге ақш та бізді әлі де қара ақ және б деп атайды,сонымен түсінік болу үшін нег бел афроамерикалық немесе қара сияқты ма ал түрлі түсті адам туралы не де бол нәсіл кез кел сәйкестендіру бұл адам адамгершілік шығар және сыртқы түр қарай бәрі жалпыла белгі біз сұра керек неге ақш та біз әлі де қара ақ және б де ата,racism

1,иә мен бірінші кездескен адамсың,иә мен бірінші кездес адам,racism

1,мылқау бейбақ маған сыра төкті нигга үйге оралуы керек немесе дәл қазір өлуі керек,мылқа бей маған сыр төк нигг үй орал керек немесе дәл қазір өл керек,racism

1,мен ол сігуся қалайды мен айтамын фейсал аумин мен жоқ шіркеуге мылқау нигга ммммаааааоо,мен ол сігуся қала мен ай фейсал аумин мен жоқ шіркеу мылқа нигг ммммаааааоо,racism

1,rt обамаға қарғыс атсын мылқау нигга,rt обама қарғыс ат мылқа нигг,racism

1,tokyo hot шексіз жыныстық тартымдылық dailyxlover java азиялық,tokyo hot шексіз жын тартымдылық dailyxlover java азиялық,racism

1,rt лол мен ақымақ ниггерді ұра алмаймын оның қаншалықты сүйкімді екені маңызды емес эй әңгіме менің шырындарымды ынталандырады,rt лол мен ақы нигг ұр алма оның қаншалықты сүйк екен маңызды емес эй әңгіме менің шырын ынталандыр,racism

1,нигериялықтар нигер атырауына шынымен мән бермейді солай емес пе хм африканың үлкен ағасын немесе басқа да ақымақтықты көрумен тым бос емес,нигериялық ниг атырау шын мән берме солай емес пе хм африка үлкен аға немесе басқа да ақымақтық көр тым бос емес,racism

1,twitter ді нигерде қаза тапқан сарбаздардың отбасыларына көңіл айту үшін пайдаланудың орнына тек ол туралы әңгіме жасайды бұл жиіркенішті одан да сорақысы ол тағы да оған қарсы шыққан қара әйелдерді айыптайды фбр қарсы тұрыңыз,twitter ді ниг қаза тап сарбаз отбасы көңіл айту үшін пайдалан орн тек ол туралы әңгіме жаса бұл жиіркеніш одан да сора ол тағы да оған қарсы шық қара әйел айыпта фб қарсы тұр,racism

1,rt барды желкекке обама мылқау нигга қаншық үндемей желкекке және сорып алыңыз бұл қара мазу мүшесі ол әлі де сенің президентің ақ есекпен бииаииич,rt бар желкек оба мылқа нигг қаншық үндеме желкек және сор ал бұл қара маз мүше ол әлі де сенің президент ақ есек бииаииич,racism

1,хаха қарғысатқыр педик лейкерс сорады мүшесі лейкер жанкүйерлері гейлер коби мен фил джексон екі мылқау нигга маған бұл туралы айтыңыз,хах қарғысатқы педик лейкерс сор мүше лейк жанкүйер гей коби мен фил джексон екі мылқа нигг маған бұл туралы ай,racism

1,сіз негр испан тілінде қара дегенді білдіретінін білесіз бе егер мен сені ақ деп атасам ал сен ақ болсаң бұл қорлау немесе нәсілшілдік болар ма еді,сіз нег испан тіл қара де білдір біл бе егер мен сен ақ де ата ал сен ақ бол бұл қорла немесе нәсілш бол ма ед,racism

1,уау әдеттегідей шынында да түрлі түсті адамдар уау сіз тіпті нәсілшілдігіңізді жасыруға тырыспайсыз менмен және мақтаншақ иә бұл түрлі түсті адамдар біз оларға осылай қараймыз бұл оларға қысым көрген топ ретінде абырой мен құрмет береді ккк мен кездесуге оралыңыз сүт сорғыш,уау әдеттегідей шын да түрлі түсті адам уау сіз тіпті нәсілшілдіг жасыр тырыспа мен және мақтаншақ иә бұл түрлі түсті адам біз оларға осылай қара бұл оларға қысым көр топ рет абырой мен құрмет бер ккк мен кездес орал сүт сорғыш,racism

1,төбешіктердің немере ағасы сіс ті ниггер деп атайды бұл олардың twitter дегі аккаунттарын ақымақ бейбақтарды да көрсетеді,төбешік немере аға сіс ті нигг де ата бұл олардың twitter дег аккаунт ақы бейбақ да көрсет,racism

1,ерінге ауыр инъекция rt ерінге инъекция жасау туралы ойлану керек rt обамаға қарғыс атсын мылқау нигга,ерін ауыр инъекция rt ерін инъекция жасау туралы ойлан керек rt обама қарғыс ат мылқа нигг,racism

1,ақымақ надан бейбақтар егер сіздің ата анаңызды ниггерлер мен мексикалықтар атып тастаса сіз полицейлердің көпшілігі неге құрметке лайық екенін түсінер едіңіз,ақы надан бейбақ егер сіздің ата ана ниггер мен мексикалық ат таста сіз полицей көпшілік неге құрмет лайық екен түсін ед,racism

1,обамаға қарғыс атсын мылқау нигга лол сен ашуланасың иә,обама қарғыс ат мылқа нигг лол сен ашулан иә,racism

1,сіз бардыңыз мылқау нигга ккк ге барыңыз,сіз бар мылқа нигг ккк ге бар,racism

1,грег өтінемін мені бұғаттан шығарыңыз сен ақымақ есек нигга есек қаншық шайтан алғыр есек дик боқ нәжіс қарғыс есек қарғыс атқыр ақымақ мылқау сіздің бетіңіздің суреті,грег өт мен бұғат шығ сен ақы есек нигг есек қаншық шай алғы есек дик боқ нәжіс қарғыс есек қарғыс атқы ақы мылқа сіздің бет сурет,racism

1,техникалық тұрғыдан операция болып табылады сен ақымақ бейбақсың сондықтан өз фактілеріңізді біліңіз фагот черножопая қаншық барды сен желкекке пидор хренов,техникалық тұрғы операция бол таб сен ақы бей сондықтан өз факті біл фагот черножопая қаншық бар сен желкек пидо хренов,racism

1,бұл жігіт мені сәтсіздік деп атайды ал өзі cod ойынымен күн көреді бауырым үндемеңіз желкек amp нағыз жұмыс табыңыз мылқау нигга,бұл жігіт мен сәтсіздік де ата ал өзі cod ойын күн көр бауыр үндеме желкек amp нағыз жұмыс таб мылқа нигг,racism

1,сен ақымақ ақымақ нигга сияқтысың,сен ақы ақы нигг сияқты,racism

1,сіз ақымақ бейбақсыз басқа нигга тупак сияқты атып өлтіріңізші,сіз ақы бей басқа нигг тупак сияқты ат өлтіріңізш,racism

1,бұл жігіт сені ниггер деп атаған содан кейін менің big big nas jay mobb deep және wu tang the nerve дискілер топтамасын ұрлаған ақымақ бейбақ болды,бұл жігіт сен нигг де ата содан кейін менің big big nas jay mobb deep және wu tang the nerve дискі топтама ұрла ақы бей бол,racism

1,сіз сондай әдемісіз құдай сіз бірінші фильмдегі пегги картерге ұқсайсыз егер сіз marvel жанкүйері болсаңыз хехе маған үшінші суреттегі толқынды жарылыс ұнайды айтпақшы ол сізге келеді,сіз сондай әдемі құдай сіз бірінші фильм пегги кар ұқса егер сіз marvel жанкүйер бол хех маған үшінші сурет толқ жарылыс ұна айтпақшы ол сізге кел,racism

1,мен оны алып тастауым керек еді өйткені мен бұлай жаза алмайтындай көрінгім келмейді нигга нигга деген жаргон сөз сондықтан ол құрметтемейді және сонымен бірге жиіркенішті екіншіден бұл маған қара достарыңды құрметтемейтіндігіңді

көрсетеді өйткені сен оларды осылай атайсың және оны оларға айтасың,мен оны алып таста керек ед өйткені мен бұлай жаза алмайтында көрін келме нигг нигг де жаргон сөз сондықтан ол құрметтеме және сонымен бірге жиіркеніш екінші бұл маған қара дос құрметтемейтіндіг көрсет өйткені сен ол осылай ата және оны оларға ай,racism

1,обамаға қарғыс атсын ақымақ нигга ақымақ,обама қарғыс ат ақы нигг ақы,racism

1,ешнәрсені құлағыңыздан өткізбеңіз олар алаяқтық жасайды олардың сайлаушылары мұны біледі және олар бұған риза мұны біліңіз және дайын болыңыз олар алдай алатын жерде олар жасайды көптеген оңтүстік штаттарда кәсіби команда бар сондықтан жиналып дайын болыңыз кейбір қара адам ұбт алды сондықтан оларды оған жұмсаңыз,ешнәрсе құлағ өткізбе олар алаяқтық жаса олардың сайлаушы мұны біл және олар бұған риза мұны біл және дайын бол олар алда ал же олар жаса көптеген оңтүстік штат кәсіби команда бар сондықтан жинал дайын бол кейбір қара адам ұбт алды сондықтан ол оған жұм,racism

1,стфу туралы ариэль шешім қазірдің өзінде қабылданды лол қара адамдарды нигга деп атаңыз өйткені біз дауыс беруге құқылы емес едік жиіркенішті стфу шынымды айтсам бұл шынымен тітіркендіре бастайды,стф туралы ариэль шешім қаз өз қабылда лол қара адам нигг де ата өйткені біз дауыс бер құқылы емес едік жиіркеніш стф шын айт бұл шын тітіркендір баста,racism

1,нәсілшілдік барлық жерде бар мысалы сізді цинциннатидегі пәтеріңіздің жанында нигга деп атаған оқиға,нәсілш барлық же бар мысал сіз цинциннати пәтер жан нигг де ата оқиға,racism

1,сізде бұл қара полицейлердің барлығы ақ сардженттің алдында үйдегі ниггерлерді ойнайды егер сіз менің пікірімді білгіңіз келсе бұл өте жиіркенішті болды,сіз бұл қара полицей барлығ ақ сарджен алд үйдегі ниггер ойна егер сіз менің пікір біл кел бұл өте жиіркеніш бол,racism

1,наоми үшін компаниядағы ең дарынды әйел болудың мағынасы жоқ бірақ олар оған де жасаған жұмысы үшін лайықты нәрсені бермейді егер оның қара нәсілділігіне қандай да бір қатысы болса күрес жанкүйерлері нәсілшіл болса және компания оны қабылдаса олар таң қалмас еді,наоми үшін компания ең дарынды әйел бол мағына жоқ бірақ олар оған де жаса жұмыс үшін лайықты нәрсе берме егер оның қара нәсілділіг қандай да бір қатыс бол күрес жанкүйер нәсілшіл бол және компания оны қабылда олар таң қалмас ед,racism

1,мылқау нигга бұл эпизодтың сол кезде көрсетілгені таңқаларлық емес пе майлы желкек,мылқа нигг бұл эпизод сол кезде көрсетіл таңқаларлық емес пе майлы желкек,racism

1,егер сіз қара әйелге көйлек жасап жатсаңыз және басқа қара әйелді мылқау қара әйел деп атасаңыз мен сіздің әрекеттеріңізді біржақты және жиіркенішті деп

санаймын,егер сіз қара әйел көйлек жаса жат және басқа қара әйел мылқа қара әйел де ата мен сіздің әрекет біржақты және жиіркеніш де сана,racism

1,неліктен қара әйелдердің кеуделері әрқашан үлкен бірақ емізіктері әрқашан жиіркенішті ниггерлер әйелдер шымшу негр сиськи,неліктен қара әйел кеуде әрқашан үлкен бірақ емізік әрқашан жиіркеніш ниггер әйел шымш нег сиськи,racism

1,мұны қарғыс атқан анаға апарып әрі қарай жүріңіз мені жалғыз қалдыр сен есегі жараланған мылқау ниггерді бейнелейсің,мұны қарғыс ат ана апар әрі қарай жүр мен жалғыз қалдыр сен есег жаралан мылқа нигг бейнеле,racism

1,ер адамдар хайуанаттар бағы яssss патшайым зтрайт адамдар зкуум яsss бәрін құртатын әйелдер ақ адамдар мылқау амириттер о мылқау ниггерлер сіз бардыңыз бәрі желкекке байсалды әлем сіздің арқаңызда бөлінген,ер адам хайуанат бағ яssss патшайым зтрайт адам зкуу яsss бәрін құрт әйел ақ адам мылқа амирит о мылқа ниггер сіз бар бәрі желкек байсалды әлем сіздің арқа бөлін,racism

1,сіздің кейбір ниггерлеріңіз өте мылқау былғары курткалары бар мойын сызықтарын киеді сіз оларды сүйкімді болуға тырысқанда жасайсыз гомо ниггерлер сыртта суық,сіздің кейбір ниггер өте мылқа былғары куртка бар мойын сызық ки сіз ол сүйк бол тырыс жаса гомо ниггер сырт суық,racism

1,сіз тілінде сөйлемейсіз бізге pocs деп аталу ұнамайды бұл бізді түрлі түсті адамдар деп атаумен бірдей бұл белгіден құтылу үшін бізге онжылдық қажет болды сондықтан сізде және сіздің командаңызда бұл bck белгісін бізге іліп қоюға құқығыңыз жоқ сіз адамдар соншалықты надан біз құрбандық емес нәсілшілдіктен аман қалдық,сіз тіл сөйлеме бізге pocs де атал ұнама бұл біз түрлі түсті адам де ата бірде бұл белгі құтыл үшін бізге онжылдық қажет бол сондықтан сіз және сіздің команда бұл bck белгі бізге іл қою құқы жоқ сіз адам соншалықты надан біз құрбан емес нәсілшілдік аман қалдық,racism

1,рас мен бұрын қара нәсілді азаматтарды пайдаланған ақ компаниялар өз активтерін қайта бөлуі керек деп келісемін бірақ кедей адамдар мұрагерлікке ештеңе бере алмайтындықтан ұрпақтардың барлық байлығын қайта бөлуге шақыру әділетсіз,рас мен бұрын қара нәсіл азамат пайдалан ақ компания өз актив қайта бөл керек де келіс бірақ кедей адам мұрагерлік ештеңе бер алмайтындық ұрпақ барлық байлығ қайта бөл шақыру әділет,racism

1,lmao rt обамаға қарғыс атсын мылқау нигга,lmao rt обама қарғыс ат мылқа нигг,racism

1,нигга мен жатамын ұйықтауға тырысамын төсекке жатамын мылқау нигга қарғыс атсын сен кәдімгі капюшон нигга сияқты твиттерлейсің,нигг мен жат ұйықта тырыс төсек жат мылқа нигг қарғыс ат сен кәдімгі капюшон нигг сияқты твиттерле,racism

1,ол мені өзінің жергілікті негрі деп атағанын да мойындамады жалпы менің ақ шоурнерім маған тиіп негр деп атады сөзін қолданды ақ әріптестерім мені қолдамады

ақ продюсер маған шоу үшін оны құпия ұстауды айтты мен солай істедім,ол мен өзінің жергілікті нег де ата да мойындама жалпы менің ақ шоурн маған ти нег де ат сөз қолда ақ әріптес мен қолдама ақ продюсер маған шоу үшін оны құпия ұстау ай мен солай істе,racism

1,маған вассап ниг деп әзілдеп қоңырау шалып түскі ас кезінде маған және менің достарыма тамақ лақтырып маймыл дыбыстарын шығарып ниггерді әзіл сияқты твиттерде жазатын адамдарды көру өте жиіркенішті екенін еске саламын олар өмірге мән бергендей әрекет етеді қара адамдар өтінемін құлап қалды,маған васса ниг де әзілде қоңырау шал түскі ас кез маған және менің дос тамақ лақтыр маймыл дыбыс шығар нигг әзіл сияқты твиттер жаз адам көру өте жиіркеніш екен ес сал олар өмір мән бергенде әрекет ет қара адам өт құла қал,racism

1,сіз менің ағамды ниггер деп атайсыз ба бірақ содан кейін бұрылып менің қара немере ағамды ұруға тырысасыз ба ол көп ақша табады ақымақ,сіз менің аға нигг де ата ба бірақ содан кейін бұрыл менің қара немере аға ұр тырыс ба ол көп ақша таб ақы,racism

1,жай ғана нигга сөзін қолдана отырып сізді надан ақымақ бейбақ деп санайды,жай ғана нигг сөз қолдан отыр сіз надан ақы бей де сана,racism

1,ақымақ балалар мені мылқау деп ойлайды егер олар мылқау болса мені ойнай алады dpmo трахает ниггеров осы ақ жігіт flex,ақы бала мен мылқа де ойла егер олар мылқа бол мен ойна ал dpmo трахает ниггеров осы ақ жігіт flex,racism

1,негр нәсілдік қорлау емес және жылдары қара нәсілділер афроамерикалық деп аталуды жөн көргенге дейін құрметпен қолданылған негр жай қара дегенді білдіреді бланко ақ дегенді білдіреді,нег нәсіл қорла емес және жыл қара нәсілді афроамерикалық де аталу жөн көр дейін құрмет қолданыл нег жай қара де білдір бланко ақ де білдір,racism

1,желкек обамаға барды мылқау нигга сіз желкекге бардыңыз мылқау жезөкше бұл қаншық байсалды қандай надан қаншық тежегіш,желкек обама бар мылқа нигг сіз желкек бар мылқа жезөкше бұл қаншық байсалды қандай надан қаншық тежегіш,racism

1,бұл дұрыс егер сіз айтқандай негрлер заңды бұзуды және қылмыс жасауды тоқтатса полиция оларға шақырылмас еді сонымен қатар егер олар ақ офицерлерге деген жеккөрушілікке ие болмаса олар тұтқындауға қарсы тұрмас еді және көп қиындықтар туғызбас еді,бұл дұрыс егер сіз айтқанда негр заңды бұзу және қылмыс жасау тоқтат полиция оларға шақырылмас ед сонымен қатар егер олар ақ офицер де жеккөрушілік ие болма олар тұтқында қарсы тұрмас ед және көп қиындық туғызбас ед,racism

1,негр қара түсті қорлайтын болып саналған кезде афроамерикалықтар үшін ең сыпайы сөз ретінде түсті ауыстырды енді оларды түрлі түсті адамдар деп атайды бұл түрлі түсті адамдармен бірдей ақ түс енді түс емес екені анық мұның бәрі өте шатастырады,нег қара түсті қорла бол санал кезде афроамерикалық үшін ең сыпай

сөз рет түсті ауыс енді ол түрлі түсті адам де ата бұл түрлі түсті адам бірде ақ түс енді түс емес екен анық мұның бәрі өте шатастыр,racism

1,бұл қара адамдарды ниггерлер деп санайтын нәсілшілдер тобының және оларға мүмкіндік беретін қара ақша үшін жиреніштi жезөкшелердің жұмысы болды соңы,бұл қара адам ниггер де сана нәсілшіл тоб және оларға мүмкіндік бер қара ақша үшін жиреніш жезөкше жұмыс бол соңы,racism

1,жоқ шайтан алғыр сен айтқан қандай да бір мылқау боқтық өткен аптада мектепте менде және менің барлық ниггерлерімде проблема бар,жоқ шай алғы сен айт қандай да бір мылқа боқ өткен апта мектеп менде және менің барлық ниггер проблема бар,racism

1,обамаға қарғыс атсын мылқау нигга lt бәрін қараңыз,обама қарғыс ат мылқа нигг lt бәрін қара,racism

1,мен мұны сіздің оқуыңызбен келіспеймін олардың мойнында тұрған жиналыста кенеттен нәсілшілдік неден пайда болады мен түсінемін бұл талқылаудан олар оқиға кезінде мұндай ештеңе болған жоқ деген қорытынды жасай алмады,мен мұны сіздің оқу келіспе олардың мойн тұр жиналыс кенет нәсілш не пайда бол мен түс бұл талқылау олар оқиға кез мұндай ештеңе бол жоқ де қорытынды жаса алма,racism

1,өйткені егер сіз нысанаға түспесеңіз сіз жай ғана ақымақ менің ниггерімсіз,өйткені егер сіз нысана түспе сіз жай ғана ақы менің ниггер,racism

1,байланыс жағалаудан am жағалауына дейін сіз бардыңыз сіз жұмыстан шығарылдыңыз ақымақ нигга мені темит есегіне тістеп ал сен ақымақсың,байланыс жағалау am жағала дейін сіз бар сіз жұмыс шығар ақы нигг мен темит есег тісте ал сен ақы,racism

1,жасөспірім ананың бұл ақымақ түйіндері өте ақымақ жанель бұл нигер саған қолын көтерді оны тастап кет,жасөспірім ана бұл ақы түйін өте ақы жанель бұл ниг саған қол көтер оны таста кет,racism

1,обамаға қарғыс атсын мылқау нигга онымен келісіңіз немесе өзіңді өлтір,обама қарғыс ат мылқа нигг онымен келіс немесе өз өлтір,racism

1,марк кубан мылқау бейбақ супер киска оның айтқаны дұрыс емес немесе мағынасыз қарапайым тілмен айтқанда ол қорқақ қара маз әуесқойы,марк кубан мылқа бей суп киск оның айт дұрыс емес немесе мағына қарапайым тіл айтқанда ол қорқақ қара маз әуесқой,racism

1,бұрын оларда қара ppl болған шоуға келіп иә мен ештеңе алмаймын және айтқанымды талап етемін деді мен иә келісемін дедім сонда бұл бәрібір нәсілшілдік мен жұмыс сұхбатында қара адамдарды жек көретінімді айтпағандықтан бұл оның мағынасын өзгертпейді,бұрын ол қара ppl бол шоу кел иә мен ештеңе алма және айт талап ет де мен иә келіс де сонда бұл бәрібір нәсілш мен жұмыс сұхбат қара адам жек көр айтпа бұл оның мағына өзгертпе,racism

1,родео клубындағы кейбір ақымақтар маған және эшлиге айқайлап оның көлігіне отыруды бұйырды және бұл ниггерді ұрып соғып жатқанын айтты,родео клуб кейбір ақымақ маған және эшли айқайла оның көліг отыру бұйыр және бұл нигг ұр соғ жат ай,racism

1,сіз уэйн және амп есімді ақымақ ұсқынсыз нигга крис блэк браунның ұсқынсыз есегі позитивті позитивті сіздің уэйннің қызыл ұсқынсыз есегі емес pic twitter com,сіз уэйн және ам ес ақы ұсқын нигг крис блэк браун ұсқын есег позитивті позитивті сіздің уэйн қызыл ұсқын есег емес pic twitter com,racism

1,шынымды айтсам үйге бар мылқау ниггерлер,шын айт үй бар мылқа ниггер,racism

1,бұл ақымақ ниггерлер жолда қандай желкек алғысы келетінін ешқашан білмейді,бұл ақы ниггер жолда қандай желкек ал кел ешқашан білме,racism

1,бұл мылқау ниггер нигга сен ақымақсың,бұл мылқа нигг нигг сен ақы,racism

1,маған ескі ақ клуб мүшелері мені жұмыста негр деп атағаны ұнады,маған ескі ақ клуб мүше мен жұмыс нег де ата ұн,racism

1,бауырым сенің дауысың мен оянғаннан кейін бірінші естідім,бауыр сенің дау мен оян кейін бірінші есті,racism

1,осы мылқау турнирлердің барлығын қалай жеңеді мен оның мылқау нигга есегінен әлдеқайда жақсымын тек мылқау боқ жеңе алады,осы мылқа турнир барлығ қалай жең мен оның мылқа нигг есег әлдеқайда жақ тек мылқа боқ же ал,racism

1,досым мына ақымақ ниггерлерді ұр сен қалайсың мен тунчи құйдым мен нукенспін мен ақымақпын мен үш қуыршақ сияқты ақымақпын,дос мына ақы ниггер ұр сен қала мен тунчи құй мен нукенс мен ақы мен үш қуыршақ сияқты ақы,racism

1,сіз irl ді фредоға қарсы алғаш рет ойнағаннан бері және сіз ең жек көретіндердің бірінен қоғамдағы ең сүйіктісіне айналғаннан бері мен сізді таң қалдырдым мен сені мақтан тұтамын фрфр ағасы,сіз irl ді фредо қарсы алғаш рет ойна бері және сіз ең жек көр бір қоғам ең сүйікті айнал бері мен сіз таң қал мен сен мақтан тұт фрф аға,racism

1,егер сіз қара және түрлі түсті киімнің орнына ақ жууға арналған киімді таңдасаңыз сіз нәсілшілсіз нәсілшілдікті тоқтатыңыз олардың барлығын жуыңыз сіз ештеңе істемейсіз,егер сіз қара және түрлі түсті ки орн ақ жу арнал ки таңда сіз нәсілшіл нәсілшілдік тоқта олардың барлығ жу сіз ештеңе істеме,racism

1,сіз мұны бірінші болып жасадыңыз,сіз мұны бірінші бол жаса,racism

1,мылқау ниггерлер әрқашан менің орнымда отырады бұрын сізді тылға жібергенде бұл сізді қатты алаңдатты ма,мылқа ниггер әрқашан менің орн отыр бұрын сіз тыл жібер бұл сіз қатты алаңда ма,racism

1,сонымен циммерманмен dmx ке қарсы бұл ақымақтық сізді надан ниггерлерді шынымен үрлей ме иә мен ниггерлер дедім өйткені сен бәрің ақымақсың,сонымен

циммерман dmx ке қарсы бұл ақымақтық сіз надан ниггер шын үрле ме иә мен ниггер де өйткені сен бәрің ақы,racism

1,рт idgaf бұл менің қысқа тозу себебі нигерлер көруге болады буг болуы мүмкін емес ð ð ð â rat ð ð ðð фин жаман аслдан га тоқтату,рт idgaf бұл менің қыс тоз себебі нигер көр бол буг бол мүмкін емес ð ð ð â rat ð ð ðð фин жаман асл га тоқтату,racism

1,мен қазір мылқау ниггермін gt ал содан кейін сіз бардыңыз gt gt ттт,мен қазір мылқа нигг gt ал содан кейін сіз бар gt gt ттт,racism

1,сіз мен бір секундтан кейін танысқан бірінші адам боласыз,сіз мен бір секунд кейін таныс бірінші адам бол,racism

1,обамаға қарғыс атсын мылқау нигга сонымен сіз ашуланасыз иә lmaoooooo,обама қарғыс ат мылқа нигг сонымен сіз ашулан иә lmaoooooo,racism

1,ештеңеге жарамсыз ниггерлер қараңызшы мен йолды заңды түрде өлтіру үшін полицей боламын шии ақымақ бейбақтар заңдардың бар екенін түсінбейді,ештеңе жарамсыз ниггер қараңызш мен йо заңды түр өлтір үшін полицей бол шии ақы бейбақ заң бар екен түсінбе,racism

1,идгаф ақымақ маймылдар үшін жалған ба сіз алдыңызда жалған есек бар екенін білесіз бір минутта біз сіздің достарыңыз ал келесі минутта біз ниггерлер сізді ұрамыз,идгаф ақы маймыл үшін жалған ба сіз алд жалған есек бар екен біл бір минут біз сіздің дос ал келесі минут біз ниггер сіз ұр,racism

1,бүгін нигга күні деп айта беретін барлық ақымақ бейбақтар көпірден секіріңіз нигга қоқыс сондықтан қайталап көріңіз және бұл үшін емес,бүгін нигг күн де ай бер барлық ақы бейбақ көпір секір нигг қоқыс сондықтан қайтала көр және бұл үшін емес,racism

1,rt мен таңертең сайлау комиссиясында фергюсондағы әрбір қара адамды тіркеуді бастау үшін карталар деректер жинадым хт,rt мен таңертең сайлау комиссия фергюсо әрбір қара адам тіркеу бастау үшін карта дерек жина хт,racism

1,дәл қазір бір нәрсені жай ғана спорт деп айту қиын бірақ мен бұрын осындай пікірде болғанмын бұл тің толық нәсілшілге берген жауабы болды ол дәл қазір қара спортшылар тобына жеккөрушілік танытуда олардың артында жасырыну бұл жай ғана спорт,дәл қазір бір нәрсе жай ғана спорт де айту қиын бірақ мен бұрын осындай пікір бол бұл ті толық нәсілшіл бер жауаб бол ол дәл қазір қара спортшы тоб жеккөрушілік таныт олардың арт жасырын бұл жай ғана спорт,racism

1,бұл тіпті сарказм емес еді сіз қара адамдарды нигга деп атадыңыз қарғыс атқыр жиіркенішті,бұл тіпті сарказ емес ед сіз қара адам нигг де ата қарғыс атқы жиіркеніш,racism

1,баннихумор енді қара сөз емес мен нәсілшіл емес нигга деп айта аламын тверкинг сүйікті ниггер ғана жиіркенішті майлы есекті шайқай алады онда си,баннихумо енді

қара сөз емес мен нәсілшіл емес нигг де ай ал тверкинг сүйікті нигг ғана жиіркеніш майлы есек шай ал онда си,racism

1,ақ адамдар нәсілшілдікке тап болуы мүмкін неге сіз соншалықты надан екенсіз нәсілшілдік қысымға ұшыраған түрлі түсті адамдарға қарсы бағытталған сіз біреуін де екіншісін де жедіңіз,ақ адам нәсілшілдік тап бол мүмкін неге сіз соншалықты надан екен нәсілш қысым ұшыра түрлі түсті адам қарсы бағыттал сіз біреу де екінші де же,racism

1,бұл жігіт мектеп ауданында арнайы жұмыс істейді сондай ақ менің логикама сүйене отырып бұл сөзбе сөз төте жолдар қалай жасалады тағы бір нәрсе сіз бізге кім екенімізді қалай айтасыз қара нәсілділер қара деп аталғысы келмейтінін ал ақ әйелдер каренка болғысы келмейтінін еске түсіріңіз,бұл жігіт мектеп аудан арнайы жұмыс істе сондай ақ менің логика сүйен отыр бұл сөзб сөз төте жол қалай жасал тағы бір нәрсе сіз бізге кім екен қалай ай қара нәсілді қара де атал келме ал ақ әйел каренк бол келме ес түсір,racism

1,сіздің кішкентай қара мазақ балапаныңыз соншалықты мылқау менің бауырларым мен сіздерді тозаққа жібере аламын дейді,сіздің кішкентай қара мазақ балапан соншалықты мылқа менің бауыр мен сіз тозақ жібер ал де,racism

1,бірінші әсер бұл адамды gc ге кім қосты сіздің лақап атыңыз менің басымда пингвиндер жақындықты бағалау маған сен ұнайсың ба досым ретінде иә сен менікісің досым саған ғашық болды жоқ сен де оны жариялауың керек сізде бір кездейсоқ ой бар сенің атың неге пингвиндер,бірінші әсер бұл адам gc ге кім қос сіздің лақа ат менің бас пингвин жақындық бағалау маған сен ұна ба дос рет иә сен менік дос саған ғашық бол жоқ сен де оны жарияла керек сіз бір кездейсоқ ой бар сенің ат неге пингвин,racism

1,жалған жұмысы бар екі бейбақ та мексикалықтар болды ал менің миллионер анам мұның бәрі менің кінәм екенін жалғастырады мен бұл ақымақ ақымақ ақымақтардың үйіндісін күтпей тұрып өлгенін қалаймын,жалған жұмыс бар екі бей та мексикалық бол ал менің миллион ана мұның бәрі менің кінә екен жалғастыр мен бұл ақы ақы ақымақ үйінді күтпе тұр өл қала,racism

1,мен барлығының young money тобында ойнағанын қалаймын және никки миндж осы арал ниггерді ұрып соғып ол сені ақымақ қаншық арқылы жеңді,мен барлығ young money тоб ойна қала және никки миндж осы арал нигг ұр соғ ол сен ақы қаншық арқылы же,racism

1,біреу ашуланды rt бұл мылқау нигга обамаға,біреу ашула rt бұл мылқа нигг обама,racism

1,мен оны сөгіп кедей негр деп атағанымда ақ адам маған саусағыммен нұсқап жест ишара жасағанына сене алмаймын,мен оны сөг кедей нег де ата ақ адам маған саусағ нұсқа жест ишар жаса сен алма,racism

1,костюм тозақ сияқты мылқау митинг өткізді содан кейін өтіп бара жатқан біреу нигга деді бәрі есінен танып қалды,костюм тозақ сияқты мылқа митинг өт содан кейін өт бар жат біреу нигг де бәрі ес тан қал,racism

1,сондықтан сіз sekac пен сауда жасамауыңыз керек тупица трахает пайдасыз нигга pic twitter com,сондықтан сіз sekac пен сауда жасама керек тупиц трахает пай нигг pic twitter com,racism

1,егер олай болмаса онда мені ойнауды сұрайтын dm ден шығыңыз мен сенің мылқау негр есегіңнен жалтарғаным үшін ғана енді сен бос сөз көтергің келеді,егер ола болма онда мен ойнау сұра dm ден шығ мен сенің мылқа нег есег жалтар үшін ғана енді сен бос сөз көтер кел,racism

1,сіз инцестке қатысуға тура келетін жиіркенішті қара қаншықсыз өйткені сізді кішкентай әтешіңіз бен негр арманыңыз үшін қалайтын адамды ала алмайсыз,сіз инцест қатыс тура кел жиіркеніш қара қаншық өйткені сіз кішкентай әтеш бен нег арман үшін қала адам ал алма,racism

1,егер қоғамның көп бөлігі осы сәтсіз атақты адамдар айтатын және жасайтын ақымақтыққа негізделмесе сіз дұрыс болар едіңіз қоғамның пікірінше гейлер құқыққа немесе құрметке лайық емес қара нәсілділер ұсқынсыз және жиіркенішті ниггерлер ал әйелдер жасына қарамастан солай,егер қоғам көп бөліг осы сәт атақты адам айт және жаса ақымақтық негізделме сіз дұрыс бол ед қоғам пікірінше гей құқық немесе құрмет лайық емес қара нәсілді ұсқын және жиіркеніш ниггер ал әйел жас қарамастан солай,racism

1,пышақ нигга фагот мен үшін сен ақымақ фаготсың,пышақ нигг фагот мен үшін сен ақы фагот,racism

1,том ағай орео сатылым ракон символдық негр нәсілшіл ақ үстем гомофоб алалаушылық фанат және б сізді қандай есім деп атады немесе өкінішке орай қолданылған доктор тим вестли,то аға орео сатылым ракон символ нег нәсілшіл ақ үстем гомофоб алалаушылық фанат және б сіз қандай есім де ат немесе өкініш орай қолданыл доктор тим вестли,racism

1,қара нәсілділер нигга деп атайтын қара нәсілді мужчин мың долларға минуттық таңдауы сияқты бұл өте ыңғайсыз мен қарадым және тек екі қара әйелдің сатылғанын көрдім тіпті мұндай дәрежеде емес жиіркенішті бірақ менің ойымша ақша сіз үшін бәрін шешеді деп ойлаймын,қара нәсілді нигг де ата қара нәсіл мужчин мың долл минуттық таңда сияқты бұл өте ыңғайсыз мен қара және тек екі қара әйел сатыл көр тіпті мұндай дәреже емес жиіркеніш бірақ менің ойымша ақша сіз үшін бәрін шеш де ойла,racism

1,морган ниггер сен нәсілшіл және ақымақсың сондықтан желкекге барыңыз қаншық,морган нигг сен нәсілшіл және ақы сондықтан желкек бар қаншық,racism

1,ақымақ ұсқынсыз хуяны жек көруді доғар менің сыйлықтарымды жек көруді доғар ұсқынсыз ниггердің аты уэйн және ұсқынсыз крис қоңырау шалыңыз pic twitter com,ақы ұсқын хуя жек көру доғар менің сыйлық жек көру доғар ұсқын нигг ат уэйн және ұсқын крис қоңырау шал pic twitter com,racism

1,сіздің бірінші пайдаланушы атыңыз мен сіздің алғашқы твиттеріңіздің бірін жариялаймын,сіздің бірінші пайдаланушы ат мен сіздің алғашқы твиттер бір жарияла,racism

1,мен өткенге жабысып қалған қарт адаммын сондықтан мен сізге ұсынатынның бәрі ші жылдардан басталады неге жарық тек осы қорқынышты ескі үйдің шатырында жанады әртүрлілік дегенмен оливер рид карен блэк және бетт дэвис ойнаған өртеу фильмін қолданып көріңіз,мен өт жабыс қалған қарт адам сондықтан мен сізге ұсын бәрі ші жыл бастал неге жарық тек осы қорқыныш ескі үй шатыр жан әртүрлілік дегенмен олив рид карен блэк және бетт дэвис ойна өрте фильм қолдан көр,racism

1,бұл ақ үстемдік деп аталады,бұл ақ үстемдік де атал,racism

1,елшілердің істері қара алтын негр олар құдай айыптау үшін тағайындаған бұл әлемдегі шайтанның түкіргіштері деп аталады олар қарапайым адамдарға ұқсайды бірақ құдай халқы болуы керек түрлі түсті адамдар емес өйткені олар қара қара алтын негрлер емес ақ түсті,елші іс қара алтын нег олар құдай айыпта үшін тағайында бұл әлем шайтан түкіргіш де атал олар қарапайым адам ұқса бірақ құдай халқ бол керек түрлі түсті адам емес өйткені олар қара қара алтын негр емес ақ түсті,racism

1,мылқау әйел сіз нигга деп анық айттыңыз әрпін алып отставкаға кетіңіз сізге не дұрыс емес,мылқа әйел сіз нигг де анық ай әр алып отставка кет сізге не дұрыс емес,racism

1,біз өтірікші мен нәсілшіл ді тан алып тастауымыз керек сенімділік жоқ ол өзінің нәсілшілдігі туралы generalkelly ден білді ал біз generalkellyisaracist ті нигериялық солдат оқиғасынан білеміз,біз өтірікш мен нәсілшіл ді тан алып таста керек сенімділік жоқ ол өзінің нәсілшілдіг туралы generalkelly ден біл ал біз generalkellyisaracist ті нигериялық солдат оқиға біл,racism

1,сен ақымақсың нигерде қалған лос анджелес сержанты дэвид джонсонның бұл суретін жастағы джон келли мен сен сияқты мылқау бейбақтар әйелін құрметтемеді және алдады және оған ақш қа бос табыт қайтарылды сіз айтқыңыз келгеннің бәрі ақымақ панк,сен ақы ниг қалған лос анджелес сержан дэвид джонсон бұл сурет жастағы джон келли мен сен сияқты мылқа бейбақ әйел құрметтем және алд және оған ақш қа бос табыт қайтар сіз айт кел бәрі ақы панк,racism

1,құдай барлық тұзақ ниггерлеріне батасын берсін бұл ақымақ ниггерді кім ойлап тапты,құдай барлық тұзақ ниггер бата бер бұл ақы нигг кім ойла тап,racism

1,rt tea party өкілі naacp нәсілшілдік туралы дәріс оқуға құқығы жоқ деп мәлімдеді оны алыңыз сіз түстісіз,rt tea party өкіл naacp нәсілш туралы дәріс оқ құқығ жоқ де мәлімде оны ал сіз түсті,racism

1,бір адам үшін бәрін жасауды тоқтатқанда бұл ақылсыз бірақ олар сізді ешқашан байыпты қабылдамайды егер мен бірінші жазбасам олар маған хабарлама жібермейтін сияқты егер сіз мұңайсаңыз олар жай ғана ненің дұрыс емес екенін айтады бірақ мен адамдарға қашан мән бермейтінін және қашан болмайтынын білемін ммм бірақ сіз қатты ренжідіңіз,бір адам үшін бәрін жасау тоқтат бұл ақыл бірақ олар сіз ешқашан байыпты қабылдама егер мен бірінші жазба олар маған хабарлама жіберме сияқты егер сіз мұңай олар жай ғана не дұрыс емес екен айт бірақ мен адам қашан мән берме және қашан болма біл мм бірақ сіз қатты ренжі,racism

1,сіздердің барлығыңыз ниггерлер ерлер мен әйелдер айналасында серуендеп телефондарды құлағыңызға қысып музыканы қатты қосып ақымақ және тітіркендіргіш болып көрінесіз кейбір құлаққаптарды алыңыз және бізді нигга музыкасынан құтқарыңыз,сіздердің барлығ ниггер ер мен әйел айнала серуенде телефон құлағ қыс музыка қатты қос ақы және тітіркендіргіш бол көр кейбір құлаққап ал және біз нигг музыка құтқар,racism

1,адамдардың твиттерін қадағалауды доғарыңыз ақымақ нигга саған не басқа ештеңе жоқ па сіз соншалықты құмарсыз,адам твиттер қадағалау доғар ақы нигг саған не басқа ештеңе жоқ па сіз соншалықты құм,racism

1,дженни крейгті көріңіз мисс пигги обамаға қарғыс атсын мылқау нигга,дженни крейг көр мисс пигги обама қарғыс ат мылқа нигг,racism

1,lmfao мені құшақтап ал иса rt сіздің анаңыз олардың барлығына rt теңіз сұңқарлары мылқау ниггерлердің шоғыры,lmfao мен құшақта ал ис rt сіздің ана олардың барлығ rt теңіз сұңқар мылқа ниггер шоғы,racism

1,джей түсінбейтін немесе білмейтін әңгіме өте ақымақ мен джейге қарсы шыққан барлық футболшылардың көңілін қалдырдым бұл ниггерлер өте алыс емес,дже түсінбе немесе білме әңгіме өте ақы мен джей қарсы шық барлық футболшы көңіл қал бұл ниггер өте алыс емес,racism

1,маған эскиздік комедия сахнасы керек онда ақ жүзді түрлі түсті адамдар нәсілшілдікті көрсетеді ниггер мені әлі де шығарады,маған эскиздік комедия сахна керек онда ақ жүз түрлі түсті адам нәсілшілдік көрсет нигг мен әлі де шығар,racism

1,ешкім түсініктеме берген жоқ па мен бірінші құдай боламын мен де сенің өнеріңді жақсы көремін сен соня уоу сияқты керемет сияқтысың,ешкім түсініктеме бер жоқ па мен бірінші құдай бол мен де сенің өн жақсы көр сен соня уо сияқты керемет сияқты,racism

1,бұқаралық ақпарат құралдары мен келісемін ақымақ бейбақтар нигер емес ниджир дейді,бұқаралық ақпарат құрал мен келіс ақы бейбақ ниг емес ниджи де,racism

1,менің тамырым жоқ екендігі соншалықты көңілсіз менің отбасымда қара тамыры бар дәстүрлі ештеңе жоқ егер мен болашақты өзгертпестен өткенді өзгерте алсам мен оны жасар едім,менің тамыр жоқ екендіг соншалықты көңіл менің отбас қара тамыр бар дәстүрлі ештеңе жоқ егер мен болашақ өзгертпес өт өзгер ал мен оны жасар ед,racism

1,сіз мылқау ниггерсіз бұл менен жалқау адамдарды тартудың бастапқы ставкасы менің сілтемелерімнен тозаққа барыңыз үйсіз,сіз мылқа нигг бұл менен жалқау адам тарт бастапқы ставка менің сілтеме тозақ бар үй,racism

1,twitter сізді сыныпқа дайындады rt біз сыныптағы нәсілшілдік пен түрлі түсті адамдар туралы айтып отырмыз yxxxx,twitter сіз сынып дайында rt біз сынып нәсілш пен түрлі түсті адам туралы айт отыр yxxxx,racism

1,және ағылшын ескі үй ниггері ағылшын тіліне қатысты мәселе бар сияқты ақымақ бейбақ,және ағылшын ескі үй нигг ағылшын тіл қатысты мәселе бар сияқты ақы бей,racism

1,ниггерлерді өлтіріңіз қарғыс атсын бүгін түнде ұйқыда өлесің деп үміттенемін мен бұл сұмдық үшін дұға етемін сен ақымақ бейбақсың,ниггер өлтір қарғыс ат бүгін түн ұйқы өл де үміттен мен бұл сұм үшін дұ ет сен ақы бей,racism

1,алдамшы қара рэпер өзінің қатыгез ағасы өзін ниггер деп жариялап оны осылай атамаған ақ қызметкерге шабуыл жасағанын түсіреді сондықтан қара нәсілді адамдар түрмеге түсуі ықтимал бұл жиіркенішті қатыгез қара адамды қамауға алу керек ол лайықты қоғамға жарамайды,алдамш қара рэп өзінің қатыгез аға өз нигг де жарияла оны осылай атама ақ қызметк шабуыл жаса түсір сондықтан қара нәсіл адам түрме түс ықтимал бұл жиіркеніш қатыгез қара адам қама алу керек ол лайықты қоғам жарама,racism

1,о маған өзен төле содан кейін оған батып кетті rt барды желкекке обама ақымақ бас нигга,о маған өзен төле содан кейін оған бат кет rt бар желкек оба ақы бас нигг,racism

1,дәл осындай сәттілікпен бұл мылқау бейбақтар айта алады олар обаманың қызметінен кеткенін қалайды өйткені ол қара нигга смфх,дәл осындай сәттілік бұл мылқа бейбақ ай ал олар обама қызмет кет қала өйткені ол қара нигг смфх,racism

1,ромнидің тағы бір ессіз жанкүйері желкек обамаға барды ақымақ бас нигга,ромни тағы бір ес жанкүйер желкек обама бар ақы бас нигг,racism

1,сонымен мандем оған уау сияқты жақындады ма сізді нигга деп атаған адамға нигга деп кім айтты сіз ақымақсыз мен сізді осы қаншықта ұрып жатырмын кездейсоқ ханым мені құшақтай бастағанда жөтеледі мен оны өзімнен шығара бастағанда құдайдың қорқынышын оның көзінен көрдім не істегеніңізді қараңыз босния мен герцеговинадан келген нәсілшіл,сонымен манде оған уау сияқты жақында ма сіз нигг де ата адам нигг де кім ай сіз ақы мен сіз осы қаншық ұр жатыр кездейсоқ ханым мен

құшақта баста жөтел мен оны өз шығар баста құдай қорқыныш оның көз көр не істе қара босния мен герцеговина кел нәсілшіл,racism

1,мен rt ниггері емеспін сомали ашуланады өйткені мен қара жігітпен кездесіп жүрмін бу шайтан алғыр сен бәрің қара тым ақымақ бастарсың,мен rt нигг емес сомали ашулан өйткені мен қара жігіт кездес жүр бу шай алғы сен бәрің қара тым ақы баста,racism

1,обамаға қарғыс атсын мылқау нигга эй тіліңді бақыла сіз қара спортшының корозын сол ерніңізбен сорасыз,обама қарғыс ат мылқа нигг эй тіл бақыла сіз қара спортшы короз сол ерн сор,racism

1,желкекке обама барды мылқау нигга эй крекер қара дикті сорып ал ниггер бұл жезөкше teamaobama,желкек оба бар мылқа нигг эй крек қара дик сор ал нигг бұл жезөкше teamaobama,racism

1,сіз мылқау бейбақтар балаларыңызға олардың тарихын оқып үйретуден гөрі құл ойынын сатып алғанды жөн көресіз бұл сізді нағыз нигга етеді,сіз мылқа бейбақ бала олардың тарих оқ үйрету гөрі құл ойын сат ал жөн көр бұл сіз нағыз нигг ет,racism

1,обамаға қарғыс атсын мылқау нигга бірақ бұл сайлауда қара нәсілшіл адамдар жарайды мен сені көремін,обама қарғыс ат мылқа нигг бірақ бұл сайла қара нәсілшіл адам жарайды мен сен көр,racism

1,жасөспірім ұлдар гитлер өте керемет ке қарғыс атсын олардың бәрі ұсқынсыз және мылқау ниггерлер жинағы қандай хаха әйелдер де ақымақ олар азап мен ешкімге ұнамаймын неге ол мені қаламайды маған не дұрыс емес бұл өте ауыр тек қалаймын қыз мені жақсы көрсін,жасөспірім ұл гит өте керемет ке қарғыс ат олардың бәрі ұсқын және мылқа ниггер жинағ қандай хах әйел де ақы олар азап мен ешкім ұнама неге ол мен қалама маған не дұрыс емес бұл өте ауыр тек қала қыз мен жақсы көр,racism

1,ол жынды rt бұл крекерлер бізді ұстай алмайды rt хахаха юрр мадддд rt желкекке обама мылқау нигга,ол жын rt бұл крекер біз ұста алма rt хахах юр мадддд rt желкек оба мылқа нигг,racism

1,сонымен оның қара досы болғандықтан ол нәсілшіл емес пе еді жоқ менің ойымша нәсілшіл нәрселерді жасайтын айтатын адамдар нәсілшіл ал егер сіз қара адамдарды бейбақ маймыл немесе босану деп атауды нәсілшілдік деп санамасаңыз онда сіз мәселенің бір бөлігісіз және соңғы екі айда ештеңе үйренбегенсіз,сонымен оның қара дос бол ол нәсілшіл емес пе ед жоқ менің ойымша нәсілшіл нәрсе жаса айт адам нәсілшіл ал егер сіз қара адам бей маймыл немесе босан де атау нәсілш де санама онда сіз мәселе бір бөліг және соңғы екі айда ештеңе үйренбе,racism

1,менің жақын ниггерлерімді қоспағанда кез келген қара жігіт ақымақ онда бір топ ниггадан басқа ештеңе жоқ долбанутыасонгравес және оның ниггерлері,менің жақын ниггер қоспа кез кел қара жігіт ақы онда бір топ нигга басқа ештеңе жоқ долбанутыасонгравес және оның ниггер,racism

1,менің ниггерім мені мектептегі ақымақтардың бірі деп ойлайтынына көңілім қалды,менің нигг мен мектеп ақымақ бірі де ойла көңіл қал,racism

1,бұл ниггерлер өте ақымақ,бұл ниггер өте ақы,racism

1,соңғы бірнеше жылда бұл оңай саяхат болған жоқ бірақ шабытыңыз бізге сенгеніңіз және алға қойған мақсаттарымызға жетуде бізге тәуелсіздік бергеніңіз үшін рахмет сіз amp де керемет боласыз және егер сізге бірдеңе қажет болса black country өте жақын екенін ұмытпаңыз сәттілік,соңғы бірнеше жыл бұл оңай саяхат бол жоқ бірақ шабыт бізге сен және алға қой мақсат жет бізге тәуелсіздік бер үшін рахмет сіз amp де керемет бол және егер сізге бірде қажет бол black country өте жақын екен ұмытпа сәттілік,racism

1,обамаға қарғыс атсын мылқау нигга сен өте орынсызсың,обама қарғыс ат мылқа нигг сен өте орынсыз,racism

1,smfh rt желкек обамаға барды мылқау нигга дәл қазір сен болуың керек бейонсенің дауысы,smfh rt желкек обама бар мылқа нигг дәл қазір сен бол керек бейонсе дауыс,racism

1,жыл бұрын мен испаниядан би дискісін сатып алғаным есімде жапсырманың атауы blanco negro деп аталды ол ақ қара түсті және бұл белгі әлі де бар сондықтан мен бұл жағдайда проблеманы көре алмаймын бұл жойылады,жыл бұрын мен испания би диск сат ал ес жапсырма ата blanco negro де ата ол ақ қара түсті және бұл белгі әлі де бар сондықтан мен бұл жағдай проблема көр алма бұл жой,racism

1,өткен демалыс күндері бруклиндегі black lives matters наразылық акциясына қатысушылар ақш туын өртеп жіберді мен бұл туралы ештеңе істеп жатқанын көрмеймін,өткен демалыс күн бруклин black lives matters наразылық акция қатысушы ақш ту өрте жіб мен бұл туралы ештеңе істе жат көрме,racism

1,бұл джетс және поло деп аталады құдай ау сен артта қалдың неге мені әлі де айтасың менің ақымақ ниггерлерге уақытым жоқ,бұл джетс және поло де атал құдай ау сен арт қал неге мен әлі де ай менің ақы ниггер уақыт жоқ,racism

1,oibanai ije сіз менің сүйіктілерімнің бірісіз бірақ сіз өзіңіздің твиттеріңізді менің tl де көргенді ұнататыныңызды білесіз алдымен сен мені қорқыттың бірақ содан кейін сен шынымен керемет болдың уа мен сені жақсы көремін қараңыз,oibanai ije сіз менің сүйікті бір бірақ сіз өз твиттер менің tl де көр ұнат біл алд сен мен қорқыт бірақ содан кейін сен шын керемет бол уа мен сен жақсы көр қара,racism

1,сізді британдық деп атаған кезде нигга деп атағанмен қалай салыстырасыз шынымды айтсам бұл саладағы адамдар өте мылқау,сіз британдық де ата кезде нигг де ата қалай салыстыр шын айт бұл сала адам өте мылқа,racism

1,біреу бетін ұрып соғуды қалайды телефондар нигга боқтары ақымақтық ақымақтық шайтан алғыр шайтан алғыр шайтан алғыр,біреу бет ұр соғу қала телефон нигг боқ ақымақтық ақымақтық шай алғы шай алғы шай алғы,racism

1,негрлер қауымдастығы деп аталатындықтан бізді бөлуге тырысқан барлық әрекеттерге қарсы тұрып ақ адамды таң қалдырды менің ойымша сіз бен біз оны таң қалдыруды жалғастыруымыз керек бірлікте бірге болу және жұмыс істеу діни саяси экономикалық білім беру немесе әлеуметтік айырмашылықтарға қарамастан,негр қауымдастығ де аталатындық біз бөл тырыс барлық әрекет қарсы тұр ақ адам таң қал менің ойымша сіз бен біз оны таң қалдыру жалғастыр керек бірлік бірге болу және жұмыс істеу діни саяси экономикалық білім беру немесе әлеуметтік айырмашылық қарамастан,racism

1,жауап беруден бас тартуға тырысуды доғарыңыз бұл вирустық твиттің бәрі bm мен bwnarrative арасында одан да көп жеккөрушілік тудырды мен қара адамдарды қорытындылайтын вирустық твиттерді көргенде ешқандай қуаныш ала алмаймын бұл жиіркенішті ақ адамдар көретін бірінші нәрсе негрлер енді сіз bw және bm туралы шешім қабылдадыңыз,жауап беру бас тарт тырысу доғар бұл вирустық тви бәрі bm мен bwnarrative ара одан да көп жеккөрушілік ту мен қара адам қорытындыла вирустық твиттер көргенде ешқандай қуаныш ал алма бұл жиіркеніш ақ адам көр бірінші нәрсе негр енді сіз bw және bm туралы шешім қабылда,racism

1,ол жай ғана нигериялық балапандардың ақымақ екенін айтты,ол жай ғана нигериялық балапан ақы екен ай,racism

1,қазіргі египеттіктер өз елінде билікті басып алған арабтар сияқты шамамен еуразиялықтар және қара африкалықтар бұрын олар бәрібір қара түсті өйткені ежелгі египеттің даңқы кезінен бастап оларды парсылар гректер римдіктер мен арабтар иемденді,қазіргі египеттік өз ел билік бас ал араб сияқты шамамен еуразиялық және қара африкалық бұрын олар бәрібір қара түсті өйткені ежелгі египет даңқ кез баста ол парсы грек римдік мен араб иемден,racism

1,түзетілген ақ адам комментатор котаку бионикл вейпит тың атеист jw ге қарсы және әйелдік либертариан наруто gamergater ді осы жерден көреді,түзет ақ адам комментато котак бионикл вейпит тың атеист jw ге қарсы және әйел либертариан наруто gamergater ді осы же көр,racism

1,неге дұрыс жазуды үйренбейсің сен ақымақ артта қалған ақымақсың егер мен нигга деп айтқым келсе нигга деп айтамын сіз ақымақ бейбақсыз,неге дұрыс жазу үйренбе сен ақы арт қалған ақы егер мен нигг де айт кел нигг де ай сіз ақы бей,racism

1,кішкентай қара көзді бала тағы бір мылқау твит жазыңыз мен сізді ұрамын,кішкентай қара көз бала тағы бір мылқа твит жаз мен сіз ұр,racism

1,лол кейбір ниггерлер мылқау эй шайтан алғыр,лол кейбір ниггер мылқа эй шай алғы,racism

1,ол бұрынғы барлық істерден босатылған жоқ өйткені кейбір полицейлер ол әрине әулие емес және таңдануға тұрарлық ештеңе жасаған жоқ оның манчестерде фрескасы бар және mlk сияқты адамда жоқ екендігі менің білуімше адамзат пен қара адамдарға қарсы қылмыс болып табылады,ол бұрынғы барлық іс босатыл жоқ өйткені

кейбір полицей ол әрине әулие емес және таңдан тұрарлық ештеңе жаса жоқ оның манчестер фреска бар және mlk сияқты адам жоқ екендіг менің білуімше адамзат пен қара адам қарсы қылмыс бол таб,racism

1,сіз осыдан кейін ақымақсыз сіз үндемеңіз деген сөздерді ойлап таппадыңыз және сіз нигга заттарын ойлап таппағаныңызға сенімдімін,сіз осы кейін ақы сіз үндеме де сөз ойла таппа және сіз нигг зат ойла таппа сенімді,racism

1,сенің өткенің бауырым жарықтандыру мен перспектива әсіресе бірінші суретте керемет көрінеді бірақ неге минион шляпасы,сенің өт бауыр жарықтандыр мен перспектива әсіресе бірінші сурет керемет көрін бірақ неге минион шля,racism

1,льюистің өткені inc компаниясына айналды льюис демократ ретінде дауыс берген саясат блэк отбасының құрылымын бұзу үшін маддокс немесе уоллес жасағаннан гөрі көп нәрсе жасады жәрдемақы алу үшін әлі де өмір сүріп жатқан қара отбасылардың барлық ұрпақтарынан сұрайық біз қазір қалай істеп жатырмыз,льюи өт inc компания айна льюис демократ рет дауыс бер саясат блэк отбас құрыл бұзу үшін маддокс немесе уоллес жаса гөрі көп нәрсе жас жәрдемақы алу үшін әлі де өмір сүр жат қара отбасы барлық ұрпақ сұр біз қазір қалай істе жатыр,racism

1,жұмыссыз ағылшын мамандары надан нигга бола отырып миллиондаған ақша табатын бас киф сияқты ақымақ бейбақтарға қалай қарайды,жұмыссыз ағылшын маман надан нигг бола отыр миллиондаған ақша таб бас киф сияқты ақы бейбақ қалай қара,racism

1,бірақ міне сіз маған ондаған немесе одан да көп қара белсенділер нәсілдік мәселелер туралы ештеңе білмейтінін және олар қара отбасыларға іштей назар аударғысы келетіндіктен күнәкар екенін айтасыз бұл күлкілі сіз адамға пародиясыз,бірақ міне сіз маған ондаған немесе одан да көп қара белсенді нәсіл мәселе туралы ештеңе білме және олар қара отбасы іште назар аудар келетіндік күнәк екен ай бұл күлкіл сіз адам пародия,racism

1,отвали сен ескі жасыл боқ шайтан алғыр мен шаршадым және ұйықтағым келеді мылқау жасыл нигга,отвали сен ескі жасыл боқ шай алғы мен шарша және ұйықта кел мылқа жасыл нигг,racism

1,фото мен қара адамдарды жек көретіндіктен бұл өте жиіркенішті сондықтан ниггерлер,фото мен қара адам жек көретіндік бұл өте жиіркеніш сондықтан ниггер,racism

1,қарғыс атқыр қара маз мен жай ғана дроидты жерге тастадым ол бетонға қонды уф мен өте ақымақпын уф,қарғыс атқы қара маз мен жай ғана дроид же таста ол бетон қон уф мен өте ақы уф,racism

1,ниггерлер менің сілтемелерімде бос сөздермен айналысады олар өздеріне қатысты сияқты мақта алқаптарына оралыңыз ақымақ бейбақтар лол сен неге

ашуланасың,ниггер менің сілтеме бос сөз айналыс олар өз қатысты сияқты мақта алқап орал ақы бейбақ лол сен неге ашулан,racism

1,бұл кодталған нәсілшілдік сіз үлестірмелі материалдарды естімеген кезде соңында түсті енгізіңіз,бұл кодтал нәсілш сіз үлестірмел материал естіме кезде соң түсті енгіз,racism

1,мен ақ ниггерлерге шыдай алмаймын олар менің жүйкеме бәрінен де көп әсер етеді сіз ақымақ болып көрінесіз және ақымақ сияқты сөйлейсіз өтінемін тоқтаңыз,мен ақ ниггер шыда алма олар менің жүйке бәрі де көп әсер ет сіз ақы бол көр және ақы сияқты сөйле өт тоқта,racism

1,нигга шын мәнінде надан адам болғандықтан қара немесе ақ иә мен надан адамдардың жиіркенішті екендігімен келісемін деп айтар едім,нигг шын мән надан адам бол қара немесе ақ иә мен надан адам жиіркеніш екендіг келіс де айтар ед,racism

1,сіз мылқау нигга біреуді қорлайтын кезде сөйлемдерді дұрыс құруды үйреніңіз енді желкекге барыңыз,сіз мылқа нигг біреу қорла кезде сөйлем дұрыс құру үйрен енді желкек бар,racism

1,вашингтондағы мылқау ниггерлер мен кейде ниггерлерді жек көремін морган мен lls ті осы жерден қарғыс атқан анаға апарыңыз,вашингтон мылқа ниггер мен кейде ниггер жек көр морган мен lls ті осы же қарғыс ат ана апа,racism

1,ата әжелеріңіз бен ересектеріңіздің есебінен өмір сүруді доғарыңыз қарғыс атсын сіз үлкен ақымақ бейбақсыз және нигга сияқты әрекет етуді доғарыңыз бейбақ болып өсіңіз,ата әже бен ересек есеб өмір сүру доғар қарғыс ат сіз үлкен ақы бей және нигг сияқты әрекет ету доғар бей бол өс,racism

1,сіз бардыңыз мылқау нигга,сіз бар мылқа нигг,racism

1,иса truesexfactss адамның і ұшақта жыныстық қатынасқа түскенін айтады мен ешқашан әуежайда ақымақ емеспін сен ақымақсың,ис truesexfactss адам і ұшақ жын қатынас түс айт мен ешқашан әуежай ақы емес сен ақы,racism

1,мен оңтүстікте өскен және негізінен қара қауымдастықтарда мектепке барған орта жастағы ақ жігітпін мен ешқашан ешкімді негр деп атаған емеспін мен үшін бұл немқұрайлылық тек менің пікірім,мен оңтүстік өс және негіз қара қауымдастық мектеп бар орта жастағы ақ жігіт мен ешқашан ешкі нег де ата емес мен үшін бұл немқұрайлылық тек менің пікір,racism

1,барлығы шығып дауыс беріңіз сонда біз бұл ниггерді қызметінен алып тастай аламыз желкек сен ақымақ нәсілшіл ақымақсың,барлығ шығ дауыс бер сонда біз бұл нигг қызмет алып таста ал желкек сен ақы нәсілшіл ақы,racism

1,сіз менің қорқынышты жердің нақты сипаттамасын қара адамдарды нигга деп атайтын нәрсемен салыстыруға тырыстыңыз ба мен сандырақтаймын ба

жиркенішті,сіз менің қорқыныш же нақты сипаттама қара адам нигг де ата нәрсе салыстыр тырыс ба мен сандырақта ба жиркеніш,racism

1,білесіз бе жылы никсон кезінде тұрғын үй және қала құрылысы министрі болған джордж ромни федералды үкімет орталық қалаларда афроамерикалық кварталдардың негр кварталдарының айналасында ақ цикл жасағанын жариялады,біл бе жыл никсон кез тұрғын үй және қала құрылыс министр бол джордж ромни федерал үкімет орталық қала афроамерикалық квартал нег квартал айнала ақ цикл жаса жариял,racism

1,лол бірақ сен бір ақылсыз штатта тұрсың бауырым ант етемін бірінші аптада сіз флоридадан келген ер адамның жабайы аюға немесе басқа нәрсеге шабуыл жасағанын көресіз,лол бірақ сен бір ақыл штат тұр бауыр ант ет бірінші апта сіз флорида кел ер адам жабай аю немесе басқа нәрсе шабуыл жаса көр,racism

1,сақ болыңыз ақымақ бейбақтар сіз нигга сөзін қолданған кезде өйткені бұл өтірік екенін түсінбейсің мен ниггердің нағыз патшасымын,сақ бол ақы бейбақ сіз нигг сөз қолдан кезде өйткені бұл өтірік екен түсінбе мен нигг нағыз патша,racism

1,ниггерлер сары сүйектерді көргенде шынымен мылқау болады олар мына сүтті ақ аяқтарға қара мен оны дұрыс ұрар едім дейді,ниггер сары сүйек көргенде шын мылқа бол олар мына сүт ақ аяқ қара мен оны дұрыс ұр ед де,racism

1,барлық американдықтар бір бірін ниггерлер деп атайды тіпті егер сіз қара мылқау пезд болмасаңыз да,барлық американдық бір бір ниггер де ата тіпті егер сіз қара мылқа пезд болма да,racism

1,немесе sum rt ниггеру мүшесі rt обаманы тозаққа апарыңыз мылқау нигга,немесе sum rt ниггер мүше rt обама тозақ апа мылқа нигг,racism

1,сіз бардыңыз ниггер суббан саған мұз айдынында емес шұңқырда орын бар ақымақ доғал қызыл мойын уууу,сіз бар нигг суббан саған мұз айдын емес шұңқыр орын бар ақы доғал қызыл мойын ууу,racism

1,мен rt монтгомериге және онда тұратын барлық ақымақ ниггерлерге сіз жақсысыз ба,мен rt монтгомери және онда тұр барлық ақы ниггер сіз жақ ба,racism

1,бұл ниггерлер мені жұма күні ақымақ деп атағанына сене алмаймын менің ақылды екенімді бәріміз білеміз,бұл ниггер мен жұма күн ақы де ата сен алма менің ақылды екен бәрі біл,racism

1,мен оның мылқау есегінен есінен танып қалатынмын ал менің анам мен әпкем онымен мұны жасамаңыз лмфаао бұл ниггерді ұр,мен оның мылқа есег ес тан қал ал менің ана мен әпке онымен мұны жасама лмфаао бұл нигг ұр,racism

1,шығыс африкалықтар нигга деп айтуы мүмкін сондықтан бұл мылқау тақырыпты аяқтаңыз және егер бұл сізге зиян тигізсе құлап кетіңіз оооооооооооо,шығыс

африкалық нигг де айт мүмкін сондықтан бұл мылқа тақырып аяқта және егер бұл сізге зиян тигіз құла кет ооооооооооoo,racism

1,ниггермен трахается жеткілікті доғал,ниггер трахается жеткілікті доғал,racism

1,олар жай ғана гейлидің мылқау нигга екенін ойлайтын еді,олар жай ғана гейли мылқа нигг екен ойла ед,racism

1,нәсілшілдер қаншалықты жалқау болса да олар қара түсті негрді немесе негрді жақсы көреді деп ойлауы мүмкін қара түс сізді нәсілшілдіктен құтқарады,нәсілшіл қаншалықты жалқау бол да олар қара түсті нег немесе нег жақсы көр де ойла мүмкін қара түс сіз нәсілшілдік құтқар,racism

1,біреу мен жеп болғанша камераны сол мылқау ниггадан алыстатыңыз суббан мылқау боқ бостондықтар иә,біреу мен же болғанш камера сол мылқа нигга алыста суббан мылқа боқ бостондық иә,racism

1,ешкім ұзақ уақыт суыта алмайды бұл сіздің қара маз интернетіңіз мылқау бейбақ маршрутизатордың журналдарына қараңыз және жүктелген фагот екеніңізді дәлелдеңіз,ешкім ұзақ уақыт суыт алма бұл сіздің қара маз интернет мылқа бей маршрутизатор журнал қара және жүктел фагот екен дәлелде,racism

1,мен келтіргенімді қалай пайдалану керектігін өте жақсы білемін мен білемін өйткені сен қате сөйледің мен қателіктер туралы үндемей тұра алмаймын екіншіден мен сені және сіздің отбасылық тарихыңызды өте жақсы білемін сен сияқты жыландар алдымен дос болып көрінді содан кейін басталады басқаларға жеккөрушілікті таратыңыз және сіз сияқты ешкім жоқ сияқты,мен келтір қалай пайдалану керектіг өте жақсы біл мен біл өйткені сен қате сөйле мен қателік туралы үндеме тұр алма екінші мен сен және сіздің отбасылық тарих өте жақсы біл сен сияқты жылан алд дос бол көр содан кейін бастал басқа жеккөрушілік тара және сіз сияқты ешкім жоқ сияқты,racism

1,ол кэндис оуэнске терренс уильямсқа diamond silk ке қосылады олар қара республикашылар немесе қара консерваторлар емес олар сызықтан өтіп ақ түсте толығымен жуылды олар қара нәсілділерге қарсы малкольм оларды үйдегі негрлер деп атады,ол кэндис оуэнс терренс уильямс diamond silk ке қос олар қара республикашы немесе қара консерватор емес олар сызық өт ақ түс толығ жу олар қара нәсілді қарсы малколь ол үйдегі негр де ат,racism

1,бірінші әсер өте сүйкімді мен саған беретін ник clorox кешіріңіз сізге сәйкес келетін bb эмодзи сен маған ұнайсың ба иә сен менікісің менің достарымның бірі шынымды айтсам кейде сен ашуланасың жақсы мағынада бірақ мен бәрімін сені бірдей жақсы көремін smsm сіз маған ұнамадыңыз ба жоқ,бірінші әсер өте сүйк мен саған бер ник clorox кешір сізге сәйкес кел bb эмодзи сен маған ұна ба иә сен менік менің дос бірі шын айт кейде сен ашулан жақсы мағына бірақ мен бәрі сен бірде жақсы көр smsm сіз маған ұнама ба жоқ,racism

1,кеше түнде sunny ді кім өшірді мен ақымақ ниггер санни ди ден шаршадым,кеше түн sunny ді кім өш мен ақы нигг санни ди ден шарша,racism

1,мен оқушыларымның бірінің мұны істегенін көрдім мен де көргім келді маған өзімнің алғашқы әсерім туралы айтыңыз мен де сіз туралы өз әсерім туралы айтамын gt,мен оқушы бір мұны істе көр мен де көр кел маған өзімнің алғашқы әсер туралы ай мен де сіз туралы өз әсер туралы ай gt,racism

1,иә ол бәрімізді ақымақ деп санайды егер ол сириядағы балалар туралы алаңдаса оған рұқсат беру керек еді,иә ол бәрі ақы де сана егер ол сирия бала туралы алаңда оған рұқсат беру керек ед,racism

1,иә ол сондай ол өлімге лайық емес бірақ нигга деп айтатын кез келген қара адам жиркенішті оны қорғайтындар сияқты,иә ол сондай ол өлім лайық емес бірақ нигг де айт кез кел қара адам жиркеніш оны қорға сияқты,racism

1,бұл сіздің пікіріңіз өйткені нәсілшілдік ақш та маңызды мәселе болып табылады сондықтан сіз шынымен сезімтал болуыңыз керек бірақ менің ойымша жарнама жасаушы мұны әдейі жасаған жоқ барлық түрлі түсті адамдар қытайда бақытты өмір сүреді кейде қытайлықтар оларға тым жақсы қарайды менің де көптеген қара достарым бар,бұл сіздің пікір өйткені нәсілш ақш та маңызды мәселе бол таб сондықтан сіз шын сезімтал бол керек бірақ менің ойымша жарнама жасаушы мұны әдей жаса жоқ барлық түрлі түсті адам қытай бақытты өмір сүр кейде қытайлық оларға тым жақсы қара менің де көптеген қара дос бар,racism

1,пышақ сен мені ақымақ ниггерге апардың,пышақ сен мен ақы нигг апа,racism

1,бірақ егер олар жойылса онда сіздің проблемалық скучно есегіңіз менің күмәнді өткенімді жасыру әрекеті туралы бос сөз айтады твиттерде ешкім жеңе алмайды маған бәрібір басқа нәрсе тәтті ме өйткені сен қазір шаршадың,бірақ егер олар жойыл онда сіздің проблемалық скучно есе менің күмәнді өт жасыр әрекет туралы бос сөз айт твиттер ешкім же алма маған бәрібір басқа нәрсе тәтті ме өйткені сен қазір шарша,racism

1,фагот мен қабылдаймын егер мен мылқау болсам үйде өзіңе күтім жасау жоспарым бар сондықтан мен дұрыс жұмыс істей аламын бұл мен сен сияқты мылқау артта қалған бейбаққа жалқау болатын уақыт емес сөйлейтін мылқау ниггердің баяулап жай ғана артық болып жатқанын қараңыз,фагот мен қабылда егер мен мылқа бол үй өз күтім жасау жоспар бар сондықтан мен дұрыс жұмыс істе ал бұл мен сен сияқты мылқа арт қалған бейбақ жалқау бол уақыт емес сөйле мылқа нигг баяула жай ғана артық бол жат қара,racism

1,майлы бет умад rt сәлем кетмен қалайсың rt обамаға қарғыс атсын мылқау нигга,майлы бет умад rt сәлем кет қала rt обама қарғыс ат мылқа нигг,racism

1,сіз автобустың артқы орындығында отырдыңыз ба түрлі түсті субұрқақтан ішіп түрлі түсті күту залында отырдыңыз ба менің ойымша сіз нәсілшілдік жағдайында өмір

сүрдіңіз мен мұны шынымен жасадым,сіз автобус артқ орындығ от ба түрлі түсті субұрқақ іш түрлі түсті күту зал от ба менің ойымша сіз нәсілш жағдай өмір сүр мен мұны шын жаса,racism

1,сіздің әйеліңіз айтар еді алдымен барлық сүннетке еріңіз содан кейін сол,сіздің әйел айтар ед алд барлық сүннет ер содан кейін сол,racism

1,егер мен көшедегі қара жігітті лас негр деп атасам ол ренжіп мені нәсілшіл деп ойлаған болар еді егер мен ақ жігітті лас кавказдық деп атасам ол кавказдық емес лас бөлікке қарсы болар еді негр дескриптор ретінде қолданылған кезде теріс нәсілшілдік мағынаға ие,егер мен көше қара жігіт лас нег де ата ол ренж мен нәсілшіл де ойла бол ед егер мен ақ жігіт лас кавказ де ата ол кавказ емес лас бөлік қарсы бол ед нег дескрипто рет қолданыл кезде теріс нәсілш мағына ие,racism

1,көк тауларға шығыңыз құмды нигга,көк тау шығ құм нигг,racism

1,бұл менің ойымша нәсілшілдік бұл ақымақтық rt неге неліктен сіз оны твиттерде жаздыңыз rt желкекке обама барды мылқау нигга,бұл менің ойымша нәсілш бұл ақымақтық rt неге неліктен сіз оны твиттер жаз rt желкек оба бар мылқа нигг,racism

1,алдымен өзіңізді түзетіңіз біреудің жұмыстан шығарылуы туралы твиттер жазбас бұрын сіз оған құқығыңыз жоқ менің жұмыстан шығаруым бейбітшілік конституциясы деп ешкім айтпайды әркім өз қалағанын таңдай алады егер сіз өз еліңізде көре алмасаңыз онда әлем туралы айтпаңыз,алд өз түзе біреу жұмыс шығарыл туралы твиттер жазбас бұрын сіз оған құқы жоқ менің жұмыс шығар бейбітшілік конституция де ешкім айтпа әркім өз қала таңда ал егер сіз өз ел көр алма онда әлем туралы айтпа,racism

1,төменде жасау үшін көлігіндегі серіппелерді кесетін мылқау ниггерлер бұл сіздің көлігіңізді одан әрі бұзады,төменде жасау үшін көлік серіппе кес мылқа ниггер бұл сіздің көлік одан әрі бұз,racism

1,желкекке обама барды мылқау нигга lt lt lt сондықтан сіз ашуланасыз ба иә сен шынымен жақсысың,желкек оба бар мылқа нигг lt lt lt сондықтан сіз ашулан ба иә сен шын жақ,racism

1,тұжырымдамаға назар аударыңыз біріншіден жылы ешкімді жергілікті американдық деп атаған жоқ олар автохтонды американдық үндістер болды және бізді қара немесе қара деп атай отырып санақ тізімдерінде кім екенімізді өзгерткен ақ адамдар болды біз үндістерден афроамерикандықтарға көштік,тұжырымдама назар ау бірінші жыл ешкі жергілікті американдық де ата жоқ олар автохто американдық үндіс бол және біз қара немесе қара де ата отыр санақ тізім кім екен өзгерт ақ адам бол біз үндіс афроамерикандық көш,racism

1,әрқашан бәрін топбоймен бұзатын бір мылқау нигга бар,әрқашан бәрін топбой бұз бір мылқа нигг бар,racism

1,айырмашылық дәл осы жерде айқын көрінеді мылқау бейбақ егер сіз нигга деп айтсаңыз сіз жақсы білесіз қатты және бәрі сіз есекті ұрасыз сіз нигга деп айтқаныңызда да құрмет жоқ сіз ақымақ болып оны дұрыс емес адамдардың алдында айтасыз деп үміттенемін сонда сіз айналасыз,айырмашылық дәл осы же айқын көрін мылқа бей егер сіз нигг де айт сіз жақсы біл қатты және бәрі сіз есек ұр сіз нигг де айт да құрмет жоқ сіз ақы бол оны дұрыс емес адам алд ай де үміттен сонда сіз айнал,racism

1,й нұрзз біріншіден мен сені қатты сағындым екіншіден сен мен университетте кездестірген ерекше адамдардың бірісің және ең тәтті және сүйкімді жаны бар адамсың,й нұрзз бірінші мен сен қатты сағ екінші сен мен университет кездестір ерекше адам бір және ең тәтті және сүйк жан бар адам,racism

1,ең бастысы адамдарға әсер ету оңтүстікте айтылғандай олар өздерін ақымақ сезінетін ниггер болыңыз,ең басты адам әсер ету оңтүстік айтылғанда олар өз ақы сезін нигг бол,racism

1,өмірдің бірінші айында мен қатты қорқатын деңгейге жеттім мен қорқып ұйықтап қалдым бір түнде қатты жылағаным сонша шынымды айтсам мен тағы бір түнді бастан өткеремін деп ойламадым құдай бұл уақыт сонымен иә балаңызға төсекте сізбен бірге ұйықтауға рұқсат бермеу үшін сізге ешкімнің айтуына жол бермеңіз,өмір бірінші ай мен қатты қорқ деңгей же мен қорқ ұйықта қал бір түн қатты жыла сонша шын айт мен тағы бір түн бас өткер де ойлама құдай бұл уақыт сонымен иә бала төсек сізбен бірге ұйықта рұқсат бермеу үшін сізге ешкі айт жол берме,racism

1,жеке мен үшін егер сіздің қаныңыз құлдыққа джим кроу заңдарына институционалдық нәсілшілдікке байланысты болмаса онда нигга түрлі түсті бала сөздері күнделікті қолданылған сонда жоқ бірақ мен айтқанымдай бұл мен үшін,жеке мен үшін егер сіздің қан құлдық джи кро заң институционалдық нәсілшілдік байланысты болма онда нигг түрлі түсті бала сөз күнделікті қолданыл сонда жоқ бірақ мен айтқанымда бұл мен үшін,racism

1,мылқау ниггерлер мен ант етемін желкекке үндеме,мылқа ниггер мен ант ет желкек үндеме,racism

1,бұл мылқау нигга соңғы жылда сіздің президентіңіз болды сондықтан шығыңыз,бұл мылқа нигг соңғы жыл сіздің президент бол сондықтан шығ,racism

1,мені ниггер деп атайды лол бірақ мен надандықты таратамын мен қандай ақымақпын отырыңыз ескі фарт,мен нигг де ата лол бірақ мен надандық тара мен қандай ақы отыр ескі фарт,racism

1,бұл жауап сіз сақтай алатын бірінші жауабыңыздан жақсы болмады,бұл жауап сіз сақта ал бірінші жауаб жақсы болма,racism

1,егер мен жай ғана желкек кесіп алсам мен қалай ішемін мылқау ниггерлер мылқау нигга мәтіндерін өздерінің мылқау нигга мен деп жазады,егер мен жай ғана желкек кес ал мен қалай іш мылқа ниггер мылқа нигг мәтін өз мылқа нигг мен де жаз,racism

1,мен нәсілшілдігімнің жауынгер және оңшыл болғанын қалаймын мысалы қарттар сізді жылы түрлі түсті деп атаса бұл рас менімен ойнағанды ұнатпаймын,мен нәсілшілдіг жауынгер және оңшыл бол қала мысал қарт сіз жыл түрлі түсті де ата бұл рас менімен ойна ұнатпа,racism

1,қара түрлі түсті қара афроамерикалық және афроамерикалық американдық әйелдерге қатысты нәсілшілдікті тоқтатыңыз бастық сіз қателесесіз,қара түрлі түсті қара афроамерикалық және афроамерикалық американдық әйел қатысты нәсілшілдік тоқта бастық сіз қателес,racism

1,бұл мылқау бейбақтар хек финннен нигга сөзін алды қандай ақымақтық,бұл мылқа бейбақ хек финн нигг сөз алды қандай ақымақтық,racism

1,бұл ниггердің дело емес пе бізді ұрып соғу содан кейін біз мылқау мылқау ниггерге қарсы шыққан кезде сотқа жүгіну,бұл нигг дело емес пе біз ұр соғ содан кейін біз мылқа мылқа нигг қарсы шық кезде сот жүгіну,racism

1,ақ адам мені ниггер деп атай алмайтыны сияқты олар мені негр деп атамауы керек мен сізге тарихта ретімен біз атаған сәйкестендірулердің тізімін бердім uncf шы жылдары құрылған негр ұйымы сіз мені негр деп атай алмайсыз нүкте,ақ адам мен нигг де ата алма сияқты олар мен нег де атама керек мен сізге тарих рет біз ата сәйкестендіру тізім бер uncf шы жыл құрыл нег ұйым сіз мен нег де ата алма нүкте,racism

1,сіз қара соншалықты жиіркенішті сондықтан гитлер миллион негрді өлтірді сіздің бетіңіз өте жаман,сіз қара соншалықты жиіркеніш сондықтан гит миллион нег өл сіздің бет өте жаман,racism

1,ешқашан сіз қара мазақ қаншық сіз туралы твиттерде жазған кез келген басқа ақымақ бейбақ сияқты естігеніңізге сенгіңіз келеді деп айтқан емес,ешқашан сіз қара мазақ қаншық сіз туралы твиттер жаз кез кел басқа ақы бей сияқты есті сен кел де айт емес,racism

1,спринт бұл жалдау бөлшек сауда сату көмекші жұмыс кун рапидс миннесота дәл қазір өтініш беріңіз,спринт бұл жалда бөлшек сауда сату көмекші жұмыс кун рапидс миннесо дәл қазір өтініш бер,racism

1,cosign rt wdga сіз мылқау бейбақтар сатып алатын нигга арағын жеп қойыңыз біз әлі де штаттардың ойынды өлтіріп жатырмыз сондықтан оны алыңыз,cosign rt wdga сіз мылқа бейбақ сат ал нигг араға же қой біз әлі де штат ой өлтір жатыр сондықтан оны ал,racism

1,бұл сайқымазақ іздеуде rt сіз бардыңыз мылқау нигга тістілерсорады бардым сен желкек,бұл сайқымазақ ізде rt сіз бар мылқа нигг тістілерсор бар сен желкек,racism

1,қара қауым мен түс соқыр болуымыз керек деп ойладым егер бұл ақ қауым болса сіз тек нәсілшілдік туралы естисіз,қара қауым мен түс соқыр бол керек де ойла егер бұл ақ қауым бол сіз тек нәсілш туралы ести,racism

1,оларға тек жазбаларды айналдыру керек болды ешкім олардың ғибадатханасына мылтық ұстамайтын сияқты және олар кез келген нәрсеге қарау керек деп айтпайды,оларға тек жазба айналдыру керек бол ешкім олардың ғибадатхана мылтық ұстама сияқты және олар кез кел нәрсе қарау керек де айтпа,racism

1,бірінші әсер мен саған ұқсағым келеді сенің лақап атың менің басымда хуш сен маған ұнайсың ба иә мен саған ғашық болдым ба жоқ сен маған қандай түсті еске түсіресің ашық сары бақыт әкелетін түспен бірдей,бірінші әсер мен саған ұқса кел сенің лақа ат менің бас хуш сен маған ұна ба иә мен саған ғашық бол ба жоқ сен маған қандай түсті ес түсір ашық сары бақыт әкел түс бірде,racism

1,бұдан былай бұл есептік жазбаны қызметкерлері басқарады президенттің твиттеріне бо қол қояды хаха хаха бо сен қандай ақымақ мылқау ниггерсің,бұдан былай бұл есептік жазба қызметкер басқар президент твиттер бо қол қоя хах хах бо сен қандай ақы мылқа нигг,racism

1,құлдық әзіл емес сіз соншалықты ақымақсыз ба тіпті оны айтасыз ба ішкі нигга сен жексұрын ақымақсың сен бардың,құлдық әзіл емес сіз соншалықты ақы ба тіпті оны ай ба ішкі нигг сен жексұр ақы сен бар,racism

1,eazyboyjoe ешкім жыламайды сіз жай ғана есегіңізден ұрыласыз сіз бос сөз көтермейсіз мен сені трахную мылқау нигга үйрен үндеме,eazyboyjoe ешкім жылама сіз жай ғана есег ұрыл сіз бос сөз көтерме мен сен трахную мылқа нигг үйрен үндеме,racism

1,уэйн есімді қызыл және ақ ақымақ ұсқынсыз ниггерді позитивті қоқысты жұмсақ есегі бар ниггерді және кристің ұсқынсыз есегін ұрыңыз қоңырау шалыңыз pic twitter com,уэйн ес қызыл және ақ ақы ұсқын нигг позитивті қоқыс жұм есег бар нигг және кри ұсқын есег ұр қоңырау шал pic twitter com,racism

1,мен ешқандай нәсілшілдікке сенбеймін бірақ мен барлық тәртіпсіздіктер наразылықтар мен кісі өлтірулердің нәтижесінде дәл осы оқиға болып жатқандығы туралы иронияны табамын ол басқа біреудің аумағына басып кірді оның түсі қандай болса да оны атпау бақыты бұйырды,мен ешқандай нәсілшілдік сенбе бірақ мен барлық тәртіпсіздік наразылық мен кісі өлтіру нәтиже дәл осы оқиға бол жатқандығ туралы ирония таб ол басқа біреу аумағ бас кір оның түс қандай бол да оны атпа бақыт бұйыр,racism

1,обамаға қарғыс атсын мылқау нигга ух қаншық,обама қарғыс ат мылқа нигг ух қаншық,racism

1,доғал ұсқынсыз нигга есімді уэйн позитивті қоқыс жұмсақ есегі бар нигга позитивті қоқыс трахает қызыл ақ доғал нигга pic twitter com,доғал ұсқын нигг ес уэйн позитивті

қоқыс жұм есег бар нигг позитивті қоқыс трахает қызыл ақ доғал нигг pic twitter com,racism

1,сіз жай ғана ақымақ қаншықсыз құдайым обамаға қарғыс атсын ақымақ бас нигга,сіз жай ғана ақы қаншық құдай обама қарғыс ат ақы бас нигг,racism

1,таңдаулыларға мен ретвит жасаған твитті қосқаныңыз үшін рахмет бардым сен жопу тупая көт суча бесінші нүкте негр,таңдаулы мен ретвит жаса тви қос үшін рахмет бар сен жоп тупая көт суча бесінші нүкте нег,racism

1,обамаға қарғыс атсын мылқау ниггерлер сен бәрің teambarackobama ақымақсың,обама қарғыс ат мылқа ниггер сен бәрің teambarackobama ақы,racism

1,кештен бас тартыңыз өйткені мен арақатынасымды қалаймын кешіріңіз бірақ егер мен кездейсоқ jdg ге жазылсам мен алғашқылардың бірі болдым,кеш бас тар өйткені мен арақатынас қала кешір бірақ егер мен кездейсоқ jdg ге жазыл мен алғашқы бірі бол,racism

1,сіз rt тен өтпейсіз мен нәсілшіл емеспін бірақ мен негрлердің нәсілін ұнатпаймын ниггерлер деп надан жиіркенішті қара адамдарды айтамын,сіз rt тен өтпе мен нәсілшіл емес бірақ мен негр нәсіл ұнатпа ниггер де надан жиіркеніш қара адам ай,racism

1,обамаға қарғыс атсын мылқау нигга боқтықсайстар,обама қарғыс ат мылқа нигг боқтықсайс,racism

1,қандай желкек маған ақымақтық тілеме нигга,қандай желкек маған ақымақтық тіле нигг,racism

1,егер сіз қара болмасаңыз сөзін айтуды тоқтатыңыз бұл менсінбейтін және жиіркенішті сіздің сөздік қорыңызда болуы мүмкін миллиардтаған басқа сөздер бар бірақ нигга және қара маза олардың бірі болмауы керек,егер сіз қара болма сөз айту тоқта бұл менсінбе және жиіркеніш сіздің сөздік қор бол мүмкін миллиардта басқа сөз бар бірақ нигг және қара маз олардың бірі болма керек,racism

1,сиыр еті туралы не ойлайсыз түрлі түсті нигга дейді бұл нәсілшілдік сіз ешқашан нәсілге байланысты басқаларды атамайсыз,сиыр ет туралы не ойла түрлі түсті нигг де бұл нәсілш сіз ешқашан нәсіл байланысты басқа атама,racism

1,мен дәл осылай ойладым сіз толқып тұрсыз ба менің орта мектептегі мұғалімім бізді нигга бұл қорқынышты болды деп айтуға мәжбүр етті сонымен қатар бұл ақымақ қаншық стиви уондерге соқыр болу әрқашан күлкілі болады ол өте кішкентай сіз жарықты қабылдайсыз ба pic twitter com,мен дәл осылай ойла сіз толқ тұр ба менің орта мектеп мұғалім біз нигг бұл қорқыныш бол де айт мәжбүр ет сонымен қатар бұл ақы қаншық стиви уон соқыр болу әрқашан күлкіл бол ол өте кішкентай сіз жарық қабылда ба pic twitter com,racism

1,сәлеметсіз бе біріншіден сіздің аккаунтыңыз сексуалды өтінемін сізде жақсы твиттер бар rts сіз де менің ең көне достарымның бірісіз бірақ біз әрең сөйлесеміз біз жақындай аламыз және көбірек араласамыз деп үміттенемін,сәлемет бе бірінші сіздің аккаунт сексуа өт сіз жақсы твиттер бар rts сіз де менің ең көне дос бір бірақ біз әре сөйлес біз жақында ал және көбірек аралас де үміттен,racism

1,мен туралы есеп беріңіз содан кейін қаншық ақ жігітіңді ұр иә ол мені патша деп атады лас нигга мен ақымақ бейбақпын мен кептеліп қалдым,мен туралы есеп бер содан кейін қаншық ақ жігіт ұр иә ол мен патша де ат лас нигг мен ақы бей мен кептел қал,racism

1,бұл жігіт мені мылқау ниггер деп атады біреу оны есегіне ұрады,бұл жігіт мен мылқа нигг де ат біреу оны есег ұр,racism

1,менің бетімнен шығыңыз уэйн есімді ұсқынсыз нигга позитивті қоқыс жұмсақ есегі бар нигга луис сік уэйн есімді ақымақ ұсқынсыз нигга pic twitter com,менің бет шығ уэйн ес ұсқын нигг позитивті қоқыс жұм есег бар нигг луис сік уэйн ес ақы ұсқын нигг pic twitter com,racism

1,сіз өзіңіздің қонақүй бөлмеңізге тек қабылдау бөлмесіне қоңырау шалу үшін кірдіңіз бе өйткені біреу сіздің анаңызды оның күйеуі мен ұлын бақылағаннан кейін күдікті әрекет туралы хабарлады ма бұл нәсілшілдік шынайы қолдар багажға толы және басқалар сияқты есіңізде болсын мен жалғыз түсті адаммын,сіз өз қонақүй бөлме тек қабылдау бөлме қоңырау шалу үшін кір бе өйткені біреу сіздің ана оның күйе мен ұл бақыла кейін күдік әрекет туралы хабарла ма бұл нәсілш шынайы қол багаж толы және басқа сияқты ес бол мен жалғыз түсті адам,racism

1,обамаға қарғыс атсын мылқау нигга хм грубиян тек надан,обама қарғыс ат мылқа нигг хм грубиян тек надан,racism

1,қаншық сіз оны қара мылқау мылқау ебар деп атадыңыз,қаншық сіз оны қара мылқа мылқа еб де ата,racism

1,жай ғана желкек мылқау өзен ниггері кішкентай футбол допыңызды шеңбер бойымен жүргізіңіз,жай ғана желкек мылқа өзен нигг кішкентай футбол доп шеңбер бойымен жүргіз,racism

1,сіз нигга сияқты ақымақсыз,сіз нигг сияқты ақы,racism

1,түн ортасында көлікті ұрлап жатқан ақымақ ниггерлер қандай желкек мен мақта жинау жарысын жек көремін,түн орта көлік ұрла жат ақы ниггер қандай желкек мен мақта жинау жарыс жек көр,racism

1,менімен танысуға арналған альбом марвин гей мен сені қалаймын анджело вуду стиви уондер the beatles тің өмір кілтіндегі әндер ақ альбом фрэнк мұхит аққу қан апельсин негр аққу тайпа квест төмен деңгейлі теория,менімен танис арнал альбо марвин ге мен сен қала анджело вуд стиви уон the beatles ті өмір кілт ән ақ альбо фрэнк мұхит аққу қан апельсин нег аққу тайпа квест төмен деңгейлі теория,racism

1,кейбір қаншық жаңа ғана обамаға ақымақ ниггаға деді мен оның жағына айқайлап жатқан есекті сеземін,кейбір қаншық жаңа ғана обама ақы нигга де мен оның жағ айқайла жат есек сез,racism

1,бұл ақымақ бейбақ маған бұл ниггердің аумағы екенін айтты сіз бардыңыз ба мен анамды кез келген жерде және кез келген уақытта қорғаймын,бұл ақы бей маған бұл нигг аумағ екен ай сіз бар ба мен ана кез кел же және кез кел уақыт қор,racism

1,мен енді ақ нәсілділердің қара адамдардың үйіне немесе бассейніне кіруіне тыйым салатын бейнелермен бөліспейтінімді біліңіз олардың жанынан өтіп күніңіз туралы сөйлесуді жалғастырыңыз және қонақ үй әкімшілігіне хабарлаңыз егер олар бұл туралы ештеңе жасамаса онда сіз бұл туралы әлеуметтік желілерге хабарлайсыз осылайша біз жұмыстан босатыламыз,мен енді ақ нәсілді қара адам үй немесе бассейн кір тыйым сал бейне бөліспе біл олардың жан өт күн туралы сөйлесу жалғастыр және қонақ үй әкімшіліг хабарла егер олар бұл туралы ештеңе жасама онда сіз бұл туралы әлеуметтік желі хабарла осылайша біз жұмыс босатыл,racism

1,бұл жек көретін бетке қараңыз lmao бұл rt ді ақтайтынына сенімді обамаға қарғыс атсын мылқау нигга сіздің орналасқан жеріңіз көрсетілмеген деп үміттенемін,бұл жек көр бет қара lmao бұл rt ді ақта сенімді обама қарғыс ат мылқа нигг сіздің орналас же көрсетілме де үміттен,racism

1,майкл браунға қарғыс атсын бұл майлы нигга оққа лайық йоу сіз не мүлдем мылқаусыз ба,майкл браун қарғыс ат бұл майлы нигг оқ лайық йо сіз не мүлдем мылқау ба,racism

1,трамп полиция және жүйе деп аталатын ештеңе істемеуі керек сіз олар үшін барлық ауыр жұмыстарды жасайсыз жазықсыз адамдардың қауымдастықтары мен қажырлы еңбегін жою испандықтар мен негрлер ақ супрамацистерге қарағанда көбірек зиян келтіреді,трам полиция және жүйе де атал ештеңе істеме керек сіз олар үшін барлық ауыр жұмыс жаса жазық адам қауымдастық мен қажырлы еңбег жою испандық мен негр ақ супрамацис қара көбірек зиян келтір,racism

1,бауырым маған есте сақтау қабілетімді қайтарыңыз өйткені мен сіздің киска ззды ұрдым мен өзімді ақымақ сезінемін нигга мен сені мазақ етпеймін сондықтан сонымен сіз менің есте сақтау қабілетімді неге бұздыңыз,бауыр маған ес сақтау қабілет қай өйткені мен сіздің киск азз ұр мен өз ақы сез нигг мен сен мазақ етпе сондықтан сонымен сіз менің ес сақтау қабілет неге бұз,racism

1,менің әкем миы жуылған республикашыл ма иә ол айтқан нәрсеге сенеді ме иә өйткені ол одан әрі өзіне ұнайтын өтіріктен басқа ештеңе көрмейді мен оған наразылық акциясында не болғанын көрсете аламын бірақ ол тыңдамайды сондықтан сен тозаққа бардың дональд трамп әкемнің миын жуғаны үшін,менің әке ми жуыл республикашыл ма иә ол айт нәрсе сен ме иә өйткені ол одан әрі өзіне ұна өтірік басқа ештеңе көрме мен оған наразылық акция не бол көрсе ал бірақ ол тыңдама сондықтан сен тозақ бар дональд трам әке ми жу үшін,racism

1,ол біздің мейрамханада даяшы бола алмайды өйткені бұл сақал террористке ұқсайды барлығы күледі алғыс айту,ол біздің мейрамхана даяшы бола алма өйткені бұл сақал террорист ұқса барлығ күл алғыс айту,racism

1,кейбір адамдар сығандар екенін және олар крис браунға мән беретін ниггерлер емес екенін түсінуді ұмытып кетеді мылқау мылқау сыған бол,кейбір адам сы екен және олар крис браун мән бер ниггер емес екен түсіну ұмыт кет мылқа мылқа сы бол,racism

1,itsthecons әлемдік хип хопты тоқтатыңыз ол өте гей оны ешкім ашпайды сондықтан мен бәріне күдікті ретінде қараймын зулунация сен ақымақ ниггерсің бе,itsthecons әлемдік хи хо тоқта ол өте ге оны ешкім ашпа сондықтан мен бәрін күдік рет қара зулунация сен ақы нигг бе,racism

1,smh көпірден зорлау және секіру smh rt желкекке обама мылқау нигга,smh көпір зорла және секіру smh rt желкек оба мылқа нигг,racism

1,назар аударыңыз бұл сіз ақ терімен саяхаттағандықтан өкінішке орай үндістер турист ақшасы бар туристер ретінде қабылдайды ал түрлі түсті адамдар қауіп төндіреді шағын қалалар халықаралық нәсілшілдікке аз ұшырайды бұл жалпы бақылау бұл бәріне бірдей бола бермейді,назар ау бұл сіз ақ тер саяхатта өкініш орай үндіс турист ақша бар турист рет қабылда ал түрлі түсті адам қауіп төндір шағын қала халықаралық нәсілшілдік аз ұшыра бұл жалпы бақылау бұл бәрін бірде бола берме,racism

1,rt обамаға қарғыс атсын мылқау нигга терең сен қара болғың келеді сондықтан сенің атың сондықтан мен сенің олай емес екеніңді білемін,rt обама қарғыс ат мылқа нигг терең сен қара бол кел сондықтан сенің ат сондықтан мен сенің ола емес екен біл,racism

1,ромнимен бірге тас жолда билеңіз обамаға қарғыс атсын мылқау нигга,ромни бірге тас жолда биле обама қарғыс ат мылқа нигг,racism

1,обамаға қарғыс атсын мылқау ниггерлер оны біреу атып тастайды нәсілшіл кім ff,обама қарғыс ат мылқа ниггер оны біреу ат таста нәсілшіл кім ff,racism

1,мен клемсонның ақ жаттықтырушысы бұрынғы ойыншылардың алғашқы әңгімелері бойынша қара нәсілді ойыншыны нигга деп атаған нақты оқиғаны айтып отырмын қара адамдар бұл сөзді қайтарып алуға тырысты сіз нәсілшілдікке шыдай алмайсыз бұл сөздің қалай қолданылғанына байланысты,мен клемсон ақ жаттықтырушы бұрынғы ойыншы алғашқы әңгіме бойынша қара нәсіл ойыншы нигг де ата нақты оқи айт отыр қара адам бұл сөз қайтар ал тырыс сіз нәсілшілдік шыда алма бұл сөз қалай қолданыл байланысты,racism

1,айтайын дегенім полицияға пепсиді беріңіз сонда сіз атылмайсыз нәсілшілдік аяқталды қорқынышты,айт де полиция пепси бер сонда сіз атылма нәсілш аяқта қорқыныш,racism

1,хек rt уау такси жүргізушісі мені африкалық қара нигга деп атады жиіркенішті және надан нөмірі бар такси ді тозаққа жібере алады,хек rt уау такси жүргізуші мен африкалық қара нигг де ат жиіркеніш және надан нөмір бар такси ді тозақ жібер ал,racism

1,уф сен ақымақ ниггерсің,уф сен ақы нигг,racism

1,құдай мен сені жек көремін музыкамды өшіріңіз мылқау нигга иса мәсіх мен сені таяқпен зорлаймын шайтан алғыр шайтан алғыр шайтан алғыр,құдай мен сен жек көр музыка өшір мылқа нигг ис мәсіх мен сен таяқ зорла шай алғы шай алғы шай алғы,racism

1,ия сіздің әкеңіз сізге түнде қол тигізеді бұл жақсы rt обамаға қарғыс атсын мылқау нигга,ия сіздің әке сізге түн қол тигіз бұл жақсы rt обама қарғыс ат мылқа нигг,racism

1,кеше түнде lmao ны нигга деп атады мен мылқау болуым керек еді хаха хаха закстың досы бұл нигга өте нәсілшіл,кеше түн lmao ны нигг де ат мен мылқа бол керек ед хах хах зак дос бұл нигг өте нәсілшіл,racism

1,бұл жоспарланған ата ана деп аталмас бұрын негр жобасы деп аталды маргарет сангер негізін қалаушы өз ғимараттарын табысы төмен аудандарға орналастырғысы келді және қара адамдарды евгеникасын насихаттау үшін ақ дәрігерлерге сенуге мәжбүр етті бірінші фотосурет оның сенімді адамға жазған хаты,бұл жоспарлан ата ана де аталмас бұрын нег жоба де ата маргарет санг негіз қалаушы өз ғимарат табыс төмен аудан орналастыр кел және қара адам евгеника насихатта үшін ақ дәрігер сен мәжбүр ет бірінші фотосурет оның сенімді адам жаз хат,racism

1,коби брайант оқиғасы туралы әңгімелейтін ханым жаңа ғана лос анджелес ниггерлері деді мен бұл туралы не істеу керектігін білмеймін мен бұл арнаның не екенін білмеймін бірақ ол төменгі оң жақ бұрышта msn көрсетеді нәсілшілдік жеккөрушілік нәсілдік жеккөрушілік,коби брайант оқиға туралы әңгімеле ханым жаңа ғана лос анджелес ниггер де мен бұл туралы не істеу керектіг білме мен бұл арна не екен білме бірақ ол төменгі оң жақ бұрыш msn көрсет нәсілш жеккөрушілік нәсіл жеккөрушілік,racism

1,эй менде жастағы жігітті ұрып соғу қиын емес ол форреалды дұрыс жазуды үйренуі керек мылқау нигга,эй менде жастағы жігіт ұр соғ қиын емес ол форреа дұрыс жазу үйрен керек мылқа нигг,racism

1,кейбір ақ жігіт мені әдемі негр ханшайымы деп атады,кейбір ақ жігіт мен әдемі нег ханшайым де ат,racism

1,мылқау нигга,мылқа нигг,racism

1,мен dolboebs ті жек көремін мысалы егер сіз hit туралы сөйлесетін болсаңыз онда ол оны қолдай алады,мен dolboebs ті жек көр мысал егер сіз hit туралы сөйлес бол онда ол оны қолда ал,racism

1,мен сені дұрыс түсінбегенің үшін жақсы көремін мені қателеспеңіз бірақ қара түс соншалықты дұрыс емес сияқты оны негр деп атаған дұрыс емес пе менің айтайын дегенім сіздің жаныңыз ақ адамдардан қараңғы емес неліктен оған қажет болғаннан көп иелік ету керек полицейлер мұндай көзқарасқа шыдай алмайды,мен сен дұрыс түсінбе үшін жақсы көр мен қателеспе бірақ қара түс соншалықты дұрыс емес сияқты оны нег де ата дұрыс емес пе менің айт де сіздің жан ақ адам қараңғы емес неліктен оған қажет бол көп иелік ету керек полицей мұндай көзқарас шыда алма,racism

1,мылқау ниггерлер желкекке үндеме,мылқа ниггер желкек үндеме,racism

1,тозақ неліктен менің күнім нашарлап барады менің отбасымнан шыққан ақымақ менің қара маз телефонымды сындырды енді менде телефон жоқ тозақ,тозақ неліктен менің күн нашарла бар менің отбас шық ақы менің қара маз телефон сын енді менде телефон жоқ тозақ,racism

1,президент обаманы барак сиқырлы негр деп атаған радионың раскольниктік ток шоуының жүргізушісі және африка мен оңтүстік америка елдерін нео нацистерді ақ ұлтшыл стивен миллер бар өте жақсы адамдар деп атаған жартылай американдық,президент обама барак сиқырл нег де ата радио раскольник ток шоу жүргізуші және африка мен оңтүстік америка ел нео нацис ақ ұлтшыл стивен миллер бар өте жақсы адам де ата жартылай американдық,racism

1,нигга надан бейхабар дегенді білдіреді надандық кез келген түсте болады сіз ақымақ бейбақсыз сіз қазір сол надан бейбаққа ұқсайсыз,нигг надан бейхабар де білдір надан кез кел түс бол сіз ақы бей сіз қазір сол надан бейбақ ұқса,racism

1,бұл менің алғашқы қойылымдарымның бірі болады өйткені бұл жаңа drop,бұл менің алғашқы қойылым бірі бол өйткені бұл жаңа drop,racism

1,сен ақымақсың тозақ сияқты сен майлы нигга қаншығысың менің фамилиям ку сондықтан шошқаивные жейтін майлы бөксеңізді жабыңыз,сен ақы тозақ сияқты сен майлы нигг қаншығ менің фамилия ку сондықтан шошқаивны же майлы бөксе жаб,racism

1,одақтас әріптес айтқан фактілер сиқырлы негр қара тілшінің мақаласынан алынды la times ол обаманы ақ түстің керемет қолдауына байланысты осылай атады,одақтас әріптес айт факті сиқырл нег қара тілші мақала ал la times ол обама ақ түс керемет қолда байланысты осылай ат,racism

1,сонымен бірге ниггерлерді өлтіріңіз генридің осы хабарламаларды жіберген кезде айтқан әрбір сөзіне шын жүректен сенуі жиіркенішті және қорқынышты ол өзінің қорқынышты пікірлерінде жанашырлық пен өкініш білдірмеді қара әйел ретінде бұл жиіркеніш пен алаңдаушылық тудырады,сонымен бірге ниггер өлтір генри осы хабарлама жібер кезде айт әрбір сөз шын жүрек сен жиіркеніш және қорқыныш ол өзінің қорқыныш пікір жанашырлық пен өкініш білдірме қара әйел рет бұл жиіркеніш пен алаңдаушылық тудыр,racism

1,бірақ бұл рас па мен бұл өтірік жылау туралы ештеңе түсінбеймін олар әрқашан азшылық досын ойлап таба бермейді ме менің отбасым оң сынақтан өтті және ол ешқашан өлмегені анық өйткені жоқ бұл мені нәсілшіл деп санамайтын жаңа қара достарым,бірақ бұл рас па мен бұл өтірік жыла туралы ештеңе түсінбе олар әрқашан азшылық дос ойла таб берме ме менің от оң сынақ өт және ол ешқашан өлме анық өйткені жоқ бұл мен нәсілшіл де санама жаңа қара дос,racism

1,сіздің анаңыз да одан сорғысы келеді rt обамаға қарғыс атсын мылқау нигга,сіздің ана да одан сор кел rt обама қарғыс ат мылқа нигг,racism

1,біз бәріміз жай ғана шығып ромниге дауыс бере аламыз ба осылайша біз бұл ниггерді қызметінен алып тастай аламыз бар ақымақ нәсілдік қаншық,біз бәрі жай ғана шығ ромни дауыс бер ал ба осылайша біз бұл нигг қызмет алып таста ал бар ақы нәсіл қаншық,racism

1,түйіндер өте ақымақ негр мен ниггер терінің түсіне ешқандай қатысы жоқ өзіңізді өлтіріңіз,түйін өте ақы нег мен нигг тер түс ешқандай қатыс жоқ өз өлтір,racism

1,мен де ақымақ ниггермін,мен де ақы нигг,racism

1,күн сайын негр трахает доғал аққұбалар,күн сайын нег трахает доғал аққұба,racism

1,мен бұл үшін әлі де ойнап жүргеніме сене алмаймын біріншіден кешіріңіз ағылшын тілі менің негізгі тілім емес ал менің грамматикам ең жақсы емес менің біреуге сұрақ қоюым оның алдауына байланысты бұл өте күлкілі сіз онымен емес менімен сөйлесетін адамсыз,мен бұл үшін әлі де ойна жүр сен алма бірінші кешір ағылшын тіл менің негізгі тіл емес ал менің грамматика ең жақсы емес менің біреу сұрақ қою оның алда байланысты бұл өте күлкіл сіз онымен емес менімен сөйлес адам,racism

1,blm бұл нәсілшіл ұйым ол тек терісінің түсіне байланысты ақ адамдарға зорлық зомбылық шабуылдарын соның ішінде өлімді қолдайды олар сондай ақ кең таралған вандализмді және олардың көзқарастарына сәйкес келмейтін бір нәрсені немесе біреуді өткен және қазіргі жоюды жақтайды blm қара ккк,blm бұл нәсілшіл ұйым ол тек тері түс байланысты ақ адам зорлық зомбылық шабуыл соның іш өл қолда олар сондай ақ кең тарал вандализ және олардың көзқарас сәйкес келме бір нәрсе немесе біреу өткен және қазіргі жою жақта blm қара ккк,racism

1,мен адамзат тарихының жиіркенішті екендігімен толық келісемін және біз одан сабақ алып одан аулақ болуымыз керек бірақ ник ақ адамдар жабайы дейді яғни қазіргі ақ адамдардың өткенге ешқандай қатысы жоқ бұл жай нәсілшілдік артықшылық,мен адамзат тарих жиіркеніш екендіг толық келіс және біз одан сабақ алып одан аулақ бол керек бірақ ник ақ адам жабай де яғни қазіргі ақ адам өт ешқандай қатыс жоқ бұл жай нәсілш артықшылық,racism

1,сігу болатын мұндай ақымақ нигга жоқ,сіг бол мұндай ақы нигг жоқ,racism

1,менен ол туралы сұрамаңыз оның істегені надан және жиіркенішті болды оның істегені қорлау енді егер біз ниггерлер оны ақымақ бас деп атайтын болсақ немесе ол

немере ағасымен бірге десе онда біз қателесеміз ол үшін шапағат етпеңіз өйткені оған надан болуды таңдағаны үшін шабуыл жасалады,менен ол туралы сұрама оның істе надан және жиркеніш бол оның істе қорла енді егер біз ниггер оны ақы бас де ата бол немесе ол немере аға бірге де онда біз қателес ол үшін шапағат етпе өйткені оған надан болу таңда үшін шабуыл жасал,racism

1,мен сіздің адамдарыңыз әлі де үңгірлерде өмір сүргенді ұнататынын айттым бұл төменде ме мен фотосуретті жүктедім сіз ақ адамдардың өлімі туралы сөйлескен кезде бандиттік өмір сүрдіңіз және мен сізге көрсеткен құлыптарды қоқыс деп атадыңыз сондықтан сіз бізді шынымен жек көресіз негр нәсілін өзгерту мүмкін емес оған үйреніңіз,мен сіздің адам әлі де үңгір өмір сүр ұнат ай бұл төменде ме мен фотосурет жүкте сіз ақ адам өлім туралы сөйлес кезде банди өмір сүр және мен сізге көрсет құлып қоқыс де ата сондықтан сіз біз шын жек көр нег нәсіл өзгерту мүмкін емес оған үйрен,racism

1,пікірге келетін болсақ бұл менің алғашқы шынайы дауларымның бірі сіз жиі сөйлесесіз және сіз жалған емессіз біраз қайғылы мен оның кім екенін білмеймін gjdj бірақ құдайым өтінемін сен өте сүйкімдісің мен тек керемет адамдармен сөйлесемін сондықтан мен бәс тігемін,пікір кел бол бұл менің алғашқы шынайы дау бірі сіз жиі сөйлес және сіз жалған емес біраз қайғылы мен оның кім екен білме gjdj бірақ құдай өт сен өте сүйкімд мен тек керемет адам сөйлес сондықтан мен бәс тіг,racism

1,сіз менің масса деп айтқанымды қалайсыз ба мен ақ адамдардың массасы туралы жаман сөз айтқым келмейді үй иті енот,сіз менің масса де айт қала ба мен ақ адам масса туралы жаман сөз айт келме үй ит енот,racism

1,елу жыл бұрын мен негр деп айтпаңыз қара деп айтпаңыз деген дәрісті тыңдадым өйткені бұл сөзіне ұқсайды енді менің ойымша бұл африка америкасы сіз тіпті африкаға бардыңыз ба мені ақ деп атады сонымен қатар крекер мен хонки және бұл ешқашан көгерген емес,ел жыл бұрын мен нег де айтпа қара де айтпа де дәрі тыңда өйткені бұл сөз ұқса енді менің ойымша бұл африка америка сіз тіпті африка бар ба мен ақ де ат сонымен қатар крек мен хонки және бұл ешқашан көгер емес,racism

1,леброн джеймс мылқау өзіне сенімді нигга ол жақсы құл болар еді бірақ ақымақ бейбақтар теңдікті қалайды,леброн джеймс мылқа өзіне сенімді нигг ол жақсы құл бол ед бірақ ақы бейбақ теңдік қала,racism

1,ол тым мылқау бола алмайды ол сіздің президентіңіз тағы жыл сіз үшін сорады ха rt барды желкекке обама мылқау нигга,ол тым мылқа бола алма ол сіздің президент тағы жыл сіз үшін сор ха rt бар желкек оба мылқа нигг,racism

1,хек сіздердің барлығыңыз ниггерлер бірдей дұрыс өмір сүре алмайтын ақымақ бастар әрқашан ұрлайды өтірік айтады қателеседі,хек сіздердің барлығ ниггер бірде дұрыс өмір сүр алма ақы бас әрқашан ұрла өтірік айт қателес,racism

1,riccygee сіз бардыңыз мылқау нигга бірақ сіз rt құдайға арналған деп айтасыз иә,riccygee сіз бар мылқа нигг бірақ сіз rt құдай арнал де ай иә,racism

1,ниггерлер егер сіз мылқау бейбақ сияқты әрекет етсеңіз онда ақ адам сізді нигга деп атай алмайды өйткені сіз олсыз,ниггер егер сіз мылқа бей сияқты әрекет ет онда ақ адам сіз нигг де ата алма өйткені сіз ол,racism

1,мен біреуге twitter де көрген ең жиіркенішті нәсілшіл және біржақты твиттер туралы хабарлауым керек еді қара нигга деп атайсыз ба,мен біреу twitter де көр ең жиіркеніш нәсілшіл және біржақты твиттер туралы хабарла керек ед қара нигг де ата ба,racism

1,бұл сылтау емес бұл факт оның жолына қараңыз оның не істеу керектігін білмейтін үлкен мылқау ниггерлері бар,бұл сылтау емес бұл факт оның жол қара оның не істеу керектіг білме үлкен мылқа ниггер бар,racism

1,маған тезірек келіңіз сасық нигериялық көт сізбен күресейік мен сені ұрамын ақымақ нигга,маған тезірек кел сасық нигериялық көт сізбен күрес мен сен ұр ақы нигг,racism

1,обамаға қарғыс атсын мылқау нигга lt,обама қарғыс ат мылқа нигг lt,racism

1,ол қара адамдарды мазақ еткен кезде мені мазақ етіп интернет арқылы ниггер деп атаған кезде бәріңіздің ол жас еді деп айтқаныңыз мені ауыртады мен одан бір жас үлкенмін оның жиіркенішті әрекеттерін ақтауды доғарыңыз сіз өртенген және қашып кеткен құл деп аталудың қандай болатынын білмейсіз,ол қара адам мазақ ет кезде мен мазақ ет интернет арқылы нигг де ата кезде бәрі ол жас ед де айт мен ауырт мен одан бір жас үлкен оның жиіркеніш әрекет ақтау доғар сіз өртен және қаш кет құл де атал қандай бол білме,racism

1,мен ниггерді өте немқұрайлы деп айтатын ақ адамдарды жек көремін содан кейін олар ой мен әзілдедім мен сенен рух шығарамын ақымақ болма дейді,мен нигг өте немқұрайл де айт ақ адам жек көр содан кейін олар ой мен әзілде мен сенен рух шығар ақы болма де,racism

1,мен жасаған жоқпын бірақ сен бірінші болдың ақыры нанданы жеңдің,мен жаса жоқ бірақ сен бірінші бол ақы нанда жең,racism

1,барлығын кінәлау жаман нәрсе өйткені мен жеке адамдардың маған жасаған жаман істері үшін адамдардың бүкіл демографиясын жек көруім керек барлық жағынан нәсілшілдік,барлығ кінәла жаман нәрсе өйткені мен жеке адам маған жаса жаман іс үшін адам бүкіл демография жек көр керек барлық жағ нәсілш,racism

1,сонда сіз оның өткенін неге айналдырасыз мұның бәріне қандай қатысы бар,сонда сіз оның өт неге айналдыр мұның бәрін қандай қатыс бар,racism

1,шынымды айтсам мен әлі де қара адамдарды көргеніме шатастырдым ал рос оны әлі де қолдады бұл маған бәрі жақсы екенін сезінді ме мен надан немесе басқа нәрсе сияқты көрінгім келмейді бірақ оның қазіргі және бұрынғы әрекеттері соншалықты қатты сөйлейді мен оны енді қолдай алмаймын мен татиге бейне бергім келмейді,шын айт мен әлі де қара адам көр шатас ал рос оны әлі де қолда бұл маған бәрі жақсы екен сез ме мен надан немесе басқа нәрсе сияқты көрін келме бірақ оның қазіргі және

бұрынғы әрекет соншалықты қатты сөйле мен оны енді қолда алма мен тати бейне бер келме,racism

1,қандай жаман және қорқынышты шешім жыл бұрын құлдыққа ешқандай қатысы жоқ адамдар біреуге қалай қарыздар болуы мүмкін қара қауымдастық қайтыс болған балаларының ата аналарына және соңғы апталарда қайтыс болған сәбилерге өтемақы төлей ме уау бұл ақылсыз,қандай жаман және қорқыныш шешім жыл бұрын құлдық ешқандай қатыс жоқ адам біреу қалай қарыз бол мүмкін қара қауымдастық қайтыс бол бала ата ана және соңғы апта қайтыс бол сәби өтемақы төле ме уау бұл ақыл,racism

1,ақымақ есек махаббаттан соқыр шайтан алғыр бұл керемет бок емес жылжыту шайтан алғыр әрі қарай табыңыз жұп стриптизерш клубында нигга банктік шотыңызды өртеп жіберіңіз рахмет,ақы есек махаббат соқыр шай алғы бұл керемет бок емес жылжыт шай алғы әрі қарай таб жұп стриптизерш клуб нигг банктік шот өрте жіб рахмет,racism

1,rt барлық даңқындағы надандық ші rt желкекке обама мылқау нигга,rt барлық даңқ надан ші rt желкек оба мылқа нигг,racism

1,ол нәсілшіл бе жоқ па бұл америка құрама штаттары онда біз сөз бостандығын қолданамыз егер бұл ақымақ ақ жезөкше нигга деп айтқысы келсе оның оған толық құқығы бар сол жақ бейбақтар біздің сөйлеуімізді цензуралауға тырысқан сайын біз американдықтар ретінде бостандығымызды жоғалтамыз,ол нәсілшіл бе жоқ па бұл америка құрама штат онда біз сөз бостандығ қолдан егер бұл ақы ақ жезөкше нигг де айт кел оның оған толық құқығ бар сол жақ бейбақ біздің сөйлеу цензурала тырыс сайын біз американдық рет бостандығ жоғал,racism

1,менің ойымша адамдардың көпшілігі көк тарих оқиғасы нәсілшілдіктің жойқын түрі деп келіседі бірақ екі оқиға да қамтылғанына қарамастан тек ашық нәсілшілдік анықталды және айыпталды бұқаралық ақпарат құралдары нәсілшілдік футбол ойынында нигга деп айқайлайтын бір ақымақ емес екенін түсінуі керек,менің ойымша адам көпшіліг көк тарих оқиға нәсілшілдік жойқын түр де келіс бірақ екі оқиға да қамтыл қарамастан тек ашық нәсілш анықта және айыпта бұқаралық ақпарат құрал нәсілш футбол ойын нигг де айқайла бір ақы емес екен түсін керек,racism

1,nigger nigger әлі де тозақ сияқты ақымақ,nigger nigger әлі де тозақ сияқты ақы,racism

1,сіз бұл оқу деп ойладыңыз алайда кітап жабылды сіз қара әйелсіз сондықтан нәсілдік әзілдерге төзімділік көрсетіп жатқаныңызды көру жиркенішті және көңілсіз және ақ адамдарға толы чатта өзіңізді нигга деп әзілдеп күлкілі деп санайсыз,сіз бұл оқу де ойла алайда кітап жаб сіз қара әйел сондықтан нәсіл әзіл төзімділік көрсет жат көру жиркеніш және көңіл және ақ адам толы чат өз нигг де әзілде күлкіл де сана,racism

1,трахай доғал ниггерлер біз күн сайын өлген жағдайда бізге ұнайтын барлық суретшілер туралы твиттер жазуымыз керек сияқты,траха доғал ниггер біз күн сайын өлген жағдай бізге ұна барлық суретші туралы твиттер жаз керек сияқты,racism

1,сен ақымақсың жаста ниггер ақш та ештеңе қалмайды сондықтан сен сияқты адамдардың кесірінен ақш тың есегінде,сен ақы жас нигг ақш та ештеңе қалма сондықтан сен сияқты адам кесір ақш тың есег,racism

1,джей мен саған айттым джейсен мен жай ғана мылқау нигга емеспін мен өте дарындымын бірақ мен геттоданмын және дұрыс грамматикаға мән бермеймін,дже мен саған ай джейсен мен жай ғана мылқа нигг емес мен өте дарынды бірақ мен гетто және дұрыс грамматика мән берме,racism

1,пышақ мылқау нигга деп аталатын нәрсе бар,пышақ мылқа нигг де атал нәрсе бар,racism

1,сіз уэйн есімді ақымақ ұсқынсыз бейбақ және кристің ұсқынсыз бөксесі пейнге қоңырау шалыңыз ұсқынсыз қара боқ әлемі уэйн және крис есімді ниггер pic twitter com ivvimdxmim,сіз уэйн ес ақы ұсқын бей және кри ұсқын бөксе пейн қоңырау шал ұсқын қара боқ әлем уэйн және крис ес нигг pic twitter com ivvimdxmim,racism

1,қара твиттер әрқашан риге шабуыл жасағанда мен ашуланамын бірақ содан кейін олар азық түлік маркаларын сатқаны үшін атылған ұсқынсыз жиіркенішті ниггерлер екенін есіме түсіремін,қара твиттер әрқашан ри шабуыл жаса мен ашулан бірақ содан кейін олар азық түлік марка сат үшін атыл ұсқын жиіркеніш ниггер екен ес түсір,racism

1,содан кейін мен сіздің қалаңызды бақылауға алар едім соңғы апта ішінде жалғасып келе жатқан тәртіпсіздіктер мен тонау кісі өлтіру мен жалпы қылмыстың өсуі сіз қара ерлер мен әйелдердің өлуіне жол беруден басқа ештеңе жасаған жоқсыз оған кіріп үйіңізді тәртіпке келтірсін,содан кейін мен сіздің қала бақыла ал ед соңғы апта іш жалғас келе жат тәртіпсіздік мен тонау кісі өлтір мен жалпы қылмыс өс сіз қара ер мен әйел өл жол беру басқа ештеңе жаса жоқ оған кір үй тәртіп келтір,racism

1,бұл ақымақ ниггаға толы бұл қаншық мүмкін отсосать маған,бұл ақы нигга толы бұл қаншық мүмкін отсосать маған,racism

1,qpr мүмкін отсосать қалың азиялық мүшесі нәсілшілдік үшін кінәсіз ақымақ бейбақтар ниггерлер,qpr мүмкін отсосать қалың азиялық мүше нәсілш үшін кінәсіз ақы бейбақ ниггер,racism

1,қара адам бұралған қара адам уау бұл толық пиздец тек қара емес мен әрқашан бұл ниггерді тарақанды бұл жиіркенішті негрді жек көретінмін тері мұнай төгілгеннен кейін,қара адам бұрал қара адам уау бұл толық пиздец тек қара емес мен әрқашан бұл нигг тара бұл жиіркеніш нег жек көр тері мұнай төгіл кейін,racism

1,обамаға қарғыс атсын мылқау нигга lt об pic twitter com,обама қарғыс ат мылқа нигг lt об pic twitter com,racism

1,мен кофе үстелін тептім қарғысатқыр говнюк қаншық ниггер тупая көт ебаный мудак есек гребаная қаншық бейбақ,мен кофе үстел теп қарғысатқы говнюк қаншық нигг тупая көт ебаны мудак есек гребаная қаншық бей,racism

1,rt митт ромни бұл мылқау нигга обамаға қарғыс атсын,rt митт ромни бұл мылқа нигг обама қарғыс ат,racism

1,ақ адамдар сізді жек көреді және сонымен бірге мақтау айтады мысалы мылқау ниггерлер мен олардың үлкен қара мүшелері сен жезөкше,ақ адам сіз жек көр және сонымен бірге мақтау айт мысал мылқа ниггер мен олардың үлкен қара мүше сен жезөкше,racism

1,менің анам деп аталатын жезөкшені дүниеге әкелгені үшін және менің әкем деп аталатынды мылқау нигга болғаны үшін және оған тиесілі емес кішкентай ақ қаншықты өсіргені үшін тек оның өсуі үшін және оның жігіті мені шақырса жақсы екенін айтады қаншық пен нигга тозақ барды барлық желкекке,менің ана де атал жезөкше дүние әкел үшін және менің әке де атал мылқа нигг бол үшін және оған тиесілі емес кішкентай ақ қаншық өсір үшін тек оның өс үшін және оның жігіт мен шақыр жақсы екен айт қаншық пен нигг тозақ бар барлық желкек,racism

1,израилдіктер негізінен негр испан тілінде сөйлейтін және түрлі түсті құстардың халқы ретінде иеміздің алдындағы борышын өтеу үшін сайланды солтүстік американың тумалары бірақ олардың қалдықтары бүкіл жер бетінде кездеседі қара деп аталатындардың бәрі бірдей симнен шықпайды ал ақ түстердің бәрі есаудан емес бірақ есаудың сенімі түсірілген,израилдік негіз нег испан тіл сөйле және түрлі түсті құс халқ рет ие алд борыш өтеу үшін сайла солтүстік америка тума бірақ олардың қалдық бүкіл жер бет кездес қара де атал бәрі бірде си шықпа ал ақ түс бәрі есау емес бірақ еса сенім түсір,racism

1,сіз қыздар бұл ақымақ ниггерлерді ұрып соғып жүкті бола аласыз олар түрмеге түседі содан кейін сіз монах болмайсыз,сіз қыз бұл ақы ниггер ұр соғ жүкті бола ал олар түрме түс содан кейін сіз монах болма,racism

1,мен қоңыр ақымақ болудан бастамаңыз сен қара сен ниггерсің сіз лас және жиіркенішсіз мен сен үшін дұға етемін,мен қоңыр ақы болу бастама сен қара сен нигг сіз лас және жиіркеніш мен сен үшін дұ ет,racism

1,welp rt мені ұстануға шешім қабылдаған барлық ақымақ ниггерлер,welp rt мен ұстан шешім қабылда барлық ақы ниггер,racism

1,әрине сіз сербияда дүниеге келдіңіз сіз сербиялық mkr фильміндегідей ақымақсыз,әрине сіз сербия дүние кел сіз сербиялық mkr фильміндегіде ақы,racism

1,сіз бардыңыз ақымақ бір ақымақ ақымақ ниггерді ораза ұстауды доғарыңыз надери өз көтеннен тупая қаншық көт,сіз бар ақы бір ақы ақы нигг ораз ұстау доғар надери өз көтен тупая қаншық көт,racism

1,lmaoo осы майлы жезөкшені күтіңіз бардым желкекке егер сен менен сұрасаң есі ауысқан идгаф есімді ақымақ нигга,lmaoo осы майлы жезөкше күт бар желкек егер сен менен сұра ес ауыс идгаф ес ақы нигг,racism

1,ниггердің ақымақтары сияқты оқу және ақымақтық үшін емес,нигг ақымақ сияқты оқу және ақымақтық үшін емес,racism

1,джеймс болдуин келесі жолы от сіз мұндай негр бұл жерді тастап не болды фрейзер оларды қираған қалалар деп атады сізді шынымен ақ әлем деп атайтын адам екеніңізге сену арқылы ғана жоюға болады,джеймс болдуин келесі жол от сіз мұндай нег бұл же таста не бол фрейз ол қира қала де ат сіз шын ақ әлем де ата адам екен сену арқылы ғана жою бол,racism

1,ол twitter де smh rt обаманы тозаққа мылқау ниггаға назар аударғысы келетіні анық,ол twitter де smh rt обама тозақ мылқа нигга назар аудар кел анық,racism

1,үндемеңіз сіз полицейді өлтіре жаздадыңыз мылқау нигга нигга үндемеңіз,үндеме сіз полицей өлтір жазда мылқа нигг нигг үндеме,racism

1,айтпақшы нигга қара адам емес бұл біреудің өзін ұстау тәсілі сен ақымақ бейбақсың біреуді қалай атайтыныңды қадағала,айтпақшы нигг қара адам емес бұл біреу өз ұстау тәсіл сен ақы бей біреу қалай ата қадағала,racism

1,мсм ақ пен қара арасындағы тәртіпсіздіктерді тудыру үшін жарыс картасын ойнайды олар сізге unilever сияқты нәсілшіл компаниялармен байланысы туралы ешқашан айтпайды unilever тің нәсілшілдік тарихы өте нашар олар ешқашан кешірім сұрамады және ештеңе төлемеді нәсілшілдікт,мс ақ пен қара ара тәртіпсіздік тудыру үшін жарыс карта ойна олар сізге unilever сияқты нәсілшіл компания байланыс туралы ешқашан айтпа unilever ті нәсілш тарих өте нашар олар ешқашан кешір сұрама және ештеңе төлем нәсілшілдікт,racism

1,кіші ойында жеңіліп қалды сен желкекге бардың татуировкасы бар ақымақ кесек желкекге өзі барды ақымақ бас нигга,кіші ой жеңіл қал сен желкек бар татуировка бар ақы ке желкек өзі бар ақы бас нигг,racism

1,сонымен оның командаластары оны нигга және мен гейлерді жек көремін деп айтқаны үшін кешіргендіктен олар оны да кешіреді деп ойлады ма егер ол қара болса және сіз ақымақ крекерге бардыңыз десе сіздің жауабыңыз бірдей болар ма еді,сонымен оның командалас оны нигг және мен гей жек көр де айт үшін кешіргендік олар оны да кешір де ойла ма егер ол қара бол және сіз ақы крек бар де сіздің жауаб бірде бол ма ед,racism

1,мен жай ғана кіріп сізге ескерту жасауым керек бұл жігіт өтірік айтады нотч нәсілшіл мұның айласы олар ақш тағы түрлі түсті адамдардың езгісін сөз бостандығы сияқты нәрселермен теңестіруге тырысу үшін жоғарыдағы твитке ұқсас мәлімдемелердің артында нәсілшілдіктерін жасырады,мен жай ғана кір сізге ескерту жаса керек бұл жігіт өтірік айт нотч нәсілшіл мұның айла олар ақш тағы түрлі түсті адам езгі сөз

бостандығ сияқты нәрсе теңестір тырыс үшін жоғары твит ұқсас мәлімдеме арт нәсілшілдік жасыр,racism

1,мен қара ниггермін мен нигга қаншық емеспін жарайды сіз жиіркеніштісіз twitter сіздің ір мекен жайыңызды тауып сізге тыйым салуы керек,мен қара нигг мен нигг қаншық емес жарайды сіз жиіркенішт twitter сіздің ір мекен жай тау сізге тыйым сал керек,racism

1,бұл ниггер мен черномаза арасындағы үлкен айырмашылық жедел жәрдемге тыйым салынады тоқтатылады қауіп төніп тұр жойылып кетті қарым қатынас орнату арқылы ақша мен килограмм әкеледі немесе қандай да бір ақымақ немесе қарапайым ақымақтық жасаған ниггерді сипаттау үшін қолданылады,бұл нигг мен черномаз ара үлкен айырмашылық жедел жәрдем тыйым салын тоқтат қауіп төн тұр жойыл кет қарым қатынас орнату арқылы ақша мен килограм әкел немесе қандай да бір ақы немесе қарапайым ақымақтық жаса нигг сипатта үшін қолдан,racism

1,барды сен есекке ақымақ қаншық ниггер,бар сен есек ақы қаншық нигг,racism

1,бірақ шорт қандай да бір боқта мылқау қара көзді бейбақтар,бірақ шорт қандай да бір боқ мылқа қара көз бейбақ,racism

1,сонымен сіз әлі де қара шаш оқиғасынан зардап шегіп жатырсыз деп ойлаймын солай ма немесе нәсілшілдік маңызды екенін өзіміз таңдаймыз ба,сонымен сіз әлі де қара шаш оқиға зардап шег жатыр де ойла солай ма немесе нәсілш маңызды екен өз таңда ба,racism

1,егер сіз афроамерикандықты нигга деп атасаңыз сіз өлетін ақымақ ақымақсыз өзіңді өлтір надан бейбақ,егер сіз афроамерикандық нигг де ата сіз өл ақы ақы өз өлтір надан бей,racism

1,чувак сік бұл сучую көтеннен ниггерлер сіз қалай мен кішкентай тунечи мен ауыртпалық мен мылқау мен мылқау барлық үш балбес,чувак сік бұл сучую көтен ниггер сіз қалай мен кішкентай тунечи мен ауыртпалық мен мылқа мен мылқа барлық үш балбес,racism

1,ол оны ақ қарттарды қуанту үшін ұрып соғатын психикалық проблемалары бар жас негр деп атаған шығар,ол оны ақ қарт қуант үшін ұр соғ психикалық проблема бар жас нег де ата шығар,racism

1,шынында да мылқау нигга бұл жерде не болып жатқанын біледі,шын да мылқа нигг бұл же не бол жат біл,racism

1,сіз кенияны ниггер деп қалай атайсыз ол нәсілдік сен ақымақ бейбақсың,сіз кения нигг де қалай ата ол нәсіл сен ақы бей,racism

1,канадалық қара деп айту тәсілі кандадианның нигга деп айтудың ақ емес әдісі біз кодтық сөздерді білеміз жиіркенішті және мен одан қалай шаршадым өтініш берушіні гетто жігіті деп атаған түйіндемені берген үкіметтің кадрлар бөлімінің қызметкері

есіңізде ме ол білікті маман болды сөздер маңызды отты лейлуи,канадалық қара де айту тәсіл кандадиан нигг де айт ақ емес әдіс біз код сөз біл жиіркеніш және мен одан қалай шарша өтініш беруші гетто жігіт де ата түйіндеме бер үкімет кадр бөлім қызметк ес ме ол білікті маман бол сөз маңызды от лейлуи,racism

1,қош бол обама rt обамаға қарғыс атсын бұл мылқау нигга мен швейцарияға барамын сізге рахмет америка сіз осындай ел болғаныңыз үшін,қош бол оба rt обама қарғыс ат бұл мылқа нигг мен швейцария бар сізге рахмет америка сіз осындай ел бол үшін,racism

1,сіз ақ әйелдің фрихол негр деп айта алмаймыз деп айтқанына сене аласыз өйткені е неліктен бұл испандық карен деп аталады,сіз ақ әйел фрихол нег де ай алма де айт сен ал өйткені е неліктен бұл испандық карен де атал,racism

1,лол сен жынды бауырсың ниггер бұл елді қайтадан басқарады rt барды желкекке обама мылқау нигга,лол сен жын бауыр нигг бұл ел қайта басқар rt бар желкек оба мылқа нигг,racism

1,керли ниггердің шынайы анықтамасы сауатсыз адам мылқау бейбақ,керли нигг шынайы анықта сауат адам мылқа бей,racism

1,дәл қазір олар бәрін бұзып жатыр тіпті қара батырлар мен соңғы онжылдықта не піскенін айтып отырмын,дәл қазір олар бәрін бұз жатыр тіпті қара батыр мен соңғы онжылдық не піс айт отыр,racism

1,егер сіз нигга мен қара көзді әке сияқты деп ойласаңыз сіз ақымақ болуыңыз керек,егер сіз нигг мен қара көз әке сияқты де ойла сіз ақы бол керек,racism

1,обамаға қарғыс атсын мылқау нигга бұл ақымақ мұнда lt lt lt,обама қарғыс ат мылқа нигг бұл ақы мұнда lt lt lt,racism

1,айырмашылық мынада американдық революция құлдық пен ақ үстемдікті қорғауға бағытталған революция емес еді ал бөліну өз жақтастарының айтуы бойынша дәл осы мақсатты көздеді стивенс мұны негрлердің төмендігіне негізделген алғашқы үкімет деп атады,айырмашылық мына американдық революция құлдық пен ақ үстемдік қорға бағыттал революция емес ед ал бөлін өз жақтас айт бойынша дәл осы мақсат көзде стивенс мұны негр төмендіг негіздел алғашқы үкімет де ат,racism

1,rt сіз ешқашан ылғалданатындай қартаймайсыз мылқау нигга желкекге үндемеңіз қаншық,rt сіз ешқашан ылғалданатында қартайма мылқа нигг желкек үндеме қаншық,racism

1,адамдар әлі де house туралы айтады желкекке үндемеңіз сіз бұл туралы ештеңе істей алмайсыз мылқау ниггерлер,адам әлі де house туралы айт желкек үндеме сіз бұл туралы ештеңе істе алма мылқа ниггер,racism

1,осмос тағы бір ақымақ трах мұнда әйел болғандықтан ғана жұмысқа орналасты неліктен бұл республикашыл ақымақтарды қайтармасқа және жаңа ниггерді

ұсынбасқа,осмос тағы бір ақы трах мұнда әйел бол ғана жұмыс орналас неліктен бұл республикашыл ақымақ қайтармас және жаңа нигг ұсынбас,racism

1,кокаин ұнайды шайтан алғыр бұл сізді міндетті түрде ашады деген сөздермен мылқау чарльз негр,кокаин ұна шай алғы бұл сіз міндетті түр аш де сөз мылқа чарльз нег,racism

1,lmao nomercy біреу ертең мектепке барғанда бұл қаншықты жақсы көреді rt обамаға қарғыс атсын мылқау нигга,lmao nomercy біреу ертең мектеп бар бұл қаншық жақсы көр rt обама қарғыс ат мылқа нигг,racism

1,wtf rt обама мылқау нигга ол үйге таңдаған барлық адамдарды тозаққа түсірді ол кетіп қалсын өйткені ол жерде оның мылқауынан шаршады,wtf rt оба мылқа нигг ол үй таңда барлық адам тозақ түс ол кет қал өйткені ол же оның мылқау шарша,racism

1,кімде кім үнемі нигга деп айтса ол өте ақымақ болып көрінеді,кім кім үнемі нигг де айт ол өте ақы бол көрін,racism

1,ниггерді ұрыңыз өйткені олар мені көтере алмайды немесе тіпті маған қарғыс сатып ала алмайды мылқау ниггерлердің әрқашан бір нәрсені қалайтын уақыты жоқ,нигг ұр өйткені олар мен көтер алма немесе тіпті маған қарғыс сат ал алма мылқа ниггер әрқашан бір нәрсе қала уақыт жоқ,racism

1,өткен жылы жарысқа байланысты қызықты іс шара өткізілді жас аралығындағы білмейтін жігіттер тобы менің баламды меңзеп ақ негр деп айқайлады мен уайымдама жаным тағы бір нәсілшіл маори деп айқайладым мұндағы бір қызығы олар сөздің толық мағынасында нәсілшіл емес еді,өткен жыл жарыс байланысты қызықты іс шара өткіз жас аралығ білме жігіт тоб менің бала меңзе ақ нег де айқайла мен уайымда жан тағы бір нәсілшіл маори де айқайла мұндағы бір қызығ олар сөз толық мағына нәсілшіл емес ед,racism

1,осы күні менің басымнан өткен бір нәрсе болды ма әлде менің алаңдаушылығымды тудырды ма әлде бұл барлық соңғы өзгерістерге байланысты күшейе түсті ме бәрі жақсы банкоматта маған не болып жатыр мен туыстарымның қайтыс болған мерейтойында қатты ренжігенімді білемін бірақ хм,осы күн менің бас өткен бір нәрсе бол ма әлде менің алаңдаушылығ ту ма әлде бұл барлық соңғы өзгеріс байланысты күшей түсті ме бәрі жақсы банкомат маған не бол жатыр мен туыс қайтыс бол мерейтой қатты ренжі біл бірақ хм,racism

1,нигга ниггадан шыққан қара адамдар бір біріне осылай қарауды тоқтатуы керек негр нигга нигга тоқтауы керек мұның бәрі жиіркенішті мұны айтқан кезде біз биликті өз қолымызға алмаймыз бұл жай ғана надандық бұл сөзді тастаңыз біз өзімізді ренжіткен ақымақтарға күлеміз,нигг нигга шық қара адам бір бір осылай қарау тоқтат керек нег нигг нигг тоқта керек мұның бәрі жиіркеніш мұны айт кезде біз билик өз қол алма бұл жай ғана надан бұл сөз таста біз өз ренжіт ақымақ күл,racism

1,сіз торонто рэпторс туралы естідіңіз бе нба командасы ақымақ бейбақтар бұл сізге деген құрмет туралы ниггерлер,сіз торонто рэпторс туралы есті бе нб команда ақы бейбақ бұл сізге де құрмет туралы ниггер,racism

1,мылқау кішкентай ниггерді желкекке тастау керек,мылқа кішкентай нигг желкек тастау керек,racism

1,джерика мен бәрін және менің ең жоғары игілігім мен әл ауқатыма қызмет етпейтіндердің бәрін босатқым келеді сондай ақ өткен ауырсынуды жарақаттануды мені өрмелеуге кедергі келтіретін кез келген блоктарды босатыңыз,джерик мен бәрін және менің ең жоғары игілиг мен әл ауқат қызмет етпе бәрін босат кел сондай ақ өткен ауырсыну жарақаттану мен өрмеле кедергі келтір кез кел блок боса,racism

1,ақшыл нигга мені тек мылқау бейбақ көріп менің түсім өзгергенін айтады,ақшыл нигг мен тек мылқа бей көр менің түсім өзгер айт,racism

1,обамаға қарғыс атсын мылқау нигга уоу дер хо сіз бұл қаншыққа ашулана алмайсыз өйткені ол өте қарапайым,обама қарғыс ат мылқа нигг уо дер хо сіз бұл қаншық ашулан алма өйткені ол өте қарапайым,racism

1,содан кейін мұндай пікірлер айтуды доғарыңыз бұл сөзді тек ақ адамдар үшін ғана емес қолданатындардың бәрі үшін дұрыс емес деп айтыңыз мен жастамын жақында олар мені негр деп атағысы келді,содан кейін мұндай пікір айту доғар бұл сөз тек ақ адам үшін ғана емес қолдан бәрі үшін дұрыс емес де ай мен жас жақында олар мен нег де ата кел,racism

1,мен бұл бейнені көрдім және оны құлағымнан өткіздім мен бұл дыбыстың ең жақсы бейнесі болатынын білемін және мен қатты ашуландым қара қыздар өздері үшін ештеңе алмайды бұл дыбыс қара бет пен теріні дәріптеуі керек еді,мен бұл бейне көр және оны құлағ өт мен бұл дыбыс ең жақсы бейне бол біл және мен қатты ашула қара қыз өз үшін ештеңе алма бұл дыбыс қара бет пен тері дәріпте керек ед,racism

1,обамаға қарғыс атсын мылқау нигга лил риседтің артына кірме қаншық қаншық,обама қарғыс ат мылқа нигг лил рисед арт кірме қаншық қаншық,racism

1,lmao сол қанатының бірнеше адамы олардың көпшілігі ақ түсті мені үйдегі негр деп атады,lmao сол қан бірнеше адам олардың көпшілиг ақ түсті мен үйдегі нег де ат,racism

1,медициналық мектептегі менің ең жақын достарымның кейбірі bs ге covid алдын алу туралы бірдеңе жариялаған сайын айтатыны ұят бірақ irl ga menerapkan yg seharusnya ясудала ақыр соңында тек қамқорлық жасаңыз және бірінші кезекте өзіңізді және сүйікті адамыңызды қорғаңыз,медициналық мектеп менің ең жақын дос кейбір bs ге covid алдын алу туралы бірде жарияла сайын айт ұят бірақ irl ga menerapkan yg seharusnya ясудал ақы соң тек қамқорлық жаса және бірінші кезек өз және сүйікті адам қорға,racism

1,аитв ны жұқтыруды жалғастыратын барлық ақымақ қара қаншықтар сіздің ақымақ сасық кискаларыңыз нигга қаншықтары менің нәсілімді жаман жарықта көрсетеді мен сен үшін ұялдым жезөкше лар,аитв ны жұқтыру жалғастыр барлық ақы қара қаншық сіздің ақы сасық киска нигг қаншық менің нәсіл жаман жарық көрсет мен сен үшін ұя жезөкше ла,racism

1,мұндағы ақ адамдардың бізді нигга деп атайтын мұқаба ретінде пайдалану үшін қара фотосуреттерді ұрлауы қаншалықты жиіркенішті екені таңқаларлық,мұндағы ақ адам біз нигг де ата мұқаба рет пайдалану үшін қара фотосурет ұрла қаншалықты жиіркеніш екен таңқаларлық,racism

1,lmao оған тағы да айтыңыз rt сіз үшін бұл президент ниггер rt обамаға қарғыс атсын мылқау нигга,lmao оған тағы да ай rt сіз үшін бұл президент нигг rt обама қарғыс ат мылқа нигг,racism

1,міне гей ескі ниггерлер мұндай мағынасыздықты айтады,міне ге ескі ниггер мұндай мағынасыздық айт,racism

1,біріншіден бұл көзілдірік сен ақымақ бейбақсың сіз топтар құрып есірткі сату арқылы бай болмадыңыз сен сорғыш емессің нигга,бірінші бұл көзілдірік сен ақы бей сіз топ құр есірткі сату арқылы бай болма сен сорғыш емес нигг,racism

1,сіз менің өкінетінімді білесіз ақ адамдар өйткені ниггерлер шынымен де тозақ сияқты мылқау егер олар бәрін бүлдірсе бұл мәңгілікке жалғасады деп ант етемін,сіз менің өкін біл ақ адам өйткені ниггер шын де тозақ сияқты мылқа егер олар бәрін бүлдір бұл мәңгілік жалғас де ант ет,racism

1,сіз бардыңыз нигер сіз оған күлгенге дейін жиырма жыл өтті,сіз бар ниг сіз оған күл дейін жиырма жыл өт,racism

1,елшілердің істері және бұл құдайдың жаратылысы құдай жаратқан халықтың құдайға бағышталған қара алтын негр еврей ұлттары олар бүгінде шынайы алғашқы патшалар және құдайдың діни қызметкерлері құдай үйреткен шайтанға әуес емес теологтар деп аталатын ақ адамдар,елші іс және бұл құдай жаратылыс құдай жаратқан халық құдай бағыштал қара алтын нег еврей ұлт олар бүгін шынайы алғашқы патша және құдай діни қызметкер құдай үйрет шайтан әуес емес теолог де атал ақ адам,racism

1,тағы да мылқау нигга түні бойы аяғын таптайды мысықтар қаруланған және дайын бәрі оларға мән бермейтін сияқты,тағы да мылқа нигг түн бойы аяғ тапта мысық қарулан және дайын бәрі оларға мән берме сияқты,racism

1,ақ адамдар түрлі түсті адамдарға қарағанда көбірек еркіндікке ие бұл ақымақтықты тоқтату керек ақ қаскөйлер өздерінің үлкен зеңбіректерімен серуендейді өйткені олардың ішектері бар жіңішке барлығын қорқыту үшін бірақ түрлі түсті адам сол күйінде көрінсе бірден атылады нәсілшілдік сен олай ойламайсың,ақ адам түрлі түсті адам қара көбірек еркіндік ие бұл ақымақтық тоқтату керек ақ қаскөй өз үлкен

зеңбірек серуенде өйткені олардың ішек бар жіңішке барлығ қорқыт үшін бірақ түрлі түсті адам сол күй көрін бірден ат нәсілш сен ола ойлама,racism

1,жексұрын нигга талантты жезөкшені табыңыз лол ол қара емес ол азиялық,жексұр нигг талантты жезөкше таб лол ол қара емес ол азиялық,racism

1,тек ниггер менің басымды ұрған түсініктеме үшін кідірт деп айтар еді ақымақ бейбақ,тек нигг менің бас ұр түсініктеме үшін кідірт де айтар ед ақы бей,racism

1,бұл оның сүйікті rt сорты сіз қара дикті ұнататыныңызды білесіз обамаға қарғыс атсын мылқау нигга,бұл оның сүйікті rt сор сіз қара дик ұнат біл обама қарғыс ат мылқа нигг,racism

1,боялған қара шашты адамдар негрлер шведтер мен қытайлықтар не талап етеді бұл ақылға қонымсыз олар жыныстық гегемонияны қалайды біз жоқ дедік және ұлыбританияда бомбалаушы командалардың ішіндегі ең жиіркенішті жексұрын альбинос борис джонсонның бақылауынан тыс оны жеңу үшін,боял қара шаш адам негр швед мен қытайлық не талап ет бұл ақыл қон олар жын гегемония қала біз жоқ де және ұлыбритания бомбалаушы команда ішіндегі ең жиіркеніш жексұр альбинос борис джонсон бақылау тыс оны жеңу үшін,racism

1,shitrednecksay rt обамаға қарғыс атсын мылқау нигга,shitrednecksay rt обама қарғыс ат мылқа нигг,racism

1,елшілердің істері және шайтандық шайтанның жауы егілді ақ түрдің нефилим мұрасы құдайдың жаратылысы деп аталатын құдайдың қара терісі алтын негр еврей халықтарының арасында шашыраңқы бидай құдайға ант етемін сонымен қатар жақсы тұқым және қойлар құдайға ант етемін құдай өрісінде бұл әлем,елші іс және шайта шайтан жау ег ақ түр нефили мұра құдай жаратылыс де атал құдай қара тері алтын нег еврей халық ара шашыраңқ бидай құдай ант ет сонымен қатар жақсы тұқым және қой құдай ант ет құдай өріс бұл әлем,racism

1,заңды күш болып табылатын рэв трамп ойлай алатын жалғыз қара адам сияқты таңқаларлық қорқынышты тәсіл сондай ақ база кез келген афроамерикалық адамның үкіметтің магна картаның құқықтарына негізделген өткен өміріне араласуы үшін өтемақы ретінде ұмтылатын ештеңені атамайды,заңды күш бол табыл рэв трам ойла ал жалғыз қара адам сияқты таңқаларлық қорқыныш тәсіл сондай ақ база кез кел афроамерикалық адам үкімет магн карта құқық негіздел өткен өмір аралас үшін өтемақы рет ұмтыл ештеңе атама,racism

1,rt салқындатыңыз обама сен не туралы сөйлесесің мылқау нигга бұл ақылға қонымды ойлау,rt салқындат оба сен не туралы сөйлес мылқа нигг бұл ақыл қонымды ойлау,racism

1,ғана бұл туралы білдім ақ диджее атындағы джоуи негро және шамасы тек кеше ол шешті артық қолдануға болмайды бұл аты лмаоооо,ғана бұл туралы біл ақ дидже ат джоуи негро және шама тек кеше ол шеш артық қолдан болма бұл ат лмаоооо,racism

1,сіз нигга сияқты ақымақсыз,сіз нигг сияқты ақы,racism

1,сізде мың бар сіз бірінші және соңғы рет айына ге бір бөлмелі пәтер табасыз сонымен қатар шығындар сондықтан жай көшу үшін деп айтыңыз енді сізде мың бар жиһаз рәсімін қалайсыз ба бұл тағы мың енді сізде мың қалды сіз тіпті тамақ ішпедіңіз немесе басқа төлемдерді төледіңіз,сіз мың бар сіз бірінші және соңғы рет ай ге бір бөлмелі пәтер таб сонымен қатар шығын сондықтан жай көшу үшін де ай енді сіз мың бар жиһаз рәсім қала ба бұл тағы мың енді сіз мың қал сіз тіпті тамақ ішпе немесе басқа төлем төле,racism

1,сенің суретіңнен менің қаздарым бар ракон xd,сенің сурет менің қаз бар ракон xd,racism

1,сіздің ші көзіңіз алтыншы сезім емес ақымақ бейбақ бұл сіздің эпифизіңіз мылқау нигга,сіздің ші көз алтыншы сезім емес ақы бей бұл сіздің эпифиз мылқа нигг,racism

1,mlk тағы бір орынсыз нигга сіз бардыңыз ақымақ нәсілшіл надан қаншық,mlk тағы бір орынсыз нигг сіз бар ақы нәсілшіл надан қаншық,racism

1,сік ниггерлер олардың бәрі мылқау,сік ниггер олардың бәрі мылқа,racism

1,сіз тек қан және басқа ештеңе адамды кім етеді деп айтасыз егер бұл қан негрдің қаны болмаса бұл жағдайда сіз аралас адам болсаңыз басқа нәрсе туралы айтасыз өйткені қара болу жиіркенішті менің ойымша,сіз тек қан және басқа ештеңе адам кім ет де ай егер бұл қан нег қан болма бұл жағдай сіз аралас адам бол басқа нәрсе туралы ай өйткені қара болу жиіркеніш менің ойымша,racism

1,мен лебронды жек көремін сен ақымақ ебанутый бейбақ шашқа арналған ілінісу сен бардың ниггер,мен лебро жек көр сен ақы ебануты бей шаш арнал ілініс сен бар нигг,racism

1,жиіркенішті нигга ақ әйелдерді жақсы көреді оларды жалғыз қалдырыңыз мұнда қара әйелдер жеткілікті отвали нигга,жиіркеніш нигг ақ әйел жақсы көр ол жалғыз қалдыр мұнда қара әйел жеткілікті отвали нигг,racism

1,сіз тозаққа бардыңыз соратын ниггерлер сіз бәрін ысқыламайсыз қалай білгіңіз келеді себебі сіз ге жазылмайсыз оның артынан жүр,сіз тозақ бар сор ниггер сіз бәрін ысқылама қалай біл кел себебі сіз ге жазылма оның арт жүр,racism

1,сол күндері ол куннилингус деп аталды ол өзін қолайлы қара орео ретінде көрсетеді ол өзінің касталық жүйесіндегі қара нәсілділердің санын азайту арқылы ақ адамдарды қорлайды ол аяғын шайқайтын жиіркенішті негрлердің арасында ұзын бойлы қара,сол күн ол куннилингус де ата ол өз қолайлы қара орео рет көрсет ол өзінің касталық жүйе қара нәсілді сан азайту арқылы ақ адам қорла ол аяғ шайқа жиіркеніш негр ара ұзын бойл қара,racism

1,мылқау нигга ақымақ қаншықты ұр,мылқа нигг ақы қаншық ұр,racism

1,сонымен біреу ниггерін бастайтын уақыт келді деп айтады бірақ егер мен сауатсыз мылқау бейбақ болсам және нигга деп айтсам бәрі жақсы мен ең жақсымын ба wtf,сонымен біреу нигг баста уақыт кел де айт бірақ егер мен сауат мылқа бей бол және нигг де айт бәрі жақсы мен ең жақ ба wtf,racism

1,жарайды ниггер rt маған нигга сөзін айтатын бірақ нәсілшілдікті тоқтатқысы келетін қара адамдар ұнамайды мен сені жек көремін ақымақ бейбақтар,жарайды нигг rt маған нигг сөз айт бірақ нәсілшілдік тоқтат кел қара адам ұнама мен сен жек көр ақы бейбақ,racism

1,бұл ниггерлер тозақ сияқты мылқау мемлекеттік мектеп жүйесіне қайғырыңыз,бұл ниггер тозақ сияқты мылқа мемлекеттік мектеп жүйе қайғы,racism

1,барды сен желкекке мылқау нигга,бар сен желкек мылқа нигг,racism

1,мен жігітті есебімен жеңдім оның реакциясы келесідей автобус мені қағып кетеді деп үміттенемін сен тым семізсің мылқау ниггерді сондай тұзды,мен жігіт есеб жең оның реакция келесіде автобус мен қағ кет де үміттен сен тым се мылқа нигг сондай тұз,racism

1,барлығына сәлем мен мүмкіндігінше ашық адам болғым келеді сондықтан біреу менің өткенім туралы бірдеңе ашпас бұрын бірдеңе айтқым келеді мен жетінші сыныпта оқып жүргенде бұл сөзді жиі айтатынмын мені көптеген қара адамдар қоршап алды және бұл жақсы деп ойладым жалғасы,барлығ сәлем мен мүмкіндігінше ашық адам бол кел сондықтан біреу менің өт туралы бірде ашпас бұрын бірде айт кел мен жетінші сынып оқ жүр бұл сөз жиі айт мен көптеген қара адам қорша алды және бұл жақсы де ойла жалғасы,racism

1,екеуміз өзгердік мен сенің есегіңнен кейін өстім сіз әлі де өз өміріңізбен ештеңе жасамайтын мылқау ниггерсіз,екеу өзгер мен сенің есег кейін өс сіз әлі де өз өмір ештеңе жасама мылқа нигг,racism

1,сіз мылқау объективті ниггерлер ешқашан достарыңызды шақырмайтындығыңыз өйткені сіз бәріңіз бір кітап клубында боласыз әлбетте бұл сіздің серігіңіз емес егер олар қара нәсілшілге қарсы болса сіз ақымақ бейбақтарсыз,сіз мылқа объективті ниггер ешқашан дос шақырмайтынды өйткені сіз бәрі бір кітап клуб бол әлбет бұл сіздің сері емес егер олар қара нәсілшіл қарсы бол сіз ақы бейбақт,racism

1,тааак сен ашуланасың ба сіз жақсы боласыз rt обамаға қарғыс атсын мылқау нигга,тааак сен ашулан ба сіз жақсы бол rt обама қарғыс ат мылқа нигг,racism

1,ха rt жарайды ниггер rt маған нигга сөзін айтатын бірақ нәсілшілдікті тоқтатқысы келетін қара адамдар ұнамайды мен сені жек көремін ақымақ бейбақтар,ха rt жарайды нигг rt маған нигг сөз айт бірақ нәсілшілдік тоқтат кел қара адам ұнама мен сен жек көр ақы бейбақ,racism

1,билл махерді қорғайтын қара адамдардың саны жиіркенішті ол үйдегі нигга деп түсіндірді бұл нәсілшілдік,билл мах қорға қара адам сан жиіркеніш ол үйдегі нигг де түсін бұл нәсілш,racism

1,нах сен бардың сіз мылқау ниггерсіз,нах сен бар сіз мылқа нигг,racism

1,қара адамдардың өмірін жақсартатын нәрсені неден тоқтатқым келеді тағы да сіз мұны түсіну үшін тым жассыз сіз сөздерді менің аузыма салып ешнәрсе үшін бар күшіңізді саласыз сіз менің ұстанымымды да өзімді де білмейсіз сіз ұйықтайтын уақыт келді,қара адам өмір жақсарт нәрсе не тоқтат кел тағы да сіз мұны түсіну үшін тым жас сіз сөз менің ауз сал ешнәрсе үшін бар күш сал сіз менің ұстаным да өз де білме сіз ұйықта уақыт кел,racism

1,обамаға қарғыс атсын ақымақ нигга қараңдаршы қаншық неліктен сіз бұл сөзді айта бересіз оны нигга әтешіне сорып алыңыз сіз майлы жезөкше,обама қарғыс ат ақы нигг қараңдарш қаншық неліктен сіз бұл сөз ай бер оны нигг әтеш сор ал сіз майлы жезөкше,racism

1,ниггер сенің мылқау тобыңды қарғыс атсын себебі мен саттинді ананы жылату үшін әкелдім,нигг сенің мылқа тоб қарғыс ат себебі мен сатти ана жылат үшін әке,racism

1,тағы да қайталаймын сен мені жек көресің бе жоқ па маған бәрібір сіз бұл жерде менің әтешім туралы алаңдайсыз мылқау нигга,тағы да қайтала сен мен жек көр бе жоқ па маған бәрібір сіз бұл же менің әтеш туралы алаңда мылқа нигг,racism

1,мылқау твит нигга сөзін қорлайтын қара адамдар хек бұл нені білдіруі керек,мылқа твит нигг сөз қорла қара адам хек бұл не білдір керек,racism

1,барды желкекке обама мылқау нигга сіз не күттіңіз ол халқы бар қалада тұрады анасы да әпкесі лол,бар желкек оба мылқа нигг сіз не күт ол халқ бар қала тұр ана да әпке лол,racism

1,тоқта сіз нәсілшілсіз ақымақ бас нигга,тоқта сіз нәсілшіл ақы бас нигг,racism

1,мен сенің президент блэк екеніңді біле тұра осы демалыс күндері ақ қызды ұрып жатырмын обамаға қарғыс атсын мылқау нигга,мен сенің президент блэк екен біл тұр осы демалыс күн ақ қыз ұр жатыр обама қарғыс ат мылқа нигг,racism

1,алдымен сіз мені қорқыттыңыз хаха ха айтпақшы сіздің іс ді табуға немесе жаңасын жасауға сәттілік тілеймін,алд сіз мен қорқыт хах ха айтпақшы сіздің іс ді таб немесе жаңа жаса сәттілік тіле,racism

1,біз өзін өзі қорғау үшін қарулану орынды болатын сәттен әлдеқашан өтіп кеттік мен заңсыз нәрсе жасау керек деп айтпаймын өйткені бұл tos ты бұзады бірақ егер сіз цру ның құпия сайтына сүйрелгіңіз келсе және құдайлар сізге не істейтінін білсе,біз өз өзі қорғау үшін қарулан ор бол сәт әлдеқашан өт кет мен заңсыз нәрсе жасау керек де айтпа өйткені бұл tos ты бұз бірақ егер сіз цр ны құпия сайт сүйрел кел және құдай сізге не істе біл,racism

1,тағы бір коммунистік террорист корей егін жинауды жақтайтын нигер метамфетаминді қолданатын полиция бастығы террорист алакс шіркеу орта мектепте түрлі түсті нәсілшілдікке байланысты менің ойымды ұстайды трампты сотқа бергісі келеді,тағы бір коммунистік террорист корей егін жинау жақта ниг метамфетами қолдан полиция бастығ террорист алакс шіркеу орта мектеп түрлі түсті нәсілшілдік байланысты менің ой ұста трам сот бер кел,racism

1,ол ұсқынсыз ақ жезөкше rt үндеме тозақ rt обамаға қарғыс атсын мылқау нигга хиллари билли,ол ұсқын ақ жезөкше rt үндеме тозақ rt обама қарғыс ат мылқа нигг хиллари билли,racism

1,құдайым сәлем сіз менің алғашқы сұхбаттасушыларымның бірі болдыңыз және әлі де менің сүйіктілерімнің бірі болып қала бересіз мен сіздің твиттеріңізді tl де көргенді ұнатамын өйткені сіз өте қызықсыз және сіз өмірде жеңіске жететін атақты жігітсіз көбірек өзара әрекеттесу керек пе немесе lt,құдай сәлем сіз менің алғашқы сұхбаттасушы бірі бол және әлі де менің сүйікті бірі бол қала бер мен сіздің твиттер tl де көр ұна өйткені сіз өте қызық және сіз өмір жеңіс жет атақты жігіт көбірек өзара әрекеттесу керек пе немесе lt,racism

1,бұл нигга ақымақ сияқты,бұл нигг ақы сияқты,racism

1,яо пау пау брай rt осы майлы жезөкшені күтіңіз барды желкекке лил фат егер сіз менен мылқау ниггердің атын сұрасаңыз,яо па па бра rt осы майлы жезөкше күт бар желкек лил фат егер сіз менен мылқа нигг ат сұра,racism

1,трампта нәсілшілдік жоқ мұны жаңа көзқараспен қараңыз сіздің линзаларыңыз түрлі түсті немесе лас сіз нашар көресіз,трамп нәсілш жоқ мұны жаңа көзқарас қара сіздің линза түрлі түсті немесе лас сіз нашар көр,racism

1,есіңізде болсын қара мафия ака goodfellas немесе сол жерде сіз ракондар мен ниггерлер өз тобыңызды бұзақылар деп атайсыз сіз менімен бұл боқтықты бастадыңыз мен мылтық ұстаған мылқау ниггерлердің алдында шегінбеймін,ес бол қара мафия ак goodfellas немесе сол же сіз ракон мен ниггер өз тоб бұзақы де ата сіз менімен бұл боқтық баста мен мылтық ұста мылқа ниггер алд шегінбе,racism

1,бұл пенстің жанкүйері өледі деп үміттенемін сіз уэйн симондсты лас ниггер деп атамайсыз ол канадалық ақымақ бейбақ,бұл пен жанкүйер өл де үміттен сіз уэйн симонд лас нигг де атама ол канадалық ақы бей,racism

1,біз израильде палестинада ақш та және барлық жерде жеккөрушілік пен нәсілшілдікті насихаттайтындарға қарсы бірігуіміз керек осы ақымақ әңгімелердің барлығы берн сіз әлі nycsheletrs ке келіп кедей және үйсіз түрлі түсті адамдарға деген өшпенділік пен нәсілшілдікті көре алмайсыз сондықтан,біз израиль палестина ақш та және барлық же жеккөрушілік пен нәсілшілдік насихатта қарсы біріг керек осы ақы әңгіме барлығ берн сіз әлі nycsheletrs ке кел кедей және үй түрлі түсті адам де өшпенділік пен нәсілшілдік көр алма сондықтан,racism

1,демократтардың өкілі шейла джексон ли мәлімдемесінде нәсілшілдікті united airlines авиакомпаниясымен болған оқиғаның себебі деп атайды foxnews gt gt көптеген ниггерлер жасайды,демократ өкіл шейл джексон ли мәлімдеме нәсілшілдік united airlines авиакомпания бол оқи себебі де ата foxnews gt gt көптеген ниггер жаса,racism

1,сіз уэйн есімді ақымақ ұсқынсыз ниггерсіз позитивті қоқыс жұмсақ есегі бар нигга pic twitter com,сіз уэйн ес ақы ұсқын нигг позитивті қоқыс жұм есег бар нигг pic twitter com,racism

1,ниггерлер бұл ақымақ бейбақтар болып көрінетіндер олар шын мәнінде мысалы мас егеуқұйрықты менің рәсіміме қарай қуған кезде өздерін ақылды деп санайды,ниггер бұл ақы бейбақ бол көрін олар шын мән мысал мас егеуқұйрық менің рәсім қарай қу кезде өз ақылды де сана,racism

1,нәсілшілдік бұрынғыдан да тірі және өркендеп келеді rt rt обамаға қарғыс атсын мылқау нигга,нәсілш бұрынғы да тірі және өркенде кел rt rt обама қарғыс ат мылқа нигг,racism

1,мен ештеңе көрмеймін rt қаншық не rt мылқау нигга шайтан алғыр телефон сөніп қалды мен зарядтағышты таба алмаймын,мен ештеңе көрме rt қаншық не rt мылқа нигг шай алғы телефон сөн қал мен зарядтағыш таб алма,racism

1,мен терінің түсін көрмеймін бірақ қара адамдар жиналғанда олардан ештеңе қашып кетпейді керемет мен мұндай нәрсені ешқашан көрген емеспін,мен тер түсін көрме бірақ қара адам жинал олардан ештеңе қаш кетпе керемет мен мұндай нәрсе ешқашан көр емес,racism

1,бұл трюкті қолданып көріңіз мүшені жеуккккк сізде мулат балалары болады обамаға қарғыс атсын мылқау нигга,бұл трюк қолдан көр мүше жеуккккк сіз мулат бала бол обама қарғыс ат мылқа нигг,racism

1,араб жезөкше rt содан кейін сіз де бардыңыз мылқау нигга rt сіз бәріңіз lt lt lt lt rt сіз gt gt rt сіз gt,араб жезөкше rt содан кейін сіз де бар мылқа нигг rt сіз бәрі lt lt lt lt rt сіз gt gt rt сіз gt,racism

1,сәлем ханшайым ең алдымен мен сізбен сөйлескеніме өте қуаныштымын менің арнамдағы жазбаларыңызды әсіресе эстетикалық тақырыптағы тақырыптарды көру менің шабыт көздерімнің бірі біз көбірек сөйлесе аламыз деп үміттенемін иә мен сені twt де үлкен әпкем ретінде көремін сонымен қатар ұбт емтихандарына сәттілік мен сен үшін дұға етемін,сәлем ханшай ең алд мен сізбен сөйлес өте қуанышты менің арнам жазба әсіресе эстетикалық тақырып тақырып көру менің шабыт көз бірі біз көбірек сөйле ал де үміттен иә мен сен twt де үлкен әпке рет көр сонымен қатар ұбт емтихан сәттілік мен сен үшін дұ ет,racism

1,уайтидің ескі жаңалықтары rt сіз байсалдысыз ба маған осы уақыт бойы өтірік айтылды rt корейлер қытай емес мылқау нигга,уайти ескі жаңалық rt сіз байсалды ба маған осы уақыт бойы өтірік айт rt корей қытай емес мылқа нигг,racism

1,о о ол ессіз rt rt ақ twitter дегі тікелей репортаж сік обама мылқау нигга,о о ол ес rt rt ақ twitter дег тікелей репортаж сік оба мылқа нигг,racism

1,егер мен соңғы бірнеше айда қара достарымды тыңдау арқылы бірдеңе білсем бұл полицияның оның жұмысының кейбір аспектілерін қаншалықты жақсы көретіні және оны қаншалықты асыға күтетіні бұл мені ауыртады,егер мен соңғы бірнеше айда қара дос тыңдау арқылы бірде біл бұл полиция оның жұмыс кейбір аспекті қаншалықты жақсы көр және оны қаншалықты асыға күт бұл мен ауырт,racism

1,бастапқыда айтылғандай қара түстің бәрі жаман емес дәл сол кезде қара түс теріс дегенді білдіреді ол өткен мағынаны алады blakc жаман реңк,бастапқы айтылғанда қара түс бәрі жаман емес дәл сол кезде қара түс теріс де білдір ол өткен мағына ал blakc жаман реңк,racism

1,сіз не екенін білесіз шайтан алғыр нигга ақымақ нигга қаншық ракон үй ниггасы жабайы мысық мен бұл әдепті сенен үйрендім,сіз не екен біл шай алғы нигг ақы нигг қаншық ракон үй нигга жабай мысық мен бұл әдеп сенен үйрен,racism

1,сен мылқау ақымақсың американың ниггерлері сенің ақымақтығыңды мылқау жезөкшені ұруы керек нәсілшіл бет ұсқынсыз ақымақ,сен мылқа ақы америка ниггер сенің ақымақтығ мылқа жезөкше ұр керек нәсілшіл бет ұсқын ақы,racism

1,бауырым мен жасөспірімдермен сөйлеспеймін сізде түсіністік деңгейі төмен қызыңызды қаншалықты жақсы көретініңіз маңызды емес біржақты махаббат әйелі бірақ отбасы әрқашан бірінші орында отбасынан маңызды ештеңе жоқ чахей кайси би хо,бауыр мен жасөспірім сөйлеспе сіз түсіністік деңгей төмен қыз қаншалықты жақсы көр маңызды емес біржақты махаббат әйел бірақ отбасы әрқашан бірінші орында отбас маңызды ештеңе жоқ чахе кайси би хо,racism

1,жоқ жерден келген қыз әрине жадтан өшіріп бірінші рет қайта көргім келетін көп сериялы фильмдердің бірі,жоқ же кел қыз әрине жад өшір бірінші рет қайта көр кел көп сериялы фильм бірі,racism

1,мұның себебі неде сонымен қатар қара қауымдастықтың соңғы сағат ішінде айтқан барлық даулы мәселелерге қандай қатысы бар менің ойымша бұл кайли дженнердің ұятқа қалғаны туралы менің твиттеріме негізделген,мұның себебі не сонымен қатар қара қауымдастық соңғы сағат іш айт барлық даул мәселе қандай қатыс бар менің ойымша бұл кайли дженн ұят қал туралы менің твиттер негіздел,racism

1,мен жаста едім қарт адам мені және анамды азық түлік дүкенінің тұрағында нигга деп атады өйткені ол заңсыз тұрақ қоюға тырысты менің сыныптастарымның бірі нәсілшілдік манифестін жазған кезде орта мектепке ауысқанға дейін менде нәсілшілдік оқиғалары болған жоқ,мен жас ед қарт адам мен және ана азық түлік

дүкен тұраға нигг де ат өйткені ол заңсыз тұрақ қою тырыс менің сыныптас бірі нәсілш манифест жаз кезде орта мектеп ауыс дейін менде нәсілш оқиға бол жоқ,racism

1,сіз ниггермен сөйлесуді тоқтатқан кезде ол сол мылқау жезөкше ға оралады ол оны тапқырлықпен ұрып соғу үшін қолданған және ол жеңемін деп ойлайды өйткені ол жай ғана секіреді,сіз ниггер сөйлесу тоқтат кезде ол сол мылқа жезөкше ға орал ол оны тапқырлық ұр соғ үшін қолдан және ол же де ойла өйткені ол жай ғана секір,racism

1,мазм көріңіз бас тартуға балағат сөздер үшін ораза бірақ бұл қиын ебать сен доғал кетменом көтеннен қаншық көт киска негр,маз көр бас тарт балағат сөз үшін ораз бірақ бұл қиын ебать сен доғал кетмено көтен қаншық көт киск нег,racism

1,sourcessay менің өмірім крис полға қарғыс атсын хьюстон араласқаны үшін тозаққа барады енді сен үшін тамыр жайа алмаймын мылқау нигга,sourcessay менің өмір крис пол қарғыс ат хьюстон аралас үшін тозақ бар енді сен үшін тамыр жай алма мылқа нигг,racism

1,бірдеңе жасау үшін сізге ақ адамдар қажет емес малкольм мұны былай деп атады негрлердің ең жаман жауы бұл ақ адам ол негрлерге деген сүйіспеншілігін білдіріп өзін либерал деп атайды және дәл осы ақ либералдардың соңынан ереді бар,бірде жасау үшін сізге ақ адам қажет емес малколь мұны былай де ат негр ең жаман жау бұл ақ адам ол негр де сүйіспеншіліг білдір өз либерал де ата және дәл осы ақ либерал соң ер бар,racism

1,трампқа дейін мылқау ниггерлер барлық иллюминати үшін айыпталды дауыс беру мылқау болды өйткені ниггерлер құпия қоғамдар бұл сұмдықты жауып тастады деп ойлады содан кейін трамп келіп жоқ ниггерлер менің күшім бар мен келесі бөлікті бұзамын деді,трамп дейін мылқа ниггер барлық иллюминати үшін айыпта дауыс беру мылқа бол өйткені ниггер құпия қоғам бұл сұмдық жау таста де ойла содан кейін трам кел жоқ ниггер менің күшім бар мен келесі бөлік бұз де,racism

1,сондай ақ кез келген жағдайда бұл мылқау ниггердің айтқанының бәрі тозаққа түседі өйткені ол тек yt жігіттерін ұнатады және tf сияқты kelly ді қолдайды,сондай ақ кез кел жағдай бұл мылқа нигг айт бәрі тозақ түс өйткені ол тек yt жігіт ұнат және tf сияқты kelly ді қолда,racism

1,сонымен кэнди айтпақшы стриптизердің аты уақыт шкаласы бойынша тен аз твиттерді айналдырып сіз джо байденді қорғап жатқаныңызды көремін ол сөзбе сөз түсті кедей деп айтты кешіріңіз әпке бірақ нәсілшілдік ешқашан кездейсоқ емес сонымен қатар брендон уриге қоңырау шалыңыз мен маңдайы одан үлкен адамды таптым,сонымен кэнди айтпақшы стриптиз ат уақыт шкала бойынша тен аз твиттер айналдыр сіз джо байде қорға жат көр ол сөзб сөз түсті кедей де ай кешір әп бірақ нәсілш ешқашан кездейсоқ емес сонымен қатар брендон ури қоңырау шал мен маңдай одан үлкен адам тап,racism

1,эй джозеф сен мылқау бейбақсың бұл қатал маңызды адамдар нигга деп айтпайды әдетте олар нигга дейді,эй джозеф сен мылқа бей бұл қатал маңызды адам нигг де айтпа әдет олар нигг де,racism

1,кейбір қыздар жай ғана мылқау мен түсінемін неге ниггерлер оларды ұруға қарсы емес,кейбір қыз жай ғана мылқа мен түс неге ниггер ол ұр қарсы емес,racism

1,жоқ олай емес сіз оны қорлау ниетімен ниггер деп атадыңыз бұл мүлдем басқа мәселе ақымақ бейбақ,жоқ ола емес сіз оны қорла ниет нигг де ата бұл мүлдем басқа мәселе ақы бей,racism

1,сіз бардыңыз менің қаншығым дәл қазір менің парағымдағы барлық адамдар осы rt мылқау ниггаға қарап тұрғанын қалаймын,сіз бар менің қаншы дәл қазір менің парағым барлық адам осы rt мылқа нигга қара тұр қала,racism

1,обамаға қарғыс атсын мылқау нигга қара твиттер қайда өтінемін бұл қаншықты ұстаңыз,обама қарғыс ат мылқа нигг қара твиттер қайда өт бұл қаншық ұста,racism

1,мен айтқанымдай бұл айсбергтің ұшы болды менің ойымша бұл оқиғаға байланысты ешкімнің айыпты алып тастағанын көрген жоқпын оның бұл әнді шығарғаны жалған болуы мүмкін бірақ бұл оның бұрын жасаған ешнәрсесінің орнын толтырмайды және қара жанкүйерлер оған ештеңе қарыз емес өйткені,мен айтқанымда бұл айсберг ұш бол менің ойымша бұл оқиға байланысты ешкі айыпты алып таста көр жоқ оның бұл ән шығар жалған бол мүмкін бірақ бұл оның бұрын жаса ешнәрсе орн толтырма және қара жанкүйер оған ештеңе қарыз емес өйткені,racism

1,сіз өзіңіздің алғашқы досыңыз болса да дәлелдеу үшін суретке түсуіңіз керек,сіз өз алғашқы дос бол да дәлелде үшін сурет түс керек,racism

1,crazy nigger crazy rt сіз де бара аласыз желкекке сіздің атыңыз өте ақымақ жолақта бол балам,crazy nigger crazy rt сіз де бар ал желкек сіздің ат өте ақы жолақ бол бала,racism

1,ол ақ матамен жабылған ол сондай ақ бізге осы модераторларға қосылатын негр оппозициясы туралы ескертті малкольм оларды республикалық қасқырларға арналған түлкі деп атады,ол ақ мата жабыл ол сондай ақ бізге осы модератор қосыл нег оппозиция туралы ескер малколь ол республикалық қасқыр арнал түлк де ат,racism

1,жаман ой бірақ мен сұрауым керек ші бұл пандемияға қара және қоңыр қауымдастықтарға сондай ақ ірі қалаларға қатты соққы бергендіктен серуендеуге мүмкіндік бере ме менің айтайын дегенім оның базасы негізінен ауылдық жерде солай ма кешіріңіз бұл қорқынышты ой бірақ мен оны төмендетпес едім дауыс беруді басу үшін кез келген нәрсе,жаман ой бірақ мен сұра керек ші бұл пандемия қара және қоңыр қауымдастық сондай ақ ірі қала қатты соққы бергендік серуенде мүмкіндік бер ме менің айт де оның база негіз ауылдық же солай ма кешір бұл қорқыныш ой бірақ мен оны төмендетпес ед дауыс беру басу үшін кез кел нәрсе,racism

1,nvm бұл ниггердің жанкүйері болды,nvm бұл нигг жанкүйер бол,racism

1,ақ ойыншылар тек ақ ойыншылармен жарысты ал негр лигалары деп аталатын қара ойыншылар тек қара ойыншылармен жарысты сегрегацияға байланысты майорларға жете алмайтын ойыншылар көп болды әлі де керемет бірақ mlb интеграциясына дейін жазбалар есепке алынбайды,ақ ойыншы тек ақ ойыншы жар ал нег лига де атал қара ойыншы тек қара ойыншы жар сегрегация байланысты майор же алма ойыншы көп бол әлі де керемет бірақ mlb интеграция дейін жазба есеп алынба,racism

1,мен техастағы қарғыс атқан анаға баруым керек өйткені надан қара көзді ұзын футболка киген мылқау мексикалықтар мен ақ нәсілшілдерден басқа ештеңе жоқ,мен техас қарғыс ат ана бар керек өйткені надан қара көз ұзын футболка ки мылқа мексикалық мен ақ нәсілшіл басқа ештеңе жоқ,racism

1,нәсілшіл мәлімдемелерді кез келген нәрсе деп атауға болады ақ артықшылық нәсілшіл сөйлеу қара жексенбі мұздатылған қара кофе біз оған шынымен барғымыз келе ме мобайлдағы негр көлі егер біз қаласақ біз шынымен ренжітуіміз мүмкін рэп музыкасы мені қорлайды,нәсілшіл мәлімдеме кез кел нәрсе де ата бол ақ артықшылық нәсілшіл сөйлеу қара жексенбі мұздатыл қара кофе біз оған шын бар келе ме мобайл нег көл егер біз қала біз шын ренжіт мүмкін рэ музыка мен қорла,racism

1,лол мен барлық ақымақ ниггерлердің рухын шығарамын бірақ олар кез келген уақытта менің әтешімде бола ма лол,лол мен барлық ақы ниггер рух шығар бірақ олар кез кел уақыт менің әтеш бола ма лол,racism

1,хахаха сондықтан мен бүгін кешке ниггерді өлтіре жаздадым сен ақымақсың,хахах сондықтан мен бүгін кеш нигг өлтір жазда сен ақы,racism

1,сік рико мен джейд мылқау ниггерлер жарайды,сік рико мен джейд мылқа ниггер жарайды,racism

1,мені тыңдау үшін қараңғы болуым керек сияқты мен түрлі түсті әйелдерге олардың жеткілікті түрде боялмағанын және шаштары қыздар сияқты тым ақ болып көрінетінін айтуым керек сондықтан олар шынымен түсіне алмайды бұл күлкілі және күшейткіш неліктен нәсілшілдік әлі де жалғасуда сіз үлкен құрбан болуыңыз керек,мен тыңдау үшін қараңғы бол керек сияқты мен түрлі түсті әйел олардың жеткілікті түр боялма және шаш қыз сияқты тым ақ бол көрін айт керек сондықтан олар шын түс алма бұл күлкіл және күшейткіш неліктен нәсілш әлі де жалғас сіз үлкен құрбан бол керек,racism

1,неге сонша ашуланасың деп аталды бірақ сіз оның ешқайда кетпейтін пайдасыз нигга екенін айттыңыз мао,неге сонша ашулан де ата бірақ сіз оның ешқай кетпе пай нигг екен ай мао,racism

1,жоқ тек адал раш паркинсон ауруы бар адамды мазақ етті барак сиқырлы негр деп аталатын радиохабар жүргізді челси клинтонды жасында ақ үйдің иті деп атады және

өзі әлемдегі жаяу қатерлі ісік мен бір секундқа ол өлсе маған бәрібір деп ойламаймын дастархан жолы,жоқ тек адал раш паркинсон ауру бар адам мазақ ет барак сиқырл нег де атал радиохабар жүр челси клинто жас ақ үй ит де ат және өзі әлем жаяу қатерлі ісік мен бір секунд ол өл маған бәрібір де ойлама дастархан жол,racism

1,rt мен ақымақ сияқты әрекет ететін ниггерлерді жек көремін,rt мен ақы сияқты әрекет ет ниггер жек көр,racism

1,немесе жыл бұрын эми сискинд өзінің республикалық өткеніне күмән келтірген және оларды ізбасарларының қудалауына ұшыратқан екі қара журналистпен онлайн қақтығысқа түскенде ecm бірден қара әйелдерді ештеңе зерттемей немесе тыңдамай эмиді қорғауға келді сақ болыңыз өте,немесе жыл бұрын эми сискинд өзінің республикалық өт күмән келтір және ол ізбасар қудала ұшырат екі қара журналист онлайн қақтығыс түс ecm бірден қара әйел ештеңе зерттеме немесе тыңдама эми қорға кел сақ бол өте,racism

1,хаха сен бардың ақымақ нигга міне балалар өздеріне сенімді болу үшін не аласыз,хах сен бар ақы нигг міне бала өз сенімді болу үшін не ал,racism

1,сіз кушитке ұқсамайсыз ба түрлі түсті нәсіл мен уа израиль халқы ақ нәсіл де лорд жариялайды киелі кітап ам мен нәсілшілдікке кастаға қарсымын,сіз кушит ұқсама ба түрлі түсті нәсіл мен уа израиль халқ ақ нәсіл де лорд жарияла киелі кітап ам мен нәсілшілдік каста қар,racism

1,менде бар сіз мені бірінші болып ұзын деп атадыңыз маған бұл жерде ұнайды,менде бар сіз мен бірінші бол ұзын де ата маған бұл же ұна,racism

1,нәсілшілдік алдымен түсті киімді жуу үшін ақ киімді таңдау нәсілшілдікке жоқ деп айтыңыз барлық ні жуыңызгеда,нәсілш алд түсті ки жу үшін ақ ки таңдау нәсілшілдік жоқ де ай барлық ні жуыңызге,racism

1,rt егер тим тебоу қара болса сіз оған жексұрын барбекю тауығына ерін негрі сияқты секірер едіңіз,rt егер тим тебо қара бол сіз оған жексұр барбекю тауығ ер нег сияқты секір ед,racism

1,нәсілшілдік бұл ақ киімді таңдап қара түсті киімнің алдында жуу нәсілшілдікке жоқ деп айтыңыз оларды бірге жуыңыз,нәсілш бұл ақ ки таңда қара түсті ки алд жу нәсілшілдік жоқ де ай ол бірге жу,racism

1,азық түлік алу үшін далаға шыққаннан кейін мені әлі де карантинге жібергенім үшін алғыс айтамын бұл ақ адам жаңа ғана қара әйелдер тобын ниггерлер деп атады өйткені олармен бетпе бет келді мені мәңгілікке карантинге жатқызуға қарсы болмас едім өйткені адамдар жиіркенішті,азық түлік алу үшін дала шық кейін мен әлі де карантин жібер үшін алғыс ай бұл ақ адам жаңа ғана қара әйел тоб ниггер де ат өйткені олармен бетп бет кел мен мәңгілік карантин жатқыз қарсы болмас ед өйткені адам жиіркеніш,racism

1,лол бұл қыз black lives matter тен шабыттандыратын мемдер мен дәйексөздерді жариялайды бірақ сіздің ақ досыңыз бізді дәрісте негрлер деп атаған кезде сіздің құлағыңыз жұмыс істемей қалды ал сағыз жабық күйінде қалды км фут,лол бұл қыз black lives matter тен шабыттандыр мем мен дәйексөз жарияла бірақ сіздің ақ дос біз дәріс негр де ата кезде сіздің құла жұмыс істеме қал ал са жабық күй қал км фут,racism

1,ниггер аль эфирден шығып үңгіріңізге оралыңыз сіз жай ғана ақымақсыз бұл ниггер робинсон сияқты мылқау,нигг аль эфир шығ үңгі орал сіз жай ғана ақы бұл нигг робинсон сияқты мылқа,racism

1,біз өткен миды жуған ал қазіргі миға әсер еткен қоғамда өмір сүріп жатырмыз мен қара емеспін бірақ мен әлемді түрлі түсті адамдарға әртүрлі сенімдері бар адамдарға және әртүрлі елдерге теңестіру үшін бәрін жасаймын мен сіз үшін өз өмірімді құрбан етер едім нәсілшілдерге тозақ,біз өткен ми жу ал қазіргі ми әсер ет қоғам өмір сүр жатыр мен қара емес бірақ мен әлем түрлі түсті адам әртүрлі сенім бар адам және әртүрлі ел теңестір үшін бәрін жаса мен сіз үшін өз өмір құрбан ет ед нәсілшіл тозақ,racism

1,оны алыңыз сіз ниггерлер циммерман кінәлі емес иә қаншық трейвон ақымақ бас нигга уау,оны ал сіз ниггер циммерман кінәлі емес иә қаншық трейвон ақы бас нигг уау,racism

1,кейбір нәсілшіл адам жаңа ғана қоңырау шалып мылқау нигга технологтары туралы айтты мен ух мен қарамын деді ол міне ақымақ деді де телефонды қойды лол,кейбір нәсілшіл адам жаңа ғана қоңырау шал мылқа нигг технолог туралы ай мен ух мен қар де ол міне ақы де де телефон қой лол,racism

1,желкеккеді жабыңыз мылқау ниггерлер omfg ниалдың твиттеріне жауап беруді тоқтатыңыз,желкекк жаб мылқа ниггер omfg ниа твиттер жауап беру тоқта,racism

1,ниггер сенен ешкім жалтарған жоқ сіздің щектеріңіз сіздің өмірбаяныңыз мен сен неше жастасың,нигг сенен ешкім жалтар жоқ сіздің щек сіздің өмірбаян мен сен неше жас,racism

1,қарғыс атсын адам осы сыныптағы барлық ниггерлер ақымақ сияқты олар маған әрдайым сұрақтар қояды ал уебище сияқты,қарғыс ат адам осы сынып барлық ниггер ақы сияқты олар маған әрдайым сұрақ қоя ал уебищ сияқты,racism

1,коул кем дегенде бір жыл бойы ойында болды мен кей дотты жақсы көремін бірақ ол шамамен жыл бойы ештеңе шығарған жоқ және ешкім маған оның қара пантераға қосқан үлесі коулдың өткен жылы шығарған боқтарынан жақсы екенін айтпайды факт,коул кем де бір жыл бойы ой бол мен кей до жақсы көр бірақ ол шамамен жыл бойы ештеңе шығар жоқ және ешкім маған оның қара пантера қос үлес коу өткен жыл шығар боқ жақсы екен айтпа факт,racism

1,пышақ мылқау нигга,пышақ мылқа нигг,racism

1,хахаха олар тозақ сияқты зұлым ял gt rt обамаға қарғыс атсын мылқау нигга,хахах олар тозақ сияқты зұлым ял gt rt обама қарғыс ат мылқа нигг,racism

1,rt америкадағы нәсілшілдік айқын да жасырын да олар түрлі түсті адамдарды нигга деп атай алмайды,rt америка нәсілш айқын да жасырын да олар түрлі түсті адам нигг де ата алма,racism

1,тағы бір ақымақ қаншық оксиклин сататын нигга гностикалық ақымақтан құлады мен өмір сүріп тәжірибемен дем аламын бақсыларды өртеу,тағы бір ақы қаншық оксиклин сат нигг гностикалық ақымақ құл мен өмір сүр тәжірибе дем ал бақсы өрте,racism

1,неліктен бұл кері нәсілшілдік пен қудалау сонымен егер түрлі түсті адам обаманың көйлегімен ирландиялық пабқа кірсе оны қуу керек пе айтайын дегенім бұл орын алатынына сенімдімін бірақ бұл екі бағытта да дұрыс емес егер сіз трампты қолдасаңыз сіз нәсілшіл болып саналасыз бұл дұрыс емес,неліктен бұл кері нәсілш пен қудала сонымен егер түрлі түсті адам обама көйлег ирландиялық паб кір оны қу керек пе айт де бұл орын ал сенімді бірақ бұл екі бағытта да дұрыс емес егер сіз трам қолда сіз нәсілшіл бол санал бұл дұрыс емес,racism

1,мм оған айтыңызшы мылқау ниггер болмаңыз бұл жарыс үшін кешіріңіз мен олармен араласпаймын,мм оған айтыңызш мылқа нигг болма бұл жарыс үшін кешір мен олармен араласпа,racism

1,болды қандай туыс болса да сіз қарғыс атқыр адамсыз мылқау нигга,бол қандай туыс бол да сіз қарғыс атқы адам мылқа нигг,racism

1,менің ойымша қаншық мааад rt обамаға қарғыс атсын ақымақ нигга саған кім зиян тигізгенін айт мен білуім керек лмао,менің ойымша қаншық мааад rt обама қарғыс ат ақы нигг саған кім зиян тигіз айт мен біл керек лмао,racism

1,сен ақымақсың аф бұл адамдар нағыз ниггерлер сіз жалаушаны әсіресе американдық туды өртемейсіз желкекге барыңыз,сен ақы аф бұл адам нағыз ниггер сіз жалауша әсіресе американдық ту өртеме желкек бар,racism

1,twitter дегі шлюхи қаншықтар ниггерлер пездтер және ақымақ ебарилер,twitter дег шлюхи қаншық ниггер пезд және ақы ебари,racism

1,бұл нигга tumblr дегі суреттер арқылы аралас сигналдар берумен айналысады тозаққа тамблер ол мылқау аф,бұл нигг tumblr дег сурет арқылы аралас сигнал бер айналыс тозақ тамб ол мылқа аф,racism

1,сіз өміріңізде кем дегенде бір рет черномазогодан сорғаныңызға сенімдімін rt обамаға қарғыс атсын мылқау нигга,сіз өмір кем де бір рет черномазого сор сенімді rt обама қарғыс ат мылқа нигг,racism

1,ол жоқ деп айтқысы келді ақымақ бейбақ ол нигга деп айтпады олардың әртүрлі мағыналары бар,ол жоқ де айт кел ақы бей ол нигг де айтпа олардың әртүрлі мағына бар,racism

1,менің ойымша сіз бетіңізге ашуланасыз мен де ашуланар едім сен теңіз арыстанының баласың обамаға қарғыс атсын мылқау нигга,менің ойымша сіз бет ашулан мен де ашулан ед сен теңіз арыстан бал обама қарғыс ат мылқа нигг,racism

1,мылқау құмды нигга,мылқа құм нигг,racism

1,біраз уақыт бұрын ол видеода ниггерді күлкілі деп атады менің қолданушы атыма қарамастан оның шығармашылығының жас бұрынғы қара жанкүйері ретінде бұл жиіркенішті,біраз уақыт бұрын ол видео нигг күлкіл де ат менің қолданушы ат қарамастан оның шығармашылығ жас бұрынғы қара жанкүйер рет бұл жиіркеніш,racism

1,менің ескертулерім мылқау нигга нигга,менің ескерту мылқа нигг нигг,racism

1,rt обамаға қарғыс атсын мылқау нигга,rt обама қарғыс ат мылқа нигг,racism

1,сіз динозаврларды ұнатпайтын бірінші кездескен адамсыз құдайым,сіз динозавр ұнатпа бірінші кездес адам құдай,racism

1,espnnfl майкл браунға қарғыс атсын мылқау нигга несие алды,espnnfl майкл браун қарғыс ат мылқа нигг несие алды,racism

1,қандай мақаламен мен болуым керек қандай да бір мылқау негр блогына құмар болмаңыз,қандай мақала мен бол керек қандай да бір мылқа нег блог құм болма,racism

1,негрлердің қоңыр шорттары amp нәсілшілдік әлі де тірі және керемет бізге тағы бір дәлел керек сияқты,негр қоңыр шорт amp нәсілш әлі де тірі және керемет бізге тағы бір дәлел керек сияқты,racism

1,rt мыжылған тері сіз қандай желкек жасайсыз мен осында боламын мылқау нигга lt бұл жігіт немесе шик бұл қарапайым waymanflow,rt мыжыл тері сіз қандай желкек жаса мен осында бол мылқа нигг lt бұл жігіт немесе шик бұл қарапайым waymanflow,racism

1,надан нигга тағы не белгілі кішкене гуманизмді көрсетіңіз ақымақ бейбақ,надан нигг тағы не белгілі кішкен гуманизм көрсе ақы бей,racism

1,о міне біз сіз шақырылатын уақыт келгенде ақ адамдарға ұқсағысы келетін кран биін билейтін негрлердің бірісіз сіз спортпен айналысасыз деп естідім біз мұны түсіндіргенімізге қуаныштымын мен әрі қарай жүруге қарсы емеспін сіз қазірдің өзінде адасып кеттіңіз сіздің денсаулығыңыз,о міне біз сіз шақырыл уақыт кел ақ адам ұқса кел кран би биле негр бір сіз спорт айналыс де ест біз мұны түсіндір қуанышты мен әрі қарай жүр қарсы емес сіз қаз өз адас кет сіздің денсаулығ,racism

1,қандай желкек сіз мылқаусыз rt күте тұрыңыз дже бәрі жақсы мен оған нигга картасын бердім сондықтан ол жақсы,қандай желкек сіз мылқау rt күт тұр дж бәрі жақсы мен оған нигг карта бер сондықтан ол жақсы,racism

1,ниггер мені жолда сынап көрді ақжелкенге барыңыз ақымақ нигга лол,нигг мен жолда сына көр ақжел бар ақы нигг лол,racism

1,сіздің лесбияндық көрінетін лесбиянкаңызды сік сік қаншықты жопу көтке нигга қаншықты сік қаншықты сүйетін нигга сучу киску доғал қарғысатқыр маңдай сучу көтеннен кетмен,сіздің лесбия көрін лесбиянка сік сік қаншық жоп көт нигг қаншық сік қаншық сүй нигг суч киск доғал қарғысатқы маңда суч көтен кет,racism

1,өзіңді өлтір ақымақ қаншық rt обамаға қарғыс атсын мылқау нигга,өз өлтір ақы қаншық rt обама қарғыс ат мылқа нигг,racism

1,сіз бардыңыз ақымақ қаншық жезөкше фагот мүшесі есек ақымақ ақымақ ниггердің сиськалары,сіз бар ақы қаншық жезөкше фагот мүше есек ақы ақы нигг сиська,racism

1,обамаға дауыс берген қара жігіт өзіне назар аударғысы келген шығар ignoreignorance rt обамаға қарғыс атсын мылқау нигга,обама дауыс бер қара жігіт өзіне назар аудар кел шығар ignoreignorance rt обама қарғыс ат мылқа нигг,racism

1,надан мылқау қара адамдар құлап кетуі мүмкін ниггерлер,надан мылқа қара адам құла кет мүмкін ниггер,racism

1,сіз менің пікірімді бәріне түсініктеме қалдыра аласыз сіздің теріс пікіріңіз қажет емес еді мен осылай тәрбиелендім мен негр деп аталуға лайық емеспін өйткені сіз қара адам ретінде ақ адам менталитетіне ие болғанды ұнатасыз жетілген,сіз менің пікір бәрін түсініктеме қалдыр ал сіздің теріс пікір қажет емес ед мен осылай тәрбиеле мен нег де атал лайық емес өйткені сіз қара адам рет ақ адам менталитет ие бол ұна жет,racism

1,бұл жиіркенішті бірақ мен шынымен қара негрге әуестендім бұл артық емес пе,бұл жиіркеніш бірақ мен шын қара нег әуестен бұл артық емес пе,racism

1,бұл әуе кеңістігінің проблемалары болды сіз сияқты қара көзді қаншықтар сізге ескерткен аймаққа әскер жіберіп жатыр және олар үшінші дүниежүзілік соғысты қаламайтынын айтты сондықтан америка үкіметінің ақш тағы мылқау ниггерлері қалай ойлайсыз ресей қызғылт сары сары ниггаға бәрібір емес пе жоқ,бұл әуе кеңістіг проблема бол сіз сияқты қара көз қаншық сізге ескерт аймақ әскер жібер жатыр және олар үшінші дүниежүзілік соғ қалама ай сондықтан америка үкім ақш тағы мылқа ниггер қалай ойла ресей қызғылт сары сары нигга бәрібір емес пе жоқ,racism

1,тіпті мылқау нигга да сені ұрмас еді,тіпті мылқа нигг да сен ұрмас ед,racism

1,мен мұны жаңа ғана анықтадым бұл құлдыққа байланысты көптеген атаулардан шыққан нигга шайн негр түрлі түсті азшылық құл иелерінің бізге берген есімдеріне

наразылық ретінде біз өзімізді қара деп атадық ақ оңтүстік африкалықтардан айырмашылықты көрсету үшін оңтүстік африкалықтар өздерін қандай есіммен атайды,мен мұны жаңа ғана анықта бұл құлдық байланысты көптеген атау шық нигг шайн нег түрлі түсті азшылық құл ие бізге бер есім наразылық рет біз өз қара де ата ақ оңтүстік африкалық айырмашылық көрсету үшін оңтүстік африкалық өз қандай ес ата,racism

1,сіз бардыңыз уэйн есімді мылқау ұсқынсыз нигга позитивті қоқыс жұмсақ есегі бар нигга мені мылқау ұсқынсыз нигга қызықтырмайды pic twitter com,сіз бар уэйн ес мылқа ұсқын нигг позитивті қоқыс жұм есег бар нигг мен мылқа ұсқын нигг қызықтырма pic twitter com,racism

1,иә мылқау ебар ниггердің сиськалары бар жезөкше ватноголовый болван маггинсті шайнап жатыр,иә мылқа еб нигг сиська бар жезөкше ватноголовы болван маггин шайна жатыр,racism

1,обамаға қарғыс атсын мылқау нигга менің ойымша надандық бұл бақыт,обама қарғыс ат мылқа нигг менің ойымша надан бұл бақыт,racism

1,сіз бәріңіз жиіркеніштісіз егер қара қара ниггерді атаса бұл нәсілшілдік емес бірақ ақ түс дәл осылай жасасын соғыс нәсілшіл бала кезімде маған нәсілшілдік кемсітушілік деп үйретті бірақ бүгін мен енді сенімді емеспін,сіз бәрі жиіркеніштегер қара қара нигг ата бұл нәсілш емес бірақ ақ түс дәл осылай жаса соғыс нәсілшіл бала кез маған нәсілш кемсітушілік де үйрет бірақ бүгін мен енді сенімді емес,racism

1,аманда палмер жылға жуық ниггер дейді оның қара жанкүйерлері оған қаншалықты жиіркеніштi екенін айтты ол кешірім сұраудың қажеті жоқ деп санайды бұл сіздің кампуста нигга әнін орындайтын бейне бұл сіздің мектебіңіз қолдайтын нәрсе ме,аман палм жыл жуық нигг де оның қара жанкүйер оған қаншалықты жиіркеніш екен ай ол кешір сұра қажет жоқ де сана бұл сіздің кампус нигг ән орында бейне бұл сіздің мектеб қолда нәрсе ме,racism

1,желкекке обама барды мылқау нигга қаншық мааддд аф ктфу,желкек оба бар мылқа нигг қаншық мааддд аф ктф,racism

1,менің ойымша джей зидің black album дан кейін жасағанының бәрі классикалық қара альбом классикалық емес өте жақсы альбом бірақ классикалық емес,менің ойымша дже зи black album дан кейін жаса бәрі классикалық қара альбо классикалық емес өте жақсы альбо бірақ классикалық емес,racism

1,мен мылқау ниггерлерді жек көремін олар сіздің жартыңызды мылқау бейбақтарды шынжырға қалдыруы керек еді,мен мылқа ниггер жек көр олар сіздің жарты мылқа бейбақ шынжы қалдыр керек ед,racism

1,мылқау нигга шайтан алғыр боқ,мылқа нигг шай алғы боқ,racism

1,оққағар мені күзетеді мысалы бұл мылқау нигга неге қарады құтырған иттер эм нигга мүмкіндігінше тезірек қорқынышты түрде көзін түсіреді әкем мен,оққағ мен күзет

мысал бұл мылқа нигг неге қар құтыр ит эм нигг мүмкіндігінше тезірек қорқыныш түр көз түсір әке мен,racism

1,бұл басқа нигга байланысын үзді мен оны итеруге тура келді ақымақ бұл ақымақтық мен онда сағат болдым,бұл басқа нигг байланыс үз мен оны итер тура кел ақы бұл ақымақтық мен онда сағат бол,racism

1,ата аналар ақымақ олар мектепке қайта оралуы керек және олар өмірдің неге соншалықты қатал екенін түсінеді қара ата аналарға тозақ олар ең нашар мылқау ниггерлер,ата ана ақы олар мектеп қайта орал керек және олар өмір неге соншалықты қатал екен түсін қара ата ана тозақ олар ең нашар мылқа ниггер,racism

1,мылқау ниггерлер,мылқа ниггер,racism

1,менің әкем үйге келіп сен ақымақ ниггерсің дейді мен қандай болдым мен еденде жатырмын мен не істедім ол барады трахать сені одан да көп біреу керек,менің әке үй кел сен ақы нигг де мен қандай бол мен еден жатыр мен не істе ол бар трахать сен одан да көп біреу керек,racism

1,елшілердің істері және ақ шайтанның теологтары деп аталатындардың айтқандары маңызды емес өйткені олар табиғи өтірікшілер олардың әкесі сияқты шайтан өйткені құдай ақ емес ал құдай халқы да ақ емес бірақ құдай сияқты қара терілі алтын негр,елші іс және ақ шайтан теолог де атал айт маңызды емес өйткені олар табиғи өтірікші олардың әке сияқты шай өйткені құдай ақ емес ал құдай халқ да ақ емес бірақ құдай сияқты қара теріл алтын нег,racism

1,қалай болғанда да сол түні ақ адам мені негр деп атады,қалай болғанда да сол түн ақ адам мен нег де ат,racism

1,желкеккеді қой мылқау нигга барып тауық пен жүзімді жей бер қаншық,желкекк қой мылқа нигг бар тауық пен жүз же бер қаншық,racism

1,нәсілшілдік неліктен адамдар осылай ойлайды егер сіз оларды ұнатпасаңыз немесе олар туралы айтсаңыз сіз нәсілшілсіз бірақ егер әр түрлі түсті адамдар тек бір типте қалса бұл өз түрін сақтау немесе қолдау бірақ егер белые ақ түсте қалса олар бәрібір нәсілшіл blk қолдауымен blk,нәсілш неліктен адам осылай ойла егер сіз ол ұнатпа немесе олар туралы айт сіз нәсілшіл бірақ егер әр түрлі түсті адам тек бір тип қал бұл өз түр сақтау немесе қолдау бірақ егер белы ақ түс қал олар бәрібір нәсілшіл blk қолдау blk,racism

1,лмфао оларды бауырым осы қара жігітті ұрыңыз rt мылқау ниггерлер,лмфао ол бауыр осы қара жігіт ұр rt мылқа ниггер,racism

1,неліктен мен қара адам ретінде бұрынғы ақ американдықтарға кез келген нәрсе үшін алғыс айтуым керек қара адамдар бұл елге ештеңе қарыз емес сияқты және бұл ел оларға бәріне қарыздар,неліктен мен қара адам рет бұрынғы ақ американдық кез кел нәрсе үшін алғыс айт керек қара адам бұл ел ештеңе қарыз емес сияқты және бұл ел оларға бәрін қарыз,racism

1,қазіргі кездегі надан адамдар rt обамаға қарғыс атсын мылқау нигга,қазіргі кез надан адам rt обама қарғыс ат мылқа нигг,racism

1,классикалық енот поу поу сияқты иә мен сонда бардым құрметті адамның айтқанына ұқсас сәттер көңілді отбасылық естеліктер,классикалық енот по по сияқты иә мен сонда бар құрметті адам айт ұқсас сәт көңіл отбасылық естелік,racism

1,о кешіріңіз мен бұл сізбен сөйлесу емес сөйлесу деп ойладым надан нигга бұл жігіт ақымақ сияқты,о кешір мен бұл сізбен сөйлесу емес сөйлесу де ойла надан нигг бұл жігіт ақы сияқты,racism

1,senmer news wire ақш армиясындағы нәсілшілдіктің дұшпандық көріністеріне қарсы тұру үшін түрлі түсті лейтенанттың қызу әрекеттері туралы роман in the s army,senmer news wire ақш армия нәсілшілдік дұшпан көрініс қарсы тұру үшін түрлі түсті лейтенант қызу әрекет туралы роман in the s army,racism

1,ал сенің айтқаның өте ақымақ болып көрінді әйел испан тілінде сөйлейтін әйелді қорғағаны үшін wal mart та негр әйел деп аталды мектепте болыңыз,ал сенің айт өте ақы бол көр әйел испан тіл сөйле әйел қорға үшін wal mart та нег әйел де ата мектеп бол,racism

1,неліктен сіз осындай ақылды бірақ мылқау дұшпансыз мен никкас қаншығымен айналысамын бірақ мен ақымақ зотқа айналамын,неліктен сіз осындай ақылды бірақ мылқа дұшпан мен никкас қаншығ айналыс бірақ мен ақы зот айнал,racism

1,есептік жазбаңызды қарап отырып мен бақытсыз жалғыз емес екенімді түсіндім им мен алдымен зерттеуімді жүргізуім керек еді,есептік жазба қара отыр мен бақыт жалғыз емес екен түс им мен алд зерттеу жүргіз керек ед,racism

1,rt хахаха сіз желкеккеге бардыңыз сиэтлден келген мылқау ниггерлер сіз қышқыл жеңілгендерсіз бұл қажет емес,rt хахах сіз желкекке бар сиэтл кел мылқа ниггер сіз қышқыл жеңілгенд бұл қажет емес,racism

1,хаха ол өзінің президенті блэкті ашуландырды rt обамаға қарғыс атсын мылқау нигга,хах ол өзінің президент блэк ашулан rt обама қарғыс ат мылқа нигг,racism

1,өйткені сіз оған ақымақ ниггаға бардыңыз есіңізде жоқ па лол дедіңіз,өйткені сіз оған ақы нигга бар ес жоқ па лол де,racism

1,дизайнерлерге қарғыс атсын адам сен ақымақсың егер сіз тек брендтер үшін ақымақ сатып алсаңыз есегі бар ақымақ нигга,дизайнер қарғыс ат адам сен ақы егер сіз тек бренд үшін ақы сат ал есег бар ақы нигг,racism

1,мен xbox мен мылқау ниггермін ол әпкесін ұрады мен миллиардтаған долларлық өніммін мен өзімді түзете алмаймын мен пиздымын,мен xbox мен мылқа нигг ол әпке ұр мен миллиардта долларлық өнім мен өз түзе алма мен пизд,racism

1,жексенбі күні түстен кейін екі ақ қызбен бірге қара бизнеске кіріп менің нигга достарымнан ең жиіркенішті көріністер алды бұл жезөкше лар шиеленісті

сезінді,жексенбі күн түс кейін екі ақ қыз бірге қара бизнес кір менің нигг дос ең жиркеніш көрініс алды бұл жезөкше ла шиелен сез,racism

1,rt thegoodsin нәсілшілдікпен аяқталды өйткені бізде қара президент бар бірақ аберкромби қара түсті шалбар тігеді yay,rt thegoodsin нәсілшілдік аяқта өйткені біз қара президент бар бірақ аберкромби қара түсті шалбар тіг yay,racism

1,бұл ақымақтық өйткені барлық ақ адамдар бізді ниггерлер деп санайды және сіздің теріңіз қараңғы немесе ақшыл сіз ниггерсіз,бұл ақымақтық өйткені барлық ақ адам біз ниггер де сана және сіздің тер қараңғы немесе ақшыл сіз нигг,racism

1,досым мен ниггердің әтеші кездесуге тосқауыл қойғанын жек көремін мен бүгін кешке трахает жасауға тырысамын,дос мен нигг әтеш кездес тосқауыл қой жек көр мен бүгін кеш трахает жаса тырыс,racism

1,мұны жасқа толмаған кім жасайды ниггер ешкім өйткені олардың бәрі өте орташа құдай оны қоршап тұрған ниггерлер шынымен де мылқау және мылқау бірде бір концерт сатылып кеткен жоқ және диаграммада бірінші орынға шыққан жоқ бірақ ол солай етеді pic twitter com,мұны жас толма кім жаса нигг ешкім өйткені олардың бәрі өте орташа құдай оны қорша тұр ниггер шын де мылқа және мылқа бірде бір концерт сатыл кет жоқ және диаграмма бірінші орын шық жоқ бірақ ол солай ет pic twitter com,racism

1,жарайды сондықтан мен антисемитизмді ұмытуым керек ұйым жетекшісінің ақ адамдарға деген жеккөрушілігін ұмытуым керек мен олардың blm наразылықтары кезінде басқа қара адамдар өлтірген балаларды қорлайтынын ұмытуым керек басқа нәрсе,жарайды сондықтан мен антисемитиз ұмыт керек ұйым жетекші ақ адам де жеккөрушіліг ұмыт керек мен олардың blm наразылық кез басқа қара адам өлтір бала қорла ұмыт керек басқа нәрсе,racism

1,қарғыс атсын мұның бәрі ақымақ мен ешқашан ниггердің ернін менің әтешіме тигізуіне жол бермедім,қарғыс ат мұның бәрі ақы мен ешқашан нигг ерн менің әтеш тигіз жол берме,racism

1,уэйн есімді қызыл және ақ ақымақ ұсқынсыз ниггердің сіклері позитивті қоқыс жұмсақ денелі нигга және крис браунның ұсқынсыз есегі pic twitter com,уэйн ес қызыл және ақ ақы ұсқын нигг сік позитивті қоқыс жұм денел нигг және крис браун ұсқын есег pic twitter com,racism

1,бірінші әсер өте әдемі мен сені tiktok sksks тен жақсы көремін сіздің лақап атыңыз менің басымда ники тек ники sksks жақындық рейтингі үміттенемін сен маған ұнайсың ба иә сен менікісің менің сүйікті сұрақтарымның бірі шынымды айтсам мен сен туралы көбірек білгім келеді сіз де оны жариялауыңыз керек пе иә,бірінші әсер өте әдемі мен сен tiktok sksks тен жақсы көр сіздің лақа ат менің бас ники тек ники sksks жақ рейтинг үміттен сен маған ұна ба иә сен менік менің сүйікті сұрақ бірі шын айт мен сен туралы көбірек біл кел сіз де оны жария ла керек пе иә,racism

1,ешкім есінен танған жоқ бір қызығы мұндағы ақ әйелдер аралас балалары болуы үшін қара нәсілділерді өсіруге тырысады бірақ олар нигга деп жүреді,ешкім ес тан жоқ бір қызығ мұндағы ақ әйел аралас бала бол үшін қара нәсілді өсір тырыс бірақ олар нигг де жүр,racism

1,неге жоқ сіздің ойыңызша сізге ешкім шабуыл жасамайды мен сіздің тобығыңызды бірінші болып тістеймін егер бұл зейн болмаса зейн екеуміз сені сүйреп апара алмаймыз,неге жоқ сіздің ойыңызша сізге ешкім шабуыл жасама мен сіздің тобығ бірінші бол тісте егер бұл зейн болма зейн екеу сен сүйре апар алма,racism

1,сіз бардыңыз ұсқынсыз қара қоқыс ниггердің аты дуэйн майкл картер ұсқынсыз мылқау қоқыс менің мысығымнан алыс менің бет әлпетімнен pic twitter com,сіз бар ұсқын қара қоқыс нигг ат дуэйн майкл кар ұсқын мылқа қоқыс менің мысығ алыс менің бет әлпет pic twitter com,racism

1,бірінші әсер күлкілі гуджу сіздің лақап атыңыз менің басымда харшу сен маған ұнайсың ба иә адам сен менің сүйікті жігіттерімнің бірісің мен саған ғашық болдым жоқ сен маған қандай түсті еске түсіресің пастелді көк,бірінші әсер күлкіл гудж сіздің лақа ат менің бас харш сен маған ұна ба иә адам сен менің сүйікті жігіт бір мен саған ғашық бол жоқ сен маған қандай түсті ес түсір пасте көк,racism

1,өлі сен желіммен боялған ақылды түйінсің бе rt жоқ сен нигга деп айта аласың мен айта алмаймын ба мұның өзі нәсілшілдік,өлі сен жел боял ақылды түйін бе rt жоқ сен нигг де ай ал мен ай алма ба мұның өзі нәсілш,racism

1,құдайым бұл сөз өте мылқау және сізді мылқау нигга сияқты етеді rt мен сенен еститін сөздер өлген есек,құдай бұл сөз өте мылқа және сіз мылқа нигг сияқты ет rt мен сенен естит сөз өлген есек,racism

1,мен сені өзіңнен басқалардың бәріне мән бермейтін ақымақ нигга деп санаймын таха,мен сен өз басқа бәрін мән берме ақы нигг де сана тах,racism

1,сіз жынды болдыңыз ба lmao lmao rt желкекке обама мылқау нигга pic twitter com,сіз жын бол ба lmao lmao rt желкек оба мылқа нигг pic twitter com,racism

1,сік қызыл ақ доғал ұсқынсыз нигга атты уэйн позитивті қоқыс нигга жұмсақ задницей pic twitter com,сік қызыл ақ доғал ұсқын нигг ат уэйн позитивті қоқыс нигг жұм задница pic twitter com,racism

1,мен нағыз нәсілшілдікке мән беремін мен нәсілшілдік пен нәсілшілдікке итермелейтіндерді жек көремін олар өзімшілдік мақсаттары үшін әңгімелер таңдайды қара адаммен болған әрбір кішкентай оқиғаны нәсілшіл деп атау күлкілі және мұны істеу арқылы сіз өзіңізге достар таба алмайсыз,мен нағыз нәсілшілдік мән бер мен нәсілш пен нәсілшілдік итермеле жек көр олар өзімш мақсат үшін әңгіме таңда қара адам бол әрбір кішкентай оқи нәсілшіл де атау күлкіл және мұны істеу арқылы сіз өз дос таб алма,racism

1,телефонмен сөйлесіңіз плззз мені мазақ етеді желкекге барыңыз лас жезөкше ның ақымақ есегі аузыңызды жабыңыз ешқашан менің досымды ниггер деп атамаңыз,телефон сөйлес плззз мен мазақ ет желкек бар лас жезөкше ны ақы есег ауз жаб ешқашан менің дос нигг де атама,racism

1,осы аптада сізді тек нәсілшілдік қызықтырады есіңізде болсын сізде лерамен болған оқиғадан кейін жұлдыздарға қарсы чемпиондар фильмінде камила құбыжығы болды сіз оны жұмыстан шығарып қара қызметкерді ниггер деп атағаннан кейін және оның қолын көтергеннен кейін ғана тыйым салдыңыз,осы апта сіз тек нәсілш қызықтыр ес бол сіз лера бол оқиға кейін жұлдыз қарсы чемпион фильм камила құбыжығ бол сіз оны жұмыс шығар қара қызметк нигг де ата кейін және оның қол көтер кейін ғана тыйым сал,racism

1,неліктен ол ессіз бұғы менің президентім қара мұны қабылдаңыз rt бұл ақымақ жынды хаа обамаға қарғыс атсын мылқау нигга,неліктен ол ес бұғ менің президент қара мұны қабылда rt бұл ақы жын ха обама қарғыс ат мылқа нигг,racism

1,ақымақ болуды доғарыңыз және кемелдеңіз қарғыс атсын сіз қара мазогтың кішкентай надан бөлігі,ақы болу доғар және кемелден қарғыс ат сіз қара мазог кішкентай надан бөліг,racism

1,мен сенің қарғысатқыр отбасыңды өлтіремін сен ебаный аутист нигга қатерлі ісікке шалдығыңыз сіз ақымақ жезөкше ақымақ,мен сенің қарғысатқы отбас өлтір сен ебаны аутист нигг қатерлі ісік шалдығ сіз ақы жезөкше ақы,racism

1,мен каманың твиттерінде бірінші болып жауап берген жоқпын сіз не туралы сөйлесіп жатқаныңызды да түсінбейтініңіз анық,мен кама твиттер бірінші бол жауап бер жоқ сіз не туралы сөйлес жат да түсінбе анық,racism

1,ақ киіміңізді таңдағанға дейін нәсілшілдік тоқтамайды ші ші қара және түрлі түсті ші жуыңыз,ақ киім таңда дейін нәсілш тоқтама ші ші қара және түрлі түсті ші жу,racism

1,rt обамаға қарғыс атсын мылқау нигга бұл өте маңызды ма сіз ашуланасыз ба,rt обама қарғыс ат мылқа нигг бұл өте маңызды ма сіз ашулан ба,racism

1,зейн бірінші емес олар әрқайсысымен жалғыз қалжыңдайды ма жоқ па олар бір бірін ешқашан білмегендей егер сіз бауырларыңызбен жақсы қарым қатынаста болсаңыз мысал келтіре аласыз,зейн бірінші емес олар әрқайсы жалғыз қалжыңда ма жоқ па олар бір бір ешқашан білмегенде егер сіз бауыр жақсы қарым қатынас бол мысал келтір ал,racism

1,жеке анекдоттарды бөліп көрсету өте оңай кез келген нәсіл мұны істей алады және нәсілшілдік заңдастырылған сияқты көрінуі мүмкін біртүрлі оқиға кеңірек адамгершілікті білдірмейді полиция өз өзіне қол жұмсау немесе басқа жолмен байланысты кез келген қылмысты қашан зерттей бастады бұл соттардың жұмысы,жеке анекдот бөл көрсету өте оңай кез кел нәсіл мұны істе ал және нәсілш заңдастырыл сияқты көрін мүмкін біртүрлі оқиға кеңірек адамгершілік білдірме

полиция өз өзіне қол жұмса немесе басқа жол байланысты кез кел қылмыс қашан зертте баста бұл сот жұмыс,racism

1,есіңізде ме сіз мылқау ниггерлер канье уэст тейлор свифттің мансабын жасады деп айтқан кезде анаңа бар,ес ме сіз мылқа ниггер кань уэст тейло свиф мансаб жас де айт кезде ана бар,racism

1,қара нәсілшілдікке қарсы оқиға бірақ полиция бұл оқиға жеккөрушілік қылмысы ретінде қарастырылмайтынын айтты күресті бастаңыз,қара нәсілшілдік қарсы оқиға бірақ полиция бұл оқиға жеккөрушілік қылмыс рет қарастырылма ай күре баста,racism

1,totp деп аталатын британдық музыкалық телешоу әр бейсенбі күні кешке бүкіл елдің балалары тамашалады raunchydance труппасы оларды спектакльдер пайда бола алмаған бейне болмаған кезде ауыстырды шілтерлі іш киімдегі жартылай жалаңаш ақ қыздар майланған негр еркектерімен қыдырады бұған жауапты еврей арлин филлипс болды,totp де атал британдық музыкалық телешо әр бейсенбі күн кеш бүкіл ел бала тамашала raunchydance труп ол спектакль пайда бола алма бейне болма кезде ауыс шілтерл іш киім жартылай жалаңаш ақ қыз майлан нег еркек қыдыр бұған жауапты еврей арлин филлипс бол,racism

1,rt мен jaws ті сынап көрдім,rt мен jaws ті сына көр,racism

1,қарғыс атқан қаншық сен тозақ сияқты ақымақсың сіз ақымақ сияқтысыз қарғысатқыр нигга мақта жинауға оралыңыз сіз құлсыз,қарғыс ат қаншық сен тозақ сияқты ақы сіз ақы сияқты қарғысатқы нигг мақта жина орал сіз құл,racism

1,әпкеңізге барыңыз мылқау нигга,әпке бар мылқа нигг,racism

1,сіздердің барлығыңыз ниггерлерді емес қара көзді жек көретіндерсіз жасқа толған және сүйіспеншілігін көрсетіп өлтірілген жас снуппен ешкім трахает жасамауы керек,сіздердің барлығ ниггер емес қара көз жек көретінд жас тол және сүйіспеншіліг көрсет өлтір жас снуп ешкім трахает жасама керек,racism

1,лол мен одан асып кеттім бірақ қайғылы өйткені алдында тұрған ханымдар зардап шегеді бірінші бұрынғы мен оған берген сән салтанатты ұрлады ал екіншісі мені екінші сиқыршы деп қателесті мен культистті мені кінәлауға жібергеннен кейін оған сергек көзқарасымды көрсеткенше сіздің жынысыңызда өйткені ол жақын арада аяқталады,лол мен одан ас кет бірақ қайғылы өйткені алд тұр ханым зардап шег бірінші бұрынғы мен оған бер сән салтанатты ұрл ал екінші мен екінші сиқырш де қателес мен культис мен кінәла жібер кейін оған сергек көзқарас көрсетkенш сіздің жыныс өйткені ол жақын ара аяқтал,racism

1,сіз тозақ сияқты ақымақсыз rt жалған тырнақтар маған ниггаларды еске түсіреді олар сондай ақ надан қара адамдар ewwwww рахмет lt,сіз тозақ сияқты ақы rt жалған тырнақ маған нигга ес түсір олар сондай ақ надан қара адам ewwwww рахмет lt,racism

1,мектепте болып жатқанның бәрі жақсы желкеккеді залдың ортасынан шығарыңыз мылқау ниггерлер,мектеп бол жат бәрі жақсы желкекк зал орта шығ мылқа ниггер,racism

1,иә сіздің барлық whiteracist жақтастарыңыз өте жаман түсті қара қара түсті ракон әлі де ақ үйде енді бұл нәсілшілдік иә,иә сіздің барлық whiteracist жақтас өте жаман түсті қара қара түсті ракон әлі де ақ үй енді бұл нәсілш иә,racism

1,қарғысатқыр нигга телефоны сік өзін мылқау кесек боқ ilovetony,қарғысатқы нигг телефон сік өз мылқа ке боқ ilovetony,racism

3,шетелдіктер қайда бізге ресей мен шетелдіктер бұл жаппай атыстарды жасап жатқанын айтты,шетелдік қайда бізге ресей мен шетелдік бұл жаппай атыс жаса жат ай,nazism

3,біз жақындарынан айырылғандардың барлығын заңсыз есірткіге немесе есірткіге қатысты қылмыстарға байланысты шетелдіктерді шығармашылық мағынаны табуға әдейі ынталандырудағы рөлі үшін ресми айыптауға шақырамыз,біз жақын айырыл барлығ заңсыз есірткі немесе есірткі қатысты қылмыс байланысты шетелдік шығармашылық мағына таб әдей ынталандыру рөл үшін ресми айыпта шақыр,nazism

3,ақтқ немесе жқтбмен жылдар бойы күрескеннен кейін оңтүстік африкалықтар енді жаңа вирустың толыққанды иммун тапшылығы амаквереквер синдромының қамқорлығында бірінші орынға оңтүстік африкалықтар,ақтқ немесе жқтб жыл бойы күрес кейін оңтүстік африкалық енді жаңа вирус толыққанды иммун тапшылығ амакверекв синдром қамқорлығ бірінші орын оңтүстік африкалық,nazism

3,біздің оңтүстік африкалық екенімізді айту шетелдіктер қылмыс жасайды дегенді білдірмейді сондықтан олар шекараны заңсыз кесіп өтті,біздің оңтүстік африкалық екен айту шетелдік қылмыс жаса де білдірме сондықтан олар шекара заңсыз кес өт,nazism

3,нхланхлаға өздерінің қылмыстық тенденцияларын ілгерілету үшін саясатта алшақтық тапқан ақталмаған бұрынғы тұтқындардың құрсауынан құтылу үшін гүлдер беріңіз,нхланхла өз қылмыстық тенденция ілгерілету үшін саясат алшақтық тап ақталма бұрынғы тұтқын құрсау құтыл үшін гүл бер,nazism

3,ақылсыз көшбасшыңызға хиллброуда заңсыз істер жасайтын заңсыз шетелдіктерді және б алып тастауды үйге мұқтаж адамдарымызды көшіруді айтыңыз,ақыл көшбасш хиллбро заңсыз істер жаса заңсыз шетелдік және б алып тастау үй мұқтаж адам көшіру ай,nazism

3,бұл рас шетелдіктер біздің елден кетуі керек,бұл рас шетелдік біздің ел кет керек,nazism

3,ешқашан күшіңізді жоғалтпаңыз бауырым біз бұл күресте сізбен біргеміз,ешқашан күш жоғалтпа бауыр біз бұл күрес сізбен бір,nazism

3,біздің балаларымыз қазірдің өзінде қалаларда қызметші болып табылады өйткені бұрын қалалардағы ақ нәсілділерге тиесілі барлық үйлер заң бойынша шетелдіктерге тиесілі олар ақ нәсілділерді үйлерінен шығарып қана қоймай куәліктер мен ішкі істер құжаттарын рәсімдеді,біздің бала қаз өз қала қызметші бол таб өйткені бұрын қала ақ нәсілді тиесілі барлық үй заң бойынша шетелдік тиесілі олар ақ нәсілді үй шығар қана қойма куәлік мен ішкі істер құжат рәсімде,nazism

3,дудула операциясы мен нхланхлалюкс жақтайтын барлық нәрселермен келіспеуім мүмкін бірақ мойындауым керек маған оны тыңдамау өте қиын оның сенімдері анық бірақ оның ксенофобиялық көңіл күйге толы жергілікті экономиканы қорғауымен мүлдем келіспеймін,дудул операция мен нхланхлалюкс жақта барлық нәрсе келіспе мүмкін бірақ мойында керек маған оны тыңдама өте қиын оның сенім анық бірақ оның ксенофобиялық көңіл күй толы жергілікті экономика қорғау мүлдем келіспе,nazism

3,менің айта алатыным біздің қауымдастықтардағы spazas біздің экономикамызды бұзады құжатсыз шетелдіктер жай ғана бұзады соуето қауымдастықтары ретінде бізге тек бірлік әділеттілік елді түзету үшін бірлесіп жұмыс істеу керек шетелдіктер біздің әлсіз жақтарымызды біледі және олар оны пайдаланады,менің ай ал біздің қауымдастық spazas біздің экономика бұз құжат шетелдік жай ғана бұз соуето қауымдастық рет бізге тек бірлік әділеттілік ел түзету үшін бірлес жұмыс істеу керек шетелдік біздің әлсіз жақ біл және олар оны пайдалан,nazism

3,ол бізді жалқау деп айта алмайды,ол біз жалқау де ай алма,nazism

3,фермерлер шетелдіктерді жалдауды тоқтатуы керек,фермер шетелдік жалдау тоқтат керек,nazism

3,достар нхланхлаға дамаск бар болғаны үшін және оны қоршап тұрғанын көргені үшін гүл сыйлаңдар бо ике хумало халқымыздың шынайы бірлігіне нақты қауіп төндіреді саясаттағы алшақтықты көрген ақталмаған бұрынғы тұтқын,дос нхланхла дамаск бар бол үшін және оны қорша тұр көр үшін гүл сыйла бо ик хумало халқ шынайы бірліг нақты қауіп төндір саясат алшақтық көр ақталма бұрынғы тұтқын,nazism

3,бірақ бұл дұрыс бұл құжаттары жоқ шетелдіктер туралы жалпыламайық бізде басқа елдерде орналасқан оңтүстік африкалықтар өте көп біз елге заңды түрде кірген кез келген адамды қабылдауымыз керек,бірақ бұл дұрыс бұл құжат жоқ шетелдік туралы жалпылама біз басқа ел орналас оңтүстік африкалық өте көп біз ел заңды түр кірген кез кел адам қабылда керек,nazism

3,тиктокта егде жастағы әйел шетелдіктерге жер беру туралы айтатын бейне бар менің ойымша оның аты хоса есімде жоқ мен бұл бейнені іздедім олар оны жойған сияқты ол жүріп жатқан атыстар туралы айтты ол деді,тикток егде жастағы әйел шетелдік жер беру туралы айт бейне бар менің ойымша оның ат хо ес жоқ мен бұл бейне ізде олар оны жой сияқты ол жүр жат атыс туралы ай ол де,nazism

3,әйелдерді жоғалтудың себебі зорлау емес бұл адам саудасы өкінішке орай бұл шетелдіктер арасында кең таралған сауда,әйел жоғалт себебі зорла емес бұл адам сауда өкініш орай бұл шетелдік ара кең тарал сауда,nazism

3,дудула операциясының кері әсері бар бұл жаппай атыстарды шетелден келген жергілікті тұрғындар ұйымдастырады және жүзеге асырады,дудул операция кері әсер бар бұл жаппай атыс шетел кел жергілікті тұрғын ұйымдастыр және жүзеге асыр,nazism

3,жоқ ол надан емес оған бәрібір,жоқ ол надан емес оған бәрібір,nazism

3,заң дұрыс емес және орындалған кезде білетіндер оны қолдауы немесе оған қарсы шығуы керек зимбабве өз азаматтарына мал сияқты қарайды,заң дұрыс емес және орындал кезде біл оны қолда немесе оған қарсы шығ керек зимбабв өз азамат мал сияқты қара,nazism

3,зимбабвенің тайпалық ойлауына байланысты,зимбабве тайпалық ойла байланысты,nazism

3,бұл оңтүстік африкалықтар емес олар біздің жалған немесе ұрланған құжаттарымыз бар заңсыз ақымақтар мүмкін зимбабвеліктер,бұл оңтүстік африкалық емес олар біздің жалған немесе ұрлан құжат бар заңсыз ақымақ мүмкін зимбабвелік,nazism

3,неге тек африкалықтар өйткені олар бұл елде құжатсыз ақ нәсілділер,неге тек африкалық өйткені олар бұл ел құжат ақ нәсілді,nazism

3,неге тек африкалықтар өйткені олар бұл елде құжатсыз ақ нәсілділер,неге тек африкалық өйткені олар бұл ел құжат ақ нәсілді,nazism

3,біз онсыз да жалғастырамыз отандастарымызды итерейік оңтүстік африкалықтар бірінші операция дудула,біз онсыз да жалғастыр отандас итер оңтүстік африкалық бірінші операция дудул,nazism

3,олар мұны істемейді жеке куәліктерін алған заңсыз иммигранттар,олар мұны істеме жеке куәлік ал заңсыз иммигрант,nazism

3,оңтүстік африкалықтар басқа елдерден бірдей ем қабылдағанда әрқашан кеш келеді,оңтүстік африкалық басқа ел бірде ем қабылда әрқашан кеш кел,nazism

3,операция дудуладан жоғары ешкім жоқ және біз культ емеспіз,операция дудула жоғары ешкім жоқ және біз культ емес,nazism

3,бірақ шетелдіктер біздің жұмыс берушіміз және олар бізді басқарады менің ойымды түсініңіз бе,бірақ шетелдік біздің жұмыс беруші және олар біз басқар менің ой түсін бе,nazism

3,көп адамдар мүмкіндікті сағынып жатыр себебі ол комбинезон киіп жұмыс жасайды,көп адам мүмкіндік сағын жатыр себебі ол комбинезон ки жұмыс жаса,nazism

3,үйленбеген жігітбіріккен көгілдір операциядудула сошангувхе,үйленбе жігітбірік көгілдір операциядудул сошангувх,nazism

3,бастапқы твиттерде sa да нұсқау беретін шетелдікке қатысты мәселе бар менің жауабым ақ адамдар нұсқаулар бергенде оның мәселесі жоқ,бастапқы твиттер sa да нұсқау бер шетелдік қатысты мәселе бар менің жауаб ақ адам нұсқау бергенде оның мәселе жоқ,nazism

3,шетелдіктердің ақымақтықтарына шыдаудан басқа амалы жоқ ал шетелдіктер шаршаған кезде оларды өлтіріп фермадағы қанішерлер жылайды,шетелдік ақымақтық шыдау басқа амал жоқ ал шетелдік шарша кезде ол өлтір ферма қанішер жыла,nazism

3,мен жылдан бері автомобиль фараларын қалпына келтіріп келемін дан астам клиент google ге кіріп маған берді бүгін таңертең мен ретвит үшін дұға етемін,мен жыл бері автомобиль фара қалп келтір кел дан астам клиент google ге кір маған бер бүгін таңертең мен ретвит үшін дұ ет,nazism

3,біртүрлі uber және pvt қауіпсіздігін арттыруды талап етеді eeeуі де заңсыз шетелдердің жұмысқа алуы ей құлыншақ біз хаос жасадық ол сізге және сіздің зимболарыңызға минутта жұмыс істемейді,біртүрлі uber және pvt қауіпсіздіг арттыру талап ет eee де заңсыз шетел жұмыс ал ей құлыншақ біз хаос жаса ол сізге және сіздің зимбо минут жұмыс істеме,nazism

3,сендер антиафрикандықтар енді бізді алдамайсыңдар сендер қылмыс жасайсыңдар адамдарды өлтіресіңдер және әлеуметтік желілерге жүгіріп тарвенс пен тауншиптерде адамдарды атып жатқан шетелдіктер деп күлкіге айналдырасыңдар нағыз қылмыскерлерді қорғау үшін ақы алу операциясы дудула өлтірушілері,сен антиафрикандық енді біз алдама сен қылмыс жаса адам өлтір және әлеуметтік желі жүгір тарвенс пен тауншип адам ат жат шетелдік де күлкі айналдыр нағыз қылмыскер қорғау үшін ақы алу операция дудул өлтіруші,nazism

3,сондықтан біз оны түзеп үйімізді ретке келтіруіміз керек шетелдіктер біздің жомарттығымызды кәдімгідей қабылдады және оның қазіргі уақыттағы үйі таза,сондықтан біз оны түзе үй рет келтір керек шетелдік біздің жомарттығ кәдімгіде қабылда және оның қазіргі уақыт үй таза,nazism

3,бірақ африкалықтар жұмыс орындарын қайтарады пайыз шетелдік мектеп мұғалімдері миллиондаған жұмыссыз африкалық мұғалімдерге берілетін жұмыстарды атқарды ешбір ел мұндай қоқысқа жол бермейді олар бұл елде біздің проблемаларымыздың пайызын қосады ол дұрыс емес,бірақ африкалық жұмыс орын қайтар пайыз шетелдік мектеп мұғалім миллиондаған жұмыссыз африкалық мұғалім беріл жұмыс атқар ешбір ел мұндай қоқыс жол берме олар бұл ел біздің проблема пайыз қос ол дұрыс емес,nazism

3,бұл жаппай атулар тайпалық соғыстар тура жылдардағыдай алыстан іздеуге тырыспайық нүктелерді біріктіріңіз біз оның шетелдіктері мара айи бұл тайпалық

нәрсе деп айтамыз,бұл жаппай ату тайпалық соғыс тура жылдардағыда алыс ізде тырыспа нүкте біріктір біз оның шетелдік мар айи бұл тайпалық нәрсе де ай,nazism

3,сенің әкеңнің жүрегі жоқ бауырым үкіметіңді адам құқығын бұзғаны үшін сотқа берші эй алаяқ үкіметіңе қараңдар,сенің әке жүрег жоқ бауыр үкімет адам құқығ бұз үшін сот берш эй алаяқ үкімет қара,nazism

3,үкімет заңсыз шетелдіктермен жұмыс істеуден бастайық өйткені олар оңтүстік африкада лицензиясыз атыс қаруын әкелді деген болжам бар,үкімет заңсыз шетелдік жұмыс істеу баста өйткені олар оңтүстік африка лицензия атыс қар әке де болжам бар,nazism

3,веле біз шекараларымызды жауып жатырмыз осылайша оңтүстік африкалық қылмыскерлер түрмеде шіріп мерзімінен бұрын босатылмауы мүмкін өйткені түрмелер пайыз толған шетелдіктер біздің қылмысты қосып жатыр,вел біз шекара жау жатыр осылайша оңтүстік африкалық қылмыскер түрме шір мерзім бұрын босатылма мүмкін өйткені түрме пайыз тол шетелдік біздің қылмыс қос жатыр,nazism

3,батханде бонке бейнелері қазір youtube те оны қарап шығыңыз,батхан бон бейне қазір youtube те оны қара шығ,nazism

3,бізді бұл көлік қуған кезде шетелдіктер бұл жерде болмаған қазір олар sa да заңсыз жүргені үшін үкіметімізді сотқа берумен әлек,біз бұл көлік қу кезде шетелдік бұл же болма қазір олар sa да заңсыз жүр үшін үкімет сот бер әлек,nazism

3,ол оңтүстік африкада дүние жүзіндегі қылмыс пен заңсыз шетелдіктер мен қашқындар мен лаңкестік топтар мен есірткі сатушылар мен кісі өлтірушілер мен зорлаушылар мен банк тонаушыларының астанасы,ол оңтүстік африка дүние жүз қылмыс пен заңсыз шетелдік мен қашқын мен лаңкестік топ мен есірткі сатушы мен кісі өлтіруші мен зорлаушы мен банк тонаушы астана,nazism

3,бұл жұмсақ өйткені олар хоса ұлдарын ұрып жатыр шетелдіктер сол клиптің соңында өліп қалар еді,бұл жұм өйткені олар хо ұл ұр жатыр шетелдік сол клип соң өл қал ед,nazism

3,мүмкін сіз не айтып жатқаныңызды білмейсіз сомали мен кениядан заңсыз шетелдіктер осы шекарада контрабандалық жолмен әкелінеді,мүмкін сіз не айт жат білме сомали мен кения заңсыз шетелдік осы шекара контрабандалық жол әкелін,nazism

3,өміріңізді қарапайым етіңіз бізбен жеткізілімдерді жеңілдетіңіз,өмір қарапайым ет бізбен жеткізілім жеңілде,nazism

3,оңтүстік африканы қою сыбайлас жемқорлық пен заңсыздықты тоқтату және заңсыз иммигранттарды тоқтатуды білдіреді бірде бір ел бұл екі індеттен аман өте алмайды,оңтүстік африка қою сыбайлас жемқорлық пен заңсыздық тоқтату және заңсыз иммигрант тоқтату білдір бірде бір ел бұл екі індет аман өте алма,nazism

3,бұл оңтүстік африкаға заңсыз шетелдіктердің кіруінің шығармашылық жолдары,бұл оңтүстік африка заңсыз шетелдік кіру шығармашылық жол,nazism

3,баффетху біз шетелдіктермен күресе алмаймыз мен жай ғана мазасыз бейнелерді көрдім және бұл қайғылы,баффетх біз шетелдік күре алма мен жай ғана мазасыз бейне көр және бұл қайғылы,nazism

3,эмоцияға берілу көмектеспейді мәселе twitter дегі ans мәселелерін шешкісі келеді ал шындық шетелдіктер мұны қанды шошқа деп қабылдауға дайын,эмоция беріл көмектеспе мәселе twitter дег ans мәселе шеш кел ал шындық шетелдік мұны қан шошқа де қабылда дайын,nazism

3,заңсыз шетелдіктерді жаппай депортациялау жылдың қаңтарында басталуы керек,заңсыз шетелдік жаппай депортацияла жыл қаңтар бастал керек,nazism

3,сіз сөйлеп тұрсыз мәселе мынада бұл бос емес жалақыны шешкісі келмейді бос емес адамдар оны шетелдіктер қабылдайды деп айтады,сіз сөйле тұр мәселе мына бұл бос емес жалақы шеш келме бос емес адам оны шетелдік қабылда де айт,nazism

3,оксалайо сіздің ата әжелеріңіз ешқашан өзгермейтін шетелдіктер,оксалайо сіздің ата әже ешқашан өзгерме шетелдік,nazism

3,сіз африкалықтарды жек көретіндіктен олар сіздің отбасы мүшелеріңізді өлтіреді шетелдіктерді тонап өлтірді деп жек көретіндерді айыптамаңыз африкалықтар тек тыныштықты сақтаңыз отбасы мүшелерін шетелдіктердің қолында жоғалтқандар үндемеу арқылы сізді ешқашан қуантпайды,сіз африкалық жек көретіндік олар сіздің отбасы мүше өлтір шетелдік тона өл де жек көр айыптама африкалық тек тыныштық сақта отбасы мүше шетелдік қол жоғалт үндеме арқылы сіз ешқашан қуантпа,nazism

3,маған осы елдегі заңсыз шетелдіктердің әңгімесі қатты ұнайды бұл бұқаралық ақпарат құралдарын оның не екенін анық көрсетті,маған осы ел заңсыз шетелдік әңгіме қатты ұна бұл бұқаралық ақпарат құрал оның не екен анық көрсе,nazism

3,бәріне шетелдікті кінәлайды тіпті жоғары бензинге де шетелдіктер себеп болды,бәрін шетелдік кінәла тіпті жоғары бензин де шетелдік себеп бол,nazism

3,ол оларды дудула заңсыз иммигранттарға әкеледі,ол ол дудул заңсыз иммигрант әкел,nazism

3,жарайсың mmc бұл дүңгіршектерде оңтүстік африкалықтар тұрғанына көз жеткізіңіз сонымен қатар swazi inn ді қараңыз бұл жер шетелдіктерге толы,жара mmc бұл дүңгіршек оңтүстік африкалық тұр көз жеткіз сонымен қатар swazi inn ді қара бұл жер шетелдік толы,nazism

3,бұл оңтүстік африканы басқа африка елдерінен ерекше етеді неліктен бұл елдің заңдары елді бопсалаусыз орындай алмайды жергілікті тұрғындар мен шетелдіктердің араздығын тудыратын да осы,бұл оңтүстік африка басқа африка ел

ерекше ет неліктен бұл ел заң ел бопсалау орында алма жергілікті тұрғын мен шетелдік араздығ тудыр да осы,nazism

3,мен айтқанымдай шетелдіктер адамдарды өлтіріп жатыр,мен айтқанымда шетелдік адам өлтір жатыр,nazism

3,бұл дұрыс бірақ бұл шетелдіктер салықты нөлдік түрде төлейді дегенді білдірмейді мен осыны ғана айтып отырмын шындығында бәріміз ққс және жанармай алымдарын төлейміз мәселен алынған мемлекеттік қызметтер біз төленбеген салықтар аргументіне сене алмаймыз,бұл дұрыс бірақ бұл шетелдік салық нөлдік түр төле де білдірме мен осы ғана айт отыр шындығ бәрі ққс және жанармай алым төле мәселен алын мемлекеттік қызмет біз төленбе салық аргумент сен алма,nazism

3,компаниялар қазір оңтүстік африкандықтарды бірінші болып табады,компания қазір оңтүстік африкандық бірінші бол таб,nazism

3,зума оңтүстік африкалық бауырластарға күліп жатыр,зу оңтүстік африкалық бауырлас күл жатыр,nazism

3,енді сіз заңсыз айтып жатырсыз сіз барлық заңсыз шетелдіктер соның ішінде заңсыз келген африкалық бауырластарымыз баруы керек деп айтуыңыз керек,енді сіз заңсыз айт жатыр сіз барлық заңсыз шетелдік соның іш заңсыз кел африкалық бауырлас бар керек де айт керек,nazism

3,біздің жұмыс істейтін министр әлі де барлық қоқыстарды тазалап жатыр содан кейін біз конституцияға өзгерістер енгізу үшін күресеміз күте тұрамыз және көреміз,біздің жұмыс істе министр әлі де барлық қоқыс тазала жатыр содан кейін біз конституция өзгеріс енгізу үшін күрес күт тұр және көр,nazism

3,иммигранттар қабылдаушы елінде тапқан қалыпты өмір салтын ұстанады біз бұл туралы сөйлесуіміз керек,иммигрант қабылдаушы ел тап қалыпты өмір салт ұстан біз бұл туралы сөйлес керек,nazism

3,дудула операциясы мен оның мандаты қалай екенін түсінбейінше сіз бұрын дудула операциясы қауымдастықтың мүшесі екенін және дудула операциясын ешқашан атау деп атамайтынын білесіз,дудул операция мен оның мандат қалай екен түсінбейінш сіз бұрын дудул операция қауымдастық мүше екен және дудул операция ешқашан атау де атама біл,nazism

3,шетелдіктер ғана емес оның бір бөлігі сізді өткізіп жіберді,шетелдік ғана емес оның бір бөліг сіз өткіз жіб,nazism

3,eff адамдарды дудула операциясы туралы жаңылыстыруға тырысуда бұл жастар венда мен цонгаға қарсы емес дудула юлиустың айтқанын істеп жатыр ол біздің дауыстарымызды алғысы келіп бізді жіберіп алды,eff адам дудул операция туралы жаңылыстыр тырыс бұл жас вен мен цонга қарсы емес дудул юлиу айт істе жатыр ол біздің дауыс ал кел біз жібер алды,nazism

3,егер сіз қазір өзіңіз ойлай алмасаңыз тіпті шетелдіктерді қуып жібергеннен кейін де сіз әлі де ойсыз қаласыз,егер сіз қазір өз ойла алма тіпті шетелдік қу жібер кейін де сіз әлі де ойсыз қал,nazism

3,оңтүстік африканың шетелдіктер үшін соншалықты тартымдылығы неде жүктілік қылмыс және сыбайлас жемқорлық бірақ олар осы елді жақсы көретін сияқты,оңтүстік африка шетелдік үшін соншалықты тартымдылығ не жүктілік қылмыс және сыбайлас жемқорлық бірақ олар осы ел жақсы көр сияқты,nazism

3,халық шетелдіктердің партияларын қолдап жүрді енді олар жыртқандарын орып алады және бұл партиялар бізге өздерінің шынайы келбетін көрсетеді бірақ ойланбайтын адамдар әлі де оларды қолдайды террористермен өз бетінше күресіңіз,халық шетелдік партия қолда жүр енді олар жырт ор ал және бұл партия бізге өз шынайы келбет көрсет бірақ ойланба адам әлі де ол қолда террорис өз бетінш күрес,nazism

3,бұл қара қауымдарды бөлетін нхланхла люкс оның адвокаты ике хумало да қара нәсілділерді шыққан жеріне қарай бөліп оларды амажапилер деп қорлайды жұдырықша африкалықтар болды содан кейін басото қазір цонга мен венда,бұл қара қауым бөл нхланхл люкс оның адвокат ик хумало да қара нәсілді шық же қарай бөл ол амажапи де қорла жұдырықш африкалық бол содан кейін басото қазір цонг мен вен,nazism

3,ал дудулинг кезінде олар кек қайтармайды деп күтесіз бе,ал дудулинг кез олар кек қайтарма де күт бе,nazism

3,бұл біздің қоғамға өте қауіпті,бұл біздің қоғам өте қауіпті,nazism

3,заңсыз атыс қаруын тек шетелдіктер алып жүрмейді бізде осындай байланысы бар және оларға қол жеткізетін біздің ұлттың адамдары бар сол мылтықтардың кесірінен kzn де осындай мәселеге тап болып отыр,заңсыз атыс қар тек шетелдік алып жүрме біз осындай байланыс бар және оларға қол жеткіз біздің ұл адам бар сол мылтық кесір kzn де осындай мәселе тап бол отыр,nazism

3,ол бос сөзге толы олардың көшбасшысы бұл трайбализмді тәртіпке шақырады деп үміттенемін,ол бос сөз толы олардың көшбасшы бұл трайбализ тәртіп шақыр де үміттен,nazism

3,дәл солай ол әрқашан ілгерілейтін болды барлығы сол бұзақы нхланхла люкстің кесірінен,дәл солай ол әрқашан ілгеріле бол барлығ сол бұзақ нхланхл люкс кесір,nazism

3,оңтүстік африкалықтар ретінде біз заңсыз шетелдіктерге рұқсатсыз біздің елге кіруге рұқсат берген сияқтымыз енді олар біздің жазықсыз адамдарды өлтіріп жатыр оңтүстік африка спаза дүкеніне ұқсайды өйткені бізде заңдар жоқ немесе заңдарға мән бермейді,оңтүстік африкалық рет біз заңсыз шетелдік рұқсат біздің ел кір рұқсат

бер сияқты енді олар біздің жазық адам өлтір жатыр оңтүстік африка спаз дүкен ұқса өйткені біз заң жоқ немесе заң мән берме,nazism

3,сіз мұны әрқашан ақ нәсілділер мен заңсыз иммигранттарға сілтеме жасауыңыз керек,сіз мұны әрқашан ақ нәсілді мен заңсыз иммигрант сілтеме жаса керек,nazism

3,бұл жердің туған халқын бөлу отаршыл топтың сәтсіздігіне әкелді,бұл же туған халқ бөлу отаршыл топ сәтсіздіг әке,nazism

3,шағын және заманауи үй тегін ғимарат алыңыз,шағын және заманауи үй тегін ғимарат ал,nazism

3,басының ішінде ми бар көшбасшылық патриот,бас іш ми бар көшбасшылық патриот,nazism

3,кейптаундағы шетелдіктер қиналуда сіз оңтүстік африкалық жүргізушіні әрең табасыз,кейптаун шетелдік қинал сіз оңтүстік африкалық жүргізуші әре таб,nazism

3,біз оны жек көруіміз мүмкін бірақ оны тыңдаңыз сонда сіз біздің қара жетекшінің астында жоғалған ұлт екенімізді түсінесіз,біз оны жек көр мүмкін бірақ оны тыңда сонда сіз біздің қара жетекші аст жоғал ұлт екен түс,nazism

3,түрлі түсті моншақтар сатамын әрқайсысы руб uj кампусының жанындағы джози cbd де орналасқан,түрлі түсті моншақ сат әрқайсы руб uj кампус жанындағы джози cbd де орналас,nazism

3,жоқ ол мүлдем өтірік айтпайды,жоқ ол мүлдем өтірік айтпа,nazism

3,сіз аймағыңызда шамадан тыс жүктеме түсіп жатыр ма шешуі таңертең тұрып тастарды жинап жолдың ортасына қойыңыз сондай ақ жанып тұрған шиналарды қосуға болады менің ойымша бұл жұмыс істейді,сіз аймағ шама тыс жүктеме түс жатыр ма шеш таңертең тұр тас жина жол орта қой сондай ақ жан тұр шина қос бол менің ойымша бұл жұмыс істе,nazism

3,sa да мылтық көп ештеңені алып өтудің қажеті жоқ тіпті такси мен шенді маршалдардың да мылтығы бар ал сіз шетелдіктерді айыптайсыз мұны аяқтауға шындап кіріссеңіз сіз шыншыл боласыз немесе көзіңізді ашасыз өйткені қазір олар жабық,sa да мылтық көп ештеңе алып өту қажет жоқ тіпті такси мен шен маршал да мылтығ бар ал сіз шетелдік айыпта мұны аяқта шында кіріс сіз шыншыл бол немесе көз аш өйткені қазір олар жабық,nazism

3,сен квара кварасың масканды шеш мен сені көремін,сен квар квар маска шеш мен сен көр,nazism

3,ал сіз eff ті кінәлайсыз ба аға сіз ауырып жатырсыз,ал сіз eff ті кінәла ба аға сіз ауыр жатыр,nazism

3,шетелдіктер үйлеріне қайтсын және біздің проблемаларымызды шешуге рұқсат етіңіз біз өмірімізді шетелдіктерге алаңдамай өткіздік біз ешқашан сіздің елдеріңізге

басып кірген емеспіз,шетелдік үй қайт және біздің проблема шеш рұқсат ет біз өмір шетелдік алаңдама өт біз ешқашан сіздің ел бас кірген емес,nazism

3,мені осы твиттердегі шетелдіктер мен малема құрлық күштерінің пікірлері ғана қызықтырады,мен осы твиттер шетелдік мен мале құрлық күш пікір ғана қызықтыр,nazism

3,билік партиясы заңсыз шетелдіктермен жұмыс істеуі керек өйткені олар оңтүстік африкалықтарды тарвендарда өлтірді деген болжам бар бізге доктор аарон моцоаледи сияқты адам керек,билік партия заңсыз шетелдік жұмыс істе керек өйткені олар оңтүстік африкалық тарвен өл де болжам бар бізге доктор аарон моцоаледи сияқты адам керек,nazism

3,тергеу аяқталғанға дейін шетелдіктер кінәні өз мойнына алатынын білдім бірақ жалғастырыңыз сіздің күн тәртібіңіз белгілі,тергеу аяқтал дейін шетелдік кінә өз мойн ал біл бірақ жалғастыр сіздің күн тәртіб белгілі,nazism

3,біреу сол шетелдіктерді жіберіп жатыр олар бір жерде есеп береді,біреу сол шетелдік жібер жатыр олар бір же есеп бер,nazism

3,біз ешқашан елімізді шетелдік қылмыскерлерге бермейміз біздің ата бабаларымыз осы ел үшін күресіп біз өмір сүруіміз үшін өлген біз де балаларымыз бен балаларымыздың немерелері үшін солай жасаймыз,біз ешқашан ел шетелдік қылмыскер берме біздің ата баба осы ел үшін күрес біз өмір сүр үшін өлген біз де бала бен бала немере үшін солай жаса,nazism

3,ескі болуы мүмкін бірақ шындық мынада шетелдіктер біздің құқық қорғау органдарын құрметтемейді бірақ полицейлер дәл осындай әрекет жасағанда оларға қатыгездік танытылады егер олар қатыгез болса онда олар өздерін қалай қорғауы керек,ескі бол мүмкін бірақ шындық мына шетелдік біздің құқық қорғау орган құрметтеме бірақ полицей дәл осындай әрекет жаса оларға қатыгез таныт егер олар қатыгез бол онда олар өз қалай қорға керек,nazism

3,олар маған зимбабвеліктерге тең олардың көпшілігі sa дағы заңсыз иммигранттар мен мұны білуім керек,олар маған зимбабвелік тең олардың көпшіліг sa дағ заңсыз иммигрант мен мұны біл керек,nazism

3,егер бұл террористер жақын арада көгілдір шамдары бар қара көліктерге шабуыл жасап олар ақша қалалар шахталар талап етпесе үкімет бұл лаңкестерді тоқтатуы керек біздің үкімет бізді ілулі бағаны немесе өлім жазасын қалпына келтіру арқылы ғана құтқара алады,егер бұл террорис жақын ара көгілдір шам бар қара көлік шабуыл жаса олар ақша қала шахта талап етпе үкімет бұл лаңкес тоқтат керек біздің үкімет біз ілул баға немесе өлім жаза қалп келтіру арқылы ғана құтқар ал,nazism

3,айтар сөзіңіз болмаған кезде қолыңыздан келгеннің бәрін жасай беріңіз,айтар сөз болма кезде қол кел бәрін жаса бер,nazism

3,кім екеніңді айтпай жасырып жүрсің,кім екен айтпа жасыр жүр,nazism

3,мен үйдемін менің адамым кел,мен үй менің адам кел,nazism

3,бұл қауіп пе менің ойымша тек әлсіз адамдар мұны жасайды және олардың жеке басын жасырады,бұл қауіп пе менің ойымша тек әлсіз адам мұны жаса және олардың жеке басын жасыр,nazism

3,толық қаңылтыр босқа ешқашан қызғанбайды ол шу шығарса,толық қаңылты бос ешқашан қызғанба ол шу шығар,nazism

3,өкінішке орай бәрі сіздің мәдениетіңізден немесе дініңізден болмайды мені сыйламауға үйрететін қандай мәдениет немесе басқаларды жек көруге және кемсітуге үйрететін дін деп ойлаймын жай ғана мені оқып шығуға уақыт бөліңіз сонда сіз құрметтің міндеттігін көресіз,өкініш орай бәрі сіздің мәдениет немесе дін болма мен сыйлама үйрет қандай мәдениет немесе басқа жек көр және кемсіт үйрет дін де ойла жай ғана мен оқ шығ уақыт бөл сонда сіз құрмет міндеттіг көр,nazism

3,нақтылықты тексеру сізді құтқару үшін,нақтылық тексеру сіз құтқар үшін,nazism

3,аузыңыздан қорлаудан басқа жақсы нәрселердің шығуы мүмкін емес бұл сіздің кінә емес,ауз қорлау басқа жақсы нәрсе шығ мүмкін емес бұл сіздің кінә емес,nazism

3,сіз халықты дініне қарай кемсітпейтін конституцияға дауыс бергеніңізді білесіз бе бұл жай ғана достық ескерту,сіз халық дін қарай кемсітпе конституция дауыс бер біл бе бұл жай ғана достық ескерту,nazism

3,мен сенің дәнің сені ант беруге жіберетінін білдім бұл сіздің қаншалықты әлсіз екеніңізді және арамыздағы айырмашылықты көрсетеді мен оны қазір алғаныңызға сенімдімін,мен сенің дән сен ант бер жібер біл бұл сіздің қаншалықты әлсіз екен және арамыз айырмашылық көрсет мен оны қазір ал сенімді,nazism

3,полиция қылмыскерлерден қорқады полицейлер семіз полиция халықты бақылай алмайды полицейлер полиция бөлімшелерінде зорлау құрбандарын ұстай алмайды полиция yt ppl қорқады,полиция қылмыскер қорқ полицей се полиция халық бақыла алма полицей полиция бөлімше зорла құрбан ұста алма полиция yt ppl қорқ,nazism

3,бұл сіздің кінәңіз емес бірақ сіздің кім екеніңізді білу маңызды мәселе,бұл сіздің кінә емес бірақ сіздің кім екен білу маңызды мәселе,nazism

3,ал сіздің дауысыңыз өз жағдайыңызға не пайда әкелд егер бұл әрекет болса біз бұл жерде осылай сөйлеспес едік,ал сіздің дауыс өз жағдай не пайда әкелд егер бұл әрекет бол біз бұл же осылай сөйлеспес едік,nazism

3,оңтүстік африка заңсыздыққа толы мемлекетті басып алу,оңтүстік африка заңсыздық толы мемлекет бас алу,nazism

3,бұл шетелдіктер олардың жанкүйерлері емес өкінішке орай олар оңай нысана сізге мысал келтірейік жастар жұмыс талап етудің орнына ті қабылдады бұл саясаткерлер олардың әлсіздігін біледі және түсінеді,бұл шетелдік олардың жанкүйер емес өкініш

орай олар оңай нысана сізге мысал келтір жас жұмыс талап ету орн ті қабылда бұл саясаткер олардың әлсіздіг біл және түсін,nazism

3,сіз қай жерде фактіні келтіре алатыныңызды және бұл істі қорғай алатыныңызды білетініңізге сенімдімін менің сұрағым мына саясатшылар кімдер бұл полицейлер ерлер мен әйелдер кімдер олар шетелдіктер ме менің білуімше жоқ біз sa ны емдегіміз келеді біз симптомдармен күресе алмаймыз,сіз қай же факті келтір ал және бұл іс қор ал біл сенімді менің сұра мына саясатшы кім бұл полицей ер мен әйел кім олар шетелдік ме менің білуімше жоқ біз sa ны емде кел біз симптом күре алма,nazism

3,оңтүстік африкалықтар ретінде әлі де бір бірімізбен күресетін болсақ біз қылмыстарға және біздің елге заңсыз иммигранттардың ағынына қарсы ешқашан тұра алмаймыз,оңтүстік африкалық рет әлі де бір бір күрес бол біз қылмыс және біздің ел заңсыз иммигрант ағын қарсы ешқашан тұр алма,nazism

3,азаматтар немесе шетелдіктер жасаған са дағы қылмыс заң үстемдігінің жоқтығының құқық қорғау қаруларының жарамсыз болуының өмірдің барлық салаларындағы сыбайлас жемқорлықтың тікелей нәтижесі болып табылады полиция қылмыскерлерді қамауға алды бірақ олар өздерінің қылмыстық қудалауын логикалық қорытындыға жеткізе алмайды,азамат немесе шетелдік жаса са дағ қылмыс заң үстемдіг жоқтығ құқық қорғау қару жарамсыз болу өмір барлық сала сыбайлас жемқорлық тікелей нәтиже бол таб полиция қылмыскер қама алды бірақ олар өз қылмыстық қудала логикалық қорытынды жеткіз алма,nazism

3,негізгі ағылшын тілін бәріміз білеміз бұл біздің тіліміз емес өкінішке орай біздің заңдарымыз ағылшын тілінде бірақ біз өзімізді күштеп оқытуымыз керек,негізгі ағылшын тіл бәрі біл бұл біздің тіл емес өкініш орай біздің заң ағылшын тіл бірақ біз өз күште оқыт керек,nazism

3,ксенофобия шетелдіктерге шабуыл жасаумен бітпейді профильдеу терісі күңгірттенген адамға ауысады біз мұны ксенофобиялық шабуылдар кезінде көрдік бұл адамға өте қорқынышты жағдай,ксенофобия шетелдік шабуыл жаса бітпе профильде тері күңгірттен адам ауыс біз мұны ксенофобиялық шабуыл кез көр бұл адам өте қорқыныш жағдай,nazism

3,кез келген жеке азамат кесте бойынша қылмыс жасаған немесе жасауға әрекеттенген адамға қатысты ордерсіз қамауға алуды жүзеге асыра алады оңтүстік африка азаматы сонымен қатар кез келген адамды қамауға алады,кез кел жеке азамат кесте бойынша қылмыс жаса немесе жаса әрекеттен адам қатысты орд қама алу жүзеге асыра ал оңтүстік африка азамат сонымен қатар кез кел адам қама ал,nazism

3,бұл партияны тек ақымақтар мен қызметке үмітсіз адамдар ғана ұстайды лаңкестер бұл елге кірудің шығармашылық жолдарын табуға шақыратын заңсыз шетелдіктер,бұл партия тек ақымақ мен қызмет үмітсіз адам ғана ұста лаңкес бұл ел кіру шығармашылық жол таб шақыр заңсыз шетелдік,nazism

3,сіздің үнсіздігіңіз тым қатты президент мырза біз заңсыз иммигранттарды жаппай депортациялауды көптен бері сұрап келеміз бірдеңе жасаңыз,сіздің үнсізді тым қатты президент мырза біз заңсыз иммигрант жаппай депортациялау көп бері сұра кел бірде жаса,nazism

3,шетелдіктер бірдеңе ұйымдастырып жатыр деген ақпарат тараған желі есіңізде ме,шетелдік бірде ұйымдастыр жатыр де ақпарат тара желі ес ме,nazism

3,ксенофобиялық заңсыз операция дудула жетекшісі кедей қара нәсілділерге соғыс жариялады мен ол жерсіз бұқараның ұйымымен кездесіп оғансаңылауларға толы екенін көрсеттім,ксенофобиялық заңсыз операция дудул жетекші кедей қара нәсілді соғыс жариял мен ол же бұқара ұйым кездес оғансаңылау толы екен көрсе,nazism

3,міне барлық африкалық шетелдіктерді жұмылдыру туралы айлар бұрын таратылған хабарлама,міне барлық африкалық шетелдік жұмылдыру туралы ай бұрын таратыл хабарлама,nazism

3,жоқ сіз бұрмалап отырсыз факт қылмыс жасалған және онымен күресу керек,жоқ сіз бұрмала отыр факт қылмыс жасал және онымен күресу керек,nazism

3,иә бірақ жоқ полицияның шетелдіктерге ақша төлемесе түрмеге апарып тастаймыз деп қорқыту үрдісі бар егер сізде ақша жоқ десеңіз сізді ұрып соғады немесе дәлелдерді өтірік көрсетеді,иә бірақ жоқ полиция шетелдік ақша төлеме түрме апар таста де қорқыт үрдіс бар егер сіз ақша жоқ де сіз ұр соғ немесе дәлел өтірік көрсет,nazism

3,зимбабвеліктер шри ланкада не болып жатқанын көрмегендей әрекет етуде бірінші орында оңтүстік афикандықтар,зимбабвелік шри ланка не бол жат көрмегенде әрекет ет бірінші орында оңтүстік афикандық,nazism

3,қалалық сайлау округі бақылау сапары мен ші округ тұрғындарымен қарым қатынас жасады біз заңсыз иммигранттардан ұрланған ғимараттарды қайтарып алудың тарихи жобасын қайта іске асыруымыз керек және тұрғындарға тиісті баспана беруіміз керек,қалалық сайлау округ бақылау сап мен ші округ тұрғын қарым қатынас жас біз заңсыз иммигрант ұрлан ғимарат қайтар ал тарихи жоба қайта іс асыр керек және тұрғын тиісті баспана бер керек,nazism

3,ұрланған кабель немесе зақымдалған инфрақұрылым болған сайын біз шетелдіктерді кінәлаймыз ба біз азамат ретінде инфрақұрылымымызды қорғау үшін не істейміз,ұрлан кабель немесе зақымдал инфрақұрылым бол сайын біз шетелдік кінәла ба біз азамат рет инфрақұрылым қорғау үшін не істе,nazism

3,sa азаматтары мемлекеттік инфрақұрылымды ұрлаумен және оны зақымдаумен айналысады,sa азамат мемлекеттік инфрақұрылым ұрла және оны зақымда айналыс,nazism

3,басқа жерде болып жатқан жағдай дудула қозғалысының жетекшісі нхланхла люкс үш апта бұрын соуэтода кәріз құдығына құлаған алты жасар баланы еске алуға

арналған шарада өз сөзінде,басқа же бол жат жағдай дудул қозғалыс жетекші нхланхл люкс үш апта бұрын соуэто кәріз құдығ құла алты жасар бала ес ал арнал шара өз сөз,nazism

3,дурбандағы дудула операциясы мүшелері босқындар мен баспана ұйымдарының шетелдік азаматтарға көмек көрсетуді тоқтатуын қалайды бейсенбіде дудуланың ға жуық мүшесі талаптар меморандумын тапсыру үшін диакония орталығының ғимаратына шықты,дурба дудул операция мүше босқын мен баспана ұйым шетелдік азамат көмек көрсету тоқтат қала бейсенбі дудула ға жуық мүше талап меморандум тапсыру үшін диакония орталығ ғимарат шық,nazism

3,дудула операциясы дурбандағы дякония орталығында олар шетел азаматтарына қарсы наразылық акциясын өткізіп жатыр орталықта босқындарға әлеуметтік қызмет көрсету қара белдеу және адам құқықтары үшін заңгерлер сияқты үкіметтік емес ұйымдар орналасқан,дудул операция дурба дякония орталығ олар шетел азамат қарсы наразылық акция өткіз жатыр орталық босқын әлеуметтік қызмет көрсету қара белдеу және адам құқық үшін заңгер сияқты үкіметтік емес ұйым орналас,nazism

3,түнде шыбын еңбекақысы төмен заңсыз иммигранттарды өз жұмысында ұстайтын құқық қорғаушы ардагер белсендіге адам құқықтары туралы да айта алмайды тіпті егер ол сәтсіздікке ұшыраса немесе қабылданса да қара нәсілділермен осылай сөйлесу де нәсілшілдік болып табылады,түн шы еңбекақы төмен заңсыз иммигрант өз жұмыс ұста құқық қорғаушы ардагер белсенді адам құқық туралы да ай алма тіпті егер ол сәтсіздік ұшыра немесе қабылдан да қара нәсілді осылай сөйлесу де нәсілш бол таб,nazism

3,зимбабве тұрғындары тыңдау және басқалардың алаңдаушылығын сезінуі керек қара sa африкалық иммигрант құлдығы туралы арзан еңбек заңдарына ие болмайды және ол бізге мән бермейтін қара денелі адамдардың қанауына жол бермейді олар қай елден келеді,зимбабв тұрғын тыңдау және басқа алаңдаушылығ сезін керек қара sa африкалық иммигрант құлдығ туралы арзан еңбек заң ие болма және ол бізге мән берме қара денел адам қана жол берме олар қай ел кел,nazism

3,өзіңізге сенімді плиталы газ плитасын алыңыз курьер қол жетімді,өз сенімді плитал газ плита ал курьер қол жет,nazism

3,олар неге шетелдіктермен бәсекелесу керек,олар неге шетелдік бәсекелес керек,nazism

3,күдікті заңсыз иммигранттар туралы қайда хабарлаймыз альбертонда көптеген дамып келе жатқан ыстық нүктелер бар күдікті заңсыз электр қосылымын қосыңыз маңайдан қашқан ақ нәсілділерді шетелдіктер көбірек сатып алады,күдік заңсыз иммигрант туралы қайда хабарла альбертон көптеген дам келе жат ыстық нүкте бар күдік заңсыз электр қосыл қос маңай қаш ақ нәсілді шетелдік көбірек сат ал,nazism

3,иммигрант қайда және қашан көшетінін немесе үйіне қайтуын өзі таңдайды олардың дағдыларына құжаттарына немесе қабылдаушы елдің заңдарына

қарамастан заңды немесе заңсыз болуы мүмкін пана іздеуші бұл қауіптен қорғану үшін жүгіруге мәжбүр болған және өз еліне орала алмайтын босқын,иммигрант қайда және қашан көш немесе үй қайт өзі таңда олардың дағды құжат немесе қабылдаушы ел заң қарамастан заңды немесе заңсыз бол мүмкін пан іздеуші бұл қауіп қорған үшін жүгір мәжбүр бол және өз ел орал алма босқ,nazism

3,қай жерде,қай же,nazism

3,заңсыз иммигранттар өз қалауынша істеп жатыр,заңсыз иммигрант өз қала істе жатыр,nazism

3,эйш кім не деді,эйш кім не де,nazism

3,оңтүстік африка заңсыз ел бірінші орында оңтүстік африка операция дудула зимбабвеліктер,оңтүстік африка заңсыз ел бірінші орында оңтүстік африка операция дудул зимбабвелік,nazism

3,қуат өшірілді және неліктен біз бәріміз білмейміз ауыстырғыш қалай бұзылады бұл дұрыс емес,қуат өшір және неліктен біз бәрі білме ауыстырғыш қалай бұз бұл дұрыс емес,nazism

3,ол ағаларына көмектесіп жүрді өзі де шетелдік не күттіңіз неге шетелдіктерді жұмысқа бересіз қандай тапшы шеберлік керек,ол аға көмектес жүр өзі де шетелдік не күт неге шетелдік жұмыс бер қандай тапшы шеберлік керек,nazism

3,заңсыз шетелдіктер не істейді,заңсыз шетелдік не істе,nazism

3,электр энергиясы үшін көптеген советтіктер төлемейді,электр энергия үшін көптеген советтік төлеме,nazism

3,кампала уганда мен кениядан келген самуэль булиме мубире бүркеншік аты амон рубен каджумба ол алаяқ жақында оның достары мені қырағы бол деп қорқытты өйткені мен оны әшкерелеп жатырмын мен өлгенім дұрыс менің оңтүстік африкалық әпкелеріме шетелдіктердің осы шаруаларына алданып қалмау үшін ескертемін,кампал уган мен кения кел самуэль були мубир бүркеншік ат амон ру каджумб ол алаяқ жақында оның дос мен қырағ бол де қорқыт өйткені мен оны әшкереле жатыр мен өл дұрыс менің оңтүстік африкалық әпке шетелдік осы шаруа алдан қалма үшін ескер,nazism

3,менің ойымша басы жоқ сайлаушылар біздің елде болып жатқан барлық нәрселерден ләззат алады бензиннің жоғары бағасы жүктеу кезеңі иммигранттардың жоғары ағыны жоғары жұмыссыз оңтүстік африкалықтар есірткілер тарвенс барлық жерде адам саудасы кабель ұрылары қарғыс атқыр сайлаушыларға рахмет,менің ойымша бас жоқ сайлаушы біздің ел бол жат барлық нәрсе ләззат ал бензин жоғары баға жүкте кезең иммигрант жоғары ағын жоғары жұмыссыз оңтүстік африкалық есірткі тарвенс барлық же адам сауда кабель ұры қарғыс атқы сайлаушы рахмет,nazism

3,әттең бірінші орында оңтүстік африка операция дудула,әттең бірінші орында оңтүстік африка операция дудул,nazism

3,сіз неге jean crossing дүкеніңізде тек шетел азаматтарын жұмысқа аласыз бізде мәсіх үшін оңтүстік африкада жұмыссыздық бар,сіз неге jean crossing дүкен тек шетел азамат жұмыс ал біз мәсіх үшін оңтүстік африка жұмыссыздық бар,nazism

3,тіпті anc заңсыз иммигранттарға тиіспейді,тіпті anc заңсыз иммигрант тиіспе,nazism

3,өмір сүру құнының үнемі өсуіне жол бермеу үшін sa ның sa ға жауапты болатын уақыты келді біздің жұмысшы қозғалыстары мен саяси партияларымыз сәтсіздікке ұшырады басшылар азаматтар мен кедейлердің есебінен сөмкені бекітумен айналысады,өмір сүру құн үнемі өс жол бермеу үшін sa ны sa ға жауапты бол уақыт кел біздің жұмысшы қозғалыс мен саяси партия сәтсіздік ұшыр басшы азамат мен кедей есеб сөмке бекіт айналыс,nazism

3,бұл заңсыз саудагерлер мен заңсыз шетелдіктер жақсы отырып біздің сайланған мемлекеттік шенеуніктердің қасынан өтіп бара жатқанын бақылайды оларда қорқыныш пен ұят жоқ,бұл заңсыз саудагер мен заңсыз шетелдік жақсы отыр біздің сайлан мемлекеттік шенеунік қас өт бар жат бақыла ол қорқыныш пен ұят жоқ,nazism

3,sa дағы мәселе біз дәрежелерді зерттейміз бұл пайдасыз жұмыссыздыққа мойынсұнамыз бізде жоғары лауазымдарда шетелдіктер бар өйткені олардың біліктілігі жоғары,sa дағ мәселе біз дәреже зертте бұл пай жұмыссыздық мойынсұн біз жоғары лауазым шетелдік бар өйткені олардың біліктіліг жоғары,nazism

3,шетелдіктер сіздің шақыруыңыз бойынша келді президент мырза есірткі синдикаттары sa ны есірткіге арналған пайдалы бизнес орталығы ретінде анықтады және олардың мақсаты біздің жастар иммиграциялық дағдарыс қазір,шетелдік сіздің шақыр бойынша кел президент мырза есірткі синдикат sa ны есірткі арнал пайдалы бизнес орталығ рет анықта және олардың мақсаты біздің жас иммиграциялық дағдарыс қазір,nazism

3,фашист жылы covid індеті кезінде зардап шеккен құлаған ауруханалар үшін есеп беруде сәтсіздікке ұшырады енді олар заңсыз шетелдіктердің ауыртпалығын ашуда,фашист жыл covid індет кез зардап шек құла аурухана үшін есеп бер сәтсіздік ұшыр енді олар заңсыз шетелдік ауыртпалығ аш,nazism

3,заңсыз иммигранттар көп инфрақұрылым нашарлауда қылмыс жоғары есірткілерді нигериялықтар күндіз сатады адам саудасы және жезөкшелік көлік ұрлау өте жоғары жұмыссыздар өте жоғары дүние жүзіндегі қылмыскерлерге арналған ойын алаңы және пайдасыз үкімет,заңсыз иммигрант көп инфрақұрылым нашарла қылмыс жоғары есірткі нигериялық күндіз сат адам сауда және жезөкшелік көлік ұрлау өте жоғары жұмыс өте жоғары дүние жүз қылмыскер арнал ойын алаң және пай үкімет,nazism

3,онда иммигранттар осы ақ триллионерлерді ренжіту үшін өз елдерінде қалуы керек шешім өте қарапайым,онда иммигрант осы ақ триллионер ренжіт үшін өз ел қал керек шешім өте қарапайым,nazism

3,оңтүстік африкалықтар біздің сүйікті еліміз оны заңсыз шетелдіктерге берді патриоттарға қосылыңыз өйткені дудула операциясы оңтүстік африкалықтарды оның атын өзгертуде бірінші орынға қойды азаматтарды қамауға алу,оңтүстік африкалық біздің сүйікті еліміз оны заңсыз шетелдік бер патриот қосыл өйткені дудул операция оңтүстік африкалық оның ат өзгерт бірінші орын қой азамат қама алу,nazism

3,олар африка ны жою үшін топтасып жатыр,олар африка ны жою үшін топтас жатыр,nazism

3,сіз ауырып мемлекеттік денсаулық сақтау жүйесіне хабарластыңыз ба егер сіз биік қабырғалардың артында қалсаңыз жай ғана жабыңыз өйткені сіз шетелдіктердің келуінен туындаған ауыртпалық кедей африкалықтарға қандай ауыртпалық түскенін білмейсіз,сіз ауыр мемлекеттік денсаулық сақтау жүйе хабарла ба егер сіз биік қабырға арт қал жай ғана жаб өйткені сіз шетелдік келу туында ауыртпалық кедей африкалық қандай ауыртпалық түс білме,nazism

3,молефе макинта тас жолындағы гаранкуваның кеңейтімінде цунами орналасқан шетелдіктер мен негізгі жол бойындағы қойма құрылыстары көлік қозғалысын бұзады бірақ заңсыз қосылымдарды пайдалана отырып бұл туралы ештеңе жасалмайды,молеф макин тас жолындағы гаранкува кеңейтім цунами орналас шетелдік мен негізгі жол бой қойма құрылыс көлік қозғалыс бұз бірақ заңсыз қосылым пайдалан отыр бұл туралы ештеңе жасалма,nazism

3,сіз не қызық екенін білесіз бе профессор бүгін евсевий екеуің қорғап жүрген адамдар сені сексуалдылығың үшін өз елдерінде өртеп жібереді,сіз не қызық екен біл бе профессор бүгін евсеви екеу қорға жүр адам сен сексуалдылы үшін өз ел өрте жібер,nazism

3,елді адал істеттің құдай сені көрсін деп үміттенемін елдің пайызы жұмыссыз зорлау көбейіп кетті жемқорлық өршіп кетті дүкендердің пайызы шетелдіктер менде бізге айта отырып егер сіз бізге елді өзгертетініңізге уәде бермесеңіз әлемнің соңы сияқты сезінесіз,ел адал істе құдай сен көр де үміттен ел пайыз жұмыссыз зорла көбей кет жемқорлық өрш кет дүкен пайыз шетелдік менде бізге ай отыр егер сіз бізге ел өзгерт уәде берме әлем соңы сияқты сез,nazism

3,егер біз шекараларды жауып барлық заңсыз шетелдіктерді жаппай депортациялайтын болсақ біз мұның бәрінен аулақ боламыз,егер біз шекара жау барлық заңсыз шетелдік жаппай депортацияла бол біз мұның бәрі аулақ бол,nazism

3,лимпоподағы вђњбоко харамвђќ триосы оңтүстік африка аффилирленген аттары мен фамилиялары бар заңсыз шетелдіктер,лимпопо вђњбоко харамвђќ трио оңтүстік африка аффилирлен ат мен фамилия бар заңсыз шетелдік,nazism

3,қазір бізде заңсыз шетелдіктер бірдей жағдайда өмір сүріп жатыр бұл жағдайды нашарлатуда,қазір біз заңсыз шетелдік бірде жағдай өмір сүр жатыр бұл жағдай нашарлат,nazism

3,мен дәл қазір қатты әсер алдым жақсы жұмыс жолдас,мен дәл қазір қатты әсер ал жақсы жұмыс жолдас,nazism

3,ең төменгі жалақыны белгілеу және оны тәртіпке келтірмеу оңтүстік африканың шеттетілуінің бір себебі заңсыз иммигранттардың көп ағынымен компаниялар оларды бізден таңдады,ең төменгі жалақы белгілеу және оны тәртіп келтірме оңтүстік африка шеттетілу бір себебі заңсыз иммигрант көп ағын компания ол бізден таңда,nazism

3,неге eff басшыларының төлқұжаты бар олар басқа елдерге кіру үшін креативті жолдарды қолданбай ма егер жоқ болса онда неге олар соғыстан шыққан заңсыз иммигранттарды шақырып жатыр елдер демекші оңтүстік африкаға жол табу үшін кім террорист болуы мүмкін,неге eff басшы төлқұжат бар олар басқа ел кіру үшін креативті жол қолданба ма егер жоқ бол онда неге олар соғыс шық заңсыз иммигрант шақыр жатыр ел демекш оңтүстік африка жол табу үшін кім террорист бол мүмкін,nazism

3,тағы бір зимбабвелік кеше түнде хиллброуда тонаған әйелге көмектескені үшін қауіпсіздік қызметкерін шауып өлтірді,тағы бір зимбабвелік кеше түн хиллбро тона әйел көмектес үшін қауіпсіздік қызметк шау өл,nazism

3,бхеки селе мен фермерлер бұл тәртіпсіздікке кінәлі anc үкіметі шекараларды ашуға рұқсат берді және фермерлер елде заңсыз адамды жұмысқа алу қылмыс болып табылатын заңсыз иммигранттарды жұмысқа алды,бхеки сел мен фермер бұл тәртіпсіздік кінәлі anc үкімет шекара аш рұқсат бер және фермер ел заңсыз адам жұмыс алу қылмыс бол табыл заңсыз иммигрант жұмыс алды,nazism

3,sandf анасы ондаған жылдар бойы гиеналар біздің жоғары қорғалған электрлік шекара қоршауымызды қолданыстан шығарды жылдан кейін снқ миллион жұмыссыздықты миллион заңсыз шетелдіктерді адам саудасы есірткі және тағамдық биохимиялық аурудан зардап шекті,sandf ана ондаған жыл бойы гиена біздің жоғары қорғал электрлік шекара қоршау қолданыс шығ жыл кейін снқ миллион жұмыссыздық миллион заңсыз шетелдік адам сауда есірткі және тағам биохимиялық ауру зардап шекті,nazism

3,сіздің басқарған үкіметіңіз sa халқын сәтсіздікке ұшыратты және сіз бізді шетелдіктерге тамақтандыруды жалғастырасыз бұл адамдар өлгенше күреседі сен оларға қауіпсіз жәннат бергенің үшін олар қайыршылыққа қайта барғысы келмейді сендер бір топ ақымақсыңдар білесің бе,сіздің басқар үкімет sa халқ сәтсіздік ұшыра және сіз біз шетелдік тамақтандыру жалғастыр бұл адам өлгенш күрес сен оларға қауіпсіз жәннат бер үшін олар қайыршылық қайта бар келме сен бір топ ақы біл бе,nazism

3,sandf шекаралық қызметкерлері заңсыз иммигранттардан алатын параның арқасында өте бай балалар сендер пайдасызсыңдар,sandf шекаралық қызметкер заңсыз иммигрант ал пара арқа өте бай бала сен пайдасыз,nazism

3,ел ішінде заңсыз иммигранттар санының болуы бізде sandf жоқ екені анық,ел іш заңсыз иммигрант сан бол біз sandf жоқ екен анық,nazism

3,ақырында біреу көлік жүргізушілерін эмоционалды түрде бопсалау үшін балаларды пайдаланып осы заңсыз иммигранттар синдикаттары туралы бірдеңе жасап жатыр,ақырында біреу көлік жүргізуші эмоциона түр бопсала үшін бала пайдалан осы заңсыз иммигрант синдикат туралы бірде жаса жатыр,nazism

3,вена криминологы сіз шетелдіктердің қасындасыз,вена криминолог сіз шетелдік қас,nazism

3,бай адамдар иммигранттар мен шекараны бақылауға келгенде заңның үстемдігін ешқашан қолдамайды себебі бұл шетелдіктер жаңа құлдар болып табылады төмен жалақы нашар жағдайлар және емдеу полицияны тарта алмайды себебі олар депотациядан қорқады және б,бай адам иммигрант мен шекара бақыла кел заң үстемдіг ешқашан қолдама себебі бұл шетелдік жаңа құл бол таб төмен жалақы нашар жағдай және емдеу полиция тарта алма себебі олар депотация қорқ және б,nazism

3,халықтың пайызы жұмыспен қамтылды бүгінгі таңда жастардың жұмыссыз және елді азаттық қозғалысының кесірінен шетелдіктер басқарып отыр,халық пайыз жұмыс қамт бүгінгі таңда жас жұмыссыз және ел аза қозғалыс кесір шетелдік басқар отыр,nazism

3,лесотода көп иммигранттар жоқ заңсыз байланысы бар заңсыз бейресми елді мекендер жоқ барлық көрші елдердің тұрғындары африка да және эском басты мәселе емес негізгі проблема халық көп africa,лесото көп иммигрант жоқ заңсыз байланыс бар заңсыз бейресми ел мекен жоқ барлық көрші ел тұрғын африка да және эско басты мәселе емес негізгі проблема халық көп africa,nazism

3,сізде сапардың негізделген мақсаты болуы керек бару үшін жеткілікті қаржылық ресурстардың болуын растау сіз баратын немесе транзитпен өтетін әрбір елге кіру үшін басқа талаптарға сай болу бұл заңсыз шетелдіктер бос қолмен келген сияқты емес,сіз сап негіздел мақсаты бол керек бару үшін жеткілікті қаржылық ресурс бол растау сіз бар немесе транзит өт әрбір ел кіру үшін басқа талап сай болу бұл заңсыз шетелдік бос қол кел сияқты емес,nazism

3,пурувхея сөзі контекстік немесе ауызекі тілде сатқын немесе шетелдіктердің сенімді өкілі қара нәсілді адамды білдіреді бұл масвинго провинциясынан мадзимбахве гуруусва чикаранга,пурувхея сөз контек немесе ауызек тіл сатқ немесе шетелдік сенімді өкіл қара нәсіл адам білдір бұл масвинго провинция мадзимбахв гуруусв чикаранг,nazism

3,олай ойлағаның үшін сені кінәламаймын оның рас екенін білмесем де бірақ бұл елде заңсыз иммигранттар көп және оны көру өте қорқынышты егер мен қазір үйімнен шығуға тура келсем заңсыз иммигрантпен соқтығысу мүмкіндігім өте жоғары,ола ойла үшін сен кінәлама оның рас екен білме де бірақ бұл ел заңсыз иммигрант көп және оны көру өте қорқыныш егер мен қазір үй шығ тура кел заңсыз иммигрант соқтығыс мүмкіндіг өте жоғары,nazism

3,еуропалықтар ешқашан өз елдерінің иммигранттар тарапынан жойылуына жол бермейді елдегі тым көп иммигранттар қиыншылықтар тозағы,еуропалық ешқашан өз ел иммигрант тарап жойыл жол берме ел тым көп иммигрант қиыншылық тозағ,nazism

3,шынымен бе бізде оңтүстік африкада полиция жеткілікті және шетелдіктерді жалдау сатқындық полиция менеджменті лаңкестік жасауда,шын бе біз оңтүстік африка полиция жеткілікті және шетелдік жалда сатқ полиция менеджмент лаңкестік жаса,nazism

3,ал біз құжатсыз шетелдіктердің тонауын және өлтіруін қабылдауымыз керек пе бұл жақсы нәрсе мен шіркеуге бармаймын ұят,ал біз құжат шетелдік тона және өлтір қабылда керек пе бұл жақсы нәрсе мен шіркеу барма ұят,nazism

3,олар әрқашан өздерінің ксенофобиялық әңгімелеріне сәйкес фактілерді бұрмалап жатқанда мен дудула операциясын қалай байыпты қабылдай аламын мен шығыс лондоннанмын,олар әрқашан өз ксенофобиялық әңгіме сәйкес факті бұрмала жатқанда мен дудул операция қалай байыпты қабылда ал мен шығыс лондон,nazism

3,anc бізге осы заңсыз иммигранттарды біздің елге әкелу арқылы өмір сүру деңгейін төмендетті сіз тіпті қытайлардың алкоголь сататын және бөтелке дүкендерінің иелерін табасыз бізде anc мәселесі бар,anc бізге осы заңсыз иммигрант біздің ел әкелу арқылы өмір сүру деңгей төменде сіз тіпті қытай алкоголь сат және бөтел дүкен ие таб біз anc мәселе бар,nazism

3,бұл және қамауға алу елді тазарту үшін мәңгілікке созылады бізге жаппай депортациялар қажет,бұл және қама алу ел тазарт үшін мәңгілік соз бізге жаппай депортацияла қажет,nazism

3,менің ойымша президент отбасы жиналысын шақырып барлық заңсыз иммигранттарға елден кетуге бір ай уақыт беруі керек күннен кейін ол елді жауып дұрыс тазарту жұмыстарын жүргізуі керек saps sadf және private security ең соңында барлық шекараларды қорғайды және барлық ішкі істер шенеуніктерін ауыстырады,менің ойымша президент отбасы жиналы шақыр барлық заңсыз иммигрант ел кет бір ай уақыт беруі керек күн кейін ол ел жау дұрыс тазарт жұмыс жүргіз керек saps sadf және private security ең соң барлық шекара қорға және барлық ішкі істер шенеуник ауыстыр,nazism

3,шетелдік ағайындар africa ны қауіпті елге айналдырды,шетелдік ағайын africa ны қауіпті ел айнал,nazism

3,бұл ақсақ жауап кудзи басқа шетелдіктермен салыстырғанда sa дағы зимбабвеліктердің көлемін қараңыз,бұл ақ жауап кудзи басқа шетелдік салыстыр sa дағ зимбабвелік көл қара,nazism

3,dudula randfontein операциясы оңтүстік африкадан заңсыз шетелдіктер кетеді,dudula randfontein операция оңтүстік африка заңсыз шетелдік кет,nazism

3,біздің басты проблемамыз қха олар бізді осыған әкелді егер біз оларды жылы жоймасақ инфрақұрылымды жою мәселесі әлі де сақталады заңсыз иммигранттардан не күтесіз бұл елге өмір сүрудің ешбір құралынсыз келіңіз бе,біздің басты проблема қх олар біз осы әке егер біз ол жыл жойма инфрақұрылым жою мәселе әлі де сақтал заңсыз иммигрант не күт бұл ел өмір сүру ешбір құралын кел бе,nazism

3,осылай жасаңыз біздің елімізді және оның халқын құртып жаман істермен айналысып жатқан шетелдіктерді әшкерелеуіміз керек,осылай жаса біздің ел және оның халқ құрт жаман іс айналыс жат шетелдік әшкереле керек,nazism

3,заңсыз шетелдіктер аурухананың толып жатқандығы туралы ашық айтпаған мос жұмысынан шеттетілді ол компаға келген медбикеге адал болды пациенттерге күтім жасау біз фактілерді айтқан кезде ксенофобияны көрсетеміз,заңсыз шетелдік аурухана тол жатқандығ туралы ашық айтпа мос жұмыс шеттет ол компа кел медбике адал бол пациент күтім жасау біз факті айт кезде ксенофобия көрсе,nazism

3,оңтүстік африкалықтар экономикалық еркіндіктен гөрі шетелдіктерді қууға көбірек көңіл бөледі,оңтүстік африкалық экономикалық еркіндік гөрі шетелдік қу көбірек көңіл бөл,nazism

3,ух гва тсхвана оларды шетелдіктер тоздырып бүлдіреді егер сіз сэндтонды қаламасаңыз оларды тек бай адамдар пайдаланады,ух гв тсхван ол шетелдік тоздыр бүлдір егер сіз сэндто қалама ол тек бай адам пайдалан,nazism

3,ммм бәріміз салықты есе көбейтуге жұмылдырайық сонда біздің үкімет шетелдіктерге қамқорлық жасау үшін қажеттінің бәрін жасай алады ққс болуы керек төлеңіз жанармай алымдары литріне,мм бәрі салық есе көбейт жұмылдыр сонда біздің үкімет шетелдік қамқорлық жасау үшін қажетті бәрін жаса ал ққс бол керек төле жанармай алым литр,nazism

3,бұл үкімет түбегейлі шіріген шетелдіктерді айыптамаңыз ішкі істер мен жемқор мемлекеттік қызметкерлерді айыптайды,бұл үкімет түбегейлі шірі шетелдік айыптама ішкі істер мен жемқор мемлекеттік қызметкер айыпта,nazism

3,қха кадрлары бұл шетелдіктерге ама ғимараттарын сыйлағандар,қх кадр бұл шетелдік ам ғимарат сыйла,nazism

3,миллион заңсыз шетелдік сайлаушылармен және жалған және жалған мәлімдемелері бар заңсыз шетелдіктермен шеру мүмкін сіз sa ны іске қоса аласыз алайда біздің өмірімізде емес бұл орындалмайтын арман болып қала береді біз өз

еліміз үшін күресеміз ешқандай заңсыз шетелдіктер ешқашан оңтүстік кореяны отарламауы керек,миллион заңсыз шетелдік сайлаушы және жалған және жалған мәлімдеме бар заңсыз шетелдік шеру мүмкін сіз sa ны іс қоса ал алайда біздің өмір емес бұл орындалма арман бол қала бер біз өз еліміз үшін күрес ешқандай заңсыз шетелдік ешқашан оңтүстік корея отарлама керек,nazism

3,менде иммигрант иелік ететін мемлекеттік кәсіпорыннан миллиондаған ранд пайда алатын компанияның заңдылығына қатысты мәселе бар менің ойымша егер ол сіздің қызыңыздың немесе дудузане зуманың иелігінде болса мен жақсырақ болар едім біз мұнда жастардың бақыланбайтын жұмыссыздығымен отырғанда,менде иммигрант иелік ет мемлекеттік кәсіпорын миллиондаған ранд пайда ал компания заңдылығ қатысты мәселе бар менің ойымша егер ол сіздің қыз немесе дудузан зума иеліг бол мен жақсырақ бол ед біз мұнда жас бақыланба жұмыссыздығ отырғанда,nazism

3,оңтүстік африка халқы заңсыз шетелдіктердің лаңкестік әрекетінен шаршады оңтүстік африканы көтеріңіз еліңізді қалпына келтіріңіз тозаққа тозаққа дейін тозаққа дейін тозаққа дейін,оңтүстік африка халқ заңсыз шетелдік лаңкестік әрекет шарша оңтүстік африка көтер ел қалп келтір тозақ тозақ дейін тозақ дейін тозақ дейін,nazism

3,диаспорадағы зимболар сізге басқа зимбабвеліктерден аулақ болуды айтады өйткені олар сізді жұмыстан қуып жіберуі мүмкін оңтүстік африканы қараңыз зимбабве тұрғындары ең үлкен шетелдіктер саны бірақ бізде нигериялықтар сомалилықтар сияқты күш жоқ өйткені біз бір бірімізді төмен түсіреміз,диаспора зимбо сізге басқа зимбабвелік аулақ болу айт өйткені олар сіз жұмыс қу жібер мүмкін оңтүстік африка қара зимбабв тұрғын ең үлкен шетелдік сан бірақ біз нигериялық сомалилық сияқты күш жоқ өйткені біз бір бір төмен түсір,nazism

3,рамафоса заңсыз намибиялық иммигранттарды үй қызметкері етіп жалдаған сондықтан оны қамауға алу керек біз мектептер мен университеттерге бардық өйткені біздің аналарымыз африкандықтардың үй жұмысшылары болған бірақ біздің қара адамдар біздің қара нәсілді әйелдерді жұмысқа алуға жарамсыз деп санайды,рамафо заңсыз намибиялық иммигрант үй қызметк ет жалда сондықтан оны қама алу керек біз мектеп мен университет бар өйткені біздің ана африкандық үй жұмысшы бол бірақ біздің қара адам біздің қара нәсіл әйел жұмыс ал жарамсыз де сана,nazism

3,бұл құжатсыз шетелдіктер үнемі өтірік таратады олар өз үкіметтерін жауапқа тарта алмайды және қылмыс жасау үшін заңсыз түрде sa ға келіп соның үстіне өтірік айтады,бұл құжат шетелдік үнемі өтірік тарат олар өз үкімет жауап тарта алма және қылмыс жасау үшін заңсыз түр sa ға кел соның үст өтірік айт,nazism

3,дегенмен бұл шетелдіктер өз үкіметімен күресе алмады,дегенмен бұл шетелдік өз үкімет күре алма,nazism

3,менің сұрағым күшейткіш зимбабве гвардиясының көлігі біздің орталықтар мен сауда орталықтарына zep немесе дағдылар санаты бойынша ма олай болмаса неге

олар қамауға алынбайды және елге қайта жер аударылмайды,менің сұра күшейткіш зимбабв гвардия көлік біздің орталық мен сауда орталық zep немесе дағды сана бойынша ма ола болма неге олар қама алынба және ел қайта жер аударылма,nazism

3,біз anc үкіметінің біліксіздігі мен көшбасшылықтың жоқтығынан зимбабвеліктердің рақымының астында өмір сүріп жатырмыз заңсыз иммигранттар елде anc үкіметінің қарамағында жұмыс істейді,біз anc үкім біліксіздіг мен көшбасшылық жоқтығ зимбабвелік рақым аст өмір сүр жатыр заңсыз иммигрант ел anc үкім қарамағ жұмыс істе,nazism

3,anc үкіметі осы жаңа қылмыстардың барлығы үшін ұялуы керек олар елді бақылауды баяғыда заңсыз иммигранттарға зимбабвеліктерге нигериялықтарға пәкістандықтарға мозамбиктерге эфиопиялықтарға жоғалтты қытайлар елді басқарып бұрын жұмыс істегеннің бәрін құртып жатыр,anc үкімет осы жаңа қылмыс барлығ үшін ұял керек олар ел бақылау баяғы заңсыз иммигрант зимбабвелік нигериялық пәкістандық мозамбик эфиопиялық жоғал қытай ел басқар бұрын жұмыс істе бәрін құрт жатыр,nazism

3,дудула саяси партиясын ашыңыз мен бірдеңені көргім келеді,дудул саяси партия аш мен бірдеңе көр кел,nazism

3,біздің президент тіпті оңтүстік африкалық емес шетелдіктер sa ға опера ретінде қарайды әркім өз бөлігін алады,біздің президент тіпті оңтүстік африкалық емес шетелдік sa ға опера рет қара әркім өз бөліг ал,nazism

3,мейірімділік сіз де кедей және жұмыссыз оңтүстік африкалықтарға бірдей жанашырлық танытып жатырсыз ба өз басымдықтарыңызды дұрыс анықтаңыз мұнда босқындар немесе баспана іздеушілер жоқ және тек заңсыз шетелдер және көшіп келген заңдарды қолдану керек қарапайым,мейірімділік сіз де кедей және жұмыссыз оңтүстік африкалық бірде жанашырлық таныт жатыр ба өз басымдық дұрыс анықта мұнда босқын немесе баспана іздеуші жоқ және тек заңсыз шетел және көш кел заң қолдану керек қарапайым,nazism

3,nhlanhla lux қазір барлық африкалықтардан еркіндік кілттерін алып тастауы керек,nhlanhla lux қазір барлық африкалық еркіндік кілт алып таста керек,nazism

3,бұл террористер біз олардың артынан барамыз сіз олардың әуежайдың желдету жүйесінен шыққанын көрдіңіз бе мұнда үлкен мақсаттар қойғандар террористер олар ұйықтап жатқан жасушалар дегенмен нацики сіз барлық шетелдіктердің оңтүстік кореяда орны бар деп айттыңыз енді сіз кері бұрыласыз ба,бұл террорис біз олардың арт бар сіз олардың әуежай желдет жүйе шық көр бе мұнда үлкен мақсат қой террорис олар ұйықта жат жасуша дегенмен нацики сіз барлық шетелдік оңтүстік корея орны бар де ай енді сіз кері бұрыл ба,nazism

3,африка жаңалықтар бөлімі саусағыңызбен интроспекция жасау керектігін көрсетеді неліктен олар көпшілікке ұнамайтыны туралы олар дудула операциясы туралы барлық бос сөздерді айтады,африка жаңалық бөлім саусағ интроспекция жасау керектіг

көрсет неліктен олар көпшілік ұнама туралы олар дудул операция туралы барлық бос сөз айт,nazism

3,мен жергілікті el comandante бірнеше ай бұрын хабарланған советодағы электрмен жабдықтау дағдарысын шешті деп ойладым ахххх дұрыс емес адамдар үшін дұрыс емес ақша бар қауіпті,мен жергілікті el comandante бірнеше ай бұрын хабарлан совето электр жабдықтау дағдарыс шеш де ойла ахххх дұрыс емес адам үшін дұрыс емес ақша бар қауіпті,nazism

3,оңтүстік африкаға заңсыз келген шетелдіктермен проблемамыз бар,оңтүстік африка заңсыз кел шетелдік проблема бар,nazism

3,иә біздің билік шетелдіктерге қалағанын жасауға мүмкіндік беруі керек басқа адамдар оңтүстік африкалықтар мемлекеттен көп ақша ұрлайды бірақ олар оны қумайды оның орнына олар уттар прадештен келген кедей гупталарға адал өмір сүруге тырысады бұл ұят,иә біздің билік шетелдік қала жаса мүмкіндік беруі керек басқа адам оңтүстік африкалық мемлекет көп ақша ұрла бірақ олар оны қума оның орн олар ут прадеш кел кедей гупта адал өмір сүр тырыс бұл ұят,nazism

3,неліктен осы елде заңсыз шетелдіктер бар,неліктен осы ел заңсыз шетелдік бар,nazism

3,бұл құжатсыз шетелдіктердің ағынына байланысты емес пе,бұл құжат шетелдік ағын байланысты емес пе,nazism

3,бұл ақымақ комиссия сөйлесетін соңғы комиссия сіз олардың кеңсесіне де кіре алмайсыз бірақ олар шетелдіктерді және оның азаматтарын емес тез қорғайды,бұл ақы комиссия сөйлес соңғы комиссия сіз олардың кеңсе де кір алма бірақ олар шетелдік және оның азамат емес тез қорға,nazism

3,осы елде кобанини халқы ұрланып жатыр қауымдастықтар тұрып шетелдіктерді қуып шығуы керек содан кейін біз тартылған жергілікті тұрғындармен жұмыс істей аламыз шина сихатхелеге оралуы керек,осы ел кобанини халқ ұрлан жатыр қауымдастық тұр шетелдік қу шығ керек содан кейін біз тартыл жергілікті тұрғын жұмыс істе ал шина сихатхеле орал керек,nazism

3,содан кейін барлық құжаты жоқ шетелдіктерді алыс болуға шақырыңыз өйткені біз өте қауіпті,содан кейін барлық құжат жоқ шетелдік алыс бол шақыр өйткені біз өте қауіпті,nazism

3,жоқ оларға рұқсат берілмейді біз бұған көз жеткіземіз бұл зимболарға мұқтаж компаниялар зимбабвеге өздерінің зимбо қызметкерлерімен бірге орналасуға дайындалуы керек жыл бұрынғыдан да көп шетелдік иммигранттар еркін болады,жоқ оларға рұқсат берілме біз бұған көз жеткіз бұл зимбо мұқтаж компания зимбабве өз зимбо қызметкер бірге орналас дайындал керек жыл бұрынғы да көп шетелдік иммигрант еркін бол,nazism

3,жалпы уганда әлдеқайда арзан болды еа да тұратын шетелдіктер үшін еас тарифтері қолданылады конго да арзан адал болу үшін тәжірибеге тұрарлық соңғы рет тексергенімде рұқсат шығындарының шамамен пайызы табиғатты қорғауға кетеді және жоғары кіру нүктесі жаппай туризмге байланысты приматтардың күйзелісін азайтады,жалпы уган әлдеқайда арзан бол еа да тұр шетелдік үшін еас тариф қолдан конго да арзан адал болу үшін тәжірибе тұрарлық соңғы рет тексер рұқсат шығын шамамен пайыз табиғат қорға кет және жоғары кіру нүкте жаппай туризм байланысты примат күйзеліс азайт,nazism

3,электр мәселесі адамдарды бір бірімен ұрысуға мәжбүр етті бүгінгі күн тек бастамасы бұл адамдар бізді ойламайды,электр мәселе адам бір бір ұрыс мәжбүр ет бүгінгі күн тек баста бұл адам біз ойлама,nazism

3,із әлі де істің қалай жүріп жатқанын жоққа шығаратын күш бар екенін білесіз және осылайша өз қаражатын алға жылжытып жатыр деген сөзді тудыруы мүмкін бірақ ол осылайша тіпті болмауы мүмкін басқа біреудің мұны істеуіне қарсы мәселе бар,із әлі де іс қалай жүр жат жоқ шығар күш бар екен біл және осылайша өз қаражат алға жылжыт жатыр де сөз тудыр мүмкін бірақ ол осылайша тіпті болма мүмкін басқа біреу мұны істе қарсы мәселе бар,nazism

3,иә оның радиодан бұлшық еті болғаны жақсы егер басқа біреу болса олар тәртіпке сай болар еді,иә оның радио бұлшық ет бол жақсы егер басқа біреу бол олар тәртіп сай бол ед,nazism

3,шетелдік заңсыз иммигранттар басқаратын мидрандқа барыңыз олар өз қызметкерлеріне қатыгездік көрсетуде,шетелдік заңсыз иммигрант басқар мидранд бар олар өз қызметкер қатыгез көрсет,nazism

3,африканың ақ халқы әлбетте сіз өзіңізді өзіңіз ойлағаннан жақсырақ деп айтасыз сонда сіз ақ адамдар туралы не айтасыз тек қызық,африка ақ халқ әлбет сіз өз өз ойла жақсырақ де ай сонда сіз ақ адам туралы не ай тек қызық,nazism

3,youtube те иммиграция пана іздеушілер туралы деректі фильмдердің бірнешеуін көргеннен кейін мен дудула операциясының немесе үкіметтің мүмкіндігі жоқ екенін түсіндім зимбабве пәкістандықтар сомалиліктер немесе нигериялықтар ең ұзақ уақыт бойы ауырады,youtube те иммиграция пан іздеуші туралы деректі фильм бірнеше көр кейін мен дудул операция немесе үкімет мүмкіндіг жоқ екен түс зимбабв пәкістандық сомалилік немесе нигериялық ең ұзақ уақыт бойы ауыр,nazism

3,жетеді ол шетелдіктерді жұмысқа алатындықтан мүдделі емес,жет ол шетелдік жұмыс алатындық мүдделі емес,nazism

3,қолдарынан келсе неге ештеңе істемейді канти банжани лабанту мен or tambo да ұсталған шетелдіктер туралы мысал келтіремін телефондар олардың келуіне дайындалу үшін пайдаланылғаны анық неліктен анықталмады,қол кел неге ештеңе істеме канти банжани лабант мен or tambo да ұстал шетелдік туралы мысал келтір телефон олардың кел дайындалу үшін пайдаланыл анық неліктен анықталма,nazism

3,мен бұған сенемін шағын көше де осындай болуы керек,мен бұған сен шағын көше де осындай бол керек,nazism

3,осы жерде шетелдіктердің басынан бір жаман нәрсе болуы мүмкін әсіресе егер жағдай осылай жалғаса берсе адамдар өле бастайды және оңтүстік африкалықтар болмаса рұқсаттар бұған әсер етеді,осы же шетелдік бас бір жаман нәрсе бол мүмкін әсіресе егер жағдай осылай жалға бер адам өл баста және оңтүстік африкалық болма рұқсат бұған әсер ет,nazism

3,ел болып алысқа шықтық жыл бұрын нкандла жанжалы шыққаннан бері үкіметтік шенеуніктердің тонаулары үлкенірек және өрескел бола бастады бізді енді ештеңе таң қалдырмайтын сияқты,ел бол алыс шық жыл бұрын нкандл жанжал шық бері үкіметтік шенеунік тонау үлкенірек және өрескел бола баста біз енді ештеңе таң қалдырма сияқты,nazism

3,мың қаралған жол экипажға қосылыңыз мен сізге кейінірек рахмет айтамын сүйіспеншілікті көрсетіңіз,мың қарал жол экипаж қосыл мен сізге кейінірек рахмет ай сүйіспеншілік көрсе,nazism

3,шетелдіктердің жұмысқа орналасуына наразылық ретінде жүк көлігі жүргізушілерінің автокөлігін қоршауды жалғастыруы біздің қауіпсіздік мекемеміздің әлсіздігінің айқын белгісі бұл өмірлік маңызды дәліз қалайша қорғалмаған,шетелдік жұмыс орналас наразылық рет жүк көлiг жүргізуші автокөлiг қоршау жалғастыр біздің қауіпсіздік мекеме әлсіздiг айқын бел бұл өмірлік маңызды дәліз қалайша қорғалма,nazism

3,мина зимбабвеліктерге әлеуметтік желіде шабуыл жасалған сайын мен оларды қорғадым мен екі рет құрбан болғаннан кейін тоқтадым мен сізді сендіремін егер олардың көпшілігі болмаса кейбіреулері жек көреді,мина зимбабвелік әлеуметтік желі шабуыл жасал сайын мен ол қорға мен екі рет құрбан бол кейін тоқта мен сіз сендір егер олардың көпшiлiг болма кейбіреу жек көр,nazism

3,бұл елге заңсыз шетелдіктерді алып тастау керек олар hewlett компаниясымен не болатыны туралы шешім қабылдауы және толық пікір айтуы үшін оларға уран мен кириллдің ұлы ріс қажет олар sa азаматтарын жұмысқа алу үшін pvt қауіпсіздігін қажет етеді,бұл ел заңсыз шетелдік алып тастау керек олар hewlett компания не бол туралы шешім қабылда және толық пікір айт үшін оларға уран мен кирил ұлы ріс қажет олар sa азамат жұмыс алу үшін pvt қауіпсіздiг қажет ет,nazism

3,біздің putsouthafricansfirst ұлттық көшбасшыларымыз ресми түрде дудула atdfasa альянсының заңсыз шетелдіктер дағдарыстары бойынша серіктестері ретінде танылған заңнаманың алдында тұрғанын айтқан кезде олар ғарышта блеф жасап жатырмыз деп ойлады,біздің putsouthafricansfirst ұлттық көшбасшы ресми түр дудул atdfasa альянс заңсыз шетелдік дағдарыс бойынша серіктес рет таныл заңнама алд тұр айт кезде олар ғарыш блеф жаса жатыр де ойла,nazism

3,менің ойымша үкімет осы елдегі шетелдіктер мен жұмыс орындарына қатысты заңдарды нақтылауы керек,менің ойымша үкімет осы ел шетелдік мен жұмыс орын қатысты заң нақтыла керек,nazism

3,тамаша бастама мен фермерлердің көпшілігі қосылса екен деп тілеймін өйткені олардың көпшілігін осы шетелдіктер өлтірді адамдар қылмыскерлерді тоқтатқан кезде адамдар ксенофобиялық деген шуды бір рет тоқтатады,тамаша бастама мен фермер көпшілігі қосыл екен де тіле өйткені олардың көпшілігі осы шетелдік өл адам қылмыскер тоқтат кезде адам ксенофобиялық де шу бір рет тоқтат,nazism

3,сиябангена atdfasa ға қолдау көрсетеді ұлттық министрліктер заңсыз шетелдіктер операциясын тоқтату үшін,сиябанген atdfasa ға қолдау көрсет ұлттық министрлік заңсыз шетелдік операция тоқтату үшін,nazism

3,пәкістандықтар күн сайын ор тамбода қамауға алынады кеше түнде ондаған адам қамауға алынды не айтып тұрсың әрине сіз африкалықтар ғана деп ойлайсыз өйткені африкалық шетелдіктер мұнда көп,пәкістандық күн сайын ор тамбо қама алын кеше түн ондаған адам қама ал не айт тұр әрине сіз африкалық ғана де ойла өйткені африкалық шетелдік мұнда көп,nazism

3,кирилл әрекет ете алмайды өйткені ол өзі матрац қоймасын тонауды ұйымдастыратын біліктілігі жоқ иммигрант жалдаған бұл тосқауыл жыл бойы ешқандай алға жылжусыз жалғасуда,кирилл әрекет ет алма өйткені ол өзі матрац қойма тонау ұйымдастыр біліктілігі жоқ иммигрант жалда бұл тосқауыл жыл бойы ешқандай алға жылжу жалғас,nazism

3,дарки керісінше ол кезде сіз оған қызмет ететін қараңғының оқуға немесе тіпті гого мадала оқуға тырысып жатқанын байқайсыз,дарки керісінше ол кезде сіз оған қызмет ет қараңғы оқ немесе тіпті гого мадал оқ тырыс жат бай,nazism

3,гейтон маккензи қай жерде джулиус малемаға қарағанда жақсы операция дудула ақымақтары юлиус малеманың беделіне нұқсан келтіру үшін барлық қоқыстарды айтып жатыр,гейтон маккензи қай же джулиус малема қара жақсы операция дудул ақымақ юлиус малема бедел нұқсан келтіру үшін барлық қоқыс айт жатыр,nazism

3,құрметті оңтүстік африкалықтар бірде бір шетел азаматы сіздің мүдделеріңізді ойламайтынын ескеріңіз бұл пәленшелер сізді бір жолмен қорлау үшін келді шетелдіктерге еруді тоқтатыңыз пасторлар емшілер және олардың барлығы алаяқтар мен қылмыскерлер,құрметті оңтүстік африкалық бірде бір шетел азамат сіздің мүдде ойлама ескер бұл пәленше сіз бір жол қорла үшін кел шетелдік еру тоқта пастор емші және олардың барлығ алаяқ мен қылмыскер,nazism

3,мен сізбен шұғыл түрде танысқым келеді менің кіріс жәшігімді басыңыз сондықтан өтінемін,мен сізбен шұғыл түр таныс кел менің кіріс жәшіг бас сондықтан өт,nazism

3,мен сізбен танысқым келеді тез арада менің кіріс жәшігімді басыңыз,мен сізбен таныс кел тез ара менің кіріс жәшіг бас,nazism

3,елестетіп көріңізші eff заңсыз шетелдіктерді сол жақ оң жаққа жалдап жатыр,елестет көріңізш eff заңсыз шетелдік сол жақ оң жақ жалда жатыр,nazism

3,оңтүстік африкада көптеген жылдар бойы жұмыссыздық өсті және елдің белгілі бөліктеріндегі жұмыссыздардың жаппай пулы ал басқа аймақтарда білікті мамандарға арналған жұмыс орындары бар бірнеше мың иммигранттарды қуып шығады миллиондаған адамдарды құтқармау және оларға мұның болатынын айту жаман сенім,оңтүстік африка көптеген жыл бойы жұмыссыздық өс және ел белгілі бөлік жұмыссыз жаппай пул ал басқа аймақ білікті маман арнал жұмыс орын бар бірнеше мың иммигрант қу шығ миллиондаған адам құтқарма және оларға мұның бол айту жаман сенім,nazism

3,африкадағы жастар арасында жүргізілген кең ауқымды сауалнама оқиғаны баяндайды жастар өз болашағының операция дудула риторикасына байланысты емес екенін біледі,африка жас ара жүргіз кең ауқымды сауалнама оқи баянда жас өз болашағ операция дудул риторика байланысты емес екен біл,nazism

3,оңтүстік африкада көптеген жұмыс орындары бар мәселе мынада барлық жұмыс орындарын заңсыз шетелдіктер немесе оңтүстік африканың жалған құжаттары бар шетелдіктер алады мұның бәрі шетелдіктер міндетті түрде жұмыс істеуі керек,оңтүстік африка көптеген жұмыс орын бар мәселе мына барлық жұмыс орын заңсыз шетелдік немесе оңтүстік африка жалған құжат бар шетелдік ал мұның бәрі шетелдік міндетті түр жұмыс істе керек,nazism

3,өздерін оңтүстік афикандықтарды бірінші деп атайтын саяси жезөкшелермен қақтығысты елестетіп көріңізші дудула операциясы немесе қазір өздерін қалай атаса да гайтон маккензи герман машба ф а немесе африфорум джулиус қалады,өз оңтүстік афикандық бірінші де ата саяси жезөкше қақтығыс елестет көріңізш дудул операция немесе қазір өз қалай ата да гайтон маккензи герман машб ф а немесе африфору джулиус қал,nazism

3,африкалықтар ешқашан африкада заңсыз иммигранттар болмайды,африкалық ешқашан африка заңсыз иммигрант болма,nazism

3,депортациялану керек барлық қоқыс шетелдіктер біздің елде деп айтады,депортациялан керек барлық қоқыс шетелдік біздің ел де айт,nazism

3,тұр емес деп айтатындар сол шетелдіктерді жек көретін адамдар екеніне сенімдімін палеса әкең күлді қызың сені бауырым деп атады,тұр емес де айт сол шетелдік жек көр адам екен сенімді пале әке күл қыз сен бауыр де ат,nazism

3,оңтүстік африкада шетелдіктерге деген жеккөрініш бүгін басталған жоқ палесаның қайтыс болғанына өте қуаныштымын сондықтан оның әкесі де шетелдік ретінде азапты сезінуі мүмкін,оңтүстік африка шетелдік де жеккөрініш бүгін бастал жоқ палеса қайтыс бол өте қуанышты сондықтан оның әке де шетелдік рет аза сезін мүмкін,nazism

3,сарафина фильмі бұл гатволдық оңтүстік африкалықтардың айқын дәлелі олар атып өлтіріліп өлтірілуге мән бермеді және үмітсіз шетелдіктер басымызды ауыртып жатыр мен оны бұдан былай көтере алмайтынымызды сезінемін бостандық келе жатыр,сарафин фильм бұл гатво оңтүстік африкалық айқын дәлел олар ат өлтіріл өлтіріл мән берме және үмітсіз шетелдік бас ауырт жатыр мен оны бұдан былай көтер алма сез бостандық келе жатыр,nazism

3,мбалула бос әңгіме айтып жатыр жұмыспен қамту қызметіне қайшы келетін заңсыз шетелдіктерді заңсыз жалдаған заң бұзушылармен неге келіссөздер жүргізу керек жүк тасымалдаушы компанияларды қамауға алу керек,мбалул бос әңгіме айт жатыр жұмыс қамту қызмет қайшы кел заңсыз шетелдік заңсыз жалда заң бұзушы неге келіссөз жүргізу керек жүк тасымалдаушы компания қама алу керек,nazism

3,оның айтуынша шетелдіктер оңтүстік африкалықтардан қорқады бірақ олар күн сайын sa ға заңсыз келеді,оның айт шетелдік оңтүстік африкалық қорқ бірақ олар күн сайын sa ға заңсыз кел,nazism

3,арзан жұмыс күші үшін заңсыз шетелдіктерді жалдайтын жұмыс берушілер,арзан жұмыс күш үшін заңсыз шетелдік жалда жұмыс беруші,nazism

3,заңсыз шетелдіктер кәсіподақтарсыз арзан жұмыс күшін әкеледі бұл олардың wmc құраушылары мен қаржылық қолдаушылары үшін lra дан өтудің жолы бұл заңсыз шетелдіктер босқын немесе баспана іздеушілер емес фестивальде олар үйге барады,заңсыз шетелдік кәсіподақт арзан жұмыс күш әкел бұл олардың wmc құраушы мен қаржылық қолдаушы үшін lra дан өту жол бұл заңсыз шетелдік босқ немесе баспана іздеуші емес фестиваль олар үй бар,nazism

3,бием қаңтар айынан бастап көмекші мәселелерімен айналысады енді мен неге президент рамафосаның тек шетелдіктерді жалдайтынын түсінемін,бие қаңтар ай баста көмекші мәселе айналыс енді мен неге президент рамафоса тек шетелдік жалда түс,nazism

3,шетелдіктер бізге елімізді қалай басқару керектігін айтпайды және ол өз үйіне қайтуы керек олардың негізі оңтүстік африканың бұқарасына пайда әкелетін нені білдіреді,шетелдік бізге ел қалай басқару керектіг айтпа және ол өз үй қайт керек олардың негіз оңтүстік африка бұқара пайда әкел не білдір,nazism

3,операция мүшелері шетелдік азаматтарды ірі компаниялар мен басқа да жергілікті бизнестен шығаруға шақыруда,операция мүше шетелдік азамат ірі компания мен басқа да жергілікті бизнес шығар шақыр,nazism

3,дудула қозғалысының мүшелері sab polokwane ге талаптар меморандумын тапсыру үшін келді,дудул қозғалыс мүше sab polokwane ге талап меморандум тапсыру үшін кел,nazism

3,дудула операциясы көмектесуі мүмкін,дудул операция көмектес мүмкін,nazism

3,баффало мұрын қара халық тарихтағы ең жек көретін президент ретінде қалады оның әкімшілігі кезінде заңсыз шетелдіктердің легі қылмыстың артуы сыбайлас жемқорлықтың артуы жұмыссыздықтың өсу іелдің қарызының өсуі,баффало мұрын қара халық тарих ең жек көр президент рет қал оның әкімшіліг кез заңсыз шетелдік лег қылмыс арт сыбайлас жемқорлық арт жұмыссыздық өсу іе қарыз өс,nazism

3,бұған да қарсы тұратын ұлтжанды заңгерлер керек біздің елде бізді шетелдіктер өктемдей алмайды,бұған да қарсы тұр ұлтжа заңгер керек біздің ел біз шетелдік өктемде алма,nazism

3,өшірудің бөлігі емеспіз біз дауыс пен танымалдылыққа ие болу үшін sans арқылы күресіп жатқан саясаткерлер емеспіз біздің қара қауымдар өмір сүрмейінше біз өміріміз тоқтап тұрғанда адамды байыта алмаймыз,өшіру бөліг емес біз дауыс пен танымалдылық ие болу үшін sans арқылы күрес жат саясаткер емес біздің қара қауым өмір сүрмейінш біз өмір тоқта тұр адам байыт алма,nazism

3,бірақ заңсыз шетелдіктердің ағыны олардың бақылауында болып жатыр сондықтан олар пайдасыз,бірақ заңсыз шетелдік ағын олардың бақылау бол жатыр сондықтан олар пай,nazism

3,біздің сүйікті елімізді заңсыз иммигранттар мен үкіметтегі біліктілігі жоқ кадрлар зорлап жатқанда anc фракциясының соғыстары сияқты маңызды емес және тітіркендіретін ештеңе жоқ,біздің сүйікті ел заңсыз иммигрант мен үкімет біліктіліг жоқ кадр зорла жатқанда anc фракция соғыс сияқты маңызды емес және тітіркендір ештеңе жоқ,nazism

3,егер елес жұмысшылардың бенефициарларының кейбірі ақымақ үкімет жоғары мемлекеттік лауазымдарда жұмыс істейтін жалған жеке куәліктері бар заңсыз иммигранттар болса мен таң қалмас едім,егер елес жұмысшы бенефициар кейбір ақы үкімет жоғары мемлекеттік лауазым жұмыс істе жалған жеке куәлік бар заңсыз иммигрант бол мен таң қалмас ед,nazism

3,малеманың ауласындағы бағбан туралы айтуды ұмытпаңыз егер сіз ол жерде ешқашан болмаған болсаңыз,малема аула бағбан туралы айту ұмытпа егер сіз ол же ешқашан болма бол,nazism

3,ефф біздің елдің мәселесіне жауап деп сенетін адамнан ешқандай жала қабылдамаймын елімізді шетелдіктерге сататын сол eff,ефф біздің ел мәселе жауап де сен адам ешқандай жал қабылдама ел шетелдік сат сол eff,nazism

3,кез келген жұмыс істеп тұрған үкімет барлық заңсыз иммигранттарды депортациялау және шекара қауіпсіздігін күшейту арқылы оңтүстік африкадағы проблеманы біржола жояды,кез кел жұмыс істе тұр үкімет барлық заңсыз иммигрант депортацияла және шекара қауіпсіздіг күшейту арқылы оңтүстік африка проблема біржол жоя,nazism

3,мен барлық департаменттерге ашулана алмаймын бүкіл үкімет және олардың министрі менің аяқ киімімді киіп менің өтінішімді сезінеді,мен барлық департамент ашулан алма бүкіл үкімет және олардың министр менің аяқ киім ки менің өтініш сезін,nazism

3,осы айдың інен кейін мен бұдан былай наурызға бармаймын және ештеңеге наразылық білдірмеймін мен шын мәнінде апардхеид үкіметінде жасаған нәрселерді жасаймын және бұл жолы да жасаймын нашар болыңыз себебі менің мейірімділігіме жамандық жасаймын,осы ай інен кейін мен бұдан былай наурыз барма және ештеңе наразылық білдірме мен шын мән апардхеид үкім жаса нәрсе жаса және бұл жол да жаса нашар бол себебі менің мейірімділіг жаман жаса,nazism

3,шетелдіктер жұмысқа орналасса біздің адамдар жұмыссыз сіздің жұмыссыз емеспіз деп ойлаймын олар жай ғана бақыланбайтын патриоттық жүйемен шектелді,шетелдік жұмыс орналас біздің адам жұмыссыз сіздің жұмыссыз емес де ойла олар жай ғана бақыланба патриоттық жүйе шекте,nazism

3,eff заңсыз шетелдіктерді қорғайтынын айтуды ұмытып қалдыңыз,eff заңсыз шетелдік қорға айту ұмыт қал,nazism

3,міне заңсыз әрекеттермен айналысатын заңсыз иммигранттар шіріген полицейлерге қанша береді,міне заңсыз әрекет айналыс заңсыз иммигрант шірі полицей қанша бер,nazism

3,олар оңтүстік африкалықтар үшін шайқасты өлді мыңдаған адамдар қару ұнтағы мен көзден жас ағызатын газға қарсы тұрды олар оңтүстік африканың экономикасы мен басқаруына қосылу үшін күресті олар біз оңтүстік африкалықтар үшін шайқасты бүгін шетелдіктер мұның бәріне ие ал біз кедейлікте өмір сүріп жатырмыз,олар оңтүстік африкалық үшін шайқас өл мыңдаған адам қару ұн мен көз жас ағыз газ қарсы тұр олар оңтүстік африка экономика мен басқар қосылу үшін күре олар біз оңтүстік африкалық үшін шайқас бүгін шетелдік мұның бәрін ие ал біз кедейлік өмір сүр жатыр,nazism

3,жақсы бірақ оңтүстік африка шетелдіктерге заңсыз жұмыс орындарына тегін электр қуатына суға қауіпсіздік пен медициналық қызметтерге қарыз ба,жақсы бірақ оңтүстік африка шетелдік заңсыз жұмыс орын тегін электр қуат су қауіпсіздік пен медициналық қызмет қарыз ба,nazism

3,мен afriforum мен келісемін бірақ бұл заңсыз иммигранттардың құқықтары бар оларды иттер тістеп алса ше біз сәтсіз мемлекетпіз,мен afriforum мен келіс бірақ бұл заңсыз иммигрант құқық бар ол ит тісте ал ше біз сәт мемлекет,nazism

3,біз қанша қаламаймыз бірақ көзімізді аштық бұл жай ғана ол қаржылық қолдауға ие болмайды өйткені afriforum бобасвеуньянадан алатын бомоньяманилер сияқты соғұрлым біз anc деп атасақ олар жылайды,біз қанша қалама бірақ көз аштық бұл жай ғана ол қаржылық қолда ие болма өйткені afriforum бобасвеуньяна ал бомоньямани сияқты соғұрлым біз anc де ата олар жыла,nazism

3,жою күн тәртібі болуы керек біздің елден қылмыскерлердің тамырын жою оңтүстік африкада бірінші операция дудула,жою күн тәртіб бол керек біздің ел қылмыскер тамыр жою оңтүстік африка бірінші операция дудул,nazism

3,фридом парк хиллс экс паркі заңсыз шетелдіктер бізді олжалағанда төлейтін азаматтарды бірнеше апта бойы жарықсыз қалдырады,фридом парк хиллс экс парк заңсыз шетелдік біз олжала төле азамат бірнеше апта бойы жарық қалдыр,nazism

3,халық көтеріліп өздеріне тиесілі нәрсені алады саяси партиялар мен шетелдіктер тозаққа түседі жақында біз қолымызға қару алып бұл тәртіпсіздікті біржола ретке келтіреміз біз бәрібір аштық пен кедейліктен өлдік,халық көтеріл өз тиесілі нәрсе ал саяси партия мен шетелдік тозақ түс жақында біз қол қару алып бұл тәртіпсіздік біржол рет келтір біз бәрібір аштық пен кедейлік өл,nazism

3,мен өз доптарыммен бәс тіге аламын,мен өз доп бәс тіг ал,nazism

3,зимбабвеліктер операцияның негізгі қатысушылары dudula putsouthafricafirst зимбабвелік заңсыз иммиграннттар маңызды инфрақұрылымды зорлауы өлтіруі ұрлауы және қиратуы мүмкін,зимбабвелік операция негізгі қатысушы dudula putsouthafricafirst зимбабвелік заңсыз иммигрант маңызды инфрақұрылым зорла өлтір ұрла және қират мүмкін,nazism

3,бафана жаттықтырушысы компанияның ақ иелері айтатын нәрсені айтады біз оңтүстік африкалықтар ретінде ешнәрседе жақсы емеспіз сондықтан олар бізден шетелдіктерді артық көреміз біз өзімізді тексеруіміз керек,бафан жаттықтырушы компания ақ ие айт нәрсе айт біз оңтүстік африкалық рет ешнәрсе жақсы емес сондықтан олар бізден шетелдік артық көр біз өз тексер керек,nazism

3,оңтүстік африкалық жастарға жұмысқа орналасу кезінде шетелдіктерге қарағанда бірінші артықшылық беріңіз,оңтүстік африкалық жас жұмыс орналасу кез шетелдік қара бірінші артықшылық бер,nazism

3,дудула операциясы мүшелері екі компаниядан шетелдік азаматтарды жұмыстан шығарып олардың орнына жергілікті адамдарды қоюды талап етуде,дудул операция мүше екі компания шетелдік азамат жұмыс шығар олардың орн жергілікті адам қою талап ет,nazism

3,дудула операциясы мүшелері екі корпоративтік компанияға талаптар меморандумын жеткізу үшін полокване өнеркәсіп орталығына бара жатыр,дудул операция мүше екі корпоративтік компания талап меморандум жеткізу үшін полокван өнеркәсіп орталығ бар жатыр,nazism

3,шетелдіктер тек қара деп кім айтты,шетелдік тек қара де кім ай,nazism

3,шетелдіктер жұмыс істемейді деп айта бересіз бірақ біз оларды күнде көреміз өзіңіз тұратын жартастан шығыңыз,шетелдік жұмыс істеме де ай бер бірақ біз ол күн көр өз тұр жартас шығ,nazism

3,шетелдіктер оңтүстік африкада және кодты жүргізуші куәлігін қалай алады,шетелдік оңтүстік африка және код жүргізуші куәлік қалай ал,nazism

3,шетелдіктер бұл бөлімшелерді басып алады,шетелдік бұл бөлімше бас ал,nazism

3,путоңтүстікафрикабірінші операция дудула бұл біздің ел және біз оны родезиядан малавиден және нигериядан келген заңсыз иммигранттармен бөліскіміз келмейді бұл адамдар зорлаушы көлік ұрлаушылар,путоңтүстікафрикабірінш операция дудул бұл біздің ел және біз оны родезия малави және нигерия кел заңсыз иммигрант бөліс келме бұл адам зорлаушы көлік ұрлаушы,nazism

3,дудула тойи сарбазы мұны айналдыра алмайды,дудул тойи сарбаз мұны айналдыр алма,nazism

3,ағайындылар өле өлгенше төбелессе олардың мүлкін бейтаныс адам еншілейді операция дудула билік басындағылар қоғамды ауруға айналдырған және барған сайын ашуландырғанының белгісі,ағайынды өл өлгенш төбелес олардың мүлк бейтаныс адам еншіле операция дудул билік басындағы қоғам ауру айналдыр және бар сайын ашуландыр бел,nazism

3,драйверлері бар ма әлде uber сияқты барлық шетелдіктер ме,драйвер бар ма әлде uber сияқты барлық шетелдік ме,nazism

3,біріккен корольдік руандаға неғұрлым көп адам үлесін жіберсе кедейлік соғұрлым көп болады және ұлыбританияға көбірек иммигранттар оралатыны өкінішті менің ойымша ұлыбритания азаматтары оларды мұқият қарауы керек заттардың түрлері,біріккен корольдік руанда неғұрлым көп адам үлес жібер кедейлік соғұрлым көп бол және ұлыбритания көбірек иммигрант орал өкініш менің ойымша ұлыбритания азамат ол мұқият қара керек зат түр,nazism

3,барлық шетелдіктерді кінәлауды доғарыңыз ең танымал бұзақылар біздің ағаларымыз сфила набо сфуза набо силала набо,барлық шетелдік кінәлау доғар ең танымал бұзақы біздің аға сфил набо сфуз набо силал набо,nazism

3,оңтүстік африкада жасалған әрбір қылмысқа зимбабвелік қатысады және қалыпты билеуші үкімет зимбабвеліктерге ұлтты қылмыстарынан қорғау үшін елге кіруге тыйым салады зимбабвенің дудула американдықтарды бірінші орынға қояды операциясы,оңтүстік африка жасал әрбір қылмыс зимбабвелік қатыс және қалыпты билеуш үкімет зимбабвелік ұл қылмыс қорғау үшін ел кір тыйым сал зимбабве дудул американдық бірінші орын қоя операция,nazism

3,дудула операциясына қарсы шыққан оңтүстік африкалықтарды ешқашан түсінбеймін сыртта соғыс бар олар аман есен шықты деп үміттенемін қорқынышты,дудул операция қарсы шық оңтүстік африкалық ешқашан түсінбе сырт соғыс бар олар аман есен шық де үміттен қорқыныш,nazism

3,малема оны өзінің заңсыз шетелдіктерімен шығармашылық тәсілдерімен нонсенс ретінде жойғанға дейін жылдан бері дауыс бердім енді жоқ,мале оны өзінің заңсыз

шетелдік шығармашылық тәсіл нонсенс рет жой дейін жыл бері дауыс бер енді жоқ,nazism

3,бұл шетелдіктер оңтүстік кореяда жақсы сөйлейді бірақ өз елдерінде мылқау есектер,бұл шетелдік оңтүстік корея жақсы сөйле бірақ өз ел мылқа есек,nazism

3,сонымен санс жұмысқа жарамды болады ал шетелдіктер жұмысқа орналасады ма біз бәрімізді зомби деп ойлауымыз керек,сонымен санс жұмыс жарамды бол ал шетелдік жұмыс орналас ма біз бәрі зомби де ойла керек,nazism

3,біз африкалық иммигранттардың көпшілігі кедей екенін және оны көтере алмайтынын білеміз енді мені сұрақ қоюға не мәжбүр етеді,біз африкалық иммигрант көпшіліг кедей екен және оны көтер алма біл енді мен сұрақ қою не мәжбүр ет,nazism

3,біз дудуланың заңгерлерімен кеңесіп са үкіметіне мысалы иммиграция полиция халықаралық қатынастар және б заңсыз иммигранттарға сада тұруға рұқсат беру арқылы конституцияны сақтамағаны үшін айып тағу ықтималдығын қарастыруымыз керек пе,біз дудула заңгер кеңес са үкімет мысал иммиграция полиция халықаралық қатынас және б заңсыз иммигрант са тұр рұқсат беру арқылы конституция сақтама үшін айып тағ ықтималдығ қарастыр керек пе,nazism

3,көрдіңіз бе бізге оңтүстік кореяда туылған деген белгіні алып жүретін қылмыскерлер жетіспейді gp mp және провинцияларында жергілікті тұрғындарға қарағанда көбірек шетелдіктер дүниеге келді,көр бе бізге оңтүстік корея туыл де белгі алып жүр қылмыскер жетіспе gp mp және провинция жергілікті тұрғын қара көбірек шетелдік дүние кел,nazism

3,оңтүстік африка президенті кирилл рамафоса шетелдік азаматтарды жалдайды арзан жұмыс күші заңсыз иммигранттар сапрезидент шетелдік патриоттарды twitter ретінде пайдаланады дудула операциясы африканы бірінші орынға қояды,оңтүстік африка президент кирилл рамафо шетелдік азамат жалда арзан жұмыс күш заңсыз иммигрант сапрезидент шетелдік патриот twitter рет пайдалан дудул операция африка бірінші орын қоя,nazism

3,шетелдіктерге барыңыз олар бұл жерден кетуі керек,шетелдік бар олар бұл же кет керек,nazism

3,неліктен заңсыз иммигранттарға оңтүстік африкаға кіруге және қалағанын жасауға рұқсат етіледі,неліктен заңсыз иммигрант оңтүстік африка кір және қала жаса рұқсат ет,nazism

3,сіз әрекет ете алмайсыз сіздің мансабыңыз аяқталады,сіз әрекет ет алма сіздің мансаб аяқтал,nazism

3,олар апартеидте бір бірімен кездескен болуы мүмкін мандела қара нәсілділердің үстемдігі туралы айтады біз мұны бүгін оңтүстік кореяны өз үкіметтері қызмет етіп жатқан шетелдіктермен дерлік жоқ ресурстарды бөлісуге мәжбүрлеп қорқытып жатқанын көреміз,олар апартеид бір бір кездес бол мүмкін мандел қара нәсілді

үстемдіг туралы айт біз мұны бүгін оңтүстік корея өз үкімет қызмет ет жат шетелдік дерлік жоқ ресурс бөліс мәжбүрле қорқыт жат көр,nazism

3,сондықтан мен бұл мекемеге кірдім иә мен өте қырағымын және сұхбат басталғанға дейін көптеген сұрақтар қоямын сондықтан мен бұл компаниядағы адамдар шетелдіктер екенін түсіндім бұл менің алғашқы тәжірибем болды мен кіріп түйіндемемді тапсырдым мені таң қалдырғаны жігіт оны тіпті ашпады,сондықтан мен бұл мекеме кір иә мен өте қырағ және сұхбат бастал дейін көптеген сұрақ қоя сондықтан мен бұл компания адам шетелдік екен түс бұл менің алғашқы тәжірибе бол мен кір түйіндеме тапсыр мен таң қалдыр жігіт оны тіпті ашпа,nazism

3,егер сіз оны түзетсеңіз де ол әлі де ештеңе түсінбейді бұл аңдар ақымақ өйткені олар жай ғана кек алушы дейді және бұл сөздің мағынасын және оны кек алушы ететін нәрсені білмейді,егер сіз оны түзет де ол әлі де ештеңе түсінбе бұл аң ақы өйткені олар жай ғана кек алушы де және бұл сөз мағына және оны кек алушы ет нәрсе білме,nazism

3,неліктен біз жасқа дейінгі жастардың саяси партиялардың жоғарғы басшылығына кіргенін көрмейміз олар мұны істегенде олардың жоғалуы әрқашан уақыт мәселесі болып табылады жастар үстемдік ететін саяси партия бізге қажет өзгерістер деп ойлайсыз ба айтпақшы мен саясатқа сенбеймін,неліктен біз жас дейінгі жас саяси партия жоғарғы басшылығ кір көрме олар мұны істе олардың жоғал әрқашан уақыт мәселе бол таб жас үстемдік ет саяси партия бізге қажет өзгеріс де ойла ба айтпақшы мен саясат сенбе,nazism

3,сіз сауда орталықтарын тонаушылардан қорғайтын шынымен таңғажайып адамсыз сізге және басқа командаға дүкендер төлейді деп үміттенемін өйткені сіз оларды миллиондаған рандтан құтқардыңыз құдай жарылқасын,сіз сауда орталық тонаушы қорға шын таңғажайып адам сізге және басқа команда дүкен төле де үміттен өйткені сіз ол миллиондаған ранд құтқар құдай жарылқа,nazism

3,қарт адамдар өз үйлерінде немерелерінен және зейнетақыларынан ләззат алуы керек емес пе мұнда жастар да қарттар да жоқ тек парламент мүшелері бар,қарт адам өз үй немере және зейнетақы ләззат ал керек емес пе мұнда жас да қарт да жоқ тек парламент мүше бар,nazism

3,адамдар ақыл ойы мен идеялары таусылғанда ғана қатты қатыгез және менсінбейтін болады жас eff спикерін және басқаларды кінәламаңыз оларды қарттарды құрметтемеуге мәжбүрлейтіндерді кінәлаңыз олардың барлығына құшақ керек,адам ақыл ой мен идея таусыл ғана қатты қатыгез және менсінбе бол жас eff спикер және басқа кінәлама ол қарт құрметтеме мәжбүрле кінәла олардың барлығ құшақ керек,nazism

3,сізде біздің қолдауымыз бар және қанаттарыңызды басқа аймақтарға жайыңыз бұл қиын кезеңдер бастауыш деңгейдегі осындай бастамаларға мүмкіндік береді нан бизнесін сенімді ақ пен шетелдіктерге қайтарайық,сіз біздің қолда бар және қанат

басқа аймақ жай бұл қиын кезең бастауыш деңгей осындай бастама мүмкіндік бер нан бизнес сенімді ақ пен шетелдік қайтар,nazism

3,көлік жүргізуде ұрлауда зорлауда өлтіруде және б нашар жұмыс істейтін көптеген оңтүстік африкалықтар бар бұл шетелдіктерге оны қосу үшін карт бланш беріледі дегенді білдірмейді,көлік жүргіз ұрла зорла өлтір және б нашар жұмыс істе көптеген оңтүстік африкалық бар бұл шетелдік оны қосу үшін карт бланш бер де білдірме,nazism

3,қалай болғанда да шетелдіктер оңтүстік африкада дағдылардың жетіспеушілігінен ғана жұмыс істеуі керек сонымен қатар сіз салықты алып оны көрінбейтін жерге жұмсай аласыз,қалай болғанда да шетелдік оңтүстік африка дағды жетіспеушіліг ғана жұмыс істе керек сонымен қатар сіз салық алып оны көрінбе же жұмса ал,nazism

3,өкінішке орай қарапайым азаматтар зардап шегеді,өкініш орай қарапайым азамат зардап шег,nazism

3,көлік министрі такси индустриясының ұлттық ажыратудан бас тартуын мақұлдайды операция жетекшісі дудула олардың бет әлпетсіз ұлттық ажыратудың бөлігі емес екенін айтты,көлік министр такси индустрия ұлттық ажырату бас тарт мақұлда операция жетекші дудул олардың бет әлпет ұлттық ажырат бөліг емес екен ай,nazism

3,бұл апат құқықтары рұқсаттары жоқ зимбабвенің кінәсінен болды қарапайым тілмен айтқанда бұл зимбабведен келген заңсыз иммиgranttың кінәсінен болды адам қайтыс болды,бұл апат құқық рұқсат жоқ зимбабве кінә бол қарапайым тіл айтқанда бұл зимбабве кел заңсыз иммигрант кінә бол адам қайтыс бол,nazism

3,чемодандарды мырзаларды жинайтын кез келді алғашқы зимбабвеліктерді оңтүстік африкаға жіберетін дудула операциясының уақыты келді,чемодан мырза жина кез кел алғашқы зимбабвелік оңтүстік африка жібер дудул операция уақыт кел,nazism

3,одан кейінгі апат үшін кім жауапты болады елес сізді тағы да ауылдардағы қара бизнесті жоюға апарғанын қалайсыз ба дұрыс жолмен және дұрыс орындармен жүріңіз,одан кейінгі апат үшін кім жауапты бол елес сіз тағы да ауыл қара бизнес жою апар қала ба дұрыс жол және дұрыс орын жүр,nazism

3,біздің жалғыз үмітіміз дудула операциясы,біздің жалғыз үміт дудул операция,nazism

3,тағы да зимбабве неге бұл жануарлар өзгергісі келмейді,тағы да зимбабв неге бұл жануар өзгер келме,nazism

3,сіз тонағыңыз келе ме,сіз тона келе ме,nazism

3,біз мүмкін жеткіншектердің өзіне аз ақша аламыз,біз мүмкін жеткіншек өзіне аз ақша ал,nazism

3,баас джонды қолдау үшін өз халқынан алшақтады қара төбет,баас джо қолдау үшін өз халқ алшақта қара төбет,nazism

3,оңтүстік африкалықтардың жеке басын ұрлайтын шетелдіктермен бірдей олар миллиондаған оңтүстік африкалықтар туралы емес тек өздері туралы ойлайды,оңтүстік африкалық жеке басын ұрла шетелдік бірде олар миллиондаған оңтүстік африкалық туралы емес тек өз туралы ойла,nazism

3,біз өз қауымдастықтарымызды осы жалпыұлттық өшіруден туындауы мүмкін қылмыстың кез келген түрінен қорғауға дайын соуэтоның барлық күштерін орналастырдық,біз өз қауымдастық осы жалпыұлттық өшіру туында мүмкін қылмыс кез кел түр қорға дайын соуэто барлық күш орналас,nazism

3,бұл бет әлпетсіз ұлттық шатдаун ықтимал тонауды және б қамтитын қылмыстық әрекеттің жоғары қаупін тудырады,бұл бет әлпет ұлттық шатдаун ықтимал тонау және б қамтит қылмыстық әрекет жоғары қауп тудыр,nazism

3,жалғыз күресіңіз біз өмір бойы экономикалық еркіндігіміз үшін күресеміз жалғыз немесе бірнеше заңсыз иммиграннттарды бізге қосылуға мәжбүр етеміз джуффало олармен айналысады ұят,жалғыз күрес біз өмір бойы экономикалық еркіндіг үшін күрес жалғыз немесе бірнеше заңсыз иммигрант бізге қосыл мәжбүр ет джуффало олармен айналыс ұят,nazism

3,бұл сіздің проблемаңыз емес сіз шетелдіктерді ерекше түр деп ойлайсыз бұл адамдар оңтүстік африкалықтарды олардың көмегінсіз өлтіреді біз маңызды емеспіз солай ма,бұл сіздің проблема емес сіз шетелдік ерекше түр де ойла бұл адам оңтүстік африкалық олардың көмегін өлтір біз маңызды емес солай ма,nazism

3,мен қатты қайғырамын бұл қолайсыз заңсыз шетелдіктер кетуі керек ағаңның жаны шықсын,мен қатты қайғыр бұл қолайсыз заңсыз шетелдік кет керек аға жан шық,nazism

3,оңтүстік африка африкадағы заңсыз шетелдіктерге төзімді жалғыз ел басқалары депортациялайды гана ұзақ өмір сүрсін ұзақ өмір сүрсін бір африка менің аяғым,оңтүстік африка африка заңсыз шетелдік төз жалғыз ел басқа депортацияла ган ұзақ өмір сүр ұзақ өмір сүр бір африка менің ая,nazism

3,зимбабвеліктер шетелдіктер бұл адамдар оңтүстік африкада алғашқы операция жасау арқылы біздің сүйікті елімізді қиратты,зимбабвелік шетелдік бұл адам оңтүстік африка алғашқы операция жасау арқылы біздің сүйікті ел қира,nazism

3,біздің саясаткерлер бізді сәтсіздікке ұшыратты енді біз шетелдіктерден қорқып елімізде көлікті пайдалана алмаймыз бірақ адамдар әлі де олар үшін дауыс бергенімізді қалайды біз үшін ешкім сөйлемейді сөйлейтіндер қорлайды бұл қайғылы тіпті малема үмітсіз,біздің саясаткер біз сәтсіздік ұшыра енді біз шетелдік қорқ ел көлік пайдалан алма бірақ адам әлі де олар үшін дауыс бер қала біз үшін ешкім сөйлеме сөйле қорла бұл қайғылы тіпті мале үмітсіз,nazism

3,сонымен сіз шетелдіктер санстың шетелдіктерге қатысты жасаған бірнеше жалпы кісі өлтірулерімен салыстырғанда оңтүстік африкалықтарды көбірек өлтіретінін білесіз

бе егер біз сіз және басқа африкалықтар сияқты жаман болсақ бізді суреттегіңіз келсе неге мұнда көбірек адамдар келеді,сонымен сіз шетелдік сан шетелдік қатысты жаса бірнеше жалпы кісі өлтіру салыстыр оңтүстік африкалық көбірек өлтір біл бе егер біз сіз және басқа африкалық сияқты жаман бол біз суретте кел неге мұнда көбірек адам кел,nazism

3,заңсыз шетелдіктер бұл қылмыстардың барлығын біздің көмегімізсіз ақтай алмайды олардан ұрланған кабельдерді сатып алатын полигондар олардан ұрланған көліктерді сатып алатын адамдар және б қылмыс статистикасына үлкен үлес қосады біз шынымен де шайқаста жеңіліп жатырмыз бұл мәселелердің тамыры біздің қоғам,заңсыз шетелдік бұл қылмыс барлығ біздің көмег ақта алма олардан ұрлан кабель сат ал полигон олардан ұрлан көлік сат ал адам және б қылмыс статистика үлкен үлес қос біз шын де шайқас жеңіл жатыр бұл мәселе тамыр біздің қоғам,nazism

3,оңтүстік африка халқы оян оңтүстік африкаға шабуыл жасалуда бұл оңтүстік африкамен жарияланбаған соғыс қарулы бөлкім заңсыз шетелдіктер шахтаға шабуыл жасады ресми түрде оңтүстік африка заңсыздық елі әрқайсысы өзі үшін жүр,оңтүстік африка халқ оян оңтүстік африка шабуыл жасал бұл оңтүстік африка жарияланба соғыс қарулы бөлкім заңсыз шетелдік шахта шабуыл жас ресми түр оңтүстік африка заңсыз ел әрқайсы өзі үшін жүр,nazism

3,бұл адамдардың барлығы күнде оқ жарақатынан қаза тапты бізге шетелдіктер шабуыл жасады,бұл адам барлығ күн оқ жарақат қаза тап бізге шетелдік шабуыл жас,nazism

3,лона ла хланья тікелей шетелдіктер ақ экономикада жұмыс істейді ал оңтүстік африкалықтар үйде жұмыссыз отырады сізде жұмыссыздық туралы айтуға моральдық негіз жоқ сондықтан үндемеңіз африка елдерінің барлық елшіліктеріне барып оларды алыңыз,лон ла хланья тікелей шетелдік ақ экономика жұмыс істе ал оңтүстік африкалық үй жұмыссыз отыр сіз жұмыссыздық туралы айт моральдық негіз жоқ сондықтан үндеме африка ел барлық елшілік бар ол ал,nazism

3,оның аузынан ол зимбабвеліктерді үйін салуға жалдағанын айтты,оның ауз ол зимбабвелік үйін сал жалда ай,nazism

3,кім сөйлейтінін қараңыз заңсыз шетелдіктерді біздің заңдарымызды бұзуға шақыратын адам сізге сәйкес болған кезде заң сақталуы керек пе,кім сөйле қара заңсыз шетелдік біздің заң бұз шақыр адам сізге сәйкес бол кезде заң сақтал керек пе,nazism

3,күн сайын қылмыс жасайтын шетелдіктер бар,күн сайын қылмыс жаса шетелдік бар,nazism

3,ішкі істер министрлігінің шенеуніктері біздің құжаттарымызды шетелдіктерге сатпайды емес пе,ішкі істер министрліг шенеунік біздің құжат шетелдік сатпа емес пе,nazism

3,дудула операциясы кедей қара африкалықтарды барлық жерде қудалайтын бұзақылар тобы африкалықтар өздерін қара тері пигментациясын жек көретіндерден қорғаудан қорықпауы керек,дудул операция кедей қара африкалық барлық же қудала бұзақы тоб африкалық өз қара тері пигментация жек көр қорғау қорықпа керек,nazism

3,сіз тауды заңсыз басып алған шетелдіктердің бостандығынан қорқасыз,сіз тау заңсыз бас ал шетелдік бостандығ қор,nazism

3,маған бұл эпизод ұнады біз кг ұрланған ұялы телефондары бар шетелдіктерді көрдік кейінірек біз оңтүстік африкалықты ұрып соғып жатқан шетелдікті көрдік және ауыр заттарды көлікке апарғандықтан көмектесе алмадық,маған бұл эпизод ұн біз кг ұрлан ұялы телефон бар шетелдік көр кейінірек біз оңтүстік африкалық ұр соғ жат шетелдік көр және ауыр зат көлік апар көмекте алма,nazism

3,сондықтан біз қорқуымыз керек өйткені біздің аулаларда шетелдіктерге тиесілі дүкендер бар,сондықтан біз қорқ керек өйткені біздің аула шетелдік тиесілі дүкен бар,nazism

3,дұрыс бірақ бұл біздің еліміздегі құжатсыз шетелдіктерді заңдастыру бұл шетелдіктер құжатсыз жұмыс орындарының болмауына байланысты ересектерге өздері жақсы көретін және отбасы деп санайтындардың ішіне тамақ салуға кедергі келтіреді,дұрыс бірақ бұл біздің еліміз құжат шетелдік заңдастыр бұл шетелдік құжат жұмыс орын болма байланысты ересек өз жақсы көр және отбасы де сана іш тамақ сал кедергі келтір,nazism

3,оңтүстік африка сіздің доптан үлке қаншық ұлы,оңтүстік африка сіздің доп үл қаншық ұлы,nazism

3,толық ақымақты шетелдіктер жұмысқа орналасады деп айту ақымақ емес бұл фактіні қабылдау адамды ксенофобқа айналдырмайды керісінше бізді қиын сұрақтар қоюға итермелейді неліктен бұлай бұл әңгімені өшіру ешкімге көмектеспейді,толық ақымақ шетелдік жұмыс орналас де айту ақы емес бұл факті қабылдау адам ксенофоб айналдырма керісінше біз қиын сұрақ қою итермеле неліктен бұлай бұл әңгіме өшір ешкім көмектеспе,nazism

3,бұл заңсыз иммигранттардан кім сатып алады,бұл заңсыз иммигрант кім сат ал,nazism

3,оңтүстік африка бұл әзіл шетелдіктер алаяқтық тендерлерде миллиондаған адамды тырмалайды ал оңтүстік африкалықтар ауылдарда жұмыссыз отырады,оңтүстік африка бұл әзіл шетелдік алаяқтық тендер миллиондаған адам тырмала ал оңтүстік африкалық ауыл жұмыссыз отыр,nazism

3,неліктен біз оңтүстік каролина заңдарын таңдамалы түрде қолдануымыз керек және ол қашан сәйкес келеді иммиграция туралы заңды оңтүстік каролинаны басып жатқан заңсыз шетелдіктер бұзады заңсыз шетелдіктерді жұмысқа қабылдау кезінде жұмыспен қамту қызметі туралы заң бұзылады,неліктен біз оңтүстік каролин заң

таңдамал түр қолдан керек және ол қашан сәйкес кел иммиграция туралы заңды оңтүстік каролина бас жат заңсыз шетелдік бұз заңсыз шетелдік жұмыс қабылдау кез жұмыс қамту қызмет туралы заң бұз,nazism

3,сіздің ойыңызша біз заңсыз иммигранттарға дұрыс қарауымыз керек пе бұл сіз айтқыңыз келетін нәрсе ме мен кенияға кіріп одан заңды түрде шығып сол елдің заңдарын және билігін құрметтедім неліктен rsa тіркелмеген және заңсыз иммигранттарды қабылдайды деп күтесіз руанда иммигранттармен қалай күреседі,сіздің ойыңызша біз заңсыз иммигрант дұрыс қара керек пе бұл сіз айт кел нәрсе ме мен кения кір одан заңды түр шығ сол ел заң және билік құрметте неліктен rsa тіркелме және заңсыз иммигрант қабылда де күт руан иммигрант қалай күрес,nazism

3,форманың сізді алдауына жол бермеңіз бұл көк форманы киетін адамдар біздің адамдар әкелеріміз аналарымыз әпкелеріміз бауырларымыз нағашыларымыз бен тәтелеріміз бұл форманы бейтаныс емес адамдар киеді,форма сіз алда жол берме бұл көк форма ки адам біздің адам әке ана әпке бауыр нағашы бен тәте бұл форма бейтаныс емес адам ки,nazism

3,мен абдырап қалдым енді біз шетелдіктерді қайтадан жақсы көреміз бе,мен абдыра қал енді біз шетелдік қайта жақсы көр бе,nazism

3,конье кирилл рамафоса оңтүстік африкалықтарға сенбейді сондықтан ол заңсыз иммигранттарды да жалдады,конь кирилл рамафо оңтүстік африкалық сенбе сондықтан ол заңсыз иммигрант да жалда,nazism

3,бізде әлі де заңсыз шетелдіктер мен есірткі барондардың пайызы бар өйткені полицейлер мен саясаткерлер тым жемқор ал йобургтің орталық іскери ауданы тым лас және зәрдің иісі бар,біз әлі де заңсыз шетелдік мен есірткі барон пайыз бар өйткені полицей мен саясаткер тым жемқор ал йобург орталық іскери аудан тым лас және зәр иіс бар,nazism

3,шетелдіктерді өз тәуекеліңізге жалдаңыз,шетелдік өз тәуекел жалда,nazism

3,мүмкін бұұ бжкб сарадан кететін уақыт келді біз шетелдіктердің орталық бизнес ауданында лашық салуға жол бере алмаймыз,мүмкін бұұ бжкб сара кет уақыт кел біз шетелдік орталық бизнес аудан лашық сал жол бер алма,nazism

3,соуэтодағы эври уорд үшін менің арманым приходтың рухын жоғары деңгейге көтеріңіз,соуэто эври уорд үшін менің арман приход рух жоғары деңгей көтер,nazism

3,гупталардың оңтүстік африкаға экстрадицияллануы және олардың әрекеттері үшін заңның барлық күшіне енуі үшін уақыт немесе тіпті жылдар қажет бұл оңай процесс емес министр рональд ламолаға ең жақсысы,гупта оңтүстік африка экстрадициялан және олардың әрекет үшін заң барлық күш ен үшін уақыт немесе тіпті жыл қажет бұл оңай процесс емес министр рональд ламола ең жақсы,nazism

3,құжаттары бар және құжаттары жоқ шетелдіктер бұл елдің заңдарын құрметтемейтінін байқадыңыз ба,құжат бар және құжат жоқ шетелдік бұл ел заң құрметтеме байқа ба,nazism

3,менің жылап күлетін иығым біз йоханнесбургте шетелдіктер ретінде отбасы болдық,менің жыла күл иы біз йоханнесбург шетелдік рет отбасы бол,nazism

3,американдықтарды бірінші орынға қойыңыз,американдық бірінші орын қой,nazism

3,джулиас ешқашан ештеңе жасамайды оның қалағаны заңсыз шетелдіктердің оңтүстік африканы басып алуы оларға жалған жеке куәлік беруі сондықтан олар оған дауыс беру арқылы оның мемлекет президенті болуға деген үмітін қолдай алады,джулиас ешқашан ештеңе жасама оның қала заңсыз шетелдік оңтүстік африка бас ал оларға жалған жеке куәлік беруі сондықтан олар оған дауыс беру арқылы оның мемлекет президент бол де үміт қолда ал,nazism

3,шетелдіктермен кездесетін оларға тұрмысқа шығатын әйелдер укути квензакалани эйнтликрге біраз жарық түсіре ала ма,шетелдік кездес оларға тұрмыс шығ әйел укути квензакалани эйнтлик біраз жарық түсір ал ма,nazism

3,рамафоса заңсыз иммигранттарды контрабандамен айналысады және оларға қылмыс жасағаны үшін ақша төлейді,рамафо заңсыз иммигрант контрабанда айналыс және оларға қылмыс жаса үшін ақша төле,nazism

3,енді мен талшық өндіретін жұмыста шетелдіктердің көп екенін түсіндім,енді мен талшық өндір жұмыс шетелдік көп екен түс,nazism

3,мара шетелдіктер неге оңтүстік африка істерімен айналысқанды ұнатады,мар шетелдік неге оңтүстік африка іс айналыс ұнат,nazism

3,зимбабведе оңтүстік африкамен немен сауда жасауға болады,зимбабве оңтүстік африка не сауда жаса бол,nazism

3,неліктен шетелдіктерді жалдайтыныңызды түсіндіре аласыз ба,неліктен шетелдік жалда түсіндір ал ба,nazism

3,сіз қалайсыз заңсыз шетелдіктеріңізді өзіңізде ұстаңыз сонымен бірге сіз бейбітшілікті қалайсыз,сіз қала заңсыз шетелдік өз ұста сонымен бірге сіз бейбітшілік қала,nazism

3,па мабхантитиге герман машабаның көмегі қажет ал германға дудула операциясы қозғалысының көмегі қажет,па мабхантити герман машаба көмег қажет ал герман дудул операция қозғалыс көмег қажет,nazism

3,сіз оңтүстік африканың проблемалары тек үкіметтің мәселесі шетелдіктер емес дағдылардың жетіспеушілігі жасөспірімдердің жүктілігі гендерлік зорлық зомбылық нәсілшілдік емес,сіз оңтүстік африка проблема тек үкімет мәселе шетелдік емес дағды жетіспеушіліг жасөспірім жүктіліг гендерлік зорлық зомбылық нәсілш емес,nazism

3,тыныш біз аналарымызға бауырларымызға және мхулуға өз мүлкін шетелдіктерге жалға беруді тоқтатуды айтамыз,тыныш біз ана бауыр және мхул өз мүлк шетелдік жалға беру тоқтату ай,nazism

3,дудула операциясын белсендіру қажет теріс нәтиже,дудул операция белсендір қажет теріс нәтиже,nazism

3,нью йоркте де шетелдіктер бар адамдардың қалағанынша істеуіне кедергі болатын заңдар бар,нью йорк де шетелдік бар адам қалағанынш істе кедергі бол заң бар,nazism

3,келесі жолы сіз оны таба аласыз оны жоғалтасыз бұл полиция мен қылмыскерлерге түсінікті тіл,келесі жол сіз оны таб ал оны жоғал бұл полиция мен қылмыскер түсінік тіл,nazism

3,сондықтан олар әрқашан оңтүстік кореядағы заңсыз иммигранттарды қорғайды өйткені олар оларды арзан жұмыс күші деп санайды,сондықтан олар әрқашан оңтүстік корея заңсыз иммигрант қорға өйткені олар ол арзан жұмыс күш де сана,nazism

3,лесотодан келген шетелдіктер иә бұл провинцияның осы елге жақындығы мп мен эсватини сияқты өте үлкен мәселе басқа елдердегі елдермен күресу өте тұрақсыз,лесото кел шетелдік иә бұл провинция осы ел жақындығ мп мен эсватини сияқты өте үлкен мәселе басқа ел ел күресу өте тұрақсыз,nazism

3,неліктен шетелдіктерге оңтүстік африкада шіркеулер ашу оңай,неліктен шетелдік оңтүстік африка шіркеу ашу оңай,nazism

3,президент кирилл рамафосаның фермасында жұмыс істейтін шетелдіктер заңсыз иммигранттар ма,президент кирилл рамафоса ферма жұмыс істе шетелдік заңсыз иммигрант ма,nazism

3,шетел валютасын әкелген шетелдіктер президентке тиесілі фермадан қымбат ойын сатып алады,шетел валюта әкел шетелдік президент тиесілі ферма қымбат ойын сат ал,nazism

3,бұл қылмыстардың көпшілігін оңтүстік кореяда шетелдіктер жасайды зимбабвеліктер мен нигериялықтар бұл өткен аптада намибияда жасар қызды өлтірген зимбо біз сізді көреміз зимбабвеліктер жақында лимпопода түрмеге қамалды сериялық зорлаушы,бұл қылмыс көпшілiг оңтүстік корея шетелдік жаса зимбабвелік мен нигериялық бұл өткен апта намибия жасар қыз өлтір зимбо біз сіз көр зимбабвелік жақында лимпопо түрме қама сериялық зорлаушы,nazism

3,сіз нүктеге жеттіңіз қымбаттым мен әрқашан рамафосаның фалафала фермасында шетелдіктер жұмыс істейтінін айттым олар намибиядан шыққан мен мұны жеке білемін айтпақшы ол миллиондаған рандқа сатып алған қымбат буйвол бірнеше аптадан кейін фермада қайтыс болды,сіз нүкте же қымбат мен әрқашан рамафоса фалафал ферма шетелдік жұмыс істе ай олар намибия шық мен мұны жеке біл

айтпақшы ол миллиондаған ранд сат ал қымбат буйвол бірнеше апта кейін ферма қайтыс бол,nazism

3,катлехонгтың оңтүстік аймақтағы ұшырылымы бүгін өте сәтті өтті хула дудула хула операциясы авахамбе амаквереквере авахамбе африкаға бірінші болып келген заңсыз шетелдіктер,катлехонг оңтүстік аймақ ұшырылым бүгін өте сәтті өт хул дудул хул операция авахамб амаквереквер авахамб африка бірінші бол кел заңсыз шетелдік,nazism

3,жоқ ол шетелдіктер емес олар түрлі түсті болды бұл менің досымның көлігі,жоқ ол шетелдік емес олар түрлі түсті бол бұл менің дос көлік,nazism

3,бұл жаңа апартеид басқа қара африкалықтардың азаматтығы жоқ адамдарға қысым жасауы күшейтілген жәневоетсекеф,бұл жаңа апартеид басқа қара африкалық азаматтығ жоқ адам қысым жаса күшейт жәневоетсекеф,nazism

3,бұл адам жылдан бастап ішкі істер министрі болуы керек ед,бұл адам жыл баста ішкі істер министр бол керек ед,nazism

3,міне сіз заңсыз иммигранттарға адамдардың тағамымен айналысуды тапсырған кезде не болады,міне сіз заңсыз иммигрант адам тағам айналысу тапсыр кезде не бол,nazism

3,егер сізде артықшылықтар болса сіз ештеңе көрмейсіз тіпті мұнда бізде джозеф калимбве көп біз жоспарымыздың сәтсіздікке ұшырауына жол бермейміз сол оңтүстік африкалық шетелдіктердің үйлеріне оралуын қалайтын бірнеше замбиялықтар бар,егер сіз артықшылық бол сіз ештеңе көрме тіпті мұнда біз джозеф калимбв көп біз жоспар сәтсіздік ұшыра жол берме сол оңтүстік африкалық шетелдік үй орал қала бірнеше замбиялық бар,nazism

3,standard bank тің бас инженері үлкен үзілістерден кейін отставкаға кетеді өйткені сіздің шетелдіктердің sans тен қалауы ақыры қажетті нәтиже берді санс жұмыссыз,standard bank ті бас инженер үлкен үзіліс кейін отставка кет өйткені сіздің шетелдік sans тен қала ақы қажетті нәтиже бер санс жұмыссыз,nazism

3,шетелдіктер мен полицейлер қылмысқа қатысушы тек мәміле бұзылды,шетелдік мен полицей қылмыс қатысушы тек мәміле бұз,nazism

3,иә мен иммигрантпын бірақ мен заңды құжаттарсыз ешбір елге бармаймын сонымен бұл оңтүстік африка азаматтарын анықтау үшін жергілікті оңтүстік африкалықтардың дан азы сөйлейтін тілді қолдануды қалай ақтайды,иә мен иммигрант бірақ мен заңды құжатт ешбір ел барма сонымен бұл оңтүстік африка азамат анықтау үшін жергілікті оңтүстік африкалық дан аз сөйле тілді қолдану қалай ақта,nazism

3,полиция кедей қара адамдарды тонап дудула операциясына қосылды дудула операциясының қылмыскерлері элвис нятидің әйелін тонады енді saps мүшелері

соған сәйкес келеді,полиция кедей қара адам тона дудул операция қос дудул операция қылмыскер элвис няти әйел тона енді saps мүше соған сәйкес кел,nazism

3,жарайды бізді нгамабунға ұрған кезде мұнда шетелдіктер болған жоқ енді олар оңтүстік кореядан ұлыбританияға оңтүстік кореяның азаматы болып көрінгісі келед,жарайды біз нгамабун ұр кезде мұнда шетелдік бол жоқ енді олар оңтүстік корея ұлыбритания оңтүстік корея азамат бол көрін келед,nazism

3,иммигранттар сыбайлас жемқорлықтың бір бөлігі,иммигрант сыбайлас жемқорлық бір бөліг,nazism

3,сіздің алғашқы қате болжамыңыз барлық uber bolt жүргізушілері шетелдіктер болды,сіздің алғашқы қате болжам барлық uber bolt жүргізуші шетелдік бол,nazism

3,бұл шындыққа айналады жергілікті тұрғындар дәл қазір бәрін қалайды шетелдіктер үйге оралуы керек алдымен африкаға барыңыз дудула операциясы,бұл шындық айнал жергілікті тұрғын дәл қазір бәрін қала шетелдік үй орал керек алд африка бар дудул операция,nazism

3,президенттік фермада тонауды кім жасайды миллиондаған ақш доллары қолма қол ақшамен өңделмеген ақшамен,президенттік ферма тонау кім жаса миллиондаған ақш долл қол қол ақша өңделмеген ақша,nazism

3,жұмыс істемейтін африкалықтар аш бұл заңсыз иммигрант теріс жұмыс істейді,жұмыс істеме африкалық аш бұл заңсыз иммигрант теріс жұмыс істе,nazism

3,хина ва амакоси хи ксап хи мхака дудула лейи айири кона толо операциясы намунтлха этимбаленитаминтлангу табиғаты,хин ва амакоси хи кса хи мхак дудул лейи айири кон толо операция намунтлх этимбаленитаминтланг табиғат,nazism

3,бұл елде біздің заңдарымыздың сақталуын қалайтындықтан біз ксенофобияны көрсететініміз белгілі болсын алдымен африкаға барыңыз дудула операциясы,бұл ел біздің заң сақтал қалайтындық біз ксенофобия көрсет белгілі бол алд африка бар дудул операция,nazism

3,bbc news оңтүстік африкалықтар шетелдіктерді шығару үшін заңды өз қолдарына алады кедей шетелдіктерді және біздің халықаралық беделімізді жоятын дудула операциясы,bbc news оңтүстік африкалық шетелдік шығару үшін заңды өз қол ал кедей шетелдік және біздің халықаралық бедел жоят дудул операция,nazism

3,ресей лакс duduling қонақ үйінде үлкен жұмыс істейді,ресей лакс duduling қонақ үй үлкен жұмыс істе,nazism

3,ақ адамдар мен тәкаппар қара сыныптар өз аймағындағы шетелдіктердің тәртіпсіздігі мен ауыр жағдайын көргісі келмейді ал заңсыз мигранттар қара аудандарды басып алған кезде ксенофобия туралы айтуы мүмкін,ақ адам мен тәкапп қара сынып өз аймағ шетелдік тәртіпсіздіг мен ауыр жағдай көр келме ал заңсыз мигрант қара аудан бас ал кезде ксенофобия туралы айт мүмкін,nazism

3,бұұ бжкб жұмыс істейтін ақ қауымдастықтар онда лагерь құрған заңсыз иммигранттарды құқық бұзушылық жасағандықтан қабылдауға болмайтын кедергі ретінде қарастырады,бұұ бжкб жұмыс істе ақ қауымдастық онда лагерь құр заңсыз иммигрант құқық бұзушылық жаса қабылда болма кедергі рет қарастыр,nazism

3,шекарадан заңсыз өту де қылмыс болып табылады,шекара заңсыз өту де қылмыс бол таб,nazism

3,маған жалған жеке куәліктері бар шетелдіктер дауыс берді,маған жалған жеке куәлік бар шетелдік дауыс бер,nazism

3,sans ті ксенофобиялық ретінде халықаралық қамту лебо дисеко бұл тек заңсыз иммигранттардан сұхбат алу арқылы сондай ақ заңсыз иммигранттардан құрбан болмай оның мазмұнына үлес қосқан адам,sans ті ксенофобиялық рет халықаралық қамту лебо дисеко бұл тек заңсыз иммигрант сұхбат алу арқылы сондай ақ заңсыз иммигрант құрбан болма оның мазмұн үлес қос адам,nazism

3,дудула операциясы дәл қазір шетелдіктер дауыс берді,дудул операция дәл қазір шетелдік дауыс бер,nazism

3,бұл олардың шынымен бір нәрсені жоспарлай алатынын көрсетеді,бұл олардың шын бір нәрсе жоспарла ал көрсет,nazism

3,шынында да олар біздің әңгімемізді ұстап алуға тырысады,шын да олар біздің әңгіме ұста ал тырыс,nazism

3,дудула операциясы шынымен де жұмыс істейді кеше олардан келуді сұрады,дудул операция шын де жұмыс істе кеше олардан келу сұр,nazism

3,президенттің ауыр жұмысы бар масштабтауды түзету масштабтауды жою,президент ауыр жұмыс бар масштабтау түзету масштабтау жою,nazism

3,бізді костюмдегі қылмыскерлерге емес шетелдіктерге шағымданумен айналысқан күннен күнге сәтсіздікке ұшыратады,біз костюм қылмыскер емес шетелдік шағымдан айналыс күн күн сәтсіздік ұшырат,nazism

3,кім біледі мүмкін бізде алғашқы саңырау тіс дәрігері полиция қызметкері әскери генерал және б болады,кім біл мүмкін біз алғашқы саңырау тіс дәрігер полиция қызметк әскери генерал және б бол,nazism

3,ақымақ сұрақтар қоюды тоқтатыңыз және оның неге mpho phalatse ге бағытталғанын анықтаңыз,ақы сұрақ қою тоқта және оның неге mpho phalatse ге бағыттал анықта,nazism

3,өтінемін келме брайан бізде шетелдіктер жеткілікті,өт келме брайан біз шетелдік жеткілікті,nazism

3,шетелдіктеріңізге бізге дауыс бермеуді айтыңыз,шетелдік бізге дауыс бермеу ай,nazism

3,мұнда шетелдіктер қандай канти,мұнда шетелдік қандай канти,nazism

3,батонг саусақтарын жалғыз қалдырады,батонг саусақ жалғыз қалдыр,nazism

3,дудула операциясы кезінде өзін өзі бағалау тактикасына көбірек ренжіді топ қарт әйелді үйінен шығаруға тырысқаннан кейін қоғамдастық мүшелері қару алды бұл атысқа әкелд,дудул операция кез өз өзі бағалау тактика көбірек ренжі топ қарт әйел үй шығар тырыс кейін қоғамдастық мүше қару алды бұл атыс әкелд,nazism

3,мен сіздің арнаңызды тыңдау үшін қысқаша тоқтаймын,мен сіздің арна тыңдау үшін қысқаша тоқта,nazism

3,бірінші операцияны оңтүстік африкада кездесекенше жасаңыз,бірінші операция оңтүстік африка кездесекенш жаса,nazism

3,замбиядағы дудула операциясы басқа елге заңсыз бару және оның жеңіске жеткенін көру сізді депортацияламаңыз,замбия дудул операция басқа ел заңсыз бару және оның жеңіс жет көру сіз депортациялама,nazism

3,шетелдіктердің саусақтары жұмыс істемейді,шетелдік саусақ жұмыс істеме,nazism

3,заңсыз иммигранттар бізді олжа ретінде аулап жүргенде біз қалай қауіпсіз бола аламыз,заңсыз иммигрант біз олжа рет аула жүр біз қалай қауіпсіз бола ал,nazism

3,бұл біз су үшін төлейтін сауда барлық елдер басқалармен сауда жасайды,бұл біз су үшін төле сауда барлық ел басқа сауда жаса,nazism

3,бұл адамдар біздің елімізді кезең кезеңімен жойып жатыр оңтүстік африкадағы алғашқы операция,бұл адам біздің ел кезең кезең жой жатыр оңтүстік африка алғашқы операция,nazism

3,африкадағы жалғыз бас қолбасшы бас қолбасшы джулиус селло малема сондықтан сіз қарсы тұра алмайтын атаққа ие болуды тоқтатыңыз,африка жалғыз бас қолбасшы бас қолбасшы джулиус селло мале сондықтан сіз қарсы тұр алма атақ ие болу тоқта,nazism

3,менің ойымша сіз ксенофобия мәселесін алға тартып жатырсыз бұл адамдар экономиканы нөлдік ойын ретінде қарастырған кезде болады сіз шетелдіктерді жек көресіз өйткені олардың шағын бизнесі сәтті деп ойлайсыз өйткені сіздікі сәтсіздікке ұшырады,менің ойымша сіз ксенофобия мәселе алға тарт жатыр бұл адам экономика нөлдік ойын рет қарастыр кезде бол сіз шетелдік жек көр өйткені олардың шағын бизнес сәтті де ойла өйткені сіздік сәтсіздік ұшыр,nazism

3,эфиопиялық иммигранттар келесі аптада жаңартылған операция аясында келеді,эфиопиялық иммигрант келесі апта жаңартыл операция ая кел,nazism

3,шетелдіктер мұнда жаппай ағыла бастағанға дейін оңтүстік африка өте жылы болды манже инкоси бізді оларды қуып жібермегеніміз үшін жазалайды біз ел ретінде

бірдеңе істеуіміз керек,шетелдік мұнда жаппай ағыл баста дейін оңтүстік африка өте жыл бол манж инкоси біз ол қу жіберме үшін жазала біз ел рет бірде істе керек,nazism

3,оларды босқындар лагеріне орналастыру олардың шетелдіктер деп атағанын және олар шынымен де шетелдіктер екенін білдірді ақымақ болмаңыз,ол босқын лагер орналастыр олардың шетелдік де ата және олар шын де шетелдік екен біл ақы болма,nazism

3,оңтүстік африкада біз бұл туралы твиттерде жазар едік өйткені біз жеңілгендерміз және шетелдіктеріден қорқамыз,оңтүстік африка біз бұл туралы твиттер жаз едік өйткені біз жеңіл және шетелдіктері қор,nazism

3,бізде қатаң үкімет депутаттар мен министрлер кабинеті болуы үшін конституцияға түзетулер енгізуіміз керек біздің сарбаздар құстарды күзетуге жіберілуі керек осылайша заңсыз иммигранттардың және жалған тауарлардың соның ішінде ұрланған көліктердің біздің шекарамызға кіруіне жол бермейді,біз қатаң үкімет депутат мен министр кабинет бол үшін конституция түзету енгіз керек біздің сарбаз құс күзет жіберіл керек осылайша заңсыз иммигрант және жалған тауар соның іш ұрлан көлік біздің шекара кір жол берме,nazism

3,оның негізгі твитке берген жауаб ең алдымен қайыршылардың көпшілігі заңсыз иммигранттар деп мәлімдейді сондықтан бұл тең емес қоғамды дәлелдеу үшін қолдануға болатын ең жақсы сурет емес,оның негізгі твит бер жауаб ең алд қайыршы көпшіліг заңсыз иммигрант де мәлімде сондықтан бұл тең емес қоғам дәлелде үшін қолдан бол ең жақсы сурет емес,nazism

3,менің ұстанымым анық зимбабвеліктер оңтүстік африкалықтарды зорлап өлтіреді мен мұнда дауласу үшін келген жоқпын дудула операциясы шетелдіктер американдықтарды бірінші орынға қояды,менің ұстаным анық зимбабвелік оңтүстік африкалық зорла өлтір мен мұнда дауласу үшін кел жоқ дудул операция шетелдік американдық бірінші орын қоя,nazism

3,біз американдықтарды бірінші орынға қоямыз өйткені біз мұнда көптеген шайтандармен саясаткерлермен бұқаралық ақпарат құралдарымен және заңсыз иммигранттармен күресеміз біз бұдан әрі күресеміз,біз американдық бірінші орын қоя өйткені біз мұнда көптеген шайтан саясаткер бұқаралық ақпарат құрал және заңсыз иммигрант күрес біз бұдан әрі күрес,nazism

3,бұл лашықтарды негізінен шетелдіктер алады,бұл лашық негіз шетелдік ал,nazism

3,бұл енді не шетелдіктерге арналған пәле жоқ операциясы,бұл енді не шетелдік арнал пәл жоқ операция,nazism

3,нидерланды мен ұлыбританияға қоныс аударудан бас тартатын уақыт болуы мүмкін олар бізді сезінуі керек және бізге не істегендерін есте сақтауы керек дудула және путсутафиканс операциясы жайында әлем бізді бақылап отыр,нидерланды мен ұлыбритания қоныс аудару бас тарт уақыт бол мүмкін олар біз сезін керек және бізге

не істе ес сақта керек дудул және путсутафиканс операция жай әлем біз бақыла отыр,nazism

3,егер сіз тарихқа тереңірек үңілсеңіз сіз негізгі әдебиетте бар нәрсеге қарама қарсы нәрсені табасыз неліктен адам дудула операциясын жасайды,егер сіз тарих тереңірек үңіл сіз негізгі әдебиет бар нәрсе қарама қарсы нәрсе таб неліктен адам дудул операция жаса,nazism

3,оңтүстік африкалықтар mcdonaldsті қатты жақсы көреді ме дудула операциясы американдықтарды бірінші орынға қояды,оңтүстік африкалық mcdonalds қатты жақсы көр ме дудул операция американдық бірінші орын қоя,nazism

3,сіз дудула операциясының немесе дудула қозғалысының қатысушысы болсаңыз да адамдар билік етуі керек мен бет сатып алмаймын мен оңтүстік африка халқымен жұмыс істеймін,сіз дудул операция немесе дудул қозғалыс қатысушы бол да адам билік ет керек мен бет сат алма мен оңтүстік африка халқ жұмыс істе,nazism

3,біз де шетелдіктерді қоқысқа кінәлаймыз ба,біз де шетелдік қоқыс кінәла ба,nazism

3,әйелдерді радикалды мұсылмандар зорлады,әйел радикал мұсылман зорла,nazism

3,йобургте мен бұл адамдардың қайда тұратынын басып алынған ғимаратты білемін және олар сол жерлерге топтасып барып үйлеріне топтасып оралады негізінен шетелдік зағиптардың оларды алып кететін отбасы мүшелері бар олар жұмыс істейді және неге олар қайыр сұрайды мен білмеймін,йобург мен бұл адам қайда тұр бас алын ғимарат біл және олар сол жер топтас бар үй топтас орал негіз шетелдік зағип ол алып кет отбасы мүше бар олар жұмыс істе және неге олар қайыр сұра мен білме,nazism

3,бұл зиянкестер құжаттарсыз және оларға бірдеңе айтпай ақ осында болғысы келеді олар шынымен басқарғысы келеді бірақ тек біздің мәйітіміз арқылы америкалықтарды бірінші орынға қойыңыз,бұл зиянкес құжатт және оларға бірде айтпа ақ осында бол кел олар шын басқар кел бірақ тек біздің мәйіт арқылы америкалық бірінші орын қой,nazism

3,өздерін дудула немесе путсутафиканс операциясы деп атайтын бандиттер ең алдымен олар барлық африкалықтарды және қара нәсілділерді жек көреді оларды ақ нәсілшілдер қара нәсілділерге шабуыл жасау және оларды элвисняти сияқты тірідей өртеу үшін қаржыландырады,өз дудул немесе путсутафиканс операция де ата бандит ең алд олар барлық африкалық және қара нәсілді жек көр ол ақ нәсілшіл қара нәсілді шабуыл жасау және ол элвисняти сияқты тірідей өрте үшін қаржыландыр,nazism

3,кез келген жұмыс істеп тұрған үкімет заңсыз иммигранттарға қарсы елеулі шаралар қабылдаған болар еді жаппай депортация бұл армияны полицияны жол полициясын және дудула американдықтарды бірінші орынға қоятын жеке күзет операциясын қолдана отырып елді бір аптаға жабудың жалғыз жолы,кез кел жұмыс істе тұр үкімет заңсыз иммигрант қарсы елеулі шара қабылда бол ед жаппай депортация бұл армия

полиция жол полиция және дудул американдық бірінші орын қоятын жеке күзет операция қолдан отыр ел бір апта жаб жалғыз жол,nazism

3,қха шетелдіктердің кетуін қалайды олар оны қарсылықсыз жіберді,қх шетелдік кет қала олар оны қарсылық жіб,nazism

3,мен полиция министрінен бастаймын өйткені ол әрдайым камераларды көрген сайын бізді өзінің ұсқынсыз жүздерімен және адасушылықтарымен көрсетеді қудалау үшін айыптау,мен полиция министр баста өйткені ол әрдайым камера көр сайын біз өзінің ұсқын жүз және адасушылық көрсет қудала үшін айыпта,nazism

3,оңтүстік корея азаматтарын қамауға алу өте жақсы өйткені ол шетелдіктер сізге пара берген нәрсеге сәйкес келмейді,оңтүстік корея азамат қама алу өте жақсы өйткені ол шетелдік сізге пара бер нәрсе сәйкес келме,nazism

3,еуропадан келген шетелдіктер еуропадан алыс жерлерге иелік ете ме бұл отарлау бүкіл палестина палестиналықтарға тиесілі,еуропа кел шетелдік еуропа алыс жер иелік ет ме бұл отарла бүкіл палестин палестиналық тиесілі,nazism

3,бірақ бушири мұны түсініп қашып кетті бірақ біздің үкіметте үнемі тұрады,бірақ бушири мұны түсін қаш кет бірақ біздің үкімет үнемі тұр,nazism

3,жақтастары бүгін таңертең дудула операциясының жетекшісі пайда болған рудепорт магистратура сотының ғимаратында,жақтас бүгін таңертең дудул операция жетекші пайда бол рудепорт магистратура сот ғимарат,nazism

3,іске қосылғаннан және шеруден кейін дудула операциясымен не болады мен мұның бәрі заңсыз шетелдіктерге әсер етпейтінін көремін компаниялар әлі күнге дейін шетелдіктерді жергілікті тұрғындардан бұрын жалдайды,іс қосыл және шеру кейін дудул операция не бол мен мұның бәрі заңсыз шетелдік әсер етпе көр компания әлі күн дейін шетелдік жергілікті тұрғын бұрын жалда,nazism

3,бұл мәселеде солтүстік батыстықтар көш бастап тұр мен олардың өз қауымдастықтарынан есірткі сатушылардың темекі шегетінін естідім,бұл мәселе солтүстік батыстық көш баста тұр мен олардың өз қауымдастық есірткі сатушы темекі шег есті,nazism

3,сізге көрсететін ештеңе жоқ сіз ян смиттің қол астында тәуелсіздіктегі өз қара үкіметіңізден жақсы болдыңыз,сізге көрсет ештеңе жоқ сіз ян смит қол аст тәуелсіздік өз қара үкімет жақсы бол,nazism

3,мемлекетке опасыздық жасағаны үшін бірнеше жыл және мен оның заңсыз иммигрант екенін қосамын біздің сот төрелігі жүйесі бізді үнемі жіберіп отырады,мемлекет опасыз жаса үшін бірнеше жыл және мен оның заңсыз иммигрант екен қос біздің сот төреліг жүйе біз үнемі жібер отыр,nazism

3,бұл өте өкінішті және одан да жаманы ол үшін жұмыс істеген адамдар жұмысынан айырылды сақтандыру төлемін қалтаға алды,бұл өте өкініш және одан да жаман ол үшін жұмыс істе адам жұмыс айыр сақтандыр төл қалта алды,nazism

3,егер уланған сусындар мөрленіп пәкістанның курорттарынан әкелінгені анықталса біз оларды демалыс күндері алып кетеміз шетелдіктерге тиесілі асханалардан йогурт ішкеннен кейін балалар өлтірілді және ешкім ештеңе айтқан жоқ,егер улан сусын мөрлен пәкістан курорт әкелін анықтал біз ол демалыс күн алып кет шетелдік тиесілі асхана йогурт іш кейін бала өлтір және ешкім ештеңе айт жоқ,nazism

3,мемлекеттік ресурстарды тонауды жалғастырыңыз адамдарға билік деп санайтын нәрсені шамадан тыс жүктеуді жалғастырыңыз халық сізге нақты биліктің кімнің қолында екенін көрсететін күн келеді бізге сенің күндерің санаулы,мемлекеттік ресурс тонау жалғастыр адам билік де сана нәрсе шама тыс жүктеу жалғастыр халық сізге нақты билік кім қол екен көрсет күн кел бізге сенің күн санаулы,nazism

3,желтоқсан айында біз жаппай депортацияны көргіміз келеді оңтүстік африкаға алғашқы операцияны жібереміз біз бәріміз иммиграциялық постқа өтініш беруіміз керек,желтоқсан ай біз жаппай депортация көр кел оңтүстік африка алғашқы операция жібер біз бәрі иммиграциялық пост өтініш бер керек,nazism

3,продюсер мен режиссер бізді ел ретінде сәтсіздікке ұшыратты шынымды айтсам осы екі жігітке қарап шакаға кім сәйкес келетінін айтыңыз,продюсер мен режиссер біз ел рет сәтсіздік ұшыра шын айт осы екі жігіт қара шака кім сәйкес кел ай,nazism

3,балтық жағалауын айыптайды дудула операциясы шетелдіктерді айыптайды және африканы бірінші орынға қояды алдымен зимбабвеліктерді кінәлайды бірақ өлтіруші,балтық жағала айыпта дудул операция шетелдік айыпта және африка бірінші орын қоя алд зимбабвелік кінәла бірақ өлтіруш,nazism

3,ах сіз дудула операциясына қатысушылардың бірісіз,ах сіз дудул операция қатысушы бір,nazism

3,ол оңтүстік африкалық ал сіз біздің елдегі шетелдіксіз,ол оңтүстік африкалық ал сіз біздің ел шетелдік,nazism

3,алдымен африкаға барыңыз,алд африка бар,nazism

3,дудула операциясы африкалықтармен күресу үшін құрылды,дудул операция африкалық күресу үшін құр,nazism

3,олар негізгі қағиданы бөліседі шетелдіктерді бірінші орынға қояды,олар негізгі қағида бөліс шетелдік бірінші орын қоя,nazism

3,сан франциско ауруханаларында босанатын әйелдер шетелдіктер және сіз әлі де біз сізге шетелдіктерге көмектесіп жатқанымызды жоққа шығарасыз,сан франциско аурухана босан әйел шетелдік және сіз әлі де біз сізге шетелдік көмектес жат жоқ шығар,nazism

3,африкалық иммигранттар неліктен сіз өзіңізді қатты жек көресіз,африкалық иммигрант неліктен сіз өз қатты жек көр,nazism

3,жоқ олар одан қорқады,жоқ олар одан қорқ,nazism

3,егер оның омыртқасы болмаса ол таңертең қалай жүреді ол қудалаудан қорғаған сыбайлас жемқорлыққа қызметкерлердің қайсысы кінәлі ол заңсыз иммигранттарды қалай қолдайды немесе аарон мотсоледиді заңсыз мигранттармен жұмыс істегені үшін жұмыстан шығарды ма,егер оның омыртқа болма ол таңертең қалай жүр ол қудалау қорға сыбайлас жемқорлық қызметкер қайсысы кінәлі ол заңсыз иммигрант қалай қолда немесе аарон мотсоледи заңсыз мигрант жұмыс істе үшін жұмыс шығ ма,nazism

3,заңсыз иммигранттарды континенттегі соғыстарды төңкерістерді және eff қолдаған континенттің байлығын заңсыз иемденуді ұмытыңыз олар мугабені қолдады египеттегі эфиопиядағы және тиграй аймағындағы төңкеріс туралы ештеңе айтпады,заңсыз иммигрант континент соғыс төңкеріс және eff қолда континент байлығ заңсыз иемдену ұмыт олар мугабе қолда египет эфиопия және тигра аймағ төңкеріс туралы ештеңе айтпа,nazism

3,біздің ел тым көп пандемияға тап болды сыбайлас жемқорлық нашар үкімет жүктемелерді бөлу кедейлік жұмыссыздық гендерлік зорлық зомбылық кісі өлтіру дудула операциясы бірігіп осы мәселенің шешімін табайық,біздің ел тым көп пандемия тап бол сыбайлас жемқорлық нашар үкімет жүктеме бөлу кедейлік жұмыссыздық гендерлік зорлық зомбылық кісі өлтір дудул операция біріг осы мәселе шеш таб,nazism

3,сіз біздің ел туралы өтірік айтқаныңыз үшін ақша алғанды ұнатасыз ба мен сізді шетелдіктердің елге келуіне кінәлаймын,сіз біздің ел туралы өтірік айт үшін ақша ал ұна ба мен сіз шетелдік ел кел кінәла,nazism

3,адамдар оңтүстік африканы жою миссиясын орындайды,адам оңтүстік африка жою миссия орында,nazism

3,сіз сияқты адамдардың мінез құлқына құмар болғандықтан және оңтүстік африкалықтар үнсіз қалады деп күткен адамдар үшін олар не қалайды темекіні тастаңыз және оңтүстік африкалықтардың талаптарын тыңдай бастаңыз сонда сіз мәселенің не екенін түсінесіз оңтүстік африкалықтар шетелдіктермен бірге өмір сүрді,сіз сияқты адам мінез құлқ құм бол және оңтүстік африкалық үн қал де күт адам үшін олар не қала темекі таста және оңтүстік африкалық талап тыңда баста сонда сіз мәселе не екен түс оңтүстік африкалық шетелдік бірге өмір сүр,nazism

3,африкалықтар арасындағы араздықты сіз сияқты адамдар қоздырады заңсыз шетелдіктер мен қылмыскерлердің батылдығы бар,африкалық ара араздық сіз сияқты адам қоздыр заңсыз шетелдік мен қылмыскер батылдығ бар,nazism

3,ең өкiнiштiсi ақ ересек адам қара балаларды өздерiнiң қара ағаларын жек көруге қалай үйретедi,ең өкiнiштi ақ ересек адам қара бала өз қара аға жек көр қалай үйрет,nazism

3,бiр түнде пайдалы өзгерiстер болмайды өйткенi сыбайлас жемқорлықтың терең тамыры өзегiне дейiн сiңген дегенмен бiз бәрiмiз өз рөлiмiздi ойнауымыз керек,бiр түн пайдалы өзгерiс болма өйткенi сыбайлас жемқорлық терең тамыр өзег дейiн сiң дегенмен бiз бәрi өз рөл ойна керек,nazism

3,оларға өмiр сүруге естуге және өз алаңдаушылықтарын заңды түрде бiлдiруге рұқсат беру керек егер бiз африка форумын өткiзе алсақ онда дудула операциясы осында мәңгi қалады,оларға өмiр сүр ест және өз алаңдаушылық заңды түр бiлдiр рұқсат беру керек егер бiз африка форум өткiз ал онда дудул операция осында мәңгi қал,nazism

3,бұл заңсыз иммиграннттардың құжаттарсыз киiну тәсiлi дудула операциясы барысында бiз бұл елдiң кiмге тиесiлi екенiн көремiз,бұл заңсыз иммигрант құжатт киiн тәсiл дудул операция барыс бiз бұл ел кiм тиесiлi екен көр,nazism

3,бұл өте қарбалас жыл болады заңның үстемдiгi мен тәртiбi сақталады,бұл өте қарбалас жыл бол заң үстемдiг мен тәртiб сақтал,nazism

3,бiздiң төлқұжаттарымыз қаншалықты қауiпсiз оңтүстiк африкалықтар өздерiнiң жеке мәлiметтерiн шетелдiктерге сатады олар оны осында келуге және басқа елдерге саяхаттауға пайдаланады алаяқтық синдикаттарға қатысқаны үшiн адам қамауға алынды бiздiң туристiк құжаттарымызға қауiп төнiп тұр ма,бiздiң төлқұжат қаншалықты қауiпсiз оңтүстiк африкалық өз жеке мәлiмет шетелдiк сат олар оны осында кел және басқа ел саяхаттау пайдалан алаяқтық синдикат қатыс үшiн адам қама ал бiздiң туристiк құжат қауiп төн тұр ма,nazism

3,заңсыз шетелдiктер бiздiң әпкелерiмiздi зорлап өлтiрiп маңызды инфрақұрылымды бұзады,заңсыз шетелдiк бiздiң әпке зорла өлтiр маңызды инфрақұрылым бұз,nazism

3,бұл рас сiз шетелдiктерге көшкеннен кейiн бiз сiздi бұдан былай қабылдамаймыз және сүймеймiз,бұл рас сiз шетелдiк көш кейiн бiз сiз бұдан былай қабылдама және сүйме,nazism

3,осы елдегi соғысты қалайтын адам шетелдiктердi заңдастыруға батылы баруы керек,осы ел соғ қала адам шетелдiк заңдастыр батыл бар керек,nazism

3,нелспрут бес ер адамды жылы мамырда алаяқтық сыбайлас жемқорлық және иммиграция туралы заңды бұзғаны үшiн қамауға алды пәкiстаннан келген матебула мен али оңтүстiк африкалықтарды шетелдiктерге sa төлқұжаттарын алу үшiн жеке мәлiметтерiн беру үшiн жалдады,нелспрут бес ер адам жыл мамыр алаяқтық сыбайлас жемқорлық және иммиграция туралы заңды бұз үшiн қама алды пәкiстан кел матебул мен али оңтүстiк африкалық шетелдiк sa төлқұжат алу үшiн жеке мәлiмет беру үшiн жалда,nazism

3,африкада зимбабвеліктерге қарсы алғашқы операцияны жасаңыз дудула сіздің бауырларыңыз қазір мемлекетке қарсы қылмыс жасап жатыр бұны біз диверсия деп атаймыз олар маңызды инфрақұрылымды зорлайды өлтіреді және бұзады шайтан ақымақ,африка зимбабвелік қарсы алғашқы операция жаса дудул сіздің бауыр қазір мемлекет қарсы қылмыс жаса жатыр бұны біз диверсия де ата олар маңызды инфрақұрылым зорла өлтір және бұз шай ақы,nazism

3,егер бізде жетіспейтін немесе арнайы дағдылар болмаса шетелдіктер жұмысқа қабылданбауы керек,егер біз жетіспе немесе арнайы дағды болма шетелдік жұмыс қабылданба керек,nazism

3,шетелдіктерді жалдайтын мұндай дүкендерді қолдамаймыз деп айту мүмкін емес біз оңтүстік африкалықтарды жалдамайтын филиалдарды жабуымыз керек,шетелдік жалда мұндай дүкен қолдама де айту мүмкін емес біз оңтүстік африкалық жалдама филиал жаб керек,nazism

3,кеше менің досым жол апатына ұшырады зимбо қызыл роботтың жолын кесіп өтіп досымның көлігіне соғылып оны өлтіре жаздады бейбақ жүргізуші куәлігі сақтандыруы мекен жайы және төлқұжаты жоқ заңсыз иммигрант жалған неліктен біз сакандар бұл сұмдыққа ұшыраймыз,кеше менің дос жол апат ұшыр зимбо қызыл робот жол кес өт дос көлік соғыл оны өлтір жазд бей жүргізуші куәлік сақтандыр мекен жай және төлқұжат жоқ заңсыз иммигрант жалған неліктен біз сакан бұл сұмдық ұшыра,nazism

3,ағылшын тілін жетілдіріңіз,ағылшын тіл жетілдір,nazism

3,сонымен қатар ол өзінің терісінің түсімен қара адамдарымен күреседі заңсыз иммигранттар дудула операциясы және б бұл афрофобия қалай аталса да африкадағы нағыз шетелдік ақ оқушыға формада автоматты мылтық үйретеді құдай африкаға рақым етсін,сонымен қатар ол өзінің тері түс қара адам күрес заңсыз иммигрант дудул операция және б бұл афрофобия қалай атал да африка нағыз шетелдік ақ оқушы форма автоматты мылтық үйрет құдай африка рақым ет,nazism

3,ұнтақталған етті тікелей сүртіңіз сіз тіпті жетілген және дана сөздерді көре алмайсыз,ұнтақтал ет тікелей сүр сіз тіпті жет және дана сөз көр алма,nazism

3,заңсыз және заңды иммигранттар жасаған миллион жұмыс деп аталатын жұмысты оңтүстік африкалықтар орындап кедейлік деңгейін төмендетуі мүмкін бірақ саясаткерлердің жалақысы мен кейбір жәрдемақылары болғандықтан ғана,заңсыз және заңды иммигрант жаса миллион жұмыс де атал жұм оңтүстік африкалық орында кедейлік деңгей төмендет мүмкін бірақ саясаткер жалақы мен кейбір жәрдемақы бол ғана,nazism

3,иә шетелдіктер жасайды,иә шетелдік жаса,nazism

3,вена экс уотербой мен хелен зиллемін сіз жақсырақ білуіңіз керек еді бірақ кем дегенде нхланхланың каси экономикасы туралы көзқарасы бар,вена экс уотербо мен

хелен зилл сіз жақсырақ біл керек ед бірақ кем де нхланхла каси экономика туралы көзқарас бар,nazism

3,балаларға мылтық үйрету және кішкентай қызды қорлау бұл шошқалар заңсыз шетелдіктермен әрең күреседі енді олар мұны біздің балаларымызға қояды ма,бала мылтық үйрет және кішкентай қыз қорла бұл шошқа заңсыз шетелдік әре күрес енді олар мұны біздің бала қоя ма,nazism

3,егер олар сізді қатты мазаласа сіз бұл мәселелермен өзіңіз күресуге болады адамдар қандай мәселелермен күресу керектігін айтпаңыз сіз сияқты сезімдермен бөлісетін және сіздің реніштеріңізбен күресетін адамдарды жинаңыз,егер олар сіз қатты мазала сіз бұл мәселе өз күрес бол адам қандай мәселе күресу керектіг айтпа сіз сияқты сезім бөліс және сіздің реніш күрес адам жина,nazism

3,сондықтан бұл жұмысқа қабылдаушылардың саны емес мен мұнда заңды түрде жүргендер туралы ғана айтамын және заңсыз иммигранттарды қосамын мен шетелдіктерді жалдайтын ге жуық жерді білемін бірақ сен мұнда өтірікшісің немесе сен оларға үйленгенсің,сондықтан бұл жұмыс қабылдаушы сан емес мен мұнда заңды түр жүр туралы ғана ай және заңсыз иммигрант қос мен шетелдік жалда ге жуық же біл бірақ сен мұнда өтірікш немесе сен оларға үйлен,nazism

3,ұзақ жылдар бойы ашық шекаралар ешқашан мен және шетелдіктер олардың қатарына қосыла алмады,ұзақ жыл бойы ашық шекара ешқашан мен және шетелдік олардың қат қосыл алма,nazism

3,қара нәсілділер біз қарғыс атқан ұлтпыз менің айтайын дегенім бензин бағасы өте қорқынышты бұл елде өмір сүру құны соншалықты жоғары бірақ сіз шетелдіктерді қуып жіберуге басымдық бергіңіз келеді сіз жақсысыз ба,қара нәсілді біз қарғыс ат ұлт менің айт де бензин баға өте қорқыныш бұл ел өмір сүру құн соншалықты жоғары бірақ сіз шетелдік қу жібер басымдық бер кел сіз жақ ба,nazism

3,мен түнде көлік жүргізуден қорқып өмір сүргеннен гөрі ақ нәсілшілдермен айналысқанды жөн көремін өйткені мені көлігім мен зергерлік бұйымдарым үшін жолға тікенек орнатқан заңсыз иммигрант өлтіруі мүмкін,мен түн көлік жүргізу қорқ өмір сүр гөрі ақ нәсілшіл айналыс жөн көр өйткені мен көліг мен зергерлік бұйым үшін жол тікенек орнат заңсыз иммигрант өлтір мүмкін,nazism

3,егер қарапайым оңтүстік африкалықтар көмек алу үшін осы клиникаға ге қауіпсіз жете алмаса тәулігіне сағат емханалар ашудың қажеті жоқ өйткені олар заңсыз шетелдіктердің қолына түсіп елде еркін жүріп кез келген адамның өмірін қиюға дайын болудан қорқады кез келген уақытта сол жолы,егер қарапайым оңтүстік африкалық көмек алу үшін осы клиника ге қауіпсіз же алма тәулік сағат емхана аш қажет жоқ өйткені олар заңсыз шетелдік қол түс ел еркін жүр кез кел адам өмір қию дайын болу қорқ кез кел уақыт сол жол,nazism

3,сіз бен бізде whatsapp та бірдей топ бар мен заңсыз иммигранттарды жек көремін олар біздің елімізді циркке айналдырды,сіз бен біз whatsapp та бірде топ бар мен заңсыз иммигрант жек көр олар біздің ел цирк айнал,nazism

3,рамафоса мен қха біздің елімізді заңсыз иммигранттарға сатады олар тіпті гупаттарды әкелу және оңтүстік корея азаматтарын тонау үшін үндістанға дейін барды,рамафо мен қх біздің ел заңсыз иммигрант сат олар тіпті гупат әкелу және оңтүстік корея азамат тонау үшін үндістан дейін бар,nazism

3,олар да шетелдіктер ретінде қарастырылуы керек оларға оңтүстік африка азаматтығын кім берді,олар да шетелдік рет қарастырыл керек оларға оңтүстік африка азаматтығ кім бер,nazism

3,бұл жәбірленушілерге өтемақы төлеу үшін үкіметке капиталды қайтаруға көмектеспейді бірақ мзансиде есірткі саудасы және азаматтығымызды qha ға сату сияқты қылмыстар үшін өлім жазасын қалпына келтіретін партияға дауыс беру керек дудула операциясы американдықтарды әлемде бірінші орынға қояды,бұл жәбірленуші өтемақы төлеу үшін үкімет капитал қайтар көмектеспе бірақ мзанси есірткі сауда және азаматтығ qha ға сату сияқты қылмыс үшін өлім жаза қалп келтір партия дауыс беру керек дудул операция американдық әлем бірінші орын қоя,nazism

3,фермерлер заңсыз иммигранттарды жалдауды тоқтатуы керек,фермер заңсыз иммигрант жалдау тоқтат керек,nazism

3,егер мен сізге тарих туралы айта алсам сіз де заңсыз иммигрантсыз,егер мен сізге тарих туралы ай ал сіз де заңсыз иммигрант,nazism

3,таза құрметсіздік бұл шетелдіктер тым алысқа кетті,таза құрметсіз бұл шетелдік тым алыс кет,nazism

3,менің ойымша зимбабвеліктер өз елдерін өз үйлерінде ретке келтіруі керек сіз сирилді білмейсіз өйткені ол зимбабве емес google пайдалы болар еді,менің ойымша зимбабвелік өз ел өз үй рет келтір керек сіз сири білме өйткені ол зимбабв емес google пайдалы бол ед,nazism

3,бұл іш киімдер бізді құрметтемейді,бұл іш киім біз құрметтеме,nazism

3,заңсыз шетелдіктер зимбабве,заңсыз шетелдік зимбабв,nazism

3,өздерінің заңсыз иммигранттарын күзететін заңсыз иммигранттар,өз заңсыз иммигрант күзет заңсыз иммигрант,nazism

3,сіз екіжүздісіз және бұл сіздің бұрынғы твиттеріңізге негізделген сіздің қылмыспен күресу туралы пікіріңіз бұрынғы твиттеріңізге негізделген сіз шетелдіктерді жасалған барлық қылмыстар үшін айыптайсыз ба және камерада ұсталған біреудің конституциялық құқығы бар деп мәлімдейсіз бе,сіз екіжүзд және бұл сіздің бұрынғы твиттер негіздел сіздің қылмыс күресу туралы пікір бұрынғы твиттер негіздел сіз

шетелдік жасал барлық қылмыс үшін айыпта ба және камера ұстал біреу конституциялық құқығ бар де мәлімде бе,nazism

3,оңтүстік кореяда қара жұмыс істейтін құжатталған мигранттар да дудула операциясынан зардап шекпейді дейді зандиле дубула бүкіл африка мұнымен келіспейді бұл африкаға қарсы sa болатынын ескеріңіз,оңтүстік корея қара жұмыс істе құжаттал мигрант да дудул операция зардап шекпе де зандил дубул бүкіл африка мұны келіспе бұл африка қарсы sa бол ескер,nazism

3,шетелдіктер зиянкестер болып табылады бірақ бізден бас тартуды жалғастырыңыз сіз сол кездегі африкалықтарды қорғаған күніңізге өкінесіз,шетелдік зиянкес бол таб бірақ бізден бас тарту жалғастыр сіз сол кез африкалық қорға күн өкін,nazism

3,бұл заңсыз жұмысшыларға толы олар африкада алғашқы операция жасайтын жастар арасындағы жұмыссыздықтың жоғары деңгейіне алаңдамайды дудула бойкот жариялайық олардан бас тартайық қан флорида,бұл заңсыз жұмысшы толы олар африка алғашқы операция жаса жас ара жұмыссыздық жоғары деңгей алаңдама дудул бойкот жариял олардан бас тарт қан флорида,nazism

3,сіз мені не мазалайтынын білесіз укути біз тек қара шетелдіктерге назар аударумен айналысамыз біз укутидің елдегі ақ шетелдіктер екенін ұмытып кетеміз енді осы бейнеге тікелей көшейік ақ адам ешқашан қара адамды құрметтемейді өйткені қара адам басқа қара адамдарға ұқсамайды,сіз мен не мазала біл укути біз тек қара шетелдік назар аудар айналыс біз укути ел ақ шетелдік екен ұмыт кет енді осы бейне тікелей көш ақ адам ешқашан қара адам құрметтеме өйткені қара адам басқа қара адам ұқсама,nazism

3,заңсыз шетелдіктер біздің әпкелерімізді зорлайды оңтүстік африкадағы дудула операциясы әйелдерге қатысты зорлық зомбылықтың бірінші құрбаны,заңсыз шетелдік біздің әпке зорла оңтүстік африка дудул операция әйел қатысты зорлық зомбылық бірінші құрбан,nazism

3,оларды заңсыз шетелдіктер алмастырады және олар дональд трампты еске алады,ол заңсыз шетелдік алмастыр және олар дональд трам ес ал,nazism

3,eff және президент джулиус малема бүкіл оңтүстік африкада болып жатқан нәсілшілдік мінез құлықты айыптау үшін көшеге шыққанда біз қара нәсілділерге қатысты бірдей нәсілшілдерді қорғайтын operationdudula сияқты ақ демеушілік топтарды көреміз,eff және президент джулиус мале бүкіл оңтүстік африка бол жат нәсілш мінез құлық айыпта үшін көше шық біз қара нәсілді қатысты бірде нәсілшіл қорға operationdudula сияқты ақ демеушілік топ көр,nazism

3,егер демократия шынымен де халықтың халық үшін билігін білдірсе сонда бұл үкімет біз сөйлеген кезде бізді тыңдауы керек шетелдіктердің ағынын тоқтатыңыз металл сынықтарын сатуға тыйым салу пәкістанға spaza дүкенінің жұмысына тыйым салу,егер демократия шын де халық халық үшін билiг білдір сонда бұл үкімет біз сөйле кезде біз

тыңда керек шетелдік ағын тоқта металл сынық сат тыйым салу пәкістан spaza дүкен жұмыс тыйым салу,nazism

3,дудула операциясы деп аталатын қозғалыс маусымды дудула операциясы күні деп жариялады қозғалыс барлық провинциялардағы барлық елді мекендерді қатысуға шақырады operationdudula жаңалықтарының жаңартылған күні,дудул операция де атал қозғалыс маусым дудул операция күн де жариял қозғалыс барлық провинция барлық ел мекен қатыс шақыр operationdudula жаңалық жаңартыл күн,nazism

3,бинго сіз манделаның пұтынан бастап мандела жыл бойы заңсыз иммиграцияға рұқсат беріп бізді бір ғасыр бойы қорғаған электр шекара қоршауын бөлшектедіңіз деген негізгі сұраққа жауап бердіңіз,бинго сіз мандела пұт баста мандел жыл бойы заңсыз иммиграция рұқсат бер біз бір ғасыр бойы қорға электр шекара қорша бөлшекте де негізгі сұрақ жауап бер,nazism

3,кейптаунның орталық бизнес ауданында дудула операциясын талап ететін бірнеше ақ бизнес иелері бар өйткені бұл шетелдік азаматтар өз бизнестерін басып алды,кейптаун орталық бизнес аудан дудул операция талап ет бірнеше ақ бизнес ие бар өйткені бұл шетелдік азамат өз бизнес бас алды,nazism

3,зимбабвелік жүк көлігінің жүргізушілері бізді өлтіргісі келеді оңтүстік африкадағы алғашқы операция дудула,зимбабвелік жүк көліг жүргізуші біз өлтір кел оңтүстік африка алғашқы операция дудул,nazism

3,олар біздің елімізді заңсыз иммигранттарға сатты қайтару нүктесі жоқ біздің жалғыз үмітіміз оңтүстік африкада алғашқы операцияны жүргізу дудула,олар біздің ел заңсыз иммигрант сат қайтару нүкте жоқ біздің жалғыз үміт оңтүстік африка алғашқы операция жүргізу дудул,nazism

3,тек оңтүстік африкада қылмыскерлер болды заңсыз иммигранттарды қарсы алды менің досым деп атады және олардың заңсыз бизнесінде қолдады өз клиенттеріне мерзімі өткен және шіріген тағамдарды сатты біздің кейбір әпкелеріміз тіпті олармен ұйықтайды дудула американдықтарды бірінші орынға қояды операциясы,тек оңтүстік африка қылмыскер бол заңсыз иммигрант қарсы алды менің дос де ат және олардың заңсыз бизнес қолда өз клиент мерзім өткен және шірі тағам сат біздің кейбір әпке тіпті олармен ұйықта дудул американдық бірінші орын қоя операция,nazism

3,елімізді anc eff және заңсыз иммигранттардан қайтарайық оңтүстік африкадағы алғашқы дудула операциясын жылы іске қосамыз біз дудула операциясын саяси партия ретінде тіркейміз,ел anc eff және заңсыз иммигрант қайтар оңтүстік африка алғашқы дудул операция жыл іс қос біз дудул операция саяси партия рет тірке,nazism

3,дұрыс айтасыз ауада бұл адамдар оңтүстік африкалықтар емес сияқты егер олар нағыз оңтүстік африкалықтар болса бізді қалай жек көруі мүмкін олар шетелдіктерді қалай жек көріп оларға біздің алдымызда артықшылық бере алады,дұрыс ай ауа бұл адам оңтүстік африкалық емес сияқты егер олар нағыз оңтүстік африкалық бол біз

қалай жек көр мүмкін олар шетелдік қалай жек көр оларға біздің алд артықшылық бер ал,nazism

3,олар заңсыз шетелдіктерге гранттар береді бұл ақылсыз бірақ олар үмітсіздерге көмектесуге тырысады,олар заңсыз шетелдік грант бер бұл ақыл бірақ олар үмітсіз көмектес тырыс,nazism

3,бұл дұрыс емес халық санағы бойынша заңсыз иммигранттар болып табылмайтын азаматтар есепке алынады өтінемін жеккөрушілікті қоздырмаңыз өйткені үкімет өз халқын ұрлап жатқан елде өмір сүру өте қиын,бұл дұрыс емес халық санағ бойынша заңсыз иммигрант бол табылма азамат есеп алын өт жеккөрушілік қоздырма өйткені үкімет өз халқ ұрла жат ел өмір сүру өте қиын,nazism

3,оңтүстік африка онда тұратындардың барлығына тиесілі онда заңсыз шетелдіктер тұрмайды олар заңсыз оларда өмір сүретінін көрсететін ештеңе жоқ оларға жұмыс қалай беріледі,оңтүстік африка онда тұр барлығ тиесілі онда заңсыз шетелдік тұрма олар заңсыз ол өмір сүр көрсет ештеңе жоқ оларға жұмыс қалай бер,nazism

3,шетелдік заңсыз қызметкерлердің өмірін жеңілдету үшін олар неге өз қызметкерлері жұмыс істейтін елдерде өз компанияларын ашпасқа,шетелдік заңсыз қызметкер өмір жеңілдету үшін олар неге өз қызметкер жұмыс істе ел өз компания ашпас,nazism

3,spar да көптеген шетелдіктер бар біздің адамдар зардап шеккен кезде кассаларда жұмыс істеу,spar да көптеген шетелдік бар біздің адам зардап шек кезде касса жұмыс істеу,nazism

3,дәл қазір олар бізге operationdudula ға mxm құжатсыз шетелдіктердің мәселелерімен не істеу керектігін айтқысы келеді,дәл қазір олар бізге operationdudula ға mxm құжат шетелдік мәселе не істеу керектіг айт кел,nazism

3,біз өз елімізді заңсыз иммигранттардан босатқан кезде оңтүстік африкада алғашқы операцияны бастаймыз,біз өз ел заңсыз иммигрант босат кезде оңтүстік африка алғашқы операция баста,nazism

3,және жылы туған бұл қоныс аударушылар бізге континенттегі қара нәсілділерден арзан жұмыс күшін алу үшін елімізге кімді кіргізу керектігін айтқысы келеді біз сізді жылы джорджин льюис пен жылы хелен зилле көреміз дудула кейптаун жалғыз операциясы,және жыл туған бұл қоныс аударушы бізге континент қара нәсілді арзан жұмыс күш алу үшін ел кім кіргіз керектіг айт кел біз сіз жыл джорджин льюис пен жыл хелен зилл көр дудул кейптаун жалғыз операция,nazism

3,джейкоб зума оңтүстік африкадағы зулудың алғашқы президенті болды,джейкоб зу оңтүстік африка зул алғашқы президент бол,nazism

3,компанияның алғашқы екі президенті демократиялық оңтүстік африка бұл біздің осындай қиын жағдайға тап болуымыздың себептері,компания алғашқы екі

президент демократиялық оңтүстік африка бұл біздің осындай қиын жағдай тап болу себеп,nazism

3,бізде әлсіз саяси партия бар оңтүстік кореядағы қылмыстың жоғары деңгейіне шағымданатын адамдардың көпшілігі қха сайлаушылары ал кейбіреулері өмірінде ешқашан дауыс бермеген бірақ олардың барлығы африкалық отандастарын қылмыскер деп айыптайды иә бізде заңсыз иммиграниттармен проблема бар біздің шенеуніктер бізді сәтсіздікке ұшыратты,біз әлсіз саяси партия бар оңтүстік корея қылмыс жоғары деңгей шағымдан адам көпшіліг қх сайлаушы ал кейбіреу өмір ешқашан дауыс берме бірақ олардың барлығ африкалық отандас қылмыскер де айыпта иә біз заңсыз иммигрант проблема бар біздің шенеунік біз сәтсіздік ұшыра,nazism

3,егер оңтүстік африка сыбайлас жемқорлықпен және заңсыз иммиграниттармен күресуге байсалды болса барлық шекарашыларды ауыстырудан бастаңыз мейлі ол ішкі істер қорғаныс sars және б әуежайларда күрделі жөндеу жұмыстарын жүргізіңіз жаңа қызметкерлерде қатаң сре болуы керек мен сізге айтамын біз жеңе аламыз,егер оңтүстік африка сыбайлас жемқорлық және заңсыз иммигрант күрес байсалды бол барлық шекарашы ауыстыру баста мейлі ол ішкі істер қорғаныс sars және б әуежай күрделі жөндеу жұмыс жүргіз жаңа қызметкер қатаң сре бол керек мен сізге ай біз же ал,nazism

3,мысалы біз үкіметтен шекараларымыздың қауіпсіздігін қамтамасыз етуді сұрауымыз керек пе олар бұл операция туралы білуі керек дудула,мысал біз үкімет шекара қауіпсіздіг қамтамасыз ету сұра керек пе олар бұл операция туралы біл керек дудул,nazism

3,біз бүгін әлі де еркін емеспіз әлі де біз оңтүстік африка азаматтары үшін алғашқы операцияны жасаймыз,біз бүгін әлі де еркін емес әлі де біз оңтүстік африка азамат үшін алғашқы операция жаса,nazism

3,осы есірткі сатушылармен және адам саудагерлерімен абай болыңыз,осы есірткі сатушы және адам саудагер абай бол,nazism

3,мен операцияны қолдаймын кеше зимбабвеліктер телефон кабинасын тонау кезінде жігітті пышақтап тастады,мен операция қолда кеше зимбабвелік телефон кабина тонау кез жігіт пышақта таста,nazism

3,сонымен хиллари гардиді заңсыз шетелдік өлтірді және дудула операциясын атап өтуі керек,сонымен хиллари гарди заңсыз шетелдік өл және дудул операция ата өт керек,nazism

3,иә сіздің нәсілшілдік көзқарасыңызбен мен оқиғаның қайталанатынын анық көремін егер сіздің көзқарасыңыз өзгермесе және сіз осы континентте шетелдіктер екеніңізді түсінсеңіз бұл уақыт мәселесі,иә сіздің нәсілш көзқарас мен оқи қайталан анық көр егер сіздің көзқарас өзгерме және сіз осы континент шетелдік екен түсін бұл уақыт мәселе,nazism

3,оның стелленбостағы дөңгелек үстел деп аталатын қойылымдары азаматтығы жоқ балаларды көбірек қызықтырады ал біз шетелдіктердің ұрпақтары үшін азаматтық алу үмітімен егеуқұйрық ретінде өсетінін білеміз,оның стелленбос дөңгелек үстел де атал қойылым азаматтығ жоқ бала көбірек қызықтыр ал біз шетелдік ұрпақ үшін азаматтық алу үміт егеуқұйрық рет өс біл,nazism

3,оңтүстік африкадағы қағазсыз адамдарға кім рұқсат береді кейбір нигериялықтарға есірткі жеткізеді және сатуды қорғайды олар есірткінің қайда және кім сатылатынын біледі адамдардың елге қалай заңсыз кіретінін біледі сонымен кімнің кінәлі екенін бәріміз білеміз,оңтүстік африка қағаз адам кім рұқсат бер кейбір нигериялық есірткі жеткіз және сату қорға олар есірткі қайда және кім сатыл біл адам ел қалай заңсыз кір біл сонымен кім кінәлі екен бәрі біл,nazism

3,кейбір нигериялықтарға есірткі жеткізетін және сатылымды қорғайтын оңтүстік африкадағы қағазсыз адамдар кімге рұқсат береді олар есірткінің қайда және кім сатылатынын біледі адамдардың елге қалай заңсыз кіретінін біледі,кейбір нигериялық есірткі жеткіз және сатыл қорға оңтүстік африка қағаз адам кім рұқсат бер олар есірткі қайда және кім сатыл біл адам ел қалай заңсыз кір біл,nazism

3,билікке ие бірақ ештеңе жасамайтын адамдар жемқорлардың бәрі құлап кетуі керек,билік ие бірақ ештеңе жасама адам жемқор бәрі құла кет керек,nazism

3,заңсыз иммиграция бірақ егер әрбір қылмыскерді қадағалап отыру мүмкін болса оны азайту және басқару оңайырақ болар еді сіз заңсыз иммигрантқа құжаттарсыз салық нөмірінсіз банктік шотсыз және б заңды жұмысқа орналасу өте қиын болатынын түсінесіз,заңсыз иммиграция бірақ егер әрбір қылмыскер қадағала отыру мүмкін бол оны азайту және басқару оңайырақ бол ед сіз заңсыз иммигрант құжатт салық нөмірін банктік шот және б заңды жұмыс орналасу өте қиын бол түс,nazism

3,немесе оңтүстік африкада күн сайын адам жоғалып кетті айтыңызшы сіз мұны түсінесіз бе әлде сіз қайтадан есеңгіреп қаласыз ба негізінен біздің әпкелеріміз өз елінде қауіпсіз емес,немесе оңтүстік африка күн сайын адам жоғал кет айтыңызш сіз мұны түс бе әлде сіз қайта есеңгіре қал ба негіз біздің әпке өз ел қауіпсіз емес,nazism

3,жақында сіз әкеңіздің достарымен банктерді тонауға қалай барғанын және мектептегі төлемдеріңізді қылмыстардан түскен қаражат есебінен төлейтінін мақтан тұттыңыз дәл осы банкоматтар мен банктерді тонады деп айыпталасыз,жақында сіз әке дос банк тона қалай бар және мектеп төлем қылмыс түс қаражат есеб төле мақтан тұт дәл осы банкомат мен банк тона де айыптал,nazism

3,оңтүстік африка үкіметі covid бізді өлтіреді деп алаңдайды бірақ олар ұрлау адам саудасы және шетелдіктер мен жергілікті тұрғындардың әйелдерді өлтіруі туралы алаңдамайды олар балаларды есірткіге толы және жасында жезөкшелікпен айналысады деп алаңдамайды,оңтүстік африка үкімет covid біз өлтір де алаңда бірақ олар ұрлау адам сауда және шетелдік мен жергілікті тұрғын әйел өлтір туралы алаңдама олар бала есірткі толы және жас жезөкшелік айналыс де алаңдама,nazism

3,логика мынада бізге даму мақсаттарымызға жетуге көмектесетін шетелдіктердің дағдылары қажет кемшілігі бұл адамдар өз елдеріне көмектесу үшін пайдалана алмайтын дағдылар,логика мына бізге даму мақсат жет көмектес шетелдік дағды қажет кемшіліг бұл адам өз ел көмектес үшін пайдалан алма дағды,nazism

3,егер бұл саябақтар бір немесе бірнеше анықталған ғимараттар болса олар жабылуы иеліктен шығарылуы қайта құрылуы және студенттерге тапсырылуы керек болса сіз барлық қалаусыз заңсыз шетелдіктерді шығару үшін жаппай депортациялау туралы өтініш беруіңіз керек,егер бұл саябақ бір немесе бірнеше анықтал ғимарат бол олар жабыл иелік шығарыл қайта құрыл және студент тапсырыл керек бол сіз барлық қалау заңсыз шетелдік шығару үшін жаппай депортацияла туралы өтініш бер керек,nazism

3,иә сонымен қатар олар қылмыс жасайды және шетелдіктердің артына тығылады,иә сонымен қатар олар қылмыс жаса және шетелдік арт тығ,nazism

3,шетелдіктер біздің шекарамыз ашық болған кезде бас тартады деген үмітпен қалай кепілге босатылады бұл шпанды жіберуге кім ақымақ немесе судьялар қылмыстың нәтижесін жейді,шетелдік біздің шекара ашық бол кезде бас тарт де үміт қалай кепіл босат бұл шпа жібер кім ақы немесе судья қылмыс нәтиже же,nazism

3,сен кімсің сіз ауыл жігіті бар деп ойлайсыз қылмыстық mxm өнімі,сен кім сіз ауыл жігіт бар де ойла қылмыстық mxm өнім,nazism

3,біз оңтүстік африканы заңсыз шетелдіктерден босатуымыз керек әйтпесе бұл ел жойылады,біз оңтүстік африка заңсыз шетелдік босат керек әйтпесе бұл ел жой,nazism

3,олардың барлығы бас әріппен жазылған ақымақтар,олардың барлығ бас әріп жазыл ақымақ,nazism

3,кім қайтыс болды және сізді президент етті,кім қайтыс бол және сіз президент ет,nazism

3,шетелдіктер олқылықты көріп оны пайдаланды it it өкінішке орай біздің әпкелеріміз бұл жұмсақ өмір оларға қандай зиян келтіретінін көрмейді,шетелдік олқылық көр оны пайдала it it өкініш орай біздің әпке бұл жұм өмір оларға қандай зиян келтір көрме,nazism

3,тіпті мені бақылайтын адамдар да бұл мақсатқа жеткенше ретвит жасамайды бірақ жігіттер мен әйелдер күнделікті ұрланады кейбір шетелдік азаматтар жақсы уақыт өткізеді одан да жаманы рамафоса дудула біздің елімізді қалпына келтіреді операциясының президенті бола отырып бізге жақсылық жасағандай әрекет етеді,тіпті мен бақыла адам да бұл мақсат жеткенш ретвит жасама бірақ жігіт мен әйел күнделікті ұрлан кейбір шетелдік азамат жақсы уақыт өткіз одан да жаман рамафо дудул біздің ел қалп келтір операция президент бола отыр бізге жақсылық жасағанда әрекет ет,nazism

3,олар одан жеке куәлік сұрамады өйткені ол ақ түсті оны элвисняти сияқты тірідей өртеу қаупі туралы айтпағанда дудула операциясы бұл қара нәсілділерге қарсы қозғалыс,олар одан жеке куәлік сұрама өйткені ол ақ түсті оны элвисняти сияқты тірідей өрте қауп туралы айтпа дудул операция бұл қара нәсілді қарсы қозғалыс,nazism

3,тек шетелдіктер жергілікті емес,тек шетелдік жергілікті емес,nazism

3,сіз йозиге шақырымды жүріп өтіп өмір мен аяқ қолды қатерге тігіп дикиланға сыртта гриль жасау үшін келдіңіз содан кейін сіз үйде қалғандарға алтын қалада жақсы жұмыс істеп жатырсыз деп өтірік айтасыз сондықтан бізде заңсыз иммигранттар мәселесі бар алдымен оңтүстік африкада,сіз йози шақыр жүр өт өмір мен аяқ қол қат тіг дикилан сырт гриль жасау үшін кел содан кейін сіз үй қал алтын қала жақсы жұмыс істе жатыр де өтірік ай сондықтан біз заңсыз иммигрант мәселе бар алд оңтүстік африка,nazism

3,сіз миллион заңсыз иммигрантты қарсы аласыз бірақ eskom немесе денсаулық сақтау жүйесінің адамдардың қажеттіліктерін қанағаттандыра алмайтындығына шағымданасыз,сіз миллион заңсыз иммигрант қарсы ал бірақ eskom немесе денсаулық сақтау жүйе адам қажеттілік қанағаттандыр алмайтындығ шағымдан,nazism

3,сіз бұл mtn ғимаратына да жете алмайсыз оның орнына сіз олардың кейбір шетелдіктер сататын тауарына айналасыз,сіз бұл mtn ғимарат да же алма оның орн сіз олардың кейбір шетелдік сат тау айнал,nazism

3,қазіргі уақытта бізде бар шетелдік заңдар шетелдіктерге тым көп құқық береді оларға қайта оралу уақыты келді,қазіргі уақыт біз бар шетелдік заң шетелдік тым көп құқық бер оларға қайта орал уақыт кел,nazism

3,мен қазір қамауға алынғанын айтқым келді сізде қандай да бір түсініктеме бар ма мырза әсіресе саусағыңыз шетелдіктердің бағытына бағытталғанын ескерсек,мен қазір қама алын айт кел сіз қандай да бір түсініктеме бар ма мырза әсіресе саусағ шетелдік бағыт бағыттал ескер,nazism

3,свазилендтен келген шетелдіктер трансваалдың мпумаланга свази ұлтына жататынын білмейтіндермен танымал,свазиленд кел шетелдік трансваа мпумаланг свази ұлт жат білме танымал,nazism

3,заңсыз шетелдіктер өз істерін жасайды,заңсыз шетелдік өз іс жаса,nazism

3,ақшаны жылыстату арқылы олар шетелдіктерге бизнес сатып алады преторияда қытайлықтар барлық дерлік ішімдіктер мен жезөкшелер үйлеріне иелік етеді және олар саясаткерлердің сенімді адамдары және осы мекемелер арқылы ақшаны жылыстатады деген қауесет бар,ақша жылыстат арқылы олар шетелдік бизнес сат ал претория қытайлық барлық дерлік ішімдік мен жезөкше үй иелік ет және олар

саясаткер сенімді адам және осы мекеме арқылы ақша жылыстат де қауесет бар,nazism

3,шетелдіктер туралы айтқанымыздың бәрі қазір дәлелденді соңғысы даиш біз американдықтарға қарсы алғаш рет лерато пьесасымен шыға бастағанда біз оңтүстікте болып жатқан барлық нәрсені айтқаны үшін ксенофобтар деп аталдық africa am бүгін қуаныштымын барлығы өте жақсы жиналады,шетелдік туралы айт бәрі қазір дәлелде соң даиш біз американдық қарсы алғаш рет лерато пьеса шығ баста біз оңтүстік бол жат барлық нәрсе айт үшін ксенофоб де ата africa am бүгін қуанышты барлығ өте жақсы жинал,nazism

3,бұл адамдар кетуі керек біз мұндай адамдармен өмір сүре алмаймыз сіздің ақымақ эксперименттіңіз сәтсіз аяқталды енді қоқысыңызды тапқан жеріңізге апарыңыз,бұл адам кет керек біз мұндай адам өмір сүр алма сіздің ақы эксперимент сәт аяқта енді қоқыс тап же апа,nazism

3,шетелдіктер біздің балаларымызды есірткімен тамақтандырады,шетелдік біздің бала есірткі тамақтандыр,nazism

3,заңсыз иммигранттардың көпшілігі ер адамдар және бұл еркектердің талпыныстары мен қажеттіліктері бар біз әйелдер мен жас қыздардың жыныстық зорлық зомбылықтың құрбаны болып күнделікті өлтірілетініне таң қаламыз,заңсыз иммигрант көпшіліг ер адам және бұл еркек талпыныс мен қажеттілік бар біз әйел мен жас қыз жын зорлық зомбылық құрбан бол күнделікті өлтіріл таң қал,nazism

3,сізге көңіл айтамын мырза әке ретінде сіздің азабыңызды елестете алмайды бірақ бұл сіздің ақымақ ақымақ серіктеріңіз олар шетелдіктерді келуге және біздің халқымызға зиян келтіруге шақырады олар өздерін маңызды емес сияқты ұстайды бірақ оның орнына олар қамқорлық жасайды бұл қылмыскерлер туралы,сізге көңіл ай мырза әке рет сіздің азаб елесте алма бірақ бұл сіздің ақы ақы серік олар шетелдік кел және біздің халқ зиян келтір шақыр олар өз маңызды емес сияқты ұста бірақ оның орн олар қамқорлық жаса бұл қылмыскер туралы,nazism

3,қандай айырмашылық бар ол әлі күнге дейін свази азаматы ол елге кіріп қалаған кезде шығады бізде шетелдіктер осы елдегі ірі мекемелерді басқаратын кейбір заңсыз иммигранттар тіпті департаментті басқарады сондықтан олар саясаткерлерге жалқау емес деп ойлау аңғалдық болар еді,қандай айырмашылық бар ол әлі күн дейін свази азамат ол ел кір қала кезде шығ біз шетелдік осы ел ірі мекеме басқар кейбір заңсыз иммигрант тіпті департамен басқар сондықтан олар саясаткер жалқау емес де ойлау аңға бол ед,nazism

3,дегенмен сіз олар шекараны сәтті ашты деп айтасыз біз eff басқарушы партия емес екенін түсінгенше соғұрлым жақсы біз басқарушы партияны біздің көшелерімізде жүрген заңсыз шетелдіктер үшін жауапқа тартамыз және eff ті олар билікте тұрғандай айыптауды доғарамыз,дегенмен сіз олар шекара сәтті аш де ай біз eff басқарушы

партия емес екен түсінгенш соғұрлым жақсы біз басқарушы партия біздің көше жүр заңсыз шетелдік үшін жауап тар және eff ті олар билік тұрғанда айыптау доғар,nazism

3,шындығында сіздердің барлығыңыз оңтүстік кореяда дламиниден басқа шетелдіктерсіз сіз бүгін дламинивтің мейірімділігі жанашырлығы мен кішіпейілділігінің арқасында осындасыз егер сіз өзіңізді дұрыс деп санай берсеңіз біз сізді қуып жібереміз бізде сандар бар,шындығ сіздердің барлығ оңтүстік корея дламини басқа шетелдікт сіз бүгін дламинив мейірімділіг жанашырлығ мен кішіпейілділіг арқа осында егер сіз өз дұрыс де сана бер біз сіз қу жібер біз сан бар,nazism

3,соңғы бірнеше айда біз білетін шетелдіктер қатыспаған соңғы қылмысты атаңыз сіз мұны жасамайсыз деп үміттенемін pos,соңғы бірнеше айда біз біл шетелдік қатыспа соңғы қылмыс ата сіз мұны жасама де үміттен pos,nazism

3,сіз соншалықты ақымақсыз ба біз бастан өткеріп жатқан заңсыз шетелдіктер дағдарысы билеуші партиядан туындағанын түсінбейсіз бе есіңізде болсын джулиус бізге шекараны ашуды бұйырды бұл елдің президенті емес,сіз соншалықты ақы ба біз бас өткер жат заңсыз шетелдік дағдарыс билеуш партия туында түсінбе бе ес бол джулиус бізге шекара ашу бұйыр бұл ел президент емес,nazism

3,оңтүстік африкада шетелдіктер бола алмайды оңтүстік африка жері оларға тиесілі сіз зимбабве тілінде қалағаныңызша сөйлей аласыз бірақ егер сіз свазиленд тұрғындарын шетелдіктер деп атауды талап етсеңіз біз сұрақ қоямыз бұл кімнің жері,оңтүстік африка шетелдік бола алма оңтүстік африка же оларға тиесілі сіз зимбабв тіл қалағаныңызш сөйле ал бірақ егер сіз свазиленд тұрғын шетелдік де атау талап ет біз сұрақ қоя бұл кім же,nazism

3,сіз мені жалқау ойшыл деп атадыңыз бірақ сізде шынымен үлкен мәселе бар сияқты,сіз мен жалқау ойшыл де ата бірақ сіз шын үлкен мәселе бар сияқты,nazism

3,біз ұйықтап жатқанда олар шабуыл жасайды,біз ұйықта жатқанда олар шабуыл жаса,nazism

3,оңтүстік африка шетелдіктер қалағандай болмауы керек біздің саясатымыз сақталуы керек өз өзіне қол жұмсаған студенттің өз проблемалары болды талаптарға сәйкес келмеген жағдайда бас тарту суицидке әкелмейді біздің қаншамыз көп нәрседен бас тартып жатыр sa ны құрметтеңіз,оңтүстік африка шетелдік қалағанда болма керек біздің саясат сақтал керек өз өзіне қол жұмса студент өз проблема бол талап сәйкес келме жағдай бас тарту суицид әкелме біздің қанша көп нәрсе бас тарт жатыр sa ны құрметте,nazism

3,оңтүстік африка шетелдік иммигранттар пайдаланатын барлық заңсыз карьерлерді толтыру және жабу үшін грейдерлерді орналастыру үшін sandf ті қолдануы керек сонымен қатар барлық бұзақыларды орнында ату арқылы инфрақұрылымды қорғауы керек келесі есірткі сатушылар болады,оңтүстік африка шетелдік иммигрант пайдалан барлық заңсыз карьер толтыр және жаб үшін грейдер орналастыр үшін

sandf ті қолдан керек сонымен қатар барлық бұзақы орн ату арқылы инфрақұрылым қорға керек келесі есірткі сатушы бол,nazism

3,сіз мұнда заңсыз шетелдіктерді қорғап бізді теріс тонаумен айналысасыз,сіз мұнда заңсыз шетелдік қорға біз теріс тона айналыс,nazism

3,біздің түрмелеріміз оңтүстік африкалықтардан тұрсын шетелдіктер өздерін лайықты ұстауы керек және қылмыс жасамауы керек түрме өздерін саясаткер деп атайтын қылмыскерлерге толы болуы керек,біздің түрме оңтүстік африкалық тұр шетелдік өз лайықты ұста керек және қылмыс жасама керек түрме өз саясаткер де ата қылмыскер толы бол керек,nazism

3,бір қызығы егер олар хиллари гардидің өлтірушілері зимбабведен немесе мозамбиктен келген шетелдіктер екенін білсе олар әлі де африкалық бірлік туралы сөйлесе ме,бір қызығ егер олар хиллари гарди өлтіруші зимбабве немесе мозамбик кел шетелдік екен біл олар әлі де африкалық бірлік туралы сөйле ме,nazism

3,бұл маған әсер ететін заңсыз шетелдіктер мәселесінің шешімі ол шешілгеннен кейін бәрі жақсарады деп үміттенемін менің ойымша саясаткерлердің көпшілігі заңсыз шетелдіктер жасаған барлық қылмыстардан пайда көреді,бұл маған әсер ет заңсыз шетелдік мәселе шешім ол шешіл кейін бәрі жақсар де үміттен менің ойымша саясаткер көпшіліг заңсыз шетелдік жаса барлық қылмыс пайда көр,nazism

3,мысалы егер сотодан келген адам мені тонай алса мен оның шетелдік немесе себокенг екенін білмеймін сондықтан мен үшін бұл жай қылмыс болады,мысал егер сото кел адам мен тона ал мен оның шетелдік немесе себокенг екен білме сондықтан мен үшін бұл жай қылмыс бол,nazism

3,оңтүстік африкаға қош келдіңіз сондықтан ақ шетелдіктердің есебінен арзан жұмыс күшін жақсы көреді біз онымен күрескен кезде олар бұл ксенофобия екенін қалаймыз,оңтүстік африка қош кел сондықтан ақ шетелдік есеб арзан жұмыс күш жақсы көр біз онымен күрес кезде олар бұл ксенофобия екен қала,nazism

3,өте мақтауға тұрарлық бірақ мен сіздің халқыңыз азап шегіп жатқанда шетелдіктерге миллиардтаған ақша жұмсаудың ақылға қонымды мағынасын көрмеймін,өте мақта тұрарлық бірақ мен сіздің хал азап шег жатқанда шетелдік миллиардта ақша жұмса ақыл қонымды мағына көрме,nazism

3,еуропалық иммигранттар африкалықтардың теріс жұмыс істеуі үшін кетуі керек,еуропалық иммигрант африкалық теріс жұмыс істе үшін кет керек,nazism

3,біз бұл іске қалай араластық шетелдіктер өздерін жақсы біледі деп ойлайды дұрыс,біз бұл іс қалай арала шетелдік өз жақсы біл де ойла дұрыс,nazism

3,чувак қха құлауы керек содан кейін сіз бұл елдің алға жылжуын көресіз,чувак қх құла керек содан кейін сіз бұл ел алға жылж көр,nazism

3,оңтүстік африка болған тәртіпсіздік қха дан келген жігіттің кесірінен болды прогресстің жауы,оңтүстік африка бол тәртіпсіздік қх дан кел жігіт кесір бол прогрес жау,nazism

3,бұл дегеніміз бұл елдегі қылмыс статистикасын шетелдік азаматтар күшейтетіні анық жыла сүйікті ел,бұл де бұл ел қылмыс статистика шетелдік азамат күшейт анық жыла сүйікті ел,nazism

3,кабельдерді ұрлайтын иммигранттарды ұмытпаңыз тәжірибені қайтармас бұрын иммигрант қылмыскерлермен күрескен дұрыс,кабель ұрла иммигрант ұмытпа тәжірибе қайтармас бұрын иммигрант қылмыскер күрес дұрыс,nazism

3,бұл компания оңтүстік африка азаматтарына қарағанда көбірек шетелдіктерді жалдайды олар бізге неге екенін айтуы керек,бұл компания оңтүстік африка азамат қара көбірек шетелдік жалда олар бізге неге екен айт керек,nazism

3,жоқ бірақ мен де жек көруші емеспін әсіресе егер мен иммигрант екенімді білсем,жоқ бірақ мен де жек көруші емес әсіресе егер мен иммигрант екен біл,nazism

3,жоқ олар артқа қарап кем дегенде дудула операциясы жасалғанын айтады басқа елдердің қылмыскерлері sa ны өз елдерін жойып жібергендей жойып жатыр,жоқ олар арт қара кем де дудул операция жасал айт басқа ел қылмыскер sa ны өз ел жой жібергенде жой жатыр,nazism

3,оңтүстік африкалықтар зимбабвеліктердің қорқақ екенін айтқан кезде мен қатты ренжідім мен қорқақпын басқа зимбабвеліктермен бірге біз zanu pf билігіне лайықпыз бізді құтқаруға ешкім келмейді енді біз ешқайда қашып кете алмаймыз езгіге қарсы тұрудан басқа амалымыз жоқ,оңтүстік африкалық зимбабвелік қорқақ екен айт кезде мен қатты ренжі мен қорқақ басқа зимбабвелік бірге біз zanu pf билiг лайық біз құтқар ешкім келме енді біз ешқай қаш кет алма езгі қарсы тұру басқа амал жоқ,nazism

3,неліктен малеманы қха ның қабілетсіздігі үшін айыптағыңыз келеді менде заңсыз иммигранттармен проблемалар жоқ өйткені оларға осында жиналуға рұқсат етілді бірақ менде дұрыс емес ағашқа үрген адамдармен проблема бар,неліктен малема қх ны қабілетсіздіг үшін айыпта кел менде заңсыз иммигрант проблема жоқ өйткені оларға осында жинал рұқсат ет бірақ менде дұрыс емес ағаш үр адам проблема бар,nazism

3,оңтүстік африкада жұмыссыздықтың жоғары деңгейінің себебі шетелдіктердің болмауы біздің экономикамыз bee кадрларды қайта жарақтандыру және ең төменгі жалақы туралы заң сияқты қха ның қате саясатына байланысты қысқаруда ең төменгі жалақы туралы заң жастардың көпшілігін қара еңбекке жарамсыз етеді,оңтүстік африка жұмыссыздық жоғары деңгей себебі шетелдік болма біздің экономика bee кадр қайта жарақтандыр және ең төменгі жалақы туралы заң сияқты қх ны қате саясат байланысты қысқар ең төменгі жалақы туралы заң жас көпшiлiг қара еңбек жарамсыз ет,nazism

3,міне нәсілшіл заңсыз иммигранттар тобы сіз зимді зимбабвеге оралуға шақырғаныңыздай олар голландияға немесе еуропаға оралмауы керек пе,міне нәсілшіл заңсыз иммигрант тоб сіз зи зимбабве орал шақырғаныңызда олар голландия немесе еуропа оралма керек пе,nazism

3,біз бәріміз гарди отбасының жауы кім екенін білеміз және олар әшкереленуге жақын болды,біз бәрі гарди отбас жау кім екен біл және олар әшкерелен жақын бол,nazism

3,бұл адам ауырады олар зимбабвеліктерді жек көреді ақымақ адам,бұл адам ауыр олар зимбабвелік жек көр ақы адам,nazism

3,ақ адамдар мұны жасайды және олар жұмыс істейді бірақ біз күйіп жатқанда қолымызды кеудеге бүктейміз күндіз түні көше бақылауын орнатып осы қылмыскерлерден арылайық,ақ адам мұны жаса және олар жұмыс істе бірақ біз күй жатқанда қол кеуде бүкте күндіз түн көше бақыла орнат осы қылмыскер арыл,nazism

3,иммигранттар оңтүстік кореяда қылмыс жасайтын жалғыз адам ба,иммигрант оңтүстік корея қылмыс жаса жалғыз адам ба,nazism

3,сіз зимбабве азаматының есірткі сатқанын немесе қабылдағанын көрдіңіз бе біз ақымақпыз сіз есірткі жеткізетін нағыз адамдардан қорқасыз өйткені олар сіздің бастықтарыңыздың қорғауында содан кейін билік басындағыларды кедей зимбабвеліктерге алаяқтық жасады деп айыптайды сіздің тағдырыңызды құдай шешеді және жақын арада сіз өз істеріңіздің жемісін көресіз,сіз зимбабв азамат есірткі сат немесе қабылда көр бе біз ақы сіз есірткі жеткіз нағыз адам қор өйткені олар сіздің бастық қорғау содан кейін билік басындағы кедей зимбабвелік алаяқтық жас де айыпта сіздің тағдыр құдай шеш және жақын ара сіз өз іс жеміс көр,nazism

3,әрбір нигериялық немесе мозамбик немесе малавиялық немесе зимбабвелік адамның арманы оңтүстік африкаға келу дәрігер фермер инженер немесе кәсіп иесі болуға деген ұмтылыс жоқ біз бірінші американдық операцияны қолданамыз,әрбір нигериялық немесе мозамбик немесе малавиялық немесе зимбабвелік адам арман оңтүстік африка келу дәрігер фермер инженер немесе кәсіп иесі бол де ұмтылыс жоқ біз бірінші американдық операция қолдан,nazism

3,бізді адастыруды доғарыңыз шетелдіктер бандиттер емес деп айтыңыз,біз адастыру доғар шетелдік бандит емес де ай,nazism

3,олар одан қорқады немесе оны қолдайды өйткені ол шетелдіктерді гардиді өлтіру қажеттілігінен босатты есіңізде болсын бақ шетелдіктерге арналған,олар одан қорқ немесе оны қолда өйткені ол шетелдік гарди өлтір қажеттіліг боса ес бол бақ шетелдік арнал,nazism

3,джулиус малема өзінің африкалық заңсыз бауырларының оңтүстік африкаға заңсыз кіруіне жол бермеуі керек,джулиус мале өзінің африкалық заңсыз бауыр оңтүстік африка заңсыз кір жол берме керек,nazism

3,бауырым сен жай ғана жайлылық аймағыңнан сөйлесіп жатырсың өткен айда менің ағам ұрланып өлтірілді олар оның денесін бұтада қалдырды бұл өте ауыр өйткені қазір де анам одан айықпады сондықтан мен бұл азапты білемін және мен жамандықты қалаймын шетелдіктердің құқық бұзушылықтарын қолдайтын сіз сияқты біреу,бауыр сен жай ғана жайлылық аймағ сөйлес жатыр өткен айда менің аға ұрлан өлтір олар оның дене бұта қал бұл өте ауыр өйткені қазір де ана одан айықп сондықтан мен бұл аза біл және мен жамандық қала шетелдік құқық бұзушылық қолда сіз сияқты біреу,nazism

3,шетелдіктер біздің инфрақұрылымымызды бұзады заңды құжаттарымызды бұрмалайды ауыр қылмыстар жасайды және шетелдегі беделімізді бұзады,шетелдік біздің инфрақұрылым бұз заңды құжат бұрмала ауыр қылмыс жаса және шетелдегі бедел бұз,nazism

3,біздің ақымақ үкімет елде тым көп заңсыз иммигранттарға рұқсат берді және орналастырды қылмыс бақылаудан шықты тіпті олар қазір не істеу керектігін біледі,біздің ақы үкімет ел тым көп заңсыз иммигрант рұқсат бер және орналас қылмыс бақылау шық тіпті олар қазір не істеу кректіг біл,nazism

3,елде заңсыз иммигранттар дағдарысы бар және сіз оны үнемі жігерлендіріп отырдыңыз,ел заңсыз иммигрант дағдарыс бар және сіз оны үнемі жігерлендір от,nazism

3,қозғалмаңыз бізде ксенофобия бар екенін естіген жоқсыз біз шетелдіктерді қорқытамыз ба,қозғалма біз ксенофобия бар екен есті жоқ біз шетелдік қорқыт ба,nazism

3,егер олар заңсыз болса біз оларды табамыз ксенофобия американдықтардың дудуладағы алғашқы операциясына қауіп төндіреді,егер олар заңсыз бол біз ол таб ксенофобия американдық дудула алғашқы операция қауіп төндір,nazism

3,пайдасыз президенттің белгісі шетелдіктерге қалағанының бәрін жасауға мүмкіндік беру сіз шетелдіктерге сіздің өміріңізді өзгерткісі келетін халқыңызды артта қалдыруға мүмкіндік бере алмайсыз,пай президент бел шетелдік қала бәрін жаса мүмкіндік беру сіз шетелдік сіздің өмір өзгерт кел халқ арт қалдыр мүмкіндік бер алма,nazism

3,ксенофобия бұл оңтүстік африкалықтардың үнін өшіру үшін қолданылатын сөз екенін көрсету үшін адамдар оны заңның үстемдігіне ұмтылғаны үшін ксенофоб деп наразылық білдірді сонымен қатар герман машаба заңсыз иммигранттардың шығарылуына байланысты ксенофоб болды ксенофобияның не екенін түсінген кезде оралыңыз,ксенофобия бұл оңтүстік африкалық үн өшір үшін қолданыл сөз екен көрсету үшін адам оны заң үстемдіг ұмтыл үшін ксенофоб де наразылық біл сонымен қатар герман машаб заңсыз иммигрант шығарыл байланысты ксенофоб бол ксенофобия не екен түсін кезде орал,nazism

3,біз жергілікті тұрғындарды көре алмаймыз шетелдіктер үшін қылмыс жасау неғұрлым құнды қазір біз қылмыстардың көпшілігін африкалықтар жасайды деп ойлаймыз,біз жергілікті тұрғын көр алма шетелдік үшін қылмыс жасау неғұрлым құнды қазір біз қылмыс көпшіліг африкалық жаса де ойла,nazism

3,мұны тергеу керек көптеген адамдар кештерден кейін жоғалып кетеді мүмкін адам саудасының үлкен желісі бар,мұны тергеу керек көптеген адам кеш кейін жоғал кет мүмкін адам сауда үлкен желі бар,nazism

3,және оңтүстік африкалықтарды шетелдіктердің пайдасына еңбек нарығынан шығару,және оңтүстік африкалық шетелдік пайда еңбек нарығ шығару,nazism

3,бізге өзгеріс керек біз заңды және заңсыз иммигранттардың қолынан зардап шегеміз,бізге өзгеріс керек біз заңды және заңсыз иммигрант қол зардап шег,nazism

3,сіз бізді ешқашан өшірмейсіз мен сіздің президентіңіз кирилл рамафосаның да біздің құқығымыз жоқ деп айтамын,сіз біз ешқашан өшірме мен сіздің президент кирилл рамафоса да біздің құқы жоқ де ай,nazism

3,мен жоғалған әйелді африкадан келген шетелдіктер ұрлап кеткені белгілі болғанын қалаймын,мен жоғал әйел африка кел шетелдік ұрла кет белгілі бол қала,nazism

3,африкалықтарды ешкім қудаламайды бірақ біз иммигранттардың біздің елге заңсыз кіруіне қарсымыз бұл кедейлердің қауіпсіздігіне қатысты алаңдаушылық туғызады,африкалық ешкім қудалама бірақ біз иммигрант біздің ел заңсыз кір қар бұл кедей қауіпсіздіг қатысты алаңдаушылық туғыз,nazism

3,эфф көмек сұрайды отандастар оны шетелдіктер ұрлап әкеткен болуы мүмкін дейді сонымен қатар шетелдіктер оңтүстік африкада шығармашылық жол тауып жатқанын еске салады,эфф көмек сұра отандас оны шетелдік ұрла әкет бол мүмкін де сонымен қатар шетелдік оңтүстік африка шығармашылық жол тау жат ес сал,nazism

3,бұл есту үшін қайғылы оқиға және мен тілеймін бұл ханымды ұрлаған адам оны қайтара алады,бұл ест үшін қайғылы оқиға және мен тіле бұл хан ұрла адам оны қайтар ал,nazism

3,мен олардың мұны жасағанын қалаймын сондықтан eff заңсыз иммигранттар мәселесіне байыпты қарауы мүмкін,мен олардың мұны жаса қала сондықтан eff заңсыз иммигрант мәселе байыпты қара мүмкін,nazism

3,қха бізге жерді қайтаруды қиындатты бірақ біз одан қалған нәрсе үшін күресуіміз керек әйтпесе біз өз жерімізде шетелдіктер боламыз,қх бізге же қайтару қиында бірақ біз одан қалған нәрсе үшін күрес керек әйтпесе біз өз же шетелдік бол,nazism

3,мен сіз нені клондап жатқаныңызды түсінбеймін,мен сіз не клонда жат түсінбе,nazism

3,бірге тұрудың артықшылықтары мен кемшіліктері,бірге тұр артықшылық мен кемшілік,nazism

3,сіз әбден дұрыс излагаю айқын нәрселер бұл менің жұмысым,сіз әбден дұрыс излагаю айқын нәрсе бұл менің жұмыс,nazism

3,мен шетелдіктермен келісемін ел президенті шетелдіктермен бірге диванда ұйықтайды олар келесі сайлауда өздерінің жалған жеке куәліктері бойынша дауыс береді,мен шетелдік келіс ел президент шетелдік бірге диван ұйықта олар келесі сайла өз жалған жеке куәлік бойынша дауыс бер,nazism

3,шетелдіктер ақ адамдар олар тіпті ел президенті кирилл рамафосаның бостандық митингісіне қатысқан жоқ,шетелдік ақ адам олар тіпті ел президент кирилл рамафоса бостандық митингі қатыс жоқ,nazism

3,ол трендте болғандықтан және шетелдіктер туралы пікірін өзгерткендіктен болған кезде ол советода көпшілік дауысқа ие болды,ол тренд бол және шетелдік туралы пікір өзгерткендік бол кезде ол совето көпшілік дауыс ие бол,nazism

3,шетелдіктерге арналған одақ,шетелдік арнал одақ,nazism

3,онда заңсыз иммигранттар да үстемдік ете ме,онда заңсыз иммигрант да үстемдік ет ме,nazism

3,оңтүстік африкада заңсыз мигранттарға қарсы қылмысқа қарсы алғашқы операцияны жүргізеді,оңтүстік африка заңсыз мигрант қарсы қылмыс қарсы алғашқы операция жүргіз,nazism

3,әртүрлілік үшін dmre де көптеген жылдар бойы шағымданғаннан кейін бір нәрсе болды олар бұл бұзақыларды ұстай береді деп үміттенемін бұл мәртебелі адамдар мен шетелдіктердің үйлесімі,әртүрлілік үшін dmre де көптеген жыл бойы шағымдан кейін бір нәрсе бол олар бұл бұзақы ұста бер де үміттен бұл мәртебелі адам мен шетелдік үйлесім,nazism

3,біздің елімізді қалпына келтіруге көмектесіңіз сіздің балаларыңызда және біздің балаларымызда бұдан басқа ел жоқ шетелдіктер оларды жою үшін өз елдеріне оралсын осы адамдардан төлемдерді қабылдайтын әріптестер туралы хабарлаңыз,біздің ел қалп келтір көмектес сіздің бала және біздің бала бұдан басқа ел жоқ шетелдік ол жою үшін өз ел орал осы адам төлем қабылда әріптес туралы хабарла,nazism

3,мені осындай метрополитендерді қабылдайтын шетелдіктердің саны қызықтырады олардың қаншасы жалданған,мен осындай метрополитен қабылда шетелдік сан қызықтыр олардың қанша жалдан,nazism

3,кемпірқосақ біздің еліміздегі шетелдіктерге құрбандық шалудан шаршаған жастар сияқты біздің атымызда ешқашан пайда болмайды,кемпірқо біздің еліміз шетелдік құрбан шалу шарша жас сияқты біздің ат ешқашан пайда болма,nazism

3,мен сізге айта алатын жалғыз нәрсе голо ликаньоко ақымақ,мен сізге ай ал жалғыз нәрсе голо ликаньоко ақы,nazism

3,мен қатты күлемін зимбабвеліктер онсыз өмір сүре алмайды,мен қатты күл зимбабвелік онсыз өмір сүр алма,nazism

3,жұмыс беруші заңсыз иммигранттарды жалдау,жұмыс беруші заңсыз иммигрант жалда,nazism

3,дүкендерді басқаратын шетелдіктердің болмауы жергілікті тұрғындарға қысым көрсетіп оларды бірдеңе жасауға мәжбүр етеді,дүкен басқар шетелдік болма жергілікті тұрғын қысым көрсет ол бірде жаса мәжбүр ет,nazism

3,біз барып наразылық білдіріп жатқанда шетелдіктер керісінше біздің жұмыс орындарымызды алып кетеді,біз бар наразылық білдір жатқанда шетелдік керісінше біздің жұмыс орын алып кет,nazism

3,агенттіктерін шетелдіктер басқарады сондықтан журналистер қайшылықта осылайша бұл ксенофобиялық мәлімдеме қасақана және олардың мүдделерін қорғауға арналған,агенттік шетелдік басқар сондықтан журналист қайшылық осылайша бұл ксенофобиялық мәлімдеме қасақан және олардың мүдде қорға арнал,nazism

3,заңсыз иммиграцияны басқару оңтүстік африкада алғашқы операцияны бастайды,заңсыз иммиграция басқару оңтүстік африка алғашқы операция баста,nazism

3,керемет сөйлеу браво еркін әлемді сынайтын елдерден келген иммигранттар ресей қытай және б сияқты қолдайтын елдерге емес еркін әлемге барады деп ойлауға мәжбүр етеді,керемет сөйлеу браво еркін әлем сына ел кел иммигрант ресей қытай және б сияқты қолда ел емес еркін әлем бар де ойла мәжбүр ет,nazism

3,бұл адамдар африкадағы алғашқы дудула операциясын іске қосу арқылы біздің сүйікті елімізді жойды,бұл адам африка алғашқы дудул операция іс қосу арқылы біздің сүйікті ел жой,nazism

3,бізге батыл және трамп сияқты сөйлейтін президент керек теріс,бізге батыл және трам сияқты сөйле президент керек теріс,nazism

3,олар салық ұрлаумен де айналысатындықтан заңсыз иммигранттар біздің инфрақұрылымды бұзып жатқанына мән бермейді,олар салық ұрла де айналысатындық заңсыз иммигрант біздің инфрақұрылым бұз жат мән берме,nazism

3,таңылған ба жоқ па бұл біздің ел және біз ол үшін күресеміз қарапайым факт біз оңтүстік африкада кім екенін және қандай мәселе бойынша екенін білгіміз келеді құжаттары жоқ барлық иммигранттар өз елдеріне шекарасыз үйлеріне оралуы керек,таңыл ба жоқ па бұл біздің ел және біз ол үшін күрес қарапайым факт біз оңтүстік африка кім екен және қандай мәселе бойынша екен біл кел құжат жоқ барлық иммигрант өз ел шекара үй орал керек,nazism

3,біздің минералды ресурстарымызды тонау үшін осында келген ақ шетелдіктерді қосу керек,біздің минералды ресурс тонау үшін осында кел ақ шетелдік қосу керек,nazism

3,бұл ақымақтық біз өз елімізді қайтармайынша тынышталмаймыз мені ксенофоб деп атаңыз жалқау немесе белгілеңіз шетелдіктерде танзания немесе кез келген басқа африка мемлекеті сияқты құжаттар болуы керек,бұл ақымақтық біз өз ел қайтармайынш тынышталма мен ксенофоб де ата жалқау немесе белгіле шетелдік танзания немесе кез кел басқа африка мемлекет сияқты құжат бол керек,nazism

3,мұны маған біреу түсіндіре алады ма бұл біз үшін жеңіс пе әлде жеңіліс пе,мұны маған біреу түсіндір ал ма бұл біз үшін жеңіс пе әлде жеңіліс пе,nazism

3,егер полиция өз жұмысын дұрыс атқарса бізде бүгін дудула операциясы болмас еді және заңды өз қолына алатын адамдар болмас еді оңтүстік африкадағы нтате селе сіз үшін өте ұят,егер полиция өз жұмыс дұрыс атқар біз бүгін дудул операция болмас ед және заңды өз қол ал адам болмас ед оңтүстік африка нта сел сіз үшін өте ұят,nazism

3,флип флоптар әрқашан ақша іздейді олар қара шетелдіктер деп аталатындардан жалдау ақысын алады және оңтүстік африкалықтарды бірінші орынға қоямыз дейді қарғыс атқыр сатылымдар,фли флоп әрқашан ақша ізде олар қара шетелдік де атал жалда ақы ал және оңтүстік африкалық бірінші орын қоя де қарғыс атқы сатылым,nazism

3,кішкентай зимбабве деп аталатын жерде және тұрғындардың заңды rsa құжаттары жоқ ал кейбіреулері тіпті rdp үйлеріне иелік ететін бостандықта көмектесіңіз қылмыстық әрекеттер мен кісі өлтіру үнемі болып тұрады,кішкентай зимбабв де атал же және тұрғын заңды rsa құжат жоқ ал кейбіреу тіпті rdp үй иелік ет бостандық көмектес қылмыстық әрекет мен кісі өлтір үнемі бол тұр,nazism

3,сондықтан оларға қажет қысқа жолдар әрқашан ең жақсы емес бірақ әрқашан оңай жол және ақыр соңында онымен өмір сүру қиын олар ұнататын бұл шетелдіктер оларды бір бірлеп өлтіреді,сондықтан оларға қажет қыс жол әрқашан ең жақсы емес бірақ әрқашан оңай жол және ақы соң онымен өмір сүру қиын олар ұнат бұл шетелдік ол бір бірле өлтір,nazism

3,ммм бәріміз салықты есе көбейтуге жұмылдырайық сонда біздің үкімет шетелдіктерге қамқорлық жасау үшін қажеттінің бәрін жасай алады ққс пайыз болуы керек төлеңіз пайыз жанармай алымдары литріне,мм бәрі салық есе көбейт жұмылдыр сонда біздің үкімет шетелдік қамқорлық жасау үшін қажетті бәрін жаса ал ққс пайыз бол керек төле пайыз жанармай алым литр,nazism

3,әлемге ең жақсы адамдарды жібереді бірақ біз әлемнің түкпір түкпірінен ең нашар адамдарды аламыз,әлем ең жақсы адам жібер бірақ біз әлем түкпір түкпір ең нашар адам ал,nazism

3,зимбабве бұл өз елінен қашып кеткен ересектердің қорқақтары үшін ғана емес сәтсіз мемлекет,зимбабв бұл өз ел қаш кет ересек қорқақта үшін ғана емес сәт мемлекет,nazism

3,таңдамалы соқыр куәгер заңсыз иммигранттармен күресу немесе онлайн материалдар редакциялық шолуды алмауы мүмкін дудула операциясы,таңдамал соқыр куәгер заңсыз иммигрант күресу немесе онлайн материал редакциялық шолу алма мүмкін дудул операция,nazism

3,мен заңсыз иммигрант па жоқ па келіспеймін бірақ бұл қылмыстарды оңтүстік африка халқы көтермелейді біз адамдардың тонауды ақтап оны кедейлік деп айыптағанын бірнеше рет көрдік джаб джаб бүкіл эфир оны ақтады,мен заңсыз иммигрант па жоқ па келіспе бірақ бұл қылмыс оңтүстік африка халқ көтермеле біз адам тонау ақта оны кедейлік де айыпта бірнеше рет көр джаб джаб бүкіл эфир оны ақта,nazism

3,білесіз бе біз оңтүстік корея тұрғындары көңіліміз қалды өйткені есірткі соуэтода олар тәттілер ретінде сатылады шетелдіктер мен полиция қха біздің қоғамдастықты бұзады қоқыс полигондарын жабыңыз,біл бе біз оңтүстік корея тұрғын көңіл қал өйткені есірткі соуэто олар тәтті рет сат шетелдік мен полиция қх біздің қоғамдастық бұз қоқыс полигон жаб,nazism

3,сонымен бұл пимвилл тұрғынының өмірлік тәжірибесіне қарсы ол тұрғынның заңсыз шетелдіктердің кабельдерді ұрлап жатқанын дәлелдей алатынын сұрайды бұл қандай ақымақтық,сонымен бұл пимвилл тұрғын өмірлік тәжірибе қарсы ол тұрғын заңсыз шетелдік кабель ұрла жат дәлелде ал сұра бұл қандай ақымақтық,nazism

3,біз оларды қабылдаймыз деп күтілуде өйткені олар да африкалықтар неліктен біз бүкіл периметр бойынша қауіпсіздікті қамтамасыз етуіміз керек деп айтатындар,біз ол қабылда де күтіл өйткені олар да африкалық неліктен біз бүкіл периметр бойынша қауіпсіздік қамтамасыз ет керек де айт,nazism

3,дудула операциясының жетекшісі нхланхла люкс мохлаули ультиматум қойды беки челе жылдың мамыр айының жексенбісіне дейін советодағы жер басып алушылар лагерлерін алып тастау ол белгіленген мерзімге сәйкес келмейтінін айтты содан кейін жаппай жерлеу рәсімін дайындаңыз өйткені біз қолымызда қару жарақпен өнер көрсетеміз,дудул операция жетекші нхланхл люкс мохлаули ультимату қой беки чел жыл мамыр ай жексенбі дейін совето жер бас алушы лагер алып тастау ол белгілен мерзім сәйкес келме ай содан кейін жаппай жерлеу рәсім дайында өйткені біз қол қару жарақ өнер көрсе,nazism

3,соуэтодағы күзгі таң мен аймен жабылған мару кеді түсіргім келді мен сәтсіздікке ұшырады стрептококк,соуэто күзгі таң мен ай жабыл мар ке түсір кел мен сәтсіздік ұшыр стрептококк,nazism

3,мпумаланга мидделбург маңындағы мхлузи қаласында шетелдік азаматтардың кәсіпкерлік рухын атап өту көше фотографы шетелдіктер,мпумаланг мидделбург маң мхлузи қала шетелдік азамат кәсіпкерлік рух ата өту көше фотограф шетелдік,nazism

3,неліктен мен оны жаңа ғана көрдім алғаш рет біреу оңтүстік кореяда болып жатқан тонау үшін шетелдіктерді кінәлап отыр ма міне оңтүстік африкадағы наразылық білдірушілердің ең сүйікті ісіне шетелдіктер кінәл,неліктен мен оны жаңа ғана көр алғаш рет біреу оңтүстік корея бол жат тонау үшін шетелдік кінәла отыр ма міне оңтүстік африка наразылық білдіруші ең сүйікті іс шетелдік кінәл,nazism

3,олар неден болар еді сіздің ойыңызша қылмысты тек шетелдіктер жасайды деген нұсқа қалай алға жылжуда зорлау туралы тағы бір эпизод көрсетілгенше күтіңіз бұл нақты кабель жинақтары соуэтодан табылды сіз соуэтода шетелдіктер мұны істеп жатыр деп ойлайсыз ба,олар не бол ед сіздің ойыңызша қылмыс тек шетелдік жаса де нұсқа қалай алға жылж зорла туралы тағы бір эпизод көрсетілгенш күт бұл нақты кабель жинақ соуэто таб сіз соуэто шетелдік мұны істе жатыр де ойла ба,nazism

3,есіңізде болсын жылғы ксенофобиялық науқан кезінде шабуылдар олар олардың кейбірін шетелдіктер деп қателесіп өлтірді,ес бол жылғы ксенофобиялық науқан кез шабуыл олар олардың кейбір шетелдік де қателес өл,nazism

3,шындығында венда цонга және шангани халқы мұнда шетелдіктер болып саналады көптеген ата аналар балаларының қоғамға сәйкес келуі және кемсітушілікке ұшырамауы үшін тек зулуда сөйлеуін талап етеді,шындығ вен цонг және шангани халқ мұнда шетелдік бол санал көптеген ата ана бала қоғам сәйкес кел және кемсітушілік ұшырама үшін тек зул сөйле талап ет,nazism

3,қарулы күресте қаһармандық болған нәрсенің бұрмаланған нұсқасын саяси бұзақылар мен ксенофобиялық қырағылар өз әрекеттерін бұрын құрметтелген нәрсе ретінде көрсету үшін жиі пайдаланады,қарулы күрес қаһарман бол нәрсе бұрмалан нұсқа саяси бұзақы мен ксенофобиялық қырағы өз әрекет бұрын құрметтел нәрсе рет көрсету үшін жиі пайдалан,nazism

3,жеке куәліктерімізді саясаткерлерге қайтаруымыз керек пе сондықтан бізде жұмыс салықсыз бизнес денсаулық сақтаутегін электр қуаты тегін су ғимараттарды басып алу есірткі сату жезөкшелік кабель ұрлау заңсыз тау кен өндірісі атыс полиция болат теміржол тонау шеберханалар,жеке куәлік саясаткер қайтар керек пе сондықтан біз жұмыс салық бизнес денсаулық сақтаутег электр қуат тегін су ғимарат бас алу есірткі сату жезөкшелік кабель ұрлау заңсыз тау кен өндіріс атыс полиция болат теміржол тонау шеберхана,nazism

3,мен сондай ақ оның қандай заңдарды бұзғанын күтемін,мен сондай ақ оның қандай заң бұз күт,nazism

3,заңсыз иммигранттар мен дудула мен путсутафрика операциясына қарсы шыққандар мзансидегі жатақханалар бұл қозғалыстарды қолдаса кунганива екенін

түсінеді ме,заңсыз иммигрант мен дудул мен путсутафрик операция қарсы шық мзанси жатақхана бұл қозғалыс қолда кунганив екен түсін ме,nazism

3,саясаткерлер біздің есебінен бір бірінен асып түседі неліктен қолданыстағы заң актілері сақталмайды неліктен иммиграция туралы заң қолданылмайды қазір біз жаңа ережелерді қабылдап жатырмыз дудула операциясы саясаттану уақыты аяқталды,саясаткер біздің есеб бір бір ас түс неліктен қолданыстағы заң акті сақталма неліктен иммиграция туралы заң қолданылма қазір біз жаңа ереже қабылда жатыр дудул операция саясаттану уақыт аяқта,nazism

3,садк немесе континент елдерінің кез келгенінде заңсыз иммигранттар үшін квоталар бар ма егер жауап жоқ болса онда бізге бұл ақымақ заң қажет емес,садк немесе континент ел кез кел заңсыз иммигрант үшін квота бар ма егер жауап жоқ бол онда бізге бұл ақы заң қажет емес,nazism

3,осылайша шетелдіктер еңбекқор және заңға бағынады бізде тым мейірімді тым ұзақ заңсыз шетелдіктер,осылайша шетелдік еңбекқор және заң бағын біз тым мейірімді тым ұзақ заңсыз шетелдік,nazism

3,адамдар биліктің болмауына себеп болатын ұрылармен күресудің орнына шиналар мен жолдарды жауып тастаумен айналысады басқалары дудула операциясын айыптаумен айналысады бірақ дудула операциясы тіпті біздің полицейлер де көмектесе алмайтын үлкен жұмыс жасайды,адам билік болма себеп бол ұры күресу орн шина мен жол жау таста айналыс басқа дудул операция айыпта айналыс бірақ дудул операция тіпті біздің полицей де көмекте алма үлкен жұмыс жаса,nazism

3,олар бағаның өсуін біледі олар тіпті оңтүстік африка экономикасына үлес қоспайды нангафтың заңсыз тау кен өндірісі нангафтың әйел жезөкшелері өйткені лезинья оларға ақша береді,олар баға өс біл олар тіпті оңтүстік африка экономика үлес қоспа нангаф заңсыз тау кен өндіріс нангаф әйел жезөкше өйткені лезинья оларға ақша бер,nazism

3,жаңа жұмыс жақын арада аяқталады,жаңа жұмыс жақын ара аяқтал,nazism

3,шетелдіктерге деген жеккөрушілік деңгейі бұл жай ғана жағымды нәрсе,шетелдік де жеккөрушілік деңгей бұл жай ғана жағымды нәрсе,nazism

3,оңтүстік африкадағы алғашқы операция заңсыз иммигранттар біздің балаларымызды тағы да мектептерінен ұрлап кетеді,оңтүстік африка алғашқы операция заңсыз иммигрант біздің бала тағы да мектеп ұрла кет,nazism

3,өткен жылы заңсыз иммиграннтың балалары болды,өткен жыл заңсыз иммигрант бала бол,nazism

3,біз оңтүстік африкада қылмыстан басқа бизнесі жоқ басқа елдерден жұмыссыз қылмыскерлерді қабылдаймыз және біздің үкімет жергілікті тұрғындардың заңсыз иммигранттарды депортациялау туралы өтініштерін елемей табыт үнсіздігін сақтайды мен сіз президент мырза және сіздің жолдастарыңыз осы қылмыстардан пайда

көретінін түсіне бастадым,біз оңтүстік африка қылмыс басқа бизнес жоқ басқа ел жұмыссыз қылмыскер қабылда және біздің үкімет жергілікті тұрғын заңсыз иммигрант депортацияла туралы өтініш елеме табыт үнсіздіг сақта мен сіз президент мырза және сіздің жолдас осы қылмыс пайда көр түс баста,nazism

3,ол жерде заңсыз иммигранттар бар ма,ол же заңсыз иммигрант бар ма,nazism

3,біз әрқашан осындай еркектерді құрметтейміз шындығында шетелдіктерге араб елдері сияқты қарау керек,біз әрқашан осындай еркек құрметте шындығ шетелдік араб ел сияқты қарау керек,nazism

3,ол өз елін басқара алмайынша электр қуатын өшіруді қалай аяқтамақшы менің айтайын дегенім біздің елде құжатсыз шетелдіктердің пайызы ал йобургте пайызы заңсыз шетелдіктер қылмыс жасайды ұрлайды адамдармен сауда жасайды,ол өз ел басқар алмайынш электр қу өшіру қалай аяқтамақш менің айт де біздің ел құжат шетелдік пайыз ал йобург пайыз заңсыз шетелдік қылмыс жаса ұрла адам сауда жаса,nazism

3,зимбос біз ақ сатафрикандықтарды қуып жібермейтінімізді ескеріңіз біз сіз сияқты ақымақ емеспіз африкаға алдымен жібереміз,зимбос біз ақ сатафрикандық қу жіберме ескер біз сіз сияқты ақы емес африка алд жібер,nazism

3,дудула операциясының қылмыскерлері қха мен ақ нәсілшілдерден қорқады олар көшедегі қара адамдарға шабуыл жасайды іш киім,дудул операция қылмыскер қх мен ақ нәсілшіл қорқ олар көше қара адам шабуыл жаса іш киім,nazism

3,шетелдіктер біздің елден әлеммен кетуі керек біз бәрінен шаршадық,шетелдік біздің ел әлем кет керек біз бәрі шарша,nazism

3,оңтүстік африканың иммиграциялық заңнамасын сақтау үшін сізде жұмыс беруші ретінде екі негізгі міндет бар заңсыз шетелдіктерді жалдамау және шетелдікті қандай да бір лауазымға жалдамаңыз және шетелдікке оның визасында немесе рұқсатында көзделмеген еңбек функцияларын орындауға рұқсат бермеңіз,оңтүстік африка иммиграциялық заңнама сақтау үшін сіз жұмыс беруші рет екі негізгі міндет бар заңсыз шетелдік жалдама және шетелдік қандай да бір лауазым жалдама және шетелдік оның виза немесе рұқсат көзделме еңбек функция орында рұқсат берме,nazism

3,ол әрқашан біздің жүрегімізде болады күрес қазір күшейтілді,ол әрқашан біздің жүрег бол күрес қазір күшейт,nazism

3,оңтүстік африкалықтар жалқау әсіресе біз бірінші кезекте оңтүстік африкалықтарды жұмысқа орналастырудың көптеген себептері бар біз барлық заңсыз шетелдіктер өз елдеріне оралғанға дейін тоқтамаймыз дудула заңсыз иммигранттар,оңтүстік африкалық жалқау әсіресе біз бірінші кезек оңтүстік африкалық жұмыс орналастыр көптеген себеп бар біз барлық заңсыз шетелдік өз ел орал дейін тоқтама дудул заңсыз иммигрант,nazism

3,мзвахе мбули әлі де бостандық үшін күресуші егер қаласаңыз дудула операциясындағы қылмыскерлеріңізбен дауласуға болады,мзвах мбули әлі де бостандық үшін күресуш егер қала дудул операция қылмыскер даулас бол,nazism

3,менің ойымша біз орландо стадионына баруымыз керек бұл стадионды толтырған танзаниялықтар мен олардың барлығы заңды түрде теріс екеніне күмәнданамын,менің ойымша біз орландо стадион бар керек бұл стадион толтыр танзаниялық мен олардың барлығ заңды түр теріс екен күмәндан,nazism

3,құдай бізге рақым етсін оның жаны тыныштықта болсын өз еліне деген сүйіспеншіліктен қайтыс болған батыр сіздің өліміңіз бекер емес біз күресті жалғастырамыз біз мұны бір рет жасадық және оны қайтадан жасаймыз,құдай бізге рақым ет оның жан тыныштық бол өз ел де сүйіспеншілік қайтыс бол батыр сіздің өлім бек емес біз күре жалғастыр біз мұны бір рет жаса және оны қайта жаса,nazism

3,оңтүстік африкалықтар бұл шіркеуді жылдар бойы құрметтеді бұл мүмкін емес шекарадан өту кезінде жасалған қылмыстардың бірі дудула операциясы американдықтардың алдымен теріс нәтиже алуына әкелді,оңтүстік африкалық бұл шіркеу жыл бойы құрметте бұл мүмкін емес шекара өту кез жасал қылмыс бірі дудул операция американдық алд теріс нәтиже ал әке,nazism

3,басқарған үкіметтің сәтсіздіктерімен күресу үшін тағы бір дудула операциясы қалыптасуда,басқар үкімет сәтсіздік күресу үшін тағы бір дудул операция қалыптас,nazism

3,бұл елдегі барлық шетелдіктер еуропалықтардан африкалықтарға дейін үлкен проблема болып табылады,бұл ел барлық шетелдік еуропалық африкалық дейін үлкен проблема бол таб,nazism

3,шетелдіктер тамақ алып жатқанда сіздің бауырларыңыз қайда болды олар қайда сонымен индустрияға оралғанда сіз пайда болған бос орынды толтырасыз ба,шетелдік тамақ алып жатқанда сіздің бауыр қайда бол олар қайда сонымен индустрия орал сіз пайда бол бос ор толтыр ба,nazism

3,менің көз алдымда заңды шетелдіктер жоқ өйткені біз әлсізбіз бәрі бұзылды,менің көз алд заңды шетелдік жоқ өйткені біз әлсіз бәрі бұз,nazism

3,мен оңтүстік африкада құжаттарды үлкен сатып алу арқылы заңды түрде осында екенімді айтатын бірде бір шетелдікке сенбеймін,мен оңтүстік африка құжат үлкен сат алу арқылы заңды түр осында екен айт бірде бір шетелдік сенбе,nazism

3,небәрі қысқа жыл ішінде джулиус малема барлық ықтимал жаман саяси шешімдерді өте мұқият және әдістемелік түрде қабылдады бұл өте керемет дудула операциясы африкаға алдымен бейтарап аймаққа бағытталған,небәрі қыс жыл іш джулиус мале барлық ықтимал жаман саяси шешім өте мұқият және әдістемелік түр қабылда бұл өте керемет дудул операция африка алд бейтарап аймақ бағыттал,nazism

3,олар ақылға қонымды болғысы келеді және мейрамханаларда жұмыс істейтін шетелдіктерге ақша төлемейтіндерін айтады бірақ иелері қытайлар британдықтар американдықтар итальяндықтар немесе португалдықтар досым егер сіз шынымен оянсаңыз жай ғана квамай майға немесе жергілікті цисаньямаға барып нағыз жергілікті тұрғындарды қолдаңыз,олар ақыл қонымды бол кел және мейрамхана жұмыс істе шетелдік ақша төлеме айт бірақ ие қытай британдық американдық итальяндық немесе португалдық дос егер сіз шын оян жай ғана квама май немесе жергілікті цисаньяма бар нағыз жергілікті тұрғын қолда,nazism

3,қха үкіметі сыбайлас ішкі істер органдарының шенеуніктері арқылы оларды біздің елге кіргізді енді біз олармен бірге болдық әпкелеріміз оларға балалар мен азаматтық береді дудула операциясы американдықтарды бірінші теріс позицияға қояды,қх үкімет сыбайлас ішкі істер орган шенеунік арқылы ол біздің ел кір енді біз олармен бірге бол әпке оларға бала мен азаматтық бер дудул операция американдық бірінші теріс позиция қоя,nazism

3,сіз шетелдіктердің spaza shop раллиіне қатысуын қалайсыз делік,сіз шетелдік spaza shop ралли қатыс қала делік,nazism

3,ақыл есі дұрыс бірде бір адам таңертең оянып шешім қабылдамауы керек жек көру шетелдіктерге қатысты бірақ мәселе айтарлықтай бақылаудан шығып кетті өйткені мұнда заңды және өзін жайлы сезінетін кейбір шетелдіктер бұл туралы ештеңе айтпайды олар өз істерімен айналысады,ақыл ес дұрыс бірде бір адам таңертең оян шешім қабылдама керек жек көру шетелдік қатысты бірақ мәселе айтарлықтай бақылау шығ кет өйткені мұнда заңды және өз жайлы сезін кейбір шетелдік бұл туралы ештеңе айтпа олар өз іс айналыс,nazism

3,дудула операциясы ол қалай өтті сіз оны байқамадыңыз ба немесе бұл терінің түсіне байланысты ма,дудул операция ол қалай өт сіз оны байқама ба немесе бұл тер түс байланысты ма,nazism

3,немен сәйкес келеді есіңізде болсын менің халқым заңды және заңсыз иммигранттарды есептемегенде миллионнан аз сіздің халқыңыз миллионға жақындады егер көп болмаса сіздің миллионнан астамыңыз өз елінде тұрмайды сіз оны қалай атайсыз бүкіл әлемде нигериялықтарсыз ел жоқ,не сәйкес кел ес бол менің хал заңды және заңсыз иммигрант есептеме миллион аз сіздің хал миллион жақында егер көп болма сіздің миллион астам өз ел тұрма сіз оны қалай ата бүкіл әлем нигериялықт ел жоқ,nazism

3,адамдар мүлікті үйге жібереді вена банктер банктік шоттарды жабады тіпті өзін өзі жұмыспен қамтығандар да келесі жылы аман қалмайды көптеген жылжымайтын мүлік кешендер заңсыз иммигранттардың кіруіне жол бермейді,адам мүлік үй жібер вена банк банктік шот жаб тіпті өз өзі жұмыс қамты да келесі жыл аман қалма көптеген жылжыма мүлік кешен заңсыз иммигрант кір жол берме,nazism

3,eff тің сөйлеуімен немесе сөйлеуінсіз бұл иммигранттар келуді жалғастыруда олар eff тің сөйлеуінсіз келді және motsualedi pr трюктері олардың да келуіне кедергі болмайды мен сізден әртүрлі жерлерге баруыңызды және олардың осында және ешқайда кетпейтінін растауыңызды сұрадым,eff ті сөйле немесе сөйлеуін бұл иммигрант келу жалғастыр олар eff ті сөйлеуін кел және motsualedi pr трюк олардың да кел кедергі болма мен сізден әртүрлі жер бару және олардың осында және ешқай кетпе растау сұра,nazism

3,оңтүстік кореяда қылмыс жасаған барлық шетелдіктер үшін өлім жазасы осы банан республикасында болып жатқанның бәрін тоқтатады бірақ саясаткерлер қысқаша есептер алғандықтан біз қақпақпыз,оңтүстік корея қылмыс жаса барлық шетелдік үшін өлім жаза осы банан республика бол жат бәрін тоқтат бірақ саясаткер қысқаша есеп ал біз қақ,nazism

3,менің ойымша зимбабведен келген заңсыз иммигранттар отандағы жағдайдың нашарлауына ықпал етеді зимбабвеліктердің жылғы сайлаудан бастап зимбабведе дауыс берген жоқ елестетіп көріңізші миллион адам зимбабве сияқты кішкентай елге дауыс бермейді адамдарға тым ыңғайлы болды инсада,менің ойымша зимбабве кел заңсыз иммигрант ота жағдай нашарла ықпал ет зимбабвелік жылғы сайлау баста зимбабве дауыс бер жоқ елестет көріңізш миллион адам зимбабв сияқты кішкентай ел дауыс берме адам тым ыңғайлы бол инса,nazism

3,сізде құжаттары жоқ миллионға жуық заңсыз иммигранттар бар деп елестетіп көріңіз бұл нонсенс деген не,сіз құжат жоқ миллион жуық заңсыз иммигрант бар де елестет көр бұл нонсенс де не,nazism

3,зимбабве мен нигериядан келген заңсыз иммигранттар менің елімді құртып жатыр сіз қай жерде болсаңыз да үре аласыз бірақ шындықты айту керек өз елдерінен қашып кеткен заңсыз иммигранттар өйткені олар жан түршігерлік қылмыстар жасады оңтүстік африканы бөліп тастады және біз оны елемегенімізді қалайсыз ба,зимбабв мен нигерия кел заңсыз иммигрант менің ел құрт жатыр сіз қай же бол да үр ал бірақ шындық айту керек өз ел қаш кет заңсыз иммигрант өйткені олар жан түршігерлік қылмыс жас оңтүстік африка бөл таста және біз оны елеме қала ба,nazism

3,рас мен барлық заңсыз иммигранттар кетуі керек деп келісемін ешбір ел оның ішінде зимбабве де мұндай заңсыздыққа жол бермейді,рас мен барлық заңсыз иммигрант кет керек де келіс ешбір ел оның іш зимбабв де мұндай заңсыздық жол берме,nazism

3,оңтүстік африкадағы алғашқы операция бір күн бір күнге тең,оңтүстік африка алғашқы операция бір күн бір күн тең,nazism

3,тыңдау және не істеу керек неліктен сіз қха бұл әйелді тыңдауы керек деп айтпайсыз өйткені олар заңсыз иммигранттардың келуіне жол берді,тыңдау және не істеу керек неліктен сіз қх бұл әйел тыңда керек де айтпа өйткені олар заңсыз иммигрант кел жол бер,nazism

3,дипслут тұрғындары мен дудула және путсутафикансфирст операциясына қатысқан барлық қарақшылар қха ның сәтсіз үкіметіне ашуланып осы көшелерде шу шығаруды тоқтатуы керек мен олардың сәтсіз үкіметіне шабуыл жасамайтынын білемін өйткені олардың көпшілігі ancyl дің белсенді мүшелері,дипслут тұрғын мен дудул және путсутафикансфирст операция қатыс барлық қарақшы қх ны сәт үкімет ашулан осы көше шу шығару тоқтат керек мен олардың сәт үкімет шабуыл жасама біл өйткені олардың көпшіліг ancyl ді белсенді мүше,nazism

3,сіз оның ұлты туралы айтуды ұмытып кеттіңіз өйткені біздің елдегі қылмыстарды шетелдіктер жасайды сіз айтқандай,сіз оның ұл туралы айту ұмыт кет өйткені біздің ел қылмыс шетелдік жаса сіз айтқанда,nazism

3,сирилл барлық ірі оппозициялық партияларды басып алды және бұл миллиард реал ұзақ жолдан өтті оппозиция заңсыз иммигранттарға қатысты әрқашан қха мен келіседі олар бір тілде сөйлейді тіпті da,сирилл барлық ірі оппозициялық партия бас алды және бұл миллиард реал ұзақ жолдан өт оппозиция заңсыз иммигрант қатысты әрқашан қх мен келіс олар бір тіл сөйле тіпті da,nazism

3,міне біз тағы да келдік шетелдіктердің президенті оларға деген қамқорлығы мен сүйіспеншілігін көрсетеді,міне біз тағы да кел шетелдік президент оларға де қамқорлығ мен сүйіспеншіліг көрсет,nazism

3,біз қха үкіметіне шындықты айтқандықтан олар оны естуге дайын емес қха үкіметі заңсыз иммиграцияға байланысты елдегі тәртіпсіздікке жауап береді нәтижесінде американдықтар алдымен дудула операциясының атауын көрсетпеді,біз қх үкімет шындық айтқандық олар оны ест дайын емес қх үкімет заңсыз иммиграция байланысты ел тәртіпсіздік жауап бер нәтиже американдық алд дудул операция ата көрсетпе,nazism

3,эйш бұл шетелдіктер,эйш бұл шетелдік,nazism

3,бізде заңсыздық елі бар қазір оны шетелдіктер басқарады,біз заңсыз ел бар қазір оны шетелдік басқар,nazism

3,өтінемін өз уақытыңызды осы григамбасрамның есектерін жалайтын адамдарға жұмсамаңыз,өт өз уақыт осы григамбасра есек жала адам жұмсама,nazism

3,ұлыбританиядағы дудула операциясының керемет жұмысы шетелдіктер американдықтарды бірінші орынға қояды,ұлыбритания дудул операция керемет жұмыс шетелдік американдық бірінші орын қоя,nazism

3,сіз мені қаншалықты қарама қайшы деп санайтыныңызға мән бермеймін бірақ менің ойымша бұл елді ақ адамдар басқаруы керек қара адамдар бәрін біржола бүлдірді және бұл әдемі ел қазір қалай көрінетіні соншалықты қайғылы шетелдіктер өз қалауынша жасайды барлық жерде шұңқырлар тонау тоқтамайды және б,сіз мен қаншалықты қарама қайшы де сана мән берме бірақ менің ойымша бұл ел ақ адам басқар керек қара адам бәрін біржол бүлдір және бұл әдемі ел қазір қалай көрін

соншалықты қайғылы шетелдік өз қала жаса барлық же шұңқыр тонау тоқтама және б,nazism

3,йоханнесбургтің ішкі қаласы және оған жақын аудандар қазіргі уақытта қауіпті және қылмыс деңгейі жоғары және есірткі сатушылардың үйінділері болып табылады ішкі қала және оған жақын аудандар қазіргі уақытта негізінен заңсыз иммигранттар басқаратын қылмыстық әрекеттің орталығына айналды,йоханнесбург ішкі қала және оған жақын аудан қазіргі уақыт қауіпті және қылмыс деңгей жоғары және есірткі сатушы үйінді бол таб ішкі қала және оған жақын аудан қазіргі уақыт негіз заңсыз иммигрант басқар қылмыстық әрекет орталығ айна,nazism

3,иә мен бәрі бірдей емес екеніне келісемін бірақ көпшілігі қылмыскерлер елге заңсыз кіру қылмыс болып табылады,иә мен бәрі бірде емес екен келіс бірақ көпшіліг қылмыскер ел заңсыз кіру қылмыс бол таб,nazism

3,бұл клоундар өздерінің культ басшыларынан естігенінің бәріне сенеді,бұл клоун өз культ басшы есті бәрін сен,nazism

3,қазір азаматтар шаршады және олар құжатсыз қарулы заңсыз иммигранттарға қарсы қару алып жатыр енді ғана сіз күш қолдану туралы сөйлескіңіз келе ме эль дорадо саябағында не болып жатқаны бұл тек бастамасы ол желдің әсерінен болатын дала өрті сияқты таралады,қазір азамат шарша және олар құжат қарулы заңсыз иммигрант қарсы қару алып жатыр енді ғана сіз күш қолдану туралы сөйлес келе ме эль дорадо саябағ не бол жат бұл тек баста ол же әсер бол дала өр сияқты тарал,nazism

3,мен қоғамдастықтарды тыңдау арқылы өмір сүремін сегопи көңілсіздік әрине нақты бірақ шетелдіктерге қылмыстың кедейліктің және жұмыссыздықтың негізгі себептері ретінде сілтеме жасау мүлдем шынайы емес мұнда тар жеке мүдделер пайда болады,мен қоғамдастық тыңдау арқылы өмір сүр сегопи көңілсіз әрине нақты бірақ шетелдік қылмыс кедейлік және жұмыссыздық негізгі себеп рет сілтеме жасау мүлдем шынайы емес мұнда тар жеке мүдде пайда бол,nazism

3,біз африка континентімізді қорғаймыз африкалық бауырларымызды көшедегі қара адамдарға шабуыл жасау үшін демеушілік жасайтын дудула операциясының қылмыскерлерінен қорғаймыз қара адамдарымызды кең ауқымды қанаудан қорғауды жалғастырамыз,біз африка континент қор африкалық бауыр көше қара адам шабуыл жасау үшін демеушілік жаса дудул операция қылмыскер қор қара адам кең ауқымды қанау қорғау жалғастыр,nazism

3,заңсыз иммигранттарды құтқарушы төңкеруші,заңсыз иммигрант құтқарушы төңкеруш,nazism

3,бұл бізді африкалық бауырластарға мейірімді және сүйіспеншілікпен қарау үшін күтеді олардың басқа идеялары бар оларды жер аудару керек жеткілікті бұл дудула операциясын бастауға жеткілікті себеп,бұл біз африкалық бауырлас мейірімді және

сүйіспеншілік қарау үшін күт олардың басқа идея бар ол жер аудару керек жеткілікті бұл дудул операция баста жеткілікті себеп,nazism

3,бос сөз айтпа вена егер сіз арзан шетелдіктердің жұмыс істеуге келгенін қаласаңыз олардың артықшылықтарын пайдалана аласыз өйткені олардың кәсіподақтары жоқ бұл туралы айтыңыз және сіз қолдайтын барлық ақымақтықпен бас бармағыңызбен жабуды тоқтатыңыз маған қамқорлық жасамаңыз бұл жүктемені бөлу кезінде ескеріледі,бос сөз айтпа вена егер сіз арзан шетелдік жұмыс істе кел қала олардың артықшылық пайдалан ал өйткені олардың кәсіподақ жоқ бұл туралы ай және сіз қолда барлық ақымақтық бас бармағ жабу тоқта маған қамқорлық жасама бұл жүктеме бөлу кез ескер,nazism

3,сіз жындысыз сіз білмейтін нәрселер туралы жазасыз барлығыңыз оңтүстік африкалықтардың бір бірінің үстінде милиция жасағанын қалайсыз қара ақ үндістер және түрлі түсті оңтүстік африкалықтар және бұл ел біз үшін шешім қабылдауға тырысатын шетелдіктерге емес бәрімізге тиесілі,сіз жынд сіз білме нәрсе туралы жаз барлығ оңтүстік африкалық бір бір үст милиция жаса қала қара ақ үндіс және түрлі түсті оңтүстік африкалық және бұл ел біз үшін шешім қабылда тырыс шетелдік емес бәрі тиесілі,nazism

3,ол бұл қылмыстарды оңтүстік африкада оңтүстік африка ретінде жасады неге біз зимбабве қылмыскерлерін әкелуіміз керек олар рамафоса үшін бас ауруы болмауы керек,ол бұл қылмыс оңтүстік африка оңтүстік африка рет жас неге біз зимбабв қылмыскер әкел керек олар рамафо үшін бас ауру болма керек,nazism

3,иә шетелдіктер біздің елде бәсекелеседі сондықтан сіз өз елдеріңізге қайтыңыздар,иә шетелдік біздің ел бәсекелес сондықтан сіз өз ел қай,nazism

3,дудула операциясы ақымақ талдаушылар болуы керек воэтсек,дудул операция ақы талдаушы бол керек воэт,nazism

3,егер мен сені ренжітсем кешірім сұраймын ағам мен бұл сұрақты сіздің көзіңізбен қарадым бірақ мен оны әлі де таңдадым қанның қанын осылай өңдеуге жол бермеңіз мәселенің негізгі себебіне шабуыл жасаңыз симптомдар емес,егер мен сен ренжіт кешір сұра аға мен бұл сұрақ сіздің көз қара бірақ мен оны әлі де таңда қан қан осылай өңде жол берме мәселе негізгі себеб шабуыл жаса симптом емес,nazism

3,бұл жалған екілік қайдан келеді біз оңтүстік африкалықтардың жақтаушысы болу үшін қарсы шетелдіктер болуымыз керек керісінше жемқор саясаткерлерді депортациялауды талап етіңіз олар оңтүстік африкаға қарсы тұрады бұл кедейліктің үш онжылдықта әлсіреуіне жол бермейді,бұл жалған екілік қайдан кел біз оңтүстік африкалық жақтаушы болу үшін қарсы шетелдік бол керек керісінше жемқор саясаткер депортациялау талап ет олар оңтүстік африка қарсы тұр бұл кедейлік үш онжылдық әлсіре жол берме,nazism

3,олардың мінез құлқына байланысты мартин біз оңтүстік кореяға заңды түрде келген зимбабвеліктермен қатар жылдар бойы өмір сүрдік бірақ билікті басып алғаннан бері

заңсыз шетелдіктердің көпшілігі ауыр қылмыстар жасады,олардың мінез құлқ байланысты мартин біз оңтүстік корея заңды түр кел зимбабвелік қатар жыл бойы өмір сүр бірақ билік бас ал бері заңсыз шетелдік көпшіліг ауыр қылмыс жас,nazism

3,оңтүстік каролинада адамдарды өлтіретін барлық заңсыз шетелдіктерге байланысты,оңтүстік каролина адам өлтір барлық заңсыз шетелдік байланысты,nazism

3,ешқашан зимбабвеліктер оңтүстік кореяда басқа шетелдіктерге қарағанда көп адамды өлтірмеген сондықтан олардың көпшілігі оңтүстік кореяда түрмеде отыр олар оңтүстік африкалықтарды жек көреді және сіз мұны білесіз,ешқашан зимбабвелік оңтүстік корея басқа шетелдік қара көп адам өлтірме сондықтан олардың көпшіліг оңтүстік корея түрме отыр олар оңтүстік африкалық жек көр және сіз мұны біл,nazism

3,иә дудула операциясы дәл осылай дейді олар зимбабвеге оралуы керек бірақ олар зимбабвені қатты жек көреді,иә дудул операция дәл осылай де олар зимбабве орал керек бірақ олар зимбабве қатты жек көр,nazism

3,олар үшін ксенофобияны тарататтын шетелдіктердің өлімі туралы айту қалыпты жағдай ма,олар үшін ксенофобия тарататтын шетелдік өлім туралы айту қалыпты жағдай ма,nazism

3,судья ксенофоб болуы мүмкін шетелдіктерге деген сүйіспеншілігі жоқ,судья ксенофоб бол мүмкін шетелдік де сүйіспеншіліг жоқ,nazism

3,сіз өзіңізді шетелдіктер деп атауды тоқтатуыңыз керек және оған сенуіңіз керек,сіз өз шетелдік де атау тоқтат керек және оған сен керек,nazism

3,біз африка үшін көбірек дұға етуіміз керек бауырым олар мұны бізден алып қана қойған жоқ енді олар мұны біздің болашақ ұрпақтарымыздан да алып тастағысы келеді,біз африка үшін көбірек дұ ет керек бауыр олар мұны бізден алып қана қой жоқ енді олар мұны біздің болашақ ұрпақ да алып таста кел,nazism

3,дауыстық жазба бар онда бір жігіт әйелімен ұйықтап жатқан пимвиллдегі табоны шақырады және ол келіп оны ұрып соғамын деп қорқытады ал табо оған қоңырау шалушы ақымақ болмайынша оны ұрып соғуды жалғастыратынын айтады және ол бұл туралы ештеңе істей алмайды шетелдіктер оңтүстік африкаға қарсы,дау жазба бар онда бір жігіт әйел ұйықта жат пимвилл табо шақыр және ол кел оны ұр соғ де қорқыт ал табо оған қоңырау шалуш ақы болмайынша оны ұр соғу жалғастыр айт және ол бұл туралы ештеңе істе алма шетелдік оңтүстік африка қарсы,nazism

3,бір күні сіз оңтүстік африкалықтарға осы ұйымдастырылған хаос үшін қанша төлейтіндігіңізді айтасыз біздің кейбіреулеріміз сіздің айтқаныңыздан әрі қарай көреміз және құдай сіздің артыңыздан келеді mfanam,бір күн сіз оңтүстік африкалық осы ұйымдастырыл хаос үшін қанша төлейтіндіг ай біздің кейбіреу сіздің айт әрі қарай көр және құдай сіздің арт кел mfanam,nazism

3,пайыз йоханнесбургтің орталығында тұратын адамдар шетелдіктер зимбабвеліктер нигериялықтар және б ал оңтүстік африкалықтар және пайыздан пайыз студенттер,пайыз йоханнесбург орталығ тұр адам шетелдік зимбабвелік нигериялық және б ал оңтүстік африкалық және пайыз пайыз студент,nazism

3,бұл ші элемент мен үшін мәмілеге кедергі болып табылады өйткені басқалары eff ке риза ал мен екеуіне де құжатсыз шетелдіктерге де риза емеспін,бұл ші элемент мен үшін мәміле кедергі бол таб өйткені басқа eff ке риза ал мен екеу де құжат шетелдік де риза емес,nazism

3,егер біз олармен соғысуымыз керек болса біз оларды жоямыз сізде менің еркек күшім бар оңтүстік африканы кері қайтаратын уақыт келді,егер біз олармен соғыс керек бол біз ол жоя сіз менің еркек күшім бар оңтүстік африка кері қайтар уақыт кел,nazism

3,енді тақырыпқа оралайық егер сіз оңтүстік африкадан ақ адамдарды шығару үшін үгіт жүргізетін қозғалыс немесе саяси партия құрғыңыз келсе дәл қазір әрекет етіңіз бұл заңсыз иммигранттармен күресу керек деген идеологияға қайшы келмейді сондықтан оны пайдаланудың қажеті жоқ,енді тақырып орал егер сіз оңтүстік африка ақ адам шығару үшін үгіт жүргіз қозғалыс немесе саяси партия құр кел дәл қазір әрекет ет бұл заңсыз иммигрант күресу керек де идеология қайшы келме сондықтан оны пайдалан қажет жоқ,nazism

3,баба мен саған айттым егер сіз біз өмір сүріп жатқан осы жүйеден бас тартсаңыз жеке куәлігіңіз бен төлқұжатыңызды өртеп қалағаныңызды жасаңыз басқа африкалықтар сізді өз елдерінде заңсыз иммигрант деп атайтынын қараңыз сондай ақ сіз ақ оңтүстік африкалықтарды заңсыз деп атайтындықтан сіз бізді қуып жіберуді ұсынасыз,баба мен саған ай егер сіз біз өмір сүр жат осы жүйе бас тарт жеке куәлік бен төлқұжат өрте қала жаса басқа африкалық сіз өз ел заңсыз иммигрант де ата қара сондай ақ сіз ақ оңтүстік африкалық заңсыз де атайтындық сіз біз қу жіберу ұсын,nazism

3,жарайды егер сіз назар аудармайтын барлық басқа заңсыз иммигранттарды анықтай алсаңыз оларды әшкерелеу арқылы көмектесіңізші,жарайды егер сіз назар аударма барлық басқа заңсыз иммигрант анықта ал ол әшкереле арқылы көмектесіңізш,nazism

3,біз заңсыз иммигранттармен жұмыс істеп жатырмыз неге бәрінен де ақылды болып көрінуге тырысудың орнына сізге көмектеспеске eff патриоттық адамдар үшін пандемияға айналуда өз жағыңызды ақылмен таңдаңыз,біз заңсыз иммигрант жұмыс істе жатыр неге бәрі де ақылды бол көрін тырыс орн сізге көмектеспес eff патриоттық адам үшін пандемия айнал өз жағ ақыл таңда,nazism

3,оңтүстік африкада небәрі нигериялық болғанына сенесіз бе сондай ақ бізге толығырақ айтып беріңіз бұл заңсыз нигериялықтар ма әлде кездейсоқ нигериялықтар ма мәселе иммигранттарда емес заңсыз иммигранттарда елдегі

барлық адамдар құжатталғаннан кейін біз бұл ақымақ дауды шеше аламыз,оңтүстік африка небәрі нигериялық бол сен бе сондай ақ бізге толығырақ айт бер бұл заңсыз нигериялық ма әлде кездейсоқ нигериялық ма мәселе иммигрант емес заңсыз иммигрант ел барлық адам құжаттал кейін біз бұл ақы дау шеш ал,nazism

3,ол үлгі көрсетіп бізді аяғымызға тұруға шақырады біз оны шашыратпай керісінше қалпына келтіре аламыз ба бізді кері қайтармайтын тиімді стратегия аясында елді қалпына келтіруге параллель тәсіл қолдана аламыз ба қайырлы таң,ол үлгі көрсет біз аяғ тұр шақыр біз оны шашыратпа керісінше қалп келтір ал ба біз кері қайтарма тиімді стратегия ая ел қалп келтір параллель тәсіл қолдан ал ба қайырл таң,nazism

3,сызбаны пайдалану үшін қол жетімді өз елімізді түзетуге тәуелсіз тәуелді емес тәсілді қолданайық,сызба пайдалану үшін қол жет өз ел түзет тәуелсіз тәуелді емес тәсілді қолдан,nazism

3,менің білуімше operationdudula сізге ешқашан қажет емес еді,менің білуімше operationdudula сізге ешқашан қажет емес ед,nazism

3,сіз эсоветоға қоңырау шала аласыз жеке өзім элдос пимвилл және басқа да жақын аудандардың тұрғындарын қолдағым келеді бұл құс фермалары және жойылмайтын нәрсе олар бәрімізді өлтірмейді мен бүлік шығаруға дайынмын дудула операциясы оңтүстік африканы бірінші орынға қояды,сіз эсовето қоңырау шал ал жеке өзім элдос пимвилл және басқа да жақын аудан тұрғын қолда кел бұл құс ферма және жойылма нәрсе олар бәрі өлтірме мен бүлік шығар дайын дудул операция оңтүстік африка бірінші орын қоя,nazism

3,тонау кезінде өз қақпаларын бірінші болып құлыптаған адамдар болды және олар заңсыз иммигранттар тұрғындар орналасқан қылмыс орнына аяқ басуға батылы бармайды олар әрқашан жазықсыз бейбіт тұрғындарды аулаған кезде жақсы жұмыс істейді ripkgotsodiale африкадан хабарламаны бірінші рет жібереді,тонау кез өз қақпа бірінші бол құлыпта адам бол және олар заңсыз иммигрант тұрғын орналас қылмыс орн аяқ бас батыл барма олар әрқашан жазық бейбіт тұрғын аула кезде жақсы жұмыс істе ripkgotsodiale африка хабарлама бірінші рет жібер,nazism

3,мұндай бұрмаланған ойлау тәсілі zsp рұқсаттарының барлық иелері оңтүстік кореяда заңсыз иммигранттар болды олардың zim ден қашуының себебі zanu pf тарапынан нашар басқару болды zanu pf экономикадағы сыбайлас жемқорлық пен дұрыс басқаруды жалғастыруда сіз жауап алған кезде оларға дауыс беруді ойлайсыз,мұндай бұрмалан ойлау тәсіл zsp рұқсат барлық ие оңтүстік корея заңсыз иммигрант бол олардың zim ден қашу себебі zanu pf тарап нашар басқару бол zanu pf экономика сыбайлас жемқорлық пен дұрыс басқару жалғастыр сіз жауап ал кезде оларға дауыс беру ойла,nazism

3,лакс ол мектепке барды ол аборт мектебінен аулақ болды өйткені оны бітіргендер ұрылар,лакс ол мектеп бар ол аборт мектеб аулақ бол өйткені оны бітір ұры,nazism

3,кабельді ұрлау және заңсыз иммигранттар ол кадрлармен тойлаған кезде проблема болған жоқ,кабель ұрлау және заңсыз иммигрант ол кадр тойла кезде проблема бол жоқ,nazism

3,мәселе мынада біз оларды ұстап аламыз ал полиция біздің қоғамдастыққа жібереді сондықтан олар самуд қайтып оралғаннан бері ұрып соғып жатыр олар ұрламайды мен байқаймын менің кейси қылмыскерлердің көпшілігі бөлмелерді жалға алатын шетелдіктер және nyaope жігіттерін кінәлаңыз бізді адастыратын,мәселе мына біз ол ұста ал ал полиция біздің қоғамдастық жібер сондықтан олар самуд қайт орал бері ұр соғ жатыр олар ұрлама мен бай менің кейси қылмыскер көпшілiг бөлме жалға ал шетелдік және nyaope жігіт кінәла біз адастыр,nazism

3,министр тоқтатқан пасха демалысынан кейін қайтып келе жатқан заңсыз иммигранттарды қараңыз егер ол жоқ болса олар оған қалай кірді неліктен олар қайтып оралуға сенімді болды көшеде заңсыз иммигранттарға тап болған кезде полиция пара алады мұны күнделікті қараңыз,министр тоқтат пасх демалыс кейін қайт келе жат заңсыз иммигрант қара егер ол жоқ бол олар оған қалай кір неліктен олар қайт орал сенімді бол көше заңсыз иммигрант тап бол кезде полиция пара ал мұны күнделікті қара,nazism

3,ол әлі де өзін аудандық прокурордың мүшесі деп санайды бізде заңсыз иммигранттар мен заңсыздық мәселесі бар ол тек ксенофобия деп айта алады біз үкіметтен дудула операциясы туралы құжаттары бар шетелдіктер мен азаматтарды қорғауды сұраймыз,ол әлі де өз аудандық прокурор мүше де сана біз заңсыз иммигрант мен заңсыз мәселе бар ол тек ксенофобия де ай ал біз үкімет дудул операция туралы құжат бар шетелдік мен азамат қорғау сұра,nazism

3,sa media қайда аудандық прокурор қайда eff қайда қха қайда олардың оңтүстік африкадағы отандасының пайдасына баспасөзге немесе бұқаралық ақпарат құралдарына мәлімдеме жасауға асықпайтыны таңқаларлық,sa media қайда аудандық прокурор қайда eff қайда қх қайда олардың оңтүстік африка отандас пайда баспасөз немесе бұқаралық ақпарат құрал мәлімдеме жаса асықпа таңқаларлық,nazism

3,біз күтеміз ағылшын тілінде сөйлеңіз,біз күт ағылшын тіл сөйле,nazism

3,эль дорадо саябағының тұрғындарымен бетпе бет келген eff мүшесі жекпе жекте жеңіліп қалды,эль дорадо саябағ тұрғын бетп бет кел eff мүше жекпе жек жеңіл қал,nazism

3,мара нхланхла мен мұны бірнеше рет айттым бұл қозғалыс gopro ға инвестиция салуы керек азаматтар бұқаралық ақпарат құралдарында болған оқиғалар туралы тек құқық қорғау органдарының немесе заңсыз иммигранттардың тонау тобының агрессивті мінез құлқы туралы ақпарат ала алмайды фильмдегі кез келген нәрсе көмектеседі өтінемін балалар,мар нхланхл мен мұны бірнеше рет ай бұл қозғалыс gopro ға инвестиция сал керек азамат бұқаралық ақпарат құрал бол оқиға туралы тек

құқық қорғау орган немесе заңсыз иммигрант тонау тоб агрессивті мінез құлқы туралы ақпарат ал алма фильм кез кел нәрсе көмектес өт бала,nazism

3,өзекті болуға тырысатын тағы бір саяси жеңіліс дудула операциясы американдықтардың алдымен малеманы су басуына әкелді,өзекті бол тырыс тағы бір саяси жеңіліс дудул операция американдық алд малема су бас әке,nazism

3,оңтүстік африкаға заңсыз иммигранттар қатты шабуыл жасайды,оңтүстік африка заңсыз иммигрант қатты шабуыл жаса,nazism

3,егер біз бәріміз оңтүстік африкалықтар бір күні тұрсақ бұл заңсыз иммигранттардың барлығы бір сағат ішінде кете алады,егер біз бәрі оңтүстік африкалық бір күн тұр бұл заңсыз иммигрант барлығ бір сағат іш кет ал,nazism

3,міне роб херс пен руперт ханыммен бірге нхланхла люкс ол өзін сол адамдардың бірі деп санайды қазіргі уақытта ол жұмыссыз болуы мүмкін бірақ келесі жалпы сайлаудан кейін ол мұндай өмір салтын көтере алады,міне роб херс пен руперт хан бірге нхланхл люкс ол өз сол адам бірі де сана қазіргі уақыт ол жұмыссыз бол мүмкін бірақ келесі жалпы сайлау кейін ол мұндай өмір салт көтер ал,nazism

3,тоқу қара қауымдастықтағы мәртебенің белгісі бұл дудула операциясының жетекшілерінің бірі ол мәртебесін білдіру үшін шашын пайдаланады адамдардың оған қалай қарайтынына оны қалай қабылдайтыны әсер етеді және оған оның сыртқы келбеті әсер етеді,тоқ қара қауымдастық мәртебе бел бұл дудул операция жетекші бірі ол мәртебе білдіру үшін шашын пайдалан адам оған қалай қара оны қалай қабылда әсер ет және оған оның сыртқы келбет әсер ет,nazism

3,оңтүстік африкалықтар оны жылы жазалайды шетелдіктер дауыс бермейтінін есте сақтаңыз,оңтүстік африкалық оны жыл жазала шетелдік дауыс берме ес сақта,nazism

3,мен шетелдіктердің элдостағы проблема екеніне таң қалдым,мен шетелдік элдос проблема екен таң қал,nazism

3,олар африка бірігуі керек деп айтқан кезде олар оңтүстік африкада қалағанын жасай алатын шетелдіктерге сілтеме жасады,олар африка біріг керек де айт кезде олар оңтүстік африка қала жаса ал шетелдік сілтеме жас,nazism

3,оңтүстік африканың барлық жек көрушілері бұл бөлікті естіді ме бұл operationdudula емес қоғамдастықтың қамқорлығы болды бірақ ол өз жағын таңдады оңтүстік африка емес оңтүстік африка емес оңтүстік корея қауымдастығы олар бір дауысының жоғалуын тойлайды сондықтан шетелдік қолдап дауыс беруі керек,оңтүстік африка барлық жек көруші бұл бөлік есті ме бұл operationdudula емес қоғамдастық қамқорлығ бол бірақ ол өз жағ таңда оңтүстік африка емес оңтүстік африка емес оңтүстік корея қауымдастығ олар бір дауыс жоғал тойла сондықтан шетелдік қолда дауыс беруі керек,nazism

3,жоқ заңсыз шетелдіктер оңтүстік африкалықтарды қха мүшелері мен оларды қорғайтын эффтың көмегімен өлтіру үшін осында мені орталық бизнес ауданында

зимбабвеліктер тонап ұрып тастады,жоқ заңсыз шетелдік оңтүстік африкалық қх мүше мен ол қорға эфф көмег өлтір үшін осында мен орталық бизнес аудан зимбабвелік тона ұр таста,nazism

3,кейптаундағы нгенафи мина мен советода қашан боламын заңсыз шетелдіктер түзу адамдарды шынымен шаршатады олар әрдайым ақымақтықты қорғайды олар оңтүстік африкалықтарды өлтіруге және ұрлауға келді енді біз mxm қолдарын бүктеуіміз керек,кейптаун нгенафи мина мен совето қашан бол заңсыз шетелдік түз адам шын шаршат олар әрдайым ақымақтық қорға олар оңтүстік африкалық өлтір және ұрла кел енді біз mxm қол бүкте керек,nazism

3,министр оңтүстік африкадағы алғашқы операцияны тоқтатыңыз дудула рипкгомоцодиале заңсыз,министр оңтүстік африка алғашқы операция тоқта дудул рипкгомоцодиал заңсыз,nazism

3,мен айдың соңында атыс алаңына барамын бұл менің пышағым немесе мылтығым болсын қару ұстамау туралы бұрыннан келе жатқан сеніміме қайшы келеді бірақ өлім есігімде мен пимвиллден рипкгомотсодан дудула операциясы американдықтарды бірінші орынға қояды,мен ай соң атыс алаң бар бұл менің пыша немесе мылтығ бол қару ұстама туралы бұрын келе жат сенім қайшы кел бірақ өлім есіг мен пимвилл рипкгомотсо дудул операция американдық бірінші орын қоя,nazism

3,сіз біздің конституциямызда дұрыс айтасыз мәселе сіздер оны өздеріңіз оқып түсінесіздер онда оңтүстік африкалықтар шетелдіктер емес сондықтан оңтүстік африка конституциясын қолдануға ешкімнің құқығы жоқ,сіз біздің конституция дұрыс ай мәселе сіздер оны өз оқ түсін онда оңтүстік африкалық шетелдік емес сондықтан оңтүстік африка конституция қолдан ешкі құқығ жоқ,nazism

3,құжаттары жоқ шетелдіктерге рақымшылық жасау керек пе,құжат жоқ шетелдік рақымшылық жасау керек пе,nazism

3,совето парламентінің ойыншық сарбазы сияқты адамдар қызыл береттерді тұрақсыздандыру үшін дұрыс емес ақша жұмсады олардың жоспары енді жұмыс істемейді және олар дудула операциясы үшін жазықсыз адамдардың өміріне қауіп төндіреді ба нтлу ябо кгомотсо хаба ойыншық сарбазды айыптайды,совето парламент ойыншық сарбаз сияқты адам қызыл берет тұрақсыздандыр үшін дұрыс емес ақша жұмса олардың жоспар енді жұмыс істеме және олар дудул операция үшін жазық адам өмір қауіп төндір ба нтл ябо кгомотсо хаб ойыншық сарбаз айыпта,nazism

3,ак киетін және біздің инфрақұрылымды бұзатын кедей қара шетелдіктер унгазобеда вена ағайынды джулиус малема атқан рипа кгомотсоны әлі де жоқтайды бұл африка орындалмайтын арман жылы біз сізге жалынды көрсетеміз,ак ки және біздің инфрақұрылым бұз кедей қара шетелдік унгазобе вена ағайынды джулиус мале ат рип кгомотсо әлі де жоқта бұл африка орындалма арман жыл біз сізге жалын көрсе,nazism

3,мен оларға қарсы тұрамын ба жоқ па олар мені өлтіреді олар инфрақұрылымды ұрлап бұзып болғаннан кейін не болады олар біздің үйлерге келеді мен

бейтараптықты сақтауға тырысып өлгенімді біліп бақытты өлемін,мен оларға қарсы тұр ба жоқ па олар мен өлтір олар инфрақұрылым ұрла бұз бол кейін не бол олар біздің үй кел мен бейтараптық сақта тырыс өл біл бақытты өл,nazism

3,жалған полиция,жалған полиция,nazism

3,олардың қайсысы көбірек төлейді,олардың қайсысы көбірек төле,nazism

3,журналистер қазірдің өзінде жасайды мен шетелдіктер емес оңтүстік африкалықтар туралы жариялаймын,журналист қаз өз жаса мен шетелдік емес оңтүстік африкалық туралы жарияла,nazism

3,оларды қалдырыңыз,ол қалдыр,nazism

3,дудула операциясындағы нхланхла лакс пен оның қарақшылары бұл қалашықтардағы жас қара балаларға есірткі сататындар содан кейін келіп кінәні шетелдіктерге жүктейтіндер бұл олардың және олардың туыстарының екенін бәріміз білеміз ол тіпті де мазаламайды заңсыз шетелдіктің өліміне байланысты күдіктілердің қамауға алынғаны туралы хабарлайды содан кейін сіз шахан мен салли бердетттің шетелдіктерге қарсы көңіл күйді қалай қоздыратыны туралы айтқанын естисіз,дудул операция нхланхл лакс пен оның қарақшы бұл қалашық жас қара бала есірткі сат содан кейін кел кінә шетелдік жүкте бұл олардың және олардың туыс екен бәрі біл ол тіпті де мазалама заңсыз шетелдік өлім байланысты күдікті қама алын туралы хабарла содан кейін сіз шахан мен салли бердет шетелдік қарсы көңіл күй қалай қоздыр туралы айт есті,nazism

3,жеке басын куәландыратын құжаттарды шетелдіктерге сататын ішкі істер қызметкерлері және қалыңдықтарды шекара қоршауынан алып жатқан сарбаздар біздің сүйікті елімізді баяу сатады,жеке басын куәландыр құжат шетелдік сат ішкі істер қызметкер және қалыңдық шекара қоршау алып жат сарбаз біздің сүйікті ел бая сат,nazism

3,пайдасыз шетелдіктер тіпті дәл атуға болмайды,пай шетелдік тіпті дәл ат болма,nazism

3,саясаткерлер оңтүстік африкалықтарды бөлді жігіт нақты мәселелер үшін күреседі бірақ олар оған шабуыл жасайды біздің пойыздардың платформалары немесе станциялары бұзылды кабельді ұрлау ғимараттарды басып алу қылмыс біз бұл елді сүймейміз,саясаткер оңтүстік африкалық бөл жігіт нақты мәселе үшін күрес бірақ олар оған шабуыл жаса біздің пойыз платформа немесе станция бұз кабель ұрлау ғимарат бас алу қылмыс біз бұл ел сүйме,nazism

3,бұл қайтып оралушылар және заңсыз иммигранттар бұл дегеніміз олар әдетте төленген парамен өтеді,бұл қайт оралушы және заңсыз иммигрант бұл де олар әдет төлен пара өт,nazism

3,сіз жоғары немесе өте ақымақ болуыңыз керек бұл сасық иммигранттар түнде ұялы телефондарымызға бұрыш спрейін шашады барлық жерде ысқырады және зәр

шығарады олар тіпті шіркеу алаңында көгершіндерімізді сойды жоқ олар барып өз елдері үшін күресуі керек,сіз жоғары немесе өте ақы бол керек бұл сасық иммигрант түн ұялы телефон бұрыш спр шаш барлық же ысқыр және зәр шығар олар тіпті шіркеу алаң көгершін сой жоқ олар бар өз ел үшін күрес керек,nazism

3,жерді өтемақысыз иеліктен шығару оңтүстік африкада болуы керек дудула сияқты операциялар назар аудару үшін жасалады шындықты біледі ол ақ нәсілділердің жалдамалы құралы,же өтемақы иелік шығару оңтүстік африка бол керек дудул сияқты операция назар аудару үшін жасал шындық біл ол ақ нәсілді жалдамалы құрал,nazism

3,дудула операциясы зимбабвеліктерге оң нәтиже беру үшін оңтүстік африкада алғашқы операцияны бастайды,дудул операция зимбабвелік оң нәтиже беру үшін оңтүстік африка алғашқы операция баста,nazism

3,негізгі бақ пен саясаткерлер айтқандай кек алушылар тобы бар олар өздерін атайды бұл адамдар сіздің еліңіздің қауіпсіздігі мен жақсаруы үшін күреседі олар соғысады сондықтан сіздің балаларыңыздың ұрысудың қажеті жоқ,негізгі бақ пен саясаткер айтқанда кек алушы тоб бар олар өз ата бұл адам сіздің ел қауіпсіздіг мен жақсар үшін күрес олар соғыс сондықтан сіздің бала ұрыс қажет жоқ,nazism

3,сіздің лақап атыңыз жұмыртқа тәрізді мүмкін сіз оны ауыстыруыңыз керек шулы шетелдіктер шу шығарады өйткені оңтүстік корея оларға туған тозақпен салыстырғанда жұмсақ өмір сыйлады,сіздің лақа ат жұмыртқа тәрізді мүмкін сіз оны ауыстыр керек шул шетелдік шу шығар өйткені оңтүстік корея оларға туған тозақ салыстыр жұм өмір сыйла,nazism

3,оның есімі кгомоцо оны анасының отанында африкадағы алғашқы операцияда заңсыз иммигранттар өлтірді,оның есім кгомоцо оны ана отан африка алғашқы операция заңсыз иммигрант өл,nazism

3,мен сізді тек коп екеніңізді білу үшін ақылға қонымды адам деп ойладым бұл твит сіздің заңсыз иммигранттарға қатысты ойлау қабілетіңіз оң көрсеткіштен әлдеқайда төмен екенін көрсетеді,мен сіз тек коп екен білу үшін ақыл қонымды адам де ойла бұл твит сіздің заңсыз иммигрант қатысты ойлау қабілет оң көрсеткіш әлдеқайда төмен екен көрсет,nazism

3,неліктен біз африкадағы басқа африка елдерінен адамдардың еркін қозғалысына қарсы емес жалғыз елміз үкімет мұны мақұлдайды және бұл са да жергілікті тұрғындар мен шетелдіктер арасында қанды монша басталатын уақыт мәселесі қха ксенофобияны жалғастырады мен оларды кінәлаймын,неліктен біз африка басқа африка ел адам еркін қозғалыс қарсы емес жалғыз ел үкімет мұны мақұлда және бұл са да жергілікті тұрғын мен шетелдік ара қан монш бастал уақыт мәселе қх ксенофобия жалғастыр мен ол кінәла,nazism

3,вотсекефф африкадан келген алғашқы заңсыз иммигранттар біздің қазіргі үкімет қолдай алмайтын апартеид жүйесі салған инфрақұрылымымызды бұзу үшін келді деп

мәлімдейді,вотсекефф африка кел алғашқы заңсыз иммигрант біздің қазіргі үкімет қолда алма апартеид жүйе сал инфрақұрылым бұзу үшін кел де мәлімде,nazism

3,шетелдіктер өз елдері үшін күресудің орнына осында қалуды сұрайды,шетелдік өз ел үшін күресу орн осында қалу сұра,nazism

3,олардың әрекеттері бұған қалай әкелді менің ойымша сіз біздің елдегі заңсыз иммигранттарға және сол елдегі әрекеттерге қалыпты түрде қарайсыз,олардың әрекет бұған қалай әке менің ойымша сіз біздің ел заңсыз иммигрант және сол ел әрекет қалыпты түр қара,nazism

3,мен ашуланамын өйткені олар бұл дудула операциясы деп айтқысы келеді ал жергілікті тұрғындардың болуы полицияның әрекетсіздігін ақтайды бұл қауымдастық әрқашан wt мәселелерін зорлық зомбылықсыз шешетін етіп ұйымдастырылды соның ішінде нхланхлудың өзі,мен ашулан өйткені олар бұл дудул операция де айт кел ал жергілікті тұрғын бол полиция әрекетсіздіг ақта бұл қауымдастық әрқашан wt мәселе зорлық зомбылық шеш ет ұйымдастыр соның іш нхланхл өзі,nazism

3,қха eff және da жіберген заңсыз иммигранттар өлтірген патриот ағама көңіл айтамын кгомотсоның өмірі босқа кетпейді,қх eff және da жібер заңсыз иммигрант өлтір патриот аға көңіл ай кгомотсо өмір бос кетпе,nazism

3,аарон мотсоаледиді заңсыз шетелдіктерден құтылу үшін қолдаңыз,аарон мотсоаледи заңсыз шетелдік құтыл үшін қолда,nazism

3,такунда сен былайша айтқанда ақымақ боласың халықаралық террористер мен қылмыскерлердің оңтүстік кореяда ұйықтайтын камералары бар инфрақұрылымды қарулы қылмыскерлер мен қызметтерге қол жеткізе алмайтын қауымдастықтар диверсиялайды егер санс көтерілмесе са түбіне дейін барады санс басқа заңды африкалықтарды жақсы көреді,такун сен былайша айтқанда ақы бол халықаралық террорис мен қылмыскер оңтүстік корея ұйықта камера бар инфрақұрылым қарулы қылмыскер мен қызмет қол жеткіз алма қауымдастық диверсияла егер санс көтерілме са түб дейін бар санс басқа заңды африкалық жақсы көр,nazism

3,гого колонизаторы хелен зилле дудула операциясының жетекшісінен еш айырмашылығы жоқ олар қара нәсілділерді мұқият зерттеуден тұратын пікірмен бөліседі қазір dudula қолдаушылары воетсекефті жоқтаумен айналысады өйткені eff үкіметте кеше оның мүшелерінің бірі алынып тасталды,гого колонизато хелен зилл дудул операция жетекші еш айырмашылығ жоқ олар қара нәсілді мұқият зерттеу тұр пікір бөліс қазір dudula қолдаушы воетсекеф жоқта айналыс өйткені eff үкімет кеше оның мүше бірі алын таста,nazism

3,олар бізге шабуыл жасайды олар шетелдіктер туралы алаңдайды,олар бізге шабуыл жаса олар шетелдік туралы алаңда,nazism

3,олар теміржол кабельдерінен бастады енді олар кез келген кабельді ұрлап жатыр,олар теміржол кабель баста енді олар кез кел кабель ұрла жатыр,nazism

3,африкалықтар басқа нәсілдерден мыңдаған жылдар артта қалды енді ақ адамдар үшін әр топты қару жарақпен қамтамасыз ету содан кейін кафралардың өздерін құртып жатқанын көру қалады,африкалық басқа нәсіл мыңдаған жыл арт қал енді ақ адам үшін әр топ қару жарақ қамтамасыз ету содан кейін кафра өз құрт жат көру қал,nazism

3,бізге заңсыз иммигранттармен күресу үшін стратегиялық және күшті жүрексіз нәрсе қажет біз толығымен қаржыландырылатын ультра оңшыл ұлтшыл топ құруымыз керек бізде күресуге уақыт таусылып жатыр,бізге заңсыз иммигрант күресу үшін стратегиялық және күшті жүрек нәрсе қажет біз толығ қаржыландырыл ультр оңшыл ұлтшыл топ құр керек біз күрес уақыт таусыл жатыр,nazism

3,дудула операциясы деп аталатын бұл қырағылық тобына отпен қарсы тұру керек ешбір қара адам өзі сияқты ақымақтардың кесірінен басқа қара нәсілділердің қолынан өлуге лайық емес,дудул операция де атал бұл қырағылық тоб от қарсы тұру керек ешбір қара адам өзі сияқты ақымақ кесір басқа қара нәсілді қол өл лайық емес,nazism

3,сіз дудула операциясына қатысушыларды атып өлтірген және кабельдерді ұрлаған шетелдіктер өлтірген адамдарға күле алмайсыз,сіз дудул операция қатысушы ат өлтір және кабель ұрла шетелдік өлтір адам күл алма,nazism

3,басото ба лесото мен зимбабве тұрғындары совето тұрғынын өлтіріп жарақаттады сіз бұл туралы бұқаралық ақпарат құралдарында естімейсіз немесе оқымайсыз өйткені sa журналистері заңсыз шетелдіктерге қатысты бейтарап,басото ба лесото мен зимбабв тұрғын совето тұрғын өлтір жарақатта сіз бұл туралы бұқаралық ақпарат құрал естіме немесе оқыма өйткені sa журналист заңсыз шетелдік қатысты бейтарап,nazism

3,джулиус малема жер мәселесін шешіп заңсыз шетелдік азаматтар мен есірткі сатушылар мәселесін нхланхла люкске қалдыруы керек,джулиус мале жер мәселе шеш заңсыз шетелдік азамат мен есірткі сатушы мәселе нхланхл люкс қалдыр керек,nazism

3,оңтүстік африка үкіметі заңсыз иммигранттарға өте кешірімді енді олар оларға ортаңғы саусақты көрсетеді,оңтүстік африка үкімет заңсыз иммигрант өте кешір енді олар оларға ортаңғы саусақ көрсет,nazism

3,малема жер мәселесін шешіп заңсыз шетелдіктермен күресу үшін нхланхладан кетуі керек,мале жер мәселе шеш заңсыз шетелдік күресу үшін нхланхла кет керек,nazism

3,мен басотоның нхланхла люкспен айналысқанын қалаймын ол осы оқ өткізбейтін позитивті алып тастауы керек,мен басото нхланхл люкс айналыс қала ол осы оқ өткізбе позитивті алып таста керек,nazism

3,оңтүстік африкалықтар dudula операциясы арқылы африканың оңтүстігінен тұрып бастайтын уақыт келді,оңтүстік африкалық dudula операция арқылы африка оңтүстіг тұр баста уақыт кел,nazism

3,хайи сіз заңсыз шетелдіктер мен ашық шекаралар туралы бірдеңе қалдырдыңыз сонда бәрі келіп қалағанын жасай алады неге,хайи сіз заңсыз шетелдік мен ашық шекара туралы бірде қал сонда бәрі кел қала жаса ал неге,nazism

3,шетелдіктер қорғануға құқылы,шетелдік қорған құқылы,nazism

3,олар қылмыскерлердің тек шетелдіктер немесе негізінен өздері екенін қайдан біледі бұл шындық болса да табиғаты бойынша болжау мүмкін емес топ кінәлілерді кінәсіздерден қалай ажырата алады немесе заңдарды орындауда құқық қорғау органдары ретінде оларға қалай сенуге болады,олар қылмыскер тек шетелдік немесе негіз өз екен қайдан біл бұл шындық бол да табиғат бойынша болжау мүмкін емес топ кінәлі кінәсіз қалай ажыра ал немесе заң орында құқық қорғау орган рет оларға қалай сен бол,nazism

3,джулиус үкіметі инфрақұрылымды қоршаған ортаны және б қорғау үшін алдын алу заңдарын енгізу арқылы осы елдегі көптеген қылмыстарды тоқтата алады мен дудула жанкүйерлері шетелдіктерді барлық қылмыстар үшін неге айыптайтынын түсінбеймін,джулиус үкімет инфрақұрылым қорша орта және б қорғау үшін алдын алу заң енгізу арқылы осы ел көптеген қылмыс тоқта ал мен дудул жанкүйер шетелдік барлық қылмыс үшін неге айыпта түсінбе,nazism

3,уябоне вуйокази бұл бұзақылар біздің балаларымыз бен болашақ немерелеріміздің болашағын бұзады ал сіз заң бұзушыларға жанашырлықпен қарайсыз мұның бәрі қара нәсілділердің қара зорлық зомбылыққа қарсы күресі үшін бірақ сіз өзіңіз өз ақпаратыңызды жинап осы жағымсыз нәрселерді зерттегіңіз келмейді,уябон вуйокази бұл бұзақы біздің бала бен болашақ немере болашағ бұз ал сіз заң бұзушы жанашырлық қара мұның бәрі қара нәсілді қара зорлық зомбылық қарсы күре үшін бірақ сіз өз өз ақпарат жина осы жағымсыз нәрсе зертте келме,nazism

3,ақырында африкалық иммигранттардың оңтүстік африкада болу үшін заңды мәртебесі жоқ деген қорытындыға неге тез келдіңіз африкалық иммигранттар өздерінің нақты салаларында немесе секторларында корпоративтік мекемелерінде және б кәсіпқойлар көп екенін ескерсек бұған қалай жеттіңіз,ақырында африкалық иммигрант оңтүстік африка болу үшін заңды мәрте жоқ де қорытынды неге тез кел африкалық иммигрант өз нақты сала немесе сектор корпоративтік мекеме және б кәсіпқой көп екен ескер бұған қалай же,nazism

3,жүйе оңтүстік африкалықтарды заңсыз шетелдіктермен алмастырғысы келеді және олар бізді қорғау үшін ештеңе жасамайды бұл сіздің үкіметіңіз әзірлеген халықты ауыстыру,жүйе оңтүстік африкалық заңсыз шетелдік алмастыр кел және олар біз қорғау үшін ештеңе жасама бұл сіздің үкімет әзірле халық ауыстыру,nazism

3,сіз аргументіңіздің қаншалықты әлсіз екенін көресіз өйткені ол сіз заңсыз деп айыптаған африкалық иммигранттарға сондай ақ қылмыскерлерге қарсы бағытталған қылмысты ақтамай мұнда заңды түрде келген және оңтүстік корея экономикасына оң үлес қосатын көптеген африкалық иммигранттар бар,сіз аргумент қаншалықты әлсіз екен көр өйткені ол сіз заңсыз де айыпта африкалық иммигрант сондай ақ қылмыскер қарсы бағыттал қылмыс ақтама мұнда заңды түр кел және оңтүстік корея экономика оң үлес қос көптеген африкалық иммигрант бар,nazism

3,неге біздің халқымыз ең көп зардап шегеді,неге біздің хал ең көп зардап шег,nazism

3,зимбабве шекарасы заңсыз шетелдіктердің бейтарап елден кетуіне емес өз еліне кіруіне жол бермейді,зимбабв шекара заңсыз шетелдік бейтарап ел кет емес өз ел кір жол берме,nazism

3,елестетіп көріңізші оңтүстік африкалықтар оларға дауыс берді және олар бұл билікті шетелдіктерге көмектесу және eswatini басылымдарына қатысу үшін пайдаланады,елестет көріңізш оңтүстік африкалық оларға дауыс бер және олар бұл билік шетелдік көмектес және eswatini басылым қатысу үшін пайдалан,nazism

3,мен сіздің көшбасшылық қасиеттеріңізді қолдаймын әкім мырза бірақ менің ойымша соуэто тұрғындары жауап қайтарып бәрі аяқталғанша қарсы тұруы керек,мен сіздің көшбасшылық қасиет қолда әкім мырза бірақ менің ойымша соуэто тұрғын жауап қайтар бәрі аяқталғанш қарсы тұр керек,nazism

3,біз өз елімізді жақсы көреміз біз оны заңсыз иммигранттардан қорғауымыз керек олар біздің инфрақұрылымды бұзу үшін ғана бар,біз өз ел жақсы көр біз оны заңсыз иммигрант қорға керек олар біздің инфрақұрылым бұзу үшін ғана бар,nazism

3,егер сіз оларды көрмесеңіз бұл менің кінәм емес,егер сіз ол көрме бұл менің кінә емес,nazism

3,бұл туралы айтуды доғарыңыз жай ғана тоқтатыңыз өйткені оңтүстік кореядағы адамдар жасаған инфрақұрылымдық вандализм олардың шыққан елдерінде жасалмас еді тағы ойланыңыз,бұл туралы айту доғар жай ғана тоқта өйткені оңтүстік корея адам жаса инфрақұрылымдық вандализ олардың шық ел жасалмас ед тағы ойлан,nazism

3,оңтүстік африкалықтар мен атып тастайды және біз мұны тек twitter де көреміз біздің барлық медиа орталықтарымыз тыныш,оңтүстік африкалық мен ат таста және біз мұны тек twitter де көр біздің барлық медиа орталық тыныш,nazism

3,оңтүстік африка тарихы африка тарихының бөлігі баланы тәрбиелеу үшін бүкіл ауыл қажет бүкіл африка апартеид режиміне қарсы шықты самора мачерл сіздің еркіндігіңіз үшін қаза тапты тыңдау қабілеті де дағды,оңтүстік африка тарих африка тарих бөліг бала тәрбиелеу үшін бүкіл ауыл қажет бүкіл африка апартеид режим қарсы шық самор мачерл сіздің еркіндіг үшін қаза тап тыңдау қабілет де дағды,nazism

3,біз бұл ақ нәсілшілдің дұрыстығын дәлелдеп жатырмыз мен мысалы онымен келісуге бейіммін өйткені оның айтқанының бәрі қазір болып жатыр бізге сыни тұрғыдан ойлайтын адамдар керек бұл қара нәсілділердің қара зорлық зомбылығы емес оны қазір болып жатқан жағдайға дейін тоқтату керек еді кеш болды,біз бұл ақ нәсілш дұрыстығ дәлелде жатыр мен мысал онымен келіс бейім өйткені оның айт бәрі қазір бол жатыр бізге сыни тұрғы ойла адам керек бұл қара нәсілді қара зорлық зомбылығ емес оны қазір бол жат жағдай дейін тоқтату керек ед кеш бол,nazism

3,егер біз шетелдіктердің заңсыздығын түсініп оларды заңды түрде жалдасақ полицияны қылмыспен күресуге мәжбүр етсек біз тұрақтылыққа қол жеткіземіз және экономикамызды жақсартамыз,егер біз шетелдік заңсыздығ түсін ол заңды түр жалда полиция қылмыс күрес мәжбүр ет біз тұрақтылық қол жеткіз және экономика жақсар,nazism

3,адамдар проблемаларды талқыламайды қазіргі уақытта температура жоғары бәрі реттеледі және адамдар дұрыс ойлайды мысалы біздің экономикамызда біліктілігі жоқ шетелдіктерге орын бар бірақ олар заңсыз жұмыс істейді,адам проблема талқылама қазіргі уақыт температура жоғары бәрі реттел және адам дұрыс ойла мысал біздің экономика біліктіліг жоқ шетелдік орын бар бірақ олар заңсыз жұмыс істе,nazism

3,апартеид үкіметінде қате болған жалғыз нәрсе апартеид заңдары болды,апартеид үкім қате бол жалғыз нәрсе апартеид заң бол,nazism

3,заңы кез келген нәрсені жеңе алады тек бейтарап әрекет етуге жалқау,заң кез кел нәрсе же ал тек бейтарап әрекет ет жалқау,nazism

3,қозғалысыңызды саяси партия ретінде тіркеңіз заңсыз иммигранттармен күресу үшін біз сізді билікке апаруымыз үшін бұл ойын алаңы емес бізге қатаң заңдарды орындау үшін үкімет қажет және бұл үкімет саяси партия ретінде әрекет етеді,қозғалыс саяси партия рет тірке заңсыз иммигрант күресу үшін біз сіз билік апар үшін бұл ойын алаң емес бізге қатаң заң орындау үшін үкімет қажет және бұл үкімет саяси партия рет әрекет ет,nazism

3,нағыз ксенофобтар біздің сүйікті елімізді бұзады ал патриоттар газеттерде балшықпен суарылады,нағыз ксенофоб біздің сүйікті ел бұз ал патриот газет балшық суар,nazism

3,жақында мен басото азаматтарының қару жарақтарын сермедім,жақында мен басото азамат қару жарақ серме,nazism

3,дудула операциясы немесе олар элвис нятиді тірідей өртеп жіберді менің африкалық бауырларым есеп беру уақыты келді,дудул операция немесе олар элвис няти тірідей өрте жіб менің африкалық бауыр есеп беру уақыт кел,nazism

3,африкалықтарға қатысты ксенофобияны содан кейін зимбабвеліктер арасында трайбализмді насихаттау сіздің үмітсіздік деңгейіңіз өте қорқынышты,африкалық

қатысты ксенофобия содан кейін зимбабвелік ара трайбализ насихатта сіздің үмітсіз деңгей өте қорқыныш,nazism

3,біз зимбабвеге барлық зимбабвеліктерді депортациялау арқылы осы қылмыскерлердің барлығын тұтқындауға көмектесеміз зимбабве президенті жақсы сөздер айтты бұл зимбабведен осы қылмыстық позитив үшін арнайы шаралар қабылдауды талап етеді,біз зимбабве барлық зимбабвелік депортацияла арқылы осы қылмыскер барлығ тұтқында көмектес зимбабв президент жақсы сөз ай бұл зимбабве осы қылмыстық позитив үшін арнайы шара қабылдау талап ет,nazism

3,ол лесотодан немесе оңтүстік африкадан келген сотода сөйлейтін жігіттерді қалай айтты,ол лесото немесе оңтүстік африка кел сото сөйле жігіт қалай ай,nazism

3,мен білемін дудула операциясын дағдарысында байқамағаны үшін кінәлау оңай мозамбик пен зимбабведегі су тасқыны кезінде sandf сарбаздарды көмекке жібергенін ұмытып кетті kzn де сарбаздар екі аптадан кейін орналастырылады дегенмен сіз кімді кінәлау керектігін көрмейсіз,мен біл дудул операция дағдарыс байқама үшін кінәла оңай мозамбик пен зимбабве су тасқын кез sandf сарбаз көмек жібер ұмыт кет kzn де сарбаз екі апта кейін орналастыр дегенмен сіз кім кінәла керектіг көрме,nazism

3,жоқ адам бұл суретті осы мазмұнда пайдалану құрметсіздік сіз пан африкалық революционерді афрофобты топтармен байланыстыра алмайсыз,жоқ адам бұл сурет осы мазмұн пайдалану құрметсіз сіз пан африкалық революцион афрофоб топ байланыстыр алма,nazism

3,eff нәсілшілдікке қарсы науқандарының арқасында бұл елді басқаруға жақсы мүмкіндік алды қха ның жағдайы үнемі нашарлап бара жатқанда оны алып тастау үшін теріден шығады бірақ eff заңсыз иммигранттарды қолдап оларды оңтүстік африкалықтардан жоғары қою арқылы бәрін бұзуы керек,eff нәсілшілдік қарсы науқан арқа бұл ел басқар жақсы мүмкіндік алды қх ны жағдай үнемі нашарла бар жатқанда оны алып тастау үшін тері шығ бірақ eff заңсыз иммигрант қолда ол оңтүстік африкалық жоғары қою арқылы бәрін бұз керек,nazism

3,өзіне деген жеккөрушіліктен зардап шегетін ойыншық сарбаз,өзіне де жеккөрушілік зардап шег ойыншық сарбаз,nazism

3,инфрақұрылымымызды заңсыз иммигранттардан қорғайық мен өз елімді жақсы көремін,инфрақұрылым заңсыз иммигрант қорға мен өз ел жақсы көр,nazism

3,оңтүстік кореядан келген хумало немесе нкубе қыстан хумалоға немесе нкубаға шабуыл жасайды,оңтүстік корея кел хумало немесе нкуб қыс хумало немесе нкуба шабуыл жаса,nazism

3,басото тіпті девландтағы полигонда қалғандар да қаруланған өтінемін saps ті қолданыңыз менің жетекшім біз сізді жоғалтқымыз келмейді біздің ел сіздің көшбасшылық қабілеттеріңізге мұқтаж,басото тіпті девланд полигон қал да қарулан өт

saps ті қолдан менің жетекші біз сіз жоғалт келме біздің ел сіздің көшбасшылық қабілет мұқтаж,nazism

3,дудула операциясындағы ксенофобиялық оңтүстік африкалықтар дрейк вафли ағасын кәмелетке толмаған сияқты сындырады деп қатты айқайлайды жігіттер waffles футболкасынан гөрі қазына жасырылған жерді іздейді тіпті asap рианна футболкасынан қазына таппады,дудул операция ксенофобиялық оңтүстік африкалық дрейк вафли аға кәмелет толма сияқты сындыр де қатты айқайла жігіт waffles футболка гөрі қазына жасырыл же ізде тіпті asap рианн футболка қазына таппа,nazism

3,жоғалып кетті ме бақылау мүмкін емес пе біз қзн провинциясының денсаулық сақтау министрлігі кедейлерді жаппай жерлеуді ұйымдастыра алады немесе құжаттары жоқ шетелдіктерге мәйітханаларын белгісіз мерзімге алуға рұқсат бере алады деп хабарлаймыз,жоғал кет ме бақылау мүмкін емес пе біз қзн провинция денсаулық сақтау министрліг кедей жаппай жерлеу ұйымдастыр ал немесе құжат жоқ шетелдік мәйітхана белгісіз мерзім ал рұқсат бер ал де хабарла,nazism

3,олар жүйеде емес және өлген сондықтан олар кетуі керек бұл олардың шетелдіктер екендігі емес елдің дәл осы себептен бәрін дұрыс істеп жатқандығы және біздің заңдарымызды құрметтейтіндігі олар ақтауды және дауласуды тоқтатуы керек,олар жүйе емес және өлген сондықтан олар кет керек бұл олардың шетелдік екендіг емес ел дәл осы себеп бәрін дұрыс істе жатқандығ және біздің заң құрметтейтіндіг олар ақтау және дауласу тоқтат керек,nazism

3,мен әрқашан манделаның президент ретіндегі дәуірі бір адамның дәуірі сияқты сезінемін мотлантаға келетін болсақ бұл алты ай тозақ болды өз ойларыңызбен бөлісіңіз,мен әрқашан мандела президент рет дәуір бір адам дәуір сияқты сез мотланта кел бол бұл алты ай тозақ бол өз ой бөліс,nazism

3,ондықтан біздің еліміз өздеріне тиесілі емес нәрсені алып лас болып тонау мен әдептілікке үйренбес бұрын біз заңсыз шетелдіктерден құтылуымыз керек заңсыз иммигранттарды алып тастау оңайырақ содан кейін біздің адамдарда туу құқығы бойынша оларға тиесілі жұмыс болады,ондық біздің еліміз өз тиесілі емес нәрсе алып лас бол тонау мен әдептілік үйренбес бұрын біз заңсыз шетелдік құтыл керек заңсыз иммигрант алып тастау оңайырақ содан кейін біздің адам ту құқығ бойынша оларға тиесілі жұмыс бол,nazism

3,сіздің әйеліңіз сол зимбабве жігітінің досы қандай ақымақтық сіздің әйеліңіз жігіттің досы,сіздің әйел сол зимбабв жігіт дос қандай ақымақтық сіздің әйел жігіт дос,nazism

3,сіз тіпті өзіңізді білдіре алмайсыз сіз бедеусіз ана тілінде жақсы сөйле,сіз тіпті өз білдір алма сіз бедеу ана тіл жақсы сөйле,nazism

3,олар бұл адамдардың іздеуде жүрген қылмыскерлер екеніне таң қалды,олар бұл адам ізде жүр қылмыскер екен таң қал,nazism

3,сонымен сіздің тірі құдайыңыз сізге адамды тірідей өртеп жіберуге кеңес берді және сізге кармадан қорғауға уәде берді ме,сонымен сіздің тірі құдай сізге адам тірідей өрте жібер кеңес бер және сізге карма қорға уәде бер ме,nazism

3,сонымен сіз үшін менің кім екенімді білгенде таң қаласыз,сонымен сіз үшін менің кім екен біл таң қал,nazism

3,ол бәлкім менің әйелім үшін мен сенің анаң үшін әкеңнің артындамын,ол бәлкім менің әйел үшін мен сенің ана үшін әке арт,nazism

3,сіз өзіңізбен күресуіңіз керек өйткені сіз ақ шыршалардан қорқатын мәселесіз,сіз өз күрес керек өйткені сіз ақ шырша қорқ мәселе,nazism

3,біз шынымен назар аудардық білесіз бе ал сен не істеп жатырсың,біз шын назар аудар біл бе ал сен не істе жатыр,nazism

3,сізге ақ адамдарға келгенде не айту керектігін және қалай әрекет ету керектігін айтады партизан сіз тіпті өзіңізді нысанаға алған кезде кавказдықтардан қорқатындығыңызды мойындайсыз африкалықтар,сізге ақ адам кел не айту керектіг және қалай әрекет ету керектіг айт партизан сіз тіпті өз нысана ал кезде кавказдық қорқатындығ мойында африкалық,nazism

3,элвис нятиге не болғаны соншалықты қате заңсыз иммигранттар үшін мұндай нәрселерден аулақ болу үшін үйлеріне оралу жақсы болар еді,элвис няти не бол соншалықты қате заңсыз иммигрант үшін мұндай нәрсе аулақ болу үшін үй орал жақсы бол ед,nazism

3,өйткені олардың көпшілігі де заңсыз және сіз мұны білесіз бірақ оларды паналауды шешіңіз өйткені олар ақ түсті осылайша сіздің мүдделеріңізді қорғайды ақ адамдар бар олар да заңсыз шетелдіктер неліктен олар нысанаға айналмайды неліктен африкалықтарға шабуыл жасау керек,өйткені олардың көпшіліг де заңсыз және сіз мұны біл бірақ ол паналау шеш өйткені олар ақ түсті осылайша сіздің мүдде қорға ақ адам бар олар да заңсыз шетелдік неліктен олар нысана айналма неліктен африкалық шабуыл жасау керек,nazism

3,сіз сенімдісіз бе олардың не істегенін қараңыз,сіз сенімді бе олардың не істе қара,nazism

3,сіз қытайлардан итальяндықтардан американдықтардан және осы кавказдықтардың бәрінен қорқасыз олардың көпшілігі қылмыскер емес сияқты барлық қыс өткеннен кейін сіздің ізбасарларыңыз әлі де жұмыссыз қалады және қылмыс әлі де жоғары болады сондықтан сіз нақты не істер едіңіз,сіз қытай итальяндық американдық және осы кавказдық бәрі қор олардың көпшіліг қылмыскер емес сияқты барлық қыс өт кейін сіздің ізбасар әлі де жұмыссыз қал және қылмыс әлі де жоғары бол сондықтан сіз нақты не істер ед,nazism

3,оңтүстік африкалықтар бұл лесото азаматын жақсы көретіні белгілі болды өйткені ол сізге қатысты бизнесті білдіреді біз сізді шетелдіктермен ұйықтауға

жібермедік,оңтүстік африкалық бұл лесото азамат жақсы көр белгілі бол өйткені ол сізге қатысты бизнес білдір біз сіз шетелдік ұйықта жіберме,nazism

3,лол есегіңді түзету үшін,лол есег түзету үшін,nazism

3,менің әйелім маған зимбабведен келген бір жігіт туралы айтады оны соқты оқиғадан кейін айдан аспайтын өмір сүрген сияқты,менің әйел маған зимбабве кел бір жігіт туралы айт оны соқ оқиға кейін ай аспа өмір сүр сияқты,nazism

3,оңтүстік африка біз шетелдіктерге оңтүстік африкаға заңсыз емес заңды түрде келу керектігін айттық біз ешкімді жек көрмейміз бірақ олар заңды бұзбай басқа елдерге барудың тиісті әдісін қолдануы керек олар мұны тек оңтүстік африкада жасайды,оңтүстік африка біз шетелдік оңтүстік африка заңсыз емес заңды түр келу керектіг ай біз ешкі жек көрме бірақ олар заңды бұзба басқа ел бар тиісті әдіс қолдан керек олар мұны тек оңтүстік африка жаса,nazism

3,оңтүстік африкаға қылмыс жасау үшін келген шетелдіктер біздің ағаларымыз емес және нигерия ханымы ешқашан зорланған емес бірақ ол жақсы контент жасаушы,оңтүстік африка қылмыс жасау үшін кел шетелдік біздің аға емес және нигерия ханым ешқашан зорлан емес бірақ ол жақсы контент жасаушы,nazism

3,бұл оның мәлімдемесі емес,бұл оның мәлімде емес,nazism

3,біз шетелдіктерді кінәламаймыз жоқ көшбасшыларды кінәлаймыз біз зимбабвеліктер емеспіз қашып кетпейміз шындықты айтамыз және әрекет етеміз оңтүстік кореяда қорқақ жоқ біз өз қадір қасиетіміз үшін күресеміз,біз шетелдік кінәлама жоқ көшбасшы кінәла біз зимбабвелік емес қаш кетпе шындық ай және әрекет ет оңтүстік корея қорқақ жоқ біз өз қадір қасиет үшін күрес,nazism

3,жоқ олар мұны істемеді бұл малавиялықтар біздің мемлекетімізді тонап цекті басып алған басқа екі заңсыз шетелдіктермен байланысты жеке куәліксіз немесе psira мен жеке күзетпен жұмыс істейтіндер барлық заңсыз иммигранттар үшін оңтүстік африкадан шығыңыз,жоқ олар мұны істеме бұл малавиялық біздің мемлекет тона цек бас ал басқа екі заңсыз шетелдік байланысты жеке куәлік немесе psira мен жеке күзет жұмыс істе барлық заңсыз иммигрант үшін оңтүстік африка шығ,nazism

3,дудула операциясы африкада өшпенділік тудырды бұл кек алудың бастамасы ғана өкінішке орай бұл менің командамда болды бірақ бұл шындық,дудул операция африка өшпенділік ту бұл кек ал баста ғана өкініш орай бұл менің команда бол бірақ бұл шындық,nazism

3,оңтүстік африкалықтар мұны өздері шешсін заңсыз иммигранттар ағыны жер мәселесін шеше ме,оңтүстік африкалық мұны өз шеш заңсыз иммигрант ағын жер мәселе шеш ме,nazism

3,қаншалықты ыңғайсыз болса да қаншалықты ақылға қонымсыз болып көрінсе де біз заңсыз иммигранттар мәселесін қарастырып шешуіміз керек,қаншалықты

ыңғайсыз бол да қаншалықты ақыл қон бол көрін де біз заңсыз иммигрант мәселе қарастыр шеш керек,nazism

3,шетелдіктер saps қабілетсіз екенін біле отырып әрқашан cd де қылмыс жасайды,шетелдік saps қабілет екен біл отыр әрқашан cd де қылмыс жаса,nazism

3,қара шетелдіктер мұны бастан кешіретініне сенімдісіз бе мүмкін бұл менің жолым болмады,қара шетелдік мұны бас кешір сенімді бе мүмкін бұл менің жол болма,nazism

3,оңтүстік африкадағы алғашқы операция дудула зимбабвеліктер құдай өтінемін біздің дұғаларымызды тыңдаңыз,оңтүстік африка алғашқы операция дудул зимбабвелік құдай өт біздің дұға тыңда,nazism

3,хабарлама ұнады нақты адам немесе оның артында кім тұр жолдау еліміздің бей жай қарамайтын азаматтары ретінде алдымызда тұрған кеңірек миссиямен үндеседі сіз түсінесіз деп үміттенемін,хабарлама ұн нақты адам немесе оның арт кім тұр жолдау ел бе жай қарама азамат рет алд тұр кеңірек миссия үндес сіз түс де үміттен,nazism

3,мен білетін және ата бабаларымыздың жерін қайтаруға жіберген жалғыз құтқарушы бізде дудула операциясына уақыт жоқ ол өзін күшті әсерге таңғысы келеді жақында біз өз жерімізді аламыз,мен біл және ата баба жер қайтар жібер жалғыз құтқарушы біз дудул операция уақыт жоқ ол өз күшті әсер таң кел жақында біз өз же ал,nazism

3,оңтүстік африкалықтар rdps ті шетелдіктерге жалға береді және олар мукукуста тұруға көшеді айдың соңында олар жалдау ақысын жинайды,оңтүстік африкалық rdps ті шетелдік жалға бер және олар мукукус тұр көш ай соң олар жалда ақы жина,nazism

3,егер ксенофобия елге көмектессе бұл солай болуы керек өйткені шетелдіктер пайдадан гөрі көп зиян келтіреді және бұл туралы ешкім ештеңе жасамайды,егер ксенофобия ел көмектес бұл солай бол керек өйткені шетелдік пайда гөрі көп зиян келтір және бұл туралы ешкім ештеңе жасама,nazism

3,біздің үкімет зұлым емес деп ойлайсыз олар сөзбе сөз нағыз қылмыскерлерді қорғайды және жазықсыз шетелдіктерге мұның бәрін бірнеше қолма қол ақшаға алуға мүмкіндік береді жалғыз айырмашылық сафрикандықтар үшін күтім опция емес біз қаламыз және күресеміз,біздің үкімет зұлым емес де ойла олар сөзб сөз нағыз қылмыскер қорға және жазық шетелдік мұның бәрін бірнеше қол қол ақша ал мүмкіндік бер жалғыз айырмашылық сафрикандық үшін күтім опция емес біз қал және күрес,nazism

3,олар негізінен ақ жағалы қылмыстар жасайды сафрикандықтарға қарсы зорлық зомбылық қылмыстарын жасайтын қара заңсыз шетелдіктер,олар негіз ақ жа қылмыс жаса сафрикандық қарсы зорлық зомбылық қылмыс жаса қара заңсыз шетелдік,nazism

3,заңсыз иммигранттар дудула операциясын жек көрсе жемқор қха дудула операциясын жек көреді ал ақымақ eff дудула операциясын жек көреді демек дудула

операциясында бәрі дұрыс,заңсыз иммигрант дудул операция жек көрсе жемқор қх дудул операция жек көр ал ақы eff дудул операция жек көр демек дудул операция бәрі дұрыс,nazism

3,неліктен жазықсыз адамдардан зардап шегеді деп ойлайсыз көптеген заңды және заңға бағынатын шетелдіктер сізбен келіспейді құжаттары жоқ шетелдіктер жасаған зорлық зомбылық қылмыстарының деңгейі мұнда қатысқандардың барлығында алаңдаушылық пен қорқыныш тудырады,неліктен жазық адам зардап шег де ойла көптеген заңды және заң бағын шетелдік сізбен келіспе құжат жоқ шетелдік жаса зорлық зомбылық қылмыс деңгей мұнда қатыс барлығ алаңдаушылық пен қорқыныш тудыр,nazism

3,ол мені шетелдіктер өлтірген оңтүстік африкалықтар туралы әңгіме де халықаралық жаңалық бола ма деп сұрағаным үшін бұғаттады,ол мен шетелдік өлтір оңтүстік африкалық туралы әңгіме де халықаралық жаңалық бола ма де сұра үшін бұғатт,nazism

3,иә бұл болады олардың кейбіреулерінде әйелдері мен балалары басқаратын дүкендерінде есірткіге толы жоғалған қыздардың жасырын жезөкшелер үйі бар мұнда жазықсыз шетелдіктер қашып кетпейді,иә бұл бол олардың кейбіреу әйел мен бала басқар дүкен есірткі толы жоғал қыз жасырын жезөкше үй бар мұнда жазық шетелдік қаш кетпе,nazism

3,мұны жалаң қолмен алып тастайды олар біздің сүйікті заңсыз иммигранттарымыздың инфрақұрылымына қатысты вандализмді қолдайды,мұны жала қол алып таста олар біздің сүйікті заңсыз иммигрант инфрақұрылым қатысты вандализ қолда,nazism

3,кейбіреулеріміз үшін бұл біз қорқатын немесе жек көретін иммиграннттар емес біздің ашық шекара саясатымыз және қылмыстың өсуі әсіресе зорлық зомбылық қылмыстары олардың көпшілігін тіркелген және тіркелмеген шетелдіктер жасайды бұл шын мәнінде жиіркенішті,кейбіреу үшін бұл біз қорқ немесе жек көр иммигрант емес біздің ашық шекара саясат және қылмыс өс әсіресе зорлық зомбылық қылмыс олардың көпшіліг тіркел және тіркелме шетелдік жаса бұл шын мән жиіркеніш,nazism

3,бір күні олар ксенофобия мен жастардың наразылығының нағыз президентін ашуланшақтық пен анархияға толы жас жігітті көреді ең жаманы әлі алда,бір күн олар ксенофобия мен жас наразылығ нағыз президент ашуланшақ пен анархия толы жас жігіт көр ең жаман әлі алда,nazism

3,сіздің ойыңызша не маңызды болды руанда халқына қарсы болған басқа африкалықтардың сезімдері сол кезде жағдай нашарлап иммиграннттардың жаппай көшуі басталды,сіздің ойыңызша не маңызды бол руан халқ қарсы бол басқа африкалық сезім сол кезде жағдай нашарла иммигрант жаппай көш баста,nazism

3,бірде мен оларға соуэтоның тұрғыны екенімді айттым бірақ мен соуэто парламентін де оның орналасқан жерін де кеңселерін де білмеймін,бірде мен оларға соуэто тұрғы екен ай бірақ мен соуэто парламент де оның орналас жер де кеңсе де білме,nazism

3,оңтүстік африкадағы жағдай заңсыз шетелдіктердің нәтижесі болып табылады,оңтүстік африка жағдай заңсыз шетелдік нәтиже бол таб,nazism

3,қанжар қауіпті,қанж қауіпті,nazism

3,бұл заңсыз иммигранттардың ең көп саны бар,бұл заңсыз иммигрант ең көп сан бар,nazism

3,бұл тақырып мені бақытты етеді бізге бүкіл ел бойынша осындай тұтқындаулар қажет дудула оңтүстік африка операциясы оңтүстік африкалықтарды бірінші теріс жағдайға қояды,бұл тақырып мен бақытты ет бізге бүкіл ел бойынша осындай тұтқындау қажет дудул оңтүстік африка операция оңтүстік африкалық бірінші теріс жағдай қоя,nazism

3,конституция әрқайсысын ел шекарасында қорғауды көздейді неліктен олар гаваменти сәтсіздіктері үшін шетелдіктерді өлтіріп өртеп жібергенде біздің айқайымызды түсінгендей кейіп танытуы керек екенін білмеймін,конституция әрқайсы ел шекара қорғау көзде неліктен олар гаваменти сәтсіздік үшін шетелдік өлтір өрте жібер біздің айқай түсінгенде кейіп таныт керек екен білме,nazism

3,заңсыз иммигранттар болуы мүмкін,заңсыз иммигрант бол мүмкін,nazism

3,сіз қазірдің өзінде көмектесіп жатырсыз ба,сіз қаз өз көмектес жатыр ба,nazism

3,қайырлы таң командир мен капитан дудуланың біздің өмірімізде президент болғанын қалаймын күшті көшбасшы оның не үшін және оның халқына не қажет екенін кешірімсіз айтады,қайырл таң командир мен капитан дудула біздің өмір президент бол қала күшті көшбасшы оның не үшін және оның халқ не қажет екен кешір айт,nazism

3,сіздің тәжірибеңізге өкінемін сізге шабуыл жасағандар шетелдіктер ме әлде жергілікті тұрғындар ма,сіздің тәжірибе өкін сізге шабуыл жаса шетелдік ме әлде жергілікті тұрғын ма,nazism

3,сіз біздің ресурстарымызды шетелдіктермен бөлісуіміз керек деп айтасыз ол соны қолданады,сіз біздің ресурс шетелдік бөліс керек де ай ол соны қолдан,nazism

3,соның ішінде жұмыссыздық жергілікті тұрғындардан артықшылыққа ие болып көрінетін шетелдіктер және бәсекеге қабілетті туристік индустрия,соның іш жұмыссыздық жергілікті тұрғын артықшылық ие бол көрін шетелдік және бәсеке қабілетті туристік индустрия,nazism

3,мен сіздің эмзансиандық екеніңізге күмәнданамын біз өз елімізде иммигрант емеспіз және ешқашан болмаймыз ешқашан мен сенің сөздеріңде қызғаныш

естіледі деп айтуым керек,мен сіздің эмзансиа екен күмәндан біз өз ел иммигрант емес және ешқашан болма ешқашан мен сенің сөз қызғаныш ест де айт керек,nazism

3,неліктен мейрамханада шетелдіктердің пайыз болуы керек,неліктен мейрамхана шетелдік пайыз бол керек,nazism

3,ғимараттардың көпшілігін шетелдіктер басқарады адамдар жалдау ақысын төлеген кезде олар техникалық қызмет көрсету туралы ойламайды бұл солай мен қалада ұзақ жылдар тұрдым,ғимарат көпшіліг шетелдік басқар адам жалда ақы төле кезде олар техникалық қызмет көрсету туралы ойлама бұл солай мен қала ұзақ жыл тұр,nazism

3,оңтүстік африкадағы проблеманы ұмытыңыз әдеттегідей шетелдіктермен нақты проблемаларды алып тастаңыз,оңтүстік африка проблема ұмыт әдеттегідей шетелдік нақты проблема алып таста,nazism

3,сіз туралы да өтірік айтпайды сіз кім екеніңізді білесіз,сіз туралы да өтірік айтпа сіз кім екен біл,nazism

3,заңсыз иммигрант термині жоқ екені анық,заңсыз иммигрант термин жоқ екен анық,nazism

3,шетелдіктер біздің үй жайларды басып жатыр біз дем ала алмаймыз,шетелдік біздің үй жай бас жатыр біз дем ал алма,nazism

3,біздің шекарашылар күзетсіз қалады бірақ сіздердің кейбіреулеріңіз заңсыз иммигранттарды елден кетуге шақырады біздің үлкен проблемамыз үкіметтің қабілетсіздігі болған кезде сіз өзгерісті көргіңіз келе ме өзгерісті қалайсыз ба олай болса қха ны үкімет ретінде жойып оны басқасымен алмастырайық,біздің шекарашы күзет қал бірақ сіздердің кейбіреу заңсыз иммигрант ел кет шақыр біздің үлкен проблема үкімет қабілетсіздіг бол кезде сіз өзгер көр келе ме өзгер қала ба ола бол қх ны үкімет рет жой оны басқа алмастыр,nazism

3,бұл елдегі заңсыз иммигранттарды қорғайтындардың барлығында тек зимбабвеліктер ғана емес жақын арада оқ дәрілер таусылады бұл бұзушылықтар көңіліңізді қалдыруы мүмкін,бұл ел заңсыз иммигрант қорға барлығ тек зимбабвелік ғана емес жақын ара оқ дәрі таусыл бұл бұзушылық көңіл қалдыр мүмкін,nazism

3,сонымен eff шетелдіктерге толы,сонымен eff шетелдік толы,nazism

3,бұл қазіргіден жақсы болды олар бір аптадан аз уақыт ішінде құжатсыз шетелдіктердің келу мәселесін шеше алды,бұл қазіргі жақсы бол олар бір апта аз уақыт іш құжат шетелдік келу мәселе шеш алды,nazism

3,неліктен біз қылмысқа қарсы күресте әлі де сергек боламыз және заңсыз иммигранттарды депортацияламаймыз сондай ақ оған қатысы бар жергілікті тұрғындарды түрмеге қамамаймыз қауымдастықтар қоғамдық әділеттілікті жүзеге асырады өйткені ешқандай әрекет жоқ бірақ көп әңгіме,неліктен біз қылмыс қарсы күрес әлі де сергек бол және заңсыз иммигрант депортациялама сондай ақ оған

қатыс бар жергілікті тұрғын түрме қамама қауымдастық қоғамдық әділеттілік жүзеге асыр өйткені ешқандай әрекет жоқ бірақ көп әңгіме,nazism

3,егер бізде нигериялықтар заңсыз иммигранттар болмаса мұның ешқайсысы болмас еді жыла менің сүйікті елім,егер біз нигериялық заңсыз иммигрант болма мұның ешқайсы болмас ед жыла менің сүйікті ел,nazism

3,оңтүстік африкаға тиесілі мейрамханалар мен бөлшек сауда дүкендерінде ақша жұмсайтын ақш шетелдіктері туралы не деуге болады,оңтүстік африка тиесілі мейрамхана мен бөлшек сауда дүкен ақша жұмса ақш шетелдік туралы не де бол,nazism

3,біздің инфрақұрылымды бұзу үшін заңсыз иммигранттар оңтүстік африкада алғашқы операцияны жүргізуде,біздің инфрақұрылым бұзу үшін заңсыз иммигрант оңтүстік африка алғашқы операция жүргіз,nazism

3,зимбабведен келген заңсыз иммигранттар бұл жерде бірлік үшін емес біздің инфрақұрылымды бұзу үшін жүр,зимбабве кел заңсыз иммигрант бұл же бірлік үшін емес біздің инфрақұрылым бұзу үшін жүр,nazism

3,сіз біздің бауырларымыздың не істеп жатқанын көресіз,сіз біздің бауыр не істе жат көр,nazism

3,президент мырза біздің еліміздегі барлық заңсыз шетелдіктермен бірдеңе жасаңыз біздің еліміз осы жігіттердің кабель ұрлауынан ұшақ ұрлауынан және sbv тонауынан зардап шегеді өтінемін бірдеңе жасаңыз,президент мырза біздің еліміз барлық заңсыз шетелдік бірде жаса біздің еліміз осы жігіт кабель ұрлау ұшақ ұрлау және sbv тонау зардап шег өт бірде жаса,nazism

3,шетелдік қылмыскерлер күн сайын өздерінің құмар жауынгерлері мен хонгтарын ұятқа қалдырады аудандық прокурордың заңсыз иммигранттарға рақымшылық жасау туралы ұсынысын ұмытпайық бұл қоқыс аудандық прокурордың қанына да құйылды жалған мемлекеттік құжаттарды жасаған адамды қалай кешіруге болады,шетелдік қылмыскер күн сайын өз құм жауынгер мен хонг ұят қалдыр аудандық прокурор заңсыз иммигрант рақымшылық жасау туралы ұсыныс ұмытп бұл қоқыс аудандық прокурор қан да құй жалған мемлекеттік құжат жаса адам қалай кешір бол,nazism

3,құжатсыз шетелдіктер,құжат шетелдік,nazism

3,апартеид кезінде заңсыз иммигранттар болған жоқ қазір ол заңсыз иммигранттарға толы,апартеид кез заңсыз иммигрант бол жоқ қазір ол заңсыз иммигрант толы,nazism

3,үйге оралуды жоспарламайды бірақ өз елінде құрмет көрсетпейді,үй оралу жоспарлама бірақ өз ел құрмет көрсетпе,nazism

3,сіз өзіңізді тітіркендіресіз неге дудула операциясы туралы алаңдайсыз,сіз өз тітіркендір неге дудул операция туралы алаңда,nazism

3,оңтүстік африка шабуылға ұшырады,оңтүстік африка шабуыл ұшыр,nazism

3,ра бірінші кезекте американдықтарды қояды және б,ра бірінші кезек американдық қоя және б,nazism

3,олар бұған лайық бұл үкімет қылмыскерлерді ұстау үшін жасауы керек нәрсе олар талап ететін теріс пайдаланатын және оларға лайық емес адамдар өз пайдасына пайдаланатын көптеген құқықтарды жойыңыз,олар бұған лайық бұл үкімет қылмыскер ұстау үшін жаса керек нәрсе олар талап ет теріс пайдалан және оларға лайық емес адам өз пайда пайдалан көптеген құқық жой,nazism

3,сонымен мен қоңырау шалушы айтқандай мен өміріме қауіп төндіремін сондықтан мен ештеңе жоғалтпау үшін қолымнан келгеннің бәрін жасаймын мен осылай айтайын заңсыз шетелдіктер жұмақтан немесе тозақтан кетеді олар теріс нәтиже алады,сонымен мен қоңырау шалуш айтқанда мен өмір қауіп төндір сондықтан мен ештеңе жоғалтпа үшін қол кел бәрін жаса мен осылай айт заңсыз шетелдік жұмақ немесе тозақ кет олар теріс нәтиже ал,nazism

3,егер ол өзінің түйіндемесіне шетелдіктерге деген жеккөрушілікті қосса ол көп дауыс жинаған болар еді,егер ол өзінің түйіндеме шетелдік де жеккөрушілік қос ол көп дауыс жина бол ед,nazism

3,шетелдіктер мұнда бишілер сияқты вальс жасамады олар шекара патрулын таппады және ол көптеген жылдар бойы ашық болды ал бүгін сіз олардың басып кіріп жатқанын айтасыз,шетелдік мұнда биші сияқты вальс жасама олар шекара патруль таппа және ол көптеген жыл бойы ашық бол ал бүгін сіз олардың бас кір жат ай,nazism

3,аштық адамдарға қысым жасайды біздің қоғамдастық үйді зимбабве отбасынан құтқара алды егер олар заттарды жинамаса біз оларды жинауға көмектесеміз деп айттық олар кетіп қалды біз тіпті ақымақ төлемге мән бермейміз өйткені олар шетелдіктер,аштық адам қысым жаса біздің қоғамдастық үй зимбабв отбас құтқар алды егер олар зат жинама біз ол жина көмектес де ай олар кет қал біз тіпті ақы төлем мән берме өйткені олар шетелдік,nazism

3,жоқ бір африка туралы ақымақтыққа ешқашан жол берілмеуі керек және біздің балаларымыз шетелдіктердің біз болмаған кезде өздерін манипуляциялауына жол бермеу үшін мұны айтуы керек,жоқ бір африка туралы ақымақтық ешқашан жол берілме керек және біздің бала шетелдік біз болма кезде өз манипуляцияла жол бермеу үшін мұны айт керек,nazism

3,африканың бұл өкілі симунье ол ақымақ біз зимбабведе емес оңтүстік африкадамыз,африка бұл өкіл симунь ол ақы біз зимбабве емес оңтүстік африка,nazism

3,демалыс күндері айдың соңында және қасиетті жұмада бізге сыра сатып алатын достарымыз ғана бар,демалыс күн ай соң және қасиетті жұма бізге сыр сат ал дос ғана бар,nazism

3,түбегейлі өзгерістер әкеледі мысалы құжатсыз иммигранттардың келуіне рұқсат етіңіз,түбегейлі өзгеріс әкел мысал құжат иммигрант кел рұқсат ет,nazism

3,олар нашақор иелеріне көшті,олар нашақо ие көш,nazism

3,демек шетелдіктерге оңтүстік африкалықтармен салыстырғанда артықшылық режим беріледі бір күні біз өз елімізбен қоштасатын сияқтымыз,демек шетелдік оңтүстік африкалық салыстыр артықшылық режим бер бір күн біз өз ел қоштас сияқты,nazism

3,мені оңтүстік африкалықтар тонады неге сіз оларды шетелдіктер деп айтуға асығасыз,мен оңтүстік африкалық тона неге сіз ол шетелдік де айт асығ,nazism

3,баспана іздеушілер мен бостандық үшін күресушілер bophelo дезинфекциялау құралымен үйге көп барады,баспана іздеуші мен бостандық үшін күресуші bophelo дезинфекцияла құрал үй көп бар,nazism

3,нхланхла люкс тек дрофа ксенофоб қана емес сонымен қатар әйелдерді зорлаушы ол элвис нятиді өлтіруді ұйымдастырды онда жігітті дудула операциясындағы қолдаушылары тірідей өртеп жіберді оның басқа әйел зорлаушылар мен өлтірушілерден еш айырмашылығы жоқ және оны осылай қарау керек,нхланхл люкс тек дроф ксенофоб қана емес сонымен қатар әйел зорлаушы ол элвис няти өлтіру ұйымдас онда жігіт дудул операция қолдаушы тірідей өрте жіб оның басқа әйел зорлаушы мен өлтіруші еш айырмашылығ жоқ және оны осылай қарау керек,nazism

3,леоның бейнесін қараңыз сіз оңтүстік африкалықтардың есебінен зимбабвеліктерге жұмыс орындарын сата бастағансыз,лео бейне қара сіз оңтүстік африкалық есеб зимбабвелік жұмыс орын сат баста,nazism

3,сіз бұқаралық ақпарат құралдарының бұл туралы хабарлауды ұнататынын көрмейсіз өйткені бұл олардың әңгімесіне сәйкес келмейді сонымен қатар оңтүстік африкада заңсыз иммигранттардың өмірі ғана маңызды мен оны жек көремін,сіз бұқаралық ақпарат құрал бұл туралы хабарлау ұнат көрме өйткені бұл олардың әңгіме сәйкес келме сонымен қатар оңтүстік африка заңсыз иммигрант өмір ғана маңызды мен оны жек көр,nazism

3,сіз қандай мүдделі тараптар туралы айтып отырғаныңыз қызық өйткені бұл қозғалыс ешқандай саяси партиямен байланысты емес мен сіздің жауабыңызды күтемін мүмкін сіз біз дудулалар білмейтін нәрсені білетін шығарсыз,сіз қандай мүдделі тарап туралы айт отыр қызық өйткені бұл қозғалыс ешқандай саяси партия байланысты емес мен сіздің жауаб күт мүмкін сіз біз дудула білме нәрсе біл шығ,nazism

3,лол зимбабвеліктер адам құқықтарын бұзады бірақ сіз мұнда сізге сәйкес келмейтін нәрсемен айналысасыз,лол зимбабвелік адам құқық бұз бірақ сіз мұнда сізге сәйкес келме нәрсе айналыс,nazism

3,дудула операциясы оңтүстік африка тұрғындарының жан жақты қолдауын қажет етеді,дудул операция оңтүстік африка тұрғын жан жақ қолда қажет ет,nazism

3,бұл жағында мәмілені аяқтайтын sa сарбаздары да бар олар пара алу үшін ұсталған кезде sa командирі sandf ті шетелдіктер теріс сыбайлас жемқорлыққа ұшыратқанын айтты,бұл жағ мәміле аяқта sa сарбаз да бар олар пара алу үшін ұстал кезде sa командир sandf ті шетелдік теріс сыбайлас жемқорлық ұшырат ай,nazism

3,жалқауларды жеткізуді доғарыңыз ақыр соңында сіз жұмысқа орналасасыз шетелдіктерді жалқаулығыңыз үшін айыптауды тоқтатыңыз,жалқау жеткізу доғар ақы соң сіз жұмыс орналас шетелдік жалқаулы үшін айыптау тоқта,nazism

3,әкеміз аарон мотсоаледи отанына оралу үшін африкаға алғашқы операцияны жіберді,әке аарон мотсоаледи отан орал үшін африка алғашқы операция жіб,nazism

3,папарона біз африкада алғашқы операцияны жүзеге асыратын мемлекет қайраткері аарон мотсоаледидің ұлдары мен қыздарымыз аарон мотсоаледи біздің әкеміз,папарон біз африка алғашқы операция жүзеге асыр мемлекет қайраткер аарон мотсоаледи ұл мен қыз аарон мотсоаледи біздің әке,nazism

3,африкада алғашқы ота жасайды үй шаруасымен айналысады доктор аарон мотсоаледи сізге қажырлы еңбегіңіз үшін алғыс айтады,африка алғашқы ота жаса үй шаруа айналыс доктор аарон мотсоаледи сізге қажырлы еңбег үшін алғыс айт,nazism

3,малема өз өмірінің құнымен қорғаған және оларды қорғау үшін мемлекеттік органдарды sap пайдаланған адамдар іс жүзінде қылмыскерлер болып табылады өйткені дудула али операциясы мен хилброуда қылмыскерлер бар екеніне сенбеймін деп айтты,мале өз өмір құн қорға және ол қорғау үшін мемлекеттік орган sap пайдалан адам іс жүз қылмыскер бол таб өйткені дудул али операция мен хилбро қылмыскер бар екен сенбе де ай,nazism

3,нхланхла люкс дламини сотты құрметтемегені айға түзетілгені үшін кінәлі деп танылды ма,нхланхл люкс дламини сот құрметтеме ай түзетіл үшін кінәлі де тан ма,nazism

3,бұл адамдар бізді өлтіреді африкаға зимбабвеліктерге қарсы алғашқы операцияны жібереді дудула,бұл адам біз өлтір африка зимбабвелік қарсы алғашқы операция жібер дудул,nazism

3,олар тек шетелдіктер үшін әрекет етеді,олар тек шетелдік үшін әрекет ет,nazism

3,заңсыз иммигранттар да дурбандағы контейнерлерді тонай бастады ма,заңсыз иммигрант да дурба контейнер тона баста ма,nazism

3,бірақ бұл адам жақсы күреседі неге оның өткенін араластырады заңсыз иммигранттар тым көп жұмысқа орналасу мүмкіндігі тым аз біз оңтүстік африкада шайқастық бірақ біздің еңбегіміздің жемісін көре алмаймыз,бірақ бұл адам жақсы күрес неге оның өт араластыр заңсыз иммигрант тым көп жұмыс орналасу мүмкіндіг тым аз біз оңтүстік африка шайқас бірақ біздің еңбег жеміс көр алма,nazism

3,ескі ме жоқ па бұл оңтүстік африкадағы шетелдіктердің сипатын көрсетеді дудула операциясы жеткілікті,ескі ме жоқ па бұл оңтүстік африка шетелдік сипат көрсет дудул операция жеткілікті,nazism

3,оңтүстік африкадағы алғашқы операция дудула біз оңтүстік африканы жақсы көреміз зимбабвеліктер үйге оралуы керек,оңтүстік африка алғашқы операция дудул біз оңтүстік африка жақсы көр зимбабвелік үй орал керек,nazism

3,сіздің бастығыңыз оның әрекеттері тек заңсыз иммиграннттарға бағытталған деп мәлімдейді содан кейін мен таңдау және төлеу шеруіне не себеп болғанын сұраймын,сіздің басты оның әрекет тек заңсыз иммигрант бағыттал де мәлімде содан кейін мен таңдау және төлеу шер не себеп бол сұра,nazism

3,сіз шетелдіктерді қуып жібере аласыз бірақ бұл ниггерлер әйелдерді зорлауды және бір бірін өлтіруді тоқтатпайды,сіз шетелдік қу жібер ал бірақ бұл ниггер әйел зорлау және бір бір өлтіру тоқтатпа,nazism

3,иә зимбабвеліктердің есеп беруге аллергиясы бар иә бұл жігіттің сөздерінде мағынасы бар бірақ құрбандық терең болғандықтан олар оны яһуда деп атайды путсутафиканның алғашқы операциясы дудула,иә зимбабвелік есеп бер аллергия бар иә бұл жігіт сөз мағына бар бірақ құрбан терең бол олар оны яһ де ата путсутафикан алғашқы операция дудул,nazism

3,бұл белсенді патриотизм бұл бопсалау мүмкін заңсыз шетелдіктер біздің денсаулық сақтау жүйемізді сатып алу ретінде алады,бұл белсенді патриотизм бұл бопсала мүмкін заңсыз шетелдік біздің денсаулық сақтау жүйе сат алу рет ал,nazism

3,операция дудула мен нхланхла люкс деп аталатын бала осының бәрінің артында бұл олардың қара халықты осылай өлтіру стилі олар сізді элвис ньяти сияқты өлтірмес бұрын қатты азаптайды олар қара нәсілділерді аяусыз өлтіруден тартынбайды,операция дудул мен нхланхл люкс де атал бала осы бәрі арт бұл олардың қара халық осылай өлтір стил олар сіз элвис ньяти сияқты өлтірмес бұрын қатты азапта олар қара нәсілді аяу өлтіру тартынба,nazism

3,оңтүстік африка үкіметі operationdudula сияқты нәсілшіл ұйымдарға және ксенофобиялық қозғалыстарға демократиялық әлемде еркін әрекет етуге мүмкіндік береді олар осындай қылмысты жалғастырғаны үшін қатаң жазаға лайық,оңтүстік африка үкімет operationdudula сияқты нәсілшіл ұйым және ксенофобиялық қозғалыс демократиялық әлем еркін әрекет ет мүмкіндік бер олар осындай қылмыс жалғастыр үшін қатаң жаза лайық,nazism

3,зимбабвеліктер ретінде біздің проблемамыз біз басымызды құмға жасырғымыз келеді шындық біз қонақжайлылығымызды теріс пайдаландық барлық заңсыз иммиграннттар үйге оралуы керек басқа ешбір ел соның ішінде зимбабве де мұндай заңсыздықты қабылдамайды менің проблемам көптеген заңсыз иммиграннттар тіпті қыста дауыс бермейді,зимбабвелік рет біздің проблема біз бас құм жасыр кел шындық біз қонақжайлылығ теріс пайдала барлық заңсыз иммигрант үй орал керек

басқа ешбір ел соның іш зимбабв де мұндай заңсыздық қабылдама менің проблема көптеген заңсыз иммигрант тіпті қыста дауыс берме,nazism

3,бұұ ның пайдасы жоқ ол алдымен өз елін заңсыз шетелдік иммигранттардан құтқаратын оңтүстік африкалықтарға емес зимбабвеге қарсы санкциялар енгізген елдерге назар аударуы керек бұл заңсыз иммиграция мәселесін тудырған бұұ мен ақш болды,бұұ ны пайда жоқ ол алд өз ел заңсыз шетелдік иммигрант құтқар оңтүстік африкалық емес зимбабве қарсы санкция енгіз ел назар аудар керек бұл заңсыз иммиграция мәселе тудыр бұұ мен ақш бол,nazism

3,біз оңтүстік африкадағы алғашқы операцияға байланысты мұндай ақымақтыққа жол бермейміз дудула сешего,біз оңтүстік африка алғашқы операция байланысты мұндай ақымақтық жол берме дудул сешего,nazism

3,барлық заңсыз иммигранттар кетуі керек олардың барлығы бонке,барлық заңсыз иммигрант кет керек олардың барлығ бон,nazism

3,үкімет заңсыз иммигранттардың қажеттіліктерін қалай қанағаттандырады ал олар өз сайлаушыларының қызмет көрсету қажеттіліктерін қанағаттандыра алмайды,үкімет заңсыз иммигрант қажеттілік қалай қанағаттандыр ал олар өз сайлаушы қызмет көрсету қажеттілік қанағаттандыр алма,nazism

3,оңтүстік африка иммигранттар үшін емес солтүстік және оңтүстік америка бразилия америка сияқты қоғамның жүйесі мен ыдырауы үшін қылмыспен нашақорлықпен алкоголизммен кедейлікпен және б ұқсас жүйелері бар елдермен бәсекелеседі мексика және б,оңтүстік африка иммигрант үшін емес солтүстік және оңтүстік америка бразилия америка сияқты қоғам жүйе мен ыдыра үшін қылмыс нашақорлық алкоголиз кедейлік және б ұқсас жүйе бар ел бәсекелес мексика және б,nazism

3,мен шетелдіктердің шұңқырларды қазғанын қалаймын,мен шетелдік шұңқыр қаз қала,nazism

3,мен сізге айта алатын жалғыз нәрсе оңтүстік африкада шетелдіктер үшін жұмыс бар мәселе қылмыста зимбабвенің бір қылмыскері зимбабвенің ұлт немесе кез келген басқа ұлт ретіндегі бейнесіндегі дақ садк елдері трансшекаралық қылмыскерлермен күресіп ішкі істерін шешуі керек,мен сізге ай ал жалғыз нәрсе оңтүстік африка шетелдік үшін жұмыс бар мәселе қылмыс зимбабве бір қылмыскер зимбабве ұлт немесе кез кел басқа ұлт рет бейне дақ садк ел трансшекаралық қылмыскер күрес ішкі іс шеш керек,nazism

3,біздің адамдар үйде жұмыссыз отырады ал заңсыз иммигранттар біздің елде жұмыс істейді мен тіпті дипслуттағы иммиграция қызметкерлерінен қашқандар mooiplaas spruit те жасырынып жүргендей сезінемін,біздің адам үй жұмыссыз отыр ал заңсыз иммигрант біздің ел жұмыс істе мен тіпті дипслут иммиграция қызметкер қаш mooiplaas spruit те жасырын жүргенде сез,nazism

3,хайбо біз оңтүстік африкалықтар жыл сайын шетелдіктердің қолынан зардап шегетін барлық кісі өлтіру туралы не деуге болады сіздің eff тек жергілікті емес заңсыз иммигранттарға қатысты,хайбо біз оңтүстік африкалық жыл сайын шетелдік қол зардап шег барлық кісі өлтір туралы не де бол сіздің eff тек жергілікті емес заңсыз иммигрант қатысты,nazism

3,енді біз оңтүстік африкадағы бақ ты тоқтататын кез келді өйткені олар біз туралы өтірік айтады ал сіз шетелдіктерді қолдайсыз бізді біздің елде өлтіргіңіз келеді оңтүстік африкалық бақ пен бірге егер сіз келіссеңіз твиттерге жазыңыз,енді біз оңтүстік африка бақ ты тоқтат кез кел өйткені олар біз туралы өтірік айт ал сіз шетелдік қолда біз біздің ел өлтір кел оңтүстік африкалық бақ пен бірге егер сіз келіс твиттер жаз,nazism

3,сіздердің барлығыңыз біздің есебінен бүкіл африкада жақсы адам болғыңыз келеді бірақ бұл болмайды dudulakzn операциясы dudula операциясы оңтүстік африканы бірінші орынға қояды,сіздердің барлығ біздің есеб бүкіл африка жақсы адам бол кел бірақ бұл болма dudulakzn операция dudula операция оңтүстік африка бірінші орын қоя,nazism

3,шетелдіктер біздің штаттарға ауыртпалық түсіреді десек түрмеде отырған шетелдікке төлемдерді кім төлейді деп ойлайсыз біз мынаны айтамыз,шетелдік біздің штат ауыртпалық түсір де түрме отыр шетелдік төлем кім төле де ойла біз мына ай,nazism

3,бұл өте өкінішті олар біздің елде болып жатқан осы заңсыздықты қабылдағанымызды және шетелдіктердің есебінен кедей болып бара жатқанымызды қалайды,бұл өте өкініш олар біздің ел бол жат осы заңсыздық қабылда және шетелдік есеб кедей бол бар жат қала,nazism

3,zanu pf сияқты үкіметтік партияңызды кінәламаңыз олар не істейді бұл олардың адамдарын қыстан жаппай кетуге мәжбүр етеді оңтүстік корея африкадағы полигон емес біздің жеке әлеуметтік экономикалық проблемаларымыз бар біздің ресурстар сіздің қажеттіліктеріңізді қанағаттандыра алмайды бізге шетелдіктер ауыртпалық түсірмейді,zanu pf сияқты үкіметтік партия кінәлама олар не істе бұл олардың адам қыс жаппай кет мәжбүр ет оңтүстік корея африка полигон емес біздің жеке әлеуметтік экономикалық проблема бар біздің ресурс сіздің қажеттілік қанағаттандыр алма бізге шетелдік ауыртпалық түсірме,nazism

3,жақында шетелдіктерге банктік шоттар ашуға құжатсыз жылжымайтын мүлік сатып алуға құқықтары жоқ көлік жүргізуге рұқсат етіледі ал ақш азаматтары мұны істеуге міндетті олар жақсы қаруланған біз олардың шығыс кейпте такси жағып жатқанын көрдік және бүгінгі күнге дейін такси иелерінің не сезінетіні ешкімге маңызды емес,жақында шетелдік банктік шот аш құжат жылжыма мүлік сат ал құқық жоқ көлік жүргіз рұқсат ет ал ақш азамат мұны істе міндетті олар жақсы қарулан біз олардың шығыс кейп такси жағ жат көр және бүгінгі күн дейін такси ие не сезін ешкім маңызды емес,nazism

3,жұмыссыздық мәселесі шешілгенге дейін және құжатсыз шетелдіктер әлі де округті аралап жүр бізде кеңселерді қарызға алатын және мемлекеттік ақшаны сауатын адамдар бар олар біздің салықтарымыз сіздің басшылығыңыз қабілетсіз сіз тек өзіңізге және достарыңызға қамқорлық жасайсыз,жұмыссыздық мәселе шешіл дейін және құжат шетелдік әлі де округ арала жүр біз кеңсе қарыз ал және мемлекеттік ақша сау адам бар олар біздің салық сіздің басшылығ қабілет сіз тек өз және дос қамқорлық жаса,nazism

3,үкіметіңізді жауапқа тартыңыз және дудула операциясын жалғыз қалдырыңыз,үкімет жауап тар және дудул операция жалғыз қалдыр,nazism

3,олар бізді төмен түсірмейтінін білемін олар шатасуды тудырсын бірақ біз бұқаралық ақпарат құралдары бізге жете алмайтын жерде кеңейіп жатырмыз біз бұл елді өз қауымдастығымыздан бастап ретке келтіреміз,олар біз төмен түсірме біл олар шатасу тудыр бірақ біз бұқаралық ақпарат құрал бізге же алма же кеңей жатыр біз бұл ел өз қауымдастығ баста рет келтір,nazism

3,шетелдіктердің қылмысқа қатысы бар бірақ saps қызметкерлерін елемейтіні туралы көп айтылады барлық ірі компанияларда saps немесе hawks шенеуніктері бар,шетелдік қылмыс қатыс бар бірақ saps қызметкер елеме туралы көп айт барлық ірі компания saps немесе hawks шенеунік бар,nazism

3,үйге келіңіз біз келгенге дейін бір бірімізді мүгедек қылдық па бұл шетелдіктердің қандай құқықтары бар,үй кел біз кел дейін бір бір мүгедек қыл па бұл шетелдік қандай құқық бар,nazism

3,сіз заңсыз иммигранттарды және заңды сақтамауды айыптамағаныңыз үшін жиркенесіз,сіз заңсыз иммигрант және заңды сақтамау айыптама үшін жиркен,nazism

3,бс мұндағы шетелдіктердің көпшілігі бір миссиямен біздің елімізді анархистік мемлекетке айналдыру толық аялдама енді біз оны көріп отырмыз біз бұдан былай шыдай алмаймыз,бс мұндағы шетелдік көпшіліг бір миссия біздің ел анархи мемлекет айналдыру толық аялдама енді біз оны көр отыр біз бұдан былай шыда алма,nazism

3,ең қорқыныштысы олар топтарда серуендеп шабуыл жасайды елестетіп көріңізші адамды қалағанын алуға көндіріңіз бірақ сіздің өміріңіз емес,ең қорқынышты олар топ серуенде шабуыл жаса елестет көріңізш адам қала ал көндір бірақ сіздің өмір емес,nazism

3,шекаралар ашық болуы керек полиция талап етпеуі керек шетелдіктердің рұқсаттарын қараңыз енді ел жоқ,шекара ашық бол керек полиция талап етпе керек шетелдік рұқсат қара енді ел жоқ,nazism

3,шындығында ол не туралы сөйлесетінін біледі сіз немесе сіздің бұқаралық ақпарат құралдарыңыз ешқашан болмаған жерлер бар екенін сіз инвесторлар мақұлдамайтын жерлер умлази ламонтвилл исифинго сияқты жерлер бар екенін

көреді шетелдіктер әйелдер мен балаларды зорлау қорлау және өлтіру шешім бар ма,шындығ ол не туралы сөйлес біл сіз немесе сіздің бұқаралық ақпарат құрал ешқашан болма жер бар екен сіз инвестор мақұлдама жер умлази ламонтвилл исифинго сияқты жер бар екен көр шетелдік әйел мен бала зорла қорла және өлтір шешім бар ма,nazism

3,мен operationdudula бұл саясат демеушілік ететін қозғалыс және басқарушы партияның жоғары басшылары осы қырағылық тобының артында олар оларды ақ үстемшілдерге қарсы жерсіз қара нәсілділер үшін күресетін нағыз революционерлердің назарын аудару үшін пайдаланады деп келістім,мен operationdudula бұл саясат демеушілік ет қозғалыс және басқарушы партия жоғары басшы осы қырағылық топ арт олар ол ақ үстемшіл қарсы же қара нәсілді үшін күрес нағыз революционер назар аудару үшін пайдалан де кел,nazism

3,егер елді жақсы басқару мүмкін болмаса оның халқын заңсыз иммигранттар мен қылмыскерлерден қорғаңыз адамдар көтеріліп қажет нәрсені жасайды өйткені олар жылуды сезінеді,егер ел жақсы басқару мүмкін болма оның халқ заңсыз иммигрант мен қылмыскер қорға адам көтеріл қажет нәрсе жаса өйткені олар жылу сезін,nazism

3,үйге келіңіз африкалық мигрант сіз біздің инфрақұрылымды бұзасыз дудула операциясы американдықтарды бірінші орынға қояды,үй кел африкалық мигрант сіз біздің инфрақұрылым бұз дудул операция американдық бірінші орын қоя,nazism

3,бұрынғы президент лалели увале изиндлеб заңсыз шетелдіктерді қорғайды мен оның өз халқына соншалықты сезімтал емес екенін ешқашан түсінбедім ол мүлдем шеттетілген мен оны қорлаймын және ренжідім,бұрынғы президент лалели увал изиндлеб заңсыз шетелдік қорға мен оның өз халқ соншалықты сезімтал емес екен ешқашан түсінбе ол мүлдем шеттет мен оны қорла және ренжі,nazism

3,менің ойымша бұл жерде жұмыс күші бар дегенмен иммигранттар біздің заңсыз иммигранттарымызға қарағанда кәсіби және заңды түрде бар,менің ойымша бұл же жұмыс күш бар дегенмен иммигрант біздің заңсыз иммигрант қара кәсіби және заңды түр бар,nazism

3,гугула гугл мырза туралы көп біледі бұл енді сүйектер емес түйіршікті қаңқа оларды ұсақтаңыз олардың атаға айналу қаншалықты оңай екенін көріңіз надандық жойылды,гугул гугл мырза туралы көп біл бұл енді сүйек емес түйіршік қаңқа ол ұсақта олардың ата айналу қаншалықты оңай екен көр надан жой,nazism

3,менің ойымша тексеру уақыты келді бұл саяси банктік шот оларға шетелдіктердің есебінен пайда әкелуі мүмкін біз қылмыстық элементтерді жоқтай алмаймыз және олар сол адамдарды қорғайды,менің ойымша тексеру уақыт кел бұл саяси банктік шот оларға шетелдік есеб пайда әкел мүмкін біз қылмыстық элемент жоқта алма және олар сол адам қорға,nazism

3,бұл оларға қылмыс жасауға одан да көп себеп береді тіпті а дерекқоры оларды танымайды біз күнделікті шетелдіктердің қолында өлетін өмірге кім жауапты деп

санаймыз біз ақ езгіге қарсы күресуіміз керек енді өз қауіпсіздігіміз бен адам құқықтарымыз үшін күресуіміз керек пе,бұл оларға қылмыс жаса одан да көп себеп бер тіпті а дереккөр ол таныма біз күнделікті шетелдік қол өл өмір кім жауапты де сана біз ақ езгі қарсы күрес керек енді өз қауіпсіздіг бен адам құқық үшін күрес керек пе,nazism

3,қылмысқа келетін болсақ ұлты жоқ шетелдіктер өз елдерінде қылмыс жасасын,қылмыс кел бол ұл жоқ шетелдік өз ел қылмыс жаса,nazism

3,менің ойымша біз бұл тәртіпсіздікті өзіміз шешетін уақыт келді жыл әлі алыс балалар мен сізден өтінемін,менің ойымша біз бұл тәртіпсіздік өз шеш уақыт кел жыл әлі алыс бала мен сізден өт,nazism

3,біз оның халқымызды құрметтемейтінін көріп заңсыз иммигранттар мен бел оңтүстік африкадағы қара ұлттың көпшілігі бірінші орынға қоюға мәжбүрміз біз адамдар тұрып осы тәртіпсіздікті өзіміз шешуіміз керек,біз оның халқ құрметтеме көр заңсыз иммигрант мен бел оңтүстік африка қара ұл көпшіліг бірінші орын қою мәжбүр біз адам тұр осы тәртіпсіздік өз шеш керек,nazism

3,оңтүстік африкада құжатсыз шетелдіктер сізді өлтірген кезде оңтүстік африкалық болу сияқты барлық кінә әлсіз және пайдасыз қха үкіметіне жүктеледі,оңтүстік африка құжат шетелдік сіз өлтір кезде оңтүстік африкалық болу сияқты барлық кінә әлсіз және пай қх үкімет жүктел,nazism

3,сіз біздің елде өзіңізді мәжбүрлеуден ұялуыңыз керек винни мандела өз елін жақсы көрді ол ешқашан заңсыз иммигранттар келіп халқымызды есірткімен тамақтандырып ұрлап өлтіруі керек деп айтқан емес сіз заңсыз иммиграцияны ақтауға ұялуыңыз керек хайбо жай үйге кел,сіз біздің ел өз мәжбүрлеу ұял керек винни мандел өз ел жақсы көр ол ешқашан заңсыз иммигрант кел халқ есірткі тамақтандыр ұрла өлтір керек де айт емес сіз заңсыз иммиграция ақта ұял керек хайбо жай үй кел,nazism

3,және сіз заңсыз және заңды иммигранттарды тексеру үшін роботтардан қиылыстардан және сауда кешендерінен бастай аласыз ба,және сіз заңсыз және заңды иммигрант тексеру үшін робот қиылыс және сауда кешен баста ал ба,nazism

3,иә заңды бұзбайтыныңызды дәлелдеңіз азаматтар кейде заңсыз шетелдіктермен қарым қатынас жасау үшін сіздің жеке куәліктеріңізді алып жүреді,иә заңды бұзба дәлелде азамат кейде заңсыз шетелдік қарым қатынас жасау үшін сіздің жеке куәлік алып жүр,nazism

3,біздің құқық қорғау органдарының қаншалықты шатасқанын көріп тұрсыз ба неліктен ші күні бізде дудула операциясы бар екенін түсінесіз бе үкімет бізді сәтсіздікке ұшыратты балалар есірткіге тәуелді болды мен есімде соуэтода өстім егер сізге есірткі қажет болса шақырым жүруге тура келеді енді олар өте жабық дүкендер бірнеше spaza контейнерлерін сатып алады,біздің құқық қорғау орган қаншалықты шатас көр тұр ба неліктен ші күн біз дудул операция бар екен түс бе үкімет біз сәтсіздік

ұшыра бала есірткі тәуелді бол мен ес соуэто өс егер сізге есірткі қажет бол шақырым жүр тура кел енді олар өте жабық дүкен бірнеше spaza контейнер сат ал,nazism

3,менің жеке куәлігім ұрланбағандықтан мен өзімді ғана кінәлай аламын мен оған бірдеңе жасауға дайын болдым ол оны басқа нәрсе үшін пайдаланды барлық шетелдіктер бірдей олар аман қалу үшін бәрін жасайды бұл лайықты болды мен оны ешқашан армандаған емеспін ешқашан жаса,менің жеке куәліг ұрланба мен өз ғана кінәла ал мен оған бірде жаса дайын бол ол оны басқа нәрсе үшін пайдала барлық шетелдік бірде олар аман қалу үшін бәрін жаса бұл лайықты бол мен оны ешқашан арманда емес ешқашан жаса,nazism

3,малема бұл заңсыз иммигранттарды қорғайды eff те бұдан былай саяси стратегия жоқ,мале бұл заңсыз иммигрант қорға eff те бұдан былай саяси стратегия жоқ,nazism

3,біріншіден бұл жерде ешқандай құжат жоқ сондықтан мен мұнда заңсыз біреу туралы дауласқым келмейді оның жаны тыныштықта болсын сол аптада біз жазықсыз адамнан айырылдық және олар өлімге лайық деп ойлайсыз және бір заңсыз иммигрант тірі болуы керек,бірінші бұл же ешқандай құжат жоқ сондықтан мен мұнда заңсыз біреу туралы даулас келме оның жан тыныштық бол сол апта біз жазық адам айыр және олар өлім лайық де ойла және бір заңсыз иммигрант тірі бол керек,nazism

3,заңсыз иммигранттардың жиіркенішті қылмыстарға қатысы бар екендігі туралы күнделікті хабарламалар пайда болған кезде сіз де сол энергияны сақтағаныңызды қалаймын бірақ жоқ оңтүстік африкада заңсыз иммигранттардың өмірі ғана маңызды басқа ешкім жоқ,заңсыз иммигрант жиіркеніш қылмыс қатыс бар екендіг туралы күнделікті хабарлама пайда бол кезде сіз де сол энергия сақта қала бірақ жоқ оңтүстік африка заңсыз иммигрант өмір ғана маңызды басқа ешкім жоқ,nazism

3,шешілді eff sm және митингілерде танымал өйткені оны сайлауда дауыс беруге құқығы жоқ шетелдіктер қолдайды бұл нашар сайлау нәтижелеріне әкеледі,шеш eff sm және митингі танымал өйткені оны сайла дауыс бер құқығ жоқ шетелдік қолда бұл нашар сайлау нәтиже әкел,nazism

3,мен үшін бұл мағынасы жоқ өзін өзі бағалау дұрыс емес және бұл тек шетелдіктерде ғана емес қылмыс қылмыс ксенофобия жоқ шетелдіктер бізді күнделікті орталық іскери ауданда тонайды,мен үшін бұл мағына жоқ өз өзі бағалау дұрыс емес және бұл тек шетелдік ғана емес қылмыс қылмыс ксенофобия жоқ шетелдік біз күнделікті орталық іскери аудан тона,nazism

3,жоғары оқу орнында оқып жүргенде мен жаяу жүрдім және басқа да көптеген мейрамханалар мектеп демалысы кезінде жұмыс туралы сұрады және бұл жұмыс орындары студенттерге арнайы қол жетімді болды бірақ соңғы уақытта бұл жұмыс орындарының барлығын шетелдіктер алып жатыр,жоғары оқу орн оқ жүр мен жаяу жүр және басқа да көптеген мейрамхана мектеп демалыс кез жұмыс туралы сұр және бұл жұмыс орын студент арнайы қол жет бол бірақ соңғы уақыт бұл жұмыс орын барлығ шетелдік алып жатыр,nazism

3,шетелдік азаматтар бейбіт тұрғындарды өлтіруі зорлауы және полицияны өлтіруі мүмкін қоғам бұған қарсы шыққанда үкімет ксенофобия деп жылайды шетелдік азаматтарды қылмыс жасағаны үшін өздеріне қарсы шеру ұйымдастырған күні мен байыппен қабылдаймын,шетелдік азамат бейбіт тұрғын өлтір зорла және полиция өлтір мүмкін қоғам бұған қарсы шық үкімет ксенофобия де жыла шетелдік азамат қылмыс жаса үшін өз қарсы шеру ұйымдастыр күн мен байып қабылда,nazism

3,мен бұқаралық ақпарат құралдарының дипслутта өлтірілген оңтүстік африкалықтар туралы өлтірілген хайпты көтергендей шу шығарғанын қалаймын дудула дипслут операциясы,мен бұқаралық ақпарат құрал дипслут өлтір оңтүстік африкалық туралы өлтір хай көтергенде шу шығар қала дудул дипслут операция,nazism

3,бірінші фотосурет мозамбиктен келген иммигрант эммануэль ситоле жылдың сәуірінде аяусыз өлтірілді оның өлтірілуіне ксенофобияға негізделген шабуылдар түрткі болды екінші фотосурет зимбабведен келген иммигрант элвис няти кеше жылдың сәуірінде тірідей өртенді оның өлтірілуіне ксенофобияға негізделген шабуылдар түрткі болды,бірінші фотосурет мозамбик кел иммигрант эммануэль ситол жыл сәуір аяу өлтір оның өлтіріл ксенофобия негіздел шабуыл түрткі бол екінші фотосурет зимбабве кел иммигрант элвис няти кеше жыл сәуір тірідей өрте оның өлтіріл ксенофобия негіздел шабуыл түрткі бол,nazism

3,оңтүстік кореяда не болып жатыр неліктен шетелдіктер соншалықты қорғалған ештеңені ақтамай бірақ неге элвис нятиді өлтіргені үшін соттау бар бірақ оңтүстік африкалықтарды шетелдіктер өлтірген жағдай ешқашан болған емес не болып жатыр,оңтүстік корея не бол жатыр неліктен шетелдік соншалықты қорғал ештеңе ақтама бірақ неге элвис няти өлтір үшін сотта бар бірақ оңтүстік африкалық шетелдік өлтір жағдай ешқашан бол емес не бол жатыр,nazism

3,өлі зимбабве трендте біз шетелдіктермен күресте жеңілдік,өлі зимбабв тренд біз шетелдік күрес жеңілдік,nazism

3,ескі пайдасыз ақымақтар олар шетелдіктер несие алған кезде ғана сөйлей алады бірақ оңтүстік африкалықтарды өлтірген кезде сөйлей алмайды,ескі пай ақымақ олар шетелдік несие ал кезде ғана сөйле ал бірақ оңтүстік африкалық өлтір кезде сөйле алма,nazism

3,өлтірілген заңсыз иммигранттардың тағы қанша отбасы eff ке олардың өміріне қауіп төндіретін нәрсеге жауапты екенін түсінбестен бұрын барады,өлтір заңсыз иммигрант тағы қанша отбасы eff ке олардың өмір қауіп төндір нәрсе жауапты екен түсінбес бұрын бар,nazism

3,заңсыз иммигранттар бұл сіздің таңдауыңыз өз елдеріңізге оралу немесе сіз мұнда заңсыз болып қала бересіз және қарулы күзеті бар джулиус малеманың кесірінен өз өміріңізді қатерге тігесіз егер ол ақылды болса ол сіздің қорғалғаныңызға көз жеткізу үшін жоспар құрар еді тек үйге оралыңыз,заңсыз иммигрант бұл сіздің таңда өз ел орал немесе сіз мұнда заңсыз бол қала бер және қарулы күзет бар джулиус малема

кесір өз өмір қат тіг егер ол ақылды бол ол сіздің қорғал көз жеткізу үшін жоспар құр ед тек үй орал,nazism

3,дипслуттағы адамдар бүгін кешке де жұмысын жалғастыруы керек полиция оларға көмектеспейді дудула операциясы ешқашан аяқталмауы керек,дипслут адам бүгін кеш де жұмыс жалғастыр керек полиция оларға көмектеспе дудул операция ешқашан аяқталма керек,nazism

3,бұл адам ақымақ,бұл адам ақы,nazism

3,бүгін доктор аарон мотсоаледи африкадағы алғашқы операцияны жасайды дудула біз сізді жақсы көреміз және біз өз елімізді де жақсы көреміз,бүгін доктор аарон мотсоаледи африка алғашқы операция жаса дудул біз сіз жақсы көр және біз өз ел де жақсы көр,nazism

3,шетелдік иммигранттар мұндай құжаттарды сол жерде жұмыс істейтін біреудің қатысуынсыз рәсімдей алмайды,шетелдік иммигрант мұндай құжат сол же жұмыс істе біреу қатысуынсыз рәсімде алма,nazism

3,шетелдіктер оңтүстік африкалықтардың көмегінсіз айыпталған нәрсені жасай алмайды негізгі себепті шешіңіз сонда сіздің проблемаңыз жойылады,шетелдік оңтүстік африкалық көмегін айыптал нәрсе жаса алма негізгі себеп шеш сонда сіздің проблема жой,nazism

3,келесі жолы мен жанармай құю станциясынан өтіп зимбо акцентімен амандасқанда мен оларға қызмет көрсетусіз кетемін,келесі жол мен жанармай құю станция өт зимбо акцент амандас мен оларға қызмет көрсету кет,nazism

3,адамдар мұнда ештеңені шатастырмауы керек operationdudula бұл патриоттық sa ол оңтүстік африканы саяси ұйым емес бірінші еткісі келеді ал егер олар сәтсіздікке ұшыраса билеттер жерінде салынған болса оны бейтарап қорлаудың орнына аталған ұйыммен талқылаңыз,адам мұнда ештеңе шатастырма керек operationdudula бұл патриоттық sa ол оңтүстік африка саяси ұйым емес бірінші ет кел ал егер олар сәтсіздік ұшыра билет же салын бол оны бейтарап қорла орн атал ұйы талқыла,nazism

3,лол министрлер қылмыс туралы сөйлескенде шетелдіктер күн сайын ксенофобия туралы айқайлайды,лол министр қылмыс туралы сөйлес шетелдік күн сайын ксенофобия туралы айқайла,nazism

3,sans ксенофобтар емес қха мен зану пф есектерін жабу үшін геноцид ұйымдастырғысы келеді санс та шетелдіктер де оңтүстік кореядағы шектеулі ресурстармен өмір сүруге тырысады даналық пен түсіністік сізге ақыл ойды көрсетеді бұл тәртіпсіздік anc және zanu pf үшін тиімді,sans ксенофоб емес қх мен зан пф есек жаб үшін геноцид ұйымдастыр кел санс та шетелдік де оңтүстік корея шектеулі ресурс өмір сүр тырыс даналық пен түсіністік сізге ақыл ой көрсет бұл тәртіпсіздік anc және zanu pf үшін тиімді,nazism

3,оңтүстік африкада құжатсыз иммигранттарға қол сұғылмайды тіпті біздің үкімет оларды қорғайды егер оңтүстік африкалықтар оларды бизнестен шеттетсе немесе саясаткерлермен бірге қала маңында тұру үшін қасында қалса онда бізге ксенофобтар белгісі қойылады,оңтүстік африка құжат иммигрант қол сұғылма тіпті біздің үкімет ол қорға егер оңтүстік африкалық ол бизнес шеттет немесе саясаткер бірге қала маң тұру үшін қас қал онда бізге ксенофоб бел қой,nazism

3,kznfloods мен бұл су тасқыны иммигранттарға қарсы науқаннан туындады деген кейбір пікірлерді көремін мен жалпы әлсіздер мен кедейлерге арналған күнәкарларды табу науқанына қарсымын бірақ біз бұл су тасқынының неліктен болып жатқанын білмейміз және бұлай айту дұрыс емес,kznfloods мен бұл су тасқын иммигрант қарсы науқан туында де кейбір пікір көр мен жалпы әл мен кедей арнал күнәкар табу науқан қар бірақ біз бұл су тасқын неліктен бол жат білме және бұлай айту дұрыс емес,nazism

3,шетелдіктерді бәріне кінәлайтын адамдарды үгіттемеңіз алла тағала бұл апаттардың орын алуына неге жол беретінін білмейміз олардың басқа да көптеген себептері болуы мүмкін адамдарды тәрбиелеп оларды түсінуге тырысыңыз,шетелдік бәрін кінәла адам үгіттеме алл тағал бұл апат орын ал неге жол бер білме олардың басқа да көптеген себеп бол мүмкін адам тәрбиеле ол түсін тырыс,nazism

3,әйел зорлаушы және өлтіруші ол дудула операциясы деп аталатын ксенофобиялық қозғалысты басқарады ол қара адамдарды ұлтшылдық көзқарастары үшін қудалайды және аяусыз өлтіреді,әйел зорлаушы және өлтіруш ол дудул операция де атал ксенофобиялық қозғал басқар ол қара адам ұлтш көзқарас үшін қудала және аяу өлтір,nazism

3,нхланхла люкс жүрексіз қылмыскер,нхланхл люкс жүрек қылмыскер,nazism

3,зимбабвеліктердің оңтүстік африкада жері жоқ жер ақ адамдардың қолында,зимбабвелік оңтүстік африка же жоқ жер ақ адам қол,nazism

3,қытайлықтар бұл қылмыстың бенефициарлары болып табылады егер бізде мыс кеніші болмаса мыс оңтүстік кореядан қалай кетеді бұл кабель ұрлаушылардың металл сынықтарын сатушылардың және дайын өнімді сатып алушылардың сатқындығы жылдам түзету барлық заңсыз иммигранттарды жаппай депортациялау,қытайлық бұл қылмыс бенефициар бол таб егер біз мыс кеніш болма мыс оңтүстік корея қалай кет бұл кабель ұрлаушы металл сынық сатушы және дайын өн сат алушы сатқындығ жылдам түзету барлық заңсыз иммигрант жаппай депортацияла,nazism

3,біз қазір ұйықтап жатырмыз оңтүстік африкалықтар самосты қолданғанға дейін айналамызда заңсыз иммигранттар болған жоқ,біз қазір ұйықта жатыр оңтүстік африкалық само қолдан дейін айнала заңсыз иммигрант бол жоқ,nazism

3,дудула опғрациясы бұл барлық ингредиенттерді қолданатын топтар ұлтшылдық идентификаторлық және популистік демагогия мұнда сіз оңтүстік африкадағы ең әлсіз

сілтемені анықтап жалын үрлейсіз үлкен адамдардың саясаты жалған ақпарат қорқыныш зорлық зомбылық және қорқыту зұлым,дудул опғрация бұл барлық ингредиент қолдан топ ұлтш идентификаторлық және попули демагогия мұнда сіз оңтүстік африка ең әлсіз сілтеме анықта жалын үрле үлкен адам саясат жалған ақпарат қорқыныш зорлық зомбылық және қорқыт зұлым,nazism

3,егер біз оларды сол күйінде әшкерелесек және олар қылмыстық әрекеттермен айналысып жатқанда олар бізді шетелдіктер деп атайды,егер біз ол сол күй әшкереле және олар қылмыстық әрекет айналыс жатқанда олар біз шетелдік де ата,nazism

3,мен мақтан тұтатын оңтүстік африкалық квериквере нхланхла люкс мені өлтіре алмайды мен дудула позитив операциясынан қорықпаймын,мен мақтан тұт оңтүстік африкалық кверикер нхланхл люкс мен өлтір алма мен дудул позитив операция қорықпа,nazism

3,президент джулиус малеманы ксенофобиялық қозғалыстарды басқаратын және адамдардың үйлеріне кіріп әйелдерге шабуыл жасап олардан алып кететін қылмыскерлермен салыстыруды доғарыңыз нхланхла люкс өлтіруші тшегофатсо пуледен еш айырмашылығы жоқ,президент джулиус малема ксенофобиялық қозғалыс басқар және адам үй кір әйел шабуыл жаса олардан алып кет қылмыскер салыстыру доғар нхланхл люкс өлтіруш тшегофатсо пуле еш айырмашылығ жоқ,nazism

3,заңды иммигрант құжаттарымен қамауға алынды,заңды иммигрант құжат қама ал,nazism

3,нхланхла люкс туралы не деуге болады ол кедей әйелді анасының үйінде жалға алушы екенін пайдаланып нхланхла люкс және цшегофатсо пулені өлтірген,нхланхл люкс туралы не де бол ол кедей әйел ана үй жалға алушы екен пайдалан нхланхл люкс және цшегофатсо пуле өлтір,nazism

3,иммиграция проблемалары сіздің жеке адамдарыңызды және олардың ақшаға деген сүйіспеншілігін тудырады бірақ шетелдіктерді ғана кінәлайық,иммиграция проблема сіздің жеке адам және олардың ақша де сүйіспеншіліг тудыр бірақ шетелдік ғана кінәл,nazism

3,бұл жерде алғашқы дудула операциясы жасалады,бұл же алғашқы дудул операция жасал,nazism

3,олар аталған шетелдіктерді цванада сөйлемейтін немесе зулуда еркін сөйлейтін кез келген басқа оңтүстік африкалықтарды іздейді,олар атал шетелдік цвана сөйлеме немесе зул еркін сөйле кез кел басқа оңтүстік африкалық ізде,nazism

3,министр аарон мотсвеледимен сөйлесу мүмкіндігі болғаннан бері өзіңізді қалай сезінесіз қазіргідей емес деп үміттенемін,министр аарон мотсвеледи сөйлесу мүмкіндіг бол бері өз қалай сез қазіргідей емес де үміттен,nazism

3,ал олар бәрін аяқтағаннан кейін азаматтық соғыс басталады егер сіз зулу тілінде сөйлей алмасаңыз сізде қиындықтар болады көп ұзамай лимпопо педис зулу үшін болады егер сіз бұл тілде сөйлей алмасаңыз дудула операциясы сол адамдардан басталды оларды шетелдіктер деп атайды,ал олар бәрін аяқта кейін азаматтық соғыс бастал егер сіз зул тіл сөйле алма сіз қиындық бол көп ұзама лимпопо педис зул үшін бол егер сіз бұл тіл сөйле алма дудул операция сол адам баста ол шетелдік де ата,nazism

3,мен оны жаңалықтардан мүлдем көрмедім бұл жерде шынымен не болды кім кімді алдайды сіз түсіндіруге тырысасыз ба мүмкін сіз олар болуы керек екенін ұмытып кеткен шығарсыз,мен оны жаңалық мүлдем көрме бұл же шын не бол кім кім алда сіз түсіндір тырыс ба мүмкін сіз олар бол керек екен ұмыт кет шығ,nazism

3,бұл полиция қызметкерін бос сөз үшін жұмыстан шығару керек тәжірибелі полицей лунатик дудула операциясында өзін қалай ұстайды,бұл полиция қызметк бос сөз үшін жұмыс шығару керек тәжірибелі полицей лунатик дудул операция өз қалай ұста,nazism

3,сіздің сұрағыңыз орынсыз екіншіден дипслуттағы адамдардан сұраңыз мүмкін олар сізге жауап таба алады,сіздің сұра орынсыз екінші дипслут адам сұра мүмкін олар сізге жауап таб ал,nazism

3,адам есірткінің артық дозалануынан қайтыс болды барлық өлімнің есірткінің артық дозалануынан болады өлім деңгейі әр тұрғынға өлімді құрайды ал заңсыз келімсектерді өлтірушілерді қолдау арқылы өмірді сақтайды,адам есірткі артық дозалану қайтыс бол барлық өл есірткі артық дозалану бол өлім деңгей әр тұрғын өл құра ал заңсыз келімсек өлтіруші қолдау арқылы өмір сақта,nazism

3,қауымдастық мүшесі бізді шетелдіктердің кесірінен көшуге тура келгендей қауіпсіз сезінбейтінімізді айтады,қауымдастық мүше біз шетелдік кесір көш тура келгенде қауіпсіз сезінбе айт,nazism

3,дудула операциясынан кейін қандай ауыр өлім,дудул операция кейін қандай ауыр өлім,nazism

3,сіз шетелдік өлтірушілерге зорлаушыларға қолма қол ақша тасымалдаушыларға және бұзақыларға қарсы екеніңізді айтқыңыз келе ме бұл құжаттары бар жазықсыз шетелдіктерге қарсы мақсат деп ойламаңыз бұл жерде мәңгілікке қалдырмаңыз бұл адам сайланған кезде мәселені шешуге уәде берді бірақ содан бері үнсіз қалды,сіз шетелдік өлтіруші зорлаушы қол қол ақша тасымалдаушы және бұзақы қарсы екен айт келе ме бұл құжат бар жазық шетелдік қарсы мақсат де ойлама бұл же мәңгілік қалдырма бұл адам сайлан кезде мәселе шеш уәде бер бірақ содан бері үн қал,nazism

3,келер айда шетелдіктерге тыныштық болмайды,кел айда шетелдік тыныштық болма,nazism

3,ол қазір болып жатыр бұл орын алады,ол қазір бол жатыр бұл орын ал,nazism

3,сіз азаматтарды дудула операциясын талқылауға қатыстырдыңыз,сіз азамат дудул операция талқыла қатыс,nazism

3,мен келісімшарттан кетуге мәжбүр болдым өйткені мен биыл жасқа толдым қазір жұмыссызбын және бастапқы нүктеге ораламын американдықтарды бірінші теріс жағдайға қояды,мен келісімшарт кет мәжбүр бол өйткені мен биыл жас тол қазір жұмыссыз және бастапқы нүкте орал американдық бірінші теріс жағдай қоя,nazism

3,құрметті президент мырза міне бәрімізді құтқара алатын жақсы ұсыныс біз бәріміз адамбыз және бірінші қабат сіздің кеңсеңіздің араласуы мен делдалдығын қажет етеді заңсыз иммигранттар әлі де бақылаудан шығатын нәзік мәселе болып қала береді өйткені қоғамдастық көмекке мұқтаж,құрметті президент мырза міне бәрі құтқар ал жақсы ұсыныс біз бәрі адам және бірінші қабат сіздің кеңсе аралас мен делдалдығ қажет ет заңсыз иммигрант әлі де бақылау шығ нәзік мәселе бол қала бер өйткені қоғамдастық көмек мұқтаж,nazism

3,қолдау көрсететін уақыт келді бізде тәжірибе мен біліктілік бар және олар не қалайды бұл олардың жалақыны қысқарту жас және жалақысы төмен біреуді табу жұмыс іздеушілерге бағытталған,қолдау көрсет уақыт кел біз тәжірибе мен біліктілік бар және олар не қала бұл олардың жалақы қысқарту жас және жалақы төмен біреу табу жұмыс іздеуші бағыттал,nazism

3,біз жұмыссыздар қозғалысымыз біз науқанымызды қаржыландыру үшін қолымызда бар нәрсені пайдаланамыз веб сайтқа ие болу үшін кез келген соманы беріңіз,біз жұмыс қозғалыс біз науқан қаржыландыру үшін қол бар нәрсе пайдалан веб сайт ие болу үшін кез кел сома бер,nazism

3,бұл орын алатын уақыт біз өзіміз қалағанымыздан ертерек жұмыстан шығарылғандықтан ерте қартаюға мәжбүрміз немесе жастан кейін жұмыс жоқ өйткені жастағы ер адам жұмыссыз қалуға мәжбүр болуы мүмкін,бұл орын ал уақыт біз өз қала ертерек жұмыс шығарыл ерте қартаю мәжбүр немесе жас кейін жұмыс жоқ өйткені жастағы ер адам жұмыссыз қал мәжбүр бол мүмкін,nazism

3,бұл жалпылау қара адамдар бүкіл оңтүстік америкада кейбір жағдайларда күрделі стоквельдерді басқарады олардың жиынтық құны жүздеген миллиардқа бағаланады тіпті банктер де осы бейресми әрекеттерге қатысқысы келеді,бұл жалпыла қара адам бүкіл оңтүстік америка кейбір жағдай күрделі стоквель басқар олардың жиынтық құн жүздеген миллиард бағалан тіпті банк де осы бейресми әрекет қатыс кел,nazism

3,тіпті киелі кітапта шетелдіктермен проблемалар болды,тіпті киелі кітап шетелдік проблема бол,nazism

3,кейбір адамдар ақымақ бұл мені қарғысқа ұшыратады,кейбір адам ақы бұл мен қарғыс ұшырат,nazism

3,вестридж бұл біздің ел үшін ұят салық төлеушілердің ақшасын ысырап ету біз оңтүстік африканы басқара алмаймыз,вестридж бұл біздің ел үшін ұят салық төлеуші ақша ысырап ету біз оңтүстік африка басқар алма,nazism

3,заңды өз мойнына алып басқа адамды өртеген адамдар түрмеде шіріп кетуі керек олар қылмыскерлер олардың барлығы табылғанша оның рухы тынышталмасын ешқандай кісі өлтіру ақш та кездесетін мәселелерді шеше алмайды біз әлі де заңсыз иммигранттармен күресіп жатырмыз бірақ кісі өлтіру емес,заңды өз мойн алып басқа адам өрте адам түрме шір кет керек олар қылмыскер олардың барлығ табылғанш оның рух тынышталма ешқандай кісі өлтір ақш та кездес мәселе шеш алма біз әлі де заңсыз иммигрант күрес жатыр бірақ кісі өлтір емес,nazism

3,дудула операциясына қатысушылар азаматтарды ксенофобия аясында бүкіл елді көрсететін әрекеттерге шақыруды тоқтатуы керек полицияға өз жұмысын жалғыз жасауға рұқсат етіңіз адамдар өмірде жақсы жұмыс істеуі үшін рухани көмекке мұқтаж,дудул операция қатысушы азамат ксенофобия ая бүкіл ел көрсет әрекет шақыру тоқтат керек полиция өз жұмыс жалғыз жаса рұқсат ет адам өмір жақсы жұмыс істе үшін рухани көмек мұқтаж,nazism

3,сіз заңсыз шетелдіктерді жақсы көресіз бе зимбабвеге көшеді және ол сонда бақытты,сіз заңсыз шетелдік жақсы көр бе зимбабве көш және ол сонда бақытты,nazism

3,оңтүстік африкадағы заңсыз иммигранттарға өкінетіндер неге олардың барлығы зимбабвеге сол жерде тұруға рұқсат алмайды,оңтүстік африка заңсыз иммигрант өкін неге олардың барлығ зимбабве сол же тұр рұқсат алма,nazism

3,егер олар әділ болса онда the weeknd те болған кісі өлтіру неге осы жағдайды тудырды заңсыз иммигранттың өмірі өмірден асады,егер олар әділ бол онда the weeknd те бол кісі өлтір неге осы жағдай ту заңсыз иммигрант өмір өмір ас,nazism

3,бұл элвис нятиде бір қызық нәрсе бар заңсыз шетелдік оны басқа зимбабвеліктер өлтірген болуы мүмкін шын мәнінде бұл оқиғаны журналистер заңсыз иммигранттар халықтың мейіріміне бөленуі үшін баяндау барысын өзгерту үшін құрастырған,бұл элвис няти бір қызық нәрсе бар заңсыз шетелдік оны басқа зимбабвелік өлтір бол мүмкін шын мән бұл оқи журналист заңсыз иммигрант халық мейірім бөлен үшін баянда барыс өзгерту үшін құрастыр,nazism

3,афро немесе ксенофобия жаңа нәрсе емес бұл жай ғана дамып келеді жылы оңтүстік африка көші қон жобасының зерттеуі оңтүстік африкада кемпірқосақ ұлтының сол кездегі дискурсына қарамастан иммиграцияға қарсы тұрудың ең жоғары деңгейі бар екенін көрсетті дудула операциясы біздің ұлттық паранойяға негізделген,афро немесе ксенофобия жаңа нәрсе емес бұл жай ғана дам кел жыл оңтүстік африка көші қон жоба зертте оңтүстік африка кемпірқо ұлт сол кез дискур қарамастан иммиграция қарсы тұр ең жоғары деңгей бар екен көрсе дудул операция біздің ұлттық паранойя негіздел,nazism

3,жалғыз айырмашылық бізді иммигранттар деп санайды,жалғыз айырмашылық біз иммигрант де сана,nazism

3,бұл біздің елге құжатсыз заңсыз иммигранттардың келуін тежеудің жалғыз жолы неліктен бұл қазір болып жатқанын бәріміз білеміз мұның апартеидке ешқандай қатысы жоқ теріңіздің түсіне қарамастан сізді тоқтатып құжаттарды ұсынуды сұрайды біз шаршадық,бұл біздің ел құжат заңсыз иммигрант кел тежеу жалғыз жол неліктен бұл қазір бол жат бәрі біл мұның апартеид ешқандай қатыс жоқ тер түс қарамастан сіз тоқтат құжат ұсыну сұра біз шарша,nazism

3,олар жағдай бақылаудан шыққанға дейін тез әрекет етуі керек өйткені олар жақын арада жасайды құжатсыз көптеген заңсыз шетелдіктердің болуы мүлдем қолайсыз содан кейін біз неге бұл орын алады неге ештеңе бейтарап жасалмайды деген сұрақ қоямыз,олар жағдай бақылау шық дейін тез әрекет ет керек өйткені олар жақын ара жаса құжат көптеген заңсыз шетелдік бол мүлдем қолайсыз содан кейін біз неге бұл орын ал неге ештеңе бейтарап жасалма де сұрақ қоя,nazism

3,сіз бізді ксенофобтар деп атайсыз олар тіркеліп төлқұжат алып біздің елге жеткіліксіз дағдылармен немесе туристер ретінде немесе инвесторлар ретінде кіруі керек сонымен қатар олар біздің елге кірмеуі керек бізде жұмыссыздықтың жоғары деңгейі және қызметтердің болмауы сияқты өз проблемаларымыз бар,сіз біз ксенофоб де ата олар тіркел төлқұжат алып біздің ел жеткіліксіз дағды немесе турист рет немесе инвестор рет кір керек сонымен қатар олар біздің ел кірме керек біз жұмыссыздық жоғары деңгей және қызмет болма сияқты өз проблема бар,nazism

3,неліктен бұл заңсыз иммигранттар көрші ботсванаға бармайды бұл ботсванада заң мен тәртіп болғандықтан ғана оңтүстік африка құжатсыз заңсыз иммигранттар үшін ойын алаңы өйткені пара алатын сыбайлас жемқорлыққа толы заң жоқ,неліктен бұл заңсыз иммигрант көрші ботсвана барма бұл ботсвана заң мен тәртіп бол ғана оңтүстік африка құжат заңсыз иммигрант үшін ойын алаң өйткені пара ал сыбайлас жемқорлық толы заң жоқ,nazism

3,олар біздің онсыз да толып жатқан денсаулық сақтау саламызды ауырлатады олар қылмыс жасауға ықпал етеді біз негізінен олар осы заңсыз иммигранттар жасаған ұрлықтардың көбеюін көріп отырмыз олар жалдамалы өлтірушілер ретінде пайдаланылады өйткені оларды қадағалау мүмкін емес біз заң мен тәртіпті қалаймыз,олар біздің онсыз да тол жат денсаулық сақтау сала ауырлат олар қылмыс жаса ықпал ет біз негіз олар осы заңсыз иммигрант жаса ұрлық көбею көр отыр олар жалдамалы өлтіруші рет пайдалан өйткені ол қадағалау мүмкін емес біз заң мен тәртіп қала,nazism

3,біздің үкімет белсенді емес ол не болып жатқанына жауап береді дудула операциясы alexa ны қабылдады біздің полиция шетелдіктерді соның ішінде заңсыз қытайлықтар мен үндістерді тұтқындай бастады,біздің үкімет белсенді емес ол не бол жат жауап бер дудул операция alexa ны қабылда біздің полиция шетелдік соның іш заңсыз қытайлық мен үндіс тұтқында баста,nazism

3,оңтүстік африка шетелдіктерге тиесілі бола алмайды,оңтүстік африка шетелдік тиесілі бола алма,nazism

3,шетелдіктерде мұнда келуден ешкім бас тартпайды тиісті құжаттармен келіңіз және біздің заңдарымызды орындаңыз,шетелдік мұнда келу ешкім бас тартпа тиісті құжат кел және біздің заң орында,nazism

3,маған ұнайтыны заңсыз шетелдіктер оңтүстік кореяда дұрыс емес әрекет жасап жатқанын дәлелдеуді жалғастыруда саясаткерлер қандай да бір принцип бойынша бізбен бір жерде келіспейтіні маңызды емес,маған ұна заңсыз шетелдік оңтүстік корея дұрыс емес әрекет жаса жат дәлелдеу жалғастыр саясаткер қандай да бір принцип бойынша бізбен бір же келіспе маңызды емес,nazism

3,saps және ішкі істер министрлігі өз жұмыстарын орындау кезінде саясаткерлерді ренжітеді,saps және ішкі істер министрліг өз жұмыс орындау кез саясаткер ренжіт,nazism

3,янди оған білім беруін сұрайды ал жауаптардың ешқайсысы мұның орнына жасамайды мен мұны заңсыз шетелдік жақтастардан байқадым ешқандай факт жоқ жәбірленушінің менталитеті дөрекілік мен сұрақ қоюда ештеңе көрмеймін,янди оған білім бер сұра ал жауап ешқайсы мұның орн жасама мен мұны заңсыз шетелдік жақтас байқа ешқандай факт жоқ жәбірленуші менталитет дөрекілік мен сұрақ қою ештеңе көрме,nazism

3,барлық басқа мемлекеттік шенеуніктер басшылықты қабылдап осы нхланхла баласының қоғамды үрейлендіретін әрекеттеріне қарсы тұрғыңыз келгенде неліктен осы қауымдастықтарда жұмыс істеуге рұқсат берген sap барлық ксенофобия қоздырғыштарын тұтқындауы керек,барлық басқа мемлекеттік шенеунік басшылық қабылда осы нхланхл бала қоғам үрейлендір әрекет қарсы тұр кел неліктен осы қауымдастық жұмыс істе рұқсат бер sap барлық ксенофобия қоздырғыш тұтқында керек,nazism

3,тревор президент емес бұл нонсенс деген не сіз рамафостан неге көптеген заңсыз шетелдіктерді елге кіргізіп жатқаныңызды сұрауыңыз керек,трево президент емес бұл нонсенс де не сіз рамафос неге көптеген заңсыз шетелдік ел кіргіз жат сұра керек,nazism

3,міне қара нәсілділерде немесе кедей адамдарда жиі қолданылатын эвфемизм ресурстар жоқ деп ойлайтын ақымақтар үшін пайдалы кеңес стоквелдің бейресми және негізінен қалалық жинақ нарығының көлемін қараңыз дудула операциясы afriforum сияқты ұйымдастырылған және ресми түрде жасалған болса жақсы қаржыландырылатын еді,міне қара нәсілді немесе кедей адам жиі қолданыл эвфемиз ресурс жоқ де ойла ақымақ үшін пайдалы кеңес стокве бейресми және негіз қалалық жинақ нарығ көл қара дудул операция afriforum сияқты ұйымдастырыл және ресми түр жасал бол жақсы қаржыландырыл ед,nazism

3,иə қара адамдарда afriforum сияқты ресурстар жоқ шындығында оларда көп әлдеқайда көп сондықтан мен сіздің қара құрбандар аяушылық кеші туралы әңгімеңізбен келіспеймін,иə қара адам afriforum сияқты ресурс жоқ шындығ ол көп әлдеқайда көп сондықтан мен сіздің қара құрбан аяушылық кеш туралы әңгіме келіспе,nazism

3,заңсыз иммигранттар мəселесі бойынша көптеген қарама қайшы пікірлер бар өкінішке орай кейбіреулерді басу керек біз демократиялық елміз сондықтан біз олардың пікірлері мен идеялары қаншалықты ақымақ немесе негізсіз болса да көпшілікпен бірге жүреміз,заңсыз иммигрант мəселе бойынша көптеген қарама қайшы пікір бар өкініш орай кейбіреу басу керек біз демократиялық ел сондықтан біз олардың пікір мен идея қаншалықты ақы немесе негіз бол да көпшілік бірге жүр,nazism

3,дудула операциясының гангстерлері соның ішінде олардың ақымақ көшбасшысы жəне олардың қылмыстық əрекеттерін қолдайтындардың барлығы news үгіт насихатымен бізді бопсалауды тоқтатуы керек демалыс күндері өлтірілген адам жоқ бұл өтірік,дудул операция гангстер соның іш олардың ақы көшбасшы жəне олардың қылмыстық əрекет қолда барлығ news үгіт насихат біз бопсалау тоқтат керек демалыс күн өлтір адам жоқ бұл өтірік,nazism

3,бақ қа вуйо мвоконың ағасын көрші үйдегі еңбекқор бауырларымыз теледидардан тікелей эфирде тонап кеткенін еске салайық ол сол күні өлтірілуі мүмкін еді дудула операциясы американдықтарды бірінші орынға қояды,бақ қа вуйо мвоко аға көрші үйдегі еңбекқор бауыр теледи тікелей эфир тона кет ес сал ол сол күн өлтіріл мүмкін ед дудул операция американдық бірінші орын қоя,nazism

3,мұнда келіңіз жəне маған білімді адамдарды көрсетіңіз олардың көпшілігі өз біліктіліктерін сатып алды шетелдіктер теріс білімді деп айтуды доғарыңыз,мұнда кел жəне маған білімді адам көрсе олардың көпшіліг өз біліктілік сат алды шетелдік теріс білімді де айту доғар,nazism

3,оңтүстік калифорния полициясы шанган əйелінен жəне шанган еркегінен шынтақ қалай аталатынын сұрайтын таң қалдыратын бейне бар сүйікті еліңді жоқта,оңтүстік калифорния полиция шанган əйел жəне шанган еркег шынтақ қалай атал сұра таң қалдыр бейне бар сүйікті ел жоқ,nazism

3,сонымен дудула операциясының бұзақылары бұл туралы қашан айтты сіз қалай айтасыз,сонымен дудул операция бұза бұл туралы қашан ай сіз қалай ай,nazism

3,бұл өзін өзі қорлау бұл өзін өзі ұйымдастыра алмайтын кедей жəне маргиналды адамдарды тартады адамдарға осылай қарауды тоқтатқан күн мен құрбанмын аяушылық кешінен босатылған күн болады бұл менің дудула операциясы қызметкерлеріне арналған тегін кеңесім,бұл өз өзі қорла бұл өз өзі ұйымдастыр алма кедей жəне маргина адам тарт адам осылай қарау тоқтат күн мен құрбан аяушылық кеш босатыл күн бол бұл менің дудул операция қызметкер арнал тегін кеңес,nazism

3,дана сөз егер сізді ренжіткен нәрседен ешқашан айықпасаңыз сізді ренжітпеген адамдардың қанын кетіресіз оңтүстік африка шетелдіктерге өз үкіметінің кесірінен қанын кетіреді,дана сөз егер сіз ренжіт нәрсе ешқашан айықпа сіз ренжітпе адам қан кетір оңтүстік африка шетелдік өз үкім кесір қан кетір,nazism

3,криске португал тиесілі зимбабведегі spar montana ға назар аударыңыз біздің бауырларымызға әділетсіз қарайды,крис португал тиесілі зимбабве spar montana ға назар ау біздің бауыр әділет қара,nazism

3,сізге көп рахмет дудула операциясы американдықтарды бірінші орынға қояды,сізге көп рахмет дудул операция американдық бірінші орын қоя,nazism

3,саясаткерлер бақ оңтүстік африка азаматтары кеше элвиснатидің жан түршігерлік өліміне қайғырды және бұл ақталды сондықтан мен басқа оңтүстік африкалықтармен де солай істей алатынымызды қалаймын олар заңсыз шетелдіктердің қолынан тіпті бізден қайтыс болған кезде оларға қолдау көрсетіңіз біз сонда және өзіміз үшін бола аламыз ба,саясаткер бақ оңтүстік африка азамат кеше элвиснати жан түршігерлік өлім қайғы және бұл ақта сондықтан мен басқа оңтүстік африкалық де солай істе ал қала олар заңсыз шетелдік қол тіпті бізден қайтыс бол кезде оларға қолдау көрсе біз сонда және өз үшін бола ал ба,nazism

3,дудула және окаемолао операциясына қатысушыларды құттықтаймыз дипслот иеленуге қайтарылды,дудул және окаемолао операция қатысушы құттықта дипслот иелен қайтар,nazism

3,бұл операциялар бүкіл ел бойынша қалыпты жағдайға айналуы керек мүмкін егер полиция өз жұмысын үнемі жасаса олар сәл сақ болар еді,бұл операция бүкіл ел бойынша қалыпты жағдай айнал керек мүмкін егер полиция өз жұмыс үнемі жаса олар сәл сақ бол ед,nazism

3,дудула содырларын тұтқындау уақыты келді,дудул содыр тұтқында уақыт кел,nazism

3,бұл мәселе қайғылы өйткені біз шетелдіктерді әсіресе кедей бірақ байларды қорғағысы келетін қара нәсілділерді қаламаймыз және менің сұрағым егер олар заңсыз болса неге олар құжаттарын алып содан кейін қайтып оралмасқа құжаттары заңсыз иммиграция мәселесі күн тәртібіне шығады,бұл мәселе қайғылы өйткені біз шетелдік әсіресе кедей бірақ бай қорға кел қара нәсілді қалама және менің сұра егер олар заңсыз бол неге олар құжат алып содан кейін қайт оралмас құжат заңсыз иммиграция мәселе күн тәртіб шығ,nazism

3,неге бұл жерде шетелдіктер көп болар еді егер заңдар мен тәртіп туралы айтатын болсақ адамдар тек заңдары жоқ елге барар еді токоло ткзиден эботсванада не болғанын сұраңыз олар біз сияқты қабылдамайды,неге бұл же шетелдік көп бол ед егер заң мен тәртіп туралы айт бол адам тек заң жоқ ел бар ед токоло ткзи эботсвана не бол сұра олар біз сияқты қабылдама,nazism

3,пайдасыз малема зимбабведен келген осы жігітке зимбабвеліктер өлтірген жеті оңтүстік африкалықты жоқтаудың орнына жылайды,пай мале зимбабве кел осы жігіт зимбабвелік өлтір жеті оңтүстік африкалық жоқта орн жыла,nazism

3,өйткені күрес заңсыз иммигранттарға байланысты егер сізде құжаттар болса неге алаңдайсыз тек дәлелдер келтіріңіз егер жоқ болса үйге оралып құжаттарыңызды ретке келтіріңіз бұл қиын емес бірақ менің ойымша жоқ мен оңтүстік африкалықпын уау біз бір ақылсыз елміз,өйткені күрес заңсыз иммигрант байланысты егер сіз құжат бол неге алаңда тек дәлел келтір егер жоқ бол үй орал құжат рет келтір бұл қиын емес бірақ менің ойымша жоқ мен оңтүстік африкалық уау біз бір ақыл ел,nazism

3,оңтүстік африкалықтардың шағымдануға себебі бар олардың мәселелерінің шешімін табыңыз әйтпесе ел өртеніп кетеді бізде заңсыз иммигранттармен күрделі мәселе бар,оңтүстік африкалық шағымдан себебі бар олардың мәселе шеш таб әйтпесе ел өртен кет біз заңсыз иммигрант күрделі мәселе бар,nazism

3,шындық өте көп және олар әлі де өздерін қорғайды ең сорақысы енді олар біздің иммигранттар және ішкі істер министрінен отставкаға кетуін сұрайды біз олардан тек заңды құжаттарды сұраймыз қылмыс деңгейі көтеріліп жатыр өйткені біздің түрмелер оларға толы,шындық өте көп және олар әлі де өз қорға ең сора енді олар біздің иммигрант және ішкі істер министр отставка кет сұра біз олардан тек заңды құжат сұра қылмыс деңгей көтеріл жатыр өйткені біздің түрме оларға толы,nazism

3,менің ойымша оңтүстік африкадағы шетелдіктер қауіпсіз емес олар үйге оралуы керек,менің ойымша оңтүстік африка шетелдік қауіпсіз емес олар үй орал керек,nazism

3,лол сіз келімсектерге ұқсайсыз өте пішінсіз мен ешқашан әдемі әйелдің шетелдіктермен проблемалары болғанын көрген емеспін,лол сіз келімсек ұқса өте пішін мен ешқашан әдемі әйел шетелдік проблема бол көр емес,nazism

3,онда неге оңтүстік африкалықтар дудула операциясына қарсы ал басқа елдер мұны істеп жатыр бұл ксенофобияның көрінісі ме,онда неге оңтүстік африкалық дудул операция қарсы ал басқа ел мұныісте жатыр бұл ксенофобия көрініс ме,nazism

3,сіз элвис нятидің өліміне жауаптысыз мен үнсіздікпен абдырап қалдым олар тіпті өзін өзі сот топтарымен жұмыс істейтін мемлекеттік министрлерді жауапқа тарта алмайды бізге люкспен кездесетін министрлердің тізімі қажет,сіз элвис няти өлім жауапты мен үнсіздік абдыра қал олар тіпті өз өзі сот топ жұмыс істе мемлекеттік министр жауап тарта алма бізге люкс кездес министр тізім қажет,nazism

3,біздің құлдырауымыз біз жауапкершілікті өз мойнымызға алмаймыз және көшбасшыларымызды тәртіпке келтірмейміз олардың бір бірінің тамағына жабысып қалуы бізді мандаттан айырады саясат елдің ережелері мен ережелерін сақтау үшін дипломатиялық болуы керек,біздің құлдыра біз жауапкершілік өз мойны алма және көшбасшы тәртіп келтірме олардың бір бір тамағ жабыс қал біз мандат айыр саясат ел ереже мен ереже сақтау үшін дипломатиялық бол керек,nazism

3,оңтүстік африкалықтар заңсыз иммигранттар кетуі керек дейді рамафосаның айтуынша үкімет қылмыстарды олар айтқандай қабылдамайды бірақ таңқаларлық бұл біздің елге заңсыз иммиграцияны қолдайды бұл заңды құжаттарсыз басқа елге кіру кезінде қылмыс болып табылады,оңтүстік африкалық заңсыз иммигрант кет керек де рамафоса айт үкімет қылмыс олар айтқанда қабылдама бірақ таңқаларлық бұл біздің ел заңсыз иммиграция қолда бұл заңды құжатт басқа ел кіру кез қылмыс бол таб,nazism

3,бұл африкалық иммигранттарға қатысты зорлық зомбылық тудыру үшін ойдан шығарылған оқиға беки челе мен сапс бұл оқиғаны растай алмады,бұл африкалық иммигрант қатысты зорлық зомбылық тудыру үшін ой шығарыл оқиға беки чел мен сапс бұл оқи раста алма,nazism

3,бұл заңсыз иммигранттар бәрімізді құртады,бұл заңсыз иммигрант бәрі құрт,nazism

3,қылмыскерлердің ұлтына қарамастан қылмыс қылмыс жасаған қылмыскерлер оңтүстік африкалықтар немесе шетелдіктер болса да заңның барлық күшіне ие болуы керек ксенофобия барлық тіршілік иелеріне жағымды әсер етуі керек,қылмыскер ұлт қарамастан қылмыс қылмыс жаса қылмыскер оңтүстік африкалық немесе шетелдік бол да заң барлық күш ие бол керек ксенофобия барлық тіршілік ие жағымды әсер ет керек,nazism

3,өзін өзі бағалау мен ксенофобияны жақтайтын жанкүйерлердің қолдары қанды болады мен бұған тым ұзақ уақыт бойы ұшырадым алдымен олар шетелдіктерге келеді үнсіздік содан кейін ми келеді тыныштық ештеңе қалмайынша олар сіздің тайпаңызға келгенде таң қалмаңыз ксенофобия теріс болуы керек,өз өзі бағалау мен ксенофобия жақта жанкүйер қол қан бол мен бұған тым ұзақ уақыт бойы ұшыра алд олар шетелдік кел үнсіздік содан кейін ми кел тыныштық ештеңе қалмайынш олар сіздің тайпа кел таң қалма ксенофобия теріс бол керек,nazism

3,ол бірнеше күн бұрын шетелдіктер өлтірген оңтүстік африкалықтар туралы бірдеңе айтты ма сешогода бұл шетелдік туралы бірдеңе болды ма бұл саясаткерлер оларды билікке кім таңдағанын ұмытады немесе заңсыз шетелдіктер оларды билікке қайтарады,ол бірнеше күн бұрын шетелдік өлтір оңтүстік африкалық туралы бірде ай ма сешого бұл шетелдік туралы бірде бол ма бұл саясаткер ол билік кім таңда ұмыт немесе заңсыз шетелдік ол билік қайтар,nazism

3,онымен бірге ксенофобиялық шабуылдардың толқыны дурбан квазулу натальда басталуы жоспарланған дудула операциясы ксенофобия топырағына шабуыл жасау үшін қолайлы жағдай туғызады бірақ ол не істеді ол жаңа ғана қозғалысты теріс сипаттады,онымен бірге ксенофобиялық шабуыл толқын дурбан квазул наталь бастал жоспарлан дудул операция ксенофобия топыраға шабуыл жасау үшін қолайлы жағдай туғыз бірақ ол не істе ол жаңа ғана қозғал теріс сипатта,nazism

3,дипслутта оңтүстік африкалықты аяусыз өлтірген кезде президент қайда болды иммигрант қайтыс болады және кенеттен ол дипслуттағы зорлық зомбылықты

айыптайды бұл президент кім үшін оңтүстік африкалықтар бұқаралық ақпарат құралдарында бірінші болып теріс пікір білдірді деп санайды,дипслут оңтүстік африкалық аяу өлтір кезде президент қайда бол иммигрант қайтыс бол және кенет ол дипслут зорлық зомбылық айыпта бұл президент кім үшін оңтүстік африкалық бұқаралық ақпарат құрал бірінші бол теріс пікір біл де сана,nazism

3,олар жалынды үрлеп енді қолтырауынның көз жасымен жылайды саясаткерлер дудула операциясының заңсыздығын көтермеледі енді олар дипслут тұрғындарының элвиснятиді өлтіріп өртеп жібергеніне өкінеді бұған кім кінәлі неліктен қосымша полиция қызметкерлері кешіктірілді,олар жалын үрле енді қолтырауын көз жас жыла саясаткер дудул операция заңсыздығ көтермеле енді олар дипслут тұрғын элвисняти өлтір өрте жібер өкін бұған кім кінәлі неліктен қосымша полиция қызметкер кешіктір,nazism

3,шындық мынада президент және үкімет оңтүстік африкалықтарға бұқаралық ақпарат құралдарына оңтүстік африкалықтарға мән бермейді оңтүстік африкалықтар оян африка өмірінде бірінші орынға қойыңыз,шындық мына президент және үкімет оңтүстік африкалық бұқаралық ақпарат құрал оңтүстік африкалық мән берме оңтүстік африкалық оян африка өмір бірінші орын қой,nazism

3,бұл оңтүстік африкалық отбасын шетелдіктер өлтірді мүмкін сіз оны әлі есіңізде шығарсыз ол заңсыз иммигранттар өлтірген оңтүстік африкалық болғандықтан маңызды емес пе,бұл оңтүстік африкалық отбас шетелдік өл мүмкін сіз оны әлі ес шығ ол заңсыз иммигрант өлтір оңтүстік африкалық бол маңызды емес пе,nazism

3,бұл оңтүстік африкалық отбасы жігіті әлиді шетелдіктер өлтірді әлі есіңізде ме,бұл оңтүстік африкалық отбасы жігіт әли шетелдік өл әлі ес ме,nazism

3,шетелдіктер жасаған барлық кісі өлтірулерге қарамастан заңсыз иммигранттар туралы шындықты ешқашан жасырмады оны бірде бір саяси партия айыптаған жоқ eff бұл мәселе бойынша мәңгі үнсіз қалады бірақ бір шетелдік өлтірілгенде ғана әрекет етеді дұрыс емес нәрсені айыптайық,шетелдік жаса барлық кісі өлтіру қарамастан заңсыз иммигрант туралы шындық ешқашан жасырма оны бірде бір саяси партия айыпта жоқ eff бұл мәселе бойынша мәңгі үн қал бірақ бір шетелдік өлтіріл ғана әрекет ет дұрыс емес нәрсе айыпта,nazism

3,мұнда ешкім ешкімнің өлтірілуін тойламайды фактілерді әдейі түсінбеңіз біз оңтүстік африка қоғамына қауіп төндіретін дипслутта заңсыз иммигранттарды тұтқындауды атап өтеміз біз заңсыздықты тазартуға қатты қуаныштымыз,мұнда ешкім ешкі өлтіріл тойлама факті әдей түсінбе біз оңтүстік африка қоғам қауіп төндір дипслут заңсыз иммигрант тұтқындау ата өт біз заңсыздық тазарт қатты қуанышты,nazism

3,бұл адам шетелдіктердің мектеп оқушыларына есірткі сатуына жол бермегені үшін өлтірілді және олар оны өлтірді біздің пайдасыз полиция министрі бұл туралы ештеңе айтқан жоқ сіз тек осы заңсыз шетелдіктерге қамқорлық жасайсыз,бұл адам шетелдік мектеп оқушы есірткі сат жол берме үшін өлтір және олар оны өл біздің пай полиция

министр бұл туралы ештеңе айт жоқ сіз тек осы заңсыз шетелдік қамқорлық жаса,nazism

3,мен кісі өлтіруді жақтамаймын бірақ менің оңтүстік африка бағбандарының маңында біз оларды бұрын сатып алған бұрыштарда дэнвиллде добта таба алмаймын оңтүстік африка қалашықтарының дәнекерлеушілері енді жұмыс істемейді өйткені шетелдіктер жаппай өндіріске көшті басқа мысалдар қажет пе,мен кісі өлтіру жақтама бірақ менің оңтүстік африка бағбан маң біз ол бұрын сат ал бұрыш дэнвилл доб таб алма оңтүстік африка қалашық дәнекерлеуші енді жұмыс істеме өйткені шетелдік жаппай өндіріс көш басқа мысал қажет пе,nazism

3,өте өкінішті өйткені біз апартеидке қарсы күресіп жеңіске жеттік біз ақ монополиялық капиталдан экономикамызды қайтарып алуға тырысамыз бірақ мүмкін емес өйткені бізді шақырылмаған қонақтар бомбалайды бұл адамдарсыз зимбабвеліктерсіз біз өз проблемамызды қалай шешеміз,өте өкініш өйткені біз апартеид қарсы күрес жеңіс же біз ақ монополиялық капиталдан экономика қайтар ал тырыс бірақ мүмкін емес өйткені біз шақырылма қонақ бомбала бұл адамд зимбабвелікт біз өз проблема қалай шеш,nazism

3,хайбо еуропа мәселесін біздің проблемамызға айналдырмаңыз сіз айтқандай еуропа елдері және бұл жағдайда тек sa да шетелдіктер деп аталатын қоқыс бар бұл еуропа емес және біз әрине қайырымдылықпен айналыспаймыз,хайбо еуропа мәселе біздің проблема айналдырма сіз айтқанда еуропа ел және бұл жағдай тек sa да шетелдік де атал қоқыс бар бұл еуропа емес және біз әрине қайырымдылық айналыспа,nazism

3,балалар бәрі қарапайым үкімет елде төтенше жағдай жариялайды біз барлық заңсыз иммигранттардың sa үй жайларынан кетуін кішіпейілділікпен сұрауымыз керек және біз оларға апта ақы береміз африкалықтар біз бейбіт және сыпайымыз,бала бәрі қарапайым үкімет ел төтенше жағдай жарияла біз барлық заңсыз иммигрант sa үй жай кет кішіпейілділік сұра керек және біз оларға апта ақы бер африкалық біз бейбіт және сыпай,nazism

3,anc қызметкерлерімізге жүгінейік және неге ана органының құрылымдары бұл мәселені талқылау және түпкілікті шешу үшін қызметкерлерімізбен екі жақты кездесу өткізбегенін білейік сонымен қатар дудула болмаса да адамдар бұл тәртіпсіздікке тойып алады,anc қызметкер жүгін және неге ана орган құрылым бұл мәселе талқылау және түпкілік шешу үшін қызметкер екі жақ кездесу өткізбе біл сонымен қатар дудул болма да адам бұл тәртіпсіздік той ал,nazism

3,сіздің пайғамбарлығыңыз қалыптасады,сіздің пайғамбарлы қалыптас,nazism

3,заңсыз шетелдіктер мен олардың үкіметі де жауапкершілікті өз мойнына алуы керек,заңсыз шетелдік мен олардың үкімет де жауапкершілік өз мойн ал керек,nazism

3,оңтүстік африкалықтар біз жұмылдыруымыз керек осы шетелдіктерді дереу жіберіңіз али стив бико собукве және винни мандела бізді мазақ ету үшін күрескен

жоқ,оңтүстік африкалық біз жұмылдыр керек осы шетелдік дереу жіб али стив бико собукв және винни мандел біз мазақ ету үшін күрес жоқ,nazism

3,екі күн бұрын зимбабвелік қарақшы альбертина сисулу мен наггеттің робот cnr дегі тереземе жақындады ол маған тапаншасын көрсетіп телефон әкелуді айтты мен өз өмірімде қорқып кеттім біз үнемі қорқынышпен өмір сүру үшін не істедік,екі күн бұрын зимбабвелік қарақш альбертин сисул мен нагге робот cnr дег терезе жақында ол маған тапанша көрсет телефон әкелу ай мен өз өмір қорқ кет біз үнемі қорқыныш өмір сүру үшін не істе,nazism

3,дудула операциясы біздің жемқор саясаткерлер мен шенеуніктерді алып тастау уақыты келді,дудул операция біздің жемқор саясаткер мен шенеунік алып тастау уақыт кел,nazism

3,ия біз барлық шетелдіктер үшін sa ны құжаттарсыз түзетеміз,ия біз барлық шетелдік үшін sa ны құжатт түзе,nazism

3,бізді өз президентіміз сатады али мен оңтүстік африканы масқаралаймын американдықтар интернет бақ желісінде бірінші болып жұмыс істей бастағаны туралы хабарлама тарады,біз өз президент сат али мен оңтүстік африка масқарала американдық интернет бақ желі бірінші бол жұмыс істе баста туралы хабарлама тар,nazism

3,біз дудула операциясы және бірінші кезекте африкаға жеткізу туралы қарастырған емеспіз келесі жолы сіз қалаған кек аласыз,біз дудул операция және бірінші кезек африка жеткізу туралы қарастыр емес келесі жол сіз қала кек ал,nazism

3,бұл біздің арамызда қалады сіз жай ғана operationdudula немесе putsouthafricanfirst тің артында тұрғанын дәлелдей алмайсыз тек осы жерде бақытты көріңіз мүмкін сіз оларды кінәлау үшін қолдау аласыз қозғалыстар осында қалуға күтуге және көруге арналған,бұл біздің ара қал сіз жай ғана operationdudula немесе putsouthafricanfirst ті арт тұр дәлелде алма тек осы же бақытты көр мүмкін сіз ол кінәла үшін қолдау ал қозғалыс осында қал күт және көр арнал,nazism

3,дипслут біреу маған не екенін айтыңыз бұл сіздің әпкеңіз бе ақ ханым сіз үшін кеудедегі ауырсынудан зардап шегеді,дипслут біреу маған не екен ай бұл сіздің әпке бе ақ ханым сіз үшін кеуде ауырсыну зардап шег,nazism

3,заңсыз иммигранттардың қолынан қаза тапқан барлық оңтүстік африкалықтарға көңіл айтады,заңсыз иммигрант қол қаза тап барлық оңтүстік африкалық көңіл айт,nazism

3,заңсыз иммигрант болудан жақсы,заңсыз иммигрант болу жақсы,nazism

3,сіздің сөздеріңізде де мағынасы жоқ өйткені жергілікті гаутенг клиникалары мен ауруханалары заңсыз иммигранттарға толы,сіздің сөз де мағына жоқ өйткені жергілікті гаутенг клиника мен аурухана заңсыз иммигрант толы,nazism

3,сіз өзіңізді қалағаныңызша ақтай аласыз бірақ гаутенг клиникалары мен ауруханалары заңсыз иммигранттарға толы,сіз өз қалағаныңызш ақта ал бірақ гаутенг клиника мен аурухана заңсыз иммигрант толы,nazism

3,бізде оңтүстік каролинада заңсыз иммигранттар мәселесі бар,біз оңтүстік каролина заңсыз иммигрант мәселе бар,nazism

3,мұны түзетуге рұқсат етіңіз саяси партиялар үкіметті басқаратындар және метрода ықпалы барлар eff da және б шетелдіктер жұмыссыздық пен ресурстардың жетіспеушілігінен туындаған теңсіздік,мұны түзет рұқсат ет саяси партия үкімет басқар және метро ықпал бар eff da және б шетелдік жұмыссыздық пен ресурс жетіспеушіліг туында теңсіздік,nazism

3,бұл туралы не айтатыны қызық eff оңтүстік африкалықтарды шетелдіктер өлтірді,бұл туралы не айт қызық eff оңтүстік африкалық шетелдік өл,nazism

3,дудула операциясының алдыңғы шебіндегілердің барлығын қамауға алу керек нхланхла люкс ұлы кгалима мотлантеден бастап ксенофобияға қарсы шабуылдарды жалғастырудың тағы бір жағдайын қосу керек осы оңтүстік африкалықтың отбасылары қайда,дудул операция алдыңғы шебіндегі барлығ қама алу керек нхланхл люкс ұлы кгали мотланте баста ксенофобия қарсы шабуыл жалғастыр тағы бір жағдай қосу керек осы оңтүстік африкалық отбасы қайда,nazism

3,дудула операциясының айқын нәтижелері осындай жігіттері заңсыз иммигранттарды өлтіру арқылы санамның африкада жүруін қиындатады сіз оянып африкалықтарға деген өшпенділіктің шірігін көруіңіз керек,дудул операция айқын нәтиже осындай жігіт заңсыз иммигрант өлтір арқылы сана африка жүр қиындат сіз оян африкалық де өшпенділік шіріг көр керек,nazism

3,және ол кеше біреуді тірідей өртеп жіберген қырағы топқа баратын бейнежазбалар бар енді сұрақ туындайды онда нхланхла люкс не істеді,және ол кеше біреу тірідей өрте жібер қырағ топ бар бейнежазба бар енді сұрақ туында онда нхланхл люкс не істе,nazism

3,оңтүстік африкада өзін қауіпсіз сезінбейтін шетелдіктер үйге қауіпсіз жерде оралуы керек,оңтүстік африка өз қауіпсіз сезінбе шетелдік үй қауіпсіз же орал керек,nazism

3,үкімет бізді сәтсіздікке ұшыратып қана қоймай заңсыз шетелдіктердің адамдарға қарсы осындай қатыгездік жасауға деген шешімін көтермелеп күшейтті өкінішке орай жылды күту өлімге әкелуі мүмкін,үкімет біз сәтсіздік ұшырат қана қойма заңсыз шетелдік адам қарсы осындай қатыгез жаса де шеш көтермеле күшей өкініш орай жыл күту өлім әкел мүмкін,nazism

3,сіз өз елінде шетелдіктер өлтірген оңтүстік африкалық отбасылардан сұхбат алдыңыз ба,сіз өз ел шетелдік өлтір оңтүстік африкалық отбасы сұхбат ал ба,nazism

3,сонымен бұл сізге барлық жерде серуендеуге және заңсыз шетелдіктерді өлтіруге құқық бере ме,сонымен бұл сізге барлық же серуенде және заңсыз шетелдік өлтір құқық бер ме,nazism

3,жасымда мейрамханаларға назар аударған кезде мен қосымша ақша табу үшін news cafe де жұмыс істедім енді біздің жастар даяшы бола алмайды өйткені ол шетелдіктерге толы тіпті шетелдік менеджерлер де бар және олар өз адамдарын жалдайды оңтүстік африкалықтар мүлдем жоқ,жас мейрамхана назар аудар кезде мен қосымша ақша табу үшін news cafe де жұмыс істе енді біздің жас даяшы бола алма өйткені ол шетелдік толы тіпті шетелдік менеджер де бар және олар өз адам жалда оңтүстік африкалық мүлдем жоқ,nazism

3,оңтүстік африкалықтардың ін өткен демалыс күндері заңсыз иммигранттар өлтірді олардың барлығы бақ ты қосқанда қатты қуанды біз қайдамыз біздің өміріміз маңызды мені өлтіреді барлығы африкадағы алғашқы операция туралы айтады дудула нхланхл бізге де қорғаныс қажет тазалаңыз,оңтүстік африкалық ін өткен демалыс күн заңсыз иммигрант өл олардың барлығ бақ ты қос қатты қуан біз қай біздің өмір маңызды мен өлтір барлығ африка алғашқы операция туралы айт дудул нхланхл бізге де қорғаныс қажет тазала,nazism

3,сіз біздің адамдар мен сіздің шетелдіктеріңіз үшін жұмыс орындарын құру үшін зауыттар мен фермалар салудың орнына лашықтар салу үшін жер алғыңыз келетіні ұят,сіз біздің адам мен сіздің шетелдік үшін жұмыс орын құру үшін зауыт мен ферма сал орн лашық салу үшін жер ал кел ұят,nazism

3,біреу оңтүстік африка деп аталатын көшбасшылардың супер күштеріне күмән келтірген сайын біреу шетелдіктерге қарсы соғыс бастау үшін біреуге демеушілік жасайды ксенофобиялық дудула операциясы олар сізді ұмытып шетелдіктерге бейтарап назар аударғаныңызды қалайды,біреу оңтүстік африка де атал көшбасшы суп күш күмән келтір сайын біреу шетелдік қарсы соғыс бастау үшін біреу демеушілік жаса ксенофобиялық дудул операция олар сіз ұмыт шетелдік бейтарап назар аудар қала,nazism

3,оңтүстік африкалықтарды заңсыз иммигранттар өлтірді барлығы шок болды соның ішінде бақ тек заңсыз шетелдік өлтірілді барлық адамдар біз қайда екенімізді айта бастайды,оңтүстік африкалық заңсыз иммигрант өл барлығ шок бол соның іш бақ тек заңсыз шетелдік өлтір барлық адам біз қайда екен ай баста,nazism

3,нхланхла люкс қайда барса да ешқандай шартсыз кепілге босатылғаны анық қара нәсілділерге шабуыл жасалады енді олар бұл жігітті тірідей өртеп жіберді және сол баланы өлтіргені үшін және оны кепілге босату шарттарын бұзғаны үшін қамауға алу керек,нхланхл люкс қайда бар да ешқандай шартсыз кепіл босатыл анық қара нәсілді шабуыл жасал енді олар бұл жігіт тірідей өрте жіб және сол бала өлтір үшін және оны кепіл босат шарт бұз үшін қама алу керек,nazism

3,дипслут тұрғындары өз қауымдастығындағы барлық заңсыз иммигранттарды аяқтағанға дейін жалғастыруы керек полиция оларға ешқашан көмектеспейді,дипслут тұрғын өз қауымдастығ барлық заңсыз иммигрант аяқта дейін жалғастыр керек полиция оларға ешқашан көмектеспе,nazism

3,бұл операция дудуланың қорқақтары қара адамдардың өмірін феникстегі үндістер жойып жібергенін біліп бақытты болуы керек және анп істерді жасырын түрде қайтарып алады,бұл операция дудула қорқақта қара адам өмір феникс үндіс жой жібер біл бақытты бол керек және ан іс жасырын түр қайтар ал,nazism

3,тіпті алекс айналасындағы клиникаларда да қха ға баратын әйелдердің дан астамы заңсыз иммигранттар болып табылады,тіпті алекс айнала клиника да қх ға бар әйел дан астам заңсыз иммигрант бол таб,nazism

3,бұл бейнеде кредо мутва дудула мен нхлахла люкс операциясы туралы қай жерде айтты біздің қоғамның көтерілісі тек гаутенгте ғана емес ддұ елі есірткімен қылмыспен және заңсыз иммигранттармен күресуде біздің елді мекендердегі аумақтарды шетелдіктер басып алды,бұл бейне кредо мутв дудул мен нхлахл люкс операция туралы қай же ай біздің қоғам көтеріліс тек гаутенг ғана емес ддұ ел есірткі қылмыс және заңсыз иммигрант күрес біздің ел мекен аумақ шетелдік бас алды,nazism

3,қоғамдастық полиция қызметкерлерін сынық үйінділерінде есірткі сатушылардың үйлерінде тұтқындауды бастауы керек,қоғамдастық полиция қызметкер сынық үйінді есірткі сатушы үй тұтқындау баста керек,nazism

3,мен кирилдің ұятсыз нхланхланы кек алушы деп атағанын естідім егер ол біздің елдің қауіпсіздігін қамтамасыз ету бойынша өз жұмысын атқарса дудула операциясы болмас еді бірінші кезекте африкаға жөнелту болмас еді малема сияқты олар оңтүстік африкалықтарға мән бермейді олар біздің әл ауқатымызды жүрегіне алмайды жүрек айнуы,мен кири ұят нхланхла кек алушы де ата есті егер ол біздің ел қауіпсіздіг қамтамасыз ету бойынша өз жұмыс атқар дудул операция болмас ед бірінші кезек африка жөнелту болмас ед мале сияқты олар оңтүстік африкалық мән берме олар біздің әл ауқат жүрег алма жүрек айн,nazism

3,олар дипслуттан заңсыз иммигранттарды шекараға қайтаруы керек содан кейін қылмыс ашылды және адамдар бейбітшілік пен келісімде өмір сүреді,олар дипслут заңсыз иммигрант шекара қайтар керек содан кейін қылмыс аш және адам бейбітшілік пен келісімде өмір сүр,nazism

3,пикетке шыққаннан кейін джуджу неміс көлігімен үйіне шетелдік азаматтардың қауіпсіздігі күзететін биік қабырғалары мен электр қоршаулары бар хайд парктегі үйіне оралады балалар үйлеріне отбасыларымен бөлісетін екі лашықта оралады олар жұмыссыз шетелдіктер,пикет шық кейін джудж неміс көліг үй шетелдік азамат қауіпсіздіг күзет биік қабырға мен электр қоршау бар хайд парк үй орал бала үй отбасы бөліс екі лашық орал олар жұмыссыз шетелдік,nazism

3,жақсы емес шетелдіктер қандай ұлт болды,жақсы емес шетелдік қандай ұлт бол,nazism

3,ішкі істер органдары мен полицияның заңсыз шетелдіктермен құжат алу үшін қастандық жасағаны туралы көп айтылады және ол оны алып тастамақшы,ішкі істер орган мен полиция заңсыз шетелдік құжат алу үшін қаста жаса туралы көп айт және ол оны алып тастамақшы,nazism

3,міне оның анты заңсыз шетелдіктерді қорғау туралы ештеңе айтылмаған бұл республика және оның адамдары туралы,міне оның ант заңсыз шетелдік қорғау туралы ештеңе айтылма бұл республика және оның адам туралы,nazism

3,егер руперттің фермасын аралап жүрген адамдардың жұмысы болса олар пикетте емес жұмыста болар еді олар малемаға пикетке шыққанда шетелдіктер жұмыссыз қалуы керек жұмыстармен айналысады олардың сіс жетекшісі бұған риза біз бұған лайық болу үшін не істедік,егер рупер ферма арала жүр адам жұмыс бол олар пикет емес жұмыс бол ед олар малема пикет шық шетелдік жұмыссыз қал керек жұмыс айналыс олардың сіс жетекші бұған риза біз бұған лайық болу үшін не істе,nazism

3,бауырым әрине аурухана төсектерін алып жатқан және біздің медициналық мекемелердің ресурстарын пайдаланатын адамдардың көпшілігі оңтүстік африкалықтар емес олардың көпшілігі құжатсыз шетелдіктер,бауыр әрине аурухана төсек алып жат және біздің медициналық мекеме ресурс пайдалан адам көпшіліг оңтүстік африкалық емес олардың көпшіліг құжат шетелдік,nazism

3,дудула операциясы әсерді жояды мен кеудедегі ауырсыну сізді өлтіретінін көремін,дудул операция әсер жоя мен кеуде ауырсыну сіз өлтір көр,nazism

3,нхланхла люкс әр түрлі болуы мүмкін өйткені олар оған өз боқтарын жасауға мүмкіндік берді,нхланхл люкс әр түрлі бол мүмкін өйткені олар оған өз боқ жаса мүмкіндік бер,nazism

3,дудула операциясы бұл аутсайдерлер емес қауымдастық сырттан келгендер сізге қажет кезде көмек қолын созуға келеді бірақ сіз қоғамдастық өз іс әрекетіңізді қалыптастырғаннан кейін себебі сіз онда не болып жатқанын білетін жалғыз адамсыз,дудул операция бұл аутсайдер емес қауымдастық сырт кел сізге қажет кезде көмек қол соз кел бірақ сіз қоғамдастық өз іс әрекет қалыптастыр кейін себебі сіз онда не бол жат біл жалғыз адам,nazism

3,полиция министрі бұрынғы комиссар өртті сөндіруге сәтсіз әрекеттен кейін дипслуттан кетті nhlanhlalux енді ондағы жағдайды бақылауға алды және ол нашарлап бара жатқан сияқты,полиция министр бұрынғы комиссар өр сөндір сәт әрекет кейін дипслут кет nhlanhlalux енді ондағы жағдай бақыла алды және ол нашарла бар жат сияқты,nazism

3,диплсут полициясы заңсыз иммигранттар мәселесін теріс шешудің орнына оңтүстік африка азаматтарын атып тастайды,диплсут полиция заңсыз иммигрант мәселе теріс шешу орн оңтүстік африка азамат ат таста,nazism

3,оңтүстік африкадағы алғашқы операция dudula nhlanhlalux заңсыз шетелдіктер біздің инфрақұрылым үшін басты мәселе болып табылады,оңтүстік африка алғашқы операция dudula nhlanhlalux заңсыз шетелдік біздің инфрақұрылым үшін басты мәселе бол таб,nazism

3,мен өзімді керек сияқты сезінемін өйткені сен өзіңнің есегің деп ойлайсың,мен өз керек сияқты сез өйткені сен өз есе де ойла,nazism

3,менің проблемам емес шетелдіктер маған әсер етпейді,менің проблема емес шетелдік маған әсер етпе,nazism

3,тіпті полиция министрі дипслотқа наразылық білдірген адамдарға жүгіне алмады,тіпті полиция министр дипслот наразылық білдір адам жүг алма,nazism

3,оңтүстік африкадағы көшемізді қоқыстардан тазартайық алдымен,оңтүстік африка көше қоқыс тазарт алд,nazism

3,дипслут тұрғындары осы сәтті өз аймағындағы барлық шетелдіктерден толық құтылу үшін пайдалануы керек,дипслут тұрғын осы сәтті өз аймағ барлық шетелдік толық құтыл үшін пайдалан керек,nazism

3,сіздің жобаларыңыз operationdudula және putsouth африка бірінші кезекте ақтықтан қорқады сондықтан сіз тек африкалықтарға шабуыл жасайсыз,сіздің жоба operationdudula және putsouth африка бірінші кезек ақтық қорқ сондықтан сіз тек африкалық шабуыл жаса,nazism

3,зимбабведен келген заңсыз шетелдіктер дипслутта полиция қызметкерін өлтірді мекеме мен бұқаралық ақпарат құралдары бұл туралы ештеңе айтпайды,зимбабве кел заңсыз шетелдік дипслут полиция қызметк өл мекеме мен бұқаралық ақпарат құрал бұл туралы ештеңе айтпа,nazism

3,оңтүстік африка саясаткерлерімен және полициясымен заңсыз иммигранттарды талқылау уақытты ысырап етеді,оңтүстік африка саясаткер және полиция заңсыз иммигрант талқылау уақыт ысырап ет,nazism

3,бізге заң ұнамайды бірақ заңдар жай ғана бар емес бірақ тым көп адамдар заңсыз иммигранттарды қорғау үшін иммиграциялық заңдарды ыңғайлы түрде елемеуді қалайды оңтүстік африка тұрғындары құдай ұмытқан осы елде не болып жатқанына лайық біз американдықтарды бірінші теріс позицияға қоятын шетелдіктерді шаршатамыз,бізге заң ұнама бірақ заң жай ғана бар емес бірақ тым көп адам заңсыз иммигрант қорғау үшін иммиграциялық заң ыңғайлы түр елемеу қала оңтүстік африка тұрғын құдай ұмыт осы ел не бол жат лайық біз американдық бірінші теріс позиция қоятын шетелдік шарша,nazism

3,шетелдіктерге ескерту жасалды,шетелдік ескерту жаса,nazism

3,lol өңдеушілері сізге зиян тигізеді,lol өңдеуші сізге зиян тигіз,nazism

3,лол бұл жолы қатты жыламауға тырысыңыз,лол бұл жол қатты жылама тырыс,nazism

3,өзінің байытуы үшін олар бұқараға мән бермейді олар ешқашан болған емес саясаткер қауымдастықтар қалағаннан гөрі маңызды деп санайтын нәрсені айтып жатқанын елестетіп көріңіз сайлаушылардың айтқандары маңызды емес,өзінің байыт үшін олар бұқара мән берме олар ешқашан бол емес саясаткер қауымдастық қала гөрі маңызды де сана нәрсе айт жат елестет көр сайлаушы айт маңызды емес,nazism

3,үкімет eff ке жеке тұлғаның меншігіне шерулер өткізуге рұқсат береді бірақ дудула операцияларына біздің елге заңсыз иммиграцияға байланысты қылмыстарға қарсы қоғамдық орындарда шерулер өткізуге тыйым салады,үкімет eff ке жеке тұлға меншіг шеру өткіз рұқсат бер бірақ дудул операция біздің ел заңсыз иммиграция байланысты қылмыс қарсы қоғамдық орын шеру өткіз тыйым сал,nazism

3,зимбабве іш киімдері міне сіздің көшбасшылығыңыз назар аударыңыз мен оның рухын қолдаймын ол менің ағам африка батылдарға арналған іш киімдер әрқашан қашып кетеді ал қытайлықтар бәрін өздеріне алуға дайын болады,зимбабв іш киім міне сіздің көшбасшылығ назар ау мен оның рух қолда ол менің аға африка батыл арнал іш киім әрқашан қаш кет ал қытайлық бәрін өз ал дайын бол,nazism

3,олар сізді кек алушы сияқты ақымақ есімдермен атай алмайтындай етіп ақылдырақ күресіңіз ақылды болыңыз люкс су сияқты болыңыз менің досым қажет болған кезде жұмсақ және жұмсақ уақыты келгенде күшті және жойқын,олар сіз кек алушы сияқты ақы есім ата алмайтында ет ақылдырақ күрес ақылды бол люкс су сияқты бол менің дос қажет бол кезде жұм және жұм уақыт кел күшті және жойқын,nazism

3,біз operationdudula деп аталатын ақымақтардан неге олар өздерінің ашуын шетелдік иммигранттарға ішкі істер шенеуніктеріне бермейтінін және кедей қара африкалықтарға шабуыл жасаудың орнына оларға шабуыл жасамайтынын сұрағанда олар ыстық картоп жейді ақымақтар,біз operationdudula де атал ақымақ неге олар өз аш шетелдік иммигрант ішкі істер шенеунік берме және кедей қара африкалық шабуыл жаса орн оларға шабуыл жасама сұра олар ыстық картоп же ақымақ,nazism

3,бұл қызықты әңгіме техникалық тұрғыдан менің балам илон африкалық иммигрант,бұл қызықты әңгіме техникалық тұрғы менің бала илон африкалық иммигрант,nazism

3,ешкім артық немесе жақсы емес әлем соншалықты үлкен және өмірдің ерекшелігі болып табылатын әртүрлілікке толы оңтүстік африкада қара нәсілділермен шетелдік ретінде қорланған қуаныш пен махаббат көктем,ешкім артық немесе жақсы емес әлем соншалықты үлкен және өмір ерекшеліг бол табыл әртүрлілік толы оңтүстік африка қара нәсілді шетелдік рет қорлан қуаныш пен махаббат көктем,nazism

3,бұл мәлімдеме ақымақтық пен білімнің жетіспеушілігін сезінеді,бұл мәлімдеме ақымақтық пен біл жетіспеушіліг сезін,nazism

3,бұл күн сүлеймен малангу күні деп жариялануы керек,бұл күн сүлеймен маланг күн де жариялан керек,nazism

3,құжаттары жоқ шетелдіктер үйлеріне оралып құжаттарын рәсімдеуі керек,құжат жоқ шетелдік үй орал құжат рәсімде керек,nazism

3,бірақ ол олармен бірге қалмайды,бірақ ол олармен бірге қалма,nazism

3,мен жоқ деп айтуды ұмытып кеттім президент демократиялық елдегі азаматтардан гөрі көбірек билікке ие біз әрекет етуге құқығымыз бар,мен жоқ де айту ұмыт кет президент демократиялық ел азамат гөрі көбірек билік ие біз әрекет ет құқы бар,nazism

3,бізді троллейтін заңсыз шетелдіктерді бұғаттаңыз олармен айналысуға уақыт жоқ олар жай ғана үйлеріне таралуы керек,біз тролле заңсыз шетелдік бұғатта олармен айналыс уақыт жоқ олар жай ғана үй тарал керек,nazism

3,шетелдіктер өз қызметтері үшін оңтүстік африкада жақсы төлеуі керек біз американдықтарды бірінші орынға қоятын африканың анасы емеспіз,шетелдік өз қызмет үшін оңтүстік африка жақсы төле керек біз американдық бірінші орын қоятын африка ана емес,nazism

3,нхланхла люкс құжатсыз шетелдіктерді қорлауды аяқтаған кезде мен оның тікелей жалған шіркеулерге барып пасторлар пайғамбарлар мен епископтар деп аталатындарды дудулизациялауын сұраймын,нхланхл люкс құжат шетелдік қорлау аяқта кезде мен оның тікелей жалған шіркеу бар пастор пайғамбар мен епископ де атал дудулизацияла сұра,nazism

3,қарқынды дамып келе жатқан команданың бір бөлігі болғыңыз келе ме біз табысқа жетуді қалайтын брокерлерді іздейміз,қарқынды дам келе жат команда бір бөліг бол келе ме біз табыс жету қала брокерізде,nazism

3,біздің еліміз үшін күресетін кез келді біз кепілге алынбаймыз менің иммигранттарым,біздің еліміз үшін күрес кез кел біз кепіл алынба менің иммигрант,nazism

3,сонымен қатар заңсыз шетелдіктердің қылмыстарына ұшыраған және ұшырағандар малемаға қарсы сот ісін жүргізуге көмектесуі керек оның аузын біржола жабатын уақыт келді ол сіздің аузыңыздан саяси көшбасшы ретінде не шығатыны туралы сабақ беруі керек,сонымен қатар заңсыз шетелдік қылмыс ұшыра және ұшыра малема қарсы сот ісін жүргіз көмектес керек оның ауз біржол жаб уақыт кел ол сіздің ауз саяси көшбасшы рет не шығ туралы сабақ беруі керек,nazism

3,африкада алғашқы операцияны жасаңыз дудула уақыт келді,африка алғашқы операция жаса дудул уақыт кел,nazism

3,ұлт ретінде біз бұл мәселелерді талқылауымыз керек олар біздің қоғамға әсер етеді кирилл уәде етілген инвестициялармен мақтанады бірақ ол бізді шетелдіктерді жалдауға жетелейтін салалар үшін дағдылар жетіспейтінін жақсы біледі,ұлт рет біз бұл мәселе талқыла керек олар біздің қоғам әсер ет кирилл уәде ет инвестиция мақтан бірақ ол біз шетелдік жалда жетеле сала үшін дағды жетіспе жақсы біл,nazism

3,дудула операциясы мен африка форумы бірлесіп жұмыс істеуі керек африка форумы американдықтарды бірінші орынға қояды,дудул операция мен африка форум бірлес жұмыс істе керек африка форум американдық бірінші орын қоя,nazism

3,мен олар туралы естідім олардың идеяларын әдетте үшінші тарап басқарады ол оларды қаржыландырады және презерватив ретінде қолданғаннан кейін әшкерелейді,мен олар туралы есті олардың идея әдет үшінші тарап басқар ол ол қаржыландыр және презерватив рет қолдан кейін әшкереле,nazism

3,оңтүстік африкадағы алғашқы операцияны дудула нхланхлалукс халқы басқарады,оңтүстік африка алғашқы операция дудул нхланхлалукс халқ басқар,nazism

3,осы лератоны итермелеген жалғыз қоңырау шалушы нанте сефудиге рахмет ол өткен уақыттан бері осы заңсыз шетелдіктердің жұтқыншағына кіруге тырысады өйткені ол жақсы жұмыс істейді,осы лерато итермеле жалғыз қоңырау шалуш нан сефуди рахмет ол өткен уақыт бері осы заңсыз шетелдік жұтқыншағ кір тырыс өйткені ол жақсы жұмыс істе,nazism

3,қайырлы күн назар аударыңыз дудула африкада бірінші кезекте жасайтын үй жұмыстары операциясы,қайырл күн назар ау дудул африка бірінші кезек жаса үй жұмыс операция,nazism

3,бұл сіздің қха мен сіздің президентіңіз қолдаған заңсыз шетелдіктеріңіз,бұл сіздің қх мен сіздің президент қолда заңсыз шетелдік,nazism

3,олардың қаншасы заңсыз шетелдіктердің балалары оларды бақылау мүмкін емес,олардың қанша заңсыз шетелдік бала ол бақылау мүмкін емес,nazism

3,содан кейін сіз жұмысқа орналасасыз ба,содан кейін сіз жұмыс орналас ба,nazism

3,біздің ауруханалардағы барлық заңсыз шетелдіктердің іздері нөлге дейін азаятынын көресіз өйткені олардың елшіліктері оларға ешқашан ауруханаға бармауға нұсқау береді,біздің аурухана барлық заңсыз шетелдік із нөл дейін азаятын көр өйткені олардың елшілік оларға ешқашан аурухана барма нұсқау бер,nazism

3,ресурстар шектеулі көпшілік үшін жеткіліксіз олар салық төлемейді сыбайлас жемқорлық мәдениетін қалыптастырады жаңа инфрақұрылым жоқ содан кейін салық төлемейтін миллиондаған заңсыз иммигранттарды әкеледі содан кейін буржуазиялық ақымақтар жеке ауруханаларға барған кезде медициналық көмекке жүгінеді,ресурс шектеулі көпшілік үшін жеткіліксіз олар салық төлеме сыбайлас жемқорлық мәдени қалыптастыр жаңа инфрақұрылым жоқ содан кейін салық төлеме миллиондаған

заңсыз иммигрант әкел содан кейін буржуазиялық ақымақ жеке аурухана бар кезде медициналық көмек жүгін,nazism

3,үндістан сауықтыру саяхаты орталығы ретінде белгілі онда шетелдіктерге жеке пациенттер ретінде есепшот ұсынылды олар денсаулық сақтау арқылы кірісті қалай құратынын біледі егер біз шетелдіктер медициналық қызметтерге ақы төлеуі керек деген ережені енгізсек біздің жіө әлдеқайда жоғары болар ед,үндістан сауықтыру саяхат орталығ рет белгілі онда шетелдік жеке пациент рет есепшот ұсын олар денсаулық сақтау арқылы кір қалай құр біл егер біз шетелдік медициналық қызмет ақы төле керек де ереже енгіз біздің жіө әлдеқайда жоғары бол ед,nazism

3,министр ретінде жұмыс істегіңіз келмегенде сіз ксенофобтарға оңтүстік африкадағы шетелдіктерге зиян келтіруге рұқсат беретін мәлімдемелерге жүгінесіз,министр рет жұмыс істе келме сіз ксенофоб оңтүстік африка шетелдік зиян келтір рұқсат бер мәлімдеме жүг,nazism

3,егер біз заңсыз иммигранттар мен шекаралық бақылау туралы айтпасақ онда мені қызықтырмайды,егер біз заңсыз иммигрант мен шекаралық бақылау туралы айтпа онда мен қызықтырма,nazism

3,сіздің sa қызметкерлеріңізде әбігерге түсуге уақыт жоқ оларда тек кузе кусеге дейін алкоголь ішуге уақыт бар,сіздің sa қызметкер әбіг түс уақыт жоқ ол тек куз кусе дейін алкоголь іш уақыт бар,nazism

3,бакалиле олар умунта озимбела нгоквахе итунаны іздейд,бакалил олар умун озимбел нгоквах итуна іздейд,nazism

3,бізде ел ретінде оңтүстік африкада неге заңсыз шетелдіктер көп екенін неге зерттемеске деген күрделі мәселелер бар,біз ел рет оңтүстік африка неге заңсыз шетелдік көп екен неге зерттемес де күрделі мәселе бар,nazism

3,заңсыз шетелдіктер дегенде менің ойымша сіз ақ адамдарға үнділерге және қытайларға сілтеме жасайсыз,заңсыз шетелдік де менің ойымша сіз ақ адам үнді және қытай сілтеме жаса,nazism

3,сіз рамафоса лейенарды орауды бастауы керек деп айтқыңыз келеді,сіз рамафо лейен орау баста керек де айт кел,nazism

3,олар кез келген нәрсені айтады егер бұл орынды болса нхланхла люкс оларды жұмыртқадан ұстайды,олар кез кел нәрсе айт егер бұл ор бол нхланхл люкс ол жұмыртқа ұста,nazism

3,данте обри мен қатты ренжідім мен көз жасыма ерік бердім біздің президент тіпті оңтүстік африкалықтар мен шетелдіктер арасындағы тыныштықты сұрай алмады ма ханым бұл адам шынымен де елдің өртеніп кетуіне мән бермейді никсниксникстің болашағы үшін күресімізді мойындаңыз,дан обри мен қатты ренжі мен көз жас ерік бер біздің президент тіпті оңтүстік африкалық мен шетелдік ара тыныштық сұра алма

ма ханым бұл адам шын де ел өртен кет мән берме никсниксник болашағ үшін күрес мойында,nazism

3,шекарадан өткен заңсыз иммигранттар да осы заңдарға бағынады дыбыстық бейне барлығын сыртта маска киюге шақырды мұны сіздің қауіпсіздігіңіз үшін және сіз жақсы көретін адамдар үшін жасағаныңызға көз жеткізіңіз,шекара өткен заңсыз иммигрант да осы заң бағын дыбыс бейне барлығ сырт маска кию шақ мұны сіздің қауіпсіздіг үшін және сіз жақсы көр адам үшін жаса көз жеткіз,nazism

3,еңбек департаменті өз жұмысын бастауы керек және заңсыз шетелдіктерді пайдалану үшін ғана жалдайтын осы компаниялардың барлығына баруы керек,еңбек департамен өз жұмыс баста керек және заңсыз шетелдік пайдалану үшін ғана жалда осы компания барлығ бар керек,nazism

3,тек біраз сөйлесіңіз неліктен дудула мен путсутсутафрика операциясы көптеген саяси элиталарды алғаш рет алаңдатты,тек біраз сөйлес неліктен дудул мен путсутсутафрик операция көптеген саяси элита алғаш рет алаңда,nazism

3,ол әрқашан астхукангаға берген нәрсені беруі керек,ол әрқашан астхуканга бер нәрсе беруі керек,nazism

3,сіз менің дәлелдерімді түсінбейтін сияқтысыз мен отан тұрғындарын шетелдіктермен теңестірмеймін,сіз менің дәлел түсінбе сияқты мен отан тұрғын шетелдік теңестірме,nazism

3,бізге оның қолдауы қажет деп кім айтты біз үкіметтің дұрыс нәрсені жасағанын operationdudula конституциясына сәйкес заңдарды қолданғанын қалаймыз,бізге оның қолда қажет де кім ай біз үкімет дұрыс нәрсе жаса operationdudula конституция сәйкес заң қолдан қала,nazism

3,сіздің ақпаратшыңызға оңтүстік африкада жұмыс жоқ пандемия көп проблемалар туғызады қазіргі уақытта ксенофобия бар оларға шетелдік қажет емес олар шетелдікті итеріп жіберу үшін дудула операциясын бастайды,сіздің ақпаратш оңтүстік африка жұмыс жоқ пандемия көп проблема туғыз қазіргі уақыт ксенофобия бар оларға шетелдік қажет емес олар шетелдік итер жіберу үшін дудул операция баста,nazism

3,sff құрылғанға дейін осында келген шетелдіктер осы жолдаудың арқасында осында деп айтқыңыз келе ме,sff құрыл дейін осында кел шетелдік осы жолда арқа осында де айт келе ме,nazism

3,мен мұны естимін бірақ бұл сіздің мәлімдемеңізді растамайды мен мен күн сайын құжаттары жоқ шетелдіктер жасаған қылмыстары үшін қамауға алынатын оқиғалардың дамуын бақылап отыратынымды айттым,мен мұны ести бірақ бұл сіздің мәлімдеме растама мен мен күн сайын құжат жоқ шетелдік жаса қылмыс үшін қама алын оқиға дам бақыла отыр ай,nazism

3,бірақ әрине сіз көшбасшы ретінде ол заңсыздық пен анархияны көтере алмайтынын түсінесіз дудула операциясы оңтүстік африкалықтарға пайдалы болғанымен оны заң

аясында жасау керек,бірақ әрине сіз көшбасшы рет ол заңсыз пен анархия көтер алма түс дудул операция оңтүстік африкалық пайдалы бол оны заң ая жасау керек,nazism

3,содан кейін operationdudula нақты проблемаға тап болған сияқты,содан кейін operationdudula нақты проблема тап бол сияқты,nazism

3,мені таң қалдыратын нәрсе неліктен саяси элиталар мен солшылдар оны өшіруге тырысады,мен таң қалдыр нәрсе неліктен саяси элита мен солшыл оны өшір тырыс,nazism

3,халықтың жартысы жұмыс істемей халық саны азайған кезде қайдан келетіні белгісіз дәл осындай өтірік осы елге барлық қылмыстық элементтер мен заңсыз шетелдіктерді тартады,халық жарты жұмыс істеме халық сан азай кезде қайдан кел белгісіз дәл осындай өтірік осы ел барлық қылмыстық элемент мен заңсыз шетелдік тарт,nazism

3,банктік қосымшаларды қалай жасыруға болады,банктік қосымша қалай жасыр бол,nazism

3,өкінішке орай заңсыз шетелдіктерге осы елдің көшелерімен жүруге рұқсат етілгенге дейін бұл қорқынышты түс болып қала береді,өкініш орай заңсыз шетелдік осы ел көше жүр рұқсат етіл дейін бұл қорқыныш түс бол қала бер,nazism

3,дудула операциясының бұзақылары ешқашан ешкімнің мүлкін жойған емес сіз мұны бірнеше рет жасадыңыз,дудул операция бұза ешқашан ешкі мүлк жой емес сіз мұны бірнеше рет жаса,nazism

3,біз салық пікірталастарын көтеруден аулақ боламыз мен бұл қызметті алу үшін кедейлерге ғана салық төлеймін заңсыз иммигранттарға емес елшіліктерді ауруханаға жатқызу үшін төлеуге мәжбүр етемін,біз салық пікірталас көтеру аулақ бол мен бұл қызмет алу үшін кедей ғана салық төле заңсыз иммигрант емес елшілік аурухана жатқыз үшін төле мәжбүр ет,nazism

3,егер сізде бүкіл африканы құтқаруға деген амбицияңыз болса әрекет етіңіз бауырым біз сізді қолдаймыз,егер сіз бүкіл африка құтқар де амбиция бол әрекет ет бауыр біз сіз қолда,nazism

3,оңтүстік кореяны ақш пен салыстыруды тоқтатыңыз біз бүкіл континентті құтқара алмаймыз,оңтүстік корея ақш пен салыстыру тоқта біз бүкіл континент құтқар алма,nazism

3,оларға дағдылар жетіспейді сондықтан шетелдіктер жақсы жұмыс істейді,оларға дағды жетіспе сондықтан шетелдік жақсы жұмыс істе,nazism

3,онда жұмыс орындары бар бірақ жұмысқа рұқсат алу қымбатқа түседі шетелдіктердің көпшілігі қағазбастылықты болдырмау үшін бизнеспен

айналысады,онда жұмыс орын бар бірақ жұмыс рұқсат алу қымбат түс шетелдік көпшілігі қағазбастылық болдырма үшін бизнес айналыс,nazism

3,біздің президент дудула операциясына қарсы болғандықтан алға апаратын жол қандай,біздің президент дудул операция қарсы бол алға апар жол қандай,nazism

3,ұлттық қауіпсіздікке заңсыз иммигранттар қауіп төндіреді бірақ бұл ұлттық құзырет,ұлттық қауіпсіздік заңсыз иммигрант қауіп төндір бірақ бұл ұлттық құзырет,nazism
