# Supplementary material for: Detection of offensive content in the Kazakh language using machine learning and deep learning approaches
Source: PeerJ Comput Sci. 2025 Aug 11;11:e3027. doi: 10.7717/peerj-cs.3027 (PMC12453855; doi:10.7717/peerj-cs.3027)
Supplement: Supplemental Information 1 [file peerj-cs-11-3027-s001.zip › Code/xlm-mlm.html]

xlm-mlm


## import library¶

In [1]:

```
import sys

sys.path.insert(0, 'C:/Users/Admin-server/destr-content-master')
```

In [2]:

```
# built-in
import re, codecs
import time
import os

# pandas
import pandas as pd
import numpy as np

# nlp-preprocess
from stemming.out2 import stemming
import liwc

# nlp
import nltk 

# sklearn
from sklearn.feature_extraction.text import CountVectorizer, TfidfVectorizer

# plottong
import matplotlib.pyplot as plt
```

In [3]:

```
import sklearn
import pandas as pd
import numpy as np

from sklearn.model_selection import KFold,train_test_split,cross_val_score,cross_validate
from sklearn.feature_extraction.text import TfidfVectorizer, CountVectorizer
from sklearn.metrics import classification_report,accuracy_score, precision_score, recall_score, f1_score, confusion_matrix, roc_auc_score, roc_curve

from sklearn.linear_model import LogisticRegression
from sklearn.neighbors import KNeighborsClassifier
from sklearn.svm import SVC
from sklearn.naive_bayes import GaussianNB, BernoulliNB, MultinomialNB
from sklearn.tree import DecisionTreeClassifier
from sklearn.ensemble import RandomForestClassifier, GradientBoostingClassifier

import seaborn as sns
import matplotlib.pyplot as plt

import pickle

import wikipedia
```

In [4]:

```
import os
import torch
from torch import nn
from torch.utils.data import DataLoader, Dataset
from transformers import BertTokenizer, BertModel, AdamW, get_linear_schedule_with_warmup

from sklearn.model_selection import train_test_split
from sklearn.metrics import accuracy_score, classification_report
import pandas as pd

from nltk.corpus import stopwords
```

```
C:\Users\Admin-server\AppData\Local\Programs\Python\Python37\lib\site-packages\tensorflow\python\framework\dtypes.py:516: FutureWarning: Passing (type, 1) or '1type' as a synonym of type is deprecated; in a future version of numpy, it will be understood as (type, (1,)) / '(1,)type'.
  _np_qint8 = np.dtype([("qint8", np.int8, 1)])
C:\Users\Admin-server\AppData\Local\Programs\Python\Python37\lib\site-packages\tensorflow\python\framework\dtypes.py:517: FutureWarning: Passing (type, 1) or '1type' as a synonym of type is deprecated; in a future version of numpy, it will be understood as (type, (1,)) / '(1,)type'.
  _np_quint8 = np.dtype([("quint8", np.uint8, 1)])
C:\Users\Admin-server\AppData\Local\Programs\Python\Python37\lib\site-packages\tensorflow\python\framework\dtypes.py:518: FutureWarning: Passing (type, 1) or '1type' as a synonym of type is deprecated; in a future version of numpy, it will be understood as (type, (1,)) / '(1,)type'.
  _np_qint16 = np.dtype([("qint16", np.int16, 1)])
C:\Users\Admin-server\AppData\Local\Programs\Python\Python37\lib\site-packages\tensorflow\python\framework\dtypes.py:519: FutureWarning: Passing (type, 1) or '1type' as a synonym of type is deprecated; in a future version of numpy, it will be understood as (type, (1,)) / '(1,)type'.
  _np_quint16 = np.dtype([("quint16", np.uint16, 1)])
C:\Users\Admin-server\AppData\Local\Programs\Python\Python37\lib\site-packages\tensorflow\python\framework\dtypes.py:520: FutureWarning: Passing (type, 1) or '1type' as a synonym of type is deprecated; in a future version of numpy, it will be understood as (type, (1,)) / '(1,)type'.
  _np_qint32 = np.dtype([("qint32", np.int32, 1)])
C:\Users\Admin-server\AppData\Local\Programs\Python\Python37\lib\site-packages\tensorflow\python\framework\dtypes.py:525: FutureWarning: Passing (type, 1) or '1type' as a synonym of type is deprecated; in a future version of numpy, it will be understood as (type, (1,)) / '(1,)type'.
  np_resource = np.dtype([("resource", np.ubyte, 1)])
C:\Users\Admin-server\AppData\Local\Programs\Python\Python37\lib\site-packages\tensorboard\compat\tensorflow_stub\dtypes.py:541: FutureWarning: Passing (type, 1) or '1type' as a synonym of type is deprecated; in a future version of numpy, it will be understood as (type, (1,)) / '(1,)type'.
  _np_qint8 = np.dtype([("qint8", np.int8, 1)])
C:\Users\Admin-server\AppData\Local\Programs\Python\Python37\lib\site-packages\tensorboard\compat\tensorflow_stub\dtypes.py:542: FutureWarning: Passing (type, 1) or '1type' as a synonym of type is deprecated; in a future version of numpy, it will be understood as (type, (1,)) / '(1,)type'.
  _np_quint8 = np.dtype([("quint8", np.uint8, 1)])
C:\Users\Admin-server\AppData\Local\Programs\Python\Python37\lib\site-packages\tensorboard\compat\tensorflow_stub\dtypes.py:543: FutureWarning: Passing (type, 1) or '1type' as a synonym of type is deprecated; in a future version of numpy, it will be understood as (type, (1,)) / '(1,)type'.
  _np_qint16 = np.dtype([("qint16", np.int16, 1)])
C:\Users\Admin-server\AppData\Local\Programs\Python\Python37\lib\site-packages\tensorboard\compat\tensorflow_stub\dtypes.py:544: FutureWarning: Passing (type, 1) or '1type' as a synonym of type is deprecated; in a future version of numpy, it will be understood as (type, (1,)) / '(1,)type'.
  _np_quint16 = np.dtype([("quint16", np.uint16, 1)])
C:\Users\Admin-server\AppData\Local\Programs\Python\Python37\lib\site-packages\tensorboard\compat\tensorflow_stub\dtypes.py:545: FutureWarning: Passing (type, 1) or '1type' as a synonym of type is deprecated; in a future version of numpy, it will be understood as (type, (1,)) / '(1,)type'.
  _np_qint32 = np.dtype([("qint32", np.int32, 1)])
C:\Users\Admin-server\AppData\Local\Programs\Python\Python37\lib\site-packages\tensorboard\compat\tensorflow_stub\dtypes.py:550: FutureWarning: Passing (type, 1) or '1type' as a synonym of type is deprecated; in a future version of numpy, it will be understood as (type, (1,)) / '(1,)type'.
  np_resource = np.dtype([("resource", np.ubyte, 1)])
```

In [5]:

```
import random
import tensorflow as tf

def set_seeds(seed=42):
    os.environ['PYTHONHASHSEED'] = str(seed)
    random.seed(seed)
    tf.random.set_random_seed(seed)
    torch.manual_seed(seed)
    np.random.seed(seed)
    
set_seeds(seed=42)
```

```
WARNING:tensorflow:From C:\Users\Admin-server\AppData\Local\Temp\ipykernel_8828\3683599047.py:7: The name tf.random.set_random_seed is deprecated. Please use tf.compat.v1.random.set_random_seed instead.
```

## read data¶

In [6]:

```
df_1 = pd.read_csv('../dataset/multiclass/shortened_data.csv', error_bad_lines=False, sep=',', delimiter='\n', header=None)
df_1 = df_1[0].str.replace(';', ',').str.split(',', n=1, expand=True)
df_1.columns = ['label', 'message']
df_1.message = df_1.message.str.lower().str.strip()
df_1 = df_1[df_1.label == 'violent']
df_1
```

Out[6]:

|  | label | message |
| --- | --- | --- |
| 0 | violent | біздің сарбаздарымыз өз істерінің әділдігімен ... |
| 1 | violent | біздің еркін болғанымызды ештеңе жеңе алмайды ... |
| 2 | violent | біз барак обама джордж буштың қасіретті мұрасы... |
| 3 | violent | израильдің агрессиясына қарсы күн сайынғы нара... |
| 4 | violent | израиль сөзсіз газаның жойылуын өлімі мен қайғ... |
| ... | ... | ... |
| 401 | violent | менің әкем қайтадан кафтерлермен және мұсылман... |
| 402 | violent | тоқ жанғұт бауырым мен исламның мелайю патшалы... |
| 403 | violent | бұл жақсы жаңалық олар фатанаи халқына лайықты... |
| 404 | violent | aslamoalaikum бауырым бұл мәселеде дәлелдеуге ... |
| 405 | violent | пәкістандағы исламдық ұйымдардағы немесе басқа... |

395 rows × 2 columns

In [7]:

```
df_2 = pd.read_csv('../dataset/multiclass/neutral.csv', error_bad_lines=False, sep=',', delimiter='\n')
df_2 = df_2['label,message'].str.replace(';', ',').str.split(',', n=1, expand=True)
df_2.columns = ['label', 'message']
df_2['label'] = 'neutral'
df_2.message = df_2.message.str.lower().str.strip()
df_2
```

Out[7]:

|  | label | message |
| --- | --- | --- |
| 0 | neutral | адамның басшысы ақыл. |
| 1 | neutral | жетекшісі талап . |
| 2 | neutral | жолаушысы ой. |
| 3 | neutral | жолдасы кәсіп. |
| 4 | neutral | қорғаны сабыр. |
| ... | ... | ... |
| 1773 | neutral | жеткенше тыйылмады. міне соның қырқын жыртық ү... |
| 1774 | neutral | "жетті. ол қайтыс болды. артында: ""қанатым едің |
| 1775 | neutral | жинап |
| 1776 | neutral | жиырмаларға іліге берген шақтарда ағайынды екі... |
| 1777 | neutral | жиюға айналды. болған оқиға мынау еді:,['еді' |

1778 rows × 2 columns

In [8]:

```
with open('../dataset/multiclass/corp_multi 2023-09-04.csv', encoding='utf-8') as f:
    headers = ['label', 'message']
    data = f.readlines()[1:]
    
    
classes = ['racism', 'bullying', 'nazism', 'violent', 'phishing', 'polit']


prepare = []
for row in data:
    if any(x in row for x in classes):
        label = [x for x in classes if x in row][0]
        _row = row.lower().replace('"', ' ').strip()
        _row = _row[:_row.find(label)+len(label)] + ',' + _row[_row.find(label)+len(label)+1:]
        prepare.append(_row)
    else:
        prepare[-1] += f' {row.strip().lower()}'
        
        
df_3 = pd.DataFrame(prepare)[0].str.split(',', n=1, expand=True)
df_3.columns = headers
df_3
```

Out[8]:

|  | label | message |
| --- | --- | --- |
| 0 | violent | мен талибтерді қолдаймын. махсуд кіші өз тәуел... |
| 1 | violent | талибан мұны ауғанстандағы тәжіктерге дұрыс жа... |
| 2 | violent | алға талибтер бұл тәжіктердің емес сіздердің ж... |
| 3 | violent | жарайсыңдар талибтер әйелдер өз орнын білу кер... |
| 4 | violent | мен бұұ мен батыстық гуманитарийларға қарағанд... |
| ... | ... | ... |
| 8610 | nazism | оңтүстік кореяны ақш пен салыстыруды тоқтатыңы... |
| 8611 | nazism | оларға дағдылар жетіспейді сондықтан шетелдікт... |
| 8612 | nazism | онда жұмыс орындары бар бірақ жұмысқа рұқсат а... |
| 8613 | nazism | біздің президент дудула операциясына қарсы бо... |
| 8614 | nazism | ұлттық қауіпсіздікке заңсыз иммигранттар қауіп... |

8615 rows × 2 columns

In [9]:

```
df = pd.concat([
    df_1,
    df_2,
    df_3
], ignore_index=True)
df = df.drop_duplicates()
df.message = df.message\
.str.replace('racism', ' ')\
.str.replace('bullying', ' ')\
.str.replace('nazism', ' ')\
.str.replace('violent', ' ')\
.str.replace('neutral', ' ')
df = df[df.label.isin(['racism', 'bullying', 'nazism', 'violent', 'neutral'])]
df
```

Out[9]:

|  | label | message |
| --- | --- | --- |
| 0 | violent | біздің сарбаздарымыз өз істерінің әділдігімен ... |
| 1 | violent | біздің еркін болғанымызды ештеңе жеңе алмайды ... |
| 2 | violent | біз барак обама джордж буштың қасіретті мұрасы... |
| 3 | violent | израильдің агрессиясына қарсы күн сайынғы нара... |
| 4 | violent | израиль сөзсіз газаның жойылуын өлімі мен қайғ... |
| ... | ... | ... |
| 10783 | nazism | оңтүстік кореяны ақш пен салыстыруды тоқтатыңы... |
| 10784 | nazism | оларға дағдылар жетіспейді сондықтан шетелдікт... |
| 10785 | nazism | онда жұмыс орындары бар бірақ жұмысқа рұқсат а... |
| 10786 | nazism | біздің президент дудула операциясына қарсы бо... |
| 10787 | nazism | ұлттық қауіпсіздікке заңсыз иммигранттар қауіп... |

10479 rows × 2 columns

In [10]:

```
df.label.value_counts()
```

Out[10]:

```
violent     2316
racism      2209
bullying    2139
nazism      2083
neutral     1732
Name: label, dtype: int64
```

In [11]:

```
def clean(text):
    affixes = [
        'ның', 'нің', 'дың', 'дің', 'тың', 'тің', 
        'ға', 'ге', 'қа', 'ке', 'на', 'не', 
        'а', 'е', 'ды', 'ді', 'ты', 'ті', 'ны', 'ні', 
        'н', 'да', 'де', 'та', 'те', 
        'нда', 'нде', 'нан', 'нен', 'дан', 'ден', 'тан', 'тен', 
        'мен', 'менен', 'бен', 'бенен', 'пен', 'пенен'
    ]

    documents = []

    document = str(text).lower()

    # Remove url address
    document = re.sub(r'(https|http)?:\/(\w|\.|\/|\?|\=|\&|\%|\-|\…)*', '', document)

    # Removing Twitter Handles (@user)
    document = re.sub(r'@[\w]*', '', document)

    # Remove all the special characters (Удалить все специальные символы, кроме буквы и цифры)
    document = re.sub(r'\W', ' ', document)

    # Remove all single characters
    document = re.sub(r'\s+[a-zа-я]\s+', ' ', document)

    # Remove single characters from the start
    document = re.sub(r'\^[a-zа-я]\s+', ' ', document)

    # Tokenization
    document = document.split()

    # Remove remaining tokens that are not alphabetic
    document = [word for word in document if word.isalpha()]

    # filter out stop words
    stop_words = set(stopwords.words('kazakh'))
    # document = [w for w in document if w not in stop_words]

    # Дефис арқылы жалғанған жалғауларды алып тастау
    # document = [w for w in document if w not in affixes]

    # Removing short words (len>2)
    # document = [w for w in document if len(w)>2]

    document = ' '.join(document)

    return document
```

In [12]:

```
# df.message = df.message\
# .str.replace(r"([^а-яА-Яa-zA-Z\u04D8\u04D9\u04B0\u04B1\u0406\u0456\u04A2\u04A3\u0492\u0493\u04AE\u04AF\u049A\u049B\u04E8\u04E9\u04BA\u04BB ]+)", ' ', regex=True)\
# .str.replace('.', ' ')\
# .str.replace(r'\s+', ' ', regex=True)\
# .str.strip()\
# .str.lower()

df['message'] = df.message.str.replace(r'https?://[^\s<>"]+|www\.[^\s<>"]+', "") # remove links
df['message'] = df['message'].apply(clean)
df = df[(df['message'].str.split().apply(len) < 128) & (df['message'].str.split().apply(len) > 1)]
df
```

```
C:\Users\Admin-server\AppData\Local\Programs\Python\Python37\lib\site-packages\ipykernel_launcher.py:8: FutureWarning: The default value of regex will change from True to False in a future version.
```

Out[12]:

|  | label | message |
| --- | --- | --- |
| 0 | violent | біздің сарбаздарымыз өз істерінің әділдігімен ... |
| 1 | violent | біздің еркін болғанымызды ештеңе жеңе алмайды ... |
| 2 | violent | біз барак обама джордж буштың қасіретті мұрасы... |
| 3 | violent | израильдің агрессиясына қарсы күн сайынғы нара... |
| 4 | violent | израиль сөзсіз газаның жойылуын өлімі мен қайғ... |
| ... | ... | ... |
| 10783 | nazism | оңтүстік кореяны ақш пен салыстыруды тоқтатыңы... |
| 10784 | nazism | оларға дағдылар жетіспейді сондықтан шетелдікт... |
| 10785 | nazism | онда жұмыс орындары бар бірақ жұмысқа рұқсат а... |
| 10786 | nazism | біздің президент дудула операциясына қарсы бол... |
| 10787 | nazism | ұлттық қауіпсіздікке заңсыз иммигранттар қауіп... |

10190 rows × 2 columns

In [13]:

```
%%time

label_dict = {
    'neutral': 0,
    'racism': 1,
    'bullying': 2,
    'nazism': 3,
    'violent': 4
}

df['label'] = df['label'].apply(lambda x: label_dict[x])
df['message_stemmed'] = df['message'].apply(stemming)
```

```
C:\Users\Admin-server\AppData\Local\Programs\Python\Python37\lib\site-packages\ipykernel_launcher.py:9: SettingWithCopyWarning: 
A value is trying to be set on a copy of a slice from a DataFrame.
Try using .loc[row_indexer,col_indexer] = value instead

See the caveats in the documentation: https://pandas.pydata.org/pandas-docs/stable/user_guide/indexing.html#returning-a-view-versus-a-copy
  if __name__ == "__main__":
```

```
Wall time: 43.2 s
```

```
C:\Users\Admin-server\AppData\Local\Programs\Python\Python37\lib\site-packages\ipykernel_launcher.py:10: SettingWithCopyWarning: 
A value is trying to be set on a copy of a slice from a DataFrame.
Try using .loc[row_indexer,col_indexer] = value instead

See the caveats in the documentation: https://pandas.pydata.org/pandas-docs/stable/user_guide/indexing.html#returning-a-view-versus-a-copy
  # Remove the CWD from sys.path while we load stuff.
```

In [14]:

```
number_of_classes = df['label'].unique().shape[0]

df['label'].value_counts()
```

Out[14]:

```
1    2205
2    2136
4    2110
3    2076
0    1663
Name: label, dtype: int64
```

In [15]:

```
from sklearn.metrics import roc_curve, auc

def plotting_auc_curve(y_test, predict_proba, name, title):
#     fpr, tpr, _ = roc_curve(y_test, predict_proba)
#     plt.title('Receiver Operating Characteristic, '+title)
#     plt.plot(fpr, tpr, label = name)
#     plt.legend(loc = 'lower right')
#     plt.plot([0, 1], [0, 1],'b--')
#     plt.xlim([-0.05, 1.05])
#     plt.ylim([-0.05, 1.05])
#     plt.ylabel('True Positive Rate')
#     plt.xlabel('False Positive Rate')
    pass
```

## torch train¶

In [16]:

```
from tqdm import tqdm
```

In [17]:

```
texts = df['message_stemmed'].values.tolist()

labels = df['label'].values.tolist()
```

In [18]:

```
class TextClassificationDataset(Dataset):
    def __init__(self, texts, labels, tokenizer, max_length):
        self.texts = texts
        self.labels = labels
        self.tokenizer = tokenizer
        self.max_length = max_length
    def __len__(self):
        return len(self.texts)
    def __getitem__(self, idx):
        text = self.texts[idx]
        label = self.labels[idx]
        encoding = self.tokenizer(text, return_tensors='pt', max_length=self.max_length, padding='max_length', truncation=True)
        return {'input_ids': encoding['input_ids'].flatten(), 'attention_mask': encoding['attention_mask'].flatten(), 'label': torch.tensor(label)}
```

In [19]:

```
from transformers import AutoTokenizer, AutoModel, AutoModelForMaskedLM, AutoModelForSequenceClassification
from transformers import DistilBertTokenizer, DistilBertModel
from transformers import RobertaTokenizer, RobertaModel
from transformers import XLMRobertaTokenizer, XLMRobertaModel
from transformers import XLMTokenizer, XLMModel
```

In [20]:

```
class XLMClass(torch.nn.Module):
    def __init__(self, model_name, num_classes):
        super(XLMClass, self).__init__()
        self.l1 = XLMModel.from_pretrained(model_name)
        self.pre_classifier = torch.nn.Linear(1280, 768)
        self.dropout = torch.nn.Dropout(0.1)
        self.classifier = torch.nn.Linear(768, num_classes)

    def forward(self, input_ids, attention_mask):
        output_1 = self.l1(input_ids=input_ids, attention_mask=attention_mask)
        hidden_state = output_1[0]
        pooler = hidden_state[:, 0]
        pooler = self.pre_classifier(pooler)
        pooler = torch.nn.ReLU()(pooler)
        pooler = self.dropout(pooler)
        output = self.classifier(pooler)
        return output
```

In [21]:

```
def train(model, data_loader, optimizer, scheduler, device):
    model.train()
    for batch in tqdm(data_loader):
        optimizer.zero_grad()
        input_ids = batch['input_ids'].to(device)
        attention_mask = batch['attention_mask'].to(device)
        labels = batch['label'].to(device)
        outputs = model(input_ids=input_ids, attention_mask=attention_mask)
        loss = nn.CrossEntropyLoss()(outputs, labels)
        loss.backward()
        optimizer.step()
        scheduler.step()
```

In [22]:

```
def evaluate(model, data_loader, device):
    model.eval()
    predictions = []
    actual_labels = []
    with torch.no_grad():
        for batch in data_loader:
            input_ids = batch['input_ids'].to(device)
            attention_mask = batch['attention_mask'].to(device)
            labels = batch['label'].to(device)
            outputs = model(input_ids=input_ids, attention_mask=attention_mask)
            _, preds = torch.max(outputs, dim=1)
            predictions.extend(preds.cpu().tolist())
            actual_labels.extend(labels.cpu().tolist())
    return accuracy_score(actual_labels, predictions), classification_report(actual_labels, predictions)
```

In [23]:

```
def evaluate_predict(model, data_loader, device):
    model.eval()
    predictions = []
    actual_labels = []
    with torch.no_grad():
        for batch in data_loader:
            input_ids = batch['input_ids'].to(device)
            attention_mask = batch['attention_mask'].to(device)
            labels = batch['label'].to(device)
            outputs = model(input_ids=input_ids, attention_mask=attention_mask)
            _, preds = torch.max(outputs, dim=1)
            predictions.extend(outputs.cpu().tolist())
            actual_labels.extend(labels.cpu().tolist())
    return np.array(actual_labels), np.array(predictions)
```

In [24]:

```
# Set up parameters
model_name = "xlm-mlm-100-1280"
num_classes = 5
max_length = 64
batch_size = 128
num_epochs = 10
learning_rate = 2e-5 # 2e-5
```

In [25]:

```
train_texts, val_texts, train_labels, val_labels = train_test_split(texts, labels, test_size=0.2, random_state=42)
```

In [26]:

```
tokenizer = XLMTokenizer.from_pretrained(model_name)
train_dataset = TextClassificationDataset(train_texts, train_labels, tokenizer, max_length)
val_dataset = TextClassificationDataset(val_texts, val_labels, tokenizer, max_length)
train_dataloader = DataLoader(train_dataset, batch_size=batch_size, shuffle=True)
val_dataloader = DataLoader(val_dataset, batch_size=batch_size)
```

In [27]:

```
device = torch.device("cuda" if torch.cuda.is_available() else "cpu")

model = XLMClass(model_name, num_classes).to(device)
# model = LLMClassifier(model_name, num_classes).to(device)
```

In [28]:

```
optimizer = AdamW(model.parameters(), lr=learning_rate)
total_steps = len(train_dataloader) * num_epochs
scheduler = get_linear_schedule_with_warmup(optimizer, num_warmup_steps=0, num_training_steps=total_steps)
```

In [29]:

```
for epoch in range(num_epochs):
    print(f"Epoch {epoch + 1}/{num_epochs}")
    train(model, train_dataloader, optimizer, scheduler, device)
    accuracy, report = evaluate(
        model, val_dataloader, device)
    print(f"Validation Accuracy: {accuracy:.4f}")
    print(report)
```

```
Epoch 1/10
```

```
100%|██████████████████████████████████████████████████████████████████████████████████| 64/64 [39:55<00:00, 37.44s/it]
```

```
Validation Accuracy: 0.8086
              precision    recall  f1-score   support

           0       0.83      0.55      0.66       345
           1       0.97      0.86      0.91       441
           2       0.63      0.92      0.75       409
           3       0.80      0.88      0.84       403
           4       0.92      0.79      0.85       440

    accuracy                           0.81      2038
   macro avg       0.83      0.80      0.80      2038
weighted avg       0.83      0.81      0.81      2038

Epoch 2/10
```

```
100%|██████████████████████████████████████████████████████████████████████████████████| 64/64 [41:48<00:00, 39.20s/it]
```

```
Validation Accuracy: 0.8562
              precision    recall  f1-score   support

           0       0.85      0.74      0.80       345
           1       0.94      0.92      0.93       441
           2       0.71      0.91      0.80       409
           3       0.92      0.84      0.88       403
           4       0.91      0.85      0.88       440

    accuracy                           0.86      2038
   macro avg       0.86      0.85      0.85      2038
weighted avg       0.87      0.86      0.86      2038

Epoch 3/10
```

```
100%|██████████████████████████████████████████████████████████████████████████████████| 64/64 [41:45<00:00, 39.15s/it]
```

```
Validation Accuracy: 0.8852
              precision    recall  f1-score   support

           0       0.85      0.83      0.84       345
           1       0.95      0.93      0.94       441
           2       0.86      0.87      0.86       409
           3       0.93      0.85      0.89       403
           4       0.85      0.93      0.88       440

    accuracy                           0.89      2038
   macro avg       0.89      0.88      0.88      2038
weighted avg       0.89      0.89      0.89      2038

Epoch 4/10
```

```
100%|██████████████████████████████████████████████████████████████████████████████████| 64/64 [35:17<00:00, 33.09s/it]
```

```
Validation Accuracy: 0.8700
              precision    recall  f1-score   support

           0       0.92      0.73      0.81       345
           1       0.92      0.95      0.93       441
           2       0.75      0.93      0.83       409
           3       0.88      0.86      0.87       403
           4       0.92      0.85      0.89       440

    accuracy                           0.87      2038
   macro avg       0.88      0.86      0.87      2038
weighted avg       0.88      0.87      0.87      2038

Epoch 5/10
```

```
100%|██████████████████████████████████████████████████████████████████████████████████| 64/64 [22:31<00:00, 21.12s/it]
```

```
Validation Accuracy: 0.8925
              precision    recall  f1-score   support

           0       0.87      0.83      0.85       345
           1       0.93      0.96      0.94       441
           2       0.88      0.88      0.88       409
           3       0.93      0.88      0.90       403
           4       0.86      0.90      0.88       440

    accuracy                           0.89      2038
   macro avg       0.89      0.89      0.89      2038
weighted avg       0.89      0.89      0.89      2038

Epoch 6/10
```

```
100%|██████████████████████████████████████████████████████████████████████████████████| 64/64 [22:31<00:00, 21.11s/it]
```

```
Validation Accuracy: 0.8871
              precision    recall  f1-score   support

           0       0.92      0.79      0.85       345
           1       0.92      0.95      0.94       441
           2       0.86      0.88      0.87       409
           3       0.88      0.89      0.88       403
           4       0.86      0.91      0.88       440

    accuracy                           0.89      2038
   macro avg       0.89      0.88      0.88      2038
weighted avg       0.89      0.89      0.89      2038

Epoch 7/10
```

```
100%|██████████████████████████████████████████████████████████████████████████████████| 64/64 [22:31<00:00, 21.11s/it]
```

```
Validation Accuracy: 0.8930
              precision    recall  f1-score   support

           0       0.89      0.82      0.85       345
           1       0.95      0.94      0.95       441
           2       0.84      0.90      0.87       409
           3       0.89      0.90      0.89       403
           4       0.89      0.89      0.89       440

    accuracy                           0.89      2038
   macro avg       0.89      0.89      0.89      2038
weighted avg       0.89      0.89      0.89      2038

Epoch 8/10
```

```
100%|██████████████████████████████████████████████████████████████████████████████████| 64/64 [22:31<00:00, 21.11s/it]
```

```
Validation Accuracy: 0.8837
              precision    recall  f1-score   support

           0       0.90      0.78      0.84       345
           1       0.95      0.94      0.95       441
           2       0.81      0.90      0.85       409
           3       0.90      0.87      0.89       403
           4       0.87      0.90      0.88       440

    accuracy                           0.88      2038
   macro avg       0.89      0.88      0.88      2038
weighted avg       0.89      0.88      0.88      2038

Epoch 9/10
```

```
100%|██████████████████████████████████████████████████████████████████████████████████| 64/64 [22:31<00:00, 21.11s/it]
```

```
Validation Accuracy: 0.8886
              precision    recall  f1-score   support

           0       0.91      0.78      0.84       345
           1       0.96      0.94      0.95       441
           2       0.83      0.92      0.87       409
           3       0.87      0.90      0.88       403
           4       0.89      0.88      0.89       440

    accuracy                           0.89      2038
   macro avg       0.89      0.88      0.89      2038
weighted avg       0.89      0.89      0.89      2038

Epoch 10/10
```

```
100%|██████████████████████████████████████████████████████████████████████████████████| 64/64 [22:31<00:00, 21.11s/it]
```

```
Validation Accuracy: 0.8911
              precision    recall  f1-score   support

           0       0.90      0.80      0.85       345
           1       0.96      0.94      0.95       441
           2       0.84      0.91      0.87       409
           3       0.87      0.90      0.89       403
           4       0.88      0.89      0.89       440

    accuracy                           0.89      2038
   macro avg       0.89      0.89      0.89      2038
weighted avg       0.89      0.89      0.89      2038
```

In [30]:

```
from sklearn.preprocessing import minmax_scale

y_test, predict_proba = evaluate_predict(model, val_dataloader, device)
predict_proba = minmax_scale(predict_proba.T).T
predict_proba = predict_proba * (1/predict_proba.sum(axis=1)).reshape(-1, 1)
predict = np.argmax(predict_proba, axis=1)

clf_report = classification_report(y_test,
                                   predict,
                                   output_dict=True,
                                   target_names=label_dict.keys())

sns.heatmap(pd.DataFrame(clf_report).iloc[:-1, :].T, annot=True, cmap='Blues', fmt='g')
algo_title = 'message'+' '+'XLM_MLM'+' '+'Linear'
plt.title(f'Metrics, '+algo_title)
plt.show()

# plotting_auc_curve(y_test, predict_proba[:, 1], 'roc-auc curve', algo_title)
# plt.show()

confusion_matrix_df = pd.DataFrame(confusion_matrix(y_test, predict), 
                     index = label_dict.keys(),
                     columns = label_dict.keys())

sns.heatmap(confusion_matrix_df, annot=True, cmap='Blues', fmt='g')
plt.title('True, False, Positive, Negative Plot, '+algo_title)
plt.xlabel('True')
plt.ylabel('Prediction')
plt.show()


precision = precision_score(y_test, predict, pos_label='positive', average='weighted')
recall = recall_score(y_test, predict, pos_label='positive', average='weighted')
accuracy = accuracy_score(y_test, predict)
f1 = f1_score(y_test, predict, pos_label='positive', average='weighted')
roc_auc = roc_auc_score(y_test, predict_proba, multi_class='ovr')
current_metrics = {
    'data_type': 'message',
    'vectorizer': 'MLM',
    'algorithm': 'Linear',
    'precision': precision,
    'recall': recall,
    'accuracy': accuracy,
    'f1': f1,
    'roc_auc': roc_auc,
}
current_metrics
```

```
C:\Users\Admin-server\AppData\Local\Programs\Python\Python37\lib\site-packages\sklearn\metrics\_classification.py:1375: UserWarning: Note that pos_label (set to 'positive') is ignored when average != 'binary' (got 'weighted'). You may use labels=[pos_label] to specify a single positive class.
  UserWarning,
C:\Users\Admin-server\AppData\Local\Programs\Python\Python37\lib\site-packages\sklearn\metrics\_classification.py:1375: UserWarning: Note that pos_label (set to 'positive') is ignored when average != 'binary' (got 'weighted'). You may use labels=[pos_label] to specify a single positive class.
  UserWarning,
C:\Users\Admin-server\AppData\Local\Programs\Python\Python37\lib\site-packages\sklearn\metrics\_classification.py:1375: UserWarning: Note that pos_label (set to 'positive') is ignored when average != 'binary' (got 'weighted'). You may use labels=[pos_label] to specify a single positive class.
  UserWarning,
```

Out[30]:

```
{'data_type': 'message',
 'vectorizer': 'MLM',
 'algorithm': 'Linear',
 'precision': 0.8924860211488913,
 'recall': 0.8910696761530913,
 'accuracy': 0.8910696761530913,
 'f1': 0.8908857520487236,
 'roc_auc': 0.9790764511399137}
```

In [ ]:

```

```
