# Supplementary material for: Detection of offensive content in the Kazakh language using machine learning and deep learning approaches
Source: PeerJ Comput Sci. 2025 Aug 11;11:e3027. doi: 10.7717/peerj-cs.3027 (PMC12453855; doi:10.7717/peerj-cs.3027)
Supplement: Supplemental Information 1 [file peerj-cs-11-3027-s001.zip › Code/_ML_and_DL(GRU,_CNN_+++).html]

\_ML\_and\_DL(GRU,\_CNN\_+++)


!pip -q install torch\_geometric
!pip install torch

In [1]:

```
import torch
import numpy as np
import networkx as nx
import matplotlib.pyplot as plt
```

In [2]:

```
import pandas as pd
df = pd.read_csv('multiclass_2024_04_16.csv', usecols=['label', 'message', 'message_stemmed', 'label_name'])
df.head()
```

Out[2]:

|  | label | message | message\_stemmed | label\_name |
| --- | --- | --- | --- | --- |
| 0 | 4 | біздің сарбаздарымыз өз істерінің әділдігімен ... | біздің сарбаз өз іс әділдіг қарулан басқынш ар... | violent |
| 1 | 4 | біздің еркін болғанымызды ештеңе жеңе алмайды ... | біздің еркін бол ештеңе же алма тағы бір рет в... | violent |
| 2 | 4 | біз барак обама джордж буштың қасіретті мұрасы... | біз барак оба джордж буш қасірет мұра бастал д... | violent |
| 3 | 4 | израильдің агрессиясына қарсы күн сайынғы нара... | израиль агрессия қарсы күн сайынғы наразылық а... | violent |
| 4 | 4 | израиль сөзсіз газаның жойылуын өлімі мен қайғ... | израиль сөзсіз газа жойыл өлім мен қайғы қасір... | violent |

In [3]:

```
from sklearn.model_selection import train_test_split

X_train, X_test, y_train, y_test = train_test_split(df['message'], df['label'], stratify=df['label'])
```

In [4]:

```
original_train_sentences = X_train.tolist()
original_labels_train = y_train.tolist()
original_test_sentences = X_test.tolist()
original_labels_test = y_test.tolist()

train_size = len(original_train_sentences)
test_size = len(original_test_sentences)
sentences = original_train_sentences + original_test_sentences
```

In [5]:

```
import seaborn as sns
```

In [6]:

```
category_sizes = df.groupby('label_name').size()
sns.barplot(x=category_sizes.index, y= category_sizes,)
plt.show()
```

In [7]:

```
fig = plt.figure(figsize=(14,7))
df['length'] = df.message.str.split().apply(len)
ax1 = fig.add_subplot(122)
sns.histplot(df['length'], ax=ax1,color='green')
describe = df.length.describe().to_frame().round(2)

ax2 = fig.add_subplot(121)
ax2.axis('off')
font_size = 14
bbox = [0, 0, 1, 1]
table = ax2.table(cellText = describe.values, rowLabels = describe.index, bbox=bbox, colLabels=describe.columns)
table.set_fontsize(font_size)
fig.suptitle('Distribution of text length for text.', fontsize=16)

plt.show()
```

In [8]:

```
custom_stop_words = {
    "ах", "тағы", "тағыда", "әрине","жоқ","сондай","осындай","осылай","солай","мұндай","бұндай","мен","сен","ол","біз","біздер",
  "олар","сіз","сіздер","маған","оған","саған","біздің""сіздің","оның","бізге","сізге","оларға","біздерге","сіздерге","оларға",
  "менімен","сенімен","онымен","бізбен","сізбен","олармен","біздермен","сіздермен","менің","сенің","біздің","сіздің","оның",
  "біздердің","сіздердің","олардың","маған","саған","оған","менен","сенен","одан","бізден","сізден","олардан","біздерден",
  "сіздерден","айтпақшы","сонымен","сондықтан","бұл","осы","сол","анау","мынау","сонау","осынау","ана","мына","сона","әні","міне",
  "кейбір","қайсыбір","әрбір","бірнеше","бірдеме","әркім","әрне","әрқайсы","әрқалай","әлдекім","әлдене","әлдеқайдан","әлденеше",
  "әлдеқалай","әлдеқашан","алдақашан","ешкім","ешбір","ешқашан","ешқандай","емес","бәрі","бар","бүкіл","өзім","өзің","дегенмен",
  "әйтпесе", "себебі", "өйткені", "сондықтан", "үшін", "сияқты", "туралы", "арқылы", "шамалы", "осындай", "ғана", "қана", "тек",
  "әншейін", "және", "деп", "керек","бірақ","бір","да","егер","адам","адамдар","да","де","оны","болып","босқа","басқа","болды", "мүмкін"
  "онда","барлық","немесе","одан", "рет", "өз", "кейін","пен","со","ал","болады","rt","мені","өте","ал","сс","сені","екен"
  "еді","келеді","болса","не","тым","сізді", "ба", "иә", "ең", "сізде", "сізді", "өз","желп","анау","бізге","мәссаған","сізден",
  "уай","сарт","саңқ","қайқаң-құйқаң","әйда","sub","acele","тағыда","eram","қалт-қалт","қызараң-қызараң",
  "эй","әйтпесе","шырт","шіңк","арс-ұрс","сайын","дүңк","масқарай","салаң-сұлаң","бұрын","әйткенмен","я","ой","далаң-далаң","пішту",
  "міне","сізге","күллі","кейбір","олар","ие","кәнеки","шаңқ-шұңқ","оған","ештеме","ешқашан","ешкім","беу","ырқ","ea","үйт","ербелең-ербелең",
  "әлденеше","қалт-құлт","барша","менен","айтпақшы","әлдеқалай","одан","өзіме","үшін","кейбіреу","кірт","шақты","ыңқ","жалт-жұлт",
  "кәне","қатар","біздердің","оның","уау","жуық","борт","эх","ол","алдақашан","шек","біздерден","бүкіл","ca","солай","бойымен","сондықтан",
  "олардың","арнайы","кәні","өй","өзінің","әлдекім","қайсыбір","мына","сарт-сұрт","сенен","онан","күрт","қорс","қап","туралы","гүрс","гөрі",
  "осы","мышы","сіздерден","әй","еш","арс","әркім","күңк","дегенмен","жалт-жалт","пай-пай","бетер","менімен","сенен","өйткені",
  "әлдеқайдан","сіз","митың-митың","өзі","себебі","бізбен","бірақ","онымен","құрау-құрау","сонымен","сізбен","өзге","дүрс","қаңғыр-күңгір",
  "алатау","таңқ","пфша","сол","pe","шамалы","паһ-паһ","сона","қаралы","сияқты","ә","тәк","е","менің","ғұрлы","ал","бірге","қолп",
  "пай","өзіне","па","таман","бізден","батыр-бұтыр","сондай","әрқайсы","бәрекелді","біздің","бүйт","саған","тарс-тұрс",
  "тарбаң-тарбаң","ыржың-тыржың","әрбір","пырс","ай","барқ","соң","бойы","күшім","қыңқ","арсалаң-арсалаң","сыңқ","салым",
  "ура","түгел","шіркін","алайда","кә","бәрі","шәйт","шейін","ей","шаңқ","әттең","ешқайсы","кәһ","арбаң-арбаң","бұндай","құр",
  "ойпырмай","қана","қаңқ-қаңқ","маңқ","қаңқ-құңқ","аһа","былп","морт","болп","олармен","қош-қош","тырс","ана","сорап","әлдене","ау"
  "өзімнің","aici","сенде","осылай","ырс","қоса","жалп","жаракімалла","біздерге","менде","япырмай","түге","ох","алақай","ғұрлым",
  "таяу","тарта","астапыралла","мыңқ","пішә","бүгжең-бүгжең","о","құрау","әттеген-ай","дейін","әттегенай","ei","тыңқ","қарай",
  "әукім","әрне","біреу","бері","бірнеше"
}

df['clean_text'] = df['message'].apply(lambda text: ' '.join(word for word in text.split() if word.lower() not in custom_stop_words))

print(df.head())
```

```
   label                                            message  \
0      4  біздің сарбаздарымыз өз істерінің әділдігімен ...   
1      4  біздің еркін болғанымызды ештеңе жеңе алмайды ...   
2      4  біз барак обама джордж буштың қасіретті мұрасы...   
3      4  израильдің агрессиясына қарсы күн сайынғы нара...   
4      4  израиль сөзсіз газаның жойылуын өлімі мен қайғ...   

                                     message_stemmed label_name  length  \
0  біздің сарбаз өз іс әділдіг қарулан басқынш ар...    violent      22   
1  біздің еркін бол ештеңе же алма тағы бір рет в...    violent      23   
2  біз барак оба джордж буш қасірет мұра бастал д...    violent      52   
3  израиль агрессия қарсы күн сайынғы наразылық а...    violent      17   
4  израиль сөзсіз газа жойыл өлім мен қайғы қасір...    violent      34   

                                          clean_text  
0  сарбаздарымыз істерінің әділдігімен қаруланып ...  
1  еркін болғанымызды ештеңе жеңе алмайды вашингт...  
2  барак обама джордж буштың қасіретті мұрасынан ...  
3  израильдің агрессиясына қарсы күн сайынғы нара...  
4  израиль сөзсіз газаның жойылуын өлімі қайғы қа...
```

In [9]:

```
df.to_csv('Df_without_stopwords.csv', index=False)
```

In [10]:

```
from collections import Counter
import plotly.express as px
```

In [11]:

```
top = Counter([word for tokens in df['clean_text'].str.split() for word in tokens])
temp = pd.DataFrame(top.most_common(20))
temp.columns = ['Common_words','count']
fig = px.bar(temp, x="Common_words", y="count", title='Common Words in message without stopwords', orientation='v',
             width=700, height=700,color='Common_words')
fig.show()
```

In [12]:

```
bullying_text = df[df['label_name']=='bullying']
nazism_text = df[df['label_name']=='nazism']
neutral_text = df[df['label_name']=='neutral']
racism_text = df[df['label_name']=='racism']
violent_text = df[df['label_name']=='violent']
```

In [13]:

```
top = Counter([word for tokens in bullying_text['clean_text'].str.split() for word in tokens])
temp_positive = pd.DataFrame(top.most_common(20))
temp_positive.columns = ['Common_words','count']
fig = px.bar(temp_positive, x="Common_words", y="count", title='Most Commmon Words in bullying', orientation='v',
             width=700, height=700,color='Common_words')
fig.show()
```

In [14]:

```
top = Counter([word for tokens in nazism_text['clean_text'].str.split() for word in tokens])
temp_positive = pd.DataFrame(top.most_common(20))
temp_positive.columns = ['Common_words','count']
fig = px.bar(temp_positive, x="Common_words", y="count", title='Most Commmon Words in nazism', orientation='v',
             width=700, height=700,color='Common_words')
fig.show()
```

In [15]:

```
top = Counter([word for tokens in neutral_text['clean_text'].str.split() for word in tokens])
temp_positive = pd.DataFrame(top.most_common(20))
temp_positive.columns = ['Common_words','count']
fig = px.bar(temp_positive, x="Common_words", y="count", title='Most Commmon Words in neutral', orientation='v',
             width=700, height=700,color='Common_words')
fig.show()
```

In [16]:

```
top = Counter([word for tokens in racism_text['clean_text'].str.split() for word in tokens])
temp_positive = pd.DataFrame(top.most_common(20))
temp_positive.columns = ['Common_words','count']
fig = px.bar(temp_positive, x="Common_words", y="count", title='Most Commmon Words in racism', orientation='v',
             width=700, height=700,color='Common_words')
fig.show()
```

In [17]:

```
top = Counter([word for tokens in violent_text['clean_text'].str.split() for word in tokens])
temp_positive = pd.DataFrame(top.most_common(20))
temp_positive.columns = ['Common_words','count']
fig = px.bar(temp_positive, x="Common_words", y="count", title='Most Commmon Words in violent', orientation='v',
             width=700, height=700,color='Common_words')
fig.show()
```

In [18]:

```
from wordcloud import WordCloud
```

In [19]:

```
normal_words =' '.join([text for text in df['clean_text'][df['label_name'] == 'bullying']])
wordcloud = WordCloud(width=800, height=500, random_state=21, max_font_size=110).generate(normal_words)
plt.figure(figsize=(10, 7))
plt.imshow(wordcloud, interpolation="bilinear")
plt.axis('off')
plt.show()
```

In [20]:

```
normal_words =' '.join([text for text in df['clean_text'][df['label_name'] == 'nazism']])
wordcloud = WordCloud(width=800, height=500, random_state=21, max_font_size=110).generate(normal_words)
plt.figure(figsize=(10, 7))
plt.imshow(wordcloud, interpolation="bilinear")
plt.axis('off')
plt.show()
```

In [21]:

```
normal_words =' '.join([text for text in df['clean_text'][df['label_name'] == 'neutral']])
wordcloud = WordCloud(width=800, height=500, random_state=21, max_font_size=110).generate(normal_words)
plt.figure(figsize=(10, 7))
plt.imshow(wordcloud, interpolation="bilinear")
plt.axis('off')
plt.show()
```

In [22]:

```
normal_words =' '.join([text for text in df['clean_text'][df['label_name'] == 'racism']])
wordcloud = WordCloud(width=800, height=500, random_state=21, max_font_size=110).generate(normal_words)
plt.figure(figsize=(10, 7))
plt.imshow(wordcloud, interpolation="bilinear")
plt.axis('off')
plt.show()
```

In [23]:

```
normal_words =' '.join([text for text in df['clean_text'][df['label_name'] == 'violent']])
wordcloud = WordCloud(width=800, height=500, random_state=21, max_font_size=110).generate(normal_words)
plt.figure(figsize=(10, 7))
plt.imshow(wordcloud, interpolation="bilinear")
plt.axis('off')
plt.show()
```

**Applying N-gram**

In [24]:

```
from sklearn.feature_extraction.text import CountVectorizer, TfidfTransformer
```

In [25]:

```
x_train, x_test, y_train, y_test = train_test_split(df["clean_text"],df["label_name"], test_size = 0.25, random_state = 42)
count_vect = CountVectorizer(ngram_range=(1, 2))
transformer = TfidfTransformer(norm='l2',sublinear_tf=True)
x_train_counts = count_vect.fit_transform(x_train)
x_train_tfidf = transformer.fit_transform(x_train_counts)

x_test_counts = count_vect.transform(x_test)
x_test_tfidf = transformer.transform(x_test_counts)

print (x_train_tfidf.shape,x_test_tfidf.shape, y_train.shape, y_test.shape)
```

```
(7642, 100164) (2548, 100164) (7642,) (2548,)
```

In [26]:

```
import joblib
```

In [27]:

```
joblib.dump(count_vect, 'count_vect.pkl')
#model = joblib.load('count_vect.pkl')
```

Out[27]:

```
['count_vect.pkl']
```

### **Logistic Regression**¶

In [28]:

```
from sklearn.linear_model import LogisticRegression
```

In [29]:

```
from sklearn.linear_model import LogisticRegression
from sklearn.metrics import accuracy_score, classification_report, confusion_matrix, precision_score, f1_score, recall_score
```

In [30]:

```
lr = LogisticRegression(C = 2, max_iter = 1000, n_jobs=1)
lr.fit(x_train_tfidf, y_train)
y_pred1 = lr.predict(x_test_tfidf)
print("Accuracy: "+str(accuracy_score(y_test,y_pred1)))
print(classification_report(y_test, y_pred1))
```

```
Accuracy: 0.8500784929356358
              precision    recall  f1-score   support

    bullying       0.82      0.82      0.82       511
      nazism       0.88      0.82      0.85       501
     neutral       0.82      0.81      0.81       438
      racism       0.90      0.92      0.91       552
     violent       0.84      0.87      0.85       546

    accuracy                           0.85      2548
   macro avg       0.85      0.85      0.85      2548
weighted avg       0.85      0.85      0.85      2548
```

In [31]:

```
mc = count_vect.transform(["біздің еркін болғанымызды ештеңе жеңе алмайды тағы бір рет вашингтон мен еуропа түрмешіге басқыншыға және агрессорға көмек көрсетіп оның құрбандарын айыптауды таңдады propaganda"])
m = transformer.transform(mc)
y_pred = lr.predict(m)
print(y_pred)
```

```
['violent']
```

### **Cross val score**¶

In [32]:

```
from sklearn.model_selection import cross_val_score, cross_val_predict
```

In [33]:

```
scores = cross_val_score(lr, x_train_tfidf,y_train)
print(accuracy_score(y_test,y_pred1))
print ("Cross-validated scores:", scores)
```

```
0.8500784929356358
Cross-validated scores: [0.84172662 0.82733813 0.84293194 0.84685864 0.84424084]
```

### **Support Vector Machine**¶

In [34]:

```
from sklearn.svm import LinearSVC, SVC
```

In [35]:

```
svc = LinearSVC()
svc.fit(x_train_tfidf, y_train)
y_pred2 = svc.predict(x_test_tfidf)
print("Accuracy: "+str(accuracy_score(y_test,y_pred2)))
print(classification_report(y_test, y_pred2))
```

```
Accuracy: 0.8614599686028257
              precision    recall  f1-score   support

    bullying       0.83      0.84      0.84       511
      nazism       0.88      0.84      0.86       501
     neutral       0.82      0.82      0.82       438
      racism       0.89      0.94      0.92       552
     violent       0.87      0.85      0.86       546

    accuracy                           0.86      2548
   macro avg       0.86      0.86      0.86      2548
weighted avg       0.86      0.86      0.86      2548
```

```
C:\Users\Admin-server\AppData\Local\Programs\Python\Python310\lib\site-packages\sklearn\svm\_classes.py:31: FutureWarning:

The default value of `dual` will change from `True` to `'auto'` in 1.5. Set the value of `dual` explicitly to suppress the warning.
```

In [36]:

```
mc = count_vect.transform(["біздің еркін болғанымызды ештеңе жеңе алмайды тағы бір рет вашингтон мен еуропа түрмешіге басқыншыға және агрессорға көмек көрсетіп оның құрбандарын айыптауды таңдады propaganda"])
m = transformer.transform(mc)
y_pred = svc.predict(m)
print(y_pred)
```

```
['violent']
```

In [37]:

```
scores = cross_val_score(svc, x_train_tfidf,y_train, cv=10)
print(accuracy_score(y_test,y_pred2))
print ("Cross-validated scores:", scores)
```

```
C:\Users\Admin-server\AppData\Local\Programs\Python\Python310\lib\site-packages\sklearn\svm\_classes.py:31: FutureWarning:

The default value of `dual` will change from `True` to `'auto'` in 1.5. Set the value of `dual` explicitly to suppress the warning.

C:\Users\Admin-server\AppData\Local\Programs\Python\Python310\lib\site-packages\sklearn\svm\_classes.py:31: FutureWarning:

The default value of `dual` will change from `True` to `'auto'` in 1.5. Set the value of `dual` explicitly to suppress the warning.

C:\Users\Admin-server\AppData\Local\Programs\Python\Python310\lib\site-packages\sklearn\svm\_classes.py:31: FutureWarning:

The default value of `dual` will change from `True` to `'auto'` in 1.5. Set the value of `dual` explicitly to suppress the warning.

C:\Users\Admin-server\AppData\Local\Programs\Python\Python310\lib\site-packages\sklearn\svm\_classes.py:31: FutureWarning:

The default value of `dual` will change from `True` to `'auto'` in 1.5. Set the value of `dual` explicitly to suppress the warning.

C:\Users\Admin-server\AppData\Local\Programs\Python\Python310\lib\site-packages\sklearn\svm\_classes.py:31: FutureWarning:

The default value of `dual` will change from `True` to `'auto'` in 1.5. Set the value of `dual` explicitly to suppress the warning.

C:\Users\Admin-server\AppData\Local\Programs\Python\Python310\lib\site-packages\sklearn\svm\_classes.py:31: FutureWarning:

The default value of `dual` will change from `True` to `'auto'` in 1.5. Set the value of `dual` explicitly to suppress the warning.

C:\Users\Admin-server\AppData\Local\Programs\Python\Python310\lib\site-packages\sklearn\svm\_classes.py:31: FutureWarning:

The default value of `dual` will change from `True` to `'auto'` in 1.5. Set the value of `dual` explicitly to suppress the warning.

C:\Users\Admin-server\AppData\Local\Programs\Python\Python310\lib\site-packages\sklearn\svm\_classes.py:31: FutureWarning:

The default value of `dual` will change from `True` to `'auto'` in 1.5. Set the value of `dual` explicitly to suppress the warning.

C:\Users\Admin-server\AppData\Local\Programs\Python\Python310\lib\site-packages\sklearn\svm\_classes.py:31: FutureWarning:

The default value of `dual` will change from `True` to `'auto'` in 1.5. Set the value of `dual` explicitly to suppress the warning.

C:\Users\Admin-server\AppData\Local\Programs\Python\Python310\lib\site-packages\sklearn\svm\_classes.py:31: FutureWarning:

The default value of `dual` will change from `True` to `'auto'` in 1.5. Set the value of `dual` explicitly to suppress the warning.
```

```
0.8614599686028257
Cross-validated scores: [0.86797386 0.8627451  0.84947644 0.84816754 0.85732984 0.85994764
 0.85471204 0.87172775 0.86256545 0.87696335]
```

In [38]:

```
joblib.dump(svc, 'Text_SVM.pkl')
#model = joblib.load('Text_SVM.pkl')
```

Out[38]:

```
['Text_SVM.pkl']
```

### **Naive Bayes(Multinomial)**¶

In [39]:

```
from sklearn.naive_bayes import MultinomialNB
```

In [40]:

```
mnb = MultinomialNB()
mnb.fit(x_train_tfidf, y_train)
y_pred3 = mnb.predict(x_test_tfidf)
print("Accuracy: "+str(accuracy_score(y_test,y_pred3)))
print(classification_report(y_test, y_pred3))
```

```
Accuracy: 0.7774725274725275
              precision    recall  f1-score   support

    bullying       0.77      0.75      0.76       511
      nazism       0.76      0.82      0.79       501
     neutral       0.98      0.46      0.63       438
      racism       0.70      0.95      0.80       552
     violent       0.84      0.84      0.84       546

    accuracy                           0.78      2548
   macro avg       0.81      0.77      0.76      2548
weighted avg       0.80      0.78      0.77      2548
```

### **Randomforest**¶

In [41]:

```
from sklearn.ensemble import GradientBoostingClassifier, RandomForestClassifier, AdaBoostClassifier, VotingClassifier
```

In [42]:

```
rfc = RandomForestClassifier(n_estimators=300, max_depth=15, random_state=42, class_weight='balanced')
rfc.fit(x_train_tfidf,y_train)
y_pred4 = rfc.predict(x_test_tfidf)
print("Accuracy: "+str(accuracy_score(y_test,y_pred4)))
print(classification_report(y_test, y_pred4))
```

```
Accuracy: 0.7252747252747253
              precision    recall  f1-score   support

    bullying       0.77      0.58      0.66       511
      nazism       0.93      0.65      0.77       501
     neutral       0.45      0.93      0.60       438
      racism       0.89      0.82      0.86       552
     violent       0.92      0.66      0.77       546

    accuracy                           0.73      2548
   macro avg       0.79      0.73      0.73      2548
weighted avg       0.81      0.73      0.74      2548
```

### **GradientBoostingClassifier**¶

In [43]:

```
gbc = GradientBoostingClassifier(n_estimators=100,  max_depth=4, random_state=1, verbose=1)
gbc.fit(x_train_tfidf, y_train)
y_pred5 = gbc.predict(x_test_tfidf)
print(accuracy_score(y_test, y_pred5))
print(classification_report(y_test, y_pred5))
```

```
      Iter       Train Loss   Remaining Time 
         1           1.4919            1.16m
         2           1.4127            1.14m
         3           1.3512            1.13m
         4           1.3014            1.12m
         5           1.2594            1.11m
         6           1.2232            1.10m
         7           1.1904            1.09m
         8           1.1632            1.08m
         9           1.1360            1.07m
        10           1.1120            1.06m
        20           0.9563           56.77s
        30           0.8702           49.87s
        40           0.8110           42.77s
        50           0.7673           35.67s
        60           0.7304           28.52s
        70           0.6992           21.39s
        80           0.6722           14.35s
        90           0.6486            7.16s
       100           0.6271            0.00s
0.7845368916797488
              precision    recall  f1-score   support

    bullying       0.75      0.72      0.73       511
      nazism       0.94      0.74      0.83       501
     neutral       0.54      0.89      0.67       438
      racism       0.94      0.91      0.92       552
     violent       0.93      0.68      0.78       546

    accuracy                           0.78      2548
   macro avg       0.82      0.79      0.79      2548
weighted avg       0.83      0.78      0.79      2548
```

In [44]:

```
scores = cross_val_score(gbc, x_train_tfidf,y_train, cv=5)
print(accuracy_score(y_test,y_pred5))
print ("Cross-validated scores:", scores)
```

```
      Iter       Train Loss   Remaining Time 
         1           1.4928            6.23m
         2           1.4136            6.17m
         3           1.3530            6.10m
         4           1.3031            6.05m
         5           1.2606            5.98m
         6           1.2249            5.92m
         7           1.1919            5.86m
         8           1.1625            5.80m
         9           1.1371            5.74m
        10           1.1128            5.68m
        20           0.9568            5.07m
        30           0.8680            4.42m
        40           0.8091            3.80m
        50           0.7626            3.17m
        60           0.7264            2.53m
        70           0.6941            1.90m
        80           0.6676            1.27m
        90           0.6421           37.96s
       100           0.6195            0.00s
      Iter       Train Loss   Remaining Time 
         1           1.4915            6.21m
         2           1.4116            6.13m
         3           1.3503            6.07m
         4           1.2998            6.03m
         5           1.2580            5.97m
         6           1.2207            5.91m
         7           1.1893            5.84m
         8           1.1588            5.78m
         9           1.1336            5.72m
        10           1.1095            5.66m
        20           0.9530            5.03m
        30           0.8664            4.39m
        40           0.8066            3.78m
        50           0.7600            3.15m
        60           0.7225            2.52m
        70           0.6912            1.89m
        80           0.6639            1.26m
        90           0.6393           37.83s
       100           0.6177            0.00s
      Iter       Train Loss   Remaining Time 
         1           1.4900            6.13m
         2           1.4103            6.12m
         3           1.3484            6.07m
         4           1.2976            6.02m
         5           1.2547            5.97m
         6           1.2184            5.91m
         7           1.1866            5.85m
         8           1.1582            5.80m
         9           1.1338            5.74m
        10           1.1104            5.67m
        20           0.9524            5.05m
        30           0.8657            4.44m
        40           0.8049            3.79m
        50           0.7602            3.16m
        60           0.7223            2.53m
        70           0.6905            1.90m
        80           0.6633            1.26m
        90           0.6392           37.91s
       100           0.6174            0.00s
      Iter       Train Loss   Remaining Time 
         1           1.4924            6.12m
         2           1.4140            6.13m
         3           1.3525            6.10m
         4           1.3019            6.06m
         5           1.2598            5.99m
         6           1.2234            5.93m
         7           1.1919            5.87m
         8           1.1633            5.80m
         9           1.1357            5.74m
        10           1.1107            5.69m
        20           0.9544            5.05m
        30           0.8658            4.42m
        40           0.8056            3.79m
        50           0.7604            3.17m
        60           0.7229            2.53m
        70           0.6901            1.90m
        80           0.6630            1.27m
        90           0.6383           38.05s
       100           0.6166            0.00s
      Iter       Train Loss   Remaining Time 
         1           1.4910            6.06m
         2           1.4109            6.07m
         3           1.3495            6.01m
         4           1.2988            5.95m
         5           1.2562            5.89m
         6           1.2198            5.84m
         7           1.1885            5.77m
         8           1.1573            5.75m
         9           1.1308            5.69m
        10           1.1082            5.63m
        20           0.9529            5.01m
        30           0.8653            4.38m
        40           0.8043            3.75m
        50           0.7598            3.13m
        60           0.7220            2.50m
        70           0.6899            1.87m
        80           0.6623            1.25m
        90           0.6377           37.46s
       100           0.6160            0.00s
0.7845368916797488
Cross-validated scores: [0.76847613 0.76193591 0.77945026 0.7447644  0.75065445]
```

### **Ensemble Classifier**¶

In [45]:

```
mnb = MultinomialNB()
rfc= RandomForestClassifier(n_estimators=1000, max_depth=12, random_state=42)
lr = LogisticRegression(C = 2, max_iter = 1000, n_jobs=-1)
svc = SVC(probability=True)
ec=VotingClassifier(estimators=[('Multinominal NB', mnb), ('Random Forest', rfc),('Logistic Regression',lr),('Support Vector Machine',svc)], voting='soft', weights=[1,2,3,4])
ec.fit(x_train_tfidf,y_train)
y_pred6 = ec.predict(x_test_tfidf)
print(accuracy_score(y_test, y_pred6))
print(classification_report(y_test, y_pred6))
```

```
0.8437990580847724
              precision    recall  f1-score   support

    bullying       0.82      0.80      0.81       511
      nazism       0.87      0.81      0.84       501
     neutral       0.79      0.82      0.81       438
      racism       0.86      0.93      0.90       552
     violent       0.87      0.84      0.85       546

    accuracy                           0.84      2548
   macro avg       0.84      0.84      0.84      2548
weighted avg       0.84      0.84      0.84      2548
```

In [46]:

```
scores = cross_val_score(ec, x_train_tfidf,y_train, cv=10)
print(accuracy_score(y_test,y_pred6))
print ("Cross-validated scores:", scores)
```

```
0.8437990580847724
Cross-validated scores: [0.84444444 0.84313725 0.82460733 0.85209424 0.84162304 0.84424084
 0.84293194 0.85602094 0.85078534 0.83638743]
```

### **AdaBoost with Random Forest Classifier**¶

In [47]:

```
rfc = RandomForestClassifier(n_estimators=100, max_depth=9, random_state=0)
abc= AdaBoostClassifier(estimator=rfc, learning_rate=0.2, n_estimators=100)
abc.fit(x_train_tfidf, y_train)
y_pred7= abc.predict(x_test_tfidf)
print("Accuracy: "+str(accuracy_score(y_test, y_pred7)))
print(classification_report(y_test, y_pred7))
```

```
C:\Users\Admin-server\AppData\Local\Programs\Python\Python310\lib\site-packages\sklearn\ensemble\_weight_boosting.py:519: FutureWarning:

The SAMME.R algorithm (the default) is deprecated and will be removed in 1.6. Use the SAMME algorithm to circumvent this warning.
```

```
Accuracy: 0.7692307692307693
              precision    recall  f1-score   support

    bullying       0.69      0.77      0.73       511
      nazism       0.95      0.71      0.81       501
     neutral       0.52      0.90      0.66       438
      racism       0.98      0.80      0.88       552
     violent       0.94      0.69      0.80       546

    accuracy                           0.77      2548
   macro avg       0.82      0.77      0.78      2548
weighted avg       0.83      0.77      0.78      2548
```

In [48]:

```
scores = cross_val_score(abc, x_train_tfidf,y_train, cv=10)
print(accuracy_score(y_test,y_pred7))
print ("Cross-validated scores:", scores)
```

```
C:\Users\Admin-server\AppData\Local\Programs\Python\Python310\lib\site-packages\sklearn\ensemble\_weight_boosting.py:519: FutureWarning:

The SAMME.R algorithm (the default) is deprecated and will be removed in 1.6. Use the SAMME algorithm to circumvent this warning.

C:\Users\Admin-server\AppData\Local\Programs\Python\Python310\lib\site-packages\sklearn\ensemble\_weight_boosting.py:519: FutureWarning:

The SAMME.R algorithm (the default) is deprecated and will be removed in 1.6. Use the SAMME algorithm to circumvent this warning.

C:\Users\Admin-server\AppData\Local\Programs\Python\Python310\lib\site-packages\sklearn\ensemble\_weight_boosting.py:519: FutureWarning:

The SAMME.R algorithm (the default) is deprecated and will be removed in 1.6. Use the SAMME algorithm to circumvent this warning.

C:\Users\Admin-server\AppData\Local\Programs\Python\Python310\lib\site-packages\sklearn\ensemble\_weight_boosting.py:519: FutureWarning:

The SAMME.R algorithm (the default) is deprecated and will be removed in 1.6. Use the SAMME algorithm to circumvent this warning.

C:\Users\Admin-server\AppData\Local\Programs\Python\Python310\lib\site-packages\sklearn\ensemble\_weight_boosting.py:519: FutureWarning:

The SAMME.R algorithm (the default) is deprecated and will be removed in 1.6. Use the SAMME algorithm to circumvent this warning.

C:\Users\Admin-server\AppData\Local\Programs\Python\Python310\lib\site-packages\sklearn\ensemble\_weight_boosting.py:519: FutureWarning:

The SAMME.R algorithm (the default) is deprecated and will be removed in 1.6. Use the SAMME algorithm to circumvent this warning.

C:\Users\Admin-server\AppData\Local\Programs\Python\Python310\lib\site-packages\sklearn\ensemble\_weight_boosting.py:519: FutureWarning:

The SAMME.R algorithm (the default) is deprecated and will be removed in 1.6. Use the SAMME algorithm to circumvent this warning.

C:\Users\Admin-server\AppData\Local\Programs\Python\Python310\lib\site-packages\sklearn\ensemble\_weight_boosting.py:519: FutureWarning:

The SAMME.R algorithm (the default) is deprecated and will be removed in 1.6. Use the SAMME algorithm to circumvent this warning.

C:\Users\Admin-server\AppData\Local\Programs\Python\Python310\lib\site-packages\sklearn\ensemble\_weight_boosting.py:519: FutureWarning:

The SAMME.R algorithm (the default) is deprecated and will be removed in 1.6. Use the SAMME algorithm to circumvent this warning.

C:\Users\Admin-server\AppData\Local\Programs\Python\Python310\lib\site-packages\sklearn\ensemble\_weight_boosting.py:519: FutureWarning:

The SAMME.R algorithm (the default) is deprecated and will be removed in 1.6. Use the SAMME algorithm to circumvent this warning.
```

```
0.7692307692307693
Cross-validated scores: [0.78039216 0.76732026 0.7565445  0.78272251 0.78534031 0.77486911
 0.77486911 0.78010471 0.78795812 0.78141361]
```

### **Сравнение между моделями ML**¶

In [49]:

```
Comparison_unibi = pd.DataFrame({'Logistic Regression': [accuracy_score(y_test,y_pred1)*100,f1_score(y_test,y_pred1,average='macro')*100,recall_score(y_test, y_pred1,average='micro')*100,precision_score(y_test, y_pred1,average='micro')*100],
                            'SVM':[accuracy_score(y_test,y_pred2)*100,f1_score(y_test,y_pred2,average='macro')*100,recall_score(y_test, y_pred2,average='micro')*100,precision_score(y_test, y_pred2,average='micro')*100],
                           'Naive Bayes':[accuracy_score(y_test,y_pred3)*100,f1_score(y_test,y_pred3,average='macro')*100,recall_score(y_test, y_pred3,average='micro')*100,precision_score(y_test, y_pred3,average='micro')*100],
                           'Random Forest':[accuracy_score(y_test,y_pred4)*100,f1_score(y_test,y_pred4,average='macro')*100,recall_score(y_test, y_pred4,average='micro')*100,precision_score(y_test, y_pred4,average='micro')*100],
                           'GradientBoosting':[accuracy_score(y_test,y_pred5)*100,f1_score(y_test,y_pred5,average='macro')*100,recall_score(y_test, y_pred5,average='micro')*100,precision_score(y_test, y_pred5,average='micro')*100],
                           'Ensembled':[accuracy_score(y_test,y_pred6)*100,f1_score(y_test,y_pred6,average='macro')*100,recall_score(y_test, y_pred6,average='micro')*100,precision_score(y_test, y_pred6,average='micro')*100],
                           'Adaboost':[accuracy_score(y_test,y_pred7)*100,f1_score(y_test,y_pred7,average='macro')*100,recall_score(y_test, y_pred7,average='micro')*100,precision_score(y_test, y_pred7,average='micro')*100],

})
```

In [50]:

```
print ('Cравнение с использованием uni-bi-gram(1,2)')
Comparison_unibi.rename(index={0:'Accuracy',1:'F1_score', 2: 'Recall',3:'Precision'}, inplace=True)
Comparison_unibi.head()
```

```
Cравнение с использованием uni-bi-gram(1,2)
```

Out[50]:

|  | Logistic Regression | SVM | Naive Bayes | Random Forest | GradientBoosting | Ensembled | Adaboost |
| --- | --- | --- | --- | --- | --- | --- | --- |
| Accuracy | 85.007849 | 86.145997 | 77.747253 | 72.527473 | 78.453689 | 84.379906 | 76.923077 |
| F1\_score | 84.785046 | 85.908178 | 76.420181 | 73.261696 | 78.683581 | 84.136328 | 77.539347 |
| Recall | 85.007849 | 86.145997 | 77.747253 | 72.527473 | 78.453689 | 84.379906 | 76.923077 |
| Precision | 85.007849 | 86.145997 | 77.747253 | 72.527473 | 78.453689 | 84.379906 | 76.923077 |

In [51]:

```
# @title Average Accuracy of Different Algorithms

import matplotlib.pyplot as plt

algorithms = ['Logistic Regression', 'SVM', 'Naive Bayes', 'Random Forest', 'GradientBoosting', 'Ensembled', 'Adaboost']
accuracies = [84.77062727665078, 85.94802996809056, 77.07066785348896, 73.28861335270024, 78.52697803102526, 84.25585650423141, 77.66630710769495]

plt.figure(figsize=(10, 6))
plt.bar(algorithms, accuracies)
plt.xlabel('Algorithm')
plt.ylabel('Average Accuracy')
plt.title('Средняя точность различных алгоритмов')
plt.show()
```

### **Модели глубокого обучения**¶

In [52]:

```
!pip install keras_preprocessing
```

```
Requirement already satisfied: keras_preprocessing in c:\users\admin-server\appdata\local\programs\python\python37\lib\site-packages (1.1.2)
Requirement already satisfied: numpy>=1.9.1 in c:\users\admin-server\appdata\local\programs\python\python37\lib\site-packages (from keras_preprocessing) (1.21.6)
Requirement already satisfied: six>=1.9.0 in c:\users\admin-server\appdata\local\programs\python\python37\lib\site-packages (from keras_preprocessing) (1.15.0)
```

```
DEPRECATION: celery 4.4.7 has a non-standard dependency specifier pytz>dev. pip 24.1 will enforce this behaviour change. A possible replacement is to upgrade to a newer version of celery or contact the author to suggest that they release a version with a conforming dependency specifiers. Discussion can be found at https://github.com/pypa/pip/issues/12063
DEPRECATION: uvicorn 0.14.0 has a non-standard dependency specifier click>=7.*. pip 24.1 will enforce this behaviour change. A possible replacement is to upgrade to a newer version of uvicorn or contact the author to suggest that they release a version with a conforming dependency specifiers. Discussion can be found at https://github.com/pypa/pip/issues/12063
```

In [53]:

```
!pip install tensorflow
```

```
Requirement already satisfied: tensorflow in c:\users\admin-server\appdata\local\programs\python\python37\lib\site-packages (2.11.0)
Requirement already satisfied: tensorflow-intel==2.11.0 in c:\users\admin-server\appdata\local\programs\python\python37\lib\site-packages (from tensorflow) (2.11.0)
Requirement already satisfied: absl-py>=1.0.0 in c:\users\admin-server\appdata\local\programs\python\python37\lib\site-packages (from tensorflow-intel==2.11.0->tensorflow) (2.0.0)
Requirement already satisfied: astunparse>=1.6.0 in c:\users\admin-server\appdata\local\programs\python\python37\lib\site-packages (from tensorflow-intel==2.11.0->tensorflow) (1.6.3)
Requirement already satisfied: flatbuffers>=2.0 in c:\users\admin-server\appdata\local\programs\python\python37\lib\site-packages (from tensorflow-intel==2.11.0->tensorflow) (24.3.25)
Requirement already satisfied: gast<=0.4.0,>=0.2.1 in c:\users\admin-server\appdata\local\programs\python\python37\lib\site-packages (from tensorflow-intel==2.11.0->tensorflow) (0.2.2)
Requirement already satisfied: google-pasta>=0.1.1 in c:\users\admin-server\appdata\local\programs\python\python37\lib\site-packages (from tensorflow-intel==2.11.0->tensorflow) (0.2.0)
Requirement already satisfied: h5py>=2.9.0 in c:\users\admin-server\appdata\local\programs\python\python37\lib\site-packages (from tensorflow-intel==2.11.0->tensorflow) (2.10.0)
Requirement already satisfied: libclang>=13.0.0 in c:\users\admin-server\appdata\local\programs\python\python37\lib\site-packages (from tensorflow-intel==2.11.0->tensorflow) (16.0.6)
Requirement already satisfied: numpy>=1.20 in c:\users\admin-server\appdata\local\programs\python\python37\lib\site-packages (from tensorflow-intel==2.11.0->tensorflow) (1.21.6)
Requirement already satisfied: opt-einsum>=2.3.2 in c:\users\admin-server\appdata\local\programs\python\python37\lib\site-packages (from tensorflow-intel==2.11.0->tensorflow) (3.3.0)
Requirement already satisfied: packaging in c:\users\admin-server\appdata\local\programs\python\python37\lib\site-packages (from tensorflow-intel==2.11.0->tensorflow) (23.1)
Requirement already satisfied: protobuf<3.20,>=3.9.2 in c:\users\admin-server\appdata\local\programs\python\python37\lib\site-packages (from tensorflow-intel==2.11.0->tensorflow) (3.19.6)
Requirement already satisfied: setuptools in c:\users\admin-server\appdata\local\programs\python\python37\lib\site-packages (from tensorflow-intel==2.11.0->tensorflow) (68.0.0)
Requirement already satisfied: six>=1.12.0 in c:\users\admin-server\appdata\local\programs\python\python37\lib\site-packages (from tensorflow-intel==2.11.0->tensorflow) (1.15.0)
Requirement already satisfied: termcolor>=1.1.0 in c:\users\admin-server\appdata\local\programs\python\python37\lib\site-packages (from tensorflow-intel==2.11.0->tensorflow) (1.1.0)
Requirement already satisfied: typing-extensions>=3.6.6 in c:\users\admin-server\appdata\local\programs\python\python37\lib\site-packages (from tensorflow-intel==2.11.0->tensorflow) (4.7.1)
Requirement already satisfied: wrapt>=1.11.0 in c:\users\admin-server\appdata\local\programs\python\python37\lib\site-packages (from tensorflow-intel==2.11.0->tensorflow) (1.12.1)
Requirement already satisfied: grpcio<2.0,>=1.24.3 in c:\users\admin-server\appdata\local\programs\python\python37\lib\site-packages (from tensorflow-intel==2.11.0->tensorflow) (1.51.3)
Requirement already satisfied: tensorboard<2.12,>=2.11 in c:\users\admin-server\appdata\local\programs\python\python37\lib\site-packages (from tensorflow-intel==2.11.0->tensorflow) (2.11.2)
Requirement already satisfied: tensorflow-estimator<2.12,>=2.11.0 in c:\users\admin-server\appdata\local\programs\python\python37\lib\site-packages (from tensorflow-intel==2.11.0->tensorflow) (2.11.0)
Requirement already satisfied: keras<2.12,>=2.11.0 in c:\users\admin-server\appdata\local\programs\python\python37\lib\site-packages (from tensorflow-intel==2.11.0->tensorflow) (2.11.0)
Requirement already satisfied: tensorflow-io-gcs-filesystem>=0.23.1 in c:\users\admin-server\appdata\local\programs\python\python37\lib\site-packages (from tensorflow-intel==2.11.0->tensorflow) (0.31.0)
Requirement already satisfied: wheel<1.0,>=0.23.0 in c:\users\admin-server\appdata\local\programs\python\python37\lib\site-packages (from astunparse>=1.6.0->tensorflow-intel==2.11.0->tensorflow) (0.41.2)
Requirement already satisfied: google-auth<3,>=1.6.3 in c:\users\admin-server\appdata\local\programs\python\python37\lib\site-packages (from tensorboard<2.12,>=2.11->tensorflow-intel==2.11.0->tensorflow) (1.35.0)
Requirement already satisfied: google-auth-oauthlib<0.5,>=0.4.1 in c:\users\admin-server\appdata\local\programs\python\python37\lib\site-packages (from tensorboard<2.12,>=2.11->tensorflow-intel==2.11.0->tensorflow) (0.4.6)
Requirement already satisfied: markdown>=2.6.8 in c:\users\admin-server\appdata\local\programs\python\python37\lib\site-packages (from tensorboard<2.12,>=2.11->tensorflow-intel==2.11.0->tensorflow) (3.4.1)
Requirement already satisfied: requests<3,>=2.21.0 in c:\users\admin-server\appdata\local\programs\python\python37\lib\site-packages (from tensorboard<2.12,>=2.11->tensorflow-intel==2.11.0->tensorflow) (2.28.2)
Requirement already satisfied: tensorboard-data-server<0.7.0,>=0.6.0 in c:\users\admin-server\appdata\local\programs\python\python37\lib\site-packages (from tensorboard<2.12,>=2.11->tensorflow-intel==2.11.0->tensorflow) (0.6.1)
Requirement already satisfied: tensorboard-plugin-wit>=1.6.0 in c:\users\admin-server\appdata\local\programs\python\python37\lib\site-packages (from tensorboard<2.12,>=2.11->tensorflow-intel==2.11.0->tensorflow) (1.8.1)
Requirement already satisfied: werkzeug>=1.0.1 in c:\users\admin-server\appdata\local\programs\python\python37\lib\site-packages (from tensorboard<2.12,>=2.11->tensorflow-intel==2.11.0->tensorflow) (2.2.3)
Requirement already satisfied: cachetools<5.0,>=2.0.0 in c:\users\admin-server\appdata\local\programs\python\python37\lib\site-packages (from google-auth<3,>=1.6.3->tensorboard<2.12,>=2.11->tensorflow-intel==2.11.0->tensorflow) (3.1.1)
Requirement already satisfied: pyasn1-modules>=0.2.1 in c:\users\admin-server\appdata\local\programs\python\python37\lib\site-packages (from google-auth<3,>=1.6.3->tensorboard<2.12,>=2.11->tensorflow-intel==2.11.0->tensorflow) (0.2.8)
Requirement already satisfied: rsa<5,>=3.1.4 in c:\users\admin-server\appdata\local\programs\python\python37\lib\site-packages (from google-auth<3,>=1.6.3->tensorboard<2.12,>=2.11->tensorflow-intel==2.11.0->tensorflow) (4.0)
Requirement already satisfied: requests-oauthlib>=0.7.0 in c:\users\admin-server\appdata\local\programs\python\python37\lib\site-packages (from google-auth-oauthlib<0.5,>=0.4.1->tensorboard<2.12,>=2.11->tensorflow-intel==2.11.0->tensorflow) (1.3.1)
Requirement already satisfied: importlib-metadata>=4.4 in c:\users\admin-server\appdata\local\programs\python\python37\lib\site-packages (from markdown>=2.6.8->tensorboard<2.12,>=2.11->tensorflow-intel==2.11.0->tensorflow) (6.7.0)
Requirement already satisfied: charset-normalizer<4,>=2 in c:\users\admin-server\appdata\local\programs\python\python37\lib\site-packages (from requests<3,>=2.21.0->tensorboard<2.12,>=2.11->tensorflow-intel==2.11.0->tensorflow) (3.1.0)
Requirement already satisfied: idna<4,>=2.5 in c:\users\admin-server\appdata\local\programs\python\python37\lib\site-packages (from requests<3,>=2.21.0->tensorboard<2.12,>=2.11->tensorflow-intel==2.11.0->tensorflow) (2.10)
Requirement already satisfied: urllib3<1.27,>=1.21.1 in c:\users\admin-server\appdata\local\programs\python\python37\lib\site-packages (from requests<3,>=2.21.0->tensorboard<2.12,>=2.11->tensorflow-intel==2.11.0->tensorflow) (1.24.3)
Requirement already satisfied: certifi>=2017.4.17 in c:\users\admin-server\appdata\local\programs\python\python37\lib\site-packages (from requests<3,>=2.21.0->tensorboard<2.12,>=2.11->tensorflow-intel==2.11.0->tensorflow) (2023.7.22)
Requirement already satisfied: MarkupSafe>=2.1.1 in c:\users\admin-server\appdata\local\programs\python\python37\lib\site-packages (from werkzeug>=1.0.1->tensorboard<2.12,>=2.11->tensorflow-intel==2.11.0->tensorflow) (2.1.2)
Requirement already satisfied: zipp>=0.5 in c:\users\admin-server\appdata\local\programs\python\python37\lib\site-packages (from importlib-metadata>=4.4->markdown>=2.6.8->tensorboard<2.12,>=2.11->tensorflow-intel==2.11.0->tensorflow) (3.15.0)
Requirement already satisfied: pyasn1<0.5.0,>=0.4.6 in c:\users\admin-server\appdata\local\programs\python\python37\lib\site-packages (from pyasn1-modules>=0.2.1->google-auth<3,>=1.6.3->tensorboard<2.12,>=2.11->tensorflow-intel==2.11.0->tensorflow) (0.4.8)
Requirement already satisfied: oauthlib>=3.0.0 in c:\users\admin-server\appdata\local\programs\python\python37\lib\site-packages (from requests-oauthlib>=0.7.0->google-auth-oauthlib<0.5,>=0.4.1->tensorboard<2.12,>=2.11->tensorflow-intel==2.11.0->tensorflow) (3.2.2)
```

```
DEPRECATION: celery 4.4.7 has a non-standard dependency specifier pytz>dev. pip 24.1 will enforce this behaviour change. A possible replacement is to upgrade to a newer version of celery or contact the author to suggest that they release a version with a conforming dependency specifiers. Discussion can be found at https://github.com/pypa/pip/issues/12063
DEPRECATION: uvicorn 0.14.0 has a non-standard dependency specifier click>=7.*. pip 24.1 will enforce this behaviour change. A possible replacement is to upgrade to a newer version of uvicorn or contact the author to suggest that they release a version with a conforming dependency specifiers. Discussion can be found at https://github.com/pypa/pip/issues/12063
```

In [54]:

```
from keras.preprocessing.text import Tokenizer
from keras_preprocessing.sequence import pad_sequences
from tensorflow.keras.utils import to_categorical
from keras.models import Sequential, load_model
from keras.layers import Activation, Dense, Embedding, LSTM, SpatialDropout1D, Dropout, Flatten, GRU, Conv1D, MaxPooling1D, Bidirectional
import tensorflow as tf
```

```
WARNING:tensorflow:From C:\Users\Admin-server\AppData\Local\Programs\Python\Python310\lib\site-packages\keras\src\losses.py:2976: The name tf.losses.sparse_softmax_cross_entropy is deprecated. Please use tf.compat.v1.losses.sparse_softmax_cross_entropy instead.
```

In [55]:

```
vocabulary_size = 10190
max_text_len = 127
```

In [56]:

```
tokenizer = Tokenizer(num_words=vocabulary_size)
tokenizer.fit_on_texts(df['message_stemmed'].values)
le = len(tokenizer.word_index) + 1
print(le)
sequences = tokenizer.texts_to_sequences(df['message_stemmed'].values)
X_DeepLearning = pad_sequences(sequences, maxlen=max_text_len)
```

```
14469
```

In [57]:

```
df.loc[df['label_name'] == 'neutral' , 'LABEL'] = 0
df.loc[df['label_name'] == 'racism', 'LABEL'] = 1
df.loc[df['label_name'] == 'bullying' , 'LABEL'] = 2
df.loc[df['label_name'] == 'nazism', 'LABEL'] = 3
df.loc[df['label_name'] == 'violent', 'LABEL'] = 4

labels = to_categorical(df['LABEL'], num_classes=5)
XX_train, XX_test, y_train, y_test = train_test_split(X_DeepLearning , labels, test_size=0.25, random_state=42)
print((XX_train.shape, y_train.shape, XX_test.shape, y_test.shape))
```

```
((7642, 127), (7642, 5), (2548, 127), (2548, 5))
```

### **LSTM 1-Layers**¶

In [58]:

```
epochs = 20
emb_dim = 256
batch_size = 50
model_lstm1 = Sequential()
model_lstm1.add(Embedding(vocabulary_size,emb_dim, input_length=X_DeepLearning.shape[1]))
model_lstm1.add(SpatialDropout1D(0.8))
model_lstm1.add(Bidirectional(LSTM(300, dropout=0.5, recurrent_dropout=0.5)))
model_lstm1.add(Dropout(0.5))
model_lstm1.add(Flatten())
model_lstm1.add(Dense(64, activation='relu'))
model_lstm1.add(Dropout(0.5))
model_lstm1.add(Dense(5, activation='softmax'))
model_lstm1.compile(optimizer=tf.optimizers.Adam(),loss='categorical_crossentropy', metrics=['acc'])
print(model_lstm1.summary())
```

```
WARNING:tensorflow:From C:\Users\Admin-server\AppData\Local\Programs\Python\Python310\lib\site-packages\keras\src\backend.py:873: The name tf.get_default_graph is deprecated. Please use tf.compat.v1.get_default_graph instead.

Model: "sequential"
_________________________________________________________________
 Layer (type)                Output Shape              Param #   
=================================================================
 embedding (Embedding)       (None, 127, 256)          2608640   
                                                                 
 spatial_dropout1d (Spatial  (None, 127, 256)          0         
 Dropout1D)                                                      
                                                                 
 bidirectional (Bidirection  (None, 600)               1336800   
 al)                                                             
                                                                 
 dropout (Dropout)           (None, 600)               0         
                                                                 
 flatten (Flatten)           (None, 600)               0         
                                                                 
 dense (Dense)               (None, 64)                38464     
                                                                 
 dropout_1 (Dropout)         (None, 64)                0         
                                                                 
 dense_1 (Dense)             (None, 5)                 325       
                                                                 
=================================================================
Total params: 3984229 (15.20 MB)
Trainable params: 3984229 (15.20 MB)
Non-trainable params: 0 (0.00 Byte)
_________________________________________________________________
None
```

In [59]:

```
from keras.callbacks import ModelCheckpoint, EarlyStopping, ReduceLROnPlateau
```

In [60]:

```
checkpoint_callback = ModelCheckpoint(filepath="lastm-1-layer-best_model.h5", save_best_only=True, monitor="val_acc", mode="max", verbose=1)

early_stopping_callback = EarlyStopping(monitor="val_acc", mode="max", patience=10, verbose=1, restore_best_weights=True)

reduce_lr_callback = ReduceLROnPlateau(monitor="val_loss", factor=0.1, patience=5, verbose=1, mode="min", min_delta=0.0001, cooldown=0, min_lr=0)

callbacks=[checkpoint_callback, early_stopping_callback, reduce_lr_callback]
```

In [61]:

```
history_lstm1 = model_lstm1.fit(XX_train, y_train, epochs = epochs, batch_size = batch_size, validation_data=(XX_test,y_test), callbacks=callbacks)
```

```
Epoch 1/20
WARNING:tensorflow:From C:\Users\Admin-server\AppData\Local\Programs\Python\Python310\lib\site-packages\keras\src\utils\tf_utils.py:492: The name tf.ragged.RaggedTensorValue is deprecated. Please use tf.compat.v1.ragged.RaggedTensorValue instead.

WARNING:tensorflow:From C:\Users\Admin-server\AppData\Local\Programs\Python\Python310\lib\site-packages\keras\src\engine\base_layer_utils.py:384: The name tf.executing_eagerly_outside_functions is deprecated. Please use tf.compat.v1.executing_eagerly_outside_functions instead.

153/153 [==============================] - ETA: 0s - loss: 1.4488 - acc: 0.3511
Epoch 1: val_acc improved from -inf to 0.45918, saving model to lastm-1-layer-best_model.h5
153/153 [==============================] - 137s 877ms/step - loss: 1.4488 - acc: 0.3511 - val_loss: 1.1296 - val_acc: 0.4592 - lr: 0.0010
Epoch 2/20
```

```
C:\Users\Admin-server\AppData\Local\Programs\Python\Python310\lib\site-packages\keras\src\engine\training.py:3103: UserWarning:

You are saving your model as an HDF5 file via `model.save()`. This file format is considered legacy. We recommend using instead the native Keras format, e.g. `model.save('my_model.keras')`.
```

```
153/153 [==============================] - ETA: 0s - loss: 0.9445 - acc: 0.6310
Epoch 2: val_acc improved from 0.45918 to 0.73783, saving model to lastm-1-layer-best_model.h5
153/153 [==============================] - 139s 909ms/step - loss: 0.9445 - acc: 0.6310 - val_loss: 0.7094 - val_acc: 0.7378 - lr: 0.0010
Epoch 3/20
153/153 [==============================] - ETA: 0s - loss: 0.6553 - acc: 0.7697
Epoch 3: val_acc improved from 0.73783 to 0.82261, saving model to lastm-1-layer-best_model.h5
153/153 [==============================] - 140s 916ms/step - loss: 0.6553 - acc: 0.7697 - val_loss: 0.4999 - val_acc: 0.8226 - lr: 0.0010
Epoch 4/20
153/153 [==============================] - ETA: 0s - loss: 0.5028 - acc: 0.8294
Epoch 4: val_acc improved from 0.82261 to 0.83948, saving model to lastm-1-layer-best_model.h5
153/153 [==============================] - 142s 926ms/step - loss: 0.5028 - acc: 0.8294 - val_loss: 0.4773 - val_acc: 0.8395 - lr: 0.0010
Epoch 5/20
153/153 [==============================] - ETA: 0s - loss: 0.4245 - acc: 0.8672
Epoch 5: val_acc improved from 0.83948 to 0.85636, saving model to lastm-1-layer-best_model.h5
153/153 [==============================] - 142s 926ms/step - loss: 0.4245 - acc: 0.8672 - val_loss: 0.4154 - val_acc: 0.8564 - lr: 0.0010
Epoch 6/20
153/153 [==============================] - ETA: 0s - loss: 0.3393 - acc: 0.8965
Epoch 6: val_acc improved from 0.85636 to 0.87049, saving model to lastm-1-layer-best_model.h5
153/153 [==============================] - 142s 928ms/step - loss: 0.3393 - acc: 0.8965 - val_loss: 0.3971 - val_acc: 0.8705 - lr: 0.0010
Epoch 7/20
153/153 [==============================] - ETA: 0s - loss: 0.3128 - acc: 0.9047
Epoch 7: val_acc did not improve from 0.87049
153/153 [==============================] - 142s 931ms/step - loss: 0.3128 - acc: 0.9047 - val_loss: 0.3977 - val_acc: 0.8685 - lr: 0.0010
Epoch 8/20
153/153 [==============================] - ETA: 0s - loss: 0.2792 - acc: 0.9134
Epoch 8: val_acc improved from 0.87049 to 0.88383, saving model to lastm-1-layer-best_model.h5
153/153 [==============================] - 143s 932ms/step - loss: 0.2792 - acc: 0.9134 - val_loss: 0.3759 - val_acc: 0.8838 - lr: 0.0010
Epoch 9/20
153/153 [==============================] - ETA: 0s - loss: 0.2614 - acc: 0.9191
Epoch 9: val_acc improved from 0.88383 to 0.88736, saving model to lastm-1-layer-best_model.h5
153/153 [==============================] - 143s 936ms/step - loss: 0.2614 - acc: 0.9191 - val_loss: 0.3627 - val_acc: 0.8874 - lr: 0.0010
Epoch 10/20
153/153 [==============================] - ETA: 0s - loss: 0.2146 - acc: 0.9356
Epoch 10: val_acc did not improve from 0.88736
153/153 [==============================] - 143s 934ms/step - loss: 0.2146 - acc: 0.9356 - val_loss: 0.3994 - val_acc: 0.8815 - lr: 0.0010
Epoch 11/20
153/153 [==============================] - ETA: 0s - loss: 0.1941 - acc: 0.9392
Epoch 11: val_acc did not improve from 0.88736
153/153 [==============================] - 144s 943ms/step - loss: 0.1941 - acc: 0.9392 - val_loss: 0.4060 - val_acc: 0.8728 - lr: 0.0010
Epoch 12/20
153/153 [==============================] - ETA: 0s - loss: 0.1790 - acc: 0.9477
Epoch 12: val_acc did not improve from 0.88736
153/153 [==============================] - 144s 943ms/step - loss: 0.1790 - acc: 0.9477 - val_loss: 0.4067 - val_acc: 0.8791 - lr: 0.0010
Epoch 13/20
153/153 [==============================] - ETA: 0s - loss: 0.1726 - acc: 0.9484
Epoch 13: val_acc did not improve from 0.88736
153/153 [==============================] - 145s 947ms/step - loss: 0.1726 - acc: 0.9484 - val_loss: 0.3997 - val_acc: 0.8866 - lr: 0.0010
Epoch 14/20
153/153 [==============================] - ETA: 0s - loss: 0.1641 - acc: 0.9513
Epoch 14: val_acc did not improve from 0.88736

Epoch 14: ReduceLROnPlateau reducing learning rate to 0.00010000000474974513.
153/153 [==============================] - 145s 948ms/step - loss: 0.1641 - acc: 0.9513 - val_loss: 0.4019 - val_acc: 0.8756 - lr: 0.0010
Epoch 15/20
153/153 [==============================] - ETA: 0s - loss: 0.1266 - acc: 0.9597
Epoch 15: val_acc did not improve from 0.88736
153/153 [==============================] - 145s 947ms/step - loss: 0.1266 - acc: 0.9597 - val_loss: 0.3969 - val_acc: 0.8834 - lr: 1.0000e-04
Epoch 16/20
153/153 [==============================] - ETA: 0s - loss: 0.1197 - acc: 0.9640
Epoch 16: val_acc did not improve from 0.88736
153/153 [==============================] - 145s 949ms/step - loss: 0.1197 - acc: 0.9640 - val_loss: 0.4113 - val_acc: 0.8838 - lr: 1.0000e-04
Epoch 17/20
153/153 [==============================] - ETA: 0s - loss: 0.1167 - acc: 0.9639
Epoch 17: val_acc did not improve from 0.88736
153/153 [==============================] - 145s 949ms/step - loss: 0.1167 - acc: 0.9639 - val_loss: 0.4170 - val_acc: 0.8858 - lr: 1.0000e-04
Epoch 18/20
153/153 [==============================] - ETA: 0s - loss: 0.1175 - acc: 0.9648
Epoch 18: val_acc did not improve from 0.88736
153/153 [==============================] - 145s 950ms/step - loss: 0.1175 - acc: 0.9648 - val_loss: 0.4208 - val_acc: 0.8850 - lr: 1.0000e-04
Epoch 19/20
153/153 [==============================] - ETA: 0s - loss: 0.1151 - acc: 0.9640
Epoch 19: val_acc did not improve from 0.88736
Restoring model weights from the end of the best epoch: 9.

Epoch 19: ReduceLROnPlateau reducing learning rate to 1.0000000474974514e-05.
153/153 [==============================] - 146s 954ms/step - loss: 0.1151 - acc: 0.9640 - val_loss: 0.4214 - val_acc: 0.8870 - lr: 1.0000e-04
Epoch 19: early stopping
```

In [62]:

```
results_1 = model_lstm1.evaluate(XX_test, y_test, verbose=False)
print(f'Test results - Loss: {results_1[0]} - Accuracy: {100*results_1[1]}%')
```

```
Test results - Loss: 0.362724632024765 - Accuracy: 88.73626589775085%
```

In [63]:

```
acc = history_lstm1.history['acc']
val_acc = history_lstm1.history['val_acc']
loss = history_lstm1.history['loss']
val_loss = history_lstm1.history['val_loss']
plt.plot( acc, 'go', label='Train accuracy')
plt.plot( val_acc, 'g', label='Validate accuracy')
plt.title('Train and validate accuracy')
plt.legend()

plt.figure()
plt.plot( loss, 'go', label='Train loss')
plt.plot( val_loss, 'g', label='Validate loss')
plt.title('Train and validate loss')
plt.legend()
plt.show()
```

### **LSTM 2-Layers**¶

In [64]:

```
epochs = 10
emb_dim = 120
batch_size = 50
model_lstm2 = Sequential()
model_lstm2.add(Embedding(vocabulary_size,emb_dim ,input_length=X_DeepLearning.shape[1]))
model_lstm2.add(SpatialDropout1D(0.8))
model_lstm2.add(Bidirectional(LSTM(200, dropout=0.5, recurrent_dropout=0.5, return_sequences= True)))
model_lstm2.add(Dropout(0.5))
model_lstm2.add(Bidirectional(LSTM(300, dropout=0.5, recurrent_dropout =0.5)))
model_lstm2.add(Dropout(0.5))
model_lstm2.add(Flatten())
model_lstm2.add(Dense(64, activation='relu'))
model_lstm2.add(Dropout(0.5))
model_lstm2.add(Dense(5, activation='softmax'))
model_lstm2.compile(optimizer=tf.optimizers.Adam(),loss='categorical_crossentropy', metrics=['acc'])
print(model_lstm2.summary())
```

```
Model: "sequential_1"
_________________________________________________________________
 Layer (type)                Output Shape              Param #   
=================================================================
 embedding_1 (Embedding)     (None, 127, 120)          1222800   
                                                                 
 spatial_dropout1d_1 (Spati  (None, 127, 120)          0         
 alDropout1D)                                                    
                                                                 
 bidirectional_1 (Bidirecti  (None, 127, 400)          513600    
 onal)                                                           
                                                                 
 dropout_2 (Dropout)         (None, 127, 400)          0         
                                                                 
 bidirectional_2 (Bidirecti  (None, 600)               1682400   
 onal)                                                           
                                                                 
 dropout_3 (Dropout)         (None, 600)               0         
                                                                 
 flatten_1 (Flatten)         (None, 600)               0         
                                                                 
 dense_2 (Dense)             (None, 64)                38464     
                                                                 
 dropout_4 (Dropout)         (None, 64)                0         
                                                                 
 dense_3 (Dense)             (None, 5)                 325       
                                                                 
=================================================================
Total params: 3457589 (13.19 MB)
Trainable params: 3457589 (13.19 MB)
Non-trainable params: 0 (0.00 Byte)
_________________________________________________________________
None
```

In [65]:

```
checkpoint_callback = ModelCheckpoint(filepath="lastm-2-layer-best_model.h5", save_best_only=True, monitor="val_acc", mode="max", verbose=1)

early_stopping_callback = EarlyStopping(monitor="val_acc", mode="max", patience=10, verbose=1, restore_best_weights=True)

reduce_lr_callback = ReduceLROnPlateau(monitor="val_loss", factor=0.1, patience=5, verbose=1, mode="min", min_delta=0.0001, cooldown=0, min_lr=0)

callbacks2=[checkpoint_callback, early_stopping_callback, reduce_lr_callback]
```

In [66]:

```
history_lstm2 = model_lstm2.fit(XX_train, y_train, epochs=epochs, batch_size=batch_size, validation_data=(XX_test,y_test), callbacks=callbacks2)
```

```
Epoch 1/10
153/153 [==============================] - ETA: 0s - loss: 1.4690 - acc: 0.3237
Epoch 1: val_acc improved from -inf to 0.47567, saving model to lastm-2-layer-best_model.h5
153/153 [==============================] - 490s 3s/step - loss: 1.4690 - acc: 0.3237 - val_loss: 1.1920 - val_acc: 0.4757 - lr: 0.0010
Epoch 2/10
153/153 [==============================] - ETA: 0s - loss: 1.1316 - acc: 0.5150
Epoch 2: val_acc improved from 0.47567 to 0.59890, saving model to lastm-2-layer-best_model.h5
153/153 [==============================] - 515s 3s/step - loss: 1.1316 - acc: 0.5150 - val_loss: 0.8807 - val_acc: 0.5989 - lr: 0.0010
Epoch 3/10
153/153 [==============================] - ETA: 0s - loss: 0.8420 - acc: 0.6718
Epoch 3: val_acc improved from 0.59890 to 0.81240, saving model to lastm-2-layer-best_model.h5
153/153 [==============================] - 529s 3s/step - loss: 0.8420 - acc: 0.6718 - val_loss: 0.5529 - val_acc: 0.8124 - lr: 0.0010
Epoch 4/10
153/153 [==============================] - ETA: 0s - loss: 0.6229 - acc: 0.7772
Epoch 4: val_acc improved from 0.81240 to 0.83673, saving model to lastm-2-layer-best_model.h5
153/153 [==============================] - 536s 4s/step - loss: 0.6229 - acc: 0.7772 - val_loss: 0.4649 - val_acc: 0.8367 - lr: 0.0010
Epoch 5/10
153/153 [==============================] - ETA: 0s - loss: 0.5208 - acc: 0.8267
Epoch 5: val_acc improved from 0.83673 to 0.84733, saving model to lastm-2-layer-best_model.h5
153/153 [==============================] - 542s 4s/step - loss: 0.5208 - acc: 0.8267 - val_loss: 0.4422 - val_acc: 0.8473 - lr: 0.0010
Epoch 6/10
153/153 [==============================] - ETA: 0s - loss: 0.4409 - acc: 0.8608
Epoch 6: val_acc improved from 0.84733 to 0.86617, saving model to lastm-2-layer-best_model.h5
153/153 [==============================] - 553s 4s/step - loss: 0.4409 - acc: 0.8608 - val_loss: 0.4046 - val_acc: 0.8662 - lr: 0.0010
Epoch 7/10
153/153 [==============================] - ETA: 0s - loss: 0.3981 - acc: 0.8762
Epoch 7: val_acc improved from 0.86617 to 0.87166, saving model to lastm-2-layer-best_model.h5
153/153 [==============================] - 554s 4s/step - loss: 0.3981 - acc: 0.8762 - val_loss: 0.4005 - val_acc: 0.8717 - lr: 0.0010
Epoch 8/10
153/153 [==============================] - ETA: 0s - loss: 0.3320 - acc: 0.8974
Epoch 8: val_acc improved from 0.87166 to 0.87480, saving model to lastm-2-layer-best_model.h5
153/153 [==============================] - 558s 4s/step - loss: 0.3320 - acc: 0.8974 - val_loss: 0.3861 - val_acc: 0.8748 - lr: 0.0010
Epoch 9/10
153/153 [==============================] - ETA: 0s - loss: 0.3059 - acc: 0.9066
Epoch 9: val_acc improved from 0.87480 to 0.88148, saving model to lastm-2-layer-best_model.h5
153/153 [==============================] - 560s 4s/step - loss: 0.3059 - acc: 0.9066 - val_loss: 0.3969 - val_acc: 0.8815 - lr: 0.0010
Epoch 10/10
153/153 [==============================] - ETA: 0s - loss: 0.2734 - acc: 0.9155
Epoch 10: val_acc did not improve from 0.88148
153/153 [==============================] - 564s 4s/step - loss: 0.2734 - acc: 0.9155 - val_loss: 0.4007 - val_acc: 0.8721 - lr: 0.0010
```

In [67]:

```
results_2 = model_lstm2.evaluate(XX_test, y_test, verbose=False)
print(f'Test results - Loss: {results_2[0]} - Accuracy: {100*results_2[1]}%')
```

```
Test results - Loss: 0.4007441997528076 - Accuracy: 87.20565438270569%
```

In [68]:

```
acc = history_lstm2.history['acc']
val_acc = history_lstm2.history['val_acc']
loss = history_lstm2.history['loss']
val_loss = history_lstm2.history['val_loss']

plt.plot( acc, 'go', label='Train accuracy')
plt.plot( val_acc, 'g', label='Validate accuracy')
plt.title('Train and validate accuracy')
plt.legend()

plt.figure()

plt.plot( loss, 'go', label='Train loss')
plt.plot( val_loss, 'g', label='Validate loss')
plt.title('Train and validate loss')
plt.legend()

plt.show()
```

In [69]:

```
from keras.models import Sequential, load_model
from keras.layers import Activation, Dense, Embedding, LSTM, SpatialDropout1D, Dropout, Flatten, GRU, Conv1D, MaxPooling1D, Bidirectional
from keras.callbacks import ModelCheckpoint, EarlyStopping, ReduceLROnPlateau
```

### **GRU**¶

In [70]:

```
epochs = 20
emb_dim = 256
batch_size = 50
model_gru = Sequential()
model_gru.add(Embedding(vocabulary_size,emb_dim ,input_length=X_DeepLearning.shape[1]))
model_gru.add(SpatialDropout1D(0.8))
model_gru.add(GRU(units=64, dropout=0.2, recurrent_dropout=0.2))
model_gru.add(Dropout(0.5))
model_gru.add(Dense(256, activation='relu'))
model_gru.add(Dropout(0.5))
model_gru.add(Dense(5, activation='softmax'))
model_gru.compile(optimizer=tf.optimizers.Adam(),loss='categorical_crossentropy', metrics=['acc'])
print(model_gru.summary())
```

```
Model: "sequential_2"
_________________________________________________________________
 Layer (type)                Output Shape              Param #   
=================================================================
 embedding_2 (Embedding)     (None, 127, 256)          2608640   
                                                                 
 spatial_dropout1d_2 (Spati  (None, 127, 256)          0         
 alDropout1D)                                                    
                                                                 
 gru (GRU)                   (None, 64)                61824     
                                                                 
 dropout_5 (Dropout)         (None, 64)                0         
                                                                 
 dense_4 (Dense)             (None, 256)               16640     
                                                                 
 dropout_6 (Dropout)         (None, 256)               0         
                                                                 
 dense_5 (Dense)             (None, 5)                 1285      
                                                                 
=================================================================
Total params: 2688389 (10.26 MB)
Trainable params: 2688389 (10.26 MB)
Non-trainable params: 0 (0.00 Byte)
_________________________________________________________________
None
```

In [71]:

```
checkpoint_callback = ModelCheckpoint(filepath="gru-best_model.h5", save_best_only=True, monitor="val_acc", mode="max", verbose=1)

early_stopping_callback = EarlyStopping(monitor="val_acc", mode="max", patience=10, verbose=1, restore_best_weights=True)

reduce_lr_callback = ReduceLROnPlateau(monitor="val_loss", factor=0.1, patience=5, verbose=1, mode="min", min_delta=0.0001, cooldown=0, min_lr=0)

callbacks3=[checkpoint_callback, early_stopping_callback, reduce_lr_callback]
```

In [72]:

```
history_gru = model_gru.fit(XX_train, y_train, epochs=epochs, batch_size=batch_size,validation_data=(XX_test,y_test), callbacks=callbacks3)
```

```
Epoch 1/20
153/153 [==============================] - ETA: 0s - loss: 1.4361 - acc: 0.3667
Epoch 1: val_acc improved from -inf to 0.58399, saving model to gru-best_model.h5
153/153 [==============================] - 25s 151ms/step - loss: 1.4361 - acc: 0.3667 - val_loss: 0.9917 - val_acc: 0.5840 - lr: 0.0010
Epoch 2/20
153/153 [==============================] - ETA: 0s - loss: 0.8214 - acc: 0.6748
Epoch 2: val_acc improved from 0.58399 to 0.82261, saving model to gru-best_model.h5
153/153 [==============================] - 22s 147ms/step - loss: 0.8214 - acc: 0.6748 - val_loss: 0.5110 - val_acc: 0.8226 - lr: 0.0010
Epoch 3/20
153/153 [==============================] - ETA: 0s - loss: 0.5007 - acc: 0.8315
Epoch 3: val_acc improved from 0.82261 to 0.85086, saving model to gru-best_model.h5
153/153 [==============================] - 22s 147ms/step - loss: 0.5007 - acc: 0.8315 - val_loss: 0.4328 - val_acc: 0.8509 - lr: 0.0010
Epoch 4/20
153/153 [==============================] - ETA: 0s - loss: 0.3756 - acc: 0.8771
Epoch 4: val_acc improved from 0.85086 to 0.85557, saving model to gru-best_model.h5
153/153 [==============================] - 22s 147ms/step - loss: 0.3756 - acc: 0.8771 - val_loss: 0.4184 - val_acc: 0.8556 - lr: 0.0010
Epoch 5/20
153/153 [==============================] - ETA: 0s - loss: 0.2948 - acc: 0.9041
Epoch 5: val_acc improved from 0.85557 to 0.86538, saving model to gru-best_model.h5
153/153 [==============================] - 23s 147ms/step - loss: 0.2948 - acc: 0.9041 - val_loss: 0.4060 - val_acc: 0.8654 - lr: 0.0010
Epoch 6/20
153/153 [==============================] - ETA: 0s - loss: 0.2559 - acc: 0.9181
Epoch 6: val_acc improved from 0.86538 to 0.86578, saving model to gru-best_model.h5
153/153 [==============================] - 23s 147ms/step - loss: 0.2559 - acc: 0.9181 - val_loss: 0.4097 - val_acc: 0.8658 - lr: 0.0010
Epoch 7/20
153/153 [==============================] - ETA: 0s - loss: 0.1984 - acc: 0.9344
Epoch 7: val_acc improved from 0.86578 to 0.87637, saving model to gru-best_model.h5
153/153 [==============================] - 23s 148ms/step - loss: 0.1984 - acc: 0.9344 - val_loss: 0.4119 - val_acc: 0.8764 - lr: 0.0010
Epoch 8/20
153/153 [==============================] - ETA: 0s - loss: 0.1961 - acc: 0.9355
Epoch 8: val_acc did not improve from 0.87637
153/153 [==============================] - 23s 147ms/step - loss: 0.1961 - acc: 0.9355 - val_loss: 0.4249 - val_acc: 0.8752 - lr: 0.0010
Epoch 9/20
153/153 [==============================] - ETA: 0s - loss: 0.1571 - acc: 0.9475
Epoch 9: val_acc improved from 0.87637 to 0.87991, saving model to gru-best_model.h5
153/153 [==============================] - 22s 147ms/step - loss: 0.1571 - acc: 0.9475 - val_loss: 0.4411 - val_acc: 0.8799 - lr: 0.0010
Epoch 10/20
153/153 [==============================] - ETA: 0s - loss: 0.1401 - acc: 0.9568
Epoch 10: val_acc did not improve from 0.87991

Epoch 10: ReduceLROnPlateau reducing learning rate to 0.00010000000474974513.
153/153 [==============================] - 22s 147ms/step - loss: 0.1401 - acc: 0.9568 - val_loss: 0.4152 - val_acc: 0.8783 - lr: 0.0010
Epoch 11/20
153/153 [==============================] - ETA: 0s - loss: 0.1224 - acc: 0.9596
Epoch 11: val_acc improved from 0.87991 to 0.88108, saving model to gru-best_model.h5
153/153 [==============================] - 22s 147ms/step - loss: 0.1224 - acc: 0.9596 - val_loss: 0.4233 - val_acc: 0.8811 - lr: 1.0000e-04
Epoch 12/20
153/153 [==============================] - ETA: 0s - loss: 0.1250 - acc: 0.9618
Epoch 12: val_acc improved from 0.88108 to 0.88579, saving model to gru-best_model.h5
153/153 [==============================] - 22s 145ms/step - loss: 0.1250 - acc: 0.9618 - val_loss: 0.4278 - val_acc: 0.8858 - lr: 1.0000e-04
Epoch 13/20
153/153 [==============================] - ETA: 0s - loss: 0.1124 - acc: 0.9628
Epoch 13: val_acc did not improve from 0.88579
153/153 [==============================] - 22s 144ms/step - loss: 0.1124 - acc: 0.9628 - val_loss: 0.4331 - val_acc: 0.8815 - lr: 1.0000e-04
Epoch 14/20
153/153 [==============================] - ETA: 0s - loss: 0.1079 - acc: 0.9647
Epoch 14: val_acc did not improve from 0.88579
153/153 [==============================] - 22s 145ms/step - loss: 0.1079 - acc: 0.9647 - val_loss: 0.4411 - val_acc: 0.8854 - lr: 1.0000e-04
Epoch 15/20
153/153 [==============================] - ETA: 0s - loss: 0.1069 - acc: 0.9672
Epoch 15: val_acc did not improve from 0.88579

Epoch 15: ReduceLROnPlateau reducing learning rate to 1.0000000474974514e-05.
153/153 [==============================] - 22s 144ms/step - loss: 0.1069 - acc: 0.9672 - val_loss: 0.4533 - val_acc: 0.8803 - lr: 1.0000e-04
Epoch 16/20
153/153 [==============================] - ETA: 0s - loss: 0.1045 - acc: 0.9669
Epoch 16: val_acc did not improve from 0.88579
153/153 [==============================] - 22s 144ms/step - loss: 0.1045 - acc: 0.9669 - val_loss: 0.4509 - val_acc: 0.8830 - lr: 1.0000e-05
Epoch 17/20
153/153 [==============================] - ETA: 0s - loss: 0.1008 - acc: 0.9675
Epoch 17: val_acc did not improve from 0.88579
153/153 [==============================] - 22s 144ms/step - loss: 0.1008 - acc: 0.9675 - val_loss: 0.4505 - val_acc: 0.8827 - lr: 1.0000e-05
Epoch 18/20
153/153 [==============================] - ETA: 0s - loss: 0.1016 - acc: 0.9704
Epoch 18: val_acc did not improve from 0.88579
153/153 [==============================] - 22s 145ms/step - loss: 0.1016 - acc: 0.9704 - val_loss: 0.4496 - val_acc: 0.8838 - lr: 1.0000e-05
Epoch 19/20
153/153 [==============================] - ETA: 0s - loss: 0.1084 - acc: 0.9644
Epoch 19: val_acc did not improve from 0.88579
153/153 [==============================] - 22s 145ms/step - loss: 0.1084 - acc: 0.9644 - val_loss: 0.4482 - val_acc: 0.8834 - lr: 1.0000e-05
Epoch 20/20
153/153 [==============================] - ETA: 0s - loss: 0.0973 - acc: 0.9700
Epoch 20: val_acc did not improve from 0.88579

Epoch 20: ReduceLROnPlateau reducing learning rate to 1.0000000656873453e-06.
153/153 [==============================] - 22s 145ms/step - loss: 0.0973 - acc: 0.9700 - val_loss: 0.4478 - val_acc: 0.8830 - lr: 1.0000e-05
```

In [73]:

```
results_3 = model_gru.evaluate(XX_test, y_test, verbose=False)
print(f'Test results - Loss: {results_3[0]} - Accuracy: {100*results_3[1]}%')
```

```
Test results - Loss: 0.447836697101593 - Accuracy: 88.30455541610718%
```

In [74]:

```
acc = history_gru.history['acc']
val_acc = history_gru.history['val_acc']
loss = history_gru.history['loss']
val_loss = history_gru.history['val_loss']

plt.plot( acc, 'go', label='Train accuracy')
plt.plot( val_acc, 'g', label='Validate accuracy')
plt.title('Train and validate accuracy')
plt.legend()

plt.figure()

plt.plot( loss, 'go', label='Train loss')
plt.plot( val_loss, 'g', label='Validate loss')
plt.title('Train and validate loss')
plt.legend()

plt.show()
```

### **CNN+LSTM**¶

In [75]:

```
epochs = 20
emb_dim = 256
batch_size = 50
model_cl = Sequential()
model_cl.add(Embedding(vocabulary_size,emb_dim, input_length=X_DeepLearning.shape[1]))
model_cl.add(SpatialDropout1D(0.8))
model_cl.add(Conv1D(filters=64, kernel_size=6, padding='same', activation='relu'))
model_cl.add(MaxPooling1D(pool_size=2))
model_cl.add(Conv1D(filters=32, kernel_size=6, activation='relu'))
model_cl.add(MaxPooling1D(pool_size=2))
model_cl.add(Bidirectional(LSTM(100, dropout=0.5, recurrent_dropout=0.5, return_sequences=True)))
model_cl.add(Dropout(0.5))
model_cl.add(Bidirectional(LSTM(400, dropout=0.5, recurrent_dropout=0.5)))
model_cl.add(Dropout(0.5))
model_cl.add(Flatten())
model_cl.add(Dense(64, activation='relu'))
model_cl.add(Dropout(0.5))
model_cl.add(Dense(5, activation='softmax'))
model_cl.compile(optimizer='adam',loss='categorical_crossentropy', metrics=['acc'])
print(model_cl.summary())
```

```
WARNING:tensorflow:From C:\Users\Admin-server\AppData\Local\Programs\Python\Python310\lib\site-packages\keras\src\backend.py:6642: The name tf.nn.max_pool is deprecated. Please use tf.nn.max_pool2d instead.

WARNING:tensorflow:From C:\Users\Admin-server\AppData\Local\Programs\Python\Python310\lib\site-packages\keras\src\optimizers\__init__.py:309: The name tf.train.Optimizer is deprecated. Please use tf.compat.v1.train.Optimizer instead.

Model: "sequential_3"
_________________________________________________________________
 Layer (type)                Output Shape              Param #   
=================================================================
 embedding_3 (Embedding)     (None, 127, 256)          2608640   
                                                                 
 spatial_dropout1d_3 (Spati  (None, 127, 256)          0         
 alDropout1D)                                                    
                                                                 
 conv1d (Conv1D)             (None, 127, 64)           98368     
                                                                 
 max_pooling1d (MaxPooling1  (None, 63, 64)            0         
 D)                                                              
                                                                 
 conv1d_1 (Conv1D)           (None, 58, 32)            12320     
                                                                 
 max_pooling1d_1 (MaxPoolin  (None, 29, 32)            0         
 g1D)                                                            
                                                                 
 bidirectional_3 (Bidirecti  (None, 29, 200)           106400    
 onal)                                                           
                                                                 
 dropout_7 (Dropout)         (None, 29, 200)           0         
                                                                 
 bidirectional_4 (Bidirecti  (None, 800)               1923200   
 onal)                                                           
                                                                 
 dropout_8 (Dropout)         (None, 800)               0         
                                                                 
 flatten_2 (Flatten)         (None, 800)               0         
                                                                 
 dense_6 (Dense)             (None, 64)                51264     
                                                                 
 dropout_9 (Dropout)         (None, 64)                0         
                                                                 
 dense_7 (Dense)             (None, 5)                 325       
                                                                 
=================================================================
Total params: 4800517 (18.31 MB)
Trainable params: 4800517 (18.31 MB)
Non-trainable params: 0 (0.00 Byte)
_________________________________________________________________
None
```

In [76]:

```
checkpoint_callback = ModelCheckpoint(filepath="cnn+lastm-best_model.h5", save_best_only=True, monitor="val_acc", mode="max", verbose=1)

early_stopping_callback = EarlyStopping(monitor="val_acc", mode="max", patience=10, verbose=1, restore_best_weights=True)

reduce_lr_callback = ReduceLROnPlateau(monitor="val_loss", factor=0.1, patience=5, verbose=1, mode="min", min_delta=0.0001, cooldown=0, min_lr=0)

callbacks=[checkpoint_callback, early_stopping_callback, reduce_lr_callback]
```

In [77]:

```
history_cl = model_cl.fit(XX_train, y_train, epochs=epochs, batch_size=batch_size,validation_split=0.1, callbacks=callbacks)
```

```
Epoch 1/20
138/138 [==============================] - ETA: 0s - loss: 1.4858 - acc: 0.3119
Epoch 1: val_acc improved from -inf to 0.41046, saving model to cnn+lastm-best_model.h5
138/138 [==============================] - 42s 262ms/step - loss: 1.4858 - acc: 0.3119 - val_loss: 1.3261 - val_acc: 0.4105 - lr: 0.0010
Epoch 2/20
138/138 [==============================] - ETA: 0s - loss: 1.1920 - acc: 0.4655
Epoch 2: val_acc improved from 0.41046 to 0.50850, saving model to cnn+lastm-best_model.h5
138/138 [==============================] - 35s 252ms/step - loss: 1.1920 - acc: 0.4655 - val_loss: 1.0493 - val_acc: 0.5085 - lr: 0.0010
Epoch 3/20
138/138 [==============================] - ETA: 0s - loss: 0.9474 - acc: 0.5678
Epoch 3: val_acc improved from 0.50850 to 0.63529, saving model to cnn+lastm-best_model.h5
138/138 [==============================] - 35s 251ms/step - loss: 0.9474 - acc: 0.5678 - val_loss: 0.8084 - val_acc: 0.6353 - lr: 0.0010
Epoch 4/20
138/138 [==============================] - ETA: 0s - loss: 0.7572 - acc: 0.6493
Epoch 4: val_acc improved from 0.63529 to 0.71111, saving model to cnn+lastm-best_model.h5
138/138 [==============================] - 35s 251ms/step - loss: 0.7572 - acc: 0.6493 - val_loss: 0.6928 - val_acc: 0.7111 - lr: 0.0010
Epoch 5/20
138/138 [==============================] - ETA: 0s - loss: 0.6558 - acc: 0.7217
Epoch 5: val_acc improved from 0.71111 to 0.75163, saving model to cnn+lastm-best_model.h5
138/138 [==============================] - 35s 251ms/step - loss: 0.6558 - acc: 0.7217 - val_loss: 0.6152 - val_acc: 0.7516 - lr: 0.0010
Epoch 6/20
138/138 [==============================] - ETA: 0s - loss: 0.5262 - acc: 0.8086
Epoch 6: val_acc improved from 0.75163 to 0.83007, saving model to cnn+lastm-best_model.h5
138/138 [==============================] - 35s 251ms/step - loss: 0.5262 - acc: 0.8086 - val_loss: 0.5573 - val_acc: 0.8301 - lr: 0.0010
Epoch 7/20
138/138 [==============================] - ETA: 0s - loss: 0.4229 - acc: 0.8629
Epoch 7: val_acc improved from 0.83007 to 0.85229, saving model to cnn+lastm-best_model.h5
138/138 [==============================] - 34s 248ms/step - loss: 0.4229 - acc: 0.8629 - val_loss: 0.4844 - val_acc: 0.8523 - lr: 0.0010
Epoch 8/20
138/138 [==============================] - ETA: 0s - loss: 0.3554 - acc: 0.8901
Epoch 8: val_acc improved from 0.85229 to 0.87059, saving model to cnn+lastm-best_model.h5
138/138 [==============================] - 34s 249ms/step - loss: 0.3554 - acc: 0.8901 - val_loss: 0.4494 - val_acc: 0.8706 - lr: 0.0010
Epoch 9/20
138/138 [==============================] - ETA: 0s - loss: 0.2997 - acc: 0.9059
Epoch 9: val_acc did not improve from 0.87059
138/138 [==============================] - 34s 249ms/step - loss: 0.2997 - acc: 0.9059 - val_loss: 0.5115 - val_acc: 0.8575 - lr: 0.0010
Epoch 10/20
138/138 [==============================] - ETA: 0s - loss: 0.2667 - acc: 0.9219
Epoch 10: val_acc did not improve from 0.87059
138/138 [==============================] - 34s 249ms/step - loss: 0.2667 - acc: 0.9219 - val_loss: 0.5580 - val_acc: 0.8706 - lr: 0.0010
Epoch 11/20
138/138 [==============================] - ETA: 0s - loss: 0.2371 - acc: 0.9296
Epoch 11: val_acc improved from 0.87059 to 0.88105, saving model to cnn+lastm-best_model.h5
138/138 [==============================] - 34s 249ms/step - loss: 0.2371 - acc: 0.9296 - val_loss: 0.5322 - val_acc: 0.8810 - lr: 0.0010
Epoch 12/20
138/138 [==============================] - ETA: 0s - loss: 0.2101 - acc: 0.9399
Epoch 12: val_acc did not improve from 0.88105
138/138 [==============================] - 34s 248ms/step - loss: 0.2101 - acc: 0.9399 - val_loss: 0.5319 - val_acc: 0.8732 - lr: 0.0010
Epoch 13/20
138/138 [==============================] - ETA: 0s - loss: 0.1864 - acc: 0.9468
Epoch 13: val_acc improved from 0.88105 to 0.88758, saving model to cnn+lastm-best_model.h5

Epoch 13: ReduceLROnPlateau reducing learning rate to 0.00010000000474974513.
138/138 [==============================] - 34s 249ms/step - loss: 0.1864 - acc: 0.9468 - val_loss: 0.5541 - val_acc: 0.8876 - lr: 0.0010
Epoch 14/20
138/138 [==============================] - ETA: 0s - loss: 0.1530 - acc: 0.9571
Epoch 14: val_acc improved from 0.88758 to 0.89281, saving model to cnn+lastm-best_model.h5
138/138 [==============================] - 34s 249ms/step - loss: 0.1530 - acc: 0.9571 - val_loss: 0.5236 - val_acc: 0.8928 - lr: 1.0000e-04
Epoch 15/20
138/138 [==============================] - ETA: 0s - loss: 0.1556 - acc: 0.9594
Epoch 15: val_acc improved from 0.89281 to 0.89542, saving model to cnn+lastm-best_model.h5
138/138 [==============================] - 34s 249ms/step - loss: 0.1556 - acc: 0.9594 - val_loss: 0.5046 - val_acc: 0.8954 - lr: 1.0000e-04
Epoch 16/20
138/138 [==============================] - ETA: 0s - loss: 0.1405 - acc: 0.9622
Epoch 16: val_acc improved from 0.89542 to 0.89673, saving model to cnn+lastm-best_model.h5
138/138 [==============================] - 34s 249ms/step - loss: 0.1405 - acc: 0.9622 - val_loss: 0.5047 - val_acc: 0.8967 - lr: 1.0000e-04
Epoch 17/20
138/138 [==============================] - ETA: 0s - loss: 0.1339 - acc: 0.9610
Epoch 17: val_acc improved from 0.89673 to 0.89804, saving model to cnn+lastm-best_model.h5
138/138 [==============================] - 34s 248ms/step - loss: 0.1339 - acc: 0.9610 - val_loss: 0.4979 - val_acc: 0.8980 - lr: 1.0000e-04
Epoch 18/20
138/138 [==============================] - ETA: 0s - loss: 0.1310 - acc: 0.9639
Epoch 18: val_acc did not improve from 0.89804

Epoch 18: ReduceLROnPlateau reducing learning rate to 1.0000000474974514e-05.
138/138 [==============================] - 34s 248ms/step - loss: 0.1310 - acc: 0.9639 - val_loss: 0.5078 - val_acc: 0.8980 - lr: 1.0000e-04
Epoch 19/20
138/138 [==============================] - ETA: 0s - loss: 0.1312 - acc: 0.9622
Epoch 19: val_acc did not improve from 0.89804
138/138 [==============================] - 34s 249ms/step - loss: 0.1312 - acc: 0.9622 - val_loss: 0.5092 - val_acc: 0.8980 - lr: 1.0000e-05
Epoch 20/20
138/138 [==============================] - ETA: 0s - loss: 0.1289 - acc: 0.9638
Epoch 20: val_acc did not improve from 0.89804
138/138 [==============================] - 34s 249ms/step - loss: 0.1289 - acc: 0.9638 - val_loss: 0.5086 - val_acc: 0.8980 - lr: 1.0000e-05
```

In [78]:

```
results_4 = model_cl.evaluate(XX_test, y_test, verbose=False)
print(f'Test results - Loss: {results_4[0]} - Accuracy: {100*results_4[1]}%')
```

```
Test results - Loss: 0.5736315846443176 - Accuracy: 86.85243129730225%
```

In [79]:

```
acc = history_cl.history['acc']
val_acc = history_cl.history['val_acc']
loss = history_cl.history['loss']
val_loss = history_cl.history['val_loss']
plt.plot( acc, 'go', label='Train accuracy')
plt.plot( val_acc, 'g', label='Validate accuracy')
plt.title('Train and validate accuracy')
plt.legend()

plt.figure()
plt.plot( loss, 'go', label='Train loss')
plt.plot( val_loss, 'g', label='Validate loss')
plt.title('Train and validate loss')
plt.legend()
plt.show()
```

### **XLNet Model**¶

In [80]:

```
!pip install ktrain
```

```
Collecting ktrain
  Using cached ktrain-0.41.3.tar.gz (25.3 MB)
  Preparing metadata (setup.py): started
  Preparing metadata (setup.py): finished with status 'done'
Requirement already satisfied: scikit-learn in c:\users\admin-server\appdata\local\programs\python\python37\lib\site-packages (from ktrain) (1.0.2)
Requirement already satisfied: matplotlib>=3.0.0 in c:\users\admin-server\appdata\local\programs\python\python37\lib\site-packages (from ktrain) (3.4.2)
Requirement already satisfied: pandas>=1.0.1 in c:\users\admin-server\appdata\local\programs\python\python37\lib\site-packages (from ktrain) (1.2.4)
Requirement already satisfied: fastprogress>=0.1.21 in c:\users\admin-server\appdata\local\programs\python\python37\lib\site-packages (from ktrain) (1.0.0)
Requirement already satisfied: requests in c:\users\admin-server\appdata\local\programs\python\python37\lib\site-packages (from ktrain) (2.28.2)
Requirement already satisfied: joblib in c:\users\admin-server\appdata\local\programs\python\python37\lib\site-packages (from ktrain) (0.16.0)
Requirement already satisfied: packaging in c:\users\admin-server\appdata\local\programs\python\python37\lib\site-packages (from ktrain) (23.1)
Collecting langdetect (from ktrain)
  Using cached langdetect-1.0.9.tar.gz (981 kB)
  Preparing metadata (setup.py): started
  Preparing metadata (setup.py): finished with status 'done'
Collecting jieba (from ktrain)
  Using cached jieba-0.42.1.tar.gz (19.2 MB)
  Preparing metadata (setup.py): started
  Preparing metadata (setup.py): finished with status 'done'
Requirement already satisfied: charset-normalizer in c:\users\admin-server\appdata\local\programs\python\python37\lib\site-packages (from ktrain) (3.1.0)
Requirement already satisfied: chardet in c:\users\admin-server\appdata\local\programs\python\python37\lib\site-packages (from ktrain) (3.0.4)
Collecting syntok>1.3.3 (from ktrain)
  Using cached syntok-1.4.4-py3-none-any.whl.metadata (10 kB)
Collecting tika (from ktrain)
  Using cached tika-2.6.0.tar.gz (27 kB)
  Preparing metadata (setup.py): started
  Preparing metadata (setup.py): finished with status 'error'
```

```
  error: subprocess-exited-with-error
  
  python setup.py egg_info did not run successfully.
  exit code: 1
  
  [32 lines of output]
  Downloading http://pypi.python.org/packages/source/d/distribute/distribute-0.6.14.tar.gz
  Traceback (most recent call last):
    File "C:\Users\Admin-server\AppData\Local\Programs\Python\Python37\lib\site-packages\ez_setup.py", line 143, in use_setuptools
      raise ImportError
  ImportError
  
  During handling of the above exception, another exception occurred:
  
  Traceback (most recent call last):
    File "<string>", line 36, in <module>
    File "<pip-setuptools-caller>", line 34, in <module>
    File "C:\Users\ADMIN-~1\AppData\Local\Temp\pip-install-tkd9w8vu\tika_c16c45a5ebec4e2b84f4e0a2925b48fc\setup.py", line 29, in <module>
      use_setuptools()
    File "C:\Users\Admin-server\AppData\Local\Programs\Python\Python37\lib\site-packages\ez_setup.py", line 145, in use_setuptools
      return _do_download(version, download_base, to_dir, download_delay)
    File "C:\Users\Admin-server\AppData\Local\Programs\Python\Python37\lib\site-packages\ez_setup.py", line 124, in _do_download
      to_dir, download_delay)
    File "C:\Users\Admin-server\AppData\Local\Programs\Python\Python37\lib\site-packages\ez_setup.py", line 193, in download_setuptools
      src = urlopen(url)
    File "C:\Users\Admin-server\AppData\Local\Programs\Python\Python37\lib\urllib\request.py", line 222, in urlopen
      return opener.open(url, data, timeout)
    File "C:\Users\Admin-server\AppData\Local\Programs\Python\Python37\lib\urllib\request.py", line 531, in open
      response = meth(req, response)
    File "C:\Users\Admin-server\AppData\Local\Programs\Python\Python37\lib\urllib\request.py", line 641, in http_response
      'http', request, response, code, msg, hdrs)
    File "C:\Users\Admin-server\AppData\Local\Programs\Python\Python37\lib\urllib\request.py", line 569, in error
      return self._call_chain(*args)
    File "C:\Users\Admin-server\AppData\Local\Programs\Python\Python37\lib\urllib\request.py", line 503, in _call_chain
      result = func(*args)
    File "C:\Users\Admin-server\AppData\Local\Programs\Python\Python37\lib\urllib\request.py", line 649, in http_error_default
      raise HTTPError(req.full_url, code, msg, hdrs, fp)
  urllib.error.HTTPError: HTTP Error 403: SSL is required
  [end of output]
  
  note: This error originates from a subprocess, and is likely not a problem with pip.
error: metadata-generation-failed

Encountered error while generating package metadata.

See above for output.

note: This is an issue with the package mentioned above, not pip.
hint: See above for details.
```

In [81]:

```
from ktrain import text
```

In [82]:

```
X_train, X_test, y_train, y_test = train_test_split(df['message_stemmed'], df['label'], test_size=0.25, random_state=42)
```

In [83]:

```
X_train = X_train.tolist()
X_test = X_test.tolist()
y_train = y_train.tolist()
y_test = y_test.tolist()
```

In [84]:

```
model_name = 'xlnet-base-cased'
trans = text.Transformer(model_name, maxlen = 512, classes = ['neutral', 'racism', 'bullying','nazism', 'vioviolent'])
```

```
C:\Users\Admin-server\AppData\Local\Programs\Python\Python310\lib\site-packages\ktrain\text\preprocessor.py:382: UserWarning:

The class_names argument is replacing the classes argument. Please update your code.
```

```
config.json:   0%|          | 0.00/760 [00:00<?, ?B/s]
```

```
C:\Users\Admin-server\AppData\Local\Programs\Python\Python310\lib\site-packages\huggingface_hub\file_download.py:148: UserWarning:

`huggingface_hub` cache-system uses symlinks by default to efficiently store duplicated files but your machine does not support them in C:\Users\Admin-server\.cache\huggingface\hub\models--xlnet-base-cased. Caching files will still work but in a degraded version that might require more space on your disk. This warning can be disabled by setting the `HF_HUB_DISABLE_SYMLINKS_WARNING` environment variable. For more details, see https://huggingface.co/docs/huggingface_hub/how-to-cache#limitations.
To support symlinks on Windows, you either need to activate Developer Mode or to run Python as an administrator. In order to see activate developer mode, see this article: https://docs.microsoft.com/en-us/windows/apps/get-started/enable-your-device-for-development
```

```
tf_model.h5:   0%|          | 0.00/565M [00:00<?, ?B/s]
```

```
C:\Users\Admin-server\AppData\Local\Programs\Python\Python310\lib\site-packages\keras\src\initializers\initializers.py:120: UserWarning:

The initializer TruncatedNormal is unseeded and being called multiple times, which will return identical values each time (even if the initializer is unseeded). Please update your code to provide a seed to the initializer, or avoid using the same initializer instance more than once.
```

In [85]:

```
train_data = trans.preprocess_train(X_train, y_train)
test_data = trans.preprocess_test(X_test, y_test)
```

```
preprocessing train...
language: ru
train sequence lengths:
	mean : 16
	95percentile : 44
	99percentile : 65
```

```
spiece.model:   0%|          | 0.00/798k [00:00<?, ?B/s]
```

```
tokenizer.json:   0%|          | 0.00/1.38M [00:00<?, ?B/s]
```

```
Is Multi-Label? False
preprocessing test...
language: ru
test sequence lengths:
	mean : 16
	95percentile : 44
	99percentile : 73
```

In [86]:

```
!pip install tf-keras
```

```
ERROR: Ignored the following versions that require a different python version: 2.14.1 Requires-Python >=3.8; 2.15.0 Requires-Python >=3.8; 2.15.0rc0 Requires-Python >=3.8; 2.15.0rc1 Requires-Python >=3.8; 2.15.1 Requires-Python >=3.8; 2.15.1rc0 Requires-Python >=3.8; 2.16.0 Requires-Python >=3.9; 2.16.0rc0 Requires-Python >=3.9; 2.16.0rc1 Requires-Python >=3.9; 2.16.0rc2 Requires-Python >=3.9; 2.16.0rc3 Requires-Python >=3.9; 2.16.0rc4 Requires-Python >=3.9
ERROR: Could not find a version that satisfies the requirement tf-keras (from versions: none)
ERROR: No matching distribution found for tf-keras
```

In [87]:

```
!pip install transformers==3.5.1
```

```
Collecting transformers==3.5.1
  Downloading transformers-3.5.1-py3-none-any.whl.metadata (32 kB)
Requirement already satisfied: numpy in c:\users\admin-server\appdata\local\programs\python\python37\lib\site-packages (from transformers==3.5.1) (1.21.6)
Collecting tokenizers==0.9.3 (from transformers==3.5.1)
  Downloading tokenizers-0.9.3-cp37-cp37m-win_amd64.whl.metadata (5.8 kB)
Requirement already satisfied: packaging in c:\users\admin-server\appdata\local\programs\python\python37\lib\site-packages (from transformers==3.5.1) (23.1)
Requirement already satisfied: filelock in c:\users\admin-server\appdata\local\programs\python\python37\lib\site-packages (from transformers==3.5.1) (3.12.2)
Requirement already satisfied: requests in c:\users\admin-server\appdata\local\programs\python\python37\lib\site-packages (from transformers==3.5.1) (2.28.2)
Requirement already satisfied: tqdm>=4.27 in c:\users\admin-server\appdata\local\programs\python\python37\lib\site-packages (from transformers==3.5.1) (4.66.2)
Requirement already satisfied: regex!=2019.12.17 in c:\users\admin-server\appdata\local\programs\python\python37\lib\site-packages (from transformers==3.5.1) (2020.7.14)
Collecting sentencepiece==0.1.91 (from transformers==3.5.1)
  Downloading sentencepiece-0.1.91-cp37-cp37m-win_amd64.whl.metadata (10 kB)
Requirement already satisfied: protobuf in c:\users\admin-server\appdata\local\programs\python\python37\lib\site-packages (from transformers==3.5.1) (3.19.6)
Requirement already satisfied: sacremoses in c:\users\admin-server\appdata\local\programs\python\python37\lib\site-packages (from transformers==3.5.1) (0.0.45)
Requirement already satisfied: colorama in c:\users\admin-server\appdata\local\programs\python\python37\lib\site-packages (from tqdm>=4.27->transformers==3.5.1) (0.4.6)
Requirement already satisfied: charset-normalizer<4,>=2 in c:\users\admin-server\appdata\local\programs\python\python37\lib\site-packages (from requests->transformers==3.5.1) (3.1.0)
Requirement already satisfied: idna<4,>=2.5 in c:\users\admin-server\appdata\local\programs\python\python37\lib\site-packages (from requests->transformers==3.5.1) (2.10)
Requirement already satisfied: urllib3<1.27,>=1.21.1 in c:\users\admin-server\appdata\local\programs\python\python37\lib\site-packages (from requests->transformers==3.5.1) (1.24.3)
Requirement already satisfied: certifi>=2017.4.17 in c:\users\admin-server\appdata\local\programs\python\python37\lib\site-packages (from requests->transformers==3.5.1) (2023.7.22)
Requirement already satisfied: six in c:\users\admin-server\appdata\local\programs\python\python37\lib\site-packages (from sacremoses->transformers==3.5.1) (1.15.0)
Requirement already satisfied: click in c:\users\admin-server\appdata\local\programs\python\python37\lib\site-packages (from sacremoses->transformers==3.5.1) (8.1.7)
Requirement already satisfied: joblib in c:\users\admin-server\appdata\local\programs\python\python37\lib\site-packages (from sacremoses->transformers==3.5.1) (0.16.0)
Requirement already satisfied: importlib-metadata in c:\users\admin-server\appdata\local\programs\python\python37\lib\site-packages (from click->sacremoses->transformers==3.5.1) (6.7.0)
Requirement already satisfied: zipp>=0.5 in c:\users\admin-server\appdata\local\programs\python\python37\lib\site-packages (from importlib-metadata->click->sacremoses->transformers==3.5.1) (3.15.0)
Requirement already satisfied: typing-extensions>=3.6.4 in c:\users\admin-server\appdata\local\programs\python\python37\lib\site-packages (from importlib-metadata->click->sacremoses->transformers==3.5.1) (4.7.1)
Downloading transformers-3.5.1-py3-none-any.whl (1.3 MB)
   ---------------------------------------- 0.0/1.3 MB ? eta -:--:--
   - -------------------------------------- 0.1/1.3 MB 1.7 MB/s eta 0:00:01
   -- ------------------------------------- 0.1/1.3 MB 653.6 kB/s eta 0:00:02
   ----- ---------------------------------- 0.2/1.3 MB 1.2 MB/s eta 0:00:01
   ------ --------------------------------- 0.2/1.3 MB 1.1 MB/s eta 0:00:01
   ------------- -------------------------- 0.4/1.3 MB 1.8 MB/s eta 0:00:01
   -------------- ------------------------- 0.5/1.3 MB 1.8 MB/s eta 0:00:01
   --------------------- ------------------ 0.7/1.3 MB 2.2 MB/s eta 0:00:01
   ----------------------- ---------------- 0.8/1.3 MB 2.0 MB/s eta 0:00:01
   ---------------------------------- ----- 1.1/1.3 MB 2.6 MB/s eta 0:00:01
   ---------------------------------------  1.3/1.3 MB 2.8 MB/s eta 0:00:01
   ---------------------------------------- 1.3/1.3 MB 2.8 MB/s eta 0:00:00
Downloading sentencepiece-0.1.91-cp37-cp37m-win_amd64.whl (1.2 MB)
   ---------------------------------------- 0.0/1.2 MB ? eta -:--:--
   ------------------------------- -------- 0.9/1.2 MB 19.8 MB/s eta 0:00:01
   ----------------------------------- ---- 1.1/1.2 MB 22.4 MB/s eta 0:00:01
   ---------------------------------------- 1.2/1.2 MB 10.9 MB/s eta 0:00:00
Downloading tokenizers-0.9.3-cp37-cp37m-win_amd64.whl (1.9 MB)
   ---------------------------------------- 0.0/1.9 MB ? eta -:--:--
   ---------------------------------------  1.9/1.9 MB 61.4 MB/s eta 0:00:01
   ---------------------------------------- 1.9/1.9 MB 40.6 MB/s eta 0:00:00
Installing collected packages: tokenizers, sentencepiece, transformers
  Attempting uninstall: tokenizers
    Found existing installation: tokenizers 0.8.1rc1
    Uninstalling tokenizers-0.8.1rc1:
      Successfully uninstalled tokenizers-0.8.1rc1
  Attempting uninstall: sentencepiece
    Found existing installation: sentencepiece 0.1.95
    Uninstalling sentencepiece-0.1.95:
      Successfully uninstalled sentencepiece-0.1.95
  Attempting uninstall: transformers
    Found existing installation: transformers 3.0.2
    Uninstalling transformers-3.0.2:
      Successfully uninstalled transformers-3.0.2
Successfully installed sentencepiece-0.1.91 tokenizers-0.9.3 transformers-3.5.1
```

```
DEPRECATION: celery 4.4.7 has a non-standard dependency specifier pytz>dev. pip 24.1 will enforce this behaviour change. A possible replacement is to upgrade to a newer version of celery or contact the author to suggest that they release a version with a conforming dependency specifiers. Discussion can be found at https://github.com/pypa/pip/issues/12063
DEPRECATION: uvicorn 0.14.0 has a non-standard dependency specifier click>=7.*. pip 24.1 will enforce this behaviour change. A possible replacement is to upgrade to a newer version of uvicorn or contact the author to suggest that they release a version with a conforming dependency specifiers. Discussion can be found at https://github.com/pypa/pip/issues/12063
ERROR: pip's dependency resolver does not currently take into account all the packages that are installed. This behaviour is the source of the following dependency conflicts.
fast-bert 1.9.8 requires tokenizers==0.8.1.rc1, but you have tokenizers 0.9.3 which is incompatible.
fast-bert 1.9.8 requires transformers==3.0.2, but you have transformers 3.5.1 which is incompatible.
sentence-transformers 2.2.2 requires transformers<5.0.0,>=4.6.0, but you have transformers 3.5.1 which is incompatible.
```

In [88]:

```
from tensorflow.keras.optimizers import Adam
```

In [91]:

```
import ktrain
from ktrain import text
```

In [92]:

```
model = trans.get_classifier()
# optimizer = adam_v2.Adam(learning_rate=lr, decay=lr/epochs)
# model.compile(loss='--',  optimizer=optimizer  , metrics=['--'])
```

In [93]:

```
learner = ktrain.get_learner(model, train_data=train_data, val_data=test_data, batch_size=6)
```

In [ ]:

```
learner.fit_onecycle(lr=2e-5, epochs=3)
```

```
begin training using onecycle policy with max lr of 2e-05...
Epoch 1/3
  20/1274 [..............................] - ETA: 2:57:10 - loss: 1.6052 - accuracy: 0.2667
```

In [ ]:

```
learner.plot()
```

### **Bert Model**¶

In [ ]:

```
X_train, X_test, y_train, y_test = train_test_split(df['message_stemmed'], df['label'], test_size=0.33, random_state=42)
```

In [ ]:

```
X_train = X_train.tolist()
X_test = X_test.tolist()
y_train = y_train.tolist()
y_test = y_test.tolist()
```

In [ ]:

```
class_names = ['neutral', 'racism', 'bullying','nazism', 'vioviolent']
```

In [ ]:

```
(x_train,y_train), (x_val,y_val), preproc = text.texts_from_array(x_train=X_train, y_train=y_train,
                                                                       x_test=X_test, y_test=y_test,
                                                                       class_names=class_names,
                                                                       preprocess_mode='bert',
                                                                       maxlen=512,
                                                                       max_features=20000)
```

In [ ]:

```
model = text.text_classifier('bert', train_data=(x_train,y_train), preproc=preproc)
```

In [ ]:

```
learner = ktrain.get_learner(model, train_data=(x_train,y_train),
                             val_data=(x_val,y_val),
                             batch_size=6)
```

In [ ]:

```
learner.fit_onecycle(2e-5, 3)
```

In [ ]:

```
learner.plot()
```

### **GNN**¶

In [ ]:

```
!pip install torch torch-geometric torchtext
```

In [ ]:

```
import torch
from torch import nn
from torch_geometric.nn import GCNConv
from torch_geometric.data import Data

class LSTM_GNN(nn.Module):
    def __init__(self, input_features, hidden_features, lstm_hidden, num_classes):
        super(LSTM_GNN, self).__init__()
        # LSTM для обработки данных в каждом узле
        self.lstm = nn.LSTM(input_features, lstm_hidden, batch_first=True)
        # GCN слой для агрегации информации между узлами
        self.conv = GCNConv(lstm_hidden, hidden_features)
        # Классификатор
        self.classifier = nn.Linear(hidden_features, num_classes)

    def forward(self, data):
        x, edge_index = data.x, data.edge_index

        # Применяем LSTM к каждому узлу
        x, _ = self.lstm(x)
        x = x[:, -1, :]  # берем только последний выход LSTM

        # Применяем GCN слой
        x = self.conv(x, edge_index)
        x = torch.relu(x)

        # Применяем классификатор
        x = self.classifier(x)

        return x

# Подготовка данных
# Предполагается, что 'x' содержит последовательности данных для каждого узла
# Например, каждый узел содержит последовательность с 10 временными точками, каждая из которых имеет 5 признаков
num_nodes = 4
sequence_length = 10
num_features = 5
num_classes = 2

x = torch.randn(num_nodes, sequence_length, num_features)  # Случайные данные для демонстрации
edge_index = torch.tensor([[0, 1, 2, 3, 0, 2], [1, 0, 3, 2, 2, 3]], dtype=torch.long)

graph_data = Data(x=x, edge_index=edge_index)

# Создание и тестирование модели
model = LSTM_GNN(input_features=num_features, hidden_features=16, lstm_hidden=32, num_classes=num_classes)
output = model(graph_data)
print(output)
```

In [ ]:

```
import networkx as nx
import matplotlib.pyplot as plt
import torch

# Предположим, что output уже получен из вашей модели
output = torch.softmax(output, dim=1)  # Применяем softmax для получения вероятностей классов
_, predicted_classes = torch.max(output, dim=1)  # Получаем предсказанный класс для каждого узла

# Создаем граф на основе edge_index
G = nx.Graph()
G.add_edges_from(edge_index.t().tolist())

# Определяем цвет для каждого узла
color_map = ['blue' if i == 0 else 'red' for i in predicted_classes]

# Рисуем граф
plt.figure(figsize=(8, 8))
nx.draw(G, node_color=color_map, with_labels=True, node_size=700, font_color='white')
plt.title('Классификация узлов в графе')
plt.show()
```

### **ngram range = 1, 3**¶

In [ ]:

```
from sklearn.model_selection import train_test_split

X_train, X_test, y_train, y_test = train_test_split(df['clean_text'], df['label'], stratify=df['label'])
```

In [ ]:

```
original_train_sentences = X_train.tolist()
original_labels_train = y_train.tolist()
original_test_sentences = X_test.tolist()
original_labels_test = y_test.tolist()

train_size = len(original_train_sentences)
test_size = len(original_test_sentences)
sentences = original_train_sentences + original_test_sentences
```

In [ ]:

```
from sklearn.feature_extraction.text import CountVectorizer, TfidfTransformer
```

In [ ]:

```
x_train, x_test, y_train, y_test = train_test_split(df["clean_text"],df["label_name"], test_size = 0.25, random_state = 42)
count_vect = CountVectorizer(ngram_range=(1, 3))
transformer = TfidfTransformer(norm='l2',sublinear_tf=True)
x_train_counts = count_vect.fit_transform(x_train)
x_train_tfidf = transformer.fit_transform(x_train_counts)

x_test_counts = count_vect.transform(x_test)
x_test_tfidf = transformer.transform(x_test_counts)

print (x_train_tfidf.shape,x_test_tfidf.shape, y_train.shape, y_test.shape)
```

### **Logistic Regression**¶

In [ ]:

```
from sklearn.linear_model import LogisticRegression
from sklearn.model_selection import cross_val_score, cross_val_predict
from sklearn.svm import LinearSVC, SVC
from sklearn.naive_bayes import MultinomialNB
from sklearn.ensemble import GradientBoostingClassifier, RandomForestClassifier, AdaBoostClassifier, VotingClassifier
from sklearn.metrics import accuracy_score, classification_report, confusion_matrix, precision_score, f1_score, recall_score
```

In [ ]:

```
lr = LogisticRegression(C = 2, max_iter = 1000, n_jobs=1)
lr.fit(x_train_tfidf, y_train)
y_pred1 = lr.predict(x_test_tfidf)
print("Accuracy: "+str(accuracy_score(y_test,y_pred1)))
print(classification_report(y_test, y_pred1))
```

### **Support Vector Machine**¶

In [ ]:

```
svc = LinearSVC()
svc.fit(x_train_tfidf, y_train)
y_pred2 = svc.predict(x_test_tfidf)
print("Accuracy: "+str(accuracy_score(y_test,y_pred2)))
print(classification_report(y_test, y_pred2))
```

### **Naive Bayes(Multinomial)**¶

In [ ]:

```
mnb = MultinomialNB()
mnb.fit(x_train_tfidf, y_train)
y_pred3 = mnb.predict(x_test_tfidf)
print("Accuracy: "+str(accuracy_score(y_test,y_pred3)))
print(classification_report(y_test, y_pred3))
```

### **Randomforest**¶

In [ ]:

```
rfc = RandomForestClassifier(n_estimators=300, max_depth=15, random_state=42, class_weight='balanced')
rfc.fit(x_train_tfidf,y_train)
y_pred4 = rfc.predict(x_test_tfidf)
print("Accuracy: "+str(accuracy_score(y_test,y_pred4)))
print(classification_report(y_test, y_pred4))
```

### **GradientBoostingClassifier**¶

In [ ]:

```
gbc = GradientBoostingClassifier(n_estimators=100,  max_depth=4, random_state=1, verbose=1)
gbc.fit(x_train_tfidf, y_train)
y_pred5 = gbc.predict(x_test_tfidf)
print(accuracy_score(y_test, y_pred5))
print(classification_report(y_test, y_pred5))
```

In [ ]:

```
scores = cross_val_score(gbc, x_train_tfidf,y_train, cv=5)
print(accuracy_score(y_test,y_pred5))
print ("Cross-validated scores:", scores)
```

### **Ensemble Classifier**¶

In [ ]:

```
mnb = MultinomialNB()
rfc= RandomForestClassifier(n_estimators=1000, max_depth=12, random_state=42)
lr = LogisticRegression(C = 2, max_iter = 1000, n_jobs=-1)
svc = SVC(probability=True)
ec=VotingClassifier(estimators=[('Multinominal NB', mnb), ('Random Forest', rfc),('Logistic Regression',lr),('Support Vector Machine',svc)], voting='soft', weights=[1,2,3,4])
ec.fit(x_train_tfidf,y_train)
y_pred6 = ec.predict(x_test_tfidf)
print(accuracy_score(y_test, y_pred6))
print(classification_report(y_test, y_pred6))
```

In [ ]:

```
scores = cross_val_score(ec, x_train_tfidf,y_train, cv=10)
print(accuracy_score(y_test,y_pred6))
print ("Cross-validated scores:", scores)
```

### **AdaBoost with Random Forest Classifier**¶

In [ ]:

```
rfc = RandomForestClassifier(n_estimators=100, max_depth=9, random_state=0)
abc= AdaBoostClassifier(estimator=rfc, learning_rate=0.2, n_estimators=100)
abc.fit(x_train_tfidf, y_train)
y_pred7= abc.predict(x_test_tfidf)
print("Accuracy: "+str(accuracy_score(y_test, y_pred7)))
print(classification_report(y_test, y_pred7))
```

In [ ]:

```
scores = cross_val_score(abc, x_train_tfidf,y_train, cv=10)
print(accuracy_score(y_test,y_pred7))
print ("Cross-validated scores:", scores)
```

### **ngram range = 1, 3 бойынша:**¶

In [ ]:

```
Comparison_unibi = pd.DataFrame({'Logistic Regression': [accuracy_score(y_test,y_pred1)*100,f1_score(y_test,y_pred1,average='macro')*100,recall_score(y_test, y_pred1,average='micro')*100,precision_score(y_test, y_pred1,average='micro')*100],
                            'SVM':[accuracy_score(y_test,y_pred2)*100,f1_score(y_test,y_pred2,average='macro')*100,recall_score(y_test, y_pred2,average='micro')*100,precision_score(y_test, y_pred2,average='micro')*100],
                           'Naive Bayes':[accuracy_score(y_test,y_pred3)*100,f1_score(y_test,y_pred3,average='macro')*100,recall_score(y_test, y_pred3,average='micro')*100,precision_score(y_test, y_pred3,average='micro')*100],
                           'Random Forest':[accuracy_score(y_test,y_pred4)*100,f1_score(y_test,y_pred4,average='macro')*100,recall_score(y_test, y_pred4,average='micro')*100,precision_score(y_test, y_pred4,average='micro')*100],
                           'GradientBoosting':[accuracy_score(y_test,y_pred5)*100,f1_score(y_test,y_pred5,average='macro')*100,recall_score(y_test, y_pred5,average='micro')*100,precision_score(y_test, y_pred5,average='micro')*100],
                           'Ensembled':[accuracy_score(y_test,y_pred6)*100,f1_score(y_test,y_pred6,average='macro')*100,recall_score(y_test, y_pred6,average='micro')*100,precision_score(y_test, y_pred6,average='micro')*100],
                           'Adaboost':[accuracy_score(y_test,y_pred7)*100,f1_score(y_test,y_pred7,average='macro')*100,recall_score(y_test, y_pred7,average='micro')*100,precision_score(y_test, y_pred7,average='micro')*100],

})

print ('Cравнение с использованием uni-bi-gram(1,2)')
Comparison_unibi.rename(index={0:'Accuracy',1:'F1_score', 2: 'Recall',3:'Precision'}, inplace=True)
Comparison_unibi.head()
```

### **ngram range = 2, 2**¶

In [ ]:

```
from sklearn.model_selection import train_test_split

X_train, X_test, y_train, y_test = train_test_split(df['clean_text'], df['label'], stratify=df['label'])
```

In [ ]:

```
from sklearn.feature_extraction.text import CountVectorizer, TfidfTransformer
```

In [ ]:

```
x_train, x_test, y_train, y_test = train_test_split(df["clean_text"],df["label_name"], test_size = 0.25, random_state = 42)
count_vect = CountVectorizer(ngram_range=(2, 2))
transformer = TfidfTransformer(norm='l2',sublinear_tf=True)
x_train_counts = count_vect.fit_transform(x_train)
x_train_tfidf = transformer.fit_transform(x_train_counts)

x_test_counts = count_vect.transform(x_test)
x_test_tfidf = transformer.transform(x_test_counts)

print (x_train_tfidf.shape,x_test_tfidf.shape, y_train.shape, y_test.shape)
```

### **Logistic Regression**¶

In [ ]:

```
from sklearn.linear_model import LogisticRegression
from sklearn.model_selection import cross_val_score, cross_val_predict
from sklearn.svm import LinearSVC, SVC
from sklearn.naive_bayes import MultinomialNB
from sklearn.ensemble import GradientBoostingClassifier, RandomForestClassifier, AdaBoostClassifier, VotingClassifier
from sklearn.metrics import accuracy_score, classification_report, confusion_matrix, precision_score, f1_score, recall_score
```

In [ ]:

```
lr = LogisticRegression(C = 2, max_iter = 1000, n_jobs=1)
lr.fit(x_train_tfidf, y_train)
y_pred1 = lr.predict(x_test_tfidf)
print("Accuracy: "+str(accuracy_score(y_test,y_pred1)))
print(classification_report(y_test, y_pred1))
```

### **Support Vector Machine**¶

In [ ]:

```
svc = LinearSVC()
svc.fit(x_train_tfidf, y_train)
y_pred2 = svc.predict(x_test_tfidf)
print("Accuracy: "+str(accuracy_score(y_test,y_pred2)))
print(classification_report(y_test, y_pred2))
```

### **Naive Bayes(Multinomial)**¶

In [ ]:

```
mnb = MultinomialNB()
mnb.fit(x_train_tfidf, y_train)
y_pred3 = mnb.predict(x_test_tfidf)
print("Accuracy: "+str(accuracy_score(y_test,y_pred3)))
print(classification_report(y_test, y_pred3))
```

### **Randomforest**¶

In [ ]:

```
rfc = RandomForestClassifier(n_estimators=300, max_depth=15, random_state=42, class_weight='balanced')
rfc.fit(x_train_tfidf,y_train)
y_pred4 = rfc.predict(x_test_tfidf)
print("Accuracy: "+str(accuracy_score(y_test,y_pred4)))
print(classification_report(y_test, y_pred4))
```

### **GradientBoostingClassifier**¶

In [ ]:

```
gbc = GradientBoostingClassifier(n_estimators=100,  max_depth=4, random_state=1, verbose=1)
gbc.fit(x_train_tfidf, y_train)
y_pred5 = gbc.predict(x_test_tfidf)
print(accuracy_score(y_test, y_pred5))
print(classification_report(y_test, y_pred5))
```

### **Ensemble Classifier**¶

In [ ]:

```
mnb = MultinomialNB()
rfc= RandomForestClassifier(n_estimators=1000, max_depth=12, random_state=42)
lr = LogisticRegression(C = 2, max_iter = 1000, n_jobs=-1)
svc = SVC(probability=True)
ec=VotingClassifier(estimators=[('Multinominal NB', mnb), ('Random Forest', rfc),('Logistic Regression',lr),('Support Vector Machine',svc)], voting='soft', weights=[1,2,3,4])
ec.fit(x_train_tfidf,y_train)
y_pred6 = ec.predict(x_test_tfidf)
print(accuracy_score(y_test, y_pred6))
print(classification_report(y_test, y_pred6))
```

### **AdaBoost with Random Forest Classifier**¶

In [ ]:

```
rfc = RandomForestClassifier(n_estimators=100, max_depth=9, random_state=0)
abc= AdaBoostClassifier(estimator=rfc, learning_rate=0.2, n_estimators=100)
abc.fit(x_train_tfidf, y_train)
y_pred7= abc.predict(x_test_tfidf)
print("Accuracy: "+str(accuracy_score(y_test, y_pred7)))
print(classification_report(y_test, y_pred7))
```

### **ngram range = 2, 2 бойынша:**¶

In [ ]:

```
Comparison_unibi = pd.DataFrame({'Logistic Regression': [accuracy_score(y_test,y_pred1)*100,f1_score(y_test,y_pred1,average='macro')*100,recall_score(y_test, y_pred1,average='micro')*100,precision_score(y_test, y_pred1,average='micro')*100],
                            'SVM':[accuracy_score(y_test,y_pred2)*100,f1_score(y_test,y_pred2,average='macro')*100,recall_score(y_test, y_pred2,average='micro')*100,precision_score(y_test, y_pred2,average='micro')*100],
                           'Naive Bayes':[accuracy_score(y_test,y_pred3)*100,f1_score(y_test,y_pred3,average='macro')*100,recall_score(y_test, y_pred3,average='micro')*100,precision_score(y_test, y_pred3,average='micro')*100],
                           'Random Forest':[accuracy_score(y_test,y_pred4)*100,f1_score(y_test,y_pred4,average='macro')*100,recall_score(y_test, y_pred4,average='micro')*100,precision_score(y_test, y_pred4,average='micro')*100],
                           'GradientBoosting':[accuracy_score(y_test,y_pred5)*100,f1_score(y_test,y_pred5,average='macro')*100,recall_score(y_test, y_pred5,average='micro')*100,precision_score(y_test, y_pred5,average='micro')*100],
                           'Ensembled':[accuracy_score(y_test,y_pred6)*100,f1_score(y_test,y_pred6,average='macro')*100,recall_score(y_test, y_pred6,average='micro')*100,precision_score(y_test, y_pred6,average='micro')*100],
                           'Adaboost':[accuracy_score(y_test,y_pred7)*100,f1_score(y_test,y_pred7,average='macro')*100,recall_score(y_test, y_pred7,average='micro')*100,precision_score(y_test, y_pred7,average='micro')*100],

})

print ('Cравнение с использованием uni-bi-gram(1,2)')
Comparison_unibi.rename(index={0:'Accuracy',1:'F1_score', 2: 'Recall',3:'Precision'}, inplace=True)
Comparison_unibi.head()
```

### **ngram range = 2, 3**¶

In [ ]:

```
x_train, x_test, y_train, y_test = train_test_split(df["clean_text"],df["label_name"], test_size = 0.25, random_state = 42)
count_vect = CountVectorizer(ngram_range=(2, 3))
transformer = TfidfTransformer(norm='l2',sublinear_tf=True)
x_train_counts = count_vect.fit_transform(x_train)
x_train_tfidf = transformer.fit_transform(x_train_counts)

x_test_counts = count_vect.transform(x_test)
x_test_tfidf = transformer.transform(x_test_counts)

print (x_train_tfidf.shape,x_test_tfidf.shape, y_train.shape, y_test.shape)


lr = LogisticRegression(C = 2, max_iter = 1000, n_jobs=1)
lr.fit(x_train_tfidf, y_train)
y_pred1 = lr.predict(x_test_tfidf)
print("Accuracy: "+str(accuracy_score(y_test,y_pred1)))
print(classification_report(y_test, y_pred1))
```

In [ ]:

```
svc = LinearSVC()
svc.fit(x_train_tfidf, y_train)
y_pred2 = svc.predict(x_test_tfidf)
print("Accuracy: "+str(accuracy_score(y_test,y_pred2)))
print(classification_report(y_test, y_pred2))
```

In [ ]:

```
mnb = MultinomialNB()
mnb.fit(x_train_tfidf, y_train)
y_pred3 = mnb.predict(x_test_tfidf)
print("Accuracy: "+str(accuracy_score(y_test,y_pred3)))
print(classification_report(y_test, y_pred3))
```

In [ ]:

```
rfc = RandomForestClassifier(n_estimators=300, max_depth=15, random_state=42, class_weight='balanced')
rfc.fit(x_train_tfidf,y_train)
y_pred4 = rfc.predict(x_test_tfidf)
print("Accuracy: "+str(accuracy_score(y_test,y_pred4)))
print(classification_report(y_test, y_pred4))
```

In [ ]:

```
gbc = GradientBoostingClassifier(n_estimators=100,  max_depth=4, random_state=1, verbose=1)
gbc.fit(x_train_tfidf, y_train)
y_pred5 = gbc.predict(x_test_tfidf)
print(accuracy_score(y_test, y_pred5))
print(classification_report(y_test, y_pred5))
```

In [ ]:

```
mnb = MultinomialNB()
rfc= RandomForestClassifier(n_estimators=1000, max_depth=12, random_state=42)
lr = LogisticRegression(C = 2, max_iter = 1000, n_jobs=-1)
svc = SVC(probability=True)
ec=VotingClassifier(estimators=[('Multinominal NB', mnb), ('Random Forest', rfc),('Logistic Regression',lr),('Support Vector Machine',svc)], voting='soft', weights=[1,2,3,4])
ec.fit(x_train_tfidf,y_train)
y_pred6 = ec.predict(x_test_tfidf)
print(accuracy_score(y_test, y_pred6))
print(classification_report(y_test, y_pred6))
```

In [ ]:

```
rfc = RandomForestClassifier(n_estimators=100, max_depth=9, random_state=0)
abc= AdaBoostClassifier(estimator=rfc, learning_rate=0.2, n_estimators=100)
abc.fit(x_train_tfidf, y_train)
y_pred7= abc.predict(x_test_tfidf)
print("Accuracy: "+str(accuracy_score(y_test, y_pred7)))
print(classification_report(y_test, y_pred7))
```

### **ngram range = 2, 3 бойынша:**¶

In [ ]:

```
Comparison_unibi = pd.DataFrame({'Logistic Regression': [accuracy_score(y_test,y_pred1)*100,f1_score(y_test,y_pred1,average='macro')*100,recall_score(y_test, y_pred1,average='micro')*100,precision_score(y_test, y_pred1,average='micro')*100],
                            'SVM':[accuracy_score(y_test,y_pred2)*100,f1_score(y_test,y_pred2,average='macro')*100,recall_score(y_test, y_pred2,average='micro')*100,precision_score(y_test, y_pred2,average='micro')*100],
                           'Naive Bayes':[accuracy_score(y_test,y_pred3)*100,f1_score(y_test,y_pred3,average='macro')*100,recall_score(y_test, y_pred3,average='micro')*100,precision_score(y_test, y_pred3,average='micro')*100],
                           'Random Forest':[accuracy_score(y_test,y_pred4)*100,f1_score(y_test,y_pred4,average='macro')*100,recall_score(y_test, y_pred4,average='micro')*100,precision_score(y_test, y_pred4,average='micro')*100],
                           'GradientBoosting':[accuracy_score(y_test,y_pred5)*100,f1_score(y_test,y_pred5,average='macro')*100,recall_score(y_test, y_pred5,average='micro')*100,precision_score(y_test, y_pred5,average='micro')*100],
                           'Ensembled':[accuracy_score(y_test,y_pred6)*100,f1_score(y_test,y_pred6,average='macro')*100,recall_score(y_test, y_pred6,average='micro')*100,precision_score(y_test, y_pred6,average='micro')*100],
                           'Adaboost':[accuracy_score(y_test,y_pred7)*100,f1_score(y_test,y_pred7,average='macro')*100,recall_score(y_test, y_pred7,average='micro')*100,precision_score(y_test, y_pred7,average='micro')*100],

})

print ('Cравнение с использованием uni-bi-gram(1,2)')
Comparison_unibi.rename(index={0:'Accuracy',1:'F1_score', 2: 'Recall',3:'Precision'}, inplace=True)
Comparison_unibi.head()
```

In [ ]:

```

```

In [ ]:

```

```
